# Alternating modified CAPOX/CAPIRI plus bevacizumab in untreated unresectable metastatic colorectal cancer: a phase 2 trial

**DOI:** 10.1038/s41392-024-02048-z

**Published:** 2024-12-11

**Authors:** Sheng Li, Xiaoyou Li, Hanfeng Xu, Jiayuan Huang, Jingni Zhu, Ying Peng, Jun Bao, Liangjun Zhu

**Affiliations:** 1 Department of Medical Oncology, The Affiliated Cancer Hospital of Nanjing Medical University & Jiangsu Cancer Hospital & Jiangsu Institute of Cancer Research, Nanjing, PR China; 2 Department of Oncology, The Second Hospital of Nanjing, Nanjing University of Chinese Medicine, Nanjing, PR China; 3 Senior Health Care Office, Jiangsu Provincial Health Commission, Nanjing, PR China

**Keywords:** Gastrointestinal cancer, Drug development

## Abstract

Previous studies showed encouraging efficacy of alternating FOLFOX/FOLFIRI for metastatic colorectal cancer (mCRC). This phase 2 trial (NCT04324476) aimed to evaluate efficacy and safety of alternating modified CAPOX (capecitabine and oxaliplatin)/modified CAPIRI (capecitabine and irinotecan) plus bevacizumab (anti-VEGF-A antibody) in untreated unresectable mCRC. Induction treatment included capecitabine 1000 mg/m^2^ bid D2–8 and D16–22, oxaliplatin 85 mg/m^2^ D1, irinotecan 150 mg/m^2^ D15, and bevacizumab 5 mg/kg D1 and 15 for 28-day cycles (up to six cycles). Capecitabine 1000 mg/m^2^ bid D2–15 and bevacizumab 7.5 mg/kg D1 for 21-day cycles were used as maintenance treatment. 52 patients were included. Median follow-up was 25.0 months. Median progression-free survival (PFS; the primary endpoint) was 11.0 months (95% CI 9.0–12.4). Subgroup analyses showed patients with neutrophil-to-lymphocyte ratio<5 or *RAS* wild-type disease had longer PFS (both P < 0.05). Objective response and disease control were obtained in 38 (73%; 95% CI 59%–84%) and 49 (94%; 95% CI 84%–99%), respectively. Mean depth of response, conversion and no evidence of disease rates were 46.0% ± 26.3%, 23% and 19%, respectively. Median overall survival was 28.1 months (18.4–34.0). Grade 3–4 treatment-related adverse events (TRAE) occurred in 17 (33%) patients. No treatment-related death was reported. The most common grade 3–4 TRAE were hypertension (13 [25%]), neutrophil count decreased (three [6%]), and hand-foot syndrome (two [4%]). In addition, grade 3–4 TRAE of diarrhea reported in one [2%] patient and no grade 3–4 peripheral neuropathy occurred. Thus, alternating modified CAPOX/CAPIRI plus bevacizumab had promising efficacy and acceptable safety. The regimen may be a novel option for untreated unresectable mCRC.

## Introduction

Colorectal cancer (CRC) stands as a remarkable global health challenge. According to the data from Global Cancer Statistics 2022, new cases of CRC was nearly 1.9 million, which accounts for 9.6% of all sites of cancers, and death cases of CRC reached approximately 0.9 million, representing 9.36% of all sites of cancers. These data indicate that CRC is the third most common malignant tumor and the second leading cause of cancer-related deaths worldwide.^1^ The incidence of CRC has been steadily rising, particularly in developing countries that are adopting Western lifestyles, with obesity, sedentary behavior, red meat, alcohol, and tobacco consumption as key contributing factors.^2^ In spite of advances in early detection and treatment leading to reduced mortality, the overall burden of CRC continues to grow, with an estimated 3.2 million new cases predicted for 2040.^3^ There is an urgent need of novel effective treatments. For patients with unresectable metastatic CRC (mCRC), the current standard of care involves a range of chemotherapy options, often combined with targeted therapies to enhance efficacy. First-line treatments for mCRC typically include triplet chemotherapy regimens, such as fluorouracil (with leucovorin), oxaliplatin, and irinotecan (i.e., FOLFOXIRI) with or without bevacizumab (an anti-vascular endothelial growth factor antibody), doublet chemotherapy regimens, such as fluorouracil (with leucovorin), and oxaliplatin (i.e., FOLFOX) with or without bevacizumab and fluorouracil (with leucovorin), and irinotecan (i.e., FOLFIRI) with or without bevacizumab, etc.^4,5^ For patients with certain subtypes of mCRC, chemotherapy in combination with panitumumab is also a treatment choice.^4,5^ Triplet chemotherapy regimens plus bevacizumab significantly improve survival, compared with doublet chemotherapy regimens plus bevacizumab. Nevertheless, this improvement comes with disadvantages of increased toxicity and expense, highlighting the need for a balance between therapeutic benefit, safety profiles, and cost.^6^

To explore new chemotherapy regimens, some single-arm studies investigated alternating schedules of fluoropyrimidines (fluorouracil or tegafur/uracil) plus oxaliplatin and fluoropyrimidines plus irinotecan without bevacizumab for the treatment of mCRC in the first-line setting.^7–9^ These regimens consist of three chemotherapy drugs. Thus, these combinations were considered to be variants of conventional triplet chemotherapy regimen. The results of these studies were promising, with the alternating regimens demonstrating both encouraging efficacy and acceptable safety profiles. In terms of efficacy, the median progression-free survival (PFS) or time to progression (TTP) were 8.8 to 13 months, and median OS ranged from 17.3 to 26.1 months. indicating that such alternating chemotherapy schedules could be potential first-line treatment options for patients with mCRC. In theory, addition of bevacizumab to these alternating schedules of chemotherapy could achieve better efficacy. However, there was no efficacy and safety data of alternating chemotherapy in combination with bevacizumab on mCRC.

Capecitabine is an oral fluoropyrimidine, which is absorbed from the gastrointestinal tract and converted enzymatically to the active metabolite, fluorouracil.^10^ Capecitabine, alone or combined with other agents, is also recommended for unresectable mCRC treatment.^4,5^ Capecitabine offers a convenient administration route, while a peripherally inserted central catheter or implanted port is required for fluorouracil administration. Given its established role in mCRC treatment, it is reasonable to consider that capecitabine-based alternating chemotherapy schedules in combination with bevacizumab would be a novel potent regimen for patients with unresectable mCRC.

Based on the aforementioned research findings, we have designed a new chemotherapy plus anti-angiogenic regimen: alternating schedule of modified 2-week capecitabine and oxaliplatin (i.e., mCAPOX) in combination with bevacizumab and modified 2-week capecitabine and irinotecan (i.e., mCAPIRI) in combination with bevacizumab. The regimen has the benefit of capecitabine’s convenience, synergistic therapeutic potential of an alternating chemotherapy schedule combined with bevacizumab, and lower total doses of drugs compared with triplets plus bevacizumab. The present study is designed to bridge this gap in knowledge by evaluating the efficacy and safety of the regimen in unresectable mCRC patients with no prior systemic treatment.

## Results

### Patients

Between Apr 2020 and May 2023, a total of 52 patients were enrolled, all of whom were included in the full analysis set (FAS; Supplementary Fig. S1). Mean age was 58.5 ± 9.1 years, with 38 (73%) patients <65 years old. 28 (54%) patients were male, and 24 (46%) were female. Tumor located in right colon in 19 (37%) patients. *RAS*, *BRAF*^V600E^, and *BRAF*^non-V600E^ mutations were detected in 30 (58%), 8 (15%), and 2 (4%) patients, respectively. 29 (56%) had received prior surgery, and 6 (12%) had received prior surgery adjuvant or neoadjuvant therapy. Radiotherapy had been performed in 1 (2%) patient before enrollment. Patients’ characteristics were summarized in Table 1. As of cut-off date on Dec 30, 2023, median follow-up was 25.0 months (95% confidence interval [CI] 19.6–32.8). Median numbers of cycles were 6.0 (interquartile range [IQR] 5.0–6.0) for capecitabine, oxaliplatin, and irinotecan, and 5.5 (IQR 4.0–6.0) for bevacizumab during induction treatment. Median numbers of cycles were both 4.0 (IQR 3.0–7.0) for capecitabine and bevacizumab during maintenance treatment. Secondary treatments were shown in Supplementary Table S1.

**Table 1:** Baseline demographic and tumor characteristics

Characteristics	Results (n = 52)
Age, years	58.5 ± 9.1
<65	38 (73%)
≥65	14 (27%)
Sex	
Male	28 (54%)
Female	24 (46%)
ECOG performance status	
0	33 (63%)
1	19 (37%)
2	0
Neutrophil-to-lymphocyte ratio^a^	
<5	45 (87%)
≥5	5 (10%)
Differentiation grade^b^	
Low	3 (6%)
Medium-low	13 (25%)
Medium	31 (60%)
High-medium	1 (2%)
Primary tumor location	
Right colon	19 (37%)
Left colon or rectum	33 (63%)
Time to metastasis	
Synchronous	37 (71%)
Metachronous	15 (29%)
Number of metastatic sites	
1	30 (58%)
2	15 (29%)
≥3	7 (13%)
Liver metastasis	38 (73%)
Mutations	
* RAS*	30 (58%)
* BRAF*^V600E^	8 (15%)
* BRAF*^non-V600E^	2 (4%)
Primary tumor location and mutation	
Right colon and *RAS* and *BRAF* wild-type	6 (11%)
Right colon and *RAS*-mutant	9 (17%)
Right colon and *BRAF*-mutant	4 (8%)
Left colon or rectum and *RAS* and *BRAF* wild-type	7 (13%)
Left colon or rectum and *RAS*-mutant	21 (40%)
Left colon or rectum and *BRAF*-mutant	6 (11%)
Microsatellite status^a^	
Microsatellite stability	50 (96%)
Microsatellite instability	0
Prior treatments	
Resection of primary tumor	29 (56%)
Adjuvant or neoadjuvant therapy	6 (12%)
Radiotherapy	1 (2%)

Data are expressed as mean ± standard deviation or n (%)

*ECOG* Eastern Cooperative Oncology Group

^a^Data were missing in two (4%) patients

^b^Data were missing in four (8%) patients

### Efficacy

**Fig. 1:**
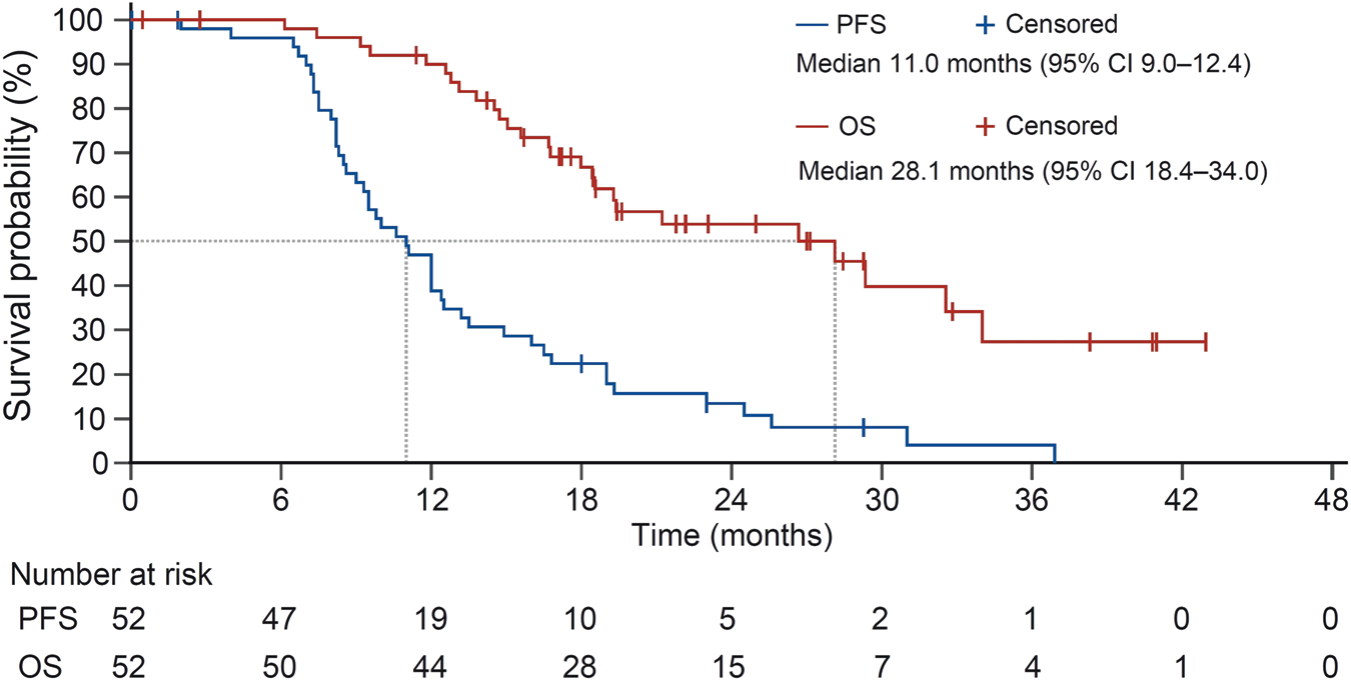
Kaplan-Meier curves of PFS and OS. Median PFS and OS were 11.0 months (95% CI 9.0–12.4) and 28.1 months (95% CI 18.4–34.0), respectively. PFS progression-free survival, OS overall survival, CI confidence interval

**Fig. 2:**
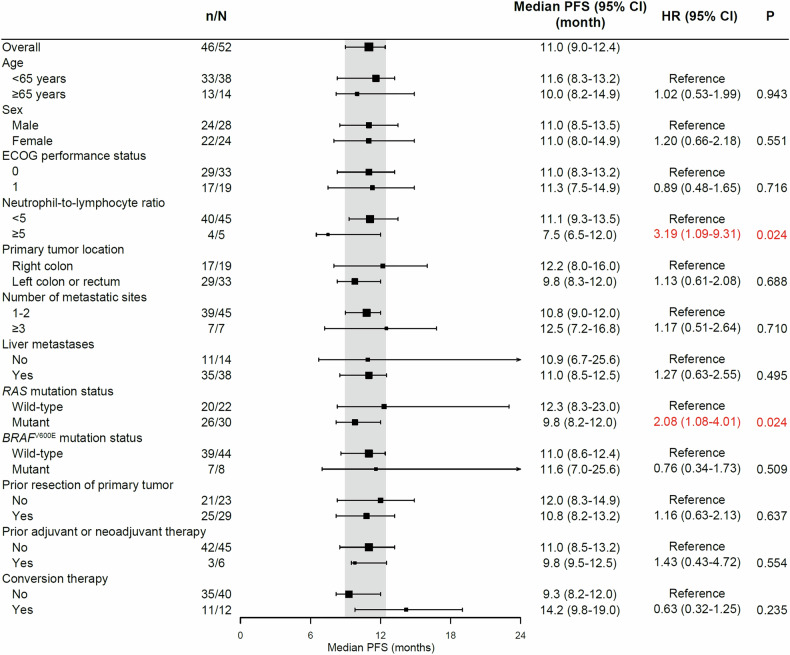
Forest plot of subgroup analyses on PFS. Red text indicates significant differences. HR for patients with neutrophil-to-lymphocyte ratio ≥5 vs <5 was 3.19 (95% CI 1.09–9.31; P = 0.024). HR for patients with *RAS*-mutant diseases vs *RAS* wild-type diseases was 2.08 (95% CI 1.08–4.01; P = 0.024). PFS progression-free survival, CI confidence interval, HR hazard ratio, ECOG Eastern Cooperative Oncology Group

Median PFS reached 11.0 months (95% CI 9.0–12.4; Fig. 1 and Table 2). Subgroup analyses demonstrated mCAPOX/mCAPIRI plus bevacizumab had similar PFS benefits in patients with different ages, sex, performance status, prior treatments, tumor locations or metastases, *BRAF* mutations, or with conversion therapy or not. However, patients with neutrophil-to-lymphocyte ratio (NLR) < 5 or *RAS* wild-type diseases showed better prognosis. Median PFS were 7.5 (95% CI 6.5–12.0) and 11.1 (95% CI 9.3–13.5) in patients with NLR ≥ 5 and <5, respectively (hazard ratio [HR] 3.19; 95% CI 1.09–9.31; P = 0.024). *RAS*-mutant patients had median PFS of 9.8 months (95% CI 8.2–12.0), while median PFS in *RAS* wild-type patients was 12.3 months (95% CI 8.3–23.0). HR was 2.08 (95% CI 1.08–4.01; P = 0.024; Fig. 2).

**Table 2:** Efficacy endpoints

Endpoints	Results (n = 52)
Progression-free survival	
Events	46 (88%)
Median, months	11.0 (95% CI 9.0–12.4)
Best overall response	
Complete response	4 (8%)
Partial response	34 (65%)
Stable disease	11 (21%)
Progressive disease	1 (2%)
Not evaluable	2 (4%)
Overall response	38 (73%; 95% CI 59%–84%)
Disease control	49 (94%; 95% CI 84%–99%)
Depth of response	46.0% ± 26.3%
Conversion	12 (23%)
No evidence of disease	10 (19%)
Median time to response, months	2.0 (95% CI 1.9–2.0)
Duration of response	
Events, n/N responders (%)	34/38 (89%)
Median, months	9.8 (95% CI 7.6–13.9)
Overall survival	
Events	26 (50%)
Median, months	28.1 (95% CI 18.4–34.0)

Data are expressed as n (%), n (%; 95% CI), or mean ± standard deviation unless otherwise specified

*CI* confidence interval

**Fig. 3:**
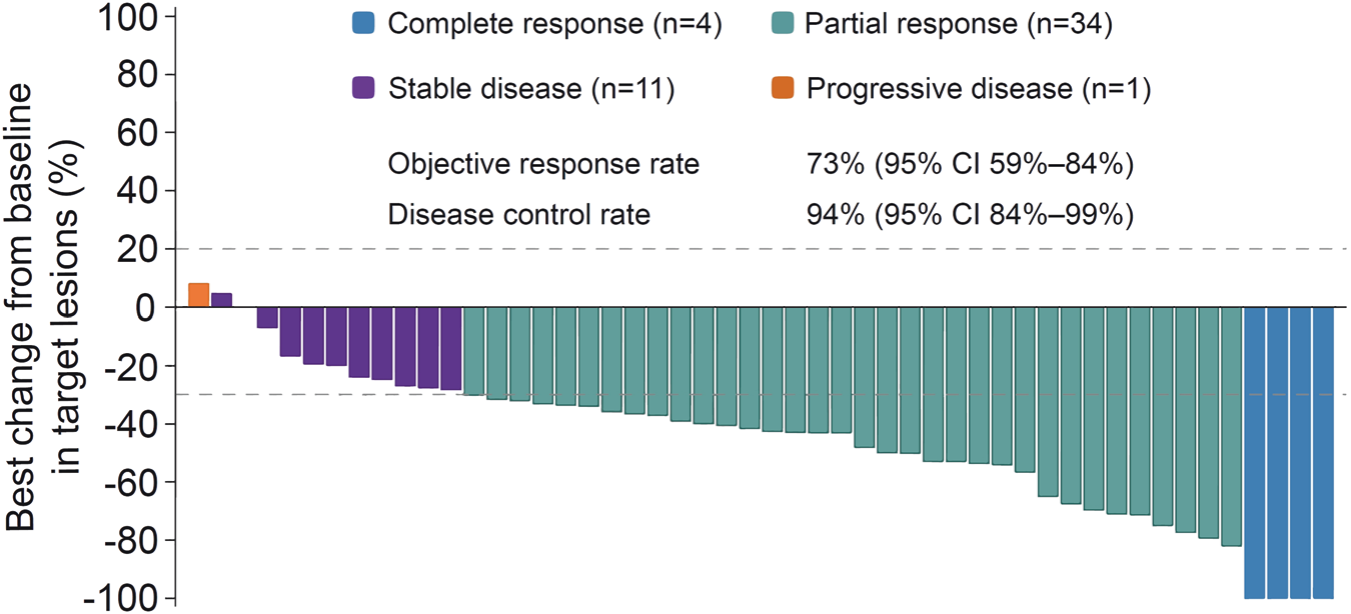
Waterfall plot. The dashed line at 20% is the cut-off value for progressive disease, and the dashed line at −30% is the cut-off value for partial response. Two (4%) patients were not evaluable. Objective response rate and disease control rate were 73% (95% CI 59–84%) and 94% (95% CI 84–99%), respectively. CI confidence interval

Four (8%) and 34 (65%) patients reached complete response and partial response, respectively. Overall response was achieved in 38 patients, and objective response rate (ORR) was 73% (95% CI 59–84%). 11 (21%) had stable disease. Thus, disease control rate (DCR) was 94% (95% CI 84–99%). Mean depth of response (DpR) was 46.0% ± 26.3% (Fig. 3). Conversion and no evidence of disease (NED) were reached in 12 (23%) and 10 (19%) patients, respectively (Table 2).

Median time to response (TTR) was 2.0 months (95% CI 1.9–2.0). In 38 patients with overall response, median duration of response (DoR) reached 9.8 months (95% CI 7.6–13.9). For overall survival (OS), 26 (50%) patients were censored, and the median was 28.1 months (95% CI 18.4–34.0; Fig. 1 and Table 2).

### Safety

The safety set (SS) included all 52 patients. Any grade and grade 3–4 treatment-related adverse events (TRAE) occurred in 50 (96%) and 17 (33%) patients, respectively. The most common grade TRAE were anemia (29 [56%]), neutrophil count decreased (25 [48%]), hypertension (24 [46%]), proteinuria (22 [42%]), and hand-foot syndrome (21 [40%]). Grade 3 to 4 TRAE with the highest incidence rates were hypertension (13 [25%]), neutrophil count decreased (three [6%]), and hand-foot syndrome (two [4%]; Table 3). Additionally, TRAE leading to dose reduction of any drug occurred in four (8%) patients, and no treatment-related death was reported.

**Table 3:** Safety summary (n = 52)

Treatment-related adverse events	Any grade	Grade 3–4
All	50 (96%)	17 (33%)
Anemia	29 (56%)	1 (2%)
Neutrophil count decreased	25 (48%)	3 (6%)
Hypertension	24 (46%)	13 (25%)
Proteinuria	22 (42%)	0
Hand-foot syndrome	21 (40%)	2 (4%)
Platelet count decreased	20 (38%)	1 (2%)
Hypoalbuminaemia	20 (38%)	1 (2%)
White blood cell count decreased	17 (33%)	1 (2%)
Alanine aminotransferase increased	17 (33%)	1 (2%)
Hyponatraemia	10 (19%)	0
Asthenia	6 (12%)	0
Peripheral neuropathy	6 (12%)	0
Diarrhea	5 (10%)	1 (2%)
Abdominal pain	5 (10%)	0
Nausea	4 (8%)	0
Gingival pain	4 (8%)	0
Epistaxis	3 (6%)	0
Vomiting	3 (6%)	0
Decreased appetite	3 (6%)	0
Alopecia	1 (2%)	0
Periodontal disease	1 (2%)	0

Data are expressed as n (%)

## Discussion

The present single-arm phase 2 study showed favorable efficacy and acceptable safety profiles of the alternating schedule of mCAPOX/mCAPIRI plus bevacizumab for unresectable mCRC patients with no prior systemic treatment.

The combination regimen of alternating mCAPOX/mCAPIRI plus bevacizumab resulted in a median PFS of 11.0 months (95% CI 9.0–12.4) and a median OS of 28.1 months (95% CI 18.4–34.0). In previous studies of alternating schedules of doublet chemotherapy without bevacizumab for unresectable mCRC in the first-line setting, the median PFS or time to progression (TTP) ranged from 8.8 to 13 months, and the median OS ranged from 17.3 to 26.1 months.^7–9^ For the first-line triplet chemotherapy (FOLFOXIRI) without bevacizumab in patients with no prior systemic treatment, the median PFS or TTP was 8.4 to 10.8 months, and the median OS was 21.5 to 28.4 months.^11–13^ A meta-analysis involved studies of first-line triplet chemotherapy (FOLFOXIRI) in combination with bevacizumab and doublet chemotherapy (FOLFOX or FOLFIRI) in combination with bevacizumab.^6^ Compared with the pooled data in the meta-analysis, the present study included a higher proportion of patients with *BRAF* mutations (19% vs 8%), which may result in worse prognosis. The pooled median PFS in the meta-analysis was 12.2 months for triplet chemotherapy plus bevacizumab and 9.9 months for doublet chemotherapy plus bevacizumab. The meta-analysis also provided a pooled median OS of 28.9 months for triplet chemotherapy combined with bevacizumab and 24.5 months for doublet chemotherapy combined with bevacizumab. Additionally, in the present study, patients who received the alternating schedule of mCAPOX/mCAPIRI combined with bevacizumab reached an ORR of 73% (95% CI 59–84%) and a DpR of 46.0% ± 26.3%. While ORR was 64.5% for triplet chemotherapy plus bevacizumab and 53.6% for doublet chemotherapy plus bevacizumab, and DpR were 43.4% for triplet chemotherapy combined with bevacizumab and 37.8% for doublet chemotherapy combined with bevacizumab.^6,14^ Thus, it is suggested that the alternating schedule of mCAPOX/mCAPIRI plus bevacizumab may have promising anti-tumor activity comparable with triplet FOLFOXIRI plus bevacizumab. We speculate that the alternating nature of chemotherapy may be less likely to lead to drug resistance, compared with conventional doublet chemotherapy regimens. Of note, due to a lack of head-to-head randomized controlled trials, the results of each study should be interpreted with caution.

Subgroup analyses for PFS indicated that the alternating schedule of chemotherapy in combination with bevacizumab brought better benefits in mCRC patients with NLR < 5 or *RAS* wild-type diseases. As a systemic inflammation indicator, increased NLR was found associated with poorer prognosis in CRC, irrespective of tumor in early stage or advanced stage.^15–17^ The role of systemic inflammation in tumor progression is well-established,^18^ and the interaction between alternating schedules of chemotherapy and inflammation is worthy to be further investigated. In previous studies, it was found patients with *RAS* wild-type mCRC had better outcome than those with *RAS* mutation,^19,20^ which was consistent with the findings in the present study. Chemotherapy in combination with anti-epidermal growth factor receptor (EGFR) antibody (e.g., panitumumab) may be more effective than chemotherapy plus bevacizumab for patients with unresectable *RAS* wild-type mCRC.^21,22^ Further studies should evaluate the efficacy and safety of alternating chemotherapy plus bevacizumab vs (alternating or conventional) chemotherapy plus anti-EGFR antibody. In treatment-naïve mCRC patients with *RAS* mutation, median PFS for alternating schedules of chemotherapy plus bevacizumab, triplet chemotherapy plus bevacizumab, and doublet chemotherapy plus bevacizumab were 9.8, 11.5 to 12.5, and 8.1 to 9.7 months, respectively.^19,20,23–25^ In addition, the alternating regimen in combination with bevacizumab had encouraging efficacy in mCRC patients with *BRAF*^V600E^ mutation. Median PFS for the regimen reached 11.6 months, which is better than triplet chemotherapy plus bevacizumab and doublet chemotherapy plus bevacizumab (median PFS 7.5 to 10.7 and 5.5 to 6.6 months, respectively).^19,25–27^ The mechanisms of alternating schedule of chemotherapy treating *RAS*- or *BRAF*-mutant tumors are also a direction for future research.

In terms of safety, although the incidence rate of any grade TRAE was 96%, only 33% of patients suffered grade 3 to 4 TRAE. The most frequent grade 3 to 4 TRAE included hypertension, neutrophil count decreased, and hand-foot syndrome, all of which were manageable. Further, no patient died due to TRAE. There was no unexpected TRAE reported, either. In detail, compared with regimens consisting of fluorouracil, capecitabine-based chemotherapy had a higher incidence rate of hand-foot syndrome.^28,29^ The present study had a similar result. Further, it was noteworthy that the alternating chemotherapy schedule regimen may have obviously lower risks of neutrophil count decreased, peripheral neuropathy, and diarrhea than triplet and doublet chemotherapy.^6^ The reason was considered that the dose intensity of each oxaliplatin and irinotecan in the alternating regimen was half of that in conventional chemotherapies. With regard to capecitabine, the dose in the alternating chemotherapy (1000 mg/m^2^ twice daily for a total of 14 days in a 28-day cycle) was also lower than that in the conventional chemotherapy regimen (1000 mg/m^2^ twice daily for a total of 14 days in a 21-day cycle).

As an alternating schedule, the doses of oxaliplatin and irinotecan in mCAPOX/mCAPIRI plus bevacizumab regimen were half of those in triplet chemotherapy plus bevacizumab, which means the regimen may cost less than triplets and reduce the disease burden. Additionally, capecitabine is administered orally, while fluorouracil needs to be given using a peripherally inserted central catheter or implanted port. Thus, the administration of capecitabine is more convenient with less expense.

Indeed, there are several limitations in the present study. First, the efficacy in subgroups should be interpreted with caution because that the sample sizes are small. Then, head-to-head comparison of alternating mCAPOX/mCAPIRI chemotherapy combined with bevacizumab and triplet chemotherapy combined with bevacizumab in the first-line setting was not conducted in this study. The regimen of mCAPOX/mCAPIRI plus bevacizumab needs to be evaluated in randomized controlled trials in future.

In conclusion, the alternating schedule of mCAPOX/mCAPIRI chemotherapy in combination with bevacizumab had promising efficacy in unresectable mCRC patients with no prior systemic treatment. The toxicities were acceptable and manageable, with no new safety signal found. The regimen may be a novel option for unresectable mCRC in the first-line setting. International randomized controlled trials are warranted.

## Methods

### Study design

This single-arm phase II trial was conducted at Jiangsu Cancer Hospital, Nanjing, China. The protocol was approved by the ethics committee of Jiangsu Cancer Hospital (approval number: 2020-006) and registered on ClinicalTrials.gov (NCT04324476). The study was conducted in accordance with the Declaration of Helsinki and Good Clinical Practice. All patients provided written informed consent before participation.

### Eligible criteria

Key eligible criteria were 1) 18–75 years old; 2) had histologically confirmed mCRC with unresectable metastases; 3) without prior systemic treatment for metastatic diseases; (Adjuvant chemotherapy was allowed.) 4) with ≥one measurable lesion via Response Evaluation Criteria in Solid Tumours (RECIST) v1.1; 5) Eastern Cooperative Oncology Group performance status of 0–2; 6) adequate organ function; and 7) life expectancy ≥3 months. Patients were excluded if they had 1) uncontrolled symptomatic brain metastases; 2) intestinal obstructions; 3) severe cardiac diseases (e.g., heart failure and myocardial infarction); 4) uncontrolled hypertension; 5) severe proteinuria with dipstick 2+ or more; or 6) severe infections.

### Treatment

Participants received the alternating schedule of mCAPOX/mCAPIRI plus bevacizumab as the induction treatment, including oral capecitabine 1000 mg/m^2^ bid on D2–8 and D16–22, intravenous oxaliplatin 85 mg/m^2^ on D1, irinotecan 150 mg/m^2^ on D15, and bevacizumab 5 mg/kg on D1 and 15. The treatment was administered every 28 days for up to six cycles. Then, maintenance treatment was given, including capecitabine 1000 mg/m^2^ bid on D2–15 and bevacizumab 7.5 mg/kg on D1 for a 21-day cycle, until disease progression, intolerable toxicity, withdrawal of consent, loss of follow-up, start of other antitumor treatment, or death. Dose interruption and reduction of chemotherapy and dose interruption of bevacizumab were allowed when grade ≥3 adverse events occurred or at the discretion of investigators. However, dose reduction of bevacizumab was not permitted.

### Study assessment and endpoints

Tumors were evaluated using computed tomography or magnetic resonance imaging according to RECIST v1.1 within four weeks before study treatment initiation, every eight weeks during induction treatment, and every six weeks during maintenance treatment until disease progression, withdrawal of consent, loss of follow-up, start of other antitumor treatment, or death. After the study treatment discontinuation, survival follow-up was performed every 12 weeks. AE was classified and graded according to the Common Terminology Criteria for Adverse Events v4.03 from study treatment initiation throughout the treatment period and four weeks after the end of treatment.

The primary endpoint was investigator-assessed PFS (defined as the time from study treatment initiation to the first time of disease progression or death from any cause, whichever occurred earlier). Secondary endpoints included investigator-assessed ORR (defined as the proportion of patients with confirmed complete response [CR] or partial response [PR]), OS (defined as the time from study treatment initiation to death from any cause), and safety. DCR (defined as the proportion of patients with CR, PR, or stable disease [SD]), DpR (defined as the percentage change in the sum of longest diameters of RECIST target lesions at the nadir compared with baseline), conversion rate (defined as the proportion of patients receiving surgeries after study treatment), NED rate (defined as the proportion of patients with CR or R0 resection of all lesions), TTR (defined as the time from study treatment initiation to the first CR or PR, whichever occurred earlier), and DoR (defined as the time from the first CR or PR to disease progression or death from any cause, whichever occurred earlier) were also analyzed.

### Statistical analysis

To reject the null hypothesis of a median PFS ≤ 9.0 months with a two-sided alpha level of 0.1 and a power of 0.6, assuming a median PFS of 12.0 months, 46 patients were needed. Considering a dropout rate of 10%, a total of 52 patients are required in the study.

The efficacy and safety endpoints were evaluated in the FAS and SS, respectively. The FAS included all patients who received at least one dose of the study drug. The SS included patients who received at least one dose of study drug and one post-baseline safety evaluation.

Continuous data were presented as mean ± standard deviation or median (interquartile range). Categorical data were presented as number (percentage). Median PFS and OS and corresponding 95% CI were calculated using Kaplan-Meier method. 95% CI for ORR and DCR were estimated using Clopper–Pearson method. Post-hoc subgroup analyses on PFS were based on age, sex, tumor location, mutation status, etc. HR and 95% CI between subgroups were estimated using Cox proportional hazards model, and log-rank test was used for comparison. A two-sided P < 0.05 was considered statistically significant. Statistical analysis was performed using SAS version 9.4 (SAS Institute Inc., Cary, NC, USA), R software version 4.3.0 (R Core Team), and R package “forestplot”.

## Supplementary information

Supplementary Material-clean

study protocol

appendix I

appendix II

appendix III

appendix IV

appendix V

## Data Availability

Data are available from the corresponding author upon reasonable request.
