# Supplementary material for: Alternating modified CAPOX/CAPIRI plus bevacizumab in untreated unresectable metastatic colorectal cancer: a phase 2 trial
Source: Signal Transduct Target Ther. 2024 Dec 11;9:346. doi: 10.1038/s41392-024-02048-z (PMC11631963; doi:10.1038/s41392-024-02048-z)
Supplement: Supplementary file 1 — Supplementary Material-clean [file 41392_2024_2048_MOESM1_ESM.docx]

Supplementary Materials for

Alternating modified CAPOX/CAPIRI plus bevacizumab in untreated unresectable metastatic colorectal cancer: a phase 2 trial

Sheng Li^1,*^, Xiaoyou Li^1,*^, Hanfeng Xu^2^, Jiayuan Huang^1^, Jingni Zhu^1^, Ying Peng^1^, Jun Bao^3,**^, Liangjun Zhu^1,**^

Correspondence to: [baojun@jszlyy.com.cn](mailto:baojun@jszlyy.com.cn) and [zhulj98@foxmail.com](mailto:zhulj98@foxmail.com)

**This PDF file includes:**

Figures. S1

Tables S1


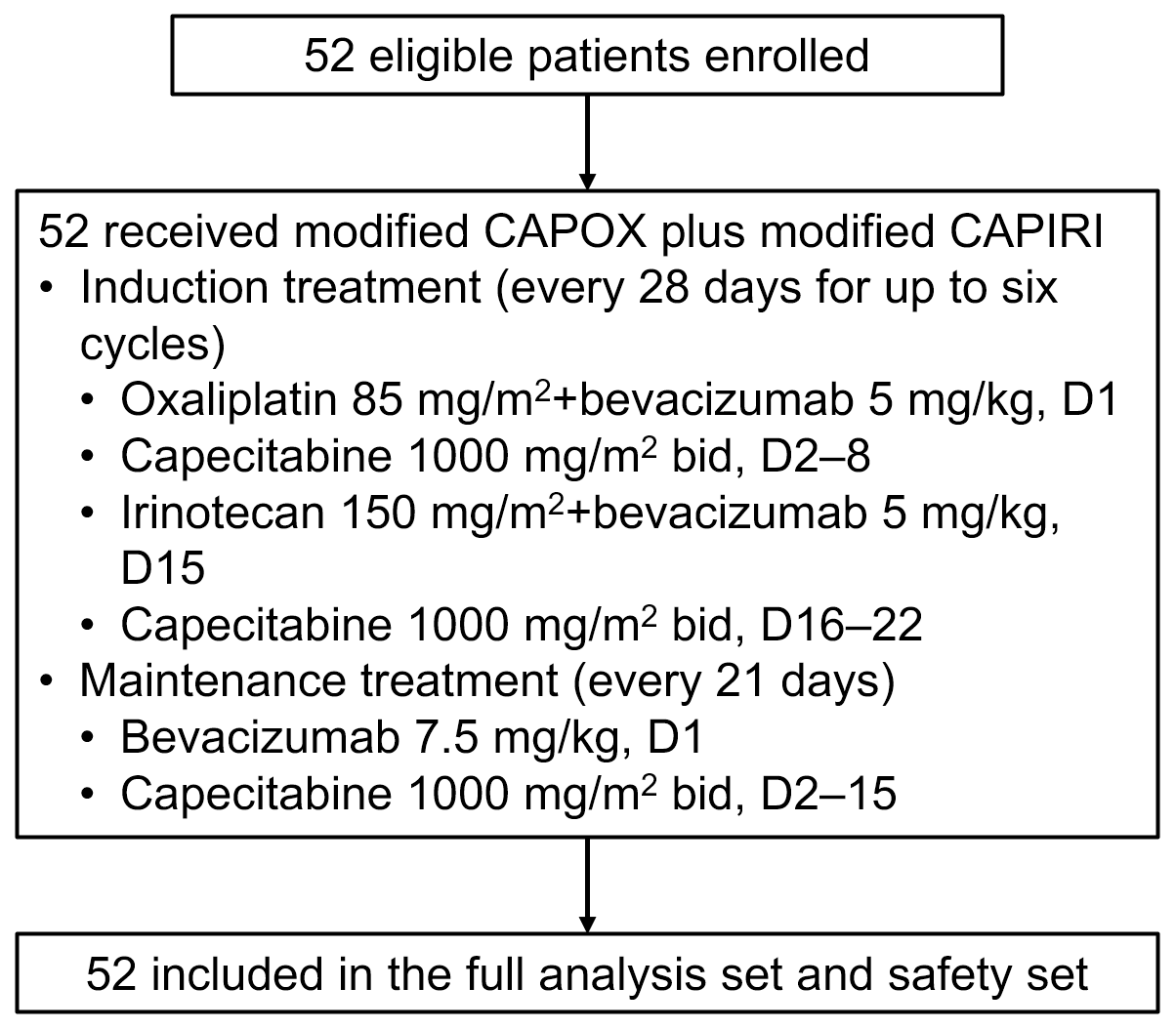


Figure. S1.

Flow chart. A total of 52 patients were enrolled. Alternating schedule of modified CAPOX and modified CAPIRI plus bevacizumab were given as induction treatment. Capecitabine and bevacizumab were given as maintenance treatment. All 52 patients were included for analysis.

Table S1. Summary of second-line treatments.

| Status and second-line treatments | Numbers of patients |
| --- | --- |
| Alive after disease progression | 46/52 (88%) |
| Any second-line therapy | 40/46 (87%) |
| Chemotherapy plus bevacizumab | 10 (25%) |
| Irinotecan-based doublet | 6 (15%) |
| Oxaliplatin-based doublet | 2 (5%) |
| FOLFOXIRI | 1 (2%) |
| Alternating chemotherapy rechallenge | 1 (2%) |
| Fruquintinib alone | 10 (25%) |
| Regorafenib plus PD-1 inhibitor | 9 (22%) |
| Regorafenib alone | 3 (8%) |
| Chemotherapy plus cetuximab | 3 (8%) |
| Raltitrexed | 1 (2%) |
| Irinotecan | 2 (5%) |
| Chemotherapy alone (capecitabine) | 1 (2%) |
| Other | 4 (10%) |

Data are expressed as n/N (%) or n (%).

*FOLFOXIRI* fluorouracil/leucovorin, oxaliplatin, and irinotecan, *PD-1* programmed cell death protein 1
