# Supplementary material for: Alternating modified CAPOX/CAPIRI plus bevacizumab in untreated unresectable metastatic colorectal cancer: a phase 2 trial
Source: Signal Transduct Target Ther. 2024 Dec 11;9:346. doi: 10.1038/s41392-024-02048-z (PMC11631963; doi:10.1038/s41392-024-02048-z)
Supplement: Supplementary file 2 — study protocol [file 41392_2024_2048_MOESM2_ESM.pdf]

A MULTICENTER PHASE II CLINICAL STUDY OF BEVACIZUMAB COMBINED WITH BIWEEKLY XELOX/XELIRI ALTERNATING REGIMEN IN THE FIRST-LINE TREATMENT OF UNRESECTABLE ADVANCED COLORECTAL CANCER

CLINICAL STUDY PROTOCOL

Clinical Trial Protocol Number: JS-GI1902

Clinical Study Leading Site:

Jiangsu Cancer Hospital (Number: 01)

Cooperating Organization: Jiangsu Cooperative Group for Clinical Study of Gastrointestinal Cancer

Date of Protocol Finalization: Sep. 13, 2019

INVESTIGATORS AND CLINICAL RESEARCH PERSONNEL

Principal Investigator:

Jiangsu Cancer Hospital Liangjun Zhu

Data Management and Statistical Analysis: Department of Medical Oncology, Jiangsu Cancer Hospital

Study Team Members:

Rui Wang, Chunrong Zhu, Zhixiang Zhuang, Mi Yang, Guoxin Mao, Yiyuan Wan, Li Wan, Chunbin Wang, Runjie Wang, Yingwei Zhu, Xiaomin Zhong, Jun Liu, Juanying Xu, Qing Sun, Sheng Li, Wenguang Li, Yu Li, Yan Yang, Huiling Shen, Haizhu Song, Yusong Zhang, Zhengrong Zhang, Xizhi Zhang, Youwei Zhang, Xianwen Zhang, Zhisheng Zhang, Yue Zhang, Yong Mao, Yu Fan, Wenbiao Zhou, Lili Zhou, Wenwei Hu, Nan Hu, Jing Hu, Hanfeng Xu, Lin Cui, Hua Jiang, Gaohua Han, Jiandong Tong, Maohuai Cai, Feng Xiong, Jing Sun, Yan Sun, Quan'an Zhang, Jiawei Zhang, Chao Li, Tongshan Wang, Jun Wu, Ping Chen, Xia Zhao, Yuan Yuan, Keyang Qian, Zhengxiang Han, Yan Ge

TABLE OF CONTENTS

I STUDY BACKGROUND
II ISTUDY OBJECTIVES
III STUDY DESIGN
IV SELECTION OF STUDY POPULATION
V DOSE MODIFICATION AND DISCONTINUATION
VI CONCOMITANT THERAPY
VII OBSERVATION INDICATORS AND EXAMINATION TIME
VIII CLINICAL EVALUATION
IX ADVERSE EVENT RECORDING AND REPORTING
X DATA MANAGEMENT AND STATISTICAL ANALYSIS
XI ETHICAL STANDARDS AND INFORMED CONSENT
XII DATA STORAGE
XIII STUDY PROGRESS AND COMPLETION TIMELINE
Appendix I: ECOG Performance Status Criteria
Appendix II: AJCC TNM Staging System for Colorectal Cancer (8th Edition)
Appendix III: National Cancer Institute (NCI) Common Terminology Criteria for Adverse Events (CTCAE) v5.0
Appendix IV: Response Criteria in Solid Tumors (RECIST) v1.1
Appendix V: European Organization for Research and Treatment of Cancer Quality of Life Questionnaire Core 30 (EORTC QLQ-C30)

I STUDY BACKGROUND

Worldwide, colorectal cancer ranks third in incidence and second in mortality, second only to lung cancer. In China, the incidence of colorectal cancer has increased greatly in recent years. According to the latest statistics from GLOBOCAN 2018, the incidence is second only to lung cancer, and the mortality rate ranks fifth[1]. Although most newly diagnosed patients can undergo radical resection of the primary intestinal lesion, due to the hidden symptoms of some patients and the need for invasive examination for diagnosis, 34% of patients are in the advanced stage at the time of initial diagnosis, and some patients undergoing radical resection will develop distant metastases. Most of these advanced cases are patients with unresectable metastases[2-3]. Both conversion therapy and palliative therapy are particularly important for these patients. Improving objective response rate (ORR) and prolonging overall survival (OS) are important indicators of achieving the goals. Therefore, a large number of clinical studies have been conducted to further optimize treatment regimens based on stratification by left vs. right side and genetic status.

In 2006 and 2007, Greek and Italian researchers reported studies on irinotecan, oxaliplatin, and fluorouracil in combination as the first-line treatment for advanced metastatic colorectal cancer, respectively[4-5]. In addition, the Italian GONO study group showed overall survival benefits, and the fever of the three-drug combination therapy has gradually risen since then. After entering the era of targeted therapy, the combination of three drugs with the targeted drug bevacizumab has further consolidated the status of this combination regimen[6-7], and recently, anti-EGFR monoclonal antibodies cetuximab and panitumumab have also joined the ranks of three-drug regimens[8-9]. The three-drug combination with bevacizumab has gained favor among clinicians due to its high response rate and significant prolongation of overall survival. Furthermore, for specific types of intestinal cancer, such as those with BRAF mutations, the benefits are even more pronounced. However, the fatal weakness of three-drug regimens is their unoptimistic safety. Severe gastrointestinal and hematological toxicities are observed in a considerable number of patients in clinical practice in China, leading to decreased quality of life and early withdrawal from the plan.

In studies exploring drug regimens, there is also another drug alternating mode. As early as 2005, Spanish researchers reported the study of alternating FOLFOX and FOLFIRI as the first-line treatment for advanced colorectal cancer. The short-term response rate reached 54%, the mPFS was 13 months, and the Grade 3–4 toxicities were significantly reduced compared with FOLFOXIRI. In addition, 100% of the patients received irinotecan or oxaliplatin[10].

Continuous infusion of fluorouracil is a traditional dosing method. Standard FOLFOX, FOLFIRI, and FOLFOXIRI use this infusion method, but this infusion method usually requires deep vein catheterization, and the treatment time is relatively long, which brings inconvenience to the patient's life. Capecitabine, an oral fluorouracil derivative, is equivalent to continuous infusion of fluorouracil in the treatment of advanced intestinal cancer. XELOX is a commonly used chemotherapy regimen, while the XELIRI regimen has also gained a place in second-line treatment. However, both regimens adopt a 3-weekly schedule, in which the single dose of oxaliplatin and irinotecan is high, exerting significant gastrointestinal and hematological toxicities. Therefore, there are also modified biweekly regimens in clinical practice that increase the frequency and reduce the single dose[11-14].

Based on the above background, considering both the efficacy of a three-drug regimen and the control of toxic reactions, and aiming to expose patients to three chemotherapy drugs as soon as possible, this study adopts a bevacizumab combined with biweekly XELOX/XELIRI alternating regimen in the first-line treatment of unresectable advanced colorectal cancer to explore its efficacy and safety.

REFERENCES

[1] LOBOCAN 2018

[2] Schmoll HJ, Van Cutsem E, Stein A, et al. ESMO Consensus Guidelines for management of patients with colon and rectal cancer. a personalized approach to clinical decision making. Ann Oncol. 2012;23:2479–2516.

[3] Van Cutsem E, Nordlinger B, Adam R, et al.Towards a pan-European consensus on the treatment of patients with colorectal liver metastases.Eur J Cancer. 2006 Sep;42(14):2212-21.

[4] Souglakos J, Androulakis N, Syrigos K, et al. FOLFOXIRI (folinic acid, 5-fluorouracil, oxaliplatin and irinotecan) vs FOLFIRI (folinic acid, 5-fluorouracil and irinotecan) as first-line treatment in metastatic colorectal cancer (MCC): a multicentre randomised phase III trial from the Hellenic Oncology Research Group (HORG) Br J Cancer. 2006;94(6):798–805.

[5] Falcone A, Ricci S, Brunetti I, et al. Phase III trial of infusional fluorouracil, leucovorin, oxaliplatin, and irinotecan (FOLFOXIRI) compared with infusional fluorouracil, leucovorin, and irinotecan (FOLFIRI) as first-line treatment for metastatic colorectal cancer: the Gruppo Oncologico Nord Ovest. J Clin Oncol. 2007;25(13):1670–1676.

[6] Loupakis F, Cremolini C, Masi G, et al. Initial therapy with FOLFOXIRI and bevacizumab for metastatic colorectal cancer. N Engl J Med. 2014;371(17):1609–1618.

[7] Cremolini C, Loupakis F, Antoniotti C, et al. FOLFOXIRI plus bevacizumab versus FOLFIRI plus bevacizumab as first-line treatment of patients with metastatic colorectal cancer: Updated overall survival and molecular subgroup analyses of the open-label, phase 3 TRIBE study. Lancet Oncol. 2015;16:1306–1315.

[8] Garufi C, Torsello A, Tumolo S, et al. Cetuximab plus chronomodulated irinotecan, 5-fluorouracil, leucovorin and oxaliplatin as neoadjuvant chemotherapy in colorectal liver metastases: POCHER trial. Br. J. Cancer. 2010;103:1542–1547.

[9] Geissler M, et al. 2018 ESMO Abstract 453PD.

[10] Aparicio J, Fernandez-Martos C, Vincent JM, et al. FOLFOX alternated with FOLFIRI as first-line chemotherapy for metastatic colorectal cancer. Clin Colorectal Cancer. 2005;5:263–7.

[11] García-Alfonso P, Muñoz-Martin AJ, Alvarez-Suarez S, et al. Bevacizumab in combination with biweekly capecitabine and irinotecan, as first-line treatment for patients with metastatic colorectal cancer. Br J Cancer. 2010;103:1524–8.

[12] Mizushima T, Fukunaga M, Sueda T,et al.Phase I/II study of bi-weekly XELIRI plus bevacizumab treatment in patients with metastatic colorectal cancer resistant to oxaliplatin-based first-line chemotherapy.Cancer Chemother Pharmacol. 2017 Jul;80(1):81-90.

[13] Soda H, Maeda H, Hasegawa J,et al.Multicenter Phase II study of FOLFOX or biweekly XELOX and Erbitux (cetuximab) as first-line therapy in patients with wild-type KRAS/BRAF metastatic colorectal cancer: The FLEET study.BMC Cancer. 2015 Oct 14;15:695.

[14] Hurwitz H, Mitchell EP, Cartwright T,et al.A randomized, phase II trial of standard triweekly compared with dose-dense biweekly capecitabine plus oxaliplatin plus bevacizumab as first-line treatment for metastatic colorectal cancer: XELOX-A-DVS (dense versus standard).Oncologist. 2012;17(7):937-46

II ISTUDY OBJECTIVES

1. Primary Objective

To evaluate the progression-free survival (PFS1) of bevacizumab combined with biweekly XELOX/XELIRI alternating regimen in the first-line treatment of unresectable advanced colorectal cancer.

2. Secondary Objective

To evaluate the second progression-free survival (PFS2), objective response rate (ORR), overall survival (OS), and safety of bevacizumab combined with biweekly XELOX/XELIRI alternating regimen in the first-line treatment of unresectable advanced colorectal cancer.

III STUDY DESIGN

1. This is a single-arm, open-label, multi-center study evaluating the efficacy and safety of bevacizumab combined with biweekly XELOX/XELIRI alternating regimen in the first-line treatment of unresectable advanced colorectal cancer.

2. Study Protocol

a Dosing regimen: Bevacizumab combined with biweekly XELOX/XELIRI alternating regimen;

Induction chemotherapy regimen: Bevacizumab 5 mg/kg, ivgtt, D1 and D15;

Oxaliplatin 85 mg/m^2, ivgtt, D1;

Irinotecan 150 mg/m^2, ivgtt, D15;

Capecitabine 1000 mg/m^2, orally, BID, D2–D8 and D16–D22.

28-day cycles

Maintenance chemotherapy regimen: Bevacizumab 7.5 mg/kg, ivgtt, D1;

Capecitabine 1000 mg/m^2, orally, BID, D2–D15.

21-day cycles

Efficacy was evaluated every 2 cycles (8 weeks) of chemotherapy during the induction phase and every 2 cycles (6 weeks) of chemotherapy during the maintenance phase.

b Surgery:

For patients with successful conversion after treatment, resection of the primary lesion and metastases can be considered. One cycle of bevacizumab should be interrupted before surgery, and 6 months of perioperative treatment should be completed after surgery, followed by maintenance therapy with bevacizumab + capecitabine. However, the total course of postoperative treatment should not exceed 6 months.

3 Study Drugs

(1) Bevacizumab, 100 mg/vial, Shanghai Roche;

Dosing method: Under sterile conditions, slowly inject the required volume of the drug into a 100 mL normal saline (0.9% sodium chloride) infusion bag, and the first intravenous infusion should last for at least 90 min. If the first infusion is well tolerated, the duration of the second infusion can be shortened to 60 min and 30 min for subsequent infusions.

(2) Capecitabine, 500 mg/tablet, Shanghai Roche;

Dosing method: The dose is calculated based on the body surface area and should be taken orally half an hour after a meal.

(3) Oxaliplatin for injection, 50 mg/vial, Jiangsu Hengrui;

Dosing method: Dissolve oxaliplatin in 250–500 mL of 5% glucose solution and infuse it continuously via a peripheral or central vein over 2–6 h.

(4) Irinotecan for injection, 100 mg/vial, Jiangsu Hengrui;

Dosing method: Dissolve irinotecan in 250 mL of normal saline and infuse it continuously via a peripheral or central vein over 0.5–1.5 h.

4 Sample Size

This is an exploratory trial with a sample size of 50 subjects.

5 Study Endpoints

Primary endpoint:

Progression-free survival (PFS1).

Secondary endpoints:

Second progression-free survival (PFS2), objective response rate (ORR), overall survival (OS), and safety.

Collection of biomarkers, including RAS, BRAF, MMR, HER2, etc.

6 Study Process

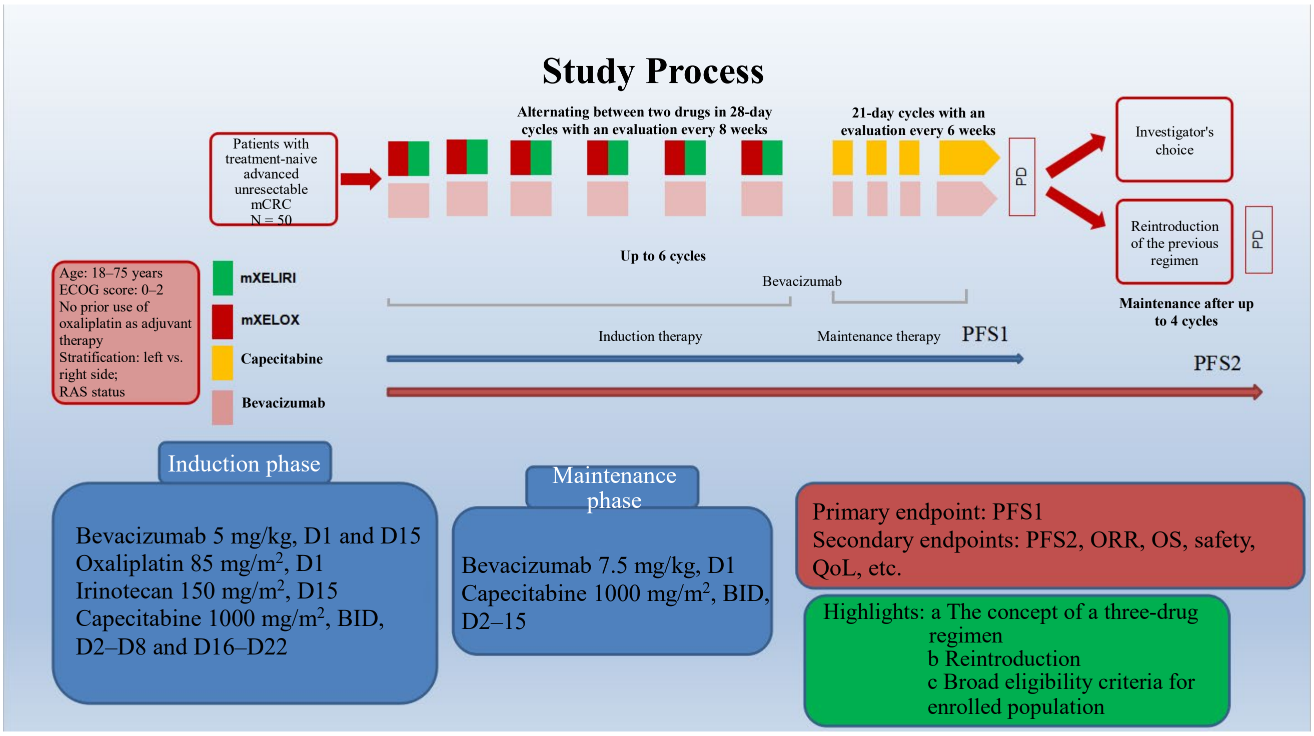

IV SELECTION OF STUDY POPULATION

1. Inclusion Criteria

a. Subjects aged 18–75 years;

b. Subjects with histologically confirmed treatment-naive advanced colorectal adenocarcinoma, or those who have developed metastases more than 12 months after radical surgery and whose metastatic lesions are unresectable;

c. Subjects with an ECOG score of ≤ 2 and a life expectancy of ≥ 3 months;

d. White blood cells ≥ 3.5 × 10^9/L, neutrophils ≥ 1.5 × 10^9/L, hemoglobin ≥ 100 g/L, platelets ≥ 80 × 10^9/L; serum liver enzymes in subjects without liver metastasis are not higher than 2.5 times the upper limit of normal (ULN); serum liver enzymes in subjects with liver metastasis are not higher than 5 times the ULN; serum bilirubin level is not higher than 1.5 times the ULN; serum creatinine level is not higher than 1.5 times the ULN;

e. Presence of at least one measurable lesion by CT or MRI;

f. No history of other malignant tumors;

g. Willing to take contraceptive measures if of childbearing potential;

h. Signing of written informed consent form.

2. Exclusion Criteria

a. Subjects with allergies or hypersensitivity to study drugs, and those with autoimmune diseases;

b. Subjects with only non-measurable lesions, such as pleural effusion, ascites, lymphangitis carcinomatosa, diffuse liver invasion, and bone metastasis; those with no measurable lesions or those with non-evaluable lesions;

c. Pregnant or lactating women, or those of childbearing potential who refuse to take contraceptive measures;

d. Subjects with uncontrolled symptomatic brain metastases or mental disorders that prevent accurate expression of subjective symptoms;

e. Subjects with failure of vital organs;

f. Subjects with factors affecting dose administration, absorption, distribution, metabolism, and excretion; uncontrolled epilepsy, central nervous system disorders, or loss of insight due to psychosis; physiological or pathological malnutrition, chronic diarrhea, or cachexia;

g. Subjects with complete or incomplete bowel obstruction.

h. Subjects with serious heart disease or a history of heart disease, including a documented history of congestive heart failure, high-risk uncontrolled arrhythmia, angina requiring medication, clinically defined valvular heart disease, a history of severe myocardial infarction, and refractory hypertension;

i. Subjects with severe uncontrolled infection;

j. Subjects with alcohol and/or drug abuse or poor compliance as judged by the investigator.

3. Withdrawal/Termination Criteria

a. The investigator may stop the trial or stop the subject's participation in the trial for any reason;

b. Unable to receive treatment as required by the study protocol;

c. Occurrence of allergic reactions or serious adverse events;

d. Disease progression during treatment;

e. Subject requests withdrawal;

f. If half of the subjects experience dose-limiting adverse reactions, the trial should be terminated;

g. Subjects may require other types of cancer treatment for any reason. In this case, the subject's participation in the trial should be terminated immediately once a new treatment is initiated, except for those undergoing local anti-cancer treatment for non-target lesions;

h. The subject is pregnant or not using adequate contraception.

4. Removal Criteria

a. Concurrent treatment with other local therapies at efficacy-evaluable sites;

b. Concomitant use of other foods or drugs that affect the judgment of tolerability;

c. Violation of the study protocol requirements, not receiving the drugs at the doses and durations specified in the protocol.

d. Poor quality of data recording, with incomplete or inaccurate information.

V DOSE MODIFICATION AND DISCONTINUATION

a. In principle, all subjects should receive the planned chemotherapy dose, and if necessary, the dose can be modified based on the most severe hematological or other toxicities. Any subject who requires a dose reduction will continue to receive the reduced dose in subsequent treatment cycles. If a subject experiences multiple toxicities with differing principles for dose modification among them, the minimum dose will be selected. If a subject has already undergone 2 dose reductions, the chemotherapy must be discontinued when a third dose reduction is required due to toxicity;

b. Hematological toxicity: modifications to subsequent treatment. Subjects must have neutrophils of ≥ 1.5 × 10^9/L and platelets of ≥ 75 × 10^9/L to receive subsequent cycles of chemotherapy. Chemotherapy should not be delayed for more than 2 weeks; otherwise, it should be discontinued in principle, unless the investigator considers it necessary to continue. According to the nadir blood cell count in the previous cycle, the following dose modifications will be made for subsequent chemotherapy cycles (Table 1):

Table 1. Dose modifications of chemotherapy drugs for subjects with hematological toxicity.

Platelet (10^9/L)		Neutrophil (10^9/L)	Oxaliplatin (%)	Irinotecan (%)	Capecitabine (%)
≥ 50	and	≥ 0.5	100	100	100
≥ 50	and	< 0.5	75	75	75
< 50	and	≥ 0.5	75	75	75
< 50	or	< 0.5	75	75	75

c. Non-hematological toxicity (excluding alopecia, nausea, and vomiting);

d. Renal toxicity: As long as the subject's creatinine clearance is ≥ 45 mL/min, the full dose of chemotherapy should be given; if the creatinine clearance is < 45 mL/min, the chemotherapy should be delayed and the full dose of chemotherapy should be given until the creatinine clearance is recovered to ≥ 45 mL/min. The chemotherapy should not be delayed for more than 2 weeks; otherwise, it should be discontinued in principle, unless the investigator considers it necessary to continue. If a subject develops Grade ≥ 3 nephrotoxicity, the treatment should be discontinued and the subject should withdraw from the study in principle;

e. Neurotoxicity: If a subject develops Grade ≥ 4 neurotoxicity, the treatment should be discontinued and the subject should withdraw from the study in principle. If the subject develops Grade ≤ 3 neurotoxicity, chemotherapy should be delayed until the toxicity is recovered to CTC Grade ≤ 1. Chemotherapy should not be delayed for more than 2 weeks; otherwise, it should be discontinued in principle, unless the investigator considers it necessary to continue;

Other non-hematological toxicities: If a subject develops Grade ≥ 3 non-hematological toxicity (excluding neurotoxicity and nephrotoxicity), chemotherapy should be delayed until the toxicity is recovered to CTC Grade ≤ 2. Chemotherapy should not be delayed for more than 2 weeks; otherwise, it should be discontinued in principle, unless the investigator considers it necessary to continue.

VI CONCOMITANT THERAPY

a. Other drugs related to cancer treatment should not be used during the study, and all drugs used should be recorded on the case report form (CRF) with explanations;

b. Colony-stimulating factor;

c. Treatment for febrile neutropenia;

d. Other drugs that can be used concomitantly;

e. Subjects with other concomitant diseases may continue taking the relevant drugs as needed or on time during the study observation period;

f. For subjects with chemotherapy-induced vomiting, antiemetics may be given;

g. When adverse events caused by the investigational product are treated, symptomatic treatment may be given.

VII OBSERVATION INDICATORS AND EXAMINATION TIME

1. Safety Endpoints

   a. Symptoms;

   b. Vital signs and physical examination;

   c. Laboratory tests: hematology, urinalysis, routine stool, and blood biochemistry;

   d. Auxiliary examinations: ECG, chest CT, and abdominal CT or MRI.

2. Anti-Tumor Efficacy Endpoints

   a. Symptoms and physical examination: pay attention to changes in symptoms related to the tumor;

   b. Imaging examination: it includes CT and MRI primarily.

3. Examination Time

   a. Before enrollment: Imaging examination related to tumor response evaluation will be completed within 2 weeks before enrollment in the clinical trial, and safety evaluation endpoints will be evaluated within 1 week after the start of treatment;

   b. During the study: Routine hematology and blood biochemistry tests will be performed every week; efficacy evaluation will be performed every 2 cycles during the induction phase and every 3 cycles during the maintenance phase;

   c. Follow-up period: Safety evaluation should last until 4 weeks after the last efficacy evaluation.

VIII CLINICAL EVALUATION

a. Safety evaluation: The intensity of adverse events will be graded according to the grading system of the US National Cancer Institute (NCI) Common Terminology Criteria for Adverse Events Version 5.0 (NCI CTCAE 5.0).

b. Anti-tumor efficacy evaluation: According to the Response Evaluation Criteria in Solid Tumors (RECIST v1.1).

c. Quality of life improvement in patients with colorectal cancer: Refer to the Quality of Life Questionnaire Core 30 for patients with tumors (EORTC QLQ-C30) score.

IX ADVERSE EVENT RECORDING AND REPORTING

Any unintended treatment-related or unrelated medical event that occurs after the use of a drug in subjects in a clinical trial is an adverse event. It can be any unfavorable and unintended signs, symptoms, or abnormal findings including abnormal laboratory findings, whether or not related to the drug. All adverse events should be recorded on the CRFs. The intensity of adverse events will be graded according to the grading method recommended by the NCI Common Toxicity Criteria.

X DATA MANAGEMENT AND STATISTICAL ANALYSIS

1. Case Report Form Filling

Design a standard CRF, complete the CRF in strict accordance with the filling instructions, and designate a clinical research associate to review it every 2 months.

2. Data Entry and Modification

A designated manager is responsible for data entry and management. The data manager will use SPSS 13.0 statistical software for data entry and management. In order to ensure the accuracy of the data, two entry personnel independently enter and proofread the data.

3. Data Locking

After data review and database establishment, a statistician designated by the Department of Medical Oncology, Jiangsu Cancer Hospital will lock the data. No modifications shall be made to the locked data. The problems found after data locking will be corrected in the statistical analysis procedure.

4. Statistical Analysis

All statistical analyses in this study use SPSS 13.0 software as the platform. Measurement data will be described in general and enumeration data will be statistically described by frequency. If a subject drops out during the study, the requirement for the number of subjects of the study should be still met after the subject who drops out is excluded.

Non-parametric tests such as chi-square test will be used for the evaluation of short-term tumor response, and a multivariate Cox regression model will be used for the analysis of survival.

XI ETHICAL STANDARDS AND INFORMED CONSENT

1. Ethical standards: The clinical trial complies with the "Declaration of Helsinki" (1996 edition), "Good Clinical Practice (GCP)" issued by SFDA (now NMPA), and other relevant regulations;
2. Informed consent: Before each patient is enrolled in this study, the study physician is responsible for providing the patient or his/her legal representative with a complete and comprehensive introduction to the study objectives, drug properties, and possible toxic and side effects and risks. The patient should be informed of their rights, risks, and benefits. Patients should sign the informed consent form prior to enrollment and remain it in the CRF.

XII DATA STORAGE

The study data will be stored in the Department of Medical Oncology, Jiangsu Cancer Hospital.

XIII STUDY PROGRESS AND COMPLETION TIMELINE

Sep. 2019–Jan. 2020, project opening argument and ethical approval of the study;

Feb. 2020–Jul. 2021, study subject enrollment, intervention measures taking, and clinical data collection;

Aug. 2021–Jun. 2022, follow-up, data collection, paper writing and publication, and result summary.

APPENDICES

Appendix I: ECOG Performance Status Criteria

Appendix II: AJCC TNM Staging System for Colorectal Cancer (8th Edition)

Appendix III: National Cancer Institute (NCI) Common Terminology Criteria for Adverse Events (CTCAE) v5.0

Appendix IV: Response Criteria in Solid Tumors (RECIST)v1.1

Appendix V: European Organization for Research and Treatment of Cancer Quality of Life Questionnaire Core 30 (EORTC QLQ-C30)
