# Supplementary material for: Alternating modified CAPOX/CAPIRI plus bevacizumab in untreated unresectable metastatic colorectal cancer: a phase 2 trial
Source: Signal Transduct Target Ther. 2024 Dec 11;9:346. doi: 10.1038/s41392-024-02048-z (PMC11631963; doi:10.1038/s41392-024-02048-z)
Supplement: Supplementary file 3 — appendix I [file 41392_2024_2048_MOESM3_ESM.pdf]

ECOG PERFORMANCE STATUS

Grade	Description
0	Fully active, able to carry on all predisease performance without restriction
1	Restricted in physically strenuous activity but ambulatory and able to carry out work of a light or sedentary nature, eg, light housework, office work
2	Ambulatory and capable of all self-care but unable to carry out any work activities. Up and about > 50% of waking hours
3	Capable of only limited self-care, confined to bed or chair > 50% of waking hours
4	Completely disabled. Cannot carry on any self-care. Totally confined to bed or chair
5	Dead

Source: Oken et al, 1982. Eastern Cooperative Oncology Group, Robert Comis MD, Group Chair.
