# Supplementary material for: Alternating modified CAPOX/CAPIRI plus bevacizumab in untreated unresectable metastatic colorectal cancer: a phase 2 trial
Source: Signal Transduct Target Ther. 2024 Dec 11;9:346. doi: 10.1038/s41392-024-02048-z (PMC11631963; doi:10.1038/s41392-024-02048-z)
Supplement: Supplementary file 5 — appendix III [file 41392_2024_2048_MOESM5_ESM.pdf]

Common Terminology Criteria for Adverse Events (CTCAE)

Version 5.0

Published: November 27, 2017

U.S. DEPARTMENT OF HEALTH AND HUMAN SERVICES

National Institutes of Health

National Cancer Institute

Common Terminology Criteria for Adverse Events (CTCAE) v5.0
Publish Date: November 27, 2017

Introduction
The NCI Common Terminology Criteria for Adverse Events is a descriptive terminology which can be utilized for Adverse Event (AE) reporting. A grading (severity) scale is provided for each AE term.

SOC
System Organ Class (SOC), the highest level of the MedDRA[1] hierarchy, is identified by anatomical or physiological system, etiology, or purpose (e.g., SOC Investigations for laboratory test results). CTCAE terms are grouped by MedDRA Primary SOCs. Within each SOC, AEs are listed and accompanied by descriptions of severity (Grade).

CTCAE Terms
An Adverse Event (AE) is any unfavorable and unintended sign (including an abnormal laboratory finding), symptom, or disease temporally associated with the use of a medical treatment or procedure that may or may not be considered related to the medical treatment or procedure. An AE is a term that is a unique representation of a specific event used for medical documentation and scientific analyses. Each CTCAE v4.0 term is a MedDRA LLT (Lowest Level Term).

Grades
Grade refers to the severity of the AE. The CTCAE displays Grades 1 through 5 with unique clinical descriptions of severity for each AE based on this general guideline:

Grade 1 Mild; asymptomatic or mild symptoms; clinical or diagnostic observations only; intervention not indicated.
Grade 2 Moderate; minimal, local or noninvasive intervention indicated; limiting age-appropriate instrumental ADL*.
Grade 3 Severe or medically significant but not immediately life-threatening; hospitalization or prolongation of hospitalization indicated; disabling; limiting self care ADL**.
Grade 4 Life-threatening consequences; urgent intervention indicated.
Grade 5 Death related to AE.

A Semi-colon indicates 'or' within the description of the grade.

A single dash (-) indicates a Grade is not available. Not all Grades are appropriate for all AEs. Therefore, some AEs are listed with fewer than five options for Grade selection.

Grade 5
Grade 5 (Death) is not appropriate for some AEs and therefore is not an option.

Definitions
A brief Definition is provided to clarify the meaning of each AE term. A single dash (-) indicates a Definition is not available.

Navigational Notes
A Navigational Note is used to assist the reporter in choosing a correct AE. It may list other AEs that should be considered in addition to or in place of the AE in question. A single dash (-) indicates a Navigational Note has not been defined for the AE term.

Activities of Daily Living (ADL)
*Instrumental ADL refer to preparing meals, shopping for groceries or clothes, using the telephone, managing money, etc.
**Self care ADL refer to bathing, dressing and undressing, feeding self, using the toilet, taking medications, and not bedridden.

[1] CTCAE v5.0 incorporates certain elements of the MedDRA terminology. For further details on MedDRA refer to the MedDRA MSSO Web site (https://www.meddra.org/).

Table of Contents

Blood and lymphatic system disorders 3
Cardiac disorders 5
Congenital, familial and genetic disorders 11
Ear and labyrinth disorders 12
Endocrine disorders 14
Eye disorders 17
Gastrointestinal disorders 22
General disorders and administration site conditions 40
Hepatobiliary disorders 44
Immune system disorders 47
Infections and infestations 49
Injury, poisoning and procedural complications 64
Investigations 78
Metabolism and nutrition disorders 84
Musculoskeletal and connective tissue disorders 88
Neoplasms benign, malignant and unspecified (incl cysts and polyps) 96
Nervous system disorders 97
Pregnancy, puerperium and perinatal conditions 107
Psychiatric disorders 108
Renal and urinary disorders 112
Reproductive system and breast disorders 116
Respiratory, thoracic and mediastinal disorders 123
Skin and subcutaneous tissue disorders 134
Social circumstances 141
Surgical and medical procedures 142
Vascular disorders 143

Blood and lymphatic system disorders					
**CTCAE Term**	**Grade 1**	**Grade 2**	**Grade 3**	**Grade 4**	**Grade 5**
Anemia	Hemoglobin (Hgb) <LLN - 10.0 g/dL; <LLN - 6.2 mmol/L; <LLN - 100 g/L	Hgb <10.0 - 8.0 g/dL; <6.2 - 4.9 mmol/L; <100 - 80g/L	Hgb <8.0 g/dL; <4.9 mmol/L; <80 g/L; transfusion indicated	Life-threatening consequences; urgent intervention indicated	Death
**Definition:** A disorder characterized by a reduction in the amount of hemoglobin in 100 ml of blood. Signs and symptoms of anemia may include pallor of the skin and mucous membranes, shortness of breath, palpitations of the heart, soft systolic murmurs, lethargy, and fatigability.<br>**Navigational Note:** -					
Bone marrow hypocellular	Mildly hypocellular or <=25% reduction from normal cellularity for age	Moderately hypocellular or >25 - <50% reduction from normal cellularity for age	Severely hypocellular or >50 - <=75% reduction cellularity from normal for age	Aplastic persistent for longer than 2 weeks	Death
**Definition:** A disorder characterized by the inability of the bone marrow to produce hematopoietic elements.<br>**Navigational Note:** -					
Disseminated intravascular coagulation	-	Laboratory findings with no bleeding	Laboratory findings and bleeding	Life-threatening consequences; urgent intervention indicated	Death
**Definition:** A disorder characterized by systemic pathological activation of blood clotting mechanisms which results in clot formation throughout the body. There is an increase in the risk of hemorrhage as the body is depleted of platelets and coagulation factors.<br>**Navigational Note:** -					
Eosinophilia	>ULN and >Baseline	-	Steroids initiated	-	-
**Definition:** A disorder characterized by laboratory test results that indicate an increased number of eosinophils in the blood.<br>**Navigational Note:** -					
Febrile neutropenia	-	-	ANC <1000/mm3 with a single temperature of >38.3 degrees C (101 degrees F) or a sustained temperature of >=38 degrees C (100.4 degrees F) for more than one hour	Life-threatening consequences; urgent intervention indicated	Death
**Definition:** A disorder characterized by an ANC <1000/mm3 and a single temperature of >38.3 degrees C (101 degrees F) or a sustained temperature of >=38 degrees C (100.4 degrees F) for more than one hour.<br>**Navigational Note:** -					
Hemolysis	Laboratory evidence of hemolysis only (e.g., direct antiglobulin test; DAT; Coombs'; schistocytes; decreased haptoglobin)	Evidence of hemolysis and >=2 g decrease in hemoglobin	Transfusion or medical intervention indicated (e.g., steroids)	Life-threatening consequences; urgent intervention indicated	Death
**Definition:** A disorder characterized by laboratory test results that indicate widespread erythrocyte cell membrane destruction.<br>**Navigational Note:** -					

Blood and lymphatic system disorders					
**CTCAE Term**	**Grade 1**	**Grade 2**	**Grade 3**	**Grade 4**	**Grade 5**
Hemolytic uremic syndrome	-	-	Laboratory findings with clinical consequences (e.g., renal insufficiency, petechiae)	Life-threatening consequences, (e.g., CNS hemorrhage or thrombosis/embolism or renal failure)	Death
**Definition:** A disorder characterized by a form of thrombotic microangiopathy with renal failure, hemolytic anemia, and severe thrombocytopenia.<br>**Navigational Note:** -					
Leukocytosis	-	-	>100,000/mm3	Clinical manifestations of leucostasis; urgent intervention indicated	Death
**Definition:** A disorder characterized by laboratory test results that indicate an increased number of white blood cells in the blood.<br>**Navigational Note:** -					
Lymph node pain	Mild pain	Moderate pain; limiting instrumental ADL	Severe pain; limiting self care ADL	-	-
**Definition:** A disorder characterized by a sensation of marked discomfort in a lymph node.<br>**Navigational Note:** -					
Methemoglobinemia	-	>ULN	Requiring urgent intervention	Life-threatening consequences	Death
**Definition:** A disorder characterized by laboratory test results that indicate increased methemoglobin in the blood.<br>**Navigational Note:** -					
Thrombotic thrombocytopenic purpura	-	-	Laboratory findings with clinical consequences (e.g., renal insufficiency, petechiae)	Life-threatening consequences, (e.g., CNS hemorrhage or thrombosis/embolism or renal failure)	Death
**Definition:** A disorder characterized by the presence of microangiopathic hemolytic anemia, thrombocytopenic purpura, fever, renal abnormalities and neurological abnormalities such as seizures, hemiplegia, and visual disturbances. It is an acute or subacute condition.<br>**Navigational Note:** -					
Blood and lymphatic system disorders - Other, specify	Asymptomatic or mild symptoms; clinical or diagnostic observations only; intervention not indicated	Moderate; minimal, local or noninvasive intervention indicated; limiting age-appropriate instrumental ADL	Severe or medically significant but not immediately life-threatening; hospitalization or prolongation of existing hospitalization indicated; limiting self care ADL	Life-threatening consequences; urgent intervention indicated	Death
**Definition:** -<br>**Navigational Note:** -					

Cardiac disorders					
**CTCAE Term**	**Grade 1**	**Grade 2**	**Grade 3**	**Grade 4**	**Grade 5**
Aortic valve disease	Asymptomatic valvular thickening with or without mild valvular regurgitation or stenosis by imaging	Asymptomatic; moderate regurgitation or stenosis by imaging	Symptomatic; severe regurgitation or stenosis by imaging; symptoms controlled with medical intervention	Life-threatening consequences; urgent intervention indicated (e.g., valve replacement, valvuloplasty)	Death
**Definition:** A disorder characterized by a defect in aortic valve function or structure.<br>**Navigational Note:** -					
Asystole	Periods of asystole; non-urgent medical management indicated	-	-	Life-threatening consequences; urgent intervention indicated	Death
**Definition:** A disorder characterized by a dysrhythmia without cardiac electrical activity. Typically, this is accompanied by cessation of the pumping function of the heart.<br>**Navigational Note:** -					
Atrial fibrillation	Asymptomatic, intervention not indicated	Non-urgent medical intervention indicated	Symptomatic, urgent intervention indicated; device (e.g., pacemaker); ablation; new onset	Life-threatening consequences; embolus requiring urgent intervention	Death
**Definition:** A disorder characterized by a dysrhythmia without discernible P waves and an irregular ventricular response due to multiple reentry circuits. The rhythm disturbance originates above the ventricles.<br>**Navigational Note:** -					
Atrial flutter	Asymptomatic, intervention not indicated	Non-urgent medical intervention indicated	Symptomatic, urgent intervention indicated; device (e.g., pacemaker); ablation	Life-threatening consequences; embolus requiring urgent intervention	Death
**Definition:** A disorder characterized by a dysrhythmia with organized rhythmic atrial contractions with a rate of 200-300 beats per minute. The rhythm disturbance originates in the atria.<br>**Navigational Note:** -					
Atrioventricular block complete	-	Non-urgent intervention indicated	Symptomatic and incompletely controlled medically, or controlled with device (e.g., pacemaker); new onset	Life-threatening consequences; urgent intervention indicated	Death
**Definition:** A disorder characterized by a dysrhythmia with complete failure of atrial electrical impulse conduction through the AV node to the ventricles.<br>**Navigational Note:** -					
Atrioventricular block first degree	Asymptomatic, intervention not indicated	Non-urgent intervention indicated	-	-	-
**Definition:** A disorder characterized by a dysrhythmia with a delay in the time required for the conduction of an electrical impulse through the atrioventricular (AV) node beyond 0.2 seconds; prolongation of the PR interval greater than 200 milliseconds.<br>**Navigational Note:** -					

Cardiac disorders					
**CTCAE Term**	**Grade 1**	**Grade 2**	**Grade 3**	**Grade 4**	**Grade 5**
Cardiac arrest	-	-	-	Life-threatening consequences; urgent intervention indicated	Death
**Definition:** A disorder characterized by cessation of the pumping function of the heart.<br>**Navigational Note:** -					
Chest pain - cardiac	Mild pain	Moderate pain; pain on exertion; limiting instrumental ADL; hemodynamically stable	Pain at rest; limiting self care ADL; cardiac catheterization; new onset cardiac chest pain; unstable angina	-	-
**Definition:** A disorder characterized by substernal discomfort due to insufficient myocardial oxygenation e.g., angina pectoris.<br>**Navigational Note:** Also consider Cardiac disorders: Myocardial infarction.					
Conduction disorder	Mild symptoms; intervention not indicated	Non-urgent medical intervention indicated	Symptomatic, urgent intervention indicated	Life-threatening consequences	Death
**Definition:** A disorder characterized by pathological irregularities in the cardiac conduction system.<br>**Navigational Note:** -					
Cyanosis	-	Present	-	-	-
**Definition:** A disorder characterized by a bluish discoloration of the skin and/or mucous membranes.<br>**Navigational Note:** -					
Heart failure	Asymptomatic with laboratory (e.g., BNP [B-Natriuretic Peptide]) or cardiac imaging abnormalities	Symptoms with moderate activity or exertion	Symptoms at rest or with minimal activity or exertion; hospitalization; new onset of symptoms	Life-threatening consequences; urgent intervention indicated (e.g., continuous IV therapy or mechanical hemodynamic support)	Death
**Definition:** A disorder characterized by the inability of the heart to pump blood at an adequate volume to meet tissue metabolic requirements, or, the ability to do so only at an elevation in the filling pressure.<br>**Navigational Note:** If left sided use Cardiac disorders: Left ventricular systolic dysfunction; also consider Cardiac disorders: Restrictive cardiomyopathy, Investigations: Ejection fraction decreased.					
Left ventricular systolic dysfunction	-	-	Symptomatic due to drop in ejection fraction responsive to intervention	Refractory or poorly controlled heart failure due to drop in ejection fraction; intervention such as ventricular assist device, intravenous vasopressor support, or heart transplant indicated	Death
**Definition:** A disorder characterized by failure of the left ventricle to produce adequate output.<br>**Navigational Note:** Also consider Investigations: Ejection fraction decreased.					

Cardiac disorders					
**CTCAE Term**	**Grade 1**	**Grade 2**	**Grade 3**	**Grade 4**	**Grade 5**
Mitral valve disease	Asymptomatic valvular thickening with or without mild valvular regurgitation or stenosis by imaging	Asymptomatic; moderate regurgitation or stenosis by imaging	Symptomatic; severe regurgitation or stenosis by imaging; symptoms controlled with medical intervention	Life-threatening consequences; urgent intervention indicated (e.g., valve replacement, valvuloplasty)	Death
**Definition:** A disorder characterized by a defect in mitral valve function or structure.<br>**Navigational Note:** -					
Mobitz (type) II atrioventricular block	Asymptomatic, intervention not indicated	Symptomatic; medical intervention indicated	Symptomatic and incompletely controlled medically, or controlled with device (e.g., pacemaker); new onset	Life-threatening consequences; urgent intervention indicated	Death
**Definition:** A disorder characterized by a dysrhythmia with relatively constant PR interval prior to the block of an atrial impulse. This is the result of intermittent failure of atrial electrical impulse conduction through the atrioventricular (AV) node to the ventricles.<br>**Navigational Note:** -					
Mobitz type I	Asymptomatic, intervention not indicated	Symptomatic; medical intervention indicated	Symptomatic and incompletely controlled medically, or controlled with device (e.g., pacemaker)	Life-threatening consequences; urgent intervention indicated	Death
**Definition:** A disorder characterized by a dysrhythmia with a progressively lengthening PR interval prior to the blocking of an atrial impulse. This is the result of intermittent failure of atrial electrical impulse conduction through the atrioventricular (AV) node to the ventricles.<br>**Navigational Note:** -					
Myocardial infarction	-	Asymptomatic and cardiac enzymes minimally abnormal and no evidence of ischemic ECG changes	Severe symptoms; cardiac enzymes abnormal; hemodynamically stable; ECG changes consistent with infarction	Life-threatening consequences; hemodynamically unstable	Death
**Definition:** A disorder characterized by gross necrosis of the myocardium; this is due to an interruption of blood supply to the area.<br>**Navigational Note:** -					
Myocarditis	-	Symptoms with moderate activity or exertion	Severe with symptoms at rest or with minimal activity or exertion; intervention indicated; new onset of symptoms	Life-threatening consequences; urgent intervention indicated (e.g., continuous IV therapy or mechanical hemodynamic support)	Death
**Definition:** A disorder characterized by inflammation of the muscle tissue of the heart.<br>**Navigational Note:** -					

Cardiac disorders					
**CTCAE Term**	**Grade 1**	**Grade 2**	**Grade 3**	**Grade 4**	**Grade 5**
Palpitations	Mild symptoms; intervention not indicated	Intervention indicated	-	-	-
**Definition:** A disorder characterized by an unpleasant sensation of irregular and/or forceful beating of the heart.					
**Navigational Note:** -					
Paroxysmal atrial tachycardia	Asymptomatic, intervention not indicated	Non-urgent medical intervention indicated	Symptomatic, urgent intervention indicated; ablation	Life-threatening consequences; incompletely controlled medically; cardioversion indicated	Death
**Definition:** A disorder characterized by a dysrhythmia with abrupt onset and sudden termination of atrial contractions with a rate of 150-250 beats per minute. The rhythm disturbance originates in the atria.					
**Navigational Note:** -					
Pericardial effusion	-	Asymptomatic effusion size small to moderate	Effusion with physiologic consequences	Life-threatening consequences; urgent intervention indicated	Death
**Definition:** A disorder characterized by fluid collection within the pericardial sac, usually due to inflammation.					
**Navigational Note:** -					
Pericardial tamponade	-	-	-	Life-threatening consequences; urgent intervention indicated	Death
**Definition:** A disorder characterized by an increase in intrapericardial pressure due to the collection of blood or fluid in the pericardium.					
**Navigational Note:** -					
Pericarditis	Asymptomatic, ECG or physical findings (e.g., rub) consistent with pericarditis	Symptomatic pericarditis (e.g., chest pain)	Pericarditis with physiologic consequences (e.g., pericardial constriction)	Life-threatening consequences; urgent intervention indicated	Death
**Definition:** A disorder characterized by irritation to the layers of the pericardium (the protective sac around the heart).					
**Navigational Note:** -					
Pulmonary valve disease	Asymptomatic valvular thickening with or without mild valvular regurgitation or stenosis by imaging	Asymptomatic; moderate regurgitation or stenosis by imaging	Symptomatic; severe regurgitation or stenosis by imaging; symptoms controlled with medical intervention	Life-threatening consequences; urgent intervention indicated (e.g., valve replacement, valvuloplasty)	Death
**Definition:** A disorder characterized by a defect in pulmonary valve function or structure.					
**Navigational Note:** -					
Restrictive cardiomyopathy	Imaging findings only	Symptomatic without signs of heart failure	Symptomatic heart failure or other cardiac symptoms, responsive to intervention; new onset of symptoms	Refractory heart failure or other poorly controlled cardiac symptoms	Death
**Definition:** A disorder characterized by an inability of the ventricles to fill with blood because the myocardium (heart muscle) stiffens and loses its flexibility.					
**Navigational Note:** -					

Cardiac disorders					
**CTCAE Term**	**Grade 1**	**Grade 2**	**Grade 3**	**Grade 4**	**Grade 5**
Right ventricular dysfunction	Asymptomatic with laboratory (e.g., BNP [B-Natriuretic Peptide]) or cardiac imaging abnormalities	Symptoms with moderate activity or exertion	Severe symptoms, associated with hypoxia, right heart failure; oxygen indicated	Life-threatening consequences; urgent intervention indicated (e.g., ventricular assist device); heart transplant indicated	Death
**Definition:** A disorder characterized by impairment of right ventricular function associated with low ejection fraction and a decrease in motility of the right ventricular wall.<br>**Navigational Note:** -					
Sick sinus syndrome	Asymptomatic, intervention not indicated	Symptomatic, intervention not indicated; change in medication initiated	Symptomatic, intervention indicated	Life-threatening consequences; urgent intervention indicated	Death
**Definition:** A disorder characterized by a dysrhythmia with alternating periods of bradycardia and atrial tachycardia accompanied by syncope, fatigue and dizziness.<br>**Navigational Note:** -					
Sinus bradycardia	Asymptomatic, intervention not indicated	Symptomatic, intervention not indicated; change in medication initiated	Symptomatic, intervention indicated	Life-threatening consequences; urgent intervention indicated	Death
**Definition:** A disorder characterized by a dysrhythmia with a heart rate less than 60 beats per minute that originates in the sinus node.<br>**Navigational Note:** -					
Sinus tachycardia	Asymptomatic, intervention not indicated	Symptomatic; non-urgent medical intervention indicated	Urgent medical intervention indicated	-	-
**Definition:** A disorder characterized by a dysrhythmia with a heart rate greater than 100 beats per minute that originates in the sinus node.<br>**Navigational Note:** -					
Supraventricular tachycardia	Asymptomatic, intervention not indicated	Non-urgent medical intervention indicated	Symptomatic, urgent intervention indicated	Life-threatening consequences	Death
**Definition:** A disorder characterized by a dysrhythmia with a heart rate greater than 100 beats per minute that originates above the ventricles.<br>**Navigational Note:** -					
Tricuspid valve disease	Asymptomatic valvular thickening with or without mild valvular regurgitation or stenosis	Asymptomatic; moderate regurgitation or stenosis by imaging	Symptomatic; severe regurgitation or stenosis; symptoms controlled with medical intervention	Life-threatening consequences; urgent intervention indicated (e.g., valve replacement, valvuloplasty)	Death
**Definition:** A disorder characterized by a defect in tricuspid valve function or structure.<br>**Navigational Note:** -					
Ventricular arrhythmia	Asymptomatic, intervention not indicated	Non-urgent medical intervention indicated	Urgent intervention indicated	Life-threatening consequences; hemodynamic compromise	Death
**Definition:** A disorder characterized by a dysrhythmia that originates in the ventricles.<br>**Navigational Note:** -					

Cardiac disorders					
**CTCAE Term**	**Grade 1**	**Grade 2**	**Grade 3**	**Grade 4**	**Grade 5**
Ventricular fibrillation	-	-	-	Life-threatening consequences; hemodynamic compromise	Death
**Definition:** A disorder characterized by a dysrhythmia without discernible QRS complexes due to rapid repetitive excitation of myocardial fibers without coordinated contraction of the ventricles.<br>**Navigational Note:** -					
Ventricular tachycardia	-	Non-urgent medical intervention indicated	Symptomatic, urgent intervention indicated	Life-threatening consequences; hemodynamic compromise	Death
**Definition:** A disorder characterized by a dysrhythmia with a heart rate greater than 100 beats per minute that originates distal to the bundle of His.<br>**Navigational Note:** -					
Cardiac disorders - Other, specify	Asymptomatic or mild symptoms; clinical or diagnostic observations only; intervention not indicated	Moderate; minimal, local or noninvasive intervention indicated; limiting age-appropriate instrumental ADL	Severe or medically significant but not immediately life-threatening; hospitalization or prolongation of existing hospitalization indicated; limiting self care ADL	Life-threatening consequences; urgent intervention indicated	Death
**Definition:** -<br>**Navigational Note:** -					

Congenital, familial and genetic disorders					
**CTCAE Term**	**Grade 1**	**Grade 2**	**Grade 3**	**Grade 4**	**Grade 5**
Congenital, familial and genetic disorders - Other, specify	Asymptomatic or mild symptoms; clinical or diagnostic observations only; intervention not indicated	Moderate; minimal, local or noninvasive intervention indicated; limiting age-appropriate instrumental ADL	Severe or medically significant but not immediately life-threatening; hospitalization or prolongation of existing hospitalization indicated; limiting self care ADL	Life-threatening consequences; urgent intervention indicated	Death
**Definition:** -					
**Navigational Note:** -					

Ear and labyrinth disorders					
**CTCAE Term**	**Grade 1**	**Grade 2**	**Grade 3**	**Grade 4**	**Grade 5**
Ear pain	Mild pain	Moderate pain; limiting instrumental ADL	Severe pain; limiting self care ADL	-	-
**Definition:** A disorder characterized by a sensation of marked discomfort in the ear.<br>**Navigational Note:** -					
External ear pain	Mild pain	Moderate pain; limiting instrumental ADL	Severe pain; limiting self care ADL	-	-
**Definition:** A disorder characterized by a sensation of marked discomfort in the external ear region.<br>**Navigational Note:** -					
Hearing impaired	**Adults** enrolled on a Monitoring Program (on a 1, 2, 4, 3, 6, and 8 kHz audiogram): Threshold shift of 15 - 25 dB averaged at 2 contiguous test frequencies in at least one ear;<br><br>**Adults** not enrolled on a Monitoring Program: Subjective change in hearing in the absence of documented hearing loss;<br><br>**Pediatric** (on a 1, 2, 3, 4, 6, and 8 kHz audiogram): Threshold shift >20 dB hearing loss (HL) (i.e., 25 dB HL or greater); sensorineural hearing loss (SNHL) above 4 kHz (i.e., 6 or 8 kHz) in at least one ear	**Adults** enrolled on a Monitoring Program (on a 1, 2, 3, 4, 6, and 8 kHz audiogram): Threshold shift of >25 dB averaged at 2 contiguous test frequencies in at least one ear;<br><br>**Adults** not enrolled on a Monitoring Program: Hearing loss with hearing aid or intervention not indicated; limiting instrumental ADL;<br><br>**Pediatric** (on a 1, 2, 3, 4, 6, and 8 kHz audiogram): Threshold shift >20 dB at 4 kHz in at least one ear	**Adults** enrolled on a Monitoring Program (on a 1, 2, 3, 4, 6, and 8 kHz audiogram): Threshold shift of >25 dB averaged at 3 contiguous test frequencies in at least one ear; therapeutic intervention indicated;<br><br>**Adults** not enrolled on a Monitoring Program: Hearing loss with hearing aid or intervention indicated; limiting self care ADL;<br><br>**Pediatric** (on a 1, 2, 3, 4, 6, and 8 kHz audiogram): Hearing loss sufficient to indicate therapeutic intervention, including hearing aids; threshold shift >20 dB at 2 to < 4 kHz in at least one ear	**Adults:** Decrease in hearing to profound bilateral loss (absolute threshold >80 dB HL at 2 kHz and above); nonservicable hearing<br><br>**Pediatric:** Audiologic indication for cochlear implant; > 40 dB HL (i.e., 45 dB HL or more); SNHL at 2 kHz and above	-
**Definition:** A disorder characterized by partial or complete loss of the ability to detect or understand sounds resulting from damage to ear structures.<br>**Navigational Note:** -					
Middle ear inflammation	Serous otitis	Serous otitis, medical intervention indicated	Mastoiditis; necrosis of canal soft tissue or bone	Life-threatening consequences; urgent intervention indicated	Death
**Definition:** A disorder characterized by inflammation (physiologic response to irritation), swelling and redness to the middle ear.<br>**Navigational Note:** -					

Ear and labyrinth disorders					
**CTCAE Term**	**Grade 1**	**Grade 2**	**Grade 3**	**Grade 4**	**Grade 5**
Tinnitus	Mild symptoms; intervention not indicated	Moderate symptoms; limiting instrumental ADL	Severe symptoms; limiting self care ADL	-	-
**Definition:** A disorder characterized by noise in the ears, such as ringing, buzzing, roaring or clicking.<br>**Navigational Note:** -					
Vertigo	Mild symptoms	Moderate symptoms; limiting instrumental ADL	Severe symptoms; limiting self care ADL	-	-
**Definition:** A disorder characterized by a sensation as if the external world were revolving around the patient (objective vertigo) or as if he himself were revolving in space (subjective vertigo).<br>**Navigational Note:** -					
Vestibular disorder	-	Symptomatic; limiting instrumental ADL	Severe symptoms; limiting self care ADL	-	-
**Definition:** A disorder characterized by dizziness, imbalance, nausea, and vision problems.<br>**Navigational Note:** -					
Ear and labyrinth disorders - Other, specify	Asymptomatic or mild symptoms; clinical or diagnostic observations only; intervention not indicated	Moderate; minimal, local or noninvasive intervention indicated; limiting age-appropriate instrumental ADL	Severe or medically significant but not immediately life-threatening; hospitalization or prolongation of existing hospitalization indicated; limiting self care ADL	Life-threatening consequences; urgent intervention indicated	Death
**Definition:** -<br>**Navigational Note:** -					

Endocrine disorders					
**CTCAE Term**	**Grade 1**	**Grade 2**	**Grade 3**	**Grade 4**	**Grade 5**
Adrenal insufficiency	Asymptomatic; clinical or diagnostic observations only; intervention not indicated	Moderate symptoms; medical intervention indicated	Severe symptoms; hospitalization indicated	Life-threatening consequences; urgent intervention indicated	Death
**Definition:** A disorder characterized by the adrenal cortex not producing enough of the hormone cortisol and in some cases, the hormone aldosterone. It may be due to a disorder of the adrenal cortex as in Addison's disease or primary adrenal insufficiency.<br>**Navigational Note:** -					
Cushingoid	Mild symptoms; intervention not indicated	Moderate symptoms; medical intervention indicated	Severe symptoms, medical intervention or hospitalization indicated	-	-
**Definition:** A disorder characterized by signs and symptoms that resemble Cushing's disease or syndrome: buffalo hump obesity, striations, adiposity, hypertension, diabetes, and osteoporosis, usually due to exogenous corticosteroids.<br>**Navigational Note:** -					
Delayed puberty	-	No breast development by age 13 yrs for females; testes volume of <3 cc or no Tanner Stage 2 development by age 14.5 yrs for males	No breast development by age 14 yrs for females; no increase in testes volume or no Tanner Stage 2 by age 16 yrs for males; hormone replacement indicated	-	-
**Definition:** A disorder characterized by unusually late sexual maturity.<br>**Navigational Note:** -					
Growth accelerated	-	>= +2 SD (standard deviation) above mid parental height or target height	-	-	-
**Definition:** A disorder characterized by greater growth than expected for age.<br>**Navigational Note:** -					
Hyperparathyroidism	Mild symptoms; intervention not indicated	Moderate symptoms; medical intervention indicated	-	-	-
**Definition:** A disorder characterized by an increase in production of parathyroid hormone by the parathyroid glands. This results in hypercalcemia (abnormally high levels of calcium in the blood).<br>**Navigational Note:** -					
Hyperthyroidism	Asymptomatic; clinical or diagnostic observations only; intervention not indicated	Symptomatic; thyroid suppression therapy indicated; limiting instrumental ADL	Severe symptoms; limiting self care ADL; hospitalization indicated	Life-threatening consequences; urgent intervention indicated	Death
**Definition:** A disorder characterized by excessive levels of thyroid hormone in the body. Common causes include an overactive thyroid gland or thyroid hormone overdose.<br>**Navigational Note:** -					

Endocrine disorders					
**CTCAE Term**	**Grade 1**	**Grade 2**	**Grade 3**	**Grade 4**	**Grade 5**
Hypoparathyroidism	Asymptomatic; clinical or diagnostic observations only; intervention not indicated	Moderate symptoms; medical intervention indicated	Severe symptoms; medical intervention or hospitalization indicated	Life-threatening consequences; urgent intervention indicated	Death
**Definition:** A disorder characterized by a decrease in production of parathyroid hormone by the parathyroid glands.					
**Navigational Note:** -					
Hypophysitis	Asymptomatic or mild symptoms; clinical or diagnostic observations only; intervention not indicated	Moderate; minimal, local or noninvasive intervention indicated; limiting age-appropriate instrumental ADL	Severe or medically significant but not immediately life-threatening; hospitalization or prolongation of existing hospitalization indicated; limiting self care ADL	Life-threatening consequences; urgent intervention indicated	Death
**Definition:** A disorder characterized by inflammation and cellular infiltration of the pituitary gland.					
**Navigational Note:** -					
Hypopituitarism	Asymptomatic or mild symptoms; clinical or diagnostic observations only; intervention not indicated	Moderate; minimal, local or noninvasive intervention indicated; limiting age-appropriate instrumental ADL	Severe or medically significant but not immediately life-threatening; hospitalization or prolongation of existing hospitalization indicated; limiting self care ADL	Life-threatening consequences; urgent intervention indicated	Death
**Definition:** A disorder characterized by a decrease in production of hormones from the pituitary gland.					
**Navigational Note:** -					
Hypothyroidism	Asymptomatic; clinical or diagnostic observations only; intervention not indicated	Symptomatic; thyroid replacement indicated; limiting instrumental ADL	Severe symptoms; limiting self care ADL; hospitalization indicated	Life-threatening consequences; urgent intervention indicated	Death
**Definition:** A disorder characterized by a decrease in production of thyroid hormone by the thyroid gland.					
**Navigational Note:** -					
Precocious puberty	Physical signs of puberty with no biochemical markers for females <8 years and males <9 years	Physical signs and biochemical markers of puberty for females <8 years and males <9 years	-	-	-
**Definition:** A disorder characterized by unusually early development of secondary sexual features; the onset of sexual maturation begins usually before age 8 for girls and before age 9 for boys.					
**Navigational Note:** -					
Testosterone deficiency	Asymptomatic; mild symptoms with no intervention indicated	Replacement therapy initiated	-	-	-
**Definition:** A disorder characterized by low testosterone.					
**Navigational Note:** -					

Back to TOC

Endocrine disorders					
**CTCAE Term**	**Grade 1**	**Grade 2**	**Grade 3**	**Grade 4**	**Grade 5**
Virilization	Mild symptoms; intervention not indicated	Moderate symptoms; medical intervention indicated	-	-	-
**Definition:** A disorder characterized by inappropriate masculinization occurring in a female or prepubertal male.					
**Navigational Note:** -					
Endocrine disorders - Other, specify	Asymptomatic or mild symptoms; clinical or diagnostic observations only; intervention not indicated	Moderate; minimal, local or noninvasive intervention indicated; limiting age-appropriate instrumental ADL	Severe or medically significant but not immediately life-threatening; hospitalization or prolongation of existing hospitalization indicated; limiting self care ADL	Life-threatening consequences; urgent intervention indicated	Death
**Definition:** -					
**Navigational Note:** -					

Eye disorders					
**CTCAE Term**	**Grade 1**	**Grade 2**	**Grade 3**	**Grade 4**	**Grade 5**
Blurred vision	Intervention not indicated	Symptomatic; moderate decrease in visual acuity (best corrected visual acuity 20/40 and better or 3 lines or less decreased vision from known baseline); limiting instrumental ADL	Symptomatic with marked decrease in visual acuity (best corrected visual acuity worse than 20/40 or more than 3 lines of decreased vision from known baseline, up to 20/200); limiting self care ADL	Best corrected visual acuity of 20/200 or worse in the affected eye	-
**Definition:** A disorder characterized by visual perception of unclear or fuzzy images.<br>**Navigational Note:** -					
Cataract	Asymptomatic; clinical or diagnostic observations only; intervention not indicated	Symptomatic; moderate decrease in visual acuity (best corrected visual acuity 20/40 and better or 3 lines or less decreased vision from known baseline); glare symptoms affecting instrumental ADL	Symptomatic with marked decrease in visual acuity (best corrected visual acuity worse than 20/40 or more than 3 lines of decreased vision from known baseline, up to 20/200); limiting self care ADL	Best corrected visual acuity of 20/200 or worse in the affected eye	-
**Definition:** A disorder characterized by partial or complete opacity of the crystalline lens of one or both eyes. This results in a decrease in visual acuity and eventual blindness if untreated.<br>**Navigational Note:** -					
Corneal ulcer	-	-	Corneal ulcer without perforation in the affected eye	Perforation in the affected eye	-
**Definition:** A disorder characterized by an area of epithelial tissue loss on the surface of the cornea. It is associated with inflammatory cells in the cornea and anterior chamber.<br>**Navigational Note:** -					
Dry eye	Asymptomatic; clinical or diagnostic observations only; symptoms relieved by lubricants	Symptomatic; moderate decrease in visual acuity (best corrected visual acuity 20/40 and better or 3 lines or less decreased vision from known baseline)	Symptomatic with marked decrease in visual acuity (best corrected visual acuity worse than 20/40 or more than 3 lines of decreased vision from known baseline, up to 20/200); limiting self care ADL	-	-
**Definition:** A disorder characterized by dryness of the cornea and conjunctiva.<br>**Navigational Note:** If corneal ulcer is present, grade under Eye disorders: Corneal ulcer.					
Extraocular muscle paresis	Asymptomatic; clinical or diagnostic observations only	Unilateral paresis without double vision	Bilateral paresis or unilateral paresis causing double vision in peripheral gaze, but not in central gaze	Bilateral paresis requiring head turning to see beyond central 60 degrees or double vision in central gaze	-
**Definition:** A disorder characterized by incomplete paralysis of an extraocular muscle.<br>**Navigational Note:** -					

Eye disorders					
**CTCAE Term**	**Grade 1**	**Grade 2**	**Grade 3**	**Grade 4**	**Grade 5**
Eye pain	Mild pain	Moderate pain; limiting instrumental ADL	Severe pain; limiting self care ADL	-	-
**Definition:** A disorder characterized by a sensation of marked discomfort in the eye.<br>**Navigational Note:** -					
Eyelid function disorder	Asymptomatic; clinical or diagnostic observations only; intervention not indicated	Symptomatic; nonoperative intervention indicated; limiting instrumental ADL	Limiting self care ADL; operative intervention indicated	-	-
**Definition:** A disorder characterized by impaired eyelid function.<br>**Navigational Note:** -					
Flashing lights	Symptomatic but not limiting ADL	Limiting instrumental ADL	Limiting self care ADL	-	-
**Definition:** A disorder characterized by a sudden or brief burst of light.<br>**Navigational Note:** Also consider Eye disorders: Retinal tear or Retinal detachment					
Floaters	Symptomatic but not limiting ADL	Limiting instrumental ADL	Limiting self care ADL	-	-
**Definition:** A disorder characterized by an individual seeing spots before their eyes. The spots are shadows of opaque cell fragments in the vitreous humor or lens.<br>**Navigational Note:** Also consider Eye disorders: Retinal tear or Retinal detachment					
Glaucoma	Less than 8 mmHg of elevated intraocular pressure (EIOP); no visual field deficit	EIOP which can be reduced to 21 mmHg or under with topical medications and no visual field deficit	EIOP causing visual field deficits	Visual field deficit within the central 10 degrees of the visual field in the affected eye	-
**Definition:** A disorder characterized by an increase in pressure in the eyeball due to obstruction of the aqueous humor outflow.<br>**Navigational Note:** -					
Keratitis	Asymptomatic; clinical or diagnostic observations only; intervention not indicated	Symptomatic; moderate decrease in visual acuity (best corrected visual acuity 20/40 and better or 3 lines or less decreased vision from known baseline)	Symptomatic with marked decrease in visual acuity (best corrected visual acuity worse than 20/40 or more than 3 lines of decreased vision from known baseline, up to 20/200); corneal ulcer; limiting self care ADL	Perforation; best corrected visual acuity of 20/200 or worse in the affected eye	-
**Definition:** A disorder characterized by inflammation to the cornea of the eye.<br>**Navigational Note:** Also consider Eye disorders: Corneal ulcer					

Eye disorders					
**CTCAE Term**	**Grade 1**	**Grade 2**	**Grade 3**	**Grade 4**	**Grade 5**
Night blindness	Symptomatic but not limiting ADL	Symptomatic; moderate decrease in visual acuity (best corrected visual acuity 20/40 and better or 3 lines or less decreased vision from known baseline); limiting instrumental ADL	Symptomatic with marked decrease in visual acuity (best corrected visual acuity worse than 20/40 or more than 3 lines of decreased vision from known baseline, up to 20/200); limiting self care ADL	Best corrected visual acuity of 20/200 or worse in the affected eye	-
**Definition:** A disorder characterized by an inability to see clearly in dim light.<br>**Navigational Note:** -					
Optic nerve disorder	Asymptomatic; clinical or diagnostic observations only	Moderate decrease in visual acuity (best corrected visual acuity 20/40 and better or 3 lines or less decreased vision from known baseline)	Marked decrease in visual acuity (best corrected visual acuity worse than 20/40 or more than 3 lines of decreased vision from known baseline, up to 20/200)	Best corrected visual acuity of 20/200 or worse in the affected eye	-
**Definition:** A disorder characterized by involvement of the optic nerve (second cranial nerve).<br>**Navigational Note:** -					
Papilledema	Asymptomatic; no visual field deficit	Symptomatic; moderate decrease in visual acuity (best corrected visual acuity 20/40 and better or 3 lines or less decreased vision from known baseline)	Symptomatic with marked decrease in visual acuity (best corrected visual acuity worse than 20/40 or more than 3 lines of decreased vision from known baseline, up to 20/200)	Best corrected visual acuity of 20/200 or worse in the affected eye	-
**Definition:** A disorder characterized by swelling around the optic disc.<br>**Navigational Note:** -					
Periorbital edema	Soft or non-pitting	Indurated or pitting edema; topical intervention indicated	Edema associated with visual disturbance; increased intraocular pressure, glaucoma or retinal hemorrhage; optic neuritis; diuretics indicated; operative intervention indicated	-	-
**Definition:** A disorder characterized by swelling due to an excessive accumulation of fluid around the orbits of the face.<br>**Navigational Note:** -					
Photophobia	Symptomatic but not limiting ADL	Limiting instrumental ADL	Limiting self care ADL	-	-
**Definition:** A disorder characterized by fear and avoidance of light.<br>**Navigational Note:** -					

Eye disorders					
**CTCAE Term**	**Grade 1**	**Grade 2**	**Grade 3**	**Grade 4**	**Grade 5**
Retinal detachment	-	-	Macular sparing rhegmatogenous detachment	Macula-off rhegmatogenous retinal detachment	-
**Definition:** A disorder characterized by the separation of the inner retina layers from the underlying pigment epithelium.					
**Navigational Note:** -					
Retinal tear	No retinal detachment and treatment not indicated	No retinal detachment and treatment indicated	-	-	-
**Definition:** A disorder characterized by a small laceration of the retina, this occurs when the vitreous separates from the retina. Symptoms include flashes and floaters.					
**Navigational Note:** If retinal detachment is present, grade under Eye disorders: Retinal detachment					
Retinal vascular disorder	-	Retinal vascular disorder without neovascularization	Retinal vascular disorder with neovascularization	-	-
**Definition:** A disorder characterized by pathological retinal blood vessels that adversely affects vision.					
**Navigational Note:** If vitreous hemorrhage is present, report under Eye disorders: Vitreous hemorrhage.					
Retinopathy	Asymptomatic; clinical or diagnostic observations only	Symptomatic; moderate decrease in visual acuity (best corrected visual acuity 20/40 and better or 3 lines or less decreased vision from known baseline); limiting instrumental ADL	Symptomatic with marked decrease in visual acuity (best corrected visual acuity worse than 20/40 or more than 3 lines of decreased vision from known baseline, up to 20/200); limiting self care ADL	Best corrected visual acuity of 20/200 or worse in the affected eye	-
**Definition:** A disorder involving the retina.					
**Navigational Note:** If vitreous hemorrhage is present, report under Eye disorders: Vitreous hemorrhage.					
Scleral disorder	No change in vision from baseline	Symptomatic; moderate decrease in visual acuity (best corrected visual acuity 20/40 and better or 3 lines or less decreased vision from known baseline); limiting instrumental ADL	Symptomatic with marked decrease in visual acuity (best corrected visual acuity worse than 20/40 or more than 3 lines of decreased vision from known baseline, up to 20/200); limiting self care ADL	Best corrected visual acuity of 20/200 or worse in the affected eye	-
**Definition:** A disorder characterized by involvement of the sclera of the eye.					
**Navigational Note:** -					
Uveitis	Anterior uveitis with trace cells	Anterior uveitis with 1+ or 2+ cells	Anterior uveitis with 3+ or greater cells; intermediate posterior or pan-uveitis	Best corrected visual acuity of 20/200 or worse in the affected eye	-
**Definition:** A disorder characterized by inflammation to the uvea of the eye.					
**Navigational Note:** -					

Eye disorders					
**CTCAE Term**	**Grade 1**	**Grade 2**	**Grade 3**	**Grade 4**	**Grade 5**
Vision decreased	-	Moderate decrease in visual acuity (best corrected visual acuity 20/40 and better or 3 lines or less decreased vision from known baseline)	Marked decrease in visual acuity (best corrected visual acuity worse than 20/40 or more than 3 lines of decreased vision from known baseline, up to 20/200)	Best corrected visual acuity of 20/200 or worse in the affected eye	-
**Definition:** A disorder characterized by a decrease in visual acuity.<br>**Navigational Note:** If etiology is known, use a more specific CTCAE term.					
Vitreous hemorrhage	Intervention not indicated	Symptomatic; moderate decrease in visual acuity (best corrected visual acuity 20/40 and better or 3 lines or less decreased vision from known baseline); limiting instrumental ADL	Symptomatic with marked decrease in visual acuity (best corrected visual acuity worse than 20/40 or more than 3 lines of decreased vision from known baseline, up to 20/200); limiting self care ADL; vitrectomy indicated	Best corrected visual acuity of 20/200 or worse in the affected eye	-
**Definition:** A disorder characterized by bleeding into the vitreous humor.<br>**Navigational Note:** -					
Watering eyes	Intervention not indicated	Symptomatic; moderate decrease in visual acuity (best corrected visual acuity 20/40 and better or 3 lines or less decreased vision from known baseline)	Marked decrease in visual acuity (best corrected visual acuity worse than 20/40 or more than 3 lines of decreased vision from known baseline, up to 20/200)	Best corrected visual acuity of 20/200 or worse in the affected eye	-
**Definition:** A disorder characterized by excessive tearing in the eyes; it can be caused by overproduction of tears or impaired drainage of the tear duct.<br>**Navigational Note:** -					
Eye disorders - Other, specify	Asymptomatic or mild symptoms; clinical or diagnostic observations only; intervention not indicated; no change in vision	Moderate; minimal, local or noninvasive intervention indicated; limiting instrumental ADL; best corrected visual acuity 20/40 and better or 3 lines or less decreased vision from known baseline	Severe or medically significant but not immediately sight-threatening; limiting self care ADL; decrease in visual acuity (best corrected visual acuity worse than 20/40 or more than 3 lines of decreased vision from known baseline, up to 20/200)	Sight-threatening consequences; urgent intervention indicated; best corrected visual acuity of 20/200 or worse in the affected eye	-
**Definition:** -<br>**Navigational Note:** -					

Gastrointestinal disorders					
**CTCAE Term**	**Grade 1**	**Grade 2**	**Grade 3**	**Grade 4**	**Grade 5**
Abdominal distension	Asymptomatic; clinical or diagnostic observations only; intervention not indicated	Symptomatic; limiting instrumental ADL	Severe discomfort; limiting self care ADL	-	-
**Definition:** A disorder characterized by swelling of the abdomen.<br>**Navigational Note:** -					
Abdominal pain	Mild pain	Moderate pain; limiting instrumental ADL	Severe pain; limiting self care ADL	-	-
**Definition:** A disorder characterized by a sensation of marked discomfort in the abdominal region.<br>**Navigational Note:** -					
Anal fissure	Asymptomatic	Symptomatic	Invasive intervention indicated	-	-
**Definition:** A disorder characterized by a tear in the lining of the anus.<br>**Navigational Note:** -					
Anal fistula	Asymptomatic	Symptomatic, invasive intervention not indicated	Invasive intervention indicated	Life-threatening consequences; urgent intervention indicated	Death
**Definition:** A disorder characterized by an abnormal communication between the opening in the anal canal to the perianal skin.<br>**Navigational Note:** -					
Anal hemorrhage	Mild symptoms; intervention not indicated	Moderate symptoms; intervention indicated	Transfusion indicated; invasive intervention indicated; hospitalization	Life-threatening consequences; urgent intervention indicated	Death
**Definition:** A disorder characterized by bleeding from the anal region.<br>**Navigational Note:** -					
Anal mucositis	Asymptomatic or mild symptoms; intervention not indicated	Symptomatic; medical intervention indicated; limiting instrumental ADL	Severe symptoms; limiting self care ADL	-	-
**Definition:** A disorder characterized by ulceration or inflammation of the mucous membrane of the anus.<br>**Navigational Note:** Report Grade 4 and 5 as Gastrointestinal disorders: Anal ulcer					
Anal necrosis	-	-	TPN or hospitalization indicated; invasive intervention indicated	Life-threatening consequences; urgent operative intervention indicated	Death
**Definition:** A disorder characterized by a necrotic process occurring in the anal region.<br>**Navigational Note:** -					
Anal pain	Mild pain	Moderate pain; limiting instrumental ADL	Severe pain; limiting self care ADL	-	-
**Definition:** A disorder characterized by a sensation of marked discomfort in the anal region.<br>**Navigational Note:** -					

Gastrointestinal disorders					
**CTCAE Term**	**Grade 1**	**Grade 2**	**Grade 3**	**Grade 4**	**Grade 5**
Anal stenosis	Asymptomatic; clinical or diagnostic observations only; intervention not indicated	Symptomatic; altered GI function	Symptomatic and severely altered GI function; non-emergent operative intervention indicated; TPN or hospitalization indicated	Life-threatening consequences; urgent operative intervention indicated	Death
**Definition:** A disorder characterized by a narrowing of the lumen of the anal canal.<br>**Navigational Note:** -					
Anal ulcer	Asymptomatic; clinical or diagnostic observations only; intervention not indicated	Symptomatic; altered GI function	Severely altered GI function; TPN indicated; elective invasive intervention indicated	Life-threatening consequences; urgent operative intervention indicated	Death
**Definition:** A disorder characterized by a circumscribed, erosive lesion on the mucosal surface of the anal canal.<br>**Navigational Note:** -					
Ascites	Asymptomatic; clinical or diagnostic observations only; intervention not indicated	Symptomatic; medical intervention indicated	Severe symptoms; invasive intervention indicated	Life-threatening consequences; urgent operative intervention indicated	Death
**Definition:** A disorder characterized by accumulation of serous or hemorrhagic fluid in the peritoneal cavity.<br>**Navigational Note:** -					
Belching	Increase from baseline	Intervention initiated (including over the counter medications)	-	-	-
**Definition:** To expel gas noisily from the mouth.<br>**Navigational Note:** Synonym: Burping					
Bloating	No change in bowel function or oral intake	Symptomatic, decreased oral intake; change in bowel function	-	-	-
**Definition:** A disorder characterized by subject-reported feeling of uncomfortable fullness of the abdomen.<br>**Navigational Note:** -					
Cecal hemorrhage	Mild symptoms; intervention not indicated	Moderate symptoms; intervention indicated	Transfusion indicated; invasive intervention indicated; hospitalization	Life-threatening consequences; urgent intervention indicated	Death
**Definition:** A disorder characterized by bleeding from the cecum.<br>**Navigational Note:** -					
Cheilitis	Asymptomatic; clinical or diagnostic observations only; intervention not indicated	Moderate symptoms; limiting instrumental ADL	Severe symptoms; limiting self care ADL; intervention indicated	-	-
**Definition:** A disorder characterized by inflammation of the lip.<br>**Navigational Note:** -					

Back to TOC

Gastrointestinal disorders					
**CTCAE Term**	**Grade 1**	**Grade 2**	**Grade 3**	**Grade 4**	**Grade 5**
Chylous ascites	Asymptomatic; clinical or diagnostic observations only; intervention not indicated	Symptomatic; medical intervention indicated (e.g., fat-restricted diet); paracentesis or tube drainage indicated	Severe symptoms; elective operative intervention indicated	Life-threatening consequences; urgent operative intervention indicated	Death
**Definition:** A disorder characterized by accumulation of milky fluid in the peritoneal cavity.<br>**Navigational Note:** -					
Colitis	Asymptomatic; clinical or diagnostic observations only; intervention not indicated	Abdominal pain; mucus or blood in stool	Severe abdominal pain; peritoneal signs	Life-threatening consequences; urgent intervention indicated	Death
**Definition:** A disorder characterized by inflammation of the colon.<br>**Navigational Note:** -					
Colonic fistula	Asymptomatic	Symptomatic, invasive intervention not indicated	Invasive intervention indicated	Life-threatening consequences; urgent intervention indicated	Death
**Definition:** A disorder characterized by an abnormal communication between the large intestine and another organ or anatomic site.<br>**Navigational Note:** -					
Colonic hemorrhage	Mild symptoms; intervention not indicated	Moderate symptoms; intervention indicated	Transfusion indicated; invasive intervention indicated; hospitalization	Life-threatening consequences; urgent intervention indicated	Death
**Definition:** A disorder characterized by bleeding from the colon.<br>**Navigational Note:** -					
Colonic obstruction	Asymptomatic; clinical or diagnostic observations only; intervention not indicated	Symptomatic; altered GI function	Hospitalization indicated; invasive intervention indicated	Life-threatening consequences; urgent operative intervention indicated	Death
**Definition:** A disorder characterized by blockage of the normal flow of the intestinal contents in the colon.<br>**Navigational Note:** -					
Colonic perforation	-	Invasive intervention not indicated	Invasive intervention indicated	Life-threatening consequences; urgent intervention indicated	Death
**Definition:** A disorder characterized by a rupture in the colonic wall.<br>**Navigational Note:** -					
Colonic stenosis	Asymptomatic; clinical or diagnostic observations only; intervention not indicated	Symptomatic; altered GI function	Severely altered GI function; tube feeding or hospitalization indicated; elective operative intervention indicated	Life-threatening consequences; urgent operative intervention indicated	Death
**Definition:** A disorder characterized by a narrowing of the lumen of the colon.<br>**Navigational Note:** -					

Gastrointestinal disorders					
**CTCAE Term**	**Grade 1**	**Grade 2**	**Grade 3**	**Grade 4**	**Grade 5**
Colonic ulcer	Asymptomatic; clinical or diagnostic observations only; intervention not indicated	Symptomatic; altered GI function	Severely altered GI function; TPN indicated; elective invasive intervention indicated	Life-threatening consequences; urgent operative intervention indicated	Death
**Definition:** A disorder characterized by a circumscribed, erosive lesion on the mucosal surface of the colon.<br>**Navigational Note:** -					
Constipation	Occasional or intermittent symptoms; occasional use of stool softeners, laxatives, dietary modification, or enema	Persistent symptoms with regular use of laxatives or enemas; limiting instrumental ADL	Obstipation with manual evacuation indicated; limiting self care ADL	Life-threatening consequences; urgent intervention indicated	Death
**Definition:** A disorder characterized by irregular and infrequent or difficult evacuation of the bowels.<br>**Navigational Note:** -					
Dental caries	One or more dental caries, not involving the root	Dental caries involving the root	Dental caries resulting in pulpitis or periapical abscess or resulting in tooth loss	-	-
**Definition:** A disorder characterized by the decay of a tooth, in which it becomes softened, discolored and/or porous.<br>**Navigational Note:** -					
Diarrhea	Increase of <4 stools per day over baseline; mild increase in ostomy output compared to baseline	Increase of 4 - 6 stools per day over baseline; moderate increase in ostomy output compared to baseline; limiting instrumental ADL	Increase of >=7 stools per day over baseline; hospitalization indicated; severe increase in ostomy output compared to baseline; limiting self care ADL	Life-threatening consequences; urgent intervention indicated	Death
**Definition:** A disorder characterized by an increase in frequency and/or loose or watery bowel movements.<br>**Navigational Note:** -					
Dry mouth	Symptomatic (e.g., dry or thick saliva) without significant dietary alteration; unstimulated saliva flow >0.2 ml/min	Moderate symptoms; oral intake alterations (e.g., copious water, other lubricants, diet limited to purees and/or soft, moist foods); unstimulated saliva 0.1 to 0.2 ml/min	Inability to adequately aliment orally; tube feeding or TPN indicated; unstimulated saliva <0.1 ml/min	-	-
**Definition:** A disorder characterized by reduced salivary flow in the oral cavity.<br>**Navigational Note:** -					
Duodenal fistula	Asymptomatic	Symptomatic, invasive intervention not indicated	Invasive intervention indicated	Life-threatening consequences; urgent intervention indicated	Death
**Definition:** A disorder characterized by an abnormal communication between the duodenum and another organ or anatomic site.<br>**Navigational Note:** -					

Gastrointestinal disorders					
**CTCAE Term**	**Grade 1**	**Grade 2**	**Grade 3**	**Grade 4**	**Grade 5**
Duodenal hemorrhage	Mild symptoms; intervention not indicated	Moderate symptoms; intervention indicated	Transfusion indicated; invasive intervention indicated; hospitalization	Life-threatening consequences; urgent intervention indicated	Death
**Definition:** A disorder characterized by bleeding from the duodenum.<br>**Navigational Note:** -					
Duodenal obstruction	Asymptomatic; clinical or diagnostic observations only; intervention not indicated	Symptomatic; altered GI function	Hospitalization indicated; invasive intervention indicated	Life-threatening consequences; urgent operative intervention indicated	Death
**Definition:** A disorder characterized by blockage of the normal flow of stomach contents through the duodenum.<br>**Navigational Note:** -					
Duodenal perforation	-	Invasive intervention not indicated	Invasive intervention indicated	Life-threatening consequences; urgent operative intervention indicated	Death
**Definition:** A disorder characterized by a rupture in the duodenal wall.<br>**Navigational Note:** -					
Duodenal stenosis	Asymptomatic; clinical or diagnostic observations only; intervention not indicated	Symptomatic; altered GI function	Severely altered GI function; tube feeding or hospitalization indicated; elective operative intervention indicated	Life-threatening consequences; urgent operative intervention indicated	Death
**Definition:** A disorder characterized by a narrowing of the lumen of the duodenum.<br>**Navigational Note:** -					
Duodenal ulcer	Asymptomatic; clinical or diagnostic observations only; intervention not indicated	Symptomatic; medical intervention indicated; limiting instrumental ADL	Severely altered GI function; TPN indicated; elective invasive intervention indicated; limiting self care ADL	Life-threatening consequences; urgent operative intervention indicated	Death
**Definition:** A disorder characterized by a circumscribed, erosive lesion on the mucosal surface of the duodenal wall.<br>**Navigational Note:** -					
Dyspepsia	Mild symptoms; intervention not indicated	Moderate symptoms; medical intervention indicated	Severe symptoms; operative intervention indicated	-	-
**Definition:** A disorder characterized by an uncomfortable, often painful feeling in the stomach, resulting from impaired digestion. Symptoms include burning stomach, bloating, heartburn, nausea and vomiting.<br>**Navigational Note:** -					

Gastrointestinal disorders					
**CTCAE Term**	**Grade 1**	**Grade 2**	**Grade 3**	**Grade 4**	**Grade 5**
Dysphagia	Symptomatic, able to eat regular diet	Symptomatic and altered eating/swallowing	Severely altered eating/swallowing; tube feeding, TPN, or hospitalization indicated	Life-threatening consequences; urgent intervention indicated	Death
**Definition:** A disorder characterized by difficulty in swallowing.<br>**Navigational Note:** -					
Enterocolitis	Asymptomatic; clinical or diagnostic observations only; intervention not indicated	Abdominal pain; mucus or blood in stool	Severe or persistent abdominal pain; fever; ileus; peritoneal signs	Life-threatening consequences; urgent intervention indicated	Death
**Definition:** A disorder characterized by inflammation of the small and large intestines.<br>**Navigational Note:** If reporting a known abnormality of the colon, use Gastrointestinal disorders: Colitis. If reporting a documented infection, use Infections and infestations: Enterocolitis infectious.					
Enterovesical fistula	Asymptomatic	Symptomatic, invasive intervention not indicated	Invasive intervention indicated	Life-threatening consequences; urgent intervention indicated	Death
**Definition:** A disorder characterized by an abnormal communication between the urinary bladder and the intestine.<br>**Navigational Note:** -					
Esophageal fistula	Asymptomatic	Symptomatic, invasive intervention not indicated	Invasive intervention indicated	Life-threatening consequences; urgent intervention indicated	Death
**Definition:** A disorder characterized by an abnormal communication between the esophagus and another organ or anatomic site.<br>**Navigational Note:** -					
Esophageal hemorrhage	Mild symptoms; intervention not indicated	Moderate symptoms; intervention indicated	Transfusion indicated; invasive intervention indicated; hospitalization	Life-threatening consequences; urgent intervention indicated	Death
**Definition:** A disorder characterized by bleeding from the esophagus.<br>**Navigational Note:** -					
Esophageal necrosis	-	-	Inability to aliment adequately by GI tract; invasive intervention indicated	Life-threatening consequences; urgent operative intervention indicated	Death
**Definition:** A disorder characterized by a necrotic process occurring in the esophageal wall.<br>**Navigational Note:** -					
Esophageal obstruction	Asymptomatic; clinical or diagnostic observations only; intervention not indicated	Symptomatic; altered GI function; limiting instrumental ADL	Hospitalization indicated; invasive intervention indicated; limiting self care ADL	Life-threatening consequences; urgent intervention indicated	Death
**Definition:** A disorder characterized by blockage of the normal flow of the contents in the esophagus.<br>**Navigational Note:** -					

Gastrointestinal disorders					
**CTCAE Term**	**Grade 1**	**Grade 2**	**Grade 3**	**Grade 4**	**Grade 5**
Esophageal pain	Mild pain	Moderate pain; limiting instrumental ADL	Severe pain; limiting self care ADL	-	-
**Definition:** A disorder characterized by a sensation of marked discomfort in the esophageal region.<br>**Navigational Note:** -					
Esophageal perforation	-	Invasive intervention not indicated	Invasive intervention indicated	Life-threatening consequences; urgent operative intervention indicated	Death
**Definition:** A disorder characterized by a rupture in the wall of the esophagus.<br>**Navigational Note:** -					
Esophageal stenosis	Asymptomatic; clinical or diagnostic observations only; intervention not indicated	Symptomatic; altered GI function	Severely altered GI function; tube feeding or hospitalization indicated; elective operative intervention indicated	Life-threatening consequences; urgent operative intervention indicated	Death
**Definition:** A disorder characterized by a narrowing of the lumen of the esophagus.<br>**Navigational Note:** -					
Esophageal ulcer	Asymptomatic; clinical or diagnostic observations only; intervention not indicated	Symptomatic; altered GI function; limiting instrumental ADL	Severely altered GI function; TPN indicated; elective invasive intervention indicated; limiting self care ADL	Life-threatening consequences; urgent operative intervention indicated	Death
**Definition:** A disorder characterized by a circumscribed, erosive lesion on the mucosal surface of the esophageal wall.<br>**Navigational Note:** -					
Esophageal varices hemorrhage	-	Self-limited; intervention not indicated	Transfusion indicated; invasive intervention indicated; hospitalization	Life-threatening consequences; urgent intervention indicated	Death
**Definition:** A disorder characterized by bleeding from esophageal varices.<br>**Navigational Note:** -					
Esophagitis	Asymptomatic; clinical or diagnostic observations only; intervention not indicated	Symptomatic; altered eating/swallowing; oral supplements indicated	Severely altered eating/swallowing; tube feeding, TPN, or hospitalization indicated	Life-threatening consequences; urgent operative intervention indicated	Death
**Definition:** A disorder characterized by inflammation of the esophageal wall.<br>**Navigational Note:** -					
Fecal incontinence	Occasional use of pads required	Daily use of pads required	Severe symptoms; elective operative intervention indicated	-	-
**Definition:** A disorder characterized by inability to control the escape of stool from the rectum.<br>**Navigational Note:** -					

Gastrointestinal disorders					
**CTCAE Term**	**Grade 1**	**Grade 2**	**Grade 3**	**Grade 4**	**Grade 5**
Flatulence	Mild symptoms; intervention not indicated	Moderate; persistent; psychosocial sequelae	-	-	-
**Definition:** A disorder characterized by a discharge of excessive gas from the lower GI tract.<br>**Navigational Note:** -					
Gastric fistula	Asymptomatic	Symptomatic, invasive intervention not indicated	Invasive intervention indicated	Life-threatening consequences; urgent intervention indicated	Death
**Definition:** A disorder characterized by an abnormal communication between the stomach and another organ or anatomic site.<br>**Navigational Note:** -					
Gastric hemorrhage	Mild symptoms; intervention not indicated	Moderate symptoms; intervention indicated	Transfusion indicated; invasive intervention indicated; hospitalization	Life-threatening consequences; urgent intervention indicated	Death
**Definition:** A disorder characterized by bleeding from the gastric wall.<br>**Navigational Note:** -					
Gastric necrosis	-	-	Inability to aliment adequately by GI tract; invasive intervention indicated	Life-threatening consequences; urgent operative intervention indicated	Death
**Definition:** A disorder characterized by a necrotic process occurring in the gastric wall.<br>**Navigational Note:** -					
Gastric perforation	-	Invasive intervention not indicated	Invasive intervention indicated	Life-threatening consequences; urgent operative intervention indicated	Death
**Definition:** A disorder characterized by a rupture in the stomach wall.<br>**Navigational Note:** -					
Gastric stenosis	Asymptomatic; clinical or diagnostic observations only; intervention not indicated	Symptomatic; altered GI function	Severely altered GI function; tube feeding or hospitalization indicated; elective operative intervention indicated	Life-threatening consequences; urgent operative intervention indicated	Death
**Definition:** A disorder characterized by a narrowing of the lumen of the stomach.<br>**Navigational Note:** -					
Gastric ulcer	Asymptomatic; clinical or diagnostic observations only; intervention not indicated	Symptomatic; altered GI function; medical intervention indicated; limiting instrumental ADL	Severely altered GI function; TPN indicated; elective invasive intervention indicated; limiting self care ADL	Life-threatening consequences; urgent operative intervention indicated	Death
**Definition:** A disorder characterized by a circumscribed, erosive lesion on the mucosal surface of the stomach.<br>**Navigational Note:** -					

Gastrointestinal disorders					
**CTCAE Term**	**Grade 1**	**Grade 2**	**Grade 3**	**Grade 4**	**Grade 5**
Gastritis	Asymptomatic; clinical or diagnostic observations only; intervention not indicated	Symptomatic; altered GI function; medical intervention indicated	Severely altered eating or gastric function; TPN or hospitalization indicated	Life-threatening consequences; urgent operative intervention indicated	Death
**Definition:** A disorder characterized by inflammation of the stomach.<br>**Navigational Note:** -					
Gastroesophageal reflux disease	Mild symptoms; intervention not indicated	Moderate symptoms; medical intervention indicated	Severe symptoms; operative intervention indicated	-	-
**Definition:** A disorder characterized by reflux of the gastric and/or duodenal contents into the distal esophagus. It is chronic in nature and usually caused by incompetence of the lower esophageal sphincter, and may result in injury to the esophageal mucosal. Symptoms include heartburn and acid indigestion.<br>**Navigational Note:** -					
Gastrointestinal fistula	Asymptomatic	Symptomatic, invasive intervention not indicated	Invasive intervention indicated	Life-threatening consequences; urgent intervention indicated	Death
**Definition:** A disorder characterized by an abnormal communication between any part of the gastrointestinal system and another organ or anatomic site.<br>**Navigational Note:** -					
Gastrointestinal pain	Mild pain	Moderate pain; limiting instrumental ADL	Severe pain; limiting self care ADL	-	-
**Definition:** A disorder characterized by a sensation of marked discomfort in the gastrointestinal region.<br>**Navigational Note:** -					
Gastroparesis	Mild nausea, early satiety and bloating, able to maintain caloric intake on regular diet	Moderate symptoms; able to maintain nutrition with dietary and lifestyle modifications; may need pharmacologic intervention	Weight loss >=20% from baseline; tube feeding or TPN indicated; elective operative intervention indicated	Life-threatening consequences; urgent intervention indicated	-
**Definition:** A disorder characterized by an incomplete paralysis of the muscles of the stomach wall resulting in delayed emptying of the gastric contents into the small intestine.<br>**Navigational Note:** -					
Gingival pain	Mild pain	Moderate pain interfering with oral intake	Severe pain; inability to aliment orally	-	-
**Definition:** A disorder characterized by a sensation of marked discomfort in the gingival region.<br>**Navigational Note:** -					
Hemorrhoidal hemorrhage	Mild symptoms; intervention not indicated	Moderate symptoms; intervention indicated	Transfusion indicated; invasive intervention indicated; hospitalization	Life-threatening consequences; urgent intervention indicated	Death
**Definition:** A disorder characterized by bleeding from the hemorrhoids.<br>**Navigational Note:** -					

Gastrointestinal disorders					
**CTCAE Term**	**Grade 1**	**Grade 2**	**Grade 3**	**Grade 4**	**Grade 5**
Hemorrhoids	Asymptomatic; clinical or diagnostic observations only; intervention not indicated	Symptomatic; banding or medical intervention indicated	Severe symptoms; invasive intervention indicated	-	-
**Definition:** A disorder characterized by the presence of dilated veins in the rectum and surrounding area.<br>**Navigational Note:** -					
Ileal fistula	Asymptomatic	Symptomatic, invasive intervention not indicated	Invasive intervention indicated	Life-threatening consequences; urgent intervention indicated	Death
**Definition:** A disorder characterized by an abnormal communication between the ileum and another organ or anatomic site.<br>**Navigational Note:** -					
Ileal hemorrhage	Mild symptoms; intervention not indicated	Moderate symptoms; intervention indicated	Transfusion indicated; invasive intervention indicated; hospitalization	Life-threatening consequences; urgent intervention indicated	Death
**Definition:** A disorder characterized by bleeding from the ileal wall.<br>**Navigational Note:** -					
Ileal obstruction	Asymptomatic; clinical or diagnostic observations only; intervention not indicated	Symptomatic; altered GI function; limiting instrumental ADL; naso-gastric tube indicated	Hospitalization indicated; invasive intervention indicated; limiting self care ADL; long intestinal tube indicated	Life-threatening consequences; urgent operative intervention indicated	Death
**Definition:** A disorder characterized by blockage of the normal flow of the intestinal contents in the ileum.<br>**Navigational Note:** -					
Ileal perforation	-	Invasive intervention not indicated	Invasive intervention indicated	Life-threatening consequences; urgent operative intervention indicated	Death
**Definition:** A disorder characterized by a rupture in the ileal wall.<br>**Navigational Note:** -					
Ileal stenosis	Asymptomatic; clinical or diagnostic observations only; intervention not indicated	Symptomatic; altered GI function	Severely altered GI function; tube feeding or hospitalization indicated; elective operative intervention indicated	Life-threatening consequences; urgent operative intervention indicated	Death
**Definition:** A disorder characterized by a narrowing of the lumen of the ileum.<br>**Navigational Note:** -					

Gastrointestinal disorders					
**CTCAE Term**	**Grade 1**	**Grade 2**	**Grade 3**	**Grade 4**	**Grade 5**
Ileal ulcer	Asymptomatic; clinical or diagnostic observations only; intervention not indicated	Symptomatic; altered GI function	Severely altered GI function; TPN indicated; elective invasive intervention indicated	Life-threatening consequences; urgent operative intervention indicated	Death
**Definition:** A disorder characterized by a circumscribed, erosive lesion on the mucosal surface of the ileum.<br>**Navigational Note:** -					
Ileus	Asymptomatic and radiologic observations only	Symptomatic; altered GI function; bowel rest indicated	Severely altered GI function; TPN indicated; tube placement indicated	Life-threatening consequences; urgent intervention indicated	Death
**Definition:** A disorder characterized by failure of the ileum to transport intestinal contents.<br>**Navigational Note:** -					
Intra-abdominal hemorrhage	-	Moderate symptoms; intervention indicated	Transfusion indicated; invasive intervention indicated; hospitalization	Life-threatening consequences; urgent intervention indicated	Death
**Definition:** A disorder characterized by bleeding in the abdominal cavity.<br>**Navigational Note:** -					
Jejunal fistula	Asymptomatic	Symptomatic, invasive intervention not indicated	Invasive intervention indicated	Life-threatening consequences; urgent intervention indicated	Death
**Definition:** A disorder characterized by an abnormal communication between the jejunum and another organ or anatomic site.<br>**Navigational Note:** -					
Jejunal hemorrhage	Mild symptoms; intervention not indicated	Moderate symptoms; intervention indicated	Transfusion indicated; invasive intervention indicated; hospitalization	Life-threatening consequences; urgent intervention indicated	Death
**Definition:** A disorder characterized by bleeding from the jejunal wall.<br>**Navigational Note:** -					
Jejunal obstruction	Asymptomatic; clinical or diagnostic observations only; intervention not indicated	Symptomatic; altered GI function; limiting instrumental ADL	Hospitalization indicated; invasive intervention indicated; limiting self care ADL	Life-threatening consequences; urgent operative intervention indicated	Death
**Definition:** A disorder characterized by blockage of the normal flow of the intestinal contents in the jejunum.<br>**Navigational Note:** -					
Jejunal perforation	-	Invasive intervention not indicated	Invasive intervention indicated	Life-threatening consequences; urgent operative intervention indicated	Death
**Definition:** A disorder characterized by a rupture in the jejunal wall.<br>**Navigational Note:** -					

Gastrointestinal disorders					
**CTCAE Term**	**Grade 1**	**Grade 2**	**Grade 3**	**Grade 4**	**Grade 5**
Jejunal stenosis	Asymptomatic; clinical or diagnostic observations only; intervention not indicated	Symptomatic; altered GI function	Severely altered GI function; tube feeding or hospitalization indicated; elective operative intervention indicated	Life-threatening consequences; urgent operative intervention indicated	Death
**Definition:** A disorder characterized by a narrowing of the lumen of the jejunum.<br>**Navigational Note:** -					
Jejunal ulcer	Asymptomatic; clinical or diagnostic observations only; intervention not indicated	Symptomatic; altered GI function	Severely altered GI function; TPN indicated; elective invasive intervention indicated	Life-threatening consequences; urgent operative intervention indicated	Death
**Definition:** A disorder characterized by a circumscribed, erosive lesion on the mucosal surface of the jejunum.<br>**Navigational Note:** -					
Lip pain	Mild pain	Moderate pain; limiting instrumental ADL	Severe pain; limiting self care ADL	-	-
**Definition:** A disorder characterized by a sensation of marked discomfort of the lip.<br>**Navigational Note:** -					
Lower gastrointestinal hemorrhage	Mild symptoms; intervention not indicated	Moderate symptoms; intervention indicated	Transfusion indicated; invasive intervention indicated; hospitalization	Life-threatening consequences; urgent intervention indicated	Death
**Definition:** A disorder characterized by bleeding from the lower gastrointestinal tract (small intestine, large intestine, and anus).<br>**Navigational Note:** -					
Malabsorption	-	Altered diet; oral intervention indicated	Inability to aliment adequately; TPN indicated	Life-threatening consequences; urgent intervention indicated	Death
**Definition:** A disorder characterized by inadequate absorption of nutrients in the small intestine. Symptoms include abdominal marked discomfort, bloating and diarrhea.<br>**Navigational Note:** -					
Mucositis oral	Asymptomatic or mild symptoms; intervention not indicated	Moderate pain or ulcer that does not interfere with oral intake; modified diet indicated	Severe pain; interfering with oral intake	Life-threatening consequences; urgent intervention indicated	Death
**Definition:** A disorder characterized by ulceration or inflammation of the oral mucosal.<br>**Navigational Note:** -					
Nausea	Loss of appetite without alteration in eating habits	Oral intake decreased without significant weight loss, dehydration or malnutrition	Inadequate oral caloric or fluid intake; tube feeding, TPN, or hospitalization indicated	-	-
**Definition:** A disorder characterized by a queasy sensation and/or the urge to vomit.<br>**Navigational Note:** -					

Gastrointestinal disorders					
**CTCAE Term**	**Grade 1**	**Grade 2**	**Grade 3**	**Grade 4**	**Grade 5**
Obstruction gastric	Asymptomatic; clinical or diagnostic observations only; intervention not indicated	Symptomatic; altered GI function; limiting instrumental ADL	Hospitalization indicated; invasive intervention indicated; limiting self care ADL	Life-threatening consequences; urgent operative intervention indicated	Death
**Definition:** A disorder characterized by blockage of the normal flow of the contents in the stomach.<br>**Navigational Note:** -					
Oral cavity fistula	Asymptomatic	Symptomatic, invasive intervention not indicated	Invasive intervention indicated	Life-threatening consequences; urgent intervention indicated	Death
**Definition:** A disorder characterized by an abnormal communication between the oral cavity and another organ or anatomic site.<br>**Navigational Note:** -					
Oral dysesthesia	Mild discomfort; not interfering with oral intake	Moderate pain; interfering with oral intake	Disabling pain; tube feeding or TPN indicated	-	-
**Definition:** A disorder characterized by a burning or tingling sensation on the lips, tongue or entire mouth.<br>**Navigational Note:** -					
Oral hemorrhage	Mild symptoms; intervention not indicated	Moderate symptoms; intervention indicated	Transfusion indicated; invasive intervention indicated; hospitalization	Life-threatening consequences; urgent intervention indicated	Death
**Definition:** A disorder characterized by bleeding from the mouth.<br>**Navigational Note:** -					
Oral pain	Mild pain	Moderate pain; limiting instrumental ADL	Severe pain; limiting self care ADL	-	-
**Definition:** A disorder characterized by a sensation of marked discomfort in the mouth, tongue or lips.<br>**Navigational Note:** -					
Pancreatic duct stenosis	Asymptomatic; clinical or diagnostic observations only; intervention not indicated	Symptomatic; altered GI function	Severely altered GI function; tube feeding or hospitalization indicated; elective operative intervention indicated	Life-threatening consequences; urgent operative intervention indicated	Death
**Definition:** A disorder characterized by a narrowing of the lumen of the pancreatic duct.<br>**Navigational Note:** -					
Pancreatic fistula	Asymptomatic	Symptomatic, invasive intervention not indicated	Invasive intervention indicated	Life-threatening consequences; urgent intervention indicated	Death
**Definition:** A disorder characterized by an abnormal communication between the pancreas and another organ or anatomic site.<br>**Navigational Note:** -					

Gastrointestinal disorders					
**CTCAE Term**	**Grade 1**	**Grade 2**	**Grade 3**	**Grade 4**	**Grade 5**
Pancreatic hemorrhage	Mild symptoms; intervention not indicated	Moderate symptoms; intervention indicated	Transfusion indicated; invasive intervention indicated; hospitalization	Life-threatening consequences; urgent intervention indicated	Death
**Definition:** A disorder characterized by bleeding from the pancreas.					
**Navigational Note:** -					
Pancreatic necrosis	-	-	Tube feeding or TPN indicated; invasive intervention indicated	Life-threatening consequences; urgent operative intervention indicated	Death
**Definition:** A disorder characterized by a necrotic process occurring in the pancreas.					
**Navigational Note:** -					
Pancreatitis	-	Enzyme elevation; radiologic findings only	Severe pain; vomiting; medical intervention indicated (e.g., analgesia, nutritional support)	Life-threatening consequences; urgent intervention indicated	Death
**Definition:** A disorder characterized by inflammation of the pancreas with no documented pancreas infection.					
**Navigational Note:** -					
Periodontal disease	Gingival recession or gingivitis; limited bleeding on probing; mild local bone loss	Moderate gingival recession or gingivitis; multiple sites of bleeding on probing; moderate bone loss	Spontaneous bleeding; severe bone loss with or without tooth loss; osteonecrosis of maxilla or mandible	-	-
**Definition:** A disorder in the gingival tissue around the teeth.					
**Navigational Note:** -					
Peritoneal necrosis	-	-	Tube feeding or TPN indicated; invasive intervention indicated	Life-threatening consequences; urgent operative intervention indicated	Death
**Definition:** A disorder characterized by a necrotic process occurring in the peritoneum.					
**Navigational Note:** -					
Proctitis	Rectal discomfort, intervention not indicated	Symptomatic (e.g., rectal discomfort, passing blood or mucus); medical intervention indicated; limiting instrumental ADL	Severe symptoms; fecal urgency or stool incontinence; limiting self care ADL	Life-threatening consequences; urgent intervention indicated	Death
**Definition:** A disorder characterized by inflammation of the rectum.					
**Navigational Note:** -					
Rectal fissure	Asymptomatic	Symptomatic	Invasive intervention indicated	-	-
**Definition:** A disorder characterized by a tear in the lining of the rectum.					
**Navigational Note:** -					

Gastrointestinal disorders					
**CTCAE Term**	**Grade 1**	**Grade 2**	**Grade 3**	**Grade 4**	**Grade 5**
Rectal fistula	Asymptomatic	Symptomatic, invasive intervention not indicated	Invasive intervention indicated	Life-threatening consequences; urgent intervention indicated	Death
**Definition:** A disorder characterized by an abnormal communication between the rectum and another organ or anatomic site.<br>**Navigational Note:** -					
Rectal hemorrhage	Mild symptoms; intervention not indicated	Moderate symptoms; intervention indicated	Transfusion indicated; invasive intervention indicated; hospitalization	Life-threatening consequences; urgent intervention indicated	Death
**Definition:** A disorder characterized by bleeding from the rectal wall and discharged from the anus.<br>**Navigational Note:** -					
Rectal mucositis	Asymptomatic or mild symptoms; intervention not indicated	Symptomatic; medical intervention indicated; limiting instrumental ADL	Severe symptoms; limiting self care ADL	Life-threatening consequences; urgent operative intervention indicated	Death
**Definition:** A disorder characterized by ulceration or inflammation of the mucous membrane of the rectum.<br>**Navigational Note:** -					
Rectal necrosis	-	-	Tube feeding or TPN indicated; invasive intervention indicated	Life-threatening consequences; urgent operative intervention indicated	Death
**Definition:** A disorder characterized by a necrotic process occurring in the rectal wall.<br>**Navigational Note:** -					
Rectal obstruction	Asymptomatic; clinical or diagnostic observations only; intervention not indicated	Symptomatic; altered GI function; limiting instrumental ADL	Hospitalization indicated; invasive intervention indicated; limiting self care ADL	Life-threatening consequences; urgent operative intervention indicated	Death
**Definition:** A disorder characterized by blockage of the normal flow of the intestinal contents in the rectum.<br>**Navigational Note:** -					
Rectal pain	Mild pain	Moderate pain; limiting instrumental ADL	Severe pain; limiting self care ADL	-	-
**Definition:** A disorder characterized by a sensation of marked discomfort in the rectal region.<br>**Navigational Note:** -					
Rectal perforation	-	Invasive intervention not indicated	Invasive intervention indicated	Life-threatening consequences; urgent operative intervention indicated	Death
**Definition:** A disorder characterized by a rupture in the rectal wall.<br>**Navigational Note:** -					

Gastrointestinal disorders					
**CTCAE Term**	**Grade 1**	**Grade 2**	**Grade 3**	**Grade 4**	**Grade 5**
Rectal stenosis	Asymptomatic; clinical or diagnostic observations only; intervention not indicated	Symptomatic; altered GI function	Severely altered GI function; tube feeding or hospitalization indicated; elective operative intervention indicated	Life-threatening consequences; urgent operative intervention indicated	Death
**Definition:** A disorder characterized by a narrowing of the lumen of the rectum.<br>**Navigational Note:** -					
Rectal ulcer	Asymptomatic; clinical or diagnostic observations only; intervention not indicated	Symptomatic; altered GI function (e.g., altered dietary habits, vomiting, diarrhea)	Severely altered GI function; TPN indicated; elective invasive intervention indicated	Life-threatening consequences; urgent operative intervention indicated	Death
**Definition:** A disorder characterized by a circumscribed, erosive lesion on the mucosal surface of the rectum.<br>**Navigational Note:** -					
Retroperitoneal hemorrhage	-	Self-limited; intervention indicated	Transfusion indicated; invasive intervention indicated; hospitalization	Life-threatening consequences; urgent intervention indicated	Death
**Definition:** A disorder characterized by bleeding from the retroperitoneal area.<br>**Navigational Note:** -					
Salivary duct inflammation	Slightly thickened saliva; slightly altered taste (e.g., metallic)	Thick, ropy, sticky saliva; markedly altered taste; alteration in diet indicated; secretion-induced symptoms; limiting instrumental ADL	Acute salivary gland necrosis; severe secretion-induced symptoms (e.g., thick saliva/oral secretions or gagging); tube feeding or TPN indicated; limiting self care ADL	Life-threatening consequences; urgent intervention indicated	Death
**Definition:** A disorder characterized by inflammation of the salivary duct.<br>**Navigational Note:** -					
Salivary gland fistula	Asymptomatic	Symptomatic, invasive intervention not indicated	Invasive intervention indicated	Life-threatening consequences; urgent intervention indicated	Death
**Definition:** A disorder characterized by an abnormal communication between a salivary gland and another organ or anatomic site.<br>**Navigational Note:** -					
Small intestinal mucositis	Asymptomatic or mild symptoms; intervention not indicated	Symptomatic; medical intervention indicated; limiting instrumental ADL	Severe pain; interfering with oral intake; tube feeding, TPN or hospitalization indicated; limiting self care ADL	Life-threatening consequences; urgent intervention indicated	Death
**Definition:** A disorder characterized by ulceration or inflammation of the mucous membrane of the small intestine.<br>**Navigational Note:** -					

Gastrointestinal disorders					
**CTCAE Term**	**Grade 1**	**Grade 2**	**Grade 3**	**Grade 4**	**Grade 5**
Small intestinal obstruction	Asymptomatic; clinical or diagnostic observations only; intervention not indicated	Symptomatic; altered GI function; limiting instrumental ADL	Hospitalization indicated; invasive intervention indicated; limiting self care ADL	Life-threatening consequences; urgent operative intervention indicated	Death
**Definition:** A disorder characterized by blockage of the normal flow of the intestinal contents of the small intestine.					
**Navigational Note:** -					
Small intestinal perforation	-	Invasive intervention not indicated	Invasive intervention indicated	Life-threatening consequences; urgent operative intervention indicated	Death
**Definition:** A disorder characterized by a rupture in the small intestine wall.					
**Navigational Note:** -					
Small intestinal stenosis	Asymptomatic; clinical or diagnostic observations only; intervention not indicated	Symptomatic; altered GI function	Symptomatic and severely altered GI function; tube feeding, TPN or hospitalization indicated; non-emergent operative intervention indicated	Life-threatening consequences; urgent operative intervention indicated	Death
**Definition:** A disorder characterized by a narrowing of the lumen of the small intestine.					
**Navigational Note:** -					
Small intestine ulcer	Asymptomatic; clinical or diagnostic observations only; intervention not indicated	Symptomatic; altered GI function; limiting instrumental ADL	Severely altered GI function; TPN indicated; elective invasive intervention indicated; limiting self care ADL	Life-threatening consequences; urgent operative intervention indicated	Death
**Definition:** A disorder characterized by a circumscribed, erosive lesion on the mucosal surface of the small intestine.					
**Navigational Note:** -					
Stomach pain	Mild pain	Moderate pain; limiting instrumental ADL	Severe pain; limiting self care ADL	-	-
**Definition:** A disorder characterized by a sensation of marked discomfort in the stomach.					
**Navigational Note:** -					
Tooth development disorder	Asymptomatic; hypoplasia of tooth or enamel	Impairment correctable with oral surgery	Maldevelopment with impairment not surgically correctable; limiting self care ADL	-	-
**Definition:** A disorder characterized by a pathological process of the teeth occurring during tooth development.					
**Navigational Note:** -					
Tooth discoloration	Surface stains	-	-	-	-
**Definition:** A disorder characterized by a change in tooth hue or tint.					
**Navigational Note:** -					

Gastrointestinal disorders					
**CTCAE Term**	**Grade 1**	**Grade 2**	**Grade 3**	**Grade 4**	**Grade 5**
Toothache	Mild pain	Moderate pain; limiting instrumental ADL	Severe pain; limiting self care ADL	-	-
**Definition:** A disorder characterized by a sensation of marked discomfort in the tooth.<br>**Navigational Note:** -					
Typhlitis	-	-	Symptomatic (e.g., abdominal pain, fever, change in bowel habits with ileus); peritoneal signs	Life-threatening consequences; urgent operative intervention indicated	Death
**Definition:** A disorder characterized by necrotizing enterocolitis in neutropenic patients.<br>**Navigational Note:** Also report Investigations: Neutrophil count decreased					
Upper gastrointestinal hemorrhage	Mild symptoms; intervention not indicated	Moderate symptoms; intervention indicated	Transfusion indicated; invasive intervention indicated; hospitalization	Life-threatening consequences; urgent intervention indicated	Death
**Definition:** A disorder characterized by bleeding from the upper gastrointestinal tract (oral cavity, pharynx, esophagus, and stomach).<br>**Navigational Note:** -					
Visceral arterial ischemia	-	Brief (<24 hrs) episode of ischemia managed medically and without permanent deficit	Prolonged (>=24 hrs) or recurring symptoms and/or invasive intervention indicated	Life-threatening consequences; evidence of end organ damage; urgent operative intervention indicated	Death
**Definition:** A disorder characterized by a decrease in blood supply due to narrowing or blockage of a visceral (mesenteric) artery.<br>**Navigational Note:** -					
Vomiting	Intervention not indicated	Outpatient IV hydration; medical intervention indicated	Tube feeding, TPN, or hospitalization indicated	Life-threatening consequences	Death
**Definition:** A disorder characterized by the reflexive act of ejecting the contents of the stomach through the mouth.<br>**Navigational Note:** -					
Gastrointestinal disorders - Other, specify	Asymptomatic or mild symptoms; clinical or diagnostic observations only; intervention not indicated	Moderate; minimal, local or noninvasive intervention indicated; limiting age-appropriate instrumental ADL	Severe or medically significant but not immediately life-threatening; hospitalization or prolongation of existing hospitalization indicated; limiting self care ADL	Life-threatening consequences; urgent intervention indicated	Death
**Definition:** -<br>**Navigational Note:** -					

General disorders and administration site conditions					
**CTCAE Term**	**Grade 1**	**Grade 2**	**Grade 3**	**Grade 4**	**Grade 5**
Chills	Mild sensation of cold; shivering; chattering of teeth	Moderate tremor of the entire body; narcotics indicated	Severe or prolonged, not responsive to narcotics	-	-
**Definition:** A disorder characterized by a sensation of cold that often marks a physiologic response to sweating after a fever.<br>**Navigational Note:** -					
Death neonatal	-	-	-	Neonatal loss of life	-
**Definition:** Newborn death occurring during the first 28 days after birth.<br>**Navigational Note:** -					
Death NOS	-	-	-	-	Death
**Definition:** Death that cannot be attributed to a CTCAE term associated with Grade 5.<br>**Navigational Note:** If death is due to an AE (ex., Cardiac disorders: Cardiac arrest), report as a Grade 5 event under that AE.					
Disease progression	-	-	-	-	Death
**Definition:** Death due to disease progression that cannot be attributed to a CTCAE term associated with Grade 5.<br>**Navigational Note:** If death is due to an AE (ex., Cardiac disorders: Cardiac arrest), report as a Grade 5 event under that AE.					
Edema face	Localized facial edema	Moderate localized facial edema; limiting instrumental ADL	Severe swelling; limiting self care ADL	-	-
**Definition:** A disorder characterized by swelling due to excessive fluid accumulation in facial tissues.<br>**Navigational Note:** -					
Edema limbs	5 - 10% inter-limb discrepancy in volume or circumference at point of greatest visible difference; swelling or obscuration of anatomic architecture on close inspection	>10 - 30% inter-limb discrepancy in volume or circumference at point of greatest visible difference; readily apparent obscuration of anatomic architecture; obliteration of skin folds; readily apparent deviation from normal anatomic contour; limiting instrumental ADL	>30% inter-limb discrepancy in volume; gross deviation from normal anatomic contour; limiting self care ADL	-	-
**Definition:** A disorder characterized by swelling due to excessive fluid accumulation in the upper or lower extremities.<br>**Navigational Note:** -					

General disorders and administration site conditions					
**CTCAE Term**	**Grade 1**	**Grade 2**	**Grade 3**	**Grade 4**	**Grade 5**
Edema trunk	Swelling or obscuration of anatomic architecture on close inspection	Readily apparent obscuration of anatomic architecture; obliteration of skin folds; readily apparent deviation from normal anatomic contour; limiting instrumental ADL	Gross deviation from normal anatomic contour; limiting self care ADL	-	-
**Definition:** A disorder characterized by swelling due to excessive fluid accumulation in the trunk area.<br>**Navigational Note:** -					
Facial pain	Mild pain	Moderate pain; limiting instrumental ADL	Severe pain; limiting self care ADL	-	-
**Definition:** A disorder characterized by a sensation of marked discomfort in the face.<br>**Navigational Note:** -					
Fatigue	Fatigue relieved by rest	Fatigue not relieved by rest; limiting instrumental ADL	Fatigue not relieved by rest, limiting self care ADL	-	-
**Definition:** A disorder characterized by a state of generalized weakness with a pronounced inability to summon sufficient energy to accomplish daily activities.<br>**Navigational Note:** -					
Fever	38.0 - 39.0 degrees C (100.4 - 102.2 degrees F)	>39.0 - 40.0 degrees C (102.3 - 104.0 degrees F)	>40.0 degrees C (>104.0 degrees F) for <=24 hrs	>40.0 degrees C (>104.0 degrees F) for >24 hrs	Death
**Definition:** A disorder characterized by elevation of the body's temperature above the upper limit of normal.<br>**Navigational Note:** -					
Flu like symptoms	Mild flu-like symptoms present	Moderate symptoms; limiting instrumental ADL	Severe symptoms; limiting self care ADL	-	-
**Definition:** A disorder characterized by a group of symptoms similar to those observed in patients with the flu. It includes fever, chills, body aches, malaise, loss of appetite and dry cough.<br>**Navigational Note:** Synonym: Flu, Influenza					
Gait disturbance	Mild change in gait (e.g., wide-based, limping or hobbling)	Moderate change in gait (e.g., wide-based, limping or hobbling); assistive device indicated; limiting instrumental ADL	Limiting self care ADL	-	-
**Definition:** A disorder characterized by walking difficulties.<br>**Navigational Note:** -					
Generalized edema	Noted on exam; 1+ pitting edema	Interfering with instrumental ADLs; oral therapy initiated	Interferes with self care ADL; intravenous therapy indicated; skin breakdown	Life-threatening consequences	-
**Definition:** A disorder characterized by fluid accumulation in the tissues of the body including the skin.<br>**Navigational Note:** -					

General disorders and administration site conditions					
**CTCAE Term**	**Grade 1**	**Grade 2**	**Grade 3**	**Grade 4**	**Grade 5**
Hypothermia	-	35 - >32 degrees C; 95 - >89.6 degrees F	32 - >28 degrees C; 89.6 - >82.4 degrees F	<=28 degrees C; 82.4 degrees F; life-threatening consequences (e.g., coma, hypotension, pulmonary edema, acidemia, ventricular fibrillation)	Death
**Definition:** A disorder characterized by an abnormally low body temperature. Treatment is required when the body temperature is 35C (95F) or below.<br>**Navigational Note:** -					
Infusion site extravasation	Painless edema	Erythema with associated symptoms (e.g., edema, pain, induration, phlebitis)	Ulceration or necrosis; severe tissue damage; operative intervention indicated	Life-threatening consequences; urgent intervention indicated	Death
**Definition:** A disorder characterized by leakage of the infusion into the surrounding tissue. Signs and symptoms may include induration, erythema, swelling, burning sensation and marked discomfort at the infusion site.<br>**Navigational Note:** -					
Injection site reaction	Tenderness with or without associated symptoms (e.g., warmth, erythema, itching)	Pain; lipodystrophy; edema; phlebitis	Ulceration or necrosis; severe tissue damage; operative intervention indicated	Life-threatening consequences; urgent intervention indicated	Death
**Definition:** A disorder characterized by an intense adverse reaction (usually immunologic) developing at the site of an injection.<br>**Navigational Note:** -					
Localized edema	Localized to dependent areas, no disability or functional impairment	Moderate localized edema and intervention indicated; limiting instrumental ADL	Severe localized edema and intervention indicated; limiting self care ADL	-	-
**Definition:** A disorder characterized by swelling due to excessive fluid accumulation at a specific anatomic site.<br>**Navigational Note:** Prior to using this term consider specific edema areas: General disorders and administration site conditions: Edema face, Edema limbs, Edema trunk, or Edema neck; Nervous system disorders: Edema cerebral; Reproductive system and breast disorders: Genital edema; Respiratory, thoracic and mediastinal disorders: Laryngeal edema or Pulmonary edema; Skin and subcutaneous tissue disorders: Periorbital edema; Vascular disorders: Lymphedema					
Malaise	Uneasiness or lack of well being	Uneasiness or lack of well being limiting instrumental ADL	Uneasiness or lack of well being limiting self-care ADL	-	-
**Definition:** A disorder characterized by a feeling of general discomfort or uneasiness, an out-of-sorts feeling.<br>**Navigational Note:** -					
Multi-organ failure	-	-	Shock with azotemia and acid-base disturbances; significant coagulation abnormalities	Life-threatening consequences (e.g., vasopressor dependent and oliguric or anuric or ischemic colitis or lactic acidosis)	Death
**Definition:** A disorder characterized by progressive deterioration of the lungs, liver, kidney and clotting mechanisms.<br>**Navigational Note:** -					

General disorders and administration site conditions					
**CTCAE Term**	**Grade 1**	**Grade 2**	**Grade 3**	**Grade 4**	**Grade 5**
Neck edema	Asymptomatic localized neck edema	Moderate neck edema; slight obliteration of anatomic landmarks; limiting instrumental ADL	Generalized neck edema (e.g., difficulty in turning neck); limiting self care ADL	Vascular or respiratory impairment requiring urgent intervention	-
**Definition:** A disorder characterized by swelling due to an accumulation of excessive fluid in the neck.					
**Navigational Note:** -					
Non-cardiac chest pain	Mild pain	Moderate pain; limiting instrumental ADL	Severe pain; limiting self care ADL	-	-
**Definition:** A disorder characterized by a sensation of marked discomfort in the chest unrelated to a heart disorder.					
**Navigational Note:** -					
Pain	Mild pain	Moderate pain; limiting instrumental ADL	Severe pain; limiting self care ADL	-	-
**Definition:** A disorder characterized by the sensation of marked discomfort, distress or agony.					
**Navigational Note:** Prior to using this term consider using a specific body part pain term found throughout the CTCAE (over 40 different pain terms).					
Sudden death NOS	-	-	-	-	Death
**Definition:** An unexpected death that cannot be attributed to a CTCAE term associated with Grade 5.					
**Navigational Note:** If death is due to an AE (ex., Cardiac disorders: Cardiac arrest), report as a Grade 5 event under that AE.					
Vaccination site lymphadenopathy	Local lymph node enlargement	Localized ulceration; generalized lymph node enlargement	-	-	-
**Definition:** A disorder characterized by lymph node enlargement after vaccination.					
**Navigational Note:** -					
General disorders and administration site conditions - Other, specify	Asymptomatic or mild symptoms; clinical or diagnostic observations only; intervention not indicated	Moderate; minimal, local or noninvasive intervention indicated; limiting age-appropriate instrumental ADL	Severe or medically significant but not immediately life-threatening; hospitalization or prolongation of existing hospitalization indicated; limiting self care ADL	Life-threatening consequences; urgent intervention indicated	Death
**Definition:** -					
**Navigational Note:** -					

Hepatobiliary disorders					
**CTCAE Term**	**Grade 1**	**Grade 2**	**Grade 3**	**Grade 4**	**Grade 5**
Bile duct stenosis	Asymptomatic; clinical or diagnostic observations only; intervention not indicated	Symptomatic; altered GI function; IV fluids indicated <24 hrs	Severely altered GI function; invasive intervention indicated	Life-threatening consequences; urgent operative intervention indicated	Death
**Definition:** A disorder characterized by a narrowing of the lumen of the bile duct.<br>**Navigational Note:** -					
Biliary fistula	-	Symptomatic, invasive intervention not indicated	Invasive intervention indicated	Life-threatening consequences; urgent intervention indicated	Death
**Definition:** A disorder characterized by an abnormal communication between the bile ducts and another organ or anatomic site.<br>**Navigational Note:** -					
Budd-Chiari syndrome	-	Medical management indicated	Severe or medically significant but not immediately life-threatening; hospitalization or prolongation of existing hospitalization indicated; asterixis; mild encephalopathy	Life-threatening consequences; moderate to severe encephalopathy; coma	Death
**Definition:** A disorder characterized by occlusion of the hepatic veins and typically presents with abdominal pain, ascites and hepatomegaly.<br>**Navigational Note:** -					
Cholecystitis	-	Symptomatic; medical intervention indicated	Severe symptoms; invasive intervention indicated	Life-threatening consequences; urgent operative intervention indicated	Death
**Definition:** A disorder characterized by inflammation involving the gallbladder. It may be associated with the presence of gallstones.<br>**Navigational Note:** -					
Gallbladder fistula	Asymptomatic	Symptomatic, invasive intervention not indicated	Invasive intervention indicated	Life-threatening consequences; urgent intervention indicated	Death
**Definition:** A disorder characterized by an abnormal communication between the gallbladder and another organ or anatomic site.<br>**Navigational Note:** -					
Gallbladder necrosis	-	-	-	Life-threatening consequences; urgent invasive intervention indicated	Death
**Definition:** A disorder characterized by a necrotic process occurring in the gallbladder.<br>**Navigational Note:** -					

Hepatobiliary disorders					
**CTCAE Term**	**Grade 1**	**Grade 2**	**Grade 3**	**Grade 4**	**Grade 5**
Gallbladder obstruction	Asymptomatic; clinical or diagnostic observations only; intervention not indicated	Symptomatic; altered GI function; IV fluids indicated <24 hrs	Symptomatic and severely altered GI function; tube feeding, TPN or hospitalization indicated; non-emergent operative intervention indicated	Life-threatening consequences; urgent operative intervention indicated	Death
**Definition:** A disorder characterized by blockage of the normal flow of the contents of the gallbladder.<br>**Navigational Note:** -					
Gallbladder pain	Mild pain	Moderate pain; limiting instrumental ADL	Severe pain; limiting self care ADL	-	-
**Definition:** A disorder characterized by a sensation of marked discomfort in the gallbladder region.<br>**Navigational Note:** -					
Gallbladder perforation	-	-	-	Life-threatening consequences; urgent intervention indicated	Death
**Definition:** A disorder characterized by a rupture in the gallbladder wall.<br>**Navigational Note:** -					
Hepatic failure	-	-	Asterixis; mild encephalopathy; drug-induced liver injury (DILI); limiting self care ADL	Life-threatening consequences; moderate to severe encephalopathy; coma	Death
**Definition:** A disorder characterized by the inability of the liver to metabolize chemicals in the body. Laboratory test results reveal abnormal plasma levels of ammonia, bilirubin, lactic dehydrogenase, alkaline phosphatase, aminotransferase, and/or prolongation of prothrombin time (INR.) Drug-induced liever injury (DILI) as defined by Hy's Law.<br>**Navigational Note:** -					
Hepatic hemorrhage	Mild symptoms; intervention not indicated	Moderate symptoms; intervention indicated	Transfusion indicated; invasive intervention indicated; hospitalization	Life-threatening consequences; urgent intervention indicated	Death
**Definition:** A disorder characterized by bleeding from the liver.<br>**Navigational Note:** -					
Hepatic necrosis	-	-	-	Life-threatening consequences; urgent invasive intervention indicated	Death
**Definition:** A disorder characterized by a necrotic process occurring in the hepatic parenchyma.<br>**Navigational Note:** -					
Hepatic pain	Mild pain	Moderate pain; limiting instrumental ADL	Severe pain; limiting self care ADL	-	-
**Definition:** A disorder characterized by a sensation of marked discomfort in the liver region.<br>**Navigational Note:** -					

Hepatobiliary disorders					
**CTCAE Term**	**Grade 1**	**Grade 2**	**Grade 3**	**Grade 4**	**Grade 5**
Perforation bile duct	-	-	Invasive intervention indicated	Life-threatening consequences; urgent operative intervention indicated	Death
**Definition:** A disorder characterized by a rupture in the wall of the extrahepatic or intrahepatic bile duct.<br>**Navigational Note:** -					
Portal hypertension	-	Decreased portal vein flow	Reversal/retrograde portal vein flow; associated with varices and/or ascites	Life-threatening consequences; urgent intervention indicated	Death
**Definition:** A disorder characterized by an increase in blood pressure in the portal venous system.<br>**Navigational Note:** -					
Portal vein thrombosis	-	Intervention not indicated	Medical intervention indicated	Life-threatening consequences; urgent intervention indicated	Death
**Definition:** A disorder characterized by the formation of a thrombus (blood clot) in the portal vein.<br>**Navigational Note:** -					
Sinusoidal obstruction syndrome	-	Blood bilirubin 2-5 mg/dL; minor interventions required (i.e., blood product, diuretic, oxygen)	Blood bilirubin >5 mg/dL; coagulation modifier indicated (e.g., defibrotide); reversal of flow on ultrasound	Life-threatening consequences (e.g., ventilatory support, dialysis, plasmapheresis, peritoneal drainage)	Death
**Definition:** A disorder characterized by severe hepatic injury as a result of the blood vessels of the liver becoming inflamed and/or blocked.<br>**Navigational Note:** -					
Hepatobiliary disorders - Other, specify	Asymptomatic or mild symptoms; clinical or diagnostic observations only; intervention not indicated	Moderate; minimal, local or noninvasive intervention indicated; limiting age-appropriate instrumental ADL	Severe or medically significant but not immediately life-threatening; hospitalization or prolongation of existing hospitalization indicated; limiting self care ADL	Life-threatening consequences; urgent intervention indicated	Death
**Definition:** -<br>**Navigational Note:** -					

Immune system disorders					
**CTCAE Term**	**Grade 1**	**Grade 2**	**Grade 3**	**Grade 4**	**Grade 5**
Allergic reaction	Systemic intervention not indicated	Oral intervention indicated	Bronchospasm; hospitalization indicated for clinical sequelae; intravenous intervention indicated	Life-threatening consequences; urgent intervention indicated	Death
**Definition:** A disorder characterized by an adverse local or general response from exposure to an allergen.<br>**Navigational Note:** If related to infusion, use Injury, poisoning and procedural complications: Infusion related reaction. Do not report both.					
Anaphylaxis	-	-	Symptomatic bronchospasm, with or without urticaria; parenteral intervention indicated; allergy-related edema/angioedema; hypotension	Life-threatening consequences; urgent intervention indicated	Death
**Definition:** A disorder characterized by an acute inflammatory reaction resulting from the release of histamine and histamine-like substances from mast cells, causing a hypersensitivity immune response. Clinically, it presents with breathing difficulty, dizziness, hypotension, cyanosis and loss of consciousness and may lead to death.<br>**Navigational Note:** -					
Autoimmune disorder	Asymptomatic; serologic or other evidence of autoimmune reaction, with normal organ function; intervention not indicated	Evidence of autoimmune reaction involving a non-essential organ or function (e.g., hypothyroidism)	Autoimmune reactions involving major organ (e.g., colitis, anemia, myocarditis, kidney)	Life-threatening consequences; urgent intervention indicated	Death
**Definition:** A disorder characterized by loss of function or tissue destruction of an organ or multiple organs, arising from humoral or cellular immune responses of the individual to his own tissue constituents.<br>**Navigational Note:** Prior to using this term consider specific autoimmune AEs					
Cytokine release syndrome	Fever with or without constitutional symptoms	Hypotension responding to fluids; hypoxia responding to <40% O2	Hypotension managed with one pressor; hypoxia requiring ≥ 40% O2	Life-threatening consequences; urgent intervention indicated	Death
**Definition:** A disorder characterized by fever, tachypnea, headache, tachycardia, hypotension, rash, and/or hypoxia caused by the release of cytokines.<br>**Navigational Note:** Also consider reporting other organ dysfunctions including neurological toxicities such as: Psychiatric disorders: Hallucinations or Confusion; Nervous system disorders: Seizure, Dysphasia, Tremor, or Headache					
Serum sickness	Asymptomatic; clinical or diagnostic observations only; intervention not indicated	Moderate arthralgia; fever, rash, urticaria, antihistamines indicated	Severe arthralgia or arthritis; extensive rash; steroids or IV fluids indicated	Life-threatening consequences; pressor or ventilatory support indicated	Death
**Definition:** A disorder characterized by a delayed-type hypersensitivity reaction to foreign proteins derived from an animal serum. It occurs approximately six to twenty-one days following the administration of the foreign antigen. Symptoms include fever, arthralgias, myalgias, skin eruptions, lymphadenopathy, chest marked discomfort and dyspnea.<br>**Navigational Note:** -					

Immune system disorders					
**CTCAE Term**	**Grade 1**	**Grade 2**	**Grade 3**	**Grade 4**	**Grade 5**
Immune system disorders - Other, specify	Asymptomatic or mild symptoms; clinical or diagnostic observations only; intervention not indicated	Moderate; minimal, local or noninvasive intervention indicated; limiting age-appropriate instrumental ADL	Severe or medically significant but not immediately life-threatening; hospitalization or prolongation of existing hospitalization indicated; limiting self care ADL	Life-threatening consequences; urgent intervention indicated	Death
**Definition:** -					
**Navigational Note:** -					

Infections and infestations					
**CTCAE Term**	**Grade 1**	**Grade 2**	**Grade 3**	**Grade 4**	**Grade 5**
Abdominal infection	-	Oral intervention indicated (e.g., antibiotic, antifungal, or antiviral)	IV antibiotic, antifungal, or antiviral intervention indicated; invasive intervention indicated	Life-threatening consequences; urgent intervention indicated	Death
**Definition:** A disorder characterized by an infectious process involving the abdominal cavity.<br>**Navigational Note:** -					
Anorectal infection	Localized, local intervention indicated	Oral intervention indicated (e.g., antibiotic, antifungal, or antiviral)	IV antibiotic, antifungal, or antiviral intervention indicated; invasive intervention indicated	Life-threatening consequences; urgent intervention indicated	Death
**Definition:** A disorder characterized by an infectious process involving the anal area and the rectum.<br>**Navigational Note:** -					
Appendicitis	-	-	IV antibiotic, antifungal, or antiviral intervention indicated; invasive intervention indicated	Life-threatening consequences; urgent intervention indicated	Death
**Definition:** A disorder characterized by acute inflammation to the vermiform appendix caused by a pathogenic agent.<br>**Navigational Note:** -					
Appendicitis perforated	-	-	Medical intervention indicated; operative intervention indicated	Life-threatening consequences; urgent intervention indicated	Death
**Definition:** A disorder characterized by acute inflammation to the vermiform appendix caused by a pathogenic agent with gangrenous changes resulting in the rupture of the appendiceal wall. The appendiceal wall rupture causes the release of inflammatory and bacterial contents from the appendiceal lumen into the abdominal cavity.<br>**Navigational Note:** -					
Arteritis infective	-	-	IV antibiotic, antifungal, or antiviral intervention indicated; invasive intervention indicated	Life-threatening consequences; urgent intervention indicated	Death
**Definition:** A disorder characterized by an infectious process involving an artery.<br>**Navigational Note:** -					
Bacteremia	-	Blood culture positive with no signs or symptoms	-	-	-
**Definition:** A disorder characterized by the presence of bacteria in the blood stream.<br>**Navigational Note:** Consider Infections and infestations: Sepsis (Grades 3, 4 & 5)					
Biliary tract infection	-	Oral intervention indicated (e.g., antibiotic, antifungal, or antiviral)	IV antibiotic, antifungal, or antiviral intervention indicated; invasive intervention indicated	Life-threatening consequences; urgent intervention indicated	Death
**Definition:** A disorder characterized by an infectious process involving the biliary tract.<br>**Navigational Note:** -					

Infections and infestations					
**CTCAE Term**	**Grade 1**	**Grade 2**	**Grade 3**	**Grade 4**	**Grade 5**
Bladder infection	-	Oral intervention indicated (e.g., antibiotic, antifungal, or antiviral)	IV antibiotic, antifungal, or antiviral intervention indicated; invasive intervention indicated	Life-threatening consequences; urgent intervention indicated	Death
**Definition:** A disorder characterized by an infectious process involving the bladder.<br>**Navigational Note:** -					
Bone infection	-	Oral intervention indicated (e.g., antibiotic, antifungal, or antiviral)	IV antibiotic, antifungal, or antiviral intervention indicated; invasive intervention indicated	Life-threatening consequences; urgent intervention indicated	Death
**Definition:** A disorder characterized by an infectious process involving the bones.<br>**Navigational Note:** -					
Breast infection	-	Local infection with moderate symptoms; oral intervention indicated (e.g., antibiotic, antifungal, or antiviral)	IV antibiotic, antifungal, or antiviral intervention indicated; severe infection; axillary adenitis	Life-threatening consequences; urgent intervention indicated	Death
**Definition:** A disorder characterized by an infectious process involving the breast.<br>**Navigational Note:** -					
Bronchial infection	-	Moderate symptoms; oral intervention indicated (e.g., antibiotic, antifungal, or antiviral)	IV antibiotic, antifungal, or antiviral intervention indicated; invasive intervention indicated	Life-threatening consequences; urgent intervention indicated	Death
**Definition:** A disorder characterized by an infectious process involving the bronchi.<br>**Navigational Note:** -					
Catheter related infection	-	Localized; local intervention indicated; oral intervention indicated (e.g., antibiotic, antifungal, or antiviral)	IV antibiotic, antifungal, or antiviral intervention indicated; invasive intervention indicated	Life-threatening consequences; urgent intervention indicated	Death
**Definition:** A disorder characterized by an infectious process that arises secondary to catheter use.<br>**Navigational Note:** -					
Cecal infection	-	Localized; oral intervention indicated (e.g., antibiotic, antifungal, or antiviral)	IV antibiotic, antifungal, or antiviral intervention indicated; invasive intervention indicated	Life-threatening consequences; urgent intervention indicated	Death
**Definition:** A disorder characterized by an infectious process involving the cecum.<br>**Navigational Note:** -					

Infections and infestations					
**CTCAE Term**	**Grade 1**	**Grade 2**	**Grade 3**	**Grade 4**	**Grade 5**
Cervicitis infection	-	Localized; local intervention indicated (e.g., topical antibiotic, antifungal, or antiviral)	IV antibiotic, antifungal, or antiviral intervention indicated; invasive intervention indicated	Life-threatening consequences; urgent intervention indicated	Death
**Definition:** A disorder characterized by an infectious process involving the uterine cervix.<br>**Navigational Note:** -					
Conjunctivitis	Asymptomatic or mild symptoms; intervention not indicated	Symptomatic; moderate decrease in visual acuity (best corrected visual acuity 20/40 and better or 3 lines or less decreased vision from known baseline)	Symptomatic with marked decrease in visual acuity (best corrected visual acuity worse than 20/40 or more than 3 lines of decreased vision from known baseline, up to 20/200); limiting self care ADL	Best corrected visual acuity of 20/200 or worse in the affected eye	-
**Definition:** A disorder characterized by inflammation, swelling and redness to the conjunctiva of the eye.<br>**Navigational Note:** Consider Infections and infestations: Conjunctivitis infective if caused by infection					
Conjunctivitis infective	-	Localized; local intervention indicated (e.g., topical antibiotic, antifungal, or antiviral)	IV antibiotic, antifungal, or antiviral intervention indicated; invasive intervention indicated	Life-threatening consequences; urgent intervention indicated	-
**Definition:** A disorder characterized by an infectious process involving the conjunctiva. Clinical manifestations include pink or red color in the eyes.<br>**Navigational Note:** -					
Corneal infection	-	Localized; local intervention indicated (e.g., topical antibiotic, antifungal, or antiviral)	IV antibiotic, antifungal, or antiviral intervention indicated; invasive intervention indicated	Life-threatening consequences; urgent intervention indicated	Death
**Definition:** A disorder characterized by an infectious process involving the cornea.<br>**Navigational Note:** -					
Cranial nerve infection	-	-	IV antibiotic, antifungal, or antiviral intervention indicated; invasive intervention indicated	Life-threatening consequences; urgent intervention indicated	Death
**Definition:** A disorder characterized by an infectious process involving a cranial nerve.<br>**Navigational Note:** -					

Infections and infestations					
**CTCAE Term**	**Grade 1**	**Grade 2**	**Grade 3**	**Grade 4**	**Grade 5**
Cytomegalovirus infection reactivation	Asymptomatic or mild symptoms; clinical or diagnostic observations only; intervention not indicated	Moderate symptoms; medical intervention indicated	Severe or medically significant but not immediately life-threatening; hospitalization or prolongation of existing hospitalization indicated; IV intervention indicated	Life-threatening consequences; urgent intervention indicated; blindness	Death
**Definition:** A disorder characterized by the reactivation of cytomegalovirus (CMV).<br>**Navigational Note:** Synonym: CMV					
Device related infection	-	Oral intervention indicated (e.g., antibiotic, antifungal)	IV antibiotic, antifungal, or antiviral intervention indicated; invasive intervention indicated	Life-threatening consequences; urgent intervention indicated	Death
**Definition:** A disorder characterized by an infectious process involving the use of a medical device.<br>**Navigational Note:** -					
Duodenal infection	-	Moderate symptoms; medical intervention indicated (e.g., oral antibiotics)	IV antibiotic, antifungal, or antiviral intervention indicated; invasive intervention indicated	Life-threatening consequences; urgent intervention indicated	Death
**Definition:** A disorder characterized by an infectious process involving the duodenum.<br>**Navigational Note:** -					
Encephalitis infection	-	-	IV antibiotic, antifungal, or antiviral intervention indicated; severe changes in mental status; self-limited seizure activity; focal neurologic abnormalities	Life-threatening consequences; urgent intervention indicated	Death
**Definition:** A disorder characterized by an infectious process involving the brain tissue.<br>**Navigational Note:** -					
Encephalomyelitis infection	-	-	IV antibiotic, antifungal, or antiviral intervention indicated; invasive intervention indicated	Life-threatening consequences; urgent intervention indicated	Death
**Definition:** A disorder characterized by an infectious process involving the brain and spinal cord tissues.<br>**Navigational Note:** -					
Endocarditis infective	-	-	IV antibiotic, antifungal, or antiviral intervention indicated; invasive intervention indicated	Life-threatening consequences; urgent intervention indicated	Death
**Definition:** A disorder characterized by an infectious process involving the endocardial layer of the heart.<br>**Navigational Note:** -					

Infections and infestations					
**CTCAE Term**	**Grade 1**	**Grade 2**	**Grade 3**	**Grade 4**	**Grade 5**
Endophthalmitis	-	Local intervention indicated	Systemic intervention; hospitalization indicated	Best corrected visual acuity of 20/200 or worse in the affected eye	-
**Definition:** A disorder characterized by an infectious process involving the internal structures of the eye.<br>**Navigational Note:** -					
Enterocolitis infectious	-	Passage of >3 unformed stools per 24 hrs or duration of illness >48 hrs; moderate abdominal pain; oral intervention indicated (e.g., antibiotic, antifungal, or antiviral)	IV antibiotic, antifungal, or antiviral intervention indicated; invasive intervention indicated; profuse watery diarrhea with signs of hypovolemia; bloody diarrhea; fever; severe abdominal pain; hospitalization indicated	Life-threatening consequences; urgent intervention indicated	Death
**Definition:** A disorder characterized by an infectious process involving the small and large intestines.<br>**Navigational Note:** Includes Clostridium difficile (c. diff, c. difficile).					
Epstein-Barr virus infection reactivation	Asymptomatic or mild symptoms; clinical or diagnostic observations only; intervention not indicated	Moderate symptoms; medical intervention indicated	Severe or medically significant but not immediately life-threatening; hospitalization or prolongation of existing hospitalization indicated; IV intervention indicated	Life-threatening consequences; urgent intervention indicated	Death
**Definition:** A disorder characterized by the reactivation of Epstein-Barr virus (EBV).<br>**Navigational Note:** Synonym: EBV					
Esophageal infection	-	Local intervention indicated (e.g., oral antibiotic, antifungal, antiviral)	IV antibiotic, antifungal, or antiviral intervention indicated; invasive intervention indicated	Life-threatening consequences; urgent intervention indicated	Death
**Definition:** A disorder characterized by an infectious process involving the esophagus.<br>**Navigational Note:** -					
Eye infection	-	Localized; local intervention indicated (e.g., topical antibiotic, antifungal, or antiviral)	IV antibiotic, antifungal, or antiviral intervention indicated; invasive intervention indicated	Life-threatening consequences; urgent intervention indicated; enucleation	Death
**Definition:** A disorder characterized by an infectious process involving the eye.<br>**Navigational Note:** -					

Infections and infestations					
**CTCAE Term**	**Grade 1**	**Grade 2**	**Grade 3**	**Grade 4**	**Grade 5**
Folliculitis	Covering <10% of the body surface area; no intervention indicated	Covering 10-30% of the body surface area; topical intervention initiated	>30% BSA; systemic intervention indicated	-	-
**Definition:** A disorder characterized by inflammation or infection of the hair follicles.					
**Navigational Note:** -					
Fungemia	-	Moderate symptoms; medical intervention indicated	Severe or medically significant but not immediately life-threatening; hospitalization or prolongation of existing hospitalization indicated	-	-
**Definition:** A disorder characterized by the presence of fungus in the blood stream.					
**Navigational Note:** -					
Gallbladder infection	-	Oral intervention indicated (e.g., antibiotic, antifungal, or antiviral)	IV antibiotic, antifungal, or antiviral intervention indicated; invasive intervention indicated	Life-threatening consequences; urgent intervention indicated	Death
**Definition:** A disorder characterized by an infectious process involving the gallbladder.					
**Navigational Note:** -					
Gum infection	Local therapy indicated (swish and swallow)	Moderate symptoms; oral intervention indicated (e.g., antibiotic, antifungal, or antiviral)	IV antibiotic, antifungal, or antiviral intervention indicated; invasive intervention indicated	Life-threatening consequences; urgent intervention indicated	Death
**Definition:** A disorder characterized by an infectious process involving the gums.					
**Navigational Note:** -					
Hepatic infection	-	Moderate symptoms; oral intervention indicated (e.g., antibiotic, antifungal, or antiviral)	IV antibiotic, antifungal, or antiviral intervention indicated; invasive intervention indicated	Life-threatening consequences; urgent intervention indicated	Death
**Definition:** A disorder characterized by an infectious process involving the liver.					
**Navigational Note:** -					
Hepatitis B reactivation	Asymptomatic or mild symptoms; clinical or diagnostic observations only; intervention not indicated	Moderate symptoms; medical intervention indicated	Severe or medically significant but not immediately life-threatening; hospitalization or prolongation of existing hospitalization indicated; IV intervention indicated	Life-threatening consequences; urgent intervention indicated; severe decompensated liver function (e.g., coagulopathy, encephalopathy, coma)	Death
**Definition:** A disorder characterized by the reactivation of hepatitis B virus.					
**Navigational Note:** -					

Infections and infestations					
**CTCAE Term**	**Grade 1**	**Grade 2**	**Grade 3**	**Grade 4**	**Grade 5**
Hepatitis viral	Asymptomatic, intervention not indicated	Moderate symptoms; medical intervention indicated	Symptomatic liver dysfunction; fibrosis by biopsy; compensated cirrhosis; hospitalization or prolongation of existing hospitalization indicated	Life-threatening consequences; severe decompensated liver function (e.g., coagulopathy, encephalopathy, coma)	Death
**Definition:** A disorder characterized by a viral pathologic process involving the liver parenchyma.					
**Navigational Note:** -					
Herpes simplex reactivation	Asymptomatic or mild symptoms; clinical or diagnostic observations only; intervention not indicated	Moderate symptoms; medical intervention indicated	Severe or medically significant but not immediately life-threatening; hospitalization or prolongation of existing hospitalization indicated; IV intervention indicated	Life-threatening consequences; urgent intervention indicated	Death
**Definition:** A disorder characterized by the reactivation of Herpes simplex virus.					
**Navigational Note:** -					
Infective myositis	-	Localized; local intervention indicated (e.g., topical antibiotic, antifungal, or antiviral)	IV antibiotic, antifungal, or antiviral intervention indicated; invasive intervention indicated	Life-threatening consequences; urgent intervention indicated	Death
**Definition:** A disorder characterized by an infectious process involving the skeletal muscles.					
**Navigational Note:** -					
Joint infection	-	Localized; local intervention indicated; oral intervention indicated (e.g., antibiotic, antifungal, or antiviral); needle aspiration indicated (single or multiple)	Arthroscopic intervention indicated (e.g., drainage) or arthrotomy (e.g., open surgical drainage)	Life-threatening consequences; urgent intervention indicated	Death
**Definition:** A disorder characterized by an infectious process involving a joint.					
**Navigational Note:** -					
Kidney infection	-	Oral intervention indicated (e.g., antibiotic, antifungal, or antiviral)	IV antibiotic, antifungal, or antiviral intervention indicated; invasive intervention indicated	Life-threatening consequences; urgent intervention indicated	Death
**Definition:** A disorder characterized by an infectious process involving the kidney.					
**Navigational Note:** -					

Infections and infestations					
**CTCAE Term**	**Grade 1**	**Grade 2**	**Grade 3**	**Grade 4**	**Grade 5**
Laryngitis	-	Moderate symptoms; oral intervention indicated (e.g., antibiotic, antifungal, or antiviral)	IV antibiotic, antifungal, or antiviral intervention indicated; invasive intervention indicated	Life-threatening consequences; urgent intervention indicated	Death
**Definition:** A disorder characterized by an inflammatory process involving the larynx.<br>**Navigational Note:** For symptoms and no intervention, consider Respiratory, thoracic and mediastinal disorders: Sore throat or Hoarseness.					
Lip infection	Localized, local intervention indicated	Oral intervention indicated (e.g., antibiotic, antifungal, or antiviral)	IV antibiotic, antifungal, or antiviral intervention indicated; invasive intervention indicated	-	-
**Definition:** A disorder characterized by an infectious process involving the lips.<br>**Navigational Note:** -					
Lung infection	-	Moderate symptoms; oral intervention indicated (e.g., antibiotic, antifungal, or antiviral)	IV antibiotic, antifungal, or antiviral intervention indicated; invasive intervention indicated	Life-threatening consequences; urgent intervention indicated	Death
**Definition:** A disorder characterized by an infectious process involving the lungs, including pneumonia.<br>**Navigational Note:** If infection is due to aspiration, consider reporting Respiratory, thoracic and mediastinal disorders: Apiration					
Lymph gland infection	-	Localized; oral intervention indicated (e.g., antibiotic, antifungal, or antiviral)	IV antibiotic, antifungal, or antiviral intervention indicated; invasive intervention indicated	Life-threatening consequences; urgent intervention indicated	Death
**Definition:** A disorder characterized by an infectious process involving the lymph nodes.<br>**Navigational Note:** -					
Mediastinal infection	-	-	IV antibiotic, antifungal, or antiviral intervention indicated; invasive intervention indicated	Life-threatening consequences; urgent intervention indicated	Death
**Definition:** A disorder characterized by an infectious process involving the mediastinum.<br>**Navigational Note:** -					
Meningitis	-	-	IV antibiotic, antifungal, or antiviral intervention indicated; invasive intervention indicated; focal neurologic deficit	Life-threatening consequences; urgent intervention indicated	Death
**Definition:** A disorder characterized by acute inflammation of the meninges of the brain and/or spinal cord.<br>**Navigational Note:** -					

Infections and infestations					
**CTCAE Term**	**Grade 1**	**Grade 2**	**Grade 3**	**Grade 4**	**Grade 5**
Mucosal infection	Localized, local intervention indicated	Oral intervention indicated (e.g., antibiotic, antifungal, or antiviral)	IV antibiotic, antifungal, or antiviral intervention indicated; invasive intervention indicated	Life-threatening consequences; urgent intervention indicated	Death
**Definition:** A disorder characterized by an infectious process involving a mucosal surface.<br>**Navigational Note:** -					
Myelitis	Asymptomatic; mild signs (e.g., Babinski's reflex or Lhermitte's sign)	Moderate weakness or sensory loss; limiting instrumental ADL	Severe weakness or sensory loss; limiting self care ADL	Life-threatening consequences; urgent intervention indicated	Death
**Definition:** A disorder characterized by inflammation involving the spinal cord. Symptoms include weakness, paresthesia, sensory loss, marked discomfort and incontinence.<br>**Navigational Note:** -					
Nail infection	Localized, local intervention indicated	Oral intervention indicated (e.g., antibiotic, antifungal, or antiviral)	IV antibiotic, antifungal, or antiviral intervention indicated; invasive intervention indicated	-	-
**Definition:** A disorder characterized by an infectious process involving the nail.<br>**Navigational Note:** -					
Otitis externa	Localized, local intervention indicated	Oral intervention indicated (e.g., antibiotic, antifungal, or antiviral)	IV antibiotic, antifungal, or antiviral intervention indicated; invasive intervention indicated	Life-threatening consequences; urgent intervention indicated	Death
**Definition:** A disorder characterized by an infectious process involving the outer ear and ear canal. Contributory factors include excessive water exposure (swimmer's ear infection) and cuts in the ear canal. Symptoms include fullness, itching, swelling and marked discomfort in the ear and ear drainage.<br>**Navigational Note:** Changes associated with radiation to external ear (pinnae) are graded under Injury, poisoning and procedural complications: Dermatitis radiation					
Otitis media	-	Localized; oral intervention indicated (e.g., antibiotic, antifungal, or antiviral)	IV antibiotic, antifungal, or antiviral intervention indicated; invasive intervention indicated	Life-threatening consequences; urgent intervention indicated	Death
**Definition:** A disorder characterized by an infectious process involving the middle ear.<br>**Navigational Note:** -					
Ovarian infection	-	Localized; oral intervention indicated (e.g., antibiotic, antifungal, or antiviral)	IV antibiotic, antifungal, or antiviral intervention indicated; invasive intervention indicated	Life-threatening consequences; urgent intervention indicated	Death
**Definition:** A disorder characterized by an infectious process involving the ovary.<br>**Navigational Note:** -					

Infections and infestations					
**CTCAE Term**	**Grade 1**	**Grade 2**	**Grade 3**	**Grade 4**	**Grade 5**
Pancreas infection	-	-	IV antibiotic, antifungal, or antiviral intervention indicated; invasive intervention indicated	Life-threatening consequences; urgent intervention indicated	Death
**Definition:** A disorder characterized by an infectious process involving the pancreas.<br>**Navigational Note:** -					
Papulopustular rash	Papules and/or pustules covering <10% BSA, which may or may not be associated with symptoms of pruritus or tenderness	Papules and/or pustules covering 10-30% BSA, which may or may not be associated with symptoms of pruritus or tenderness; associated with psychosocial impact; limiting instrumental ADL; papules and/or pustules covering > 30% BSA with or without mild symptoms	Papules and/or pustules covering >30% BSA with moderate or severe symptoms; limiting self-care ADL; IV antibiotics indicated	Life-threatening consequences	Death
**Definition:** A disorder characterized by an eruption consisting of papules (a small, raised pimple) and pustules (a small pus filled blister), typically appearing in face, scalp, and upper chest and back. Unlike acne, this rash does not present with whiteheads or blackheads, and can be symptomatic, with itchy or tender lesions.<br>**Navigational Note:** -					
Paronychia	Nail fold edema or erythema; disruption of the cuticle	Local intervention indicated; oral intervention indicated (e.g., antibiotic, antifungal, antiviral); nail fold edema or erythema with pain; associated with discharge or nail plate separation; limiting instrumental ADL	Operative intervention indicated; IV antibiotics indicated; limiting self care ADL	-	-
**Definition:** A disorder characterized by an infectious process involving the soft tissues around the nail.<br>**Navigational Note:** -					
Pelvic infection	-	Moderate symptoms; oral intervention indicated (e.g., antibiotic, antifungal, or antiviral)	IV antibiotic, antifungal, or antiviral intervention indicated; invasive intervention indicated	Life-threatening consequences; urgent intervention indicated	Death
**Definition:** A disorder characterized by an infectious process involving the pelvic cavity.<br>**Navigational Note:** -					

Infections and infestations					
**CTCAE Term**	**Grade 1**	**Grade 2**	**Grade 3**	**Grade 4**	**Grade 5**
Penile infection	Localized, local intervention indicated	Oral intervention indicated (e.g., antibiotic, antifungal, or antiviral)	IV antibiotic, antifungal, or antiviral intervention indicated; invasive intervention indicated	Life-threatening consequences; urgent intervention indicated	Death
**Definition:** A disorder characterized by an infectious process involving the penis.<br>**Navigational Note:** -					
Periorbital infection	-	Localized; local intervention indicated (e.g., topical antibiotic, antifungal, or antiviral)	IV antibiotic, antifungal, or antiviral intervention indicated; invasive intervention indicated	Life-threatening consequences; urgent intervention indicated	Death
**Definition:** A disorder characterized by an infectious process involving the orbit of the eye.<br>**Navigational Note:** -					
Peripheral nerve infection	-	Localized; local intervention indicated (e.g., topical antibiotic, antifungal, or antiviral)	IV antibiotic, antifungal, or antiviral intervention indicated; invasive intervention indicated	Life-threatening consequences; urgent intervention indicated	Death
**Definition:** A disorder characterized by an infectious process involving the peripheral nerves.<br>**Navigational Note:** -					
Peritoneal infection	-	-	IV antibiotic, antifungal, or antiviral intervention indicated; invasive intervention indicated	Life-threatening consequences; urgent intervention indicated	Death
**Definition:** A disorder characterized by an infectious process involving the peritoneum.<br>**Navigational Note:** -					
Pharyngitis	-	Oral intervention indicated (e.g., antibiotic, antifungal, or antiviral)	IV antibiotic, antifungal, or antiviral intervention indicated; invasive intervention indicated	Life-threatening consequences; urgent intervention indicated	Death
**Definition:** A disorder characterized by inflammation of the throat.<br>**Navigational Note:** For Grade 1 Consider Respiratory, thoracic and mediastinal disorders: Sore throat					
Phlebitis infective	Localized, local intervention indicated	Oral intervention indicated (e.g., antibiotic, antifungal, or antiviral)	IV antibiotic, antifungal, or antiviral intervention indicated; invasive intervention indicated	Life-threatening consequences; urgent intervention indicated	Death
**Definition:** A disorder characterized by an infectious process involving the vein. Clinical manifestations include erythema, marked discomfort, swelling, and induration along the course of the infected vein.<br>**Navigational Note:** -					

Infections and infestations					
**CTCAE Term**	**Grade 1**	**Grade 2**	**Grade 3**	**Grade 4**	**Grade 5**
Pleural infection	-	Oral intervention indicated (e.g., antibiotic, antifungal, or antiviral)	IV antibiotic, antifungal, or antiviral intervention indicated; invasive intervention indicated	Life-threatening consequences; urgent intervention indicated	Death
**Definition:** A disorder characterized by an infectious process involving the pleura.					
**Navigational Note:** -					
Prostate infection	-	Moderate symptoms; oral intervention indicated (e.g., antibiotic, antifungal, or antiviral)	IV antibiotic, antifungal, or antiviral intervention indicated; invasive intervention indicated	Life-threatening consequences; urgent intervention indicated	Death
**Definition:** A disorder characterized by an infectious process involving the prostate gland.					
**Navigational Note:** -					
Rash pustular	-	Localized; local intervention indicated (e.g., topical antibiotic, antifungal, or antiviral)	IV antibiotic, antifungal, or antiviral intervention indicated; invasive intervention indicated	-	-
**Definition:** A disorder characterized by a circumscribed and elevated skin lesion filled with pus.					
**Navigational Note:** Synonym: Boil					
Rhinitis infective	-	Localized; local intervention indicated	-	-	-
**Definition:** A disorder characterized by an infectious process involving the nasal mucosal.					
**Navigational Note:** -					
Salivary gland infection	-	Moderate symptoms; oral intervention indicated (e.g., antibiotic, antifungal, or antiviral)	IV antibiotic, antifungal, or antiviral intervention indicated; invasive intervention indicated	Life-threatening consequences; urgent intervention indicated	Death
**Definition:** A disorder characterized by an infectious process involving the salivary gland.					
**Navigational Note:** -					
Scrotal infection	Localized, local intervention indicated	Oral intervention indicated (e.g., antibiotic, antifungal, or antiviral)	IV antibiotic, antifungal, or antiviral intervention indicated; invasive intervention indicated	Life-threatening consequences; urgent intervention indicated	Death
**Definition:** A disorder characterized by an infectious process involving the scrotum.					
**Navigational Note:** -					
Sepsis	-	-	Blood culture positive with signs or symptoms; treatment indicated	Life-threatening consequences; urgent intervention indicated	Death
**Definition:** A disorder characterized by the presence of pathogenic microorganisms in the blood stream that cause a rapidly progressing systemic reaction that may lead to shock.					
**Navigational Note:** Includes SIRS. Also consider Infections and infestations: Bacteremia (Grade 2)					

Infections and infestations					
**CTCAE Term**	**Grade 1**	**Grade 2**	**Grade 3**	**Grade 4**	**Grade 5**
Shingles	Localized, local intervention indicated	Local infection with moderate symptoms; oral intervention indicated; limiting age-appropriate instrumental ADL	Severe or medically significant but not immediately life-threatening; hospitalization or prolongation of existing hospitalization indicated; IV intervention indicated; limiting self-care ADL	Life-threatening consequences; urgent intervention indicated	Death
**Definition:** A disorder characterized by the reactivation of herpes zoster virus.<br>**Navigational Note:** Synonym: Herpes zoster					
Sinusitis	-	Localized; oral intervention indicated (e.g., antibiotic, antifungal, or antiviral)	IV antibiotic, antifungal, or antiviral intervention indicated; invasive intervention indicated	Life-threatening consequences; urgent intervention indicated	Death
**Definition:** A disorder characterized by an infectious process involving the mucous membranes of the paranasal sinuses.<br>**Navigational Note:** -					
Skin infection	Localized, local intervention indicated	Oral intervention indicated (e.g., antibiotic, antifungal, or antiviral)	IV antibiotic, antifungal, or antiviral intervention indicated; invasive intervention indicated	Life-threatening consequences; urgent intervention indicated	Death
**Definition:** A disorder characterized by an infectious process involving the skin such as cellulitis.<br>**Navigational Note:** -					
Small intestine infection	-	Moderate symptoms; oral intervention indicated (e.g., antibiotic, antifungal, or antiviral)	IV antibiotic, antifungal, or antiviral intervention indicated; invasive intervention indicated	Life-threatening consequences; urgent intervention indicated	Death
**Definition:** A disorder characterized by an infectious process involving the small intestine.<br>**Navigational Note:** -					
Soft tissue infection	-	Localized; oral intervention indicated (e.g., antibiotic, antifungal, or antiviral)	IV antibiotic, antifungal, or antiviral intervention indicated; invasive intervention indicated	Life-threatening consequences; urgent intervention indicated	Death
**Definition:** A disorder characterized by an infectious process involving soft tissues.<br>**Navigational Note:** -					
Splenic infection	-	-	IV antibiotic, antifungal, or antiviral intervention indicated; invasive intervention indicated	Life-threatening consequences; urgent intervention indicated	Death
**Definition:** A disorder characterized by an infectious process involving the spleen.<br>**Navigational Note:** -					

Infections and infestations					
**CTCAE Term**	**Grade 1**	**Grade 2**	**Grade 3**	**Grade 4**	**Grade 5**
Stoma site infection	Localized, local intervention indicated	Oral intervention indicated (e.g., antibiotic, antifungal, or antiviral)	IV antibiotic, antifungal, or antiviral intervention indicated; invasive intervention indicated	Life-threatening consequences; urgent intervention indicated	Death
**Definition:** A disorder characterized by an infectious process involving a stoma (surgically created opening on the surface of the body).<br>**Navigational Note:** -					
Thrush	Asymptomatic; local symptomatic management	Oral intervention indicated (e.g., antifungal)	IV antifungal intervention indicated	-	-
**Definition:** A disorder characterized by a suspected candidal infection involving an oral mucosal surface.<br>**Navigational Note:** -					
Tooth infection	-	Localized; local intervention indicated (e.g., topical antibiotic, antifungal, or antiviral)	IV antibiotic, antifungal, or antiviral intervention indicated; invasive intervention indicated	Life-threatening consequences; urgent intervention indicated	Death
**Definition:** A disorder characterized by an infectious process involving a tooth.<br>**Navigational Note:** -					
Tracheitis	-	Moderate symptoms; oral intervention indicated (e.g., antibiotic, antifungal, or antiviral)	IV antibiotic, antifungal, or antiviral intervention indicated; invasive intervention indicated	Life-threatening consequences; urgent intervention indicated	Death
**Definition:** A disorder characterized by an infectious process involving the trachea.<br>**Navigational Note:** -					
Upper respiratory infection	-	Moderate symptoms; oral intervention indicated (e.g., antibiotic, antifungal, or antiviral)	IV antibiotic, antifungal, or antiviral intervention indicated; invasive intervention indicated	Life-threatening consequences; urgent intervention indicated	Death
**Definition:** A disorder characterized by an infectious process involving the upper respiratory tract (nose, paranasal sinuses, pharynx, larynx, or trachea).<br>**Navigational Note:** -					
Urethral infection	-	Localized; oral intervention indicated (e.g., antibiotic, antifungal, or antiviral)	IV antibiotic, antifungal, or antiviral intervention indicated; invasive intervention indicated	Life-threatening consequences; urgent intervention indicated	Death
**Definition:** A disorder characterized by an infectious process involving the urethra.<br>**Navigational Note:** -					
Urinary tract infection	-	Localized; oral intervention indicated (e.g., antibiotic, antifungal, or antiviral)	IV antibiotic, antifungal, or antiviral intervention indicated; invasive intervention indicated	Life-threatening consequences; urgent intervention indicated	Death
**Definition:** A disorder characterized by an infectious process involving the urinary tract, most commonly the bladder and the urethra.<br>**Navigational Note:** -					

Infections and infestations					
**CTCAE Term**	**Grade 1**	**Grade 2**	**Grade 3**	**Grade 4**	**Grade 5**
Uterine infection	-	Moderate symptoms; oral intervention indicated (e.g., antibiotic, antifungal, or antiviral)	IV antibiotic, antifungal, or antiviral intervention indicated; invasive intervention indicated	Life-threatening consequences; urgent intervention indicated	Death
**Definition:** A disorder characterized by an infectious process involving the endometrium. It may extend to the myometrium and parametrial tissues.<br>**Navigational Note:** -					
Vaginal infection	Localized, local intervention indicated	Oral intervention indicated (e.g., antibiotic, antifungal, or antiviral)	IV antibiotic, antifungal, or antiviral intervention indicated; invasive intervention indicated	Life-threatening consequences; urgent intervention indicated	Death
**Definition:** A disorder characterized by an infectious process involving the vagina.<br>**Navigational Note:** -					
Viremia	-	Moderate symptoms; medical intervention indicated	Severe or medically significant but not immediately life-threatening; hospitalization or prolongation of existing hospitalization indicated	-	-
**Definition:** A disorder characterized by the presence of a virus in the blood stream.<br>**Navigational Note:** -					
Vulval infection	Localized, local intervention indicated	Oral intervention indicated (e.g., antibiotic, antifungal, or antiviral)	IV antibiotic, antifungal, or antiviral intervention indicated; invasive intervention indicated	Life-threatening consequences; urgent intervention indicated	Death
**Definition:** A disorder characterized by an infectious process involving the vulva.<br>**Navigational Note:** -					
Wound infection	Localized, local intervention indicated	Oral intervention indicated (e.g., antibiotic, antifungal, or antiviral)	IV antibiotic, antifungal, or antiviral intervention indicated; invasive intervention indicated	Life-threatening consequences; urgent intervention indicated	Death
**Definition:** A disorder characterized by an infectious process involving the wound.<br>**Navigational Note:** -					
Infections and infestations - Other, specify	Asymptomatic or mild symptoms; clinical or diagnostic observations only; intervention not indicated	Moderate; minimal, local or noninvasive intervention indicated; limiting age-appropriate instrumental ADL	Severe or medically significant but not immediately life-threatening; hospitalization or prolongation of existing hospitalization indicated; limiting self care ADL	Life-threatening consequences; urgent intervention indicated	Death
**Definition:** -<br>**Navigational Note:** -					

Injury, poisoning and procedural complications					
**CTCAE Term**	**Grade 1**	**Grade 2**	**Grade 3**	**Grade 4**	**Grade 5**
Ankle fracture	Mild; non-operative intervention indicated	Limiting instrumental ADL; outpatient operative intervention indicated	Limiting self care ADL; elective operative intervention indicated requiring hospitalization	-	-
**Definition:** A finding of damage to the ankle joint characterized by a break in the continuity of the ankle bone. Symptoms include marked discomfort, swelling and difficulty moving the affected leg and foot.<br>**Navigational Note:** -					
Aortic injury	-	Operative intervention not indicated	Severe symptoms; limiting self care ADL; repair or revision indicated	Life-threatening consequences; evidence of end organ damage; urgent operative intervention indicated	Death
**Definition:** A finding of damage to the aorta.<br>**Navigational Note:** -					
Arterial injury	Asymptomatic diagnostic finding; intervention not indicated	Symptomatic; repair or revision not indicated	Severe symptoms; limiting self care ADL; repair or revision indicated	Life-threatening consequences; evidence of end organ damage; urgent operative intervention indicated	Death
**Definition:** A finding of damage to an artery.<br>**Navigational Note:** -					
Biliary anastomotic leak	Asymptomatic diagnostic finding; intervention not indicated	Symptomatic; medical intervention indicated	Severe symptoms; invasive intervention indicated	Life-threatening consequences; urgent operative intervention indicated	Death
**Definition:** A finding of leakage of bile due to breakdown of a biliary anastomosis (surgical connection of two separate anatomic structures).<br>**Navigational Note:** -					
Bladder anastomotic leak	Asymptomatic diagnostic finding; intervention not indicated	Symptomatic; medical intervention indicated	Severe symptoms; invasive intervention indicated	Life-threatening consequences; urgent operative intervention indicated	Death
**Definition:** A finding of leakage of urine due to breakdown of a bladder anastomosis (surgical connection of two separate anatomic structures).<br>**Navigational Note:** -					
Bruising	Localized or in a dependent area	Generalized	-	-	-
**Definition:** A finding of injury of the soft tissues or bone characterized by leakage of blood into surrounding tissues.<br>**Navigational Note:** -					

Injury, poisoning and procedural complications					
**CTCAE Term**	**Grade 1**	**Grade 2**	**Grade 3**	**Grade 4**	**Grade 5**
Burn	Minimal symptoms; intervention not indicated	Medical intervention; minimal debridement indicated	Moderate to major debridement or reconstruction indicated	Life-threatening consequences	Death
**Definition:** A finding of impaired integrity to the anatomic site of an adverse thermal reaction. Burns can be caused by exposure to chemicals, direct heat, electricity, flames and radiation. The extent of damage depends on the length and intensity of exposure and time until provision of treatment.<br>**Navigational Note:** -					
Dermatitis radiation	Faint erythema or dry desquamation	Moderate to brisk erythema; patchy moist desquamation, mostly confined to skin folds and creases; moderate edema	Moist desquamation in areas other than skin folds and creases; bleeding induced by minor trauma or abrasion	Life-threatening consequences; skin necrosis or ulceration of full thickness dermis; spontaneous bleeding from involved site; skin graft indicated	Death
**Definition:** A finding of cutaneous inflammatory reaction occurring as a result of exposure to biologically effective levels of ionizing radiation.<br>**Navigational Note:** Synonym: Radiation induced skin toxicities (CTCAE v3.0)					
Esophageal anastomotic leak	Asymptomatic diagnostic finding; intervention not indicated	Symptomatic; medical intervention indicated	Severe symptoms; invasive intervention indicated	Life-threatening consequences; urgent operative intervention indicated	Death
**Definition:** A finding of leakage due to breakdown of an esophageal anastomosis (surgical connection of two separate anatomic structures).<br>**Navigational Note:** -					
Fall	Minor with no resultant injuries; intervention not indicated	Symptomatic; noninvasive intervention indicated	Hospitalization indicated; invasive intervention indicated	-	-
**Definition:** A finding of sudden movement downward, usually resulting in injury.<br>**Navigational Note:** -					
Fallopian tube anastomotic leak	Asymptomatic; clinical or diagnostic observations only; intervention not indicated	Symptomatic; medical intervention indicated	Severe symptoms; invasive intervention indicated	Life-threatening consequences; urgent operative intervention indicated	Death
**Definition:** A finding of leakage due to breakdown of a fallopian tube anastomosis (surgical connection of two separate anatomic structures).<br>**Navigational Note:** -					
Fallopian tube perforation	-	Invasive intervention not indicated	Invasive intervention indicated	Life-threatening consequences; urgent operative intervention indicated (e.g., organ resection)	Death
**Definition:** A disorder characterized by a rupture of the fallopian tube wall.<br>**Navigational Note:** -					

Injury, poisoning and procedural complications					
**CTCAE Term**	**Grade 1**	**Grade 2**	**Grade 3**	**Grade 4**	**Grade 5**
Fracture	Asymptomatic; clinical or diagnostic observations only; intervention not indicated	Symptomatic but non-displaced; immobilization indicated	Severe symptoms; displaced or open wound with bone exposure; limiting self care ADL; operative intervention indicated	Life-threatening consequences; urgent intervention indicated	Death
**Definition:** A finding of traumatic injury to the bone in which the continuity of the bone is broken.<br>**Navigational Note:** Prior to using this term consider specific fracture areas: Injury, poisoning and procedural complications: Ankle fracture, Hip fracture, Spinal fracture, or Wrist fracture					
Gastric anastomotic leak	Asymptomatic diagnostic finding; intervention not indicated	Symptomatic; medical intervention indicated	Severe symptoms; invasive intervention indicated	Life-threatening consequences; urgent operative intervention indicated	Death
**Definition:** A finding of leakage due to breakdown of a gastric anastomosis (surgical connection of two separate anatomic structures).<br>**Navigational Note:** -					
Gastrointestinal anastomotic leak	Asymptomatic diagnostic finding; intervention not indicated	Symptomatic; medical intervention indicated	Severe symptoms; invasive intervention indicated	Life-threatening consequences; urgent operative intervention indicated	Death
**Definition:** A finding of leakage due to breakdown of a gastrointestinal anastomosis (surgical connection of two separate anatomic structures).<br>**Navigational Note:** -					
Gastrointestinal stoma necrosis	-	Superficial necrosis; intervention not indicated	Severe symptoms; hospitalization indicated; elective operative intervention indicated	Life-threatening consequences; urgent intervention indicated	Death
**Definition:** A disorder characterized by a necrotic process occurring in the gastrointestinal tract stoma.<br>**Navigational Note:** -					
Hip fracture	-	Hairline fracture; mild pain; limiting instrumental ADL; non-operative intervention indicated	Severe pain; hospitalization or intervention indicated for pain control (e.g., traction); operative intervention indicated	Life-threatening consequences; symptoms associated with neurovascular compromise	-
**Definition:** A finding of traumatic injury to the hip in which the continuity of either the femoral head, femoral neck, intertrochanteric or subtrochanteric regions is broken.<br>**Navigational Note:** -					

Injury, poisoning and procedural complications					
**CTCAE Term**	**Grade 1**	**Grade 2**	**Grade 3**	**Grade 4**	**Grade 5**
Infusion related reaction	Mild transient reaction; infusion interruption not indicated; intervention not indicated	Therapy or infusion interruption indicated but responds promptly to symptomatic treatment (e.g., antihistamines, NSAIDS, narcotics, IV fluids); prophylactic medications indicated for <=24 hrs	Prolonged (e.g., not rapidly responsive to symptomatic medication and/or brief interruption of infusion); recurrence of symptoms following initial improvement; hospitalization indicated for clinical sequelae	Life-threatening consequences; urgent intervention indicated	Death
**Definition:** A disorder characterized by adverse reaction to the infusion of pharmacological or biological substances.<br>**Navigational Note:** -					
Injury to carotid artery	-	Repair or revision not indicated	Severe symptoms; limiting self care ADL (e.g., transient cerebral ischemia); repair or revision indicated	Life-threatening consequences; urgent intervention indicated	Death
**Definition:** A finding of damage to the carotid artery.<br>**Navigational Note:** -					
Injury to inferior vena cava	Asymptomatic diagnostic finding; intervention not indicated	Symptomatic; repair or revision not indicated	Severe symptoms; limiting self care ADL; repair or revision indicated	Life-threatening consequences; urgent intervention indicated	Death
**Definition:** A finding of damage to the inferior vena cava.<br>**Navigational Note:** -					
Injury to jugular vein	-	Repair or revision not indicated	Symptomatic limiting self care ADL; repair or revision indicated	Life-threatening consequences; urgent intervention indicated	Death
**Definition:** A finding of damage to the jugular vein.<br>**Navigational Note:** -					
Injury to superior vena cava	Asymptomatic diagnostic finding; intervention not indicated	Symptomatic; repair or revision not indicated	Severe symptoms; limiting self care ADL; repair or revision indicated	Life-threatening consequences; urgent intervention indicated	Death
**Definition:** A finding of damage to the superior vena cava.<br>**Navigational Note:** -					
Intestinal stoma leak	Asymptomatic diagnostic finding; intervention not indicated	Symptomatic; medical intervention indicated	Severe symptoms; invasive intervention indicated	Life-threatening consequences; urgent operative intervention indicated	Death
**Definition:** A finding of leakage of contents from an intestinal stoma (surgically created opening on the surface of the body).<br>**Navigational Note:** -					

Injury, poisoning and procedural complications					
**CTCAE Term**	**Grade 1**	**Grade 2**	**Grade 3**	**Grade 4**	**Grade 5**
Intestinal stoma obstruction	-	Self-limited; intervention not indicated	Severe symptoms; IV fluids, tube feeding, or TPN indicated >=24 hrs; elective operative intervention indicated	Life-threatening consequences; urgent operative intervention indicated	Death
**Definition:** A disorder characterized by blockage of the normal flow of the contents of the intestinal stoma.<br>**Navigational Note:** -					
Intestinal stoma site bleeding	Minimal bleeding identified on clinical exam; intervention not indicated	Moderate bleeding; medical intervention indicated	Transfusion indicated; invasive intervention indicated	Life-threatening consequences; urgent intervention indicated	Death
**Definition:** A disorder characterized by bleeding from the intestinal stoma.<br>**Navigational Note:** -					
Intraoperative arterial injury	Primary repair of injured organ/structure indicated	Partial resection of injured organ/structure indicated	Complete resection or reconstruction of injured organ/structure indicated; limiting self care ADL	Life-threatening consequences; urgent intervention indicated	Death
**Definition:** A finding of damage to an artery during a surgical procedure.<br>**Navigational Note:** -					
Intraoperative breast injury	Primary repair of injured organ/structure indicated	Partial resection of injured organ/structure indicated	Complete resection or reconstruction of injured organ/structure indicated; limiting self care ADL	Life-threatening consequences; urgent intervention indicated	Death
**Definition:** A finding of damage to the breast parenchyma during a surgical procedure.<br>**Navigational Note:** -					
Intraoperative cardiac injury	-	-	Primary repair of injured organ/structure indicated	Life-threatening consequences; urgent intervention indicated	Death
**Definition:** A finding of damage to the heart during a surgical procedure.<br>**Navigational Note:** -					
Intraoperative ear injury	Primary repair of injured organ/structure indicated	Partial resection of injured organ/structure indicated	Complete resection or reconstruction of injured organ/structure indicated; limiting self care ADL (e.g., impaired hearing; impaired balance)	Life-threatening consequences; urgent intervention indicated	Death
**Definition:** A finding of damage to the ear during a surgical procedure.<br>**Navigational Note:** -					

Injury, poisoning and procedural complications					
**CTCAE Term**	**Grade 1**	**Grade 2**	**Grade 3**	**Grade 4**	**Grade 5**
Intraoperative endocrine injury	Primary repair of injured organ/structure indicated	Partial resection of injured organ/structure indicated	Complete resection or reconstruction of injured organ/structure indicated; limiting self care ADL	Life-threatening consequences; urgent intervention indicated	Death
**Definition:** A finding of damage to the endocrine gland during a surgical procedure. **Navigational Note:** -					
Intraoperative gastrointestinal injury	Primary repair of injured organ/structure indicated	Partial resection of injured organ/structure indicated	Complete resection or reconstruction of injured organ/structure indicated; limiting self care ADL	Life-threatening consequences; urgent intervention indicated	Death
**Definition:** A finding of damage to the gastrointestinal system during a surgical procedure. **Navigational Note:** -					
Intraoperative head and neck injury	Primary repair of injured organ/structure indicated	Partial resection of injured organ/structure indicated	Complete resection or reconstruction of injured organ/structure indicated; limiting self care ADL	Life-threatening consequences; urgent intervention indicated	Death
**Definition:** A finding of damage to the head and neck during a surgical procedure. **Navigational Note:** -					
Intraoperative hemorrhage	-	-	Postoperative invasive intervention indicated; hospitalization	Life-threatening consequences; urgent intervention indicated	Death
**Definition:** A finding of uncontrolled bleeding during a surgical procedure. **Navigational Note:** -					
Intraoperative hepatobiliary injury	Primary repair of injured organ/structure indicated	Partial resection of injured organ/structure indicated	Complete resection or reconstruction of injured organ/structure indicated; limiting self care ADL	Life-threatening consequences; urgent intervention indicated	Death
**Definition:** A finding of damage to the hepatic parenchyma and/or biliary tract during a surgical procedure. **Navigational Note:** -					
Intraoperative musculoskeletal injury	Primary repair of injured organ/structure indicated	Partial resection of injured organ/structure indicated	Complete resection or reconstruction of injured organ/structure indicated; limiting self care ADL	Life-threatening consequences; urgent intervention indicated	Death
**Definition:** A finding of damage to the musculoskeletal system during a surgical procedure. **Navigational Note:** -					

Injury, poisoning and procedural complications					
**CTCAE Term**	**Grade 1**	**Grade 2**	**Grade 3**	**Grade 4**	**Grade 5**
Intraoperative neurological injury	Primary repair of injured organ/structure indicated	Partial resection of injured organ/structure indicated	Complete resection or reconstruction of injured organ/structure indicated; limiting self care ADL	Life-threatening consequences; urgent intervention indicated	Death
**Definition:** A finding of damage to the nervous system during a surgical procedure.					
**Navigational Note:** -					
Intraoperative ocular injury	Primary repair of injured organ/structure indicated	Partial resection of injured organ/structure indicated	Complete resection or reconstruction of injured organ/structure indicated; limiting self care ADL	Life-threatening consequences; urgent intervention indicated	Death
**Definition:** A finding of damage to the eye during a surgical procedure.					
**Navigational Note:** -					
Intraoperative renal injury	Primary repair of injured organ/structure indicated	Partial resection of injured organ/structure indicated	Complete resection or reconstruction of injured organ/structure indicated; limiting self care ADL	Life-threatening consequences; urgent intervention indicated	Death
**Definition:** A finding of damage to the kidney during a surgical procedure.					
**Navigational Note:** -					
Intraoperative reproductive tract injury	Primary repair of injured organ/structure indicated	Partial resection of injured organ/structure indicated	Complete resection or reconstruction of injured organ/structure indicated; limiting self care ADL	Life-threatening consequences; urgent intervention indicated	Death
**Definition:** A finding of damage to the reproductive organs during a surgical procedure.					
**Navigational Note:** -					
Intraoperative respiratory injury	Primary repair of injured organ/structure indicated	Partial resection of injured organ/structure indicated	Complete resection or reconstruction of injured organ/structure indicated; limiting self care ADL	Life-threatening consequences; urgent intervention indicated	Death
**Definition:** A finding of damage to the respiratory system during a surgical procedure.					
**Navigational Note:** -					
Intraoperative splenic injury	-	Primary repair of injured organ/structure indicated	Resection or reconstruction of injured organ/structure indicated; limiting self care ADL	Life-threatening consequences; urgent intervention indicated	Death
**Definition:** A finding of damage to the spleen during a surgical procedure.					
**Navigational Note:** -					

Injury, poisoning and procedural complications					
**CTCAE Term**	**Grade 1**	**Grade 2**	**Grade 3**	**Grade 4**	**Grade 5**
Intraoperative urinary injury	Primary repair of injured organ/structure indicated	Partial resection of injured organ/structure indicated	Complete resection or reconstruction of injured organ/structure indicated; limiting self care ADL	Life-threatening consequences; urgent intervention indicated	Death
**Definition:** A finding of damage to the urinary system during a surgical procedure.<br>**Navigational Note:** -					
Intraoperative venous injury	Primary repair of injured organ/structure indicated	Partial resection of injured organ/structure indicated	Complete resection or reconstruction of injured organ/structure indicated; limiting self care ADL	Life-threatening consequences; urgent intervention indicated	Death
**Definition:** A finding of damage to a vein during a surgical procedure.<br>**Navigational Note:** -					
Kidney anastomotic leak	Asymptomatic diagnostic finding; intervention not indicated	Symptomatic; medical intervention indicated	Severe symptoms; invasive intervention indicated	Life-threatening consequences; urgent operative intervention indicated	Death
**Definition:** A finding of leakage of urine due to breakdown of a kidney anastomosis (surgical connection of two separate anatomic structures).<br>**Navigational Note:** -					
Large intestinal anastomotic leak	Asymptomatic diagnostic finding; intervention not indicated	Symptomatic; medical intervention indicated	Severe symptoms; invasive intervention indicated	Life-threatening consequences; urgent operative intervention indicated	Death
**Definition:** A finding of leakage due to breakdown of an anastomosis (surgical connection of two separate anatomic structures) in the large intestine.<br>**Navigational Note:** -					
Pancreatic anastomotic leak	Asymptomatic diagnostic finding; intervention not indicated	Symptomatic; medical intervention indicated	Severe symptoms; invasive intervention indicated	Life-threatening consequences; urgent operative intervention indicated	Death
**Definition:** A finding of leakage due to breakdown of a pancreatic anastomosis (surgical connection of two separate anatomic structures).<br>**Navigational Note:** -					
Pharyngeal anastomotic leak	Asymptomatic diagnostic finding; intervention not indicated	Symptomatic; medical intervention indicated	Severe symptoms; invasive intervention indicated	Life-threatening consequences; urgent operative intervention indicated	Death
**Definition:** A finding of leakage due to breakdown of a pharyngeal anastomosis (surgical connection of two separate anatomic structures).<br>**Navigational Note:** -					

Injury, poisoning and procedural complications					
**CTCAE Term**	**Grade 1**	**Grade 2**	**Grade 3**	**Grade 4**	**Grade 5**
Postoperative hemorrhage	Mild symptoms; intervention not indicated	Moderate bleeding requiring transfusion < 2 units (10 cc/kg for pediatrics) of pRBCs	Transfusion indicated of >=2 units (10 cc/kg for pediatrics) pRBCs; invasive intervention indicated; hospitalization	Life-threatening consequences; urgent intervention indicated	Death
**Definition:** A disorder characterized by bleeding occurring after a surgical procedure.<br>**Navigational Note:** -					
Postoperative thoracic procedure complication	-	Extubated within 24 - 72 hrs postoperatively	Extubated >72 hrs postoperatively, but before tracheostomy indicated	Life-threatening airway compromise; urgent intervention indicated (e.g., tracheotomy or intubation)	Death
**Definition:** A finding of a previously undocumented problem that occurs after a thoracic procedure.<br>**Navigational Note:** -					
Prolapse of intestinal stoma	Asymptomatic; reducible	Recurrent after manual reduction; local irritation or stool leakage; difficulty to fit appliance; limiting instrumental ADL	Severe symptoms; elective operative intervention indicated; limiting self care ADL	Life-threatening consequences; urgent operative intervention indicated	Death
**Definition:** A finding of protrusion of the intestinal stoma (surgically created opening on the surface of the body) above the abdominal surface.<br>**Navigational Note:** -					
Prolapse of urostomy	Asymptomatic; clinical or diagnostic observations only; intervention not indicated	Local care or maintenance; minor revision indicated	Dysfunctional stoma; elective operative intervention or major stomal revision indicated	Life-threatening consequences; urgent intervention indicated	Death
**Definition:** A finding of displacement of the urostomy.<br>**Navigational Note:** -					
Radiation recall reaction (dermatologic)	Faint erythema or dry desquamation	Moderate to brisk erythema; patchy moist desquamation, mostly confined to skin folds and creases; moderate edema	Moist desquamation in areas other than skin folds and creases; bleeding induced by minor trauma or abrasion	Life-threatening consequences; skin necrosis or ulceration of full thickness dermis; spontaneous bleeding from involved site; skin graft indicated	Death
**Definition:** A finding of acute skin inflammatory reaction caused by drugs, especially chemotherapeutic agents, for weeks or months following radiotherapy. The inflammatory reaction is confined to the previously irradiated skin and the symptoms disappear after the removal of the pharmaceutical agent.<br>**Navigational Note:** -					
Rectal anastomotic leak	Asymptomatic diagnostic finding; intervention not indicated	Symptomatic; medical intervention indicated	Severe symptoms; invasive intervention indicated	Life-threatening consequences; urgent operative intervention indicated	Death
**Definition:** A finding of leakage due to breakdown of a rectal anastomosis (surgical connection of two separate anatomic structures).<br>**Navigational Note:** -					

Injury, poisoning and procedural complications					
**CTCAE Term**	**Grade 1**	**Grade 2**	**Grade 3**	**Grade 4**	**Grade 5**
Seroma	Asymptomatic; clinical or diagnostic observations only; intervention not indicated	Symptomatic; simple aspiration indicated	Symptomatic, elective invasive intervention indicated	-	-
**Definition:** A finding of tumor-like collection of serum in the tissues.					
**Navigational Note:** -					
Small intestinal anastomotic leak	Asymptomatic diagnostic finding; intervention not indicated	Symptomatic; medical intervention indicated	Severe symptoms; invasive intervention indicated	Life-threatening consequences; urgent operative intervention indicated	Death
**Definition:** A finding of leakage due to breakdown of an anastomosis (surgical connection of two separate anatomic structures) in the small bowel.					
**Navigational Note:** -					
Spermatic cord anastomotic leak	Asymptomatic diagnostic finding; intervention not indicated	Symptomatic; medical intervention indicated	Severe symptoms; invasive intervention indicated	Life-threatening consequences; urgent operative intervention indicated	Death
**Definition:** A finding of leakage due to breakdown of a spermatic cord anastomosis (surgical connection of two separate anatomic structures).					
**Navigational Note:** -					
Spinal fracture	Mild back pain; nonprescription analgesics indicated	Moderate back pain; prescription analgesics indicated; limiting instrumental ADL	Severe back pain; hospitalization or intervention indicated for pain control (e.g., vertebroplasty); limiting self care ADL; disability	Life-threatening consequences; symptoms associated with neurovascular compromise	Death
**Definition:** A finding of traumatic injury to the spine in which the continuity of a vertebral bone is broken.					
**Navigational Note:** -					
Stenosis of gastrointestinal stoma	-	Symptomatic; IV fluids indicated <24 hrs; manual dilation at bedside	Severely altered GI function; tube feeding, TPN or hospitalization indicated; elective operative intervention indicated	Life-threatening consequences; urgent operative intervention indicated	Death
**Definition:** A finding of narrowing of the gastrointestinal stoma (surgically created opening on the surface of the body).					
**Navigational Note:** -					
Stomal ulcer	Asymptomatic; clinical or diagnostic observations only; intervention not indicated	Symptomatic; medical intervention indicated	Severe symptoms; elective operative intervention indicated	-	-
**Definition:** A disorder characterized by a circumscribed, erosive lesion on the jejunal mucosal surface close to the anastomosis site following a gastroenterostomy procedure.					
**Navigational Note:** -					

Injury, poisoning and procedural complications					
**CTCAE Term**	**Grade 1**	**Grade 2**	**Grade 3**	**Grade 4**	**Grade 5**
Tracheal hemorrhage	Mild symptoms; intervention not indicated	Moderate symptoms; intervention indicated	Transfusion indicated; invasive intervention indicated; hospitalization	Life-threatening consequences; urgent intervention indicated	Death
**Definition:** A disorder characterized by bleeding from the trachea.<br>**Navigational Note:** -					
Tracheal obstruction	Partial asymptomatic obstruction on examination (e.g., visual, radiologic or endoscopic)	Symptomatic (e.g., noisy airway breathing), no respiratory distress; medical intervention indicated (e.g., steroids); limiting instrumental ADL	Stridor or respiratory distress limiting self care ADL; invasive intervention indicated (e.g., stent, laser)	Life-threatening airway compromise; urgent intervention indicated (e.g., tracheotomy or intubation)	Death
**Definition:** A disorder characterized by blockage of the lumen of the trachea.<br>**Navigational Note:** -					
Tracheostomy site bleeding	Minimal bleeding identified on clinical exam; intervention not indicated	Moderate bleeding; medical intervention indicated	Transfusion indicated; invasive intervention indicated	Life-threatening consequences; urgent intervention indicated	Death
**Definition:** A disorder characterized by bleeding from the tracheostomy site.<br>**Navigational Note:** -					
Ureteric anastomotic leak	Asymptomatic diagnostic finding; intervention not indicated	Symptomatic; medical intervention indicated	Severe symptoms; invasive intervention indicated	Life-threatening consequences; urgent operative intervention indicated	Death
**Definition:** A finding of leakage due to breakdown of a ureteral anastomosis (surgical connection of two separate anatomic structures).<br>**Navigational Note:** -					
Urethral anastomotic leak	Asymptomatic diagnostic finding; intervention not indicated	Symptomatic; medical intervention indicated	Severe symptoms; invasive intervention indicated	Life-threatening consequences; urgent operative intervention indicated	Death
**Definition:** A finding of leakage due to breakdown of a urethral anastomosis (surgical connection of two separate anatomic structures).<br>**Navigational Note:** -					
Urostomy leak	Asymptomatic diagnostic finding; intervention not indicated	Symptomatic; medical intervention indicated	Severe symptoms; invasive intervention indicated	Life-threatening consequences; urgent operative intervention indicated	Death
**Definition:** A finding of leakage of contents from a urostomy.<br>**Navigational Note:** -					

Injury, poisoning and procedural complications					
**CTCAE Term**	**Grade 1**	**Grade 2**	**Grade 3**	**Grade 4**	**Grade 5**
Urostomy obstruction	Asymptomatic; clinical or diagnostic observations only; intervention not indicated	Symptomatic; dilation or endoscopic repair or stent placement indicated	Altered organ function (e.g., sepsis or hydronephrosis, or renal dysfunction); elective operative intervention indicated	Life-threatening consequences; organ failure; urgent operative intervention indicated	Death
**Definition:** A disorder characterized by blockage of the urostomy.					
**Navigational Note:** -					
Urostomy site bleeding	Minimal bleeding identified on clinical exam; intervention not indicated	Moderate bleeding; medical intervention indicated	Transfusion indicated; invasive intervention indicated	Life-threatening consequences; urgent intervention indicated	Death
**Definition:** A disorder characterized by bleeding from the urostomy site.					
**Navigational Note:** -					
Urostomy stenosis	-	Symptomatic but no hydronephrosis, sepsis, or renal dysfunction; dilation or endoscopic repair or stent placement indicated	Symptomatic (e.g., hydronephrosis, or renal dysfunction); elective operative intervention indicated	Life-threatening consequences; urgent operative intervention indicated	Death
**Definition:** A finding of narrowing of the opening of a urostomy.					
**Navigational Note:** -					
Uterine anastomotic leak	Asymptomatic diagnostic finding; intervention not indicated	Symptomatic; medical intervention indicated	Severe symptoms; invasive intervention indicated	Life-threatening consequences; urgent operative intervention indicated	Death
**Definition:** A finding of leakage due to breakdown of a uterine anastomosis (surgical connection of two separate anatomic structures).					
**Navigational Note:** -					
Uterine perforation	-	Invasive intervention not indicated	Invasive intervention indicated	Life-threatening consequences; urgent intervention indicated	Death
**Definition:** A disorder characterized by a rupture in the uterine wall.					
**Navigational Note:** -					
Vaccination complication	Mild pain; erythema 2.5-5cm; induration/swelling 2.5-5cm; does not interfere with activity	Moderate pain; Erythema 5.1-10 cm; Induration/swelling 5.1-10 cm; lipodystrophy; limiting instrumental ADL	Severe pain; Erythema > 10 cm; Induration/swelling > 10 cm; necrosis; limiting self care ADL	Life-threatening consequences; urgent intervention indicated	-
**Definition:** A disorder that occurs after the injection of a substance with antigenic properties, administered to activate the immune system.					
**Navigational Note:** For systemic vaccination complications, consider Immune system disorders: Allergic reaction or Anaphylaxis.					

Injury, poisoning and procedural complications					
**CTCAE Term**	**Grade 1**	**Grade 2**	**Grade 3**	**Grade 4**	**Grade 5**
Vaginal anastomotic leak	Asymptomatic diagnostic finding; intervention not indicated	Symptomatic; medical intervention indicated	Severe symptoms; invasive intervention indicated	Life-threatening consequences; urgent operative intervention indicated	Death
**Definition:** A finding of leakage due to breakdown of a vaginal anastomosis (surgical connection of two separate anatomic structures).<br>**Navigational Note:** -					
Vas deferens anastomotic leak	Asymptomatic diagnostic finding; intervention not indicated	Symptomatic; medical intervention indicated	Severe symptoms; invasive intervention indicated	Life-threatening consequences; urgent operative intervention indicated	Death
**Definition:** A finding of leakage due to breakdown of a vas deferens anastomosis (surgical connection of two separate anatomic structures).<br>**Navigational Note:** -					
Vascular access complication	TPA administration into line with no intent for systemic therapy indicated	Device dislodgement, blockage, leak, or malposition; device replacement indicated	Pulmonary embolism, deep vein or cardiac thrombosis; intervention indicated (e.g., anticoagulation, lysis, filter, invasive procedure)	Life-threatening consequences with hemodynamic or neurologic instability	Death
**Definition:** A finding of a previously undocumented problem related to the vascular access site.<br>**Navigational Note:** -					
Venous injury	Asymptomatic diagnostic finding; intervention not indicated	Symptomatic (e.g., claudication); repair or revision not indicated	Severe symptoms; limiting self care ADL; repair or revision indicated	Life-threatening consequences; evidence of end organ damage; urgent operative intervention indicated	Death
**Definition:** A finding of damage to a vein.<br>**Navigational Note:** -					
Wound complication	Observation only; topical intervention indicated	Bedside local care indicated	Operative intervention indicated	Life-threatening consequences	Death
**Definition:** A finding of development of a new problem at the site of an existing wound.<br>**Navigational Note:** Prior to using this term consider Injury, poisoning and procedural complications: Wound dehiscence or Infections and infestations: Wound infection					
Wound dehiscence	Incisional separation, intervention not indicated	Incisional separation, local care (e.g., suturing) or medical intervention indicated (e.g., analgesic)	Fascial disruption or dehiscence without evisceration; revision by operative intervention indicated	Life-threatening consequences; symptomatic hernia with evidence of strangulation; fascial disruption with evisceration; major reconstruction flap, grafting, resection, or amputation indicated	Death
**Definition:** A finding of separation of the approximated margins of a surgical wound.<br>**Navigational Note:** Also consider Infections and infestations: Wound infection					

Injury, poisoning and procedural complications					
**CTCAE Term**	**Grade 1**	**Grade 2**	**Grade 3**	**Grade 4**	**Grade 5**
Wrist fracture	Mild; non-operative intervention indicated	Limiting instrumental ADL; outpatient operative intervention indicated	Limiting self care ADL; elective operative intervention indicated requiring hospitalization	-	-
**Definition:** A finding of traumatic injury to the wrist joint in which the continuity of a wrist bone is broken.<br>**Navigational Note:** -					
Injury, poisoning and procedural complications - Other, specify	Asymptomatic or mild symptoms; clinical or diagnostic observations only; intervention not indicated	Moderate; minimal, local or noninvasive intervention indicated; limiting age-appropriate instrumental ADL	Severe or medically significant but not immediately life-threatening; hospitalization or prolongation of existing hospitalization indicated; limiting self care ADL	Life-threatening consequences; urgent intervention indicated	Death
**Definition:** -<br>**Navigational Note:** -					

Investigations					
**CTCAE Term**	**Grade 1**	**Grade 2**	**Grade 3**	**Grade 4**	**Grade 5**
Activated partial thromboplastin time prolonged	>ULN - 1.5 x ULN	>1.5 - 2.5 x ULN	>2.5 x ULN; bleeding	-	-
**Definition:** A finding based on laboratory test results in which the partial thromboplastin time is found to be greater than the control value. As a possible indicator of coagulopathy, a prolonged partial thromboplastin time (PTT) may occur in a variety of diseases and disorders, both primary and related to treatment. **Navigational Note:** -					
Alanine aminotransferase increased	>ULN - 3.0 x ULN if baseline was normal; 1.5 - 3.0 x baseline if baseline was abnormal	>3.0 - 5.0 x ULN if baseline was normal; >3.0 - 5.0 x baseline if baseline was abnormal	>5.0 - 20.0 x ULN if baseline was normal; >5.0 - 20.0 x baseline if baseline was abnormal	>20.0 x ULN if baseline was normal; >20.0 x baseline if baseline was abnormal	-
**Definition:** A finding based on laboratory test results that indicate an increase in the level of alanine aminotransferase (ALT or SGPT) in the blood specimen. **Navigational Note:** Also consider Hepatobiliary disorders: Hepatic failure					
Alkaline phosphatase increased	>ULN - 2.5 x ULN if baseline was normal; 2.0 - 2.5 x baseline if baseline was abnormal	>2.5 - 5.0 x ULN if baseline was normal; >2.5 - 5.0 x baseline if baseline was abnormal	>5.0 - 20.0 x ULN if baseline was normal; >5.0 - 20.0 x baseline if baseline was abnormal	>20.0 x ULN if baseline was normal; >20.0 x baseline if baseline was abnormal	-
**Definition:** A finding based on laboratory test results that indicate an increase in the level of alkaline phosphatase in a blood specimen. **Navigational Note:** -					
Aspartate aminotransferase increased	>ULN - 3.0 x ULN if baseline was normal; 1.5 - 3.0 x baseline if baseline was abnormal	>3.0 - 5.0 x ULN if baseline was normal; >3.0 - 5.0 x baseline if baseline was abnormal	>5.0 - 20.0 x ULN if baseline was normal; >5.0 - 20.0 x baseline if baseline was abnormal	>20.0 x ULN if baseline was normal; >20.0 x baseline if baseline was abnormal	-
**Definition:** A finding based on laboratory test results that indicate an increase in the level of aspartate aminotransferase (AST or SGOT) in a blood specimen. **Navigational Note:** Also consider Hepatobiliary disorders: Hepatic failure					
Blood antidiuretic hormone abnormal	Asymptomatic; clinical or diagnostic observations only; intervention not indicated	Symptomatic; medical intervention indicated	Hospitalization indicated	-	-
**Definition:** A finding based on laboratory test results that indicate abnormal levels of antidiuretic hormone in the blood specimen. **Navigational Note:** -					
Blood bicarbonate decreased	<LLN and no intervention initiated	-	-	-	-
**Definition:** A finding based on laboratory test results that indicate a decrease in levels of bicarbonate in a venous blood specimen. **Navigational Note:** Also consider Metabolism and nutrition disorders: Acidosis or Alkalosis					
Blood bilirubin increased	>ULN - 1.5 x ULN if baseline was normal; > 1.0 - 1.5 x baseline if baseline was abnormal	>1.5 - 3.0 x ULN if baseline was normal; >1.5 - 3.0 x baseline if baseline was abnormal	>3.0 - 10.0 x ULN if baseline was normal; >3.0 - 10.0 x baseline if baseline was abnormal	>10.0 x ULN if baseline was normal; >10.0 x baseline if baseline was abnormal	-
**Definition:** A finding based on laboratory test results that indicate an abnormally high level of bilirubin in the blood. Excess bilirubin is associated with jaundice. **Navigational Note:** Also consider Hepatobiliary disorders: Hepatic failure					

Investigations					
**CTCAE Term**	**Grade 1**	**Grade 2**	**Grade 3**	**Grade 4**	**Grade 5**
Blood corticotrophin decreased	Asymptomatic; clinical or diagnostic observations only; intervention not indicated	Symptomatic; medical intervention indicated	Hospitalization indicated	-	-
**Definition:** A finding based on laboratory test results that indicate an decrease in levels of corticotrophin in a blood specimen.<br>**Navigational Note:** -					
Blood gonadotrophin abnormal	Asymptomatic; clinical or diagnostic observations only; intervention not indicated	Symptomatic; medical intervention indicated; limiting instrumental ADL	Severe symptoms; limiting self care ADL	-	-
**Definition:** A finding based on laboratory test results that indicate abnormal levels of gonadotrophin hormone in a blood specimen.<br>**Navigational Note:** -					
Blood lactate dehydrogenase increased	>ULN	-	-	-	-
**Definition:** A finding based on laboratory test results that indicate increased levels of lactate dehydrogenase in the blood specimen.<br>**Navigational Note:** -					
Blood prolactin abnormal	Asymptomatic; clinical or diagnostic observations only; intervention not indicated	Moderate symptoms; limiting instrumental ADL	-	-	-
**Definition:** A finding based on laboratory test results that indicate abnormal levels of prolactin hormone in a blood specimen.<br>**Navigational Note:** -					
Carbon monoxide diffusing capacity decreased	3 - 5 units below LLN; for follow-up, a decrease of 3 - 5 units (ml/min/mm Hg) below the baseline value; asymptomatic and intervention not indicated	6 - 8 units below LLN; for follow-up, an asymptomatic decrease of >5 - 8 units (ml/min/mm Hg) below the baseline value; symptomatic and intervention not indicated	Asymptomatic decrease of >8 units drop; >5 units drop along with the presence of pulmonary symptoms (e.g., >Grade 2 hypoxia or >Grade 2 dyspnea); intervention indicated	-	-
**Definition:** A finding based on lung function test results that indicate a decrease in the lung capacity to absorb carbon monoxide.<br>**Navigational Note:** Also consider Respiratory, thoracic and mediastinal disorders: Respiratory failure or Dyspnea					
Cardiac troponin I increased	Levels above the upper limit of normal and below the level of myocardial infarction as defined by the manufacturer	-	Levels consistent with myocardial infarction as defined by the manufacturer	-	-
**Definition:** A finding based on laboratory test results that indicate increased levels of cardiac troponin I in a biological specimen.<br>**Navigational Note:** Also consider Cardiac disorders: Heart failure or Cardiac disorders: Myocardial infarction. Report Cardiac disorders: Heart failure or Cardiac disorders: Myocardial infarction if same grade event.					

Investigations					
**CTCAE Term**	**Grade 1**	**Grade 2**	**Grade 3**	**Grade 4**	**Grade 5**
Cardiac troponin T increased	Levels above the upper limit of normal and below the level of myocardial infarction as defined by the manufacturer	-	Levels consistent with myocardial infarction as defined by the manufacturer	-	-
**Definition:** A finding based on laboratory test results that indicate increased levels of cardiac troponin T in a biological specimen.					
**Navigational Note:** Also consider Cardiac disorders: Heart failure or Cardiac disorders: Myocardial infarction. Report Cardiac disorders: Heart failure or Cardiac disorders: Myocardial infarction if same grade event.					
CD4 lymphocytes decreased	<LLN - 500/mm3; <LLN - 0.5 x 10e9 /L	<500 - 200/mm3; <0.5 - 0.2 x 10e9 /L	<200 - 50/mm3; <0.2 x 0.05 - 10e9 /L	<50/mm3; <0.05 x 10e9 /L	-
**Definition:** A finding based on laboratory test results that indicate an decrease in levels of CD4 lymphocytes in a blood specimen.					
**Navigational Note:** -					
Cholesterol high	>ULN - 300 mg/dL; >ULN - 7.75 mmol/L	>300 - 400 mg/dL; >7.75 - 10.34 mmol/L	>400 - 500 mg/dL; >10.34 - 12.92 mmol/L	>500 mg/dL; >12.92 mmol/L	-
**Definition:** A finding based on laboratory test results that indicate higher than normal levels of cholesterol in a blood specimen.					
**Navigational Note:** -					
CPK increased	>ULN - 2.5 x ULN	>2.5 x ULN - 5 x ULN	>5 x ULN - 10 x ULN	>10 x ULN	-
**Definition:** A finding based on laboratory test results that indicate an increase in levels of creatine phosphokinase in a blood specimen.					
**Navigational Note:** Also consider Cardiac disorders: Heart failure or Cardiac disorders: Myocardial infarction. Report Cardiac disorders: Heart failure or Cardiac disorders: Myocardial infarction if same grade event.					
Creatinine increased	>ULN - 1.5 x ULN	>1.5 - 3.0 x baseline; >1.5 - 3.0 x ULN	>3.0 x baseline; >3.0 - 6.0 x ULN	>6.0 x ULN	-
**Definition:** A finding based on laboratory test results that indicate increased levels of creatinine in a biological specimen.					
**Navigational Note:** Also consider Renal and urinary disorders: Acute kidney injury					
Ejection fraction decreased	-	Resting ejection fraction (EF) 50 - 40%; 10 - 19% drop from baseline	Resting ejection fraction (EF) 39 - 20%; >=20% drop from baseline	Resting ejection fraction (EF) <20%	-
**Definition:** The percentage computed when the amount of blood ejected during a ventricular contraction of the heart is compared to the amount that was present prior to the contraction.					
**Navigational Note:** Also consider Cardiac disorders: Left ventricular systolic dysfunction. Report Cardiac disorders: Left ventricular systolic dysfunction if same grade event.					
Electrocardiogram QT corrected interval prolonged	Average QTc 450 - 480 ms	Average QTc 481 - 500 ms	Average QTc >= 501 ms; >60 ms change from baseline	Torsade de pointes; polymorphic ventricular tachycardia; signs/symptoms of serious arrhythmia	-
**Definition:** A finding of a cardiac dysrhythmia characterized by an abnormally long corrected QT interval.					
**Navigational Note:** -					

Investigations					
**CTCAE Term**	**Grade 1**	**Grade 2**	**Grade 3**	**Grade 4**	**Grade 5**
Electrocardiogram T wave abnormal	T wave flattening	Nonspecific ST segment change	-	-	-
**Definition:** A disorder characterized by Electrocardiogram T wave amplitude changes.					
**Navigational Note:** -					
Fibrinogen decreased	<1.0 - 0.75 x LLN; if abnormal, <25% decrease from baseline	<0.75 - 0.5 x LLN; if abnormal, 25 - <50% decrease from baseline	<0.5 - 0.25 x LLN; if abnormal, 50 - <75% decrease from baseline	<0.25 x LLN; if abnormal, 75% decrease from baseline; absolute value <50 mg/dL	-
**Definition:** A finding based on laboratory test results that indicate an decrease in levels of fibrinogen in a blood specimen.					
**Navigational Note:** -					
Forced expiratory volume decreased	FEV1% (percentages of observed FEV1 and FVC related to their respective predicted values) 99 - 70% predicted	FEV1 60 - 69%	50 - 59%	<= 49%	-
**Definition:** A finding based on test results that indicate a relative decrease in the fraction of the forced vital capacity that is exhaled in a specific number of seconds.					
**Navigational Note:** Also consider Respiratory, thoracic and mediastinal disorders: Respiratory failure or Dyspnea					
GGT increased	>ULN - 2.5 x ULN if baseline was normal; 2.0 - 2.5 x baseline if baseline was abnormal	>2.5 - 5.0 x ULN if baseline was normal; >2.5 - 5.0 x baseline if baseline was abnormal	>5.0 - 20.0 x ULN if baseline was normal; >5.0 - 20.0 x baseline if baseline was abnormal	>20.0 x ULN if baseline was normal; >20.0 x baseline if baseline was abnormal	-
**Definition:** A finding based on laboratory test results that indicate higher than normal levels of the enzyme gamma-glutamyltransferase in the blood specimen. GGT (gamma-glutamyltransferase) catalyzes the transfer of a gamma glutamyl group from a gamma glutamyl peptide to another peptide, amino acids or water.					
**Navigational Note:** -					
Growth hormone abnormal	Asymptomatic; clinical or diagnostic observations only; intervention not indicated	Symptomatic; medical intervention indicated; limiting instrumental ADL	-	-	-
**Definition:** A finding based on laboratory test results that indicate abnormal levels of growth hormone in a biological specimen.					
**Navigational Note:** -					
Haptoglobin decreased	<LLN	-	-	-	-
**Definition:** A finding based on laboratory test results that indicate an decrease in levels of haptoglobin in a blood specimen.					
**Navigational Note:** -					
Hemoglobin increased	Increase in >0 - 2 g/dL	Increase in >2 - 4 g/dL	Increase in >4 g/dL	-	-
**Definition:** A finding based on laboratory test results that indicate increased levels of hemoglobin above normal.					
**Navigational Note:** -					
INR increased	>1.2 - 1.5; >1 - 1.5 x baseline if on anticoagulation; monitoring only indicated	>1.5 - 2.5; >1.5 - 2.5 x baseline if on anticoagulation; dose adjustment indicated	>2.5; >2.5 x baseline if on anticoagulation; bleeding	-	-
**Definition:** A finding based on laboratory test results that indicate an increase in the ratio of the patient's prothrombin time to a control sample in the blood.					
**Navigational Note:** -					

Investigations

CTCAE Term	Grade 1	Grade 2	Grade 3	Grade 4	Grade 5
Lipase increased	>ULN - 1.5 x ULN	>1.5 - 2.0 x ULN; >2.0 - 5.0 x ULN and asymptomatic	>2.0 - 5.0 x ULN with signs or symptoms; >5.0 x ULN and asymptomatic	>5.0 x ULN and with signs or symptoms	-
**Definition:** A finding based on laboratory test results that indicate an increase in the level of lipase in a biological specimen.<br>**Navigational Note:** -					
Lymphocyte count decreased	<LLN - 800/mm3; <LLN - 0.8 x 10e9/L	<800 - 500/mm3; <0.8 - 0.5 x 10e9 /L	<500 - 200/mm3; <0.5 - 0.2 x 10e9 /L	<200/mm3; <0.2 x 10e9 /L	-
**Definition:** A finding based on laboratory test results that indicate a decrease in number of lymphocytes in a blood specimen.<br>**Navigational Note:** -					
Lymphocyte count increased	-	>4000/mm3 - 20,000/mm3	>20,000/mm3	-	-
**Definition:** A finding based on laboratory test results that indicate an abnormal increase in the number of lymphocytes in the blood, effusions or bone marrow.<br>**Navigational Note:** -					
Neutrophil count decreased	<LLN - 1500/mm3; <LLN - 1.5 x 10e9 /L	<1500 - 1000/mm3; <1.5 - 1.0 x 10e9 /L	<1000 - 500/mm3; <1.0 - 0.5 x 10e9 /L	<500/mm3; <0.5 x 10e9 /L	-
**Definition:** A finding based on laboratory test results that indicate a decrease in number of neutrophils in a blood specimen.<br>**Navigational Note:** -					
Pancreatic enzymes decreased	<LLN and asymptomatic	Increase in stool frequency, bulk, or odor; steatorrhea	Sequelae of absorption deficiency	-	-
**Definition:** A finding based on laboratory test results that indicate an decrease in levels of pancreatic enzymes in a biological specimen.<br>**Navigational Note:** -					
Platelet count decreased	<LLN - 75,000/mm3; <LLN - 75.0 x 10e9 /L	<75,000 - 50,000/mm3; <75.0 - 50.0 x 10e9 /L	<50,000 - 25,000/mm3; <50.0 - 25.0 x 10e9 /L	<25,000/mm3; <25.0 x 10e9 /L	-
**Definition:** A finding based on laboratory test results that indicate a decrease in number of platelets in a blood specimen.<br>**Navigational Note:** -					
Serum amylase increased	>ULN - 1.5 x ULN	>1.5 - 2.0 x ULN; >2.0 - 5.0 x ULN and asymptomatic	>2.0 - 5.0 x ULN with signs or symptoms; >5.0 x ULN and asymptomatic	>5.0 x ULN and with signs or symptoms	-
**Definition:** A finding based on laboratory test results that indicate an increase in the levels of amylase in a serum specimen.<br>**Navigational Note:** -					
Thyroid stimulating hormone increased	TSH increased and no intervention initiated	-	-	-	-
**Definition:** A disorder characterized by an increase in thyroid stimulating hormone.<br>**Navigational Note:** If intervention initiated or symptomatic, report as Endocrine disorders: Hypothyroidism.					

Investigations					
**CTCAE Term**	**Grade 1**	**Grade 2**	**Grade 3**	**Grade 4**	**Grade 5**
Urine output decreased	-	-	**Adult:** Oliguria (<80 ml in 8 hr);<br><br>**Infants:** < 0.5 mL/kg per hour for 24 hours;<br><br>**Children:** < 500 mL/1.73 m2 body surface area per day	**Adult:** Anuria (<240 ml in 24 hr);<br><br>**Pediatric:** No urine output over 12 hours	-
**Definition:** A finding based on test results that indicate urine production is less relative to previous output.<br>**Navigational Note:** -					
Vital capacity abnormal	90 - 75% of predicted value	<75 - 50% of predicted value; limiting instrumental ADL	<50% of predicted value; limiting self care ADL	-	-
**Definition:** A finding based on pulmonary function test results that indicate an abnormal vital capacity (amount of exhaled after a maximum inhalation) when compared to the predicted value.<br>**Navigational Note:** Also consider Investigations: Forced Expiratory Volume; Respiratory, thoracic and mediastinal disorders: Respiratory failure or Dyspnea					
Weight gain	5 - <10% from baseline	10 - <20% from baseline	>=20% from baseline	-	-
**Definition:** A finding characterized by an unexpected or abnormal increase in overall body weight; for pediatrics, greater than the baseline growth curve.<br>**Navigational Note:** Do not use Metabolism and nutrition disorders: Obesity, this term is being retired.					
Weight loss	5 to <10% from baseline; intervention not indicated	10 - <20% from baseline; nutritional support indicated	>=20% from baseline; tube feeding or TPN indicated	-	-
**Definition:** A finding characterized by a decrease in overall body weight; for pediatrics, less than the baseline growth curve.<br>**Navigational Note:** -					
White blood cell decreased	<LLN - 3000/mm3; <LLN - 3.0 x 10e9 /L	<3000 - 2000/mm3; <3.0 - 2.0 x 10e9 /L	<2000 - 1000/mm3; <2.0 - 1.0 x 10e9 /L	<1000/mm3; <1.0 x 10e9 /L	-
**Definition:** A finding based on laboratory test results that indicate an decrease in number of white blood cells in a blood specimen.<br>**Navigational Note:** -					
Investigations - Other, specify	Asymptomatic or mild symptoms; clinical or diagnostic observations only; intervention not indicated	Moderate; minimal, local or noninvasive intervention indicated; limiting age-appropriate instrumental ADL	Severe or medically significant but not immediately life-threatening; hospitalization or prolongation of existing hospitalization indicated; limiting self care ADL	Life-threatening consequences; urgent intervention indicated	Death
**Definition:** -<br>**Navigational Note:** -					

Metabolism and nutrition disorders					
**CTCAE Term**	**Grade 1**	**Grade 2**	**Grade 3**	**Grade 4**	**Grade 5**
Acidosis	pH <normal, but >=7.3	-	pH <7.3	Life-threatening consequences	Death
**Definition:** A disorder characterized by abnormally high acidity (high hydrogen-ion concentration) of the blood and other body tissues.					
**Navigational Note:** -					
Alcohol intolerance	-	Present	Severe symptoms; limiting self care ADL	Life-threatening consequences; urgent intervention indicated	Death
**Definition:** A disorder characterized by an increase in sensitivity to the adverse effects of alcohol, which can include nasal congestion, skin flushes, heart dysrhythmias, nausea, vomiting, indigestion and headaches.					
**Navigational Note:** -					
Alkalosis	pH >normal, but <=7.5	-	pH >7.5	Life-threatening consequences	Death
**Definition:** A disorder characterized by abnormally high alkalinity (low hydrogen-ion concentration) of the blood and other body tissues.					
**Navigational Note:** -					
Anorexia	Loss of appetite without alteration in eating habits	Oral intake altered without significant weight loss or malnutrition; oral nutritional supplements indicated	Associated with significant weight loss or malnutrition (e.g., inadequate oral caloric and/or fluid intake); tube feeding or TPN indicated	Life-threatening consequences; urgent intervention indicated	Death
**Definition:** A disorder characterized by a loss of appetite.					
**Navigational Note:** -					
Dehydration	Increased oral fluids indicated; dry mucous membranes; diminished skin turgor	IV fluids indicated	Hospitalization indicated	Life-threatening consequences; urgent intervention indicated	Death
**Definition:** A disorder characterized by excessive loss of water from the body. It is usually caused by severe diarrhea, vomiting or diaphoresis.					
**Navigational Note:** -					
Glucose intolerance	Asymptomatic; clinical or diagnostic observations only; intervention not indicated	Symptomatic; dietary modification or oral agent indicated	Severe symptoms; insulin indicated	Life-threatening consequences; urgent intervention indicated	Death
**Definition:** A disorder characterized by an inability to properly metabolize glucose.					
**Navigational Note:** -					
Hypercalcemia	Corrected serum calcium of >ULN - 11.5 mg/dL; >ULN - 2.9 mmol/L; Ionized calcium >ULN - 1.5 mmol/L	Corrected serum calcium of >11.5 - 12.5 mg/dL; >2.9 - 3.1 mmol/L; Ionized calcium >1.5 - 1.6 mmol/L; symptomatic	Corrected serum calcium of >12.5 - 13.5 mg/dL; >3.1 - 3.4 mmol/L; Ionized calcium >1.6 - 1.8 mmol/L; hospitalization indicated	Corrected serum calcium of >13.5 mg/dL; >3.4 mmol/L; Ionized calcium >1.8 mmol/L; life-threatening consequences	Death
**Definition:** A disorder characterized by laboratory test results that indicate an elevation in the concentration of calcium (corrected for albumin) in blood.					
**Navigational Note:** -					

Metabolism and nutrition disorders					
**CTCAE Term**	**Grade 1**	**Grade 2**	**Grade 3**	**Grade 4**	**Grade 5**
Hyperglycemia	Abnormal glucose above baseline with no medical intervention	Change in daily management from baseline for a diabetic; oral antiglycemic agent initiated; workup for diabetes	Insulin therapy initiated; hospitalization indicated	Life-threatening consequences; urgent intervention indicated	Death
**Definition:** A disorder characterized by laboratory test results that indicate an elevation in the concentration of blood sugar. It is usually an indication of diabetes mellitus or glucose intolerance.<br>**Navigational Note:** -					
Hyperkalemia	>ULN - 5.5 mmol/L	>5.5 - 6.0 mmol/L; intervention initiated	>6.0 - 7.0 mmol/L; hospitalization indicated	>7.0 mmol/L; life-threatening consequences	Death
**Definition:** A disorder characterized by laboratory test results that indicate an elevation in the concentration of potassium in the blood; associated with kidney failure or sometimes with the use of diuretic drugs.<br>**Navigational Note:** -					
Hyperlipidemia	Requiring diet changes	Requiring pharmaceutical intervention	Hospitalization; pancreatitis	Life-threatening consequences	-
**Definition:** A disorder characterized by laboratory test results that indicate an elevation in the concentration of lipids in blood.<br>**Navigational Note:** -					
Hypermagnesemia	>ULN - 3.0 mg/dL; >ULN - 1.23 mmol/L	-	>3.0 - 8.0 mg/dL; >1.23 - 3.30 mmol/L	>8.0 mg/dL; >3.30 mmol/L; life-threatening consequences	Death
**Definition:** A disorder characterized by laboratory test results that indicate an elevation in the concentration of magnesium in the blood.<br>**Navigational Note:** -					
Hypernatremia	>ULN - 150 mmol/L	>150 - 155 mmol/L; intervention initiated	>155 - 160 mmol/L; hospitalization indicated	>160 mmol/L; life-threatening consequences	Death
**Definition:** A disorder characterized by laboratory test results that indicate an elevation in the concentration of sodium in the blood.<br>**Navigational Note:** -					
Hyperphosphatemia	Laboratory finding only and intervention not indicated	Noninvasive intervention indicated	Severe or medically significant but not immediately life-threatening; hospitalization or prolongation of existing hospitalization indicated	Life-threatening consequences; urgent intervention indicated (e.g., dialysis)	Death
**Definition:** A disorder characterized by laboratory test results that indicate an elevation in the concentration of phosphate in a blood.<br>**Navigational Note:** -					
Hypertriglyceridemia	150 mg/dL - 300 mg/dL; 1.71 mmol/L - 3.42 mmol/L	>300 mg/dL - 500 mg/dL; >3.42 mmol/L - 5.7 mmol/L	>500 mg/dL - 1000 mg/dL; >5.7 mmol/L - 11.4 mmol/L	>1000 mg/dL; >11.4 mmol/L; life-threatening consequences	Death
**Definition:** A disorder characterized by laboratory test results that indicate an elevation in the concentration of triglyceride concentration in the blood.<br>**Navigational Note:** -					
Hyperuricemia	>ULN without physiologic consequences	-	>ULN with physiologic consequences	Life-threatening consequences	Death
**Definition:** A disorder characterized by laboratory test results that indicate an elevation in the concentration of uric acid.<br>**Navigational Note:** -					

Metabolism and nutrition disorders					
**CTCAE Term**	**Grade 1**	**Grade 2**	**Grade 3**	**Grade 4**	**Grade 5**
Hypoalbuminemia	<LLN - 3 g/dL; <LLN - 30 g/L	<3 - 2 g/dL; <30 - 20 g/L	<2 g/dL; <20 g/L	Life-threatening consequences; urgent intervention indicated	Death
**Definition:** A disorder characterized by laboratory test results that indicate a low concentration of albumin in the blood.<br>**Navigational Note:** -					
Hypocalcemia	Corrected serum calcium of <LLN - 8.0 mg/dL; <LLN - 2.0 mmol/L; Ionized calcium <LLN - 1.0 mmol/L	Corrected serum calcium of <8.0 - 7.0 mg/dL; <2.0 - 1.75 mmol/L; Ionized calcium <1.0 - 0.9 mmol/L; symptomatic	Corrected serum calcium of <7.0 - 6.0 mg/dL; <1.75 - 1.5 mmol/L; Ionized calcium <0.9 - 0.8 mmol/L; hospitalization indicated	Corrected serum calcium of <6.0 mg/dL; <1.5 mmol/L; Ionized calcium <0.8 mmol/L; life-threatening consequences	Death
**Definition:** A disorder characterized by laboratory test results that indicate a low concentration of calcium (corrected for albumin) in the blood.<br>**Navigational Note:** -					
Hypoglycemia	<LLN - 55 mg/dL; <LLN - 3.0 mmol/L	<55 - 40 mg/dL; <3.0 - 2.2 mmol/L	<40 - 30 mg/dL; <2.2 - 1.7 mmol/L	<30 mg/dL; <1.7 mmol/L; life-threatening consequences; seizures	Death
**Definition:** A disorder characterized by laboratory test results that indicate a low concentration of glucose in the blood.<br>**Navigational Note:** -					
Hypokalemia	<LLN - 3.0 mmol/L	Symptomatic with <LLN - 3.0 mmol/L; intervention indicated	<3.0 - 2.5 mmol/L; hospitalization indicated	<2.5 mmol/L; life-threatening consequences	Death
**Definition:** A disorder characterized by laboratory test results that indicate a low concentration of potassium in the blood.<br>**Navigational Note:** -					
Hypomagnesemia	<LLN - 1.2 mg/dL; <LLN - 0.5 mmol/L	<1.2 - 0.9 mg/dL; <0.5 - 0.4 mmol/L	<0.9 - 0.7 mg/dL; <0.4 - 0.3 mmol/L	<0.7 mg/dL; <0.3 mmol/L; life-threatening consequences	Death
**Definition:** A disorder characterized by laboratory test results that indicate a low concentration of magnesium in the blood.<br>**Navigational Note:** -					
Hyponatremia	<LLN - 130 mmol/L	125-129 mmol/L and asymptomatic	125-129 mmol/L symptomatic; 120-124 mmol/L regardless of symptoms	<120 mmol/L; life-threatening consequences	Death
**Definition:** A disorder characterized by laboratory test results that indicate a low concentration of sodium in the blood.<br>**Navigational Note:** -					
Hypophosphatemia	Laboratory finding only and intervention not indicated	Oral replacement therapy indicated	Severe or medically significant but not immediately life-threatening; hospitalization or prolongation of existing hospitalization indicated	Life-threatening consequences	Death
**Definition:** A disorder characterized by laboratory test results that indicate a low concentration of phosphates in the blood.<br>**Navigational Note:** -					

Metabolism and nutrition disorders					
**CTCAE Term**	**Grade 1**	**Grade 2**	**Grade 3**	**Grade 4**	**Grade 5**
Iron overload	-	Moderate symptoms; intervention not indicated	Severe symptoms; intervention indicated	Life-threatening consequences; urgent intervention indicated	Death
**Definition:** A disorder characterized by accumulation of iron in the tissues.					
**Navigational Note:** -					
Obesity	-	BMI 25 - 29.9 kg/m2	BMI 30 - 39.9 kg/m2	BMI >=40 kg/m2	-
**Definition:** A disorder characterized by having a high amount of body fat.					
**Navigational Note:** Use term Investigations: Weight gain					
Tumor lysis syndrome	-	-	Present	Life-threatening consequences; urgent intervention indicated	Death
**Definition:** A disorder characterized by metabolic abnormalities that result from a spontaneous or therapy-related cytolysis of tumor cells.					
**Navigational Note:** -					
Metabolism and nutrition disorders - Other, specify	Asymptomatic or mild symptoms; clinical or diagnostic observations only; intervention not indicated	Moderate; minimal, local or noninvasive intervention indicated; limiting age-appropriate instrumental ADL	Severe or medically significant but not immediately life-threatening; hospitalization or prolongation of existing hospitalization indicated; limiting self care ADL	Life-threatening consequences; urgent intervention indicated	Death
**Definition:** -					
**Navigational Note:** -					

Musculoskeletal and connective tissue disorders					
**CTCAE Term**	**Grade 1**	**Grade 2**	**Grade 3**	**Grade 4**	**Grade 5**
Abdominal soft tissue necrosis	-	Local wound care; medical intervention indicated (e.g., dressings or topical medications)	Operative debridement or other invasive intervention indicated (e.g., tissue reconstruction, flap, or grafting)	Life-threatening consequences; urgent intervention indicated	Death
**Definition:** A disorder characterized by a necrotic process occurring in the soft tissues of the abdominal wall.<br>**Navigational Note:** -					
Arthralgia	Mild pain	Moderate pain; limiting instrumental ADL	Severe pain; limiting self care ADL	-	-
**Definition:** A disorder characterized by a sensation of marked discomfort in a joint.<br>**Navigational Note:** -					
Arthritis	Mild pain with inflammation, erythema, or joint swelling	Moderate pain associated with signs of inflammation, erythema, or joint swelling; limiting instrumental ADL	Severe pain associated with signs of inflammation, erythema, or joint swelling; irreversible joint damage; limiting self care ADL	-	-
**Definition:** A disorder characterized by inflammation involving a joint.<br>**Navigational Note:** -					
Avascular necrosis	Asymptomatic; clinical or diagnostic observations only; intervention not indicated	Symptomatic; limiting instrumental ADL	Severe symptoms; limiting self care ADL; elective operative intervention indicated	Life-threatening consequences; urgent intervention indicated	Death
**Definition:** A disorder characterized by necrotic changes in the bone tissue due to interruption of blood supply. Most often affecting the epiphysis of the long bones, the necrotic changes result in the collapse and the destruction of the bone structure.<br>**Navigational Note:** Use new term: Musculoskeletal and connective tissue disorders: Osteonecrosis					
Back pain	Mild pain	Moderate pain; limiting instrumental ADL	Severe pain; limiting self care ADL	-	-
**Definition:** A disorder characterized by a sensation of marked discomfort in the back region.<br>**Navigational Note:** -					
Bone pain	Mild pain	Moderate pain; limiting instrumental ADL	Severe pain; limiting self care ADL	-	-
**Definition:** A disorder characterized by a sensation of marked discomfort in the bones.<br>**Navigational Note:** -					
Buttock pain	Mild pain	Moderate pain; limiting instrumental ADL	Severe pain; limiting self care ADL	-	-
**Definition:** A disorder characterized by a sensation of marked discomfort in the buttocks.<br>**Navigational Note:** -					

Musculoskeletal and connective tissue disorders					
**CTCAE Term**	**Grade 1**	**Grade 2**	**Grade 3**	**Grade 4**	**Grade 5**
Chest wall necrosis	-	Local wound care; medical intervention indicated (e.g., dressings or topical medications)	Operative debridement or other invasive intervention indicated (e.g., tissue reconstruction, flap, or grafting)	Life-threatening consequences; urgent intervention indicated	Death
**Definition:** A disorder characterized by a necrotic process occurring in the soft tissues of the chest wall including breast.<br>**Navigational Note:** -					
Chest wall pain	Mild pain	Moderate pain; limiting instrumental ADL	Severe pain; limiting self care ADL	-	-
**Definition:** A disorder characterized by a sensation of marked discomfort in the chest wall.<br>**Navigational Note:** -					
Exostosis	Asymptomatic; clinical or diagnostic observations only; intervention not indicated	Symptomatic; limiting instrumental ADL	Severe symptoms; limiting self care ADL; elective operative intervention indicated	-	-
**Definition:** A disorder characterized by non-neoplastic overgrowth of bone.<br>**Navigational Note:** -					
Fibrosis deep connective tissue	Mild induration, able to move skin parallel to plane (sliding) and perpendicular to skin (pinching up)	Moderate induration, able to slide skin, unable to pinch skin; limiting instrumental ADL	Severe induration; unable to slide or pinch skin; limiting joint or orifice movement (e.g., mouth, anus); limiting self care ADL	Generalized; associated with signs or symptoms of impaired breathing or feeding	Death
**Definition:** A disorder characterized by fibrotic degeneration of the deep connective tissues.<br>**Navigational Note:** -					
Flank pain	Mild pain	Moderate pain; limiting instrumental ADL	Severe pain; limiting self care ADL	-	-
**Definition:** A disorder characterized by a sensation of marked discomfort on the lateral side of the body in the region below the ribs and above the hip.<br>**Navigational Note:** -					
Generalized muscle weakness	Symptomatic; perceived by patient but not evident on physical exam	Symptomatic; evident on physical exam; limiting instrumental ADL	Limiting self care ADL	-	-
**Definition:** A disorder characterized by a reduction in the strength of muscles in multiple anatomic sites.<br>**Navigational Note:** -					

Musculoskeletal and connective tissue disorders					
**CTCAE Term**	**Grade 1**	**Grade 2**	**Grade 3**	**Grade 4**	**Grade 5**
Growth suppression	Reduction in growth velocity by 10 - 29% ideally measured over the period of a year	Reduction in growth velocity by 30 - 49% ideally measured over the period of a year or 0 - 49% reduction in growth from the baseline growth curve	Reduction in growth velocity of >=50% ideally measured over the period of a year	-	-
**Definition:** A disorder characterized by stature that is smaller than normal as expected for age.<br>**Navigational Note:** -					
Head soft tissue necrosis	-	Local wound care; medical intervention indicated (e.g., dressings or topical medications)	Operative debridement or other invasive intervention indicated (e.g., tissue reconstruction, flap, or grafting)	Life-threatening consequences; urgent intervention indicated	Death
**Definition:** A disorder characterized by a necrotic process occurring in the soft tissues of the head.<br>**Navigational Note:** -					
Joint effusion	Asymptomatic; clinical or diagnostic observations only; intervention not indicated	Symptomatic; limiting instrumental ADL	Severe symptoms; limiting self care ADL; invasive intervention indicated	-	-
**Definition:** A disorder characterized by excessive fluid in a joint, usually as a result of joint inflammation.<br>**Navigational Note:** -					
Joint range of motion decreased	<=25% loss of ROM (range of motion); decreased ROM limiting athletic activity	>25 - 50% decrease in ROM; limiting instrumental ADL	>50% decrease in ROM; limiting self care ADL	-	-
**Definition:** A disorder characterized by a decrease in joint flexibility of any joint.<br>**Navigational Note:** -					
Joint range of motion decreased cervical spine	Mild restriction of rotation or flexion between 60 - 70 degrees	Rotation <60 degrees to right or left; <60 degrees of flexion	Ankylosed/fused over multiple segments with no C-spine rotation	-	-
**Definition:** A disorder characterized by a decrease in flexibility of a cervical spine joint.<br>**Navigational Note:** -					
Joint range of motion decreased lumbar spine	Stiffness; difficulty bending to the floor to pick up a very light object but able to do athletic activity	Pain with range of motion (ROM) in lumbar spine; requires a reaching aid to pick up a very light object from the floor	<50% lumbar spine flexion; associated with symptoms of ankylosis or fused over multiple segments with no L-spine flexion (e.g., unable to reach to floor to pick up a very light object)	-	-
**Definition:** A disorder characterized by a decrease in flexibility of a lumbar spine joint.<br>**Navigational Note:** -					

Musculoskeletal and connective tissue disorders					
**CTCAE Term**	**Grade 1**	**Grade 2**	**Grade 3**	**Grade 4**	**Grade 5**
Kyphosis	Asymptomatic; clinical or diagnostic observations only; intervention not indicated	Moderate accentuation; limiting instrumental ADL	Severe accentuation; operative intervention indicated; limiting self care ADL	-	-
**Definition:** A disorder characterized by an abnormal increase in the curvature of the thoracic portion of the spine.<br>**Navigational Note:** -					
Lordosis	Asymptomatic; clinical or diagnostic observations only; intervention not indicated	Moderate accentuation; limiting instrumental ADL	Severe accentuation; operative intervention indicated; limiting self care ADL	-	-
**Definition:** A disorder characterized by an abnormal increase in the curvature of the lumbar portion of the spine.<br>**Navigational Note:** -					
Muscle cramp	Mild pain	Moderate pain; limiting instrumental ADL	Severe pain; limiting self care ADL	-	-
**Definition:** A disorder characterized by marked cramping sensation originating from a muscle or group of muscles.<br>**Navigational Note:** Synonym: Muscle spasm					
Muscle weakness lower limb	Symptomatic; perceived by patient but not evident on physical exam	Symptomatic; evident on physical exam; limiting instrumental ADL	Limiting self care ADL	-	-
**Definition:** A disorder characterized by a reduction in the strength of the lower limb muscles.<br>**Navigational Note:** -					
Muscle weakness trunk	Symptomatic; perceived by patient but not evident on physical exam	Symptomatic; evident on physical exam; limiting instrumental ADL	Limiting self care ADL	-	-
**Definition:** A disorder characterized by a reduction in the strength of the trunk muscles.<br>**Navigational Note:** -					
Muscle weakness upper limb	Symptomatic; perceived by patient but not evident on physical exam	Symptomatic; evident on physical exam; limiting instrumental ADL	Limiting self care ADL	-	-
**Definition:** A disorder characterized by a reduction in the strength of the upper limb muscles.<br>**Navigational Note:** -					
Musculoskeletal deformity	Cosmetically and functionally insignificant hypoplasia	Deformity, hypoplasia, or asymmetry able to be remediated by prosthesis (e.g., shoe insert) or covered by clothing	Significant deformity, hypoplasia, or asymmetry, unable to be remediated by prosthesis or covered by clothing; limiting self care ADL	-	-
**Definition:** A disorder characterized by a malformation of the musculoskeletal system.<br>**Navigational Note:** -					

Musculoskeletal and connective tissue disorders					
**CTCAE Term**	**Grade 1**	**Grade 2**	**Grade 3**	**Grade 4**	**Grade 5**
Myalgia	Mild pain	Moderate pain; limiting instrumental ADL	Severe pain; limiting self care ADL	-	-
**Definition:** A disorder characterized by marked discomfort sensation originating from a muscle or group of muscles.<br>**Navigational Note:** -					
Myositis	Mild pain	Moderate pain associated with weakness; pain limiting instrumental ADL	Pain associated with severe weakness; limiting self care ADL	Life-threatening consequences; urgent intervention indicated	-
**Definition:** A disorder characterized by inflammation involving the skeletal muscles.<br>**Navigational Note:** -					
Neck pain	Mild pain	Moderate pain; limiting instrumental ADL	Severe pain; limiting self care ADL	-	-
**Definition:** A disorder characterized by a sensation of marked discomfort in the neck area.<br>**Navigational Note:** -					
Neck soft tissue necrosis	-	Local wound care; medical intervention indicated (e.g., dressings or topical medications)	Operative debridement or other invasive intervention indicated (e.g., tissue reconstruction, flap, or grafting)	Life-threatening consequences; urgent intervention indicated	Death
**Definition:** A disorder characterized by a necrotic process occurring in the soft tissues of the neck.<br>**Navigational Note:** -					
Osteonecrosis	Asymptomatic; clinical or diagnostic observations only; intervention not indicated	Symptomatic; medical intervention indicated (e.g., analgesics or bisphosphonates); limiting instrumental ADL	Severe symptoms; limiting self care ADL; elective operative intervention indicated	Life-threatening consequences; urgent intervention indicated	Death
**Definition:** A disorder characterized by necrotic changes in the bone tissue due to interruption of blood supply. Most often affecting the epiphysis of the long bones, the necrotic changes result in the collapse and the destruction of the bone structure.<br>**Navigational Note:** -					
Osteonecrosis of jaw	Asymptomatic; clinical or diagnostic observations only; intervention not indicated	Symptomatic; medical intervention indicated (e.g., topical agents); limiting instrumental ADL	Severe symptoms; limiting self care ADL; elective operative intervention indicated	Life-threatening consequences; urgent intervention indicated	Death
**Definition:** A disorder characterized by a necrotic process occurring in the bone of the mandible.<br>**Navigational Note:** -					

Musculoskeletal and connective tissue disorders					
**CTCAE Term**	**Grade 1**	**Grade 2**	**Grade 3**	**Grade 4**	**Grade 5**
Osteoporosis	**Adult:** Radiologic evidence of osteoporosis or Bone Mineral Density (BMD) t-score -1 to -2.5 (osteopenia);<br><br>**Pediatric:** Radiologic evidence of low BMD with z score of <= -2.0 and no history of significant fractures	**Adult:** BMD t-score < -2.5; loss of height <2 cm; therapy to improve BMD indicated; limiting instrumental ADL;<br><br>**Pediatric:** Low BMD (z-score <= -2.0) and significant fracture history (defined as a long bone fracture of the lower extremity, vertebral compression, 2 or more long bone fractures of the upper extremities); therapy to improve BMD indicated	**Adult:** Loss of height >=2 cm; hospitalization indicated; limiting self care ADL;<br><br>**Pediatric:** Limiting self care ADL	-	-
**Definition:** A disorder characterized by reduced bone mass, with a decrease in cortical thickness and in the number and size of the trabeculae of cancellous bone (but normal chemical composition), resulting in increased fracture incidence.<br>**Navigational Note:** -					
Pain in extremity	Mild pain	Moderate pain; limiting instrumental ADL	Severe pain; limiting self care ADL	-	-
**Definition:** A disorder characterized by a sensation of marked discomfort in the upper or lower extremities.<br>**Navigational Note:** -					
Pelvic soft tissue necrosis	-	Local wound care; medical intervention indicated (e.g., dressings or topical medications)	Operative debridement or other invasive intervention indicated (e.g., tissue reconstruction, flap, or grafting)	Life-threatening consequences; urgent intervention indicated	Death
**Definition:** A disorder characterized by a necrotic process occurring in the soft tissues of the pelvis.<br>**Navigational Note:** -					
Rhabdomyolysis	Asymptomatic, intervention not indicated; laboratory findings only	Non-urgent intervention indicated	Symptomatic, urgent intervention indicated	Life-threatening consequences; dialysis	Death
**Definition:** A disorder characterized by the breakdown of muscle tissue resulting in the release of muscle fiber contents into the bloodstream.<br>**Navigational Note:** -					

Musculoskeletal and connective tissue disorders					
CTCAE Term	Grade 1	Grade 2	Grade 3	Grade 4	Grade 5
Rotator cuff injury	Asymptomatic or mild symptoms; clinical or diagnostic observations only; intervention not indicated	Moderate; minimal, local or noninvasive intervention indicated; limiting instrumental ADL	Severe or medically significant but not immediately life-threatening; hospitalization or prolongation of existing hospitalization indicated; limiting self care ADL	-	-
**Definition:** A disorder characterized by an injury of the rotator cuff.<br>**Navigational Note:** -					
Scoliosis	<20 degrees; clinically undetectable	>20 - 45 degrees; visible by forward flexion; limiting instrumental ADL	>45 degrees; scapular prominence in forward flexion; operative intervention indicated; limiting self care ADL	-	-
**Definition:** A disorder characterized by a malformed, lateral curvature of the spine.<br>**Navigational Note:** -					
Soft tissue necrosis lower limb	-	Local wound care; medical intervention indicated (e.g., dressings or topical medications)	Operative debridement or other invasive intervention indicated (e.g., tissue reconstruction, flap, or grafting)	Life-threatening consequences; urgent intervention indicated	Death
**Definition:** A disorder characterized by a necrotic process occurring in the soft tissues of the lower extremity.<br>**Navigational Note:** -					
Soft tissue necrosis upper limb	-	Local wound care; medical intervention indicated (e.g., dressings or topical medications)	Operative debridement or other invasive intervention indicated (e.g., tissue reconstruction, flap, or grafting)	Life-threatening consequences; urgent intervention indicated	Death
**Definition:** A disorder characterized by a necrotic process occurring in the soft tissues of the upper extremity.<br>**Navigational Note:** -					
Superficial soft tissue fibrosis	Mild induration, able to move skin parallel to plane (sliding) and perpendicular to skin (pinching up)	Moderate induration, able to slide skin, unable to pinch skin; limiting instrumental ADL	Severe induration; unable to slide or pinch skin; limiting joint or orifice movement (e.g., mouth, anus); limiting self care ADL	Generalized; associated with signs or symptoms of impaired breathing or feeding	Death
**Definition:** A disorder characterized by fibrotic degeneration of the superficial soft tissues.<br>**Navigational Note:** -					

Musculoskeletal and connective tissue disorders					
**CTCAE Term**	**Grade 1**	**Grade 2**	**Grade 3**	**Grade 4**	**Grade 5**
Trismus	Decreased ROM (range of motion) without impaired eating	Decreased ROM requiring small bites, soft foods or purees	Decreased ROM with inability to adequately aliment or hydrate orally	-	-
**Definition:** A disorder characterized by lack of ability to open the mouth fully due to a decrease in the range of motion of the muscles of mastication. **Navigational Note:** -					
Unequal limb length	Mild length discrepancy <2 cm	Moderate length discrepancy 2 - 5 cm; shoe lift indicated; limiting instrumental ADL	Severe length discrepancy >5 cm; limiting self care ADL; operative intervention indicated	-	-
**Definition:** A disorder characterized by a discrepancy between the lengths of the lower or upper extremities. **Navigational Note:** -					
Musculoskeletal and connective tissue disorder - Other, specify	Asymptomatic or mild symptoms; clinical or diagnostic observations only; intervention not indicated	Moderate; minimal, local or noninvasive intervention indicated; limiting age-appropriate instrumental ADL	Severe or medically significant but not immediately life-threatening; hospitalization or prolongation of existing hospitalization indicated; limiting self care ADL	Life-threatening consequences; urgent intervention indicated	Death
**Definition:** - **Navigational Note:** -					

Neoplasms benign, malignant and unspecified (incl cysts and polyps)					
**CTCAE Term**	**Grade 1**	**Grade 2**	**Grade 3**	**Grade 4**	**Grade 5**
Leukemia secondary to oncology chemotherapy	-	-	-	Present	Death
**Definition:** A disorder characterized by leukemia arising as a result of the mutagenic effect of chemotherapy agents.<br>**Navigational Note:** -					
Myelodysplastic syndrome	-	-	-	Life-threatening consequences; urgent intervention indicated	Death
**Definition:** A disorder characterized by insufficiently healthy hematapoietic cell production by the bone marrow.<br>**Navigational Note:** -					
Skin papilloma	Asymptomatic; intervention not indicated	Intervention initiated	-	-	-
**Definition:** A disorder characterized by the presence of one or more warts.<br>**Navigational Note:** -					
Treatment related secondary malignancy	-	-	Non life-threatening secondary malignancy	Acute life-threatening secondary malignancy; blast crisis in leukemia	Death
**Definition:** A disorder characterized by development of a malignancy most probably as a result of treatment for a previously existing malignancy.<br>**Navigational Note:** -					
Tumor hemorrhage	Mild symptoms; intervention not indicated	Moderate symptoms; intervention indicated	Transfusion indicated; invasive intervention indicated; hospitalization	Life-threatening consequences; urgent intervention indicated	Death
**Definition:** A disorder characterized by bleeding in a tumor.<br>**Navigational Note:** -					
Tumor pain	Mild pain	Moderate pain; limiting instrumental ADL	Severe pain; limiting self care ADL	-	-
**Definition:** A disorder characterized by a sensation of marked discomfort from a neoplasm that may be pressing on a nerve, blocking blood vessels, inflamed or fractured from metastasis.<br>**Navigational Note:** -					
Neoplasms benign, malignant and unspecified (incl cysts and polyps) - Other, specify	Asymptomatic or mild symptoms; clinical or diagnostic observations only; intervention not indicated	Moderate; minimal, local or noninvasive intervention indicated; limiting age-appropriate instrumental ADL	Severe or medically significant but not immediately life-threatening; hospitalization or prolongation of existing hospitalization indicated; limiting self care ADL	Life-threatening consequences; urgent intervention indicated	Death
**Definition:** -<br>**Navigational Note:** -					

Nervous system disorders					
**CTCAE Term**	**Grade 1**	**Grade 2**	**Grade 3**	**Grade 4**	**Grade 5**
Abducens nerve disorder	Asymptomatic; clinical or diagnostic observations only; intervention not indicated	Moderate symptoms; limiting instrumental ADL	Severe symptoms; limiting self care ADL	-	-
**Definition:** A disorder characterized by dysfunction of the abducens nerve (sixth cranial nerve).					
**Navigational Note:** -					
Accessory nerve disorder	Asymptomatic; clinical or diagnostic observations only; intervention not indicated	Moderate symptoms; limiting instrumental ADL	Severe symptoms; limiting self care ADL	-	-
**Definition:** A disorder characterized by dysfunction of the accessory nerve (eleventh cranial nerve).					
**Navigational Note:** -					
Acoustic nerve disorder NOS	Asymptomatic; clinical or diagnostic observations only; intervention not indicated	Moderate symptoms; limiting instrumental ADL	Severe symptoms; limiting self care ADL	-	-
**Definition:** A disorder characterized by dysfunction of the acoustic nerve (eighth cranial nerve).					
**Navigational Note:** -					
Akathisia	Mild restlessness or increased motor activity	Moderate restlessness or increased motor activity; limiting instrumental ADL	Severe restlessness or increased motor activity; limiting self care ADL	-	-
**Definition:** A disorder characterized by an uncomfortable feeling of inner restlessness and inability to stay still; this is a side effect of some psychotropic drugs.					
**Navigational Note:** -					
Amnesia	Mild; transient memory loss	Moderate; short term memory loss; limiting instrumental ADL	Severe; long term memory loss; limiting self care ADL	-	-
**Definition:** A disorder characterized by systematic and extensive loss of memory.					
**Navigational Note:** -					
Anosmia	Present	-	-	-	-
**Definition:** A disorder characterized by a change in the sense of smell.					
**Navigational Note:** Also consider Olfactory nerve disorder					
Aphonia	-	-	Voicelessness; unable to speak	-	-
**Definition:** A disorder characterized by the inability to speak. It may result from injuries to the vocal cords or may be functional (psychogenic).					
**Navigational Note:** -					
Arachnoiditis	Mild symptoms	Moderate symptoms; limiting instrumental ADL	Severe symptoms; limiting self care ADL	Life-threatening consequences; urgent intervention indicated	Death
**Definition:** A disorder characterized by inflammation of the arachnoid membrane and adjacent subarachnoid space.					
**Navigational Note:** -					

Nervous system disorders					
**CTCAE Term**	**Grade 1**	**Grade 2**	**Grade 3**	**Grade 4**	**Grade 5**
Ataxia	Asymptomatic; clinical or diagnostic observations only; intervention not indicated	Moderate symptoms; limiting instrumental ADL	Severe symptoms; limiting self care ADL; mechanical assistance indicated	-	-
**Definition:** A disorder characterized by lack of coordination of muscle movements resulting in the impairment or inability to perform voluntary activities.					
**Navigational Note:** -					
Brachial plexopathy	Asymptomatic; clinical or diagnostic observations only; intervention not indicated	Moderate symptoms; limiting instrumental ADL	Severe symptoms; limiting self care ADL	-	-
**Definition:** A disorder characterized by regional paresthesia of the brachial plexus, marked discomfort and muscle weakness, and limited movement in the arm or hand.					
**Navigational Note:** -					
Central nervous system necrosis	Asymptomatic; clinical or diagnostic observations only; intervention not indicated	Moderate symptoms; corticosteroids indicated	Severe symptoms; medical intervention indicated	Life-threatening consequences; urgent intervention indicated	Death
**Definition:** A disorder characterized by a necrotic process occurring in the brain and/or spinal cord.					
**Navigational Note:** -					
Cerebrospinal fluid leakage	Post-craniotomy: asymptomatic; Post-lumbar puncture: transient headache; postural care indicated	Post-craniotomy: moderate symptoms; medical intervention indicated; Post-lumbar puncture: persistent moderate symptoms; blood patch indicated	Severe symptoms; medical intervention indicated	Life-threatening consequences; urgent intervention indicated	Death
**Definition:** A disorder characterized by loss of cerebrospinal fluid into the surrounding tissues.					
**Navigational Note:** -					
Cognitive disturbance	Mild cognitive disability; not interfering with work/school/life performance; specialized educational services/devices not indicated	Moderate cognitive disability; interfering with work/school/life performance but capable of independent living; specialized resources on part time basis indicated	Severe cognitive disability; significant impairment of work/school/life performance	-	-
**Definition:** A disorder characterized by a conspicuous change in cognitive function.					
**Navigational Note:** -					
Concentration impairment	Mild inattention or decreased level of concentration	Moderate impairment in attention or decreased level of concentration; limiting instrumental ADL	Severe impairment in attention or decreased level of concentration; limiting self care ADL	-	-
**Definition:** A disorder characterized by a deterioration in the ability to concentrate.					
**Navigational Note:** -					

Nervous system disorders					
**CTCAE Term**	**Grade 1**	**Grade 2**	**Grade 3**	**Grade 4**	**Grade 5**
Depressed level of consciousness	Decreased level of alertness	Sedation; slow response to stimuli; limiting instrumental ADL	Difficult to arouse	Life-threatening consequences; coma; urgent intervention indicated	Death
**Definition:** A disorder characterized by a decrease in ability to perceive and respond.					
**Navigational Note:** -					
Dizziness	Mild unsteadiness or sensation of movement	Moderate unsteadiness or sensation of movement; limiting instrumental ADL	Severe unsteadiness or sensation of movement; limiting self care ADL	-	-
**Definition:** A disorder characterized by a disturbing sensation of lightheadedness, unsteadiness, giddiness, spinning or rocking.					
**Navigational Note:** -					
Dysarthria	Mild slurred speech	Moderate impairment of articulation or slurred speech	Severe impairment of articulation or slurred speech	-	-
**Definition:** A disorder characterized by slow and slurred speech resulting from an inability to coordinate the muscles used in speech.					
**Navigational Note:** -					
Dysesthesia	Mild sensory alteration	Moderate sensory alteration; limiting instrumental ADL	Severe sensory alteration; limiting self care ADL	-	-
**Definition:** A disorder characterized by distortion of sensory perception, resulting in an abnormal and unpleasant sensation.					
**Navigational Note:** -					
Dysgeusia	Altered taste but no change in diet	Altered taste with change in diet (e.g., oral supplements); noxious or unpleasant taste; loss of taste	-	-	-
**Definition:** A disorder characterized by abnormal sensual experience with the taste of foodstuffs; it can be related to a decrease in the sense of smell.					
**Navigational Note:** -					
Dysphasia	Awareness of receptive or expressive characteristics; not impairing ability to communicate	Moderate receptive or expressive characteristics; impairing ability to communicate spontaneously	Severe receptive or expressive characteristics; impairing ability to read, write or communicate intelligibly	-	-
**Definition:** A disorder characterized by impairment of verbal communication skills, often resulting from brain damage.					
**Navigational Note:** -					
Edema cerebral	-	-	New onset; worsening from baseline	Life-threatening consequences; urgent intervention indicated	Death
**Definition:** A disorder characterized by swelling due to an excessive accumulation of fluid in the brain.					
**Navigational Note:** -					
Encephalopathy	Mild symptoms	Moderate symptoms; limiting instrumental ADL	Severe symptoms; limiting self care ADL	Life-threatening consequences; urgent intervention indicated	Death
**Definition:** A disorder characterized by a pathologic process involving the brain.					
**Navigational Note:** -					

Nervous system disorders					
**CTCAE Term**	**Grade 1**	**Grade 2**	**Grade 3**	**Grade 4**	**Grade 5**
Extrapyramidal disorder	Mild involuntary movements	Moderate involuntary movements; limiting instrumental ADL	Severe involuntary movements or torticollis; limiting self care ADL	Life-threatening consequences; urgent intervention indicated	Death
**Definition:** A disorder characterized by abnormal, repetitive, involuntary muscle movements, frenzied speech and extreme restlessness.					
**Navigational Note:** Synonym: Restless legs					
Facial muscle weakness	Asymptomatic; clinical or diagnostic observations only; intervention not indicated	Moderate symptoms; limiting instrumental ADL	Severe symptoms; limiting self care ADL	-	-
**Definition:** A disorder characterized by a reduction in the strength of the facial muscles.					
**Navigational Note:** -					
Facial nerve disorder	Asymptomatic; clinical or diagnostic observations only; intervention not indicated	Moderate symptoms; limiting instrumental ADL	Severe symptoms; limiting self care ADL	-	-
**Definition:** A disorder characterized by dysfunction of the facial nerve (seventh cranial nerve).					
**Navigational Note:** -					
Glossopharyngeal nerve disorder	Asymptomatic; clinical or diagnostic observations only; intervention not indicated	Moderate symptoms; limiting instrumental ADL	Severe symptoms; limiting self care ADL	Life-threatening consequences; urgent intervention indicated	Death
**Definition:** A disorder characterized by dysfunction of the glossopharyngeal nerve (ninth cranial nerve).					
**Navigational Note:** -					
Guillain-Barre syndrome	Mild symptoms	Moderate symptoms; limiting instrumental ADL	Severe symptoms; limiting self care ADL	Life-threatening consequences; urgent intervention indicated; intubation	Death
**Definition:** A disorder characterized by the body's immune system attacking the peripheral nervous system causing ascending paralysis.					
**Navigational Note:** -					
Headache	Mild pain	Moderate pain; limiting instrumental ADL	Severe pain; limiting self care ADL	-	-
**Definition:** A disorder characterized by a sensation of marked discomfort in various parts of the head, not confined to the area of distribution of any nerve.					
**Navigational Note:** -					
Hydrocephalus	Asymptomatic; clinical or diagnostic observations only; intervention not indicated	Moderate symptoms; intervention not indicated	Severe symptoms or neurological deficit; intervention indicated	Life-threatening consequences; urgent intervention indicated	Death
**Definition:** A disorder characterized by an abnormal increase of cerebrospinal fluid in the ventricles of the brain.					
**Navigational Note:** -					
Hypersomnia	Mild increased need for sleep	Moderate increased need for sleep	Severe increased need for sleep	-	-
**Definition:** A disorder characterized by characterized by excessive sleepiness during the daytime.					
**Navigational Note:** -					

Nervous system disorders					
**CTCAE Term**	**Grade 1**	**Grade 2**	**Grade 3**	**Grade 4**	**Grade 5**
Hypoglossal nerve disorder	Asymptomatic; clinical or diagnostic observations only; intervention not indicated	Moderate symptoms; limiting instrumental ADL	Severe symptoms; limiting self care ADL	-	-
**Definition:** A disorder characterized by dysfunction of the hypoglossal nerve (twelfth cranial nerve).<br>**Navigational Note:** -					
Intracranial hemorrhage	Asymptomatic; clinical or diagnostic observations only; intervention not indicated	Moderate symptoms; intervention indicated	Ventriculostomy, ICP monitoring, intraventricular thrombolysis, or invasive intervention indicated; hospitalization	Life-threatening consequences; urgent intervention indicated	Death
**Definition:** A disorder characterized by bleeding from the cranium.<br>**Navigational Note:** -					
Ischemia cerebrovascular	Asymptomatic; clinical or diagnostic observations only; intervention not indicated	Moderate symptoms	-	-	-
**Definition:** A disorder characterized by a decrease or absence of blood supply to the brain caused by obstruction (thrombosis or embolism) of an artery resulting in neurological damage.<br>**Navigational Note:** Prior to using this term consider Nervous system disorder: TIA or Stroke					
Lethargy	Mild symptoms; reduced alertness and awareness	Moderate symptoms; limiting instrumental ADL	-	-	-
**Definition:** A disorder characterized by a decrease in consciousness characterized by mental and physical inertness.<br>**Navigational Note:** -					
Leukoencephalopathy	Asymptomatic; small focal T2/FLAIR hyperintensities; involving periventricular white matter or <1/3 of susceptible areas of cerebrum +/- mild increase in subarachnoid space (SAS) and/or mild ventriculomegaly	Moderate symptoms; focal T2/FLAIR hyperintensities, involving periventricular white matter extending into centrum semiovale or involving 1/3 to 2/3 of susceptible areas of cerebrum +/- moderate increase in SAS and/or moderate ventriculomegaly	Severe symptoms; extensive T2/FLAIR hyperintensities, involving periventricular white matter involving 2/3 or more of susceptible areas of cerebrum +/- moderate to severe increase in SAS and/or moderate to severe ventriculomegaly	Life-threatening consequences; extensive T2/FLAIR hyperintensities, involving periventricular white matter involving most of susceptible areas of cerebrum +/- moderate to severe increase in SAS and/or moderate to severe ventriculomegaly	Death
**Definition:** A disorder characterized by diffuse reactive astrocytosis with multiple areas of necrotic foci without inflammation.<br>**Navigational Note:** -					
Memory impairment	Mild memory impairment	Moderate memory impairment; limiting instrumental ADL	Severe memory impairment; limiting self care ADL	-	-
**Definition:** A disorder characterized by a deterioration in memory function.<br>**Navigational Note:** -					

Nervous system disorders					
**CTCAE Term**	**Grade 1**	**Grade 2**	**Grade 3**	**Grade 4**	**Grade 5**
Meningismus	Mild symptoms	Moderate symptoms; limiting instrumental ADL	Severe symptoms; limiting self care ADL	Life-threatening consequences; urgent intervention indicated	Death
**Definition:** A disorder characterized by neck stiffness, headache, and photophobia resulting from irritation of the cerebral meninges.					
**Navigational Note:** -					
Movements involuntary	Mild symptoms	Moderate symptoms; limiting instrumental ADL	Severe symptoms; limiting self care ADL	-	-
**Definition:** A disorder characterized by uncontrolled and purposeless movements.					
**Navigational Note:** -					
Muscle weakness left-sided	Symptomatic; perceived by patient but not evident on physical exam	Symptomatic; evident on physical exam; limiting instrumental ADL	Limiting self care ADL	-	-
**Definition:** A disorder characterized by a reduction in the strength of the muscles on the left side of the body.					
**Navigational Note:** -					
Muscle weakness right-sided	Symptomatic; perceived by patient but not evident on physical exam	Symptomatic; evident on physical exam; limiting instrumental ADL	Limiting self care ADL	-	-
**Definition:** A disorder characterized by a reduction in the strength of the muscles on the right side of the body.					
**Navigational Note:** -					
Myasthenia gravis	Asymptomatic or mild symptoms; clinical or diagnostic observations only; intervention not indicated	Moderate; minimal, local or noninvasive intervention indicated; limiting age-appropriate instrumental ADL	Severe or medically significant but not immediately life-threatening; hospitalization or prolongation of existing hospitalization indicated; limiting self care ADL	Life-threatening consequences; urgent intervention indicated	Death
**Definition:** A disorder characterized by weakness and rapid fatigue of any of the skeletal muscles.					
**Navigational Note:** -					
Neuralgia	Mild pain	Moderate pain; limiting instrumental ADL	Severe pain; limiting self care ADL	-	-
**Definition:** A disorder characterized by intense painful sensation along a nerve or group of nerves.					
**Navigational Note:** -					
Nystagmus	-	Moderate symptoms; limiting instrumental ADL	Severe symptoms; limiting self care ADL	-	-
**Definition:** A disorder characterized by involuntary movements of the eyeballs.					
**Navigational Note:** -					
Oculomotor nerve disorder	Asymptomatic; clinical or diagnostic observations only; intervention not indicated	Moderate symptoms; limiting instrumental ADL	Severe symptoms; limiting self care ADL	-	-
**Definition:** A disorder characterized by dysfunction of the oculomotor nerve (third cranial nerve).					
**Navigational Note:** -					

Nervous system disorders					
**CTCAE Term**	**Grade 1**	**Grade 2**	**Grade 3**	**Grade 4**	**Grade 5**
Olfactory nerve disorder	-	Moderate symptoms; limiting instrumental ADL	Severe symptoms; limiting self care ADL	-	-
**Definition:** A disorder characterized by dysfunction of the olfactory nerve (first cranial nerve).<br>**Navigational Note:** -					
Paresthesia	Mild symptoms	Moderate symptoms; limiting instrumental ADL	Severe symptoms; limiting self care ADL	-	-
**Definition:** A disorder characterized by functional disturbances of sensory neurons resulting in abnormal cutaneous sensations of tingling, numbness, pressure, cold, and/or warmth.<br>**Navigational Note:** -					
Peripheral motor neuropathy	Asymptomatic; clinical or diagnostic observations only	Moderate symptoms; limiting instrumental ADL	Severe symptoms; limiting self care ADL	Life-threatening consequences; urgent intervention indicated	Death
**Definition:** A disorder characterized by damage or dysfunction of the peripheral motor nerves.<br>**Navigational Note:** Also consider Nervous system disroders: Peripheral sensory neuropathy					
Peripheral sensory neuropathy	Asymptomatic	Moderate symptoms; limiting instrumental ADL	Severe symptoms; limiting self care ADL	Life-threatening consequences; urgent intervention indicated	-
**Definition:** A disorder characterized by damage or dysfunction of the peripheral sensory nerves.<br>**Navigational Note:** -					
Phantom pain	Mild pain	Moderate pain; limiting instrumental ADL	Severe pain; limiting self care ADL	-	-
**Definition:** A disorder characterized by a sensation of marked discomfort related to a limb or an organ that is removed from or is not physically part of the body.<br>**Navigational Note:** -					
Presyncope	-	Present (e.g., near fainting)	-	-	-
**Definition:** A disorder characterized by an episode of lightheadedness and dizziness which may precede an episode of syncope.<br>**Navigational Note:** -					
Pyramidal tract syndrome	Asymptomatic; clinical or diagnostic observations only; intervention not indicated	Moderate symptoms; limiting instrumental ADL	Severe symptoms; limiting self care ADL	Life-threatening consequences; urgent intervention indicated	Death
**Definition:** A disorder characterized by dysfunction of the corticospinal (pyramidal) tracts of the spinal cord. Symptoms include an increase in the muscle tone in the lower extremities, hyperreflexia, positive Babinski and a decrease in fine motor coordination.<br>**Navigational Note:** -					
Radiculitis	Mild symptoms	Moderate symptoms; medical intervention indicated; limiting instrumental ADL	Severe symptoms; limiting self care ADL	Life-threatening consequences; urgent intervention indicated	Death
**Definition:** A disorder characterized by inflammation involving a nerve root. Patients experience marked discomfort radiating along a nerve path because of spinal pressure on the connecting nerve root.<br>**Navigational Note:** -					

Nervous system disorders					
**CTCAE Term**	**Grade 1**	**Grade 2**	**Grade 3**	**Grade 4**	**Grade 5**
Recurrent laryngeal nerve palsy	Asymptomatic; clinical or diagnostic observations only; intervention not indicated	Moderate symptoms	Severe symptoms; medical intervention indicated (e.g., thyroplasty, vocal cord injection)	Life-threatening consequences; urgent intervention indicated	Death
**Definition:** A disorder characterized by paralysis of the recurrent laryngeal nerve.<br>**Navigational Note:** -					
Reversible posterior leukoencephalopathy syndrome	-	Moderate symptoms; limiting instrumental ADL	Severe symptoms; limiting self care ADL; hospitalization	Life-threatening consequences	Death
**Definition:** A disorder characterized by headaches, mental status changes, visual disturbances, and/or seizures associated with imaging findings of posterior leukoencephalopathy. It has been observed in association with hypertensive encephalopathy, eclampsia, and immunosuppressive and cytotoxic drug treatment. It is an acute or subacute reversible condition. Also known as posterior reversible encephalopathy syndrome (PRES).<br>**Navigational Note:** -					
Seizure	Brief partial seizure and no loss of consciousness	Brief generalized seizure	New onset seizures (partial or generalized); multiple seizures despite medical intervention	Life-threatening consequences; prolonged repetitive seizures	Death
**Definition:** A disorder characterized by a sudden, involuntary skeletal muscular contractions of cerebral or brain stem origin.<br>**Navigational Note:** -					
Somnolence	Mild but more than usual drowsiness or sleepiness	Moderate sedation; limiting instrumental ADL	Obtundation or stupor	Life-threatening consequences; urgent intervention indicated	Death
**Definition:** A disorder characterized by characterized by excessive sleepiness and drowsiness.<br>**Navigational Note:** -					
Spasticity	Mild or slight increase in muscle tone	Moderate increase in muscle tone and increase in resistance through range of motion	Severe increase in muscle tone and increase in resistance through range of motion	Life-threatening consequences; unable to move active or passive range of motion	Death
**Definition:** A disorder characterized by increased involuntary muscle tone that affects the regions interfering with voluntary movement. It results in gait, movement, and speech disturbances.<br>**Navigational Note:** -					
Spinal cord compression	-	-	Severe symptoms; limiting self care ADL	Life-threatening consequences; urgent intervention indicated	Death
**Definition:** A disorder characterized by pressure on the spinal cord.<br>**Navigational Note:** -					

Nervous system disorders					
**CTCAE Term**	**Grade 1**	**Grade 2**	**Grade 3**	**Grade 4**	**Grade 5**
Stroke	Incidental radiographic findings only	Mild to moderate neurologic deficit; limiting instrumental ADL	Severe neurologic deficit; limiting self care ADL; hospitalization	Life-threatening consequences; urgent intervention indicated	Death
**Definition:** A disorder characterized by a decrease or absence of blood supply to the brain caused by obstruction (thrombosis or embolism) of an artery resulting in neurological damage.<br>**Navigational Note:** -					
Syncope	-	-	Fainting; orthostatic collapse	-	-
**Definition:** A disorder characterized by spontaneous loss of consciousness caused by insufficient blood supply to the brain.<br>**Navigational Note:** -					
Tendon reflex decreased	Ankle reflex reduced	Ankle reflex absent; other reflexes reduced	Absence of all reflexes	-	-
**Definition:** A disorder characterized by less than normal deep tendon reflexes.<br>**Navigational Note:** Also consider Nervous system disorders: Peripheral motor neuropathy or Peripheral sensory neuropathy					
Transient ischemic attacks	Mild neurologic deficit with or without imaging confirmation	Moderate neurologic deficit with or without imaging confirmation	-	-	-
**Definition:** A disorder characterized by a brief attack (less than 24 hours) of cerebral dysfunction of vascular origin, with no persistent neurological deficit.<br>**Navigational Note:** If >24 hours, Consider Nervous system disorders: Stroke					
Tremor	Mild symptoms	Moderate symptoms; limiting instrumental ADL	Severe symptoms; limiting self care ADL	-	-
**Definition:** A disorder characterized by the uncontrolled shaking movement of the whole body or individual parts.<br>**Navigational Note:** -					
Trigeminal nerve disorder	Asymptomatic; clinical or diagnostic observations only; intervention not indicated	Moderate symptoms; limiting instrumental ADL	Severe symptoms; limiting self care ADL	-	-
**Definition:** A disorder characterized by dysfunction of the trigeminal nerve (fifth cranial nerve).<br>**Navigational Note:** -					
Trochlear nerve disorder	Asymptomatic; clinical or diagnostic observations only; intervention not indicated	Moderate symptoms; limiting instrumental ADL	Severe symptoms; limiting self care ADL	-	-
**Definition:** A disorder characterized by dysfunction of the trochlear nerve (fourth cranial nerve).<br>**Navigational Note:** -					
Vagus nerve disorder	Asymptomatic; clinical or diagnostic observations only; intervention not indicated	Moderate symptoms; limiting instrumental ADL	Severe symptoms; limiting self care ADL	Life-threatening consequences; urgent intervention indicated	Death
**Definition:** A disorder characterized by dysfunction of the vagus nerve (tenth cranial nerve).<br>**Navigational Note:** -					

Nervous system disorders					
**CTCAE Term**	**Grade 1**	**Grade 2**	**Grade 3**	**Grade 4**	**Grade 5**
Vasovagal reaction	-	-	Present	Life-threatening consequences; urgent intervention indicated	Death
**Definition:** A disorder characterized by a sudden drop of the blood pressure, bradycardia, and peripheral vasodilation that may lead to loss of consciousness. It results from an increase in the stimulation of the vagus nerve.<br>**Navigational Note:** -					
Nervous system disorders - Other, specify	Asymptomatic or mild symptoms; clinical or diagnostic observations only; intervention not indicated	Moderate; minimal, local or noninvasive intervention indicated; limiting age-appropriate instrumental ADL	Severe or medically significant but not immediately life-threatening; hospitalization or prolongation of existing hospitalization indicated; limiting self care ADL	Life-threatening consequences; urgent intervention indicated	Death
**Definition:** -<br>**Navigational Note:** -					

Pregnancy, puerperium and perinatal conditions					
**CTCAE Term**	**Grade 1**	**Grade 2**	**Grade 3**	**Grade 4**	**Grade 5**
Fetal growth retardation	-	<10% percentile of weight for gestational age	<5% percentile of weight for gestational age	<1% percentile of weight for gestational age	-
**Definition:** A disorder characterized by inhibition of fetal growth resulting in the inability of the fetus to achieve its potential weight.					
**Navigational Note:** -					
Pregnancy loss	-	-	-	Fetal loss at any gestational age	-
**Definition:** Death in utero.					
**Navigational Note:** -					
Premature delivery	Delivery of a liveborn infant at >34 to 37 weeks gestation	Delivery of a liveborn infant at >28 to 34 weeks gestation	Delivery of a liveborn infant at 24 to 28 weeks gestation	Delivery of a liveborn infant at 24 weeks of gestation or less	-
**Definition:** A disorder characterized by delivery of a viable infant before the normal end of gestation. Typically, viability is achievable between the twentieth and thirty-seventh week of gestation.					
**Navigational Note:** -					
Pregnancy, puerperium and perinatal conditions - Other, specify	Asymptomatic or mild symptoms; clinical or diagnostic observations only; intervention not indicated	Moderate; minimal, local or noninvasive intervention indicated; limiting age-appropriate instrumental ADL	Severe or medically significant but not immediately life-threatening; hospitalization or prolongation of existing hospitalization indicated; limiting self care ADL	Life-threatening consequences; urgent intervention indicated	Death
**Definition:** -					
**Navigational Note:** -					

Psychiatric disorders					
**CTCAE Term**	**Grade 1**	**Grade 2**	**Grade 3**	**Grade 4**	**Grade 5**
Agitation	Mild mood alteration	Moderate mood alteration	Severe agitation; hospitalization not indicated	Life-threatening consequences; urgent intervention indicated	-
**Definition:** A disorder characterized by a state of restlessness associated with unpleasant feelings of irritability and tension.					
**Navigational Note:** -					
Anorgasmia	Inability to achieve orgasm not adversely affecting relationship	Inability to achieve orgasm adversely affecting relationship	-	-	-
**Definition:** A disorder characterized by an inability to achieve orgasm.					
**Navigational Note:** -					
Anxiety	Mild symptoms; intervention not indicated	Moderate symptoms; limiting instrumental ADL	Severe symptoms; limiting self care ADL; hospitalization indicated	Life-threatening consequences; urgent intervention indicated	-
**Definition:** A disorder characterized by apprehension of danger and dread accompanied by restlessness, tension, tachycardia, and dyspnea unattached to a clearly identifiable stimulus.					
**Navigational Note:** -					
Confusion	Mild disorientation	Moderate disorientation; limiting instrumental ADL	Severe disorientation; limiting self care ADL	Life-threatening consequences; urgent intervention indicated	-
**Definition:** A disorder characterized by a lack of clear and orderly thought and behavior.					
**Navigational Note:** -					
Delayed orgasm	Delay in achieving orgasm not adversely affecting relationship	Delay in achieving orgasm adversely affecting relationship	-	-	-
**Definition:** A disorder characterized by sexual dysfunction characterized by a delay in climax.					
**Navigational Note:** -					
Delirium	Mild acute confusional state	Moderate and acute confusional state; limiting instrumental ADL	Severe and acute confusional state; limiting self care ADL; urgent intervention indicated; new onset	Life-threatening consequences, threats of harm to self or others; urgent intervention indicated	Death
**Definition:** A disorder characterized by the acute and sudden development of confusion, illusions, movement changes, inattentiveness, agitation, and hallucinations. Usually, it is a reversible condition.					
**Navigational Note:** -					
Delusions	-	Moderate delusional symptoms	Severe delusional symptoms; hospitalization not indicated; new onset	Life-threatening consequences, threats of harm to self or others; hospitalization indicated	Death
**Definition:** A disorder characterized by false personal beliefs held contrary to reality, despite contradictory evidence and common sense.					
**Navigational Note:** -					

Psychiatric disorders					
**CTCAE Term**	**Grade 1**	**Grade 2**	**Grade 3**	**Grade 4**	**Grade 5**
Depression	Mild depressive symptoms	Moderate depressive symptoms; limiting instrumental ADL	Severe depressive symptoms; limiting self care ADL; hospitalization not indicated	Life-threatening consequences, threats of harm to self or others; hospitalization indicated	Death
**Definition:** A disorder characterized by melancholic feelings of grief or unhappiness.<br>**Navigational Note:** -					
Euphoria	Mild mood elevation	Moderate mood elevation	Severe mood elevation (e.g., hypomania)	-	-
**Definition:** A disorder characterized by an exaggerated feeling of well-being which is disproportionate to events and stimuli.<br>**Navigational Note:** -					
Hallucinations	Mild hallucinations (e.g., perceptual distortions)	Moderate hallucinations	Severe hallucinations; hospitalization not indicated	Life-threatening consequences, threats of harm to self or others; hospitalization indicated	Death
**Definition:** A disorder characterized by a false sensory perception in the absence of an external stimulus.<br>**Navigational Note:** -					
Insomnia	Mild difficulty falling asleep, staying asleep or waking up early	Moderate difficulty falling asleep, staying asleep or waking up early	Severe difficulty in falling asleep, staying asleep or waking up early	-	-
**Definition:** A disorder characterized by difficulty in falling asleep and/or remaining asleep.<br>**Navigational Note:** -					
Irritability	Mild; easily consolable	Moderate; limiting instrumental ADL; increased attention indicated	Severe abnormal or excessive response; limiting self care ADL; inconsolable; medical or psychiatric intervention indicated	-	-
**Definition:** A disorder characterized by an abnormal responsiveness to stimuli or physiological arousal; may be in response to pain, fright, a drug, an emotional situation or a medical condition.<br>**Navigational Note:** -					
Libido decreased	Decrease in sexual interest not adversely affecting relationship	Decrease in sexual interest adversely affecting relationship	-	-	-
**Definition:** A disorder characterized by a decrease in sexual desire.<br>**Navigational Note:** -					
Libido increased	Present	-	-	-	-
**Definition:** A disorder characterized by an increase in sexual desire.<br>**Navigational Note:** -					

Psychiatric disorders					
**CTCAE Term**	**Grade 1**	**Grade 2**	**Grade 3**	**Grade 4**	**Grade 5**
Mania	Mild manic symptoms (e.g., elevated mood, rapid thoughts, rapid speech, decreased need for sleep)	Moderate manic symptoms (e.g., relationship and work difficulties; poor hygiene)	Severe manic symptoms (e.g., hypomania; major sexual or financial indiscretions); hospitalization not indicated; new onset	Life-threatening consequences, threats of harm to self or others; hospitalization indicated	Death
**Definition:** A disorder characterized by excitement of psychotic proportions manifested by mental and physical hyperactivity, disorganization of behavior and elevation of mood.<br>**Navigational Note:** -					
Personality change	Mild personality change	Moderate personality change	Severe personality change; hospitalization not indicated	Life-threatening consequences, threats of harm to self or others; hospitalization indicated	-
**Definition:** A disorder characterized by a conspicuous change in a person's behavior and thinking.<br>**Navigational Note:** -					
Psychosis	Mild psychotic symptoms	Moderate psychotic symptoms (e.g., disorganized speech; impaired reality testing)	Severe psychotic symptoms (e.g., paranoid, extreme disorganization); hospitalization not indicated; new onset	Life-threatening consequences, threats of harm to self or others; hospitalization indicated	Death
**Definition:** A disorder characterized by personality change, impaired functioning, and loss of touch with reality. It may be a manifestation of schizophrenia, bipolar disorder or brain tumor.<br>**Navigational Note:** -					
Restlessness	Mild symptoms; intervention not indicated	Moderate symptoms; limiting instrumental ADL	Severe symptoms; limiting self care ADL	-	-
**Definition:** A disorder characterized by an inability to rest, relax or be still.<br>**Navigational Note:** -					
Suicidal ideation	Increased thoughts of death but no wish to kill oneself	Suicidal ideation with no specific plan or intent	Specific plan to commit suicide without serious intent to die which may not require hospitalization	Specific plan to commit suicide with serious intent to die which requires hospitalization	-
**Definition:** A disorder characterized by thoughts of taking one's own life.<br>**Navigational Note:** -					
Suicide attempt	-	-	Suicide attempt or gesture without intent to die	Suicide attempt with intent to die which requires hospitalization	Death
**Definition:** A disorder characterized by self-inflicted harm in an attempt to end one's own life.<br>**Navigational Note:** -					

Psychiatric disorders					
**CTCAE Term**	**Grade 1**	**Grade 2**	**Grade 3**	**Grade 4**	**Grade 5**
Psychiatric disorders - Other, specify	Asymptomatic or mild symptoms; clinical or diagnostic observations only; intervention not indicated	Moderate; minimal, local or noninvasive intervention indicated; limiting age-appropriate instrumental ADL	Severe or medically significant but not immediately life-threatening; limiting self care ADL	Life-threatening consequences; hospitalization or urgent intervention indicated	Death
**Definition:** -					
**Navigational Note:** -					

Renal and urinary disorders					
**CTCAE Term**	**Grade 1**	**Grade 2**	**Grade 3**	**Grade 4**	**Grade 5**
Acute kidney injury	-	-	Hospitalization indicated	Life-threatening consequences; dialysis indicated	Death
**Definition:** A disorder characterized by the acute loss of renal function (within 2 weeks) and is traditionally classified as pre-renal (low blood flow into kidney), renal (kidney damage) and post-renal causes (ureteral or bladder outflow obstruction).					
**Navigational Note:** Also consider Investigations: Creatinine increased					
Bladder perforation	-	Invasive intervention not indicated	Invasive intervention indicated	Life-threatening consequences; organ failure; urgent operative intervention indicated	Death
**Definition:** A disorder characterized by a rupture in the bladder wall.					
**Navigational Note:** -					
Bladder spasm	Intervention not indicated	Antispasmodics indicated	Hospitalization indicated	-	-
**Definition:** A disorder characterized by a sudden and involuntary contraction of the bladder wall.					
**Navigational Note:** -					
Chronic kidney disease	eGFR (estimated Glomerular Filtration Rate) or CrCl (creatinine clearance) <LLN - 60 ml/min/1.73 m2 or proteinuria 2+ present; urine protein/creatinine >0.5	eGFR or CrCl 59 - 30 ml/min/1.73 m2	eGFR or CrCl 29 - 15 ml/min/1.73 m2	eGFR or CrCl <15 ml/min/1.73 m2; dialysis or renal transplant indicated	Death
**Definition:** A disorder characterized by gradual and usually permanent loss of kidney function resulting in renal failure.					
**Navigational Note:** -					
Cystitis noninfective	Microscopic hematuria; minimal increase in frequency, urgency, dysuria, or nocturia; new onset of incontinence	Moderate hematuria; moderate increase in frequency, urgency, dysuria, nocturia or incontinence; urinary catheter placement or bladder irrigation indicated; limiting instrumental ADL	Gross hematuria; transfusion, IV medications, or hospitalization indicated; elective invasive intervention indicated	Life-threatening consequences; urgent invasive intervention indicated	Death
**Definition:** A disorder characterized by inflammation of the bladder which is not caused by an infection of the urinary tract.					
**Navigational Note:** -					
Dysuria	Present	-	-	-	-
**Definition:** A disorder characterized by painful urination.					
**Navigational Note:** If associated with an infection, report the infection. For grades higher than Grade 1, consider Renal and urinary disorders: Bladder spasm or Cystitis noninfective; Infections and infestations: Urinary tract infection.					
Glucosuria	Present	-	-	-	-
**Definition:** A disorder characterized by laboratory test results that indicate glucose in the urine.					
**Navigational Note:** -					

Renal and urinary disorders					
**CTCAE Term**	**Grade 1**	**Grade 2**	**Grade 3**	**Grade 4**	**Grade 5**
Hematuria	Asymptomatic; clinical or diagnostic observations only; intervention not indicated	Symptomatic; urinary catheter or bladder irrigation indicated; limiting instrumental ADL	Gross hematuria; transfusion, IV medications, or hospitalization indicated; elective invasive intervention indicated; limiting self care ADL	Life-threatening consequences; urgent invasive intervention indicated	Death
**Definition:** A disorder characterized by laboratory test results that indicate blood in the urine.<br>**Navigational Note:** -					
Hemoglobinuria	Asymptomatic; clinical or diagnostic observations only; intervention not indicated	-	-	-	-
**Definition:** A disorder characterized by laboratory test results that indicate the presence of free hemoglobin in the urine.<br>**Navigational Note:** Report underlying AE if > Grade 1					
Nephrotic syndrome	-	-	Not immediately life-threatening; hospitalization or prolongation of existing hospitalization indicated; limiting self care ADL	Life-threatening consequences; urgent intervention indicated	Death
**Definition:** A disorder characterized by symptoms that include severe edema, proteinuria, and hypoalbuminemia; it is indicative of renal dysfunction.<br>**Navigational Note:** -					
Proteinuria	1+ proteinuria; urinary protein ≥ULN - <1.0 g/24 hrs	**Adult:** 2+ and 3+ proteinuria; urinary protein 1.0 - <3.5 g/24 hrs;<br><br>**Pediatric:** Urine P/C (Protein/Creatinine) ratio 0.5 - 1.9	**Adult:** Urinary protein >=3.5 g/24 hrs; 4+ proteinuria;<br><br>**Pediatric:** Urine P/C (Protein/Creatinine) ratio >1.9	-	-
**Definition:** A disorder characterized by laboratory test results that indicate the presence of excessive protein in the urine. It is predominantly albumin, but also globulin.<br>**Navigational Note:** 24-hour urine collection takes precedence over dipstick					
Renal calculi	Asymptomatic or mild symptoms; occasional use of nonprescription analgesics indicated	Symptomatic; oral antiemetics indicated; around the clock nonprescription analgesics or any oral narcotic analgesics indicated	Hospitalization indicated; IV intervention (e.g., analgesics, antiemetics); elective invasive intervention indicated	Life-threatening consequences; urgent invasive intervention indicated	Death
**Definition:** A disorder characterized by the formation of crystals/kidney stones in the pelvis of the kidney.<br>**Navigational Note:** -					

Renal and urinary disorders					
**CTCAE Term**	**Grade 1**	**Grade 2**	**Grade 3**	**Grade 4**	**Grade 5**
Renal colic	Mild pain not interfering with activity; nonprescription medication indicated	Moderate pain; limiting instrumental ADL; prescription medication indicated	Hospitalization indicated; limiting self care ADL	-	-
**Definition:** A disorder characterized by paroxysmal and severe flank marked discomfort radiating to the inguinal area. Often, the cause is the passage of crystals/kidney stones.					
**Navigational Note:** -					
Renal hemorrhage	Mild symptoms; intervention not indicated	Analgesics and hematocrit monitoring indicated	Transfusion indicated; invasive intervention indicated; hospitalization	Life-threatening consequences; urgent intervention indicated	Death
**Definition:** A disorder characterized by bleeding from the kidney.					
**Navigational Note:** -					
Urinary fistula	-	Symptomatic, invasive intervention not indicated	Invasive intervention indicated	Life-threatening consequences; urgent invasive intervention indicated	Death
**Definition:** A disorder characterized by an abnormal communication between any part of the urinary system and another organ or anatomic site.					
**Navigational Note:** -					
Urinary frequency	Present	Limiting instrumental ADL; medical management indicated	-	-	-
**Definition:** A disorder characterized by urination at short intervals.					
**Navigational Note:** -					
Urinary incontinence	Occasional (e.g., with coughing, sneezing, etc.), pads not indicated	Spontaneous; pads indicated; limiting instrumental ADL	Intervention indicated (e.g., clamp, collagen injections); operative intervention indicated; limiting self care ADL	-	-
**Definition:** A disorder characterized by inability to control the flow of urine from the bladder.					
**Navigational Note:** -					
Urinary retention	Urinary, suprapubic or intermittent catheter placement not indicated; able to void with some residual	Placement of urinary, suprapubic or intermittent catheter placement indicated; medication indicated	Elective invasive intervention indicated; substantial loss of affected kidney function or mass	Life-threatening consequences; organ failure; urgent operative intervention indicated	Death
**Definition:** A disorder characterized by accumulation of urine within the bladder because of the inability to urinate.					
**Navigational Note:** -					

Renal and urinary disorders					
**CTCAE Term**	**Grade 1**	**Grade 2**	**Grade 3**	**Grade 4**	**Grade 5**
Urinary tract obstruction	Asymptomatic; clinical or diagnostic observations only; intervention not indicated	Symptomatic but no hydronephrosis, sepsis, or renal dysfunction; urethral dilation, urinary or suprapubic catheter indicated	Altered organ function (e.g., hydronephrosis or renal dysfunction); invasive intervention indicated	Life-threatening consequences; urgent intervention indicated	Death
**Definition:** A disorder characterized by blockage of the normal flow of contents of the urinary tract.<br>**Navigational Note:** -					
Urinary tract pain	Mild pain	Moderate pain; limiting instrumental ADL	Severe pain; limiting self care ADL	-	-
**Definition:** A disorder characterized by a sensation of marked discomfort in the urinary tract.<br>**Navigational Note:** -					
Urinary urgency	Present	Limiting instrumental ADL; medical management indicated	-	-	-
**Definition:** A disorder characterized by a sudden compelling urge to urinate.<br>**Navigational Note:** -					
Urine discoloration	Present	-	-	-	-
**Definition:** A disorder characterized by a change in the color of the urine.<br>**Navigational Note:** -					
Renal and urinary disorders - Other, specify	Asymptomatic or mild symptoms; clinical or diagnostic observations only; intervention not indicated	Moderate; minimal, local or noninvasive intervention indicated; limiting age-appropriate instrumental ADL	Severe or medically significant but not immediately life-threatening; hospitalization or prolongation of existing hospitalization indicated; limiting self care ADL	Life-threatening consequences; urgent intervention indicated	Death
**Definition:** -<br>**Navigational Note:** -					

Reproductive system and breast disorders					
**CTCAE Term**	**Grade 1**	**Grade 2**	**Grade 3**	**Grade 4**	**Grade 5**
Amenorrhea	-	Present	-	-	-
**Definition:** A disorder characterized by the abnormal absence of menses for at least three consecutive menstrual cycles.					
**Navigational Note:** -					
Azoospermia	-	Absence of sperm in ejaculate	-	-	-
**Definition:** A disorder characterized by laboratory test results that indicate complete absence of spermatozoa in the semen.					
**Navigational Note:** -					
Breast atrophy	Minimal asymmetry; minimal atrophy	Moderate asymmetry; moderate atrophy	Asymmetry >1/3 of breast volume; severe atrophy	-	-
**Definition:** A disorder characterized by underdevelopment of the breast.					
**Navigational Note:** -					
Breast pain	Mild pain	Moderate pain; limiting instrumental ADL	Severe pain; limiting self care ADL	-	-
**Definition:** A disorder characterized by a sensation of marked discomfort in the breast region.					
**Navigational Note:** -					
Dysmenorrhea	Mild symptoms; intervention not indicated	Moderate symptoms; limiting instrumental ADL	Severe symptoms; limiting self care ADL	-	-
**Definition:** A disorder characterized by abnormally painful abdominal cramps during menses.					
**Navigational Note:** -					
Dyspareunia	Mild discomfort or pain associated with vaginal penetration; discomfort relieved with use of vaginal lubricants or estrogen	Moderate discomfort or pain associated with vaginal penetration; discomfort or pain partially relieved with use of vaginal lubricants or estrogen	Severe discomfort or pain associated with vaginal penetration; discomfort or pain unrelieved by vaginal lubricants or estrogen	-	-
**Definition:** A disorder characterized by painful or difficult coitus.					
**Navigational Note:** -					
Ejaculation disorder	Diminished ejaculation	Anejaculation or retrograde ejaculation	-	-	-
**Definition:** A disorder characterized by problems related to ejaculation. This category includes premature, delayed, retrograde and painful ejaculation.					
**Navigational Note:** -					

Reproductive system and breast disorders					
**CTCAE Term**	**Grade 1**	**Grade 2**	**Grade 3**	**Grade 4**	**Grade 5**
Erectile dysfunction	Decrease in erectile function (frequency or rigidity of erections) but intervention not indicated (e.g., medication or use of mechanical device, penile pump)	Decrease in erectile function (frequency/rigidity of erections), erectile intervention indicated, (e.g., medication or mechanical devices such as penile pump)	Decrease in erectile function (frequency/rigidity of erections) but erectile intervention not helpful (e.g., medication or mechanical devices such as penile pump); placement of a permanent penile prosthesis indicated (not previously present)	-	-
**Definition:** A disorder characterized by the persistent or recurrent inability to achieve or to maintain an erection during sexual activity.<br>**Navigational Note:** -					
Fallopian tube obstruction	Asymptomatic; clinical or diagnostic observations only; intervention not indicated	Symptomatic; elective intervention indicated	Severe symptoms; invasive intervention indicated	-	-
**Definition:** A disorder characterized by blockage of the normal flow of the contents in the fallopian tube.<br>**Navigational Note:** -					
Feminization acquired	Mild symptoms; intervention not indicated	Moderate symptoms; medical intervention indicated	-	-	-
**Definition:** A disorder characterized by the development of secondary female sex characteristics in males due to extrinsic factors.<br>**Navigational Note:** -					
Genital edema	Mild swelling or obscuration of anatomic architecture on close inspection	Readily apparent obscuration of anatomic architecture; obliteration of skin folds; readily apparent deviation from normal anatomic contour	Lymphorrhea; gross deviation from normal anatomic contour; limiting self care ADL	-	-
**Definition:** A disorder characterized by swelling due to an excessive accumulation of fluid in the genitals.<br>**Navigational Note:** -					
Gynecomastia	Asymptomatic	Symptomatic (e.g., pain or psychosocial impact)	Severe symptoms; elective operative intervention indicated	-	-
**Definition:** A disorder characterized by excessive development of the breasts in males.<br>**Navigational Note:** -					
Hematosalpinx	Minimal bleeding identified on imaging study or laparoscopy; intervention not indicated	Moderate bleeding; medical intervention indicated	Transfusion indicated; invasive intervention indicated	Life-threatening consequences; urgent operative intervention indicated	Death
**Definition:** A disorder characterized by the presence of blood in a fallopian tube.<br>**Navigational Note:** -					

Reproductive system and breast disorders					
**CTCAE Term**	**Grade 1**	**Grade 2**	**Grade 3**	**Grade 4**	**Grade 5**
Irregular menstruation	Intermittent/irregular menses for no more than 3 consecutive menstrual cycles	Intermittent/irregular menses for more than 3 consecutive menstrual cycles	-	-	-
**Definition:** A disorder characterized by a change in cycle or duration of menses from baseline.					
**Navigational Note:** Also consider Reproductive system and breast disorders: Premature menopause, Amenorrhea.					
Lactation disorder	Mild changes in lactation, not significantly affecting production or expression of breast milk	Changes in lactation, significantly affecting breast production or expression of breast milk	-	-	-
**Definition:** A disorder characterized by disturbances of milk secretion. It is not necessarily related to pregnancy that is observed in females and can be observed in males.					
**Navigational Note:** -					
Menorrhagia	Mild; iron supplements indicated	Moderate symptoms; medical intervention indicated (e.g., hormones)	Severe; transfusion indicated; operative intervention indicated (e.g., hysterectomy)	Life-threatening consequences; urgent intervention indicated	Death
**Definition:** A disorder characterized by abnormally heavy vaginal bleeding during menses.					
**Navigational Note:** -					
Nipple deformity	Asymptomatic; asymmetry with slight retraction and/or thickening of the nipple areolar complex	Symptomatic; asymmetry of nipple areolar complex with moderate retraction and/or thickening of the nipple areolar complex	-	-	-
**Definition:** A disorder characterized by a malformation of the nipple.					
**Navigational Note:** -					
Oligospermia	Sperm concentration > 0 to < 15 million/ml	-	-	-	-
**Definition:** A disorder characterized by a decrease in the number of spermatozoa in the semen.					
**Navigational Note:** -					
Ovarian hemorrhage	Mild symptoms; intervention not indicated	Moderate symptoms; intervention indicated	Transfusion indicated; invasive intervention indicated; hospitalization	Life-threatening consequences; urgent intervention indicated	Death
**Definition:** A disorder characterized by bleeding from the ovary.					
**Navigational Note:** -					
Ovarian rupture	Asymptomatic clinical or diagnostic observations only; intervention not indicated	Symptomatic and intervention not indicated	Transfusion; invasive intervention indicated	Life-threatening consequences; urgent intervention indicated	Death
**Definition:** A disorder characterized by tearing or disruption of the ovarian tissue.					
**Navigational Note:** -					

Reproductive system and breast disorders					
**CTCAE Term**	**Grade 1**	**Grade 2**	**Grade 3**	**Grade 4**	**Grade 5**
Ovulation pain	-	Present	-	-	-
**Definition:** A disorder characterized by a sensation of marked discomfort in one side of the abdomen between menstrual cycles, around the time of the discharge of the ovum from the ovarian follicle.<br>**Navigational Note:** -					
Pelvic floor muscle weakness	Asymptomatic; clinical or diagnostic observations only; intervention not indicated	Symptomatic, not interfering with bladder, bowel, or vaginal function; limiting instrumental ADL	Severe symptoms; limiting self care ADL	-	-
**Definition:** A disorder characterized by a reduction in the strength of the muscles of the pelvic floor.<br>**Navigational Note:** -					
Pelvic pain	Mild pain	Moderate pain; limiting instrumental ADL	Severe pain; limiting self care ADL	-	-
**Definition:** A disorder characterized by a sensation of marked discomfort in the pelvis.<br>**Navigational Note:** -					
Penile pain	Mild pain	Moderate pain; limiting instrumental ADL	Severe pain; limiting self care ADL	-	-
**Definition:** A disorder characterized by a sensation of marked discomfort in the penis.<br>**Navigational Note:** -					
Perineal pain	Mild pain	Moderate pain; limiting instrumental ADL	Severe pain; limiting self care ADL	-	-
**Definition:** A disorder characterized by a sensation of marked discomfort in the area between the genital organs and the anus.<br>**Navigational Note:** -					
Premature menopause	-	Present	-	-	-
**Definition:** A disorder characterized by premature ovarian failure. Symptoms may include hot flashes, night sweats, mood swings, and a decrease in sex drive. Laboratory findings include elevated luteinizing hormone (LH) and follicle-stimulating hormone (FSH.)<br>**Navigational Note:** -					
Prostatic hemorrhage	Mild symptoms; intervention not indicated	Moderate symptoms; intervention indicated	Transfusion indicated; invasive intervention indicated; hospitalization	Life-threatening consequences; urgent intervention indicated	Death
**Definition:** A disorder characterized by bleeding from the prostate gland.<br>**Navigational Note:** -					
Prostatic obstruction	Asymptomatic; clinical or diagnostic observations only; intervention not indicated	Symptomatic; elective intervention indicated	Severe symptoms; invasive intervention indicated	-	-
**Definition:** A disorder characterized by compression of the urethra secondary to enlargement of the prostate gland. This results in voiding difficulties (straining to void, slow urine stream, and incomplete emptying of the bladder).<br>**Navigational Note:** -					

Reproductive system and breast disorders					
**CTCAE Term**	**Grade 1**	**Grade 2**	**Grade 3**	**Grade 4**	**Grade 5**
Prostatic pain	Mild pain	Moderate pain; limiting instrumental ADL	Severe pain; limiting self care ADL	-	-
**Definition:** A disorder characterized by a sensation of marked discomfort in the prostate gland.<br>**Navigational Note:** -					
Scrotal pain	Mild pain	Moderate pain; limiting instrumental ADL	Severe pain; limiting self care ADL	-	-
**Definition:** A disorder characterized by a sensation of marked discomfort in the scrotal area.<br>**Navigational Note:** -					
Spermatic cord hemorrhage	Mild symptoms; intervention not indicated	Moderate symptoms; intervention indicated	Transfusion indicated; invasive intervention indicated; hospitalization	Life-threatening consequences; urgent intervention indicated	Death
**Definition:** A disorder characterized by bleeding from the spermatic cord.<br>**Navigational Note:** -					
Spermatic cord obstruction	Asymptomatic; clinical or diagnostic observations only; intervention not indicated	Symptomatic; elective intervention indicated	Severe symptoms; invasive intervention indicated	-	-
**Definition:** A disorder characterized by blockage of the normal flow of the contents of the spermatic cord.<br>**Navigational Note:** -					
Testicular disorder	Asymptomatic; clinical or diagnostic observations only; intervention not indicated	Symptomatic but not interfering with sexual function; intervention not indicated; limiting instrumental ADL	Severe symptoms; interfering with sexual function; limiting self care ADL; intervention indicated	Life-threatening consequences; urgent intervention indicated	-
**Definition:** A disorder characterized by abnormal function or appearance of the testis.<br>**Navigational Note:** Also consider Reproductive system and breast disorders: Genital edema or other AE terms in the Renal and urinary disorders SOC or Reproductive system and breast disorders SOC.					
Testicular hemorrhage	Mild symptoms; intervention not indicated	Moderate symptoms; intervention indicated	Transfusion indicated; invasive intervention indicated; hospitalization	Life-threatening consequences; urgent intervention indicated	Death
**Definition:** A disorder characterized by bleeding from the testis.<br>**Navigational Note:** -					
Testicular pain	Mild pain	Moderate pain; limiting instrumental ADL	Severe pain; limiting self care ADL	-	-
**Definition:** A disorder characterized by a sensation of marked discomfort in the testis.<br>**Navigational Note:** -					
Uterine fistula	Asymptomatic	Symptomatic, invasive intervention not indicated	Invasive intervention indicated	Life-threatening consequences; urgent intervention indicated	Death
**Definition:** A disorder characterized by an abnormal communication between the uterus and another organ or anatomic site.<br>**Navigational Note:** -					

Reproductive system and breast disorders					
**CTCAE Term**	**Grade 1**	**Grade 2**	**Grade 3**	**Grade 4**	**Grade 5**
Uterine hemorrhage	Mild symptoms; intervention not indicated	Moderate symptoms; intervention indicated	Transfusion indicated; invasive intervention indicated; hospitalization	Life-threatening consequences; urgent intervention indicated	Death
**Definition:** A disorder characterized by bleeding from the uterus.<br>**Navigational Note:** -					
Uterine obstruction	Asymptomatic; clinical or diagnostic observations only; intervention not indicated	Symptomatic; elective intervention indicated	Severe symptoms; invasive intervention indicated	-	-
**Definition:** A disorder characterized by blockage of the uterine outlet.<br>**Navigational Note:** -					
Uterine pain	Mild pain	Moderate pain; limiting instrumental ADL	Severe pain; limiting self care ADL	-	-
**Definition:** A disorder characterized by a sensation of marked discomfort in the uterus.<br>**Navigational Note:** -					
Vaginal discharge	Mild vaginal discharge (greater than baseline for patient)	Moderate to heavy vaginal discharge; use of perineal pad or tampon indicated	-	-	-
**Definition:** A disorder characterized by vaginal secretions. Mucus produced by the cervical glands is discharged from the vagina naturally, especially during the childbearing years.<br>**Navigational Note:** -					
Vaginal dryness	Mild vaginal dryness not interfering with sexual function	Moderate vaginal dryness interfering with sexual function or causing frequent discomfort	Severe vaginal dryness resulting in dyspareunia or severe discomfort	-	-
**Definition:** A disorder characterized by an uncomfortable feeling of itching and burning in the vagina.<br>**Navigational Note:** -					
Vaginal fistula	Asymptomatic	Symptomatic, invasive intervention not indicated	Invasive intervention indicated	Life-threatening consequences; urgent intervention indicated	Death
**Definition:** A disorder characterized by an abnormal communication between the vagina and another organ or anatomic site.<br>**Navigational Note:** -					
Vaginal hemorrhage	Mild symptoms; intervention not indicated	Moderate symptoms; intervention indicated	Transfusion indicated; invasive intervention indicated; hospitalization	Life-threatening consequences; urgent intervention indicated	Death
**Definition:** A disorder characterized by bleeding from the vagina.<br>**Navigational Note:** -					

Reproductive system and breast disorders					
**CTCAE Term**	**Grade 1**	**Grade 2**	**Grade 3**	**Grade 4**	**Grade 5**
Vaginal inflammation	Mild discomfort or pain, edema, or redness	Moderate discomfort or pain, edema, or redness; limiting instrumental ADL	Severe discomfort or pain, edema, or redness; limiting self care ADL; small areas of mucosal ulceration	Life-threatening consequences; widespread areas of mucosal ulceration; urgent intervention indicated	-
**Definition:** A disorder characterized by inflammation involving the vagina. Symptoms may include redness, edema, marked discomfort and an increase in vaginal discharge.					
**Navigational Note:** -					
Vaginal obstruction	Asymptomatic; clinical or diagnostic observations only; intervention not indicated	Symptomatic; elective intervention indicated	Severe symptoms; invasive intervention indicated	-	-
**Definition:** A disorder characterized by blockage of vaginal canal.					
**Navigational Note:** -					
Vaginal pain	Mild pain	Moderate pain; limiting instrumental ADL	Severe pain; limiting self care ADL	-	-
**Definition:** A disorder characterized by a sensation of marked discomfort in the vagina.					
**Navigational Note:** -					
Vaginal perforation	-	Invasive intervention not indicated	Invasive intervention indicated	Life-threatening consequences; urgent intervention indicated	Death
**Definition:** A disorder characterized by a rupture in the vaginal wall.					
**Navigational Note:** -					
Vaginal stricture	Asymptomatic; mild vaginal shortening or narrowing	Vaginal narrowing and/or shortening not interfering with physical examination	Vaginal narrowing and/or shortening interfering with the use of tampons, sexual activity or physical examination	-	Death
**Definition:** A disorder characterized by a narrowing of the vaginal canal.					
**Navigational Note:** -					
Reproductive system and breast disorders - Other, specify	Asymptomatic or mild symptoms; clinical or diagnostic observations only; intervention not indicated	Moderate; minimal, local or noninvasive intervention indicated; limiting age-appropriate instrumental ADL	Severe or medically significant but not immediately life-threatening; hospitalization or prolongation of existing hospitalization indicated; limiting self care ADL	Life-threatening consequences; urgent intervention indicated	Death
**Definition:** -					
**Navigational Note:** -					

Respiratory, thoracic and mediastinal disorders					
**CTCAE Term**	**Grade 1**	**Grade 2**	**Grade 3**	**Grade 4**	**Grade 5**
Adult respiratory distress syndrome	-	-	Present with radiologic findings; intubation not indicated	Life-threatening respiratory or hemodynamic compromise; intubation or urgent intervention indicated	Death
**Definition:** A disorder characterized by progressive and life-threatening pulmonary distress in the absence of an underlying pulmonary condition, usually following major trauma or surgery.<br>**Navigational Note:** -					
Allergic rhinitis	Mild symptoms; intervention not indicated	Moderate symptoms; medical intervention indicated	-	-	-
**Definition:** A disorder characterized by an inflammation of the nasal mucous membranes caused by an IgE-mediated response to external allergens. The inflammation may also involve the mucous membranes of the sinuses, eyes, middle ear, and pharynx. Symptoms include sneezing, nasal congestion, rhinorrhea and itching.<br>**Navigational Note:** -					
Apnea	-	-	Present; medical intervention indicated	Life-threatening respiratory or hemodynamic compromise; intubation or urgent intervention indicated	Death
**Definition:** A disorder characterized by cessation of breathing.<br>**Navigational Note:** -					
Aspiration	Asymptomatic; clinical or diagnostic observations only; intervention not indicated	Altered eating habits; coughing or choking episodes after eating or swallowing; medical intervention indicated (e.g., suction or oxygen)	Dyspnea and pneumonia symptoms (e.g., aspiration pneumonia); hospitalization indicated; unable to aliment orally	Life-threatening respiratory or hemodynamic compromise; intubation or urgent intervention indicated	Death
**Definition:** A disorder characterized by inhalation of solids or liquids into the lungs.<br>**Navigational Note:** -					
Atelectasis	Asymptomatic; clinical or diagnostic observations only; intervention not indicated	Symptomatic (e.g., dyspnea, cough); medical intervention indicated (e.g., chest physiotherapy, suctioning); bronchoscopic suctioning	Supplemental oxygen indicated; hospitalization or elective operative intervention indicated (e.g., stent, laser)	Life-threatening respiratory or hemodynamic compromise; intubation or urgent intervention indicated	Death
**Definition:** A disorder characterized by the collapse of part or the entire lung.<br>**Navigational Note:** -					
Bronchial fistula	Asymptomatic	Symptomatic, invasive intervention not indicated	Invasive intervention indicated	Life-threatening consequences; urgent intervention indicated	Death
**Definition:** A disorder characterized by an abnormal communication between the bronchus and another organ or anatomic site.<br>**Navigational Note:** -					

Respiratory, thoracic and mediastinal disorders					
**CTCAE Term**	**Grade 1**	**Grade 2**	**Grade 3**	**Grade 4**	**Grade 5**
Bronchial obstruction	Asymptomatic; clinical or diagnostic observations only; intervention not indicated	Symptomatic (e.g., mild wheezing); endoscopic evaluation indicated; radiographic evidence of atelectasis/lobar collapse; medical management indicated (e.g., steroids, bronchodilators)	Shortness of breath with stridor; endoscopic intervention indicated (e.g., laser, stent placement)	Life-threatening respiratory or hemodynamic compromise; intubation or urgent intervention indicated	Death
**Definition:** A disorder characterized by blockage of a bronchus passage, most often by bronchial secretions and exudates.<br>**Navigational Note:** -					
Bronchial stricture	Asymptomatic; clinical or diagnostic observations only; intervention not indicated	Symptomatic (e.g., rhonchi or wheezing) but without respiratory distress; medical intervention indicated (e.g., steroids, bronchodilators)	Shortness of breath with stridor; endoscopic intervention indicated (e.g., laser, stent placement)	Life-threatening respiratory or hemodynamic compromise; intubation or urgent intervention indicated	Death
**Definition:** A disorder characterized by a narrowing of the bronchial tube.<br>**Navigational Note:** -					
Bronchopleural fistula	Asymptomatic	Symptomatic, invasive intervention not indicated	Hospitalization; invasive intervention indicated	Life-threatening consequences; urgent intervention indicated	Death
**Definition:** A disorder characterized by an abnormal communication between a bronchus and the pleural cavity.<br>**Navigational Note:** -					
Bronchopulmonary hemorrhage	Mild symptoms; intervention not indicated	Moderate symptoms; invasive intervention not indicated	Transfusion indicated; invasive intervention indicated; hospitalization	Life-threatening consequences; intubation or urgent intervention indicated	Death
**Definition:** A disorder characterized by bleeding from the bronchial wall and/or lung parenchyma.<br>**Navigational Note:** -					
Bronchospasm	Mild symptoms; intervention not indicated	Symptomatic; medical intervention indicated; limiting instrumental ADL	Limiting self care ADL; supplemental oxygen indicated	Life-threatening respiratory or hemodynamic compromise; intubation or urgent intervention indicated	Death
**Definition:** A disorder characterized by a sudden contraction of the smooth muscles of the bronchial wall.<br>**Navigational Note:** -					
Chylothorax	Asymptomatic; clinical or diagnostic observations only; intervention not indicated	Symptomatic; medical intervention indicated (e.g., fat-restricted diet); thoracentesis or tube drainage indicated	Severe symptoms; elective operative intervention indicated	Life-threatening respiratory or hemodynamic compromise; intubation or urgent intervention indicated	Death
**Definition:** A disorder characterized by milky pleural effusion (abnormal collection of fluid) resulting from accumulation of lymph fluid in the pleural cavity.<br>**Navigational Note:** -					

Respiratory, thoracic and mediastinal disorders					
**CTCAE Term**	**Grade 1**	**Grade 2**	**Grade 3**	**Grade 4**	**Grade 5**
Cough	Mild symptoms; nonprescription intervention indicated	Moderate symptoms, medical intervention indicated; limiting instrumental ADL	Severe symptoms; limiting self care ADL	-	-
**Definition:** A disorder characterized by sudden, often repetitive, spasmodic contraction of the thoracic cavity, resulting in violent release of air from the lungs and usually accompanied by a distinctive sound.<br>**Navigational Note:** -					
Dyspnea	Shortness of breath with moderate exertion	Shortness of breath with minimal exertion; limiting instrumental ADL	Shortness of breath at rest; limiting self care ADL	Life-threatening consequences; urgent intervention indicated	Death
**Definition:** A disorder characterized by an uncomfortable sensation of difficulty breathing.<br>**Navigational Note:** -					
Epistaxis	Mild symptoms; intervention not indicated	Moderate symptoms; medical intervention indicated (e.g., nasal packing, cauterization; topical vasoconstrictors)	Transfusion; invasive intervention indicated (e.g., hemostasis of bleeding site)	Life-threatening consequences; urgent intervention indicated	Death
**Definition:** A disorder characterized by bleeding from the nose.<br>**Navigational Note:** -					
Hiccups	Mild symptoms; intervention not indicated	Moderate symptoms; medical intervention indicated; limiting instrumental ADL	Severe symptoms; interfering with sleep; limiting self care ADL	-	-
**Definition:** A disorder characterized by repeated gulp sounds that result from an involuntary opening and closing of the glottis. This is attributed to a spasm of the diaphragm.<br>**Navigational Note:** -					
Hoarseness	Mild or intermittent voice change; fully understandable; self-resolves	Moderate or persistent voice changes; may require occasional repetition but understandable on telephone; medical evaluation indicated	Severe voice changes including predominantly whispered speech	-	-
**Definition:** A disorder characterized by harsh and raspy voice arising from or spreading to the larynx.<br>**Navigational Note:** -					
Hypoxia	-	Decreased oxygen saturation with exercise (e.g., pulse oximeter <88%); intermittent supplemental oxygen	Decreased oxygen saturation at rest (e.g., pulse oximeter <88% or PaO2 <=55 mm Hg)	Life-threatening airway compromise; urgent intervention indicated (e.g., tracheotomy or intubation)	Death
**Definition:** A disorder characterized by a decrease in the level of oxygen in the body.<br>**Navigational Note:** -					

Respiratory, thoracic and mediastinal disorders					
**CTCAE Term**	**Grade 1**	**Grade 2**	**Grade 3**	**Grade 4**	**Grade 5**
Laryngeal edema	Asymptomatic; clinical or diagnostic observations only; intervention not indicated	Symptomatic; medical intervention indicated (e.g., dexamethasone, epinephrine, antihistamines)	Stridor; respiratory distress; hospitalization indicated	Life-threatening airway compromise; urgent intervention indicated (e.g., tracheotomy or intubation)	Death
**Definition:** A disorder characterized by swelling due to an excessive accumulation of fluid in the larynx.<br>**Navigational Note:** -					
Laryngeal fistula	Asymptomatic	Symptomatic, invasive intervention not indicated	Invasive intervention indicated	Life-threatening consequences; urgent intervention indicated	Death
**Definition:** A disorder characterized by an abnormal communication between the larynx and another organ or anatomic site.<br>**Navigational Note:** -					
Laryngeal hemorrhage	Mild cough or trace hemoptysis; laryngoscopic findings	Moderate symptoms; intervention indicated	Transfusion indicated; invasive intervention indicated; hospitalization	Life-threatening consequences; urgent intervention indicated (e.g., tracheotomy or intubation)	Death
**Definition:** A disorder characterized by bleeding from the larynx.<br>**Navigational Note:** -					
Laryngeal inflammation	Mild sore throat; raspy voice	Moderate sore throat; analgesics indicated	Severe throat pain; endoscopic intervention indicated	-	-
**Definition:** A disorder characterized by an inflammation involving the larynx.<br>**Navigational Note:** -					
Laryngeal mucositis	Endoscopic findings only; mild discomfort with normal intake	Moderate pain, analgesics indicated; altered oral intake; limiting instrumental ADL	Severe pain; severely altered eating/swallowing; medical intervention indicated	Life-threatening airway compromise; urgent intervention indicated (e.g., tracheotomy or intubation)	Death
**Definition:** A disorder characterized by ulceration or inflammation involving the mucous membrane of the larynx.<br>**Navigational Note:** -					
Laryngeal obstruction	Asymptomatic; clinical or diagnostic observations only; intervention not indicated	Symptomatic (e.g., noisy airway breathing), no respiratory distress; medical intervention indicated (e.g., steroids); limiting instrumental ADL	Limiting self care ADL; stridor; endoscopic intervention indicated (e.g., stent, laser)	Life-threatening consequences; urgent intervention indicated	Death
**Definition:** A disorder characterized by blockage of the laryngeal airway.<br>**Navigational Note:** -					

Respiratory, thoracic and mediastinal disorders					
**CTCAE Term**	**Grade 1**	**Grade 2**	**Grade 3**	**Grade 4**	**Grade 5**
Laryngeal stenosis	Asymptomatic; clinical or diagnostic observations only; intervention not indicated	Symptomatic (e.g., noisy airway breathing), no respiratory distress; medical intervention indicated (e.g., steroids); limiting instrumental ADL	Limiting self care ADL; stridor; endoscopic intervention indicated (e.g., stent, laser)	Life-threatening consequences; urgent intervention indicated	Death
**Definition:** A disorder characterized by a narrowing of the laryngeal airway.<br>**Navigational Note:** -					
Laryngopharyngeal dysesthesia	Mild symptoms; no anxiety; intervention not indicated	Moderate symptoms; mild anxiety, but no dyspnea; short duration of observation and/or anxiolytic indicated; limiting instrumental ADL	Severe symptoms; dyspnea and swallowing difficulty; limiting self care ADL	Life-threatening consequences	Death
**Definition:** A disorder characterized by an uncomfortable persistent sensation in the area of the laryngopharynx.<br>**Navigational Note:** -					
Laryngospasm	-	Transient episode; intervention not indicated	Recurrent episodes; noninvasive intervention indicated (e.g., breathing technique, pressure point massage)	Persistent or severe episodes associated with syncope; urgent intervention indicated (e.g., fiberoptic laryngoscopy, intubation, botox injection)	Death
**Definition:** A disorder characterized by paroxysmal spasmodic muscular contraction of the vocal cords.<br>**Navigational Note:** -					
Mediastinal hemorrhage	Mild symptoms; intervention not indicated; radiologic evidence only	Moderate symptoms; intervention indicated	Transfusion indicated; invasive intervention indicated; hospitalization	Life-threatening consequences; urgent intervention indicated	Death
**Definition:** A disorder characterized by bleeding from the mediastinum.<br>**Navigational Note:** -					
Nasal congestion	Mild symptoms; intervention not indicated	Moderate symptoms; medical intervention indicated	Associated with bloody nasal discharge or epistaxis	-	-
**Definition:** A disorder characterized by obstruction of the nasal passage due to mucosal edema.<br>**Navigational Note:** -					
Oropharyngeal pain	Mild pain	Moderate pain; altered oral intake; non-narcotics initiated; topical analgesics initiated	Severe pain; severely altered eating/swallowing; narcotics initiated; requires parenteral nutrition	-	-
**Definition:** A disorder characterized by a sensation of marked discomfort in the oropharynx.<br>**Navigational Note:** -					

Respiratory, thoracic and mediastinal disorders					
**CTCAE Term**	**Grade 1**	**Grade 2**	**Grade 3**	**Grade 4**	**Grade 5**
Pharyngeal fistula	Asymptomatic	Symptomatic, invasive intervention not indicated	Invasive intervention indicated	Life-threatening consequences; urgent intervention indicated	Death
**Definition:** A disorder characterized by an abnormal communication between the pharynx and another organ or anatomic site.<br>**Navigational Note:** -					
Pharyngeal hemorrhage	Mild symptoms; intervention not indicated	Moderate symptoms; intervention indicated	Transfusion indicated; invasive intervention indicated; hospitalization	Life-threatening consequences; intubation or urgent intervention indicated	Death
**Definition:** A disorder characterized by bleeding from the pharynx.<br>**Navigational Note:** -					
Pharyngeal mucositis	Endoscopic findings only; minimal symptoms with normal oral intake; mild pain but analgesics not indicated	Moderate pain, analgesics indicated; altered oral intake; limiting instrumental ADL	Severe pain; unable to adequately aliment or hydrate orally; limiting self care ADL	Life-threatening consequences; urgent intervention indicated	Death
**Definition:** A disorder characterized by ulceration or inflammation involving the mucous membrane of the pharynx.<br>**Navigational Note:** -					
Pharyngeal necrosis	-	-	Inability to aliment adequately by GI tract; invasive intervention indicated; tube feeding or TPN indicated	Life-threatening consequences; urgent operative intervention indicated	Death
**Definition:** A disorder characterized by a necrotic process occurring in the pharynx.<br>**Navigational Note:** -					
Pharyngeal stenosis	Asymptomatic; clinical or diagnostic observations only; intervention not indicated	Symptomatic (e.g., noisy airway breathing), no respiratory distress; medical intervention indicated (e.g., steroids); limiting instrumental ADL	Limiting self care ADL; stridor; endoscopic intervention indicated (e.g., stent, laser)	Life-threatening airway compromise; urgent intervention indicated (e.g., tracheotomy or intubation)	Death
**Definition:** A disorder characterized by a narrowing of the pharyngeal airway.<br>**Navigational Note:** -					
Pharyngolaryngeal pain	Mild pain	Moderate pain; limiting instrumental ADL	Severe pain; limiting self care ADL	-	-
**Definition:** A disorder characterized by a sensation of marked discomfort in the pharyngolaryngeal region.<br>**Navigational Note:** -					

Respiratory, thoracic and mediastinal disorders					
**CTCAE Term**	**Grade 1**	**Grade 2**	**Grade 3**	**Grade 4**	**Grade 5**
Pleural effusion	Asymptomatic; clinical or diagnostic observations only; intervention not indicated	Symptomatic; intervention indicated (e.g., diuretics or therapeutic thoracentesis)	Symptomatic with respiratory distress and hypoxia; operative intervention including chest tube or pleurodesis indicated	Life-threatening respiratory or hemodynamic compromise; intubation or urgent intervention indicated	Death
**Definition:** A disorder characterized by an increase in amounts of fluid within the pleural cavity. Symptoms include shortness of breath, cough and marked chest discomfort.<br>**Navigational Note:** -					
Pleural hemorrhage	Asymptomatic; mild hemorrhage confirmed by thoracentesis	Symptomatic or associated with pneumothorax; chest tube drainage indicated	>1000 ml of blood evacuated; persistent bleeding (150-200 ml/hr for 2 - 4 hr); persistent transfusion indicated; elective operative intervention indicated; hospitalization	Life-threatening consequences; intubation or urgent intervention indicated	Death
**Definition:** A disorder characterized by bleeding from the pleural cavity.<br>**Navigational Note:** -					
Pleuritic pain	Mild pain	Moderate pain; limiting instrumental ADL	Severe pain; limiting self care ADL	-	-
**Definition:** A disorder characterized by a sensation of marked discomfort in the pleura.<br>**Navigational Note:** -					
Pneumonitis	Asymptomatic; clinical or diagnostic observations only; intervention not indicated	Symptomatic; medical intervention indicated; limiting instrumental ADL	Severe symptoms; limiting self care ADL; oxygen indicated	Life-threatening respiratory compromise; urgent intervention indicated (e.g., tracheotomy or intubation)	Death
**Definition:** A disorder characterized by inflammation focally or diffusely affecting the lung parenchyma.<br>**Navigational Note:** -					
Pneumothorax	Asymptomatic; clinical or diagnostic observations only; intervention not indicated	Symptomatic; intervention indicated	Sclerosis and/or operative intervention indicated; hospitalization indicated	Life-threatening consequences; urgent intervention indicated	Death
**Definition:** A disorder characterized by abnormal presence of air in the pleural cavity resulting in the collapse of the lung.<br>**Navigational Note:** -					
Postnasal drip	Mild symptoms; intervention not indicated	Moderate symptoms; medical intervention indicated	-	-	-
**Definition:** A disorder characterized by excessive mucous secretion in the back of the nasal cavity or throat, causing sore throat and/or coughing.<br>**Navigational Note:** -					
Productive cough	Occasional/minimal production of sputum with cough	Moderate sputum production; limiting instrumental ADL	Persistent or copious production of sputum; limiting self care ADL	-	-
**Definition:** A disorder characterized by expectorated secretions upon coughing.<br>**Navigational Note:** -					

Respiratory, thoracic and mediastinal disorders					
**CTCAE Term**	**Grade 1**	**Grade 2**	**Grade 3**	**Grade 4**	**Grade 5**
Pulmonary edema	Radiologic findings only; minimal dyspnea on exertion	Moderate dyspnea on exertion; medical intervention indicated; limiting instrumental ADL	Severe dyspnea or dyspnea at rest; oxygen indicated; limiting self care ADL	Life-threatening respiratory compromise; urgent intervention or intubation with ventilatory support indicated	Death
**Definition:** A disorder characterized by accumulation of fluid in the lung tissues that causes a disturbance of the gas exchange that may lead to respiratory failure.<br>**Navigational Note:** -					
Pulmonary fibrosis	Radiologic pulmonary fibrosis <25% of lung volume associated with hypoxia	Evidence of pulmonary hypertension; radiographic pulmonary fibrosis 25 - 50% associated with hypoxia	Severe hypoxia; evidence of right-sided heart failure; radiographic pulmonary fibrosis >50 - 75%	Life-threatening consequences (e.g., hemodynamic/pulmonary complications); intubation with ventilatory support indicated; radiographic pulmonary fibrosis >75% with severe honeycombing	Death
**Definition:** A disorder characterized by the replacement of the lung tissue by connective tissue, leading to progressive dyspnea, respiratory failure or right heart failure.<br>**Navigational Note:** -					
Pulmonary fistula	Asymptomatic	Symptomatic, invasive intervention not indicated	Invasive intervention indicated	Life-threatening consequences; urgent intervention indicated	Death
**Definition:** A disorder characterized by an abnormal communication between the lung and another organ or anatomic site.<br>**Navigational Note:** -					
Pulmonary hypertension	Minimal dyspnea; findings on physical exam or other evaluation	Moderate dyspnea, cough; requiring evaluation by cardiac catheterization and medical intervention	Severe symptoms, associated with hypoxia, right heart failure; oxygen indicated	Life-threatening airway consequences; urgent intervention indicated (e.g., tracheotomy or intubation)	Death
**Definition:** A disorder characterized by an increase in pressure within the pulmonary circulation due to lung or heart disorder.<br>**Navigational Note:** -					
Respiratory failure	-	-	-	Life-threatening consequences; urgent intervention, intubation, or ventilatory support indicated	Death
**Definition:** A disorder characterized by impaired gas exchange by the respiratory system resulting in hypoxia and a decrease in oxygenation of the tissues that may be associated with an increase in arterial levels of carbon dioxide.<br>**Navigational Note:** -					

Respiratory, thoracic and mediastinal disorders					
**CTCAE Term**	**Grade 1**	**Grade 2**	**Grade 3**	**Grade 4**	**Grade 5**
Retinoic acid syndrome	Fluid retention; <3 kg of weight gain; intervention with fluid restriction and/or diuretics indicated	Moderate signs or symptoms; steroids indicated	Severe symptoms; hospitalization indicated	Life-threatening consequences; ventilatory support indicated	Death
**Definition:** A disorder characterized by weight gain, dyspnea, pleural and pericardial effusions, leukocytosis and/or renal failure originally described in patients treated with all-trans retinoic acid.					
**Navigational Note:** -					
Rhinorrhea	Present	-	-	-	-
**Definition:** A disorder characterized by excessive mucous secretions draining from the nose.					
**Navigational Note:** -					
Sinus disorder	Asymptomatic mucosal crusting; blood-tinged secretions	Symptomatic stenosis or edema/narrowing interfering with airflow; limiting instrumental ADL	Stenosis with significant nasal obstruction; limiting self care ADL	Necrosis of soft tissue or bone; urgent operative intervention indicated	Death
**Definition:** A disorder characterized by involvement of the paranasal sinuses.					
**Navigational Note:** -					
Sinus pain	Mild pain	Moderate pain; limiting instrumental ADL	Severe pain; limiting self care ADL	-	-
**Definition:** A disorder characterized by a sensation of marked discomfort in the face, between the eyes, or upper teeth originating from the sinuses.					
**Navigational Note:** -					
Sleep apnea	Snoring and nocturnal sleep arousal without apneic periods	Moderate apnea and oxygen desaturation; excessive daytime sleepiness; medical evaluation indicated; limiting instrumental ADL	Oxygen desaturation; associated with pulmonary hypertension; medical intervention indicated; limiting self care ADL	Cardiovascular or neuropsychiatric symptoms; urgent operative intervention indicated	Death
**Definition:** A disorder characterized by cessation of breathing for short periods during sleep.					
**Navigational Note:** -					
Sneezing	Mild symptoms; intervention not indicated	Moderate symptoms; medical intervention indicated	-	-	-
**Definition:** A disorder characterized by the involuntary expulsion of air from the nose.					
**Navigational Note:** -					
Sore throat	Mild pain	Moderate pain; limiting instrumental ADL	Severe pain; limiting self care ADL; limiting ability to swallow	-	-
**Definition:** A disorder characterized by marked discomfort in the throat.					
**Navigational Note:** -					

Respiratory, thoracic and mediastinal disorders					
**CTCAE Term**	**Grade 1**	**Grade 2**	**Grade 3**	**Grade 4**	**Grade 5**
Stridor	-	-	Respiratory distress limiting self care ADL; medical intervention indicated	Life-threatening airway compromise; urgent intervention indicated (e.g., tracheotomy or intubation)	Death
**Definition:** A disorder characterized by a high pitched breathing sound due to laryngeal or upper airway obstruction.<br>**Navigational Note:** -					
Tracheal fistula	Asymptomatic	Symptomatic, invasive intervention not indicated	Invasive intervention indicated	Life-threatening consequences; urgent intervention indicated	Death
**Definition:** A disorder characterized by an abnormal communication between the trachea and another organ or anatomic site.<br>**Navigational Note:** -					
Tracheal mucositis	Endoscopic findings only; minimal hemoptysis, pain, or respiratory symptoms	Moderate symptoms; medical intervention indicated; limiting instrumental ADL	Severe pain; hemorrhage or respiratory symptoms; limiting self care ADL	Life-threatening consequences; urgent intervention indicated	Death
**Definition:** A disorder characterized by an inflammation or ulceration involving the mucous membrane of the trachea.<br>**Navigational Note:** -					
Tracheal stenosis	Asymptomatic; clinical or diagnostic observations only; intervention not indicated	Symptomatic (e.g., noisy airway breathing), no respiratory distress; medical intervention indicated (e.g., steroids); limiting instrumental ADL	Stridor or respiratory distress limiting self care ADL; invasive intervention indicated (e.g., stent, laser)	Life-threatening airway compromise; urgent intervention indicated (e.g., tracheotomy or intubation)	Death
**Definition:** A disorder characterized by a narrowing of the trachea.<br>**Navigational Note:** -					
Voice alteration	Mild or intermittent change from normal voice	Moderate or persistent change from normal voice; still understandable	Severe voice changes including predominantly whispered speech; may require frequent repetition or face-to-face contact for understandability; may require assistive technology	-	-
**Definition:** A disorder characterized by a change in the sound and/or speed of the voice.<br>**Navigational Note:** -					
Wheezing	Detectable airway noise with minimal symptoms	Moderate symptoms; medical intervention indicated; limiting instrumental ADL	Severe respiratory symptoms limiting self care ADL; oxygen therapy or hospitalization indicated	Life-threatening consequences; urgent intervention indicated	Death
**Definition:** A disorder characterized by a high-pitched, whistling sound during breathing. It results from the narrowing or obstruction of the respiratory airways.<br>**Navigational Note:** -					

Respiratory, thoracic and mediastinal disorders					
**CTCAE Term**	**Grade 1**	**Grade 2**	**Grade 3**	**Grade 4**	**Grade 5**
Respiratory, thoracic and mediastinal disorders - Other, specify	Asymptomatic or mild symptoms; clinical or diagnostic observations only; intervention not indicated	Moderate; minimal, local or noninvasive intervention indicated; limiting age-appropriate instrumental ADL	Severe or medically significant but not immediately life-threatening; hospitalization or prolongation of existing hospitalization indicated; limiting self care ADL	Life-threatening consequences; urgent intervention indicated	Death
**Definition:** -					
**Navigational Note:** -					

Skin and subcutaneous tissue disorders					
**CTCAE Term**	**Grade 1**	**Grade 2**	**Grade 3**	**Grade 4**	**Grade 5**
Alopecia	Hair loss of <50% of normal for that individual that is not obvious from a distance but only on close inspection; a different hair style may be required to cover the hair loss but it does not require a wig or hair piece to camouflage	Hair loss of >=50% normal for that individual that is readily apparent to others; a wig or hair piece is necessary if the patient desires to completely camouflage the hair loss; associated with psychosocial impact	-	-	-
**Definition:** A disorder characterized by a decrease in density of hair compared to normal for a given individual at a given age and body location.					
**Navigational Note:** -					
Body odor	Mild odor; physician intervention not indicated; self care interventions	Pronounced odor; psychosocial impact; patient seeks medical intervention	-	-	-
**Definition:** A disorder characterized by an abnormal body smell resulting from the growth of bacteria on the body.					
**Navigational Note:** -					
Bullous dermatitis	Asymptomatic; blisters covering <10% BSA	Blisters covering 10 - 30% BSA; painful blisters; limiting instrumental ADL	Blisters covering >30% BSA; limiting self care ADL	Blisters covering >30% BSA; associated with fluid or electrolyte abnormalities; ICU care or burn unit indicated	Death
**Definition:** A disorder characterized by inflammation of the skin characterized by the presence of bullae which are filled with fluid.					
**Navigational Note:** If infectious, consider Infections and infestations: Rash pustular or other site-specific Infections and infestations term.					
Dry skin	Covering <10% BSA and no associated erythema or pruritus	Covering 10 - 30% BSA and associated with erythema or pruritus; limiting instrumental ADL	Covering >30% BSA and associated with pruritus; limiting self care ADL	-	-
**Definition:** A disorder characterized by flaky and dull skin; the pores are generally fine, the texture is a papery thin texture.					
**Navigational Note:** -					
Eczema	Asymptomatic or mild symptoms; additional medical intervention over baseline not indicated	Moderate; topical or oral intervention indicated; additional medical intervention over baseline indicated	Severe or medically significant but not immediately life-threatening; IV intervention indicated	-	-
**Definition:** A disorder characterized by skin which becomes itchy, red, inflamed, crusty, thick, scaly, and/or forms blisters.					
**Navigational Note:** -					
Erythema multiforme	Target lesions covering <10% BSA and not associated with skin tenderness	Target lesions covering 10 - 30% BSA and associated with skin tenderness	Target lesions covering >30% BSA and associated with oral or genital erosions	Target lesions covering >30% BSA; associated with fluid or electrolyte abnormalities; ICU care or burn unit indicated	Death
**Definition:** A disorder characterized by target lesions (a pink-red ring around a pale center).					
**Navigational Note:** -					

Skin and subcutaneous tissue disorders					
**CTCAE Term**	**Grade 1**	**Grade 2**	**Grade 3**	**Grade 4**	**Grade 5**
Erythroderma	-	Erythema covering >90% BSA without associated symptoms; limiting instrumental ADL	Erythema covering >90% BSA with associated symptoms (e.g., pruritus or tenderness); limiting self care ADL	Erythema covering >90% BSA with associated fluid or electrolyte abnormalities; ICU care or burn unit indicated	Death
**Definition:** A disorder characterized by generalized inflammatory erythema and exfoliation. The inflammatory process involves > 90% of the body surface area.<br>**Navigational Note:** -					
Fat atrophy	Covering <10% BSA and asymptomatic	Covering 10 - 30% BSA and associated with erythema or tenderness; limiting instrumental ADL	Covering >30% BSA; associated with erythema or tenderness; limiting self-care ADL	-	-
**Definition:** A disorder characterized by shrinking of adipose tissue.<br>**Navigational Note:** -					
Hair color changes	Present	-	-	-	-
**Definition:** A disorder characterized by change in hair color or loss of normal pigmentation.<br>**Navigational Note:** -					
Hair texture abnormal	Present	-	-	-	-
**Definition:** A disorder characterized by a change in the way the hair feels.<br>**Navigational Note:** -					
Hirsutism	In women, increase in length, thickness or density of hair in a male distribution that the patient is able to camouflage by periodic shaving, bleaching, or removal of hair	In women, increase in length, thickness or density of hair in a male distribution that requires daily shaving or consistent destructive means of hair removal to camouflage; associated with psychosocial impact	-	-	-
**Definition:** A disorder characterized by the presence of excess hair growth in women in anatomic sites where growth is considered to be a secondary male characteristic and under androgen control (beard, moustache, chest, abdomen).<br>**Navigational Note:** -					
Hyperhidrosis	Limited to one site (palms, soles, or axillae); self care interventions	Involving >1 site; patient seeks medical intervention; associated with psychosocial impact	Associated with electrolyte/hemodynamic imbalance	-	-
**Definition:** A disorder characterized by excessive sweating.<br>**Navigational Note:** Synonym: Night sweats, diaphoresis					
Hyperkeratosis	Present	-	Limiting self-care ADLs	-	-
**Definition:** A disorder characterized by a thickening of the outer layer of the skin.<br>**Navigational Note:** -					

Skin and subcutaneous tissue disorders					
**CTCAE Term**	**Grade 1**	**Grade 2**	**Grade 3**	**Grade 4**	**Grade 5**
Hypertrichosis	Increase in length, thickness or density of hair that the patient is either able to camouflage by periodic shaving or removal of hairs or is not concerned enough about the overgrowth to use any form of hair removal	Increase in length, thickness or density of hair at least on the usual exposed areas of the body [face (not limited to beard/moustache area) plus/minus arms] that requires frequent shaving or use of destructive means of hair removal to camouflage; associated with psychosocial impact	-	-	-
**Definition:** A disorder characterized by hair density or length beyond the accepted limits of normal in a particular body region, for a particular age or race.<br>**Navigational Note:** -					
Hypohidrosis	-	Symptomatic; limiting instrumental ADL	Increase in body temperature; limiting self care ADL	Heat stroke	Death
**Definition:** A disorder characterized by reduced sweating.<br>**Navigational Note:** -					
Lipohypertrophy	Asymptomatic and covering <10% BSA	Covering 10 - 30% BSA and associated tenderness; limiting instrumental ADL	Covering >30% BSA and associated tenderness and narcotics or NSAIDs indicated; lipohypertrophy; limiting self care ADL	-	-
**Definition:** A disorder characterized by hypertrophy of the subcutaneous adipose tissue at the site of multiple subcutaneous injections of insulin.<br>**Navigational Note:** -					
Nail changes	Present	-	-	-	-
**Definition:** A disorder characterized by a change in the nails.<br>**Navigational Note:** -					
Nail discoloration	Asymptomatic; clinical or diagnostic observations only	-	-	-	-
**Definition:** A disorder characterized by a change in the color of the nail plate.<br>**Navigational Note:** -					
Nail loss	Asymptomatic separation of the nail bed from the nail plate or nail loss	Symptomatic separation of the nail bed from the nail plate or nail loss; limiting instrumental ADL	-	-	-
**Definition:** A disorder characterized by loss of all or a portion of the nail.<br>**Navigational Note:** -					

Skin and subcutaneous tissue disorders					
**CTCAE Term**	**Grade 1**	**Grade 2**	**Grade 3**	**Grade 4**	**Grade 5**
Nail ridging	Asymptomatic; clinical or diagnostic observations only; intervention not indicated	-	-	-	-
**Definition:** A disorder characterized by vertical or horizontal ridges on the nails.<br>**Navigational Note:** -					
Pain of skin	Mild pain	Moderate pain; limiting instrumental ADL	Severe pain; limiting self care ADL	-	-
**Definition:** A disorder characterized by a sensation of marked discomfort in the skin.<br>**Navigational Note:** -					
Palmar-plantar erythrodysesthesia syndrome	Minimal skin changes or dermatitis (e.g., erythema, edema, or hyperkeratosis) without pain	Skin changes (e.g., peeling, blisters, bleeding, fissures, edema, or hyperkeratosis) with pain; limiting instrumental ADL	Severe skin changes (e.g., peeling, blisters, bleeding, fissures, edema, or hyperkeratosis) with pain; limiting self care ADL	-	-
**Definition:** A disorder characterized by redness, marked discomfort, swelling, and tingling in the palms of the hands or the soles of the feet. Also known as Hand-Foot Syndrome.<br>**Navigational Note:** -					
Photosensitivity	Painless erythema and erythema covering <10% BSA	Tender erythema covering 10 - 30% BSA	Erythema covering >30% BSA and erythema with blistering; photosensitivity; oral corticosteroid therapy indicated; pain control indicated (e.g., narcotics or NSAIDs)	Life-threatening consequences; urgent intervention indicated	Death
**Definition:** A disorder characterized by an increase in sensitivity of the skin to light.<br>**Navigational Note:** -					
Pruritus	Mild or localized; topical intervention indicated	Widespread and intermittent; skin changes from scratching (e.g., edema, papulation, excoriations, lichenification, oozing/crusts); oral intervention indicated; limiting instrumental ADL	Widespread and constant; limiting self care ADL or sleep; systemic corticosteroid or immunosuppressive therapy indicated	-	-
**Definition:** A disorder characterized by an intense itching sensation.<br>**Navigational Note:** -					

Skin and subcutaneous tissue disorders					
**CTCAE Term**	**Grade 1**	**Grade 2**	**Grade 3**	**Grade 4**	**Grade 5**
Purpura	Combined area of lesions covering <10% BSA	Combined area of lesions covering 10 - 30% BSA; bleeding with trauma	Combined area of lesions covering >30% BSA; spontaneous bleeding	-	-
**Definition:** A disorder characterized by hemorrhagic areas of the skin and mucous membrane. Newer lesions appear reddish in color. Older lesions are usually a darker purple color and eventually become a brownish-yellow color.					
**Navigational Note:** -					
Rash acneiform	Papules and/or pustules covering <10% BSA, which may or may not be associated with symptoms of pruritus or tenderness	Papules and/or pustules covering 10 - 30% BSA, which may or may not be associated with symptoms of pruritus or tenderness; associated with psychosocial impact; limiting instrumental ADL; papules and/or pustules covering > 30% BSA with or without mild symptoms	Papules and/or pustules covering >30% BSA with moderate or severe symptoms; limiting self-care ADL; associated with local superinfection with oral antibiotics indicated	Life-threatening consequences; papules and/or pustules covering any % BSA, which may or may not be associated with symptoms of pruritus or tenderness and are associated with extensive superinfection with IV antibiotics indicated	Death
**Definition:** A disorder characterized by an eruption of papules and pustules, typically appearing in face, scalp, upper chest and back.					
**Navigational Note:** -					
Rash maculo-papular	Macules/papules covering <10% BSA with or without symptoms (e.g., pruritus, burning, tightness)	Macules/papules covering 10 - 30% BSA with or without symptoms (e.g., pruritus, burning, tightness); limiting instrumental ADL; rash covering > 30% BSA with or without mild symptoms	Macules/papules covering >30% BSA with moderate or severe symptoms; limiting self care ADL	-	-
**Definition:** A disorder characterized by the presence of macules (flat) and papules (elevated). Also known as morbillform rash, it is one of the most common cutaneous adverse events, frequently affecting the upper trunk, spreading centripetally and associated with pruritis.					
**Navigational Note:** -					
Scalp pain	Mild pain	Moderate pain; limiting instrumental ADL	Severe pain; limiting self care ADL	-	-
**Definition:** A disorder characterized by a sensation of marked discomfort in the skin covering the top and the back of the head.					
**Navigational Note:** -					
Skin atrophy	Covering <10% BSA; associated with telangiectasias or changes in skin color	Covering 10 - 30% BSA; associated with striae or adnexal structure loss	Covering >30% BSA; associated with ulceration	-	-
**Definition:** A disorder characterized by the degeneration and thinning of the epidermis and dermis.					
**Navigational Note:** -					

Skin and subcutaneous tissue disorders					
**CTCAE Term**	**Grade 1**	**Grade 2**	**Grade 3**	**Grade 4**	**Grade 5**
Skin hyperpigmentation	Hyperpigmentation covering <10% BSA; no psychosocial impact	Hyperpigmentation covering >10% BSA; associated psychosocial impact	-	-	-
**Definition:** A disorder characterized by darkening of the skin due to excessive melanin deposition.					
**Navigational Note:** -					
Skin hypopigmentation	Hypopigmentation or depigmentation covering <10% BSA; no psychosocial impact	Hypopigmentation or depigmentation covering >10% BSA; associated psychosocial impact	-	-	-
**Definition:** A disorder characterized by loss of skin pigment (e.g., vitiligo).					
**Navigational Note:** -					
Skin induration	Mild induration, able to move skin parallel to plane (sliding) and perpendicular to skin (pinching up)	Moderate induration, able to slide skin, unable to pinch skin; limiting instrumental ADL	Severe induration; unable to slide or pinch skin; limiting joint or orifice movement (e.g., mouth, anus); limiting self care ADL	Generalized; associated with signs or symptoms of impaired breathing or feeding	Death
**Definition:** A disorder characterized by an area of hardness in the skin.					
**Navigational Note:** -					
Skin ulceration	Combined area of ulcers <1 cm; nonblanchable erythema of intact skin with associated warmth or edema	Combined area of ulcers 1 - 2 cm; partial thickness skin loss involving skin or subcutaneous fat	Combined area of ulcers >2 cm; full-thickness skin loss involving damage to or necrosis of subcutaneous tissue that may extend down to fascia	Any size ulcer with extensive destruction, tissue necrosis, or damage to muscle, bone, or supporting structures with or without full thickness skin loss	Death
**Definition:** A disorder characterized by a circumscribed, erosive lesion on the skin.					
**Navigational Note:** -					
Stevens-Johnson syndrome	-	-	Skin sloughing covering <10% BSA with associated signs (e.g., erythema, purpura, epidermal detachment, and mucous membrane detachment)	Skin sloughing covering 10 - 30% BSA with associated signs (e.g., erythema, purpura, epidermal detachment and mucous membrane detachment)	Death
**Definition:** A disorder characterized by less than 10% total body skin area separation of dermis. The syndrome is thought to be a hypersensitivity complex affecting the skin and the mucous membranes.					
**Navigational Note:** -					

Skin and subcutaneous tissue disorders					
**CTCAE Term**	**Grade 1**	**Grade 2**	**Grade 3**	**Grade 4**	**Grade 5**
Subcutaneous emphysema	Asymptomatic or mild symptoms; clinical or diagnostic observations only; intervention not indicated	Moderate; minimal, local or noninvasive intervention indicated	Severe or medically significant but not immediately life-threatening; hospitalization or prolongation of existing hospitalization indicated; limiting self care ADL	-	-
**Definition:** A disorder characterized by air in the subcutaneous tissue.					
**Navigational Note:** -					
Telangiectasia	Telangiectasias covering <10% BSA	Telangiectasias covering >=10% BSA; associated with psychosocial impact	-	-	-
**Definition:** A disorder characterized by local dilatation of small vessels resulting in red discoloration of the skin or mucous membranes.					
**Navigational Note:** -					
Toxic epidermal necrolysis	-	-	-	Skin sloughing covering >=30% BSA with associated symptoms (e.g., erythema, purpura, or epidermal detachment)	Death
**Definition:** A disorder characterized by greater than 30% total body skin area separation of dermis. The syndrome is thought to be a hypersensitivity complex affecting the skin and the mucous membranes.					
**Navigational Note:** -					
Urticaria	Urticarial lesions covering <10% BSA; topical intervention indicated	Urticarial lesions covering 10 - 30% BSA; oral intervention indicated	Urticarial lesions covering >30% BSA; IV intervention indicated	-	-
**Definition:** A disorder characterized by an itchy skin eruption characterized by wheals with pale interiors and well-defined red margins.					
**Navigational Note:** -					
Skin and subcutaneous tissue disorders - Other, specify	Asymptomatic or mild symptoms; clinical or diagnostic observations only; intervention not indicated	Moderate; minimal, local or noninvasive intervention indicated; limiting age-appropriate instrumental ADL	Severe or medically significant but not immediately life-threatening; hospitalization or prolongation of existing hospitalization indicated; limiting self care ADL	Life-threatening consequences; urgent intervention indicated	Death
**Definition:** -					
**Navigational Note:** -					

Social circumstances					
**CTCAE Term**	**Grade 1**	**Grade 2**	**Grade 3**	**Grade 4**	**Grade 5**
Social circumstances - Other, specify	Asymptomatic or mild symptoms; clinical or diagnostic observations only; intervention not indicated	Moderate; minimal, local or noninvasive intervention indicated; limiting age-appropriate instrumental ADL	Severe or medically significant but not immediately life-threatening; hospitalization or prolongation of existing hospitalization indicated; limiting self care ADL	Life-threatening consequences; urgent intervention indicated	Death
**Definition:** -					
**Navigational Note:** -					

Surgical and medical procedures					
**CTCAE Term**	**Grade 1**	**Grade 2**	**Grade 3**	**Grade 4**	**Grade 5**
Surgical and medical procedures - Other, specify	Asymptomatic or mild symptoms; clinical or diagnostic observations only; intervention not indicated	Moderate; minimal, local or noninvasive intervention indicated; limiting age-appropriate instrumental ADL	Severe or medically significant but not immediately life-threatening; hospitalization or prolongation of existing hospitalization indicated; limiting self care ADL	Life-threatening consequences; urgent intervention indicated	Death
**Definition:** -					
**Navigational Note:** -					

Vascular disorders					
**CTCAE Term**	**Grade 1**	**Grade 2**	**Grade 3**	**Grade 4**	**Grade 5**
Arterial thromboembolism	-	-	Urgent intervention indicated	Life-threatening consequences; hemodynamic or neurologic instability; organ damage; loss of extremity(ies)	Death
**Definition:** A disorder characterized by occlusion of an arterial vessel by a blood clot that develops in an artery.<br>**Navigational Note:** Consider Nervous system disorders: TIA or Stroke for CNS-related events or Cardiac disorders: Myocardial infarction					
Capillary leak syndrome	Asymptomatic	Symptomatic; medical intervention indicated	Severe symptoms; intervention indicated	Life-threatening consequences; urgent intervention indicated	Death
**Definition:** A disorder characterized by leakage of intravascular fluids into the extravascular space. This syndrome is observed in patients who demonstrate a state of generalized leaky capillaries following shock syndromes, low-flow states, ischemia-reperfusion injuries, toxemias, medications, or poisoning. It can lead to generalized edema and multiple organ failure.<br>**Navigational Note:** -					
Flushing	Asymptomatic; clinical or diagnostic observations only	Moderate symptoms; limiting instrumental ADL	Symptomatic, associated with hypotension and/or tachycardia; limiting self care ADL	-	-
**Definition:** A disorder characterized by episodic reddening of the skin, especially face, neck, or chest.<br>**Navigational Note:** -					
Hematoma	Mild symptoms; intervention not indicated	Minimally invasive evacuation or aspiration indicated	Transfusion; invasive intervention indicated	Life-threatening consequences; urgent intervention indicated	Death
**Definition:** A disorder characterized by a localized collection of blood, usually clotted, in an organ, space, or tissue, due to a break in the wall of a blood vessel.<br>**Navigational Note:** -					
Hot flashes	Mild symptoms; intervention not indicated	Moderate symptoms; limiting instrumental ADL	Severe symptoms; limiting self care ADL	-	-
**Definition:** A disorder characterized by an uncomfortable and temporary sensation of intense body warmth, flushing, sometimes accompanied by sweating upon cooling.<br>**Navigational Note:** -					

Vascular disorders					
**CTCAE Term**	**Grade 1**	**Grade 2**	**Grade 3**	**Grade 4**	**Grade 5**
Hypertension	**Adult:** Systolic BP 120 - 139 mm Hg or diastolic BP 80 - 89 mm Hg;<br><br>**Pediatric:** Systolic/diastolic BP >90th percentile but< 95th percentile;<br><br>**Adolescent:** BP ≥120/80 even if < 95th percentile	**Adult:** Systolic BP 140 - 159 mm Hg or diastolic BP 90 - 99 mm Hg if previously WNL; change in baseline medical intervention indicated; recurrent or persistent (>=24 hrs); symptomatic increase by >20 mm Hg (diastolic) or to >140/90 mm Hg; monotherapy indicated initiated;<br><br>**Pediatric and adolescent:** Recurrent or persistent (>=24 hrs) BP >ULN; monotherapy indicated; systolic and /or diastolic BP between the 95th percentile and 5 mmHg above the 99th percentile;<br><br>A**dolescent:** Systolic between 130-139 or diastolic between 80-89 even if < 95th percentile	**Adult:** Systolic BP >=160 mm Hg or diastolic BP >=100 mm Hg; medical intervention indicated; more than one drug or more intensive therapy than previously used indicated;<br><br>**Pediatric and adolescent:** Systolic and/or diastolic > 5 mmHg above the 99th percentile	**Adult and Pediatric:** Life-threatening consequences (e.g., malignant hypertension, transient or permanent neurologic deficit, hypertensive crisis); urgent intervention indicated	Death
**Definition:** A disorder characterized by a pathological increase in blood pressure.<br>**Navigational Note:** -					
Hypotension	Asymptomatic, intervention not indicated	Non-urgent medical intervention indicated	Medical intervention indicated; hospitalization indicated	Life-threatening consequences and urgent intervention indicated	Death
**Definition:** A disorder characterized by a blood pressure that is below the normal expected for an individual in a given environment.<br>**Navigational Note:** -					
Lymph leakage	-	Symptomatic; medical intervention indicated	Severe symptoms; invasive intervention indicated	Life-threatening consequences; urgent intervention indicated	Death
**Definition:** A disorder characterized by the loss of lymph fluid into the surrounding tissue or body cavity.<br>**Navigational Note:** -					

Vascular disorders					
**CTCAE Term**	**Grade 1**	**Grade 2**	**Grade 3**	**Grade 4**	**Grade 5**
Lymphedema	Trace thickening or faint discoloration	Marked discoloration; leathery skin texture; papillary formation; limiting instrumental ADL	Severe symptoms; limiting self care ADL	-	-
**Definition:** A disorder characterized by excessive fluid collection in tissues that causes swelling.<br>**Navigational Note:** -					
Lymphocele	Asymptomatic; clinical or diagnostic observations only; intervention not indicated	Symptomatic; medical intervention indicated	Severe symptoms; invasive intervention indicated	-	-
**Definition:** A disorder characterized by a cystic lesion containing lymph.<br>**Navigational Note:** -					
Peripheral ischemia	-	Brief (<24 hrs) episode of ischemia managed medically and without permanent deficit	Prolonged (>=24 hrs) or recurring symptoms and/or invasive intervention indicated	Life-threatening consequences; evidence of end organ damage; urgent operative intervention indicated	Death
**Definition:** A disorder characterized by impaired circulation to an extremity.<br>**Navigational Note:** -					
Phlebitis	-	Present	-	-	-
**Definition:** A disorder characterized by inflammation of the wall of a vein.<br>**Navigational Note:** -					
Superficial thrombophlebitis	-	Present	-	-	-
**Definition:** A disorder characterized by a blood clot and inflammation involving a superficial vein of the extremities.<br>**Navigational Note:** -					
Superior vena cava syndrome	Asymptomatic; incidental finding of SVC thrombosis	Symptomatic; medical intervention indicated (e.g., anticoagulation, radiation or chemotherapy)	Severe symptoms; multi-modality intervention indicated (e.g., anticoagulation, chemotherapy, radiation, stenting)	Life-threatening consequences; urgent multi-modality intervention indicated (e.g., lysis, thrombectomy, surgery)	Death
**Definition:** A disorder characterized by obstruction of the blood flow in the superior vena cava. Signs and symptoms include swelling and cyanosis of the face, neck, and upper arms, cough, orthopnea and headache.<br>**Navigational Note:** -					
Thromboembolic event	Medical intervention not indicated (e.g., superficial thrombosis)	Medical intervention indicated	Urgent medical intervention indicated (e.g., pulmonary embolism or intracardiac thrombus)	Life-threatening consequences with hemodynamic or neurologic instability	Death
**Definition:** A disorder characterized by occlusion of a vessel by a thrombus that has migrated from a distal site via the blood stream.<br>**Navigational Note:** Consider Nervous system disorders: TIA or Stroke for CNS-related events. Use Vascular disorders: Arterial thromboembolism for arterial thrombi.					

Vascular disorders					
**CTCAE Term**	**Grade 1**	**Grade 2**	**Grade 3**	**Grade 4**	**Grade 5**
Vasculitis	Asymptomatic, intervention not indicated	Moderate symptoms, medical intervention indicated	Severe symptoms, medical intervention indicated (e.g., steroids)	Life-threatening consequences; evidence of peripheral or visceral ischemia; urgent intervention indicated	Death
**Definition:** A disorder characterized by inflammation involving the wall of a vessel.<br>**Navigational Note:** -					
Vascular disorders - Other, specify	Asymptomatic or mild symptoms; clinical or diagnostic observations only; intervention not indicated	Moderate; minimal, local or noninvasive intervention indicated; limiting age-appropriate instrumental ADL	Severe or medically significant but not immediately life-threatening; hospitalization or prolongation of existing hospitalization indicated; limiting self care ADL	Life-threatening consequences; urgent intervention indicated	Death
**Definition:** -<br>**Navigational Note:** -					
