# Supplementary material for: Alternating modified CAPOX/CAPIRI plus bevacizumab in untreated unresectable metastatic colorectal cancer: a phase 2 trial
Source: Signal Transduct Target Ther. 2024 Dec 11;9:346. doi: 10.1038/s41392-024-02048-z (PMC11631963; doi:10.1038/s41392-024-02048-z)
Supplement: Supplementary file 6 — appendix IV [file 41392_2024_2048_MOESM6_ESM.pdf]

THE RESPONSE EVALUATION CRITERIA IN SOLID TUMORS (RECIST) GUIDELINES, VERSION 1.1

The text below was obtained from the following reference: Eisenhauer et al, 2009.

DEFINITIONS

Response and progression will be evaluated in this trial using the international criteria proposed by the Response Evaluation Criteria in Solid Tumors (RECIST) Committee (v1.1). Changes in only the largest diameter (uni-dimensional measurement) of the tumor lesions are used in the RECIST criteria.

Note: Lesions are either measurable or non-measurable using the criteria provided below. The term "evaluable" in reference to measurability will not be used because it does not provide additional meaning or accuracy.

<u>Measurable Disease</u>

Tumor lesions: Must be accurately measured in at least 1 dimension (longest diameter in the plane of measurement is to be recorded) with a minimum size of:

- 10 mm by CT scan and MRI (no less than double the slice thickness and a minimum of 10 mm)
- 10 mm caliper measurement by clinical exam (when superficial)
- 20 mm by chest X-ray (if clearly defined and surrounded by aerated lung)

Malignant lymph nodes: To be considered pathologically enlarged and measurable, a lymph node must be ≥ 15 mm in short axis when assessed by CT scan (CT scan slice thickness recommended to be no greater than 5 mm). At baseline and in follow-up, only the short axis will be measured and followed.

<u>Nonmeasurable Disease</u>

All other lesions (or sites of disease), including small lesions (longest diameter ≥ 10 to < 15 mm with conventional techniques or < 10 mm using spiral CT scan), are considered nonmeasurable disease. Leptomeningeal disease, ascites, pleural, or pericardial effusion, inflammatory breast disease, lymphangitic involvement of skin or lung, abdominal masses/abdominal organomegaly identified by physical examination that is not measurable by reproducible imaging techniques are all non-measurable.

Bone lesions:

- Bone scan, PET scan, or plain films are not considered adequate imaging techniques to measure bone lesions. However, these techniques can be used to confirm the presence or disappearance of bone lesions.
- Lytic bone lesions or mixed lytic-blastic lesions, with identifiable soft tissue components, that can be evaluated by cross sectional imaging techniques such as CT or MRI can be considered as measurable lesions if the soft tissue component meets the definition of measurability described above

- Blastic bone lesions are nonmeasurable

Cystic lesions:

- Lesions that meet the criteria for radiographically defined simple cysts should not be considered as malignant lesions (neither measurable nor non-measurable) since they are, by definition, simple cysts
- Cystic lesions thought to represent cystic metastases can be considered as measurable lesions, if they meet the definition of measurability described above. However, if non-cystic lesions are present in the same patient, these are preferred for selection as target lesions.

Lesions with prior local treatment:

- Tumor lesions situated in a previously irradiated area, or in an area subjected to other loco-regional therapy, are usually not considered measurable unless there has been demonstrated progression in the lesion. Trial protocols should detail the conditions under which such lesions would be considered measurable.

<u>Target Lesions</u>

All measurable lesions up to a maximum of 2 lesions per organ and 5 lesions in total, should be identified as target lesions and recorded and measured at baseline. Target lesions should be selected on the basis of their size (lesions with the longest diameter), be representative of all involved organ, but in addition should be those that lend themselves to reproducible repeated measurements.

Lymph nodes merit special mention since they are normal anatomical structures which may be visible by imaging even if not involved by tumor. Pathological nodes which are defined as measurable and may be identified as target lesions must meet the criterion of a short axis of ≥ 15 mm by CT scan. Only the short axis of these nodes will contribute to the baseline sum. The short axis of the node is the diameter normally used by radiologists to judge if a node is involved by solid tumor. Nodal size is normally reported as 2 dimensions in the plane in which the image is obtained (for CT scan, this is almost always the axial plane; for MRI the plane of acquisition may be axial, sagittal, or coronal). The smaller of these measures is the short axis. For example, an abdominal node which is reported as being 20 mm × 30 mm has a short axis of 20 mm and qualifies as a malignant, measurable node. In this example, 20 mm should be recorded as the node measurement. All other pathological nodes (those with short axis ≥10 mm but < 15 mm) should be considered nontarget lesions. Nodes that have a short axis < 10 mm are considered nonpathological and should not be recorded or followed.

A sum of the diameters (longest for non-nodal lesions, short axis for nodal lesions) for all target lesions will be calculated and reported as the baseline sum diameters. If lymph nodes are to be included in the sum, then as noted above, only the short axis is added into the sum. The baseline sum diameters will be used as reference to further characterize any objective tumor regression in the measurable dimension of the disease.

Nontarget Lesions

All other lesions (or sites of disease) including pathological lymph nodes should be identified as non-target lesions and should also be recorded at baseline. Measurements are not required and these lesions should be followed as "present", "absent", or in rare cases "unequivocal progression" (more details to follow). In addition, it is possible to record multiple nontarget lesions involving the same organ as a single item on the case record form (eg, "multiple enlarged pelvic lymph node" or "multiple liver metastases").

GUIDELINES FOR EVALUATION OF MEASURABLE DISEASE

All measurements should be recorded in metric notation, using calipers if clinically assessed. All baseline evaluations should be performed as close as possible to the treatment start and never more than 4 weeks before the beginning of the treatment.

The same method of assessment and the same technique should be used to characterize each identified and reported lesion at baseline and during follow-up. Imaging based evaluation should always be done rather than clinical examination unless the lesion(s) being followed cannot be imaged but are accessible by clinical examination.

Clinical lesions: Clinical lesions will only be considered measurable when they are superficial and P10 mm diameter as assessed using calipers (eg, skin nodules). For the case of skin lesions, documentation by color photography including a ruler to estimate the size of the lesion is suggested. As noted above, when lesions can be evaluated by both clinical examination and imaging, imaging evaluation should be undertaken since it is more objective and may also be reviewed at the end of the trial.

- Chest X-ray: Chest CT is preferred over chest X-ray, particularly when progression is an important endpoint, since CT is more sensitive than X-ray, particularly in identifying new lesions. However, lesions on chest X-ray may be considered measurable if they are clearly defined and surrounded by aerated lung.
- CT, MRI: CT is the best currently available and reproducible method to measure lesions selected for response assessment. This guideline has defined measurability of lesions on CT scan based on the assumption that CT slice thickness is 5 mm or less. When CT scans have slice thickness greater than 5 mm, the minimum size for a measurable lesion should be twice the slice thickness. MRI is also acceptable in certain situations (eg, for body scans).
- Ultrasound: Ultrasound is not useful in assessment of lesion size and should not be used as a method of measurement. Ultrasound examinations cannot be reproduced in their entirety for independent review at a later date and, because they are operator dependent, it cannot be guaranteed that the same technique and measurements will be taken from one assessment to the next. If new lesions are identified by ultrasound in the course of the study, confirmation by CT or MRI is advised. If there is concern about radiation exposure at CT, MRI may be used instead of CT in selected instances.

- Endoscopy, laparoscopy: The utilization of these techniques for objective tumor evaluation is not advised. However, they can be useful to confirm complete pathological response when biopsies are obtained or to determine relapse in trials where recurrence following complete response (CR) or surgical resection is an endpoint.
- Tumor markers: Tumor markers alone cannot be used to assess objective tumor response. If markers are initially above the upper normal limit, however, they must normalize for a patient to be considered in CR. Because tumor markers are disease specific, instructions for their measurement should be incorporated into protocols on a disease specific basis. Specific guidelines for both CA-125 response (in recurrent ovarian cancer) and prostate-specific antigen response (in recurrent prostate cancer), have been published. In addition, the Gynecologic Cancer Intergroup has developed CA-125 progression criteria which are to be integrated with objective tumor assessment for use in first-line trials in ovarian cancer.
- Cytology, histology: These techniques can be used to differentiate between PR and CR in rare cases if required by protocol (for example, residual lesions in tumor types such as germ cell tumors, where known residual benign tumors can remain). When effusions are known to be a potential adverse effect of treatment (eg, with certain taxane compounds or angiogenesis inhibitors), the cytological confirmation of the neoplastic origin of any effusion that appears or worsens during treatment can be considered if the measurable tumor has met criteria for response or stable disease in order to differentiate between response (or stable disease) and progressive disease.

RESPONSE CRITERIA

Evaluation of Target Lesions

- Complete Response (CR): Disappearance of all target lesions. Any pathological lymph nodes (whether target or non-target) must have reduction in short axis to < 10 mm.
- Partial Response (PR): At least a 30% decrease in the sum of diameters of target lesions, taking as reference the baseline sum diameters
- Progressive Disease (PD): At least a 20% increase in the sum of diameters of target lesions, taking as reference the smallest sum on study (this includes the baseline sum if that is the smallest on study). In addition to the relative increase of 20%, the sum must also demonstrate an absolute increase of at least 5 mm. (Note: the appearance of one or more new lesions is also considered progression).
- Stable Disease (SD): Neither sufficient shrinkage to qualify for PR nor sufficient increase to qualify for PD, taking as reference the smallest sum diameters while on study
- Lymph nodes: Lymph nodes identified as target lesions should always have the actual short axis measurement recorded (measured in the same anatomical plane as the baseline examination), even if the nodes regress to below 10 mm on study. This means that when lymph nodes are included as target lesions, the “sum” of lesions may not be zero even if CR criteria are met, since a normal lymph node is defined as

having a short axis of < 10 mm. Case report recorded in a separate section where, in order to qualify for CR, each node must achieve a short axis <10 mm. For PR, SD and PD, the actual short axis measurement of the nodes is to be included in the sum of target lesions.

- Target lesions that become "too small to measure". While on study, all lesions (nodal and non-nodal) recorded at baseline should have their actual measurements recorded at each subsequent evaluation, even when very small (eg, 2 mm). However, sometimes lesions or lymph nodes which are recorded as target lesions at baseline become so faint on CT scan that the radiologist may not feel comfortable assigning an exact measure and may report them as being "too small to measure".
  When this occurs, it is important that a value be recorded on the eCRF. If it is the opinion of the radiologist that the lesion has likely disappeared, the measurement should be recorded as 0 mm. If the lesion is believed to be present and is faintly seen but too small to measure, a default value of 5 mm should be assigned (Note: It is less likely that this rule will be used for lymph nodes since they usually have a definable size when normal and are frequently surrounded by fat such as in the retroperitoneum; however, if a lymph node is believed to be present and is faintly seen but too small to measure, a default value of 5 mm should be assigned in this circumstance as well). This default value is derived from the 5 mm CT slice thickness (but should not be changed with varying CT slice thickness). The measurement of these lesions is potentially non-reproducible, therefore providing this default value will prevent false responses or progressions based upon measurement error. To reiterate, however, if the radiologist is able to provide an actual measure, that should be recorded, even if it is below 5 mm.
- Lesions that split or coalesce on treatment: When non-nodal lesions "fragment", the longest diameters of the fragmented portions should be added together to calculate the target lesion sum. Similarly, as lesions coalesce, a plane between them may be maintained that would aid in obtaining maximal diameter measurements of each individual lesion. If the lesions have truly coalesced such that they are no longer separable, the vector of the longest diameter in this instance should be the maximal longest diameter for the "coalesced lesion".

Evaluation of Nontarget Lesions

While some nontarget lesions may actually be measurable, they need not be measured and instead should be assessed only qualitatively at the time points specified in the protocol.

- CR: Disappearance of all non-target lesions and normalization of tumor marker level. All lymph nodes must be nonpathological in size (<10 mm short axis).
- PD: Unequivocal progression (as detailed below) of existing nontarget lesions. (Note: the appearance of one or more new lesions is also considered progression.)
- Non-CR/Non-PD: Persistence of one or more nontarget lesion(s) and/or maintenance of tumor marker level above the normal limits
- When the patient also has measurable disease: In this setting, to achieve "unequivocal progression" on the basis of the nontarget disease, there must be an overall level of

substantial worsening in nontarget disease such that, even in presence of SD or PR in target disease, the overall tumor burden has increased sufficiently to merit discontinuation of therapy. A modest "increase" in the size of one or more nontarget lesions is usually not sufficient to qualify for unequivocal progression status. The designation of overall progression solely on the basis of change in nontarget disease in the face of SD or PR of target disease will therefore be extremely rare.

- <u>When the patient has only non-measurable disease:</u> This circumstance arises in some phase 3 trials when it is not a criterion of trial entry to have measurable disease. The same general concept applies here as noted above, however, in this instance there is no measurable disease assessment to factor into the interpretation of an increase in non-measurable disease burden. Because worsening in non-target disease cannot be easily quantified (by definition: if all lesions are truly non-measurable) a useful test that can be applied when assessing patients for unequivocal progression is to consider if the increase in overall disease burden based on the change in non-measurable disease is comparable in magnitude to the increase that would be required to declare PD for measurable disease: ie, an increase in tumor burden representing an additional 73% increase in "volume" (which is equivalent to a 20% increase diameter in a measurable lesion).
- Examples include an increase in a pleural effusion from "trace" to "large", an increase in lymphangitic disease from localized to widespread, or may be described in protocols as "sufficient to require a change in therapy". If "unequivocal progression" is seen, the patient should be considered to have had overall PD at that point. While it would be ideal to have objective criteria to apply to non-measurable disease, the very nature of that disease makes it impossible to do so, therefore the increase must be substantial.

<u>New Lesions</u>

The appearance of new malignant lesions denotes disease progression; therefore, some comments on detection of new lesions are important. There are no specific criteria for the identification of new radiographic lesions; however, the finding of a new lesion should be unequivocal: ie, not attributable to differences in scanning technique, change in imaging modality or findings thought to represent something other than tumor (for example, some "new" bone lesions may be simply healing or flare of pre-existing lesions). This is particularly important when the patient's baseline lesions show partial or CR. For example, necrosis of a liver lesion may be reported on a CT scan report as a "new" cystic lesion, which it is not.

A lesion identified on a follow-up trial in an anatomical location that was not scanned at baseline is considered a new lesion and will indicate disease progression. An example of this is the patient who has visceral disease at baseline and while on trial has a CT or MRI brain ordered which reveals metastases. The patient's brain metastases are considered to be evidence of PD even if he/she did not have brain imaging at baseline.

If a new lesion is equivocal, for example because of its small size, continued therapy and follow-up evaluation will clarify if it represents truly new disease. If repeat scans confirm there is definitely a new lesion, then progression should be declared using the date of the initial scan.

While fluorodeoxyglucose (FDG)-PET response assessments need additional study, it is sometimes reasonable to incorporate the use of FDG-PET scanning to complement CT scanning in assessment of progression (particularly possible "new" disease). New lesions on the basis of FDG-PET imaging can be identified according to the following algorithm:
Negative FDG-PET at baseline, with a positive FDG-PET at follow-up, is a sign of PD based on a new lesion.

- No FDG-PET at baseline and a positive FDG-PET at follow-up: If the positive FDG-PET at follow-up corresponds to a new site of disease confirmed by CT, this is PD. If the positive FDG-PET at follow-up is not confirmed as a new site of disease on CT, additional follow-up CT scans are needed to determine if there is truly progression occurring at that site (if so, the date of PD will be the date of the initial abnormal FDG-PET scan). If the positive FDG-PET at follow-up corresponds to a pre-existing site of disease on CT that is not progressing on the basis of the anatomic images, this is not PD.

Evaluation of Best Overall Response

The BOR is the best response recorded from the start of the study drug treatment until the end of treatment taking into account any requirement for confirmation. On occasion a response may not be documented until after the end of therapy so protocols should be clear if post-treatment assessments are to be considered in determination of BOR. Protocols must specify how any new therapy introduced before progression will affect best response designation. The patient's BOR assignment will depend on the findings of both target and nontarget disease and will also take into consideration the appearance of new lesions. Furthermore, depending on the nature of the trial and the protocol requirements, it may also require confirmatory measurement. Specifically, in non-randomized trials where response is the primary endpoint, confirmation of PR or CR is needed to deem either one the "best overall response".

The BOR is determined once all the data for the patient is known. Best response determination in trials where confirmation of complete or partial response IS NOT required: Best response in these trials is defined as the best response across all time points (for example, a patient who has SD at first assessment, PR at second assessment, and PD on last assessment has a BOR of PR). When SD is believed to be best response, it must also meet the protocol specified minimum time from baseline. If the minimum time is not met when SD is otherwise the best time point response, the patient's best response depends on the subsequent assessments. For example, a patient who has SD at first assessment, PD at second and does not meet minimum duration for SD, will have a best response of PD. The same patient lost to follow-up after the first SD assessment would be considered inevaluable.

Target Lesions	Non-target Lesions	New Lesions	Overall Response
CR	CR	No	CR
CR	Non-CR/non-PD	No	PR
CR	Not evaluated	No	PR
PR	Non-PD or not all evaluated	No	PR
SD	Non-PD or not all evaluated	No	SD
Not all evaluated	Non-PD	No	NE
PD	Any	Yes or No	PD
Any	PD	Yes or No	PD
Any	Any	Yes	PD

Abbreviations: CR, complete response; NE, not evaluable; PD, progressive disease; PR, partial response; SD, stable disease.

When nodal disease is included in the sum of target lesions and the nodes decrease to "normal" size (< 10 mm), they may still have a measurement reported on scans. This measurement should be recorded even though the nodes are normal in order not to overstate progression should it be based on increase in size of the nodes. As noted earlier, this means that patients with CR may not have a total sum of "zero."

In trials where confirmation of response is required, repeated "NE" time point assessments may complicate best response determination. The analysis plan for the trial must address how missing data/assessments will be addressed in determination of response and progression. For example, in most trials it is reasonable to consider a patient with time point responses of PR-NE-PR as a confirmed response.

Patients with a global deterioration of health status requiring discontinuation of treatment without objective evidence of disease progression at that time should be reported as "symptomatic deterioration". Every effort should be made to document objective progression even after discontinuation of treatment. Symptomatic deterioration is not a descriptor of an objective response: it is a reason for stopping trial therapy.

Conditions that define "early progression, early death, and inevaluability" are trial specific and should be clearly described in each protocol (depending on treatment duration, treatment periodicity).

In some circumstances it may be difficult to distinguish residual disease from normal tissue. When the evaluation of CR depends upon this determination, it is recommended that the residual lesion be investigated (fine needle aspirate/biopsy) before assigning a status of CR. FDG-PET may be used to upgrade a response to a CR in a manner similar to a biopsy in cases where a residual radiographic abnormality is thought to represent fibrosis or scarring. The use of FDG-PET in this circumstance should be prospectively described in the protocol and supported by disease specific medical literature for the indication. However, it must be acknowledged that both approaches may lead to false positive CR due to limitations of FDG-PET and biopsy resolution/ sensitivity.

For equivocal findings of progression (eg, very small and uncertain new lesions; cystic changes, or necrosis in existing lesions), treatment may continue until the next scheduled assessment. If at the next scheduled assessment, progression is confirmed, the date of progression should be the earlier date when progression was suspected.

CONFIRMATORY MEASUREMENT/DURATION OF RESPONSE

Confirmation

In nonrandomized trials where response is the primary endpoint, confirmation of PR and CR is required to ensure responses identified are not the result of measurement error. This will also permit appropriate interpretation of results in the context of historical data where response has traditionally required confirmation in such trials. However, in all other circumstances, ie, in randomized trials (phase 2 or 3) or trials where stable disease or progression are the primary endpoints, confirmation of response is not required since it will not add value to the interpretation of trial results. However, elimination of the requirement for response confirmation may increase the importance of central review to protect against bias, in particular in trials which are not blinded.

In the case of SD, measurements must have met the SD criteria at least once after trial entry at a minimum interval (in general not less than 6 weeks).

Duration of Overall Response

The duration of overall response is measured from the time measurement criteria are first met for CR/PR (whichever is first recorded) until the first date that recurrent or progressive disease is objectively documented (taking as reference for progressive disease the smallest measurements recorded on study).

The duration of overall CR is measured from the time measurement criteria are first met for CR until the first date that recurrent disease is objectively documented.

Duration of Stable Disease

Stable disease is measured from the start of the treatment (in randomized trials, from date of randomization) until the criteria for progression are met, taking as reference the smallest sum on study (if the baseline sum is the smallest, this is the reference for calculation of PD).

The clinical relevance of the duration of stable disease varies in different studies and diseases. If the proportion of patients achieving stable disease for a minimum period of time is an endpoint of importance in a particular trial, the protocol should specify the minimal time interval required between 2 measurements for determination of stable disease.

Note: The duration of response and stable disease as well as the progression-free survival are influenced by the frequency of follow-up after baseline evaluation. It is not in the scope of this guideline to define a standard follow-up frequency. The frequency should take into account many parameters including disease types and stages, treatment periodicity, and standard practice. However, these limitations of the precision of the measured endpoint should be taken into account if comparisons between trials are to be made.
