# Supplementary material for: Alternating modified CAPOX/CAPIRI plus bevacizumab in untreated unresectable metastatic colorectal cancer: a phase 2 trial
Source: Signal Transduct Target Ther. 2024 Dec 11;9:346. doi: 10.1038/s41392-024-02048-z (PMC11631963; doi:10.1038/s41392-024-02048-z)
Supplement: Supplementary file 4 — appendix II [file 41392_2024_2048_MOESM4_ESM.pdf]

AJCC
American Joint Committee on Cancer

AJCC Cancer Staging Manual

Eighth Edition

Springer

Brief Contents by Subject

Adrenal Cortical Carcinoma . . . 911
Adrenal, Neuroendocrine . . . 919
Ampulla of Vater . . . 327
Ampulla of Vater, Neuroendocrine . . . 361
Anus . . . 275
Appendix, Carcinoma . . . 237
Appendix, Neuroendocrine . . . 389
Bile Duct, Distal . . . 317
Bile Ducts, Intrahepatic . . . 295
Bile Ducts, Perihilar . . . 311
Bladder, Urinary . . . 757
Bone, Appendicular Skeleton . . . 471
Bone, Pelvis . . . 471
Bone, Spine . . . 471
Brain . . . 857
Breast . . . 589
Cervical Nodes and Unknown Primary, Head and Neck . . . 67
Cervix Uteri . . . 649
Colon . . . 251
Colon, Neuroendocrine . . . 395
Conjunctival Carcinoma . . . 787
Conjunctival Melanoma . . . 795
Corpus Uteri—Carcinoma and Carcinosarcoma . . . 661
Corpus Uteri—Sarcoma . . . 671
Cutaneous Squamous Cell Carcinoma, Head and Neck . . . 171
Duodenum, Neuroendocrine . . . 361
Esophagogastric Junction . . . 185
Esophagus . . . 185
Eyelid Carcinoma . . . 779
Fallopian Tube . . . 681
Gallbladder . . . 303
Gastrointestinal Stromal Tumor . . . 523
General Staging Rules . . . 3
Gestational Trophoblastic Neoplasms . . . 691
Head and Neck Staging Overview . . . 55
Kidney . . . 739
Lacrimal Gland Carcinoma . . . 833
Larynx . . . 149
Leukemia . . . 979
Lip . . . 79
Liver . . . 287
Lung . . . 431
Lymphoma, Hodgkin . . . 937
Lymphoma, Non-Hodgkin . . . 937
Lymphoma, Primary Cutaneous . . . 967
Melanoma of the Skin . . . 563
Melanoma, Mucosal Melanoma of the Head and Neck . . . 163
Merkel Cell Carcinoma . . . 549
Mesothelioma, Malignant Pleural . . . 457
Myeloma, Multiple Myeloma and Plasma Cell Disorders . . . 973
Nasal Cavity . . . 137
Ocular Adnexal Lymphoma . . . 849
Oral Cavity . . . 79
Orbital Sarcoma . . . 841
Ovary . . . 681
Pancreas, Exocrine . . . 337
Pancreas, Neuroendocrine . . . 407
Paranasal Sinuses . . . 137
Parathyroid . . . 903
Pediatric Hematologic Malignancies . . . 959
Penis . . . 701
Peritoneum, Primary Peritoneal Carcinoma . . . 681
Pharynx—Oropharynx, HPV-Mediated (p16+) . . . 123
Pharynx—Nasopharynx . . . 103
Pharynx—Oropharynx (p16-) . . . 123
Pharynx—Hypopharynx . . . 123
Pleural Mesothelioma, Malignant . . . 457
Prostate . . . 715
Rectum . . . 251
Rectum, Neuroendocrine . . . 395
Renal Pelvis . . . 749
Retinoblastoma . . . 819
Salivary Glands, Major . . . 95
Small Intestine . . . 221
Small Intestine, Neuroendocrine . . . 375
Soft Tissue Sarcoma . . . 489
Soft Tissue Sarcoma, Abdominal and Visceral . . . 517
Soft Tissue Sarcoma, Head and Neck . . . 499
Soft Tissue Sarcoma, Retroperitoneal . . . 531
Soft Tissue Sarcoma, Trunk and Extremity . . . 507
Soft Tissue Sarcoma, Unusual Histologies . . . 539
Spinal Cord . . . 857
Stomach . . . 203
Stomach, Neuroendocrine . . . 351
Testis . . . 727
Thymus . . . 423
Thyroid, Differentiated and Anaplastic . . . 873
Thyroid, Medullary . . . 891
Ureter . . . 749
Urethra . . . 677
Uveal Melanoma . . . 805
Vagina . . . 641
Vulva . . . 633

AJCC Cancer Staging Manual

Eighth Edition

AMERICAN JOINT COMMITTEE ON CANCER

ADMINISTRATIVE SPONSOR
American College of Surgeons

FOUNDING ORGANIZATIONS
American Cancer Society
American College of Physicians
American College of Radiology
American College of Surgeons
College of American Pathologists
National Cancer Institute

SUSTAINING MEMBER ORGANIZATIONS
American Cancer Society
American College of Surgeons
American Society of Clinical Oncology
Centers for Disease Control and Prevention
College of American Pathologists

ADDITIONAL MEMBER ORGANIZATIONS
American Association of Pathologists' Assistants
American College of Physicians
American College of Radiology
American Head and Neck Society
American Society of Colon and Rectal Surgeons
American Society for Radiation Oncology
American Urological Association
Canadian Partnership Against Cancer
International Collaboration on Cancer Reporting
National Cancer Institute
National Cancer Registrars Association
National Comprehensive Cancer Network
North American Association of Central Cancer Registries
Society of Gynecologic Oncology
Society of Surgical Oncology
Society of Urologic Oncology

EXECUTIVE OFFICE
American Joint Committee on Cancer
633 North Saint Clair Street
Chicago, IL 60611-3211
PHONE: 312-202-5205
www.cancerstaging.org
ajcc@facs.org

AJCC
American Joint Committee on Cancer

AJCC Cancer Staging Manual
8th Edition

EDITOR-IN-CHIEF
Mahul B. Amin, MD, FCAP

EDITORIAL BOARD

Stephen B. Edge, MD, FACS
7th Edition Editor-in-Chief

Frederick L. Greene, MD, FACS
6th Edition Editor-in-Chief

Richard L. Schilsky, MD, FACP, FASCO
Clinical Oncology Representative

Laurie E. Gaspar, MD, MBA, FACR
Radiation Oncology Representative

Mary Kay Washington, MD, PhD
Pathology Representative

Daniel C. Sullivan, MD
Radiology Representative

Robert K. Brookland, MD, FACR, FACRO
AJCC Vice Chair, American Cancer Society Representative

James D. Brierley, MB, FRCR, FRCR, FRCPSC
UICC Representative

Charles M. Balch, MD, FACS
Professional Organization and Corporate Relationship Core Chair

Carolyn C. Compton, MD, PhD, FCAP
Precision Medicine Core Chair

Kenneth R. Hess, PhD
Evidence Based Medicine and Statistics Core Chair

Jeffrey E. Gershenwald, MD, FACS
Content Harmonization Core Chair

J. Milburn Jessup, MD
Data Collection Core Chair

David R. Byrd, MD, FACS
AJCC Chair, Administrative Core Chair

David P. Winchester, MD, FACS
AJCC Executive Director

Martin Madera
AJCC Manager, Administrative Core Manager

Elliot A. Asare, MD
AJCC Clinical Scholar-in-Residence

MANAGING EDITOR
Laura R. Meyer, CAPM

TECHNICAL EDITOR
Donna M. Gress, RHIT, CTR

EXPERT PANEL LEADERS

Bone
Jeffrey S. Kneisl, MD, FACS, Chair
Andrew Rosenberg, MD, Vice-Chair

Breast
Gabriel N. Hortobagyi, MD, FACP, Co-Chair
Armando Giuliano, MD, Co-Chair

Central Nervous System
Edward R. Laws, Jr., MD, FACS, Chair
Walter Curran, MD, Vice-Chair

Endocrine System
Nancy Perrier, MD, Chair
Herbert Chen, MD, Vice-Chair

Female Reproductive Organs
David G. Mutch, MD, Chair
Alexander B. Olawaiye, MD, Vice-Chair

Head And Neck
Jatin P. Shah, MD, FACS, Chair
William Lydiatt, MD, FACS, Vice-Chair

Hematologic Malignancies
John P. Leonard, MD, Chair
Elaine Jaffe, MD, Vice-Chair

Hepatobiliary System
Nicolas Vauthey, MD, Chair
Timothy Pawlik, MD, Vice-Chair

Urinary System
Walter M. Stadler, MD, FACP, Chair
James M. McKiernan, MD, Vice-Chair

Lower Gastrointestinal Tract
J. Milburn Jessup, MD, Chair
Richard M. Goldberg, MD, FACP, Vice-Chair

Male Genital Organs
Daniel Lin, MD, Chair
Howard Sandler, MD, Vice-Chair

Skin – Melanoma
Jeffrey E. Gershenwald, MD, FACS, Chair
Richard Scolyer, MD, Vice-Chair

Neuroendocrine Tumors
Eugene Woltering, MD, Chair
Emily Bergsland, MD, Vice-Chair

Skin – Merkel Cell Carcinoma
Arthur Sober, MD, Chair
Timothy M. Johnson, MD, Vice-Chair

Ophthalmic Sites
Paul T. Finger, MD, FACS, Chair
Sarah Coupland, MBBS, PhD, Vice-Chair

Soft Tissue Sarcoma
Raphael Pollock, MD, PhD, FACS, Chair
Robert Maki, MD, Vice-Chair

Thorax
Valerie W. Rusch, MD, FACS, Chair
Douglas Wood, MD, Vice-Chair

Upper Gastrointestinal Tract
Wayne Hofstetter, MD, Chair
David Kelsen, MD, Vice-Chair

AJCC STAFF

Chantel Ellis
Education and Product Development Administrator

Judy Janes
AJCC Coordinator

Ashley Yannello
Electronic Production Administrator

AJCC Cancer Staging Manual

Eighth Edition

AMERICAN JOINT COMMITTEE ON CANCER
Executive Office
633 North Saint Clair Street
Chicago, IL 60611-3211

This manual was prepared and published through the support of the American College of Surgeons, the American Cancer Society, the American Society of Clinical Oncology, the College of American Pathologists, and the Centers for Disease Control and Prevention

AJCC
American Joint Committee on Cancer

Springer

Editor-in-Chief
Mahul B. Amin, MD, FCAP

Editors
Stephen B. Edge, MD, FACS
Frederick L. Greene, MD, FACS
David R. Byrd, MD, FACS
Robert K. Brookland, MD, FACR, FACRO
Mary Kay Washington, MD, PhD
Jeffrey E. Gershenwald, MD, FACS
Carolyn C. Compton, MD, PhD, FCAP
Kenneth R. Hess, PhD
Daniel C. Sullivan, MD
J. Milburn Jessup, MD
James D. Brierley, MB, FRCR, FRCR, FRCPSC
Laurie E. Gaspar, MD, MBA, FACR
Richard L. Schilsky, MD, FACP, FASCO
Charles M. Balch, MD, FACS
David P. Winchester, MD, FACS
Elliot A. Asare, MD
Martin Madera
Donna M. Gress, RHIT, CTR – Technical Editor
Laura R. Meyer, CAPM – Managing Editor

ISBN 978-3-319-40617-6 ISBN 978-3-319-40618-3 (eBook)
DOI 10.1007/978-3-319-40618-3

Library of Congress Control Number: 2016952640

© American Joint Committee on Cancer 2017
This work is subject to copyright. All rights are reserved by the Publisher, whether the whole or part of the material is concerned, specifically the rights of translation, reprinting, reuse of illustrations, recitation, broadcasting, reproduction on microfilms or in any other physical way, and transmission or information storage and retrieval, electronic adaptation, computer software, or by similar or dissimilar methodology now known or hereafter developed.
The use of general descriptive names, registered names, trademarks, service marks, etc. in this publication does not imply, even in the absence of a specific statement, that such names are exempt from the relevant protective laws and regulations and therefore free for general use.
The publisher, the authors and the editors are safe to assume that the advice and information in this book are believed to be true and accurate at the date of publication. Neither the publisher nor the authors or the editors give a warranty, express or implied, with respect to the material contained herein or for any errors or omissions that may have been made.

Printed on acid-free paper

This Springer imprint is published by Springer Nature
The registered company is Springer International Publishing AG Switzerland

Eighth Edition Dedication

The AJCC Cancer Staging Manual, 8th Edition is dedicated to all CANCER REGISTRARS in recognition of their:

- *education and unique commitment to the recording and maintenance of data that are so vital for the care of the cancer patient;*
- *professionalism in the collection of factors that are fundamental to sustaining local, state, and national cancer registries;*
- *dedication to the cataloging of information crucial to cancer research;*
- *leadership, support, and promulgation of the principles of cancer staging;*
- *AND THEIR POSITIVE IMPACT ON CANCER PATIENT OUTCOMES.*

EIGHTH EDITION
Cancer Registrars

SEVENTH EDITION
Dedicated to Irvin D. Fleming, MD

SIXTH EDITION
Dedicated to Robert V. P. Hutter, MD

FIFTH EDITION
Dedicated to Oliver Howard Beahrs, MD

FOURTH EDITION
Dedicated to the memory of Harvey Baker, MD

THIRD EDITION
Dedicated to the memory of W. A. D. Anderson, MD
Marvin Pollard, MD
Paul Sherlock, MD

SECOND EDITION
Dedicated to the memory of Murray M. Copeland, MD

Preface

The *AJCC Cancer Staging Manual*, 8th Edition: Continuing to build a bridge from a "population-based" to a more "personalized" approach

Cancer staging plays a pivotal role in the battle against cancer. First and foremost, staging provides patients with cancer and their physicians the critical benchmark and standards for defining prognosis, the likelihood of overcoming the cancer once diagnosed, and for determining the best treatment approach for the disease. Staging also forms the basis for understanding the changes in population cancer incidence, the extent of disease at initial presentation, and the overall impact of improvements in cancer treatment. Staging is the foremost classifier of cancer patients and defines groups for inclusion in clinical trials and analysis of outcomes data in clinical studies. For clinicians and scientists engaged in research, it provides consistent nomenclature, which is essential for the study of cancer from biology to clinical presentation to management.

Refining the standards by which to provide the best possible staging system is a never-ending process. Toward this end, the American Joint Committee on Cancer (AJCC) has led these efforts in the United States since 1959. A collaborative effort between the AJCC and the Union for International Cancer Control (UICC) maintains the system that is used worldwide. This system classifies the extent of disease based mostly on anatomic information related to the extent of the primary tumor, the status of regional lymph nodes, and presence or absence of distant metastases (TNM classification). The basis of this classification was developed in the 1940s by Pierre Denoix of France and formalized by the UICC in the 1950s with the formation of the Committee on Clinical Stage Classification and Applied Statistics. The AJCC was founded in 1959 to complement this work.

The AJCC published its first cancer staging manual in 1977. Since the 1980s, the work of the AJCC and UICC has been coordinated, resulting in concordant stage definitions and simultaneous publication of the *AJCC Cancer Staging Manual* and the *TNM Classification of Malignant Tumours* by the UICC. The revision cycle has been 6 to 8 years, a time frame that provides for accommodation of advances in cancer care while allowing cancer registry systems to maintain stable operations. This new edition of the *AJCC Cancer Staging Manual* being published in 2016 is effective for all cases diagnosed on or after January 1, 2017.

The ongoing work of the AJCC is made possible by the dedicated, continuous volunteer effort of hundreds, and perhaps thousands, of committed health professionals including physicians, population scientists, statisticians, cancer registrars, supporting staff, and others. When the staging manual is being updated, volunteers, representing all relevant disciplines, are organized into expert panels chaired by leading clinicians. These panels make recommendations for changes in the staging system based on available evidence supplemented with expert consensus (see Chapter 2, Organization of the *AJCC Cancer Staging Manual*). Over the years, as a result of these efforts, the TNM staging system has become the global standard for gathering, communicating, and exchanging cancer information worldwide and is widely used by clinicians, the surveillance community, registrars, researchers, medical industries, patient advocates, and patients.

The process for review and revision of the staging system has become increasingly rigorous with each new edition of the *AJCC Cancer Staging Manual*. For each subsequent edition, a development team or "expert panel" is appointed for each disease site staging system or chapter. The expert panel includes experts from all relevant medical disciplines (surgical, medical and radiation oncology, pathology, radiology, and others; plus cancer registrars, population scientists, statisticians, and other professionals). The AJCC expert panels are international in scope, and each panel consists of at least one individual representing the UICC. The expert panel revisits, restructures, validates, and researches data of the current system used in clinical practice. Based on this work, the expert panel makes appropriate revisions to the staging system, which are reflected in this publication. The AJCC, for the first time, also empaneled seven AJCC cores with multiple team members in each core, with defined functions and expertise. These cores, serving across all 18 disease site expert panels, include the Precision Medicine Core, Evidence Based Medicine and Statistics Core, Content Harmonization Core, Data Collection Core, Professional Organization and Corporate Relationship Core, and Administrative Core. For this new edition, the editorial board worked to increase the level of documentation explaining the reasons for changes and the level of evidence supporting each change. The Evidence Based Medicine and Statistics Core established a system for quantifying the level of evidence supporting each major staging recommendation (see Chapter 2). The level of evidence supporting the staging systems and their ongoing refinement during the publication of the next edition varies among disease sites.

For some diseases, particularly the less common cancers or cancers for which we are proposing a system for the first time, few outcome data may be available. In the 8th Edition, at least 12 new staging systems are presented, and these are based on single, large international cohort experiences or on other limited data that are available and supplemented by expert consensus. Although potentially imperfect, new and evolving classification schemas are critical to allow the collection of standardized data to support clinical care and for future evaluation and refinement of the system. Although the state of the science varies among staging systems, these systems nonetheless form the basis for new follow-up data and research that informs future systems. Throughout the 8th Edition, when stage definitions have been changed or new definitions provided, the AJCC has respected a principle of transparency in providing the levels of evidence that inform the changes (see Chapter 2, Organization of the *AJCC Cancer Staging Manual*, for more information on levels of evidence).

Increasingly, the expert panels of the AJCC have used existing datasets or established the necessary relationships to develop new large datasets to provide high-level evidence to support changes in the staging system. Examples include the work in melanoma that led to changes in the 7th Edition and their refinement in the 8th Edition; use of the National Cancer Data Base and Surveillance, Epidemiology, and End Results (SEER) database for evaluation of the colorectal staging system; and the use of existing datasets from the United States, Europe, and Asia in gastric cancer. Other groups have been established to collect very large international datasets to refine staging. The best examples in refining staging for the 8th Edition are the international group collecting outcome and staging data in melanoma, the collaborative group of the International Association for the Study of Lung Cancer (IASLC), the Worldwide Esophageal Cancer Collaborative (WECC), and the Eye Cancer Network's Universal Eye Cancer Database Project.

A major challenge to TNM staging is the rapid evolution of knowledge in cancer biology and the discovery and development of biologic factors that predict cancer outcome and response to treatment with better accuracy than purely anatomically based staging. These advances have led some cancer experts to conclude that TNM is obsolete or, at best, less relevant in clinical practice. Although such statements are misguided, the reality is that the anatomic extent of disease tells only part of the story for many cancer patients. The prospect of including nonanatomic prognostic factors in staging has led to intense debate about the purpose and structure of staging. The philosophy of staging by the TNM system described in the *AJCC Cancer Staging Manual*, 1st Edition states that "it is intended to provide a way by which

designation for the state of cancer at various points in time can be readily communicated to others to assist in decisions regarding treatment and to be a factor in the judgment as to prognosis. Ultimately, it provides a mechanism for comparing like and unlike groups of cases, particularly in regard to the results of different therapeutic procedures." As viewed by some, the intention of assigning a TNM stage was to understand prognosis and to judge the overall impact of improvement in cancer treatment at the population level. Needless to say, in reality, the AJCC staging system functioned as a patient classifier and began to drive paradigms to stratify patient management for different cancers. Over time, this became an important factor in clinical decision making at the bedside for each patient. As nonanatomic factors, particularly molecular markers, become more relevant in the current genomic and precision medicine era, debate continues regarding inclusion of prognostic (defining outcome) and predictive factors (predicting response to particular therapy) so that the staging system is more relevant at an individual patient level rather than only on a population level.

Beginning with the *AJCC Cancer Staging Manual*, 6th Edition, nonanatomic factors have been added judiciously to the classifications that modify stage groups. Relevant markers that are so important they are required for clinicians to make clear treatment decisions have been included gradually in stage groupings. Examples include the mitotic rate in staging gastrointestinal stromal tumors and prostate-specific antigen and Gleason score in staging prostate cancer (6th and 7th Editions). This shift away from purely anatomic information continues in the current edition. In all chapters, several new features that include nonanatomic factors have been added, such as detailed listings of prognostic factors, endorsed risk assessment models (in select cancers), and factors important for clinical trial stratification. Details of the overall approach are outlined in Chapter 2, Organization of the Cancer Staging Manual, and the 8th Edition AJCC TNM editorial board views this edition as continuing to build the important bridge from a "population-based" approach to a more "personalized" approach.

That said, it also must be clearly stated that it is critical to maintain the anatomic basis of cancer staging. Anatomic extent of disease remains the key prognostic factor, the strongest predictor of outcome, in most diseases. In addition, it is necessary to have clear links to past data to assess trends in cancer incidence and the impact of advances in screening and treatment and to be able to apply stage and compare stage worldwide in situations in which new nonanatomic factors are not or cannot be collected. Therefore, the staging algorithms in this edition of the *AJCC Cancer Staging Manual* using nonanatomic factors use them only as modifiers of the traditional anatomic TNM-based stage groups to derive a Prognostic Stage Group. These factors are not used to define the T, N, and M components, which remain purely anatomic.

The work of the 8th Edition involved many professionals in all fields in the clinical and diagnostic oncology, cancer registry, population surveillance, and statistical communities. We are very grateful and realize that this combined, synergistic, and exhaustive effort could not have occurred without the teamwork of countless individuals and their expertise, unity of purpose to make a difference in cancer care, dedication, professionalism, and time commitment, largely without remuneration. We acknowledge the leadership and support of the chairs and vice chairs of the disease site expert panels and of the cores. We also thank the very capable, dedicated, and efficient administrative staff at the AJCC: Laura Meyer, AJCC 8th Edition Project Manager and Managing Editor, for administrative oversight of the entire project; Donna Gress, AJCC Technical Specialist and Technical Editor, for reviewing staging rules and advising on data collection processes throughout the 8th Edition development process; Ashley Yannello, AJCC Electronic Production Administrator, for coordinating the illustrations and SharePoint facilities; Chantel Ellis, AJCC Education and Product Development Administrator, for planning education and promotion of the cancer staging system; Judy Janes, AJCC Coordinator, for coordinating innumerable phone and web conference calls and face-to-face meetings; and Martin Madera, AJCC Manager, for coordination of all administrative staff functions.

We believe this revised, updated, and expanded 8th Edition, along with its new and exciting electronic and print product capabilities, will be a powerful resource for patients and physi-

cians alike as they face the battle against cancer. We hope that it provides the conceptual framework and foundation for the future of cancer staging as we make further strides in this era of precision/personalized molecular oncology.

Mahul B. Amin (Editor in Chief), and members of the Editorial Board: Stephen B. Edge, Frederick L. Greene, Richard L. Schilsky, Laurie E. Gaspar, Mary Kay Washington, Daniel C. Sullivan, James D. Brierley, Charles M. Balch, Carolyn C. Compton, Kenneth R. Hess, Jeffrey E. Gershenwald, J. Milburn Jessup, Martin Madera, Elliot A. Asare, Donna M. Gress, Laura R. Meyer, David P. Winchester, Robert K. Brookland, David R. Byrd

Contents

Dedication ... vii

Preface ... ix

Introduction and Historical Overview ... xv

Part I General Information on Cancer Staging and End-Results Reporting

1. Principles of Cancer Staging ... 3
2. Organization of the AJCC Cancer Staging Manual ... 31
3. Cancer Survival Analysis ... 39
4. Risk Models for Individualized Prognosis in the Practice of Precision Oncology ... 47

Part II Head and Neck

5. Staging Head and Neck Cancers ... 55
6. Cervical Lymph Nodes and Unknown Primary Tumors of the Head and Neck ... 67
7. Lip and Oral Cavity ... 79
8. Major Salivary Glands ... 95
9. Nasopharynx ... 103
10. HPV-Mediated (p16+) Oropharyngeal Cancer ... 113
11. Oropharynx (p16–) and Hypopharynx ... 123
12. Nasal Cavity and Paranasal Sinuses ... 137
13. Larynx ... 149
14. Mucosal Melanoma of the Head and Neck ... 163
15. Cutaneous Squamous Cell Carcinoma of the Head and Neck ... 171

Part III Upper Gastrointestinal Tract

16. Esophagus and Esophagogastric Junction ... 185
17. Stomach ... 203
18. Small Intestine ... 221

Part IV Lower Gastrointestinal Tract

19. Appendix – Carcinoma ... 237
20. Colon and Rectum ... 251
21. Anus ... 275

Part V Hepatobiliary System

22. Liver ... 287
23. Intrahepatic Bile Ducts ... 295
24. Gallbladder ... 303
25. Perihilar Bile Ducts ... 311
26. Distal Bile Duct ... 317
27. Ampulla of Vater ... 327
28. Exocrine Pancreas ... 337

Part VI Neuroendocrine Tumors

29. Neuroendocrine Tumors of the Stomach ... 351
30. Neuroendocrine Tumors of the Duodenum and Ampulla of Vater ... 361
31. Neuroendocrine Tumors of the Jejunum and Ileum ... 375
32. Neuroendocrine Tumors of the Appendix ... 389
33. Neuroendocrine Tumors of the Colon and Rectum ... 395
34. Neuroendocrine Tumors of the Pancreas ... 407

Part VII Thorax

35. Thymus 423
36. Lung 431
37. Malignant Pleural Mesothelioma 457

Part VIII Bone

38. Bone 471

Part IX Soft Tissue Sarcoma

39. Introduction to Soft Tissue Sarcoma 489
40. Soft Tissue Sarcoma of the Head and Neck 499
41. Soft Tissue Sarcoma of the Trunk and Extremities 507
42. Soft Tissue Sarcoma of the Abdomen and Thoracic Visceral Organs 517
43. Gastrointestinal Stromal Tumor 523
44. Soft Tissue Sarcoma of the Retroperitoneum 531
45. Soft Tissue Sarcoma – Unusual Histologies and Sites 539

Part X Skin

46. Merkel Cell Carcinoma 549
47. Melanoma of the Skin 563

Part XI Breast

48. Breast 589

Part XII Female Reproductive Organs

49. Introduction to Female Reproductive Organs 631
50. Vulva 633
51. Vagina 641
52. Cervix Uteri 649
53. Corpus Uteri – Carcinoma and Carcinosarcoma 661
54. Corpus Uteri – Sarcoma 671
55. Ovary, Fallopian Tube, and Primary Peritoneal Carcinoma 681
56. Gestational Trophoblastic Neoplasms 691

Part XIII Male Genital Organs

57. Penis 701
58. Prostate 715
59. Testis 727

Part XIV Urinary Tract

60. Kidney 739
61. Renal Pelvis and Ureter 749
62. Urinary Bladder 757
63. Urethra 767

Part XV Ophthalmic Sites

64. Eyelid Carcinoma 779
65. Conjunctival Carcinoma 787
66. Conjunctival Melanoma 795
67. Uveal Melanoma 805
68. Retinoblastoma 819
69. Lacrimal Gland Carcinoma 833
70. Orbital Sarcoma 841
71. Ocular Adnexal Lymphoma 849

Part XVI Central Nervous System

72. Brain and Spinal Cord 857

Part XVII Endocrine System

73. Thyroid – Differentiated and Anaplastic Carcinoma 873
74. Thyroid – Medullary 891
75. Parathyroid 903
76. Adrenal Cortical Carcinoma 911
77. Adrenal – Neuroendocrine Tumors 919

Part XVIII Hematologic Malignancies

78. Introduction to Hematologic Malignancies 931
79. Hodgkin and Non-Hodgkin Lymphomas 937
80. Pediatric Hodgkin and Non-Hodgkin Lymphomas 959
81. Primary Cutaneous Lymphomas 967
82. Plasma Cell Myeloma and Plasma Cell Disorders 973
83. Leukemia 979

Additional Contributors 999

Index 1001

Introduction and Historical Overview

The *AJCC Cancer Staging Manual*, 8th Edition is a compendium of all currently available information on the staging of adult cancers for most clinically important anatomic sites. It has been developed by the American Joint Committee on Cancer (AJCC) in cooperation with the TNM Committee of the Union for International Cancer Control (UICC). The two organizations have worked together at every level to create a staging schema that is largely identical between the two organizations, although some differences exist. The current climate that supports consistency of staging worldwide has been made possible by the mutual respect and diligence of those working in the staging area for both the AJCC and the UICC.

Classification and staging of cancer allow the physician to stratify patients, enabling better management of care; permit the cancer registrar to collect essential cancer data in a uniform manner that facilitates data consolidation and analysis; and facilitate the development of a common language that supports clinical research and the development of new cancer treatment strategies. Cancer staging is widely used at the level of individual patient care, not only to inform prognosis but to determine appropriate treatment options. A common language of cancer staging is mandatory in order to harmonize important contributions from individuals and many institutions throughout the world. The need for consistent nomenclature was the driving force that led to clinical classification of cancer by the League of Nations Health Organization in 1929 and later by the UICC and its TNM Committee.

The AJCC was organized on January 9, 1959, as the American Joint Committee for Cancer Staging and End Results Reporting (AJC); in 1980, it became the American Joint Committee on Cancer (AJCC). The organization was formed to develop a system of cancer staging that was acceptable to the American medical profession. The founding organizations that came together to accomplish this goal were the American College of Surgeons, the College of American Pathologists, the American College of Physicians, the American College of Radiology, the American Cancer Society, and the National Cancer Institute. The governance of the AJCC is still overseen by designees from the founding organizations and representatives of other sponsoring organizations, including the American Society of Clinical Oncology and the Centers for Disease Control and Prevention. The Medical Director of the American College of Surgeons' Commission on Cancer (CoC) functions as the Executive Director of the AJCC. The work of the AJCC has been fostered by the volunteer work committees, called expert panels, that are focused on specific anatomic sites of cancer. In preparation for each new edition of the *AJCC Cancer Staging Manual*, expert panels are convened and serve as consensus groups to review scholarly material related to cancer staging and to make recommendations to the AJCC regarding potential changes in the staging taxonomy. For the 8th Edition, additional expert resources were added in the form of seven cross-cutting core committees, each made up of several members with relevant domain expertise. The cores provide input relevant to all disease site expert panels and assure a uniform and informed approach to their individual topic areas, such as imaging, statistics, levels of evidence, and prognostication modeling.

During the past 50 years of activity related to the AJCC, a large group of consultants and member organization representatives have worked with the AJCC leadership. In addition to representatives from AJCC's founding and sponsoring organizations, representatives are appointed by the American Association of Pathologists' Assistants, American College of

Radiology, American Head and Neck Society, American Society of Colon and Rectal Surgeons, American Society for Radiation Oncology, American Urological Association, Canadian Partnership Against Cancer, National Cancer Institute, National Cancer Registrars Association, National Comprehensive Cancer Network, North American Association of Central Cancer Registries, Society of Gynecologic Oncology, Society of Surgical Oncology, and Society of Urologic Oncology.

Chairing the AJCC have been Murray Copeland, MD (1959–1969); W. A. D. Anderson, MD (1969–1974); Oliver H. Beahrs, MD (1974–1979); David T. Carr, MD (1979–1982); Harvey W. Baker, MD (1982–1985); Robert V. P. Hutter, MD (1985–1990); Donald E. Henson, MD (1990– 1995); Irvin D. Fleming, MD (1995–2000); Frederick L. Greene, MD (2000–2004); David L. Page, MD (2004–2005); Stephen B. Edge, MD (2005–2008); and Carolyn C. Compton, MD, PhD (2008-2013). David R. Byrd, MD, FACS is the current chair.

The initial work on the clinical classification of cancer was instituted by the League of Nations Health Organization (1929), the International Commission on Stage Grouping and Presentation of Results (ICPR) of the International Congress of Radiology (1953), and the Union for International Cancer Control (UICC). The latter organization became most active in the field through its Committee on Clinical Stage Classification and Applied Statistics (1954). This committee was later known as the UICC TNM Committee, which now contains a representative from the AJCC and the current Editor-in-Chief of the *AJCC Cancer Staging Manual* and is supported by a grant from the Centers for Disease Control and Prevention. In November 1969, in a joint meeting of the AJC and UICC, the two groups agreed that they would have a discussion before obligation of a classification scheme by either group. In 1970, the AJC adopted "objectives, rules and regulations of the AJC," which resulted in the formulation and publication of systems of classification of cancer. Since its inception, the AJCC has embraced the TNM system and has used it as its foundation to describe the anatomic extent of cancer at the time of initial diagnosis and before the application of definitive treatment. In addition, a classification of the stages of cancer was used as a guide for treatment and prognosis and for comparison of outcomes in cancer management. In 1976, the AJCC sponsored a National Cancer Conference on Classification and Staging. The deliberation at this conference led directly to the development of the *AJCC Cancer Staging Manual*, 1st Edition, which was published in 1977 and allowed the AJCC to broaden its scope and recognize its leadership role in the staging of cancer for American physicians and registrars. The *AJCC Cancer Staging Manual*, 2nd Edition (1983) updated the earlier edition and included additional sites. This edition also served to enhance conformity with the staging espoused by the TNM Committee of the UICC. The expanding role of the American Joint Committee in a variety of cancer classifications suggested that the original name was no longer applicable. In June 1980, a new name, the American Joint Committee on Cancer, was selected. Since the early 1980s, the close collaboration between the AJCC and the UICC has resulted in uniform and near-identical definitions and stage groupings of cancers for all anatomic sites so that a universal system is now available. This worldwide system was espoused by Robert V. P. Hutter, MD, in his presidential address at the combined meeting of the Society of Surgical Oncology and the British Association of Surgical Oncology in London in 1987. The *AJCC Cancer Staging Manual*, 3rd Edition was published shortly thereafter in 1988.

During the 1990s, the importance of TNM staging of cancer in the United States was heightened by the mandatory requirement that CoC-approved hospitals use the AJCC TNM system as the major language for cancer reporting. This requirement has stimulated education of all physicians and registrars in the use of the TNM system, and credit goes to the Accreditation Program of the CoC for this insightful recognition. The *AJCC Cancer Staging Manual*, 4th and 5th Editions were published in 1992 and 1997.

In the 1st Edition, the editors noted astutely that "*staging of cancer is not an exact science. As new information becomes available about etiology and various methods of diagnosis and treatment, the classification and staging of cancer will change. Periodically, this manual will be revised so that it reflects the changing state of the art. However, changes will occur only at*

reasonable periods." The editors of the 2nd Edition made another erudite comment: "*At the present time the anatomic extent of the cancer is the primary basis for staging; the degree of differentiation of the tumor, the age of the patient are also factors in some cases. In the future, biologic markers and other factors may also play a part.*"

Almost two decades later, in 2002, the *AJCC Cancer Staging Manual*, 6th Edition judiciously added nonanatomic factors that modified stage groups to recognize the emerging importance of these nonanatomic factors as complementary to staging paradigms. The *AJCC Cancer Staging Manual*, 7th Edition, published in 2010, expanded the relevant nonanatomic markers to stage groups, as these factors were deemed critical to make staging more applicable for prognostication and to help make treatment decisions. The heading *Anatomic Stage and Prognostic Groups* was used instead of *Anatomic Stage* to designate this slightly modified approach to determine the stage of a cancer based on stage grouping tables.

While maintaining the anatomic extent of disease as its foundation, the *AJCC Cancer Staging Manual*, 8th Edition makes a concrete attempt to continue to build the bridge from a more traditional "population-based" approach to a more contemporary "personalized approach," one that not only is relevant as a robust classification system for population-based analyses, but also is equally powerful in the care of cancer patients on an individual level and at the bedside. Toward this end, several specific steps and new features regarding prognostic factors and their cumulative role in risk assessment and clinical trials have been added in the presentation of the 8th Edition contents in the disease-site chapter (see Chapter 2, Organization of the AJCC Cancer Staging Manual). Importantly, instead of *Anatomic Stage and Prognostic Groups*, used in the 7th Edition, the term *Prognostic Stage Groups* is consistently used in the 8th Edition to merge the two concepts (anatomic stage and prognostic groups) in tables used to determine the stage group for a particular cancer.

The AJCC recognizes that with this 8th Edition, the education of medical students, resident physicians, physicians in practice, and cancer registrars is paramount. As the 21st century unfolds, new methods of education will complement the 8th Edition and will ensure that all those who care for cancer patients and perform research to improve their lives will be trained in the language of cancer staging.

The AJCC also is pleased to deliver *AJCC Cancer Staging Manual* content by using additional methods and formats for the first time to improve ease of access and use while assuring consistency and accuracy of content. The AJCC has made a significant investment in importing the content of the 8th Edition into a Component Content Management System (CCMS), enabling the content to be organized and managed in a central location and to be distributed electronically through the AJCC's Application Programing Interface (API). Electronic health records (EHR) software vendors, cancer registry software vendors, and electronic application developers will benefit from this digitally structured content by being able to incorporate the content directly into their products when they license access to the API. Vendors who choose to incorporate the API into their software products will be ensured the highest fidelity and accuracy of the AJCC cancer staging rules.

Refining the standards by which to provide the best possible staging system is a never-ending process. This edition, which continues to build the important bridge from a more population-based to a more personalized approach, promulgates new paradigms in staging, and provides novel and exciting electronic and print product capabilities, will pave the foundation for the future of cancer staging as we make further strides in this era of precision/personalized molecular oncology.

American Joint Committee on Cancer, October 2016.

Part I

General Information on Cancer Staging and End-Results Reporting

Principles of Cancer Staging

1

Donna M. Gress, Stephen B. Edge, Frederick L. Greene, Mary Kay Washington, Elliot A. Asare, James D. Brierley, David R. Byrd, Carolyn C. Compton, J. Milburn Jessup, David P. Winchester, Mahul B. Amin, and Jeffrey E. Gershenwald

INTRODUCTION AND OVERVIEW

The extent or *stage* of cancer at the time of diagnosis is a key factor that defines prognosis and is a critical element in determining appropriate treatment based on the experience and outcomes of groups of previous patients with similar stage. In addition, cancer stage often is a key component of inclusion, exclusion, and stratification criteria for clinical trials. Indeed, accurate staging is necessary to evaluate the results of treatments and clinical trials, to facilitate the exchange and comparison of information across treatment centers and within and between cancer-specific registries, and to serve as a basis for clinical and translational cancer research. At the national and international levels, a cohesive approach to the classification of cancer provides a method of clearly conveying clinical experience to others without ambiguity.

Cancer treatment requires assessment of the extent and behavior of the tumor and patient-related factors. Several cancer staging systems are used worldwide. Differences among these systems stem from the needs and objectives of users in clinical medicine and in population surveillance. The most clinically useful staging system is the tumor, node, and metastasis (TNM) staging system developed by the American Joint Committee on Cancer (AJCC) in collaboration with the Union for International Cancer Control (UICC), herein referred to as the AJCC TNM staging system. The AJCC TNM system classifies cancers by the size and extent of the primary tumor (T), involvement of regional lymph nodes (N), and the presence or absence of distant metastases (M), supplemented in recent years by evidence-based prognostic and predictive factors. There is a TNM staging algorithm for cancers of virtually every anatomic site and histology, with the primary exception of pediatric cancers.

Philosophy of Revisions to the TNM Staging System

The AJCC and UICC periodically modify the AJCC TNM staging system in response to newly acquired clinical and pathological data and an improved understanding of cancer biology and other factors affecting prognosis. Periodic and, to the extent possible, evidence-based revision is a key feature that makes this staging system the most clinically useful among staging systems and accounts for its widespread use worldwide. However, because changes in staging systems may make it difficult to compare outcomes of patients over time, evidence-based changes to this staging system are made with deliberate care.

In general, the revision cycle for AJCC TNM staging has historically been 5 to 7 years. This approach provides sufficient time for implementation of changes in clinical management and cancer registry operations and for relevant examination and discussion of data supporting changes in staging. Table 1.1 shows the publication year for each version of the AJCC TNM system up through this current *AJCC Cancer Staging Manual*, 8th Edition. The *AJCC Cancer Staging Manual*, 7th Edition was used for cancer patients diagnosed on or after January 1, 2010. The 8th Edition published in this manual is effective for cancer patients diagnosed on or after January 1, 2017. The AJCC recognizes that rapidly evolving evidence may necessitate more frequent updates of AJCC TNM staging in the future, and anticipates providing more frequent updates for disease sites as new and validated evidence becomes available. Moreover, the AJCC also recognizes that as clinical cancer care continues to evolve and incorporates factors that are not used to determine stage but that provide key information on specific outcomes and/or expected benefit from specific therapies, new,

To access the AJCC cancer staging forms, please visit www.cancerstaging.org.

© American Joint Committee on Cancer 2017

M.B. Amin et al. (eds.), *AJCC Cancer Staging Manual, Eighth Edition*, DOI 10.1007/978-3-319-40618-3_1

Table 1.1 *AJCC Cancer Staging Manual* editions

Edition	Publication	Effective dates for cancer diagnoses
1st	1977	1978–1983
2nd	1983	1984–1988
3rd	1988	1989–1992
4th	1992	1993–1997
5th	1997	1998–2002
6th	2002	2003–2009
7th	2009	2010–2016
8th	2016	2017–

validated clinical tools will be needed to help clinicians efficiently and accurately use these important data to enhance clinical care (see Anatomic Staging and the Evolving Use of Nonanatomic Factors).

Comprehensive Analysis of Staging Rules and Nomenclature

In January 2012, the AJCC and UICC initiated a comprehensive analysis of staging nomenclature: the AJCC–UICC Lexicon Project. This effort focused on harmonization of their collective staging taxonomies with each other and with international standards. This group concluded that terminology should be categorized into four main groups: (1) anatomic stage—disease extent and timing/classification; (2) tumor profile—characterization of tumor (e.g., biomarkers, viral load); (3) patient profile—age, gender, race, and health status; and (4) environment—availability of treatment and quality of imaging. This joint project thus far has encompassed two working groups—anatomic stage and tumor profile—to thoroughly review the existing nomenclature and standard definitions. The patient profile and environment categories will be addressed in future work.

The Content Harmonization Core (CHC) is one of seven AJCC "cores" developed to inform a more uniform 8th Edition effort. The CHC had its first meeting in August 2014. Building upon the work of the AJCC–UICC Lexicon Project, its charge was to review and update the general staging rules and nomenclature (published in Chapter 1 of the 7th Edition) and to develop a more precise language of cancer to enhance the accuracy of the staging system. A goal of this effort is to standardize technical terms and concepts as well as conflicting terms and usage. Once it identified key issues, the CHC worked with thought leaders and organizations to clarify and ensure precise, standardized, and clear definitions and rules for staging to the extent possible; for some terms and concepts, however, unequivocal clarity could not be achieved (and is noted in the chapter). The work product of the CHC is reflected in this chapter, and provides overall rules for staging that apply across all tumor sites. In most cases, the rules are unchanged from previous versions of TNM; to the extent possible, ambiguities have been resolved. Although the rules generally apply across all disease sites, there are some exceptions as to how these rules are applied to specific disease sites. Wherever possible, such exceptions are noted, both in this chapter and in the appropriate disease site chapters.

Assigning Stage: Role of the Managing Physician

Staging requires the collaborative effort of many professionals, including the managing physician, pathologist, radiologist, cancer registrar, and others. The pathologist plays a central role. An accurate microscopic diagnosis is essential to the evaluation and treatment of cancer. Pathologists must also accurately report several anatomic, histologic, and morphologic characteristics of tumors, as well as key biologic features. Pathological reporting is best accomplished by using standardized nomenclature in a structured report, such as the synoptic reports or cancer protocols defined by the College of American Pathologists (CAP). In addition, for some cancers, measurements of other factors, including biochemical, molecular, genetic, immunologic, or functional characteristics of the tumor or normal tissues have become important or essential elements to improve tumor classification. Some of the growing repertoire of techniques that supplement standard histologic evaluation used to characterize tumors and their potential behavior and response to treatment include immunohistochemistry (IHC), cytogenetic analysis, and genetic characterization in the form of mutational analysis. Similarly, imaging specialists must provide concise and unambiguous reports on the extent of cancer as identified on a variety of imaging studies.

Although the pathologist and the radiologist provide important staging information, and may provide important T-, N-, and/or M-related information, stage is defined ultimately from the synthesis of an array of patient history and physical examination findings supplemented by imaging and pathology data. Only the managing physician can assign the patient's stage, because only (s)he routinely has access to all the pertinent information from physical examination, imaging studies, biopsies, diagnostic procedures, surgical findings, and pathology reports.

Related Publications to Facilitate Staging

In the interest of promoting high-quality care, and to facilitate international collaboration in cancer research and comparison of data among different clinical studies, the AJCC uses information from other organizations and publications to facilitate staging, including:

- *World Health Organization Classification of Tumours, Pathology and Genetics*. Since 1958, the World Health Organization (WHO) has had a program aimed at providing internationally accepted criteria for the histologic classification of tumors. The series contains definitions, descriptions, and illustrations of tumor types and related nomenclature (WHO: World Health Organization Classification of Tumours. Various editions. Lyon, France: IARC Press, 2000–2016).
- *WHO International Classification of Diseases for Oncology (ICD-O), 3rd edition*. ICD-O is a numeric classification and coding system by topography and morphology (WHO: ICD-O-3 International Classification of Diseases for Oncology. 3rd ed. Geneva: WHO, 2000).
- *American College of Radiology Appropriateness Criteria®*. The American College of Radiology (ACR) maintains guidelines and criteria for use of imaging and interventional radiology procedures for many aspects of cancer care. This includes the extent of imaging recommended for the diagnostic evaluation of the extent of disease of the primary tumor, nodes, and distant metastases for several cancer types. The ACR Appropriateness Criteria® are updated regularly (http://www.acr.org/ac).
- *CAP Cancer Protocols*. CAP publishes standards for pathology reporting of cancer specimens for all cancer types and cancer resection types. These specify the elements necessary for the pathologist to report the extent and characteristics of cancer specimens (http://www.cap.org).
- *National Comprehensive Cancer Network Clinical Practice Guidelines in Oncology (NCCN Guidelines®)*. The National Comprehensive Cancer Network (NCCN) provides practice guidelines for most types of cancer. These guidelines are updated at least annually. They include recommendations for diagnostic evaluation and imaging of the primary tumor and screening for metastases for each cancer type that may be useful to guide staging (http://www.nccn.org).
- *American Society of Clinical Oncology (ASCO) Guidelines*. ASCO develops guidelines and technical assessments for an array of clinical situations and tools. These include disease- and modality- specific guidelines and assessments of tools, such as the use of biomarkers in certain cancers. These guidelines may be found at the ASCO website: www.asco.org.

Anatomic Staging and the Evolving Use of Nonanatomic Factors

Historically, cancer staging has been based solely on the anatomic extent of cancer, and the 8th Edition approach remains primarily anatomic. However, an increasing number of nonanatomic cancer- and host-related factors provide critical prognostic information and may predict the benefit of specific therapies. Among factors shown to affect patient outcome and/or response to therapy are the clinical and pathological anatomic extent of disease; the reported duration of signs or symptoms; the gender, age, and health status of the patient; the tumor type and grade; and specific biological properties of the cancer and host. Clinicians often use pure anatomic extent of disease in defining treatment, but in many cases, they supplement TNM-based staging with other factors to counsel patients and offer specific treatment recommendations. As more of these and other factors are embraced, applying them in practice will become increasingly complex. This will make it essential to initiate strategies to develop clinically validated prognostic tools and incorporate them into practice to enhance patient management and overall clinical decision making, ideally while maintaining a core anatomic-based structure of staging. Such an integrated approach may reduce the potential for the de facto anatomically constrained TNM system to be rendered obsolete by fostering incorporation of an unprecedented and rapidly evolving understanding of the biology of human cancer. See also Chapter 4, Risk Assessment Models, for more information on AJCC-initiated efforts to embrace development of clinically validated tools.

As introduced in this chapter and detailed throughout this cancer staging manual, in many of the revised AJCC staging algorithms, prognostic factors have been incorporated into stage groupings for specific disease sites where indicated. Because this practice was initiated in a limited fashion in previous editions, most prognostic factors in use, if validated, have been done so only for patients with specific types of disease stratified largely by anatomic stage (e.g., Gleason score in early-stage prostate cancer and genomic profiles in women with node-negative breast cancer). It is important to recognize that even with these advances, anatomic extent of disease remains central to defining cancer prognosis. Inclusion of anatomic extent also maintains the ability to compare patients in a similar fashion across both contemporary and historical treatment regimens and eras, as well as patient populations for whom new prognostic factors cannot be obtained because of cost, available expertise, reporting systems, and/or other logistical issues.

AJCC TNM STAGING SYSTEM: CLASSIFICATIONS, CATEGORIES, AND RULES FOR STAGING

The AJCC TNM stage for each cancer type is built by defining the anatomic extent of cancer for the tumor (T), lymph nodes (N), and distant metastases (M), supplemented in some cases with nonanatomic factors. For each of the T, N, and M, there is a set of categories, most often defined by a

number (e.g., T1, N2). The description of the anatomic factors is specific for each disease site. These descriptors and the nomenclature for TNM have been developed and refined over many editions of the *AJCC Cancer Staging Manual* by experts in each disease and by cancer registrars who collect the information, taking into consideration the behavior and natural history of each type of cancer. These elements are then combined, in a fashion set forth for each cancer type, into prognostic stage groups (often called "stage groups").

Importantly, the term *stage* should be used only to describe the aggregate information resulting from T, N, and M category designations (i.e., based on T, N, and M classifications) combined with any prognostic factors relevant to the specific disease. The term *stage* should not be used to describe individual T, N, or M category designations that often are mistakenly referred to as "stage."

Assigning the T, N, and M categories follows general rules described in the tables in this chapter. These rules apply to all cancer sites, with relatively few exceptions. These exceptions are defined in the relevant disease-specific chapters.

Rules are repeated throughout this chapter to facilitate easy reference based on the topic.

Before delineating the specific rules for T, N, and M categorization and for generating prognostic stage groups, it is important to first delineate the time points, termed *classifications*, at which staging information is collected and reported.

TNM Staging Classification: Clinical, Pathological, Posttherapy, Recurrence, and Autopsy

Stage may be defined at several time points in the care of the cancer patient. To properly stage a patient's cancer, it is essential to first determine the time point in a patient's care. These points in time are termed *classifications*, and are based on time during the continuum of evaluation and management of the disease. Then, T, N, and M categories are assigned for a particular classification (clinical, pathological, posttherapy, recurrence, and/or autopsy) by using information obtained during the relevant time frame, sometimes also referred to as a *staging window*. These staging windows are unique to each particular classification and are set forth explicitly in the following tables. The prognostic stage groups then are assigned using the T, N, and M categories, and sometimes also site-specific prognostic and predictive factors.

Among these classifications, the two predominant are clinical classification (i.e., pretreatment) and pathological classification (i.e., after surgical treatment).

Clinical Classification (cTNM)

Clinical stage classification is based on patient history, physical examination, and any imaging done before initiation of treatment. Imaging study information may be used for clinical staging, but clinical stage may be assigned based on whatever information is available. No specific imaging is required to assign a clinical stage for any cancer site. When performed within this framework, biopsy information on regional lymph nodes and/or other sites of metastatic disease may be included in the clinical classification.

Clinical evaluation by physical examination often underestimates the extent of cancer burden at the time of patient presentation. Although imaging is not required to assign clinical stage, clinical imaging has become increasingly important, and for many cancer sites, imaging is essential to stage solid tumors accurately. Imaging allows assessment of the tumor's size, location, and relationship to normal anatomic structures, as well as the existence of nodal and/or distant metastatic disease. Computed tomography (CT) and magnetic resonance (MR) imaging are the most commonly used imaging modalities, although positron emission tomography (PET; often combined with CT), ultrasound, and plain film radiography also have important roles in various clinical situations. Thus, a new section was added to the disease site chapters to provide context-specific imaging information. To adequately and comprehensively communicate essential information, radiologists should use standardized nomenclature and structured report formats, such as those recommended by the Radiological Society of North America (RSNA) reporting initiative (http://www.rsna.org/Reporting_Initiative.aspx). In addition to providing key information for assigning the T, N, and M categories, clinical imaging is invaluable for guiding biopsies and surgical resections. Later in the course of a patient's treatment, imaging also often plays an important role in monitoring response to treatment.

Pathological Classification (pTNM)

Pathological stage classification is based on clinical stage information supplemented/modified by operative findings and pathological evaluation of the resected specimens. This classification is applicable when surgery is performed before initiation of adjuvant radiation or systemic therapy.

Posttherapy or Post Neoadjuvant Therapy (ycTNM and ypTNM)

Stage determined after treatment for patients receiving systemic and/or radiation therapy alone or as a component of their initial treatment, or as neoadjuvant therapy before planned surgery, is referred to as posttherapy classification. It also may be referred to as post neoadjuvant therapy classification.

Recurrence or Retreatment (rTNM)

Staging classifications at the time of retreatment for a recurrence or disease progression is referred to as recurrence classification. It also may be referred to as retreatment classification.

Autopsy (aTNM)

Staging classification for cancers identified only at autopsy is referred to as autopsy classification.

Defining T, N, M, and Prognostic Factor Categories

The T, N, and M designations are referred to as categories. The category criteria for defining anatomic extent of disease are specific for tumors at different anatomic sites and sometimes for tumors comprising different histologic types arising from similar anatomic sites. For example, the size of the tumor is a key factor in breast cancer but has no impact on prognosis in colorectal cancer, in which the depth of invasion or extent of the cancer is the primary prognostic feature. In summary, the T, N, and M category criteria are defined separately for each tumor and histologic type.

In addition to anatomic-based T, N, and M information, the AJCC recommends collection of key prognostic factors for specific cancer sites (as detailed in each site chapter) that in some cases are used to define T, N, or M and/or may be used to define stage groupings critical to prognosis and/or helpful to guide patient care and to ensure uniformity in comparative research and reporting environments.

The AJCC includes additional factors that play a role in the calculation of the AJCC Prognostic Stage Group for a disease site. If available and applicable to the disease site, so-called Prognostic Factors Required for Stage Grouping can modify the calculation of stage based only on TNM. These factors are involved in the calculation of stage in several disease sites, such as breast and prostate.

A different system for designating the extent of disease and prognosis is necessary for certain types of tumors, such as Hodgkin and other lymphomas. In these circumstances, other categories are used instead of T, N, and M, and for lymphoma, only the stage group is defined. General staging rules are presented in this chapter, and the specifics for each type of disease are detailed in the respective disease site–specific chapters.

AJCC Prognostic Stage Groups

For the purposes of tabulation and to analyze the care of patients who generally have a similar prognosis, T, N, and M are grouped into *prognostic stage groups*, commonly referred to as stage groups. As introduced earlier, a stage group is determined from aggregate information on the primary tumor (T), regional lymph nodes (N), and distant metastases (M), as well as any specified prognostic factors for certain cancer types. Stage groups are based primarily on anatomic information, supplemented by selected prognostic factors in some disease sites. Stage groups are defined for each of the classifications: clinical stage group and pathological stage group.

Documenting Cancer Stage in the Medical Record

All staging classifications—and, most importantly, clinical and pathological classifications—should be documented in the medical record. The documentation in the record should include the type of classification (e.g., clinical or pathological); T, N, and M categories; relevant prognostic factor categories; and the stage grouping. Clinical stage generally is used to define primary therapy. TNM-based clinical stage also is important because it may be the only common denominator across all cancers of a certain anatomic site and histology. Examples include lung cancer, advanced gastrointestinal tumors, and head and neck cancers, for which surgery may not be performed, and others, such as prostate cancer, for which surgical resection for limited disease may not be applicable. In such scenarios, it may be impossible to compare patients for whom information is obtained solely by clinical staging strategies with those undergoing surgical resection and for whom pathological staging is performed. The importance of clinical stage was reinforced in 2008 when the American College of Surgeons Commission on Cancer (CoC) introduced the requirement that clinical stage be documented in all cancer patients as part of its cancer program standards, as a key determinant of treatment choice. Pathological staging is used to define a more precise prognosis and to plan other therapies as required.

Many options exist for documenting staging data in the medical record. Examples of source documents in the medical record that may contain patient-specific cancer staging information include initial clinical evaluations and consultations, operative reports, imaging studies, pathology reports, discharge summaries, and follow-up reports. Physicians are encouraged to enter the stage of cancer in every record of clinical encounters with the cancer patient. Paper or electronic staging forms may be useful to record stage in the medical record as well as to facilitate communication of staging data to a cancer registry. A form for recording cancer staging data will be made available for each disease site on www.cancerstaging.org.

T, N, and M category information as well as disease site-specific prognostic factor data should be included in pathology reports whenever these data are available. Pathologists should use the appropriate AJCC-specified data elements as defined by the CAP Cancer Protocols. However, the determination of stage usually involves synthesis of

information from multiple sources, including clinical data, imaging studies, and pathology reports. Because all this information may not be available to the reporting pathologist, final T, N, and M categories and stage may not be fully assessed from pathology reports alone and should be assigned by the managing physician(s).

TNM and Prognostic Stage Group Tables

TNM information in each chapter provides precise criteria and rules for categorizing the T, N, or M of a patient for the relevant classification (e.g., clinical, pathological). This information is used to assign prognostic stage groups based on the assigned T, N, and M categories (with other prognostic factors if required for that specific cancer type).

Elements of TNM tables	Description
Classification	A lower case prefix describes the time point in a patient's cancer continuum when stage is assigned, including:<br>• c: clinical<br>• p: pathological<br>• yc: post neoadjuvant (radiation or systemic) therapy—clinical<br>• yp: post neoadjuvant (radiation or systemic) therapy—pathological<br>• r: recurrence or retreatment<br>• a: autopsy
Category	T-, N-, and M-specific data are used to assign a cancer site–specific T, N, and M category for a patient at a given classification. Generally, the higher the T, N, or M category, the greater the extent of the disease and generally the worse the prognosis.<br>*Note*: Exceptions exist in which T-, N-, or M-specific category elements may represent unique characteristics of the cancer but not necessarily worse prognosis. For example, N1c in colon cancer does not represent greater nodal disease burden than N1a or N1b, but rather a unique situation.
Subcategory	Some disease sites have subcategories devised to facilitate reporting of more detailed information and often more specific prognostic information. Examples:<br>• breast cancer: T1mi, T1a, T1b, T1c<br>• breast cancer: N2a, N2b<br>• prostate cancer: M1a, M1b, M1c<br>*Note*: If there is uncertainty in assigning a subcategory, the patient is assigned to the general category. For example, a breast cancer reported clinically as <2 cm without further specification is assigned T1 and cannot be assigned T1a, T1b, or T1c.<br>If uncertain or incomplete information precludes subcategory assignment, which may result in different stage groups or management paradigms, a subcategory assignment may still be required. In that case, the general category, the physician/managing team categorization, or the lower or less advanced subcategory should be used.
AJCC prognostic stage groups (stage groups)	AJCC prognostic stage groups are assigned based on disease site–specific T, N, and M categories and relevant prognostic factors to group patients with similar prognosis and/or treatment approach. For each cancer type in which prognostic factors are used to assign stage groups, a separate stage group may be assigned based solely on anatomic categories so as to allow stage group comparisons among patients who have and do not have available prognostic factor information.

T, N, M and Prognostic Factor Category Criteria

The three categories—T, N, and M—and the prognostic factors collectively describe, with rare exceptions, the extent of tumor, including local spread, regional nodal involvement, and distant metastasis. It is important to stress that each component (T, N, and M) is referred to as a *category*. The term *stage* is used when T, N, and M and cancer site–specific required prognostic factors are combined. The criteria for T, N, and M are defined separately for cancers in different anatomic locations and/or for different histologic types.

This category...	Is defined by...
T	The size and/or contiguous extension of the primary tumor.<br>*Note*: The roles of the size component and the extent of contiguous spread are specifically defined for each cancer site.
N	Cancer in the regional lymph nodes as defined for each cancer site, including<br>• absence or presence of cancer in regional node(s), and/or<br>• number of positive regional nodes, and/or<br>• involvement of specific regional nodal groups, and/or<br>• size of nodal metastasis or extension through the regional node capsule, and/or<br>• In-transit and satellite metastases, somewhat unique manifestations of nonnodal intralymphatic regional disease, usually found between the primary tumor site and draining nodal basins.<br>*Note*: For melanoma and Merkel cell carcinoma, nonnodal regional metastasis, such as satellites and in-transit metastases, may be included in the N categorization (see the melanoma and Merkel cell carcinoma chapters for specifics). For colorectal carcinoma, mesenteric tumor deposits without remaining nodal architecture are included in the N category.
M	The absence or presence of distant metastases in sites and/or organs outside the local tumor area and regional nodes as defined for each cancer site. For some cancer sites, the location and volume or burden of distant metastases are included.

This category...	Is defined by...
Prognostic factors required for stage grouping	The prognostic factors required for stage grouping have such a strong correlation with prognosis that they are included in the AJCC Prognostic Stage Groups table. It is important to collect these factors in cancer registries and databases to measure their impact on prognosis.

Primary Tumor (T) Categories

Primary tumor categories have specific notations to describe the existence, size, or extent of the tumor.

Tumor category...	Is assigned when there is...
TX	No information about the T category for the primary tumor, or it is unknown or cannot be assessed<br>*Note*: Use of the TX category should be minimized.
T0	No evidence of a primary tumor
Tis	Carcinoma *in situ*<br>**Examples of exceptions include**: Tis for *in situ* melanoma of the skin, germ cell neoplasia *in situ* for testis, and high-grade dysplasia in colorectal carcinoma.
T1, T2, T3, or T4	Primary invasive tumor, for which a higher category generally means<br>• an increasing size<br>• an increasing local extension, or<br>• both

Regional Lymph Node (N) Categories

Categorizing regional lymph node involvement depends on its existence and extent.

Regional node category...	Is assigned when there is...
NX	No information about the N category for the regional lymph nodes, or it is unknown or cannot be assessed<br>*Note:* Use of NX should be minimized.
N0	No regional lymph node involvement with cancer and for some disease sites, nonnodal regional disease as noted earlier
N1, N2, or N3	Evidence of regional node(s) containing cancer, with<br>• an increasing number, and/or<br>• regional nodal group involvement, and/or<br>• size of the nodal metastatic cancer deposit, or<br>• non-nodal regional disease as noted earlier for melanoma and Merkel cell carcinoma, and for colorectal carcinoma

Distant Metastasis (M) Categories

The distant metastasis category specifies whether distant metastasis is present.

Distant metastasis category...	Is assigned when there is...
M0	No evidence of distant metastasis
M1	Distant metastasis

Notes: There is no designation of MX. The absence of any clinical history or physical findings suggestive of metastases in a patient who has not undergone any imaging is sufficient to assign the clinical M0 category (cM0).
There is no designation of pM0. Biopsy or other pathological information is required to assign the pathological M1 category. Patients with a negative biopsy of a suspected metastatic site are classified as clinical M0 (cM0).

Distant Metastasis: Selected Locations

The M1 category may be specified further according to the location of distant metastases.

Location	Notation
Pulmonary	PUL
Osseous	OSS
Hepatic	HEP
Brain	BRA
Distant lymph nodes	LYM
Bone marrow	MAR
Pleura	PLE
Peritoneum	PER
Adrenal	ADR
Distant skin	SKI
Other	OTH

Unknown Designation: X

The X designation is used if information on a specific T or N category is unknown; such cases usually cannot be assigned a stage. Therefore, TX and NX should be used only if absolutely necessary. Of note, there is no MX category.

Exceptions: TX

Stage may be assigned when the TNM stage group results in Any T or Any N with M1, which includes TX or NX. These are classified as Stage IV. Examples include:

- TX NX M1, or
- TX N3 M1.

Stage may be assigned when the TNM stage group results in Any T or Any N with M0, which includes TX or NX. Examples include:

- TX N1 M0 Stage III in melanoma clinical stage
- T4 NX M0 Stage III in pancreas

MX is Not a Valid M Category

The MX category was eliminated from the AJCC and UICC TNM systems in the *AJCC Cancer Staging Manual*, 6th Edition. Unless there is clinical or pathological evidence of distant metastases, the patient is classified as clinical M0 and denoted as cM0. It is not necessary to perform any imaging or invasive studies to categorize a patient as cM0. A history and physical examination are all that is needed to assign cM0. The M category must always be known and reported to assign a stage group.

Pathologists should not report an M category unless appropriate for the specimen evaluated. CAP Cancer Protocols require documentation of distant metastases as pM1 only if present in the specimen(s) provided to the pathologist. If the pathologist does not review and report on a metastatic specimen, or if a biopsy is performed of a possible distant metastasis and the biopsy does not show cancer, then there should be no mention of the M category in the pathology report, or the pathologist should designate the M category as "not applicable." The term *MX* should not be used in the pathology report.

The managing physician should stage a patient for whom a biopsy performed for possible distant metastasis does not demonstrate cancer as cM0; there is no pM0 designation. Only the managing physician can assign cM0 after taking into account physical examination, imaging, and other information.

AJCC Prognostic Stage Groups

The purpose of defining and assigning stage groups is to generate a reproducible and easily communicated summary of staging information. The staging tables generally group patients with similar prognoses, usually with a statistically significant separation in outcomes between stage groups. Patients within a stage group generally have similar outcomes, even though their burden of disease may vary. Exceptions to this general stage group convention are noted in each chapter where relevant. For example, to retain an anatomic- and TNM-based staging system in melanoma, some prognostic overlap was allowed between patients with Stage IIC melanoma and those with Stage IIIA melanoma; many patients with Stage IIIA disease have a prognosis more favorable than that of patients with Stage IIC disease.

Stage groups are denoted by Roman numerals from I to IV with increasing extent of disease and generally with worsening overall prognosis. Stage I generally indicates cancers that are smaller or less deeply invasive without regional disease or nodes, Stages II and III define patients with increasing tumor or nodal extent, and Stage IV identifies those who present with distant metastases (M1) at diagnosis.

The term *Stage 0* is used to denote carcinoma *in situ* (or melanoma *in situ* for melanoma of the skin or germ cell neoplasia *in situ* for testicular germ cell tumors) and generally is considered to have no metastatic potential. Stage 0 is determined by microscopic examination of the primary tumor. Stage I through Stage IV subgroups are denoted by capital letters—for example, A, B, or C—according to cancer site stage grouping definitions and are used to expand the main groupings to provide more refined prognostic information.

Prognostic Factors Required for Stage Grouping

For some cancer types, in addition to T, N, and M categories, prognostic factors are required to assign a stage group. Examples include tumor grade, age at diagnosis, histologic type, mitotic rate, serum tumor markers, hormone receptors, hereditary factors, prostate-specific antigen, and Gleason score. Specifically, cancer site–specific prognostic factors populate nonanatomic categories and are defined clearly if required for a particular disease site.

These factors generally constitute categories used with the TNM categories to assign prognostic stage groups. In some cases in which factors are used in stage groups, an X category is provided for use by the managing physician if the factor is not available. Generally, in cases in which the factor is absent and X is not provided as an option, the physician's determination or lowest category (best prognosis) of the factor is used to assign the stage group.

In contrast, cancer registry data collection should record *X* or *unknown* if the prognostic factor is not available, and should not use the lowest category. This allows for accurate analysis of the data.

GENERAL STAGING RULES

These general rules apply to the application of T, N, and M categories for all anatomic sites and classifications.

Topic	Rules
Microscopic confirmation	• Microscopic confirmation is necessary for TNM classification, including clinical classification (with rare exception).<br>• In rare clinical scenarios, patients who do not have any biopsy or cytology of the tumor may be staged. This is recommended in rare clinical situations, only if the cancer diagnosis is NOT in doubt. In the absence of histologic confirmation, survival analysis may be performed separately from staged cohorts with histologic confirmation. Separate survival analysis is not required if clinical findings support a cancer diagnosis and specific site.<br>**Example**: Lung cancer diagnosed by CT scan only, that is, without a confirmatory biopsy
Time frame/staging window for determining clinical stage	Information gathered about the extent of the cancer is part of clinical classification:<br>• from date of diagnosis before initiation of primary treatment or decision for watchful waiting or supportive care to one of the following time points, whichever is shortest:<br>○ 4 months after diagnosis<br>○ to the date of cancer progression if the cancer progresses before the end of the 4 month window; data on the extent of the cancer is only included before the date of observed progression
Time frame/staging window for determining pathological stage	Information including clinical staging data and information from surgical resection and examination of the resected specimens—if surgery is performed before the initiation of radiation and/or systemic therapy—from the date of diagnosis:<br>• within 4 months after diagnosis<br>• to the date of cancer progression if the cancer progresses before the end of the 4-month window; data on the extent of the cancer is included only before the date of observed progression<br>• and includes any information obtained about the extent of cancer up through completion of definitive surgery as part of primary treatment if that surgery occurs later than 4 months after diagnosis and the cancer has not clearly progressed during the time window<br>*Note*: Patients who receive radiation and/or systemic therapy (neoadjuvant therapy) before surgical resection are not assigned a pathological category or stage, and instead are staged according to post neoadjuvant therapy criteria.
Time frame/staging window for staging post neoadjuvant therapy or posttherapy	After completion of neoadjuvant therapy, patients should be staged as:<br>• yc: posttherapy clinical<br>After completion of neoadjuvant therapy followed by surgery, patients should be staged as:<br>• yp: posttherapy pathological<br>The time frame should be such that the post neoadjuvant surgery and staging occur within a time frame that accommodates disease-specific circumstances, as outlined in the specific chapters and in relevant guidelines.<br>*Note*: Clinical stage should be assigned before the start of neoadjuvant therapy.
Progression of disease	If there is documented progression of cancer before therapy or surgery, only information obtained before the documented progression is used for clinical and pathological staging.<br>Progression does not include growth during the time needed for the diagnostic workup, but rather a major change in clinical status.<br>Determination of progression is based on managing physician judgment, and may result in a major change in the treatment plan.
Uncertainty among T, N, or M categories, and/or stage groups: rules for clinical decision making	If uncertainty exists regarding how to assign a category, subcategory, or stage group, the lower of the **two possible** categories, subcategories, or groups is assigned for<br>• T, N, or M<br>• prognostic stage group/stage group<br>Stage groups are for patient care and prognosis based on data. Physicians may need to make treatment decisions if staging information is uncertain or unclear.<br>*Note*: Unknown or missing information for T, N, M or stage group is never assigned the lower category, subcategory, or group.
Uncertainty rules do not apply to cancer registry data	If information is not available to the cancer registrar for documentation of a subcategory, the main (umbrella) category should be assigned (e.g., T1 for a breast cancer described as <2 cm in place of T1a, T1b, or T1c).<br>If the specific information to assign the stage group is not available to the cancer registrar (including subcategories or missing prognostic factor categories), the stage group should not be assigned but should be documented as unknown.
Prognostic factor category information is unavailable	If a required prognostic factor category is unavailable, the category used to assign the stage group is:<br>• X, or<br>• If the prognostic factor is unavailable, default to assigning the anatomic stage using clinical judgment.
Grade	The recommended histologic grading system for each disease site and/or cancer type, if applicable, is specified in each chapter and should be used by the pathologist to assign grade.<br>The cancer registrar will document grade for a specific site according to the coding structure in the relevant disease site chapter.

Topic	Rules
Synchronous primary tumors in a single organ: *(m)* suffix	If multiple tumors of the same histology are present in one organ:<br>• the tumor with the highest T category is classified and staged, and<br>• the *(m)* suffix is used<br>• An example of a preferred designation is: pT3(m) N0 M0.<br>• If the number of synchronous tumors is important, an acceptable alternative designation is to specify the number of tumors. For example, pT3(4) N0 M0 indicates four synchronous primary tumors.<br>*Note*: The *(m)* suffix applies to multiple invasive cancers. It is not applicable for multiple foci of *in situ* cancer or for a mixed invasive and *in situ* cancer.
Synchronous primary tumors in paired organs	Cancers occurring at the same time in each of paired organs are staged as separate cancers. Examples include breast, lung, and kidney.<br>**Exception:** For tumors of the thyroid, liver, and ovary, multiplicity is a T-category criterion, thus multiple synchronous tumors are not staged independently.
Metachronous primary tumors	Second or subsequent primary cancers occurring in the same organ or in different organs outside the staging window are staged independently and are known as metachronous primary tumors.<br>Such cancers are not staged using the *y* prefix.
Unknown primary or no evidence of primary tumor	If there is no evidence of a primary tumor, or the site of the primary tumor is unknown, staging may be based on the clinical suspicion of the organ site of the primary tumor, with the tumor categorized as T0. The rules for staging cancers categorized as T0 are specified in the relevant disease site chapters.<br>**Example**: An axillary lymph node with an adenocarcinoma in a woman, suspected clinically to be from the breast, may be categorized as T0 N1 (or N2 or N3) M0 and assigned Stage II (or Stage III).<br>**Examples of exception**: The T0 category is not used for head and neck squamous cancer sites, as such patients with an involved lymph node are staged as unknown primary cancers using the "Cervical Nodes and Unknown Primary Tumors of the Head and Neck" system (T0 remains a valid category for human papillomavirus [HPV]- and Epstein–Barr virus [EBV]-associated oropharyngeal and nasopharyngeal cancers).
Date of diagnosis	It is important to document the date of diagnosis, because this information is used for survival calculations and time periods for staging.<br>The date of diagnosis is the date a physician determines the patient has cancer. It may be the date of a diagnostic biopsy or other microscopic confirmation or of clear evidence on imaging. This rule varies by disease site and shares similarities with the earlier discussion on microscopic confirmation.

STAGE CLASSIFICATIONS

Stage classifications are determined according to the point in time of the patient's care in relation to diagnosis and treatment. The five stage classifications are clinical, pathological, posttherapy/post neoadjuvant therapy, recurrence/retreatment, and autopsy.

Classification	Designation	Details
Clinical	cTNM or TNM	**Criteria:** used for all patients with cancer identified before treatment<br>It is composed of diagnostic workup information, until first treatment, including:<br>• clinical history and symptoms<br>• physical examination<br>• imaging<br>• endoscopy<br>• biopsy of the primary site<br>• biopsy or excision of a single regional node or sentinel nodes, or sampling of regional nodes, with clinical T<br>• biopsy of distant metastatic site<br>• surgical exploration without resection<br>• other relevant examinations<br>*Note*: Exceptions exist by site, such as complete excision of primary tumor for melanoma.
Pathological	pTNM	**Criteria**: used for patients if surgery is the first definitive therapy<br>It is composed of information from:<br>• diagnostic workup from clinical staging combined with<br>• operative findings, and<br>• pathology review of resected surgical specimens

Classification	Designation	Details
Posttherapy or post neoadjuvant therapy	ycTNM and ypTNM	For purposes of posttherapy or post neoadjuvant therapy, *neoadjuvant therapy* is defined as systemic and/or radiation therapy given before surgery; primary radiation and/or systemic therapy is treatment given as definitive therapy without surgery.<br><br>**yc**<br>The yc classification is used for staging after primary systemic and/or radiation therapy, or after neoadjuvant therapy and before planned surgery<br>**Criteria**: First therapy is systemic and/or radiation therapy<br><br>**yp**<br>The yp classification is used for staging after neoadjuvant therapy and planned post neoadjuvant therapy surgery.<br>**Criteria:** First therapy is systemic and/or radiation therapy and is followed by surgery.
Recurrence or retreatment	rTNM	This classification is used for assigning stage at time of recurrence or progression until treatment is initiated.<br>**Criteria**: Disease recurrence after disease-free interval or upon disease progression if further treatment is planned for a cancer that:<br>• recurs after a disease-free interval or<br>• progresses (without a disease-free interval)<br><br>**rc**<br>Clinical recurrence staging is assigned as rc.<br><br>**rp**<br>Pathological staging information is assigned as rp for the rTNM staging classification. This classification is recorded in addition to and does not replace the original previously assigned clinical (c), pathological (p), and/or posttherapy (yc, yp) stage classifications, and these previously documented classifications are not changed.
Autopsy	aTNM	This classification is used for cancers not previously recognized that are found as an incidental finding at autopsy, and not suspected before death (i.e., this classification does not apply if an autopsy is performed in a patient with a previously diagnosed cancer).<br>**Criteria:** No cancer suspected prior to death<br>Both clinical and pathological staging information is used to assign aTNM.

Clinical Classification

Classification of T, N, and M during the diagnostic workup time frame is denoted by use of a lower case *c* prefix: cT, cN, and cM0, cM1 or pM1, or the use of no prefix: T, N, M.

Clinical stage is important to record for all patients because:

- clinical stage is essential for selecting initial therapy, and
- clinical stage is critical for comparison across patient cohorts when some have surgery as a component of initial treatment and others do not.

Clinical stage may be the only stage classification by which comparisons can be made across all patients, because not all patients will undergo surgical treatment before other therapy, and response to treatment varies. Differences in primary therapy make comparing groups of patients difficult if that comparison is based on pathological assessment. For example, it is difficult to compare patients treated with primary surgery with those treated with chemotherapy or radiotherapy without surgery or neoadjuvant therapy.

Time frame: Clinical classification is based on any information gathered about the extent of the cancer from the time of diagnosis until the initiation of primary treatment or the decision for watchful waiting or supportive care, and is based on the shorter of two periods of time:

- within 4 months after diagnosis, or
- the time of cancer progression if the cancer progresses before the end of the 4-month window; data on the extent of the cancer is included only before the date of observed progression

Criteria: All patients with cancer identified before treatment.

Clinical classification is based on:

- clinical history and symptoms
- physical examination
- imaging
- endoscopy or surgical exploration without resection
- biopsy of the primary site, biopsy or excision of a single regional node or sentinel nodes, sampling of regional nodes with clinical T, or biopsy of a distant metastatic site

Clinical classification is based on evidence acquired from the date of diagnosis until initiation of primary treatment. Examples of primary treatment include definitive surgery, radiation therapy, systemic therapy, and neoadjuvant radiation and systemic therapy.

Importantly, clinical stage groups cannot be assigned for some cancer sites if the necessary minimum information to assign a clinical stage group is not available. Although this scenario is quite uncommon, it may occur—for example, if lymph nodes cannot be examined before surgical resection or if a cancer is identified and resected incidentally during surgery for another medical condition.

Component of clinical staging	Details
Assignment of stage by managing physician	Clinical stage is assigned based on a synthesis of clinical data from multiple sources and only by the managing physician, usually a surgical or medical oncologist. As noted earlier, the assignment of clinical stage also may include pathological data from biopsies.
Known or suspected tumor	Tumor must be known or suspected and have a diagnostic workup including at least a history and physical examination to assign a clinical stage.<br>Incidental findings at the time of surgical treatment may not be assigned a clinical stage retrospectively.
Imaging studies	Imaging may be of value and useful, but imaging is not necessary to assign a clinical stage.<br>Guidelines for diagnostic evaluation of individual cancer types are found in these publications:<br>• ACR Appropriateness Criteria® http://www.acr.org/ac<br>• NCCN Guidelines® http://www.nccn.org
Impact of subsequent information	The clinical stage should not be changed based on:<br>• subsequent information obtained from the pathological examination of resected tissue, or<br>• information obtained after initiation of definitive therapy.

Clinical T (T or cT)

Assessment of the primary tumor is necessary to determine the cT category.

Component of cT	Details
Tumor size and extent	Based on physical examination, imaging, endoscopy, biopsy of the primary site (core through long axis), surgical exploration, or other relevant examinations.<br>The most accurate size should be used, as some methods may overestimate the size. Therefore, the largest size may not be the most accurate and should not be used automatically. Guidance on which imaging technique(s) may be most accurate is discussed in site-specific chapters. Physicians should document the most accurate tumor size used for staging.
Tumor size in millimeters and rounding for T-category assignment	Primary tumor size is the most accurate/largest dimension and is<br>• measured to the nearest whole millimeter, unless a smaller unit is specified in a specific disease site, and<br>• rounded up or down as appropriate for assigning T category:<br>○ down when the numerals are between 1 and 4<br>○ up when the numerals are between 5 and 9.<br>**Examples:**<br>• Tumor measured as 2.2 mm is recorded as 2 mm.<br>• Tumor measured as 1.7 mm is recorded as 2 mm.<br>• Tumor measured as 2.04 cm is recorded as 20 mm and would be grouped with ≤2 cm and not >2 cm.<br>**Nonexhaustive exceptions**:<br>• Melanoma: primary tumor measured to nearest 0.1 mm<br>• Breast cancer: primary tumor >1.0 mm to 1.4 mm rounded to 2 mm (this avoids assigning the "microinvasion" category to cancer >1.0 mm)
Surgical exploration	Observations made at surgical exploration without resection are used to assign clinical categories. Biopsies of the primary site during surgical exploration without resection of the primary tumor are used for clinical categorization.<br>**Exception**: This information also may be used for pathological T categorization if the biopsy provides histologic material corresponding to the highest possible T category for the specific cancer type, and if it meets other criteria described in stage group.

Component of cT	Details
Synchronous primary tumors in a single organ: *(m)* suffix	For multiple tumors in a single organ, T is assigned to the highest T category; the preferred designation is:<br>• *m* suffix; for example, pT3(m) N0 M0<br>If the number of tumors is important, an acceptable alternative is:<br>• number of tumors; for example, pT3(4) N0 M0<br>*Note*: The *(m)* suffix applies to multiple invasive cancers. It is not applicable to multiple foci of *in situ* cancer or a mixed invasive and *in situ* cancer.
Direct extension into an organ	Direct extension of a primary tumor into a contiguous or adjacent organ is classified as part of the tumor (T) classification and is not classified as metastasis (M).<br>**Example:** Direct extension into the liver from a primary colon cancer would be in the T category and not in the M category.
Microscopic assessment of highest T category	If microscopic assessment of the primary site or regional tissue establishes the highest T category, it is:<br>• assigned as cT, and<br>• it also may be used for assignment of pT ONLY if there is microscopic confirmation of the highest pN.<br>There must be microscopic confirmation of both the highest T and the highest N in order to assign a pathological stage group without resection of the primary site.
Unknown primary or no evidence of primary tumor	If there is no evidence of a primary tumor, or the site of the primary tumor is unknown, staging may be based on the clinical suspicion of the primary tumor, with the tumor categorized as T0. The rules for staging cancers categorized as T0 are specified in the relevant disease site chapters.<br>**Examples of exception**: The T0 category is not used for head and neck squamous cancer sites, as such patients with an involved lymph node are staged as unknown primary cancers using the cervical lymph node system (T0 remains a valid category for HPV- and EBV-associated oropharyngeal and nasopharyngeal cancers).
Tis	*In situ* neoplasia identified during the diagnostic workup on a core or incisional biopsy is assigned cTis.
Any T	*Any T* includes all T categories except Tis. This includes TX and T0.

Clinical N (N or cN)

Assessment of the regional lymph nodes is necessary to determine the cN category.

Component of cN	Details
Lymph node assessment	Clinical regional lymph node assessment may be performed by physical examination and imaging. Clinical nodal category cN0 may be assigned based solely on physical examination. Imaging to assess regional lymph nodes is not required to assign clinical stage.
Node status not required in rare circumstances	For some cancer sites in which lymph node involvement is rare, patients whose nodal status is not determined to be positive for tumor should be designated as cN0. These circumstances are identified in specific disease chapters for these sites; NX is not listed as a category.<br>**Example:** Bone and soft tissue sarcoma may use cN0 to assign the clinical stage group, that is, cT1 cN0 cM0.
Microscopic assessment for cN	Microscopic examination of regional nodes during the diagnostic workup is included in the clinical classification as cN.<br>Microscopic examination or assessment may be by:<br>• fine-needle aspiration (FNA),<br>• core biopsy,<br>• incisional biopsy,<br>• excisional biopsy, or<br>• sentinel node biopsy/procedure.<br>This information also is included in the pathological staging if the patient has surgical resection as the first course of therapy.<br>**Example**: Sentinel node biopsy performed before neoadjuvant therapy in breast cancer is designated as clinical (cN).
Sentinel lymph node	A sentinel lymph node (SLN) is a regional lymph node that receives direct afferent lymphatic drainage from a primary tumor site (e.g., breast, melanoma), and in many solid tumors it represents the regional lymph node(s) most likely to contain metastatic disease, if any are involved. More than one SLN may be present in a regional nodal basin, and some primary tumors (e.g., melanoma) may drain to more than one regional nodal basin.<br>Sentinel nodes are identified by lymphatic mapping as evidenced by nodes that concentrate a colloidal material injected near the primary tumor or in the involved organ (the most commonly used agents for sentinel node biopsy are vital stains such as isosulfan blue and/or radiotracers such as technetium-99 (^{99}Tc)-sulfur colloid). In some circumstances, the managing physician also may label regional lymph nodes that are palpably abnormal during surgery as sentinel nodes. Nodes that do not concentrate colloidal material and are resected along with other sentinel nodes are nonsentinel nodes and are considered as part of the sentinel node procedure. Their resection is not coded as a separate nodal procedure or a lymph node dissection.

Component of cN	Details
Sentinel node (sn) and FNA or core biopsy (f)	To distinguish lymph nodes identified during diagnostic evaluation by sentinel node biopsy or FNA or core biopsy from those identified by physical examination and imaging, the following suffixes are used in assigning the clinical N (cN) category:<br>If SLN biopsy is performed as part of the diagnostic workup:<br>• the cN category should have the *sn* suffix; for example, cN1(sn).<br>If an FNA or a core biopsy is performed on lymph nodes as part of the diagnostic workup:<br>• the cN category should have the *f* suffix; for example, cN1(f).
Isolated tumor cells (ITCs): use of the *(i+)* designator	ITCs include single tumor cells or small clusters of cells ≤0.2 mm in greatest diameter, generally without stromal response in the lymph node. Such cells usually are found in the subcapsular nodal sinuses but may be seen within the nodal parenchyma. Because ITCs may represent in-transit tumor cells that are not proliferating within the node, lymph nodes with only ITCs usually are categorized as N0, with some exceptions. They are denoted as N0(i+).<br>*The concepts regarding this staging rule continue to evolve, and further study is warranted. In the meantime, the staging rule serves as a guideline for uniformity and consistency in practice in recording information, and clinical judgment by the managing physician prevails.*<br>**Exception:** In melanoma and Merkel cell carcinoma, tumor cell deposits defined here as ITCs are considered positive nodes and are designated as N1 or higher.<br>*Note*: Cancer site–specific designators have been developed to identify ITCs in nodes. For example, N0(i+) in breast and gynecologic cancers applies to nodes with ITCs only.
Pathological techniques for ITCs or detection of micro-metastasis	ITCs or lymph node micro-metastases may be identified in lymph nodes by hematoxylin and eosin staining or by specialized pathological techniques, such as IHC for cytokeratin proteins for carcinomas. Specialized pathology techniques, such as IHC and molecular techniques, are not recommended for routine examination of lymph nodes.<br>*The concepts regarding this staging rule continue to evolve, and further study is warranted.*

Component of cN	Details
Nonmorphologic techniques for identifying ITCs: use of the *(mol+)* designator	Nonmorphologic techniques, including flow cytometry and reverse transcriptase polymerase chain reaction studies, may identify minimal deposits of cancer in lymph nodes.<br>These deposits usually are classified as clinically node negative and are identified with the (mol+) designator: for example, cN0(mol+).<br>*The concepts regarding this staging rule continue to evolve, and further study is warranted.*
Micro-metastases: use of the *mi* designator	Lymph node micro-metastases are defined as tumor deposits >0.2 mm but ≤2.0 mm. For certain disease sites, micro-metastases are denoted by using the *mi* designator: for example, cN1mi.<br>*Further studies are needed to determine the significance of micro-metastases across many cancer sites.*<br>*The concepts regarding this staging rule continue to evolve, and further study is warranted.*
Extranodal extension	Extranodal extension (ENE) is defined as the extension of a nodal metastasis through the lymph node capsule into adjacent tissues. *ENE* is the preferred terminology. It also is termed *extranodal spread, extracapsular extension*, or *extracapsular spread*.
Regional node metastasis invading a distant organ is ENE	A regional node extending into a distant structure or organ is categorized as ENE and is not considered distant metastatic disease.
Regional nodes when tumor involves more than one organ or structure	In rare cases in which a tumor involves more than one organ or structure, the regional nodes include the nodes of all involved structures, even if the nodes of the primary site are not involved.<br>**Example**: If a primary transverse colon cancer invades the stomach, for staging purposes, the gastric regional nodes are considered regional for the transverse colon, even if the regional nodes of the colon are not involved.
Microscopic assessment of regional node is the highest N category	If microscopic assessment of the regional node is the highest N category, it is<br>• assigned as cN, and<br>• also may be used for the assignment of pN ONLY if there is microscopic confirmation of the highest pT.<br>There must be microscopic evidence of both highest T and highest N to assign a pathological stage group without surgical resection of the primary site.
Any N	Any N includes all N categories, including NX and N0.

Clinical M Classification (cM and pM)

Assignment of the M category for clinical classification may be cM0, cM1, or pM1. The M category is based on clinical history, physical examination, any imaging results, and whether there is microscopic confirmation of the distant metastasis during the diagnostic workup. The terms pM0 and MX are NOT valid categories in the TNM system.

Component of clinical M	Details
No distant metastasis	**cM0**<br>If there are no symptoms or signs of distant metastasis, M is categorized as clinically M0 (cM0). Evaluation methods include:<br>• history and physical examination<br>• imaging studies<br>*Note*: Imaging studies may be used in assigning the M category but are not required to assign the cM0 category.
Clinical evidence of distant metastasis	**cM1**<br>If there is clinical evidence of distant metastases on physical examination, imaging studies, or invasive procedures, but no microscopic evidence of the presumed distant metastases, M is categorized as clinically M1 (cM1). Examination methods include:<br>• physical examination<br>• imaging (if performed)<br>• exploratory surgery and/or endoscopy (if performed)
Microscopic evidence of distant metastasis	**pM1**<br>If there is microscopic evidence of distant metastatic disease, M is categorized as pathological M1 (pM1).<br>Microscopic evidence includes:<br>• cytology from FNA<br>• core biopsy<br>• incisional biopsy<br>• excisional biopsy<br>• resection
Use of pM1 for multiple distant metastases	**pM1**<br>In patients who have distant metastases in multiple sites and have a cancer type for which M subcategories distinguish between one or more metastatic sites, microscopic evidence of one of these sites is necessary to assign the higher pM subcategory.<br>In general, metastases to both sides of a paired organ are considered a single metastatic site of involvement (e.g., metastases to both lungs are designated metastasis to one distant site—lung).<br>If clinical evidence of distant metastasis remains in other areas that are not or cannot be microscopically confirmed, cM1 is assigned.
pM1, both clinical and pathological Stage IV	**pM1**<br>A patient may be staged as both clinical and pathological Stage IV if:<br>• there is confirmatory microscopic evidence of a distant metastatic site during the diagnostic workup, which is categorized as pM1, and<br>• T and N are categorized only clinically.<br>**Example:** cT3 cN1 pM1 clinical Stage IV and cT3 cN1 pM1 pathological Stage IV
Circulating tumor cells and disseminated tumor cells: cM0(i+) category	**cM0(i+)**<br>Patients with:<br>• Circulating tumor cells (CTCs) in blood, or<br>• Disseminated tumor cells (DTCs) in organs and micro-metastasis in bone marrow detected by IHC or molecular techniques<br>are categorized as cM0(i+).<br>The cM0(i+) category denotes the uncertain prognostic significance of these findings.<br>*The concepts regarding this staging rule continue to evolve, and further study is warranted.*
Clinical suspicion and biopsy does not confirm distant metastatic disease	If there is clinical suspicion for distant metastases and a biopsy or excision does not confirm metastatic cancer, M is categorized as clinically M0 (cM0) or clinically M1 (cM1) based on the evaluation of other possible sites of distant metastatic disease. There is no TNM pM0 designation.<br>*Note*: pM0 is not a valid category.<br>If clinical evidence of distant metastasis remains in other areas that are not or cannot be confirmed microscopically, cM1 is assigned.
Unknown distant metastasis status	**MX does not exist**<br>MX is not a valid category and cannot be assigned. Unless there is clinical or pathologic evidence of metastases, M is categorized as clinically negative: cM0.
Direct extension into an organ not M category	Direct extension from the primary tumor or lymph nodes into a contiguous or adjacent organ is not included in the M category but is used in the T and N category assignments as noted earlier.<br>**Example**: Direct extension of a colon cancer into the liver is categorized as pT4 and cM0.
Definition of metastases timing	Metastases defined during the relevant time frame/staging window are classified as metastases (cM1/pM1) and are considered synchronous with diagnosis of the primary cancer.<br>Metastases detected after the relevant time frame/staging window are not included in the initial staging and generally are considered recurrent cancer.

Pathological Classification

Classification of T, N, and M after surgical treatment is denoted by use of a lowercase *p* prefix: pT, pN, and cM0, cM1, or pM1.

Time frame: From date of diagnosis through surgical resection in the absence of cancer progression

Criteria: Surgery is first therapy

Pathological classification is based on the:

- clinical stage information (acquired before treatment), and supplemented/modified by
- operative findings, and
- pathological evaluation of the resected specimen(s).

Pathological stage is assigned for patients first treated with surgery. The surgical resection required for assignment of this classification is specified for each disease site, and ranges from resection of the tumor to complete resection of the organ and usually includes resection of at least some of the regional lymph nodes.

The purpose of pathological classification is to provide additional precise and objective data:

- for prognosis and outcomes, and
- to guide subsequent therapy.

Criteria for assigning pathological stage

Component of pathological staging	Details
Assignment of pathological stage by managing physician	Pathological stage is based on a synthesis of clinical and pathological findings and is assigned only by the managing physician, such as a surgical, radiation, or medical oncologist.
Primary tumor surgical resection for pathological staging	The surgical resection criteria in the disease site must be met in order to assign a pathological stage. The extent of primary tumor surgical resection ranges from:<br>• resection of the tumor, up to<br>• complete resection of the organ, and<br>• usually includes resection of at least some regional lymph nodes<br>*Note*: Surgical resection criteria depend on the cancer site–specific information necessary to determine the need for adjuvant therapy and the patient's prognosis, including tumor (T) and regional nodes (N).
Basis of pathological staging	Pathological staging encompasses:<br>• clinical staging information<br>• the surgeon's operative findings<br>• pathological evaluation of the resected specimen(s)
Imaging studies used in assigning pathological stage	Imaging studies performed after surgery are included in the pathological staging if they are within the time frame or staging window.

Criteria for assigning pathological stage

Component of pathological staging	Details
Unresectable tumor and assignment of pathological stage	If the highest T and N categories or the M1 category of the tumor are confirmed microscopically, even if a primary tumor technically cannot be removed or if it is unreasonable to remove it, the criteria for pathological staging are considered satisfied without total removal of the primary tumor.<br>*Note*: Microscopic confirmation of the highest T and N does not necessarily require removal of that structure and may entail biopsy or FNA only.<br>**Example:** Supraclavicular node involvement in inflammatory breast cancer in which inflammatory carcinoma was identified on the core needle breast biopsy and the supraclavicular node involvement is documented by FNA

Pathological T (pT)

The pathological assessment of the primary tumor generally is based on resection of the primary tumor.

Component of pT	Description
Tumor size and extent	Primarily based on size and local extension of the resected specimen<br>The pathologist provides information to assign the pT category based on the specimen received, but this may not be the final pT used for staging assignment. Final pT is assigned by the managing physician and also may include clinical stage information and operative findings.
Tumor size in millimeters and rounding for T-category assignment	Primary tumor size is the most accurate/largest dimension and is:<br>• measured to the nearest whole millimeter, unless a smaller unit is specified in a specific disease site, and<br>• rounded up or down as appropriate for assigning T category:<br>○ down when the numerals are between 1 and 4<br>○ up when the numerals are between 5 and 9<br>**Examples:**<br>• Tumor measured as 2.2 mm is recorded as 2 mm.<br>• Tumor measured as 1.7 mm is recorded as 2 mm.<br>• Tumor measured as 2.04 cm is recorded as 20 mm, and would be grouped with ≤2 cm and not >2 cm<br>**Nonexhaustive exceptions**:<br>• Melanoma: primary tumor measured to nearest 0.1 mm<br>• Breast cancer: primary tumor >1.0 mm to 1.4 mm rounded to 2 mm (this avoids assigning the "microinvasion" category to cancer >1.0 mm)

Component of pT	Description
Resection specimen role in pT category	pT category optimally is based on resection of a single specimen. If resected in several partial specimens at the same or separate operative setting, a reasonable estimate of size and extension should be made.<br>The estimate of multiple specimens may be based on the best combination of gross and microscopic findings, and may include reconstruction of the tumor with the assistance of the radiologist and surgeon. See CAP Protocols for tumor-specific recommendations.
Impact on pT category of positive resection margins	The presence of microscopic cancer at the resection margin does not affect the assignment of the pT category, which is assigned based on findings in the resection specimen and at operation.<br>In situations in which the surgeon has left behind grossly identified tumor in performing a noncurative resection, the T category should be based on all available clinical and pathological information.
Pathological tumor size variance based on assessment approach	Tumor size may vary based on whether it is measured on an unfixed or a fixed specimen. Size is often reported on the fixed specimen, and gross impression of tumor size may be adjusted based on microscopic examination. The pathologist should note potential alteration in tumor size caused by fixation if it might affect staging.
Synchronous primary tumors is a single organ: *(m)* suffix	For multiple tumors in a single organ, T is assigned to the highest T category; the preferred designation is:<br>• *m* suffix; for example, pT3(m) N0 M0<br>If the number of tumors is important, an acceptable alternative is:<br>• number of tumors; for example, pT3(4) N0 M0<br>*Note*: The *(m)* suffix applies to multiple invasive cancers. It is not applicable for multiple foci of *in situ* cancer, or for a mixed invasive and *in situ* cancer.
Direct extension into regional node	If a primary tumor directly extends into a regional lymph node, it is:<br>• included in the N category as a positive regional lymph node<br>• not included as a criterion for assigning the T category
Tumor nodule in node area not considered in T category	Rounded tumor nodules with smooth-contoured capsules in the regional nodal drainage area generally represent lymph nodes completely replaced with cancer and are classified as lymph nodes, unless there is clear evidence of residual blood vessel wall to justify classification as vascular involvement. They are not considered in the T category.
Direct extension into an organ	Direct extension of a primary tumor into a contiguous or adjacent organ is classified as part of the tumor (T) classification and is not classified as metastasis (M).<br>**Example**: Direct extension of a primary colon cancer into the liver is categorized as T4 and is not in the M category.

Component of pT	Description
Unresected tumor and highest T category	The pathological T (pT) category may be assigned without tumor resection if:<br>• a biopsy of the primary tumor (cT) is performed and is adequate to evaluate the highest pT category.<br>Other criteria, such as microscopic confirmation of the highest pN, must be met in order to assign pathological staging.
Disease sites have specific rules	Some disease sites have specific rules to guide assignment of pT. Refer to specific disease site chapters for further guidance.
Unknown primary or no evidence of primary tumor	If there is no evidence of a primary tumor, or the site of the primary tumor is unknown, staging may be based on clinical suspicion of the primary tumor, with the tumor categorized as T0. The rules for staging cancers categorized as T0 are specified in the relevant disease site chapters.<br>**Examples of exception**: The T0 category is not used for head and neck squamous cancer sites, as such patients with an involved lymph node are staged as unknown primary cancers using the system for cervical nodes and unknown primary tumors of the head and neck (T0 remains a valid category for HPV- and EBV-associated oropharyngeal and nasopharyngeal cancers).
Tis and surgical resection criteria	*In situ* neoplasia identified from a surgical resection, as specified in the disease site pathological criteria, is assigned pTis.<br>*In situ* neoplasia identified microscopically during the diagnostic workup may be used to assign the pathological stage pTis if the patient had a surgical resection and no residual tumor was identified.
Any T	*Any T* includes all T categories except Tis. This includes TX and T0.

Pathological N (pN)

Pathological assessment of regional node involvement (pN) is necessary.

Component of pN	Details
Microscopic assessment for pN	Microscopic assessment of a regional node includes:<br>• FNA cytology<br>• Core biopsy<br>• Incisional biopsy<br>• Excisional biopsy<br>• SLN biopsy/procedure<br>• Regional lymph node dissection
Requirements for assigning pN category	To assign a pN category, there must be:<br>• pathological documentation of the presence or absence of cancer in at least one node, and<br>• pathological assessment of the primary tumor (pT), except in cases of an unknown primary (T0)

Component of pN	Details
	Note: It is not necessary to pathologically confirm the status of the highest N category to assign the pN. If pT is available (resection), then any microscopic evaluation of nodes is classified as pN. For example, assessment of the axillary nodes is sufficient to assign pN for breast cancer, and it is not necessary to microscopically confirm the status of supraclavicular nodes.<br>Many cancer sites have specific recommendations regarding the minimum number of lymph nodes to be removed during lymph node dissection to provide optimal prognostic information. However, pathological categorization (pN) still applies even in cases in which fewer than the recommended number of lymph nodes are resected (e.g., a colon cancer resection specimen with only four pathologically negative lymph nodes is categorized as pN0).<br>FNA and core needle biopsy of a node both satisfy the requirement that at least one regional node be microscopically examined.
Categorize N	pN generally is categorized by disease-specific rules based on:<br>• number and/or<br>• location of positive regional nodes and/or<br>• size of the largest deposit of tumor cells in the node(s)
Size of regional nodal metastasis	Size of regional nodal metastasis generally is specified in disease site chapters and may be based on:<br>• size of metastasis in the node,<br>• size of the lymph node, or<br>• size of the nodal mass, which may be a mass of matted nodes<br>For some disease sites, the size of tumor metastasis within the regional lymph node is a criterion for the N category. If the size of the tumor in the regional nodal metastasis is unknown, the size of the involved lymph node may be used.<br>The size of any mass, from a single node to a conglomerate mass of matted nodes, is used to determine the N category for some disease sites, such as head and neck.<br>*Note:* Please refer to disease site chapters for specific criteria on assessment of size of regional nodal metastasis.
Direct extension into regional node is N category	If a primary tumor directly extends into a regional lymph node, it is:<br>• included in the N category as a positive regional lymph node<br>• not included as a criterion for assigning the T category
Tumor nodule in node area not considered in T category	Rounded tumor nodules with smooth-contoured capsules in the regional nodal drainage area generally represent lymph nodes completely replaced with cancer and are classified as lymph nodes, unless there is clear evidence of residual blood vessel wall to justify classification as vascular involvement. They are not considered in the T category.
Sentinel node or regional node excision	Microscopic examination of regional nodes without resection of the primary site (during the diagnostic workup) is included in the clinical classification as cN.<br>Microscopic examination of regional nodes with surgical resection of the primary site (surgical treatment) is categorized as pN.<br>**Example**: Sentinel node biopsy performed at the time of wide re-excision for melanoma (surgical treatment) is pathological (pN).
SLN	An SLN is a regional lymph node that receives direct afferent lymphatic drainage from a primary tumor site (e.g., breast, melanoma), and in many solid tumors represents the regional lymph node(s) most likely to contain metastatic disease, if any are involved. More than one SLN may be present in a regional nodal basin, and some primary tumors (e.g., melanoma) may drain to more than one regional nodal basin.<br>Sentinel nodes are identified by lymphatic mapping, as evidenced by nodes that concentrate a colloidal material injected near the primary tumor or in the involved organ (the most commonly used agents for sentinel node biopsy are vital stains such as isosulfan blue and/or radiotracers such as ^{99}Tc-sulfur colloid). In some circumstances, the managing physician also may label regional lymph nodes that are palpably abnormal during surgery as sentinel nodes. Nodes that do not concentrate colloidal material and are resected along with other sentinel nodes are nonsentinel nodes, and are considered part of the sentinel node procedure. Their resection is not coded as a separate nodal procedure or a lymph node dissection.
Sentinel node (sn) and FNA or core biopsy (f)	If SLN biopsy is performed in the absence of complete dissection of the nodal basin:<br>• the N category should have the *sn* suffix; for example, pN0(sn).<br>If FNA or core biopsy is performed in the absence of a complete dissection of the nodal basin:<br>• the N category should have the *f* suffix; for example, pN0(f).<br>*Note*: This distinguishes it from a complete nodal dissection, for which the pN is assigned without the *(sn)* or *(f)* suffix.
ITCs: use of the (i+) designator	ITCs include single tumor cells or small clusters of cells ≤0.2 mm in greatest diameter, generally without stromal response in the lymph node. These cells usually are found in the subcapsular nodal sinuses but may be seen within the nodal parenchyma. Because ITCs may represent tumor cells that are in transit that are not proliferating within the node, lymph nodes with only ITCs usually are categorized as N0, with some exceptions. They are denoted as N0(i+).

Component of pN	Details
	The concepts regarding this staging rule continue to evolve, and further study is warranted. In the meantime, the staging rule serves as a guideline for uniformity and consistency in practice in recording information, and clinical judgment by the managing physician prevails.<br>**Exception:** In melanoma and Merkel cell carcinoma, ITCs are considered positive nodes and are designated as N1 or higher.<br>*Note*: There are cancer site–specific designators to identify ITCs in nodes.<br>**Example:** N0(i+) in breast and gynecologic cancers applies to nodes with ITCs only.
Pathological techniques for ITCs or detection of micro-metastasis	ITCs or lymph node micro-metastases may be identified in lymph nodes by hematoxylin and eosin staining or by specialized pathological techniques, such as IHC for cytokeratin proteins for carcinomas.<br>Specialized pathology techniques such as IHC and molecular techniques are not recommended for routine examination of lymph nodes.<br>*The concepts regarding this staging rule continue to evolve, and further study is warranted.*
Nonmorphologic techniques for identifying ITCs: use of *(mol+)* designator	If used, nonmorphologic techniques, including flow cytometry and reverse transcriptase polymerase chain reaction studies, may identify minimal deposits of cancer in lymph nodes.<br>These usually are classified as clinically node negative and identified with the *(mol+)* designator: for example, cN0(mol+).<br>*The concepts regarding this staging rule continue to evolve, and further study is warranted.*
Micro-metastases: use of *mi* designator	Lymph node micro-metastases are defined as tumor deposits >0.2 mm but ≤2.0 mm. For certain disease sites, micro-metastases are denoted by using the *mi* designator: for example, cN1mi. Further studies are needed to determine the significance of micro-metastases across many cancer sites.<br>*The concepts regarding this staging rule continue to evolve, and further study is warranted.*
Extranodal extension (ENE)	*ENE* is defined as the extension of a nodal metastasis through the lymph node capsule into adjacent tissues. ENE is the preferred terminology. It is sometimes also termed *extranodal spread*, *extracapsular extension*, or *extracapsular spread*.
Regional node metastasis invading a distant organ is ENE	A regional node extending into a distant structure or organ is categorized as ENE and is not considered distant metastatic disease.

Component of pN	Details
Recommended minimum number of lymph nodes	As noted in previous editions of the *AJCC Cancer Staging Manual*, as well as this 8th Edition, several cancer sites contain a recommendation regarding the minimum number of regional nodes to be surgically resected and pathologically analyzed for determination of the N category.<br>These recommendations are offered as metrics for evaluation of quality review of the extent of surgical resection and resultant pathological analysis. These minimum benchmarks should not be construed as unique indicators for additional surgical resection or adjuvant therapy if the recommended nodal count has not been met.<br>In cases in which fewer than the recommended optimal number of lymph nodes are removed, pathological node category (pN) should be assigned and complete pathological staging applied based on whatever number of nodes are reported. A suboptimal node count may lead to further dialogue between the surgeon and pathologist to support the opportunity for further evaluation (e.g., fat clearance techniques) of the node-bearing specimen to assure that a maximum node assessment is reached; however, this is not necessary to assign the pathological node category.
Node status not required in rare circumstances	For some cancer sites in which lymph node involvement is rare, patients whose nodal status is not determined to be positive for tumor should be designated as cN0. These circumstances are identified in specific disease site chapters for these sites; NX may not be listed as a category.<br>The assignment of cN0 will ensure it is not confused with a case in which the nodes were microscopically proven to not contain tumor, that is, pN0.<br>**Examples:** For bone and soft tissue sarcoma, cN0 may be used to assign the pathological stage group—that is, pT1 cN0 cM0.<br>For melanoma, cN0 may be used to assign a pathological stage group for T1 melanoma.
Regional node invading a distant organ	Tumor involving a regional node and extending into a distant structure or organ is categorized as ENE and is not considered metastatic disease.
Regional nodes when a tumor involves more than one organ or structure	In the rare occurrence in which a tumor involves more than one organ or structure, the regional nodes include those of all involved structures, even if the nodes of the primary site are not involved.<br>**Example**: If a transverse colon cancer invades the stomach, the gastric regional nodes would be considered regional for the transverse colon, even if the colon regional nodes were not involved.

Component of pN	Details
Unresectable tumor and highest N category	If the primary tumor and/or regional lymph nodes technically cannot be removed or it is clinically not indicated to remove them, the following criteria may be used to assign pathological stage:<br>• microscopically confirmed highest T category, and<br>• microscopically confirmed single node or nodes in the highest N category<br>*Note*: Microscopic confirmation of the highest T and N categories may use biopsy or FNA only.
Any N	*Any N* includes all N categories. This includes NX and N0.

Pathological M Categorization (cM and pM)

Any of the M categories (cM0, cM1, or pM1) may be used with pathological stage grouping. The terms pM0 and MX are NOT valid categories in the TNM system.

Component of M for pathological staging	Details
No distant metastasis	**cM0**<br>If there are no symptoms or signs of distant metastasis, the case is classified as clinically M0 (cM0). Evaluation includes:<br>• history and physical examination<br>• imaging studies performed<br>*Note*: Imaging studies are NOT required to assign cM0.
Clinical evidence of distant metastasis	**cM1**<br>Patients with clinical evidence of distant metastases by history, physical examination, imaging studies, or invasive procedures, but without microscopic evidence of the presumed distant metastases, are categorized as clinically M1 (cM1). Examination methods include:<br>• physical examination<br>• imaging<br>• exploratory surgery or endoscopy
Microscopic evidence of distant metastasis	**pM1**<br>Patients in whom there is microscopic evidence confirming distant metastatic disease are categorized as pathologically M1 (pM1).<br>Microscopic evidence includes:<br>• cytology from FNA<br>• core biopsy<br>• incisional biopsy<br>• excisional biopsy<br>• resection
Use of pM1 if there are multiple distant metastases	**pM1**<br>In patients who have distant metastases in multiple sites, and have a cancer type for which M subcategories distinguish between one or more metastatic sites, microscopic evidence of one of these sites is necessary to assign the higher pM subcategory.

Component of M for pathological staging	Details
	In general, metastases to both sides of a paired organ are considered a single metastatic site of involvement (e.g., metastases to both lungs are assigned as metastasis to one distant site—lung).<br>If clinical evidence of distant metastasis remains in other areas that are not or cannot be microscopically confirmed, cM1 is assigned.
pM1 may be used for both clinical and pathological Stage IV	**pM1**<br>A patient may be staged as both clinical and pathological Stage IV if there is:<br>• confirmatory microscopic evidence of a distant metastatic site during the diagnostic workup, which is categorized as pM1, and<br>• T and N may be categorized only clinically.<br>**Example:** cT3 cN1 pM1 clinical Stage IV, and cT3 cN1 pM1 pathological Stage IV
Circulating tumor cells and disseminated tumor cells: cM0(i+) category	**cM0(i+)**<br>Patients with<br>• CTCs, or<br>• DTCs in organs and micro-metastasis in bone marrow, detected by IHC or molecular techniques,<br>are categorized as cM0(i+).<br>The cM0(i+) category denotes the uncertain prognostic significance of these findings.<br>*The concepts regarding this staging rule continue to evolve, and further study is warranted.*
Clinical suspicion of metastasis, but biopsy does not confirm distant metastatic disease	If there is clinical suspicion of distant metastases and a biopsy or excision does not confirm metastatic cancer, M is classified as clinically M0 (cM0) or clinically M1 (cM1) based on the evaluation of other possible sites of distant metastatic disease. There is no TNM pM0 designation.<br>***Note:* pM0 is not a valid category**<br>If clinical evidence of distant metastasis remains in other areas that are not or cannot be microscopically confirmed, cM1 is assigned.
Unknown distant metastasis status	**MX does not exist**<br>MX is not a valid category and cannot be assigned. Unless there is clinical or pathologic evidence of metastases, M is categorized as clinically negative: cM0.
No direct extension in M category	Direct extension from the primary tumor or lymph nodes into a contiguous or adjacent organ is not included in the M category but is used in the T and N category assignments as noted earlier.<br>**Example**: Direct extension of a colon cancer into the liver is categorized as pT4 and cM0.

Component of posttherapy staging	Details
Distant metastasis	The presence of distant metastases is classified by the M status defined during the clinical classification, cM or pM, before initiation of neoadjuvant radiation and/or systemic therapy.<br>*Note:* Once distant metastasis is identified, that M category designation always remains, even if there no longer is evidence of the metastasis after neoadjuvant therapy. In this situation, the yc and yp stages always maintain the M1 category.
Complete pathological response	If a complete pathological response has occurred and the ypTNM is ypT0 ypN0 cM0, no stage group is assigned.<br>*Note*: This situation is not classified as Stage 0, because such a designation would denote *in situ* neoplasia. Nonetheless, the individual T, N, and M categories should be documented as T0, N0, M0.<br>The complete pathological response also may be documented by using the response designation.
Response to neoadjuvant therapy	It is important to record the response to neoadjuvant therapy. Consult disease site chapters for specific systems.<br>For example, some disease sites include "complete," "partial," and "no response," whereas others consist of a numerical scoring system or a "regression score."<br>If surgery is performed, it is critical to also assign the ypT and ypN for analysis of response to neoadjuvant therapy.
Mucin pools, necrosis, and other reactive changes not included in the assessment of residual cancer	Histologic confirmation of residual cancer requires identification of non-necrotic tumor cells.<br>Mucin pools, necrosis, and other degenerative and reactive changes without viable-appearing tumor cells are insufficient for a diagnosis of residual cancer. Mucin pools and necrotic cells currently play no role in assigning the ypT and ypN.

Recurrence or Retreatment Classification (rTNM)

Classification of T, N, and M for recurrence or retreatment is denoted by use of the lowercase *r* prefix: rcT, rcN, rc/rpM, and rpT, rpN, rc/rpM. The rc/rpM may include rcM0, rcM1, or rpM1.

Time frame: From identification of recurrence or progression until treatment is initiated

Criteria: Disease recurrence after disease-free interval, or disease progression

The recurrence or retreatment classification is assigned if a cancer recurs after an interval during which the patient has been considered cancer-free (disease-free interval), or if the cancer progresses and the patient has never been disease-free (even if no retreatment is planned).

Assessment of recurrence and retreatment follows specific criteria.

Recurrence/retreatment staging assessment criteria

Component of recurrence/ retreatment staging	Details
Stage at initial diagnosis is not affected by recurrence	The initially assigned clinical and pathological stages at diagnosis do not change if a cancer recurs or progresses.
Use of *r* prefix	In staging for recurrence or retreatment, the *r* prefix is applied.
Information included: r classification	All information available at the time of recurrence or retreatment should be used to determine the rTNM stage, including clinical and pathological information.<br>**Important**: Biopsy confirmation is not required but is encouraged if clinically feasible.<br>**rc**<br>The r-clinical (rc) classification is based on:<br>• clinical history and physical examination and<br>• any imaging studies, if performed<br>*Note*: Imaging studies may be considered standard practice but are NOT required to assign rc categories.<br>**rp**<br>The r-pathological (rp) classification is based on:<br>• r-clinical stage information, and supplemented/modified by<br>• operative findings, and<br>• pathological evaluation of the resected specimen.

Autopsy Classification (aTNM)

Classification of T, N, and M at autopsy is denoted by use of the lowercase *a* prefix: aT, aN, aM.

Time frame: At death

Criteria: Incidental finding of cancer at autopsy; cancer not suspected or evident before death (i.e., classification does not apply if autopsy is performed in a patent with a known cancer before death).

Autopsy assessment has specific criteria.

Component of autopsy staging	Details
Diagnosis at autopsy	Cancer must be diagnosed at autopsy.<br>No prior suspicion or evidence of cancer before death.
Information included	All clinical and pathological information is included. It is obtained:<br>• at time of death, and<br>• through postmortem examination.

Posttherapy or Post Neoadjuvant Therapy Classification (yTNM)

For purposes of posttherapy or post neoadjuvant therapy classification, *neoadjuvant therapy* is defined as systemic and/or radiation therapy given before surgery; primary radiation and/or systemic therapy is treatment given as definitive therapy without surgery.

Classification of T, N, and M after systemic or radiation treatment intended as definitive therapy, or after neoadjuvant therapy followed by surgery, is denoted by use of a lowercase *yc* or *yp* prefix, respectively: ycT, ycN, c/pM, and ypT, ypN, c/pM, respectively. The c/pM category may include cM0, cM1, or pM1.

yc

Time frame: After primary systemic and/or radiation therapy without subsequent surgical resection, or after neoadjuvant and before planned surgical resection

Criteria: First therapy is systemic and/or radiation therapy. y-clinical (yc) classification is based on the:

- clinical history and physical examination and
- any imaging studies, if performed

Note: imaging studies may be considered standard practice, but are NOT required to assign yc categories.

yp

Time frame: The yp classification is used when staging after neoadjuvant therapy and planned post neoadjuvant therapy surgery. The time frame should be such that the post neoadjuvant therapy surgery and staging occur within a period that accommodates disease-specific circumstances, as outlined in the specific chapters and in relevant guidelines.

Criteria: First therapy is systemic and/or radiation therapy followed by surgery.

y-pathological (yp) classification is based on the:

- y-clinical stage information, and supplemented/modified by
- operative findings, and
- pathological evaluation of the resected specimen.

Observed changes between the clinical classification and the posttherapy classification may provide clinicians with information regarding the response to therapy. The clinical extent of response to therapy may guide the scope of planned surgery, and the clinical and pathological extent of response to therapy may provide prognostic information and guide the use of further adjuvant radiation and/or systemic therapy.

Examples of treatments that satisfy the definition of neoadjuvant therapy for a disease site may be found in sources such as the NCCN Guidelines, ASCO guidelines, or other treatment guidelines. Systemic therapy includes chemotherapy, hormone therapy, and immunotherapy. Not all medication given to a patient meets the criteria for neoadjuvant therapy (e.g., a short course, such as a few days of endocrine therapy in breast cancer or prostate cancer that is provided for variable and often unconventional reasons, should not be categorized as neoadjuvant therapy).

The time frame should be such that the post neoadjuvant therapy surgery and staging occur within a period that accommodates disease-specific circumstances, as outlined in the specific chapters and in relevant guidelines.

The post neoadjuvant therapy assessment of the T and N (yTNM) categories uses specific criteria. In contrast, the M category for post neoadjuvant therapy classification remains the same as that assigned in the clinical stage before initiation of neoadjuvant therapy (e.g., if there is a complete clinical response to therapy in a patient previously categorized as cM1, the M1 category is used for final yc and pc staging).

Component of posttherapy staging	Details
Assignment of stage by managing physician	Posttherapy or post neoadjuvant therapy stage is based on a synthesis of clinical and pathological findings and is assigned only by the managing physician, such as a surgical, radiation, or medical oncologist.<br>Pathologists may provide T, N, and M information based on the specimens received to assist the managing physician in assigning the final stage.<br>Radiologists may provide T, N, and M information based on imaging studies to assist the managing physician in assigning the final stage.
Use of yTNM	To use the yTNM classification, the extent of disease is assessed:<br>• after systemic and/or radiation therapy as the primary treatment, and<br>• after surgery when it follows the systemic and/or radiation therapy
Use of *y* prefix	The *y* prefix is always combined with either a clinical or pathological prefix, that is, ycTNM or ypTNM.
Time frame in the patient's care for use of yc and yp	• ycTNM denotes information gathered using clinical classification rules and methods:<br>o after neoadjuvant systemic and/or radiation therapy, and<br>o before surgical resection or if no surgery is performed.<br>• ypTNM denotes information gathered using pathological classification rules and methods:<br>o after neoadjuvant systemic and/or radiation therapy, and<br>o after the surgical resection.<br>**Examples:**<br>• ycT and ycN with cM or pM<br>• ypT and ypN with cM or pM.

AJCC PROGNOSTIC STAGE GROUPS

Cancer patients with similar prognoses are grouped by using prognostic stage group tables. Clinical and pathological stage groups are defined for each case as appropriate. These disease-specific groups are composed of the following categories:

- cT, cN, and cM or pM
- pT, pN, and cM or pM
- factors for both groups, if applicable

Stage group assignment follows specific rules.

Rules for assigning prognostic stage groups (stage groups)

**Component of prognostic stage group**	**Rule(s)**
Prognostic stage groups	Prognostic stage groups are based on combinations of T, N, M, and relevant prognostic factors and usually define groups of patients with similar outcomes to help define prognosis and appropriate treatment, as well as to enable comparisons of similar groups of patients between institutions and over time.
Categories and subcategories	When a category (e.g., T1) is identified in the stage group table, it includes all subcategories (e.g., for T1, this may include T1mi, T1a, T1b, etc.).<br>However, If the specific subcategories are listed separately (e.g., T1a, T1b, N1mi), only the specific subcategory is included in the stage group.
Unknown T or N	A stage group cannot be assigned if X is used for either T or N. If a prognostic factor is X, it should be assigned based on TNM.<br>**Exception:** Stage IV is always assigned if there is:<br>• evidence of distant metastasis (cM1 or pM1), even if the T or N category is unknown (TX or NX).<br>Stage may be assigned if the TNM stage group results in Any T or Any N with M0, which includes TX or NX. Examples include:<br>• TX N1 M0 Stage III in melanoma clinical stage<br>• T4 NX M0 Stage III in pancreatic cancer
Stage documentation in the medical record	The patient's medical record should be updated with any applicable stage group information as it is available, including:<br>• clinical<br>• pathological<br>• posttherapy or post neoadjuvant therapy<br>• recurrence or retreatment<br>• autopsy<br>Once assigned according to the appropriate rules and timing, the documented stage group does not change.

Rules for assigning prognostic stage groups (stage groups)

**Component of prognostic stage group**	**Rule(s)**
Assigning stage with incomplete information	A presumptive stage to facilitate patient management may be used by the treating physician/management team. This is not a formal stage classification type in the TNM system. It is only for physician use in patient care. It should never be documented by cancer registries.<br>During the diagnostic workup, the managing physician may assign a preliminary clinical stage based on the information known at that time, and may continually update the stage as the workup progresses. This approach commonly is used for cancer conferences (tumor boards) and other medical conversations. Once the final clinical stage is determined, these preliminary stages no longer are used and are replaced by the clinical stage. The stage(s) provisionally assigned during the diagnostic workup may be referred to as the *presumptive stage(s)*.<br>In patient care, it may be appropriate for the managing physician to combine clinical and pathological T and N categories if only partial information is available in the pathological classification. Although this strategy may be used to plan treatment and to provide the patient with a stage group and prognosis, it does not represent the actual TNM stage and therefore is not used to assign a stage group.
Missing/unknown prognostic factor	If a required prognostic factor category is unavailable, the patient may still be staged. The stage group assigned is the:<br>• group containing the prognostic factor X category, or<br>• anatomic stage, assigned by default using clinical judgment
pM1 in stage groups	If a patient has microscopic confirmation of distant metastases (pM1) during the diagnostic workup, the patient may be classified as clinical Stage IV and pathological Stage IV, regardless of whether the T and N are classified by clinical or pathological means.<br>**Example:** For pM1 and cT and cN, the patient may be assigned both:<br>• clinical stage group, and<br>• pathological stage group<br>*Note*: This rule does not apply to patients with clinical metastases without microscopic confirmation. These patients may be staged only clinically.
cM or pM used in all stage groups	cM0, cM1, or pM1 may be used in any of the following stage groups:<br>• clinical stage group<br>• pathological stage group<br>• post neoadjuvant therapy or primary radiation/systemic therapy clinical stage group<br>• post neoadjuvant therapy pathological stage group<br>• recurrence or retreatment stage group

Rules for assigning prognostic stage groups (stage groups)	
**Component of prognostic stage group**	**Rule(s)**
Microscopic evaluation without resection for assigning pathological classification	If the highest T and N categories of the tumor are confirmed microscopically, the criteria for pathological staging have been satisfied. This may occur if a primary tumor technically cannot be removed or if it is unreasonable to remove it, but the criteria for pathological staging have been satisfied without total removal of the primary tumor. *Note*: Microscopic confirmation of the highest T and N does not necessarily require removal of that structure and may include biopsy or FNA only. Please refer to disease sites for specific guidelines.
In situ neoplasia, Stage 0 for clinical classification	*In situ* neoplasia identified microscopically during the diagnostic workup is assigned as cTis cN0 cM0 clinical Stage 0.
In situ neoplasia, Stage 0 does not require node evaluation for pathological classification	*In situ* neoplasia is an exception to the stage grouping guidelines that otherwise require regional lymph node evaluation for pathological classification. By definition, *in situ* neoplasia has not involved any structures in the primary organ that would allow tumor cells to spread to regional nodes or distant sites.<br>The primary tumor surgical resection criteria for pathological stage must be met in order to assign pathological Stage 0.<br>Lymph node microscopic assessment is not necessary to assign pathological Stage 0 for *in situ* neoplasia; for example, pTis cN0 cM0 is staged as pathological Stage 0.<br>*Notes*:<br>• *In situ* neoplasia includes carcinoma *in situ* (CIS) and other *in situ* neoplasia.<br>• Disease sites having two Stage 0 groups usually are denoted as 0is and 0a.
Noninvasive, Stage 0a	Ta is assigned for noninvasive papillary carcinoma in the renal pelvis and ureter, urinary bladder, and urethra. The stage group usually is 0a.<br>The same rules apply to noninvasive tumors as those for *in situ* neoplasia.<br>Noninvasive papillary carcinoma identified microscopically during the diagnostic workup is assigned as cTa cN0 cM0 clinical Stage 0a.<br>Noninvasive papillary carcinoma identified on surgical resection meeting the criteria for pathological stage is assigned as pTa cN0 cM0 pathological Stage 0a.
Tis N1–3	In rare situations, whenever the pathology fails to reveal invasive cancer and shows Tis only with nodal involvement, the stage group may be assigned by the managing physician based on the N category as available for patient care. The cancer registry should document Tis with the appropriate N category and no stage group.
	In melanoma, patients with histologically documented melanoma *in situ* disease only may develop regional metastasis. Biologically, this may represent melanoma metastasis associated with a regressed primary, which may be associated with the Tis lesion or may be a completely regressed tumor (i.e., unknown primary). The stage may be assigned by the managing physician as Tis N1-3 M0 with a stage group based on the N category as available for patient care.<br>*Note*: Rarely, patients with a resected cancer showing only *in situ* disease (Tis) have metastatic cancer in regional lymph nodes. This mostly involves breast cancer (ductal carcinoma *in situ*), although it is still rare. The common theory is that the node metastases come from an unidentified occult invasive cancer. For clarity in registry operations and to allow study of these patients in the future, such cases should be categorized as:<br>• Tis N1 (or N2/N3 as appropriate).<br>• These cases cannot be assigned a stage group in the registry database.<br>Clinicians should use careful judgment in counseling patients with this unusual finding.
Uncertainty in assigning stage group	If uncertainty exists regarding the stage group, the lower or less advanced of two possible stage groups should be assigned.<br>*Note*: This rule does not apply to situations in which not enough information is available to allow staging, such as cases with unknown T (TX) or unknown N (NX).
Complete pathological response	If a complete pathological response has occurred and the ypTNM is ypT0 ypN0 cM0, no stage group is assigned.<br>*Note*: This situation is not classified as Stage 0, because such a designation would denote *in situ* neoplasia. Nonetheless, the individual T, N, and M categories should be documented as T0, N0, M0

ADDITIONAL STAGING DESCRIPTORS AND GUIDELINES

N Suffixes: Sentinel Node Suffix (sn) and FNA or Core Biopsy (f)

Node category suffixes are used to indicate the method of assessment, which may have implications for the completeness of the pathological review.

Component of *N* suffix	Description
Sentinel node procedure indication (sn)	If a regional lymph node metastasis is identified by SLN biopsy only, and additional surgery in the form of a completion lymph node dissection is *not* performed, the N category is assigned with the addition of the *(sn)* suffix: for example, cN1(sn) or pN1(sn).
Time frame for cN(sn) and pN(sn)	If the sentinel node procedure is performed as:<br>• part of the diagnostic workup and before definitive surgical treatment, in which case the proper assignment is cN1–3(sn), or<br>• part of initial surgical management, in which case the proper assignment is pN1–3(sn).<br>*Note*: If the patient has a completion lymph node dissection performed as a component of the initial surgical management, the suffix is not used.
(sn) suffix in clinical and pathological classifications	If a sentinel node biopsy is performed as a component of the:<br>• diagnostic workup, it is assigned cN1(sn).<br>• surgical resection procedure and no additional (e.g., completion) lymph node dissection is performed, it is assigned pN1(sn).<br>• surgical resection procedure and a completion lymph node dissection is performed, it is assigned pN1.<br>• diagnostic workup, it is assigned cN1(sn) for clinical stage, and if completion lymph node dissection is performed during surgical resection of primary site, it is assigned pN1 for pathological stage.
FNA or core biopsy indication (f)	An FNA or core needle biopsy is denoted by the *(f)* suffix, if no further resection of the nodes is performed. FNA or core biopsy meets the criterion for microscopic examination of one node for assigning the pN category.
Time frame for use of *(f)* suffix	If the FNA/biopsy procedure is performed as:<br>• part of the diagnostic workup before treatment, it is assigned cN1–3(f).<br>• part of primary site surgical resection, then it is assigned pN1–3(f).<br>*Note*: If the patient subsequently undergoes a completion lymph node dissection as a component of the initial surgical management, the suffix is not used.
(f) suffix in clinical and pathological classifications	If FNA or core biopsy of regional lymph nodes is performed as a component of:<br>• diagnostic workup, it is assigned cN1(f).<br>• surgical resection of primary with no lymph node dissection performed, it is assigned pN1(f).<br>• surgical resection with lymph node dissection performed, it is assigned pN1.<br>• diagnostic workup, it is assigned cN1(f) for clinical stage; if lymph node dissection is performed as a component of the surgical resection of the primary site, it is assigned pN1 for pathological stage.

Guidelines for Primary Cancers

Multiple Primary Tumors

Multiple cancers may occur in the same organ and may be diagnosed at or about the same time (synchronous) or at separate time points (metachronous). For the purpose of staging, the following definitions apply.

Synchronous Primary Cancers

Component of synchronous cancers	Description
Timing for synchronous cancers	Cancers occurring in the same organ (including paired organs) that are identified with a diagnosis date ≤4 months apart, or that are identified at the time of surgery for the first cancer if that surgery is part of the planned first course of therapy
Multiple synchronous tumors	Multiple synchronous tumors:<br>• are cancers of the same histology<br>• occur in one organ
Synchronous primary tumors in a single organ	For multiple tumors in a single organ, T is assigned to the highest T category; the preferred designation is:<br>• *m* suffix; for example, pT3(m) N0 M0.<br>If the number of tumors is important, an acceptable alternative is:<br>• number of tumors; for example, pT3(4) N0 M0.<br>*Note*: The *(m)* suffix applies to multiple invasive cancers. It is not applicable to multiple foci of *in situ* cancer or to mixed invasive and *in situ* cancer.
Synchronous primary tumors in paired organs	Cancers occurring at the same time in each of paired organs are staged as separate cancers. Examples include breast, lung, and kidney. **Exception:** For tumors of the thyroid, liver, and ovary, multiplicity is a criterion of the T category and is not independently staged.

Multiple Synchronous Tumors, Suffix (m)

Component of T suffix	Description
(m) suffix for synchronous primary tumors in single organ	For multiple tumors in a single organ, T is assigned to the highest T category; the preferred designation is:<br>• *m* suffix; for example, pT3(m) N0 M0.<br>If the number of tumors is important, an acceptable alternative is:<br>• number of tumors; for example, pT3(4) N0 M0.<br>*Note*: The *(m)* suffix applies to multiple invasive cancers. It is not applicable to multiple foci of *in situ* cancer or to mixed invasive and *in situ* cancer.

Metachronous Primary Cancers

Component of metachronous cancers	Description
Timing for metachronous cancers	Cancers occurring in the same organ system that are identified with diagnosis dates >4 months from each other, except for cancers identified at the time of surgery for the first cancer occurring >4 months after the diagnosis of the first cancer if that surgery is part of the planned first course of therapy
Metachronous primaries	Metachronous primaries are primary cancers:<br>• occurring at different times in the same or different organs.
Staging	A metachronous primary is staged as a new cancer by using the applicable TNM disease site system.
Previous treatment of the organ	Second cancers in the same organ occurring after treatment of the original cancer are staged as new cancers and are not staged using the *y* prefix.

Cancers of Unknown Primary Site

There is no evidence of a primary tumor, but the anatomic site is suspected.

Component of T0	Description
T0	T0 is assigned if there is clinical suspicion of a primary tumor, with evidence of regional or distant metastases, but there is:<br>• no evidence of a primary tumor, or<br>• the site of the primary tumor remains unknown.<br>**Example**: T0 N1 M0 is assigned if:<br>• metastatic adenocarcinoma in axillary lymph nodes is pathologically consistent with breast cancer, and there is no apparent primary breast tumor or other primary tumor site<br>• metastatic melanoma is found in lymph nodes with no apparent primary skin lesion.<br>*Note*: The T0 category was eliminated for head and neck squamous cell cancer, except that T0 remains a valid category for HPV- and EBV-associated oropharyngeal and nasopharyngeal cancers.
cT0 and pT0	**cT0**<br>If physical examination, imaging, endoscopy, and other diagnostic procedures do not identify a primary tumor:<br>• the T category is assigned as cT0.<br>**pT0**<br>If after surgical resection of a suspected primary tumor no evidence of tumor is identified, and it was never identified on biopsy:<br>• the T category is assigned as pT0.
No information on primary tumor site of origin	T0 is not used for a cancer whose site of origin cannot be determined.<br>**Example:** Poorly differentiated carcinoma with histology that is not specific for a particular primary, and for which no actual site is identified. This is designated as an unknown primary and cannot be staged.

Histologic and Specimen Descriptors

Histopathologic Type

Histopathologic type is determined by microscopic assessment whereby a tumor is categorized according to the normal tissue type or cell type it most closely resembles (e.g., hepatocellular or cholangiocarcinoma, osteosarcoma, squamous cell carcinoma).

Component of histology	Description
Resource	The *World Health Organization Classification of Tumours*, published in numerous anatomic site-specific editions, is used most commonly for histopathologic typing.
Histologic codes for staging	Each chapter in the *AJCC Cancer Staging Manual* includes the applicable WHO and ICD-O-3 histopathologic codes. If a specific histology is not listed, the case should not be staged using the AJCC classification in that chapter.

Grade (G)

The grade of a cancer is a qualitative assessment of the degree of differentiation of the tumor. It may reflect the

extent to which a tumor resembles the normal tissue at that site. Grade may provide important information on the risk of cancer metastasis and prognosis.

Component of grade	Description
Histologic grade stratification	Historically, stratification of solid tumors has sometimes included an assessment of the overall histologic differentiation of the cancer. The most common grading schema uses numeric grades from the most or well differentiated (grade 1) to the least differentiated (grade 3 or 4). This system is still used in some cancer types, although site-specific grading systems are used more commonly.
Disease site–specific histologic grade stratification	The recommended grading system for each cancer type is specified in each chapter and is the grading system to be used by the pathologist and documented in the cancer registry.<br>For many cancer types, more precise and reproducible grading systems have been developed beyond the standard systems, and these may incorporate more specific and objective criteria based on single or multiple characteristics of the cancers. These factors include nuclear grade, number of mitoses identified microscopically (mitotic count), and measures of histologic differentiation (e.g., tubule formation in breast cancer), among others.<br>For some cancer types, these systems have been fully validated and largely implemented worldwide. Examples include the Gleason scoring system and the grade grouping for prostate cancer and the Scarff–Bloom–Richardson (Nottingham) grading system for breast cancer.
Histologic grade if more than one grade is noted	If there is evidence of more than one grade or level of differentiation of the tumor, the highest grade is recorded, assuming that the recommended grading system was used for both biopsy and resection.
Cancer registry documentation	The cancer registry must record the grade as specified in the disease site chapter, according to the rules only in this chapter and the disease site chapter.

Lymphovascular Invasion

This descriptor indicates whether microscopic lymphovascular invasion (LVI) is identified in the cancer as recorded in the pathology report. LVI includes lymphatic invasion, vascular invasion, and lymphovascular invasion. This coding convention has been developed and implemented for use in the 8th Edition for appropriate disease sites.

Component of LVI coding	Description
0	LVI not present (absent)/not identified
1	LVI present/identified, NOS
2	Lymphatic and small vessel invasion only (L)
3	Venous (large vessel) invasion only (V)
4	BOTH lymphatic and small vessel AND venous (large vessel) invasion
9	Presence of LVI unknown/ indeterminate

The concepts regarding this staging rule continue to evolve, and further study is warranted.

Residual Tumor and Surgical Margins

The absence or presence of residual tumor after treatment is described by the symbol R (capital *R*). cTNM and pTNM describe the extent of cancer in general without consideration of treatment. cTNM and pTNM may be supplemented by the R designation to categorize the absence or presence of residual tumor status after treatment.

It is important to note that the R designation is not incorporated into TNM staging itself. However, the absence or presence of residual tumor and status of the margins may provide important information that affects subsequent treatment and prognosis and may be recorded in the medical record and cancer registry.

The absence or presence of residual tumor at the primary tumor site after treatment is denoted by the symbol R. The R categories for the primary tumor site are as follows:

R	R Definition
RX	Presence of residual tumor cannot be assessed
R0	No residual tumor
R1	Microscopic residual tumor
R2	Macroscopic residual tumor at the primary cancer site or regional nodal sites (This designation is not used to indicate metastatic disease identified but not resected at surgical exploration.)

Component of residual tumor and margins	Description
Causes of residual tumor	In some patients treated with surgery and/or neoadjuvant therapy, residual tumor may persist at the primary site and/or regional sites of disease after such treatment as a result of incomplete resection (i.e., the tumor may extend beyond the limit or ability of resection).
Indications of residual tumor	The presence of residual tumor may:<br>• indicate the effect of therapy<br>• influence further therapy<br>• be a strong predictor of prognosis

Component of residual tumor and margins	Description
Indicator of risk	The presence or absence of disease at the margin of resection may be a predictor of the risk of recurrent cancer.<br>The presence of residual disease or positive margins may be more likely with more advanced T- or N-category tumors.
Margin status following tumor resection	Margin status after tumor resection is based on the pathology report (and correlation with the operative report if necessary) and should be recorded by using the following categories:<br>• negative margins (tumor not present at the surgical margin)<br>• microscopic positive margin (tumor not identified grossly at the margin, but present microscopically at the margin). For rare sites, definitions of margin positivity may vary, and relevant interpretation is specified in the respective chapter.<br>• macroscopic positive margin (tumor identified grossly at the margin)<br>• margin not assessed

Response to Neoadjuvant Therapy Assessment

Specific guidance for pathologists may assist in determining the response to neoadjuvant therapy. Additional information on reporting the response to therapy for some specific cancer types is provided in the respective disease site chapters.

Component of response to therapy	Description
Response to neoadjuvant therapy	It is important to record the response to neoadjuvant therapy. Consult disease site chapters for specific systems.<br>For example, some disease sites include "complete," "partial," and "no response," whereas others consist of a numeric scoring system or a "regression score."<br>If surgery is performed, it is critical to also assign the ypT and ypN for analysis of response to neoadjuvant therapy.
Mucin pools, necrosis and reactive changes not included in the assessment of residual cancer	Histologic confirmation of residual cancer requires identification of non-necrotic tumor cells.<br>Mucin pools, necrosis, or degenerative and reactive changes without viable-appearing tumor cells are insufficient for a diagnosis of residual cancer.<br>Mucin pools and necrotic cells currently play no role in assigning the ypT and ypN.

Organization of the AJCC Cancer Staging Manual

2

Mahul B. Amin, Stephen B. Edge, Frederick L. Greene, David R. Byrd, Robert K. Brookland, Mary Kay Washington, Jeffrey E. Gershenwald, Carolyn C. Compton, Kenneth R. Hess, Daniel C. Sullivan, J. Milburn Jessup, James D. Brierley, Laurie E. Gaspar, Richard L. Schilsky, Charles M. Balch, David P. Winchester, Elliot A. Asare, Martin Madera, Donna M. Gress, and Laura R. Meyer

VISION FOR THE *AJCC CANCER STAGING MANUAL*, 8TH EDITION

As surgical, medical, and radiation oncology therapies continue to become more sophisticated in their approach to combat cancer, much also has changed since the 2010 release of the *AJCC Cancer Staging Manual*, 7th Edition in terms of understanding the molecular landscape of cancer. With federally funded efforts, such as The Cancer Genome Atlas (TCGA) project and other scientific endeavors, the molecular underpinnings of cancer are better understood in terms of oncogenesis, progression, and resistance, and the concept of molecular classification of cancer at a clinically relevant level is now accepted as an imminent reality. It is widely believed that the new molecular classification schema will complement traditional and time-honored classifications, such as staging, histologic typing, and grading. These advances, ushering in the precision/personalized medicine era, paralleled with increasing availability of high-throughput testing, such as mutational analysis (sequencing) and microarrays (RNA, micro-RNA, single nucleotide polymorphisms), and advances in bioinformatics and computational biology, provide a transformational opportunity to positively affect the management of cancer. As these technologies catapult prognostic and predictive factor assessment in cancer, the continued discovery of new clinically relevant markers makes it necessary to include them judiciously in staging algorithms and likely will require the development of new strategies beyond those currently adopted.

The Editorial Board views this edition of the *AJCC Cancer Staging Manual* as a concrete step to continue to build the bridge from a "population-based" approach to a more "personalized" one that not only is relevant as a robust classification system for population-based analyses, but also is equally powerful in the care of cancer patients on an individual level and at the bedside. Toward this goal, we have taken several specific steps in the presentation of the 8th Edition contents per disease site chapter. We built in new chapter sections that incorporate these more novel aspects, such as a detailed listing of prognostic factors (classified separately as those *required* for stage grouping, those *recommended* for clinical care, and those that may be regarded as *emerging* prognostic factors).

In addition, for select cancers, we now endorse risk assessment models and prediction tools. After much deliberation, the AJCC (through its Precision Medicine Core) recently established guidelines that will be used to evaluate published statistical prediction models for the purpose of granting endorsement for clinical use. Although this is a monumental step toward the goal of precision medicine, these AJCC guidelines have been published only very recently; hence, specific recommendations of prediction models are available for only a few select sites, including prostate, lung, colon, breast, and soft tissue. We anticipate that the critical task of evaluating risk assessment models for cancer will continue on an ongoing basis for most if not all cancers so that those meeting the stringent AJCC requirements will be endorsed and made available to the cancer community. Finally, each disease site chapter has a listing of factors important for clinical trial stratification, which will be periodically updated at www.cancerstaging.org.

DEVELOPING THE 8TH EDITION PROJECT PLAN

The work for the 8th Edition began immediately upon publication of the *AJCC Cancer Staging Manual*, 7th Edition. Several working groups continued data collection and analysis with the plan of advising AJCC expert panels. The AJCC launched

To access the AJCC cancer staging forms, please visit www.cancerstaging.org.

© American Joint Committee on Cancer 2017

M.B. Amin et al. (eds.), *AJCC Cancer Staging Manual, Eighth Edition*, DOI 10.1007/978-3-319-40618-3_2

several user studies to evaluate the usefulness of the *Cancer Staging Manual* and to explore opportunities to modernize the delivery of cancer staging content so that this critical information is available—and accurate—at all points of care.

In 2010, the AJCC began research to develop alternative content delivery methods, including a centralized Component Content Management System (CCMS) for all the chapters and stage tables and an Application Programming Interface (API) to deliver content from a single source, maintaining the fidelity of AJCC cancer staging content and the ultimate accuracy of its use. This effort will enable content harmonization across all chapters and facilitate standardized incorporation of AJCC cancer staging content into electronic health products and other publications. This system was organized around three principles: use, utility, and maintainability.

The Union for International Cancer Control (UICC) Prognostic Factors Task Force continued performing annual reviews of literature relevant to staging. In 2012, the AJCC 8th Edition Work Group of the Education and Promotions Committee was formed to plan development of the 8th Edition. A survey of past authors formed the foundation for refining the development process. In 2013, a national search for an editor-in-chief was performed. Shortly after a strategic retreat that year, the Editorial Board was composed. In contrast to previous editions, the current board is much larger, strategically balanced to include a widely representative multidisciplinary group of specialists from surgical oncology, radiation oncology, medical oncology, anatomic pathology and molecular pathology, imaging, biostatistics, the population sciences and registrar community, and key administrative staff. The AJCC also empaneled seven core groups, each consisting of multiple team members with defined functions and expertise: the Precision Medicine Core, Evidence-Based Medicine and Statistics Core, Imaging Core, Content Harmonization Core, Data Collection Core, Professional Organization and Corporate Relationship Core, and Administrative Core.

Disease sites were reorganized into 18 expert panels. The expert panel chairs and vice chairs were carefully identified to ensure a balance in multidisciplinary representation of expertise. For the first time, the AJCC also issued an open call for contributors to the 8th Edition; 416 physicians responded, and 174 were selected. Additional contributors were nominated by the chairs and vice chairs of the respective panels with a view to ensure comprehensive and balanced inclusion of all oncologic disciplines, representation from a spectrum of academic institutions across the United States, and appropriate inclusion of international experts, as necessary. All members of the 18 expert panels and seven cores were approved by the Editorial Board. All in all, approximately 420 contributors from 181 institutions, 22 countries, and six continents participated in the massive and coordinated effort to produce the 8th Edition.

In 2014 and 2015, the disease expert panels were convened to review data and available evidence, meet and deliberate, and recommend changes to the AJCC cancer staging system. Electronic collaborative tools such as GoToMeeting and SharePoint enabled frequent web-based meetings, sharing of data, pooling of literature, documentation of minutes of meetings and conference calls, and authoring of chapters across all sites. Writing of the actual chapters commenced in late 2014 and was completed in the spring of 2016. There was editorial oversight and guidance throughout the development process for each site by specifically identified editorial board members designated as liaisons for the disease sites. All major changes were approved by the AJCC Editorial Board and the UICC; the changes reflected in this manual are adopted for application to cases diagnosed on or after January 1, 2017.

ORGANIZATION OF AJCC CANCER STAGING CONTENT

The *AJCC Cancer Staging Manual* provides several key introductory chapters and is then organized primarily by groups of similar cancer types or disease sites (e.g., head and neck, gastrointestinal). In general, the anatomic sites for cancer in this manual are listed by primary site topographical code number according to the World Health Organization (WHO) *International Classification of Diseases for Oncology*. Each disease site or region discussed is indicated by topography codes; the staging classifications are defined in a separate chapter.

Each chapter includes a discussion of information relevant to staging that cancer type, the data supporting the staging system, and the specific rationale for changes in staging. In addition, it includes definitions of key prognostic factors, including those required for stage grouping, those recommended for clinical care, and those recommended for collection in cancer registries. Each chapter ends with the specific definitions of T, N, M, and anatomic stage and prognostic stage groups (Table 2.1).

NEW FOR THIS EDITION

There are several new features and additions in this edition of the *AJCC Cancer Staging Manual*. These include a new organizational structure for consistent and synergistic development of content throughout all chapters, new approaches and paradigms to staging, new chapters, and split chapters based on reorganization (see Table 2.2 for a complete list).

Levels of Evidence for Changes to Staging

In 2013, a core team of statisticians, research methodologists, and clinicians—the AJCC 8th Edition Evidence-Based Medicine and Statistics Core—was formed to establish the levels of evidence that should be provided along with any change to a staging system. Documentation of these levels of evidence, approved by the AJCC Editorial Board, will

Table 2.1 Chapter outline for the *AJCC Cancer Staging Manual, 8th Edition*

**Chapter Summary**	Summary of major changes and applicable diseases<br>• Cancers Staged Using This Staging System<br>• Cancers Not Staged Using This Staging System<br>• Summary of Changes<br>• ICD-O-3 Topography Codes<br>• WHO Histology Codes
**Introduction**	General information on the disease site, such as background, trends, and recent discoveries
**Anatomy**	• Primary Site(s)<br>• Regional Lymph Nodes<br>• Metastatic Sites
**Rules for Classification**	• Clinical<br>◦ Imaging<br>• Pathological
**Prognostic Factors**	Identification and discussion of non-TNM prognostic factors important in each disease<br>• Prognostic Factors Required for Stage Grouping<br>• Additional Factors Recommended for Clinical Care<br>• Emerging Factors for Clinical Care (Web Only)
**Risk Assessment Models**	Prognostic and predictive models validated by the AJCC's acceptance criteria for inclusion of risk models for individualized prognosis in the practice of precision medicine<br>• Updates are available at www.cancerstaging.org.
**Recommendations for Clinical Trial Stratification**	Recommended factors for partitioning patients entering a clinical trial (web only)
**Definitions of AJCC TNM**	• Definition of Primary Tumor (T)<br>• Definition of Regional Lymph Node (N)<br>• Definition of Distant Metastasis (M)
**AJCC Prognostic Stage Groupings**	Organization of T, N, M, and any additional categories into groups.
**Registry Data Collection Variables**	Prognostic variables recommended for collection in cancer registries
**Histologic Grade (G)**	Grading system to be used
**Histopathologic Type**	Discussion or listing of histopathologic types
**Survival Data**	Survival data are the basis for anatomic stage and prognostic groups
**Illustrations**	Additional figures illustrating anatomic extent of disease
**Bibliography**	References for chapter

serve to establish a baseline for measuring how the evidence evolves over future editions of the AJCC staging system and provide transparency for the expert panel decisions.

AJCC Levels of Evidence

I. The available evidence includes consistent results from multiple large, well-designed, and well-conducted national and international studies in appropriate patient populations, with appropriate end points and appropriate treatments. Both prospective studies and retrospective population-based registry studies are acceptable; studies should be evaluated based on methodology rather than chronology.

II. The available evidence is obtained from at least one large, well-designed, and well-conducted study in appropriate patient populations with appropriate end points and with external validation.

III. The available evidence is somewhat problematic because of one or more factors, such as the number, size, or quality of individual studies; inconsistency of results across individual studies; appropriateness of the patient population used in one or more studies; or the appropriateness of outcomes used in one or more studies.

IV. The available evidence is insufficient because appropriate studies have not yet been performed.

Levels of evidence are assessed based on the quality of evidence available in support of changes to the staging system. For each change from the 7th Edition in each chapter, a level is specified (I, II, III, or IV) with published references if available. Each expert panel assigns the levels of evidence, with consultation as needed from the Evidence-Based Medicine and Statistics Core.

Although a goal of including levels of evidence is to evaluate the quality of evidence in support of proposed changes, this approach is not designed to restrict the implementation of proposed changes (e.g., even if supporting evidence is weak). Instead, the intent is to evaluate individual changes as well as the aggregate effect of all the proposed changes to a given staging system. Levels of evidence are applied to changes in AJCC TNM classification as well as to prognostic

Table 2.2 What's new in the 8th Edition

Revised organizational structure

- 18 expert panels and seven cores (420 contributors from 181 institutions, 22 countries and six continents)
- Cores: Precision Medicine, Evidence-Based Medicine and Statistics, Imaging, Content Harmonization, Professional Organization and Corporate Relationship, Data Collection, Administrative
- Expanded Editorial Board with editor-in-chief

Updates

- General staging rules (Chapter 1)
- Staging systems in several chapters
- Histologic classifications and grading systems
- WHO histology codes
- More illustrations

New paradigms

- Human papillomavirus (HPV): oropharyngeal carcinoma staging systems based on HPV status
- Separate staging systems for patients with neoadjuvant therapy (esophagus and stomach)
- Bone and soft tissue sarcoma (separate staging systems based on anatomic sites)
- Introduction of H category (TNMH) for heritable cancer trait in AJCC prognostic stage grouping of Retinoblastoma

New features

- Levels of evidence provided for revisions to staging systems
- Imaging section
- Risk Assessment Models for select cancer sites
- Recommendations for Clinical Trial Stratification
- Prognostic factors
  - Required for prognostic stage grouping
  - Recommended for clinical care
  - Emerging factors

New chapters/staging systems

- Risk Assessment Models
- Cervical Nodes and Unknown Primary Tumors of the Head and Neck
- Oropharynx, HPV-Mediated (p16+)
- Cutaneous Squamous Cell Carcinoma of the Head and Neck
- Thymus
- Bone: Appendicular Skeleton/Trunk/Skull/Face, Pelvis, and Spine
- Soft Tissue Sarcoma of the Head and Neck
- Soft Tissue Sarcoma of the Trunk and Extremities
- Soft Tissue Sarcoma of the Abdomen and Thoracic Visceral Organs
- Soft Tissue Sarcoma of the Retroperitoneum
- Soft Tissue Sarcoma—Unusual Histologies and Sites
- Parathyroid
- Leukemia

Split chapters

- Oropharynx (p16−) and Hypopharynx (previously Pharynx)
- Nasopharynx (previously Pharynx)
- Pancreas—Exocrine (previously Endocrine/Exocrine Pancreas)
- Neuroendocrine Tumors of the Pancreas (previously Endocrine/Exocrine Pancreas)
- Neuroendocrine Tumors of the Stomach
- Neuroendocrine Tumors of the Duodenum and Ampulla of Vater
- Neuroendocrine Tumors of the Jejunum and Ileum
- Neuroendocrine Tumors of the Appendix
- Neuroendocrine Tumors of the Colon and Rectum
- Thyroid—Differentiated and Anaplastic
- Thyroid—Medullary
- Adrenal Cortical Carcinoma
- Adrenal—Neuroendocrine

Merged chapters

- Ovary, Fallopian Tube, and Primary Peritoneal Carcinoma

Deleted chapters

- Cutaneous Squamous Cell Carcinoma and Other Cutaneous Carcinomas for all topographies
  - Specific system devised for cutaneous carcinomas arising in head and neck sites

Future updates at www.cancerstaging.org

- 8th Edition content is available to electronic health record vendors, registry software vendors, and other users through the API
- Cancer staging forms will be available
- Rolling updates
  - Emerging Factors for Clinical Care
  - Risk Assessment Models for additional cancers

factors to be included in prognostic stage groups and recommended for clinical care. No changes to stage definition were made on level IV evidence.

For some diseases, particularly the less common cancers or those for which we are proposing new systems for the first time, few outcome data may be available. In the 8th Edition, at least 12 new staging systems are presented, and these are based on single large international cohort experiences or other limited data that are available and supplemented by expert consensus. Although potentially imperfect, new and evolving classification schemas are critical to allow the collection of standardized data to support clinical care and for future evaluation and refinement of the system. Although the state of the science varies among staging systems, these systems nonetheless form the basis for new follow-up data and research that informs future systems.

Imaging

This manual now includes disease site–specific information about the most appropriate imaging evaluation in each disease site chapter for solid tumors. The imaging section typically describes what imaging tests are most appropriate for assessing tumor stage information (i.e., tumor size, nodal involvement, metastases) for the cancer, the temporal order in which the appropriate imaging tests are typically performed, and the specific T, N, and M information that may be extracted from imaging tests for the cancer. If a structured report format for the cancer has been developed, it also is summarized. A general format for a structured report is as follows:

a. Primary tumor: Location, size, characterization (if applicable)
b. Local extent: involved structures
c. Lymph node involvement (if assessable)
d. Distant spread
e. Other findings relevant to staging or treatment

Where appropriate, specific issues, pitfalls, cautions, and reminders for interpreting imaging stage information for the cancer also are included, along with links or references to disease- or specialty-specific society guidelines. Some chapters also mention emerging imaging methods or imaging biomarkers for the cancer for which there is not yet a significant evidence base.

In vivo imaging examinations, especially the cross-sectional imaging modalities of computed tomography (CT) and magnetic resonance (MR) imaging, are essential for the evaluation of most solid tumors. CT remains the major modality for assessing solid cancers, but MR imaging is used increasingly for many cancers as well as in patients who are either allergic to iodinated CT contrast media or who decline to have CT scans because of concerns regarding radiation exposure. Ultrasonography may be the primary imaging modality for certain tumors (e.g., thyroid cancer). Positron emission tomography (PET) scans performed with fluorine-18 (^{18}F)-fluorodeoxyglucose (FDG) are commonly used to evaluate suspicious masses seen on CT or MRI, as well as to survey the entire body for metastases. Contemporary PET scanners are manufactured with CT scanners integrated into the imaging device. Plain film radiography (e.g., "chest X-rays") may be suitable for evaluations in certain patients, such as assessing for lung metastases in patients with soft tissue sarcoma of an extremity.

The longest linear measurement on imaging studies is the most important for assigning the T category. Although measurements from imaging modalities generally are good, their accuracy and precision (reproducibility) inherently are variable and potentially imprecise. Thus, measurements of the same tumor obtained by two or more different modalities, or at two different time points, likely will differ, even if there was no biological change in the actual tumor size. There is no way to say *a priori* which one of the multiple modalities or measurements is most likely to be correct, as it probably depends on the calibration of the particular imaging device at the time the image is made and, to some extent, on the expertise of the observer making the measurement. If multiple, discordant imaging measurements of a tumor are available at the time of staging, then the longest measurement should be used in assigning the T category.

In addition to the size and extent of tumor at baseline, an important prognostic factor for many cancers is the extent of resection, or the amount of residual tumor after surgery. Imaging plays a critical role in this determination. As a general rule, whichever imaging modality showed the tumor best at baseline should be used for imaging the tumor postoperatively. This imaging modality also likely was the one used to obtain the T category measurement preoperatively. Thus, the image acquisition parameters on the pre- and postoperative scans should be matched as closely as possible so that the measurements and other tumor features can be compared most reliably on the sequential imaging studies.

Assessing lymph nodes for metastatic disease is difficult in cross-sectional oncologic imaging. The significant limitations of using size criteria alone for predicting lymph node involvement are well documented. A maximum short axis diameter of 1 cm generally is considered the upper limit of normal for lymph nodes, but some exceptions to this rule of upper threshold size exist for different sites or specific tumor types throughout the body. Nodes involved with tumor may be smaller than the cutoff threshold size, and reactive nodes often may be larger. However, for most anatomic sites, the use of size criteria is still accepted as the best method available. Additional, secondary criteria for judging malignant nodal involvement include the nodal shape (reniform vs. rounded), nodal margin (regular vs. irregular contour), nodal

density (homogeneous vs. heterogeneous), and asymmetric presence of small but clustered nodes. PET/CT scans or needle biopsy may be performed to reduce uncertainty.

Care should be exercised in the use of ambiguous terminology in reporting imaging results. Efforts are under way to define common terminology for reporting and recording imaging findings in medical records and cancer registries. A key effort is the work being done in 2016 to update the Commission on Cancer Facility Oncology Registry Data Standards Manual.

The imaging report should follow a structured format whenever possible. The Radiological Society of North America (RSNA) reporting initiative (http://www.rsna.org/Reporting_Initiative.aspx) created a library of clear and consistent report templates for many, but not all, cancers (http://www.radreport.org/specialty/oi). These templates make it possible to integrate evidence collected during the imaging procedure, including clinical data, coded terminology, technical parameters, measurements, annotations, and key images. The templates are free and not subject to license restrictions on their reuse.

Prognostic Factors

In this edition, the AJCC expands the use of nonanatomic prognostic factors and biomarkers in assigning stage groups. The AJCC continues to place an emphasis on changes and developments leading to improved clinical decision making and/or improved predictive accuracy in stratifying patients.

The Prognostic Factors section of each disease chapter describes factors that affect patient prognosis. Some of these factors have such a strong correlation with prognosis that they are included in defining stage groups. Other factors also may be important modifiers of stage and are used in medical decision making but are not part of stage groups. Additional factors have not yet become standard of practice for medical decision making but have sufficient evidence to support their consideration in treatment planning.

Prognostic Factors Required for Stage Grouping

The Prognostic Factors Required for Stage Grouping section describes factors that have such a strong correlation with prognosis that they are included as a category used to determine the stage group in the stage table. Levels of evidence are provided. It is important to collect these factors in cancer registries and databases in order to measure their impact on prognosis: for example, prostate-specific antigen and histologic grade group for prostate cancer, serum marker levels for testicular cancer, and age and histology in thyroid cancer. Because of the need to support staging in areas of the world where all these prognostic factors are not obtained for reasons including local practice and resource availability, and to allow comparison of outcomes worldwide, for every cancer type with prognostic factors incorporated into staging, a stage group based solely on anatomic information also may be generated.

Additional Factors Recommended for Clinical Care

The Additional Factors Recommended for Clinical Care section describes factors that are clinically significant but are not included in stage tables. These factors have a strong or growing evidence base. Levels of evidence are provided. It is important to collect these factors in cancer registries and databases in order to measure their impact on prognosis; indeed, some of these factors are critical for future prognostic and predictive model building, as well as for clinical tool development and validation. Examples include recording of carcinoembryonic antigen level for colon cancer, *KRAS* mutation status in stage IV colon cancer, and mitotic rate for melanoma.

Emerging Factors: Web Only

The Emerging Factors for Clinical Care section describes factors for which there is not a widespread significant evidence base. Some institutional and national databases abstract data on these variables. The plan is for these factors to be iteratively reevaluated as the evidence base grows.

Risk Assessment Models

For many cancer types, research groups around the world have developed models based on staging and other prognostic information that provide individual patients and their physicians with information on prognosis and potential response to therapy. To address the use of these models and guide their use in conjunction with TNM staging, the AJCC empaneled the AJCC Precision Medicine Core. This group developed a schema for potential AJCC endorsement of validated clinical tools that includes rigorous exclusion and inclusion criteria to be applied to the assessment of proposed risk assessment models. These criteria and the scope of their work are presented in Chapter 4. A section on risk assessment models is included in the disease site chapters for a few pilot sites in this edition, including lung, prostate, melanoma, breast, and colorectal cancers.

Recommendations for Clinical Trial Stratification: Web Only

Each expert panel was asked to identify key factors of value in stratifying patients in clinical trials. The goal of this section is to guide entities that design clinical trials, whether academic or commercial, regarding the most important prognostic factors of a given disease that should be built into the stratification criteria for their studies. Single-institution and cooperative group trials often include stratification factors based on prognostic features. An ongoing goal of the AJCC is to fully support clinical trial development by specifically citing these factors as issues to consider in specific disease areas. The AJCC also plans to iteratively review these site-specific factors going forward to maintain their relevance in contemporary clinical trial decision making.

CANCER STAGING DATA FORM

A form for recording cancer staging data will be made available for each disease site on www.cancerstaging.org. This printable form may be used by physicians to record data on T, N, and M categories; prognostic stage groups; additional prognostic factors; cancer grade; and other important information. This form may be useful for recording information in the medical record and for communicating information from physicians to the cancer registrar.

The staging form may be used to document cancer stage at different points in the patient's care and during the course of therapy, including before therapy begins, after surgery and completion of all staging evaluations, or at the time of recurrence. It is best to use a separate form for each of these points in time on the patient care continuum.

The cancer staging form is a document for the patient record; it is not a substitute for documentation of history, physical examination, and staging evaluation, or for documenting treatment plans or follow-up. The data forms available in conjunction with this manual may be used by individuals without permission from the AJCC or the publisher. Any other use, changes to these forms, or incorporation of these forms into institutional electronic record systems require appropriate permission from the AJCC.

Cancer Survival Analysis

3

Kenneth R. Hess

INTRODUCTION

Analysis of cancer survival data and related outcomes is necessary to assess cancer treatment programs and to monitor the progress of regional and national cancer control programs. The appropriate use of data from cancer registries for outcomes analyses requires an understanding of the correct application of appropriate quantitative tools and the limitations of the analyses imposed by the source of data, the degree to which the available data represent the population, and the quality and completeness of registry data. In this chapter, the most common survival analysis methodology is illustrated, basic terminology is defined, and basic concepts are introduced. Although the underlying principles are applicable to both, the focus of this discussion is on the use of survival analysis to describe data typically available in cancer registries rather than to analyze research data obtained from clinical trials. Discussion of statistical principles and methodology is limited. Readers interested in statistical underpinnings or research applications are referred to textbooks that explore these topics at length.[1–5]

BASIC CONCEPTS

A *survival probability* is a statistical index that represents a patient group's probability of surviving at a particular point in time. A *survival curve* is a summary display of the pattern of survival probabilities over time. The basic concept is simple. For example, for a certain category of patient, one might ask what proportion is likely to be alive at the end of a specified interval, such as 5 years. The greater the proportion surviving, the lower the *risk of dying* for this category of patients. Survival analysis, however, is somewhat more complicated than it first might appear. If one were to measure the length of time between diagnosis and death or record the vital status when last observed for every patient in a selected patient group, one might be tempted to describe the survival of the group as the proportion alive at the end of the period under investigation. This simple measure is informative only if all of the patients were observed for the same length of time.

In most real situations, not all members of the group are observed for the same amount of time. Patients diagnosed near the end of the study period are more likely to be alive at last contact and will have been followed for less time than those diagnosed earlier. Although it was not possible to follow these patients as long as the others, their survival might eventually prove to be just as long or longer. Although we do not know the complete survival time for these individuals, we do know a minimum survival time (time from diagnosis to last known contact date), and this information is still valuable in estimating survival. Similarly, it is usually not possible to know the outcome status of all the patients who were in the group at the beginning. People may be lost to follow-up for many reasons: they may move, change names, or change physicians. Some of these individuals may have died and others might be still living. Thus, if a survival probability is to describe the outcomes for an entire group accurately, there must be some means to deal with the fact that different people in the group are observed for different lengths of time and that for others, their vital status is not known at the time of analysis. In the language of survival analysis, subjects who are observed until they reach the end point of interest (e.g., recurrence or death) are called *uncensored* cases, and those who survive beyond the end of the follow-up or who are lost to follow-up at some point are termed *censored* cases.

Two basic survival procedures that enable one to determine overall group survival, taking into account both censored and uncensored observations, are the life table method and the Kaplan–Meier method.[6,7] The life table method was

To access the AJCC cancer staging forms, please visit www.cancerstaging.org.

© American Joint Committee on Cancer 2017

M.B. Amin et al. (eds.), *AJCC Cancer Staging Manual, Eighth Edition*, DOI 10.1007/978-3-319-40618-3_3

the first method generally used to describe cancer survival results, and it came to be known as the actuarial method because of its similarity to the work done by actuaries in the insurance industry. It is most useful when data are available only at specified time intervals (e.g., annually), as described in the next section. The Kaplan–Meier estimate uses individual survival times for each patient and is preferable when data are available in this form. The specific method of computation, that is, life table or Kaplan–Meier, used for a specific study should always be clearly indicated in the report to avoid any confusion associated with the use of less precise terminology.

The concepts of survival analysis are illustrated in this chapter. These illustrations are based on data obtained from the public-use files of the National Cancer Institute's Surveillance, Epidemiology, and End Results (SEER) Program. The cases selected are a 1 % random sample of the total number for the selected sites and years of diagnosis. For this illustration, follow-up of these patients is through the end of 1999. For the earliest patients, there may be as many as 16 years of follow-up, but for those diagnosed at the end of the study period, there may be as little as 1 year of follow-up. These data are used both because they are realistic in terms of the actual survival estimates they yield and because they encompass a number of cases that might be seen in a single large tumor registry over a comparable number of years. They are intended only to illustrate the methodology and concepts of survival analysis. SEER results from 1975 to 2012 are described more fully elsewhere.[8] These illustrations are not intended and should not be used or cited as an analysis of patterns of survival in breast and lung cancer in the United States.

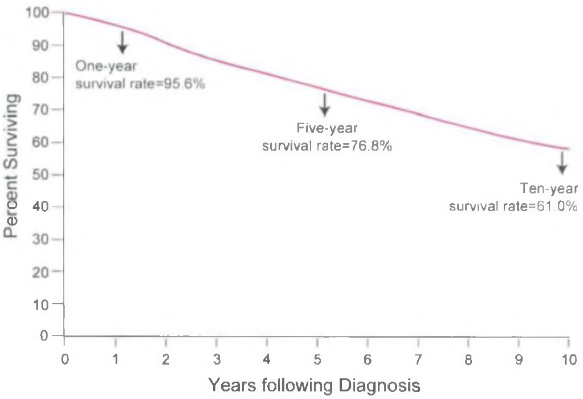

Fig. 3.1 Survival of 2,819 breast cancer patients from the SEER Program of the National Cancer Institute, 1983 to 1998, calculated by the life table method

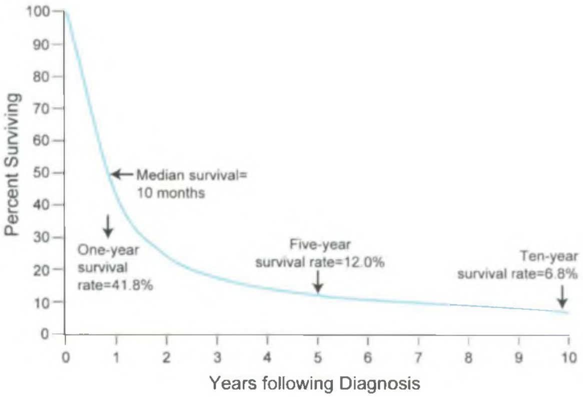

Fig. 3.2 Survival of 2,347 lung cancer patients from the SEER Program of the National Cancer Institute, 1983 to 1998, calculated by the life table method

THE LIFE TABLE METHOD

The life table method involves dividing the total period during which a group is observed into fixed intervals, usually months or years. For each interval, the proportion surviving to the end of the interval is calculated on the basis of the number known to have experienced the end point event (e.g., death) during the interval and the number estimated to have been at risk at the start of the interval. For each succeeding interval, a cumulative survival estimate may be calculated. The cumulative survival estimate is the probability of surviving the most recent interval multiplied by the probabilities of surviving all the previous intervals. Thus, if the percentage of patients surviving the first interval is 90 % and is the same for the second and third intervals, the cumulative survival percentage is 72.9 % ($0.9 \times 0.9 \times 0.9 = 0.729$).

Results from the life table method for calculating survival for the breast cancer illustration are shown in Fig. 3.1. This illustration shows that 2,819 patients diagnosed between 1983 and 1998 were followed up through 1999. Based on the life table calculation method for each year after diagnosis, the 1-year survival estimate is 95.6 %. The 5-year cumulative survival estimate is 76.8 %. At 10 years, the cumulative survival is 61.0 %.

The lung cancer data show a much different survival pattern (Fig. 3.2). At 1 year following diagnosis, the survival estimate is only 41.8 %. By 5 years, it has fallen to 12.0 %, and only 6.8 % of lung cancer patients are estimated to have survived for 10 years following diagnosis. For lung cancer patients, the *median survival time* is 10.0 months. Median survival time is the point on the time axis at which the survival curve crosses 50 %. If the survival curve does not fall below 50 %, it is not possible to estimate median survival from the data, as is the case in the breast cancer data.

In the case of breast cancer, the 10-year survival estimate is important because such a large proportion of patients live more than 5 years past their diagnosis. The 10-year time frame for lung cancer is less meaningful because such a large proportion of this patient group dies well before that much time passes.

An important assumption of these survival methods is that censored cases do not differ from the entire collection of uncensored cases in any systematic manner that would affect their survival. For example, if the more recently diagnosed cases in Fig. 3.1, that is, those who were most likely not to have died yet, tended to be detected with earlier-stage disease than the uncensored cases or if they were treated differently, the assumption about comparability of censored and uncensored cases would not be met, and the result for the group as a whole would be inaccurate. Thus, it is important, when patients are included in a life table analysis, that one be reasonably confident that differences in the amount of information available about survival are not related to differences that might affect survival.

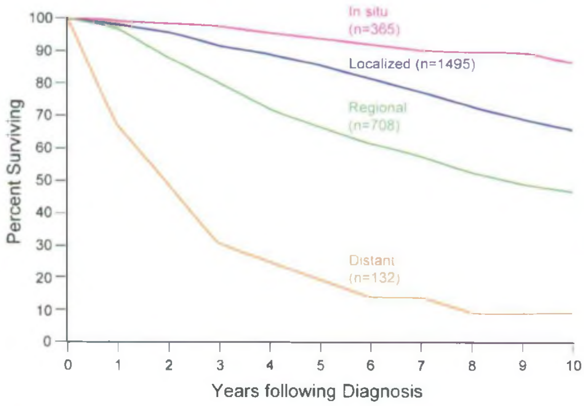

Fig. 3.3 Survival of 2,819 breast cancer patients from the SEER Program of the National Cancer Institute, 1983 to 1998, calculated by the life table method and stratified by historic stage of disease. *Note*: Excludes 119 patients with unknown stage of disease. SEER uses extent of disease (EOD) staging

THE KAPLAN–MEIER METHOD

If individual patient data are available, these same data may be analyzed using the Kaplan–Meier method.[7] It is similar to the life table method but calculates the proportion surviving to each point that a death occurs, rather than at fixed intervals. The principal difference evident in a survival curve computed with the Kaplan–Meier method is that the stepwise changes in cumulative survival appear more frequently and more irregularly. Where available, this method provides a more accurate estimate of the survival curve.

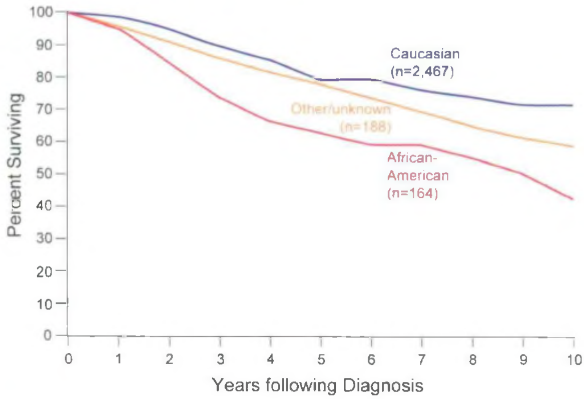

Fig. 3.4 Survival of 2,819 breast cancer patients from the SEER Program of the National Cancer Institute, 1983 to 1998, calculated by the life table method and stratified by race

PATIENT-, DISEASE-, AND TREATMENT-SPECIFIC SURVIVAL

Although overall group survival is informative, comparisons of the overall survival between two groups often are confounded by differences in the patients, their tumors, or the treatments they received. For example, it would be misleading to compare the overall survival depicted in Fig. 3.1 for the sample of all breast cancer cases with the overall survival for a sample of breast cancer patients who were diagnosed with more advanced disease, whose survival would be presumed to be poorer. The simplest approach to accounting for possible differences between groups is to provide survival results that are specific to the categories of patient, disease, or treatment that may affect results. In most cancer applications, the most important variable by which survival results should be subdivided is the stage of disease. Figure 3.3 shows the *stage-specific* 10-year survival curves of the same breast cancer patients described earlier. These data show that breast cancer patient survival differs markedly according to the stage of the tumor at the time of diagnosis.

Almost any variable may be used to subclassify survival data, but some variables are more meaningful than others. For example, it would be possible to provide season-of-diagnosis–specific (i.e., spring, summer, winter, and fall) survival curves, but the season of diagnosis probably has no biologic association with the length of a breast cancer patient's survival. On the other hand, the race-specific and age-specific survival estimates shown in Figs. 3.4 and 3.5 suggest that both these variables are related to breast cancer survival. Caucasians have the highest survival estimates and African Americans the lowest. In the case of age, these data

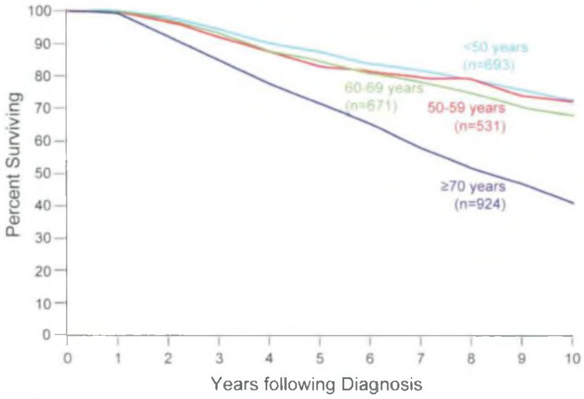

Fig. 3.5 Survival of 2,819 breast cancer patients from the SEER Program of the National Cancer Institute, 1983 to 1998, calculated by the life table method and stratified by age at diagnosis

suggest that only the oldest patients experience poor survival and that it would be helpful to consider the effects of other causes of death that affect older persons using the adjustments to be described.

Although the factors that affect survival may be unique to each type of cancer, it is common for a basic description of survival for a specific cancer to include stage-, age-, and race-specific survival results. Treatment is a factor by which survival commonly is subdivided, but it must be kept in mind that selection of treatment usually is related to other factors that exert influence on survival. For example, in cancer care, the choice of treatment often depends on the stage of disease at diagnosis. Comparison of survival curves by treatment is accomplished most appropriately within the confines of randomized clinical trials.

CAUSE-ADJUSTED SURVIVAL

The survival estimates depicted in the illustrations account for all deaths, regardless of cause. This is known as the *observed survival*. Although observed survival is a true reflection of total mortality in the patient group, we frequently are interested in describing mortality attributable only to the disease under investigation. In the past, this was most often calculated using *cause-adjusted survival*, defined as the proportion of the initial patient group that escaped death due to a specific cause (e.g., cancer) if no other cause of death was operating. This technique requires that reliable information on cause of death is available and makes an adjustment for deaths due to causes other than the disease under study. This was accomplished by treating patients who died without the disease of interest as censored observations.

COMPETING RISKS/CUMULATIVE INCIDENCE

The treatment of deaths from other causes as censored is controversial, because statistical methods used in survival analysis settings assume that censoring is independent of outcome. This means that if the patient were followed longer, one might eventually observe the outcome of interest. This makes sense for patients lost to follow-up (if we located them, we might eventually observe their true survival time). However, if a patient dies from another cause, we will never observe his or her death due to the cancer of interest. Estimation of the adjusted survival, as described previously, does not appropriately distinguish between patients who are still alive at the last known contact date and those known to have died from another cause. These latter events are called *competing risks*.[9]

When competing risks are present, an alternative to the Kaplan–Meier estimate is the cumulative incidence method. This technique is similar to the Kaplan–Meier estimate in its treatment of censored observations and is identical to the Kaplan–Meier estimate if there are no competing risks. However, in the presence of competing risks, the other causes of death are handled in a different manner.[9,10]

RELATIVE SURVIVAL

Information on cause of death is sometimes unavailable or unreliable. Under such circumstances, it is not possible to compute cause-adjusted survival. However, it is possible to adjust partially for differences in the risk of dying from causes other than the disease under study. This may be done by means of *relative survival*, which is the ratio of the observed survival to the expected survival for a group of people in the general population similar to the patient group with respect to race, sex, and age. The relative survival estimate is calculated using a procedure described by Ederer et al.[11]

The relative survival estimate represents the likelihood that a patient will not die from causes associated specifically with the cancer at some specified time after diagnosis. It is always greater than the observed survival estimate for the same group of patients. If the group is sufficiently large and the patients are roughly representative of the US population (taking race, sex, and age into account), the relative survival estimate provides a useful estimate of the probability of escaping death from the specific cancer under study. However, if reliable information on cause of death is available, it is preferable to use the cause-adjusted estimate. This is particularly true if the series is small or if the patients are largely drawn from a particular socioeconomic segment of the population. Relative survival estimates may be derived from life table or Kaplan–Meier results.

STANDARD ERROR OF A SURVIVAL ESTIMATE

Survival estimates that describe the experience of a specific group of patients are frequently used to generalize to larger populations. The existence of true population values is postulated, and these values are estimated from the group under study, which is only a sample of the larger population. If a survival estimate were calculated from a second sample taken from the same population, it is unlikely that the results would be exactly the same. The difference between the two results is called the sampling variation (chance variation or sampling error). The *standard error* is a measure of the extent to which sampling variation influences the computed survival estimate. In repeated observations under the same conditions, the true or population survival probability will lie within the range of two standard errors on either side of the computed estimate approximately 95 times in 100. This range is called the *95% confidence interval.*

COMPARISON OF SURVIVAL BETWEEN PATIENT GROUPS

In comparing survival estimates for two patient groups, the statistical significance of the observed difference is often of interest. The essential question is, What is the probability that the observed difference may have occurred by chance? The standard error of the survival estimate provides a simple means for answering this question. If the 95% confidence intervals of two survival estimates do not overlap, the observed difference would customarily be considered statistically significant, that is, unlikely to be the result of chance. This latter statement generally is true, although it is possible for a formal statistical test to yield a significant difference even with overlapping confidence intervals. Moreover, comparisons at any single time point must be made with care; if a specific time (e.g., 5 years) is known to be of interest when the study is planned, such a comparison may be valid; however, identification of a time based on inspection of the curves and selection of the widest difference make any formal assessment of difference invalid.

It is possible that the differences between two groups at each comparable time of follow-up do not differ significantly but that when the survival curves are considered in their entirety, the individual insignificant differences combine to yield a significantly different pattern of survival. The most common statistical test that examines the whole pattern of differences between survival curves is the *log-rank test.*[12] This test equally weights the effects of differences occurring throughout the follow-up and is the appropriate choice for most situations. Other tests weight the differences according to the numbers of persons at risk at different points and may yield different results depending on whether deaths tend to occur earlier or later in the follow-up.

Care must be exercised in interpreting tests of statistical significance. For example, if differences exist in the patient and disease characteristics of two treatment groups, a statistically significant difference in survival results may primarily reflect differences between the two patient series, rather than differences in efficacy of the treatment regimens. The more definitive approach to therapy evaluation requires a randomized clinical trial that helps ensure comparability of the patient and disease characteristics of the two treatment groups.

Regression Methods

Examining survival within specific patient, disease, or treatment categories is the simplest way to study factors possibly associated with survival. This approach, however, is limited to factors into which patients may be broadly grouped. This approach does not lend itself to studying the effects of measures that vary on an interval scale or studying the effects of multiple factors simultaneously. There are many examples of interval-scaled variables in cancer, such as age, number of positive lymph nodes, tumor size, and laboratory marker values. If the patient population were to be divided into each interval value, too few subjects would be in each analysis to be meaningful. In addition, if more than one factor is considered, the number of curves that result provides so many comparisons that the effects of the factors defy interpretation.

Conventional multiple regression analysis investigates the joint effects of multiple variables on a single outcome, but it is incapable of dealing with censored observations. For this reason, other statistical methods are used to assess the relationship of survival time to several variables simultaneously. The most commonly used is the Cox proportional hazards regression model.[13] This model provides a method for estimating the influence of multiple covariates on the survival distribution from data that include censored observations. Covariates are the multiple factors to be studied in association with survival. In the Cox proportional hazards regression model, the covariates may be categorical variables such as race or interval measures such as age or laboratory test results.

The specifics of these methods are beyond the scope of this chapter. Fortunately, many readily accessible computer packages for statistical analysis now permit these methods to be applied quite easily by the knowledgeable analyst. Although much useful information may be derived from multivariable survival models, these models generally require additional assumptions about the nature of the effects of the covariates on survival. One must always examine the appropriateness of the model that is used relative to the assumptions required.

Defining Survival Starting Point

The appropriate starting time for determining survival of patients depends on the nature of the study. For example, the starting time for studying the natural history of a particular cancer might be defined in reference to the appearance of the first symptoms. Various reference dates are commonly used as starting times for evaluating survival. These include (1) date of diagnosis, (2) date of first visit to the physician or clinic, (3) date of hospital admission, (4) date of treatment initiation, (5) date of randomization in a clinical trial evaluating treatment efficacy, and (6) others. The specific reference date used should be documented clearly in every report. It is also important that any variables used to stratify survival have values that are known as of this reference date.

Vital Status

At any given time, the vital status of each patient is defined as alive, dead, or unknown (i.e., lost to follow-up). The end point of each patient's participation in the study is (1) a specified *terminal event* such as death, (2) survival to the completion of the study, or (3) loss to follow-up. In each case, the observed follow-up time is the time from the starting point to the terminal event, to the end of the study, or to the date of last observation. This observed follow-up may be described further in terms of patient status at the end point, such as the following:

- Alive; tumor-free; no recurrence
- Alive with persistent, recurrent, or metastatic disease
- Dead; tumor-free; no recurrence
- Dead; with cancer (primary, recurrent, or metastatic disease)
- Unknown; lost to follow-up

Completeness of the follow-up is crucial in any study of survival, because even a small number of patients lost to follow-up may lead to inaccurate or biased results. The maximum possible effect of bias from patients lost to follow-up may be ascertained by calculating a maximum survival curve, assuming that all lost patients lived to the end of the study. A minimum survival curve may be calculated by assuming that all patients lost to follow-up died at the time they were lost.

Time Intervals

The total survival time is often divided into intervals in units of weeks, months, or years. The survival curve for these intervals provides a description of the population under study with respect to the dynamics of survival over a specified time. The time interval used should be selected with regard to the natural history of the disease under consideration. In diseases with a long natural history, the duration of study might be 5 to 20 years, and survival intervals of 6 to 12 months will provide a meaningful description of the survival dynamics. If the population being studied has a very poor prognosis (e.g., patients with carcinoma of the esophagus or pancreas), the total duration of study may be 2 to 3 years, and the survival intervals may be described in terms of 1 to 3 months. In interpreting survival estimates, one also must take into account the number of individuals entering a survival interval (which also is reflected in the standard error of the survival estimate).

SUMMARY

This chapter reviews the rudiments of survival analysis as it often is applied to cancer registry data and to the analysis of data from clinical trials. Complex analysis of data and exploration of research hypotheses demand greater knowledge and expertise than can be conveyed here. Survival analysis is now performed automatically in many different registry data management and statistical analysis programs available for use on personal computers. Persons with access to these programs are encouraged to explore the different analysis features available to demonstrate for themselves the insight on cancer registry data that survival analysis can provide and to understand the limitations of these analyses and how their validity is affected by the characteristics of the patient cohorts and the quality and completeness of data.

Bibliography

1. Cox DR, Oakes D. *Analysis of survival data*. Vol 21: CRC Press; 1984.
2. Collett D. *Modelling survival data in medical research*. 3rd Edition. CRC press; 2015.
3. Kalbfleisch JD, Prentice RL. Relative risk (Cox) regression models. *The Statistical Analysis of Failure Time Data, Second Edition*. 2002:95–147.
4. Klein JP, Moeschberger ML. *Survival analysis: techniques for censored and truncated data*. Springer Science & Business Media; 2005.
5. Kleinbaum DG, Klein JP. *Survival Analysis: A Self-Learning Text, Third Edition*. 3 ed: Springer-Verlag New York; 2012.
6. Berkson J, Gage RP. Calculation of survival rates for cancer. *Proceedings of the staff meetings. Mayo Clinic*. May 24 1950;25(11):270–286.
7. Kaplan EL, Meier P. Nonparametric estimation from incomplete observations. *Journal of the American statistical association*. 1958;53(282):457–481.
8. Howlader N, Noone AM, Krapcho M, et al. SEER Cancer Statistics Review, 1975-2012 National Cancer Institute. Bethesda, MD. http://seer.cancer.gov/csr/1975_2012/, based on November 2014

SEER data submission, posted to the SEER web site, April 2015. Accessed 2/19/16.
9. Pintilie M. *Competing risks: a practical perspective.* John Wiley & Sons; 2006.
10. Gooley TA, Leisenring W, Crowley J, Storer BE. Estimation of failure probabilities in the presence of competing risks: new representations of old estimators. *Statistics in medicine.* Mar 30 1999;18(6):695–706.
11. Ederer F, Axtell LM, Cutler SJ. The relative survival rate: a statistical methodology. *Natl Cancer Inst Monogr.* Sep 1961;6:101–121.
12. Mantel N. Evaluation of survival data and two new rank order statistics arising in its consideration. *Cancer Chemother Rep.* Mar 1966;50(3):163–170.
13. Cox DR. Regression models and life tables. *Journal of the Royal Statistical Society*, B. 1972;74:187–220.

Risk Models for Individualized Prognosis in the Practice of Precision Oncology

4

Carolyn C. Compton, Kenneth R. Hess, Susan Halabi, Ulysses G.J. Balis, Jeffrey E. Gershenwald, Phyllis A. Gimotty, Justin Guinney, Alexander J. Lazar, Ying Lu, Alyson L. Mahar, Angela Mariotto, Karel GM Moons, Snehal G. Patel, Daniel J. Sargent, Martin R. Weiser, and Michael W. Kattan

BACKGROUND

Since its inception, the AJCC has had the core mission of developing and maintaining state-of-the-science anatomic staging systems for cancers and is the global leader in this endeavor.[1] The AJCC TNM staging system codifies the anatomic extent of disease at diagnosis, which has long been the most accurate predictor of outcome for solid malignancies and the most commonly used classifier for patients. However, survival prediction based on AJCC TNM staging is calculated from patient population data and represents a range of overall survival (OS) for patients within a given stage grouping. Thus, survival prediction for an individual cancer patient cannot be precisely determined from AJCC TNM stage grouping alone.

The AJCC has recognized the growing need for more accurate and probabilistic individualized outcome prediction to include additional prognostic factors beyond those related to the anatomic extent of disease. Such factors may derive from clinical information about the patient or pathological information related to the tumor. More recently, the vision of precision medicine has created even greater urgency to improve prognostication for cancer patients and allow more accurate and specific patient-related decision making for both clinical management and clinical research.

Since 2002 (AJCC Cancer Staging Manual, 6th Edition), nonanatomic factors that modified stage groupings and improved outcome predictions have been judiciously included for some cancer sites.[2] However, the capacity to include additional prognostic factors into the inelastic mathematical bin model on which the AJCC TNM stage groupings are based is severely limited. It has been recognized for some time that new approaches to prognostic calculation are needed to allow the incorporation of relevant and validated factors and increase prediction accuracy.

To help address this need and aid the cancer community, in 2008 the AJCC convened a group of experts, known as the Molecular Modelers Working Group, to identify and review existing prognostication calculation tools that incorporate multiple prognostic factors in addition to stage to predict outcome for cancer patients. Included in this process were prognostication tools for five major cancers: lung cancer, colorectal cancer, melanoma, breast cancer, and prostate cancer. After an intensive search of the scientific literature and online resources, a total of 176 prognostication tools were identified in the form of equations, equations and risk scores, equations and calculators, nomograms, risk scores, and other presentations. The review process revealed wide variation in the quality and content of the prognostication tool landscape. However, the overall evaluation process was agnostic and focused on comparing and contrasting the specific features of identified tools. In March 2016, detailed assessment results for the tools designed for lung cancer and cutaneous melanoma were published,[3,4] and as of press time, those for colorectal, breast, and prostate cancer are in preparation.

This work laid the foundation for the Precision Medicine Core (PMC) of the AJCC Cancer Staging Manual, 8th Edition project. The PMC, composed of members with disease-specific knowledge as well as special expertise in biostatistics and prediction modeling for cancer, has taken the additional step of updating the list of tools and gauging the quality and accessibility of all prognostication tools (i.e., models, calculators, and algorithms) for these five cancers as well as for those brought forward by expert panels during the 8th Edition development process. The goal of the PMC was to identify, for the cancer community, all readily available outcome probability models of specifically defined quality and type that include nonanatomic prognostic factors. It was reasoned

To access the AJCC cancer staging forms, please visit www.cancerstaging.org.

© American Joint Committee on Cancer 2017

M.B. Amin et al. (eds.), *AJCC Cancer Staging Manual, Eighth Edition*, DOI 10.1007/978-3-319-40618-3_4

that such models would allow for more individualized prognosis for patients whose disease falls within a given AJCC TNM stage grouping, building on and extending the prognostication power of anatomic staging.

The vast majority of prognostication tools are based on models that combine information from multiple patient and tumor characteristics (predictors) to yield probability estimates for experiencing a particular event (outcome) in a specified time period.[5] Most such models are based on multivariable statistical regression models that form a mathematical equation to predict outcome probabilities for individual patients based on a weighted average of their predictor values.[6] Increasingly, however, prognostication models are based on machine learning predictive analytic methods.[7,8]

In contrast to prognostication models that provide individualized probability estimates (i.e., risk calculators), some prognostication tools classify patients into ordered risk groups either directly or based on cut-points for individual probability estimates. The TNM staging system is an example of such a classification tool yielding – at the least granular level – ordered classes (I, II, III, IV) of increasingly poor prognosis. While such prognostic stratification is useful, it is limited by the number of categories that are manageable, by the complexity of combining information from multiple predictors to form discrete ordered categories in a transparent manner and by the inherent variability of patient prognosis in a given risk class. In its contributions to the 8th Edition, the AJCC PMC focused its attention on prognostication models rather than prognostic classifiers with the belief that individualized predictions are more accurate and more useful for clinical decision making.

SEARCH STRATEGY FOR PROGNOSTICATION TOOLS

The search for clinical prognostication tools and information on their validity was performed via two mechanisms: a search of the peer-reviewed published literature, including both a systematic literature review and cited reference search, and a search of the online scientific community. Prognostication tools were defined as any nomogram, risk classification system, equation, risk score, electronic calculator, or other statistical regression model–based tool developed with the purpose of predicting time to death for use in clinical practice.

A separate systematic search of the scientific literature was performed to identify clinical prediction tools used in the following cancer sites: colorectal, lung, melanoma, breast, and prostate. The search strategy (outlined here) was supplemented by a cited reference search of included articles to ensure completeness of the literature search. A search of the online scientific community to identify tools available online (detailed here) also was conducted for each cancer site.

Searches were performed in Medline, Embase, and HealthStar from 1996 through 2015. Searches of the online scientific community also were performed using Google and search terms such as "clinical prediction tool cancer," "online calculator cancer," and "nomogram cancer." Seemingly eligible studies were excluded if they met any of the following a priori exclusion criteria: (1) assessment of the prognostic impact of a single factor; (2) inappropriate analytic purpose (e.g., multivariate modeling not aimed at prognostication, development of novel statistical methods); (3) not specific to breast, colorectal, lung, melanoma, or prostate cancer patients; (4) not original data/research (e.g., editorial, review); or (5) outcome other than survival. Eligible survival end points included all time-to-death analyses (e.g., OS, cause-specific survival), as well as vital status analyses (e.g., probability of being dead 5 years following diagnosis).

THE EVALUATION PROCESS

Citations identified through the search process were assessed first as titles/abstracts and then as full articles by a single reviewer and independently reevaluated by a blinded second reviewer. Overall, the percent agreement was high between reviewers. All eligible tools were then evaluated against a previously agreed upon set of inclusion/exclusion criteria for AJCC endorsement that was established by the PMC for the AJCC and published by the group. During the evaluation process, discrepancies related to either conformity with individual inclusion criteria or overall endorsement recommendations were resolved by consensus.

Ultimately, the total number of prognostication models identified and evaluated by the PMC for the 8th Edition is as follows: 27 for breast cancer, 37 for colorectal cancer, 16 for prostate cancer, 27 for lung cancer, 7 for melanoma, 4 for head and neck cancer, 4 for soft tissue sarcoma, and 19 for selected hematologic malignancies. It is envisioned that PMC identification and evaluation of prognostication models for other cancer types/sites will continue over the life of the edition, and new findings will be made available on www.cancerstaging.org.

ESTABLISHING ENDORSEMENT CRITERIA FOR PROGNOSTICATION MODELS

Recognizing that assessment of the quality and acceptability of a risk model is complex, the PMC determined that a well-defined set of quality criteria for prognostication tools was required. It was agreed that such criteria should reflect the current state of the science of prediction modeling, meet the highest standards for data quality, and have demonstrated clinical validity. On January 23 and 24, 2015, the AJCC PMC met in Phoenix, Arizona, to develop an approach to evaluate a statistical model of high quality that would meet these requirements; would be

both useful and usable by the oncology community, including cancer patients; and would include tumor-related and patient-related prognostic factors in additional to anatomic stage. The PMC envisioned that a prognostication system for each cancer type/site, which covered all stages of disease, could eventually be constructed as a comprehensive web-based tool and could possibly be built in modular fashion from existing and/or newly developed high-quality models created for stage-specific use.

The key issue for the PMC was to decide on the precise criteria for endorsing any probability or risk model, either existing or to be built in the future, that reflects the AJCC's commitment to quality and reliability. The philosophy of the PMC was that validated predictive accuracy of a risk calculator or model is paramount. The PMC also recognized the complexity of both validation[9] and generalization,[10] each of which has various levels/types, such that true validation requires multiple datasets over various levels of time and place.

The PMC independently established and published the criteria it would use to judge existing risk models or would require of newly built models.[11] The final result was a checklist of 16 items—13 inclusion and 3 exclusion criteria—necessary for AJCC endorsement of a risk model. It was unanimously decided that all inclusion criteria must be met for endorsement and any exclusion criterion would eliminate the model from consideration. The emphasis centers on performance metrics, implementation clarity, and clinical relevance. Recognizing that high-quality personalized probabilistic prediction calculators hold tremendous promise for cancer care and research, it also was hoped that these criteria would facilitate and accelerate their development.

ENDORSEMENT OF PROGNOSTICATION MODELS MEETING AJCC CRITERIA

The PMC assumed that any published prediction or risk model would be eligible for consideration for AJCC endorsement. The first step in the evaluation process was to briefly characterize the prediction model with respect to the following elements.

Model Description

A. The cancer that the patients being modeled have (e.g., clinically localized prostate cancer) and whatever inclusion/exclusion criteria should be applied when using the model (e.g., no prior cancer, untreated for prostate cancer)
B. The diagnosis or treatment that defines the baseline or prognostic time zero—that is, at which time the outcome prediction will be calculated in future patients (e.g., preoperatively, before potential treatment, at start of treatment)
C. The predictors measured at baseline and how they were measured (e.g., prostate-specific antigen by the Hybritech assay [nanograms per milliliter], stage by AJCC , 6th Edition TNM)
D. The end point being predicted: OS or disease-specific survival (DSS)
E. The horizon time point being predicted: how far in time from baseline (e.g., 10-year survival probability)
F. The perceived impact of this prediction on clinical practice. For example, addressing patient queries (patients always ask about this outcome before choosing a specific treatment), or providing decision support (if the prediction is <X, this treatment would not be recommended).

The PMC subsequently engaged in an intensive process of assessment and adjudication of assessments of prognostication tools existing in the public domain through December 2015 to identify tools for AJCC endorsement at the time of publication of the 8th Edition. A short summary of the review process for each site-specific set of tools is included in the relevant disease chapter. Tools meeting AJCC criteria are specified, along with their web addresses and an itemized list of the specific prognostic factors needed to use each model. The entire list of models reviewed by the PMC, along with the specifically identified exclusions or deviations from the AJCC criteria, will be made available in the supplementary online materials that accompany the 8th Edition. The PMC regards the process of prognostication tool evaluation on behalf of the AJCC and the cancer community as ongoing into the future. The committee also anticipates that future interaction with the authors/developers of tools that initially failed to meet the AJCC criteria may result in reassessment and possible endorsement if the issues in question are addressed.

Inclusion Criteria

An endorsed model must have all of the following characteristics:

1. OS or DSS must be the outcome predicted. OS (freedom from death by any cause) is the end point consistent with the prior work of the AJCC and has the fewest methodologic issues. Although clinicians and patients may be more interested in DSS, the potential difficulty in reliably assigning cause of death makes this a less reliable end point. DSS can be calculated either by censoring patients who die from other causes or by using alternative methods based on competing risks.[12,13]

4

The exclusion of progression-free, disease-free, or recurrence-free survival type as end points for AJCC endorsement is a narrow perspective that might need to be reconsidered in the future, but would introduce additional complexities, such as definitions of progression and frequency of assessment.

2. The model should address a clinically relevant question—a prediction of relevance to physicians and patients. This is a somewhat subjective criterion best assessed by clinicians with disease management expertise. However, the following considerations are examples of issues pertinent to this criterion. Is the treatment assumed by the prediction model relevant today? Do the inclusion and exclusion criteria in place for this model define a patient population of interest to the clinician? Is the end point important and relevant to predict?
3. At face value, the model should include the relevant predictors of outcome for a cancer, or explain why something relevant was not included. The AJCC will not endorse a prognostication model that lacks predictors that most clinicians would expect to see in this context. If the missing predictor is evaluated by the modeling team but is removed because it lacks incremental predictive ability, this circumstance would be considered acceptable and would not constitute a reason to withhold endorsement. Ultimately, this checklist item is likely best judged by clinicians with disease management expertise.
4. The model validation study should specify precisely which patients were used to evaluate the model and what inclusion/exclusion criteria were imposed upon the validation dataset. The end user of an endorsed prediction model needs to know whether the model is applicable to his or her patient. Therefore, clear understanding of how patients were chosen for inclusion in the validation dataset(s) is needed and is the only way to determine whether the end user's patient would have qualified for the validation study.
5. The model should be assessed for generalizability and external validation. The key assumption being made with endorsement is that the risk model is valid for future patients. Because validation first requires patient follow-up and data analysis, validation cannot be undertaken immediately and relies on presently available studies and data. Those studies should separate reproducibility (i.e., simply new patients) from transportability (i.e., patients who differ in one or more ways from the development dataset).[10] In general, true validity assessment likely will require multiple studies over time. In the interim, state-of-the-art internal and internal–external validation procedures are recommended.[14]
6. The model should have a well-defined prognostic time zero (i.e., what event/observation starts the clock for its prediction calculation?). A prediction is calculated at a certain time point in a patient's course of illness. This time point must be obvious and explicit for any model. Examples include immediately following diagnosis, before a certain treatment, or immediately following a particular treatment.
7. All predictors must be known at time zero and sufficiently defined for accurate use by another user. The end user needs to know exactly which factors to enter into the risk model and, if applicable, which measurement units to use.
8. Sufficient detail must be available to implement the model (i.e., the equation itself is needed, not a derived version or a simplified, unvalidated score) OR the author must allow free access to the model. If a prediction model is otherwise a candidate for endorsement, the AJCC typically requires access to the underlying equation/formula. However, this would not be required if the developer provided a free online risk model along with publication of the underlying equation/formula. Note that *availability* or *access* refers to the actual model that was validated; the developer must notify AJCC if the online model or calculator is modified from the published validation.
9. A measure of discrimination must be reported. This is often measured as the concordance index[15] and must be assessed on the validation dataset(s).
10. Calibration in the small must be assessed (from the external validation dataset) and provided. Calibration in the small is a plot of predicted probability versus observed proportion. The objective is to demonstrate that the observed proportion of deaths closely resembles the predicted probability in the external dataset across the spectrum of predictions.
11. The model should be validated over a time frame and practice setting relevant to contemporary patients with disease. The validation dataset should be reflective of a patient being evaluated today. The treatment(s) applied in the validation dataset should be similar to those used today, and the clinician user should be comfortable that the disease reflected in the validation dataset resembles the disease he or she sees today. Furthermore, the setting in which the validation was performed should be similar to the setting of interest to the clinician today.
12. It should be clear which initial treatment(s), if any, were applied and with what frequency. The initial treatment need not be a specific predictor, nor must the model be restricted to a single treatment. In other words, the prediction model does not need to include the treatment type as a predictor, but it may. The rele-

vant requirement is knowledge of how patients in the validation dataset were treated (i.e., the proportions who received each form of therapy if patients treated with different initial therapies were included in the dataset). Adjuvant treatments should be described but ignored as predictors in the model.

13. The development and/or the validation of the prediction model must be reported as a peer-reviewed journal article. The reference(s) for the published model is/are required.

Exclusion Criteria

Any one of the following criteria excludes a model from consideration:

1. A substantial proportion of patients in the dataset have little to no follow-up (i.e., follow-up is missing entirely, is arbitrarily truncated, or is censored). This criterion is intentionally subjective. The concern is that selection bias may be introduced by excluding many patients because of missing outcome and that such patients may be quite different from the rest of the patients who were included. Those with missing follow-up must be compared with the rest of the patients.
2. No information is available on the number of missing values in the validation dataset. Virtually any dataset will have missing values. When a dataset appears to have no missing values for any variables, it is often the case that those observations with missing values were excluded previously, prior to analysis. This may create substantial bias. Thus, the true time frame for patient accrual into the validation dataset must be transparent in order to evaluate this issue.
3. The number of events in the validation dataset is small. There is relatively little literature on which to base a firm definition of "small"; however, 100 events may be the minimum needed.[16]

CONSIDERATIONS FOR USE OF PROGNOSTICATION MODELS

The goal of the AJCC PMC checklist is to establish criteria for the endorsement of an online risk model or calculator. Satisfying the checklist does not establish when or how the risk model should be used. The checklist sets minimum quality and accessibility criteria for a risk model to be considered further for the possible application to comprehensive prognostication tools applicable to all TNM stages of disease that may be developed by the AJCC. The AJCC checklist is not useful for comparing one risk model with another, because all models must meet all criteria (i.e., all endorsed risk models would be considered equivalent according to these criteria).

The AJCC considers individualized prognostication that builds on but extends beyond TNM staging to be essential for the practice of precision medicine and for the clinical research that will continue to inform the practice of cancer medicine. The AJCC is committed to supporting the overall concept of prediction modeling and the development of high-quality prognostication tools. As a service to patients and the entire cancer community, the AJCC plans to continue to evaluate new prognostication models as they become available and to review endorsed models for contemporary relevance. The AJCC may consider building new models and/or partnering with model developers as necessary to fill gaps in the compendium of endorsed prognostication tools. All endorsed models will be made available through the AJCC website and online resources for the 8th Edition and beyond.

Worthy of mention is that the AJCC inclusion/exclusion criteria complement the recently published transparent reporting of a multivariable prediction model for individual prognosis or diagnosis (TRIPOD) document.[17] The TRIPOD statement sets forth the details deemed necessary for reporting on what was done in a prediction modeling study, development, or validation. The elements of the AJCC checklist mirror these requirements but are intended to define the minimal requirements for a cancer prognostication model to be endorsed by end users.

ACKNOWLEDGMENTS

The authors thank Donna M. Gress, RHIT, CTR, and Laura R. Meyer, CAPM, for their support of the work of the AJCC Precision Medicine Core and their administrative support of the AJCC Personalized Medicine Core Committee Meeting held in Phoenix, Arizona, January 2015.

Bibliography

1. Gospodarowicz M, Benedet L, Hutter RV, Fleming I, Henson DE, Sobin LH. History and international developments in cancer staging. *Cancer prevention & control : CPC = Prevention & controle en cancerologie : PCC.* Dec 1998;2(6):262-268.
2. Greene FL, Page DL, Fleming ID, et al. *AJCC Cancer Staging Manual.* 6th ed. New York, NY: Springer; 2011.
3. Mahar AL, Compton C, Halabi S, Gershenwald J, Scolyer RA, Groome PA. Critical assessment of clinical prognostic tools in melanoma. *Annals of surgical oncology.* 2016;(in press).
4. Mahar AL, Compton C, McShane LM, et al. Refining Prognosis in Lung Cancer: A Report on the Quality and Relevance of Clinical Prognostic Tools. *J Thorac Oncol.* Nov 2015;10(11):1576-1589.

5. Steyerberg EW, Moons KG, van der Windt DA, et al. Prognosis Research Strategy (PROGRESS) 3: prognostic model research. *PLoS medicine.* 2013;10(2):e1001381.
6. Harrell FE, Jr., Lee KL, Mark DB. Multivariable prognostic models: issues in developing models, evaluating assumptions and adequacy, and measuring and reducing errors. *Statistics in medicine.* Feb 28 1996;15(4):361-387.
7. Kourou K, Exarchos TP, Exarchos KP, Karamouzis MV, Fotiadis DI. Machine learning applications in cancer prognosis and prediction. *Comput Struct Biotechnol J.* 2015;13:8-17.
8. Cruz JA, Wishart DS. Applications of machine learning in cancer prediction and prognosis. *Cancer informatics.* 2006;2.
9. Reilly BM, Evans AT. Translating clinical research into clinical practice: impact of using prediction rules to make decisions. *Annals of internal medicine.* 2006;144(3):201-209.
10. Justice AC, Covinsky KE, Berlin JA. Assessing the generalizability of prognostic information. *Annals of internal medicine.* Mar 16 1999;130(6):515-524.
11. Kattan MW, Hess KR, Amin MB, et al. American Joint Committee on Cancer acceptance criteria for inclusion of risk models for individualized prognosis in the practice of precision medicine. *CA: a cancer journal for clinicians.* Jan 19 2016.
12. Austin PC, Lee DS, Fine JP. Introduction to the Analysis of Survival Data in the Presence of Competing Risks. *Circulation.* Feb 9 2016;133(6):601-609.
13. Koller MT, Raatz H, Steyerberg EW, Wolbers M. Competing risks and the clinical community: irrelevance or ignorance? *Statistics in medicine.* May 20 2012;31(11-12):1089-1097.
14. Steyerberg EW, Harrell FE, Jr. Prediction models need appropriate internal, internal-external, and external validation. *Journal of clinical epidemiology.* Jan 2016;69:245-247.
15. Harrell FE, Jr., Califf RM, Pryor DB, Lee KL, Rosati RA. Evaluating the yield of medical tests. *JAMA.* May 14 1982;247(18):2543-2546.
16. Collins GS, Ogundimu EO, Altman DG. Sample size considerations for the external validation of a multivariable prognostic model: a resampling study. *Statistics in medicine.* Jan 30 2016;35(2):214-226.
17. Moons KG, Altman DG, Reitsma JB, et al. Transparent Reporting of a multivariable prediction model for Individual Prognosis or Diagnosis (TRIPOD): explanation and elaboration. *Annals of internal medicine.* Jan 6 2015;162(1):W1-73.

Part II

Head and Neck

Members of the Head and Neck Expert Panel

Robert J. Baatenburg deJong, MD, PhD
Margaret Brandwein-Gensler, MD
David M. Brizel, MD
Joseph A. Califano, MD
Amy Y. Chen, MD, MPH, FACS
A. Dimitrios Colevas, MD
Stephen B. Edge, MD, FACS – Editorial Board Liaison
Matthew Fury, MD, PhD
Ronald A. Ghossein, MD
Christine M. Glastonbury, MBBS
Robert Haddad, MD
Bruce H. Haughey, MBChB, FACS, FRACS
Dennis H. Kraus, MD, FACS
Quynh Thu X. Le, MD
Anne W.M. Lee, MD
William M. Lydiatt, MD – Vice Chair
Suresh K. Mukherji, MD, FACR
Kishwer S. Nehal, MD
Brian O'Sullivan, MD, FRCPC – UICC Representative
Snehal G. Patel, MD
John A. Ridge, MD, PhD, FACS
Simon N. Rogers, MD, FRCS
Chrysalyne Schmults, MD, MSCE
Raja R. Seethala, MD – CAP Representative
Jatin P. Shah, MD, PhD(Hon), FACS, FRCS(Hon) – Chair
Erich M. Sturgis, MD, MPH, FACS
Randal Scott Weber, MD
Bruce M. Wenig, MD

Staging Head and Neck Cancers

5

William M. Lydiatt, Snehal G. Patel, John A. Ridge,
Brian O'Sullivan, and Jatin P. Shah

INTRODUCTION AND OVERVIEW OF KEY CONCEPTS

Cancers of the head and neck may arise from any of the mucosal surfaces of the upper aerodigestive tract. The American Joint Committee on Cancer (AJCC) Cancer Staging Manual, 8th Edition (8th Edition) introduces a number of significant changes. These include a separate staging algorithm for human papilloma virus–associated cancer, restructuring of the head- and neck- specific cutaneous malignancy chapter, division of the pharynx staging system into three components, changes to the tumor (T) categories, addition of depth of invasion as a T characteristic in oral cancer, and the addition of extranodal tumor extension to the node (N) category.

Maintaining a balance between hazard discrimination, hazard consistency, desirable spread in outcomes, prediction of cure, and the highest possible compliance was paramount.[1] As the world moves toward personalized medicine, demand will increase for individualized prediction of risk and outcomes, which may ultimately eclipse the traditional groupings of cancers used in staging. The complexity associated with defining individual risk, prognosis, and benefit from treatment is undeniable but it may eventually be mitigated by relying on computerized algorithms presented in a user-friendly format on the handheld devices so ubiquitous in modern society.[2] These may be applied through nomograms relying on key anatomic, biologic, and clinical factors. Nonetheless, a feasible pretreatment approach (i.e., clinical TNM) applicable in all medical settings and to all patients should be maintained both for defining treatment and for evaluating the effect of treatment across populations and time. Thus, the 8th Edition represents a compromise between a very accurate, but very complex system (where compliance would be low) and a very simple system, which would permit high compliance at the expense of reduced predictive capacity.

Cancer staging is used worldwide in countries with widely varying levels of resources. Assuring harmony between the AJCC and Union for International Cancer Control (UICC) staging systems was an important goal and it could not have been accomplished without the wisdom, collegiality, and sense of purpose displayed by members of the UICC Head and Neck Committee. We are especially indebted to the tremendous work ethic and attention to detail exhibited by members of the AJCC Head and Neck Expert Panel and its many subcommittees and disease site leaders. Both groups have balanced the need for a worldwide system with the need for incremental improvement.

KEY CHANGES TO HEAD AND NECK CANCER STAGING

The major changes in the 8th Edition for head and neck cancer reflect the changing environment of head and neck oncology. The areas highlighted in this section include general changes and additions to cancer staging that apply across most Head and Neck sites. The specific changes for each site are detailed in the respective chapters.

New and Restructured Chapters

A major addition is a restructured head- and neck-specific cutaneous malignancy chapter. This acknowledges the increasing need for head and neck oncologists to stage cutaneous malignancies.

The Pharynx chapter has been divided into three separate anatomic regions that better reflect the different diseases arising in the pharynx.

- Nasopharynx has its own chapter recognizing the unique biology and etiology of this disease.

To access the AJCC cancer staging forms, please visit www.cancerstaging.org.

© American Joint Committee on Cancer 2017

M.B. Amin et al. (eds.), *AJCC Cancer Staging Manual, Eighth Edition*, DOI 10.1007/978-3-319-40618-3_5

- HPV-negative oropharynx and hypopharynx remain together in view of their shared biology and typical risk factors.
- A new chapter describing the staging of human papilloma virus–associated (HPV) oropharyngeal cancers (OPC) has been added.

The rapidly increasing incidence of high-risk HPV (HR-HPV) associated cancers of the tonsil and tongue base has posed numerous challenges in diagnosis, management strategies, and outcomes reporting.[3] The AJCC Cancer Staging Manual, 7th Edition (7th Edition) TNM staging of OPC tumors lacks hazard discrimination, hazard consistency, and capacity to predict outcome. HR-HPV associated cancers occur more often in younger, healthier individuals with little or no tobacco exposure. HPV16/18 are the most commonly detected transcriptionally active HR-HPV types. The established staging criteria defined in the 7th Edition are insufficient to adequately stage and define the biology of this emerging disease. Immunohistochemistry for p16 overexpression has emerged as a robust surrogate biomarker for HPV-mediated carcinogenesis and as an independent positive prognosticator in this specific context.[4] Direct detection of HPV is not used as a defining factor due to limited availability of the test, cost, and lack of additional ability to predict survival compared with p16 overexpression. p16 overexpression was chosen as the best identifier of disease because of its low cost, universal applicability, and ease of reading compared with other HPV identifiers. It is important to remind clinicians, however, that p16 overexpression is context-specific and currently applicable only to the tonsillar and base of tongue regions of the oropharynx. Designation of p16 overexpression should occur only when there is significant staining following established criteria.[4] To increase the utility of staging and acknowledge the emergence of a distinct disease, the p16+ and p16– OPCs are now separately described. OPC with p16 expression of weak intensity or limited distribution (<75% of cells) should be staged using oropharyngeal carcinoma p16– guidelines.

The 8th Edition TNM staging system for HPV-related cancers of the oropharynx provides better discrimination between stages. T categories remain the same for both p16+ and p16–, except that the p16+ classification includes no Tis category and no T4b, and p16– oropharynx, like other non-HPV associated cancers in the head and neck, includes no T0 category.

The p16+ clinical TNM classification is applicable to all cases before treatment (both surgically and non-surgically treated cases). The pathological classification is confined to cases managed with surgery (following examination of the resected specimens, as with all other pathologically staged tumors).

A unique and potentially perplexing feature of pathological staging in HPV+ is that in the data set, pN3 category behaves as Stage I while pN2 behaves as Stage II. This finding is unprecedented, and only prospective collection of data will help clarify this apparent paradox.[5]

Rules for Classification

Because a fundamental difference in outcome was observed for cases based on the number of nodes confirmed pathologically and the clinical presence of contralateral nodes and nodes larger than 6 cm, two systems were developed for these two clinical scenarios. Pathological TNM (pTNM) applies only if the patient undergoes surgery and uses the pathological characteristics of the primary tumor and the number of positive nodes obtained from pathological examination of the surgically resected tissue. Clinical TNM (cTNM) utilizes information from available history, physical examination, and whatever imaging is performed. It is clear that to accurately enumerate clinically involved lymph nodes is not possible for a worldwide pretreatment clinical stage classification; that parameter, therefore, is confined to pTNM. It is recommended that clinical staging data be collected on ALL patients for the purposes of pretreatment assessment, providing a uniform standard for comparing cases across treatment centers around the world and for treatment planning, including selection of postsurgical treatment and prediction of prognosis.

Definition of Primary Tumor (T): Changes

Throughout the head and neck chapters, the Primary Tumor (T) categories (for size and extent of the primary tumor) are generally similar, with changes in the skin, nasopharynx, and oral cavity chapters. A key change from prior editions of the TNM system is the elimination of the T0 category in sites other than nasopharynx and HPV+ oropharynx. Specific changes include the following:

- T categorization for skin cancer recognizes the critical importance of depth of invasion beyond 6 mm and perineural invasion, both of which upstage a lesion to T3.
- In nasopharynx, the previous T4 criteria "masticator space" and "infratemporal fossa" are replaced by a specific description of soft tissue involvement to avoid ambiguity. Adjacent muscle involvement (including medial pterygoid, lateral pterygoid, and prevertebral muscles) is designated as T2.
- The biggest change in T category is for the oral cavity. Depth of invasion (DOI) has been added as a modification to T to enhance the distinction between the superficial or exophytic tumors and those that are more invasive. Clinicians have long recognized the very dif-

ferent biological behaviors between these types of lesions, and this is now acknowledged by increasing the T category for every 5-mm increase in DOI in three categories: less than or equal to 5 mm; greater than 5 mm, but not greater than 10 mm; and greater than 10 mm). It is important to recognize the distinction between tumor thickness and true DOI. It has been recognized since the early work of Spiro and co-workers, in the mid-1980s, that prognosis of oral cancers worsens as the tumor grows thicker, as with skin malignancies.[6] The somewhat more sophisticated measure of DOI has been recorded for oral cavity cancers since the AJCC Cancer Staging Manual, 6th Edition (6th Edition). Clinicians experienced with head and neck cancer generally will have few problems identifying a superficial and less invasive lesion (≤5 mm) from those of moderate depth (>5 mm and ≤ 10 mm) or deeply invasive cancers (>10 mm) through clinical examination alone. Such experts have estimated the maximum dimensions for complicated lesions of the tonsil or palate for many years. In applying the DOI modifier, if there are doubts or ambiguity, the clinician should apply the general TNM uncertainty staging rule of using the lower attribute for a category (in this case a lower DOI categorization).

- Extrinsic muscle infiltration is no longer a staging criterion for T4 designation in oral cavity, because depth of invasion supersedes it and extrinsic muscle invasion is difficult to assess (both clinically and pathologically).
- An additional change is the elimination of the T0 category for all oral cavity, skin, larynx, salivary gland, HPV– oropharynx, hypopharynx, and sinus. This change affects cases where a cervical lymph node has metastatic squamous cell carcinoma, but no primary tumor is identified despite thorough history, examination, and available imaging studies. Assigning these cases to a specific head and neck site is not possible. Previous editions of TNM staging included a T0 category in each of these disease sites. However, it is seldom used and, if it is, the cancer could not be assigned to a stage group. Therefore, for the 8th Edition, the Expert Panel eliminated the T0 category from the head and neck staging systems. A separate staging system for those cases with an involved cervical node without a known primary tumor has been added to the chapter entitled Cervical Node and Unknown Primary of the Head and Neck. These cases should be classified under the TNM rules for a cancer of unknown primary outlined in that chapter.
- The two exceptions where T0 continues to be used as a T-category are HPV-associated cancer and Epstein-Barr Virus (EBV) associated cancers. For HPV-associated cancers identified in a cervical lymph node (defined as p16 positive), the case is staged using the p16 positive oropharynx system, which continues to include a "T0" category. EBV-positive cancers identified in a cervical node with no obvious primary are staged using the EBV-related nasopharynx system, in which the "T0" category is maintained.

Regional Lymph Node (N) Category: Introduction of Extranodal Extension

The 8th Edition introduces the use of extranodal extension (ENE) in categorizing the "N" category for metastatic cancer to neck nodes. The effect of ENE on prognosis in head and neck cancers is profound, except for those tumors associated with HR-HPV.[7] Including this important prognostic feature was considered critical for improving staging.

Most of the evidence supporting ENE as an adverse prognostic factor is based on histopathological characterization of ENE, especially the distinction between microscopic and macroscopic (or gross) ENE. For clinical staging only, the presence of unquestionable ENE as determined by physical examination and supported by radiological evidence is to be used. As per the standard rules of cancer staging, if there is uncertainty or ambiguity for categorization of T, N, or M, the lower category is assigned.

Stringent criteria for clinical examination must be met to assign a diagnosis of ENE. Current imaging modalities have significant limitations in their ability to accurately identify ENE.[8] This mandates that radiological evidence alone is insufficient. Unambiguous evidence of gross ENE on clinical examination (e.g., invasion of skin, infiltration of musculature or dense tethering to adjacent structures, or cranial nerve, brachial plexus, sympathetic trunk, or phrenic nerve invasion with dysfunction) supported by strong radiographic evidence is required to permit clinical classification of disease as ENE(+).

Pathological ENE also must be clearly defined as extension of metastatic tumor (tumor present within the confines of the lymph node and extending through the lymph node capsule into the surrounding connective tissue, with or without associated stromal reaction). Again, when there is doubt or ambiguity in the reporting, the lesser or lower category is assigned, in this case ENE(−).

PRINCIPLES OF STAGING

All staging systems presented in this section are for clinical staging, based on the best possible estimate of the extent of disease before initiation of first treatment. Imaging tech-

niques—computed tomography (CT), magnetic resonance (MR) imaging, positron emission tomography (PET), and ultrasonography—may be employed, but clinical examination is sufficient to assign clinical stage. In advanced tumor stages, imaging studies add to the accuracy of primary tumor (T) and nodal (N) categorization, especially in the nasopharyngeal and paranasal sinuses primary sites, as well as for regional lymph nodes. Endoscopic evaluation of the primary tumor and examination under anesthesia when appropriate may be needed for accurate T categorization. These may be supplemented by needle biopsy (most often fine-needle aspiration biopsy [FNAB]) to confirm the presence of tumor and its histopathologic nature, while recognizing that a negative needle biopsy cannot rule out the presence of tumor. When imaging studies are not done or not available, as in low-resource regions, clinical stage may be based solely on careful history, physical examination, and/or endoscopy.

Clinical stage should be reported for all cases, as well as pathological stage when surgery is performed. Any diagnostic information that contributes to the overall accuracy of the pretreatment assessment should be considered in clinical staging and treatment planning. When surgical treatment is carried out, cancer of the head and neck can be staged pathologically (pTNM) using all information available from clinical assessment, as well as from the surgeon's operative findings and pathological study of the resected specimen.

With the exception of p16+ OPC (a recently recognized disease entity), no major changes in the stage groupings are recommended. The current stage groupings reflect recent practices, clinical relevance, and contemporary data. T4 tumors continue to be subdivided into moderately advanced (T4a) and very advanced (T4b) categories. Stage IV disease is divided into moderately advanced, local/regional disease (Stage IVA), very advanced local/regional disease (Stage IVB), and distant metastatic disease (Stage IVC).

The following chapters present the staging classification for nine major head and neck sites and disease entities:

- Lip and Oral Cavity
- Major Salivary Glands
- Nasopharynx
- HPV-Mediated (p16+) Oropharyngeal Cancer
- Oropharynx (p16−) and Hypopharynx
- Nasal Cavity and Paranasal Sinuses
- Larynx
- Mucosal Melanoma of the Head and Neck
- Cutaneous Squamous Cell Carcinoma of the Head and Neck
- An additional chapter explaining Cervical Lymph Nodes and Unknown Primary Tumors of the Head and Neck is included for additional clarity and staging information.

COLLECTION OF KEY PATIENT AND TUMOR FACTORS

An ongoing effort to evaluate the effect on prognosis of both tumor and non-tumor-related factors is underway. Cancer registrars should continue to perform chart abstraction to collect information regarding specific factors related to prognosis as outlined in each chapter. These data will be used to enhance the predictive power of the staging system in future revisions. Key domains of information to collect include the following:

- Comorbidity: In addition to the importance of the TNM factors outlined previously, the overall health or presence of comorbid conditions of patients influences outcome. Comorbidity can be assessed and semi-quantified using validated standardized tools applied by review of medical records.[9] Accurate reporting of all major illnesses in the patient's medical record is essential. General performance measures are helpful in predicting survival.
- Performance Status: In addition, the AJCC strongly recommends that the clinician report performance status using the Eastern Cooperative Oncology Group (ECOG), Zubrod, or Karnofsky performance measures, along with standard staging information.
- Lifestyle factors: Tobacco and alcohol abuse adversely influence survival. Accurate recording of smoking (in pack-years) and alcohol consumption (in number of days drinking per week and number of drinks per day) will provide important data for future analysis. However, exactly how these should be incorporated in staging remains undefined. Smoking is known to have a deleterious effect on prognosis, but valid data are insufficient to allow it to be readily introduced into the staging systems. Smoking history should be collected as an important element of the demographics and may be included in Prognostic Groups in the future. For practicality, the minimum standard should classify smoking history as follows: never, ≤ 10 pack-years, > 10 but ≤ 20 pack-years, > 20 pack-years.
- Nutrition: Nutrition also is important to prognosis and can be indirectly measured by weight loss of > 10 % of body weight over 3 months.[10]
- Depression: Depression adversely impacts quality of life and survival.[11, 12] A previous or current diagnosis of depression should be recorded in the medical record.

REGIONAL LYMPH NODES

The status of the regional lymph nodes in head and neck cancer is of such prognostic importance that the cervical nodes must be assessed for each patient and tumor. The lymph

nodes in the head and neck are subdivided into specific anatomic subsites and grouped into seven levels for ease of description (Fig. 5.1, Tables 5.1 and 5.2).

In addition to the standard groups listed in Tables 5.1 and 5.2, other lymph node groups are defined by their specific anatomic location. Numbers of nodes are counted toward N category, but they are listed separately using the following descriptors.

- Suboccipital
- Retropharyngeal
- Parapharyngeal
- Buccinator (facial)
- Preauricular
- Periparotid and intraparotid

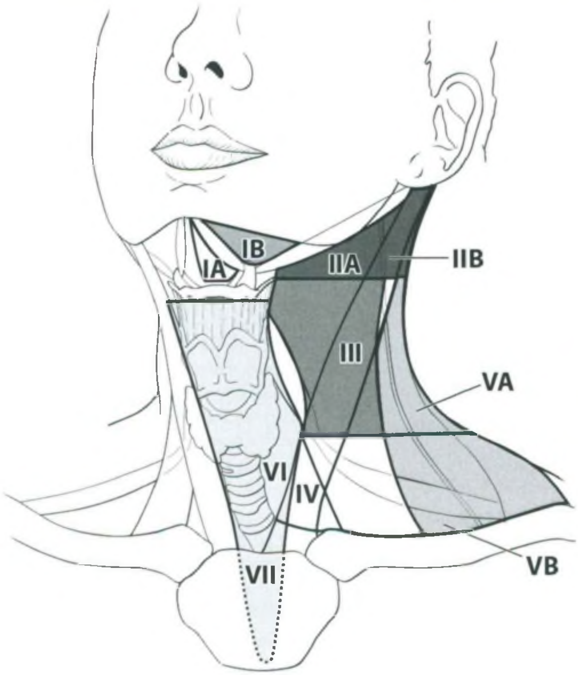

Fig. 5.1 Schematic indicating the location of the lymph node levels in the neck as described in Table 5.1

Other General Rules for Assessing Regional Lymph Nodes

Histopathologic examination is necessary to exclude the presence of tumor in lymph nodes for pathological categorization (pN0). No imaging study (as yet) can identify microscopic tumor foci in regional nodes or distinguish between small reactive nodes and small malignant nodes.

Table 5.1 Anatomical structures defining the boundaries of the neck levels and sublevels

Boundary Level	Superior	Inferior	Anterior (medial)	Posterior (lateral)
IA	Symphysis of the mandible	Body of the hyoid	Anterior belly of the contralateral digastric muscle	Anterior belly of the ipsilateral digastric muscle
IB	Body of the mandible	Posterior belly of the diagastric muscle	Anterior belly of the digastric muscle	Stylohyoid muscle
IIA	Skull base	Horizontal plane defined by the inferior border of the hyoid bone	Stylohyoid muscle	Vertical plane defined by the spinal accessory nerve
IIB	Skull base	Horizontal plane defined by the inferior body of the hyoid bone	Vertical plane defined by the spinal accessory nerve	Lateral border of the sternocleidomastoid muscle
III	Horizontal plane defined by the inferior body of the hyoid	Horizontal plane defined by the inferior border of the cricoid cartilage	Lateral border of the sternohyoid muscle	Lateral border of the sternocleidomastoid or sensory branches of the cervical plexus
IV	Horizontal plane defined by the inferior border of the cricoid cartilage	Clavicle	Lateral border of the sternohyoid muscle	Lateral border of the sternocleidomastoid or sensory branches of the cervical plexus
VA	Apex of the convergence of the sternocleidomastoid and trapezius muscles	Horizontal plane defined by the lower border of the cricoid cartilage	Posterior border of the sternocleidomastoid muscle or sensory branches of the cervical plexus	Anterior border of the trapezius muscle
VB	Horizontal plane defined by the lower border of the cricoid cartilage	Clavicle	Posterior border of the sternocleidomastoid muscle	Anterior border of the trapezius muscle
VI	Hyoid bone	Suprasternal notch	Common carotid artery	Common carotid artery
VII	Suprasternal notch	Innominate artery	Sternum	Trachea, esophagus, and prevertebral fascia

Modified from Robbins KT, Clayman G, Levine PA, et al.,[13] with permission from the American Medical Association.

Table 5.2 Lymph node groups found within the seven levels and sublevels of the neck

Lymph node group	Description
Submental (sublevel IA)	Lymph nodes within the triangular boundary of the anterior belly of the digastric muscles and the hyoid bone. These nodes are at greatest risk for harboring metastases from cancers arising from the floor of mouth, anterior oral tongue, anterior mandibular alveolar ridge, and lower lip.
Submandibular (sublevel IB)	Lymph nodes within the boundaries of the anterior and posterior bellies of the digastric muscle, the stylohyoid muscle, and the body of the mandible. These include the preglandular and the postglandular nodes and the prevascular and postvascular nodes. The submandibular gland is included in the specimen when the lymph nodes within the triangle are removed. These nodes are at greatest risk for harboring metastases from cancers arising from the oral cavity, anterior nasal cavity, skin, and soft tissue structures of the midface, as well as from the submandibular gland.
Upper Jugular (sublevels IIA and IIB)	Lymph nodes located around the upper third of the internal jugular vein and adjacent spinal accessory nerve, extending from the level of the skull base (above) to the level of the inferior border of the hyoid bone (below). The anterior (medial) boundary is stylohyoid muscle (the radiologic correlate is the vertical plane defined by the posterior surface of the submandibular gland) and the posterior (lateral) boundary is the posterior border of the sternocleidomastoid muscle. Sublevel IIA nodes are located anterior (medial) to the vertical plane defined by the spinal accessory nerve. Sublevel IIB nodes are located posterior lateral to the vertical plane defined by the spinal accessory nerve. (The radiologic correlate is the lateral border of the internal jugular on a contrast-enhanced CT scan.) The upper jugular nodes are at greatest risk for harboring metastases from cancers arising from the oral cavity, nasal cavity, nasopharynx, oropharynx, hypopharynx, larynx, and parotid gland.
Middle Jugular (level III)	Lymph nodes located around the middle third of the internal jugular vein, extending from the inferior border of the hyoid bone (above) to the inferior border of the cricoid cartilage (below). The anterior (medial) boundary is the lateral border of the sternohyoid muscle, and the posterior (lateral) boundary is the posterior border of the sternocleidomastoid muscle. These nodes are at greatest risk for harboring metastases from cancers arising from the oral cavity, nasopharynx, oropharynx, hypopharynx, and larynx.
Lower Jugular (level IV)	Lymph nodes located around the lower third of the internal jugular vein, extending from the inferior border of the cricoid cartilage (above) to the clavicle below. The anterior (medial) boundary is the lateral border of the sternohyoid muscle and the posterior (lateral) boundary is the posterior border of the sternocleidomastoid muscle. These nodes are at greatest risk for harboring metastases from cancers arising from the hypopharynx, thyroid, cervical esophagus, and larynx.
Posterior Triangle (sublevels VA and VB)	This group is composed predominantly of the lymph nodes located along the lower half of the spinal accessory nerve and the transverse cervical artery. The supraclavicular nodes also are included in the posterior triangle group. The superior boundary is the apex formed by the convergence of the sternocleidomastoid and trapezius muscles; the inferior boundary is the clavicle; the anterior (medial) boundary is the posterior border of the sternocleidomastoid muscle; and the posterior (lateral) boundary is the anterior border of the trapezius muscle. Thus, sublevel VA includes the spinal accessory nodes, whereas sublevel VB includes the nodes following the transverse cervical vessels and the supraclavicular nodes, with the exception of the Virchow node, which is located in level IV. The posterior triangle nodes are at greatest risk for harboring metastases from cancers arising from the nasopharynx, oropharynx, and cutaneous structures of the posterior scalp and neck.
Anterior Compartment (level VI)	Lymph nodes in this compartment include the pretracheal and paratracheal nodes, precricoid (Delphian) node, and the perithyroidal nodes, including the lymph nodes along the recurrent laryngeal nerves. The superior boundary is the hyoid bone; the inferior boundary is the suprasternal notch; and the lateral boundaries are the common carotid arteries. These nodes are at greatest risk for harboring metastases from cancers arising from the thyroid gland, glottic and subglottic larynx, apex of the piriform sinus, and cervical esophagus.
Superior Mediastinal (level VII)	Lymph nodes in this group include pretracheal, paratracheal, and esophageal groove lymph nodes, extending from the level of the suprasternal notch cephalad and up to the innominate artery caudad. These nodes are at greatest risk of involvement by thyroid cancer and cancer of the esophagus.

Modified from Robbins KT, Clayman G, Levine PA, et al.,[13] with permission from the American Medical Association.

When enlarged lymph nodes are detected, the actual size (measured as the maximum dimension in any direction) of the nodal mass(es) should be recorded. Pathological examination is necessary for documentation of tumor extent in terms of the location or level of the lymph node(s) involved and the number of nodes that contain metastases. The pathological presence or absence of ENE should be designated as ENE(+) or ENE(−).

Definition of Regional Lymph Nodes (N)

Clinical N (cN)

N Category	N Criteria
NX	Regional lymph nodes cannot be assessed
N0	No regional lymph node metastasis
N1	Metastasis in a single ipsilateral lymph node, 3 cm or smaller in greatest dimension and ENE(−)

N Category	N Criteria
N2	Metastasis in a single ipsilateral lymph node larger than 3 cm but not larger than 6 cm in greatest dimension and ENE(−); *or* metastases in multiple ipsilateral lymph nodes, none larger than 6 cm in greatest dimension and ENE(−); *or* in bilateral or contralateral lymph nodes, none larger than 6 cm in greatest dimension, ENE(−)
N2a	Metastasis in single ipsilateral or contralateral node larger than 3 cm but not larger than 6 cm in greatest dimension and ENE(−)
N2b	Metastasis in multiple ipsilateral nodes, none larger than 6 cm in greatest dimension and ENE(−)
N2c	Metastasis in bilateral or contralateral lymph nodes, none larger than 6 cm in greatest dimension and ENE(−)
N3	Metastasis in a lymph node larger than 6 cm in greatest dimension and ENE(−); *or* metastasis in a single ipsilateral node ENE(+) *or* multiple ipsilateral, contralateral, or bilateral nodes, any with ENE(+)
N3a	Metastasis in a lymph node larger than 6 cm in greatest dimension and ENE(−)
N3b	Metastasis in a single ipsilateral node ENE(+) *or* multiple ipsilateral, contralateral, or bilateral nodes, any with ENE(+)

Note: A designation of "U" or "L" may be used for any N category to indicate metastasis above the lower border of the cricoid (U) or below the lower border of the cricoid (L).
Similarly, clinical and pathological ENE should be recorded as ENE(−) or ENE(+).

Pathological N (pN)

N Category	N Criteria
NX	Regional lymph nodes cannot be assessed
N0	No regional lymph node metastasis
N1	Metastasis in a single ipsilateral lymph node, 3 cm or smaller in greatest dimension and ENE(−)
N2	Metastasis in a single ipsilateral lymph node, 3 cm or smaller in greatest dimension and ENE(+); *or* larger than 3 cm but not larger than 6 cm in greatest dimension and ENE(−); *or* metastases in multiple ipsilateral lymph nodes, none larger than 6 cm in greatest dimension and ENE(−); *or* in bilateral or contralateral lymph nodes, none larger than 6 cm in greatest dimension, ENE(−)
N2a	Metastasis in single ipsilateral or contralateral node 3 cm or smaller in greatest dimension and ENE(+) *or* a single ipsilateral node larger than 3 cm but not larger than 6 cm in greatest dimension and ENE(−)
N2b	Metastasis in multiple ipsilateral nodes, none larger than 6 cm in greatest dimension and ENE(−)
N2c	Metastasis in bilateral or contralateral lymph nodes, none larger than 6 cm in greatest dimension and ENE(−)
N3	Metastasis in a lymph node larger than 6 cm in greatest dimension and ENE(−) *or* metastasis in a single ipsilateral node larger than 3 cm in greatest dimension and ENE(+) *or* multiple ipsilateral, contralateral, or bilateral nodes, any with ENE(+)
N3a	Metastasis in a lymph node larger than 6 cm in greatest dimension and ENE(−)
N3b	Metastasis in a single ipsilateral node larger than 3 cm in greatest dimension and ENE(+) *or* multiple ipsilateral, contralateral, or bilateral nodes, any with ENE(+)

Note: A designation of "U" or "L" may be used for any N category to indicate metastasis above the lower border of the cricoid (U) or below the lower border of the cricoid (L).
Similarly, clinical and pathological ENE should be recorded as ENE(−) or ENE(+).

DISTANT METASTASES

The most common sites of distant spread are in the lungs and bones; hepatic and brain metastases occur less often. Mediastinal lymph node metastases are considered distant metastases, except level VII lymph nodes (anterior superior mediastinal lymph nodes cephalad to the innominate artery).

SURVIVAL DATA

No large database registry systems have collected data with sufficient validation on ENE to demonstrate long-term survival. However, abundant data support its adverse prognostic effect.[7] The inclusion of ENE in defining the N category based on these data will allow future validation and modification as necessary.

Anatomic sites and histologic types for Head and Neck cancers are coded according to the 3rd Edition of the International Classification of Diseases for Oncology (ICD-O-3). The subsites included in each analysis were chosen on the basis of those listed in the AJCC Cancer Staging Manual, 5th Edition (5th Edition).

Treatment paradigms influence the type and quality of data available for use in prognostication and staging. Cancers that are largely treated using nonsurgical modalities (e.g., nasopharynx) will obviously not have pathological data comparable to cancers that are treated surgically (e.g., oral cavity cancer). Therefore, in these disease sites, parameters that require pathological examination, such as number of involved lymph nodes or microscopic ENE, cannot be evaluated in a significant proportion of patients with head and neck cancer.

Oral cavity cancer represents the most common situation for which histopathologic data are commonly available for relatively large numbers of patients and, therefore, is the one anatomic site that has undergone significant revisions in T category. Additionally, the N categorization for all sites was heavily influenced by the data from oral cavity cancer. Oral cavity cancer outcomes were analyzed using a large data set of patients treated at two tertiary cancer care centers in North America comprising patients treated with a common staging and treatment strategy (Table 5.3).[14] Comparable cancer registry data sets on oral cancer are not available, so these revisions based on single institutional data have not yet been validated on large populations. The following description of the process of stage revision of oral cancer vividly illustrates the importance of high-fidelity data collection and the need for comparable cancer registry data for future iterations of the staging system.

The T criteria for oral cancer patients were modified based on the DOI of the primary tumor, which has long been recognized as an important predictor of outcome. The basis for this modification is the report from the International Consortium for Outcomes Research in Head and Neck Cancer.[14] Outcomes based on the revised T criteria are shown in Fig. 5.2 and Table 5.4.

The N category criteria also were revised for the 8th Edition to incorporate the influence of ENE on prognosis. The preliminary analysis of the influence of ENE on prognosis was performed on a data set from the National Cancer Data Base (NCDB) that included patients treated in 2010–2011. The data on ENE from other sites also have been published widely in institutional data sets and support inclusion in the 8th Edition (Fig. 5.3).

These new N criteria were then validated using the Memorial Sloan Kettering Cancer Center–Princess Margaret Hospital (MSKCC-PMH) institutional data set (Fig. 5.4 and Table 5.5).[15]

After validation of the N criteria, the next step was to examine the interplay of these new T and N criteria for stage grouping. The NCDB data could not be used for this purpose because of the lack of information on depth of information of the primary tumor. The MSKCC-PMH data set was therefore used for stage group analysis using 7th Edition AJCC/UICC criteria (Fig. 5.5 and Table 5.6).[15]

As seen in Fig. 5.5, the 7th Edition stage groupings were unable to discriminate between Stage II and Stage III, an effect that may largely be attributable to the redistribution of prognostic weight introduced by the addition of DOI and the relatively lower prognostic impact of low-volume nodal metastatic disease, especially with the addition of adjuvant treatment. In recognition of these newer prognostic factors, the MSKCC-PMH data were re-interrogated after appropriate adjustment of the stage groups, resulting in better discrimination of stage groups (Fig. 5.6 and Table 5.7).

However, these stage groupings could not be validated on cancer registry data because comparable data on DOI of the primary tumor and ENE were not recorded for 7th Edition AJCC/UICC staging. Therefore, although institutional data that support restructuring stage groups for oral cavity cancer exist, the stage groupings will remain unchanged pending future validation.

Table 5.3 Characteristics of patients with oral cavity cancer (MSKCC = Memorial Sloan Kettering Cancer Center, NY; PMH = Princess Margaret Hospital, Toronto)[14]

	Combined	MSKCC, NY	PMH, Toronto
**Total Number of Patients**	1788	1119	669
**Follow-up in months - median (range)**	44.23 (0.03-307.75)	51.02 (0.13-307.75)	37.11 (0.03-197.61)
**Years treated**	1985-2012	1985-2012	1993-2011
**Age – Median (Range)**	60 (15-96)	60 (16-96)	61 (15-89)
**Gender: Male – Number (%)**	1063 (59 %)	642 (57 %)	421 (63 %)

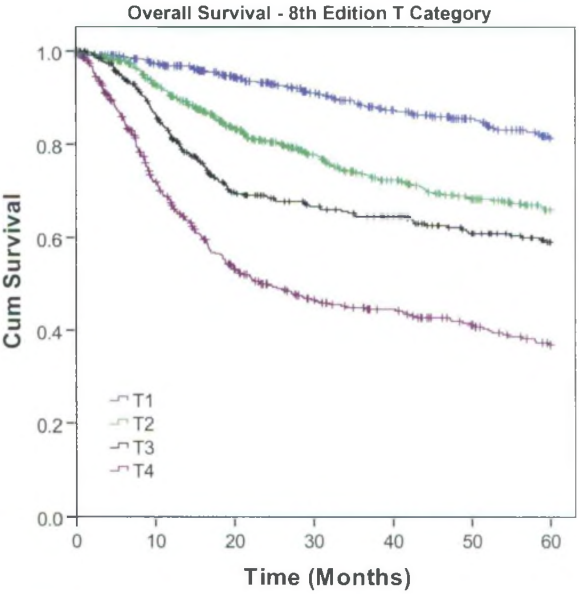

Fig. 5.2 Overall Survival based on 8th edition T category criteria. Kaplan Meier methods were used to perform cancer-specific analyses predicting overall survival as the endpoint on a population of oral cavity cancer patients from MSKCC and PMH

Table 5.4 Overall survival based on 8th Edition T category criteria (MSKCC-PMH Data)

# of pts at Risk	0 Months	12 Months	24 Months	36 Months	48 Months	60 Months
**T1**	429	376	313	262	222	179
**T2**	563	459	344	275	232	190
**T3**	376	285	205	179	150	120
**T4**	420	255	165	132	107	83

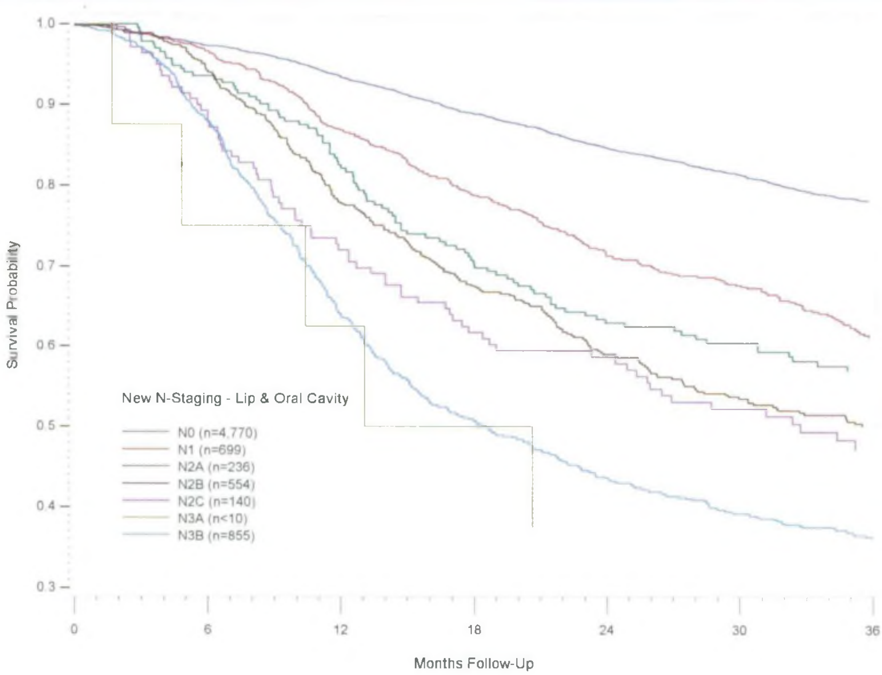

Fig. 5.3 Overall Survival based on 8th edition N category criteria that incorporate ENE as a prognostic factor. Kaplan Meier methods were used to perform cancer-specific analyses predicting overall survival as the endpoint on a population of lip and oral cavity cancer patients from NCDB[14]

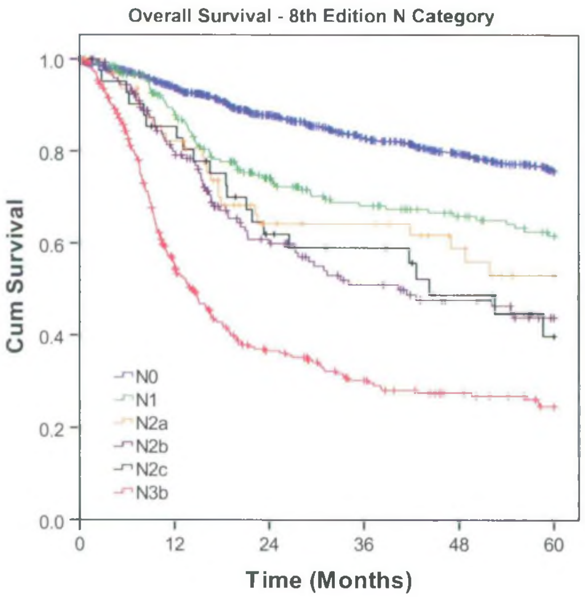

Fig. 5.4 Overall Survival based on 8th edition N category criteria that incorporate ENE as a prognostic factor. Kaplan Meier methods were used to perform cancer-specific analyses predicting overall survival as the endpoint on a population of oral cavity cancer patients from MSKCC and PMH

Table 5.5 Overall survival based on 8th Edition N category criteria (MSKCC-PMH Data)

# of pts at Risk	0 Months	12 Months	24 Months	36 Months	48 Months	60 Months
**N0**	1018	870	710	596	513	421
**N1**	211	168	119	97	82	70
**N2a**	66	50	30	28	21	13
**N2b**	148	107	65	49	39	29
**N2c**	42	34	22	19	12	8
**N3b**	303	146	81	59	43	31

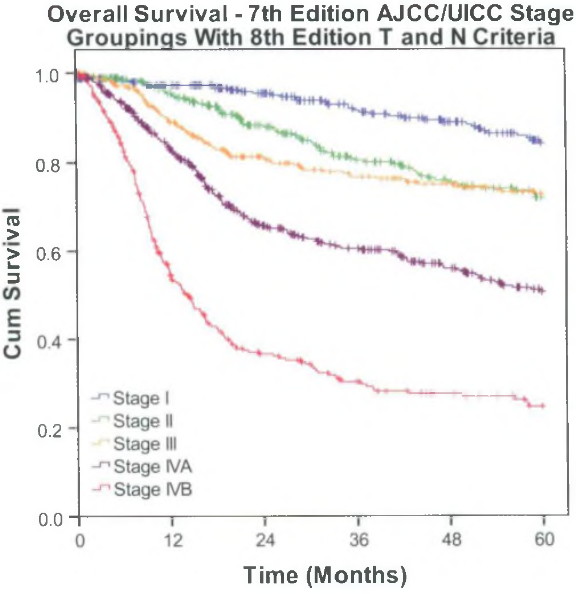

Fig. 5.5 Overall Survival based on 7th Edition AJCC/UICC stage groupings using 8th edition T and N criteria. Kaplan Meier methods were used to perform cancer-specific analyses predicting overall survival as the endpoint on a population of oral cavity cancer patients from MSKCC and PMH[15]

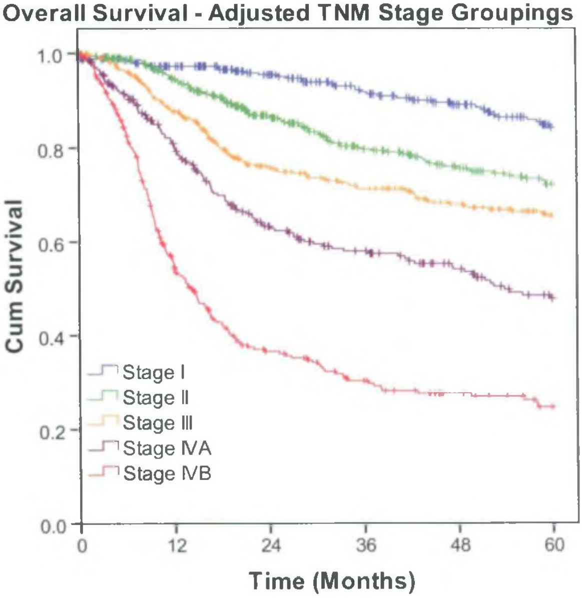

Fig. 5.6 Overall Survival based on Kaplan Meier methods were used to perform cancer-specific analyses predicting overall survival as the endpoint on a population of oral cavity cancer patients from MSKCC and PMH[15]

Table 5.6 Overall survival based on 7th edition AJCC/UICC Stage groupings with 8th edition T and N criteria

# of pts at Risk	0 Months	12 Months	24 Months	36 Months	48 Months	60 Months
I	338	295	252	215	184	147
II	349	303	240	191	161	137
III	346	288	224	195	173	145
IVA	452	343	230	188	150	112
IVB	303	146	81	59	43	31

Table 5.7 Overall Survival in oral Cancer patients after adjustment of stage groups (MSKCC-PMH Data)

# of pts at Risk	0 Months	12 Months	24 Months	36 Months	48 Months	60 Months
Stage I	338	295	252	215	184	147
Stage II	462	398	309	250	210	182
Stage III	414	339	247	211	179	141
Stage IVA	271	197	138	113	92	71
Stage IVB	303	146	81	59	43	31

Bibliography

1. Lydiatt WM, Shah JP, Hoffman HT. AJCC stage groupings for head and neck cancer: should we look at alternatives? A report of the Head and Neck Sites Task Force. *Head & neck.* 2001;23(8):607-612.
2. Patel SG, Lydiatt WM. Staging of head and neck cancers: is it time to change the balance between the ideal and the practical? *Journal of surgical oncology.* 2008;97(8):653-657.
3. Mehanna H, Jones TM, Gregoire V, Ang KK. Oropharyngeal carcinoma related to human papillomavirus. *Bmj.* 2010;340:c1439.
4. El-Naggar AK, Westra WH. p16 expression as a surrogate marker for HPV-related oropharyngeal carcinoma: A guide for interpretative relevance and consistency. *Head & neck.* 2012;34(4):459-461.
5. Haughey BH. Personal Communication. In: Lydiatt W, Shah JP, eds2015.
6. Spiro RH, Huvos AG, Wong GY, Spiro JD, Gnecco CA, Strong EW. Predictive value of tumor thickness in squamous carcinoma confined to the tongue and floor of the mouth. *American journal of surgery.* Oct 1986;152(4):345-350.
7. Wreesmann VB, Katabi N, Palmer FL, et al. Influence of extracapsular nodal spread extent on prognosis of oral squamous cell carcinoma. *Head & neck.* Oct 30 2015.
8. Prabhu RS, Magliocca KR, Hanasoge S, et al. Accuracy of computed tomography for predicting pathologic nodal extracapsular extension in patients with head-and-neck cancer undergoing initial surgical resection. *International journal of radiation oncology, biology, physics.* Jan 1 2014;88(1):122-129.
9. Piccirillo JF. Inclusion of comorbidity in a staging system for head and neck cancer. *Oncology (Williston Park).* Sep 1995;9(9):831-836; discussion 841, 845-838.
10. Couch ME, Dittus K, Toth MJ, et al. Cancer cachexia update in head and neck cancer: Pathophysiology and treatment. *Head & neck.* Jul 2015;37(7):1057-1072.
11. Lazure KE, Lydiatt WM, Denman D, Burke WJ. Association between depression and survival or disease recurrence in patients with head and neck cancer enrolled in a depression prevention trial. *Head & neck.* 2009;31(7):888-892.
12. Lydiatt WM, Bessette D, Schmid KK, Sayles H, Burke WJ. Prevention of depression with escitalopram in patients undergoing treatment for head and neck cancer: randomized, double-blind, placebo-controlled clinical trial. *JAMA otolaryngology– head & neck surgery.* Jul 2013;139(7):678-686.
13. Robbins KT, Clayman G, Levine PA, et al. Neck dissection classification update: revisions proposed by the American Head and Neck Society and the American Academy of Otolaryngology-Head and Neck Surgery. *Archives of otolaryngology–head & neck surgery.* Jul 2002;128(7):751-758.
14. International Consortium for Outcome Research in Head and Neck Cancer, Ebrahimi A, Gil Z, et al. Primary tumor staging for oral cancer and a proposed modification incorporating depth of invasion: an international multicenter retrospective study. *JAMA otolaryngology– head & neck surgery.* Dec 2014;140(12):1138-1148.
15. Patel S. Personal Communication. In: Lydiatt W, Shah JP, eds2015.

Cervical Lymph Nodes and Unknown Primary Tumors of the Head and Neck

6

Snehal G. Patel, William M. Lydiatt, John A. Ridge, Christine M. Glastonbury, Suresh K. Mukherji, Ronald A. Ghossein, Margaret Brandwein-Gensler, Raja R. Seethala, A. Dimitrios Colevas, Bruce H. Haughey, Brian O'Sullivan, and Jatin P. Shah

CHAPTER SUMMARY

Cancers Staged Using This Staging System

Squamous cell carcinoma and salivary gland carcinoma of all head and neck sites *except* HPV-related oropharynx cancer, nasopharynx cancer, melanoma, thyroid carcinoma, and sarcoma. Staging of the patient who presents with an occult primary tumor and EBV-unrelated and HPV-unrelated metastatic cervical lymphadenopathy is also included.

Cancers Not Staged Using This Staging System

These histopathologic types of cancer...	Are staged according to the classification for...	And can be found in chapter...
Nasopharyngeal cancer	Nasopharynx	9
HPV-related oropharynx cancer	HPV-mediated (p16+) oropharyngeal cancer	10
Melanoma	Melanoma of the skin	47
Mucosal melanoma	Mucosal melanoma of the head and neck	14
Thyroid carcinoma	Thyroid carcinoma	73–74
Soft tissue sarcoma	Soft tissue sarcoma of the head and neck	40
Eyelid	Eyelid carcinoma	64

Summary of Changes

Change	Details of Change	Level of Evidence
Definition of Regional Lymph Node (N)	Separate N staging approaches have been described for HPV-related and HPV-unrelated cancers.	II[1,2]
Definition of Regional Lymph Node (N)	Separate N category approaches have been described for patients treated without cervical lymph node dissection (clinical N) and patients treated with cervical lymph node dissection (pathological N).	II[1,2]
Definition of Regional Lymph Node (N)	Extranodal extension (ENE) is introduced as a descriptor in all HPV-unrelated cancers.	II[2]
ENE in HPV-negative cancers	Only clinically and radiographically overt ENE should be used for cN.	II[2]

To access the AJCC cancer staging forms, please visit www.cancerstaging.org.

© American Joint Committee on Cancer 2017
M.B. Amin et al. (eds.), *AJCC Cancer Staging Manual, Eighth Edition*, DOI 10.1007/978-3-319-40618-3_6

Change	Details of Change	Level of Evidence
ENE in HPV-negative cancers	Any pathologically detected ENE is considered ENE(+) and is used for pN.	II[2]
ENE in HPV-negative cancers	Presence of ENE is designated pN2a for a single ipsilateral node < 3 cm and pN3b for all other node(s).	II[2]
Classification of ENE	Clinically overt ENE is classified as ENE_c and is considered ENE(+) for cN.	III[3]
Classification of ENE	Pathologically detected ENE is classified as either ENE_{mi} ($\leq$2 mm) or ENE_{ma} (>2 mm) for data collection purposes only but both are considered ENE(+) for definition of pN.	III[3]
Occult Primary Tumor	Staging of the patient who presents with EBV-unrelated and HPV-unrelated metastatic cervical lymphadenopathy is now included in this chapter.	IV

ICD-O-3 Topography Codes

Code	Description
C00.0	External upper lip
C00.1	External lower lip
C00.2	External lip, NOS
C00.3	Mucosa of upper lip
C00.4	Mucosa of lower lip
C00.5	Mucosa of lip, NOS
C00.6	Commissure of lip
C00.8	Overlapping lesion of lip
C00.9	Lip, NOS
C01.9	Base of tongue, NOS
C02.0	Dorsal surface of tongue, NOS
C02.1	Border of tongue
C02.2	Ventral surface of tongue, NOS
C02.3	Anterior 2/3 of tongue, NOS
C02.4	Lingual tonsil
C02.8	Overlapping lesion of tongue
C02.9	Tongue, NOS
C03.0	Upper gum
C03.1	Lower gum
C03.9	Gum, NOS
C04.0	Anterior floor of mouth
C04.1	Lateral floor of mouth
C04.8	Overlapping lesion of floor of mouth
C04.9	Floor of mouth, NOS
C05.0	Hard palate
C05.1	Soft palate, NOS
C05.2	Uvula
C05.8	Overlapping lesion of palate
C05.9	Palate, NOS
C06.0	Cheek mucosa
C06.1	Vestibule of mouth
C06.2	Retromolar area
C06.8	Overlapping lesion of other and unspecified parts of mouth
C06.9	Mouth, NOS
C07.9	Parotid gland
C08.0	Submandibular gland
C08.1	Sublingual gland
C08.8	Overlapping lesion of major salivary glands
C08.9	Major salivary gland, NOS
C09.0	Tonsillar fossa
C09.1	Tonsillar pillar
C09.8	Overlapping lesion of tonsil
C09.9	Tonsil, NOS
C10.0	Vallecula
C10.1	Anterior surface of epiglottis
C10.2	Lateral wall of oropharynx
C10.3	Posterior wall of oropharynx
C10.4	Branchial cleft
C10.8	Overlapping lesions of oropharynx
C10.9	Oropharynx, NOS
C12.9	Pyriform sinus
C13.0	Postcricoid region
C13.1	Hypopharyngeal aspect of aryepiglottic fold
C13.2	Posterior wall of hypopharynx
C13.8	Overlapping lesion of hypopharynx
C13.9	Hypopharynx, NOS
C14.0	Pharynx, NOS
C14.2	Waldeyer's ring
C14.8	Overlapping lesion of lip, oral cavity, and pharynx
C30.0	Nasal cavity
C30.1	Middle ear
C31.0	Maxillary sinus
C31.1	Ethmoid sinus
C31.2	Frontal sinus
C31.3	Sphenoid sinus
C31.8	Overlapping lesion of accessory sinuses
C31.9	Accessory sinus, NOS
C32.0	Glottis
C32.1	Supraglottis
C32.2	Subglottis
C32.3	Laryngeal cartilage
C32.8	Overlapping lesion of larynx

Code	Description
C32.9	Larynx, NOS
C44.0	Skin of lip, NOS
C44.2	External ear
C44.3	Skin of other and unspecified parts of face
C44.4	Skin of scalp and neck
C44.8	Overlapping lesion of skin
C80.9	Unknown primary site

WHO Classification of Tumors

Code	Description
8051	Verrucous carcinoma, NOS
8051	Condylomatous carcinoma
8051	Verrucous squamous cell carcinoma
8051	Verrucous epidermoid carcinoma
8051	Warty carcinoma
8052	Papillary squamous cell carcinoma
8052	Papillary epidermoid carcinoma
8070	Squamous cell carcinoma, NOS
8070	Epidermoid carcinoma, NOS
8070	Squamous carcinoma
8070	Squamous cell epithelioma
8071	Squamous cell carcinoma, keratinizing, NOS
8071	Squamous cell carcinoma, large cell, keratinizing
8071	Epidermoid carcinoma, keratinizing
8072	Squamous cell carcinoma, large cell, nonkeratinizing, NOS
8072	Squamous cell carcinoma, nonkeratinizing, NOS
8072	Epidermoid carcinoma, large cell, nonkeratinizing
8073	Squamous cell carcinoma, small cell, nonkeratinizing
8073	Epidermoid carcinoma, small cell, nonkeratinizing
8074	Squamous cell carcinoma, spindle cell
8074	Epidermoid carcinoma, spindle cell
8074	Squamous cell carcinoma, sarcomatoid
8082	Lymphoepithelial carcinoma
8082	Lymphoepithelioma
8082	Lymphoepithelioma-like carcinoma
8082	Schmincke's tumor (C11._)
8083	Basaloid squamous cell carcinoma
8084	Squamous cell carcinoma, clear cell type
8121	Schneiderian carcinoma (C30.0, C31._)
8121	Cylindrical cell carcinoma (C30.0, C31._)
8147	Basal cell adenocarcinoma
8200	Adenoid cystic carcinoma
8200	Adenocystic carcinoma
8200	Cylindroma, NOS (except cylindroma of skin M-8200/0)
8200	Adenocarcinoma, cylindroid
8430	Mucoepidermoid carcinoma
8450	Papillary cystadenocarcinoma, NOS
8525	Polymorphous low-grade adenocarcinoma
8550	Acinar cell carcinoma
8562	Epithelial-myoepithelial carcinoma
8310	Clear cell adenocarcinoma, NOS
8480	Mucinous adenocarcinoma
8140	Adenocarcinoma, NOS
8941	Carcinoma in pleomorphic adenoma

Barnes L, Eveson JW, Reichart P, Sidransky D, eds. World Health Organization Classification of Tumours Pathology and Genetics of Head and Neck Tumours. Lyon: IARC; 2005.

INTRODUCTION

The presence of cervical lymph node metastases is the most important adverse prognostic feature for most cancers of the head and neck. However, the degree of impact on prognosis varies depending on host and tumor characteristics such as interplay of Human Papilloma Virus-initiation and smoking status in cancers of the oropharynx, and the presence of extranodal extension (ENE) for most other sites. The natural history and response to treatment of cervical nodal metastases from Epstein-Barr virus-associated nasopharynx (EBV-related) and HPV-related oropharynx primary sites are different in terms of their impact on prognosis, so they warrant separate N classification schemes. This difference also has led to reclassification of the T0 category (occult primary tumor) based on the EBV and HPV status of the metastatic cervical nodes. Current understanding of other nodal features—such as the size of the largest metastatic node, number of metastatic nodes, and laterality—has improved based on an analysis of large multi-institutional datasets for oral cavity cancer. The American Joint Committee on Cancer (AJCC) Cancer Staging Manual, 8th Edition (8th Edition) incorporates these data while striving to maintain the balance between increased complexity and ease of use.

Two major changes are instituted in the 8th Edition for staging cervical nodal metastasis.

1. For the first time, different clinical and pathological N classifications are proposed for definition of regional lymph node metastasis. This breaks with tradition in head and neck cancer, although distinct clinical and pathological classifications have been promulgated for other tumor sites (e.g., breast cancer). It is helpful to consider that clinical TNM (cTNM) and pathological TNM (pTNM) have different purposes. cTNM classification is required for all patients, including those undergoing surgery. pTNM classification provides additional information, but only for patients undergoing surgery; this guides the use of adjuvant treatment based on factors that become available after histopathological examination of the specimen (e.g., high-risk features of the primary site, ENE, laterality, and volume of metastatic nodal disease).

2. A second significant change is in the use of ENE in categorizing metastatic cancer to neck nodes. The effect of ENE in prognosis in head and neck cancers is profound, except for those tumors associated with HPV. Most of the data supporting ENE as an adverse prognostic factor is based on histopathological characterization of ENE, especially the distinction between microscopic and macroscopic ENE. Only unquestionable clinically evident ENE that is supported by radiological evidence is to be used for clinical staging. The guiding principle is to assign the lesser attribute (ENE negative) if there are doubts in a particular case to avoid stage migration, in accordance with the uncertain rule, which articulates the concept that whenever there is a question, the lesser stage should be chosen. For clinical ENE, the known limitations of current imaging modalities to define ENE accurately demand that stringent criteria be met prior to assigning a clinical diagnosis of ENE if the patient is treated with nonsurgical therapy for neck metastasis. However, unambiguous evidence of gross ENE on clinical examination (e.g., invasion of skin, infiltration of musculature, dense tethering or fixation to adjacent structures, or cranial nerve, brachial plexus, sympathetic trunk, or phrenic nerve invasion with dysfunction) supported by strong radiographic evidence permits classification of disease as ENEc. Pathological ENE also will be clearly defined as extension of metastatic tumor (beyond the confines of the lymph node, through the lymph node capsule into the surrounding connective tissue, with or without associated stromal reaction). Again when there is doubt, recall the uncertain rule and assign a lower stage.

ANATOMY

Regional Lymph Nodes

The lymph nodes in the neck may be subdivided into specific anatomic subsites and grouped into seven levels for ease of description (Fig. 6.1, Tables 6.1 and 6.2).

Other groups not included in these levels are

- Suboccipital
- Retropharyngeal
- Parapharyngeal
- Buccinator (facial)
- Preauricular
- Periparotid and intraparotid

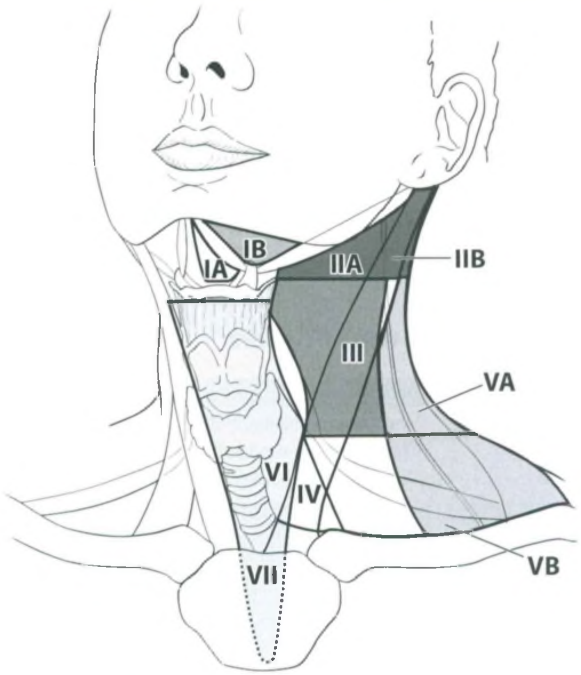

Fig. 6.1 Schematic indicating the location of the lymph node levels in the neck as described in Table 6.1

RULES FOR CLASSIFICATION

Clinical Classification

When enlarged lymph nodes are detected, the size of the nodal mass(es) should be measured and their location should be recorded in terms of the levels of the neck (Tables 6.1 and 6.2). The presence of ENE can be diagnosed clinically by the presence of involvement of overlying skin, fixity to adjacent soft tissue, or clinical signs of cranial nerve or brachial plexus, sympathetic chain or phrenic nerve invasion. The presence of clinically evident gross ENE is designated cENE(+).

Pathological confirmation of metastasis by a fine-needle aspirate, needle biopsy, excisional biopsy of a lymph node, or a sentinel node procedure should be assigned cN.

The Occult Primary Tumor (T0)

The head and neck region is unique among solid tumor sites because several different staging classifications are predicated upon anatomic site of the primary tumor. The AJCC Cancer Staging Manual, 7th Edition, T classifications for head and neck sites included T0 (primary site cannot be identified). This concept is not consistent with anatomic site staging and represents a problem if a primary

Table 6.1 Anatomical structures defining the boundaries of the neck levels and sublevels

Boundary Level	Superior	Inferior	Anterior (medial)	Posterior (lateral)
IA	Symphysis of the mandible	Body of the hyoid	Anterior belly of the contralateral digastric muscle	Anterior belly of the ipsilateral digastric muscle
IB	Body of the mandible	Posterior belly of the diagastric muscle	Anterior belly of the digastric muscle	Stylohyoid muscle
IIA	Skull base	Horizontal plane defined by the inferior border of the hyoid bone	Stylohyoid muscle	Vertical plane defined by the spinal accessory nerve
IIB	Skull base	Horizontal plane defined by the inferior body of the hyoid bone	Vertical plane defined by the spinal accessory nerve	Lateral border of the sternocleidomastoid muscle
III	Horizontal plane defined by the inferior body of the hyoid	Horizontal plane defined by the inferior border of the cricoid cartilage	Lateral border of the sternohyoid muscle	Lateral border of the sternocleidomastoid or sensory branches of the cervical plexus
IV	Horizontal plane defined by the inferior border of the cricoid cartilage	Clavicle	Lateral border of the sternohyoid muscle	Lateral border of the sternocleidomastoid or sensory branches of the cervical plexus
VA	Apex of the convergence of the sternocleidomastoid and trapezius muscles	Horizontal plane defined by the lower border of the cricoid cartilage	Posterior border of the sternocleidomastoid muscle or sensory branches of the cervical plexus	Anterior border of the trapezius muscle
VB	Horizontal plane defined by the lower border of the cricoid cartilage	Clavicle	Posterior border of the sternocleidomastoid muscle	Anterior border of the trapezius muscle
VI	Hyoid bone	Suprasternal notch	Common carotid artery	Common carotid artery
VII	Suprasternal notch	Innominate artery	Sternum	Trachea, esophagus, and prevertebral fascia

Modified from Robbins KT, Clayman G, Levine PA, et al.,[4] with permission from the American Medical Association

Table 6.2 Lymph node groups found within the seven levels and sublevels of the neck

Lymph node group	Description
Submental (sublevel IA)	Lymph nodes within the triangular boundary of the anterior belly of the digastric muscles and the hyoid bone. These nodes are at greatest risk for harboring metastases from cancers arising from the floor of mouth, anterior oral tongue, anterior mandibular alveolar ridge, and lower lip.
Submandibular (sublevel IB)	Lymph nodes within the boundaries of the anterior and posterior bellies of the digastric muscle, the stylohyoid muscle, and the body of the mandible. These include the preglandular and the postglandular nodes and the prevascular and postvascular nodes. The submandibular gland is included in the specimen when the lymph nodes within the triangle are removed. These nodes are at greatest risk for harboring metastases from cancers arising from the oral cavity, anterior nasal cavity, skin, and soft tissue structures of the midface, as well as from the submandibular gland.
Upper Jugular (sublevels IIA and IIB)	Lymph nodes located around the upper third of the internal jugular vein and adjacent spinal accessory nerve, extending from the level of the skull base (above) to the level of the inferior border of the hyoid bone (below). The anterior (medial) boundary is stylohyoid muscle (the radiologic correlate is the vertical plane defined by the posterior surface of the submandibular gland) and the posterior (lateral) boundary is the posterior border of the sternocleidomastoid muscle. Sublevel IIA nodes are located anterior (medial) to the vertical plane defined by the spinal accessory nerve. Sublevel IIB nodes are located posterior lateral to the vertical plane defined by the spinal accessory nerve. (The radiologic correlate is the lateral border of the internal jugular on a contrast-enhanced CT scan.) The upper jugular nodes are at greatest risk for harboring metastases from cancers arising from the oral cavity, nasal cavity, nasopharynx, oropharynx, hypopharynx, larynx, and parotid gland.
Middle Jugular (level III)	Lymph nodes located around the middle third of the internal jugular vein, extending from the inferior border of the hyoid bone (above) to the inferior border of the cricoid cartilage (below). The anterior (medial) boundary is the lateral border of the sternohyoid muscle, and the posterior (lateral) boundary is the posterior border of the sternocleidomastoid muscle. These nodes are at greatest risk for harboring metastases from cancers arising from the oral cavity, nasopharynx, oropharynx, hypopharynx, and larynx.
Lower Jugular (level IV)	Lymph nodes located around the lower third of the internal jugular vein, extending from the inferior border of the cricoid cartilage (above) to the clavicle below. The anterior (medial) boundary is the lateral border of the sternohyoid muscle and the posterior (lateral) boundary is the posterior border of the sternocleidomastoid muscle. These nodes are at greatest risk for harboring metastases from cancers arising from the hypopharynx, thyroid, cervical esophagus, and larynx.

(Continued)

6

Table 6.2 (Continued)

Lymph node group	Description
Posterior Triangle (sublevels VA and VB)	This group is composed predominantly of the lymph nodes located along the lower half of the spinal accessory nerve and the transverse cervical artery. The supraclavicular nodes also are included in the posterior triangle group. The superior boundary is the apex formed by the convergence of the sternocleidomastoid and trapezius muscles; the inferior boundary is the clavicle; the anterior (medial) boundary is the posterior border of the sternocleidomastoid muscle; and the posterior (lateral) boundary is the anterior border of the trapezius muscle. Thus, sublevel VA includes the spinal accessory nodes, whereas sublevel VB includes the nodes following the transverse cervical vessels and the supraclavicular nodes, with the exception of the Virchow node, which is located in level IV. The posterior triangle nodes are at greatest risk for harboring metastases from cancers arising from the nasopharynx, oropharynx, and cutaneous structures of the posterior scalp and neck.
Anterior Compartment (level VI)	Lymph nodes in this compartment include the pretracheal and paratracheal nodes, precricoid (Delphian) node, and the perithyroidal nodes, including the lymph nodes along the recurrent laryngeal nerves. The superior boundary is the hyoid bone; the inferior boundary is the suprasternal notch; and the lateral boundaries are the common carotid arteries. These nodes are at greatest risk for harboring metastases from cancers arising from the thyroid gland, glottic and subglottic larynx, apex of the piriform sinus, and cervical esophagus.
Superior Mediastinal (level VII)	Lymph nodes in this group include pretracheal, paratracheal, and esophageal groove lymph nodes, extending from the level of the suprasternal notch cephalad and up to the innominate artery caudad. These nodes are at greatest risk of involvement by thyroid cancer and cancer of the esophagus.

Modified from Robbins KT, Clayman G, Levine PA, et al.,[4] with permission from the American Medical Association

tumor cannot be identified on clinical examination and with currently available radiographic imaging techniques. This dilemma of the occult primary has been partially resolved by improved understanding of tumorigenesis and availability of cytologic and histologic methods to identify EBV- and HPV-related tumors, which are known to predominantly arise in the nasopharynx and oropharynx, respectively. In spite of modern technology, the origin of the primary tumor does remain unknown in all other patients whose primary tumor is clinically and radiographically occult and who present with EBV-negative and HPV-negative metastatic cervical node(s). Furthermore, T0 will still exist in salivary gland primary sites based on histology of the lymph node.

Three separate approaches are employed to stage patients who present with an occult primary tumor. The primary T category is described as T0 and the N category is designated according to the respective anatomic site based on EBV and HPV status: (1) patients with EBV-related cervical adenopathy are staged according to Chapter 9 (Nasopharynx); (2) patients with HPV-related cervical adenopathy are staged according to Chapter 10 (HPV-mediated oropharyngeal cancer (p16+)); and (3) all other patients with EBV-unrelated and HPV-unrelated cervical adenopathy are staged according to the N category described in this chapter. The stage groupings for these specific patients with occult primary tumors (T0) take into account the varying prognostic impact of metastatic cervical adenopathy for their different diseases. The stage groupings for EBV-related nasopharynx and HPV-related oropharynx cancer are described separately in the relevant chapters. Stage grouping for the patient with EBV-unrelated HPV-unrelated occult primary tumor is described in AJCC Prognostic Stage Groups in this chapter.

Imaging

Abnormal lymph nodes should be described according to the level of the neck that is involved, using the standard imaging-based classification (Table 6.1).

Ultrasonography (US) is a convenient and commonly used modality for assessment of the neck. However, interpretation is observer dependent and certain areas, such as the retropharyngeal nodes and mediastinal nodes, cannot be assessed with US. Currently, its utility is being defined in the assessment of ENE. Computed tomography (CT) or magnetic resonance (MR) imaging can be used for evaluating all nodal levels for metastatic involvement, with particular attention to the expected drainage pattern of the primary tumor site. Fluorodeoxyglucose (FDG) positron emission tomography (PET) may increase sensitivity and specificity over cross-sectional imaging alone for nodal detection, although small and cystic nodes may be falsely negative while reactive nodes may be falsely positive.

A maximum short-axis diameter of 10 mm for lymph nodes is commonly used to describe abnormal nodes, although this results in a high false-negative rate. Because size criteria alone are not reliable, small nodes, particularly in the expected drainage levels of the primary site, should be carefully evaluated. Enlarged or round nodes with loss of normal oval contour and/or loss of the fatty hilus, and nodes with focal nodal inhomogeneity suggestive of necrosis or cystic change should be sought.

Cross-sectional imaging (CT or MR imaging) generally has low sensitivity (65–80%) but high specificity (86–93%) for the detection of ENE. US appears to be less accurate in assessment of ENE than CT and MR imaging, and its utility is currently being defined. ENE is suggested by interrupted or undefined nodal contours with high-resolution US imaging. Several CT or MR imaging features suggest ENE, such as indistinct nodal margins and irregular nodal capsular

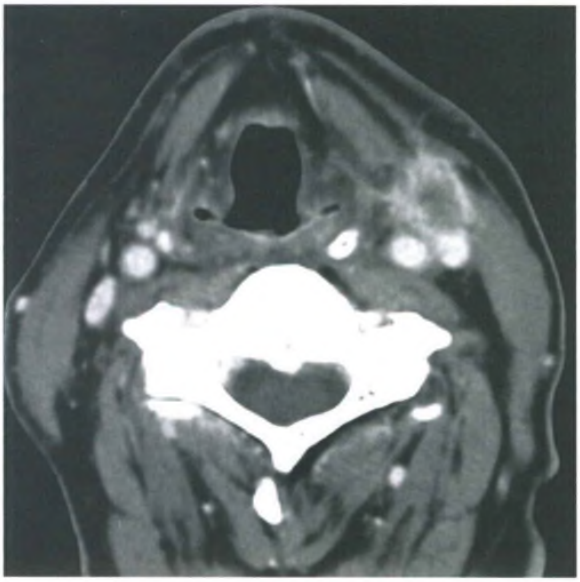

Fig. 6.2 Axial contrast enhanced CT scan in a patient with a left level III enlarged heterogeneous node with ill-defined margins, infiltrating the adjacent fat and sternocleidomastoid muscle

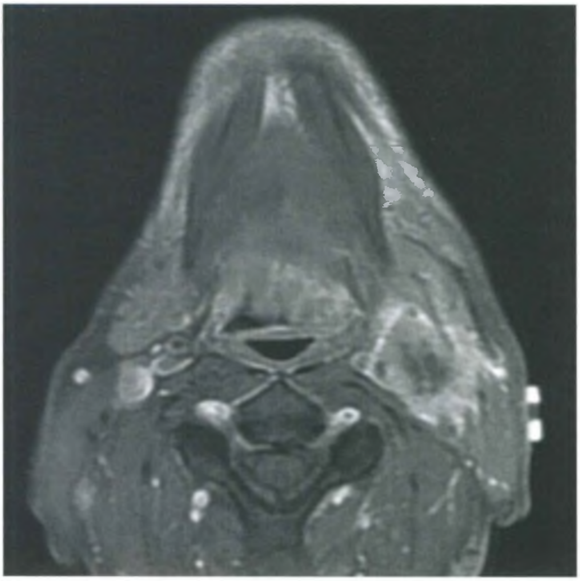

Fig. 6.3 Axial T1 post-contrast fat-saturated MR imaging demonstrates a large heterogeneous and enhancing left level II mass with ill-defined margins and infiltration into the adjacent fat and sternocleidomastoid muscle. The primary left base of tongue squamous cell carcinoma is also evident on this image

enhancement; however, the strongest imaging feature supporting the clinical diagnosis for ENE is clear infiltration into the adjacent fat or muscle. (Figs. 6.2 and 6.3) This limitation of current technology limits the definitive nonsurgical diagnosis of ENE to only those patients who have clinically obvious signs described above. As noted, the presence of clinically obvious ENE is designated ENE$_c$.

Pathological Classification

Histopathological examination is necessary to exclude the presence of tumor in lymph nodes because no imaging study as yet is accurate enough to identify microscopic tumor foci in regional nodes or distinguish between small reactive nodes and small malignant nodes. When a histopathologically involved node is identified, pN is designated based on measurement of the largest dimension of the metastatic deposit and not of the entire lymph node.

An excisional biopsy of a lymph node does not qualify for full evaluation of the pN category and should be assigned cN.

Pathological examination is necessary for documentation of tumor extent in terms of the location or level of the lymph node(s) involved, the number of nodes that contain metastases, and the presence, absence, and extent of ENE.

Minimum number of lymph nodes harvested in an adequate neck dissection

For assessment of pN, an adequate neck dissection ordinarily will include 15 or more lymph nodes in a previously untreated patient. Examination of fewer tumor-free nodes in a neck dissection specimen still mandates a pN0 designation.

Sentinel node(s)

Sentinel lymph nodes (SLN) are described as the first nodes directly draining lymph from the primary tumor. SLN biopsy has been used as a staging procedure for the clinically negative neck in certain sites, such as oral cancer. Negative histopathological examination of SLNs justifies cN0 when sentinel node biopsy is part of the diagnostic workup and also is used for pathological categorization (pN0) in those cases that meet other criteria for pathological staging (e.g., resection of the primary tumor). The positive sentinel node also may be used to classify as pN1, though patients with positive SLNs usually undergo lymphadenectomy, and pN status is assigned based on assessment of the neck dissection specimen if performed.

Micrometastases

Micrometastases are labeled as pN1(mi), pN2b(mi), or pN2c(mi) for $\leq$ 2 mm deposits detected in single or multiple nodes detected exclusively on histopathological examination. These nodes are considered positive for the definition of pN. Although this designation will not influence staging, it is recommended for data collection and future analysis of the impact of incidentally detected micrometastases on outcomes.

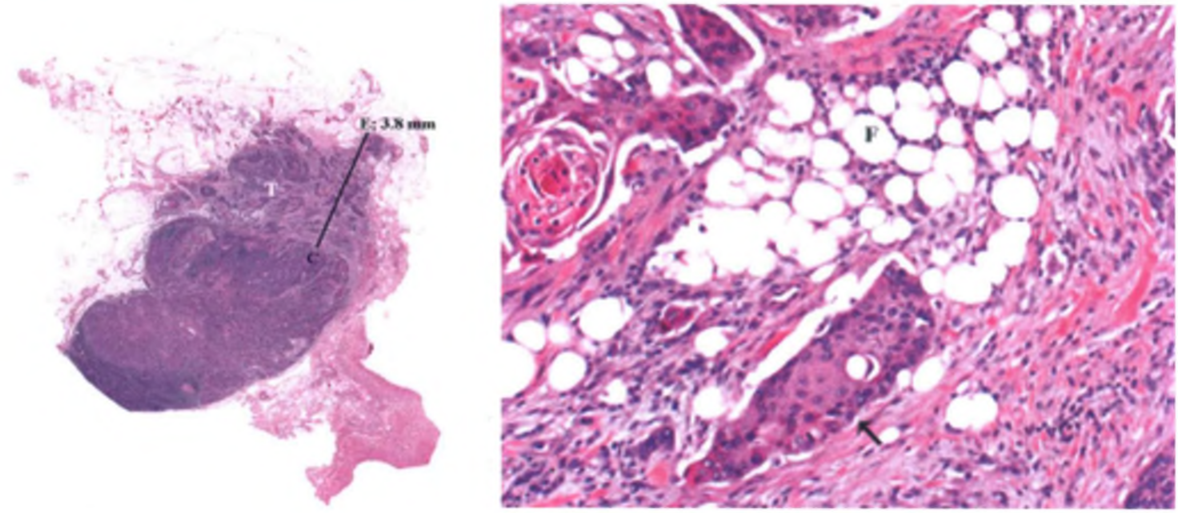

Fig. 6.4 Histologic appearance of major extranodal extension (ENE). (A) Lymph node with metastatic tumor (T) invading perinodal fat. The extent of ENE (3.8 mm) is measured (solid bar) from the outer aspect of the lymph node capsule (C) to the most distant point of perinodal invasion (E). A 1-mm bar is shown for size comparison. (B) Higher power showing the squamous cell carcinoma (arrow) infiltrating in between adipose cells (F). From Wreesmann VB, et. al.[3] with permission

Terminology for disease extension outside lymph nodes

Although terms such as extracapsular spread (ECS), extracapsular extension (ECE), or extranodal involvement (ENI) have been used to denote tumor extension outside the capsule of a metastatic node, extranodal extension (ENE) is the preferred wording.

Definition of ENE and description of its extent

All surgically resected metastatic nodes should be examined for the presence and extent of ENE. The precise definition of ENE has varied in the literature over the course of time. The College of American Pathologists defines ENE as extension of metastatic tumor, present within the confines of the lymph node, through the lymph node capsule into the surrounding connective tissue, with or without associated stromal reaction.

Gross ENE that is evident on clinical examination is designated ENE_c and qualifies as ENE(+) for definition of cN. ENE detected on histopathologic examination is designated as ENE_{mi} (microscopic $ENE \leq 2$ mm) or ENE_{ma} (major $ENE > 2$ mm). Both ENE_{mi} and ENE_{ma} qualify as ENE(+) for definition of pN. These descriptors of ENE will not be required for current pN definition, but data collection is recommended to allow standardization of data collection and future analysis.

Tumor deposits in the lymph drainage area of a primary carcinoma without histological evidence of residual lymph node tissue may represent a lymph node totally replaced by metastatic tumor. Such a nodule should be recorded as a positive lymph node with ENE(+).

Extent of ENE is defined as the maximal distance in millimeters between the outer aspect of the intact or reconstructed nodal capsule and the farthest point of invasion into the extranodal tissue. Figure 6.4 illustrates the method for measurement of extent of ENE.

DEFINITION OF REGIONAL LYMPH NODES (N)

Clinical N (cN)

For patients who are treated with primary nonsurgical treatment without a cervical lymph node dissection.

N Category	N Criteria
NX	Regional lymph nodes cannot be assessed
N0	No regional lymph node metastasis
N1	Metastasis in a single ipsilateral lymph node, 3 cm or smaller in greatest dimension and ENE(−)
N2	Metastasis in a single ipsilateral node larger than 3 cm but not larger than 6 cm in greatest dimension and ENE(−);<br>*or* metastases in multiple ipsilateral lymph nodes, none larger than 6 cm in greatest dimension and ENE(−);<br>*or* in bilateral or contralateral lymph nodes, none larger than 6 cm in greatest dimension, ENE(−)
N2a	Metastasis in a single ipsilateral node larger than 3 cm but not larger than 6 cm in greatest dimension and ENE(−)
N2b	Metastasis in multiple ipsilateral nodes, none larger than 6 cm in greatest dimension and ENE(−)
N2c	Metastasis in bilateral or contralateral lymph nodes, none larger than 6 cm in greatest dimension and ENE(−)
N3	Metastasis in a lymph node larger than 6 cm in greatest dimension and ENE(−);<br>*or* metastasis in any node(s) with clinically overt ENE(+) (ENE_c)[2]

N Category	N Criteria
N3a	Metastasis in a lymph node larger than 6 cm in greatest dimension and ENE(−)
N3b	Metastasis in any node(s) with clinically overt ENE(+) (ENE_c)[2]

Notes:
1. Midline nodes are considered ipsilateral nodes
2. ENE_c is defined as invasion of skin, infiltration of musculature, dense tethering or fixation to adjacent structures, or cranial nerve, brachial plexus, sympathetic trunk, or phrenic nerve invasion with dysfunction
Note: A designation of "U" or "L" may be used for any N category to indicate metastasis above the lower border of the cricoid (U) or below the lower border of the cricoid (L)
Similarly, clinical and pathological ENE should be recorded as ENE(−) or ENE(+)

Pathological N (pN)

For patients who are treated surgically with a cervical lymph node dissection.

N Category	N Criteria
NX	Regional lymph nodes cannot be assessed
N0	No regional lymph node metastasis
N1	Metastasis in a single ipsilateral lymph node, 3 cm or smaller in greatest dimension and ENE(−)
N2	Metastasis in a single ipsilateral or contralateral lymph node, 3 cm or smaller in greatest dimension and ENE(+);<br>*or* single ipsilateral node larger than 3 cm but not larger than 6 cm in greatest dimension and ENE(−);<br>*or* metastases in multiple ipsilateral lymph nodes, none larger than 6 cm in greatest dimension and ENE(−);<br>*or* in bilateral or contralateral lymph nodes, none larger than 6 cm in greatest dimension and ENE(−)
N2a	Metastasis in a single ipsilateral or contralateral node 3 cm or less in greatest dimension and ENE(+);<br>*or* a single ipsilateral node larger than 3 cm but not larger than 6 cm in greatest dimension and ENE(−)
N2b	Metastasis in multiple ipsilateral nodes, none larger than 6 cm in greatest dimension and ENE(−)
N2c	Metastasis in bilateral or contralateral lymph nodes, none larger than 6 cm in greatest dimension and ENE(−)
N3	Metastasis in a lymph node larger than 6 cm in greatest dimension and ENE(−);<br>*or* metastasis in a single ipsilateral node larger than 3 cm in greatest dimension and ENE(+);<br>*or* multiple ipsilateral, contralateral, or bilateral nodes any size and ENE(+) in any node
N3a	Metastasis in a lymph node larger than 6 cm in greatest dimension and ENE(−)
N3b	Metastasis in a single ipsilateral node larger than 3 cm in greatest dimension and ENE(+);<br>*or* multiple ipsilateral, contralateral, or bilateral nodes any size and ENE(+) in any node

Notes:
1. Midline nodes are considered ipsilateral nodes
2. ENE detected on histopathologic examination is designated as ENE_{mi} (microscopic ENE ≤ 2 mm) or ENE_{ma} (major ENE > 2 mm)
Both ENE_{mi} and ENE_{ma} qualify as ENE(+) for definition of pN.
Note: A designation of "U" or "L" may be used for any N category to indicate metastasis above the lower border of the cricoid (U) or below the lower border of the cricoid (L)
Similarly, clinical and pathological ENE should be recorded as ENE(−) or ENE(+)

AJCC PROGNOSTIC STAGE GROUPS

Prognostic Stage Groups for metastatic cervical adenopathy and unknown primary tumor *except* for EBV-related and HPV-related tumors.

When T is...	And N is...	And M is...	Then the stage group is...
T0	N1	M0	III
T0	N2	M0	IVA
T0	N3	M0	IVB
T0	Any N	M1	IVC

6

REGISTRY DATA COLLECTION VARIABLES

1. Extranodal extension for all anatomic sites with the exception of HPV-related oropharynx cancer, nasopharynx cancer, melanoma, sarcoma, and thyroid carcinoma
2. Size of largest metastatic node
3. Number of metastatic lymph nodes
4. Laterality of metastatic nodes; note that midline nodes are considered ipsilateral nodes.
5. Level of nodal involvement
6. ENE clinical (+ or −)
7. ENE pathological (+ or −)

SURVIVAL DATA

The data underpinning inclusion of ENE in the staging system are derived from histopathological examination of neck dissection specimens in patients treated surgically for their head and neck cancer. The modification is based on analysis of a large National Cancer Data Base (NCDB) data set, including cases with squamous cell carcinoma of the head and neck, with the exception of HPV-related oropharynx cancer and nasopharynx cancer (Fig. 6.5). The new N category was then tested for validation on another large collaborative data set from Memorial Sloan Kettering Cancer Center, New York, and Princess Margaret Hospital, Toronto, for surgically treated oral cancer patients (Fig. 6.6 and Table 6.3). The lack of ENE data in patients treated by nonsurgical modalities is problematic because currently available radiographic techniques are

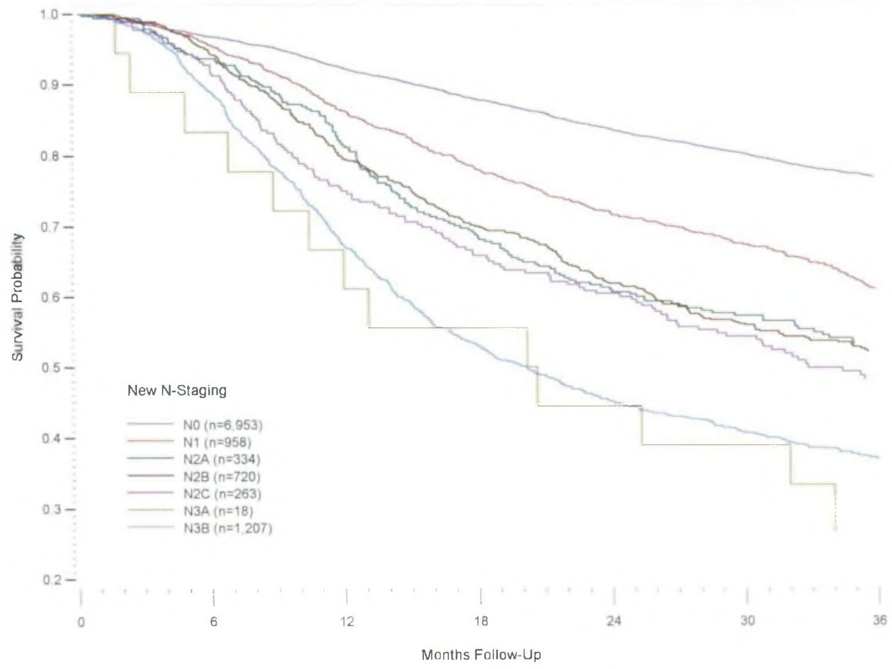

Fig. 6.5 Overall Survival in squamous cell carcinoma of the head and neck based on the 8th edition N category criteria that incorporate ENE as a prognostic factor. Kaplan Meier methods were used to perform cancer-specific analyses predicting overall survival as the endpoint on a population of lip and oral cavity cancer patients from NCDB

not sensitive enough to detect microscopic or less than gross ENE. Therefore, only clinically obvious ENE should be used for definition of cN when the patient is treated with nonsurgical therapy. This inability of current technology to reliably identify minimal or microscopic ENE without pathological examination of lymph node dissection specimens was the basis for separate cN and pN approaches for staging the neck. Clinically overt ENE (ENE_c) will be designated cN3b irrespective of any other nodal characteristic in patients treated without neck dissection. Histologically identified ENE (ENE_{mi} or ENE_{ma}) in a neck dissection specimen will be used in conjunction with node size and laterality for pN: Histopathologically confirmed ENE in a single ipsilateral or contralateral metastatic node 3 cm or smaller in largest dimension upstages the patient to pN2a, while all other nodes with histopathologically detected ENE are categorized pN3b.

Up-to-date cancer registry data on the influence of the new N criteria on outcomes are not available because ENE is only now being introduced into the nodal staging system. Limited data are available, however, from the NCDB for patients treated in 2010–11 for squamous cell carcinoma at sites other than nasopharynx and HPV-related oropharynx cancer (Fig. 6.5). The proposed new N classification was then validated in a large dataset of patients with oral cancer treated at two tertiary cancer care centers in North America. (Fig. 6.6 and Table 6.3).

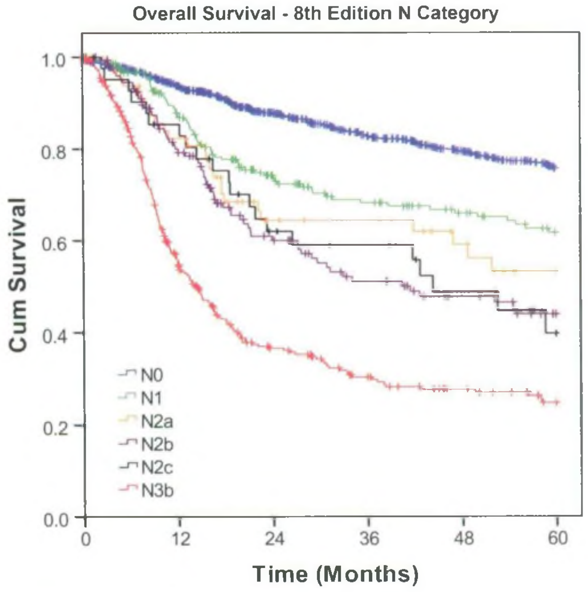

Fig. 6.6 Overall Survival based on 8th edition N criteria that incorporate ENE as a prognostic factor. Kaplan Meier methods were used to perform cancer-specific analyses predicting overall survival as the endpoint on a population of oral cavity cancer patients from MSKCC and PMH

Table 6.3 Overall survival based on 8th Edition N category criteria (MSKCC-PMH Data)

# of pts at Risk	0 Months	12 Months	24 Months	36 Months	48 Months	60 Months
**N0**	1018	870	710	596	513	421
**N1**	211	168	119	97	82	70
**N2a**	66	50	30	28	21	13
**N2b**	148	107	65	49	39	29
**N2c**	42	34	22	19	12	8
**N3b**	303	146	81	59	43	31

Bibliography

1. O'Sullivan B, Huang SH, Su J, et al. Development and validation of a staging system for HPV-related oropharyngeal cancer by the International Collaboration on Oropharyngeal cancer Network for Staging (ICON-S): a multicentre cohort study. *The lancet oncology.* Feb 26 2016
2. Patel S. Personal Communication. In: Lydiatt W, Shah JP, eds.2015
3. Wreesmann VB, Katabi N, Palmer FL, et al. Influence of extracapsular nodal spread extent on prognosis of oral squamous cell carcinoma. *Head & neck.* Oct 30 2015
4. Robbins KT, Clayman G, Levine PA, et al. Neck dissection classification update: revisions proposed by the American Head and Neck Society and the American Academy of Otolaryngology-Head and Neck Surgery. *Archives of otolaryngology–head & neck surgery.* Jul 2002;128(7):751–758.
5. Agrawal A, Civantos FJ, Brumund KT, et al. [99mTc] Tilmanocept Accurately Detects Sentinel Lymph Nodes and Predicts Node Pathology Status in Patients with Oral Squamous Cell Carcinoma of the Head and Neck: Results of a Phase III Multi-institutional Trial. *Annals of surgical oncology.* 2015:1–8
6. Alkureishi LW, Ross GL, Shoaib T, et al. Sentinel node biopsy in head and neck squamous cell cancer: 5-year follow-up of a European multicenter trial. *Annals of surgical oncology.* Sep 2010;17(9):2459–2464.
7. Civantos FJ, Zitsch RP, Schuller DE, et al. Sentinel lymph node biopsy accurately stages the regional lymph nodes for T1-T2 oral squamous cell carcinomas: results of a prospective multi-institutional trial. *J Clin Oncol.* Mar 10 2010;28(8):1395–1400.
8. Curtin HD, Ishwaran H, Mancuso AA, Dalley RW, Caudry DJ, McNeil BJ. Comparison of CT and MR imaging in staging of neck metastases. *Radiology.* Apr 1998;207(1):123–130.
9. de Juan J, Garcia J, Lopez M, et al. Inclusion of extracapsular spread in the pTNM classification system: a proposal for patients with head and neck carcinoma. *JAMA otolaryngology– head & neck surgery.* May 2013;139(5):483–488.

0. Ebrahimi A, Gil Z, Amit M, et al. The prognosis of N2b and N2c lymph node disease in oral squamous cell carcinoma is determined by the number of metastatic lymph nodes rather than laterality: evidence to support a revision of the American Joint Committee on Cancer staging system. *Cancer.* 2014;120(13):1968–1974.

1. Ebrahimi A GZ, Amit M, Yen TC, Liao CT, Chatturvedi P, Agarwal J, Kowalski L, Kreppel M, Cernea C, Brandao J, Bachar G, Villaret AB, Fliss D, Fridman E, Robbins KT, Shah J, Patel S, Clark J; International Consortium for Outcome Research (ICOR) in Head and Neck Cancer. Comparison of the American Joint Committee on Cancer N1 versus N2a nodal categories for predicting survival and recurrence in patients with oral cancer: Time to acknowledge an arbitrary distinction and modify the system. *Head and neck pathology.* 2014

12. Gregoire V, Ang K, Budach W, et al. Delineation of the neck node levels for head and neck tumors: a 2013 update. DAHANCA, EORTC, HKNPCSG, NCIC CTG, NCRI, RTOG, TROG consensus guidelines. *Radiotherapy and oncology : journal of the European Society for Therapeutic Radiology and Oncology.* Jan 2014;110(1):172–181.
13. Hoang JK, Vanka J, Ludwig BJ, Glastonbury CM. Evaluation of cervical lymph nodes in head and neck cancer with CT and MRI: tips, traps, and a systematic approach. *AJR. American journal of roentgenology.* Jan 2013;200(1):W17–25.
14. Jones A, Roland N, Field J, Phillips D. The level of cervical lymph node metastases: their prognostic relevance and relationship with head and neck squamous carcinoma primary sites. *Clinical Otolaryngology & Allied Sciences.* 1994;19(1):63–69.
15. Jose J, Moor JW, Coatesworth AP, Johnston C, MacLennan K. Soft tissue deposits in neck dissections of patients with head and neck squamous cell carcinoma: prospective analysis of prevalence, survival, and its implications. *Archives of otolaryngology–head & neck surgery.* Feb 2004;130(2):157–160.
16. King AD, Tse GM, Yuen EH, et al. Comparison of CT and MR imaging for the detection of extranodal neoplastic spread in metastatic neck nodes. *Eur J Radiol.* Dec 2004;52(3):264–270.
17. Kyzas PA, Evangelou E, Denaxa-Kyza D, Ioannidis JP. 18F-fluorodeoxyglucose positron emission tomography to evaluate cervical node metastases in patients with head and neck squamous cell carcinoma: a meta-analysis. *Journal of the National Cancer Institute.* May 21 2008;100(10):712–720.
18. Lodder WL, Lange CA, van Velthuysen M-LF, et al. Can extranodal spread in head and neck cancer be detected on MR imaging. *Oral oncology.* 2013;49(6):626–633.
19. Medina JE. A rational classification of neck dissections. *Otolaryngology–head and neck surgery: official journal of American Academy of Otolaryngology-Head and Neck Surgery.* Mar 1989;100(3):169–176.
20. Patel SG, Amit M, Yen TC, et al. Lymph node density in oral cavity cancer: results of the International Consortium for Outcomes Research. *Br J Cancer.* Oct 15 2013;109(8):2087–2095.
21. Prabhu RS, Magliocca KR, Hanasoge S, et al. Accuracy of computed tomography for predicting pathologic nodal extracapsular extension in patients with head-and-neck cancer undergoing initial surgical resection. *International journal of radiation oncology, biology, physics.* Jan 1 2014;88(1):122–129.
22. Saindane AM. Pitfalls in the staging of cervical lymph node metastasis. *Neuroimaging Clin N Am.* Feb 2013;23(1):147–166.
23. Shah JP. Patterns of cervical lymph node metastasis from squamous carcinomas of the upper aerodigestive tract. *American journal of surgery.* Oct 1990;160(4):405–409.

24. Shah JP, Medina JE, Shaha AR, Schantz SP, Marti JR. Cervical lymph node metastasis. *Curr Probl Surg.* Mar 1993;30(3):1–335.
25. Som PM, Curtin HD, Mancuso AA. An imaging-based classification for the cervical nodes designed as an adjunct to recent clinically based nodal classifications. *Archives of Otolaryngology–Head & Neck Surgery.* 1999;125(4):388–396.
26. Url C, Schartinger VH, Riechelmann H, et al. Radiological detection of extracapsular spread in head and neck squamous cell carcinoma (HNSCC) cervical metastases. *Eur J Radiol.* Oct 2013;82(10):1783–1787.
27. van den Brekel MW, Lodder WL, Stel HV, Bloemena E, Leemans CR, van der Waal I. Observer variation in the histopathologic assessment of extranodal tumor spread in lymph node metastases in the neck. *Head & neck.* Jun 2012;34(6):840–845.

Lip and Oral Cavity

7

John A. Ridge, William M. Lydiatt, Snehal G. Patel,
Christine M. Glastonbury, Margaret Brandwein-Gensler,
Ronald A. Ghossein, and Jatin P. Shah

CHAPTER SUMMARY

Cancers Staged Using This Staging System

Epithelial and minor salivary gland cancers of the lip and oral cavity

Cancers Not Staged Using This Staging System

These histopathologic types of cancer...	Are staged according to the classification for...	And can be found in chapter...
Nonepithelial tumors of lymphoid tissue	Hematologic malignancies	78–83
Nonepithelial tumors of soft tissue	Soft tissue sarcoma of the head and neck	40
Nonepithelial tumors of bone and cartilage	Bone	38
Mucosal melanoma	Mucosal melanoma of the head and neck	14
Cutaneous squamous cell carcinoma of the vermilion lip	Cutaneous squamous cell carcinoma of the head and neck	15

Summary of Changes

Change	Details of Change	Level of Evidence
Anatomy – Primary Site(s)	Occult Primary Tumor: Staging of the patient who presents with EBV-unrelated and HPV-unrelated metastatic cervical lymphadenopathy is not included in this chapter.	IV
Definition of Primary Tumor (T)	Clinical and pathological depth of invasion (DOI) are now used to increase the T category.	III
Definition of Primary Tumor (T)	Extrinsic tongue muscle invasion is no longer used in T4 because this is a feature of DOI.	III
Definition of Regional Lymph Node (N)	Separate N staging approaches have been developed for HPV-related and HPV-unrelated cancers.	II[1,2]
Definition of Regional Lymph Node (N)	Separate N category approaches have been developed for patients treated without cervical lymph node dissection (clinical cN) and patients treated with cervical lymph neck dissection (pathological pN).	II[1,2]
Definition of Regional Lymph Node (N)	Extranodal extension (ENE) is introduced as a descriptor in all HPV-unrelated cancers.	II[2]
Definition of Regional Lymph Node (N)	ENE in HPV negative cancers: Only clinically and radiographically overt ENE—ENE(+)—should be used for cN.	II[2]
Definition of Regional Lymph Node (N)	ENE in HPV negative cancers: Any pathologically detected ENE is considered ENE(+) and is used for pN.	II[2]

To access the AJCC cancer staging forms, please visit www.cancerstaging.org.

© American Joint Committee on Cancer 2017

M.B. Amin et al. (eds.), *AJCC Cancer Staging Manual, Eighth Edition*, DOI 10.1007/978-3-319-40618-3_7

Change	Details of Change	Level of Evidence
Definition of Regional Lymph Node (N)	ENE in HPV-negative cancers: Presence of ENE is designated pN2a for a single ipsilateral node < 3 cm and pN3b for all other node(s).	II[2]
Definition of Regional Lymph Node (N)	Classification of ENE: Clinically overt ENE is classified as ENE_c and is considered ENE(+) for cN.	III[3]
Definition of Regional Lymph Node (N)	Classification of ENE: Pathologically detected ENE is classified as either ENE_{mi} ($\leq$2 mm) or ENE_{ma} (>2 mm) for data collection purposes only, but both are considered ENE(+) for definition of pN.	III[3]

ICD-O-3 Topography Codes

Code	Description
C00.0	External upper lip (exclude vermilion border)
C00.1	External lower lip (exclude vermilion border)
C00.2	External lip NOS (exclude vermilion border)
C00.3	Mucosa of upper lip
C00.4	Mucosa of lower lip
C00.5	Mucosa of lip, NOS
C00.6	Commissure of lip
C00.8	Overlapping lesion of lip
C00.9	Lip, NOS
C02.0	Dorsal surface of tongue, NOS
C02.1	Border of tongue
C02.2	Ventral surface of tongue, NOS
C02.3	Anterior two-thirds of tongue, NOS
C02.8	Overlapping lesion of tongue
C02.9	Tongue, NOS
C03.0	Upper gum
C03.1	Lower gum
C03.9	Gum, NOS
C04.0	Anterior floor of mouth
C04.1	Lateral floor of mouth
C04.8	Overlapping lesion of floor of mouth
C04.9	Floor of mouth, NOS
C05.0	Hard palate
C05.8	Overlapping lesion of palate
C05.9	Palate, NOS
C06.0	Cheek mucosa
C06.1	Vestibule of mouth
C06.2	Retromolar area
C06.8	Overlapping lesion of other and unspecified parts of mouth
C06.9	Mouth, NOS

WHO Classification of Tumors

Code	Description
8070	Squamous cell carcinoma, conventional
8075	Acantholytic squamous cell carcinoma
8560	Adenosquamous carcinoma
8083	Basaloid squamous cell carcinoma
8051	Carcinoma cuniculatum
8052	Papillary squamous cell carcinoma
8074	Spindle cell squamous carcinoma
8051	Verrucous carcinoma
8082	Lymphoepithelial carcinoma
8550	Acinic cell carcinoma
8430	Mucoepidermoid carcinoma
8200	Adenoid cystic carcinoma
8525	Polymorphous adenocarcinoma
8147	Basal cell adenocarcinoma
8562	Epithelial-myoepithelial carcinoma
8310	Clear cell carcinoma
8480	Mucinous adenocarcinoma
8290	Oncocytic carcinoma
8500	Salivary duct carcinoma
8982	Myoepithelial carcinoma

Barnes L, Eveson JW, Reichart P, Sidransky D, eds. World Health Organization Classification of Tumours Pathology and Genetics of Head and Neck Tumours. Lyon: IARC; 2005.

INTRODUCTION

Cancers of the oral cavity continue to represent a major problem worldwide. The American Joint Committee on Cancer (AJCC) Cancer Staging Manual, 8th Edition (8th Edition), makes two significant changes based upon enhanced understanding of the behavior of these malignancies.

The first modification is in T categorization incorporating depth of invasion (DOI). It is important to recognize the distinction between tumor thickness and true DOI. A detailed description of how this should be measured is included in this chapter. It has been recognized since the early work of Spiro and colleagues, in the mid-1980s, that prognosis of oral cancers worsens as the tumor grows thicker, similar to skin malignancies.[4,5] The somewhat more sophisticated measure of DOI has been recorded for oral cavity cancers since the AJCC Cancer Staging Manual, 6th Edition. Extrinsic muscle infiltration is no longer a staging criterion for T4 designation because DOI supersedes it and extrinsic muscle invasion is difficult to assess (clinically and pathologically). Clinicians experienced with head and neck

cancer will generally have few problems identifying a superficial and less invasive lesion ($\leq$5 mm) from those of moderate depth (from>5 to$\leq$10 mm) or deeply invasive cancers (>10 mm) through examination alone. Such experts have estimated the maximum dimensions for complicated lesions of the tonsil or palate for many years. The guiding principle, if there are doubts, is to select the less ominous attribute (a lesser depth) in a given case to avoid stage migration (according to the uncertain rule of the AJCC/Union for International Cancer Control (UICC) TNM, as defined in Chapter 1).

A second significant change is in the use of extranodal extension (ENE) in categorizing metastatic cancer to neck nodes. The effect of ENE on prognosis in head and neck cancers is profound, except for those tumors associated with HPV.[5] Including this important prognostic feature was considered critical in revising staging. Most of the data supporting ENE as an adverse prognostic factor is based on histopathological characterization of ENE, especially the distinction between microscopic and macroscopic ENE.[6–8] Only unquestionable ENE is to be used for clinical staging (as in the uncertain rule above). For clinical ENE, the known limitations of current imaging modalities to define ENE accurately demand that stringent criteria be met prior to assigning a clinical diagnosis of ENE. However, unambiguous evidence of gross ENE on clinical examination (e.g., multiple matted nodes, invasion of skin, infiltration of musculature/dense tethering to adjacent structures, or cranial nerve, brachial plexus, sympathetic trunk, or phrenic nerve invasion with dysfunction) supported by strong radiographic evidence permits classification of disease as ENE(+). Pathological ENE also will be clearly defined as extension of metastatic tumor (present within the confines of the lymph node, through the lymph node capsule into the surrounding connective tissue, with or without associated stromal reaction). Again if there is doubt or uncertainty of the presence of ENE, the case should be categorized as ENE(−).

A staging system revision should address and respond to new information that influences patient outcome. An appropriate balance between complexity and utility (ease-of-use) is necessary for universal acceptance. The TNM system for oral cancers has strongly predicted prognosis and is applied worldwide. The introduction of two new parameters in oral cavity staging, DOI and ENE, better fits the prognostic modeling from large datasets. However, it must be balanced by the ability to derive accurate information from clinicians caring for patients with head and neck cancer in many different environments. Therefore, thorough descriptions of ENE and DOI are included in this chapter.

Effectively, DOI will increase the T category by 1 for each 5 mm of tumor depth (until$\geq$10 mm). Pathological ENE(+) will increase the nodal category by 1.

ANATOMY

Primary Site(s)

The oral cavity extends from the skin–vermilion junction of the lips to the junction of the hard and soft palate above, to the line of circumvallate papillae below, and to the anterior tonsillar pillars laterally. It is additionally divided into multiple specific sites listed below (Figs. 7.1–7.4).

Mucosal Lip

The lip begins at the junction of the vermilion border with the skin and includes only the vermilion surface or that portion of the lip that comes into contact with the opposed lip. The remainder of the vermillion is staged using the skin chapter (Chapter 47). It is subdivided into an upper and lower lip, joined at the commissures of the mouth.

Buccal Mucosa

The buccal mucosa includes all the mucous membrane lining of the inner surface of the cheeks and lips from the line of contact of the opposing lips to the line of attachment of mucosa of the alveolar ridge (upper and lower) and pterygomandibular raphe.

Lower Alveolar Ridge

The lower alveolar ridge refers to the mucosa overlying the alveolar process of the mandible, which extends from the line of attachment of mucosa in the lower gingivobuccal sulcus to the line of attachment of free mucosa of the floor of the mouth. Posteriorly, it extends to the ascending ramus of the mandible.

Upper Alveolar Ridge

The upper alveolar ridge refers to the mucosa overlying the alveolar process of the maxilla, which extends from the line of attachment of mucosa in the upper gingivobuccal sulcus to the junction of the hard palate. Its posterior margin is the upper end of the pterygopalatine arch.

Retromolar Gingiva (Retromolar Trigone)

The retromolar gingiva, or retromolar trigone, is the attached mucosa overlying the ascending ramus of the mandible from the level of the posterior surface of the last lower molar tooth to the apex superiorly, adjacent to the tuberosity of the maxilla.

Floor of the Mouth

The floor of the mouth is a crescentic surface overlying the mylohyoid and hyoglossus muscles, extending from the inner surface of the lower alveolar ridge to the undersurface of the tongue. Its posterior boundary is the base of the anterior pillar of the tonsil. It is divided into two sides by the

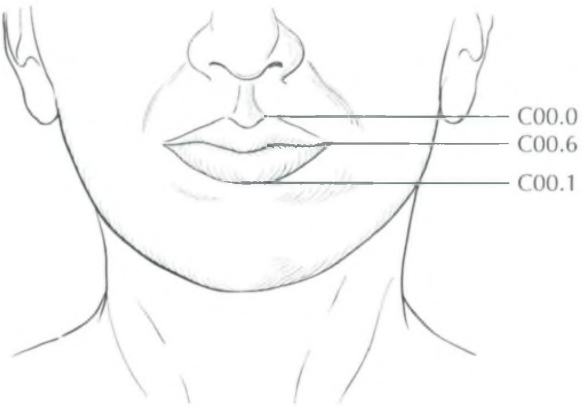

Fig. 7.1 Anatomical subsites of the lip

frenulum of the tongue and harbors the ostia of the submandibular and sublingual salivary glands.

Hard Palate

The hard palate is the semilunar area between the upper alveolar ridge and the mucous membrane covering the palatine process of the maxillary palatine bones. It extends from the inner surface of the superior alveolar ridge to the posterior edge of the palatine bone.

Anterior Two-Thirds of the Tongue (Oral Tongue)

The anterior two-thirds of the tongue is the freely mobile portion of the tongue that extends anteriorly from the line of circumvallate papillae to the undersurface of the tongue at the junction with the floor of the mouth. It is composed of four areas: the tip, the lateral borders, the dorsum, and the undersurface (nonvillous ventral surface of the tongue). The undersurface of the tongue is considered a separate category by the World Health Organization (WHO).

Regional Lymph Nodes

The risk of regional metastasis is generally related to the T category. In general, cervical lymph node involvement from oral cavity primary sites is predictable and orderly, spreading from the primary to upper, then middle, and subsequently lower cervical nodes. Any previous treatment of the neck, through surgery or radiation, may alter normal lymphatic drainage patterns and result in unusual dissemination of disease to the cervical lymph nodes. Cancer of the lip, with a low metastatic risk, initially involves adjacent submental and submandibular nodes, then jugular nodes. Cancers of the hard palate likewise have a low metastatic potential and involve buccinator, pre-vascular facial and submandibular, jugular, and, occasionally, retropharyngeal nodes. Other oral cancers spread primarily to submandibular and jugular nodes and uncommonly to posterior triangle/supraclavicular nodes. Cancer of the anterior oral tongue may occasionally spread directly to lower jugular nodes. The closer the primary is to the midline, the greater the propensity for bilateral cervical nodal spread. Although patterns of regional lymph node metastases are typically predictable and sequential, disease in the anterior oral cavity also may spread directly to bilateral or mid-cervical lymph nodes.

Metastatic Sites

The lungs are the most common site of distant metastases; skeletal and hepatic metastases occur less often. Mediastinal lymph node metastases are considered distant metastases, except level VII lymph nodes (anterior superior mediastinal lymph nodes cephalad of the innominate artery).

RULES FOR CLASSIFICATION

Clinical Classification

Clinical staging for Lip and Oral Cavity cancers is predicated most strongly upon the history and physical examination. Biopsy is necessary to confirm diagnosis and is typically done of the primary. Nodal biopsy is done by fine needle aspiration when indicated. Results from diagnostic biopsy of the primary tumor, regional nodes, and distant metastases can be included in clinical classification.

Inspection of the lip and oral cavity typically reveals the greatest diameter of a cancer, though palpation is essential to assess DOI and submucosal extension. The mucosal extent of the cancer usually reflects its true linear dimension. Induration surrounding a cancer typically is due to peritumoral inflammation. DOI should be distinguished from tumor thickness, and its determination is predicated on invasion beneath the plane defined by surrounding normal mucosa. Any exophytic character should be noted, but assignment of stage is determined by what transpires at or beneath the surface (defined by adjacent normal mucosa). Clinical evidence of bone destruction should be noted and its depth estimated (e.g., into bone versus through cortex into the marrow space). Thick lesions often are defined by computed tomography (CT) or magnetic resonance (MR) imaging, but the difference between thickness and DOI must be observed. Lesions located near the midline more often involve the contralateral side of the neck than well-lateralized cancers. Dysphagia is suggestive of a tumor with sufficient invasion of oral structures to engender dysfunction. It is seldom present when cancers have little DOI. Similarly,

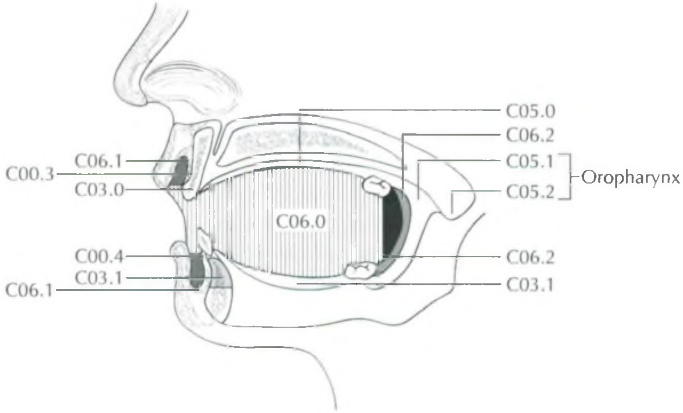

Fig. 7.2 Anatomical sites and subsites of the oral cavity

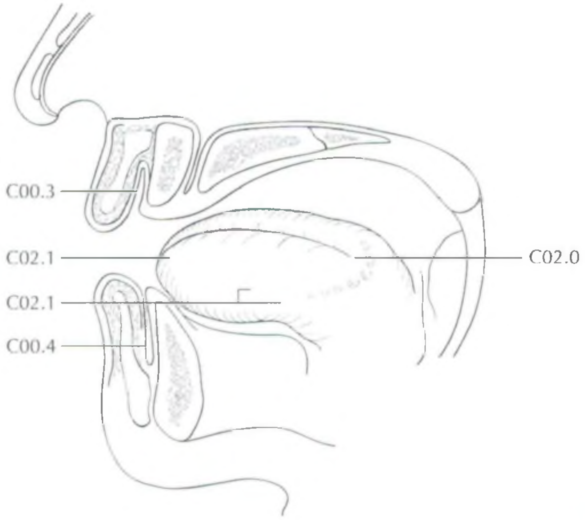

Fig. 7.3 Anatomical sites and subsites of the oral cavity

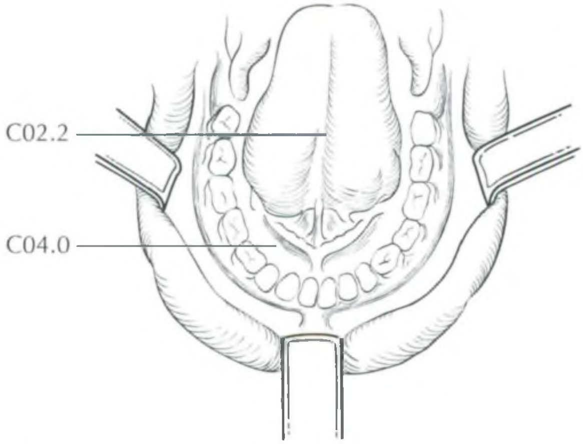

Fig. 7.4 Anatomical sites and subsites of the oral cavity

drooling or the inability to swallow liquids without difficulty suggests a tumor with substantial DOI. Trismus, when not caused by pain, is consistent with a deeply invasive lesion. Complaints of numbness of the lip and/or teeth are commonly associated with nerve invasion. The distinction between 4 mm DOI and 6 mm DOI (for example) may not be possible on clinical grounds. The stage should only be raised on the basis of DOI if the differences are clear.

Evidence of cranial nerve dysfunction should be sought (testing sensation and motion to command) and skin should be examined for evidence of invasion by underlying nodes. Palpable neck nodes should be considered in terms of their location (level in the neck), size, number, character (smooth or irregular), attachment to other nodes, and mobility. Nodes that do not move in all directions may be invading nearby structures. Invasion of the sternomastoid muscle and/or cranial nerves is associated with lateral motion with restricted ability to move the node along the cranial-caudal axis. Inability to move the node at all (without moving the head) is worrisome for ENE, though the suspicion should be tempered for smaller nodes with limited mobility in level II. Assignment of ENE should be based almost entirely upon the physical examination, rather than upon imaging studies; gross ENE is required to raise the stage beyond the assignment based upon node size and number, and this may be overestimated with current imaging modalities.

Clinical or radiographic extranodal extension

ENE worsens the adverse outcome associated with nodal metastasis. The presence of ENE can be diagnosed clinically by the presence of a "matted" mass of nodes, involvement of overlying skin, adjacent soft tissue, or clinical signs

of cranial nerve or brachial plexus, sympathetic chain or phrenic nerve invasion. Cross-sectional imaging (CT or MR) generally has low sensitivity (65–80 %) but high specificity (86–93 %) for the detection of ENE. The most reliable imaging signs are an indistinct nodal margin, irregular nodal capsular enhancement or infiltration into the adjacent fat or muscle, with the latter finding on CT and MR imaging as the most specific sign of ENE. Ultrasound appears to be less accurate than CT and MR imaging, but ENE is suggested by interrupted or undefined nodal contours with high-resolution ultrasound imaging. The absence or presence of clinical/radiologic ENE is designated ENE(−) or ENE(+), respectively.

Imaging

Cross-sectional imaging of the oral cavity may be performed with either CT or MR imaging, depending on availability, patient imaging tolerance, contrast allergies, and cost. With either modality, the coronal plane view—either as direct MR imaging or from reformats obtained from axially acquired thin-slice CT—allows excellent evaluation of the floor of the mouth.[9] CT offers some advantage over MR imaging in the evaluation of cortical bone erosion, although MR imaging appears to be more sensitive but less specific for the detection of bone marrow invasion by tumor.[10,11] MR imaging offers the additional advantage of evaluation of perineural tumor spread, which for oral cavity tumors is primarily along the inferior alveolar nerve (CNV3) of the mandible and the greater and lesser palatine nerves (CNV2) of the maxilla. Gadolinium contrast is always recommended unless contraindicated by prior reaction or very poor renal function. Positron emission tomography (PET)/CT is primarily done for nodal staging of disease or when distant metastases are suspected, unless the CT component is performed as a post-contrast examination with dedicated neck imaging. Ultrasound does not allow adequate evaluation of the oral cavity primary tumor site, but it may be supplementary for nodal evaluation with otherwise equivocal nodal imaging findings.

As small but clinically evident mucosal tumors may be subtle on imaging, it is important to review the imaging exam with knowledge of the tumor site. T1, T2, and T3 tumors are distinguished only by size and depth of invasion. The former is better determined by clinical examination, although a radiologic measurement should be given as part of the imaging report. The radiologist's more important role during tumor staging is to determine deep tissue involvement and assess for nodal and/or distant metastases. T4 disease entails deep tissue invasion, which varies according to the specific subsite of the oral cavity. For alveolar ridge, floor of mouth, retromolar triangle, hard palate, and large lip tumors, careful attention should be paid to the cortex and marrow space of the adjacent maxilla or mandible, because such invasion denotes T4a disease. In the AJCC Cancer Staging Manual, 7th Edition, oral tongue tumors were designated T4a when there was deep invasion into the extrinsic muscles of the tongue and/or the floor of the mouth. DOI will supersede muscle invasion in the 8th Edition. Depth is frequently better evaluated in the coronal plane and/or sagittal plane. More posterior extensive spread of tumor—such as buccal tumors invading into the muscles of mastication, or spreading to the pterygoid plates or superiorly to the skull base—denotes T4b tumor. Additionally, posterolateral tumor spread to surround the internal carotid artery is also T4b disease.

Both CT and MR imaging allow evaluation of nodal morphology to determine possible tumor involvement. Levels IA, IB, and IIA are the most frequently involved sites, and these levels should be scrutinized specifically with concern for rounded contour, heterogeneous texture including cystic or necrotic change, enlargement, and ill-defined margins. It also is important to be cognizant that nodal spread may be bilateral, particularly with anterior and/or midline oral cavity tumors. Skip nodal metastases (level IV without level III involvement) while described with lateral tongue tumors, appear to be rare. As previously described, PET/CT may also be used to improve predictive yield for nodal metastases by the addition of physiologic information, and ultrasound may be an additive tool for evaluation of indeterminate nodes. PET/CT is the only modality to allow whole-body evaluation of distant metastatic spread, and the upper lungs and bone should always be reviewed as potential metastatic sites on any staging neck CT or MR imaging.

The risk of distant metastasis is more dependent on the N than on the T status of the head and neck cancer. In addition to the node size, number, and presence of ENE, regional lymph nodes also should be described according to the level of the neck that is involved. The level of involved nodes in the neck is prognostically significant for oral cavity (caudad nodal disease is worse), as is the presence of ENE metastatic tumor from individual nodes. Midline nodes are considered ipsilateral. Imaging studies showing amorphous spiculated margins of involved nodes or involvement of internodal fat resulting in loss of normal oval-to-round nodal shape strongly suggest extranodal extension; however, pathological examination is necessary to prove its presence. No imaging study can currently identify microscopic foci of cancer in regional nodes or distinguish between small reactive nodes and small nodes with metastatic deposits (in the absence of central radiographic inhomogeneity).

Pathological Classification

Complete resection of the primary site and/or regional lymph node dissections, followed by pathological examination of

the resection specimen allows for the use of this designation for pT and/or pN, respectively. Resections after radiation or chemotherapy should be identified and considered in context. pT is derived from the actual measurement of the unfixed tumor in the surgical specimen. It should be noted, however, that up to 30% shrinkage of soft tissues may occur in resected specimen after formalin fixation. Pathological staging represents additional and important information and should be included as such in staging, but it does not supplant clinical staging as the primary staging scheme. Metastasis found on imaging is considered cM1. Biopsy-proven metastasis is considered pM1.

Pathological assessment of ENE

Resected positive lymph nodes require examination for the presence and extent of ENE. ENE_{mi} is defined as microscopic ENE ≤ 2 mm. Macroscopic ENE (ENE_{ma}) is defined as either extranodal extension apparent to the naked eye at the time of dissection or extension > 2 mm beyond the lymph node capsule microscopically. Only ENE_{ma} is used to define pathological ENE(+) nodal status.

For assessment of pN, a selective neck dissection will ordinarily include 10 or more lymph nodes, and a comprehensive neck dissection (radical or modified radical neck dissection) will ordinarily include 15 or more lymph nodes. Examination of fewer tumor-free nodes still mandates a pN0 designation.

PROGNOSTIC FACTORS

Prognostic Factors Required for Stage Grouping

Beyond the factors used to assign T, N, or M categories, no additional prognostic factors are required for stage grouping.

Additional Factors Recommended for Clinical Care

Extranodal Extension

ENE is defined as extension of metastatic carcinoma within lymph node, through the capsule, and into the surrounding connective tissue, regardless of associated stromal reaction. Histopathologic designations for ENE are as follows:

- ENE_n (none)
- ENE_{mi} (microscopic ENE ≤ 2 mm)
- ENE_{ma} (ENE > 2 mm or gross ENE)

Only ENE_{ma} is used to define pathological ENE nodal status (Fig. 7.5). ENE_{mi} versus ENE_n will not affect current nodal staging, but data collection is recommended to allow standardization of data collection and future analysis.

Depth of Invasion

DOI assesses the invasiveness of a carcinoma, regardless of any exophytic component. It is measured by first finding the "horizon" of the basement membrane of the adjacent squamous mucosa (Fig. 7.6). A perpendicular "plumb line" is established from this horizon to the deepest point of tumor invasion, which represents DOI. The DOI is recorded in millimeters. Measurements in millimeters can easily be accomplished by printing rulers on acetate sheets, which can be overlaid onto glass slides. Figure 7.7 demonstrates DOI of an ulcerated carcinoma.

Resection Margins

The ideal manner of intraoperative margin assessment is the "specimen driven approach."[12,13] Direct discussion between surgeon and pathologist at specimen hand-off allows for correct anatomic orientation and identification of any intraoperative non-margin tissue tears or cuts. The pathologist maps the specimen, paints the different margin planes with unique colors, and documents the designations. In the event of non-margin tissue tears, these non-margins should be inked first using a unique color (e.g., yellow). This obviates the problem of ink running. The pathologist then makes multiple cuts into the margins at 5- to 10-mm intervals perpendicular to the resection plane. Initial gross assessment yields important preliminary information. This is followed by targeted microscopic examination of margins of interest. The margin sections should be taken perpendicular to the resection plane. The distance between carcinoma and resection margin should be reported in millimeters (Fig. 7.8).

Worst Pattern of Invasion

Worst pattern of invasion (WPOI) is a validated outcome predictor for oral cavity squamous carcinoma patients in multivariate analysis.[14–16] To simplify prognostication and enhance adaptation, the only cutpoint recommended for assessment is whether or not WPOI-5 is present. WPOI-5 is defined as tumor dispersion of ≥ 1 mm between tumor satellites. With respect to low-stage oral cavity squamous carcinomas > 4 mm DOI, the presence of WPOI-5 is significantly predictive of locoregional recurrence and disease-specific survival ($p = 0.0008$, HR 2.55, 95% CI 1.48, 4.41, and $p = 0.0001$, HR 6.34, 95% CI 2.50, 16.09, respectively) and the probability of developing locoregional recurrence is almost 42%. Figures 7.8 and 7.9 illustrate examples of WPOI-5. Tumor dispersion is assessed at the advancing tumor edge. The most common WPOI-5 phenotype is tumor dispersion through soft tissue. Dispersed extratumoral perineural invasion, or extratumoral lymphovascular invasion, also can qualify for classification as WPOI-5.

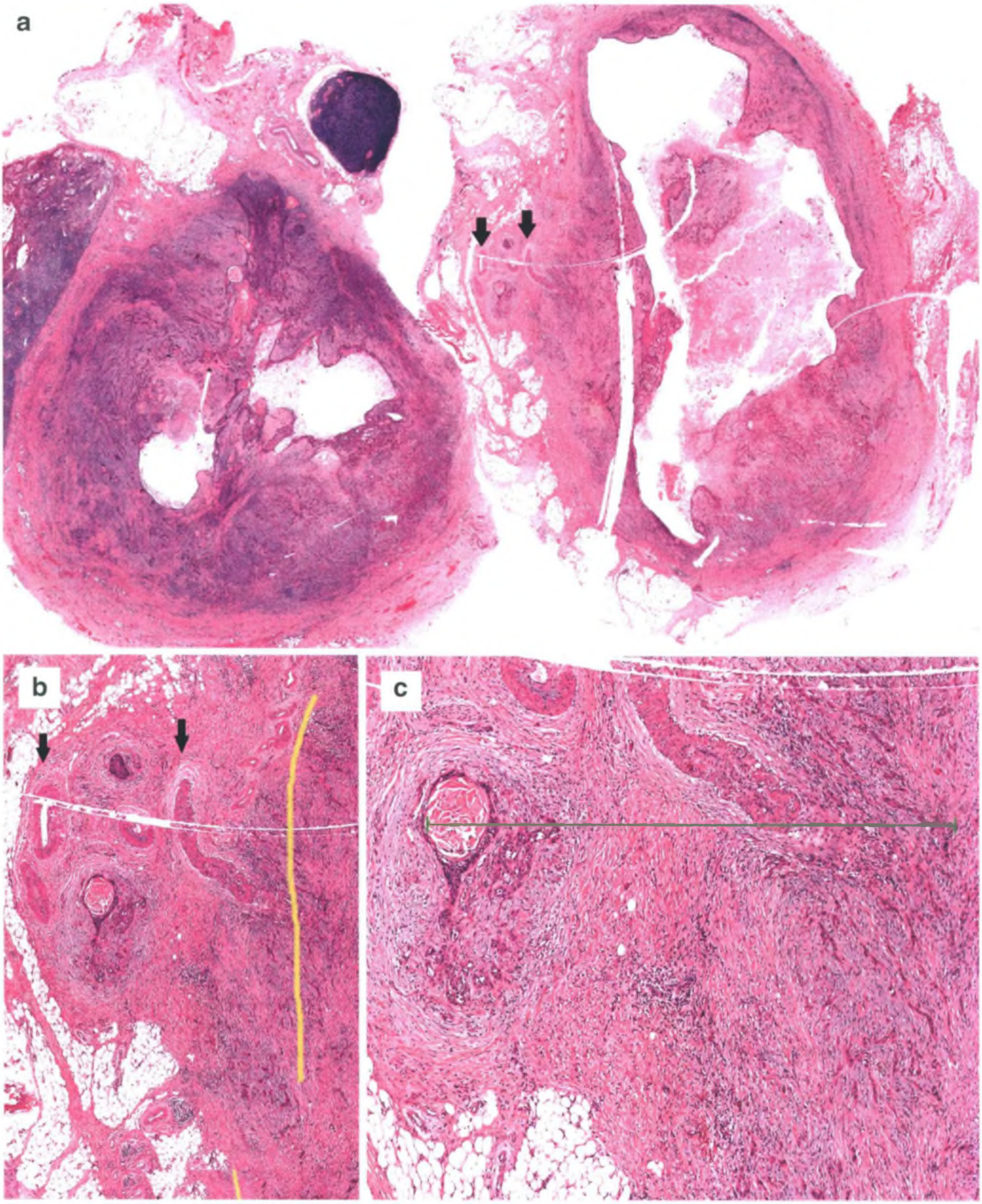

Fig. 7.5 (**a**) Extranodal extension of metastatic carcinoma, low-power. The large vessels (*black arrows*) are extranodal in location. (**b**) The direction of the collagen and the location of vessels guide the estimation of the natural lymph node boundary (*yellow line*). (**c**) This carcinoma extends > 2 mm from the estimated lymph node boundary (green line) and should be classified as ENE_{ma}

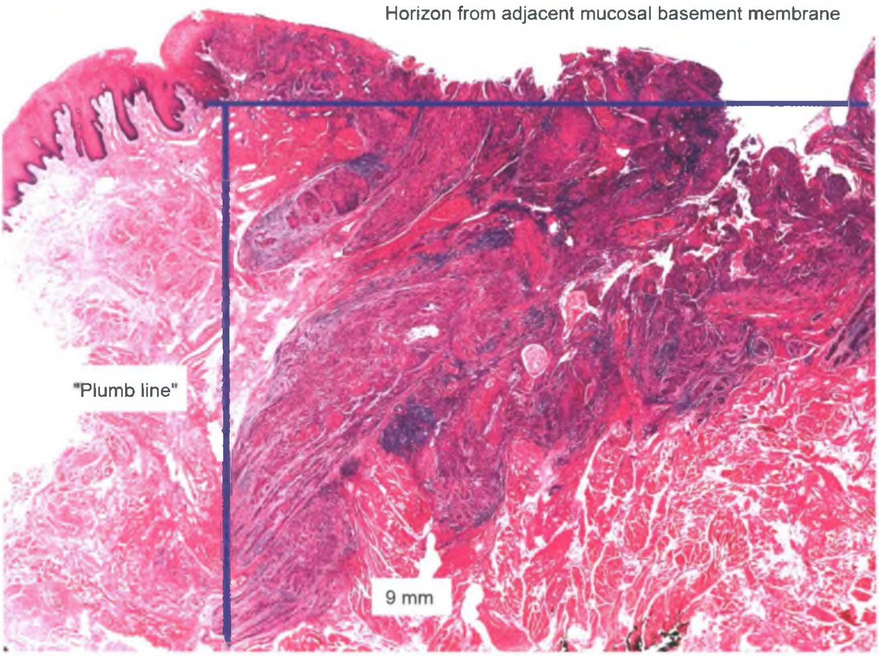

Fig. 7.6 Depth of invasion (DOI). The horizon is established at the level of the basement membrane relative to the closest intact squamous mucosa. The greatest DOI is measured by dropping a "plumb line" from the horizon

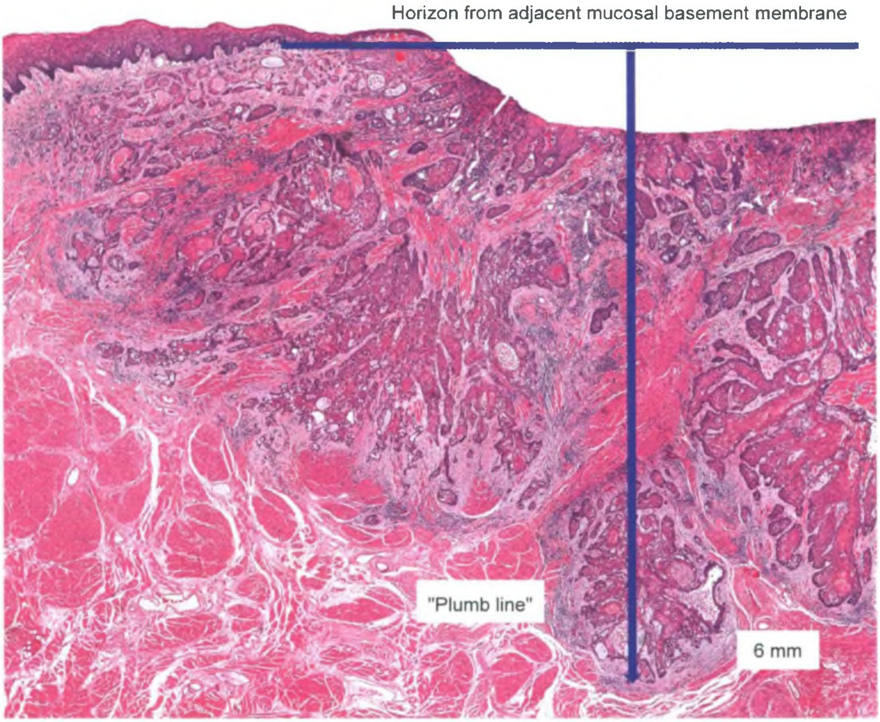

Fig. 7.7 Depth of invasion (DOI) in an ulcerated carcinoma. Notice how "tumor thickness" would be deceptively thinner than DOI

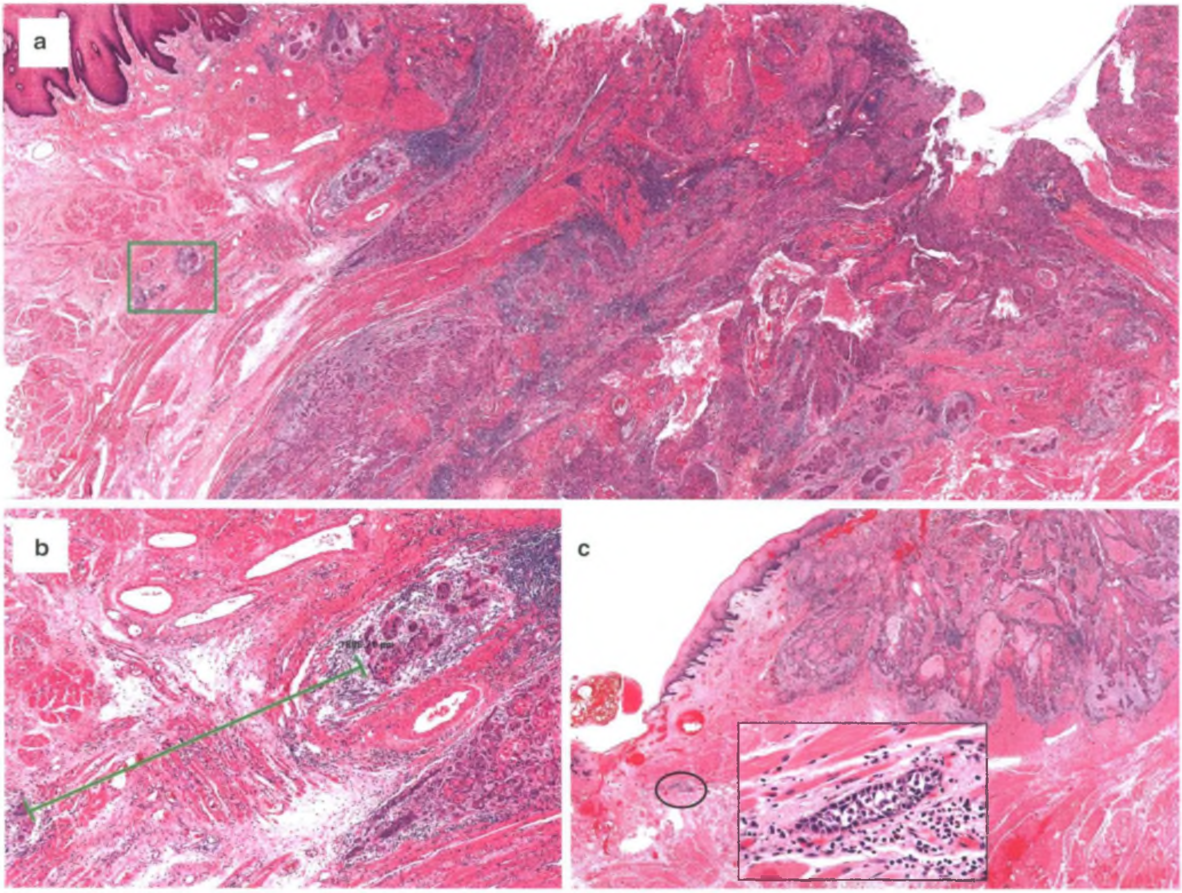

Fig. 7.8 "WPOI-5" describes a dispersed tumor pattern of invasion which is significantly predictive of worst outcome. Carcinomas are classifiable as WPOI-5 when satellite dispersion is ≥ 1 mm from neighboring satellites. (**a**) Low-power overview demonstrating a context of generalized tumor dispersion. Tumor dispersion is measured at the advancing tumor edge. Carcinoma satellites in the green box are shown in panel (**b**), lower edge. The green line measures dispersion of almost 2 mm. (**c**) This carcinoma reveals only few dispersed satellites fulfilling this criteria, likely due to extratumoral lymphovascular emboli

Perineural Invasion

Perineural invasion (PNI) should be subclassified as either intratumoral or extratumoral (Fig. 7.10). Involvement of *named* nerves should be specifically reported.[17] PNI should be subclassified as focal or multifocal. Extensive multifocal PNI is usually extratumoral and frequently associated with a "strand-like" tumor phenotype. The largest nerve diameter should be reported for multifocal, extratumoral PNI.

Lymphovascular invasion

Lymphovascular invasion should be reported as either intratumoral or extratumoral, as well as focal or multifocal.

Overall Health

In addition to the importance of the TNM factors outlined previously, the overall health of the patient clearly influences outcome (Level III). An ongoing effort to better assess prognosis using both tumor and nontumor-related factors is underway. Chart abstraction will continue to be performed by cancer registrars to obtain important information regarding specific factors related to prognosis. These data will then be used to further hone the predictive power of the staging system in future revisions.

Comorbidity

Comorbidity can be classified by specific measures of additional medical illnesses.[18] Accurate reporting of all illnesses in the patients' medical record is essential to assessment of these parameters. General performance measures are helpful in predicting survival. The AJCC strongly recommends the clinician report performance status using the Eastern Cooperative Oncology Group (ECOG), Zubrod, or Karnofsky performance measures, along with stan dard staging information. An interrelationship between each of the major performance tools exists. AJCC Level of Evidence: II

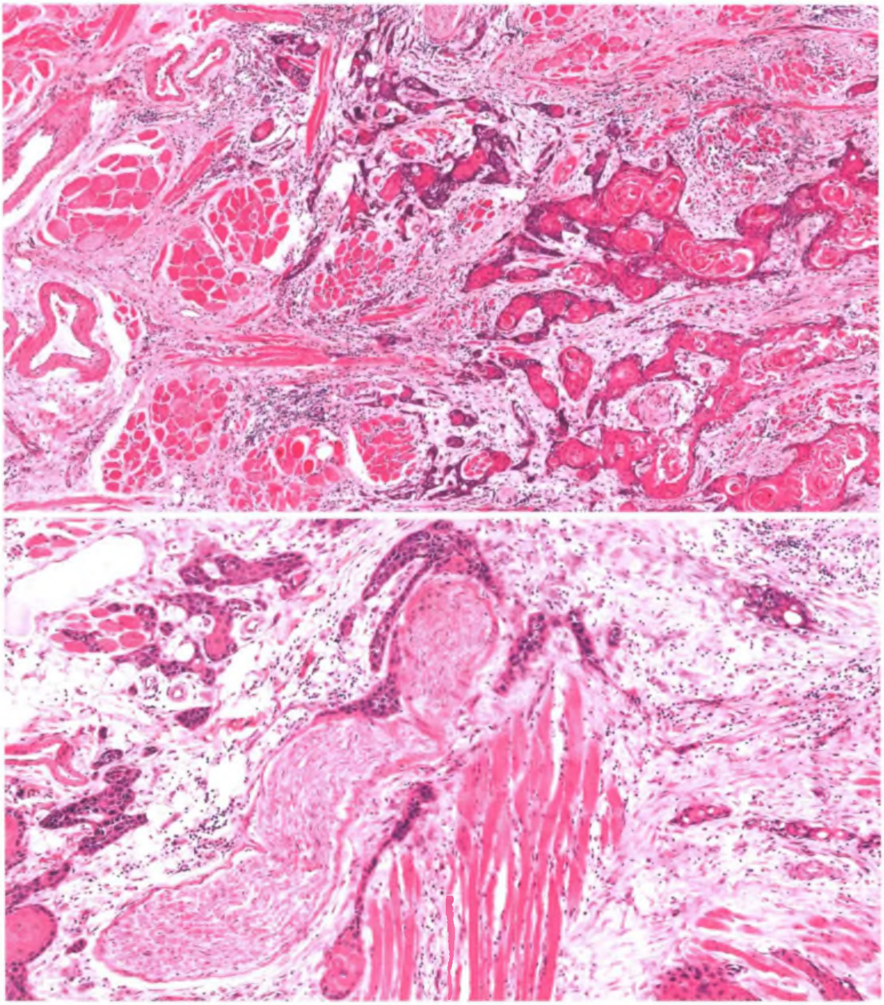

Fig. 7.9 Top: A "strandy" pattern with intervening skeletal muscle observable at low-power is often classifiable as WPOI-5. Bottom: This strand pattern is also often associated with perineural invasion

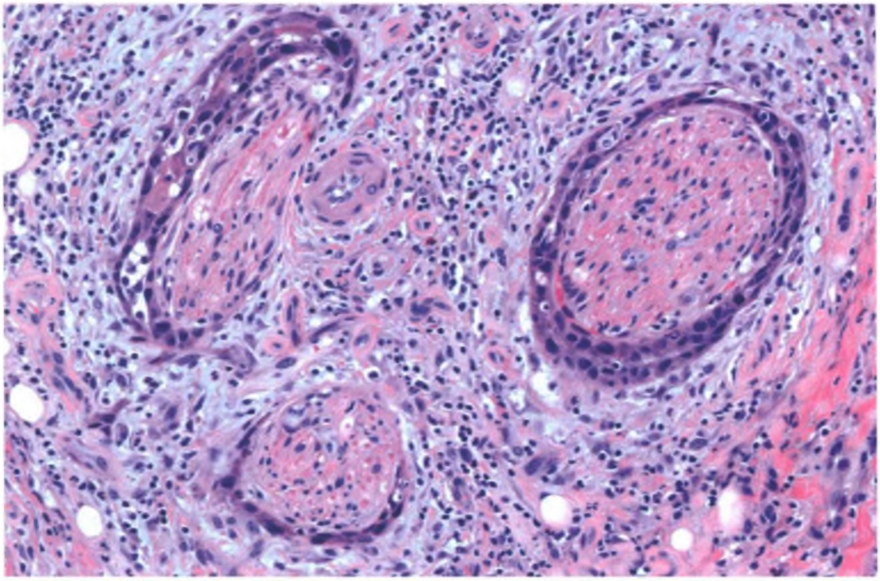

Fig. 7.10 Carcinoma should demonstrate a specific relationship with nerve, such as wrapping around nerves, in order to be classified as perineural invasion (PNI). Merely "bumping" into a nerve does not constitute PNI

Zubrod/ECOG Performance Scale	
0	Fully active, able to carry out all predisease activities without restriction (Karnofsky 90–100)
1	Restricted in physically strenuous activity but ambulatory and able to carry work of a light or sedentary nature. For example, light housework, office work (Karnofsky 70–80)
2	Ambulatory and capable of all self-care but unable to carry out any work activities. Up and about more than 50% of waking hours (Karnofsky 50–60)
3	Capable of only limited self-care, confined to bed or chair 50% or more of waking hours (Karnofsky 30–40)
4	Completely disabled. Cannot carry on self-care. Totally confined to bed (Karnofsky 10–20)
5	Death (Karnofsky 0)

Lifestyle Factors

Lifestyle factors such as tobacco and alcohol abuse negatively influence survival. Accurate recording of smoking in pack years and alcohol in number of days drinking per week and number of drinks per day will provide important data for future analysis. Nutrition is important to prognosis and will be indirectly measured by weight loss of > 5% of body weight in the previous 6 months.[19] Depression adversely impacts quality of life and survival. Notation of a previous or current diagnosis of depression should be recorded in the medical record.[20] AJCC Level of Evidence: III

Tobacco Use

The role of tobacco as a negative prognostic factor is well established. However, exactly how this could be codified in the staging system is less clear. At this time, smoking is known to have a deleterious effect on prognosis but it is hard to accurately apply it to the staging system. AJCC Level of Evidence: III

Smoking history should be collected as an important element of the demographics and may be included in Prognostic Groups in the future. For practicality, the minimum standard should classify smoking history as never, ≤ 10 pack-years, > 10 but ≤ 20 pack-years, or > 20 pack-years.

RISK ASSESSMENT MODELS

The AJCC recently established guidelines that will be used to evaluate published statistical prediction models for the purpose of granting endorsement for clinical use.[21] Although this is a monumental step toward the goal of precision medicine, this work was published only very recently. Therefore, the existing models that have been published or may be in clinical use have not yet been evaluated for this cancer site by the Precision Medicine Core of the AJCC. In the future, the statistical prediction models for this cancer site will be evaluated, and those that meet all AJCC criteria will be endorsed.

DEFINITIONS OF AJCC TNM

Definition of Primary Tumor (T)

T Category	T Criteria
TX	Primary tumor cannot be assessed
Tis	Carcinoma *in situ*
T1	Tumor ≤ 2 cm, ≤ 5 mm depth of invasion (DOI)<br>DOI is depth of invasion and not tumor thickness.
T2	Tumor ≤ 2 cm, DOI > 5 mm and ≤ 10 mm<br>*or* tumor > 2 cm but ≤ 4 cm, and ≤ 10 mm DOI
T3	Tumor > 4 cm<br>*or* any tumor > 10 mm DOI
T4	Moderately advanced or very advanced local disease
T4a	Moderately advanced local disease<br>(lip) Tumor invades through cortical bone or involves the inferior alveolar nerve, floor of mouth, or skin of face (i.e., chin or nose)<br>(oral cavity) Tumor invades adjacent structures only (e.g., through cortical bone of the mandible or maxilla, or involves the maxillary sinus or skin of the face)<br>Note: Superficial erosion of bone/tooth socket (alone) by a gingival primary is not sufficient to classify a tumor as T4.
T4b	Very advanced local disease<br>Tumor invades masticator space, pterygoid plates, or skull base and/or encases the internal carotid artery

Definition of Regional Lymph Node (N)

Clinical N (cN)

N Category	N Criteria
NX	Regional lymph nodes cannot be assessed
N0	No regional lymph node metastasis
N1	Metastasis in a single ipsilateral lymph node, 3 cm or smaller in greatest dimension ENE(−)
N2	Metastasis in a single ipsilateral node larger than 3 cm but not larger than 6 cm in greatest dimension and ENE(−);<br>*or* metastases in multiple ipsilateral lymph nodes, none larger than 6 cm in greatest dimension and ENE(−);<br>*or* in bilateral or contralateral lymph nodes, none larger than 6 cm in greatest dimension, and ENE(−)
N2a	Metastasis in a single ipsilateral node larger than 3 cm but not larger than 6 cm in greatest dimension, and ENE(−)
N2b	Metastasis in multiple ipsilateral nodes, none larger than 6 cm in greatest dimension, and ENE(−)
N2c	Metastasis in bilateral or contralateral lymph nodes, none larger than 6 cm in greatest dimension, and ENE(−)
N3	Metastasis in a lymph node larger than 6 cm in greatest dimension and ENE(−);<br>*or* metastasis in any node(s) and clinically overt ENE(+)
N3a	Metastasis in a lymph node larger than 6 cm in greatest dimension and ENE(−)
N3b	Metastasis in any node(s) and clinically overt ENE(+)

Note: A designation of "U" or "L" may be used for any N category to indicate metastasis above the lower border of the cricoid (U) or below the lower border of the cricoid (L)
Similarly, clinical and pathological ENE should be recorded as ENE(−) or ENE(+)

Pathological N (pN)

N Category	N Criteria
NX	Regional lymph nodes cannot be assessed
N0	No regional lymph node metastasis
N1	Metastasis in a single ipsilateral lymph node, 3 cm or smaller in greatest dimension and ENE(−)
N2	Metastasis in a single ipsilateral lymph node, 3 cm or smaller in greatest dimension and ENE(+); *or* larger than 3 cm but not larger than 6 cm in greatest dimension and ENE(−); *or* metastases in multiple ipsilateral lymph nodes, none larger than 6 cm in greatest dimension and ENE(−); *or* in bilateral or contralateral lymph nodes, none larger than 6 cm in greatest dimension, ENE(−)
N2a	Metastasis in single ipsilateral or contralateral node 3 cm or smaller in greatest dimension and ENE(+); *or* a single ipsilateral node larger than 3 cm but not larger than 6 cm in greatest dimension and ENE(−)
N2b	Metastasis in multiple ipsilateral nodes, none larger than 6 cm in greatest dimension and ENE(−)
N2c	Metastasis in bilateral or contralateral lymph nodes, none larger than 6 cm in greatest dimension and ENE(−)
N3	Metastasis in a lymph node larger than 6 cm in greatest dimension and ENE(−); *or* in a single ipsilateral node larger than 3 cm in greatest dimension and ENE(+); *or* multiple ipsilateral, contralateral or bilateral nodes any with ENE(+)
N3a	Metastasis in a lymph node larger than 6 cm in greatest dimension and ENE(−)
N3b	Metastasis in a single ipsilateral node larger than 3 cm in greatest dimension and ENE(+); *or* multiple ipsilateral, contralateral or bilateral nodes any with ENE(+)

Note: A designation of "U" or "L" may be used for any N category to indicate metastasis above the lower border of the cricoid (U) or below the lower border of the cricoid (L)
Similarly, clinical and pathological ENE should be recorded as ENE(−) or ENE(+)

Definition of Distant Metastasis (M)

M Category	M Criteria
M0	No distant metastasis
M1	Distant metastasis

AJCC PROGNOSTIC STAGE GROUPS

When T is...	And N is...	And M is...	Then the stage group is...
T1	N0	M0	I
T2	N0	M0	II
T3	N0	M0	III
T1,2,3	N1	M0	III
T4a	N0,1	M0	IVA
T1,2,3,4a	N2	M0	IVA
Any T	N3	M0	IVB
T4b	Any N	M0	IVB
Any T	Any N	M1	IVC

REGISTRY DATA COLLECTION VARIABLES

1. Lip location (external lip or vermilion border)
2. ENE clinical (presence or absence)
3. ENE pathological (presence or absence)
4. Extent of microscopic ENE (distance of extension from the native lymph node capsule to the farthest point of invasion in the extranodal tissue)
5. Perineural invasion
6. Lymphovascular invasion
7. p16/HPV status
8. Performance status
9. Tobacco use and pack-year
10. Alcohol use
11. Depression diagnosis
12. Depth of invasion (mm)
13. Margin status (grossly involved, microscopic involvement)
14. Distance of tumor (or moderate/severe dysplasia) from closest margin
15. WPOI-5

7

HISTOLOGIC GRADE (G)

G	G Definition
GX	Cannot be assessed
G1	Well differentiated
G2	Moderately differentiated
G3	Poorly differentiated

HISTOPATHOLOGIC TYPE

Squamous cell carcinoma (conventional, variants), carcinoma of minor salivary gland (acinic cell, adenoid cystic, adenocarcinoma, NOS, basal cell adenocarcinoma, carcinoma ex-pleomorphic adenoma, carcinoma type cannot be

determined, carcinosarcoma, clear cell adenocarcinoma, cystadenocarcinoma, epithelial-myoepithelial carcinoma, mammary analogue secretory carcinoma, mucoepidermoid carcinoma, mucinous carcinoma, myoepithelial carcinoma, oncocytic carcinoma, polymorphous low-grade adenocarcinoma, salivary duct carcinoma), non-salivary gland adenocarcinoma, neuroendocrine carcinoma (typical carcinoid, atypical carcinoid, large cell, small cell, composite small cell-other type), mucosal melanoma, carcinoma type cannot be determined.

ILLUSTRATIONS

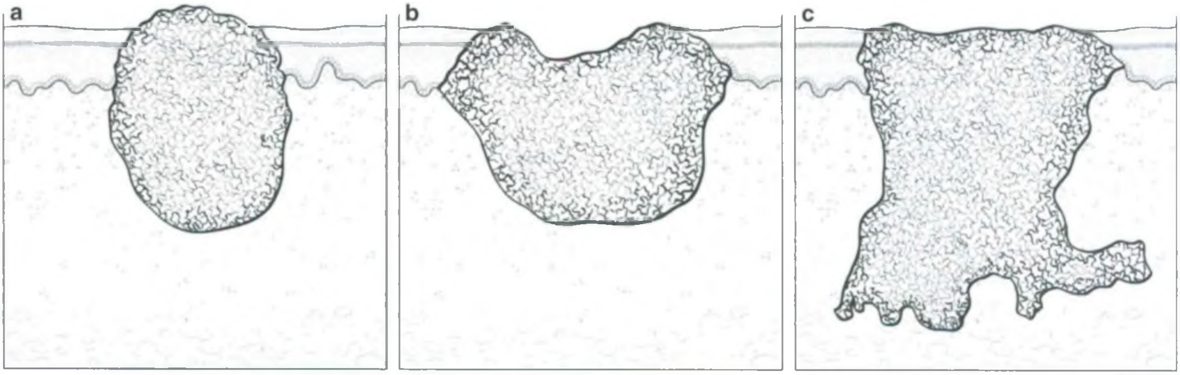

Fig. 7.11 Characteristics of lip and oral cavity tumors. (**a**) Exophytic. (**b**) Ulcerated. (**c**) Endophytic

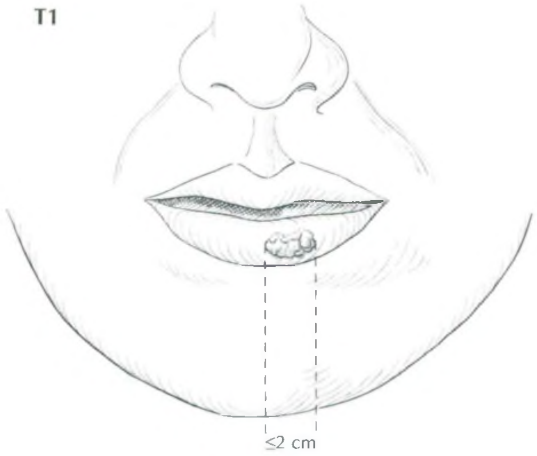

Fig. 7.12 T1 is defined as a tumor 2 cm or smaller in greatest dimension

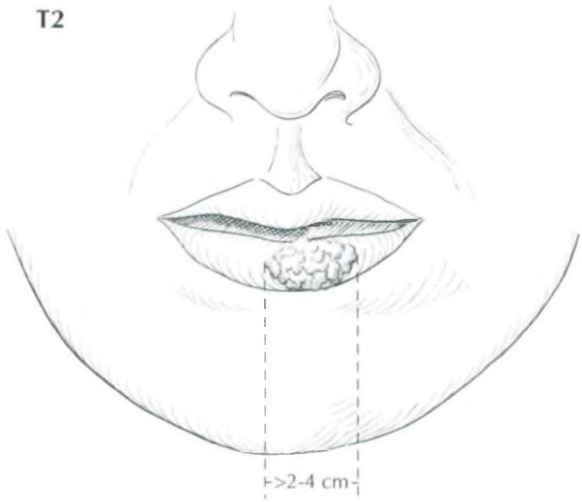

Fig. 7.13 T2 is defined as a tumor larger than 2 cm but not larger than 4 cm in greatest dimension

T3

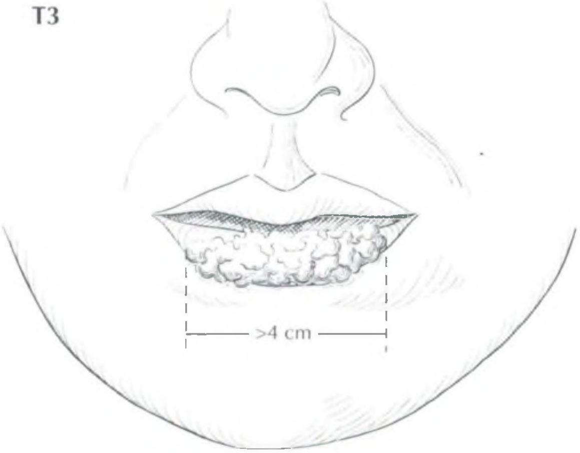

Fig. 7.14 T3 is defined as tumor larger than 4 cm in greatest dimension

T4b

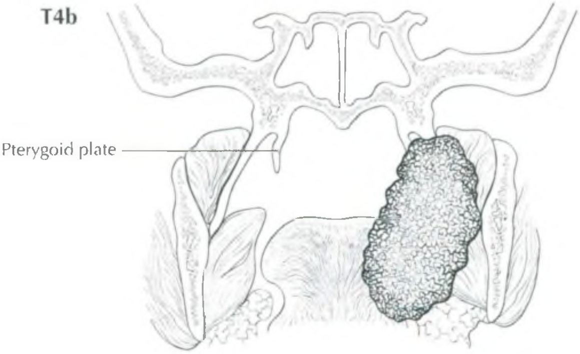

Fig. 7.16 T4b is defined as very advanced local disease, tumor involves masticator space, pterygoid plates (as shown), or skull base and/or encases internal carotid artery

T4a (Lip)

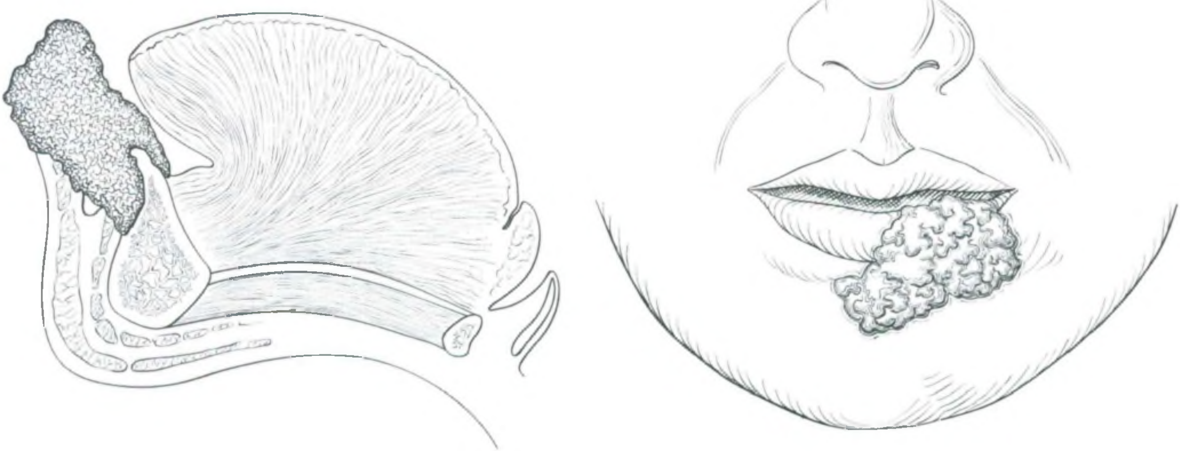

Fig. 7.15 T4a (Lip) is defined as moderately advanced local disease, tumor invading through cortical bone, inferior alveolar nerve, floor of mouth, or skin of face, i.e., chin or nose (as shown)

Bibliography

1. O'Sullivan B, Huang SH, Su J, et al. Development and validation of a staging system for HPV-related oropharyngeal cancer by the International Collaboration on Oropharyngeal cancer Network for Staging (ICON-S): a multicentre cohort study. *The lancet oncology*. Feb 26 2016.
2. Patel S. Personal Communication. In: Lydiatt W, Shah JP, eds2015.
3. Wreesmann VB, Katabi N, Palmer FL, et al. Influence of extracapsular nodal spread extent on prognosis of oral squamous cell carcinoma. *Head & neck*. Oct 30 2015.
4. Spiro RH, Huvos AG, Wong GY, Spiro JD, Gnecco CA, Strong EW. Predictive value of tumor thickness in squamous carcinoma confined to the tongue and floor of the mouth. *American journal of surgery*. Oct 1986;152(4):345–350.
5. Ebrahimi A, Gil Z, Amit M. International Consortium for Outcome Research (ICOR) in Head and Neck Cancer. Primary tumor staging for oral cancer and a proposed modification incorporating depth of invasion: an international multicenter retrospective study. *JAMA otolaryngology– head & neck surgery*. 2014;140(12):1138–1148.
6. Ebrahimi A, Clark JR, Amit M, et al. Minimum nodal yield in oral squamous cell carcinoma: defining the standard of care in a multicenter international pooled validation study. *Annals of surgical oncology*. Sep 2014;21(9):3049–3055.
7. Prabhu RS, Hanasoge S, Magliocca KR, et al. Extent of pathologic extracapsular extension and outcomes in patients with nonoropharyngeal head and neck cancer treated with initial surgical resection. *Cancer*. May 15 2014;120(10):1499–1506.
8. Dunne AA, Muller HH, Eisele DW, Kessel K, Moll R, Werner JA. Meta-analysis of the prognostic significance of perinodal spread in head and neck squamous cell carcinomas (HNSCC) patients. *European journal of cancer*. Aug 2006;42(12):1863–1868.
9. Landry D, Glastonbury CM. Squamous cell carcinoma of the upper aerodigestive tract: a review. *Radiol Clin North Am*. Jan 2015; 53(1):81–97.
10. Li C, Yang W, Men Y, Wu F, Pan J, Li L. Magnetic resonance imaging for diagnosis of mandibular involvement from head and neck cancers: a systematic review and meta-analysis. *PloS one*. 2014;9(11):e112267.
11. Gu DH, Yoon DY, Park CH, et al. CT, MR, 18F-FDG PET/CT, and their combined use for the assessment of mandibular invasion by squamous cell carcinomas of the oral cavity. *Acta Radiologica*. 2010;51(10):1111–1119.
12. Maxwell JH, Thompson LD, Brandwein-Gensler MS, et al. Early Oral Tongue Squamous Cell Carcinoma: Sampling of Margins From Tumor Bed and Worse Local Control. *JAMA otolaryngology– head & neck surgery*. Dec 1 2015;141(12):1104–1110.
13. Hinni ML, Ferlito A, Brandwein-Gensler MS, et al. Surgical margins in head and neck cancer: a contemporary review. *Head & neck*. Sep 2013;35(9):1362–1370.
14. Brandwein-Gensler M, Smith RV, Wang B, et al. Validation of the histologic risk model in a new cohort of patients with head and neck squamous cell carcinoma. *The American journal of surgical pathology*. May 2010;34(5):676–688.
15. Brandwein-Gensler M, Teixeira MS, Lewis CM, et al. Oral squamous cell carcinoma: histologic risk assessment, but not margin status, is strongly predictive of local disease-free and overall survival. *The American journal of surgical pathology*. Feb 2005;29(2):167–178.
16. Li Y, Bai S, Carroll W, et al. Validation of the risk model: high-risk classification and tumor pattern of invasion predict outcome for patients with low-stage oral cavity squamous cell carcinoma. *Head and neck pathology*. Sep 2013;7(3):211–223.
17. Chinn SB, Spector ME, Bellile EL, et al. Impact of perineural invasion in the pathologically N0 neck in oral cavity squamous cell carcinoma. *Otolaryngology–head and neck surgery : official journal of American Academy of Otolaryngology-Head and Neck Surgery*. Dec 2013;149(6):893–899.
18. Piccirillo JF. Inclusion of comorbidity in a staging system for head and neck cancer. *Oncology (Williston Park)*. Sep 1995;9(9):831–836; discussion 841, 845–838.
19. Marion E. Couch MD P, MBA1,*, Kim Dittus MD, PhD2, Michael J. Toth PhD3, Monte S. Willis MD, PhD4, Denis C. Guttridge PhD5, Jonathan R. George MD6, Eric Y. Chang7, Christine G. Gourin MD8 andHirak Der-Torossian MD, MPH1 Cancer cachexia update in head and neck cancer: Pathophysiology and treatment *Head & neck surgery*. 2015;37(7):1057–1072.
20. Lazure KE, Lydiatt WM, Denman D, Burke WJ. Association between depression and survival or disease recurrence in patients with head and neck cancer enrolled in a depression prevention trial. *Head & neck*. 2009;31(7):888–892.
21. Kattan MW, Hess KR, Amin MB, et al. American Joint Committee on Cancer acceptance criteria for inclusion of risk models for individualized prognosis in the practice of precision medicine. *CA: a cancer journal for clinicians*. Jan 19 2016.
22. Chai RL, Rath TJ, Johnson JT, et al. Accuracy of computed tomography in the prediction of extracapsular spread of lymph node metastases in squamous cell carcinoma of the head and neck. *JAMA otolaryngology– head & neck surgery*. Nov 2013;139(11):1187–1194.
23. Dillon JK, Glastonbury CM, Jabeen F, Schmidt BL. Gauze padding: a simple technique to delineate small oral cavity tumors. *AJNR. American journal of neuroradiology*. May 2011;32(5):934–937.
24. Feng Z, Li JN, Niu LX, Guo CB. Supraomohyoid neck dissection in the management of oral squamous cell carcinoma: special consideration for skip metastases at level IV or V. *Journal of Oral and Maxillofacial Surgery*. 2014;72(6):1203–1211.
25. Henrot P, Blum A, Toussaint B, Troufleau P, Stines J, Roland J. Dynamic maneuvers in local staging of head and neck malignancies with current imaging techniques: principles and clinical applications. *Radiographics : a review publication of the Radiological Society of North America, Inc*. Sep-Oct 2003;23(5): 1201–1213.
26. Hoang JK, Glastonbury CM, Chen LF, Salvatore JK, Eastwood JD. CT mucosal window settings: a novel approach to evaluating early T-stage head and neck carcinoma. *AJR. American journal of roentgenology*. Oct 2010;195(4):1002–1006.
27. Kann BH, Buckstein M, Carpenter TJ, et al. Radiographic extracapsular extension and treatment outcomes in locally advanced oropharyngeal carcinoma. *Head & neck*. Dec 2014;36(12):1689–1694.
28. Katayama I, Sasaki M, Kimura Y, et al. Comparison between ultrasonography and MR imaging for discriminating squamous cell carcinoma nodes with extranodal spread in the neck. *European journal of radiology*. 2012;81(11):3326–3331.
29. Kimura Y, Sumi M, Sakihama N, Tanaka F, Takahashi H, Nakamura T. MR imaging criteria for the prediction of extranodal spread of metastatic cancer in the neck. *AJNR. American journal of neuroradiology*. Aug 2008;29(7):1355–1359.
30. King AD, Tse GM, Yuen EH, et al. Comparison of CT and MR imaging for the detection of extranodal neoplastic spread in metastatic neck nodes. *Eur J Radiol*. Dec 2004;52(3):264–270.
31. Lodder WL, Lange CA, van Velthuysen M-LF, et al. Can extranodal spread in head and neck cancer be detected on MR imaging. *Oral oncology*. 2013;49(6):626–633.
32. Prabhu RS, Magliocca KR, Hanasoge S, et al. Accuracy of computed tomography for predicting pathologic nodal extracapsular extension in patients with head-and-neck cancer undergoing initial surgical resection. *International journal of radiation oncology, biology, physics*. Jan 1 2014;88(1):122–129.
33. Randall DR, Lysack JT, Hudon ME, et al. Diagnostic utility of central node necrosis in predicting extracapsular spread among oral cavity squamous cell carcinoma. *Head & neck*. 2015;37(1):92–96.
34. Weissman JL, Carrau RL. "Puffed-cheek" CT improves evaluation of the oral cavity. *AJNR. American journal of neuroradiology*. Apr 2001;22(4):741–744.

Major Salivary Glands

8

William M. Lydiatt, Suresh K. Mukherji, Brian O'Sullivan, Snehal G. Patel, and Jatin P. Shah

CHAPTER SUMMARY

Cancers Staged Using This Staging System

All malignancies arising in the major salivary glands are staged by the rules outlined in this chapter.

Cancers Not Staged Using This Staging System

These histopathologic types of cancer…	Are staged according to the classification for…	And can be found in chapter…
Lymphoma	Hodgkin and Non-Hodgkin Lymphoma	79
Minor salivary gland tumors	The primary site in which they arise, staged similar to squamous cell carcinoma	N/A

Summary of Changes

Change	Details of Change	Level of Evidence
Definition of Regional Lymph Node (N)	Separate N staging approaches have been described for human papilloma virus (HPV)-related and HPV-unrelated cancers.	II[1,2]
Definition of Regional Lymph Node (N)	Separate N category approaches have been described for patients treated without cervical lymph node dissection (clinical N) and patients treated with cervical lymph neck dissection (pathological N).	II[1,2]
Definition of Regional Lymph Node (N)	Extranodal extension (ENE) is introduced as a descriptor in all HPV-unrelated cancers.	II[2]
Definition of Regional Lymph Node (N)	ENE in HPV negative cancers: Only clinically and radiographically overt ENE should be used for cN.	II2
Definition of Regional Lymph Node (N)	ENE in HPV negative cancers: Any pathologically detected ENE is considered ENE(+) and is used for pN.	II[2]
Definition of Regional Lymph Node (N)	ENE in HPV-negative cancers: Presence of ENE is designated pN2a for a single ipsilateral node<3 cm and pN3b for all other node(s).	II[2]
Definition of Regional Lymph Node (N)	Classification of ENE: Clinically overt ENE is classified as ENE_c and is considered ENE(+) for cN.	III[3]
Definition of Regional Lymph Node (N)	Classification of ENE: Pathologically detected ENE is classified as either ENE_{mi} ($\leq$2 mm) or ENE_{ma} (>2 mm) for data collection purposes only, but both are considered ENE(+) for definition of pN.	III[3]

To access the AJCC cancer staging forms, please visit www.cancerstaging.org.

© American Joint Committee on Cancer 2017
M.B. Amin et al. (eds.), *AJCC Cancer Staging Manual, Eighth Edition*, DOI 10.1007/978-3-319-40618-3_8

ICD-O-3 Topography Codes

Code	Description
C07.9	Parotid gland
C08.0	Submandibular gland
C08.1	Sublingual gland
C08.8	Overlapping lesion of major salivary glands
C08.9	Major salivary gland, NOS

WHO Classification of Tumors

Code	Description
8550	Acinic cell carcinoma
8430	Mucoepidermoid carcinoma
8200	Adenoid cystic carcinoma
8525	Polymorphous adenocarcinoma
8562	Epithelial-myoepithelial carcinoma
8310	Clear cell carcinoma
8147	Basal cell adenocarcinoma
8410	Sebaceous carcinoma
8140	Adenocarcinoma
8500	Salivary duct carcinoma
8982	Myoepithelial carcinoma
8941	Carcinoma ex pleomorphic adenoma
8980	Carcinosarcoma
8041	Small cell carcinoma
8012	Large cell carcinoma
8082	Lymphoepithelial carcinoma
8070	Squamous cell carcinoma
8290	Oncocytic carcinoma
8974	Sialoblastoma

Barnes L, Eveson JW, Reichart P, Sidransky D, eds. World Health Organization Classification of Tumours Pathology and Genetics of Head and Neck Tumours. Lyon: IARC; 2005.

INTRODUCTION

Salivary gland malignancies represent a large variety of histological entities. The staging of major salivary gland malignancies is described in this chapter. Minor salivary gland tumors are staged similar to squamous cell carcinoma, according to the site in which they arise (e.g., oral cavity, pharynx, sinuses, etc.).

Staging of major salivary gland malignancies is important in defining their natural history and in predicting prognosis. The varied prognosis associated with the histological type also is a major factor in prognosis, in addition to stage, and should always be taken into consideration when determining treatment options.4 Because of the relative rarity of salivary gland cancers and the wide histological varieties, general principles of staging are used—such as size, involvement of facial nerve, skin, or sensory nerve involvement—because these factors have a negative effect on local-regional control and survival. Nodal involvement also is an important negative prognostic feature, just as it is elsewhere in the head and neck.

A staging system should address and respond to new information that influences patient outcome. An appropriate balance between complexity and compliance (ease-of-use) is necessary for worldwide adoption. The TNM system has strongly predicted prognosis over the years and is adopted worldwide. The introduction of the new parameter of ENE better fits the prognostic modeling from large datasets. However, it must be balanced by the ability to derive accurate information from clinicians caring for patients with head and neck cancer in many different environments. Thorough descriptions of ENE are included in this chapter. Effectively, ENE will increase the nodal category by 1 (as described in the section "Definitions of AJCC TNM"). Although the data for salivary gland are less complete, retrospective reviews suggest ENE is a negative factor and extrapolation to salivary gland is deemed warranted.

ANATOMY

Primary Site(s)

The major salivary glands include the parotid, submandibular, and sublingual glands (Fig. 8.1). Tumors arising in minor salivary glands (mucus-secreting glands in the lining membrane of the upper aerodigestive tract) are staged according to the anatomic site of origin (e.g., oral cavity, sinuses, etc.).[4]

Primary tumors of the parotid constitute the largest proportion of major salivary gland tumors. The parotid is a paired gland that constitutes the majority of the salivary gland tissue and thus harbors the majority of salivary gland tumors, although the majority are benign. Submandibular glands also are paired and lie on the mylohyoid muscle anteriorly and the hyoglossus muscle posteriorly. Relatively more malignancies arise in the submandibular glands than the parotids. Sublingual primary cancers are rare and may be difficult to distinguish with certainty from minor salivary gland primary tumors of the anterior floor of the mouth.

Regional Lymph Nodes

Regional lymphatic spread from salivary gland cancer varies according to the histology and size of the primary tumor. Most nodal metastases will be clinically apparent on initial evaluation. Low-grade tumors rarely metastasize to regional nodes, whereas the risk of regional spread is substantially higher from high-grade cancers. Regional dissemination tends to be orderly, progressing from intraglandular to

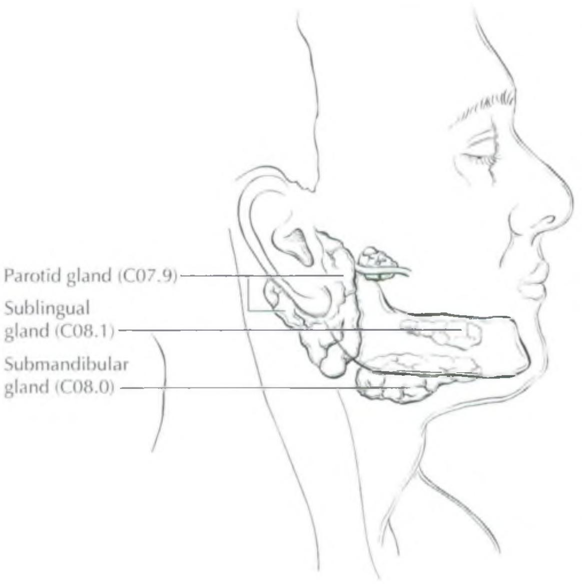

Fig. 8.1 Major salivary glands include the parotid, submandibular, and sublingual glands

adjacent (periparotid, submandibular) nodes, then to upper and midjugular nodes, apex of the posterior triangle (level VA) nodes, and occasionally to retropharyngeal nodes. Bilateral lymphatic spread is rare.

Metastatic Sites

Distant metastatic spread is most frequently to the lungs.

RULES FOR CLASSIFICATION

Clinical Classification

The assessment of primary salivary gland tumors includes a pertinent history (pain, trismus, etc.), inspection, palpation, and evaluation of the cranial nerves. Assessment is based primarily on inspection and palpation of salivary glands, skin, and neck nodes. Biopsy is typically by fine-needle aspiration or by surgical resection of the tumor. Biopsy results may be included as part of clinical classification. The presence of fixation and trismus should be noted. Neurologic evaluation of all cranial nerves is required, but special attention to the facial nerve is mandatory. Careful assessment for pain and hypesthesia of the skin is important.

In clinical evaluation, the maximum size of any nodal mass should be measured. The three categories of clinically involved nodes are N1, N2, and N3. Midline nodes are considered ipsilateral nodes. Superior mediastinal lymph nodes are considered regional lymph nodes (level VII). In addition to the components to describe the N category, regional lymph nodes also should be described according to the level of the neck that is involved. Provide a description or map of the regional lymph nodes and node groups that the cancer affects. Unambiguous evidence of gross extranodal extension (ENE)—which is defined as invasion of skin, infiltration of musculature/fixation to adjacent structures on clinical examination, or cranial nerve, brachial plexus, or sympathetic trunk or phrenic nerve invasion with dysfunction—is a sufficiently high threshold to classify these as clinical ENE(+). Note that the results of a diagnostic biopsy of regional nodes can be included as part of clinical classification.

ENE worsens the adverse outcome associated with nodal metastasis.[3,5–8] Cross-sectional imaging using computed tomography (CT) or magnetic resonance (MR) generally has low sensitivity (65–80 %) but high specificity (86–93 %) for the detection of ENE. The most reliable imaging signs are an indistinct nodal margin, irregular nodal capsular enhancement, or infiltration into the adjacent fat or muscle; the latter finding on CT and MR imaging is the most specific sign of ENE. Ultrasound appears to be less accurate than CT and MR imaging, but ENE is suggested by interrupted or undefined nodal contours with high-resolution ultrasound imaging. The absence or presence of clinical/radiologic ENE is designated ENE(−) or ENE(+), respectively.

Only unquestionable ENE is to be used for clinical staging. As per the "uncertain rule" of the American Joint Committee on Cancer (AJCC)/Union for International Cancer Control (UICC) staging, which mandates that the lower category always be assigned in ambiguous cases, a case should be categorized as ENE(−) unless there is unquestionable ENE. For clinical ENE, the known inability of current imaging modalities to define ENE accurately mandated that stringent criteria be met prior to assigning a clinical diagnosis of ENE.[9] However, unambiguous evidence of gross ENE on clinical examination (e.g., invasion of skin, infiltration of musculature/dense tethering to adjacent structures, or cranial nerve, brachial plexus, sympathetic trunk, or phrenic nerve invasion with dysfunction) supported by strong radiographic evidence permits classification of disease as ENE(+). Pathological ENE will be clearly defined in this chapter. Again, if there is doubt or uncertainty of the presence of ENE, the case should be categorized as ENE(−).

Imaging

Both CT and MR imaging are beneficial and may occasionally be complimentary for staging of major salivary gland tumors. MR imaging provides better tissue characterization and is more accurate than CT for properly identifying pleomorphic adenomas or acinic cell carcinomas. There is no

defined role of positron emission tomography–CT or other nuclear medicine studies for the initial evaluation of salivary gland malignancies. Technetium pertechnetate studies have been reported to be beneficial in identifying Warthin tumors. However, the clinical benefit is debatable if biopsy is being considered.

Both modalities can be used to measure the lesion and help assess tumor size and extension. Imaging can be helpful for lesions that cannot be fully assessed on clinical examination, locally advanced disease, or symptomatic patients. Both modalities also are helpful in determining if the tumor has spread through the surrounding capsule of the salivary gland into the extraparenchymal tissues. Both modalities can be used to evaluate for carotid encasement. MR imaging is superior to CT for assessing for perineural spread. Specifically, MR imaging should be performed to evaluate for the presence of retrograde perineural spread along the facial nerve at the skull base, extending through the stylomastoid foramen to involve the mastoid segment of the facial nerve. CT is superior to MR imaging for early cortical skull base involvement, but MR imaging is superior to CT for bone marrow invasion. The role of imaging in evaluating nodal metastases is discussed in Chapter 6, Cervical Lymph Nodes and Unknown Primary Tumors of the Head and Neck.

Radiology reports should include information on the following:

1. Primary Tumor: Primary site and local spread, with specific mention of structures that would change staging to T4a or T4b
2. Status of lymph node metastases
3. Presence of distant spread

Pathological Classification

Complete resection of the primary site and/or regional nodal dissections, followed by pathological examination of the resected specimen(s), allows the use of this designation for pT and/or pN, respectively. Specimens that are resected after radiation or chemotherapy need to be identified and considered in context, and use yp instead of p. pT is derived from the actual measurement of the unfixed tumor in the surgical specimen. It should be noted, however, that up to 30% shrinkage of soft tissues may occur in resected specimen after formalin fixation. Pathological staging represents additional and important information and should be included as such in staging, but it does not supplant clinical staging as the primary staging scheme.

For pN, a selective neck dissection will ordinarily include 10 or more lymph nodes, and a radical or modified radical neck dissection will ordinarily include 15 or more lymph nodes. Negative pathological examination of a smaller number of nodes still mandates a pN0 designation.

Definition of ENE and Description of Its Extent

All surgically resected metastatic nodes should be examined for the presence and extent of ENE. The precise definition of ENE has varied in the literature over time. The American College of Pathologists defines ENE as extension of metastatic tumor, present within the confines of the lymph node, through the lymph node capsule into the surrounding connective tissue, with or without associated stromal reaction. Gross ENE (Eg) is defined as tumor apparent to the naked eye beyond the confines of the nodal capsule. Microscopic ECS (Em) is defined as extension of metastatic tumor, present within the confines of the lymph node, through the lymph node capsule into the surrounding connective tissue, with or without associated stromal reaction. Only gross ENE is used to define pathological ENE(+) nodal status.

ENE detected on histopathologic examination is designated as ENE_{mi} (microscopic ENE ≤ 2 mm) or ENE_{ma} (major ENE > 2 mm). Both ENE_{mi} and ENE_{ma} qualify as ENE(+) for definition of pN. These descriptors of ENE will not be required for current pN definition, but data collection is recommended to allow standardization of data collection and future analysis.

PROGNOSTIC FACTORS

Prognostic Factors Required For Stage Grouping

Beyond the factors used to assign T, N, or M categories, no additional prognostic factors are required for stage grouping.

Additional Factors Recommended for Clinical Care

Extranodal Extension

ENE is defined as extension of metastatic tumor, present within the confines of the lymph node, through the lymph node capsule into the surrounding connective tissue, with or without associated stromal reaction. Unambiguous evidence of gross ENE (defined as invasion of skin, infiltration of musculature/fixation to adjacent structures on clinical examination, or cranial nerve, brachial plexus, sympathetic trunk or phrenic nerve invasion with dysfunction) is a sufficiently high threshold to classify these tumors as clinical ENE(+). AJCC Level of Evidence: III

Overall Health

In addition to the importance of the TNM factors outlined previously, the overall health of the patient clearly influences outcome. An ongoing effort to better assess prognosis using both tumor and nontumor-related factors is underway. Chart abstraction will continue to be performed by cancer registrars to obtain important information regarding specific factors related to prognosis. These data then will be used to further hone the predictive power of the staging system in future revisions. AJCC Level of Evidence: III

Comorbidity

Comorbidity can be classified by specific measures of additional medical illnesses.[10] Accurate reporting of all illnesses in the patient's medical record is essential to assessment of these parameters. General performance measures are helpful in predicting survival. The AJCC strongly recommends the clinician report performance status using the Eastern Cooperative Oncology Group (ECOG), Zubrod, or Karnofsky performance measures, along with standard staging information. An interrelationship between each of the major performance tools exists. AJCC Level of Evidence: II

Zubrod/ECOG Performance Scale	
0	Fully active, able to carry on all predisease activities without restriction (Karnofsky 90–100)
1	Restricted in physically strenuous activity but ambulatory and able to carry out work of a light or sedentary nature; for example, light housework, office work (Karnofsky 70–80)
2	Ambulatory and capable of all self-care but unable to carry out any work activities; up and about more than 50% of waking hours (Karnofsky 50–60)
3	Capable of only limited self-care, confined to bed or chair 50% or more of waking hours (Karnofsky 30–40)
4	Completely disabled; cannot carry on self-care; totally confined to bed or chair (Karnofsky 10–20)
5	Death (Karnofsky 0)

Lifestyle Factors

Lifestyle factors such as tobacco and alcohol abuse negatively influence survival. Accurate recording of smoking in pack-years and alcohol in number of days drinking per week and number of drinks per day will provide important data for future analysis. Nutrition is important to prognosis and will be measured indirectly by weight loss of>5% of body weight in the previous 6months.11 Depression adversely affects quality of life and survival. Notation of a previous or current diagnosis of depression should be recorded in the medical record.12 AJCC Level of Evidence: III

Tobacco Use

The role of tobacco as a negative prognostic factor is well established. Exactly how this could be codified in the staging system, however, is less clear. At this time, smoking is known to have a deleterious effect on prognosis but it is difficult to accurately apply it to the staging system. AJCC Level of Evidence: III

Smoking history should be collected as an important element of the demographics and may be included in Prognostic Groups in the future. For practicality, the minimum standard should classify smoking history as never, ≤ 10 pack-years, > 10 but ≤ 20 pack-years, or > 20 pack years.

RISK ASSESSMENT MODELS

The AJCC recently established guidelines that will be used to evaluate published statistical prediction models for the purpose of granting endorsement for clinical use.13 Although this is a monumental step toward the goal of precision medicine, this work was published only very recently. Therefore, the existing models that have been published or may be in clinical use have not yet been evaluated for this cancer site by the Precision Medicine Core of the AJCC. In the future, the statistical prediction models for this cancer site will be evaluated, and those that meet all AJCC criteria will be endorsed.

DEFINITIONS OF AJCC TNM

Definition of Primary Tumor (T)

T Category	T Criteria
TX	Primary tumor cannot be assessed
T0	No evidence of primary tumor
Tis	Carcinoma *in situ*
T1	Tumor 2 cm or smaller in greatest dimension without extraparenchymal extension*
T2	Tumor larger than 2 cm but not larger than 4 cm in greatest dimension without extraparenchymal extension*
T3	Tumor larger than 4 cm and/or tumor having extraparenchymal extension*
T4	Moderately advanced or very advanced disease
T4a	Moderately advanced disease<br>Tumor invades skin, mandible, ear canal, and/or facial nerve
T4b	Very advanced disease<br>Tumor invades skull base and/or pterygoid plates and/or encases carotid artery

* Extraparenchymal extension is clinical or macroscopic evidence of invasion of soft tissues. Microscopic evidence alone does not constitute extraparenchymal extension for classification purposes

Definition of Regional Lymph Node (N)

Clinical N (cN)

N Category	N Criteria
NX	Regional lymph nodes cannot be assessed
N0	No regional lymph node metastasis
N1	Metastasis in a single ipsilateral lymph node, 3 cm or smaller in greatest dimension and ENE(−)
N2	Metastasis in a single ipsilateral node larger than 3 cm but not larger than 6 cm in greatest dimension and ENE(−); *or* metastases in multiple ipsilateral lymph nodes, none larger than 6 cm in greatest dimension and ENE(−); *or* in bilateral or contralateral lymph nodes, none larger than 6 cm in greatest dimension and ENE(−)
N2a	Metastasis in a single ipsilateral node larger than 3 cm but not larger than 6 cm in greatest dimension and ENE(−)
N2b	Metastasis in multiple ipsilateral nodes, none larger than 6 cm in greatest dimension and ENE(−)
N2c	Metastasis in bilateral or contralateral lymph nodes, none larger than 6 cm in greatest dimension and ENE(−)
N3	Metastasis in a lymph node larger than 6 cm in greatest dimension and ENE(−); *or* metastasis in any node(s) with clinically overt ENE(+)
N3a	Metastasis in a lymph node larger than 6 cm in greatest dimension and ENE(−)
N3b	Metastasis in any node(s) with clinically overt ENE(+)

Note: A designation of "U" or "L" may be used for any N category to indicate metastasis above the lower border of the cricoid (U) or below the lower border of the cricoid (L)
Similarly, clinical and pathological ENE should be recorded as ENE(−) or ENE(+)

Pathological N (pN)

N Category	N Criteria
NX	Regional lymph nodes cannot be assessed
N0	No regional lymph node metastasis
N1	Metastasis in a single ipsilateral lymph node, 3 cm or smaller in greatest dimension and ENE(−)
N2	Metastasis in a single ipsilateral lymph node, 3 cm or smaller in greatest dimension and ENE(+); *or* larger than 3 cm but not larger than 6 cm in greatest dimension and ENE(−); *or* metastases in multiple ipsilateral lymph nodes, none larger than 6 cm in greatest dimension and ENE(−); *or* in bilateral or contralateral lymph nodes, none larger than 6 cm in greatest dimension and ENE(−)
N2a	Metastasis in single ipsilateral or contralateral node 3 cm or smaller in greatest dimension and ENE(+) *or* a single ipsilateral node larger than 3 cm but not larger than 6 cm in greatest dimension and ENE(−)
N2b	Metastasis in multiple ipsilateral nodes, none larger than 6 cm in greatest dimension and ENE(−)
N2c	Metastasis in bilateral or contralateral lymph nodes, none larger than 6 cm in greatest dimension and ENE(−)
N3	Metastasis in a lymph node larger than 6 cm in greatest dimension and ENE(−); *or* in a single ipsilateral node larger than 3 cm in greatest dimension and ENE(+); *or* multiple ipsilateral, contralateral, or bilateral nodes any with ENE(+)
N3a	Metastasis in a lymph node larger than 6 cm in greatest dimension and ENE(−)
N3b	Metastasis in a single ipsilateral node larger than 3 cm in greatest dimension and ENE(+); *or* multiple ipsilateral, contralateral, or bilateral nodes any with ENE(+)

Note: A designation of "U" or "L" may be used for any N category to indicate metastasis above the lower border of the cricoid (U) or below the lower border of the cricoid (L)
Similarly, clinical and pathological ENE should be recorded as ENE(−) or ENE(+)

Definition of Distant Metastasis (M)

M Category	M Criteria
M0	No distant metastasis
M1	Distant metastasis

AJCC PROGNOSTIC STAGE GROUPS

When T is...	And N is...	And M is...	Then the stage group is...
Tis	N0	M0	0
T1	N0	M0	I
T2	N0	M0	II
T3	N0	M0	III
T0, T1, T2, T3	N1	M0	III
T4a	N0, N1	M0	IVA
T0, T1, T2, T3, T4a	N2	M0	IVA
Any T	N3	M0	IVB
T4b	Any N	M0	IVB
Any T	Any N	M1	IVC

REGISTRY DATA COLLECTION VARIABLES

1. ENE clinical presence or absence
2. ENE pathological presence or absence
3. Extent of microscopic ENE (distance of extension from the native lymph node capsule to the farthest point of invasion in the extranodal tissue)
4. Perineural invasion

5. Lymphovascular invasion
6. p16/HPV status
7. Performance status
8. Tobacco use and pack-years
9. Alcohol use
10. Depression diagnosis

HISTOLOGIC GRADE (G)

There is no uniform grading system for salivary gland.

HISTOPATHOLOGIC TYPE

The exact classification of salivary tumors can be challenging, especially in limited material, given the potential for phenotypic overlap.[14] The histology table in this chapter reflects the 2017 World Health Organization classification of salivary malignancies. Some tumors are routinely graded by three-tiered schema, some by two-tiered schema, some may develop a "dedifferentiated" or abrupt high-grade transformation, and others are not graded. Ductal carcinomas are modified by the presence or absence of invasion plus grade. The additional criteria of "minimally invasive" is a modifier for carcinoma ex pleomorphic adenoma.

Bibliography

1. O'Sullivan B, Huang SH, Su J, et al. Development and validation of a staging system for HPV-related oropharyngeal cancer by the International Collaboration on Oropharyngeal cancer Network for Staging (ICON-S): a multicentre cohort study. *The lancet oncology.* Feb 26 2016.
2. Patel S. Personal Communication. In: Lydiatt W, Shah JP, eds2015.
3. Wreesmann VB, Katabi N, Palmer FL, et al. Influence of extracapsular nodal spread extent on prognosis of oral squamous cell carcinoma. *Head & neck.* Oct 30 2015.
4. Boukheris H, Curtis RE, Land CE, Dores GM. Incidence of carcinoma of the major salivary glands according to the WHO classification, 1992 to 2006: a population-based study in the United States. *Cancer epidemiology, biomarkers & prevention : a publication of the American Association for Cancer Research, cosponsored by the American Society of Preventive Oncology.* Nov 2009;18(11):2899–2906.
5. Ebrahimi A GZ AM, Yen TC, Liao CT, Chatturvedi P, Agarwal J, Kowalski L, Kreppel M, Cernea C, Brandao J, Bachar G, Villaret AB, Fliss D, Fridman E, Robbins KT, Shah J, Patel S, Clark J: . International Consortium for Outcome Research (ICOR) in Head and Neck Cancer. Comparison of the American Joint Committee on Cancer N1 versus N2a nodal categories for predicting survival and recurrence in patients with oral cancer: Time to acknowledge an arbitrary distinction and modify the system. *Head and neck pathology.* 2014.
6. de Juan J, Garcia J, Lopez M, et al. Inclusion of extracapsular spread in the pTNM classification system: a proposal for patients with head and neck carcinoma. *JAMA otolaryngology– head & neck surgery.* May 2013;139(5):483–488.
7. Prabhu RS, Hanasoge S, Magliocca KR, et al. Extent of pathologic extracapsular extension and outcomes in patients with nonoropharyngeal head and neck cancer treated with initial surgical resection. *Cancer.* May 15 2014;120(10):1499–1506.
8. Dunne AA, Muller HH, Eisele DW, Kessel K, Moll R, Werner JA. Meta-analysis of the prognostic significance of perinodal spread in head and neck squamous cell carcinomas (HNSCC) patients. *European journal of cancer.* Aug 2006;42(12):1863–1868.
9. Prabhu RS, Magliocca KR, Hanasoge S, et al. Accuracy of computed tomography for predicting pathologic nodal extracapsular extension in patients with head-and-neck cancer undergoing initial surgical resection. *International journal of radiation oncology, biology, physics.* Jan 1 2014;88(1):122–129.
10. Piccirillo JF. Inclusion of comorbidity in a staging system for head and neck cancer. *Oncology (Williston Park).* Sep 1995;9(9):831–836; discussion 841, 845–838.
11. Couch ME, Dittus K, Toth MJ, et al. Cancer cachexia update in head and neck cancer: Pathophysiology and treatment. *Head & neck.* Jul 2015;37(7):1057–1072.
12. Lazure KE, Lydiatt WM, Denman D, Burke WJ. Association between depression and survival or disease recurrence in patients with head and neck cancer enrolled in a depression prevention trial. *Head & neck.* 2009;31(7):888–892.
13. Kattan MW, Hess KR, Amin MB, et al. American Joint Committee on Cancer acceptance criteria for inclusion of risk models for individualized prognosis in the practice of precision medicine. *CA: a cancer journal for clinicians.* Jan 19 2016
14. Nagao T. "Dedifferentiation" and high-grade transformation in salivary gland carcinomas. *Head and neck pathology.* Jul 2013;7 Suppl 1:S37–47.

8

Nasopharynx

9

Anne W.M. Lee, William M. Lydiatt, A. Dimitrios Colevas,
Christine M. Glastonbury, Quynh Thu X. Le,
Brian O'Sullivan, Randal Scott Weber, and Jatin P. Shah

CHAPTER SUMMARY

Cancers Staged Using This Staging System

Epithelial tumors of the nasopharynx are staged using this staging system.

Cancers Not Staged Using This Staging System

These histopathologic types of cancer…	Are staged according to the classification for…	And can be found in chapter…
Mucosal melanoma	Mucosal melanoma of the head and neck	14
Lymphoma	Hodgkin and non-Hodgkin lymphoma	79
Sarcoma of soft tissue	Soft tissue sarcoma of the head and neck	40
Bone and cartilage	Bone	38

Summary of Changes

Change	Details of Change	Level of Evidence
Definition of Primary Tumor (T)	T0 is added for Epstein-Barr virus (EBV) positive unknown primary with cervical lymph node involvement. The stage group is defined in the same way as T1 (or TX).	III
Definition of Primary Tumor (T)	Adjacent muscles involvement (including medial pterygoid, lateral pterygoid, and prevertebral muscles) is now designated as T2.	II
Definition of Primary Tumor (T)	The previous T4 criteria "masticator space" and "infratemporal fossa" is now replaced by specific description of soft tissue involvement to avoid ambiguity.	II
Definition of Regional Lymph Node (N)	The previous N3b criterion of supraclavicular fossa is now changed to lower neck (as defined by nodal extension below the caudal border of the cricoid cartilage).	II
Definition of Regional Lymph Node (N)	N3a and N3b are merged into a single N3 category, which is now defined as unilateral or bilateral metastasis in cervical lymph node(s), larger than 6 cm in greatest dimension, and/or extension below the caudal border of cricoid cartilage.	II
AJCC Prognostic Stage Groups	The previous Sub-Stages IVA (T4 N0-2 M0) and IVB (any T N3, M0) are now merged to form IVA.	II
AJCC Prognostic Stage Groups	The previous IVC (any T any N M1) is now upstaged to IVB.	II

To access the AJCC cancer staging forms, please visit www.cancerstaging.org.

© American Joint Committee on Cancer 2017

M.B. Amin et al. (eds.), *AJCC Cancer Staging Manual, Eighth Edition*, DOI 10.1007/978-3-319-40618-3_9

ICD-O-3 Topography Codes

Code	Description
C11.0	Superior wall of nasopharynx
C11.1	Posterior wall of nasopharynx
C11.2	Lateral wall of nasopharynx
C11.3	Anterior wall of nasopharynx
C11.8	Overlapping lesion of nasopharynx
C11.9	Nasopharynx, NOS

WHO Classification of Tumors

Code	Description
8070	Squamous cell carcinoma *in situ*, NOS
8010	Carcinoma, NOS
8020	Carcinoma, undifferentiated, NOS
8071	Squamous cell carcinoma, keratinizing, NOS
8072	Squamous cell carcinoma, large cell, nonkeratinizing, NOS
8073	Squamous cell carcinoma, small cell, nonkeratinizing
8083	Basaloid squamous cell carcinoma

Barnes L, Eveson JW, Reichart P, Sidransky D, eds. World Health Organization Classification of Tumours Pathology and Genetics of Head and Neck Tumours. Lyon: IARC; 2005.

INTRODUCTION

An accurate staging system is crucial in cancer management for predicting prognosis, guiding clinicians in treatment decisions for different risk groups, and sharing experience on results of treatment between centers. Prognostic significance of staging system changes with advances in investigation and treatment methods. Evaluation of staging systems to ensure continual suitability and exploration for further improvement is essential.

This chapter focuses on TNM staging for epithelial tumors of the nasopharynx. Nonepithelial tumors such as mucosal melanoma, lymphoma, and sarcoma of soft tissue, bone, and cartilage are not included. Nasopharyngeal carcinoma (NPC) has a very skewed geographic and ethnic distribution, with 80% of the global burden in Asian countries. The natural behavior and therapeutic consideration for NPC are different from other head and neck cancers. The adoption of a customized system for NPC in the American Joint Committee on Cancer (AJCC) Cancer Staging Manual, 5th Edition, by the AJCC and the Union for International Cancer Control (UICC) was a milestone.[1,2] The staging criteria were developed by merging the strengths of the AJCC/UICC, 4th Edition, and the Ho's System from Hong Kong.[3,4] This development has gained global acceptance as studies from different countries (endemic and nonendemic) consistently showed substantial improvement as compared with prior systems. Almost all countries, except China, had adopted this international system.

No change was recommended in the AJCC Cancer Staging Manual, 6th Edition[5,6] except for addition of the term "masticator space" as a synonym for "infratemporal fossa" (one of the T4 criteria) because although the intended extent was described in the staging handbook, the latter was not a clearly defined space with universal acceptance. Both terms were retained as T4 criteria in the AJCC Cancer Staging Manual, 7th Edition (7th Edition);[7,8] however, the term "masticator space" was described using the boundaries stated in classical anatomy textbooks instead of the demarcation used for "infratemporal fossa." Additional changes included down-shifting of tumors with extension to nasal fossa/oropharynx without parapharyngeal extension (previously T2a) to T1[9] and clear definition of retropharyngeal lymph node(s) involvement (unilateral or bilateral) as N1.[10]

The management of NPC has undergone substantial evolution in the past two decades. More accurate imaging methods have allowed better delineation of tumor extent and early detection of occult metastases. The advances in radiotherapy technique has led to increasing conformity of tumor coverage and sparing of noninvolved structures. The use of combination chemotherapy has further improved tumor control and cure rates, especially for advanced locoregional disease. It is therefore important that the new staging system be based on data from patients managed with contemporary methods.

Extensive literature review showed that there are four major issues for consideration of improvement: (1) the controversy about the significance of "masticator space",[11–16] (2) uncertainty about the significance of prevertebral muscle invasion,[17–19] (3) the possibility of replacing supraclavicular fossa (SCF)[3] with anatomic nodal "levels,"[20–25] and (4) simplification by elimination of unnecessary subgroups.[25,26] These suggestions were validated by a large series of patients who were staged with magnetic resonance (MR) imaging and treated with intensity-modulated radiotherapy ± chemotherapy from two major centers (in Hong Kong and Fujian, China),[27] before attaining consensus among international multidisciplinary experts. The strengths of the 7th Edition and the Chinese 2008 staging system[23,24] are incorporated in developing the staging criteria in this AJCC Cancer Staging Manual, 8th Edition (8th Edition).

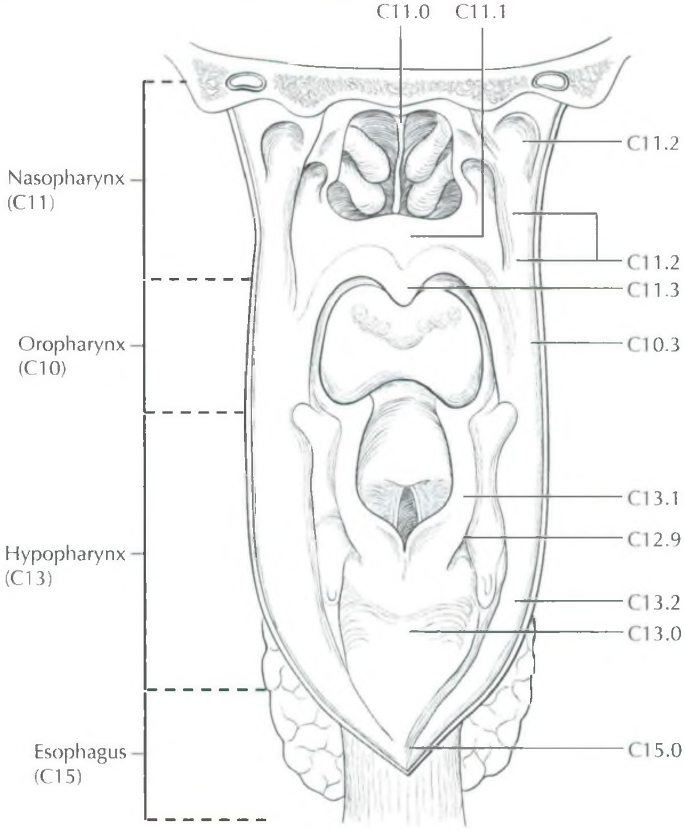

Fig. 9.1 Anatomical sites and subsites of the nasopharynx, oropharynx, hypopharynx, and esophagus

ANATOMY

Primary Site(s)

The pharynx is divided into three regions: nasopharynx, oropharynx, and hypopharynx (Fig. 9.1). The specific anatomic site of nasopharynx and regional lymphatics are described in this section.

The nasopharynx begins anteriorly at the posterior choana and extends along the plane of the airway to the level of the free border of the soft palate. It includes the superior wall, the posterior wall, and the lateral walls, which include the fossae of Rosenmuller and the mucosa covering the torus tubaris forming the Eustachian tube orifice. The floor is the superior surface of the soft palate. The posterior margins of the choanal orifices and of the nasal septum are included in the nasal fossa.

Nasopharyngeal tumors extending to the nasal cavity or oropharynx in the absence of parapharyngeal space involvement do not have a significantly worse outcome than tumors confined to the nasopharynx. Involvement of the parapharyngeal space is defined as posterolateral infiltration from the nasopharynx beyond the buccopharyngeal fascia into the triangular space lateral to the pharynx.

Regional Lymph Nodes

Nasopharyngeal carcinoma often presents with early lymphatic spread. The retropharyngeal nodes and the cervical nodes (both jugular and spinal accessory chains) are involved, often bilaterally. The lymphatic spread in NPC follows a predictable and orderly pattern from upper to lower neck; "skip" metastasis is rare.[21,28]

In clinical evaluation, the maximum dimension (in any direction) of the nodal mass, the laterality, and the lowest level of neck involvement should be assessed. Midline nodes are considered ipsilateral nodes. Nodal size larger than 6 cm in greatest dimension and/or extension below the caudal border of the cricoid cartilage are associated with the worst prognosis.

Metastatic Sites

Nasopharyngeal carcinoma is notorious for a high risk of distant metastasis. The most common sites include lung, bone, liver, and distant lymph nodes. Involvement of lymph nodes below the clavicle (including mediastinum, infraclavicular region, axilla, or groin) is considered as distant metastases.

RULES FOR CLASSIFICATION

Clinical Classification

Clinical staging is employed for NPC. Assessment is based primarily on thorough history, physical examination, indirect or direct endoscopy, and imaging. Physical examination should include neurologic evaluation of all cranial nerves, palpation of neck nodes (greatest dimension, laterality, location, and lowest extent of nodal involvement), and exclusion of gross signs of distant metastases. Indirect or direct endoscopy should assess the extent of anterior involvement into the nasal cavities and inferior infiltration into the oropharynx and hypopharynx. Biopsy should be taken for histological confirmation. Routine testing for complete blood picture, renal, and liver functions (including alkaline phosphatase) are indicated.

Imaging

Cross-sectional imaging studies covering the nasopharyngeal and cervical regions are essential for clinical staging of NPC. Magnetic resonance (MR) imaging is the study of choice because of its multiplanar capability, superior soft tissue contrast, and sensitivity for detecting skull base and intracranial tumor spread. Computed tomography (CT) imaging with axial and coronal thin section technique with contrast is an alternative. Regional nodal status (greatest dimension in any direction, laterality, location, and lowest extent of nodal involvement) should be assessed; measurement of the maximal diameter of nodal disease should not be confined to the axial radiological plane only.

Metastatic workup is recommended for patients with node-positive or locally advanced (T3–4) disease, those with symptoms, signs, and/or biochemical tests suggestive of distant metastasis. Whole body 18F-fluorodeoxyglucose (18F-FDG) positron emission tomography (PET) coupled with CT is increasingly used because of its sensitivity for detecting distant metastases and second primary malignancy, the possibility of its supplementing MR imaging in assessing nodal status,[29] and its use of the maximal standard uptake values (SUVmax) as an additional independent prognostic predictor.[30,31] Assessment by CT thorax and upper abdomen (or chest X-ray and abdominal ultrasound) and bone scan is an alternative.

PATHOLOGICAL CLASSIFICATION

Unlike other head and neck cancer, NPC is primarily treated by radiotherapy, with or without chemotherapy, with no resection of the primary cancer. This makes pathological classification largely irrelevant. Surgery to primary or neck nodes is used only for recurrence.

PROGNOSTIC FACTORS

Prognostic Factors Required for Stage Grouping

Although additional factors may contribute to refining prognostication, none have an adequate level of evidence and consistent cut-off value that attain consensus for incorporation as staging criteria.

Additional Factors Recommended for Clinical Care

Overall Health

In addition to the importance of the TNM factors, the overall health of these patients clearly influences outcome. An ongoing effort to better assess prognosis using both tumor and nontumor-related factors is underway. Chart abstraction will continue to be performed by cancer registrars to obtain important information regarding specific factors related to prognosis. These data will then be used to further hone the predictive power of the staging system in future revisions. AJCC Level of Evidence: II

Comorbidity

Comorbidity can be classified by specific measures of additional medical illnesses. Accurate reporting of all illnesses in the patients' medical record is essential to assessment of these parameters. General performance measures are helpful

in predicting survival. The AJCC strongly recommends that the clinician report performance status using the Eastern Cooperative Oncology Group (ECOG), Zubrod, or Karnofsky performance measures, along with standard staging information. An interrelationship between each of the major performance tools exists. AJCC Level of Evidence: II

Zubrod/ECOG Performance Scale	
0	Fully active, able to carry out all predisease activities without restriction (Karnofsky 90–100)
1	Restricted in physically strenuous activity but ambulatory and able to carry out work of a light or sedentary nature. For example, light housework, office work. (Karnofsky 70–80)
2	Ambulatory and capable of all self-care but unable to carry out any work activities. Up and about more than 50 % of waking hours. (Karnofsky 50–60)
3	Capable of only limited self-care, confined to bed or chair 50 % or more of waking hours (Karnofsky 30–40)
4	Completely disabled. Cannot carry out self-care. Totally confined to bed. (Karnofsky 10–20)
5	Death (Karnofsky 0)

Lifestyle Factors

Lifestyle factors such as tobacco and alcohol abuse negatively influence survival. Accurate recording of smoking in pack-years and alcohol in number of days drinking per week and number of drinks per day will provide important data for future analysis. Nutrition is important to prognosis and will be indirectly measured by weight loss of > 10 % of body weight. Depression adversely impacts quality of life and survival. Notation of a previous or current diagnosis of depression should be recorded in the medical record. AJCC Level of Evidence: III

The role of tobacco as a negative prognostic factor is well established. However, exactly how this could be codified in the staging system is less clear. At this time, smoking is known to have a deleterious effect on prognosis but is hard to accurately apply to the staging system. Smoking history should be collected as an important element of the demographics and may be included in Prognostic Groups in the future. For practicality, the minimum standard should classify smoking history as never, ≤ 10 pack-years, > 10 but ≤ 20 pack-years, or > 20 pack-years.

RISK ASSESSMENT MODELS

The AJCC recently established guidelines that will be used to evaluate published statistical prediction models for the purpose of granting endorsement for clinical use.[32] Although this is a monumental step toward the goal of precision medicine, this work was published only very recently. Therefore, the existing models that have been published or may be in clinical use have not yet been evaluated for this cancer site by the Precision Medicine Core of the AJCC. In the future, the statistical prediction models for this cancer site will be evaluated, and those that meet all AJCC criteria will be endorsed.

DEFINITIONS OF AJCC TNM

Definition of Primary Tumor (T)

T Category	T Criteria
TX	Primary tumor cannot be assessed
T0	No tumor identified, but EBV-positive cervical node(s) involvement
T1	Tumor confined to nasopharynx, or extension to oropharynx and/or nasal cavity without parapharyngeal involvement
T2	Tumor with extension to parapharyngeal space, and/or adjacent soft tissue involvement (medial pterygoid, lateral pterygoid, prevertebral muscles)
T3	Tumor with infiltration of bony structures at skull base, cervical vertebra, pterygoid structures, and/or paranasal sinuses
T4	Tumor with intracranial extension, involvement of cranial nerves, hypopharynx, orbit, parotid gland, and/or extensive soft tissue infiltration beyond the lateral surface of the lateral pterygoid muscle

9

Definition of Regional Lymph Node (N)

N Category	N Criteria
NX	Regional lymph nodes cannot be assessed
N0	No regional lymph node metastasis
N1	Unilateral metastasis in cervical lymph node(s) and/or unilateral or bilateral metastasis in retropharyngeal lymph node(s), 6 cm or smaller in greatest dimension, above the caudal border of cricoid cartilage
N2	Bilateral metastasis in cervical lymph node(s), 6 cm or smaller in greatest dimension, above the caudal border of cricoid cartilage
N3	Unilateral or bilateral metastasis in cervical lymph node(s), larger than 6 cm in greatest dimension, and/or extension below the caudal border of cricoid cartilage

Definition of Distant Metastasis (M)

M Category	M Criteria
M0	No distant metastasis
M1	Distant metastasis

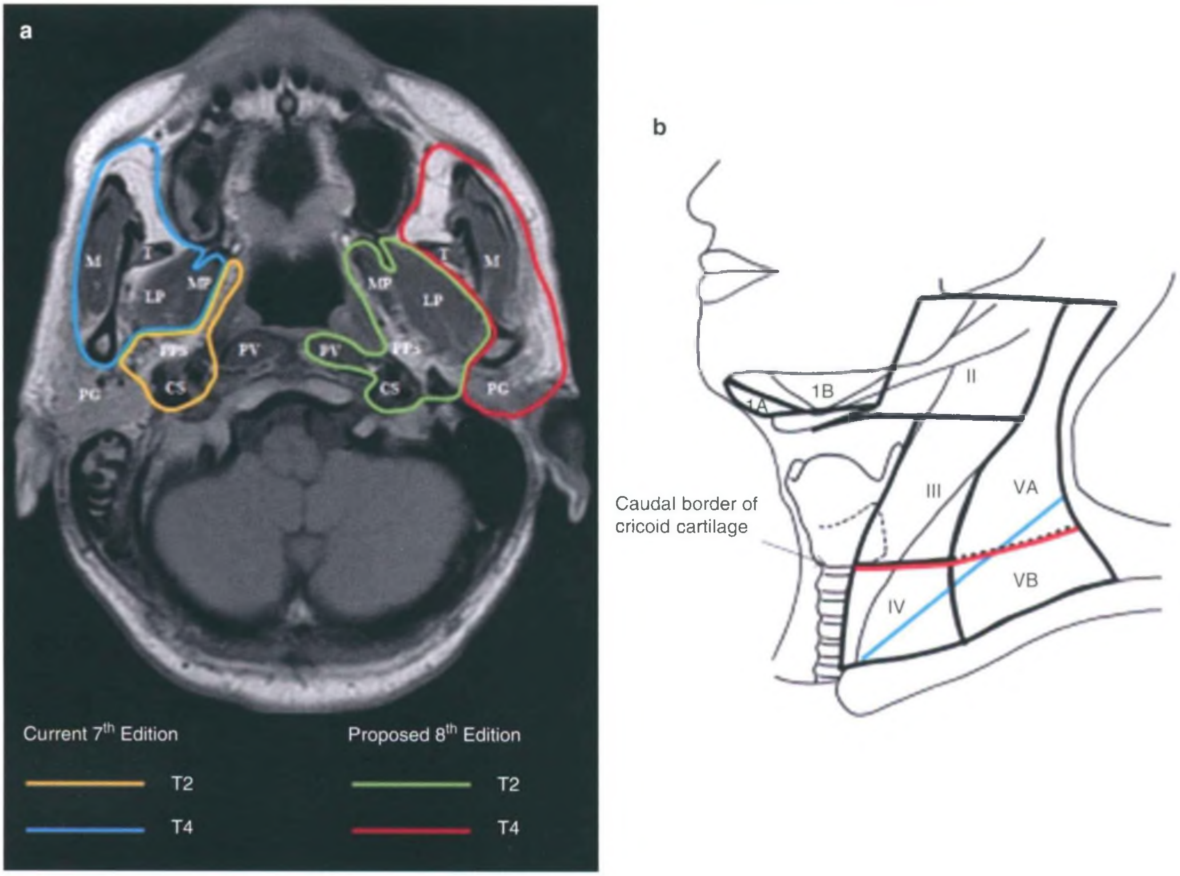

Fig. 9.2 Differences in defining criteria between the 7th Edition and the 8th Edition for staging of NPC: (**a**) changing the extent of soft tissue involvement as T2 and T4 criteria. Abbreviation: CS = carotid space, LP = lateral pterygoid muscle, M = masseter muscle, MP = medial pterygoid muscle, PG = parotid gland, PPS = parapharyngeal space, PV = prevertebral muscle, T = temporalis muscle, (**b**) replacing supraclavicular fossa (*blue*) by lower neck, i.e., below caudal border of cricoid cartilage (*red*) as N3 criteria. From Pan et al.,[27] with permission

AJCC PROGNOSTIC STAGE GROUPS

When T is...	And N is...	And M is...	Then the stage group is...
Tis	N0	M0	Stage 0
T1	N0	M0	Stage I
T1, T0	N1	M0	Stage II
T2	N0	M0	Stage II
T2	N1	M0	Stage II
T1, T0	N2	M0	Stage III
T2	N2	M0	Stage III
T3	N0	M0	Stage III
T3	N1	M0	Stage III
T3	N2	M0	Stage III
T4	N0	M0	Stage IVA
T4	N1	M0	Stage IVA
T4	N2	M0	Stage IVA
Any T	N3	M0	Stage IVA
Any T	Any N	M1	Stage IVB

REGISTRY DATA COLLECTION VARIABLES

None

HISTOLOGIC GRADE (G)

A grading system is not used for NPCs.

HISTOPATHOLOGIC TYPE

The World Health Organization (WHO) classification system[33] is recommended for histopathologic classification, and the following histopathologic types are covered by the staging system (Table 9.1).

Table 9.1 Classification of NPC

WHO classification	Former terminology
Keratinizing squamous cell carcinoma	WHO Type I (squamous cell carcinoma)
Nonkeratinizing carcinoma	
Differentiated	WHO Type II (transitional cell carcinoma)
Undifferentiated	WHO Type III (lymphoepithelial carcinoma)
Basaloid squamous cell carcinoma	No synonym exists (recently described)

SURVIVAL DATA

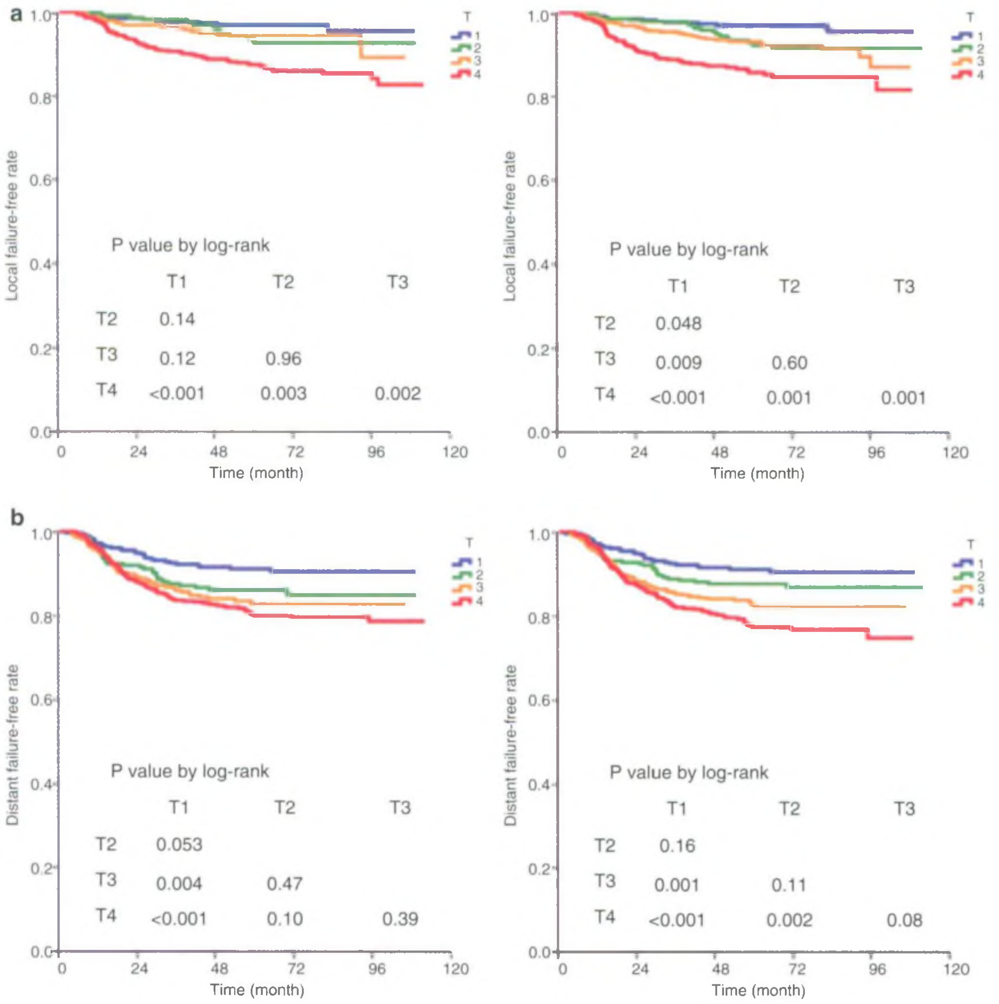

Fig. 9.3 Differences in prognostication of overall survival between the 7th Edition (*Left*) and the 8th Edition (*Right*) by (**a**) T-category, (**b**) N-category, and (**c**) stage group for NPC. From Pan et al., with permission[27]

9

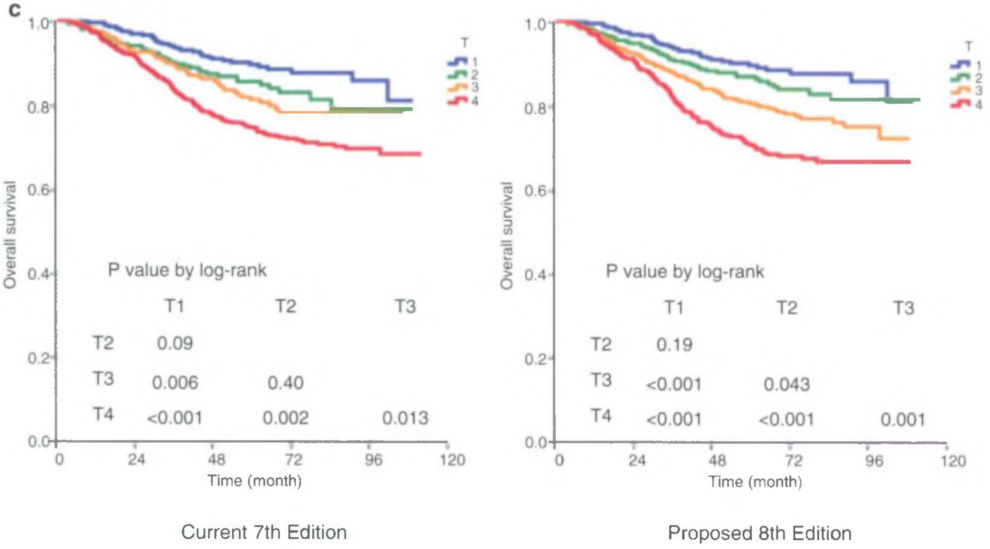

Fig. 9.3 (continued)

Bibliography

1. Fleming I, Cooper J, Henson D, et al. American Joint Committee on Cancer. AJCC cancer staging manual. 5. Lippincott-Raven, Philadelphia: 1997.
2. Sobin LH, Fleming ID. TNM Classification of Malignant Tumors, fifth edition (1997). Union Internationale Contre le Cancer and the American Joint Committee on Cancer. *Cancer.* Nov 1 1997;80(9):1803-1804.
3. Ho J. Stage classification of nasopharyngeal carcinoma: a review. *IARC scientific publications.* 1977(20):99-113.
4. Lee AW, Foo W, Law SC, et al. Staging of nasopharyngeal carcinoma: from Ho's to the new UICC system. *Int J Cancer.* Apr 20 1999;84(2):179-187.
5. Greene FL. *AJCC cancer staging manual.* Vol 1: Springer Science & Business Media; 2002.
6. Sobin LH WC. TNM classification of malignant tumours. *International Union Against Cancer (UICC): 6th edition. New York: Wiley-Liss; 2002.* 2002.
7. Edge SB, Byrd DR, Compton CC, Fritz AG, Greene FL, Trotti A. *AJCC cancer staging manual.* Vol 649: Springer New York; 2010.
8. Sobin L, Gospodarowicz M, Wittekind C. TNM classification of malignant tumors. Hoboken, NJ: Wiley-Blackwell; 2009.
9. Lee AW, Au JS, Teo PM, et al. Staging of nasopharyngeal carcinoma: suggestions for improving the current UICC/AJCC Staging System. *Clinical oncology.* Jun 2004;16(4):269-276.
10. Tang L, Li L, Mao Y, et al. Retropharyngeal lymph node metastasis in nasopharyngeal carcinoma detected by magnetic resonance imaging: prognostic value and staging categories. *Cancer.* Jul 15 2008;113(2):347-354.
11. Tang LL, Li WF, Chen L, et al. Prognostic value and staging categories of anatomic masticator space involvement in nasopharyngeal carcinoma: a study of 924 cases with MR imaging. *Radiology.* Oct 2010;257(1):151-157.
12. Chen L, Liu L-Z, Chen M, et al. Prognostic value of subclassification using MRI in the t4 classification nasopharyngeal carcinoma intensity-modulated radiotherapy treatment. *International Journal of Radiation Oncology* Biology* Physics.* 2012;84(1):196-202.
13. Luo DH, Yang J, Qiu HZ, et al. A new T classification based on masticator space involvement in nasopharyngeal carcinoma: a study of 742 cases with magnetic resonance imaging. *BMC cancer.* 2014;14(1):653.
14. Zhang GY, Huang Y, Cai XY, et al. Prognostic value of grading masticator space involvement in nasopharyngeal carcinoma according to MR imaging findings. *Radiology.* Oct 2014;273(1):136-143.
15. Sze H, Chan LL, Ng W, et al. Should all nasopharyngeal carcinoma with masticator space involvement be staged as T4? *Oral oncology.* 2014;50(12):1188-1195.
16. Xiao Y, Pan J, Chen Y, et al. The prognosis of nasopharyngeal carcinoma involving masticatory muscles: a retrospective analysis for revising T subclassifications. *Medicine (Baltimore).* Jan 2015;94(4):e420.
17. Feng AC, Wu MC, Tsai SY, et al. Prevertebral muscle involvement in nasopharyngeal carcinoma. *International journal of radiation oncology, biology, physics.* Jul 15 2006;65(4):1026-1035.
18. Lee CC, Chu ST, Chou P, Lee CC, Chen LF. The prognostic influence of prevertebral space involvement in nasopharyngeal carcinoma. *Clin Otolaryngol.* Oct 2008;33(5):442-449.
19. Zhou GQ, Mao YP, Chen L, et al. Prognostic value of prevertebral space involvement in nasopharyngeal carcinoma based on intensity-modulated radiotherapy. *International journal of radiation oncology, biology, physics.* Mar 1 2012;82(3):1090-1097.
20. Som PM, Curtin HD, Mancuso AA. Imaging-based nodal classification for evaluation of neck metastatic adenopathy. *AJR. American journal of roentgenology.* Mar 2000;174(3):837-844.
21. Ng WT, Lee AW, Kan WK, et al. N-staging by magnetic resonance imaging for patients with nasopharyngeal carcinoma: pattern of nodal involvement by radiological levels. *Radiotherapy and oncology : journal of the European Society for Therapeutic Radiology and Oncology.* Jan 2007;82(1):70-75.
22. Li W-F, Sun Y, Mao Y-P, et al. Proposed lymph node staging system using the International Consensus Guidelines for lymph node levels is predictive for nasopharyngeal carcinoma patients from endemic areas treated with intensity modulated radiation therapy. *International Journal of Radiation Oncology* Biology* Physics.* 2013;86(2):249-256.

23. Pan J XY, Qiu S, et al. A Comparison Between the Chinese 2008 and the 7th Edition AJCC Staging Systems for Nasopharyngeal Carcinoma. *American journal of clinical oncology.* 2013;23:192-198.
24. OuYang PY, Su Z, Ma XH, Mao YP, Liu MZ, Xie FY. Comparison of TNM staging systems for nasopharyngeal carcinoma, and proposal of a new staging system. *Br J Cancer.* Dec 10 2013;109(12):2987-2997.
25. Yue D, Xu Y-F, Zhang F, et al. Is replacement of the supraclavicular fossa with the lower level classification based on magnetic resonance imaging beneficial in nasopharyngeal carcinoma? *Radiotherapy and Oncology.* 2014;113(1):108-114.
26. Lee AW, Ng WT, Chan LK, et al. The strength/weakness of the AJCC/UICC staging system (7th edition) for nasopharyngeal cancer and suggestions for future improvement. *Oral oncology.* Oct 2012;48(10):1007-1013.
27. Pan JJ, Ng WT, Zong JF, et al. Proposal for the 8th edition of the AJCC/UICC staging system for nasopharyngeal cancer in the era of intensity-modulated radiotherapy. *Cancer.* Nov 20 2015.
28. Ho FC, Tham IW, Earnest A, Lee KM, Lu JJ. Patterns of regional lymph node metastasis of nasopharyngeal carcinoma: a meta-analysis of clinical evidence. *BMC cancer.* 2012;12(1):98.
29. Ng SH, Chan SC, Yen TC, et al. Staging of untreated nasopharyngeal carcinoma with PET/CT: comparison with conventional imaging work-up. *European journal of nuclear medicine and molecular imaging.* Jan 2009;36(1):12-22.
30. Lee S-w, Nam SY, Im KC, et al. Prediction of prognosis using standardized uptake value of 2-[18F] fluoro-2-deoxy-d-glucose positron emission tomography for nasopharyngeal carcinomas. *Radiotherapy and Oncology.* 2008;87(2):211-216.
31. Liu WS, Wu MF, Tseng HC, et al. The role of pretreatment FDG-PET in nasopharyngeal carcinoma treated with intensity-modulated radiotherapy. *International journal of radiation oncology, biology, physics.* Feb 1 2012;82(2):561-566.
32. Kattan MW, Hess KR, Amin MB, et al. American Joint Committee on Cancer acceptance criteria for inclusion of risk models for individualized prognosis in the practice of precision medicine. *CA: a cancer journal for clinicians.* Jan 19 2016.
33. Chan JKC PB, Kuo TT, Wenig BM, Lee AWM. Tumours of the nasopharynx. In: Eveson BL, JW RP, Sidransky D, editors. World Health Organization classification of tumour, pathology and genetics. Head and neck tumours. *Lyon: IARC Press;.* 2005: 815–897.

9

active HR-HPV types. Immunohistochemistry for p16 overexpression has emerged as a robust surrogate biomarker for HR-HPV–mediated carcinogenesis.[3] It is a surrogate marker for HPV DNA testing because it detects p16 cyclin-dependent kinase inhibitor. p16 is upregulated when HPV16 (and to lesser extent HPV18) oncoproteins degrade p53 and pRB. p16 overexpression is used as the proxy for HPV-associated cancers. All oropharyngeal cancers should be tested for p16. Those that do not overexpress p16, or for which p16 testing is not performed, are staged using the staging system in Chapter 11, the separate chapter for p16 negative oropharyngeal cancer.

Human papillomavirus (HPV)-related squamous cell carcinoma of the oropharynx (HPV-OPSCC, Table 10.1) is caused by HR-HPV and originates from the reticulated epithelium lining the crypts of the lingual and palatine tonsils. It represents an epidemiologically, pathologically, and clinically distinct form of head and neck squamous cell carcinoma.

Cervical metastasis of an unknown primary site in level II/III that is p16 positive with histology consistent with HPV-mediated oropharyngeal carcinoma (OPC) also should be staged with this system. Additional testing for HR-HPV and Epstein–Barr virus-encoded small RNAs (EBER) by *in situ* hybridization is recommended to confirm HPV mediation and to exclude Epstein-Barr virus (EBV) origin, which suggests nasopharyngeal origin. The "T0" category has been eliminated for most head and neck squamous cell cancers, and those with cervical nodes without a known primary cancer are staged using the cervical node system. However, cases of HPV-associated (p16 positive cancers) cervical node with no apparent primary are an exception. These cases are staged using the system defined in this chapter and using the "T0" category.

Table 10.1 Synonyms for HPV-Mediated Oropharyngeal Cancer

Synonyms for HPV-Mediated Oropharyngeal Cancer
HPV positive oropharyngeal squamous cell carcinoma
p16-positive oropharyngeal squamous cell carcinoma
non-keratinizing oropharyngeal squamous cell carcinoma

Current TNM staging of HPV-related OPC tumors is non-discriminating due to the unique nature of the disease. To enhance the accuracy reflecting the biological behavior of these cancers and to increase utility of staging, OPC staging will be separately described as either p16 overexpressing (p16+), or p16 negative (p16-). Usually, p16+ is either diffuse and strong or entirely negative. The cut point for determining p16+ by immunohistochemistry is nuclear expression with $\geq$ +2/+3 intensity and $\geq$ 75% distribution (Fig. 10.1). Cytoplasmic staining is common but does not impact determination of p16 status. Oropharyngeal cancers with p16 expression of weak intensity or limited distribution should be staged using OPC p16- guidelines (see Chapter 11). Additionally, p16+ presently is considered only for oropharyngeal cancer; the role of p16+ at other anatomic sites is unclear as yet. Direct detection of HPV will not be used as a defining factor due to its difficulty in universal availability and applicability, cost, and failure to stratify survival as well as p16 overexpression.

A staging system that recognizes the rising incidence of epidemic proportions of HPV-related cancers of the oropharynx is needed for better discrimination between stages. T categories will remain the same as traditional OPC for both p16+ and p16-, except that the p16+ classification will not include a Tis category, and there will not be a T4b within T4.

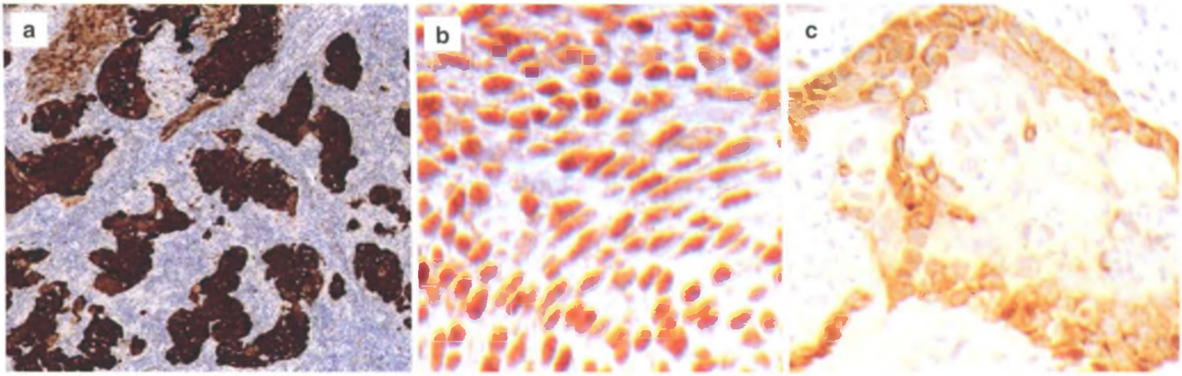

Fig. 10.1 In the context of oropharyngeal cancer, the optimal cut-off for p16 overexpression as a surrogate HR-HPV biomarker is $\geq$+2/+3 nuclear staining intensity (+/- cytoplasmic staining) with $\geq$75% distribution.[4] (**a**) The typical p16 overexpression phenotype is greater than this threshold. Here diffuse, robust nuclear and cytoplasmic overexpression is seen. (**b**) A rarer positive pattern is that of p16 overexpression limited to nuclei. (**c**) Nonspecific cytoplasmic p16 expression is usually associated with limited staining distribution. Carcinomas with this staining pattern are excluded from this chapter

HPV-Mediated (p16+) Oropharyngeal Cancer

10

Brian O'Sullivan, William M. Lydiatt, Bruce H. Haughey, Margaret Brandwein-Gensler, Christine M. Glastonbury, and Jatin P. Shah

CHAPTER SUMMARY

Cancers Staged Using This Staging System

Human papillomavirus (HPV)-related squamous cell carcinoma of the oropharynx (HPV-OPSCC)

Cancers Not Staged Using This Staging System

These histopathologic types of cancer...	Are staged according to the classification for...	And can be found in chapter...
p16- cancers of the oropharynx	Oropharynx (p16-) and hypopharynx	11

Summary of Changes

This is a new classification for the AJCC Cancer Staging Manual, 8th Edition (8th Edition).

ICD-O-3 Topography Codes

Code	Description
C01.9	Base of tongue, NOS
C02.4	Lingual tonsil
C05.1	Soft palate, NOS
C05.2	Uvula
C09.0	Tonsillar fossa
C09.1	Tonsillar pillar
C09.8	Overlapping lesion of tonsil
C09.9	Tonsil, NOS
C10.0	Vallecula
C10.2	Lateral wall of oropharynx
C10.3	Posterior pharyngeal wall
C10.8	Overlapping lesion of oropharynx
C10.9	Oropharynx, NOS
C11.1	Pharyngeal tonsils

WHO Classification of Tumors

Code	Description
8070	Squamous cell carcinoma, nonkeratinizing
8070	HPV-mediated squamous carcinoma
8070	p16+ squamous carcinoma
8083	Basaloid squamous carcinoma

Barnes L, Eveson JW, Reichart P, Sidransky D, eds. World Health Organization Classification of Tumours Pathology and Genetics of Head and Neck Tumours. Lyon: IARC; 2005.

INTRODUCTION

The meteoric rise in the incidence of high-risk HPV (HR-HPV)–associated cancers of the tonsil and tongue base has posed numerous challenges in its diagnosis, management strategies, and outcomes reporting.[1] Due to its unique biological behavior, the established staging criteria and staging system reported in the AJCC Cancer Staging Manual, 7th Edition (7th Edition), is felt to be inadequate to accurately define the natural history of this disease.[2] This disease occurs more often in younger, healthier individuals with little or no tobacco exposure, and has a better prognosis than traditional HPV-unrelated cancers in this site. HPV16/18 are the most commonly detected transcriptionally

To access the AJCC cancer staging forms, please visit www.cancerstaging.org.

© American Joint Committee on Cancer 2017

M.B. Amin et al. (eds.), *AJCC Cancer Staging Manual, Eighth Edition*, DOI 10.1007/978-3-319-40618-3_10

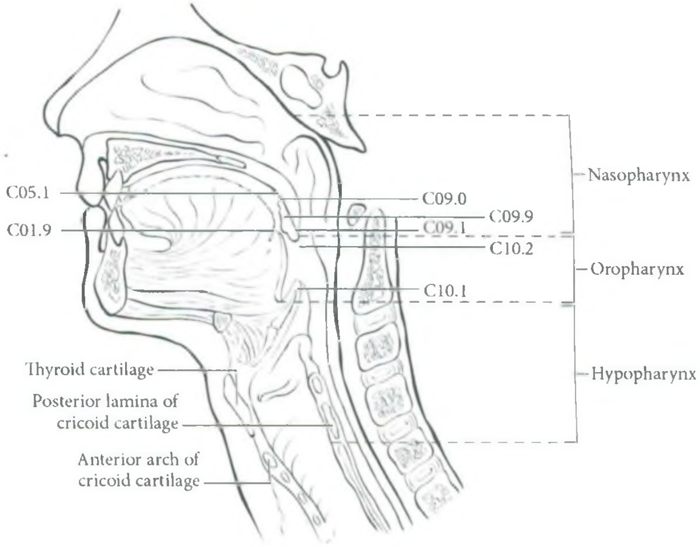

Fig. 10.2 Sagittal view of the face and neck depicting the subdivisions of the pharynx

Like all other pathologically staged tumors, p16+ also has a clinical TNM classification applicable to all cases before treatment (surgically and nonsurgically treated cases) and a pathological classification confined to cases managed with surgery following examination of the primary tumor and neck dissection specimens.

ANATOMY

Primary Site(s)

The oropharynx is the portion of the continuity of the pharynx extending from the plane of the superior surface of the soft palate to the superior surface of the hyoid bone (or vallecula). It includes the base of the tongue, including the lingual tonsil; the inferior (anterior) surface of the soft palate and the uvula; the anterior and posterior tonsillar pillars with the palatine tonsils; the glossotonsillar sulci; and the lateral and posterior pharyngeal walls. (Fig. 10.2)

HPV-mediated cancers most commonly arise in the lymphatic tissue of the palatine and lingual tonsil but may arise in any of the regions of the oropharynx.

Regional Lymph Nodes

Oropharyngeal cancers usually involve upper and midjugular lymph nodes and (less commonly) submental/submandibular nodes. Base-of-tongue cancers commonly manifest bilateral lymphatic drainage.

Unknown primary (T0) nodal positive disease should be staged using this section if a node is p16+ and no primary site is identified.

Metastatic Sites

The most common site of distant metastasis is the lung followed by bone.

RULES FOR CLASSIFICATION

Clinical Classification

Clinical stage is based on reports from a large dataset from five North American and two European Centers, the International Collaboration for Oropharyngeal Cancer Network for Staging (ICON-S). The ICON-S is a validation study following the Princess Margaret Hospital report and contains approximately 3,000 cases, about 2,000 of which are HPV+.[5]

This study showed that traditional N0 to N2b nodal stages are homogeneous for outcome within T1 and T2 categories (creating Stage I). N2c and T3 have an intermediate stage (Stage II), and T4 and N3 are the least favorable group (Stage III). Stage IV is reserved for distant metastases. T categories are the same for clinical and pathological staging.

Clinical staging is generally employed for squamous cell carcinomas of the pharynx. Assessment is based primarily on inspection: indirect and direct endoscopy, as well as imaging. Palpation of sites (when feasible) and of neck nodes

is essential. Diagnostic biopsy of the primary and/or distant metastatic sites and the neck are part of clinical staging. Neurologic evaluation of all cranial nerves is required.

In clinical evaluation, the maximum size of any nodal mass should be measured. There are three categories of clinically involved nodes for the oropharynx: N1, N2, and N3. Midline nodes are considered ipsilateral nodes. Superior mediastinal lymph nodes are considered regional lymph nodes (level VII). In addition to the components to describe the N category, regional lymph nodes also should be described according to the level of the neck that is involved. Provide a description or map of the regional lymph nodes and node groups that the cancer affects. The role of extranodal extension is less obvious in p16+ oropharyngeal cancer. It is not a factor in staging this disease.

Imaging

Contrast-enhanced computed tomography (CT) is often the first imaging study of choice with a new neck mass that is suspected to be adenopathy, or when either metastatic nodal disease or a primary oropharyngeal tumor is detected. Magnetic resonance (MR) imaging is the alternate cross-sectional modality which may be employed for initial staging, post-treatment evaluation or surveillance. Both modalities allow excellent evaluation of the primary oropharyngeal tumor and of nodal drainage sites. Positron emission tomography (PET)/CT is being used with greater frequency for staging, treatment evaluation, and surveillance. It may offer an advantage in the detection of an otherwise clinically occult primary tumor and in exclusion of a viable tumor in residual enlarged nodes after chemoradiation.

Imaging staging of a known oropharyngeal tumor requires a systematic review of the primary tumor, nodal drainage sites, and imaged potential sites of spread. For this purpose, structured reporting may offer advantages to the radiologist for ensuring that critical information is not omitted.

Primary tumor

The subsite of the oropharynx should be clarified (palatine/lingual tonsil, tonsillar pillar, posterior pharyngeal wall, soft palate). The size of the primary tumor should be measured along its longest diameter to determine whether this is T1 (≤2 cm), T2 (> 2 cm and ≤ 4 cm) or T3 (> 4 cm). It may be difficult to be certain by imaging whether a base-of-tongue tumor is extending along the mucosal surface of the lingual aspect of the epiglottis (also T3) or whether the exophytic tumor is abutting against this surface. In these situations, clarification by direct clinical observation is essential. T4 disease is determined by invasion (1) anteriorly to the extrinsic muscles of the tongue in the floor of the mouth or from the soft palate anteriorly to the hard palate, (2) laterally into the pterygoid muscles or mandible, (3) inferiorly to the larynx, or (4) superiorly to the skull base or beyond. Skull base involvement also necessitates careful evaluation for perineural and intracranial spread of the tumor. Description of the extent of invasion of these specific structures with T4 tumors is important for radiation planning and should be clearly articulated in the report.

CT and MR imaging afford some view of the lungs for metastases, albeit with only a small volume of the lungs being imaged and with low sensitivity for MR imaging. A complete staging report includes evaluation of the lung apices for potential metastases and of the bones of the skull and cervical spine for metastatic disease. PET/CT allows more accurate and complete evaluation for distant metastatic disease, which is most often to the lung, bone, and liver with HPV-related squamous cell carcinoma (SCCa).

Specific pitfalls in imaging relate to the imaging modality being used or to strict interpretation of the definitions of tumor involvement. Detection of metastatic involvement of nodes with either CT or MR imaging requires careful evaluation of multiple morphological features: size, shape, density (intensity on MR imaging), necrosis, and extranodal extension of tumor. It is important to review all criteria and not merely the size of a lymph node. This also should be performed in conjunction with knowledge of the expected drainage pattern of the tumor. As stated previously, oropharyngeal tumors most often drain to the upper- and midjugular nodes (levels II and III, respectively), and bilateral drainage is frequent. These nodal sites should be scrutinized carefully for abnormal shape, size, contour, and texture. The retropharyngeal nodes (RPN) also should be evaluated, particularly when a posterior pharyngeal wall tumor is present. Keep in mind that unless cystic or necrotic, RPN frequently appear isodense to the adjacent prevertebral muscles on CT, making them readily overlooked.

A common pitfall of PET/CT that is particularly pertinent to the evaluation of HPV-related SCCa is the absence of elevated fluorodeoxyglucose (FDG) uptake in cystic nodes. For this reason, it is imperative that the CT component of the staging examination be carefully evaluated for cystic (low-density) nodes, which have too little solid tissue to demonstrate elevated FDG activity. With this in mind, PET/CT for head and neck malignancies are best performed with contrast-enhanced CT portions of the examination and preferably with dedicated smaller field of view (FOV) neck images. When iodinated contrast is not employed for PET/CT, it also is difficult to evaluate for specific sites of disease involvement, which might upstage a tumor. For example, determining the presence of medial or lateral pterygoid muscle involvement (both T4) by a tonsillar carcinoma is difficult when relying on noncontrast large FOV CT for correlation with FDG-PET uptake.

Some subtleties of tumor involvement for staging also require clarification. The palatoglossus muscle forms the muscle bulk of the anterior tonsillar pillar and may be

invaded with even a relatively small tonsillar or tonsillar pillar tumor. Although palatoglossus is defined as an extrinsic muscle of the tongue, involvement of this muscle *within the oropharynx* does not denote a significantly poorer prognosis for the patient and does not change the T category. A final imaging pitfall for consideration is during the imaging evaluation of a carcinoma of unknown primary (CUP). Most often, the small primary tumor is determined to be in the ipsilateral pharyngeal or lingual tonsil, but particularly with HPV-related tumors, it can be subtle and readily overlooked. It is important to evaluate carefully the CT or MR imaging for asymmetric size of ipsilateral tonsillar tissue and for heterogeneous tonsillar enhancement. PET/CT is an alternate modality if the lesion is not found on CT or MR images, and is best performed prior to oropharyngeal biopsies to avoid a false-positive FDG uptake.

PET/MR imaging is an emerging modality; currently, there are few sites where these scanners are available for clinical imaging. MR images of the primary tumor and nodes and measurements of FDG activity are obtained simultaneously, resulting in fused PET/MR images. There presently is little literature on PET/MR imaging for head and neck tumors and none yet demonstrating increased accuracy of PET/MR imaging over PET/CT for the staging, followup, or surveillance of head and neck tumors. There is reduced ionizing radiation to the patient with PET/MR imaging as compared to PET/CT.

Pathological Classification

T categories are the same for clinical and pathological staging. However, a different pathological N category is proposed for p16+ OPC, based on data from the Washington University School of Medicine.[6–8] High metastatic node number rather than extracapsular spread, laterality, or nodal size is the key prognosticator in surgically resected, neck-dissected p16+ oropharynx cancer.[7]

One interesting finding in this surgically managed dataset is that N3 disease behaves unusually well and is equivalent to N1; therefore, N3 is eliminated from pN categorization. The favorable outcome for N3 is not apparent in the clinical dataset. Unlike other head and neck sites, extranodal extension (ENE) may not have the same prognostic significance, provided that adjuvant treatments are administered according to conventional practice. Additionally, N0 did not discriminate between the N1 category but has been left in the neck category as N0 for data collection and historical reasons.

Primary tumor resection is required to assign pathological stage and may be performed using transoral or conventional techniques. Current trends strongly favor transoral approaches, because they diminish morbidity significantly. For pN, a selective neck dissection will ordinarily include 10 or more lymph nodes, and a radical or modified radical neck dissection will ordinarily include 15 or more ymph nodes. Negative pathologic examination of a lesser number of nodes still mandates a pN0 designation.

Histopathology

Standard nomenclature is unsatisfactory in describing HPV-mediated p16+ oropharyngeal cancers. The descriptor of "poorly differentiated," which is based on a high nuclear/cytoplasmic ratio, is at odds with the known improved prognosis. Therefore, tumor classification as "poorly differentiated oropharyngeal carcinoma" should be avoided. "Basaloid squamous carcinoma" nosology implies palisading tumor cells, basement membrane deposition, and cribriform-like morphology, which also is not the typical phenotype of HPV-mediated p16+ oropharyngeal cancer. The terminology of "oropharyngeal squamous carcinoma, nonkeratinzing-type" is recommended, acknowledging that "nonkeratinizing" is a low-power descriptor. Observation at higher power confirms that individual cell keratinization and tumor maturation is quite common and compatible with the "nonkeratinizing" descriptor. Grading is not relevant in this context.

The histopathology of HPV-mediated p16+ oropharyngeal cancers is characteristic and easily recognizable. The carcinoma may form nests, islands, or ribbons of tumor cells with either limited cytoplasm ("basaloid"), moderate amount of cytoplasm (epithelioid), or individual cell keratinization. If a ribbon-like pattern is present (transitional pattern), then tumor maturation and flattened keratinizing cells are seen. Frank tumor keratinization and keratin pearls usually predict no association with either HPV-mediated carcinogenesis or p16 overexpression. In the event that a keratinizing phenotype does demonstrate p16 overexpression, it should be staged in this chapter.

The "inside out" pattern of maturation is another characteristic feature; keratinizing tumor cells are localized to the periphery of tumor islands, and proliferating tumor cells are centrally located (Fig. 10.3) This is the converse of the maturation pattern usually seen in non-HPV-mediated keratinizing squamous carcinoma. Anaplastic tumor cells with multinucleated or bizarre nuclei can be seen in HPV-mediated p16+ oropharyngeal cancers, and are a poor prognosticator.[8]

Cervical lymph node metastases to level II/III from an unknown primary are staged in this chapter if p16 overexpression is documented and the histology is consistent with HPV-mediated carcinogenesis. These metastatic carcinomas are invariably cystic and include the other findings enumerated above (Fig. 10.4). "Normalization" or maturation of metastatic squamous carcinoma to the point of mimicking a benign cyst is a well-recognized phenomenon; thus the diagnosis of "malignant transformation of a branchial cleft cyst"

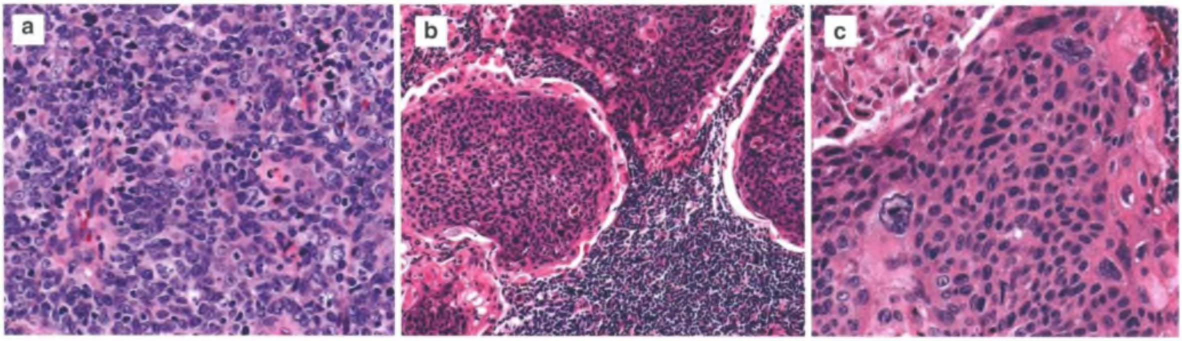

Fig. 10.3 In the context of oropharyngeal cancer, these histologies are predictive of HR-HPV-mediated carcinogenesis and p16 overexpression.[4] (**a**) These cancers typically are composed of basaloid tumor cells with minimal cytoplasm. Individual cell keratinization may be observed at high power; however, overt keratinization is usually not seen at low-power observation. (**b**) "Inside out" maturation—The peripheral rim of the tumor islands is composed of flattened keratinizing tumor cells, while the proliferating more immature cells are centrally located. This is distinct from the usual non-HPV-mediated keratinizing squamous carcinoma, which reveals central keratinization, and proliferating tumor cells at the periphery of tumor islands. (**c**) Anaplastic tumor cells, as well as multinucleated tumor cells can be seen in these carcinomas

Fig. 10.4 HR-HPV-mediated oropharyngeal cancer can form cystic metastases in cervical lymph nodes, whereas the primary carcinoma is usually solid.[4] It is unnecessary to document extranodal extension in this context. This phenotype, plus p16 overexpression, can be especially useful for tumors of unknown primaries. (*Top*) Uniloculated cystic metastases. (*Bottom*) The metastatic carcinoma forms a ribbon-like (transitional) pattern with variable tumor maturation appearing as flattened keratinizing cells

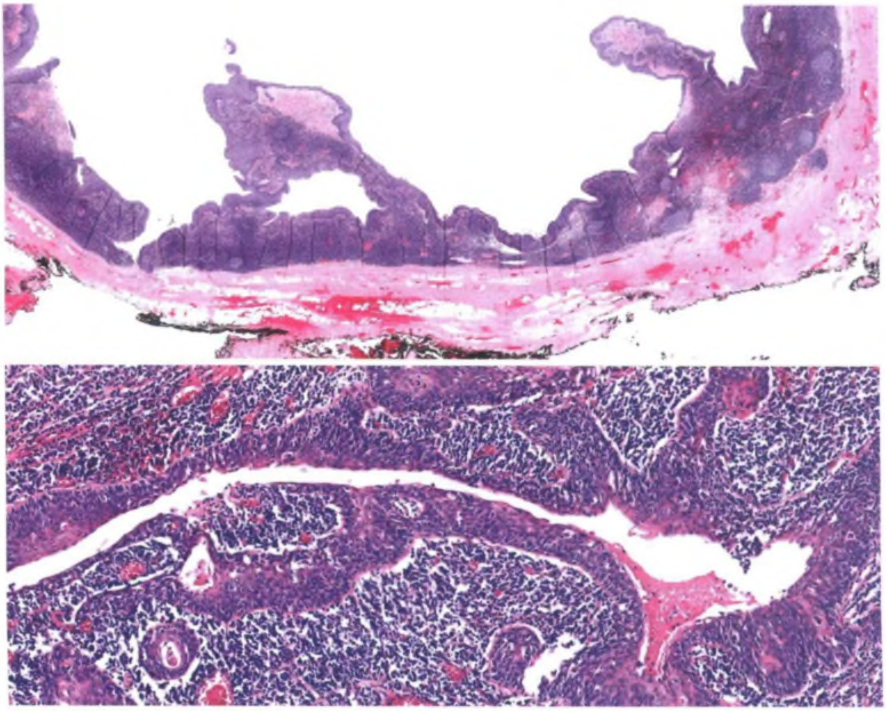

should be rejected. In these cases, additional viral *in situ* hybridization studies (HR-HPV, EBER) are recommended. Ciliated squamous carcinoma cells have been observed within both cystic primary and metastatic HPV-mediated cancers, which may also cause diagnostic confusion.[9]

Finally, there is the potential for confusing nonaggressive pattern of tumor invasion with noninvasive carcinoma. The epithelial crypts of Waldeyer's ring are lined by specialized reticulated epithelium, which is thinner than stratified squamous epithelium and characterized by abundant intraepithelial lymphocytes and mononuclear cells. The basement membrane in this region is composed of fine, discontinuous fibers, thus enabling lymphocyte trafficking.[10,11] Because of the lack of a complete, well-defined basement membrane, the idea of *in situ* carcinoma within these crypts is incorrect. HPV-mediated oropharyngeal carcinoma most often reveals

a nonaggressive pattern of invasion (e.g., transitional pattern, which is reminiscent of urinary bladder carcinoma). The smooth, ribbon-like contours of this pattern plus the lack of desmoplastic response might lead the pathologist toward a mistaken diagnosis of *in situ* carcinoma (Fig. 10.4).

PROGNOSTIC FACTORS

Prognostic Factors Required for Stage Grouping

p16 Testing

p16 immunotesting is mandatory to use this staging system for HPV-associated cancer. HPV by *in situ* hybridization (ISH) may be done as an alternative. If a case of oropharyngeal cancer does not have p16 or HPV by ISH, then the case is staged by the p16- negative system (Chapter 11).

Additional Prognostic Factors Recommended for Clinical Care

Overall Health

In addition to the importance of the TNM factors outlined previously, the overall health of these patients clearly influences outcome. An ongoing effort to better assess prognosis using both tumor- and nontumor-related factors is underway. Chart abstraction will continue to be performed by cancer registrars to obtain important information regarding specific factors related to prognosis. These data will then be used to further hone the predictive power of the staging system in future revisions. AJCC Level of Evidence: III

Comorbidity

Comorbidity can be classified by specific measures of additional medical illnesses.[12] Accurate reporting of all illnesses in the patients' medical record is essential to assess these parameters. General performance measures are helpful in predicting survival. The AJCC strongly recommends that the clinician report performance status using the Eastern Cooperative Oncology Group (ECOG)/Zubrod or Karnofsky performance measures, along with standard staging information. An interrelationship between each of the major performance tools exists. AJCC Level of Evidence: II

Zubrod/ECOG Performance Scale	
0	Fully active, able to carry on all predisease activities without restriction (Karnofsky 90–100)
1	Restricted in physically strenuous activity but ambulatory and able to carry out work of a light or sedentary nature; for example, light housework, office work (Karnofsky 70–80)
2	Ambulatory and capable of all self-care but unable to carry out any work activities; up and about more than 50% of waking hours (Karnofsky 50–60)
3	Capable of only limited self-care, confined to bed or chair 50% or more of waking hours (Karnofsky 30–40)
4	Completely disabled; cannot carry on self-care; totally confined to bed or chair (Karnofsky 10–20)
5	Death (Karnofsky 0)

Lifestyle Factors

Lifestyle factors such as tobacco and alcohol abuse negatively influence survival. Accurate recording of smoking in pack-years and alcohol in number of days drinking per week and number of drinks per day will provide important data for future analysis. Nutrition is important to prognosis and will be indirectly measured by weight loss of >5% of body weight in the previous 6 months.[13] Depression adversely affects quality of life and survival. Notation of a previous or current diagnosis of depression should be recorded in the medical record.[14] AJCC Level of Evidence: III

Tobacco Use

The role of tobacco as a negative prognostic factor is well established. However, exactly how this could be codified in the staging system is less clear. At this time, smoking is known to have a deleterious effect on prognosis but is hard to apply accurately to the staging system. AJCC Level of Evidence: III

Smoking history should be collected as an important element of the demographics and may be included in Prognostic Groups in the future. For practicality, the minimum standard should classify smoking history as never, ≤ 10 pack-years, > 10 but ≤ 20 pack-years, or > 20 pack-years.

RISK ASSESSMENT MODELS

The AJCC recently established guidelines that will be used to evaluate published statistical prediction models for the purpose of granting endorsement for clinical use.[15] Although this is a monumental step toward the goal of precision medicine, this work was published only very recently. Therefore, the existing models that have been published or may be in clinical use have not yet been evaluated for this cancer site by the Precision Medicine Core of the AJCC. In the future, the statistical prediction models for this cancer site will be evaluated, and those that meet all AJCC criteria will be endorsed.

DEFINITIONS OF AJCC TNM

Definition of Primary Tumor (T)

T Category	T Criteria
T0	No primary identified
T1	Tumor 2 cm or smaller in greatest dimension
T2	Tumor larger than 2 cm but not larger than 4 cm in greatest dimension
T3	Tumor larger than 4 cm in greatest dimension or extension to lingual surface of epiglottis
T4	Moderately advanced local disease<br>Tumor invades the larynx, extrinsic muscle of tongue, medial pterygoid, hard palate, or mandible or beyond*

*Mucosal extension to lingual surface of epiglottis from primary tumors of the base of the tongue and vallecula does not constitute invasion of the larynx.

Definition of Regional Lymph Node (N)

Clinical N (cN)

N Category	N Criteria
NX	Regional lymph nodes cannot be assessed
N0	No regional lymph node metastasis
N1	One or more ipsilateral lymph nodes, none larger than 6 cm
N2	Contralateral or bilateral lymph nodes, none larger than 6 cm
N3	Lymph node(s) larger than 6 cm

Pathological N (pN)

N Category	N Criteria
NX	Regional lymph nodes cannot be assessed
pN0	No regional lymph node metastasis
pN1	Metastasis in 4 or fewer lymph nodes
pN2	Metastasis in more than 4 lymph nodes

Definition of Distant Metastasis (M)

M Category	M Criteria
M0	No distant metastasis
M1	Distant metastasis

AJCC PROGNOSTIC STAGE GROUPS

Clinical

When T is...	And N is...	And M is...	Then the stage group is...
T0, T1 or T2	N0 or N1	M0	I
T0, T1 or T2	N2	M0	II
T3	N0, N1 or N2	M0	II
T0, T1, T2, T3 or T4	N3	M0	III
T4	N0, N1, N2 or N3	M0	III
Any T	Any N	M1	IV

Pathological

When T is...	And N is...	And M is...	Then the stage group is...
T0, T1 or T2	N0, N1	M0	I
T0, T1 or T2	N2	M0	II
T3 or T4	N0, N1	M0	II
T3 or T4	N2	M0	III
Any T	Any N	M1	IV

REGISTRY DATA COLLECTION VARIABLES

1. Tumor location (posterior wall nasopharynx or pharyngeal tonsils)
2. Number and size of nodes
3. Perineural invasion
4. Extranodal extension gross ≥2 cm or microscopic
5. Smoking history and pack years

HISTOLOGIC GRADE (G)

No grading system exists for HPV-mediated oropharyngeal tumors.

HISTOPATHOLOGIC TYPE

The histopathology of HPV-mediated p16+ oropharyngeal cancers is characteristic and easily recognizable.

Bibliography

1. Mehanna H, Jones TM, Gregoire V, Ang KK. Oropharyngeal carcinoma related to human papillomavirus. *Bmj*. 2010;340:c1439.
2. Straetmans JM, Olthof N, Mooren JJ, de Jong J, Speel EJ, Kremer B. Human papillomavirus reduces the prognostic value of nodal involvement in tonsillar squamous cell carcinomas. *The Laryngoscope*. Oct 2009;119(10):1951-1957.
3. El-Naggar AK, Westra WH. p16 expression as a surrogate marker for HPV-related oropharyngeal carcinoma: A guide for interpretative relevance and consistency. *Head & neck*. 2012;34(4):459-461.
4. Schlecht NF, Brandwein-Gensler M, Nuovo GJ, et al. A comparison of clinically utilized human papillomavirus detection methods in head and neck cancer. *Modern pathology : an official journal of the United States and Canadian Academy of Pathology, Inc*. Oct 2011;24(10):1295-1305.
5. O'Sullivan B, Huang SH, Su J, et al. Development and validation of a staging system for HPV-related oropharyngeal cancer by the International Collaboration on Oropharyngeal cancer Network for Staging (ICON-S): a multicentre cohort study. *The lancet oncology*. Feb 26 2016.
6. Sinha P, Kallogjeri D, Gay H, et al. High metastatic node number, not extracapsular spread or N-classification is a node-related prognosticator in transorally-resected, neck-dissected p16-positive oropharynx cancer. *Oral oncology*. May 2015;51(5):514-520.
7. Haughey BH. Personal Communication. In: Lydiatt W, Shah JP, eds2015.
8. Lewis Jr JS, Scantlebury JB, Luo J, Thorstad WL. Tumor cell anaplasia and multinucleation are predictors of disease recurrence in oropharyngeal squamous cell carcinoma, including among just the human papillomavirus-related cancers. *The American journal of surgical pathology*. 2012;36(7):1036.
9. Bishop JA, Westra WH. Ciliated HPV-related Carcinoma: A Well-differentiated Form of Head and Neck Carcinoma That Can Be Mistaken for a Benign Cyst. *The American journal of surgical pathology*. Nov 2015;39(11):1591-1595.
10. Gloghini A, Colombatti A, Bressan G, Carbone A. Basement membrane components in lymphoid follicles: immunohistochemical demonstration and relationship to the follicular dendritic cell network. *Human pathology*. Oct 1989;20(10):1001-1007.
11. Perry ME. The specialised structure of crypt epithelium in the human palatine tonsil and its functional significance. *J Anat*. Aug 1994;185(Pt 1):111-127.
12. Piccirillo JF. Inclusion of comorbidity in a staging system for head and neck cancer. *Oncology (Williston Park)*. Sep 1995;9(9):831-836; discussion 841, 845-838.
13. Couch ME, Dittus K, Toth MJ, et al. Cancer cachexia update in head and neck cancer: Pathophysiology and treatment. *Head & neck*. Jul 2015;37(7):1057-1072.
14. Lazure KE, Lydiatt WM, Denman D, Burke WJ. Association between depression and survival or disease recurrence in patients with head and neck cancer enrolled in a depression prevention trial. *Head & neck*. 2009;31(7):888-892.
15. Kattan MW, Hess KR, Amin MB, et al. American Joint Committee on Cancer acceptance criteria for inclusion of risk models for individualized prognosis in the practice of precision medicine. *CA: a cancer journal for clinicians*. Jan 19 2016.

10

Oropharynx (p16-) and Hypopharynx

11

William M. Lydiatt, John A. Ridge, Snehal G. Patel,
David M. Brizel, Bruce H. Haughey,
Christine M. Glastonbury, Margaret Brandwein-Gensler,
Brian O'Sullivan, and Jatin P. Shah

CHAPTER SUMMARY

Cancers Staged Using This Staging System

p16 negative (p16-) squamous cancers of the oropharynx; oropharyngeal cancers without a p16 immunostain performed; and all cancers of the hypopharynx. Minor salivary cancers and neuroendocrine carcinomas of the oropharynx and hypopharynx. p16 assessment is necessary only for squamous carcinomas. Typically, p16-negative squamous carcinomas are keratinizing.

Cancers Not Staged Using This Staging System

These histopathologic types of cancer…	Are staged according to the classification for…	And can be found in chapter…
P16-positive (p16+) oropharyngeal cancers	Human papillomavirus (HPV)-mediated (p16+) oropharyngeal cancer	10
Nasopharyngeal cancers	Nasopharynx	9

Summary of Changes

Change	Details of Change	Level of Evidence
Anatomy – Primary Site(s)	Occult Primary Tumor: Staging of the patient who presents with Epstein-Barr virus (EBV)-unrelated and HPV-unrelated metastatic cervical lymphadenopathy is not included in this chapter.	IV
Definition of Regional Lymph Node (N)	Separate approaches have been described for N categorization for HPV-related and HPV-unrelated cancers.	II[1,2]
Definition of Regional Lymph Node (N)	Separate N category approaches have been described for patients treated without cervical lymph node dissection (clinical N (cN)) and patients treated with cervical lymph neck dissection (pathological N (pN)).	II[1,2]
Definition of Regional Lymph Node (N)	Extranodal extension (ENE) is introduced as a descriptor in N categorization for all HPV-unrelated cancers.	II[1]
Definition of Regional Lymph Node (N)	ENE in HPV-negative cancers: Only clinically and radiographically overt ENE should be used for cN.	II[1]
Definition of Regional Lymph Node (N)	ENE in HPV-negative cancers: Any pathologically detected ENE is considered ENE(+) and is used for pN.	II[1]

To access the AJCC cancer staging forms, please visit www.cancerstaging.org.

© American Joint Committee on Cancer 2017

M.B. Amin et al. (eds.), *AJCC Cancer Staging Manual, Eighth Edition*, DOI 10.1007/978-3-319-40618-3_11

Change	Details of Change	Level of Evidence
Definition of Regional Lymph Node (N)	ENE in HPV-negative cancers: Presence of ENE is designated pN2a for a single ipsilateral node <3 cm and pN3b for all other node(s).	II[1]
Definition of Regional Lymph Node (N)	Classification of ENE: Clinically overt ENE is classified as ENE_c and is considered ENE(+) for cN.	III[3]
Definition of Regional Lymph Node (N)	Classification of ENE: Pathologically detected ENE is classified as either ENE_{mi} (≤2 mm) or ENE_{ma} (> 2 mm) for data collection purposes only, but both are considered ENE(+) for definition of pN.	III[3]

ICD-O-3 Topography Codes

Code	Description
C12.9	Pyriform sinus
C13.0	Postcricoid region
C13.1	Hypopharyngeal aspect of aryepiglottic fold
C13.2	Posterior wall of hypopharynx
C13.8	Overlapping lesion of hypopharynx
C13.9	Hypopharynx, NOS
C01.9	Base of tongue, NOS
C02.4	Lingual tonsil
C05.1	Soft palate, NOS
C05.2	Uvula
C09.0	Tonsillar fossa
C09.1	Tonsillar pillar
C09.8	Overlapping lesion of tonsil
C09.9	Tonsil, NOS
C10.0	Vallecula
C10.2	Lateral wall of oropharynx
C10.3	Posterior pharyngeal wall
C10.8	Overlapping lesion of oropharynx
C10.9	Oropharynx, NOS
C11.1	Pharyngeal tonsils

WHO Classification of Tumors

Code	Description
8070	Squamous cell carcinoma, conventional
8075	Acantholytic squamous cell carcinoma
8560	Adenosquamous carcinoma
8083	Basaloid squamous cell carcinoma
8051	Carcinoma cuniculatum
8052	Papillary squamous cell carcinoma
8074	Spindle cell squamous carcinoma
8051	Verrucous carcinoma
8082	Lymphoepithelial carcinoma (non-nasopharyngeal)

Barnes L, Eveson JW, Reichart P, Sidransky D, eds. World Health Organization Classification of Tumours Pathology and Genetics of Head and Neck Tumours. Lyon: IARC; 2005.

INTRODUCTION

The epidemic of HPV-mediated oropharyngeal cancer and its significantly different behavior and natural history has resulted in the need to develop two different staging systems for oropharynx cancer: one for HPV-positive and another for HPV-negative cancers.[4,5] Historically, the pharynx was covered in a single chapter, but in the AJCC Cancer Staging Manual, 8th Edition (8th Edition), the recognition of the unique nature of the two virally mediated diseases of the nasopharynx and oropharynx have resulted in dividing one chapter into three. This chapter deals with p16- oropharyngeal cancers and all mucosally based hypopharyngeal cancers. The staging of these two sites will be similar to the AJCC Cancer Staging Manual, 7th Edition (7th Edition), with the exception of the N category and the removal of T0. T0 will be used only when a node is p16+, and therefore staged in the HPV-associated oropharynx chapter, EBV-associated and staged in the nasopharynx chapter, or staged in the cervical node chapter. ENE has such a profound effect on outcome that it has been incorporated into the determination of N.[6]

The effect of ENE on prognosis of head and neck cancers not caused by HPV is profound. Accounting for this important prognostic feature was considered critical in revising staging. ENE will increase the pathological nodal category by 1 (as described in the section "Definitions of AJCC TNM").

Most of the data supporting ENE as an adverse prognostic factor is based on histopathological characterization of ENE, especially the distinction between microscopic and macroscopic ENE.[3,7,8] Therefore, only unquestionable ENE is to be used for clinical staging. As per the "uncertain rule" of the AJCC/Union for International Cancer Control (UICC) staging, which mandates that the lower category should always be assigned in ambiguous cases, a case should be categorized as ENE(−) unless there is unquestionable ENE. For clinical ENE, the known inability of current imaging modalities to define ENE accurately mandated that stringent criteria are met prior to

assigning a clinical diagnosis of ENE.[9] However, unambiguous evidence of gross ENE on clinical examination (e.g., invasion of skin; infiltration of musculature/dense tethering to adjacent structures; or cranial nerve, brachial plexus, sympathetic trunk, or phrenic nerve invasion with dysfunction) supported by strong radiographic evidence permit classification of disease as ENE(+). Pathological ENE also will be clearly defined below. Again, if there is doubt or uncertainty of the presence of ENE, the case should be categorized as ENE(−).

TNM staging of cancers of the oropharynx not associated with HPV and cancers of the hypopharynx is critical to understanding their natural history. The stage groupings provide stratification for prognosis that is valuable to the clinician and the individual patient. Taking the p16+, HPV-mediated cancers out of this staging increases its utility and accuracy.

ANATOMY

Primary Site(s)

Cancers arise in the mucosa of the oropharynx and hypopharynx (Fig. 11.1). They invade into neighboring structures as they advance.

Oropharynx

The oropharynx is the portion of the continuity of the pharynx extending from the plane of the superior surface of the soft palate to the superior surface of the hyoid bone (or vallecula). It includes the base of the tongue, the inferior (anterior) surface of the soft palate and the uvula, the anterior and posterior tonsillar pillars, the glossotonsillar sulci, the pharyngeal tonsils, and the lateral and posterior pharyngeal walls.

Hypopharynx

The hypopharynx is that portion of the pharynx extending from the plane of the superior border of the hyoid bone (or vallecula) to the plane corresponding to the lower border of the cricoid cartilage. It includes the pyriform sinuses (right and left), the lateral and posterior hypopharyngeal walls, and the postcricoid region. The postcricoid area extends from the level of the arytenoid cartilages and connecting folds to the plane of the inferior border of the cricoid cartilage. It connects the two pyriform sinuses, thus forming the anterior wall of the hypopharynx. The pyriform sinus extends from the pharyngoepiglottic fold to the upper end of the esophagus at the lower border of the cricoid cartilage and is bounded laterally by the lateral pharyngeal wall and medially by the lateral surface of the aryepiglottic fold and the arytenoid and cricoid cartilages. The posterior pharyngeal wall extends from the level of the superior surface of the hyoid bone (or vallecula) to the inferior border of the cricoid cartilage and from the apex of one pyriform sinus to the other.

Regional Lymph Nodes

The risk of regional nodal spread from cancers of the pharynx is high. Oropharyngeal cancers usually involve upper and mid-jugular lymph nodes and (less commonly) submental/submandibular nodes. Base of tongue cancers often manifest bilateral lymphatic drainage. Hypopharyngeal cancers spread to adjacent parapharyngeal, paratracheal, and mid- and lower jugular nodes. Bilateral lymphatic drainage is common. In clinical evaluation, the maximum size of the nodal mass should be measured. Midline nodes are considered ipsilateral nodes. Superior mediastinal lymph nodes are considered regional

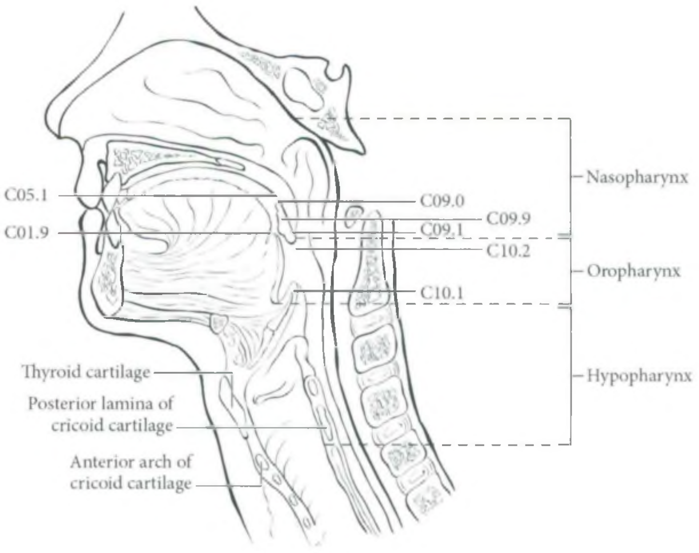

Fig. 11.1 Sagittal view of the face and neck depicting the subdivisions of the pharynx

lymph nodes (level VII). In addition to the components to describe the N category, regional lymph nodes should also be described according to the level of the neck that is involved.

Metastatic Sites

The lungs are the most common site of distant metastases; skeletal or hepatic metastases occur less often. Mediastinal lymph node metastases are considered distant metastases, except level VII lymph nodes.

RULES FOR CLASSIFICATION

Clinical Classification

Clinical staging is essential for squamous cell carcinomas of the oropharynx and hypopharynx. Assessment is based primarily on inspection and on indirect and direct endoscopy. Palpation of sites (when feasible) and of neck nodes is essential. Neurologic evaluation of all cranial nerves is required. Complete endoscopy, usually under general anesthesia, is performed after completion of other staging studies, to assess the surface extent of the tumor accurately and to assess deep involvement by palpation for muscle invasion and to facilitate biopsy. A careful search for other primary tumors of the upper aerodigestive tract may be indicated because of the potential of multiple independent primary tumors occurring simultaneously.

In clinical evaluation, the maximum size of any nodal mass should be measured. There are three categories of clinically involved nodes for the oropharynx: N1, N2, and N3. Midline nodes are considered ipsilateral nodes. Superior mediastinal lymph nodes are considered regional lymph nodes (level VII). In addition to the components to describe the N category, regional lymph nodes should also be described according to the level of the neck that is involved. Provide a description or map of the regional lymph nodes and node groups that the cancer affects. Unambiguous evidence of gross ENE extension (i.e., defined as invasion of skin, infiltration of musculature/fixation to adjacent structures on clinical examination, cranial nerve, brachial plexus, sympathetic trunk or phrenic nerve invasion with dysfunction) is a sufficiently high threshold to classify these as clinical ENE (ENE_c) and qualifies as ENE(+) for definition of cN.

Evidence of cranial nerve dysfunction should be sought (testing sensation and motion to command) and skin should be examined for evidence of invasion by underlying nodes. Palpable neck nodes should be considered in terms of their location (level in the neck), size, number, character (smooth or irregular), attachment to other nodes, and mobility. Nodes that do not move in all directions may be invading nearby structures. Invasion of the sternomastoid muscle and or cranial nerves is associated with lateral motion but restricted ability to move the node along the cranial-caudal axis. Inability to move the node at all (without moving the head) is worrisome for ENE, though the suspicion should be tempered for smaller nodes with limited mobility in level II. Assignment of ENE should be based almost entirely upon the physical examination, rather than upon imaging studies; gross ENE is required to raise the stage beyond the assignment based upon node size and number and this may be overestimated with current imaging modalities.

The known lack of ability for radiographic imaging accurately to define ENE suggests the need for stringent criteria prior to assigning ENE on a clinical basis. Imaging studies showing amorphous spiculated margins of involved nodes or involvement of internodal fat resulting in loss of normal oval-to-round nodal shape suggest extranodal spread of tumor but are not sufficient without corresponding evidence on physical examination. Pathological examination is necessary for documentation of such disease extent. No imaging study (as yet) can identify microscopic foci in regional nodes or distinguish between small reactive nodes and small malignant nodes (unless central radiographic inhomogeneity is present).

Imaging

Both contrast-enhanced computed tomography (CT) and magnetic resonance (MR) imaging allow excellent evaluation of the primary oropharyngeal or hypopharyngeal tumor and of nodal drainage sites. Positron emission tomography (PET)/CT is being used with greater frequency for staging, treatment evaluation, and surveillance. It may offer an advantage in detection of an otherwise clinically occult primary tumor.

Imaging staging of a known oropharyngeal or hypopharyngeal tumor requires a systematic review of the primary tumor, nodal drainage sites and imaged potential sites of spread. For this purpose, structured reporting may offer advantages to the radiologist for ensuring that critical information is not omitted.

Primary Tumor

The subsite of the oropharynx should be clarified (palatine/lingual tonsil, tonsillar pillar, posterior pharyngeal wall, soft palate). The size of the primary tumor should be measured along its longest diameter to determine whether this is T1 ($\leq$ 2 cm), T2 (> 2 cm and $\leq$ 4 cm) or T3 (> 4 cm). It may be difficult to be certain by imaging whether a base of tongue tumor is extending along the mucosal surface of the lingual aspect of the epiglottis (also T3), or whether the exophytic tumor is abutting against this surface. In these situations, clarification by direct clinical observation is essential. T4 disease is determined by invasion anteriorly to the extrinsic muscles of the tongue in the floor of the mouth or from the soft palate anteriorly to the hard palate, laterally into the pterygoid muscles or

mandible, inferiorly to the larynx or superiorly to the skull base or beyond. Skull base involvement also necessitates careful evaluation for perineural and intracranial spread of tumor. Cross-sectional imaging of hypopharyngeal carcinoma is recommended to define the extent of the primary tumor, particularly its deep extent in relationship to adjacent structures (i.e., larynx, cricoid and thyroid cartilage, cervical vertebrae, and carotid sheath). CT is preferred currently because it entails less motion artifact than MR imaging.

CT and MR imaging afford some view of the lungs for metastases, albeit with only a small volume of the lungs being imaged and with low sensitivity for MR imaging. A complete staging report includes evaluation of the lung apices for potential metastases and of the bones of the skull and cervical spine for metastatic disease. PET/CT allows more accurate and complete evaluation for distant metastatic disease, which is most often to the lung, bone, and liver.

There are specific pitfalls in imaging that relate to the imaging modality being used or to strict interpretation of the definitions of tumor involvement. Detection of metastatic involvement of nodes with either CT or MR imaging requires careful evaluation of multiple morphological features: size, shape, density (intensity on MR imaging), necrosis, and extranodal spread of tumor. It is important to review all criteria and not merely the size of a lymph node. This should also be performed in conjunction with knowledge of the expected drainage pattern of the tumor. As stated previously, oropharyngeal tumors most often drain to the upper and mid jugular nodes (levels 2 and 3 respectively), and bilateral drainage is frequent. These nodal sites should be carefully scrutinized for abnormal shape, size, contour, and texture. The retropharyngeal nodes (RPN) should also be evaluated, particularly when a posterior pharyngeal wall tumor is present. Keep in mind that unless cystic or necrotic, RPN frequently appear isodense to the adjacent prevertebral muscles on CT making them readily overlooked. Imaging studies showing amorphous spiculated margins of involved nodes or involvement of internodal fat resulting in loss of normal oval-to-round nodal shape suggest extranodal spread of tumor but are not sufficient without corresponding evidence on physical examination to classify as ENE(+).

PET/CT for head and neck malignancies are best performed with contrast-enhanced CT portions of the examination and preferably with dedicated smaller field of view (FOV) neck images. When iodinated contrast is not employed for PET/CT, it is also difficult to evaluate for specific sites of disease involvement that might upstage a tumor. For example, determining the presence of medial or lateral pterygoid muscle involvement (both T4) by a tonsillar carcinoma is difficult when relying on non-contrast large FOV CT for correlation with 18F-fluorodeoxyglucose-PET uptake.

Some subtleties of tumor involvement for staging also require clarification. The palatoglossus muscle forms the muscle bulk of the anterior tonsillar pillar and may be invaded with even a relatively small tonsillar or tonsillar pillar tumor. Although palatoglossus is defined as an extrinsic muscle of the tongue, involvement of this muscle *within the oropharynx* does not denote a significantly poorer prognosis for the patient and does not change the T category.

Pathological Classification

Complete resection of the primary site and/or regional nodal dissections, followed by pathological examination of the resected specimen(s), allows the use of this designation for pT and/or pN, respectively. Specimens that are resected after radiation or chemotherapy need to be identified and considered in context, and use yp instead of p. pT is derived from the actual measurement of the unfixed tumor in the surgical specimen. It should be noted, however, that up to 30% shrinkage of soft tissues may occur in the resected specimen after formalin fixation. Pathological staging represents additional and important information and should be included as such in staging, but it does not supplant clinical staging as the primary staging scheme.

For pN, a selective neck dissection will ordinarily include 10 or more lymph nodes, and a radical or modified radical neck dissection will ordinarily include 15 or more lymph nodes. Negative pathological examination of a smaller number of nodes still mandates a pN0 designation.

Definition of ENE and Description of its extent

All surgically resected metastatic nodes should be examined for the presence and extent of ENE. The precise definition of ENE has also varied in the literature over the course of time. The College of American Pathologists defines ENE as extension of metastatic tumor, present within the confines of the lymph node, through the lymph node capsule into the surrounding connective tissue, with or without associated stromal reaction.

ENE detected on histopathologic examination is designated as ENE_{mi} (microscopic ENE $\leq$2 mm) or ENE_{ma} (major ENE>2 mm). Both ENE_{mi} and ENE_{ma} qualify as ENE(+) for definition of pN. These descriptors of ENE will not be required for current pN definition, but data collection is recommended to allow standardization of data collection and future analysis.

PROGNOSTIC FACTORS

Prognostic Factors Required for Stage Grouping

p16 Immunotesting

Testing for p16 is mandatory for all oropharyngeal squamous carcinomas but not for hypopharyngeal cancers. If p16 testing is not performed, that case is staged according to this system for p16- cancers. AJCC Level of Evidence: II

mandible, inferiorly to the larynx or superiorly to the skull base or beyond. Skull base involvement also necessitates careful evaluation for perineural and intracranial spread of tumor. Cross-sectional imaging of hypopharyngeal carcinoma is recommended to define the extent of the primary tumor, particularly its deep extent in relationship to adjacent structures (i.e., larynx, cricoid and thyroid cartilage, cervical vertebrae, and carotid sheath). CT is preferred currently because it entails less motion artifact than MR imaging.

CT and MR imaging afford some view of the lungs for metastases, albeit with only a small volume of the lungs being imaged and with low sensitivity for MR imaging. A complete staging report includes evaluation of the lung apices for potential metastases and of the bones of the skull and cervical spine for metastatic disease. PET/CT allows more accurate and complete evaluation for distant metastatic disease, which is most often to the lung, bone, and liver.

There are specific pitfalls in imaging that relate to the imaging modality being used or to strict interpretation of the definitions of tumor involvement. Detection of metastatic involvement of nodes with either CT or MR imaging requires careful evaluation of multiple morphological features: size, shape, density (intensity on MR imaging), necrosis, and extranodal spread of tumor. It is important to review all criteria and not merely the size of a lymph node. This should also be performed in conjunction with knowledge of the expected drainage pattern of the tumor. As stated previously, oropharyngeal tumors most often drain to the upper and mid jugular nodes (levels 2 and 3 respectively), and bilateral drainage is frequent. These nodal sites should be carefully scrutinized for abnormal shape, size, contour, and texture. The retropharyngeal nodes (RPN) should also be evaluated, particularly when a posterior pharyngeal wall tumor is present. Keep in mind that unless cystic or necrotic, RPN frequently appear isodense to the adjacent prevertebral muscles on CT making them readily overlooked. Imaging studies showing amorphous spiculated margins of involved nodes or involvement of internodal fat resulting in loss of normal oval-to-round nodal shape suggest extranodal spread of tumor but are not sufficient without corresponding evidence on physical examination to classify as ENE(+).

PET/CT for head and neck malignancies are best performed with contrast-enhanced CT portions of the examination and preferably with dedicated smaller field of view (FOV) neck images. When iodinated contrast is not employed for PET/CT, it is also difficult to evaluate for specific sites of disease involvement that might upstage a tumor. For example, determining the presence of medial or lateral pterygoid muscle involvement (both T4) by a tonsillar carcinoma is difficult when relying on non-contrast large FOV CT for correlation with 18F-fluorodeoxyglucose-PET uptake.

Some subtleties of tumor involvement for staging also require clarification. The palatoglossus muscle forms the muscle bulk of the anterior tonsillar pillar and may be invaded with even a relatively small tonsillar or tonsillar pillar tumor. Although palatoglossus is defined as an extrinsic muscle of the tongue, involvement of this muscle *within the oropharynx* does not denote a significantly poorer prognosis for the patient and does not change the T category.

Pathological Classification

Complete resection of the primary site and/or regional nodal dissections, followed by pathological examination of the resected specimen(s), allows the use of this designation for pT and/or pN, respectively. Specimens that are resected after radiation or chemotherapy need to be identified and considered in context, and use yp instead of p. pT is derived from the actual measurement of the unfixed tumor in the surgical specimen. It should be noted, however, that up to 30% shrinkage of soft tissues may occur in the resected specimen after formalin fixation. Pathological staging represents additional and important information and should be included as such in staging, but it does not supplant clinical staging as the primary staging scheme.

For pN, a selective neck dissection will ordinarily include 10 or more lymph nodes, and a radical or modified radical neck dissection will ordinarily include 15 or more lymph nodes. Negative pathological examination of a smaller number of nodes still mandates a pN0 designation.

Definition of ENE and Description of its extent

All surgically resected metastatic nodes should be examined for the presence and extent of ENE. The precise definition of ENE has also varied in the literature over the course of time. The College of American Pathologists defines ENE as extension of metastatic tumor, present within the confines of the lymph node, through the lymph node capsule into the surrounding connective tissue, with or without associated stromal reaction.

ENE detected on histopathologic examination is designated as ENE_{mi} (microscopic ENE $\leq$2 mm) or ENE_{ma} (major ENE>2 mm). Both ENE_{mi} and ENE_{ma} qualify as ENE(+) for definition of pN. These descriptors of ENE will not be required for current pN definition, but data collection is recommended to allow standardization of data collection and future analysis.

PROGNOSTIC FACTORS

Prognostic Factors Required for Stage Grouping

p16 Immunotesting

Testing for p16 is mandatory for all oropharyngeal squamous carcinomas but not for hypopharyngeal cancers. If p16 testing is not performed, that case is staged according to this system for p16- cancers. AJCC Level of Evidence: II

11

Additional Factors Recommended for Clinical Care

Extranodal Extension

ENE is defined as extension of metastatic tumor, present within the confines of the lymph node, through the lymph node capsule into the surrounding connective tissue, with or without associated stromal reaction. Unambiguous evidence of gross ENE (i.e., defined as invasion of skin, infiltration of musculature/fixation to adjacent structures on clinical examination, cranial nerve, brachial plexus, sympathetic trunk or phrenic nerve invasion with dysfunction) is a sufficiently high threshold to classify these as clinical ENE(+). AJCC Level of Evidence: III

Overall Health

In addition to the importance of the TNM factors outlined previously, the overall health of these patients clearly influences outcome. An ongoing effort to better assess prognosis using both tumor and nontumor-related factors is underway. Chart abstraction will continue to be performed by cancer registrars to obtain important information regarding specific factors related to prognosis. These data will then be used to further hone the predictive power of the staging system in future revisions. AJCC Level of Evidence: II

Comorbidity

Comorbidity can be classified by specific measures of additional medical illnesses.[10] Accurate reporting of all illnesses in the patients' medical record is essential to assessment of these parameters. General performance measures are helpful in predicting survival. The AJCC strongly recommends the clinician report performance status using the Eastern Cooperative Oncology Group (ECOG), Zubrod, or Karnofsky performance measures along with standard staging information. An interrelationship between each of the major performance tools exists. AJCC Level of Evidence: II

Zubrod/ECOG Performance Scale	
0	Fully active, able to carry on all predisease activities without restriction (Karnofsky 90–100)
1	Restricted in physically strenuous activity but ambulatory and able to carry out work of a light or sedentary nature. For example, light housework, office work (Karnofsky 70–80)
2	Ambulatory and capable of all self-care but unable to carry out any work activities. Up and about more than 50 % of waking hours (Karnofsky 50–60)
3	Capable of only limited self-care, confined to bed or chair 50 % or more of waking hours (Karnofsky 30–40)
4	Completely disabled; cannot carry on self-care; totally confined to bed or chair (Karnofsky 10–20)
5	Death (Karnofsky 0)

Lifestyle Factors

Lifestyle factors such as tobacco and alcohol abuse negatively influence survival. Accurate recording of smoking in pack-years and alcohol in number of days drinking per week and number of drinks per day will provide important data for future analysis. Nutrition is important to prognosis and will be indirectly measured by weight loss of > 5 % of body weight in the previous 6 months.[11] Depression adversely affects quality of life and survival. Notation of a previous or current diagnosis of depression should be recorded in the medical record.[12] AJCC Level of Evidence: III

Tobacco Use

The role of tobacco as a negative prognostic factor is well established. However, exactly how this could be codified in the staging system is less clear. At this time, smoking is known to have a deleterious effect on prognosis but is hard to apply accurately to the staging system. AJCC Level of Evidence: III

Smoking history should be collected as an important element of the demographics and may be included in Prognostic Groups in the future. For practicality, the minimum standard should classify smoking history as never, ≤ 10 pack-years, > 10 but ≤ 20 pack-years, or > 20 pack years.

RISK ASSESSMENT MODELS

The AJCC recently established guidelines that will be used to evaluate published statistical prediction models for the purpose of granting endorsement for clinical use.[13] Although this is a monumental step toward the goal of precision medicine, this work was published only very recently. Therefore, the existing models that have been published or may be in clinical use have not yet been evaluated for this cancer site by the Precision Medicine Core of the AJCC. In the future, the statistical prediction models for this cancer site will be evaluated, and those that meet all AJCC criteria will be endorsed.

DEFINITIONS OF AJCC TNM

Definition of Primary Tumor (T)

Oropharynx (p16-)

T Category	T Criteria
TX	Primary tumor cannot be assessed
Tis	Carcinoma *in situ*
T1	Tumor 2 cm or smaller in greatest dimension
T2	Tumor larger than 2 cm but not larger than 4 cm in greatest dimension
T3	Tumor larger than 4 cm in greatest dimension or extension to lingual surface of epiglottis

T Category	T Criteria
T4	Moderately advanced or very advanced local disease
T4a	Moderately advanced local disease<br>Tumor invades the larynx, extrinsic muscle of tongue, medial pterygoid, hard palate, or mandible*
T4b	Very advanced local disease<br>Tumor invades lateral pterygoid muscle, pterygoid plates, lateral nasopharynx, or skull base or encases carotid artery

*Note: Mucosal extension to lingual surface of epiglottis from primary tumors of the base of the tongue and vallecula does not constitute invasion of the larynx

Hypopharynx

T Category	T Criteria
TX	Primary tumor cannot be assessed
Tis	Carcinoma *in situ*
T1	Tumor limited to one subsite of hypopharynx and/or 2 cm or smaller in greatest dimension
T2	Tumor invades more than one subsite of hypopharynx or an adjacent site, or measures larger than 2 cm but not larger than 4 cm in greatest dimension without fixation of hemilarynx
T3	Tumor larger than 4 cm in greatest dimension or with fixation of hemilarynx or extension to esophagus
T4	Moderately advanced and very advanced local disease
T4a	Moderately advanced local disease<br>Tumor invades thyroid/cricoid cartilage, hyoid bone, thyroid gland, or central compartment soft tissue*
T4b	Very advanced local disease<br>Tumor invades prevertebral fascia, encases carotid artery, or involves mediastinal structures

*Note: Central compartment soft tissue includes prelaryngeal strap muscles and subcutaneous fat

Definition of Regional Lymph Node (N)

Clinical N (cN) - Oropharynx (p16-) and Hypopharynx

N Category	N Criteria
NX	Regional lymph nodes cannot be assessed
N0	No regional lymph node metastasis
N1	Metastasis in a single ipsilateral lymph node, 3 cm or smaller in greatest dimension and ENE(−)
N2	Metastasis in a single ipsilateral node larger than 3 cm but not larger than 6 cm in greatest dimension and ENE(−);<br>*or* metastases in multiple ipsilateral lymph nodes, none larger than 6 cm in greatest dimension and ENE(−);<br>*or* in bilateral or contralateral lymph nodes, none larger than 6 cm in greatest dimension and ENE(−)
N2a	Metastasis in a single ipsilateral node larger than 3 cm but not larger than 6 cm in greatest dimension and ENE(−)
N2b	Metastasis in multiple ipsilateral nodes, none larger than 6 cm in greatest dimension and ENE(−)
N2c	Metastasis in bilateral or contralateral lymph nodes, none larger than 6 cm in greatest dimension and ENE(−)
N3	Metastasis in a lymph node larger than 6 cm in greatest dimension and ENE(−);<br>*or* metastasis in any node(s) and clinically overt ENE(+)
N3a	Metastasis in a lymph node larger than 6 cm in greatest dimension and ENE(−)
N3b	Metastasis in any node(s) and clinically overt ENE(+)

Note: A designation of "U" or "L" may be used for any N category to indicate metastasis above the lower border of the cricoid (U) or below the lower border of the cricoid (L).
Similarly, clinical and pathological ENE should be recorded as ENE(−) or ENE(+)

Pathological N (pN) – Oropharynx (p16-) and Hypopharynx

N Category	N Criteria
NX	Regional lymph nodes cannot be assessed
N0	No regional lymph node metastasis
N1	Metastasis in a single ipsilateral lymph node, 3 cm or smaller in greatest dimension and ENE(−)
N2	Metastasis in a single ipsilateral lymph node, 3 cm or smaller in greatest dimension and ENE(+);<br>*or* larger than 3 cm but not larger than 6 cm in greatest dimension and ENE(−);<br>*or* metastases in multiple ipsilateral lymph nodes, none larger than 6 cm in greatest dimension and ENE(−);<br>*or* in bilateral or contralateral lymph nodes, none larger than 6 cm in greatest dimension and ENE(−)
N2a	Metastasis in single ipsilateral or contralateral node 3 cm or smaller in greatest dimension and ENE(+);<br>*or* a single ipsilateral node larger than 3 cm but not larger than 6 cm in greatest dimension and ENE(−)
N2b	Metastasis in multiple ipsilateral nodes, none larger than 6 cm in greatest dimension and ENE(−)
N2c	Metastasis in bilateral or contralateral lymph nodes, none larger than 6 cm in greatest dimension and ENE(−)
N3	Metastasis in a lymph node larger than 6 cm in greatest dimension and ENE(−);<br>*or* in a single ipsilateral node larger than 3 cm in greatest dimension and ENE(+);<br>*or* multiple ipsilateral, contralateral or bilateral nodes, any with ENE(+)
N3a	Metastasis in a lymph node larger than 6 cm in greatest dimension and ENE(−)
N3b	Metastasis in a single ipsilateral node larger than 3 cm in greatest dimension and ENE(+);<br>*or* multiple ipsilateral, contralateral or bilateral nodes, any with ENE(+)

Note: A designation of "U" or "L" may be used for any N category to indicate metastasis above the lower border of the cricoid (U) or below the lower border of the cricoid (L).
Similarly, clinical and pathological ENE should be recorded as ENE(−) or ENE(+)

Definition of Distant Metastasis (M)

Oropharynx (p16-) and Hypopharynx

M Category	M Criteria
M0	No distant metastasis
M1	Distant metastasis

AJCC PROGNOSTIC STAGE GROUPS

When T is...	And N is...	And M is...	Then the stage group is...
Tis	N0	M0	0
T1	N0	M0	I
T2	N0	M0	II
T3	N0	M0	III
T1,T2,T3	N1	M0	III
T4a	N0,1	M0	IVA
T1,T2,T3,T4a	N2	M0	IVA
Any T	N3	M0	IVB
T4b	Any N	M0	IVB
Any T	Any N	M1	IVC

REGISTRY DATA COLLECTION VARIABLES

1. ENE clinical: ENE(+) or ENE(−)
2. ENE pathological: ENE(+) or ENE(−)
3. Extent of microscopic ENE (distance of extension from the native lymph node capsule to the farthest point of invasion in the extranodal tissue)
4. Perineural invasion
5. Lymphovascular invasion
6. p16/HPV status
7. Performance status
8. Tobacco use and pack-years
9. Alcohol use
10. Depression diagnosis

HISTOLOGIC GRADE (G)

G	G Definition
GX	Grade cannot be assessed
G1	Well differentiated
G2	Moderately differentiated
G3	Poorly differentiated
G4	Undifferentiated

HISTOPATHOLOGIC TYPE

The predominant cancer is squamous cell carcinoma. The staging guidelines are applicable to all forms of carcinoma, including those arising from minor salivary glands. Other nonepithelial tumors such as those of lymphoid tissue, soft tissue, bone, and cartilage (i.e., lymphoma and sarcoma) are not included. Histologic confirmation of diagnosis is required. Histopathologic grading of squamous carcinoma is recommended. The grade is subjective and uses a descriptive as well as numerical form (i.e., well differentiated, moderately differentiated, and poorly differentiated), depending on the degree of closeness to or deviation from squamous epithelium in mucosal sites. Also recommended where feasible is a quantitative evaluation of depth of invasion of the primary tumor and the presence or absence of vascular invasion and perineural invasion. Although the grade of tumor does not enter into the staging of the tumor, it should be recorded. The pathological description of any lymphadenectomy specimen should describe the size, number, and position of the involved node(s) and the presence or absence of ENE.

ILLUSTRATIONS

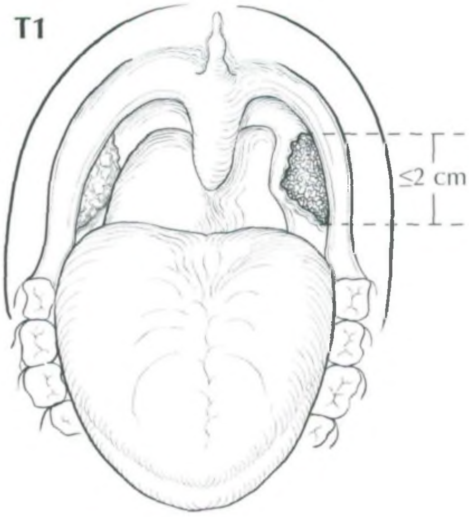

Fig. 11.2 T1 tumors of the oropharynx are 2 cm or smaller in greatest dimension

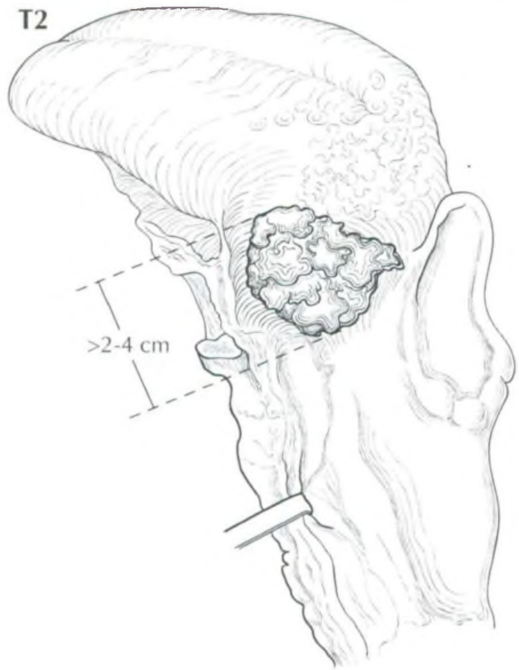

Fig. 11.3 T2 tumors of the oropharynx measure larger than 2 cm but not larger than 4 cm

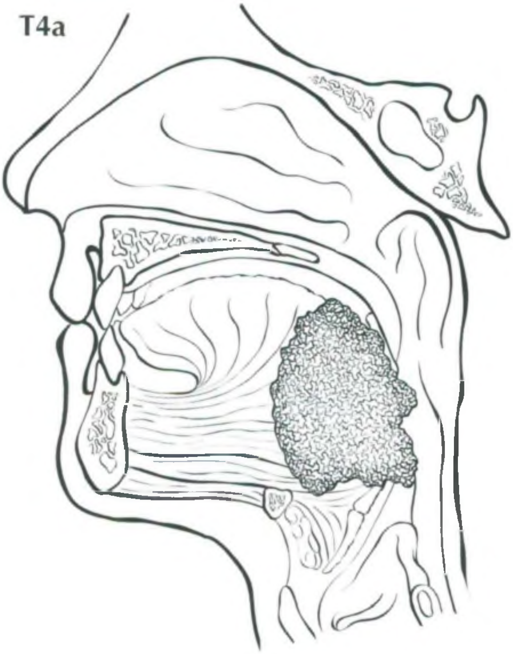

Fig. 11.5 T4a tumor of the oropharynx is described as moderately advanced local disease, a tumor that invades the larynx, extrinsic muscle or tongue, medial pterygoid, hard palate, or mandible

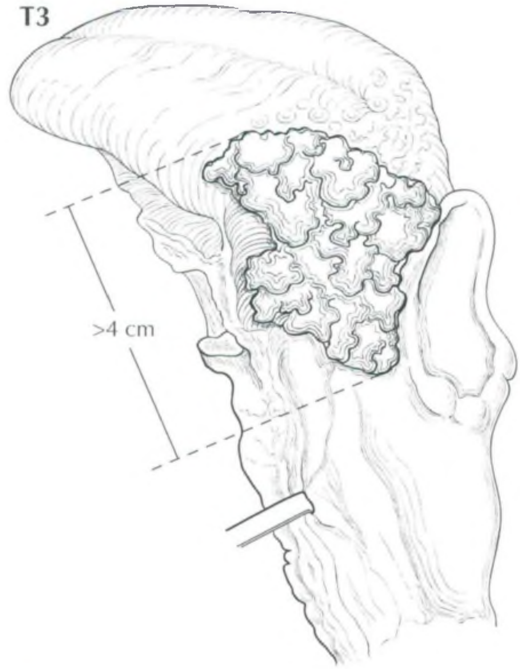

Fig. 11.4 T3 tumors of the oropharynx are larger than 4 cm in greatest dimension or have extension to lingual surface of epiglottis

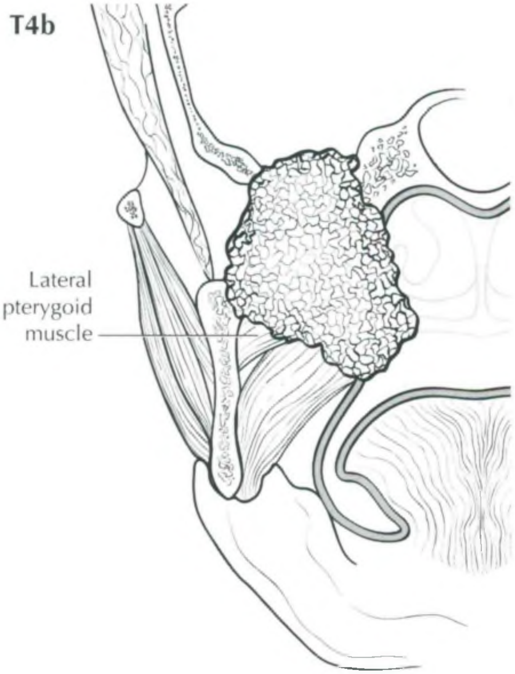

Fig. 11.6 T4b tumor of the oropharynx is a tumor described as very advanced local disease, a tumor that invades lateral pterygoid muscle, pterygoid plates, lateral nasopharynx, or skull base or encases carotid artery

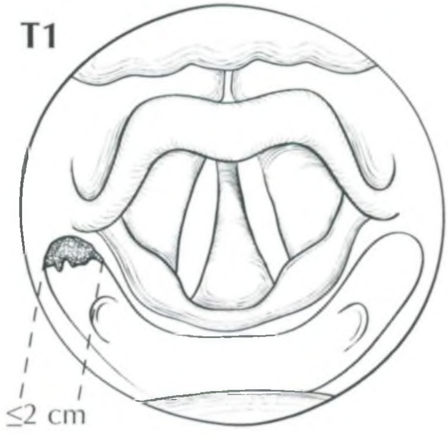

Fig. 11.7 T1 tumor of the hypopharynx with involvement of the pyriform sinus

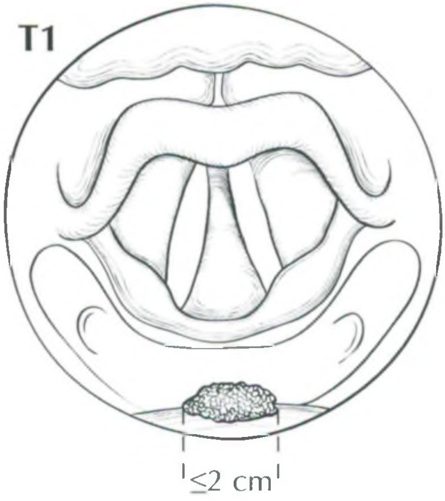

Fig. 11.8 T1 tumor of the hypopharynx with involvement of the posterior wall

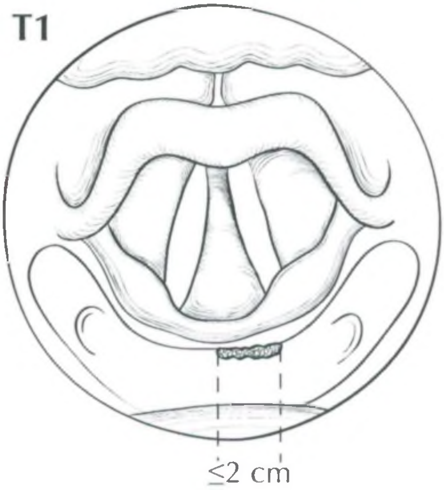

Fig. 11.9 T1 tumor of the hypopharynx with involvement of the postcricoid area

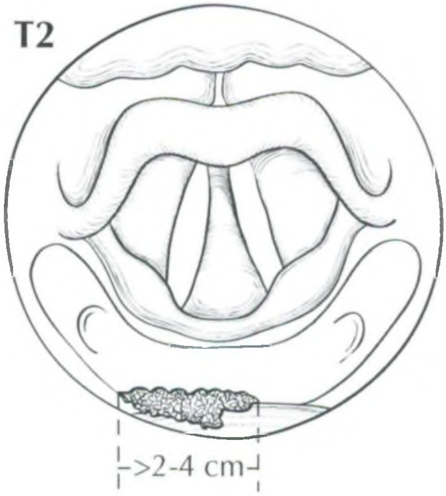

Fig. 11.10 T2 tumor of the hypopharynx with involvement of the posterior wall of the hypopharynx

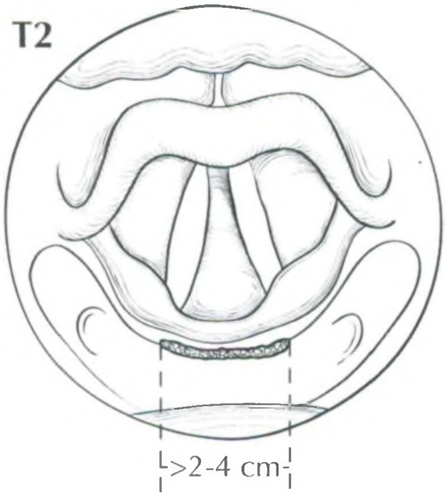

Fig. 11.11 T2 tumor of the hypopharynx with involvement of the post-cricoid area

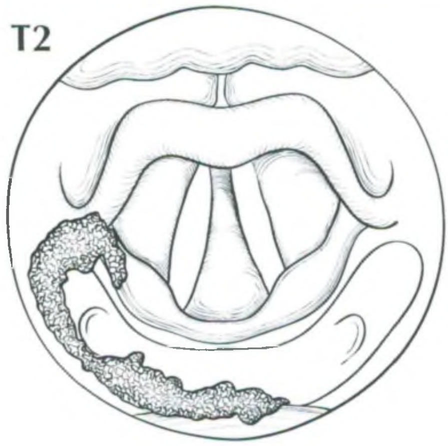

Fig. 11.13 T2 tumor of the hypopharynx with involvement of the pyriform sinus and the posterior wall

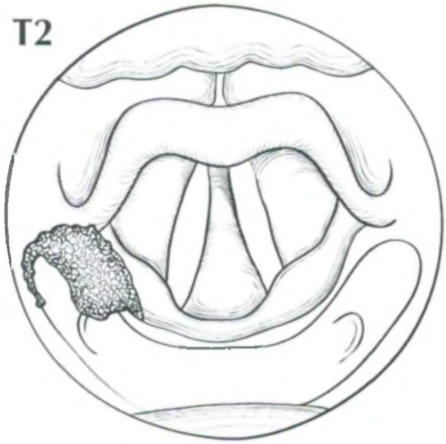

Fig. 11.12 T2 tumor of the hypopharynx with involvement of the pyriform sinus and the aryepiglottic fold

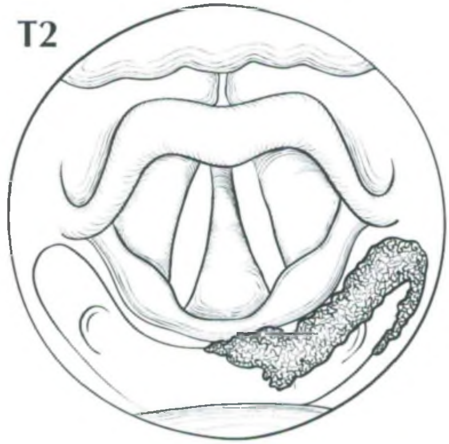

Fig. 11.14 T2 tumor of the hypopharynx with involvement of the pyriform sinus and the post-cricoid area

11

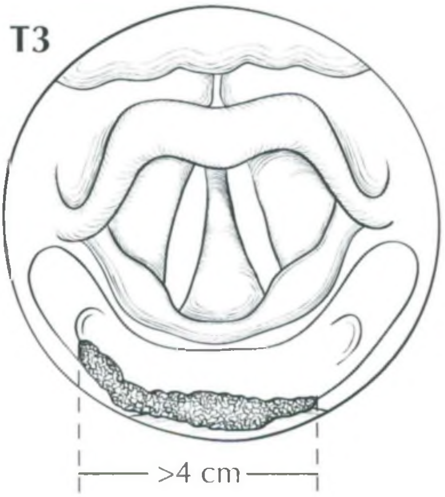

Fig. 11.15 T3 tumor of the hypopharynx larger than 4 cm in diameter and with involvement of the posterior wall

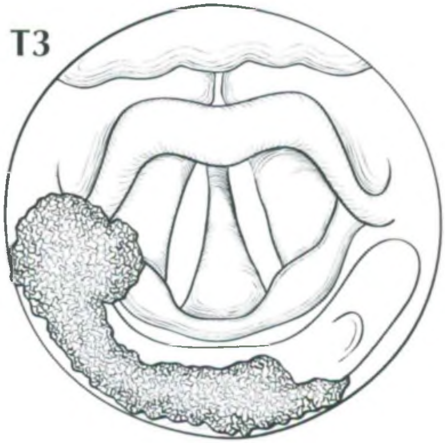

Fig. 11.16 T3 tumor of the hypopharynx with fixation of the hemilarynx and invasion of the pyriform sinus, aryepiglottic fold, and posterior wall

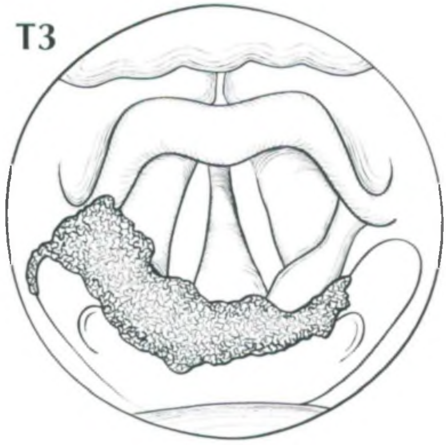

Fig. 11.17 T3 tumor of the hypopharynx with fixation of the hemilarynx with invasion of the pyriform sinus and post-cricoid area

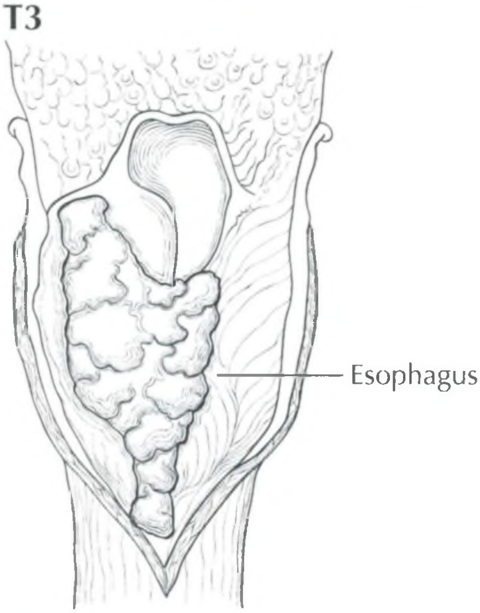

Fig. 11.18 T3 tumor of the hypopharynx with invasion of the esophagus

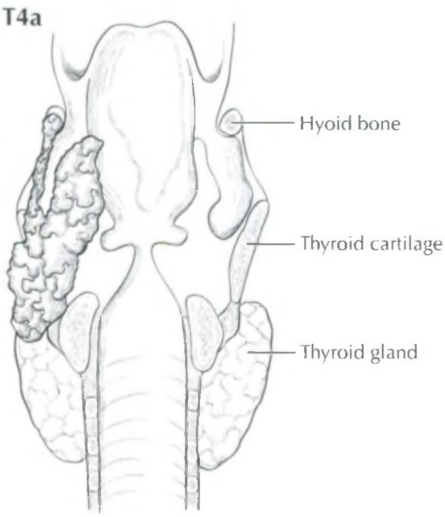

Fig. 11.19 T4a tumor of the hypopharynx that is moderately advanced local disease, with invasion of the hyoid bone, thyroid/cricoid cartilage, thyroid gland, or central compartment soft tissue

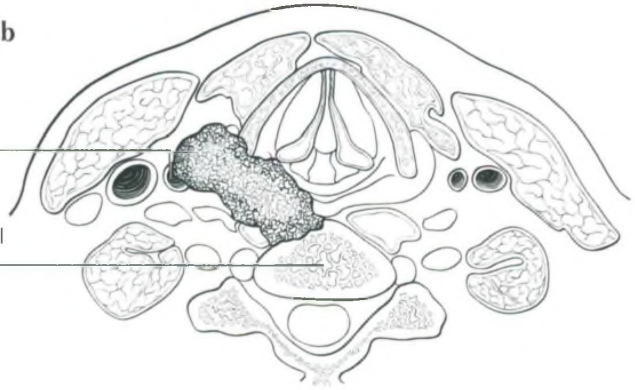

Fig. 11.20 T4b tumor the hypopharynx that is very advanced local disease, with invasion of the prevertebral fascia, encases carotid artery, or involves mediastinal structures

Bibliography

1. Patel S. Personal Communication. In: Lydiatt W, Shah JP, eds2015.
2. O'Sullivan B, Huang SH, Su J, et al. Development and validation of a staging system for HPV-related oropharyngeal cancer by the International Collaboration on Oropharyngeal cancer Network for Staging (ICON-S): a multicentre cohort study. *The lancet oncology*. Feb 26 2016.
3. Wreesmann VB, Katabi N, Palmer FL, et al. Influence of extracapsular nodal spread extent on prognosis of oral squamous cell carcinoma. *Head & neck*. Oct 30 2015.
4. Ang KK, Harris J, Wheeler R, et al. Human papillomavirus and survival of patients with oropharyngeal cancer. *N Engl J Med*. Jul 1 2010;363(1):24-35.
5. Ebrahimi A GZ, Amit M, Yen TC, Liao CT, Chatturvedi P, Agarwal J, Kowalski L, Kreppel M, Cernea C, Brandao J, Bachar G, Villaret AB, Fliss D, Fridman E, Robbins KT, Shah J, Patel S, Clark J; International Consortium for Outcome Research (ICOR) in Head and Neck Cancer. Comparison of the American Joint Committee on Cancer N1 versus N2a nodal categories for predicting survival and recurrence in patients with oral cancer: Time to acknowledge an arbitrary distinction and modify the system. . *Head and neck pathology*. 2014.
6. de Juan J, Garcia J, Lopez M, et al. Inclusion of extracapsular spread in the pTNM classification system: a proposal for patients with head and neck carcinoma. *JAMA otolaryngology– head & neck surgery*. May 2013;139(5):483-488.
7. Prabhu RS, Hanasoge S, Magliocca KR, et al. Extent of pathologic extracapsular extension and outcomes in patients with nonoropharyngeal head and neck cancer treated with initial surgical resection. *Cancer*. May 15 2014;120(10):1499-1506.
8. Dunne AA, Muller HH, Eisele DW, Kessel K, Moll R, Werner JA. Meta-analysis of the prognostic significance of perinodal spread in head and neck squamous cell carcinomas (HNSCC) patients. *European journal of cancer*. Aug 2006;42(12): 1863-1868.
9. Prabhu RS, Magliocca KR, Hanasoge S, et al. Accuracy of computed tomography for predicting pathologic nodal extracapsular extension in patients with head-and-neck cancer undergoing initial surgical resection. *International journal of radiation oncology, biology, physics*. Jan 1 2014;88(1):122-129.
10. Piccirillo JF. Inclusion of comorbidity in a staging system for head and neck cancer. *Oncology (Williston Park)*. Sep 1995;9(9):831-836; discussion 841, 845-838.
11. Couch ME, Dittus K, Toth MJ, et al. Cancer cachexia update in head and neck cancer: Pathophysiology and treatment. *Head & neck*. Jul 2015;37(7):1057-1072.
12. Lazure KE, Lydiatt WM, Denman D, Burke WJ. Association between depression and survival or disease recurrence in patients with head and neck cancer enrolled in a depression prevention trial. *Head & neck*. 2009;31(7):888-892.
13. Kattan MW, Hess KR, Amin MB, et al. American Joint Committee on Cancer acceptance criteria for inclusion of risk models for individualized prognosis in the practice of precision medicine. *CA: a cancer journal for clinicians*. Jan 19 2016.

Nasal Cavity and Paranasal Sinuses

12

Dennis H. Kraus, William M. Lydiatt, Snehal G. Patel,
Brian O'Sullivan, Ronald A. Ghossein, Suresh K. Mukherji,
and Jatin P. Shah

CHAPTER SUMMARY

Cancers Staged Using This Staging System

Malignancies that arise in the epithelial lining of the paranasal sinuses and nasal cavity, with the exception of lymphoma and sarcoma, are staged according to this system.

Cancers Not Staged Using This Staging System

These histopathologic types of cancer…	Are staged according to the classification for…	And can be found in chapter…
Mucosal melanoma of the nasal cavity and paranasal sinuses	Mucosal melanoma of the head and neck	14

Summary of Changes

Change	Details of Change	Level of Evidence
Anatomy – Primary Site(s)	Occult Primary Tumor: Staging of the patient who presents with EBV-unrelated and HPV-unrelated metastatic cervical lymphadenopathy is not included in this chapter.	IV
Definition of Regional Lymph Node (N)	Separate N staging approaches have been described for HPV-related and HPV-unrelated cancers.	II[1,2]
Definition of Regional Lymph Node (N)	Separate N category approaches have been described for patients treated without cervical lymph node dissection (clinical N) and patients treated with cervical lymph neck dissection (pathologic N).	II[2]
Definition of Regional Lymph Node (N)	Extranodal extension (ENE) is introduced as a descriptor in all HPV-unrelated cancers.	II[1]
Definition of Regional Lymph Node (N)	ENE in HPV negative cancers: Only clinically and radiographically overt ENE [ENE(+)] should be used for cN.	II[1]
Definition of Regional Lymph Node (N)	ENE in HPV negative cancers: Any pathologically detected ENE is considered ENE(+) and is used for pN.	II[1]
Definition of Regional Lymph Node (N)	ENE in HPV-negative cancers: Presence of ENE is designated pN2a for a single ipsilateral node < 3 cm and pN3b for all other node(s).	II[1]
Definition of Regional Lymph Node (N)	Classification of ENE: Clinically overt ENE is classified as ENE_c and is considered ENE(+) for cN.	III[3]
Definition of Regional Lymph Node (N)	Classification of ENE: Pathologically detected ENE is classified as either ENE_{mi} ($\leq$2 mm) or ENE_{ma} (>2 mm) for data collection purposes only, but both are considered ENE(+) for definition of pN.	III[3]

To access the AJCC cancer staging forms, please visit www.cancerstaging.org.

© American Joint Committee on Cancer 2017

M.B. Amin et al. (eds.), *AJCC Cancer Staging Manual, Eighth Edition*, DOI 10.1007/978-3-319-40618-3_12

ICD-O-3 Topography Codes

Code	Description
C30.0	Nasal cavity
C31.0	Maxillary sinus
C31.1	Ethmoid sinus

WHO Classification of Tumors

Code	Description
8070	Squamous cell carcinoma
8075	Acantholytic squamous cell carcinoma
8560	Adenosquamous carcinoma
8083	Basaloid squamous cell carcinoma
8052	Papillary squamous cell carcinoma
8074	Spindle cell squamous carcinoma
8051	Verrucous carcinoma
8082	Lymphoepithelial carcinoma (non-nasopharyngeal)
8020	Sinonasal undifferentiated carcinoma
8144	Intestinal-type adenocarcinoma
8140	Non-intestinal-type adenocarcinoma
8200	Adenoid cystic carcinoma
8430	Mucoepidermoid carcinoma
8562	Epithelial-myoepithelial carcinoma
8310	Clear cell carcinoma
8982	Myoepithelial carcinoma
8941	Carcinoma ex pleomorphic adenoma
8525	Polymorphous adenocarcinoma
8240	Typical carcinoid
8429	Atypical carcinoid
8041	Small cell carcinoma, neuroendocrine type

Barnes L, Eveson JW, Reichart P, Sidransky D, eds. World Health Organization Classification of Tumours Pathology and Genetics of Head and Neck Tumours. Lyon: IARC; 2005.

INTRODUCTION

Malignancies that arise in the epithelial lining of the paranasal sinuses and nasal cavity, with the exception of lymphoma and sarcoma, are staged according to this system. Many histologic subtypes exist and also are a factor in determining prognosis.[4] T category is identical to the American Joint Committee on Cancer (AJCC) Cancer Staging Manual, 7th Edition (7th Edition). As in other head and neck sites, the N category has been expanded to incorporate extranodal extension (ENE).[5]

The role of ENE in prognosis of head and neck cancers is profound. Accounting for this important prognostic feature was considered critical in revising staging. Most of the data supporting ENE as an adverse prognostic factor are based on histopathological characterization of ENE, especially the distinction between microscopic and macroscopic or gross ENE.[3,6,7] As per the "uncertain rule" of the AJCC/Union for International Cancer Control (UICC) staging, which mandates that the lower category should always be assigned in ambiguous cases, a case should be categorized as ENE(−) unless there is unquestionable ENE. For clinical ENE, the known inability of current imaging modalities to define ENE accurately mandated that stringent criteria be met prior to assigning a clinical diagnosis of ENE.[8] However, unambiguous evidence of gross ENE on clinical examination (e.g., invasion of skin, infiltration of musculature/dense tethering to adjacent structures, or cranial nerve, brachial plexus, sympathetic trunk, or phrenic nerve invasion with dysfunction) supported by strong radiographic evidence permit classification of disease as ENE(+). Pathological ENE also will be clearly defined in this chapter. Again, if there is doubt or uncertainty of the presence of ENE, the case should be categorized as ENE(−).

Cancer of the maxillary sinus is the most common of the sinonasal malignancies. Ethmoid sinus and nasal cavity cancers are equal in frequency, but considerably less common than maxillary sinus cancers. Tumors of the sphenoid and frontal sinuses are rare.

ANATOMY

Primary Site(s)

The location and the extent of the mucosal lesion within the maxillary sinus have prognostic significance. Historically, a plane connecting the medial canthus of the eye to the angle of the mandible, represented by Ohngren's line, is used to divide the maxillary sinus into an anteroinferior portion (infrastructure), which is associated with a good prognosis, and a posterosuperior portion (suprastructure), which has a poor prognosis (Fig. 12.1). The poorer outcome associated with suprastructure cancers reflects early invasion by these tumors to critical structures, including the orbit, skull base, pterygoid plates, and infratemporal fossa.

For the purpose of staging, the nasoethmoidal complex is divided into two sites: nasal cavity and ethmoid sinuses. The ethmoids are further subdivided into two subsites: left and right, separated by the nasal septum (perpendicular plate of ethmoid). The nasal cavity is divided into four subsites: the septum, floor, lateral wall, and the edge of naris to mucocutaneous junction.

Site	Subsite(s)
Maxillary sinus	Left/right
Nasal cavity	Septum
	Floor
	Lateral wall
	Edge of naris to mucocutaneous junction
Ethmoid sinus	Left/right

Regional Lymph Nodes

Regional lymph node spread from cancer of nasal cavity and paranasal sinuses is relatively uncommon. Involvement of buccinator, prevascular facial, submandibular, upper jugular, and (occasionally) retropharyngeal nodes may occur with advanced maxillary sinus cancer, particularly those extending beyond the sinus walls to involve adjacent structures, including soft tissues of the cheek, upper alveolus, palate, and buccal mucosa or overlying skin. Ethmoid sinus cancers are less prone to regional lymphatic spread. When only one side of the neck is involved, it should be considered ipsilateral. Bilateral spread may occur with advanced primary cancer, particularly with spread of the primary beyond the midline.

Metastatic Sites

Distant spread usually occurs to lungs, but occasionally there is spread to bone.

RULES FOR CLASSIFICATION

Clinical Classification

The assessment of primary maxillary sinus, nasal cavity, and ethmoid tumors is based on inspection and palpation, including examination of the orbits, nasal and oral cavities, and

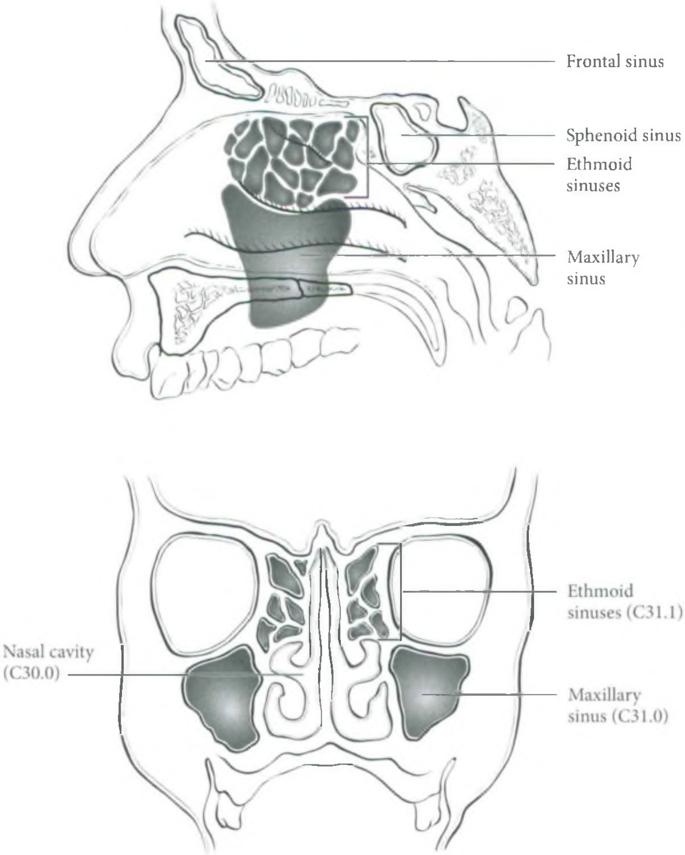

Fig. 12.1 Primary sites of the paranasal sinuses

12

nasopharynx, and neurologic evaluation of the cranial nerves. Nasal endoscopy with rigid or fiberoptic flexible instruments is recommended for inspection and biopsy.

Neck nodes are assessed by palpation. In clinical evaluation, the maximum size of any nodal mass should be measured. Biopsy, when indicated, is done with a fine needle and not open approach and is included in clinical classification if done. The three categories of clinically involved nodes for the nasal cavity and paranasal sinus are N1, N2, and N3. Midline nodes are considered ipsilateral nodes. Superior mediastinal lymph nodes are considered regional lymph nodes (level VII). In addition to the components to describe the N category, regional lymph nodes should also be described according to the level of the neck that is involved. Provide a description or map of the regional lymph nodes and node groups that the cancer affects. Unambiguous evidence of gross ENE (i.e., defined as invasion of skin, infiltration of musculature/fixation to adjacent structures on clinical examination, cranial nerve, brachial plexus, sympathetic trunk or phrenic nerve invasion with dysfunction) is a sufficiently high threshold to classify these as clinical ENE(+). Examinations for distant metastases include appropriate imaging, blood chemistries, blood count, and other routine studies as indicated. Biopsy is typically needed to confirm metastasis, although a risk-to-benefit ratio is always weighed and is included in clinical stage as pM1 if done.

Imaging studies showing amorphous spiculated margins of involved nodes or involvement of internodal fat resulting in loss of normal oval-to-round nodal shape strongly suggest extracapsular (extranodal) tumor spread. No imaging study (as yet) can identify microscopic foci in regional nodes or distinguish between small reactive nodes and small malignant nodes without central radiographic inhomogeneity.

Imaging

Imaging is beneficial for lesions that cannot be fully assessed on clinical examination, locally advanced disease, or symptomatic patients. Computed tomography (CT) and magnetic resonance (MR) imaging are complementary imaging studies for staging patients with cancers involving the nasal cavity and/or the paranasal sinuses. There is no indication for plain films or positron emission tomography (PET)-CT for staging of the primary site. PET-CT may be helpful for assessing nodal metastases; however, lymph node staging is discussed in Chapter 6.

CT is superior to MR imaging for identifying bone erosion of thin walls and septa of the paranasal sinuses. CT is superior to MR imaging for identifying involvement of the hard palate. Either CT or MR imaging may be performed for tumor extending outside of the nose and/or paranasal sinuses to involve the adjacent structures, including the orbital apex (T4b). MR imaging, especially with T2-weighted images, is helpful for tumor mapping and for distinguishing between tumor extension and obstructed secretions. Tumors may obstruct the frontal recess or sphenoethmoidal recess, which may result in obstruction of the frontal or sphenoid sinus, respectively. Differential between tumor extension and proteinaceous obstructed secretions is best performed with MR imaging, which will help accurately identify T4a tumors. Both CT and MR imaging may be used to evaluate for posterior spread to the pterygopalantine fossa. However, MR imaging is superior to CT for evaluating for retrograde perineural spread along V2 through foramen rotundum or V3. CT is superior to MR imaging for early cortical involvement, but MR imaging is superior to CT for bone marrow invasion. MR imaging also is superior to CT for identifying dural involvement or other types of intracranial extension (T4b).

Pathological Classification

Complete resection of the primary site and/or regional nodal dissections, followed by pathological examination of the resected specimen(s), allows the use of this designation for pT and/or pN, respectively. Specimens that are resected after radiation or chemotherapy need to be identified and considered in context, and use yp instead of p. pT is derived from the invasion of bone, orbit, dura, and presence of disease in multiple subsites. Pathological staging represents additional and important information and should be included as such in staging, but it does not supplant clinical staging as the primary staging scheme.

For pN, a selective neck dissection will ordinarily include 10 or more lymph nodes, and a radical or modified radical neck dissection will ordinarily include 15 or more lymph nodes. Negative pathological examination of a smaller number of nodes still mandates a pN0 designation.

Definition of ENE and Description of Its Extent

All surgically resected metastatic nodes should be examined for the presence and extent of ENE. The precise definition of ENE has varied in the literature over the course of time. The

American College of Pathologists defines ENE as extension of metastatic tumor, present within the confines of the lymph node, through the lymph node capsule into the surrounding connective tissue, with or without associated stromal reaction.

ENE detected on histopathologic examination is designated as ENE_{mi} (microscopic ENE ≤ 2 mm) or ENE_{ma} (major ENE > 2 mm). Both ENE_{mi} and ENE_{ma} qualify as ENE(+) for definition of pN. These descriptors of ENE will not be required for current pN definition, but data collection is recommended to allow standardization of data collection and future analysis.

PROGNOSTIC FACTORS

Prognostic Factors Required for Stage Grouping

Beyond the factors used to assign T, N, or M categories, no additional prognostic factors are required for stage grouping.

Additional Factors Recommended for Clinical Care

Extranodal Extension (ENE)

ENE is defined as extension of metastatic tumor, present beyond the confines of the lymph node, through the lymph node capsule into the surrounding connective tissue, with or without associated stromal reaction. Unambiguous evidence of gross ENE extension (i.e., defined as invasion of skin, infiltration of musculature/fixation to adjacent structures on clinical examination, cranial nerve, brachial plexus, sympathetic trunk, or phrenic nerve invasion with dysfunction) is a sufficiently high threshold to classify these as clinical ENE(+). AJCC Level of Evidence: III

Overall Health

In addition to the importance of the TNM factors outlined previously, the overall health of the patient clearly influences outcome. An ongoing effort to better assess prognosis using both tumor and nontumor-related factors is underway. Chart abstraction will continue to be performed by cancer registrars to obtain important information regarding specific factors related to prognosis. These data will then be used to further hone the predictive power of the staging system in future revisions.

Comorbidity

Comorbidity can be classified by specific measures of additional medical illnesses.[9] Accurate reporting of all illnesses in the patient's medical record is essential to the assessment of these parameters. General performance measures are helpful in predicting survival. The AJCC strongly recommends the clinician report performance status using the Eastern Cooperative Oncology Group (ECOG), Zubrod, or Karnofsky performance measures, along with standard staging information. An interrelationship between each of the major performance tools exists. AJCC Level of Evidence: III

Zubrod/ECOG Performance Scale	
0	Fully active, able to carry out all predisease activities without restriction (Karnofsky 90–100)
1	Restricted in physically strenuous activity but ambulatory and able to carry out work of a light or sedentary nature. For example, light housework, office work (Karnofsky 70–80)
2	Ambulatory and capable of all self-care but unable to carry out any work activities. Up and about more than 50 % of waking hours (Karnofsky 50–60)
3	Capable of only limited self-care, confined to bed or chair 50 % or more of waking hours (Karnofsky 30–40)
4	Completely disabled. Cannot carry out self-care. Totally confined to bed (Karnofsky 10–20)
5	Death (Karnofsky 0)

Lifestyle Factors

Lifestyle factors such as tobacco and alcohol abuse negatively influence survival. Accurate recording of smoking in pack-years and alcohol in number of days drinking per week and number of drinks per day will provide important data for future analysis. Nutrition is important to prognosis and will be indirectly measured by weight loss of > 5 % of body weight in the previous 6 months.[10] Depression adversely impacts quality of life and survival. Notation of a previous or current diagnosis of depression should be recorded in the medical record.[11] AJCC Level of Evidence: III

The role of tobacco as a negative prognostic factor is well established. However, exactly how this could be codified in the staging system is less clear. At this time, smoking is known to have a deleterious effect on prognosis but is hard to accurately apply to the staging system.

12

Smoking history should be collected as an important element of the demographics and may be included in Prognostic Groups in the future. For practicality, the minimum standard should classify smoking history as never, ≤ 10 pack-years, > 10 but ≤ 20 pack-years, or > 20 pack-years.

RISK ASSESSMENT MODELS

The AJCC recently established guidelines that will be used to evaluate published statistical prediction models for the purpose of granting endorsement for clinical use.[12] Although this is a monumental step toward the goal of precision medicine, this work was published only very recently. Therefore, the existing models that have been published or may be in clinical use have not yet been evaluated for this cancer site by the Precision Medicine Core of the AJCC. In the future, the statistical prediction models for this cancer site will be evaluated, and those that meet all AJCC criteria will be endorsed.

DEFINITIONS OF AJCC TNM

Definition of Primary Tumor (T)

Maxillary Sinus

T Category	T Criteria
TX	Primary tumor cannot be assessed
Tis	Carcinoma *in situ*
T1	Tumor limited to maxillary sinus mucosa with no erosion or destruction of bone
T2	Tumor causing bone erosion or destruction including extension into the hard palate and/or middle nasal meatus, except extension to posterior wall of maxillary sinus and pterygoid plates
T3	Tumor invades any of the following: bone of the posterior wall of maxillary sinus, subcutaneous tissues, floor or medial wall of orbit, pterygoid fossa, ethmoid sinuses
T4	Moderately advanced or very advanced local disease
T4a	Moderately advanced local disease<br>Tumor invades anterior orbital contents, skin of cheek, pterygoid plates, infratemporal fossa, cribriform plate, sphenoid or frontal sinuses
T4b	Very advanced local disease<br>Tumor invades any of the following: orbital apex, dura, brain, middle cranial fossa, cranial nerves other than maxillary division of trigeminal nerve (V2), nasopharynx, or clivus

Nasal Cavity and Ethmoid Sinus

**T Category**	**T Criteria**
TX	Primary tumor cannot be assessed
Tis	Carcinoma *in situ*
T1	Tumor restricted to any one subsite, with or without bony invasion
T2	Tumor invading two subsites in a single region or extending to involve an adjacent region within the nasoethmoidal complex, with or without bony invasion
T3	Tumor extends to invade the medial wall or floor of the orbit, maxillary sinus, palate, or cribriform plate
T4	Moderately advanced or very advanced local disease
T4a	Moderately advanced local disease<br>Tumor invades any of the following: anterior orbital contents, skin of nose or cheek, minimal extension to anterior cranial fossa, pterygoid plates, sphenoid or frontal sinuses
T4b	Very advanced local disease<br>Tumor invades any of the following: orbital apex, dura, brain, middle cranial fossa, cranial nerves other than (V2), nasopharynx, or clivus

Definition of Regional Lymph Node (N)

Clinical N (cN)

**N Category**	**N Criteria**
NX	Regional lymph nodes cannot be assessed
N0	No regional lymph node metastasis
N1	Metastasis in a single ipsilateral lymph node, 3 cm or smaller in greatest dimension and ENE(−)
N2	Metastasis in a single ipsilateral node larger than 3 cm but not larger than 6 cm in greatest dimension and ENE(−);<br>*or* metastases in multiple ipsilateral lymph nodes, none larger than 6 cm in greatest dimension and ENE(−);<br>*or* in bilateral or contralateral lymph nodes, none larger than 6 cm in greatest dimension and ENE(−)
N2a	Metastasis in a single ipsilateral node larger than 3 cm but not larger than 6 cm in greatest dimension and ENE(−)
N2b	Metastasis in multiple ipsilateral nodes, none larger than 6 cm in greatest dimension and ENE(−)
N2c	Metastasis in bilateral or contralateral lymph nodes, none larger than 6 cm in greatest dimension and ENE(−)

N Category	N Criteria
N3	Metastasis in a lymph node larger than 6 cm in greatest dimension and ENE(−); *or* metastasis in any node(s) with clinically overt ENE(+)
N3a	Metastasis in a lymph node larger than 6 cm in greatest dimension and ENE(−)
N3b	Metastasis in any node(s) with clinically overt ENE (ENE$_c$)

Note: A designation of "U" or "L" may be used for any N category to indicate metastasis above the lower border of the cricoid (U) or below the lower border of the cricoid (L).
Similarly, clinical and pathological ENE should be recorded as ENE(−) or ENE(+).

Pathological N (pN)

N Category	N Criteria
NX	Regional lymph nodes cannot be assessed
N0	No regional lymph node metastasis
N1	Metastasis in a single ipsilateral lymph node, 3 cm or smaller in greatest dimension and ENE(−)
N2	Metastasis in a single ipsilateral lymph node, 3 cm or smaller in greatest dimension and ENE(+); *or* larger than 3 cm but not larger than 6 cm in greatest dimension and ENE(−); *or* metastases in multiple ipsilateral lymph nodes, none larger than 6 cm in greatest dimension and ENE(−); *or* in bilateral or contralateral lymph nodes, none larger than 6 cm in greatest dimension and ENE(−)
N2a	Metastasis in single ipsilateral or contralateral node 3 cm or less in greatest dimension and ENE(+); *or* a single ipsilateral node larger than 3 cm but not larger than 6 cm in greatest dimension and ENE(−)
N2b	Metastasis in multiple ipsilateral nodes, none larger than 6 cm in greatest dimension and ENE(−)
N2c	Metastasis in bilateral or contralateral lymph nodes, none larger than 6 cm in greatest dimension and ENE(−)
N3	Metastasis in a lymph node larger than 6 cm in greatest dimension and ENE(−); *or* in a single ipsilateral node larger than 3 cm in greatest dimension and ENE(+); *or* multiple ipsilateral, contralateral or bilateral nodes, any with ENE(+)
N3a	Metastasis in a lymph node larger than 6 cm in greatest dimension and ENE(−)
N3b	Metastasis in a single ipsilateral node larger than 3 cm in greatest dimension and ENE(+); *or* multiple ipsilateral, contralateral or bilateral nodes, any with ENE(+)

Note: A designation of "U" or "L" may be used for any N category to indicate metastasis above the lower border of the cricoid (U) or below the lower border of the cricoid (L).
Similarly, clinical and pathological ENE should be recorded as ENE(−) or ENE(+).

Definition of Distant Metastasis (M)

M Category	M Criteria
M0	No distant metastasis (no pathologic M0; use clinical M to complete stage group)
M1	Distant metastasis

AJCC PROGNOSTIC STAGE GROUPS

When T is...	And N is...	And M is...	Then the stage group is...
Tis	N0	M0	0
T1	N0	M0	I
T2	N0	M0	II
T3	N0	M0	III
T1,T2,T3	N1	M0	III
T4a	N0,N1	M0	IVA
T1,T2,T3,T4a	N2	M0	IVA
Any T	N3	M0	IVB
T4b	Any N	M0	IVB
Any T	Any N	M1	IVC

REGISTRY DATA COLLECTION VARIABLES

1. ENE clinical status: ENE(−) or ENE(+)
2. ENE pathological status: ENE(−) or ENE(+)
3. The extent of microscopic ENE (distance of extension from the native lymph node capsule to the farthest point of invasion in the extranodal tissue)
4. Perineural invasion
5. Lymphovascular invasion
6. Performance status
7. Tobacco use
8. Alcohol use
9. Depression diagnosis

HISTOLOGIC GRADE (G)

G	G Definition
GX	Grade cannot be assessed
G1	Well differentiated
G2	Moderately differentiated
G3	Poorly differentiated

HISTOPATHOLOGIC TYPE

The predominant cancer is squamous cell carcinoma. The staging guidelines are applicable to all forms of carcinoma, including those arising from minor salivary glands. Other nonepithelial tumors such as those of lymphoid tissue, soft tissue, bone, and cartilage (i.e., lymphoma and sarcoma) are not included. Histologic confirmation of diagnosis is required. Histopathologic grading of squamous carcinoma is recommended. The grade is subjective and uses a descriptive, as well as numerical, form (i.e., well differentiated, moderately differentiated, and poorly differentiated), depending on the degree of closeness to or deviation from squamous epithelium in mucosal sites. Also recommended where feasible is a quantitative evaluation of depth of invasion of the primary tumor and the presence or absence of vascular invasion and perineural invasion. Although the grade of tumor does not enter into the staging of the tumor, it should be recorded. The pathological description of any lymphadenectomy specimen should describe the size, number, and position of the involved node(s) and the presence or absence of ENE.

ILLUSTRATIONS

T1

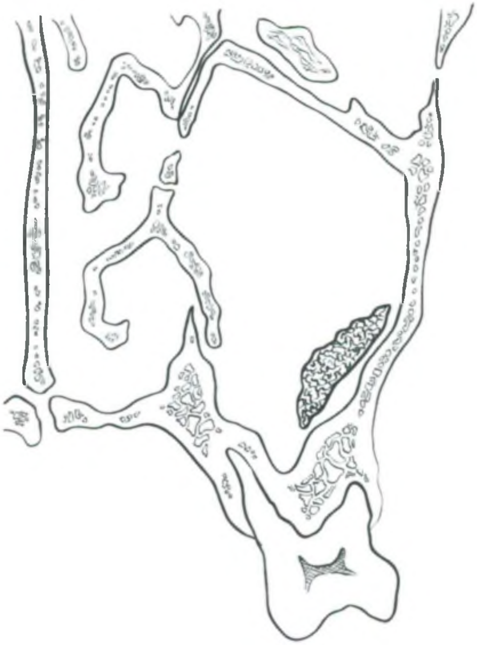

Fig. 12.2 T1 in the maxillary sinus is limited to the maxillary sinus mucosa with no erosion or destruction of bone

T2

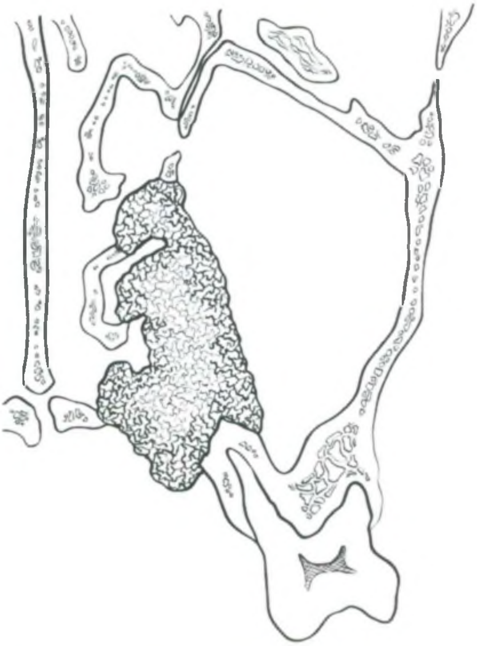

Fig. 12.3 T2 in the maxillary sinus causes bone erosion or destruction including extension into the hard palate and/or middle nasal meatus, with the exception of extension to posterior wall of maxillary sinus and pterygoid plates

T3 T3

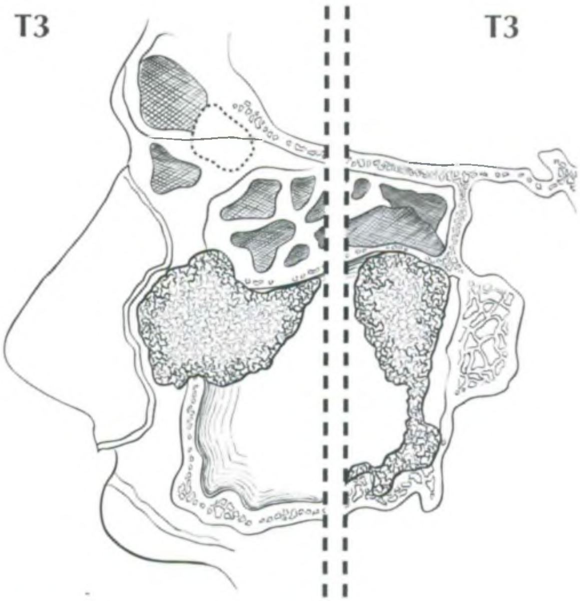

Fig. 12.4 Two views of T3 in the maxillary sinus. Tumor invades any of the following: bone of the posterior wall of maxillary sinus, subcutaneous tissues, floor or medial wall of orbit, pterygoid fossa, ethmoid sinuses

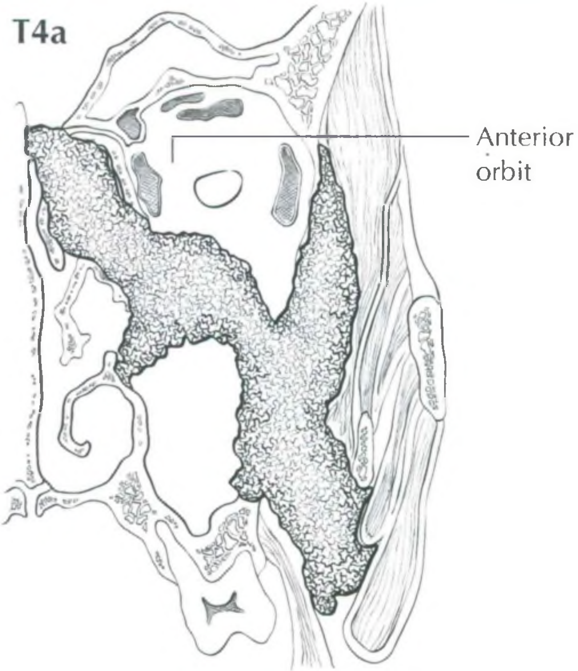

Fig. 12.5 T4a in the maxillary sinus is moderately advanced local disease, showing tumor invasion of anterior orbital contents

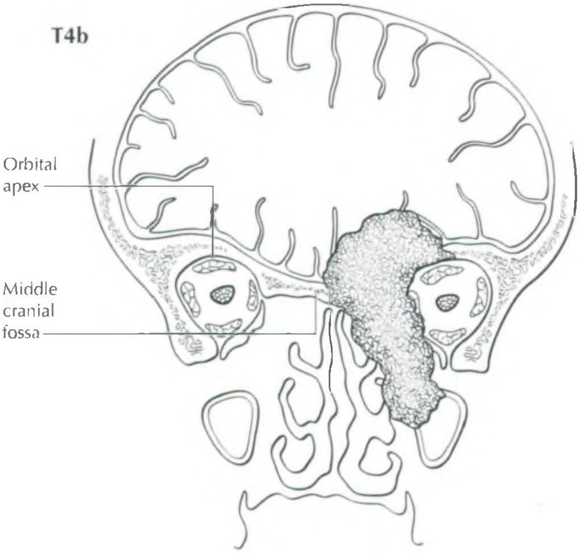

Fig. 12.7 Coronal view of T4b in the maxillary sinus, very advanced local disease, shows tumor invades orbital apex

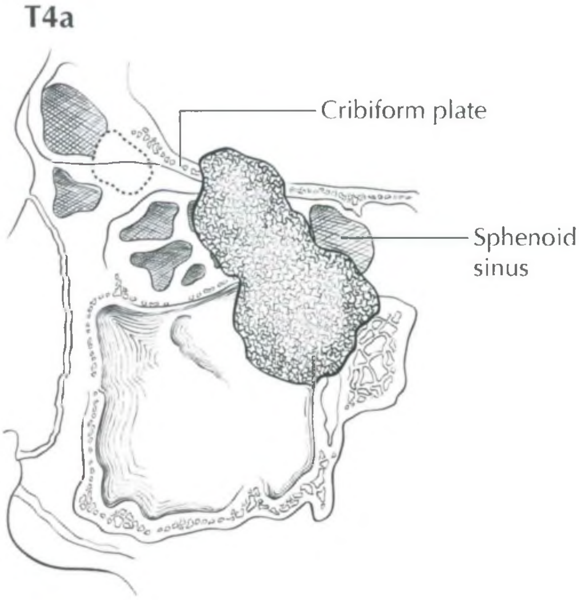

Fig. 12.6 T4a in the maxillary sinus is moderately advanced local disease, showing tumor invasion of sphenoid sinus and cribriform plate

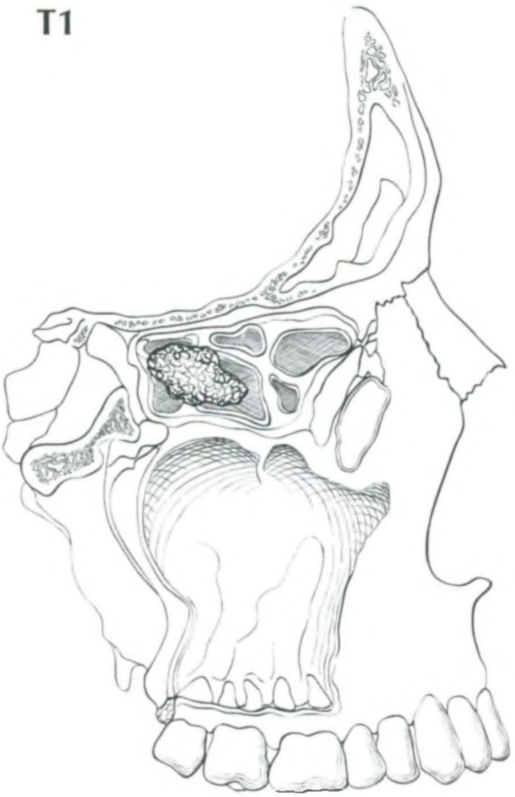

Fig. 12.8 In the nasal cavity and ethmoid sinus, T1 is defined as tumor restricted to any one subsite, with or without bony invasion

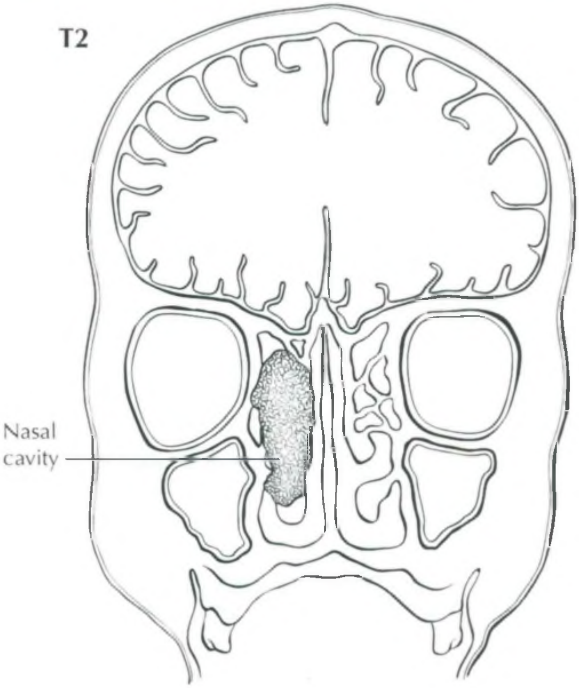

Fig. 12.9 T2 in the nasal cavity and ethmoid sinus is defined as invading two subsites in a single region or extending to involve an adjacent region within the nasoethmoidal complex, here the nasal cavity, with or without bony invasion

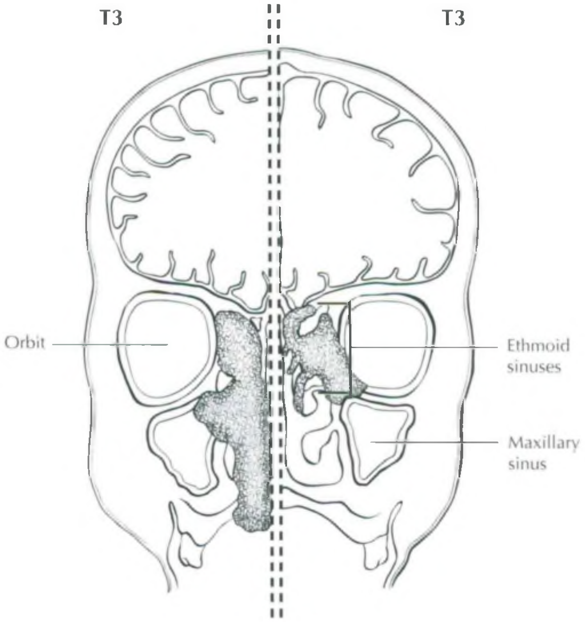

Fig. 12.10 Two views of T3 in the nasal cavity and ethmoid sinus showing tumor invading maxillary sinus and palate (*left*) and extending to the floor of the orbit (*right*)

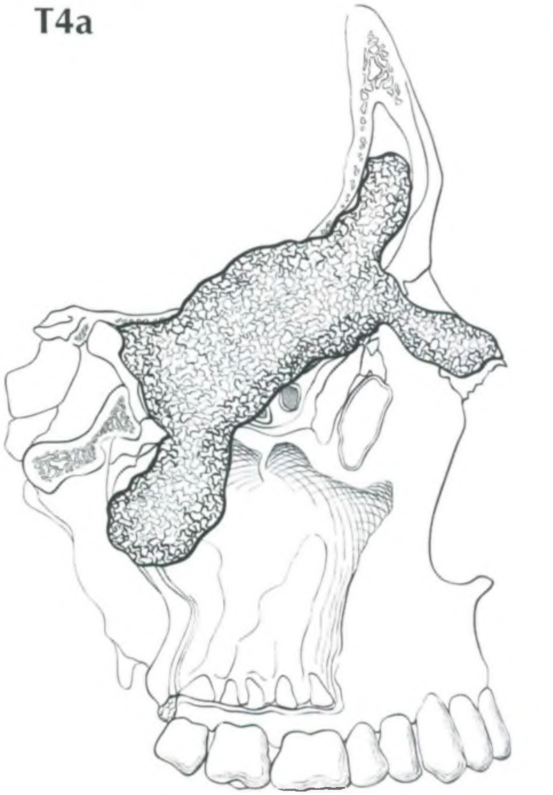

Fig. 12.11 T4a in the nasal cavity and ethmoid sinus is moderately advanced local disease, and invades any of the following: anterior orbital contents, skin of nose or cheek, minimal extension to anterior cranial fossa, pterygoid plates, sphenoid, or frontal sinuses

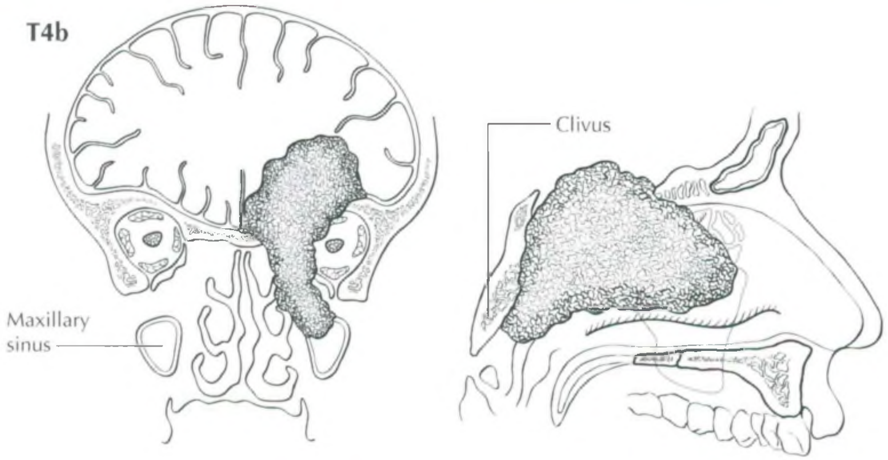

Fig. 12.12 Two views of T4b in the nasal cavity and ethmoid sinus. This is very advanced local disease, and the coronal view on the *left* shows invasion in the orbital apex and brain. On the *right*, tumor invades the clivus

Bibliography

1. Patel S. Personal Communication. In: Lydiatt W, Shah JP, eds2015.
2. O'Sullivan B, Huang SH, Su J, et al. Development and validation of a staging system for HPV-related oropharyngeal cancer by the International Collaboration on Oropharyngeal cancer Network for Staging (ICON-S): a multicentre cohort study. *The lancet oncology.* Feb 26 2016.
3. Wreesmann VB, Katabi N, Palmer FL, et al. Influence of extracapsular nodal spread extent on prognosis of oral squamous cell carcinoma. *Head & neck.* Oct 30 2015.
4. Harbo G, Grau C, Bundgaard T, et al. Cancer of the nasal cavity and paranasal sinuses. A clinico-pathological study of 277 patients. *Acta oncologica.* 1997;36(1):45-50.
5. de Juan J, Garcia J, Lopez M, et al. Inclusion of extracapsular spread in the pTNM classification system: a proposal for patients with head and neck carcinoma. *JAMA otolaryngology– head & neck surgery.* May 2013;139(5):483-488.
6. Dunne AA, Muller HH, Eisele DW, Kessel K, Moll R, Werner JA. Meta-analysis of the prognostic significance of perinodal spread in head and neck squamous cell carcinomas (HNSCC) patients. *European journal of cancer.* Aug 2006;42(12):1863-1868.
7. Prabhu RS, Hanasoge S, Magliocca KR, et al. Extent of pathologic extracapsular extension and outcomes in patients with nonoropharyngeal head and neck cancer treated with initial surgical resection. *Cancer.* May 15 2014;120(10):1499-1506.
8. Prabhu RS, Magliocca KR, Hanasoge S, et al. Accuracy of computed tomography for predicting pathologic nodal extracapsular extension in patients with head-and-neck cancer undergoing initial surgical resection. *International journal of radiation oncology, biology, physics.* Jan 1 2014;88(1):122-129.
9. Piccirillo JF. Inclusion of comorbidity in a staging system for head and neck cancer. *Oncology (Williston Park).* Sep 1995;9(9):831-836; discussion 841, 845-838.
10. Couch ME, Dittus K, Toth MJ, et al. Cancer cachexia update in head and neck cancer: Pathophysiology and treatment. *Head & neck.* Jul 2015;37(7):1057-1072.
11. Lazure KE, Lydiatt WM, Denman D, Burke WJ. Association between depression and survival or disease recurrence in patients with head and neck cancer enrolled in a depression prevention trial. *Head & neck.* 2009;31(7):888-892.
12. Kattan MW, Hess KR, Amin MB, et al. American Joint Committee on Cancer acceptance criteria for inclusion of risk models for individualized prognosis in the practice of precision medicine. *CA: a cancer journal for clinicians.* Jan 19 2016.

Larynx

13

Snehal G. Patel, William M. Lydiatt,
Christine M. Glastonbury, Suresh K. Mukherji,
Ronald A. Ghossein, Margaret Brandwein-Gensler,
Brian O'Sullivan, and Jatin P. Shah

CHAPTER SUMMARY

Cancers Staged Using This Staging System

Staging of carcinoma of the supraglottic, glottic, and subglottic larynx should use this system.

Cancers Not Staged Using This Staging System

These histopathologic types of cancer...	Are staged according to the classification for...	And can be found in chapter...
Nonepithelial tumors of lymphoid tissue	Hematologic malignancies	78–83
Nonepithelial tumors of soft tissue	Soft tissue sarcoma of the head and neck	40
Nonepithelial tumors of bone and cartilage	Bone	38
Mucosal melanoma of the lip and oral cavity	Mucosal melanoma of the head and neck	14

Summary of Changes

Change	Details of Change	Level of Evidence
Anatomy – Primary Site(s)	Occult Primary Tumor: Staging of the patient who presents with Epstein-Barr virus (EBV)-unrelated and human papilloma virus (HPV)-unrelated metastatic cervical lymphadenopathy is not included in this chapter.	IV
Definition of Regional Lymph Node (N)	There now are separate N staging approaches for HPV-related and HPV-unrelated cancers.	II[1,2]
Definition of Regional Lymph Node (N)	There now are separate N category approaches for patients treated without cervical lymph node dissection (clinical N) and patients treated with cervical lymph neck dissection (pathological N).	II[1,2]
Definition of Regional Lymph Node (N)	Extranodal extension (ENE) is introduced as a descriptor in all HPV-unrelated cancers.	II[2]
Definition of Regional Lymph Node (N)	ENE in HPV negative cancers: Only clinically and radiographically overt ENE should be used for cN.	II[2]
Definition of Regional Lymph Node (N)	ENE in HPV negative cancers: Any pathologically detected ENE is considered ENE(+) and is used for pN.	II[2]

To access the AJCC cancer staging forms, please visit www.cancerstaging.org.

© American Joint Committee on Cancer 2017

M.B. Amin et al. (eds.), *AJCC Cancer Staging Manual, Eighth Edition*, DOI 10.1007/978-3-319-40618-3_13

Change	Details of Change	Level of Evidence
Definition of Regional Lymph Node (N)	ENE in HPV-negative cancers: Presence of ENE is designated pN2a for a single ipsilateral node < 3 cm and pN3b for all other node(s).	II[2]
Definition of Regional Lymph Node (N)	Classification of ENE: Clinically overt ENE is classified as ENE_c and is considered ENE(+) for cN.	III[3]
Definition of Regional Lymph Node (N)	Classification of ENE: Pathologically detected ENE is classified as either ENE_{mi} (≤2 mm) or ENE_{ma} (>2 mm) for data collection purposes only, but both are considered ENE+ for definition of pN.	III[3]

ICD-O-3 Topography Codes

Code	Description
C10.1	Anterior (lingual) surface of epiglottis
C32.0	Glottis
C32.1	Supraglottis (laryngeal surface)
C32.2	Subglottis
C32.8	Overlapping lesion of larynx*<br>*Stage by location of tumor bulk or epicenter
C32.9	Larynx, NOS*<br>*Stage by location of tumor bulk or epicenter

WHO Classification of Tumors

Code	Description
8070	Squamous cell carcinoma
8075	Acantholytic squamous cell carcinoma
8560	Adenosquamous carcinoma
8083	Basaloid squamous cell carcinoma
8052	Papillary squamous cell carcinoma
8074	Spindle cell squamous carcinoma
8051	Verrucous carcinoma
8082	Lymphoepithelial carcinoma
8430	Mucoepidermoid carcinoma
8200	Adenoid cystic carcinoma
8240	Typical carcinoid
8249	Atypical carcinoid
8041	Small cell carcinoma, neuroendocrine type
8045	Combined small cell carcinoma, neuroendocrine type

Barnes L, Eveson JW, Reichart P, Sidransky D, eds. World Health Organization Classification of Tumours Pathology and Genetics of Head and Neck Tumours. Lyon: IARC; 2005.

INTRODUCTION

Cancers of the larynx are staged using the TNM system. The T category is unchanged from the American Joint Committee on Cancer (AJCC) Cancer Staging Manual, 7th Edition. N category, however, now includes extranodal extension (ENE) in categorizing metastatic cancer to neck nodes. The effect of ENE in prognosis on head and neck cancers not caused by human papilloma virus (HPV) is profound.[4] Including this important prognostic feature was considered critical in revising staging. Most of the data supporting ENE as an adverse prognostic factor are based on histopathological characterization of ENE, especially the distinction between microscopic and macroscopic (or gross) ENE.[3,5,6] Therefore, only unquestionable ENE is used for clinical staging. For clinical ENE, the known inability of current imaging modalities to define ENE accurately mandated stringent criteria for assigning a clinical diagnosis of ENE. However, unambiguous evidence of gross ENE on clinical examination (e.g., invasion of skin, infiltration of musculature/dense tethering to adjacent structures, or cranial nerve, brachial plexus, sympathetic trunk, or phrenic nerve invasion with dysfunction) supported by strong radiographic evidence permits classification of disease as N3b. Pathological ENE also will be clearly defined in this chapter. As per the "uncertain rule" of the AJCC/UICC staging, which mandates that the lower category always be assigned in ambiguous cases, a case should be categorized as ENE(-) unless there is unquestionable ENE.

A staging system should address and respond to new information that influences patient outcome. An appropriate balance between complexity and compliance (ease-of-use) is necessary for worldwide adoption. The TNM system has strongly predicted prognosis over the years and is adopted worldwide. The introduction of the new parameter of ENE better fits the prognostic modeling from large datasets. It must be balanced, however, by the ability to derive accurate information from clinicians caring for patients with head and neck cancer in many different environments. Thorough descriptions of ENE are included in the Definition of ENE and Description of its Extent section of this chapter. Effectively, ENE will increase the nodal category by 1 (demonstrated in this chapter).

ANATOMY

Primary Site(s)

The following anatomic definition of the larynx allows classification of carcinomas arising in the encompassed mucous

membranes but excludes cancers arising on the lateral or posterior pharyngeal wall, pyriform fossa, postcricoid area, or base of tongue.

The anterior limit of the larynx is composed of the anterior or lingual surface of the suprahyoid epiglottis, the thyrohyoid membrane, the anterior commissure, and the anterior wall of the subglottic region, which is composed of the thyroid cartilage, the cricothyroid membrane, and the anterior arch of the cricoid cartilage.

The posterior and lateral limits include the laryngeal aspect of the aryepiglottic folds, the arytenoid region, the interarytenoid space, and the posterior surface of the subglottic space, represented by the mucous membrane covering the surface of the cricoid cartilage.

The superolateral limits are composed of the tip and the lateral borders of the epiglottis. The inferior limits are made up of the plane passing through the inferior edge of the cricoid cartilage.

For purposes of this clinical stage classification, the larynx is divided into three regions: supraglottis, glottis, and subglottis (Fig. 13.1). The supraglottis is composed of the epiglottis (both its lingual and laryngeal aspects), aryepiglottic folds (laryngeal aspect), arytenoids, and ventricular bands (false cords) (Fig. 13.2). The epiglottis is divided for staging purposes into suprahyoid and infrahyoid portions by a plane at the level of the hyoid bone. The inferior boundary of the supraglottis is a horizontal plane passing through the lateral margin of the ventricle at its junction with the superior surface of the vocal cord. The glottis is composed of the superior and inferior surfaces of the true vocal cords, including the anterior and posterior commissures (Fig. 13.2). It occupies a horizontal plane 1 cm in thickness, extending inferiorly from the lateral margin of the ventricle. The subglottis is the region extending from the lower boundary of the glottis to the lower margin of the cricoid cartilage.

The division of the larynx is summarized as follows:

Site	Subsite(s)
Supraglottis	Suprahyoid epiglottis
	Infrahyoid epiglottis
	Aryepiglottic folds (laryngeal aspect); arytenoids
	Ventricular bands (false cords)
Glottis	True vocal cords, including anterior and posterior commissures
Subglottis	Subglottis

Regional Lymph Nodes

The risk of regional metastasis generally is related to the T category. The incidence and distribution of cervical nodal metastases from cancer of the larynx vary with the site of origin and the T category of the primary tumor. The true vocal cords are nearly devoid of lymphatics, and tumors limited to the glottis alone rarely spread to regional nodes. By contrast, the supraglottis has a rich and bilaterally interconnected lymphatic network, so primary supraglottic cancers commonly are accompanied by regional lymph node spread. Advanced glottic tumors may spread directly to adjacent soft tissues, to prelaryngeal, pretracheal, paralaryngeal, and paratracheal nodes, as well as to upper, mid, and lower jugular nodes. Supraglottic tumors

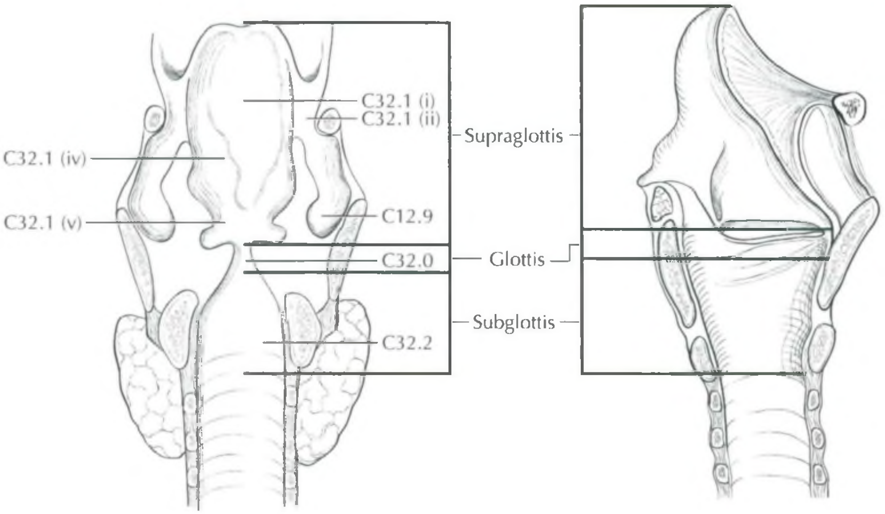

Fig. 13.1 Anatomical sites and subsites of the three regions of the larynx: supraglottis, glottis, and subglottis. Supraglottis (C32.1) subsites include suprahyoid epiglottis (i), aryepiglottic fold, laryngeal aspect (ii), infrahyoid epiglottis (iv), and ventricular bands or false cords (v)

13

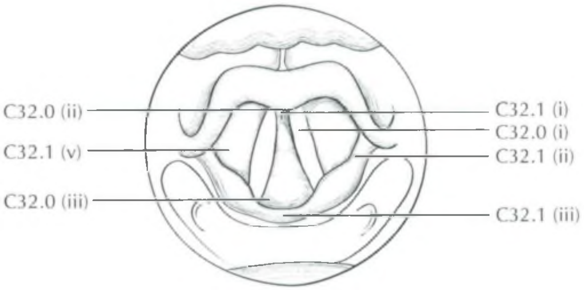

Fig. 13.2 Anatomical sites and subsites of the supraglottis and glottis. Supraglottis (C32.1) subsites include suprahyoid epiglottis (i), aryepiglottic fold, laryngeal aspect (ii), arytenoids (iii), and ventricular bands or false cords (v). Glottis (C32.0) subsites include vocal cords (i), anterior commissure (ii), and posterior commissure (iii)

commonly spread to upper and mid jugular nodes, considerably less commonly to submental or submandibular nodes, and occasionally to retropharyngeal nodes. The rare subglottic primary tumors spread first to adjacent soft tissues and prelaryngeal, pretracheal, paralaryngeal, and paratracheal nodes, then to mid and lower jugular nodes. Contralateral lymphatic spread is common. Any previous manipulation to the neck, through surgery or radiation, may alter normal lymphatic drainage patterns, resulting in unusual distribution of regional spread of disease to the cervical lymph nodes.

Metastatic Sites

Distant spread is common only for patients who have bulky regional lymphadenopathy. When distant metastases occur, spread to the lungs is most common; skeletal or hepatic metastases occur less often. Mediastinal lymph node metastases are considered distant metastases, except Level VII lymph nodes (in the anterior superior mediastinum, cephalad to the innominate artery).

RULES FOR CLASSIFICATION

Clinical Classification

Clinical staging is essential for squamous cell carcinomas of the larynx. Assessment is primarily based on inspection of the larynx via indirect and direct endoscopy. Palpation of sites (when feasible) and of neck nodes is essential. Neurologic evaluation of all cranial nerves is required. Complete endoscopy, usually under general anesthesia, is performed after completion of other staging studies, to assess the surface extent of the tumor accurately, assess for vocal cord mobility, and facilitate biopsy. A careful search for other primary tumors of the upper aerodigestive tract may be indicated because of the potential of multiple independent primary tumors occurring simultaneously.

Primary site clinical staging for supraglottic carcinoma is based on involvement of various subsites of the supraglottic larynx adjacent regions and vocal cord mobility. Imaging may be helpful to identify occult submucosal transglottic extension. Imaging criteria that define T3 lesions are extension into the preepiglottic space (paralaryngeal fat) or tumors that erode the inner cortex of the thyroid cartilage. Tumors that erode the outer cortex of the thyroid cartilage are defined as T4a tumors.

In clinical evaluation of the neck, the maximum size of any nodal mass should be measured. There are three categories of clinically involved nodes for the larynx: N1, N2, and N3. Midline nodes are considered ipsilateral nodes. Superior mediastinal lymph nodes are considered regional lymph nodes (Level VII). In addition to the components to describe the N category, regional lymph nodes also should be described according to the level of the neck that is involved. Provide a description or map of the regional lymph nodes and node groups that the cancer affects. Unambiguous evidence of gross ENE (i.e., invasion of skin, infiltration of musculature/fixation to adjacent structures on clinical examination, or invasion of cranial nerve, brachial plexus, sympathetic trunk, or phrenic nerve with dysfunction) is a sufficiently high threshold to classify these as clinical ENE (ENE_c) and qualifies as ENE(+) for definition of cN.

The known lack of ability for radiographic imaging to define ENE accurately suggests the need for stringent criteria prior to assigning ENE on a clinical basis. Imaging studies showing amorphous spiculated margins of involved nodes or involvement of internodal fat resulting in loss of normal oval-to-round nodal shape suggest extranodal spread of tumor but are not sufficient without corresponding evidence on physical examination. Pathological examination is necessary for documentation of such disease extent. No imaging study (as yet) can identify microscopic foci in regional nodes or distinguish between small reactive nodes and small malignant nodes (unless central radiographic inhomogeneity is present).

Imaging

Both contrast-enhanced computed tomography (CT) and magnetic resonance (MR) imaging allow excellent evaluation of the primary tumor and of nodal drainage sites.[7] Positron emission tomography (PET)/CT is being used with greater frequency for staging, treatment evaluation, and surveillance. It may offer an advantage in detection of an otherwise clinically occult primary tumor.

For T1 and T2 tumors of the glottic larynx, cross-sectional imaging may be used to ensure that the clinical diagnosis of early stage lesions is correct. Imaging may be used as an important adjunct to identify the presence of submucosal extension, especially at the anterior commissure where lesions may spread anteriorly along Broyle's ligament to involve the inner cortex of the thyroid cartilage. Imaging also may identify glottic carcinomas that have occult transglottic or subglottic spread. The normal *paraglottic* space often is difficult to routinely detect at the level of the true vocal cord due to the close apposition of the lateral thyroarytenoid muscle to the inner cortex of the thyroid cartilage. Tumor erosion limited to the inner cortex of the thyroid cartilage indicates a T3 lesion, whereas carcinomas that erode the outer cortex of the thyroid cartilage define a T4a tumor. Stage T4 (a and b) is difficult to identify based on clinical examination alone, because the majority of the criteria cannot be assessed by endoscopy and palpation.

Pathological Classification

Pathological staging requires the use of all information obtained in clinical staging and in histologic study of the surgically resected specimen. The surgeon's evaluation of gross unresected residual tumor also must be included.

Complete resection of the primary site and/or regional nodal dissections, followed by pathological examination of the resected specimen(s), allows the use of this designation for pT and/or pN, respectively. Specimens that are resected after radiation or chemotherapy need to be identified and considered in context, and use yp instead of p. pT is derived from the actual measurement of the unfixed tumor in the surgical specimen. It should be noted, however, that up to 30% shrinkage of soft tissues may occur in resected specimen after formalin fixation. Pathological staging represents additional and important information and should be included as such in staging, but it does not supplant clinical staging as the primary staging scheme.

Histopathologic grading of squamous carcinoma is recommended. The grade is subjective and uses a descriptive, as well as a numerical, form (i.e., well differentiated, moderately differentiated, and poorly differentiated), depending on the degree of closeness to or deviation from squamous epithelium in mucosal sites. Also recommended, where feasible, is a quantitative evaluation of depth of invasion of the primary tumor and the presence or absence of vascular invasion and perineural invasion. Although the grade of tumor does not enter into the staging of the tumor, it should be recorded.

The pathological description of any lymphadenectomy specimen should describe the size, number, and location/level of the involved node(s) and the presence or absence of ENE. For pN, a selective neck dissection ordinarily will include 10 or more lymph nodes, and a radical or modified radical neck dissection ordinarily will include 15 or more lymph nodes. Negative pathological examination of a smaller number of nodes still mandates a pN0 designation.

Definition of ENE and Description of its Extent

All surgically resected metastatic nodes should be examined for the presence and extent of ENE. The precise definition of ENE also has varied in the literature over time. The American College of Pathologists defines ENE as extension of metastatic tumor, present within the confines of the lymph node, through the lymph node capsule into the surrounding connective tissue, with or without associated stromal reaction. Pathological examination is necessary for documentation of such disease extent.

ENE detected on histopathologic examination is designated as ENE_{mi} (microscopic ENE $\leq$ 2 mm) or ENE_{ma} (major ENE > 2 mm). Both ENE_{mi} and ENE_{ma} qualify as ENE(+) for definition of pN. These descriptors of ENE will not be required for current pN definition, but data collection is recommended to allow standardization of data collection and future analysis.

PROGNOSTIC FACTORS

Prognostic Factors Required for Stage Grouping

Beyond the factors used to assign T, N, or M categories, no additional prognostic factors are required for stage grouping.

Additional Factors Recommended for Clinical Care

In addition to the importance of the TNM factors outlined previously, the overall health of these patients clearly influences outcome. An ongoing effort to better assess prognosis using both tumor and nontumor-related factors is underway. Chart abstraction will continue to be performed by cancer registrars to obtain important information regarding specific factors related to prognosis. These data then will be used to further hone the predictive power of the staging system in future revisions.

Extranodal Extension

ENE is defined as extension of metastatic tumor, present within the confines of the lymph node, through the lymph node capsule into the surrounding connective tissue, with or without associated stromal reaction. Unambiguous evidence of gross ENE (i.e., defined as invasion of skin, infiltration of musculature/fixation to adjacent structures on clinical examination, or invasion of cranial nerve, brachial plexus, sympathetic trunk, or phrenic nerve with dysfunction) is a sufficiently high threshold to classify these as clinical ENE+. AJCC Level of Evidence: III

Comorbidity

Comorbidity can be classified by specific measures of additional medical illnesses. Accurate reporting of all illnesses in the patient's medical record is essential to assessment of these parameters. General performance measures are helpful in predicting survival. The AJCC strongly recommends the clinician report performance status using the Eastern Cooperative Oncology Group (ECOG), Zubrod, or Karnofsky performance measures, along with standard staging information. An interrelationship between each of the major performance tools exists. AJCC Level of Evidence: II[8]

Zubrod/ECOG Performance Scale	
0	Fully active, able to carry out all predisease activities without restriction (Karnofsky 90–100)
1	Restricted in physically strenuous activity but ambulatory and able to carry out work of a light or sedentary nature; for example, light housework, office work (Karnofsky 70–80)
2	Ambulatory and capable of all self-care but unable to carry out any work activities; up and about more than 50 % of waking hours (Karnofsky 50–60)
3	Capable of only limited self-care, confined to bed or chair 50 % or more of waking hours (Karnofsky 30–40)
4	Completely disabled; cannot carry on self-care; totally confined to bed or chair (Karnofsky 10–20)
5	Death (Karnofsky 0)

Lifestyle factors

Lifestyle factors such as tobacco and alcohol abuse negatively influence survival. Accurate recording of smoking in pack-years and alcohol in number of days drinking per week and number of drinks per day will provide important data for future analysis. Nutrition is important to prognosis and will be measured indirectly by weight loss of $>5\%$ of body weight in the previous 6 months.[9] Depression adversely impacts quality of life and survival. Notation of a previous or current diagnosis of depression should be recorded in the medical record.[10] AJCC Level of Evidence: III

Tobacco Use

The role of tobacco as a negative prognostic factor is well established. Exactly how this could be codified in the staging system, however, is less clear. At this time, smoking is known to have a deleterious effect on prognosis but it is difficult to accurately apply it to the staging system. AJCC Level of Evidence: III

Smoking history should be collected as an important element of the demographics and may be included in Prognostic Groups in the future. For practicality, the minimum standard should classify smoking history as never, ≤ 10 pack-years, >10 but ≤ 20 pack-years, or >20 pack-years.

RISK ASSESSMENT MODELS

The AJCC recently established guidelines that will be used to evaluate published statistical prediction models for the purpose of granting endorsement for clinical use.[11] Although this is a monumental step toward the goal of precision medicine, this work was published only very recently. Therefore, the existing models that have been published or may be in clinical use have not yet been evaluated for this cancer site by the Precision Medicine Core of the AJCC. In the future, the statistical prediction models for this cancer site will be evaluated, and those that meet all AJCC criteria will be endorsed.

DEFINITIONS OF AJCC TNM

Definition of Primary Tumor (T)

Supraglottis

T Category	T Criteria
TX	Primary tumor cannot be assessed
Tis	Carcinoma *in situ*
T1	Tumor limited to one subsite of supraglottis with normal vocal cord mobility
T2	Tumor invades mucosa of more than one adjacent subsite of supraglottis or glottis or region outside the supraglottis (e.g., mucosa of base of tongue, vallecula, medial wall of pyriform sinus) without fixation of the larynx
T3	Tumor limited to larynx with vocal cord fixation and/or invades any of the following: postcricoid area, preepiglottic space, paraglottic space, and/or inner cortex of thyroid cartilage
T4	Moderately advanced or very advanced
T4a	Moderately advanced local disease Tumor invades through the outer cortex of the thyroid cartilage and/or invades tissues beyond the larynx (e.g., trachea, soft tissues of neck including deep extrinsic muscle of the tongue, strap muscles, thyroid, or esophagus)
T4b	Very advanced local disease<br>Tumor invades prevertebral space, encases carotid artery, or invades mediastinal structures

Glottis

T Category	T Criteria
TX	Primary tumor cannot be assessed
Tis	Carcinoma *in situ*
T1	Tumor limited to the vocal cord(s) (may involve anterior or posterior commissure) with normal mobility
T1a	Tumor limited to one vocal cord
T1b	Tumor involves both vocal cords
T2	Tumor extends to supraglottis and/or subglottis, and/or with impaired vocal cord mobility
T3	Tumor limited to the larynx with vocal cord fixation and/or invasion of paraglottic space and/or inner cortex of the thyroid cartilage
T4	Moderately advanced or very advanced
T4a	Moderately advanced local disease<br>Tumor invades through the outer cortex of the thyroid cartilage and/or invades tissues beyond the larynx (e.g., trachea, cricoid cartilage, soft tissues of neck including deep extrinsic muscle of the tongue, strap muscles, thyroid, or esophagus)
T4b	Very advanced local disease<br>Tumor invades prevertebral space, encases carotid artery, or invades mediastinal structures

Subglottis

T Category	T Criteria
TX	Primary tumor cannot be assessed
Tis	Carcinoma *in situ*
T1	Tumor limited to the subglottis
T2	Tumor extends to vocal cord(s) with normal or impaired mobility
T3	Tumor limited to larynx with vocal cord fixation and/or invasion of paraglottic space and/or inner cortex of the thyroid cartilage
T4	Moderately advanced or very advanced
T4a	Moderately advanced local disease<br>Tumor invades cricoid or thyroid cartilage and/or invades tissues beyond the larynx (e.g., trachea, soft tissues of neck including deep extrinsic muscles of the tongue, strap muscles, thyroid, or esophagus)
T4b	Very advanced local disease<br>Tumor invades prevertebral space, encases carotid artery, or invades mediastinal structures

Definition of Regional Lymph Nodes (N)

Clinical N (cN)

N Category	N Criteria
NX	Regional lymph nodes cannot be assessed
N0	No regional lymph node metastasis
N1	Metastasis in a single ipsilateral lymph node, 3 cm or smaller in greatest dimension and ENE(−)
N2	Metastasis in a single ipsilateral node, larger than 3 cm but not larger than 6 cm in greatest dimension and ENE(−);<br>*or* metastases in multiple ipsilateral lymph nodes, none larger than 6 cm in greatest dimension and ENE(−);<br>*or* metastasis in bilateral or contralateral lymph nodes, none larger than 6 cm in greatest dimension and ENE(−)
N2a	Metastasis in a single ipsilateral node, larger than 3 cm but not larger than 6 cm in greatest dimension and ENE(−)
N2b	Metastases in multiple ipsilateral nodes, none larger than 6 cm in greatest dimension and ENE(−)
N2c	Metastasis in bilateral or contralateral lymph nodes, none larger than 6 cm in greatest dimension and ENE(−)
N3	Metastasis in a lymph node, larger than 6 cm in greatest dimension and ENE(−);<br>*or* metastasis in any lymph node(s) with clinically overt ENE(+)
N3a	Metastasis in a lymph node, larger than 6 cm in greatest dimension and ENE(−)
N3b	Metastasis in any lymph node(s) with clinically overt ENE(+)

Note: A designation of "U" or "L" may be used for any N category to indicate metastasis above the lower border of the cricoid (U) or below the lower border of the cricoid (L)
Similarly, clinical and pathological ENE should be recorded as ENE(−) or ENE(+)

Pathological N (pN)

N Category	N Criteria
NX	Regional lymph nodes cannot be assessed
N0	No regional lymph node metastasis
N1	Metastasis in a single ipsilateral lymph node, 3 cm or smaller in greatest dimension and ENE(−)
N2	Metastasis in a single ipsilateral lymph node, 3 cm or smaller in greatest dimension and ENE(+); *or* metastasis in a single ipsilateral lymph node, larger than 3 cm but not larger than 6 cm in greatest dimension and ENE(−); *or* metastases in multiple ipsilateral lymph nodes, none larger than 6 cm in greatest dimension and ENE(−); *or* metastasis in bilateral or contralateral lymph nodes, none larger than 6 cm in greatest dimension and ENE(−)
N2a	Metastasis in a single ipsilateral or contralateral node, 3 cm or smaller in greatest dimension and ENE(+); *or* metastasis in a single ipsilateral node, larger than 3 cm but not larger than 6 cm in greatest dimension and ENE(−)
N2b	Metastases in multiple ipsilateral nodes, none larger than 6 cm in greatest dimension and ENE(−)
N2c	Metastasis in bilateral or contralateral lymph nodes, none larger than 6 cm in greatest dimension and ENE(−)
N3	Metastasis in a lymph node, larger than 6 cm in greatest dimension and ENE(−); *or* metastasis in a single ipsilateral node, larger than 3 cm in greatest dimension and ENE(+); *or* metastases in multiple ipsilateral, contralateral, or bilateral lymph nodes and any with ENE(+)
N3a	Metastasis in a lymph node, larger than 6 cm in greatest dimension and ENE(−)
N3b	Metastasis in a single ipsilateral node, larger than 3 cm in greatest dimension and ENE(+); *or* metastases in multiple ipsilateral, contralateral, or bilateral lymph nodes and any with ENE(+)

Note: A designation of "U" or "L" may be used for any N category to indicate metastasis above the lower border of the cricoid (U) or below the lower border of the cricoid (L)
Similarly, clinical and pathological ENE should be recorded as ENE(−) or ENE(+)

Definition of Distant Metastasis (M)

M Category	M Criteria
M0	No distant metastasis
M1	Distant metastasis

AJCC PROGNOSTIC STAGE GROUPS

When T is...	And N is...	And M is...	Then the stage group is...
Tis	N0	M0	0
T1	N0	M0	I
T2	N0	M0	II
T3	N0	M0	III
T1, T2, T3	N1	M0	III
T4a	N0, N1	M0	IVA
T1, T2, T3, T4a	N2	M0	IVA
Any T	N3	M0	IVB
T4b	Any N	M0	IVB
Any T	Any N	M1	IVC

REGISTRY DATA COLLECTION VARIABLES

1. ENE clinical presence or absence
2. ENE pathological presence or absence
3. Extent of microscopic ENE (distance of extension from the native lymph node capsule to the farthest point of invasion in the extranodal tissue)
4. Perineural invasion
5. Lymphovascular invasion
6. Performance status
7. Tobacco use and pack-year
8. Alcohol use
9. Depression diagnosis

HISTOLOGIC GRADE (G)

G	G Definition
GX	Grade cannot be assessed
G1	Well differentiated
G2	Moderately differentiated
G3	Poorly differentiated

HISTOPATHOLOGIC TYPE

The predominant cancer is squamous cell carcinoma. The staging guidelines are applicable to all forms of epithelial carcinoma, including those arising from minor salivary glands. Other nonepithelial tumors—such as those of lymphoid tissue, soft tissue, bone, and cartilage (i.e., lymphoma and sarcoma)—are not included. Histologic confirmation of diagnosis is required.

ILLUSTRATIONS

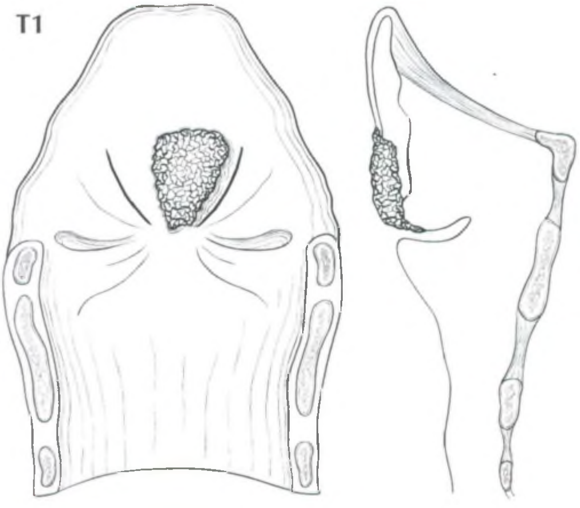

Fig. 13.3 T1 for the supraglottis is defined as tumor limited to one subsite of supraglottis (shown here in the epiglottis) with normal vocal cord mobility

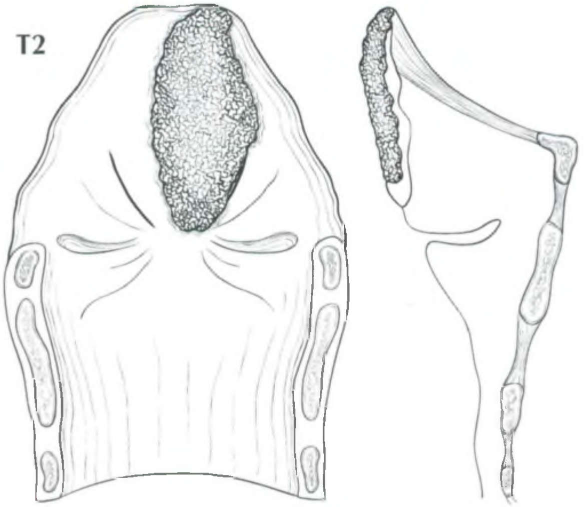

Fig. 13.5 T2 for the supraglottis is defined as tumor invading the mucosa of more than one adjacent subsite of supraglottis or glottis or region outside the supraglottis (e.g., mucosa of base of tongue, vallecula, and medial wall of pyriform sinus) without fixation of the larynx (shown here with tumor involvement in the suprahyoid and mucosa of the infrahyoid epiglottis)

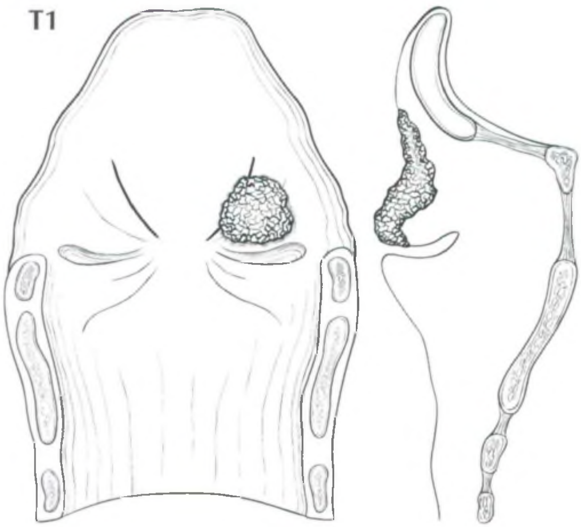

Fig. 13.4 T1 for the supraglottis is defined as tumor limited to one subsite of supraglottis (shown here in the ventricular bands) with normal vocal cord mobility

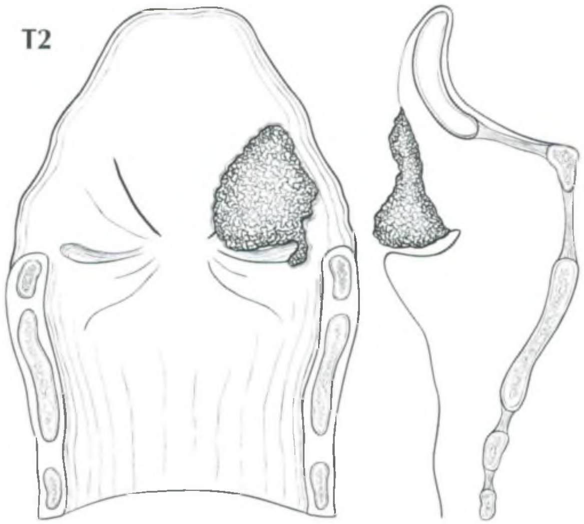

Fig. 13.6 T2 for the supraglottis with invasion of ventricular bands (false cords) and the epiglottis

13

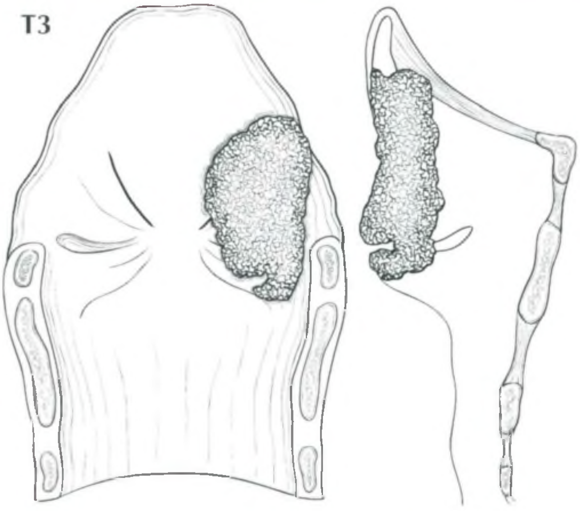

Fig. 13.7 T3 for the supraglottis is defined as tumor limited to larynx with vocal cord fixation and/or invading any of the following: postcricoid area, preepiglottic tissues, paraglottic space, and/or inner cortex of thyroid cartilage (shown here with invasion of the supraglottis and vocal cord with vocal cord fixation)

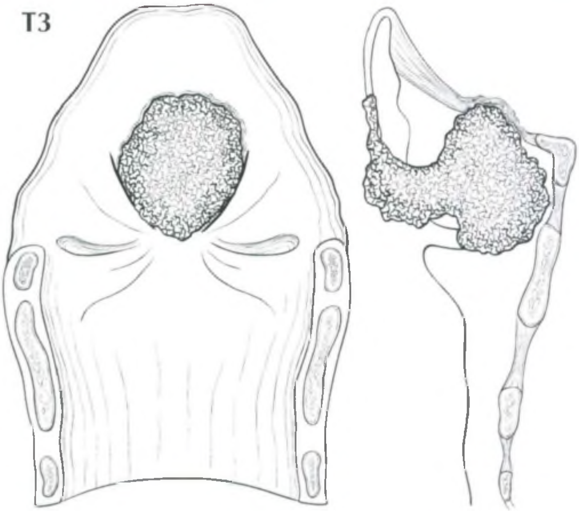

Fig. 13.8 T3 for the supraglottis with invasion of the preepiglottic tissues with vocal cord fixation

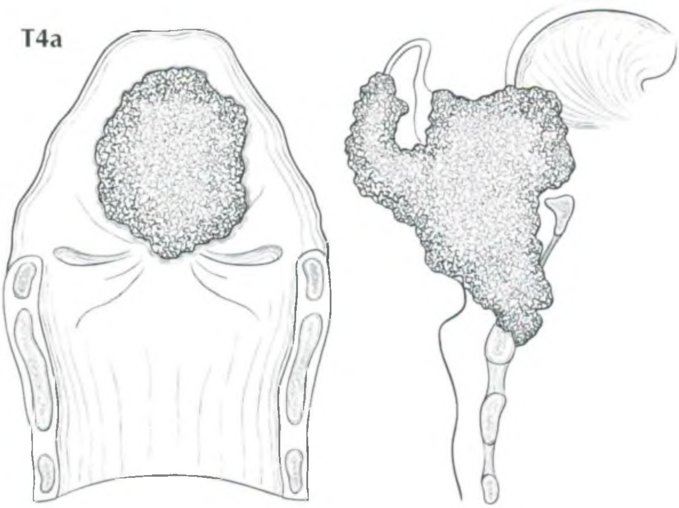

Fig. 13.9 T4a for the supraglottis is defined as moderately advanced local disease, tumor invading through the thyroid cartilage and/or invading tissues beyond the larynx (e.g., trachea, soft tissues of neck, including deep extrinsic muscle of the tongue, strap muscles, thyroid, or esophagus). Here, tumor has invaded beyond the larynx into the vallecula and base of the tongue, as well as into soft tissues of the neck

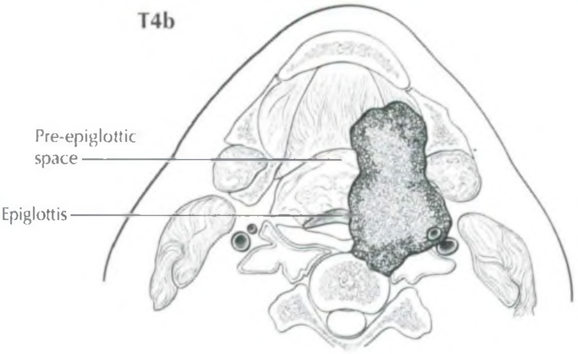

Fig. 13.10 Cross-sectional illustration of T4b tumor for the supraglottis, which is defined as very advanced local disease, invading prevertebral space, encasing the carotid artery (shown), or invading mediastinal structures

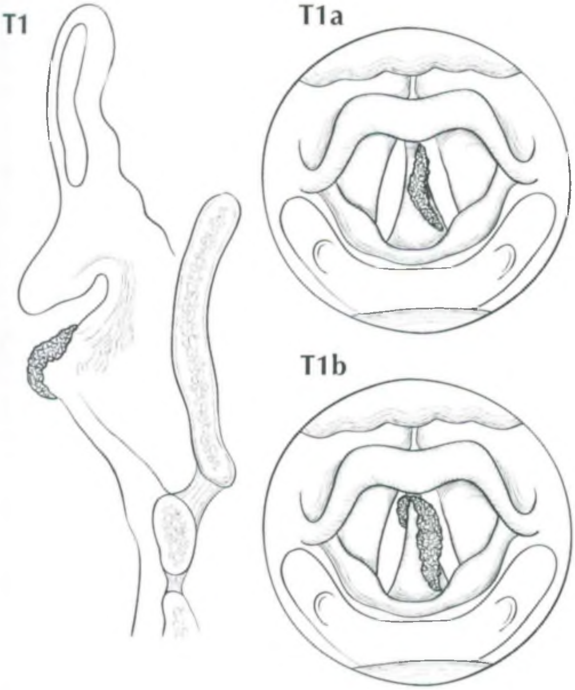

Fig. 13.11 T1 tumors of the glottis are limited to the vocal cord(s) with normal mobility (may involve anterior or posterior commissure). T1a tumors are limited to one vocal cord (top right) and T1b tumors involve both vocal cords (bottom right)

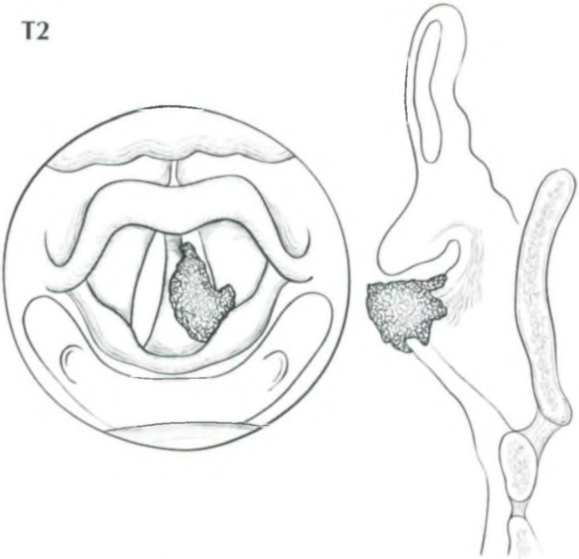

Fig. 13.12 T2 tumors of the glottis extend to supraglottis and/or subglottis, and/or with impaired vocal cord mobility

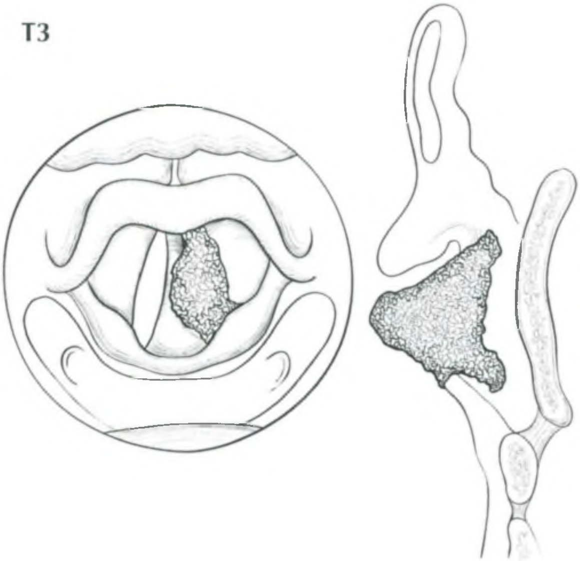

Fig. 13.13 T3 tumors of the glottis are limited to the larynx with vocal cord fixation (shown), and/or invade paraglottic space and/or inner cortex of the thyroid cartilage

13

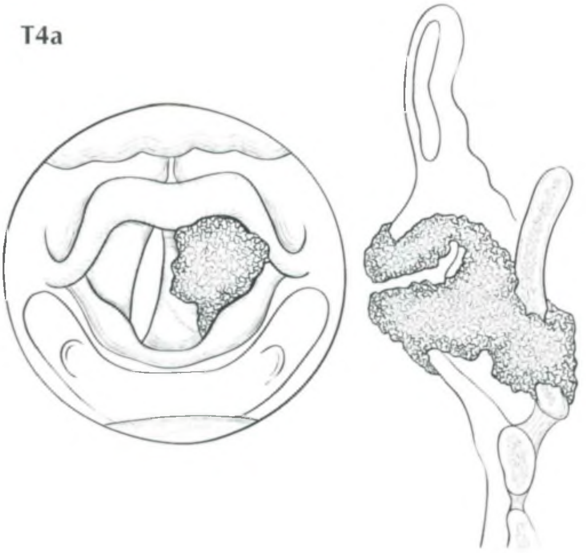

Fig. 13.14 T4a tumors of the glottis are moderately advanced local disease, and invade through the outer cortex of the thyroid cartilage and/or invade tissues beyond the larynx (e.g., trachea, soft tissues of neck, including deep extrinsic muscle of the tongue, strap muscles, thyroid, or esophagus)

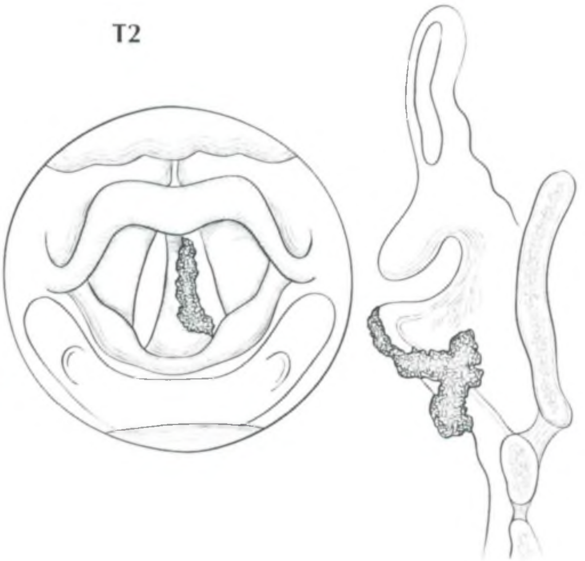

Fig. 13.16 T2 tumors of the subglottis extend to the vocal cord(s), with normal or impaired mobility

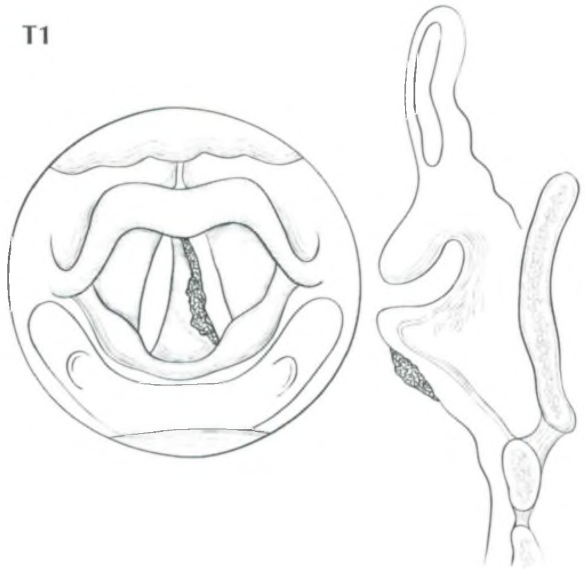

Fig. 13.15 T1 tumors of the subglottis are limited to the subglottis

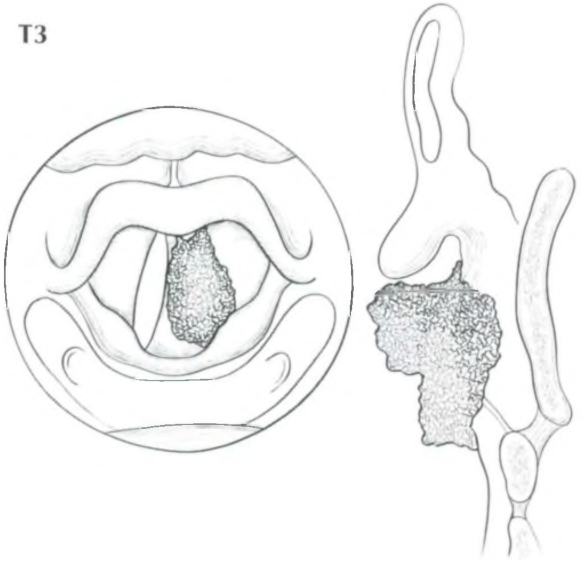

Fig. 13.17 T3 tumors of the subglottis are limited to the larynx with vocal cord fixation

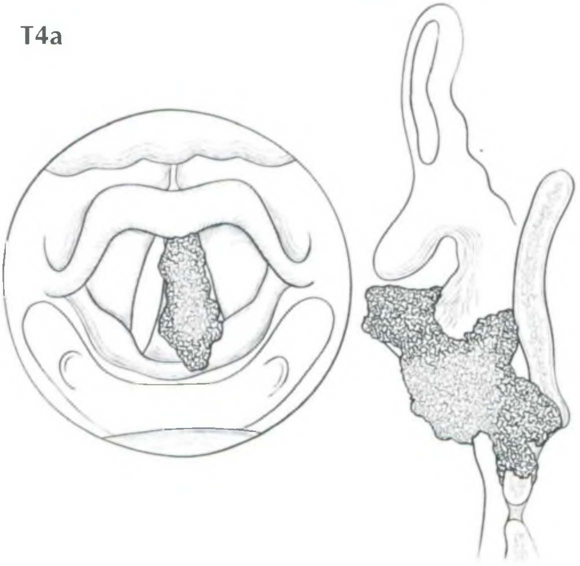

Fig. 13.18 T4a tumors of the subglottis are moderately advanced local disease, and invade cricoid or thyroid cartilage and/or invade tissues beyond the larynx (e.g., trachea, soft tissues of neck, including deep extrinsic muscles of the tongue, strap muscles, thyroid, or esophagus)

Bibliography

1. O'Sullivan B, Huang SH, Su J, et al. Development and validation of a staging system for HPV-related oropharyngeal cancer by the International Collaboration on Oropharyngeal cancer Network for Staging (ICON-S): a multicentre cohort study. *The lancet oncology*. Feb 26 2016.
2. Patel S. Personal Communication. In: Lydiatt W, Shah JP, eds. 2015.
3. Wreesmann VB, Katabi N, Palmer FL, et al. Influence of extracapsular nodal spread extent on prognosis of oral squamous cell carcinoma. *Head & neck*. Oct 30 2015.
4. de Juan J, Garcia J, Lopez M, et al. Inclusion of extracapsular spread in the pTNM classification system: a proposal for patients with head and neck carcinoma. *JAMA otolaryngology– head & neck surgery*. May 2013;139(5):483-488.
5. Prabhu RS, Hanasoge S, Magliocca KR, et al. Extent of pathologic extracapsular extension and outcomes in patients with nonoropharyngeal head and neck cancer treated with initial surgical resection. *Cancer*. May 15 2014;120(10):1499-1506.
6. Dunne AA, Muller HH, Eisele DW, Kessel K, Moll R, Werner JA. Meta-analysis of the prognostic significance of perinodal spread in head and neck squamous cell carcinomas (HNSCC) patients. *European journal of cancer*. Aug 2006;42(12):1863-1868.
7. Prabhu RS, Magliocca KR, Hanasoge S, et al. Accuracy of computed tomography for predicting pathologic nodal extracapsular extension in patients with head-and-neck cancer undergoing initial surgical resection. *International journal of radiation oncology, biology, physics*. Jan 1 2014;88(1):122-129.
8. Piccirillo JF. Inclusion of comorbidity in a staging system for head and neck cancer. *Oncology (Williston Park)*. Sep 1995;9(9):831-836; discussion 841, 845-838.
9. Marion E. Couch MD P, MBA1,*, Kim Dittus MD, PhD2, Michael J. Toth PhD3, Monte S. Willis MD, PhD4, Denis C. Guttridge PhD5, Jonathan R. George MD6, Eric Y. Chang7, Christine G. Gourin MD8 andHirak Der-Torossian MD, MPH1 Cancer cachexia update in head and neck cancer: Pathophysiology and treatment *Head & neck surgery*. 2015;37(7):1057–1072.
10. Lazure KE, Lydiatt WM, Denman D, Burke WJ. Association between depression and survival or disease recurrence in patients with head and neck cancer enrolled in a depression prevention trial. *Head & neck*. 2009;31(7):888-892.
11. Kattan MW, Hess KR, Amin MB, et al. American Joint Committee on Cancer acceptance criteria for inclusion of risk models for individualized prognosis in the practice of precision medicine. *CA: a cancer journal for clinicians*. Jan 19 2016.

Mucosal Melanoma of the Head and Neck

14

William M. Lydiatt, Margaret Brandwein-Gensler, Dennis H. Kraus, Suresh K. Mukherji, John A. Ridge, and Jatin P. Shah

CHAPTER SUMMARY

Cancers Staged Using This Staging System

Mucosal melanoma (MM) arising in the nasal cavity, paranasal sinuses, oral cavity, oropharynx, nasopharynx, larynx, and hypopharynx are addressed in this chapter.

Summary of Changes

There are no changes to this staging system.

ICD-O-3 Topography Codes

Code	Description
C00.0	External upper lip
C00.1	External lower lip
C00.2	External lip, NOS
C00.3	Mucosa of upper lip
C00.4	Mucosa of lower lip
C00.5	Mucosa of lip, NOS
C00.6	Commissure of lip
C00.8	Overlapping lesion of lip
C00.9	Lip, NOS
C01.9	Base of tongue, NOS
C02.0	Dorsal surface of tongue, NOS
C02.1	Border of tongue
C02.2	Ventral surface of tongue, NOS
C02.3	Anterior two-thirds of tongue, NOS
C02.4	Lingual tonsil
C02.8	Overlapping lesion of tongue
C02.9	Tongue, NOS
C03.0	Upper gum
C03.1	Lower gum
C03.9	Gum, NOS
C04.0	Anterior floor of mouth
C04.1	Lateral floor of mouth
C04.8	Overlapping lesion of floor of mouth
C04.9	Floor of mouth, NOS
C05.0	Hard palate
C05.1	Soft palate, NOS
C05.2	Uvula
C05.8	Overlapping lesion of palate
C05.9	Palate, NOS
C06.0	Cheek mucosa
C06.1	Vestibule of mouth
C06.2	Retromolar area
C06.8	Overlapping lesion of other and unspecified parts of mouth
C06.9	Mouth, NOS
C09.0	Tonsillar fossa
C09.1	Tonsillar pillar
C09.8	Overlapping lesion of tonsil
C09.9	Tonsil, NOS
C10.0	Vallecula
C10.1	Anterior (lingual) surface of epiglottis
C10.2	Lateral wall of oropharynx
C10.3	Posterior pharyngeal wall
C10.8	Overlapping lesion of oropharynx
C10.9	Oropharynx, NOS
C11.0	Superior wall of nasopharynx
C11.1	Posterior wall of nasopharynx

To access the AJCC cancer staging forms, please visit www.cancerstaging.org.

© American Joint Committee on Cancer 2017

M.B. Amin et al. (eds.), *AJCC Cancer Staging Manual, Eighth Edition*, DOI 10.1007/978-3-319-40618-3_14

Code	Description
C11.2	Lateral wall of nasopharynx
C11.3	Anterior wall of nasopharynx
C11.8	Overlapping lesion of nasopharynx
C11.9	Nasopharynx, NOS
C12.9	Pyriform sinus
C13.0	Postcricoid region
C13.1	Hypopharyngeal aspect of aryepiglottic fold
C13.2	Posterior wall of hypopharynx
C13.8	Overlapping lesion of hypopharynx
C13.9	Hypopharynx, NOS
C14.0	Pharynx, NOS
C14.2	Waldeyer's ring
C14.8	Overlapping lesion of lip, oral cavity, and pharynx
C30.0	Nasal cavity
C31.0	Maxillary sinus
C31.1	Ethmoid sinus
C32.0	Glottis
C32.1	Supraglottis (laryngeal surface)
C32.2	Subglottis
C32.8	Overlapping lesion of larynx
C32.9	Larynx, NOS

WHO Classification of Tumors

Code	Description
8720	Melanoma, NOS
8722	Balloon cell melanoma
8770	Mixed epithelioid and spindle cell melanoma
8771	Epithelioid cell melanoma
8772	Spindle cell melanoma

Barnes L, Eveson JW, Reichart P, Sidransky D, eds. World Health Organization Classification of Tumours Pathology and Genetics of Head and Neck Tumours. Lyon: IARC; 2005.

INTRODUCTION

Approximately 55 % of all mucosal melanomas (MMs) arise in the head and neck region. This disease represents less than 1 % of all melanomas.[1] MM is an aggressive neoplasm that exhibits unique features relative to other paranasal sinus and head and neck malignancies, as well as features distinct from cutaneous melanoma. Approximately two thirds of these lesions arise in the nasal cavity and paranasal sinuses, one quarter are found in the oral cavity, and the remainder occur sporadically in other mucosal sites of the head and neck.

MM is an aggressive neoplasm with staging introduced in the American Joint Committee on Cancer (AJCC) Cancer Staging Manual, 7th Edition for separate consideration from other mucosal-based lesions. The utility of this new system has been confirmed.[2–5]

The staging system of Ballantyne showed its utility and emerged as the first staging system utilized specifically for MM.[6] The TNM system for paranasal sinus cancer was not designed for and did not discriminate differences in prognosis between the various stages in MM. It also did not provide a staging system for MMs of the other potential sites where disease arose in the head and neck. Therefore, in the 7th Edition, AJCC and the Union for International Cancer Control (UICC) adopted a novel system for MM using only T3, T4a, and T4b categories to characterize the local extent of disease. The lack of clear discrimination in outcomes based on the number and size of nodal metastases resulted in adopting a dichotomous categorization of N0 versus N+. Thus, the four stages of disease for MM are represented by III, IVA, IVB, and IVC. The system omits T1 and T2 categories, justified by the overall poor prognosis for even small superficial lesions. Stratification into these stages assists the clinician in treatment decision making. In Stage III disease, the role of radiation still is not completely certain, but should be strongly considered according to National Comprehensive Cancer Network (NCCN) recommendations; in Stage IVA, local radiation is important and confers a survival benefit.[7]

Stage IVB denotes extensive local invasion for which treatment often is a nonsurgical approach for local palliation. Stage IVC denotes distant metastatic disease.[7] This stage designation allows patients to understand their prognosis. Furthermore, it provides a starting point for worldwide data collection and analysis. At this time, key genetic mutations such as *BRAF* are rarely seen in MM, thus making systemic treatment with targeted agents problematic.[1]

ANATOMY

Primary Site(s)

MMs occur throughout the mucosa of the upper aerodigestive tract. For a description of anatomy, refer to the appropriate anatomic site chapter based on the location of the mucosal melanoma (e.g., paranasal sinus and oral cavity).

MM originates from benign intramucosal melanocytes that reside in the mucosa of the upper aerodigestive tract (paranasal sinuses, oral cavity, pharynx, and larynx).

There is no T0 category for MM, because melanoma of unknown primary is unlikely to arise from the mucosal surfaces and far more likely to arise from skin.

Regional Lymph Nodes

The cervical nodes are the primary lymphatic drainage, and those at risk are in the basin that corresponds to the anatomic site where the tumor arises. Due to the rarity of the disease, the role of nodal metastasis is confined to either present (N+) or absent (N0). At this time, the role of extranodal extension (ENE) is unknown and this modifier is not incorporated into the system for MM.

Metastatic Sites

Distant metastases are common at some point in the course of the disease. The most common sites are lung and liver.[8]

RULES FOR CLASSIFICATION

Clinical Classification

MM tends to occur in older patients, as compared with cutaneous melanoma. MM can occur in any mucosal surface of the head and neck. The majority arise, however, in the paranasal sinuses and nasal cavity, with the remainder primarily in the oral cavity. Presenting symptoms depend on the tumor site of origin. Nasal obstruction, bleeding, and a polypoid mass are the most common symptoms. In the oral cavity, a painless pigmented mass, often on the hard palate or alveolus is the typical presenting finding.[1] Up to 40 % of head and neck MMs may be amelanotic. Nodal disease occurs in up to 15 % of patients with oral cavity MM.

Clinical staging of MM is done through clinical examination, appropriate imaging, and histological confirmation of disease. Pathological staging is done after surgical resection. Even small MMs behave aggressively, with high rates of recurrence and death. Because even superficial MMs exhibit this aggressive behavior, there is no T1 or T2 category in the MM staging system. Thus, primary cancers limited to the mucosa and underlying soft tissue are considered T3 lesions. Advanced MMs are classified as T4a and T4b. The anatomic extent criteria to define moderately advanced (T4a) and very advanced (T4b) disease are given below. *In situ* MMs are extremely rare and are excluded from staging.

Imaging

Imaging recommendations for MM differ from those for other head and neck cancers. T3 disease is defined as mucosa and immediately adjacent soft tissue. Mucosal lesions often are superficial and may be evaluated easily by direct visualization and palpation or endoscopy. Superficial mucosal lesions that are assessed easily and confidently may not require any imaging. Computed tomography (CT) and magnetic resonance (MR) imaging studies can be performed or reconstructed in planes that are orthogonal to the tumor and can potentially assess the depth of tumor invasion.

Imaging can be helpful for lesions that cannot be fully assessed on clinical examination and for locally advanced disease or symptomatic patients. Either CT or MR imaging may be performed for determining soft tissue involvement (T4a). CT is superior to MR imaging to identify early cortical involvement, but MR imaging is superior to CT for bone marrow invasion. Both CT and MR imaging may be used to evaluate for spread to the masticator, carotid, or pre-vertebral space (T4b).MR imaging, however, is superior to CT for identifying involvement of the skull base, dura, or other types of intracranial extension (T4b). MR imaging also is superior to CT to evaluate for perineural spread of "named" nerves, which should be distinguished from microscopic "perineural invasion." Positron emission tomography (PET) using 2-deoxy-2[^{18}F]-fluoro-D-glucose (FDG) is not very useful to evaluate the primary site or locoregional spread. However, PET-FDG may be helpful to screen for distant metastases in patients with local advanced disease. The role of imaging in evaluating nodal metastases is discussed in Chapter 6, Cervical Lymph Nodes and Unknown Primary Tumors of the Head and Neck.

Radiology reports should include information on the following:

1. Primary tumor: primary site and locoregional spread with specific mention of structures that would change staging to T4a or T4b
2. Status of lymph node metastases
3. Presence of distant spread

Pathological Classification

Pathological staging is assigned after surgical resection. Margin status and invasion of bone, cartilage, dura, and other resected tissue should be documented. If a lymph node dissection is performed, the number of lymph nodes resected, the size and number of positive lymph nodes, and the presence of soft tissue invasion should be noted.

PROGNOSTIC FACTORS

Prognostic Factors Required for Stage Grouping

Beyond the factors used to assign T, N, or M categories, no additional prognostic factors are required for stage grouping.

Additional Factors Recommended for Clinical Care

As with all cancers, the overall frailty and comorbidities of the patient are important determinants of prognosis. MM has few defined disease-specific prognostic factors. The site of origin in the head and neck is one of the only clear prognostic factors. Disease in the oral cavity has a higher rate of cervical nodal metastasis than those arising in the paranasal sinuses. Overall 5-year survival is 15–30 % for nasal cavity, 12 % for oral cavity, and 0–5 % for paranasal sinus disease.[9–11] Other series have demonstrated slightly better outcomes, but the relative proportion of survival remains best for nasal cavity and worst for paranasal sinus.

14

Prasad and colleagues proposed a microstaging system for MM. They reported that findings of vascular invasion, polymorphous tumor population, and necrosis conferred a worse prognosis.[12] Others, however, have not confirmed these findings and suggest high mitotic index and other findings are more salient. At this time, it appears that no clear prognostic factors exist for MM, although many promising candidates exist; collection of these data for future editions is advantageous.

In addition to the importance of the TNM factors, the overall health of these patients clearly influences outcome. An ongoing effort to better assess prognosis using both tumor and nontumor-related factors is underway. Chart abstraction will continue to be performed by cancer registrars to obtain important information regarding specific factors related to prognosis. These data then will be used to further hone the predictive power of the staging system in future revisions.

Comorbidity

Comorbidity can be classified by specific measures of additional medical illnesses. Accurate reporting of all illnesses in the patient's medical record is essential to assessment of these parameters. General performance measures are helpful in predicting survival. The AJCC strongly recommends the clinician report performance status using the Eastern Cooperative Oncology Group (ECOG), Zubrod, or Karnofsky performance measures, along with standard staging information. An interrelationship between each of the major performance tools exists. AJCC Level of Evidence: II[13]

Zubrod/ECOG Performance Scale	
0	Fully active, able to carry out all predisease activities without restriction (Karnofsky 90–100)
1	Restricted in physically strenuous activity but ambulatory and able to carry out work of a light or sedentary nature; for example, light housework, office work (Karnofsky 70–80)
2	Ambulatory and capable of all self-care, but unable to carry out any work activities; up and about more than 50% of waking hours (Karnofsky 50–60)
3	Capable of only limited self-care; confined to bed or chair 50% or more of waking hours (Karnofsky 30–40)
4	Completely disabled; cannot carry on self-care; totally confined to bed (Karnofsky 10–20)
5	Death (Karnofsky 0)

Lifestyle Factors

Lifestyle factors such as tobacco and alcohol abuse negatively influence survival. Accurate recording of smoking in pack-years and alcohol in number of days drinking per week and number of drinks per day will provide important data for future analysis. Nutrition is important to prognosis and will be indirectly measured by weight loss of > 5% of body weight in the previous 6 months.[14] Depression adversely affects quality of life and survival. Notation of a previous or current diagnosis of depression should be recorded in the medical record.[15] AJCC Level of Evidence: III

Tobacco Use

The role of tobacco as a negative prognostic factor is well established. Exactly how this could be codified in the staging system, however, is less clear. At this time, smoking is known to have a deleterious effect on prognosis but it is difficult to accurately apply this to the staging system. AJCC Level of Evidence: III

Smoking history should be collected as an important element of the demographics and may be included in Prognostic Groups in the future. For practicality, the minimum standard should classify smoking history as never, ≤ 10 pack-years, > 10 but ≤ 20 pack-years, or > 20 pack-years.

RISK ASSESSMENT MODELS

The AJCC recently established guidelines that will be used to evaluate published statistical prediction models for the purpose of granting endorsement for clinical use.[16] Although this is a monumental step toward the goal of precision medicine, this work was published only very recently. Therefore, the existing models that have been published or may be in clinical use have not yet been evaluated for this cancer site by the Precision Medicine Core of the AJCC. In the future, the statistical prediction models for this cancer site will be evaluated, and those that meet all AJCC criteria will be endorsed.

DEFINITIONS OF AJCC TNM

Definition of Primary Tumor (T)

T Category	T Criteria
T3	Tumors limited to the mucosa and immediately underlying soft tissue, regardless of thickness or greatest dimension; for example, polypoid nasal disease, pigmented or nonpigmented lesions of the oral cavity, pharynx, or larynx
T4	Moderately advanced or very advanced
T4a	Moderately advanced disease<br>Tumor involving deep soft tissue, cartilage, bone, or overlying skin
T4b	Very advanced disease<br>Tumor involving brain, dura, skull base, lower cranial nerves (IX, X, XI, XII), masticator space, carotid artery, prevertebral space, or mediastinal structures

Definition of Regional Lymph Node (N)

N Category	N Criteria
NX	Regional lymph nodes cannot be assessed
N0	No regional lymph node metastases
N1	Regional lymph node metastases present

Definition of Distant Metastasis (M)

M Category	M Criteria
M0	No distant metastasis
M1	Distant metastasis present

AJCC PROGNOSTIC STAGE GROUPS

No prognostic stage grouping is proposed at this time.

REGISTRY DATA COLLECTION VARIABLES

1. Size of lymph nodes
2. Extracapsular extension from lymph node for head and neck
3. Head and neck lymph nodes levels I–III
4. Head and neck lymph nodes levels IV–V
5. Head and neck lymph nodes levels VI–VII
6. Other lymph node group
7. Clinical location of cervical nodes
8. ENE clinical
9. ENE pathological
10. Tumor thickness

HISTOLOGIC GRADE (G)

There is no recommended histologic grading system at this time.

HISTOPATHOLOGIC TYPE

Currently, there is no clear ability to determine prognosis based on histological differences.

SURVIVAL DATA

Figure 14.1 shows 24-month follow-up of patients older than 18 years of age, diagnosed with MM of the head and neck, lip and oral cavity, pharynx, larynx, and nasal cavity and paranasal sinuses using the AJCC Cancer Staging Manual, 7th Edition. The cases were diagnosed in 2010–12. The curves indicate a reasonable hazard discrimination and distribution. They also suggest good prognostic discrimination.

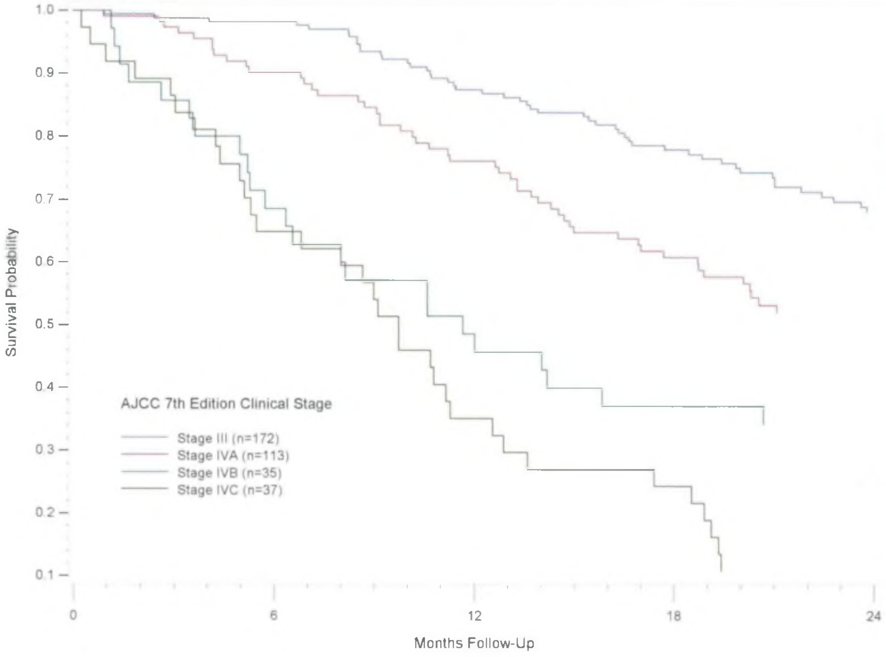

Fig. 14.1 24-month follow-up of patients older than 18 years of age, diagnosed with MM of the head and neck, lip and oral cavity, pharynx, larynx, and nasal cavity and paranasal sinuses using the AJCC Cancer Staging Manual, 7th Edition. The cases were diagnosed in 2010–12

ILLUSTRATIONS

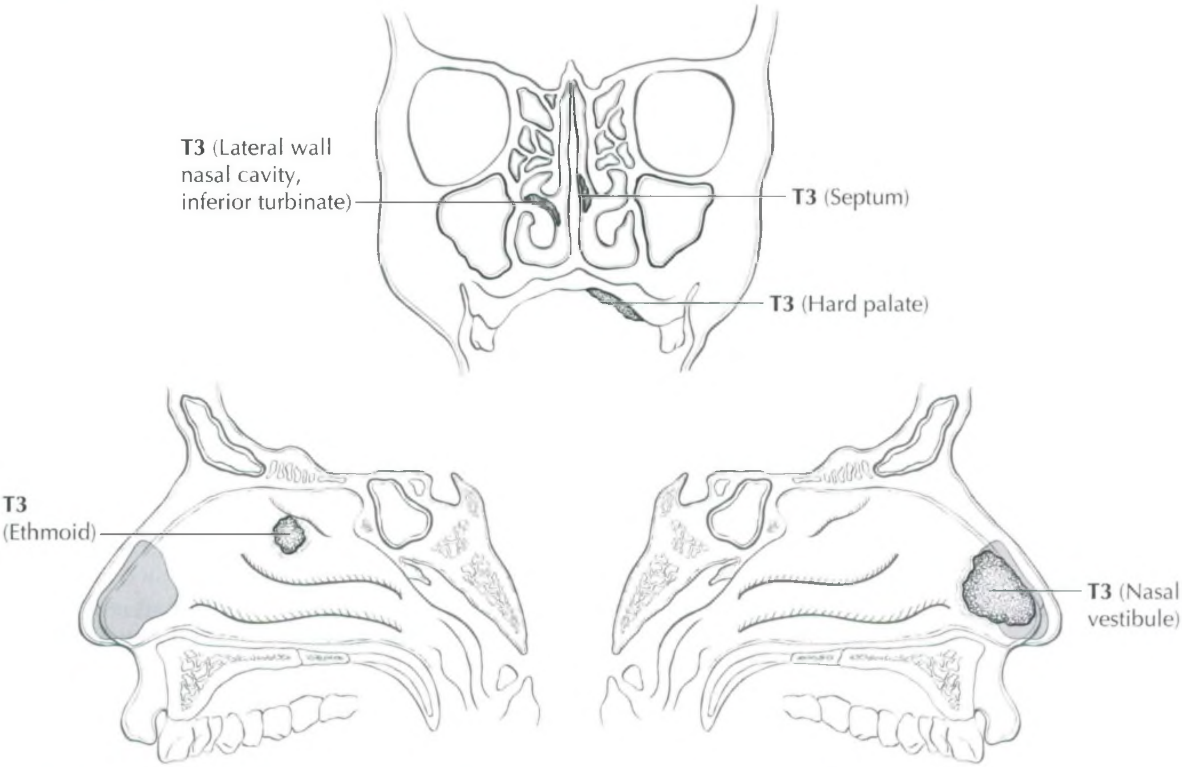

Fig. 14.2 T3 is defined as mucosal disease. Involvement of the lateral wall nasal cavity, inferior turbinate is illustrated, as well as septum, hard palate, ethmoid, and nasal vestibule

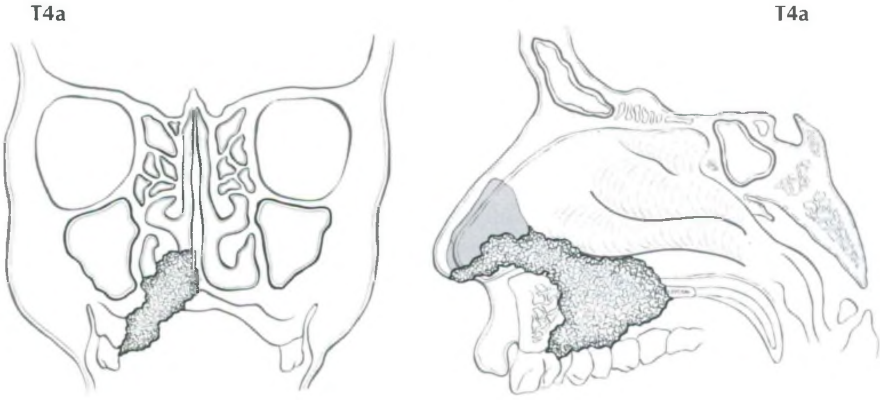

Fig. 14.3 T4a is defined as moderately advanced disease, with tumor involving deep soft tissue, cartilage, bone, or overlying skin

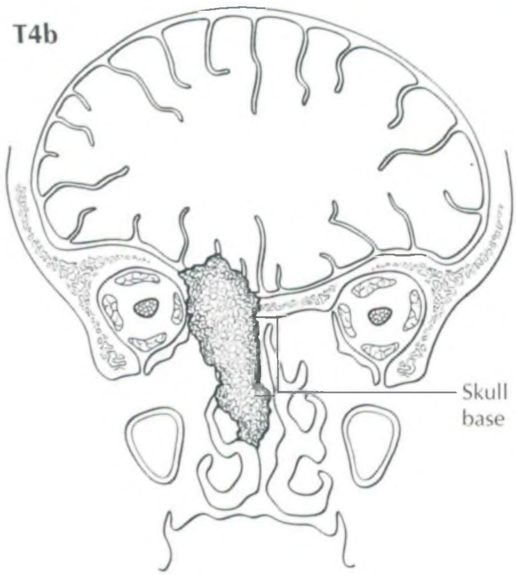

Fig. 14.4 T4b is defined as very advanced disease, with tumor involving the brain as illustrated, or also involving dura, lower cranial nerves (IX, X, XI, XII), masticator space, carotid artery, prevertebral space, or mediastinal structures

Bibliography

1. Carvajal RD, Spencer SA, Lydiatt W. Mucosal melanoma: a clinically and biologically unique disease entity. *Journal of the National Comprehensive Cancer Network : JNCCN.* Mar 2012;10(3):345-356.
2. Edge SB, Compton CC. The American Joint Committee on Cancer: the 7th edition of the AJCC cancer staging manual and the future of TNM. *Annals of surgical oncology.* Jun 2010;17(6):1471-1474.
3. Sobin L GM, Wittekind C, eds. . International Union Against Cancer (UICC). TNM Classification of Malignant Tumors. 7th edition. West Sussex, UK: Wiley-Blackwell;. *UICC.* 2009.
4. Koivunen P, Back L, Pukkila M, et al. Accuracy of the current TNM classification in predicting survival in patients with sinonasal mucosal melanoma. *The Laryngoscope.* Aug 2012;122(8): 1734-1738.
5. Shuman AG, Light E, Olsen SH, et al. Mucosal melanoma of the head and neck: predictors of prognosis. *Archives of otolaryngology–head & neck surgery.* Apr 2011;137(4):331-337.
6. Ballantyne AJ. Malignant melanoma of the skin of the head and neck. An analysis of 405 cases. *American journal of surgery.* Oct 1970;120(4):425-431.
7. National Comprehensive Cancer Network. NCCN Clinical Practice Guidelines in Oncology (NCCN Guidelines) Head and Neck Cancers (Version I.2015). http://www.nccn.org/professionals/physician_gls/pdf/head-and-neck.pdf. Accessed January 20, 2016.
8. O'Regan K, Breen M, Ramaiya N, et al. Metastatic mucosal melanoma: imaging patterns of metastasis and recurrence. *Cancer Imaging.* 2013;13(4):626-632.
9. Patel SG, Prasad ML, Escrig M, et al. Primary mucosal malignant melanoma of the head and neck. *Head & neck.* Mar 2002;24(3): 247-257.
10. Benlyazid A, Thariat J, Temam S, et al. Postoperative radiotherapy in head and neck mucosal melanoma: a GETTEC study. *Archives of otolaryngology–head & neck surgery.* Dec 2010;136(12):1219-1225.
11. Wu AJ, Gomez J, Zhung JE, et al. Radiotherapy after surgical resection for head and neck mucosal melanoma. *American journal of clinical oncology.* Jun 2010;33(3):281-285.
12. Prasad ML, Patel S, Hoshaw-Woodard S, et al. Prognostic factors for malignant melanoma of the squamous mucosa of the head and neck. *The American journal of surgical pathology.* Jul 2002;26(7): 883-892.
13. Piccirillo JF. Inclusion of comorbidity in a staging system for head and neck cancer. *Oncology (Williston Park).* Sep 1995;9(9):831-836; discussion 841, 845-838.
14. Marion E. Couch MD P, MBA1,*, Kim Dittus MD, PhD2, Michael J. Toth PhD3, Monte S. Willis MD, PhD4, Denis C. Guttridge PhD5, Jonathan R. George MD6, Eric Y. Chang7, Christine G. Gourin MD8 andHirak Der-Torossian MD, MPH1 Cancer cachexia update in head and neck cancer: Pathophysiology and treatment *Head & neck surgery.* 2015;37(7):1057–1072.
15. Lazure KE, Lydiatt WM, Denman D, Burke WJ. Association between depression and survival or disease recurrence in patients with head and neck cancer enrolled in a depression prevention trial. *Head & neck.* 2009;31(7):888-892.
16. Kattan MW, Hess KR, Amin MB, et al. American Joint Committee on Cancer acceptance criteria for inclusion of risk models for individualized prognosis in the practice of precision medicine. *CA: a cancer journal for clinicians.* Jan 19 2016.

Cutaneous Squamous Cell Carcinoma of the Head and Neck

15

Joseph A. Califano, William M. Lydiatt, Kishwer S. Nehal, Brian O'Sullivan, Chrysalyne Schmults, Raja R. Seethala, Randal S. Weber, and Jatin P. Shah

CHAPTER SUMMARY

Cancers Staged Using This System

Cutaneous squamous cell carcinoma (CSCC) of the head and neck and all other nonmelanoma skin carcinomas of the head and neck (except Merkel cell carcinoma [MCC]). Anatomic site of vermilion lip is included (and is excluded from Oral Cavity Carcinoma) because its etiology is primarily based on ultraviolet (UV) exposure, like other nonmelanoma cancers.

Cancers Not Staged Using This Staging System

These histopathologic types of cancer...	Are staged according to the classification for...	And can be found in chapter...
Carcinoma of the eyelid	Eyelid Carcinoma	64
Carcinoma of the vulva	Vulva	50
Carcinoma of the penis	Penis	57
Perianal carcinoma	Anus	21
Cutaneous squamous cell carcinoma and basal cell carcinoma of the skin outside the head and neck	No AJCC staging system	N/A

Summary of Changes

This is a new chapter encompassing nonmelanoma cutaneous cancers of the head and neck.

ICD-O-3 Topography Codes

Code	Description
C00.0	Vermilion border of upper lip (excludes external upper lip)
C00.1	Vermilion border of lower lip (excludes external lower lip)
C00.2	Vermilion border of lip, NOS (excludes external lip, NOS)
C44.0	Skin of lip, NOS
C44.2	External ear
C44.3	Skin of other and unspecified parts of the face
C44.4	Skin of scalp and neck
C44.8	Overlapping lesion of skin

WHO Classification of Tumors

Code	Description
8070	Squamous cell carcinoma
8211	Tubular carcinoma
8407	Microcystic adnexal carcinoma
8409	Porocarcinoma
8403	Spiradenocarcinoma
8940	Malignant mixed tumor
8400	Hidradenocarcinoma
8480	Mucinous carcinoma

To access the AJCC cancer staging forms, please visit www.cancerstaging.org.

© American Joint Committee on Cancer 2017

M.B. Amin et al. (eds.), *AJCC Cancer Staging Manual, Eighth Edition*, DOI 10.1007/978-3-319-40618-3_15

Code	Description
8408	Digital papillary carcinoma
8200	Adenoid cystic carcinoma
8401	Apocrine carcinoma
8540	Paget disease of breast
8542	Extramammary Paget disease
8110	Pilomatriceal carcinoma
8103	Proliferating trichilemmal tumor
8410	Sebaceous carcinoma
8982	Myoepithelial carcinoma

LeBoit PE, Burg G, Weedon D, Sarasin A, eds. World Health Organization Classification of Tumours. Pathology and Genetics of Skin Tumours. Lyon: IARC Press; 2006.

INTRODUCTION

This chapter continues the multidisciplinary effort that the American Joint Committee on Cancer (AJCC) began with the AJCC Cancer Staging Manual, 7th Edition (7th Edition) to provide a mechanism for staging nonmelanoma skin cancers. In total, seven board-certified disciplines collaborated to develop this chapter: Dermatology, Otolaryngology-Head and Neck Surgery, Surgical Oncology, Dermatopathology, Oncology, Radiation Oncology, and Plastic Surgery. The title of this chapter reflects the scope of the data, which are focused on and may be staged according to the CSCC) staging system. In the absence of cancer registry data for nonmelanoma skin cancer (owing to its commonness and subsequent unfeasibility of tracking all cases) the T category is based on tumor risk factors that have been shown to be independent prognostic factors for poor outcomes (local recurrence, nodal or regional metastasis, distant metastasis, or disease-specific death) in studies employing multivariate analysis. Several such studies have been published since the 7th Edition. T4 category is reserved for bony extension or involvement, perineural invasion of the skull base or foramena, or presence of four or more of the risk factors mentioned above. Nodal (N) category has been completely revised to reflect published evidence-based data demonstrating that survival decreases with increasing nodal size and number of nodes involved. Because the majority of CSCC tumors occur on the head and neck, the AJCC Cancer Staging Manual, 8th Edition (8th Edition) staging system for CSCC and other cutaneous carcinomas was developed by the AJCC Head and Neck Expert Panel. This staging system applies to head and neck cutaneous carcinomas.

The term nonmelanoma skin carcinoma (NMSC) includes approximately 82 types of skin malignancies with wide variability in prognosis, ranging from those that generally portend a poor prognosis, such as MCC which subsequently has its own separate staging system (Chapter 46), to the far more frequent and clinically favorable basal cell carcinoma (BCC) and cutaneous squamous cell carcinoma (CSCC). Although the discussion in this chapter focuses primarily on CSCC, the staging system applies to all NMSC of the head and neck except MCC. Recently published data regarding prognostic factors have been utilized as the basis for this new and revised staging system.

The incidence of CSCC and other carcinomas of the skin varies globally, but it is thought to have been increasing overall since the 1960s at a rate of 3–8% per year.[1] In the United States, NMSC is the most frequently diagnosed cancer.[2] Although the vast majority of these tumors present at Stage I and Stage II, CSCC is responsible for the majority of NMSC deaths[3] and accounts for approximately 20% of all skin cancer-related deaths.[4] Due to lack of registry data, the precise number of deaths due to CSCC is unknown but has been estimated to be between 3,900 and 8,800 in the United States annually.[5] The high incidence of CSCC and BCC is thought to be mostly the result of sun exposure and mutagenic effects of UV light.[6] BCC and CSCC tumors are far more common in light-skinned individuals (those who sunburn readily, e.g., Fitzpatrick types I–III) and are typically located on anatomic areas exposed to the sun, such as the head, neck, or extremities. The incidence varies with geographic latitude, as well as with regions of atmospheric ozone depletion, with a high incidence in such areas as Australia and New Zealand.[1,7–14] Other risk factors for developing NMSC include advanced age and induced or acquired immunosuppression, seen after solid organ transplantation[15–17] or in patients diagnosed and treated for leukemia or lymphoma, particularly chronic lymphocytic leukemia.[18,19] Male gender is a well-described risk factor for the development of CSCC.[6]

A revised staging system is described herein, along with operational definitions of the T, N, and M categories. This new staging system was based on published data demonstrating a significantly increased risk of recurrence or death associated with specific clinical and histologic features. This revised version of CSCC staging more accurately reflects the prognosis and natural history of CSCC and therefore will be more applicable to treatment planning and design of clinical trials for carcinomas of the skin. Because a significant number of NMSC primaries occur on the head and neck, the revised staging system was developed within the Head and Neck Carcinoma Staging Expert Panel. The major differences between the new chapter entitled "Cutaneous Squamous Cell Carcinoma and Other Cutaneous Carcinomas" and the chapter found in the 7th Edition are summarized in the section on Additional Factors Recommended for Clinical Care.

ANATOMY

Primary Site(s)

CSCC and other carcinomas can occur anywhere on the skin. CSCC and BCC most commonly arise on anatomic sites that have been exposed to sunlight.[6] CSCC can also arise in skin that was previously scarred or ulcerated, that is, at the sites of burns and chronic ulcers (chronic inflammation). All of the components of the skin (epidermis, dermis, and adnexal structures) can give rise to malignant neoplasms.

Nonaggressive NMSC, such as BCC, usually grow solely by local extension, both horizontally and vertically. Continued local extension may result in growth into deep structures, including adipose tissue, cartilage, muscle, and bone. Perineural extension is a particularly insidious form of local extension, as this is often clinically occult. If neglected for an extended length of time, nodal metastasis can occur with otherwise nonaggressive NMSC.

Aggressive NMSC, including CSCC and some types of sebaceous and eccrine neoplasms, also grow by local lateral and vertical extension early in their natural history. Once deeper extension occurs, growth may become discontinuous, resulting in deeper local extension, in-transit metastasis, and nodal metastasis. In more advanced cases, CSCC and other tumors can extend along cranial foramina through the skull base into the cranial vault. Uncommon types of NMSC vary considerably in their propensity for metastasis.

Regional Lymph Nodes

When deep invasion and eventual metastasis occurs, local and regional lymph nodes are the most common sites of metastasis. Nodal metastasis usually occurs in an orderly manner, initially in a single node, which expands in size. Eventually, multiple nodes become involved with metastasis. Metastatic disease may spread to secondary nodal basins, including contralateral nodes when advanced. Uncommonly, nodal metastases may bypass a primary nodal basin.

Metastatic Sites

Nonaggressive NMSC more often involves deep tissue by direct extension than by metastasis. After metastasizing to nodes, CSCC may spread to visceral sites, including lung. Unlike most other forms of cancer, the majority of deaths from CSCC (81 %) appear to result from uncontrolled loco-regional recurrence, rather than from distant organ metastasis.[20]

RULES FOR CLASSIFICATION

Clinical Classification

The clinical staging of skin cancer is based on inspection and palpation of the involved area and the regional lymph nodes. Imaging studies may be important to stage CSCC for which there is clinical suspicion for nodal metastasis or bone invasion. Information from biopsies of the primary tumor, lymph nodes, and distant metastases can be included in the clinical classification.

Patients with CSCC *in situ* are categorized as Tis. Carcinomas that are indeterminate or cannot be staged should be category TX. Small primary tumors <2 cm with no high-risk features are categorized as T1, and tumors ≥ 2 cm and <4 cm as T2. Clinical high-risk features defining primary tumors as T3 include (1) depth of invasion (DOI) beyond the subcutaneous fat or ≥ 6 mm (as measured from the granular layer of adjacent normal epidermis to the base of the tumor); (2) perineural invasion defined as clinical or radiographic involvement of named nerves without skull base invasion or transgression or tumor cells within the nerve sheath of a nerve lying deeper than the dermis or measuring ≥ 0.1 mm in caliber; and/or (3) minor bone erosion. T4a includes tumors demonstrating gross cortical bone erosion with marrow invasion, and T4b includes tumors with skull base invasion and/or skull base foramen involvement.

Local and regional metastases most commonly present in the regional lymph nodes. The actual status of nodal metastases identified by clinical inspection or imaging and the status and number of positive and total nodes by pathologic analysis must be reported for staging purposes. In instances where lymph node status is not recorded, a category of NX is used. A solitary parotid or regional lymph node metastasis measuring 3 cm or smaller in size is categorized as N1. In clinical evaluation, the greatest diameter of the nodal mass should be measured. The three categories of clinically positive nodes are N1, N2, and N3. Midline nodes are considered ipsilateral nodes.

Imaging studies showing amorphous spiculated margins of involved nodes or involvement of internodal fat resulting in loss of normal oval-to-round nodal shape strongly suggest extranodal tumor spread; however, pathologic examination is necessary to prove its presence. No imaging study can currently identify microscopic foci of cancer in regional nodes or distinguish between small reactive nodes and small malignant nodes (in the absence of central radiographic inhomogeneity).

The effect of extranodal extension (ENE) on prognosis of head and neck cancers is profound. Accounting for this important prognostic feature was considered critical in revis-

ing staging. Most of the data supporting ENE as an adverse prognostic factor are based on histopathological characterization of ENE, especially the distinction between microscopic and macroscopic or gross ENE. Therefore, only unquestionable ENE is to be used for clinical staging, as in the uncertain rule of the TNM staging that mandates that the lower category for any given situation should be selected in ambiguous cases. For clinical ENE, the known inability of current imaging modalities to define ENE accurately mandated that stringent criteria be met prior to assigning a clinical diagnosis of ENE. However, unambiguous evidence of gross ENE on clinical examination (e.g., invasion of skin, infiltration of musculature/dense tethering to adjacent structures, or cranial nerve, brachial plexus, sympathetic trunk, or phrenic nerve invasion with dysfunction) supported by strong radiographic evidence permit classification of disease as ENE(+). Pathological ENE is clearly defined in the section on Pathological Classification. Again, if there is doubt or uncertainty of the presence of ENE, the case should be categorized as ENE(−).

Distant metastases are staged primarily by the presence (M1) or absence (M0) of metastases in distant organs or sites outside of the regional lymph nodes.

Imaging

Primary CSCC of the head and neck are present on sun-exposed areas of the skin; therefore, assessment of size is usually derived in a straightforward manner from clinical examination and does not require imaging. T1 and T2 tumors rarely exhibit nodal metastasis and are staged primarily by clinical examination without additional imaging. However, the presence of adverse prognostic factors noted after excision of the primary tumor, including those that increase T stage, is often an indicator of aggressive behavior and may indicate additional imaging to assess occult nodal metastasis. These imaging modalities may include computed tomography (CT) of the neck and/or magnetic resonance (MR) imaging with contrast enhancement, as well as other modalities. Stage III–IV cancers routinely undergo imaging prior to therapy, including a neck CT and/or MR imaging with contrast enhancement, as well as other modalities, such as a positron emission tomography (PET)–CT scan. Imaging with chest X-ray, chest CT, or PET-CT may be employed for clinical Stage III–IV cancer to screen for the presence of distant metastatic spread.

Information derived from these imaging tests includes T category based on size and DOI of tumor, as well as the presence of perineural invasion that can be noted on MR imaging because of involvement of named cranial nerves. In addition, the presence, size, and number of cervical nodal metastases and presence of ENE may be defined by contrast-enhanced neck CT, MR imaging, or PET-CT. A suggested structure for reporting in the medical record is as follows:

- Primary tumor: Location, size, characterization (when applicable).
- Local extent: involved structures
- Perineural spread
- Lymph node involvement (if assessable) and location by level and anatomic site
- Presence of ENE
- Distant spread
- Other findings relevant to staging or treatment

Pathological Classification

Complete resection of the primary tumor site is required for accurate pathological staging and for cure. Surgical resection of lymph node tissue is necessary when involvement is suspected. Pathologists should report key histologic characteristics of the tumor, particularly depth, grade/differentiation, and perineural invasion. Low-grade tumors show considerable cell differentiation, uniform cell size, infrequent cellular mitoses and nuclear irregularity, and intact intercellular bridges. High-grade tumors show poor differentiation, spindle cell characteristics, necrosis, high mitotic activity, and deep invasion. Depth of CSCC invasion, as measured by both Breslow millimeter depth[21] (measured from granular layer of adjacent normal skin to base of tumor so as to exclude the exophytic component) and tissue level depth, correlates with metastatic potential.

For assessment of pathological node status (pN), a selective neck dissection often is required and ordinarily should include 10 or more lymph nodes. A comprehensive (radical or modified radical neck dissection) ordinarily will include 15 or more lymph nodes. Examination of a smaller number of tumor-free nodes still mandates a pN0 designation.

Surgically resected metastatic nodes should be examined for the presence and extent of ENE. ENE detected on histopathologic examination is designated as ENEmi (microscopic ENE $\leq$ 2 mm) or ENEma (major ENE > 2 mm). Both ENEmi and ENEma qualify as ENE(+) for definition of pN.

PROGNOSTIC FACTORS

Prognostic Factors Required for Stage Grouping

Beyond the factors used to assign T, N, or M categories, no additional prognostic factors are required for stage grouping.

Additional Factors Recommended for Clinical Care

Most studies that analyze early-stage CSCC are retrospective in nature and therefore classified as level II evidence. However, several recent studies have included multivariate analysis, including one prospective level I evidence investigation.[21] The revision of the staging system for Stage I, II, and III CSCC was primarily based on consensus opinion of the CSCC Expert Panel. Poor prognosis for recurrence and metastasis has been correlated with multiple factors, such as anatomic site, tumor diameter, poor differentiation, perineural invasion, and DOI. These prognostic factors are discussed in detail; they apply primarily to CSCC and an aggressive subset of NMSC, but rarely to BCC. The following rationale determined the multiple factors used for the T and N categories.

Extranodal Extension

The presence or absence of ENE is required to assign N category. ENE is defined as extension through the lymph node capsule into the surrounding connective tissue, with or without associated stromal reaction.[22–25] Unambiguous evidence of gross ENE (i.e., defined as invasion of skin, infiltration of musculature/fixation to adjacent structures on clinical examination, cranial nerve, brachial plexus, sympathetic trunk or phrenic nerve invasion with dysfunction) is a sufficiently high threshold to classify these as clinical ENE(+). AJCC Level of Evidence: III

Tumor Diameter

Tumor diameter refers to the maximum clinical diameter of the CSCC lesion (preoperatively based on physical exam). Tumor diameter larger than 2 cm changes the T category to T2. Multiple studies corroborate a correlation between tumor diameter and more biologically aggressive disease, including local recurrence and metastasis in multivariate analysis.[20,21,26–29] Two of these studies point toward size of 2 cm as a threshold beyond which tumors are more likely to metastasize to lymph nodes.[20,21] A 2.1-fold risk of nodal metastasis for tumors larger than 2 cm was noted when prospectively reviewing risk factors for poor prognosis in 615 patients with CSCC.[21] Another study of 985 CSCC patients found that tumors 2 cm or larger were associated with a 5.6-fold higher risk of local recurrence, a 7.0-fold higher risk of nodal metastasis, and a 15.9-fold higher risk of death from CSCC.[20] The 2 -cm threshold was decided upon for assigning a T2 category because of the existing published data that a clinical diameter larger than 2 cm is associated with a poor prognosis. In addition, this breakpoint allows continued congruence between CSCC and Head and Neck Staging. A further cut point of 4 cm was included for assigning a T3 category because one study showed this to be predictor of particularly poor outcomes, with a 4.5-fold increase in disease-specific death in tumors 4 cm or larger in diameter.[28]

Although 2 cm is recognized by many to be an important size cutoff, the metastatic potential of tumors smaller than 2 cm cannot be ignored, as they too can metastasize. Multiple studies have identified several other factors independently associated with elevated risks of recurrence, metastasis, and/or death. These factors are weighted on an equal basis with size of 2 cm or larger because there are no clear means of differentiating the significance of prognostic factors.

Depth of Tumor

Recent studies show that both tumor thickness (measured in millimeters) and the tissue level of invasion are important variables for the prognosis of CSCC. Prospective studies show that increasing tumor thickness[21,30] and anatomic DOI[30] correlate with an increased risk of metastases. In one study, no metastases were present with primary tumors less than 2 mm in depth (tumor thickness), but 16 % of cases with tumors greater than 6 mm in depth had metastases.[21] This study also found that tumors greater than 6 mm in depth had a 6.0-fold higher risk of local recurrence and nodal metastasis on multivariate analysis.[21] Another study reported increasing metastatic rates as tumor invasion progressed from dermis (0.0 % risk) to subcutaneous adipose tissue (4.1 % risk), to muscle or bone (12.5 % risk).[30] A 5–20 % increase in nodal metastasis risk has been reported for each 1-mm increase in tumor thickness.[27,31]

Traditional Breslow depth is measured from the granular layer to the base of the tumor. In CSCC, however, the granular layer is lost and simply measuring from the surface of the tumor to the base may overestimate prognostic impact because the dead keratotic surface of tumors may contribute little prognostically, and some exophytic CSCCs, such as keratoacanthomas, have a low risk of metastases. Thus, the authors recommend that millimeter depth be measured from the granular layer of adjacent normal skin to the base of the tumor to avoid these issues. Such measurement is assumed in the staging system herein.

Two studies employing multivariate analysis have shown DOI past the subcutaneous fat to be associated with poor outcomes.[20,29] Invasion past subcutaneous fat was associated with a 9.3-fold increased risk of nodal metastasis and a 13.0-fold increased risk of death from CSCC. A smaller study of 256 patients with high-risk CSCC (defined as those with one of the following risk factors: perineural or lymphovascular invasion, poorly differentiated histology, depth beyond subcutaneous fat, diameter of at least 2 cm, location on the ear, or location on the vermilion lip) found that invasion past subcutaneous fat was associated with 7.2-fold higher risk of nodal metastasis and 4.1-fold higher risk of death from CSCC.[29]

Based on the data above, the 8th Edition CSCC staging system incorporates deep invasion, defined as either greater than 6 mm depth as measured above or invasion past subcutaneous fat (to fascia, muscle, perichondrium, periosteum, etc.), as one of the high-risk features in the T category. Differentiation between the prognostic contributions of millimeter thickness versus tissue level of invasion will depend on future studies.

Anatomic Site

Specific anatomic locations including the lip (vermilion and hair-bearing), ear, temple, and cheek have an increased risk of local recurrence and metastatic potential in multivariate studies and thus have been categorized as high risk in the this staging system.[20,21,26] Location is not part of T categorization because studies have varied in how location was classified. In a large retrospective study of about 9,000 CSCC patients, tumors located on the ear/cheek and lip were 3.0 and 4.8 times more likely, respectively, to result in nodal metastasis than tumors located on other anatomic sites.[26] A prospective study found similar results with a 3.6-fold increased risk of nodal metastasis for tumors located on the ear.[21] Another study found that location on ear or temple was independently associated with an increased risk of local recurrence, nodal metastasis, and death from CSCC.[20]

Perineural Invasion

Four studies have shown perineural invasion (PNI, defined as tumor cells within the nerve sheath) is an independent factor associated with poor outcomes.[20,26,28,32] Two additional studies showed small-caliber PNI (involving nerves < 0.1 mm in caliber) to have no independent association with poor outcomes, indicating that invasion of small dermal nerve fibers alone may not significantly affect prognosis.[33,34] In a study of 114 CSCC cases with PNI, the risk of nodal metastasis CSCC was significantly higher in patients with large-caliber (risk 17%) versus small-caliber (risk 4%) PNI.[33] Another study found that the risk of nodal metastasis and CSCC death in cases with large-caliber PNI was 18% and 22%, respectively.[34] Thus, larger caliber (≥0.1 mm) nerve invasion is a risk factor in the 8th Edition staging system. Because most nerves deep to the dermis are > 0.1 mm in caliber, nerve invasion beyond the dermis also is a risk factor.

Histopathologic Grade or Differentiation and Desmoplasia

Early studies recognized that the histological grade or degree of differentiation of a CSCC affects prognosis: the more well-differentiated, the less aggressive the clinical course.[35,36] In 1978, Mohs, in his review of "microscopically controlled surgery," reported significant differences in cure rates for well-differentiated tumors (99.4%) compared with poorly differentiated tumors (42.1%).[37] More recently, three studies have confirmed poor differentiation to be independently associated with recurrence, metastasis, and death.[20,26,29] Patients with poorly differentiated CSCCs have a 2.5-fold to 3.0-fold[20,29] higher risk of local recurrence and a 3.3-fold to 6.1-fold[20,26,29] higher risk of nodal metastasis than patients with well-differentiated CSCCs. Death due to CSCC is also higher in poorly differentiated CSCCs with a 4.1–6.7 times higher risk reported.[20,29]

Other studies have found desmoplasia to be associated with poor outcomes.[21,38,39] Desmoplasia, single-cell spread, and poor or sarcomatoid differentiation can often occur together and are all suggestive of an aggressive tumor phenotype. Thus, CSCC staging in the 8th Edition continues to include aggressive histologic features (poorly differentiated tumors) as one of the several high-risk features and expands that definition to include desmoplasia and sarcomatoid presentations. Specific associations of these histologic subtypes independent of other risk factors are not definitive and, therefore, they have not been included as determinants of T categorization.

Extension to Bony Structures

In the AJCC Cancer Staging Manual, 6th Edition (6th Edition), the T4 designation was used for tumors that "invaded extradermal structures." The most common and important instances of deep anatomic extension for CSCC involve extension to bone and perineural extension to skull base. Based on these considerations, in the 7th Edition, T3 and T4 were reserved for these presentations of locally advanced disease consistent with data from several head and neck studies suggesting that CSCC extending to skull base is associated with poor prognosis, similar to advanced lymph node disease.[9–12,40–42] Subsequent cohort studies, however, have shown that although these presentations do connote a poor prognosis, they are very rare for primary CSCC and thus few tumors are in the T3 and T4 categories as designated by the 7th Edition staging system. This resulted in most cases of poor outcomes occurring in what the 7th Edition staged as T2 cases.[29,34] To improve upon this, the 8th Edition CSCC staging groups all bone and skull base invasion in T4 because they are likely similar in their poor prognosis.

Nodal Disease

Since the 6th Edition, multiple studies have examined the outcomes in patients with CSCC and regional lymph node metastasis.[10,12,40,41] These studies show that the number of nodes involved and the size of lymph node metastasis correlate with poor prognosis.

Based on data from O'Brien, et al.,[9] the NMSC Expert Panel decided that sufficient evidence exists to stage patients according to increasing nodal disease. Although preliminary data exist to suggest that cervical nodal disease may portend a worse prognosis than similar disease in the parotid, the data

are insufficient to support this separation at this time. Separating facial nerve involvement or involvement of the skull base (now T4) from extensive parotid disease will further clarify the prognosis of these patients.

Immunosuppression and Advanced Disease

It is well known that immunosuppressed patients are at risk for developing malignancies, especially CSCCs. Organ transplant recipients develop squamous cell carcinoma 65 times more frequently than age-matched controls.[43,44] The CSCCs in immunocompromised patients are more numerous and tend to recur and metastasize at a higher rate.[15,16,45–51] It has been reported that immunocompromised patients have a 7.2 times increased risk of local recurrence and a 5.3 times increased risk of any recurrence of disease.[52] Mortality also is increased with skin cancer, the fourth most common cause of death in a renal transplant cohort.[53] In transplant recipients, CSCC develops 10–30 years earlier than in immunocompetent hosts.[3,4] Strong consideration was given therefore toward including immunosuppression as a risk factor. However, in studies employing multivariate analysis, only a single study showed immunosuppression independently associated with poor outcomes,[21] possibly because immunosuppression is a broad category with varying degrees of associated immune dysfunction and variable prognostic effects. It is therefore not part of the staging system. This factor (including type or cause of immunosuppression) should be collected by cancer registries and investigators as a potentially important prognostic factor. Centers collecting such data and performing studies may designate immunosuppressed status with an "I" after the staging designation.

Overall Health

In addition to the importance of the TNM factors outlined previously, the overall health of these patients clearly influences outcome. An ongoing effort to better assess prognosis using both tumor and nontumor-related factors is underway. Chart abstraction will continue to be performed by cancer registrars to obtain important information regarding specific factors related to prognosis. These data will then be used to further hone the predictive power of the staging system in future revisions. AJCC Level of Evidence: III

Comorbidity

Comorbidity can be classified by specific measures of additional medical illnesses.[54] Accurate reporting of all illnesses in the patient's medical record is essential to assessment of these parameters. General performance measures are helpful in predicting survival. The AJCC strongly recommends the clinician report performance status using the Eastern Cooperative Oncology Group (ECOG), Zubrod, or Karnofsky performance measures, along with standard staging information. An interrelationship between each of the major performance tools exists. AJCC Level of Evidence: II

Zubrod/ECOG Performance Scale	
0	Fully active, able to carry on all pre-disease activities without restriction (Karnofsky 90–100)
1	Restricted in physically strenuous activity, but ambulatory and able to carry out work of a light or sedentary nature. For example, light housework, office work (Karnofsky 70–80)
2	Ambulatory and capable of all self-care but unable to carry out any work activities. Up and about more than 50 % of waking hours (Karnofsky 50–60)
3	Capable of only limited self-care, confined to bed or chair 50 % or more of waking hours (Karnofsky 30–40)
4	Completely disabled. Cannot carry on self-care. Totally confined to bed (Karnofsky 10–20)
5	Death (Karnofsky 0)

Lifestyle Factors

Lifestyle factors, such as tobacco and alcohol abuse, negatively influence survival. Accurate recording of smoking in pack-years and alcohol in number of days drinking per week and number of drinks per day will provide important data for future analysis. Nutrition is important to prognosis and will be indirectly measured by weight loss of > 5 % of body weight in the previous 6 months.[55] Depression adversely affects quality of life and survival. Notation of a previous or current diagnosis of depression should be recorded in the medical record.[56] AJCC Level of Evidence: III

Tobacco Use

The role of tobacco as a negative prognostic factor is well established. However, exactly how this could be codified in the staging system is less clear. At this time, smoking is known to have a deleterious effect on prognosis but that effect is hard to accurately apply to the staging system. AJCC Level of Evidence: III

Smoking history should be collected as an important element of the demographics and may be included in Prognostic Groups in the future. For practicality, the minimum standard should classify smoking history as never, ≤ 10 pack-years, > 10 but ≤20 pack-years, or > 20 pack-years.

15

RISK ASSESSMENT MODELS

The AJCC recently established guidelines that will be used to evaluate published statistical prediction models for the purpose of granting endorsement for clinical use.[57] Although this is a monumental step toward the goal of precision medicine, this work was published only very recently. Therefore, the existing models that have been published or may be in clinical

use have not yet been evaluated for this cancer site by the Precision Medicine Core of the AJCC. In the future, the statistical prediction models for this cancer site will be evaluated, and those that meet all AJCC criteria will be endorsed.

DEFINITIONS OF AJCC TNM

Definition of Primary Tumor (T)

T Category	T Criteria
TX	Primary tumor cannot be identified
Tis	Carcinoma *in situ*
T1	Tumor smaller than 2 cm in greatest dimension
T2	Tumor 2 cm or larger, but smaller than 4 cm in greatest dimension
T3	Tumor 4 cm or larger in maximum dimension or minor bone erosion or perineural invasion or deep invasion*
T4	Tumor with gross cortical bone/marrow, skull base invasion and/or skull base foramen invasion
T4a	Tumor with gross cortical bone/marrow invasion
T4b	Tumor with skull base invasion and/or skull base foramen involvement

*Deep invasion is defined as invasion beyond the subcutaneous fat or >6 mm (as measured from the granular layer of adjacent normal epidermis to the base of the tumor); perineural invasion for T3 classification is defined as tumor cells within the nerve sheath of a nerve lying deeper than the dermis or measuring 0.1 mm or larger in caliber, or presenting with clinical or radiographic involvement of named nerves without skull base invasion or transgression.

Definition of Regional Lymph Node (N)

Clinical N (cN)

N Category	N Criteria
NX	Regional lymph nodes cannot be assessed
N0	No regional lymph node metastasis
N1	Metastasis in a single ipsilateral lymph node, 3 cm or smaller in greatest dimension and ENE(−)
N2	Metastasis in a single ipsilateral node larger than 3 cm but not larger than 6 cm in greatest dimension and ENE(−);<br>*or* metastases in multiple ipsilateral lymph nodes, none larger than 6 cm in greatest dimension and ENE(−);<br>*or* in bilateral or contralateral lymph nodes, none larger than 6 cm in greatest dimension and ENE(−)
N2a	Metastasis in a single ipsilateral node larger than 3 cm but not larger than 6 cm in greatest dimension and ENE(−)
N2b	Metastasis in multiple ipsilateral nodes, none larger than 6 cm in greatest dimension and ENE(−)
N2c	Metastasis in bilateral or contralateral lymph nodes, none larger than 6 cm in greatest dimension and ENE(−)
N3	Metastasis in a lymph node larger than 6 cm in greatest dimension and ENE(−);<br>*or* metastasis in any node(s) and clinically overt ENE [ENE(+)]
N3a	Metastasis in a lymph node larger than 6 cm in greatest dimension and ENE(−)
N3b	Metastasis in any node(s) and ENE(+)

Note: A designation of "U" or "L" may be used for any N category to indicate metastasis above the lower border of the cricoid (U) or below the lower border of the cricoid (L).
Similarly, clinical and pathological ENE should be recorded as ENE(−) or ENE(+).

Pathological N (pN)

N Category	N Criteria
NX	Regional lymph nodes cannot be assessed
N0	No regional lymph node metastasis
N1	Metastasis in a single ipsilateral lymph node, 3 cm or smaller in greatest dimension and ENE(−)
N2	Metastasis in a single ipsilateral lymph node, 3 cm or smaller in greatest dimension and ENE(+);<br>*or* larger than 3 cm but not larger than 6 cm in greatest dimension and ENE(−);<br>*or* metastases in multiple ipsilateral lymph nodes, none larger than 6 cm in greatest dimension and ENE(−);<br>*or* in bilateral or contralateral lymph nodes, none larger than 6 cm in greatest dimension, ENE(−)
N2a	Metastasis in single ipsilateral or contralateral node 3 cm or smaller in greatest dimension and ENE(+);<br>*or* a single ipsilateral node larger than 3 cm but not larger than 6 cm in greatest dimension and ENE(−)
N2b	Metastasis in multiple ipsilateral nodes, none larger than 6 cm in greatest dimension and ENE(−)
N2c	Metastasis in bilateral or contralateral lymph nodes, none larger than 6 cm in greatest dimension and ENE(−)
N3	Metastasis in a lymph node larger than 6 cm in greatest dimension and ENE(−);<br>*or* in a single ipsilateral node larger than 3 cm in greatest dimension and ENE(+);<br>*or* multiple ipsilateral, contralateral, or bilateral nodes, any with ENE(+)
N3a	Metastasis in a lymph node larger than 6 cm in greatest dimension and ENE(−)
N3b	Metastasis in a single ipsilateral node larger than 3 cm in greatest dimension and ENE(+);<br>*or* multiple ipsilateral, contralateral, or bilateral nodes, any with ENE(+)

Note: A designation of "U" or "L" may be used for any N category to indicate metastasis above the lower border of the cricoid (U) or below the lower border of the cricoid (L).
Similarly, clinical and pathological ENE should be recorded as ENE(−) or ENE(+).

Definition of Distant Metastasis (M)

M Category	M Criteria
M0	No distant metastasis
M1	Distant metastasis

AJCC PROGNOSTIC STAGE GROUPS

When T is...	And N is...	And M is...	Then the stage group is...
Tis	N0	M0	0
T1	N0	M0	I
T2	N0	M0	II
T3	N0	M0	III
T1	N1	M0	III
T2	N1	M0	III
T3	N1	M0	III
T1	N2	M0	IV
T2	N2	M0	IV
T3	N2	M0	IV
Any T	N3	M0	IV
T4	Any N	M0	IV
Any T	Any N	M1	IV

REGISTRY DATA COLLECTION VARIABLES

1. Lip location (vermilion border or external lip)
2. ENE clinical presence or absence
3. ENE pathological presence or absence
4. Preoperative clinical tumor diameter in millimeters
5. Tumor thickness in mm (as measured from the granular layer of adjacent normal epidermis to the base of the tumor) and/or tissue level
6. Presence/absence of perineural invasion including millimeter
7. Primary site location on ear, temple, lip (hair-bearing vs. vermilion), or cheek
8. High-risk histologic features (poor differentiation, desmoplasia, sarcomatoid differentiation, undifferentiated)
9. Immune status (immunosuppressed or not) and cause of immunosuppression, if present
10. Depression
11. Comorbidities

HISTOLOGIC GRADE (G)

G	G Definition
GX	Grade cannot be assessed
G1	Well differentiated
G2	Moderately differentiated
G3	Poorly differentiated
G4	Undifferentiated

HISTOPATHOLOGIC TYPE

The classification applies only to carcinomas of the skin, primarily CSCC and other carcinomas. It also applies to the adenocarcinomas that develop from eccrine or sebaceous glands and to the spindle cell variant of CSCC. Microscopic verification is necessary to group by histologic type. One form of *in situ* CSCC or intraepidermal CSCC often is referred to as Bowen's disease. This lesion should be assigned Tis.

Bibliography

1. Diepgen TL, Mahler V. The epidemiology of skin cancer. *Br J Dermatol.* Apr 2002;146 Suppl 61(s61):1-6.
2. Housman TS, Feldman SR, Williford PM, et al. Skin cancer is among the most costly of all cancers to treat for the Medicare population. *Journal of the American Academy of Dermatology.* 2003;48(3):425-429.
3. Alam M, Ratner D. Cutaneous squamous-cell carcinoma. *N Engl J Med.* Mar 29 2001;344(13):975-983.
4. Rowe DE, Carroll RJ, Day CL, Jr. Prognostic factors for local recurrence, metastasis, and survival rates in squamous cell carcinoma of the skin, ear, and lip. Implications for treatment modality selection. *Journal of the American Academy of Dermatology.* Jun 1992;26(6):976-990.
5. Karia PS, Han J, Schmults CD. Cutaneous squamous cell carcinoma: estimated incidence of disease, nodal metastasis, and deaths from disease in the United States, 2012. *Journal of the American Academy of Dermatology.* Jun 2013;68(6):957-966.
6. Preston DS, Stern RS. Nonmelanoma cancers of the skin. *N Engl J Med.* Dec 3 1992;327(23):1649-1662.
7. Zak-Prelich M, Narbutt J, Sysa-Jedrzejowska A. Environmental risk factors predisposing to the development of basal cell carcinoma. *Dermatologic surgery : official publication for American Society for Dermatologic Surgery [et al.].* Feb 2004;30(2 Pt 2):248-252.
8. Nolan RC, Chan MT, Heenan PJ. A clinicopathologic review of lethal nonmelanoma skin cancers in Western Australia. *Journal of the American Academy of Dermatology.* Jan 2005;52(1):101-108.
9. O'Brien CJ, McNeil EB, McMahon JD, Pathak I, Lauer CS, Jackson MA. Significance of clinical stage, extent of surgery, and pathologic findings in metastatic cutaneous squamous carcinoma of the parotid gland. *Head & neck.* May 2002;24(5):417-422.
10. Palme CE, O'Brien CJ, Veness MJ, McNeil EB, Bron LP, Morgan GJ. Extent of parotid disease influences outcome in patients with metastatic cutaneous squamous cell carcinoma. *Archives of otolaryngology–head & neck surgery.* Jul 2003;129(7):750-753.
11. Veness MJ, Palme CE, Smith M, Cakir B, Morgan GJ, Kalnins I. Cutaneous head and neck squamous cell carcinoma metastatic to cervical lymph nodes (nonparotid): a better outcome with surgery and adjuvant radiotherapy. *The Laryngoscope.* Oct 2003;113(10):1827-1833.
12. Andruchow JL, Veness MJ, Morgan GJ, et al. Implications for clinical staging of metastatic cutaneous squamous carcinoma of the head and neck based on a multicenter study of treatment outcomes. *Cancer* Mar 1 2006;106(5):1078-1083.

15

13. Veness MJ, Palme CE, Morgan GJ. High-risk cutaneous squamous cell carcinoma of the head and neck: results from 266 treated patients with metastatic lymph node disease. *Cancer.* Jun 1 2006;106(11):2389-2396.
14. Veness MJ, Ong C, Cakir B, Morgan G. Squamous cell carcinoma of the lip. Patterns of relapse and outcome: Reporting the Westmead Hospital experience, 1980-1997. *Australasian radiology.* May 2001;45(2):195-199.
15. Ulrich C, Schmook T, Sachse MM, Sterry W, Stockfleth E. Comparative epidemiology and pathogenic factors for nonmelanoma skin cancer in organ transplant patients. *Dermatologic surgery : official publication for American Society for Dermatologic Surgery [et al.].* Apr 2004;30(4 Pt 2):622-627.
16. Ramsay HM, Fryer AA, Hawley CM, Smith AG, Nicol DL, Harden PN. Factors associated with nonmelanoma skin cancer following renal transplantation in Queensland, Australia. *Journal of the American Academy of Dermatology.* Sep 2003;49(3):397-406.
17. Veness MJ, Quinn DI, Ong CS, et al. Aggressive cutaneous malignancies following cardiothoracic transplantation: the Australian experience. *Cancer.* Apr 15 1999;85(8):1758-1764.
18. Mehrany K, Weenig RH, Lee KK, Pittelkow MR, Otley CC. Increased metastasis and mortality from cutaneous squamous cell carcinoma in patients with chronic lymphocytic leukemia. *Journal of the American Academy of Dermatology.* Dec 2005;53(6): 1067-1071.
19. Velez NF, Karia PS, Vartanov AR, Davids MS, Brown JR, Schmults CD. Association of advanced leukemic stage and skin cancer tumor stage with poor skin cancer outcomes in patients with chronic lymphocytic leukemia. *JAMA dermatology.* Mar 2014;150(3): 280-287
20. Schmults CD, Karia PS, Carter JB, Han J, Qureshi AA. Factors predictive of recurrence and death from cutaneous squamous cell carcinoma: a 10-year, single-institution cohort study. *JAMA dermatology.* May 2013;149(5):541-547.
21. Brantsch KD, Meisner C, Schonfisch B, et al. Analysis of risk factors determining prognosis of cutaneous squamous-cell carcinoma: a prospective study. *The lancet oncology.* Aug 2008;9(8):713-720.
22. Prabhu RS, Hanasoge S, Magliocca KR, et al. Extent of pathologic extracapsular extension and outcomes in patients with nonoropharyngeal head and neck cancer treated with initial surgical resection. *Cancer.* May 15 2014;120(10):1499-1506.
23. Dunne AA, Muller HH, Eisele DW, Kessel K, Moll R, Werner JA. Meta-analysis of the prognostic significance of perinodal spread in head and neck squamous cell carcinomas (HNSCC) patients. *European journal of cancer.* Aug 2006;42(12):1863-1868.
24. Wreesmann VB, Katabi N, Palmer FL, et al. Influence of extracapsular nodal spread extent on prognosis of oral squamous cell carcinoma. *Head & neck.* Oct 30 2015.
25. Prabhu RS, Magliocca KR, Hanasoge S, et al. Accuracy of computed tomography for predicting pathologic nodal extracapsular extension in patients with head-and-neck cancer undergoing initial surgical resection. *International journal of radiation oncology, biology, physics.* Jan 1 2014;88(1):122-129.
26. Brougham ND, Dennett ER, Cameron R, Tan ST. The incidence of metastasis from cutaneous squamous cell carcinoma and the impact of its risk factors. *Journal of surgical oncology.* Dec 2012;106(7):811-815.
27. Roozeboom MH, Lohman BG, Westers-Attema A, et al. Clinical and histological prognostic factors for local recurrence and metastasis of cutaneous squamous cell carcinoma: analysis of a defined population. *Acta dermato-venereologica.* Jul 6 2013;93(4):417-421.
28. Clayman GL, Lee JJ, Holsinger FC, et al. Mortality risk from squamous cell skin cancer. *J Clin Oncol.* Feb 1 2005;23(4):759-765.
29. Jambusaria-Pahlajani A, Kanetsky PA, Karia PS, et al. Evaluation of AJCC tumor staging for cutaneous squamous cell carcinoma and a proposed alternative tumor staging system. *JAMA dermatology.* Apr 2013;149(4):402-410.
30. Breuninger H, Black B, Rassner G. Microstaging of squamous cell carcinomas. *Am J Clin Pathol.* Nov 1990;94(5):624-627.
31. Moore BA, Weber RS, Prieto V, et al. Lymph node metastases from cutaneous squamous cell carcinoma of the head and neck. *The Laryngoscope.* Sep 2005;115(9):1561-1567.
32. Kyrgidis A, Tzellos TG, Kechagias N, et al. Cutaneous squamous cell carcinoma (SCC) of the head and neck: risk factors of overall and recurrence-free survival. *European journal of cancer.* Jun 2010;46(9):1563-1572.
33. Carter JB, Johnson MM, Chua TL, Karia PS, Schmults CD. Outcomes of primary cutaneous squamous cell carcinoma with perineural invasion: an 11-year cohort study. *JAMA dermatology.* Jan 2013;149(1):35-41.
34. Karia PS, Jambusaria-Pahlajani A, Harrington DP, Murphy GF, Qureshi AA, Schmults CD. Evaluation of American Joint Committee on Cancer, International Union Against Cancer, and Brigham and Women's Hospital tumor staging for cutaneous squamous cell carcinoma. *J Clin Oncol.* Feb 1 2014;32(4):327-334.
35. Broders AC. Squamous-Cell Epithelioma of the Skin: A Study of 256 Cases. *Annals of surgery.* Feb 1921;73(2):141-160.
36. Eroğlu A, Berberoğlu U, Berreroğlu S. Risk factors related to locoregional recurrence in squamous cell carcinoma of the skin. *Journal of surgical oncology.* 1996;61(2):124-130.
37. F. M. Chemosurgery: microscopically controlled surgery for skin cancer. Springfield IL: Charles C. Thomas; . 1978.
38. Breuninger H, Schaumburg-Lever G, Holzschuh J, Horny HP. Desmoplastic squamous cell carcinoma of skin and vermilion surface: a highly malignant subtype of skin cancer. *Cancer.* Mar 1 1997;79(5):915-919.
39. Quaedvlieg PJ, Creytens DH, Epping GG, et al. Histopathological characteristics of metastasizing squamous cell carcinoma of the skin and lips. *Histopathology.* Sep 2006;49(3):256-264.
40. Audet N, Palme CE, Gullane PJ, et al. Cutaneous metastatic squamous cell carcinoma to the parotid gland: analysis and outcome. *Head & neck.* Aug 2004;26(8):727-732.
41. Ch'ng S, Maitra A, Allison RS, et al. Parotid and cervical nodal status predict prognosis for patients with head and neck metastatic cutaneous squamous cell carcinoma. *Journal of surgical oncology.* Aug 1 2008;98(2):101-105.
42. Garcia-Serra A, Hinerman RW, Mendenhall WM, et al. Carcinoma of the skin with perineural invasion. *Head & neck.* Dec 2003; 25(12):1027-1033.
43. Jensen P, Hansen S, Møller B, Leivestad T, Pfeffer P, Fauchald P. Are renal transplant recipients on CsA-based immunosuppressive regimens more likely to develop skin cancer than those on azathioprine and prednisolone? Paper presented at: Transplantation proceedings1999.
44. Jensen P, Hansen S, Moller B, et al. Skin cancer in kidney and heart transplant recipients and different long-term immunosuppressive therapy regimens. *Journal of the American Academy of Dermatology.* Feb 1999;40(2 Pt 1):177-186.
45. Berg D, Otley CC. Skin cancer in organ transplant recipients: Epidemiology, pathogenesis, and management. *Journal of the American Academy of Dermatology.* Jul 2002;47(1):1-17; quiz 18-20.
46. Bordea C, Wojnarowska F, Millard P, Doll H, Welsh K, Morris P. Skin cancers in renal-transplant recipients occur more frequently than previously recognized in a temperate climate. *Transplantation.* 2004;77(4):574-579.
47. Fortina AB, Piaserico S, Caforio AL, et al. Immunosuppressive level and other risk factors for basal cell carcinoma and squamous cell carcinoma in heart transplant recipients. *Arch Dermatol.* Sep 2004;140(9):1079-1085.
48. Herrero JI EA, Quiroga J, et al. Nonmelanoma skin cancer after liver transplantation. Study of risk factors. Liver Transplant. 2005;11:1100-1106.
49. Jemec GB, Holm EA. Nonmelanoma skin cancer in organ transplant patients. *Transplantation.* Feb 15 2003;75(3):253-257.

50. Moloney FJ, Comber H, O'Lorcain P, O'Kelly P, Conlon PJ, Murphy GM. A population-based study of skin cancer incidence and prevalence in renal transplant recipients. *Br J Dermatol.* Mar 2006;154(3):498-504.
51. Patel MJ, Liegeois NJ. Skin cancer and the solid organ transplant recipient. *Current treatment options in oncology.* Dec 2008;9(4-6): 251-258.
52. Southwell KE, Chaplin JM, Eisenberg RL, McIvor NP, Morton RP. Effect of immunocompromise on metastatic cutaneous squamous cell carcinoma in the parotid and neck. *Head & neck.* Mar 2006;28(3):244-248.
53. Marcen R, Pascual J, Tato A, et al. Influence of immunosuppression on the prevalence of cancer after kidney transplantation. Paper presented at: Transplantation proceedings2003.
54. Piccirillo JF. Inclusion of comorbidity in a staging system for head and neck cancer. *Oncology (Williston Park).* Sep 1995;9(9):831-836; discussion 841, 845-838.
55. Couch ME, Dittus K, Toth MJ, et al. Cancer cachexia update in head and neck cancer: Pathophysiology and treatment. *Head & neck.* Jul 2015;37(7):1057-1072.
56. Lazure KE, Lydiatt WM, Denman D, Burke WJ. Association between depression and survival or disease recurrence in patients with head and neck cancer enrolled in a depression prevention trial. *Head & neck.* 2009;31(7):888-892.
57. Kattan MW, Hess KR, Amin MB, et al. American Joint Committee on Cancer acceptance criteria for inclusion of risk models for individualized prognosis in the practice of precision medicine. *CA: a cancer journal for clinicians.* Jan 19 2016.

Part III

Upper Gastrointestinal Tract

Members of the Upper Gastrointestinal Tract Expert Panel
Jaffer A. Ajani, MD
Adam J. Bass, MD
Shanda H. Blackmon, MD, MPH, FACS
Arthur W. Blackstock Jr., MD
Eugene H. Blackstone, MD
James D. Brierley, BSc, MB, FRCP, FRCR, FRCP(C) – UICC Representative
Björn L.D.M. Brücher, MD, PhD, FRCS, FACS
Daniel G. Coit, MD, FACS
Jeremy J. Erasmus, MD
Mark K. Ferguson, MD, FACS
Laurie E. Gaspar, MD, FASTRO, FACR, MBA – Editorial Board Liaison
Hans Gerdes, MD
John Goldblum, MD
Wayne L. Hofstetter, MD – Chair
Haejin In, MD, MBA, MPH
Hemant Ishwaran, PhD
David Kelsen, MD – Vice Chair
Richard A. Malthaner, MD
Paul F. Mansfield, MD
Bruce D. Minsky, MD
Robert D. Odze, MD
Deepa T. Patil, MD
Thomas William Rice, MD
Cathy Rimmer, BA, MDIV, CTR – Data Collection Core Representative
Takeshi Sano, MD, PhD
Roderich E. Schwarz, MD, PhD, FACS
Laura H. Tang, MD, PhD – CAP Representative
Christian W. Wittekind, MD – UICC Representative

Esophagus and Esophagogastric Junction

16

Thomas William Rice, David Kelsen,
Eugene H. Blackstone, Hemant Ishwaran, Deepa T. Patil,
Adam J. Bass, Jeremy J. Erasmus, Hans Gerdes,
and Wayne L. Hofstetter

CHAPTER SUMMARY

Cancers Staged Using This Staging System

Epithelial cancers including squamous cell carcinoma, adenocarcinoma, adenosquamous carcinoma, undifferentiated carcinoma, neuroendocrine cancers, and adenocarcinoma with neuroendocrine features are staged.

Cancers Not Staged Using This Staging System

These histopathologic types of cancer…	Are staged according to the classification for…	And can be found in chapter…
Sarcomas, nonepithelial cancers	Soft tissue sarcoma of the trunk and extremities	41
Gastrointestinal stromal tumor	Gastrointestinal stromal tumor	43

Summary of Changes

Squamous Cell Carcinoma

Change	Details of Change	Level of Evidence
Anatomy—Primary Site(s)	Anatomic boundary between esophagus and stomach: tumors involving the esophagogastric junction (EGJ) with epicenter no more than 2 cm into the promixal stomach are staged as esophageal cancers; tumors with epicenter located greater than 2 cm into the proximal stomach are staged as stomach cancers even if EGJ involved.	III
AJCC Prognostic Stage Groups	pT1a and pT1b are now incorporated into stage groupings.	II
AJCC Prognostic Stage Groups	pT2–pT3 was separated into pT2 and pT3 for Stages I–III	II
AJCC Prognostic Stage Groups	Unique cTNM prognostic stage groupings are based on clinically determined TNM.	II
AJCC Prognostic Stage Groups	Unique ypTNM prognostic stage groupings are based on patients who have received preoperative treatment and surgical resection.	II

Adenocarcinoma

Change	Details of Change	Level of Evidence
Anatomy—Primary Site(s)	Anatomic boundary between esophagus and stomach: tumors involving the EGJ with epicenter no more than 2 cm into the proximal stomach are staged as esophageal cancers; tumors with epicenter located greater than 2 cm into the proximal stomach are staged as stomach cancers even if EGJ involved	III
AJCC Prognostic Stage Groups	pT1a and pT1b are now incorporated into stage groupings.	II
AJCC Prognostic Stage Groups	Unique cTNM prognostic stage groupings are based on clinically determined TNM.	II
AJCC Prognostic Stage Groups	Unique ypTNM prognostic stage groupings are based on patients who have received preoperative treatment and surgical resection.	II

To access the AJCC cancer staging forms, please visit www.cancerstaging.org.

© American Joint Committee on Cancer 2017

M.B. Amin et al. (eds.), *AJCC Cancer Staging Manual, Eighth Edition*, DOI 10.1007/978-3-319-40618-3_16

ICD-O-3 Topography Codes

Code	Description
C15.0	Cervical esophagus
C15.1	Thoracic esophagus
C15.2	Abdominal esophagus
C15.3	Upper third of esophagus
C15.4	Middle third of esophagus
C15.5	Lower third of esophagus
C15.8	Overlapping lesion of esophagus
C15.9	Esophagus, NOS
C16.0	Cardia, esophagogastric junction*

*Tumors of the EGJ with ≤2 cm of proximal stomach involvement are staged as esophageal cancers.

WHO Classification of Tumors

Code	Description
	**Squamous**
8077	Squamous intraepithelial neoplasia (dysplasia), high grade
8070	Squamous cell carcinoma
8083	Basaloid squamous cell carcinoma
8560	Adenosquamous carcinoma
8074	Spindle cell (squamous) carcinoma
8051	Verrucous (squamous) carcinoma
**8020**	**Undifferentiated carcinoma with squamous component** (If there is any squamous component, use squamous carcinoma staging system.)
	**Adenocarcinoma**
8148	Glandular dysplasia (intraepithelial neoplasia), high grade
8140	Adenocarcinoma
8200	Adenoid cystic carcinoma
8430	Mucoepidermoid carcinoma
8244	Mixed adenoneuroendocrine carcinoma
**8020**	**Undifferentiated carcinoma with glandular component** (If there is absence of a squamous component and the presence of any glandular component, use adenocarcinoma staging system.)
	**Other Histologies** (To be categorized using TNM, but do not use stage grouping for prognosis.)
8240	Neuroendocrine tumor (NET) G1 (carcinoid)
8249	NET G2
8246	Neuroendocrine carcinoma (NEC)
8013	Large cell NEC
8041	Small cell NEC

Bosman FT, Carneiro F, Hruban RH, Theise ND, eds. World Health Organization Classification of Tumours of the Digestive System. Lyon: IARC; 2010.

INTRODUCTION

The AJCC Cancer Staging Manual, 8th Edition esophageal cancer staging chapter is based on updated data, with a significantly increased sample size and number of risk adjustment variables compared with the AJCC Cancer Staging Manual, 7th Edition. The stage groupings were determined by using a risk-adjusted random survival forest analysis of collated data from 33 esophageal centers spanning six continents and including 22,654 patients.[1] All-cause mortality—a hard end point—was used because after risk adjustment, the residual information regarding death may be attributed to esophageal cancer.[1–6]

Stage groupings for the 8th Edition are not based on an orderly increase in T category followed by number of involved nodes. The unique lymphatic anatomy of the esophagus results in the possibility of regional lymph node metastasis even with superficial (T1) cancers; therefore, patients with regional lymph node metastasis (pN+) from superficial cancers may have a prognosis similar to that of patients with deeper (greater than pT1) pN0 cancers. Similarly, deeper cancers (greater than pT1) with a few positive nodes may have a prognosis similar to that of superficial cancers (pT1) with more positive nodes. Possibly as a reflection of the genomic alterations of esophageal cancers, histologic grade (G) modulates stage such that the prognosis of well-differentiated (G1) deeper cancers is similar to that of less well-differentiated (G2–G3) superficial cancers. Staging recommendations in the 7th Edition partially separated histopathologic type for early-stage cancers. The larger dataset used for this edition has allowed for better separation of squamous cell carcinoma and adenocarcinoma staging. It is evident in the recent survival analysis that, except for advanced-stage cancers, the survival of squamous cell carcinoma patients is worse than that of patients with adenocarcinoma when comparing similarly grouped patients. Although at first glance these multiple trade-offs seem to create a less orderly arrangement of TNM categories within and among stage groupings compared with previous stage groupings, when viewed from the perspective of the interplay of these important prognostic factors, the new staging system becomes biologically compelling.

In an effort to overcome the limitations of the 7th Edition, which was based entirely on patients treated by esophagectomy alone (without preoperative or postoperative chemotherapy and/or chemoradiotherapy), the dataset used to develop the 8th Edition TNM stage groupings included patients who had received preoperative induction therapy (neoadjuvant) and/or postoperative adjuvant therapy. The availability of these data led to the ability to explicitly define cTNM and ypTNM cohorts and stages.[1,3,5–6] These data reflect the difficult landscape of clinical staging for esophageal cancer and the current preference for treating locally advanced esophageal cancer

with neoadjuvant therapy. In comparison with previous editions, analysis of this large dataset illuminated significant differences in outcome when comparing the same stage groups between patients receiving neoadjuvant therapy versus those treated with surgery alone. Therefore, it was necessary to construct a distinct composition of stage groupings for ypTNM.[5–6]

The clinical modalities currently available for pretreatment staging are often inaccurate, resulting in frequent understaging and overstaging. This ultimately leads to the potential for suboptimal treatment of esophageal cancers. When comparing survival of clinically staged patients with that of patients with equivalent pathological stage, it is evident that prognoses are not equivalent.[1–4] The prognosis for clinically staged early cancers is clearly worse, indicating that cTNM for these cancers is understaged compared with pTNM. Conversely, apparently advanced cTNM cancers carry a somewhat better prognosis than equivalent pTNM cancers. In part, this may be the result of earlier cancers being overstaged and in part because of the random effect of neoadjuvant and adjuvant therapy on more advanced-stage cancers. Although this approach may change in the future, the 8th Edition TNM staging system reflects the widespread use of neoadjuvant therapy.

There are limitations in the data that were available to construct cTNM cohorts and clinical stage groups for this edition. The exact modalities used to arrive at a clinical stage before the initiation of therapy were not available for analysis. Patients not offered surgery, deemed inoperable, or undergoing exploratory surgery without esophagectomy were relatively poorly represented in the data. In addition, patients undergoing surgery alone with pT4 and/or M1 cancers represent a select population; placing these categories into stage groups, therefore, required either combining some categories or using consensus to arrive at stage grouping, noting that in general, their prognosis was poor.

ANATOMY

Primary Site(s)

The esophagus traverses three anatomic compartments: cervical, thoracic, and abdominal. The thoracic esophagus is divided arbitrarily into equal thirds: upper, middle, and lower (Table 16.1). However, the clinical importance of the primary site of an esophageal cancer is related less to its position in the esophagus than to its relation to adjacent structures (Fig. 16.1).

The esophageal wall has three layers: mucosa, submucosa, and muscularis propria (Fig. 16.2). The *mucosa* is composed of epithelium, lamina propria, and muscularis mucosae. A basement membrane isolates the epithelium from the rest of the esophageal wall. In the columnar-lined esophagus, the muscularis mucosae may be a two-layered (duplicated) structure. The clinical importance of this duplicate layer is questionable.[7,8] The outer layer is considered the true boundary. The mucosal division may be classified as m1 (epithelium), m2 (lamina propria), or m3 (muscularis mucosae).[9] The *submucosa* has no landmarks, but it may be divided into inner (sm1), middle (sm2), and outer (sm3) thirds.[9] The *muscularis propria* has inner circular and outer longitudinal muscle layers. There is no serosa; rather, *adventitia* (periesophageal connective tissue) lies directly on the muscularis propria.

Location

Cervical Esophagus

Cancers located in the cervical esophagus are staged as upper thoracic esophageal cancers, not as head and neck cancers.

Anatomically, the cervical esophagus lies in the neck, bordered superiorly by the hypopharynx and inferiorly by the thoracic inlet, which lies at the level of the sternal notch. It is subtended by the trachea, carotid sheaths, and vertebrae. Although the length of the esophagus differs somewhat with body habitus, gender, and age, typical endoscopic measurements for the cervical esophagus measured from the incisors are from 15 to <20 cm (Fig. 16.1). If esophagoscopy is not available, location may be assessed by computed tomography (CT). If the epicenter of the tumor begins above the sternal notch, the location is defined as cervical esophagus.

Upper Thoracic Esophagus

The upper thoracic esophagus is bordered superiorly by the thoracic inlet and inferiorly by the lower border of the azygos

Table 16.1 Primary site of esophageal cancer based on proximal edge of tumor

Anatomic name	Compartment ICD-O-3	Esophageal location ICD-O-3	Esophageal location Name	Anatomic boundaries	Typical esophagectomy, cm
Cervical	C15.0	C15.3	Upper	Hypopharynx to sternal notch	15 to <20
Thoracic	C15.1	C15.3	Upper	Sternal notch to azygos vein	20 to <25
		C15.4	Middle	Lower border of azygos vein to inferior pulmonary vein	25 to <30
		C15.5	Lower	Lower border of inferior pulmonary vein to EGJ	30 to <40
Abdominal	C15.2	C15.5	Lower	EGJ to 2 cm below EGJ	40 to 45
		C16.0	EGJ/cardia	EGJ to 2 cm below EGJ	40 to 45

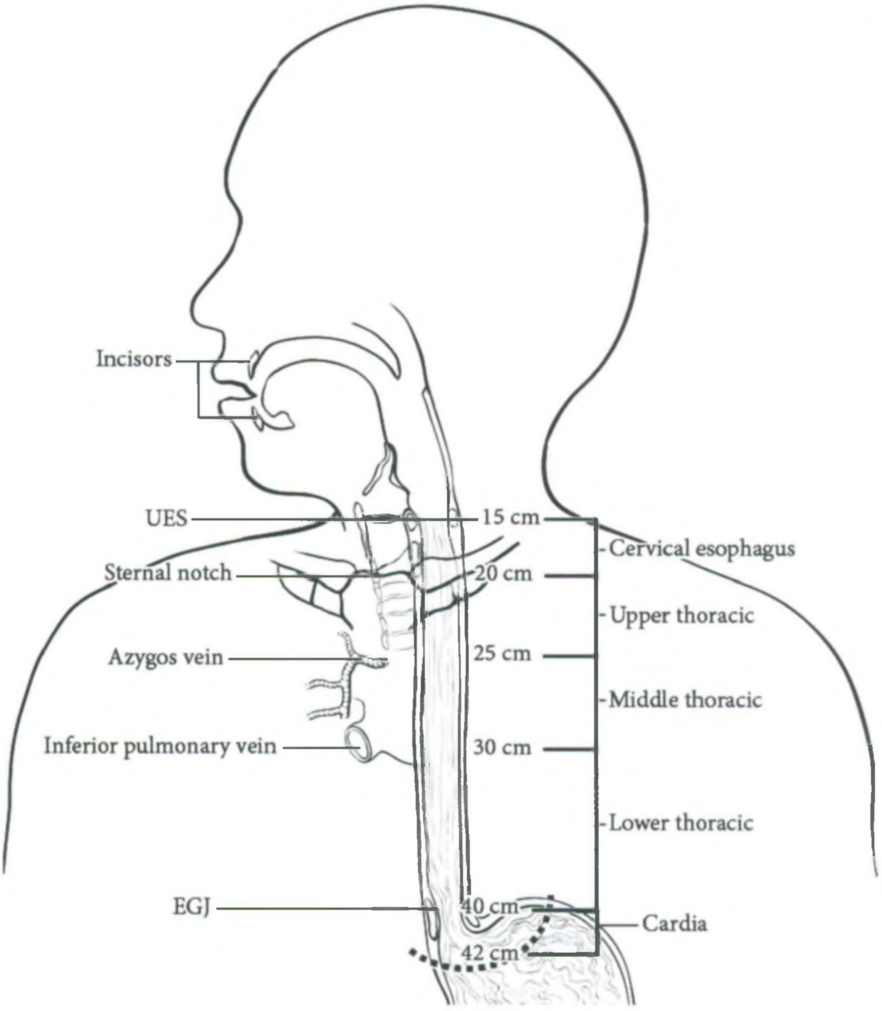

Fig. 16.1 Anatomy of esophageal cancer primary site, including typical endoscopic measurements of each region measured from the incisors. Exact measurements depend on body size and height. Location of cancer primary site is defined by cancer epicenter. EGJ, esophagogastric junction; LES, lower esophageal sphincter; UES, upper esophageal sphincter

vein. Anterolaterally, it is surrounded by the trachea, aortic arch, and great vessels, posteriorly by the vertebrae. Typical endoscopic measurements from the incisor teeth are from 20 to <25 cm (Fig. 16.1). On CT, to determine the location, the epicenter of an upper thoracic cancer is visible between the sternal notch and the azygos vein.

Middle Thoracic Esophagus

The middle thoracic esophagus is bordered superiorly by the lower border of the azygos vein and inferiorly by the lower border of the inferior pulmonary vein. It is sandwiched between the pulmonary hilum anteriorly, descending thoracic aorta on the left, and vertebrae posteriorly; on the right, it lies freely on the pleura. Typical endoscopic measurements from the incisors are from 25 to <30 cm (Fig. 16.1). On CT, to determine the location, the epicenter of a middle thoracic cancer is between the azygos vein and the inferior pulmonary vein.

Lower Thoracic Esophagus/Esophagogastic Junction (EGJ)

The lower thoracic esophagus is bordered superiorly by the lower border of the inferior pulmonary vein and inferiorly by the stomach. It is bordered anteriorly by the pericardium, posteriorly by vertebrae, and on the left by the descending thoracic aorta. It normally passes through the diaphragm to reach the stomach, but there is a variable intra-abdominal portion, and in the presence of a hiatal hernia, this portion may be absent. Typical endoscopic measurements from the incisors are from 30 to 40 cm (Fig. 16.1). On CT, to determine the location, the epicenter of a lower thoracic esophagus/EGJ cancer is below the inferior pulmonary vein. The

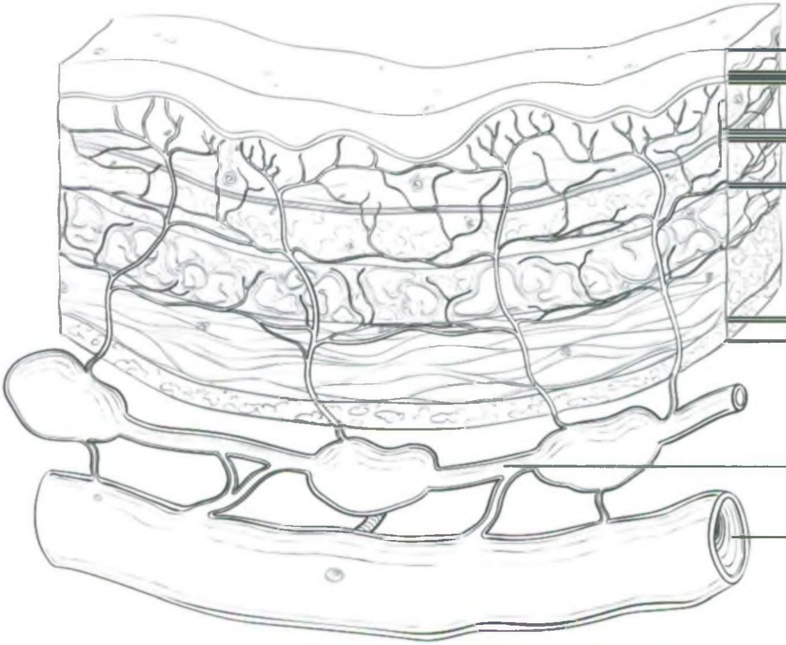

Fig. 16.2 Esophageal wall

abdominal esophagus is included in the lower thoracic esophagus. Cancers involving the EGJ that have their epicenter within the proximal 2 cm of the cardia (Siewert types I/II) are to be staged as esophageal cancers. Cancers whose epicenter is more than 2 cm distal from the EGJ, even if the EGJ is involved, will be staged using the stomach cancer TNM and stage groupings (see Chapter 17).

Regional Lymph Nodes

Esophageal lymphatic drainage is intramural and longitudinal. The lymphatic network within the esophagus is concentrated in the submucosa, although lymphatic channels also are present in the lamina propria. This arrangement may permit lymphatic metastases early in the course of the disease from otherwise superficial cancers.[10] Lymphatic drainage of the muscularis propria is more limited, but lymphatic channels pierce this layer to drain into regional lymphatic channels and lymph nodes in the periesophageal fat. Up to 43 % of autopsy dissections demonstrate direct drainage from the submucosal plexus into the thoracic duct, which facilitates systemic metastases.[11–13] The longitudinal nature of the submucosal lymphatic plexus permits lymphatic metastases orthogonal to depth of tumor invasion.[14] The implication of the longitudinal nature of lymphatic drainage is that the anatomic site of the cancer and the lymph nodes to which lymphatics drain from that site may not be the same (Fig. 16.3).

Therefore it follows, and analysis of data supports, that regional lymph nodes for all locations in the esophagus discussed in this chapter extend from periesophageal cervical nodes to celiac nodes (Figs. 16.3 and 16.4). The nomenclature for thoracic and abdominal regional lymph nodes is listed in Fig. 16.3. The nomenclature for cervical regional lymph nodes follows that of head and neck chapters (see Chapter 6) and are located in periesophageal levels VI and VII. Lymph nodes in continuity with the esophagus would be considered regional.

The specific regional lymph nodes are as follows:

- Right lower cervical paratracheal nodes: between the supraclavicular paratracheal space and apex of the lung
- Left lower cervical paratracheal nodes: between the supraclavicular paratracheal space and apex of the lung
- Right upper paratracheal nodes: between the intersection of the caudal margin of the brachiocephalic artery with the trachea and the apex of the lung
- Left upper paratracheal nodes: between the top of the aortic arch and apex of the lung
- Right lower paratracheal nodes: between the intersection of the caudal margin of the brachiocephalic artery with the trachea and cephalic border of the azygos vein
- Left lower paratracheal nodes: between the top of the aortic arch and the carina
- Subcarinal nodes: caudal to the carina of the trachea

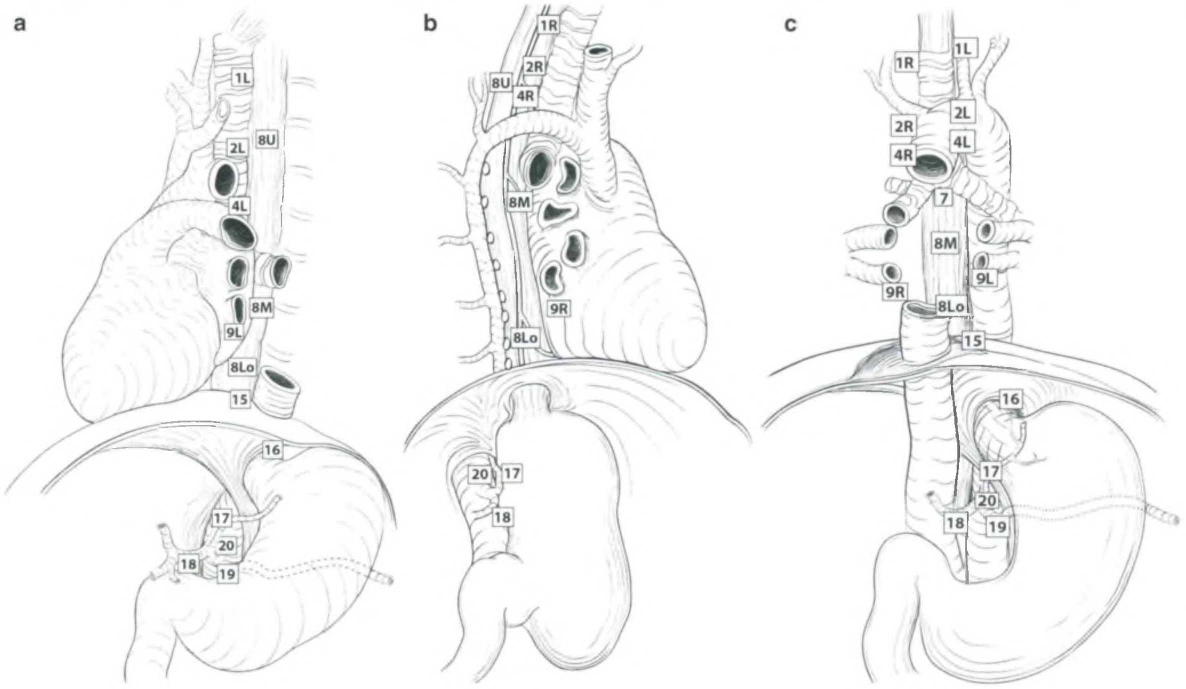

Fig. 16.3 (**A–C**) Lymph node maps for esophageal cancer. Regional lymph node stations for staging esophageal cancer from left (**A**), right (**B**), and anterior (**C**). 1R, Right lower cervical paratracheal nodes, between the supraclavicular paratracheal space and apex of the lung. 1L, Left lower cervical paratracheal nodes, between the supraclavicular paratracheal space and apex of the lung. 2R, Right upper paratracheal nodes, between the intersection of the caudal margin of the brachiocephalic artery with the trachea and the apex of the lung. 2L, Left upper paratracheal nodes, between the top of the aortic arch and the apex of the lung. 4R, Right lower paratracheal nodes, between the intersection of the caudal margin of the brachiocephalic artery with the trachea and cephalic border of the azygos vein. 4L, Left lower paratracheal nodes, between the top of the aortic arch and the carina. 7, Subcarinal nodes, caudal to the carina of the trachea. 8U, Upper thoracic paraesophageal lymph nodes, from the apex of the lung to the tracheal bifurcation. 8M, Middle thoracic paraesophageal lymph nodes, from the tracheal bifurcation to the caudal margin of the inferior pulmonary vein. 8Lo, Lower thoracic paraesophageal lymph nodes, from the caudal margin of the inferior pulmonary vein to the EGJ. 9R, Pulmonary ligament nodes, within the right inferior pulmonary ligament. 9L, Pulmonary ligament nodes, within the left inferior pulmonary ligament. 15, Diaphragmatic nodes, lying on the dome of the diaphragm and adjacent to or behind its crura. 16, Paracardial nodes, immediately adjacent to the gastroesophageal junction. 17, Left gastric nodes, along the course of the left gastric artery. 18, Common hepatic nodes, immediately on the proximal common hepatic artery. 19, Splenic nodes, immediately on the proximal splenic artery. 20, Celiac nodes, at the base of the celiac artery

- Upper thoracic paraesophageal lymph nodes: from the apex of the lung to the tracheal bifurcation
- Middle thoracic paraesophageal lymph nodes: from the tracheal bifurcation to the caudal margin of the inferior pulmonary vein
- Lower thoracic paraesophageal lymph nodes: from the caudal margin of the inferior pulmonary vein to the EGJ
- Pulmonary ligament nodes: within the right inferior pulmonary ligament
- Pulmonary ligament nodes: within the left inferior pulmonary ligament
- Diaphragmatic nodes: lying on the dome of the diaphragm and adjacent to or behind its crura
- Paracardial nodes: immediately adjacent to the gastroesophageal junction
- Left gastric nodes: along the course of the left gastric artery
- Common hepatic nodes: immediately on the proximal common hepatic artery
- Splenic nodes: immediately on the proximal splenic artery
- Celiac nodes: at the base of the celiac artery
- Cervical periesophageal level VI lymph nodes (see Chapter 6)
- Cervical periesophageal level VII lymph nodes (see Chapter 6)

Metastatic Sites

Sites of distant metastases are those not in direct continuity with the esophagus, and include nonregional lymph nodes (M1).

Fig. 16.4 Celiac lymph node

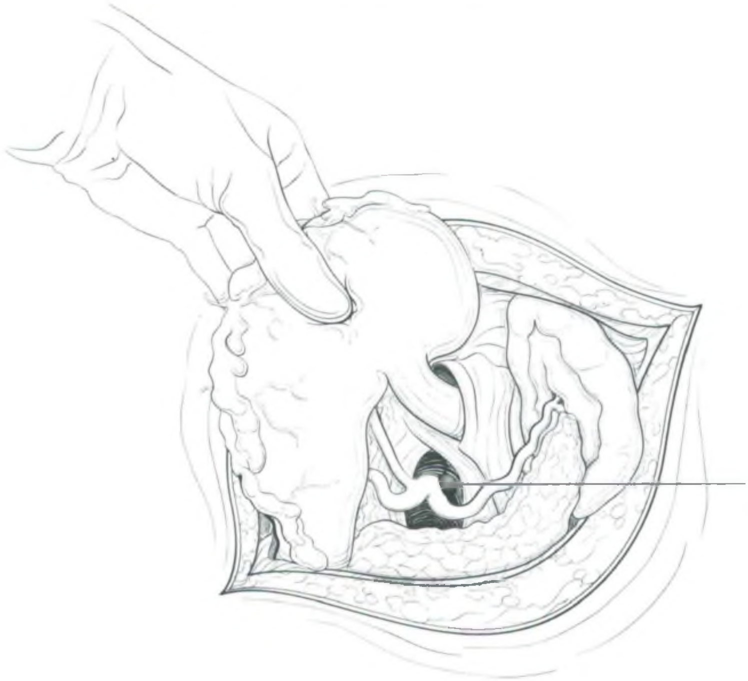

RULES FOR CLASSIFICATION

T

Malignant cells confined to the esophageal epithelium are categorized as Tis (high-grade dysplasia). Cancers confined to the mucosa are T1a (intramucosal), and those that invade beyond, but are confined to the submucosa, are T1b (submucosal). Cancers confined to the muscularis propria are T2. Cancers invading the adventitia are T3. Cancers invading adjacent structures are T4, which are subcategorized into T4a and T4b (See Fig. 16.5).

N

The data on which this chapter is based demonstrate that the total number of lymph nodes containing metastases (positive nodes) is an important prognostic factor. In classifying N, the data support convenient coarse groupings of number of positive nodes (zero, one to two, three to six, seven or more). These groups have been designated N1 (one to two), N2 (three to six), and N3 (seven or more) (Fig. 16.5). Nevertheless, there are no sharp cut points; rather, each additional positive node reduces survival. Clinical determination of the number of positive lymph nodes is possible and correlates with survival.[15–17]

M

If there is no evidence of metastasis to distant sites, the category is M0. If metastases to distant sites are evident, these are categorized as M1 (Fig. 16.5).

Classifications

Staging recommendations presented in this chapter for both squamous cell carcinoma and adenocarcinoma of the esophagus and EGJ apply to clinical staging (cTNM; newly diagnosed, not yet treated patients), pathological staging (pTNM) for patients directly undergoing resection without prior treatment, and patients who have received preoperative therapy (ypTNM).

Clinical Classification (c, yc)

Clinical assessment begins with a patient's history and physical examination. The recent onset of dysphagia and weight loss often heralds at least locally advanced disease. Abnormal physical findings suggesting distant metastasis, such as palpable lymphadenopathy or subcutaneous masses, should prompt immediate definition of the cause via imaging, aspiration cytology, biopsy, or other methods.

16

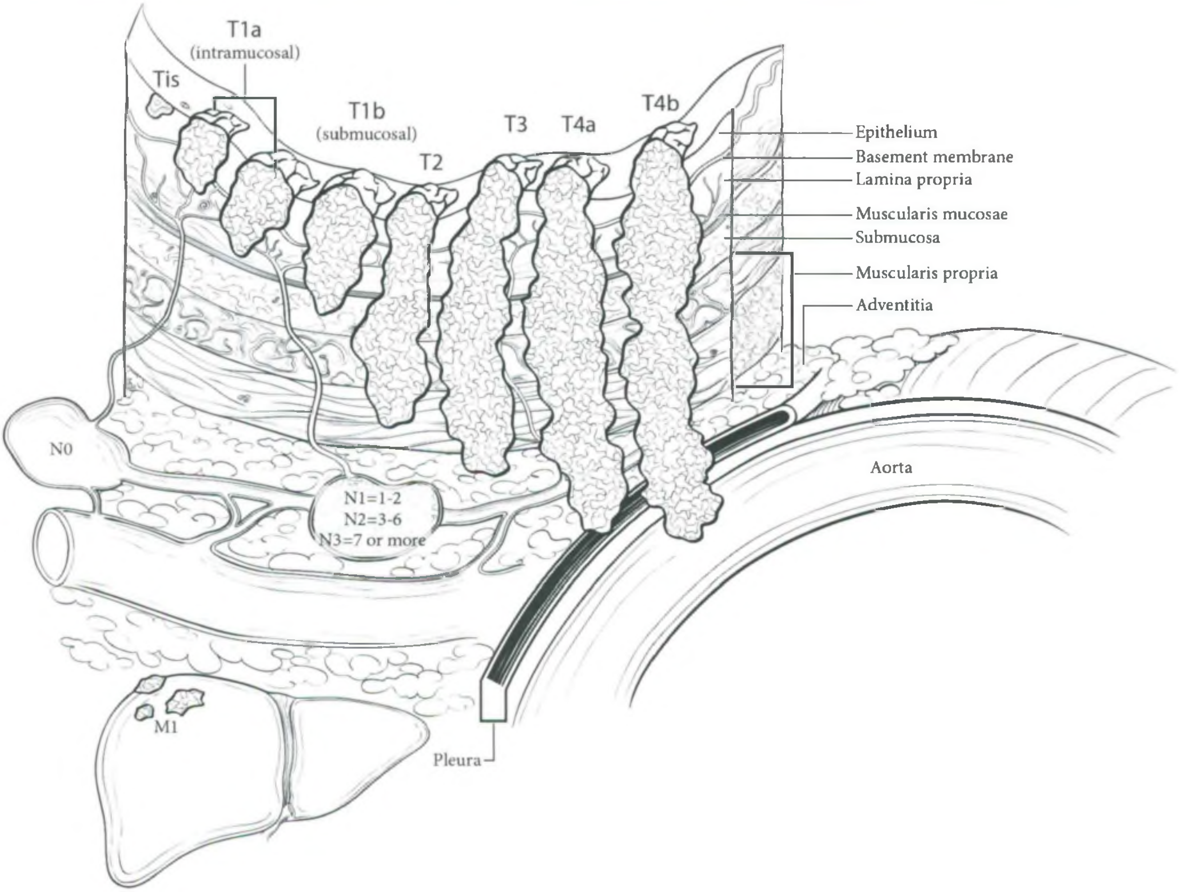

Fig. 16.5 T, N, and M categories. Primary tumor (T) is classified by depth of tumor invasion. Regional lymph node categories are determined by metastatic burden. Distant metastatic sites are designated M1

Imaging and endoscopy currently are critical components of clinical staging. This section describes current recommendations for studies to define T, N, and M. Blood-based assays and tumor genomics analysis so far have not identified validated biomarkers to inform staging.

Imaging (cN,cM)

Given the disparity in outcomes when comparing esophageal cTNM with pTNM staging, there clearly is a need for more accurate and precise clinical staging modalities. It is important for physicians to clearly indicate in the medical record the modalities used to determine clinical stage (e.g., endoscopy with or without biopsy, endoscopic resection, CT, fluorine-18 fluoro-2-deoxy-D-glucose [FDG] positron emission tomography [PET]/CT, endoscopic ultrasound [EUS] with or without fine-needle aspiration [FNA]). These data will inform future clinical staging systems.

CT of the chest and abdomen with oral and intravenous contrast frequently is the initial imaging modality used to determine the proximity of the tumor to other structures, as well as the cN and cM categories. PET/CT with FDG is used to further refine cN category away from the primary tumor, and is more sensitive than CT for determining cM category.[18–26] Some of these studies suggest that FDG PET/CT may also be useful in estimating the extent of gastric tumor extension for lower EGJ tumors, especially in obstructing tumors of the esophagus (Fig. 16.1).

CT of the chest and abdomen with intravenous and oral contrast and FDG PET/CT imaging may be used to describe the primary cancer in terms of location in the cervical, upper thoracic, middle thoracic, lower thoracic, or abdominal esophagus, as well as its orientation to other structures. Determination of locoregional involvement with regard to adjacent structures is important in treatment planning. However, CT of the chest and abdomen and FDG PET/CT have a limited role in determining primary tumor category (cT). The inability to differentiate between cT1, cT2, and cT3 and invasion of adjacent structures (cT4) is a major limitation in the use of CT for the primary tumor category (cT). Additionally, although the intensity of FDG uptake

and cT category are positively related, this association is weak.[18,27,28]

CT of the chest and abdomen with intravenous and oral contrast and FDG PET/CT imaging may be used to describe locoregional (cN) lymph nodes. Unfortunately, CT and FDG PET/CT imaging are not optimal for detecting locoregional nodal metastasis because of their low accuracy.[18,19,21–23,26] In clinical practice, locoregional nodes generally are suspicious for tumor involvement when round and/or >10 mm in short axis diameter. The portocaval lymph node, however, is an exception to these criteria. This lymph node has an elongated shape with a long transverse diameter and small anterior posterior diameter, and relying on measurement alone would result in frequent false positive interpretations. Additionally, the diagnostic benefit of FDG PET/CT is especially limited in patients with an early T category (pT1) because of the low prevalence of nodal and distant metastases and the high rate of false positive PET findings.[27,29] Because the criteria for cN category have not been defined rigorously in peer-reviewed literature, the current cN category requires evaluation of the size, shape, and number of abnormal lymph nodes in determining the cN category by imaging. As we make an effort to make clinical stage more accurate, obtaining histologic samples through various endoscopic techniques (endobronchial ultrasound, EUS-FNA) also should be considered.

CT of the chest and abdomen with intravenous and oral contrast and FDG PET/CT imaging are useful in detecting distant metastasis (cM). The addition of FDG PET/CT imaging to conventional clinical staging improves the detection of distant metastases missed or not visualized on CT of the chest and abdomen. However, a potential pitfall is the poor detection of hepatic metastases when the CT component of the FDG PET/CT is performed without intravenous contrast. An additional pitfall is the high rate of false positive PET findings that may result in unnecessary additional investigations.[23,25–27,29,30] Furthermore, the diagnostic benefit of performing FDG PET/CT may be limited if comprehensive conventional staging, including CT of the chest, abdomen and pelvis; EUS; and sonography of the neck, is performed.

Recent improvements in magnetic resonance (MR) imaging techniques have resulted in better imaging quality and improved determination of cT and cN categories.[31–33] In addition, whole-body MR imaging with or without diffusion weighting may have a role in cM categorization. However, a current limitation is that because MR imaging is not commonly performed in the staging of patients with esophageal cancer, the studies indicating its utility in staging are small, and the ultimate role of MR imaging in staging is uncertain.

Endoscopy (cT, cN, c/pM, G, L)

Esophagoscopy with multiple biopsies provides information on cancer location (L) and tissue to determine the cell type and histologic grade (G) of the tumor. Location of the primary tumor in relation to the EGJ should always be documented for purposes of appropriate staging and therapy. The presence of skip lesions (multiple discrete lesions) should be recorded and included in the overall length of the tumor. This requires the suffix *m*: T(m).

The clinical assessment of depth of tumor invasion and nodal involvement, as well as some limited areas of distant disease, may be facilitated by the use of EUS or EUS-FNA. Esophageal staging is best performed with the use of commercially available ultrasound endoscopes with multi-frequency (5-, 7.5-, 10-, and 12-MHz) radial transducers.[34]

Sonographic evaluation is performed as the instrument is withdrawn starting at the pylorus. Orienting the images in an anteroposterior axis enables careful assessment of anatomic landmarks to permit correlation with the location of the tumor, lymph nodes, and surrounding organs. The individual layers of the gastrointestinal wall are visualized throughout the examination, to correlate the extent of the tumor relative to the alternating bright and dark layers seen on ultrasound. On the basis of in vitro studies, the first two layers (bright and dark starting at the lumen) correspond to the acoustical interface and mucosa, the third (bright) layer corresponds to the submucosa, the fourth (dark) layer to the muscularis propria, and the fifth (bright) layer to the adventitia.[35] Alterations in thickness of individual layers are identified, permitting an estimate of depth of tumor invasion (cT).

The presence of a mass in the esophagus usually is diagnosed as a hypoechoic or dark thickening in one or more layers, or loss of the usual layer pattern.[34,35] The first bright layer, which represents a transition echo layer, rarely is lost or thickened. Thickening of the second layer, or the inner dark layer, suggests a cT1 tumor. Although at higher EUS frequencies of 10 or 12 MHz one should be able to distinguish tumors limited to the mucosa (cT1a) from those extending into the submucosa (cT1b), most studies have shown poor accuracy.[36–39] A dark thickening extending from the second to the third layer (mucosa and submucosa) but not reaching the fourth layer (muscularis propria) is evidence of a T1b tumor. A dark thickening extending to the fourth layer with a smooth outer border is associated with a cT2 tumor.

Suspicious nodules or lesions known to be malignant that are identified on endoscopy as potentially superficial should be excised by endoscopic resection to provide the best available determination of tumor depth in early carcinomas. Ultimately, a cancer that is completely removed by endoscopic resection (negative deep margin designated by a pathologist) should be designated as pT. The final stage designation of a patient who has undergone endoscopic resection followed by esophagectomy must take into account all pathology results, using the deepest point of invasion for the final pT category.

Complete loss of all the layers, associated with an irregular outer surface, indicates penetration beyond the muscularis propria, consistent with a cT3 tumor in the esophagus. If the dark thickening extends to the pleura, pericardium,

azygos vein, diaphragm, or peritoneum, the tumor is categorized as cT4a. Extension through the muscularis propria with loss of the echogenic stripe separating the esophagus from surrounding structures, such as the aorta, heart, lung parenchyma, or other adjacent structure, indicates a cT4b tumor.

The lymphatic drainage areas routinely investigated are both regional and nonregional (cN, cM), including the peritumoral, paratracheal, subcarinal, crural, celiac axis, splenic vein, portacaval, and gastrohepatic ligament areas. The presence of hypoechoic, rounded, sharply demarcated structures in these areas is considered diagnostic of malignant lymph nodes.[34,36,37] Histologic confirmation of nodal disease (cN) by EUS-FNA is strongly encouraged.[39,40] Since the 7th Edition of AJCC staging, clinical nodal staging in these areas has required documentation of the number and location of suspicious nodes. The appropriate nodal staging by EUS should include reporting of the number of suspicious nodes seen during the examination, followed by interpretation of the categorization according to AJCC N criteria: no suspicious nodes, N0; one or two suspicious nodes, N1; three to six suspicious nodes, N2; and seven or more suspicious nodes, N3.

Parts of the liver are readily seen with EUS with the endoscope positioned in the antrum and along the lesser curvature and cardia, permitting the identification of liver metastases (M1). Similarly, the presence of ascites adjacent to the stomach raises suspicion for peritoneal metastases, if other causes of ascites are ruled out.[41,42] This, however, has not been shown to be a reliable indicator of M1 disease. If the site of distant metastases is seen on imaging or on EUS without histologic confirmation, the metastases should be considered clinically determined (cM1). If a biopsy is performed (strongly encouraged) and there is pathological confirmation of cancer, then it is assigned pM1 for the clinical classification.[43]

Pathological Classification (p, yp)

Comparing the survival of patients receiving surgery alone (pTNM) with that of patients receiving neoadjuvant therapy (ypTNM) with equivalent pathological classifications, it is evident that prognostic implications for neoadjuvant stage classifications differ from those of equivalent pathological stage classifications (pTNM).[2,4–6] Survival of node-negative patients receiving neoadjuvant therapy (ypN0) is worse than that of equivalently pathologically staged patients undergoing esophagectomy alone (pN0); the prognosis of node-positive patients receiving neoadjuvant therapy (ypN+) is either worse or no better than that of equivalently staged patients receiving esophagectomy alone (pN+). Therefore, separate stage groupings for p and yp groupings are needed to stage patients more accurately within each treatment algorithm.

Accurate pathological staging requires careful examination of the gross specimen in terms of tumor size, shape, configuration, location, distance from margins (proximal, distal, and radial/circumferential), and nodal dissection. Amalgamation with clinical data is critical for pretreatment length or for final depth determination in patients who have undergone previous endoscopic resection. Pretreatment clinical M category (cM) would be included in the definition of ypTNM unless upstaged from cM0 to pM1 after resection (ypTypNcM).

Adjacent Structures

In close proximity to the esophagus lie the pleura, peritoneum, pericardium, azygos vein, and diaphragm. Cancers invading these structures are subcategorized as T4a. The aorta, arch vessels, airway, and vertebral body also are nearby, but cancers invading these structures are subcategorized as T4b.

Regional Lymph Node Assessment

Data demonstrate that in general, the more lymph nodes resected, the better the survival, which may be the result of either improved N categorization or a therapeutic effect of lymphadenectomy. Based on worldwide data, the adequacy of lymphadenectomy depends on T categorization. For pT1, approximately 10 nodes must be resected to maximize survival; for pT2, 20 nodes; and for pT3 or pT4, 30 nodes or more.[44] Based on different data and analysis methods that focus on maximizing sensitivity, others have suggested that an adequate lymphadenectomy requires resecting 12 to 23 nodes.[45,46] However, to determine pN category adequately, paradoxically more nodes must be resected for early-stage cancers than for advanced-stage cancers.[47] Overall, it is desirable to resect as many regional lymph nodes as possible, balancing the extent of lymph node resection necessary to accurately determine pN and maximize survival without unnecessarily increasing the morbidity of radical lymphadenectomy.

Optimal lymph node yield and staging depend on the amount of nodal tissue resected by the surgeon as well as specimen handling by pathology personnel. The periesophageal soft tissue should be dissected thoroughly to maximize the lymph node yield. In cases in which lymph node tissue is submitted so that nodes may be individually counted, the number of lymph nodes should be documented in the pathology report. In cases in which the nodal specimens are received in multiple fragments, accurate lymph node count may not be possible, and this finding should be documented. However, in such cases, the surgeon should note the number of lymph nodes submitted in the fragmented specimen.

In patients who have received neoadjuvant therapy, lymph nodes may undergo atrophy and may be difficult to recognize macroscopically. Extent of lymphadenectomy may not

be as related to survival as in pTNM.[4,5] In these cases, histologic assessment of most of the periesophageal soft tissue is helpful to retrieve grossly impalpable lymph nodes.

Following neoadjuvant treatment, the lymph node parenchyma shows fibrosis, lymphoid depletion, and acellular mucin lakes. Lymph nodes with these changes, and without any viable tumor cells, should be considered negative for metastasis. Immunohistochemical stains, such as cytokeratin AE1/AE3, may be used to confirm the presence of rare residual tumor cells. However, as false positive results may occur, they should be interpreted in conjunction with morphologic findings.

Distant Metastasis

The categorization of distant metastasis for pathological staging may be cM0, cM1, or pM1. Extensive imaging is not required to assign cM0. Distant metastasis identified on imaging or during surgery but not biopsied is assigned cM1. Histologic evidence of distant metastasis is categorized as pM1.

In postneoadjuvant therapy staging (yp), the M category is identified during clinical staging and is not changed based on the response to therapy, unless upstaged from cM0 to pM1.

PROGNOSTIC FACTORS

Prognostic Factors Required for Stage Grouping

Histopathologic cell type is an important prognostic factor for all staging efforts in esophageal cancer. Recent genomic alteration analyses demonstrated that gastroesophageal adenocarcinomas may be classified molecularly into different subgroups, and that squamous cell and adenocarcinomas of the esophagus and EGJ are genomically distinct.[48] Extensive data analysis also indicates that survival by stage is distinctly different for squamous cell carcinoma and adenocarcinoma, requiring a separate stage grouping system. Therefore, each major cell type is given its own section.

Squamous Cell Carcinoma

Squamous cell carcinoma is defined as a squamous neoplasm arising from the esophageal squamous epithelium that penetrates the epithelial basement membrane and infiltrates the lamina propria or deeper layers of the esophageal wall. It is characterized by a variable amount of keratinization, which is visualized in the form of dense eosinophilic, opaque cytoplasm. Higher-grade lesions show increased cytologic atypia and a progressively decreasing amount of nests with keratinization.

Histologic grade and location are required for staging esophageal squamous cell cancer.

Histologic Grade (G)

Histologic grade for squamous cell carcinoma is defined as follows:

G	G Definition
G1	Well-differentiated squamous cell carcinoma. In well-differentiated squamous cell carcinoma, there is prominent keratinization and a minor component of nonkeratinizing basal-like cells. The keratin component shows squamous pearls akin to the appearance of nonneoplastic squamous epithelium (normal esophageal squamous epithelium does not keratinize). Tumor cells are arranged in sheets, and mitotic counts are low compared with those for moderately and poorly differentiated tumors.[49]
G2	Moderately differentiated squamous cell carcinoma. This is the most common histologic type, demonstrating variable histologic features, ranging from parakeratotic to poorly keratinizing lesions. Generally, squamous pearl formation is absent. However, definite histologic criteria for moderately differentiated squamous cell carcinoma are not established, thus grading is affected by interobserver variability.[49]
G3	Poorly differentiated squamous cell carcinoma. This consists predominantly of basal-like cells forming large and small nests with frequent central necrosis. The nests consist of sheets or pavement-like arrangements of tumor cells, and occasionally are punctuated by small numbers of parakeratotic or keratinizing cells.[49] Note that every effort should be made to avoid signing out a histologic grade as "undifferentiated." If this cannot be resolved, the cancer should be staged as a G3 squamous cell carcinoma.

Grading of cancers based on biopsy specimens follows the aforementioned guidelines that are applicable to resection specimens. Every attempt must be made to grade tumors on preoperative specimens, because this may be the only available material for cTNM, pTNM, and ypTNM staging. The overall grade is assigned based on the foci with the highest grade within the specimen.

In the posttreatment setting, therapy-related changes often preclude accurate grading of tumors. This is problematic especially in cases in which the residual tumor cells are dispersed as single, atypical cells within the esophageal wall. In such situations, the cancer may be upstaged inaccurately to poorly differentiated carcinoma.[50]

If grade is not available, it should be recorded as GX. See AJCC Prognostic Stage Groups for instructions on incorporating GX in the pathological stage group. AJCC Level of Evidence: II

Location (L)

See Anatomy—Primary Site(s) in this chapter for a description of the cervical esophagus, upper thoracic esophagus, middle thoracic esophagus, and lower thoracic esophagus/EGJ. AJCC Level of Evidence: II

Adenocarcinoma

Adenocarcinoma is defined as a neoplasm composed of atypical glands in which the epithelial cells breach the basement membrane of the glands and infiltrate the surrounding lamina propria or muscularis mucosae (intramucosal

adenocarcinoma). Deeply invasive adenocarcinoma is defined as infiltration of neoplastic glands into the submucosa or deeper layers of the esophageal wall. AJCC Level of Evidence: I

Grade is required for staging esophageal adenocarcinoma.

Definition of Histologic Grade (G)

Grading of adenocarcinoma is based on the proportion of tumor that is composed of glands.[51]

G	G Definition
G1	Well-differentiated adenocarcinoma. In these tumors, >95 % of the tumor is composed of well-formed glands.
G2	Moderately differentiated adenocarcinoma. In these tumors, 50–95 % of the tumor shows gland formation. Most adenocarcinomas are categorized as moderately differentiated tumors.
G3	Poorly differentiated adenocarcinoma. These tumors are composed predominantly of nests and sheets of neoplastic cells. Only <50 % of the tumor shows gland formation.

In biopsy specimens of well-differentiated tumors, the infiltrating component may be difficult to recognize as invasive. Grading of cancers on biopsy specimens follows the aforementioned guidelines that are applicable to resection specimens. The overall grade is assigned based on the foci with the highest grade within the specimen.

Note that every effort should be made to avoid signing out a histologic grade as "undifferentiated." If this cannot be resolved, the cancer should be staged as a G3 squamous cell carcinoma. AJCC Level of Evidence: II

Adenosquamous Carcinoma

Adenosquamous carcinoma is defined as a neoplasm composed of elements of adenocarcinoma and squamous cell carcinoma, which remain clearly distinguishable within the tumor. These are to be staged as squamous cell cancers. AJCC Level of Evidence: I

Additional Factors Recommended for Clinical Care

Tumor Length

Tumor length may be a strong surrogate benchmark for the presence or absence of nodal disease in early- to intermediate-stage esophageal cancer. If skip lesions are present (multiple discrete lesions), these should be considered in overall length so that length is measured from the top of the highest lesion to the bottom of the lowest. The suffix *m*—T(m)—is required in this instance. AJCC Level of Evidence: II

Lymphovascular Invasion

Lymphovascular invasion (LVI) refers to the presence of malignant cells within an endothelial-lined space, and correlates with the ability of the cancer to metastasize. It therefore is an important predictor of outcome. The presence or absence of LVI in preoperative biopsies, as well as resection specimens, should be documented. Whenever possible, invasion of lymphatic vessels should be reported separately from vascular invasion, as this may portend a difference in prognosis. AJCC Level of Evidence: II

Histoviability

Neoadjuvant therapy induces a spectrum of changes within the tumor and nonneoplastic tissue of the esophagus. Residual cancer cells often are present only in the form of small nests or as single cells dispersed within the esophageal wall. The residual cancer is admixed with fibrosis and elastosis. Fibrosis causes significant obliteration of the histologic boundaries and hampers accurate assessment of depth of invasion.[50]

The tumor regression grading system described by Mandard et al.[52] appears to be the most widely used system to assess response to therapy.[53] AJCC Level of Evidence: II

Surgical Margin: R Category

Assessment of the surgical margin (R category) applies only to a surgically resected specimen. In addition to proximal and distal margins of resection, the status of the radial or circumferential margin of resection determines whether the tumor has been excised completely. The surgical margin is based on a combination of intraoperative assessment by the surgeon and pathological evaluation of the resected specimen. R0 indicates no evidence of residual tumor. R1 indicates presence of microscopic tumor at margins, as defined by College of American Pathologists (CAP); however, the Royal College of Pathologists (RCP) R1 definition includes tumors within a 1-mm margin. Macroscopically visible tumor at margins is classified as R2. Presence of tumor cells at the inked radial margin constitutes a positive margin by CAP criteria.

Tumors undergoing endoscopic resection should be assessed at the deepest (vertical) margin. Lateral margins typically are not useful in piecemeal mucosal resection cases and should not be considered in R designation. Lateral margins may be considered important in cases in which endoscopic submucosal dissection has been performed, and there is one complete resection specimen. AJCC Level of Evidence: I

Extranodal Extension

Extranodal extension, or extracapsular lymph node invasion, is the extension of tumor cells through the lymph node cap-

sule into the perinodal soft tissue. It is encountered more frequently in patients with node-positive adenocarcinoma than in those with node-positive squamous cell carcinoma.[54] AJCC Level of Evidence: II

HER2 (Adenocarcinoma Only)

Overexpression or amplification of *HER2* in an adenocarcinoma tumor specimen directs the choice of systemic therapy for patients with advanced, incurable disease, but is not yet validated as a prognostic biomarker. AJCC Level of Evidence: II

At this time, there are no validated serum biomarkers that direct staging or therapy for squamous cell carcinoma of the esophagus.

RISK ASSESSMENT MODELS

The AJCC recently established guidelines that will be used to evaluate published statistical prediction models for the purpose of granting endorsement for clinical use.[55] Although this is a monumental step toward the goal of precision medicine, this work was published only very recently. Therefore, the existing models that have been published or may be in clinical use have not yet been evaluated for this cancer site by the Precision Medicine Core of the AJCC. In the future, the statistical prediction models for this cancer site will be evaluated, and those that meet all AJCC criteria will be endorsed.

DEFINITIONS OF AJCC TNM

Definition of Primary Tumor (T)

Squamous Cell Carcinoma and Adenocarcinoma

T Category	T Criteria
TX	Tumor cannot be assessed
T0	No evidence of primary tumor
Tis	High-grade dysplasia, defined as malignant cells confined to the epithelium by the basement membrane
T1	Tumor invades the lamina propria, muscularis mucosae, or submucosa
T1a	Tumor invades the lamina propria or muscularis mucosae
T1b	Tumor invades the submucosa
T2	Tumor invades the muscularis propria
T3	Tumor invades adventitia
T4	Tumor invades adjacent structures
T4a	Tumor invades the pleura, pericardium, azygos vein, diaphragm, or peritoneum
T4b	Tumor invades other adjacent structures, such as the aorta, vertebral body, or airway

Definition of Regional Lymph Nodes (N)

Squamous Cell Carcinoma and Adenocarcinoma

N Category	N Criteria
NX	Regional lymph nodes cannot be assessed
N0	No regional lymph node metastasis
N1	Metastasis in one or two regional lymph nodes
N2	Metastasis in three to six regional lymph nodes
N3	Metastasis in seven or more regional lymph nodes

Definition of Distant Metastasis (M)

Squamous Cell Carcinoma and Adenocarcinoma

M Category	M Criteria
M0	No distant metastasis
M1	Distant metastasis

Definition of Histologic Grade (G)

Squamous Cell Carcinoma and Adenocarcinoma

G	G Definition
GX	Grade cannot be assessed
G1	Well differentiated
G2	Moderately differentiated
G3	Poorly differentiated, undifferentiated

Definition of Location (L)

Squamous Cell Carcinoma

Location plays a role in the stage grouping of esophageal squamous cancers.

Location Category	Location Criteria
X	Location unknown
Upper	Cervical esophagus to lower border of azygos vein
Middle	Lower border of azygos vein to lower border of inferior pulmonary vein
Lower	Lower border of inferior pulmonary vein to stomach, including gastroesophageal junction

Note: Location is defined by the position of the epicenter of the tumor in the esophagus.

16

AJCC PROGNOSTIC STAGE GROUPS

Squamous Cell Carcinoma

In addition to anatomic tumor depth, nodal status, and metastasis (see Definitions of AJCC TNM), other prognostic factors-grade (G) and location (L)-affect outcome, and therefore staging, of squamous cell carcinoma.

Clinical (cTNM) (Fig. 16.6)

When cT is...	And cN is...	And M is...	Then the stage group is...
Tis	N0	M0	0
T1	N0–1	M0	I
T2	N0–1	M0	II
T3	N0	M0	II
T3	N1	M0	III
T1–3	N2	M0	III
T4	N0–2	M0	IVA
Any T	N3	M0	IVA
Any T	Any N	M1	IVB

Pathological (pTNM) (Fig. 16.7)

When pT is...	And pN is...	And M is	And G is...	And location is...	Then the stage group is...
Tis	N0	M0	N/A	Any	0
T1a	N0	M0	G1	Any	IA
T1a	N0	M0	G2–3	Any	IB
T1a	N0	M0	GX	Any	IA
T1b	N0	M0	G1–3	Any	IB
T1b	N0	M0	GX	Any	IB
T2	N0	M0	G1	Any	IB
T2	N0	M0	G2–3	Any	IIA
T2	N0	M0	GX	Any	IIA
T3	N0	M0	Any	Lower	IIA
T3	N0	M0	G1	Upper/middle	IIA
T3	N0	M0	G2–3	Upper/middle	IIB
T3	N0	M0	GX	Any	IIB
T3	N0	M0	Any	Location X	IIB
T1	N1	M0	Any	Any	IIB
T1	N2	M0	Any	Any	IIIA
T2	N1	M0	Any	Any	IIIA
T2	N2	M0	Any	Any	IIIB
T3	N1–2	M0	Any	Any	IIIB
T4a	N0–1	M0	Any	Any	IIIB
T4a	N2	M0	Any	Any	IVA
T4b	N0–2	M0	Any	Any	IVA
Any T	N3	M0	Any	Any	IVA
Any T	Any N	M1	Any	Any	IVB

Postneoadjuvant Therapy (ypTNM) (Fig. 16.8)

When yp T is...	And yp N is...	And M is...	Then the stage group is...
T0–2	N0	M0	I
T3	N0	M0	II
T0–2	N1	M0	IIIA
T3	N1	M0	IIIB
T0–3	N2	M0	IIIB
T4a	N0	M0	IIIB
T4a	N1–2	M0	IVA
T4a	NX	M0	IVA
T4b	N0–2	M0	IVA
Any T	N3	M0	IVA
Any T	Any N	M1	IVB

Adenocarcinoma

The requirements and rules for staging esophageal adenocarcinoma are similar to those for squamous cell carcinoma with regard to determining primary tumor stage, nodal status, and metastasis (see Definitions of AJCC TNM and G for squamous cell carcinoma). Whereas location of tumor is not a prognostic variable in adenocarcinoma of the esophagus, grade significantly affects outcome and therefore staging.

Clinical (cTNM) (Fig. 16.9)

When cT is...	And cN is...	And M is...	Then the stage group is...
Tis	N0	M0	0
T1	N0	M0	I
T1	N1	M0	IIA
T2	N0	M0	IIB
T2	N1	M0	III
T3	N0–1	M0	III
T4a	N0–1	M0	III
T1–4a	N2	M0	IVA
T4b	N0–2	M0	IVA
Any T	N3	M0	IVA
Any T	Any N	M1	IVB

Pathological (pTNM) (Fig. 16.10)

When pT is...	And pN is...	And M is...	And G is...	Then the stage group is...
Tis	N0	M0	N/A	0
T1a	N0	M0	G1	IA
T1a	N0	M0	GX	IA
T1a	N0	M0	G2	IB
T1b	N0	M0	G1–2	IB
T1b	N0	M0	GX	IB
T1	N0	M0	G3	IC
T2	N0	M0	G1–2	IC
T2	N0	M0	G3	IIA
T2	N0	M0	GX	IIA
T1	N1	M0	Any	IIB
T3	N0	M0	Any	IIB
T1	N2	M0	Any	IIIA
T2	N1	N0	Any	IIIA
T2	N2	M0	Any	IIIB
T3	N1–2	M0	Any	IIIB
T4a	N0–1	M0	Any	IIIB
T4a	N2	M0	Any	IVA

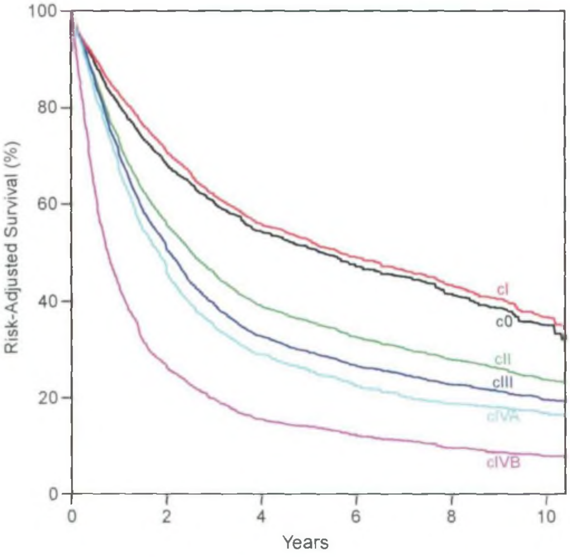

Fig. 16.6 Risk-adjusted survival after treatment decision for clinically staged (c) squamous cell carcinoma of the esophagus based on Worldwide Esophageal Cancer Collaboration (WECC) data [1,3]

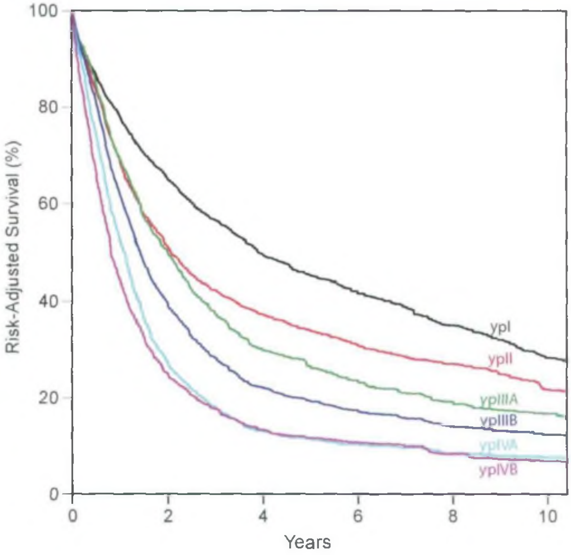

Fig. 16.8 Risk-adjusted survival after treatment decision for postneoadjuvant pathologically staged (yp) squamous cell carcinoma of the esophagus based on WECC data.[5,6]

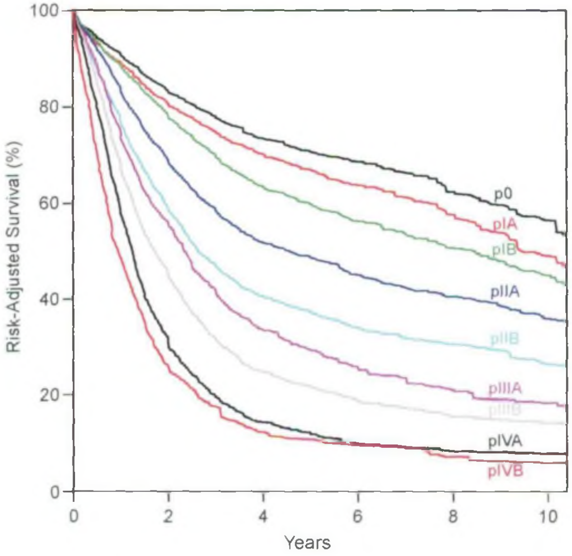

Fig. 16.7 Risk-adjusted survival after treatment decision for pathologically staged (p) squamous cell carcinoma of the esophagus based on WECC data [2,4]

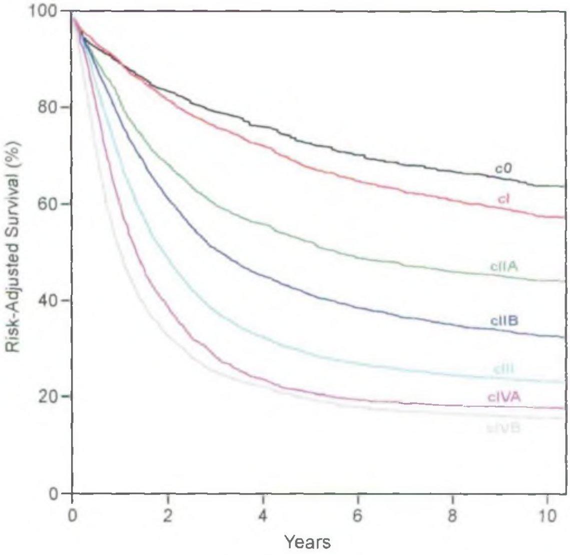

Fig. 16.9 Risk-adjusted survival after treatment decision for clinically staged (c) adenocarcinoma of the esophagus based on WECC data [1,3]

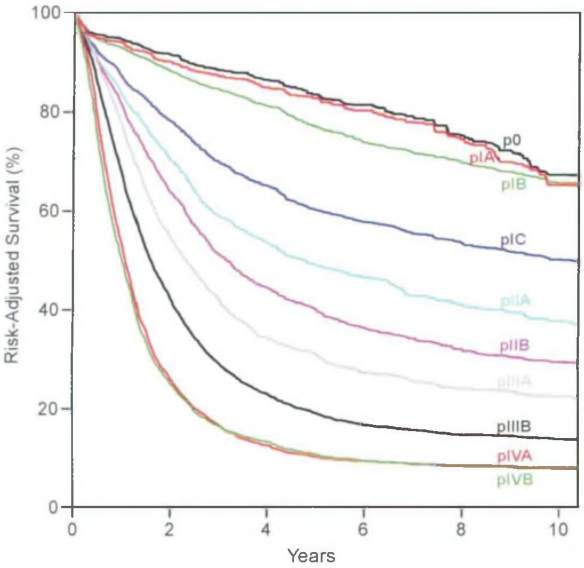

Fig. 16.10 Risk-adjusted survival after treatment decision for pathologically staged (p) adenocarcinoma of the esophagus based on WECC data [2,4]

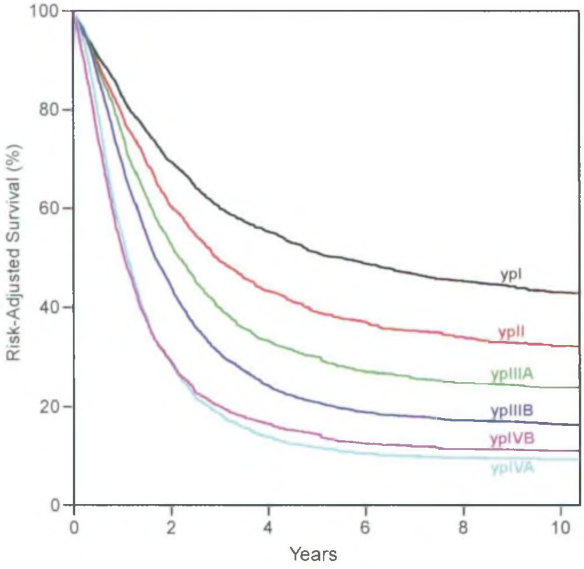

Fig. 16.11 Risk-adjusted survival after treatment decision for post-neoadjuvant pathologically staged (yp) adenocarcinoma of the esophagus based on WECC data [5,6]

Bibliography

1. Rice TW, Apperson-Hansen C, DiPaola LM, et al. Worldwide Esophageal Cancer Collaboration: clinical staging data. *Dis Esophagus* (in press).
2. Rice TW, Chen L-Q, Hofstetter WL, et al. Worldwide Esophageal Cancer Collaboration: pathologic staging data. *Dis Esophagus* (in press).
3. Rice TW, Ishwaran H, Blackstone EH, et al. Recommendations for clinical staging (cTNM) of cancer of the esophagus and esophagogastric junction for the 8th edition AJCC/UICC staging manuals. *Dis Esophagus* (in press).
4. Rice TW, Ishwaran H, Hofstetter WL, et al. Recommendations for pathologic staging (pTNM) of cancer of the esophagus and esophagogastric junction for the 8th edition AJCC/UICC staging manuals. *Dis Esophagus* (in press).
5. Rice TW, Ishwaran H, Kelsen DP, et al. Recommendations for neoadjuvant stage grouping (ypTNM) of cancer of the esophagus and esophagogastric junction for the 8th edition AJCC/UICC staging manuals. *Dis Esophagus* (in press).
6. Rice TW, Lerut TEMR, Orringer MB, et al. Worldwide Esophageal Cancer Collaboration: neoadjuvant staging data. *Dis Esophagus* (in press).
7. Abraham SC, Krasinskas AM, Correa AM, et al. Duplication of the muscularis mucosae in Barrett esophagus: an underrecognized feature and its implication for staging of adenocarcinoma. *Am J Surg Pathol.* 2007;31(11):1719–1725.
8. Kaneshiro DK, Post JC, Rybicki L, Rice TW, Goldblum JR. Clinical significance of the duplicated muscularis mucosae in Barrett esophagus-related superficial adenocarcinoma. *Am J Surg Pathol.* 2011;35(5):697–700.
9. Kodama M, Kakegawa T. Treatment of superficial cancer of the esophagus: a summary of responses to a questionnaire on superficial cancer of the esophagus in Japan. *Surgery.* 1998; 123(4):432-439.
10. Rice TW, Blackstone EH, Goldblum JR, et al. Superficial adenocarcinoma of the esophagus. *J Thorac Cardiovasc Surg.* 2001; 122(6):1077–1090.
11. Riquet M, Saab M, Le Pimpec Barthes F, Hidden G. Lymphatic drainage of the esophagus in the adult. *Surg Radiol Anat.* 1993;15(3):209–211.
12. Murakami G, Sato I, Shimada K, Dong C, Kato Y, Imazeki T. Direct lymphatic drainage from the esophagus into the thoracic duct. *Surg Radiol Anat.* 1994;16(4):399–407.
13. Kuge K, Murakami G, Mizobuchi S, Hata Y, Aikou T, Sasaguri S. Submucosal territory of the direct lymphatic drainage system to the thoracic duct in the human esophagus. *J Thorac Cardiovasc Surg.* 2003;125(6):1343–1349.
14. Akiyama H, Tsurumaru M, Kawamura T, Ono Y. Principles of surgical treatment for carcinoma of the esophagus: analysis of lymph node involvement. *Ann Surg.* 1981;194(4):438–446.
15. Natsugoe S, Yoshinaka H, Shimada M, et al. Number of lymph node metastases determined by presurgical ultrasound and endoscopic ultrasound is related to prognosis in patients with esophageal carcinoma. *Ann Surg.* 2001;234(5):613–618.
16. Chen J, Xu R, Hunt GC, Krinsky ML, Savides TJ. Influence of the number of malignant regional lymph nodes detected by endoscopic ultrasonography on survival stratification in esophageal adenocarcinoma. *Clin Gastroenterol Hepatol.* 2006;4(5):573–579.
17. Twine CP, Roberts SA, Rawlinson CE, et al. Prognostic significance of the endoscopic ultrasound defined lymph node metastasis count in esophageal cancer. *Dis Esophagus.* 2010;23(8): 652–659.
18. Kato H, Kuwano H, Nakajima M, et al. Comparison between positron emission tomography and computed tomography in the use of the assessment of esophageal carcinoma. *Cancer.* 2002;94(4): 921–928.
19. Lowe VJ, Booya F, Fletcher JG, et al. Comparison of positron emission tomography, computed tomography, and endoscopic ultrasound in the initial staging of patients with esophageal cancer. *Mol Imaging Bio.* 2005;7(6):422–430.
20. van Westreenen HL, Heeren PA, van Dullemen HM, et al. Positron emission tomography with F-18-fluorodeoxyglucose in a combined staging strategy of esophageal cancer prevents unnecessary surgical explorations. *J Gastrointest Surg.* 2005;9(1):54–61.

21. Takizawa K, Matsuda T, Kozu T, et al. Lymph node staging in esophageal squamous cell carcinoma: a comparative study of endoscopic ultrasonography versus computed tomography. *J Gastroenterol Hepatol.* 2009;24(10):1687–1691.
22. Choi J, Kim SG, Kim JS, Jung HC, Song IS. Comparison of endoscopic ultrasonography (EUS), positron emission tomography (PET), and computed tomography (CT) in the preoperative locoregional staging of resectable esophageal cancer. *Surg Endosc.* 2010;24(6):1380–1386.
23. Walker AJ, Spier BJ, Perlman SB, et al. Integrated PET/CT fusion imaging and endoscopic ultrasound in the pre-operative staging and evaluation of esophageal cancer. *Mol Imaging Biology.* 2011;13(1):166–171.
24. You JJ, Wong RK, Darling G, Gulenchyn K, Urbain JL, Evans WK. Clinical utility of 18F-fluorodeoxyglucose positron emission tomography/computed tomography in the staging of patients with potentially resectable esophageal cancer. *J Thorac Oncol.* 2013;8(12):1563–1569.
25. Purandare NC, Pramesh CS, Karimundackal G, et al. Incremental value of 18F-FDG PET/CT in therapeutic decision-making of potentially curable esophageal adenocarcinoma. *Nucl Med Commun.* 2014;35(8):864–869.
26. Findlay JM, Bradley KM, Maile EJ, et al. Pragmatic staging of oesophageal cancer using decision theory involving selective endoscopic ultrasonography, PET and laparoscopy. *Br J Surg.* 2015;102(12):1488–1499.
27. Cuellar SL, Carter BW, Macapinlac HA, et al. Clinical staging of patients with early esophageal adenocarcinoma: does FDG–PET/CT have a role? *J Thorac Oncol.* 2014;9(8):1202–1206.
28. Omloo JM, Sloof GW, Boellaard R, et al. Importance of fluorodeoxyglucose-positron emission tomography (FDG-PET) and endoscopic ultrasonography parameters in predicting survival following surgery for esophageal cancer. *Endoscopy.* 2008;40(6):464–471.
29. Little SG, Rice TW, Bybel B, et al. Is FDG-PET indicated for superficial esophageal cancer? *Eur J Cardio-thorac Surg.* 2007;31(5):791–796.
30. Adams HL, Jaunoo SS. Clinical significance of incidental findings on staging positron emission tomography for oesophagogastric malignancies. *Ann R Coll Surg Engl.* 2014;96(3):207–210.
31. Malik V, Harmon M, Johnston C, et al. Whole body MRI in the staging of esophageal cancer - a prospective comparison with whole body 18F-FDG PET-CT. *Dig Surg.* 2015;32(5):397–408.
32. Yamada I, Miyasaka N, Hikishima K, et al. Ultra-high-resolution MR imaging of esophageal carcinoma at ultra-high field strength (7.0T) ex vivo: correlation with histopathologic findings. *Magn Reson Imaging.* 2015;33(4):413–419.
33. Yamada I, Hikishima K, Miyasaka N, et al. Esophageal carcinoma: evaluation with q-space diffusion-weighted MR imaging ex vivo. *Magn Reson Med.* 2015;73(6):2262–2273.
34. Botet JF, Lightdale CJ, Zauber AG, Gerdes H, Urmacher C, Brennan MF. Preoperative staging of esophageal cancer: comparison of endoscopic US and dynamic CT. *Radiology.* 1991;181(2):419–425.
35. Kimmey MB, Martin RW, Haggitt RC, Wang KY, Franklin DW, Silverstein FE. Histologic correlates of gastrointestinal ultrasound images. *Gastroenterology.* 1989;96(2 Pt 1):433–441.
36. Barbour AP, Rizk NP, Gerdes H, et al. Endoscopic ultrasound predicts outcomes for patients with adenocarcinoma of the gastroesophageal junction. *J Am Coll Surg.* 2007;205(4):593–601.
37. Blackshaw G, Lewis WG, Hopper AN, et al. Prospective comparison of endosonography, computed tomography, and histopathological stage of junctional oesophagogastric cancer. *Clin Radiol.* 2008;63(10):1092–1098.
38. Murata Y, Napoleon B, Odegaard S. High-frequency endoscopic ultrasonography in the evaluation of superficial esophageal cancer. *Endoscopy.* 2003;35(5):429–435; discussion 436.
39. Puli SR, Reddy JB, Bechtold ML, Antillon D, Ibdah JA, Antillon MR. Staging accuracy of esophageal cancer by endoscopic ultrasound: a meta-analysis and systematic review. *World J Gastroenterol.* 2008;14(10):1479–1490.
40. Eloubeidi MA, Wallace MB, Reed CE, et al. The utility of EUS and EUS-guided fine needle aspiration in detecting celiac lymph node metastasis in patients with esophageal cancer: a single-center experience. *Gastrointest Endosc.* 2001;54(6):714–719.
41. Lee YT, Ng EK, Hung LC, et al. Accuracy of endoscopic ultrasonography in diagnosing ascites and predicting peritoneal metastases in gastric cancer patients. *Gut.* 2005;54(11):1541–1545.
42. Sultan J, Robinson S, Hayes N, Griffin SM, Richardson DL, Preston SR. Endoscopic ultrasonography-detected low-volume ascites as a predictor of inoperability for oesophagogastric cancer. *Br J Surg.* 2008;95(9):1127–1130.
43. tenBerge J, Hoffman BJ, Hawes RH, et al. EUS-guided fine needle aspiration of the liver: indications, yield, and safety based on an international survey of 167 cases. *Gastrointest Endosc.* 2002;55(7):859–862.
44. Rizk NP, Ishwaran H, Rice TW, et al. Optimum lymphadenectomy for esophageal cancer. *Ann Surg.* 2010;251(1):46–50.
45. Chen YJ, Schultheiss TE, Wong JY, Kernstine KH. Impact of the number of resected and involved lymph nodes on esophageal cancer survival. *J Surg Oncol.* 2009;100(2):127–132.
46. Peyre CG, Hagen JA, DeMeester SR, et al. The number of lymph nodes removed predicts survival in esophageal cancer: an international study on the impact of extent of surgical resection. *Ann Surg.* 2008;248(4):549–556.
47. Rice TW, Ishwaran H, Hofstetter WL, et al. Esophageal cancer: association with pN+. *Ann Surg (in press).*
48. Cancer Genome Atlas Research Network. Comprehensive molecular characterization of gastric adenocarcinoma. *Nature.* 2014;513(7517):202–209.
49. Montgomery E, Field JK, Boffetta P, et al. Squamous cell carcinoma of the oesophagus. In: Bosman FT, Carneiro F, Hruban RH, and Theise ND, eds. *WHO Classification of Tumors of the Digestive System. 4th ed.* Lyon, France: International Agency for Research and Cancer (IARC) 2010:18–24.
50. Chang F, Deere H, Mahadeva U, George S. Histopathologic examination and reporting of esophageal carcinomas following preoperative neoadjuvant therapy: practical guidelines and current issues. *Am J Clin Pathol.* 2008;129(2):252–262.
51. Fléjou J, Odze R, Montgomery E, et al. Adenocarcinoma of the oesophagus. In: Bosman FT, Carneiro F, Hruban RH, and Theise ND, eds. *WHO Classification of Tumors of the Digestive System. 4th ed.* Lyon, France: International Agency for Research and Cancer (IARC) 2010:25–31.
52. Mandard AM, Dalibard F, Mandard JC, et al. Pathologic assessment of tumor regression after preoperative chemoradiotherapy of esophageal carcinoma. Clinicopathologic correlations. *Cancer.* 1994;73(11):2680–2686.
53. Ryan R, Gibbons D, Hyland JM, et al. Pathological response following long-course neoadjuvant chemoradiotherapy for locally advanced rectal cancer. *Histopathology.* 2005;47(2):141–146.
54. Nafteux PR, Lerut AM, Moons J, et al. International multicenter study on the impact of extracapsular lymph node involvement in primary surgery adenocarcinoma of the esophagus on overall survival and staging systems. *Ann Surg.* 2015;262(5):809–815; discussion 815–806.
55. Kattan MW, Hess KR, Amin MB, et al. American Joint Committee on Cancer acceptance criteria for inclusion of risk models for individualized prognosis in the practice of precision medicine. *CA Cancer J Clin.* Jan 19 2016 [Epub ahead of print].

Stomach

17

Jaffer A. Ajani, Haejin In, Takeshi Sano, Laurie E. Gaspar, Jeremy J. Erasmus, Laura H. Tang, Mary Kay Washington, Hans Gerdes, Christian W. Wittekind, Paul F. Mansfield, Cathy Rimmer, Wayne L. Hofstetter, and David Kelsen

CHAPTER SUMMARY

Cancers Staged Using This Staging System

The staging system applies to all primary carcinomas that arise in the stomach. Adenocarcinoma is the most common histologic type, whereas other histologic types are observed less frequently.

Cancers Not Staged Using This Staging System

These histopathologic types of cancer…	Are staged according to the classification for…	And can be found in chapter…
Gastrointestinal stromal tumors	Gastrointestinal stromal tumors	43
Other sarcomas	Soft tissue sarcoma of the abdomen and thoracic visceral organs	42
Lymphomas	Hodgkin and non-Hodgkin lymphomas	79
Well-differentiated neuroendocrine tumors (G1 and G2)	Neuroendocrine tumors of the stomach	29

Summary of Changes

Change	Details of Change	Level of Evidence
Anatomy—Primary Site(s)	Anatomic boundary between esophagus and stomach: tumors involving the esophagogastric junction (EGJ) with the tumor epicenter no more than 2 cm into the proximal stomach are staged as esophageal cancers; EGJ tumors with their epicenter located greater than 2 cm into the proximal stomach are staged as stomach cancers. Cardia cancer not involving the EGJ is staged as stomach cancer.	III
Definition of Regional Lymph Node (N)	N3 has been subdivided into N3a and N3b.	II
AJCC Prognostic Stage Groups	cTNM: stage groupings for cTNM differ from those of pTNM. New cTNM groupings and their cooresponding prognostic information are presented in this edition.	III
AJCC Prognostic Stage Groups	ypTNM: stage groupings are the same as those for pTNM; however, prognostic information is presented using only the four broad stage categrories (Stages I–IV).	III
AJCC Prognostic Stage Groups	In pathological classification (pTNM), T4aN2 and T4bN0 are now classified as Stage IIIA.	II

To access the AJCC cancer staging forms, please visit www.cancerstaging.org.

© American Joint Committee on Cancer 2017

M.B. Amin et al. (eds.), *AJCC Cancer Staging Manual, Eighth Edition*, DOI 10.1007/978-3-319-40618-3_17

ICD-O-3 Topography Codes

Code	Description
C16.0	Cardia, esophagogastric junction*
C16.1	Fundus of stomach
C16.2	Body of stomach
C16.3	Gastric antrum
C16.4	Pylorus
C16.5	Lesser curvature of stomach, NOS
C16.6	Greater curvature of stomach, NOS
C16.8	Overlapping lesion of stomach
C16.9	Stomach, NOS

* Tumors that involve the esophagastric junction with their tumor epicenter >2 cm into the proximal stomach are staged as stomach cancers.

WHO Classification of Tumors

Code	Description
8148	Intraepithelial neoplasia (dysplasia), high grade
8140	Adenocarcinoma, NOS
8144	Adenocarcinoma, intestinal type
8145	Carcinoma, diffuse type
8260	Papillary adenocarcinoma
8211	Tubular adenocarcinoma
8480	Mucinous adenocarcinoma
8214	Parietal cell carcinoma
8260	Papillary adenocarcinoma
8211	Tubular adenocarcinoma
8490	Signet ring cell carcinoma
8490	Poorly cohesive carcinoma
8255	Mixed adenocarcinoma
8560	Adenosquamous carcinoma
8512	Carcinoma with lymphoid stroma
8576	Hepatoid adenocarcinoma
8070	Squamous cell carcinoma, NOS
8082	Lymphoepithelial carcinoma
8510	Medullary carcinoma, NOS
8020	Undifferentiated carcinoma
8246	Neuroendocrine carcinoma
8013	Large cell neuroendocrine carcinoma
8041	Small cell neuroendocrine carcinoma
8244	Mixed adenoneuroendocrine carcinoma

Bosman FT, Carneiro F, Hruban RH, Theise ND, eds. World Health Organization Classification of Tumours of the Digestive System. Lyon: IARC; 2010.

INTRODUCTION

Gastric adenocarcinoma is rampant around the world[1] and often is diagnosed in advanced stages.[1] Earlier stages (Stage I or less) are treated either endoscopically or surgically, but the intermediate stages (Stages II and III) are treated with multimodality therapies.[2] Stage IV gastric adenocarcinoma is uniformly incurable, and therapies are palliative. Some progress has been made in the treatment of gastric adenocarcinoma, including laparoscopic surgery, better techniques for endoscopic resection of early tumors, the establishment of postoperative adjuvant therapy, a better understanding of molecular subtypes, and further understanding of carcinogenesis and prevention.

This edition of gastric adenocarcinoma provides additional resources that were not available in the AJCC Cancer Staging Manual, 7th Edition. When patients are diagnosed with gastric adenocarcinoma, they invariably undergo various diagnostic/staging tests to establish a "clinical stage." This clinical information guides the treating physician(s) in making initial therapy decisions. Because of the lack of an official clinical stage classification in the past, treating physicians have used the pathological stage (pStage) classification proposed in previous AJCC classification versions to clinically stage patients. However, application of pStage to designate a clinical stage may not be appropriate. Additionally, the use of pStage to establish the clinical stage has not been validated. Moreover, one is almost always uncertain about the type of treatment that should be given to a patient (e.g., he or she may not receive surgery, may receive preoperative therapy, or may develop metastatic tumor within weeks of initial evaluation); therefore, applying pStage to each of these patients is problematic. The assumption that pTNM can be used for clinical stage grouping may lead to inappropriate therapies. Therefore, to avoid the continued use of pTNM groups for clinical staging, we added clinical stage groups based on two datasets: the National Cancer Data Base (NCDB), representing patients diagnosed in the United States (surgical and nonsurgical), and the Shizuoka Cancer Center dataset, representing surgically treated patients in Japan, for a total of 4,091 patients. Clinical stage groupings are different from stage groupings used for pathological or postneoadjuvant therapy (ypTNM). In particular, prognosis for cT4bNXM0 was found to be extremely poor, likely reflecting understaging in the clinical setting, and is designated as clinical Stage IV.

The stage groupings and prognostic information for pathological stage are now based on >25,000 gastric adenocarcinoma patients in the International Gastric Cancer Association (IGCA) database, which includes both Asian and Western patients who had gastric cancer surgery with adequate lymph node removal and pathological assessments and were followed up for a minimum of 5 years.[3] Patients treated neoadjuvantly with chemotherapy or radiation before surgery were not included in this analysis.[3]

During the past several years, there has been increasing awareness of the documented benefits from preoperative

therapy in patients with localized gastric cancer.[4] As a result, more patients are receiving preoperative (neoadjuvant) therapy. Because there has been no postneoadjuvant therapy classification for stage grouping, pTNM groupings have been applied in such patients; however, this practice is not validated and may not be appropriate. Therefore, in the AJCC Cancer Staging Manual, 8th Edition staging classification, we provide meaningful prognostic information that may be used for patients who have received therapy before surgery (see Survival Data in this chapter). Because of the limited number of patients available for this analysis (n=~700), broad stage groupings were created to provide prognostic information. This ypTNM stage grouping system fulfills an unmet need in the clinics.

In this edition, there is a modification regarding tumors located at the esophagogastric junction (EGJ) and in the cardia of the stomach. If a tumor involves the EGJ and its epicenter is $\leq$2 cm into the proximal stomach (i.e, $\leq$2 cm distal to the EGJ), we recommend using the esophageal cancer schema for stage groupings. Tumors involving the EGJ with their epicenter >2 cm into the proximal stomach (i.e., >2 cm distal to the EGJ) are now classified using the stomach schema. Cardia cancers that do not invade the EGJ should be classified based on the stomach cancer schema for stage groupings. Thus, determining the exact location of the EGJ and whether it is involved by the tumor is critically important for assessing tumors in this region.

In summary, the current classifications provide more comprehensive tools (cTNM, ypTNM, and pTNM) for stage grouping of gastric cancer patients under different circumstances. The three classifications should provide the resources needed for the stage grouping required in the clinic and may prove more useful than the one staging system applied to different situations that was provided previously. Some deficiencies are acknowledged, such as a lack of uniformity in initial clinical stage assessments (including nonstandardized radiology reports and endoscopic assessments/descriptions), the lack of a uniform surgical approach (particularly in the United States), and pathology assessments of yp categories. However, we will make a concerted effort to improve these assessments and evaluate their value based on the emerging data for future revisions of the staging system for gastric adenocarcinoma.

ANATOMY

Primary Site(s)

The stomach is the first division of the abdominal portion of the alimentary tract, beginning at the EGJ and extending to the pylorus. The proximal stomach is located immediately below the diaphragm and is termed the *cardia*. The remaining portions are the fundus and body of the stomach, and the distal portion of the stomach is known as the antrum. The pylorus is a muscular ring that controls the flow of ingested contents from the stomach into the first portion of the duodenum. The medial and lateral curvatures of the stomach are known as the lesser and greater curvatures, respectively. Histologically, the wall of the stomach has five layers: mucosal, submucosal, muscular, subserosal, and serosal.

In the 8th Edition, cancers crossing the EGJ with their epicenter in the proximal 2 cm of the stomach are incorporated into the esophagus chapter, whereas cancers crossing the EGJ with their epicenter in the proximal 2 to 5 cm of the stomach are addressed in the stomach chapter. All tumors in the stomach that do not cross the EGJ are classified in the stomach chapter (Figs. 17.1 and 17.2).

Regional Lymph Nodes

Several groups of regional lymph nodes drain the wall of the stomach. Perigastric nodes are found along the lesser and greater curvatures (Fig. 17.3). Other major nodal groups follow the main arterial and venous vessels from the celiac artery and its branches and the portal circulation. Adequate nodal dissection of these regional nodal areas is important to ensure appropriate designation of the N (ypN or pN) category.[5] Although it is suggested that at least 16 regional nodes be removed/assessed pathologically, removal/evaluation of more nodes ($\geq$30) is desirable.[2]

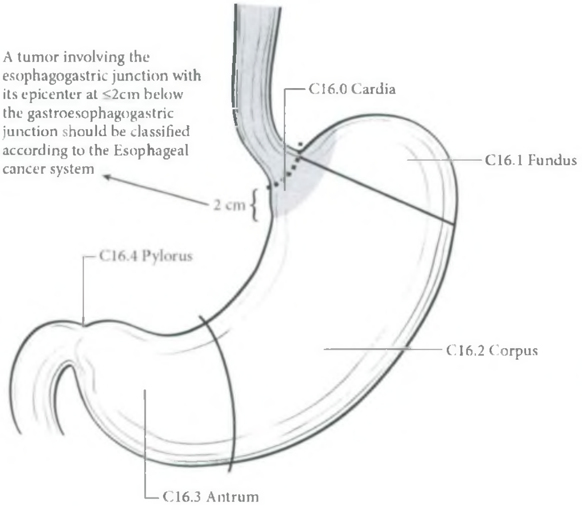

Fig. 17.1 Anatomic subsites of the stomach

17

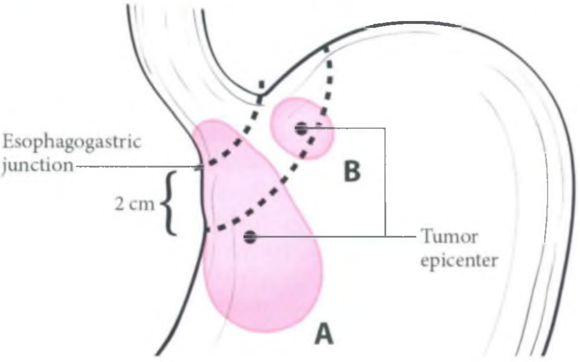

A tumor that has its epicenter located >2 cm from esophagogastric junction (A) or a tumor located within 2 cm of the esophagogastric junction (B) but does not involve the esophagogastric junction is classified as stomach cancer.

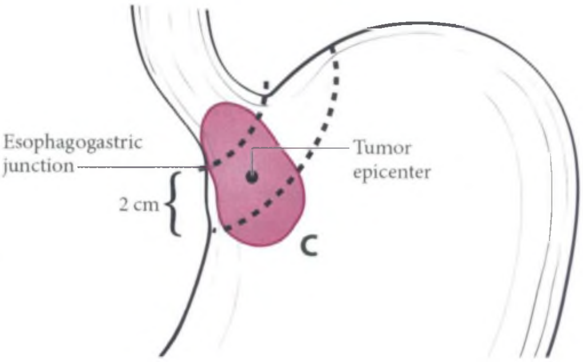

A tumor that has its epicenter located within 2 cm of esophagogastric junction and involves the esophagogatric junction (C) is classified as esophageal cancer.

Fig. 17.2 (A) EGJ tumors with their epicenter located >2 cm into the proximal stomach are staged as stomach cancers. (B) Cardia cancers not involving the EGJ are staged as stomach cancers. (C) Tumors involving the EGJ with thier epicenter <2 cm into the proximal stomach are staged as esophageal cancers

The specific regional nodal areas are as follows:

- Perigastric along the greater curvature (including greater curvature, greater omental)
- Perigastric along the lesser curvature (including lesser curvature, lesser omental)
- Right and left paracardial (cardioesophageal)
- Suprapyloric (including gastroduodenal)
- Infrapyloric (including gastroepiploic)
- Left gastric artery
- Celiac artery
- Common hepatic artery
- Hepatoduodenal (along the proper hepatic artery, including portal)
- Splenic artery
- Splenic hilum

Metastatic Sites

The most common metastatic distribution is to the liver, peritoneal surfaces, and nonregional/distant lymph nodes. Central nervous system and pulmonary metastases occur but are less frequent. Tumors found in these locations are considered metastatic disease (M1). In contrast, direct extension of bulky tumors to the liver, transverse colon, pancreas, and/or undersurface of the diaphragm is considered as tumor invading adjacent structures/organs (T4b) not M1. Positive peritoneal cytology is classified as metastatic disease (M1).

Distant Nodal Groups. Involvement of other (nonregional) intra-abdominal lymph nodes, such as the retropancreatic, pancreaticoduodenal, peripancreatic, superior mesenteric, middle colic, para-aortic, or retroperitoneal nodes, translates into a patient having metastatic disease (M1).

RULES FOR CLASSIFICATION

Clinical Classification

Designated as cTNM, clinical stage is based on evidence of extent of disease present before therapy is instituted. It includes physical examination, laboratory testing, radiologic imaging, endoscopy (possibly including endoscopic ultrasonography [EUS] with fine-needle aspiration [FNA] for cytologic assessment), and biopsy (for histologic confirmation), and may include diagnostic laparoscopy with peritoneal washings for cytologic/histologic assessment.

Clinical T Category

Staging of primary gastric adenocarcinoma depends on the depth of penetration of the primary tumor. The T1 designation is subdivided into T1a (invasion of the lamina propria or muscularis mucosae) and T1b (invasion of the submucosa). T2 is invasion of the muscularis propria, and T3 is invasion of the subserosal connective tissue without invasion of adjacent structures or the serosa (visceral peritoneum). T4 tumors penetrate the serosa (T4a) or invade adjacent structures/organs (T4b). EUS correlates highly with cT category. For

Fig. 17.3 Regional lymph nodes of the stomach

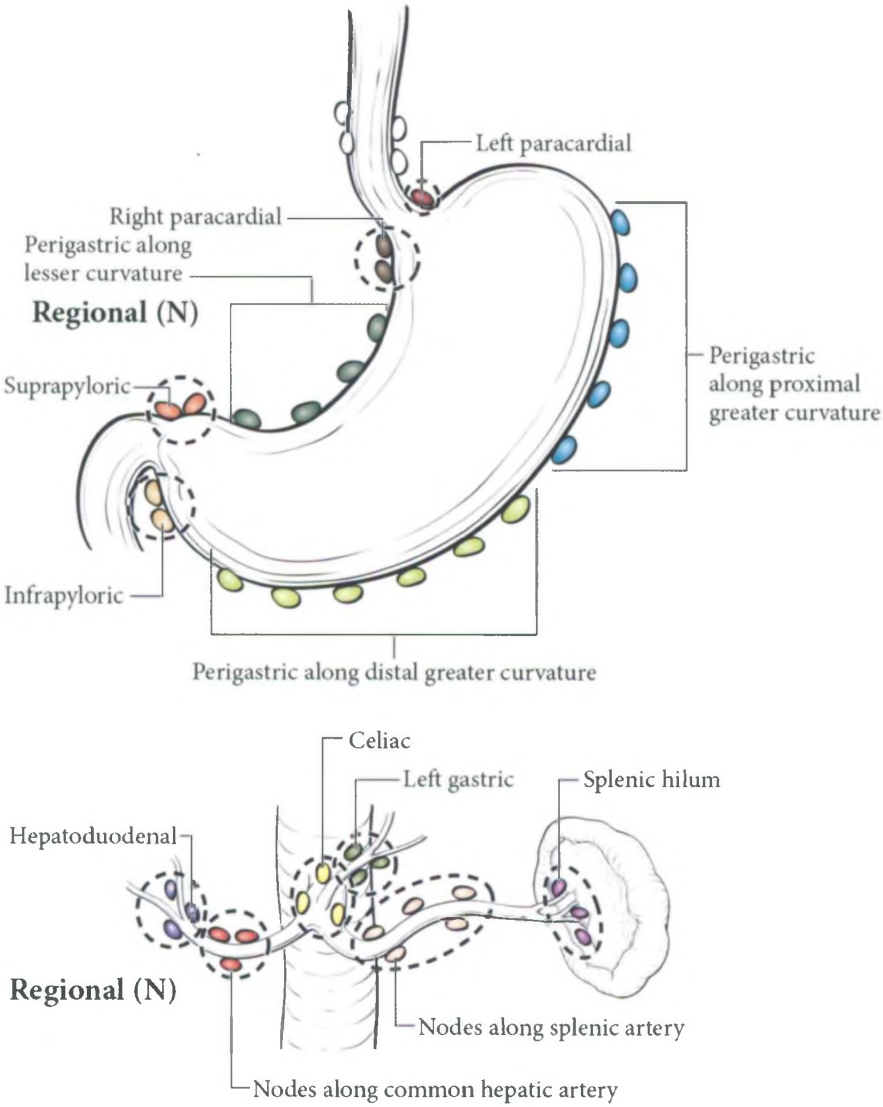

tumors close to adjacent structures, radiologic imaging and EUS may help determine whether the adjacent anatomic structures are invaded (cT4a or cT4b).

Clinical N Category

Radiologic reports should document the number of enlarged nodes (with malignant features), and a multidisciplinary tumor board should review the images to document the number of nodes that appear malignant (see clinical staging methods in the next section). EUS also may be useful in identifying enlarged or malignant-appearing lymph nodes to determine cN category and may provide an opportunity to perform FNA or biopsy for cytologic assessment).

Clinical M Category

Imaging-based detection of organ metastasis (including peritoneal) is assigned cM1. This may be confirmed with tissue diagnosis, after which pM1 is assigned for the clinical stage. Confirmation of peritoneal metastasis by diagnostic laparoscopy or peritoneal washings performed as part of the staging workup is considered positive metastasis. Specifically, gross evidence of metastasis seen during diagnostic laparoscopy is cTcNcM1, whereas positive washings obtained during diagnostic laparoscopy without evidence of gross metastasis is considered cTcNpM1, which is clinical stage IV and pathological stage IV. See the detailed discussion in Chapter 1.

Endoscopy and Imaging

EUS and computed tomography (CT) of the chest, abdomen, and pelvis with oral and intravenous contrast are the initial imaging modalities used to determine clinical primary tumor (cT), node (cN), and metastasis (cM) categories. Positron emission tomography (PET)/CT with fluorine-18 (^{18}F)-fluoro-2-deoxy-D-glucose (FDG) and magnetic resonance (MR) imaging may be used to further refine cN category and cM category in locoregionally advanced cTcN.

Endoscopic Evaluations

Clinical assessment of depth of invasion and nodal involvement, as well as some limited areas of distant disease, may be facilitated by the use of EUS. Gastric cancer staging is best performed by using commercially available ultrasound endoscopes with multifrequency (5-, 7.5-, 10-, and 12-MHz) radial transducers. Sonographic evaluation is performed as the instrument is withdrawn, starting at the pylorus. Orienting the images in an anteroposterior axis permits careful assessment of anatomic landmarks to allow correlation with the location of the tumor, nodes, and surrounding organs.

The individual layers of the gastrointestinal wall are visualized throughout the examination to correlate the extent of the tumor relative to the alternating bright and dark layers seen on ultrasound. On the basis of *in vitro* studies, the first two layers (bright and dark starting at the lumen) correspond to the superficial and deep mucosa, the third (bright) layer corresponds to the submucosa, the fourth (dark) layer to the muscularis propria, and the fifth (bright) layer to the adventitia or serosa.[6]

Alterations in the thickness of individual layers are easily and reproducibly identified, allowing one to determine the depth of tumor invasion. The presence of a mass in the stomach usually is diagnosed as a hypoechoic or dark thickening in one or more layers, or a loss of the usual layer pattern.[7] The first bright layer, which represents a transition echo layer, rarely is lost or thickened. Thickening of the second, or inner dark, layer suggests a T1 tumor. Although at higher EUS frequencies of 10 or 12 MHz one should be able to distinguish tumors limited to the mucosa (T1a) from those extending into the submucosa (T1b), some studies have shown reduced accuracy with T1 staging.[8–11] A dark thickening extending from the second to the third layer (mucosa and submucosa), but not reaching the fourth layer (muscularis propria), is evidence of a T1b tumor. A dark thickening extending to but not completely through the fourth layer with a preserved smooth outer echogenic border is associated with a T2 tumor. Complete loss of all the layers, associated with a smooth white outer layer representing the serosal surface, suggests penetration through the muscularis propria into the subserosal fat, consistent with a T3 tumor in the stomach. If there is extension of the dark wall thickening with loss of the serosal echogenic stripe, the tumor is staged T4a. The distinction between T3 and T4a by EUS may be very challenging, as the actual thickness of the subserosal fat may vary and the serosa is very thin and likely not seen clearly on EUS. Full transmural extension with loss of the echogenic stripe separating the stomach from surrounding structures, such as the aorta, pancreas, liver, or other adjacent structure, indicates a T4b tumor.

The lymphatic drainage areas routinely investigated are the lower periesophageal, right and left paracardial, lesser curve (gastrohepatic ligament), greater curve, and supra- and infrapyloric regions, and areas along major vessels, including the celiac axis, splenic and hepatic artery, porta hepatis, and splenic hilum. Opinions vary regarding the specific cutoff for what is considered malignant. The presence of hypoechoic, rounded, sharply demarcated structures >10 mm on EUS may be considered diagnostic of malignant nodes.[12] Histologic confirmation of nodal disease by EUS-guided FNA is strongly encouraged if it can be achieved without traversing an area of tumor.[13] Although not necessary for the current prognostic staging, the number of nodes remains important for future prognostic efforts; therefore, we encourage careful counting and reporting of suspicious nodes along with interpretation of clinical T category.

Parts of the liver are seen readily with EUS with the scope positioned in the antrum and along the lesser curvature and cardia, allowing identification of liver metastases (M1), which may be confirmed by EUS-FNA.[14] Similarly, the presence of ascites adjacent to the stomach raises suspicion for peritoneal metastases, if other causes of ascites are ruled out. However, this finding has not been shown to be a reliable indicator of M1 disease.[15,16]

Imaging

Primary Tumor (cT)

EUS may be ideal for cT categorization of the primary tumor. With advances in multidetector CT scanners and greater attention to improving gastric distention, together with the use of negative oral contrast, dynamic intravenous contrast enhancement, and multiplanar reformatting of images, the accuracy of cT category has improved.[17–19] In this regard, CT of the chest, abdomen, and pelvis with intravenous and oral contrast is used to describe cT in terms of location and degree of local invasion. However, overall, CT of the chest, abdomen, and pelvis still has a limited role in the cT category, especially for cT1, cT2, and cT3 tumors, although limitations also exist in identifying invasion of adjacent structures (cT4) unless gross invasion is present.[20,21] FDG PET/CT generally is not useful in cT categorization because of the normal increased background uptake of FDG in the gastric mucosa together with the general lack of FDG uptake by signet ring cells and/or poorly differentiated adenocarcinomas.[22–24] Although MR imaging has better contrast resolution than CT and has been reported in small studies to be comparable to CT, overall the imaging evaluation of cT categorization is limited.

Regional Lymph Nodes (cN)

CT of the chest and abdomen with intravenous and oral contrast and FDG PET/CT imaging are used to describe locoregional (cN) lymph nodes. In clinical practice, locoregional nodes generally are suspicious for tumor involvement if round and/or >10 mm in short axis diameter. The portocaval lymph node, however, is an exception to these criteria. This lymph node has an elongated shape with a long transverse diameter and small anterior posterior diameter, and relying on measurement alone for this node results in frequent false positive interpretations. CT and FDG PET/CT are not optimal in detecting locoregional nodal metastasis.[25–30] Additionally, the diagnostic benefit of FDG PET/CT is especially limited in patients with an early T classification (pT1) because of the low prevalence of nodal and distant metastases and the high rate of false positive PET findings.[31,32] Because the criteria for cN classification have not been rigorously defined in peer-reviewed literature, the current cN classification requires evaluation of the size, shape, and number of abnormal lymph nodes in determining the cN classification. The diagnostic benefit of FDG PET/CT in detecting nodal metastasis is of limited value because of the high rate of false negative PET findings due to its inability to detect microscopic disease and because of poor FDG uptake by signet ring cells and/or poorly differentiated adenocarcinomas.

Metastatic Disease (cM)

CT of the chest, abdomen, and pelvis with intravenous and oral contrast can detect distant metastasis (cM1). Because advanced gastric adenocarcinoma has a propensity to metastasize to the peritoneum, ovaries, and retrovesicle pouch, pelvic CT imaging is useful in cM categorization. MR imaging is used less frequently as a primary imaging modality. The addition of FDG PET/CT imaging to conventional clinical staging improves the detection of metastases missed or not visualized on CT of the chest, abdomen, and pelvis.

Diagnostic Laparoscopy and Peritoneal Washing Evaluations

Diagnostic peritoneal staging laparoscopy is used to identify occult metastates not detected by imaging (or by EUS) and is recommended for all patients with tumor depth cT3 or greater, or in the presence of clinically suspicious nodes in the absence of distant metastases on imaging (CT or PET). It generally is performed as a separate surgical procedure and allows detection of gross metastases on the peritoneal surface and visceral organs as well as of microscopic malignant cells in the washings. Clinical studies have found positive peritoneal cytology to be an independent prognosticator,[33,34] and this finding is considered pM1 for both the clinical and pathological classification M categories, resulting in clinical Stage IV and pathological Stage IV.

Peritoneal washing generally involves instilling ~200 mL of normal saline into the different quadrants of the abdominal cavity. The fluid is dispersed by gentle stirring of the area and then is aspirated from different portions of the body. Areas typically include the right and left subphrenic space and the pouch of Douglas. Ideally, >50 mL of washings should be retrieved for cytologic assessment.

Residual Disease (R Classification)

Assessment of the R classification applies only to a surgically resected specimen. In addition to proximal and distal margins of resection, the status of the radial or circumferential margin of resection determines whether the tumor was excised completely. The R classification is based on a combination of intraoperative assessment by the surgeon and pathological evaluation of the resected specimen. R0 indicates no evidence of residual tumor. R1 indicates the presence of microscopic tumor at the margins, as defined by the College of American Pathologists (CAP); however, the Royal College of Pathologists (RCP) definition of R1 includes tumors within a 1-mm margin. Macroscopically visible tumor at margins is classified as R2. The presence of tumor cells at the inked radial margin constitutes a positive margin according to CAP criteria.

Early malignant lesions removed endoscopically by either an endoscopic submucosal dissection or an endoscopic mucosal resection should be assessed for pT designation along with the assessment of deep and lateral margins. The pathological assessment also should include the presence/absence of lymphovascular and/or perineural invasion, maximum tumor diameter, and the final histologic grade (including the presence of high-grade dysplasia). This information may assist in making future therapeutic decisions, including surveillance strategies.

Pathological Classification

Pathological staging depends on data acquired clinically, together with findings from subsequent surgical exploration and examination of resected tissue (specimen). Surgical resections that qualify for pathological staging include total, near-total, subtotal, partial, proximal, and distal gastrectomy and antrectomy.

Primary Tumor (pT)

Depth of tumor invasion should be based on examination of the surgical specimen after resection. Tumor distance from closest margin or involvement of margin should be documented. Histologic classification, as well as grade and presence of lymphovascular or perineural invasion, should be documented according to CAP guidelines. The specimen should be tested for the presence of *Helicobacter pylori* infection.

Regional Lymph Nodes (pN)

Pathological assessment of regional lymph nodes entails the removal and histologic examination of nodes to evaluate the total number removed, as well as how many contain tumor. The number of nodes, as well as the number of positive nodes, should be documented.

Metastatic tumor deposits in the subserosal fat adjacent to a gastric carcinoma, without evidence of residual lymph node tissue, are considered regional lymph node metastases for purposes of gastric cancer staging. Tumor deposits are defined as discrete tumor nodules within the lymph drainage area of the primary carcinoma without identifiable lymph node tissue or identifiable vascular or neural structure. Shape, contour, and size of the deposit are not considered in these designations.

Metastatic Disease (pM)

Pathologically confirmed metastatic tissue obtained from a site outside of what is considered local or regional for gastric cancer is considered pM1. This designation includes tumor identified in distant nodal stations in the surgical resection specimen and tissue samples obtained from other organs (including peritoneum) showing malignant cells in peritoneal washings or implants.

When recording pathological stage, clinical stage M category (cM) may be used for final pathological Stage IV, such as pTpNcM0–1.

Post–Neoadjuvant Therapy Classification

Although grading systems for tumor response have not been established for this disease, response of tumor to preoperative (neoadjuvant) therapy should be reported using the post–neoadjuvant therapy classification staging system. The assessment of pathological response to neoadjuvant therapy involves both the gross and the microscopic examination of the resected surgical specimen. At the microscopic level, a positive treatment-related effect is observed as abolition of the malignant epithelium and replacement by dense fibrosis or fibroinflammation. The pathological response to treatment is determined by the amount of residual viable carcinoma in relation to areas of fibrosis or fibroinflammation within the gross lesion. This relationship may be expressed as the inverse percentage of a favorable treatment response. Thus, a 100% treatment response indicates fibrosis or fibroinflammation within an entire gross lesion, without microscopic evidence of carcinoma, whereas a 0% response represents an unaffected tumor in the absence of any fibrosis or fibroinflammation. The presence of residual tumor cells suggests an incomplete response. Acellular mucin is regarded as a form of positive treatment response, not as residual tumor. The ypT category of the residual carcinoma is based on the deepest focus of residual malignant epithelium of the gastric wall. Positive lymph nodes are defined as having at least one focus of residual tumor cells in the lymph nodes.[35] The pathology report should include ypT and ypN based on the submitted specimen. If no further diagnostic tests are performed after neoadjuvant therapy, the M designation should remain the same as cM. If further diagnostic tests are performed after neoadjuvant therapy, then ypM also should reflect these new tests. Pathologically confirmed metastatic tissue obtained from a site outside of what is considered local or regional for gastric cancer after neoadjuant therapy is considered ypM1.

PROGNOSTIC FACTORS

Prognostic Factors Required for Stage Grouping

Beyond the factors used to assign T, N, or M categories, no additional prognostic factors are required for stage grouping.

Additional Factors Recommended for Clinical Care

Carcinoembryonic Antigen

Elevated carcinoembryonic antigen (CEA) levels are not shown to have independent prognostic value. Treatment decisions should not be altered based on baseline levels of CEA. However, monitoring of CEA levels may be useful in the surveillance period. AJCC Level of Evidence: III

Cancer Antigen 19-9

Elevated cancer antigen (CA) 19-9 levels are not shown to have independent prognostic value. Treatment decisions should not be altered based on baseline levels of CA 19-9. However, monitoring of CA 19-9 levels may be useful in the surveillance period. AJCC Level of Evidence: III

HER2

The biomarker HER2 is examined directly on tumor tissue. There are mixed reports regarding the prognostic value of this biomarker; it is not shown to be independently prognostic. If the tumor is HER2 positive, HER2-directed therapy should be considered. AJCC Level of Evidence: III

Microsatellite Instabiity

Microsatellite instability (MSI) is examined directly on the tumor tissue. The level of evidence is based on a limited number of patients. Patients with high MSI (MSI-H) tend to have a better overall prognosis. However, the independent prognostic value of MSI-H is not yet established. AJCC Level of Evidence: III

RISK ASSESSMENT MODELS

The AJCC recently established guidelines that will be used to evaluate published statistical prediction models for the purpose of granting endorsement for clinical use.[36] Although this is a monumental step toward the goal of precision medicine, this work was published only very recently. Therefore, the existing models that have been published or may be in clinical use have not yet been evaluated for this cancer site by the Precision Medicine Core of the AJCC. In the future, the statistical prediction models for this cancer site will be evaluated, and those that meet all AJCC criteria will be endorsed.

DEFINITIONS OF AJCC TNM

Definition of Primary Tumor (T)

T Category	T Criteria
TX	Primary tumor cannot be assessed
T0	No evidence of primary tumor
Tis	Carcinoma *in situ*: intraepithelial tumor without invasion of the lamina propria, high-grade dysplasia
T1	Tumor invades the lamina propria, muscularis mucosae, or submucosa
T1a	Tumor invades the lamina propria or muscularis mucosae
T1b	Tumor invades the submucosa
T2	Tumor invades the muscularis propria*
T3	Tumor penetrates the subserosal connective tissue without invasion of the visceral peritoneum or adjacent structures**,***
T4	Tumor invades the serosa (visceral peritoneum) or adjacent structures **,***
T4a	Tumor invades the serosa (visceral peritoneum)
T4b	Tumor invades adjacent structures/organs

* A tumor may penetrate the muscularis propria with extension into the gastrocolic or gastrohepatic ligaments, or into the greater or lesser omentum, without perforation of the visceral peritoneum covering these structures. In this case, the tumor is classified as T3. If there is perforation of the visceral peritoneum covering the gastric ligaments or the omentum, the tumor should be classified as T4.
** The adjacent structures of the stomach include the spleen, transverse colon, liver, diaphragm, pancreas, abdominal wall, adrenal gland, kidney, small intestine, and retroperitoneum.
*** Intramural extension to the duodenum or esophagus is not considered invasion of an adjacent structure, but is classified using the depth of the greatest invasion in any of these sites.

Definition of Regional Lymph Node (N)

N Category	N Criteria
NX	Regional lymph node(s) cannot be assessed
N0	No regional lymph node metastasis
N1	Metastasis in one or two regional lymph nodes
N2	Metastasis in three to six regional lymph nodes
N3	Metastasis in seven or more regional lymph nodes
N3a	Metastasis in seven to 15 regional lymph nodes
N3b	Metastasis in 16 or more regional lymph nodes

Definition of Distant Metastasis (M)

M Category	M Criteria
M0	No distant metastasis
M1	Distant metastasis

AJCC PROGNOSTIC STAGE GROUPS

Clinical (cTNM)

When T is...	And N is...	And M is...	Then the stage group is...
Tis	N0	M0	0
T1	N0	M0	I
T2	N0	M0	I
T1	N1, N2, or N3	M0	IIA
T2	N1, N2, or N3	M0	IIA
T3	N0	M0	IIB
T4a	N0	M0	IIB
T3	N1, N2, or N3	M0	III
T4a	N1, N2, or N3	M0	III
T4b	Any N	M0	IVA
Any T	Any N	M1	IVB

Pathological (pTNM)

When T is...	And N is...	And M is...	Then the stage group is...
Tis	N0	M0	0
T1	N0	M0	IA
T1	N1	M0	IB
T2	N0	M0	IB
T1	N2	M0	IIA
T2	N1	M0	IIA
T3	N0	M0	IIA
T1	N3a	M0	IIB
T2	N2	M0	IIB
T3	N1	M0	IIB
T4a	N0	M0	IIB
T2	N3a	M0	IIIA
T3	N2	M0	IIIA
T4a	N1	M0	IIIA
T4a	N2	M0	IIIA
T4b	N0	M0	IIIA
T1	N3b	M0	IIIB
T2	N3b	M0	IIIB

When T is...	And N is...	And M is...	Then the stage group is...
T3	N3a	M0	IIIB
T4a	N3a	M0	IIIB
T4b	N1	M0	IIIB
T4b	N2	M0	IIIB
T3	N3b	M0	IIIC
T4a	N3b	M0	IIIC
T4b	N3a	M0	IIIC
T4b	N3b	M0	IIIC
Any T	Any N	M1	IV

Post-Neoadjuvant Therapy (ypTNM)

When T is...	And N is...	And M is...	Then the stage group is...
T1	N0	M0	I
T2	N0	M0	I
T1	N1	M0	I
T3	N0	M0	II
T2	N1	M0	II
T1	N2	M0	II
T4a	N0	M0	II
T3	N1	M0	II
T2	N2	M0	II
T1	N3	M0	II
T4a	N1	M0	III
T3	N2	M0	III
T2	N3	M0	III
T4b	N0	M0	III
T4b	N1	M0	III
T4a	N2	M0	III
T3	N3	M0	III
T4b	N2	M0	III
T4b	N3	M0	III
T4a	N3	M0	III
Any T	Any N	M1	IV

REGISTRY DATA COLLECTION VARIABLES

1. Tumor location (needed because C16.0 is both cardia and EGJ)
2. Serum CEA
3. Serum CA 19-9
4. Clinical staging modalities (endoscopy and biopsy, EUS, EUS-FNA, CT, PET/CT)
5. Tumor length
6. Depth of invasion
7. Number of suspicious malignant lymph nodes on baseline radiologic images
8. Number of suspicious malignant lymph nodes by EUS assessment
9. Location of suspicious nodes (clinical)
10. Location of malignant nodes (pathological)
11. Number of tumor deposits
12. Lymphovascular invasion
13. Neural invasion
14. Extranodal extension
15. HER2 status (positive or negative)
16. MSI
17. Surgical margin (negative, microscopic, macroscopic)
18. Sites of metastasis, if applicable
19. Type of surgery

HISTOLOGIC GRADE (G)

G	G Definition
GX	Grade cannot be assessed
G1	Well differentiated
G2	Moderately differentiated
G3	Poorly differentiated, undifferentiated

HISTOPATHOLOGIC TYPE

The staging system applies to all primary carcinomas that arise in the stomach, but it excludes sarcomas, lymphomas, and neuroendocrine tumors. Adenocarcinoma is the most common histologic type, whereas other histologic types are observed less frequently.

Adenocarcinomas may be divided into general subtypes. In addition, the histologic terms *intestinal*, *diffuse*, and *mixed* may be applied. Mixed glandular/neuroendocrine carcinomas should be staged using the gastric carcinoma staging system described in this chapter, not the staging system for well-differentiated neuroendocrine tumors of the stomach.

SURVIVAL DATA

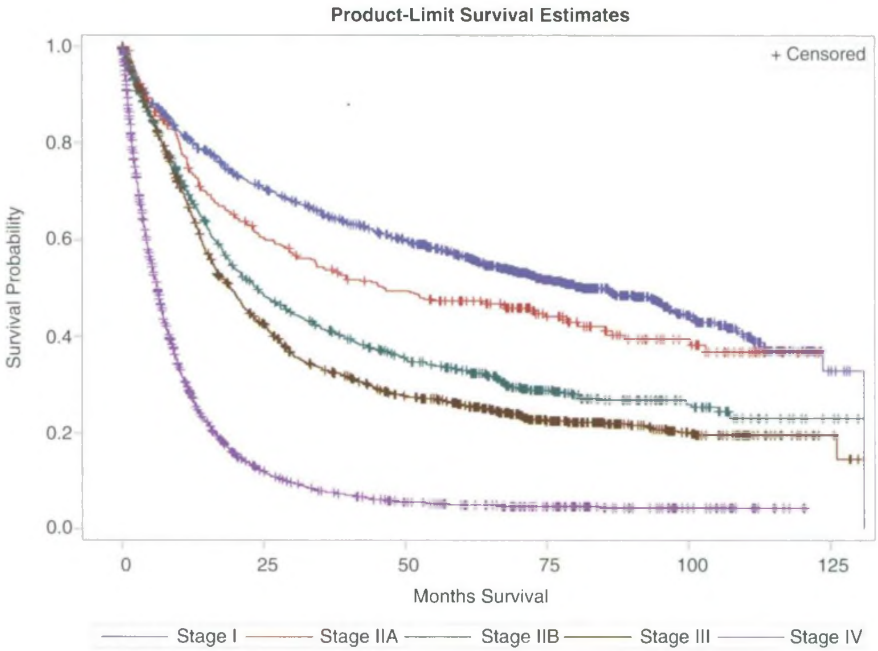

Fig. 17.4 Clinical stage (cTNM) and overall survival in patients diagnosed with gastric cancer, stratified by clinical stage groupings, based on NCDB data (2004–2008; median follow-up, 12 months; $n = 7{,}306$)

Table 17.1 Clinical stage and 1-, 3-, and 5-year and median overall survivals in patients diagnosed with gastric cancer, stratified by clinical stage groupings, based on NCDB data

Clinical stage group	Patients, n	1-y Survival, %	3-y Survival, %	5-y Survival, %	Median survival, mo
I (T1/2, N0)	1,418	80.6	64.9	56.7	84.93
IIA (T1/2, N+)	296	74.2	53.7	47.3	46.06
IIB (T3/T4a, N0)	783	68.9	41.4	33.1	23.82
III (T3/T4a, N+)	1,427	66.4	33.1	25.9	19.12
IV (T4b & M+)	3,382	28.3	7.8	5.0	6.24

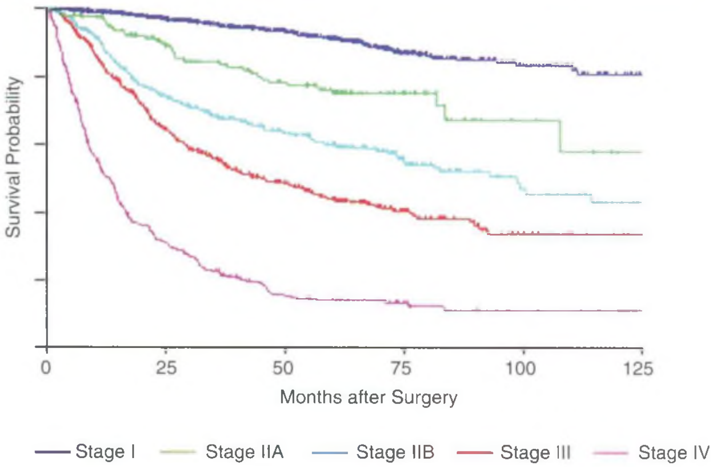

Fig. 17.5 Clinical stage and overall survival in patients with gastric cancer who received curative or palliative surgery, stratified by clinical stage groupings, based on Shizuoka Cancer Center data (2002–2015; median follow-up, 47 months; n=4,091)

Table 17.2 Clinical stage and 1-, 3-, and 5-year and median overall survivals in patients with gastric cancer who received curative or palliative surgery, stratified by clinical stage groupings, based on Shizuoka Cancer Center data

Clinical stage group	Patients, n	1-y Survival, %	3-y Survival, %	5-y Survival, %	Median survival
I (T1/2, N0)	2,318	98.9	95.0	90.2	Not reached
IIA (T1/2, N+)	161	96.8	83.6	75.2	Not reached
IIB (T3/T4a, N0)	566	87.8	67.7	59.3	98.73 mo
III (T3/T4a, N+)	758	82.9	55.1	43.4	45.07 mo
IV (T4b & M+)	288	51.7	22.1	14.1	13.3 mo

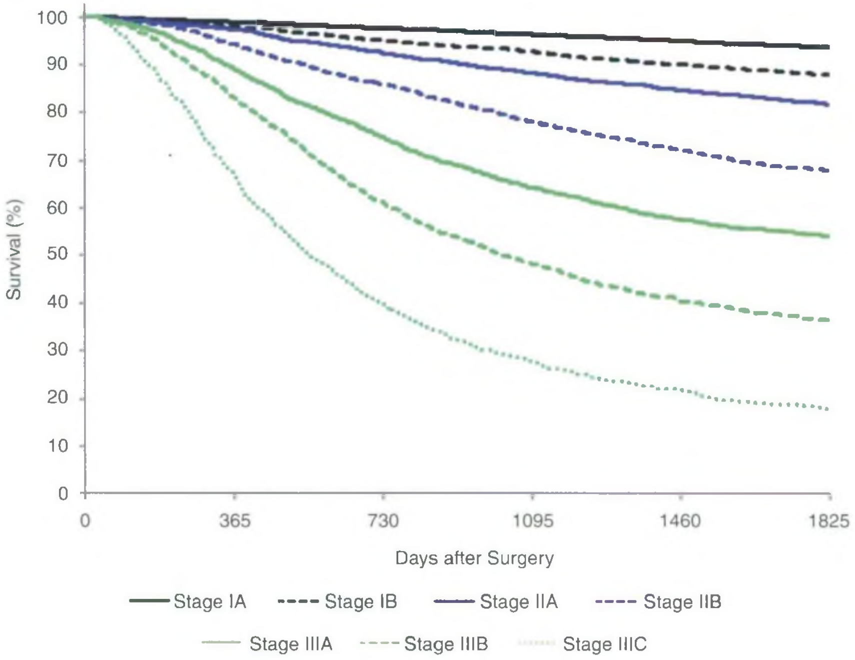

Fig. 17.6 Pathological stage (pTNM) and overall survival in gastric cancer patients who underwent surgical resection with adequate lymphadenectomy (D2) without prior chemotherapy or radiation therapy, stratified by pathological stage groupings, based on IGCA data (2000–2004; only patients with complete 5-year follow-up were included, $n=25{,}411$)

Table 17.3 Pathological stage and 1-, 3-, and 5-year and median overall survivals in patients with gastric cancer who received curative surgery, stratified by pathological stage groupings, based on IGCA data[3]

Pathological stage group	Patients, n	1-y Survival, %	3-y Survival, %	5-y Survival, %	Median survival
IA	10,606	99	96.30	93.60	Not reached
IB	2,606	98	92.80	88	Not reached
IIA	2,291	97.40	88.30	81.80	Not reached
IIB	2,481	94.30	78.20	68	Not reached
IIIA	3,044	89	64.40	54.20	Not reached
IIIB	2,218	83.10	48.20	36.20	32.8 mo
IIIC	1,350	66.80	27.70	17.90	18.5 mo

17

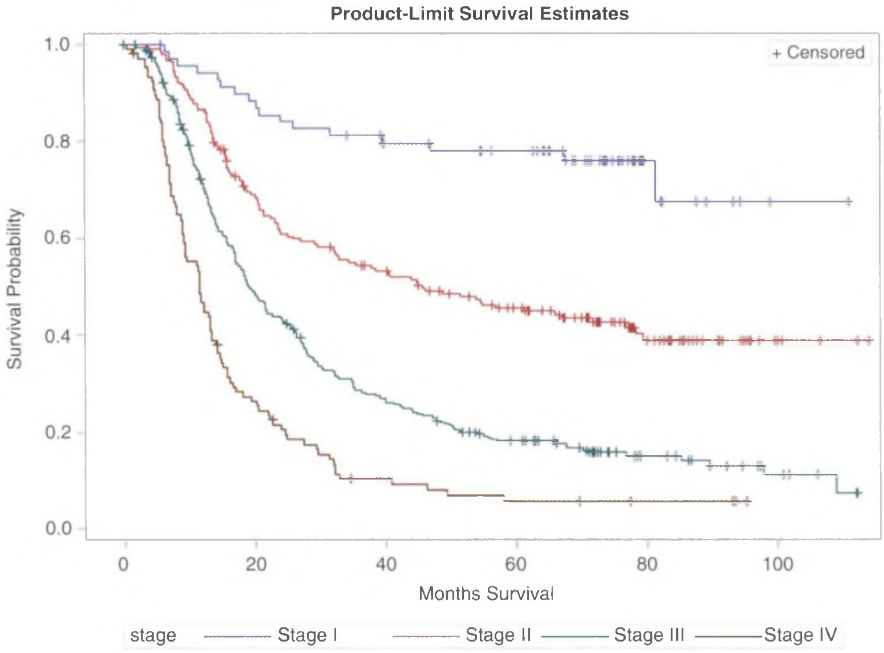

Fig. 17.7 Post–neoadjuvant therapy stage (ypTNM) and overall survival in patients who underwent surgical resection and were given chemotherapy and/or radiation therapy before surgery, stratified by ypStage groupings, based on NCDB data (2004–2008; median follow-up, 23 months; $n=683$)

Table 17.4 Post–neoadjuvant therapy stage (ypTNM) and 1-, 3-, and 5-year and median overall survivals in patients with gastric cancer, stratified by ypStage groupings, based on NCDB data

Posttreatment stage group	Patients, n	1-y Survival, %	3-y Survival, %	5-y Survival, %	Median survival, mo
I	70	94.3	81.4	76.5	117.8
II	195	86.7	54.8	46.3	46.0
III	301	71.7	28.8	18.3	19.2
IV	117	46.7	10.2	5.7	11.6

ILLUSTRATIONS

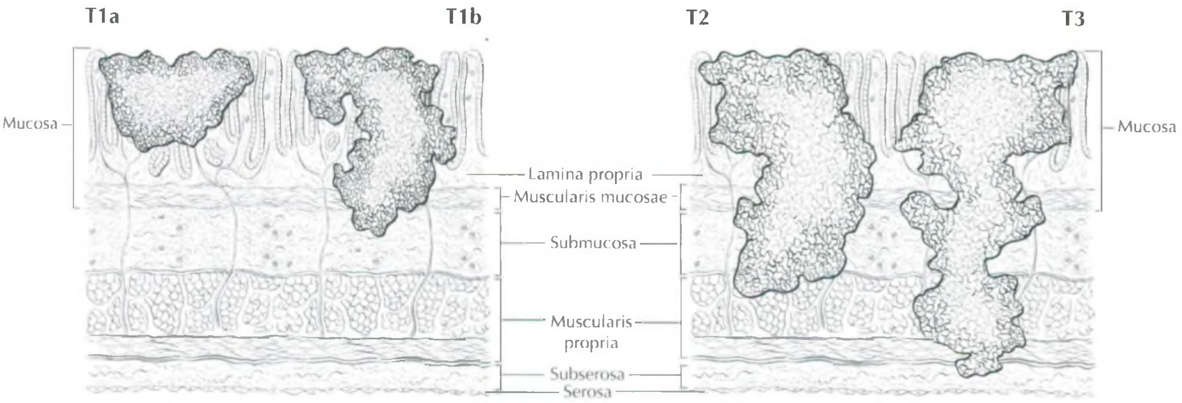

Fig. 17.8 T1a is defined as tumor that invades the lamina propria. T1b is defined as tumor that invades the submucosa. T2 is defined as tumor that invades the muscularis propria, whereas T3 is defined as tumor that extends through the muscularis propria into the subserosal tissue

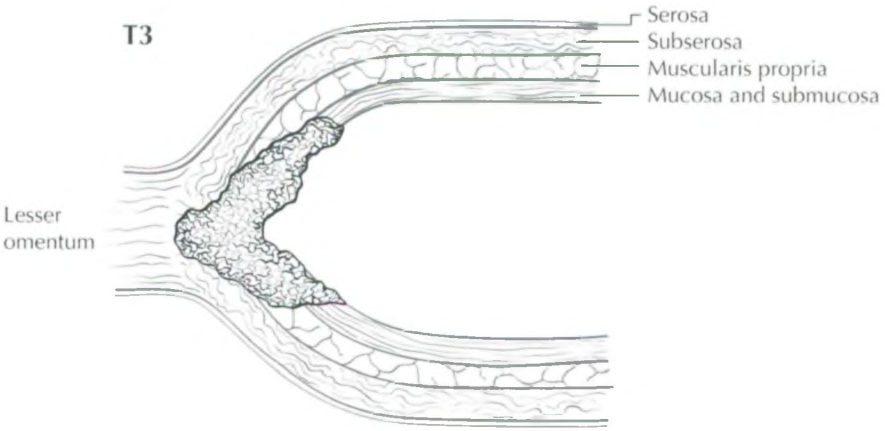

Fig. 17.9 T3 is defined as tumor that invades the subserosa, shown here invading the lesser omentum without involvement of the serosa (visceral peritoneum)

17

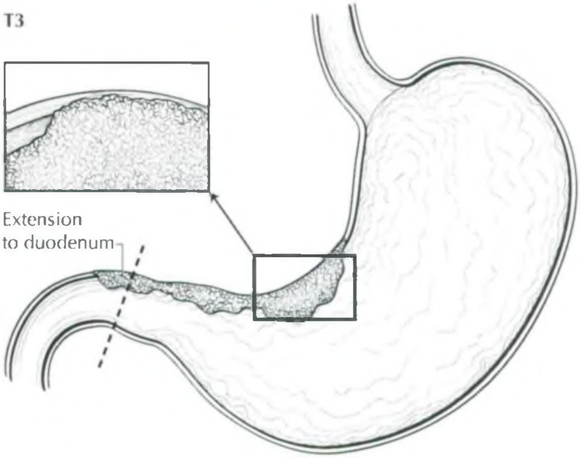

Fig. 17.10 Distal extension to duodenum does not affect the T3 category

Fig. 17.11 T4a is defined as tumor that penetrates the serosa (visceral peritoneum) without invasion of adjacent structures

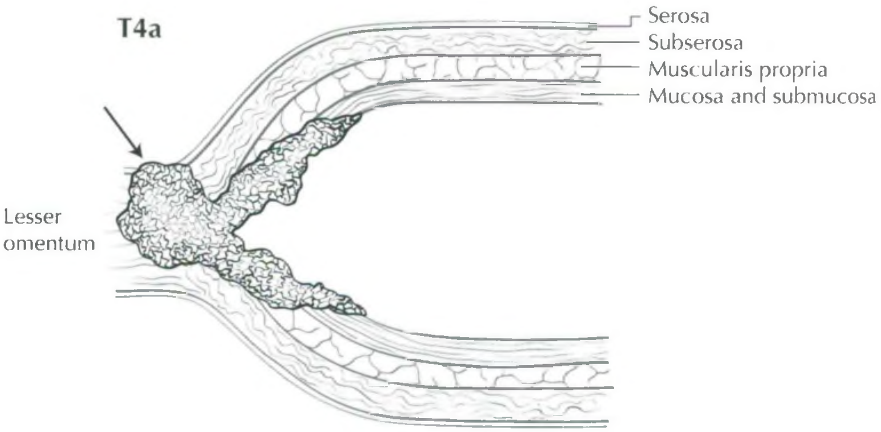

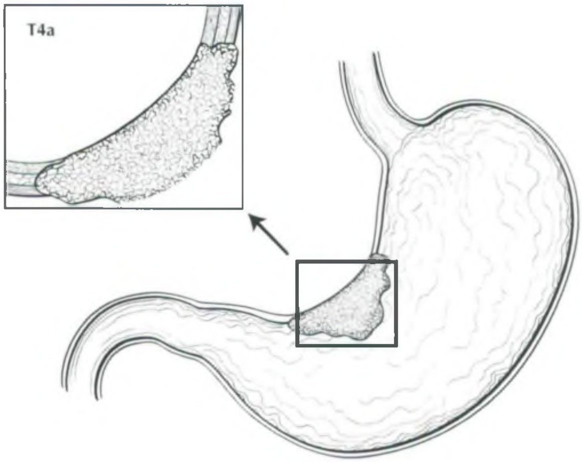

Fig. 17.12 T4a is defined as tumor that penetrates the serosa (visceral peritoneum) without invasion of adjacent structures

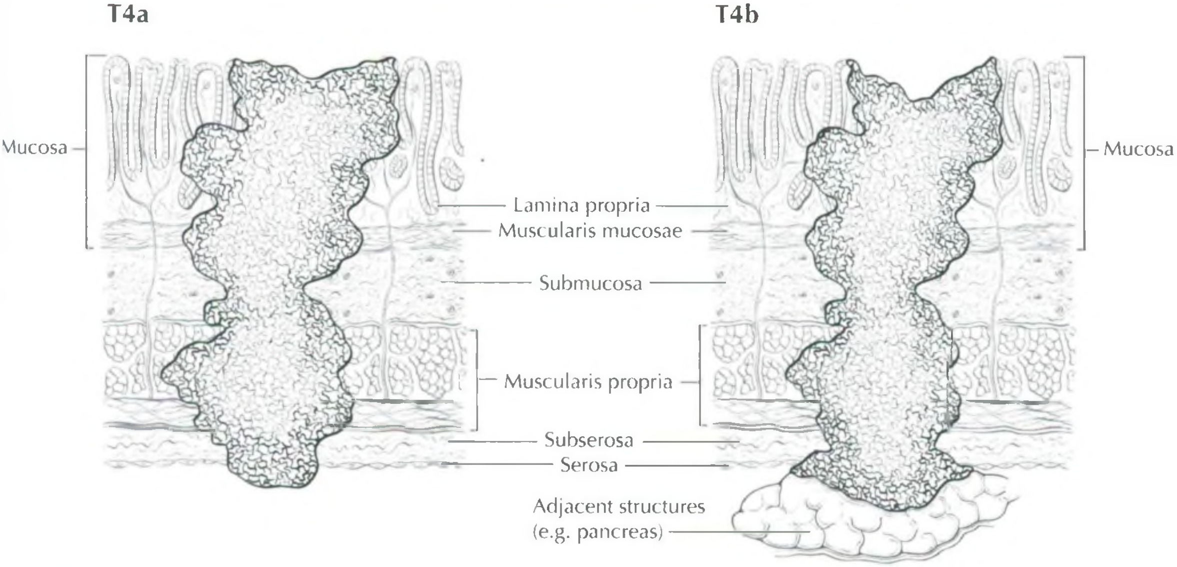

Fig. 17.13 T4a is defined as tumor that penetrates the serosa (visceral peritoneum) without invasion of adjacent structures, whereas T4b is defined as tumor that radially invades adjacent structures, shown here invading the pancreas

Bibliography

1. Torre LA, Bray F, Siegel RL, Ferlay J, Lortet-Tieulent J, Jemal A. Global cancer statistics, 2012. *CA: a cancer journal for clinicians.* Mar 2015;65(2):87–108.
2. Ajani JA, Bentrem DJ, Besh S, et al. Gastric cancer, version 2.2013: featured updates to the NCCN Guidelines. *Journal of the National Comprehensive Cancer Network : JNCCN.* May 1 2013;11(5):531–546.
3. Sano T, Coit DG, Kim HH, et al. Proposal of a new stage grouping of gastric cancer for TNM classification: International Gastric Cancer Association staging project. *Gastric cancer : official journal of the International Gastric Cancer Association and the Japanese Gastric Cancer Association.* Feb 20 2016.
4. Choi AH, Kim J, Chao J. Perioperative chemotherapy for resectable gastric cancer: MAGIC and beyond. *World journal of gastroenterology : WJG.* Jun 28 2015;21(24):7343–7348.
5. Association JGC. Japanese gastric cancer treatment guidelines 2010 (ver. 3). *Gastric cancer : official journal of the International Gastric Cancer Association and the Japanese Gastric Cancer Association.* 2011;14(2):113–123.
6. Kimmey MB, Martin RW, Haggitt RC, Wang KY, Franklin DW, Silverstein FE. Histologic correlates of gastrointestinal ultrasound images. *Gastroenterology.* Feb 1989;96(2 Pt 1):433–441.
7. Botet JF, Lightdale CJ, Zauber AG, et al. Preoperative staging of gastric cancer: comparison of endoscopic US and dynamic CT. *Radiology.* Nov 1991;181(2):426–432.
8. Barbour AP, Rizk NP, Gerdes H, et al. Endoscopic ultrasound predicts outcomes for patients with adenocarcinoma of the gastroesophageal junction. *Journal of the American College of Surgeons.* Oct 2007;205(4):593–601.
9. Blackshaw G, Lewis WG, Hopper AN, et al. Prospective comparison of endosonography, computed tomography, and histopathological stage of junctional oesophagogastric cancer. *Clin Radiol.* Oct 2008;63(10):1092–1098.
10. Murata Y, Napoleon B, Odegaard S. High-frequency endoscopic ultrasonography in the evaluation of superficial esophageal cancer. *Endoscopy.* May 2003;35(5):429–435; discussion 436.
11. Mocellin S, Pasquali S. Diagnostic accuracy of endoscopic ultrasonography (EUS) for the preoperative locoregional staging of primary gastric cancer. *Cochrane Database Syst Rev.* 2015;2:CD009944.
12. Catalano MF, Sivak MV, Jr., Rice T, Gragg LA, Van Dam J. Endosonographic features predictive of lymph node metastasis. *Gastrointestinal endoscopy.* Jul-Aug 1994;40(4):442–446.
13. Puli SR, Reddy JB, Bechtold ML, Antillon D, Ibdah JA, Antillon MR. Staging accuracy of esophageal cancer by endoscopic ultrasound: a meta-analysis and systematic review. *World journal of gastroenterology : WJG.* Mar 14 2008;14(10):1479–1490.
14. tenBerge J, Hoffman BJ, Hawes RH, et al. EUS-guided fine needle aspiration of the liver: indications, yield, and safety based on an international survey of 167 cases. *Gastrointestinal endoscopy.* Jun 2002;55(7):859–862.
15. Lee YT, Ng EK, Hung LC, et al. Accuracy of endoscopic ultrasonography in diagnosing ascites and predicting peritoneal metastases in gastric cancer patients. *Gut.* Nov 2005;54(11):1541–1545.
16. Sultan J, Robinson S, Hayes N, Griffin SM, Richardson DL, Preston SR. Endoscopic ultrasonography-detected low-volume ascites as a predictor of inoperability for oesophagogastric cancer. *The British journal of surgery.* Sep 2008;95(9):1127–1130.
17. Chen CY, Hsu JS, Wu DC, et al. Gastric cancer: preoperative local staging with 3D multi-detector row CT–correlation with surgical and histopathologic results. *Radiology.* Feb 2007;242(2):472–482.
18. Park HS, Lee JM, Kim SH, et al. Three-dimensional MDCT for preoperative local staging of gastric cancer using gas and water distention methods: a retrospective cohort study. *AJR. American journal of roentgenology.* Dec 2010;195(6):1316–1323.
19. Anzidei M, Napoli A, Zaccagna F, et al. Diagnostic performance of 64-MDCT and 1.5-T MRI with high-resolution sequences in the T staging of gastric cancer: a comparative analysis with histopathology. *Radiol Med.* Oct 2009;114(7):1065–1079.

20. Kim YH, Lee KH, Park SH, et al. Staging of T3 and T4 gastric carcinoma with multidetector CT: added value of multiplanar reformations for prediction of adjacent organ invasion. *Radiology*. Mar 2009;250(3):767–775.
21. Fujikawa H, Yoshikawa T, Hasegawa S, et al. Diagnostic value of computed tomography for staging of clinical T1 gastric cancer. *Annals of surgical oncology*. Sep 2014;21(9):3002–3007.
22. Yun M, Lim JS, Noh SH, et al. Lymph node staging of gastric cancer using (18)F-FDG PET: a comparison study with CT. *Journal of nuclear medicine : official publication, Society of Nuclear Medicine*. Oct 2005;46(10):1582–1588.
23. Chen J, Cheong JH, Yun MJ, et al. Improvement in preoperative staging of gastric adenocarcinoma with positron emission tomography. *Cancer*. Jun 1 2005;103(11):2383–2390.
24. Namikawa T, Okabayshi T, Nogami M, Ogawa Y, Kobayashi M, Hanazaki K. Assessment of (18)F-fluorodeoxyglucose positron emission tomography combined with computed tomography in the preoperative management of patients with gastric cancer. *International journal of clinical oncology*. Aug 2014;19(4):649–655.
25. Kato H, Kuwano H, Nakajima M, et al. Comparison between positron emission tomography and computed tomography in the use of the assessment of esophageal carcinoma. *Cancer*. Feb 15 2002;94(4):921–928.
26. Lowe VJ, Booya F, Fletcher JG, et al. Comparison of positron emission tomography, computed tomography, and endoscopic ultrasound in the initial staging of patients with esophageal cancer. *Molecular imaging and biology : MIB : the official publication of the Academy of Molecular Imaging*. Nov-Dec 2005;7(6):422–430.
27. Takizawa K, Matsuda T, Kozu T, et al. Lymph node staging in esophageal squamous cell carcinoma: a comparative study of endoscopic ultrasonography versus computed tomography. *J Gastroenterol Hepatol*. Oct 2009;24(10):1687–1691.
28. Choi J, Kim SG, Kim JS, Jung HC, Song IS. Comparison of endoscopic ultrasonography (EUS), positron emission tomography (PET), and computed tomography (CT) in the preoperative locoregional staging of resectable esophageal cancer. *Surg Endosc*. Jun 2010;24(6):1380–1386.
29. Walker AJ, Spier BJ, Perlman SB, et al. Integrated PET/CT fusion imaging and endoscopic ultrasound in the pre-operative staging and evaluation of esophageal cancer. *Molecular imaging and biology : MIB : the official publication of the Academy of Molecular Imaging*. Feb 2011;13(1):166–171.
30. Findlay JM, Bradley KM, Maile EJ, et al. Pragmatic staging of oesophageal cancer using decision theory involving selective endoscopic ultrasonography, PET and laparoscopy. *The British journal of surgery*. Nov 2015;102(12):1488–1499.
31. Cuellar SL, Carter BW, Macapinlac HA, et al. Clinical staging of patients with early esophageal adenocarcinoma: does FDG-PET/CT have a role? *J Thorac Oncol*. Aug 2014;9(8):1202–1206.
32. Little SG, Rice TW, Bybel B, et al. Is FDG-PET indicated for superficial esophageal cancer? *European journal of cardio-thoracic surgery : official journal of the European Association for Cardio-thoracic Surgery*. May 2007;31(5):791–796.
33. Shiozaki H, Elimova E, Slack RS, et al. Prognosis of gastric adenocarcinoma patients with various burdens of peritoneal metastases. *Journal of surgical oncology*. 2016;113(1):29–35.
34. Mezhir JJ, Shah MA, Jacks LM, Brennan MF, Coit DG, Strong VE. Positive peritoneal cytology in patients with gastric cancer: natural history and outcome of 291 patients. *Annals of surgical oncology*. Dec 2010;17(12):3173–3180.
35. Mansour JC, Tang L, Shah M, et al. Does graded histologic response after neoadjuvant chemotherapy predict survival for completely resected gastric cancer? *Annals of surgical oncology*. 2007;14(12):3412–3418.
36. Kattan MW, Hess KR, Amin MB, et al. American Joint Committee on Cancer acceptance criteria for inclusion of risk models for individualized prognosis in the practice of precision medicine. *CA: a cancer journal for clinicians*. Jan 19 2016.

Small Intestine

18

Daniel G. Coit, David Kelsen, Laura H. Tang,
Jeremy J. Erasmus, Hans Gerdes, and Wayne L. Hofstetter

CHAPTER SUMMARY

Cancers Staged Using This Staging System

Carcinomas of the nonampullary duodenum, jejunum, and ileum. Only adenocarcinomas are assigned a stage group.

Cancers Not Staged Using This Staging System

These histopathologic types of cancer…	Are staged according to the classification for…	And can be found in chapter…
Well-differentiated neuroendocrine tumor (carcinoid)	Neuroendocrine tumors of the duodenum and ampulla of Vater	30
Well-differentiated neuroendocrine tumor (carcinoid)	Neuroendocrine tumors of the jejunum and ileum	31
Visceral sarcoma	Soft tissue sarcoma of the abdomen and thoracic visceral organs	42
Gastrointestinal stromal tumors	Gastrointestinal stromal tumors	43
Lymphoma	Hodgkin and non-Hodgkin lymphomas	79

Summary of Changes

Change	Details of Change	Level of Evidence
Definition of Primary Tumor (T)	For T3 and T4, the description of extent of penetration into the retroperitoneum was omitted. It is not reliably reported in the pathology assessment and is not a validated prognostic factor.	IV
Definition of Regional Lymph Node (N)	N1 was redefined as one or two positive nodes and N2 as more than two positive nodes. This change harmonizes N1 staging with the rest of the upper gastrointestinal tumors and provides improved stage-specific discrimination based on a new National Cancer Data Base query.	III
AJCC Prognostic Stage Groups	All histologies are assigned TNM, but prognostic stage grouping is only for adenocarcinoma.	III

To access the AJCC cancer staging forms, please visit www.cancerstaging.org.

© American Joint Committee on Cancer 2017

M.B. Amin et al. (eds.), *AJCC Cancer Staging Manual, Eighth Edition*, DOI 10.1007/978-3-319-40618-3_18

ICD-O-3 Topography Codes

Code	Description
C17.0	Duodenum
C17.1	Jejunum
C17.2	Ileum
C17.8	Overlapping lesion of small intestine
C17.9	Small intestine, NOS

WHO Classification of Tumors

Code	Description
	**Adenocarcinoma**
8140	Adenocarcinoma, NOS
8480	Mucinous adenocarcinoma
8481	Mucin-producing adenocarcinoma
8210	Adenocarcinoma in adenomatous polyp
8261	Adenocarcinoma in villous adenoma
8263	Adenocarcinoma in tubulovillous adenoma
8490	Signet ring cell adenocarcinoma
8010	Carcinoma, NOS
	**Other**
8560	Adenosquamous carcinoma
8070	Squamous cell carcinoma
8013	Large cell neuroendocrine carcinoma
8041	Small cell neuroendocrine carcinoma
8244	Mixed adenoneuroendocrine carcinoma
8020	Undifferentiated carcinoma
8148	Dysplasia (intraepithelial neoplasia), high grade
8510	Medullary carcinoma

Bosman FT, Carneiro F, Hruban RH, Theise ND, eds. World Health Organization Classification of Tumours of the Digestive System. Lyon: IARC; 2010.

INTRODUCTION

Although the small intestine accounts for one of the largest surface areas in the human body, it is one of the least common cancer sites in the digestive system, accounting for less than 3% of all malignant tumors in the gastrointestinal (GI) tract.[1] In 2016, approximately 10,090 cases of malignant tumors in the small intestine will be diagnosed in the United States.[2] A variety of tumors occur in the small intestine, with approximately 25–50% of the primary malignant tumors being adenocarcinomas, depending on the population surveyed. The natural history of small bowel adenocarcinoma has been well described.[3–15] The 1,330 deaths predicted to occur from small intestinal cancer in 2016 are divided almost equally between men and women.[2] More than 60% of tumors occur in the duodenum, followed by the jejunum (20%) and ileum (15%). An increased incidence of second malignancies has been noted in patients with primary small bowel adenocarcinoma, a finding related in part to the significantly increased risk for this malignancy in patients with hereditary nonpolyposis colorectal cancer, familial adenomatosis polyposis, and Peutz–Jeghers syndrome.[1,16–18] Crohn's disease and celiac disease also are associated with an increased risk for small intestinal adenocarcinomas and lymphomas.[19–21]

The patterns of local, regional, and metastatic spread for adenocarcinomas of the small intestine are comparable with those of similar histologic malignancies in other areas of the GI tract. The most common sites of metastatic disease include the regional lymph nodes, peritoneal cavity, and liver.

The classification and stage grouping described in this chapter are used for both clinical and pathological staging of adenocarcinomas of the small bowel, and do not apply to other types of malignant small bowel tumors.

The staging system described in this chapter is supported by an analysis of a dataset extracted from the National Cancer Data Base (NCDB) of 3,141 patients with nonampullary duodenal adenocarcinoma and 3,807 patients with nonduodenal small bowel adenocarcinoma, treated in the interval from 1998 to 2008.

ANATOMY

Primary Site(s)

This classification applies to adenocarcinomas arising in the duodenum, jejunum, and ileum. It does not apply to carcinomas arising in the ileocecal valve or to carcinomas that may arise in a Meckel diverticulum. Carcinomas arising in the ampulla of Vater are staged according to the system described in Chapter 27.

Duodenum. About 10 inches (25 cm) in length, the duodenum extends from the pyloric sphincter of the stomach to the jejunum. It usually is divided anatomically into four parts, with the common bile duct and pancreatic duct opening into the second part at the ampulla of Vater (Fig. 18.1).

Jejunum and Ileum. The jejunum (approximately 8 ft or 240 cm in length) and ileum (approximately 12 ft or 360 cm in length) extend from the junction with the duodenum proximally to the ileocecal valve distally. The division point between the jejunum and the ileum is arbitrary. As a general rule, the jejunum includes the proximal 40% and the ileum includes the distal 60% of the small intestine, excluding the duodenum (Fig. 18.1).

General. A fold of the peritoneum containing the blood supply and the regional lymph nodes, the mesentery, supports

the jejunal and ileal portions of the small intestine. The shortest segment, the duodenum, has no real mesentery and is covered only by peritoneum anteriorly. The wall of all parts of the nonduodenal small intestine has five layers: mucosal, submucosal, muscular, subserosal, and serosal. A very thin layer of smooth muscle cells, the muscularis mucosae, separates the mucosa from the submucosa. The small intestine is ensheathed entirely by peritoneum, except for a narrow strip of bowel attached to the mesentery and the retroperitoneal portion of the duodenum.

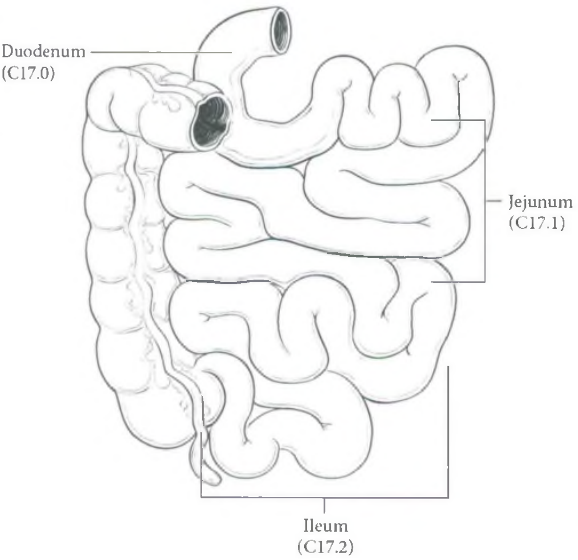

Fig. 18.1 Anatomic sites of the small intestine

Regional Lymph Nodes

Regional lymph nodes for the nonampullary duodenum include the following groups of peripancreaticoduodenal nodes (Figs. 18.2 and 18.3):

- Retropancreatic
- Hepatic artery
- Inferior pancreaticoduodenal
- Superior mesenteric

Regional lymph nodes for a tumor of the nonduodenal small intestine are those distributed along the mesenteric vessels extending to the base of the mesentery (Fig. 18.4):

- Cecal (terminal ileum only)
- Ileocolic (terminal ileum only)
- Superior mesenteric
- Mesenteric, NOS

Metastatic Sites

Adenocarcinoma of the small intestine metastasizes primarily to the regional lymph nodes, liver, and peritoneal cavity, although metastases to any organ may be seen. Invasion of adjacent structures is not uncommon. Involvement of nonregional celiac and para-aortic nodes is considered M1 distant disease for adenocarcinomas of the duodenum, jejunum, and ileum.

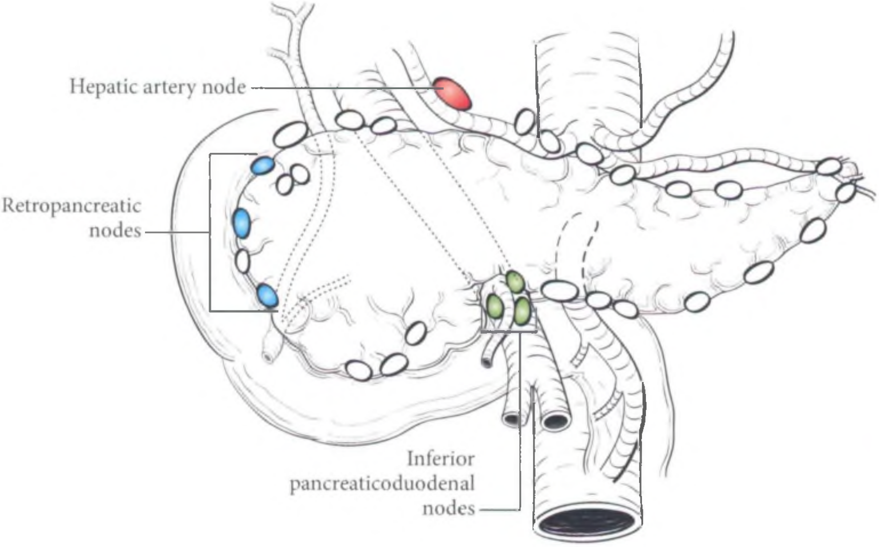

Fig. 18.2 The regional lymph nodes of the duodenum

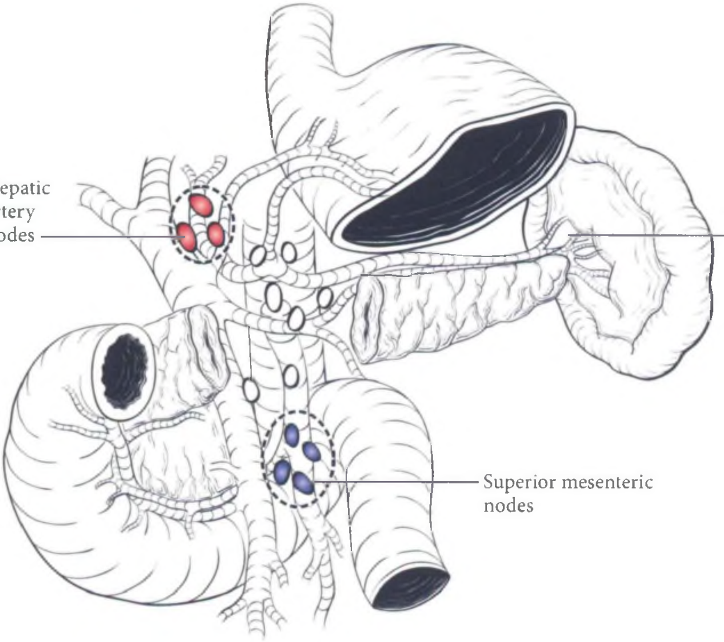

Fig. 18.3 The regional lymph nodes of the duodenum

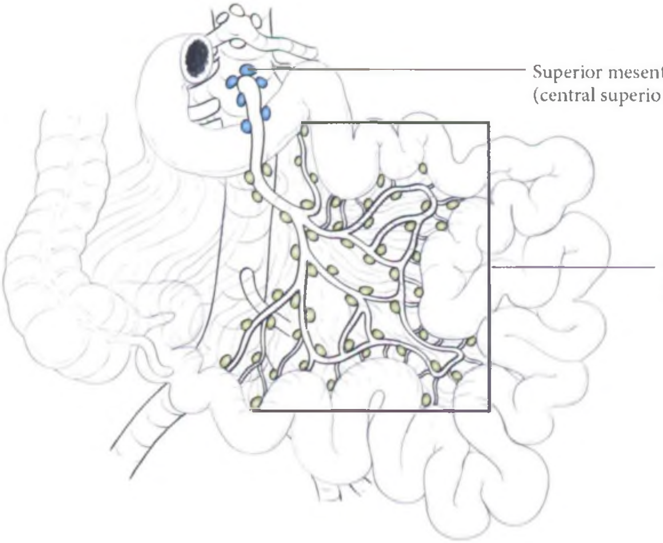

Fig. 18.4 The regional lymph nodes of the ileum and jejunum

RULES FOR CLASSIFICATION

Clinical Classification

Clinical staging represents risk assessment before initiating definitive therapy. It is performed much more often in nonampullary duodenal adenocarcinoma, in which the diagnosis is usually known before initial treatment, than in non-duodenal small intestinal adenocarcinoma, in which the diagnosis is usually established at or after initial treatment of symptomatic patients, especially in patients with resectable disease. In general, clinical staging is based on initial biopsy, cross-sectional imaging, and, when appropriate, endoscopic ultrasound (EUS).

Imaging

The following paragraphs review and clarify aspects of the imaging performed in the clinical staging of patients with

adenocarcinoma of the nonampullary duodenum and nonduodenal small intestine. These are rare tumors, and as such, virtually all information pertaining to cross-sectional imaging is derived from small retrospective studies (usually spanning decades, during which imaging technology evolved substantially) or is extrapolated from data derived from the study of more common GI tract tumors. There is a small literature describing experience with cross-sectional imaging to detect, characterize, and locate tumors in the small intestine. However, few if any data are available to specifically address the relative contributions of different imaging modalities to the accurate clinical staging of adenocarcinoma of the nonampullary duodenum and the nonduodenal small intestine.

Cross-sectional Imaging

Computed tomography (CT) of the chest, abdomen, and pelvis with oral and intravenous contrast is the initial imaging modality used for clinical staging of adenocarcinoma of the nonampullary duodenum and nonduodenal small intestine.[22–24] Positron emission tomography (PET)/CT with fluorine-18 fluoro-2-deoxy-D-glucose (FDG) may be used to further refine cN category and may best define cM category in patients with locoregionally advanced primary tumors.[25]

CT of the abdomen with intravenous and oral contrast may be helpful in describing the location of a primary tumor in the small intestine.[26] CT enteroclysis with water or oral contrast is reported to be superior to routine CT scanning in characterizing the type and location of small bowel malignancy.[27,28] However, CT of the abdomen has a limited role in the cT classification because it cannot distinguish a specific cT stage.

CT of the abdomen and pelvis with intravenous and oral contrast may be used to describe locoregional (cN) lymph nodes.[22] Although there are no good prospective data correlating the size of regional nodes as seen on CT scan with the presence of malignancy, consistent with other sites in the GI tract, locoregional nodes generally are considered positive if they are round and/or $\geq$10 mm in mean diameter and adjacent to the primary malignancy. The portocaval lymph node is a potential false positive finding in cN categorization. This node normally has an elongated shape with a long transverse diameter and small anterior posterior diameter, and simple measurement may result in a false positive interpretation. Nodes felt to be equivocal on CT scan might be characterized further with PET/CT.

CT of the chest, abdomen, and pelvis with intravenous and oral contrast and FDG PET/CT imaging may be most useful in detecting distant metastasis (cM), including liver, remote node, and significant omental or peritoneal metastases. Ascites may be an indicator of subradiologic peritoneal metastases.

Magnetic resonance (MR) imaging with or without gadolinium may have a role in clarifying the nature of lesions in the liver that are characterized as indeterminate on contrast-enhanced CT scan. MR imaging enteroclysis has been reported to characterize the type and location of small bowel malignancies,[29,30] but has a limited role in staging these tumors.

PET/CT is unlikely to help further define an accurate cT categorization. Although there are no prospective data to define the incremental value of PET/CT in more accurately defining cN or cM category after contrast-enhanced CT, this would be a reasonable investigation to help clarify indeterminate findings seen on CT scan. Use of PET/CT must be tempered with an awareness of its limitations in detecting low-volume metastatic disease (false negative) and the high rate of false positive findings that might lead to further, unnecessary investigations.

EUS

In contrast to its utility in gastric and gastroesophageal junction tumors, EUS has a relatively limited role in the clinical staging of adenocarcinoma of the nonampullary duodenum, and virtually no role in staging adenocarcinoma of the nonduodenal small intestine. Most of our understanding of the utility of EUS in staging nonampullary duodenal adenocarcinoma is extrapolated from retrospective studies examining its accuracy and utility in patients with ampullary carcinomas.[31–35] The length of ultrasound endoscopes generally limits their utility to tumors in the first, second, or proximal third portions of the duodenum. Tumors beyond this point usually cannot be reached. EUS can assess the depth of primary tumor invasion, regional nodal involvement, and limited areas of distant disease for these proximally located tumors. EUS is best performed with the use of commercially available ultrasound endoscopes with multifrequency (5-, 7.5-, 10-, and 12-MHz) radial transducers.

The individual layers of the gastrointestinal wall are visualized throughout the examination to correlate the extent of tumor penetration relative to the alternating bright and dark layers seen on ultrasound, as described for tumors of the esophagus and gastroesophageal junction.

EUS can routinely visualize and characterize the periduodenal, mesenteric, retroperitoneal, celiac axis, splenic vein, portal vein, and gastrohepatic ligament lymphatic areas. The presence of hypoechoic, rounded, sharply demarcated structures in these areas is considered diagnostic of malignant nodes. Histologic confirmation of nodal disease by EUS-guided fine-needle aspiration may be performed if clinically indicated. Clinical nodal categorization in these areas is enhanced by careful counting and reporting of the number of suspicious nodes.

Parts of the liver are readily seen with EUS with the scope positioned in the proximal duodenum, in the gastric antrum, and along the lesser curvature and cardia, permitting the identification of liver metastases (M1). Similarly, the

presence of intra-abdominal ascites, on EUS or cross-sectional imaging, raises suspicion for peritoneal metastases, and this observation usually prompts further investigation with diagnostic (image-guided) paracentesis or laparoscopy.

Capsule Endoscopy

For patients presenting with an occult GI bleed and negative upper/lower endoscopy, capsule endoscopy may be helpful in identifying the site of a nonobstructing small bowel malignancy. Capsule endoscopy is not helpful in establishing the specific diagnosis or in staging the primary tumor.

Pathological Classification

Pathological staging represents risk assessment after initial primary surgical treatment, along with all clinical staging information, including imaging and the surgeon's operative assessment. In the absence of preoperative neoadjuvant treatment, this information informs a p stage; following neoadjuvant therapy, this information informs a yp stage.

The primary tumor is staged according to its depth of penetration into or through the bowel wall and the involvement of adjacent structures. Lateral spread within the duodenum, jejunum, or ileum is not considered in this categorization. Most cases of adenocarcinoma of the nonampullary duodenum and small intestine arise in association with a precursor neoplasm, such as tubular/tubulovillous adenoma or flat epithelial dysplasia in association with Crohn's disease or celiac disease. If present in the biopsy specimen, these preinvasive lesions are classified as low-grade dysplasia or high-grade dysplasia (including *in situ* carcinoma). Early invasive carcinoma or intramucosal carcinoma is defined by tumor invasion into the lamina propria or muscularis mucosae (T1a). If the entire tumor is present in a polypectomy specimen in an attempt at complete removal, tumor (T) category should be provided by the extent of invasion. Furthermore, to facilitate clinical decision making regarding subsequent surgical intervention after endoscopic polypectomy, it also is important to provide information on tumor differentiation, the presence of lymphovascular invasion, and margin clearance.

Although the two are similar, differences between this small intestine staging system and that of the colon should be noted, particularly for early-stage tumors. In the colon, pTis applies to intraepithelial as well as to intramucosal lesions (invasion within the lamina propria and muscularis mucosae) as *in situ* carcinoma. In the small intestine, intramucosal spread within the lamina propria or muscularis mucosae is staged as pT1a instead of pTis. In this regard, the pT1 definition for the small bowel is essentially the same as the pT1 definition for adenocarcinoma of the stomach. Invasion into and through the bowel wall is staged the same as for colon cancer.

Regional nodal disease is staged according to the number of involved lymph nodes resected, if primary resection is performed. Recording of both the number of involved nodes and the total number of nodes resected/examined is strongly encouraged.

Discontinuous hematogenous, nonregional nodal, or peritoneal metastases are all assigned as M1.

The final pathological stage after surgery should include the essential elements for T, N, and M categories, that is, depth of tumor invasion, number of positive lymph nodes, and documentation of discontinuous tumor deposits/implants identified as potential metastatic disease. In addition, other pathological factors associated with disease management and prognosis (tumor site, size, subtype, and differentiation; total lymph nodes retrieved; lymphovascular invasion; large vessel invasion; and resection margin status) should be reported.

For segmental small bowel resections, margin assessment should include the proximal, distal, and mesenteric margins of resection. For all small bowel segments, except the retroperitoneal portion of the duodenum, which is completely unencased by peritoneum, the mesenteric resection margin is the only pertinent radial margin, and may be partially or completely encased by peritoneum (Fig. 18.4). For pancreaticoduodenectomy specimens of carcinomas of the duodenum, the nonperitonealized surface constitutes a deep radial (nonperitonealized soft tissue) margin.

The pathological extent of the primary tumor's penetration into/through the bowel wall and regional lymph node involvement are the two strongest indicators of outcome if the tumor can be resected. Prognosis in cases of incomplete removal and in patients who do not undergo cancer-directed surgery, both of which are often associated with the presence of distant metastatic disease, is poor.

PROGNOSTIC FACTORS

Prognostic Factors Required for Stage Grouping

Beyond the factors used to assign T, N, or M categories, no additional prognostic factors are required for stage grouping.

Additional Factors Recommended for Clinical Care

Primary Tumor Site (Duodenum versus Nonduodenum)

Based on our review of data from the NCDB to prepare for this staging update, 3,141 patients with nonampullary duodenal adenocarcinoma had a worse stage-specific survival than 3,807 patients with nonduodenal small intestine adenocarcinoma. Although other investigators have observed this,[36] another recent review of 2,772 patients from the Surveillance, Epidemiology, and End Results (SEER)

database found survival in patients with nonampullary duodenal adenocarcinoma to be similar to that seen in patients with nonduodenal small intestine adenocarcinoma.[36] AJCC Level of Evidence: II

Number of Lymph Nodes Examined

Although there are limited data to support an optimal number of lymph nodes to be examined and reported for accurate pathological nodal staging of small intestine adenocarcinoma, two recent analyses of the SEER database suggest that optimal staging would be achieved by examining at least five nodes for nonampullary duodenal adenocarcinoma and at least nine nodes for small intestinal adenocarcinomas.[37,38] Overman et al.[39] examined a pooled set of patients in the SEER database with adenocarcinoma of the duodenum and small intestine and found that the number of nodes to be examined for optimal staging was eight or more. If the lymph nodes are reported to be negative but fewer than the specified number of nodes are examined, pN0 should be assigned. Because the SEER review suggests that the ratio of positive nodes to total nodes examined also is important for prognosis, both the number of lymph nodes examined and the number of positive lymph nodes should be recorded. AJCC Level of Evidence: III

Presurgical Carcinoembryonic Antigen

There are insufficient data to assess the impact of serum tumor markers, but it is logical to believe that the effect of those factors would be similar to that observed for adenocarcinoma elsewhere in the GI tract. Recording of pretreatment carcinoembryonic antigen (CEA) is encouraged. AJCC Level of Evidence: III

Lymphovascular Invasion

Lymphovascular invasion (LVI) is an important independent predictor of outcome in adenocarcinomas elsewhere in the GI tract. Current data are insufficient to characterize the significance of LVI in small bowel adenocarcinoma. Prospective collection of this data point is encouraged. AJCC Level of Evidence: III

Microsatellite Instability

Ancillary studies for mismatch repair proteins may be performed in young patients or in patients with carcinomas with morphologic features that suggest microsatellite instability, such as increased intraepithelial lymphocytes, medullary carcinoma, and the presence of tumor heterogeneity. This is a predictive and prognostic marker for adenocarcinomas elsewhere in the GI tract and should be recorded if available to help define its significance in small bowel adenocarcinoma. AJCC Level of Evidence: IV

Tumor Histologic Grade (G)

Histologic grade has emerged inconsistently as a significant predictor of outcome in several multivariate analyses. As the quality of datasets for this rare tumor improve, prospective collection of information on primary tumor grade may help clarify its role in the prognosis of small bowel adenocarcinoma. AJCC Level of Evidence: III

Presence of Crohn's Disease

The presence of Crohn's disease is associated with poorer outcomes in patients with small bowel adenocarcinoma. This association may be related to the difficulty of early diagnosis of small bowel malignancy in the setting of preexisting abnormal Crohn's-related symptoms and radiographic findings. AJCC Level of Evidence: IV

Personal or Family History of Familial GI Malignancies (Familial Adenomatous Polyposis, Lynch Syndrome, Peutz–Jeghers Syndrome)

The influence of genetic predisposition on small bowel adenocarcinoma is not known to affect survival. Prospective collection of these data should help clarify this point. AJCC Level of Evidence: IV

RISK ASSESSMENT MODELS

The AJCC recently established guidelines that will be used to evaluate published statistical prediction models for the purpose of granting endorsement for clinical use.[40] Although this is a monumental step toward the goal of precision medicine, this work was published only very recently. Therefore, the existing models that have been published or may be in clinical use have not yet been evaluated for this cancer site by the Precision Medicine Core of the AJCC. In the future, the statistical prediction models for this cancer site will be evaluated, and those that meet all AJCC criteria will be endorsed.

DEFINITIONS OF AJCC TNM

Definition of Primary Tumor (T)

T Category	T Criteria
TX	Primary tumor cannot be assessed
T0	No evidence of primary tumor
Tis	High-grade dysplasia/carcinoma *in situ*
T1	Tumor invades the lamina propria or submucosa
T1a	Tumor invades the lamina propria
T1b	Tumor invades the submucosa
T2	Tumor invades the muscularis propria
T3	Tumor invades through the muscularis propria into the subserosa, or extends into nonperitonealized perimuscular tissue (mesentery or retroperitoneum) without serosal penetration*

18

T Category	T Criteria
T4	Tumor perforates the visceral peritoneum or directly invades other organs or structures (e.g., other loops of small intestine, mesentery of adjacent loops of bowel, and abdominal wall by way of serosa; for duodenum only, invasion of pancreas or bile duct)

**Note*: For T3 tumors, the nonperitonealized perimuscular tissue is, for the jejunum and ileum, part of the mesentery and, for the duodenum in areas where serosa is lacking, part of the interface with the pancreas (Fig. 18.5).

Definition of Regional Lymph Node (N)

N Category	N Criteria
NX	Regional lymph nodes cannot be assessed
N0	No regional lymph node metastasis
N1	Metastasis in one or two regional lymph nodes
N2	Metastasis in three or more regional lymph nodes

Definition of Distant Metastasis (M)

M Category	M Criteria
M0	No distant metastasis
M1	Distant metastasis present

AJCC PROGNOSTIC STAGE GROUPS

Adenocarcinoma

When T is...	And N is...	And M is...	Then the stage group is...
Tis	N0	M0	0
T1–2	N0	M0	I
T3	N0	M0	IIA
T4	N0	M0	IIB
Any T	N1	M0	IIIA
Any T	N2	M0	IIIB
Any T	Any N	M1	IV

REGISTRY DATA COLLECTION VARIABLES

1. Primary tumor site (duodenum, jejunum, ileum)
2. Number of lymph nodes examined
3. Presurgical CEA
4. LVI
5. Microsatellite instability
6. Tumor grade
7. Presence of disease
8. Personal or family history of familial GI malignancies (familial adenomatous polyposis, Lynch syndrome, Peutz–Jeghers syndrome)

HISTOLOGIC GRADE (G)

G	G Definition
GX	Grade cannot be assessed
G1	Well differentiated
G2	Moderately differentiated
G3	Poorly differentiated
G4	Undifferentiated

HISTOPATHOLOGIC TYPE

This staging classification applies only to adenocarcinomas arising in the nonampullary duodenum and small intestine. Nonadenocarcinomas arising in the small intestine should have a TNM assigned but are not assigned a stage classification according to the criteria in this chapter.

Lymphomas, well-differentiated neuroendocrine (carcinoid) tumors, and visceral sarcomas are not included. Primary lymphomas of the small intestine are staged as extranodal lymphomas. Well-differentiated neuroendocrine (carcinoid) tumors of the small intestine are staged as described in Chapters 30 (duodenum) and 31 (jejunum and ileum). Less common malignant tumors include gastrointestinal stromal tumors (GISTs), angiosarcoma, leiomyosarcoma, and metastatic tumors. GISTs are classified using factors described in Chapter 43.

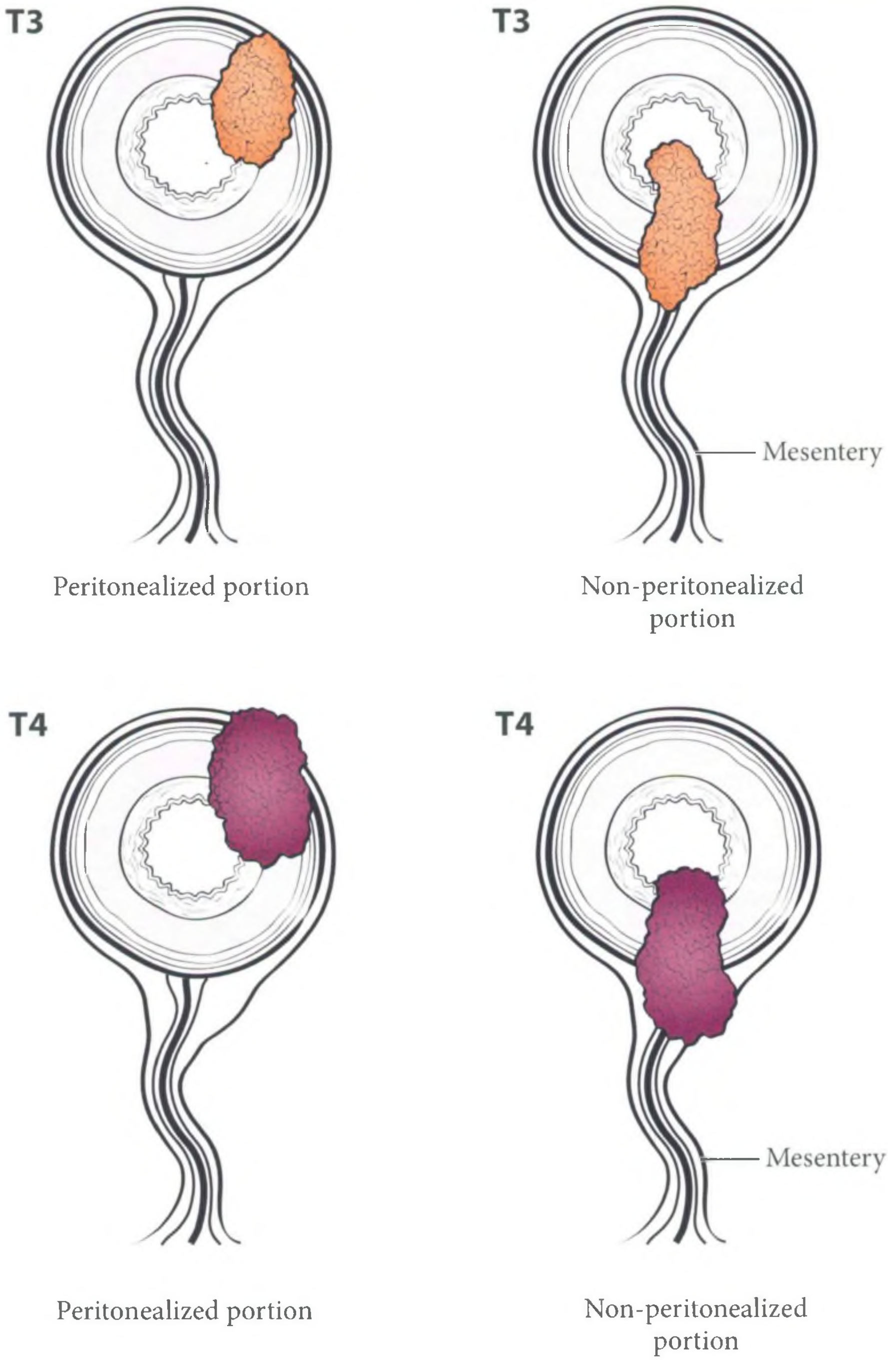

Fig. 18.5 T3 is defined as a tumor that invades through the muscularis propria into the subserosa, whereas T4 is defined as a tumor that perforates the visceral peritoneum. T3 is defined as extension into nonperitonealized perimuscular tissue (mesentery or retroperitoneum) without serosal penetration, whereas T4 is defined as tumor that directly invades other organs or structures (including other loops of small intestine, mesentery, and abdominal wall by way of serosa; for duodenum only, invasion of the pancreas or bile duct)

SURVIVAL DATA

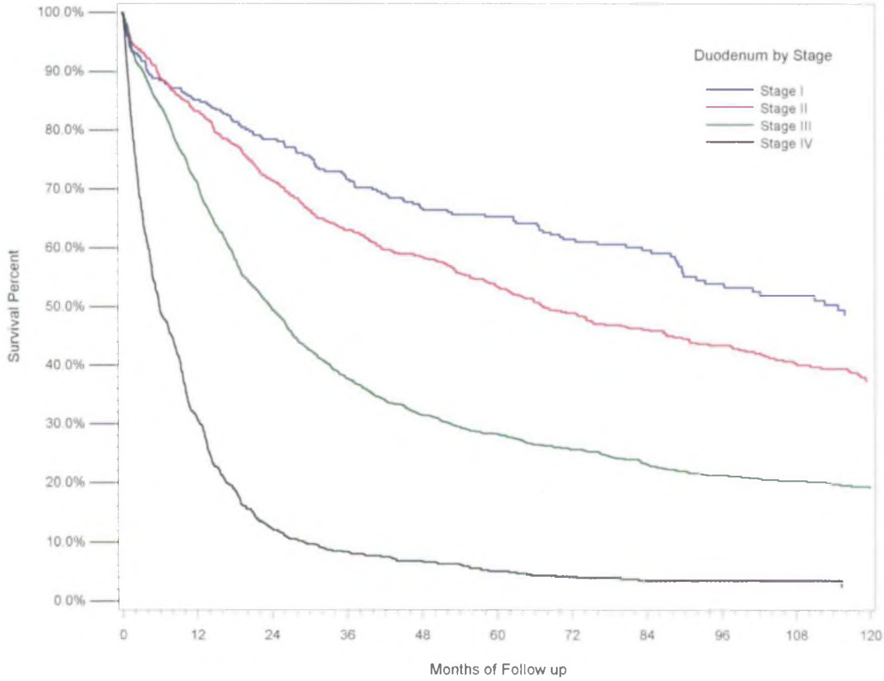

Fig. 18.6 Survival in small bowel–duodenum cancer by stage, diagnosis years 1998 to 2008. Overall survival, nonampullary duodenum, by pathological stage group: I, $n=309$; IIA, $n=443$; IIB, $n=307$; IIIA, $n=804$; IIIB, $n=606$; IV, $n=672$

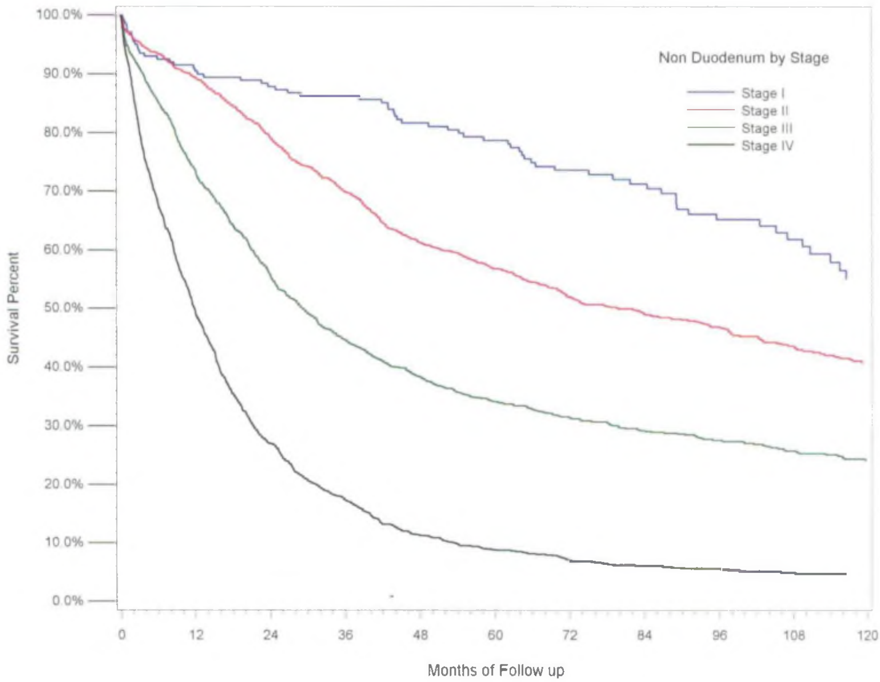

Fig. 18.7 Survival in small bowel–nonduodenum cancer by stage, diagnosis years 1998 to 2008. Overall survival, nonduodenal small bowel, by pathological stage group: I, $n=210$; IIA, $n=850$; IIB, $n=286$; IIIA, $n=676$; IIIB, $n=562$; IV, $n=1,223$

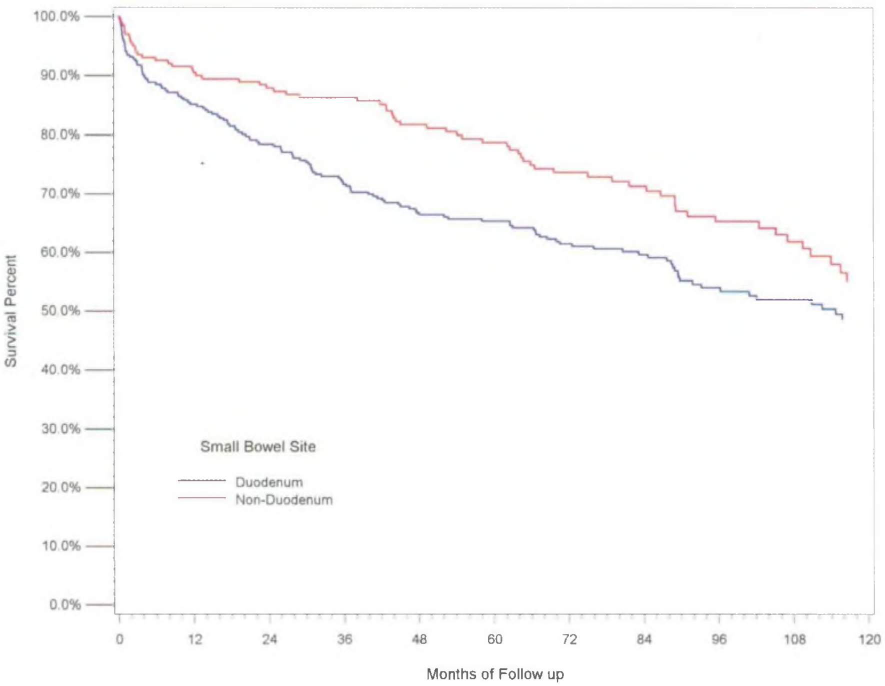

Fig. 18.8 Survival in Stage I small bowel cancer, duodenal versus nonduodenal, diagnosis years 1998 to 2008. Overall survival, duodenum versus nonduodenum, pathological Stage I: duodenum, $n=309$; nonduodenum, $n=210$ ($p=0.01$)

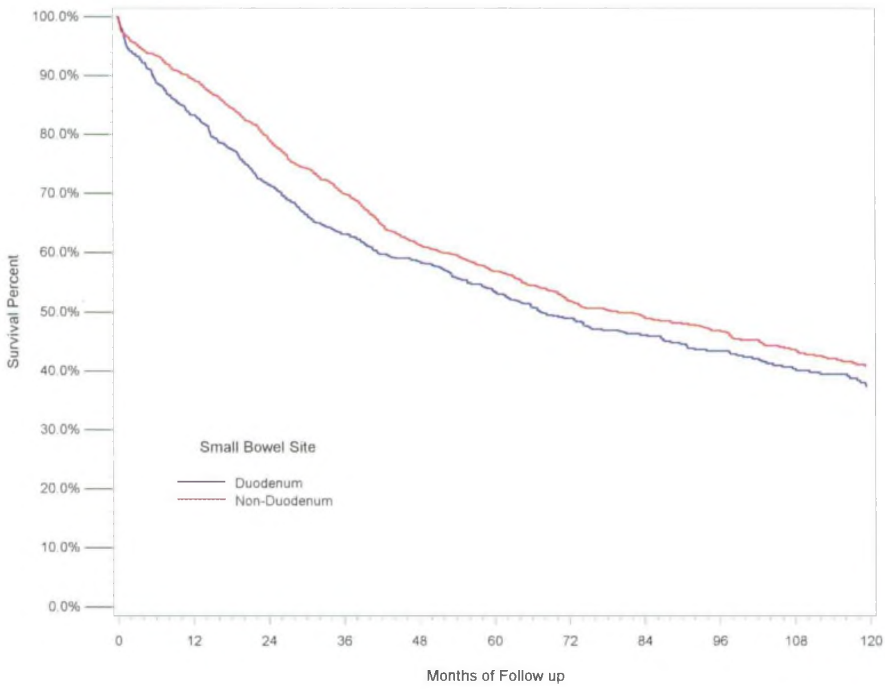

Fig. 18.9 Survival in Stage II small bowel cancer, duodenal versus nonduodenal, diagnosis years 1998 to 2008. Overall survival, duodenum versus nonduodenum, pathological Stage II: duodenum, $n=750$; nonduodenum, $n=1,136$ ($p=0.04$)

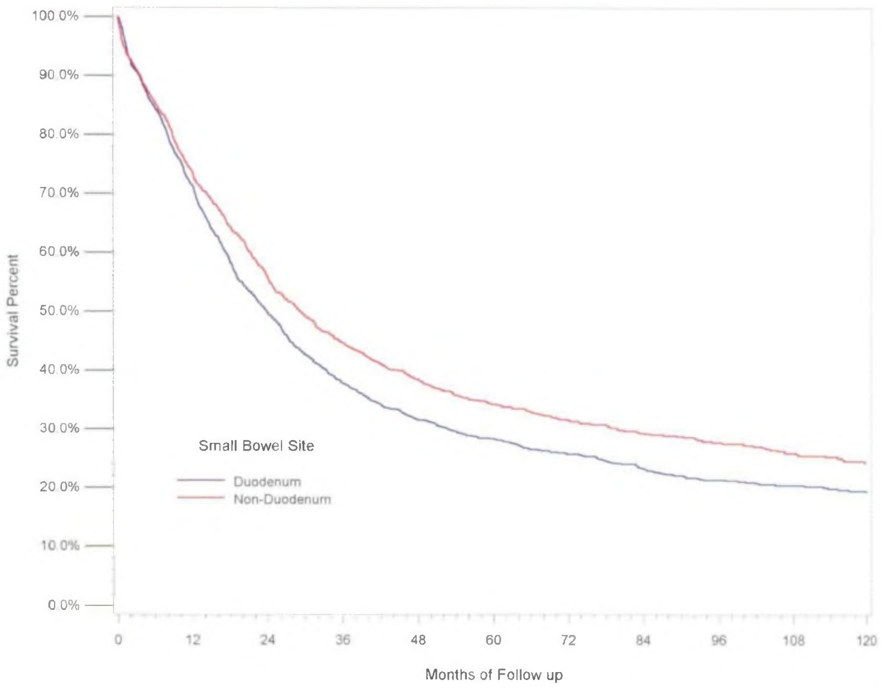

Fig. 18.10 Survival in Stage III small bowel cancer, duodenal versus nonduodenal, diagnosis years 1998 to 2008. Overall survival, duodenum versus nonduodenum, pathological Stage III: duodenum, $n = 1{,}410$; nonduodenum, $n = 1{,}238$ ($p = 0.0003$)

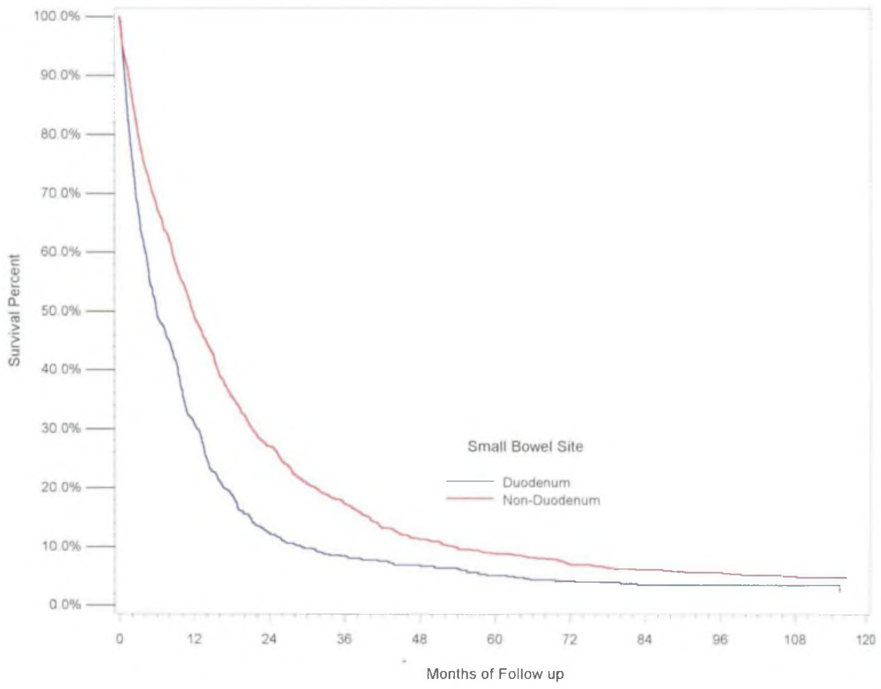

Fig. 18.11 Survival in Stage IV small bowel cancer, duodenal versus nonduodenal, diagnosis years 1998 to 2008. Overall survival, duodenum versus nonduodenum, pathological Stage IV: duodenum, $n = 672$; nonduodenum, $n = 1{,}223$ ($p < 0.0001$)

Bibliography

1. Chamberlain RS, Mahendraraj K, Shah SA. Cancer of the small bowel. In: DeVita VT, Lawrence TS, Rosenberg SA, eds. *Principles and Practice of Oncology*. 10th ed. Philadelphia, PA: Wolters Kluwer; 2015:734–744.
2. Siegel RL, Miller KD, Jemal A. Cancer statistics, 2016. *CA: a cancer journal for clinicians*. Jan 2016;66(1):7–30.
3. Bilimoria KY, Bentrem DJ, Wayne JD, Ko CY, Bennett CL, Talamonti MS. Small bowel cancer in the United States: changes in epidemiology, treatment, and survival over the last 20 years. *Annals of surgery*. Jan 2009;249(1):63–71.
4. Howe JR, Karnell LH, Menck HR, Scott-Conner C. The American College of Surgeons Commission on Cancer and the American Cancer Society. Adenocarcinoma of the small bowel: review of the National Cancer Data Base, 1985-1995. *Cancer* Dec 15 1999; 86(12):2693–2706.
5. Halfdanarson TR, McWilliams RR, Donohue JH, Quevedo JF. A single-institution experience with 491 cases of small bowel adenocarcinoma. *American journal of surgery*. Jun 2010;199(6): 797–803.
6. Hatzaras I, Palesty JA, Abir F, et al. Small-bowel tumors: epidemiologic and clinical characteristics of 1260 cases from the connecticut tumor registry. *Archives of surgery*. Mar 2007;142(3): 229–235.
7. Overman MJ, Hu C-Y, Kopetz S, Abbruzzese JL, Wolff RA, Chang GJ. A population-based comparison of adenocarcinoma of the large and small intestine: insights into a rare disease. *Annals of surgical oncology*. 2012;19(5):1439–1445.
8. Raghav K, Overman MJ. Small bowel adenocarcinomas [mdash] existing evidence and evolving paradigms. *Nature Reviews Clinical Oncology*. 2013;10(9):534–544.
9. Dabaja BS, Suki D, Pro B, Bonnen M, Ajani J. Adenocarcinoma of the small bowel: presentation, prognostic factors, and outcome of 217 patients. *Cancer*. Aug 1 2004;101(3):518–526.
10. Ugurlu MM, Asoglu O, Potter DD, Barnes SA, Harmsen WS, Donohue JH. Adenocarcinomas of the jejunum and ileum: a 25-year experience. *Journal of gastrointestinal surgery : official journal of the Society for Surgery of the Alimentary Tract*. Nov 2005;9(8): 1182–1188.
11. Verma D, Stroehlein JR. Adenocarcinoma of the small bowel: a 60-yr perspective derived from MD Anderson Cancer Center Tumor Registry. *The American journal of gastroenterology*. 2006;101(7):1647–1654.
12. Young JI, Mongoue-Tchokote S, Wieghard N, et al. Treatment and Survival of Small-bowel Adenocarcinoma in the United States: A Comparison With Colon Cancer. *Diseases of the colon and rectum*. Apr 2016;59(4):306–315.
13. Zouhairi ME, Venner A, Charabaty A, Pishvaian MJ. Small bowel adenocarcinoma. *Current treatment options in oncology*. Dec 2008;9(4-6):388–399.
14. Hutchins RR, Bani Hani A, Kojodjojo P, Ho R, Snooks SJ. Adenocarcinoma of the small bowel. *ANZ journal of surgery*. Jul 2001;71(7):428–437.
15. Aparicio T, Zaanan A, Svrcek M, et al. Small bowel adenocarcinoma: epidemiology, risk factors, diagnosis and treatment. *Dig Liver Dis*. Feb 2014;46(2):97–104.
16. Bonadona V, Bonaiti B, Olschwang S, et al. Cancer risks associated with germline mutations in MLH1, MSH2, and MSH6 genes in Lynch syndrome. *JAMA*. Jun 8 2011;305(22):2304–2310.
17. Schulmann K, Brasch FE, Kunstmann E, et al. HNPCC-associated small bowel cancer: clinical and molecular characteristics. *Gastroenterology*. Mar 2005;128(3):590–599.
18. Giardiello FM, Brensinger JD, Tersmette AC, et al. Very high risk of cancer in familial Peutz–Jeghers syndrome. *Gastroenterology*. 2000;119(6):1447–1453.
19. Jess T, Loftus EV, Jr., Velayos FS, et al. Risk of intestinal cancer in inflammatory bowel disease: a population-based study from olmsted county, Minnesota. *Gastroenterology*. Apr 2006;130(4): 1039–1046.
20. Shaukat A, Virnig DJ, Howard D, Sitaraman SV, Liff JM, Lederle FA. Crohn's disease and small bowel adenocarcinoma: a population-based case-control study. *Cancer epidemiology, biomarkers & prevention : a publication of the American Association for Cancer Research, cosponsored by the American Society of Preventive Oncology*. Jun 2011;20(6):1120–1123.
21. Weber NK, Fletcher JG, Fidler JL, et al. Clinical characteristics and imaging features of small bowel adenocarcinomas in Crohn's disease. *Abdom Imaging*. Jun 2015;40(5):1060–1067.
22. Buckley JA, Siegelman SS, Jones B, Fishman EK. The accuracy of CT staging of small bowel adenocarcinoma: CT/pathologic correlation. *Journal of computer assisted tomography*. Nov-Dec 1997; 21(6):986–991.
23. Sailer J, Zacherl J, Schima W. MDCT of small bowel tumours. *Cancer Imaging*. 2007;7:224–233.
24. Horton KM, Fishman EK. The current status of multidetector row CT and three-dimensional imaging of the small bowel. *Radiol Clin North Am*. Mar 2003;41(2):199–212.
25. Das CJ, Manchanda S, Panda A, Sharma A, Gupta AK. Recent Advances in Imaging of Small and Large Bowel. *PET clinics*. Jan 2016;11(1):21–37.
26. Anzidei M, Napoli A, Zini C, Kirchin M, Catalano C, Passariello R. Malignant tumours of the small intestine: a review of histopathology, multidetector CT and MRI aspects. *The British journal of radiology*. 2014.
27. Pilleul F, Penigaud M, Milot L, Saurin JC, Chayvialle JA, Valette PJ. Possible small-bowel neoplasms: contrast-enhanced and water-enhanced multidetector CT enteroclysis. *Radiology*. Dec 2006; 241(3):796–801.
28. Paulsen SR, Huprich JE, Fletcher JG, et al. CT enterography as a diagnostic tool in evaluating small bowel disorders: review of clinical experience with over 700 cases 1. *Radiographics : a review publication of the Radiological Society of North America, Inc*. 2006;26(3):641–657.
29. Masselli G, Polettini E, Casciani E, Bertini L, Vecchioli A, Gualdi G. Small-bowel neoplasms: prospective evaluation of MR enteroclysis. *Radiology*. Jun 2009;251(3):743–750.
30. Wiarda BM, Kuipers EJ, Houdijk LP, Tuynman HA. MR enteroclysis: imaging technique of choice in diagnosis of small bowel diseases. *Digestive diseases and sciences*. Jun 2005;50(6):1036–1040.
31. Trikudanathan G, Njei B, Attam R, Arain M, Shaukat A. Staging accuracy of ampullary tumors by endoscopic ultrasound: meta-analysis and systematic review. *Digestive endoscopy : official journal of the Japan Gastroenterological Endoscopy Society*. Sep 2014;26(5):617–626.
32. Chen CH, Yang CC, Yeh YH, Chou DA, Nien CK. Reappraisal of endosonography of ampullary tumors: correlation with transabdominal sonography, CT, and MRI. *Journal of clinical ultrasound : JCU*. Jan 2009;37(1):18–25.
33. Skordilis P, Mouzas IA, Dimoulios PD, Alexandrakis G, Moschandrea J, Kouroumalis E. Is endosonography an effective method for detection and local staging of the ampullary carcinoma? A prospective study. *BMC surgery*. 2002;2(1):1.
34. Ridtitid W, Schmidt SE, Al-Haddad MA, et al. Performance characteristics of EUS for locoregional evaluation of ampullary lesions. *Gastrointestinal endoscopy*. Feb 2015;81(2):380–388.

35. Artifon EL, Couto D, Jr., Sakai P, da Silveira EB. Prospective evaluation of EUS versus CT scan for staging of ampullary cancer. *Gastrointestinal endoscopy*. Aug 2009;70(2):290–296.
36. Bakaeen FG, Murr MM, Sarr MG, et al. What prognostic factors are important in duodenal adenocarcinoma? *Archives of surgery*. 2000;135(6):635–642.
37. Tran TB, Qadan M, Dua MM, Norton JA, Poultsides GA, Visser BC. Prognostic relevance of lymph node ratio and total lymph node count for small bowel adenocarcinoma. *Surgery*. Aug 2015;158(2):486–493.
38. Wilhelm A, Müller SA, Steffen T, Schmied BM, Beutner U, Warschkow R. Patients with Adenocarcinoma of the Small Intestine with 9 or More Regional Lymph Nodes Retrieved Have a Higher Rate of Positive Lymph Nodes and Improved Survival. *Journal of Gastrointestinal Surgery*. 2016;20(2):401–410.
39. Overman MJ, Hu CY, Wolff RA, Chang GJ. Prognostic value of lymph node evaluation in small bowel adenocarcinoma: analysis of the surveillance, epidemiology, and end results database. *Cancer*. Dec 1 2010;116(23):5374–5382.
40. Kattan MW, Hess KR, Amin MB, et al. American Joint Committee on Cancer acceptance criteria for inclusion of risk models for individualized prognosis in the practice of precision medicine. *CA: a cancer journal for clinicians*. Jan 19 2016.

Part IV

Lower Gastrointestinal Tract

Members of the Lower Gastrointestinal Tract Expert Panel

Elliot A. Asare, MD
Al B. Benson III, MD, FACP, FASCO
James D. Brierley, BSc, MB, FRCP, FRCR, FRCP(C) – UICC Representative
Vivien W. Chen, PhD – Data Collection Core Representative
Robert Coffey, MD
Carolyn C. Compton, MD, PhD, FCAP
Paola De Nardi, MD
Richard M. Goldberg, MD – Vice Chair
Karyn A. Goodman, MD
Leonard L. Gunderson, MD, MS
Stanley R. Hamilton, MD, FCAP, AGAF
Nader N. Hanna, MD, FACS
J. Milburn Jessup, MD – Chair
Sanjay Kakar, MD – CAP Representative
Lauren A. Kosinski, MD, FASCRS
Shuji Ogino, MD, PhD, MS
Michael J. Overman, MD
Philip Quirke, BM, PhD, FRCPath
Eric Rohren, MD, PhD
Daniel J. Sargent, PhD – Precision Medicine Core Representative
Lynne T. Schumacher-Penberthy, MD, MPH
David Shibata, MD, FACS, FASCRS
Scott R. Steele, MD, FACS
Alexander Stojadinovic, MD, FACS
Sabine Tejpar, MD, PhD
Mary Kay Washington, MD, PhD – Editorial Board Liaison
Martin R. Weiser, MD – Precision Medicine Core Representative
Mark Lane Welton, MD, MHCM, FACS, FASCRS

Appendix — Carcinoma

19

Michael J. Overman, Elliot A. Asare, Carolyn C. Compton,
Nader N. Hanna, Sanjay Kakar, Lauren A. Kosinski,
Mary Kay Washington, Martin R. Weiser,
and J. Milburn Jessup

HAPTER SUMMARY

ancers Staged Using This Staging System

arcinomas of the appendix, including high-grade neuroendocrine carcinomas, mixed adenoneuroendocrine carcinomas, ıd goblet cell carcinoids, are staged using this system.

ancers Not Staged Using This Staging System

These histopathologic types of cancer…	Are staged according to the classification for…	And can be found in chapter…
Vell-differentiated neuroendocrine tumor carcinoid)	Neuroendocrine tumors of the appendix	32

ummary of Changes

Change	Details of Change	Level of Evidence
ntroduction	The introduction was revised to clarify the heterogeneity in behavior of appendiceal cancers.	III
Definition of Primary Tumor (T)	Tis(LAMN) category: A T category was created for low-grade appendiceal mucinous neoplasms (LAMNs) that invade or push into the muscularis propria. This change allows T category to be determined for these lesions, which previously were recorded as TX or unstaged.	III
Definition of Primary Tumor (T)	T4 redefined: *Right lower quadrant* was deleted from the T4 category.	III
Definition of Distant Metastasis (M)	M1 categorization: Intraperitoneal acellular mucin is now designated as M1a. M1a and M1b definitions from the AJCC Cancer Staging Manual, 7th Edition are now M1b and M1c, respectively.	III
Definition of Distant Metastasis (M)	M1 definition: The term *pseudomyxoma peritonei*, a clinical syndrome, was removed from the M1a category definition and is now discussed in the text.	III

o access the AJCC cancer staging forms, please visit www.cancerstaging.org.

American Joint Committee on Cancer 2017

1.B. Amin et al. (eds.), *AJCC Cancer Staging Manual, Eighth Edition*, DOI 10.1007/978-3-319-40618-3_19

Change	Details of Change	Level of Evidence
Pathological Classification	This section was updated to clarify the changes to T and M staging. The description for nodal staging has been harmonized with the colon and rectum chapter.	III
AJCC Prognostic Stage Groups	Stage 0: A Tis(LAMN) category for LAMN was added to stage 0. Acellular mucin and LAMNs were incorporated into the staging system.	III
AJCC Prognostic Stage Groups	Stage IV: The mucinous grading system of high and low grade was replaced by a three-tiered grading system (well, moderately, and poorly differentiated).	II
AJCC Prognostic Stage Groups	Stage IVa was revised to include intraperitoneal acellular mucin (M1a) and intraperitoneal (M1b) grade 1 tumors.	II

ICD-O-3 Topography Codes

Code	Description
C18.1	Appendix

WHO Classification of Tumors

Code	Description
8148	Dysplasia (intraepithelial neoplasia), high grade
8480	Low-grade appendiceal mucinous neoplasm
8140	Adenocarcinoma
8480	Mucinous adenocarcinoma (>50 % extracellular mucin)
8490	Signet ring cell carcinoma (>50 % signet ring cells)
8073	Squamous cell carcinoma
8560	Adenosquamous carcinoma
8510	Medullary carcinoma
8020	Undifferentiated carcinoma
8243	Goblet cell carcinoid
8243	Mixed goblet cell carcinoid/adenocarcinoma (adenocarcinoma ex goblet cell carcinoid)
8246	Neuroendocrine carcinoma
8013	Large cell neuroendocrine carcinoma
8041	Small cell neuroendocrine carcinoma
8244	Mixed adenoneuroendocrine carcinoma

Bosman FT, Carneiro F, Hruban RH, Theise ND, eds. World Health Organization Classification of Tumours of the Digestive System. Lyon: IARC; 2010.

INTRODUCTION

Appendiceal adenocarcinomas represent a heterogeneous group of neoplasms with outcomes that strongly depend on stage, histologic grade, and histologic subtype (mucinous, nonmucinous, or signet ring cell; Fig. 19.3).[1–4] Mucinous adenocarcinomas are common, comprising 50 % of appendiceal adenocarcinomas, compared with only 10 % of colonic adenocarcinomas.

Outcomes for Stage IV disease markedly differ based on mucinous versus nonmucinous histologic types (Fig. 19.3).[1,5] In particular, low-grade mucinous appendiceal adenocarcinomas with peritoneal involvement demonstrate a unique pathological feature characterized by massive accumulation of extracellular mucin within the peritoneal cavity, which corresponds to the clinical syndrome of pseudomyxoma peritonei.[6,7] Treatment with cytoreductive surgery for these neoplasms results in a 5-year survival of approximately 50–70 %, in contrast to 5-year survival rates of <10 % for nonmucinous appendiceal adenocarcinomas.[1,8,9] This differential clinical behavior has justified the stratification of Stage IV disease by both grade and mucinous histology, with Stage IVA disease designed to primarily well-differentiated mucinous adenocarcinomas with peritoneum as the only site of metastatic spread.

Several different terms and grading systems have been used for mucinous neoplasms of the appendix, reflecting the challenge of grading a highly mucinous tumor with limited tumor cellularity. In the context of an appendiceal neoplasm with peritoneum as the only site of metastatic involvement, terms such as *disseminated peritoneal adenomucinosis* (DPAM) and *low-grade appendiceal mucinous neoplasm* (LAMN) with peritoneal involvement have been used; these lesions are included within the category of well-differentiated (G1) appendiceal adenocarcinoma, Stage IVA.[6,10–12]

Goblet cell carcinoids demonstrate both endocrine and exocrine differentiation. Because the biologic behavior of goblet cell carcinoids is closer to that of adenocarcinomas, these cancers should be staged using the system for adenocarcinomas as opposed to appendiceal carcinoids (well-differentiated neuroendocrine tumors).[3,13,14]

Low-grade neuroendocrine (carcinoid) tumors of the appendix are classified according to the TNM staging system for low-grade neuroendocrine tumors, as described in Chapter 32.

Appendix — Carcinoma

19

Michael J. Overman, Elliot A. Asare, Carolyn C. Compton, Nader N. Hanna, Sanjay Kakar, Lauren A. Kosinski, Mary Kay Washington, Martin R. Weiser, and J. Milburn Jessup

CHAPTER SUMMARY

Cancers Staged Using This Staging System

Carcinomas of the appendix, including high-grade neuroendocrine carcinomas, mixed adenoneuroendocrine carcinomas, and goblet cell carcinoids, are staged using this system.

Cancers Not Staged Using This Staging System

These histopathologic types of cancer…	Are staged according to the classification for…	And can be found in chapter…
Well-differentiated neuroendocrine tumor (carcinoid)	Neuroendocrine tumors of the appendix	32

Summary of Changes

Change	Details of Change	Level of Evidence
Introduction	The introduction was revised to clarify the heterogeneity in behavior of appendiceal cancers.	III
Definition of Primary Tumor (T)	Tis(LAMN) category: A T category was created for low-grade appendiceal mucinous neoplasms (LAMNs) that invade or push into the muscularis propria. This change allows T category to be determined for these lesions, which previously were recorded as TX or unstaged.	III
Definition of Primary Tumor (T)	T4 redefined: *Right lower quadrant* was deleted from the T4 category.	III
Definition of Distant Metastasis (M)	M1 categorization: Intraperitoneal acellular mucin is now designated as M1a. M1a and M1b definitions from the AJCC Cancer Staging Manual, 7th Edition are now M1b and M1c, respectively.	III
Definition of Distant Metastasis (M)	M1 definition: The term *pseudomyxoma peritonei*, a clinical syndrome, was removed from the M1a category definition and is now discussed in the text.	III

To access the AJCC cancer staging forms, please visit www.cancerstaging.org.

© American Joint Committee on Cancer 2017
M.B. Amin et al. (eds.), *AJCC Cancer Staging Manual, Eighth Edition*, DOI 10.1007/978-3-319-40618-3_19

ANATOMY

Primary Site(s)

The appendix is a tubular structure that arises from the base of the cecum (Fig. 19.1). Its length varies but is about 10 mm. It is connected to the ileal mesentery by the mesoappendix, through which its blood supply passes from the ileocolic artery.

Regional Lymph Nodes

Lymphatic drainage passes into the ileocolic chain of lymph nodes (Fig. 19.2). Well-differentiated mucinous adenocarcinomas commonly demonstrate peritoneal spread in the absence of lymph node metastasis.[1,4,15,16] In contrast, poorly differentiated mucinous and nonmucinous appendiceal adenocarcinomas often have lymph node metastases in conjunction with metastatic spread.[1,4]

Metastatic Sites

All adenocarcinomas of the appendix demonstrate a predilection for peritoneal spread. Even poorly differentiated or signet ring cell metastatic adenocarcinomas demonstrate near-universal involvement of the peritoneal cavity.[17] In a manner similar to that of appendiceal adenocarcinomas, goblet cell carcinoids demonstrate a predilection for peritoneal metastases.[14]

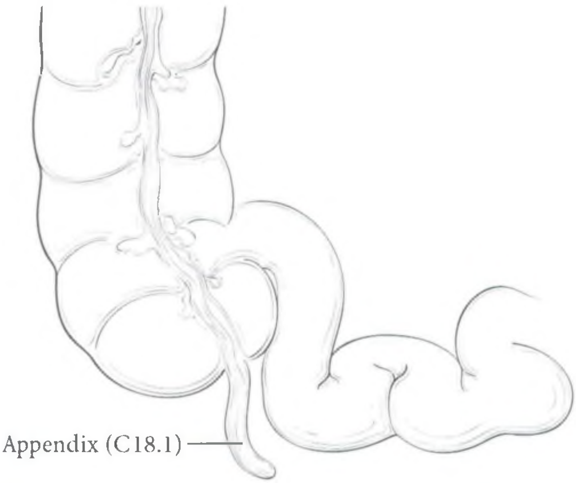

Fig. 19.1 Anatomic location of the appendix

RULES FOR CLASSIFICATION

Clinical Classification

Clinical assessment is based on medical history, physical examination, and imaging. Because peritoneal metastases represent such a common form of dissemination, it is highly recommended that a thorough visual inspection of the peritoneal cavity for peritoneal metastases be conducted during exploratory abdominal surgery. In the case of peritoneal metastases from mucinous neoplasms, it is recommended that pathological assessment from multiple locations be performed to optimally determine tumor cellularity.

The term *pseudomyxoma peritonei* is not a histologically based term and thus is not used in the staging of appendiceal neoplasms. This term represents the clinical syndrome of an appendiceal neoplasm with diffuse mucinous peritoneal involvement.

Imaging

Clinical staging with cross-sectional imaging is designed to identify the presence of regional lymph node metastases

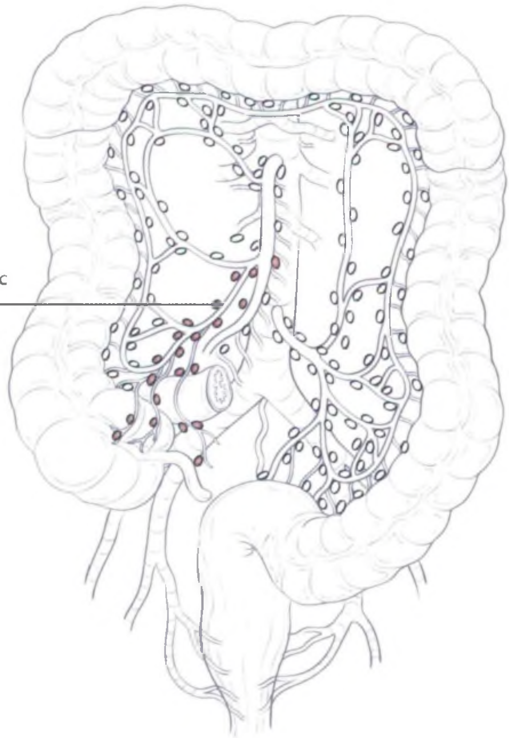

Fig. 19.2 The regional lymph nodes of the appendix

and, more importantly, the presence of peritoneal and extra-peritoneal metastases. Computed tomography (CT; abdomen, pelvis, and chest), magnetic resonance (MR) imaging, and positron emission tomography (PET) or fused PET/CT scans may be used. For highly mucinous neoplasms, PET imaging is not recommended because of the high likelihood of a false negative finding.[18] In addition, for optimal assessment of the peritoneal cavity with CT imaging, it is recommended that both oral and intravenous contrast be used. Chest radiographs may be obtained to evaluate the thoracic cavity.

Pathological Classification

Complete pathological staging includes evaluation of the primary appendiceal cancer with associated regional lymph nodes. In addition to samples from the appendix, specimens may be obtained from the cecum and peritoneum.

For premalignant lesions, the term *high-grade dysplasia* is used if the neoplastic cells are confined to the crypts and do not invade the lamina propria. Intramucosal adenocarcinoma (IMC) invades the lamina propria with or without extension into but not through the muscularis mucosae. IMC is regarded as a premalignant process in the appendix, similar to colorectal lesions, and is assigned the category Tis. T1, T2, and T3 are classified as extension into the submucosa, penetration through the submucosa into but not through the muscularis propria, and penetration through the muscularis propria, respectively. These T categories apply to invasive carcinomas of the appendix, including nonmucinous adenocarcinoma, mucinous adenocarcinoma, signet ring cell carcinoma, and goblet cell carcinoids.

LAMN is a unique histologic subtype of mucinous appendiceal neoplasm.[19] Typical adenocarcinomas show invasion into the appendiceal wall, evidenced by infiltrative growth, confluent cribriform, destructive invasion with desmoplasia, and/or tumor cells floating in extracellular mucin. In contrast, LAMNs extend into and sometimes through the appendiceal wall with a pushing front and do not demonstrate overt features of invasion, such as desmoplasia. By definition, LAMNs are associated with obliteration of the muscularis mucosae; a LAMN confined to the mucosa with intact muscularis mucosae is categorized as appendiceal adenoma.[19] An infiltrative growth pattern, destructive invasion, or stromal desmoplastic reaction is not present in LAMNs, and lymph node involvement is exceedingly rare. For LAMNs confined to the appendix, the depth of appendiceal wall involvement is not a significant risk factor for recurrence.[19] Hence, LAMNs that have not penetrated the muscularis propria are now assigned to the newly created Tis(LAMN) category. Involvement of the subserosa and beyond is assigned the T3 or T4 category, as other invasive carcinomas, respectively. In occasional cases, tumors with architectural features of LAMN may have areas of high-grade cytologic features. These are designated as high-grade appendiceal mucinous neoplasm (HAMN) or mucinous adenocarcinoma without invasion. Although outcome data for these tumors are limited, the staging system for invasive adenocarcinomas should be used for HAMN because of its higher risk of recurrence.[19]

Tumors (including acellular mucin) that involve the serosal surface (visceral peritoneum) or directly invade adjacent organs or structures are assigned to the T4 category. T4a tumors are characterized by localized involvement of the serosal surface (visceral peritoneum) in the area of the primary tumor by acellular mucin or cellular tumor. Serosal involvement of the appendix by acellular mucin may demonstrate an excellent outcome with only localized surgical resection.[6,20] In view of the small risk of recurrence, this localized involvement is categorized as T4a along with tumors with cellular mucinous involvement of appendiceal serosa. Tumors with perforation in which tumor cells or acellular/cellular mucin is continuous with the serosal surface through inflammation also are considered T4a.

Tumors that directly invade other organs or structures are categorized as T4b. However, luminal or mural spread into adjacent parts of the bowel (e.g., appendiceal tumor extending into the cecum through the lumen or wall) is not considered T4b and should be categorized by the deepest area of invasion. Direct invasion of other segments of the colorectum via the serosa (e.g., invasion of adherent ileum) is considered T4b. A tumor grossly adherent to other organs or structures is classified as cT4b; however, if no tumor is identified on pathological examination of the adhesion, the T category is assigned based on the depth of wall invasion observed on microscopic examination (typically pT1–3).

Lymph node involvement is classified as N1 or N2 according to the number of nodes involved with metastatic tumor. Involvement of one to three nodes is pN1, and the presence of four or more nodes involved with tumor metastasis is considered pN2. Histologic examination of a right hemicolectomy specimen ordinarily includes 12 or more lymph nodes. If the lymph nodes are negative but fewer than 12 lymph nodes are retrieved, the N category should be classified as pN0. In accordance with the general rules of staging, the presence of acellular mucin within lymph nodes or tumor deposits is not considered involvement and is categorized as pN0.

Tumor deposits are defined as discrete tumor nodules within the lymph drainage area of the primary carcinoma without identifiable lymph node tissue or identifiable vascular or neural structure. Shape, contour, and size of the deposit are not considered in this designation. If the vessel wall or its remnant is identifiable on hematoxylin and eosin, elastin, or any other stain, the lesion should be classified as lymphovascular invasion (LVI) present. Further documentation should subclassify LVI as small vessel invasion ("L" positive for either lymphatic or small venule involvement) or venous invasion ("V" positive for tumor within an endothelial-lined space containing red cells or surrounded by smooth muscle. These definitions are similar to those for large vessel invasion on page 122 of the AJCC Cancer Staging Manual, 6th Edition.[21] If neural structures are identifiable, the lesion should be classified as perineural invasion. Individual one to four deposits or five or more tumor deposits without involvement of lymphatic, venous, or neural structures within the lymph drainage area of the primary carcinoma should be recorded.

Although the prognostic relevance of tumor deposits for appendiceal adenocarcinomas has not been well studied, tumor deposits represent a proven poor prognostic factor for adenocarcinomas of the colorectum, and collection as a separate data element is warranted. In cases with tumor deposits but no identified lymph node metastases, the N1c category is used and applicable to all T categories. The presence of tumor deposits does not change the primary tumor T category; however, it does change the node status (N) to N1c if all regional lymph nodes are pathologically negative. The number of tumor deposits is not added to the number of positive regional lymph nodes if one or more lymph nodes contain cancer.

Histopathologic parameters such as mucinous histology and grade are used for appropriate staging of Stage IV patients. Peritoneal spread is common in appendiceal tumors and is categorized as M1b if limited to the peritoneum. Peritoneal implants involving abdominopelvic organs, such as the serosa of the small or large bowel and the surfaces of the ovary, spleen, or liver, should be classified as M1b disease, regardless of whether implants demonstrate infiltration of underlying tissue, manifested as invasion. Nonperitoneal metastasis, such as pleuropulmonary involvement, is rare and would be categorized as M1c (Stage IVC).

Peritoneal involvement by a well-differentiated (G1) adenocarcinoma, without dissemination to other organs, is staged as IVA. This category includes various proposed terms, such as *LAMN with peritoneal involvement*, *DPAM*, and low-grade mucinous carcinoma peritonei.[6,10–12] In some instances, the peritoneal disease comprises acellular mucin only. Although the outcome for patients with peritoneal dissemination of acellular as compared with cellular mucin is better and aggressive surgical resection is not required in all cases, a subset will demonstrate disease progression.[6,11,22] Given this unique behavior, peritoneal involvement by acellular mucin is now recognized by the new category of M1a and is staged as IVA.

pTNM Pathological Classification

The pT, pN, and c/pM categories correspond to the T, N, and c/pM categories.

Staging following Neoadjuvant Treatment

The *y* prefix is used for cancers classified after neoadjuvant pretreatment (ypTNM).

Restaging following Disease-free Interval

For appendiceal carcinomas, the *r* prefix is used for recurrent tumor status (rTNM) following a disease-free interval after treatment.

PROGNOSTIC FACTORS

Prognostic Factors Required for Stage Grouping

Histologic Grade

Determination of histologic grade has prognostic significance and is needed to define Stage IV tumors as either IVA or IVB. Well-differentiated mucinous appendiceal adenocarcinomas that are metastatic (IVA) have a markedly improved outcome (Fig. 19.3). Poorly differentiated mucinous or signet ring cell adenocarcinomas (G3), defined as having more than 50% signet ring cells, that have metastasized to the peritoneal cavity demonstrate a markedly worse outcome, which is similar to that of nonmucinous appendiceal adenocarcinomas.[1,17,23] The rate of extraperitoneal spread of well-differentiated mucinous appendiceal adenocarcinomas is extremely low. Given this peritoneal predilection and more indolent biologic behavior, cytoreductive surgery should be considered as the initial treatment modality for Stage IVA patients.[24,25,26] The role of cytoreductive surgery for moderately or poorly differentiated mucinous appendiceal adenocarcinomas is not well delineated in the literature. However, based on the Surveillance, Epidemiology, and End Results (SEER) database and the National Cancer Data Base (NCDB), moderately differentiated mucinous appendiceal adenocarcinomas have demonstrated an improved natural history that appears more similar to that of well-differentiated than poorly differentiated mucinous appendiceal adenocarcinomas.[1,4] AJCC Level of Evidence: I

Grading of Mucinous Adenocarcinomas

Although a two-tier grading system (low and high grade) was used for mucinous appendiceal neoplasms in several series,[7,10,12,27–29] other studies have demonstrated the prognostic significance of a three-tier grading scheme.[1,5,30–32] The NCDB also supports grading mucinous carcinomas into three tiers: well-, moderately, and poorly differentiated categories.[4] Because conventional grading schemes based on gland formation are difficult to apply in mucinous tumors, a three-tier grading scheme based on cytologic features, tumor cellularity, and signet ring component is recommended.[31,32] The grade of the appendiceal and peritoneal tumors is concordant in most instances, but some cases may show discordant grades in the appendix and peritoneum.[10–12]

Well-differentiated (G1) mucinous tumors often are composed of tall columnar cells, show low-grade cytologic atypia, and do not contain signet ring cells. In the appendix, these tumors lack typical features of invasion and usually are classified as LAMNs. If these tumors involve the peritoneum, they show acellular mucin or low cellularity (typically <20%) and lack infiltrative invasion of the peritoneum or other organs.[32] Involvement of organs with pushing front without desmoplasia is not considered true invasion and may occur in well-differentiated tumors. Aggressive features such as perineural invasion and lymphovascular invasion are not seen.[32] Mucinous G1 tumors with peritoneal involvement may be categorized as LAMN with peritoneal involvement, and as T4a, M1a, or M1b, depending on the extent of disease and the presence of tumor cells in peritoneal mucin deposits.

Moderately differentiated (G2) mucinous tumors show a mix of low- and high-grade cytologic atypia or diffuse high-grade cytologic atypia, but no signet ring cell component.[31,32] In the appendix, most of these tumors show features of invasion (at least focally). In rare instances, invasion may not be present, and these tumors have been referred to as high-grade mucinous appendiceal neoplasms or mucinous adenocarcinomas without destructive invasion.[10,11,32] If these tumors involve the peritoneum, they often show high cellularity (typically >20%).[32] Infiltrative invasion into the peritoneum or other organs, perineural invasion, and lymphovascular invasion may be present.[32]

Poorly differentiated (G3) mucinous tumors are high-grade, invasive tumors that usually have a signet ring cell component.[31,32] Peritoneal involvement typically is accompanied by high cellularity (typically >20%), and other adverse histologic features, such as infiltrative invasion into the peritoneum or other organs, perineural invasion, and lymphovascular invasion, may be present.[32]

Most mucinous G1 tumors with peritoneal involvement would correspond to tumors that variously have been referred to as LAMN with peritoneal involvement, low-grade mucinous carcinoma peritonei, and DPAM. Most mucinous G2 and G3 tumors with peritoneal involvement would correspond to highgrade mucinous carcinoma peritoneii and peritoneal mucinous adenocarcinoma.

Grading of Nonmucinous Adenocarcinomas

These tumors are graded as well-differentiated (G1, >95% gland formation), moderately differentiated (G2, 50–95% gland formation), and poorly differentiated (G3, <50% gland formation), similar to grading of colorectal cancers.

Additional Factors Recommended for Clinical Care

Mucinous Histology

Appendiceal mucinous adenocarcinomas (those with >50% of the tumor mass consisting of extracellular mucin) that spread to the peritoneum have a much better prognosis than nonmucinous tumors.[1,3] Although outcomes for localized (Stage I, II, or III) mucinous and nonmucinous appendiceal adenocarcinomas are similar, there is a marked difference in outcomes for Stage IV disease (Fig. 19.4).[1,4] Determination of mucinous versus nonmucinous histology is needed to differentiate between Stage IVA and IVB tumors. AJCC Level of Evidence: I

Additional Factors

The prognostic utility of additional factors for appendiceal neoplasms has not been well studied. Several factors are used in clinical care based on extrapolation from treatment of colorectal cancer, and information regarding these factors may be found in the colon and rectum chapter. These factors include:

- Preoperative/pretreatment tumor markers: carcinoembryonic antigen (CEA), cancer antigen (CA) 19-9, or CA 125
- Tumor deposits
- Lymphovascular invasion
- Perineural invasion
- Microsatellite instability

RISK ASSESSMENT MODELS

The AJCC recently established guidelines that will be used to evaluate published statistical prediction models for the purpose of granting endorsement for clinical use.[33] Although this is a monumental step toward the goal of precision medicine, this work was published only very recently. Therefore, the existing models that have been published or

may be in clinical use have not yet been evaluated for this cancer site by the Precision Medicine Core of the AJCC. In the future, the statistical prediction models for this cancer site will be evaluated, and those that meet all AJCC criteria will be endorsed.

DEFINITIONS OF AJCC TNM

Definition of Primary Tumor (T)

T Category	T Criteria
TX	Primary tumor cannot be assessed
T0	No evidence of primary tumor
Tis	Carcinoma *in situ* (intramucosal carcinoma; invasion of the lamina propria or extension into but not through the muscularis mucosae)
Tis(LAMN)	Low-grade appendiceal mucinous neoplasm confined by the muscularis propria. Acellular mucin or mucinous epithelium may invade into the muscularis propria.<br>T1 and T2 are not applicable to LAMN. Acellular mucin or mucinous epithelium that extends into the subserosa or serosa should be classified as T3 or T4a, respectively.
T1	Tumor invades the submucosa (through the muscularis mucosa but not into the muscularis propria)
T2	Tumor invades the muscularis propria
T3	Tumor invades through the muscularis propria into the subserosa or the mesoappendix
T4	Tumor invades the visceral peritoneum, including the acellular mucin or mucinous epithelium involving the serosa of the appendix or mesoappendix, and/or directly invades adjacent organs or structures
T4a	Tumor invades through the visceral peritoneum, including the acellular mucin or mucinous epithelium involving the serosa of the appendix or serosa of the mesoappendix
T4b	Tumor directly invades or adheres to adjacent organs or structures

Definition of Regional Lymph Node (N)

N Category	N Criteria
NX	Regional lymph nodes cannot be assessed
N0	No regional lymph node metastasis
N1	One to three regional lymph nodes are positive (tumor in lymph node measuring $\geq$0.2 mm) or any number of tumor deposits is present, and all identifiable lymph nodes are negative
N1a	One regional lymph node is positive
N1b	Two or three regional lymph nodes are positive
N1c	No regional lymph nodes are positive, but there are tumor deposits in the subserosa or mesentery
N2	Four or more regional lymph nodes are positive

Definition of Distant Metastasis (M)

M Category	M Criteria
M0	No distant metastasis
M1	Distant metastasis
M1a	Intraperitoneal acellular mucin, without identifiable tumor cells in the disseminated peritoneal mucinous deposits
M1b	Intraperitoneal metastasis only, including peritoneal mucinous deposits containing tumor cells
M1c	Metastasis to sites other than peritoneum

Note: For specimens containing acellular mucin without identifiable tumor cells, efforts should be made to obtain additional tissue for thorough histologic examination to evaluate for cellularity

HISTOLOGIC GRADE (G)

G	G Definition
GX	Grade cannot be assessed
G1	Well differentiated
G2	Moderately differentiated
G3	Poorly differentiated

AJCC PROGNOSTIC STAGE GROUPS

When T is...	And N is...	And M is...	And grade is...	Then the stage group is...
Tis	N0	M0		0
Tis(LAMN)	N0	M0		0
T1	N0	M0		I
T2	N0	M0		I
T3	N0	M0		IIA
T4a	N0	M0		IIB
T4b	N0	M0		IIC
T1	N1	M0		IIIA
T2	N1	M0		IIIA
T3	N1	M0		IIIB
T4	N1	M0		IIIB
Any T	N2	M0		IIIC
Any T	N0	M1a		IVA
Any T	Any N	M1b	G1	IVA
Any T	Any N	M1b	G2, G3, or GX	IVB
Any T	Any N	M1c	Any G	IVC

REGISTRY DATA COLLECTION VARIABLES

1. Grade
2. CEA levels
3. Tumor deposits
4. Lymphovascular invasion
5. Perineural invasion

HISTOPATHOLOGIC TYPE

- Adenocarcinoma *in situ*
- Low-grade appendiceal mucinous neoplasm
- Adenocarcinoma
- Mucinous carcinoma (>50% mucinous carcinoma)
- Signet ring cell carcinoma (>50% signet ring cell)
- Undifferentiated carcinoma
- Goblet cell carcinoid
- Mixed adenoneuroendocrine carcinoma
- Carcinoma, NOS

RESIDUAL TUMOR (R)

R	R Definition
R0	Complete resection, margins histologically negative, no residual tumor left after resection
R1	Incomplete resection, margins histologically involved, microscopic tumor remains after resection of gross disease (relevant to resection margins that are microscopically involved by tumor)
R2	Incomplete resection, margins involved, or gross disease remains

Note: The importance of acellular mucin at the resection edge in appendiceal tumors has not been studied extensively. At present, acellular mucin at the resection edge should not be used to determine resection margin status because its impact on recurrence is uncertain [20,34]

SURVIVAL DATA

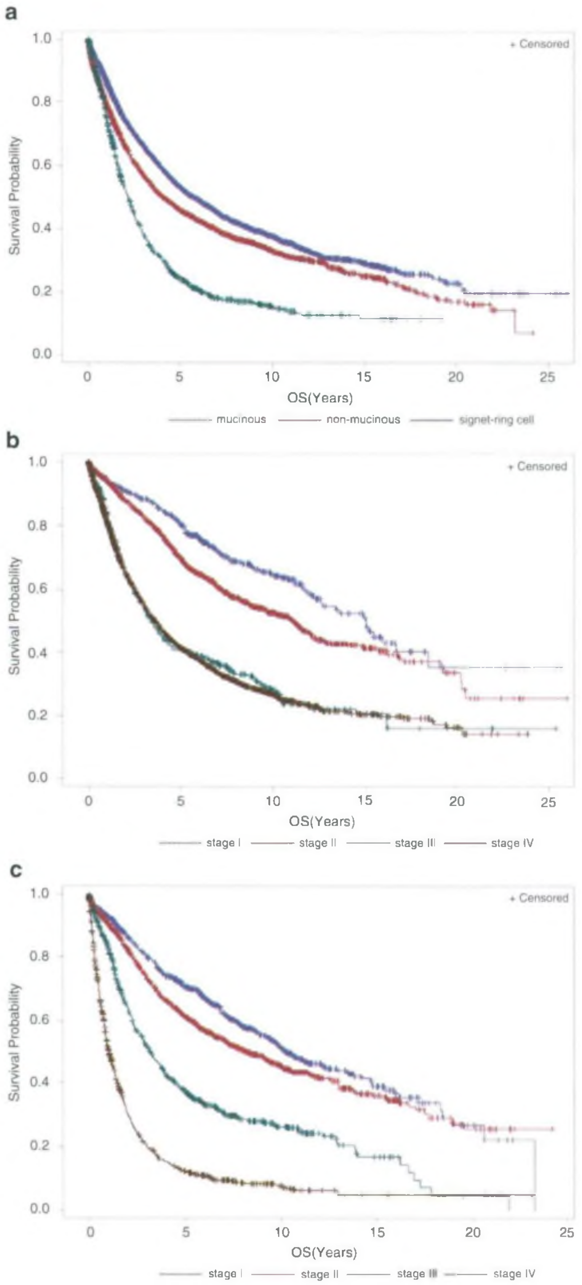

Fig. 19.3 Overall survival from the NCDB, stratified by histologic type (*top*), stage stratified for mucinous histology (*middle*), and stage stratified for nonmucinous histology (*bottom*). Observed survival calculated using the Kaplan–Meier method (Adapted from Asare et al.[4])

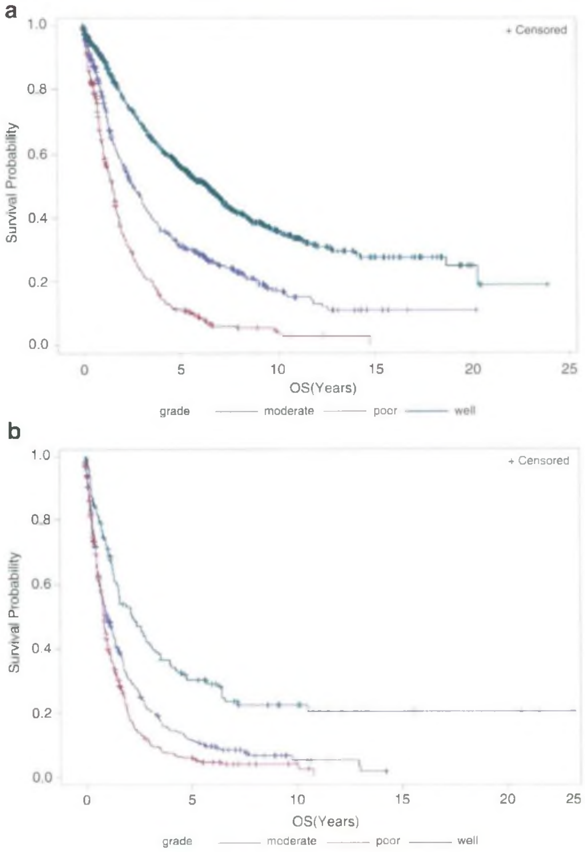

Fig. 19.4 Overall survival from the NCDB for Stage IV, stratified by histological grade for (*top*) mucinous and (*bottom*) nonmucinous histology. Observed survival calculated using the Kaplan–Meier method (Adapted from Asare et al.[4])

ILLUSTRATIONS

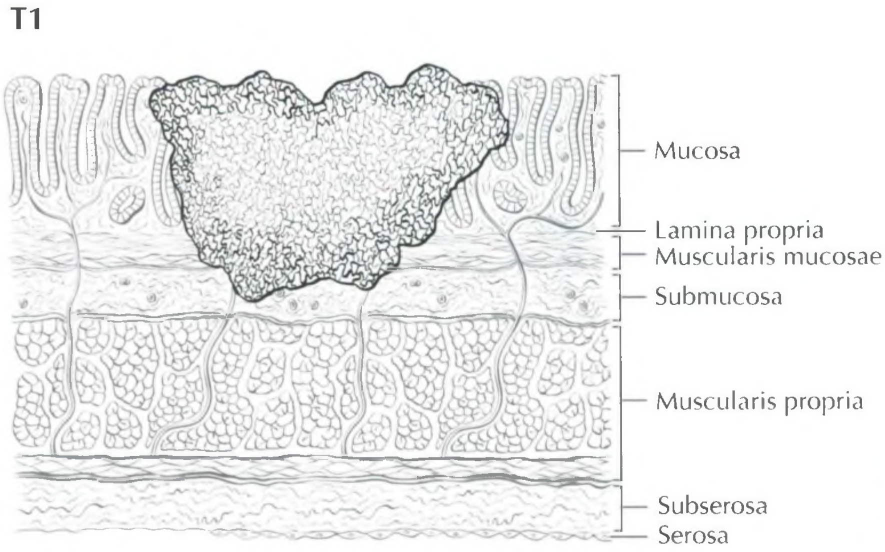

Fig. 19.5 T1 is defined as tumor that invades the submucosa (through the muscularis mucosa but not into the muscularis propria)

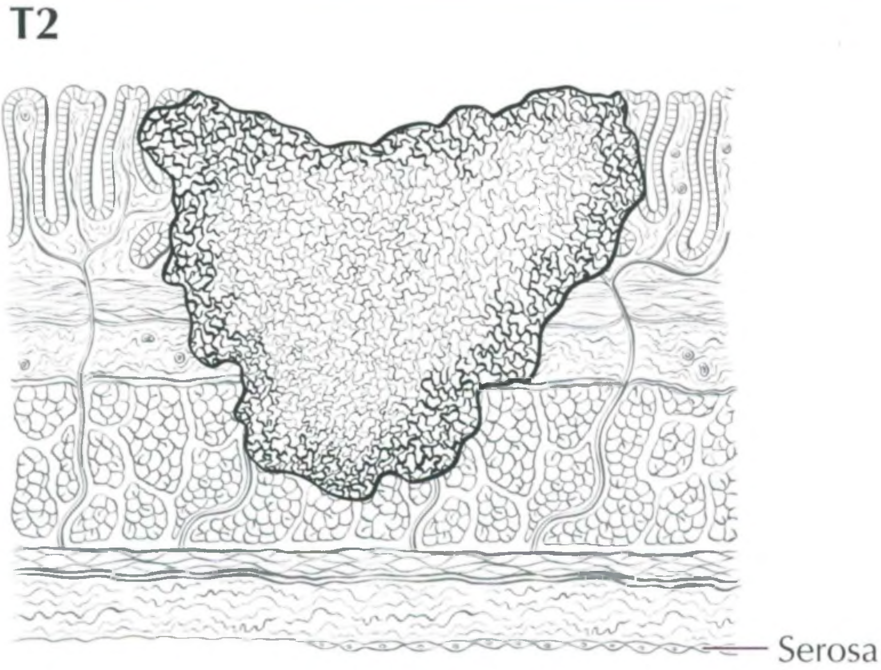

Fig. 19.6 T2 is defined as tumor that invades muscularis propria

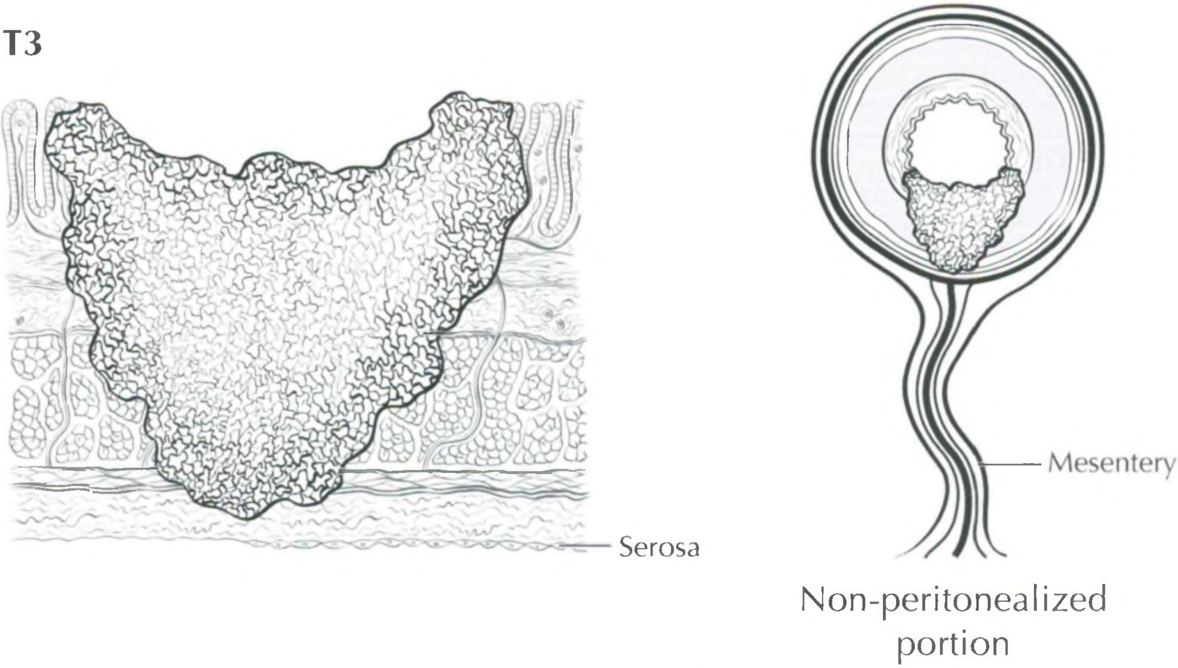

Fig. 19.7 T3 is defined as tumor that invades through the muscularis propria into the subserosa or the mesoappendix

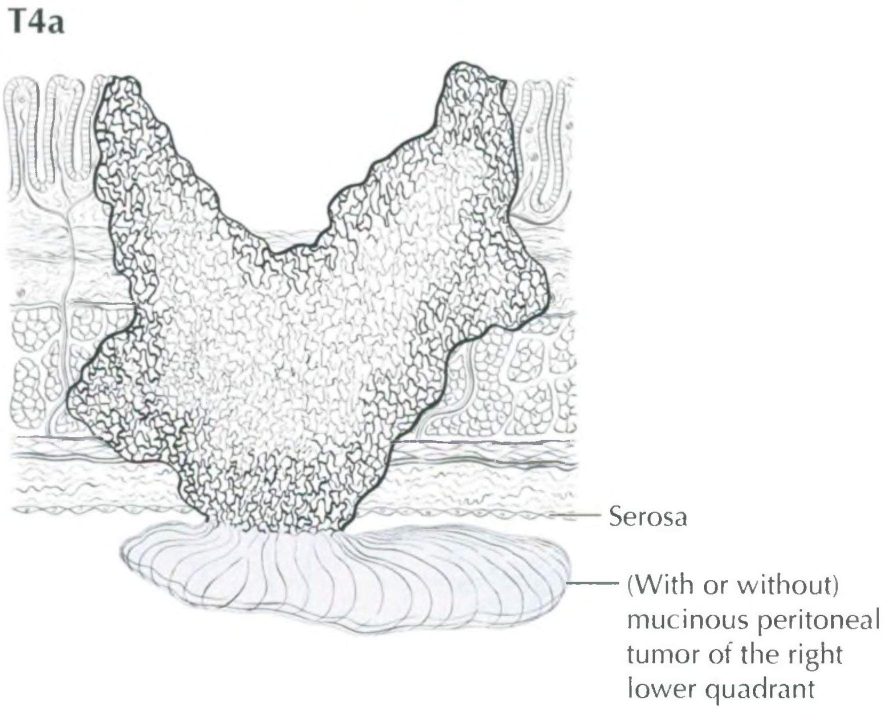

Fig. 19.8 T4a is defined as tumor that invades through the visceral peritoneum, including the acellular mucin or mucinous epithelium involving the serosa of the appendix or serosa of the mesoappendix

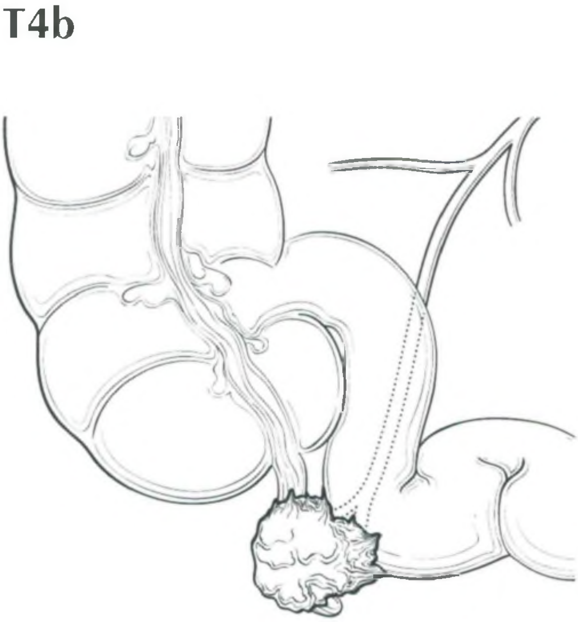

Fig. 19.9 T4b is defined as tumor that directly invades or adheres to adjacent organs or structures

Fig. 19.10 N1 is defined as metastasis to one to three regional lymph nodes (tumor in lymph node measuring ≥0.2 mm) or any number of tumor deposits is present, and all identifiable lymph nodes are negative

N1

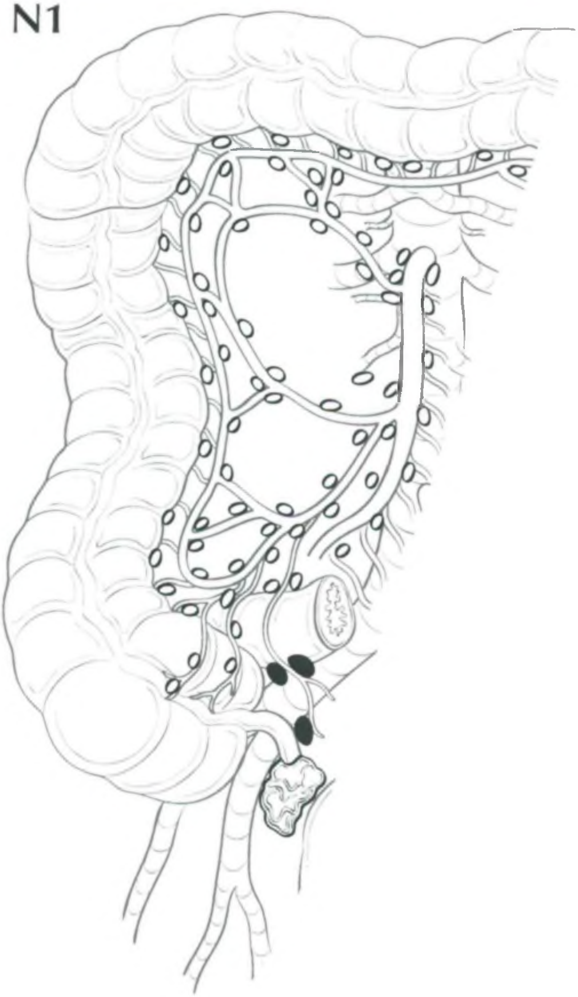

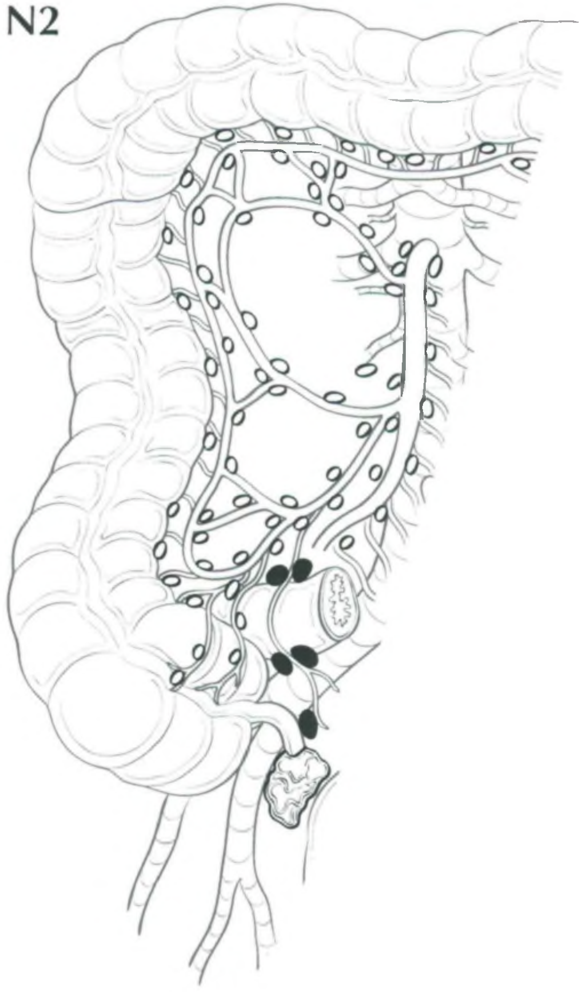

Fig. 19.11 N2 is defined as metastasis in 4 or more regional lymph nodes

Bibliography

1. Overman MJ, Fournier K, Hu CY, et al. Improving the AJCC/TNM staging for adenocarcinomas of the appendix: the prognostic impact of histological grade. *Annals of surgery*. Jun 2013;257(6):1072–1078.
2. Turaga KK, Pappas SG, Gamblin T. Importance of histologic subtype in the staging of appendiceal tumors. *Annals of surgical oncology*. May 2012;19(5):1379–1385.
3. McCusker ME, Cote TR, Clegg LX, Sobin LH. Primary malignant neoplasms of the appendix: a population-based study from the surveillance, epidemiology and end-results program, 1973-1998. *Cancer*. Jun 15 2002;94(12):3307–3312.
4. Asare EA, Compton CC, Hanna NN, et al. The impact of stage, grade, and mucinous histology on the efficacy of systemic chemotherapy in adenocarcinomas of the appendix: Analysis of the National Cancer Data Base. *Cancer*. Jan 15 2016;122(2):213–221.
5. Loungnarath R, Causeret S, Bossard N, et al. Cytoreductive surgery with intraperitoneal chemohyperthermia for the treatment of pseudomyxoma peritonei: a prospective study. *Diseases of the colon and rectum*. Jul 2005;48(7):1372–1379.
6. Carr NJ, McCarthy WF, Sobin LH. Epithelial noncarcinoid tumors and tumor-like lesions of the appendix. A clinicopathologic study of 184 patients with a multivariate analysis of prognostic factors. *Cancer*. Feb 1 1995;75(3):757–768.
7. Bradley RF, Stewart JHt, Russell GB, Levine EA, Geisinger KR. Pseudomyxoma peritonei of appendiceal origin: a clinicopathologic analysis of 101 patients uniformly treated at a single institution, with literature review. *The American journal of surgical pathology*. May 2006;30(5):551–559.
8. Sugarbaker PH, Alderman R, Edwards G, et al. Prospective morbidity and mortality assessment of cytoreductive surgery plus perioperative intraperitoneal chemotherapy to treat peritoneal dissemination of appendiceal mucinous malignancy. *Annals of surgical oncology*. May 2006;13(5):635–644.
9. Gough DB, Donohue JH, Schutt AJ, et al. Pseudomyxoma peritonei. Long-term patient survival with an aggressive regional approach. *Annals of surgery*. Feb 1994;219(2):112–119.
10. Misdraji J, Yantiss RK, Graeme-Cook FM, Balis UJ, Young RH. Appendiceal mucinous neoplasms: a clinicopathologic analysis of 107 cases. *The American journal of surgical pathology*. Aug 2003;27(8):1089–1103.

11. Pai RK, Beck AH, Norton JA, Longacre TA. Appendiceal mucinous neoplasms: clinicopathologic study of 116 cases with analysis of factors predicting recurrence. *The American journal of surgical pathology.* Oct 2009;33(10):1425–1439.
12. Ronnett BM, Zahn CM, Kurman RJ, Kass ME, Sugarbaker PH, Shmookler BM. Disseminated peritoneal adenomucinosis and peritoneal mucinous carcinomatosis. A clinicopathologic analysis of 109 cases with emphasis on distinguishing pathologic features, site of origin, prognosis, and relationship to "pseudomyxoma peritonei". *The American journal of surgical pathology.* Dec 1995;19(12):1390–1408.
13. Burke AP, Sobin LH, Federspiel BH, Shekitka KM, Helwig EB. Goblet cell carcinoids and related tumors of the vermiform appendix. *Am J Clin Pathol.* Jul 1990;94(1):27–35.
14. Tang LH, Shia J, Soslow RA, et al. Pathologic classification and clinical behavior of the spectrum of goblet cell carcinoid tumors of the appendix. *The American journal of surgical pathology.* Oct 2008;32(10):1429–1443.
15. Gonzalez-Moreno S, Sugarbaker PH. Right hemicolectomy does not confer a survival advantage in patients with mucinous carcinoma of the appendix and peritoneal seeding. *The British journal of surgery.* Mar 2004;91(3):304–311.
16. Turaga KK, Pappas S, Gamblin TC. Right hemicolectomy for mucinous adenocarcinoma of the appendix: just right or too much? *Annals of surgical oncology.* Apr 2013;20(4):1063–1067.
17. Lieu CH, Lambert LA, Wolff RA, et al. Systemic chemotherapy and surgical cytoreduction for poorly differentiated and signet ring cell adenocarcinomas of the appendix. *Ann Oncol.* Mar 2012;23(3):652–658.
18. Rohani P, Scotti SD, Shen P, et al. Use of FDG-PET imaging for patients with disseminated cancer of the appendix. *The American surgeon.* Dec 2010;76(12):1338–1344.
19. Bosman FT, Carneiro F, Hruban RH, Theise ND. *WHO classification of tumours of the digestive system.* World Health Organization; 2010.
20. Yantiss RK, Shia J, Klimstra DS, Hahn HP, Odze RD, Misdraji J. Prognostic significance of localized extra-appendiceal mucin deposition in appendiceal mucinous neoplasms. *The American journal of surgical pathology.* Feb 2009;33(2):248–255.
21. Greene FL. *AJCC cancer staging manual.* Vol 1: Springer Science & Business Media; 2002.
22. Jackson SL, Fleming RA, Loggie BW, Geisinger KR. Gelatinous ascites: a cytohistologic study of pseudomyxoma peritonei in 67 patients. *Modern pathology : an official journal of the United States and Canadian Academy of Pathology, Inc.* Jul 2001;14(7):664–671.
23. Shapiro JF, Chase JL, Wolff RA, et al. Modern systemic chemotherapy in surgically unresectable neoplasms of appendiceal origin: a single-institution experience. *Cancer.* Jan 15 2010;116(2):316–322.
24. Glehen O, Gilly FN, Boutitie F, et al. Toward curative treatment of peritoneal carcinomatosis from nonovarian origin by cytoreductive surgery combined with perioperative intraperitoneal chemotherapy: a multi-institutional study of 1,290 patients. *Cancer.* Dec 15 2010;116(24):5608–5618.
25. Miner TJ, Shia J, Jaques DP, Klimstra DS, Brennan MF, Coit DG. Long-term survival following treatment of pseudomyxoma peritonei: an analysis of surgical therapy. *Annals of surgery.* Feb 2005;241(2):300–308.
26. Sugarbaker PH, Chang D. Results of treatment of 385 patients with peritoneal surface spread of appendiceal malignancy. *Annals of surgical oncology.* Dec 1999;6(8):727–731.
27. Carr NJ, Finch J, Ilesley IC, et al. Pathology and prognosis in pseudomyxoma peritonei: a review of 274 cases. *Journal of clinical pathology.* Oct 2012;65(10):919–923.
28. Chua TC, Moran BJ, Sugarbaker PH, et al. Early- and long-term outcome data of patients with pseudomyxoma peritonei from appendiceal origin treated by a strategy of cytoreductive surgery and hyperthermic intraperitoneal chemotherapy. *J Clin Oncol.* Jul 10 2012;30(20):2449–2456.
29. Baratti D, Kusamura S, Nonaka D, Cabras AD, Laterza B, Deraco M. Pseudomyxoma peritonei: biological features are the dominant prognostic determinants after complete cytoreduction and hyperthermic intraperitoneal chemotherapy. *Annals of surgery.* Feb 2009;249(2):243–249.
30. Smeenk RM, Verwaal VJ, Antonini N, Zoetmulder FA. Survival analysis of pseudomyxoma peritonei patients treated by cytoreductive surgery and hyperthermic intraperitoneal chemotherapy. *Annals of surgery.* Jan 2007;245(1):104–109.
31. Shetty S, Natarajan B, Thomas P, Govindarajan V, Sharma P, Loggie B. Proposed classification of pseudomyxoma peritonei: influence of signet ring cells on survival. *The American surgeon.* Nov 2013;79(11):1171–1176.
32. Davison JM, Choudry HA, Pingpank JF, et al. Clinicopathologic and molecular analysis of disseminated appendiceal mucinous neoplasms: identification of factors predicting survival and proposed criteria for a three-tiered assessment of tumor grade. *Modern pathology : an official journal of the United States and Canadian Academy of Pathology, Inc.* Nov 2014;27(11):1521–1539.
33. Kattan MW, Hess KR, Amin MB, et al. American Joint Committee on Cancer acceptance criteria for inclusion of risk models for individualized prognosis in the practice of precision medicine. *CA: a cancer journal for clinicians.* Jan 19 2016
34. Arnason T, Kamionek M, Yang M, Yantiss RK, Misdraji J. Significance of proximal margin involvement in low-grade appendiceal mucinous neoplasms. *Arch Pathol Lab Med.* Apr 2015;139(4):518–521.

Colon and Rectum

20

J. Milburn Jessup, Richard M. Goldberg, Elliot A. Asare, Al B. Benson III, James D. Brierley, George J. Chang, Vivien Chen, Carolyn C. Compton, Paola De Nardi, Karyn A. Goodman, Donna Gress, Justin Guinney, Leonard L. Gunderson, Stanley R. Hamilton, Nader N. Hanna, Sanjay Kakar, Lauren A. Kosinski, Serban Negoita, Shuji Ogino, Michael J. Overman, Philip Quirke, Eric Rohren, Daniel J. Sargent, Lynne T. Schumacher-Penberthy, David Shibata, Frank A. Sinicrope, Scott R. Steele, Alexander Stojadinovic, Sabine Tejpar, Martin R. Weiser, Mark Lane Welton, and Mary Kay Washington

CHAPTER SUMMARY

Cancers Staged Using This Staging System

Adenocarcinomas, high-grade neuroendocrine carcinomas, and squamous carcinomas of the colon and rectum are covered by this staging system.

Cancers Not Staged Using This Staging System

These histopathologic types of cancer…	Are staged according to the classification for…	And can be found in chapter…
Appendiceal carcinomas	Appendix—carcinoma	19
Anal carcinomas	Anus	21
Well-differentiated neuroendocrine tumors (carcinoids)	Well-differentiated neuroendocrine tumors of the colon and rectum	33

Summary of Changes

Change	Details of Change	Level of Evidence
Definition of Distant Metastasis (M)	Introduced M1c, which details peritoneal carcinomatosis as a poor prognostic factor	I
Definition of Regional Lymph Node (N)	Clarified the definition of tumor deposits	II
Additional Factors Recommended for Clinical Care	Lymphovascular invasion: reintroduced the L and V elements to better identify lymphatic and vessel invasion	I
Additional Factors Recommended for Clinical Care	Microsatellite instability (MSI): clarified the importance of MSI as a prognostic and predictive factor	I
Additional Factors Recommended for Clinical Care	Identified *KRAS*, *NRAS*, and *BRAF* mutations as critical prognostic factors that are also predictive	I and II

To access the AJCC cancer staging forms, please visit www.cancerstaging.org.

© American Joint Committee on Cancer 2017

M.B. Amin et al. (eds.), *AJCC Cancer Staging Manual, Eighth Edition*, DOI 10.1007/978-3-319-40618-3_20

ICD-O-3 Topography Codes

Code	Description
C18.0	Cecum
C18.2	Ascending colon
C18.3	Hepatic flexure of colon
C18.4	Transverse colon
C18.5	Splenic flexure of colon
C18.6	Descending colon
C18.7	Sigmoid colon
C18.8	Overlapping lesion of colon
C18.9	Colon, NOS
C19.9	Rectosigmoid junction
C20.9	Rectum, NOS

WHO Classification of Tumors

Code	Description
8140	Adenocarcinoma *in situ*
8140	Adenocarcinoma
8510	Medullary carcinoma
8480	Mucinous carcinoma (colloid type; >50% extracellular mucinous carcinoma)
8490	Signet ring cell carcinoma
8070	Squamous cell carcinoma
8560	Adenosquamous carcinoma
8246	Neuroendocrine carcinoma
8041	Small cell neuroendocrine carcinoma
8013	Large cell neuroendocrine carcinoma
8020	Undifferentiated carcinoma
8010	Carcinoma, NOS

Bosman FT, Carneiro F, Hruban RH, Theise ND, eds. World Health Organization Classification of Tumours of the Digestive System. Lyon: IARC; 2010.

INTRODUCTION

Adenocarcinoma of the colon and rectum is the second most lethal cancer in the United States; its treatment is determined primarily by TNM staging. The advent of detailed molecular characterization of colorectal carcinoma has led to better understanding of not only the etiology of the malignancy but also how the disease responds to stage-specific treatment. The molecular characteristics provide a set of prognostic factors that likely will become more important in the future. In addition, histopathologic analysis of primary carcinomas, as well as the development of immune checkpoint inhibitors, has led to an increasing appreciation of the role of host immunity in improving survival. This approach involves the current development of a histologic prognostic and predictive scoring system, called Immunoscore, that may improve TNM staging after it is validated. It also involves early trials of inhibitors of checkpoint proteins such as PD-1, PD-L1, and CTL4. These recent molecular and immune findings portend exciting future methods for treating colorectal carcinoma.

The AJCC Cancer Staging Manual, 8th Edition is very similar to the AJCC Cancer Staging Manual, 7th Edition. The colorectal disease team of the Lower GI Expert Panel has attempted to clarify the issues that have perplexed some experts in the last several editions. This has has led to the recommendation that small vessel and large venous involvement be collected as registry data items in addition to tumor deposits. We also present data that validate the division of T4 colon or rectal cancer primaries into T4a and T4b categories in a dataset independent from the one used in the 7th Edition. We have strengthened the evidence for collecting molecular data such as microsatellite instability (MSI) status and BRAF mutations as prognostic factors, as well as mutations in BRAF, KRAS, and NRAS as predictive factors.

ANATOMY

Primary Site(s)

The large intestine (colon and rectum) extends from the terminal ileum to the anal canal. Excluding the vermiform appendix and rectum, the colon is divided into four parts: the right or ascending colon, the middle or transverse colon, the left or descending colon, and the sigmoid colon. The sigmoid colon is continuous with the rectum, which terminates at the anal canal (Figs. 20.1 and 20.2).

The ascending colon begins with the cecum, a 6- to 9-cm pouch that arises as the proximal segment of the right colon at the end of the terminal ileum. It is covered with a visceral peritoneum (serosa). The ascending colon continues from the cecum and measures about 15 to 20 cm in length. The posterior surface of the ascending (and descending) colon lacks peritoneum and thus is in direct contact with the retroperitoneum. In contrast, the anterior and lateral surfaces of the ascending (and descending) colon have serosa and are intraperitoneal. The ascending colon ends at the hepatic flexure, which transitions the ascending colon into the transverse colon, passing just inferior to the liver and anterior to the duodenum.

The transverse colon is entirely intraperitoneal, about 18 to 22 cm long, and supported on a mesentery that is attached to the pancreas. Anteriorly, its serosa is continuous with the gastrocolic ligament. The transverse colon ends at the splenic flexure, which transitions into the descending colon.

The descending colon passes inferior to the spleen and anterior to the tail of the pancreas. As noted earlier, the pos-

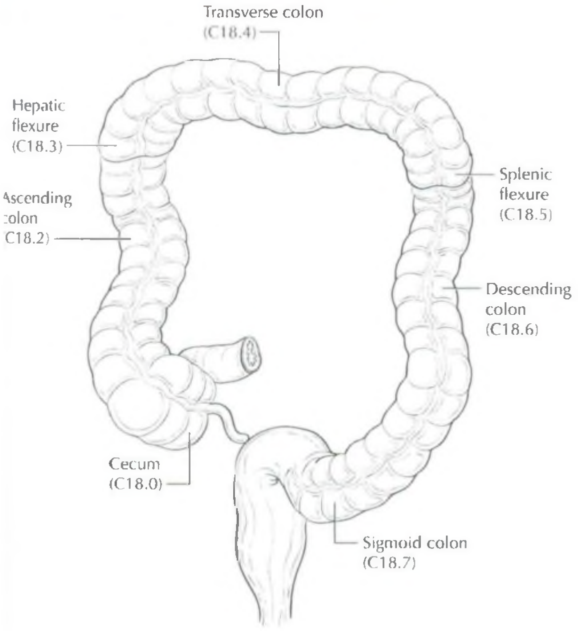

Fig. 20.1 Anatomic subsites of the colon

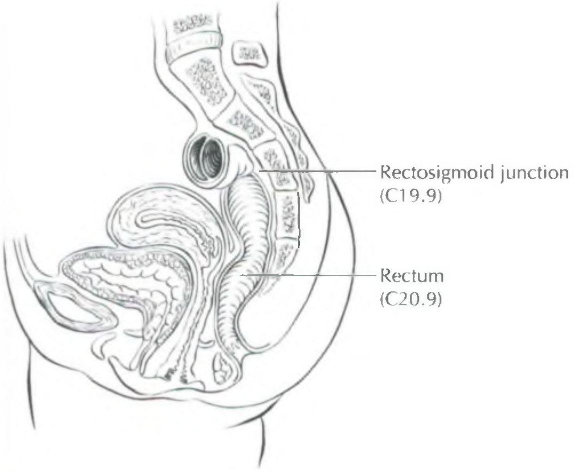

Fig. 20.2 Anatomic subsites of the rectum

erior aspect of the descending colon lacks serosa and is in direct contact with the retroperitoneum, whereas the lateral and anterior surfaces have serosa and are intraperitoneal. The descending colon measures about 10 to 15 cm in length.

The sigmoid colon is completely intraperitoneal, once again with a mesentery that develops at the medial border of the left psoas major muscle and extends to the rectum. The transition from sigmoid colon to rectum is marked by the fusion of the taenia of the sigmoid colon to the circumferential longitudinal muscle of the rectum. The sigmoid colon is approximately 15 to 20 cm long.

The proximal rectum is defined by the fusion of the taenia, which typically occurs at the level of the sacral promontory. The distal boundary of the rectal reservoir or ampulla is the puborectalis ring, which is palpable as the anorectal ring on digital rectal examination. The rectal mucosa extends below this ring into the functional anal canal to the dentate line. This feature is critical to understanding how rectal cancer may occur within the functional ("surgical") anal canal. The rectum is approximately 12 to 16 cm in length. It is covered by peritoneum in front and on both sides in its upper third and only on the anterior wall in its middle third. The peritoneum is reflected laterally from the rectum to form the perirectal fossa and, anteriorly, the uterine or rectovesical fold. Depending on body habitus and gender, this fossa may be widely variable, and may extend to the pelvic floor.

In general, the lower third of the rectum (the reservoir or ampulla) does not have a peritoneal covering. This extraperitoneal rectum is encircled by a variably thick fatty sheath containing perirectal lymph nodes and enveloped circumferentially by the fascia propria, with separation posteriorly from the sacrum by Waldeyer's fascia, from the pelvic sidewalls by the pelvic parietal fascia, and anteriorly from the prostate or vagina by Denonvilliers' fascia. The mesorectum tapers distally so that no fatty sheath surrounds the rectal wall at the puborectalis sling. The rectum has semilunar transverse rectal folds, also known as the valves of Houston. Most commonly, there are three folds, although two or four may be present. These boundaries are described further in the evaluation of total mesorectal excision (TME) specimens.

The mucosa of the colon and rectum comprises a single layer of epithelial cells arranged in invaginations called the crypts of Lieberkühn, separated by the lamina propria, a loose connective tissue stroma investing the crypts. The base of the mucosa is separated from the submucosa by a thin but distinct muscle layer, the muscularis mucosae. The thicker, separate, and deeper layer of smooth muscle is the muscularis propria. The area between the two muscle layers is the submucosa. The layer of connective tissue beyond the muscularis propria is the pericolorectal connective tissue, which alternatively may be called subserosal tissue when covered by a peritoneal lining, or adventia, in areas lacking peritoneal lining. Histologically, the colorectal mucosa extends to the dentate line, which is the superior boundary of the anal mucosa.

Two definitions of the anal canal exist, one based on function (the "surgical" anal canal) and the other on embryologic development. The functional anal canal is defined by the anal

sphincter, which measures 3 to 5 cm in length and whose boundaries are the puborectalis sling cephalad and the intersphincteric groove (the anal verge) caudad on digital rectal examination. The superior border of the embryologic anal canal is the dentate line, which is visible but not palpable and coincides with the midpoint of the functional anal canal, approximately 1 to 2 cm distal to the puborectalis sling (Fig. 20.3). The embryologic anal canal shares the same distal boundary as the functional anal canal: the anal verge. The common practice of reporting rectal tumor level relative to the anal verge and the variable length of the sphincter complex contribute to challenges in relating the measurement of the distal extent of the rectal tumor to the probability of sphincter preservation. Distinguishing the origin of distal rectal carcinomas from anal carcinomas is sometimes difficult, because the rectal mucosa may extend within 1 to 2 cm of the anal verge (Fig. 20.3).

Regional Lymph Nodes

Regional nodes are located 1) along the course of the major vessels supplying the colon and rectum, 2) along the vascular arcades of the marginal artery, and 3) adjacent to the colon—that is, along the mesocolic borders of the colon. Specifically, the regional lymph nodes are termed *pericolic* and *perirectal/mesorectal* and also are found along the ileocolic, right colic, middle colic, left colic, inferior mesenteric, superior rectal (hemorrhoidal), and internal iliac arteries (Fig. 20.4).

The regional lymph nodes for each segment of the large bowel are designated as follows:

Segment	Regional lymph nodes
Cecum	Pericolic, ileocolic, right colic
Ascending colon	Pericolic, ileocolic, right colic, right branch of the middle colic
Hepatic flexure	Pericolic, ileocolic, right colic, middle colic
Transverse colon	Pericolic, middle colic
Splenic flexure	Pericolic, middle colic, left colic
Descending colon	Pericolic, left colic, sigmoid, inferior mesenteric
Sigmoid colon	Pericolic, sigmoid, superior rectal (hemorrhoidal), inferior mesenteric
Rectosigmoid	Pericolic, sigmoid, superior rectal (hemorrhoidal), inferior mesenteric
Rectum	Mesorectal, superior rectal (hemorrhoidal), inferior mesenteric, internal iliac, inferior rectal (hemorrhoidal)

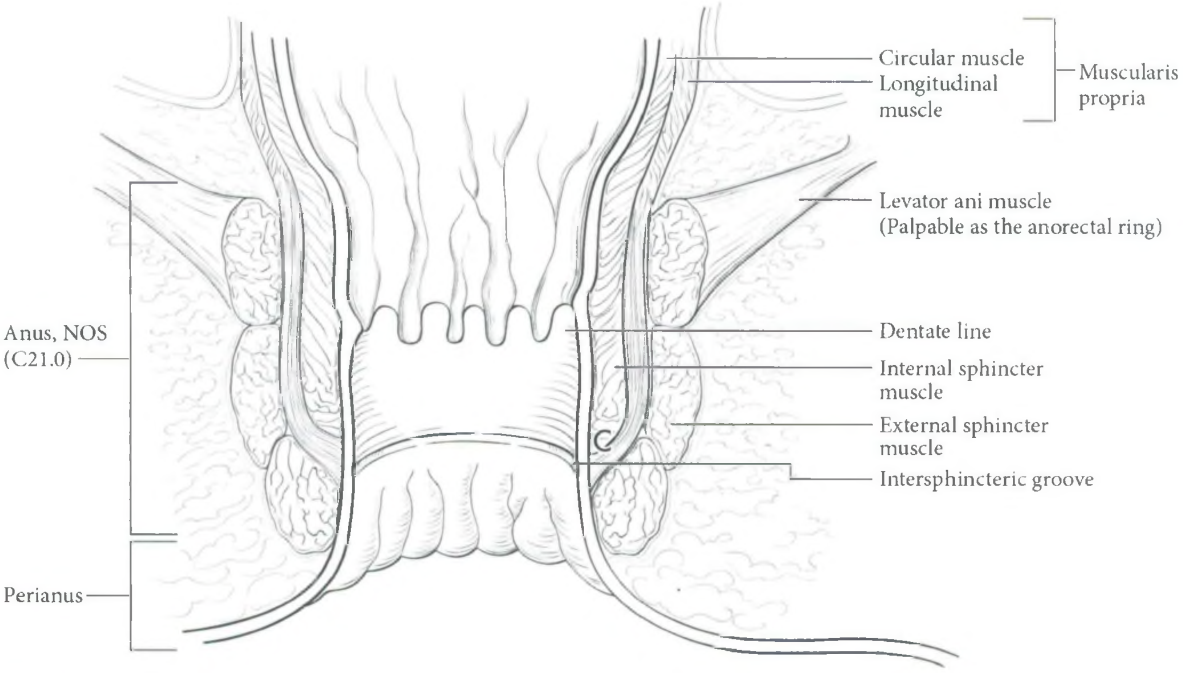

Fig. 20.3 The anal canal extends from the proximal aspect of the external sphincter to the anal verge at the intersphincteric groove

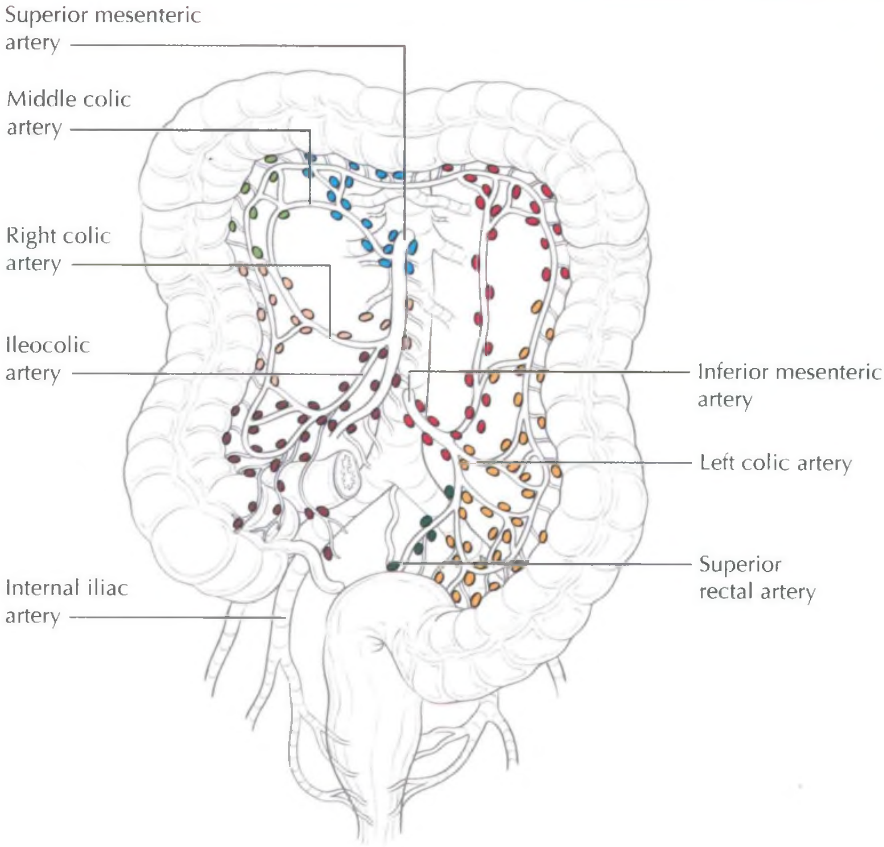

Fig. 20.4 The regional lymph nodes of the colon and rectum

Metastatic Sites

Although carcinomas of the colon and rectum can metastasize to almost any organ, the liver and lungs are most commonly affected. Seeding of other segments of the colon, small intestine, or peritoneum also may occur.

RULES FOR CLASSIFICATION

Clinical Classification

Clinical assessment is based on medical history, physical examination, radiology, and endoscopy with biopsy. Radiologic examinations designed to demonstrate the presence of extrarectal or extracolonic metastasis may include chest radiographs, computed tomography (CT; abdomen, pelvis, chest), magnetic resonance (MR) imaging, positron emission tomography (PET), or fused PET/CT scans. Clinical stage (cTNM) then may be assigned. Pathological stage (pTNM) is assigned based on the resection specimen. Preoperative measurement of carcinoembryonic antigen (CEA) is recommended, as CEA level reflects the likelihood that subclinical or clinical liver or lung metastases are present. In the event of recurrence or synchronous metastases, it now is recommended that the status of the genes *KRAS*, *NRAS*, and *BRAF* be evaluated and MSI or mismatch repair (MMR) be measured.

Primary Site(s)

Carcinoma arising at the ileocecal valve should be classified as colonic cancer. For staging purposes, adenocarcinomas should be classified as rectal cancers if proximal to the dentate line or anorectal ring on digital examination. Squamous carcinomas should be staged as anal canal cancers if they are distal to the dentate line or the anorectal ring. However, there are instances of rectal squamous carcinomas and anal adenocarcinomas in this area. The former may need to be treated according to anal squamous carcinoma regimens, whereas the anal adenocarcinomas may require surgery in addition to chemotherapy and radiation. For rectal cancers that extend

beyond the dentate line, as for anal canal cancers, the superficial inguinal lymph nodes are among the regional nodal groups at risk for metastatic spread and are included in cN/pN analysis.

Carcinomas that arise in the colon or rectum spread by direct invasion into the mucosa, submucosa, muscularis propria, and subserosal tissue (or adventitia) of the bowel wall, and each level of penetration is annotated by a T category. Primary tumors also spread by invading lymphatics and blood vessels to form metastases in lymph nodes or distant sites; this is annotated by the N and M categories, respectively. In addition, carcinomas may spread and grow in the adventitia as discrete nodules of cells called tumor deposits. These characteristics, along with further description of the T categories, are described in detail later in the chapter.

For patients with rectal cancer, the pelvic extent of disease (cT and cN categories), combined with the status of extrapelvic metastasis (cM) and patient symptoms, determines whether preoperative adjuvant treatment is appropriate. The primary imaging modalities to assess the pelvic extent of disease are endoscopic ultrasound (EUS) and pelvic MR imaging. To improve the accuracy of nodal staging, EUS may be augmented with fine-needle aspiration of lymph nodes suspicious for metastasis, but microscopic evidence of tumor by such a procedure is part of the clinical TNM (cTNM) staging. It is especially important that patients who will receive preoperative adjuvant treatment or neoadjuvant therapy be assigned a pretreatment clinical stage based on disease extent before beginning treatment (cTNM).[1–4] Pathological stage is assigned if the patient undergoes resection, and a modified pathological stage is generated if the patient undergoes neoadjuvant therapy (ypTNM).

For carcinomas of the colon or rectum, the number of metastatic sites involved is an important prognostic factor and is reflected in the subdivision of M1, as described in greater detail later in the chapter. Metastases to both ovaries or both lobes of the lungs are considered involvement of a single site by themselves. Peritoneal carcinomatosis with or without blood-borne metastasis to visceral organs has a worse prognosis.

Imaging

As stated elsewhere in this chapter, several imaging studies may be performed in newly diagnosed colon or rectal carcinoma patients. The National Comprehensive Cancer Network (NCCN) guidelines for evaluation of colon[1] or rectal[3] carcinoma cases recommend that a CT scan with intravenous and oral contrast be performed on the chest, abdomen, and pelvis. If the CT scan cannot be performed because of contrast sensitivity or the images are not adequate, then MR imaging with contrast may be performed with a noncontrast CT scan.[1] PET/CT is recommended only if there is an equivocal finding on a contrast-enhanced CT scan or in the case of sensitivity to CT contrast. If synchronous metastases or distant metastases appear later and resection seems possible, then PET/CT should be considered to further delineate the extent of disease.[1,3]

Pathological Classification

Most cancers of the colon and many cancers of the rectum are pathologically staged after microscopic examination of the resected specimen (pTNM) resulting from surgical exploration of the abdomen and cancer-directed surgical resection.

Primary Tumor

Tis and T1. Regarding the colorectum, pathologists apply the term *high-grade dysplasia* to lesions that are confined to the epithelial layer of crypts and lack invasion through the basement membrane into the lamina propria. The term *intraepithelial carcinoma* is synonymous with *high-grade dysplasia* but rarely is used to apply to the colorectum. High-grade dysplasia should not be assigned to the Tis category or recorded in cancer registries, because these lesions lack potential for tumor spread. However, Tis is assigned to lesions confined to the mucosa in which cancer cells invade into the lamina propria and may involve but not penetrate through the muscularis mucosa. (These lesions are more correctly termed *intramucosal carcinoma.*) Although invasion through the basement membrane in all gastrointestinal sites is considered invasive, in colorectal tumors, invasion of the lamina propria without penetration through the muscularis mucosa (intramucosal carcinoma) is designated Tis, as it is associated with a negligible risk for metastasis. Because there is potential for missing deeper invasion because of sampling, such lesions should be recorded in the cancer registry. The term *invasive adenocarcinoma* is used for colorectal cancer if the tumor extends through the muscularis mucosae into the submucosa or beyond (Fig. 20.5).

Carcinoma in a Polyp. These lesions are classified according to the pT definitions adopted for colorectal carcinomas. For instance, invasive carcinoma limited to the muscularis mucosae and/or lamina propria is classified as pTis, whereas tumor that has invaded through the muscularis mucosae and has entered the submucosa of the polyp head or stalk is classified as pT1. pTis in a polyp resected with a clear margin during endoscopy is a Stage 0 carcinoma with nodal and metastatic status unknown, but with a sufficiently low probability of nodal involvement that node resection is not justified. The probability of metastasis is similarly low.

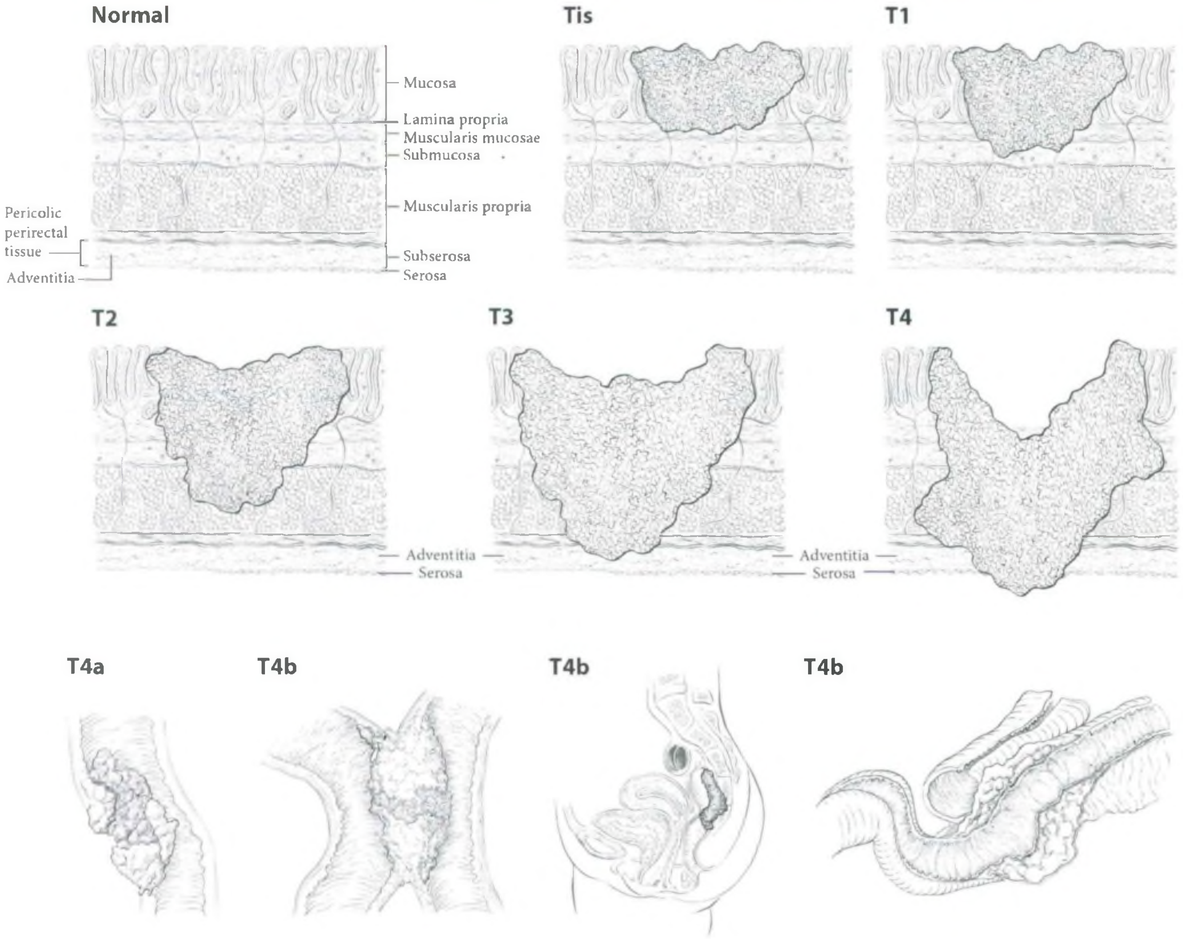

Fig. 20.5 T1–T3 as defined in Definition of Primary Tumor (T). T4 is a tumor that penetrates or perforates the visceral peritoneum in the parts of the colon or rectum covered only by peritoneum (T4a) or that invades an adjacent structure or organ (T4b)

Haggitt levels and submucosal depth of invasion categories may be used to classify polyps for their malignant potential, but reporting of these parameters is optional.[5–10] Guidelines from several organizations[1,3,4,11,12] and authors[8,12,13] recommend surgical resection for polyps that contain high-grade carcinoma, have invasive carcinoma at or less than 1 mm from the resection margin, or have lymphatic/venous vessel invasion.

T1, T2, and T3. As in previous AJCC editions, these tumors are defined as involvement of the submucosa, penetration through the submucosa into but not through the muscularis propria, and penetration through the muscularis propria, respectively.[14,15]

T4. Tumors that involve the serosal surface (visceral peritoneum) or directly invade adjacent organs or structures are assigned to the T4 category. For both colon and rectum, T4 is divided into two categories (T4a and T4b) based on different outcomes shown in expanded datasets[16,17] (Figs. 20.6 and 20.7). T4a tumors are characterized by involvement of the serosal surface (visceral peritoneum) by direct tumor extension. Tumors with perforation in which the tumor cells are continuous with the serosal surface through inflammation also are considered T4a. The significance of tumors that are <1 mm from the serosal surface and accompanied by serosal reaction is unclear, with some[18] but not all studies[19] indicating a higher risk for peritoneal relapse. Multiple-level sections and/or additional tissue blocks of the tumor should be examined in these cases to detect serosal surface involvement. If the latter is not present after additional evaluation, the tumor should be assigned to the pT3 category. In portions

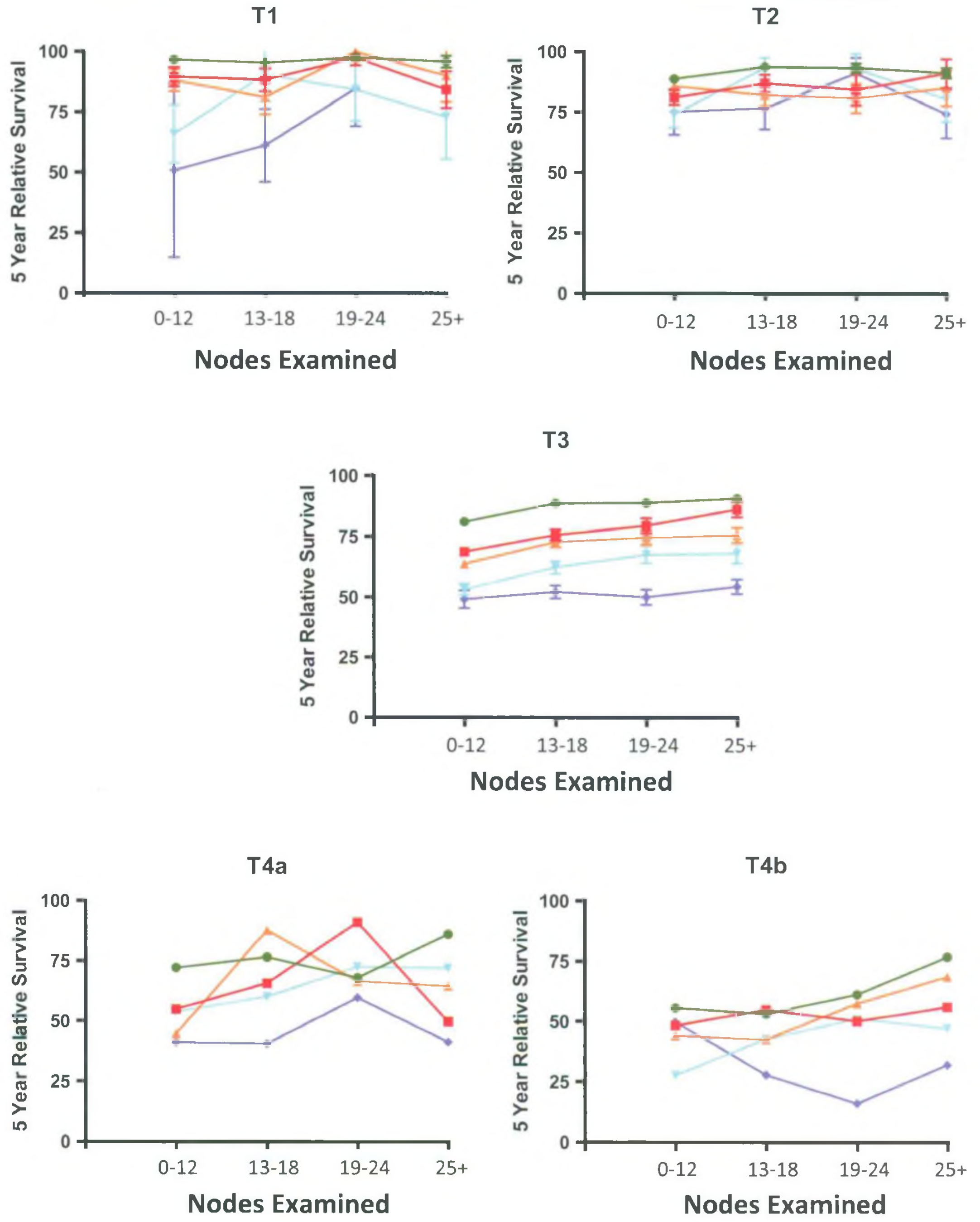

Fig. 20.6 Impact of positive nodes, the total number of nodes examined, and the depth of primary tumor invasion in a population-based cohort of rectal carcinoma. Data from 30,202 patients diagnosed with rectal carcinoma between 2004 and 2010 and included in the population-based Surveillance, Epidemiology, and End Results (SEER) database were analyzed for 5-year relative survival. Each T category is presented separately. The N categories are color coded as follows: green, N0 (all nodes negative); red, N1a (one positive node); orange, N1b (two or three positive nodes); light blue, N2a (four to six positive nodes); and dark blue, N2b (seven or more positive nodes). The N1c category is not represented because there is not yet a mature cohort in SEER with 5-year relative survival. The lines for N category levels spread across the numbers of nodes examined. All patients were free of clinical metastases by surgical and clinical staging; that is, they were in stage groups I to III. Results are mean±SEM of 5-year relative survival except for T4a and T4b, for which the number of patients is small and the error is large. The results suggest that the examination of more nodes for a given N category is associated with increasing survival. N0(i+) and N0mi are not included because they were not recorded previously for colorectal carcinoma

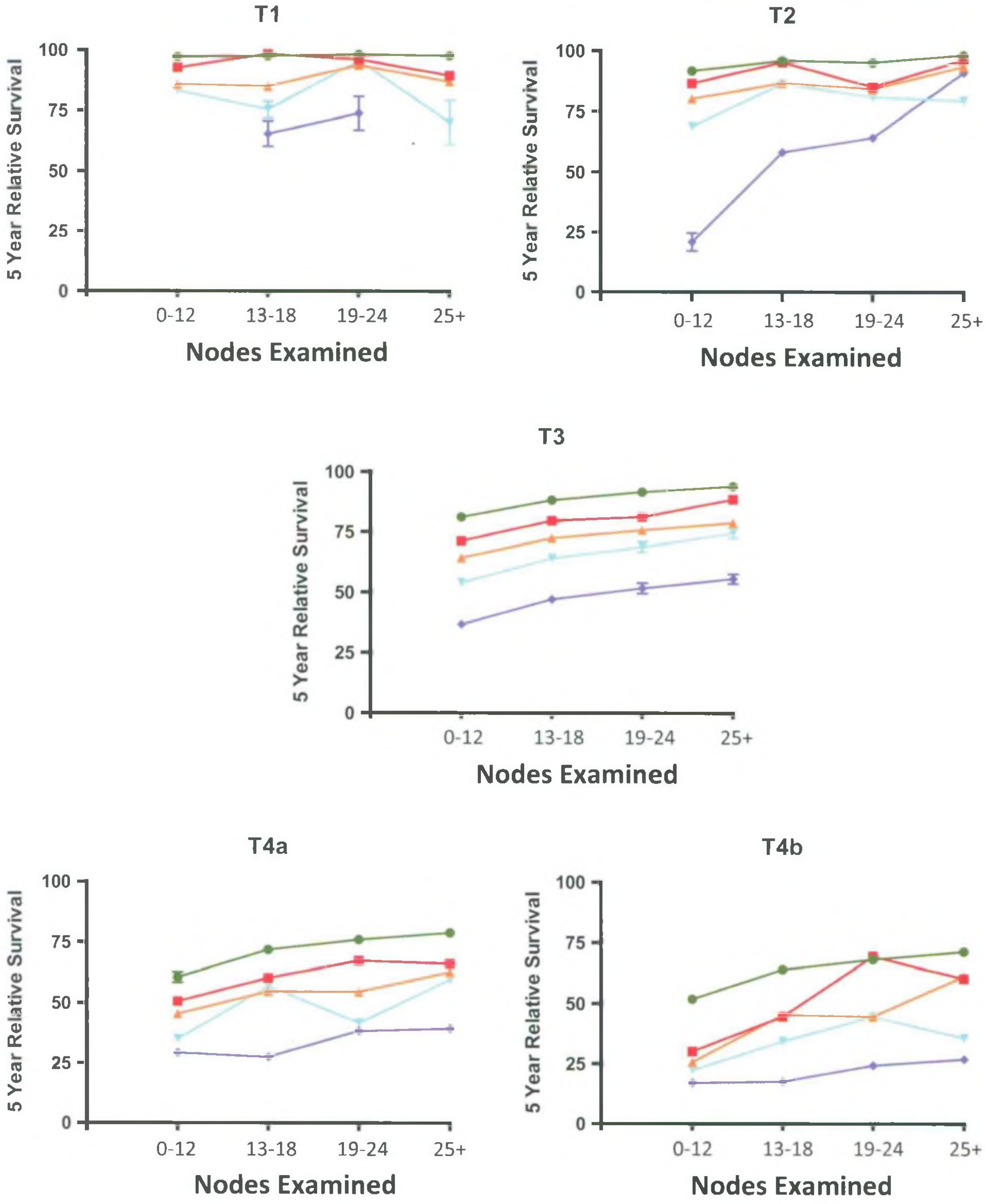

Fig. 20.7 Impact of positive nodes, the total number of nodes examined, and the depth of primary tumor invasion in a population-based cohort of colon carcinoma. Data from 144,744 patients diagnosed with colon carcinoma between 2004 and 2011 and included in the population-based SEER database were analyzed for 5-year relative survival. Each T category is presented separately. The N categories are color-coded as follows: green, N0 (all nodes negative); red, N1a (one positive node); orange, N1b (two or three positive nodes); light blue, N2a (four to six positive nodes); and dark blue, N2b (seven or more positive nodes). N1c category is not represented because there is not yet a mature cohort in SEER with 5-year relative survival. The lines for N category levels spread across the numbers of nodes examined. All patients were free of clinical metastases by surgical and clinical staging; that is, they were in stage groups I to III. Results are mean ± SEM of 5-year relative survival. Results suggest that the examination of more nodes for a given N category is associated with increasing survival. N0(i+) and N0mi are not included because they were not recorded previously for colorectal carcinoma

Table 20.1 Grading of quality and completeness of the mesorectum in a total mesorectal excision

	Mesorectum	Defects	Coning	CRM
Complete	Intact, smooth	Not deeper than 5 mm	None	Smooth, regular
Nearly complete	Moderate bulk, irregular	No visible muscularis propria	Moderate	Irregular
Incomplete	Little bulk	Down to muscularis propria	Moderate/marked	Irregular

Both the specimen as a whole (fresh) and cross-sectional slices (fixed) are examined to make an adequate interpretation (Adapted from Parfitt and Driman[23] with permission).
CRM, circumferential resection margin.

of the colorectum that are not peritonealized (e.g., posterior aspects of the ascending and descending colon, lower portion of the rectum), the T4a category is not applicable.

Treatment of Primary Colorectal Carcinomas. Colon carcinomas are resected according to guidelines by several organizations.[1–4] The standard of care for primary treatment of rectal carcinoma should include sharp dissection with an intact fascia propria of the mesorectum. The extent of mesorectal excision may be total (TME) or partial, as in the setting of proximal tumors located more than 5 cm from the distal extent of the mesorectum (tumor-specific mesorectal excision [TSME]). For the purpose of discussion throughout the rest of this chapter, *TME* is used to indicate either TME or TSME. The rate of local recurrence is inversely proportional to the completeness of the excision and the distance from the tumor to the circumferential resection margin (CRM).[20–22] Thus, macroscopic assessment of the quality and completeness of the excision of the mesorectum and the state of the fascia propria should be reported for rectal cancers. As suggested by Parfitt and Driman,[23] the College of American Pathologists (CAP) guidelines support a three-tiered evaluation system of a) complete, b) nearly complete, and c) incomplete (Table 20.1). The site-specific factor of the CRM is related to, but separate from, the grading of the TME specimen, as TME grading includes an assessment of the mesorectum for bulk, presence of surgical defects, degree of coning, and perforation. The CRM is described further elsewhere in the chapter and applies to the status of the nonserosal margin closest to the deepest point of penetration by the primary cancer throughout the colorectum.[11] As indicated in the current CAP guidelines[11] and in a systematic review,[24] patients with an incomplete mesorectum after rectal resection have a worse outcome than those with a complete mesorectum. For those with a negative CRM, the quality of the TME specimen is especially important, because the recurrence rate is higher in those with incomplete specimens.

Tumor Regression after Neoadjuvant Preoperative Radiotherapy and/or Chemotherapy for Rectal Carcinoma

The pathological response to preoperative radiotherapy and/or chemotherapy is an important prognostic factor for rectal carcinoma. This response is assessed by the pathologist and is reported with the prefix *y* before a pT and pN. Both a large institutional study[25] and a randomized phase III trial[26] demonstrated that complete eradication of the tumor, as detected by microscopic examination of the resected specimen, is associated with a better prognosis than an incomplete or poor response to neoadjuvant therapy. Failure of the tumor to respond to neoadjuvant treatment is an adverse prognostic factor. Parallel associations are observed in breast, esophageal, and pancreatic carcinomas. The US Food and Drug Administration (FDA) has approved the use of complete pathological response to neoadjuvant therapy as such a strong positive prognostic factor in breast cancer that it may be used as an end point for evaluating drug responses. In addition, measures of tumor response to therapy such as minimal residual disease in leukemias after induction therapy and necrosis after neoadjuvant treatment of osteosarcomas have similar prognostic impact.

According to the CAP guidelines for recording tumor regression, the resection specimen must be evaluated and recorded by the pathologist (see CAP's "Protocol for the Examination of Specimens from Patients with Carcinomas of the Colon and Rectum"[11,12]), because neoadjuvant chemoradiation in rectal cancer often is associated with significant tumor response and downstaging. Therefore, specimens from patients receiving neoadjuvant chemoradiation should be examined thoroughly at the primary tumor site, in regional nodes, and for peritumoral satellite nodules or deposits in the remainder of the specimen. It is especially important to evaluate the resection specimen for lymph node metastases, because nodal metastasis is an important poor prognostic factor.[26]

Lymph Nodes

In the assessment of pN, the number of lymph nodes sampled should be recorded. The number of nodes removed and retrieved from an operative specimen has been reported to correlate with improved survival, possibly because of increased accuracy in staging. For nodal sampling to be accurate, it is important to obtain and examine at least 12 lymph nodes in radical colon and rectum resections in patients who undergo surgery for cure. In cases in which tumor is resected for palliation, or in patients who have received preoperative radiation or chemoradiation, fewer

lymph nodes may be present. In all cases, however, it is essential that the total number of regional lymph nodes recovered from the resection specimen be recorded, because that number is prognostically important. A pN0 determination is assigned if all nodes are histologically negative, even if fewer than the recommended number of nodes have been analyzed.

Regional lymph nodes are classified as N1 or N2 according to the number involved by metastatic tumor. Involvement of one to three nodes by metastasis is pN1; involvement of four or more nodes by tumor metastasis is pN2. The number of nodes involved with metastasis influences outcome in both the N1 and N2 groups (Figs. 20.5 and 20.6). pN1 is subdivided further into pN1a (metastasis in one regional lymph node) and pN1b (metastasis in two or three regional lymph nodes), and pN2 is subdivided into pN2a (metastasis in four to six regional lymph nodes) and pN2b (metastasis in seven or more regional lymph nodes), because each subgroup represents roughly half the population of N1 or N2, and the subgroups with fewer positive nodes have better survival than those with more positive nodes within the N1 and N2 categories[16,17] (Figs. 20.6 and 20.7). Lymph nodes outside the regional drainage area of the primary tumor should be categorized as distant metastases; for example, external iliac or common iliac node involvement in a rectosigmoid carcinoma would be M1a.

Controversy exists regarding whether isolated tumor cells or micrometastases in regional nodes are prognostically important. The literature is contradictory because some authors have defined *isolated tumor cells* as not only individual tumor cells in the subcapsular or marginal sinus but clumps of up to 20 tumor cells.[27] *Micrometastases* have been defined as clusters of 10 to 20 tumor cells or clumps of tumor on cut section that measure ≥0.2 mm in diameter. These cell clusters indicate that tumor cells have entered a node and replicated and are not merely isolated dormant cells. A recent meta-analysis[28] demonstrated that micrometastases, defined as clusters of tumor cells greater than 0.2 mm in diameter, are a significant poor prognostic factor. Lymph nodes harboring such clusters therefore should be considered positive. Although these micrometastases may be designated as N1mi, it may be better to consider these as standard positive nodes with the corresponding number, as pathologists likely have considered these to be positive nodes in the past.

In a recent multicenter prospective trial, nearly 200 patients with Stage I or II disease had negative nodes based on standard hematoxylin and eosin (H&E) staining but were found to have tumor cells <0.2 mm in diameter that stained positively with a pan-keratin antibody (N0i+). These patients had a 10% decrease in overall survival compared with patients with N0 disease.[29] This decrease in survival occurred in patients with T3–T4 primary tumors but not in those with T1 or T2 primary tumors.[29] Further research into N0(i+) detected by pan-keratin staining in otherwise negative H&E-stained lymph nodes may be warranted, especially in patients with T3 or T4 primary carcinomas.

N1c—Tumor Deposits

Tumor deposits are defined as discrete tumor nodules within the lymph drainage area of the primary carcinoma without identifiable lymph node tissue or identifiable vascular or neural structure. The shape, contour, and size of the deposit are not considered in these designations.

If the vessel wall or its remnant is identifiable on H&E, elastin, or any other stain, the lesion should be classified as lymphovascular invasion (LVI) present (a CAP-required data element). Further documentation should subclassify LVI as small vessel invasion ("L" positive for either lymphatic or small venule involvement) or venous invasion ("V" positive for tumor within an endothelial-lined space that contains red cells or is surrounded by smooth muscle [adapted from the RCPath Colorectal Cancer Dataset 2014]).[30] These definitions are similar to those for large vessel invasion on page 122 of the AJCC Cancer Staging Manual, 6th Edition,[14] and LVI is a new data item to be collected. If neural structures are identifiable, the lesion should be classified as perineural invasion.

One to four individual tumor deposits or five or more deposits without involvement of lymphatic, venous, or neural structures within the lymph drainage area of the primary carcinoma should be recorded. In the evaluation of tumors pretreated with radiation and/or chemotherapy, it is important for the pathologist to assess whether tumor nodules represent tumor deposits as defined earlier or discontinuous eradication of the original tumor so that he or she can record the appropriate ypT and ypN categories.

As reported by Quirke, Nagtagaal, and others,[31–35] tumor deposits are associated with poor overall survival. A recent population-based study[36] demonstrated that approximately 10% of primary colon or rectal carcinomas have tumor deposits and that 2.5% of colon and 3.3% of rectal cases have tumor deposits with otherwise histologically negative lymph nodes. The strength of tumor deposits as a negative prognostic factor in the absence of any nodal metastases resulted in the introduction of the N1c category.

In cases with tumor deposits but no identified lymph node metastases, the N1c category is used and is applicable to all T categories. The presence of tumor deposits does not change the primary tumor T category, but does change the node status (N) to N1c if all regional lymph nodes are pathologically negative. The number of tumor deposits is not added to the number of positive regional lymph nodes if one or more lymph nodes contain cancer.

Metastasis

Metastasis to only one site/solid organ (e.g., liver, lung, ovaries, nonregional lymph node) should be recorded as M1a. Multiple metastases within only one organ, even if the organ is paired (e.g., the ovaries or lungs), is still M1a disease. Metastases to multiple sites or solid organs distant from the primary site is M1b, excluding peritoneal carcinomatosis. Peritoneal carcinomatosis with or without blood-borne metastasis to visceral organs is designated as M1c, because recent studies suggest that this occurs in 1–4% of patients[37,38] and that the prognosis for peritoneal disease is worse than that for visceral metastases to one or more solid organs.[39,40] The pathologist should not assign pM0 because M0 is a global designation referring to the absence of detectable metastasis anywhere in the body.

Anastomotic Recurrence

If the tumor recurs at the site of surgery, it is anatomically assigned to the proximal segment of the anastomosis (unless that segment is the small intestine, in which case the colonic or rectal segment should be designated as appropriate) and restaged by the TNM classification. The *r* prefix is used for the recurrent tumor stage (rTNM).

Colorectal Carcinoma Found at Death

The *a* prefix is used for cancer discovered as an incidental finding during autopsy and not suspected before death.

Molecular Advances That Will Lead to Future Markers and Therapies

In the past few years, there has been an explosion in the understanding of the molecular pathology of colorectal carcinoma. The Cancer Genome Atlas (TCGA) project provided a reference against which alterations that occur in cancer may be compared. The TCGA study in primary colorectal carcinomas[41] identified several different pathways that may lead to colorectal carcinoma, including chromosomal instability characterized by truncating mutations in the *APC* gene; MSI due to loss of function by somatic mutation or promoter hypermethylation of at least one of the DNA MMR genes (*MLH1*, *MSH2*, *MSH6*, *PMS2*) with or without *POLE* mutations and/or the presence of hypermutation; and the CpG island methylator ohenotype (CIMP) pathway caused by epigenetic alterations. The various pathways provide several molecular alterations that will create not only prognostic and predictive markers but also opportunities for therapy to be developed in coming years. As stated, hypermutated carcinomas show frequent deficiency in DNA MMR as well as specific mutations associated with this subset.[42] Their high rate of somatic mutation may form neoantigens that may induce an antitumor immune response, leading to a better prognosis.[43] The emergence of the Immunoscore project, which quantitates host immune infiltrating cells in colorectal carcinoma,[44] may be standardized in the future. Currently, however, its lack of standardization prevents it from being added to TNM staging.

Presently, the only major molecular alterations validated as significant markers with level I evidence are high levels of MSI/defective MMR (MSH-H/dMMR; good prognosis) and mutation in the *BRAF* gene (poor prognosis). Predictive markers are mutations in *KRAS*, the related *NRAS*, and *BRAF* genes that cause resistance to therapies using monoclonal antibodies to epidermal growth factor receptor (EGFR)[45] and possibly to vascular endothelial growth factor A (VEGFA)[46] in advanced colorectal carcinoma. The prognostic effects of mutations in the *RAS* genes appear to be small; this is discussed further in the site-specific factor section. Mutations in the gene *PIK3CA*[47] also may be prognostic and may predict lack of response to therapy against EGFR in advanced colorectal carcinoma, but their level of evidence currently is low.

PROGNOSTIC FACTORS

Prognostic Factors Required for Stage Grouping

Beyond the factors used to assign T, N, or M categories, no additional prognostic factors are required for stage grouping.

Additional Factors Recommended for Clinical Care

Tumor Deposits

A tumor deposit is a discrete nodule of cancer in pericolic/perirectal fat or in adjacent mesentery (mesocolic or rectal fat) within the lymph drainage area of the primary carcinoma, without identifiable lymph node tissue or identifiable vascular structure. If the vessel wall or its remnant is identifiable on H&E, elastic, or any other stain, it should be classified as vascular (venous) invasion. Similarly, if neural structures are identifiable, the lesion should be classified as perineural invasion.

If tumor deposits are present in the absence of any regional nodes involved with carcinoma, then the nodal classification is N1c, regardless of T category, and the tumor deposits should be recorded on the staging form. Evidence indicates that tumor deposits are equivalent to positive nodes as a negative prognostic factor and that adjuvant therapy is warranted in patients whose stage group would otherwise be I or II. The level of evidence supporting this marker is I.[1,3]

Tumor deposits are identified during pathological review of the resection specimen, as described in Pathological Classification. Venous invasion (V+) with lymphatic invasion (L+) or perineural invasion or spread (PNI) should be separated from tumor deposits based on histologic review and should not be categorized as N1c. If tumor deposits are observed in lesions that otherwise would be classified as pN0, then the primary tumor classification is not changed, but the nodule is recorded as a tumor deposit and categorized as N1c. The number of tumor deposits should be recorded on the staging form as one to four individual tumor deposits or five or more tumor deposits. If preoperative or neoadjuvant therapy has been administered, it is important to consider whether the potential tumor nodule near a partially responding tumor represents residual primary carcinoma or a true tumor deposit. Nagtegaal and Quirke[32] provide guidance in evaluating potential tumor deposits after preoperative therapy. Currently, the level of evidence for tumor deposits as a factor is II, but it must be included in data collection because it is required for staging node-negative carcinoma of the colon or rectum.

Factors Important to Consider in Making Decisions about Treatment

Eight prognostic factors now are judged to be clinically significant in colorectal carcinoma and should be considered when physicians and patients are deciding on what treatments to use. These factors, which have varying degrees of usefulness depending on disease stage, are as follows:

1. Serum CEA levels in patients who are to undergo surgery for potential cure (e.g., patients with Stage I–III colorectal carcinoma or Stage IV patients undergoing metastectomy) and changes in CEA as a response marker during chemotherapy for Stage IV disease. Data must be recorded as XXXX.X ng/mL
2. Tumor regression score in rectal carcinoma, which quantitates the pathological response to neoadjuvant therapy (similar to the pathological complete response measurement in breast cancer, which is FDA approved as a marker for drug development)
3. Circumferential resection margin (CRM), measured in millimeters from the edge of the tumor to the nearest dissected margin of the surgical resection in rectal cancer and retroperitoneal regions of the colon
4. Lymphovascular invasion (LVI) in all colorectal specimens, to identify small vessel or venous invasion
5. Perineural invasion (PNI), which provides histologic evidence of invasion of nerves or perineural spaces by the primary tumor and may have a negative prognostic impact similar to that of LVI
6. Microsatellite instability (MSI), which not only is a prognostic factor but also predicts lack of response to 5-fluorouracil (5-FU) chemotherapy
7. KRAS and NRAS mutation status
8. BRAF mutation, which along with KRAS and NRAS mutation is important because mutation in these genes is associated with lack of response to treatment with monoclonal antibodies directed against the epidermal growth factor receptor (EGFR) in patients with Stage IV colorectal carcinoma

Serum CEA Levels

Carcinoembryonic antigen (CEA), now known as CEACAM5, belongs to a 35-member family of related molecules that are part of the immunoglobulin supergene family.[48] Produced by epithelial cells that line the gut,[49] this 185-kDa glycoprotein is produced by almost all adenocarcinomas from all sites, as well as by many squamous cell carcinomas of the lung and other sites. CEA is cleared from the blood only by cells in the liver and lung[50]; therefore, it is an important marker for the presence of subclinical hepatic or pulmonary metastases, even before such lesions are detectable by current imaging modalities.[51] Although CEA has been considered a glycoprotein without specific function, research shows that it can promote metastasis in human xenograft models[52] through increased cell adhesion,[53–55] induction of cytokines that promote cancer cell survival,[56] inhibition of inflammatory responses,[57] and inhibition of programmed cell death, or apoptosis.[58,59] These functions of CEA are important for its ability to cause treatment resistance. The level of evidence supporting CEA as a prognostic marker is I.[1,51]

The ability of CEA to increase cell–cell adhesion, decrease reactive oxygen and nitrogen radical formation, and block apoptosis makes it a significant molecule for metastasis and response to therapy.

CEA levels may be measured in blood, plasma, or serum, primarily by an enzyme-linked immunosorbent assay (ELISA)-based method in a laboratory accredited by the Clinical Laboratory Improvement Amendments (CLIA). It is recommended that a preoperative level be obtained before potentially curative resection surgery (e.g., for clinical Stage I–III disease), then every 3 to 6 months for 2 years and annually thereafter until 5 years after first treatment. CEA levels also may be used as a response marker for treatment of Stage IV disease when measured at monthly intervals.

Tumor Regression Score

The pathological response to preoperative radiotherapy (rectal cancer), chemoradiation (rectal cancer), or chemotherapy (colon or rectal cancer) is an important prognostic factor. This response is assessed by the pathologist and is reported as a score based on resection specimens from patients who have undergone preoperative radiotherapy and/or chemotherapy.

Strong observational evidence exists that complete eradication of the tumor, as detected by pathological examination of the resected specimen, is associated with a better prognosis. Moreover, the degree of response appears to be associated with the degree of improvement in prognosis. Patients with minimal or no residual disease after therapy have a better prognosis than those with gross residual disease. Failure of the tumor to respond to neoadjuvant treatment is an adverse prognostic factor. Parallel associations are observed in breast and pancreatic carcinomas. The FDA has approved the use of complete pathological response to neoadjuvant therapy as such a strong positive prognostic factor in breast cancer that it may be used as an end point for evaluating drug responses. In addition, similar measures of tumor response to therapy have similar prognostic impacts, including minimal residual disease in leukemias after induction therapy and necrosis after neoadjuvant treatment of osteosarcomas. The level of evidence is II.

The pathologist must evaluate and record the resection specimen according to the CAP guidelines for recording tumor regression (see CAP's "Protocol for the Examination of Specimens from Patients with Carcinomas of the Colon and Rectum"[11,12]). Neoadjuvant chemoradiation in rectal cancer is often associated with significant tumor response, including the potential for sterilization of involved mesorectal lymph nodes. Therefore, specimens from patients receiving neoadjuvant chemoradiation should be examined thoroughly at the primary tumor site, in regional nodes, and for peritumoral satellite nodules or deposits in the remainder of the specimen. Sterilizing the primary tumor site does not assure sterilization of the regional lymph nodes. Although several different scoring systems for tumor regression have been advocated, a four-point tumor regression score is used to assess response; this system is similar to that of Ryan et al.,[11,12,60] except the score for complete absence of viable tumor is recorded as 0 (Table 20.2).

Acellular pools of mucin in specimens from patients receiving neoadjuvant therapy are considered to represent completely eradicated tumor and are not used to assign pT category or counted as positive lymph nodes.

Circumferential Resection Margins (CRM)

The CRM is the distance in millimeters between the deepest point of tumor invasion in the primary cancer and the margin of resection in the retroperitoneum or mesentery (Fig. 20.8). The longer this distance is, the better the prognosis for rectal carcinomas and for colon carcinomas arising in areas of the colon that partially lack a peritoneal lining. The CRM is produced by surgical resection of pericolic or perirectal fibroadipose tissue or pelvic structures. The term does not apply to the anatomic serosa of the colon or rectum that is peritonealized.

Table 20.2 Modified Ryan scheme for tumor regression score

Description	Tumor regression score
No viable cancer cells (complete response)	0
Single cells or rare small groups of cancer cells (near-complete response)	1
Residual cancer with evident tumor regression, but more than single cells or rare small groups of cancer cells (partial response)	2
Extensive residual cancer with no evident tumor regression (poor or no response)	3

(Adapted from Ryan et al[11,12,60] with permission).

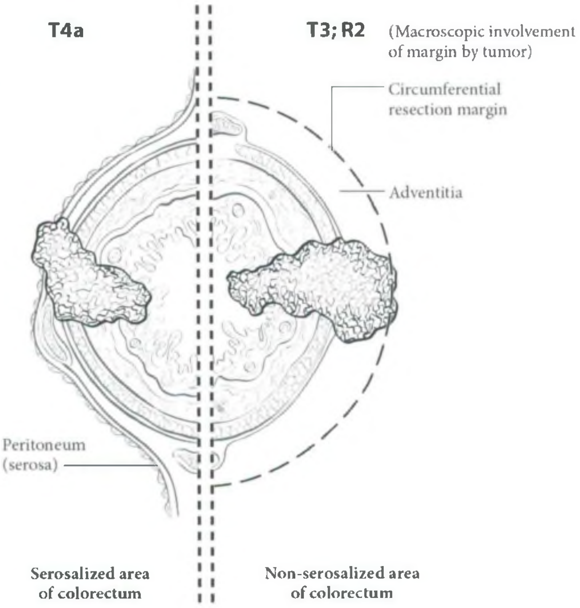

Fig. 20.8 Depiction of T4a lesions and the importance of the circumferential margin

Strong evidence exists that CRM status is one of the most important determinants of local control in colon and rectal cancers.[61] Distance to the CRM is an important prognostic indicator, and tumors with a CRM distance of 0 to 1 mm are at high risk for recurrence. In addition, involvement of the CRM is associated with decreased survival. If the CRM distance is 1 cm or more, the chance of local recurrence is significantly reduced and survival is improved. AJCC Level of Evidence: I

The circumferential surface of surgical resection specimens from the ascending colon, descending colon, or upper rectum is only partially peritonealized, and the demarcation between the peritonealized surface and the nonperitonealized surface (corresponding to the CRM) of such specimens is not always easily appreciated on pathological examina-

tion. The nonperitonealized resection margin may occur on an adjacent structure beyond the fascia propria that has been resected en bloc to achieve a clear CRM. Therefore, the surgeon is encouraged to mark the peritoneal reflection and/or the area of deepest tumor penetration adjacent to a nonperitonealized surface with a clip or suture or to specifically orient the specimen for the pathologist so that the CRM may be identified and evaluated accurately.

For mid- and distal rectal cancers (below the peritoneal reflection), the entire surface of the resection specimen (anterior, posterior, medial, and lateral) is a CRM. For proximal rectal or retroperitoneal colon cancers (ascending and descending colon and cecum), surgically dissected margins include those that lie in a retroperitoneal or subperitoneal location (Fig. 20.8). For segments of the colon covered completely by visceral peritoneum (transverse, sigmoid, sometimes cecum), the only radial margin dissected surgically is the mesenteric margin, unless the cancer adheres to or invades an adjacent organ or structure. Therefore, for cancers of the cecum, transverse colon, or sigmoid colon that extend to the cut edge of the mesentery, assignment of a positive CRM is appropriate.

For rectal cancer, the quality of the surgical technique is a key factor in the success of surgical outcomes relative to local recurrence and possibly long-term survival. Numerous nonrandomized studies demonstrated that total mesorectal excision (TME) with adequate surgical clearance around the penetrating edge of the tumor decreases the rate of local relapse. The TME technique entails precise sharp dissection within the areolar plane of loose connective tissue outside (lateral to) the visceral mesorectal fascia in order to remove the rectum. With this approach, all mesorectal soft tissues encasing the rectum, including the mesentery and all mesorectal and mesenteric lymph nodes, are removed *en bloc*. Thus, the circumferential surface (CRM) of TME resection specimens is the mesorectal fascia. The CRM of extended resections beyond the mesorectal fascial plane corresponds to the resection plane of the extended resection. Rectal resection performed by less precise techniques may be associated with incomplete excision of the mesorectum.

It is critical that the analysis of the surgical specimen follow the CAP guidelines that refer to examination of the TME specimen. In addition, it is essential that the distance between the closest leading edge of the tumor and the CRM (known as the surgical clearance) be measured pathologically and recorded in millimeters in the CRM field on the staging form. A margin greater than 1 mm is considered a negative margin. Because surgical clearance of 1 mm or less is associated with a significantly increased risk of local recurrence, margins less than 1 mm should be classified as positive[61] (Fig. 20.8).

Lymphovascular Invasion (LVI)

Invasion of either small or large vessels by the primary tumor is an important poor prognostic factor. Small vessel invasion is involvement by tumor of thin-walled structures lined by endothelium, without an identifiable smooth muscle layer or elastic lamina. These thin-walled structures include lymphatics, capillaries, and postcapillary venules. Large vessel invasion is defined by tumor involving endothelium-lined spaces that have an elastic lamina and/or smooth muscle layer. Circumscribed tumor nodules surrounded by an elastic lamina on H&E or elastic stain also are considered venous invasion and may be extramural (beyond the muscularis propria) or intramural (submucosa or muscularis propria).

LVI has been recognized as a category I factor since 1999 (summarized with recommendation by Compton et al.[62]). Although formal meta-analyses have not been performed, the depth of data in the literature indicates that involvement of either small or large vessels is a significant sign of poor prognosis. As such, it should be considered a prognostic factor with level I evidence. Small vessel invasion is associated with lymph node metastasis, and several studies found it to be an independent indicator of adverse outcome.[63–65] Multivariate analysis demonstrated that extramural venous invasion was an independent adverse prognostic factor in multiple studies and is a risk factor for liver metastasis.[66–68] The significance of intramural venous invasion is less clear.

Analysis of resected specimens and indications for using special stains should follow CAP guidelines.[11,12]

Perineural Invasion (PNI)

Invasion of the nerves within or adjacent to the primary tumor by colorectal carcinoma is a negative prognostic factor that may be as important as invasion of lymphatics or blood vessels. However, it often is overlooked and may be present in as many as 20% of primary colonic or rectal carcinomas.[69]

Carcinoma invasion of peripheral nerves, including perineural spaces within the regional drainage area of the primary tumor, is an adverse prognostic factor, as identified in multiple institutional studies,[69–71] and indicates an especially aggressive carcinoma. The level of evidence for this factor is I.

If present, PNI usually is apparent on standard H&E staining of formalin-fixed tissues. Its presence in any field is sufficient to warrant a positive or present designation on a pathology report, and this should be so designated on the staging form.

Microsatellite Instability (MSI)

One form of genetic instability is manifested by changes in the length of repeated single- to six-nucleotide sequences (known as DNA microsatellite sequences), which are

caused by a functional defect in DNA MMR.[72] High levels of MSI (MSI-H) occur in about 15% of colorectal carcinomas and are associated with right-sided colon carcinomas, frequently with poorly differentiated and mucinous histology but good prognosis.[73] This MSI is a hallmark of hereditary nonpolyposis colorectal carcinoma (HNPCC), or Lynch syndrome.[74] The vast majority of MSI tumors are sporadic because of epigenetic inactivation of the *MLH1* gene, whereas the others occur in patients with a mutation in a DNA MMR gene. Frequently, the mutation in the latter group is in the germline and confers Lynch syndrome. Thus, new patients with MSI tumors should be screened for Lynch syndrome in accordance with current NCCN guidelines.[1,3]

MSI-H is important because it not only is a good prognostic factor, but it also predicts a poor response to 5-FU chemotherapy.[75] However, the addition of oxaliplatin (in the FOLFOX regimens) negates the adverse effects of MSI-H.[76,77] Recent data suggest that mutation in *BRAF* is associated with MSI-H tumors and that colon carcinomas with both gene alterations have a significantly, although modestly, worse prognosis in Stage III and IV colon carcinomas.[78] MSI-H also is associated with Lynch syndrome/HNPCC.[79]

MSI-H is identified by DNA polymerase chain reaction (PCR) amplification of known DNA microsatellites to show that the length of the microsatellites is greater in tumor than in normal colon. In addition, immunohistochemistry may be used to detect loss of expression of DNA MMR proteins, including MLH1, MSH2, MSH6, and PMS2. The most common cause of MSI-H is sporadic nonfamilial loss of expression of MLH1 due to somatic promoter hypermethylation. MSI-H results in hypermutated colorectal cancers with large numbers of mutated genes, especially in those that contain microsatellites.

The NCCN[3,4] and Spanish Society of Pathology[80] recommend testing for MSI in patients younger than 70 years, especially those with high-grade right-sided colon carcinomas, mucinous histology, or Crohn's disease–like peritumoral lymphoid follicles, which are features of MSI-H cancers. Alternatively, several reports indicate that the sensitivity and specificity of identifying MSI-H tumors increases if all tumors are analyzed,[81] as opposed to following either the Bethesda guidelines[82] or the MSI-like morphology described here. All these considerations make MSI a level I factor to be collected.

KRAS and NRAS Mutation

KRAS and *NRAS* are important signaling intermediates in the growth receptor pathway, which controls cell proliferation and survival. These genes are activated when EGFR binds EGF or similar growth factors and then activate the RAF or PIK3CA proteins. Both KRAS and NRAS may be constitutively activated through mutation during colorectal carcinogenesis so that they continuously stimulate cell proliferation and prevent cell death. Activating mutations are most likely to occur in codons 12 and 13, but codons 61, 146, and, less frequently, other codons in *KRAS* also may be mutated.[47] Similarly, codons in *NRAS* may have activating mutations. *KRAS* may be activated by somatic mutation in up to 40% of colorectal carcinomas and *NRAS* in about 7%.[47] Activation of either *RAS* gene is a modestly poor prognostic factor in Stage III and IV disease.[45,83,84] More importantly, *RAS* activation predicts a poor response to monoclonal anti-EGFR antibody therapy in advanced colorectal carcinoma.[45] KRAS mutation also may predict a poor response to anti-VEGF therapy in advanced colorectal carcinoma.[46] The level of evidence for the poor predictive effect of *KRAS* mutation is I in advanced colorectal carcinoma and II as a prognostic factor in Stage II–IV disease. The level of evidence for *NRAS* mutation as a prognostic factor currently is II.

FDA-approved kits now are available for detecting *KRAS* mutations. There is no consensus regarding the level of mutation necessary to suspect a tumor of containing an activated *RAS* gene; however, the level often is considered to be around 5% of the amount of KRAS gene expression, as detected by PCR or a sequencing method.

BRAF Mutation

The BRAF oncoprotein is a serine–threonine kinase that transmits cell growth and proliferation signals from KRAS or NRAS to other enzymes, leading to cell proliferation and growth. An activating point mutation at *BRAF V600E* may be detected in 6–10% of colorectal carcinomas,[41] which constitutively stimulate these other enzymes to promote continuous cell growth. This stimulation abrogates the ability of EGFR inhibitors to block cell proliferation and growth.

Multiple studies in Stage IV colorectal cancers and more recent data in Stage III disease indicate that the *BRAF V600E* mutation is associated with significantly worse prognosis, including survival after tumor recurrence.[85] MSI status and BRAF mutation are prognostic factors that interact significantly. Although *BRAF* mutation is associated with poor prognosis, the presence of MSI may attenuate its adverse impact. MSI is an established good prognostic factor.[73–76] The concurrent presence of a *BRAF* mutation may attenuate survival slightly.[85] MSI without BRAF is a good prognostic factor, whereas MSI-H with BRAF mutation portends slightly worse survival.[83,84] Both patient groups have better survival than those with microsatellite stability (MSS) without BRAF mutation, who in turn have better survival than those with MSS tumors with BRAF mutation.[83–87] BRAF V600E mutation in colorectal carcinomas blocks the effect of anti-EGFR antibodies on disease progression in Stage IV colorectal carcinoma.[50] The level of evidence for these

effects of BRAF is I (meta-analysis) for blocking the effect of anti-EGFR antibody therapy and II for the prognostic effect on survival.

BRAF V600E mutation may be determined by an assay approved by the FDA for detecting the mutation in melanoma. In addition, laboratory-developed tests that involve standard genotyping or next-generation sequencing may be used to measure the level of this mutation in colorectal carcinoma specimens. As with other somatic mutations, the cutoff or threshold for assigning a positive result is under consideration, but a common standard is that 5% of the alleles or gene expression should be mutated for a test to be considered positive. Immunohistochemistry for mutated BRAF V600E protein is not recommended for use in colorectal carcinomas, because it is not as sensitive and concordant with genomic sequencing as it is in melanomas.[88]

RISK ASSESSMENT MODELS

Prognostic models will continue to play an important role in 21st century medicine for several reasons.[89] First, by identifying which factors predict outcomes, clinicians gain insight into the biology and natural history of the disease. Second, treatment strategies may be optimized based on the outcome risks of the individual patient. Third, because of the heterogeneity of disease in most cancers, prognostic models will play a critical role in the design, conduct, and analysis of clinical trials in oncology.[89] If developed and validated appropriately, these models will become part of routine patient care, decision making, and trial design and conduct.

The AJCC Precision Medicine Core (PMC) developed and published criteria for critical evaluation of prognostic tool quality,[90] which are presented and discussed in Chapter 4. Although developed independently by the PMC, the AJCC quality criteria corresponded fully to the recently developed Cochrane CHARMS tool for critical appraisal in systematic reviews of prediction modeling studies.[91]

Existing prognostic models for colon and rectum cancer meeting all the AJCC inclusion/exclusion criteria and meriting AJCC endorsement are presented in this section. A full list of the evaluated models and their adherence to the quality criteria is available at www.cancerstaging.org.

The PMC performed a systematic search of published literature for prognostic models/tools in colon and rectum cancer from January 2011 to December 2015. The search strategy is provided in Chapter 4. The PMC defined *prognostic model* as a multivariable model in which factors predict a clinical outcome that will occur in the future. Each tool identified was compared against the quality criteria developed by the PMC as guidelines for AJCC recommendation for prognostication models (see Chapter 4).

Twenty-nine prognostication tools[92–120] for colon or colorectal cancer were identified: 14 for patients with resected liver metastases,[96–99,102,105–110,116,120] two for patients with unresectable liver metastases,[92,114] four in the adjuvant (Stage I/II/III) setting,[94,112,118,119] seven for patients with metastatic disease,[93,95,101,103,104,111,113] one for patients with resected pulmonary metastases,[100] one for patients with locally advanced rectal cancer,[117] and one across all disease stages.[115]

Of the 14 models for patients with resected liver metastases, none met all the predefined criteria. Most were excluded because their development used single-institution series lacking sufficient external validation,[97,98,102,106–109] made predictions generated from data not reflecting current clinical standards of treatment,[96,99,100,105,110,120] or lacked external validation.[102,116] Both tools for patients with unresectable liver metastases were excluded because they were from single-institution series and were too small to be reliably generalizable.[92,114] Of the tools for patients with metastatic disease, Elias et al. (2014)[95] lacked external validation; Peng et al.[111] and Shitara et al. (2011)[113] were from a single institution or the patient set was too small to be generalizable; and Chibaudel et al.[93], Kato et al. (2005),[101] Kobayashi et al. (2013),[103] and Kohne et al. (2002)[104] were felt to be based on datasets not reflective of current treatment paradigms for patients with metastatic disease (although the Kohne tool[104] was of very high quality).

Among the four models for patients in the adjuvant setting, two met all inclusion criteria.[112, 118] We note that the Numeracy model[94] was excluded because it was replaced by ACCENT.[112] Weiser et al.[119] was excluded because it predicted only recurrence, although it met all other criteria. The model in Stojadinovic et al. (2013)[115] was considered very promising; however, it lacked sufficient detail for it to be implemented in practice. The final model, which predicted outcomes in patients with locally advanced rectal cancer,[102] met all criteria and is endorsed by the committee for this somewhat limited treatment setting.

Twenty-nine models for prognostication in colon or colorectal cancer were identified, but only three models, two for adjuvant disease[112,118] and one for local advanced rectal cancer,[117] met all predefined AJCC inclusion and exclusion criteria and therefore are endorsed by the AJCC.[112,117,118] Table 20.3 presents the models meeting the AJCC quality criteria. The two models in the adjuvant setting were developed using very different datasets. Renfro et al.[112] was based on a large collection of completed randomized clinical trials, whereas Weiser et al.[118] was built using SEER data. However, both models were externally validated. The third endorsed tool, by Valentini et al.[117] in locally advanced rectal cancer, also was developed using data from completed clinical trials.

Table 20.3 Prognostic tools for colon and rectum cancer meeting all AJCC quality criteria

Approved prognostic tool	Web address	Factors Included in the model
ACCENT-based web calculators to predict recurrence and overall survival in Stage III colon cancer[112]	http://www.mayoclinic.org/medical-professionals/cancer-prediction-tools/colon-cancer	Age, sex, race, BMI, performance status (PS), T category, lymph node ratio, grade, treatment group, location
Predicting survival after curative colectomy for cancer: indvidualizing colon cancer staging[118]	https://www.mskcc.org/nomograms/colorectal/overall-survival-probability	T category, N category, age, sex, tumor differentiation/grade, number of regional lymph nodes evaluated, number of regional lymph nodes positive
Nomograms predicting local recurrence, distant metastases, and overall survival for patients with locally advanced rectal cancer on the basis of European randomized clinical trials[117]	http://www.predictcancer.org/Main.php?page=RectumFollowUpModel	Age, sex, T category, radiotherapy dose, concomitant chemotherapy, surgical procedure, pT category, pN category, adjuvant chemotherapy

In the interest of precision medicine and informed individualized care for patients, the AJCC supports the appropriate use of both high-value patient classifiers (prognostic factors) and prognostication tools (risk calculators). Both are valuable. Prognostication tools (i.e., risk calculators) provide individualized probability estimates, whereas patient classifiers group patients into ordered risk strata (either directly or based on cut-points for individual probability estimates). The TNM staging system is an example of such a classification tool, yielding at the least granular level ordered classes (I, II, III, IV) of increasingly poor prognosis. Strata based on prognostic factors (e.g., a gene signature) are other examples.

While such stratification is useful, it also limited by the number of categories that are manageable, by the complexity of combining information from multiple predictors to form discrete ordered categories in a transparent manner, and by the inherent variability of prognosis of patients in a given risk class. Risk calculators, in contrast, are designed to deliver a more precise estimate of outcome for an individual patient through computational integration of a variety of patient-specific data elements.

DEFINITIONS OF AJCC TNM

Definition of Primary Tumor (T)

T Category	T Criteria
TX	Primary tumor cannot be assessed
T0	No evidence of primary tumor
Tis	Carcinoma *in situ*, intramucosal carcinoma (involvement of lamina propria with no extension through muscularis mucosae)
T1	Tumor invades the submucosa (through the muscularis mucosa but not into the muscularis propria)
T2	Tumor invades the muscularis propria
T3	Tumor invades through the muscularis propria into pericolorectal tissues
T4	Tumor invades the visceral peritoneum or invades or adheres to adjacent organ or structure
T4a	Tumor invades through the visceral peritoneum (including gross perforation of the bowel through tumor and continuous invasion of tumor through areas of inflammation to the surface of the visceral peritoneum)
T4b	Tumor directly invades or adheres to adjacent organs or structures

Definition of Regional Lymph Node (N)

N Category	N Criteria
NX	Regional lymph nodes cannot be assessed
N0	No regional lymph node metastasis
N1	One to three regional lymph nodes are positive (tumor in lymph nodes measuring ≥0.2 mm), or any number of tumor deposits are present and all identifiable lymph nodes are negative
N1a	One regional lymph node is positive
N1b	Two or three regional lymph nodes are positive
N1c	No regional lymph nodes are positive, but there are tumor deposits in the<br>• subserosa<br>• mesentery<br>• or nonperitonealized pericolic, or perirectal/mesorectal tissues.
N2	Four or more regional nodes are positive
N2a	Four to six regional lymph nodes are positive
N2b	Seven or more regional lymph nodes are positive

Definition of Distant Metastasis (M)

M Category	M Criteria
M0	No distant metastasis by imaging, etc.; no evidence of tumor in distant sites or organs (This category is not assigned by pathologists.)
M1	Metastasis to one or more distant sites or organs or peritoneal metastasis is identified

M Category	M Criteria
M1a	Metastasis to one site or organ is identified without peritoneal metastastis
M1b	Metastasis to two or more sites or organs is identified without peritoneal metastasis
M1c	Metastasis to the peritoneal surface is identified alone or with other site or organ metastases

AJCC PROGNOSTIC STAGE GROUPS

When T is...	And N is...	And M is...	Then the stage group is...
Tis	N0	M0	0
T1, T2	N0	M0	I
T3	N0	M0	IIA
T4a	N0	M0	IIB
T4b	N0	M0	IIC
T1–T2	N1/N1c	M0	IIIA
T1	N2a	M0	IIIA
T3–T4a	N1/N1c	M0	IIIB
T2–T3	N2a	M0	IIIB
T1–T2	N2b	M0	IIIB
T4a	N2a	M0	IIIC
T3–T4a	N2b	M0	IIIC
T4b	N1–N2	M0	IIIC
Any T	Any N	M1a	IVA
Any T	Any N	M1b	IVB
Any T	Any N	M1c	IVC

REGISTRY DATA COLLECTION VARIABLES

1. Tumor deposits
2. CEA levels: preoperative blood level recorded in nanograms per milliliter with fixed decimal point and five numbers (XXXX.X ng/mL)
3. Tumor regression score
4. Circumferential resection margin
5. Lymphovascular invasion
6. Perineural invasion
7. Microsatellite instability
8. KRAS and NRAS mutation
9. BRAF mutation

HISTOLOGIC GRADE (G)

G	G Definition
GX	Grade cannot be assessed
G1	Well differentiated
G2	Moderately differentiated
G3	Poorly differentiated
G4	Undifferentiated

HISTOPATHOLOGIC TYPE

Adenocarcinoma *in situ*
Adenocarcinoma
Medullary carcinoma
Mucinous carcinoma (colloid type; >50% extracellular mucinous carcinoma)
Signet ring cell carcinoma
Squamous cell (epidermoid) carcinoma
Adenosquamous carcinoma
High-grade neuroendocrine carcinoma (small cell carcinoma and large cell neuroendocrine carcinoma)
Undifferentiated carcinoma
Carcinoma, NOS

SURVIVAL DATA

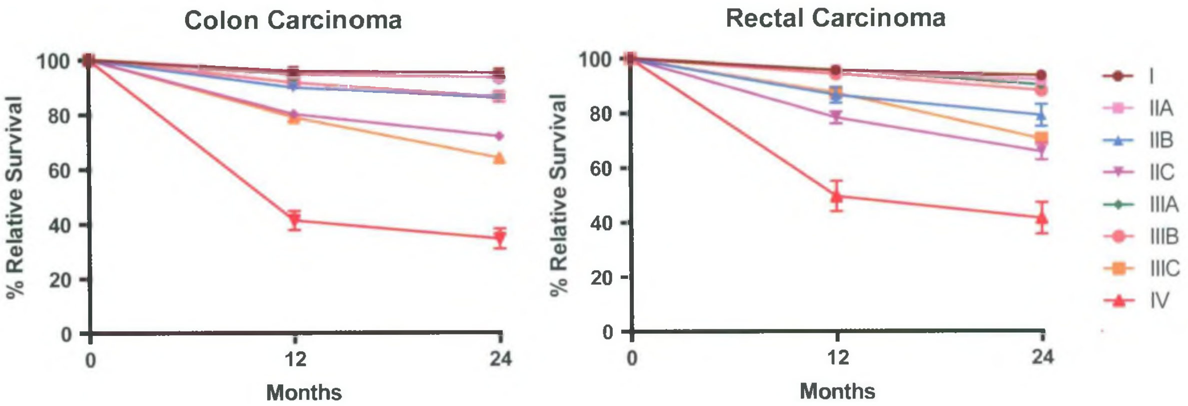

Fig. 20.9 Relative survival of colon and rectal carcinoma patients in the SEER database since the TNM 7th Edition was introduced in January 2010. The relative survival of colon (42,435) and rectal cancer (18,540) patients was calculated by the Kaplan–Meier method for the 2 years during which there was sufficient follow-up. Results are percentage of relative survival mean ± SEM

ILLUSTRATIONS

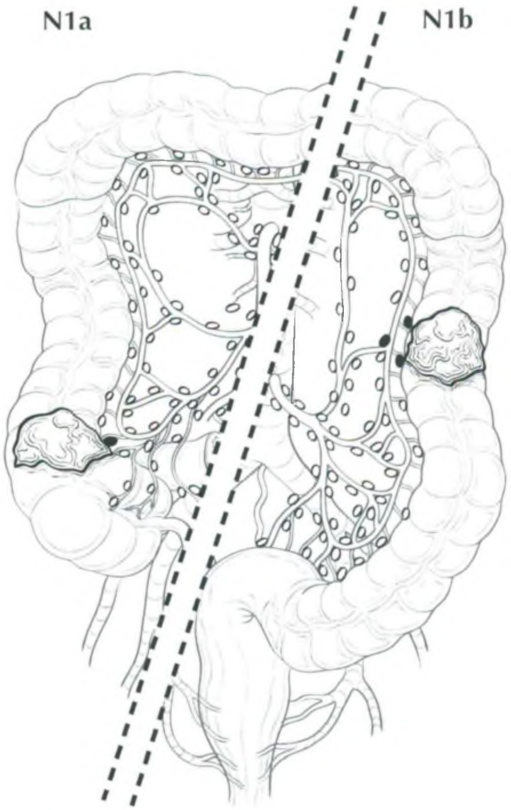

Fig. 20.10 N1a is defined as metastasis in one regional lymph node. N1b is defined as metastasis in 2 to 3 regional lymph nodes

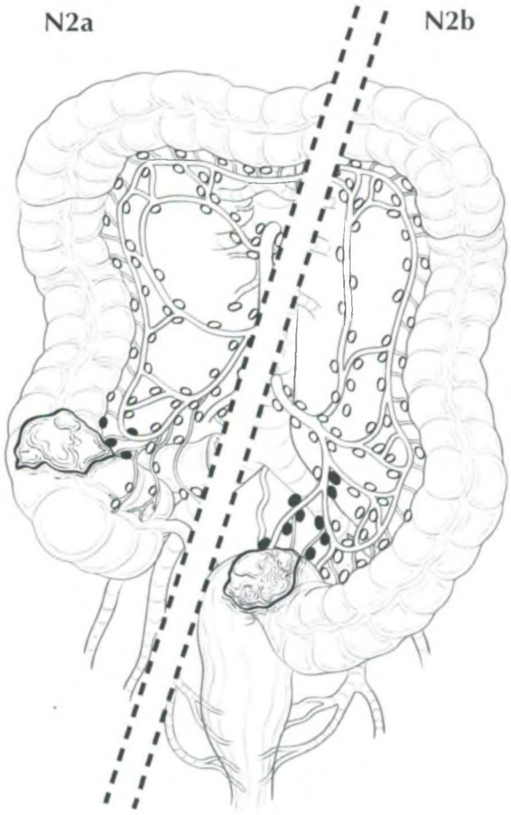

Fig. 20.11 N2a is defined as metastasis in 4 to 6 regional lymph nodes. N2b is defined as metastasis in seven or more regional lymph nodes

N2b

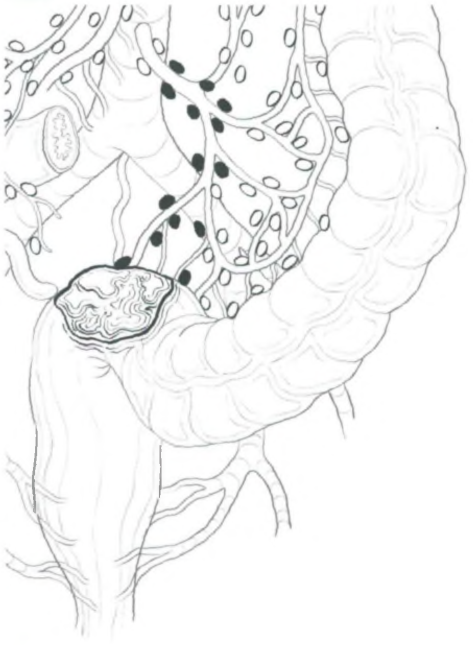

Fig. 20.12 N2b showing nodal masses in more than 7 regional lymph nodes

Bibliography

1. NCCN colon carcinoma treatment guidelines. http://www.nccn.org/professionals/physician_gls/pdf/colon.pdf. Accessed 5/29/15.
2. Chang GJ, Kaiser AM, Mills S, et al. Practice parameters for the management of colon cancer. *Diseases of the colon and rectum*. Aug 2012;55(8):831-843.
3. NCCN rectal carcinoma treatment guidelines. http://www.nccn.org/professionals/physician_gls/pdf/rectal.pdf. Accessed 5/29/15.
4. Monson JR, Weiser MR, Buie WD, et al. Practice parameters for the management of rectal cancer (revised). *Diseases of the colon and rectum*. May 2013;56(5):535-550.
5. Tominaga K, Nakanishi Y, Nimura S, Yoshimura K, Sakai Y, Shimoda T. Predictive histopathologic factors for lymph node metastasis in patients with nonpedunculated submucosal invasive colorectal carcinoma. *Diseases of the colon and rectum*. Jan 2005;48(1):92-100.
6. Choi DH, Sohn DK, Chang HJ, Lim SB, Choi HS, Jeong SY. Indications for subsequent surgery after endoscopic resection of submucosally invasive colorectal carcinomas: a prospective cohort study. *Diseases of the colon and rectum*. Mar 2009;52(3):438-445.
7. Hassan C, Zullo A, Risio M, Rossini FP, Morini S. Histologic risk factors and clinical outcome in colorectal malignant polyp: a pooled-data analysis. *Diseases of the colon and rectum*. Aug 2005;48(8):1588-1596.
8. Nivatvongs S, Rojanasakul A, Reiman HM, et al. The risk of lymph node metastasis in colorectal polyps with invasive adenocarcinoma. *Diseases of the colon & rectum*. 1991;34(4):323-328.
9. Nivatvongs S. Surgical management of malignant colorectal polyps. *Surg Clin North Am*. Oct 2002;82(5):959-966.
10. Aarons CB, Shanmugan S, Bleier JI. Management of malignant colon polyps: current status and controversies. *World journal of gastroenterology : WJG*. Nov 21 2014;20(43):16178-16183.
11. Tang L, al. E. Protocol for the Examination of Speciments From Patients with Primary Carcinoma of the Colon and Rectum. *CAP Cancer Protocol Templates* 2016; http://www.cap.org/ShowProperty?nodePath=/UCMCon/Contribution%20Folders/WebContent/pdf/cp-colon-16protocol-3400.pdf. Accessed 3/23/16, 2016.
12. Washington MK, Berlin J, Branton PA, et al. Protocol for the examination of specimens from patients with primary carcinomas of the colon and rectum. *Arch Pathol Lab Med*. Jul 2008;132(7):1182-1193.
13. Cooper HS, Deppisch LM, Gourley WK, et al. Endoscopically removed malignant colorectal polyps: clinicopathologic correlations. *Gastroenterology*. Jun 1995;108(6):1657-1665.
14. Greene FL. *AJCC cancer staging manual*. Vol 1: Springer Science & Business Media; 2002.
15. Edge SB, Compton CC. The American Joint Committee on Cancer: the 7th edition of the AJCC cancer staging manual and the future of TNM. *Annals of surgical oncology*. Jun 2010;17(6):1471-1474.
16. Gunderson LL, Jessup JM, Sargent DJ, Greene FL, Stewart A. Revised tumor and node categorization for rectal cancer based on surveillance, epidemiology, and end results and rectal pooled analysis outcomes. *Journal of clinical oncology*. 2010;28(2):256-263.
17. Gunderson LL, Jessup JM, Sargent DJ, Greene FL, Stewart AK. Revised TN categorization for colon cancer based on national survival outcomes data. *Journal of clinical oncology*. 2010;28(2):264-271.
18. Panarelli NC, Schreiner AM, Brandt SM, Shepherd NA, Yantiss RK. Histologic features and cytologic techniques that aid pathologic stage assessment of colonic adenocarcinoma. *The American journal of surgical pathology*. Aug 2013;37(8):1252-1258.
19. Shepherd NA, Baxter KJ, Love SB. The prognostic importance of peritoneal involvement in colonic cancer: a prospective evaluation. *Gastroenterology*. Apr 1997;112(4):1096-1102.
20. Arbman G, Nilsson E, Hallbook O, Sjodahl R. Local recurrence following total mesorectal excision for rectal cancer. *The British journal of surgery*. Mar 1996;83(3):375-379.
21. Kapiteijn E, Marijnen CA, Nagtegaal ID, et al. Preoperative radiotherapy combined with total mesorectal excision for resectable rectal cancer. *N Engl J Med*. Aug 30 2001;345(9):638-646.
22. Marr R, Birbeck K, Garvican J, et al. The modern abdominoperineal excision: the next challenge after total mesorectal excision. *Annals of surgery*. Jul 2005;242(1):74-82.
23. Partitt JR, Driman DK. The total mesorectal excision specimen for rectal cancer: a review of its pathological assessment. *Journal of clinical pathology*. Aug 2007;60(8):849-855.
24. How P, Shihab O, Tekkis P, et al. A systematic review of cancer related patient outcomes after anterior resection and abdominoperineal excision for rectal cancer in the total mesorectal excision era. *Surgical oncology*. Dec 2011;20(4):e149-155.
25. Park IJ, You YN, Agarwal A, et al. Neoadjuvant treatment response as an early response indicator for patients with rectal cancer. *J Clin Oncol*. May 20 2012;30(15):1770-1776.
26. Fokas E, Liersch T, Fietkau R, et al. Tumor regression grading after preoperative chemoradiotherapy for locally advanced rectal carcinoma revisited: updated results of the CAO/ARO/AIO-94 trial. *J Clin Oncol*. May 20 2014;32(15):1554-1562.
27. Mescoli C, Albertoni L, Pucciarelli S, et al. Isolated tumor cells in regional lymph nodes as relapse predictors in stage I and II colorectal cancer. *J Clin Oncol*. Mar 20 2012;30(9):965-971.
28. Sloothaak DA, Sahami S, van der Zaag-Loonen HJ, et al. The prognostic value of micrometastases and isolated tumour cells in histologically negative lymph nodes of patients with colorectal cancer: a systematic review and meta-analysis. *European journal of*

surgical oncology : the journal of the European Society of Surgical Oncology and the British Association of Surgical Oncology. Mar 2014;40(3):263-269.
29. Protic M, Stojadinovic A, Nissan A, et al. Prognostic Effect of Ultra-Staging Node-Negative Colon Cancer Without Adjuvant Chemotherapy: A Prospective National Cancer Institute-Sponsored Clinical Trial. *Journal of the American College of Surgeons.* Sep 2015;221(3):643-651.
30. Loughrey MB, Quirke P, Shepherd NA. *Dataset for colorectal cancer histopathology reports.* London July 2014 2014.
31. Ueno H, Mochizuki H, Hashiguchi Y, et al. Extramural Cancer Deposits Without Nodal Structure in Colorectal Cancer Optimal Categorization for Prognostic Staging. *American journal of clinical pathology.* 2007;127(2):287-294.
32. Nagtegaal I, Quirke P. Colorectal tumour deposits in the mesorectum and pericolon; a critical review. *Histopathology.* 2007;51(2):141-149.
33. Puppa G, Ueno H, Kayahara M, et al. Tumor deposits are encountered in advanced colorectal cancer and other adenocarcinomas: an expanded classification with implications for colorectal cancer staging system including a unifying concept of in-transit metastases. *Modern pathology.* 2009;22(3):410-415.
34. Tong L-l, Gao P, Wang Z-n, et al. Is the seventh edition of the UICC/AJCC TNM staging system reasonable for patients with tumor deposits in colorectal cancer? *Annals of surgery.* 2012;255(2):208-213.
35. Jin M, Roth R, Rock JB, Washington MK, Lehman A, Frankel WL. The Impact of Tumor Deposits on Colonic Adenocarcinoma AJCC TNM Staging and Outcome. *The American journal of surgical pathology.* 2015;39(1):109-115.
36. Chen VW, Hsieh MC, Charlton ME, et al. Analysis of stage and clinical/prognostic factors for colon and rectal cancer from SEER registries: AJCC and collaborative stage data collection system. *Cancer.* 2014;120(S23):3793-3806.
37. Lemmens VE, Klaver YL, Verwaal VJ, Rutten HJ, Coebergh JWW, de Hingh IH. Predictors and survival of synchronous peritoneal carcinomatosis of colorectal origin: A population-based study. *International Journal of Cancer.* 2011;128(11): 2717-2725.
38. Segelman J, Granath F, Holm T, Machado M, Mahteme H, Martling A. Incidence, prevalence and risk factors for peritoneal carcinomatosis from colorectal cancer. *British Journal of Surgery.* 2012;99(5):699-705.
39. Cao CQ, Yan TD, Liauw W, Morris DL. Comparison of optimally resected hepatectomy and peritonectomy patients with colorectal cancer metastasis. *Journal of surgical oncology.* 2009;100(7):529-533.
40. Franko J, Shi Q, Goldman CD, et al. Treatment of colorectal peritoneal carcinomatosis with systemic chemotherapy: a pooled analysis of north central cancer treatment group phase III trials N9741 and N9841. *Journal of Clinical Oncology.* 2012;30(3):263-267.
41. Cancer Genome Atlas Network. Comprehensive molecular characterization of human colon and rectal cancer. *Nature.* 2012;487(7407):330-337.
42. Donehower LA, Creighton CJ, Schultz N, et al. MLH1-silenced and non-silenced subgroups of hypermutated colorectal carcinomas have distinct mutational landscapes. *The Journal of pathology.* 2013;229(1):99-110.
43. Brown SD, Warren RL, Gibb EA, et al. Neo-antigens predicted by tumor genome meta-analysis correlate with increased patient survival. *Genome research.* 2014;24(5):743-750.
44. Angelova M, Charoentong P, Hackl H, et al. Characterization of the immunophenotypes and antigenomes of colorectal cancers reveals distinct tumor escape mechanisms and novel targets for immunotherapy. *Genome biology.* 2015;16(1):64.
45. Allegra CJ, Jessup JM, Somerfield MR, et al. American Society of Clinical Oncology provisional clinical opinion: testing for KRAS gene mutations in patients with metastatic colorectal carcinoma to predict response to anti–epidermal growth factor receptor monoclonal antibody therapy. *Journal of Clinical Oncology.* 2009;27(12):2091-2096.
46. Petrelli F, Coinu A, Cabiddu M, Ghilardi M, Barni S. KRAS as prognostic biomarker in metastatic colorectal cancer patients treated with bevacizumab: a pooled analysis of 12 published trials. *Medical oncology.* 2013;30(3):1-8.
47. Ciardiello F, Normanno N, Maiello E, et al. Clinical activity of FOLFIRI plus cetuximab according to extended gene mutation status by next-generation sequencing: findings from the CAPRI-GOIM trial. *Annals of Oncology.* 2014;25(9):1756-1761.
48. Pavlopoulou A, Scorilas A. A comprehensive phylogenetic and structural analysis of the carcinoembryonic antigen (CEA) gene family. *Genome biology and evolution.* 2014;6(6):1314-1326.
49. Gold P, Freedman SO. Specific carcinoembryonic antigens of the human digestive system. *The Journal of experimental medicine.* 1965;122(3):467-481.
50. Thomas P, Petrick AT, Toth CA, Fox ES, Elting JJ, Steele G. A peptide sequence on carcinoembryonic antigen binds to a 80kD protein on Kupffer cells. *Biochemical and biophysical research communications.* 1992;188(2):671-677.
51. Locker GY, Hamilton S, Harris J, et al. ASCO 2006 update of recommendations for the use of tumor markers in gastrointestinal cancer. *Journal of Clinical Oncology.* 2006;24(33):5313-5327.
52. Hostetter RB, Augustus LB, Mankarious R, et al. Carcinoembryonic antigen as a selective enhancer of colorectal cancer metastasis. *Journal of the National Cancer Institute.* 1990;82(5):380-385.
53. Benchimol S, Fuks A, Jothy S, Beauchemin N, Shirota K, Stanners CP. Carcinoembryonic antigen, a human tumor marker, functions as an intercellular adhesion molecule. *Cell.* 1989;57(2):327-334.
54. Jessup J, Kim J, Thomas P, et al. Adhesion to carcinoembryonic antigen by human colorectal carcinoma cells involves at least two epitopes. *International journal of cancer.* 1993;55(2):262-268.
55. Zhou H, Fuks A, Alcaraz G, Bolling TJ, Stanners CP. Homophilic adhesion between Ig superfamily carcinoembryonic antigen molecules involves double reciprocal bonds. *The Journal of cell biology.* 1993;122(4):951-960.
56. Gangopadhyay A, Bajenova O, Kelly TM, Thomas P. Carcinoembryonic antigen induces cytokine expression in Kupffer cells: implications for hepatic metastasis from colorectal cancer. *Cancer research.* 1996;56(20):4805-4810.
57. Jessup JM, Laguinge L, Lin S, et al. Carcinoembryonic antigen induction of IL-10 and IL-6 inhibits hepatic ischemic/reperfusion injury to colorectal carcinoma cells. *International journal of cancer.* 2004;111(3):332-337.
58. Ordoñez C, Screaton RA, Ilantzis C, Stanners CP. Human carcinoembryonic antigen functions as a general inhibitor of anoikis. *Cancer Research.* 2000;60(13):3419-3424.
59. Samara RN, Laguinge LM, Jessup JM. Carcinoembryonic antigen inhibits anoikis in colorectal carcinoma cells by interfering with TRAIL-R2 (DR5) signaling. *Cancer research.* 2007;67(10): 4774-4782.
60. Ryan R, Gibbons D, Hyland J, et al. Pathological response following long-course neoadjuvant chemoradiotherapy for locally advanced rectal cancer. *Histopathology.* 2005;47(2):141-146.
61. Wittekind C, Compton C, Quirke P, et al. A uniform residual tumor (R) classification. *Cancer.* 2009;115(15):3483-3488.
62. Compton CC, Fielding LP, Burgart LJ, et al. Prognostic factors in colorectal cancer. *Arch Pathol Lab Med.* 2000;124(7):979-994.
63. Di Fabio F, Nascimbeni R, Villanacci V, et al. Prognostic variables for cancer-related survival in node-negative colorectal carcinomas. *Digestive surgery.* 2004;21(2):128-133.

64. Santos C, López-Doriga A, Navarro M, et al. Clinicopathological risk factors of stage II colon cancer: results of a prospective study. *Colorectal Disease.* 2013;15(4):414-422.
65. Lim S-B, Yu CS, Jang SJ, Kim TW, Kim JH, Kim JC. Prognostic significance of lymphovascular invasion in sporadic colorectal cancer. *Diseases of the colon & rectum.* 2010;53(4):377-384.
66. Betge J, Pollheimer MJ, Lindtner RA, et al. Intramural and extramural vascular invasion in colorectal cancer. *Cancer.* 2012;118(3):628-638.
67. Compton C. Colorectal cancer. In: Gospodarowicz M, ed. *Prognostic factors in cancer.* New York, NY: Wiley-Liss; 2006:133-137.
68. Blenkinsopp W, Stewart-Brown S, Blesovsky L, Kearney G, Fielding L. Histopathology reporting in large bowel cancer. *Journal of clinical pathology.* 1981;34(5):509-513.
69. Liebig C, Ayala G, Wilks J, et al. Perineural invasion is an independent predictor of outcome in colorectal cancer. *Journal of clinical oncology.* 2009;27(31):5131-5137.
70. Quah H-M, Chou JF, Gonen M, et al. Identification of patients with high-risk stage II colon cancer for adjuvant therapy. *Diseases of the Colon & Rectum.* 2008;51(5):503-507.
71. Fujita S, Shimoda T, Yoshimura K, Yamamoto S, Akasu T, Moriya Y. Prospective evaluation of prognostic factors in patients with colorectal cancer undergoing curative resection. *Journal of surgical oncology.* 2003;84(3):127-131.
72. Ogino S, Meyerhardt JA, Irahara N, et al. KRAS mutation in stage III colon cancer and clinical outcome following intergroup trial CALGB 89803. *Clinical Cancer Research.* 2009;15(23):7322-7329.
73. Thibodeau S, Bren G, Schaid D. Microsatellite instability in cancer of the proximal colon. *Science.* 1993;260(5109):816-819.
74. Eshleman JR, Markowitz SD. Microsatellite instability in inherited and sporadic neoplasms. *Current opinion in oncology.* 1995;7(1):83-89.
75. Sargent DJ, Marsoni S, Monges G, et al. Defective mismatch repair as a predictive marker for lack of efficacy of fluorouracil-based adjuvant therapy in colon cancer. *Journal of Clinical Oncology.* 2010;28(20):3219-3226.
76. Zaanan A, Cuilliere-Dartigues P, Guilloux A, et al. Impact of p53 expression and microsatellite instability on stage III colon cancer disease-free survival in patients treated by 5-fluorouracil and leucovorin with or without oxaliplatin. *Annals of oncology.* 2010;21(4):772-780.
77. Zaanan A, Fléjou J-F, Emile J-F, et al. Defective mismatch repair status as a prognostic biomarker of disease-free survival in stage III colon cancer patients treated with adjuvant FOLFOX chemotherapy. *Clinical Cancer Research.* 2011;17(23):7470-7478.
78. Ooki A, Akagi K, Yatsuoka T, et al. Combined microsatellite instability and BRAF gene status as biomarkers for adjuvant chemotherapy in stage III colorectal cancer. *Journal of surgical oncology.* 2014;110(8):982-988.
79. Aaltonen LA, Peltomaki P, Leach FS, et al. Clues to the pathogenesis of familial colorectal cancer. *Science.* 1993;260(5109):812-816.
80. García-Alfonso P, Salazar R, García-Foncillas J, et al. Guidelines for biomarker testing in colorectal carcinoma (CRC): a national consensus of the Spanish Society of Pathology (SEAP) and the Spanish Society of Medical Oncology (SEOM). *Clinical and Translational Oncology.* 2012;14(10):726-739.
81. Moreira L, Balaguer F, Lindor N, et al. Identification of Lynch syndrome among patients with colorectal cancer. *Jama.* 2012;308(15):1555-1565.
82. Pérez-Carbonell L, Ruiz-Ponte C, Guarinos C, et al. Comparison between universal molecular screening for Lynch syndrome and revised Bethesda guidelines in a large population-based cohort of patients with colorectal cancer. *Gut.* 2011:gutjnl-2011-300041.
83. Lochhead P, Kuchiba A, Imamura Y, et al. Microsatellite instability and BRAF mutation testing in colorectal cancer prognostication. *Journal of the National Cancer Institute.* 2013:djt173.
84. Sinicrope FA, Shi Q, Smyrk TC, et al. Molecular markers identify subtypes of stage III colon cancer associated with patient outcomes. *Gastroenterology.* 2015;148(1):88-99.
85. Gavin PG, Colangelo LH, Fumagalli D, et al. Mutation profiling and microsatellite instability in stage II and III colon cancer: an assessment of their prognostic and oxaliplatin predictive value. *Clinical cancer research.* 2012;18(23):6531-6541.
86. Souglakos J, Philips J, Wang R, et al. Prognostic and predictive value of common mutations for treatment response and survival in patients with metastatic colorectal cancer. *British journal of cancer.* 2009;101(3):465-472.
87. Pietrantonio F, Petrelli F, Coinu A, et al. Predictive role of BRAF mutations in patients with advanced colorectal cancer receiving cetuximab and panitumumab: A meta-analysis. *European journal of cancer.* 2015;51(5):587-594.
88. Estrella JS, Tetzlaff MT, Bassett RL, Jr., et al. Assessment of BRAF V600E Status in Colorectal Carcinoma: Tissue-Specific Discordances between Immunohistochemistry and Sequencing. *Mol Cancer Ther.* Dec 2015;14(12):2887-2895.
89. Halabi S, Owzar K. The importance of identifying and validating prognostic factors in oncology. Paper presented at: Seminars in oncology2010.
90. Kattan MW, Hess KR, Amin MB, et al. American Joint Committee on Cancer acceptance criteria for inclusion of risk models for individualized prognosis in the practice of precision medicine. *CA: a cancer journal for clinicians.* Jan 19 2016.
91. Moons KG, de Groot JA, Bouwmeester W, et al. Critical appraisal and data extraction for systematic reviews of prediction modelling studies: the CHARMS checklist. *PLoS medicine.* Oct 2014;11(10):e1001744.
92. Adam R, Delvart V, Pascal G, et al. Rescue surgery for unresectable colorectal liver metastases downstaged by chemotherapy: a model to predict long-term survival. *Annals of surgery.* Oct 2004;240(4):644-657; discussion 657-648.
93. Chibaudel B, Bonnetain F, Tournigand C, et al. Simplified prognostic model in patients with oxaliplatin-based or irinotecan-based first-line chemotherapy for metastatic colorectal cancer: a GERCOR study. *The oncologist.* 2011;16(9):1228-1238.
94. Mayo Clinic. Numeracy: Adjuvant systemic therapy for resected colon cancer. http://www.mayoclinic.com/calcs/colon/input.cfm?CFID=6391747&CFTOKEN=28314080&jsessionid=8630241 41fdb6e5fff40189664216522c4e4 Accessed 1/21/15.
95. Elias D, Faron M, Goere D, et al. A simple tumor load-based nomogram for surgery in patients with colorectal liver and peritoneal metastases. *Annals of surgical oncology.* Jun 2014;21(6):2052-2058.
96. Fong Y, Fortner J, Sun RL, Brennan MF, Blumgart LH. Clinical score for predicting recurrence after hepatic resection for metastatic colorectal cancer: analysis of 1001 consecutive cases. *Annals of surgery.* Sep 1999;230(3):309-318; discussion 318-321.
97. Hill CR, Chagpar RB, Callender GG, et al. recurrence following hepatectomy for metastatic colorectal cancer: development of a model that predicts patterns of recurrence and survival. *Annals of surgical oncology.* Jan 2012;19(1):139-144.
98. Iwatsuki S, Dvorchik I, Madariaga JR, et al. Hepatic resection for metastatic colorectal adenocarcinoma: a proposal of a prognostic scoring system. *Journal of the American College of Surgeons.* Sep 1999;189(3):291-299.
99. Kanemitsu Y, Kato T. Prognostic models for predicting death after hepatectomy in individuals with hepatic metastases from colorectal cancer. *World journal of surgery.* Jun 2008;32(6):1097-1107.

100. Kanemitsu Y, Kato T, Hirai T, Yasui K. Preoperative probability model for predicting overall survival after resection of pulmonary metastases from colorectal cancer. *The British journal of surgery.* Jan 2004;91(1):112-120.
101. Kato H, Yoshimatsu K, Ishibashi K, et al. A new staging system for colorectal carcinoma with liver metastasis. *Anticancer research.* Mar-Apr 2005;25(2B):1251-1255.
102. Kattan MW, Gonen M, Jarnagin WR, et al. A nomogram for predicting disease-specific survival after hepatic resection for metastatic colorectal cancer. *Annals of surgery.* Feb 2008;247(2): 282-287.
103. Kobayashi H, Kotake K, Sugihara K. Prognostic scoring system for stage IV colorectal cancer: is the AJCC sub-classification of stage IV colorectal cancer appropriate? *International journal of clinical oncology.* Aug 2013;18(4):696-703.
104. Kohne CH, Cunningham D, Di Costanzo F, et al. Clinical determinants of survival in patients with 5-fluorouracil-based treatment for metastatic colorectal cancer: results of a multivariate analysis of 3825 patients. *Ann Oncol.* Feb 2002;13(2):308-317.
105. Konopke R, Kersting S, Distler M, et al. Prognostic factors and evaluation of a clinical score for predicting survival after resection of colorectal liver metastases. *Liver international : official journal of the International Association for the Study of the Liver.* Jan 2009;29(1):89-102.
106. Lee WS, Kim MJ, Yun SH, et al. Risk factor stratification after simultaneous liver and colorectal resection for synchronous colorectal metastasis. *Langenbeck's archives of surgery/Deutsche Gesellschaft fur Chirurgie.* Jan 2008;393(1):13-19.
107. Lise M, Bacchetti S, Da Pian P, Nitti D, Pilati P. Patterns of recurrence after resection of colorectal liver metastases: prediction by models of outcome analysis. *World journal of surgery.* May 2001;25(5):638-644.
108. Malik HZ, Prasad KR, Halazun KJ, et al. Preoperative prognostic score for predicting survival after hepatic resection for colorectal liver metastases. *Annals of surgery.* Nov 2007;246(5):806-814.
109. Nanashima A, Sumida Y, Abo T, et al. A modified grading system for post-hepatectomy metastatic liver cancer originating from colorectal carcinoma. *Journal of surgical oncology.* Oct 1 2008;98(5):363-370.
110. Nordlinger B, Guiguet M, Vaillant JC, et al. Surgical resection of colorectal carcinoma metastases to the liver. A prognostic scoring system to improve case selection, based on 1568 patients. Association Francaise de Chirurgie. *Cancer.* Apr 1 1996;77(7):1254-1262.
111. Peng J, Ding Y, Tu S, et al. Prognostic nomograms for predicting survival and distant metastases in locally advanced rectal cancers. *PloS one.* 2014;9(8):e106344.
112. Renfro LA, Grothey A, Xue Y, et al. ACCENT-based web calculators to predict recurrence and overall survival in stage III colon cancer. *Journal of the National Cancer Institute.* Dec 2014;106(12).
113. Shitara K, Matsuo K, Yokota T, et al. Prognostic factors for metastatic colorectal cancer patients undergoing irinotecan-based second-line chemotherapy. *Gastrointestinal cancer research : GCR.* Sep 2011;4(5-6):168-172.
114. Stang A, Oldhafer KJ, Weilert H, Keles H, Donati M. Selection criteria for radiofrequency ablation for colorectal liver metastases in the era of effective systemic therapy: a clinical score based proposal. *BMC cancer.* 2014;14:500.
115. Stojadinovic A, Bilchik A, Smith D, et al. Clinical decision support and individualized prediction of survival in colon cancer: bayesian belief network model. *Annals of surgical oncology.* Jan 2013;20(1):161-174.
116. Tan MC, Castaldo ET, Gao F, et al. A prognostic system applicable to patients with resectable liver metastasis from colorectal carcinoma staged by positron emission tomography with [18F]fluoro-2-deoxy-D-glucose: role of primary tumor variables. *Journal of the American College of Surgeons.* May 2008;206(5):857-868; discussion 868-859.
117. Valentini V, van Stiphout RG, Lammering G, et al. Nomograms for predicting local recurrence, distant metastases, and overall survival for patients with locally advanced rectal cancer on the basis of European randomized clinical trials. *J Clin Oncol.* Aug 10 2011;29(23):3163-3172.
118. Weiser MR, Gonen M, Chou JF, Kattan MW, Schrag D. Predicting survival after curative colectomy for cancer: individualizing colon cancer staging. *J Clin Oncol.* Dec 20 2011;29(36):4796-4802.
119. Weiser MR, Landmann RG, Kattan MW, et al. Individualized prediction of colon cancer recurrence using a nomogram. *J Clin Oncol.* Jan 20 2008;26(3):380-385.
120. Yamaguchi T, Mori T, Takahashi K, Matsumoto H, Miyamoto H, Kato T. A new classification system for liver metastases from colorectal cancer in Japanese multicenter analysis. *Hepato-gastroenterology.* Jan-Feb 2008;55(81):173-178.

Anus

21

Mark Lane Welton, Scott R. Steele, Karyn A. Goodman,
Leonard L. Gunderson, Elliot A. Asare, James D. Brierley,
Carolyn C. Compton, Paola De Nardi,
Richard M. Goldberg, Donna Gress,
Mary Kay Washington, and J. Milburn Jessup

CHAPTER SUMMARY

Cancers Staged Using This Staging System

This staging system applies to all carcinomas arising in the anal canal, including carcinomas that arise within anorectal fistulas and those arising in the perianal area. High-grade neuroendocrine carcinomas (small cell neuroendocrine carcinoma and large cell neuroendocrine carcinoma) are staged using this system.

Cancers Not Staged Using This Staging System

These histopathologic types of cancer…	Are staged according to the classification for…	And can be found in chapter…
Sarcomas	Soft tissue sarcoma of the abdomen and thoracic visceral organs	42
Mucosal melanoma of the anus	No AJCC staging system	N/A
Well-differentiated neuroendocrine tumors	No AJCC staging system	N/A

Summary of Changes

Change	Details of Change	Level of Evidence
Anatomy—Primary Site(s)	Landmarks that define anal and perianal tumors have been clarified and made consistent with terminology in the colon and rectum chapter.	IV
Anatomy—Primary Site(s)	Landmarks that define vulvar and perianal areas are discussed, and a classification that differentiates the two is proposed to allow collection of data for subsequent review.	IV
Anatomy—Regional Lymph Nodes	New terminology referring to regional lymph nodes draining the region has been made consistent with terminology used in the colon and rectum chapter.	IV
Definition of Regional Lymph Node (N)	N2 and N3 categories were removed, and new categories of N1a, N1b, and N1c are defined.	II
AJCC Prognostic Stage Groups	Stage groups were revised to accommodate the new N categories.	II

To access the AJCC cancer staging forms, please visit www.cancerstaging.org.

© American Joint Committee on Cancer 2017

M.B. Amin et al. (eds.), *AJCC Cancer Staging Manual, Eighth Edition*, DOI 10.1007/978-3-319-40618-3_21

ICD-O-3 Topography Codes

Code	Description
C21.0	Anus, NOS
C21.1	Anal canal
C21.8	Overlapping lesion of rectum, anus, and anal canal

WHO Classification of Tumors

Code	Description
8070	Squamous cell carcinoma, NOS
8077	Squamous intraepithelial neoplasia, high grade (formerly Bowen disease)
8051	Verrucous carcinoma, NOS
8020	Carcinoma, undifferentiated, NOS
8140	Adenocarcinoma, NOS
8542	Paget disease, extramammary
8480	Mucinous adenocarcinoma
8090	Basal cell carcinoma, NOS
8246	Neuroendocrine carcinoma
8041	Small cell neuroendocrine carcinoma
8013	Large cell neuroendocrine carcinoma
8244	Mixed adenoneuroendocrine carcinoma

Bosman FT, Carneiro F, Hruban RH, Theise ND, eds. World Health Organization Classification of Tumours of the Digestive System. Lyon: IARC; 2010.

INTRODUCTION

This classification applies to carcinomas of the anal canal and perianus (formerly anal margin). The landmarks that define the anal canal and perianus are discussed and illustrated in the anatomy section. This chapter also covers the pathological classification and staging of carcinomas of the perianal region.

Anal cancer is rare, representing only 0.4 % of all new cancer cases in the United States. The incidence is higher in women than men, 2.0 versus 1.5 per 100,000, respectively, with an overall rate of 1.8 per 100,000 persons. The incidence of anal cancer in both men and women has been rising steadily in the United States over the past decade, increasing roughly 2.2 % each year.[1] In 2014 alone, 7,210 new anal cancer cases were estimated in the United States, with 950 deaths.[1,2] In comparison, only approximately 27,000 cases of anal cancer were diagnosed worldwide in 2008.

A focus of this chapter is to further refine the TNM staging system to clarify what constitutes anal canal versus perianal cancer and to specify additional considerations that may be of value for cancers in the region of the perianus and vulva. For instance, squamous cell carcinomas (SCCs) overlying the perineal body may be classified as perianal or vulvar, and the treatment plans may be quite dissimilar. For this reason, we recommend the following: lesions that clearly arise from the vulva and extend onto the perineum and potentially involve the anus should be classified as vulvar. Similarly, lesions that clearly arise from the distal anal mucosa and extend onto the perineum should be classified as perianal. Lesions localized to the perineum that are not clearly arising from either the vulva or the anus should be categorized based on the clinician's clinical impression. Thus, we recommend the following terminology: perineum favor vulva and perineum favor perianus. We also recommend consulting with colleagues in gynecologic oncology, colorectal or general surgery, or surgical oncology, because classification has a significant impact on treatment.

Most carcinomas of the anal canal and perianal region are SCCs. The terms *transitional cell* and *cloacogenic carcinoma* have been abandoned, because these tumors are now recognized as nonkeratinizing types of SCC.

Squamous Cell Carcinoma

The predominant histologic type of malignancy arising in the anal canal is SCC. Estimates suggest that 95 % of these cancers are caused by oncogenic human papillomavirus (HPV) types, with HPV 16 associated with 89 % of cases.[3–6] Higher-risk populations are men who have sex with men and people who are immunosuppressed. Mortality rates are rising as well, with an average increase of 1.7 % each year from 2001 to 2010. Only 65.5 % of patients survive 5 years or more.[1] Nonsquamous anal cancers include adenocarcinomas, basal cell carcinomas (BCCs), and melanomas (not discussed here).

Carcinomas of the anal canal typically are staged clinically according to the size and extent of the untreated primary tumor. Patients with cancer of the anal canal typically are staged at the time of presentation with inspection, palpation, and biopsy of the mass; palpation (and biopsy as needed) of regional lymph nodes; and radiologic imaging of the chest, abdomen, and pelvis.

High-grade squamous intraepithelial lesion (HSIL) is not a malignancy and should not be coded as such. Direct evidence supports the progression of HSIL to SCC, but the impact of treatment on the progression to carcinoma is being studied in the ANCHOR trial, a multi-institutional randomized prospective trial comparing observation with excision/destruction.[7]

The primary management of SCCs of the anal canal is nonoperative, involving combined chemotherapy and radiation therapy. Fortunately, localized SCC of the anal canal has been associated with excellent outcomes with combined-modality therapy, with a 5-year overall survival rate of 78 % in the positive arm of a US phase III randomized trial, Radiation Therapy Oncology Group (RTOG) 98-11.[8]

In contrast, the management of perianal carcinomas remains mixed, with both operative and nonoperative treatments used selectively, based on involvement of adjacent structures, tumor size, and histology of the primary lesion. Complete pathological staging is possible for a perianal primary tumor treated surgically. The remainder of the staging of regional lymph nodes and distant disease in perianal tumors is as described for anal cancers.

Other Histologic Types

Verrucous Carcinoma

Verrucous carcinoma historically has been distinguished from ordinary condyloma acuminatum by its apparent combination of exophytic and endophytic growth.[9–11] However, the appearance of endophytic growth may, in fact, represent growth along preexisting cryptoglandular fistulous disease rather than actual endophytic invasion, as convincing evidence of actual invasion is rare. Some verrucous carcinomas contain HPV, the most common types being HPV 6 and HPV 11. This is another distinction from SCCs, which demonstrate predominantly HPV 16. Treatment typically focuses on local control in the absence of invasion. However, any histologic evidence of invasive disease or metastasis should lead to the diagnosis of SCC and appropriate therapy.

Basal Cell Carcinoma

BCC of the perianus is an uncommon entity, with a reported incidence of about 0.1 % of all BCCs and <1 % of all anorectal neoplasms.[12–14] Differentiating basaloid SCC from BCC may be challenging, as they have similar histologic features. However, SCCs arise from a known precursor lesion (anal squamous intraepithelial neoplasia), whereas BCCs do not have a well-defined precursor. SCCs may be distinguished further from BCCs in that they may arise from the anal mucosa or the perianal skin, whereas BCCs usually arise in the perianal skin. Rarely, BCCs may extend from the perianal skin into the anal canal. In general, BCCs are associated with a low recurrence rate (0–29 %), and wide local excision with negative margins remains the standard of care, especially for smaller lesions. Electrodesiccation and curettage, Mohs micrographic surgery, and external beam irradiation also have been reported with successful results.[15] Radiation and/or abdominal–perineal resection may be required for large lesions and recurrent tumors.

Table 21.1 Surveillance, Epidemiology, and End Results (SEER) 5-year OS for cohort stratified by histologic type and stage (KM method)

Stage	Squamous OS (%)	Nonsquamous OS (%)
I	76.9	71
II	66.7	58.7
IIIA	57.7	50.1
IIIB	50.7	34.6
IV	15.2	6.8

Adenocarcinoma

Adenocarcinomas of the anus and perianus are rare and generally fall into three categories: those that extend down from above the dentate line, those that originate from the underlying anorectal glands or longstanding fistulous disease, and those arising primarily from the anal mucosa or perianal skin.[16–18] Lesions in the perianal skin may be amenable to wide local excision in select cases. Most perianal, anal, and distal rectal lesions extending into the anal mucosa are treated with abdominal perineal excision with or without neoadjuvant chemoradiation. The outcomes for all other histologic types, including adenocarcinomas, are poorer, stage for stage, than for SCC (Table 21.1).

The presumed precursor lesion for perianal adenocarcinoma, extramammary Paget disease, is adenocarcinoma *in situ*. Anal Paget disease may be divided into two classes. About half the cases are associated with synchronous or metachronous colorectal malignancies; the other subset does not appear to be associated with internal malignancies but is associated with a high local recurrence rate and is more likely to progress to invasive cancer.[19] Perianal Paget disease may be treated with wide local excision.[20,21]

ANATOMY

Primary Site(s)

The anatomic subsites of the anal canal are illustrated in Fig. 21.1. The anal canal begins where the rectum enters the puborectalis sling at the apex of the anal sphincter complex (palpable as the anorectal ring on digital rectal examination and approximately 1 to 2 cm proximal to the dentate line) and ends with the squamous mucosa blending with the

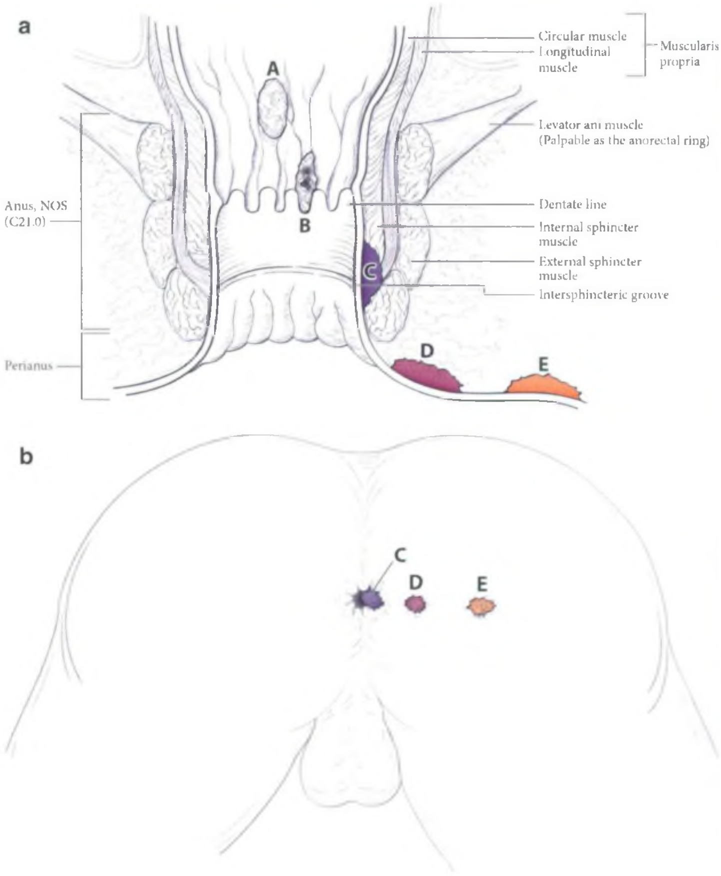

Fig. 21.1a-b Anal cancer (**A–C**), perianal cancer (**D**), and skin cancer (**E**) as visualized with gentle traction placed on the buttocks

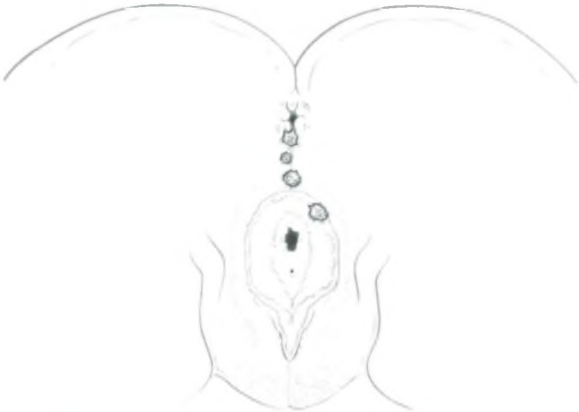

Fig. 21.2 Perineal and vulvar lesions, from top to bottom. The lesion at the top is perianal, the middle two lesions are perineal, and the lesion at the bottom is considered vulvar

perianal skin, which coincides roughly with the palpable intersphincteric groove or the outermost boundary of the internal sphincter muscle, easily visualized on endoanal ultrasound. The anus encompasses true mucosa of three different histologic types: glandular, transitional, and squamous (proximal to distal, respectively). The most proximal aspect of the anal canal is lined by colorectal mucosa in which squamous metaplasia may occur. When involved by metaplasia, this zone also may be referred to as the transformation zone. Immediately proximal to the macroscopically visible dentate line, a narrow zone of multilayered transitional mucosa is variably present. In the region of the dentate line, anal glands are subjacent to the mucosa, often penetrating through the internal sphincter into the intersphincteric plane. The distal zone of the anal canal extends from the dentate line to the mucocutaneous junction with the perianal skin and is lined by a nonkeratinizing squamous epithelium devoid of epidermal appendages (hair follicles, apocrine glands, and sweat glands).

Tumors that develop from mucosa (of any of the three types) that cannot be visualized in their entirety while gentle traction is placed on the buttocks are termed *anal cancers*, whereas those that arise within the skin at or distal to the squamous mucocutaneous junction, can be seen in their entirety with gentle traction placed on the buttocks, and are within 5 cm of the anus are termed *perianal cancers*.

Fig. 21.3 Regional lymph nodes of the anal canal

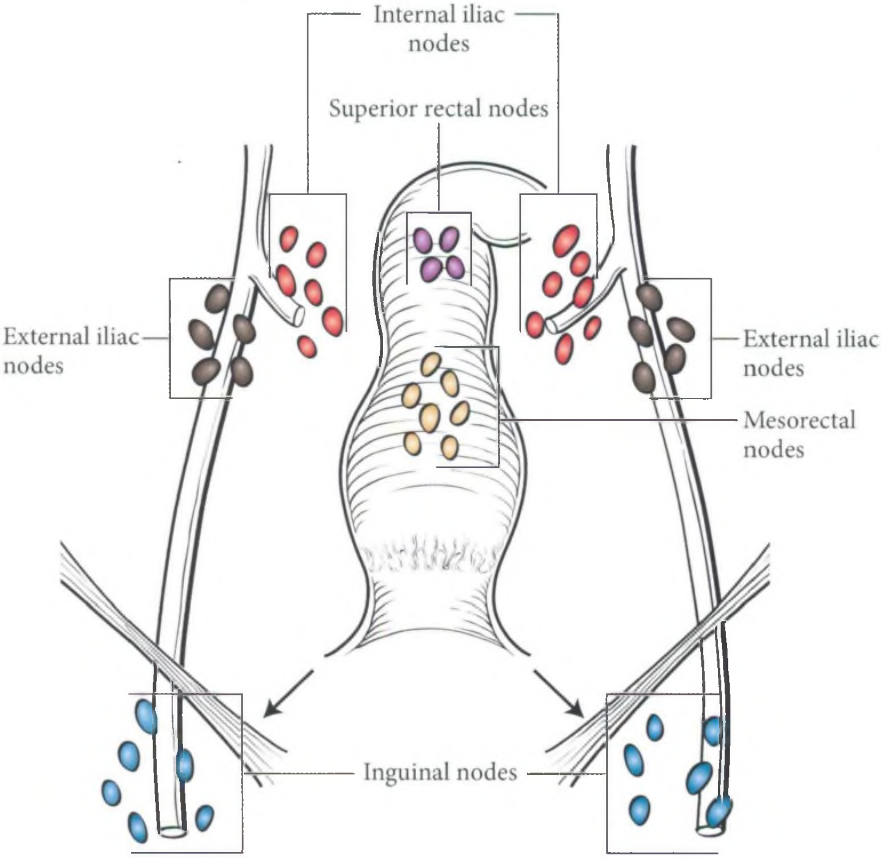

Regional Lymph Nodes

Lymphatic drainage and nodal involvement of anal cancers often depend on the location of the primary tumor. Tumors above the dentate line spread primarily to the mesorectal and internal iliac nodes, whereas tumors below the dentate line also may spread to the superficial inguinal and external iliac (deep inguinal) nodes.

The regional lymph nodes are as follows (Fig. 21.3):

- Mesorectal
- Inguinal: superficial, deep
- Superior rectal (hemorrhoidal)
- External iliac
- Internal iliac (hypogastric)

All other nodal groups represent sites of distant metastasis.

Metastatic Sites

Cancers of the anus may metastasize to any organ, but the liver and lungs are the distal organs involved most frequently.

RULES FOR CLASSIFICATION

Clinical Classification

Clinical assessment is based on medical history, physical examination, radiology, and endoscopy, with biopsy for histologic confirmation of malignancy. HIV testing should be performed in patients with established risk factors. Given patterns of spread, a thorough examination of lymph node areas, including inguinofemoral regions, should be performed. If possible, suspicious inguinal lymphadenopathy should be biopsied, generally through fine-needle aspiration, to further facilitate diagnosis and tumor staging, particularly in the HIV-positive population, in whom reactive nodes are common. In female patients, vaginal examination should be performed to rule out posterior vaginal invasion and fistula, and the cervix should be evaluated to rule out gynecologic malignancy.

Imaging

Radiologic examinations may include chest radiographic films, computed tomography (CT, of the abdomen, pelvis, and chest), magnetic resonance imaging, and positron emission

Table 21.2 Impact of TN category on survival, relapse, and colostomy failure in US GI Intergroup RTOG 98-11 phase III chemoradiation trial*

		OS		DFS		LRF		DM		CF	
**TN category**	**Patients, n**	**TD**	**5-y, %****	**TF**	**5-y, %**	**TF**	**5-y, %**	**TF**	**5-y, %**	**TF**	**3-y, %**
T2N0	323	76	82	110	72	57	17	38	10	36	11
T3N0	96	30	74	45	61	17	18	13	14	15	13
T4N0	31	14	57	16	50	11	37	7	21	8	26
T2N1–3	99	38	70	50	57	26	26	28	27	11	11
T3N1–3	46	20	57	29	38	20	44	11	24	12	27
T4N1–3	25	16	42	18	31	15	60	6	24	6	24
p* value**		<0.0001		<0.0001		<0.0001		0.0011		0.01	

Abbreviations: DM, distant metastasis; CF, colostomy failure; DFS, disease-free survival; LRF, locoregional failure; OS, overall survival; TD, total deaths; TF, total failures; 5-y, 5-year; 3-y, 3-year

*Modified from Gunderson LL et al. Int J Radiat Oncol Biol Phys 2013; 87:638-645

*******Note*: Some 5-year estimates might be unstable because of small sample sizes; therefore, too few patients are at risk at 5 years

***Log-rank test for OS and DFS; Gray's test for LRF, DM, and CF

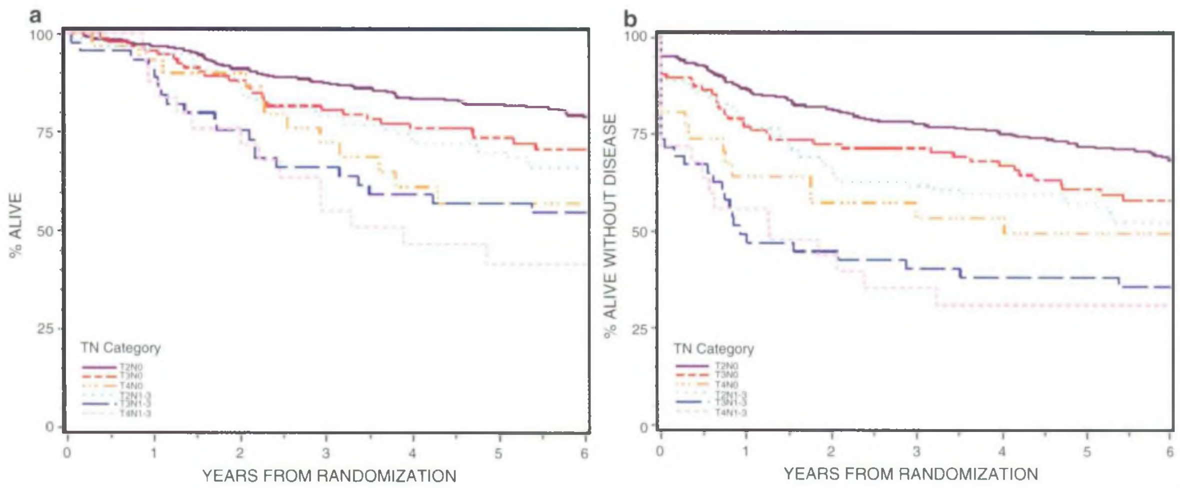

Fig. 21.4a-b Kaplan–Meier curves demonstrating the impact of TN category on survival in US GI Intergroup RTOG phase III chemoradiation trial: (**a**) OS ($p<0.0001$) and (**b**) DFS ($p<0.0001$) (From Gunderson et al.[22])

tomography (PET)/CT scans. These are obtained simultaneously during the initial evaluation. Of note, HIV-positive patients may have falsely positive nodes on PET imaging.

Imaging is the primary modality for diagnosing nonpalpable nodal disease and distant metastatic disease. Nonregional nodes or involvement of extrapelvic sites on imaging studies are considered M1 disease.

Pathological Classification

Because the primary treatment for anal canal SCC usually is combined chemoradiation, a surgical resection specimen is not commonly available for complete evaluation by the pathologist (pTNM), unless there is persistent or recurrent local tumor after chemoradiation. For the latter group of patients who were assigned a clinical stage (cTNM) before beginning primary chemoradiation, a modified pathological stage is determined after surgical resection (usually combined abdominoperineal resection) and annotated by the *y* prefix (ypTNM). For tumors treated by surgery and without neoadjuvant chemoradiation, and for those not clinically staged before surgery, pTN is assigned on pathological examination of the resection specimen, in consultation with the surgeon, who may comment on residual disease status and assign the c/pM.

PROGNOSTIC FACTORS

Regarding SCC of the anal canal, based on data from the National Cancer Data Base (NCDB) for 2003 to 2006, the 5-year observed survival rates for each of the stage groups are as follows: Stage I ($n=1{,}516$), 76.9 %; Stage II ($n=3{,}214$), 66.7 %; Stage IIIA ($n=735$), 57.7 %; Stage IIIB ($n=1{,}117$), 50.7 %; Stage IV ($n=364$), 15.2 %. Stage-related survival is shown in Table 21.1. These figures reflect in-patient data; therefore, because most early tumors are not treated in the in-patient setting, the reported data might be skewed.

The TN category of disease is shown to have an impact on overall and disease-free survival (OS and DFS), the need for colostomy, and disease relapse (locoregional failure [LRF] and distant metastasis [DM]) in patients with SCC treated with primary chemoradiation.[22] In the most recent analysis of the US GI Intergroup phase III trial RTOG 98-11, 620 of 682 randomly assigned patients were analyzable for outcomes by TN category, and six TN categories were compared (T2N0, T3N0, T4N0, T2N1–3, T3N1–3, and T4N1–3). All end points showed statistically significant differences among the TN categories of disease (Table 21.2 and Figs. 21.4a and b). The best OS, DFS, and LRF outcomes were found in patients with T2N0 and T3N0 categories and the poorest outcomes in those with T4N0 and T3–4N+ disease. Survival and LRF outcomes for T2N+ patients were intermediate between T3N0 and T4N0 patients, as observed in previous TN outcome analyses in patients with colon and rectal cancer.[22] The need for colostomy was lowest for T2N0 and T2N+ disease (both 11%) and worst for T4N0, T3N+, and T4N+ (26%, 27%, and 24%, respectively). In summary, the prognosis of T2N+ category tumors is more akin to T2–3 N0 tumors, and that of T3N+ tumors to T4N0 and T4N+.

The most important risk factor for squamous cell anal cancer is HPV infection, primarily types 16 and 18. Viral proteins in these high-risk HPV types mediate oncogenic transformation of the anal squamous epithelia.[23,24] Recent evidence indicates that virtually every anal SCC is related to HPV, although uncertainty continues to exist regarding the impact of various subclassifications of the virus.[6] A recent meta-analysis suggests that HPV 16 is found more frequently (75%) and HPV 18 less frequently (10%) in anal carcinomas than in cervical carcinomas.[25] In contrast to squamous carcinomas of the head and neck, there do not appear to be two major categories with entirely different biologies and prognoses based on the HPV status.

Other known risk factors for anal SCC include a history of sexually transmitted disease, a history of multiple sexual partners, and anal intercourse, all of which may be associated with a higher incidence of HPV infection.[26,27] SCCs of the anus are more common in women; however, men have a worse prognosis.[2,27] The poorer prognosis in males also was confirmed in three phase III chemoradiation trials (US GI Intergroup 98-11[8,28], European Organisation for Research and Treatment of Cancer [EORTC] 22861,[29] and United Kingdom ACT I[30]). Chronic immunosuppression, which may be related to HIV-positive status or a history of organ transplantation, and tobacco use also are important risk factors for anal cancer.[26,31–33]

Nonsquamous histologies of anal canal carcinomas, including adenocarcinoma, mucinous adenocarcinoma, high-grade neuroendocrine carcinoma, and undifferentiated carcinoma, are associated with worse 5-year survival (OS) rates than SCCs of the anal canal. At each stage, survival rates for patients with SCCs are better than those for patients with nonsquamous tumors, as shown in Table 21.1. However, historically recognized histologic SCC variants, such as large cell keratinizing, large cell nonkeratinizing, and basaloid subtypes, have no associated prognostic differences. Therefore, the World Health Organization recommends that the generic term *squamous cell carcinoma* be used for all squamous tumors of the anal canal. As previously stated, BCCs of the perianal region tend to have a better prognosis, with lower risk of relapse.

Prognostic Factors Required for Stage Grouping

Beyond the factors used to assign T, N, or M categories, no additional prognostic factors are required for stage grouping.

Additional Factors Recommended for Clinical Care

Tumor Location

Tumor location defines nodal drainage fields at risk and treatment approach. For anal canal lesions, external beam irradiation fields differ from those for perianal lesions. Perianal cancers would be treated as skin cancers elsewhere in the body with focused irradiation fields without inclusion of regional nodes, unless there was deep invasion of the tumor, necessitating inguinal node coverage. For cancers of the anal canal, irradiation fields commonly include the primary tumor plus all regional node groups felt to be at risk (inguinal, mesorectal/superior rectal, internal iliac, external iliac). Tumor location is reported as anal, perianal, or perineal and left/right/anterior/posterior/lateral, and it is documented in and abstracted from the medical record. AJCC Level of Evidence: I

HIV Status

The impact of HIV status on prognosis remains incompletely defined. The literature remains controversial regarding the perception that HIV-positive patients tolerate chemoradiation therapy less well and have poorer outcomes compared with HIV-negative patients. Patients whose HIV is well controlled with highly active antiretroviral therapy appear to do as well as HIV-negative patients. Better documentation of HIV status is needed.[34–36] HIV status is reported as positive or negative, and it is documented in and abstracted from the medical record. AJCC Level of Evidence: I

Gender

Male gender has a negative impact on prognosis. Although SCCs of the anus are more common in women, men have a worse prognosis.[2,27] The poorer prognosis in males also was confirmed in three phase III chemoradiation trials (US GI Intergroup 98-11[8,28], EORTC 22861,[29] and United Kingdom ACT I[30]). Gender is reported as male or female, and it is documented in and abstracted from the medical record. AJCC Level of Evidence: I

Grade

High-grade (poorly differentiated) squamous carcinomas or adenocarcinomas of the anus have a worse prognosis than low-grade tumors. Grade is reported as well- (G1), moderately (G2), or poorly differentiated (G3), or undifferentiated (G4) carcinoma, and it is documented in and abstracted from the medical record. AJCC Level of Evidence: I

HPV Status and p16 or p18 Expression

The most important risk factor for squamous cell anal cancer is infection of the anus, cervix, or vulva with HPV, primarily types 16 and 18. Recent evidence indicates that virtually every anal SCC is related to HPV, although uncertainty continues to exist regarding the impact of various subclassifications of the virus.[6] A recent meta-analysis suggests that HPV 16 is found more frequently (75%) and HPV 18 less frequently (10%) in anal carcinomas than in cervical carcinomas.[25] This factor is reported as HPV type, and it is documented in and abstracted from the medical record. AJCC Level of Evidence: I

RISK ASSESSMENT MODELS

The AJCC recently established guidelines that will be used to evaluate published statistical prediction models for the purpose of granting endorsement for clinical use.[37] Although this is a monumental step toward the goal of precision medicine, this work was published only very recently. Therefore, the existing models that have been published or may be in clinical use have not yet been evaluated for this cancer site by the Precision Medicine Core of the AJCC. In the future, the statistical prediction models for this cancer site will be evaluated, and those that meet all AJCC criteria will be endorsed.

DEFINITIONS OF AJCC TNM

Definition of Primary Tumor (T)

T Category	T Criteria
TX	Primary tumor not assessed
T0	No evidence of primary tumor
Tis	High-grade squamous intraepithelial lesion (previously termed carcinoma *in situ*, Bowen disease, anal intraepithelial neoplasia II–III, high-grade anal intraepithelial neoplasia)
T1	Tumor ≤2 cm
T2	Tumor >2 cm but ≤5 cm
T3	Tumor >5 cm
T4	Tumor of any size invading adjacent organ(s), such as the vagina, urethra, or bladder

Definition of Regional Lymph Node (N)

N Category	N Criteria
NX	Regional lymph nodes cannot be assessed
N0	No regional lymph node metastasis
N1	Metastasis in inguinal, mesorectal, internal iliac, or external iliac nodes
N1a	Metastasis in inguinal, mesorectal, or internal iliac lymph nodes
N1b	Metastasis in external iliac lymph nodes
N1c	Metastasis in external iliac with any N1a nodes

Definition of Distant Metastasis (M)

M Category	M Criteria
M0	No distant metastasis
M1	Distant metastasis

AJCC PROGNOSTIC STAGE GROUPS

When T is...	And N is...	And M is...	Then the stage group is...
Tis	N0	M0	0
T1	N0	M0	I
T1	N1	M0	IIIA
T2	N0	M0	IIA
T2	N1	M0	IIIA
T3	N0	M0	IIB
T3	N1	M0	IIIC
T4	N0	M0	IIIB
T4	N1	M0	IIIC
Any T	Any N	M1	IV

REGISTRY DATA COLLECTION VARIABLES

1. Tumor location: anal, perianal, or perineal, and left/right/anterior/posterior/lateral
2. HIV status
3. Gender
4. Grade
5. HPV status and p16 or p18 expression

HISTOLOGIC GRADE (G)

G	G Definition
GX	Grade cannot be determined
G1	Well differentiated (low grade)
G2	Moderately differentiated (low grade)
G3	Poorly differentiated (high grade)
G4	Undifferentiated (high grade)

HISTOPATHOLOGIC TYPE

Squamous cell carcinoma
Adenocarcinoma
Basal cell carcinoma
Verrucous carcinoma
Mucinous adenocarcinoma
Carcinoma, undifferentiated
High-grade neuroendocrine carcinoma
    Small cell neuroendocrine carcinoma
    Large cell neuroendocrine carcinoma

Bibliography

1. National Cancer Institute. SEER Stat Fact Sheets: Anal Cancer. 2015; http://seer.cancer.gov/statfacts/html/anus.html.
2. American Cancer Society. Anal Cancer: Key Statistics. 2015; http://www.cancer.org/cancer/analcancer/detailedguide/anal-cancer-what-is-key-statistics. Accessed Oct 16, 2015.
3. Forman D, de Martel C, Lacey CJ, et al. Global burden of human papillomavirus and related diseases. *Vaccine*. Nov 20 2012;30 Suppl 5:F12-23.
4. Parkin DM, Bray F. Chapter 2: The burden of HPV-related cancers. *Vaccine*. Aug 31 2006;24 Suppl 3:S3/11-25.
5. Abramowitz L, Jacquard AC, Jaroud F, et al. Human papillomavirus genotype distribution in anal cancer in France: the EDiTH V study. *Int J Cancer*. Jul 15 2011;129(2):433-439.
6. Baricevic I, He X, Chakrabarty B, et al. High-sensitivity human papilloma virus genotyping reveals near universal positivity in anal squamous cell carcinoma: different implications for vaccine prevention and prognosis. *European journal of cancer*. Apr 2015;51(6):776-785.
7. Berry JM, Jay N, Cranston RD, et al. Progression of anal high-grade squamous intraepithelial lesions to invasive anal cancer among HIV-infected men who have sex with men. *Int J Cancer*. Mar 1 2014;134(5):1147-1155.
8. Gunderson LL, Winter KA, Ajani JA, et al. Long-term update of US GI intergroup RTOG 98-11 phase III trial for anal carcinoma: survival, relapse, and colostomy failure with concurrent chemoradiation involving fluorouracil/mitomycin versus fluorouracil/cisplatin. *J Clin Oncol*. Dec 10 2012;30(35):4344-4351.
9. Bertram P, Treutner KH, Rubben A, Hauptmann S, Schumpelick V. Invasive squamous-cell carcinoma in giant anorectal condyloma (Buschke-Lowenstein tumor). *Langenbecks Arch Chir*. 1995;380(2):115-118.
10. Longacre TA, Kong CS, Welton ML. Diagnostic problems in anal pathology. *Adv Anat Pathol*. Sep 2008;15(5):263-278.
11. Welton ML, Lambert R, Bosman FT. Tumours of the anal canal. In: Bosman FT, Carneiro F, Hruban RH, Theise ND, eds. *WHO classification of tumours of the digestive system*. 4th ed: International Agency for Research on Cancer; 2010.
11. Patil DT, Goldblum JR, Billings SD. Clinicopathological analysis of basal cell carcinoma of the anal region and its distinction from basaloid squamous cell carcinoma. *Modern pathology : an official journal of the United States and Canadian Academy of Pathology, Inc*. Oct 2013;26(10):1382-1389.
12. Paterson CA, Young-Fadok TM, Dozois RR. Basal cell carcinoma of the perianal region: 20-year experience. *Diseases of the colon and rectum*. Sep 1999;42(9):1200-1202.
13. Moore HG, Guillem JG. Anal neoplasms. *Surg Clin North Am*. Dec 2002;82(6):1233-1251.
14. Gibson GE, Ahmed I. Perianal and genital basal cell carcinoma: A clinicopathologic review of 51 cases. *Journal of the American Academy of Dermatology*. Jul 2001;45(1):68-71.
15. Abel ME, Chiu YS, Russell TR, Volpe PA. Adenocarcinoma of the anal glands. Results of a survey. *Diseases of the colon and rectum*. Apr 1993;36(4):383-387.
16. Basik M, Rodriguez-Bigas MA, Penetrante R, Petrelli NJ. Prognosis and recurrence patterns of anal adenocarcinoma. *American journal of surgery*. Feb 1995;169(2):233-237.
17. Anthony T, Simmang C, Lee EL, Turnage RH. Perianal mucinous adenocarcinoma. *Journal of surgical oncology*. Mar 1997;64(3):218-221.
18. Goldblum JR, Hart WR. Perianal Paget's disease: a histologic and immunohistochemical study of 11 cases with and without associated rectal adenocarcinoma. *The American journal of surgical pathology*. Feb 1998;22(2):170-179.
19. Marchesa P, Fazio VW, Oliart S, Goldblum JR, Lavery IC, Milsom JW. Long-term outcome of patients with perianal Paget's disease. *Annals of surgical oncology*. Sep 1997;4(6):475-480.
20. McCarter MD, Quan SH, Busam K, Paty PP, Wong D, Guillem JG. Long-term outcome of perianal Paget's disease. *Diseases of the colon and rectum*. May 2003;46(5):612-616.
21. Gunderson LL, Moughan J, Ajani JA, et al. Anal carcinoma: impact of TN category of disease on survival, disease relapse, and colostomy failure in US Gastrointestinal Intergroup RTOG 98-11 phase 3 trial. *International journal of radiation oncology, biology, physics*. Nov 15 2013;87(4):638-645.
22. Munoz N, Bosch FX, de Sanjose S, et al. Epidemiologic classification of human papillomavirus types associated with cervical cancer. *N Engl J Med*. Feb 6 2003;348(6):518-527.
23. Organization WH. *IARC monograph on the evaluation of carcinogenic risks to humans: human papillomaviruses, 1995*. Lyons, France2000.
24. Machalek DA, Poynten M, Jin F, et al. Anal human papillomavirus infection and associated neoplastic lesions in men who have sex with men: a systematic review and meta-analysis. *The lancet oncology*. May 2012;13(5):487-500.
25. Palefsky JM, Holly EA, Ralston ML, Da Costa M, Greenblatt RM. Prevalence and risk factors for anal human papillomavirus infection in human immunodeficiency virus (HIV)-positive and high-risk HIV-negative women. *J Infect Dis*. Feb 1 2001;183(3):383-391.
26. Moscicki AB, Darragh TM, Berry-Lawhorn JM, et al. Screening for Anal Cancer in Women. *J Low Genit Tract Dis*. Jul 2015;19(3 Suppl 1):S27-42.
27. Ajani JA, Winter KA, Gunderson LL, et al. Prognostic factors derived from a prospective database dictate clinical biology of anal cancer: the intergroup trial (RTOG 98-11). *Cancer*. Sep 1 2010;116(17):4007-4013.
28. Bartelink H, Roelofsen F, Eschwege F, et al. Concomitant radiotherapy and chemotherapy is superior to radiotherapy alone in the treatment of locally advanced anal cancer: results of a phase III randomized trial of the European Organization for Research and Treatment of Cancer Radiotherapy and Gastrointestinal Cooperative Groups. *J Clin Oncol*. May 1997;15(5):2040-2049.
29. Glynne-Jones R, Sebag-Montefiore D, Adams R, et al. Prognostic factors for recurrence and survival in anal cancer: generating hypotheses from the mature outcomes of the first United Kingdom Coordinating Committee on Cancer Research Anal Cancer Trial (ACT I). *Cancer*. Feb 15 2013;119(4):748-755.
30. Penn I. Cancers of the anogenital region in renal transplant recipients. Analysis of 65 cases. *Cancer*. Aug 1 1986;58(3):611-616.
31. Sillman FH, Sedlis A. Anogenital papillomavirus infection and neoplasia in immunodeficient women: an update. *Dermatol Clin*. Apr 1991;9(2):353-369.
32. Holly EA, Whittemore AS, Aston DA, Ahn DK, Nickoloff BJ, Kristiansen JJ. Anal cancer incidence: genital warts, anal fissure or

21

fistula, hemorrhoids, and smoking. *Journal of the National Cancer Institute*. Nov 15 1989;81(22):1726-1731.

33. Marcus JL, Chao C, Leyden WA, et al. Survival among HIV-infected and HIV-uninfected individuals with common non-AIDS-defining cancers. *Cancer epidemiology, biomarkers & prevention : a publication of the American Association for Cancer Research, cosponsored by the American Society of Preventive Oncology*. Aug 2015;24(8):1167-1173.
34. Coghill AE, Shiels MS, Suneja G, Engels EA. Elevated Cancer-Specific Mortality Among HIV-Infected Patients in the United States. *J Clin Oncol*. Jul 20 2015;33(21):2376-2383.
35. Fraunholz IB, Haberl A, Klauke S, Gute P, Rodel CM. Long-term effects of chemoradiotherapy for anal cancer in patients with HIV infection: oncological outcomes, immunological status, and the clinical course of the HIV disease. *Diseases of the colon and rectum*. Apr 2014;57(4):423-431.
36. Kattan MW, Hess KR, Amin MB, et al. American Joint Committee on Cancer acceptance criteria for inclusion of risk models for individualized prognosis in the practice of precision medicine. *CA: a cancer journal for clinicians*. Jan 19 2016.

Part V

Hepatobiliary System

Members of the Hepatobiliary System Expert Panel
Ghassan K. Abou-Alfa, MD
Peter J. Allen, MD
Thomas Aloia, MD, FACS
Yun Shin Chun, MD, FACS
Elijah Dixon, MD, BSc, MSc, FRCSC, FACS
Tomoki Ebata, MD
Jean-Francois H. Geschwind, MD, FSIR
Joseph M. Herman, MD, MSc
Sanjay Kakar, MD – CAP Representative
David A. Kooby, MD, FACS
Alyssa Krasinskas, MD
Nipun B. Merchant, MD, FACS
Mari Mino-Kenudson, MD
David M. Nagorney, MD
Timothy M. Pawlik, MD, MPH, PhD – Vice Chair
Laura Rubbia-Brandt, MD, PhD
Tracey E. Schefter, MD
Junichi Shindoh, MD, PhD
Eric P. Tamm, MD
Bachir Taouli, MD
Jean-Nicolas Vauthey, MD, FACS – Chair
Mary Kay Washington, MD, PhD
Christian W. Wittekind, MD – UICC Representative
Andrew X. Zhu, MD, PhD

Liver

22

Ghassan K. Abou-Alfa, Timothy M. Pawlik,
Junichi Shindoh, and Jean-Nicolas Vauthey

CHAPTER SUMMARY

Cancers Staged Using This Staging System

Hepatocellular carcinoma (HCC), Fibrolamellar carcinoma (fibrolamellar variant of HCC)

Cancers Not Staged Using This Staging System

These histopathologic types of cancer…	Are staged according to the classification for…	And can be found in chapter…
Intrahepatic cholangiocarcinoma	Intrahepatic bile ducts	23
Combined hepatocellular-cholangiocarcinoma	Intrahepatic bile ducts	23
Sarcomas of the liver	Soft tissue sarcoma of the abdomen and thoracic visceral organs	42

Summary of Changes

Change	Details of Change	Level of Evidence
Definition of Primary Tumor (T)	T1 is now divided into two subcatgories: T1a, solitary tumor ≤2 cm; and T1b, solitary tumor without vascular invasion, >2 cm.	II
Definition of Primary Tumor (T)	T2 now includes solitary tumor with vascular invasion >2 cm, or multiple tumors, none >5 cm.	II
Definition of Primary Tumor (T)	T3a is now recategorized as T3.	III
Definition of Primary Tumor (T)	T3b: Tumors involving a major branch of the portal vein or hepatic vein formerly were categorized as T3b and are now categorized as T4.	III

ICD-O-3 Topography Codes

Code	Description
C22.0	Liver

WHO Classification of Tumors

Code	Description
8170	Hepatocellular carcinoma
8171	Fibrolamellar carcinoma
8172	Scirrhous hepatocellular carcinoma
8173	Sarcomatoid hepatocellular carcinoma

Bosman FT, Carneiro F, Hruban RH, Theise ND, eds. World Health Organization Classification of Tumours of the Digestive System. Lyon: IARC; 2010.

To access the AJCC cancer staging forms, please visit www.cancerstaging.org.

© American Joint Committee on Cancer 2017
M.B. Amin et al. (eds.), *AJCC Cancer Staging Manual, Eighth Edition*, DOI 10.1007/978-3-319-40618-3_22

INTRODUCTION

Hepatocellular carcinoma (HCC) is the sixth most common malignancy in the world and is responsible for 600,000 deaths annually. HCC is an etiology-driven malignancy, mainly the result of cirrhosis that is attributed to hepatitis B, hepatitis C, alcohol, nonalcoholic steatohepatitis, and many genetically inherited metabolic diseases, the most common of which is hemochromatosis. These entities may be associated with HCC in the absence of cirrhosis. Chronic hepatitis C infection contributes the most to the incidence of HCC in the United States. The advent of novel protease inhibitors as curative therapies for hepatitis C is expected to reduce hepatitis C incidence and prevalence and, thus, hepatitis C–related HCC. However, the continued rise in morbid obesity and diabetes most likely will lead to a continued increase in nonalcoholic steatohepatitis–related HCC.[1]

Cirrhosis is a major component of the clinical presentation and a key determinant of prognosis. Anatomic stage is the other major determinant of outcome and prognosis. TNM staging helps determine curative resectability, as well as the presence and extent of vascular invasion, a key determinant of potential cure versus no cure. However, staging HCC remains a challenge, as incorporating components of the anatomic extent of the disease and the presence of cirrhosis have led to multiple staging and scoring systems, on which opinions continue to differ.[2] The lack of consensus regarding HCC staging is driven mainly by the etiology-specific prognostic factors. Nonetheless, TNM staging will continue to serve as the backbone for the anatomic description of disease extent as part of most other staging and scoring systems.

ANATOMY

Primary Site(s)

The liver has a dual blood supply from the hepatic artery and portal vein. Tumors are fed by the arterial blood supply. The liver is divided into right and left hemilivers by a plane called the Rex–Cantlie line, which projects between the gallbladder fossa and the vena cava and is defined by the middle hepatic vein. Couinaud refined knowledge about the functional anatomy of the liver and proposed dividing the liver into four sectors and eight segments. In this nomenclature, the liver is divided by vertical and oblique planes, or scissurae, defined by the three main hepatic veins, and a transverse plane or scissura that follows a line drawn through the right and left portal branches, making the four sectors (right paramedian, right lateral, left paramedian, and left lateral), which are further divided into segments by the transverse scissura (Fig. 22.1). The eight segments are numbered clockwise in the frontal plane. Recent advances in hepatic surgery have enabled anatomic (also called systematic) resections along these planes.

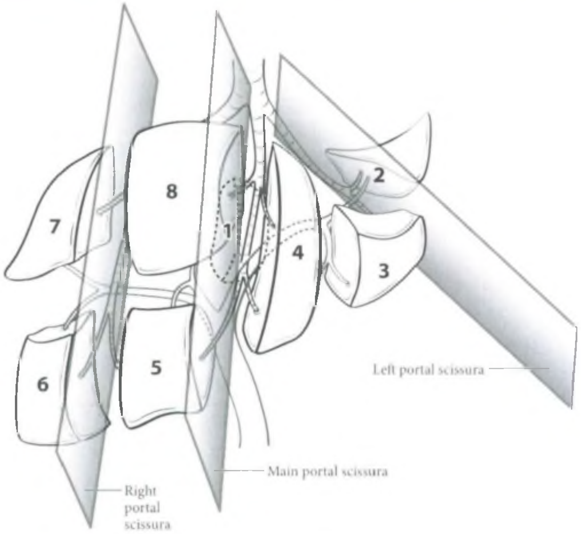

Fig. 22.1 Couinaud's segmental anatomy of the liver. The liver is divided into two hemilivers and eight segments according to the portal venous ramification pattern. Three major hepatic veins represent the position of scissural planes

Histologically, the liver is divided into lobules, each of which is drained by central veins. The portal triads between the lobules contain the intrahepatic bile ducts and the blood supply, which consists of small branches of the hepatic artery and portal vein and intrahepatic lymphatic channels.

HCC may spread through capsular invasion, extracapsular invasion, vascular invasion, and/or intrahepatic metastases. Tumors may extend through the liver capsule to adjacent organs (adrenal gland, diaphragm, and colon) or may rupture, causing acute hemorrhage and peritoneal metastasis.

Regional Lymph Nodes

The regional lymph nodes are the hilar, hepatoduodenal ligament, inferior phrenic, and caval lymph nodes, among which the most prominent are the hepatic artery and portal vein lymph nodes.

Metastatic Sites

The most common sites of extrahepatic dissemination are the lungs and bones. Recent data show that up to 28 % of patients with HCC may have bone metastases as the first site of extrahepatic disease. Intrahepatic venous dissemination remains

poorly understood, and it remains difficult to differentiate between satellitosis and multifocal tumors.

RULES FOR CLASSIFICATION

Clinical Classification

Clinical manifestations may include malaise, anorexia, and abdominal pain. A mass effect or cirrhosis-related ascites may cause abdominal fullness. Spontaneous rupture, causing acute abdominal pain and distension, represents a potentially fatal event that warrants prompt diagnosis and management. Hepatitis serologic studies—hepatitis B surface antigen, hepatitis B core antibody, and hepatitis C antibody—are warranted. If applicable, a polymerase chain reaction quantitative viral load assay also should be performed. An assessment of liver function and degree of cirrhosis is key; the Child–Pugh scoring system is used most commonly (Table 22.1). In patients treated with systemic therapy, liver biopsy is important for translational research to elucidate key signaling pathways that may be targeted with novel therapies. Liver biopsy is a comparatively safe and well-tolerated procedure.

The T classification is based primarily on the results of a multicenter international study of pathological factors affecting prognosis after resection of HCC.[3] The classification considers the presence or absence of vascular invasion (as assessed radiographically or microscopically), the number of tumor nodules (single vs. multiple), and the size of the largest tumor. The simplified classification adopted in the AJCC Cancer Staging Manual, 6th Edition and 7th Edition, stratifies patient survival well (Fig. 22.2). This staging system subsequently was validated in multiple studies after liver resection[4–10] and in a large multicenter series after liver transplantation (Fig. 22.3).[11]

In a recent study of 1,109 patients with solitary HCC measuring up to 2 cm, neither microvascular invasion nor histologic grade had an impact on long-term survival (Fig. 22.4).[12] Based on these data, the AJCC Cancer Staging Manual, 8th Edition divides T1 disease into two subcategories: T1a, for patients with solitary HCC ≤2 cm irrespective of microvascular invasion, and T1b for patients with solitary HCC >2 cm without microvascular invasion. The survival curve for solitary HCC >2 cm with microvascular invasion was similar to that for multiple HCCs ≤5 cm. Therefore, these two groups were classified together in a revised T2 category.

In another long-term survival study of 754 patients, there was no survival difference between patients with T3a and those with T3b tumors ($p=0.073$), or between patients with T3b and those with T4 tumors ($p=0.227$).[13] Thus, the revised 8th Edition reclassifies T3a as T3 and adds T3b to the T4 category.

Major vascular invasion is defined as invasion of the branches of the main portal vein (right or left portal vein, excluding the sectoral and segmental branches),[3] one or more of the three hepatic veins (right, middle, or left),[3] or the main branches of the proper hepatic artery (right or left hepatic artery).

Multiple tumors include satellitosis, multifocal tumors, and intrahepatic metastases. Assessment of lymph node involvement by clinical or radiographic means is a challenge, as reactive lymph nodes may be present. Invasion of adjacent organs other than the gallbladder or perforation of the visceral peritoneum is considered T4.

Imaging

Several imaging modalities have relatively high sensitivity and specificity for diagnosis or staging of HCC, although test performance is suboptimal for small or well-differentiated HCC. Computed tomography (CT) and magnetic resonance (MR) imaging with intravenous contrast are the preferred examinations to detect HCC, and constitute key elements in defining the TNM stage.[14–16] CT scanning should be performed with hepatic arterial, portal venous,

Table 22.1 Child-Pugh Score

	Points		
	1	2	3
Albumin (g/dL)	>3.5	2.8–3.5	<2.8
Bilirubin (mg/dL)	<2.0	2.0–3.0	>3.0
Prothrombin time			
Seconds	<4	4–6	>6
INR	<1.7	1.7–2.3	>2.3
Ascites	None	Moderate	Severe
Encephalopathy	None	Grade I–II	Grade III–VI
Child–Pugh class A	5–6 points		
Child–Pugh class B	7–9 points		
Child–Pugh class C	10–15 points		

and delayed venous phases. Similarly, if MR imaging is used, precontrast, arterial, venous, and delayed phases are essential. CT scanning frequently is the first examination, particularly if MR imaging is not available or is contraindicated. Ultrasound has lower sensitivity for detection of HCC, although it may be used to evaluate for vascular invasion of the portal and hepatic veins through color Doppler imaging.

Suggested Report Format

1. Liver morphology: describe whether cirrhotic or noncirrhotic
2. Portal hypertension: spleen size, ascites, varices
3. Tumor
   a. Primary tumor
   b. Number
   c. Size (centimeters)
   d. Location: involved segments
   e. Characterization (enhancement, pseudocapsule, fat on in- and opposed-phase T1-weighted MR imaging, calcification)
   f. Satellite lesion(s)
4. Local extent
   a. If present, describe vascular involvement.
5. Regional lymph nodes
   a. If present, describe abnormal or suspicious nodes, especially those in the porta hepatis, periceliac, and portacaval spaces.
6. Distant metastases
   a. If present, describe metastatic lesions seen on CT, MR imaging, PET/CT, or bone scans.

Pathological Classification

Complete pathological staging consists of evaluation of the primary tumor, including histologic grade, regional lymph node status, and underlying liver disease. Tumor size, number, and margin add to the critical prognostic data. Portal venous tumor thrombus should be clearly documented, as it carries a poor prognosis. Tumor grade is based on the degree of nuclear pleomorphism, as described by Edmonson and Steiner. Because of the prognostic significance of underlying liver disease in HCC, it is recommended that the results of the histopathologic analysis of the adjacent (nontumorous) liver be reported. Advanced fibrosis/cirrhosis (modified Ishak score of 5–6) is associated with a worse prognosis than absence of or moderate fibrosis (modified Ishak score of 0–4). Although grade and underlying liver disease have prognostic significance, they are not included in the current staging system.

Regional lymph node involvement is rare (5%). Positive lymph nodes are classified as Stage IV because they carry the same prognosis as cases with distant metastases. For pathological classification, vascular invasion includes gross as well as microscopic involvement of vessels.

PROGNOSTIC FACTORS

Prognostic Factors Required for Stage Grouping

Beyond the factors used to assign T, N, or M categories, no additional prognostic factors are required for stage grouping.

Additional Factors Recommended for Clinical Care

Cirrhosis

Although there is clear agreement on the prognostic value of the extent of hepatic fibrosis, how to incorporate it into clinically relevant prognostic systems remains a controversy.[2] Child–Pugh remains the most commonly used scoring system for assessing prognosis of cirrhosis and has been used in most clinical trials. The Okuda staging system[17] was the first clinical system to join tumor extent parameters with cirrhosis-related ones. Other systems include the Cancer of the Liver Italian Program (CLIP),[18] the Chinese University Prognostic Index (CUPI) scoring system, the Groupe d'Etude et de Traitement du Carcinoma Hepatocellulaire (GETCH) staging system, the Japan Integrated Staging (JIS) system, and the Barcelona Clinic Liver Cancer (BCLC) classification system.[19] The BCLC couples prognosis with treatment assignment. AJCC Level of Evidence: II

Fibrosis Score

Multiple fibrosis scoring systems have been described for use in pathological evaluation of liver disease. The system most commonly used by US pathologists is the Batts–Ludwig system[20]; other systems include the modified Ishak scoring system[21] and the METAVIR score.[22] The latter is used more widely in Europe than in the United States.

The Ishak scoring system uses a 0–6 scale.

F0	Fibrosis score 0–4 (no to moderate fibrosis)
F1	Fibrosis score 5–6 (severe fibrosis or cirrhosis)

The Batts-Ludwig system uses a 0-4 scale, with a score of 3 defined as fibrous septa with architectural distortion but no obvious cirrhosis, and a score of 4 defined as cirrhosis. AJCC Level of Evidence: II

α-Fetoprotein

α-Fetoprotein (AFP) is a nonspecific serum protein that generally is elevated in the setting of HCC, especially hepatitis B–related HCC.[23] It has been an integral part of different scoring and staging systems, including the CLIP and CUPI. However, because of its nonspecificity, levels should be interpreted in the context of other findings, such as results of imaging studies. AFP is reported to be useful as a predictive marker for response to therapy; however, this application requires prospective study evaluation.[24] AJCC Level of Evidence: II

Model for End-stage Liver Disease Score

Model for End-stage Liver Disease (MELD) scoring is useful in determining prognosis and prioritizing for receipt of a liver transplant.[25] MELD uses serum bilirubin, serum creatinine, and international normalized ratio (INR) to predict survival. MELD is used by the United Network for Organ Sharing (UNOS) to help allocate livers for transplant. AJCC Level of Evidence: II

RISK ASSESSMENT MODELS

The AJCC recently established guidelines that will be used to evaluate published statistical prediction models for the purpose of granting endorsement for clinical use.[26] Although this is a monumental step toward the goal of precision medicine, this work was published only very recently. Therefore, the existing models that have been published or may be in clinical use have not yet been evaluated for this cancer site by the Precision Medicine Core of the AJCC. In the future, the statistical prediction models for this cancer site will be evaluated, and those that meet all AJCC criteria will be endorsed.

DEFINITIONS OF AJCC TNM

Definition of Primary Tumor (T)

T Category	T Criteria
TX	Primary tumor cannot be assessed
T0	No evidence of primary tumor
T1	Solitary tumor ≤2 cm, or >2 cm without vascular invasion
T1a	Solitary tumor ≤2 cm
T1b	Solitary tumor >2 cm without vascular invasion
T2	Solitary tumor >2 cm with vascular invasion, or multiple tumors, none >5 cm
T3	Multiple tumors, at least one of which is >5 cm
T4	Single tumor or multiple tumors of any size involving a major branch of the portal vein or hepatic vein, or tumor(s) with direct invasion of adjacent organs other than the gallbladder or with perforation of visceral peritoneum

Definition of Regional Lymph Node (N)

N Category	N Criteria
NX	Regional lymph nodes cannot be assessed
N0	No regional lymph node metastasis
N1	Regional lymph node metastasis

Definition of Distant Metastasis (M)

M Category	M Criteria
M0	No distant metastasis
M1	Distant metastasis

AJCC PROGNOSTIC STAGE GROUPS

When T is...	And N is...	And M is...	Then the stage group is...
T1a	N0	M0	IA
T1b	N0	M0	IB
T2	N0	M0	II
T3	N0	M0	IIIA
T4	N0	M0	IIIB
Any T	N1	M0	IVA
Any T	Any N	M1	IVB

REGISTRY DATA COLLECTION VARIABLES

1. AFP
2. Fibrosis score
3. Hepatitis serology
4. Creatinine (part of the MELD score)
5. Bilirubin (part of the MELD score)
6. Prothrombin time (INR; part of the MELD score)

HISTOLOGIC GRADE (G)

G	G Definition
GX	Grade cannot be assessed
G1	Well differentiated
G2	Moderately differentiated
G3	Poorly differentiated
G4	Undifferentiated

HISTOPATHOLOGIC TYPE

Fibrolamellar carcinoma, previously known as fibrolamellar variant of HCC, lacks a specific staging system; thus, the current HCC staging system should be used. Lymph node involvement is much more common in fibrolamellar carci-

22

noma than in HCC. In view of the common involvement of lymph nodes in fibrolamellar carcinoma, lymphadenectomy commonly is considered part of its surgical treatment.

The staging classification does not apply to biliary tumors, specifically intrahepatic cholangiocarcinomas, including combined hepatocellular-cholangiocarcinoma, which are considered in a separate staging system (see Chapter 23). It also does not apply to primary sarcoma or metastatic tumors.

SURVIVAL DATA

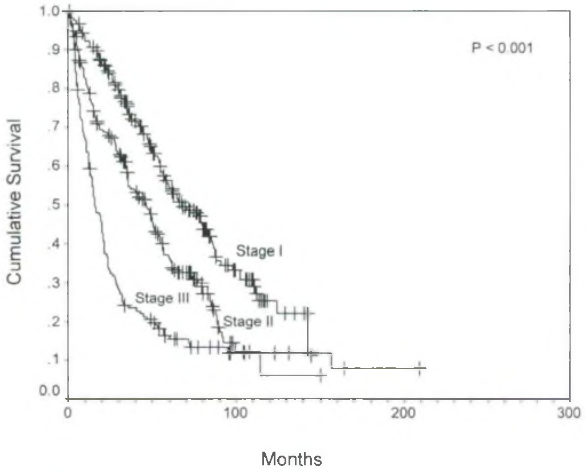

Fig. 22.2 Survival after resection for HCC according to stage grouping. Data from Vauthey et al.[3]

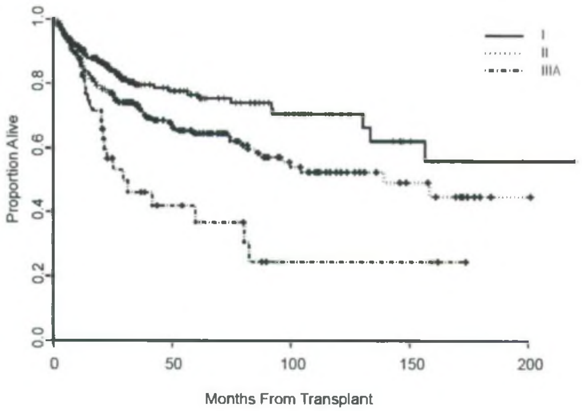

Fig. 22.3 Survival after liver transplantation for HCC according to stage grouping. Data from Vauthey et al.[11]

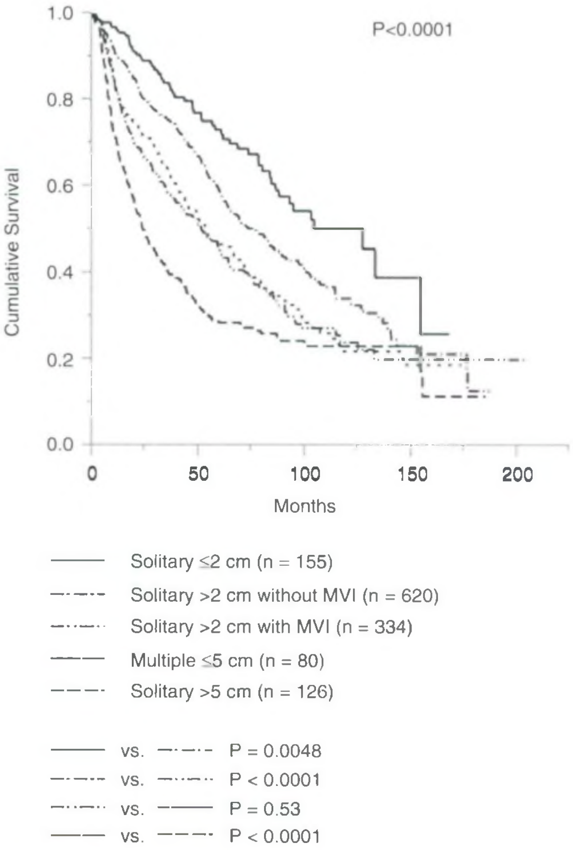

Fig. 22.4 Comparison of the new classification for solitary tumor and the 7th Edition classification for multiple HCC. Data from Shindoh et al.[12]

Bibliography

1. Abou-Alfa GK, Jarnagin W, Lowery M, D'Angelica M, Brown K, Ludwig E. Liver and bile duct cancer. In: Niederhuber J, Armitage J, Doroshow J, Kastan M, Tepper J, eds. *Abeloff's Clinical Oncology*. 5 ed. Philadelphia, PA: Churchill Livingstone Elsevier; 2013.
2. Huitzil-Melendez FD, Capanu M, O'Reilly EM, et al. Advanced hepatocellular carcinoma: which staging systems best predict prognosis? *J Clin Oncol*. Jun 10 2010;28(17):2889-2895.
3. Vauthey JN, Lauwers GY, Esnaola NF, et al. Simplified staging for hepatocellular carcinoma. *J Clin Oncol*. Mar 15 2002;20(6): 1527-1536.
4. Cheng CH, Lee CF, Wu TH, et al. Evaluation of the new AJCC staging system for resectable hepatocellular carcinoma. *World journal of surgical oncology*. 2011;9:114.
5. Kee KM, Wang JH, Lee CM, et al. Validation of clinical AJCC/UICC TNM staging system for hepatocellular carcinoma: analysis of 5,613 cases from a medical center in southern Taiwan. *Int J Cancer*. Jun 15 2007;120(12):2650–2655.
6. Lei HJ, Chau GY, Lui WY, et al. Prognostic value and clinical relevance of the 6th Edition 2002 American Joint Committee on Cancer staging system in patients with resectable hepatocellular carcinoma. *Journal of the American College of Surgeons*. Oct 2006;203(4):426–435.
7. Poon RT, Fan ST. Evaluation of the new AJCC/UICC staging system for hepatocellular carcinoma after hepatic resection in Chinese patients. *Surg Oncol Clin N Am*. Jan 2003;12(1):35–50, viii.
8. Ramacciato G, Mercantini P, Cautero N, et al. Prognostic evaluation of the new American Joint Committee on Cancer/International Union Against Cancer staging system for hepatocellular carcinoma: analysis of 112 cirrhotic patients resected for hepatocellular carcinoma. *Annals of surgical oncology*. Apr 2005;12(4):289–297.
9. Varotti G, Ramacciato G, Ercolani G, et al. Comparison between the fifth and sixth editions of the AJCC/UICC TNM staging systems for hepatocellular carcinoma: multicentric study on 393 cirrhotic resected patients. *European journal of surgical oncology : the journal of the European Society of Surgical Oncology and the British Association of Surgical Oncology*. Sep 2005;31(7):760–767.
10. Wu CC, Cheng SB, Ho WM, Chen JT, Liu TJ, P'Eng F K. Liver resection for hepatocellular carcinoma in patients with cirrhosis. *The British journal of surgery*. Mar 2005;92(3):348–355.
11. Vauthey JN, Ribero D, Abdalla EK, et al. Outcomes of liver transplantation in 490 patients with hepatocellular carcinoma: validation of a uniform staging after surgical treatment. *Journal of the American College of Surgeons*. May 2007;204(5):1016–1027; discussion 1027–1018.
12. Shindoh J, Andreou A, Aloia TA, et al. Microvascular invasion does not predict long-term survival in hepatocellular carcinoma up to 2 cm: reappraisal of the staging system for solitary tumors. *Annals of surgical oncology*. 2013;20(4):1223–1229.
13. Chan AC, Fan ST, Poon RT, et al. Evaluation of the seventh edition of the American Joint Committee on Cancer tumour-node-metastasis (TNM) staging system for patients undergoing curative resection of hepatocellular carcinoma: implications for the development of a refined staging system. *HPB : the official journal of the International Hepato Pancreato Biliary Association*. Jun 2013;15(6):439–448.
14. Choi JY, Lee JM, Sirlin CB. CT and MR imaging diagnosis and staging of hepatocellular carcinoma: part I. Development, growth, and spread: key pathologic and imaging aspects. *Radiology*. Sep 2014;272(3):635–654.
15. Choi JY, Lee JM, Sirlin CB. CT and MR imaging diagnosis and staging of hepatocellular carcinoma: part II. Extracellular agents, hepatobiliary agents, and ancillary imaging features. *Radiology*. Oct 2014;273(1):30–50.
16. Cruite I, Tang A, Sirlin CB. Imaging-based diagnostic systems for hepatocellular carcinoma. *AJR. American journal of roentgenology*. Jul 2013;201(1):41–55.
17. Okuda K, Ohtsuki T, Obata H, et al. Natural history of hepatocellular carcinoma and prognosis in relation to treatment. Study of 850 patients. *Cancer*. Aug 15 1985;56(4):918–928.
18. The Cancer of the Liver Italian Program Investigators. A new prognostic system for hepatocellular carcinoma: a retrospective study of 435 patients: the Cancer of the Liver Italian Program (CLIP) investigators. *Hepatology*. Sep 1998;28(3):751–755.
19. Llovet JM, Bru C, Bruix J. Prognosis of hepatocellular carcinoma: the BCLC staging classification. *Seminars in liver disease*. 1999;19(3):329–338.
20. Batts KP, Ludwig J. Chronic hepatitis. An update on terminology and reporting. *The American journal of surgical pathology*. Dec 1995;19(12):1409–1417.
21. Ishak K, Baptista A, Bianchi L, et al. Histological grading and staging of chronic hepatitis. *J Hepatol*. Jun 1995;22(6):696–699.
22. Bedossa P. Intraobserver and interobserver variations in liver biopsy interpretation in patients with chronic hepatitis C. *Hepatology*. 1994;20(1):15–20.
23. Leung TW, Tang AM, Zee B, et al. Construction of the Chinese University Prognostic Index for hepatocellular carcinoma and comparison with the TNM staging system, the Okuda staging system, and the Cancer of the Liver Italian Program staging system: a study based on 926 patients. *Cancer*. Mar 15 2002;94(6):1760–1769.
24. Zhu AX, Rosmorduc O, Evans TR, et al. SEARCH: a phase III, randomized, double-blind, placebo-controlled trial of sorafenib plus erlotinib in patients with advanced hepatocellular carcinoma. *J Clin Oncol*. Feb 20 2015;33(6):559–566.
25. Wiesner R, Edwards E, Freeman R, et al. Model for end-stage liver disease (MELD) and allocation of donor livers. *Gastroenterology*. Jan 2003;124(1):91–96.
26. Kattan MW, Hess KR, Amin MB, et al. American Joint Committee on Cancer acceptance criteria for inclusion of risk models for individualized prognosis in the practice of precision medicine. *CA: a cancer journal for clinicians*. Jan 19 2016.

Intrahepatic Bile Ducts

23

Thomas Aloia, Timothy M. Pawlik, Bachir Taouli,
Laura Rubbia-Brandt, and Jean-Nicolas Vauthey

CHAPTER SUMMARY

Cancers Staged Using This Staging System

This staging system applies to primary carcinomas of the intrahepatic bile ducts, including the following:

- Intrahepatic cholangiocarcinoma (IHCC)
- Combined hepatocellular–cholangiocarcinoma (mixed hepatocholangiocarcinomas)
- Primary neuroendocrine tumors of the liver

Cancers Not Staged Using This Staging System

These histopathologic types of cancer…	Are staged according to the classification for…	And can be found in chapter…
Primary sarcomas of the liver	Soft tissue sarcoma of the abdomen and thoracic visceral organs	42
Pure hepatocellular carcinoma	Liver	22
Hilar cholangiocarcinoma	Perihilar bile ducts	25
Gallbladder carcinoma	Gallbladder	24

Summary of Changes

Change	Details of Change	Level of Evidence
Definition of Primary Tumor (T)	The T1 category was revised to account for the prognostic impact of tumor size (T1a: ≤5 cm vs. T1b: >5 cm).	II
Definition of Primary Tumor (T)	The T2 category is modified to reflect the equivalent prognostic value of vascular invasion and tumor multifocality.	II
Definition of Primary Tumor (T)	The AJCC Cancer Staging Manual, 7th Edition T4 category describing the tumor growth pattern was eliminated from staging but is still recommended for data collection.	III

ICD-O-3 Topography Codes

Code	Description
C22.1	Intrahepatic bile ducts

WHO Classification of Tumors

Code	Description
8160	Intrahepatic cholangiocarcinoma
8148	Biliary intraepithelial neoplasia, grade 3 (high-grade dysplasia)
8180	Combined hepatocellular–cholangiocarcinoma
8980	Carcinosarcoma

To access the AJCC cancer staging forms, please visit www.cancerstaging.org.

© American Joint Committee on Cancer 2017

M.B. Amin et al. (eds.), *AJCC Cancer Staging Manual, Eighth Edition*, DOI 10.1007/978-3-319-40618-3_23

Code	Description
8161	Intraductal papillary neoplasm with an associated invasive carcinoma
8470	Mucinous cystic neoplasm with an associated invasive carcinoma
8246	Neuroendocrine carcinoma
8013	Large cell neuroendocrine carcinoma
8041	Small cell neuroendocrine carcinoma
8503	Intraductal papillary neoplasm with high-grade dysplasia

Bosman FT, Carneiro F, Hruban RH, Theise ND, eds. World Health Organization Classification of Tumours of the Digestive System. Lyon: IARC; 2010.

INTRODUCTION

This is the first revision to a previously novel staging system (7th Edition) that remains independent of the staging systems for both hepatocellular carcinoma and extrahepatic bile duct malignancy, including hilar bile duct cancers.

Primary hepatobiliary malignancy includes tumors of the hepatocytes (hepatocellular carcinoma), bile ducts (cholangiocarcinoma/primary neuroendocrine), gallbladder, and interstitium of the liver (sarcoma). This TNM classification applies only to cancers arising in the intrahepatic bile ducts, including pure intrahepatic cholangiocarcinomas, mixed hepatocholangiocarcinomas, and primary neuroendocrine liver tumors. Pure hepatocellular carcinoma and extrahepatic bile duct tumors, including perihilar bile duct and gallbladder carcinomas, are classified separately.

Tumors of the bile ducts may be subdivided anatomically into three categories: intrahepatic, perihilar, and distal cholangiocarcinoma. Tumors of intrahepatic bile duct origin represent 15–20% of all primary liver malignancies and account for approximately 20% of cholangiocarcinoma/gallbladder malignancies.[1]

Clinically, these primary intrahepatic tumors may be difficult to differentiate from extrahepatic adenocarcinomas that metastasize to the liver from other primary sites. The etiologic factors that predispose to development of intrahepatic cholangiocarcinoma include primary sclerosing cholangitis, hepatobiliary parasitosis, intrahepatic lithiasis, and chronic viral hepatitis.[2] The overall incidence of intrahepatic cholangiocarcinoma is 0.7 cases per 100,000 adults in the United States.[3,4] The incidence of intrahepatic cholangiocarcinoma is age dependent, with a progressive increase in cases starting in the sixth decade of life and peaking in the ninth decade.[1] Although less common than either hepatocellular carcinoma or hilar bile duct cancer, the incidence of intrahepatic cholangiocarcinoma is increasing.[3,4]

On radiologic imaging, it may be difficult to determine the local extent of disease. However, the major prognostic factors included in the staging system (tumor size, tumor number, vascular invasion, perforation of the visceral peritoneum, and regional lymph node involvement) often may be determined from high-resolution cross-sectional imaging, analysis of image-guided biopsy tissue, and/or surgical exploration.

ANATOMY

Primary Site(s)

At the hilar plate, the right and left hepatic bile ducts enter the liver parenchyma (Fig. 23.1). Histologically, the bile ducts are internally lined by a single layer of tall uniform columnar cells. The mucosa usually forms irregular pleats or small longitudinal folds. The walls of the bile ducts have a layer of subepithelial connective tissue and muscle fiber. However, within the hepatic parenchyma, bile duct muscle fibers typically are sparse or absent. The periductal tissue does contain a neural network and a rich lymphatic plexus, frequently providing a means for longitudinal tumor spread along the bile ducts.

Intrahepatic cholangiocarcinoma tumor growth patterns include the mass-forming type, the periductal infiltrating type, and a mixed type. Mass-forming intrahepatic cholangiocarcinoma shows a radial growth pattern invading into the adjacent liver parenchyma. Histopathologic examination reveals nodular sclerotic masses with distinct borders. In contrast, the periductal infiltrating type of cholangiocarcinoma demonstrates a diffuse and often ill-defined longitudinal growth pattern along the bile duct.

The purely mass-forming type is estimated to be present in 60% of all patients with intrahepatic cholangiocarcinoma, whereas the purely periductal infiltrating type and the mixed type each represent 20% of cases. The prognostic value of growth pattern remains controversial, and the significance of this variable has not been compared with that of other prognostic factors.[5,6] Either growth pattern may invade vascular structures, with the mass-forming intrahepatic cholangiocarcinomas frequently involving the retrohepatic vena cava. Anatomically, the intrahepatic bile ducts extend from the periphery of the liver to the second-order bile ducts. Therefore, it may be difficult to distinguish central intrahepatic from hilar cholangiocarcinoma, particularly in the presence of a periductal infiltrating growth pattern.

Regional Lymph Nodes

Compared with primary hepatocellular carcinoma, regional lymph node metastases are more commonly associated with intrahepatic cholangiocarcinoma. The pattern of lymph node drainage from the intrahepatic bile ducts is uniquely lateral.[7]

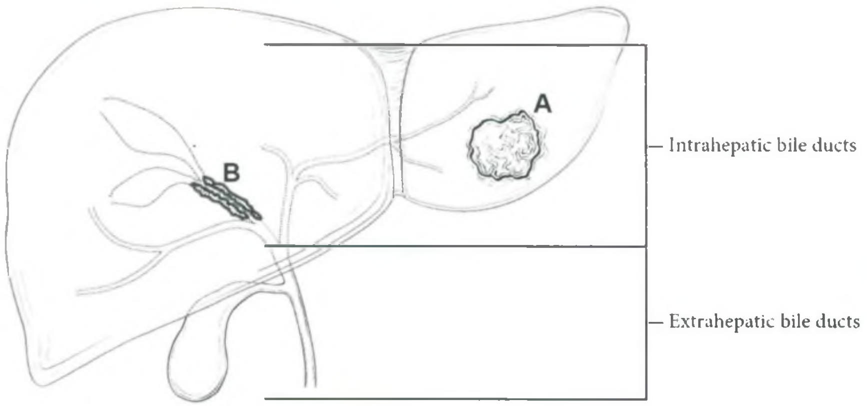

Fig. 23.1 Liver diagram differentiating intrahepatic bile ducts from extrahepatic bile ducts and mass-forming growth pattern (A) from periductal infiltrating growth pattern (B), with associated intrahepatic biliary dilatation

Tumors in the left liver may preferentially drain to inferior phrenic lymph nodes and lymph nodes along the lesser curvature of the stomach, subsequently involving the celiac nodal basin. In contrast, intrahepatic cholangiocarcinomas arising from the right liver have a lymphatic drainage pattern similar to that of gallbladder cancers, primarily draining to right-sided hilar lymph nodes and subsequently to portocaval lymph nodes (Fig. 23.2).

For left liver intrahepatic cholangiocarcinomas, regional lymph nodes include inferior phrenic, hilar (common bile duct, hepatic artery, portal vein, and cystic duct), and gastrohepatic lymph nodes. For right liver intrahepatic cholangiocarcinomas, the regional lymph nodes include the hilar, periduodenal and peripancreatic lymph node areas.

Metastatic Sites

Common extrahepatic sites of metastatic disease include the peritoneum, bone, lungs, and pleura (classified in the M1 category as distant metastasis). Extraregional abdominal nodal involvement also constitutes M1 status. For all intrahepatic cholangiocarcinomas, spread to the celiac, periaortic, and/or pericaval lymph nodes is considered distant metastatic disease (M1).

RULES FOR CLASSIFICATION

Clinical Classification

Clinical staging relies on imaging procedures designed to demonstrate the tumor growth pattern of intrahepatic cholangiocarcinoma, the size and number of intrahepatic masses, and the presence or absence of major vascular invasion. In the presence of cirrhosis, the patient's Child–Pugh class and Model for End-stage Liver Disease (MELD) score should be considered. Radiologic assessment for the presence or absence of distant metastases before surgical exploration is warranted.

Intrahepatic cholangiocarcinoma frequently spreads to other intrahepatic locations (classified in the T2 category as multiple tumors).

Validation of T1a, T1b, T2, T3, T4, and N1 categories is based on multivariate analyses of outcome and survival data of single- and multi-institution studies of patients with intrahepatic cholangiocarcinoma (Fig. 23.3).

Imaging[8-11]

Imaging techniques of choice include multiphasic contrast-enhanced CT and MR imaging with MR cholangiopancreatography (MRCP). Both techniques are equally valuable in detecting tumors larger than 2 cm and determining portal vein and arterial involvement. However, MR imaging with MRCP may provide additional information regarding extent of disease.

Ultrasound is less accurate in assessing disease burden and tumor resectability, although it is useful in evaluating vascular invasion and the degree of biliary involvement, especially in patients who already have biliary stents traversing the tumor.

Cholangiography may be performed by percutaneous transhepatic cholangiography, by MRCP, or endoscopically with endoscopic retrograde cholangiopancreatography. All these techniques allow evaluation of the biliary tree and may be useful for defining the extent of ductal involvement.

Extra-abdominal staging may include chest CT and PET. Positron emission tomography (PET)/CT may be used to detect occult metastatic disease.

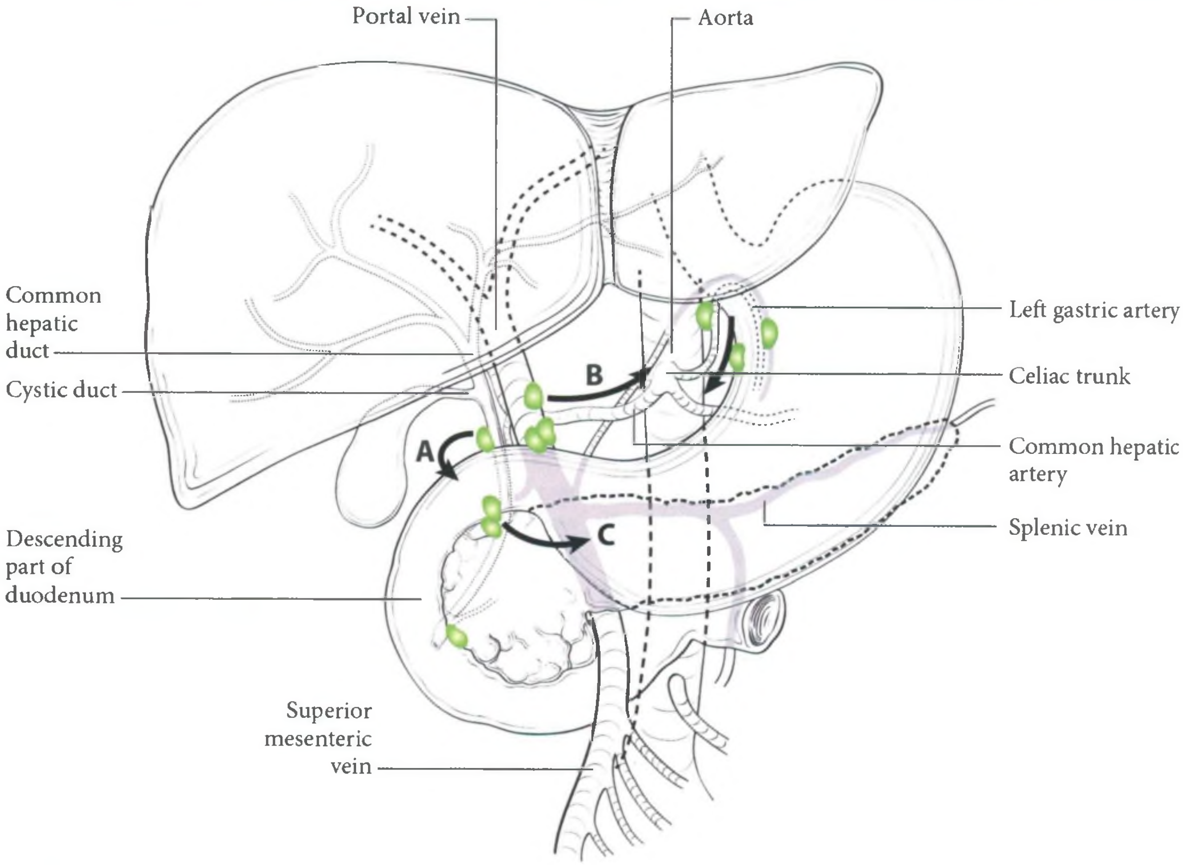

Fig. 23.2 Differential lymphatic drainage patterns for left and right liver intrahepatic cholangiocarcinomas. Right liver tumors drain to right portal (A) and then portocaval (C) nodal basins, while left liver tumors drain to left gastric and celiac (B) nodal basins[7]

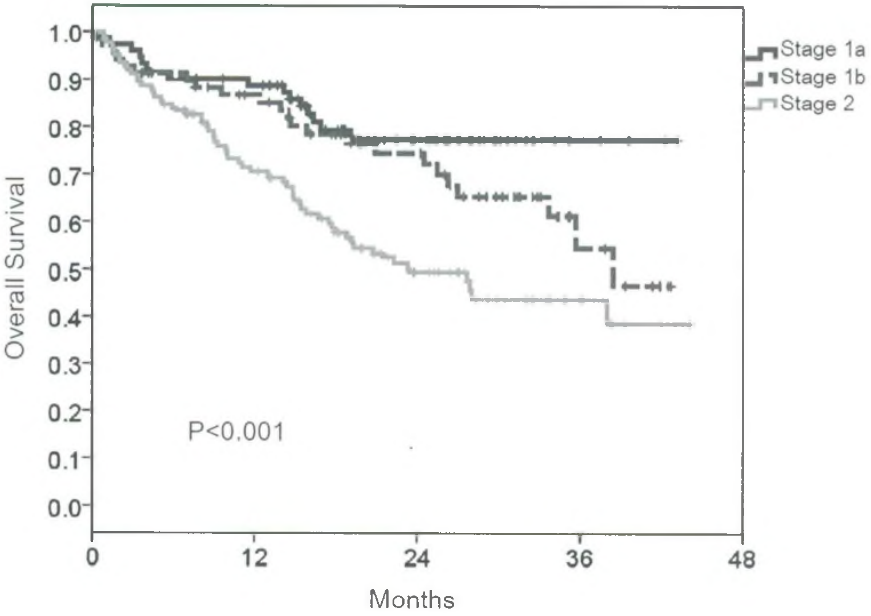

Fig. 23.3 Stratification of survival for 861 N0,M0 patients with confirmed intrahepatic cholangiocarcinoma based on new stage IA, IB, and II classification using National Cancer Data Base (NCDB) registry data

Suggested Report Format

1. Primary tumor (T)
   a. Size of tumor: bidimensional
   b. Location
   c. Morphology
   d. Number of tumors
   e. Associated liver atrophy
2. Local extent, adjacent structure involvement, and vascular invasion
3. Regional lymph node (N)
   a. If present, describe abnormal or suspicious nodes, especially those in the relevant nodal groups.
4. Metastasis (M)
   a. If present, describe metastatic lesions seen on CT, MR imaging, PET/CT, or bone scans.

Pathological Classification

Intraductal papillary bile duct tumors with high-grade dysplasia not invading beyond the basement membrane may be identified in some patients with unilateral biliary obstruction and are classified as *in situ* tumors (Tis). The T classification of invasive intrahepatic cholangiocarcinoma is determined primarily by the number of tumors present (solitary vs. multiple), the presence of vascular invasion, and the presence of visceral peritoneal perforation, with or without direct involvement of local extrahepatic structures. Solitary tumors without vascular invasion are subclassified by tumor size (T1a vs. T1b).

Vascular invasion includes major hepatic vessel invasion (defined as invasion of the first- and second-order branches of the portal veins or hepatic arteries, or as invasion of one or more of the three hepatic veins [right, middle, or left]) and/or microscopic invasion of smaller intraparenchymal vascular structures identified on histopathologic examination (T2). The definition of the term *multiple tumors* includes satellitosis, multifocal tumors, and intrahepatic "metastasis" (T2). Invasion through the liver capsule in the absence of adjacent organ involvement is classified as T3, whereas direct invasion of adjacent organs and structures, including the colon, duodenum, stomach, common bile duct, retrohepatic vena cava, abdominal wall, and diaphragm, is considered T4 disease.

In addition to the various prognostic factors included in the T classification, the presence of regional nodal involvement (N1) and/or involvement of extraregional abdominal lymph nodes and other distant metastatic sites (M1) is a strong predictor of survival.

Complete pathological staging consists of evaluation of the primary tumor, including tumor size and tumor number, the presence or absence of vascular invasion, and involvement of locoregional lymph nodes. Nontumoral hepatic parenchymal fibrosis/cirrhosis should be reported using a standard grading system.[12,13]

pT Classification

Lesions classified as carcinoma *in situ* should meet histologic criteria for biliary intraepithelial neoplasia grade 3 (BilIN-3) or for high-grade dysplasia in an intraductal papillary lesion or mucinous cystic lesion. These lesions usually show pseudopapillary or micropapillary architecture and display cytologic features of carcinoma without invasion. Solitary tumors confined to the liver without gross or microscopic vascular invasion are subclassified as T1a if they are ≤5 cm and T1b if they are >5 cm. T2 is defined by either a solitary vascular invasive tumor or multifocal tumors within the liver. T3 classification is reserved for tumors that perforate the visceral peritoneum without invasion of extrahepatic structures, and T4 denotes direct tumor extension to local extrahepatic structures, including the retrohepatic vena cava, hepatoduodenal ligament, and visceral structures (e.g., colon, duodenum).

pN Classification

For complete pathological staging, recovery of at least six lymph nodes from appropriate nodal stations is recommended. Presence of disease in at least one regional node constitutes N1 status.

PROGNOSTIC FACTORS

Prognostic Factors Required for Stage Grouping

Beyond the factors used to assign T, N, or M categories, no additional prognostic factors are required for stage grouping.

Additional Factors Recommended for Clinical Care

In addition to the factors embedded in the staging system, additional clinical factors predictive of decreased survival include the following:

Presence of Nontumoral Hepatic Parenchymal Fibrosis/Cirrhosis

Evidence of parenchymal fibrosis or cirrhosis of the nontumorous liver as defined in the surgical pathology report. Fibrosis is defined by the Ishak staging scale using a 0–6 scoring system: a fibrosis score of 0–4 (F0) denotes no to moderate fibrosis, and a score of 5–6 indicates severe fibrosis or cirrhosis. AJCC Level of Evidence: II

Primary Sclerosing Cholangitis

Primary sclerosing cholangitis denotes a chronic autoimmune inflammation of the bile ducts that leads to scar formation and narrowing of the ducts over time. As scarring increases, the ducts become injured and blocked. The chronic inflammation and injury to the ducts may predispose a patient to IHCC. AJCC Level of Evidence: II

Serum Carbohydrate Antigen 19-9 Level (>200 U/mL)

The serum marker carbohydrate antigen (CA) 19-9 may have prognostic significance. The CA 19-9 value is obtained in the preoperative period and reportedly is associated with long-term outcomes. Although the exact value associated with outcomes is not established, >200 U/mL in the absence of hyperbilirubinemia has been proposed as a relevant cutoff value. AJCC Level of Evidence: II

RISK ASSESSMENT MODELS

The AJCC recently established guidelines that will be used to evaluate published statistical prediction models for the purpose of granting endorsement for clinical use.[32] Although this is a monumental step toward the goal of precision medicine, this work was published only very recently. Therefore, the existing models that have been published or may be in clinical use have not yet been evaluated for this cancer site by the Precision Medicine Core of the AJCC. In the future, the statistical prediction models for this cancer site will be evaluated, and those that meet all AJCC criteria will be endorsed.

DEFINITIONS OF AJCC TNM

Definition of Primary Tumor (T)

T Category	T Criteria
TX	Primary tumor cannot be assessed
T0	No evidence of primary tumor
Tis	Carcinoma *in situ* (intraductal tumor)
T1	Solitary tumor without vascular invasion, ≤5 cm or >5 cm
T1a	Solitary tumor ≤5 cm without vascular invasion
T1b	Solitary tumor >5 cm without vascular invasion
T2	Solitary tumor with intrahepatic vascular invasion or multiple tumors, with or without vascular invasion
T3	Tumor perforating the visceral peritoneum
T4	Tumor involving local extrahepatic structures by direct invasion

Definition of Regional Lymph Node (N)

N Category	N Criteria
NX	Regional lymph nodes cannot be assessed
N0	No regional lymph node metastasis
N1	Regional lymph node metastasis present

Definition of Distant Metastasis (M)

M Category	M Criteria
M0	No distant metastasis
M1	Distant metastasis present

AJCC PROGNOSTIC STAGE GROUPS

When T is...	And N is...	And M is...	Then the stage group is...
Tis	N0	M0	0
T1a	N0	M0	IA
T1b	N0	M0	IB
T2	N0	M0	II
T3	N0	M0	IIIA
T4	N0	M0	IIIB
Any T	N1	M0	IIIB
Any T	Any N	M1	IV

REGISTRY DATA COLLECTION VARIABLES

1. Presence of nontumoral hepatic parenchymal fibrosis/cirrhosis
2. Primary sclerosing cholangitis
3. Serum CA 19-9 level
4. Tumor growth pattern

HISTOLOGIC GRADE (G)

The histologic grade should be reported using the following scheme:

G	G Definition
GX	Grade cannot be assessed
G1	Well differentiated
G2	Moderately differentiated
G3	Poorly differentiated

HISTOPATHOLOGIC TYPE

This staging system applies to primary carcinomas of the intrahepatic bile ducts, including:

- Intrahepatic cholangiocarcinoma
  - Mass-forming tumor growth pattern
  - Periductal infiltrating tumor growth pattern
  - Mixed mass-forming/periductal infiltrating growth pattern
- Mixed hepatocholangiocarcinomas
- Primary neuroendocrine carcinoma of the liver

Bibliography

1. El Rassi ZE, Partensky C, Scoazec JY, Henry L, Lombard-Bohas C, Maddern G. Peripheral cholangiocarcinoma: presentation, diagnosis, pathology and management. *European journal of surgical oncology: the journal of the European Society of Surgical Oncology and the British Association of Surgical Oncology*. Aug 1999;25(4):375-380.
2. Shaib YH, El-Serag HB, Nooka AK, et al. Risk factors for intrahepatic and extrahepatic cholangiocarcinoma: a hospital-based case-control study. *Am J Gastroenterol*. May 2007;102(5): 1016-1021.
3. McGlynn KA, Tarone RE, El-Serag HB. A comparison of trends in the incidence of hepatocellular carcinoma and intrahepatic cholangiocarcinoma in the United States. *Cancer epidemiology, biomarkers & prevention: a publication of the American Association for Cancer Research, cosponsored by the American Society of Preventive Oncology*. Jun 2006;15(6):1198-1203.
4. Patel T. Increasing incidence and mortality of primary intrahepatic cholangiocarcinoma in the United States. *Hepatology*. Jun 2001;33(6):1353-1357.
5. Hirohashi K, Uenishi T, Kubo S, et al. Macroscopic types of intrahepatic cholangiocarcinoma: clinicopathologic features and surgical outcomes. *Hepato-gastroenterology*. Mar-Apr 2002;49(44): 326-329.
6. Yamasaki S. Intrahepatic cholangiocarcinoma: macroscopic type and stage classification. *Journal of hepato-biliary-pancreatic surgery*. 2003;10(4):288-291.
7. Rouvière H. Anatomie des lymphatiques de l'homme. Vol 1. Paris: Mason; 1932.
8. Blechacz B, Komuta M, Roskams T, Gores GJ. Clinical diagnosis and staging of cholangiocarcinoma. *Nature reviews. Gastroenterology & hepatology*. Sep 2011;8(9):512-522.
9. Baheti AD, Tirumani SH, Rosenthal MH, Shinagare AB, Ramaiya NH. Diagnosis and management of intrahepatic cholangiocarcinoma: a comprehensive update for the radiologist. *Clin Radiol*. Dec 2014;69(12):e463-470.
10. Weber SM, Ribero D, O'Reilly EM, Kokudo N, Miyazaki M, Pawlik TM. Intrahepatic cholangiocarcinoma: expert consensus statement. *HPB: the official journal of the International Hepato Pancreato Biliary Association*. Aug 2015;17(8):669-680.
11. Ringe KI, Wacker F. Radiological diagnosis in cholangiocarcinoma: Application of computed tomography, magnetic resonance imaging, and positron emission tomography. *Best practice & research. Clinical gastroenterology*. Apr 2015;29(2):253-265.
12. Bedossa P, Poynard T. An algorithm for the grading of activity in chronic hepatitis C. The METAVIR Cooperative Study Group. *Hepatology*. Aug 1996;24(2):289-293.
13. Ishak K, Baptista A, Bianchi L, et al. Histological grading and staging of chronic hepatitis. *J Hepatol*. Jun 1995;22(6):696-699.
14. Berry JL, Jubran R, Kim JW, et al. Long-term outcomes of Group D eyes in bilateral retinoblastoma patients treated with chemoreduction and low-dose IMRT salvage. *Pediatric blood & cancer*. Apr 2013;60(4):688-693.
15. Nozaki Y, Yamamoto M, Ikai I, et al. Reconsideration of the lymph node metastasis pattern (N factor) from intrahepatic cholangiocarcinoma using the International Union Against Cancer TNM staging system for primary liver carcinoma. *Cancer*. Nov 1 1998;83(9):1923-1929.
16. Shimada M, Yamashita Y, Aishima S, Shirabe K, Takenaka K, Sugimachi K. Value of lymph node dissection during resection of intrahepatic cholangiocarcinoma. *The British journal of surgery*. Nov 2001;88(11):1463-1466.
17. Kim Y, Spolverato G, Amini N, et al. Surgical Management of Intrahepatic Cholangiocarcinoma: Defining an Optimal Prognostic Lymph Node Stratification Schema. *Annals of surgical oncology*. Aug 2015;22(8):2772-2778.
18. Yamamoto M, Takasaki K, Yoshikawa T. Extended resection for intrahepatic cholangiocarcinoma in Japan. *Journal of hepato-biliary-pancreatic surgery*. 1999;6(2):117-121.
19. Valverde A, Bonhomme N, Farges O, Sauvanet A, Flejou JF, Belghiti J. Resection of intrahepatic cholangiocarcinoma: a Western experience. *Journal of hepato-biliary-pancreatic surgery*. 1999;6(2):122-127.
20. Uenishi T, Yamazaki O, Yamamoto T, et al. Serosal invasion in TNM staging of mass-forming intrahepatic cholangiocarcinoma. *Journal of hepato-biliary-pancreatic surgery*. 2005;12(6):479-483.
21. Robles R, Figueras J, Turrion VS, et al. Spanish experience in liver transplantation for hilar and peripheral cholangiocarcinoma. *Annals of surgery*. Feb 2004;239(2):265-271.
22. Okabayashi T, Yamamoto J, Kosuge T, et al. A new staging system for mass-forming intrahepatic cholangiocarcinoma: analysis of preoperative and postoperative variables. *Cancer*. Nov 1 2001;92(9): 2374-2383.
23. Ohtsuka M, Ito H, Kimura F, et al. Results of surgical treatment for intrahepatic cholangiocarcinoma and clinicopathological factors influencing survival. *The British journal of surgery*. Dec 2002; 89(12):1525-1531.
24. Lieser MJ, Barry MK, Rowland C, Ilstrup DM, Nagorney DM. Surgical management of intrahepatic cholangiocarcinoma: a 31-year experience. *Journal of hepato-biliary-pancreatic surgery*. 1998;5(1):41-47.
25. Mavros MN, Economopoulos KP, Alexiou VG, Pawlik TM. Treatment and Prognosis for Patients With Intrahepatic Cholangiocarcinoma: Systematic Review and Meta-analysis. *JAMA surgery*. Jun 2014;149(6):565-574.
26. Hyder O, Marques H, Pulitano C, et al. A nomogram to predict long-term survival after resection for intrahepatic cholangiocarcinoma: an Eastern and Western experience. *JAMA surgery*. May 2014;149(5):432-438.
27. Li T, Qin LX, Zhou J, et al. Staging, prognostic factors and adjuvant therapy of intrahepatic cholangiocarcinoma after curative resection. *Liver international: official journal of the International Association for the Study of the Liver*. Jul 2014;34(6):953-960.
28. Wang Y, Li J, Xia Y, et al. Prognostic nomogram for intrahepatic cholangiocarcinoma after partial hepatectomy. *J Clin Oncol*. Mar 20 2013;31(9):1188-1195.
29. Dhanasekaran R, Hemming AW, Zendejas I, et al. Treatment outcomes and prognostic factors of intrahepatic cholangiocarcinoma. *Oncology reports*. Apr 2013;29(4):1259-1267.
30. Farges O, Fuks D, Le Treut YP, et al. AJCC 7th edition of TNM staging accurately discriminates outcomes of patients with resectable intrahepatic cholangiocarcinoma: By the AFC-IHCC-2009 study group. *Cancer*. May 15 2011;117(10):2170-2177.

31. Spolverato G, Vitale A, Cucchetti A, et al. Can hepatic resection provide a long-term cure for patients with intrahepatic cholangiocarcinoma? *Cancer*. Nov 15 2015;121(22):3998-4006.
32. Kattan MW, Hess KR, Amin MB, et al. American Joint Committee on Cancer acceptance criteria for inclusion of risk models for individualized prognosis in the practice of precision medicine. *CA: a cancer journal for clinicians*. Jan 19 2016.
33. Bagante F, Gani F, Spolverato G, et al. Intrahepatic Cholangiocarcinoma: Prognosis of Patients Who Did Not Undergo Lymphadenectomy. *Journal of the American College of Surgeons*. Dec 2015;221(6):1031-1040 e1031-1034.
34. de Jong MC, Nathan H, Sotiropoulos GC, et al. Intrahepatic cholangiocarcinoma: an international multi-institutional analysis of prognostic factors and lymph node assessment. *J Clin Oncol*. Aug 10 2011;29(23):3140-3145.
35. Zhu AX, Borger DR, Kim Y, et al. Genomic profiling of intrahepatic cholangiocarcinoma: refining prognosis and identifying therapeutic targets. *Annals of surgical oncology*. Nov 2014;21(12):3827-3834.

Gallbladder

24

Andrew X. Zhu, Timothy M. Pawlik, David A. Kooby,
Tracey E. Schefter, and Jean-Nicolas Vauthey

CHAPTER SUMMARY

Cancers Staged Using This Staging System

Gallbladder carcinoma

Cancers Not Staged Using This Staging System

These histopathologic types of cancer…	Are staged according to the classification for…	And can be found in chapter…
Well-differentiated neuroendocrine tumors	No AJCC staging system	N/A
Sarcomas	Soft tissue sarcoma of the abdomen and thoracic visceral organs	42

Summary of Changes

Change	Details of Change	Level of Evidence
Definition of Primary Tumor (T)	T2 disease is now subdivided into two groups: T2 tumors on the peritoneal side (T2a) and those on the hepatic side (T2b) of the gallbladder.	II
Definition of Regional Lymph Node (N)	Changed from location-based definitions to number-based N category assessment. N categories have been revised to define N1 as one to three positive nodes and N2 as four or more positive nodes. The recommendation that six or more nodes be harvested and evaluated has been added.	III

ICD-O-3 Topography Codes

Code	Description
C23.9	Gallbladder
C24.0	Cystic duct only

WHO Classification of Tumors

Code	Description
8010	Carcinoma *in situ*
8148	Biliary intraepithelial neoplasia, high grade (BilIN-3)
8503	Intracystic papillary neoplasm with high-grade intraepithelial neoplasia
8470	Mucinous cystic neoplasm with high-grade intraepithelial neoplasia
8140	Adenocarcinoma

To access the AJCC cancer staging forms, please visit www.cancerstaging.org.

© American Joint Committee on Cancer 2017
M.B. Amin et al. (eds.), *AJCC Cancer Staging Manual, Eighth Edition*, DOI 10.1007/978-3-319-40618-3_24

Code	Description
8140	Adenocarcinoma, biliary type
8144	Adenocarcinoma, intestinal type
8140	Adenocarcinoma, gastric foveolar type
8480	Mucinous adenocarcinoma
8310	Clear cell adenocarcinoma
8490	Signet ring cell carcinoma
8070	Squamous cell carcinoma
8560	Adenosquamous carcinoma
8020	Undifferentiated carcinoma
8246	High-grade neuroendocrine carcinoma
8041	Small cell neuroendocrine carcinoma
8013	High-grade neuroendocrine carcinoma
8244	Mixed adenoneuroendocrine carcinoma
8503	Intraductal papillary neoplasm with an associated invasive carcinoma
8470	Mucinous cystic neoplasm with an associated invasive carcinoma

Bosman FT, Carneiro F, Hruban RH, Theise ND, eds. World Health Organization Classification of Tumours of the Digestive System. Lyon: IARC; 2010.

INTRODUCTION

Cancers of the gallbladder are staged according to their depth of invasion into the gallbladder wall and extent of spread to surrounding structures and lymph nodes. The liver is a common site of involvement; thus, liver invasion affects the primary tumor (T) classification. Other surrounding structures, such as the duodenum and transverse colon, are at risk of direct tumor extension. Invasion of hilar structures (common bile duct, hepatic artery, portal vein) usually renders these tumors locally unresectable. Development of jaundice suggests hilar involvement and is associated with unresectability and poor prognosis. In as many as 50% of cases, gallbladder cancers are discovered at pathological examination after simple cholecystectomy for presumed gallstone disease.[1] Five-year survival is 50% for patients with T1 tumors. Patients with T2 tumors have a 5-year survival rate of 29%, which appears to improve with more radical resection.

Cholelithiasis is associated with carcinoma of the gallbladder in most cases. Many of these cancers are found incidentally following cholecystectomy, either during surgery or on final histologic analysis of the specimen. Tumors encountered this way may have a better prognosis if they are amenable to definitive surgical resection, either at the time of cholecystectomy or at a subsequent operation. As many as 50% of resected gallbladder cancers undergo definitive resection at a second operation, with the gallbladder removed previously for presumed benign disease. Cystic duct involvement merits consideration of formal bile duct resection at the time of the definitive operation to achieve negative margin status.

ANATOMY

Primary Site(s)

The gallbladder is a pear-shaped, saccular organ located under the liver, situated in line with the physiologic division of the right and left lobes of the liver (Cantlie's line). It straddles Couinaud segments IVB and V. The organ may be divided into three parts: the fundus, body, and neck, which tapers into the cystic duct (Fig. 24.1). The wall is considerably thinner than that of other hollow organs and lacks a submucosal layer. The layers of the gallbladder consist of mucosa, a muscular layer, perimuscular connective tissue, and serosa on one side (serosa is lacking on the side of the gallbladder embedded in the liver).

Regional Lymph Nodes

The lymph node locations include nodes along the common bile duct, hepatic artery, portal vein, and cystic duct.[2,3]

Metastatic Sites

Cancers of the gallbladder usually metastasize to the peritoneum as well as liver, and occasionally to the lungs and pleura.

RULES FOR CLASSIFICATION

Clinical Classification

Clinical staging for suspected or proven gallbladder cancer is based on high-quality, contrast-enhanced cross-sectional imaging to evaluate regional nodal and systemic metastases, vascular invasion, and surgical resectability. Diagnostic laparoscopy is recommended to identify radiologically occult metastases, particularly peritoneal implants.[4] Gallbladder cancers are staged primarily on the basis of surgical exploration or resection, but not all patients with gallbladder cancer undergo surgical resection. Many *in situ* and early-stage carcinomas are not recognized grossly. They usually are staged pathologically on histologic examination of the resected specimen. The T category depends on the depth of tumor penetration into the wall of the gallbladder; the presence or absence of tumor invasion into the liver, hepatic artery, or portal vein; and the presence or absence of adjacent organ involvement.

Tumor confined to the gallbladder is classified as either T1 or T2, depending on the depth of invasion. T2 is classified as T2a if the tumor is on the peritoneal side and T2b if it is on the hepatic side of the gallbladder, given the worse prognosis of the latter (Fig. 24.2).[5] The serosal surface of the gall-

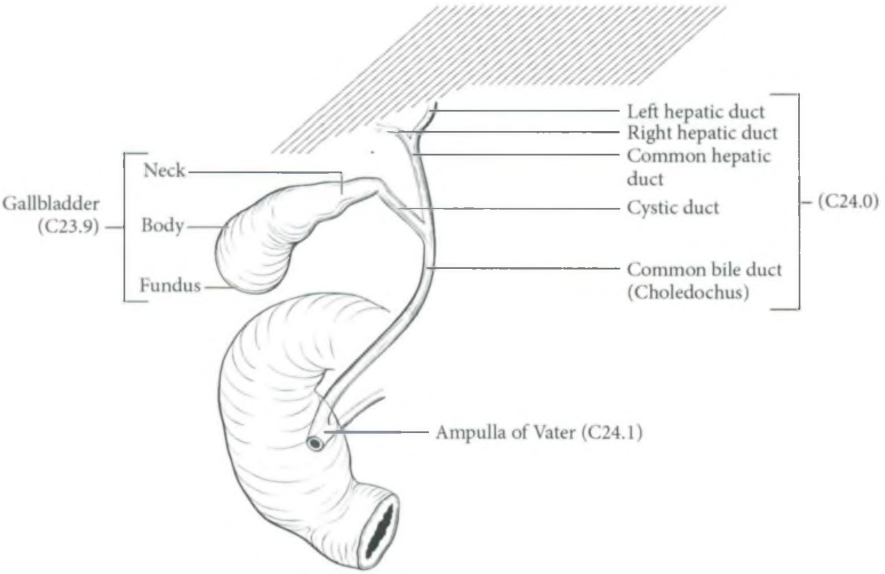

ig. 24.1 Schematic of the gallbladder in relation to the liver and biliary tract

ladder on the side attached to the liver is absent; thus, a mple cholecystectomy may not remove a T2 tumor com- letely, even though such tumors are considered to be con- ned to the gallbladder. Direct tumor extension into the liver not considered distant metastasis. Likewise, direct inva- on of other adjacent organs, including the colon, duode- um, stomach, common bile duct, abdominal wall, and iaphragm, is not considered distant metastasis but is classi- ed as T3 or T4, depending on the extent of tumor. Validation f stage grouping is based on multivariate analyses of out- ome and survival data from the National Cancer Database otaling 10,705 patients nationwide).[6]

naging

t imaging, gallbladder cancer may appear as focal or dif- ise thickening of the gallbladder wall, an intraluminal gall- ladder wall mass, or a mass involving both the gallbladder nd adjacent liver. Gallstones typically are present.

Ultrasound most frequently is the initial diagnostic tudy when gallbladder disease is suspected. However, it ften fails to detect any abnormality in early gallbladder ancer. In advanced disease, ultrasound is useful in provid- ig staging information by defining the extent of biliary ee involvement and confirming the presence of vascular ivasion. Multiphasic contrast-enhanced computed tomog- aphy (CT) and magnetic resonance (MR) imaging are the naging techniques of choice for local staging. These imag- ig modalities may detect liver, vascular, or biliary tree ivasion, lymphadenopathy, and involvement of the adja- ent organs.

Endoscopic ultrasound (EUS) allows precise imaging and acquisition of a fine-needle aspiration biopsy sample. Newer technologies include contrast-enhanced harmonic EUS to characterize gallbladder polyps.

Noncontrast chest CT is used to assess for distant metastasis. Positron emission tomography (PET) or PET/CT scanning may be useful in diagnosing ambiguous primary lesions and occult metastatic disease.[7–9]

Suggested Report Format

1. Primary tumor (T)
   a. Size of tumor: bidimensional, if measurable
   b. Location: fundus/body/neck
   c. Morphology: For example, if present, describe mural wall thickening, polypoid mass within the gallbladder, or solid mass replacing the gallbladder.
   d. Local extent: If present, describe invasion of the main portal vein and/or hepatic artery, hepatic veins, liver, or other adjacent organ or structures.
2. Lymph nodes (N)
   a. If present, describe abnormal or suspicious nodes along the cystic duct, common bile duct, hepatic artery, and/or portal vein.
3. Metastasis (M)
   a. If present, describe metastatic lesions seen on CT, MR imaging, or PET/CT scans.
   b. If present, describe abnormal or suspicious periaortic, pericaval, superior mesentery artery, and/or celiac artery lymph nodes.

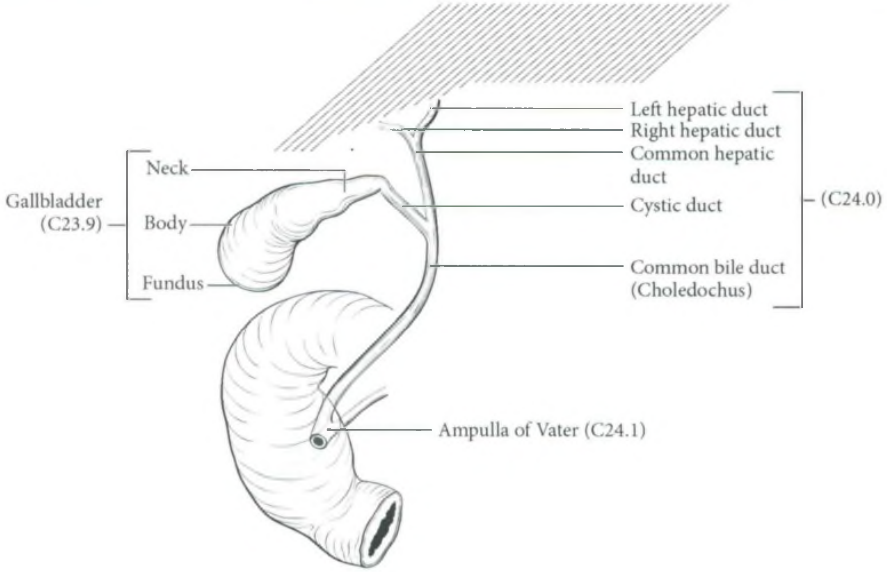

Fig. 24.1 Schematic of the gallbladder in relation to the liver and biliary tract

bladder on the side attached to the liver is absent; thus, a simple cholecystectomy may not remove a T2 tumor completely, even though such tumors are considered to be confined to the gallbladder. Direct tumor extension into the liver is not considered distant metastasis. Likewise, direct invasion of other adjacent organs, including the colon, duodenum, stomach, common bile duct, abdominal wall, and diaphragm, is not considered distant metastasis but is classified as T3 or T4, depending on the extent of tumor. Validation of stage grouping is based on multivariate analyses of outcome and survival data from the National Cancer Database (totaling 10,705 patients nationwide).[6]

Imaging

At imaging, gallbladder cancer may appear as focal or diffuse thickening of the gallbladder wall, an intraluminal gallbladder wall mass, or a mass involving both the gallbladder and adjacent liver. Gallstones typically are present.

Ultrasound most frequently is the initial diagnostic study when gallbladder disease is suspected. However, it often fails to detect any abnormality in early gallbladder cancer. In advanced disease, ultrasound is useful in providing staging information by defining the extent of biliary tree involvement and confirming the presence of vascular invasion. Multiphasic contrast-enhanced computed tomography (CT) and magnetic resonance (MR) imaging are the imaging techniques of choice for local staging. These imaging modalities may detect liver, vascular, or biliary tree invasion, lymphadenopathy, and involvement of the adjacent organs.

Endoscopic ultrasound (EUS) allows precise imaging and acquisition of a fine-needle aspiration biopsy sample. Newer technologies include contrast-enhanced harmonic EUS to characterize gallbladder polyps.

Noncontrast chest CT is used to assess for distant metastasis. Positron emission tomography (PET) or PET/CT scanning may be useful in diagnosing ambiguous primary lesions and occult metastatic disease.[7–9]

Suggested Report Format

1. Primary tumor (T)
   a. Size of tumor: bidimensional, if measurable
   b. Location: fundus/body/neck
   c. Morphology: For example, if present, describe mural wall thickening, polypoid mass within the gallbladder, or solid mass replacing the gallbladder.
   d. Local extent: If present, describe invasion of the main portal vein and/or hepatic artery, hepatic veins, liver, or other adjacent organ or structures.
2. Lymph nodes (N)
   a. If present, describe abnormal or suspicious nodes along the cystic duct, common bile duct, hepatic artery, and/or portal vein.
3. Metastasis (M)
   a. If present, describe metastatic lesions seen on CT, MR imaging, or PET/CT scans.
   b. If present, describe abnormal or suspicious periaortic, pericaval, superior mesentery artery, and/or celiac artery lymph nodes.

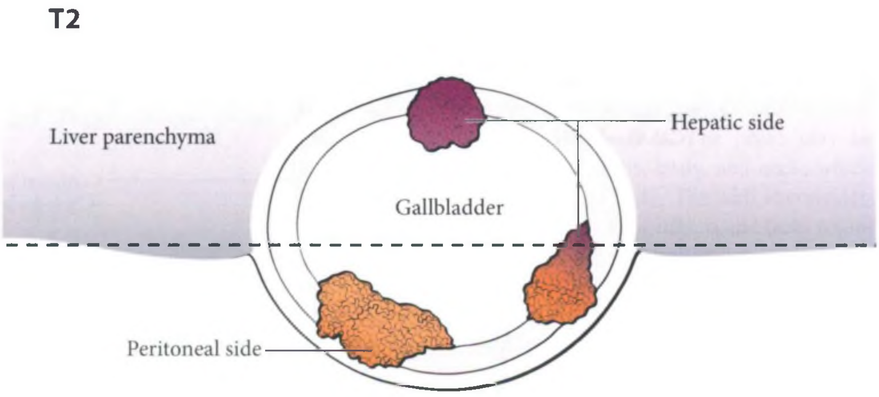

Fig. 24.2 Definition of tumor location for T2a and T2b. T2 tumors invade the perimuscular connective tissue along the free peritoneal side of the gallbladder, T2a, or adjacent to the liver, T2b

Pathological Classification

Pathological staging is based on examination of the gallbladder; in patients undergoing radical cholecystectomy, it is based on examination of the liver parenchyma adjacent to the gallbladder fossa and on regional lymphadenectomy. The extent of resection (R0, complete resection with grossly and microscopically negative margins of resection; R1, grossly negative but microscopically positive margins of resection; R2, grossly and microscopically positive margins of resection) is a descriptor in the TNM staging system and is the most important stage-independent prognostic factor.[10] It should be reported in all cases.

An important anatomic consideration is that the serosa along the liver edge is absent and the perimuscular connective tissue at this interface is densely adherent to the liver (cystic plate), and much of this often is left behind at the time of cholecystectomy. For this reason, partial hepatic resection incorporating portions of segments IVb and V is undertaken for some cases (typically T1b and higher).

Patients with T1b–T3 cancers discovered at pathological analysis usually are offered a second surgery for radical resection of residual tumor. This operation may include nonanatomic resection of the gallbladder bed (segments IVB and V of the liver) or more formal anatomic resection, such as a right hepatectomy. Resection of the biliary tree depends on surgical decision making at the time of the definitive procedure and may be based on cystic duct margin status.[11]

Comment should be made as to whether the primary tumor was located on the free peritoneal (T2a) or the hepatic side (T2b) of the gallbladder, as tumors on the hepatic side carry a worse prognosis (Fig. 24.3).[5]

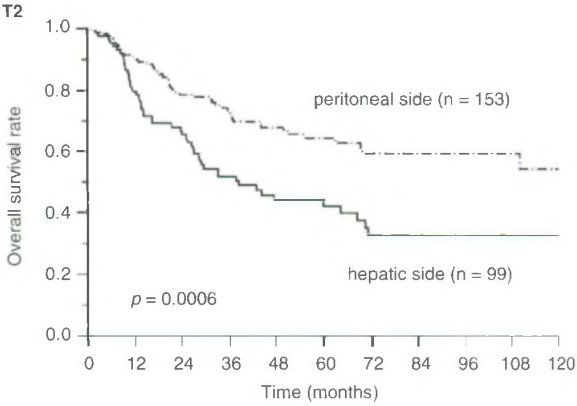

Fig. 24.3 Impact of tumor location relative to hepatic and peritoneal surfaces, by T category. Data from Shindoh et al.[5]

For accurate staging, all nodes removed at operation should be assessed for metastasis. The number of lymph node assessment, rather than the location of the lymph nodes, will dictate the nodal category.[12] It is recommended that at least six lymph nodes be harvested and evaluated.[3,13] Nodal categories are defined as N1 (one to three positive lymph nodes) and N2 (four or more positive lymph nodes).[14]

Patients with lymph node metastases (Stage IIIB or higher) or locally advanced tumors (Stage IVA or higher) rarely experience long-term survival (Fig. 24.4).[5,15]

Peritoneal involvement is common, and diagnostic laparoscopy at the time of surgery usually is advised. Systemic therapeutic options are limited, making prognosis for patients with unresectable disease extremely poor. Survival correlates with stage of disease.

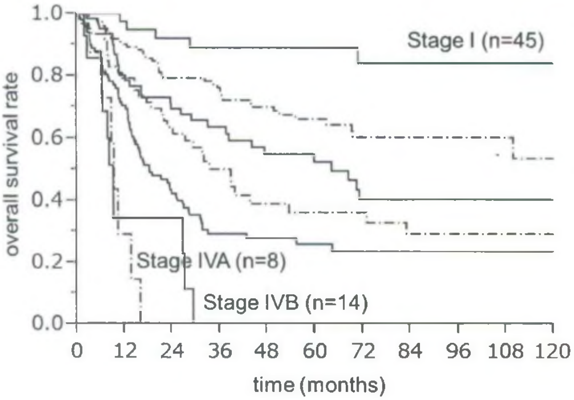

Fig. 24.4 Survival after resection of gallbladder cancer, according to AJCC staging. Data from Shindoh et al.[5]

PROGNOSTIC FACTORS

Prognostic Factors Required for Stage Grouping

Beyond the factors used to assign T, N, or M categories, no additional prognostic factors are required for stage grouping.

Additional Factors Recommended for Clinical Care

Histologic Grade

Histologic grade has been shown to be an independent prognostic factor for overall and disease-specific survival, with reported median disease-specific survival of 69 months in patients with well- or moderately differentiated gallbladder cancer, compared with 28 months in those with worse differentiated tumors ($p < 0.001$).[13] AJCC Level of Evidence: II

Histologic Subtype

Papillary carcinomas are uncommon, representing 5% of all gallbladder cancers, and have a favorable prognosis.[9] Squamous and adenosquamous carcinomas also are rare but are associated with worse survival than adenocarcinomas. AJCC Level of Evidence: II

Lymphovascular Invasion

Lymphatic infiltration is associated with a 5-year overall survival of 4%. Microscopic vascular invasion is associated with a similarly poor prognosis, with a 5-year survival of 0% in a large series by Roa et al.[16] AJCC Level of Evidence: II

Resection Margin and Extent of Resection

R0 resection was associated with improved survival in multiple series.[10,17] Multiple studies have shown that major hepatectomy and routine bile duct resection are not associated with improved survival.[4] Hepatoduodenal lymphadenectomy and nonanatomic hepatic resection of the gallbladder bed, with the goal of achieving an R0 resection, are recommended. AJCC Level of Evidence: II

RISK ASSESSMENT MODELS

The AJCC recently established guidelines that will be used to evaluate published statistical prediction models for the purpose of granting endorsement for clinical use.[18] Although this is a monumental step toward the goal of precision medicine, this work was published only very recently. Therefore, the existing models that have been published or may be in clinical use have not yet been evaluated for this cancer site by the Precision Medicine Core of the AJCC. In the future, the statistical prediction models for this cancer site will be evaluated, and those that meet all AJCC criteria will be endorsed.

DEFINITIONS OF AJCC TNM

Definition of Primary Tumor (T)

T Category	T Criteria
TX	Primary tumor cannot be assessed
T0	No evidence of primary tumor
Tis	Carcinoma *in situ*
T1	Tumor invades the lamina propria or muscular layer
T1a	Tumor invades the lamina propria
T1b	Tumor invades the muscular layer
T2	Tumor invades the perimuscular connective tissue on the peritoneal side, without involvement of the serosa (visceral peritoneum)<br>Or tumor invades the perimuscular connective tissue on the hepatic side, with no extension into the liver
T2a	Tumor invades the perimuscular connective tissue on the peritoneal side, without involvement of the serosa (visceral peritoneum)
T2b	Tumor invades the perimuscular connective tissue on the hepatic side, with no extension into the liver
T3	Tumor perforates the serosa (visceral peritoneum) and/or directly invades the liver and/or one other adjacent organ or structure, such as the stomach, duodenum, colon, pancreas, omentum, or extrahepatic bile ducts
T4	Tumor invades the main portal vein or hepatic artery or invades two or more extrahepatic organs or structures

Definition of Regional Lymph Node (N)

N Category	N Criteria
NX	Regional lymph nodes cannot be assessed
N0	No regional lymph node metastasis
N1	Metastases to one to three regional lymph nodes
N2	Metastases to four or more regional lymph nodes

Definition of Distant Metastasis (M)

M Category	M Criteria
M0	No distant metastasis
M1	Distant metastasis

AJCC PROGNOSTIC STAGE GROUPS

When T is…	And N is…	And M is…	Then the stage group is…
Tis	N0	M0	0
T1	N0	M0	I
T2a	N0	M0	IIA
T2b	N0	M0	IIB
T3	N0	M0	IIIA
T1–3	N1	M0	IIIB
T4	N0–1	M0	IVA
Any T	N2	M0	IVB
Any T	Any N	M1	IVB

REGISTRY DATA COLLECTION VARIABLES

1. Specimen type
2. Extent of liver resection
3. Free peritoneal side versus hepatic side for T2 tumors

HISTOLOGIC GRADE (G)

G	G Definition
GX	Grade cannot be assessed
G1	Well differentiated
G2	Moderately differentiated
G3	Poorly differentiated

HISTOPATHOLOGIC TYPE

Papillary carcinomas have the most favorable prognosis. Unfavorable histologic types include small cell carcinomas and undifferentiated carcinomas.

Bibliography

1. Shih SP, Schulick RD, Cameron JL, et al. Gallbladder cancer: the role of laparoscopy and radical resection. *Annals of surgery*. Jun 2007;245(6):893–901.
2. Chijiiwa K, Noshiro H, Nakano K, et al. Role of surgery for gallbladder carcinoma with special reference to lymph node metastasis and stage using western and Japanese classification systems. *World journal of surgery*. 2000;24(10):1271–1277.
3. Liu GJ, Li XH, Chen YX, Sun HD, Zhao GM, Hu SY. Radical lymph node dissection and assessment: Impact on gallbladder cancer prognosis. *World journal of gastroenterology: WJG*. Aug 21 2013;19(31):5150–5158.
4. Aloia TA, Jarufe N, Javle M, et al. Gallbladder cancer: expert consensus statement. *HPB: the official journal of the International Hepato Pancreato Biliary Association*. Aug 2015;17(8):681–690.
5. Shindoh J, de Aretxabala X, Aloia TA, et al. Tumor location is a strong predictor of tumor progression and survival in t2 gallbladder cancer: an international multicenter study. *Annals of surgery*. 2015;261(4):733–739.
6. Fong Y, Wagman L, Gonen M, et al. Evidence-based gallbladder cancer staging: changing cancer staging by analysis of data from the National Cancer Database. *Annals of surgery*. Jun 2006; 243(6):767–771; discussion 771–764.
7. Annunziata S, Pizzuto DA, Caldarella C, Galiandro F, Sadeghi R, Treglia G. Diagnostic accuracy of fluorine-18-fluorodeoxyglucose positron emission tomography in gallbladder cancer: A meta-analysis. *World journal of gastroenterology: WJG*. Oct 28 2015;21(40):11481–11488.
8. D'Hondt M, Lapointe R, Benamira Z, et al. Carcinoma of the gallbladder: patterns of presentation, prognostic factors and survival rate. An 11-year single centre experience. *European journal of surgical oncology: the journal of the European Society of Surgical Oncology and the British Association of Surgical Oncology*. Jun 2013;39(6):548–553.
9. Kanthan R, Senger JL, Ahmed S, Kanthan SC. Gallbladder Cancer in the 21st Century. *J Oncol*. 2015;2015:967472.
10. Dixon E, Vollmer Jr CM, Sahajpal A, et al. An aggressive surgical approach leads to improved survival in patients with gallbladder cancer: a 12-year study at a North American Center. *Annals of surgery*. 2005;241(3):385.
11. Adsay NV, Bagci P, Tajiri T, et al. Pathologic staging of pancreatic, ampullary, biliary, and gallbladder cancers: pitfalls and practical limitations of the current AJCC/UICC TNM staging system and opportunities for improvement. *Seminars in diagnostic pathology*. Aug 2012;29(3):127–141.
12. Sakata J, Shirai Y, Wakai T, Ajioka Y, Hatakeyama K. Number of positive lymph nodes independently determines the prognosis after resection in patients with gallbladder carcinoma. *Annals of surgical oncology*. Jul 2010;17(7):1831–1840.

13. Ito H, Ito K, D'Angelica M, et al. Accurate staging for gallbladder cancer: implications for surgical therapy and pathological assessment. *Annals of surgery*. Aug 2011;254(2):320 325.
14. Amini N, Spolverato G, Kim Y, et al. Lymph node status after resection for gallbladder adenocarcinoma: prognostic implications of different nodal staging/scoring systems. *Journal of surgical oncology*. Mar 2015;111(3):299 305.
15. Wakabayashi H, Ishimura K, Hashimoto N, Otani T, Kondo A, Maeta H. Analysis of prognostic factors after surgery for stage III and IV gallbladder cancer. *European journal of surgical oncology: the journal of the European Society of Surgical Oncology and the British Association of Surgical Oncology*. Oct 2004;30(8):842 846.
16. Roa I, Ibacache G, Munoz S, de Aretxabala X. Gallbladder cancer in Chile: Pathologic characteristics of survival and prognostic factors: analysis of 1,366 cases. *Am J Clin Pathol*. May 2014;141(5): 675 682.
17. Hari DM, Howard JH, Leung AM, Chui CG, Sim MS, Bilchik AJ. A 21-year analysis of stage I gallbladder carcinoma: is cholecystectomy alone adequate? *HPB*. 2013;15(1):40 48.
18. Kattan MW, Hess KR, Amin MB, et al. American Joint Committee on Cancer acceptance criteria for inclusion of risk models for individualized prognosis in the practice of precision medicine. *CA: a cancer journal for clinicians*. Jan 19 2016.

Perihilar Bile Ducts

25

David M. Nagorney, Timothy M. Pawlik, Yun Shin Chun, Tomoki Ebata, and Jean-Nicolas Vauthey

CHAPTER SUMMARY

Cancers Staged Using This Staging System

Perihilar cholangiocarcinoma or bile duct cancer, hilar cholangiocarcinoma, Klatskin tumor

Cancers Not Staged Using This Staging System

These histopathologic types of cancer…	Are staged according to the classification for…	And can be found in chapter…
Sarcoma	Soft tissue sarcoma of the abdomen and thoracic visceral organs	42
Well-differentiated neuroendocrine tumor (carcinoid)	No AJCC staging system	N/A

Summary of Changes

Change	Details of Change	Level of Evidence
Definition of Primary Tumor (T)	The definition of Tis has been expanded to include high-grade biliary intraepithelial neoplasia (BilIn-3). High-grade dysplasia (BilIn-3), a noninvasive neoplastic process, is synonymous with carcinoma *in situ* at this site.	N/A
Definition of Primary Tumor (T)	Bilateral second-order biliary radical invasion (Bismuth–Corlette type IV) has been removed from T4 category.	II
Definition of Regional Lymph Node (N)	N category was reclassified based on number of positive nodes to N1 (one to three positive nodes) and N2 (four or more positive nodes).	II
AJCC Prognostic Stage Groups	The stage group for T4 tumors was changed from Stage IVA to Stage IIIB	II
AJCC Prognostic Stage Groups	N1 category was changed from Stage IIIB to IIIC, and N2 category is classified as Stage IVA.	II

To access the AJCC cancer staging forms, please visit www.cancerstaging.org.

© American Joint Committee on Cancer 2017

M.B. Amin et al. (eds.), *AJCC Cancer Staging Manual, Eighth Edition*, DOI 10.1007/978-3-319-40618-3_25

ICD-O-3 Topography Codes

Code	Description
C24.0	Proximal or perihilar bile ducts only

WHO Classification of Tumors

Code	Description
8010	Carcinoma *in situ*
8148	Biliary intraepithelial neoplasia, high grade (BilIn-3)
8503	Intraductal papillary neoplasim with high-grade dysplasia
8470	Mucinous cystic neoplasm with high-grade intraepithelial neoplasia
8140	Adenocarcinoma
8140	Adenocarcinoma, biliary type
8140	Adenocarcinoma, gastric foveolar type
8144	Adenocarcinoma, intestinal type
8310	Clear cell adenocarcinoma
8480	Mucinous carcinoma
8490	Signet ring cell carcinoma
8070	Squamous cell carcinoma
8560	Adenosquamous carcinoma
8020	Undifferentiated carcinoma
8246	High-grade neuroendocrine carcinoma
8041	Small cell neuroendocrine carcinoma
8031	High-grade neuroendocrine carcinoma
8503	Intraductal papillary neoplasm with an associated invasive carcinoma
8470	Mucinous cystic neoplasm with an associated invasive carcinoma

Bosman FT, Carneiro F, Hruban RH, Theise ND, eds. World Health Organization Classification of Tumours of the Digestive System. Lyon: IARC; 2010.

INTRODUCTION

Proximal or perihilar cholangiocarcinomas involve the main biliary confluence of the right and left hepatic ducts and comprise 50–70% of all cases of bile duct carcinomas. They are uncommon cancers, with an incidence of 1 to 2 per 100,000 in the United States. Complete resection with histopathologically negative margins is the most robust predictor of long-term survival. However, the apposition of perihilar cholangiocarcinoma to adjacent hepatic arterial and portal venous branches and hepatic parenchyma technically complicates complete resection.

Recent advances in dimensional imaging, perioperative care, and operative technique have increased rates of resectability. Specifically, the understanding that perihilar cholangiocarcinoma extends proximally to involve intrahepatic bile ducts, with or without direct hepatic invasion and lobar hepatic atrophy, has led to routine incorporation of major hepatectomy, whether lobar, extended lobar, or total hepatectomy with transplantation, as an essential component of resection. These approaches have resulted in increased rates of margin-negative resection and improved overall survival.[1–4]

Before the AJCC Cancer Staging Manual, 7th Edition, perihilar and distal cholangiocarcinomas were grouped together as extrahepatic bile duct cancer. The prognostic accuracy of the separate perihilar cholangiocarcinoma TNM staging was validated independently.[5]

ANATOMY

Primary Site(s)

Cholangiocarcinoma develops anywhere within the biliary tree and arises from the most proximal intrahepatic bile ducts to the most distal intraduodenal bile duct. Extrahepatic cholangiocarcinoma was separated traditionally into perihilar, mid-duct, and distal cholangiocarcinoma. However, mid-duct cholangiocarcinomas do not comprise a separate site for staging. The AJCC Cancer Staging Manual, 8th Edition, affirms the prior stratification of cholangiocarcinoma into proximal and distal cholangiocarcinoma.

Perihilar cholangiocarcinoma is defined as arising predominantly in the main lobar extrahepatic bile ducts distal to segmental bile ducts and proximal to the cystic duct. Perihilar cholangiocarcinoma is characterized predominantly by local and regional growth patterns. Perineural invasion is typical for perihilar cholangiocarcinoma, and spread through periductal lymphatic channels is common. Cholangiocarcinoma may extend intrahepatically or proximally with involvement of the lobar sectoral and segmental bile ducts. Cholangiocarcinoma may extend radially with involvement of the hepatic parenchyma and hepatic arterial or portal venous vasculature, or both.

Regional Lymph Nodes

Hilar, cystic duct, choledochal, portal, hepatic arterial, and posterior pancreaticoduodenal lymph nodes are classified as regional lymph nodes.

Metastatic Sites

Lymph node metastasis distant to the hepatoduodenal ligament is classified as distant disease. Unilateral portal venous obstruction results in hepatic lobar atrophy, reflecting locally advanced disease, and increases the prevalence of distant disease. Peritoneum and liver are the most common sites of distant metastases. Other sites include lung, bone, brain, and skin.

RULES FOR CLASSIFICATION

Clinical Classification

Most patients diagnosed with perihilar cholangiocarcinoma ıre older than 60 years, with peak incidence in the eighth lecade of life.[6] Risk factors for developing perihilar cholan-giocarcinoma include hepatolithiasis, biliary parasites, and :holedochal cysts. In the United States, the most common dentifiable risk factor is primary sclerosing cholangitis, an ıutoimmune disease that predisposes the entire biliary tree to he development of malignancy. Most cases of perihilar chol-ıngiocarcinoma are sporadic, without identifiable risk actors.

Early symptoms are nonspecific and include constitutional ;ymptoms of abdominal discomfort, anorexia, and weight oss. Symptoms and signs from bile duct obstruction, with aundice, acholic stools, dark urine, and pruritus, occur fre-quently, regardless of disease stage.[7] Diagnosis of perihilar :holangiocarcinoma may be challenging, with frequent inde-erminate or false negative results from bile duct biopsies and ›iliary brushing cytology. Elevated serum cancer antigen 19-9 CA 19-9) levels >100 U/mL lend support to the diagnosis.[8] Fluorescence in situ hybridization (FISH) analysis increases he sensitivity of cytology in diagnosing perihilar cholangio-:arcinoma. In a patient with a resectable, malignant-appearing ;tricture involving the proximal biliary tree, pathological diag-ıosis of cancer is not compulsory before surgical exploration.

Most patients with perihilar cholangiocarcinoma have ocoregional extension or distant metastasis that precludes esection and thus are treated, and do not qualify for pathologi-:al staging. A single TNM classification must apply to both :linical and pathological staging. Therefore, in most patients vith perihilar cholangiocarcinoma, the basis for TNM staging s high-quality cross-sectional imaging. Peritoneal metastases nay be radiographically occult and in patients undergoing sur-;ery, identified only at time of staging laparoscopy.

The 7th edition of the AJCC Cancer Staging Manual eclassified adjacent hepatic parenchymal invasion as T2 but maintained unilateral vascular involvement as T3. The current edition affirms findings supporting that classification.[9]

The 7th edition of the AJCC Cancer Staging Manual defined T4 cholangiocarcinoma as cholangiocarcinoma with bilateral involvement of hepatic arterial or portal vasculature, bilateral ductal extension into the secondary or segmental bile ducts (Bismuth-Corlette type IV), and ductal extension into the secondary or segmental bile ducts with contralateral involvement of the hepatic vasculature. The current edition of AJCC Cancer Staging Manual eliminates bilateral ductal extension into the secondary or segmental bile ducts (Bismuth-Corlette type IV) alone from T4 cholangiocarcinoma. Thus, the current T category definitions exclude any Bismuth-Corlette typing. Such tumors were previously classified as Stage IVA disease and now are distributed by other T and N criteria into overall disease stage. The modified T categories have resulted in improved stratification of overall survival (Fig. 25.1).[10]

Lobar hepatic atrophy of variable extent is often associated with perihilar cholangiocarcinoma. Atrophy typically is associated with an advanced T category and ipsilateral portal venous obstruction. Because hepatic atrophy in advanced degrees reduces resectability, it has been proposed as a group component.[4] However, because the spectrum of hepatic atrophy is based on radiographic and gross clinical findings and not by histopathological criteria, atrophy is not incorporated into the current staging system.

Imaging

Clinical evaluation usually depends on the results of duplex ultrasound, computed tomography (CT), and magnetic resonance cholangiopancreatography (MRCP). Patients typically present with jaundice and undergo ultrasound as their first imaging modality. High-quality multidetector CT should demonstrate the level of biliary obstruction, vascular involvement, liver atrophy, and presence of nodal and distant metastases. The biliary extent of disease is assessed with percutaneous transhepatic cholangiography or MRCP. CT and/or MRCP should be performed before placement of biliary stents, which can obscure anatomic detail.

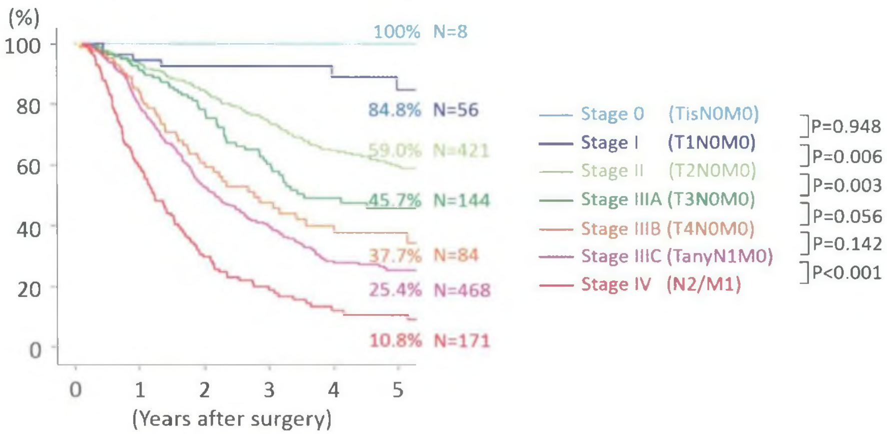

Fig. 25.1 Overall survival after surgical resection of perihilar cholangiocarcinoma at Nagoya University, Japan. Changes from the 7th Edition include removal of Bismuth–Corlette type IV tumors from the T4 category and downstaging of T4 tumors from Stage IVA to IIIB. Data from Ebata et al.[10]

25

Cross-sectional imaging can also demonstrate the presence of lobar atrophy, which indicates the presence of biliary and/or vascular involvement and represents a gross and significant reduction of expected standard liver volume of the involved liver. Lobar atrophy is an important consideration before surgery, since an inadequate liver remnant volume can preclude hepatic resection or require preoperative portal vein embolization to induce hypertrophy of the remnant liver.

Clinical staging also may be based on findings from surgical exploration when the main tumor mass is not resected.

Suggested Report Format

1. Primary tumor (T)
   a. Size of tumor: bidimensional
   b. Location
      i. Proximal common hepatic duct
      ii. Confluence of the left and right hepatic ducts
      iii. Left or right hepatic duct
   c. Morphology: growth type
2. Local extent, if present describe:
   a. Segmental duct involvement on each side, including Bismuth–Corlette type; mention biliary variant anatomy, if present
   b. Lobar atrophy
   c. Vascular involvement (left, right, or main portal vein or hepatic artery on each side)
3. Regional lymph nodes (N)
   a. If present, describe abnormal or suspicious nodes along the hilus, cystic duct, extrahepatic bile duct, head of pancreas, proximal duodenum, hepatic artery, and portal vein.
4. Metastasis (M): if present, describe metastatic lesions seen on CT, MR imaging or PET/CT scans in the noncontiguous liver, peritoneum, lung, brain, bone, or other areas
   a. If present, describe abnormal or suspicious periaortic, pericaval, superior mesenteric, or celiac artery nodes

Pathological Classification

Macroscopically, perihilar cholangiocarcinoma is classified into three subtypes: papillary, nodular, and sclerosing.[14] Sclerosing cholangiocarcinoma, the most frequent subtype, is characterized by periductal infiltration and desmoplasia. The nodular subtype is characterized by local irregular infiltration into the bile duct. Often, features of both nodular and sclerosing subtypes are observed together. Papillary tumors account for 5–10% of cases and frequently are soft and friable, with limited mural invasion. Papillary cholangiocarcinoma is more often surgically resectable and has a better prognosis than nodular and sclerosing subtypes.

Tumors classified as Tis cytologically resemble carcinoma, with diffuse, severe distortion of cellular polarity, but invasion through the basement membrane is absent.[15]

Complete resection of perihilar cholangiocarcinoma requires en bloc resection of the liver (usually major anatomic hepatectomy), extrahepatic bile duct, and hepatoduodenal lymph nodes. If involved, the portal vein and/or hepatic artery may need resection and reconstruction. The extent of resection (R0, complete resection with grossly and microscopically negative margins of resection; R1, grossly negative but microscopically positive margins of resection; R2, grossly and microscopically positive margins of resection) is a descriptor in the TNM staging system, is the most important stage-independent prognostic factor, and should be reported.

Patients who undergo surgical resection for localized perihilar cholangiocarcinoma have a median survival of approximately 3 years and a 5-year survival rate of 20–40%. In carefully selected patients with primary sclerosing cholangitis and locally unresectable lymph node–negative perihilar cholangiocarcinoma, excellent survival has been reported after neoadjuvant chemoradiation and liver transplantation.

Extended hepatic resections (trisectorectomy) with resection and reconstruction of the hepatic remnant portal vein and hepatic artery have been used increasingly, with promising early outcomes. Complete resection with negative histopathologic margins is the major predictor of outcome. Invasive, but not *in situ*, carcinoma at the margin of resection adversely affects survival. Hepatic resection is considered integral to achieving negative proximal intrahepatic margins. Factors adversely associated with survival include high tumor grade, vascular invasion, and lymph node metastasis.

The prevalence of lymphatic metastases increases directly with T categories and ranges overall from 30–53% by site. Nodal involvement adversely correlates with survival.[16] Accurate localization of the site of lymph nodes in the hepatoduodenal ligament is difficult. Because the total number of metastatically involved lymph nodes correlates with survival, the number of positive lymph nodes has been added to classify N categories. Regional lymph node involvement is stratified into three N groupings: N0 (no lymph node involvement), N1 (one to three positive lymph nodes), and N2 (four or more positive lymph nodes).

PROGNOSTIC FACTORS

Prognostic Factors Required for Stage Grouping

Beyond the factors used to assign T, N, or M categories, no additional prognostic factors are required for stage grouping.

Additional Factors Recommended for Clinical Care

Tumor Location and Extent

The Bismuth–Corlette classification describes the location and extent of biliary infiltration by tumor. Bismuth–Corlette type IV tumors, defined as tumor invasion of second-order biliary radicals bilaterally, are associated with a higher rate of positive surgical margins and significantly poorer 5-year overall survival after resection than Bismuth–Corlette types I to III.[10] AJCC Level of Evidence: II

Papillary Histology

Papillary tumors account for approximately one quarter of hilar cholangiocarcinomas in surgical series. They are characterized by an intraductal growth pattern, are more often well-differentiated, and confer a higher median disease-specific survival after resection: 58 months, compared with 36 months for nonpapillary tumors ($p = 0.01$).[4] AJCC Level of Evidence: II

Primary Sclerosing Cholangitis

Primary sclerosing cholangitis is an idiopathic chronic liver disease characterized by inflammation and fibrosis of the entire biliary tree. The chronic inflammation and injury to ducts may lead to cirrhosis and predispose to cholangiocarcinomas at any site in the biliary tree. Patients with primary sclerosing cholangitis are advised to receive neoadjuvant chemoradiation and liver transplantation.[14] AJCC Level of Evidence: II

Risk Assessment Models

The AJCC recently established guidelines that will be used to evaluate published statistical prediction models for the purpose of granting endorsement for clinical use.[17] Although this is a monumental step toward the goal of precision medicine, this work was published only very recently. Therefore, the existing models that have been published or may be in clinical use have not yet been evaluated for this cancer site by the Precision Medicine Core of the AJCC. In the future, the statistical prediction models for this cancer site will be evaluated, and those that meet all AJCC criteria will be endorsed.

Table 25.1 Bismuth–Corlette classification

Type	Definition
I	Tumor is limited to the common hepatic duct, below the level of the confluence of the right and left hepatic ducts
II	Tumor involves the confluence of the right and left hepatic ducts
IIIa	Tumor with type II involvement plus extension to the right 2nd-order ducts
IIIb	Tumor with type II involvement plus extension to the left 2nd-order ducts
IV	Tumor extends into both right and left 2nd-order ducts

DEFINITIONS OF AJCC TNM

Definition of Primary Tumor (T)

T Category	T Criteria
TX	Primary tumor cannot be assessed
T0	No evidence of primary tumor
Tis	Carcinoma *in situ*/high-grade dysplasia
T1	Tumor confined to the bile duct, with extension up to the muscle layer or fibrous tissue
T2	Tumor invades beyond the wall of the bile duct to surrounding adipose tissue, or tumor invades adjacent hepatic parenchyma
T2a	Tumor invades beyond the wall of the bile duct to surrounding adipose tissue
T2b	Tumor invades adjacent hepatic parenchyma
T3	Tumor invades unilateral branches of the portal vein or hepatic artery
T4	Tumor invades the main portal vein or its branches bilaterally, or the common hepatic artery; or unilateral second-order biliary radicals with contralateral portal vein or hepatic artery involvement

Definition of Regional Lymph Node (N)

N Category	N Criteria
NX	Regional lymph nodes cannot be assessed
N0	No regional lymph node metastasis
N1	One to three positive lymph nodes typically involving the hilar, cystic duct, common bile duct, hepatic artery, posterior pancreatoduodenal, and portal vein lymph nodes
N2	Four or more positive lymph nodes from the sites described for N1

Definition of Distant Metastasis (M)

M Category	M Criteria
M0	No distant metastasis
M1	Distant metastasis

25

AJCC PROGNOSTIC STAGE GROUPS

When T is...	And N is...	And M is...	Then the stage group is...
Tis	N0	M0	0
T1	N0	M0	I
T2a–b	N0	M0	II
T3	N0	M0	IIIA
T4	N0	M0	IIIB
Any T	N1	M0	IIIC
Any T	N2	M0	IVA
Any T	Any N	M1	IVB

REGISTRY DATA COLLECTION VARIABLES

1. Tumor location and extent according to Bismuth–Corlette classification
2. Papillary histology
3. Primary sclerosing cholangitis

HISTOLOGIC GRADE (G)

G	G Definition
GX	Grade cannot be assessed
G1	Well differentiated
G2	Moderately differentiated
G3	Poorly differentiated

HISTOPATHOLOGIC TYPE

Adenocarcinoma that is not further subclassified is the most common histologic type.

Bibliography

1. Nagino M, Ebata T, Yokoyama Y, et al. Evolution of surgical treatment for perihilar cholangiocarcinoma: a single-center 34-year review of 574 consecutive resections. *Annals of surgery*. Jul 2013;258(1):129-140.
2. Natsume S, Ebata T, Yokoyama Y, et al. Clinical significance of left trisectionectomy for perihilar cholangiocarcinoma: an appraisal and comparison with left hepatectomy. *Annals of surgery*. Apr 2012;255(4):754-762.
3. Croome KP, Rosen CB, Heimbach JK, Nagorney DM. Is Liver Transplantation Appropriate for Patients with Potentially Resectable De Novo Hilar Cholangiocarcinoma? *Journal of the American College of Surgeons*. Jul 2015;221(1):130-139.
4. Matsuo K, Rocha FG, Ito K, et al. The Blumgart preoperative staging system for hilar cholangiocarcinoma: analysis of resectability and outcomes in 380 patients. *Journal of the American College of Surgeons*. Sep 2012;215(3):343-355.
5. Juntermanns B, Sotiropoulos GC, Radunz S, et al. Comparison of the sixth and the seventh editions of the UICC classification for perihilar cholangiocarcinoma. *Annals of surgical oncology*. Jan 2013;20(1):277-284.
6. Carriaga MT, Henson DE. Liver, gallbladder, extrahepatic bile ducts, and pancreas. *Cancer*. Jan 1 1995;75(1 Suppl):171-190.
7. Razumilava N, Gores GJ. Classification, diagnosis, and management of cholangiocarcinoma. *Clin Gastroenterol Hepatol*. Jan 2013;11(1):13-21 e11; quiz e13-14.
8. Blechacz B, Komuta M, Roskams T, Gores GJ. Clinical diagnosis and staging of cholangiocarcinoma. *Nature reviews. Gastroenterology & hepatology*. Sep 2011;8(9):512-522.
9. Ito T, Ebata T, Yokoyama Y, et al. The Pathologic Correlation Between Liver and Portal Vein Invasion in Perihilar Cholangiocarcinoma: Evaluating the Oncologic Rationale for the American Joint Committee on Cancer Definitions of T2 and T3 Tumors. *World journal of surgery*. 2014;38(12): 3215-3221.
10. Ebata T, Kosuge T, Hirano S, et al. Proposal to modify the International Union Against Cancer staging system for perihilar cholangiocarcinomas. *The British journal of surgery*. Jan 2014;101(2):79-88.
11. Rizvi S, Gores GJ. Current diagnostic and management options in perihilar cholangiocarcinoma. *Digestion*. 2014;89(3):216-224.
12. Deoliveira ML, Schulick RD, Nimura Y, et al. New staging system and a registry for perihilar cholangiocarcinoma. *Hepatology*. Apr 2011;53(4):1363-1371.
13. Engelbrecht MR, Katz SS, van Gulik TM, Laméris JS, van Delden OM. Imaging of perihilar cholangiocarcinoma. *American Journal of Roentgenology*. 2015;204(4):782-791.
14. Zaydfudim VM, Rosen CB, Nagorney DM. Hilar cholangiocarcinoma. *Surg Oncol Clin N Am*. Apr 2014;23(2):247-263.
15. Zen Y, Adsay NV, Bardadin K, et al. Biliary intraepithelial neoplasia: an international interobserver agreement study and proposal for diagnostic criteria. *Modern pathology: an official journal of the United States and Canadian Academy of Pathology, Inc*. Jun 2007;20(6):701-709.
16. Aoba T, Ebata T, Yokoyama Y, et al. Assessment of nodal status for perihilar cholangiocarcinoma: location, number, or ratio of involved nodes. *Annals of surgery*. Apr 2013;257(4):718-725.
17. Kattan MW, Hess KR, Amin MB, et al. American Joint Committee on Cancer acceptance criteria for inclusion of risk models for individualized prognosis in the practice of precision medicine. *CA: a cancer journal for clinicians*. Jan 19 2016.

Distal Bile Duct

26

Alyssa Krasinskas, Timothy M. Pawlik,
Mari Mino-Kenudson, and Jean-Nicolas Vauthey

CHAPTER SUMMARY

Cancers Staged Using This Staging System

Bile duct adenocarcinoma, distal cholangiocarcinoma, biliary intraepithelial neoplasia, high-grade neuroendocrine carcinoma, and papillary carcinoma

Cancers Not Staged Using This Staging System

These histopathologic types of cancer…	Are staged according to the classification for…	And can be found in chapter…
Tumors arising in the ampulla of Vater	Ampulla of Vater	27
Sarcoma	Soft tissue sarcoma of the abdomen and thoracic visceral organs	42
Well-differentiated neuroendocrine tumor (carcinoid)	Neuroendocrine tumors of the duodenum and ampulla of Vater	30

Summary of Changes

Change	Details of Change	Level of Evidence
Definition of Primary Tumor (T)	The definition of Tis has been expanded to include high-grade biliary intraepithelial neoplasia (BilIn-3). High-grade dysplasia (BilIn-3), a noninvasive neoplastic process, is synonymous with carcinoma *in situ* at this site.	N/A
Definition of Primary Tumor (T)	Definitions of T1, T2, and T3 have been revised based on measured depth of invasion (<5 mm, 5–12 mm, >12 mm). The descriptive extent of invasion also should still be reported. Depth of tumor invasion is better than the descriptive extent of tumor invasion at predicting patient outcomes.	II
Definition of Regional Lymph Node (N)	N categories have been expanded (N1, one to three positive lymph nodes; N2, four or more positive lymph nodes). The number of involved lymph nodes appears to be useful in predicting patient outcomes.	II
WHO Classification of Tumors	The histologic type of high-grade neuroendocrine carcinoma has been added for consistency with other gastrointestinal and hepatobiliary neuroendocrine carcinoma designations. Large cell and small cell neuroendocrine carcinomas fall under this subtype.	N/A
WHO Classification of Tumors	The histologic types have been updated to match current World Health Organization terminology.	N/A

To access the AJCC cancer staging forms, please visit www.cancerstaging.org.

© American Joint Committee on Cancer 2017

M.B. Amin et al. (eds.), *AJCC Cancer Staging Manual, Eighth Edition*, DOI 10.1007/978-3-319-40618-3_26

ICD-O-3 Topography Codes

Code	Description
C24.0	Distal bile duct only

WHO Classification of Tumors

Code	Description
8010	Carcinoma *in situ*
8148	Biliary intraepithelial neoplasia, high grade (BilIN-3)
8503	Intraductal papillary neoplasm with high-grade intraepithelial neoplasia
8470	Mucinous cystic neoplasm with high-grade intraepithelial neoplasia
8140	Adenocarcinoma
8140	Adenocarcinoma, biliary type
8144	Adenocarcinoma, intestinal type
8140	Adenocarcinoma, gastric foveolar type
8480	Mucinous adenocarcinoma
8310	Clear cell adenocarcinoma
8490	Signet ring cell carcinoma
8070	Squamous cell carcinoma
8560	Adenosquamous carcinoma
8020	Undifferentiated carcinoma
8246	High grade neuroendocrine carcinoma
8041	Small cell neuroendocrine carcinoma
8013	High-grade neuroendocrine carcinoma
8244	Mixed adenoneuroendocrine carcinoma
8503	Intraductal papillary neoplasm with an associated invasive carcinoma
8470	Mucinous cystic neoplasm with an associated invasive carcinoma

Bosman FT, Carneiro F, Hruban RH, Theise ND, eds. World Health Organization Classification of Tumours of the Digestive System. Lyon: IARC; 2010.

INTRODUCTION

Malignant tumors may develop anywhere along the extrahepatic bile ducts. Given the differences in anatomy of the bile duct and consideration of local factors related to resectability, extrahepatic bile duct carcinomas have been divided into proximal (perihilar) and distal bile duct tumors. This TNM classification applies to the 20–30% of bile duct tumors that arise in the distal bile duct, including malignant tumors that develop in congenital choledochal cysts. Currently, the TNM classification is the only staging scheme for distal bile duct cancers. All malignant tumors of the extrahepatic bile ducts inevitably cause partial or complete ductal obstruction. Because the bile ducts have a small diameter, the signs and symptoms of obstruction usually occur while tumors are relatively small. Most tumors involve the intrapancreatic portion of the common bile duct, and a primary tumor in the intrapancreatic portion of the common bile duct may be misclassified as pancreatic cancer if surgical resection is not performed. In such cases, it often is impossible to determine (from radiographic images or endoscopy) whether a tumor arises from the intrapancreatic portion of the bile duct, the ampulla of Vater, or the pancreas. Tumors of the pancreas and ampulla of Vater are staged separately.

ANATOMY

Primary Site(s)

The cystic duct connects to the gallbladder and joins the common hepatic duct to form the common bile duct, which passes posterior to the first part of the duodenum, traverses the head of the pancreas, and then enters the second part of the duodenum through the ampulla of Vater. Tumors with their center located between the confluence of the cystic duct and common hepatic duct and the Ampulla of Vater (excluding ampullary carcinoma) are considered distal bile duct tumors (Fig. 26.1). Histologically, the bile ducts are lined by a single layer of tall, uniform columnar calls. The mucosa usually forms irregular pleats or small longitudinal folds. The walls of the bile ducts have a layer of subepithelial connective tissue and muscle fibers. It should be noted that the muscle fibers are most prominent in the distal segment of the common bile duct. The extrahepatic ducts lack a serosa but are surrounded by varying amounts of adventitial adipose tissue. Adipose tissue surrounding the fibromuscular wall is not considered part of the bile duct mural anatomy.

Carcinomas that arise in the distal segment of the common bile duct may spread directly into the pancreas, duodenum, gallbladder, colon, stomach, or omentum.

Regional Lymph Nodes

The regional lymph nodes are the same as those resected for cancers of the head of the pancreas: nodes along the common bile duct and hepatic artery, the posterior and anterior pancreaticoduodenal nodes, and the nodes along the right lateral wall of the superior mesenteric artery.

Metastatic Sites

Distant metastases usually occur late in the course of the disease and most often are found in the liver, lungs, and peritoneum.

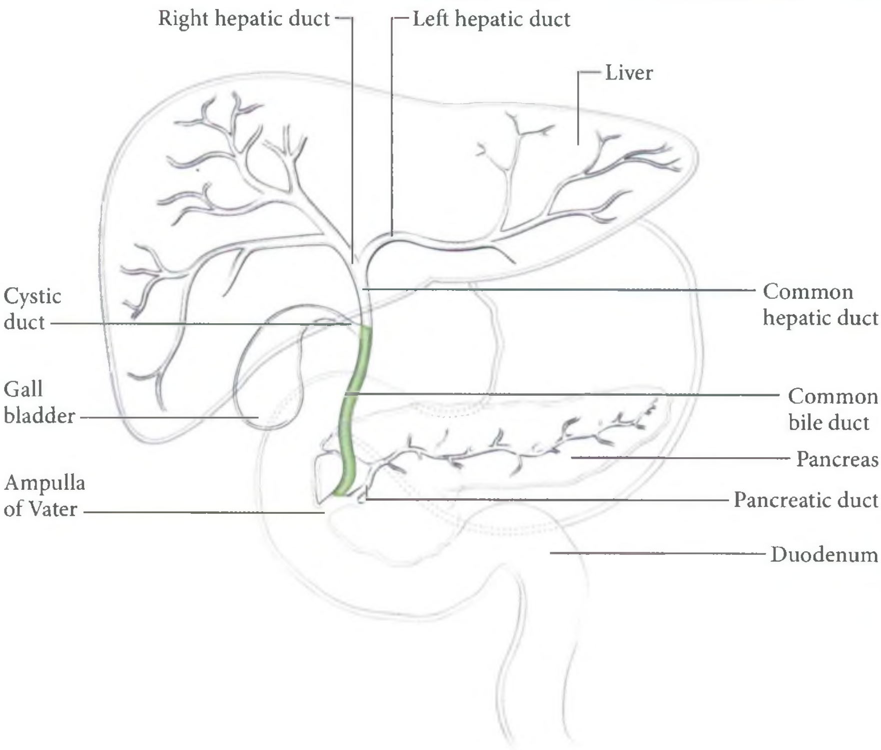

Fig. 26.1 Diagram highlighting the location of tumors to be staged as distal bile duct tumors. These tumors have an epicenter located between the confluence of the cystic duct and common hepatic duct and the ampulla of Vater (highlighted) (Modified from the College of American Pathologists)

RULES FOR CLASSIFICATION

Clinical Classification

Most patients with distal bile duct cancer present with biliary symptoms such as painless jaundice and have abnormal liver function tests. Subsequent imaging often detects a biliary obstruction or abnormality. The ideal workup of the stricture includes direct visualization of the bile duct with targeted biopsies. Endoscopic ultrasound (EUS) may help define the lesion or bile duct wall thickening and may help direct biopsies. Delayed-contrast computed tomography (CT), magnetic resonance (MR) imaging, or MR cholangiopancreatography (MRCP) is used to further assess the lesion, adjacent vessels, and nearby lymph nodes and to detect metastatic disease. Endoscopic retrograde cholangiopancreatography (ERCP) may allow for bile duct brushings and stenting for unresectable disease. Serologic studies (carcinoembryonic antigen [CEA] and cancer antigen [CA] 19-9) may be considered. Clinical staging also may be based on findings from surgical exploration if the main tumor mass is not resected. The initial surgical assessment should rule out distant metastatic disease and determine local resectability. The presence of a dominant stricture may be a diagnostic feature of distal bile duct cancer. Positive biopsy, cytology, and/or polysomy on fluorescent *in situ* hybridization confirms the diagnosis.[1]

Most often, patients are staged following surgery and pathological examination. In one third to half of the cases, surgical resection is not attempted because of local/regional extension, and patients are treated without pathological staging. A single TNM classification applies to both clinical and pathological staging. With advances in imaging, integrated radiologic and pathological staging of patients may be achieved satisfactorily.

Imaging[2-19]

Cross-sectional imaging, either contrast-enhanced, multiphasic, thin-section MR imaging or CT, typically is the preferred examination for assessing the stage of pancreatic cancers, ampullary tumors, and distal common bile duct tumors and should be performed before any interventions (e.g., biopsy, stent placement). The choice of MR imaging or CT should be based on the imaging equipment available, the expertise of the radiologists performing and interpreting the studies, and whether there are confounding issues, such as allergies to intravenous contrast or renal insufficiency (in the

latter case, unenhanced MR imaging is preferred to unenhanced CT because of MR imaging's superior soft tissue contrast). As noted, imaging should be performed before interventions (e.g., stent placement, biopsy) to avoid the effects of potential postprocedure pancreatitis interfering with staging assessments.

If intravenous contrast is used, dynamic imaging (MR or CT) should be performed both during the phase of peak pancreatic enhancement ("pancreatic parenchymal" or "late arterial" phase), to enhance the conspicuity of tumor against the background pancreas (regardless of ampullary, pancreatic, or distal biliary origin), and during the portal venous phase of liver enhancement (peak liver enhancement), when veins are fully opacified, to judge extrapancreatic extent of tumor, involvement of vasculature, and the possibility of liver metastases, as liver metastases from these tumors typically are hypodense against uninvolved liver. Thin-section imaging (e.g., 2–3 mm for CT) is particularly important for judging vascular involvement and to assess for potential small sites of metastatic disease. In the setting of preoperative therapy, this technique is important, not only at baseline but also following therapy, to determine whether patients are still surgical candidates and to follow up borderline suspicious findings.

Endoscopic ultrasound may be used in conjunction with CT/MR imaging to assist in locoregional staging; however, EUS has limited utility in assessing for distant disease such as liver metastases, peritoneal implants, or adenopathy outside the surgical field. EUS and EUS/fine-needle aspiration also should be performed before ERCP, as pancreatitis may degrade the ability of EUS to visualize the tumor and stent placement makes it impossible to identify sites of duct cutoff that may be useful in guiding biopsies. ERCP subsequently may be helpful in the setting of duct abnormalities, both for treatment (stent placement) and for diagnosis (brushings).

TNM Categories of Staging by Imaging

The relationship of the tumor to relevant vessels should be reported, specifically the relation of the tumor to arteries, such as the superior mesenteric, celiac, splenic, and common hepatic arteries, as well as the aorta if the tumor extends posteriorly into the retroperitoneum. The relationship of the tumor to relevant veins, including the portal vein, splenic vein, splenoportal confluence, and superior mesenteric vein, as well as to branch vessels, such as the gastrocolic trunk, first jejunal vein, and ileocolic branches, also should be recorded.

The relationship of the tumor to the vessels should be described using terms commonly understood by the clinical community, such as degrees of circumferential involvement and the terms *abutment* (i.e., up to and including 180° of involvement of a given vessel by tumor) and *encasement* (i.e., greater than 180° of circumferential vessel involvement by tumor). Multiplanar reconstructions for CT and direct multiplanar imaging for MR imaging may be particularly helpful in visualizing the circumferential relationship of the tumor to relevant vasculature. It also is important to describe the relationship of the tumor to adjacent structures such as the stomach, spleen, colon, small bowel, and adrenal glands.

Assessment of N category (nodal) status may be a challenge for all imaging modalities, because preoperative imaging is limited and cannot detect microscopic metastatic disease. Nevertheless, it is important to fully identify the location of visibly suspicious nodes. Nodes are considered suspicious for metastatic involvement if they are greater than 1 cm in short axis or have abnormal morphology (e.g., are rounded, hypodense, or heterogeneous; have irregular margins; involve adjacent vessels or structures). Lymph nodes outside the usual surgical field, such as retroperitoneal nodes, pelvic nodes, and lymph nodes within the jejunal mesentery or ileocolic mesentery, also should be evaluated and reported if abnormal.

The most common sites of metastatic disease include the liver, peritoneum, and lung. Evaluation for potential metastatic disease is best done with contrast-enhanced CT or MR imaging.

Suggested Radiology Report Format

Tumor involvement with adjacent vasculature should be reported with terms generally understood by the oncology community, such as degrees of circumferential involvement by tumor of a given vessel, and the terms *abutment* and *encasement*, as defined earlier.

The radiology report should include detailed descriptions of the following:

1. Primary tumor: location, size, characterization and effect on ducts (common bile duct and main pancreatic duct). Details regarding any findings suspicious for superimposed acute pancreatitis, which may distort findings relevant to staging, or chronic pancreatitis/autoimmune pancreatitis also should be reported, as these diseases may closely mimic malignancy and may be associated with duct strictures.
2. Local extent: the relationship of the tumor, with reference to degrees of circumferential involvement using commonly understood terms, such as *abutment* and *encasement*, and occlusion with regard to adjacent arterial structures (celiac, superior mesenteric, hepatic, and splenic arteries and the aorta) and venous structures (portal, splenic, and superior mesenteric veins,

and if relevant, inferior vena cava). The following observations also should be noted:

a. How much of the vascular involvement is related to solid tumor versus stranding, and whether vessel involvement is related to direct involvement by tumor or is separate from the tumor
b. Narrowing of vasculature, vascular thrombi, and the length of tumor involvement with the vasculature
c. Enlarged collaterals or varices
d. Involvement of branch vessels, such as the gastrocolic, first jejunal, and ileocolic branches of the superior mesenteric vein

3. Relevant arterial variants: This is particularly important with regard to hepatic arterial variants, such as those arising from the superior mesenteric artery, and the nature of the variant (e.g., accessory right hepatic vs. common hepatic artery arising from the superior mesenteric artery). Confounding factors, such as narrowing of the celiac origin by arcuate ligament syndrome or atherosclerotic disease of the celiac and superior mesenteric arteries, as well as their effects on adjacent vasculature, also are important for treatment planning.
4. Lymph node involvement: Suspicious nodes should be documented, particularly those greater than 1 cm in short axis or morphologically abnormal (e.g., rounded nodes, hypodense/heterogeneous/necrotic nodes, nodes with irregular margins); suspicious nodes outside the typical surgical field, such as retroperitoneal, pelvic, and mesenteric nodes, also should be recorded.
5. Distant spread: Evaluation of the liver, peritoneum (including whether ascites is present or absent), bone, and lung should be recorded.

a. Ascites should be noted, as it may indicate peritoneal metastases.

Pathological Classification

Pathological staging depends on surgical resection and pathological examination of the specimen and associated lymph nodes. The College of American Pathologists (CAP) Protocol for the Examination of Specimens from Patients with Carcinoma of the Distal Extrahepatic Bile Ducts is recommended as a guideline for the pathological evaluation of resection specimens for distal bile duct cancer (www.cap.org).

As for the T category, assessment of tumor extension may be difficult because the extrahepatic biliary tree lacks uniform smooth muscle distribution along its length, with scattered or no muscle fibers in the wall of the proximal ducts as compared with the distal bile duct.[20,21] In addition to the problem created by the lack of discrete tissue boundaries, inflammatory changes in the bile ducts and desmoplastic stromal reaction to tumor may cause distortion of the bile duct wall. To overcome these difficulties, the measurement of tumor depth has been adopted in the new classification.[22] This system, however, requires careful perpendicular or longitudinal sectioning of the bile duct so that the deepest tumor invasion (from the basal lamina of adjacent normal or dysplastic epithelium) can be identified and measured. If the depth of invasion is difficult to measure, a best estimate should be given. The level of invasion also should be reported separately (tumor confined to the bile duct, tumor invading beyond the bile duct wall, or tumor extending into an adjacent organ, such as the pancreas, gallbladder, duodenum, or other adjacent organ).

Depth of tumor invasion using the cutoff values of 0.5 cm and 1.2 cm was more powerful than the descriptive extent of tumor invasion in predicting patient outcomes (Fig. 26.2) in several single-institution studies.[20,22] Tumor depth should be measured from the basement membrane of adjacent normal or dysplastic epithelium to the point of deepest tumor invasion in appropriately oriented and sectioned specimens.[22] AJCC Level of Evidence: II

An effort should be made to distinguish a tumor that arises in the intrapancreatic portion of the common bile duct from

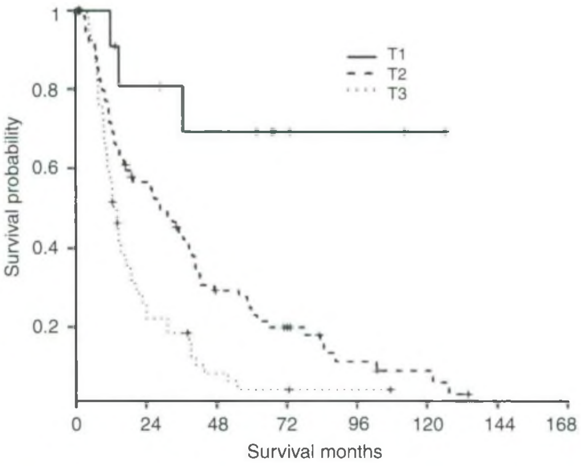

Fig. 26.2 Survival based on T category. Results from 147 US patients who underwent resection of distal bile duct carcinoma confirm earlier study results from 222 Korean patients regarding the use of depth of tumor invasion to predict prognosis and stratify T category. Data from Hong et al.[22]

26

pancreatic cancer, given the differences in tumor biology, patient outcomes, availability of clinical trials, and staging classifications that apply to each clinical entity. Making this distinction, however, may be challenging because of the intimate association of the bile duct with the pancreas and identical immunophenotypic features.[23] Tumors growing symmetrically around the common bile duct are more likely to be distal bile duct carcinomas, whereas an eccentric tumor mass with an epicenter away from the intrapancreatic bile duct more likely is a pancreatic cancer. Another helpful feature that points to distal bile duct origin is the finding of an *in situ* component, such as prominent biliary intraepithelial neoplasia or a biliary intraductal tubular/tubulopapillary neoplasm.[23]

The N category for distal bile duct cancer mirrors that of pancreatic cancer. Specifically, patients are categorized as having no regional lymph node metastasis (N0), metastasis in one to three regional lymph nodes (N1), or metastasis in four or more regional lymph nodes (N2). Tumor involvement of other nodal groups outside the region is considered distant metastasis. Although the minimal number of lymph nodes to be examined for accurate staging has not been determined, examination of at least 12 lymph nodes is recommended.

Accurate pathological staging requires that all lymph nodes that are removed be analyzed. Published studies on optimal histologic examination of a pancreaticoduodenectomy specimen for pancreatic adenocarcinoma support analysis of a minimum of 12 lymph nodes.[24] If the resected lymph nodes do not contain metastatic disease but fewer than 12 are retrieved, pN0 should still be assigned. Anatomic division of regional lymph nodes is not necessary; however, separately submitted lymph nodes should be reported as submitted.

The extent of resection (R0, R1, R2) is an important stage-independent prognostic factor and should be reported.[25,26] Extrahepatic bile duct carcinomas may be multifocal; thus, microscopic foci of carcinoma or intraepithelial neoplasia may be found at the margin(s) and should be reported.

PROGNOSTIC FACTORS

Prognostic Factors Required for Stage Grouping

Beyond the factors used to assign T, N, or M categories, no additional prognostic factors are required for stage grouping.

Additional Factors Recommended for Clinical Care

Extent of Resection

Resection status (R0, complete resection with grossly and microscopically negative margins of resection; R1, grossly negative but microscopically positive margins of resection; R2, grossly and microscopically positive margins of resection) is not part of the TNM staging system, but complete surgical resection with microscopically negative surgical margins is an important predictor of outcome for distal bile duct cancers.[25,26] It is important to confirm complete resection in intraoperative consultation, but prominent inflammation and reactive change of the surface epithelium or within the intramural mucous glands secondary to stent insertion and/or biliary obstruction may hamper the evaluation of margins on frozen section. AJCC Level of Evidence: II

Invasion of Adjacent Organs

Invasion of adjacent organs should be described. Carcinomas that arise in the distal segment of the common bile duct may spread directly into the pancreas, duodenum, gallbladder, colon, stomach, or omentum. In particular, invasion of adjacent pancreas occurs frequently but loses prognostic significance after an adjustment is made for depth of tumor invasion.[22] AJCC Level of Evidence: II

Histologic Parameters

Histologic features have a less significant impact on prognosis than stage. Nevertheless, several histologic parameters, such as high grade (poorly differentiated), perineural invasion, and lymphovascular invasion, are associated with unfavorable patient outcomes and should be noted in the pathology report.[27] High-grade tumors, such as signet ring cell carcinomas, undifferentiated carcinomas, and high-grade neuroendocrine carcinomas, are associated with unfavorable patient outcomes. AJCC Level of Evidence: II

Tumor Markers CEA and CA 19-9

CEA and CA 19-9 are not sensitive enough to be used as screening markers. In addition, these markers are not specific for bile duct cancer, as they may be elevated in other malignancies (e.g., pancreatic, gastric) and in nonneoplastic conditions (e.g., hepatolithiasis, cholangitis). An elevated CA 19-9 level, however, has been associated with unfavorable patient outcomes.[25] AJCC Level of Evidence: III

RISK ASSESSMENT MODELS

The AJCC recently established guidelines that will be used to evaluate published statistical prediction models for the purpose of granting endorsement for clinical use.[28] Although this is a monumental step toward the goal of precision medicine, this work was published only very recently. Therefore, the existing models that have been published or may be in clinical use have not yet been evaluated for this cancer site by the Precision Medicine Core of the AJCC. In the future, the statistical prediction models for this cancer site will be evaluated, and those that meet all AJCC criteria will be endorsed.

DEFINITIONS OF AJCC TNM

Definition of Primary Tumor (T)

T Category	T Criteria
TX	Primary tumor cannot be assessed
Tis	Carcinoma *in situ*/high-grade dysplasia
T1	Tumor invades the bile duct wall with a depth less than 5 mm
T2	Tumor invades the bile duct wall with a depth of 5–12 mm
T3	Tumor invades the bile duct wall with a depth greater than 12 mm
T4	Tumor involves the celiac axis, superior mesenteric artery, and/or common hepatic artery

Definition of Regional Lymph Node (N)

N Category	N Criteria
NX	Regional lymph nodes cannot be assessed
N0	No regional lymph node metastasis
N1	Metastasis in one to three regional lymph nodes
N2	Metastasis in four or more regional lymph nodes

Definition of Distant Metastasis (M)

M Category	M Criteria
M0	No distant metastasis
M1	Distant metastasis

AJCC PROGNOSTIC STAGE GROUPS

When T is...	And N is...	And M is...	Then the stage group is...
Tis	N0	M0	0
T1	N0	M0	I
T1	N1	M0	IIA
T1	N2	M0	IIIA
T2	N0	M0	IIA
T2	N1	M0	IIB
T2	N2	M0	IIIA
T3	N0	M0	IIB
T3	N1	M0	IIB
T3	N2	M0	IIIA
T4	N0	M0	IIIB
T4	N1	M0	IIIB
T4	N2	M0	IIIB
Any T	Any N	M1	IV

REGISTRY DATA COLLECTION VARIABLES

1. Tumor location (ICD code lacks specificity): cystic duct, perihilar bile ducts, or distal bile duct
2. CEA
3. CA 19-9

HISTOLOGIC GRADE (G)

The following grading system is recommended for distal bile duct carcinomas.

G	G Definition
GX	Grade cannot be assessed
G1	Well differentiated
G2	Moderately differentiated
G3	Poorly differentiated

HISTOPATHOLOGIC TYPE

The staging system applies to all carcinomas that arise in the distal extrahepatic bile ducts. Sarcomas, lymphomas, and well-differentiated neuroendocrine tumors are excluded. Adenocarcinoma without specific subtype features is the most common histologic type. Carcinomas account for more than 98% of cancers of the distal extrahepatic bile ducts.

SURVIVAL DATA

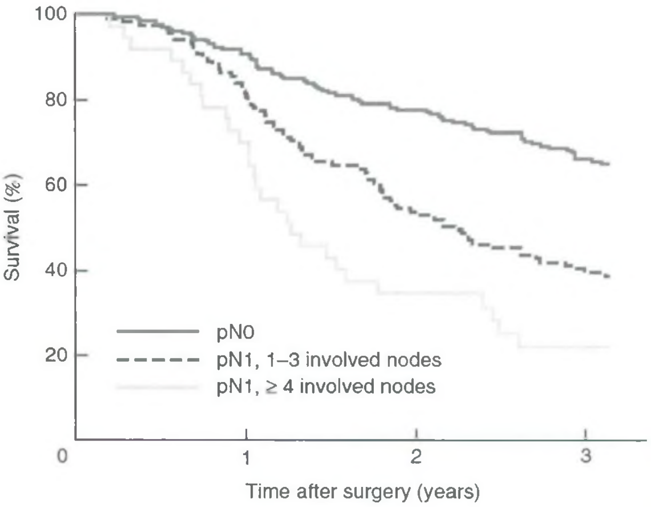

No. at risk				
pN0	213	190	159	131
pN1, 1–3 involved nodes	120	100	65	48
pN1, ≥ 4 involved nodes	37	27	11	7

Fig. 26.3 Survival based on N category. Data from Kiriyama et al.[29]

Bibliography

1. Blechacz B, Komuta M, Roskams T, Gores GJ. Clinical diagnosis and staging of cholangiocarcinoma. *Nature reviews. Gastroenterology & hepatology*. Sep 2011;8(9):512-522.
2. Al-Hawary MM, Francis IR, Chari ST, et al. Pancreatic ductal adenocarcinoma radiology reporting template: consensus statement of the Society of Abdominal Radiology and the American Pancreatic Association. *Radiology*. Jan 2014;270(1):248-260.
3. Al-Hawary MM, Kaza RK, Wasnik AP, Francis IR. Staging of pancreatic cancer: role of imaging. *Seminars in roentgenology*. Jul 2013;48(3):245-252.
4. Tamm EP, Balachandran A, Bhosale PR, et al. Imaging of pancreatic adenocarcinoma: update on staging/resectability. *Radiol Clin North Am*. May 2012;50(3):407-428.
5. Brook OR, Brook A, Vollmer CM, Kent TS, Sanchez N, Pedrosa I. Structured reporting of multiphasic CT for pancreatic cancer: potential effect on staging and surgical planning. *Radiology*. Feb 2015;274(2):464-472.
6. Marcal LP, Fox PS, Evans DB, et al. Analysis of free-form radiology dictations for completeness and clarity for pancreatic cancer staging. *Abdom Imaging*. Oct 2015;40(7):2391-2397.
7. Gottlieb R. CT Onco Primary Pancreas Mass. *RSNA Radiology Reporting Templates* 2012. Accessed 8/13/2015, 2015.
8. Tempero MA, Malafa MP, Asbun H, et al. NCCN Guidelines Version 2.2015 Pancreatic Adenocarcinoma. *NCCN Guidelines* [pdf]. 2015; http://www.nccn.org/professionals/physician_gls/pdf/pancreatic.pdf. Accessed 10/16/2015, 2015.
9. Varadhachary GR, Tamm EP, Abbruzzese JL, et al. Borderline resectable pancreatic cancer: definitions, management, and role of preoperative therapy. *Annals of surgical oncology*. Aug 2006;13(8):1035-1046.
10. Katz MH, Crane CH, Varadhachary G. Management of borderline resectable pancreatic cancer. *Semin Radiat Oncol*. Apr 2014;24(2):105-112.
11. Valls C, Andia E, Sanchez A, et al. Dual-phase helical CT of pancreatic adenocarcinoma: assessment of resectability before surgery. *AJR. American journal of roentgenology*. Apr 2002;178(4):821-826.
12. Tamm EP, Loyer EM, Faria S, et al. Staging of pancreatic cancer with multidetector CT in the setting of preoperative chemoradiation therapy. *Abdom Imaging*. Sep-Oct 2006;31(5):568-574.
13. Cassinotto C, Cortade J, Belleannee G, et al. An evaluation of the accuracy of CT when determining resectability of pancreatic head adenocarcinoma after neoadjuvant treatment. *Eur J Radiol*. Apr 2013;82(4):589-593.
14. DeWitt J, Devereaux B, Chriswell M, et al. Comparison of endoscopic ultrasonography and multidetector computed tomography for detecting and staging pancreatic cancer.[see comment][summary for patients in Ann Intern Med. 2004 Nov 16;141(10):146; PMID: 15545671]. *Annals of internal medicine*. 2004;141(10):753-763.
15. Tamm EP, Loyer EM, Faria SC, Evans DB, Wolff RA, Charnsangavej C. Retrospective analysis of dual-phase MDCT and follow-up EUS/EUS-FNA in the diagnosis of pancreatic cancer. *Abdom Imaging*. Sep-Oct 2007;32(5):660-667.
16. Nikolaidis P, Hammond NA, Day K, et al. Imaging features of benign and malignant ampullary and periampullary lesions. *Radiographics: a review publication of the Radiological Society of North America, Inc*. May-Jun 2014;34(3):624-641.
17. Kim JH, Park SH, Yu ES, et al. Visually isoattenuating pancreatic adenocarcinoma at dynamic-enhanced CT: frequency, clinical and pathologic characteristics, and diagnosis at imaging examinations. *Radiology*. Oct 2010;257(1):87-96.
18. Raman SP, Fishman EK. Abnormalities of the distal common bile duct and ampulla: diagnostic approach and differential diagnosis

using multiplanar reformations and 3D imaging. *AJR. American journal of roentgenology*. Jul 2014;203(1):17-28.

9. Motosugi U, Ichikawa T, Morisaka H, et al. Detection of pancreatic carcinoma and liver metastases with gadoxetic acid-enhanced MR imaging: comparison with contrast-enhanced multi-detector row CT. *Radiology*. Aug 2011;260(2):446-453.

:0. Hong SM, Cho H, Moskaluk CA, Yu E. Measurement of the invasion depth of extrahepatic bile duct carcinoma: An alternative method overcoming the current T classification problems of the AJCC staging system. *The American journal of surgical pathology*. Feb 2007;31(2):199-206.

21. Hong SM, Kim MJ, Pi DY, et al. Analysis of extrahepatic bile duct carcinomas according to the New American Joint Committee on Cancer staging system focused on tumor classification problems in 222 patients. *Cancer*. Aug 15 2005;104(4):802-810.

22. Hong SM, Pawlik TM, Cho H, et al. Depth of tumor invasion better predicts prognosis than the current American Joint Committee on Cancer T classification for distal bile duct carcinoma. *Surgery*. 2009;146(2):250-257.

23. Bledsoe JR, Shinagare SA, Deshpande V. Difficult Diagnostic Problems in Pancreatobiliary Neoplasia. *Arch Pathol Lab Med*. Jul 2015;139(7):848-857.

24. Adsay NV, Basturk O, Altinel D, et al. The number of lymph nodes identified in a simple pancreatoduodenectomy specimen: comparison of conventional vs orange-peeling approach in pathologic assessment. *Modern pathology: an official journal of the United States and Canadian Academy of Pathology, Inc*. Jan 2009;22(1):107-112.

25. Chung YJ, Choi DW, Choi SH, Heo JS, Kim DH. Prognostic factors following surgical resection of distal bile duct cancer. *J Korean Surg Soc*. Nov 2013;85(5):212-218.

26. DeOliveira ML, Cunningham SC, Cameron JL, et al. Cholangiocarcinoma: thirty-one-year experience with 564 patients at a single institution. *Annals of surgery*. 2007;245(5):755.

27. He P, Shi JS, Chen WK, Wang ZR, Ren H, Li H. Multivariate statistical analysis of clinicopathologic factors influencing survival of patients with bile duct carcinoma. *World journal of gastroenterology: WJG*. Oct 2002;8(5):943-946.

28. Kattan MW, Hess KR, Amin MB, et al. American Joint Committee on Cancer acceptance criteria for inclusion of risk models for individualized prognosis in the practice of precision medicine. *CA: a cancer journal for clinicians*. Jan 19 2016.

29. Kiriyama M, Ebata T, Aoba T, et al. Prognostic impact of lymph node metastasis in distal cholangiocarcinoma. *The British journal of surgery*. Mar 2015;102(4):399-406.

Ampulla of Vater

27

Joseph M. Herman, Timothy M. Pawlik,
Nipun B. Merchant, Eric P. Tamm, and Jean-Nicolas Vauthey

CHAPTER SUMMARY

Cancers Staged Using This Staging System

This staging system applies to all primary carcinomas that arise in the ampulla or on the duodenal papilla. Adenocarcinomas are the most common histologic type. This AJCC staging and classification does not apply to well-differentiated neuroendocrine (carcinoid) tumors but does apply to high-grade neuroendocrine carcinomas, such as small cell carcinoma and large cell neuroendocrine carcinoma.

Cancers Not Staged Using This Staging System

These histopathologic types of cancer…	Are staged according to the classification for…	And can be found in chapter…
Well-differentiated neuroendocrine tumor (carcinoid)	Neuroendocrine tumors of the duodenum and ampulla of Vater	30

Summary of Changes

Change	Details of Change	Level of Evidence
Definition of Primary Tumor (T)	T1 tumors have been subdivided into T1a and T1b.<br>T1a: tumor limited to ampulla of Vater or sphincter of Oddi<br>T1b: tumor invades beyond the sphincter of Oddi (perisphincteric invasion) and/or into the duodenal submucosa	III
Definition of Primary Tumor (T)	The T2 definition has been revised to define T2 as invasion into the muscularis propria of the duodenum.	III
Definition of Primary Tumor (T)	T3 tumors have been subdivided into T3a and T3b.<br>T3a: tumor directly invades the pancreas (up to 0.5 cm)<br>T3b: tumor extends more than 0.5 cm into the pancreas or extends into peripancreatic or periduodenal tissue or duodenal serosa, but without involvement of the celiac axis or superior mesenteric artery	III
Definition of Primary Tumor (T)	The T4 definition has been revised to be consistent with the staging system for exocrine pancreas:<br>tumor with vascular involvement of the superior mesenteric artery, celiac axis, and/or common hepatic artery (consistent with pancreas staging).	III
Definition of Regional Lymph Node (N)	N1 is defined as one to three positive regional lymph nodes.	II
Definition of Regional Lymph Node (N)	N2 is defined as metastasis to four or more regional lymph nodes.	II

To access the AJCC cancer staging forms, please visit www.cancerstaging.org.

© American Joint Committee on Cancer 2017

M.B. Amin et al. (eds.), *AJCC Cancer Staging Manual, Eighth Edition*, DOI 10.1007/978-3-319-40618-3_27

ICD-O-3 Topography Codes

Code	Description
C24.1	Ampulla of Vater

WHO Classification of Tumors

Code	Description
8010	Carcinoma *in situ*
8140	Adenocarcinoma
8144	Adenocarcinoma, invasive intestinal type
8163	Adenocarcinoma, pancreatobiliary type
8310	Clear cell adenocarcinoma
8576	Hepatoid adenocarcinoma
8480	Mucinous carcinoma
8490	Signet ring cell carcinoma
8070	Squamous cell carcinoma
8560	Adenosquamous carcinoma
8246	Neuroendocrine carcinoma
8013	Large cell neuroendocrine carcinoma
8041	Small cell neuroendocrine carcinoma
8244	Mixed adenoneuroendocrine carcinoma
8020	Undifferentiated carcinoma
8035	Undifferentiated carcinoma with osteoclast-like giant cells
8163	Noninvasive pancreaticobiliary papillary neoplasm with high-grade dysplasia
8260	Papillary carcinoma, invasive

Bosman FT, Carneiro F, Hruban RH, Theise ND, eds. World Health Organization Classification of Tumours of the Digestive System. Lyon: IARC; 2010.

INTRODUCTION

The ampulla of Vater is the common channel formed by the confluence of the pancreatic and common bile ducts. Most tumors that arise in this small structure obstruct the common bile duct, causing jaundice, abdominal pain, bleeding, and occasionally pancreatitis. Clinically and pathologically, carcinomas of the ampulla may be difficult to differentiate from those arising in the duodenum, the head of the pancreas, or the distal segment of the common bile duct. Primary cancers of the ampulla are not common, accounting for roughly 6% of neoplasms arising in the periampullary region, although these lesions constitute a high proportion of malignant tumors occurring in the duodenum.

The staging of ampullary cancers is highly challenging because of the marked anatomic complexity of the area, the unfamiliarity with the three-dimensional spread patterns of tumors occurring in this region, and the lack of a standardized approach in grossing of ampullary tumors.

Carcinomas of the ampulla of Vater may arise in the mucosa of the confluence of the pancreatic and common bile ducts or in the epithelium covering the papilla of Vater. The proposed changes for staging in the AJCC Cancer Staging Manual, 8th Edition account for the three-dimensional spread patterns of these tumors. T category now accounts for the local extension of the tumor and clarifies the degree and depth of extension into adjacent structures. For example, the definition of T4 is now harmonized with that of other periampullary cancers, such as pancreatic cancer, in which direct extension into arterial structures correlates with locally advanced disease. Based on reviews of pathological staging of ampullary cancers since the last edition, the revised TNM staging more accurately correlates with survival. However, given the rarity of this malignancy, this is not based on level I evidence, and further validation is needed.[1]

In addition to TNM staging, several studies have focused on characterizing ampullary cancers into intestinal or pancreaticobiliary subtypes.[2–4] Although some studies have suggested a significantly worse survival outcome for pancreaticobiliary subtypes, validation of these histologic subtypes as an independent prognostic variable for survival has not been firmly established. A combination of hematoxylin and eosin (H&E) staining, immunohistochemistry, and molecular profiling is needed to characterize these subtypes more accurately before they are officially incorporated into AJCC staging.[5] However, we recommend that these histologic subtypes should be characterized for patient care purposes, as this information may help guide the use of adjuvant therapy based on a pancreaticobiliary regimen versus a gastrointestinal one.

ANATOMY

Primary Site(s)

As the common channel formed by the confluence of the pancreatic and common bile ducts, the ampulla of Vater is composed of four histologically and physiologically distinct anatomic structures: the common bile duct, the pancreatic duct, the duodenum, and the adjoining papilla of Vater. A small dilated duct less than 1.5 cm long, the ampulla is formed by the duodenal aspect of the sphincter of Oddi muscle, which surrounds the confluence of the distal common bile duct and main pancreatic duct, as well as the papilla of Vater, and a mucosal papillary mound at the distal insertion of these ducts on the medial wall of the duodenum. In 42% of individuals, however, the ampulla is the termination of the common bile duct only, whereas the pancreatic duct has its own distinct entrance into the duodenum adjacent to the ampulla. In these individuals, the ampulla may be difficult to locate or may even be nonexistent.

ICD-O-3 Topography Codes

Code	Description
C24.1	Ampulla of Vater

WHO Classification of Tumors

Code	Description
8010	Carcinoma *in situ*
8140	Adenocarcinoma
8144	Adenocarcinoma, invasive intestinal type
8163	Adenocarcinoma, pancreatobiliary type
8310	Clear cell adenocarcinoma
8576	Hepatoid adenocarcinoma
8480	Mucinous carcinoma
8490	Signet ring cell carcinoma
8070	Squamous cell carcinoma
8560	Adenosquamous carcinoma
8246	Neuroendocrine carcinoma
8013	Large cell neuroendocrine carcinoma
8041	Small cell neuroendocrine carcinoma
8244	Mixed adenoneuroendocrine carcinoma
8020	Undifferentiated carcinoma
8035	Undifferentiated carcinoma with osteoclast-like giant cells
8163	Noninvasive pancreaticobiliary papillary neoplasm with high-grade dysplasia
8260	Papillary carcinoma, invasive

Bosman FT, Carneiro F, Hruban RH, Theise ND, eds. World Health Organization Classification of Tumours of the Digestive System. Lyon: IARC; 2010.

INTRODUCTION

The ampulla of Vater is the common channel formed by the confluence of the pancreatic and common bile ducts. Most tumors that arise in this small structure obstruct the common bile duct, causing jaundice, abdominal pain, bleeding, and occasionally pancreatitis. Clinically and pathologically, carcinomas of the ampulla may be difficult to differentiate from those arising in the duodenum, the head of the pancreas, or the distal segment of the common bile duct. Primary cancers of the ampulla are not common, accounting for roughly 6% of neoplasms arising in the periampullary region, although these lesions constitute a high proportion of malignant tumors occurring in the duodenum.

The staging of ampullary cancers is highly challenging because of the marked anatomic complexity of the area, the unfamiliarity with the three-dimensional spread patterns of tumors occurring in this region, and the lack of a standardized approach in grossing of ampullary tumors.

Carcinomas of the ampulla of Vater may arise in the mucosa of the confluence of the pancreatic and common bile ducts or in the epithelium covering the papilla of Vater. The proposed changes for staging in the AJCC Cancer Staging Manual, 8th Edition account for the three-dimensional spread patterns of these tumors. T category now accounts for the local extension of the tumor and clarifies the degree and depth of extension into adjacent structures. For example, the definition of T4 is now harmonized with that of other periampullary cancers, such as pancreatic cancer, in which direct extension into arterial structures correlates with locally advanced disease. Based on reviews of pathological staging of ampullary cancers since the last edition, the revised TNM staging more accurately correlates with survival. However, given the rarity of this malignancy, this is not based on level I evidence, and further validation is needed.[1]

In addition to TNM staging, several studies have focused on characterizing ampullary cancers into intestinal or pancreaticobiliary subtypes.[2–4] Although some studies have suggested a significantly worse survival outcome for pancreaticobiliary subtypes, validation of these histologic subtypes as an independent prognostic variable for survival has not been firmly established. A combination of hematoxylin and eosin (H&E) staining, immunohistochemistry, and molecular profiling is needed to characterize these subtypes more accurately before they are officially incorporated into AJCC staging.[5] However, we recommend that these histologic subtypes should be characterized for patient care purposes, as this information may help guide the use of adjuvant therapy based on a pancreaticobiliary regimen versus a gastrointestinal one.

ANATOMY

Primary Site(s)

As the common channel formed by the confluence of the pancreatic and common bile ducts, the ampulla of Vater is composed of four histologically and physiologically distinct anatomic structures: the common bile duct, the pancreatic duct, the duodenum, and the adjoining papilla of Vater. A small dilated duct less than 1.5 cm long, the ampulla is formed by the duodenal aspect of the sphincter of Oddi muscle, which surrounds the confluence of the distal common bile duct and main pancreatic duct, as well as the papilla of Vater, and a mucosal papillary mound at the distal insertion of these ducts on the medial wall of the duodenum. In 42% of individuals, however, the ampulla is the termination of the common bile duct only, whereas the pancreatic duct has its own distinct entrance into the duodenum adjacent to the ampulla. In these individuals, the ampulla may be difficult to locate or may even be nonexistent.

The ampulla opens into the duodenum, usually on the osteromedial wall, through a small mucosal elevation, the luodenal papilla, which is also called the papilla of Vater. Although carcinomas may arise either in the ampulla or in he mucosa on the papilla, tumors most commonly arise near he junction of the mucosa of the ampulla with that of the apilla. Many times, it may not be possible to determine the xact site of origin for larger tumors; however, tumors of the mpulla must be differentiated from those arising in the second part of the duodenum that invade the ampulla. Identifying he midpoint of duodenal masses and evaluating the presence or absence of involvement of the mucosa of the ampulla nay help make this distinction.

The sphincter of Oddi, a landmark important to recognize or staging purposes, is the collection of delicate muscle ibers that invest the bile and pancreatic ducts as they pass hrough the wall of the duodenum and into the papilla of Vater. The muscle fibers of the sphincter of Oddi are thinner ind more poorly organized than those of the muscularis proria of the duodenum.

Regional Lymph Nodes

A rich lymphatic network surrounds the pancreas and periampullary region, and accurate tumor staging requires that all lymph nodes removed be analyzed. The regional lymph nodes are the peripancreatic lymph nodes, which also include the lymph nodes along the hepatic artery and portal vein (Fig. 27.1).

Metastatic Sites

Metastatic disease is found most commonly in the liver and peritoneum and less commonly in the lungs, pleura, and other organs.

RULES FOR CLASSIFICATION

Clinical Classification

Patients with neoplasms of the ampulla of Vater may present with right upper quadrant pain, obstructive jaundice, weight loss, bleeding, or pancreatitis. The lesions also may be detected incidentally during routine imaging for other indications. Endoscopic ultrasonography (EUS) and computed tomography (CT) are effective in preoperative staging and in evaluating the resectability of ampullary carcinomas. Magnetic resonance (MR) imaging with MR cholangiopancreatography (MRCP) may be helpful, especially in the setting of complete obstruction of the pancreatic duct. Fluorodeoxyglucose positron emission tomography (PET) has not proven useful in the initial evaluation of ampullary neoplasms, although it may be useful in detecting metastatic disease. Laparoscopy occasionally is performed for patients who are believed to have localized, potentially resectable tumors to exclude peritoneal metastases and small metastases on the surface of the liver.

Clinical staging is achieved with radiographic imaging. Ampullary carcinomas may be difficult to distinguish from

27

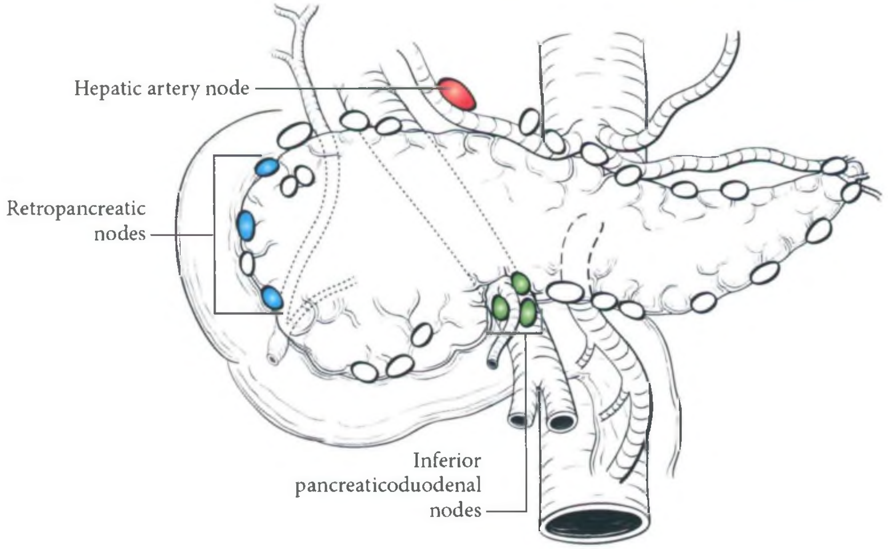

Fig. 27.1 Anatomic distribution of hepatic artery, retropancreatic, and inferior pancreaticoduodenal lymph nodes (regional nodes)

other periampullary malignancies on radiographic imaging (CT and MR imaging), especially if the tumor is advanced or large. The three-dimensional architecture of the tumor also may be difficult to interpret on imaging. EUS may help delineate the origin and extent of the tumor; however, the final diagnosis often relies on the surgically resected specimen (see later). Of note, the tumor markers carcinoembryonic antigen (CEA) and carbohydrate antigen 19-9 (CA 19-9) are not specific to ampullary cancer and cannot be used reliably to distinguish an ampullary cancer from other periampullary tumors.

Imaging[6–23]

Cross-sectional imaging, either contrast-enhanced, multiphasic, thin-section MR imaging or CT, is typically the preferred examination for assessing the stage of pancreatic cancers, ampullary tumors, and distal common bile duct tumors and should be performed before any interventions (e.g., biopsy, stent placement). The choice of MR imaging or CT should be based on the imaging equipment available, the expertise of the radiologists performing and interpreting the studies, and whether there are confounding issues such as allergies to intravenous contrast or renal insufficiency (in the latter case, unenhanced MR imaging is preferred to unenhanced CT because of MR imaging's superior soft tissue contrast). As noted, imaging should be performed before interventions (e.g., stent placement, biopsy) to avoid the effects of potential postprocedure pancreatitis interfering with staging assessments.

If intravenous contrast is used, dynamic imaging (MR imaging or CT) should be performed both during the phase of peak pancreatic enhancement ("pancreatic parenchymal" or "late arterial" phase) to enhance the conspicuity of tumor against the background pancreas (regardless of ampullary, pancreatic, or distal biliary origin) and during the portal venous phase of liver enhancement (peak liver enhancement), when veins are fully opacified, to assess extrapancreatic extent of tumor, involvement of vasculature, and the possibility of liver metastases, as liver metastases from these tumors typically are hypodense against uninvolved liver. Thin-section imaging (2–3 mm for CT, thicker but as thin as reasonably possible for MR imaging) is particularly important for judging vascular involvement and to assess for potential small sites of metastatic disease. In the setting of preoperative therapy, this technique is important, not only at baseline but also following therapy, to determine whether patients are still surgical candidates and to follow up borderline suspicious findings.

EUS may be used next after CT/MR imaging. Its ability to detect small tumors and to guide biopsy is particularly helpful for tumors that may appear isodense to, and therefore indistinguishable from, background pancreas on CT and/or MR imaging. For ampullary tumors and periampullary duodenal tumors, endoscopic evaluation is particularly helpful, as CT and MR imaging have limited utility in evaluating the intraluminal component of tumors. EUS and EUS/fine-needle aspiration also should be performed before ERCP, because pancreatitis may degrade the ability of EUS to visualize tumor and stent placement eliminates the ability to identify sites of duct cutoff that may be useful to guide biopsies. ERCP may be helpful in the setting of duct abnormalities, both for treatment (stent placement) and for diagnosis (brushings).

TNM Categories of Staging by Imaging

The relationship of the tumor to relevant vessels should be reported, specifically its relation to arteries such as the superior mesenteric, celiac, splenic, and common hepatic arteries, as well as the aorta if the tumor extends posteriorly into the retroperitoneum. The relationship of the tumor to relevant veins, including the portal vein, splenic vein, splenoportal confluence, and superior mesenteric vein, as well as to branch vessels such as the gastrocolic trunk, first jejunal vein, and ileocolic branches, also should be recorded.

The relationship of the tumor to the vessels should be described using terms commonly understood by the clinical community, such as degrees of circumferential involvement, or the terms *abutment* (i.e., up to and including 180° of involvement of a given vessel by tumor) and *encasement* (i.e., >180° of circumferential vessel involvement by tumor). Multiplanar reconstructions for CT, as well as direct multiplanar imaging for MR imaging, may be particularly helpful in visualizing the circumferential relationship of the tumor to relevant vasculature. It also is important to describe the relationship of the tumor to adjacent structures, such as the stomach, spleen, colon, small bowel, and adrenal glands.

Assessment of N category (nodal) status may be a challenge for all imaging modalities, because preoperative imaging is limited and cannot detect microscopic metastatic disease. Nevertheless, it is important to fully identify the location of visibly suspicious nodes. Nodes are considered suspicious for metastatic involvement if they are greater than 1 cm in short axis or have an abnormal morphology (e.g., they are rounded, are hypodense or heterogeneous, have irregular margins, involve adjacent vessels or structures). Lymph nodes outside the usual surgical field—for example, retroperitoneal nodes, pelvic nodes, or lymph nodes within the jejunal mesentery or ileocolic mesentery—also should be evaluated and reported if abnormal.

The most common sites of metastatic disease include the liver, peritoneum, and lung. Evaluation for potential metastatic disease is best done with contrast-enhanced CT or MR imaging.

Suggested Radiology Report Format

Tumor involvement with adjacent vasculature should be reported with terms generally understood by the oncology community, such as degrees of circumferential involvement by tumor of a given vessel or the terms *abutment* and *encasement*, as defined earlier.

The radiology report should include detailed descriptions of the following:

1. Primary tumor: location, size, characterization and effect on ducts (common bile duct and main pancreatic duct). Details regarding any findings suspicious for superimposed acute pancreatitis, which may distort findings relevant to staging, or chronic pancreatitis/autoimmune pancreatitis also should be reported, as these diseases may closely mimic malignancy and may be associated with duct strictures.
2. Local extent: the relationship of the tumor, with reference to degrees of circumferential involvement using commonly understood terms such as *abutment* and *encasement*, and occlusion with regard to adjacent arterial structures (celiac, superior mesenteric, hepatic, and splenic arteries and the aorta) and venous structures (portal, splenic, and superior mesenteric veins, and if relevant, inferior vena cava). The following should be noted:
   a. How much of the vascular involvement is related to solid tumor versus stranding, and whether vessel involvement is related to direct involvement by tumor or is separate from the tumor
   b. Narrowing of vasculature, the presence of vascular thrombi, and the length of tumor involvement with the vasculature
   c. The presence of enlarged collaterals or varices
   d. Involvement of branch vessels, such as the gastrocolic, first jejunal, and ileocolic branches of the superior mesenteric vein
3. Relevant arterial variants: This is particularly important with regard to hepatic arterial variants, such as those arising from the superior mesenteric artery, and the nature of the variant (e.g., accessory right hepatic vs. common hepatic artery arising from the superior mesenteric artery). Confounding factors such as narrowing of the celiac origin by arcuate ligament syndrome or atherosclerotic disease of the celiac and superior mesenteric arteries, as well as their effects on adjacent vasculature, also are important to note for treatment planning.
4. Lymph node involvement: Suspicious nodes should be documented, particularly those greater than 1 cm in short axis and those that are morphologically abnormal (e.g., are rounded or hypodense/heterogeneous/necrotic or have irregular margins); suspicious nodes outside the typical surgical field, such as retroperitoneal, pelvic, and mesenteric nodes, also should be recorded.
5. Distant spread: Evaluation of the liver, peritoneum (including whether ascites is present or absent), bone, and lung should be recorded.
   a. Ascites should be noted, as it may indicate peritoneal metastases.

Pathological Classification

Pathological staging depends on surgical resection, typically a pancreaticoduodenectomy, with pathological examination of the specimen and associated lymph nodes. The finding of metastatic disease in the regional lymph nodes has a significant adverse impact on survival, with 5-year overall survival decreasing from 63% for patients who had no nodal metastasis to as low as 40% for patients with at least one node with metastatic disease. Some studies have reported no long-term survivors among patients who have four or more metastatic lymph nodes.[24,25] Removal of ≥12 lymph nodes has been shown to correlate with survival among patients with resected ampullary cancer.[26,27] The completeness of resection (R0, complete resection with no residual tumor; R1, microscopic residual tumor; R2, macroscopic residual tumor) is not part of the TNM staging system but is of prognostic importance. Although tumor size is not part of the T category, the size of the component invading the surrounding duodenum and/or pancreas should be documented and distinguished from the total size of the lesion, which may include both noninvasive and invasive components.[1]

Survival analyses based on the previous T category classification noted an improved survival for patients with T2 tumors versus those with T1 tumors.[1] However, additional analysis of subsets of T1, T2, and T3 lesions suggests further prognostic variability. Therefore, a more clinically relevant reclassification of T1 and T2 tumors is proposed in the 8th Edition. T1a tumors now are defined as those limited to the ampulla of Vater or sphincter of Oddi, whereas T1b tumors invade beyond the sphincter of Oddi (perisphincteric invasion) and/or into the duodenal submucosa. T2 tumors are reclassified as those that invade into the muscularis propria of the duodenum.[28] T3 tumors are now subdivided into T3a lesions, which show direct pancreas invasion up to 0.5 cm, and T3b lesions, which extend more than 0.5 cm into the pancreas or extend into peripancreatic or periduodenal tissue or duodenal serosa without involving the celiac axis or superior mesenteric artery. To harmonize with pancreatic cancer staging, T4 tumors are reclassified to those that involve the celiac axis, superior mesenteric artery, and/or common hepatic artery.

Tumors arising from the papilla of Vater (the edge of the ampulla neighboring the duodenum) that do not invade into the muscularis propria of the duodenum should be classified as T1a. Heterotopic lobules of pancreatic tissue found on the wall of the ampulla and duodenum should not be classified as T3 unless there is true pancreatic invasion.

Anatomic division of regional lymph nodes is not necessary. However, separately submitted lymph nodes should be reported separately. Optimal histologic examination of a pancreaticoduodenectomy specimen should include analysis of a minimum of 12 lymph nodes.[1] The number of lymph nodes sampled and the number of involved lymph nodes should be recorded. If the resected lymph nodes are negative, but the minimum number of 12 is not met, pN0 should still be assigned.

Lymph node metastasis in patients with adenocarcinoma of the ampulla of Vater is consistently reported to be a predictor of poor outcome, although it does not appear to be as powerful a predictor of disease recurrence or decreased survival as it is for pancreatic adenocarcinoma.[25,29–32] A Surveillance, Epidemiology, and End Results (SEER) analysis published in 2014 showed significant differences in survival rates when nodal metastases were categorized as N0 (no metastatic lymph node), N1 (one or two metastatic lymph nodes), and N2 (three or more metastatic lymph nodes).[27,33] AJCC Level of Evidence: II

PROGNOSTIC FACTORS

Prognostic Factors Required for Stage Grouping

Beyond the factors used to assign T, N, or M categories, no additional prognostic factors are required for stage grouping.

Additional Factors Recommended for Clinical Care

Tumor Size

Although tumor size is not part of the TNM classification, the size of the tumor and that of the invasive component should be reported carefully.[34,35] Even among patients who undergo a potentially curative resection, the size of the tumor and presence of tumor invasion into the pancreas are associated with a less favorable outcome.[35,36] Histologic evidence of tumor extension from the ampulla into the pancreatic parenchyma appears to reflect the extent of both local and regional disease.[29,35,36] Furthermore, lymphovascular and perineural invasion also are adverse pathological factors.[25,35,37] AJCC Level of Evidence: II

Margin Status

Tumor involvement (R1, R2) of resection margins repeatedly has been demonstrated to be an adverse prognostic factor.[31,38,39] As such, surgical margin status (R0, R1, or R2) should be reported, and if malignant disease is at the margins, then the margin that is specifically involved should be specified (e.g., bile duct, pancreatic neck). If the margins are negative, it is recommended that the distance to the nearest margin be reported. AJCC Level of Evidence: II

Histologic Differentiation

High histopathologic grade (poor vs. well- or moderately differentiated tumors) has been shown to correlate with adverse survival outcomes in patients with ampullary malignancies.[2,29] Furthermore, tumors with papillary histology have a better outcome than nonpapillary tumors.[2] Some investigators have suggested that ampullary cancer is simply an extension of duodenal or pancreatic cancer into the ampulla, and therefore, survival likely is dictated more by the characteristics of these tumors (intestinal or pancreaticobiliary histologic subtype). As a result, some have suggested that ampullary tumors be categorized as part of pancreas or duodenal staging as opposed to its own entity. Further research on the pathogenesis and genetic profile of these tumors will lead to more consistent staging of ampullary cancers in the future. AJCC Level of Evidence: II

Histologic Subtypes

It has been suggested that histologic subtyping has prognostic significance and may guide treatment, although studies are not conclusive and the topic remains controversial. Analysis of CK20, CDX2, MUC1, and MUC2 immunohistochemistry in conjunction with H&E study allows for a dichotomous classification of pancreaticobiliary and intestinal subtypes in 92% of cases.[5] Several retrospective studies reported improved survival in patients with intestinal subtypes and an increased risk of recurrence in those with the pancreaticobiliary type.[3,4] However, a prospective randomized cooperative group study exploring the role of adjuvant therapy in periampullary cancers found no significant improvement in the pancreaticobiliary type compared with the intestinal type.[40] AJCC Level of Evidence: III

Preoperative or Pretreatment Serum CEA and CA 19-9

The serum markers CEA and CA 19-9 may have prognostic significance but are not specific for ampullary cancer. Obtaining these values before surgery or the onset of treatment may be useful in assessing treatment response.[3,41–43] However, few studies have explored the value of CEA and CA 19-9 levels in ampullary tumors. CEA appears to be an

important predictor of recurrence in patients with the intestinal subtype of ampullary cancer, whereas CA 19-9 levels ≤37 U/mL were predictive of prolonged disease-free survival.[3,41] AJCC Level of Evidence: III

Adjuvant Therapy

Data supporting the role of adjuvant chemotherapy are limited because of the lack of prospective studies as well as the grouping of ampullary carcinomas with other periampullary cancers in many prospective studies.[44] Heterogeneity in staging, patient population, and treatment regimens makes it difficult to determine the relative benefit of therapy in this rare group of patients. However, a prospective randomized study (ESPAC-3) with the largest number of ampullary cancers showed that adjuvant chemotherapy may play a role in improving survival outcomes in ampullary malignancies.[40] AJCC Level of Evidence: III

RISK ASSESSMENT MODELS

The AJCC recently established guidelines that will be used to evaluate published statistical prediction models for the purpose of granting endorsement for clinical use.[45] Although this is a monumental step toward the goal of precision medicine, this work was published only very recently. Therefore, the existing models that have been published or may be in clinical use have not yet been evaluated for this cancer site by the Precision Medicine Core of the AJCC. In the future, the statistical prediction models for this cancer site will be evaluated, and those that meet all AJCC criteria will be endorsed.

DEFINITIONS OF AJCC TNM

Definition of Primary Tumor (T)

T Category	T Criteria
TX	Primary tumor cannot be assessed
T0	No evidence of primary tumor
Tis	Carcinoma *in situ*
T1	Tumor limited to ampulla of Vater or sphincter of Oddi or tumor invades beyond the sphincter of Oddi (perisphincteric invasion) and/or into the duodenal submucosa
T1a	Tumor limited to ampulla of Vater or sphincter of Oddi
T1b	Tumor invades beyond the sphincter of Oddi (perisphincteric invasion) and/or into the duodenal submucosa
T2	Tumor invades into the muscularis propria of the duodenum
T3	Tumor directly invades the pancreas (up to 0.5 cm) or tumor extends more than 0.5 cm into the pancreas, or extends into peripancreatic or periduodenal tissue or duodenal serosa without involvement of the celiac axis or superior mesenteric artery
T3a	Tumor directly invades pancreas (up to 0.5 cm)
T3b	Tumor extends more than 0.5 cm into the pancreas, or extends into peripancreatic tissue or duodenal serosa without involvement of the celiac axis or superior mesenteric artery
T4	Tumor involves the celiac axis, superior mesenteric artery, and/or common hepatic artery, irrespective of size

Definition of Regional Lymph Node (N)

N Category	N Criteria
NX	Regional lymph nodes cannot be assessed
N0	No regional lymph node metastasis
N1	Metastasis to one to three regional lymph nodes
N2	Metastasis to four or more regional lymph nodes

Definition of Distant Metastasis (M)

M Category	M Criteria
M0	No distant metastasis
M1	Distant metastasis

AJCC PROGNOSTIC STAGE GROUPS

When T is...	And N is...	And M is...	Then the stage group is...
Tis	N0	M0	0
T1a	N0	M0	IA
T1a	N1	M0	IIIA
T1b	N0	M0	IB
T1b	N1	M0	IIIA
T2	N0	M0	IB
T2	N1	M0	IIIA
T3a	N0	M0	IIA
T3a	N1	M0	IIIA
T3b	N0	M0	IIB
T3b	N1	M0	IIIA
T4	Any N	M0	IIIB
Any T	N2	M0	IIIB
Any T	Any N	M1	IV

REGISTRY DATA COLLECTION VARIABLES

1. Tumor size
2. Lymph node status
3. Margin status
4. Histologic differentiation
5. Histologic subtype
6. Preoperative or pretreatment CEA
7. Preoperative or pretreatment CA 19-9
8. Adjuvant therapy

HISTOLOGIC GRADE (G)

G	G Definition
GX	Grade cannot be assessed
G1	Well differentiated
G2	Moderately differentiated
G3	Poorly differentiated

HISTOPATHOLOGIC TYPE

The histology of ampullary carcinomas more often resembles that of adenomas and adenocarcinomas of intestinal origin rather than those of pancreaticobiliary origin. Of 170 pure adenocarcinoma histologic subtypes, most include intestinal (47%) followed by pancreaticobiliary (24%) and less commonly poorly differentiated adenocarcinoma (13%), intestinal–mucinous (8%), or invasive papillary (5%) carcinomas.[2]

Bibliography

1. Adsay NV, Bagci P, Tajiri T, et al. Pathologic staging of pancreatic, ampullary, biliary, and gallbladder cancers: pitfalls and practical limitations of the current AJCC/UICC TNM staging system and opportunities for improvement. Paper presented at: Seminars in diagnostic pathology2012.
2. Ruemmele P, Dietmaier W, Terracciano L, et al. Histopathologic features and microsatellite instability of cancers of the papilla of vater and their precursor lesions. *The American journal of surgical pathology*. 2009;33(5):691–704.
3. Kim WS, Choi DW, Choi SH, Heo JS, You DD, Lee HG. Clinical significance of pathologic subtype in curatively resected ampulla of vater cancer. *Journal of surgical oncology*. Mar 2012;105(3): 266–272.
4. Perysinakis I, Margaris I, Kouraklis G. Ampullary cancer--a separate clinical entity? *Histopathology*. May 2014;64(6):759–768.
5. Ang DC, Shia J, Tang LH, Katabi N, Klimstra DS. The utility of immunohistochemistry in subtyping adenocarcinoma of the ampulla of vater. *The American journal of surgical pathology*. Oct 2014;38(10):1371–1379.
6. Al-Hawary MM, Francis IR, Chari ST, et al. Pancreatic ductal adenocarcinoma radiology reporting template: consensus statement of the Society of Abdominal Radiology and the American Pancreatic Association. *Radiology*. Jan 2014;270(1):248–260.
7. Al-Hawary MM, Kaza RK, Wasnik AP, Francis IR. Staging of pancreatic cancer: role of imaging. *Seminars in roentgenology*. Jul 2013;48(3):245–252.
8. Tamm EP, Balachandran A, Bhosale PR, et al. Imaging of pancreatic adenocarcinoma: update on staging/resectability. *Radiol Clin North Am*. May 2012;50(3):407–428.
9. Brook OR, Brook A, Vollmer CM, Kent TS, Sanchez N, Pedrosa I. Structured reporting of multiphasic CT for pancreatic cancer: potential effect on staging and surgical planning. *Radiology*. Feb 2015;274(2):464–472.
10. Marcal LP, Fox PS, Evans DB, et al. Analysis of free-form radiology dictations for completeness and clarity for pancreatic cancer staging. *Abdom Imaging*. Oct 2015;40(7):2391–2397.
11. Gottlieb R. CT Onco Primary Pancreas Mass. *RSNA Radiology Reporting Templates* 2012. Accessed 8/13/2015, 2015.
12. Tempero MA, Malafa MP, Asbun H, et al. NCCN Guidelines Version 2.2015 Pancreatic Adenocarcinoma. *NCCN Guidelines* [pdf]. 2015; http://www.nccn.org/professionals/physician_gls/pdf/pancreatic.pdf. Accessed 10/16/2015, 2015.
13. Varadhachary GR, Tamm EP, Abbruzzese JL, et al. Borderline resectable pancreatic cancer: definitions, management, and role of preoperative therapy. *Annals of surgical oncology*. Aug 2006;13(8): 1035–1046.
14. Katz MH, Crane CH, Varadhachary G. Management of borderline resectable pancreatic cancer. *Semin Radiat Oncol*. Apr 2014;24(2): 105–112.
15. Valls C, Andia E, Sanchez A, et al. Dual-phase helical CT of pancreatic adenocarcinoma: assessment of resectability before surgery. *AJR. American journal of roentgenology*. Apr 2002;178(4): 821–826.
16. Tamm EP, Loyer EM, Faria S, et al. Staging of pancreatic cancer with multidetector CT in the setting of preoperative chemoradiation therapy. *Abdom Imaging*. Sep-Oct 2006;31(5):568–574.
17. Cassinotto C, Cortade J, Belleannee G, et al. An evaluation of the accuracy of CT when determining resectability of pancreatic head adenocarcinoma after neoadjuvant treatment. *Eur J Radiol*. Apr 2013;82(4):589–593.
18. DeWitt J, Devereaux B, Chriswell M, et al. Comparison of endoscopic ultrasonography and multidetector computed tomography for detecting and staging pancreatic cancer.[see comment][summary for patients in Ann Intern Med. 2004 Nov 16;141(10):I46; PMID: 15545671]. *Annals of internal medicine*. 2004;141(10):753–763.
19. Tamm EP, Loyer EM, Faria SC, Evans DB, Wolff RA, Charnsangavej C. Retrospective analysis of dual-phase MDCT and follow-up EUS/EUS-FNA in the diagnosis of pancreatic cancer. *Abdom Imaging*. Sep-Oct 2007;32(5):660–667.
20. Nikolaidis P, Hammond NA, Day K, et al. Imaging features of benign and malignant ampullary and periampullary lesions. *Radiographics : a review publication of the Radiological Society of North America, Inc*. May-Jun 2014;34(3):624–641.
21. Kim JH, Park SH, Yu ES, et al. Visually isoattenuating pancreatic adenocarcinoma at dynamic-enhanced CT: frequency, clinical and pathologic characteristics, and diagnosis at imaging examinations. *Radiology*. Oct 2010;257(1):87–96.
22. Raman SP, Fishman EK. Abnormalities of the distal common bile duct and ampulla: diagnostic approach and differential diagnosis using multiplanar reformations and 3D imaging. *AJR. American journal of roentgenology*. Jul 2014;203(1):17–28.
23. Motosugi U, Ichikawa T, Morisaka H, et al. Detection of pancreatic carcinoma and liver metastases with gadoxetic acid-enhanced MR imaging: comparison with contrast-enhanced multi-detector row CT. *Radiology*. Aug 2011;260(2):446–453.
24. Narang AK, Miller RC, Hsu CC, et al. Evaluation of adjuvant chemoradiation therapy for ampullary adenocarcinoma: the Johns Hopkins Hospital-Mayo Clinic collaborative study. *Radiation oncology*. 2011;6:126.

5. Winter JM, Cameron JL, Olino K, et al. Clinicopathologic analysis of ampullary neoplasms in 450 patients: implications for surgical strategy and long-term prognosis. *Journal of gastrointestinal surgery : official journal of the Society for Surgery of the Alimentary Tract*. Feb 2010;14(2):379–387.
6. Partelli S, Crippa S, Capelli P, et al. Adequacy of lymph node retrieval for ampullary cancer and its association with improved staging and survival. *World journal of surgery*. Jun 2013;37(6):1397–1404.
7. Balci S, Basturk O, Saka B, et al. Substaging Nodal Status in Ampullary Carcinomas has Significant Prognostic Value: Proposed Revised Staging Based on an Analysis of 313 Well-Characterized Cases. *Annals of surgical oncology*. 2015:1–10.
8. You D, Heo J, Choi S, Choi D, Jang K-T. Pathologic t1 subclassification of ampullary carcinoma with perisphincteric or duodenal submucosal invasion: is it t1b? *Archives of pathology & laboratory medicine*. 2014;138(8):1072.
9. Hsu HP, Yang TM, Hsieh YH, Shan YS, Lin PW. Predictors for patterns of failure after pancreaticoduodenectomy in ampullary cancer. *Annals of surgical oncology*. Jan 2007;14(1):50–60.
0. Roder J, Schneider P, Stein H, Siewert J. Number of lymph node metastases is significantly associated with survival in patients with radically resected carcinoma of the ampulla of Vater. *British journal of surgery*. 1995;82(12):1693–1696.
1. Howe JR, Klimstra DS, Moccia RD, Conlon KC, Brennan MF. Factors predictive of survival in ampullary carcinoma. *Annals of surgery*. Jul 1998;228(1):87–94.
2. Qiao QL, Zhao YG, Ye ML, et al. Carcinoma of the ampulla of Vater: factors influencing long-term survival of 127 patients with resection. *World journal of surgery*. Jan 2007;31(1):137–143; discussion 144–136.
3. Kang HJ, Eo S-H, Kim SC, et al. Increased number of metastatic lymph nodes in adenocarcinoma of the ampulla of Vater as a prognostic factor: A proposal of new nodal classification. *Surgery*. 2014;155(1):74–84.
4. Klempnauer J, Ridder GJ, Pichlmayr R. Prognostic factors after resection of ampullary carcinoma: multivariate survival analysis in comparison with ductal cancer of the pancreatic head. *The British journal of surgery*. Dec 1995;82(12):1686–1691.
5. Nakai T, Koh K, Kawabe T, Son E, Yoshkawa H, Yasutomi M. Importance of microperineural invasion as a prognostic factor in ampullary carcinoma. *British journal of Surgery*. 1997;84(10):1399–1401.
6. Willett CG, Warshaw AL, Convery K, Compton CC. Patterns of failure after pancreaticoduodenectomy for ampullary carcinoma. *Surg Gynecol Obstet*. Jan 1993;176(1):33–38.
37. Carter JT, Grenert JP, Rubenstein L, Stewart L, Way LW. Tumors of the ampulla of vater: histopathologic classification and predictors of survival. *Journal of the American College of Surgeons*. Aug 2008;207(2):210–218.
38. Todoroki T, Koike N, Morishita Y, et al. Patterns and predictors of failure after curative resections of carcinoma of the ampulla of Vater. *Annals of surgical oncology*. Dec 2003;10(10):1176–1183.
39. Allema JH, Reinders ME, van Gulik TM, et al. Results of pancreaticoduodenectomy for ampullary carcinoma and analysis of prognostic factors for survival. *Surgery*. Mar 1995;117(3):247–253.
40. Neoptolemos JP, Moore MJ, Cox TF, et al. Effect of adjuvant chemotherapy with fluorouracil plus folinic acid or gemcitabine vs observation on survival in patients with resected periampullary adenocarcinoma: the ESPAC-3 periampullary cancer randomized trial. *JAMA*. Jul 11 2012;308(2):147–156.
41. Klein F, Jacob D, Bahra M, et al. Prognostic factors for long-term survival in patients with ampullary carcinoma: the results of a 15-year observation period after pancreaticoduodenectomy. *HPB surgery : a world journal of hepatic, pancreatic and biliary surgery*. 2014;2014:970234.
42. Yamaguchi K, Enjoji M, Tsuneyoshi M. Pancreatoduodenal carcinoma: a clinicopathologic study of 304 patients and immunohistochemical observation for CEA and CA19-9. *Journal of surgical oncology*. Jul 1991;47(3):148–154.
43. Berger AC, Winter K, Hoffman JP, et al. Five year results of US intergroup/RTOG 9704 with postoperative CA 19-9 </=90 U/mL and comparison to the CONKO-001 trial. *International journal of radiation oncology, biology, physics*. Nov 1 2012;84(3):e291–297.
44. Jabbour SK, Mulvihill D. Defining the Role of Adjuvant Therapy: Ampullary and Duodenal Adenocarcinoma. Paper presented at: Seminars in radiation oncology2014.
45. Kattan MW, Hess KR, Amin MB, et al. American Joint Committee on Cancer acceptance criteria for inclusion of risk models for individualized prognosis in the practice of precision medicine. *CA: a cancer journal for clinicians*. Jan 19 2016.
46. Balachandran P, Sikora SS, Kapoor S, et al. Long-term survival and recurrence patterns in ampullary cancer. *Pancreas*. May 2006;32(4):390–395.
47. Talamini MA, Moesinger RC, Pitt HA, et al. Adenocarcinoma of the ampulla of Vater. A 28-year experience. *Annals of surgery*. May 1997;225(5):590–599; discussion 599–600.

Exocrine Pancreas

28

Sanjay Kakar, Timothy M. Pawlik, Peter J. Allen, and Jean-Nicolas Vauthey

CHAPTER SUMMARY

Cancers Staged Using This Staging System

Pancreatic ductal adenocarcinoma, acinar cell carcinoma, intraductal papillary mucinous neoplasm with associated invasive carcinoma, intraductal tubulopapillary neoplasm with associated invasive carcinoma, colloid carcinoma, mucinous cystic neoplasm with associated invasive carcinoma, solid pseudopapillary neoplasm, large cell neuroendocrine carcinoma, small cell neuroendocrine carcinoma, pancreaticoblastoma

Cancers Not Staged Using This Staging System

These histopathologic types of cancer…	Are staged according to the classification for…	And can be found in chapter…
Well-differentiated neuroendocrine tumor	Neuroendocrine tumors of the pancreas	34

Summary of Changes

Change	Details of Change	Level of Evidence
Definition of Primary Tumor (T)	T1 are subcategorized into T1a, T1b, and T1c based on size.<br>Rationale: Size-based categorization of small invasive tumors that have been characterized as "minimally invasive" and have better outcome.	III
Definition of Primary Tumor (T)	T2 and T3 categories are now based on size of invasive tumor; extrapancreatic extension is no longer part of the definition.<br>Rationale: Size-based definitions are more objective as it is difficult to determine extrapancreatic extension. These definitions show better correlation with survival.	II
Definition of Primary Tumor (T)	T4 categorization is now based on involvement of arteries; resectability has been removed from the definition.<br>Rationale: Resectability is subjective, and the T category is better defined by extent of invasion.	II
Definition of Regional Lymph Node (N)	Node-positive disease N1 has been subdivided into N1 and N2, based on number of positive lymph nodes.<br>Rationale: Better prognostic stratification is provided based on number of positive lymph nodes.	II

To access the AJCC cancer staging forms, please visit www.cancerstaging.org.

© American Joint Committee on Cancer 2017

M.B. Amin et al. (eds.), *AJCC Cancer Staging Manual, Eighth Edition*, DOI 10.1007/978-3-319-40618-3_28

ICD-O-3 Topography Codes

Code	Description
C25.0	Head of pancreas
C25.1	Body of pancreas
C25.2	Tail of pancreas
C25.3	Pancreatic duct
C25.7	Other specified part of pancreas
C25.8	Overlapping lesion of pancreas
C25.9	Pancreas, NOS

WHO Classification of Tumors

Code	Description
8148	Pancreatic intraepithelial neoplasia, high grade (PanIN-3)
8453	Intraductal papillary mucinous neoplasm with high-grade dysplasia
8503	Intraductal tubulopapillary neoplasm with high-grade dysplasia
8470	Mucinous cystic neoplasm with high-grade dysplasia
8500	Ductal adenocarcinoma
8560	Adenosquamous carcinoma
8576	Hepatoid carcinoma
8510	Medullary carcinoma
8480	Mucinous noncystic carcinoma (colloid carcinoma)
8490	Signet ring cell carcinoma
8020	Undifferentiated carcinoma
8035	Undifferentiated carcinoma with osteoclast-like giant cells
8550	Acinar cell carcinoma
8551	Acinar cell cystadenocarcinoma
8453	Intraductal papillary mucinous neoplasm with associated invasive carcinoma
8503	Intraductal tubulopapillary neoplasm with associated invasive carcinoma
8470	Mucinous cystic neoplasm with associated invasive carcinoma
8971	Pancreaticoblastoma
8441	Serous cystadenocarcinoma
8452	Solid pseudopapillary neoplasm
8246	High-grade neuroendocrine carcinoma
8041	Small cell neuroendocrine carcinoma
8013	Large cell neuroendocrine carcinoma
8552	Mixed acinar–ductal carcinoma
8154	Mixed acinar–neuroendocrine carcinoma
8154	Mixed acinar–neuroendocrine–ductal carcinoma
8154	Mixed ductal–neuroendocrine carcinoma

Bosman FT, Carneiro F, Hruban RH, Theise ND, eds. World Health Organization Classification of Tumours of the Digestive System. Lyon: IARC; 2010.

INTRODUCTION

In the United States, pancreatic cancer is the second most common malignant tumor of the gastrointestinal tract and the fourth leading cause of cancer-related death in adults. The American Cancer Society estimated nearly 49,000 new cases in the United States and approximately 41,000 deaths in 2015. Based on Surveillance, Epidemiology, and End Results (SEER) data from 2005 to 2011, the 5-year survival is 7.2%: 27.1% for localized disease, 10.7% for regional disease, and 2.4% for metastatic disease. Most pancreatic cancers are ductal adenocarcinomas. Surgical resection remains the only potentially curative approach, although multimodal therapy consisting of systemic agents, and often radiation, may improve survival. Staging of pancreatic cancers depends on the size and extent of the primary tumor and the presence and extent of metastasis.

ANATOMY

Primary Site(s)

The pancreas is a coarsely lobulated gland that lies transversely across the posterior abdomen and extends from the duodenum to the splenic hilum. The organ is divided into a head with an uncinate process, a neck, a body, and a tail (Fig. 28.1). The anterior aspect of the body of the pancreas is in direct contact with the posterior wall of the stomach; posteriorly, the pancreas extends to the inferior vena cava, superior mesenteric vein, splenic vein, and left kidney. The uncinate process is part of the pancreatic head and may extend posterior to the superior mesenteric vein and artery. The pancreatic head accounts for 60–70% of pancreatic adenocarcinomas, whereas 20–25% arise in the body and tail and 10–20% diffusely involve the pancreas.[1]

Regional Lymph Nodes

A rich lymphatic network surrounds the pancreas, and accurate tumor staging requires resection of peripancreatic lymph nodes for pathological assessment. The standard regional lymph node basins and soft tissues resected for tumors located in the head and neck of the pancreas include lymph nodes along the common bile duct, common hepatic artery, portal vein, pyloric, posterior and anterior pancreatoduodenal arcades, and along the superior mesenteric vein and right lateral wall of the superior mesenteric artery. For cancers located in the body and tail, regional lymph node basins include lymph nodes along the common hepatic artery, celiac axis, splenic artery, and splenic hilum. Tumor involvement of other nodal groups is considered distant metastasis.

Metastatic Sites

More than half of all patients with pancreatic cancer have distant metastases at presentation. The most commonly

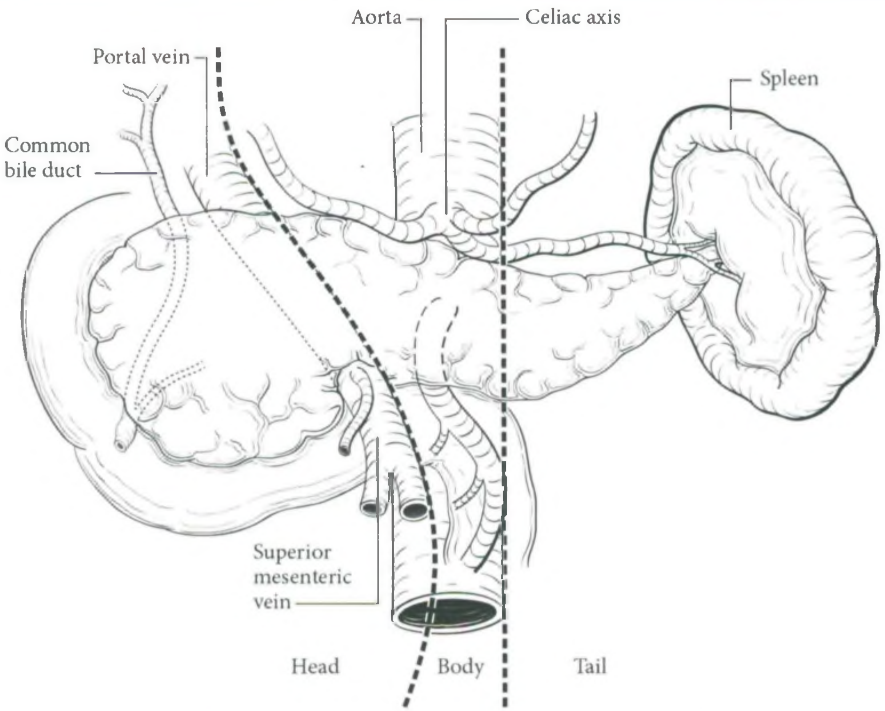

Fig. 28.1 Tumors of the head of the pancreas are those arising to the right of the superior mesenteric-portal vein confluence. Tumors of the body of the pancreas are those arising between the left border of the superior mesenteric vein and the left border of the aorta. Tumors of the tail of the pancreas are those arising between the left border of the aorta and the hilum of the spleen

involved sites are the liver, peritoneal cavity, and lungs. Metastases to other sites, such as the brain, bone, umbilicus, skin, and gastrointestinal sites, are uncommon.

RULES FOR CLASSIFICATION

Clinical Classification

Information necessary for the clinical staging of pancreatic cancer may be obtained from physical examination and three-dimensional radiographic imaging studies, which include triphasic, contrast-enhanced multislice computed tomography (CT) or magnetic resonance (MR) imaging. Endoscopic ultrasonography (if done by experienced gastroenterologists) also provides information helpful for clinical staging and is the procedure of choice for performing fine-needle aspiration (FNA) biopsy of the pancreas. The standard radiographic assessment of resectability includes evaluation for peritoneal or hepatic metastases; the patency of the superior mesenteric vein and portal vein and the relationship of these vessels and their tributaries to the tumor; and the relationship of the tumor to the superior mesenteric artery, celiac axis, and hepatic artery. If the appropriate clinical and radiographic findings are present, preoperative biopsy is not necessary before resection, but a core biopsy or an endoscopic ultrasound (EUS)-guided fine-needle aspiration biopsy specimen should be obtained to confirm the diagnosis for patients undergoing neoadjuvant therapy. Serum IgG4 levels may be helpful in identifying autoimmune pancreatitis if a tissue diagnosis has not been obtained.

Laparoscopy may be performed in patients believed to have localized, potentially resectable tumors to exclude peritoneal metastases and small metastases on the surface of the liver. Laparoscopy will reveal small (<1 cm) peritoneal or liver metastases and upstage (to Stage IV) approximately 10% of patients with tumors in the pancreatic head, and probably a greater percentage of patients with tumors in the body and tail. A significantly elevated preoperative cancer antigen (CA) 19-9 level (>250 U/mL) increases the yield of staging laparoscopy.

Pancreatic cancers that are locally advanced have a low likelihood of resectability and a high chance of incomplete removal if resected. This scenario has given rise to the term *borderline resectable tumor*, which has been defined by the Americas Hepato-Pancreato-Biliary Association/Society of Surgical Oncology/Society for Surgery of the Alimentary Tract, the International Study Group of Pancreatic Surgery (ISGPS), and the National Comprehensive Cancer Network (NCCN).[2,3] The superior mesenteric artery may have tumor

abutment up to but not more than 180° of the circumference of the vessel and may have short segment involvement of the hepatic artery that does not extend onto the celiac axis. Venous involvement of the superior mesenteric vein/portal vein is defined as tumor abutment with or without impingement and narrowing of the lumen or short-segment venous occlusion resulting from either tumor thrombus or encasement but with suitable vessel proximal and distal to the area of vessel involvement. In general, these locally advanced cancers should be treated as part of a clinical trial or receive neoadjuvant therapy.

Imaging[4-21]

Cross-sectional imaging, either contrast-enhanced, multiphasic, thin-section MR imaging or CT, typically is the preferred examination for assessing the stage of pancreatic cancers, ampullary tumors, and distal common bile duct tumors and should be performed before any interventions (e.g., biopsy, stent placement). The choice of MR imaging or CT should be based on the imaging equipment available, the expertise of the radiologists performing and interpreting the studies, and whether there are confounding issues, such as allergies to intravenous contrast or renal insufficiency (in the latter case, unenhanced MR imaging is preferred to unenhanced CT because of MR imaging's superior soft tissue contrast). Imaging should be performed before interventions (e.g. stent placement, biopsy) to avoid the effects of potential postprocedure pancreatitis interfering with staging assessments.

If intravenous contrast is used, dynamic imaging (MR imaging or CT) should be performed both during the phase of peak pancreatic enhancement ("pancreatic parenchymal" or "late arterial" phase), to enhance the conspicuity of tumor against the background pancreas (regardless of ampullary, pancreatic, or distal biliary origin), and during the portal venous phase of liver enhancement (peak liver enhancement) and when veins are fully opacified, to judge extrapancreatic extent of tumor, involvement of vasculature, and the possibility of liver metastases, as liver metastases from these tumors typically are hypodense against uninvolved liver. Thin-section imaging (2–3 mm for CT, thicker but as thin as reasonably possible for MR imaging) is particularly important for judging vascular involvement and to assess for potential small sites of metastatic disease. In the setting of preoperative therapy, such technique is important not only at baseline but also following therapy to determine whether patients are still surgical candidates and to follow up borderline suspicious findings.

Endoscopic ultrasound may be used next after CT/MR imaging for problem-solving, given that it is a relatively invasive technique and has limited utility for assessing for distant disease such as liver metastases, peritoneal implants, or adenopathy outside the surgical field. However, the ability of EUS to detect small tumors and guide biopsy is particularly helpful for tumors that may appear isodense to, and therefore indistinguishable from, background pancreas on CT and/or MR imaging. EUS and EUS-FNA also should be performed before ERCP, as pancreatitis may degrade the ability of EUS to visualize tumor and the placement of stents eliminates the ability to identify sites of duct cutoff that may be useful in guiding biopsies. ERCP subsequently may be helpful in the setting of duct abnormalities, both for treatment (stent placement) and for diagnosis (brushings).

TNM Categories of Staging by Imaging

The T category is assessed by measuring the largest diameter of the tumor in the axial plane, whether by CT or by MR imaging. With regard to MR imaging, the measurement should be made on the sequence that best delineates the tumor. Note should be made if there is suspicion that pancreatitis may be present, which may alter the apparent size of the tumor.

The relationship of tumor to relevant vessels should be reported, including its relationship to arteries such as the superior mesenteric, celiac, splenic, and common hepatic arteries, as well as the aorta if the tumor extends sufficiently posteriorly into the retroperitoneum. The tumor's relationship to relevant veins includes the portal vein, splenic vein, splenoportal confluence, and superior mesenteric vein, as well as branch vessels such as the gastrocolic trunk, first jejunal vein, and ileocolic branches. The goal is to delineate tumor extent sufficiently to provide useful information for potential en bloc resection with vascular graft placement.

The relationship of tumor to vessels should be described using terms commonly understood by the clinical community, such as degrees of circumferential involvement and the terms *abutment* (i.e., ≤180° of involvement of a given vessel by tumor) and *encasement* (i.e., >180° of circumferential vessel involvement by tumor). Multiplanar reconstructions for CT and direct multiplanar imaging for MR imaging may be particularly helpful in visualizing the circumferential relationship of tumor to relevant vasculature. It also is important to describe the relationship of tumor to adjacent structures, such as the stomach, spleen, colon, small bowel, and adrenal glands.

Assessment of N category (nodal) status is a challenge for all imaging modalities, because all are limited with regard to detection of microscopic metastatic disease to nodes. Nevertheless, it is important to fully identify the location of visibly suspicious nodes. Nodes are considered suspicious for metastatic involvement if they are greater than 1 cm in short axis or have abnormal morphology (e.g., are rounded, are hypodense or heterogeneous, have irregular margins, involve adjacent vessels or structures).

The most common sites of metastatic disease include the liver, peritoneum, lung, and bone, with metastases to the lat-

ter two sites usually occurring late in the disease. Evaluations for potential metastases are best done with contrast-enhanced CT and MR imaging; MR imaging likely provides superior capability for assessing potential liver metastases and involvement of bone, whereas CT is better for evaluating potential lung metastases.

Suggested Radiology Report Format

With the development of neoadjuvant therapy and the category of borderline resectable disease, it is particularly important that radiology reports use commonly understood terminology and that borderline suspicious findings, whether of tumor involvement of vasculature or of potential metastatic disease, such as to the liver, be noted so that they can be monitored on follow-up.

Details of the radiology report should include descriptions of:

1. Primary tumor: location, size, characterization (enhancement pattern, e.g., hypodense, hyperdense, cystic, or mixed), and effect on ducts (common bile duct and main pancreatic duct). It also should be noted whether there are findings suspicious for superimposed acute pancreatitis, which may distort findings relevant to staging, or chronic pancreatitis/autoimmune pancreatitis, because these diseases may closely mimic malignancy and may be associated with duct strictures.
2. Local extent: the relationship of tumor, with reference to degrees of circumferential involvement using commonly understood terms such as *abutment* and *encasement*, and occlusion with regard to adjacent arterial structures (celiac, superior mesenteric, hepatic, and splenic arteries and the aorta) and venous structures (portal, splenic, and superior mesenteric veins, and if relevant, inferior vena cava).
   a. It also should be noted how much of the vascular involvement is related to solid tumor versus stranding, and whether vessel involvement is related to direct involvement by tumor or is distinctly separate from the prior tumor.
   b. Other descriptors that should be reported include narrowing of the vasculature, vascular thrombi, and potentially, the length of involvement by tumor.
   c. In the case of borderline resectable disease and tumor involvement of the common hepatic artery, it should be noted whether there is sparing of the origin of the common hepatic artery from the celiac, as well as the length of that sparing, because vascular grafting may be considered.
   d. The presence of enlarged collaterals or varices should be noted.
   e. The involvement of branch vessels such as the gastrocolic, first jejunal, and ileocolic branches of the superior mesenteric vein should be noted. These findings are particularly relevant in planning the extent and feasibility of venous vascular grafts.
3. Relevant arterial variants: This information is particularly important with regard to hepatic arterial variants, such as those arising from the superior mesenteric artery, and to the nature of the variant (e.g., accessory right hepatic vs. common hepatic artery arising from the superior mesenteric artery). Confounding factors, such as narrowing of the celiac origin by arcuate ligament syndrome or atherosclerotic disease of the celiac and superior mesenteric arteries, and their effects on adjacent vasculature also are important for treatment planning.
4. Lymph node involvement: Suspicious nodes should be documented, particularly if they are greater than 1 cm in short axis or morphologically abnormal (e.g., are rounded, are hypodense/heterogeneous/necrotic, have irregular margins).
5. Distant spread: Evaluation should include the liver, peritoneum (including whether ascites is present or absent), bone, and lung. Note should be taken of indeterminate lesions, particularly if they are too small to characterize, because they may be monitored on follow-up imaging to assess for growth or resolution.
   a. Ascites should be noted because it may indicate peritoneal metastases; however, it should be addressed in the context of whether there are confounding secondary causes of ascites, such as superior mesenteric vein or portal vein narrowing or occlusion.
6. Unexpected but notable other findings relevant to management should be noted and described as well.

PATHOLOGICAL CLASSIFICATION

Partial resection (pancreaticoduodenectomy or distal pancreatectomy) or complete resection of the pancreas, including the tumor and associated regional lymph nodes, provides the information necessary for pathological staging. In pancreaticoduodenectomy specimens, the bile duct, pancreatic parenchymal, uncinate, proximal (duodenal or gastric), and distal duodenal margins should be evaluated. The pancreatic parenchymal margin is also referred to as the pancreatic duct margin, pancreatic neck margin, and distal pancreatic resection margin. The uncinate margin has also been termed the superior mesenteric artery margin, retroperitoneal margin, mesopancreatic margin, posterior–inferior margin, deep margin, and radial margin. All margins except the pancreatic parenchymal margin should

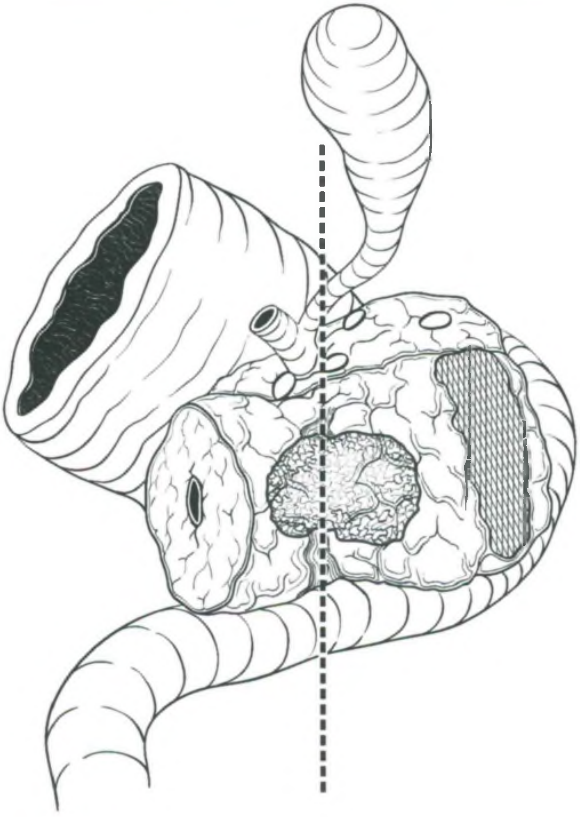

Fig. 28.2 The retroperitoneal pancreatic margin (hatched area; also referred to as the mesenteric or uncinated margin) consists of soft tissue that often contains perineural tissue adjacent to the superior mesenteric artery

be assessed in total pancreatectomy specimens. The College of American Pathologists (CAP) Checklist for Exocrine Pancreatic Tumors is recommended as a guide for the pathological evaluation of pancreatic resection specimens (www.cap.org).

Most local recurrences arise in the pancreatic bed in the region of the uncinate margin. The soft tissue in this area is richly innervated and is adjacent to the right lateral aspect of the superior mesenteric artery (Fig. 28.2). The uncinate margin should be inked as part of the gross evaluation of the specimen; the specimen is then cut perpendicular to the inked margin for histologic analysis. The closest microscopic approach of the tumor to the margin should be recorded. The smooth area adjacent to the uncinate process corresponds to the superior mesenteric and portal veins, and is referred to as the vascular groove or vascular bed. This area, as well as the posterior surface (including the nonuncinate posterior surface of the pancreatic head) and anterior surface (corresponding to the peritoneum), is not considered a true surgical margin in the CAP protocol, although this practice is not universally accepted. Histologic assessment of these areas for tumor is recommended but not mandated by the CAP protocol.

The T categories are based on tumor size. For invasive carcinomas in association with intraductal mucinous neoplasm, intraductal tubulopapillary neoplasm, and mucinous cystic neoplasm, the T category should be determined by the size of the invasive component. The invasive carcinomas in this setting often are small and have a favorable outcome. These tumors have been referred to as *minimally invasive* carcinomas, with various criteria being used to define this term. T1 subcategories (T1a, T1b, and T1c) provide objective criteria for describing small invasive tumors, justifying the incorporation of these strata into the current system. The current cut-off points of ≤2 cm, >2 to 4 cm, and >4 cm for definitions of T1 to T3 are based on recent reviews of large databases.[1,22-26] T4 for pancreatic cancer is defined as involvement of the superior mesenteric artery, celiac axis, and/or common hepatic artery, which in most cases renders the tumor unresectable. This situation usually is determined by radiographic and endoscopic findings. Hence, T4 category is not determined by pathological examination of surgical resection specimens. Adenocarcinomas of the head of the pancreas often show direct extension into the ampulla of Vater, intrapancreatic portion of the common bile duct, duodenum, peritoneum, and peripancreatic soft tissue. Adenocarcinomas of the body and tail of the pancreas may directly invade the stomach, spleen, left adrenal gland, and peritoneum. In the absence of arterial involvement (celiac axis, superior mesenteric artery, common hepatic artery), the T category is based on size, regardless of invasion of adjacent organs or veins. Extrapancreatic extension may be difficult to determine because the pancreas does not have a capsule, and the distinction between pancreas and extrapancreatic soft tissue often is obscured by fibrosis as part of the tumor or chronic pancreatitis. This parameter is no longer included in the definition of T categories.

Nodal involvement, regardless of direct extension or metastases to peripancreatic nodes, has been associated with unfavorable outcomes in multiple studies.[27] Thus, it is important to identify and properly assess as many regional lymph nodes in the specimen as possible. Based on survival data and a review of the number of lymph nodes that can be practically obtained from resection specimens, evaluation of a minimum of 12 lymph nodes is recommended to accurately stage N0 tumors.[28,29] Recent studies show that the total number of positive lymph nodes and/or lymph node ratio (LNR) are also strong prognostic predictors.[30-33] The total number of positive lymph nodes outperformed LNR in studies with sufficient numbers of lymph nodes obtained and evaluated.[32,34] Thus, lymph node–positive categories based on the number of positive lymph nodes have been added to the N category classification of the pancreas, similar to other gastrointestinal sites. Although different cutoffs have been used in differ-

ent studies,[31,32,34] the cutoffs of zero versus one to three versus four or more have been adopted in the current staging scheme based on available data.[34,35] Anatomic division of regional lymph nodes is not necessary. However, separately submitted lymph nodes should be reported as labeled by the surgeon. Seeding of the peritoneum (even if limited to the lesser sac region), as well as peritoneal fluid with microscopic evidence of carcinoma, is considered M1.

Patients who undergo surgical resection for localized nonmetastatic adenocarcinoma of the pancreas have a long-term survival rate of approximately 27% and a median survival of 12–20 months. Patients with resectable tumors showing regional lymph node involvement and without distant metastasis have a 5-year survival of approximately 11% and a median survival of 6–10 months. Patients with metastatic disease have a short survival (3–6 months), the length of which depends on the extent of disease, performance status, and response to systemic therapy.

PROGNOSTIC FACTORS

Prognostic Factors Required for Stage Grouping

Beyond the factors used to assign T, N, or M categories, no additional prognostic factors are required for stage grouping.

Additional Factors Recommended for Clinical Care

Involvement of Visceral Arteries

Recent improvements in vascular surgery have led to a profusion of small reports on arterial resection and reconstruction for T4 pancreatic cancers.[36,37] These reports demonstrate two main points. First, these operations are still associated with a much higher rate of morbidity and mortality compared with resections without vascular involvement or resections associated with venous resection and reconstruction. Additionally, in patients surviving the perioperative period, the long-term (1-, 3-, and 5-year) survival is not as good as that of patients who have undergone resection without arterial involvement or those who have had venous resection and reconstruction. However, their long-term survival is better than that of patients with similar arterial involvement who do not undergo resection. Therefore, involvement of the celiac and/or superior mesenteric artery remains, at a minimum, a relative contraindication to resection. If contemplated, resection with arterial resection and reconstruction should be performed at expert centers. AJCC Level of Evidence: II

Preoperative CA 19-9 Levels

The only serum biomarker approved by the US Food and Drug Administration for pancreatic ductal adenocarcinoma is CA 19-9. This marker, however, has limitations in its specificity and sensitivity, and an estimated 15% of the population cannot produce the CA 19-9 (sialyl Lewis a) antigen. Despite these limitations, CA 19-9 may be a useful prognostic marker in the settings of both localized and metastatic disease.[38,39] Several reports have noted that an elevated preoperative CA 19-9 level is associated with an increased likelihood of radiographically occult metastatic disease being found at staging laparoscopy in patients about to undergo resection.[40] Preoperative CA 19-9 level also is a strong predictor of resectability in the absence of metastatic disease. Many studies also have shown that an increased CA 19-9 level is associated with higher pathological stage and decreased survival.[41] In addition, postresection CA 19-9 levels have been associated with postresection survival and have been used as a stratification variable in randomized trials of adjuvant therapy. AJCC Level of Evidence: II

Completeness of Resection

The uncinate margin represents the plane of abutment of the uncinate process with the superior mesenteric artery. Because only a scant buffer of connective tissue separates the uncinate process from the superior mesenteric artery and the neural and lymphatic plexus around the celiac trunk, this margin is at highest risk for residual disease in tumors involving the pancreatic head.[42] The margin is considered positive if the tumor is at or within 1 mm of the margin. Several studies showed that the recurrence rates are similar in both these situations.[43-46] Incomplete resection resulting in a grossly positive uncinate margin provides no survival advantage with surgical resection (compared with chemoradiation and no surgery). The resection status is not part of the TNM staging system, but because of its prognostic significance, it should be recorded in the pathology report as follows: complete resection with grossly and microscopically negative margins of resection (R0), grossly negative but microscopically positive margin(s) of resection (R1), or grossly and microscopically positive margin(s) of resection (R2). The nonuncinate posterior surface, anterior surface, and the vascular groove (corresponding to the superior mesenteric and portal veins) are regarded as resection margins by some groups but are not considered true resection margins by CAP or AJCC. Because involvement of these surfaces may have prognostic significance, it is recommended that this information be included in the pathology report. AJCC Level of Evidence: II

Tumor Regression after Neoadjuvant Therapy

Given the increasing number of studies investigating the use of neoadjuvant treatment for ductal adenocarcinoma of the pancreas, it is important to assess the response of tumor to

preoperative chemotherapy and/or radiation therapy. Several grading schemes for the extent of residual tumor in posttreatment pancreatectomy specimens have been proposed. The CAP protocol recommends a four-tiered grading system (modified Ryan scheme) similar to that for the rectum.[47] AJCC Level of Evidence: II

Histologic Features (Grade, Perineural Invasion, Lymphovascular Invasion)

Histologic features have less impact on outcome than stage. Several histologic parameters, such as high grade (poorly differentiated), perineural invasion, lymphovascular invasion, and involvement of muscular vessels, have been shown to adversely affect survival and should be noted in pathology reports.[48-50] Perineural and lymphovascular invasion also are important prognostic factors after neoadjuvant therapy.[51] AJCC Level of Evidence: II

RISK ASSESSMENT MODELS

The AJCC recently established guidelines that will be used to evaluate published statistical prediction models for the purpose of granting endorsement for clinical use.[52] Although this is a monumental step toward the goal of precision medicine, this work was published only very recently. Therefore, the existing models that have been published or may be in clinical use have not yet been evaluated for this cancer site by the Precision Medicine Core of the AJCC. In the future, the statistical prediction models for this cancer site will be evaluated, and those that meet all AJCC criteria will be endorsed.

DEFINITIONS OF AJCC TNM

Definition of Primary Tumor (T)

T Category	T Criteria
TX	Primary tumor cannot be assessed
T0	No evidence of primary tumor
Tis	Carcinoma *in situ*<br>This includes high-grade pancreatic intraepithelial neoplasia (PanIn-3), intraductal papillary mucinous neoplasm with high-grade dysplasia, intraductal tubulopapillary neoplasm with high-grade dysplasia, and mucinous cystic neoplasm with high-grade dysplasia.
T1	Tumor ≤2 cm in greatest dimension
T1a	Tumor ≤0.5 cm in greatest dimension
T1b	Tumor >0.5 cm and <1 cm in greatest dimension
T1c	Tumor 1–2 cm in greatest dimension
T2	Tumor >2 cm and ≤4 cm in greatest dimension
T3	Tumor >4 cm in greatest dimension
T4	Tumor involves celiac axis, superior mesenteric artery, and/or common hepatic artery, regardless of size

Definition of Regional Lymph Node (N)

N Category	N Criteria
NX	Regional lymph nodes cannot be assessed
N0	No regional lymph node metastases
N1	Metastasis in one to three regional lymph nodes
N2	Metastasis in four or more regional lymph nodes

Definition of Distant Metastasis (M)

M Category	M Criteria
M0	No distant metastasis
M1	Distant metastasis

AJCC PROGNOSTIC STAGE GROUPS

When T is...	And N is...	And M is...	Then the stage group is...
Tis	N0	M0	0
T1	N0	M0	IA
T1	N1	M0	IIB
T1	N2	M0	III
T2	N0	M0	IB
T2	N1	M0	IIB
T2	N2	M0	III
T3	N0	M0	IIA
T3	N1	M0	IIB
T3	N2	M0	III
T4	Any N	M0	III
Any T	Any N	M1	IV

REGISTRY DATA COLLECTION VARIABLES

1. Preoperative CA 19-9
2. Preoperative carcinoembryonic antigen (CEA)

HISTOLOGIC GRADE (G)

For ductal adenocarcinomas, the grading scheme recommended by the World Health Organization (Kloeppel grading scheme)[59] is based on glandular differentiation, mucin

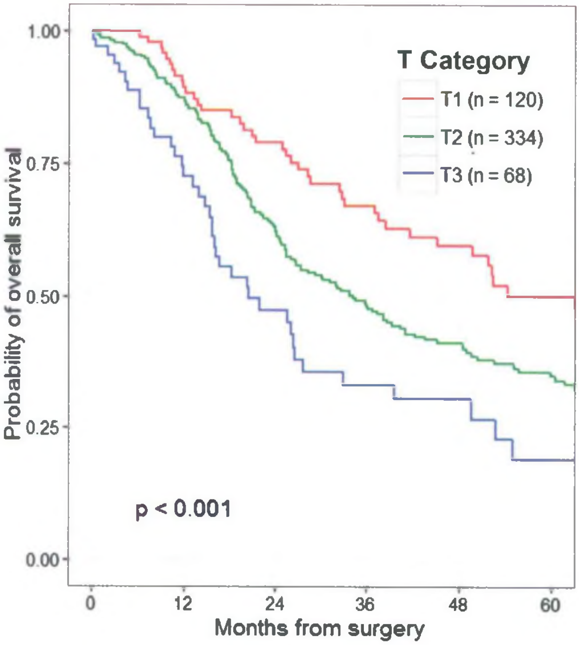

Fig. 28.3 Survival by T category of 525 patients who underwent resection for node-negative pancreatic cancer stratified by proposed AJCC 8th Edition criteria. Data from Allen et al.[26]

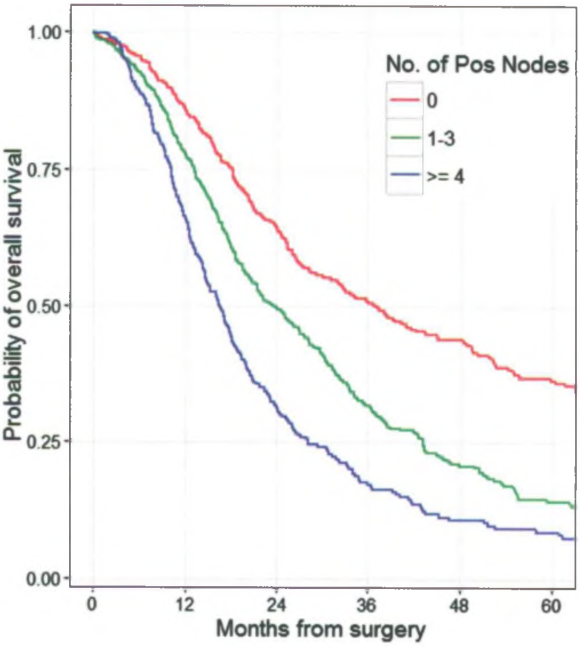

Fig. 28.4 Survival by number of positive nodes for all patients who underwent R0 resection (n=1551) stratified by proposed AJCC 8th Edition criteria. Data from Allen et al.[26]

production, mitoses, and nuclear pleomorphism. Variation in these features is common within the same tumor, and the highest grade is reported. Histologic grade has been shown to have prognostic significance, with grade 3 being an unfavorable prognostic factor.[53-55] Other grading schemes have been proposed but have not been adopted widely.

G	G Definition
GX	Grade cannot be assessed
G1	Well differentiated
G2	Moderately differentiated
G3	Poorly differentiated

HISTOPATHOLOGIC TYPE

Infiltrating ductal adenocarcinoma of the pancreas is characterized by invasive neoplastic glands, desmoplastic reaction, and frequent perineural and vascular invasion. Acinar cell carcinomas account for less than 2% of all pancreatic cancers and are composed of cells with acinar differentiation. Invasive carcinomas associated with intraductal papillary mucinous neoplasms range from ductal adenocarcinomas to colloid carcinomas.

Bibliography

1. McIntyre CA, Winter JM. Diagnostic Evaluation and Staging of Pancreatic Ductal Adenocarcinoma. Paper presented at: Seminars in oncology; 2015.
2. Vauthey JN, Dixon E. AHPBA/SSO/SSAT Consensus Conference on Resectable and Borderline Resectable Pancreatic Cancer: rationale and overview of the conference. *Annals of surgical oncology*. Jul 2009;16(7):1725–1726.
3. Callery MP, Chang KJ, Fishman EK, Talamonti MS, William Traverso L, Linehan DC. Pretreatment assessment of resectable and borderline resectable pancreatic cancer: expert consensus statement. *Annals of surgical oncology*. Jul 2009;16(7):1727–1733.
4. Al-Hawary MM, Francis IR, Chari ST, et al. Pancreatic ductal adenocarcinoma radiology reporting template: consensus statement of the Society of Abdominal Radiology and the American Pancreatic Association. *Radiology*. Jan 2014;270(1):248–260.
5. Al-Hawary MM, Kaza RK, Wasnik AP, Francis IR. Staging of pancreatic cancer: role of imaging. *Seminars in roentgenology*. Jul 2013;48(3):245–252.
6. Tamm EP, Balachandran A, Bhosale PR, et al. Imaging of pancreatic adenocarcinoma: update on staging/resectability. *Radiol Clin North Am*. May 2012;50(3):407–428.
7. Brook OR, Brook A, Vollmer CM, Kent TS, Sanchez N, Pedrosa I. Structured reporting of multiphasic CT for pancreatic cancer: potential effect on staging and surgical planning. *Radiology*. Feb 2015;274(2):464–472.
8. Marcal LP, Fox PS, Evans DB, et al. Analysis of free-form radiology dictations for completeness and clarity for pancreatic cancer staging. *Abdom Imaging*. Oct 2015;40(7):2391–2397.

9. Gottlieb R. CT Onco Primary Pancreas Mass. *RSNA Radiology Reporting Templates* 2012. Accessed 8/13/2015, 2015.
10. Tempero MA, Malafa MP, Asbun H, et al. NCCN Guidelines Version 2.2015 Pancreatic Adenocarcinoma. *NCCN Guidelines* [pdf]. 2015; http://www.nccn.org/professionals/physician_gls/pdf/pancreatic.pdf. Accessed 10/16/2015, 2015.
11. Varadhachary GR, Tamm EP, Abbruzzese JL, et al. Borderline resectable pancreatic cancer: definitions, management, and role of preoperative therapy. *Annals of surgical oncology*. Aug 2006;13(8):1035–1046.
12. Katz MH, Crane CH, Varadhachary G. Management of borderline resectable pancreatic cancer. *Semin Radiat Oncol*. Apr 2014;24(2):105–112.
13. Valls C, Andia E, Sanchez A, et al. Dual-phase helical CT of pancreatic adenocarcinoma: assessment of resectability before surgery. *AJR. American journal of roentgenology*. Apr 2002;178(4):821–826.
14. Tamm EP, Loyer EM, Faria S, et al. Staging of pancreatic cancer with multidetector CT in the setting of preoperative chemoradiation therapy. *Abdom Imaging*. Sep-Oct 2006;31(5):568–574.
15. Cassinotto C, Cortade J, Belleannee G, et al. An evaluation of the accuracy of CT when determining resectability of pancreatic head adenocarcinoma after neoadjuvant treatment. *Eur J Radiol*. Apr 2013;82(4):589–593.
16. DeWitt J, Devereaux B, Chriswell M, et al. Comparison of endoscopic ultrasonography and multidetector computed tomography for detecting and staging pancreatic cancer.[see comment][summary for patients in Ann Intern Med. 2004 Nov 16;141(10):I46; PMID: 15545671]. *Annals of internal medicine*. 2004;141(10):753–763.
17. Tamm EP, Loyer EM, Faria SC, Evans DB, Wolff RA, Charnsangavej C. Retrospective analysis of dual-phase MDCT and follow-up EUS/EUS-FNA in the diagnosis of pancreatic cancer. *Abdom Imaging*. Sep-Oct 2007;32(5):660–667.
18. Nikolaidis P, Hammond NA, Day K, et al. Imaging features of benign and malignant ampullary and periampullary lesions. *Radiographics: a review publication of the Radiological Society of North America, Inc*. May-Jun 2014;34(3):624–641.
19. Kim JH, Park SH, Yu ES, et al. Visually isoattenuating pancreatic adenocarcinoma at dynamic-enhanced CT: frequency, clinical and pathologic characteristics, and diagnosis at imaging examinations. *Radiology*. Oct 2010;257(1):87–96.
20. Raman SP, Fishman EK. Abnormalities of the distal common bile duct and ampulla: diagnostic approach and differential diagnosis using multiplanar reformations and 3D imaging. *AJR. American journal of roentgenology*. Jul 2014;203(1):17–28.
21. Motosugi U, Ichikawa T, Morisaka H, et al. Detection of pancreatic carcinoma and liver metastases with gadoxetic acid-enhanced MR imaging: comparison with contrast-enhanced multi-detector row CT. *Radiology*. Aug 2011;260(2):446–453.
22. Adsay NV, Bagci P, Tajiri T, et al. Pathologic staging of pancreatic, ampullary, biliary, and gallbladder cancers: pitfalls and practical limitations of the current AJCC/UICC TNM staging system and opportunities for improvement. Paper presented at: Seminars in diagnostic pathology; 2012.
23. Winter JM, Jiang W, Basturk O, et al. Recurrence and Survival After Resection of Small Intraductal Papillary Mucinous Neoplasm-associated Carcinomas (</=20-mm Invasive Component): A Multi-institutional Analysis. *Annals of surgery*. Apr 2016;263(4): 793–801.
24. Oliva I, Bandyopadhyay S, Coban I, et al. Peripancreatic soft tissue involvement by pancreatic ductal adenocarcinomas: incidence, patterns and significance. *Laboratory Investigation*. Jan 2009;89(Supplement 1s) Supp:318A–319A.
25. Saka B, Balci S, Basturk O, et al. Pancreatic Ductal Adenocarcinoma is Spread to the Peripancreatic Soft Tissue in the Majority of Resected Cases, Rendering the AJCC T-Stage Protocol (7th Edition) Inapplicable and Insignificant: A Size-Based Staging System (pT1: </=2, pT2: >2-</=4, pT3: >4 cm) is More Valid and Clinically Relevant. *Annals of surgical oncology*. Jan 29 2016.
26. Allen PJ, Kuk D, Fernandez-Del Castillo C, et al. Multi-Institutional validation study of the American Joint Commission on Cancer (8th edition) changes for T and N staging in patients with pancreatic adenocarcinoma. *Annals of surgery*. May 2016; http://www.ncbi.nlm.nih.gov/pubmed/?term=allen+kuk+castillo+2016 [Epub ahead of print].
27. Konstantinidis IT, Deshpande V, Zheng H, et al. Does the mechanism of lymph node invasion affect survival in patients with pancreatic ductal adenocarcinoma? *Journal of Gastrointestinal Surgery*. 2010;14(2):261–267.
28. Schwarz RE, Smith DD. Extent of lymph node retrieval and pancreatic cancer survival: information from a large US population database. *Annals of surgical oncology*. Sep 2006;13(9):1189–1200.
29. Tomlinson JS, Jain S, Bentrem DJ, et al. Accuracy of staging node-negative pancreas cancer: a potential quality measure. *Archives of surgery*. Aug 2007;142(8):767–723; discussion 773–764.
30. Berger AC, Watson JC, Ross EA, Hoffman JP. The Metastatic/Examined Lymph Node Ratio Is an Important Prognostic Factor After Pancreaticoduodenectomy for Pancreatic Adenocarcinoma/DISCUSSION. *The American surgeon*. 2004;70(3):235.
31. Riediger H, Keck T, Wellner U, et al. The lymph node ratio is the strongest prognostic factor after resection of pancreatic cancer. *Journal of gastrointestinal surgery*. 2009;13(7):1337–1344.
32. Murakami Y, Uemura K, Sudo T, et al. Number of metastatic lymph nodes, but not lymph node ratio, is an independent prognostic factor after resection of pancreatic carcinoma. *Journal of the American College of Surgeons*. 2010;211(2):196–204.
33. Hartwig W, Hackert T, Hinz U, et al. Pancreatic cancer surgery in the new millennium: better prediction of outcome. *Annals of surgery*. Aug 2011;254(2):311–319.
34. Strobel O, Hinz U, Gluth A, et al. Pancreatic adenocarcinoma: number of positive nodes allows to distinguish several N categories. *Annals of surgery*. 2015;261(5):961–969.
35. Olca B, Burcu S, Serdar B, al. E. Substaging of lymph node status in resected pancreatic ductal adenocarcinoma has strong prognostic correlations: proposal for a revised N classification for TNM staging. *Annals of surgical oncology*. In press.
36. Mollberg N, Rahbari NN, Koch M, et al. Arterial resection during pancreatectomy for pancreatic cancer: a systematic review and meta-analysis. *Annals of surgery*. Dec 2011;254(6):882–893.
37. Gurusamy KS, Kumar S, Davidson BR, Fusai G. Resection versus other treatments for locally advanced pancreatic cancer. *Cochrane Database Syst Rev*. 2014;2:CD010244.
38. Tempero MA, Uchida E, Takasaki H, Burnett DA, Steplewski Z, Pour PM. Relationship of carbohydrate antigen 19-9 and Lewis antigens in pancreatic cancer. *Cancer Res*. Oct 15 1987;47(20):5501–5503.
39. Humphris JL, Chang DK, Johns AL, et al. The prognostic and predictive value of serum CA19.9 in pancreatic cancer. *Ann Oncol*. Jul 2012;23(7):1713–1722.
40. Maithel SK, Maloney S, Winston C, et al. Preoperative CA 19-9 and the yield of staging laparoscopy in patients with radiographically resectable pancreatic adenocarcinoma. *Annals of surgical oncology*. Dec 2008;15(12):3512–3520.
41. Ferrone CR, Finkelstein DM, Thayer SP, Muzikansky A, Fernandez-del Castillo C, Warshaw AL. Perioperative CA19-9 levels can predict stage and survival in patients with resectable pancreatic adenocarcinoma. *Journal of clinical oncology*. 2006;24(18):2897–2902.
42. Evans DB, Farnell MB, Lillemoe KD, Vollmer C, Jr., Strasberg SM, Schulick RD. Surgical treatment of resectable and borderline

resectable pancreas cancer: expert consensus statement. *Annals of surgical oncology*. Jul 2009;16(7):1736–1744.
43. Campbell F, Smith RA, Whelan P, et al. Classification of R1 resections for pancreatic cancer: the prognostic relevance of tumour involvement within 1 mm of a resection margin. *Histopathology*. 2009;55(3):277–283.
44. Van den Broeck A, Sergeant G, Ectors N, Van Steenbergen W, Aerts R, Topal B. Patterns of recurrence after curative resection of pancreatic ductal adenocarcinoma. *European journal of surgical oncology: the journal of the European Society of Surgical Oncology and the British Association of Surgical Oncology*. Jun 2009;35(6):600–604.
45. Verbeke CS, Menon KV. Redefining resection margin status in pancreatic cancer. *HPB: the official journal of the International Hepato Pancreato Biliary Association*. Jun 2009;11(4):282–289.
46. Schlitter AM, Esposito I. Definition of microscopic tumor clearance (r0) in pancreatic cancer resections. *Cancers (Basel)*. 2010;2(4):2001–2010.
47. Ryan R, Gibbons D, Hyland JM, et al. Pathological response following long-course neoadjuvant chemoradiotherapy for locally advanced rectal cancer. *Histopathology*. Aug 2005;47(2):141–146.
48. Garcea G, Dennison AR, Ong SL, et al. Tumour characteristics predictive of survival following resection for ductal adenocarcinoma of the head of pancreas. *European journal of surgical oncology: the journal of the European Society of Surgical Oncology and the British Association of Surgical Oncology*. Sep 2007;33(7):892–897.
49. Chen JW, Bhandari M, Astill DS, et al. Predicting patient survival after pancreaticoduodenectomy for malignancy: histopathological criteria based on perineural infiltration and lymphovascular invasion. *HPB: the official journal of the International Hepato Pancreato Biliary Association*. Mar 2010;12(2):101–108.
50. Chatterjee D, Katz MH, Rashid A, et al. Perineural and Intra-neural Invasion in Posttherapy Pancreaticoduodenectomy Specimens Predicts Poor Prognosis in Patients with Pancreatic Ductal Adenocarcinoma. *The American journal of surgical pathology*. 2012;36(3):409.
51. Chatterjee D, Rashid A, Wang H, et al. Tumor invasion of muscular vessels predicts poor prognosis in patients with pancreatic ductal adenocarcinoma who have received neoadjuvant therapy and pancreaticoduodenectomy. *The American journal of surgical pathology*. Apr 2012;36(4):552–559.
52. Kattan MW, Hess KR, Amin MB, et al. American Joint Committee on Cancer acceptance criteria for inclusion of risk models for individualized prognosis in the practice of precision medicine. *CA: a cancer journal for clinicians*. Jan 19 2016.
53. Giulianotti PC, Boggi U, Fornaciari G, et al. Prognostic value of histological grading in ductal adenocarcinoma of the pancreas. Kloppel vs TNM grading. *International journal of pancreatology: official journal of the International Association of Pancreatology*. Jun 1995;17(3):279–289.
54. Adsay NV, Basturk O, Bonnett M, et al. A proposal for a new and more practical grading scheme for pancreatic ductal adenocarcinoma. *The American journal of surgical pathology*. Jun 2005;29(6):724–733.
55. Bosman FT, Carneiro F, Hruban RH, Theise ND. *WHO classification of tumours of the digestive system*. World Health Organization; 2010.
56. Robinson S, Rahman A, Haugk B, et al. Metastatic lymph node ratio as an important prognostic factor in pancreatic ductal adenocarcinoma. *European Journal of Surgical Oncology (EJSO)*. 2012;38(4):333–339.

Part VI

Neuroendocrine Tumors

Members of the Neuroendocrine Tumors Expert Panel
Elliot Asare, MD
Emily K. Bergsland, MD, FCAP – Vice Chair
James Brierley, BSc, MB, FRCP, FRCR, FRCP(C) – UICC Representative
David Bushnell, MD
Robert Jensen, MD
Michelle Kim, MD, MSc
David Klimstra, MD
Eric Liu, MD
Eric Nakakura, MD, PhD, FACS
Thomas O'Dorisio, MD
Rodney Pommier, MD
John Ramage, MD, PhD
Diane Reidy-Lagunes, MD
Guido Rindi, MD, PhD
Frances Ross, CTR – Data Collection Core Representative
Richard Schilsky, MD, FACP, FASCO – Editorial Board Liaison
Jonathan Strosberg, MD
Laura Tang, MD, PhD – CAP Representative
Aaron Vinik, MD, PhD, FCP, MACP, FACE
Yi-Zarn Wang, DDS, MD, FACS
Edward Wolin, MD
Eugene Woltering, MD, FACS – Chair
Rebecca Wong, MBChB, MSc, FRCP

Neuroendocrine Tumors of the Stomach

29

Eugene A. Woltering, Emily K. Bergsland, David T. Beyer, Thomas M. O'Dorisio, Guido Rindi, David S. Klimstra, Laura H. Tang, Diane Reidy-Lagunes, Jonathan R. Strosberg, Edward M. Wolin, Aaron I. Vinik, Eric K. Nakakura, Elliot A. Asare, David L. Bushnell, Richard L. Schilsky, Yi-Zarn Wang, Michelle K. Kim, Eric H. Liu, Robert T. Jensen, Rebecca K.S. Wong, John K. Ramage, and Rodney F. Pommier

CHAPTER SUMMARY

Cancers Staged Using This Staging System

This section includes gastric "carcinoid" tumors (NET G1 and G2, and rare well-differentiated G3).

Cancers Not Staged Using This Staging System

These histopathologic types of cancer…	Are staged according to the classification for…	And can be found in chapter…
High-grade neuroendocrine carcinoma (NEC)	Stomach	17
Mixed adenoneuroendocrine carcinoma	Stomach	17

Summary of Changes

Change	Details of Change	Level of Evidence
AJCC Prognostic Stage Groups	Stages I–IV have been condensed; i.e., no substages A and B.	II
Additional Factors Recommended for Clinical Care	Gastrin has been added as an additional factor recommended for clinical care.	II
Emerging Factors for Clinical Care	Pancreastatin has added as an emerging prognostic factor for clinical care.	II

ICD-O-3 Topography Codes

Code	Description
C16.0	Cardia
C16.1	Fundus of stomach
C16.2	Body of stomach
C16.3	Gastric antrum
C16.4	Pylorus
C16.5	Lesser curvature of stomach
C16.6	Greater curvature of stomach
C16.8	Overlapping lesion of stomach
C16.9	Stomach, NOS

WHO Classification of Tumors

Code	Description
8240	Neuroendocrine tumor (NET) G1 (carcinoid)
8249	NET G2

Bosman FT, Carneiro F, Hruban RH, Theise ND, eds. World Health Organization Classification of Tumours of the Digestive System. Lyon: IARC; 2010.

To access the AJCC cancer staging forms, please visit www.cancerstaging.org.

© American Joint Committee on Cancer 2017

M.B. Amin et al. (eds.), *AJCC Cancer Staging Manual, Eighth Edition*, DOI 10.1007/978-3-319-40618-3_29

INTRODUCTION

Queries to the Surveillance Epidemiology and End Results (SEER) database, 1973–2012, demonstrate that the incidence of well-differentiated gastric neuroendocrine tumors (NETs) in the US population in 2012 was 0.4/100,000.[1] Since 1973, the annual increase in incidence has been approximately 9% per year. The reason for the marked increase in incidence of these tumors probably represents an increased awareness by pathologists and clinicians as well as the availability of more sophisticated diagnostic tools. Overall, NETs are slightly more common in women (55%); however, gastric NETs exhibit a more pronounced female predominance (64.3%). Gastric NETs also may occur as a component of familial syndromes such as multiple endocrine neoplasia (MEN).[2,3] Measurement of circulating gastrin and gastric pH may help further subdivide the types of gastric NETsGastric NETs may be subdivided into NET types I–III.

I. Type I gastric NETs (approximately 80–90%) originate in a hypergastrinemic milieu, rarely metastasize (approximately 1–3%), and have a 5-year survival of approximately 100%. Type I gastric NETs are associated with gastric pHs that reflect hypochlorhydria or achlorhydria (pH near neutral).[4,5]
II. Type II gastric NETs are rare (5–7%), may occur in the context of MEN1 in a hypergastrinemic milieu and exhibit a more aggressive neoplastic phenotype (10–30% metastasis, and a 5-year survival of 60–90%). Because type II gastric NETs are variants of the Zollinger–Ellison syndrome, the gastric pH measured at the time of endoscopy typically is extremely low (high acidity).[4,5] Somatostatin analogs may be useful to control hypergastrenemia in type I or type II gastric NETs.[6,7]
III. Type III gastric NETs occur in a normogastrinemic environment and constitute approximately 10–15% of gastric NETS. They have a higher tendency to metastasize (50%) and have a 5-year survival of less than 50%.[2,8] Little biological information exists regarding the mechanisms responsible for human enterochromaffin-like (ECL) cell transformation. Gastrin and gastric pH in type III gastric NETs are typically normal.[4]

ANATOMY

Primary Site(s)

Most gastric NETs (particularly type I and type II) arise from the fundic gastric glands. Among 13,601 gastroenteropancreatic NETs (GEP-NETs) in the SEER database, 13% were classified as gastric NETs.[1]

Regional Lymph Nodes

A rich lymphatic network surrounds the gastrointestinal organs, and NETs exhibit an affinity for spread via the lymphatic system almost equal to spread via the bloodstream (Fig. 29.1).

Stomach

- *Greater curvature of the stomach.* Greater curvature, greater omental, gastroduodenal, gastroepiploic, pyloric, and pancreaticoduodenal nodes
- *Pancreatic and splenic areas.* Pancreaticolienal, peripancreatic, and splenic nodes
- *Lesser curvature of the stomach.* Lesser curvature, lesser omental, left gastric, cardioesophageal, common hepatic, celiac, and hepatoduodenal nodes

Metastatic Sites

The most common metastatic sites of well-differentiated gastric NETs are regional and distant lymph nodes (5.2%), liver (2.8%), lung (0.3%), and bone (0.1%).[1]

RULES FOR CLASSIFICATION

Clinical Classification

Clinical staging depends on the anatomic extent and hormonal activity of the primary tumor, which may be ascertained by clinical examination before treatment. Such an examination includes medical history, physical examination, measurement of gastric pH, and routine laboratory studies. Biochemical markers of gastric NETs include serum gastrin level and measurement of anti–parietal cell antibodies or anti–intrinsic factor antibodies. Currently, chromogranin A (CgA) is widely used as a biomarker for gastric NETs. CgA is a general NET marker that may reflect tumor load, monitor response to treatment, and correlate with a poor prognosis if elevated.[5,8] However, elevated CgA levels may occur in patients with other conditions and in the setting of proton-pump inhibitor (PPI) therapy.[3, 9, 10] Alternatively, pancreastatin may prove to be a more reliable biochemical indicator of NET disease because it is not affected by PPI use or pernicious anemia.[2,9] Pancreastatin is expected to be normal in type I gastric NETs and PPI-induced neuroendocrine cell hyperplasia and to be elevated in type II gastrinoma-associated gastric NETs. Some studies suggest

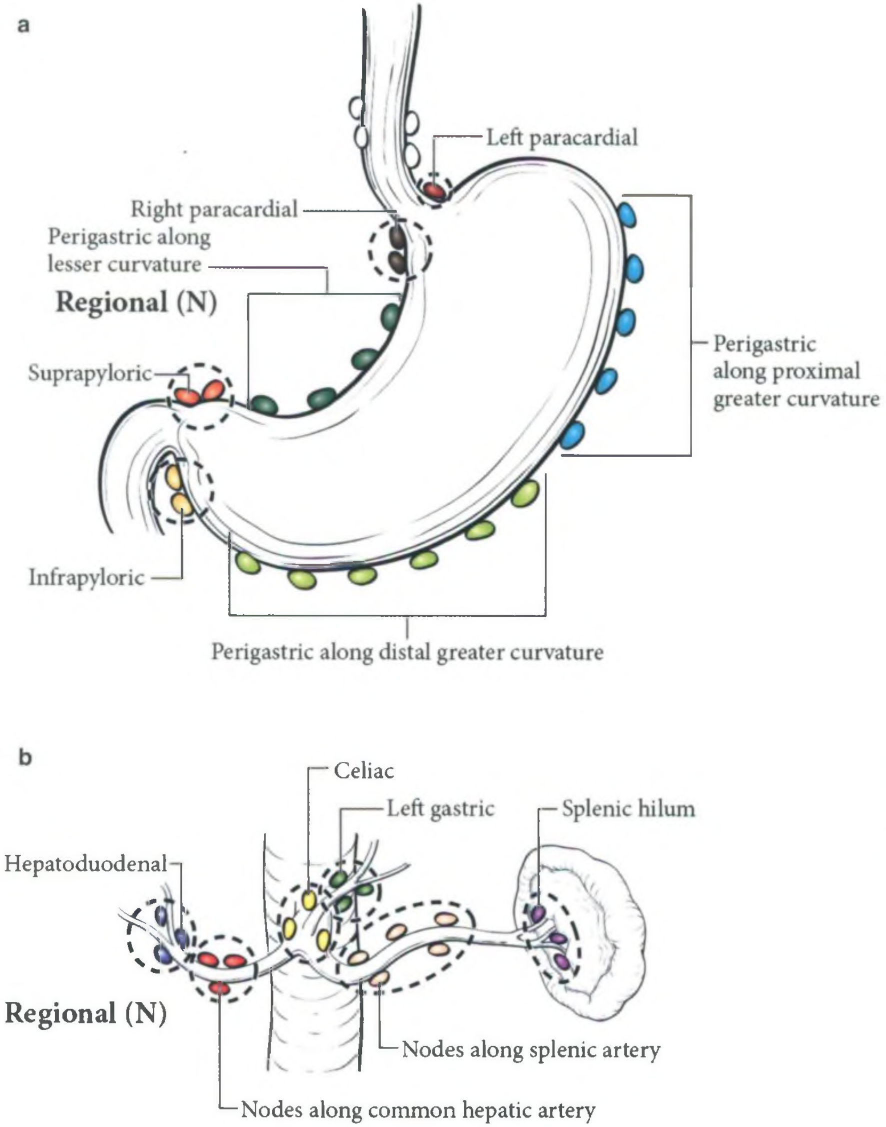

Fig. 29.1 The regional lymph nodes of the stomach for neuroendocrine tumors

that if metastasis has occurred in a type II gastric NET, pancreastatin usually is elevated.[4] The utility of pancreastatin as a biomarker of NETs, however, must be validated in future prospective trials. Measurement of gastric pH is helpful to understand the subtype of gastric NETs.

Gastroscopy can identify lesions down to the ligament of Treitz.[2] Endoscopic ultrasound (EUS) is a highly sensitive method for diagnostic and preoperative evaluation of NETs of the stomach, because it not only identifies submucosal lesions but also facilitates staging and allows fine-needle aspiration for histology.[2,8] Submucosal endoscopic resection may provide an easy method to resect small lesions (type I gastric NETs).

Imaging

For type I tumors less than 2 cm, esophagogastroduodenoscopy with EUS examination should be performed.[2,8] For type I tumors greater than 2 cm and type II and type III gastric NETs, magnetic resonance (MR) imaging and computed tomography (CT) are effective in the localization of liver metastases and extrahepatic metastases, respectively. The median detection rates and sensitivities of these diagnostic tests are approximately 80%.[2,8] Based on the sensitivity and specificity of MR imaging and CT scans for detecting liver or extrahepatic metastases, respectively, we recommend that both testing modalities be performed during staging. Somatostatin receptor imaging with ^{111}In-pentetreotide (Octreoscan™) has a high sensitivity

and specificity (80–90%) for detecting primary and metastatic tumors.[11] However, ^{68}Ga-labeled octreopeptide positron emission tomography/CT is more accurate than Octreoscan™ imaging for detecting NET sites in a variety of clinical settings.[12-14] Recently, ^{68}Ga-octreopeptide scans were approved by the FDA.

Pathological Classification

Pathological staging is based on surgical exploration with resection of at least the primary tumor, with potential resection of lymph nodes and distant metastases and examination of resected specimens.

Restaging

For gastric NETs, the *r* prefix is used for recurrent tumor status (rTNM) following a disease-free interval after treatment.

PROGNOSTIC FACTORS

Prognostic Factors Required for Stage Grouping

Beyond the factors used to assign T, N, or M categories, no additional prognostic factors are required for stage grouping.

Additional Factors Recommended for Clinical Care

Measurement of gastric pH, α-intrinsic factor, or α-parietal cell antibody is useful in diagnosing and differentiating among type I, type II, and type III gastric NETs. In addition, several prognostic factors may be useful in diagnosing, detecting recurrence, and following disease progression of well-differentiated gastric NETs.

Ki-67 Proliferative Index

Histologic tumor grade is determined by Ki-67 proliferative index and/or mitotic count. Ki-67 proliferative index is inversely correlated with patient prognosis and usually measured using MIB1 antibody as a percentage of 500 to 2,000 tumor cells that stain positive in the areas of highest nuclear labeling. AJCC Level of Evidence: I

Mitotic Count

Mitotic count is inversely correlated with patient prognosis and usually measured by determining the number of mitoses per 10 high-power fields (HPF) in the areas of highest mitotic density. At least 50 HPFs must be assessed to be consistent with World Health Organization (WHO) 2010 criteria. AJCC Level of Evidence: I

Gastrin Level

Gastrin is expected to be elevated in type I and type II gastric NETs.[8] However, gastrin is expected to be within normal ranges in type III gastric NETs.[4] Therefore, a normal gastrin level is associated with a worse prognosis in gastric NETs. PPI use (by inhibiting gastric acid production) induces hypergastrinemia as a physiologic feedback, which may be interpreted as a false positive result. A similar mechanism is responsible for the high gastrin levels observed in pernicious anemia. Multiple reference laboratories in the United States that are licensed by the Clinical Laboratory Improvement Amendments (CLIA) and accredited by the College of American Pathology (CAP) measure gastrin levels. AJCC Level of Evidence: II

Chromogranin A (CgA) Level

Chromogranin A is a 49-kDa acidic polypeptide present in the secretory granules of all neuroendocrine cells. CgA is a general NET marker, and plasma or serum CgA may be used as a marker in patients with gastric NETs. CgA has prognostic significance, with higher levels associated with a worse prognosis.[5,8] Furthermore, changes over time may be useful in assessing for recurrence after surgery or response to therapy in patients with metastatic disease.[15,16]

Despite the potential merits of monitoring CgA levels, the clinical utility of CgA is limited by the fact that it is falsely elevated in the setting of PPI use, chronic atrophic gastritis, renal failure, and other conditions.[3,9,10] Moreover, levels may fluctuate depending on the time of collection and fasting versus nonfasting states. Also, the upper limit of normal varies widely depending on the assay used and whether plasma or serum is assessed; thus, both the assay and type of sample should be considered when comparing CgA values over time.[17] As a result, routine measurement of CgA is not a consensus recommendation by the National Comprehensive Cancer Network. Multiple CLIA-licensed and CAP-accredited reference laboratories in the United States measure CgA levels. AJCC Level of Evidence: II

RISK ASSESSMENT MODELS

The AJCC recently established guidelines that will be used to evaluate published statistical prediction models for the purpose of granting endorsement for clinical use.[18] Although this is a monumental step toward the goal of precision

medicine, this work was published only very recently. Therefore, the existing models that have been published or may be in clinical use have not yet been evaluated for this cancer site by the Precision Medicine Core of the AJCC. In the future, the statistical prediction models for this cancer site will be evaluated, and those that meet all AJCC criteria will be endorsed.

DEFINITIONS OF AJCC TNM

Definition of Primary Tumor (T)

T Category	T Criteria
TX	Primary tumor cannot be assessed
T0	No evidence of primary tumor
T1*	Invades the lamina propria or submucosa and less than or equal to 1 cm in size
T2*	Invades the muscularis propria or greater than 1 cm in size
T3*	Invades through the muscularis propria into subserosal tissue without penetration of overlying serosa
T4*	Invades visceral peritoneum (serosal) or other organs or adjacent structures

Note:* For any T, add (m) for multiple tumors [TX(#) or TX(m), where X = 1–4 and # = number of primary tumors identified]; for multiple tumors with different Ts, use the highest.

***Example*: If there are two primary tumors, one of which penetrates only the subserosa, we define the primary tumor as either T3(2) or T3(m)

Definition of Regional Lymph Node (N)

N Category	N Criteria
NX	Regional lymph nodes cannot be assessed
N0	No regional lymph node metastasis
N1	Regional lymph node metastasis

Definition of Distant Metastasis (M)

M Category	M Criteria
M0	No distant metastasis
M1	Distant metastasis
M1a	Metastasis confined to liver
M1b	Metastases in at least one extrahepatic site (e.g., lung, ovary, nonregional lymph node, peritoneum, bone)
M1c	Both hepatic and extrahepatic metastases

AJCC PROGNOSTIC STAGE GROUPS

When T is...	And N is...	And M is...	Then the stage group is...
T1	N0	M0	I
T1	N1	M0	III
T1	N0, N1	M1	IV
T2	N0	M0	II
T2	N1	M0	III
T2	N0, N1	M1	IV
T3	N0	M0	II
T3	N1	M0	III
T3	N0, N1	M1	IV
T4	N0	M0	III
T4	N1	M0	III
T4	N0, N1	M1	IV

REGISTRY DATA COLLECTION VARIABLES

1. Size of tumor (value or unknown)
2. Depth of invasion
3. Nodal status and number of nodes involved, if applicable
4. Sites of metastasis, if applicable
5. Ki-67 index
6. Mitotic count
7. Histologic grading (from Ki-67 and mitotic count): G1, G2, G3
8. Preoperative pancreastatin level
9. Preoperative gastrin level
10. Preoperative CgA level
11. Type of gastric NET (I, II, or III)

29

HISTOLOGIC GRADE (G)

Cellular pleomorphism per se is not a useful feature for grading NETs. The following grading scheme has been proposed for gastrointestinal NETs.[19-21]

G	G Definition
GX	Grade cannot be assessed
G1	Mitotic count (per 10 HPF)* < 2 and Ki-67 index (%)** < 3
G2	Mitotic count (per 10 HPF) = 2–20 or Ki-67 index (%)** = 3–20
G3	Mitotic count (per 10 HPF) > 20 or Ki-67 index (%)** > 20

*10 HPF = 2 mm^2; at least 50 HPF (at 40× magnification) must be evaluated in areas of highest mitotic density in order to adhere to WHO 2010 criteria.

**MIB1 antibody; % of 500–2,000 tumor cells in areas of highest nuclear labeling.

In cases of disparity between Ki-67 proliferative index and mitotic count, the result that indicates a higher-grade tumor should be selected as the final grade. For example, a mitotic count of 1 per 10 HPF and a Ki-67 of 12% should be designated as a G2 NET.

High-grade NECs and mixed adenoneuroendocrine carcinomas are excluded from this staging system and should be staged according to guidelines for staging adenocarcinomas at this site (found in Chapter 17).

HISTOPATHOLOGIC TYPE

This staging system applies to the following gastric NETs: NET G1 and G2. On rare occasions, tumors may be classified as "well-differentiated G3 NETs." These tumors most commonly behave as well-differentiated neoplasms, as opposed to G3 tumors.

SURVIVAL DATA

Because of the short follow-up period and small study population, there are not enough data to draw reasonable conclusions about the new staging parameters for the survival of patients with gastric NETs.

ILLUSTRATIONS

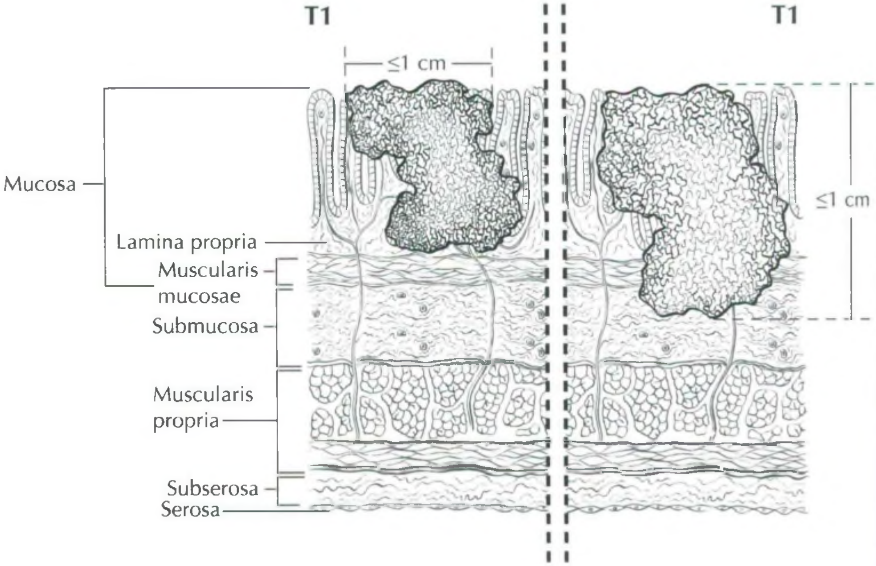

Fig. 29.2 T1 is defined as tumor that invades lamina propria (left side) or submucosa (right side) and 1 cm or less in size

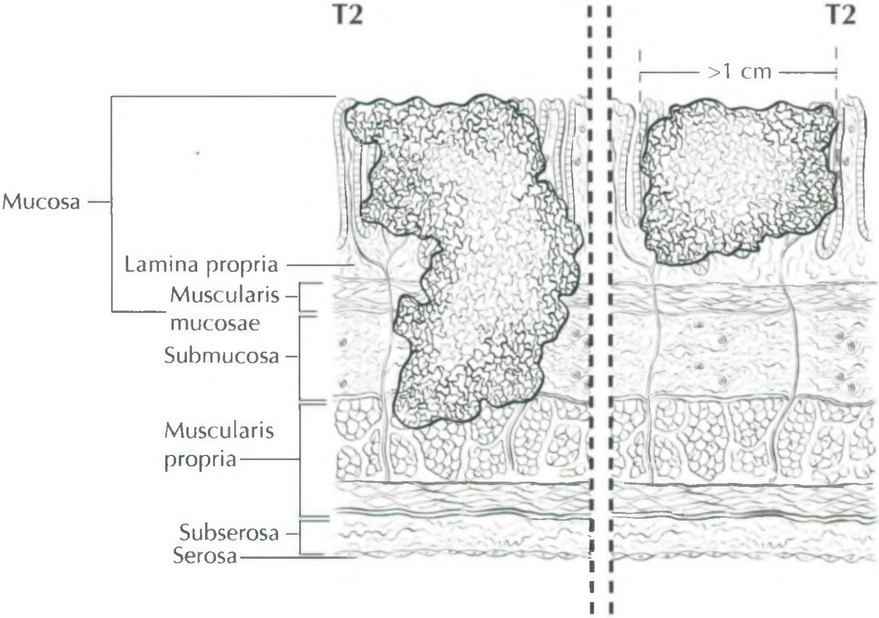

Fig. 29.3 T2 is defined as tumor that invades muscularis propria (left side) or more than 1 cm in size (right side)

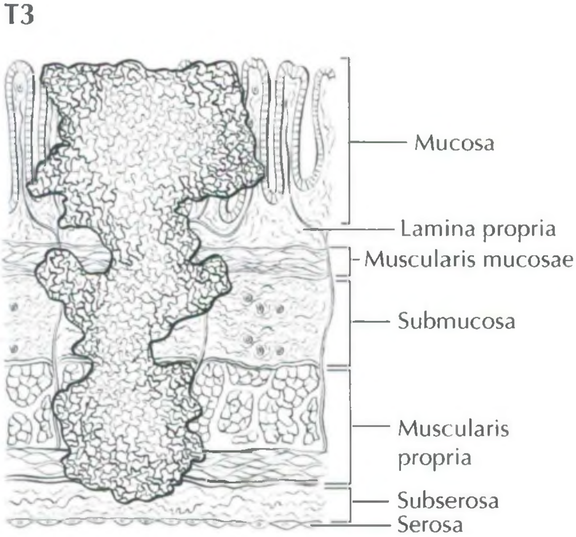

Fig. 29.4 T3 is defined as tumor through the muscularis propria into subserosal tissue without penetration of overlying serosa

29

Fig. 29.5 For stomach T4 is defined as tumor that invades visceral peritoneum (serosa) or other organs or adjacent structures

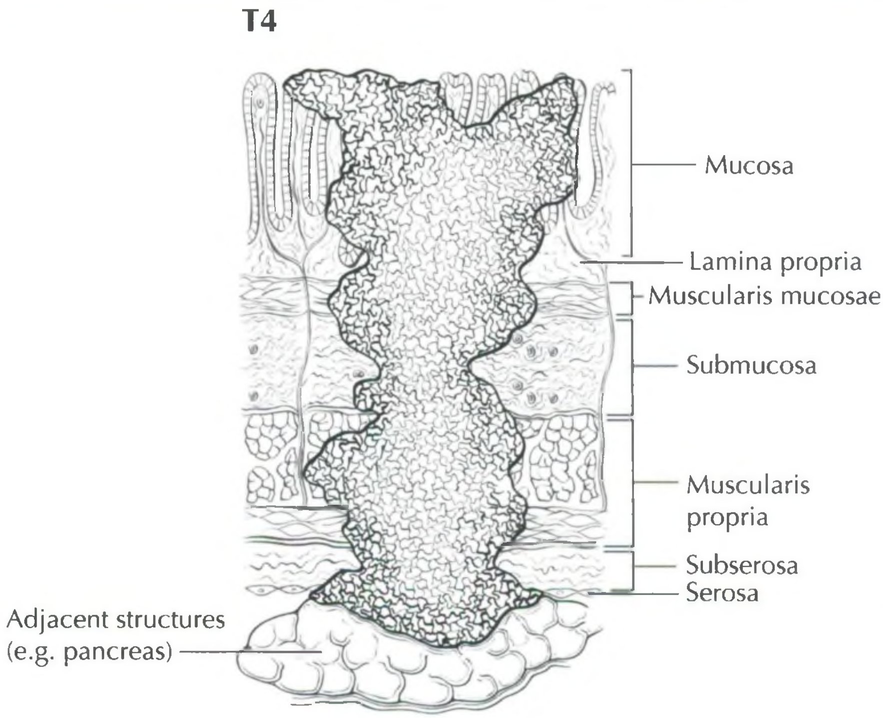

Bibliography

1. Surveillance Epidemiology and End Results (SEER) Program (www.seer.cancer.gov). SEER*Stat Database: Incidence - SEER 9 Regs Research Data, Nov 2013 Sub (1973-2011) <Katrina/Rita Population Adjustment> - Linked To County Attributes - Total U.S., 1969-2012 Counties, National Cancer Institute, DCCPS, Surveillance Research Program, Surveillance Systems Branch, based on the November 2013 submission, released April 2014.
2. Modlin IM, Kidd M, Latich I, Zikusoka MN, Shapiro MD. Current status of gastrointestinal carcinoids. *Gastroenterology*. May 2005;128(6):1717-1751.
3. Vinik A, Woltering EA, O'Dorisio T, Go V, Mamikunian G. Neuroendocrine Tumors: A Comprehensive Guide To Diagnosis And Management. *Inglewood: InterScience Institute*. 2014;5th Edition:13-14, 18-25.
4. La Rosa S, Inzani F, Vanoli A, et al. Histologic characterization and improved prognostic evaluation of 209 gastric neuroendocrine neoplasms. *Human pathology*. Oct 2011;42(10):1373-1384.
5. Rorstad O. Prognostic indicators for carcinoid neuroendocrine tumors of the gastrointestinal tract. *Journal of surgical oncology*. Mar 1 2005;89(3):151-160.
6. Tomassetti P, Migliori M, Caletti GC, Fusaroli P, Corinaldesi R, Gullo L. Treatment of type II gastric carcinoid tumors with somatostatin analogues. *N Engl J Med*. Aug 24 2000;343(8):551-554.
7. Ellison E, O'dorisio T, Woltering E, et al. Suppression of gastrin and gastric acid secretion in the Zollinger-Ellison syndrome by long-acting somatostatin (SMS 201-995). *Scandinavian Journal of Gastroenterology*. 1986;21(S119):206-211.
8. Modlin IM, Latich I, Zikusoka M, Kidd M, Eick G, Chan AK. Gastrointestinal carcinoids: the evolution of diagnostic strategies. *Journal of clinical gastroenterology*. Aug 2006;40(7):572-582.
9. Raines D, Chester M, Diebold AE, et al. A prospective evaluation of the effect of chronic proton pump inhibitor use on plasma biomarker levels in humans. *Pancreas*. May 2012;41(4):508-511.
10. Åkerström G, Norlén O, Edfeldt K, et al. A review on management discussions of small intestinal neuroendocrine tumors' midgut carcinoids'. *International Journal of Endocrine Oncology*. 2015;2(2):119-128.
11. Tan EH, Tan CH. Imaging of gastroenteropancreatic neuroendocrine tumors. *World J Clin Oncol*. Jan 10 2011;2(1):28-43.
12. Srirajaskanthan R, Kayani I, Quigley AM, Soh J, Caplin ME, Bomanji J. The role of 68Ga-DOTATATE PET in patients with neuroendocrine tumors and negative or equivocal findings on 111In-DTPA-octreotide scintigraphy. *Journal of nuclear medicine : official publication, Society of Nuclear Medicine*. Jun 2010;51(6):875-882.
13. Hofman MS, Kong G, Neels OC, Eu P, Hong E, Hicks RJ. High management impact of Ga-68 DOTATATE (GaTate) PET/CT for imaging neuroendocrine and other somatostatin expressing tumours. *J Med Imaging Radiat Oncol*. Feb 2012;56(1):40-47.
14. Prasad V, Ambrosini V, Hommann M, Hoersch D, Fanti S, Baum RP. Detection of unknown primary neuroendocrine tumours (CUP-NET) using (68)Ga-DOTA-NOC receptor PET/CT. *European journal of nuclear medicine and molecular imaging*. Jan 2010;37(1):67-77.
15. Massironi S, Rossi RE, Casazza G, et al. Chromogranin A in diagnosing and monitoring patients with gastroenteropancreatic neuroendocrine neoplasms: a large series from a single institution. *Neuroendocrinology*. 2014;100(2-3):240-249.
16. de Herder WW. Biochemistry of neuroendocrine tumours. *Best practice & research. Clinical endocrinology & metabolism*. Mar 2007;21(1):33-41.
17. Glinicki P, Jeske W, Kapuscinska R, Zgliczynski W. Comparison of chromogranin A (CgA) levels in serum and plasma (EDTA2K) and

the respective reference ranges in healthy males. *Endokrynologia Polska*. 2015;66(1):53-56.
18. Kattan MW, Hess KR, Amin MB, et al. American Joint Committee on Cancer acceptance criteria for inclusion of risk models for individualized prognosis in the practice of precision medicine. *CA: a cancer journal for clinicians*. Jan 19 2016.
19. Rindi G, Kloppel G, Couvelard A, et al. TNM staging of midgut and hindgut (neuro) endocrine tumors: a consensus proposal including a grading system. *Virchows Arch*. Oct 2007;451(4):757-762.
20. Dhall D, Mertens R, Bresee C, et al. Ki-67 proliferative index predicts progression-free survival of patients with well-differentiated ileal neuroendocrine tumors. *Human pathology*. Apr 2012;43(4):489-495.
21. Jann H, Roll S, Couvelard A, et al. Neuroendocrine tumors of midgut and hindgut origin: tumor-node-metastasis classification determines clinical outcome. *Cancer*. Aug 1 2011;117(15):3332-3341.
22. Pape UF, Jann H, Muller-Nordhorn J, et al. Prognostic relevance of a novel TNM classification system for upper gastroenteropancreatic neuroendocrine tumors. *Cancer*. Jul 15 2008;113(2):256-265.
23. Rindi G AR, Capella C, et al. . Nomenclature and classification of digestive neuroendocrine tumors. *In: Bosman F, Carneiro F, ed.^, eds. World Health Organization Classification of Tumours, Pathology and Genetics of Tumours of the Digestive System. Lyon: IARC Press*, . 2010.
24. Rodrigues M, Traub-Weidinger T, Li S, Ibi B, Virgolini I. Comparison of 111In-DOTA-DPhe1-Tyr3-octreotide and 111In-DOTA-lanreotide scintigraphy and dosimetry in patients with neuroendocrine tumours. *European journal of nuclear medicine and molecular imaging*. May 2006;33(5):532-540.

Neuroendocrine Tumors of the Duodenum and Ampulla of Vater

30

Emily K. Bergsland, Eugene A. Woltering, Guido Rindi, Thomas M. O'Dorisio, Richard L. Schilsky, Eric H. Liu, Michelle K. Kim, Eric K. Nakakura, Diane L. Reidy-Lagunes, Jonathan R. Strosberg, Laura H. Tang, Aaron I. Vinik, Yi-Zarn Wang, Elliot A. Asare, James D. Brierley, David L. Bushnell, Robert T. Jensen, Rodney F. Pommier, Edward M. Wolin, Rebecca K.S. Wong, and David S. Klimstra

CHAPTER SUMMARY

Cancers Staged Using This Staging System

This staging system applies to well-differentiated neuroendocrine tumors of the duodenum and ampulla of Vater.

Cancers Not Staged Using This Staging System

These histopathologic types of cancer…	Are staged according to the classification for…	And can be found in chapter…
Carcinomas of the ampulla of Vater, including high-grade (grade 3), poorly differentiated neuroendocrine carcinoma	Ampulla of Vater	27
Carcinomas of the duodenum, including high-grade (grade 3), poorly differentiated neuroendocrine carcinoma	Small intestine	18

Summary of Changes

Change	Details of Change	Level of evidence
New chapter	This staging system was included in the neuroendocrine tumors chapters in previous editions.	N/A
AJCC Prognostic Stage Groups	Duodenal and ampullary neuroendocrine tumors are now considered separately from jejunal and ileal tumors, as they differ in terms of underlying tumor biology and prognosis.	II
Definition of Primary Tumor (T)	The Tis distinction was eliminated. It is not relevant for tumors arising in the duodenum/ampulla.	II

To access the AJCC cancer staging forms, please visit www.cancerstaging.org.

© American Joint Committee on Cancer 2017

M.B. Amin et al. (eds.), *AJCC Cancer Staging Manual, Eighth Edition*, DOI 10.1007/978-3-319-40618-3_30

ICD-O-3 Topography Codes

Code	Description
C17.0	Duodenum
C24.1	Ampulla of Vater (periampullary duodenum, including sphincter of Oddi)

WHO Classification of Tumors

Code	Description
8153	Gastrinoma
8156	Somatostatinoma—*also known as glandular duodenal neuroendocrine tumor (as well as glandular duodenal carcinoid, ampullary somatostatinoma, or psammomatous somatostatinoma)*
8158	Endocrine tumor, functioning, NOS
8240	Neuroendocrine tumor, grade 1
8249	Neuroendocrine tumor, grade 2
8683	Gangliocytic paraganglioma

Bosman FT, Carneiro F, Hruban RH, Theise ND, eds. World Health Organization Classification of Tumours of the Digestive System. Lyon: IARC; 2010.

INTRODUCTION

Neuroendocrine tumors (NETs) arising in the duodenum represent <4% of all gastrointestinal NETs in most series; however, the incidence may be increasing because of physician awareness and a greater use of endoscopy.[1] One recent analysis of Surveillance, Epidemiology, and End Results (SEER) tumor registry data identified 1,258 patients from 1983 to 2010.[2] There was an increase in the incidence rate of duodenal NETs, from 0.27 per 100,000 in 1983 to 1.1 per 100,000 in 2010.[2] Recently diagnosed patients were more likely to present with Stage I disease (69.9% vs. 57.5%; $p < .01$). Most duodenal NETs are nonfunctioning, but gastrinomas associated with Zollinger–Ellison syndrome (ZES) occur in the duodenum, and rare duodenal NETs produce carcinoid syndrome. Histologic grade, depth of invasion, and size are associated with outcome.[3]

Most duodenal NETs are small (<2 cm) and limited to the lamina propria without lymph node involvement.1 However, gastrinomas may develop lymph node metastases if they are very small (<1 cm), and the duodenum often is the primary site in cases presenting with the bulk of disease within regional lymph nodes and an occult primary tumor. In one recent report of 949 duodenal NETs, 47% measured <1 cm, 35% were 1 to 2 cm, and 8 % were >2 cm.[2] Most tumors (76%) were associated with invasion through the lamina propria but not into the muscularis propria. Lymph node involvement was associated with age, depth of invasion (lamina propria, only 4%; invading muscularis propria, 28%; through muscularis propria, 54%; and through serosa, 57%), and size (<1 cm, 3%; 1–2 cm, 13%; and >2 cm, 40%).[2] As such, in contrast to the treatment of ileal tumors, endoscopic mucosal resection (EMR) may be adequate for small nonfunctioning duodenal tumors restricted to the lamina propria, although regional lymphadenectomy is advised for tumors >2 cm or tumors involving the muscularis propria.[2,3] The feasibility of this approach depends on accurate staging of lesions with endoscopic ultrasound (EUS). The accuracy rates for EUS staging range from 80–100%.[4,5] EUS is most helpful in evaluating the depth of NET involvement, excluding lymph node metastases, and determining appropriate candidacy for EMR. Controversy surrounds the optimal therapy for intermediate-size tumors (1–2 cm). The overall survival for duodenal NETs is excellent and on par with NETs of appendiceal origin.[2]

NETs arising in the ampulla of Vater are extremely rare. As such, our understanding of ampullary NETs historically has been based on case reports and limited single-institution case series.[5,6,8-10] Furthermore, the small size of the ampulla and its anatomic continuity with the duodenum mean that many NETs involving the ampulla extend to the adjacent duodenum as well, making clear determination of primary origin challenging. In contrast to NETs arising in other portions of the duodenum, ampulla of Vater tumors often are larger, more likely to be high grade (G3; 41% vs. 11%), more likely to metastasize at a smaller size and lower mitotic count, and associated with a worse overall survival.[6] Some studies also suggest a high risk of regional lymph node involvement.[3] Because high-grade neuroendocrine carcinomas (NECs) fare particularly poorly; grade and extent of disease must be considered carefully when reviewing data related to outcome.[7] For example, a recent analysis of 1,480 patients in the SEER database (92% with duodenal NETs and 8% with ampulla of vater NETs) demonstrated that overall survival is actually similar for patients with locally resected ampullary and duodenal NETs.[6] Therefore, although ampulla of Vater NETs historically have been treated with pancreaticoduodenectomy regardless of tumor size, endoscopic excision may be appropriate for select patients with limited disease.[8,9]

Most tumors arising in the duodenum/ampulla of Vater are nonfunctional. However, functional tumors may occur, the most common being gastrinomas (associated with ZES in one third of cases).[1,5] A gastrinoma is a NET arising in the duodenum (60–80% of cases) or pancreas that produces gastrin, causing gastric acid hypersecretion. ZES is the clinical syndrome resulting from a gastrinoma, characterized by diarrhea, severe gastroesophageal reflux disease (GERD), and refractory peptic ulcer disease (PUD). Most gastrinomas (75–85%) are located in the "gastrinoma triangle" involving the duodenum and pancreatic head.[10] However, they also may arise in the pancreas body/tail and in the fourth portion of the duodenum. Gastrinomas fre-

quently display malignant behavior (60–90%) and often metastasize to regional lymph nodes, even if the primary tumor is quite small.

Somatostatin-producing tumors (somatostatinomas) are less common, accounting for about 1% of gastrointestinal NETs. Pancreatic primaries sometimes are associated with the functional syndrome of mild diabetes mellitus, cholelithiasis, and steatorrhea.[10] However, duodenal and ampullary NETs (which often stain positive for somatostatin on immunohistochemistry [IHC]) rarely are associated with a functional clinical syndrome. Thus, the term *glandular duodenal NET*, reflecting the tumor's most characteristic histologic appearance, is preferred. Synonyms include *ampullary somatostatinoma* and *psammomatous somatostatinoma*. In addition to their glandular growth pattern, they often contain scattered psammoma bodies and may be confused with conventional adenocarcinomas.[7] Traditional carcinoid syndrome, adrenocorticotropic hormone overproduction, and other rare syndromes (including VIPomas) also occur occasionally in association with duodenal or ampullary NETs.[1]

Gangliocytic paraganglioma is a distinctive neoplasm arising in the ampulla of Vater and periampullary duodenum. Gangliocytic paragangliomas contain NET (carcinoid)-like elements but also show variable amounts of ganglion-like cells and spindled Schwann cells. The proportion and distribution of each component vary among cases. Gangliocytic paragangliomas generally are indolent neoplasms that typically do not recur after resection. However, their initial interpretation as benign tumors is now challenged by increasing reports of metastases, usually to lymph nodes. Often only the NET-like elements are found in metastases, but rarely the other two components occur as well.

The etiology of duodenal/ampulla of Vater NETs is largely unknown. Most are thought to be sporadic, although a small fraction (<10%) arise in the setting of a hereditary cancer syndrome, with multiple endocrine neoplasia type 1 (MEN1) being the most common.[11] MEN1 is caused by mutations in the *MEN1* gene located in chromosome region 11q13, thus altering transcriptional regulation, genomic stability, cell division, and cell cycle control.[12] Affected patients develop hyperplasia or tumors of multiple endocrine and nonendocrine tissues.[10] Gastrinomas (>80% duodenal) develop in 54% of MEN1 patients, and MEN1 is present in 20–30% of patients with ZES (usually involving a duodenal gastrinoma).[10] Glandular duodenal NETs arising in the ampulla or periampullary duodenum also may occur in the setting of neurofibromatosis type 1 (NF1) and Von Hippel–Lindau syndrome.[8,13] Gangliocytic paragangliomas also are reported in patients with NF1, but less commonly than glandular duodenal NETs.

The staging of duodenal and ampullary NETs depends on the size and extent of the primary tumor and whether there is lymph node involvement and/or distant metastases. Importantly, the American Joint Committee on Cancer (AJCC) TNM classification system for these tumors was first introduced in the AJCC Cancer Staging Manual, 7th Edition, at which time they were bundled with all NETs arising in the gastrointestinal tract. Given a growing acceptance that the clinical behavior of gastroenteropancreatic NETs (GEP-NETs) tends to be site specific (e.g., histologic variants, overall survival, hormone production), NETs arising in the duodenum and ampulla of Vater are discussed separately from midgut tumors in the AJCC Cancer Staging Manual, 8th Edition, also in reflection of their foregut origin.[14] Furthermore, it may be very difficult to distinguish a true ampullary NET from a periampullary duodenal NET, underscoring the value of a single staging system for NETs arising in this region.

A modification of the AJCC staging system for duodenal tumors was proposed in which 1- to 2-cm tumors confined to the lamina propria would be reclassified as T1b.[2] T1b tumors demonstrated a 4.7% risk of lymph node metastasis and a survival similar to that of T1aN0M0 patients, suggesting that local excision may be a viable option for this group. However, interpretation of these results is limited by the lack of complete information on lymph node status and grade for all patients. [2] Furthermore, the lack of difference in outcome between T1a and T1b patients calls into question the validity of changing the current staging system.

The grading scheme for all NETs arising in the pancreas and gastrointestinal tract was developed by the European Neuroendocrine Tumor Society (ENETS) and adopted by the World Health Organization (WHO) in 2010. This system consists of three grades (G1, G2, and G3) that correspond to well-differentiated NETs (G1 and G2) and poorly differentiated NECs (G3).[15-18] The vast majority of NETs arising in the duodenum are well-differentiated (G1 and G2) tumors.3 Although well-differentiated NETs predominate in the ampulla of Vater, poorly differentiated NECs are evident in up to 42% of cases (and should be staged according to carcinomas arising in this location).[6] Grade is an independent predictor of outcome.

Endoscopic or surgical resection remains the only potentially curative approach for well-differentiated (G1/G2) NETs of the duodenum and ampulla of Vater. Small duodenal NETs <1 cm may be approached with a minimally invasive, endoscopic resection. Before resection is considered, EUS should be performed to confirm NET confined to the mucosa, as well as lack of lymph node involvement. A lymphadenectomy is standard for duodenal tumors >2 cm. The optimal treatment of 1- to 2-cm tumors remains controversial; if EUS reveals tumor limited to the mucosa and without lymph node involvement, patients may be referred for initial EMR.[2] After EMR, surveillance endoscopies should be performed if the margins are negative, in accordance with established guidelines.[19] If the EMR is grossly

30

incomplete and/or the margins are positive, a definitive surgical resection should be performed. An analysis of 949 patients with duodenal NETs (86.1% of whom underwent surgery) identified in the SEER database (1983–2010) demonstrated a 5-year overall survival of 93.8%; 5-year overall survival correlated with AJCC stage: Stage I, 96.7%; Stage IIA, 95.7%; Stage IIB, 83.1%; Stage IIIA, 86.7%; and Stage IIIB, 84.3%.[2] Importantly, neither grade nor functional status was reported for this population. A similar analysis of the SEER database (1988–2009) identified 1,360 duodenal neuroendocrine neoplasms (92% of the study cohort) and 120 ampullary tumors (8% of the study cohort).[6] Of 376 cases for which information was available, 11.6% of duodenal tumors and 41.7% of ampullary tumors were poorly differentiated. Duodenal tumors most commonly presented with localized disease (77%); ampullary tumors typically presented with regional lymph node involvement (55%). The natural history of these tumors is poorly understood because of their relative rarity (4% of all "carcinoid" tumors), but demonstrated prognostic factors include location (ampulla of Vater vs. duodenum), size (duodenum), depth of invasion (duodenum), and grade/degree of differentiation.[3,6] The type of surgery performed depends on the tumor stage, location, and functional status, and ranges from EMR to pancreatoduodenectomy.[19]

Limited treatment options exist for patients with advanced, unresectable NETs arising in the duodenum or ampulla of Vater. Somatostatin analogs have cytostatic activity and may be used to treat hormone-mediated symptoms.[20-22] Chemotherapy plays a limited role in nonpancreatic NETs. Everolimus delays progression in advanced nonfunctional NETs of gastrointestinal origin and is now approved for this indication.[23] The use of liver-directed therapy or other treatments depends on several factors, including the rate of growth, extent of disease, and whether the tumor is functional. See published guidelines for additional information regarding the workup and treatment of duodenal and ampulla of Vater NETs.[19,24]

ANATOMY

Primary Site(s)

The duodenum is composed of the first segment (25 cm) of the small intestine, extending from the pyloric sphincter to the jejunum (Fig. 30.1). The duodenum is divided into four anatomic parts. The first part, or bulb, is connected to the underside of the liver by the hepatoduodenal ligament and lies adjacent to the head of the pancreas, with the common bile duct, portal vein, and gastroduodenal artery passing posteriorly. The second part also passes across the head of the pancreas; the common bile duct and pancreatic duct drain into this part through the ampulla of Vater, and the right kidney and inferior vena cava lie posteriorly. The third part runs horizontally to the left in front of the vena cava

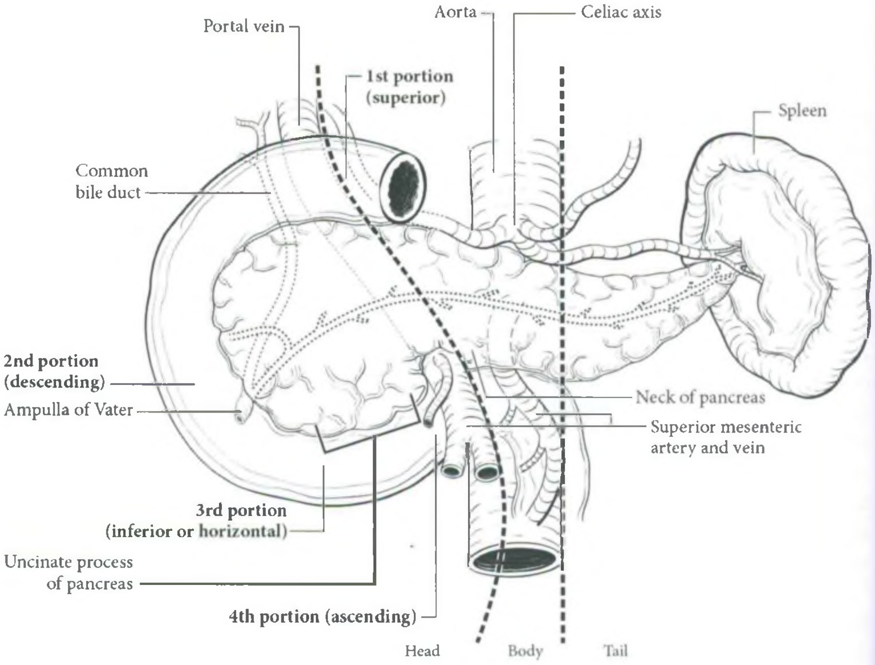

Fig. 30.1 Anatomic sites used in the staging of tumors of the duodenum and ampulla of Vater

and aorta and behind the superior mesenteric vessels. The fourth part ascends to the ligament of Treitz, where an abrupt turn demarcates the beginning of the jejunum. The microscopic anatomy of the duodenum resembles the remainder of the small bowel, with the addition of Brunner's glands, which are most numerous in the first two parts. The anterior surface is covered by peritoneum (serosa), whereas the posterior aspect lies in the retroperitoneum.

The ampulla of Vater is composed of the distal portions of the common bile duct and main pancreatic duct within the wall of the duodenum (Fig. 30.1). The ducts may fuse a variable distance below the duodenal mucosa to form a common channel, or they may remain distinct. Both are ensheathed by circumferential muscle bundles, the sphincter of Oddi, which is continuous with the duodenal muscularis propria and muscularis mucosae. The epithelium lining the distal ducts and common channel is the pancreatobiliary type; it transitions to the intestinal type at the surface of the ampulla, which forms a bulge in the duodenum known as the papilla of Vater. Because the ampulla is such a small structure, neoplasms arising within it extend to involve the adjacent duodenum or underlying pancreas while still quite small. In the case of larger neoplasms with extensive involvement of the ampulla and duodenum, it may be challenging to determine the precise site of origin, and the geographic center of the tumor often is used to define the primary site on gross evaluation. For these reasons, duodenal and ampullary neoplasms often are considered together for purposes of clinical and pathological classification, treatment, and prognosis.

Primary tumors of the first and second parts of the duodenum may invade the head of the pancreas immediately beneath the muscularis propria. Therefore, pancreatic invasion does not constitute invasion of other organs for staging purposes.

Regional Lymph Nodes

For duodenal tumors, the regional lymph nodes are duodenal, hepatic, pancreatoduodenal, infrapyloric, gastroduodenal, pyloric, superior mesenteric, and pericholedochal nodes (Fig. 30.2). Metastases to celiac nodes are considered distant metastases.

The regional nodes for the ampulla may be subdivided as follows:

- Superior: lymph nodes superior to the head and body of the pancreas
- Inferior: lymph nodes inferior to the head and body of the pancreas
- Anterior: anterior pancreatoduodenal, pyloric, and proximal mesenteric lymph nodes
- Posterior: posterior pancreatoduodenal, common bile duct or pericholedochal, and proximal mesenteric nodes

30

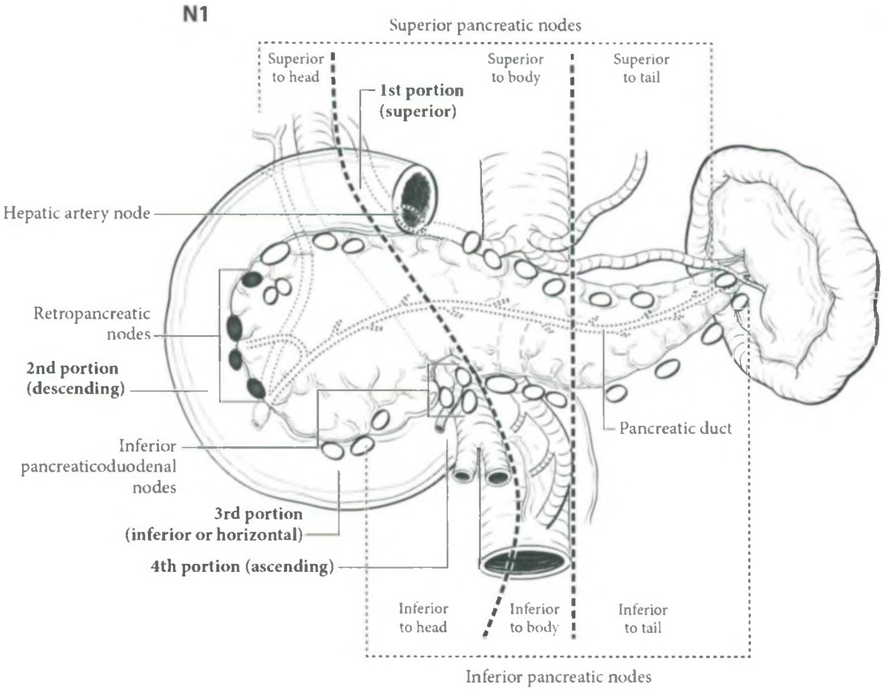

Fig. 30.2 Regional lymph nodes of the duodenum and ampulla of Vater

Metastatic Sites

Distant spread typically involves the liver. Metastases to other sites, such as the lung, bones, and peritoneum, are less common but may occur, particularly with higher-grade tumors[3,25-27] Involvement of the para-aortic or other distant lymph nodes is considered M1 disease.

RULES FOR CLASSIFICATION

Clinical Classification

The classification of NETs is based on size, functionality, site, grade, and invasion. Duodenal NETs encompass a heterogeneous group of neoplasms ranging from nonfunctional tumors to gastrinomas (regardless of whether they occur in the setting of MEN1), somatostatinomas, other functional tumors, gangliocytic paragangliomas, and tumors arising in the ampulla of Vater. The clinical presentation and workup depend on the underlying tumor subtype and level of suspicion for a functional tumor.

Guidelines for the workup of patients with duodenal/ampulla of Vater NETs have been established.[19,24] In general, patients should be evaluated by triple-phase computed tomography (CT) or magnetic resonance (MR) imaging, biochemical assessment as clinically indicated, and somatostatin scintigraphy, esophagogastroduodenoscopy (EGD)/EUS, and/or chest CT as appropriate. In the setting of nonfunctional tumors, some experts suggest using chromogranin A (CgA) as a biomarker.[24] In the setting of suspected hormone-mediated symptoms, additional testing should be performed. Results obtained from endoscopic biopsy specimens, percutaneous biopsies, and fine-needle aspirates also contribute to clinical staging.

Gastrinomas

Gastrinomas typically are single, and 90% are located in the gastrinoma triangle (first part of the duodenum including the bulb and the pancreatic head). Duodenal gastrinomas are usually small (mean, 0.93 cm), whereas the pancreatic tumors generally are larger (mean, 3.8 cm).[33] Clinically, patients present with ZES (peptic ulcerations, diarrhea, and heartburn) with a highly acidic gastric pH (<2). ZES should be suspected in cases of recurrent or severe or familial PUD, ulcers without *Helicobacter pylori* or other risk factors (e.g., use of nonsteroidal anti-inflammatory drugs, aspirin), severe GERD, and PUD resistant to treatment, and in the presence of complications (perforation, bleeding). Prominent gastric folds are seen on endoscopy in 92% of cases.[33] The diarrhea typically responds to therapy with a proton-pump inhibitor (PPI).

Atrophic gastritis (often associated with pernicious anemia), hypercalcemia, and PPI use cause hypergastrinemia. As such, making the diagnosis of gastrinoma may require switching from PPIs to H_2 receptor antagonists, which potentially may cause complications (e.g., worsening PUD, bleeding, perforation); hence, it should be done in a specialty center experienced in diagnosing ZES. A fasting serum gastrin level $\geq$1,000 ng/L (pg/mL) and gastric pH <2 in a normocalcemic patient (off PPIs) free of pyloric obstruction and with normal renal function establish the diagnosis of gastrinoma.[34]

MEN1 should be explored in all patients with ZES, in accordance with established guidelines, as it is present in 20–25% of patients.[28,29] When associated with MEN1, gastrinomas may be multiple and present at an earlier age (approximately one decade earlier).[36] A background of neuroendocrine cell hyperplasia may be found in the duodenum. [30,31]

Somatostatinoma

The term *somatostatinoma* often is used to describe glandular duodenal NETs with particular histologic characteristics (e.g., psammoma bodies, immunohistochemical staining for somatostatin). However, most of these tumors arising in the duodenum are not associated with a clear hormonal syndrome. Therefore, use of the term *somatostatinoma* should be discouraged; *glandular duodenal NET* is more appropriate. Somatostatin is a tetradecapeptide that inhibits the secretion of numerous other hormones and peptides with endocrine and exocrine function. The tumors tend to be large and located in the pancreas (60%) or duodenum/small intestine (40%).[32] Metastatic disease is present at diagnosis in 70% of cases.[32,33] In the case of truly "functional" somatostatinomas, increased blood levels of somatostatin lead to a clinical syndrome that includes diabetes (due to inhibition of insulin), steatorrhea, and gallstones (due to inhibition of cholecystokinin release). A new syndrome of polycythemia, paragangliomas, and duodenal somatostatinomas recently was identified (stemming from somatic gain-of-function hypoxia-inducible factor-2α mutations).[28]

Imaging

EGD is the procedure of choice for localizing and biopsying duodenal/ampullary NETs. EUS also provides useful information regarding clinical staging for both disease sites (to assess tumor depth and regional lymphadenopathy). Studies suggest detection rates of 90–100% for pancreatic lesions and 45–60% for tumors arising in the duodenum.[34] Information necessary for the clinical staging of functional or nonfunctional duodenal/ampullary NETs may be obtained from physical examination; three-dimensional radiographic imaging studies, including triphasic (noncontrast, arterial,

and venous) contrast-enhanced CT or MR imaging; and somatostatin receptor scintigraphy.[35] (Refer to established guidelines for details.[19,24]) Standard fluorodeoxyglucose positron emission tomography (PET) has limited value in the evaluation of well-differentiated NETs. The standard radiographic assessment of resectability includes evaluation for distant metastases (e.g., peritoneal, liver), the patency of the superior mesenteric vein and portal vein and the relationship of these vessels and their tributaries to the tumor, and the relationship of the tumor to the superior mesenteric artery, celiac axis, and hepatic artery.

NET imaging using PET with gallium-68 (^{68}Ga)-labeled somatostatin analogs appears promising (same-day results, potential for increased sensitivity, broader affinity profile, better spatial resolution, easier quantification of tracer uptake).[36-38] Studies to assess the value of ^{68}Ga-labeled somatostatin analog-based PET/CT and PET/MR imaging relative to standard somatostatin scintigraphy are ongoing. This technology represents an emerging imaging tool for NETs in the United States. A kit for the preparation of ^{68}Ga-dotatate injection for PET imaging recently received U.S. Food and Drug Administration approval.

Pathological Classification

Pathological staging is based on EMR and surgical resection specimens (segmental resection of the intestine; ampullectomy; pancreaticoduodenectomy, partial or complete, with or without partial gastrectomy; Whipple resection). The most sensitive pathological staging is obtained by examining surgically resected primary tumor(s), lymph nodes, and distant metastases according to an established minimum pathology dataset.[17,39-41] The College of American Pathologists (CAP) Protocol for Examination of Specimens from Patients with NETs of Small Intestine and Ampulla is recommended as a guideline for the evaluation of duodenal/ampulla of Vater NET pathological specimens (www.cap.org). Required elements also are outlined in the current National Comprehensive Cancer Network (NCCN) guidelines for NETs (www.nccn.org).[19]

Accurate tumor staging requires that all lymph nodes that are removed be analyzed (recognizing that lymph nodes are not assessed in the setting of EMR or ampullectomy). Anatomic division of regional lymph nodes is not necessary; however, separately submitted lymph nodes should be reported as labeled by the surgeon. Finally, an N category (N1 or N0) should be assigned as long as at least one lymph node has been assessed. NX should be applied only if no lymph nodes were assessed (e.g., because of EMR).

For tumors of the first and second portions of the duodenum, pancreaticoduodenectomy, including the tumor and associated regional lymph nodes, provides the optimal information necessary for pathological staging. In pancreatoduodenectomy specimens, the bile duct, pancreatic duct, and superior mesenteric artery margins should be evaluated grossly and microscopically. The superior mesenteric artery margin also has been termed the retroperitoneal, mesopancreatic, and unicate margin. Duodenal (with pylorus-preserving pancreaticoduodenectomy) and gastric (with standard pancreaticoduodenectomy) margin status should be included in the surgical pathology report. Reporting of margins may be facilitated by ensuring documentation of the pertinent margins: 1) common bile (hepatic) duct, 2) pancreatic neck, 3) superior mesenteric artery, 4) other soft tissue margins (i.e., posterior pancreatic, duodenum, and stomach).

IHC for hormones does not have prognostic significance and is performed only in an attempt to document that a tumor is responsible for a functional syndrome. Evidence for hormone expression by IHC does not define tumor functionality (nor does the absolute level of peptide in the blood).

Psammoma Bodies

Psammoma bodies commonly are found in duodenal NETs, especially periampullary tumors expressing somatostatin and associated with NF1 (glandular duodenal NETs).[42]

Restaging

For duodenal and ampullary NETs, the *r* prefix is used for recurrent tumor status (rTNM) following a disease-free interval after treatment.

PROGNOSTIC FACTORS

Prognostic Factors Required for Stage Grouping

Beyond the factors used to assign T, N, or M categories, no additional prognostic factors are required for stage grouping.

Additional Factors Recommended for Clinical Care

Mitotic Count

Tumor grade is determined by mitotic count and Ki-67 labeling index and correlates with outcome in ampullary and duodenal NETs.[3,6,7,25,43] Mitotic count should be assessed as the

number of mitoses per 10 high power fields (HPF): HPF = 2 mm^2, at least 50 fields (at 40× magnification) evaluated. AJCC Level of Evidence: I

Mitotic count # of mitoses per 10 HPF (specify: _____)

___ <2
___ 2 to 20
___ >20
___Not performed

Ki-67 Labeling Index

Tumor grade is determined by mitotic count and Ki-67 labeling index and correlates with outcome in ampullary and duodenal NETs.[3,6,7,43] The proliferation index, as measured by Ki-67, also correlates with these outcome measures in gastrointestinal NETs.[25] The Ki-67 labeling index typically is measured using the MIB1 antibody, by counting the number of immunolabeled tumor cells per 500 to 2,000 cells in areas of highest nuclear labeling; the Ki-67 proliferation index is expressed as a percentage. AJCC Level of evidence: I

___ Ki-67 labeling index (specify: _____)

___ <3%
___ 3% to 20%
___ >20%
___ Other (specify): ____________
___ Not performed

Associated Genetic Syndrome

GEP-NETs rarely arise in the setting of an inherited cancer syndrome characterized by a germline mutation. Patients with an inherited cancer syndrome often have multiple primary NETs, which may be associated with a better prognosis than sporadic tumors, at least in the setting of MEN1.[44,45] AJCC Level of Evidence: II

This factor should be recorded as follows:

- Familial syndrome
  - MEN1
  - Von Hippel–Lindau disease
  - NF1
  - Other syndrome
- Sporadic tumor
- Unknown/unable to assess

Chromogranin A (CgA)

CgA is a 49-kDa acidic polypeptide present in the secretory granules of all neuroendocrine cells. CgA is a general NET marker, and plasma or serum CgA may be used as a marker both in patients with functional NETs and in those with nonfunctional NETs, although specific data on the use of CgA in duodenal/ampullary NETs are unavailable.[46-48] Extrapolating from other NETs (e.g., small bowel NETs), CgA has prognostic significance in the setting of metastatic disease, with higher levels indicating a worse prognosis. Furthermore, changes over time may be useful in assessing for recurrence after surgery or response to therapy in patients with metastatic disease.[46,49,50]

Despite the potential merits of monitoring CgA levels, the clinical utility of CgA is limited by the fact that it is falsely elevated in the setting of PPI use, chronic atrophic gastritis, renal failure, and other conditions.[8] Moreover, levels may fluctuate based on the time of collection and fasting versus nonfasting states. Also, the upper limit of normal (ULN) varies widely and depends on the assay used and whether plasma or serum is assessed; thus, both the assay and the sample type should be considered when comparing CgA values over time.[51] As a result, routine measurement of CgA is not a consensus NCCN recommendation. Multiple Clinical Laboratory Improvement Amendments (CLIA)-compliant and CAP-accredited reference laboratories in the United States can measure CgA levels. AJCC Level of Evidence: II

Location in the Duodenum

Duodenal tumors are classically considered by their location (first, second, third, or fourth portion). Tumors arising in the third or fourth portion of the duodenum may behave more like those arising in other parts of the small bowel (e.g., the jejunum and ileum) and may have a better prognosis than tumors in the first or second portion of the duodenum. Furthermore, although the data are not conclusive, ampulla of Vater tumors may be more advanced at presentation and have a worse outcome than other duodenal tumors (although these differences likely are related to the higher incidence of high-grade tumors in this site of origin).[6] Collection of information about the specific location of the NET within the duodenum may contribute to future refinements to AJCC staging. Location should be categorized as follows (AJCC Level of Evidence: III):

- First portion (bulb)
- Second portion
- Third portion
- Fourth portion
- Ampulla of Vater
- Unknown

RISK ASSESSMENT MODELS

The AJCC recently established guidelines that will be used to evaluate published statistical prediction models for the purpose of granting endorsement for clinical use.[52] Although

this is a monumental step toward the goal of precision medicine, this work was published only very recently. Therefore, the existing models that have been published or may be in clinical use have not yet been evaluated for this cancer site by the Precision Medicine Core of the AJCC. In the future, the statistical prediction models for this cancer site will be evaluated, and those that meet all AJCC criteria will be endorsed.

DEFINITIONS OF AJCC TNM

Definition of Primary Tumor (T)

T Category	T Criteria
TX	Primary tumor cannot be assessed
T1	Tumor invades the mucosa or submucosa only and is ≤1 cm (duodenal tumors);<br>Tumor ≤1 cm and confined within the sphincter of Oddi (ampullary tumors)
T2	Tumor invades the muscularis propria or is >1 cm (duodenal);<br>Tumor invades through sphincter into duodenal submucosa or muscularis propria, or is >1 cm (ampullary)
T3	Tumor invades the pancreas or peripancreatic adipose tissue
T4	Tumor invades the visceral peritoneum (serosa) or other organs

Note: Multiple tumors should be designated as such (and the largest tumor should be used to assign the T category):

- If the number of tumors is known, use T(#); e.g., pT3(4)N0M0.
- If the number of tumors is unavailable or too numerous, use the suffix *m*—T(m)—e.g., pT3(m)N0M0.

Definition of Regional Lymph Node (N)

N Category	N Criteria
NX	Regional lymph nodes cannot be assessed
N0	No regional lymph node involvement
N1	Regional lymph node involvement

Definition of Distant Metastasis (M)

M Category	M Criteria
M0	No distant metastasis
M1	Distant metastases
M1a	Metastasis confined to liver
M1b	Metastases in at least one extrahepatic site (e.g., lung, ovary, nonregional lymph node, peritoneum, bone)
M1c	Both hepatic and extrahepatic metastases

AJCC PROGNOSTIC STAGE GROUPS

When T is...	And N is...	And M is...	Then the stage group is...
T1	N0	M0	I
T2	N0	M0	II
T3	N0	M0	II
T4	N0	M0	III
Any T	N1	M0	III
Any T	Any N	M1	IV

REGISTRY DATA COLLECTION VARIABLES

1. Size of tumor (value, unknown)
2. Maximum depth of invasion (microscopic tumor extension)
   a) Small intestine (including duodenum): cannot be assessed, no evidence of primary tumor, lamina propria, submucosa, muscularis propria, subserosal tissue without involvement of visceral peritoneum, penetrates serosa (visceral peritoneum), directly invades adjacent structures, penetrates visceral peritoneum and adjacent structures
   b) Ampulla of Vater: cannot be assessed, no evidence of primary tumor, tumor limited to ampulla of Vater or sphincter of Oddi, tumor invades duodenal submucosa, tumor invades duodenal muscularis propria, tumor invades pancreas, tumor invades peripancreatic soft tissues, tumor invades common bile duct, directly invades adjacent structures
3. Number of tumors (multicentric disease at primary site)
4. Lymph node status (including number of nodes assessed and number of positive nodes)
5. Grade (based on Ki-67 and mitotic count; G1, G2, G3, unknown)
6. Mitotic count (value; unknown)
7. Ki-67 Labeling Index (value; unknown)
8. Perineural invasion (Y/N)
9. Lymphovascular invasion (Y/N)
10. Margin status (+/–)
11. Functional status (Y/N, if yes, then list type of syndrome)
    a) Functional
       - Gastrininoma (ZES)
       - Somatostatinoma
       - NET causing carcinoid syndrome (5HIAA, serotonin excess)
       - Other
    b) Nonfunctional
    c) Unknown/unable to assess

30

12. Genetic syndrome (Y/N, type of syndrome)
    a) MEN1
    b) Von Hippel–Lindau disease
    c) NF1
    d) Other syndrome, NOS
13. Location in duodenum (first portion, second portion, third portion, fourth portion, ampulla of Vater)
14. Type of surgery (EMR; pancreaticoduodenectomy, partial or complete, with or without partial gastrectomy; Whipple procedure; ampullectomy; segmental resection, small intestine; unknown; other)
15. Preoperative CgA level (absolute value with ULN; unknown)
16. Preoperative pancreastatin level (absolute value with ULN; unknown)
17. Preoperative neurokinin level (absolute value with ULN; unknown)
18. Age of patient
19. Histologic variants
    a) Well-differentiated NET
    b) Glandular duodenal NET (somatostatinoma)
    c) Gangliocytic paraganglioma

HISTOLOGIC GRADE (G)

Grading of duodenal NETs is required for prognostic stratification and should be performed on all resection specimens and on biopsy samples containing enough tumor tissue to allow accurate measurement of proliferation (50 HPF for mitotic counting and 500 cells to determine the Ki-67 labeling index). If multiple disease sites are sampled (e.g., a primary tumor as well as a metastasis), the grade of each site should be recorded separately. In the event multiple foci are sampled within a single anatomic site (e.g., multiple liver metastases), the highest grade may be recorded. The following grading scheme is currently endorsed by ENETS/WHO for gastrointestinal and pancreatic neuroendocrine neoplasms:

The Ki-67 labeling index is based on the region with the highest labeling rate ("hot spot"), determined by examining the Ki-67 stain at low magnification. In the event of discordance between the grade indicated by the mitotic count and that suggested by the Ki-67 index, the higher grade should be assigned. Nuclear pleomorphism is not a useful feature for grading neuroendocrine neoplasms. Although necrosis has been regarded as a prognostic factor in some studies, its presence is not incorporated into the grading scheme.

Well-differentiated NETs are subdivided into G1 and G2 tumors based on proliferative and mitotic index. G1 and G2 refer to well-differentiated NETs displaying diffuse and intense expression of two general IHC neuroendocrine markers (i.e., CgA and synaptophysin). G3 generally indicates a poorly differentiated NEC, which should be staged using the system for duodenal and ampullary carcinomas (Chapters 18 and 27, respectively). High-grade (G3) tumors typically are characterized by a high mitotic count/Ki-67 index, extensive necrosis, and reduced CgA and synaptophysin expression.

Table 30.1 ENETS/WHO grading system for gastroenteropancreatic neuroendocrine neoplasms

G	G Definition
GX	Grade cannot be assessed
G1	Mitotic count (per 10 HPF)* <2 and Ki-67 index (%)** <3
G2	Mitotic count (per 10 HPF) = 2–20 or Ki-67 index (%)** = 3–20
G3	Mitotic count (per 10 HPF) >20 or Ki-67 index (%)** >20

*10 HPF = 2 mm^2; at least 50 HPF (at 40× magnification) must be evaluated in areas of highest mitotic density in order to match WHO 2010 criteria.
**MIB1 antibody; % of 500–2,000 tumor cells in areas of highest nuclear labeling.

In some cases, neuroendocrine neoplasms with well-differentiated histologic features have a Ki-67 index (or, more rarely, a mitotic count) within the G3 range.[53-57] Although these neoplasms currently are considered high grade in the WHO grading scheme, emerging data suggest that they are not as aggressive as poorly differentiated NECs (small cell carcinoma and large cell NEC) and that their response to therapy is more in line with other well-differentiated NETs.[54,58] Although the occurrence of G3 well-differentiated NETs has yet to be documented in the duodenum or ampulla, the concept applies to all gastrointestinal and pancreatic NETs. Progression of a well-differentiated G1 or G2 pancreatic NET to G3 also has been documented.[58] It has been proposed that these well-differentiated neuroendocrine neoplasms be classified as well-differentiated NETs, G3. As such, they should be staged using the parameters of well-differentiated NETs, rather than those of poorly differentiated carcinomas.

HISTOPATHOLOGIC TYPE

This staging system applies to well-differentiated NETs arising in the duodenum or ampulla of Vater:

- Well-differentiated NET
- Glandular duodenal NET (also known as glandular duodenal carcinoid, ampullary somatostatinoma, or psammomatous somatostatinoma)
- Gangliocytic paraganglioma

SURVIVAL DATA

Four years of data (diagnosis years 2004–2008) from the National Cancer Data Base were used to assess survival. Maximum follow-up time for these patients was 3 years.

Selection criteria included primary site codes C17.0 and C24.1; grade 1 and grade 2; histology codes 8153, 8156, 8158, 8240, 8245, 8249, 8246, and 8683; age 18 years or older; and no prior cancers. The investigators identified 609 cases of duodenal NET and only 55 cases of ampullary NET. Product limit survival curves (Kaplan–Meier) and 95% confidence intervals adjusted for age were produced; however, there were not enough cases or follow-up to assess survival accurately. Therefore, survival tables and charts are not included.

ILLUSTRATIONS

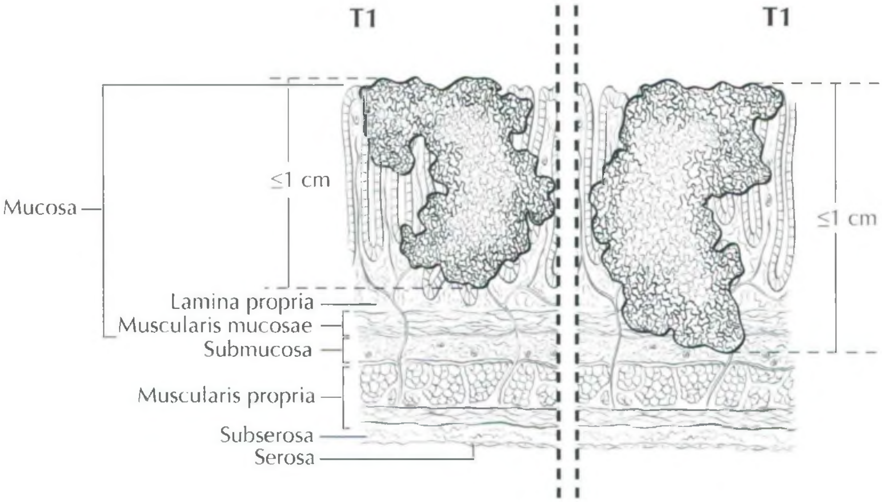

Fig. 30.3 For duodenum T1 is defined as tumor that invades lamina propria (left side) or submucosa (right side) and size 1 cm or less. For ampulla T1 is defined as tumor 1 cm or less

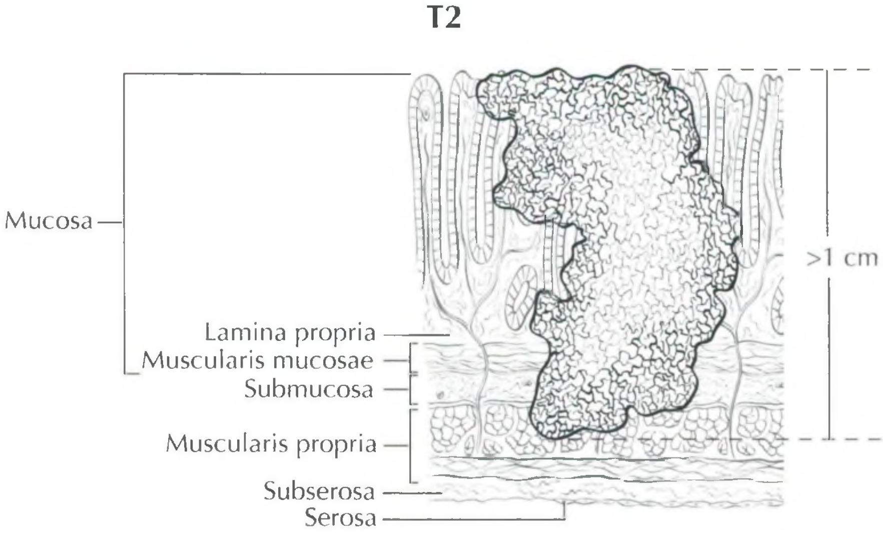

Fig. 30.4 For duodenum T2 is defined as tumor that invades muscularis propria or size > 1 cm. For ampulla T2 is defined as tumor > 1 cm (ampullary tumors)

Bibliography

1. Hoffmann KM, Furukawa M, Jensen RT. Duodenal neuroendocrine tumors: Classification, functional syndromes, diagnosis and medical treatment. *Best practice & research. Clinical gastroenterology.* Oct 2005;19(5):675–697.
2. Kachare SD, Liner KR, Vohra NA, Zervos EE, Fitzgerald TL. A modified duodenal neuroendocrine tumor staging schema better defines the risk of lymph node metastasis and disease-free survival. *The American surgeon.* Aug 2014;80(8):821–826.
3. Untch BR, Bonner KP, Roggin KK, et al. Pathologic grade and tumor size are associated with recurrence-free survival in patients with duodenal neuroendocrine tumors. *Journal of gastrointestinal surgery: official journal of the Society for Surgery of the Alimentary Tract.* Mar 2014;18(3):457-462; discussion 462–453.
4. Yoshikane H, Tsukamoto Y, Niwa Y, et al. Carcinoid tumors of the gastrointestinal tract: evaluation with endoscopic ultrasonography. *Gastrointestinal endoscopy.* May-Jun 1993;39(3):375–383.
5. Shroff SR, Kushnir VM, Wani SB, et al. Efficacy of Endoscopic Mucosal Resection for Management of Small Duodenal Neuroendocrine Tumors. *Surgical laparoscopy, endoscopy & percutaneous techniques.* Oct 2015;25(5):e134–139.
6. Randle RW, Ahmed S, Newman NA, Clark CJ. Clinical outcomes for neuroendocrine tumors of the duodenum and ampulla of Vater: a population-based study. *Journal of gastrointestinal surgery: official journal of the Society for Surgery of the Alimentary Tract.* Feb 2014;18(2):354–362.
7. Albores-Saavedra J, Hart A, Chable-Montero F, Henson DE. Carcinoids and high-grade neuroendocrine carcinomas of the ampulla of vater: a comparative analysis of 139 cases from the surveillance, epidemiology, and end results program-a population based study. *Arch Pathol Lab Med.* Nov 2010;134(11):1692–1696.
8. Clements WM, Martin SP, Stemmerman G, Lowy AM. Ampullary carcinoid tumors: rationale for an aggressive surgical approach. *Journal of gastrointestinal surgery: official journal of the Society for Surgery of the Alimentary Tract.* Sep-Oct 2003;7(6):773–776.
9. Carter JT, Grenert JP, Rubenstein L, Stewart L, Way LW. Neuroendocrine tumors of the ampulla of Vater: biological behavior and surgical management. *Archives of surgery.* Jun 2009;144(6):527–531.
10. Jensen RT, Cadiot G, Brandi ML, et al. ENETS Consensus Guidelines for the management of patients with digestive neuroendocrine neoplasms: functional pancreatic endocrine tumor syndromes. *Neuroendocrinology.* 2012;95(2):98–119.
11. Oberg K. The genetics of neuroendocrine tumors. *Semin Oncol.* Feb 2013;40(1):37–44.
12. Eriksson B, Renstrup J, Imam H, Oberg K. High-dose treatment with lanreotide of patients with advanced neuroendocrine gastrointestinal tumors: clinical and biological effects. *Ann Oncol.* Oct 1997;8(10):1041–1044.
13. Nesi G, Marcucci T, Rubio CA, Brandi ML, Tonelli F. Somatostatinoma: clinico-pathological features of three cases and literature reviewed. *J Gastroenterol Hepatol.* Apr 2008;23(4):521–526.
14. Rindi G, Kloppel G, Alhman H, et al. TNM staging of foregut (neuro)endocrine tumors: a consensus proposal including a grading system. *Virchows Arch.* Oct 2006;449(4):395–401.
15. Rindi G, Klersy C, Inzani F, et al. Grading the neuroendocrine tumors of the lung: an evidence-based proposal. *Endocrine-related cancer.* Feb 2014;21(1):1–16.
16. Rindi G AR, Bosman F, et al. Nomenclature and classification of neuroendocrine neoplasms of the digestive system. *Lyon: IARC Press: .* 2010.
17. Bosman FT, Carneiro F, Hruban RH, Theise ND. *WHO classification of tumours of the digestive system.* World Health Organization; 2010.
18. Qadan M, Ma Y, Visser BC, et al. Reassessment of the current American Joint Committee on Cancer staging system for pancreatic neuroendocrine tumors. *Journal of the American College of Surgeons.* Feb 2014;218(2):188–195.
19. Kulke MH, Shah MH, Benson AB, 3rd, et al. Neuroendocrine tumors, version 1.2015. *Journal of the National Comprehensive Cancer Network: JNCCN.* Jan 2015;13(1):78–108.
20. Caplin ME, Pavel M, Cwikla JB, et al. Lanreotide in metastatic enteropancreatic neuroendocrine tumors. *N Engl J Med.* Jul 17 2014;371(3):224–233.
21. Rinke A, Muller HH, Schade-Brittinger C, et al. Placebo-controlled, double-blind, prospective, randomized study on the effect of octreotide LAR in the control of tumor growth in patients with metastatic neuroendocrine midgut tumors: a report from the PROMID Study Group. *J Clin Oncol.* Oct 1 2009;27(28):4656–4663.
22. Oberg K. Somatostatin analog octreotide LAR in gastro-entero-pancreatic tumors. *Expert review of anticancer therapy.* May 2009;9(5):557–566.
23. Yao JC, Fazio N, Singh S, et al. Everolimus for the treatment of advanced, non-functional neuroendocrine tumours of the lung or gastrointestinal tract (RADIANT-4): a randomised, placebo-controlled, phase 3 study. Lancet. 2016;387(10022):968–977.
24. Delle Fave G, Kwekkeboom DJ, Van Cutsem E, et al. ENETS Consensus Guidelines for the management of patients with gastroduodenal neoplasms. *Neuroendocrinology.* 2012;95(2):74–87.
25. Panzuto F, Merola E, Rinzivillo M, et al. Advanced digestive neuroendocrine tumors: metastatic pattern is an independent factor affecting clinical outcome. *Pancreas.* Mar 2014;43(2):212–218.
26. Jayant M, Punia R, Kaushik R, et al. Neuroendocrine tumors of the ampulla of vater: presentation, pathology and prognosis. *JOP.* May 2012;13(3):263–267.
27. Nassar H, Albores-Saavedra J, Klimstra DS. High-grade neuroendocrine carcinoma of the ampulla of vater: a clinicopathologic and immunohistochemical analysis of 14 cases. *The American journal of surgical pathology.* May 2005;29(5):588–594.
28. Pacak K, Jochmanova I, Prodanov T, et al. New syndrome of paraganglioma and somatostatinoma associated with polycythemia. *J Clin Oncol.* May 1 2013;31(13):1690–1698.
29. Thakker RV, Newey PJ, Walls GV, et al. Clinical practice guidelines for multiple endocrine neoplasia type 1 (MEN1). *The Journal of clinical endocrinology and metabolism.* Sep 2012;97(9):2990–3011.
30. Anlauf M, Perren A, Meyer CL, et al. Precursor lesions in patients with multiple endocrine neoplasia type 1-associated duodenal gastrinomas. *Gastroenterology.* May 2005;128(5):1187–1198.
31. Anlauf M, Perren A, Kloppel G. Endocrine precursor lesions and microadenomas of the duodenum and pancreas with and without MEN1: criteria, molecular concepts and clinical significance. *Pathobiology: journal of immunopathology, molecular and cellular biology.* 2007;74(5):279–284.
32. Williamson JM, Thorn CC, Spalding D, Williamson RC. Pancreatic and peripancreatic somatostatinomas. *Ann R Coll Surg Engl.* Jul 2011;93(5):356–360.
33. Doherty GM. Rare endocrine tumours of the GI tract. *Best practice & research. Clinical gastroenterology.* Oct 2005;19(5):807–817.
34. Anderson MA, Carpenter S, Thompson NW, Nostrant TT, Elta GH, Scheiman JM. Endoscopic ultrasound is highly accurate and directs management in patients with neuroendocrine tumors of the pancreas. *The American journal of gastroenterology.* 2000;95(9):2271–2277.
35. Falconi M, Bartsch DK, Eriksson B, et al. ENETS Consensus Guidelines for the management of patients with digestive neuroendocrine neoplasms of the digestive system: well-differentiated pancreatic non-functioning tumors. *Neuroendocrinology.* 2012;95(2):120–134.

36. Lebtahi R, Cadiot G, Sarda L, et al. Clinical impact of somatostatin receptor scintigraphy in the management of patients with neuroendocrine gastroenteropancreatic tumors. *Journal of nuclear medicine: official publication, Society of Nuclear Medicine*. Jun 1997;38(6):853–858.
37. Hofman MS, Lau WF, Hicks RJ. Somatostatin receptor imaging with 68Ga DOTATATE PET/CT: clinical utility, normal patterns, pearls, and pitfalls in interpretation. *Radiographics: a review publication of the Radiological Society of North America, Inc*. Mar-Apr 2015;35(2):500–516.
38. Toumpanakis C, Kim MK, Rinke A, et al. Combination of cross-sectional and molecular imaging studies in the localization of gastroenteropancreatic neuroendocrine tumors. *Neuroendocrinology*. 2014;99(2):63–74.
39. Klimstra DS. Pathology reporting of neuroendocrine tumors: essential elements for accurate diagnosis, classification, and staging. *Semin Oncol*. Feb 2013;40(1):23–36.
40. Klimstra DS, Modlin IR, Adsay NV, et al. Pathology reporting of neuroendocrine tumors: application of the Delphic consensus process to the development of a minimum pathology data set. *The American journal of surgical pathology*. Mar 2010;34(3):300–313.
41. Travis WD, Brambilla E, Muller-Hermelink HK, Harris CC. Pathology and genetics of tumours of the lung, pleura, thymus and heart. 2004.
42. Burke AP, Sobin LH, Federspiel BH, Shekitka KM, Helwig EB. Carcinoid tumors of the duodenum. A clinicopathologic study of 99 cases. *Arch Pathol Lab Med*. Jul 1990;114(7):700–704.
43. Dumitrascu T, Dima S, Herlea V, Tomulescu V, Ionescu M, Popescu I. Neuroendocrine tumours of the ampulla of Vater: clinico-pathological features, surgical approach and assessment of prognosis. *Langenbeck's archives of surgery / Deutsche Gesellschaft fur Chirurgie*. Aug 2012;397(6):933–943.
44. Goudet P, Dalac A, Le Bras M, et al. MEN1 Disease Occurring Before 21 Years Old: A 160-Patient Cohort Study From the Groupe d'étude des Tumeurs Endocrines. *The Journal of Clinical Endocrinology & Metabolism*. 2015;100(4):1568–1577.
45. Pieterman CR, Conemans EB, Dreijerink KM, et al. Thoracic and duodenopancreatic neuroendocrine tumors in multiple endocrine neoplasia type 1: natural history and function of menin in tumorigenesis. *Endocrine-related cancer*. Jun 2014;21(3):R121–142.
46. de Herder WW. Biochemistry of neuroendocrine tumours. *Best practice & research. Clinical endocrinology & metabolism*. Mar 2007;21(1):33–41.
47. Stronge RL, Turner GB, Johnston BT, et al. A rapid rise in circulating pancreastatin in response to somatostatin analogue therapy is associated with poor survival in patients with neuroendocrine tumours. *Annals of clinical biochemistry*. Nov 2008;45(Pt 6):560–566.
48. Yang X, Yang Y, Li Z, et al. Diagnostic value of circulating chromogranin a for neuroendocrine tumors: a systematic review and meta-analysis. *PloS one*. 2015;10(4):e0124884.
49. Massironi S, Rossi RE, Casazza G, et al. Chromogranin A in diagnosing and monitoring patients with gastroenteropancreatic neuroendocrine neoplasms: a large series from a single institution. *Neuroendocrinology*. 2014;100(2-3):240–249.
50. Yao JC, Pavel M, Phan AT, et al. Chromogranin A and neuron-specific enolase as prognostic markers in patients with advanced pNET treated with everolimus. *The Journal of clinical endocrinology and metabolism*. Dec 2011;96(12):3741–3749.
51. Glinicki P, Jeske W, Kapuscinska R, Zgliczynski W. Comparison of chromogranin A (CgA) levels in serum and plasma (EDTA2K) and the respective reference ranges in healthy males. *Endokrynologia Polska*. 2015;66(1):53–56.
52. Kattan MW, Hess KR, Amin MB, et al. American Joint Committee on Cancer acceptance criteria for inclusion of risk models for individualized prognosis in the practice of precision medicine. *CA: a cancer journal for clinicians*. Jan 19 2016.
53. Basturk O, Yang Z, Tang LH, et al. The high-grade (WHO G3) pancreatic neuroendocrine tumor category is morphologically and biologically heterogenous and includes both well differentiated and poorly differentiated neoplasms. *The American journal of surgical pathology*. May 2015;39(5):683–690.
54. Sorbye H, Strosberg J, Baudin E, Klimstra DS, Yao JC. Gastroenteropancreatic high-grade neuroendocrine carcinoma. *Cancer*. Sep 15 2014;120(18):2814–2823.
55. Velayoudom-Cephise FL, Duvillard P, Foucan L, et al. Are G3 ENETS neuroendocrine neoplasms heterogeneous? *Endocrine-related cancer*. Oct 2013;20(5):649–657.
56. Hijioka S, Hosoda W, Mizuno N, et al. Does the WHO 2010 classification of pancreatic neuroendocrine neoplasms accurately characterize pancreatic neuroendocrine carcinomas? *Journal of gastroenterology*. 2014;50(5):564–572.
57. Heetfeld M, Chougnet CN, Olsen IH, et al. Characteristics and treatment of patients with G3 gastroenteropancreatic neuroendocrine neoplasms. *Endocrine-related cancer*. Aug 2015;22(4):657–664.
58. Tang LH, Untch BR, Reidy DL, et al. Well-Differentiated Neuroendocrine Tumors with a Morphologically Apparent High-Grade Component: A Pathway Distinct from Poorly Differentiated Neuroendocrine Carcinomas. *Clin Cancer Res*. Feb 15 2016;22(4):1011–1017.

Neuroendocrine Tumors of the Jejunum and Ileum

31

Eugene A. Woltering, Emily K. Bergsland, David T. Beyer, Thomas M. O'Dorisio, Guido Rindi, David S. Klimstra, Laura H. Tang, Diane Reidy-Lagunes, Jonathan R. Strosberg, Edward M. Wolin, Aaron I. Vinik, Eric K. Nakakura, Elliot A. Asare, David L. Bushnell, Richard L. Schilsky, Yi-Zarn Wang, Michelle K. Kim, Eric H. Liu, Robert T. Jensen, Rebecca K.S. Wong, John K. Ramage, Kathy Mallin, and Rodney F. Pommier

CHAPTER SUMMARY

Cancers Staged Using This Staging System

This section includes small bowel "carcinoid" tumors (NET G1 and G2, and rare well-differentiated G3) arising in the jejunum and ileum.

Cancers Not Staged Using This Staging System

These histopathologic types of cancer…	Are staged according to the classification for…	And can be found in chapter…
High-grade neuroendocrine carcinoma (NEC)	Small intestine	17
Mixed adenoneuroendocrine carcinoma	Small intestine	17
Neuroendocrine tumors of the duodenum	Neuroendocrine tumors of the duodenum and ampulla of vater	30

Summary of Changes

Change	Details of Change	Level of Evidence
Definitions of Regional Lymph Node (N)	A new classification of nodal involvement, N2, is proposed.	IV
AJCC Prognostic Stage Groups	Stages I–IV have been condensed; i.e., no substages A and B.	II
Primary Site(s)	Duodenum small bowel is no longer staged with this system and has moved to Chapter 30.	III
Emerging Prognostic Factors for Clinical Care	Neurokinin A (NKA) has been added as an emerging prognostic factor for clinical care.	III

To access the AJCC cancer staging forms, please visit www.cancerstaging.org.

© American Joint Committee on Cancer 2017
M.B. Amin et al. (eds.), *AJCC Cancer Staging Manual, Eighth Edition*, DOI 10.1007/978-3-319-40618-3_31

ICD-O-3 Topography Codes

Code	Description
C17.1	Jejunum
C17.2	Ileum

WHO Classification of Tumors

Code	Description
8240	Neuroendocrine tumor (NET) G1 (carcinoid)
8249	NET G2

Bosman FT, Carneiro F, Hruban RH, Theise ND, eds. World Health Organization Classification of Tumours of the Digestive System. Lyon: IARC; 2010.

INTRODUCTION

Queries to the Surveillance Epidemiology and End Results (SEER) database, 1973–2012, demonstrate that the incidence of well-differentiated small intestinal neuroendocrine tumors (NETs) in the US population is 1.2/100,000 in 2012.[1] Overall, NETs are slightly more common in women (55%). Small intestinal (53% in women) NETs follow this trend. The overall black-to-white ratio for gastroenteropancreatic NETs (GEP-NETs) has increased from 1.13 to 1.32 since 1973. Several treatment guidelines exist that may help guide clinical decisions in patients with NETs. These include guidelines published by the European Neuroendocrine Tumor Society (ENETS), the North American Neuroendocrine Tumor Society (NANETS), the National Comprehensive Cancer Network (NCCN), and the American Joint Committee on Cancer (AJCC).

Overall, gut-based NETs exhibit a propensity for slower growth than adenocarcinomas, but aggressive variants are not uncommon. Small bowel NETs often present a considerable diagnostic challenge, especially if covert in the segment of gut that is not examined during routine endoscopic surveillance. Because most small intestinal NETs (90%) are metastatic at diagnosis, a therapeutic management strategy is often complex and requires multispecialty input. The primary jejunal or ileal tumor is usually small but may be multicentric (2% overall but as much as 33% in the small intestine), and clinical symptoms are often absent (hence diagnosis is delayed) until the tumor has metastasized to the liver.[2] In this case, a substantial number of NETs (20% of small intestinal) exhibit a disabling hormonal-induced symptom complex (flushing, sweating, diarrhea, wheezing) traditionally defined as "carcinoid syndrome," which may be difficult to control.[2,3] Extensive local and distant fibrosis due to production of fibroblast growth factor is a common feature of small bowel NETs and may result in local problems (adhesions and obstructions). Production of factors that cause fibrosis also may induce right-sided cardiac valve damage.[3-5]

ANATOMY

Primary Site(s)

The small intestine is the most common primary location (52%).[1] The terminal ileal area is the most common tumor site, and lesions may be multicentric.[1,6] Most NETs of the ileum are composed of serotonin-producing cells. Among 13,601 GEP-NETs in the SEER database, 52% were small intestinal (of the small intestinal GEP-NETs, 8% were jejunal and 67% were ileal).[1] Approximately 70% of patients have tumors that cause symptoms, the most common of which are flushing, diarrhea, and wheezing, also known as the carcinoid syndrome.[2,3,7] Biologically, duodenal NETs behave differently from small bowel NETs; therefore, they should be staged along with ampulla of vater because of their behavioral similarities. Local spread to adjacent organs is often characterized by associated extensive fibrosis.

Regional Lymph Nodes

A rich lymphatic network surrounds the gastrointestinal organs, and NETs exhibit an affinity for spread via the lymphatic system almost equal to spread via the bloodstream.

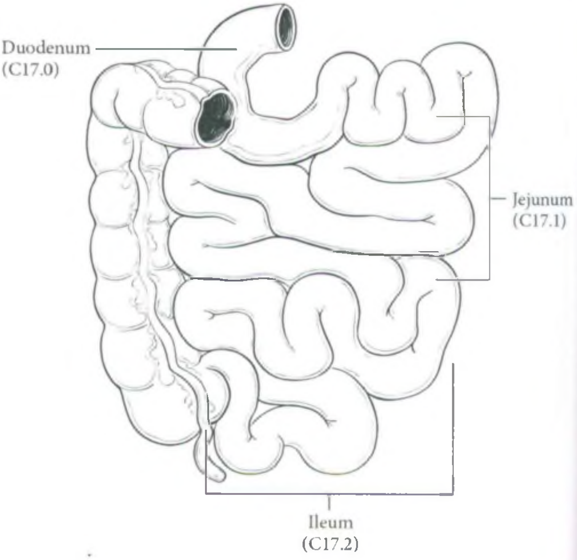

Fig. 31.1 Anatomic sites of the small intestine. This chapter stages neuroendocrine tumors of the jejunum and ileum. See chapter 30 for more information about staging neuroendocrine tumors of the duodenum

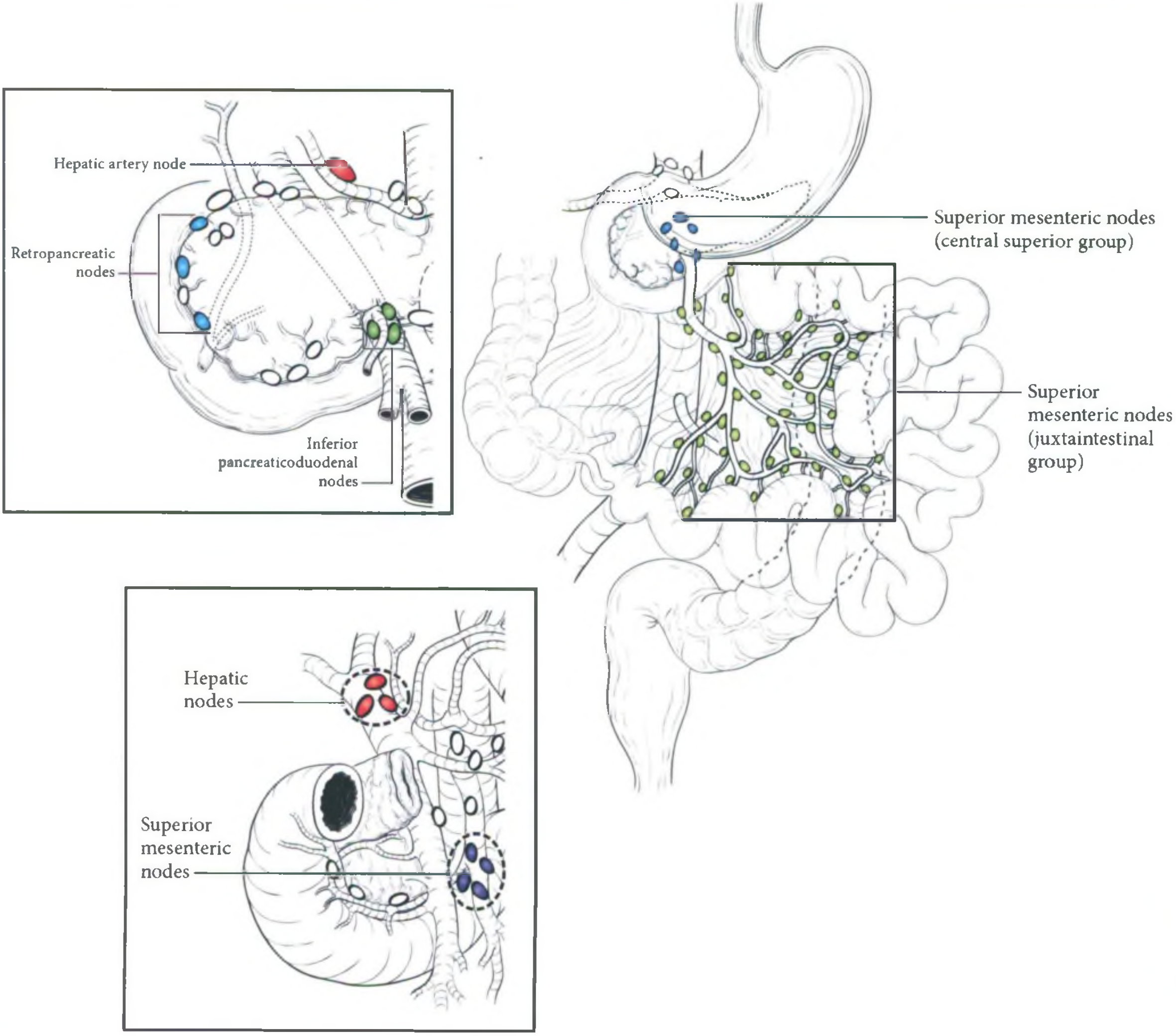

Fig. 31.2 The regional lymph nodes of the small intestine for neuroendocrine tumors

Small Intestine

- *Ileum and jejunum.* Posterior cecal (terminal ileum only), superior mesenteric, and mesenteric (NOS) nodes

Metastatic Sites

The most common metastatic sites of small bowel NETs are the regional and distant lymph nodes (72.4%), liver (19.5%), lung (0.5%), and bone (0.3%).[1] The right diaphragm is also a common site for small bowel NET metastasis.

RULES FOR CLASSIFICATION

Clinical Classification

Clinical staging depends on the anatomic extent and hormonal activity of the primary tumor. Although the extent of liver involvement is difficult to quantify, most clinicians are comfortable with estimating tumor burden in the liver via ultrasound or magnetic resonance (MR) imaging. It is not necessary to estimate the tumor burden in the liver for staging purposes. However, knowledge of the extent of tumor burden is critical for treatment planning. Clinical staging is

31

based on endoscopic biopsies, percutaneous biopsies, fine-needle aspirates, and surgical exploration. Clinical examinations include medical history, physical examination, routine laboratory studies, and biochemical markers of NET disease, including chromogranin A (CgA), plasma or urinary 5-hydroxyindoleacetic acid (5-HIAA; for hormone-secreting tumors), pancreastatin, and NKA. Currently, CgA is widely used as a biomarker for small bowel NETs. CgA is a general NET marker that may reflect tumor load, monitor response to treatment, and correlate with a poor prognosis if elevated.[2,3] However, elevated CgA levels also occur in patients with a variety of other conditions and in the setting of proton-pump inhibitor (PPI) therapy.[8-10] Pancreastatin levels are not affected by PPI use, pernicious anemia, or type I gastric NETs. NKA values greater than 50 pg/mL may portend a worse prognosis than values less than 50 pg/mL. Patients whose NKA levels exceed 50 pg/mL but fall below 50 pg/mL after treatment continue to have an excellent prognosis.[11-13] Although pancreastatin, plasma 5-HIAA, serotonin, and NKA are available for use as biomarkers for NETs, their efficacy must be validated in future prospective trials. Colonoscopy with intubation of the terminal ileum is a common way to identify terminal ileal NETs.

In addition, we propose a novel addition (N2) to the current staging of small bowel NETs. Currently, the adverse effect of large mesenteric masses (>2 cm) or encasement of the mesenteric vessels on survival is supported with level IV evidence.[14-19]

Imaging

Magnetic resonance imaging and triple-phase computed tomography (CT) are effective in the initial localization of NET metastases, with median detection rates and sensitivities of approximately 80%.[2-7] MR imaging scans are highly sensitive in detecting liver metastases, whereas CT scans are more sensitive in their ability to detect extrahepatic metastatic sites.[2] Based on the sensitivity and specificity of MR imaging and CT scans for detecting liver or extrahepatic metastases, respectively, we recommend that both testing modalities be performed during staging. Somatostatin receptor imaging with ^{111}In-pentetreotide (Octreoscan™) has a high sensitivity and specificity (80%–90%) for detecting primary and metastatic tumors.[20] However, ^{68}Ga-labeled octreopeptide positron emission tomography/CT is more accurate than Octreoscan™ imaging for detecting NET sites in a variety of clinical settings.[21-23] Recently, ^{68}Ga octreopeptide scans were approved by the FDA.

Pathological Classification

Pathological staging is based on surgical exploration with resection of at least the primary tumor, with potential resection of lymph nodes and distant metastases.[2] The pathological staging comes with an examination of surgically resected specimen(s). Significant differences may exist in the attributes of multiple primaries or in the primary tissue and its nodal or distant metastases.[24]

In addition, we propose a novel addition (N2) to the current staging of small bowel NETs. Currently, the adverse effect of large mesenteric masses (>2 cm) or encasement of the mesenteric vessels on survival is supported with level IV evidence.[14-19] This change is designed to alert physicians of the increasing incidence of large mesenteric masses and mesenteric vascular encasement, and to enable collection of this information prospectively so as to prospectively validate the utility of the N2 category as a prognostic variable; it will not affect stage groups.

Restaging

For small bowel NETs, the *r* prefix is used for recurrent tumor status (rTNM) following a disease-free interval after treatment.

PROGNOSTIC FACTORS

Prognostic Factors Required for Stage Grouping

Beyond the factors used to assign T, N, or M categories, no additional prognostic factors are required for stage grouping.

Additional Factors Recommended for Clinical Care

Measurement of the following factors is recommended for monitoring the clinical care of patients with well-differentiated small bowel NETs.

Ki-67 Proliferative Index

Histologic tumor grade is determined by Ki-67 proliferative index and/or mitotic count. Ki-67 proliferative index is inversely correlated with patient prognosis and usually measured using MIB1 antibody as a percentage of 500 to 2,000 tumor cells that stain positive in the areas of highest nuclear labeling. AJCC Level of Evidence: I

Mitotic Count

Mitotic count is inversely correlated with patient prognosis and usually measured by determining the number of mitoses per 10 high-power fields (HPF) in the areas of highest mitotic

density. A minimum of 50 HPF must be assessed to adhere to World Health Organization (WHO) 2010 criteria. AJCC Level of Evidence: I

Echocardiogram

Serial echocardiograms may be useful for early detection of carcinoid heart disease, most likely caused by the hypersecretion of vasoactive substances such as serotonin or its metabolites (5-HIAA) in patients with hormone-secreting tumors. AJCC Level of Evidence: I

Plasma or Urinary 5-HIAA Level

The vast majority of symptomatic NETs present with elevated serotonin, which may be detected with a very sensitive and specific assay for 24-hour urine metabolites of serotonin (5-HIAA). Plasma 5-HIAA (also commercially available) correlates very closely with the 24-hour urine values of 5-HIAA.[10,25,26] Multiple reference laboratories in the United States that are licensed by the Clinical Laboratory Improvement Amendments (CLIA) and accredited by the College of American Pathology (CAP) measure plasma or urinary 5-HIAA. AJCC Level of Evidence: II

Plasma Pancreastatin Level

Pancreastatin has proved to be a reliable biomarker for NETs because it is not affected by PPI use, type I gastric NETs, or pernicious anemia.[9,10] Pancreastatin levels were significantly correlated with both progression-free survival (PFS) and overall survival (OS; $P < 0.05$) when all biomarkers were characterized as categorical (binary) variables. Only elevated preoperative pancreastatin showed significant association with worse PFS and OS ($p < 0.05$).[26,27] There are at least three large reference laboratories that are CLIA licensed and CAP sanctioned and routinely measure pancreastatin. AJCC Level of Evidence: III

Plasma Serotonin Level

Repeat serotonin measurements from the same referral laboratory are excellent indicators of tumor burden and the severity of carcinoid syndrome in a patient.[25] Several CLIA-licensed and CAP-accredited reference laboratories in the United States measure whole-blood serotonin levels. These measurements are performed using isocratic, reverse-phase high-performance liquid chromatography.[28] AJCC Level of Evidence: II

Chromogranin A Level

Chromogranin A is a 49-kDa acidic polypeptide present in the secretory granules of all neuroendocrine cells. CgA is a general NET marker, and plasma or serum CgA may be used as a marker in patients with small bowel NETs. CgA has prognostic significance, with higher levels associated with a worse prognosis.[2,3] Furthermore, changes over time may be useful in assessing for recurrence after surgery or response to therapy in patients with metastatic disease.[29,30]

Despite the potential merits of monitoring CgA levels, the clinical utility of CgA is limited by the fact that it is falsely elevated in the setting of PPI use, chronic atrophic gastritis, renal failure, and other conditions.[8-10] Moreover, levels may fluctuate depending on the time of collection and fasting versus nonfasting states. Also, the upper limit of normal varies widely and depends on the assay used and whether plasma or serum is assessed; thus, both the assay and type of sample should be considered when comparing CgA values over time.[31] As a result, routine measurement of CgA is not a consensus NCCN recommendation. Multiple CLIA-licensed and CAP-accredited reference laboratories in the United States measure CgA levels. AJCC Level of Evidence: II

RISK ASSESSMENT MODELS

The AJCC has recently established guidelines that will be used to evaluate published statistical prediction models for the purpose of granting endorsement for clinical use.[32] Although this is a monumental step forward towards the goal of precision medicine, this work was only very recently published. For this reason, the existing models that have been published or may be in clinical use have not yet been evaluated for this cancer site by the Precision Medicine core of the AJCC. In the future, the statistical prediction models for this cancer site will be evaluated, and those that meet all AJCC criteria will be endorsed.

DEFINITIONS OF AJCC TNM

Definition of Primary Tumor (T)

T Category	T Criteria
TX	Primary tumor cannot be assessed
T0	No evidence of primary tumor
T1*	Invades lamina propria or submucosa and less than or equal to1 cm in size
T2*	Invades muscularis propria or greater than 1 cm in size
T3*	Invades through the muscularis propria into subserosal tissue without penetration of overlying serosa
T4*	Invades visceral peritoneum (serosal) or other organs or adjacent structures

Note:* For any T, add (m) for multiple tumors [TX(#) or TX(m), where X = 1–4, and # = number of primary tumors identified]; for multiple tumors with different T, use the highest.

***Example:* If there are two primary tumors, only one of which invades through the muscularis propria into subserosal tissue without penetration of overlying serosa (jejunal or ileal), we define the primary tumor as either T3(2) or T3(m).

Definition of Regional Lymph Node (N)

N Category	N Criteria
NX	Regional lymph nodes cannot be assessed
N0	No regional lymph node metastasis has occurred
N1	Regional lymph node metastasis less than 12 nodes
N2	Large mesenteric masses (>2 cm) and/or extensive nodal deposits (12 or greater), especially those that encase the superior mesenteric vessels

Definition of Distant Metastasis (M)

M Category	M Criteria
M0	No distant metastasis
M1	Distant metastasis
M1a	Metastasis confined to liver
M1b	Metastases in at least one extrahepatic site (e.g., lung, ovary, nonregional lymph node, peritoneum, bone)
M1c	Both hepatic and extrahepatic metastases

AJCC PROGNOSTIC STAGE GROUPS

When T is...	And N is...	And M is...	Then the stage group is...
T1	N0	M0	I
T1	N1, N2	M0	III
T1	N0, N1, N2	M1	IV
T2	N0	M0	II
T2	N1, N2	M0	III
T2	N0, N1, N2	M1	IV
T3	N0	M0	II
T3	N1, N2	M0	III
T3	N0, N1, N2	M1	IV
T4	N0	M0	III
T4	N1, N2	M0	III
T4	N0, N1, N2	M1	IV

For multiple synchronous tumors, the highest T category should be used and the multiplicity or the number of tumors should be indicated in parenthesis: e.g., T3(2) or T3(m).

REGISTRY DATA COLLECTION VARIABLES

1. Size of tumor (value or unknown)
2. Tumor focality (unifocal or multifocal)
3. Depth of Invasion
4. Nodal status and number of nodes involved, if applicable
5. Sites of metastasis, if applicable
6. NKA level
7. Pancreastatin level
8. Ki-67 index
9. Mitotic count
10. Histologic grading (from Ki-67 and mitotic count): G1, G2, G3

HISTOLOGIC GRADE (G)

Cellular pleomorphism per se is not a useful feature for grading NETs. The following grading scheme has been proposed for gastrointestinal NETs[33-35]:

G	G Definition
GX	Grade cannot be assessed
G1	Mitotic count (per 10 HPF)* < 2 and Ki-67 index (%)** < 3
G2	Mitotic count (per 10 HPF) = 2–20 or Ki-67 index (%)** = 3–20
G3	Mitotic count (per 10 HPF) > 20 or Ki-67 index (%)** > 20

*10 HPF = 2 mm^2; at least 50 HPFs (at 40× magnification) must be evaluated in areas of highest mitotic density in order to adhere to WHO 2010 criteria.

**MIB1 antibody; % of 500–2,000 tumor cells in areas of highest nuclear labeling.

In cases of disparity between Ki-67 proliferative index and mitotic count, the result indicating a higher-grade tumor should be selected as the final grade. For example, a mitotic count of 1 per 10 HPF and a Ki-67 of 12% should be designated as a G2 NET.

HISTOPATHOLOGIC TYPE

This staging system applies to the following intestinal NETs: NETs G1 and G2. On rare occasions, tumors may be classified as "well-differentiated G3 NETs." These tumors most commonly behave as well-differentiated neoplasms, as opposed to high grade neuroendocrine carcinomas.

Adenocarcinomas, high-grade neuroendocrine carcinomas, goblet cell carcinomas, and other mixed adenoneuroendocrine carcinomas are excluded from this staging system, and these tumors should be staged according to guidelines for staging carcinomas at these sites.

SURVIVAL DATA

Four years of data (diagnosis years 2010–2013) from the National Cancer Data Base were used to assess survival. Maximum follow-up time for these patients was 3 years. Product limit survival curves (Kaplan–Meier) and 95% confidence intervals adjusted for age were produced. Selection criteria included the following: primary site codes C17.1 and C17.2; histology codes 8240, 8241, 8246, and 8249; ages 18 and older; only primary or first of multiple primaries. Only patients with low-grade disease were included; *low grade* is defined by mitotic count, or if the mitotic count was missing, only those with grade 1 and grade 2 were selected.

To determine clinical stage, AJCC Cancer Staging Manual, 7th edition T, N, and M were used. Kaplan–Meier survival curves for each clinical stage are shown in Figs. 31.3 to 31.7.

For pathological N, number of nodes positive was used to determine N1 (1–11 positive nodes), and N2 (12 or more positive nodes). Kaplan–Meier survival curves for each pathological stage are shown in Figs. 31.8 to 31.12. There were 1,906 patients with clinical stage information and 3,445 patients with pathological stage information who were included in the survival analyses. The staging definitions are included in the tables in "Definitions of AJCC TNM" and "AJCC Prognostic Stage Groups." Using a 5% significance level, log-rank test results showed no significant differences between clinical Stages I and II, Stages II and III, or Stages III and IV. For pathological stage, only Stage III vs Stage IV was significantly different ($p < .001$).

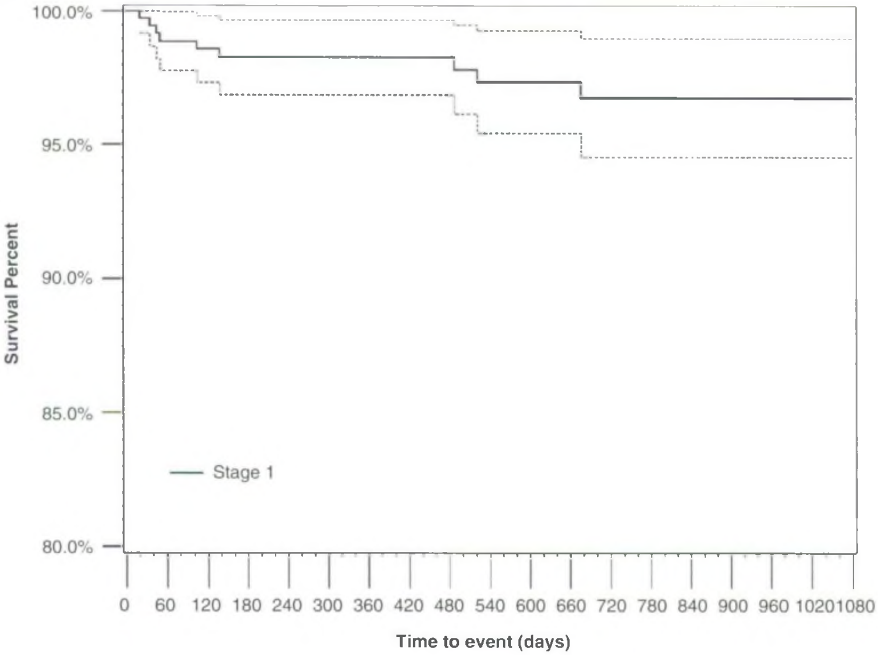

Fig. 31.3 Neuroendocrine tumors of the small bowel: Kaplan–Meier survival curves with 95% confidence intervals by the 8th Edition proposed clinical Stage I, n =269/1855. Data from the National Cancer Data Base (diagnosis years 2010–2013), adjusted for patient age

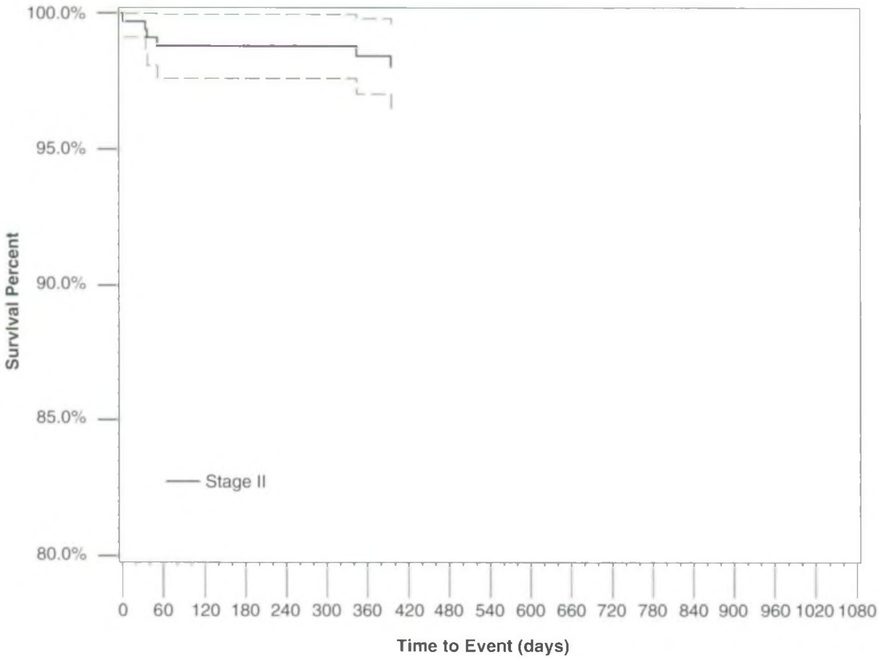

Fig. 31.4 Neuroendocrine tumors of the small bowel: Kaplan–Meier survival curves with 95% confidence intervals by the 8th Edition proposed clinical Stage II, n =240/1855. Data from the National Cancer Data Base (diagnosis years 2010–2013), adjusted for patient age

31

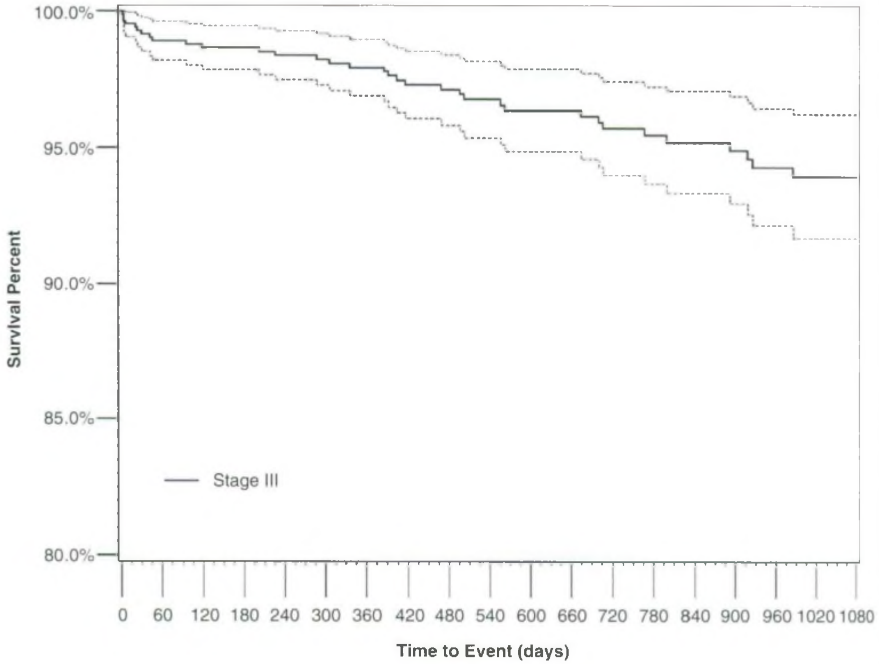

Fig. 31.5 Neuroendocrine tumors of the small bowel: Kaplan–Meier survival curves with 95% confidence intervals by the 8th Edition proposed clinical Stage III, n =617/1855. Data from the National Cancer Data Base (diagnosis years 2010–2013), adjusted for patient age

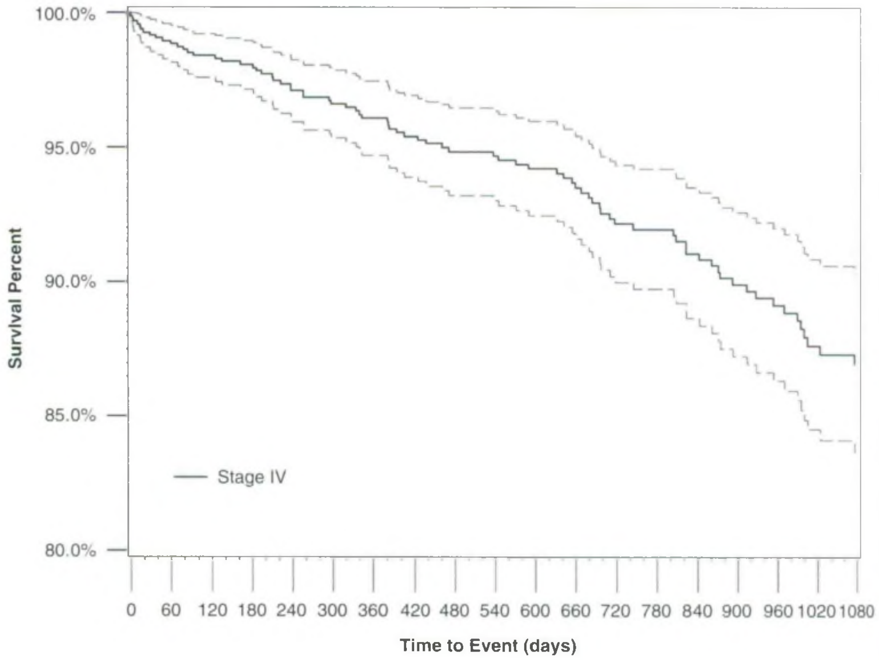

Fig. 31.6 Neuroendocrine tumors of the small bowel: Kaplan–Meier survival curves with 95% confidence intervals by the 8th Edition proposed clinical Stage IV, n =729/1855. Data from the National Cancer Data Base (diagnosis years 2010–2013), adjusted for patient age

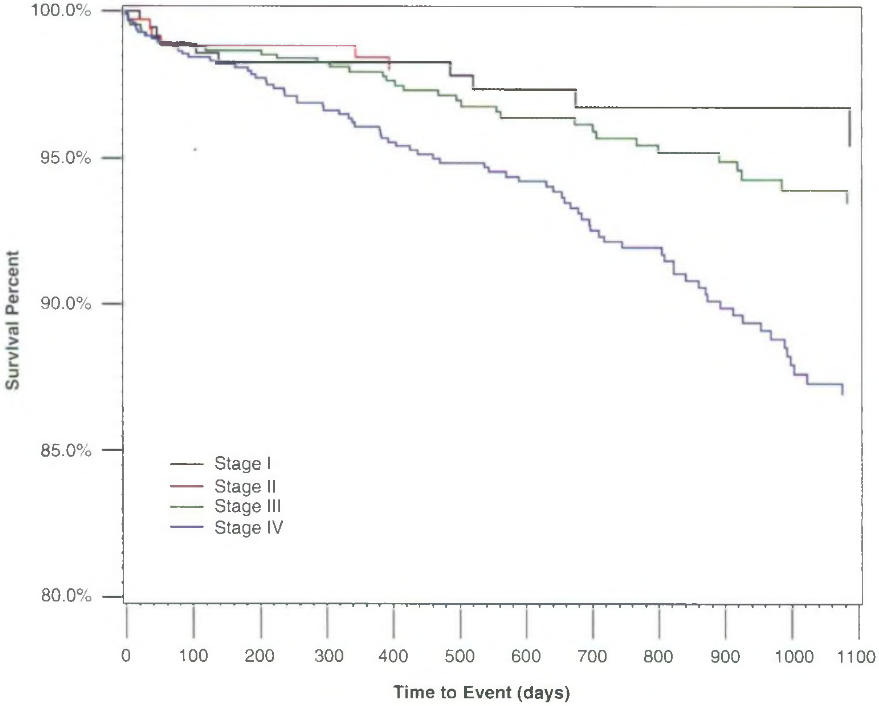

Fig. 31.7 Neuroendocrine tumors of the small bowel: Kaplan–Meier survival curves by the 8th Edition proposed clinical stage, with a follow-up time of 3 years (2010–2013) and adjusted for patient age. A total of 1,855 patients were included in the clinical stage survival calculation. Data from the National Cancer Data Base

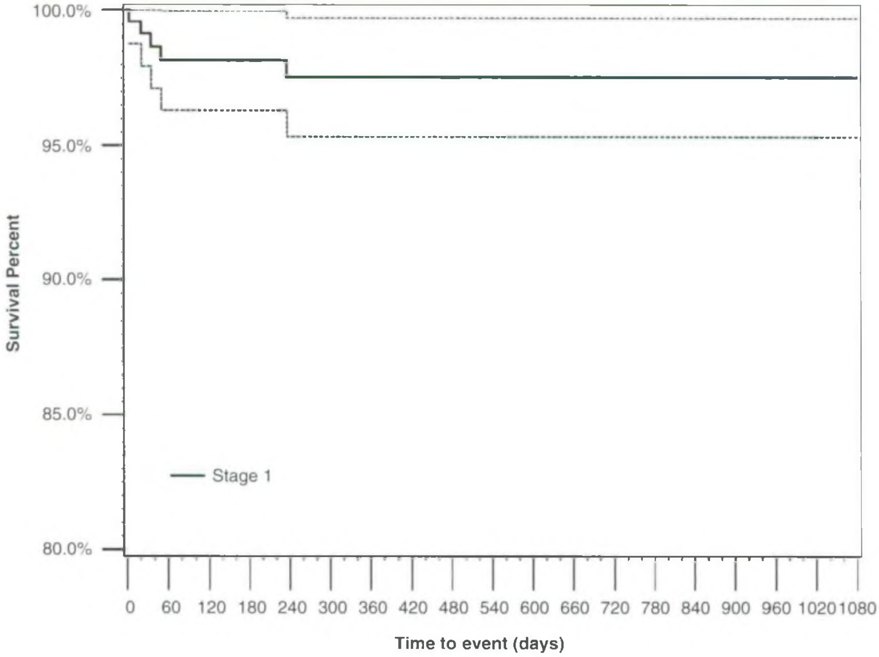

Fig. 31.8 Neuroendocrine tumors of the small bowel: Kaplan–Meier survival curves with 95% confidence intervals by 8th Edition pathological Stage I, n = 142/3366 Data from the National Cancer Data Base (diagnosis years 2010–2013), adjusted for patient age

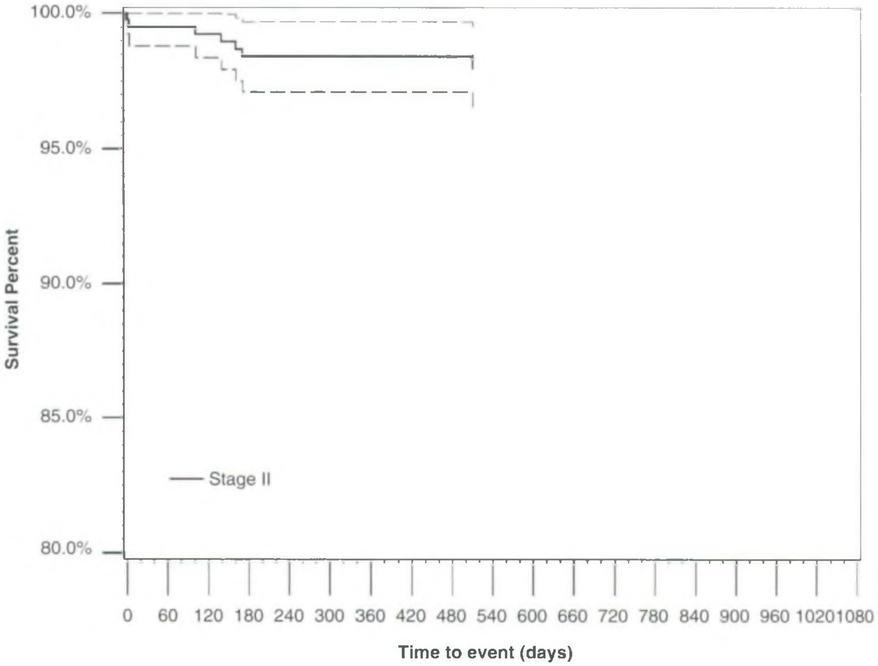

Fig. 31.9 Neuroendocrine tumors of the small bowel: Kaplan–Meier survival curves with 95% confidence intervals by 8th Edition pathological Stage II, n = 281/3366 Data from the National Cancer Data Base (diagnosis years 2010–2013), adjusted for patient age

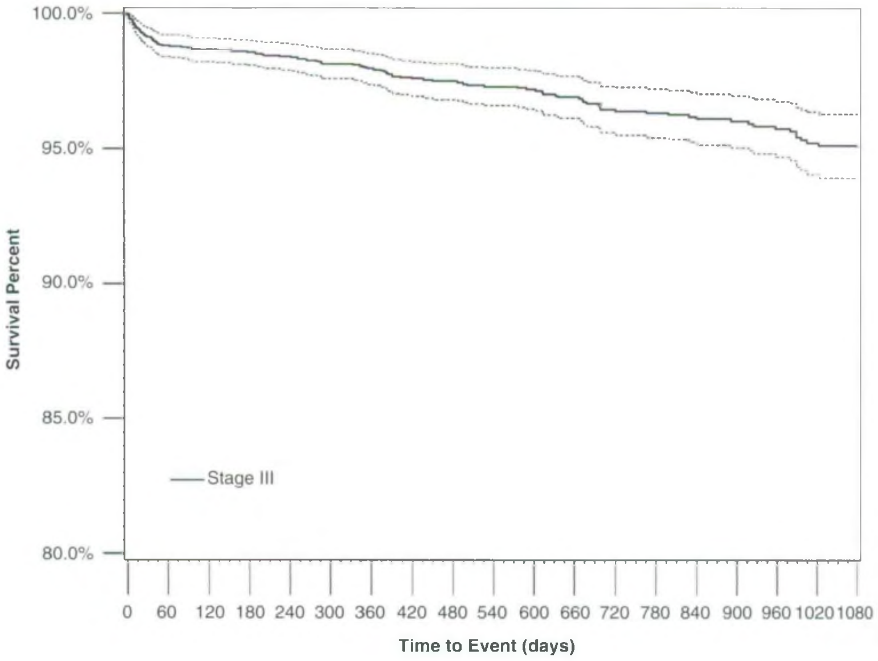

Fig. 31.10 Neuroendocrine tumors of the small bowel: Kaplan–Meier survival curves with 95% confidence intervals by 8th Edition pathological Stage III, n =2,136/3366. Data from the National Cancer Data Base (diagnosis years 2010–2013), adjusted for patient age

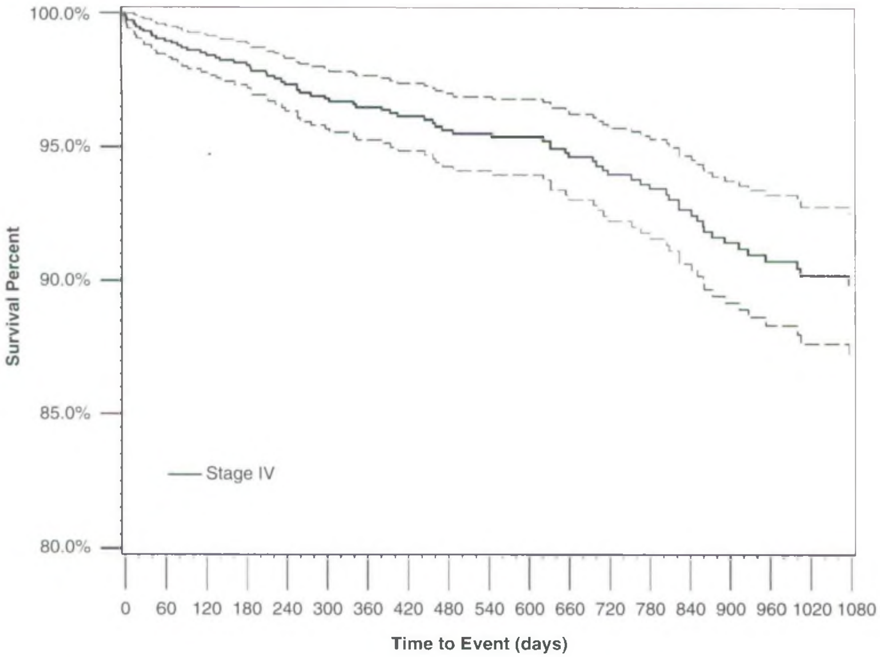

Fig. 31.11 Neuroendocrine tumors of the small bowel: Kaplan–Meier survival curves with 95% confidence intervals by 8th Edition pathological Stage IV, n =807/3366. Data from the National Cancer Data Base (diagnosis years 2010–2013), adjusted for patient age

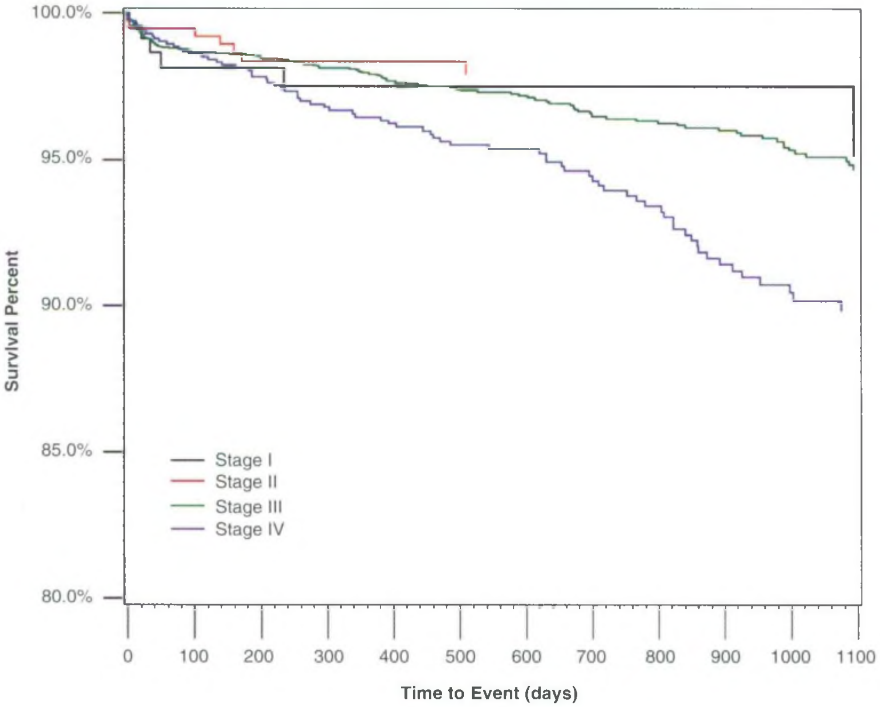

Fig. 31.12 Kaplan–Meier survival curves for each 8th Edition pathological stage for small bowel primary NETs, with a follow-up time of 3 years (2010–2013) and adjusted for patient age. A total of 3,366 patients were included in the pathological stage survival calculation. Data from the National Cancer Data Base

31

Bibliography

1. Surveillance Epidemiology and End Results (SEER) Program (www.seer.cancer.gov) SEER*Stat Database: Incidence - SEER 9 Regs Research Data, Nov 2013 Sub (1973-2011) <Katrina/Rita Population Adjustment> - Linked To County Attributes - Total U.S., 1969-2012 Counties, National Cancer Institute, DCCPS, Surveillance Research Program, Surveillance Systems Branch, based on the November 2013 submission, released April 2014.
2. Modlin IM, Latich I, Zikusoka M, Kidd M, Eick G, Chan AK. Gastrointestinal carcinoids: the evolution of diagnostic strategies. *Journal of clinical gastroenterology*. Aug 2006;40(7): 572-582.
3. Rorstad O. Prognostic indicators for carcinoid neuroendocrine tumors of the gastrointestinal tract. *Journal of surgical oncology*. Mar 1 2005;89(3):151-160.
4. Capella C AR, Klimstra DS, Klöppel G, Komminoth P, Solcia E, Rindi G. Neuoroendocrine neoplasms of the small intestine. In: Bosman F, Carneiro F, ed. eds. World Health Organization Classification of Tumours. Pathology and Genetics of Tumours of the Digestive System. Lyon: IARC Press, 2010; 2010: 102-107.
5. Norlen O, Stalberg P, Oberg K, et al. Long-term results of surgery for small intestinal neuroendocrine tumors at a tertiary referral center. *World journal of surgery*. Jun 2012;36(6):1419-1431.
6. Katona TM, Jones TD, Wang M, Abdul-Karim FW, Cummings OW, Cheng L. Molecular evidence for independent origin of multifocal neuroendocrine tumors of the enteropancreatic axis. *Cancer Res*. May 1 2006;66(9):4936-4942.
7. Modlin IM, Kidd M, Latich I, Zikusoka MN, Shapiro MD. Current status of gastrointestinal carcinoids. *Gastroenterology*. May 2005; 128(6):1717-1751.
8. Åkerström G, Norlén O, Edfeldt K, et al. A review on management discussions of small intestinal neuroendocrine tumors' midgut carcinoids'. *International Journal of Endocrine Oncology*. 2015;2(2): 119-128.
9. Raines D, Chester M, Diebold AE, et al. A prospective evaluation of the effect of chronic proton pump inhibitor use on plasma biomarker levels in humans. *Pancreas*. May 2012;41(4):508-511.
10. Vinik A WE, O'Dorisio T, Go V, Mamikunian G. Neuroendocrine Tumors: A Comprehensive Guide To Diagnosis And Management. 5th ed. Inglewood: InterScience Institute. 2014:13-14,18-25.
11. Diebold AE, Boudreaux JP, Wang YZ, et al. Neurokinin A levels predict survival in patients with stage IV well differentiated small bowel neuroendocrine neoplasms. *Surgery*. Dec 2012;152(6): 1172-1176.
12. Ardill JES JB, Turner GB, McGinty A, McCance DR. Improved prognosis in midgut carcinoid patients by treating raising circulating neurokinin A (NKA) [abstract]. . *Glasgow, UK: European Congress of Endocrinology*. 2006.
13. Turner GB, Johnston BT, McCance DR, et al. Circulating markers of prognosis and response to treatment in patients with midgut carcinoid tumours. *Gut*. Nov 2006;55(11):1586-1591.
14. Gonzalez RS, Liu EH, Alvarez JR, Ayers GD, Washington MK, Shi C. Should mesenteric tumor deposits be included in staging of well-differentiated small intestine neuroendocrine tumors&quest. *Modern Pathology*. 2014.
15. Strosberg JR, Weber JM, Feldman M, Coppola D, Meredith K, Kvols LK. Prognostic validity of the American Joint Committee on Cancer staging classification for midgut neuroendocrine tumors. *J Clin Oncol*. Feb 1 2013;31(4):420-425.
16. Woodbridge LR, Murtagh BM, Yu DF, Planche KL. Midgut neuroendocrine tumors: imaging assessment for surgical resection. *Radiographics: a review publication of the Radiological Society of North America, Inc*. Mar-Apr 2014;34(2):413-426.
17. Hellman P, Lundstrom T, Ohrvall U, et al. Effect of surgery on the outcome of midgut carcinoid disease with lymph node and liver metastases. *World journal of surgery*. Aug 2002;26(8):991-997.
18. Boudreaux JP. Surgery for gastroenteropancreatic neuroendocrine tumors (GEPNETS). *Endocrinology and metabolism clinics of North America*. Mar 2011;40(1):163-171, ix.
19. Joseph S, Wang YZ, Boudreaux JP, et al. Neuroendocrine tumors: current recommendations for diagnosis and surgical management. *Endocrinology and metabolism clinics of North America*. Mar 2011;40(1):205-231, x.
20. Tan EH, Tan CH. Imaging of gastroenteropancreatic neuroendocrine tumors. *World J Clin Oncol*. Jan 10 2011;2(1):28-43.
21. Srirajaskanthan R, Kayani I, Quigley AM, Soh J, Caplin ME, Bomanji J. The role of 68Ga-DOTATATE PET in patients with neuroendocrine tumors and negative or equivocal findings on 111In-DTPA-octreotide scintigraphy. *Journal of nuclear medicine: official publication, Society of Nuclear Medicine*. Jun 2010;51(6):875-882.
22. Hofman MS, Kong G, Neels OC, Eu P, Hong E, Hicks RJ. High management impact of Ga-68 DOTATATE (GaTate) PET/CT for imaging neuroendocrine and other somatostatin expressing tumours. *J Med Imaging Radiat Oncol*. Feb 2012;56(1):40-47.
23. Prasad V, Ambrosini V, Hommann M, Hoersch D, Fanti S, Baum RP. Detection of unknown primary neuroendocrine tumours (CUP-NET) using (68)Ga-DOTA-NOC receptor PET/CT. *European journal of nuclear medicine and molecular imaging*. Jan 2010;37(1):67-77.
24. Lindholm EB, Lyons J, Anthony CT, Boudreaux JP, Wang Y-Z, Woltering EA. Do primary neuroendocrine tumors and metastasis have the same characteristics? *Journal of Surgical Research*. 2012;174(2):200-206.
25. Halfdanarson T HJ, Haraldsdottir S, O'Dorisio T. . Circulating tumor markers in patients with neuroendocrine tumors a clinical perspective. *International Journal of Endocrine Oncology*. 2015:2:89-99.
26. Tellez MR, Mamikunian G, O'Dorisio TM, Vinik AI, Woltering EA. A single fasting plasma 5-HIAA value correlates with 24-hour urinary 5-HIAA values and other biomarkers in midgut neuroendocrine tumors (NETs). *Pancreas*. Apr 2013;42(3):405-410.
27. Sherman SK, Maxwell JE, O'Dorisio MS, O'Dorisio TM, Howe JR. Pancreastatin predicts survival in neuroendocrine tumors. *Annals of surgical oncology*. Sep 2014;21(9):2971-2980.
28. Anderson GM, Feibel FC, Cohen DJ. Determination of serotonin in whole blood, platelet-rich plasma, platelet-poor plasma and plasma ultrafiltrate. *Life Sci*. Mar 16 1987;40(11):1063-1070.
29. Massironi S, Rossi RE, Casazza G, et al. Chromogranin A in diagnosing and monitoring patients with gastroenteropancreatic neuroendocrine neoplasms: a large series from a single institution. *Neuroendocrinology*. 2014;100(2-3):240-249.
30. de Herder WW. Biochemistry of neuroendocrine tumours. *Best practice & research. Clinical endocrinology & metabolism*. Mar 2007;21(1):33-41.
31. Glinicki P, Jeske W, Kapuscinska R, Zgliczynski W. Comparison of chromogranin A (CgA) levels in serum and plasma (EDTA2K) and the respective reference ranges in healthy males. *Endokrynologia Polska*. 2015;66(1):53-56.
32. Kattan MW, Hess KR, Amin MB, et al. American Joint Committee on Cancer acceptance criteria for inclusion of risk models for individualized prognosis in the practice of precision medicine. *CA: a cancer journal for clinicians*. Jan 19 2016.
33. Rindi G, Kloppel G, Couvelard A, et al. TNM staging of midgut and hindgut (neuro) endocrine tumors: a consensus proposal including a grading system. *Virchows Arch*. Oct 2007;451(4):757-762.
34. Jann H, Roll S, Couvelard A, et al. Neuroendocrine tumors of midgut and hindgut origin: tumor-node-metastasis classification

determines clinical outcome. *Cancer*. Aug 1 2011;117(15): 3332-3341.
35. Dhall D, Mertens R, Bresee C, et al. Ki-67 proliferative index predicts progression-free survival of patients with well-differentiated ileal neuroendocrine tumors. *Human pathology*. Apr 2012;43(4):489-495.
36. Berge T, Linell F. Carcinoid tumours. Frequency in a defined population during a 12-year period. *Acta Pathol Microbiol Scand A*. Jul 1976;84(4):322-330.
37. Boudreaux JP, Wang YZ, Diebold AE, et al. A single institution's experience with surgical cytoreduction of stage IV, well-differentiated, small bowel neuroendocrine tumors. *Journal of the American College of Surgeons*. Apr 2014;218(4):837-844.
38. Kloppel G, Perren A, Heitz PU. The gastroenteropancreatic neuroendocrine cell system and its tumors: the WHO classification. *Ann N Y Acad Sci*. Apr 2004;1014:13-27.
39. Landerholm K, Zar N, Andersson RE, Falkmer SE, Jarhult J. Survival and prognostic factors in patients with small bowel carcinoid tumour. *The British journal of surgery*. Nov 2011;98(11): 1617-1624.
40. Zar N, Garmo H, Holmberg L, Rastad J, Hellman P. Long-term survival of patients with small intestinal carcinoid tumors. *World journal of surgery*. Nov 2004;28(11):1163-1168.

Neuroendocrine Tumors of the Appendix

32

Eugene A. Woltering, Emily K. Bergsland, David T. Beyer, Thomas M. O'Dorisio, Guido Rindi, David S. Klimstra, Laura H. Tang, Diane Reidy-Lagunes, Jonathan R. Strosberg, Edward M. Wolin, Aaron I. Vinik, Eric K. Nakakura, Elliot A. Asare, David L. Bushnell, Richard L. Schilsky, Yi-Zarn Wang, Michelle K. Kim, Eric H. Liu, Robert T. Jensen, Rebecca K.S. Wong, John K. Ramage, and Rodney F. Pommier

CHAPTER SUMMARY

Cancers Staged Using This Staging System

Appendiceal NETs (carcinoid) tumors (NET G1 and G2, and rare well-differentiated G3)

Cancers Not Staged Using This Staging System

These histopathologic types of cancer...	Are staged according to the classification for...	And can be found in chapter...
High-grade neuroendocrine carcinoma (NEC)	Appendix – carcinoma	19
Goblet cell carcinoid	Appendix – carcinoma	19
Mixed adenocarcinoma	Appendix – carcinoma	19
Adenocarcinoma	Appendix – carcinoma	19

Summary of Changes

Change	Details of Change	Level of Evidence
AJCC Prognostic Stage Groups	Stages I-IV have been condensed; i.e., no substages A and B	II

ICD-O-3 Topography Codes

Code	Description
C18.1	Appendix

WHO Classification of Tumors

Code	Description
8240	Neuroendocrine tumor (NET) G1 (carcinoid)
8249	NET G2

Bosman FT, Carneiro F, Hruban RH, Theise ND, eds. World Health Organization Classification of Tumours of the Digestive System. Lyon: IARC; 2010.

To access the AJCC cancer staging forms, please visit www.cancerstaging.org.

© American Joint Committee on Cancer 2017

M.B. Amin et al. (eds.), *AJCC Cancer Staging Manual, Eighth Edition*, DOI 10.1007/978-3-319-40618-3_32

INTRODUCTION

Appendiceal neuroendocrine tumors (NETs) are also commonly called appendiceal "carcinoid" tumors. Although neuroendocrine in nature, they are separately classified from other midgut NETs because of their higher incidence. Investigation of the Surveillance Epidemiology and End Results (SEER) database, 1973–2012, demonstrates that the incidence of appendiceal NETs in the US population in 2012 was 0.2/100,000.[1] Although seemingly rare, appendiceal NETs represent up to 85% of all appendiceal neoplasms.[2] These tumors are commonly diagnosed at a young age, and have behavioral differences compared with other gastrointestinal tract NETs.[3] In most cases, appendiceal NETs are found during an incidental appendectomy and are diagnosed postoperatively during histologic evaluation.[4,5] Separate staging criteria for appendiceal NETs are needed because they have no apparent *in situ* state, they may arise in deep mucosa or submucosa, and the tumor size is considered more important than the depth of invasion.[6] Tumor size and invasion of the mesoappendix are major criteria for aggressiveness in a localized tumor.[7,8] Simple appendectomy is usually adequate for treatment of appendiceal NETs smaller than 1 cm. Whether management of appendiceal NETs greater than 1 cm but less than 2 cm mandates a more aggressive surgical procedure is an ongoing debate because of the potential of appendiceal NETs to develop metastases. Some reports state that for tumors greater than 1 cm, a formal resection of the right colon is necessary, whereas others recommend this surgical procedure only for appendiceal NETs larger than 2 cm.[4,9,10] For appendiceal NETs larger than 2 cm, a more extensive surgical procedure (i.e., right hemicolectomy with lymphadenectomy) is warranted because of the higher risk of residual disease and nodal involvement.[2,9,11] Overall, patients with appendiceal NETs have an excellent prognosis, with several reports citing 10-year survival rates of up to 90%.[12]

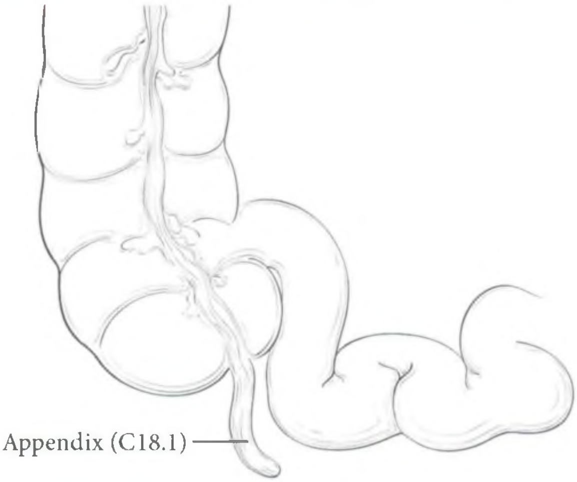

Fig. 32.1 Anatomic location of the appendix

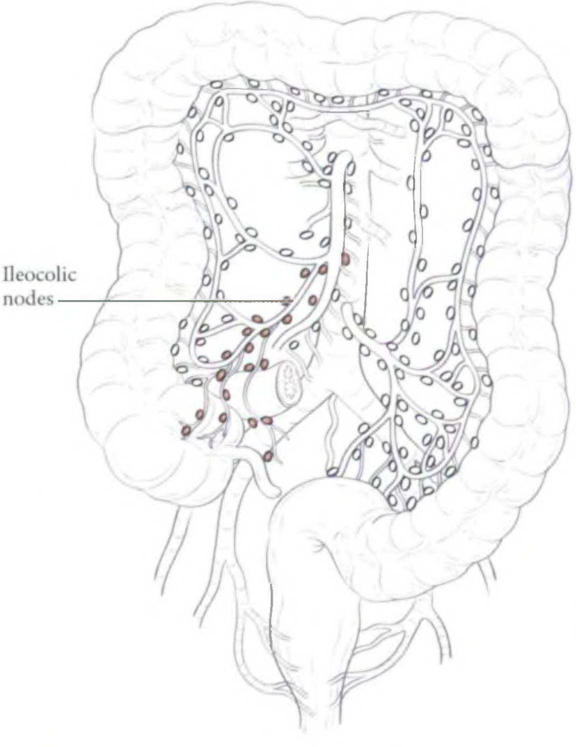

Fig. 32.2 The regional lymph nodes of the appendix

ANATOMY

Primary Site(s)

The appendix is a tubular structure that arises from the base of the cecum (Fig. 32.1). Its length varies but is about 10 cm. It is connected to the ileal mesentery by the mesoappendix, through which its blood supply passes from the ileocolic artery. Among the 13,601 gastroenteropancreatic NETs in the SEER database, 5% were classified as appendiceal NETs.[1]

Regional Lymph Nodes

Lymphatic drainage passes into the ileocolic chain of lymph nodes via the mesoappendix (Fig. 32.2).

Metastatic Sites

The most common metastatic sites of well-differentiated appendiceal NETs are regional and distant lymph nodes (15.8%), liver (0.6%), lung (0.2%), and bone (0.3%).[1]

RULES FOR CLASSIFICATION

Clinical Classification

Clinical assessment of appendiceal NETs is based on medical history, laboratory evaluation, physical examination, and imaging.[13] Currently, chromogranin A (CgA) is used as a biomarker for appendiceal NETs. CgA is a general NET marker that can reflect tumor load, monitor response to treatment, and correlate with a poor prognosis if elevated.[13,14] However, elevated CgA levels also occur in patients with a variety of other conditions and in the setting of proton-pump inhibitor therapy.[15-17] Other biomarkers, such as plasma or urinary 5-hydroxyindoleacetic acid (5-HIAA) and serotonin, may be used to identify patients with NETs of the gut or with carcinoid syndrome, but prospective trials are needed to validate their efficacy as a biomarker of appendiceal NETs.

Imaging

Examinations designed to demonstrate the presence of extra-appendiceal metastasis (M) include chest computed tomography (CT) and triple-phase, contrast-enhanced CT of the abdomen and pelvis. Magnetic resonance (MR) imaging is also useful, especially for defining the size, location, and number of liver metastases.[13] Based on the sensitivity and specificity of MR imaging and CT scans for detecting liver or extrahepatic metastases, respectively, we recommend that both testing modalities be performed during staging. Somatostatin receptor imaging with ^{111}In pentetreotide (Octreoscan™) has a high sensitivity and specificity (80-90%) for detecting primary and metastatic tumors.[18] However, ^{68}Ga-labeled octreopeptide positron emission tomography/CT is more accurate than Octreoscan™ imaging for detection of NET sites in a variety of clinical settings.[19-21] Recently, ^{68}Ga-octreopeptide scans were approved by the FDA.

Pathological Classification

Appendiceal NETs are usually staged after laparoscopic or open surgical exploration of the abdomen (often for appendicitis) and pathological examination of the resected specimen.[6] Lymph nodes with NET deposits are classified N1 regardless of the number of nodes involved.

Restaging

For appendiceal NETs, the *r* prefix is used for recurrent tumor status (rTNM) following a disease-free interval after treatment.

PROGNOSTIC FACTORS

Prognostic Factors Required for Stage Grouping

Beyond the factors used to assign T, N, or M categories, no additional prognostic factors are required for stage grouping.

Additional Factors Recommended for Clinical Care

Measurement of the following factors is recommended for monitoring the clinical care of patients with well-differentiated appendiceal NETs.

Ki-67 Proliferative Index

Histologic tumor grade is determined by Ki-67 proliferative index and/or mitotic count. Ki-67 proliferative index is inversely correlated with patient prognosis and usually measured using MIB1 antibody as a percentage of 500 to 2,000 tumor cells that stain positive in the areas of highest nuclear labeling. AJCC Level of Evidence: I

Mitotic Count

Mitotic count is inversely correlated with patient prognosis and usually measured by determining the number of mitoses per 10 high-power fields (HPF) in the areas of highest mitotic density. At least 50 HPF must be assessed to match World Health Organization (WHO) criteria for mitotic count. AJCC Level of Evidence: I

Plasma Serotonin Level

Repeat serotonin measurements from the same referral laboratory are critical indicators of tumor burden and the severity of carcinoid syndrome in a patient with metastatic disease.[22] Several reference laboratories in the United States that are licensed by the Clinical Laboratory Improvement Amendments (CLIA) and accredited by the College of American Pathology (CAP) measure whole-blood serotonin levels. These measurements are performed using isocratic, reverse-phase high-performance liquid chromatography.[23] AJCC Level of Evidence: II

Plasma or Urinary 5-HIAA Level

The vast majority of symptomatic appendiceal NETs present with elevated serotonin, which may be detected with a very sensitive and specific assay for 24-hour urine metabolites of serotonin (5-HIAA). Plasma 5-HIAA (also commercially available) correlates very closely with the 24-hour urine values of 5-HIAA.[17,23] Serial measurements of plasma or urinary 5-HIAA are useful indicators of a change in tumor volume. Multiple reference laboratories in the United States that are CLIA licensed and CAP accredited measure plasma or urinary 5-HIAA. AJCC Level of Evidence: II

Plasma CgA Level

Chromogranin A is a 49-kDa acidic polypeptide present in the secretory granules of all neuroendocrine cells. CgA is a general NET marker, and plasma or serum CgA may be used as a marker in patients with appendiceal NETs. CgA has prognostic significance, with higher levels indicating a worse prognosis.[13,14] Furthermore, changes over time may be useful for assessing for recurrence after surgery or response to therapy in patients with metastatic disease.[24,25]

Despite the potential merits of monitoring CgA levels, the clinical utility of CgA is limited by the fact that it is falsely elevated in the setting of proton-pump inhibitor use, chronic atrophic gastritis, renal failure, and other conditions.[15-17] Moreover, levels may fluctuate depending on the time of collection and fasting versus nonfasting states. Also, the upper limit of normal varies widely and depends on the assay used and whether plasma or serum is assessed; thus, both the assay and type of sample should be considered when comparing CgA values over time.[26] As a result, routine measurement of CgA is not a consensus recommendation of the National Comprehensive Cancer Network (NCCN). Multiple CLIA-licensed and CAP-accredited reference laboratories in the United States measure CgA levels. AJCC Level of Evidence: II

RISK ASSESSMENT MODELS

The AJCC recently established guidelines that will be used to evaluate published statistical prediction models for the purpose of granting endorsement for clinical use.[27] Although this is a monumental step toward the goal of precision medicine, this work was published only very recently. Therefore, the existing models that have been published or may be in clinical use have not yet been evaluated for this cancer site by the Precision Medicine Core of the AJCC. In the future, the statistical prediction models for this cancer site will be evaluated, and those that meet all AJCC criteria will be endorsed.

DEFINITIONS OF AJCC TNM

Definition of Primary Tumor (T)

T Category	T Criteria
TX	Primary tumor cannot be assessed
T0	No evidence of primary tumor
T1	Tumor 2 cm or less in greatest dimension
T2	Tumor more than 2 cm but less than or equal to 4 cm
T3	Tumor more than 4 cm or with subserosal invasion or involvement of the mesoappendix
T4	Tumor perforates the peritoneum or directly invades other adjacent organs or structures (excluding direct mural extension to adjacent subserosa of adjacent bowel), e.g., abdominal wall and skeletal muscle

Definition of Regional Lymph Node (N)

N Category	N Criteria
NX	Regional lymph nodes cannot be assessed
N0	No regional lymph node metastasis
N1	Regional lymph node metastasis

Definition of Distant Metastasis (M)

M Category	M Criteria
M0	No distant metastasis
M1	Distant metastasis
M1a	Metastasis confined to liver
M1b	Metastases in at least one extrahepatic site (e.g., lung, ovary, nonregional lymph node, peritoneum, bone)
M1c	Both hepatic and extrahepatic metastases

AJCC PROGNOSTIC STAGE GROUPS

When T is...	And N is...	And M is...	Then the stage group is...
T1	N0	M0	I
T1	N1	M0	III
T1	N0, N1	M1	IV
T2	N0	M0	II
T2	N1	M0	III
T2	N0, N1	M1	IV
T3	N0	M0	II
T3	N1	M0	III
T3	N0, N1	M1	IV
T4	N0	M0	III
T4	N1	M0	III
T4	N0, N1	M1	IV

REGISTRY DATA COLLECTION VARIABLES

1. Size of tumor
2. Depth of invasion
3. Invasion of mesoappendix
4. Number of nodes involved, mesenteric mass, mesenteric vessel encasement
5. Perineural invasion
6. Lymphovascular invasion
7. Sites of metastasis, if applicable
8. Type of surgery
9. Ki-67 proliferative index
10. Mitotic count
11. Histologic grading (from Ki-67 and mitotic count): G1, G2, G3

HISTOLOGIC GRADE (G)

Cellular pleomorphism per se is not a useful feature for grading NETs. High proliferative indices (Ki-67 index, mitotic count) have been linked to more aggressive behavior. The following grading system has been proposed for gastrointestinal NETs.[28]

G	G Definition
GX	Grade cannot be assessed
G1	Mitotic count (per 10 HPF)* < 2 and Ki-67 index (%)** < 3
G2	Mitotic count (per 10 HPF) = 2–20 or Ki-67 index (%)** = 3–20
G3	Mitotic count (per 10 HPF) > 20 or Ki-67 index (%)** > 20

*10 HPF = 2 mm^2; at least 50 HPFs (at 40× magnification) must be evaluated in areas of highest mitotic density in order to match WHO 2010 criteria.
**MIB1 atibody; % of 500–2,000 tumor cells in areas of highest nuclear labeling.

In cases of disparity between Ki-67 (proliferative index) and mitotic count, the result indicating a higher-grade tumor should be selected as the final grade. For example, a mitotic count of 1 per 10 HPF and a Ki-67 of 12% should be designated as a G2 NET.

HISTOPATHOLOGIC TYPE

This staging system applies to the following appendiceal NETs: NET G1 and G2. On rare occasions, tumors may be classified as "well-differentiated G3 NETs." These tumors most commonly behave as well-differentiated neoplasms, as opposed to G3 tumors.

High-grade NECs and mixed adenoneuroendocrine carcinomas, including "goblet cell carcinoid," "adenocarcinoid," and mixed adenoneuroendocrine carcinomas, are excluded from this staging system, and these tumors should be staged according to guidelines for staging carcinomas at this site.

SURVIVAL DATA

Because of the short follow-up period and small study population, there are not enough data to draw reasonable conclusions about the new staging parameters for the survival of patients with appendiceal NETs.

Bibliography

1. Surveillance, Epidemiology, and End Results (SEER) Program (www.seer.cancer.gov) SEER*Stat Database: Incidence - SEER 9 Regs Research Data, Nov 2013 Sub (1973-2011) <Katrina/Rita Population Adjustment> - Linked To County Attributes - Total U.S., 1969-2012 Counties, National Cancer Institute, DCCPS, Surveillance Research Program, Surveillance Systems Branch, based on the November 2013 submission, released April 2014.
2. McGory ML, Maggard MA, Kang H, O'Connell JB, Ko CY. Malignancies of the appendix: beyond case series reports. *Diseases of the colon and rectum*. Dec 2005;48(12):2264-2271.
3. Modlin IM, Kidd M, Latich I, Zikusoka MN, Shapiro MD. Current status of gastrointestinal carcinoids. *Gastroenterology*. May 2005;128(6):1717-1751.
4. Grozinsky-Glasberg S, Alexandraki K, Barak D, et al. Current size criteria for the management of neuroendocrine tumors of the appendix: are they valid? Clinical experience and review of the literature. *Neuroendocrinology*. 2013;98(1):31-37.
5. Carr NJ, Sobin LH. Neuroendocrine tumors of the appendix. *Seminars in diagnostic pathology*. May 2004;21(2):108-119.
6. Rossi G, Valli R, Bertolini F, et al. Does mesoappendix infiltration predict a worse prognosis in incidental neuroendocrine tumors of the appendix? A clinicopathologic and immunohistochemical study of 15 cases. *American journal of clinical pathology*. 2003;120(5):706-711.
7. Anderson JR, Wilson BG. Carcinoid tumours of the appendix. *The British journal of surgery*. Jul 1985;72(7):545-546.
8. Roggo A, Wood WC, Ottinger LW. Carcinoid tumors of the appendix. *Annals of surgery*. Apr 1993;217(4):385-390.
9. Goede AC, Caplin ME, Winslet MC. Carcinoid tumour of the appendix. *The British journal of surgery*. Nov 2003;90(11):1317-1322.
10. Nussbaum DP, Speicher PJ, Gulack BC, et al. Management of 1-to 2-cm Carcinoid Tumors of the Appendix: Using the National Cancer Data Base to Address Controversies in General Surgery. *Journal of the American College of Surgeons*. 2015;220(5):894-903.
11. Pape UF, Perren A, Niederle B, et al. ENETS Consensus Guidelines for the management of patients with neuroendocrine neoplasms from the jejuno-ileum and the appendix including goblet cell carcinomas. *Neuroendocrinology*. 2012;95(2):135-156.
12. Mullen JT, Savarese DM. Carcinoid tumors of the appendix: a population-based study. *Journal of surgical oncology*. Jul 1 2011;104(1):41-44.
13. Modlin IM, Latich I, Zikusoka M, Kidd M, Eick G, Chan AK. Gastrointestinal carcinoids: the evolution of diagnostic strategies. *Journal of clinical gastroenterology*. Aug 2006;40(7):572-582.
14. Rorstad O. Prognostic indicators for carcinoid neuroendocrine tumors of the gastrointestinal tract. *Journal of surgical oncology*. Mar 1 2005;89(3):151-160.

32

15. Åkerström G, Norlén O, Edfeldt K, et al. A review on management discussions of small intestinal neuroendocrine tumors' midgut carcinoids'. *International Journal of Endocrine Oncology.* 2015;2(2):119-128.
16. Raines D, Chester M, Diebold AE, et al. A prospective evaluation of the effect of chronic proton pump inhibitor use on plasma biomarker levels in humans. *Pancreas.* 2012;41(4):508-511.
17. Vinik A, Woltering EA, O'Dorisio T, Go V, Mamikunian G. Neuroendocrine Tumors: A Comprehensive Guide To Diagnosis And Management. *Inglewood: InterScience Institute.* 2014;5th Edition:13-14, 18-25.
18. Tan EH, Tan CH. Imaging of gastroenteropancreatic neuroendocrine tumors. *World J Clin Oncol.* Jan 10 2011;2(1):28-43.
19. Srirajaskanthan R, Kayani I, Quigley AM, Soh J, Caplin ME, Bomanji J. The role of 68Ga-DOTATATE PET in patients with neuroendocrine tumors and negative or equivocal findings on 111In-DTPA-octreotide scintigraphy. *Journal of nuclear medicine : official publication, Society of Nuclear Medicine.* Jun 2010;51(6): 875-882.
20. Hofman MS, Kong G, Neels OC, Eu P, Hong E, Hicks RJ. High management impact of Ga-68 DOTATATE (GaTate) PET/CT for imaging neuroendocrine and other somatostatin expressing tumours. *J Med Imaging Radiat Oncol.* Feb 2012;56(1): 40-47.
21. Prasad V, Ambrosini V, Hommann M, Hoersch D, Fanti S, Baum RP. Detection of unknown primary neuroendocrine tumours (CUP-NET) using 68Ga-DOTA-NOC receptor PET/CT. *European journal of nuclear medicine and molecular imaging.* 2010;37(1):67-77.
22. Halfdanarson TR, Howe JR, Haraldsdottir S, O'Dorisio TM. Circulating tumor markers in patients with neuroendocrine tumors–a clinical perspective. 2015.
23. Anderson GM, Feibel FC, Cohen DJ. Determination of serotonin in whole blood, platelet-rich plasma, platelet-poor plasma and plasma ultrafiltrate. *Life Sci.* Mar 16 1987;40(11):1063-1070.
24. Massironi S, Rossi RE, Casazza G, et al. Chromogranin A in diagnosing and monitoring patients with gastroenteropancreatic neuroendocrine neoplasms: a large series from a single institution. *Neuroendocrinology.* 2014;100(2-3):240-249.
25. de Herder WW. Biochemistry of neuroendocrine tumours. *Best practice & research. Clinical endocrinology & metabolism.* Mar 2007;21(1):33-41.
26. Glinicki P, Jeske W, Kapuscinska R, Zgliczynski W. Comparison of chromogranin A (CgA) levels in serum and plasma (EDTA2K) and the respective reference ranges in healthy males. *Endokrynologia Polska.* 2015;66(1):53-56.
27. Kattan MW, Hess KR, Amin MB, et al. American Joint Committee on Cancer acceptance criteria for inclusion of risk models for individualized prognosis in the practice of precision medicine. *CA: a cancer journal for clinicians.* Jan 19 2016.
28. Rindi G, Kloppel G, Couvelard A, et al. TNM staging of midgut and hindgut (neuro) endocrine tumors: a consensus proposal including a grading system. *Virchows Arch.* Oct 2007;451(4):757-762.
29. Burke AP, Sobin LH, Federspiel BH, Shekitka KM. Appendiceal carcinoids: correlation of histology and immunohistochemistry. *Modern pathology : an official journal of the United States and Canadian Academy of Pathology, Inc.* Nov 1989;2(6): 630-637.
30. Jann H, Roll S, Couvelard A, et al. Neuroendocrine tumors of midgut and hindgut origin: tumor-node-metastasis classification determines clinical outcome. *Cancer.* Aug 1 2011;117(15): 3332-3341.
31. Moertel CG, Weiland LH, Nagorney DM, Dockerty MB. Carcinoid tumor of the appendix: treatment and prognosis. *N Engl J Med.* Dec 31 1987;317(27):1699-1701.
32. Rindi G, Arnold R, Capella C, et al. Nomenclature and classification of digestive neuroendocrine tumours. *World Health Organization classification of tumours, pathology and genetics of tumours of the digestive system. IARC Press, Lyon.* 2010:10-12.

Neuroendocrine Tumors of the Colon and Rectum

33

Chanjuan Shi, Eugene Woltering, David T. Beyer,
David Klimstra, Kathy Mallin, Emily Bergsland,
and Mary Kay Washington

CHAPTER SUMMARY

Cancers Staged Using This Staging System

This section includes colonic and rectal "carcinoid" tumors (neuroendocrine tumor G1 and G2, and rare well-differentiated G3).

Cancers Not Staged Using This Staging System

These histopathologic types of cancer...	Are staged according to the classification for...	And can be found in chapter...
High-grade neuroendocrine carcinoma	Colon and rectum	20
Mixed adenoneuroendocrine carcinoma	Colon and rectum	20

Summary of Changes

Change	Details of Change	Level of evidence
New chapter	This staging system was included in the neuroendocrine tumors chapters in previous editions.	N/A

ICD-O-3 Topography Codes

Code	Description
C18.0	Cecum
C18.2	Ascending colon
C18.3	Hepatic flexure of colon
C18.4	Transverse colon
C18.5	Splenic flexure of colon
C18.6	Descending colon
C18.7	Sigmoid colon
C18.8	Overlappling lesion of colon
C18.9	Colon, NOS
C19.9	Rectosigmoid junction
C20.9	Rectum, NOS

WHO Classification of Tumors

Code	Description
8240	Neuroendocrine tumor (NET) G1 (carcinoid)
8249	NET G2

Bosman FT, Carneiro F, Hruban RH, Theise ND, eds. World Health Organization Classification of Tumours of the Digestive System. Lyon: IARC; 2010.

To access the AJCC cancer staging forms, please visit www.cancerstaging.org.

© American Joint Committee on Cancer 2017

M.B. Amin et al. (eds.), *AJCC Cancer Staging Manual, Eighth Edition*, DOI 10.1007/978-3-319-40618-3_33

INTRODUCTION

The incidence of colorectal NETs has been increasing in the past decades, partly because of screening colonoscopy.[1,2] Queries to the Surveillance, Epidemiology, and End Results (SEER) database, 1975 to 2008, demonstrate that the incidence for well-differentiated colonic and rectal neuroendocrine tumors (NETs) in the US population was 0.3 per 100,000 and 1.1 per 100,000 in 2008, respectively.[2] Colonic NETs are slightly more common in women (55%), whereas there is no significant gender predilection for rectal NETs.[2] Individuals diagnosed with rectal NETs (mean age, 55.6 years) tend to be younger than those diagnosed with small intestinal (mean age, 62.8) and colonic NETs (mean age, 63.3).[2] Rectal NETs are more common in Asians and blacks than in whites.[1,2] In addition, a more substantial increase in the incidence of rectal NETs has been seen among black patients compared with white patients.

Overall, gut-based NETs exhibit a propensity for slower growth than adenocarcinomas, but aggressive variants are not uncommon. A distinct dichotomy in behavior exists between colonic NETs, which have the worst prognosis among gastrointestinal (GI) NETs—with a 5-year survival of approximately 67%—and rectal NETs, which carry the best prognosis—with a 5-year overall survival of about 96%.[2] However, the survival rate for colonic NETs has significantly improved over time.

Most patients with rectal NETs are asymptomic. More than half of rectal NETs are discovered during routine colonoscopy[3] for colorectal cancer screening or other indications, and these asymptomatic tumors are virtually always less than 1 cm, with no lymph node or distant metastasis.[4-8] Only 4% of all rectal NETs present with distant metastasis.[2] In contrast, patients with colonic NET may present with pain, bleeding, altered bowel habits, weight loss, anorexia, or even bowel obstruction. Colonic NETs typically are larger than 2 cm,[9,10] and approximately two thirds of the cases have either regional or distant metastasis.[2] Although most small localized rectal NETs can be excised by endoscopic resection with a very low risk of distant metastasis,[5,7,8,11,12] radical surgery is recommended for large localized rectal NETs, rectal NETs with regional metastasis, and most colonic NETs.[13-15] Currently, there are no treatment recommendations that are specifically designated for advanced colorectal NETs, and current treatment modalities are based on those used for small intestinal NETs.[13,14]

Several treatment guidelines exist that may help guide clinical decisions in patients with NETs. These include guidelines published by the European Neuroendocrine Tumor Society (ENETS), the North American Neuroendocrine Tumor Society (NANETS), and the National Comprehensive Cancer Network (NCCN).

ANATOMY

Primary Site(s)

The rectum is the most common primary location for colorectal NETs. Among 19,669 gut NETs in the SEER database, 1975 to 2008, 14% were colonic (including the cecum, right colon, transverse colon, and descending colon) and 35% were rectal.[2] Most colonic NETs occur in the the cecum. Up to 4.5% of rectal NETs are multicentric.[16]

Colorectal NETs may arise from serotonin-producing enterochromaffin cells or from glucagon-like peptide-producing and pancreatic polypeptide/peptide YY (PP/PYY)-producing L cells, with most rectal NETs being L-cell phenotype.[17,18] Only a few rectal NETs produce serotonin, and carcinoid syndrome is rare in patients with rectal NETs.

Regional Lymph Nodes

Regional nodes are located 1) along the course of the major vessels supplying the colon and rectum, 2) along the vascular arcades of the marginal artery, and 3) adjacent to the colon—that is, located along the mesocolic borders of the colon (Fig. 33.1). Specifically, the regional lymph nodes are those termed *pericolic* and *perirectal/mesorectal* and those found along the ileocolic, right colic, middle colic, left colic, inferior mesenteric, superior rectal (hemorrhoidal), and internal iliac arteries (Fig. 33.1).

The regional lymph nodes for each segment of the large bowel are designated as follows:

Segment	Regional lymph nodes
Cecum	Pericolic, ileocolic, right colic
Ascending colon	Pericolic, ileocolic, right colic, right branch of the middle colic
Hepatic flexure	Pericolic, ileocolic, right colic, middle colic
Transverse colon	Pericolic, middle colic
Splenic flexure	Pericolic, middle colic, left colic
Descending colon	Pericolic, left colic, sigmoid, inferior mesenteric
Sigmoid colon	Pericolic, sigmoid, superior rectal (hemorrhoidal), inferior mesenteric
Rectosigmoid	Pericolic, sigmoid, superior rectal (hemorrhoidal), inferior mesenteric
Rectum	Mesorectal, superior rectal (hemorrhoidal), inferior mesenteric, internal iliac, inferior rectal (hemorrhoidal)

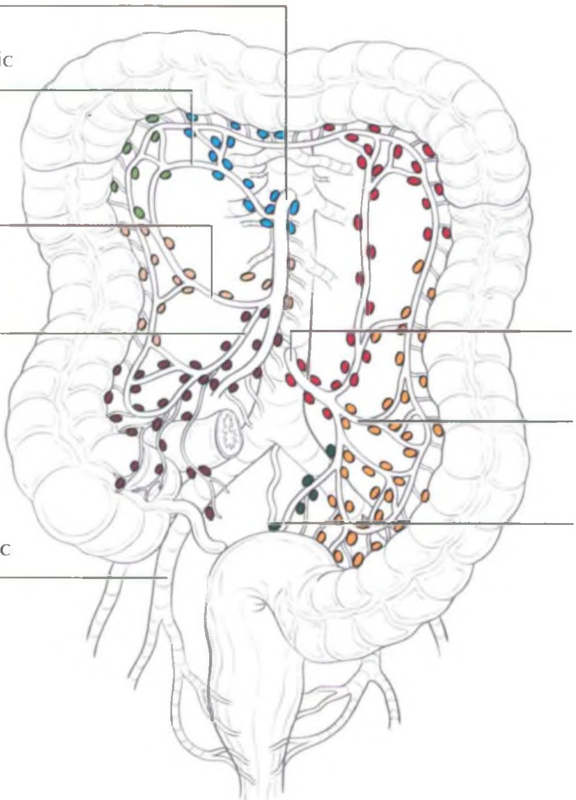

Fig. 33.1 Regional lymph nodes for NETs of the colon and rectum

Metastatic Sites

The most common metastatic sites of colorectal NETs are the regional lymph nodes, liver, mesentery, and peritoneum.[3] Rare reported metastatic sites include bone, lung, epicardium, mediastinum, and orbit.

RULES FOR CLASSIFICATION

Clinical Classification

Clinical staging depends on the local anatomic extent and regional/distant metastasis. Colonoscopy is a common way to identify colorectal NETs. Most colorectal NETs appear as polypoid lesions or submucosal nodules. The diagnosis is made after histologic examination of biopsies or polypectomies. Endoanal/rectal ultrasound may be used to assess tumor size and depth of rectal NETs.[3] Magnetic resonance (MR) imaging/computed tomography (CT) may be used to assess regional and distant metastasis.[3,13,14] Although the extent of liver involvement is difficult to quantify, most clinicians are comfortable with estimating tumor burden in the liver via ultrasound or MR imaging. It is not necessary to estimate the tumor burden in the liver for staging purposes; however, knowledge of the extent of tumor burden is critical for treatment planning.

Biochemical markers of NET disease include chromogranin A (CgA), plasma or urinary 5-hydroxyindoleacetic acid (5-HIAA; for hormone-secreting tumors), pancreastatin, and neurokinin A (NKA). Routine analysis of plasma or urinary 5-HIAA is rarely performed in monitoring colorectal NETs, as few tumors produce serotonin.[3,13,14] CgA, a general NET marker, may be used as a biomarker for metastastic colorectal NETs.[13,14,19-21] CgA levels correlate with a poor prognosis if elevated, may reflect tumor load, and may be used to monitor response to treatment.[22,23] However, elevated CgA levels also occur in patients with a variety of other conditions and in the setting of proton-pump inhibitor (PPI) therapy.[24-26] Immunohistochemistry may be performed on surgical resection or biopsy specimens for confirmation of neuroendocrine differentiation. Colonic NETs generally are positive for both CgA and

synaptophysin. Rectal NETs often do not express CgA but are positive for synaptophysin.

Pancreastatin levels are not affected by PPI usage, pernicious anemia, or type I gastric NETs. NKA values greater than 50 pg/mL may portend a worse prognosis than values less than 50 pg/mL in NETs. However, although the diagnostic and prognostic significance of pancreastatin and NKA have been studied in midgut NETs, their utility has not been demonstrated in colorectal NETs.

Imaging

Endoanal/rectal ultrasound is useful in assessing the depth of invasion and regional lymph node metastasis for rectal NETs.[27,28] It can detect rectal tumors as small as 2 mm. In adition, it can discriminate accurately between T1 and T2 tumors as well as T3 and T4 tumors.[27] Pelvic MR imaging also should be performed to assess local/regional spread for rectal NETs with tumor size >2 cm, invasion into the muscularis propria, or positive regional lymph node(s).[14]

Triple-phase CT should be used to stage all colonic NETs. Although MR imaging scans are highly sensitive in detecting liver metastases, CT scans are more sensitive in their ability to detect extrahepatic metastatic sites.[23] Based on the sensitivity and specificity of MR imaging and CT scans for detecting liver or extrahepatic metastases, respectively, we recommend that both testing modalities be performed during staging. Somatostatin receptor imaging with indium-111 pentetreotide (Octreoscan; Mallinckrodt Pharmaceuticals, Dublin, Ireland) is useful in detecting metastatic tumors; however, its utility in detecting primary tumors may be marginal.[14] Gallium-68 (^{68}Ga)-labeled octreopeptide positron emission tomography/CT is more accurate than Octreoscan imaging for detecting NET sites in a variety of clinical settings.[29-31] Recently, ^{68}Ga-octreopeptide scans were approved by the FDA.

Pathological Classification

Pathological staging is based on surgical exploration with resection of at least the primary tumor, with potential resection of lymph nodes and distant metastases.[23] The pathological staging is performed by examining the surgically resected specimen(s). Significant differences may exist in the attributes of multiple primaries or in the primary tissue and its nodal or distant metastases.[32]

Restaging

For colon and rectum NETs, the *r* prefix is used for recurrent tumor status (rTNM) following a disease-free interval after treatment.

PROGNOSTIC FACTORS

Prognostic Factors Required for Stage Grouping

Beyond the factors used to assign T, N, or M categories, no additional prognostic factors are required for stage grouping.

Additional Factors Recommended for Clinical Care

Measurement of the following factors is recommended for monitoring the clinical care of patients with well-differentiated colorectal NETs.

Ki-67 Proliferative Index

Histologic tumor grade is determined by Ki-67 proliferative index and/or mitotic count. Ki-67 proliferative index is inversely correlated with patient prognosis and usually is measured by using MIB1 antibody as a percentage of 500 to 2,000 tumor cells that stain positive in the areas of highest nuclear labeling. AJCC Level of Evidence: I

Mitotic Count

Mitotic count is inversely correlated with patient prognosis and usually measured by determining the number of mitoses per 10 high-power fields (HPF) in the areas of highest mitotic density. A minimum of 50 HPF must be assessed to adhere to World Health Organization (WHO) 2010 criteria. AJCC Level of Evidence: I

Chromogranin A (CgA) Level

CgA is a 49-kDa acidic polypeptide present in the secretory granules of all neuroendocrine cells. CgA is a general neuroendocrine tumor marker, and plasma or serum CgA may be used as a marker in patients with metastatic colonic NETs.[14] CgA has prognostic significance, with higher levels associated with a worse prognosis.[22,23] Furthermore, changes over time may be useful in assessing for recurrence after surgery or for response to therapy in patients with metastatic disease.[33,34]

Despite the potential merits of monitoring CgA levels, the clinical utility of CgA is limited by the fact that it is falsely elevated in the setting of PPI use, chronic atrophic gastritis, renal failure, and other conditions.[24-26] Moreover, levels may fluctuate based on time of collection and fasting versus non-fasting states. Also, the upper limit of normal varies widely and depends on the assay used and whether plasma or serum is assessed; thus, both the assay and type of sample should be considered when comparing CgA values over time.[35] As a result, routine measurement of CgA is not a consensus NCCN recommendation. Multiple Clinical Laboratory

Improvement Amendments (CLIA)-licensed and College of American Pathology (CAP)-accredited reference laboratories in the United States measure CgA levels. AJCC Level of Evidence: II

RISK ASSESSMENT MODELS

The AJCC recently established guidelines that will be used to evaluate published statistical prediction models for the purpose of granting endorsement for clinical use.[36] Although this is a monumental step toward the goal of precision medicine, this work was published only very recently. For this reason, the existing models that have been published or may be in clinical use have not yet been evaluated for this cancer site by the Precision Medicine Core of the AJCC. In the future, the statistical prediction models for this cancer site will be evaluated, and those that meet all AJCC criteria will be endorsed.

DEFINITIONS OF AJCC TNM

Definition of Primary Tumor (T)

T Category*	T Criteria
TX	Primary tumor cannot be assessed
T0	No evidence of primary tumor
T1	Tumor invades the lamina propria or submucosa and is ≤2 cm
T1a	Tumor <1 cm in greatest dimension
T1b	Tumor 1–2 cm in greatest dimension
T2	Tumor invades the muscularis propria or is >2 cm with invasion of the lamina propria or submucosa
T3	Tumor invades through the muscularis propria into subserosal tissue without penetration of overlying serosa
T4	Tumor invades the visceral peritoneum (serosa) or other organs or adjacent structures

Note:* For any T, add "(m)" for multiple tumors [TX(#) or TX(m), where X = 1–4 and # = number of primary tumors identified]; for multiple tumors with different T, use the highest.
****Example:* If there are two primary tumors, only one of which invades through the muscularis propria into the subserosal tissue without penetration of the overlying serosa, we define the primary tumor as either T3(2) or T3(m).

Definition of Regional Lymph Node (N)

N Category	N Criteria
NX	Regional lymph nodes cannot be assessed
N0	No regional lymph node metastasis has occurred
N1	Regional lymph node metastasis

Definition of Distant Metastasis (M)

M Category	M Criteria
M0	No distant metastasis
M1	Distant metastasis
M1a	Metastasis confined to liver
M1b	Metastases in at least one extrahepatic site (e.g., lung, ovary, nonregional lymph node, peritoneum, bone)
M1c	Both hepatic and extrahepatic metastases

AJCC PROGNOSTIC STAGE GROUPS

When T is...	And N is...	And M is...	Then the stage group is...
T1	N0	M0	I
T1	N1	M0	IIIB
T1	Any N	M1	IV
T2	N0	M0	IIA
T2	N1	M0	IIIB
T2	Any N	M1	IV
T3	N0	M0	IIB
T3	N1	M0	IIIB
T3	Any N	M1	IV
T4	N0	M0	IIIA
T4	N1	M0	IIIB
T4	Any N	M1	IV

Note: For multiple synchronous tumors, the highest T category should be used and the multiplicity or the number of tumors should be indicated in parenthesis, e.g., T3(2) or T3(m).

REGISTRY DATA COLLECTION VARIABLES

1. Tumor site
2. Size of tumor (value or unknown)
3. Depth of invasion
4. Nodal status and number of nodes involved, if applicable
5. Sites of metastasis, if applicable
6. Ki-67 index
7. Mitotic count
8. Histologic grade (from Ki-67 and mitotic count): G1, G2, G3

HISTOLOGIC GRADE (G)

Cellular pleomorphism per se is not a useful feature for grading NETs. The following grading scheme has been proposed for GI NETs[37-39]:

G	G Definition
GX	Grade cannot be assessed
G1	Mitotic count (per 10 HPF)* <2 and Ki-67 Index (%)** <3
G2	Mitotic count (per 10 HPF) = 2–20 or Ki-67 index (%)** = 3–20
G3	Mitotic count (per 10 HPF) >20 or Ki-67 index (%)** >20

*10 HPF = 2 mm^2; at least 50 HPF (at 40× magnification) must be evaluated in areas of highest mitotic density in order to adhere to WHO 2010 criteria.
**MIB1 antibody; % of 500–2,000 tumor cells in areas of highest nuclear labeling.

In cases of disparity between Ki-67 proliferative index and mitotic count, the result indicating a higher-grade tumor should be selected as the final grade. For example, a mitotic count of 1 per 10 HPF and a Ki-67 of 12% should be designated as a G2 NET.

HISTOPATHOLOGIC TYPE

This staging system applies to the following colorectal NETs: well differentiated NETs G1 and G2. On rare occasions, tumors may be classified as "well-differentiated G3 NETs." These tumors most commonly behave as well-differentiated neoplasms as opposed to high-grade neuroendocrine carcinomas (NECs).

Adenocarcinomas, high-grade NECs, goblet cell carcinoid tumors, and other mixed adenoneuroendocrine carcinomas are excluded from this staging system, and these tumors should be staged according to guidelines for staging carcinomas at these sites.

SURVIVAL DATA

Four years of data (diagnosis years 2010–2013) from the National Cancer Data Base were used to assess survival. Maximum follow-up time for these patients was 3 years. Product limit survival curves (Kaplan–Meier) and 95% confidence intervals (CIs) adjusted for age were produced. Selection criteria included the following: primary site codes C18.0, C18.2, C18.3, C18.4, C18.5, C18.6, C18.7, C18.8, C18.9., C19.9, and C20.9; histology codes 8240 and 8249; ages 18 and older; and only primary or first of multiple primaries. Only patients with low-grade disease were included. Low grade was defined by mitotic count, or if the mitotic count was missing, by selecting only grade 1 and grade 2.

To determine clinical and pathological stage, the AJCC Cancer Staging Manual, 7th Edition TNM classification was used. Kaplan–Meier survival curves for each clinical stage are shown in Fig. 33.2. Kaplan–Meier survival curves for each pathological stage are shown in Fig. 33.3. The survival analyses included 3,148 patients with clinical stage information and 1,414 patients with pathological stage information. Using a 5% significance level, log-rank test results showed significant differences between Stages I and II and between Stages III and IV for both pathological and clinical staging, but no significant differences between Stages II and III.

Table 33.1 Colorectal NET observed Kaplan–Meier 3-year survival, 95% CIs by clinical and pathological (Path) stage, diagnosis years 2010 to 2013, age ≥18, no prior cancers

Stage (7th Edition)	3-Year survival, % (95% CI)
Clinical Stage I	97.7 (96.9–i98.4)
Clinical Stage II	85.6 (77.6–94.5)
Clinical Stage III	85.1 (76.1–95.2)
Clinical Stage IV	51.2 (43.1–60.7)
Path Stage I	96.5 (94.8–98.1)
Path Stage II	81.7 (71.7–93.0)
Path Stage III	86.6 (81.9–91.4)
Path Stage IV	71.5 (62.8–81.4)

g. 33.2 Overall survival for ch clinical stage group for lorectal primary NETs. aplan–Meier survival curves. th a follow-up time of 3 years 010–2013) and adjusted for tient age, for a total of 3,148 tients included in the clinical ige survival calculation

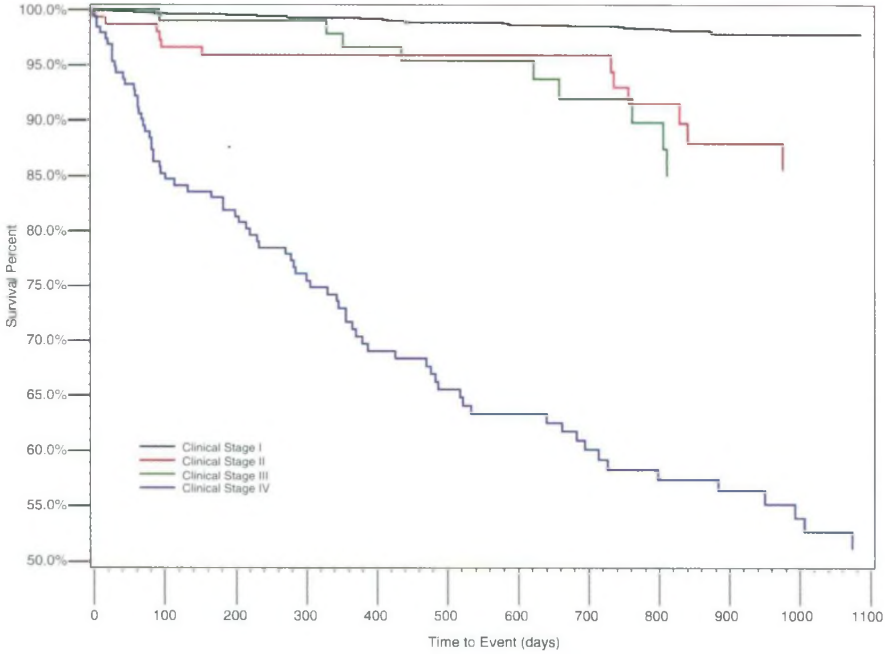

ıble 33.2 NET colorectal clinical stage, number of cases, deaths, censored 2010-2013

linical stage	Cases, n	Deaths, n	Censored, n	Censored, %
tage I	2,683	39	2,644	98.55
tage II	160	12	148	92.50
tage III	109	9	100	91.74
tage IV	196	75	121	61.73
otal	3,148	135	3,013	95.71

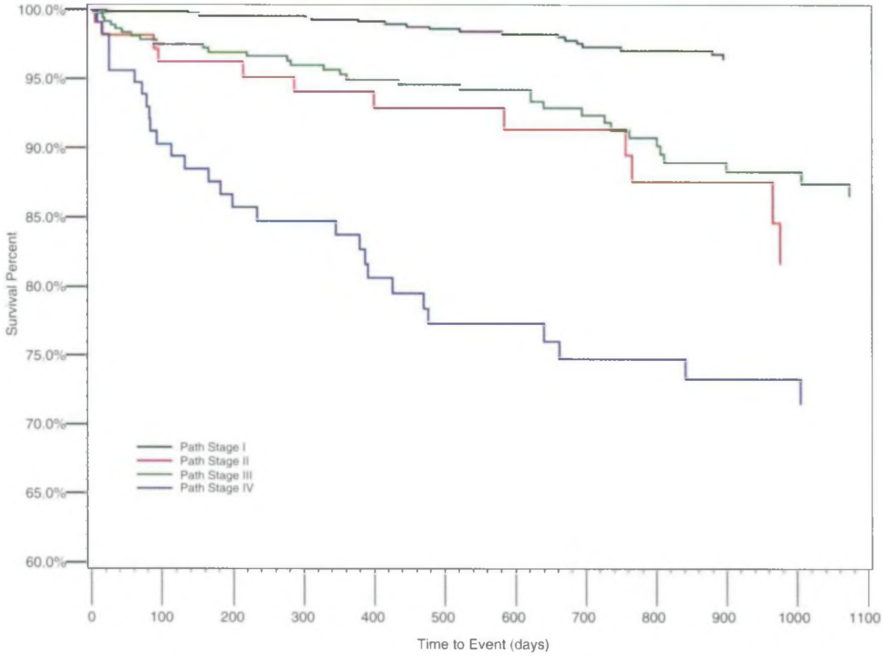

ȷ. 33.3 Overall survival for ch pathological stage group for lorectal primary NETs. ıplan–Meier survival curves, th a follow-up time of 3 years)10–2013) and adjusted for tient age, for a total of 1,414 tients included in the thological stage survival lculation

33

Table 33.3 NET colorectal pathological stage, number of cases, deaths, censored 2010–2013

Pathological stage	Cases, n	Deaths, n	Censored, n	Censored, %
Stage I	817	18	799	97.80
Stage II	110	12	98	89.09
Stage III	370	32	338	91.35
Stage IV	117	28	89	76.07
Total	1,414	90	1,324	93.64

ILLUSTRATIONS

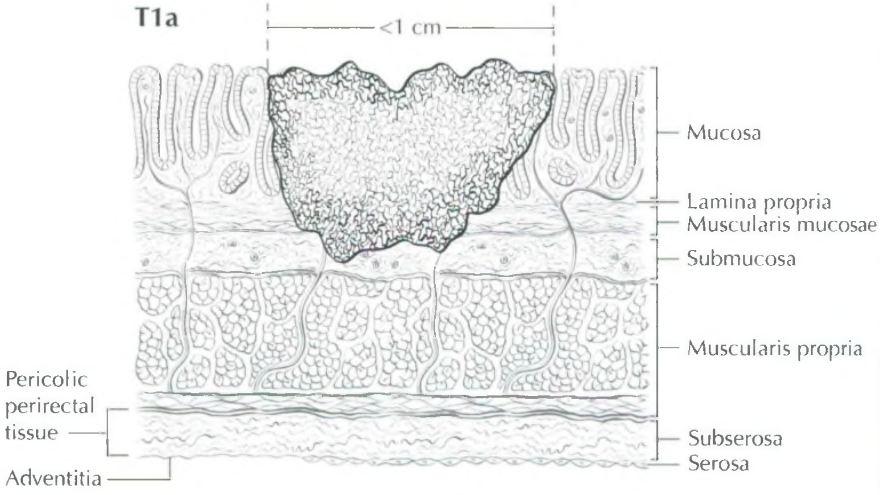

Fig. 33.4 For colon or rectum T1 is defined as tumor that invades the lamina propria or submucosa and size 2 cm or less. T1a is defined as tumor size less than 1 cm in greatest dimension

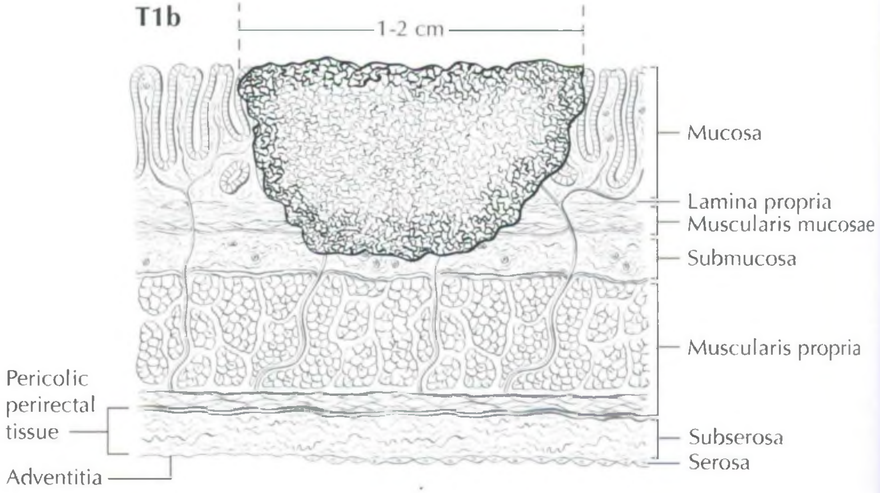

Fig. 33.5 For colon or rectum T1b is defined as tumor that is size 1–2 cm in greatest dimension

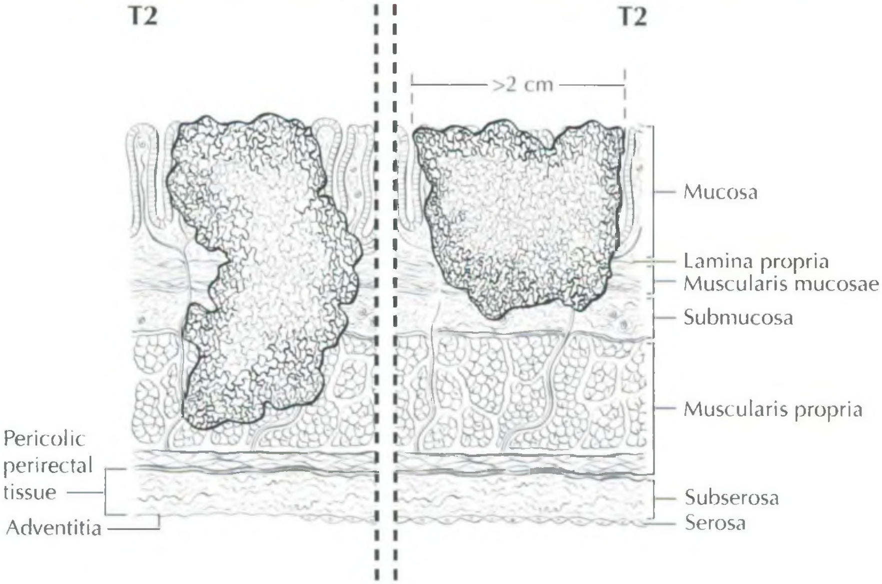

Fig. 33.6 For colon or rectum T2 is defined as tumor that invades the muscularis propria (left side) or size more than 2 cm with invasion of the lamina propria or submucosa (right side)

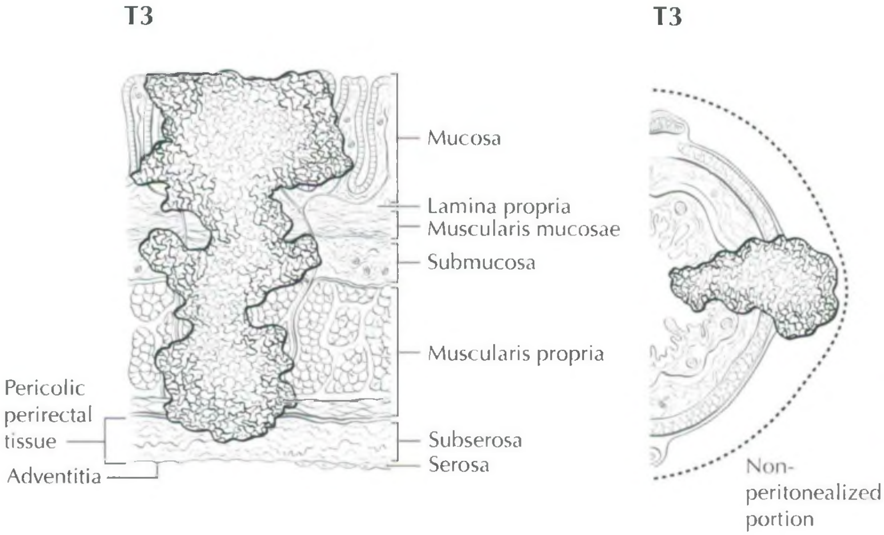

Fig. 33.7 For colon or rectum T3 is defined as tumor that invades through the muscularis propria into the subserosa (left side), or into non-peritonealized pericolic or perirectal tissues (right side)

33

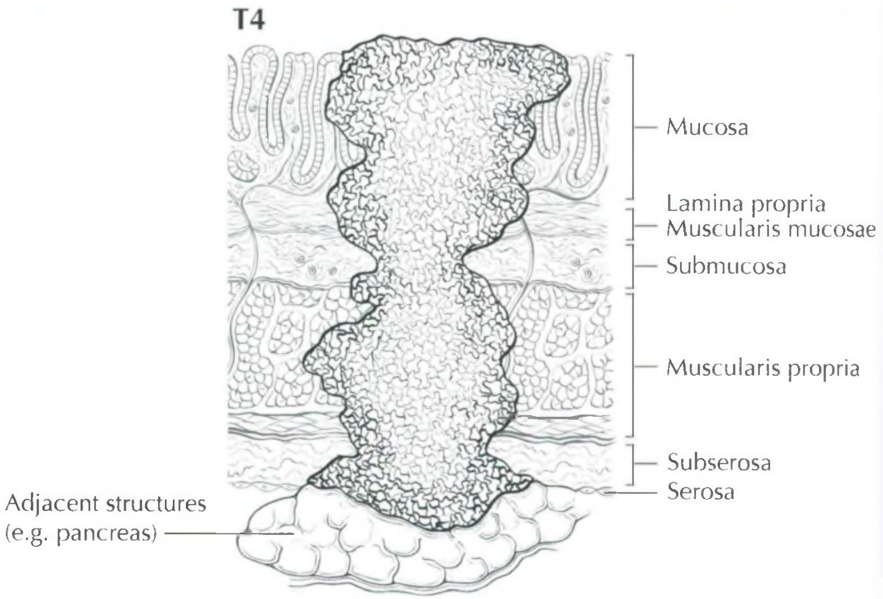

Fig. 33.8 For colon or rectum T4 is defined as tumor that invades the peritoneum or other organs

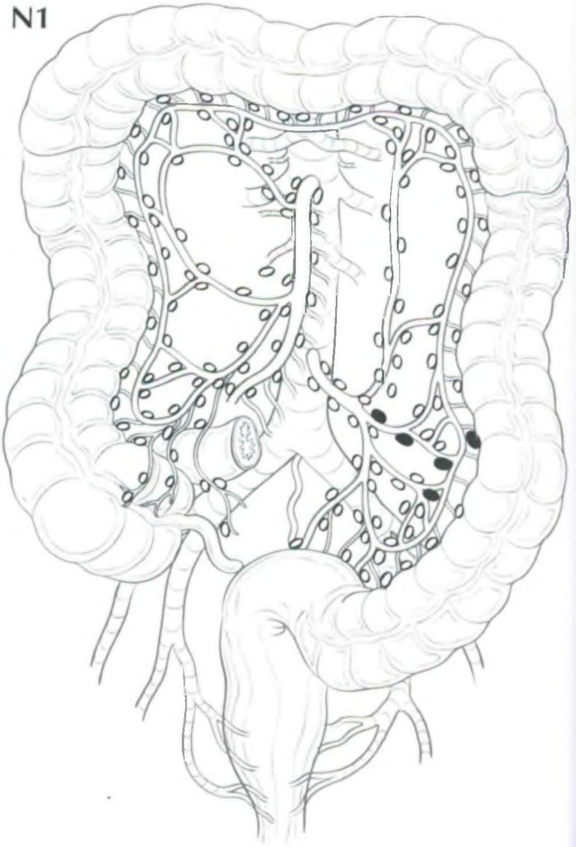

Fig. 33.9 For colon or rectum N1 is defined as regional lymph node metastasis

36. Kattan MW, Hess KR, Amin MB, et al. American Joint Committee on Cancer acceptance criteria for inclusion of risk models for individualized prognosis in the practice of precision medicine. *CA: a cancer journal for clinicians*. Jan 19 2016.
37. Rindi G, Kloppel G, Couvelard A, et al. TNM staging of midgut and hindgut (neuro) endocrine tumors: a consensus proposal including a grading system. *Virchows Arch*. Oct 2007;451(4):757–762.
38. Jann H, Roll S, Couvelard A, et al. Neuroendocrine tumors of midgut and hindgut origin: tumor-node-metastasis classification determines clinical outcome. *Cancer*. Aug 1 2011;117(15):3332–3341.
39. Dhall D, Mertens R, Bresee C, et al. Ki-67 proliferative index predicts progression-free survival of patients with well-differentiated ileal neuroendocrine tumors. *Human pathology*. Apr 2012;43(4):489–495.

Bibliography

1. Taghavi S, Jayarajan SN, Powers BD, Davey A, Willis AI. Examining rectal carcinoids in the era of screening colonoscopy: a surveillance, epidemiology, and end results analysis. *Diseases of the colon and rectum*. Aug 2013;56(8):952–959.
2. Tsikitis VL, Wertheim BC, Guerrero MA. Trends of incidence and survival of gastrointestinal neuroendocrine tumors in the United States: a seer analysis. *J Cancer*. 2012;3:292–302.
3. Mandair D, Caplin ME. Colonic and rectal NET's. *Best practice & research. Clinical gastroenterology*. Dec 2012;26(6):775–789.
4. Coloproctology CSGoKSo. Clinical characteristics of colorectal carcinoid tumors. *J Korean Soc Coloproctol*. Feb 2011;27(1):17–20.
5. Kasuga A, Chino A, Uragami N, et al. Treatment strategy for rectal carcinoids: a clinicopathological analysis of 229 cases at a single cancer institution. *J Gastroenterol Hepatol*. Dec 2012;27(12):1801–1807.
6. McDermott FD, Heeney A, Courtney D, Mohan H, Winter D. Rectal carcinoids: a systematic review. *Surg Endosc*. Jul 2014;28(7):2020–2026.
7. Park CH, Cheon JH, Kim JO, et al. Criteria for decision making after endoscopic resection of well-differentiated rectal carcinoids with regard to potential lymphatic spread. *Endoscopy*. Sep 2011;43(9):790–795.
8. Yangong H, Shi C, Shahbaz M, et al. Diagnosis and treatment experience of rectal carcinoid (a report of 312 cases). *International journal of surgery*. 2014;12(5):408–411.
9. Al Natour RH, Saund MS, Sanchez VM, et al. Tumor size and depth predict rate of lymph node metastasis in colon carcinoids and can be used to select patients for endoscopic resection. *Journal of gastrointestinal surgery: official journal of the Society for Surgery of the Alimentary Tract*. Mar 2012;16(3):595–602.
10. Murray SE, Lloyd RV, Sippel RS, Chen H. Clinicopathologic characteristics of colonic carcinoid tumors. *J Surg Res*. Sep 2013;184(1):183–188.
11. Kim DH, Lee JH, Cha YJ, et al. Surveillance strategy for rectal neuroendocrine tumors according to recurrence risk stratification. *Digestive diseases and sciences*. Apr 2014;59(4):850–856.
12. Yoon SN, Yu CS, Shin US, Kim CW, Lim S-B, Kim JC. Clinicopathological characteristics of rectal carcinoids. *International journal of colorectal disease*. 2010;25(9):1087–1092.
13. Anthony LB, Strosberg JR, Klimstra DS, et al. The NANETS consensus guidelines for the diagnosis and management of gastrointestinal neuroendocrine tumors (nets): well-differentiated nets of the distal colon and rectum. *Pancreas*. Aug 2010;39(6):767–774.
14. Caplin M, Sundin A, Nillson O, et al. ENETS Consensus Guidelines for the management of patients with digestive neuroendocrine neoplasms: colorectal neuroendocrine neoplasms. *Neuroendocrinology*. 2012;95(2):88–97.
15. de Mestier L, Brixi H, Gincul R, Ponchon T, Cadiot G. Updating the management of patients with rectal neuroendocrine tumors. *Endoscopy*. Dec 2013;45(12):1039–1046.
16. Park CS, Lee SH, Kim SB, Kim KO, Jang BI. Multiple rectal neuroendocrine tumors: report of five cases. *Korean J Gastroenterol*. Aug 2014;64(2):103–109.
17. Klimstra DS AR, Capella C, Klöppel G, Komminoth P, Solcia E, Rindi G. Neuroendocrine neoplasms of the colon and rectum. In: Bosman FT, Carneiro F, Hruban RF, Theise ND (Eds) WHO Classification of Tumours of the Digestive System. IARC: Lyon 2010:174–177.
18. Lee SH, Kim BC, Chang HJ, et al. Rectal neuroendocrine and L-cell tumors: diagnostic dilemma and therapeutic strategy. *The American journal of surgical pathology*. Jul 2013;37(7):1044–1052.
19. Ardill JE, O'Dorisio TM. Circulating biomarkers in neuroendocrine tumors of the enteropancreatic tract: application to diagnosis, monitoring disease, and as prognostic indicators. *Endocrinology and metabolism clinics of North America*. Dec 2010;39(4):777–790.
20. Janson ET, Holmberg L, Stridsberg M, et al. Carcinoid tumors: analysis of prognostic factors and survival in 301 patients from a referral center. *Ann Oncol*. Jul 1997;8(7):685–690.
21. Namwongprom S, Wong FC, Tateishi U, Kim EE, Boonyaprapa S. Correlation of chromogranin A levels and somatostatin receptor scintigraphy findings in the evaluation of metastases in carcinoid tumors. *Annals of nuclear medicine*. May 2008;22(4):237–243.
22. Rorstad O. Prognostic indicators for carcinoid neuroendocrine tumors of the gastrointestinal tract. *Journal of surgical oncology*. Mar 1 2005;89(3):151–160.
23. Modlin IM, Latich I, Zikusoka M, Kidd M, Eick G, Chan AK. Gastrointestinal carcinoids: the evolution of diagnostic strategies. *Journal of clinical gastroenterology*. Aug 2006;40(7):572–582.
24. Åkerström G, Norlén O, Edfeldt K, et al. A review on management discussions of small intestinal neuroendocrine tumors' midgut carcinoids'. *International Journal of Endocrine Oncology*. 2015;2(2):119–128.
25. Raines D, Chester M, Diebold AE, et al. A prospective evaluation of the effect of chronic proton pump inhibitor use on plasma biomarker levels in humans. *Pancreas*. May 2012;41(4):508–511.
26. Vinik A WE, O'Dorisio T, Go V, Mamikunian G. Neuroendocrine Tumors: A Comprehensive Guide To Diagnosis And Management. 5th ed. Inglewood: InterScience Institute. 2014:13–14,18–25.
27. Jurgensen C, Teubner A, Habeck JO, Diener F, Scherubl H, Stolzel U. Staging of rectal cancer by EUS: depth of infiltration in T3 cancers is important. *Gastrointestinal endoscopy*. Feb 2011;73(2):325–328.
28. Kobayashi K, Katsumata T, Yoshizawa S, et al. Indications of endoscopic polypectomy for rectal carcinoid tumors and clinical usefulness of endoscopic ultrasonography. *Diseases of the colon and rectum*. Feb 2005;48(2):285–291.
29. Srirajaskanthan R, Kayani I, Quigley AM, Soh J, Caplin ME, Bomanji J. The role of 68Ga-DOTATATE PET in patients with neuroendocrine tumors and negative or equivocal findings on 111In-DTPA-octreotide scintigraphy. *Journal of nuclear medicine: official publication, Society of Nuclear Medicine*. Jun 2010;51(6):875–882.
30. Hofman MS, Kong G, Neels OC, Eu P, Hong E, Hicks RJ. High management impact of Ga-68 DOTATATE (GaTate) PET/CT for imaging neuroendocrine and other somatostatin expressing tumours. *J Med Imaging Radiat Oncol*. Feb 2012;56(1):40–47.
31. Prasad V, Ambrosini V, Hommann M, Hoersch D, Fanti S, Baum RP. Detection of unknown primary neuroendocrine tumours (CUP-NET) using (68)Ga-DOTA-NOC receptor PET/CT. *European journal of nuclear medicine and molecular imaging*. Jan 2010;37(1):67–77.
32. Lindholm EB, Lyons J, Anthony CT, Boudreaux JP, Wang Y-Z, Woltering EA. Do primary neuroendocrine tumors and metastasis have the same characteristics? *Journal of Surgical Research*. 2012;174(2):200–206.
33. Massironi S, Rossi RE, Casazza G, et al. Chromogranin A in diagnosing and monitoring patients with gastroenteropancreatic neuroendocrine neoplasms: a large series from a single institution. *Neuroendocrinology*. 2014;100(2-3):240–249.
34. de Herder WW. Biochemistry of neuroendocrine tumours. *Best practice & research. Clinical endocrinology & metabolism*. Mar 2007;21(1):33–41.
35. Glinicki P, Jeske W, Kapuscinska R, Zgliczynski W. Comparison of chromogranin A (CgA) levels in serum and plasma (EDTA2K) and the respective reference ranges in healthy males. *Endokrynologia Polska*. 2015;66(1):53–56.

Neuroendocrine Tumors of the Pancreas

34

Emily K. Bergsland, Eugene A. Woltering, Guido Rindi, Thomas M. O'Dorisio, Richard L. Schilsky, Eric H. Liu, Michelle K. Kim, Eric K. Nakakura, Diane L. Reidy-Lagunes, Jonathan R. Strosberg, Laura H. Tang, Aaron I. Vinik, Yi-Zarn Wang, Elliot A. Asare, James D. Brierley, David L. Bushnell, Robert T. Jensen, Rodney F. Pommier, Edward M. Wolin, Rebecca K.S. Wong, and David S. Klimstra

CHAPTER SUMMARY

Cancers Staged Using This Staging System

This staging system applies to well-differentiated neuroendocrine tumors arising in the pancreas.

Cancers Not Staged Using This Staging System

These histopathologic types of cancer…	Are staged according to the classification for…	And can be found in chapter…
Carcinomas of the pancreas, including high-grade (grade 3), poorly differentiated neuroendocrine carcinoma	Exocrine pancreas	28
Well-differentiated neuroendocrine tumors arising in the duodenum (C17.0) or ampulla of Vater (C24.1)	Neuroendocrine tumors of the duodenum and ampulla of Vater	30

Summary of Changes

Change	Details of Change	Level of Evidence
New chapter	This staging system was included in the exocrine and endocrine pancreas chapters in previous editions.	N/A
AJCC Prognostic Stage Groups	Pancreatic neuroendocrine tumors are now staged using a TNM staging system predominantly based on size; the criterion of peripancreatic soft tissue invasion was eliminated.	II
Definition of Primary Tumor (T)	The Tis distinction was eliminated.	II
Definition of Distant Metastasis (M)	M1 is subdivided as follows:<br>M1a: metastasis confined to the liver<br>M1b: metastases in at least one extrahepatic site (e.g., lung, ovary, nonregional lymph node, peritoneum, bone)<br>M1c: both hepatic and extrahepatic metastases	IV

To access the AJCC cancer staging forms, please visit www.cancerstaging.org.

© American Joint Committee on Cancer 2017

M.B. Amin et al. (eds.), *AJCC Cancer Staging Manual, Eighth Edition*, DOI 10.1007/978-3-319-40618-3_34

ICD-O-3 Topography Codes

Code	Description
C25.0	Head of pancreas
C25.1	Body of pancreas
C25.2	Tail of pancreas
C25.4	Islets of Langerhans (endocrine pancreas)
C25.7	Other specified parts of the pancreas
C25.8	Overlapping lesion of the pancreas
C25.9	Pancreas, NOS

WHO Classification of Tumors

Code	Description
8150	Pancreatic endocrine tumor
8151	Insulinoma
8152	Glucagonoma
8153	Gastrinoma
8155	VIPoma
8156	Somatostatinoma
8158	Endocrine tumor, functioning, NOS
8240	Neuroendocrine tumor (NET) G1 (carcinoid)
8249	NET G2

Bosman FT, Carneiro F, Hruban RH, Theise ND, eds. World Health Organization Classification of Tumours of the Digestive System. Lyon: IARC; 2010.

INTRODUCTION

Pancreatic neuroendocrine tumors (NETs) exhibit neuroendocrine differentiation and comprise <2% of all pancreatic malignancies. Although these tumors are rare, their relatively indolent nature translates into a relatively high prevalence: approximately 10% of all pancreatic tumors.[1] An analysis of the Surveillance, Epidemiology, and End Results (SEER) database, 1973 to 2004, suggests that the incidence of pancreatic NETs is increasing.[2] In 1973, the age-adjusted incidence of pancreatic NETs in the US population was 0.18 per 100,000; in 2003, it was 0.30 per 100,000.[2] A recent analysis of the national population-based Cancer Registry of Norway revealed a similar trend.[3] The age-standardized incidence rate of pancreatic NETs in the population overall (1993–2010) was 0.47 per 100,000 (95% CI, 0.43–0.52); in 2006 to 2010, it was 0.71 per 100,000 (95% CI, 0.61–0.82). The estimated annual percentage change was +6.9%. The reason for the increasing incidence likely is multifactorial; it probably is a result, at least in part, of more accurate classification by pathologists and improved diagnostic tools (cross-sectional and functional imaging), the latter of which has led to an increase in incidentally discovered tumors.[4] Pancreatic NETs appear to be slightly more common in men (53%).[1] They may occur at any age but are most commonly detected in the fifth to eighth decades; the median age at diagnosis is 60.[2] With the exception of patients with insulinoma, patients with pancreatic NETs often present with advanced disease.[2,5] Increased detection of incidental tumors has led to a reduction in the proportion of patients diagnosed with metastatic disease at presentation.[6] Autopsy studies assessing the presence of small (<1 cm) NETs reported frequencies ranging from 0.8–10%.[7]

The grading classification scheme for pancreatic NETs has evolved over the years to encompass all NETs arising in the pancreas and gastrointestinal tract. Developed by the European Neuroendocrine Tumor Society (ENETS) and adopted by the World Health Organization (WHO) in 2010, the most common classification system consists of three grades (G1, G2, and G3), which correspond to well-differentiated (G1 and G2) and poorly differentiated neoplasms (G3).[8-11]

Grade is a significant and independent predictor of outcome.[12,13] Most NETs arising in the pancreas are well differentiated (G1 and G2) tumors, which are termed *pancreatic neuroendocrine tumors, pancreatic NETs*, or *panNETs*. Poorly differentiated neoplasms are termed *pancreatic neuroendocrine carcinomas* or *pancreatic NECs*. Although this classification implies that all high-grade tumors are poorly differentiated, recent data suggest that a significant fraction of patients with well-differentiated tumors have a ki-67 index greater than 20%, and usually less than 50%. These "well-differentiated, high-grade" tumors represent a favorable prognostic category compared with poorly differentiated NECs.[14]

Approximately 20% of pancreatic NETs are associated with a clinical syndrome due to hormone excess. These "functional" tumors (F-pancreatic NETs) thus are defined based on the clinical syndrome, as asymptomatic production of hormones also may be detected in nonfunctional tumors (NF-pancreatic NETs).[15] Among functional tumors, the most common hormones produced are insulin and gastrin (Table 34.1).[16] Overproduction of glucagon, vasoactive intestinal peptide, or proinsulin is less common.[17] Other, rarer hormone-mediated syndromes also have been reported, including pancreatic NETs secreting adrenocorticotropic hormone (ACTH), leading to Cushing's syndrome (ACTHomas); pancreatic NETs causing the carcinoid syndrome; and pancreatic NETs causing hypercalcemia (PTHrp-omas).[18,19] Pancreatic NETs associated with calcitonin or ACTH production appear to be relatively aggressive, as do those that switch from one functional syndrome to another.

More than 50% of functional tumors are located in the tail of the pancreas, the exception being gastrinomas, which are more likely (63%) to be located in the head of the pancreas (Table 34.1).[1] Interestingly, most gastrinomas (60–80%) actually arise in the duodenum; 75–85% are located in the "gastrinoma triangle" involving the duodenum and pancreatic head.[17] Insulinomas typically are small, well-circumscribed tumors that are diagnosed at an early stage as the result of symptoms associated with hypoglycemia. The vast majority do not recur after resection. Importantly, F-pancreatic NETs

Table 34.1 Clinical features of functional pancreatic neuroendocrine tumors

Name	Biologically active peptide(s)	Incidence (new cases/10^6 population/year)	Tumor location	Most common symptoms/signs
**Most common syndromes**				
Insulinoma	Insulin	1–3	Pancreas (>99%)	Hypoglycemic symptoms (Whipple's triad)
Zollinger–Ellison syndrome	Gastrin	0.5–2	Duodenum (70%); pancreas (25%); other sites (5%)	Abdominal pain, gastroesophageal reflux, diarrhea, duodenal ulcers, PUD/GERD
**Less common syndromes (additional, rarer syndromes also exist)**				
**Name**	**Biologically active peptide(s)**	**Incidence (new cases/10^6 population/year)**	**Tumor location**	**Most common symptoms/signs**
VIPoma (Verner–Morrison syndrome, pancreatic cholera, WDHA syndrome)	Vasoactive intestinal peptide	0.05–0.2	Pancreas (90%, adult); other (10%, neural, adrenal, periganglionic)	Diarrhea, hypokalemia, dehydration
Glucagonoma	Glucagon	0.01–0.1	Pancreas (100%)	Rash, glucose intolerance, weight loss
Somatostatinoma	Somatostatin	Rare	Pancreas (55%); duodenum/jejunum (44%)	Diabetes mellitus, cholelithiasis, diarrhea
ACTHoma/Cushing's Syndrome	ACTH	Rare	Pancreas (4–16% all ectopic Cushing's)	Cushing's syndrome
Pancreatic NET causing carcinoid syndrome	Serotonin	Rare	Pancreas (<1% all carcinoid syndrome)	Flushing, diarrhea
PTHrp-oma (hypercalcemia)	PTHrp, others unknown	Rare	Pancreas	Symptoms due to hypercalcemia

Abbreviations: GERD, gastroesophageal reflux disease; PTHrp, parathyroid hormone–related protein; PUD, peptic ulcer disease; WDHA, watery diarrhea, hypokalemia, and achlorhydria.
(Adapted from Jensen et al.[17])

may produce more than one hormone, and hormone production may change over the course of tumor progression.[20]

Pancreatic NETs frequently secrete several substances into the serum, including chromogranin A (CgA), pancreatic polypeptide (PP), pancreastatin, and neuron-specific enolase, without obvious clinical consequence. As such (assuming they are not secreting any additional hormones, as listed in Table 34.1), these tumors are considered NF-pancreatic NETs.[18,21-23] NF-pancreatic NETs occur at least twice as frequently as F-pancreatic NETs in most series.

The etiology of pancreatic NETs largely is unknown. Most pancreatic NETs are thought to be sporadic. Of these, approximately 43% harbor *DAXX/ATRX* mutations, 44% harbor somatic inactivating mutations of *MEN1*, and 15% contain mutations in genes encoding mTOR pathway components.[24] The prognostic significance of these mutations remains to be determined definitively.[24,25] A small fraction of pancreatic NETs (<10%) arise in the context of a hereditary cancer syndrome, the most common of which is multiple endocrine neoplasia type 1 (MEN1).[26] MEN1 is caused by mutations in the *MEN1* gene located at chromosome 11q13 region, thus altering transcriptional regulation, genomic stability, cell division, and cell cycle control.[27] Affected patients develop hyperplasia or neoplasia of multiple endocrine and nonendocrine tissues, including parathyroid adenomas (95–100%) resulting in hyperparathyroidism, pituitary adenomas (54–65%), adrenal adenomas (27–36%), various NETs (gastric, lung, thymic; 0–10%), thyroid adenomas (up to 10%), various skin tumors (80–95%), central nervous system tumors (up to 8%), and smooth muscle tumors (up to 10%).[17,28] Pancreatic NETs develop in 80–100% of MEN1 patients and are nearly always multifocal. Pancreatic NETs in this setting often are small and nonfunctional. Gastrinomas (>80% duodenal) develop in 54% of MEN1 patients, insulinomas in 18%, and glucagonomas, vasoactive intestinal peptide–secreting tumors (VIPomas), growth hormone–releasing factor–secreting tumors (GRFomas), and somatostatinomas in <5%.[17] Pancreatic NETs also occur in up to 10% of patients with von Recklinghausen's disease (also known as neurofibromatosis type 1 [NF1]), 10–17% of patients with von Hippel–Lindau (VHL) syndrome, and rarely in patients with tuberous sclerosis. The likelihood of an underlying genetic syndrome depends on the type of tumor as well as the patient's personal and family history, which should be recorded for every patient presenting with a pancreatic NET. Multifocal disease is a

risk factor, as is the type of hormone produced. MEN1 is present in 20–30% of patients with Zollinger–Ellison syndrome (ZES; usually associated with a duodenal gastrinoma) and 5% of patients with an insulinoma.[17] Given the high incidence of parathyroid adenomas in MEN1 patients, assessment of ionized calcium and serum parathyroid hormone (PTH) may be used as a screening tool in appropriate patients, with the caveat that secondary elevation of PTH may occur in the setting of vitamin D deficiency.[29,30]

The staging of pancreatic NETs depends on the size and extent of the primary tumor (including whether there is lymph node involvement and/or distant metastasis). Importantly, the American Joint Committee on Cancer (AJCC) TNM classification system for pancreatic NETs was first introduced in the AJCC Cancer Staging Manual, 7th Edition and was based on the staging algorithm for exocrine pancreatic carcinomas. Recognizing that exocrine and neuroendocrine tumors of the pancreas are distinct entities in terms of underlying tumor biology and prognosis, other staging classification systems have been proposed.[12,13,31-33] The 7th Edition AJCC staging system is significantly associated with survival, but its value is limited by the inability to discriminate between the intermediate stages (i.e., Stages II and III are prognostically indistinguishable).[12] Furthermore, some of the parameters necessary for staging (e.g., presence of extrapancreatic extension) are difficult to assess pathologically because of the expansile growth pattern common in pancreatic NETs. The system developed by ENETS in 2006 incorporates a narrower T definition. It has proven prognostic value and appears to provide superior distinction among stages (I, II, III, and IV) compared with the AJCC/Union for International Cancer Control (UICC)/WHO 2010 system.[12] Therefore, the AJCC Cancer Staging Manual, 8th Edition staging system has been modified to be consistent with the ENETS system.

Importantly, even the current ENETS staging system is imperfect; patients with Stage IIIB (any T N1 M0) disease fare better than those with Stage IIIA (T4 N0 M0) disease, and clear discrimination between stages has not been evident in all validation studies.[12,13] There are several potential reasons for this, including the generally favorable survival outcomes of patients with nonmetastatic disease, which limits the ability to distinguish prognostic groups, especially in the absence of very long-term follow-up. In addition, inconsistent lymph node sampling may underlie the conflicting findings of the prognostic importance of lymph node involvement.[34] Further refinement of the ENETS staging system is likely, although inadequate data exist for modifications at present.[12,33]

Surgical resection remains the only potentially curative treatment for well-differentiated (G1/G2) pancreatic NETs. The natural history of these tumors is poorly understood because of their relative rarity, but accepted prognostic factors include patient age, distant metastases, tumor grade, and tumor differentiation.[12] Recent studies suggest that lymph node involvement also may be an important prognostic factor.[35,36] For accurate staging, routine lymph node sampling is critical for most patients with pancreatic NETs undergoing surgery.

Well-differentiated insulinomas rarely metastasize and have a particularly good prognosis (>90% have a benign clinical course); the prognosis of other functional tumors appears to match that of nonfunctional tumors in most series, although this is not consistent in all studies.[12] The type of surgery performed depends on the tumor stage, location, and functional status, and ranges from enucleation to pancreaticoduodenectomy.[37] Because insulinomas typically are small and pursue a benign clinical course, enucleation of tumors located away from the main pancreatic duct usually is curative. For insulinomas close to the main pancreatic duct, a pancreatectomy, such as distal pancreatectomy for left-sided lesions or pancreaticoduodenectomy for right-sided lesions, may be required. Insulinomas located in the pancreatic neck region may be treated with a central pancreatectomy. Because most insulinomas are very indolent, a lymphadenectomy typically is not necessary, and spleen preservation may be considered.

In contrast, most NF-pancreatic NETs and other F-pancreatic NETs (i.e., not insulinomas) are capable of malignant behavior. The optimal treatment of incidentally identified small NF-pancreatic NETs <1.5 cm is unclear. Recent analyses suggest that surveillance, rather than surgery, is appropriate in many cases. A careful analysis of the potential risks and benefits of pancreatic resection is required, particularly in asymptomatic elderly patients with significant comorbidities.[4,38]

Most larger NF-NETs or localized pancreatic NETs with an elevated proliferative index have a higher risk of invasion and metastases; thus, resection with a lymphadenectomy should be strongly considered for these neoplasms. Even small pancreatic NETs may be associated with significant lymph node and/or liver metastases.[39] Several factors influence the choice of surgical procedure, including primary tumor size, Ki-67 labeling index, mitotic index, location, and medical comorbidities. For left-sided lesions, a distal pancreatectomy and, if necessary, an *en bloc* splenectomy should be done to ensure adequate lymphadenectomy. A pancreaticoduodenectomy (Whipple procedure) should be considered for right-sided

lesions. Rarely, a total pancreatectomy with en bloc splenectomy is required for large lesions that occupy much of the pancreas, but given the high morbidity of such a procedure, a thorough evaluation should be performed first to rule out metastatic disease. A central pancreatectomy with regional lymphadenectomy might be considered for lesions located in the pancreatic neck. If enucleation is considered for these neoplasms, a regional lymphadenectomy may be done to ensure adequate lymph node sampling for staging. Removal of a pancreatic primary tumor in the setting of resectable liver metastases may be considered, particularly if a pancreaticoduodenectomy is not required. Although complete resection and/or palliative debulking surgeries (i.e., primary tumor and liver metastases) are not necessarily curative, data from nonrandomized studies suggest they may be associated with improved hormone-mediated symptoms and improved survival in carefully selected patients.[40,41]

Several treatment options exist for patients with advanced, unresectable pancreatic NETs. Somatostatin analogs have cytostatic activity and may be used to treat hormone-mediated symptoms.[42-44] Chemotherapy has been used with some success, although the optimal regimen remains unclear.[45-47] Two targeted therapies are approved for this indication: everolimus (an inhibitor of mTOR signaling) and sunitinib (an oral inhibitor of vascular endothelial growth factor signaling) both delay progression of progressive panNETs.[48,49] The use of liver-directed therapy or other treatments depends on several factors, including the tumor's growth rate, extent of disease, and whether the tumor is functional. See published guidelines for additional information regarding the workup and treatment of panNETs.[17,37,50]

ANATOMY

Primary Site(s)

The pancreas is a long, coarsely lobulated gland that lies transversely across the posterior abdomen and extends from the duodenum to the splenic hilum. The organ is divided into a head with a small uncinate process, a neck, a body, and a tail. These are contiguous regions without sharp anatomic distinctions. The pancreas neck lies anterior to the superior mesenteric vessels. The anterior aspect of the body of the pancreas is covered by peritoneum and is in direct contact with the posterior wall of the stomach; posteriorly, the pancreas extends within the retroperitoneal soft tissue to the inferior vena cava, superior mesenteric vein, splenic vein, and left adrenal and kidney.

Pancreatic NETs are distributed throughout the pancreas.[1,11,12] Tumors of the head of the pancreas are those arising to the right of the superior mesenteric–portal vein confluence (Fig. 34.1). The uncinate process is the part of the pancreatic head that extends behind the superior mesenteric vessels. The

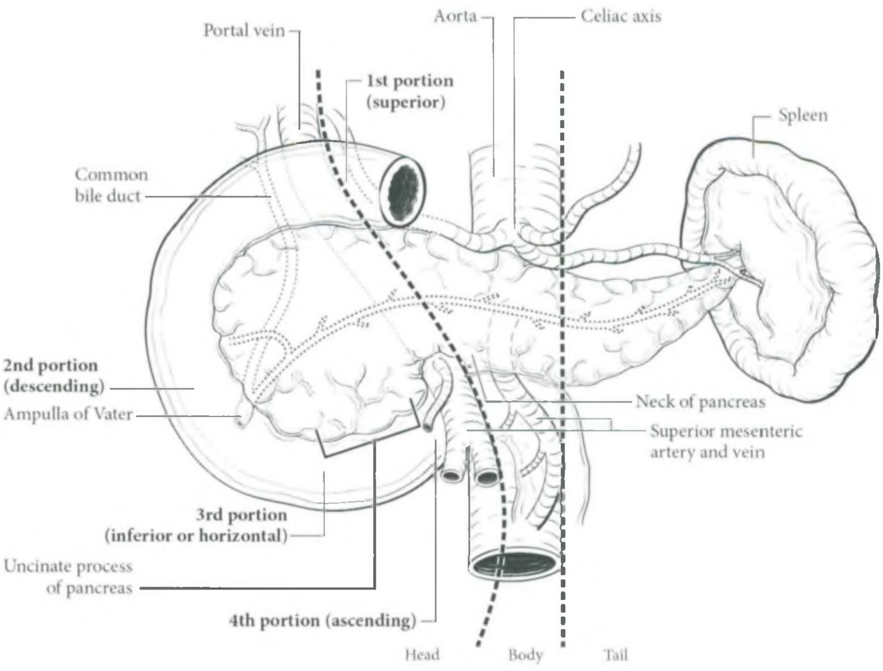

Fig. 34.1 Anatomy of the pancreas

34

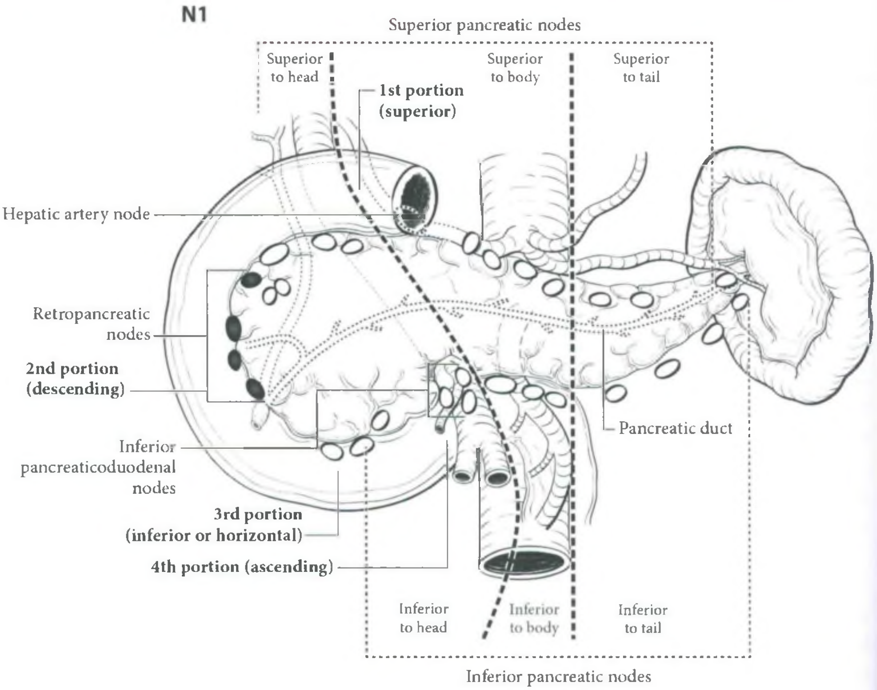

Fig. 34.2 Regional lymph nodes of the pancreas (anterior view)

neck overlies the superior mesenteric vessels. Tumors of the body of the pancreas are defined as those arising to the left of the neck. Laterally to the left side, the body becomes the tail of the pancreas without any clear junction point.

Regional Lymph Nodes

The standard regional lymph node basins and soft tissues resected for tumors located in the head and neck of the pancreas include lymph nodes along the common bile duct, common hepatic artery, portal vein, posterior and anterior pancreatoduodenal arcades, and the superior mesenteric vein and right lateral wall of the superior mesenteric artery (Fig. 34.2). For cancers located in the body and tail, regional lymph node basins include lymph nodes along the common hepatic artery, celiac axis, splenic artery, and splenic hilum (Fig. 34.2). Involvement of peripancreatic lymph nodes is considered regional disease and classified as N1.

Metastatic Sites

Distant spread is common on presentation and most commonly involves the liver. Metastases to other sites, such as lung, bones, and peritoneum, also may occur. Involvement of the para-aortic or other distant lymph nodes (i.e., retroperitoneal, retrocrural, and mesenteric lymph nodes) is considered M1 disease (see Fig. 34.2 for regional lymph nodes). Seeding of the peritoneum (even if limited to the lesser sac region) is considered M1.

RULES FOR CLASSIFICATION

Clinical Classification

Guidelines have been established for the workup of patients with pancreatic NETs.[37,50,51] In general, patients should be evaluated by multiphasic computed tomography (CT) or magnetic resonance (MR) imaging to assess 1) the proximity of the primary pancreatic NET to major vessels and 2) the clinical T, N, and M staging of the lesion before any surgical or medical therapy is considered. In addition, biochemical assessment, somatostatin receptor scintigraphy (SRS), and endoscopic ultrasonography (EUS) should be performed as appropriate. For localized tumors, a biopsy is not necessarily required before surgical resection. However, if a biopsy (e.g., endoscopic biopsy, percutaneous core needle biopsy, fine-needle aspiration) is performed, the results should be incorporated when assessing clinical stage.

By definition, patients with F-pancreatic NETs present with hormone-mediated symptoms consistent with a characteristic syndrome.[17,37] As such, the diagnosis of F-pancreatic NETs requires demonstration of a significantly elevated hormone combined with clinical signs or symptoms of overse-

cretion (e.g., ulcers in the setting of gastrinoma, hypoglycemia in the setting of insulinoma).[37] Specifically, for insulinomas, assessment of plasma insulin, proinsulin, and C-peptide is needed at the time of glucose determinations, usually during a 72-hour supervised fast.[17] For ZES, fasting serum gastrin (FSG) should be assessed, unstimulated or during a secretin provocation test.[18] The diagnosis of ZES requires demonstration of an inappropriate elevation of gastrin in the presence of hyperchlorhydria or an acidic pH (<2). FSG alone is not diagnostic, because hypergastrinemia may be caused by hypochlorhydria/achlorhydria (chronic atrophic fundic [autoimmune] gastritis, often associated with pernicious anemia) and is a common consequence of proton pump inhibitor (PPI) use. Ideally, PPIs should be stopped in order to make the diagnosis of gastrinoma, but this may be difficult in patients with severe gastroesophageal reflux disease/gastrinoma and necessitates switching to an H_2 blocker (ideally in the context of a specialty unit with experience in diagnosing ZES). Furthermore, other disorders cause hypergastrinemia with hyperchlorhydria (e.g., *Helicobacter pylori* infection, gastric outlet obstruction, renal failure, antral G-cell syndromes, G-cell hyperplasia, short bowel syndrome, retained antrum). FSG level alone cannot distinguish ZES from achlorhydric states including PPI use.

For VIPomas, the plasma vasoactive intestinal peptide level must be determined. For glucagonoma, measurement of plasma glucagon levels is appropriate. For GRFomas, plasma growth hormone and growth hormone–releasing factor levels should be measured. For Cushing's syndrome, urinary cortisol, plasma ACTH, and appropriate ACTH suppression studies should be performed. For pancreatic NET–associated hypercalcemia, measurement of both serum PTH and PTHrP levels is indicated, and for a pancreatic NET associated with carcinoid syndrome, urinary or plasma 5-hydroxyindoleacetic acid (5-HIAA) should be measured.[18,52] Assessment for hormones associated with rarer syndromes should be performed as clinically indicated.[19]

In contrast, unless incidentally discovered during a workup for an unrelated problem, NF-pancreatic NETs typically present with symptoms due to the tumor itself, including abdominal pain (40–60%), weight loss, or jaundice.[18,23,53] Although NF-pancreatic NETs do not secrete peptides causing a clinical syndrome, they characteristically secrete several other peptides, such as CgA, PP, neuron-specific enolase, and/or pancreastatin (a subunit of chromogranin), which may be helpful for the diagnosis and monitoring of affected patients.[18,23] At this time, there is insufficient evidence regarding the impact of any individual tumor marker on clinical decision making to recommend a particular assay.

Imaging

Information necessary for the clinical staging of pancreatic NETs may be obtained from physical examination; cross-sectional radiographic imaging studies, including triphasic (noncontrast, arterial, and venous) contrast-enhanced CT or MR imaging; and SRS.[5] (Refer to established guidelines for details.)[37] The detection rate of pancreatic primary tumors is in the range of 75–79% with cross-sectional imaging, and the sensitivity for detection of liver metastases is up to 80% with contrast-enhanced, multiphasic CT or MR imaging.[5,54] SRS with indium-111 pentetreotide imaging (Octreoscan; Mallinckrodt Pharmaceuticals, Dublin, Ireland) has a sensitivity of up to 90% for pancreatic NETs, depending on tumor size and type (e.g., 20–60% sensitivity for detecting insulinomas).[54] Standard fluorodeoxyglucose positron emission tomography (PET) with fluorine-18 glucose has limited value in the evaluation of well-differentiated pancreatic NETs. EUS also provides useful information for detection of small and/or multifocal pancreatic NETs and is the procedure of choice for performing fine-needle aspiration biopsy of the pancreas. Studies suggest detection rates of 90–100% for pancreatic lesions and 45–60% for tumors arising in the duodenum.[55]

Unlike its exocrine counterpart (pancreatic ductal adenocarcinoma), tumor involvement of the celiac axis or superior mesenteric artery is rare in pancreatic NETs. The standard radiographic assessment of resectability includes evaluation for distant metastases (e.g., peritoneal, liver, bone); the patency of the superior mesenteric vein and portal vein, as well as the relationship of these vessels and their tributaries to the tumor; and the relationship of the tumor to the superior mesenteric artery, celiac axis, and hepatic artery.

NET imaging using PET with gallium-68 (^{68}Ga)-labeled somatostatin analogs appears promising (same-day results, potential for increased sensitivity, broader affinity profile, better spatial resolution, easier quantification of tracer uptake).[56,57,58] Studies to assess the value of ^{68}Ga-labeled somatostatin analog–based PET/CT and PET/MR imaging relative to standard somatostatin scintigraphy are ongoing. This technology represents an emerging imaging tool for NETs in the United States; a kit for the preparation of ^{68}Ga-dotatate injection for PET imaging recently received U.S. Food and Drug Administration approval.[59]

34

Pathological Classification

Pathological staging is based on surgical resection specimens. The most sensitive pathological staging is obtained by examining surgically resected primary tumor(s), lymph nodes, and distant metastases according to an established minimum pathology dataset.[10,60-62]

Partial resection (pancreaticoduodenectomy or distal pancreatectomy) or complete resection of the pancreas, including the tumor and associated regional lymph nodes, provides the optimal information for pathological staging.

In pancreaticoduodenectomy specimens, the bile duct, pancreatic duct, and superior mesenteric artery margins should be evaluated grossly and microscopically. The superior mesenteric artery margin also has been termed the retroperitoneal, vascular, or uncinate margin. In total pancreatectomy specimens, the bile duct and retroperitoneal margins should be assessed. Duodenal (with pylorus-preserving pancreaticoduodenectomy) and gastric (with standard pancreaticoduodenectomy) margins rarely are involved, but their status should be included in the surgical pathology report. Reporting of margins may be facilitated by ensuring documentation of the pertinent margins: 1) common bile (hepatic) duct, 2) pancreatic neck, 3) superior mesenteric artery, 4) other soft tissue margins (i.e., posterior pancreatic, duodenum, and stomach).

A rich lymphatic network surrounds the pancreas, and accurate tumor staging requires analysis of all lymph nodes removed. Optimal histologic examination of a pancreaticoduodenectomy specimen should include analysis of a minimum of 12 lymph nodes. However, the number of lymph nodes removed depends on the type of surgery performed and may not be feasible in the setting of a distal pancreatectomy without *en bloc* splenectomy. Therefore, spleen preservation should not be done when there is a high chance of malignancy (i.e., for any pancreatic NET other than a small insulinoma). Furthermore, lymph nodes typically are not sampled in the setting of an enucleation procedure. The number of lymph nodes examined should be specified in the pathology report. Anatomic division of regional lymph nodes is not necessary; however, separately submitted lymph nodes should be reported as labeled by the surgeon. Finally, an N category (N1 or N0) should be assigned as long as at least one lymph node has been assessed, even if the optimal number of lymph nodes have not been examined. Nx should be applied only if no lymph nodes were assessed (e.g., if enucleation was performed). Positive peritoneal cytology is considered M1.

The pathological diagnosis of pancreatic NETs may be established by histologic evaluation alone if classic morphologic features are present. However, the morphology of pancreatic NETs is highly variable, and alternative diagnoses, such as acinar cell carcinoma (or mixed acinar NEC), solid pseudopapillary neoplasm, or ductal adenocarcinoma, may be considered in many cases. Immunolabeling for the general neuroendocrine markers chromogranin and synaptophysin is helpful to support the diagnosis of pancreatic NETs, provided other markers are performed to exclude the alternative diagnoses, some of which share expression of chromogranin or synaptophysin with pancreatic NETs.

Immunohistochemistry for hormones is optional and does not have prognostic significance. Positive immunostaining for a hormone does not necessarily indicate the presence of a hormonal syndrome.

Restaging

For pancreatic NETs, the *r* prefix is used for recurrent tumor status (rTNM) following a disease-free interval after treatment.

PROGNOSTIC FACTORS

Prognostic Factors Required for Stage Grouping

Beyond the factors used to assign T, N, or M categories, no additional prognostic factors are required for stage grouping.

Additional Factors Recommended for Clinical Care

Mitotic Count

Tumor grade is determined by mitotic count and Ki-67 labeling index and correlates with progression-free survival, overall survival, and lymph node status in pancreatic NETs.[12,63,64] Mitotic count should be assessed as the number of mitoses per 10 high-power fields (HPF): HPF = 2 mm^2, at least 50 fields (at 40× magnification) evaluated. AJCC Level of Evidence: I

Mitotic count, # of mitoses per 10 HPF (specify: _____)

___ <2
___ 2 to 20
___ >20
___Not performed

Ki-67 Labeling Index

Tumor grade is determined by mitotic count and Ki-67 labeling index and correlates with progression-free survival, overall survival, and lymph node status in pancreatic NETs. The proliferation index as measured by Ki-67 also correlates with these outcome measures in pancreatic NETs.[12,38,63,64] The Ki-67 index typically is measured using the MIB1 antibody, by counting the number of immunolabeled tumor cells per 500 to 2,000 cells in areas of highest nuclear labeling; the Ki-67 index is expressed as a percentage. AJCC Level of Evidence: I

___ Ki-67 labeling index (specify: _____)
___ <3%
___ 3% to 20%
___ >20%
___ Other (specify): ____________
___ Not performed

Associated Genetic Syndrome

Gastroenteropancreatic NETs sometimes arise in the setting of an inherited cancer syndrome characterized by a germline mutation. Tumors arising in the setting of an inherited cancer syndrome may be multiple and appear to be associated with a better prognosis than sporadic tumors, at least in the setting of MEN1.[12] AJCC Level of Evidence: II

This factor should be recorded as follows:

- Familial syndrome
  - Multiple endocrine neoplasia type 1 (alteration in *MEN1*)
  - Von Hippel–Lindau disease (mutation in *VHL* gene)
  - Neurofibromatosis type 1 (mutation in *Nf1*)
  - Tuberous sclerosis complex (mutation in *TSC1* or *TSC2*)
  - Mahvash disease (pancreatic NET caused by inactivating glucagon receptor mutation)[65]
  - Other syndrome
- Sporadic tumor
- Unknown/unable to assess

Chromogranin A (CgA)

CgA is a 49-kDa acidic polypeptide present in the secretory granules of all neuroendocrine cells. CgA is a general NET marker, and plasma or serum CgA may be used as a marker in patients with either F- or NF-pancreatic NETs.[18,66-68] CgA has prognostic significance, with higher levels indicating a worse prognosis.[69] Furthermore, changes over time may be useful in assessing for recurrence after surgery or response to therapy in patients with metastatic disease.[66,67,70,71]

Despite the potential merits of monitoring CgA levels, the clinical utility of CgA is limited by the fact that it is falsely elevated in the setting of PPI use, chronic atrophic gastritis, renal failure, severe hypertension, and other conditions.[8] Moreover, levels may fluctuate based on time of collection and fasting versus nonfasting states. Also, the upper limit of normal (ULN) varies widely depending on the assay used and whether plasma or serum is assessed; thus, both the assay and type of sample should be considered when comparing CgA values over time.[72] As a result, routine measurement of CgA is not a consensus National Comprehensive Cancer Network (NCCN) recommendation. Multiple Clinical Laboratory Improvement Amendments (CLIA)-licensed and College of American Pathology (CAP)-accredited reference laboratories in the United States can measure CgA levels. AJCC Level of evidence: II

Functionality

Tumors with hormone expression noted on immunohistochemistry but not associated with a clinically relevant syndrome or signs should be recorded as nonfunctional. Similarly, tumors associated with elevated blood levels of hormones that are not associated with clinical symptoms also should be recorded as nonfunctional. Insulinomas typically have a low risk of metastasis and thus carry a good prognosis; the outcome of other F-pancreatic NETs appears to be similar to that of nonfunctional tumors in most studies.[12,13] Importantly, the clinical manifestations and morbidity of F-pancreatic NETs may differ, and in some cases, mortality may relate to the hormonal syndrome rather than to the extent of the neoplasm. AJCC level of evidence: III

Functionality should be characterized as follows:

- Functional
  - Insulinoma
  - Gastrinoma (ZES)
  - Glucagonoma
  - VIPoma (Verner–Morrison syndrome)
  - Somatostatinoma
  - ACTHoma
  - PanNET causing carcinoid syndrome (5-HIAA, serotonin excess)
  - PanNET causing hypercalcemia (PTHrp or other)
  - Other
- Nonfunctional
- Unknown/unable to assess

RISK ASSESSMENT MODELS

The AJCC recently established guidelines that will be used to evaluate published statistical prediction models for the purpose of granting endorsement for clinical use.[73] Although this is a monumental step toward the goal of precision medicine, this work was published only very recently. Therefore, the existing models that have been published or may be in clinical use have not yet been evaluated for this cancer site by the Precision Medicine Core of the AJCC. In the future, the statistical prediction models for this cancer site will be evaluated, and those that meet all AJCC criteria will be endorsed.

DEFINITIONS OF AJCC TNM

Definition of Primary Tumor (T)

T Category	T Criteria
TX	Tumor cannot be assessed
T1	Tumor limited to the pancreas,* <2 cm
T2	Tumor limited to the pancreas,* 2–4 cm
T3	Tumor limited to the pancreas,* >4 cm; or tumor invading the duodenum or bile duct
T4	Tumor invading adjacent organs (stomach, spleen, colon, adrenal gland) or the wall of large vessels (celiac axis or the superior mesenteric artery)

**Limited to the pancreas* means there is no invasion of adjacent organs (stomach, spleen, colon, adrenal gland) or the wall of large vessels (celiac axis or the superior mesenteric artery). Extension of tumor into peripancreatic adipose tissue is NOT a basis for staging.
Note: Multiple tumors should be designated as such (the largest tumor should be used to assign T category):
• If the number of tumors is known, use T(#); e.g., pT3(4) N0 M0.
• If the number of tumors is unavailable or too numerous, use the *m* suffix, T(m); e.g., pT3(m) N0 M0.

Definition of Regional Lymph Node (N)

N Category	N Criteria
NX	Regional lymph nodes cannot be assessed
N0	No regional lymph node involvement
N1	Regional lymph node involvement

Definition of Distant Metastasis (M)

M Category	M Criteria
M0	No distant metastasis
M1	Distant metastases
M1a	Metastasis confined to liver
M1b	Metastases in at least one extrahepatic site (e.g., lung, ovary, nonregional lymph node, peritoneum, bone)
M1c	Both hepatic and extrahepatic metastases

AJCC PROGNOSTIC STAGE GROUPS

When T is...	And N is...	And M is...	Then the stage group is...
T1	N0	M0	I
T2	N0	M0	II
T3	N0	M0	II
T4	N0	M0	III
Any T	N1	M0	III
Any T	Any N	M1	IV

REGISTRY DATA COLLECTION VARIABLES

1. Size of tumor (value, unknown)
2. Presence of invasion into adjacent organs/structures (Y/N)
   a) If yes, which ones (pick all that apply):
      • Stomach (Y/N)
      • Duodenum (Y/N)
      • Spleen (Y/N)
      • Colon (Y/N)
      • Other:
   b) If yes, were multiple adjacent organs involved(Y/N)
3. Presence of necrosis
4. Number of tumors (multicentric disease at primary site)
5. Lymph node status (including number of lymph nodes assessed and number of positive nodes)
6. Grade (based on Ki-67 and/or mitotic count; G1, G2, G3, unknown)
7. Mitotic count (value; unknown)
8. Ki-67 Labeling Index (value; unknown)
9. Perineural invasion (Y/N)
10. Lymphovascular invasion (Y/N)
11. Margin status (+/−)
12. Functional status (Y/N, type of syndrome)
13. Genetic syndrome (Y/N, type of syndrome)
14. Location in pancreas (head, tail, body, junction body/tail, junction body/head, unknown)
15. Type of surgery (enucleation, distal pancreatectomy with or without splenectomy, central pancreatectomy, pancreaticoduodenectomy–Whipple procedure, unknown, other)
16. Preoperative CgA level (absolute value with ULN; unknown)
17. Preoperative pancreastatin level (absolute value with ULN; unknown)
18. Age of patient

HISTOLOGIC GRADE (G)

Grading of pancreatic NETs is required for prognostic stratification and should be performed on all resection specimens and on biopsy specimens containing sufficient tumor tissue to allow accurate measurement of proliferation (50 HPF for mitotic counting and 500 cells to determine the Ki-67 index). If multiple disease sites are sampled (e.g., a primary tumor as well as a metastasis), the grade of each site should be recorded separately. If multiple foci are sampled within a single anatomic site (e.g., multiple liver metastases), the highest grade may be recorded. The grading scheme in Table 34.2 is currently endorsed by ENETS/WHO for gastrointestinal and pancreatic neuroendocrine neoplasms.

Table 34.2 ENETS/WHO grading system for pancreatic neuroendocrine neoplasms

G	G Definition
GX	Grade cannot be assessed
G1	Mitotic count (per 10 HPF)* <2 and Ki-67 index (%)** <3
G2	Mitotic count (per 10 HPF) = 2–20 or Ki-67 index (%)** = 3–20
G3	Mitotic count (per 10 HPF) >20 or Ki-67 index (%)** >20

*10 HPF = 2 mm^2; at least 50 HPF (at 40× magnification) must be evaluated in areas of highest mitotic density in order to match WHO 2010 criteria.
**MIB1 antibody; % of 500–2,000 tumor cells in areas of highest nuclear labeling.

The Ki-67 index is based on the region with the highest labeling rate ("hot spot"), determined by examining the Ki-67 stain at low magnification. In the event of discordance between the grade indicated by the mitotic count and that suggested by the Ki-67 index, the higher grade should be assigned. Nuclear pleomorphism is not a useful feature for grading neuroendocrine neoplasms. Although necrosis has been regarded as a prognostic factor in some studies, its presence is not incorporated into the grading scheme.

Well-differentiated NETs are subdivided into G1 and G2 tumors based on proliferative and mitotic index. *G1* and *G2* refer to well-differentiated NETs displaying diffuse and intense expression of two general immunohistochemical neuroendocrine markers (i.e., CgA and synaptophysin). *G3* usually indicates a poorly differentiated NEC, which should be staged using the system for pancreatic carcinomas (Chapter 28). High-grade (G3) tumors typically are characterized by a high mitotic count/Ki-67 index, nuclear pleomorphism, and extensive necrosis. Immunohistochemical expression of CgA and/or synaptophysin may be weak.

In some cases, pancreatic neuroendocrine neoplasms with well-differentiated histologic features have a Ki-67 index (or, more rarely, a mitotic count) within the G3 range.[14,74-77] Although these neoplasms currently are considered high grade in the WHO grading scheme, emerging data suggest that they are not as aggressive as poorly differentiated NECs with undifferentiated, small cell, or large cell morphology, and their response to therapy is more in line with that of other well-differentiated pancreatic NETs.[74] Progression of a well-differentiated pancreatic NET from G1 or G2 to G3 also has been documented. It has been proposed that these well-differentiated neuroendocrine neoplasms be classified as well-differentiated NETs, G3. As such, they should be staged using the parameters of pancreatic NETs, rather than those of pancreatic carcinomas.

HISTOPATHLOGIC TYPE

This staging system applies to well-differentiated NETs arising in the pancreas.

SURVIVAL DATA

Four years of data (diagnosis years 2010–2013) from the National Cancer Data Base were used to assess survival. Maximum follow-up time for these patients was 3 years. Selection criteria included primary site codes C25.0, C25.1, C25.2, C25.4, C25.7, C25.8, and C25.9; grade 1 and grade 2; histology codes 8150, 8151, 8152, 8153, 8155, 8156, 8158, 8240, 8249, and 8246; ages 18 and older; and only primary or first of multiple primaries.

In this study, 1,174 pancreatic NETs were identified and recategorized according to the 8th Edition (ENETS) staging system as indicated: Stage 1, n =262; Stage IIA, n = 221; Stage IIB, n = 191; Stage IIIA, n = 32; Stage IIIB, n = 346; and Stage IV, n = 122). Product limit survival curves (Kaplan–Meier) and 95% confidence intervals adjusted for age were produced; however, there were not enough cases or follow-up to assess survival accurately. Therefore, survival tables and charts are not included.

Bibliography

1. Yao JC, Eisner MP, Leary C, et al. Population-based study of islet cell carcinoma. *Annals of surgical oncology*. Dec 2007;14(12):3492-3500.
2. Yao JC, Hassan M, Phan A, et al. One hundred years after "carcinoid": epidemiology of and prognostic factors for neuroendocrine tumors in 35,825 cases in the United States. *J Clin Oncol*. Jun 20 2008;26(18):3063-3072.
3. Boyar Cetinkaya R, Aagnes B, Thiis-Evensen E, Tretli S, Bergestuen DS, Hansen S. Trends in Incidence of Neuroendocrine Neoplasms in Norway: A Report of 16,075 Cases from 1993 through 2010. *Neuroendocrinology*. 2015.
4. Crippa S, Partelli S, Zamboni G, et al. Incidental diagnosis as prognostic factor in different tumor stages of nonfunctioning pancreatic endocrine tumors. *Surgery*. 2014;155(1):145-153.
5. Falconi M, Bartsch DK, Eriksson B, et al. ENETS Consensus Guidelines for the management of patients with digestive neuroendocrine neoplasms of the digestive system: well-differentiated pancreatic non-functioning tumors. *Neuroendocrinology*. 2012;95(2):120-134.
6. Zerbi A, Falconi M, Rindi G, et al. Clinicopathological features of pancreatic endocrine tumors: a prospective multicenter study in Italy of 297 sporadic cases. *Am J Gastroenterol*. Jun 2010;105(6):1421-1429.
7. Kimura W, Kuroda A, Morioka Y. Clinical pathology of endocrine tumors of the pancreas. Analysis of autopsy cases. *Digestive diseases and sciences*. Jul 1991;36(7):933-942.
8. Rindi G, Klersy C, Inzani F, et al. Grading the neuroendocrine tumors of the lung: an evidence-based proposal. *Endocrine-related cancer*. Feb 2014;21(1):1-16.
9. Rindi G, Arnold R, Capella C, et al. Nomenclature and classification of digestive neuroendocrine tumours. *World Health Organization classification of tumours, pathology and genetics of tumours of the digestive system. IARC Press, Lyon*. 2010:10-12.
10. Bosman FT, Carneiro F, Hruban RH, Theise ND. *WHO classification of tumours of the digestive system*. World Health Organization; 2010.
11. Qadan M, Ma Y, Visser BC, et al. Reassessment of the current American Joint Committee on Cancer staging system for pancreatic neuroendocrine tumors. *Journal of the American College of Surgeons*. Feb 2014;218(2):188-195.
12. Rindi G, Falconi M, Klersy C, et al. TNM staging of neoplasms of the endocrine pancreas: results from a large international cohort study. *Journal of the National Cancer Institute*. May 16 2012;104(10):764-777.
13. Strosberg JR, Cheema A, Weber J, Han G, Coppola D, Kvols LK. Prognostic validity of a novel American Joint Committee on Cancer Staging Classification for pancreatic neuroendocrine tumors. *J Clin Oncol*. Aug 1 2011;29(22):3044-3049.
14. Basturk O, Yang Z, Tang LH, et al. The high-grade (WHO G3) pancreatic neuroendocrine tumor category is morphologically and biologically heterogenous and includes both well differentiated and

poorly differentiated neoplasms. *The American journal of surgical pathology*. May 2015;39(5):683-690.

15. Choti MA, Bobiak S, Strosberg JR, et al. Prevalence of functional tumors in neuroendocrine carcinoma: An analysis from the NCCN NET database. Paper presented at: ASCO Annual Meeting Proceedings2012.
16. Vinik AI, Woltering EA, Warner RR, et al. NANETS consensus guidelines for the diagnosis of neuroendocrine tumor. *Pancreas*. Aug 2010;39(6):713-734.
17. Jensen RT, Cadiot G, Brandi ML, et al. ENETS Consensus Guidelines for the management of patients with digestive neuroendocrine neoplasms: functional pancreatic endocrine tumor syndromes. *Neuroendocrinology*. 2012;95(2):98-119.
18. Metz DC, Jensen RT. Gastrointestinal neuroendocrine tumors: pancreatic endocrine tumors. *Gastroenterology*. Nov 2008;135(5): 1469-1492.
19. Vinik AI, Chaya C. Clinical Presentation and Diagnosis of Neuroendocrine Tumors. *Hematol Oncol Clin North Am*. Feb 2016;30(1):21-48.
20. Nahmias A, Grozinsky-Glasberg S, Salmon A, Gross DJ. Pancreatic neuroendocrine tumors with transformation to insulinoma: an unusual presentation of a rare disease. *Endocrinology, diabetes & metabolism case reports*. 2015;2015:150032.
21. Panzuto F, Severi C, Cannizzaro R, et al. Utility of combined use of plasma levels of chromogranin A and pancreatic polypeptide in the diagnosis of gastrointestinal and pancreatic endocrine tumors. *Journal of endocrinological investigation*. 2004;27(1):6-11.
22. Kloppel G, Anlauf M. Epidemiology, tumour biology and histopathological classification of neuroendocrine tumours of the gastrointestinal tract. *Best practice & research. Clinical gastroenterology*. Aug 2005;19(4):507-517.
23. Oberg K, Eriksson B. Nuclear medicine in the detection, staging and treatment of gastrointestinal carcinoid tumours. *Best practice & research. Clinical endocrinology & metabolism*. Jun 2005;19(2): 265-276.
24. Jiao Y, Shi C, Edil BH, et al. DAXX/ATRX, MEN1, and mTOR pathway genes are frequently altered in pancreatic neuroendocrine tumors. *Science*. Mar 4 2011;331(6021):1199-1203.
25. Marinoni I, Kurrer AS, Vassella E, et al. Loss of DAXX and ATRX are associated with chromosome instability and reduced survival of patients with pancreatic neuroendocrine tumors. *Gastroenterology*. Feb 2014;146(2):453-460 e455.
26. Oberg K. The genetics of neuroendocrine tumors. *Semin Oncol*. Feb 2013;40(1):37-44.
27. Eriksson B, Renstrup J, Imam H, Oberg K. High-dose treatment with lanreotide of patients with advanced neuroendocrine gastrointestinal tumors: clinical and biological effects. *Ann Oncol*. Oct 1997;8(10):1041-1044.
28. Thakker RV, Newey PJ, Walls GV, et al. Clinical practice guidelines for multiple endocrine neoplasia type 1 (MEN1). *The Journal of clinical endocrinology and metabolism*. Sep 2012;97(9): 2990-3011.
29. Souberbielle J-C, Cavalier E, Cormier C. How to manage an isolated elevated PTH? Paper presented at: Annales d'endocrinologie2015.
30. Vinik AI, Silva MP, Woltering EA, Go VL, Warner R, Caplin M. Biochemical testing for neuroendocrine tumors. *Pancreas*. Nov 2009;38(8):876-889.
31. Rindi G. The ENETS guidelines: the new TNM classification system. *Tumori*. Sep-Oct 2010;96(5):806-809.
32. Martin RC, Kooby DA, Weber SM, et al. Analysis of 6,747 pancreatic neuroendocrine tumors for a proposed staging system. *Journal of gastrointestinal surgery: official journal of the Society for Surgery of the Alimentary Tract*. Jan 2011;15(1):175-183.
33. Scarpa A, Mantovani W, Capelli P, et al. Pancreatic endocrine tumors: improved TNM staging and histopathological grading permit a clinically efficient prognostic stratification of patients. *Modern pathology: an official journal of the United States and Canadian Academy of Pathology, Inc*. Jun 2010;23(6):824-833.
34. Parekh JR, Wang SC, Bergsland EK, et al. Lymph node sampling rates and predictors of nodal metastasis in pancreatic neuroendocrine tumor resections: the UCSF experience with 149 patients. *Pancreas*. Aug 2012;41(6):840-844.
35. Hashim YM, Trinkaus KM, Linehan DC, et al. Regional lymphadenectomy is indicated in the surgical treatment of pancreatic neuroendocrine tumors (PNETs). *Annals of surgery*. 2014;259(2):197.
36. Krampitz GW, Norton JA, Poultsides GA, Visser BC, Sun L, Jensen RT. Lymph nodes and survival in pancreatic neuroendocrine tumors. *Archives of surgery*. Sep 2012;147(9):820-827.
37. Kulke MH, Shah MH, Benson AB, 3rd, et al. Neuroendocrine tumors, version 1.2015. *Journal of the National Comprehensive Cancer Network: JNCCN*. Jan 2015;13(1):78-108.
38. Strosberg JR, Cheema A, Weber JM, et al. Relapse-free survival in patients with nonmetastatic, surgically resected pancreatic neuroendocrine tumors: an analysis of the AJCC and ENETS staging classifications. *Annals of surgery*. Aug 2012;256(2):321-325.
39. Massimino KP, Han E, Pommier SJ, Pommier RF. Laparoscopic surgical exploration is an effective strategy for locating occult primary neuroendocrine tumors. *The American Journal of Surgery*. 2012;203(5):628-631.
40. Chamberlain RS, Canes D, Brown KT, et al. Hepatic neuroendocrine metastases: does intervention alter outcomes? *Journal of the American College of Surgeons*. Apr 2000;190(4):432-445.
41. Mayo SC, de Jong MC, Pulitano C, et al. Surgical management of hepatic neuroendocrine tumor metastasis: results from an international multi-institutional analysis. *Annals of surgical oncology*. Dec 2010;17(12):3129-3136.
42. Caplin ME, Pavel M, Cwikla JB, et al. Lanreotide in metastatic enteropancreatic neuroendocrine tumors. *N Engl J Med*. Jul 17 2014;371(3):224-233.
43. Rinke A, Muller HH, Schade-Brittinger C, et al. Placebo-controlled, double-blind, prospective, randomized study on the effect of octreotide LAR in the control of tumor growth in patients with metastatic neuroendocrine midgut tumors: a report from the PROMID Study Group. *J Clin Oncol*. Oct 1 2009;27(28):4656-4663.
44. Oberg K. Somatostatin analog octreotide LAR in gastro-entero-pancreatic tumors. *Expert review of anticancer therapy*. May 2009;9(5):557-566.
45. Strosberg JR, Fine RL, Choi J, et al. First-line chemotherapy with capecitabine and temozolomide in patients with metastatic pancreatic endocrine carcinomas. *Cancer*. Jan 15 2011;117(2):268-275.
46. Moertel CG, Lefkopoulo M, Lipsitz S, Hahn RG, Klaassen D. Streptozocin-doxorubicin, streptozocin-fluorouracil or chlorozotocin in the treatment of advanced islet-cell carcinoma. *N Engl J Med*. Feb 20 1992;326(8):519-523.
47. Cheng PN, Saltz LB. Failure to confirm major objective antitumor activity for streptozocin and doxorubicin in the treatment of patients with advanced islet cell carcinoma. *Cancer*. Sep 15 1999;86(6): 944-948.
48. Raymond E, Dahan L, Raoul JL, et al. Sunitinib malate for the treatment of pancreatic neuroendocrine tumors. *N Engl J Med*. Feb 10 2011;364(6):501-513.
49. Yao JC, Shah MH, Ito T, et al. Everolimus for advanced pancreatic neuroendocrine tumors. *N Engl J Med*. Feb 10 2011;364(6): 514-523.
50. Kulke MH, Anthony LB, Bushnell DL, et al. NANETS treatment guidelines: well-differentiated neuroendocrine tumors of the stomach and pancreas. *Pancreas*. Aug 2010;39(6):735-752.
51. Ramage JK, Ahmed A, Ardill J, et al. Guidelines for the management of gastroenteropancreatic neuroendocrine (including carcinoid) tumours (NETs). *Gut*. Jan 2012;61(1):6-32.
52. O'Toole D, Saveanu A, Couvelard A, et al. The analysis of quantitative expression of somatostatin and dopamine receptors in gastro-

entero-pancreatic tumours opens new therapeutic strategies. *European journal of endocrinology / European Federation of Endocrine Societies*. Dec 2006;155(6):849-857.

53. Plockinger U, Rindi G, Arnold R, et al. Guidelines for the diagnosis and treatment of neuroendocrine gastrointestinal tumours. A consensus statement on behalf of the European Neuroendocrine Tumour Society (ENETS). *Neuroendocrinology*. 2004;80(6): 394-424.
54. Teunissen JJ, Kwekkeboom DJ, Valkema R, Krenning EP. Nuclear medicine techniques for the imaging and treatment of neuroendocrine tumours. *Endocrine-related cancer*. Oct 2011;18 Suppl 1:S27-51.
55. Anderson MA, Carpenter S, Thompson NW, Nostrant TT, Elta GH, Scheiman JM. Endoscopic ultrasound is highly accurate and directs management in patients with neuroendocrine tumors of the pancreas. *The American journal of gastroenterology*. 2000;95(9): 2271-2277.
56. Lebtahi R, Cadiot G, Sarda L, et al. Clinical impact of somatostatin receptor scintigraphy in the management of patients with neuroendocrine gastroenteropancreatic tumors. *Journal of nuclear medicine: official publication, Society of Nuclear Medicine*. Jun 1997;38(6):853-858.
57. Hofman MS, Lau WF, Hicks RJ. Somatostatin receptor imaging with 68Ga DOTATATE PET/CT: clinical utility, normal patterns, pearls, and pitfalls in interpretation. *Radiographics: a review publication of the Radiological Society of North America, Inc*. Mar-Apr 2015;35(2):500-516.
58. Toumpanakis C, Kim MK, Rinke A, et al. Combination of cross-sectional and molecular imaging studies in the localization of gastroenteropancreatic neuroendocrine tumors. *Neuroendocrinology*. 2014;99(2):63-74.
59. Chatalic KL, Kwekkeboom DJ, de Jong M. Radiopeptides for Imaging and Therapy: A Radiant Future. *Journal of nuclear medicine: official publication, Society of Nuclear Medicine*. Dec 2015;56(12):1809-1812.
60. Travis WD, Brambilla E, Muller-Hermelink HK, Harris CC. Pathology and genetics of tumours of the lung, pleura, thymus and heart. 2004.
61. Klimstra DS, Modlin IR, Adsay NV, et al. Pathology reporting of neuroendocrine tumors: application of the Delphic consensus process to the development of a minimum pathology data set. *The American journal of surgical pathology*. Mar 2010;34(3): 300-313.
62. Klimstra DS. Pathology reporting of neuroendocrine tumors: essential elements for accurate diagnosis, classification, and staging. *Semin Oncol*. Feb 2013;40(1):23-36.
63. Panzuto F, Boninsegna L, Fazio N, et al. Metastatic and locally advanced pancreatic endocrine carcinomas: analysis of factors associated with disease progression. *J Clin Oncol*. Jun 10 2011;29(17):2372-2377.
64. Panzuto F, Merola E, Rinzivillo M, et al. Advanced digestive neuroendocrine tumors: metastatic pattern is an independent factor affecting clinical outcome. *Pancreas*. Mar 2014;43(2):212-218.
65. Lucas MB, Yu VEYR. Mahvash disease: pancreatic neuroendocrine tumor syndrome caused by inactivating glucagon receptor mutation. *Journal of Molecular and Genetic Medicine*. 2013.
66. de Herder WW. Biochemistry of neuroendocrine tumours. *Best practice & research. Clinical endocrinology & metabolism*. Mar 2007;21(1):33-41.
67. Stronge RL, Turner GB, Johnston BT, et al. A rapid rise in circulating pancreastatin in response to somatostatin analogue therapy is associated with poor survival in patients with neuroendocrine tumours. *Annals of clinical biochemistry*. Nov 2008;45(Pt 6):560-566.
68. Yang X, Yang Y, Li Z, et al. Diagnostic value of circulating chromogranin a for neuroendocrine tumors: a systematic review and meta-analysis. *PloS one*. 2015;10(4):e0124884.
69. Han X, Zhang C, Tang M, et al. The value of serum chromogranin A as a predictor of tumor burden, therapeutic response, and nomogram-based survival in well-moderate nonfunctional pancreatic neuroendocrine tumors with liver metastases. *European journal of gastroenterology & hepatology*. May 2015;27(5):527-535.
70. Massironi S, Rossi RE, Casazza G, et al. Chromogranin A in diagnosing and monitoring patients with gastroenteropancreatic neuroendocrine neoplasms: a large series from a single institution. *Neuroendocrinology*. 2014;100(2-3):240-249.
71. Yao JC, Pavel M, Phan AT, et al. Chromogranin A and neuron-specific enolase as prognostic markers in patients with advanced pNET treated with everolimus. *The Journal of clinical endocrinology and metabolism*. Dec 2011;96(12):3741-3749.
72. Glinicki P, Jeske W, Kapuscinska R, Zgliczynski W. Comparison of chromogranin A (CgA) levels in serum and plasma (EDTA2K) and the respective reference ranges in healthy males. *Endokrynologia Polska*. 2015;66(1):53-56.
73. Kattan MW, Hess KR, Amin MB, et al. American Joint Committee on Cancer acceptance criteria for inclusion of risk models for individualized prognosis in the practice of precision medicine. *CA: a cancer journal for clinicians*. Jan 19 2016.
74. Sorbye H, Strosberg J, Baudin E, Klimstra DS, Yao JC. Gastroenteropancreatic high-grade neuroendocrine carcinoma. *Cancer*. Sep 15 2014;120(18):2814-2823.
75. Velayoudom-Cephise FL, Duvillard P, Foucan L, et al. Are G3 ENETS neuroendocrine neoplasms heterogeneous? *Endocrine-related cancer*. Oct 2013;20(5):649-657.
76. Hijioka S, Hosoda W, Mizuno N, et al. Does the WHO 2010 classification of pancreatic neuroendocrine neoplasms accurately characterize pancreatic neuroendocrine carcinomas? *Journal of gastroenterology*. 2014;50(5):564-572.
77. Heetfeld M, Chougnet CN, Olsen IH, et al. Characteristics and treatment of patients with G3 gastroenteropancreatic neuroendocrine neoplasms. *Endocrine-related cancer*. Aug 2015;22(4):657-664.

Part VII

Thorax

Members of the Thoracic Expert Panel
Hisao Asamura, MD
Kelly J. Butnor, MD
Joe Y. Chang, MD, PhD
Kari Chansky, MS
Marc de Perrot, MD
Frank C. Detterbeck, MD, FACS, FCCP
Ritu R. Gill, MD, MPH
Kirk D. Jones, MD – CAP Representative
Hedy L. Kindler, MD
Edith Marom, MD
Anna Nowak, MB, BS, PhD, W.Aust. FRACP
Harvey Pass, MD
Ramon Rami-Porta, MD, FETCS
David Rice, MB, BCh
Gregory J. Riely, MD, PhD
Kenneth E. Rosenzweig, MD
Valerie W. Rusch, MD, FACS – Chair
Lawrence H. Schwartz, MD
Daniel C. Sullivan, MD – Editorial Board Liaison
William D. Travis, MD
Theresa M. Vallerand, BGS, CTR – Data Collection Core Representative
Douglas Wood, MD, FACS, FRCSEd – Vice Chair

Thymus

35

Frank C. Detterbeck and Edith M. Marom

CHAPTER SUMMARY

Cancers Staged Using This Staging System

Thymoma, thymic carcinoma, thymic neuroendocrine tumors, combined thymic carcinoma

Summary of Changes

This is the first staging system for thymic tumors.

ICD-O-3 Topography Codes

Code	Description
C37.9	Thymus

WHO Classification of Tumors

Code	Description
8580	Thymoma, malignant, NOS
8581	Type A thymoma, including atypical variant
8582	Type AB thymoma
8583	Type B1 thymoma
8584	Type B2 thymoma
8585	Type B3 thymoma
8580	Micronodular thymoma with lymphoid stroma
8580	Metaplastic thymoma
8586	Thymic carcinoma, NOS
8070	Squamous cell carcinoma
8123	Basaloid carcinoma
8430	Mucoepidermoid carcinoma
8082	Lymphoepithelioma-like carcinoma
8310	Clear cell carcinoma
8033	Sarcomatoid carcinoma
8260	Papillary adenocarcinoma
8200	Thymic carcinoma with adenoid cystic carcinoma-like features
8480	Mucinous adenocarcinoma
8140	Adenocarcinoma, NOS
8023	NUT carcinoma
8020	Undifferentiated carcinoma
8560	Adenosquamous carcinoma
8576	Hepatoid carcinoma
8586	Thymic carcinoma, NOS
8240	Typical carcinoid tumor
8249	Atypical carcinoid tumor
8013	Large cell neuroendocrine carcinoma
8013	Combined large cell neuroendocrine carcinoma
8041	Small cell neuroendocrine carcinoma
8045	Combined small cell neuroendocrine carcinoma

Travis WD, Brambilla E, Burke AP, Marx A, Nicholson AG, eds. World Health Organization Classification of Tumours of the Lung, Pleura, Thymus and Heart. Lyon: IARC; 2015.

To access the AJCC cancer staging forms, please visit www.cancerstaging.org.

© American Joint Committee on Cancer 2017

M.B. Amin et al. (eds.), *AJCC Cancer Staging Manual, Eighth Edition*, DOI 10.1007/978-3-319-40618-3_35

INTRODUCTION

No AJCC/UICC stage classification system has existed for thymic tumors, although at least 15 different stage classification systems have been promoted by various authors.[1] Furthermore, these systems have been variously interpreted, hampering the ability to collaborate and make progress in these rare diseases. To address this problem, the International Association for the Study of Lung Cancer (IASLC) and the International Thymic Malignancies Interest Group (ITMIG) partnered to develop proposals for the AJCC Cancer Staging Manual, 8th Edition classification of TNM, founded on an international database (10,808 patients, 105 institutions, 22 countries) and a detailed statistical analysis with internal validation.[2-5] These proposals form the basis for the thymic stage classification of the 8th Edition TNM classification.

The recommendations for a TNM classification for thymic epithelial tumors are based on the analyses of a large international database collected by the ITMIG[2] and IASLC and are the result of a wide international consensus. The data elements regarding staging were analyzed by the members of the Thymic Domain of the IASLC Staging and Prognostic Factors Committee and the members of its advisory board in collaboration with the biostatisticians of Cancer Research And Biostatistics (CRAB). This analysis provided the basis for the 8th Edition of TNM, in which a stage classification of thymic tumors is included for the first time.

The stage classification is determined primarily by levels of local invasion of a thymic malignancy into surrounding mediastinal structures (T classification). Nodal involvement is less common; nodes are classified as being either in the superficial region (anterior mediastinum and lower cervical region) or a deep region (deep cervical and middle mediastinal nodes, Figs. 35.1–35.4, Table 35.1). Metastatic spread most commonly takes the form of pleural and pericardial nodules (M1a), less commonly extrathoracic or pulmonary nodules (M1b).

ANATOMY

Primary Site(s)

The thymus is located in the anterior mediastinum. A modern, computed tomography (CT)-based definition of the mediastinal compartments designates this as the prevascular compartment.[6] The lower poles of the thymus gland extend as far down as the diaphragm, whereas the upper poles extend into the lower neck up to the inferior border of the thyroid.[7] The gland itself is enclosed by a sheath consisting of thin, poorly defined connective tissue. The thymus is surrounded primarily by loose areolar and fatty tissue. The mediastinal pleura marks the lateral boundary of the pleural cavities. Although this layer can be distinguished surgically, it often is difficult to identify microscopically in the resection specimen. Adjacent structures include the pericardium, phrenic nerves, left brachiocephalic (innominate) vein, superior vena cava, lungs, and chest wall, and the aorta, arch vessels, intrapericardial pulmonary artery, and myocardium.

Regional Lymph Nodes

The incidence of lymph node involvement in thymoma is relatively low, although it also must be said that this has often not been carefully assessed. On the other hand, node involvement is reported in about a third of patients with thymic carcinoma or thymic neuroendocrine tumors.[1,5,8,9] The nodes most commonly involved are those in the anterior mediastinum and lower cervical region (~75%) and to a lesser degree, the middle mediastinum (~40%).

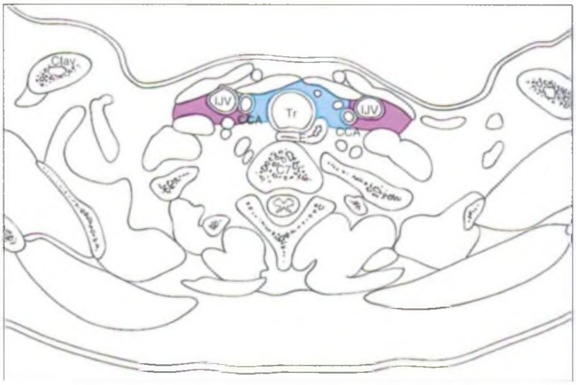

Fig. 35.1 ITMIG/IASLC lymph node map. Anterior (*blue*) and deep (*purple*) node regions as depicted on axial images. For further details, see Bhora, Chen et al.[12] Thoracic inlet is shown here. IJV, internal jugular vein; Tr, trachea

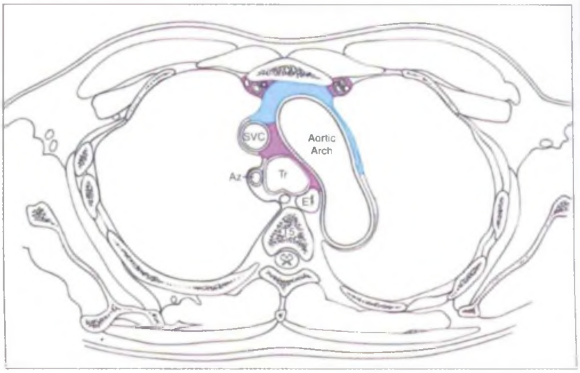

Fig. 35.2 ITMIG/IASLC lymph node map. Anterior (*blue*) and deep (*purple*) node regions as depicted on axial images. For further details, see Bhora, Chen et al.[12] Para-aortic level is shown here. Az, azygos vein; E, esophagus; SVC, superior vena cava; Tr, trachea

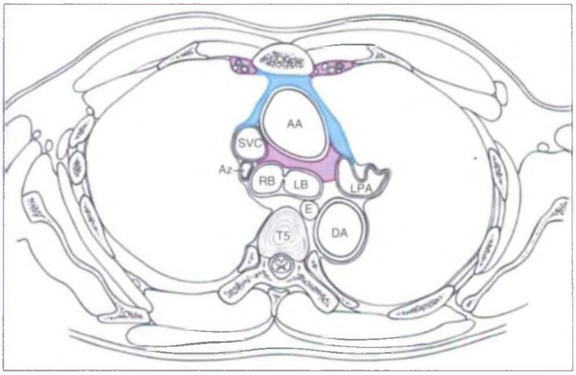

Fig. 35.3 ITMIG/IASLC lymph node map. Anterior (*blue*) and deep (*purple*) node regions as depicted on axial images. For further details, see Bhora, Chen et al.[12] Aortopulmonary window level is shown here. AA, ascending aorta; Az, azygos vein; DA, descending aorta; E, esophagus; LB, left main bronchus; LPA, left pulmonary artery; RB, right main bronchus; SVC, superior vena cava

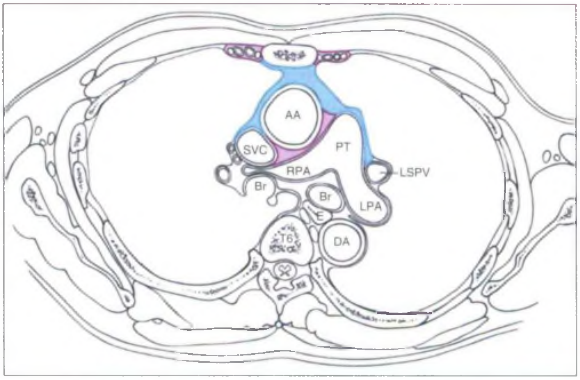

Fig. 35.4 ITMIG/IASLC lymph node map. Anterior (*blue*) and deep (*purple*) node regions as depicted on axial images. For further details, see Bhora, Chen et al.[12] Carina level is shown here. AA, ascending aorta; Br, bronchus; DA, descending aorta; E, esophagus; LPA, left pulmonary artery; LSPV, left superior pulmonary vein; PT, pulmonary trunk; RPA, right pulmonary artery; SVC, superior vena cava

Table 35.1 Lymph node regions for thymic malignancies

	Region Boundaries	Node Groups*
N1: Anterior Region	*Superior:* hyoid bone<br>*Lateral (neck):* medial border of carotid sheaths<br>*Lateral (chest):* mediastinal pleura<br>*Anterior:* Sternum<br>*Posterior (medially):* great vessels, pericardium<br>*Posterior (laterally):* phrenic nerve<br>*Inferior:* Xiphoid, diaphragm	Low anterior cervical: pretracheal, paratracheal, peri-thyroid, precricoid/delphian<br>Peri-Thymic<br>Prevascular<br>Para-aortic, Ascending Aorta, Superior Phrenics<br>Supradiaphragmatic / Inferior Phrenics / Pericardial
N2: Deep Region	*Superior:* Level of lower border of cricoid cartilage<br>*Anteromedial (neck):* lateral border of sternohyoid, medial border of carotid sheath<br>*Posterolateral (neck):* anterior border of trapezius<br>*Anterior (chest): Right* – Anterior Border of SVC; *Left* – aortic arch, aortopulmonary window<br>*Posterior (Chest):* Esophagus<br>*Lateral (chest):* pulmonary hila<br>*Inferior:* Diaphragm	Lower Jugular<br>Supraclavicular/venous angle: confluence of internal jugular & subclavian vein<br>Internal Mammary nodes<br>Upper Paratracheal<br>Lower Paratracheal<br>Subaortic / Aortopulmonary Window<br>Subcarinal<br>Hilar

*Region and node group boundaries match those established by the American Academy of Otolaryngology - Head and Neck Surgery, American Society for Head and Neck Surgery, and the International Association for the Study of Lung Cancer where applicable.
SVC, superior vena cava

The regional nodes are illustrated and described in Figs. 35.1–35.4 and Table 35.1.

Metastatic Sites

The most common pattern of metastatic dissemination of thymic malignancies is the appearance of discrete subpleural nodules (visceral or parietal), as well as pericardial nodules. Intraparenchymal pulmonary nodules (as opposed to nodules along the visceral pleura) also are seen occasionally. As with other malignant tumors, dissemination to other parts of the body may occur.

RULES FOR CLASSIFICATION

Clinical Classification

Clinical TNM classification of thymic tumors relies on assessment of whether tissues are involved based on physical examination, imaging, diagnostic biopsies, and diagnostic procedures.

However, the reliability of most clinical findings in determining involvement by a thymic malignancy is poorly defined. The correlation between recorded clinical and pathological stages (using a combination of the Masaoka and Masaoka–Koga stage classification systems) in the IASLC/ITMIG database was fair, with a weighted Kappa coefficient of 0.61. It has been difficult to clearly define actual invasion of mediastinal structures based on imaging.[10,11] Size has not correlated with higher stage (i.e., invasion) or higher rates of unresectability in the IASLC/ITMIG database.[4]

To permit accurate classification of nodal involvement for the 8th Edition of TNM staging, an ITMIG/IASLC node map was developed for thymic malignancies.[12] Nodes in the anterior mediastinum or lower cervical regions (i.e., close to the thymus gland) are classified as N1; deep cervical, supraclavicular and, middle mediastinal nodes are grouped as N2. The boundaries of these regions are illustrated and described in Figs. 35.1–35.4 and Table 35.1. More detailed descriptions are available elsewhere.[12]

Survival clearly is affected negatively by nodal involvement. However, the available data allow only partial assessment of the relative impact of the N1 and N2 node regions. The development of the node map and the stage classification system presented here sets the foundation for a better definition of the prognostic impact. Furthermore, to promote a better assessment, ITMIG suggests evaluation and resection of any suspicious N1 nodes during resection of Stage I thymoma, and a systematic sampling or node dissection of N1 and N2 regions during resection of Stage II–IVa thymomas and all thymic carcinomas or neuroendocrine tumors.[13]

The patterns of subpleural and/or pericardial nodules are designated as M1a, and are distinguished from direct pleural or pericardial invasion of the primary tumor.[5] Less commonly, involvement of extrathoracic sites or intraparenchymal pulmonary nodules is seen; such involvement is classified as M1b. Involvement of lymph nodes not included in the nodal regions described in Table 35.1. also are classified as M1b (e.g., lateral cervical, axillary, subdiaphragmatic nodes). Involvement of M1a or M1b sites is relatively uncommon (<10%); nevertheless, prolonged survival may be achieved in patients with these tumors.

Imaging

The most appropriate imaging modality for assessing tumor stage is contrast-enhanced chest CT.[14,15] Intravenous contrast should be administered if not contraindicated, as vessel evaluation is important for staging.[10,11] However, when iodinated intravenous contrast is contraindicated, magnetic resonance (MR) imaging is used for staging. Fluorine-18 fluorodeoxyglucose (FDG) positron emission tomography (PET)-CT is not used routinely in the staging of thymic epithelial tumors, because the ones with more indolent histologic features do not have much FDG uptake.[16–21] However, when CT features suggest or preoperative tissue biopsy identifies a more aggressive histology (i.e., thymic carcinoma or thymic carcinoid), FDG PET-CT is helpful and can detect unsuspected nodal or distant metastatic disease.[16]

For staging, imaging interpretation should address local invasion, nodal involvement, and distant metastatic disease.[22] An irregular vessel lumen contour, vascular encasement or obliteration, and endoluminal soft tissue that may extend into cardiac chambers are considered clear features of local vascular invasion that can be appreciated readily by contrast-enhanced chest CT or chest MR imaging.[23] However, it is very difficult to determine by imaging whether there is actual involvement when tumor is seen abutting local structures (e.g., pericardium, lung, superior vena cava). For the N category, enlarged lymph nodes (i.e., short axis >10 mm) are considered suspicious for tumor involvement, although the reliability of this finding is not defined. CT and MR imaging readily detect pleural metastases greater than 1 cm in diameter, but smaller pleural abnormalities are notoriously difficult to assess. Extrathoracic metastatic disease is highly unlikely to be present in the case of thymoma but sometimes is encountered with thymic carcinoma or thymic neuroendocrine tumors. Because the latter tumors often are FDG avid, FDG PET-CT may be helpful in detecting metastases in these patients.

Pathological Classification

The thymus is surrounded by relatively loose, amorphous anterior mediastinal tissue that is disrupted easily if the specimen is not handled gently. Furthermore, unless marked at the time of resection, the specimen may be impossible to orient by the pathologist, hampering definition of sites of potential remaining disease in the event of an incomplete resection. Therefore, it is recommended that marking stitches be placed during resection where the surrounding tissue has been disrupted by handling (i.e., does not represent a true positive margin).[13] In addition, the specimen should be oriented by the surgeon and this orientation communicated clearly to the pathologist—for example, through the use of a "mediastinal board" upon which the specimen is laid out.[13] The pathologist should ink the various aspects (e.g., anterior, posterior) and prepare the specimen in a way that allows eventual identification of the site of a positive margin.[13]

Possible invasion of adjacent mediastinal tissues must be confirmed histologically, if possible, to be counted in pathological staging.[4] Simple adhesion or close proximity without histologic evidence of invasion does not constitute

involvement. Invasion of adjacent lymph nodes by direct extension is counted as nodal involvement.[5]

The extent of disease, especially in thymoma, is characterized primarily by local invasion into adjacent structures. Extension into the surrounding loose fatty tissue is not of prognostic significance.[4] The extent of local extension is characterized by "levels" of involvement. A tumor is counted in a certain level of involvement if either one or more than one structure of that level is involved, with or without involvement of structures included in a lower level. Structures are grouped in a level based primarily on how similar or distinct the survival and recurrence outcomes are, but also take into account anatomic considerations. This approach manages the complexity of many different structures that may be involved, either alone or in combination with others.

Previous stage classification systems often distinguished whether the tumor was encapsulated. However, the "tumor capsule" is not an actual anatomic structure, and it turns out this has no prognostic significance. Involvement of the fatty tissues of the anterior mediastinum up to the mediastinal pleura carries the same excellent prognosis as encapsulated tumors. The mediastinal pleura also is not clearly of prognostic significance, but it warrants further study because of possible incomplete data collection in the past.

The pericardium is perhaps the most frequently involved adjacent structure; this is counted whether the involvement is partial or full thickness. However, actual pericardial involvement must be confirmed microscopically to be counted if the tumor has been resected. Level 3 structures include the lung, innominate vein, phrenic nerves, superior vena cava, chest wall, and sternum. It is not clear that any one of these carries a different prognosis. Involvement of multiple level 3 structures shows a trend toward worse survival compared with a single structure. Finally, level 4 structures include the aorta, arch vessels, intrapericardial pulmonary artery, and myocardium. Although available data show only a trend toward worse survival compared with involvement of level 3 structures, these data do not account for the fact that most patients with such involvement do not undergo resection and therefore are not included in the available data, which are primarily surgically based.

ITMIG recommends that anterior mediastinal nodes be removed routinely along with the thymus and encourages a systematic sampling of deep nodes (e.g., paratracheal, aortopulmonary window, subcarinal) during resection of thymomas with invasion of mediastinal structures (e.g., pericardium, lung).[5,13] For thymic carcinoma, a systematic removal of both N1 and N2 nodes is recommended during curative-intent resection.[5,13]

PROGNOSTIC FACTORS

Prognostic Factors Required for Stage Grouping

Beyond the factors used to assign T, N, or M categories, no additional prognostic factors are required for stage grouping.

Additional Factors Recommended for Clinical Care

Recent reviews show that the resection status and the histologic type are consistent additional prognostic factors supplementing stage.[24-26] A complete resection has been identified as a major prognostic factor and should be captured by the resection status classification: R0, complete resection; R1, microscopically incomplete resection; R2, grossly incomplete resection.

The histologic type of thymic malignancy also is prognostically important. Most important is the distinction between thymoma and thymic carcinoma or thymic neuroendocrine tumors.[25,26] The independent prognostic significance of the histologic subtype of thymoma previously was less clear, because the subtype is strongly associated with anatomic extent of disease.[24] The advent of larger databases has allowed more statistically sound analysis. There does not appear to be an independent effect on survival, although a minor effect is seen on recurrence. The effect on recurrence is seen in Stage I+II thymoma and for type AB versus B1, B2, or B3.[26] Another, larger multivariate study found no independent association between thymoma subtype and survival or recurrence; however, type B2–B3 thymoma was associated with a higher risk of incomplete resection.[25]

In conclusion, the type of thymic malignancy, including the histologic subtype of thymoma, should be recorded, although the subtype is of only minor independent prognostic value.

Factor	Definition	Clinical Significance	Level of Evidence
Resection status	Resection of all known tumor	Associated with survival and recurrence	I
Histologic type	As defined in the *WHO Classification of Tumours of the Lung, Pleura, Thymus and Heart*	Thymic carcinoma and neuroendocrine tumor of the thymus associated with survival and recurrence; thymoma subtype of minor prognostic significance	I

RISK ASSESSMENT MODELS

The AJCC recently established guidelines that will be used to evaluate published statistical prediction models for the purpose of granting endorsement for clinical use.[27] Although this is a monumental step toward the goal of precision medicine, this work was published only very recently. Therefore, the existing models that have been published or may be in clinical use have not yet been evaluated for this cancer site by the Precision Medicine Core of the AJCC. In the future, the statistical prediction models for this cancer site will be evaluated, and those that meet all AJCC criteria will be endorsed.

DEFINITIONS OF AJCC TNM

Definition of Primary Tumor (T)*,**

T Category	T Description
TX	Primary tumor cannot be assessed
T0	No evidence of primary tumor
T1	Tumor encapsulated or extending into the mediastinal fat; may involve the mediastinal pleura
T1a	Tumor with no mediastinal pleura involvement
T1b	Tumor with direct invasion of mediastinal pleura
T2	Tumor with direct invasion of the pericardium (either partial or full thickness)
T3	Tumor with direct invasion into any of the following: lung, brachiocephalic vein, superior vena cava, phrenic nerve, chest wall, or extrapericardial pulmonary artery or veins
T4	Tumor with invasion into any of the following: aorta (ascending, arch, or descending), arch vessels, intrapericardial pulmonary artery, myocardium, trachea, esophagus

*Involvement must be microscopically confirmed in pathological staging, if possible.
**T categories are defined by "levels" of invasion; they reflect the highest degree of invasion regardless of how many other (lower-level) structures are invaded. T1, level 1 structures: thymus, anterior mediastinal fat, mediastinal pleura; T2, level 2 structures: pericardium; T3, level 3 structures: lung, brachiocephalic vein, superior vena cava, phrenic nerve, chest wall, hilar pulmonary vessels; T4, level 4 structures: aorta (ascending, arch, or descending), arch vessels, intrapericardial pulmonary artery, myocardium, trachea, esophagus.

Definition of Regional Lymph Node (N)*

N Category	N Description
NX	Regional lymph nodes cannot be assessed
N0	No regional lymph node metastasis
N1	Metastasis in anterior (perithymic) lymph nodes
N2	Metastasis in deep intrathoracic or cervical lymph nodes

*Involvement must be microscopically confirmed in pathological staging, if possible.

Definition of Distant Metastasis (M)

M Category	M Description
M0	No pleural, pericardial, or distant metastasis
M1	Pleural, pericardial, or distant metastasis
M1a	Separate pleural or pericardial nodule(s)
M1b	Pulmonary intraparenchymal nodule or distant organ metastasis

AJCC PROGNOSTIC STAGE GROUPS

The T, N, and M categories are organized into stage groups, as shown in the table. This schema was developed based primarily on outcomes; in the lower stages, recurrence rates in patients with complete resections were judged most relevant, whereas in higher stages, survival in all patients, regardless of resection status, was weighed more heavily. Practical applicability and clinical implications also were considered. Differences among the stage groups were subjected to statistical analysis and generally were found to have a stepwise progression toward worse survival in multiple patient cohorts (e.g., R0, R-any).

When T is...	And N is...	And M is...	Then the stage group is...
T1a,b	N0	M0	I
T2	N0	M0	II
T3	N0	M0	IIIA
T4	N0	M0	IIIB
Any T	N1	M0	IVA
Any T	N0,1	M1a	IVA
Any T	N2	M0,M1a	IVB
Any T	Any N	M1b	IVB

REGISTRY DATA COLLECTION VARIABLES

The authors have not noted any registry data collection variables.

HISTOLOGIC GRADE (G)

There is no recommended histologic grading system at this time.

HISTOPATHOLOGIC TYPE

Thymoma
Thymic carcinoma
Neuroendocrine tumor of the thymus

SURVIVAL DATA

Figures and tables of outcomes are available in reference papers.[3-5]

Bibliography

1. Filosso PL, Ruffini E, Lausi PO, Lucchi M, Oliaro A, Detterbeck F. Historical perspectives: The evolution of the thymic epithelial tumors staging system. *Lung cancer*. Feb 2014;83(2):126-132.
2. Huang J, Ahmad U, Antonicelli A, et al. Development of the international thymic malignancy interest group international database: an unprecedented resource for the study of a rare group of tumors. *J Thorac Oncol*. Oct 2014;9(10):1573-1578.
3. Detterbeck FC, Stratton K, Giroux D, et al. The IASLC/ITMIG Thymic Epithelial Tumors Staging Project: proposal for an evidence-based stage classification system for the forthcoming (8th) edition of the TNM classification of malignant tumors. *J Thorac Oncol*. Sep 2014;9(9 Suppl 2):S65-72.
4. Nicholson AG, Detterbeck FC, Marino M, et al. The IASLC/ITMIG Thymic Epithelial Tumors Staging Project: proposals for the T Component for the forthcoming (8th) edition of the TNM classification of malignant tumors. *J Thorac Oncol*. Sep 2014;9(9 Suppl 2):S73-80.
5. Kondo K, Van Schil P, Detterbeck FC, et al. The IASLC/ITMIG Thymic Epithelial Tumors Staging Project: proposals for the N and M components for the forthcoming (8th) edition of the TNM classification of malignant tumors. *J Thorac Oncol*. Sep 2014;9(9 Suppl 2):S81-87.
6. Carter BW, Tomiyama N, Bhora FY, et al. A modern definition of mediastinal compartments. *J Thorac Oncol*. Sep 2014;9(9 Suppl 2):S97-101.
7. Safieddine N, Keshavjee S. Anatomy of the thymus gland. *Thorac Surg Clin*. May 2011;21(2):191-195, viii.
8. Kondo K, Monden Y. Lymphogenous and hematogenous metastasis of thymic epithelial tumors. *The Annals of thoracic surgery*. Dec 2003;76(6):1859-1864; discussion 1864-1855.
9. Ahmad U, Yao X, Detterbeck F, et al. Thymic carcinoma outcomes and prognosis: results of an international analysis. *J Thorac Cardiovasc Surg*. Jan 2015;149(1):95-100, 101 e101-102.
10. Marom EM, Milito MA, Moran CA, et al. Computed tomography findings predicting invasiveness of thymoma. *J Thorac Oncol*. Jul 2011;6(7):1274-1281.
11. Marom E. Definitions of Terms for the Clinical Stage Characterization of Thymic Malignancies. *J Thorac Oncol*.
12. Bhora FY, Chen DJ, Detterbeck FC, et al. The ITMIG/IASLC Thymic Epithelial Tumors Staging Project: A Proposed Lymph Node Map for Thymic Epithelial Tumors in the Forthcoming 8th Edition of the TNM Classification of Malignant Tumors. *J Thorac Oncol*. Sep 2014;9(9 Suppl 2):S88-96.
13. Detterbeck FC, Moran C, Huang J, et al. Which way is up? Policies and procedures for surgeons and pathologists regarding resection specimens of thymic malignancy. *Journal of Thoracic Oncology*. 2011;6(7):S1730-S1738.
14. Huang J, Detterbeck FC, Wang Z, Loehrer PJ, Sr. Standard outcome measures for thymic malignancies. *J Thorac Oncol*. Dec 2010;5(12):2017-2023.
15. Benveniste MF, Rosado-de-Christenson ML, Sabloff BS, Moran CA, Swisher SG, Marom EM. Role of imaging in the diagnosis, staging, and treatment of thymoma. *Radiographics : a review publication of the Radiological Society of North America, Inc*. Nov-Dec 2011;31(7):1847-1861; discussion 1861-1843.
16. Sung YM, Lee KS, Kim BT, Choi JY, Shim YM, Yi CA. 18F-FDG PET/CT of thymic epithelial tumors: usefulness for distinguishing and staging tumor subgroups. *Journal of nuclear medicine : official publication, Society of Nuclear Medicine*. Oct 2006;47(10):1628-1634.
17. Benveniste MF, Moran CA, Mawlawi O, et al. FDG PET-CT aids in the preoperative assessment of patients with newly diagnosed thymic epithelial malignancies. *J Thorac Oncol*. Apr 2013;8(4):502-510.
18. Igai H, Matsuura N, Tarumi S, et al. Usefulness of [18F]fluoro-2-deoxy-D-glucose positron emission tomography for predicting the World Health Organization malignancy grade of thymic epithelial tumors. *European journal of cardio-thoracic surgery : official journal of the European Association for Cardio-thoracic Surgery*. Jul 2011;40(1):143-145.
19. Kaira K, Endo M, Abe M, et al. Biologic correlation of 2-[18F]-fluoro-2-deoxy-D-glucose uptake on positron emission tomography in thymic epithelial tumors. *J Clin Oncol*. Aug 10 2010;28(23):3746-3753.
20. Kumar A, Regmi SK, Dutta R, et al. Characterization of thymic masses using 18F-FDG PET-CT. *Annals of nuclear medicine*. 2009;23(6):569-577.
21. Shibata H, Nomori H, Uno K, et al. 18F-fluorodeoxyglucose and 11C-acetate positron emission tomography are useful modalities for diagnosing the histologic type of thymoma. *Cancer*. Jun 1 2009;115(11):2531-2538.
22. Marom EM, Rosado-de-Christenson ML, Bruzzi JF, Hara M, Sonett JR, Ketai L. Standard report terms for chest computed tomography reports of anterior mediastinal masses suspicious for thymoma. *J Thorac Oncol*. Jul 2011;6(7 Suppl 3):S1717-1723.
23. Rosado-de-Christenson ML, Strollo DC, Marom EM. Imaging of thymic epithelial neoplasms. *Hematol Oncol Clin North Am*. Jun 2008;22(3):409-431.
24. Detterbeck F, Youssef S, Ruffini E, Okumura M. A review of prognostic factors in thymic malignancies. *J Thorac Oncol*. Jul 2011; 6(7 Suppl 3):S1698-1704.
25. Ruffini E, Detterbeck F, Van Raemdonck D, et al. Tumours of the thymus: a cohort study of prognostic factors from the European Society of Thoracic Surgeons database. *European journal of cardio-thoracic surgery : official journal of the European Association for Cardio-thoracic Surgery*. Sep 2014;46(3):361-368.
26. Weis CA, Yao X, Deng Y, et al. The impact of thymoma histotype on prognosis in a worldwide database. *J Thorac Oncol*. Feb 2015;10(2):367-372.
27. Kattan MW, Hess KR, Amin MB, et al. American Joint Committee on Cancer acceptance criteria for inclusion of risk models for individualized prognosis in the practice of precision medicine. *CA: a cancer journal for clinicians*. Jan 19 2016.

Lung

36

Ramon Rami-Porta, Hisao Asamura, William D. Travis, and
Valerie W. Rusch

CHAPTER SUMMARY

The changes introduced in the AJCC Cancer Staging Manual, 8th Edition of the TNM classification for lung cancer derive from analyses of the new retrospective and prospective databases of the International Association for the Study of Lung Cancer (IASLC). These databases contain information on patients diagnosed with lung cancer from 1999 to 2010 originating from 35 different databases in 16 countries around the world.[1]

Cancers Staged Using This Staging System

This classification applies to carcinomas of the lung, including non–small cell and small cell carcinomas, and bronchopulmonary carcinoid tumors.

Cancers Not Staged Using This Staging System

This classification does not apply to sarcomas or other rare tumors of the lung.

Summary of Changes

Change	Details of Change	Level of Evidence
Definition of Primary Tumor (T)	Tis: Adenocarcinoma *in situ* (AIS), Tis (AIS), added in addition to squamous carcinoma *in situ* (SCIS), Tis (SCIS)	II[2]
Definition of Primary Tumor (T)	T1mi: Addition of a new T category: minimally invasive adenocarcinoma	II[2]
Definition of Primary Tumor (T)	T1: Subdivision into T1a, T1b, and T1c at 1-cm intervals from ≤1 cm to ≤3 cm	II[3]
Definition of Primary Tumor (T)	T2: Subdivision into T2a and T2b at 1-cm intervals from >3 cm to ≤5 cm	II[3]
Definition of Primary Tumor (T)	T2: Tumors with endobronchial location <2 cm from the carina, but without involving the carina, are now included in this T category.	II[3]
Definition of Primary Tumor (T)	T2: Tumors with complete atelectasis or pneumonitis are now included in this category.	II[3]
Definition of Primary Tumor (T)	T3: Tumors >5 cm but ≤7 cm are now included in this T category.	II[3]
Definition of Primary Tumor (T)	T3: Invasion of the mediastinal pleura is no longer used as a T descriptor.	II[3]
Definition of Primary Tumor (T)	T4: Tumors >7 cm are now included in this T category.	II[3]
Definition of Primary Tumor (T)	T4: Tumors with invasion of the diaphragm are now included in this T category.	II[3]

To access the AJCC cancer staging forms, please visit www.cancerstaging.org.

© American Joint Committee on Cancer 2017

M.B. Amin et al. (eds.), *AJCC Cancer Staging Manual, Eighth Edition*, DOI 10.1007/978-3-319-40618-3_36

Change	Details of Change	Level of Evidence
Definition of Distant Metastasis (M)	M1b: The revised M1b category now includes tumors with a single extrathoracic metastasis in a single organ.	II[4]
Definition of Distant Metastasis (M)	M1c: This new M1 category includes tumors with multiple extrathoracic metastases in one or multiple organs.	II[4]
AJCC Prognostic Stage Groups	Stage IA now is divided into three stages—IA1, IA2, and IA3—to include T1aN0M0, T1bN0M0, and T1cN0M0 tumors, respectively.	II[5]
AJCC Prognostic Stage Groups	Stage IIB now includes T1aN1M0, T1bN1M0, T1cN1M0, and T2aN1M0 tumors.	II[5]
AJCC Prognostic Stage Groups	Stage IIIB now includes T3N2M0 tumors.	II[5]
AJCC Prognostic Stage Groups	Stage IIIC: This new stage includes T3N3M0 and T4N3M0 tumors.	II[5]
AJCC Prognostic Stage Groups	Stage IVA includes tumors with any T and any N, but with M1a or M1b disease.	II[5]
AJCC Prognostic Stage Groups	Stage IVB includes tumors with any T and any N, but with M1c disease.	II[5]

ICD-O-3 Topography Codes

Code	Description
C34.0	Main bronchus
C34.1	Upper lobe, lung
C34.2	Middle lobe, lung
C34.3	Lower lobe, lung
C34.8	Overlapping lesion of lung
C34.9	Lung, not otherwise specified (NOS)

WHO Classification of Tumors[6,7]

Code	Description
8140	Adenocarcinoma
8250	Lepidic adenocarcinoma
8551	Acinar adenocarcinoma
8260	Papillary adenocarcinoma
8265	Micropapillary adenocarcinoma
8230	Solid adenocarcinoma
8253	Invasive mucinous adenocarcinoma
8254	Mixed invasive mucinous and nonmucinous adenocarcinoma
8480	Colloid adenocarcinoma
8333	Fetal adenocarcinoma
8144	Enteric adenocarcinoma
	Minimally invasive adenocarcinoma
8256	Nonmucinous
8257	Mucinous
	Preinvasive lesion
8140	Adenocarcinoma *in situ*
8250	Nonmucinous
8253	Mucinous
8070	Squamous cell carcinoma
8071	Keratinizing squamous cell carcinoma
8072	Nonkeratinizing squamous cell carcinoma
8083	Basaloid squamous cell carcinoma
	Preinvasive lesion
8070	Squamous cell carcinoma *in situ*
	Neuroendocrine tumors
8041	Small cell carcinoma
8045	Combined small cell carcinoma
8013	Large cell neuroendocrine carcinoma
8013	Combined large cell neuroendocrine carcinoma
	Carcinoid tumors
8240	Typical carcinoid
8249	Atypical carcinoid
	Preinvasive lesion
8040	Diffuse idiopathic pulmonary neuroendocrine cell hyperplasia
8012	Large cell carcinoma
8560	Adenosquamous carcinoma
	Sarcomatoid carcinomas
8022	Pleomorphic carcinoma
8032	Spindle cell carcinoma
8031	Giant cell carcinoma
8980	Carcinosarcoma
8972	Pulmonary blastoma
	Other and unclassified carcinomas
8082	Lymphoepithelioma-like carcinoma
8023	NUT carcinoma
	Salivary gland–type tumors
8430	Mucoepidermoid carcinoma
8200	Adenoid cystic carcinoma
8562	Epithelial–myoepithelial carcinoma

INTRODUCTION

Lung cancer is the most frequent cancer diagnosed and the leading cause of cancer mortality in the world. The World Health Organization (WHO) reported that in 2012, more than 1.8 million lung cancers were diagnosed, for an incidence of 13%, the highest among all cancers except nonmelanoma skin cancers. In addition, more than 1.5 million patients died from the disease, for a 19.4% cancer mortality rate, again number one among all neoplasms.[8] Lung cancer is classified according to the TNM system, which codes the anatomic extent of the disease and is the most important prognosticator we have to

date. As such, the classification does not include clinical, biological, molecular, or genetic descriptors, although they may be used in combination with the TNM classification to build prognostic groups, different from stage groups, which are combinations of tumors with TNMs of similar prognosis.

For the second consecutive time, the revision of the TNM classification for lung cancer has derived from analyses of the new retrospective and prospective databases collected by the IASLC.[1] Detailed analyses of the T, N, and M components of the classification; of the stages; and of the applicability of the TNM classification to small cell lung cancer already have been published and are the basis for the recommendations for changes in the 8th Edition of the TNM classification.[3-5,9,10]

Whereas there is no alternative way to classify non–small cell lung cancer anatomically, small cell lung cancer may be classified by the dichotomous "limited and extensive disease" system and by the TNM classification. However, the analyses performed in revising the AJCC Cancer Staging Manual, 6th Edition of the classification for the AJCC Cancer Staging Manual, 7th Edition clearly showed the advantages of classifying small cell lung cancer with the TNM system at clinical and pathological staging.[11,12] The new analyses performed for the 8th Edition of the classification confirm that the TNM system works for small cell lung cancer and that its use increases our capacity to indicate prognosis, as limited disease may be subdivided in anatomic stages from IA1 to IIIC, with a wide range of 5-year survival rates (93% for Stage IA1 and 19% for Stage IIIC), progressively worsening as tumor stage increases. This prognostic refinement is lost if the dichotomous classification is used.[10] Therefore, the recommendation to use the TNM to classify small cell lung cancer is emphasized in the 8th Edition, especially to indicate prognosis and to stratify tumors in future clinical trials.

Although the TNM classification does not explain all the prognostic variability found in lung cancer, it is the strongest prognosticator. The anatomic classification may be determined by a variety of noninvasive and invasive methods. It is universally applicable in different medical settings and constitutes the basis for planning therapy and therapeutic clinical trials. Therefore, even if prognostic groups are built in the future to refine prognosis for individual patients, the TNM classification system will remain a fundamental component among other variables. Its periodic revision is relevant, because the more robust the TNM classification, the more important its contribution to prognostic groups.[13]

ANATOMY

Primary Site(s)

For the purpose of TNM classification, the lungs are not paired organs but a single organ.[14] Basically, they are formed by the bronchi and the lung parenchyma. Lung cancer is a bronchogenic neoplasm arising from the epithelial cells of the bronchial mucosa or from the cells lining the alveoli. The right lung has three lobes—upper, middle, and lower—with three, two, and five segments, respectively. The left lung has two lobes—upper and lower—with five and four segments, respectively. The segment is considered the smallest anatomic unit of the lung.

Although all lung cancers may be located in any part of the lung, squamous cell and small cell carcinomas tend to arise from the mucosa of the more central bronchi, involving the lobar origins and the main bronchi. This central location often causes bronchial obstruction and atelectasis, either lobar or complete. The natural progression of these central tumors is to invade the bronchial wall and the mediastinal structures, such as the pericardium, the phrenic nerve, the superior vena cava, and more rarely, the esophagus, the aorta, and the heart. On the other hand, adenocarcinomas tend to locate in the periphery of the lung, with extension to the visceral pleura, often causing pleural dissemination and malignant pleural effusion, and to the chest wall. The earlier adenocarcinomas, such as adenocarcinoma *in situ* and minimally invasive adenocarcinoma, also tend to be located peripherally. The fact that lung lesions do not generate pain and that lung compliance allows tumors to grow within the lung parenchyma accounts for the late diagnosis of the disease. Only when the tumor causes bronchial obstruction and subsequent atelectasis, pneumonia or dyspnea, bleeding from the bronchial mucosa, or pain due to invasion of the parietal pleura, do patients present with symptoms, and the diagnostic process begins. A high index of suspicion is needed to avoid minimizing the nonspecific symptoms and attributing them to benign diseases.

Regional Lymph Nodes

Spread to the regional lymph nodes is a common feature in lung cancer. The natural progression from the primary tumor to the intrapulmonary, hilar, mediastinal, and supraclavicular lymph nodes is not found in every patient with lung cancer and nodal disease. Some patients have mediastinal nodal disease without intrapulmonary or hilar nodal involvement, which is referred to as skip metastases. Figure 36.1 shows the regional pulmonary, mediastinal, and supraclavicular lymph nodes, and Table 36.1 describes the anatomic limits of the nodal stations and their grouping in nodal zones.[15]

Metastatic Sites

Although any organ may be the site of metastasis from primary lung cancer, the brain, bones, adrenal glands, contralateral lung, liver, pericardium, kidneys, and subcutaneous tissue are the most common sites of metastatic spread. In the absence of specific clinical findings, the staging process should focus on ruling out metastasis in these common sites.

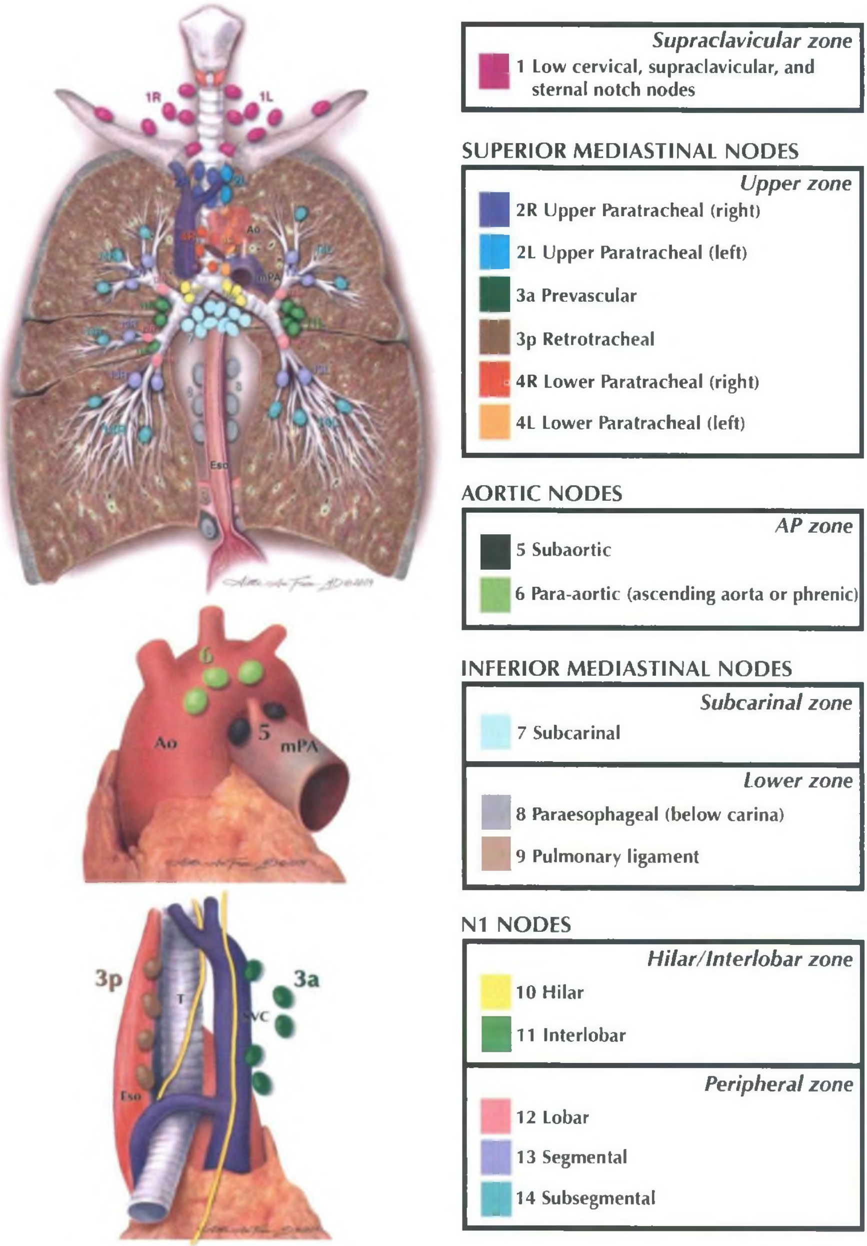

Fig. 36.1 International Association for the Study of Lung Cancer lymph node map (From Rusch et al.[15] Copyright © 2008 Aletta Ann Frazier, MD)

RULES FOR CLASSIFICATION

Clinical Classification

Clinical classification or pretreatment clinical classification, designated TNM or cTNM, is essential for selecting and evaluating therapy. It is based on the evidence found before treatment, including the results of history and physical examination, imaging studies (e.g., computed tomography [CT] and positron emission tomography [PET]), laboratory tests, and staging procedures such as bronchoscopy or esophagoscopy with or without ultrasound-guided biopsy (e.g., using endobronchial ultrasound [EBUS] or endoscopic ultrasound [EUS]), mediastinoscopy, mediastinotomy, extended cervical mediastinoscopy, thoracentesis, pleural biopsy, pericardioscopy, thoracoscopy, and video-assisted thoracoscopic surgery, as well as exploratory thoracotomy.

Table 36.1 Anatomic definitions for each lymph node station and station grouping by nodal zones in the map proposed by the IASLC.

Lymph node station number (#)	Description	Anatomic limits
		**Supraclavicular zone**
1	Low cervical, supraclavicular, and sternal notch nodes	• Upper border: lower margin of cricoid cartilage<br>• Lower border: clavicles bilaterally and, in the midline, the upper border of the manubrium. 1R designates right-sided nodes, 1L left-sided nodes in this region.<br>• For lymph node station 1, the midline of the trachea serves as the border between 1R and 1L.
		**Upper zone**
2	Upper paratracheal nodes	2R<br>• Upper border: apex of the right lung and pleural space and, in the midline, the upper border of the manubrium<br>• Lower border: intersection of caudal margin of the innominate vein with the trachea<br>• Like lymph node station 4R, 2R includes nodes extending to the left lateral border of the trachea<br>2L<br>• Upper border: apex of the lung and pleural space and, in the midline, the upper border of the manubrium<br>• Lower border: superior border of the aortic arch
3	Prevascular and retrotracheal nodes	3a: Prevascular<br>• On the right<br>○ Upper border: apex of chest<br>○ Lower border: level of carina<br>○ Anterior border: posterior aspect of sternum<br>○ Posterior border: anterior border of superior vena cava<br>• On the left<br>○ Upper border: apex of chest<br>○ Lower border: level of carina<br>○ Anterior border: posterior aspect of sternum<br>○ Posterior border: left carotid artery<br>3p: Retrotracheal<br>• Upper border: apex of chest<br>• Lower border: carina
4	Lower paratracheal nodes	4R—includes right paratracheal nodes, and pretracheal nodes extending to the left lateral border of the trachea<br>• Upper border: intersection of caudal margin of innominate vein with the trachea<br>• Lower border: lower border of the azygos vein<br>4L—includes nodes to the left of the left lateral border of the trachea, medial to the ligamentum arteriosum<br>• Upper border: upper margin of the aortic arch<br>• Lower border: upper rim of the left main pulmonary artery
		**Aortopulmonary zone**
5	Subaortic (aortopulmonary window)	Subaortic lymph nodes lateral to the ligamentum arteriosum<br>• Upper border: lower border of the aortic arch<br>• Lower border: upper rim of the left main pulmonary artery
6	Para-aortic nodes (ascending aorta or phrenic)	Lymph nodes anterior and lateral to the ascending aorta and aortic arch<br>• Upper border: a line tangential to the upper border of the aortic arch<br>• Lower border: lower border of the aortic arch
		**Subcarinal zone**
7	Subcarinal nodes	• Upper border: carina of the trachea<br>• Lower border: upper border of the lower lobe bronchus on the left; lower border of the bronchus intermedius on the right
		**Lower zone**
8	Paraesophageal nodes (below carina)	Nodes lying adjacent to the wall of the esophagus and to the right or left of the midline, excluding subcarinal nodes<br>• Upper border: upper border of the lower lobe bronchus on the left; lower border of the bronchus intermedius on the right<br>• Lower border: diaphragm
9	Pulmonary ligament nodes	Nodes lying within the pulmonary ligament<br>• Upper border: inferior pulmonary vein<br>• Lower border: diaphragm

(continued)

Table 36.5 Clinical criteria to categorize a tumor as multifocal GG/L adenocarcinoma[19]

Tumors should be considered multifocal GG/L lung adenocarcinoma if:
There are multiple subsolid nodules (either pure ground glass or part solid), at least one of which is suspected (or proven) to be cancer.
- This applies regardless of whether or not the nodules have been biopsied.
- This applies if the other nodules(s) are suspected to be AIS, MIA, or LPA.
- This applies if a nodule has become >50% solid but is judged to have arisen from a GGN, provided there are other subsolid nodules.
- GGN lesions <5 mm or lesions suspected to be AAH are not counted for TNM classification.

AAH, atypical adenomatous hyperplasia, AIS, adenocarcinoma *in situ*; GG/L, ground-glass/lepidic; GGN, ground-glass nodule; LPA, lepidic-predominant adenocarcinoma; MIA, minimally invasive adenocarcinoma
Note: A radiographically solid appearance and the specific histologic subtype of solid adenocarcinoma denote different things.

Table 36.6 Clinical criteria to categorize a tumor as pneumonic-type adenocarcinoma[19]

Tumors should be considered pneumonic-type adenocarcinoma if:
The cancer manifests in a regional distribution, similar to a pneumonic infiltrate or consolidation.
- This applies whether there is one confluent area or multiple regions of disease. The region(s) may be confined to one lobe or in multiple lobes or bilateral, but should involve a regional pattern of distribution.
- The appearance of involved areas may be ground glass, solid consolidation, or a combination thereof.
- This can be applied when there is compelling suspicion of malignancy, whether or not a biopsy has been performed of the area(s).
- This should not be applied to discrete nodules (i.e., GG/L nodules).
- This should not be applied to tumors causing bronchial obstruction resulting in obstructive pneumonia or atelectasis.

GG/L, ground-glass/lepidic.
Note: A radiographically solid appearance and the specific histologic subtype of solid adenocarcinoma denote different things.

the request for a series of explorations to confirm or rule out lung cancer. There are many imaging techniques and invasive procedures that may be used to diagnose and stage lung cancer. Whenever possible, they should be performed sequentially and with an increasing degree of invasiveness.

The IASLC recommends a three-step protocol to rationalize the use of staging procedures. Step I includes medical history and physical examination, as well as plain radiographs of the chest and blood tests (hemoglobin, leukocytes, platelets, alanine aminotransferase, aspartate aminotransferase, lactate dehydrogenase, calcium, and albumin). Step II includes more complex investigations, such as contrast-enhanced CT scan of the chest and upper abdomen, bone scan, PET scan, brain CT, and bronchoscopy. Step III includes the more invasive procedures, including surgical exploration of the mediastinum (mediastinoscopy, extended cervical mediastinoscopy, mediastinotomy, video-assisted mediastinal lymphadenectomy, transcervical extended mediastinal lymphadenectomy), the pleural space (thoracentesis, percutaneous needle biopsy, thoracoscopy, video-assisted thoracoscopic surgery), or the pericardium (pericardiocentesis, pericardioscopy).[20]

Posteroanterior and lateral chest radiography usually is the first imaging technique requested for a patient with suspected lung cancer. The minimal information to be extracted from chest X-rays is:

a. Tumor size (to assign a T category based on size)
b. Lobar and segmental location of the tumor
c. Presence of atelectasis and its extent (lobar or complete; cT2)
d. Presence of separate tumor nodules (cT3, cT4, or cM1a)
e. Evidence of lymphangitic carcinomatosis (cLy0: no radiologic evidence of lymphangitic carcinomatosis; cLy1: radiologic evidence of lymphangitic carcinomatosis confined to the area of the primary tumor; cLy2: lymphangitic carcinomatosis at a distance from the primary tumor but confined to the same lobe; cLy3: presence of lymphangitis in other ipsilateral lobes; cLy4: lymphangitis affecting the contralateral lung)
f. Relation of the primary tumor to the chest wall (contact or bone destruction [cT3]) or the mediastinum (elevated diaphragm may indicate invasion of the phrenic nerve [cT3])
g. Nodal spread: enlarged hili and abnormal mediastinum may indicate cN1, cN2, or cN3 disease
h. Intrathoracic spread: the presence of pleural or pericardial effusions (cM1a)
i. Extrathoracic spread: the integrity or involvement of the bones visible on chest X-rays—the ribs, the sternum, both scapulae, the vertebral column, the shoulder joint, and most of the length of both humeri; masses in the soft tissues of the chest wall (cM1b or cM1c)

Contrast CT of the chest and upper abdomen to include the liver and both adrenal glands is recommended for patients who are candidates for radical treatment, whether it be surgery, primarily or after induction, or definitive chemoradiation.[21] CT should confirm and refine the information obtained from the chest X-rays:

a. Tumor size in its greatest dimension, which may be assessed by axial, coronal, or sagittal measurement; therefore, if technically possible, all the different

Table 36.1 (continued)

Lymph node station number (#)	Description	Anatomic limits
		**Hilar/interlobar zone**
10	Hilar nodes	Includes nodes immediately adjacent to the mainstem bronchus and hilar vessels, including proximal portions of the pulmonary veins and main pulmonary artery<br>• Upper border: lower rim of the azygos vein on the right; upper rim of the pulmonary artery on the left<br>• Lower border: interlobar region bilaterally
11	Interlobar nodes	Between the origin of the lobar bronchi; optional notations for subcategories of station:<br>• 11s: between the upper lobe bronchus and bronchus intermedius on the right<br>• 11i: between the middle and lower bronchi on the right
		**Peripheral zone**
12	Lobar nodes	Adjacent to the lobar bronchi
13	Segmental nodes	Adjacent to the segmental bronchi
14	Subsegmental nodes	Adjacent to the subsegmental bronchi.

Adapted from Rusch et al.,[15] with permission.

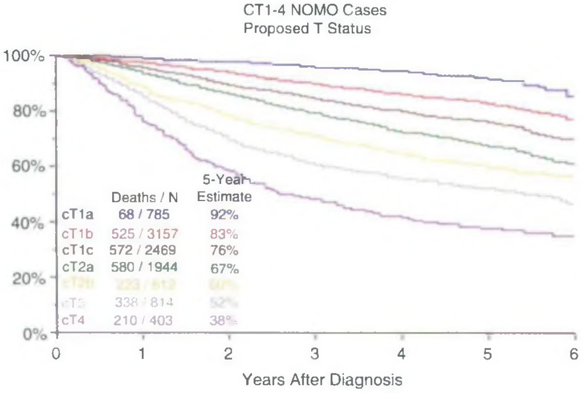

Fig. 36.2 Overall survival according to the proposed T categories for the 8th Edition of the TNM classification for clinically staged T1–T4N0M0 tumors. $p < 0.001$ (From Rami-Porta et al.[3])

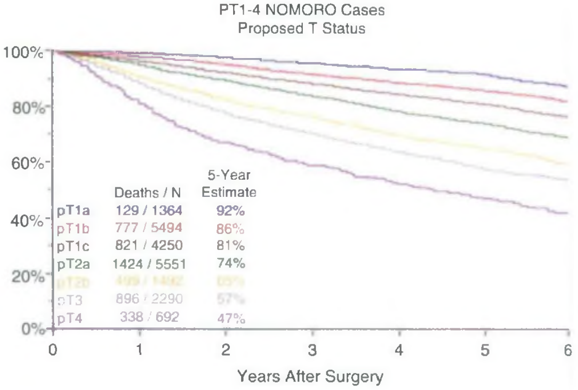

Fig. 36.3 Overall survival according to the proposed T categories for the 8th Edition of the TNM classification for pathologically staged T1–T4N0M0R0 tumors. $p < 0.001$ (From Rami Porta et al.[3])

The analyses of the new IASLC database concerning the primary tumor (T) component of the TNM classification revealed that tumor size has more prognostic relevance than was shown in previous editions. Each centimeter increase in size, from less than 1 cm to up to 5 cm, separates tumors of significantly different prognosis, and larger tumors have a worse prognosis than their assigned T categories in the 7th Edition. Regarding the descriptors of tumor invasion, the prognosis worsens if tumor invasion is more central. On the other hand, endobronchial tumors ≤2 cm from the carina, but not involving the carina, and those causing complete atelectasis or pneumonitis do not have a worse prognosis than those more than 2 cm from the carina or those causing partial atelectasis and pneumonitis. Figure 36.2 shows the survival curves of the new T categories for clinically staged tumors with no nodal involvement and no metastases, and Fig. 36.3 shows the survival curves for completely resected tumors with no nodal involvement and no metastases. In these graphs, the survival curves separate completely, with no overlapping or crossing; and survival differences between T3 and T4 tumors with no nodal involvement or metastasis are statistically significant, which was not the case in previous editions of the TNM classification.[3]

The analyses performed for the 8th Edition of the TNM classification validate the present N categories at clinical and pathological staging. Figures 36.4 and 36.5 show the survival curves according to the N categories for clinically and pathologically staged tumors, respectively. These analyses also reveal that quantification of nodal disease has prognostic impact. In the recent data analysis, quantification was based on the number of involved lymph node stations. The progressive worsening of survival is as follows: single-station N1 has the best prognosis; multiple-station N1 disease follows, but it has the same prognosis as single-station N2 disease without N1 disease (skip metastasis); single-station N2 disease with N1 disease follows; and finally,

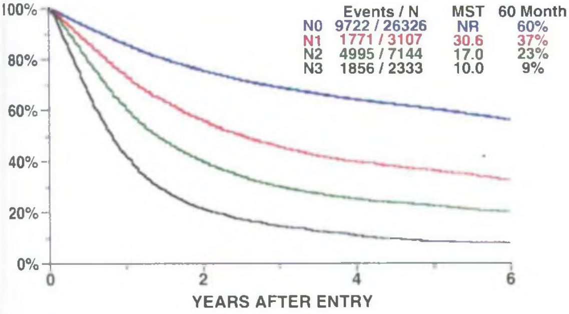

N3 vs N2 vs N1 vs N0 Comparisons Adjusted for Histology (adeno vs others), Sex, Age 60+, and Region (Cox PH regression)		
comparison	HR	P
N1 vs N0	1.68	<0.0001
N2 vs N1	1.42	<0.0001
N3 vs N2	1.38	<0.0001

Fig. 36.4 Overall survival according to the N descriptors for clinically staged tumors (From Asamura et al.[9])

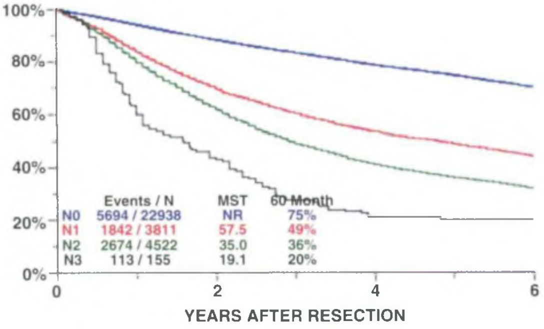

N0 vs N1 vs N2 vs N3 Comparisons Adjusted for Histology (adeno vs others), Sex, Age 60+, R0 resection, and Region. (Cox PH regression on all cases)		
comparison	HR	P
N1 vs N0	2.13	<0.0001
N2 vs N1	1.74	<0.0001
N3 vs N2	1.66	<0.0001

Fig. 36.5 Overall survival according to the N descriptors for pathologically staged tumors including all types of resection (R0, R1 and R2) (From Asamura et al.[9])

multiple-station N2 disease has the worst prognosis. These findings derived from pathological staging and could not be validated at clinical staging.[9] Therefore, they could not be used to modify the present N categories. However, knowing the prognostic impact of the number of involved nodal stations is clinically relevant, as postoperative prognosis can be refined for patients with pathological nodal disease, and it also may be useful for stratifying tumors in future clinical trials focusing on nodal disease. The subclassification of nodal disease presented in Table 36.2 is recommended for prospective registration of clinical and pathological data.

Figure 36.6 shows the survival curves according to the number of involved nodal stations in pathologically staged tumors.

The prospectively collected IASLC database through the electronic data capture (EDC) online system had nearly 4,000 patients. Their data had sufficient detail to validate the M1a categories established in the 7th Edition and to analyze extrathoracic metastatic disease (M1b) according to organ location and number of metastases. The results of these analyses show that number of metastases had more prognostic relevance than organ location and led to the recommendation to separate single extrathoracic metastasis in one organ (new M1b) from multiple extrathoracic metastases in one or several organs (new M1c). Figure 36.7 shows the survival curves of the different types of intrathoracic metastases, and Fig. 36.8 shows the survival curves of single extrathoracic metastasis, multiple extrathoracic metastases, and intrathoracic metastases. Although the prognosis of intrathoracic metastases (M1a) is similar to that of single extrathoracic metastasis, it makes sense to code them differently as they represent different anatomic extents of disease and require different diagnostic and therapeutic strategies.[4]

Table 36.2 Subclassification of nodal disease[8]

NX	Regional lymph nodes cannot be assessed
N0	No regional lymph node metastasis
N1a	Single-station N1
N1b	Multiple-station N1
N2a1	Single-station N2 without N1 (skip metastasis)
N2a2	Single-station N2 with N1
N2b	Multiple-station N2
N3	As defined in the 8th Edition

Classification of lung cancers with multiple lesions poses some problems, because the rules are sometimes ambiguous and their application may be interpreted differently in each situation. Therefore, a special subcommittee of the IASLC Staging and Prognostic Factors Committee studied the problem and made some recommendations regarding the uniform use of the classification rules depending on the pattern of disease. The subcommittee established four disease patterns: second primary tumors, lung cancers with separate tumor nodules of the same histopathological type, multiple tumors with predominant ground-glass features on CT and a lepidic pattern on pathological examination, and, finally, diffuse pneumonic-type lung cancer.[16] Three in-depth articles expand the rationale for applying the classification rules to each disease pattern.[17-19]

36

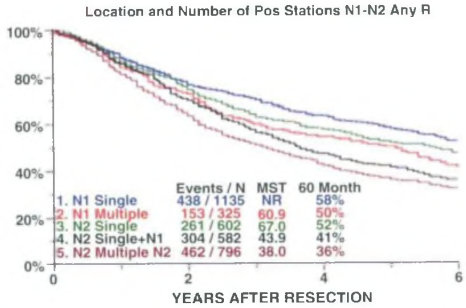

Fig. 36.6 Overall survival according to number of involved lymph node stations for pathologically staged tumors including all types of resection (R0, R1 and R2). N1a: single N1 station; N1b: multiple N1 stations; N2a1: single N2 station without N1 disease (skip metastasis); N2a2: single N2 station with N1 disease; N2b: multiple N2 stations (From Asamura et al.[9])

N1 a vs N1b vs N2a1 vs N2a2 vs N2b Comparisons
Adjusted for Histology (adeno vs others), Sex, Age 60+ , R0 Resection, and Region.
(Cox PH regression on All cases)

comparison	HR	P
N1b vs N1a	1.38	0.0005
N2a1 (skip) vs N1b	0.92	0.4331
N2a2 vs N2a1 (skip)	1.37	0.0002
N2b vs N2a2	1.21	0.0117
N2a2 vs N1b	1.26	0.0197

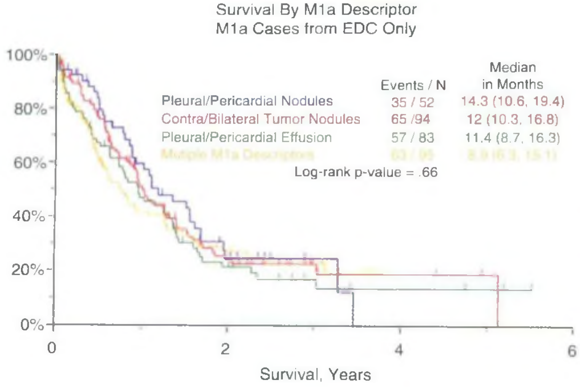

Fig. 36.7 Overall survival according to M1a descriptors. $p = 0.66$ (From Eberhardt et al.[4])

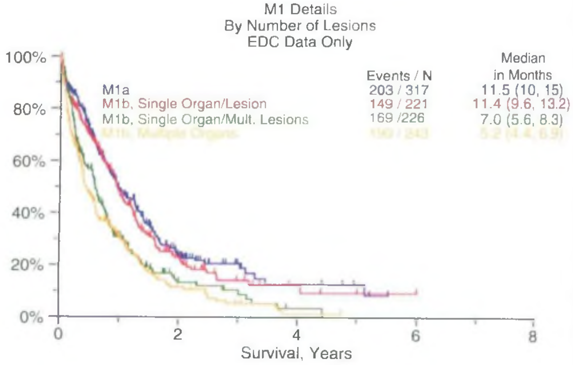

Fig. 36.8 Overall survival according to M1a and M1b descriptors. Single M1b metastasis has a prognosis similar to that of M1a but significantly different from that of M1b with multiple metastases (the new M1c category in the 8th Edition of the TNM classification). $p < 0.0001$ (From Eberhardt et al.[4])

The recommendations for classification are as follows:

a. *Second primary tumors*: Two or more synchronous or metachronous primary tumors should be classified separately, with an individual TNM for each one, regardless of whether they are in the same lobe, same lung, or contralateral lung. This rule applies to tumors identified clinically or grossly and also to those identified microscopically on pathological examination.[18]
b. *Separate tumor nodules of the same histopathologic type (intrapulmonary metastases)*: The classification of these tumors is based on their lobar location. If the separate tumor nodule(s) is(are) in the same lobe of the primary, the tumor is classified as T3. If the separate tumor nodule(s) is(are) in another ipsilateral lobe, the tumor is classified as T4. The tumor classification is M1a if the separate tumor nodule(s) is(are) in the contralateral lung. This classification applies to separate tumor nodules identified clinically or grossly and also to those identified microscopically on pathological examination.[17]
c. *Multifocal lung adenocarcinomas with ground-glass/lepidic features*: These tumors should be classified by the T category of the lesion, with the highest T followed by the number of lesions (#) or simply (m) for multiple lesions indicated in parentheses, and an N and M category that applies to all of the multiple tumor foci collectively. The tumor size should be determined

Table 36.3 Clinical criteria to distinguish second primary versus related tumors[18]*

Tumors may be considered second primary tumors if:
They clearly are of a different histologic type (e.g., squamous carcinoma and adenocarcinoma) on biopsy.
Tumors may be considered to be arising from a single tumor source if:
Exactly matching breakpoints are identified by comparative genomic hybridization.
Relative arguments that favor separate tumors:
Different radiographic appearance or metabolic uptake
Different biomarker pattern (driver gene mutations)
Different rates of growth (if previous imaging is available)
Absence of nodal or systemic metastases
Relative arguments that favor a single tumor source:
The same radiographic appearance
Similar growth patterns (if previous imaging is available)
Significant nodal or systemic metastases
The same biomarker pattern (and same histotype)

*A comprehensive histologic assessment is not included in clinical staging, as it requires the entire specimen to have been resected.

Table 36.4 Clinical criteria to categorize a lesion as a separate tumor nodule (intrapulmonary metastasis)[17]

Tumors should be considered to have a separate tumor nodule(s) if:
There is a solid lung cancer and a separate tumor nodule(s) with a similar solid appearance and with (presumed) matching histologic appearance.<br>• This applies regardless of whether or not the lesions have been biopsied, provided there is strong suspicion that the lesions are histologically identical.<br>• This applies regardless of whether or not there are sites of extrathoracic metastases.
AND provided that:
The lesions are NOT judged to be synchronous primary lung cancers.
The lesions are NOT multifocal GG/L lung cancer (multiple nodules with ground-glass/lepidic features) or pneumonic-type lung cancer.

GG/L, ground-glass/lepidic
Note: A radiographically solid appearance and the specific histologic subtype of solid adenocarcinoma denote different things.

by the largest diameter of the solid component (by CT) or the invasive component on pathological examination. The T(#/m) multifocal classification should be applied equally whether the lesions are in the same lobe or in different ipsilateral or contralateral lobes. Furthermore, this classification should be applied to grossly recognizable lesions as well as to lesions discovered only on pathological examination.[19]

d. *Diffuse pneumonic-type adenocarcinoma*: In the case of a single tumor area, a standard TNM classification based on tumor size, nodal disease, and metastasis should be applied. In cases of multiple tumor areas, the T and M categories should be based on the location of the involved areas: T3 if the disease is confined to one lobe, T4 if it involves other ipsilateral lobes, and M1a if it involves the contralateral lung. If an area of involvement extends to the adjacent lobe, a T4 category should be assigned to recognize the extension into another ipsilateral lobe. If the tumor is confined to one lobe but its size is difficult to measure, a T3 category should be assigned. The N category is selected to apply to all pulmonary sites of the primary tumor collectively. Pleural/pericardial tumor nodules or distant metastases will lead to an M1a, M1b, or M1c designation. The classification should be applied to grossly recognizable lesions as well as to lesions discovered only on histologic examination. Miliary forms of adenocarcinoma should also be classified in this way. If the miliary involvement is limited to one lobe, because measurement of tumor size often is difficult, a T3 category should be assigned.[19]

The aforementioned recommendations for classifying the different patterns of disease are the result of a multidisciplinary and international consensus as well as a thorough literature review and statistical analysis of data from the IASLC database regarding separate tumor nodules. These suggestions are meant to minimize ambiguity and to serve as a guide in classifying these tumors uniformly.

Tables 36.3 through 36.6 describe the clinical criteria used to define the different disease patterns in which lung cancers with multiple lesions may present.

Imaging

Medical history (e.g., family history of lung cancer or of any cancer, smoking history, exposure to asbestos or radon, history of passive smoking, presence of respiratory symptoms or chest pain) and physical examination findings (e.g., peripheral adenopathy, abnormal breath sounds, superior vena cava syndrome, hemoptysis, hepatomegaly) will prompt

projections should be studied to determine tumor size. Lung CT window display settings should be used when assessing images for tumor size.

b. Lobar and segmental location
c. Presence of atelectasis (partial or total; cT2) and endobronchial lesions
d. Presence of separate solid tumor nodules (cT3, cT4, or cM1a) and presence of part-solid lesions
e. Nodal spread: hilar enlargement suggests cN1 disease if it is ipsilateral to the primary tumor or cN3 disease if it is contralateral. Nodes should be measured in their short axis; those larger than 1 cm in the short axis are considered abnormal and suggest metastatic involvement. Enlarged mediastinal lymph nodes may indicate cN2 disease if they are ipsilateral or subcarinal or cN3 disease if they are contralateral or supraclavicular. The number of nodal stations involved should be determined; bulky disease should be identified if present. Mediastinal CT window display settings should be used when assessing images for mediastinal structures, pleural or pericardial effusions, etc.
f. Evidence of lymphangitic carcinomatosis (cLy0, cLy1, cLy2, cLy3, and cLy4 as defined earlier)
g. Intrathoracic spread: pleural and pericardial effusion or nodules (cM1a)
h. Extrathoracic spread: bone lesions, soft tissue masses, adrenal masses, and liver nodules may indicate cM1b disease if single or cM1c disease if multiple

CT is important for assessing the size and location of any enlarged mediastinal lymph nodes present, because in the absence of metastasis, they are the strongest indicators of prognosis. In a review of 7,368 patients with a median prevalence of mediastinal nodal disease of 30%, staging values of chest CT were as follows: sensitivity, 0.55; specificity, 0.81; positive predictive value, 0.58; and negative predictive value, 0.83.[21]

PET is indicated in patients with no clinical abnormalities and no signs of metastatic spread on CT who are candidates for treatment with curative intent. It is useful for evaluating metastatic spread, except that occurring in the brain. PET is not required in patients with ground-glass opacities or clinical stage IA tumors with no other abnormality on chest CT. Regarding mediastinal staging, in a review of 4,105 patients with a median prevalence of nodal disease of 28%, staging values were as follows: sensitivity, 0.8; specificity, 0.88; positive predictive value, 0.75; and negative predictive value, 0.91.[21] PET should provide the following information:

a. Presence of normal or abnormal uptake in the primary tumor and quantification by maximum standardized uptake value (SUV_{max})
b. Presence of normal or abnormal uptake in hilar and mediastinal nodes and quantification by SUV_{max}
c. Presence of normal or abnormal uptake in other parts of the lungs or in the rest of the body

Although SUV_{max} is subject to many intra- and interinstitutional variations, it is important to record it at initial staging to assess metabolic tumor response after treatment, especially after induction treatment to evaluate the possibility of tumor resection. SUV_{max} also has shown prognostic value, at least for Stage I–III squamous cell carcinomas and adenocarcinomas.[22]

Because PET has a poor anatomic resolution, the superimposition of PET with CT (e.g., with hybrid PET/CT scanners) may help the clinician locate the lesions with abnormal uptake. However, the mean staging values of combined PET/CT are similar to those of PET alone. In a review of 2,014 patients with a median prevalence of mediastinal nodal disease of 22%, the staging values for combined PET/CT were as follows: sensitivity, 0.62; specificity, 0.9; positive predictive value, 0.63; and negative predictive value, 0.9.[21]

The positive predictive value of PET is relatively low; therefore, histopathological confirmation of the lesions is recommended if this will affect therapy. Inflammations, granulomas, and infections may have high SUV_{max}, and if the correct histology remains unconfirmed, the patient may be excluded from radical treatment. If PET is not available, bone scanning and abdominal CT should be done to rule out metastatic spread.[21]

Magnetic resonance (MR) imaging has very specific indications in lung cancer staging. MR imaging of the brain currently is indicated in patients with Stage III and IV tumors, even if they have a negative clinical evaluation.[21] It also is indicated in patients with brain metastasis identified on CT, as MR imaging may identify more lesions.[23] It also may help define the involved anatomic structures in patients with apical (Pancoast) tumors or tumors invading the chest wall and mediastinum. MR imaging of the adrenals with in- and out-of-phase imaging may help exclude adrenal metastases in cases with indeterminate adrenal lesions on PET/CT.

The order in which the aforementioned anatomic and metabolic imaging tests are performed usually is chest X-rays first, followed by CT scan of the chest and upper abdomen, PET scan or PET/CT, and MR imaging in indicated cases.

The anatomic and metabolic imaging techniques described here provide a thorough description of the primary lesion and its local and distant spread, but do not provide its diagnosis. The TNM classification requires microscopic confirmation of malignancy[24,25] and specification of histopathological type.[14] The type of procedure used to obtain pathological confirmation of lung cancer differs depending on the location and spread of the tumor.

Sputum cytology may provide the diagnosis of lung cancer with high specificity. In a review of 29,145 patients, the

diagnostic values of sputum cytology were as follows: sensitivity, 0.66; specificity, 0.99; false positive rate, 8%; and false negative rate, 10%.[26] In certain patients with evident metastatic disease, this may be the only diagnostic test needed. However, molecular profiling of tumors is best performed on cell blocks; if these are not available in the sputum specimen, then larger samples may be needed.

Fiberoptic bronchoscopy is both a diagnostic and a staging procedure. As a diagnostic procedure including bronchial biopsy, brushings, washings, and endobronchial and transbronchial needle aspiration, its sensitivity is 0.88 and 0.78 for central and peripheral tumors, respectively.[26] As a staging procedure, it shows the endobronchial location of the tumor: T2 if the main brochus is involved, regardless of its distance to the carina, and T4 if the carina is involved. It may suggest nodal involvement if there is extrinsic compression of the bronchi. The lymph nodes may be punctured with fine needles, either blindly—the classic transbronchial needle aspiration procedure—or with the assistance of EBUS and fine-needle aspiration (EBUS-FNA) or EUS and FNA (EUS-FNA). Peripheral tumors that remain undiagnosed by fiberoptic bronchoscopy may be diagnosed by transthoracic needle aspiration or biopsy, with a sensitivity of 0.9, a specificity of 0.97, a false positive rate of 1%, and a false negative rate of 22%.[26]

Thoracentesis and cytopathologic study of the pleural fluid may be enough in patients with malignant pleural effusion. It provides a diagnosis in 72% of patients.[26] If cytology is negative, further pleural explorations with closed pleural biopsy and thoracoscopy should follow. Sensitivity and negative predictive values are both around 80% for closed pleural biopsy and greater than 80% and approximately 100%, respectively, for thoracoscopic biopsy.[26] A malignant pleural effusion or tumor nodules on the pleural surface (parietal or visceral) classify the tumor as M1a. Thoracoscopy has the advantage of allowing exploration of the pleural cavity, lung surface, and mediastinum. Video-assisted thoracoscopic surgery also allows resection of peripheral nodules and assists in their diagnosis and staging. Ipsilateral hilar and mediastinal nodes may be biopsied as well.

The American College of Chest Physicians (ACCP) and the European Society of Thoracic Surgeons (ESTS) published guidelines on the preoperative staging of mediastinal lymph nodes.[21,27] The 2013 ACCP Evidence-based Clinical Practice Guidelines favor invasive staging by needle aspiration techniques (EBUS-FNA, EUS-FNA) as the first procedures, but recommend confirmation with surgical biopsies (mediastinoscopy) if needle techniques are negative. In the absence of metastatic disease, the indications for invasive staging are as follows[21]:

a. Discrete mediastinal lymph node enlargement with or without PET uptake in mediastinal lymph nodes
b. PET activity in mediastinal lymph nodes and abnormal lymph nodes on CT
c. High suspicion of N2 or N3 disease, either by lymph node enlargement on CT or PET uptake
d. Intermediate suspicion of N2 or N3 disease by CT and PET and a central tumor or N1 disease

Invasive staging is not indicated for patients with extensive mediastinal infiltration or stage IA tumors with no suspicion of mediastinal lymph node involvement on CT or PET.[21]

The ESTS guidelines also recommend performing EBUS-FNA and EUS-FNA as the initial exploration in the following situations[27]:

a. Positive mediastinal nodes on CT and/or PET or PET/CT
b. Cases in which there is no evidence of N2–N3, but there is suspicion of N1 disease; central tumors larger than 3 cm; and adenocarcinomas with high PET uptake

Invasive staging may be avoided in patients with no evidence of mediastinal disease on CT and PET and tumors less than 3 cm in greatest dimension located peripherally, that is, in the outer third of the lung.

If needle techniques produce negative results, video-assisted mediastinoscopy is recommended to confirm the results or to identify mediastinal disease. In general, the negative predictive values of EBUS-FNA and EUS-FNA are too low, both in patients with normal and those with abnormal mediastinal lymph nodes, to make therapeutic decisions without proper confirmation by a surgical technique. In a recent article on the staging value of EBUS-FNA in patients with no mediastinal abnormalities, the sensitivity and negative predictive values for EBUS-FNA were 0.38 and 0.81, respectively, whereas they were 0.73 and 0.91 for mediastinoscopy.[28] This article clearly highlights the importance of confirming negative results of EBUS-FNA and EUS-FNS with mediastinoscopy.

Additionally, the ESTS guidelines recommend exploration of the aortopulmonary window for left lung cancers and establish minimum requirements for mediastinoscopy in clinical practice: at least the inferior right and left paratracheal lymph nodes and the subcarinal lymph nodes should be biopsied or removed; the superior right and left paratracheal lymph nodes and the hilar lymph nodes should be explored if there is evidence of involvement on CT or PET.[27]

Other invasive procedures should be performed as required, including pericardiocentesis or pericardioscopy, either transpleural or subxiphoid, for pericardial effusion; needle biopsies of liver and adrenal lesions; endoscopies of the gastrointestinal tract in cases of digestive symptoms or bleeding; and biopsy or excision of skin lesions.

These procedures should be performed sequentially from the least to most invasive: first, to rule out metastatic disease

if imaging suggests metastatic spread, as this will avoid more invasive procedures; next, to rule out supraclavicular nodal disease (N3) if there is anatomic or metabolic suspicion; and finally, to explore the mediastinum as indicated by the aforementioned guidelines.

Pathological Classification

Pathological classification or postsurgical histopathological classification, designated pTNM, is used to guide adjuvant therapy and provides useful information to estimate prognosis and calculate end results. It is based on evidence acquired before treatment, supplemented or modified by additional evidence acquired from surgery and from pathological examination of the resected specimens.

The pathological assessment of the primary tumor (pT) entails resection of the primary tumor or a biopsy specimen adequate to evaluate the highest T category. The pathological assessment of the regional lymph nodes (pN) entails removal of enough nodes to validate the absence of regional lymph node metastasis (pN0) or to evaluate the highest pN category. The pathological assessment of distant metastasis (pM) entails microscopic examination. For pathological staging, cM0 or cM1 categories also are valid.

Visceral pleural invasion is defined as invasion beyond the elastic layer or to the surface of the visceral pleura. A tumor that falls short of completely traversing the elastic layer is defined as PL0. A tumor that extends through the elastic layer is defined as PL1, and one that extends to the surface of the visceral pleura as PL2. Elastic stains should be performed in cases in which there is any uncertainty based on review of hematoxylin and eosin sections. Either PL1 or PL2 status allows classification of the primary tumor as T2. Extension of the tumor to the parietal pleura or chest wall is defined as PL3 and categorizes the primary tumor as T3. Direct tumor invasion into an adjacent ipsilateral lobe (i.e., invasion across a fissure) is classified as T2a, unless tumor size indicates a higher T category. Figure 36.9 shows the graphic representation of visceral and parietal pleura invasion.[29]

For proper pathological lymph node staging and to fulfill the requirements for complete resection and pathological N0, the IASLC recommends performing a systematic nodal dissection or a lobe-specific systematic nodal dissection. Systematic nodal dissection is the en bloc removal of the mediastinal fatty tissue, including the lymph nodes, which should be followed by hilar and intrapulmonary nodal dissection.[30] Lobe-specific systematic nodal dissection consists of the removal of certain mediastinal lymph nodes, depending on the lobar location of the primary tumor.[31] The mediastinal nodal stations that should be biopsied or removed, according to the location of the primary tumor, are as follows (based on the IASLC lymph node map in Fig. 36.1):

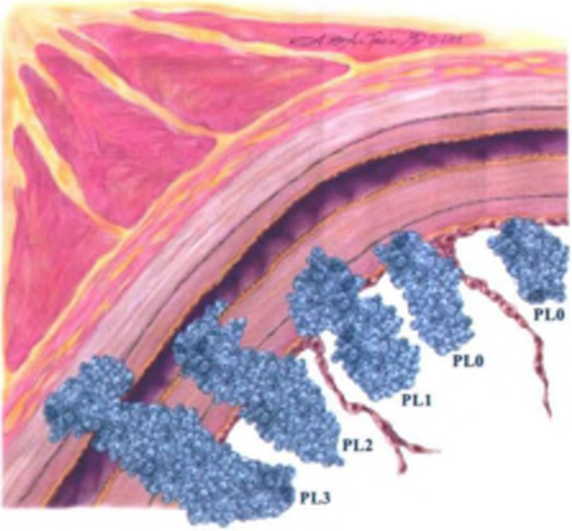

Fig. 36.9 Visceral pleura invasion. See text for definitions (From Travis et al.[29] Copyright © 2008 Aletta Ann Frazier, MD)

- Right upper and middle lobes: 7, 2R, and 4R
- Right lower lobe: 7, 4R, and 8 or 9
- Left upper lobe: 7, 5, and 6
- Left lower lobe: 7, 8, and 9

In any case, at least six lymph nodes/stations (six lymph nodes from six lymph node stations) should be removed or sampled. Three of these nodes/stations should be mediastinal, including the subcarinal nodes (nodal station #7), and three should be hilar–intrapulmonary lymph nodes/stations. The Union for International Cancer Control (UICC) and the AJCC accept that if all resected/sampled lymph nodes are negative, but the number recommended is not met, the classification is pN0. If resection has been performed, and otherwise fulfills the requirement for complete resection, the classification is R0.[14,24] However, the ACCP recommends the classificiation of pN0(un), *un* for uncertain, if the number of recommended lymph nodes removed /sampled is not met. The suffix *(un)* also should be added to pN1, and pN2 if fewer than six lymph nodes are evaluated.[32] This suffix is not added to the N categories, as it is an opinion of the ACCP that has not been discussed sufficiently at the international level or approved by the AJCC or UICC. Nevertheless, it makes sense to use it in cases in which the required standards of intraoperative lymph node examination are not met, especially when it has been proven that failure to perform a proper lymphadenectomy has had deleterious prognostic implications.[33]

Regarding tumors that underwent an attempt at resection but were not removed completely, the UICC and AJCC differ on when to consider pathological classification. According to the UICC, the following criteria should be satisfied[14]:

a. Biopsy confirmed a pT category, and there is microscopic confirmation of nodal disease at any level (pN1–3)
b. There is microscopic confirmation of the highest N category (pN3) or
c. There is microscopic confirmation of pM1

However, the AJCC requires[24]:

a. Microscopic confirmation of highest T and N categories or
b. Microscopic confirmation of M1

In view of the discrepancy and to avoid any confusion,[34,35] the ACCP recommends the use of the *p* prefix for resected tumors, as well as for the rare cases in which a tumor was not resected, but extensive biopsy samples were taken during the resection attempt. In the clinical staging context, even if there is pathological confirmation of tumor extent, the *c* prefix should be used. It seems reasonable to use the ACCP recommendation and to discontinue the use of different criteria to assign pathological classification to tumors that have not been resected.[32]

Pleural lavage cytology is an easy and inexpensive method to further study tumor extent at the time of resection. In this procedure, performed before lung manipulation, a specified amount of saline is introduced into the pleural space and then retrieved for pathological study. If the fluid is positive for malignant cells, the prognosis is invariably worse than in the case of negative cytology, regardless of the T category of the primary tumor.[36,37] Positive pleural lavage cytology is a descriptor of incomplete resection and is coded as R1(cy+).[14]

Since the 7th Edition TNM stage classification, WHO has defined new entities of adenocarcinoma *in situ* and minimally invasive adenocarcinoma. It also has histologically classified nonmucinous adenocarcinomas based on an estimate of percentages of lepidic and invasive (acinar, papillary, solid, and micropapillary) patterns. In general, the lepidic-versus-invasive patterns by histology correspond to ground-glass versus solid components by CT.

Adenocarcinoma *in situ* (AIS) is added to the category of Tis, which previously consisted only of squamous cell carcinoma *in situ* (SCIS). Because the histologic type of *in situ* carcinoma does not always match that of the associated primary lung carcinoma, it is important to specify Tis (AIS) versus Tis (SCIS). AIS is a localized small ($\leq$3-cm) adenocarcinoma with growth restricted to neoplastic cells along pre-existing alveolar structures (lepidic growth) and lacking stromal, vascular, alveolar space, or pleural invasion. Most AISs are nonmucinous, but rarely, they may be mucinous. Most AISs show a pure ground-glass nodule by CT, unless there are benign areas such as fibrous scar, inflammation, or organizing pneumonia contributing to a solid component.[6,7]

Minimally invasive adenocarcinoma (MIA) is defined as a lepidic-predominant adenocarcinoma measuring up to 3 cm with an invasive component measuring up to 0.5 cm. Most MIAs are nonmucinous, but rarely, they may be mucinous. In some nonmucinous MIA cases, there is a single, discrete focus of invasion or a solid component on CT. However, if the invasive/solid component assessed by histology/CT, respectively, consists of multiple foci, it is proposed that the percentage area of the invasive/solid be estimated and then multiplied by the total size. For example, a 2.0-cm total size with a 20% invasive or solid component on histology or CT, respectively, would have an estimated size of 0.4 cm.[2,6,7]

To measure tumor size in part-solid, nonmucinous adenocarcinomas, the recommendation is to follow the TNM rule to consider only the size of the invasive component in assigning a T category. This recommendation does not apply to other histologic types of lung cancer or to mucinous lung adenocarcinomas. Although this rule has been in place since 2001, until now it has not been applied in lung adenocarcinoma.[2,14] Therefore, a lesion consisting of a 15-mm part-solid opacity with a 7-mm solid component would be classified as a cT1a lesion, because its solid component, excluding the ground-glass component, is less than 10 mm in greatest dimension. If the lesion is resected and proves to be an adenocarcinoma with lepidic and invasive components, the measurement of the invasive component at pathological examination will be used for the pathological classification. This recommendation is based on the increasing number of studies in small lung adenocarcinomas reporting that in part-solid adenocarcinomas, it is the invasive component that correlates with prognosis.[38-40] Similar to MIA, in cases in which multiple invasive/solid areas, rather than a single, discrete focus, are observed on histology/CT, it is proposed that the percentage invasive/solid area be multiplied by the total tumor size to estimate the size of invasion.[2] It is recommended that both total size and invasive/solid size continue to be documented in radiology and pathology reports.

In special situations, tumor size is determined after induction therapy. If no viable tumor cells remain after induction therapy, the tumor is classified as ypT0. However, no rules have been established to measure tumor size in patients who have had a partial response, the degree of which has prognostic relevance. A practical way to estimate tumor size is to multiply the percentage of viable tumor cells by the size of the total mass. This formula may be applied in cases in which there is a single focus of disease or multiple foci of viable cells.[2]

Pathological classification of lung cancers with multiple lesions follows the same criteria recommended for clinical classification of the four different patterns of disease: separate primary tumors, separate tumor nodules (intrapulmonary metastasis), ground-glass/lepidid adenocarcinomas, and pneumonic-type adenocarcinomas.[16]

Tables 36.7 through 36.11 describe the pathological criteria for defining the different disease patterns in which lung cancers with multiple lesions may present.

Table 36.7 Pathological criteria (i.e., after resection) for separate versus related pulmonary tumors[18]

Tumors may be considered second primary tumors if:
They clearly are a different histologic type (e.g., squamous carcinoma and adenocarcinoma).
They clearly are different based on a comprehensive histologic assessment.
They are squamous carcinomas that have arisen from carcinoma *in situ*.
Tumors may be considered to be arising from a single tumor source if:
Exactly matching breakpoints are identified by comparative genomic hybridization.
Relative arguments that favor separate tumors (to be considered together with clinical factors):
Different biomarker pattern
Absence of nodal or systemic metastases
Relative arguments that favor a single tumor source (to be considered together with clinical factors):
Matching appearance on comprehensive histologic assessment
The same biomarker pattern
Significant nodal or systemic metastases

Table 36.8 Pathological criteria to categorize a lesion as a separate tumor nodule (intrapulmonary metastasis)[17]

Tumors should be considered to have a separate tumor nodule (intrapulmonary metastasis) if:
There is (are) a separate tumor nodule(s) of cancer in the lung with a similar histologic appearance to a primary lung cancer.
AND provided that:
The lesions are NOT judged to be synchronous primary lung cancers.
The lesions are NOT multiple foci of LPA, MIA, or AIS.

AIS, adenocarcinoma *in situ*; LPA, lepidic-predominant adenocarcinoma; MIA, minimally invasive adenocarcinoma
Note: A radiographically solid appearance and the specific histologic subtype of solid adenocarcinoma denote different things.

Table 36.9 Pathological criteria identifying multifocal ground-glass/lepidic lung adenocarcinoma[19]

Tumors should be considered multifocal GG/L lung adenocarcinoma if:
There are multiple foci of LPA, MIA, or AIS.
• This applies whether a detailed histologic assessment (i.e., proportion of subtypes, etc.) shows a matching or different appearance.
• This applies if one lesion(s) is (are) LPA, MIA, or AIS and there are other subsolid nodules that have not been biopsied.
• This applies whether the nodule(s) is (are) identified preoperatively or only on pathological examination.
• Foci of AAH are not counted for TNM classification.

AAH, atypical adenomatous hyperplasia; AIS, adenocarcinoma *in situ*; GG/L, ground-glass/lepidic; LPA, lepidic-predominant adenocarcinoma; MIA, minimally invasive adenocarcinoma
Note: A radiographically solid appearance and the specific histologic subtype of solid adenocarcinoma denote different things.

Table 36.10 Pathological criteria identifying pneumonic-type adenocarcinoma[19]

Tumors should be considered pneumonic-type adenocarcinoma if:
There is diffuse distribution of adenocarcinoma throughout a region(s) of the lung, as opposed to a single well-demarcated mass or multiple discrete well-demarcated nodules.
• This typically involves an invasive mucinous adenocarcinoma, although a mixed mucinous and nonmucinous pattern may occur.
• The tumor may show a heterogeneous mixture of acinar, papillary, and micropapillary growth patterns, although it usually is lepidic predominant.

Note: A radiographically solid appearance and the specific histologic subtype of solid adenocarcinoma denote different things.

Table 36.11 Schematic summary of disease patterns and TNM classification of patients with lung cancer with multiple pulmonary sites of involvement[16]

	Second primary lung cancer	Multifocal GG/L nodules	Pneumonic-type adenocarcinoma	Separate tumor nodule
Imaging features	Two or more distinct masses with imaging characteristic of lung cancer (e.g., spiculated)	Multiple ground-glass or part-solid nodules	Patchy areas of ground glass and consolidation	Typical lung cancer (e.g., solid, spiculated) with separate solid nodule
Pathological features	Different histotype or different morphology based on comprehensive histologic assessment	Adenocarcinomas with prominent lepidic component (typically varying degrees of AIS, MIA, LPA)	Same histology throughout (most often invasive mucinous adenocarcinoma)	Distinct masses with the same morphologic features based on comprehensive histologic assessment
TNM classification	Separate cTNM and pTNM for each cancer	T based on highest T lesion, with (#/m) indicating multiplicity; single N and M	T based on size or T3 if in single lobe, T4 or M1a if in different ipsilateral or contralateral lobes; single N and M	Location of separate nodule relative to primary site determines whether T3, T4, or M1a; single N and M
Conceptual view	Unrelated tumors	Separate tumors, albeit with similarities	Single tumor, diffuse pulmonary involvement	Single tumor with intrapulmonary metastasis

AIS, adenocarcinoma *in situ*; GG/L, ground-glass/lepidic; LPA, lepidic-predominant adenocarcinoma; MIA, minimally invasive adenocarcinoma

PROGNOSTIC FACTORS

Prognostic Factors Required for Stage Grouping

Beyond the factors used to assign T, N, or M categories, no additional prognostic factors are required for stage grouping.

Additional Factors Recommended for Clinical Care

The analyses of the database used to revise the 6th Edition of the TNM classification of lung cancer showed that prognosis beyond that indicated by the classification of anatomic extent may be refined by adding nonanatomic prognostic factors, both at clinical and pathological staging. In addition to anatomic stage, performance status, age, and gender were important prognostic factors, and their combination separated groups of tumors of significantly different prognoses.[41,42]

The following lists of nonanatomic prognostic factors are based on *Prognostic Factors in Cancer*, 3rd edition.[43]

Resectable Non–Small Cell Lung Cancer in Inoperable Patients Treated with Radiotherapy

Patient related:

- Symptoms (presence/absence)
- Performance status
- Hemoglobin

Surgically Resected Non–Small Cell Lung Cancer

Tumor related:

- Histologic type
- Differentiation grade
- Vascular invasion
- Lymphatic permeation
- Perineural invasion
- Type of visceral pleura invasion: PL1 versus PL2
- Positive pleural lavage cytology
- SUV_{max} of primary tumor
- Molecular/biologic markers

Patient related:

- Age
- Gender
- Weight loss
- Performance status
- Quality of life
- Marital status

Environment related:

- Resection margins
- Adequacy of mediastinal dissection
- Radiotherapy dose
- Adjuvant radiation
- Adjuvant chemotherapy

Locally Advanced or Metastatic Non–Small Cell Lung Cancer

Tumor related:

- Hemoglobin
- Lactate dehydrogenase (LDH)
- Albumin

Patient related:

- Gender
- Symptom burden
- Weight loss
- Performance status
- Quality of life
- Marital status
- Anxiety/depression

Environment related:

- Chemoradiotherapy
- Chemotherapy

Small Cell Lung Cancer

Tumor related:

- LDH
- Alkaline phosphatase
- Cushing syndrome
- White blood cells
- Platelet count
- Molecular/biologic markers

Patient related:

- Age
- Comorbidity
- Performance status

Environment related:

- Chemotherapy
- Thoracic radiotherapy
- Prophylactic cranial radiotherapy

A recent review on prognostic tools for non–small cell and small cell lung cancers shows that most of them lack internal and external validation. However, prognostic tools combining a series of variables may contribute to the development of personalized medicine.[44]

RISK ASSESSMENT MODELS

The AJCC recently established guidelines that will be used to evaluate published statistical prediction models for the purpose of granting endorsement for clinical use.[45] Although this is a monumental step toward the goal of precision medicine, this work was published only very recently. Therefore, the existing models that have been published or may be in clinical use have not yet been evaluated for this cancer site by the Precision Medicine Core of the AJCC. In the future, the statistical prediction models for this cancer site will be evaluated, and those that meet all AJCC criteria will be endorsed.

DEFINITIONS OF AJCC TNM

Definition of Primary Tumor (T)

T Category	T Criteria
TX	Primary tumor cannot be assessed, or tumor proven by the presence of malignant cells in sputum or bronchial washings but not visualized by imaging or bronchoscopy
T0	No evidence of primary tumor
Tis	Carcinoma *in situ*<br>Squamous cell carcinoma *in situ* (SCIS)<br>Adenocarcinoma *in situ* (AIS): adenocarcinoma with pure lepidic pattern, ≤3 cm in greatest dimension
T1	Tumor ≤3 cm in greatest dimension, surrounded by lung or visceral pleura, without bronchoscopic evidence of invasion more proximal than the lobar bronchus (i.e., not in the main bronchus)
T1mi	Minimally invasive adenocarcinoma: adenocarcinoma (≤3 cm in greatest dimension) with a predominantly lepidic pattern and ≤5 mm invasion in greatest dimension
T1a	Tumor ≤1 cm in greatest dimension. A superficial, spreading tumor of any size whose invasive component is limited to the bronchial wall and may extend proximal to the main bronchus also is classified as T1a, but these tumors are uncommon.
T1b	Tumor >1 cm but ≤2 cm in greatest dimension
T1c	Tumor >2 cm but ≤3 cm in greatest dimension
T2	Tumor >3 cm but ≤5 cm or having any of the following features:<br>• Involves the main bronchus regardless of distance to the carina, but without involvement of the carina<br>• Invades visceral pleura (PL1 or PL2)<br>• Associated with atelectasis or obstructive pneumonitis that extends to the hilar region, involving part or all of the lung<br>T2 tumors with these features are classified as T2a if ≤4 cm or if the size cannot be determined and T2b if >4 cm but ≤5 cm.
T2a	Tumor >3 cm but ≤4 cm in greatest dimension
T2b	Tumor >4 cm but ≤5 cm in greatest dimension
T3	Tumor >5 cm but ≤7 cm in greatest dimension or directly invading any of the following: parietal pleura (PL3), chest wall (including superior sulcus tumors), phrenic nerve, parietal pericardium; or separate tumor nodule(s) in the same lobe as the primary
T4	Tumor >7 cm or tumor of any size invading one or more of the following: diaphragm, mediastinum, heart, great vessels, trachea, recurrent laryngeal nerve, esophagus, vertebral body, or carina; separate tumor nodule(s) in an ipsilateral lobe different from that of the primary

36

Definition of Regional Lymph Node (N)

N Category	N Criteria
NX	Regional lymph nodes cannot be assessed
N0	No regional lymph node metastasis
N1	Metastasis in ipsilateral peribronchial and/or ipsilateral hilar lymph nodes and intrapulmonary nodes, including involvement by direct extension
N2	Metastasis in ipsilateral mediastinal and/or subcarinal lymph node(s)
N3	Metastasis in contralateral mediastinal, contralateral hilar, ipsilateral or contralateral scalene, or supraclavicular lymph node(s)

Definition of Distant Metastasis (M)

M Category	M Criteria
M0	No distant metastasis
M1	Distant metastasis
M1a	Separate tumor nodule(s) in a contralateral lobe; tumor with pleural or pericardial nodules or malignant pleural or pericardial effusion. Most pleural (pericardial) effusions with lung cancer are a result of the tumor. In a few patients, however, multiple microscopic examinations of pleural (pericardial) fluid are negative for tumor, and the fluid is nonbloody and not an exudate. If these elements and clinical judgment dictate that the effusion is not related to the tumor, the effusion should be excluded as a staging descriptor.
M1b	Single extrathoracic metastasis in a single organ (including involvement of a single nonregional node)
M1c	Multiple extrathoracic metastases in a single organ or in multiple organs

AJCC PROGNOSTIC STAGE GROUPS

When T is...	And N is...	And M is...	Then the stage group is...
TX	N0	M0	Occult carcinoma
Tis	N0	M0	0
T1mi	N0	M0	IA1
T1a	N0	M0	IA1
T1a	N1	M0	IIB
T1a	N2	M0	IIIA
T1a	N3	M0	IIIB
T1b	N0	M0	IA2
T1b	N1	M0	IIB
T1b	N2	M0	IIIA
T1b	N3	M0	IIIB
T1c	N0	M0	IA3
T1c	N1	M0	IIB
T1c	N2	M0	IIIA
T1c	N3	M0	IIIB
T2a	N0	M0	IB
T2a	N1	M0	IIB
T2a	N2	M0	IIIA
T2a	N3	M0	IIIB
T2b	N0	M0	IIA
T2b	N1	M0	IIB
T2b	N2	M0	IIIA
T2b	N3	M0	IIIB
T3	N0	M0	IIB
T3	N1	M0	IIIA
T3	N2	M0	IIIB
T3	N3	M0	IIIC
T4	N0	M0	IIIA
T4	N1	M0	IIIA
T4	N2	M0	IIIB
T4	N3	M0	IIIC
Any T	Any N	M1a	IVA
Any T	Any N	M1b	IVA
Any T	Any N	M1c	IVB

Table 36.12 Guide to uniform classification of situations beyond the standard descriptors[14]

Situation	Classification
Direct invasion of an adjacent lobe, across the fissure or directly if the fissure is incomplete, unless other criteria assign a higher T	T2a
Invasion of phrenic nerve	T3
Paralysis of the recurrent laryngeal nerve, superior vena caval obstruction, or compression of the trachea or esophagus related to direct extension of the primary tumor	T4
Paralysis of the recurrent laryngeal nerve, superior vena caval obstruction, or compression of the trachea or esophagus related to lymph node involvement	N2
Involvement of great vessels: aorta, superior vena cava, inferior vena cava, main pulmonary artery (pulmonary trunk), intrapericardial portions of the right and left pulmonary artery, intrapericardial portions of the superior and inferior right and left pulmonary veins	T4
Pancoast tumors with evidence of invasion of the vertebral body or spinal canal, encasement of the subclavian vessels, or unequivocal involvement of the superior branches of the brachial plexus (C8 or above)	T4
Pancoast tumors without the criteria for T4 classification	T3
Direct extension to parietal pericardium	T3
Direct extension to visceral pericardium	T4
Tumor extending to rib	T3
Invasion into hilar fat, unless other criteria assign a higher T	T2a
Invasion into mediastinal fat	T4
Discontinuous tumor nodules in the ipsilateral parietal or visceral pleura	M1a
Discontinuous tumor nodules outside the parietal pleura in the chest wall or in the diaphragm	M1b or M1c

REGISTRY DATA COLLECTION VARIABLES

For data collection, all T, N, and M descriptors and at least the prognostic factors considered essential and additional in Additional Factors Recommended for Clinical Care should be collected.[43]

For surgically resected non–small cell lung cancer

Patient related:

- Gender
- Age
- Weight loss
- Performance status

Environment related:

- Resection margins
- Adequacy of mediastinal dissection

For advanced non–small cell lung cancer

Tumor related:

- *EGFR* mutation
- *ALK* gene rearrangement

Patient related:

- Gender
- Symptoms
- Weight loss
- Performance status

Environment related:

- Chemoradiotherapy
- Chemotherapy

For small cell lung cancer

Patient related:

- Performance status
- Age
- Comorbidity

Environment related:

- Chemotherapy
- Thoracic radiotherapy
- Prophylactic cranial radiotherapy

HISTOLOGIC GRADE (G)

G	G Definition
GX	Grade of differentiation cannot be assessed
G1	Well differentiated
G2	Moderately differentiated
G3	Poorly differentiated
G4	Undifferentiated

HISTOPATHOLOGIC TYPE

This classification applies to carcinomas of the lung, including non–small cell and small cell carcinomas, and bronchopulmonary carcinoid tumors.

SURVIVAL DATA

Figures 36.10 and 36.11 show the survival graphs and 2- and 5-year survival rates for 8th Edition clinical and pathological stages.

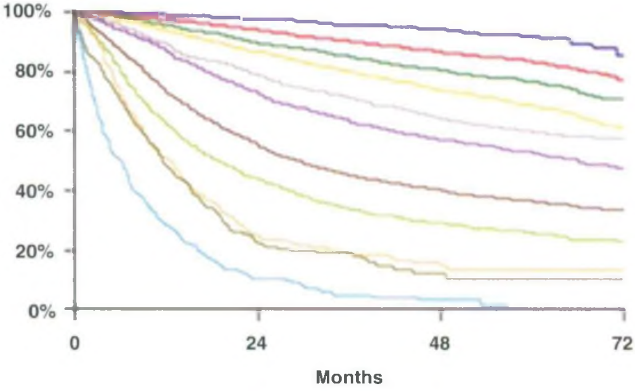

	Events/N	MST	24 months	60 months
IA1	68/781	NR	97%	92%
IA2	505/3105	NR	94%	83%
IA3	546/2417	NR	90%	77%
IB	500/1929	NR	87%	68%
IIA	215/585	NR	79%	60%
IIB	605/1453	66.0	72%	53%
IIIA	2052/3200	29.3	55%	36%
IIIB	1551/2140	19.0	44%	26%
IIIC	831/986	12.6	24%	13%
IVA	336/484	11.5	23%	10%
IVB	328/398	6.0	10%	0%

Fig. 36.10 Overall survival graph and 2- and 5-year overall survival rates for 8th Edition clinical stages (From Goldstraw P et al.[5])

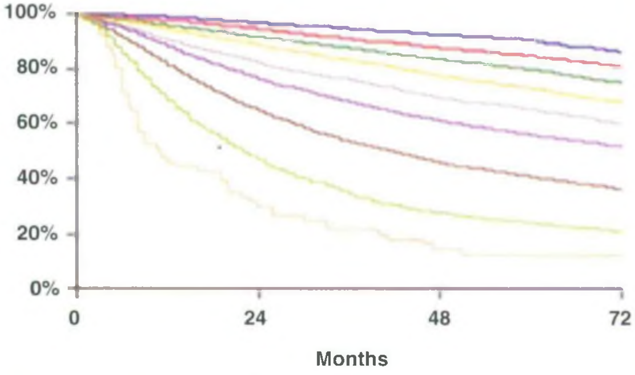

	Events/N	MST	24 months	60 months
IA1	139/1389	NR	97%	90%
IA2	823/5633	NR	94%	85%
IA3	875/4401	NR	92%	80%
IB	[illegible]	[illegible]	[illegible]	[illegible]
IIA	556/1638	NR	82%	65%
IIB	2175/5226	NR	76%	56%
IIIA	3219/5756	41.9	65%	41%
IIIB	1215/1729	22.0	47%	24%
IIIC	55/69	11.0	30%	12%

Fig. 36.11 Overall survival graph and 2- and 5-year overall survival rates for 8th Edition pathological stages (From Goldstraw P et al.[5])

ILLUSTRATIONS

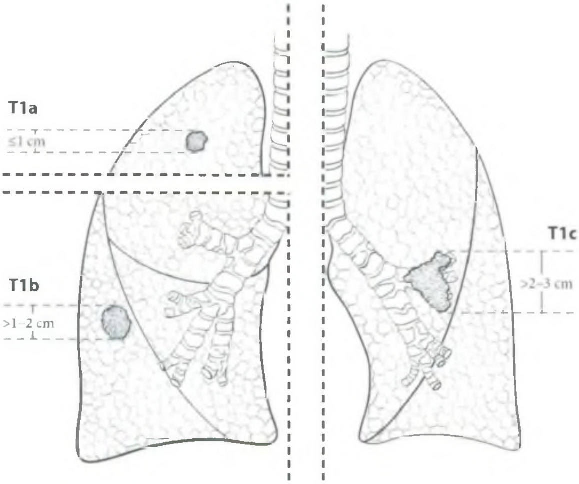

Fig. 36.12 T1 is defined as a tumor ≤3 cm in greatest dimension, surrounded by lung or visceral pleura, without bronchoscopic evidence of invasion more proximal than the lobar bronchus (i.e., not in the main bronchus). T1a is defined as tumor ≤1 cm in greatest dimension. A superficial, spreading tumor of any size whose invasive component is limited to the bronchial wall and may extend proximal to the main bronchus also is classified as T1a, but these tumors are uncommon. T1b is defined as tumor >1 cm but ≤2 cm in greatest dimension. T1c is defined as tumor >2 cm but ≤3 cm in greatest dimension

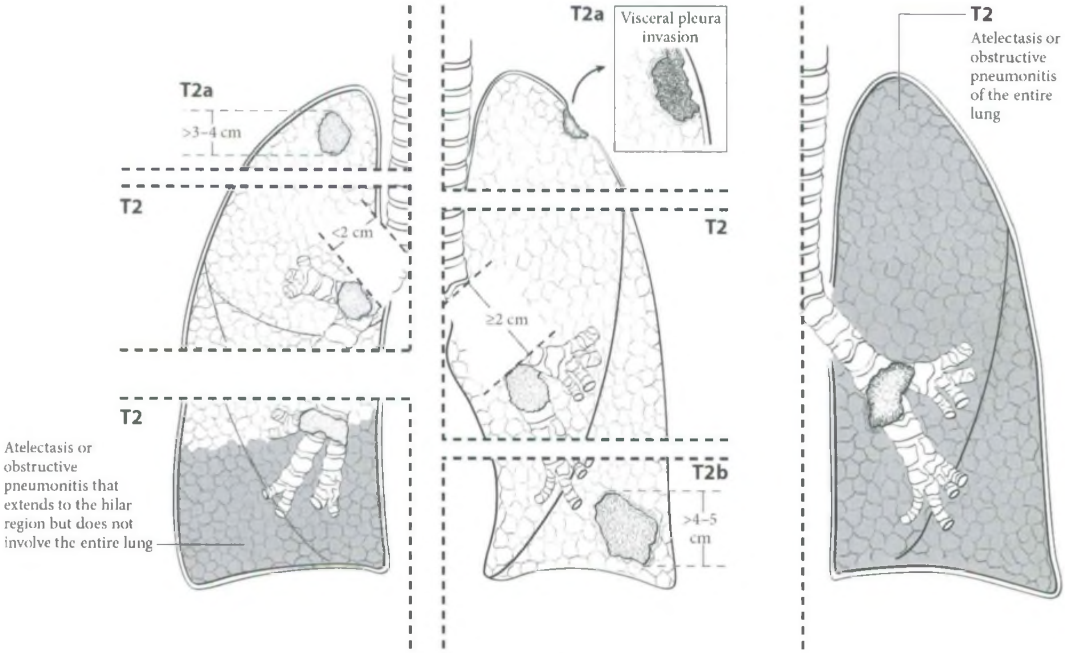

Fig. 36.13 T2 is defined as tumor >3 cm but ≤5 cm or having any of the following features: involves the main bronchus regardless of distance to the carina, but without involvement of the carina; invades visceral pleura (PL1 or PL2); associated with atelectasis or obstructive pneumonitis that extends to the hilar region, involving part or all of the lung. T2 tumors with these features are classified as T2a if ≤4 cm or if the size cannot be determined and T2b if >4 cm but ≤5 cm. T2a is defined as tumor >3 cm but ≤4 cm in greatest dimension. T2b is defined as tumor >4 cm but ≤5 cm in greatest dimension

36

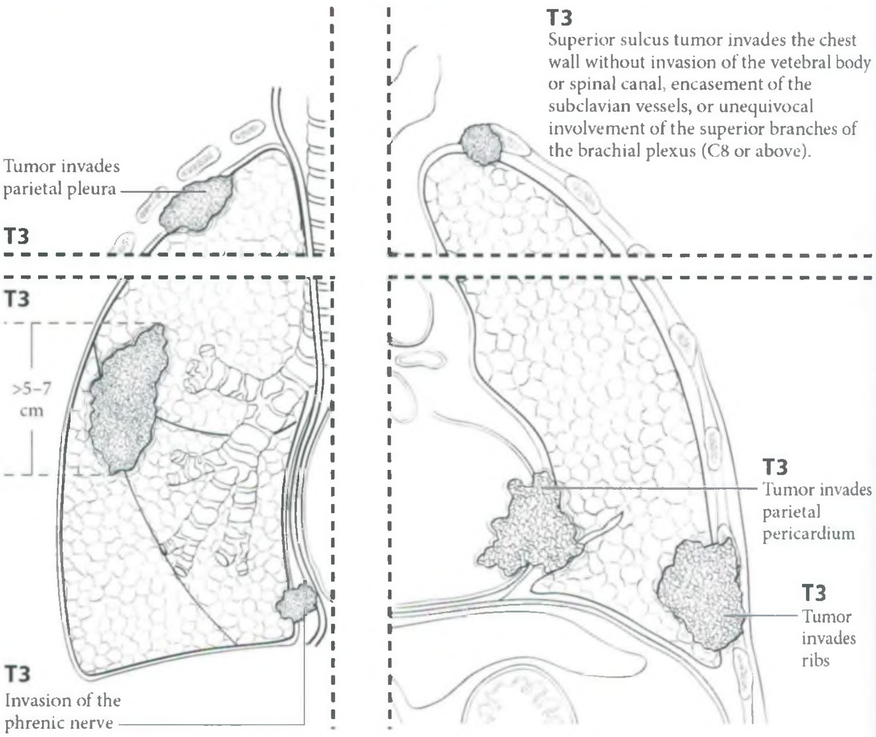

Fig. 36.14 T3 is defined as tumor >5 cm but ≤7 cm in greatest dimension or directly invading any of the following: parietal pleura (PL3), chest wall (including superior sulcus tumors), phrenic nerve, parietal pericardium; or separate tumor nodule(s) in the same lobe as the primary

Fig. 36.15 T4 is defined as tumor >7 cm or tumor of any size invading one or more of the following: diaphragm, mediastinum, heart, great vessels, trachea, recurrent laryngeal nerve, esophagus, vertebral body, or carina; separate tumor nodule(s) in an ipsilateral lobe different from that of the primary

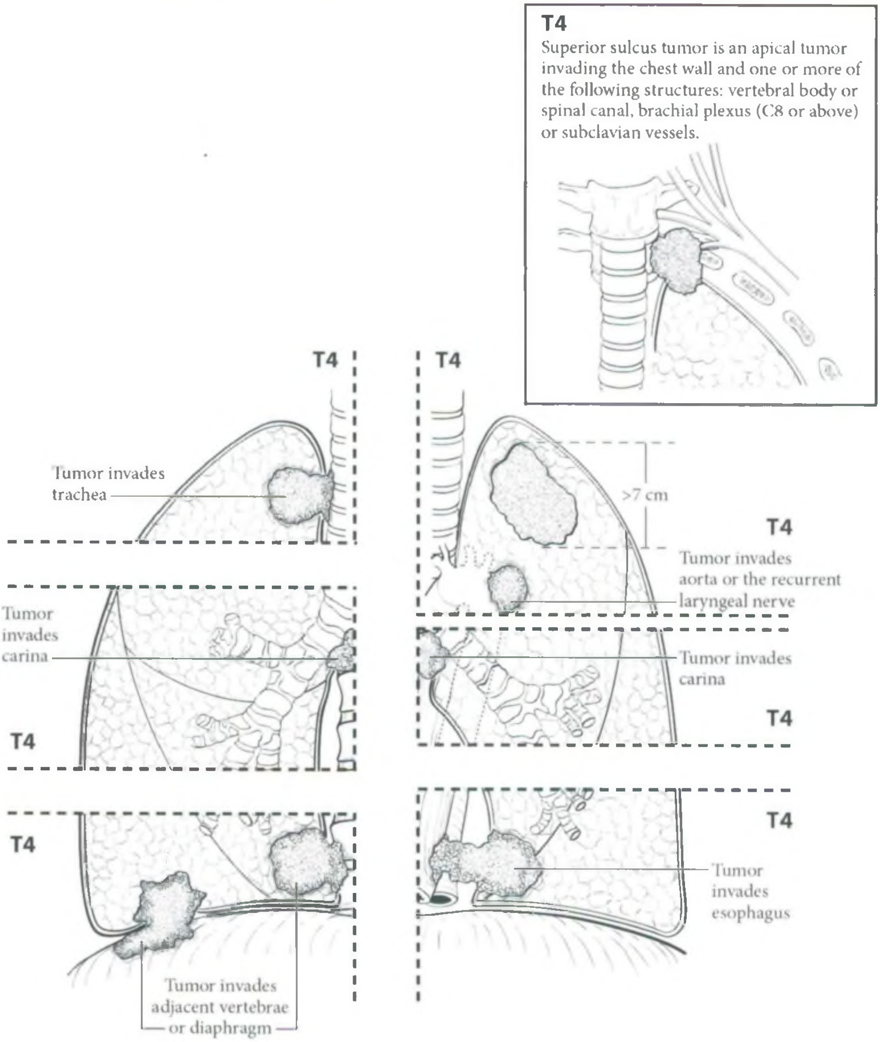

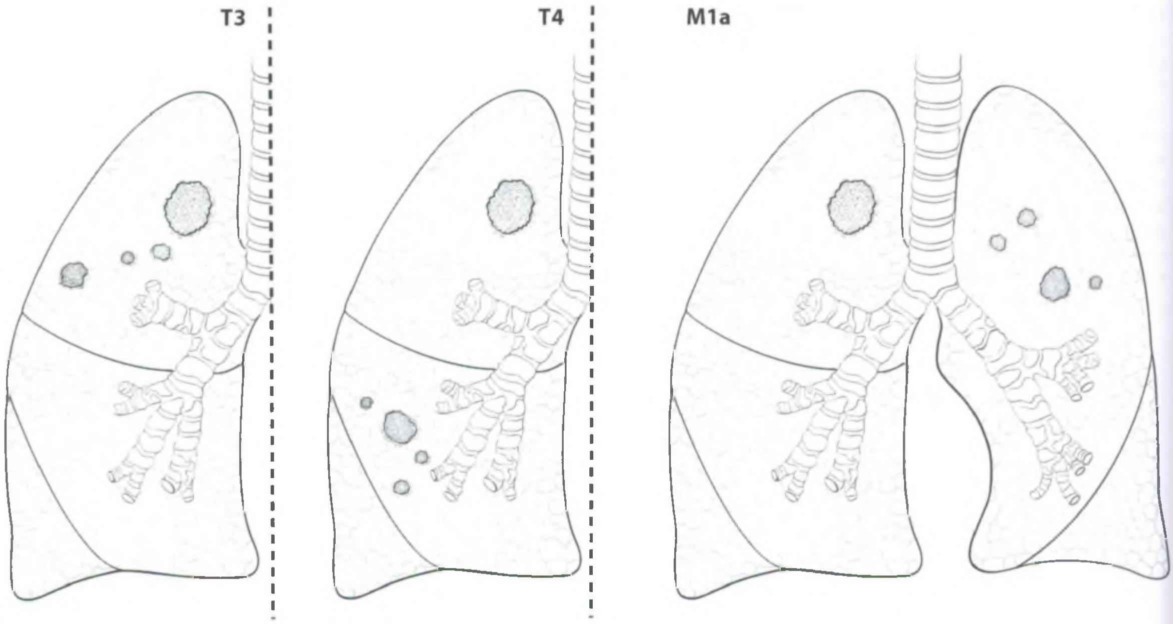

Fig. 36.16 T3 includes separate tumor nodule(s) in the same lobe as the primary. T4 includes separate tumor nodule(s) in an ipsilateral lobe different from that of the primary. M1a includes separate tumor nodule(s) in a contralateral lobe; tumor with pleural or pericardial nodules or malignant pleural or pericardial effusion

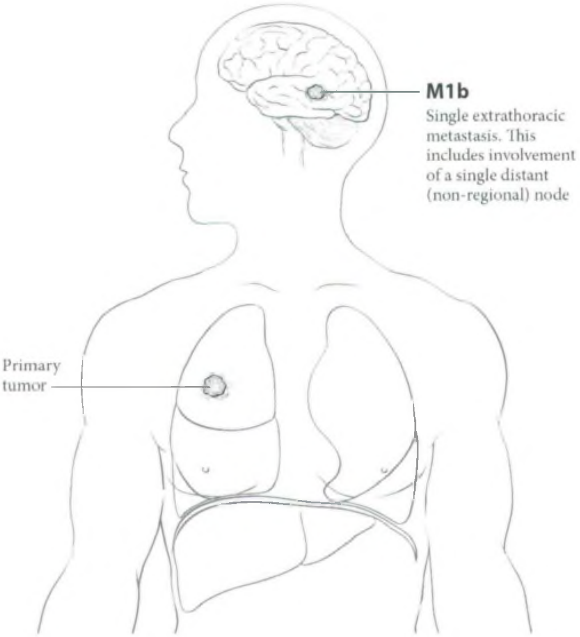

Fig. 36.17 M1b is defined as single extrathoracic metastasis in a single organ (including involvement of a single nonregional node)

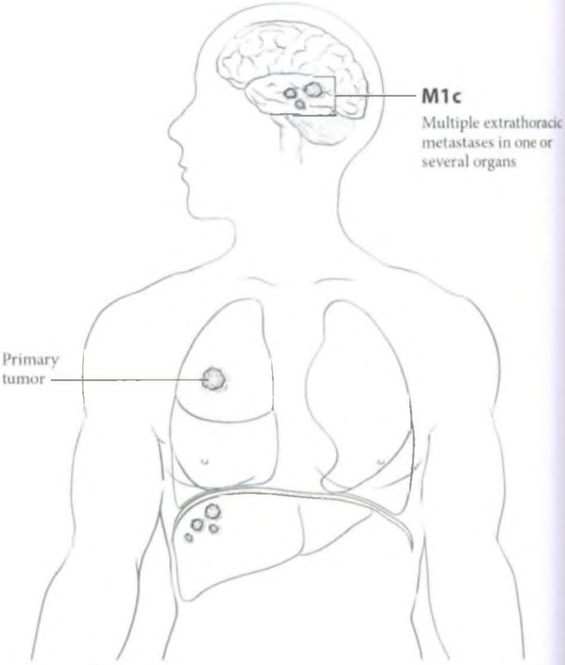

Fig. 36.18 M1c includes multiple extrathoracic metastases in a single organ or in multiple organs

Bibliography

1. Rami-Porta R, Bolejack V, Giroux DJ, et al. The IASLC lung cancer staging project: the new database to inform the eighth edition of the TNM classification of lung cancer. *J Thorac Oncol.* 2014;9(11): 1618-1624.
2. Travis WD, Asamura H, Bankier A, et al. The IASLC lung cancer staging project: proposals for coding T categories for subsolid nodules and assessment of tumor size in part-solid tumors in the forthcoming eighth edition of the TNM classification of lung cancer. *J Thorac Oncol 2015; in press.* 2015.
3. Rami-Porta R, Bolejack V, Crowley J, et al. The IASLC Lung Cancer Staging Project: Proposals for the Revisions of the T Descriptors in the Forthcoming Eighth Edition of the TNM Classification for Lung Cancer. *J Thorac Oncol.* 2015;10(7): 990–1003.
4. Eberhardt WE, Mitchell A, Crowley J, et al. The IASLC Lung Cancer Staging Project: Proposals for the Revision of the M Descriptors in the Forthcoming Eighth Edition of the TNM Classification of Lung Cancer. *J Thorac Oncol.* 2015;10(11):1515–1522.
5. Goldstraw P, Chansky K, Crowley J, et al. The IASLC Lung Cancer Staging Project: Proposals for Revision of the TNM Stage Groupings in the Forthcoming (Eighth) Edition of the TNM Classification for Lung Cancer. *J Thorac Oncol.* 2016;11(1): 39–51.
6. Travis W, Brambilla E, Burke A, Marx A, Nicholson AG. *WHO Classification of Tumours of the Lung, Pleura, Thymus and Heart.* Fourth ed. Lyon: IARC; 2015.
7. Travis WD, Brambilla E, Nicholson AG, et al. The 2015 World Health Organization Classification of Lung Tumors: Impact of Genetic, Clinical and Radiologic Advances Since the 2004 Classification. *J Thorac Oncol.* 2015;10(9):1243–1260.
8. Globocan 2012. Estimated cancer incidence, mortality and relevance worldwide in 2012. International Agency for Research on Cancer. World Health Organization. 2015.
9. Asamura H, Chansky K, Crowley J, et al. The International Association for the Study of Lung Cancer Lung Cancer Staging Project: Proposals for the Revision of the N Descriptors in the Forthcoming 8th Edition of the TNM Classification for Lung Cancer. *J Thorac Oncol.* 2015;10(12):1675–1684.
10. Nicholson AG, Chansky K, Crowley J, et al. The IASLC lung cancer staging project: proposals for the revision of the clinical and pathologic staging of small cell lung cancer in the forthcoming eighth edition of the TNM classification for lung cancer. *J Thorac Oncol 2015; in press.* 2015.
11. Shepherd FA, Crowley J, Van Houtte P, et al. The International Association for the Study of Lung Cancer lung cancer staging project: proposals regarding the clinical staging of small cell lung cancer in the forthcoming (seventh) edition of the tumor, node, metastasis classification for lung cancer. *J Thorac Oncol.* 2007;2(12): 1067–1077.
12. Vallieres E, Shepherd FA, Crowley J, et al. The IASLC Lung Cancer Staging Project: proposals regarding the relevance of TNM in the pathologic staging of small cell lung cancer in the forthcoming (seventh) edition of the TNM classification for lung cancer. *J Thorac Oncol.* 2009;4(9):1049–1059.
13. Rami-Porta R, Asamura H, Goldstraw P. Predicting the prognosis of lung cancer: the evolution of tumor, node and metastasis in the molecular age-challenges and opportunities. *Transl Lung Cancer Res.* 2015;4(4):415–423.
14. Wittekind C, Compton CC, Brierley J, et al. *TNM supplement: a commentary on uniform use.* John Wiley & Sons; 2012.
15. Rusch VW, Asamura H, Watanabe H, et al. The IASLC lung cancer staging project: a proposal for a new international lymph node map in the forthcoming seventh edition of the TNM classification for lung cancer. *J Thorac Oncol.* 2009;4(5):568–577.
16. Detterbeck FC, Nicholson AG, Franklin WA, et al. The IASLC Lung Cancer Staging Project: Summary of Proposals for Revisions of the Classification of Lung Cancers with Multiple Pulmonary Sites of Involvement in the Forthcoming Eighth Edition of the TNM Classification. *J Thorac Oncol.* 2016;11:539–650.
17. Detterbeck FC, Bolejack V, Arenberg DA, et al. The IASLC Lung Cancer Staging Project: Background Data and Proposals for the Classification of Lung Cancer with Separate Tumor Nodules in the Forthcoming Eighth Edition of the TNM Classification for Lung Cancer. *J Thorac Oncol.* 2016;11:681–692.
18. Detterbeck FC, Franklin WA, Nicholson AG, et al. The IASLC Lung Cancer Staging Project: Background Data and Proposed Criteria to Distinguish Separate Primary Lung Cancers from Metastatic Foci in Patients with Two Lung Tumors in the Forthcoming Eighth Edition of the TNM Classification for Lung Cancer. *J Thorac Oncol.* 2016;11:651–655.
19. Detterbeck FC, Marom EM, Arenberg DA, et al. The IASLC Lung Cancer Staging Project: Background Data and Proposals for the Application of TNM Staging Rules to Lung Cancer Presenting as Multiple Nodules with Ground Glass or Lepidic Features or a Pneumonic-Type of Involvement in the Forthcoming Eighth Edition of the TNM Classification. *J Thorac Oncol.* 2016;11:666–680.
20. Postmus PE, Rocmans P, Asamura H, et al. Consensus report IASLC workshop Bruges, September 2002: pretreatment minimal staging for non-small cell lung cancer. *Lung Cancer.* 2003;42 Suppl 1(2):S3-6.
21. Silvestri GA, Gonzalez AV, Jantz MA, et al. Methods for staging non-small cell lung cancer: Diagnosis and management of lung cancer, 3rd ed: American College of Chest Physicians evidence-based clinical practice guidelines. *Chest.* 2013;143(5 Suppl):e211S–250S.
22. Paesmans M, Garcia C, Wong CY, et al. Primary tumour standardised uptake value is prognostic in nonsmall cell lung cancer: a multivariate pooled analysis of individual data. *Eur Respir J.* 2015;46(6):1751–1761.
23. Davis PC, Hudgins PA, Peterman SB, Hoffman JC, Jr. Diagnosis of cerebral metastases: double-dose delayed CT vs contrast-enhanced MR imaging. *AJNR. American journal of neuroradiology.* 1991;12(2):293–300.
24. Edge SB, Byrd DR, Compton CC, et al. *The AJCC Cancer Staging Manual.* 7th ed: Springer; 2010.
25. Sobin LH, Gospodarowicz MK, Wittekind C. *TNM classification of malignant tumours.* John Wiley & Sons; 2010.
26. Rivera MP, Mehta AC, Wahidi MM. Establishing the diagnosis of lung cancer: Diagnosis and management of lung cancer, 3rd ed: American College of Chest Physicians evidence-based clinical practice guidelines. *Chest.* 2013;143(5 Suppl):e142S–165S.
27. De Leyn P, Dooms C, Kuzdzal J, et al. Revised ESTS guidelines for preoperative mediastinal lymph node staging for non-small-cell lung cancer. *Eur J Cardiothorac Surg.* 2014;45(5):787–798.
28. Dooms C, Tournoy KG, Schuurbiers O, et al. Endosonography for mediastinal nodal staging of clinical N1 non-small cell lung cancer: a prospective multicenter study. *Chest.* 2015;147(1):209–215.
29. Travis WD, Brambilla E, Rami-Porta R, et al. Visceral pleural invasion: pathologic criteria and use of elastic stains: proposal for the 7th edition of the TNM classification for lung cancer. *J Thorac Oncol.* 2008;3(12):1384–1390.
30. Goldstraw P. Report on the international workshop on intrathoracic staging. London, October 1996. *Lung Cancer.* 1997;18(1):107–111.
31. Rami-Porta R, Wittekind C, Goldstraw P. International Association for the Study of Lung Cancer Staging C. Complete resection in lung cancer surgery: proposed definition. *Lung Cancer.* 2005;49(1): 25–33.

32. Detterbeck FC, Postmus PE, Tanoue LT. The stage classification of lung cancer: Diagnosis and management of lung cancer, 3rd ed: American College of Chest Physicians evidence-based clinical practice guidelines. *Chest*. 2013;143(5 Suppl):e191S–210S.
33. Osarogiagbon RU, Allen JW, Farooq A, Berry A, Spencer D, O'Brien T. Outcome of surgical resection for pathologic N0 and Nx non-small cell lung cancer. *J Thorac Oncol*. 2010;5(2):191–196.
34. Rami-Porta R, Lopez-Encuentra A, Duque-Medina JL. Caution! The latest AJCC's rules for lung cancer classification differ from the latest UICC's. *Lung Cancer*. 2004;43(3):361–362.
35. Lopez-Encuentra A, Duque-Medina JL, Rami-Porta R. Persistent confusion on the clinical and pathologic nodal staging in lung cancer. *J Thorac Oncol*. 2010;5(2):285-286; discussion 286–287.
36. Lim E, Clough R, Goldstraw P, et al. Impact of positive pleural lavage cytology on survival in patients having lung resection for non-small-cell lung cancer: An international individual patient data meta-analysis. *J Thorac Cardiovasc Surg*. 2010;139(6):1441–1446.
37. Kameyama K, Okumura N, Miyaoka E, et al. Prognostic value of intraoperative pleural lavage cytology for non-small cell lung cancer: the influence of positive pleural lavage cytology results on T classification. *J Thorac Cardiovasc Surg*. 2014;148(6):2659–2664.
38. Tsutani Y, Miyata Y, Nakayama H, et al. Prognostic significance of using solid versus whole tumor size on high-resolution computed tomography for predicting pathologic malignant grade of tumors in clinical stage IA lung adenocarcinoma: a multicenter study. *J Thorac Cardiovasc Surg*. 2012;143(3):607–612.
39. Yoshizawa A, Motoi N, Riely GJ, et al. Impact of proposed IASLC/ATS/ERS classification of lung adenocarcinoma: prognostic subgroups and implications for further revision of staging based on analysis of 514 stage I cases. *Mod Pathol*. 2011;24(5):653–664.
40. Maeyashiki T, Suzuki K, Hattori A, Matsunaga T, Takamochi K, Oh S. The size of consolidation on thin-section computed tomography is a better predictor of survival than the maximum tumour dimension in resectable lung cancer. *Eur J Cardiothorac Surg*. 2013;43(5):915–918.
41. Sculier JP, Chansky K, Crowley JJ, et al. The impact of additional prognostic factors on survival and their relationship with the anatomical extent of disease expressed by the 6th Edition of the TNM Classification of Malignant Tumors and the proposals for the 7th Edition. *J Thorac Oncol*. 2008;3(5):457–466.
42. Chansky K, Sculier JP, Crowley JJ, et al. The International Association for the Study of Lung Cancer Staging Project: prognostic factors and pathologic TNM stage in surgically managed non-small cell lung cancer. *J Thorac Oncol*. 2009;4(7): 792–801.
43. Brundage MD MW. Lung cancer. In: Gospodarowicz MK, O'Sullivan B, Sobin LH, ed. *International Union Against Cancer Prognostic Factors in Cancer, Third Edition*. Hoboken, NJ: Wiley-Liss; 2006.
44. Mahar AL, Compton C, McShane LM, et al. Refining Prognosis in Lung Cancer: A Report on the Quality and Relevance of Clinical Prognostic Tools. *J Thorac Oncol*. 2015;10(11):1576–1589.
45. Kattan MW, Hess KR, Amin MB, et al. American Joint Committee on Cancer acceptance criteria for inclusion of risk models for individualized prognosis in the practice of precision medicine. *CA Cancer J Clin*. 2016. doi: 10.3322/caac.21339.

Malignant Pleural Mesothelioma

37

Valerie W. Rusch, Kari Chansky, Anna K. Nowak, David Rice, Hedy Kindler, Ritu R. Gill, William D. Travis, and Harvey Pass

CHAPTER SUMMARY

Cancers Staged Using This Staging System

This classification applies to diffuse malignant pleural mesotheliomas.

Cancers Not Staged Using This Staging System

These histopathologic types of cancer...	Are staged according to the classification for...	And can be found in chapter...
Localized malignant pleural mesotheliomas	No AJCC staging system	N/A
Other primary tumors of the pleura	No AJCC staging system	N/A

Summary of Changes

Change	Details of Change	Level of Evidence
Definition of Primary Tumor (T)	T1a was consolidated with T1b into T1.	II
Definition of Primary Tumor (T)	T1b was consolidated with T1a into T1.	II
Definition of Regional Lymph Node (N)	N1 was consolidated with N2 into N1.	II
Definition of Regional Lymph Node (N)	N2 was consolidated with N1 into N1.	II
Definition of Regional Lymph Node (N)	N3 was relabeled as N2.	II
Definition of Regional Lymph Node (N)	Intercostal lymph nodes were added to N1 category.	II
AJCC Prognostic Stage Groups	Stage IA now includes T1N0M0.	II
AJCC Prognostic Stage Groups	Stage IB now includes T2N0M0 and T3N0M0.	II
AJCC Prognostic Stage Groups	Stage II now includes T1N1M0 and T2N1M0.	II
AJCC Prognostic Stage Groups	Stage IIIA now includes T3N1M0.	II
AJCC Prognostic Stage Groups	Stage IIIB now includes T1–3N2 and T4 Any N.	II
AJCC Prognostic Stage Groups	Stage IV now includes Any M1.	II

To access the AJCC cancer staging forms, please visit www.cancerstaging.org.

© American Joint Committee on Cancer 2017

M.B. Amin et al. (eds.), *AJCC Cancer Staging Manual, Eighth Edition*, DOI 10.1007/978-3-319-40618-3_37

ICD-0-3 Topography Codes

Code	Description
C38.4	Pleura, NOS

WHO Classification of Tumors

Code	Description
9050	Mesothelioma, malignant
9051	Mesothelioma, sarcomatoid, malignant
9051	Mesothelioma, desmoplastic, malignant
9052	Mesothelioma, epithelioid, malignant
9053	Mesothelioma, biphasic, malignant

Travis WD, Brambilla E, Burke AP, Marx A, Nicholson AG, eds. World Health Organization Classification of Tumours of the Lung, Pleura, Thymus and Heart. Lyon: IARC; 2015.

INTRODUCTION

The changes introduced in the AJCC Cancer Staging Manual, 8th Edition of the TNM classification for malignant pleural mesothelioma (MPM) derive primarily from analyses of the International Association for the Study of Lung Cancer (IASLC) retrospective and prospective databases. These databases contain data on patients diagnosed as early as 1995, but predominantly between 2000 and 2013, originating from 29 centers around the world spanning four continents.

Background

Malignant mesotheliomas are rare and usually fatal malignancies that arise from the mesothelium lining the pleural, pericardial, and peritoneal cavities. They represent fewer than 2% of all cancers. The most common risk factor for MPM is previous exposure to asbestos. The latency period between asbestos exposure and the development of MPM is 20 years or more. Little was known about the pathophysiology and natural history of MPM until the 1960s, when epidemiologic studies of South African mine workers by Dr. Christopher Wagner[1] established the link between asbestos exposure and subsequent development of MPM. Because of the rarity of this disease and widespread nihilism regarding treatment, MPM was poorly studied for many years. From the 1970s to the 1990s, at least five staging systems were proposed for MPM,[2-5] most of which were not TNM based and none of which was generally accepted.

In 1994, at a workshop sponsored by the IASLC and the International Mesothelioma Interest Group (IMIG), MPM investigators analyzed existing surgical databases to develop a TNM-based staging system.[6] Subsequently, this proposed staging system was accepted by the AJCC and the Union for International Cancer Control (UICC) as the international MPM staging system for the 6th and 7th editions of their staging manuals and became widely used in retrospective analyses and prospective clinical trials. However, concerns existed about its broad applicability because it was derived mainly from retrospective analyses of small, single-institution surgical series. It has been difficult to apply to clinical staging and has empirically used the N categories developed for lung cancer, which may not be relevant to MPM. Multiple studies suggest that it would appropriate to consider revisions to this staging system,[7-9] but these investigations were limited by being single institution and, for the most part, by including small numbers of patients.

After the successful development of a large international database for lung cancer that informed revisions of the lung cancer staging system for the 7th editions of the AJCC and UICC staging manuals, the IASLC, in collaboration with members of the IMIG, undertook development of a similar effort in MPM. As for the lung cancer effort, biostatistical support was provided by Cancer Research and Biostatistics (CRAB) in Seattle, Washington, USA. An international database (hereafter referred to as the IASLC MPM database), the largest to date, was developed, using data on 3,101 patients submitted by 15 centers from around the world. Analyses, published in 2012,[10] identified components of the staging system that would benefit from revision. Additional analyses provided several models of supplementary prognostic variables.[11] However, this initial iteration of the IASLC database did not include sufficiently granular information about T, N, and M categories to support evidence-based revisions of the staging system. Therefore, a second MPM database was developed. Data elements of the IASLC MPM database were revised extensively to acquire more detailed TNM information, and an electronic data capture (EDC) system was established to facilitate data submission. An effort also was made to broaden the patient population by recruiting data from studies of patients managed nonsurgically.

Characteristics of the Second IASLC MPM Database

The second IASLC MPM database includes data from 3,519 MPM cases, 2,460 of which were considered eligible for analysis after data review. This provides the largest international source of data to support revisions of the MPM staging system. Data were submitted from 29 centers around the world spanning four continents. Cases diagnosed as early as 1995 were included, provided they met data quality standards, but most were diagnosed between 2000 and 2013. Cases diagnosed after June 30, 2013, were excluded, and analyses were undertaken at the end of 2014, allowing a minimum potential follow-up of 18 months. Pretreatment (clinical) staging information was available in 827 patients, postsurgical staging data

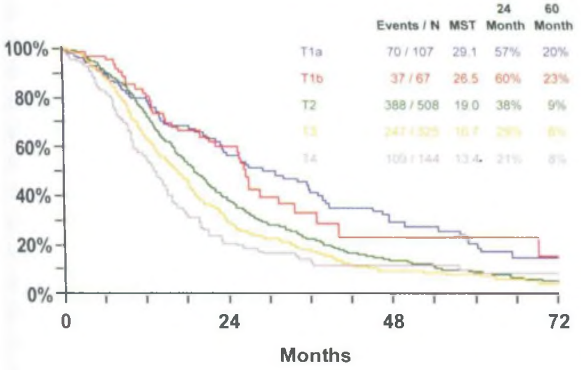

Fig. 37.1 Overall survival according to clinical T-category. AJCC 7th edition. Included cases: Clinically staged, M0 with any N category. Cases without adequate descriptors to distinguish T1a and T1b are excluded. Statistical method: Kaplan-Meier

Table 37.1 Overall survival comparisons between adjacent stage categories. Method: Cox proportional hazards regression. All comparisons are adjusted for sex and geographical region

Comparison	HR	P-Value
Clinical T-category, 7th Edition		
T1b vs. T1a	0.99	0.95
T2 vs. T1b	1.50	0.018
T3 vs. T2	1.23	0.013
T4 vs. T3	1.22	0.089
Pathological T-category, 7th Edition		
T1b vs. T1a	1.16	0.27
T2 vs. T1b	1.08	0.50
T3 vs. T2	1.01	0.87
T4 vs. T3	1.34	0.0005
Pathological N-category, 7th Edition		
N1 vs. N0	1.51	0.0063
N2 vs. N1	1.03	0.8398

in 830 patients, and both types of information in 803 cases. As in the first iteration of the database, as well as in most published series, most patients had locally advanced disease (Stage III) at diagnosis, which also is reflected in the distribution of T, N, and M components. Almost half the patients for whom clinical staging data were available either did not undergo surgery or had only an exploration without resection. Thus, relative to the initial IASLC database, the second one includes a broader patient population.

Statistical Methods

The prognostic capabilities of T and N categories according to the current system were evaluated by using Kaplan–Meier survival curves and Cox proportional hazards regression analysis with and without adjustment for type and extent of surgical management and other baseline factors. Individual T descriptors also were evaluated by Kaplan–Meier survival analysis to determine whether any anatomic factors warranted placement in a different T category based on survival prognosis in clinical and/or pathological staging. Alternative N category schemes, based on a combination of location and number of positive nodal stations, were explored using Kaplan–Meier survival analysis and Cox proportional hazards regression. M1 cases were analyzed in an exploratory fashion only because of the small number of cases with distant metastatic sites. Cases were reclassified with respect to proposed revisions to T and N categories, and a recursive partitioning and amalgamation (RPA) was applied to the reclassified cases. The RPA algorithm generates a tree-based model for the survival data using log-rank test statistics for recursive partitioning and, for selection of the important groupings, bootstrap resampling to correct for the adaptive nature of the splitting algorithm. Candidate TNM stage grouping schemes then were evaluated by assessing overall survival (OS) in clinical, pathological, and "best" stage, defined as the stage that combines clinical and pathological data. The resultant proposal for TNM stage groupings was developed based both on clinical practice and on guidance from the results of the RPA and survival analyses. Cox regression analyses were performed using the PHREG procedure in the SAS 9.4 system for Windows. RPA analyses were performed using the statistical package R, version 3.1.0.

T-Category Analyses

Current clinical (cT) and pathological (pT) T categories were analyzed in relationship to OS. By clinical staging, there was a significant difference between T1b and T2 and between T2 and T3 (Fig. 37.1 and Table 37.1). By pathological staging, there was a significant difference between T3 and T4 (Fig. 37.2 and Table 37.1). No significant difference in OS could be identified for T1a versus T1b in either clinical or pathological staging, suggesting that it is appropriate to collapse T1a and T1b into a single T1 category. To some extent, the results of the T-category analyses reflect the known inaccuracies in pretreatment staging with current clinical staging modalities and the discrepancies between clinical and pathological staging in MPM that were observed in analyses of the first IASLC MPM database.[10]

The descriptors within each of the T categories (e.g., confluent visceral pleural tumor, invasion of diaphragmatic muscle for T2, invasion of endothoracic fascia or mediastinal fat for T3) in the 7th editions of the staging manuals were examined in multiple analyses to determine whether any of these should be shifted within T categories or eliminated. No significant differences were found, suggesting that the current T categories should be maintained.

Previous publications[12-16] suggested that measurements of pleural tumor thickness and/or tumor volume may provide more accurate T categorization than the current anatomic descriptors. For this reason, measurements of pleural thickness and a qualitative description (minimal, nodular, or rind-like) of pleural appearance on computed tomography (CT) were included as data elements in the second IASLC database. Exploratory analyses were performed on the 460 cases for which such data were available, primarily from cases submitted via EDC. Both the descriptive categories and the analyses of pleural thickness were found to correlate significantly with OS. A breakpoint of 5 mm of maximal pleural thickness or 13 mm for the sum of pleural thickness measured at the upper, mid, and lower regions was found to be prognostic. Increasing pleural thickness also correlated with the presence of nodal metastases. These findings are hypothesis generating. They suggest the need for future studies to determine whether measurements of pleural thickness, which can be reproducibly performed on CT in hospitals worldwide, should replace or supplement the current anatomic descriptors for T categorization in the 9th edition of the international MPM staging system.

N-Category Analyses

Because no data were available defining the relationship between lymph node involvement and OS when the first international MPM staging system was developed in 1994, the nodal staging system and lymph node map for lung cancer were simply adopted as a matter of convenience. However, the pleura appears to have an anatomic pattern of lymphatic drainage different from that of the lung. Thus, early involvement of lymph nodes in mediastinal locations is common in MPM, and some of these locations are not seen in lung cancer, including the internal mammary, peridiaphragmatic, pericardial fat pad, and posterior intercostal lymph nodes.[16,17] Some single-institution retrospective studies suggested that OS is influenced by mediastinal nodal location (inferior vs. superior mediastinum),[7] whereas a greater number of studies reported that OS is influenced by the number of involved lymph node stations. Most studies have not shown a difference in OS for N1 versus N2 involvement.[16-22] Analyses of the first IASLC database found that OS was influenced predominantly by the presence or absence of lymph node metastases rather than by N1 versus N2 nodal involvement. Data on the number of involved lymph node stations was available in only 181 patients, and in this small cohort, no difference in OS was seen in relation to the number of involved nodes.[10]

To determine whether the N categorization should be altered for the 8th Edition of the MPM staging system, data elements for the second IASLC MPM database included detailed information about lymph node involvement according to anatomic location (including the additional mediastinal sites noted earlier) and the number of involved lymph node stations. Analyses of lymph node involvement according to lymph node stations, N1 versus N2, and the number of involved lymph node stations show a significant difference in OS only for N0 versus ipsilateral intrathoracic N(+) (Fig. 37.3). The data regarding "N3," that is, contralateral, mediastinal, or supraclavicular nodal metastases, are too sparse to analyze these reliably in relationship to OS, but such lymph nodes fall outside the usual areas for which surgery and/or radiation might be considered. This suggests that the current N categories should be collapsed into N0, N1 (any ipsilateral intrathoracic lymph node involvement irrespective of location or number of involved nodal stations), and N2 (contralateral hilar or mediastinal, or supraclavicular). Although this nodal categorization may not seem intuitive for physicians used to managing lung cancer, it is supported by most of the published data and by the analyses of both the first and second IASLC MPM databases.

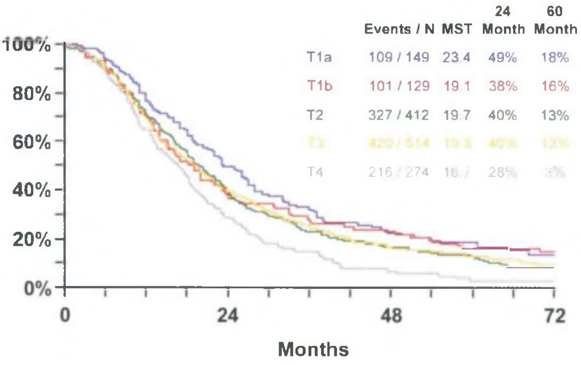

Fig. 37.2 Overall survival according to pathological T-category, AJCC 7th edition. Included cases: Pathologically staged, M0, with any N category. Cases without adequate descriptors to distinguish T1a and T1b are exlcluded. Statistical method: Kaplan-Meier

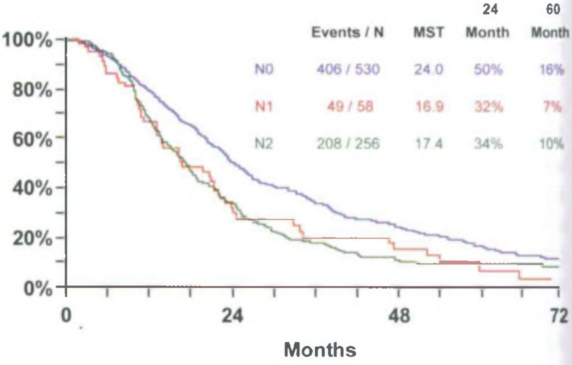

Fig. 37.3 Overall survival according to pathological N-category, AJCC 7th edition. Included cases: Pathologically staged, M0, any T category. Included cases have station-specific data necessary to confirm the N-category designation. Statistical method: Kaplan-Meier

M Categories

The current MPM staging system uses the traditional categorization of M0 (no evidence of metastatic disease) versus M1 (any evidence of metastases). The issue of subcategorizing M1 in MPM has not been well examined in the published literature, likely because most patients present with symptomatic disease localized to the ipsilateral hemithorax. The current IASLC MPM database includes data on 89 patients who had M1 disease before treatment. Consistent with previous reports, distant lymph nodes, intra-abdominal disease, and contralateral lung were the most common sites of metastatic disease.[16,23,24] Analyses of OS support the continued separation of M0 versus M1 (Fig. 37.4). The numbers of patients presenting with a solitary metastasis versus multiple metastases in a single site or in multiple sites are too small to permit valid comparisons of these categories relative to OS. These potential differences need to be examined in larger numbers of patients in the future, especially with respect to an apparently better median OS in patients with a single metastasis in a single site.

Stage Groupings

Stage groupings arranged according to the AJCC Cancer Staging Manual, 7th Edition of the MPM staging system were analyzed in relationship to OS and did not show good monotonic separation of survival curves for either clinical or pathological staging. Using the proposed new T (amalgamation of T1a and T1b, Fig. 37.5) and N (amalgamation of previous N1 and N2 into new N1, Fig. 37.6) categories shows a better distribution of OS curves for clinical, pathological, and best staging. As noted earlier, RPA was used to develop "trees" to develop new stage groupings. Accordingly, T1N0 now forms Stage IA, whereas T2–3N0 constitutes Stage IB. T1–2N1 forms Stage II, whereas T3N1 is Stage IIIA. Any T4 or N2 becomes Stage IIIB, and any M1 remains Stage IV (Fig. 37.7).

Tumor histology is reported to be the single most important prognostic factor in MPM in virtually all published literature on this topic.[10,25] The difference in OS between epithelioid and nonepithelioid (mixed/biphasic or sarcomatoid) histologic subtypes is analogous to the difference between non–small cell and small cell lung cancers. The proposed new stage groupings were examined for epithelioid versus nonepithelioid MPM and for clinical, pathological, and best staging within those two histologic subtypes. Although the separation of the survival curves is not ideally monotonic across all these analyses, the proposed stage groupings clearly show a steady decrement in OS with increasing tumor stage (Fig. 37.8). Thus, the proposed stage groupings appear to be applicable to all MPM histologic subtypes.

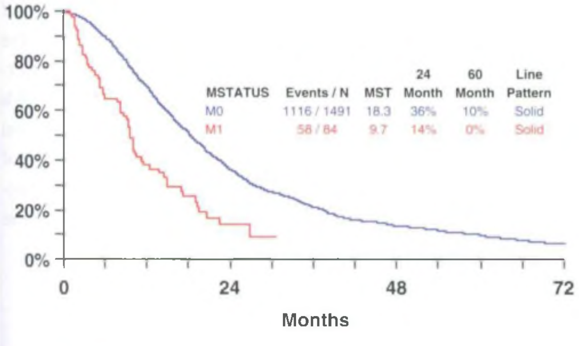

Fig. 37.4 Overall survival according to clinical M-category, AJCC 7th edition. Included: Cases with a clinical stage. Statistical method: Kaplan-Meier

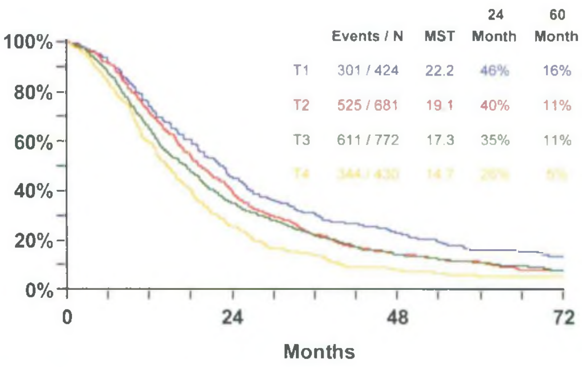

Fig. 37.5 Overall survival according to "best stage" T-category, AJCC 8th edition. Included cases: M0 with any N category. Best stage is defined as pathological stage where available, clinical stage otherwise. Cases without adequate descriptors to distinguish T1a and T1b are excluded. Statistical method: Kaplan-Meier

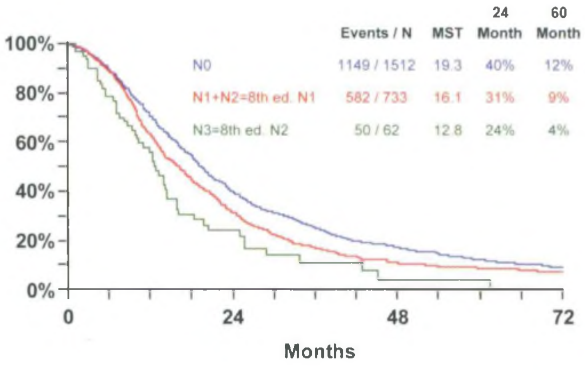

Fig. 37.6 Overall survival according to "best stage" N-category, AJCC 8th edition. Included cases: M0 with any T category. Best stage is defined as pathological stage where available, clinical stage otherwise. The "N1N2" group consists of 7th edition N1 and N2 cases, and will be classified as the new "N1." The "N3" group will be classified as "N2." Statistical method: Kaplan-Meier

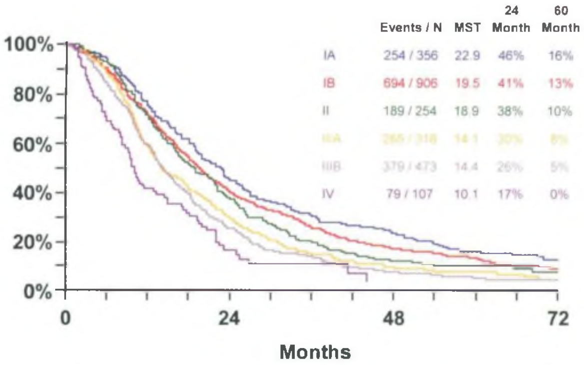

Fig. 37.7 Overall survival according to "best" overall stage, AJCC 8th edition. Included cases: All classifiable cases. Best stage is defined as pathological stage where available, clinical stage otherwise. Statistical method: Kaplan-Meier

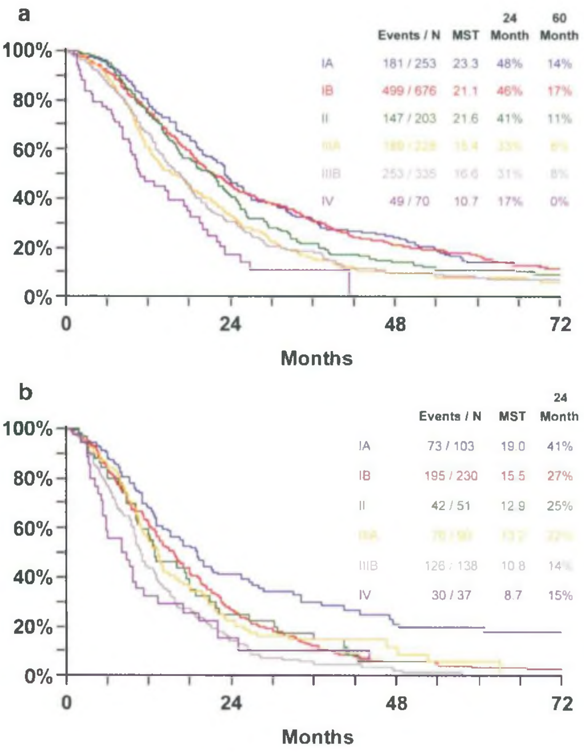

Fig. 37.8 Overall survival according to "best" overall stage, AJCC 8th edition, for a) cases with epithelioid histology, and b) cases with non-epithelioid histology. Included cases: All classifiable cases. Best stage is defined as pathological stage where available, clinical stage otherwise. Statistical method: Kaplan-Meier

Strengths and Limitations of the Proposed Changes to the International MPM Staging System

The principal strength of the proposed changes is that they are derived from the largest available international database in MPM, with data submitted from centers of excellence around the world and analyzed by a biostatistical center highly experienced in analyzing large datasets of this type. Thus, for the purposes of establishing changes for the 8th Edition of the staging system, the IASLC database represents the best resource. An attempt to obtain external validation of the proposed changes through analysis of the Surveillance, Epidemiology, and End Results (SEER) database yielded uninterpretable results (data not shown), likely because of the vagaries of diagnosis, staging, and management of this rare cancer outside centers of excellence. An effort to validate the proposed changes through analyses of the National Cancer Data Base (NCDB) housed at the American College of Surgeons is under way.

Although the proposed changes are based on analyses of the best currently available staging data in MPM, many of the results are hypothesis generating for future revisions of the staging system. The issue of whether pleural tumor thickness and/ or tumor volume should replace anatomic descriptors for T categorizing needs to be investigated further. Originally it was hypothesized that analyses of N categories in the IASLC database would allow better subdivision rather than amalgamation of N categories. Many more high-quality surgical and pathological data are needed to determine whether this amalgamation will stand the test of time. The issue of M1 subcategorization has not really been addressed previously but should be in the future as treatment options for this disease continue to improve. Examination of outcomes according to more refined histologic subtyping (e.g., defining what percentage of a tumor is epithelioid versus sarcomatoid) also may be relevant. It is hoped that the proposed new staging system for MPM not only will provide better categorization of TNM stages but will challenge investigators to examine the current limitations of the data in order to propose further changes for the AJCC Cancer Staging Manual, 9th Edition. There also is a growing body of information regarding the tumor biology of MPM. Currently, none of this information is robust enough to be considered in staging or prognostic models for this disease but may become so in the future.

ANATOMY

Primary Site(s)

The mesothelium covers the external surface of the lungs and mediastinal organs, as well as the inside of the chest wall (Fig. 37.9). It usually is composed of flat, tightly connected cells no more than one layer thick.

Regional Lymph Nodes

The regional lymph nodes include the following (Fig. 37.10):

- Intrathoracic (including internal mammary, peridiaphragmatic, pericardial fat pad, and intercostal)
- Scalene
- Supraclavicular

The regional lymph node map and nomenclature for MPM are adapted from those used for lung cancer.[25] See Chapter 36, Lung, for a detailed list of intrathoracic lymph nodes and the anatomic descriptors. In addition, MPM often metastasizes to lymph nodes not involved by lung cancers, including the internal mammary, peridiaphragmatic, pericardial fat pad, and intercostal lymph nodes. These additional lymph node regions are included in the category of intrathoracic lymph nodes, now designated as N1.

Metastatic Sites

MPM can metastasize widely, even to uncommon sites, such as the brain, thyroid, or prostate. However, the most frequent sites of metastatic disease are the contralateral pleura and lung, peritoneum, extrathoracic lymph nodes, bones, and liver.

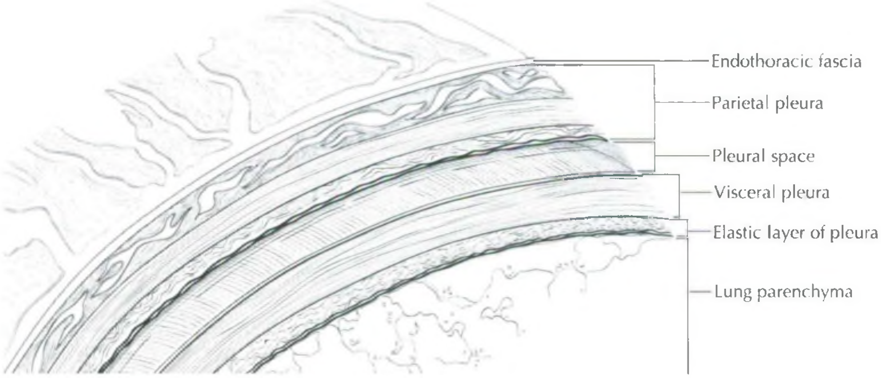

Fig. 37.9 Anatomy of the pleura

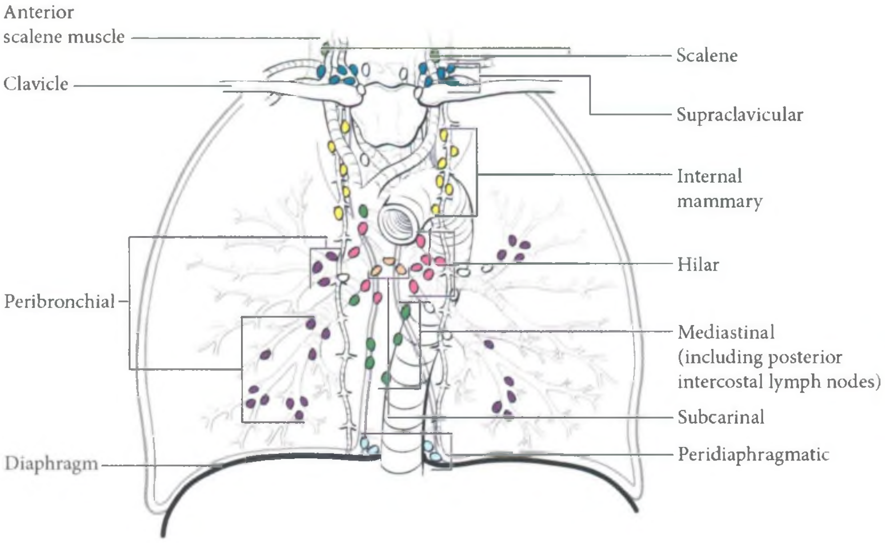

Fig. 37.10 Regional lymph nodes of the pleura

37

RULES FOR CLASSIFICATION

Clinical Classification

This staging system serves both clinical and pathological staging. Clinical staging relies primarily on imaging, most frequently CT and more recently fludeoxyglucose (FDG) positron emission tomography (PET) scanning. Some centers also use magnetic resonance (MR) imaging to assess potential invasion of the chest wall and diaphragm. As noted previously, CT volumetry assessment of tumor or simple tumor thickness measurements on CT hold promise for clinical staging, although these techniques are not yet standard care. Invasive staging procedures, including laparoscopy, extrathoracic lymph node biopsy, endobronchial ultrasound-guided transbronchial needle aspiration (EBUS-TBNA), and mediastinoscopy also are sometimes used to refine preresection staging.

Imaging

CT scan with contrast is the primary imaging modality to assess invasion of adjacent structures by MPM. MR imaging and FDG PET/CT subsequently are performed as part of the preoperative workup and initial staging strategy.[26] The trimodality approach provides complementary information and is useful in determining resectability, which, along with nuclear medicine ventilation and perfusion scans, can then be used to select the type of surgical resection optimal for the patient. Poor concordance between clinical and pathological staging tends at best to result in "ruling in or out" of early versus advanced disease[2] based on preoperative imaging. There currently are no size criteria for assigning T category, and the "T" category is based only on overall extent of invasion of adjacent structures.[26] MR imaging of the chest has proven superior to CT scans alone in identifying transdiaphragmatic extension, mediastinal invasion and multifocal chest wall invasion.[27,28] Early endothoracic fascial involvement also is best assessed by MR imaging. Although diffusion-weighted MR imaging has shown promise in predicting histologic cell type in MPM, no reliable imaging biomarker currently is available to distinguish among the histologic subtypes reliably.[29] Even good pleural biopsies may fail to yield the diagnosis, especially in patients with early tumors. Image-guided pleural biopsies are not done commonly, partly because of the risk of seeding of the needle track and the inability of such small samples to provide adequate tissue for diagnosis.[30]

Imaging tends to underestimate the overall extent of the MPM, leading to understaging both in the resectable categories (T1–T3) and in unresectable cases (T4).[29] If the tumors extend into interlobar fissures or invade the lung parenchyma or diaphragm muscle, they are categorized as T2 lesions. T3 tumors involve invasion of the endothoracic fascia and/or mediastinal fat, but do not extend through the pericardium or chest wall soft tissue along a single focus. Contiguous or direct extension of tumor invading the chest wall diffusely or at more than one foci; invasion of the brachial plexus, bone (rib or spine), mediastinal organs, or contralateral pleura; or invasion through the diaphragm or pericardium is categorized as T4.[30] Nodes greater than 1 cm on short axis in mediastinal and hilar nodal stations are considered abnormal and therefore suspicious for metastasis. The distinction between direct intra-abdominal spread and hematogenous metastasis is sometimes difficult, especially in advanced stages. Metastases to the central nervous system, bone, brain, kidney, adrenal glands, lung, and contralateral pleura have been reported but are more likely to occur in sarcomatoid tumors and advanced epithelioid or biphasic tumors.

Clinical staging for MPM is descriptive without a size criterion. Adoption of structured reporting for imaging may help improve diagnostic reports because of repetition of the same anatomic structures, such as the diaphragm or pericardium, in more than one T criterion with different degrees of invasion. Nodes smaller than 1 cm may harbor metastases and may not have uptake on FDG PET/CT; therefore, false negatives may result. On the other hand, enlarged nodes with uptake on FDG PET/CT may be reactive or inflammatory nodes and not contain metastatic tumor.[31,32] One reason for this is that many MPM patients undergo pleurodesis and other interventions, such as pleural biopsies and chest tube placement, which may cause an inflammatory response with enlarged reactive nodes, thus resulting in false positive determination of clinical N category. Additionally, accurate assessment of nodal size may be difficult because of limited tissue differentiation (insufficient contrast differential) from adjacent pleural tumor in the hilum.

Pathological Classification

Pathological staging is based on surgical resection. The extent of disease before and after resection should be documented carefully. In some cases, complete N categorization may not be possible, especially if technically unresectable tumor (T4) is found during surgical exploration and prevents access to the intrathoracic lymph nodes. Assessment of tumor in relation to the endothoracic fascia is best made by the surgeon intraoperatively, as this is difficult to appreciate histologically by pathologists.

For pN, histologic examination optimally includes lymph nodes from all these intrathoracic regions acquired by systematic dissection or lymph node sampling. Contralateral and supraclavicular nodes may be available if EBUS-TBNA

of nodes, mediastinoscopy, or surgical lymph node biopsy is performed.

PROGNOSTIC FACTORS

Prognostic Factors Required for Stage Grouping

Beyond the factors used to assign T, N, or M categories, no additional prognostic factors are required for stage grouping.

Additional Factors Recommended for Clinical Care

Several factors are reported to have prognostic significance in patients with MPM. These were analyzed most recently in the initial IASLC MPM database, which included 2,141 patients.[11] Three prognostic models were defined depending on the amount of data available. Scenario A, which included a maximal amount of information about clinical and pathological parameters, identified the following prognostic factors: pathological stage, histology, sex, age, type of surgery, adjuvant treatment, and white blood cell (WBC) and platelet count. Scenario B, in which surgical staging information was not available, identified the following as prognostic: clinical stage, histology, sex, age, type of surgery, adjuvant treatment, WBC, hemoglobin, and platelet count. Scenario C, in which only limited clinical data were available, identified the following as prognostic: histology, sex, age, WBC, hemoglobin, and platelet count. The strong prognostic influence of all the factors defined in these three models also is consistent with previously published literature on this topic. Performance status also is a reported prognostic factor, one that was not evident in the IASLC database, because most of the patients were considered for surgical intervention and therefore were more fit than patients who may be seen in daily clinical practice.

RISK ASSESSMENT MODELS

The AJCC recently established guidelines that will be used to evaluate published statistical prediction models for the purpose of granting endorsement for clinical use.[33] Although this is a monumental step toward the goal of precision medicine, this work was published only very recently. Therefore, the existing models that have been published or may be in clinical use have not yet been evaluated for this cancer site by the Precision Medicine Core of the AJCC. In the future, the statistical prediction models for this cancer site will be evaluated, and those that meet all AJCC criteria will be endorsed.

DEFINITIONS OF AJCC TNM

Definition of Primary Tumor (T)

T Category	T Criteria
TX	Primary tumor cannot be assessed
T0	No evidence of primary tumor
T1	Tumor limited to the ipsilateral parietal with or without involvement of<br>• visceral pleura<br>• mediastinal pleura<br>• diaphragmatic pleura
T2	Tumor involving each of the ipsilateral pleural surfaces (parietal, mediastinal, diaphragmatic, and visceral pleura) with at least one of the following features:<br>• involvement of diaphragmatic muscle<br>• extension of tumor from visceral pleura into the underlying pulmonary parenchyma
T3	Describes locally advanced but **potentially resectable** tumor.<br>Tumor involving all the ipsilateral pleural surfaces (parietal, mediastinal, diaphragmatic, and visceral pleura) with at least one of the following features:<br>• involvement of the endothoracic fascia<br>• extension into the mediastinal fat<br>• solitary, completely resectable focus of tumor extending into the soft tissues of the chest wall<br>• nontransmural involvement of the pericardium
T4	Describes locally advanced **technically unresectable** tumor. Tumor involving all the ipsilateral pleural surfaces (parietal, mediastinal, diaphragmatic, and visceral pleura) with at least one of the following features:<br>• diffuse extension or multifocal masses of tumor in the chest wall, with or without associated rib destruction<br>• direct transdiaphragmatic extension of tumor to the peritoneum<br>• direct extension of tumor to the contralateral pleura<br>• direct extension of tumor to mediastinal organs<br>• direct extension of tumor into the spine<br>• tumor extending through to the internal surface of the pericardium with or without a pericardial effusion; or tumor involving the myocardium

Definition of Regional Lymph Node (N)

N Category	N Criteria
NX	Regional lymph nodes cannot be assessed
N0	No regional lymph node metastases
N1	Metastases in the ipsilateral bronchopulmonary, hilar, or mediastinal (including the internal mammary, peridiaphragmatic, pericardial fat pad, or intercostal) lymph nodes
N2	Metastases in the contralateral mediastinal, ipsilateral, or contralateral supraclavicular lymph nodes

Definition of Distant Metastasis (M)

M Category	M Criteria
M0	No distant metastasis
M1	Distant metastasis present

AJCC PROGNOSTIC STAGE GROUPS

When T is...	And N is...	And M is...	Then the stage group is...
T1	N0	M0	IA
T2 or T3	N0	M0	IB
T1	N1	M0	II
T2	N1	M0	II
T3	N1	M0	IIIA
T1–3	N2	M0	IIIB
T4	Any N	M0	IIIB
Any T	Any N	M1	IV

REGISTRY DATA COLLECTION VARIABLES

For data collection, all T, N, and M categories and the prognostic factors listed for clinical trial stratification should be collected.

1. Histologic type
2. Sex
3. Age
4. Performance status
5. Laboratory parameters including WBC, platelet count, and hemoglobin
6. Surgical resection with curative intent (pleurectomy/decortications, extended pleurectomy/decortications or extrapleural pneumonectomy)
7. For patients undergoing multimodality therapy, use of chemotherapy and/or radiotherapy

HISTOLOGIC GRADE (G)

G	G Definition
GX	Grade of differentiation cannot be assessed
G1	Well differentiated
G2	Moderately differentiated
G3	Poorly differentiated
G4	Undifferentiated

HISTOPATHOLOGIC TYPE

There are four types of MPM, listed here in descending order of frequency:

1. Epithelioid
2. Biphasic (at least 10% of both epithelioid and sarcomatoid components)
3. Sarcomat
4. Desmoplastic

Pure epithelioid tumors are associated with a prognosis better than that of the biphasic or sarcomatoid tumors. The pleomorphic subtype of epithelioid MPM is reported to be associated with a survival similar to that of biphasic or sarcomatoid MPM but is still classified under the epithelioid category.[34] Despite their bland histologic appearance, desmoplastic MPMs appear to have the worst prognosis. The biology underlying these differences is not yet understood.

ILLUSTRATIONS

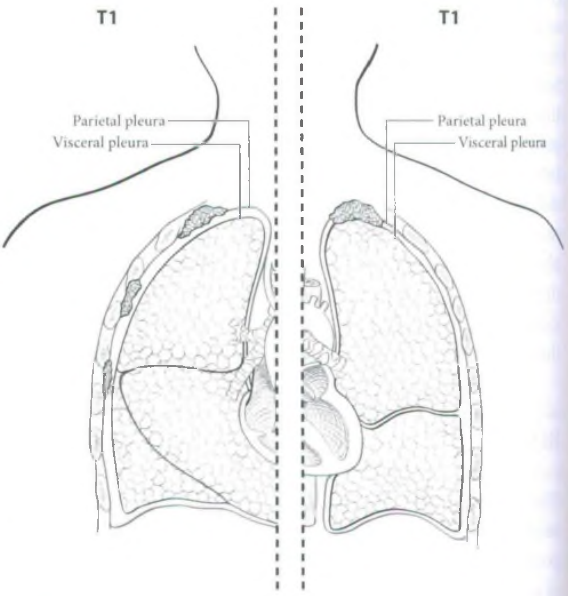

Fig. 37.11 T1 tumors may involve either the parietal pleura alone (*left*) or the parietal and visceral pleura (*right*)

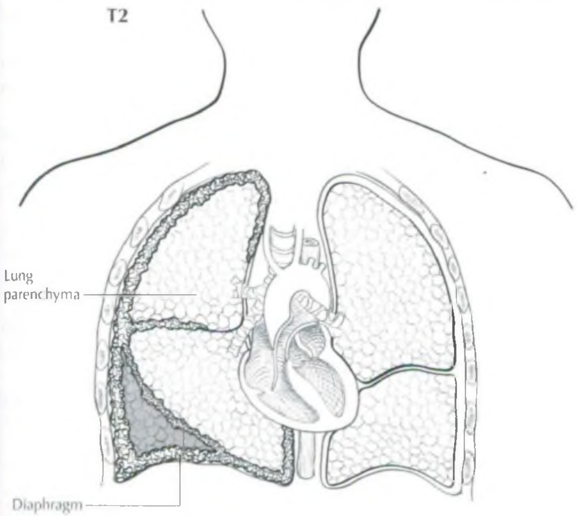

Fig. 37.12 T2 involves each of the ipsilateral pleural surfaces (parietal, mediastinal, diaphragmatic, and visceral pleura) with at least one of the following: involvement of diaphragmatic muscle (as illustrated) and/or extension of tumor from the visceral pleura into the underlying pulmonary parenchyma (as illustrated)

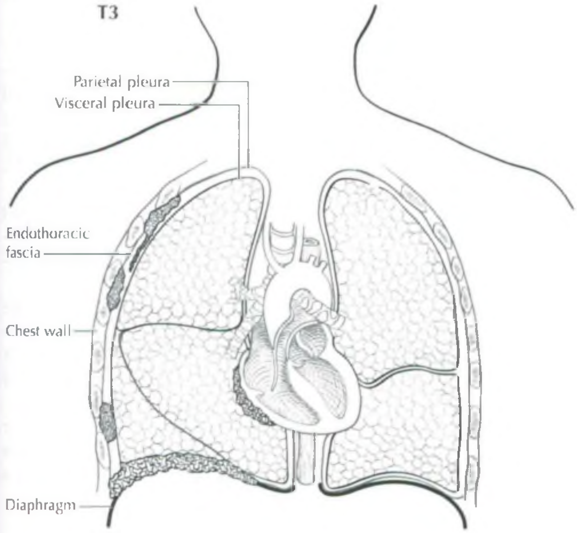

Fig. 37.13 T3 is locally advanced but potentially resectable tumor. Tumor involves all the ipsilateral pleural surfaces (parietal, mediastinal, diaphragmatic, and visceral pleura) with at least one of the following: involvement of the endothoracic fascia (as illustrated); extension into mediastinal fat; solitary, completely resectable focus of tumor extending into the soft tissues of the chest wall (as illustrated); and/or non-transmural involvement of the pericardium (as illustrated)

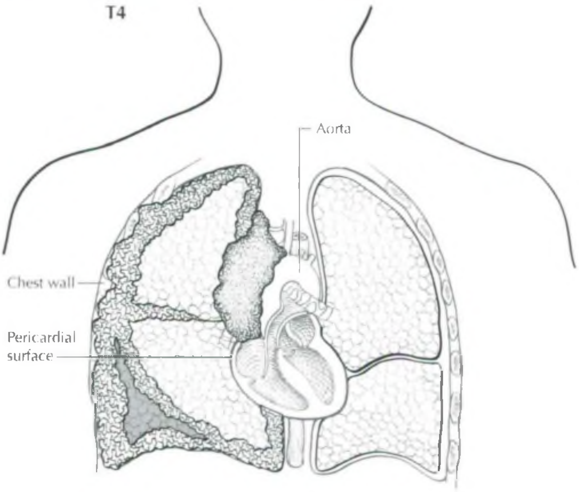

Fig. 37.14 T4 is locally advanced, technically unresectable tumor. Tumor involves all the ipsilateral pleural surfaces (parietal, mediastinal, diaphragmatic, and visceral pleura) with at least one additional parameter, such as extension through to the internal surface of the pericardium, as illustrated here, and diffuse invasion of chest wall, also illustrated here. (The full list of additional parameters is provided under Definitions of AJCC TNM.)

Bibliography

1. Wagner JC. Mesothelioma and mineral fibers. *Cancer.* May 15 1986;57(10):1905-1911.
2. Dimitrov N, McMahon S. Presentation, diagnostic methods, staging, and natural history of malignant mesothelioma. *Asbestos-related malignancy. Orlando, FL: Grune and Stratton.* 1987: 225-238.
3. Chahinian A. Therapeutic modalities in malignant pleural mesothelioma. *Diseases of the Pleura. New York, NY: Masson Publishers.* 1983.
4. Butchart EG, Ashcroft T, Barnsley WC, Holden MP. Pleuropneumonectomy in the management of diffuse malignant mesothelioma of the pleura. Experience with 29 patients. *Thorax.* Feb 1976;31(1):15-24.
5. Tammilehto L, Kivisaari L, Salminen US, Maasilta P, Mattson K. Evaluation of the clinical TNM staging system for malignant pleural mesothelioma: an assessment in 88 patients. *Lung cancer.* Mar 1995;12(1-2):25-34.
6. Rusch VW. A proposed new international TNM staging system for malignant pleural mesothelioma. From the International Mesothelioma Interest Group. *Chest.* Oct 1995;108(4):1122-1128.
7. Richards WG, Godleski JJ, Yeap BY, et al. Proposed adjustments to pathologic staging of epithelial malignant pleural mesothelioma based on analysis of 354 cases. *Cancer.* Mar 15 2010;116(6): 1510-1517.
8. Nakas A, Black E, Entwisle J, Muller S, Waller DA. Surgical assessment of malignant pleural mesothelioma: have we reached a

critical stage? *European Journal of Cardio-Thoracic Surgery.* 2010;37(6):1457-1463.

9. Cao C, Andvik SKK, Yan TD, Kennedy C, Bannon PG, McCaughan BC. Staging of patients after extrapleural pneumonectomy for malignant pleural mesothelioma–institutional review and current update. *Interactive cardiovascular and thoracic surgery.* 2011;12(5):754-757.
10. Rusch VW, Giroux D, Kennedy C, et al. Initial analysis of the international association for the study of lung cancer mesothelioma database. *J Thorac Oncol.* Nov 2012;7(11):1631-1639.
11. Pass HI, Giroux D, Kennedy C, et al. Supplementary prognostic variables for pleural mesothelioma: a report from the IASLC staging committee. *J Thorac Oncol.* Jun 2014;9(6):856-864.
12. Pass HI, Temeck BK, Kranda K, Steinberg SM, Feuerstein IR. Preoperative tumor volume is associated with outcome in malignant pleural mesothelioma. *The Journal of thoracic and cardiovascular surgery.* 1998;115(2):310-318.
13. Liu F, Zhao B, Krug LM, et al. Assessment of therapy responses and prediction of survival in malignant pleural mesothelioma through computer-aided volumetric measurement on computed tomography scans. *Journal of Thoracic Oncology.* 2010;5(6):879-884.
14. Plathow C, Klopp M, Thieke C, et al. Therapy response in malignant pleural mesothelioma-role of MRI using RECIST, modified RECIST and volumetric approaches in comparison with CT. *European radiology.* 2008;18(8):1635-1643.
15. Sensakovic WF, Armato SG, 3rd, Straus C, et al. Computerized segmentation and measurement of malignant pleural mesothelioma. *Med Phys.* Jan 2011;38(1):238-244.
16. Rusch VW, Venkatraman E. The importance of surgical staging in the treatment of malignant pleural mesothelioma. *J Thorac Cardiovasc Surg.* Apr 1996;111(4):815-825; discussion 825-816.
17. Flores RM, Routledge T, Seshan VE, et al. The impact of lymph node station on survival in 348 patients with surgically resected malignant pleural mesothelioma: implications for revision of the American Joint Committee on Cancer staging system. *J Thorac Cardiovasc Surg.* Sep 2008;136(3):605-610.
18. Gill RR, Richards WG, Yeap BY, et al. Epithelial malignant pleural mesothelioma after extrapleural pneumonectomy: stratification of survival with CT-derived tumor volume. *AJR. American journal of roentgenology.* Feb 2012;198(2):359-363.
19. de Perrot M, Uy K, Anraku M, et al. Impact of lymph node metastasis on outcome after extrapleural pneumonectomy for malignant pleural mesothelioma. *J Thorac Cardiovasc Surg.* Jan 2007;133(1):111-116.
20. Edwards JG, Stewart DJ, Martin-Ucar A, Muller S, Richards C, Waller DA. The pattern of lymph node involvement influences outcome after extrapleural pneumonectomy for malignant mesothelioma. *J Thorac Cardiovasc Surg.* May 2006;131(5):981-987.
21. Abdel Rahman AR, Gaafar RM, Baki HA, et al. Prevalence and pattern of lymph node metastasis in malignant pleural mesothelioma. *The Annals of thoracic surgery.* Aug 2008;86(2):391-395.
22. Friedberg J, Culligan M, Putt M, Hahn SM, Alley E, Simone C, et al. Posterior intercostal lymph nodes - first report of a new independent prognostic factor for malignant pleural mesothelioma. *J Thorac Oncol.* 2013;8(Suppl 2):S314.
23. Hasani A, Alvarez JM, Wyatt JM, et al. Outcome for patients with malignant pleural mesothelioma referred for Trimodality therapy in Western Australia. *J Thorac Oncol.* Aug 2009;4(8):1010-1016.
24. Rusch VW, Rosenzweig K, Venkatraman E, et al. A phase II trial of surgical resection and adjuvant high-dose hemithoracic radiation for malignant pleural mesothelioma. *J Thorac Cardiovasc Surg.* Oct 2001;122(4):788-795.
25. Flores RM, Zakowski M, Venkatraman E, et al. Prognostic factors in the treatment of malignant pleural mesothelioma at a large tertiary referral center. *Journal of Thoracic Oncology.* 2007;2(10): 957-965.
26. Gill RR, Gerbaudo VH, Sugarbaker DJ, Hatabu H. Current trends in radiologic management of malignant pleural mesothelioma. *Semin Thorac Cardiovasc Surg.* 2009;21(2):111–120.
27. Marom EM, Erasmus JJ, Pass HI, Patz Jr. EF. The role of imaging in malignant pleural mesothelioma. *Semin Oncol.* 2002;29(1): 26–35.
28. Wang ZJ, Reddy GP, Gotway MB, et al. Malignant pleural mesothelioma: evaluation with CT, MR imaging, and PET. *Radiographics: a review publication of the Radiological Society of North America, Inc.* Jan-Feb 2004;24(1):105-119.
29. Gill RR, Umeoka S, Mamata H, et al. Diffusion-weighted MRI of malignant pleural mesothelioma: preliminary assessment of apparent diffusion coefficient in histologic subtypes. *AJR. American journal of roentgenology.* Aug 2010;195(2):W125-130.
30. De Rienzo A, Dong L, Yeap BY, et al. Fine-needle aspiration biopsies for gene expression ratio-based diagnostic and prognostic tests in malignant pleural mesothelioma. *Clin Cancer Res.* Jan 15 2011;17(2):310-316.
31. Sørensen JB, Ravn J, Loft A, Brenøe J, Berthelsen AK. Preoperative staging procedures using 18F-FDG-Positron Emission Tomography-Computed Tomography fused imaging (PET-CT-Scan) and Mediastinoscopy compared to Surgical-Pathological findings in Malignant Pleural Mesothelioma undergoing Extrapleural Pneumonectomy: P1-091. *Journal of Thoracic Oncology.* 2007; 2(8):S586.
32. Erasmus JJ, Truong MT, Smythe WR, et al. Integrated computed tomography-positron emission tomography in patients with potentially resectable malignant pleural mesothelioma: Staging implications. *J Thorac Cardiovasc Surg.* Jun 2005;129(6):1364-1370.
33. Kattan MW, Hess KR, Amin MB, et al. American Joint Committee on Cancer acceptance criteria for inclusion of risk models for individualized prognosis in the practice of precision medicine. *CA Cancer J Clin.* Jan 19. doi: 10.3322/caac.21339. [Epub ahead of print].
34. Rusch VW, Asamura H, Watanabe H, et al. The IASLC lung cancer staging project: a proposal for a new international lymph node map in the forthcoming seventh edition of the TNM classification for lung cancer. *J Thorac Oncol.* May 2009;4(5):568-577.

Part VIII

Bone

Members of the Bone Expert Panel
Peter M. Anderson, MD, PhD
Cristina R. Antonescu, MD
Øyvind S. Bruland, MD, PhD
Kumarasen Cooper, MBChB, DPhil, FRCPath – CAP Representative
Frederick L. Greene, MD, FACS – Editorial Board Liaison
Ginger E. Holt, MD, FACS
Andrew E. Horvai, MD, PhD
Jeffrey S. Kneisl, MD, FACS – Chair
Mark D. Murphey, MD, FACR
Brian O'Sullivan, MD, FRCPC – UICC Representative
Shreyaskumar R. Patel, MD
Peter S. Rose, MD
Andrew E. Rosenberg, MD – Vice Chair
Daniel I. Rosenthal, MD, FACR
Paige S. Tedder, RHIT, CTR – Data Collection Core Member

Bone

38

Jeffrey S. Kneisl, Andrew E. Rosenberg,
Peter M. Anderson, Cristina R. Antonescu,
Oyvind S. Bruland, Kumarasen Cooper, Andrew E. Horvai,
Ginger E. Holt, Brian O'Sullivan, Shreyaskumar R. Patel,
and Peter S. Rose

CHAPTER SUMMARY

Cancers Staged Using This Staging System

Osteosarcoma, chondrosarcoma, Ewing's sarcoma, spindle cell sarcoma, hemangioendothelioma, angiosarcoma, fibrosarcoma/myofibroid sarcoma, chordoma, adamantinoma, and other cancers arising in the bone

Cancers Not Staged Using This Staging System

These histopathologic types of cancer…	Are staged according to the classification for…	And can be found in chapter…
Primary malignant lymphoma	Hodgkin and Non-Hodgkin Lymphoma	79
Multiple myeloma	Multiple Myeloma and Plasma Cell Disorders	82

Summary of Changes

Change	Details of Change	Level of Evidence
Definitions of AJCC TNM	Pelvis and spine each have a separate and distinct TNM classification but not a separate stage grouping.	III
AJCC Prognostic Stage Groups	Stage III is reserved for G2 and G3.	III
Histologic Grade (G)	G4 designation has been eliminated (G1, low grade; G2 and G3, high grade).	III

ICD-O-3 Topography Codes

Code	Description
	**Appendicular skeleton, trunk, skull, and facial bones**
C40.0	Long bones of upper limb, scapula, and associated joints
C40.1	Short bones of upper limb and associated joints
C40.2	Long bones of lower limb and associated joints
C40.3	Short bones of lower limb and associated joints
C40.8	Overlapping lesion of bones, joints, and articular cartilage of limbs

To access the AJCC cancer staging forms, please visit www.cancerstaging.org.

© American Joint Committee on Cancer 2017

M.B. Amin et al. (eds.), *AJCC Cancer Staging Manual, Eighth Edition*, DOI 10.1007/978-3-319-40618-3_38

Code	Description
	**Appendicular skeleton, trunk, skull, and facial bones**
C40.9	Bone of limb, NOS
C41.0	Bones of skull and face and associated joints
C41.1	Mandible
C41.3	Rib, sternum, clavicle, and associated joints
C41.8	Overlapping lesion of bones, joints, and articular cartilage
C41.9	Bone, NOS
	**Spine**
C41.2	Vertebral column
	**Pelvis**
C41.4	Pelvic bones, sacrum, coccyx, and associated joints

WHO Classification of Tumors

Code	Description
9180	Osteosarcoma
9180	Osteoblastic osteosarcoma
9181	Chondroblastic osteosarcoma
9182	Fibroblastic osteosarcoma
9183	Telangiectatic osteosarcoma
9185	Small cell osteosarcoma
9187	Intramedullary low grade
9194	Juxtacortical high grade (high grade surface osteosarcoma)
9193	Juxtacortical intermediate grade, often chondroblastic (periosteal osteosarcoma)
9192	Juxtacortical low grade (parosteal osteosarcoma)
9184	Secondary osteosarcoma
9220	Chondrosarcoma
9220	Conventional (hyaline/myxoid) chondrosarcoma
9242	Clear cell chondrosarcoma
9243	Dedifferentiated chondrosarcoma
9240	Mesenchymal chondrosarcoma
9221	Juxtacortical chondrosarcoma
9364	Un/poorly differentiated small round/spindle cell sarcoma (SR/SCT)
9364	SR/SCT translocation positive
9364	EWSR1–ETS fusions—Ewing sarcoma/primitive neuroectodermal tumor (PNET)
9364	EWSR1–non-ETS fusions—Ewing sarcoma/PNET
9364	CIC–DUX 4 fusion
9364	BCOR–CCNB3 fusion
9364	Translocation negative
9133	Hemangioendothelioma
9133	Epithelioid hemangioendothelioma
9133	Pseudomyogenic hemangioendothelioma
9133	Retiform hemangioendothelioma
9120	Angiosarcoma
9120	Conventional angiosarcoma
9120	Epithelioid angiosarcoma
8810	Fibrosarcoma/myofibrosarcoma
9370	Chordoma
9370	Conventional chordoma
9370	Dedifferentiated chordoma
9370	Poorly differentiated chordoma
9261	Adamantinoma
9261	Well differentiated—osteofibrous dysplasia-like adamantinoma
9261	Conventional adamantinoma
8850	Liposarcoma
8890	Leiomyosarcoma
8540	Malignant peripheral nerve sheath tumor
8900	Rhabdomyosarcoma
9040	Synovial sarcoma
8815	Malignant solitary fibrous tumor
8804	Epithelioid sarcoma
8830	Undifferentiated pleomorphic sarcoma
8830	Undifferentiated epithelioid sarcoma
8830	Undifferentiated spindle cell sarcoma

Fletcher CDM, Bridge JA, Hogendoorn P, Mertens F, eds. World Health Organization Classification of Tumours of Soft Tissue and Bone. Fourth Edition. Lyon: IARC; 2013.

INTRODUCTION

This classification is used for all primary malignant tumors of bone except primary malignant lymphoma and multiple myeloma. These tumors are relatively rare, representing less than 0.2% of all malignancies. Osteosarcoma (35%), chondrosarcoma (30%), and Ewing sarcoma (16%) are the three most common forms of primary bone cancer. Osteosarcoma and Ewing sarcoma develop mainly in children and young adults, whereas chondrosarcoma is usually found in middle-aged and older adults. Data from these three histologies, analyzed at multiple institutions, predominantly influence this staging system. In the staging of bone sarcomas, patients are evaluated with regard to the pathological features of the tumor as well as the local and distant extent of disease. Bone sarcomas are staged based on the histologic type, grade, size, and location of the tumor and the presence and location of metastases. The system is designed to help stratify patients according to known risk factors.

ANATOMY

Primary Site(s)

All bones of the skeleton are included in this system. The current staging system takes into account anatomic site, because anatomic site is known to influence outcome.

Site groups for bone sarcoma:

- Appendicular skeleton, trunk, skull, and facial bones
- Pelvis
- Spine

Regional Lymph Nodes

Regional lymph node metastases from primary bone tumors are extremely rare.

Metastatic Sites

Pulmonary metastases are the most frequent site for all bone sarcomas. Extrapulmonary metastases occur infrequently and may include secondary bone metastases, for example.

RULES FOR CLASSIFICATION

Clinical Classification

Clinical staging includes all relevant data prior to primary definitive therapy, including patient history and physical examination, imaging, and biopsy. It depends on the location and TNM characteristics of the identified tumor.

Patients presenting with primary bone sarcomas frequently demonstrate a mass or swelling and note crescendo pain symptoms, often occurring at night. This constellation of symptoms is most common for the appendicular skeleton, whereas patients with spine or pelvic tumors may note vague pain without mass effect because of the anatomic depth of the tumor location.

The prognosis of bone sarcoma is affected by the location of the primary tumor, which is reflected in the T classification as follows:

For extremity, trunk, skull, and facial bones, T is divided into lesions with a maximum dimension of 8 cm or less (T1) and those greater than 8 cm (T2). T3 has been redefined to include only high-grade tumors, discontinuous, within the same bone.

For tumors occurring in the spine, T is now primarily defined by the number of spinal segments involved or involvement of the spinal canal/great vessels (Fig. 38.1). T1 is defined as a tumor occupying either one or two adjacent vertebral segments, whereas T2 is defined as a tumor occupying three adjacent vertebral segments. T3 is defined as a tumor occupying four or more adjacent segments, or any tumor involving nonadjacent segments. T4 includes tumors that have invaded either the spinal canal or the great vessels.

For tumors occurring in the pelvis, T is now defined primarily by the number of pelvic segments involved, the presence of extraosseous extension, or involvement of the pelvic vessels (Fig. 38.2). T1 is defined as a tumor occupying one pelvic segment without extraosseous extension. T2 is defined as a tumor occupying one pelvic segment with extraosseous extension or two segments without extraosseous extension. T3 includes tumors that occupy two pelvic segments with extraosseous extension, whereas T4 includes tumors that span three pelvic segments,extend across the sacroiliac joint, encase the external iliac vessels, or demonstrate the presence of gross tumor within the pelvic vessels.

Imaging

Metastatic disease should be evaluated for and described. In general, lymph node metastasis from a bone sarcoma is uncommon, and a negative clinical examination for lymphadenopathy is sufficient to warrant a classification of N0.

The radiograph remains the mainstay in determining whether a bone lesion requires staging and usually is the modality that permits reliable assessment of the type of bone tumor. The minimum clinical staging workup of a bone sarcoma should include axial imaging using magnetic resonance (MR) and/or computed tomography (CT), a contrast-enhanced CT scan of the chest, and technetium scintigraphy of the entire skeleton.

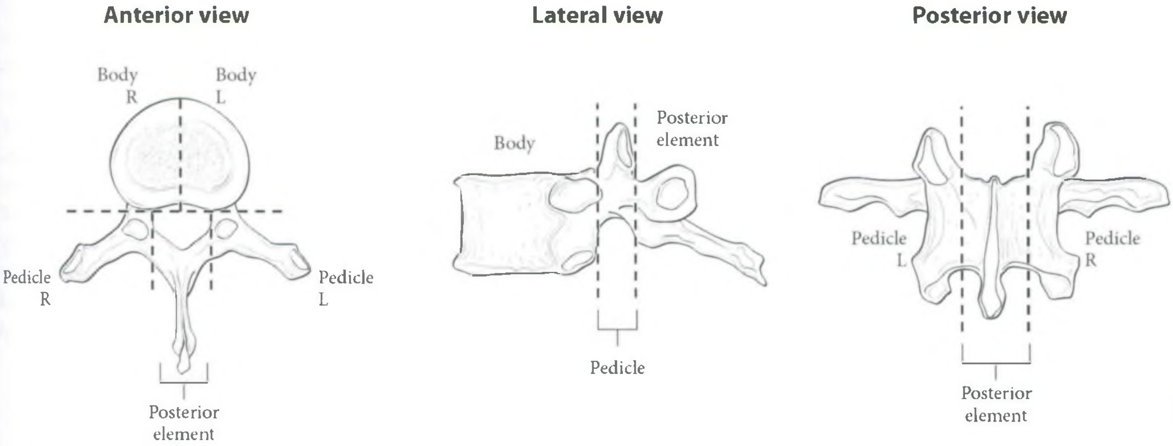

Fig. 38.1 Spine segments for staging

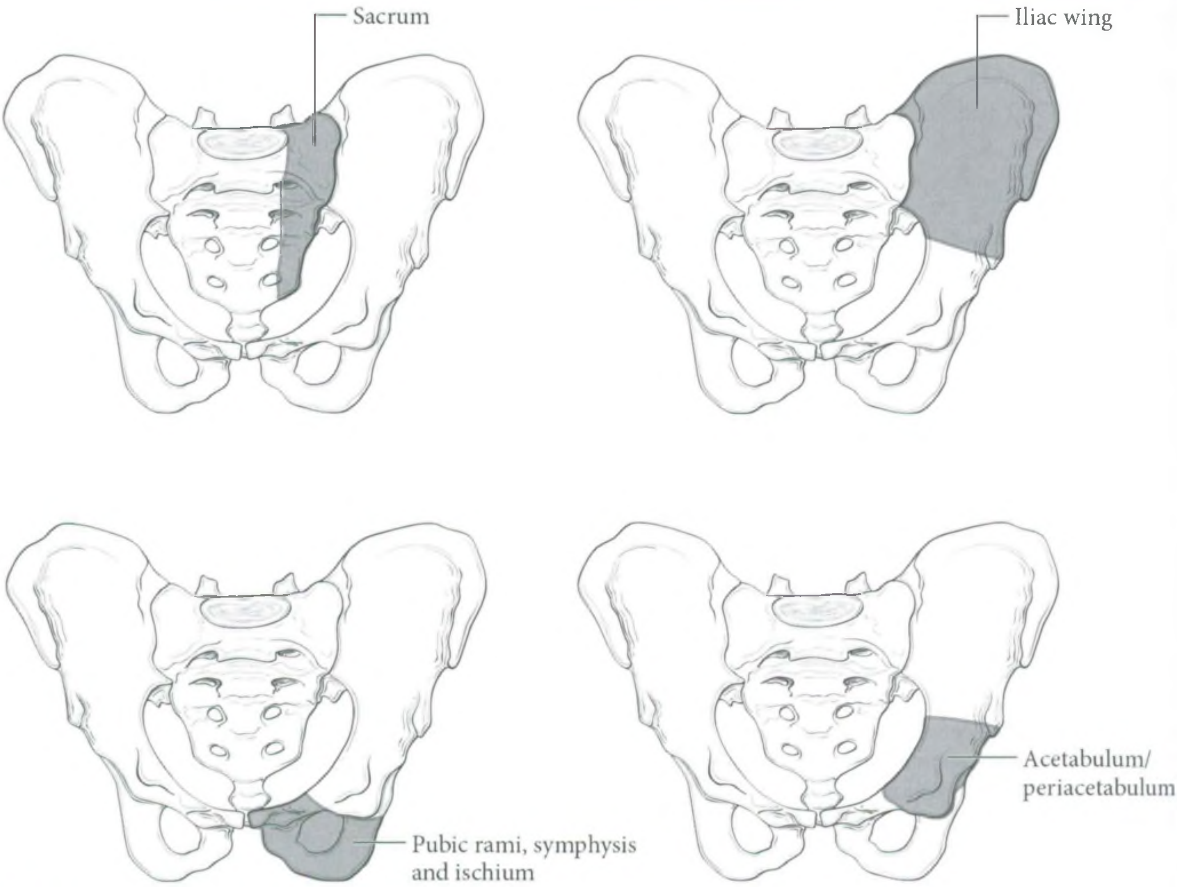

Fig. 38.2 Pelvic segments for staging

Local staging of all bone sarcomas is achieved most accurately by MR imaging. Axial imaging, complemented by either coronal or sagittal imaging planes using T1- and T2-weighted spin-echo sequences, most often provides an accurate depiction of intra- and extraosseous tumor. To improve assessment in locations such as the pelvis or vertebrae, these sequences may be augmented by fat-suppressed pulse sequences. The maximum dimension of the tumor in three dimensions must be measured before any treatment. The decision to use intravenous gadolinium contrast should be based on medical appropriateness.

CT has a limited role in local staging of primary bone tumors. Some patients have contraindications to MR imaging evaluation (e.g., an implanted cardiac pacemaker), and axial imaging is best accomplished by CT in these cases. In other situations, in which characterization of a lesion by radiography may be incomplete or difficult because of inadequate visualization of the matrix of a lesion, CT may be preferred over MR imaging. The role of CT in these circumstances is to characterize the lesion and determine whether it is potentially malignant, and the CT images obtained may suffice for local staging. Contrast-enhanced chest CT remains the examination of choice for evaluating the presence or absence of pulmonary metastases.

Technetium scintigraphy is the assessment of choice for evaluating the entire skeleton to determine whether multiple bony lesions exist. The role of positron emission tomography in the evaluation and staging of bone sarcomas remains promising, although standards for its use are still evolving and are incompletely defined. Reports indicate its usefulness in detecting extrapulmonary metastases, evaluating response to chemotherapy, and determining local recurrence adjacent to prosthetic implants.

The same staging should be used in patients who require restaging of sarcoma recurrence. Such reports should specify whether the patient has a primary lesion or lesions that were treated previously and recurred. The identification and reporting of etiologic factors such as radiation exposure and inherited or genetic syndromes are encouraged.

Biopsy

Biopsy of the tumor completes the clinical staging process. Patients with suspected primary bone sarcomas should undergo biopsy at a referral center with expertise in pathology and surgical treatment of sarcomas, if possible. In general,

appropriate core needle biopsy or planned open biopsy is preferred. The location of the biopsy must be planned carefully to allow for eventual en bloc resection of the entire biopsy tract at the time of definitive resection of a malignant primary bone neoplasm. Imaging assessment of the lesion should precede biopsy. Limited biopsy specimens may not be truly representative and this may affect both classification and grading of bone sarcomas. Imaging the tumor after biopsy may compromise the accuracy of the staging process.

Pathological Classification

The classification of bone sarcomas is accomplished by evaluating tissue retrieved from needle biopsy, open biopsy, and resection specimens. The classification scheme of bone sarcomas is based on the normal cell or tissue type that the tumors recapitulate. The vast majority of sarcomas differentiate along the cell lines or tissue types that compose the skeletal system, such as bone and cartilage; only a few have consistent and distinctive clinicopathologic features but lack a normal tissue counterpart. Further subclassification of sarcomas is based on their specific histologic characteristics, their relationship to the underlying bone, and the presence of preexisting conditions. Basic parameters of assessing a bone sarcoma are the identification of cell morphology; type of stroma, including matrix; degree of differentiation, including cytologic atypia; mitotic activity and atypical mitoses; and necrosis. If needed, ancillary studies, such as immunohistochemistry (IHC) and molecular analyses, should be performed to confirm a diagnosis. Importantly, the pathological diagnosis should be correlated with the clinical findings and imaging studies.

Pathological staging pTNM includes pathological data obtained from examination of a resected specimen, histopathologic type and grade, regional lymph nodes as appropriate, or distant metastasis. Because regional lymph node involvement from bone tumors is rare, the pathological stage grouping includes any of the following combinations: pT pN c/pM pG, pT cN c/pM pG, or cT cN pM1. Biological grade should be assigned to all bone sarcomas, and based on published outcomes data, the current staging system accommodates a two-tiered (low- vs. high-grade) system for recording grade. Histologic grading (G) uses a three-tiered system: G1 is considered low grade, and G2 and G3 are grouped together as high grade for biological grading.

PROGNOSTIC FACTORS

Prognostic Factors Required for Stage Grouping

Histologic Grade

A three-tier system of grading, similar to that used for soft tissue sarcomas, is now recommended for assessing bone sarcomas. The grade of bone sarcomas is based on a combination of histologic type, cellularity, cytologic atypia, mitotic activity, necrosis, and degree of differentiation. Some sarcomas are definitionally G3 (high grade; e.g., Ewing sarcoma), whereas others range from G1 to G3 depending on their pathological features. This classification, however, is based on level III–IV data owing to the rarity of most subtypes of bone sarcomas. Neoadjuvent therapy may affect tumor cell morphology and interfere with grading. In problematic cases, the grade of the pretreatment specimen should take precedence.

Additional Factors Recommended for Clinical Care

Known prognostic factors for malignant bone tumors are as follows:

Size and Extent of Local Spread

Smaller and anatomically confined tumors have a better prognosis than larger and more extensive ones. For both extremity and pelvic tumors, the size threshold of 8 cm is a reporting standard. For spine and pelvic tumors, T classifications now include definitions that reflect the poorer prognosis associated with a) the increased number of anatomic bone segments involved, b) extraosseous extension, and c) extension into the spinal canal or involvement of the great vessels.

Grade

Histopathologic low-grade (G1) sarcomas have a better prognosis than high-grade (G2, G3) sarcomas.

Location

Patients with tumors of the extremities have a better prognosis than those with tumors arising in the pelvis and spine. Anatomically resectable primary tumors are associated with a better outcome than those that are nonresectable.

Size, Three Dimensions of Tumor Size

Clinical staging is performed from three dimensions, as reported from either MR imaging or CT scaning. Pathological staging is based on the final pathology report on the resected specimen.

The size of ≤8 cm in greatest dimension remains a critical threshold. Ewing sarcoma patients with a tumor ≤8 cm in greatest dimension have a better prognosis than those with a tumor >8 cm. Osteosarcoma patients with a tumor ≤9 cm in greatest dimension have a better prognosis than those with a tumor >9 cm.

Stage

Patients who have a localized primary tumor have a better prognosis than those with metastases.

Metastatic Sites

Certain anatomic sites of metastases are associated with a poorer prognosis; for example, bone metastases convey a much worse prognosis than do lung metastases, and patients with solitary lung metastasis have a better prognosis than those with multiple lung lesions. Therefore, it is important to document the number of lung metastases.

Histologic Response to Chemotherapy

Patients with Ewing sarcoma or osteosarcoma whose tumors have a "good" response (i.e., ≥90% tumor necrosis) to systemic therapy have a better prognosis than those with less necrosis. Histologic response of the primary tumor to neoadjuvant chemotherapy is a prognostic factor for osteosarcoma and Ewing sarcoma. A variety of systems to stratify post-chemotherapy tumor necrosis for both osteosarcoma and Ewing sarcoma have been proposed, ranging from two to six tiers. A condensed two-tiered system in which ≥90% tumor necrosis is considered a good response is used most commonly. A cutoff of ≥90% necrosis also predicted survival in a univariate analysis in Ewing sarcoma. Sampling the tumor to assess chemotherapy response is accomplished by processing one full cross-sectional slab of tumor at its greatest cross-section area and then taking one section per centimer of tumor from the remaining hemispheres of the neoplasm. The sum of all viable areas measured microscopically is divided by the total cross-sectional area occupied by tumor to arrive at a percentage. Level II and III evidence supports these findings and cutoffs. For other types of bone sarcoma (fibrosarcoma, chondrosarcoma) treated with neoadjuvant chemotherapy, the prognostic significance of chemotherapeutic response to neoadjuvant therapy is unknown.

P16 expression by untreated osteosarcoma as assessed by IHC has been found to correlate with percentage of necrosis. Its use in pretreatment biopsies may predict which osteosarcomas will have a good response to standard neoadjuvant chemotherapy.

Pathological Fracture

Patients with osteosarcoma who experience pathological fractures may have a poorer prognosis, particularly if their fracture does not heal during chemotherapy.

RISK ASSESSMENT MODELS

The AJCC recently established guidelines that will be used to evaluate published statistical prediction models for the purpose of granting endorsement for clinical use. Although this is a monumental step toward the goal of precision medicine, this work was published only very recently. Therefore, the existing models that have been published or may be in clinical use have not yet been evaluated for this cancer site by the Precision Medicine Core of the AJCC. In the future, the statistical prediction models for this cancer site will be evaluated, and those that meet all AJCC criteria will be endorsed.

DEFINITIONS OF AJCC TNM

Definition of Primary Tumor (T)

Appendicular Skeleton, Trunk, Skull, and Facial Bones

T Category	T Criteria
TX	Primary tumor cannot be assessed
T0	No evidence of primary tumor
T1	Tumor ≤8 cm in greatest dimension
T2	Tumor >8 cm in greatest dimension
T3	Discontinuous tumors in the primary bone site

Spine

T Category	T Criteria
TX	Primary tumor cannot be assessed
T0	No evidence of primary tumor
T1	Tumor confined to one vertebral segment or two adjacent vertebral segments
T2	Tumor confined to three adjacent vertebral segments
T3	Tumor confined to four or more adjacent vertebral segments, or any nonadjacent vertebral segments
T4	Extension into the spinal canal or great vessels
T4a	Extension into the spinal canal
T4b	Evidence of gross vascular invasion or tumor thrombus in the great vessels

Pelvis

T Category	T Criteria
TX	Primary tumor cannot be assessed
T0	No evidence of primary tumor
T1	Tumor confined to one pelvic segment with no extraosseous extension
T1a	Tumor ≤8 cm in greatest dimension
T1b	Tumor >8 cm in greatest dimension
T2	Tumor confined to one pelvic segment with extraosseous extension or two segments without extraosseous extension
T2a	Tumor ≤8 cm in greatest dimension
T2b	Tumor >8 cm in greatest dimension
T3	Tumor spanning two pelvic segments with extraosseous extension
T3a	Tumor ≤8 cm in greatest dimension
T3b	Tumor >8 cm in greatest dimension
T4	Tumor spanning three pelvic segments or crossing the sacroiliac joint

T Category	T Criteria
T4a	Tumor involves sacroiliac joint and extends medial to the sacral neuroforamen
T4b	Tumor encasement of external iliac vessels or presence of gross tumor thrombus in major pelvic vessels

Definition of Regional Lymph Node (N)

N Category	N Criteria
NX	Regional lymph nodes cannot be assessed.<br>Because of the rarity of lymph node involvement in bone sarcomas, the designation NX may not be appropriate, and cases should be considered N0 unless clinical node involvement clearly is evident.
N0	No regional lymph node metastasis
N1	Regional lymph node metastasis

Definition of Distant Metastasis (M)

M Category	M Criteria
M0	No distant metastasis
M1	Distant metastasis
M1a	Lung
M1b	Bone or other distant sites

AJCC PROGNOSTIC STAGE GROUPS

Appendicular Skeleton, Trunk, Skull, and Facial Bones

When T is...	And N is...	And M is...	And grade is...	Then the stage group is...
T1	N0	M0	G1 or GX	IA
T2	N0	M0	G1 or GX	IB
T3	N0	M0	G1 or GX	IB
T1	N0	M0	G2 or G3	IIA
T2	N0	M0	G2 or G3	IIB
T3	N0	M0	G2 or G3	III
Any T	N0	M1a	Any G	IVA
Any T	N1	Any M	Any G	IVB
Any T	Any N	M1b	Any G	IVB

Spine and Pelvis

There are no AJCC prognostic stage groupings for spine and pelvis.

REGISTRY DATA COLLECTION VARIABLES

1. Grade: G1, G2, G3
2. Three dimensions of tumor size
3. Percentage of necrosis after neoadjuvant systemic therapy, from pathology report
4. Number of resected pulmonary metastases, from pathology report

HISTOLOGIC GRADE (G)

G	G Definition
GX	Grade cannot be assessed
G1	Well differentiated, low grade
G2	Moderately differentiated, high grade
G3	Poorly differentiated, high grade

HISTOPATHOLOGIC TYPE

Classification of primary malignant bone tumors:

- Osteosarcoma
  - Intramedullary high grade
    - Osteoblastic
    - Chondroblastic
    - Fibroblastic
    - Mixed
    - Small cell
    - Telangiectatic
    - Other (epithelioid, chondromyxoid fibroma-like, chondroblastoma-like, osteoblastoma-like, giant cell–rich)
  - Intramedullary low grade
  - Juxtacortical high grade (high-grade surface osteosarcoma)
  - Juxtacortical intermediate grade—often chondroblastic (periosteal osteosarcoma)
  - Juxtacortical low grade (parosteal osteosarcoma)
  - Secondary osteosarcoma
- Chondrosarcoma
  - Intramedullary and juxtacortical
    - Conventional (hyaline/myxoid)
    - Clear cell
    - Dedifferentiated
    - Mesenchymal
- Un/poorly differentiated small round/spindle cell tumor
  - Translocation positive
    - EWSR1–ETS fusions—Ewing sarcoma/PNET
    - EWSR1–non-ETS fusions—Ewing sarcoma/PNET

        - CIC–DUX4 fusion
        - BCOR–CCNB3 fusion
    - Translocation negative
- Hemangioendothelioma
    - Epithelioid
    - Pseudomyogenic
    - Retiform
- Angiosarcoma
    - Conventional
    - Epithelioid
- Fibrosarcoma/myofibrosarcoma
- Chordoma
    - Conventional
    - Dedifferentiated
    - Poorly differentiated
- Adamantinoma
    - Well differentiated—osteofibrous dysplasia-like
    - Conventional
- Other
    - Liposarcoma
    - Leiomyosarcoma
    - Malignant peripheral nerve sheath tumor
    - Rhabdomyosarcoma
    - Synovial sarcoma
    - Malignant solitary fibrous tumor
    - Epithelioid sarcoma
    - Undifferentiated pleomorphic sarcoma
    - Undifferentiated epithelioid sarcoma
    - Undifferentiated spindle cell sarcoma

SURVIVAL DATA

The survival curves presented here were generated based on the most recent National Cancer Data Base (NCDB) cohort available, with at least 60 months of follow-up time. Data are based specifically on diagnosis years 2002 to 2008. Staging was based on the AJCC Cancer Staging Manual, 6th Edition at time of accrual. The topography codes used included the following: appendicular skeletal bones (C40.0–C40.3, C40.8–C40.9, and C41.3); the pelvis, including the sacrum (C41.4); and the spine, excluding the sacrum (C41.2). The histology codes used included osteosarcoma (9180–9195), chondrosarcoma (9220, 9221, 9230, 9231, 9240, 9242, and 9243), and Ewing sarcoma/PNET (9260, 9261, and 9365). For this time period, 9,507 patients were identified, with AJCC staging available for 5,671 patients who were analyzed according to an actuarial 5-year approach by the NCDB.

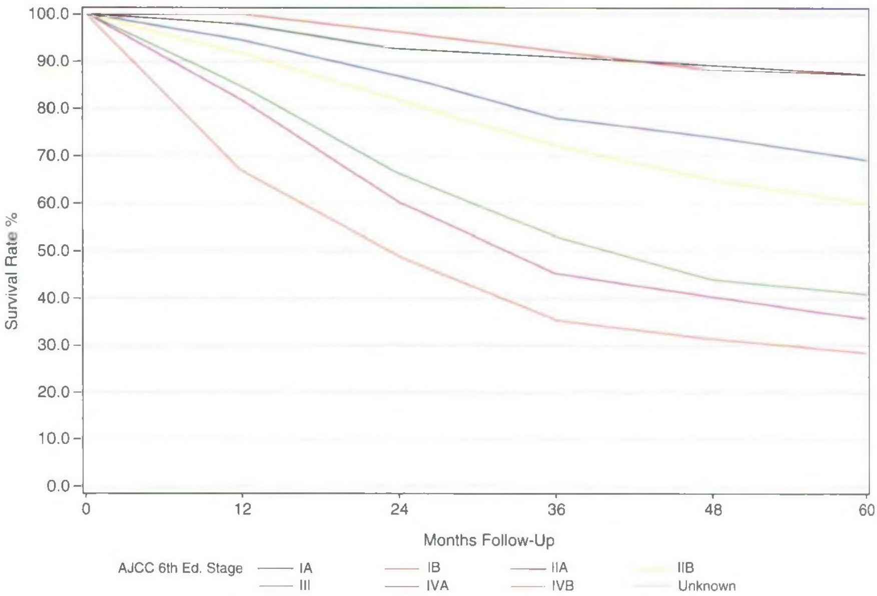

Fig. 38.3 Osteosarcoma of the appendicular skeleton by stage (6th Edition) (Data from NCDB, 2002–2008)

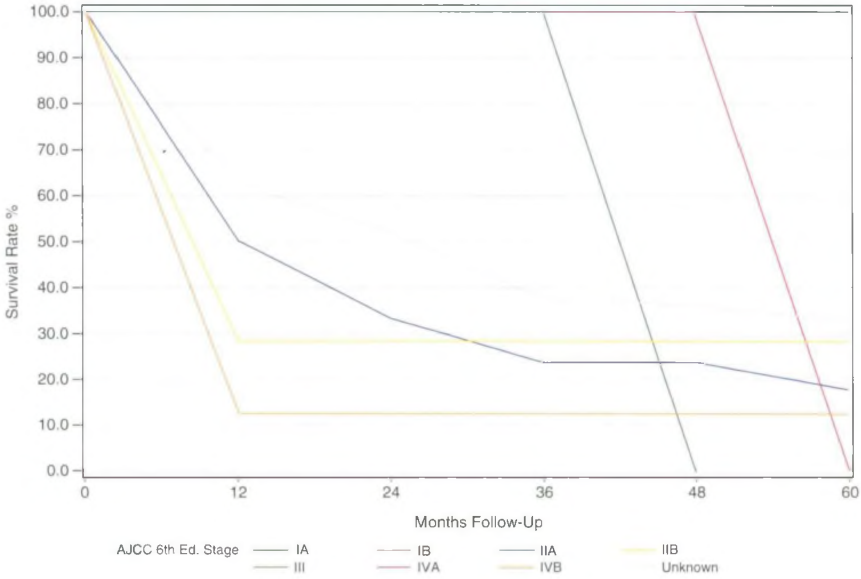

Fig. 38.4 Osteosarcoma of the spine (excluding the sacrum) by stage (6th Edition) (Data from NCDB, 2002–2008)

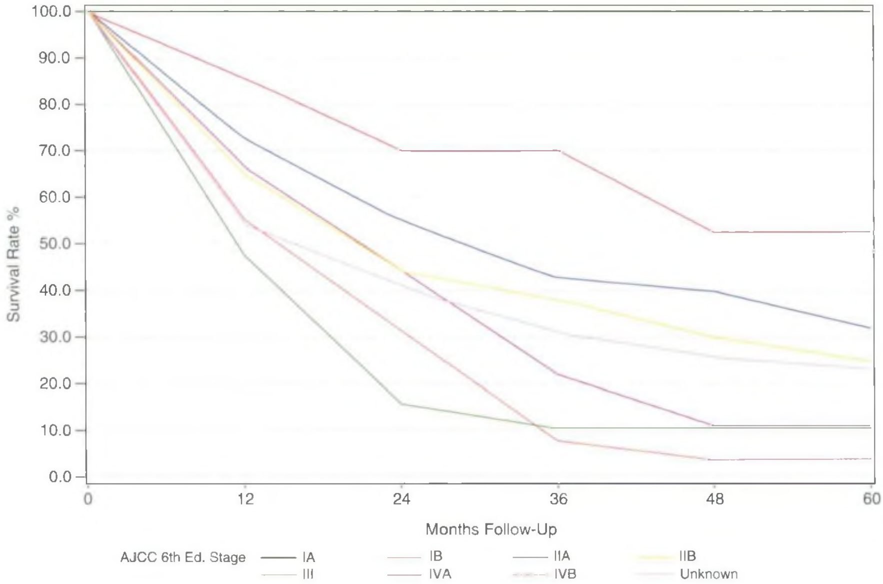

Fig. 38.5 Osteosarcoma of the pelvis (including the sacrum) by stage (6th Edition) (Data from NCDB, 2002–2008)

Fig. 38.6 Chondrosarcoma of the appendicular skeleton by stage (6th Edition) (Data from NCDB, 2002–2008)

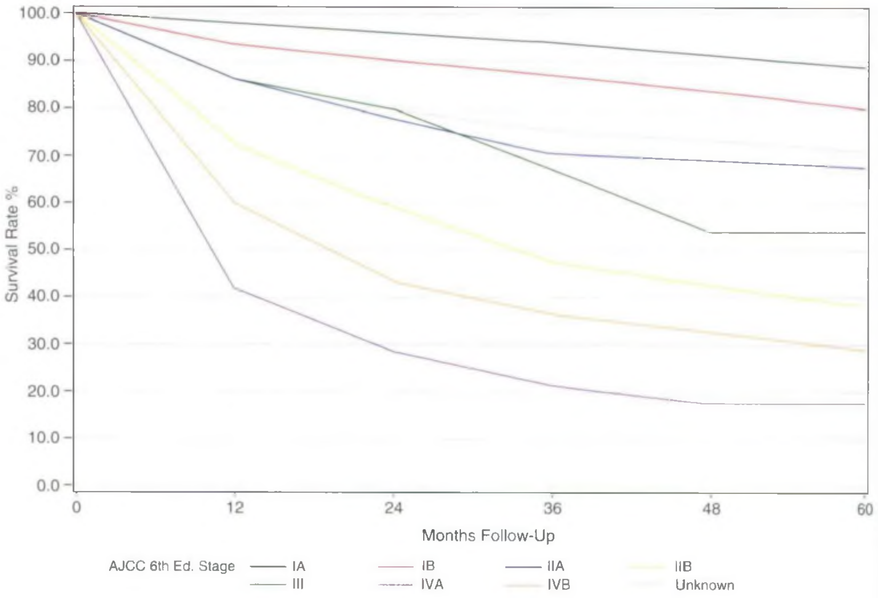

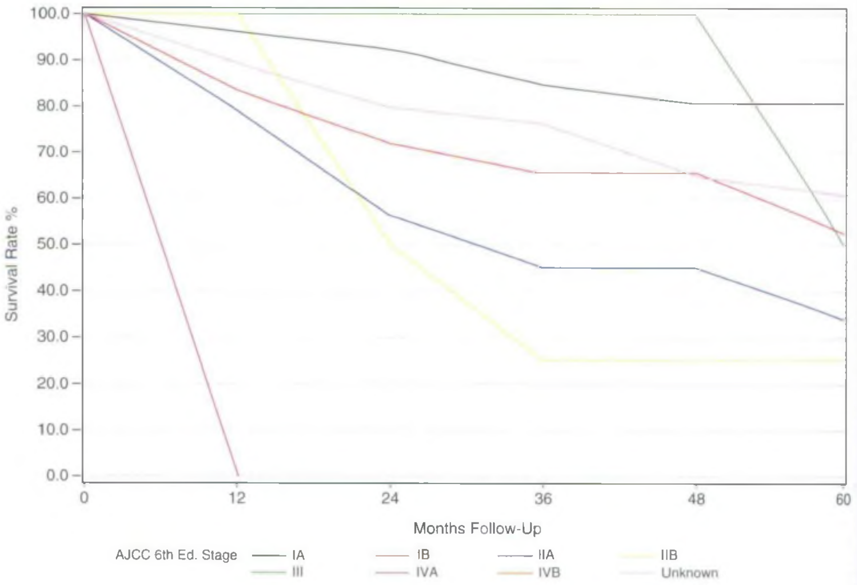

Fig. 38.7 Chondrosarcoma of the spine (excluding the sacrum) by stage (6th Edition) (Data from NCDB, 2002–2008)

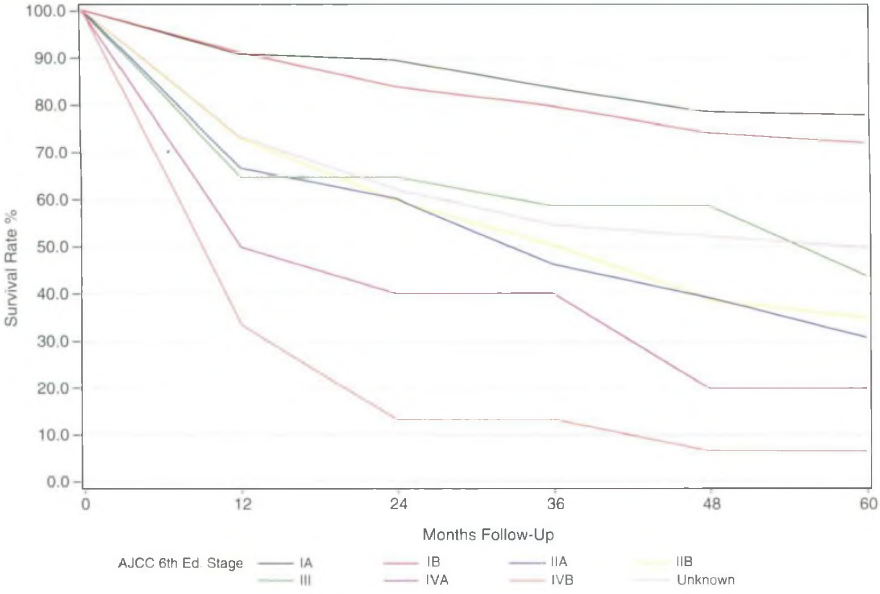

Fig. 38.8 Chondrosarcoma of the pelvis (including the sacrum) by stage (6th Edition) (Data from NCDB, 2002–2008)

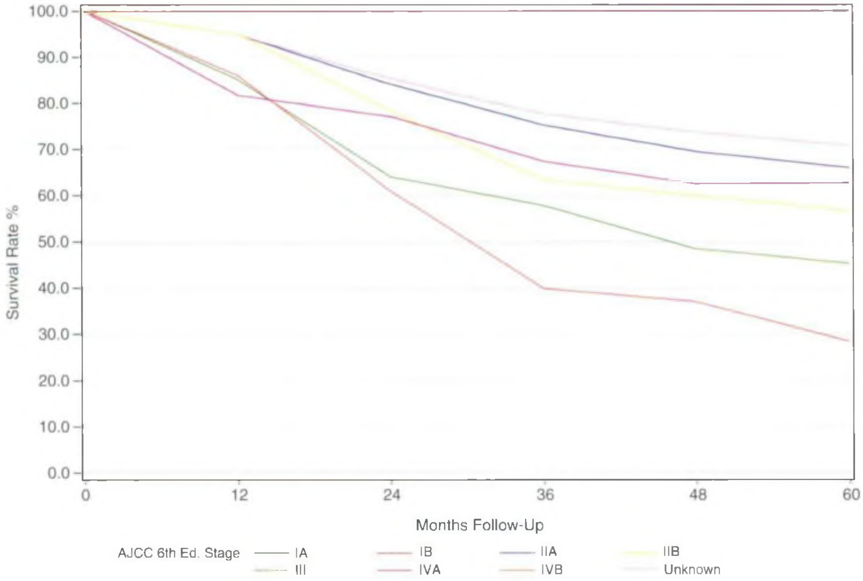

Fig. 38.9 Ewing sarcoma/PNET of the appendicular skeleton by stage (6th Edition) (Data from NCDB, 2002–2008)

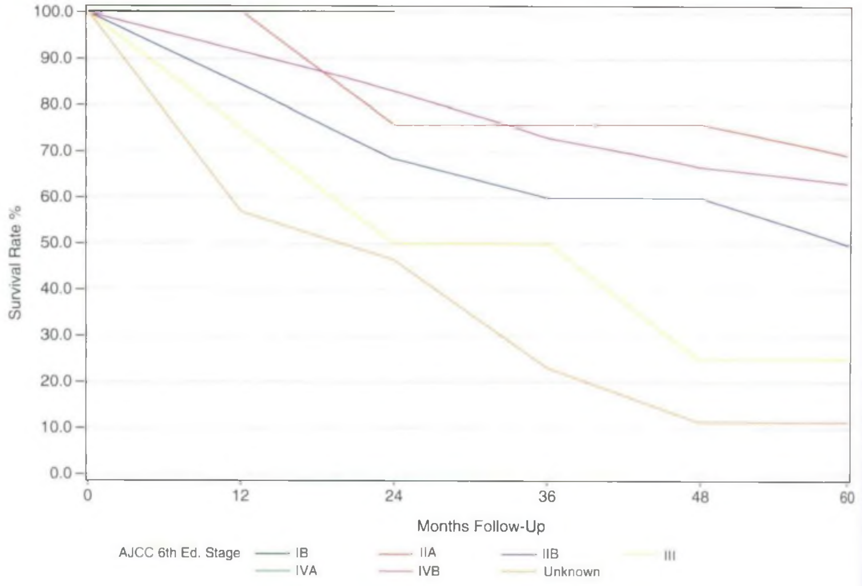

Fig. 38.10 Ewing sarcoma/PNET of the spine (excluding the sacrum) by stage (6th Edition) (Data from NCDB, 2002–2008)

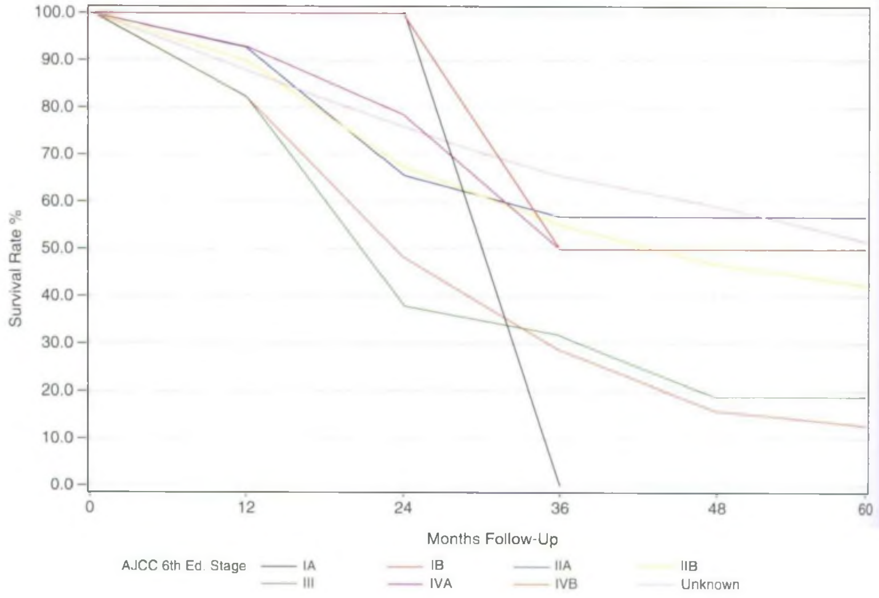

Fig. 38.11 Ewing sarcoma/PNET of the pelvis (including the sacrum) by stage (6th Edition) (Data from NCDB, 2002–2008)

ILLUSTRATIONS

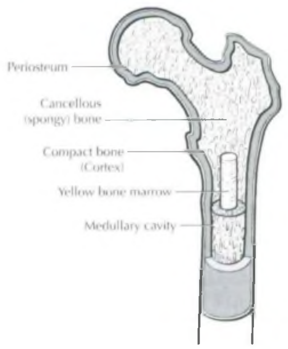

Fig. 38.12 The anatomic subsites of the bone

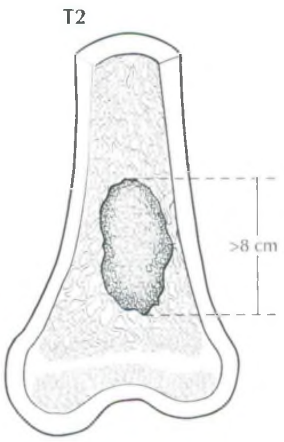

Fig. 38.14 For appendicular skeleton, trunk, skull, and facial bones, T2 is defined as tumor more than 8 cm in greatest dimension

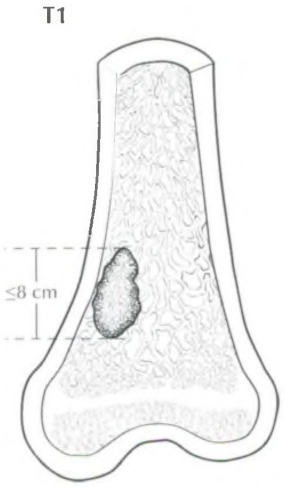

Fig. 38.13 For appendicular skeleton, trunk, skull, and facial bones, T1 is defined as tumor 8 cm or less in greatest dimension

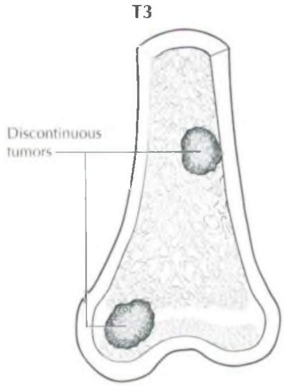

Fig. 38.15 For appendicular skeleton, trunk, skull, and facial bones, T3 is defined as discontinuous tumors in the primary bone site

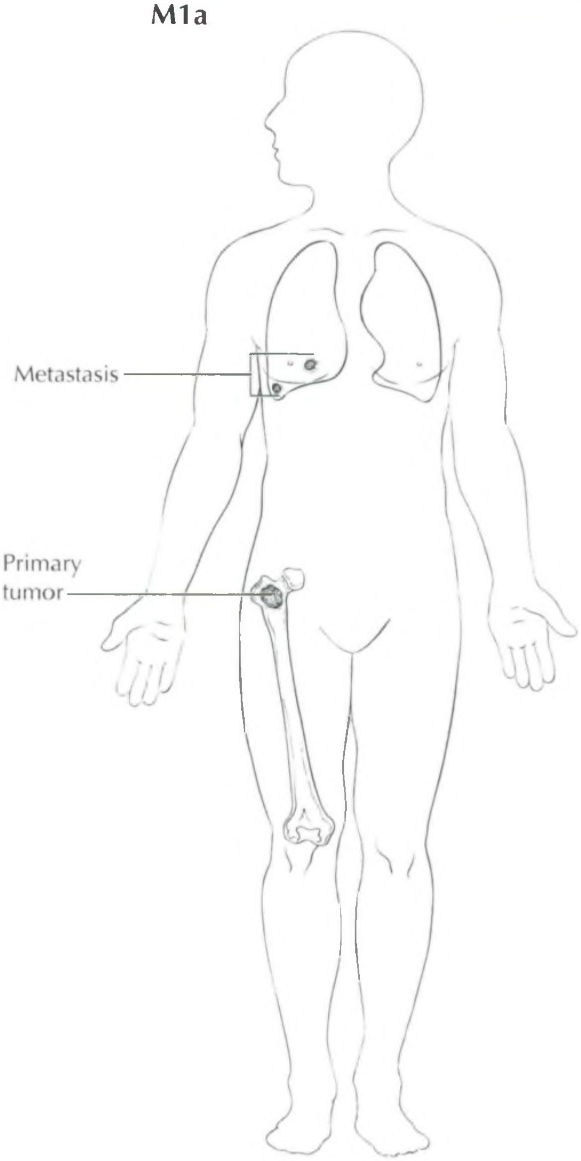

Fig. 38.16 M1a is defined as lung-only metastases

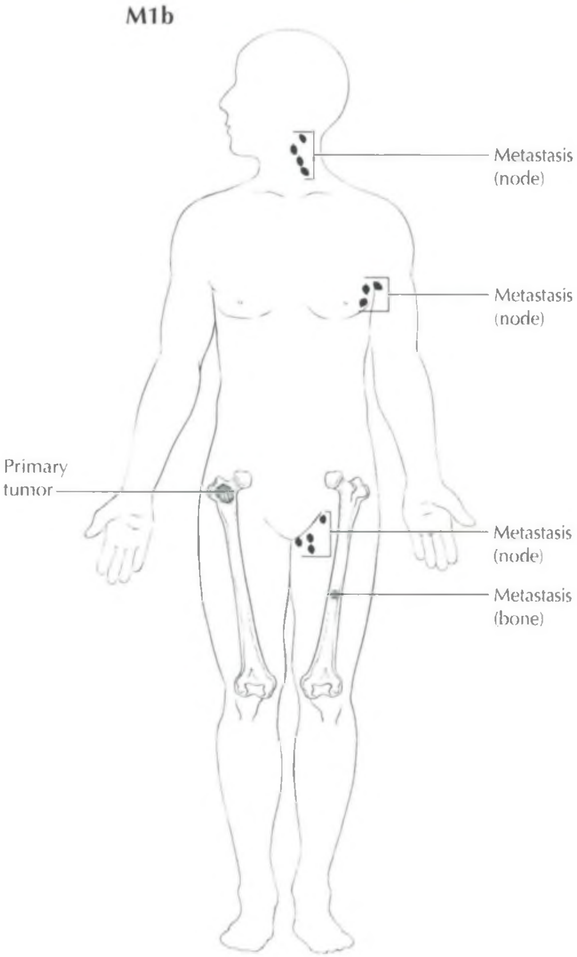

Fig. 38.17 M1b is defined as metastases to other distant sites, including lymph nodes

Bibliography

1. Anderson ME. Update on Survival in Osteosarcoma. *Orthop Clin North Am.* Jan 2016;47(1):283-292.
2. Borys D, Canter RJ, Hoch B, et al. P16 expression predicts necrotic response among patients with osteosarcoma receiving neoadjuvant chemotherapy. *Human pathology.* 2012;43(11):1948-1954.
3. Carter JM, Inwards CY, Jin L, et al. Activating GNAS mutations in parosteal osteosarcoma. *The American journal of surgical pathology.* Mar 2014;38(3):402-409.
4. Chen X, Bahrami A, Pappo A, et al. Recurrent somatic structural variations contribute to tumorigenesis in pediatric osteosarcoma. *Cell reports.* Apr 10 2014;7(1):104-112.
5. Damron TA, Ward WG, Stewart A. Osteosarcoma, chondrosarcoma, and Ewing's sarcoma: National Cancer Data Base Report. *Clinical orthopaedics and related research.* Jun 2007;459:40-47.
6. Davis AM, Bell RS, Goodwin PJ. Prognostic factors in osteosarcoma: a critical review. *J Clin Oncol.* Feb 1994;12(2):423-431.
7. Duhamel LA, Ye H, Halai D, et al. Frequency of Mouse Double Minute 2 (MDM2) and Mouse Double Minute 4 (MDM4) amplification in parosteal and conventional osteosarcoma subtypes. *Histopathology.* Jan 2012;60(2):357-359.
8. Enneking WF, Spanier SS, Goodman MA. A system for the surgical staging of musculoskeletal sarcoma. *Clinical orthopaedics and related research.* Nov-Dec 1980;153(153):106-120.
9. Gaspar N, Hawkins DS, Dirksen U, et al. Ewing Sarcoma: Current Management and Future Approaches Through Collaboration. *Journal of Clinical Oncology.* 2015;33(27):3036-3046.
10. Herrmann BG, Kispert A. The T genes in embryogenesis. *Trends in genetics : TIG.* Aug 1994;10(8):280-286.
11. Isakoff MS, Bielack SS, Meltzer P, Gorlick R. Osteosarcoma: Current Treatment and a Collaborative Pathway to Success. *J Clin Oncol.* Sep 20 2015;33(27):3029-3035.
12. Italiano A, Sung YS, Zhang L, et al. High prevalence of CIC fusion with double-homeobox (DUX4) transcription factors in EWSR1-negative undifferentiated small blue round cell sarcomas. *Genes Chromosomes Cancer.* Mar 2012;51(3):207-218.
13. Kattan MW, Hess KR, Amin MB, et al. American Joint Committee on Cancer acceptance criteria for inclusion of risk models for individualized prognosis in the practice of precision medicine. *CA: a cancer journal for clinicians.* Jan 19 2016.
14. Kelley MJ, Shi J, Ballew B, et al. Characterization of T gene sequence variants and germline duplications in familial and sporadic chordoma. *Human genetics.* Oct 2014;133(10):1289-1297.
15. Kispert A, Koschorz B, Herrmann BG. The T protein encoded by Brachyury is a tissue-specific transcription factor. *The EMBO journal.* Oct 2 1995;14(19):4763-4772.
16. Leerapun T, Hugate RR, Inwards CY, Scully SP, Sim FH. Surgical management of conventional grade I chondrosarcoma of long bones. *Clinical orthopaedics and related research.* Oct 2007;463:166-172.
17. Lin PP, Jaffe N, Herzog CE, et al. Chemotherapy response is an important predictor of local recurrence in Ewing sarcoma. *Cancer.* Feb 1 2007;109(3):603-611.
18. Liu X, Kato Y, Kaneko MK, et al. Isocitrate dehydrogenase 2 mutation is a frequent event in osteosarcoma detected by a multi-specific monoclonal antibody MsMab-1. *Cancer medicine.* 2013;2(6):803-814.
19. Ozaki T, Flege S, Liljenqvist U, et al. Osteosarcoma of the spine: experience of the Cooperative Osteosarcoma Study Group. *Cancer.* Feb 15 2002;94(4):1069-1077.
20. Peabody TD GC, Simon MA. Evaluation and staging of musculoskeletal neoplasms. *The Journal of bone and joint surgery. American volume.* 1988;80(A):1204–1218.
21. Peters TL, Kumar V, Polikepahad S, et al. BCOR-CCNB3 fusions are frequent in undifferentiated sarcomas of male children. *Modern pathology : an official journal of the United States and Canadian Academy of Pathology, Inc.* Apr 2015;28(4):575-586.
22. Pillay N, Plagnol V, Tarpey PS, et al. A common single-nucleotide variant in T is strongly associated with chordoma. *Nature genetics.* 2012;44(11):1185-1187.
23. Presneau N, Shalaby A, Ye H, et al. Role of the transcription factor T (brachyury) in the pathogenesis of sporadic chordoma: a genetic and functional-based study. *J Pathol.* Feb 2011;223(3):327-335.
24. Pring ME, Weber KL, Unni KK, Sim FH. Chondrosarcoma of the pelvis. A review of sixty-four cases. *The Journal of bone and joint surgery. American volume.* Nov 2001;83-A(11):1630-1642.
25. Reith JD, Horodyski MB, Scarborough MT. Grade 2 chondrosarcoma: stage I or stage II tumor? *Clinical orthopaedics and related research.* Oct 2003(415):45-51.
26. Rougraff BT, Simon MA, Kneisl JS, Greenberg DB, Mankin HJ. Limb salvage compared with amputation for osteosarcoma of the distal end of the femur. A long-term oncological, functional, and quality-of-life study. *The Journal of bone and joint surgery. American volume.* May 1994;76(5):649-656.
27. Saifuddin A. The accuracy of imaging in the local staging of appendicular osteosarcoma. *Skeletal radiology.* Apr 2002;31(4):191-201.
28. Salinas-Souza C, De Andrea C, Bihl M, et al. GNAS mutations are not detected in parosteal and low-grade central osteosarcomas. *Modern Pathology.* 2015;28(10):1336-1342.
29. Schoenfeld AJ, Hornicek FJ, Pedlow FX, et al. Osteosarcoma of the spine: experience in 26 patients treated at the Massachusetts General Hospital. *The spine journal : official journal of the North American Spine Society.* Aug 2010;10(8):708-714.
30. Schwab J, Gasbarrini A, Bandiera S, et al. Osteosarcoma of the mobile spine. *Spine.* Mar 15 2012;37(6):E381-386.
31. Söderstrom M, Ekfors TO, Böhling TO, Teppo LH, Vuorio EI, Aro HT. No improvement in the overall survival of 194 patients with chondrosarcoma in Finland in 1971-1990. *Acta orthopaedica.* 2003;74(3):344-350.
32. Stacy GS, Mahal RS, Peabody TD. Staging of bone tumors: a review with illustrative examples. *AJR. American journal of roentgenology.* Apr 2006;186(4):967-976.
33. Stephens PJ, Greenman CD, Fu B, et al. Massive genomic rearrangement acquired in a single catastrophic event during cancer development. *Cell.* Jan 7 2011;144(1):27-40.
34. Szymanska J, Mandahl N, Mertens F, Tarkkanen M, Karaharju E, Knuutila S. Ring chromosomes in parosteal osteosarcoma contain sequences from 12q13-15: a combined cytogenetic and comparative genomic hybridization study. *Genes Chromosomes Cancer.* May 1996;16(1):31-34.
35. Tabareau-Delalande F, Collin C, Gomez-Brouchet A, et al. Diagnostic value of investigating GNAS mutations in fibro-osseous lesions: a retrospective study of 91 cases of fibrous dysplasia and 40 other fibro-osseous lesions. *Modern pathology : an official journal of the United States and Canadian Academy of Pathology, Inc.* Jul 2013;26(7):911-921.
36. Talac R, Yaszemski MJ, Currier BL, et al. Relationship between surgical margins and local recurrence in sarcomas of the spine. *Clinical orthopaedics and related research.* 2002;397:127-132.
37. Tirode F, Surdez D, Ma X, et al. Genomic landscape of Ewing sarcoma defines an aggressive subtype with co-association of STAG2 and TP53 mutations. *Cancer Discov.* Nov 2014;4(11):1342-1353.
38. Vujovic S, Henderson S, Presneau N, et al. Brachyury, a crucial regulator of notochordal development, is a novel biomarker for chordomas. *J Pathol.* Jun 2006;209(2):157-165.

39. Wuisman P, Enneking W. Prognosis for patients who have osteosarcoma with skip metastasis. *The Journal of Bone & Joint Surgery.* 1990;72(1):60-68.
40. Yang M. Prognostic role of pathologic fracture in osteosarcoma: Evidence based on 1,677 subjects. *J Cancer Res Ther.* Apr-Jun 2015;11(2):264-267.
41. Yoshida A, Ushiku T, Motoi T, et al. MDM2 and CDK4 immunohistochemical coexpression in high-grade osteosarcoma: correlation with a dedifferentiated subtype. *The American journal of surgical pathology.* Mar 2012;36(3):423-431.
42. Yoshida A, Ushiku T, Motoi T, et al. Immunohistochemical analysis of MDM2 and CDK4 distinguishes low-grade osteosarcoma from benign mimics. *Modern pathology : an official journal of the United States and Canadian Academy of Pathology, Inc.* Sep 2010;23(9):1279-1288.
43. Morrison WB, Weissman BN, Kransdorf MJ, et al. ACR Appropriateness Criteria for Primary Bone Tumors. 2013; https://acsearch.acr.org/docs/69421/Narrative/. Accessed January 25, 2016, 2016.

Part IX

Soft Tissue Sarcoma

Members of the Soft Tissue Sarcoma Expert Panel

Mark Agulnik, MD
Elliot A. Asare, MD
Elizabeth H. Baldini, MD, MPH
Robert K. Brookland, MD, FACR, FACRO – Editorial Board Liaison
Kumarasen Cooper, MBChB, DPhil, FRCPath – CAP Representative
Ronald P. DeMatteo, MD
Andrew L. Folpe, MD
B. Ashleigh Guadagnolo, MD, MPH
Jason L. Hornick, MD, PhD
Robin L. Jones, MD
Vicki L. Keedy, MD, MSCI
David G. Kirsch, MD, PhD
Alexander J. Lazar, MD, PhD, FACP – Precision Medicine Core Representative
John E. Madewell, MD
Robert G. Maki, MD, PhD, FACP – Vice Chair
Brian O'Sullivan, MD, FRCPC – UICC Representative
David M. Panicek, MD, FACR
Snehal G. Patel, MD
Raphael E. Pollock, MD, PhD, FACS – Chair
R. Lor Randall, MD, FACS
Chandrajit P. Raut, MD, MSc, FACS
Richard F. Riedel, MD
Erich M. Sturgis, MD, MPH, FACS
Paige S. Tedder, RHIT, CTR – Data Collection Core Representative
Sam S. Yoon, MD

Introduction to Soft Tissue Sarcoma

39

Raphael E. Pollock and Robert G. Maki

CHAPTER SUMMARY

Cancers Staged In This Section

This section addresses several soft tissue sarcomas that arise in the following areas:

- Head and neck
- Extremity and trunk
- Gastrointestinal tract
- Genitourinary tract
- Viscera and retroperitoneum
- Gynecologic sites
- Breast
- Lung, pleura, and mediastinum
- Other histologies

Cancers Not Staged In This Section

These histopathologic types of cancer…	Are staged according to the classification for…	And can be found in chapter…
Desmoid tumor/deep fibromatosis	No AJCC staging system. Anatomic site, sizes and, margin status should be recorded.	N/A
Kaposi sarcoma	No AJCC staging system. AIDS Clinical Trials Group (ACTG) system TIS (tumor, immune system, systemic illness) staging may be used; however, the utility of this system appears inadequate in the era of antiretroviral therapy, although high-risk cases remain.[1]	N/A

Summary of Changes

Change	Details of Change	Level of Evidence
Multiple chapters	A greater emphasis is placed on the anatomic primary site of the soft tissue sarcoma, which has implications for local recurrence and metastatic disease.	N/A
Gastrointestinal stromal tumor (GIST)	GIST still has its own staging system and remains unchanged but is collected under sarcomas, as these are mesenchymal malignancies	N/A

(continued)

To access the AJCC cancer staging forms, please visit www.cancerstaging.org.

© American Joint Committee on Cancer 2017

M.B. Amin et al. (eds.), *AJCC Cancer Staging Manual, Eighth Edition*, DOI 10.1007/978-3-319-40618-3_39

Summary of Changes (continued)

Change	Details of Change	Level of Evidence
New retroperitoneal sarcoma staging system	More accurately reflects the biology of this tumor site; a validated nomogram may be used to help guide risk assessment in addition to traditional staging categories.	I
New head and neck sarcoma staging system	Tumors are recognized at smaller sizes than those at other sites but have higher risk on a size basis than those of other sites. Provisional TNM criteria are provided to facilitate prospective data collection.	IV
New visceral sarcoma staging system	There are no superficial tumors in this anatomic site.	IV
Definition of Primary Tumor (T)	A new size category reflects the increased risk of metastasis as primary size increases. The superficial-versus-deep distinction is less important and has been eliminated.	II
Definition of Regional Lymph Node (N)	N1 disease behaves similarly between Stage III and Stage IV disease and is captured as Stage IV disease for simplicity.	II
Unusual sites and histologies	Guidance is provided regarding some unique histologies and their biological behavior. Some sarcomas metastasize early, but patients may live with metastatic disease far longer than with other sarcoma histologies.	N/A

ICD-O-3 Topography Codes

Code	Description
C38.0	Malignant neoplasm of heart
C38.1	Malignant neoplasm of anterior mediastinum
C38.2	Malignant neoplasm of posterior mediastinum
C38.3	Malignant neoplasm of mediastinum, part unspecified
C38.4	Malignant neoplasm of pleura
C38.8	Malignant neoplasm of overlapping lesion of heart, mediastinum, and pleura
C47.0	Peripheral nerves and autonomic nervous system of head, face and neck
C47.1	Peripheral nerves and autonomic nervous system of upper limb and shoulder
C47.2	Peripheral nerves and autonomic nervous system of lower limb, including hip
C47.3	Peripheral nerves and autonomic nervous system of thorax
C47.4	Peripheral nerves and autonomic nervous system of abdomen
C47.5	Peripheral nerves and autonomic nervous system of pelvis
C47.6	Peripheral nerves and autonomic nervous system of trunk, unspecified
C47.8	Overlapping lesion of peripheral nerves and autonomic nervous system
C47.9	Peripheral nerves and autonomic nervous system, unspecified
C48.0	Malignant neoplasm of retroperitoneum
C48.1	Malignant neoplasm of specified parts of peritoneum
C48.2	Malignant neoplasm of peritoneum, unspecified
C48.8	Overlapping lesion of retroperitoneum and peritoneum
C49.0	Connective, subcutaneous, and other soft tissues of head, face, and neck
C49.1	Connective, subcutaneous, and other soft tissues of upper limb and shoulder
C49.2	Connective, subcutaneous, and other soft tissues of lower limb and hip
C49.3	Connective, subcutaneous, and other soft tissues of thorax
C49.4	Connective, subcutaneous, and other soft tissues of abdomen
C49.5	Connective, subcutaneous, and other soft tissues of pelvis
C49.6	Connective, subcutaneous, and other soft tissues of trunk, NOS
C49.8	Overlapping lesion of connective, subcutaneous, and other soft tissues
C49.9	Connective, subcutaneous, and other soft tissues, NOS

Sarcomas Arising in These Areas

Code	Description
C00-C14	Lip, oral cavity, and pharynx
C15-C26	Digestive organs
C30-C33	Respiratory system
C34-C37	Intrathoracic organs
C50	Breast
C51-C53	Female genital organs
C58	Female genital organs
C60-C63	Male genital organs
C64-C68	Urinary tract
C69.0-C69.5, C69.9	Eye
C70-72	Brain and central nervous system
C73-C75	Thyroid and other endocrine glands
C80.9	Unknown primary site

WHO Classification of Tumors

This list of sarcomas is derived from the World Health Organization (WHO) fascicle on soft tissue and bone sarcoma pathology (2013), edited to exclude benign diagnoses. The full reference contains information on the benign soft tissue and bone entities.[2]

Adipocytic Tumors

Code	Description
8850	Atypical lipomatous tumor
8850	Well-differentiated liposarcoma
8850	Liposarcoma, NOS
8858	Dedifferentiated liposarcoma
8852	Myxoid/round cell liposarcoma
8854	Pleomorphic liposarcoma

Fibroblastic/Myofibroblastic Tumors

Code	Description
8832	Dermatofibrosarcoma protuberans
8832	Fibrosarcomatous dermatofibrosarcoma protuberans
8833	Pigmented dermatofibrosarcoma protuberans
8815	Solitary fibrous tumor, malignant
8825	Inflammatory myofibroblastic tumor
8825	Low-grade myofibroblastic sarcoma
8810	Adult fibrosarcoma
8811	Myxofibrosarcoma (formerly myxoid maligant fibrous histiocytoma [myxoid MFH])
8840	Low-grade fibromyxoid sarcoma
8840	Sclerosing epithelioid fibrosarcoma

So-called Fibrohistiocytic Tumors

Code	Description
9251	Giant cell tumor of soft tissues

Smooth Muscle Tumors

Code	Description
8890	Leiomyosarcoma (excluding skin)

Pericytic (Perivascular) Tumors

Code	Description
8711	Malignant glomus tumor

Skeletal Muscle Tumors

Code	Description
8910	Embryonal rhabdomyosarcoma (including botryoid, anaplastic)
8920	Alveolar rhabdomyosarcoma (including solid, anaplastic)
8901	Pleomorphic rhabdomyosarcoma
8912	Spindle cell/sclerosing rhabdomyosarcoma

Vascular Tumors of Soft Tissue

Code	Description
9136	Retiform hemangioendothelioma
9136	Pseudomyogenic (epithelioid sarcoma-like) hemangioendothelioma
9133	Epithelioid hemangioendothelioma
9120	Angiosarcoma of soft tissue

Chondro-osseous Tumors

Code	Description
9180	Extraskeletal osteosarcoma

Gastrointestinal Stromal Tumors

Code	Description
8936	Gastrointestinal stromal tumor, malignant

Nerve Sheath Tumors

Code	Description
9540	Malignant peripheral nerve sheath tumor
9542	Epithelioid malignant peripheral nerve sheath tumor
9561	Malignant Triton tumor
9580	Malignant granular cell tumor

Tumors of Uncertain Differentiation

Code	Description
8842	Ossifying fibromyxoid tumor, malignant
8935	Stromal sarcoma, NOS
8982	Myoepithelial carcinoma
8990	Phosphaturic mesenchymal tumor, malignant
9040	Synovial sarcoma, NOS
9041	Synovial sarcoma, spindle cell
9043	Synovial sarcoma, biphasic
8804	Epithelioid sarcoma
9581	Alveolar soft part sarcoma
9044	Clear cell sarcoma of soft tissue
9231	Extraskeletal myxoid chondrosarcoma
9364	Extraskeletal Ewing sarcoma
8806	Desmoplastic small round cell tumor
8963	Extrarenal rhabdoid tumor
8714	Perivascular epithelioid cell tumor (PEComa), NOS
9137	Intimal sarcoma

Undifferentiated/Unclassified Sarcomas

Code	Description
8801	Undifferentiated spindle cell sarcoma
8802	Undifferentiated pleomorphic sarcoma
8803	Undifferentiated round cell sarcoma
8804	Undifferentiated epithelioid sarcoma
8805	Undifferentiated sarcoma, NOS

INTRODUCTION

The AJCC Cancer Staging Manual, 8th Edition staging criteria stratify risk of recurrence or death from cancer and also serve to categorize cancers for registry purposes. With those goals in mind, the staging criteria for soft tissue sarcomas have expanded significantly from previous editions in the effort to address some of the shortfalls in categorizing the more than 50 diagnoses that comprise soft tissue sarcomas. Although it would be impractical to create a staging system for each histology, commonalities among the sarcomas allow some ability to stratify risk of cancer recurrence as a group.

A greater emphasis is placed on the anatomic primary site of the soft tissue sarcoma, which has implications for local recurrence and metastatic disease. Specifically, separate chapters on staging soft tissue sarcomas of the (1) extremity and trunk, (2) retroperitoneum, (3) head and neck, and (4) visceral sites are presented. For the first two sites, outcomes are well characterized and good predictive models exist for recurrence based on staging data; however, for the latter two sites the available data are more limited, and the criteria presented herein will serve as a starting point and research tool for refining risk for these anatomic sites in future editions. The chapter regarding the most common sarcoma, GIST, is now incorporated into the soft tissue sarcoma section, and a final chapter on some of the clinical features of unusual histologies and particular anatomic primary sites is presented to provide a better understanding of some of the unusual histologies captured by the soft tissue sarcoma staging system.

Soft tissue sarcomas constitute a family of more than 50 different subtypes of cancer, as well as lesions that are locally aggressive and only infrequently or never metastasize.[3] An even greater number of subtypes are defined if specific DNA alterations are included in the characterization of the tumor. It is not clear for sarcomas other than GIST whether there is a relevant impact on outcome from a specific genetic alteration, and even in GIST, these data remain incomplete and a topic of ongoing research.

Histologic subtype, grade, and tumor size are essential for staging. Histologic grade of a sarcoma is one of the most important parameters of the staging system. Grade is based on analysis of numerous pathological features of a tumor, such as histologic subtype, degree of differentiation, mitotic activity, and necrosis. Accurate grading requires an adequate sample of well-fixed tissue for evaluation. Accurate grading is not always possible on the basis of needle biopsies or in tumors that were previously irradiated or treated with chemotherapy.

The current staging system attempts, for the first time, to distinguish anatomic primary tumor site. This is particularly applicable in sites such as head and neck and retroperitoneum, where grade (head and neck) or size (retroperitoneum) may disproportionately drive prognosis relative to other staging criteria in comparison with sarcomas arising elsewhere in the body. Primary sarcomas of the breast are another special situation in which the tumor should be staged and managed as would any comparably staged sarcoma located elsewhere in the body (e.g., staged and treated in a manner analogous to a superficial truncal sarcoma). Generic grouping of site is accepted.

The following site groups may be used for reports that include sarcomas arising in tissues other than soft tissues (such as parenchymal organs). Extremity and trunk may be combined; viscera, including the intra-abdominal viscera, also may be combined. Where enough numbers exist, these may be reported by subdivision into the various components of the gastrointestinal tract. Lung, gastrointestinal, genitourinary, and gynecologic sarcomas should be grouped separately.

Site Groups for Soft Tissue Sarcoma

- Head and neck
- Extremity and trunk
- Gastrointestinal
- Genitourinary
- Visceral retroperitoneal
- Gynecologic
- Breast
- Lung, pleura, and mediastinum
- Other

ANATOMY

Primary Site(s)

The present staging system applies to soft tissue sarcomas. Primary sarcomas may arise from a variety of soft tissues. These tissues include fibrous connective tissue (fibroblasts), fat, smooth or striated muscle, vascular tissue, peripheral neural tissue, and visceral tissue. Ewing sarcoma may arise in bone (and staged as a bone tumor) or in soft tissue (and staged as a soft tissue sarcoma).

Regional Lymph Nodes

Involvement of regional lymph nodes by soft tissue sarcomas is uncommon in adults. Specific histologies in which regional lymph node metastatic disease is most commonly observed include alveolar rhabdomyosarcoma, embryonal rhabdomyosarcoma, epithelioid sarcoma, and angiosarcoma.

Metastatic Sites

Metastatic sites for soft tissue sarcoma often depend on the original site of the primary lesion. For example, the most common site of metastatic disease for patients with extremity sarcoma is the lung, whereas retroperitoneal and gastrointestinal sarcomas often have liver as the first site of metastasis.

RULES FOR CLASSIFICATION

Clinical Classification

Clinical staging involves a definition of the sarcoma by physical examination; imaging; diagnostic biopsies of the primary, nodes, and/or potential metastatic sites; and other diagnostic procedures, such as endoscopy. It is based on characteristics of tumor (T), nodes (N), metastasis (M), and grade (G). Tumor size may be determined clinically or radiologically. Metastatic disease should be described according to the most likely sites of metastasis. In general, the minimal clinical staging workup of soft tissue sarcoma is accomplished by axial imaging of the involved site by using magnetic resonance (MR) imaging or computed tomography (CT) scan, as well as imaging of the lungs, the most likely site of occult metastatic disease, with chest CT scans. Myxoid and round cell liposarcoma metastasizes to soft tissue sites and bone marrow sites such as the pelvis and spine, and more thorough staging may be necessary for high-risk lesions. Diagnostic biopsies of the primary site, nodes, and distant metastasis are included in clinical staging.

TNM Categories of Tumor Staging

The T category is assessed by measuring the largest diameter of the tumor in any plane. The measurement should be made on whichever MR imaging pulse sequence best delineates the tumor. Some tumors, such as pleomorphic sarcoma and myxofibrosarcoma, often have tail-like projections that extend for considerable distances along fascial and neurovascular planes. Surrounding edema, if present, should not be included in the measurement.

Regional nodal metastases are uncommon with most histologic types of extremity soft tissue sarcoma. Nodes are considered suspicious for tumor involvement if enlarged, rounded, or necrotic, or if the normal fatty hilum of the node is replaced by soft tissue.

Extremity soft tissue sarcomas most commonly metastasize to lung, manifesting as sharply defined nodules. Hemorrhagic nodules, such as in angiosarcoma, may show surrounding halos of ground-glass attenuation. The metastases of some types of sarcomas, such as extraskeletal osteosarcoma or chondrosarcoma, may contain calcification at CT and should not be assumed to represent calcified granulomas.

Definition of T

Tumor size criteria vary by anatomic site. Particular emphasis should be placed on providing size measurements (or even volume determinants) in all sites. Size should be regarded as a continuous variable, with the centimeter cutoffs as arbitrary divisions that make it possible to characterize patient populations.

Depth

Because there is less impact of depth on outcome and because of the inherent inability to use depth in visceral and other sites, in the 8th Edition, depth no longer is used in the staging system. For completeness, depth has been evaluated relative to the investing fascia of the extremity and trunk. *Superficial* was defined as lack of any involvement of the superficial investing muscular fascia in extremity or trunk lesions. For staging, nonsuperficial head and neck, intrathoracic, intraabdominal, retroperitoneal, and visceral lesions were considered deep lesions.

Nodal Disease

Nodal involvement is rare in adult soft tissue sarcomas. In assigning a stage group, patients whose nodal status is not determined to be positive for tumor, either clinically or microscopically, should be designated as N0, given the rarity of involvement of this site by most sarcomas. The designation for the clinical stage is cN0. If microscopically determined for the pathological stage, it would be designated as pN0. If clinically determined by physical examination or imaging for the pathological stage, it would be designated as cN0 and not pNX.

Grade

The issue of grade continues to play an important role in ultimate sarcoma staging, especially because these cancers generally do not metastasize to lymph nodes. Thus, functionally speaking, only tumor size and the presence or absence of metastatic disease are the variables in risk assessment if grade is omitted. It is well accepted that histology is even more important than grade in many instances but that, in general, grade helps determine risk better than primary tumor size.

Grade should be assigned to all sarcomas. Historically, the AJCC soft tissue staging system used a four-grade system, but this was revised starting with the AJCC Cancer Staging Manual, 7th Edition to a three-grade system used by the two most commonly recognized staging systems. Comprehensive grading of soft tissue sarcomas is strongly correlated with disease-specific survival and incorporates differentiation (histology specific), mitotic rate, and extent of necrosis. In accordance with the College of American Pathologists (CAP) recommendations,[4] the French Federation of Cancer Centers Sarcoma

Group (FNCLCC) system[5] is preferred over the National Institutes of Health system because of its ease of use/reproducibility and slightly superior performance.

Applying histologic grading to core needle biopsies is problematic when neoadjuvant chemotherapy or radiation has been administered. However, given the importance of grade to staging and treatment, efforts are encouraged to separate sarcomas on needle biopsies as described earlier. In many instances, the type of sarcoma will permit this distinction readily (e.g., Ewing sarcoma, undifferentiated pleomorphic sarcoma), whereas in less obvious instances, the difficulty of assigning a grade should be noted. In general, multiple core needle biopsies disclosing a high-grade sarcoma may be regarded as high grade because the probability of subsequent downgrading is remote, but limited cores biopsies of low-grade sarcoma carry a risk of subsequent upgrading.

There are several subtypes not specifically defined by FNCLCC grading criteria in Table 39.1. Yet there are some helpful data on grading from the FNCLCC definitions, especially under differentiation.[6] The FNCLCC grade is determined by three parameters: differentiation, mitotic activity, and extent of necrosis. Each parameter is scored as follows: differentiation (1–3), mitotic activity (1–3), and necrosis (0–2). The scores are added to determine the grade.

Table 39.1 Histology-specific tumor differentiation score

Histologic type	Score
Atypical lipomatous tumor/well-differentiated liposarcoma	1
Myxoid liposarcoma	2
Round cell liposarcoma	3
Pleomorphic liposarcoma	3
Dedifferentiated liposarcoma	3
Fibrosarcoma	2
Myxofibrosarcoma	2
Undifferentiated pleomorphic sarcoma (formerly termed malignant fibrous histiocytoma, pleomorphic type)	3
Well-differentiated leiomyosarcoma	1
Conventional leiomyosarcoma	2
Poorly differentiated/pleomorphic/epithelioid leiomyosarcoma	3
Biphasic/monophasic synovial sarcoma	3
Poorly differentiated synovial sarcoma	3
Pleomorphic rhabdomyosarcoma	3
Mesenchymal chondrosarcoma	3
Extraskeletal osteosarcoma	3
Ewing sarcoma/primitive neuroectodermal tumor (PNET)	3
Malignant rhabdoid tumor	3
Undifferentiated sarcoma, not otherwise specified	3

Note: Grading of gastrointestinal stromal tumor, malignant peripheral nerve sheath tumor, embryonal and alveolar rhabdomyosarcoma and angiosarcoma (rapid growth, dissemination common), as well as extraskeletal myxoid chondrosarcoma, alveolar soft part sarcoma, clear cell sarcoma, and epithelioid sarcoma (slower growth, dissemination common) is not recommended under this system. The case for grading malignant peripheral nerve sheath tumor is debated. Although all these histologies have a high rate of dissemination, survival with metastatic disease varies widely.

Modified from Guillou et al.,[5] with permission

Tumor Differentiation

Tumor differentiation is histology specific and is generally scored as follows:

Differentiation Score	Definition
1	Sarcomas closely resembling normal adult mesenchymal tissue (e.g., low-grade leiomyosarcoma)
2	Sarcomas for which histologic typing is certain (e.g., myxoid/round cell liposarcoma)
3	Embryonal and undifferentiated sarcomas, sarcomas of doubtful type, synovial sarcomas, soft tissue osteosarcoma, Ewing sarcoma/primitive neuroectodermal tumor (PNET) of soft tissue

Mitotic Count

In the most mitotically active area of the sarcoma, 10 successive high-power fields (HPF; one HPF at 400× magnification = 0.1734 mm^2) are assessed using a 40× objective.

Mitotic Count Score	Definition
1	0–9 mitoses per 10 HPF
2	10–19 mitoses per 10 HPF
3	≥20 mitoses per 10 HPF

Tumor Necrosis

Evaluated on gross examination and validated with histologic sections.

Necrosis Score	Definition
0	No necrosis
1	<50% tumor necrosis
2	≥50% tumor necrosis

FNCLCC Histologic Grade

G	G Definition
GX	Grade cannot be assessed
G1	Total differentiation, mitotic count and necrosis score of 2 or 3
G2	Total differentiation, mitotic count and necrosis score of 4 or 5
G3	Total differentiation, mitotic count and necrosis score of 6, 7, or 8

Tumor differentiation score is the most subjective aspect of the FNCLCC system (Table 39.1). In addition, it is not validated for every subtype of sarcoma and is inapplicable to certain subtypes. However, this score is critical given its proportional weight such that any sarcoma assigned a differentiation score of 3 will be at least intermediate to high grade.

Although not specifically mentioned in the original FNCLCC grading system, differentiation scores may be used for sarcomas newer than those described in the original document. For example, dermatofibrosarcoma protuberans merits a differentiation score of 1. Low-grade fibromyxoid sarcoma and sclerosing epithelioid fibrosarcoma do not have a differentiation grade, because they were characterized after the FNCLCC criteria were developed. A differentiation score of 2 is suggested based on the general differentiation criteria of FNCLCC and their propensity for metastatic disease.

Imaging

MR imaging is the preferred examination for assessing primary tumor stage information. CT performed with intravenous contrast material, however, may provide similar information, particularly if MR imaging is not available or is contraindicated. MR imaging and CT also may guide the selection of an optimal site for biopsy, such as the most vascular or cellular region, and avoid nondiagnostic necrotic portions. Plain radiography may demonstrate subtle cortical involvement better than MR imaging or CT. For sarcomas with a particular propensity to metastasize to lymph nodes, scintigraphic sentinel node mapping may be performed to guide subsequent lymph node sampling. Chest CT is used to assess for pulmonary metastases, the most common site of metastasis of most soft tissue sarcomas of the extremities. In myxoid/round cell liposarcoma, spine and pelvis MR imaging is used for higher risk lesions to assess for marrow metastases, which are occult by other imaging modalities. Positron emission tomography/CT is useful for whole-body staging in rhabdomyosarcoma, given its higher risk of metastatic disease and lymph node metastases.

Radiologic Staging of Tumor

Describe the location and extent of the primary tumor, including relationship to adjoining muscles, blood vessels, nerves, bones, and joints. Neurovascular encasement and bone marrow involvement are assessed best on (non–fat-suppressed) T1-weighted images. Contact of more than 180° of the circumference of the vessel wall by tumor should be considered suspicious for encasement; lesser degrees of contact should be described as contact without encasement. Tumor margins often can be distinguished from the surrounding reactive zone (which manifests as soft tissue edema and may contain viable tumor cells) on T2-weighted or postcontrast fat-suppressed T1-weighted MR images.

Proposed Report Format

1. Primary tumor
   a. MR imaging signal or CT attenuation characteristics
   b. Extent and location of necrosis within tumor
   c. Location in extremity, including relationship to superficial fascia
   d. Presence and location of tumor tails
   e. Size (in three dimensions)
2. Local extent
   a. Invasion of muscles, bones, and joints
   b. Contact with, or encasement of, blood vessels and nerves
   c. Extension into lumen of blood vessels
   d. Presence of nearby satellite nodules
3. Regional lymph node involvement

Proposed Risk Categories

As is happening in radiology, in which specific terms are used to categorize the certainty of the assessment, we introduce here a common vocabulary for recurrence risk. It is hoped that regardless of the staging method employed, this risk terminology may be used to accurately communicate present-day best understanding of the risk of recurrence or metastatic disease, which can then aid the clinician in determining whether adjuvant therapy may or should be considered.

Table 39.2 lists the different risk categories. For example, primary soft tissue sarcomas that have a less than 10% risk of recurrence or metastatic disease are termed low risk, whereas those with a greater than 30% risk of recurrence or metastatic disease are termed high risk primary tumors.

Pathological Classification

Pathological (pTNMG) staging consists of the removal and pathological evaluation of the primary tumor and clinical/radiologic evaluation for regional and distant metastases. In circumstances in which it is not possible to obtain accurate measurements of the excised primary sarcoma specimen, it is acceptable to use radiologic assessment to assign a pT category using the dimensions of the sarcoma. In examining the primary tumor, the pathologist should subclassify the lesion and assign a histopathologic grade. Occasionally, immunohistochemistry or cytogenetics may be necessary for accurate assignment of subtype.

Staging after neoadjuvant therapy is classified as **yp**, instead of **p**. Assignment of grade may be affected by prior administration of chemotherapy and/or radiotherapy. Lesions initially assigned a high-grade status may have a less ominous appearance on microscopic examination after response to presurgical treatments; therefore, they may be

Table 39.2 Proposed risk categories for primary soft tissue sarcomas

Risk of recurrence or metastatic disease (%)	Risk category
≤10	Low
>10 to ≤30	Intermediate
>30	High

assigned a grade lower than the initial designation. Occasionally, the reverse situation is observed either because of sampling error or as the result of elimination of lower-grade cells by presurgical treatment of these typically heterogeneous tumors.

Restaging of Recurrent Tumors

The same staging should be used when a patient requires restaging of sarcoma recurrence. This classification is assigned using the prefix *r* (rTNM). Such reports should specify whether patients have primary lesions or lesions that were treated previously and have recurred. The identification and reporting of etiologic factors such as radiation exposure and inherited or genetic syndromes are encouraged. Appropriate workup for recurrent sarcoma should include cross-sectional imaging (CT or MR imaging scan) of the tumor, a CT scan of the chest, and a tissue biopsy to confirm diagnosis before therapy is initiated.

PROGNOSTIC FACTORS

Prognostic Factors Required for Stage Grouping

French Federation of Cancer Centers Sarcoma Group (FNCLCC) Grade

See Clinical Classification in this chapter.

Mitotic Rate

GIST (Chapter 43)

Additional Factors Recommended for Clinical Care

Neurovascular and Bone Invasion

In earlier staging systems, neurovascular and bone invasion by soft tissue sarcomas was included as a determinant of stage. However, it is not included in the current staging system, and no plans are proposed to add it at the present time. Nevertheless, neurovascular and bone invasion should be reported if possible, although further studies are needed to determine whether such invasion is an independent prognostic factor for clinical outcomes. AJCC Level of Evidence: III

Molecular Markers

Molecular markers and genetic abnormalities are being evaluated as determinants of outcome. At the present time, except for GIST, in which *KIT*, *PDGFRA*, or other mutation status has a prognostic and predictive impact on patient management, insufficient data exist to include specific molecular markers in the staging system.

Some of the staging issues regarding tumor grade may be supplanted by genomic tests in the future. A characteristic genetic signature of aneuploidy, termed Complexity Index in Sarcoma (CINSARC), outperforms histologic grading in soft tissue sarcomas and GIST alike[7], and in the future it might become accepted as a prognostic marker in lieu of FNCLCC sarcoma grade.[8] AJCC Level of Evidence: III

Validation

The current staging system has the capacity to discriminate the overall survival of patients with soft tissue sarcoma. Patients with Stage I lesions are at low risk for disease-related mortality, whereas Stages II and III entail progressively greater risk. In extremity and trunk sarcomas, this factor meets level I evidence for AJCC. In head and neck, retroperitoneal, and visceral sarcomas, the level of evidence is IV.

For specific information on TNMG staging of soft tissue sarcomas by anatomic site, please refer to the appropriate chapter in this section.

REGISTRY DATA COLLECTION VARIABLES

1. Bone invasion as determined by imaging
2. If pM1, source of pathological metastatic specimen
3. Additional dimensions of tumor size
4. FNCLCC grade
5. Central nervous system extension (head and neck primaries)
6. Mitotic rate for GIST
7. KIT immunohistochemistry for GIST
8. Mutational status of *KIT*, *PDGFRA* for GIST

HISTOLOGIC GRADE (G)

FNCLCC
Mitotic rate for GIST

HISTOPATHOLOGIC TYPE

Please see the WHO Classification of Tumors section in this chapter for a list of the soft tissue sarcoma histologies.

Bibliography

1. Krown SE, Metroka C, Wernz JC. Kaposi's sarcoma in the acquired immune deficiency syndrome: a proposal for uniform evaluation, response, and staging criteria. AIDS Clinical Trials Group Oncology Committee. *J Clin Oncol*. Sep 1989;7(9): 1201–1207.

2. Fletcher CDM, Bridge JA, Hogendoorn P, Mertens F, eds. *World Health Organization Classification of Tumours of Soft Tissue and Bone. Fourth Edition.* Lyon: IARC; 2013.
3. Brennan MF, Antonescu CR, Maki RG. *Management of soft tissue sarcoma.* Springer Science & Business Media; 2012.
4. Rubin BP, Cooper K, Fletcher CD, et al. Protocol for the examination of specimens from patients with tumors of soft tissue. *Arch Pathol Lab Med.* Apr 2010;134(4):e31–39.
5. Guillou L, Coindre JM, Bonichon F, et al. Comparative study of the National Cancer Institute and French Federation of Cancer Centers Sarcoma Group grading systems in a population of 410 adult patients with soft tissue sarcoma. *J Clin Oncol.* Jan 1997;15(1):350–362.
6. Coindre JM, Terrier P, Bui NB, et al. Prognostic factors in adult patients with locally controlled soft tissue sarcoma. A study of 546 patients from the French Federation of Cancer Centers Sarcoma Group. *J Clin Oncol.* Mar 1996;14(3):869–877.
7. Chibon F, Lagarde P, Salas S, et al. Validated prediction of clinical outcome in sarcomas and multiple types of cancer on the basis of a gene expression signature related to genome complexity. *Nature medicine.* Jul 2010;16(7):781–787.
8. Neuville A, Chibon F, Coindre JM. Grading of soft tissue sarcomas: from histological to molecular assessment. *Pathology.* Feb 2014; 46(2):113-120.

Soft Tissue Sarcoma of the Head and Neck

40

Brian O'Sullivan, Robert G. Maki, Mark Agulnik, Snehal G. Patel, Alexander J. Lazar, Robin L. Jones, Erich M. Sturgis, and Raphael E. Pollock

CHAPTER SUMMARY

Cancers Staged Using This Staging System

This staging system applies to all soft tissue sarcomas of the head and neck except angiosarcoma, rhabdomyosarcoma of the embryonal and alveolar subtype, Kaposi sarcoma, and dermatofibrosarcoma protuberans, which do not share the same behavior and natural history.

Cancers Not Staged Using This Staging System

These histopathologic types of cancer...	Are staged according to the classification for...	And can be found in chapter ...
Sarcoma of orbit	Orbital Sarcoma	70
Embryonal and alveolar rhabdomyosarcoma	No AJCC staging system. See pediatric staging guidelines for alveolar and embryonal rhabdomyosarcoma	N/A
Cutaneous angiosarcoma	No AJCC staging system	N/A
Kaposi sarcoma	No AJCC staging system. AIDS Clinical Trials Group (ACTG) system TIS (tumor, immune system, systemic illness) staging may be used; however, the utility of this system appears inadequate in the era of antiretroviral therapy, although high-risk cases remain.	N/A
Dermatofibrosarcoma protuberans	No AJCC staging system	N/A

Summary of Changes

Change	Details of Change	Level of Evidence
New classification	This classification is being introduced for the first time because the previous classification developed for sarcomas elsewhere is not suited to this anatomic region. It is based on the principles of TNM. Since there are only preliminary data to suggest its effectiveness, the purpose of inclusion here is to prospectively collect data.	N/A
Definition of Primary Tumor (T)	A new set of T categories (T1–T4) has been created. Traditional T1 and T2 according to the 5-cm breakpoint for soft tissue sarcoma have been eliminated in the head and neck because they are less relevant in this anatomic site than smaller tumor size cutoffs.	IV
Definition of Regional Lymph Node (N)	Follow criteria used for extremity and trunk lesions.	IV
Histologic Grade (G)	Follow criteria used for extremity and trunk lesions.	IV

To access the AJCC cancer staging forms, please visit www.cancerstaging.org.

© American Joint Committee on Cancer 2017

M.B. Amin et al. (eds.), *AJCC Cancer Staging Manual, Eighth Edition*, DOI 10.1007/978-3-319-40618-3_40

ICD-O-3 Topography Codes

Code	Description
C00.0	External upper lip
C00.1	External lower lip
C00.2	External lip, NOS
C00.3	Mucosa of upper lip
C00.4	Mucosa of lower lip
C00.5	Mucosa of lip, NOS
C00.6	Commissure of lip
C00.8	Overlapping lesion of lip
C00.9	Lip, NOS
C01.9	Base of tongue, NOS
C02.0	Dorsal surface of tongue, NOS
C02.1	Border of tongue
C02.2	Ventral surface of tongue, NOS
C02.3	Anterior two thirds of tongue, NOS
C02.4	Lingual tonsil
C02.8	Overlapping lesion of tongue
C02.9	Tongue, NOS
C03.0	Upper gum
C03.1	Lower gum
C03.9	Gum, NOS
C04.0	Anterior floor of mouth
C04.1	Lateral floor of mouth
C04.8	Overlapping lesion of floor of mouth
C04.9	Floor of mouth, NOS
C05.0	Hard palate
C05.1	Soft palate, NOS
C05.2	Uvula
C05.8	Overlapping lesion of palate
C05.9	Palate, NOS
C06.0	Cheek mucosa
C06.1	Vestibule of mouth
C06.2	Retromolar area
C06.8	Overlapping lesion of other and unspecified parts of mouth
C06.9	Mouth, NOS
C07.9	Parotid gland
C08.0	Submandibular gland
C08.1	Sublingual gland
C08.8	Overlapping lesion of major salivary glands
C08.9	Major salivary gland, NOS
C09.0	Tonsillar fossa
C09.1	Tonsillar pillar
C09.8	Overlapping lesion of tonsil
C09.9	Tonsil, NOS
C10.0	Vallecula
C10.1	Anterior surface of epiglottis
C10.2	Lateral wall of oropharynx
C10.3	Posterior wall of oropharynx
C10.4	Branchial cleft
C10.8	Overlapping lesion of oropharynx
C10.9	Oropharynx, NOS
C11.0	Superior wall of nasopharynx
C11.1	Posterior wall of nasopharynx
C11.2	Lateral wall of nasopharynx
C11.3	Anterior wall of nasopharynx
C11.8	Overlapping lesion of nasopharynx
C11.9	Nasopharynx, NOS
C12.9	Pyriform sinus
C13.0	Postcricoid region
C13.1	Hypopharyngeal aspect of aryepiglottic fold
C13.2	Posterior wall of hypopharynx
C13.8	Overlapping lesion of hypopharynx
C13.9	Hypopharynx, NOS
C14.0	Pharynx, NOS
C14.2	Waldeyer ring
C14.8	Overlapping lesion of lip, oral cavity, and pharynx
C15.0	Cervical esophagus
C15.3	Upper third of esophagus
C15.8	Overlapping lesion of esophagus
C30.0	Nasal cavity
C30.1	Middle ear
C31.0	Maxillary sinus
C31.1	Ethmoid sinus
C31.2	Frontal sinus
C31.3	Sphenoid sinus
C31.8	Overlapping lesion of accessory sinuses
C31.9	Accessory sinus, NOS
C32.0	Glottis
C32.1	Supraglottis
C32.2	Subglottis
C32.3	Laryngeal cartilage
C32.8	Overlapping lesion of larynx
C32.9	Larynx, NOS
C47.0	Peripheral nerves and autonomic nervous system of head, face, and neck
C49.0	Connective, subcutaneous, and other soft tissues of head, face, and neck
C72.2	Olfactory nerve
C72.4	Acoustic nerve
C72.5	Cranial nerve, NOS
C73.9	Thyroid
C75.0	Parathyroid gland
C75.1	Pituitary gland
C75.2	Craniopharyngeal duct
C75.3	Pineal gland
C75.4	Carotid body
C75.5	Aortic body and other paraganglia
C75.8	Overlapping lesion of endocrine glands and related structures
C75.9	Endocrine gland, NOS

WHO Classification of Tumors

Adipocytic Tumors

Code	Description
8850	Atypical lipomatous tumor
8850	Well-differentiated liposarcoma
8850	Liposarcoma, NOS
8858	Dedifferentiated liposarcoma
8852	Myxoid/round cell liposarcoma
8854	Pleomorphic liposarcoma

Fletcher CDM, Bridge JA, Hogendoorn P, Mertens F, eds. World Health Organization Classification of Tumours of Soft Tissue and Bone. Fourth Edition. Lyon: IARC; 2013.

Fibroblastic/Myofibroblastic Tumors

Code	Description
8815	Solitary fibrous tumor, malignant
8825	Inflammatory myofibroblastic tumor
8825	Low-grade myofibroblastic sarcoma
8810	Adult fibrosarcoma
8811	Myxofibrosarcoma
8840	Low-grade fibromyxoid sarcoma
8840	Sclerosing epithelioid fibrosarcoma

So-called Fibrohistiocytic Tumors

Code	Description
9251	Giant cell tumor of soft tissues

Smooth Muscle Tumors

Code	Description
8890	Leiomyosarcoma (excluding skin)

Pericytic (Perivascular) Tumors

Code	Description
8711	Malignant glomus tumor

Skeletal Muscle Tumors

Code	Description
8901	Pleomorphic rhabdomyosarcoma
8912	Spindle cell/sclerosing rhabdomyosarcoma

Vascular Tumors of Soft Tissue

Code	Description
9136	Retiform hemangioendothelioma
9136	Pseudomyogenic (epithelioid sarcoma-like) hemangioendothelioma
9133	Epithelioid hemangioendothelioma
9120	Angiosarcoma of soft tissue

Chondro-osseous Tumors

Code	Description
9180	Extraskeletal osteosarcoma

Nerve Sheath Tumors

Code	Description
9540	Malignant peripheral nerve sheath tumor
9542	Epithelioid malignant peripheral nerve sheath tumor
9561	Malignant Triton tumor
9580	Malignant granular cell tumor

INTRODUCTION

This chapter is dedicated to staging criteria for sarcomas of the head and neck. Although these sarcomas usually are found at a smaller size than those in other sites, they often have disproportionately greater risk of local recurrence compared with other sites.[1]

Head and neck soft tissue sarcomas do not differ obviously in their biology from soft tissue sarcomas of other sites. However they present unique problems from an anatomic standpoint. Tumors have variable presentations depending on the potential anatomic site of origin, whether neurovascular or bone invasion is evident, and whether swallowing and airway problems exist. Lesions may originate in the upper aerodigestive tract, paranasal sinus, and skull base with symptoms referable to these areas (e.g., nasal symptoms, including obstruction or discharge, and ocular proptosis from direct invasion in paranasal sinus tumors; cranial nerve abnormalities in lesions arising in the skull base or masticator space; alteration of voice or airway compromise for laryngeal and hypopharyngeal lesions). Tumors originating in the subcutaneous tissues of the face, neck, or scalp initially may present with a superficial mass or with bleeding.[2,3]

Unlike other sarcomas for which local salvage options often are available if disease recurs, death from head and neck sarcomas often is a consequence of uncontrolled local disease rather than metastases.[4]

Because of the anatomic location of these tumors in the head and neck, problems of an esthetic and functional nature, as well as tumor resectability, dominate their management. Tumor size and extent present challenges that may be greater compared with other anatomic sites for similarly sized tumors. Furthermore, the AJCC Soft Tissue Sarcoma Expert Panel determined that this disease area has received scant attention in recent decades. In particular, the traditional 5-cm size cut point separating T1 and T2 soft tissue sarcomas of the extremity and trunk lacks relevance for head and neck sarcomas. A literature review indicated that approximately 70% of cases in reported series involved tumors less than 5 cm in largest dimension.[5-7] Moreover, there are no reports of outcome regarding tumors less than 5 cm, presumably because no obvious classification for categorizing smaller primary tumors has been available to date.

Accordingly, and arbitrarily, it was decided that a classification system should be proposed for head and neck sarcomas and its use encouraged so that data can be collected. A T-category classification strategy was proposed based on size criteria following traditionally used size breakpoints for other head and neck malignancies. These criteria include T1 for tumors with a maximum dimension $\leq$2 cm, T2 for those >2 to $\leq$4 cm, and T3 for those >4 cm. Very extensive tumors would be categorized as T4, in line with traditional head and neck lesions.

This chapter represents the recommendations of the AJCC Soft Tissue Sarcoma Expert Panel and supported by the Head and Neck Expert Panel.

As implied, tumor size and histologic grade are essential for this new head and neck soft tissue sarcoma stage classification as is addressing the extent of disease in sufficient detail to address the criteria required for the T4 categories. As in other soft tissue sarcomas, most particularly extremity and trunk lesions, grade is based on analysis of various pathological features, including degree of differentiation, mitotic activity, and necrosis. Accurate grading requires an adequate sample of well-fixed tissue for evaluation and may not always be possible on the basis of needle biopsies or in tumors previously treated with radiation or chemotherapy.

The current head and neck staging system also does not take into account some important prognostic factors, including histologic subtype.

ANATOMY

Primary Site(s)

Primary soft tissue sarcomas are ubiquitous to all soft tissue sites of the head and neck. These include the neck (subcutaneous and deep structures, including neurovascular structures); oral cavity; upper aerodigestive tract, including laryngeal structures; pharyngeal areas; nasal cavity and paranasal sinuses; infratemporal fossa and masticator space; major salivary and thyroid/parathyroid glands; cervical esophagus and trachea; and peripheral and cranial nerves.

Sarcomas of the head and neck are rare tumors of connective tissue origin that comprise about 10% of soft tissue sarcomas overall and less than 1% of head and neck malignancies. They arise in any soft tissue of the region and may be found in patients of any age or gender.

Some sarcoma subtypes appear to exhibit certain preferences for different topographic regions in the head and neck. The four most common groups are lesions in the neck (including the larynx/pharynx), where liposarcoma, malignant peripheral nerve sheath tumor (MPNST), and synovial sarcoma predominate; the scalp and facial skin, where angiosarcoma and dermatofibrosarcoma protuberans are most frequent, although these are not being considered in this classification; the sinonasal tract, where MPNST and angiosarcoma predominate, followed by myxofibrosarcoma and rhabdomyosarcoma; and the oral cavity, which is most often affected by leiomyosarcoma and rhabdomyosarcoma. In the pediatric population, alveolar and embryonal rhabdomyosarcoma are the most common diagnoses and are staged according to pediatric guidelines.

Regional Lymph Nodes

Involvement of regional lymph nodes is uncommon for soft tissue sarcomas, except for certain subtypes, in particular epithelioid sarcoma, clear cell sarcoma, angiosarcoma, and rhabdomyosarcoma.

Metastatic Sites

The most common site of metastatic disease for a patient with head and neck sarcomas is the lung. Certain histologic subtypes, such as myxoid/round cell liposarcoma, more commonly have extrapulmonary metastases.

RULES FOR CLASSIFICATION

Clinical Classification

Clinical staging depends on characteristics of T, N, M, and grade. Tumor size may be measured clinically or radiologically. In addition to traditional inspection and palpation as the basis for clinical examination, diagnostic biopsy of the primary site, nodes, and metastatic sites is part of clinical staging. Endoscopic examination also may be helpful and is emphasized particularly for tumors adjacent to or originating in the upper airway and pharyngeal regions.

In general, the minimal clinical staging workup of soft tissue sarcoma is accomplished by axial imaging of the involved site using magnetic resonance (MR) imaging or computed tomography (CT) and by imaging of the lungs, the most likely site for occult metastatic disease, using chest CT scans.

TNM Categories of Tumor Staging

The T category is assessed by measuring the largest diameter of the tumor in any plane. The measurement should be made on whichever MR imaging sequence best delineates the tumor. Some tumors, such as undifferentiated pleomorphic sarcoma and myxofibrosarcoma, often have tail-like projections that extend for considerable distances along the fascial and neurovascular planes. Surrounding edema, if present, should not be included in the measurement.

Regional lymph nodes are considered suspicious for tumor involvement if enlarged, rounded, or necrotic, or if the normal fatty hilum of the node is replaced by soft tissue.

Like other sarcomas, head and soft tissue sarcomas most commonly metastasize to the lung, manifesting as sharply defined nodules. Hemorrhagic nodules, such as in angiosarcoma, may show surrounding halos of ground-glass attenuation.

Imaging

MR imaging is the preferred examination for assessing tumor stage information. CT performed with intravenous contrast material, however, can provide similar information, particularly if MR imaging is not available or is contraindicated, and it may be useful for evaluation of regional lymph nodes in the neck. MR imaging and CT also can guide the selection of an optimal site for biopsy, such as the most vascular or cellular region, and avoid nondiagnostic necrotic portions. Plain radiography may demonstrate subtle cortical involvement better than MR imaging or CT. For sarcomas with a particular propensity to metastasize to lymph nodes, scintigraphic sentinel node mapping may be performed to guide subsequent lymph node sampling. Chest CT is used to assess for pulmonary metastases, the most common site of metastasis of most soft tissue sarcomas.

Radiologic staging of tumor

Neurovascular encasement and bone marrow involvement are assessed best on (non–fat-suppressed) T1-weighted images. Contact of more than 180° of the circumference of the vessel wall by tumor should be considered suspicious for encasement; lesser degrees of contact should be described as contact without encasement. Tumor margins often can be distinguished from the surrounding reactive zone (which manifests as soft tissue edema and which may contain viable tumor cells) on T2-weighted or postcontrast fat-suppressed T1-weighted MR images.

Pathological Classification

Pathological (pTNMG) staging consists of the removal and pathological evaluation of the primary tumor and clinical/radiologic evaluation for regional and distant metastases. In circumstances in which it is not possible to obtain accurate measurements of the excised primary sarcoma specimen, it is acceptable to use radiologic assessment to assign a pT category by using the dimensions of the sarcoma. In examining the primary tumor, the pathologist should subclassify the lesion and assign a histopathologic grade. Occasionally, immunohistochemistry or cytogenetics may be necessary for accurate assignment of subtype.

Staging after neoadjuvant therapy is classified as **yp**, instead of **p**. Assignment of grade may be affected by prior administration of chemotherapy and/or radiotherapy. Lesions initially assigned a high-grade status may have a less ominous appearance on microscopic examination after response to presurgical treatments and therefore may be assigned a grade lower than the initial designation. Occasionally, the reverse situation is observed, either because of sampling error or as the result of elimination of lower-grade cells by presurgical treatment of these typically heterogeneous tumors. For neoadjuvant therapy, see chapter 39.

PROGNOSTIC FACTORS

Prognostic Factors Required for Stage Grouping

French Federation of Cancer Centers Sarcoma Group (FNCLCC) grade – see Histologic Grade (G).

Additional Factors Recommended for Clinical Care

The authors have not noted any additional factors for clinical care.

RISK ASSESSMENT MODELS

The AJCC recently established guidelines that will be used to evaluate published statistical prediction models for the purpose of granting endorsement for clinical use.[8] Although this is a monumental step toward the goal of precision medicine, this work was published only very recently. Therefore, the existing models that have been published or may be in clinical use have not yet been evaluated for this cancer site by the Precision Medicine Core of the AJCC. In the future, the statistical prediction models for this cancer site will be evaluated, and those that meet all AJCC criteria will be endorsed.

DEFINITIONS OF AJCC TNM

Definition of Primary Tumor (T)

T Category	T Criteria
TX	Primary tumor cannot be assessed
T1	Tumor ≤2 cm
T2	Tumor >2 to ≤4 cm
T3	Tumor >4 cm
T4	Tumor with invasion of adjoining structures
T4a	Tumor with orbital invasion, skull base/dural invasion, invasion of central compartment viscera, involvement of facial skeleton, or invasion of pterygoid muscles
T4b	Tumor with brain parenchymal invasion, carotid artery encasement, prevertebral muscle invasion, or central nervous system involvement via perineural spread

Definition of Regional Lymph Node (N)

N Category	N Criteria
N0	No regional lymph node metastases or unknown lymph node status
N1	Regional lymph node metastasis

Definition of Distant Metastasis (M)

M Category	M Criteria
M0	No distant metastasis
M1	Distant metastasis

Definition of Grade (G)

FNCLCC Histologic Grade – see Histologic Grade (G)

G	G Definition
GX	Grade cannot be assessed
G1	Total differentiation, mitotic count and necrosis score of 2 or 3
G2	Total differentiation, mitotic count and necrosis score of 4 or 5
G3	Total differentiation, mitotic count and necrosis score of 6, 7, or 8

AJCC PROGNOSTIC STAGE GROUPS

This is a new classification that needs data collection before defining a stage grouping for head and neck sarcomas.

REGISTRY DATA COLLECTION VARIABLES

1. Bone invasion as determined by imaging
2. If pM1, source of pathological metastatic specimen
3. Additional dimensions of tumor size
4. FNCLCC grade
5. Central nervous system extension (head and neck primaries)

HISTOLOGIC GRADE (G)

The FNCLCC grade is determined by three parameters: differentiation, mitotic activity, and extent of necrosis. Each parameter is scored as follows: differentiation (1–3), mitotic activity (1–3), and necrosis (0–2). The scores are added to determine the grade.

Tumor Differentiation

Tumor differentiation is histology specific (see chapter 39, table 39.1) and is generally scored as follows:

Differentiation Score	Definition
1	Sarcomas closely resembling normal adult mesenchymal tissue (e.g., low-grade leiomyosarcoma)
2	Sarcomas for which histologic typing is certain (e.g., myxoid/round cell liposarcoma)
3	Embryonal and undifferentiated sarcomas, sarcomas of doubtful type, synovial sarcomas, soft tissue osteosarcoma, Ewing sarcoma / primitive neuroectodermal tumor (PNET) of soft tissue

Mitotic Count

In the most mitotically active area of the sarcoma, 10 successive high-power fields (HPF; one HPF at 400× magnification = 0.1734 mm^2) are assessed using a 40× objective.

Mitotic Count Score	Definition
1	0–9 mitoses per 10 HPF
2	10–19 mitoses per 10 HPF
3	≥20 mitoses per 10 HPF

Tumor Necrosis

Evaluated on gross examination and validated with histologic sections.

Necrosis Score	Definition
0	No necrosis
1	<50% tumor necrosis
2	≥50% tumor necrosis

FNCLCC Histologic Grade

G	G Definition
GX	Grade cannot be assessed
G1	Total differentiation, mitotic count and necrosis score of 2 or 3
G2	Total differentiation, mitotic count and necrosis score of 4 or 5
G3	Total differentiation, mitotic count and necrosis score of 6, 7, or 8

HISTOPATHOLOGIC TYPE

Please see the WHO Classification of Tumors section in this chapter for a list of head and neck soft tissue sarcoma histologies.

Bibliography

1. Penel N, Mallet Y, Robin YM, et al. Prognostic factors for adult sarcomas of head and neck. *International journal of oral and maxillofacial surgery.* May 2008;37(5):428–432.
2. O'Sullivan B, Gupta A, Gullane P. Soft tissue and bone sarcomas of the head and neck: general principle and management. In: Harrison L, Sessions R, Kies M, eds. *Head and Neck Cancer: a multidisciplinary approach.* 4th ed: Lippincott Williams and Wilkins; 2014:838–866.
3. Shellenberger TD, Sturgis EM. Sarcomas of the head and neck region. *Curr Oncol Rep.* Mar 2009;11(2):135–142.
4. Peng KA, Grogan T, Wang MB. Head and Neck Sarcomas Analysis of the SEER Database. *Otolaryngology--Head and Neck Surgery.* 2014:0194599814545747.
5. Chang AE, Chai X, Pollack SM, et al. Analysis of Clinical Prognostic Factors for Adult Patients with Head and Neck Sarcomas. *Otolaryngology--Head and Neck Surgery.* 2014;151(6):976–983.
6. Mattavelli D, Miceli R, Radaelli S, et al. Head and neck soft tissue sarcomas: prognostic factors and outcome in a series of patients treated at a single institution. *Annals of oncology.* 2013;24(8): 2181–2189.
7. Park JT, Roh J-L, Kim S-O, et al. Prognostic Factors and Oncological Outcomes of 122 Head and Neck Soft Tissue Sarcoma Patients Treated at a Single Institution. *Annals of surgical oncology.* 2015; 22(1):248–255.
8. Kattan MW, Hess KR, Amin MB, et al. American Joint Committee on Cancer acceptance criteria for inclusion of risk models for individualized prognosis in the practice of precision medicine. *CA: a cancer journal for clinicians.* Jan 19 2016.

Soft Tissue Sarcoma of the Trunk and Extremities

41

Sam S. Yoon, Robert G. Maki, Elliot A. Asare, Kumarasen Cooper, Jason L. Hornick, Alexander J. Lazar, Vicki L. Keedy, David G. Kirsch, John E. Madewell, David M. Panicek, R. Lor Randall, Paige S. Tedder, and Raphael E. Pollock

CHAPTER SUMMARY

Cancers Staged Using This Staging System

Soft tissue sarcomas of the extremity and trunk

Summary of Changes

Change	Details of Change	Level of Evidence
New chapter	Soft tissue sarcoma was divided into chapters by anatomic site.	N/A
Definition of Primary Tumor (T)	Superficial and deep location has been removed as part of T criteria.	II
Definition of Primary Tumor (T)	T categories have been increased from two to four.	II
Definition of Primary Tumor (T)	T1 remains as tumor 5 cm or less in greatest dimension.	II
Definition of Primary Tumor (T)	T2 is now tumor more than 5 cm and less than or equal to 10 cm in greatest dimension.	II
Definition of Primary Tumor (T)	T3 is newly categorized as tumor more than 10 cm and less than or equal to 15 cm in greatest dimension.	II[1]
Definition of Primary Tumor (T)	T4 is a new category defined as tumor more than 15 cm in greatest dimension	II[1]
AJCC Prognostic Stage Groups	AJCC Prognostic Stage Groups have been changed.	II

ICD-O-3 Topography Codes

Code	Description
C47.1	Peripheral nerves and autonomic nervous system of upper limb and shoulder
C47.2	Peripheral nerves and autonomic nervous system of lower limb, including hip
C47.6	Peripheral nerves and autonomic nervous system of trunk, unspecified
C47.8	Overlapping lesion of peripheral nerves and autonomic nervous system
C47.9	Peripheral nerves and autonomic nervous system, unspecified

Code	Description
C49.1	Connective, subcutaneous, and other soft tissues of upper limb and shoulder
C49.2	Connective, subcutaneous, and other soft tissues of lower limb and hip
C49.6	Connective, subcutaneous, and other soft tissues of trunk, NOS
C49.8	Overlapping lesion of connective, subcutaneous, and other soft tissues
C49.9	Connective, subcutaneous, and other soft tissues, NOS

To access the AJCC cancer staging forms, please visit www.cancerstaging.org.

© American Joint Committee on Cancer 2017

M.B. Amin et al. (eds.), *AJCC Cancer Staging Manual, Eighth Edition*, DOI 10.1007/978-3-319-40618-3_41

WHO Classification of Tumors[2]

Adipocytic Tumors

Code	Description
8850	Atypical lipomatous tumor
8850	Well-differentiated liposarcoma
8850	Liposarcoma, NOS
8858	Dedifferentiated liposarcoma
8852	Myxoid/round cell liposarcoma
8854	Pleomorphic liposarcoma

Fibroblastic/Myofibroblastic Tumors

Code	Description
8832	Dermatofibrosarcoma protuberans
8832	Fibrosarcomatous dermatofibrosarcoma protuberans
8833	Pigmented dermatofibrosarcoma protuberans
8815	Solitary fibrous tumor, malignant
8825	Inflammatory myofibroblastic tumor
8825	Low-grade myofibroblastic sarcoma
8810	Adult fibrosarcoma
8811	Myxofibrosarcoma
8840	Low-grade fibromyxoid sarcoma
8840	Sclerosing epithelioid fibrosarcoma

So-called Fibrohistiocytic Tumors

Code	Description
9251	Giant cell tumor of soft tissues

Smooth Muscle Tumors

Code	Description
8890	Leiomyosarcoma (excluding skin)

Pericytic (Perivascular) Tumors

Code	Description
8711	Malignant glomus tumor

Skeletal Muscle Tumors

Code	Description
8910	Embryonal rhabdomyosarcoma (including botryoid, anaplastic)
8920	Alveolar rhabdomyosarcoma (including solid, anaplastic)
8901	Pleomorphic rhabdomyosarcoma
8912	Spindle cell/sclerosing rhabdomyosarcoma

Vascular Tumors of Soft Tissue

Code	Description
9136	Retiform hemangioendothelioma
9136	Pseudomyogenic (epithelioid sarcoma-like) hemangioendothelioma
9133	Epithelioid hemangioendothelioma
9120	Angiosarcoma of soft tissue

Chondro-osseous Tumors

Code	Description
9180	Extraskeletal osteosarcoma

Nerve Sheath Tumors

Code	Description
9540	Malignant peripheral nerve sheath tumor
9542	Epithelioid malignant peripheral nerve sheath tumor
9561	Malignant Triton tumor
9580	Malignant granular cell tumor

INTRODUCTION

This chapter covers the most common primary anatomic sites of soft tissue sarcomas: the extremity and trunk. Other anatomic sites for soft tissue sarcomas (STS) are addressed in other chapters but use the same TNMG staging as this section if not otherwise specified (e.g. gastrointestinal stromal tumor [GIST]).

This staging system applies to all extremity and trunk soft tissue sarcomas except desmoid tumors and Kaposi sarcoma. Data to support this staging system are based on current analyses from multiple institutions and represent the recommendations of the AJCC Soft Tissue Sarcoma Expert Panel.

Tumor size and histologic grade are essential for soft tissue sarcoma staging. Grade is based on analysis of various pathological features, including degree of differentiation, mitotic activity, and necrosis. Accurate grading requires an adequate sample of well-fixed tissue for evaluation and may not always be possible on the basis of needle biopsies or in tumors that previously were treated with radiation or chemotherapy.

ANATOMY

Primary Site(s)

About 40–50% of soft tissue sarcomas occur in the extremities, and about 10% occur in the trunk. The breast is a trunk site and often is collected as a separate anatomic site.

Regional Lymph Nodes

Involvement of regional lymph nodes by soft tissue sarcomas is uncommon for soft tissue sarcomas, except for certain subtypes, including epithelioid sarcoma, clear cell sarcoma, and alveolar and embryonal rhabdomyosarcoma.

Metastatic Sites

The most common site of metastatic disease in patients with extremity and trunk sarcomas is the lung. Certain histologic subtypes, such as myxoid/round cell liposarcoma, more commonly have extrapulmonary metastases, such as metastases to soft tissue and bone marrow sites. Brain metastases, although rare, are seen most often in leiomyosarcoma patients; angiosarcoma and alveolar soft part sarcoma appear to have a disproportionate propensity for brain metastases compared with other histologies.

RULES FOR CLASSIFICATION

Clinical Classification

Clinical staging is based on characteristics of T, N, M, and grade (G). Tumor size may be measured clinically or radiologically. Distant metastatic disease should be described according to the most likely sites of metastasis. In general, the minimal clinical staging workup for soft tissue sarcoma is accomplished by axial imaging of the involved site using magnetic resonance (MR) imaging or computed tomography (CT) scanning and by imaging of the lungs, the most likely site for occult metastatic disease, using chest CT scans. Diagnostic biopsies of the primary site, nodes, and distant metastasis are included in clinical staging.

TNM Categories of Tumor Staging

The T category is assessed by measuring the largest diameter of the tumor in any plane. The measurement should be made on whichever MR imaging pulse sequence best delineates the tumor. Some tumors, such as pleomorphic sarcoma and myxofibrosarcoma, often have tail-like projections that extend for considerable distances along fascial and neurovascular planes. Surrounding edema, if present, should not be included in the measurement.

Regional nodal metastases are uncommon in most histologic types of extremity soft tissue sarcoma. Nodes are considered suspicious for tumor involvement if enlarged, rounded, or necrotic, or if the normal fatty hilum of the node is replaced by soft tissue.

Extremity soft tissue sarcomas most commonly metastasize to the lung, manifesting as sharply defined nodules. Hemorrhagic nodules, such as in angiosarcoma, may show surrounding halos of ground-glass attenuation. The metastases of some types of sarcomas, such as extraskeletal osteosarcoma and chondrosarcoma, may contain calcification on CT and should not be assumed to represent calcified granulomas.

Definition of T

T is categorized based on greatest dimension as (1) lesions less than or equal to 5 cm, (2) lesions more than 5 cm and less than or equal to 10 cm, (3) more than 10 cm and less than or equal to 15 cm, or (4) lesions more than 15 cm.[1] Size should be regarded as a continuous variable, with 5 cm, 10 cm and 15 cm used merely as arbitrary divisions that make it possible to group patient populations.

41

Nodal Disease

Nodal involvement is rare in adult soft tissue sarcomas. In the assignment of stage group, patients whose nodal status is not determined to be positive for tumor, either clinically or pathologically, should be designed as N0. The term *NX* should not be used. The designation for the clinical stage is cN0. If microscopically determined for the pathological stage, it would be designated as pN0. If clinically determined by physical examination or imaging for the pathological stage, it would be designated as cN0 and not pNX.

Grade

The issue of grade continues to play an important role in ultimate sarcoma staging, especially because these cancers generally do not metastasize to lymph nodes. Thus, functionally speaking, only tumor size and presence or absence of metastatic disease are the variables in risk assessment if grade is omitted. It is well accepted that histology is even more important than grade in many instances but that, in general, grade helps dictate risk more than primary tumor size.

Grade should be assigned to all sarcomas. Historically the AJCC soft tissue staging system used a four-grade system, but this was revised starting with the AJCC Cancer Staging Manual, 7th Edition to the three-grade system used by the two most commonly recognized staging systems. Comprehensive grading of soft tissue sarcomas is strongly correlated with disease-specific survival and incorporates differentiation (histology-specific), mitotic rate, and extent of necrosis. In accordance with recommendations by the College of American Pathologists,[3] the French Federation of Cancer Centers Sarcoma Group (FNCLCC) system[4] is preferred over the National Institutes of Health system because of its ease of use/reproducibility and perhaps its slightly superior performance.

Applying histologic grading to core needle biopsies is problematic if neoadjuvant chemotherapy or radiation has been administered. However, given the importance of grade to staging and treatment, efforts are encouraged to separate sarcomas on needle biopsies as described earlier. In many instances, the type of sarcoma will permit this distinction readily (e.g., Ewing sarcoma, undifferentiated pleomorphic sarcoma), whereas in less obvious instances, the difficulty of assigning grade should be noted. In general, multiple core

needle biopsies disclosing a high-grade sarcoma may be regarded as high grade because the probability of subsequent downgrading is remote, but limited cores biopsies of low-grade sarcoma carry a risk of subsequent upgrading.

The FNCLCC grade is determined by three parameters: differentiation, mitotic activity, and extent of necrosis. Each parameter is scored as follows: differentiation (1–3), mitotic activity (1–3), and necrosis (0–2). The scores are added to determine the grade.

The tumor differentiation score is the most subjective aspect of the FNCLCC system. In addition, it is not validated for every subtype of sarcoma and is inapplicable to certain subtypes. However, this score is critical given its proportional weight, such that any sarcoma assigned a differentiation score of 3 will be at least intermediate to high grade.

Although they are not specifically mentioned in the original FNCLCC grading system, differentiation scores may be used for sarcomas newer than those described in the original document. For example, dermatofibrosarcoma protuberans merits a differentiation score of 1. Low-grade fibromyxoid sarcoma and sclerosing epithelioid fibrosarcoma do not have a differentiation grade, because they were characterized after the FNCLCC criteria were developed. A differentiation score of 2 is suggested based on the general differentiation criteria of FNCLCC and their propensity for metastatic disease.

Imaging

MR imaging is the preferred examination for assessing primary tumor stage information. CT performed with intravenous contrast material, however, can provide similar information, particularly if MR imaging is not available or is contraindicated. MR imaging and CT also may guide the selection of an optimal site for biopsy, such as the most vascular or cellular region, and avoid nondiagnostic necrotic portions. Radiography may demonstrate subtle cortical involvement better than MR imaging or CT. For sarcomas with a particular propensity to metastasize to lymph nodes, scintigraphic sentinel node mapping may be performed to guide subsequent lymph node sampling. Chest CT is used to assess for pulmonary metastases, the most common site of metastasis of most soft tissue sarcomas of the extremities. In myxoid/round cell liposarcoma, spine MR imaging is used to assess for marrow metastases, which are occult by other imaging modalities. Positron emission tomography/CT is useful for whole-body staging in rhabdomyosarcoma and other histologies in which overt or occult lymph node metastases are most common.

Radiological Staging of Tumor

Describe the location and extent of the primary tumor, including the tumor's relationship to adjoining muscles, blood vessels, nerves, bones, and joints. Neurovascular encasement and bone marrow involvement are assessed best on (non–fat-suppressed) T1-weighted images. Contact of more than 180° of the circumference of the vessel wall by tumor should be considered suspicious for encasement; lesser degrees of contact should be described as contact without encasement. Tumor margins often can be distinguished from the surrounding reactive zone (which manifests as soft tissue edema and which may contain viable tumor cells) on T2-weighted or postcontrast fat-suppressed T1-weighted MR images.

Proposed Report Format

1. Primary tumor
   a. MR imaging signal or CT attenuation characteristics
   b. Extent and location of necrosis within tumor
   c. Location in extremity, including relationship to superficial fascia
   d. Presence and location of tumor tails
   e. Size (in three dimensions)
2. Local extent
   a. Invasion of muscles, bones, and joints
   b. Contact with, or encasement of, blood vessels and nerves
   c. Extension into lumen of blood vessels
   d. Presence of nearby satellite nodules
3. Regional lymph node involvement

Pathological Classification

Pathological (pTNMG) staging consists of the removal and pathological evaluation of the primary tumor and clinical/radiologic evaluation for regional and distant metastases. In circumstances in which it is not possible to obtain accurate measurements of the excised primary sarcoma specimen, it is acceptable to use radiologic assessment to assign a pT category using the dimensions of the sarcoma. In examining the primary tumor, the pathologist should assign a histopathologic subtype and grade. Immunohistochemistry or genetic studies may be necessary for accurate assignment of subtype. Assignment of grade may be affected by prior administration of chemotherapy and/or radiotherapy. After response to presurgical treatments, lesions initially assigned a high-grade status may have a less ominous appearance on

microscopic examination and therefore may be assigned a grade lower than the initial designation. Occasionally, the reverse situation is observed because of either sampling error or elimination of lower-grade cells by presurgical treatment in these typically heterogeneous tumors.

PROGNOSTIC FACTORS

Prognostic Factors Required for Stage Grouping

French Federation of Cancer Centers Sarcoma Group (FNCLCC) grade – see Histologic Grade (G).

Additional Factors Recommended for Clinical Care

Neurovascular and Bone Invasion

In earlier staging systems, neurovascular and bone invasion by soft tissue sarcomas was included as a determinant of stage. It is not included in the current staging system, and currently no plans are proposed to add it. Nevertheless, neurovascular and bone invasion should be reported whenever possible, and further studies are needed to determine whether such invasion is an independent prognostic factor for clinical outcomes. AJCC Level of Evidence: III

Validation

The current staging system has the capacity to discriminate the overall survival of patients with soft tissue sarcoma. Patients with Stage I lesions are at low risk for disease-related mortality, whereas Stages II and III entail progressively greater risk. In extremity and trunk sarcomas, this meets level I evidence for AJCC.

RISK ASSESSMENT MODELS

The AJCC has recently established guidelines that will be used to evaluate published statistical prediction models for the purpose of granting endorsement for clinical use.[5] Although this is a monumental step forward towards the goal of precision medicine, this work was only very recently published. For this reason, the existing models that have been published or may be in clinical use have not yet been evaluated for this cancer site by the Precision Medicine core of the AJCC. In the future, the statistical prediction models for this cancer site will be evaluated, and those that meet all AJCC criteria will be endorsed.

DEFINITIONS OF AJCC TNM

Definition of Primary Tumor (T)

T Category	T Criteria
TX	Primary tumor cannot be assessed
T0	No evidence of primary tumor
T1	Tumor 5 cm or less in greatest dimension
T2	Tumor more than 5 cm and less than or equal to 10 cm in greatest dimension
T3	Tumor more than 10 cm and less than or equal to 15 cm in greatest dimension
T4	Tumor more than 15 cm in greatest dimension

Definition of Regional Lymph Node (N)

N Category	N Criteria
N0	No regional lymph node metastasis or unknown lymph node status
N1	Regional lymph node metastasis

Definition of Distant Metastasis (M)

M Category	M Criteria
M0	No distant metastasis
M1	Distant metastasis

Definition of Grade (G)

FNCLCC Histologic Grade – see Histologic Grade (G)

G	G Definition
GX	Grade cannot be assessed
G1	Total differentiation, mitotic count and necrosis score of 2 or 3
G2	Total differentiation, mitotic count and necrosis score of 4 or 5
G3	Total differentiation, mitotic count and necrosis score of 6, 7, or 8

AJCC PROGNOSTIC STAGE GROUPS

When T is...	And N is...	And M is...	And grade is...	Then the stage group is...
T1	N0	M0	G1, GX	IA
T2, T3, T4	N0	M0	G1, GX	IB
T1	N0	M0	G2, G3	II
T2	N0	M0	G2, G3	IIIA
T3, T4	N0	M0	G2, G3	IIIB
Any T	N1	M0	Any G	IV
Any T	Any N	M1	Any G	IV

41

REGISTRY DATA COLLECTION VARIABLES

1. Bone invasion as determined by imaging
2. If pM1, source of pathologic metastatic specimen
3. Additional dimensions of tumor size
4. FNCLCC grade

HISTOLOGIC GRADE (G)

The FNCLCC grade is determined by three parameters: differentiation, mitotic activity, and extent of necrosis. Each parameter is scored as follows: differentiation (1–3), mitotic activity (1–3), and necrosis (0–2). The scores are added to determine the grade.

Tumor Differentiation

Tumor differentiation is histology specific (see Chapter 39, Table 39.1) and is generally scored as follows:

Differentiation Score	Definition
1	Sarcomas closely resembling normal adult mesenchymal tissue (e.g., low-grade leiomyosarcoma)
2	Sarcomas for which histologic typing is certain (e.g., myxoid/round cell liposarcoma)
3	Embryonal and undifferentiated sarcomas, sarcomas of doubtful type, synovial sarcomas, soft tissue osteosarcoma, Ewing sarcoma /primitive neuroectodermal tumor (PNET) of soft tissue

Mitotic Count

In the most mitotically active area of the sarcoma, 10 successive high-power fields (HPF; one HPF at 400× magnification = 0.1734 mm^2) are assessed using a 40× objective.

Mitotic Count Score	Definition
1	0–9 mitoses per 10 HPF
2	10–19 mitoses per 10 HPF
3	≥20 mitoses per 10 HPF

Tumor Necrosis

Evaluated on gross examination and validated with histologic sections.

Necrosis Score	Definition
0	No necrosis
1	<50% tumor necrosis
2	≥50% tumor necrosis

FNCLCC Histologic Grade

G	G Definition
GX	Grade cannot be assessed
G1	Total differentiation, mitotic count and necrosis score of 2 or 3
G2	Total differentiation, mitotic count and necrosis score of 4 or 5
G3	Total differentiation, mitotic count and necrosis score of 6, 7, or 8

HISTOPATHOLOGIC TYPE

Please see the WHO Classification of Tumors section in this chapter for a list of trunk and extremity soft tissue sarcoma histologies.

SURVIVAL DATA

The justification for use of more T groups than AJCC 7th Edition comes from two primary large clinical sarcoma databases, which indicate that the risk of local recurrence continues to increase with greater primary tumor size (Figs. 41.1 and 41.3). In particular for high grade primary sarcomas, local recurrence risk increases with tumor size, but some differences are seen in the survival of patients in the 10-15 cm range. In one series, overall survival is inferior for each T group (Fig. 41.3), whereas in the other study (Fig. 41.1), sarcoma specific survival decreases with each size group to plateau at > 10 cm. Thus, factors associated with local recurrence are slightly different from those associated with sarcoma-specific death. For low grade tumors, the differences are more subtle, and captured as part of the present TNMG staging system.

The rationale for grouping N1 sarcomas as stage IV comes from one source (Fig. 41.2), in which AJCC 7th Edition T2G3N0 disease is compared to TXN1 disease. In this setting N1 disease fared worse despite the G3 nature of all tumors in the former group. The survival curve for TXN1 disease was different from that of T2G3N0 disease, but was not statistically significantly different from that of TXM1 disease. For this very rare sarcoma subset, a stage IV assignation is suggested.

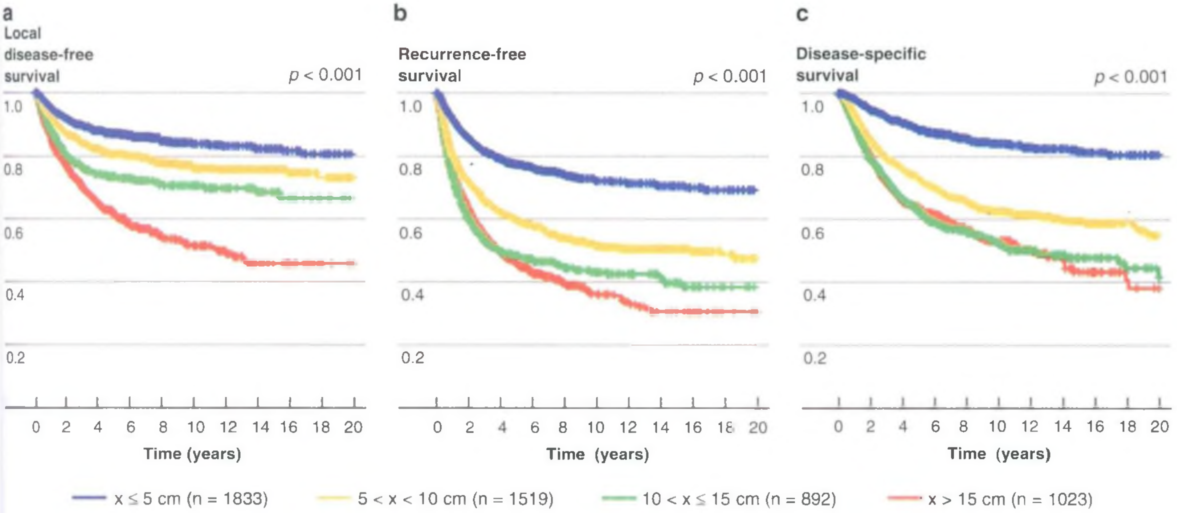

Fig. 41.1 Local recurrence-free survival (RFS), overall recurrence-free survival, and disease-specific survival (DSS) by size category, ≤5, 5–10, 10–15, and >15 cm. (**a**) Local recurrence-free survival (time from primary surgery to first local recurrence), n = 5,267 patients, excludes 75 patients with unknown size categories; log rank, p < 0.001. (**b**) Recurrence-free survival (time from primary surgery to first local or distant recurrence), n = 5,267, excludes 75 patients with unknown size categories; log rank, p < 0.001. (**c**) Disease-specific survival (time from primary surgery to death from disease), n = 5267, excludes 75 patients with unknown size categories; log rank, p < 0.001; log rank, p value = 0.91 comparing >10–15 and >15 cm groups. From Maki et al with permission[6]

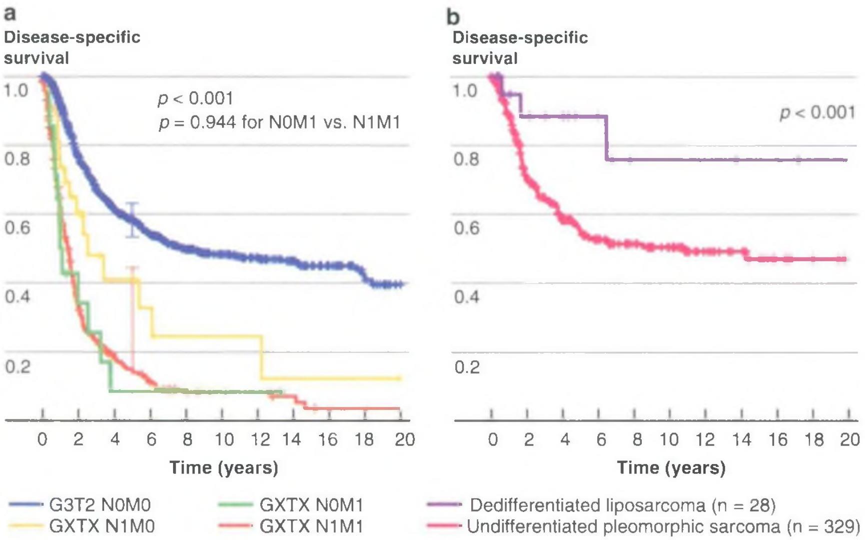

Fig. 41.2 (**a**) Disease-specific survival comparing AJCC 7th Edition G3T2N0M0 primary STS to GXTXN1M0 and GXTXN1M1 STS, n = 1440 total; G3T2N0M0 disease (n = 1123), GXTXN1M0 (n = 33), GXTXN1M1 (n = 15), and GXTXN0M1 disease (n = 269); log rank, p < 0.001. Comparing GXTXN0M1 and GXTXN1M1 patients; log rank, p = 0.944. 95% confidence intervals are noted at 5 years for the two largest groups; they are not meaningful for the smallest groups with so few events. (**b**) Disease-specific survival comparing extremity dedifferentiated liposarcoma (n = 28) and undifferentiated pleomorphic sarcoma (n = 329); log rank, p < 0.001. From Maki et al with permission[6]

Fig. 41.3 (**a**) Cumulative incidence of sarcoma-related deaths by tumor size as a function of time from initial diagnosis. (**b**) Cumulative incidence of sarcoma-related deaths by tumor grade as a function of time from initial diagnosis. (**c**) Cumulative incidence of sarcoma-related deaths by tumor size and grade as a function of time from initial diagnosis. From Lahat et al with permission[1]

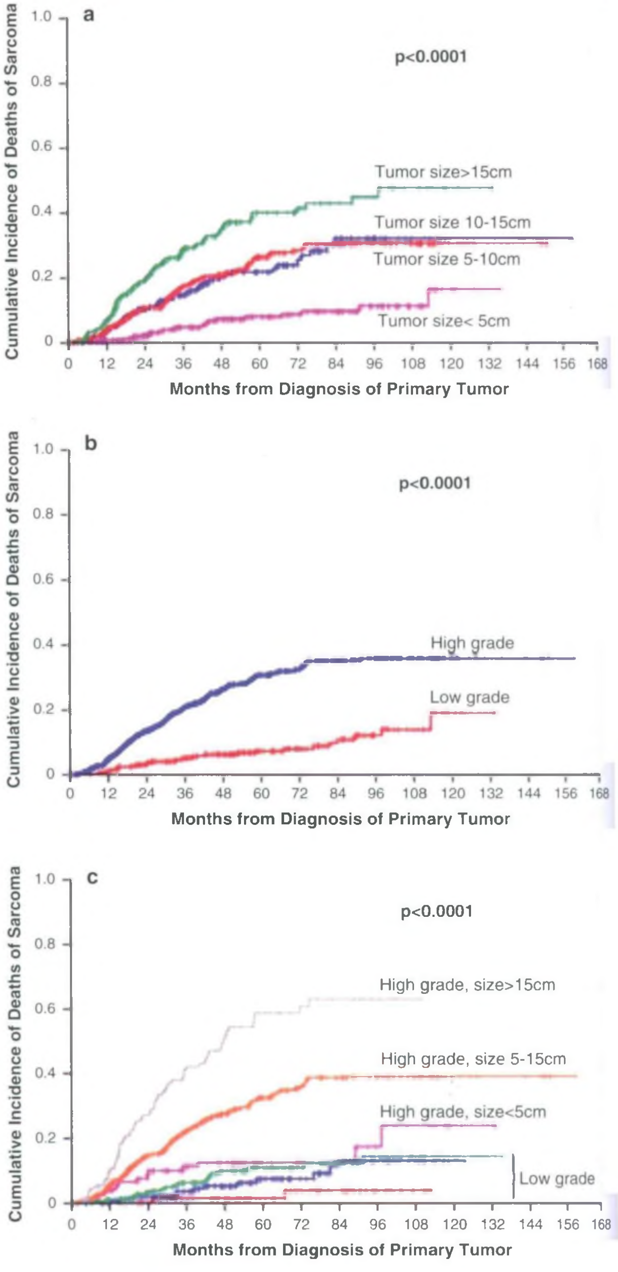

Bibliography

1. Lahat G, Tuvin D, Wei C, et al. New perspectives for staging and prognosis in soft tissue sarcoma. *Annals of surgical oncology.* Oct 2008;15(10):2739–2748.
2. Fletcher CDM, Bridge JA, Hogendoorn P, Mertens F, eds. World Health Organization Classification of Tumours of Soft Tissue and Bone. Fourth Edition. Lyon: IARC; 2013.
3. Rubin BP, Cooper K, Fletcher CD, et al. Protocol for the examination of specimens from patients with tumors of soft tissue. *Arch Pathol Lab Med.* Apr 2010;134(4):e31–39.
4. Guillou L, Coindre JM, Bonichon F, et al. Comparative study of the National Cancer Institute and French Federation of Cancer Centers Sarcoma Group grading systems in a population of 410 adult patients with soft tissue sarcoma. *J Clin Oncol.* Jan 1997;15(1):350–362.
5. Kattan MW, Hess KR, Amin MB, et al. American Joint Committee on Cancer acceptance criteria for inclusion of risk models for individualized prognosis in the practice of precision medicine. *CA: a cancer journal for clinicians.* Jan 19 2016.
6. Maki RG, Moraco N, Antonescu CR, et al. Toward better soft tissue sarcoma staging: building on american joint committee on cancer staging systems versions 6 and 7. *Annals of surgical oncology.* Oct 2013;20(11):3377–3383.
7. Brennan MF AC, Maki RG. Management of soft tissue sarcoma. New York: Springer. 2013.
8. Kattan MW, Leung DH, Brennan MF. Postoperative nomogram for 12-year sarcoma-specific death. *J Clin Oncol.* Feb 1 2002;20(3):791–796.
9. Mariani L, Miceli R, Kattan MW, et al. Validation and adaptation of a nomogram for predicting the survival of patients with extremity soft tissue sarcoma using a three-grade system. *Cancer* Jan 15 2005;103(2):402–408.
10. Panicek DM, Gatsonis C, Rosenthal DI, et al. CT and MR imaging in the local staging of primary malignant musculoskeletal neoplasms: Report of the Radiology Diagnostic Oncology Group. *Radiology.* Jan 1997;202(1):237–246.
11. Pisters PW, Leung DH, Woodruff J, Shi W, Brennan MF. Analysis of prognostic factors in 1,041 patients with localized soft tissue sarcomas of the extremities. *J Clin Oncol.* May 1996;14(5):1679–1689.
12. Schwab JH, Boland PJ, Antonescu C, Bilsky MH, Healey JH. Spinal metastases from myxoid liposarcoma warrant screening with magnetic resonance imaging. *Cancer.* Oct 15 2007;110(8):1815–1822.

Soft Tissue Sarcoma of the Abdomen and Thoracic Visceral Organs

42

Chandrajit P. Raut, Robert G. Maki, Elizabeth H. Baldini, Jason L. Hornick, Alexander J. Lazar, Richard F. Riedel, Paige S. Tedder, and Raphael E. Pollock

CHAPTER SUMMARY

Cancers Staged Using This Staging System

Soft tissue sarcomas of the abdominal and thoracic visceral organs

Cancers Not Staged Using This Staging System

These histopathologic types of cancer…	Are staged according to the classification for…	And can be found in chapter…
Desmoplastic small round cell tumor (DSRCT)	Soft tissue sarcoma—unusual histologies and sites	45
Epithelioid hemangioendothelioma (EHE)	Soft tissue sarcoma—unusual histologies and sites	45
Inflammatory myofibroblastic tumor (IMT)	No AJCC staging system	N/A
Perivascular epithelioid cell tumor (PEComa)	No AJCC staging system	N/A
Solitary fibrous tumor (SFT)	Soft tissue sarcoma of the retroperitoneum (or other appropriate anatomic site)	44
Gastrointestinal stromal tumor (GIST), gastrointestinal stromal sarcoma	Gastrointestinal stromal tumor	43
Leiomyosarcoma, uterine	Corpus uteri – sarcoma	54
Leiomyosarcoma, retroperitoneal	Soft tissue sarcoma of the retroperitoneum	44

Summary of Changes

Change	Details of Change	Level of Evidence
New staging system	A new staging system for abdominal and thoracic and visceral sarcomas is introduced.	IV
Definition of Primary Tumor (T)	A new designation for T category is proposed. The designation of deep versus superficial sarcoma does not make sense for these anatomic sites and is deleted.	IV

To access the AJCC cancer staging forms, please visit www.cancerstaging.org.

© American Joint Committee on Cancer 2017

M.B. Amin et al. (eds.), *AJCC Cancer Staging Manual, Eighth Edition*, DOI 10.1007/978-3-319-40618-3_42

Soft Tissue Sarcoma of the Abdomen and Thoracic Visceral Organs

42

Chandrajit P. Raut, Robert G. Maki, Elizabeth H. Baldini, Jason L. Hornick, Alexander J. Lazar, Richard F. Riedel, Paige S. Tedder, and Raphael E. Pollock

CHAPTER SUMMARY

Cancers Staged Using This Staging System

Soft tissue sarcomas of the abdominal and thoracic visceral organs

Cancers Not Staged Using This Staging System

These histopathologic types of cancer...	Are staged according to the classification for...	And can be found in chapter...
Desmoplastic small round cell tumor (DSRCT)	Soft tissue sarcoma—unusual histologies and sites	45
Epithelioid hemangioendothelioma (EHE)	Soft tissue sarcoma—unusual histologies and sites	45
Inflammatory myofibroblastic tumor (IMT)	No AJCC staging system	N/A
Perivascular epithelioid cell tumor (PEComa)	No AJCC staging system	N/A
Solitary fibrous tumor (SFT)	Soft tissue sarcoma of the retroperitoneum (or other appropriate anatomic site)	44
Gastrointestinal stromal tumor (GIST), gastrointestinal stromal sarcoma	Gastrointestinal stromal tumor	43
Leiomyosarcoma, uterine	Corpus uteri – sarcoma	54
Leiomyosarcoma, retroperitoneal	Soft tissue sarcoma of the retroperitoneum	44

Summary of Changes

Change	Details of Change	Level of Evidence
New staging system	A new staging system for abdominal and thoracic and visceral sarcomas is introduced.	IV
Definition of Primary Tumor (T)	A new designation for T category is proposed. The designation of deep versus superficial sarcoma does not make sense for these anatomic sites and is deleted.	IV

To access the AJCC cancer staging forms, please visit www.cancerstaging.org.

© American Joint Committee on Cancer 2017

M.B. Amin et al. (eds.), *AJCC Cancer Staging Manual, Eighth Edition*, DOI 10.1007/978-3-319-40618-3_42

ICD-O-3 Topography Codes

Code	Description
C15.1	Thoracic esophagus
C15.2	Abdominal esophagus
C15.4	Middle third of esophagus
C15.5	Lower third of esophagus
C15.9	Esophagus, NOS
C38.0	Malignant neoplasm of heart
C38.1	Malignant neoplasm of anterior mediastinum
C38.2	Malignant neoplasm of posterior mediastinum
C38.3	Malignant neoplasm of mediastinum, part unspecified
C38.4	Malignant neoplasm of pleura
C38.8	Malignant neoplasm of overlapping lesion of heart, mediastinum, and pleura
C33.9	Trachea
C47.3	Peripheral nerves and autonomic nervous system of thorax
C47.4	Peripheral nerves and autonomic nervous system of abdomen
C47.5	Peripheral nerves and autonomic nervous system of pelvis
C49.3	Connective, subcutaneous and other soft tissues of thorax
C49.4	Connective, subcutaneous and other soft tissues of abdomen
C49.5	Connective, subcutaneous and other soft tissues of pelvis
C15-C26	Digestive organs
C34-C37	Intrathoracic organs
C51-C53, C58	Female genital organs
C60-C63	Male genital organs
C64-C68	Urinary tract

WHO Classification of Tumors[1]

Adipocytic Tumors

Code	Description
8850	Atypical lipomatous tumor
8850	Well-differentiated liposarcoma
8850	Lipsarcoma, not otherwise specified
8858	Dedifferentiated liposarcoma
8852	Myxoid/round cell liposarcoma
8854	Pleomorphic liposarcoma

Fibroblastic/Myofibroblastic Tumors

Code	Description
8832	Dermatofibrosarcoma protuberans
8832	Fibrosarcomatous dermatofibrosarcoma protuberans
8833	Pigmented dermatofibrosarcoma protuberans
8815	Solitary fibrous tumor, malignant
8825	Inflammatory myofibroblastic tumor
8825	Low-grade myofibroblastic sarcoma
8810	Adult fibrosarcoma
8811	Myxofibrosarcoma
8840	Low-grade fibromyxoid sarcoma
8840	Sclerosing epithelioid fibrosarcoma

So-called Fibrohistiocytic Tumors

Code	Description
9251	Giant cell tumor of soft tissues

Smooth Muscle Tumors

Code	Description
8890	Leiomyosarcoma (excluding skin)

Pericytic (Perivascular) Tumors

Code	Description
8711	Malignant glomus tumor

Skeletal Muscle Tumors

Code	Description
8910	Embryonal rhabdomyosarcoma (including botryoid, anaplastic)
8920	Alveolar rhabdomyosarcoma (including solid, anaplastic)
8901	Pleomorphic rhabdomyosarcoma
8912	Spindle cell/sclerosing rhabdomyosarcoma

Vascular Tumors of Soft Tissue

Code	Description
9136	Retiform hemangioendothelioma
9136	Pseudomyogenic (epithelioid sarcoma-like) hemangioendothelioma
9133	Epithelioid hemangioendothelioma
9120	Angiosarcoma of soft tissue

Chondro-Osseous Tumors

Code	Description
9180	Extraskeletal osteosarcoma

Nerve Sheath Tumors

Code	Description
9540	Malignant peripheral nerve sheath tumor
9542	Epithelioid malignant peripheral nerve sheath tumor
9561	Malignant Triton tumor
9580	Malignant granular cell tumor

INTRODUCTION

Sarcomas of the abdominal and thoracic viscera represent a varied and heterogeneous group of mesenchymal neoplasms. Traditional staging algorithms have not been able to reliably prognosticate this cohort of sarcomas. This chapter provides a brief introduction to this unique group of sarcomas and proposes a new T classification system for purposes of future data collection and potential development of a specific visceral sarcoma staging algorithm.

Soft tissue sarcomas arise across a breadth of anatomic sites and tissues of origin, and display a variety of different behaviors. This chapter focuses specifically on those arising from abdominal and thoracic viscera.

ANATOMY

Primary Site(s)

Soft tissue sarcomas may arise within the abdominal and thoracic cavities, as well as within the solid and hollow visceral organs therein.

Soft tissue sarcomas, including but not limited to leiomyosarcoma and undifferentiated pleomorphic sarcoma, may arise from hollow viscera, including the esophagus (very rare; often presenting as polypoid intraluminal masses with poor prognosis), stomach, small intestine, colon, and rectum, as well as solid viscera such as the liver, kidneys, lungs, and heart.

Soft tissue sarcomas also may arise from the peritoneal or pleural surfaces or in the mediastinum.

The typical mechanism of spread is local extension within the organ or within the involved cavity and hematogenously to distant sites.

Prior staging systems do not apply to sarcomas of visceral organs or to those of the peritoneal and pleural cavities.

Regional Lymph Nodes

Involvement of regional lymph nodes by soft tissue sarcomas is uncommon in adults. Specific histologies in which regional lymph node metastatic disease is most commonly observed include alveolar rhabdomyosarcoma, embryonal rhabdomyosarcoma, epithelioid sarcoma, and angiosarcoma.

Metastatic Sites

Sarcomas of visceral origin may metastasize to liver or lung or within the involved body cavity. Other sites of spread are less common.

RULES FOR CLASSIFICATION

Clinical Classification

Tumors suspected of being sarcoma but without overt malignant features should be classified as having uncertain malignant potential.

As is recognized in other anatomic sites in which there is apparent multifocality, soft tissue sarcomas that present as true multifocal disease (but not metastatic tumors) are challenging to stage. Histologies that may present with true multifocal disease within the abdominal viscera or liver include DSRCT, EHE, and retroperitoneal well-differentiated/dedifferentiated liposarcoma. However, the presence of multifocality is not necessarily of prognostic value for all subtypes.

A new T classification is proposed for visceral sarcomas. Sarcomas arising within the peritoneal, pleural, or mediastinal cavities but not from a specific organ may be staged in a manner similar to that of retroperitoneal sarcomas.

Common sense suggests the number of sites of disease at presentation impacts outcome. With recurrences of some diagnoses, such as well differentiated/dedifferentiated liposarcoma, it is clear that having more than one site of recurrence is worse than a single site of recurrence, since this diagnosis is one that is usually treated surgically. For other diagnoses, such as epithelioid hemangioendothelioma, the utility of the multifocality designation is less clear. Furthermore, there are no criteria by which to declare multifocality vs metastatic disease. This is a determination that will be made clinically. For example, a dominant lesion with small implants elsewhere should be considered metastatic disease, whereas lesions without a dominant primary site can be considered multifocal. Multifocality data will be captured as part of the new staging system to allow further clinical research to continue into this thorny question.

Imaging

Generally, the extent of disease may be evaluated before treatment with contrast-enhanced computed tomography (CT) of the chest, abdomen, and pelvis. Magnetic resonance imaging may be helpful in specific clinical situations, such as

fixed organs, to provider better anatomic definition (e.g., for sarcomas arising in the rectum or liver). Fluorodeoxyglucose positron emission tomography, bone scan, or CT of the head is not routinely necessary for staging but may be used, along with plain films of axial sites, to evaluate areas of ambiguity on other imaging or sites of symptoms.

Pathological Classification

Accurate histologic classification by an experienced sarcoma pathologist is critical to appropriate care. Reclassification by a sarcoma pathologist has been reported in nearly a quarter of patients seen in a sarcoma specialty clinic, resulting in treatment changes in approximately two thirds of those patients.

At present, there is no role for routine genetic testing for sarcomas arising within abdominal and thoracic viscera. However, selective testing for genetic alterations together with appropriate immunohistochemistry may help distinguish ambiguous cases.

PROGNOSTIC FACTORS

Prognostic Factors Required for Stage Grouping

French Federation of Cancer Centers Sarcoma Group (FNCLCC) grade – see Histologic Grade (G). Where present, multifocality should be documented, as well as the number of involved sites.

Additional Factors Recommended for Clinical Care

There are no additional factors recommended for clinical care.

RISK ASSESSMENT MODELS

The AJCC has recently established guidelines that will be used to evaluate published statistical prediction models for the purpose of granting endorsement for clinical use.[2] Although this is a monumental step forward towards the goal of precision medicine, this work was only very recently published. For this reason, the existing models that have been published or may be in clinical use have not yet been evaluated for this cancer site by the Precision Medicine core of the AJCC. In the future, the statistical prediction models for this cancer site will be evaluated, and those that meet all AJCC criteria will be endorsed.

DEFINITIONS OF AJCC TNM

Definition of Primary Tumor (T)

T Category	T Criteria
TX	Primary tumor cannot be assessed
T1	Organ confined
T2	Tumor extension into tissue beyond organ
T2a	Invades serosa or visceral peritoneum
T2b	Extension beyond serosa (mesentery)
T3	Invades another organ
T4	Multifocal involvement
T4a	Multifocal (2 sites)
T4b	Multifocal (3–5 sites)
T4c	Multifocal (>5 sites)

Definition of Regional Lymph Node (N)

N Category	N Criteria
N0	No lymph node involvement or unknown lymph node status
N1	Lymph node involvement present

Definition of Distant Metastasis (M)

M Category	M Criteria
M0	No metastases
M1	Metastases present

Definition of Grade (G)

FNCLCC Histologic Grade – see Histologic Grade (G)

G	G Definition
GX	Grade cannot be assessed
G1	Total differentiation, mitotic count and necrosis score of 2 or 3
G2	Total differentiation, mitotic count and necrosis score of 4 or 5
G3	Total differentiation, mitotic count and necrosis score of 6, 7, or 8

AJCC PROGNOSTIC STAGE GROUPS

There is no recommended prognostic stage grouping at this time.

REGISTRY DATA COLLECTION VARIABLES

1. Bone invasion as determined by imaging
2. If pM1, source of pathological metastatic specimen
3. Additional dimensions of tumor size
4. FNCLCC grade
5. Evidence of multifocality (number of sites)

HISTOLOGIC GRADE (G)

The FNCLCC grade is determined by three parameters: differentiation, mitotic activity, and extent of necrosis. Each parameter is scored as follows: differentiation (1–3), mitotic activity (1–3), and necrosis (0–2). The scores are added to determine the grade.

Tumor Differentiation

Tumor differentiation is histology specific (see Chapter 39, Table 39.1) and is generally scored as follows:

Differentiation Score	Definition
1	Sarcomas closely resembling normal adult mesenchymal tissue (e.g., low-grade leiomyosarcoma)
2	Sarcomas for which histologic typing is certain (e.g., myxoid/round cell liposarcoma)
3	Embryonal and undifferentiated sarcomas, sarcomas of doubtful type, synovial sarcomas, soft tissue osteosarcoma, Ewing sarcoma / primitive neuroectodermal tumor (PNET) of soft tissue

Mitotic Count

In the most mitotically active area of the sarcoma, 10 successive high-power fields (HPF; one HPF at 400× magnification = 0.1734 mm^2) are assessed using a 40× objective.

Mitotic Count Score	Definition
1	0–9 mitoses per 10 HPF
2	10–19 mitoses per 10 HPF
3	≥20 mitoses per 10 HPF

Tumor Necrosis

Evaluated on gross examination and validated with histologic sections.

Necrosis Score	Definition
0	No necrosis
1	<50% tumor necrosis
2	≥50% tumor necrosis

42

FNCLCC Histologic Grade

G	G Definition
GX	Grade cannot be assessed
G1	Total differentiation, mitotic count and necrosis score of 2 or 3
G2	Total differentiation, mitotic count and necrosis score of 4 or 5
G3	Total differentiation, mitotic count and necrosis score of 6, 7, or 8

HISTOPATHOLOGIC TYPES

Please see the WHO Classification of Tumors section in this chapter for a list of abdominal and thoracic visceral sarcoma histologies.

Bibliography

1. Fletcher CDM, Bridge JA, Hogendoorn P, Mertens F, eds. World Health Organization Classification of Tumours of Soft Tissue and Bone. Fourth Edition. Lyon: IARC; 2013.
2. Kattan MW, Hess KR, Amin MB, et al. American Joint Committee on Cancer acceptance criteria for inclusion of risk models for individualized prognosis in the practice of precision medicine. *CA: a cancer journal for clinicians*. Jan 19 2016.

Gastrointestinal Stromal Tumor

43

Ronald P. DeMatteo, Robert G. Maki, Mark Agulnik, Robert K. Brookland, Jason L. Hornick, Robin L. Jones, Vicki L. Keedy, Alexander J. Lazar, Brian O'Sullivan, Chandrajit P. Raut, Paige S. Tedder, and Raphael E. Pollock

CHAPTER SUMMARY

Cancers Staged Using This Staging System

Gastrointestinal stromal tumor (GIST)

Cancers Not Staged Using This Staging System

These histopathologic types of cancer…	Are staged according to the classification for…	And can be found in chapter…
Pediatric GIST, familial GIST (germline mutant *KIT* or *PDGFRA*), or syndromic GIST	No AJCC staging system	N/A

Summary of Changes

There are no changes to this staging system.

ICD-O-3 Topography Codes

Code	Description	Code	Description
C15.0	Cervical esophagus	C16.6	Greater curvature of stomach, NOS
C15.1	Thoracic esophagus	C16.8	Overlapping lesion of stomach
C15.2	Abdominal esophagus	C16.9	Stomach, NOS
C15.3	Upper third of esophagus	C17.0	Duodenum
C15.4	Middle third of esophagus	C17.1	Jejunum
C15.5	Lower third of esophagus	C17.2	Ileum
C15.8	Overlapping lesion of esophagus	C17.8	Overlapping lesion of small intestine
C15.9	Esophagus, NOS	C17.9	Small intestine, NOS
C16.0	Cardia, NOS	C18.0	Cecum
C16.1	Fundus of stomach	C18.1	Appendix
C16.2	Body of stomach	C18.2	Ascending colon
C16.3	Gastric antrum	C18.3	Hepatic flexure of colon
C16.4	Pylorus	C18.4	Transverse colon
C16.5	Lesser curvature of stomach, NOS	C18.5	Splenic flexure of colon

To access the AJCC cancer staging forms, please visit www.cancerstaging.org.

© American Joint Committee on Cancer 2017
M.B. Amin et al. (eds.), *AJCC Cancer Staging Manual, Eighth Edition*, DOI 10.1007/978-3-319-40618-3_43

Code	Description
C18.6	Descending colon
C18.7	Sigmoid colon
C18.8	Overlapping lesion of colon
C18.9	Colon, NOS
C19.9	Rectosigmoid junction
C20.9	Rectum
C48.0	Retroperitoneum
C48.1	Specified parts of peritoneum
C48.2	Peritoneum, NOS
C48.8	Overlapping lesion of retroperitoneum and peritoneum

WHO Classification of Tumors

Code	Description
8936	Gastrointestinal stromal tumor, malignant

Fletcher CDM, Bridge JA, Hogendoorn P, Mertens F, eds. World Health Organization Classification of Tumours of Soft Tissue and Bone. Fourth Edition. Lyon: IARC; 2013.

INTRODUCTION

Gastrointestinal stromal tumor (GIST) is the most common human sarcoma and the most common mesenchymal tumor of the gastrointestinal (GI) tract. The designation of GIST refers to a specific tumor type that is almost always immunohistochemically KIT and DOG1 positive and about 85% of the time is driven by an activating mutation in either *KIT* or *PDGFRA* (Fig. 43.1). GISTs are distinct from leiomyomas and leiomyosarcomas.

In terms of biologic potential, GISTs encompass a continuum from small tumors with a low mitotic rate to large tumors with a high mitotic rate. Tumors smaller than 2 cm generally are considered indolent, and observation may be employed in most of these patients. The tyrosine kinase inhibitors imatinib, sunitinib, and regorafenib are approved by the US Food and Drug Administration (FDA) for advanced GIST.[1-3] Imatinib also is FDA approved for adjuvant therapy (Figs. 43.2 and 43.3).[4]

This staging system attempts to assist in the management of GIST by offering statistical probabilities for the development of metastasis based on tumor site, size, and mitotic rate, which are the most important and most widely studied prognostic parameters in GIST. There are two National Institutes of Health staging systems for GIST,[5,6] both of which have fewer categories and are not used widely any longer. Instead, the staging system from the AJCC Cancer Staging Manual, 7th Edition is continued in this edition because it is based on the largest retrospective series of GISTs. Use of the AJCC staging system will permit uniform collection of data, which is particularly important in an uncommon disease such as GIST.

The AJCC staging system currently does not incorporate mutation status, as its effect in the absence of tyrosine kinase inhibition has not been well defined. In addition, a nomogram to estimate the chance of recurrence after resection of a localized, primary GIST has been developed and validated in several series.

The staging system should not be applied to pediatric GIST, familial GIST (germline mutant *KIT* or *PDGFRA*), or syndromic GIST. These are rare subsets of GIST that typically have an indolent biology but eventually may be lethal. Deficiency in succinate dehydrogenase (SDH) may occur via mutation or methylation and accounts for about 85% of pediatric GISTs and about 5% of adult GISTs, all arising in the stomach. Syndromic GISTs occur in patients with neurofibromatosis

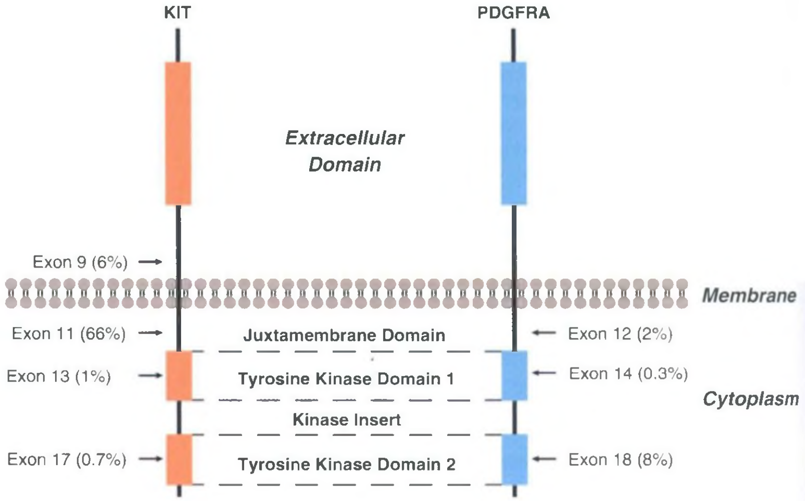

Fig. 43.1 Distribution of mutations in GIST prior to treatment with systemic therapy. Schematic of KIT and PDGFRA showing the relative percentage of mutations in various exons and domains present in primary GIST (Adapted from Joensuu et al.[13])

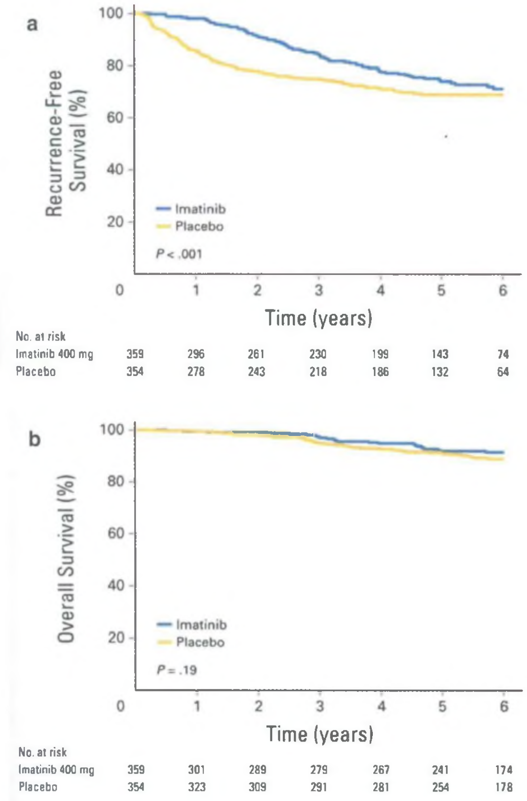

Fig. 43.2 Natural history of GIST. Kaplan–Meier analysis of recurrence-free and overall survival in patients with a GIST ≥3 cm treated on an American College of Surgeons Oncology Group (ACOSOG) intergroup trial with 1 year of imatinib versus placebo. The median follow-up was 74 months (From Corless et al.[14] with permission). (**a**) Recurrence-free survival of patients on placebo (*yellow*) or on 1 year of imatinib (*blue*). (**b**) Overall survival is essentially identical in both patients treated with adjuvant imatinib or not

type 1, Carney triad, or Carney dyad (also known as Carney–Stratakis syndrome). The Carney triad includes gastric GIST, pulmonary chondroma, and extra-adrenal paraganglioma, usually secondary to *SDHC* promoter hypermethylation. Carney dyad comprises gastric GIST and paraganglioma due to a germline *SDHB*, *-C*, or *-D* mutation.

ANATOMY

Primary Site(s)

GISTs occur throughout the GI tract. They are most common in the stomach (60%) and small intestine jejunum and ileum (30%) and are relatively rare in the duodenum (5%), rectum (3%), colon (1%), and esophagus (<1%). In some cases, they present as disseminated tumors without a known primary site, and a few GISTs may originate in the omentum or mesentery. GISTs are thought to arise from the interstitial cells of Cajal, which are pacemaker cells that form a myenteric plexus within the muscle of the GI tract. The tumors generally are enclosed by a pseudocapsule and rarely infiltrate within the wall of the GI tract. They often grow in an exophytic pattern. Like many sarcomas, GISTs tend to push and not invade surrounding organs, although GISTs may become adherent to nearby structures.

Regional Lymph Nodes

Nodal metastasis is extremely rare in GIST, except in SDH-deficient GISTs, which tend to be less aggressive than most other GISTs, despite nodal involvement.

Metastatic Sites

Metastasis most commonly occurs in the liver or on the peritoneal surface. Metastases much less commonly affect bone, lungs, or other extra-abdominal soft tissues.

RULES FOR CLASSIFICATION

Clinical Classification

Patients are assessed with a complete medical history, physical examination, laboratory tests, radiologic tests, and in some cases, endoscopy and biopsy. Clinical staging is achieved by the use of cross-sectional imaging with either computed tomography (CT) or magnetic resonance (MR) imaging of the abdomen and pelvis. Biopsy specimens may be obtained percutaneously or endoscopically. Percutaneous biopsies have a potential risk of inducing bleeding or disseminating tumor. Endoscopic biopsies sometimes are inadequate to establish a definitive diagnosis because GIST typically does not invade the mucosal surface, making it more difficult to obtain an adequate portion of tumor. Endoscopic ultrasound-guided fine-needle aspiration (EUS-FNA) may provide enough tissue for diagnosis, although not enough to assess mitotic count reliably (see Prognostic Factors Required for Stage Grouping: Mitotic Rate). In general, EUS-FNA avoids the hypothetical risk of percutaneous biopsies seeding the peritoneum while accessing tissue deeper than that from an endoscopic biopsy. Preoperatively, clinical stage (cTNM) can only be estimated because mitotic rate generally cannot be assessed adequately by FNA, and it may be estimated incorrectly with core needle biopsy. Pathological staging (pTNM) is determined after tumor removal. If preoperative tyrosine kinase inhibitor therapy is

a Recurrence-free survival: intention-to-treat population

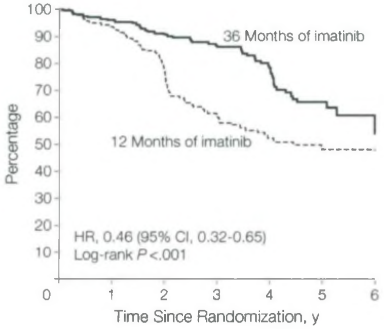

No. of patients							
36 Months of imatinib	198	184	173	133	82	39	8
12 Months of imatinib	199	177	137	88	49	27	10

b Overall survival: intention-to-treat population

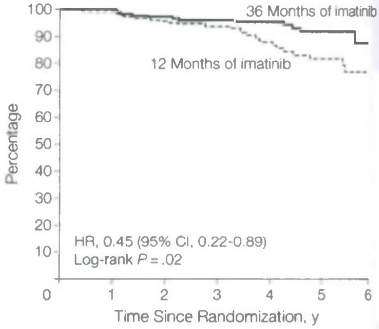

No. of patients							
36 Months of imatinib	198	192	184	152	100	56	13
12 Months of imatinib	199	188	176	140	87	46	20

Fig. 43.3 Recurrence-free and overall survival for patients treated with 1 versus 3 years of imatinib for higher-risk GIST, defined as having at least one of the following features: (1) longest tumor diameter greater than 10.0 cm, (2) mitotic count greater than 10 mitoses per 50 HPF of the microscope, (3) tumor diameter greater than 5.0 cm and mitotic count greater than 5, or (4) tumor rupture before surgery or at surgery (From Joensuu et al.[15] with permission). (**a**) Progression free survival of the intention to treat population (solid line, 3 years imatinib; dotted line, 1 year imatinib). (**b**) Overall survival of the intention to treat population (solid line, 3 years imatinib; dotted line, 1 year imatinib)

administered and there is tumor stabilization or shrinkage, determination of mitotic rate is confounded. Obviously, tumor size also may have been altered.

Tumor size

Because most GISTs are spherical or ovoid, the maximum tumor diameter is readily determined. The size thresholds of the greatest tumor diameter used in this staging system are 2, 5, and 10 cm. Depth of gastric or intestinal wall involvement is not considered in the staging criteria, because most GISTs, except the smallest ones, form transmural masses.

Regional nodal metastasis is extremely rare in GIST, except in SDH-deficient GISTs, which tend to be less aggressive than most other GISTs despite nodal involvement. Therefore, nodal dissection generally is not indicated for GIST unless there are clinically suspicious, enlarged nodes. In the absence of information on regional lymph node status, cN0 is appropriate; the specification NX should not be used.

The presence of any metastasis is designated as M1. It should be noted that a true local recurrence isolated to the exact site of resection of the primary tumor is very rare in GIST.

Imaging

Clinical staging is achieved by the use of cross-sectional imaging with either CT or MR imaging of the abdomen and pelvis. Intravenous contrast is necessary to fully evaluate the extent of the primary tumor and its relation to surrounding structures and to assess for intra-abdominal metastases. For CT scans, oral contrast also should be administered. ^{18}F-fluorodexoyglucose positron emission tomography (FDG-PET) scans are still considered a research tool and not required for adequate staging.

Pathological Classification

Tumor size

Because most GISTs are spherical or ovoid, the maximum tumor diameter is readily determined. In the case of ruptured tumors, one may have to estimate the tumor size or obtain assistance for maximum diameter measurement from radiologic studies. The size thresholds of the greatest tumor diameter used in this staging system are 2, 5, and 10 cm. Depth of gastric or intestinal wall involvement is not considered in the staging criteria, because most GISTs, except the smallest ones, form transmural masses.

Mitotic rate

Mitotic rate has a significant impact on clinical outcomes but has not been completely standardized. The mitotic rate should be obtained from an area that on screening shows the highest level of mitotic activity and be performed as consecutive high-power fields (HPF). The mitotic rate of GIST is best expressed as the number of mitoses per 5 mm^2 (using 400× magnification). This value corresponds to 50 HPF in large prognostic studies obtained with older-model microscopes using "conventional" optics (i.e., not using a wide field size). The number of fields required for 5 mm^2 should be determined for individual microscopes. Practically, this means counting mitoses in 20 to 25 HPF with modern microscopes

equipped with wide field optics in order to obtain a total area of 5 mm^2. Stringent criteria should be followed when defining a mitosis: pyknotic or dyskaryotic nuclei should not be regarded as mitoses. It is important to use this correct 5-mm^2 mitotic assessment to allow comparisons between studies. However, this change does not affect the staging of the great majority of GISTs since mitoses usually are either found readily or quite rarely, regardless of the number of fields assessed.

Distant metastasis

Distant metastasis includes spread to the liver, peritoneal surface, omentum, retroperitoneum, bone, lung, soft tissue, or any other site away from the primary tumor. Liver metastasis signifies the presence of one or more tumor nodules inside the liver parenchyma. Adherence to the liver capsule, even if extensive, as sometimes is seen in gastric GISTs, should not be construed as liver metastasis.

Detailed criteria for pathological classification have been published,[7] and the recommendations of the College of American Pathologists are available online (www.cap.org).

PROGNOSTIC FACTORS

Prognostic Factors Required for Stage Grouping

Mitotic Rate

Histologic grading is a component of sarcoma staging, but it is not well suited to GIST. This is because most of these tumors have low or relatively low mitotic rates, below the thresholds used for grading soft tissue tumors, and because GISTs often manifest aggressive features with mitotic rates below the thresholds used for soft tissue tumor grading (the lowest tier of mitotic rate for most soft tissue sarcomas is 10 mitoses per 10 HPF). In GIST staging, the grade is replaced by mitotic activity.

Although risk is stratified in the staging criteria using a cutoff of 5 mitoses per 5 mm^2, the risk by mitotic rate is a continuous variable with the cutoff being used to define sets of low-mitotic and higher-mitotic populations. AJCC Level of Evidence: I

Mitotic rate	Definition
Low	5 or fewer mitoses per 5 mm^2, or per 50 HPF
High	over 5 mitoses per 5 mm^2, or per 50 HPF

Additional Factors Recommended for Clinical Care

Tumor Rupture

Because of inadequate data, tumor rupture is not included in the staging system. Nevertheless, tumor rupture into the peritoneal cavity significantly increases the likelihood of peritoneal recurrence. AJCC Level of Evidence: III

RISK ASSESSMENT MODELS

The AJCC recently established guidelines that will be used to evaluate published statistical prediction models for the purpose of granting endorsement for clinical use.[8] Although this is a monumental step toward the goal of precision medicine, this work was published only very recently. Therefore, the existing models that have been published or may be in clinical use have not yet been evaluated for this cancer site by the Precision Medicine Core of the AJCC. In the future, the statistical prediction models for this cancer site will be evaluated, and those that meet all AJCC criteria will be endorsed.

DEFINITIONS OF AJCC TNM

Definition of Primary Tumor (T)

T Category	T Criteria
TX	Primary tumor cannot be assessed
T0	No evidence of primary tumor
T1	Tumor 2 cm or less
T2	Tumor more than 2 cm but not more than 5 cm
T3	Tumor more than 5 cm but not more than 10 cm
T4	Tumor more than 10 cm in greatest dimension

Definition of Regional Lymph Node (N)

N Category	N Criteria
N0	No regional lymph node metastasis or unknown lymph node status
N1	Regional lymph node metastasis

Definition of Distant Metastasis (M)

M Category	M Criteria
M0	No distant metastasis
M1	Distant metastasis

Definition of Mitotic Rate

Mitotic rate	Definition
Low	5 or fewer mitoses per 5 mm^2, or per 50 HPF
High	Over 5 mitoses per 5 mm^2, or per 50 HPF

AJCC PROGNOSTIC STAGE GROUPS

The staging system parallels the one used for peripheral soft tissue tumors. In addition, a numeric value for risk of metastasis is provided, based on the studies with the larg-

est follow-up.[9-12] Although T, N, and M criteria are identical for all GISTs, separate stage grouping schemes are provided for gastric and small intestinal tumors because they have different prognoses (Tables 43.1 and 43.2). There are fewer data on primary GISTs that arise in the omentum, mesentery, esophagus, colon, and rectum. Primary omental GISTs should follow the gastric GIST staging, whereas the small intestinal group staging should be used for the other sites.

Gastric and Omental GIST

When T is...	And N is...	And M is...	And mitotic rate is...	Then the stage group is...
T1 or T2	N0	M0	Low	IA
T3	N0	M0	Low	IB
T1	N0	M0	High	II
T2	N0	M0	High	II
T4	N0	M0	Low	II
T3	N0	M0	High	IIIA
T4	N0	M0	High	IIIB
Any T	N1	M0	Any rate	IV
Any T	Any N	M1	Any rate	IV

Small Intestinal, Esophageal, Colorectal, Mesenteric, and Peritoneal GIST

When T is...	And N is...	And M is...	And mitotic rate is...	Then the stage group is...
T1 or T2	N0	M0	Low	I
T3	N0	M0	Low	II
T1	N0	M0	High	IIIA
T4	N0	M0	Low	IIIA
T2	N0	M0	High	IIIB
T3	N0	M0	High	IIIB
T4	N0	M0	High	IIIB
Any T	N1	M0	Any rate	IV
Any T	Any N	M1	Any rate	IV

REGISTRY DATA COLLECTION VARIABLES

1. Tumor size
2. Tumor site (esophagus, stomach, duodenum, jejunum/ileum, rectum, or extraintestinal)
3. Tumor mitotic rate
4. Tumor rupture
5. Tumor metastasis—liver, peritoneum, other
6. Tumor KIT immunohistochemistry
7. Tumor mutational status of *KIT*, *PDGFRA* (if known)

HISTOLOGIC GRADE (G)

Grading for GIST is dependent on mitotic rate.

Mitotic Rate	Definition
Low	5 or fewer mitoses per 5 mm^2, or per 50 HPF
High	Over 5 mitoses per 5 mm^2, or per 50 HPF

HISTOPATHOLOGIC TYPE

The morphologic subtypes of GIST include spindle cell (70%), epithelioid (20%), and mixed-cell types.

SURVIVAL DATA

Table 43.1 Disease progression in gastric GISTs

Stage	Tumor size (cm)	Mitotic rate	Observed rate of progressive disease
Stage IA	≤5	Low	0–2% ·
Stage IB	>5–10	Low	3–4%
StageII	≤2	High	Insufficient data
	>2-5	High	16%
	>10	Low	12%
Stage IIIA	>5–10	High	55%
Stage IIIB	>10	High	86%

Based on Miettinen and Lasota,[10] with permission.

Table 43.2 Disease progression in small intestinal GISTs

Stage	Tumor size (cm)	Mitotic rate	Observed rate of progressive disease
Stage IA	≤5	Low	0–4%
Stage II	>5–10	Low	24%
Stage IIIA	>10	Low	52%
	≤2	High	50%
Stage IIIB	>2–5	High	73%
	>5-10	High	85%
	>10	High	90%

Based on Miettinen and Lasota,[10] with permission.

Bibliography

1. Demetri GD, Reichardt P, Kang YK, et al. Efficacy and safety of regorafenib for advanced gastrointestinal stromal tumours after failure of imatinib and sunitinib (GRID): an international, multicentre, randomised, placebo-controlled, phase 3 trial. *Lancet.* Jan 26 2013;381(9863):295-302.
2. Demetri GD, van Oosterom AT, Garrett CR, et al. Efficacy and safety of sunitinib in patients with advanced gastrointestinal stromal tumour after failure of imatinib: a randomised controlled trial. *Lancet.* Oct 14 2006;368(9544):1329-1338.
3. Demetri GD, von Mehren M, Blanke CD, et al. Efficacy and safety of imatinib mesylate in advanced gastrointestinal stromal tumors. *N Engl J Med.* Aug 15 2002;347(7):472-480.
4. Dematteo RP, Ballman KV, Antonescu CR, et al. Adjuvant imatinib mesylate after resection of localised, primary gastrointestinal stromal tumour: a randomised, double-blind, placebo-controlled trial. *Lancet.* Mar 28 2009;373(9669):1097-1104.
5. Fletcher CD, Berman JJ, Corless C, et al. Diagnosis of gastrointestinal stromal tumors: A consensus approach. *Human pathology.* May 2002;33(5):459-465.
6. Miettinen M, El-Rifai W, L HLS, Lasota J. Evaluation of malignancy and prognosis of gastrointestinal stromal tumors: a review. *Human pathology.* May 2002;33(5):478-483.
7. Hameed M, Corless C, George S, et al. Template for Reporting Results of Biomarker Testing of Specimens From Patients With Gastrointestinal Stromal Tumors. *Arch Pathol Lab Med.* Oct 2015;139(10):1271-1275.
8. Kattan MW, Hess KR, Amin MB, et al. American Joint Committee on Cancer acceptance criteria for inclusion of risk models for individualized prognosis in the practice of precision medicine. *CA: a cancer journal for clinicians.* Jan 19 2016.
9. Miettinen M, Sobin LH, Lasota J. Gastrointestinal stromal tumors of the stomach: a clinicopathologic, immunohistochemical, and molecular genetic study of 1765 cases with long-term follow-up. *The American journal of surgical pathology.* Jan 2005;29(1):52-68.
10. Miettinen M, Lasota J. Gastrointestinal stromal tumors: pathology and prognosis at different sites. *Seminars in diagnostic pathology.* May 2006;23(2):70-83.
11. Miettinen M, Makhlouf H, Sobin LH, Lasota J. Gastrointestinal stromal tumors of the jejunum and ileum: a clinicopathologic, immunohistochemical, and molecular genetic study of 906 cases before imatinib with long-term follow-up. *The American journal of surgical pathology.* Apr 2006;30(4):477-489.
12. Miettinen M, Lasota J. Gastrointestinal stromal tumors: review on morphology, molecular pathology, prognosis, and differential diagnosis. *Arch Pathol Lab Med.* Oct 2006;130(10):1466-1478.
13. Joensuu H, Rutkowski P, Nishida T, et al. KIT and PDGFRA mutations and the risk of GI stromal tumor recurrence. *J Clin Oncol.* Feb 20 2015;33(6):634-642.
14. Corless CL, Ballman KV, Antonescu CR, et al. Pathologic and molecular features correlate with long-term outcome after adjuvant therapy of resected primary GI stromal tumor: the ACOSOG Z9001 trial. *J Clin Oncol.* May 20 2014;32(15):1563-1570.
15. Joensuu H, Eriksson M, Sundby Hall K, et al. One vs three years of adjuvant imatinib for operable gastrointestinal stromal tumor: a randomized trial. *JAMA.* Mar 28 2012;307(12):1265-1272.

Soft Tissue Sarcoma of the Retroperitoneum

44

Raphael E. Pollock, Robert G. Maki, Elizabeth H. Baldini, Jason L. Hornick, Vicki L. Keedy, Alexander J. Lazar, John E. Madewell, Chandrajit P. Raut, Paige S. Tedder, and Sam S. Yoon

CHAPTER SUMMARY

Cancers Staged Using This Staging System

Common sarcomas of the retroperitoneum

Summary of Changes

Change	Details of Change	Level of Evidence
Definition of Primary Tumor (T)	Retroperitoneal sarcomas use the same revised tumor size (T) classification for extremity and trunk sarcomas	II
Definition of Primary Tumor (T)	Superficial and deep location has been removed as part of T criteria.	II
Definition of Primary Tumor (T)	T categories have been increased from two to four.	II
Definition of Primary Tumor (T)	T1 remains as tumor 5 cm or less in greatest dimension.	II
Definition of Primary Tumor (T)	T2 is now tumor more than 5 cm and less than or equal to 10 cm in greatest dimension.	II
Definition of Primary Tumor (T)	T3 is newly categorized as tumor more than 10 cm and less than or equal to 15 cm in greatest dimension.	II[1]
Definition of Primary Tumor (T)	T4 is a new category defined as tumor more than 15 cm in greatest dimension	II[1]
Risk Assessment Models	The retroperitoneum poses particular challenges to staging, especially in the context of resectable retroperitoneal sarcoma (AJCC Stage I–III; discussed later). These difficulties are particularly apparent in using the AJCC staging system to counsel patients regarding prognosis in that most resectable retroperitoneal sarcomas present as large lesions (T2) without any metastasis (N0 M0). In light of this relative lack of prognostic discrimination of the AJCC soft tissue sarcoma staging system, a prognostic nomogram is now included as a means to assess prognosis more accurately for patients bearing retroperitoneal soft tissue sarcoma.[1]	I

To access the AJCC cancer staging forms, please visit www.cancerstaging.org.

© American Joint Committee on Cancer 2017

M.B. Amin et al. (eds.), *AJCC Cancer Staging Manual, Eighth Edition*, DOI 10.1007/978-3-319-40618-3_44

ICD-O-3 Topography Codes

Code	Description
C48.0	Malignant neoplasm of retroperitoneum
C48.1	Malignant neoplasm of specified parts of peritoneum
C48.2	Malignant neoplasm of peritoneum, unspecified
C48.8	Overlapping lesion of retroperitoneum and peritoneum

WHO Classification of Tumors[2]

Adipocytic Tumors

Code	Description
8850	Atypical lipomatous tumor
8850	Well-differentiated liposarcoma
8850	Liposarcoma, NOS
8858	Dedifferentiated liposarcoma
8852	Myxoid/round cell liposarcoma
8854	Pleomorphic liposarcoma

Fibroblastic/Myofibroblastic Tumors

Code	Description
8832	Dermatofibrosarcoma protuberans
8832	Fibrosarcomatous dermatofibrosarcoma protuberans
8833	Pigmented dermatofibrosarcoma protuberans
8815	Solitary fibrous tumor, malignant
8825	Inflammatory myofibroblastic tumor
8825	Low-grade myofibroblastic sarcoma
8810	Adult fibrosarcoma
8811	Myxofibrosarcoma
8840	Low-grade fibromyxoid sarcoma
8840	Sclerosing epithelioid fibrosarcoma

So-called Fibrohistiocytic Tumors

Code	Description
9251	Giant cell tumor of soft tissues

Smooth Muscle Tumors

Code	Description
8890	Leiomyosarcoma (excluding skin)

Pericytic (Perivascular) Tumors

Code	Description
8711	Malignant glomus tumor

Skeletal Muscle Tumors

Code	Description
8910	Embryonal rhabdomyosarcoma (including botryoid, anaplastic)
8920	Alveolar rhabdomyosarcoma (including solid, anaplastic)
8901	Pleomorphic rhabdomyosarcoma
8912	Spindle cell/sclerosing rhabdomyosarcoma

Vascular Tumors of Soft Tissue

Code	Description
9136	Retiform hemangioendothelioma
9136	Pseudomyogenic (epithelioid sarcoma-like) hemangioendothelioma
9133	Epithelioid hemangioendothelioma
9120	Angiosarcoma of soft tissue

Chondro-osseous Tumors

Code	Description
9180	Extraskeletal osteosarcoma

Nerve Sheath Tumors

Code	Description
9540	Malignant peripheral nerve sheath tumor
9542	Epithelioid malignant peripheral nerve sheath tumor
9561	Malignant Triton tumor
9580	Malignant granular cell tumor

INTRODUCTION

Approximately 10% of all sarcomas occur in the retroperitoneum. Although virtually any soft tissue sarcoma histologic subtype may be seen in this anatomic location, the most common types are shown in Table 44.1. All these histologic subtypes are subject to staging.

Several recent trends in retroperitoneal sarcoma management are worthy of mention. Although surgery remains the mainstay treatment modality, a prospective randomized trial being conducted under the auspices of the European Organisation for Research and Treatment of Cancer (EORTC 62092-22092) is comparing surgery versus preoperative radiotherapy and surgery. It is hoped this first-ever rigorous evaluation will be completed over the next several years.

Controversy exists regarding the extent of resection needed for optimal control of retroperitoneal soft tissue sarcoma. The dispute focuses on the extent of contiguous organ resection. Some proponents advocate such resection in all patients, whereas others recommend it for all histologic subtypes except the generally more indolent-behaving well-differentiated liposarcoma, the most common histologic subtype in the retroperitoneum. An international prospective patient registry has been established, and it is hoped this registry will help resolve this controversy.

TNM staging is important for retroperitoneal sarcoma to help accomplish three major objectives: (1) to provide a rigorous basis by which to compare clinical results within an institution over time or to compare results using different therapeutic approaches within or between performance

sites; (2) to provide a means to perform verifiable cancer registry functions; and (3) to provide a basis for assessing prognosis on behalf of patient populations of comparable stage. With the current and future incorporation of molecular staging criteria, it may be anticipated that these three broad mandates will be increasingly challenging using a single staging system. Nonetheless, the AJCC Prognostic Stage Grouping for retroperitoneal soft tissue sarcoma retains the key elements (with modification) to satisfy at least two of these three objectives, the exception being precise prognostic assessment for individual retroperitoneal sarcoma patients.

Table 44.1 Most common and least common histologies arising in the retroperitoneum

Most common histologies	Less common histologies (not an exhaustive list)
Liposarcoma (well-differentiated and dedifferentiated)<br>Leiomyosarcoma	Pleomorphic liposarcoma<br>Undifferentiated pleomorphic sarcoma<br>Malignant peripheral nerve sheath tumor (MPNST)<br>Solitary fibrous tumor (malignant)

ANATOMY

Primary Site(s)

The retroperitoneum is a complex anatomic compartment. The presence of critical anatomy that is difficult to manage surgically (e.g., superior mesenteric vasculature), coupled with the potential for this locus to accommodate large tumors before symptoms develop, makes it particularly difficult to provide effective therapy for these tumors.

Sarcomas in the retroperitoneum may grow by direct extension; depending on histologic subtype, the leading edge of tumors in this location may be either infiltrative or "pushing" in nature. Assessing contiguous structure involvement intraoperatively may be difficult without transgressing the tumor *per se*.

Regional Lymph Nodes

Nodal metastases are rare for sarcomas, particularly so for lesions in this anatomic location. When they occur, they most commonly are found in the para-aortic and intestinal mesenteric regions. Local control is difficult to achieve, and patients usually die from locoregional rather than metastatic disease.

Metastatic Sites

Compared with the more common well-differentiated/dedifferentiated liposarcoma and its propensity for locoregional recurrence rather than metastatic disease, leiomyosarcomas arising from branches of the inferior vena cava typically present as relatively smaller tumors and have a predilection to manifest metastatic disease. Pulmonary metastasis remains the major site of dissemination when it occurs, except in the case of leiomyosarcoma, which frequently is associated with liver and lung metastases. Patients with well-differentiated/dedifferentiated liposarcoma may present an unusual circumstance in solid tumor oncology in that both histologic subtypes may be synchronously or metachronously detectable as an extreme manifestation of intratumoral heterogeneity.

44

RULES FOR CLASSIFICATION

Clinical Classification

AJCC soft tissue sarcoma staging depends on clinically and microscopically derived information, including radiologic assessment. Tumor size (T) is based on the maximum measured tumor dimension as assessed by physical examination and/or radiologic measurement. The presence or absence of nodal disease (N) or metastatic disease (M) ultimately is confirmed by biopsy-dependent microscopic verification. Grade (G) is assessed on the basis of primary tumor biopsy by using the French Federation of Cancer Centers Sarcoma Group (FNCLCC) three-tiered criteria.

The retroperitoneum poses special challenges in AJCC staging. Other than in the context of incidental discovery, the vast majority of retroperitoneal sarcomas have already grown to at least 5 cm in maximum dimension (T2 or T3) by the time they are detected; indeed, this is part of the rationale for expanding T classification into more than two criteria for this edition of the AJCC Cancer Staging Manual. Metastasis to lymph nodes is an unusual occurrence. Grade assessment, from a prognostic perspective, devolves into two basic criteria: G1, low-grade lesions, versus G2 and G3 lesions. The latter, although distinct entities on the basis of grade, actually have overlying Kaplan–Meier survival plots in most large published series. Consequently, the vast majority of resectable retroperitoneal sarcomas for which curative surgical intent is theoretically possible are T2 or T3 (N0 M0) G1 versus G2/3 lesions; therefore, staging of these tumors is based on which of the two G clusters better describes a given tumor. The obvious lack of prognostic

refinement embodied in a two-factor staging system underlies the development of the prognostic nomogram proposed in this chapter.

Imaging

A variety of imaging techniques may be used to assess retroperitoneal soft tissue sarcomas, and their selection ultimately depends on local institutional practice and available expertise. Imaging is critical for these tumors to guide the performance of the safe, image-directed biopsies needed to establish grade as well as to determine resectability, that is, the tumor's proximity to life-defining anatomy and/or nondispensable anatomic structures. Typically, patients initially undergo computed tomography (CT) scanning of the abdomen and pelvis, frequently in response to vague, nonresolving pain unrelated to eating or other vegetative functions. Magnetic resonance (MR) imaging also is very useful in this context but is used less commonly as the initial imaging modality because of local ease of access and timeliness issues. CT scanning has the additional advantage of avoiding gastrointestinal peristalsis artifacts, which may obscure pertinent anatomic detail in MR imaging scans.

Positron emission tomography (PET) scanning of retroperitoneal sarcomas is most useful in the context of anticipated neoadjuvant systemic therapies, as a means to assess metabolic tumor response to such treatments if the sarcoma is initially PET avid. Although CT scanning avoids motion artifacts due to intestinal peristalsis, MR imaging occasionally provides slightly more detail regarding the sarcoma–normal tissue interface and does not result in patient exposure to radiation sources. Either technique can provide information about the possibility of nodal disease, multifocal sarcoma presentations, and other abdominopelvic metastases, as well as information about sarcoma heterogeneity, which may be particularly pertinent in the context of synchronous well-differentiated/dedifferentiated retroperitoneal liposarcoma presentations for which neoadjuvant systemic therapy may be a consideration given the potential for dedifferentiated (but not well-differentiated) liposarcoma dissemination, particularly to the lungs.

Pathological Classification

After retroperitoneal sarcoma resection, the permanent pathological analysis of the surgical specimen, at a minimum, should include comments about piecemeal versus intact resection, maximal measured tumor dimension, histologic subtype, margin of resection status, lymph node involvement, sarcoma involvement of any adjacent contiguous organs included in the resection, assignment of grade, and other nonstandard, local descriptive findings, such as immunohistochemical interrogations and molecular determinants. Although several leading sarcoma centers worldwide are analyzing a wide range of molecular and genetic sarcoma determinants that may prove useful for future staging purposes, such information has not yet matured to the point of incorporation into AJCC soft tissue sarcoma staging systems. Please see the introductory section of this chapter for a more comprehensive discussion of the relevant pathological assessment of retroperitoneal soft tissue sarcoma in the pretherapeutic diagnostic and postresection clinical contexts.

PROGNOSTIC FACTORS

Prognostic Factors Required for Stage Grouping

French Federation of Cancer Centers Sarcoma Group (FNCLCC) grade (G), which is based on pretreatment biopsy materials. See Histologic Grade (G) in this chapter.

Additional Factors Recommended for Clinical Care

The current AJCC staging criteria for retroperitoneal sarcoma include tumor size (T), assessed as the maximal tumor dimension as measured on physical examination and/or radiologically, and the biopsy-proven presence or absence of nodal (N) or distant site (M) metastases. The possibility of generating the information needed to assess these four factors is readily available in almost all oncology centers; their utility has been validated over time in numerous prospective and retrospective studies.

This AJCC Prognostic Stage Grouping is useful for cancer registry and intra- and inter-institutional treatment outcome comparisons. However, this approach is less useful in assessing prognosis for retroperitoneal soft tissue sarcoma because G1 versus G2/3 appears to be the only applicable prognostic discriminator given that almost all resectable sarcomas are T2-3N0M0 lesions. Although histologic subtype, margin of resection status, patient age, and several histology-specific factors (e.g., percentage of round cell liposarcoma in a given myxoid/round cell liposarcoma specimen, *TP53* mutational status in some histologic subtypes) have utility in establishing prognosis, they generally have not achieved enough validation maturity for inclusion in AJCC staging for retroperitoneal soft tissue sarcoma.

In light of these issues, researchers at several European and US institutions developed a prognostic nomogram for retroperitoneal soft tissue sarcoma that incorporates database information from the Istituto Tumori, Milan, Italy; The University of Texas MD Anderson Cancer Center; and the University of California, Los Angeles. The resultant algorithms for prognosis assessment are useful in several con-

texts that cannot be determined in the current AJCC staging system for soft tissue sarcoma of the retroperitoneum, including overall survival, disease-free survival, and either primary or recurrent retroperitoneal disease status. The resulting prognostic nomogram subsequently was validated versus two large-scale independent datasets maintained at the Institute Gustave Roussey, Paris, France,[3] and more recently using a dataset from Brigham and Women's Hospital, Boston, MA.[3]

In using this nomogram, the relevant factors that must be determined to assess a specific patient's prognosis include patient age, maximal tumor dimension, tumor grade, tumor multifocality, extent of resection (R0/R1 vs. R2) and underlying tumor histologic subtype. Points are assigned for each of these individual criteria; then, the points are added to determine an overall numerical score from which 5-year and 10-year overall and disease-free survival can be predicted with a very high degree of accuracy. Note that all the input information needed already has been determined for almost all patients as part of their sarcoma-specific diagnostic workup. This approach has another advantage: as new (especially molecular and genetic) determinants become available, their utility can be verified easily by incorporating a candidate parameter into the prognostic algorithm and then observing whether the overall accuracy of the prediction is enhanced relative to the antecedent algorithm devoid of candidate new factors.

RISK ASSESSMENT MODELS

Prognostic models are important for cancer staging and treatment. Traditionally AJCC staging has been powerfully driven primarily by a small number of anatomic variables. Increasingly, it is recognized that that increasing the number of variables used for prognosis and supplementing with helpful non-anatomic variables can be extremely helpful for prognosis. In recognition of this, the AJCC endeavored to evaluate additional models to see if they might be helpful in cancer prognosis as adjuncts to traditional staging groups.

The AJCC Precision Medicine Core (PMC) developed and published clear criteria for critical evaluation of prognostic tool quality, which are presented and discussed in Chapter 4.[4] Although developed independently by the PMC, the AJCC quality criteria corresponded fully with the recently developed Cochrane CHARMS tool and TRIPOD criteria for critical appraisal in systematic reviews of prediction modeling studies.[5,6]

A prognostic model for retroperitoneal sarcoma meeting all of the AJCC inclusion/exclusion criteria and meriting AJCC endorsement is briefly presented in this section. A full list of the evaluated models for other cancer types and their adherence to the quality criteria is available on http://www.cancerstaging.org.

The AJCC Soft Tissue Sarcoma Expert Panel nominated a model predicting overall survival and disease-free survival in patients with retroperitoneal sarcoma – the Gronchi et al model.[3] This model was rigorously compared against the quality criteria developed by the PMC as guidelines for AJCC commendation for prognostication models (see Chapter 4).

In addition, the PMC performed a systematic search of published literature for prognostic models/tools in retroperitoneal sarcoma from January 2011 to December 2015. The search strategy is detailed in Chapter 4. The PMC defined "prognostic model" as a multivariable model where factors predict a defined future clinical outcome (specifically survival). No additional appropriate published models were identified.

The Gronchi et al model is based on more than 500 patients from three institutions. Nomogram factors included: patient age, tumor size, FNCLCC grade, histologic subtype, multifocality, and extent of resection to predict overall survival at 7 years. An additional validation was performed with more than 1,100 patients.[7] These are large patient cohorts for this rare disease and robustly confirm the usefulness of this model.

Table 44.2 Prognostic tools for retroperitoneal sarcoma that met all AJCC quality criteria

Approved Prognostic Tool	Web Address	Factors Included in the Model
Outcome Prediction in Primary Resected Retroperitoneal Soft Tissue Sarcoma: Histology-Specific Overall Survival and Disease-Free Survival Nomograms Built on Major Sarcoma Center Data Sets	http://www.ncbi.nlm.nih.gov/pubmed/23530096	patient age, tumor size, FNCLCC grade, histologic subtype, multifocality, extent of resection

DEFINITIONS OF AJCC TNM

Definition of Primary Tumor (T)

T Category	T Criteria
TX	Primary tumor cannot be assessed
T0	No evidence of primary tumor
T1	Tumor 5 cm or less in greatest dimension
T2	Tumor more than 5 cm and less than or equal to 10 cm in greatest dimension
T3	Tumor more than 10 cm and less than or equal to 15 cm in greatest dimension
T4	Tumor more than 15 cm in greatest dimension

44

Definition of Regional Lymph Node (N)

N Category	N Criteria
N0	No regional lymph node metastasis or unknown lymph node status
N1	Regional lymph node metastasis

Definition of Distant Metastasis (M)

M Category	M Criteria
M0	No distant metastasis
M1	Distant metastasis

Definition of Grade (G)

FNCLCC Histologic Grade – see Histologic Grade (G)

G	G Definition
GX	Grade cannot be assessed
G1	Total differentiation, mitotic count and necrosis score of 2 or 3
G2	Total differentiation, mitotic count and necrosis score of 4 or 5
G3	Total differentiation, mitotic count and necrosis score of 6, 7, or 8

AJCC PROGNOSTIC STAGE GROUPS

When T is...	And N is...	And M is...	And grade is...	Then the stage group is...
T1	N0	M0	G1, GX	IA
T2, T3, T4	N0	M0	G1, GX	IB
T1	N0	M0	G2, G3	II
T2	N0	M0	G2, G3	IIIA
T3, T4	N0	M0	G2, G3	IIIB
Any T	N1	M0	Any G	IIIB
Any T	Any N	M1	Any G	IV

REGISTRY DATA COLLECTION VARIABLES

1. Bone invasion as determined by imaging
2. If pM1, source of pathological metastatic specimen
3. Additional dimensions of tumor size
4. FNCLCC grade

HISTOLOGIC GRADE (G)

The FNCLCC grade is determined by three parameters: differentiation, mitotic activity, and extent of necrosis. Each parameter is scored as follows: differentiation (1–3), mitotic activity (1–3), and necrosis (0–2). The scores are added to determine the grade.

Tumor Differentiation

Tumor differentiation is histology specific and is generally scored as follows:

Differentiation Score	Definition
1	Sarcomas closely resembling normal adult mesenchymal tissue (e.g., low-grade leiomyosarcoma)
2	Sarcomas for which histologic typing is certain (e.g., myxoid/round cell liposarcoma)
3	Embryonal and undifferentiated sarcomas, sarcomas of doubtful type, synovial sarcomas soft tissue osteosarcoma, Ewing sarcoma / primitive neuroectodermal tumor (PNET) of soft tissue

Mitotic Count

In the most mitotically active area of the sarcoma, 10 successive high-power fields (HPF; one HPF at 400× magnification = 0.1734 mm2) are assessed using a 40× objective.

Mitotic Count Score	Definition
1	0–9 mitoses per 10 HPF
2	10–19 mitoses per 10 HPF
3	≥20 mitoses per 10 HPF

Tumor Necrosis

Evaluated on gross examination and validated with histologic sections.

Necrosis Score	Definition
0	No necrosis
1	<50% tumor necrosis
2	≥50% tumor necrosis

FNCLCC Histologic Grade

G	G Definition
GX	Grade cannot be assessed
G1	Total differentiation, mitotic count and necrosis score of 2 or 3
G2	Total differentiation, mitotic count and necrosis score of 4 or 5
G3	Total differentiation, mitotic count and necrosis score of 6, 7, or 8

HISTOPATHOLOGIC TYPE

Please see the WHO Classification of Tumors section in this chapter for a list of retroperitoneal soft tissue sarcoma histologies.

Bibliography

1. Lahat G, Tuvin D, Wei C, et al. New perspectives for staging and prognosis in soft tissue sarcoma. *Annals of surgical oncology*. Oct 2008;15(10):2739–2748.
2. Fletcher CDM, Bridge JA, Hogendoorn P, Mertens F, eds. World Health Organization Classification of Tumours of Soft Tissue and Bone. Fourth Edition. Lyon: IARC; 2013.
3. Gronchi A, Miceli R, Shurell E, et al. Outcome prediction in primary resected retroperitoneal soft tissue sarcoma: histology-specific overall survival and disease-free survival nomograms built on major sarcoma center data sets. *J Clin Oncol*. May 1 2013;31(13):1649–1655.
4. Kattan MW, Hess KR, Amin MB, et al. American Joint Committee on Cancer acceptance criteria for inclusion of risk models for individualized prognosis in the practice of precision medicine. *CA: a cancer journal for clinicians*. Jan 19 2016.
5. Moons KG, Altman DG, Reitsma JB, et al. Transparent Reporting of a multivariable prediction model for Individual Prognosis or Diagnosis (TRIPOD): explanation and elaboration. *Annals of internal medicine*. Jan 6 2015;162(1):W1–73.
6. Moons KG, de Groot JA, Bouwmeester W, et al. Critical appraisal and data extraction for systematic reviews of prediction modelling studies: the CHARMS checklist. *PLoS medicine*. Oct 2014;11(10):e1001744.
7. Raut CP, Miceli R, Strauss DC, et al. External validation of a multi-institutional retroperitoneal sarcoma nomogram. *Cancer*. Feb 24 2016.
8. Tseng WW, Madewell JE, Wei W, et al. Locoregional disease patterns in well-differentiated and dedifferentiated retroperitoneal liposarcoma: implications for the extent of resection? *Annals of surgical oncology*. Jul 2014;21(7):2136–2143.
9. Anaya DA, Lahat G, Wang X, et al. Establishing prognosis in retroperitoneal sarcoma: a new histology-based paradigm. *Annals of surgical oncology*. Mar 2009;16(3):667–675.

Soft Tissue Sarcoma – Unusual Histologies and Sites

45

Robert G. Maki, Andrew L. Folpe,
B. Ashleigh Guadagnolo, Vicki L. Keedy,
Alexander J. Lazar, R. Lor Randall, Chandrajit P. Raut,
Sam S. Yoon, and Raphael E. Pollock

CHAPTER SUMMARY

Diagnoses Discussed In This Chapter

- Alveolar soft part sarcoma
- Angiosarcoma
- Desmoplastic small round cell tumor
- Epithelioid hemangioendothelioma
- Extraskeletal myxoid chondrosarcoma
- Inflammatory myofibroblastic tumor
- Kaposi sarcoma
- Osteosarcoma of soft tissue
- Phyllodes tumor
- Rhabdomyosarcoma
- Solitary fibrous tumor

Diagnoses Not Staged Using This Staging System

It is worth noting that there is a group of connective tissue neoplasms that are locally aggressive (i.e., they may recur locally) but have either no risk of metastatic disease or an extremely low risk of such metastasis. Some of these diagnoses have recognized genomic alterations. Because they do not have the regular ability to metastasize, they are excluded from the AJCC Cancer Staging Manual, 8th Edition soft tissue sarcoma staging system. Please refer to pathology texts[1] for more details on these diagnoses. Examples of histologies not staged using the AJCC system are:

- Desmoid tumor (deep fibromatosis)
- Superficial fibromatosis (e.g., palmar fibromatosis/Dupuytren contracture, plantar fibromatosis, Peyronie disease)
- Lipofibromatosis
- Giant cell fibroblastoma
- Plexiform fibrohistiocytic tumor
- Giant cell tumor of soft tissues
- Kaposiform hemangioendothelioma
- Hemosiderotic fibrolipomatous tumor
- Atypical fibroxanthoma
- Angiomatoid fibrous histiocytoma
- Pleomorphic hyalinizing angiectatic tumor

To access the AJCC cancer staging forms, please visit www.cancerstaging.org.

© American Joint Committee on Cancer 2017

M.B. Amin et al. (eds.), *AJCC Cancer Staging Manual, Eighth Edition*, DOI 10.1007/978-3-319-40618-3_45

Summary of Changes

Change	Details of Change	Level of Evidence
New chapter	Given the difficulty of classifying more than 70 different cancers using a single staging system, this chapter discusses key histologies that are troublesome regarding their staging. Reference is made to other sections or chapters in which these diagnoses are addressed in more detail.	N/A

ICD-O-3 Topography Codes

Code	Description
C38.0	Malignant neoplasm of heart
C38.1	Malignant neoplasm of anterior mediastinum
C38.2	Malignant neoplasm of posterior mediastinum
C38.3	Malignant neoplasm of mediastinum, part unspecified
C38.4	Malignant neoplasm of pleura
C38.8	Malignant neoplasm of overlapping lesion of heart, mediastinum, and pleura
C47.0	Peripheral nerves and autonomic nervous system of head, face, and neck
C47.1	Peripheral nerves and autonomic nervous system of upper limb and shoulder
C47.2	Peripheral nerves and autonomic nervous system of lower limb, including hip
C47.3	Peripheral nerves and autonomic nervous system of thorax
C47.4	Peripheral nerves and autonomic nervous system of abdomen
C47.5	Peripheral nerves and autonomic nervous system of pelvis
C47.6	Peripheral nerves and autonomic nervous system of trunk, unspecified
C47.8	Overlapping lesion of peripheral nerves and autonomic nervous system
C47.9	Peripheral nerves and autonomic nervous system, unspecified
C48.0	Malignant neoplasm of retroperitoneum
C48.1	Malignant neoplasm of specified parts of peritoneum
C48.2	Malignant neoplasm of peritoneum, unspecified
C48.8	Overlapping lesion of retroperitoneum and peritoneum
C49.0	Connective, subcutaneous, and other soft tissues of head, face, and neck
C49.1	Connective, subcutaneous, and other soft tissues of upper limb and shoulder
C49.2	Connective, subcutaneous, and other soft tissues of lower limb and hip
C49.3	Connective, subcutaneous, and other soft tissues of thorax
C49.4	Connective, subcutaneous, and other soft tissues of abdomen
C49.5	Connective, subcutaneous, and other soft tissues of pelvis
C49.6	Connective, subcutaneous, and other soft tissues of trunk, NOS
C49.8	Overlapping lesion of connective, subcutaneous, and other soft tissues
C49.9	Connective, subcutaneous, and other soft tissues, NOS

Sarcomas Arising in These Areas

Code	Description
C00-C14	Lip, oral cavity, and pharynx
C15-C26	Digestive organs
C30-C33	Respiratory system
C34-C37	Intrathoracic organs
C50	Breast
C51-C53	Female genital organs
C56-C58	Female genital organs
C60-C63	Male genital organs
C64-C68	Urinary tract
C69.0-C69.5, C69.9	Eye
C70-72	Brain and central nervous system
C73-C75	Thyroid and other endocrine glands
C80.9	Unknown primary site

WHO Classification of Tumors[1]

Code	Description
9581	Alveolar soft part sarcoma
9120	Angiosarcoma
9044	Clear cell sarcoma of soft tissue
8806	Desmoplastic small round cell tumor
8991	Embryonal sarcoma
8931	Endometrial stromal sarcoma, low grade
8930	Endometrial stromal sarcoma, high grade
9130	Epithelioid hemangioendothelioma
8804	Epithelioid sarcoma
9231	Extraskeletal myxoid chondrosarcoma
9180	Extraskeletal osteosarcoma
9140	Kaposi sarcoma
9020	Phyllodes tumor
8920	Rhabdomyosarcoma, alveolar
8910	Rhabdomyosarcoma, embryonal
8901	Rhabdomyosarcoma, pleomorphic
8912	Rhabdomyosarcoma, spindle cell/sclerosing
8815	Solitary fibrous tumor, malignant
8805	Undifferentiated uterine sarcoma

INTRODUCTION

This chapter is written to provide guidance on a few of the uncommon histologies that are unique to specific anatomic sites or raise several questions regarding their staging.

The breadth of soft tissue sarcoma (STS) behavior has implications for understanding prognosis. Some soft tissue neoplasms are locally aggressive, with little or no risk of metastasis. These are not staged using 8th Edition criteria. Other sarcomas are addressed in specific other sections—for example, uterine sarcomas in Chapter 51. Other soft tissue neoplasms, however, very frequently metastasize or are multifocal but remain indolent, so patients may live for a decade or more with metastatic disease without intervention. In this chapter, we highlight some of the STS histologies that are difficult to stage or present unique features that have implications for patient outcomes.

The understanding of STS biology increased rapidly with the advent of genetic techniques for tumor assessment. Many STSs that arise before age 40 have specific genomic alterations, such as chromosomal translocations (e.g., synovial sarcoma, Ewing sarcoma). It is notable that beyond their specific translocation, such tumors otherwise carry few mutations. As a result, their biology is characteristic of the specific translocation of that sarcoma.

Many common sarcomas occurring after age 50, such as leiomyosarcoma and undifferentiated pleomorphic sarcoma (formerly termed malignant fibrous histiocytoma), have aneuploid karyotypes and evidence of chromothripsis, common to many malignancies more common in adults. There is more variability in the behavior of aneuploid sarcomas.

Gastrointestinal stromal tumor (GIST) is a unique sarcoma in which a single point mutation is one of the few molecular drivers for the cancer. The specific mutation, most commonly in *KIT* or *PDGFRA*, has implications for tumor biology and drug sensitivity; the mutation type is prognostic for outcome and predictive of response to systemic therapy. Regarding GIST, the data regarding outcomes based on DNA alterations are closest to reality for incorporation into the AJCC staging system; however, data regarding other STS subtypes remain much more investigational.

ANATOMY

Primary Site(s)

Soft tissue sarcomas most commonly arise in the lower extremity and in the hip musculature. However, certain histologies are more or less unique to specific anatomic sites.

By definition, endometrial stromal sarcoma (low grade, high grade) and undifferentiated uterine sarcoma are unique to the uterus, serving as two of the best examples of anatomically specific STS. However, tumors that appear microscopically similar to these sarcomas are found elsewhere in the body. Histologies and unique anatomic sites are as follows.

Histology	Anatomic site
Clear cell sarcoma	Joint tendons and aponeurosis, small bowel
Desmoplastic small round cell tumor	Peritoneum
Embryonal sarcoma*	Liver
Endometrial stromal sarcomas (low grade, high grade), undifferentiated uterine sarcoma	Uterus
Epithelioid hemangioendothelioma	Liver, lung, pleura, rarely elsewhere
Epithelioid sarcoma, proximal type	Shoulder girdle, hip musculature
Epithelioid sarcoma, distal type	Hands, feet
GIST	Gastrointestinal tract, stomach > small bowel > other site; occasional primaries are found in mesentery independent of the bowel wall
Phyllodes tumor	Breast

*Not equivalent to embryonal rhabdomyosarcoma.

Staging for these malignancies continues to follow AJCC staging principles of TNM and tumor grade.

Regional Lymph Nodes

Lymph node involvement is uncommon for most STS histologies. Those with a greater than 10% risk of regional nodal involvement include angiosarcoma, clear cell sarcoma, epithelioid sarcoma, and rhabdomyosarcoma (but not pleomorphic rhabdomyosarcoma). GISTs without *KIT* or *PDGFRA* mutations are found more commonly in children and young adults and may involve locoregional lymph nodes.

Metastatic Sites

The most common site for metastatic disease for most extremity and uterine sarcomas is the lung. GISTs have a propensity to metastasize to the peritoneum and liver, and much less commonly elsewhere. Myxoid and round cell liposarcomas metastasize to other soft tissue sites and to bone marrow, which may not be evident on fluorodeoxyglucose positron emission tomography (^{18}F-FDG PET) scans but may be seen on magnetic resonance (MR) imaging, with the spine and pelvis most commonly affected. Brain metastases are unusual in people who have STS; alveolar soft part sarcoma has a relatively higher risk of brain meta-

45

static disease than other histologies. It is unclear whether to consider some STSs multifocal or metastatic, affecting multiple sites. Examples of sarcomas undergoing the multifocality/metastasis debate include radiation-associated angiosarcoma, desmoplastic small round cell tumor, epithelioid hemangioendothelioma, GIST without *KIT* or *PDGFRA* mutation, and Kaposi sarcoma.

RULES FOR CLASSIFICATION

Clinical Classification

Key clinical features of the diagnoses that are a focus of this chapter are found in Table 45.1. The suggested initial staging of unusual sites/histologies of STS also are indicated.

Imaging

Imaging with standard computed tomography (CT) or MR may be difficult for superficial sarcomas such as angiosarcoma and Kaposi sarcoma. For both, standard cross-sectional imaging is used to evaluate for metastatic disease; however, measuring the size of the primary/primaries will more commonly requires a tape measure or calipers. As noted earlier, myxoid and round cell liposarcomas will metastasize to bone marrow and soft tissue sites, unlike the lung and liver metastatic disease more common to other primary STSs.

As with other STSs, cross-sectional imaging of the rarer STS subtypes will yield primary tumor size. It also must be restated that axial imaging may not yield the greatest diameter of tumor. Reconstruction of images on MR or CT now commonly is performed and helps determine the maximum tumor dimension. ^{18}F-FDG PET scans may be used to help in staging for nodal status in patients with high-risk tumors (angiosarcoma, clear cell sarcoma, epithelioid sarcoma, and rhabdomyosarcoma) and in patients with clinically apparent nodal disease. Staging also is expected to yield the metastatic status of the STS; in the uncommon event of metastasis outside the chest, abdomen, and pelvis, it may not be necessary to image the tumor to measure the metastatic deposit, such as skin lesions from metastatic leiomyosarcoma.

Pathological Classification

The pathologist is the gateway to patient care. Without a proper diagnosis, it is not possible to treat the patient appropriately.[3] Because many pathologists are not familiar with the entire family of STSs, review at an expert institution is recommended; indeed, in retrospective analyses, the diagnosis may have been partially or entirely incorrect for 15% or more of patients.

In some cases, assessment for mutations or translocations is necessary to seal the diagnosis. In this situation, the pathologist is best suited to determine whether the required testing is feasible at the home institution or whether it should be sent out to a reference laboratory. At present, there is no routine role for genomic mutation panel testing in the routine diagnosis of STS; the ideal person to make this decision is the pathologist.

PROGNOSTIC FACTORS

Prognostic Factors Required for Stage Grouping

French Federation of Cancer Centers Sarcoma Group (FNCLCC) grade – see Histologic Grade (G).[4]

Additional Factors Recommended for Clinical Care

Presently, there are no prognostic markers for these rarer sarcomas that will affect prognosis. More than one type of translocation may be associated with a specific sarcoma, such as *SSX1-SS18* or *SSX2-SS18* in synovial sarcoma. The specific type of translocation plays only a weak role in prognosis, if any, and presently has no impact on STS therapy.

Standard TNM and grade should be recorded for these rarer STSs. Because genomics probably will affect future treatment decisions, collection of the specific mutation or translocation in a specific tumor may help in prognostication if enough cases can be collected. Desmoplastic small round cell tumor, angiosarcoma, Kaposi sarcoma and epithelioid sarcoma often present with multifocal disease. However, the utility of the multifocality designation is unclear as a prognostic factor, since many people with these diagnoses present with multifocal disease. Furthermore, there are no criteria by which to declare multifocality vs metastatic disease. The determination of multifocality vs metastatic disease is made clinically. For example, a dominant lesion with small implants elsewhere should be considered metastatic disease, whereas lesions without a dominant primary site can be considered multifocal. Multifocality data captured over time will permit further clinical research regarding the relevance of this observation that is common in specific soft tissue sarcoma subtypes.

Table 45.1 Clinical features of unusual histologies and their implications for staging

Histology	Clinical features	Implications for staging
Angiosarcoma	May present with satellite lesions; radiation-associated may present as multifocal disease; head and neck primaries may cover an extensive area of the scalp. Over time, metastatic sites may become protean: lung, liver, bone marrow, brain, heart.	Record size of largest lesion based on multifocality guidelines. If possible, record greatest dimensions of tissue affected by tumor. Ascertain whether prior therapy, such as radiation, was administered to clarify the frequency of this clinical scenario.
Desmoplastic small round cell tumor	Typically presents as multiple masses throughout the peritoneum	Record size of largest lesion according to multifocality guidelines.
Embryonal sarcoma	Typically, large dominant mass in liver	Stage according to AJCC guidelines for visceral STS.
Endometrial stromal sarcoma, low grade	Typically, dominant mass in uterus	See chapter on staging of uterine sarcomas. Note that in the uterine sarcoma staging system, the degree of organ involvement is a surrogate for primary tumor size.
Endometrial stromal sarcoma, low grade	Typically, dominant mass in uterus	See chapter on staging of uterine sarcomas. Note that in the uterine sarcoma staging system, the degree of organ involvement is a surrogate for primary tumor size.
Epithelioid hemangioendothelioma	Typically presents as multiple masses in the liver and/or lungs, less commonly in pleura	If possible, record size of largest lesion according to multifocality guidelines.
Epithelioid sarcoma (proximal type)	May present as multifocal disease; lymph node involvement common	If possible, record size of largest lesion according to multifocality guidelines.
Extraskeletal myxoid chondrosarcoma	Frequently metastasizes, with innumerable lung metastases very common, but has a very indolent course lasting 5–10 years or longer	Stage according to AJCC guidelines for STS.
Inflammatory myofibroblastic tumor (IMT)	Multinodular intermediate-grade fibrocytic neoplasm arising from soft tissue or viscera. It most commonly affects the lung, mesentery, and omentum; may present with multifocal disease within the abdomen; and grows over 5–10 years or longer.	Difficult to stage using AJCC criteria. Ninety percent of IMTs have benign or favorable behavior. Approximately 25% of extrapulmonary IMTs recur. Malignant IMTs with a specific *ALK* gene fusion have a propensity to recur locally and may respond to ALK inhibitors, such as crizotinib. Malignant IMTs lacking an *ALK* fusion (the more common adult scenario) may have a greater propensity to metastasize.
Kaposi sarcoma	Appears in older patients from the Mediterranean basin (endemic) and in association with HIV (epidemic). Multifocal lesions commonly affect skin (feet > legs > remainder of body); visceral disease less common	Record size of largest lesion according to multifocality guidelines if possible, but many times this is not feasible given the confluency of lesions affecting extremities or the genuine innumerability of lesions. Previous staging systems involving immune status of the patients[2] no longer are as relevant, given the availability of antiretroviral therapy.
Malignant mixed Müllerian tumor (MMMT), e.g. carcinosarcoma	Some controversy remains as to whether to stage as a sarcoma or carcinoma. Recurrences are commonly of the carcinomatous component, thus most patients appear to have transdifferentiation of a uterine carcinoma. However, some patients develop purely sarcomatous recurrent or metastatic disease.	Refer to the chapters on female reproductive organs (Part XII) regarding staging.
Osteosarcoma of soft tissue	Despite this histology, this is a rare sarcoma of soft tissue.	Stage according to AJCC guidelines for STS. Ensure there is not a bone primary site of disease.

(continued)

45

Table 45.1 (continued)

Histology	Clinical features	Implications for staging
Phyllodes tumor	Appears to arise from dedifferentiation of fibroadenomas. Tumors may have varying degrees of epithelial and mesenchymal cells in the tumor mass. Lymph node metastases are observed only infrequently.	Stage according to AJCC guidelines for STS, extremity and trunk.
Rhabdomyosarcoma	Anatomic site, nodal status, and histology affect outcomes in children. Adults fare worse than children.	Stage according to AJCC guidelines for STS.*
Solitary fibrous tumor	Slowly growing mass in pleura, pelvis, or dura may result in metastatic disease to liver, lung, and bone more than a decade after initial diagnosis.	Stage according to AJCC guidelines for STS.
Undifferentiated uterine sarcoma	Typically, dominant mass in uterus	See chapter on staging of uterine sarcomas. Note that in the uterine sarcoma staging system, the degree of organ involvement is a surrogate for primary tumor size.

*Pediatric staging systems are available for risk assessment for alveolar and embryonal sybtypes, and may be required for clinical trials.

RISK ASSESSMENT MODELS

The AJCC recently established guidelines that will be used to evaluate published statistical prediction models for the purpose of granting endorsement for clinical use.[1] Although this is a monumental step toward the goal of precision medicine, this work was published only very recently. Therefore, the existing models that have been published or may be in clinical use have not yet been evaluated for this cancer site by the Precision Medicine Core of the AJCC. In the future, the statistical prediction models for this cancer site will be evaluated, and those that meet all AJCC criteria will be endorsed.

REGISTRY DATA COLLECTION VARIABLES

1. Bone invasion as determined by imaging
2. If pM1, source of pathological metastatic specimen
3. Additional dimensions of tumor size
4. FNCLCC grade
5. Multifocality and number of sites, when noted

HISTOLOGIC GRADE (G)

The FNCLCC grade is determined by three parameters: differentiation, mitotic activity, and extent of necrosis. Each parameter is scored as follows: differentiation (1–3), mitotic activity (1–3), and necrosis (0–2). The scores are added to determine the grade.[4,6]

Tumor Differentiation

Tumor differentiation is histology specific (see Chapter 39, Table 39.1) and is generally scored as follows:

Differentiation Score	Definition
1	Sarcomas closely resembling normal adult mesenchymal tissue (e.g., low-grade leiomyosarcoma)
2	Sarcomas for which histologic typing is certain (e.g., myxoid/round cell liposarcoma)
3	Embryonal and undifferentiated sarcomas, sarcomas of doubtful type, synovial sarcomas, soft tissue osteosarcoma, Ewing sarcoma /primitive neuroectodermal tumor (PNET) of soft tissue

Mitotic Count

In the most mitotically active area of the sarcoma, 10 successive high-power fields (HPF; one HPF at 400× magnification = 0.1734 mm^2) are assessed using a 40× objective.

Mitotic Count Score	Definition
1	0–9 mitoses per 10 HPF
2	10–19 mitoses per 10 HPF
3	≥20 mitoses per 10 HPF

Tumor Necrosis

Evaluated on gross examination and validated with histologic sections.

Necrosis Score	Definition
0	No necrosis
1	<50% tumor necrosis
2	≥50% tumor necrosis

FNCLCC Histologic Grade

G	G Definition
GX	Grade cannot be assessed
G1	Total differentiation, mitotic count and necrosis score of 2 or 3
G2	Total differentiation, mitotic count and necrosis score of 4 or 5
G3	Total differentiation, mitotic count and necrosis score of 6, 7, or 8

HISTOPATHOLOGIC TYPE

Please see the WHO Classification of Tumors section in this chapter for a list of unusual histologies.

Bibliography

1. Fletcher CDM, Bridge JA, Hogendoorn P, Mertens F, eds. World Health Organization Classification of Tumours of Soft Tissue and Bone. 4th ed. Lyon: IARC; 2013.
2. Krown SE, Metroka C, Wernz JC. Kaposi's sarcoma in the acquired immune deficiency syndrome: a proposal for uniform evaluation, response, and staging criteria. AIDS Clinical Trials Group Oncology Committee. *J Clin Oncol*. 1989;7(9):1201-1207.
3. Rubin BP, Cooper K, Fletcher CD, et al. Protocol for the examination of specimens from patients with tumors of soft tissue. Arch Pathol Lab Med. 2010;134(4):e31-39.
4. Coindre JM, Terrier P, Bui NB, et al. Prognostic factors in adult patients with locally controlled soft tissue sarcoma. A study of 546 patients from the French Federation of Cancer Centers Sarcoma Group. *J Clin Oncol*. 1996;14(3):869-877.
5. Kattan MW, Hess KR, Amin MB, et al. American Joint Committee on Cancer acceptance criteria for inclusion of risk models for individualized prognosis in the practice of precision medicine. CA: a cancer journal for clinicians. 2016.
6. Neuville A, Chibon F, Coindre JM. Grading of soft tissue sarcomas: from histological to molecular assessment. Pathology. 2014;46(2):113-120.
7. Brennan MF, Antonescu CR, Maki RG. Management of soft tissue sarcoma. Springer Science & Business Media; 2012.

Part X

Skin

Members of the Non-Melanoma Skin Expert Panel
Thomas P. Baker, MD – CAP Representative
Christopher K. Bichakjian, MD – Merkel Cell Carcinoma Lead
James D. Brierley, BSc, MB, FRCP, FRCR, FRCP(C) – UICC Representative
Klaus J. Busam, MD
David R. Byrd, MD, FACS – Editorial Board Liaison
Daniel G. Coit, MD, FACS
David P. Frishberg, MD
Timothy Johnson, MD – Vice Chair
Anne W.M. Lee, MD – UICC Representative
Paul Nghiem, MD, PhD – Merkel Cell Carcinoma Lead
Thomas E. Olencki, DO
Upendra Parvathaneni, MBBS, FRANZCR
M. Angelica Selim, MD
Erica M. Shantha, MD
Arthur J. Sober, MD – Chair
Vernon K. Sondak, MD, FACS
Manisha Thakuria, MD
Michael J. Veness, MB, BS, MMed, MD, FRANZCR
Chadwick L. Wright, MD, PhD
Siegrid S. Yu, MD

Members of the Melanoma Expert Panel
Michael B. Atkins, MDGer
Charles M. Balch, MD, FACS – Editorial Board Liaison
Raymond Barnhill, MD, MSc
Karl Y. Bilimoria, MD, MS
James D. Brierley, BSc, MB, FRCP, FRCR, FRCP(C) – UICC Representative
Antonio C. Buzaid, MD
David R. Byrd, MD, FACS
Paul B. Chapman, MD
Alistair J. Cochran, MD
Daniel G. Coit, MD, FACS
Alexander M. Eggermont, MD, PhD
David E. Elder, MBChB, FRCPA
Mark B. Faries, MD
Keith T. Flaherty, MD
Claus Garbe, MD
Julie M. Gardner
Jeffrey E. Gershenwald, MD, FACS – Chair
Phyllis A. Gimotty, PhD – Precision Medicine Core Representative
Allan C. Halpern, MD
Lauren E. Haydu
Kenneth R. Hess, PhD
Timothy Johnson, MD
John M. Kirkwood, MD
Alexander J. Lazar, MD, PhD, FACP – CAP Representative
Anne W.M. Lee, MD – UICC Representative
Georgina V. Long, BSc, MB, BS, PhD, FRACP
Grant A. McArthur, MBBS, BMedSc, PhD, FRACP
Martin C. Mihm Jr., MD, FACP
Victor G. Prieto, MD, PhD
Merrick I. Ross, MD
Richard A. Scolyer, BMedSci MBBS MD FRCPA FRCPath – Vice Chair
Arthur J. Sober, MD
Vernon K. Sondak, MD, FACS
John F. Thompson, MD, FRACS, FACS
Richard L. Wahl, MD
Sandra L. Wong, MD, MS, FACS

Merkel Cell Carcinoma

46

Christopher K. Bichakjian, Paul Nghiem,
Timothy Johnson, Chadwick L. Wright,
and Arthur J. Sober

CHAPTER SUMMARY

Cancers Staged Using This Staging System

Primary cutaneous neuroendocrine carcinoma (Merkel cell carcinoma)

Summary of Changes

Change	Details of Change	Level of Evidence
Definition of Regional Lymph Nodes (N)	Separation of clinical and pathological categories	IV
Definition of Regional Lymph Nodes (N)	New category N2 denotes in-transit metastasis without lymph node metastasis.	IV
Definition of Regional Lymph Nodes (N)	New category N3 denotes in-transit metastasis with lymph node metastasis.	IV
Definition of Regional Lymph Nodes (N)	New category pN1a(sn) denotes clinically occult lymph node metastasis identified by sentinel lymph node biopsy only.	IV
Definition of Regional Lymph Nodes (N)	Category pN1a now denotes clinically occult lymph node metastasis following lymph node dissection.	IV
Definition of Regional Lymph Nodes (N)	Category pN2 now denotes in-transit metastasis without lymph node metastasis.	IV
Definition of Regional Lymph Nodes (N)	New category pN3 denotes in-transit metastasis with lymph node metastasis.	IV
Definition of Distant Metastasis (M)	Separation of clinical and pathological categories	IV
Definition of Distant Metastasis (M)	Stage IV: Clinical and pathological M classifications are both subcategorized as M1a, M1b, and M1c, by location of distant metastasis.	IV
AJCC Prognostic Stage Groups	Separation of clinical and pathological stage groups	IV
AJCC Prognostic Stage Groups	Stage I: Pathological nodal status no longer determines Stage I grouping.<br>Substage groups IA and IB were eliminated.<br>Stage group I refers to category T1.	IV
AJCC Prognostic Stage Groups	Stage II: Pathological nodal status no longer determines Stage II grouping.<br>Substage group IIA refers to category T2/3.<br>Substage group IIB refers to category T4.<br>Substage group IIC was eliminated.	IV
AJCC Prognostic Stage Groups	Stage III: New *clinical* stage group III denotes categories T0–4 cN1–3 M0.	IV
AJCC Prognostic Stage Groups	Stage IIIA: New inclusion of category T0 pN1b M0 (lymph node metastasis with unknown primary tumor) in pathological Stage group IIIA.	II
AJCC Prognostic Stage Groups	Stage IIIB: Pathological Stage group IIIB refers to categories T1–4 pN1b–3 M0, *excluding* lymph node metastasis with unknown primary tumor.	II

To access the AJCC cancer staging forms, please visit www.cancerstaging.org.

© American Joint Committee on Cancer 2017

M.B. Amin et al. (eds.), *AJCC Cancer Staging Manual, Eighth Edition*, DOI 10.1007/978-3-319-40618-3_46

ICD-0-3 Topography Codes

Code	Description
C00.0	External upper lip
C00.1	External lower lip
C00.2	External lip, NOS
C00.3	Mucosa of upper lip
C00.4	Mucosa of lower lip
C00.5	Mucosa of lip, NOS
C00.6	Commissure of lip
C00.7	Overlapping lesion of lip
C00.8	Lip, NOS
C21.0	Anus, NOS
C30.0	Nasal cavity
C44.0	Skin of lip, NOS
C44.1	Skin of eyelid
C44.2	Skin of external ear
C44.3	Skin of other and unspecified parts of face
C44.4	Skin of scalp and neck
C44.5	Skin of trunk
C44.6	Skin of upper limb and shoulder
C44.7	Skin of lower limb and hip
C44.8	Overlapping lesion of skin
C44.9	Skin, NOS
C50.0	Nipple
C51.0	Labium majus
C51.1	Labium minus
C51.2	Clitoris
C51.8	Overlapping lesion of vulva
C51.9	Vulva, NOS
C60.0	Prepuce
C60.1	Glans penis
C60.2	Body of penis
C60.8	Overlapping lesion of penis
C60.9	Penis, NOS
C63.2	Scrotum, NOS
C80.9	Unknown primary site

WHO Classification of Tumors

Code	Description
8041	Small cell neuroendocrine carcinoma
8190	Trabecular carcinoma
8247	Merkel cell carcinoma

LeBoit PE, Burg G, Weedon D, Sarasin A, eds. World Health Organization Classification of Tumours. Pathology and Genetics of Skin Tumours. Lyon: IARC Press; 2006.

INTRODUCTION

Merkel cell carcinoma (MCC) is a relatively rare, potentially aggressive primary cutaneous neuroendocrine carcinoma, originally described by Tang and Toker[1] in 1978 as trabecular carcinoma. The overall mortality rate is twice that observed in cutaneous melanoma (33% vs. 15%).[2,3] Although the molecular pathogenesis remains largely unknown, the frequent association with clonal integration of Merkel cell polyomavirus (MCPyV) in MCC tumors strongly suggests that this virus is present at tumor initiation and that MCPyV viral proteins may have an important oncogenic role.[4,5] Ultraviolet (UV) radiation and immune suppression likely are significant additional predisposing factors. MCC occurs most commonly on sun-exposed skin in fair-skinned individuals older than 50 years, with a slight male predominance.[6,7] An increased incidence is observed in patients who have undergone solid organ transplantation and those with leukemia, HIV infection, or other causes of immunosuppresion.[8-10] MCC is increasing in frequency, rising from 0.22 cases per 100,000 in 1986 to 0.79 cases per 100,000 in 2011, although some of this increase may be the result of greater recognition and improved techniques for diagnosis.[11] Approximately 1,600 to 2,500 cases of MCC are diagnosed annually in the United States.[11,12] As the US population ages and improved transplantation regimens prolong the lives of organ transplant recipients, the incidence of MCC likely will continue to rise.

MCC has a nonspecific clinical presentation, although rapid growth of a firm, red to violaceous, nontender papule or nodule often is noted.[6] Diagnosis is made via biopsy, almost invariably with the aid of immunohistochemistry (IHC), classically demonstrating a paranuclear dot pattern of cytokeratin-20 staining. Most patients present with clinically localized disease.[7] However, the disease can metastasize rapidly to regional and distant sites, and the regional draining nodal basin is the most common initial site of metastasis.[13] The natural history of MCC is variable and heavily dependent on the stage at diagnosis.

In the AJCC Cancer Staging Manual, 7th Edition, published in 2010, MCC was assigned its first disease-specific chapter; previously it was included in the chapter on carcinoma of the skin. The development of a consensus staging system was a significant improvement over the five previously and concurrently used competing systems.[14] It was based on an extensive review of the literature and an analysis of 2,856 cases of MCC recorded in the National Cancer Database (NCDB).[2] In this first consensus staging system, a strong emphasis was placed on the prognostic value of staging of the regional draining lymph node basin. To accomplish this goal, both clinical and pathological TNM categories were used within the same stage, resulting in an

overlap between clinical and pathological stage groups. Specifically, Stage IA referred to a tumor ≤2 cm in diameter with a pathologically confirmed negative regional lymph node (T1 pN0), whereas Stage IB referred to a similar diameter tumor without pathological nodal evaluation (T1 cN0). However, in recent years, more consistent usé of sentinel lymph node biopsy (SLNB) in most patients with MCC has contributed to a selection bias for Stage IB (and IIB), which increasingly comprises patients in whom SLNB is not performed because of underlying comorbidities.[15-17] To eliminate this bias and achieve consistency with other malignancies, the AJCC Cancer Staging Manual, 8th Edition MCC staging system separates clinical and pathological stages and removes pathological regional lymph node status from Stages I and II. Previous TNM categories are validated and maintained in the new prognostic stage groups.

Several independent cohorts confirmed that patients presenting with metastatic MCC in a lymph node without a known primary tumor have a more favorable prognosis compared with those presenting with a primary tumor and synchronous clinically evident lymph node metastasis.[18-20] Validation and revision of the MCC staging system are based on an analysis of the largest national MCC cohort of 9,387 MCC cases from the NCDB.

ANATOMY

Primary Site(s)

MCC tumor cells have several markers in common with normal Merkel cells, neuroendocrine cells of the skin involved in light touch located in the basal layer of the epidermis.[21] However, the cell of origin of MCC, a tumor characteristically located in the dermis without overlying epidermal involvement, is unknown.[22] MCC may occur anywhere on the skin but arises most commonly in sun-exposed areas. It occurs most frequently on the head and neck, followed by the extremities.[6,7] In approximately 8–14% of cases, MCC presents as a metastasis in a lymph node (most common presentation) or visceral site, without a known primary cutaneous tumor.[6,20]

Regional Lymph Nodes

The regional lymph nodes are the most common site of metastasis (Fig. 46.1). Regional lymph node metastasis occurs relatively frequently and early, even in the absence of deep local extension or large primary tumor size. Several studies showed that even the smallest primary tumor is associated with at least a 10–20% risk of occult nodal metastasis at the time of diagnosis.[15-17] Intralymphatic "in-transit" regional metastases may occur and are defined as a tumor discontinuous from the primary lesion, located either (1) between the primary lesion and the draining regional lymph nodes or (2) distal to the primary lesion. Similar to melanoma, no subclassification of in-transit metastases based on distance from the primary tumor exists in MCC (i.e., there is no satellite metastasis classification). By convention, the term *regional nodal metastasis* refers to disease confined to one nodal basin or two contiguous nodal basins. Examples include patients with metastases to combinations of femoral/iliac, axillary/supraclavicular, or cervical/supraclavicular lymph node basins or with primary truncal MCC metastatic to axillary/femoral, bilateral axillary, or bilateral femoral basins.

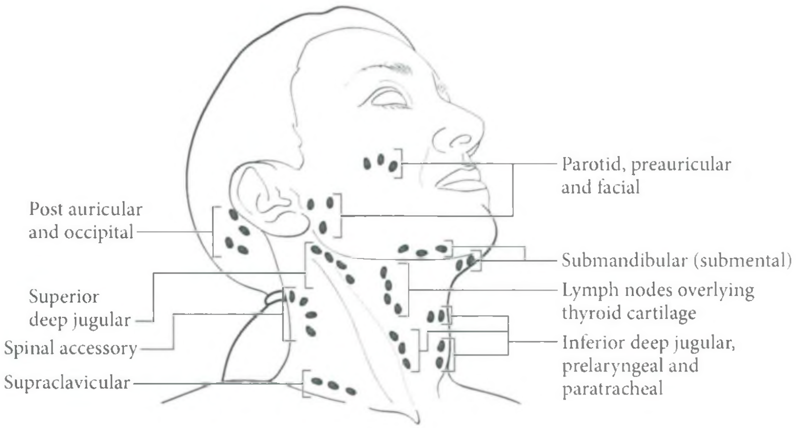

Fig. 46.1 Regional lymph nodes for skin sites of the head and neck

Metastatic Sites

MCC can metastasize to virtually any organ site. Distant metastases occur most commonly to distant skin, followed by the lung, liver, bone, and central nervous system.[23]

RULES FOR CLASSIFICATION

Clinical Classification

T classification of MCC is determined by a combination of pathological evaluation (microstaging) and clinical measurement of the largest diameter of the primary tumor. If the primary tumor cannot be assessed accurately (because of curettage or other form of destruction of the tumor), category TX is applied. In contrast, T0 refers to the inability to identify a primary tumor in the context of metastatic disease. Although quite rare, MCC *in situ* is categorized as Tis. The remainder of the T category is determined primarily by measurement of the largest clinical diameter of the primary tumor in centimeters: ≤2 cm (T1), >2 cm but ≤5 cm (T2), and >5 cm (T3). Extracutaneous invasion by the primary tumor into fascia, muscle, cartilage, or bone is classified as T4. The T category based on the clinically measured diameter is maintained from the 7th Edition and was validated by an updated NCDB analysis. (Fig. 46.2) Histologic measurement of tumor diameter is subject to underestimation due to shrinkage of formalin-fixed tissue and inaccuracy of measurement of the largest diameter of oval tumors.[7] If clinical tumor size is unavailable, histopathologic gross or microscopic measurement should be used.

Clinical N classification of MCC is based on the clinical or radiologic detection of regional draining lymph node and/or subcutaneous in-transit metastases. If the regional draining lymph nodes cannot be clinically assessed (e.g., because of previous removal for other reasons, or body habitus), category cNX is applied. N0 refers to the absence of evidence of regional lymph node or subcutaneous in-transit metastases, based on clinical and/or radiologic examination. Category N1 refers to the clinical and/or radiologic detection of regional draining lymph node metastasis. Category N2 is defined as the clinical presence of in-transit metastasis in the absence of clinically apparent lymph node metastasis. If both in-transit and lymph node metastases are clinically and/or radiologically detected, category N3 is assigned. It should be noted that diagnostic biopsies confirming nodal or in-transit metastases are included in the clinical N classification, provided that definitive surgical treatment of the primary site and/or lymph node basin has not yet been performed. Diagnostic biopsies may include sentinel, core, or open lymph node biopsy, or fine-needle aspiration. However, in clinical practice, SLNB typically is performed concurrently with definitive surgery. Physical examination of the patient should include careful inspection and palpation of the skin and soft tissues surrounding the primary tumor site, extending to and including the regional lymph node basin(s). Careful clinical examination may identify enlarged lymph nodes in conventional or in-transit basins (e.g., epitrochlear, popliteal), as well as subcutaneous in-transit metastases. For primary tumor locations on an extremity, the entire arm or leg should be examined proximally and distally to the tumor. Particularly for truncal locations, attention should be focused on possible drainage to multiple lymph node basins.

M classification for clinical staging of MCC is more limited. If a thorough history and physical examination do not identify evidence of distant metastatic disease, clinical category M0 applies. Imaging studies are not required to assign this category. If clinical and/or radiologic examination reveals lesions suspicious for distant metastasis, M classification is based on their location. Distant skin, subcutaneous, or lymph node metastases are classified as cM1a, lung metastases as cM1b, and all other distant sites of metastasis as cM1c. If a distant metastasis is confirmed microscopically during diagnostic workup, category pM1(a, b, or c) is used in clinical staging.

Imaging

For asymptomatic patients with localized disease without clinical evidence of metastasis based on thorough history and physical examination, SLNB is considered the most appropriate staging tool. In such circumstances, cross-sectional imaging should be performed only as clinically indicated based on abnormal clinical findings.[24] For clinical N1–2 disease, cross-sectional imaging is indicated to evaluate the extent of lymph node involvement and/or the presence of distant metastasis.

Some evidence suggests 2-fluoro-(fluorine-18)-deoxy-2-D-glucose (FDG) positron emission tomography (PET)/computed tomography (CT) imaging may be preferred in certain clinical circumstances.[25,26] Some advantages of FDG PET/CT imaging may include high uptake of FDG in MCC; whole-body assessment, including visualization of extremities, allowing detection of potential in-transit metastases; and early detection of distant osseous or bone marrow metastases. However, false positivity for PET/CT imaging has been reported, and tissue confirmation of metastasis is strongly recommended.[27] If PET/CT is not available, CT or magnetic resonance (MR) imaging is appropriate. MR imaging is preferred for the detection of brain metastases.

The AJCC Imaging Expert Panel suggests the use of structured reporting for the description of malignant/metastatic lesions when interpreting imaging tests. If imaging is performed, it is recommended that the sites and extent of any locoregional lymph node involvement be described, as should other distant sites of malignant/metastatic involvement (e.g., skin, lungs, liver, bone, central nervous system) that may be relevant to staging and/or therapy.

Pathological Classification

AJCC staging systems for MCC and cutaneous melanoma differ from those of other cancer types in that they include microstaging of the primary tumor in the clinical staging and T category. Pathological stage normally is assigned following definitive surgical treatment. However, a patient with MCC may not undergo additional surgery after narrow excision of the primary tumor with or without SLNB, as further treatment may consist only of radiation therapy. In such cases, provided the entire clinically apparent primary tumor has been excised (i.e., microstaged), a pathological stage may be assigned as outlined here.

Pathological N classification for MCC is determined primarily by tumor burden in the regional draining lymph nodes. By convention, pNX is assigned if the regional draining lymph nodes cannot be assessed (e.g., if they were previously removed) or if no regional draining lymph node was biopsied or removed for pathological evaluation. In contrast, category pN0 is assigned if no evidence of a regional lymph node metastasis is identified microscopically. This category may apply to three distinct clinical scenarios: (1) SLNB was performed and found to be negative (most common scenario in recent years); (2) a lymph node suspicious for metastasis was detected on clinical or radiologic examination but was microscopically negative; or (3) elective complete lymphadenectomy revealed all nodes to be negative for metastasis (standard treatment before widespread acceptance of SLNB). In terms of SLNB, the presurgical administration of intradermal radiolabeled nanocolloids around a cutaneous malignant lesion/biopsy site allows subsequent identification of sentinel lymph nodes and draining lymphatics by using gamma scintigraphy and also enables intraoperative removal of radiolabeled lymph nodes with handheld gamma probe guidance. If a clinically occult regional lymph node metastasis is identified by SLNB, and additional surgery in the form of a complete lymph node dissection is *not* performed, category pN1a(sn) is assigned. However, all patients with occult regional lymph node metastasis who have undergone lymph node dissection, following SLNB or otherwise, are assigned category pN1a. Lymph nodes containing isolated tumor cells, whether detected by standard hematoxylin and eosin or by immunohistochemical staining, should be considered positive, similar to melanoma. Category pN1b refers to a clinically or radiologically detected, and pathologically confirmed, regional nodal metastasis. A patient with in-transit metastasis confirmed by pathological evaluation is assigned category pN2 in the absence of lymph node metastasis. If both in-transit and lymph node metastases are pathologically confirmed, category pN3 is applied.

Similar to clinical classification, the M category for pathological staging is based on the location of the distant metastasis. Distant skin, subcutaneous, or lymph node metastases are classified as cM1a; lung metastases as cM1b; and all other distant sites of metastasis as cM1c if detected on clinical or radiologic examination. These clinical M categories may be used for pathological stage grouping. If a distant metastasis is microscopically confirmed, category pM1(a, b, or c) is applied. However, if a distant metastasis is clinically or radiologically suspected, but microscopically confirmed to be negative, category cM0 should be assigned.

PROGNOSTIC FACTORS

Prognostic Factors Required for Stage Grouping

Beyond the factors used to assign T, N, or M categories, no additional prognostic factors are required for stage grouping.

46

Additional Factors Recommended for Clinical Care

Prognosis in MCC is based primarily on the extent of disease at presentation, which formed the basis of the first AJCC staging system.[2] Recent analysis of overall survival in an expanded and more contemporary cohort of 9,387 patients with MCC from the NCDB validated the previous staging system and confirmed the correlation between primary tumor size, as well as extent of disease at presentation, and prognosis (Figs. 46.2 and 46.3). A significant limitation of NCDB data is the lack of disease-specific survival data and reliance on overall survival rates. Particularly in a patient population with a median age of 76, such as in MCC, overall survival is significantly influenced by the rate of death from unrelated causes. The previous analysis of NCDB data calculated relative survival by adjusting overall survival data using age- and sex-matched life expectancy information.[2] Because this adjustment was not performed in the most recent NCDB reanalysis, survival curves must be viewed in that context, rather than by comparing absolute rates. MCC-specific survival rates are expected to be more favorable than the overall survival rates shown here.

Consistent with other AJCC staging systems, the current prognostic stage groups for MCC are separated by clinical and pathological staging. The previous inclusion of the extent of pathological nodal evaluation into Stage I and II substages (i.e., IA/IB and IIA/IIB) does not apply to the current system. A patient in whom pathological evaluation of the regional lymph node basin (i.e., most commonly by SLNB) has not been performed is no longer staged as IB (or IIB if the primary tumor is >2 cm). Analogous to melanoma, this patient will be assigned category pNX and staged as prognostic stage group I

or II based on characteristics of the primary tumor. Survival rates of patients in the NCDB cohort with localized disease based on clinical staging only (i.e., those who did not undergo pathological nodal evaluation) are shown in Fig. 46.4. Survival rates of patients with clinically staged nodal or distant metastases (i.e. those who did not undergo pathological confirmation of clinically detected presumed metastatic disease) are not included due to small number of patients with inconsistent data in the NCDB dataset.

Primary Tumor Size

The clinically measured largest diameter of an MCC remains the only parameter of the primary tumor that is shown to be predictive of survival and has been independently validated by multiple cohorts[2,13,17,28] (AJCC Level of Evidence: I). Measurement is performed clinically, preferably before biopsy, by determining the largest diameter of the tumor in centimeters. Unfortunately, this measurement is subject to an inherent degree of inaccuracy and subjectivity. However, previous studies showing a correlation between tumor size and prognosis were based on this parameter, which therefore is maintained until more accurate parameters have been validated. Categories are divided into primary tumors measuring ≤2 cm (T1), those >2 cm but ≤5 cm (T2), and those >5 cm (T3). Histologic measurement of tumor diameter is subject to underestimation due to shrinkage of formalin-fixed tissue and inaccuracy in measuring the largest diameter of oval tumors.[7] If clinical tumor size is unavailable, histopathologic gross or microscopic measurement should be used for staging.

Unknown Primary Tumor

A new addition to the current staging system is based on the consistent observation in several independent cohorts that patients presenting with metastatic MCC in a lymph node, in the absence of a primary tumor (T0), have a significantly more favorable prognosis than those presenting with a primary tumor and synchronous lymph node metastasis.[18-20] Survival rates for patients presenting with a lymph node metastasis of MCC and unknown primary tumor are consistently similar to the rates for patients presenting with a primary tumor and occult nodal metastases detected by SLNB [pN1a(sn) or pN1a]. This finding is supported by analysis of the NCDB cohort (Fig. 46.5; AJCC Level of Evidence: I). Based on these data, patients presenting with a clinically or radiologically detected, pathologically confirmed, nodal MCC metastasis (pN1b) without a primary cutaneous tumor are staged in prognostic group IIIA rather than IIIB, as in the previous system. Because this represents a diagnosis of exclusion, clinical examination to rule out a primary cutaneous tumor must be thorough and should include examination of mucosal surfaces for lymph node metastases in the cervical or inguinal basins.

Regional Nodal Tumor Burden

Consistent with the previous staging system, tumor burden in the regional draining lymph nodes is maintained as a prognostic parameter for staging (AJCC Level of Evidence: I). Multiple studies have consistently shown that patients presenting with a primary cutaneous MCC and clinically or radiologically detected regional lymph node metastasis (pN1b) have a worse prognosis than those with a primary tumor and occult nodal metastasis detected by SLNB.[2,15,17,28,29] This finding is supported by analysis of the NCDB cohort (Fig. 46.5). Moreover, based on the updated NCDB analysis, patients with localized MCC and pathologically proven negative lymph node(s) (pathological stage groups I and IIA) have a more favorable prognosis than those with occult nodal metastases in the draining nodal basin (IIIA; Fig. 46.6). This finding is consistent with most other independent cohorts.[2,15,17,28,30] Finally, the 5-year overall survival advantage of pathologically staged versus clinically staged node-negative patients (stage I, 62.8% vs. 45.0%; stage IIA, 54.6% vs. 30.9%; and stage IIB, 34.8% vs. 27.3%, respectively) signifies the prognostic value of pathologic nodal evaluation for clinically node-negative patients (Figs. 46.4 and 46.6). These findings, combined with the high rate (at least 30%) of clinically occult nodal metastases in the draining regional lymph nodes, strongly support the recommendation to perform SLNB routinely in all patients with localized MCC, if clinically feasible.[2,16,17,24,30,31] Early detection likely will improve control of the regional lymph node basin and may have a favorable impact on survival.

Tumor Thickness

Tumor thickness, also known as Breslow thickness in cutaneous melanoma, is measured microscopically from the granular layer of the overlying epidermis to the deepest point of tumor invasion. Breslow thickness is the principal prognostic parameter for primary melanoma and forms the basis of the AJCC staging system. Based on the spherical shape of a primary MCC, one intuitively would expect tumor thickness to correlate with tumor diameter, an established prognostic factor currently required for the T category. Several single-institution studies demonstrated a correlation between tumor thickness and prognosis in MCC[17,32,33] (AJCC Level of Evidence: II). However, consistent synoptic reporting of various histopathologic parameters, including tumor thickness, is not performed uniformly for MCC; therefore, this information is available only sparsely in large national datasets such as the NCDB. Consistent recording is strongly encouraged to validate or refute prognostic correlations identified in smaller, single-institution cohorts.

Immunosuppression

The strong association between immunosuppression and MCC is well known[10,34,35] (AJCC Level of Evidence: I). Whether compromised immune surveillance is the result of an underlying comorbidity, such as chronic lymphocytic leukemia, or immunosuppressive medication to prevent organ rejection, it increases the risk of developing MCC and negatively influences the subsequent immune response against the tumor, resulting in a higher mortality rate. Similarly, immune senescence, the natural decline of the immune system with age, is believed to be a contributing factor in the continually increasing incidence of MCC with advancing age.[36] Because of the subjective nature of this known prognostic factor, immunosuppression is not incorporated in the staging system. However, the practitioner is strongly encouraged to maintain a higher level of suspicion for the development of MCC in an immunocompromised patient, and to bear in mind the more aggressive course of MCC in the context of suppressed immune status when considering treatment options and surveillance for such patients.[37]

RISK ASSESSMENT MODELS

The AJCC recently established guidelines that will be used to evaluate published statistical prediction models for the purpose of granting endorsement for clinical use.[38] Although this is a monumental step toward the goal of precision medicine, this work was published only very recently. Therefore, the existing models that have been published or may be in clinical use have not yet been evaluated for this cancer site by the Precision Medicine Core of the AJCC. In the future, the statistical prediction models for this cancer site will be evaluated, and those that meet all AJCC criteria will be endorsed.

DEFINITIONS OF AJCC TNM

Definition of Primary Tumor (T)

T Category	T Criteria
TX	Primary tumor cannot be assessed (e.g., curetted)
T0	No evidence of primary tumor
Tis	*In situ* primary tumor
T1	Maximum clinical tumor diameter ≤2 cm
T2	Maximum clinical tumor diameter >2 but ≤5 cm
T3	Maximum clinical tumor diameter >5 cm
T4	Primary tumor invades fascia, muscle, cartilage, or bone

Definition of Regional Lymph Node (N)

Clinical (N)

N Category	N Criteria
NX	Regional lymph nodes cannot be clinically assessed (e.g., previously removed for another reason, or because of body habitus)
N0	No regional lymph node metastasis detected on clinical and/or radiologic examination
N1	Metastasis in regional lymph node(s)
N2	In-transit metastasis (discontinuous from primary tumor; located between primary tumor and draining regional nodal basin, or distal to the primary tumor) *without* lymph node metastasis
N3	In-transit metastasis (discontinuous from primary tumor; located between primary tumor and draining regional nodal basin, or distal to the primary tumor) *with* lymph node metastasis

Pathological (pN)

pN Category	pN Criteria
pNX	Regional lymph nodes cannot be assessed (e.g., previously removed for another reason or *not* removed for pathological evaluation)
pN0	No regional lymph node metastasis detected on pathological evaluation
pN1	Metastasis in regional lymph node(s)
pN1a(sn)	Clinically occult regional lymph node metastasis identified only by sentinel lymph node biopsy
pN1a	Clinically occult regional lymph node metastasis following lymph node dissection
pN1b	Clinically and/or radiologically detected regional lymph node metastasis, microscopically confirmed
pN2	In-transit metastasis (discontinuous from primary tumor; located between primary tumor and draining regional nodal basin, or distal to the primary tumor) *without* lymph node metastasis
pN3	In-transit metastasis (discontinuous from primary tumor; located between primary tumor and draining regional nodal basin, or distal to the primary tumor) *with* lymph node metastasis

Definition of Distant Metastasis (M)

Clinical (M)

M Category	M Criteria
M0	No distant metastasis detected on clinical and/or radiologic examination
M1	Distant metastasis detected on clinical and/or radiologic examination
M1a	Metastasis to distant skin, distant subcutaneous tissue, or distant lymph node(s)
M1b	Metastasis to lung
M1c	Metastasis to all other visceral sites

Pathological (M)

M Category	M Criteria
M0	No distant metastasis detected on clinical and/or radiologic examination
pM1	Distant metastasis microscopically confirmed
pM1a	Metastasis to distant skin, distant subcutaneous tissue, or distant lymph node(s), microscopically confirmed
pM1b	Metastasis to lung, microscopically confirmed
pM1c	Metastasis to all other distant sites, microscopically confirmed

AJCC PROGNOSTIC STAGE GROUPS

Clinical Stage Group (cTNM)

When T is...	And N is...	And M is...	Then the stage group is...
Tis	N0	M0	0
T1	N0	M0	I
T2–3	N0	M0	IIA
T4	N0	M0	IIB
T0–4	N1–3	M0	III
T0–4	Any N	M1	IV

Pathological Stage Group (pTNM)

When T is...	And N is...	And M is...	Then the stage group is...
Tis	N0	M0	0
T1	N0	M0	I
T2–3	N0	M0	IIA
T4	N0	M0	IIB
T1–4	N1a(sn) or N1a	M0	IIIA
T0	N1b	M0	IIIA
T1–4	N1b–3	M0	IIIB
T0–4	Any N	M1	IV

REGISTRY DATA COLLECTION VARIABLES

1. Largest tumor diameter (in millimeters, measured clinically, or histologically if not available)
2. Regional nodal status (examined clinically, pathologically, or neither)
3. Unknown primary status (yes/no)
4. Tumor thickness (whole millimeters)
5. Excision margin status (tumor base transected or not transected)
6. Profound immunosuppression (no immunosuppressive conditions, HIV/AIDS, solid organ transplant recipient, chronic lymphocytic leukemia, non-Hodgkin lymphoma, multiple conditions, condition NOS)
7. LVI (present/absent/no comment by pathologist)
8. MCPyV-positive staining by IHC (yes/no/not applicable)
9. p63-positive staining by IHC (if applicable) (yes/no)
10. Tumor-infiltrating lymphocytes in primary tumor (not present; present, nonbrisk; present, brisk; present, NOS)
11. Growth pattern of primary tumor (circumscribed/nodular or infiltrative)
12. Extranodal extension in regional lymph node(s) (yes/no)
13. Tumor nest size in regional lymph node(s) (greatest dimension of largest aggregate in millimeters)
14. Isolated tumor cells in regional lymph node(s) (yes/no)
15. Eyelid tumor involving the upper or lower eyelid, or both
16. Eyelid tumor involving the eyelid margin, defined as the juncture of eyelid skin and tarsal plate at the lash line; if present, is the eyelid margin involvement full thickness? (no/yes, no full thickness/yes full thickness)

HISTOLOGIC GRADE (G)

There is no recommended histologic grading system at this time.

HISTOPATHOLOGIC TYPE

Although several distinct morphologic patterns have been described for MCC, none has been reproducibly found to be of prognostic significance. These histologic subtypes include intermediate type (most common), small cell type (second most common), and trabecular type (least common but most characteristic pattern of MCC).[23]

SURVIVAL DATA

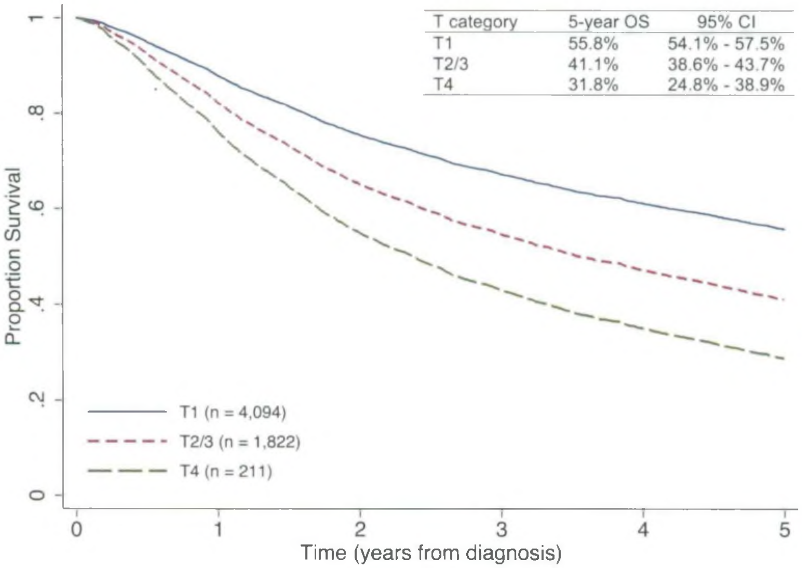

Fig. 46.2 Five-year overall survival of 6,127 patients with local MCC only (clinically and, if known, pathologically node negative) in the NCDB, stratified by T category (≤ 2 cm, T1; >2 cm, T2/3; involving fascia, muscle, cartilage, or bone, T4). Eleven patients with *in situ* disease (Tis) were excluded. Categories T2 (n = 1,511) and T3 (n = 311) were combined because of overlapping survival curves

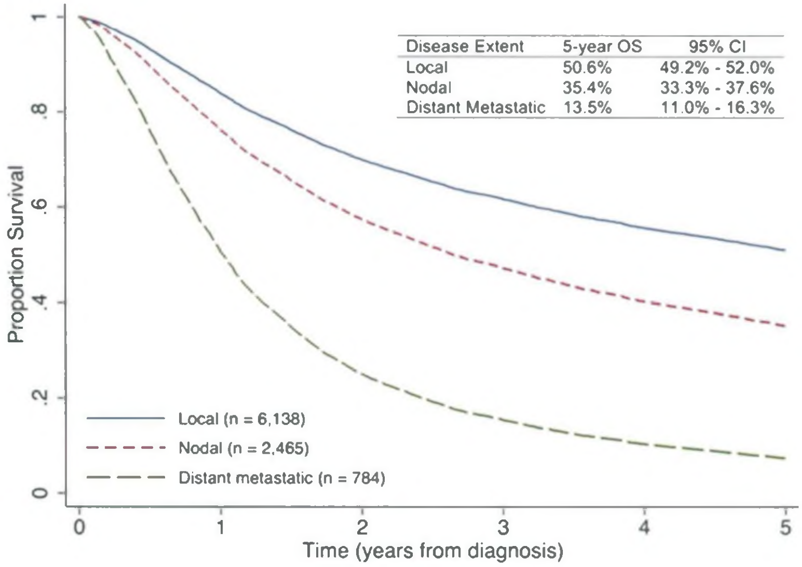

Fig. 46.3 Five-year overall survival of 9,387 patients with MCC in the NCDB, stratified by local, regional nodal, and distant metastatic disease

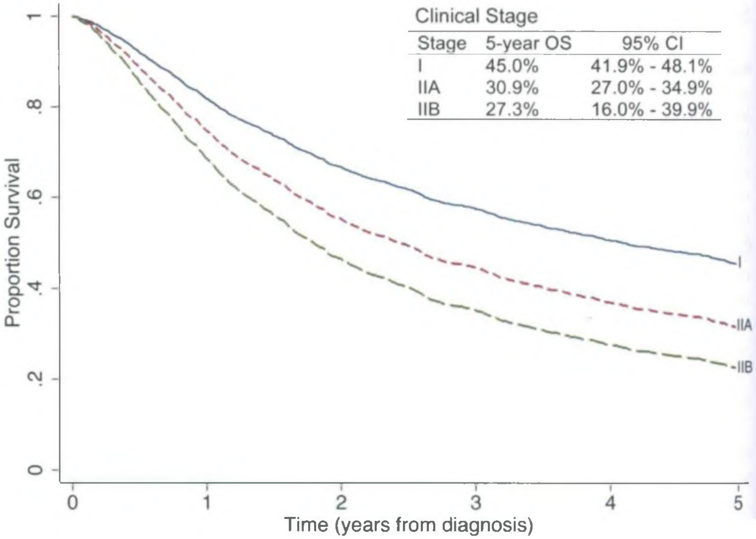

Fig. 46.4 Five-year overall survival of 2,013 patients with MCC in the NCDB with clinical staging only for local disease (cN0 pNx; i.e., pathological nodal staging was not performed). Of these patients, 1,272 presented with clinical Stage I, 675 presented with clinical Stage IIA, and 66 presented with clinical Stage IIB. Survival rates of clinically staged nodal and distant metastases are not depicted; see Additional Factors Recommended for Clinical Care for details

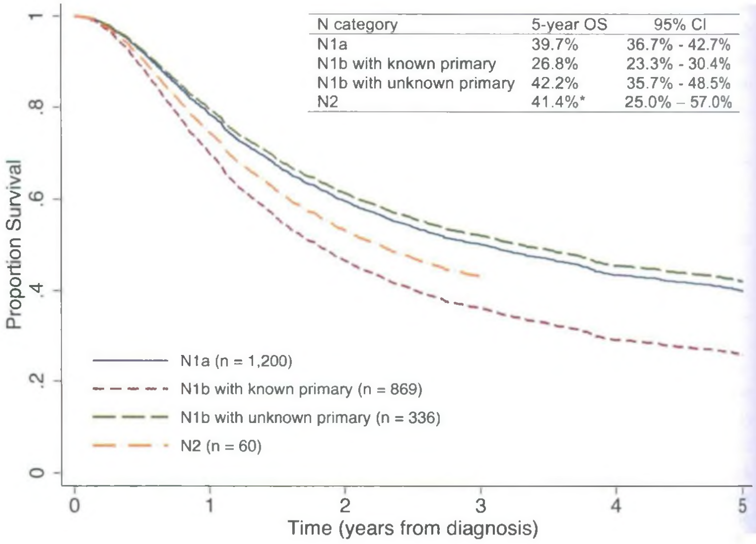

Fig. 46.5 Five-year overall survival of 2,465 MCC patients with regional lymph node metastases in the NCDB, stratified by tumor burden and primary tumor status: clinically and radiologically occult nodal metastasis (N1a) detected by SLNB or otherwise; clinically or radiologically detected and pathologically confirmed nodal metastasis (N1b) with or without the presence of a primary tumor; and in-transit disease (N2). The latter category represents 3-year overall survival because of the small sample size of 60 patients

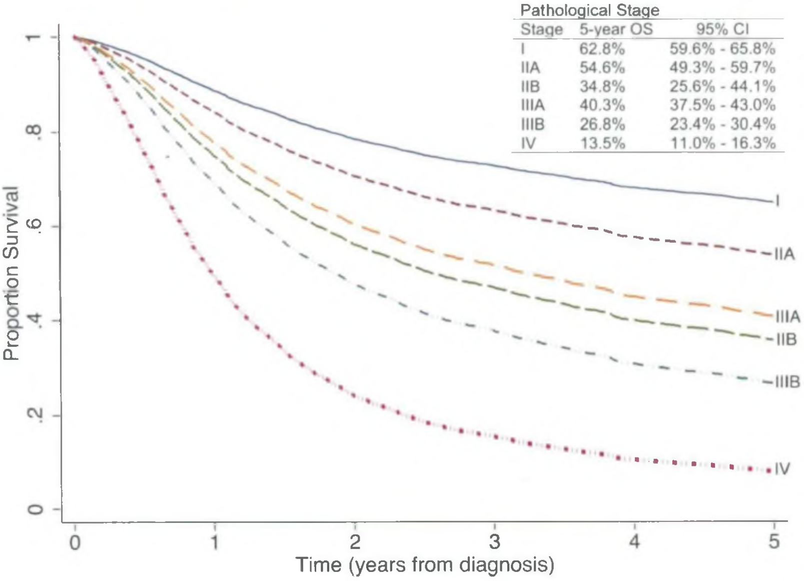

Fig. 46.6 Five-year overall survival of 5,371 patients with MCC in the NCDB with pathological staging, including 1,502 patients in stage group I, 493 in stage group IIA, 127 in stage group IIB, 1,536 in stage group IIIA, 929 in stage group IIIB, and 784 in stage group IV

ILLUSTRATIONS

Tis

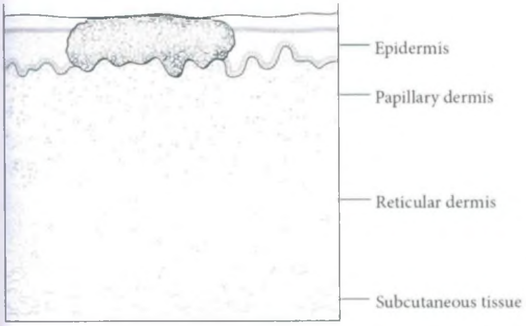

Fig. 46.7 Merkel cell carcinoma *in situ* (Tis)

T1

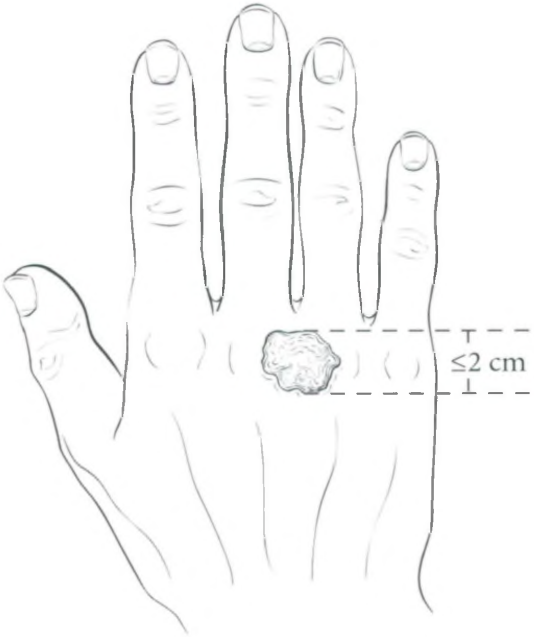

Fig. 46.8 T1 is defined as tumor with a maximum clinical diameter ≤2 cm

T2

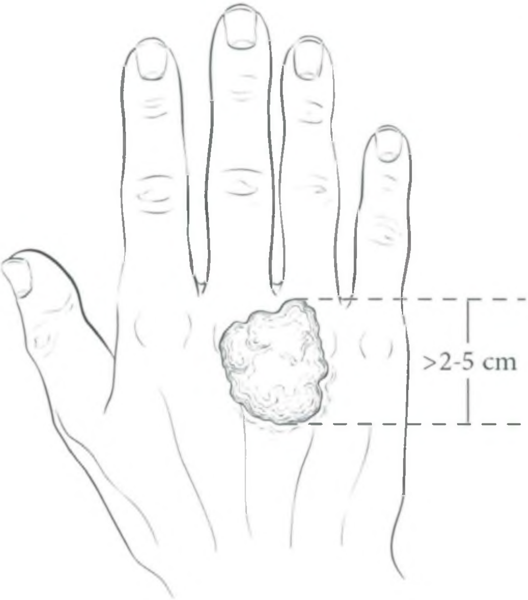

Fig. 46.9 T2 is defined as tumor with a maximum clinical diameter >2 but ≤5 cm

T3

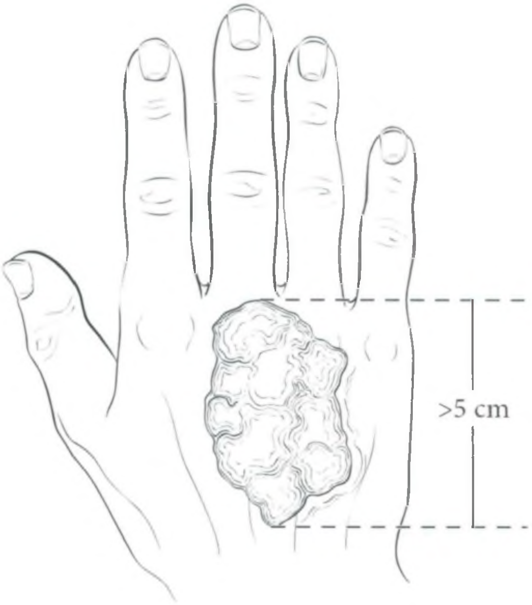

Fig. 46.10 T3 is defined as tumor with a maximum clinical diameter >5 cm

T4

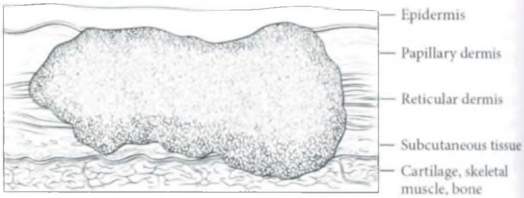

Fig. 46.11 T4 is defined as a primary tumor invading fascia, muscle, cartilage, or bone

N1a

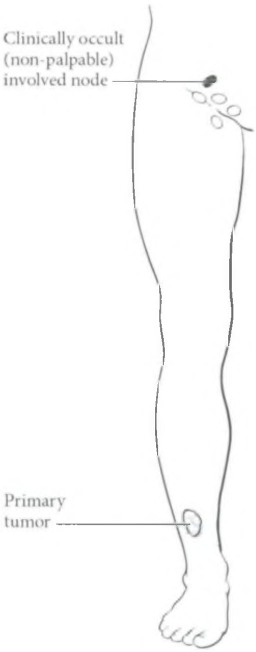

Fig. 46.12 pN1a is defined as clinically occult regional lymph node metastasis following lymph node dissection

N1b

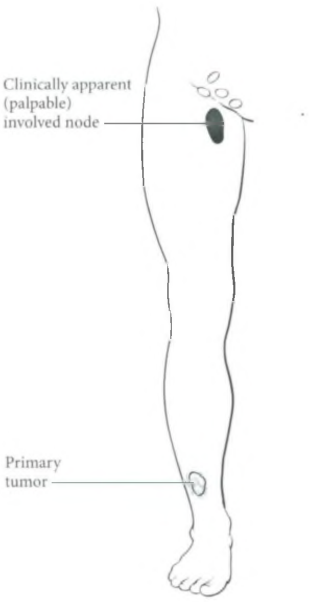

Fig. 46.13 pN1b is defined as clinically apparent and/or radiologically detected regional lymph node metastasis

N2

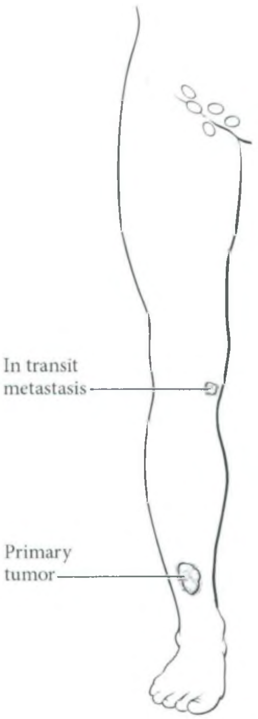

Fig. 46.14 N2 is defined as in-transit metastasis (discontinuous from primary tumor; located between primary tumor and draining regional nodal basin, or distal to the primary tumor) *without* lymph node metastasis

Bibliography

1. Tang CK, Toker C. Trabecular carcinoma of the skin: an ultrastructural study. *Cancer.* Nov 1978;42(5):2311–2321.
2. Lemos BD, Storer BE, Iyer JG, et al. Pathologic nodal evaluation improves prognostic accuracy in Merkel cell carcinoma: analysis of 5823 cases as the basis of the first consensus staging system. *Journal of the American Academy of Dermatology.* Nov 2010;63(5):751–761.
3. Balch CM, Soong SJ, Gershenwald JE, et al. Prognostic factors analysis of 17,600 melanoma patients: validation of the American Joint Committee on Cancer melanoma staging system. *J Clin Oncol.* Aug 15 2001;19(16):3622–3634.
4. Feng H, Shuda M, Chang Y, Moore PS. Clonal integration of a polyomavirus in human Merkel cell carcinoma. *Science.* Feb 22 2008;319(5866):1096–1100.
5. Verhaegen ME, Mangelberger D, Harms PW, et al. Merkel cell polyomavirus small T antigen is oncogenic in transgenic mice. *The Journal of investigative dermatology.* May 2015;135(5):1415–1424.
6. Heath M, Jaimes N, Lemos B, et al. Clinical characteristics of Merkel cell carcinoma at diagnosis in 195 patients: the AEIOU features. *Journal of the American Academy of Dermatology.* Mar 2008;58(3):375–381.
7. Schwartz JL, Bichakjian CK, Lowe L, et al. Clinicopathologic features of primary Merkel cell carcinoma: a detailed descriptive analysis of a large contemporary cohort. *Dermatologic surgery : official publication for American Society for Dermatologic Surgery [et al.].* Jul 2013;39(7):1009–1016.
8. Brewer JD, Shanafelt TD, Call TG, et al. Increased incidence of malignant melanoma and other rare cutaneous cancers in the setting of chronic lymphocytic leukemia. *Int J Dermatol.* Aug 2015;54(8):e287–293.
9. Kanitakis J, Euvrard S, Chouvet B, Butnaru AC, Claudy A. Merkel cell carcinoma in organ-transplant recipients: report of two cases with unusual histological features and literature review. *J Cutan Pathol.* Oct 2006;33(10):686–694.
10. Penn I, First MR. Merkel's cell carcinoma in organ recipients: report of 41 cases. *Transplantation.* Dec 15 1999;68(11):1717–1721.
11. Fitzgerald TL, Dennis S, Kachare SD, Vohra NA, Wong JH, Zervos EE. Dramatic Increase in the Incidence and Mortality from Merkel Cell Carcinoma in the United States. *The American surgeon.* Aug 2015;81(8):802–806.
12. Lemos B, Nghiem P. Merkel cell carcinoma: more deaths but still no pathway to blame. *The Journal of investigative dermatology.* Sep 2007;127(9):2100–2103.
13. Medina-Franco H, Urist MM, Fiveash J, Heslin MJ, Bland KI, Beenken SW. Multimodality treatment of Merkel cell carcinoma: case series and literature review of 1024 cases. *Annals of surgical oncology.* Apr 2001;8(3):204–208.

14. Moshiri AS, Nghiem P. Milestones in the staging, classification, and biology of Merkel cell carcinoma. *Journal of the National Comprehensive Cancer Network : JNCCN.* Sep 2014;12(9):1255–1262.
15. Iyer JG, Storer BE, Paulson KG, et al. Relationships among primary tumor size, number of involved nodes, and survival for 8044 cases of Merkel cell carcinoma. *Journal of the American Academy of Dermatology.* Apr 2014;70(4):637–643.
16. Schwartz JL, Griffith KA, Lowe L, et al. Features predicting sentinel lymph node positivity in Merkel cell carcinoma. *J Clin Oncol.* Mar 10 2011;29(8):1036–1041.
17. Smith FO, Yue B, Marzban SS, et al. Both tumor depth and diameter are predictive of sentinel lymph node status and survival in Merkel cell carcinoma. *Cancer.* Sep 15 2015;121(18):3252–3260.
18. Tarantola TI, Vallow LA, Halyard MY, et al. Unknown primary Merkel cell carcinoma: 23 new cases and a review. *Journal of the American Academy of Dermatology.* Mar 2013;68(3):433–440.
19. Chen KT, Papavasiliou P, Edwards K, et al. A better prognosis for Merkel cell carcinoma of unknown primary origin. *American journal of surgery.* Nov 2013;206(5):752–757.
20. Foote M, Veness M, Zarate D, Poulsen M. Merkel cell carcinoma: the prognostic implications of an occult primary in stage IIIB (nodal) disease. *Journal of the American Academy of Dermatology.* Sep 2012;67(3):395–399.
21. Maricich SM, Wellnitz SA, Nelson AM, et al. Merkel cells are essential for light-touch responses. *Science.* Jun 19 2009;324(5934):1580–1582.
22. Tilling T, Moll I. Which are the cells of origin in merkel cell carcinoma? *J Skin Cancer.* 2012;2012:680410.
23. Bichakjian CK, Lowe L, Lao CD, et al. Merkel cell carcinoma: critical review with guidelines for multidisciplinary management. *Cancer.* Jul 1 2007;110(1):1–12.
24. Bichakjian CK, Olencki T, Alam M, et al. Merkel cell carcinoma, version 1.2014. *Journal of the National Comprehensive Cancer Network : JNCCN.* Mar 1 2014;12(3):410–424.
25. Hawryluk EB, O'Regan KN, Sheehy N, et al. Positron emission tomography/computed tomography imaging in Merkel cell carcinoma: a study of 270 scans in 97 patients at the Dana-Farber/Brigham and Women's Cancer Center. *Journal of the American Academy of Dermatology.* Apr 2013;68(4):592–599.
26. Byrne K, Siva S, Chait L, et al. 15-Year Experience of 18F-FDG PET Imaging in Response Assessment and Restaging After Definitive Treatment of Merkel Cell Carcinoma. *Journal of nuclear medicine : official publication, Society of Nuclear Medicine.* Sep 2015;56(9):1328–1333.
27. Sollini M, Taralli S, Milella M, et al. Somatostatin receptor positron emission tomography/computed tomography imaging in Merkel cell carcinoma. *J Eur Acad Dermatol Venereol.* Oct 7 2015.
28. Allen PJ, Bowne WB, Jaques DP, Brennan MF, Busam K, Coit DG. Merkel cell carcinoma: prognosis and treatment of patients from a single institution. *J Clin Oncol.* Apr 1 2005;23(10):2300–2309.
29. Fields RC, Busam KJ, Chou JF, et al. Five hundred patients with Merkel cell carcinoma evaluated at a single institution. *Annals of surgery.* Sep 2011;254(3):465–473; discussion 473–465.
30. Tarantola TI, Vallow LA, Halyard MY, et al. Prognostic factors in Merkel cell carcinoma: analysis of 240 cases. *Journal of the American Academy of Dermatology.* Mar 2013;68(3):425–432.
31. Grotz TE, Joseph RW, Pockaj BA, et al. Negative Sentinel Lymph Node Biopsy in Merkel Cell Carcinoma is Associated with a Low Risk of Same-Nodal-Basin Recurrences. *Annals of surgical oncology.* Nov 2015;22(12):4060–4066.
32. Andea AA, Coit DG, Amin B, Busam KJ. Merkel cell carcinoma: histologic features and prognosis. *Cancer.* Nov 1 2008;113(9):2549–2558.
33. Fields RC, Coit DG. Is a "Merkel" just like a melanoma? The pathologic analysis of Merkel cell carcinoma specimens. *Annals of surgical oncology.* Oct 2012;19(11):3304–3306.
34. An KP, Ratner D. Merkel cell carcinoma in the setting of HIV infection. *Journal of the American Academy of Dermatology.* Aug 2001;45(2):309–312.
35. Brewer JD, Shanafelt TD, Otley CC, et al. Chronic lymphocytic leukemia is associated with decreased survival of patients with malignant melanoma and Merkel cell carcinoma in a SEER population-based study. *J Clin Oncol.* Mar 10 2012;30(8):843–849.
36. Miller RW, Rabkin CS. Merkel cell carcinoma and melanoma: etiological similarities and differences. *Cancer epidemiology, biomarkers & prevention : a publication of the American Association for Cancer Research, cosponsored by the American Society of Preventive Oncology.* Feb 1999;8(2):153–158.
37. Paulson KG, Iyer JG, Blom A, et al. Systemic immune suppression predicts diminished Merkel cell carcinoma-specific survival independent of stage. *The Journal of investigative dermatology.* Mar 2013;133(3):642–646.
38. Kattan MW, Hess KR, Amin MB, et al. American Joint Committee on Cancer acceptance criteria for inclusion of risk models for individualized prognosis in the practice of precision medicine. *CA: a cancer journal for clinicians.* Jan 19 2016.

Melanoma of the Skin

47

Jeffrey E. Gershenwald, Richard A. Scolyer, Kenneth R. Hess, John F. Thompson, Georgina V. Long, Merrick I. Ross, Alexander J. Lazar, Michael B. Atkins, Charles M. Balch, Raymond L. Barnhill, Karl Y. Bilimoria, James D. Brierley, Antonio C. Buzaid, David R. Byrd, Paul B. Chapman, Alistair J. Cochran, Daniel G. Coit, Alexander M. Eggermont, David E. Elder, Mark B. Faries, Keith T. Flaherty, Claus Garbe, Julie M. Gardner, Phyllis A. Gimotty, Allan C. Halpern, Lauren E. Haydu, Timothy Johnson, John M. Kirkwood, Anne W. M. Lee, Grant A. McArthur, Martin C. Mihm, Victor G. Prieto, Arthur J. Sober, Richard L. Wahl, Sandra L. Wong, and Vernon K. Sondak

CHAPTER SUMMARY

Cancers Staged Using This Staging System

Cutaneous melanoma

Cancers Not Staged Using This Staging System

These histopathologic types of cancer…	Are staged according to the classification for…	And can be found in chapter…
Melanoma of the conjunctiva	Conjunctival melanoma	66
Melanoma of the uvea	Uveal melanoma	67
Mucosal melanoma arising in the head and neck	Mucosal melanoma of the head and neck	14
Mucosal melanoma of the urethra, vagina, rectum, and anus	No AJCC staging system	N/A
Merkel cell carcinoma	Merkel cell carcinoma	46
Squamous cell carcinoma	Cutaneous squamous cell carcinoma of the head and neck	15

To access the AJCC cancer staging forms, please visit www.cancerstaging.org.

© American Joint Committee on Cancer 2017

M.B. Amin et al. (eds.), *AJCC Cancer Staging Manual, Eighth Edition*, DOI 10.1007/978-3-319-40618-3_47

Summary of Changes

Change	Details of Change	Level of Evidence
Definition of Primary Tumor (T)	All principal T-category tumor thickness ranges are maintained, but **T1** now is subcategorized by tumor thickness strata at 0.8 mm threshold.	I
Definition of Primary Tumor (T)	Tumor mitotic rate was removed as a staging criterion for **T1** tumors, but remains an overall important prognostic factor that should continue to be recorded for all patients with T1-T4 primary cutaneous melanoma.<br>• **T1a** melanomas now are defined as nonulcerated and $<$0.8 mm in thickness.<br>• **T1b** melanomas now are defined as 0.8 to 1.0 mm in thickness regardless of ulceration status OR ulcerated melanomas $<$0.8 mm in thickness.	II
Definition of Primary Tumor (T)	**T0** definition has been clarified. **T0** should be used if there is no evidence of a primary tumor (e.g., in a patient who presents with axillary metastasis and no known primary tumor). Staging may be based on clinical suspicion of a primary cutaneous melanoma, with the tumor categorized as **T0**. (**Tis**, not **T0**, designates melanoma *in situ*.)	N/A
Definition of Primary Tumor (T)	Tumor thickness measurements now are recorded to the nearest 0.1 mm, not the nearest 0.01 mm, because of the impracticality and imprecision of measurements, particularly for tumors $>$1 mm thick. Tumors $\leq$1 mm may be measured to the nearest 0.01 mm if practical, but should be reported rounded to the nearest 0.1 mm (e.g., melanomas measured to be in the range of 0.75 to 0.84 mm are reported as 0.8 mm in thickness; hence **T1b**).	N/A
Definition of Primary Tumor (T)	**Tis** (melanoma *in situ*), **T0** (no evidence of a primary tumor), and **TX** (tumor thickness cannot be determined) may now be used as the T category designation for stage groupings.	N/A
Definition of Regional Lymph Node (N)	Number of tumor-involved regional lymph nodes is maintained.	I
Definition of Regional Lymph Node (N)	Previously empirically defined "microscopic" and "macroscopic" descriptors are redefined as "clinically occult" (i.e., clinical Stage I–II with nodal metastasis determined at sentinel node biopsy) and "clinically detected" regional node disease (clinical Stage III), respectively.	N/A
Definition of Regional Lymph Node (N)	Sentinel node tumor burden is considered a regional disease prognostic factor that should be collected for all patients with positive sentinel nodes, but it is not used to determine N-category groupings.	I
Definition of Regional Lymph Node (N)	Non-nodal regional disease (i.e., microsatellites, satellites, and in-transit cutaneous and/or subcutaneous metastases) is more formally stratified by N category according to the number of tumor-involved lymph nodes.<br>• Presence of microsatellites, satellites, or in-transit metastases is now categorized as N1c, N2c, or N3c based on the number of tumor-involved regional lymph nodes, if any.	II
Definition of Regional Lymph Node (N)	"Gross" extranodal extension no longer is used as an N-category criterion (but the presence of "matted nodes" is retained).	N/A
Definition of Distant Metastasis (M)	**M1** is now defined by both anatomic site of distant metastatic disease and serum lactate dehydrogenase (LDH) value for all anatomic site subcategories.	II
Definition of Distant Metastasis (M)	Descriptions of distant anatomic sites of disease are clarified in M subcategories.	N/A
Definition of Distant Metastasis (M)	Descriptors were added to the **M1** subcategory designation that provide LDH status (designated as "0" for "not elevated" and "1" for "elevated" level) for all sites of distant disease; for example, skin/soft tissue/nodal metastasis with elevated LDH is now **M1a(1)** not **M1c**.	II
Definition of Distant Metastasis (M)	New **M1d** designation was added to include distant metastasis to the central nervous system (CNS) with or without any other distant sites of disease; **M1c** no longer includes CNS metastasis.	II
Definition of Distant Metastasis (M)	Elevated LDH level no longer defines **M1c**.	N/A
AJCC Prognostic Stage Groups	No overall changes were made in T subcategories, but definitions of **T1a** and **T1b** were refined. In addition, while the stage group for cT1bN0 remains clinical Stage IB, the stage group for pT1bN0 now is pathological Stage IA.	II
AJCC Prognostic Stage Groups	N category now comprises four stage groups rather than three, and these groups are based on multivariable models, including T-category elements (tumor thickness and ulceration) and N-category elements (number of tumor-involved nodes, satellites/in-transits/microsatellites), demonstrating a significant impact of primary tumor factors in assigning N stage groups.	II
AJCC Prognostic Stage Groups	Clarification is provided that Stage IV is not further stage grouped (i.e., **M1c** is Stage IV not Stage IVC).	N/A

ICD-O-3 Topography Codes

Code	Description
C44.0	Skin of lip, NOS
C44.1	Eyelid
C44.2	External ear
C44.3	Skin of other and unspecified parts of face
C44.4	Skin of scalp and neck
C44.5	Skin of trunk
C44.6	Skin of upper limb and shoulder
C44.7	Skin of lower limb and hip
C44.8	Overlapping lesion of skin
C44.9	Skin, NOS
C51.0	Labium majus
C51.1	Labium minus
C51.2	Clitoris
C51.8	Overlapping lesion of vulva
C51.9	Vulva, NOS
C60.0	Prepuce
C60.1	Glans penis
C60.2	Body of penis
C60.8	Overlapping lesion of penis
C60.9	Penis, NOS
C63.2	Scrotum, NOS

WHO Classification of Tumors

Code	Description
8720	Melanoma
8720	Nevoid melanoma
8720	Melanoma of childhood
8721	Nodular melanoma
8742	Lentigo maligna melanoma
8743	Superficial spreading melanoma
8744	Acral lentiginous melanoma
8745	Desmoplastic melanoma
8761	Melanoma arising in a giant congenital nevus
8780	Melanoma arising from a blue nevus

LeBoit PE, Burg G, Weedon D, Sarasin A, eds. World Health Organization Classification of Tumours. Pathology and Genetics of Skin Tumours. Lyon: IARC Press; 2006.

INTRODUCTION

Cutaneous melanoma represents only a minority of all skin cancers but is responsible for the vast majority of skin cancer deaths. Unlike most solid tumors for which incidence has either decreased or stabilized, the incidence of melanoma in the United States has continued to rise approximately 3% per year for the past few decades.[1,2] There is compelling evidence that melanoma is associated with overexposure to ultraviolet (UV) radiation, particularly intense intermittent exposure, in the vast majority of patients, including overexposure from the sun (natural source) and any use of UV-emitting indoor tanning devices (artificial sources).[3–5] Early-stage primary cutaneous melanoma generally is associated with an overall favorable prognosis[6–9]; however, advanced-stage melanoma has been associated with very poor survival historically.[6] For patients with primary cutaneous melanoma and clinically negative regional nodes, the risk of synchronous occult regional metastasis at initial presentation is very low in most thin (i.e., clinical T1) melanomas. This risk increases with increasing primary tumor thickness and other adverse clinicopathologic prognostic factors, to approximately 35–50% in patients who present with clinically node-negative clinical T4b primary melanoma. Among the subset of patients who have synchronous regional lymph node disease at the time of primary tumor diagnosis, clinically occult synchronous regional node involvement (identified by the technique of lymphatic mapping and sentinel lymph node [SLN] biopsy) is more common than clinically evident regional nodal disease. Presentation with synchronous distant metastasis at initial diagnosis of primary cutaneous melanoma is very uncommon. Occasionally, melanoma may present in lymph nodes or, even less commonly, at distant sites without a known associated primary cutaneous melanoma.

47

The limited treatment armamentarium for patients with regional and particularly unresectable and/or distant disease was without significant change for the first decade of the 21st century. However, based on tremendous advances in our understanding of the molecular underpinnings of melanoma and the role of the immune system in cancer, multiple new therapeutic strategies have radically transformed the care of melanoma patients, particularly those with advanced-stage disease. Examples of new treatment approaches include immunotherapy using immune system checkpoint inhibitors against cytotoxic T-lymphocyte antigen 4 (CTLA-4)[10,11] and/or programmed death 1 (PD-1)[12–14] and molecularly targeted therapy using BRAF inhibitors alone (monotherapy)[15–19] or in combination with MEK inhibitors[20–24] (for the approximately 40–50% of patients with metastatic melanoma whose melanoma harbors a BRAF V600 mutation).[25,26]

The AJCC Cancer Staging Manual, 7th Edition staging system for cutaneous melanoma has been widely adopted since its rollout in 2010.[6] In this 8th Edition, particular attention is directed at clarifying major themes, introducing some clinically relevant revisions, and importantly, investing in creation of a new contemporary international database that will empower ongoing development of robust and iteratively refined clinical prognostic models and tools that will be useful for clinical decision making. This database was interrogated, particularly for Stages I-III disease, to develop the evidence underpinning the 8th Edition of the staging system, the results of which are planned for publication elsewhere.

For Stage IV disease, findings from the 7th Edition AJCC Stage IV international melanoma database analysis,[6] together with salient observations from multiple completed clinical trials support reconsideration of the AJCC M categories and

M stage groupings. It has been well appreciated that patients with brain metastasis have a particularly poor response to therapy and survival outcome, reinforced by their exclusion from most clinical trials that tested now-approved targeted therapies (e.g., BRAF inhibitors and BRAF/MEK inhibitors) and immunotherapies (e.g., anti–CTLA-4 and anti–PD-1 antibodies) in patients with unresectable or advanced metastatic melanoma. In addition, an elevated serum lactate dehydrogenase (LDH) level was shown to be an adverse prognostic factor among patients with Stage IV melanoma in the 7th Edition AJCC analysis, which included patients treated before the era of effective targeted therapy and immunotherapy (with the caveat that only approximately 10–15% of the more than 9,000 patients had LDH data recorded by contributing institutions[6,27]) and continues to be identified as an adverse prognostic factor in multiple contemporary clinical trials.[28–32] However, few data exist to define exactly how anatomic sites of metastatic melanoma and serum LDH level interact to affect prognosis; for example, does a patient with soft tissue distant metastasis and an elevated LDH level actually have a prognosis similar to that of a patient with liver or brain metastasis and a normal LDH? Therefore, the AJCC Melanoma Expert Panel modified the Stage IV melanoma staging system in an attempt to better classify patients for the contemporary era.

For several decades, the AJCC staging system has been used widely for staging, prognostic assessment, and clinical decision making for patients diagnosed with melanoma.[6] That notwithstanding, it is well appreciated that a traditional, anatomically based TNM system is to some extent constrained by the number and richness of the factors that may be included in the actual staging system. Given the significant advances in our understanding of the biology and pathogenesis of melanoma, coupled with advances in analytic techniques and an electronic era that facilitates the incorporation of numerous factors into point-of-care analytics, the Melanoma Expert Panel will continue to explore and expand the development of improved prognostic (and predictive) models and validated clinical tools that can be applied at the individual patient level.

ANATOMY

Primary Site(s)

Primary cutaneous melanoma usually arises in sun-exposed cutaneous anatomic sites but also may occur on potentially sun-protected sites, such as the volar aspects of the hands and feet, subungual locations and genital skin. Occasionally, melanoma may be discovered in lymph nodes or, less commonly, at distant sites without a known associated primary cutaneous melanoma.

Melanoma arises from melanocytes, pigment-producing cells scattered along the basal epidermis of the skin and also present in hair follicles. Smaller numbers of melanocytes reside in mucosal membranes of the upper aerodigestive tract and genital organs, leptomeninges, and a variety of other sites, and melanocytes also populate the ocular uveal tract. The density of cutaneous melanocytes varies depending on factors such as the anatomic site and a person's age, but there usually is one melanocyte for every 5 to 10 basal keratinocytes. Melanin pigment, produced by the melanocytes, provides some protection from the toxic and well-established carcinogenic effects of UV radiation.[33] It is transported to keratinocytes and other cells of the adjacent epidermis by a network of dendritic processes that extend from melanocytes.

Most cutaneous melanomas arise from melanocytes at the dermoepidermal junction, either in previously normal skin or in association with a melanocytic nevus. Initially, melanoma cells proliferate within the epidermis; when they are restricted to the epidermis and associated adnexal structures, the neoplasm is termed *in situ* melanoma. After a variable period, the tumor (now called *invasive* melanoma) may invade the underlying dermis and deeper tissues, where it may gain access to lymphatics and blood vessels and acquire metastatic potential.

Overall, the risk of metastasis correlates with the vertical depth of tumor extension (termed *primary tumor Breslow thickness*) as well as other prognostic factors, such as the primary tumor mitotic rate and presence or absence of surface ulceration. In theory, melanoma *in situ* is thought to be incapable of metastasis, although on rare occasions, synchronous and/or metachronous nodal metastasis has been reported, possibly as a consequence of an undiscovered small focus of dermal invasive melanoma or regression of the invasive component at the primary tumor site. Regression is a phenomenon in which immune-mediated recognition and destruction of the tumor causes loss of part or all of the primary tumor.

Cutaneous melanoma may occur anywhere on the skin. In Caucasians, melanoma predominantly involves sun-exposed cutaneous sites, but may occur at almost any anatomic skin site. In individuals with more heavily pigmented skin, melanomas involving acral and mucosal sites represent a much greater proportion of all melanomas than in Caucasian populations, although the actual incidence of acral and mucosal melanomas likely is similar in all races.

Regional Lymph Nodes

Locoregional melanoma metastasis consists of regional lymph node involvement (either clinically occult metastasis detected by microscopic evaluation of an SLN biopsy or clinically detected metastasis to regional lymph node[s]) as well as non-nodal locoregional disease (i.e., pathologically detected microsatellite or clinically detected satellite and in-transit metastases). These various manifestations of N-category disease are described later in this chapter.

Regional lymph nodes are the most common site of initial metastasis in patients with cutaneous melanoma. Among patients with regional lymph node metastasis, most have clinically occult disease detected by the technique of

lymphatic mapping and SLN biopsy. Small, isolated melanoma metastases in regional lymph nodes (consisting of isolated tumor cells or aggregates of up to hundreds of tumor cells) usually are located in the subcapsular sinus region of the lymph node. Multiple or larger deposits may coalesce and also involve the parenchymal region of the lymph node; intraparenchymal deposits in the absence of subcapsular sinus metastases are uncommon. Melanoma metastases must be distinguished from benign nevus cells within lymph nodes (sometimes termed benign nevus rest cells), as the latter are not melanoma metastases and should not be assigned as such.

As discussed in the section on imaging, the regional nodal basins for any primary melanoma site are determined by its lymphatic drainage pathways and are commonly defined by preoperative lymphoscintigraphy. Experience with lymphoscintigraphy has shown that primary tumors located on the extremities usually drain to one or two regional nodal basins (e.g., only axillary or axillary and epitrochlear basins; only inguinal or inguinal and pelvic basins), but some primary melanomas located on the head and neck or trunk regions may drain to three or more regional nodal basins. Patients with either clinically occult or clinically detected nodal metastases in any combination of these regional nodal basins draining the primary tumor would be categorized as having Stage III melanoma. In cases of clinically detected nodal metastases in which preoperative lymphoscintigraphy was not performed or was not informative, the patient should be categorized as having Stage III melanoma whenever the site(s) of nodal disease are considered as basins potentially draining the primary tumor.

Local and regional metastases, probably lymphatic or possibly angiotropic in origin,[34,35] also may become clinically manifest as (1) *microsatellites*—defined as any foci of metastatic tumor cells in the skin or subcutis adjacent or deep to but discontinuous from the primary tumor (but not separated only by fibrosis or inflammation that may signify regression of the intervening tumor) detected on microscopic examination of tissue from around the primary melanoma site; (2) *satellite* metastases (defined empirically as any foci of clinically evident cutaneous and/or subcutaneous metastases occurring within 2 cm of but discontinuous from the primary melanoma); or (3) *in-transit* metastases (defined empirically as clinically evident cutaneous and/or subcutaneous metastases occurring >2 cm from the primary melanoma in the region between the primary and the regional lymph node basin). Occasionally, satellite or in-transit metastases may occur distal to the primary site.

Metastatic Sites

Melanoma may metastasize to virtually any distant site. Distant (hematogenous) metastases most commonly occur in the skin or soft tissues (including muscle), distant nodes (i.e., those beyond the regional basin), lung, liver, brain, bone, or gastrointestinal tract, particularly in the small intestine. Although most metastases are detected within a few years of diagnosis of the primary tumor, occasionally patients present with distant metastatic disease many decades later; synchronous distant metastasis at initial diagnosis of primary cutaneous melanoma is very uncommon.

RULES FOR CLASSIFICATION

The definitions of clinical versus pathological classification are based upon whether the primary site has undergone a wide reexcision, and whether regional lymph nodes are assessed by clinical/radiographic examination/diagnostic biopsies during the diagnostic workup or microscopically examined as part of the treatment.

Clinical Classification

Clinical Stages I and II are defined as patients who have no evidence of metastases, either at regional or distant sites, based on clinical, radiographic, and/or laboratory evaluation. Clinical Stage III melanoma patients are those with clinical or radiographic evidence of regional metastases, either in the regional lymph nodes or locoregional metastases manifesting as satellite or in-transit metastases, or microsatellites discovered at the time of microscopic evaluation of the primary tumor diagnostic biopsy.

By convention, clinical staging is performed after biopsy of the primary melanoma (including microstaging of the primary melanoma) with clinical or biopsy assessment of regional lymph nodes. Pathological staging uses information gained from *both* microstaging of the primary melanoma after biopsy *and* wide reexcision as well as pathological evaluation of the regional node basin after SLN biopsy (required for N categorization of all >T1 melanomas) and/or complete regional lymphadenectomy. As systemic treatment becomes more effective in patients with melanoma, it is possible that neoadjuvant systemic treatment will be offered after initial diagnosis and staging of regional lymph nodes by SLN biopsy. In this setting, consistent with AJCC convention, the pathological status of the sentinel node would constitute part of clinical staging if it is performed before initiation of systemic therapy. The Melanoma Expert Panel provides no subgroup definitions of clinically staged patients with nodal or satellite/in-transit metastases. They are all categorized as having clinical Stage III disease. Clinical Stage IV melanoma patients have metastases at a distant site or sites.

Breslow Tumor Thickness

The T category of melanoma is classified primarily by measuring the thickness of the melanoma as defined by Dr. Alexander Breslow.[36,37] Tumor thickness is measured from the top of the granular layer of the epidermis (or, if the surface overlying the entire dermal component is ulcerated,

from the base of the ulcer) to the deepest invasive cell across the broad base of the tumor (in the dermis or subcutis). Thickness should be measured by using an ocular micrometer calibrated to the magnification of the microscope used for the measurement. In accordance with consensus recommendations,[38] thickness measurements should be recorded to the nearest 0.1 mm, not the nearest 0.01 mm, because of impracticality and imprecision of measurements, particularly for tumors >1 mm thick. Tumors ≤1 mm thick may be measured to the nearest 0.01 mm if practical, but the measurement should be rounded up or down to be recorded as a single digit after the decimal (i.e., to the nearest 0.1 mm). The convention for rounding decimal values is to round down those ending in 1 to 4 and to round up for those ending in 5 to 9. For example, a melanoma measuring 0.75 mm in thickness would be recorded as 0.8 mm in thickness. A tumor measuring 0.95 mm and one measuring 1.04 mm both would be rounded to 1.0 mm (i.e., T1b).

Tumor thickness can be evaluated accurately only in sections cut perpendicular to the epidermal surface, and the T category should be recorded as TX if the thickness cannot be evaluated. Nevertheless, in some tangentially cut sections, it often is still possible to report a tangentially measured thickness. The latter may be clinically useful because it may reasonably be inferred that the true tumor thickness would be no greater than this measurement, and if appropriate, this should be stated clearly in the pathology report. At other times, particularly when the epidermis is not visualized, tumor thickness cannot be provided. When sections have been cut tangentially, it may be fruitful to melt the paraffin block and re-embed the tissue, as it may then be possible to obtain perpendicular sections for determination of the tumor thickness. If there is evidence of regression of part of an invasive melanoma, the thickness should be measured in the usual way to the deepest identifiable viable tumor cell, and the tumor should be assigned to the appropriate T category. Partially regressed melanomas should not be designated TX or T0. If a melanoma has regressed completely, the tumor should be classified as T0. Other specific recommendations for the measurement of tumor thickness in certain clinical circumstances will be detailed elsewhere in a planned separate publication on the pathological aspects of melanoma staging from the International Melanoma Pathology Study Group.

The initial biopsy sample is used for clinical T categorization purposes; both the initial biopsy and the definitive excision specimens are used for pathological staging. If the pathology of the initial biopsy sample (such as a punch or superficial shave biopsy) reveals that the tumor was transected at the base and the specimen includes only its superficial portion, the maximum thickness should be recorded and it should be stated that the tumor thickness is "at least" a certain thickness for the T clinical category. This initial maximal thickness defines the clinical T category and does not change on subsequent wide reexcision.

For primary melanoma lacking an epidermal component, the tumor thickness should be measured in the standard manner (from the top of the epidermal granular layer to the deepest invasive cell).

In the 8th Edition melanoma staging system, the T-category thresholds of melanoma thickness continue to be defined in whole-number integers (1.0, 2.0, and 4.0 mm). However, the T categories have been revised to promote consistency, with the recommendation for recording tumor thickness to the nearest 0.1 mm (e.g., T2 category is now >1.0–2.0 mm, as opposed to 1.01–2.0 mm in the 7th Edition).[6,27] Remarkably, these T-category thresholds inform substaging in patients both without and with regional disease in the 8th Edition staging system.

Melanoma *In Situ*, Indeterminate Melanomas, and Multiple Primary Melanomas

Patients with melanoma *in situ* are categorized as Tis (not T0). Those with melanoma presentations that are indeterminate or cannot be microstaged should be categorized as TX. In general, when patients present with multiple primary cutaneous melanomas, each different skin area is considered a different primary site and each is categorized separately. When patients present with multiple primary melanomas that drain to the same regional node field, if nodal metastases are present, it may be difficult or impossible to ascertain which primary melanoma gave rise to the metastasis. In this situation, the tumor with the highest T category should be assigned as the origin of the nodal metastasis, and the N categorization should reflect this. Similarly, in patients with multiple primary melanomas, it may be difficult or impossible to ascertain which primary melanoma gave rise to the distant metastasis. In this situation, the tumor with the highest N category (or the highest T category if N0) should be assigned as the origin of the distant metastasis. In patients with multiple primary melanomas, the stage is reflected by the highest stage group of any of the primary tumors.

The convention for patients with multiple primary melanomas follows the AJCC general staging rules (see Chapter 1). If there are multiple synchronous melanomas, the tumor with the highest T category is assigned, and the *m* suffix is used. For example, if a patient has three synchronous primary melanomas and is otherwise clinically without evidence of regional or distant metastasis, and the highest T category is T3, the patient would be assigned as pT3(m)N0M0. An alternate acceptable approach is designation based on the number of tumors: in this example, pT3(3)N0M0.

Primary Tumor Ulceration

The second criterion for determining T category is primary tumor ulceration, that is, the full-thickness absence of an intact epidermis with associated host reaction above the primary melanoma based on a histopathologic examination. Melanoma ulceration is characterized by the combination of

the following features: full-thickness epidermal defect (including absence of stratum corneum and basement membrane of the dermoepidermal junction), evidence of reactive changes (i.e., fibrin deposition and neutrophils), and thinning, effacement, or reactive hyperplasia of the surrounding epidermis in the absence of trauma or a recent surgical procedure.[39–43] If a lesion recently was biopsied or there is only focal loss of the epidermis, assessment of ulceration may be difficult or impossible; in this instance, it may be difficult to determine whether the epidermal deficiency is the result of true ulceration or sectioning artifact. Absence of fibrin, neutrophils, or granulation tissue from putative areas of ulceration are clues that the apparent ulceration is actually the result of sectioning of only part of the epidermis, and this should not be designated as ulceration.

If nontraumatic ("tumorigenic") ulceration is present in either an initial partial biopsy or a reexcision specimen, then the tumor should be recorded as ulcerated for staging purposes.

Microsatellites

A microsatellite is a microscopic cutaneous and/or subcutaneous metastasis adjacent or deep to a primary melanoma identified on pathological examination of the primary tumor site, usually but not always on a wide excision specimen. Microsatellites are discussed in detail in the section on pathological staging.

Metastatic Melanoma from an Unknown Primary Site

In general, the staging criteria for unknown primary metastatic melanoma should be the same as those for known primary melanomas. Potential sources might be primary cutaneous melanomas that were previously ablated by iatrogenic or non-iatrogenic procedures or have regressed, primary melanomas from mucosal or ocular primary sites, or primary melanoma arising from nevus cell rests within lymph nodes. If a patient initially presents with lymph node metastases, these should be presumed to be regional (i.e., Stage III instead of Stage IV) if an appropriate staging workup does not reveal any other sites of metastasis. Such patients have a prognosis and natural history similar to, if not more favorable than, those of patients with the same staging characteristics from a known primary cutaneous melanoma.[44–50] A careful history should be obtained, and the skin from which lymphatics drain to that nodal basin should be examined closely for previous biopsy scars or areas of depigmentation. If previous biopsies have been performed, the pathology slides should be reviewed to determine whether, in retrospect, any of these may have been a primary melanoma.

Melanoma with No Epidermal Component

Occasionally, it may be extremely difficult or even impossible based on pathological examination alone to determine definitively whether a melanoma is a primary tumor or a metastasis. This is particularly the case for a melanoma located in the dermis that lacks an *in situ* component in the overlying epidermis. In many instances, such tumors represent a primary melanoma with regression of the superficial dermal and epidermal components. The pathologist should recognize this phenomenon by the presence of some subtle clues, such as the presence of rare single atypical epidermal melanocytes, epidermal thinning with loss of rete ridges, fibrosis and vascular proliferation in the dermis overlying the lesion, a defect in the band of superficial solar elastosis, and a band-like lymphohistiocytic inflammatory cell infiltrate (which usually includes numerous melanophages). In some patients, examination of the skin with a Wood (black or UV) light reveals skin changes of a regressed primary melanoma that can be confirmed pathologically.[51] In cases in which difficulty persists, it is prudent to microscopically examine additional tissue from the lesion, including further sections cut from the original and additional tissue blocks. However, despite the best efforts, in some instances it is impossible to be certain from the pathological features whether a melanoma is primary or metastatic. In such cases, correlation with clinical information is essential, as this may provide further clues (e.g., a history of a pigmented plaque that disappeared over time, leaving a lump in the dermis). In this instance, melanomas may incorrectly be reported pathologically as metastatic melanoma. Because some apparently primary melanomas (sometimes termed *primary dermal melanomas*) may be pathologically indistinguishable from dermal melanoma metastases, pathologists should be cautious in diagnosing melanoma as metastatic if there is no clinical evidence of a previous melanoma. In fact, if an appropriate staging workup does not reveal any other sites of metastases, it is recommended that such cases be managed and staged as for a primary melanoma of a similar thickness, because their prognosis appears to reflect this.[52–54] In this instance, the tumor thickness should be measured in the usual manner (from the top of the epidermal granular layer to the deepest invasive cell), and their T category is defined by this measurement. However, for melanomas some distance away from the dermal–epidermal junction, including melanomas arising in congenital nevi or blue nevus-like melanomas, the tumor thickness (in millimeters) of the melanoma component should be reported but with the qualification that this is not a conventional Breslow thickness. If there is a wide separation from the epidermis (e.g., ≥ 1 mm), the dimensions of the tumor itself should be given as an additional guide to the tumor burden.

Distant Metastasis

Given the poor prognosis associated with the development of central nervous system (CNS) metastases (i.e., those involving the brain, spinal cord, leptomeninges, or any other component of the CNS) in melanoma patients[55,56] despite the advances in systemic drug therapies,[19,57,58] as well as the frequent exclusion from clinical trials of patients with active brain and other CNS metastases, a separate M category, M1d, was added to

specifically categorize these patients (regardless of the presence of any other metastases) in the 8th Edition of the AJCC staging system. M1c no longer includes patients with brain or other CNS metastases. In addition, given the adverse survival observed among patients who have an elevated serum LDH level in both the 7th Edition analysis and in recent clinical trials,[28–32] the revised M category now includes a suffix to signify the absence or presence of an elevated LDH. The suffix *(0)* designates patients whose LDH is not elevated, whereas the suffix *(1)* describes patients with an elevated LDH level [e.g., M1a(1), M1b(1), M1c(1), and M1d(1)]. Patients in whom the LDH level is unknown or unspecified are categorized as M1a, M1b, M1c, and M1d (i.e., without any suffix noted).

Other circumstances (i.e., metastases to a visceral site and no known primary melanoma) should be categorized as Stage IV melanoma by using the M1 category criteria reflecting metastatic site and serum LDH status (discussed in detail in Additional Factors Recommended for Clinical Care) unless there is clinical or pathological evidence to suggest that they represent examples of the rare phenomenon of primary visceral melanoma.

Imaging

Imaging for Accurate Stage Determination in Melanoma

Radiologic imaging in melanoma patients often is used to refine accurate clinical stage designations by detecting the presence and anatomic locations of regional (nodal and in-transit) and/or distant (skin/soft tissue/distant nodal/intramuscular, lung, visceral, or CNS) metastatic disease. Because assignment of the primary tumor (T category) is based entirely on pathological findings (thickness and ulceration), imaging of the primary tumor has no T category implications. Assignment of regional disease (N category) is based in part on whether regional nodal disease is clinically occult (i.e., detected by SLN biopsy) or clinically detected, including detection by ultrasound, computed tomography (CT), or positron emission tomography (PET)/CT or on physical examination. In the absence of signs or symptoms that might indicate disease spread, selective use of cross-sectional imaging based on clinical stage may identify clinically occult metastases and result in a more accurate stage determination.[59] Regardless of clinical stage, imaging modalities should be used selectively to evaluate specific signs and symptoms that may be related to metastatic disease and to define the nature and significance of ambiguous physical examination findings.

Imaging in Early-stage Melanoma

Patients with melanoma *in situ* (Stage 0) and localized invasive melanoma (clinical Stages I and II) do not require imaging for staging of their cancer above and beyond any required to ensure safe conduct of indicated surgery.[59] In selected patients with high-risk clinical Stage II melanoma (e.g., T4cN0M0), especially those with an equivocal physical examination or a body habitus that limits clinical examination of the regional lymph nodes, an ultrasound examination of the lymph node basins draining the primary site may be considered. Routine use of ultrasound before SLN biopsy, however, has not been proven effective.[60] For patients undergoing SLN biopsy, to more completely identify those who are clinically occult Stage III (generally Stages IB and II), radionuclide lymphoscintigraphy is an important tool to define the regional drainage of a primary melanoma. This is especially important for patients with melanomas arising in the trunk or head and neck, in whom the lymphatic drainage may be unpredictable in direction, and/or to multiple basins. Also, in-transit sentinel nodes (defined as minor basin nodes in the epitrochlear or popliteal basins and "interval" nodes in the soft tissues outside a major or minor nodal basin) may be encountered anywhere in the body.[61] On occasion, interval in-transit nodes are the only site of nodal metastasis. Single-photon emission CT (SPECT)/CT lymphoscintigraphy may be particularly useful for identifying the sentinel node from primaries located on the head and neck or close to a draining nodal basin on the trunk (e.g., on the shoulder).[62]

Imaging in Stage III and IV Melanoma

For patients with clinical Stage III melanoma without symptoms or signs of distant metastases, cross-sectional imaging (CT or PET/CT) frequently is used to evaluate for the possible presence of synchronous occult distant metastases.[63–65]

Any cross-sectional imaging modality selected must take into account the fact that melanoma metastases may well be found outside the thoracic and abdominal cavities. Whole-body PET/CT scans have the advantage of imaging the entire body and are useful in detecting bone metastases. The CT component of most PET/CT scans usually is a coregistry scan and may not have the high resolution of a dedicated contrast-enhanced CT scan for detecting minimal-volume metastatic disease. If CT scans are used to stage a clinical Stage III melanoma, in addition to scans of the thorax and abdomen, the pelvis should routinely be included for primary sites on the lower half of the body, whereas the neck should routinely be included for primary sites on the head and neck. Although the clinical Stage III group of patients are at high risk of developing distant metastases, the frequency of identifying occult Stage IV disease (true positives) with routine imaging at presentation is surprisingly low and no higher than the frequency of uncovering abnormalities not related to melanoma metastases (false positives).[65–68]

For stage IV patients, routine cross-sectional imaging, PET/CT in particular, can identify additional metastatic disease at stage-relevant anatomic sites, affecting the ultimate stage designation.[64,69–71] Contrast-enhanced brain magnetic resonance (MR) imaging is the most sensitive test currently available to evaluate for the presence of CNS metastasis (Stage IV M1d), and should be the imaging modality of

choice unless contraindicated (in which case, brain CT should be used), when staging patients with high-risk clinical Stage III or IV melanoma before initiating treatment.

Using Imaging Findings to Determine Stage

Staging information for melanoma in all stages is based predominantly on histologic confirmation of the presence of disease, rather than on imaging findings, with some key exceptions. The categorization of regional node disease as "clinically occult" (N1–3a) or "clinically detected" (N1–3b) may be based on the identification of lymph nodes that are abnormally large, are hypermetabolic, or have characteristic abnormalities on CT, PET/CT, or ultrasound, respectively, in patients with clinically normal regional lymph node examinations. SLN or needle biopsy usually is performed to determine the pathological staging of nodal metastases (if performed before the reexcision, it is considered part of clinical staging). Image guidance may be helpful to biopsy clinically occult regional nodes found to be suspicious on imaging; cytologic or histologic confirmation by fine-needle aspiration biopsy or core biopsy, respectively, is considered part of clinical staging.

Most patients with suspected Stage IV melanoma should undergo biopsy of at least one site of unequivocal metastatic disease (i.e., beyond the regional lymph node basin) for confirmation of metastatic disease. Once biopsy-proven metastatic disease has been established, cross-sectional imaging may be used to determine the anatomic sites involved by metastasis for the purpose of assigning M1 subcategorization. An important exception to the general recommendation for biopsy confirmation of Stage IV melanoma before treatment is patients within the new category of M1d (CNS disease); in these patients, MR or CT evidence of CNS metastasis without biopsy confirmation is acceptable to stage the patient as clinical M1d, even if the patient has no other biopsy-proven metastatic site.

One pitfall of which to be aware is the potential for "false positive" findings on imaging related to second primary malignancies (e.g., primary lung cancer in a melanoma patient may be mistaken for M1b disease) or as the result of increased metabolic activity unrelated to melanoma on PET/CT scan. Recognizing the potential for second primaries and false-positive findings, only those abnormalities on cross-sectional imaging that are considered likely to represent metastatic disease should be considered evidence of clinical Stage IV disease.

Pathological Classification

Pathological Stages I and II melanoma include patients with primary invasive cutaneous melanoma who have no evidence of regional or distant metastases, if clinically appropriate use of SLN biopsy demonstrates the absence of nodal metastases after careful pathologic examination and following wide excision of the primary melanoma. Pathological Stage III melanoma patients have pathological evidence of regional metastases, regional lymph node and/or microsatellite/satellite/in-transit metastases, and no distant metastasis.

Wide excision or reexcision of the primary site is required to assign the pT classification. If a partial biopsy of a melanoma has been performed, the maximum tumor thickness from the thicker of *either* the biopsy or definitive excision and presence of (nontraumatic) ulceration in either specimen should be recorded for pathological T categorization purposes. The quantitative classification for pathological nodal status requires careful microscopic examination of the surgically resected nodal basin and documentation of the number of lymph nodes examined, the number of nodes that contain metastases, the maximum dimension of the largest discrete metastasis, and the presence or absence of extranodal spread. Pathological Stage IV melanoma patients have clinical (cM1) and/or histologic (pM1) documentation of metastases at one or more distant sites.

Breslow Thickness

Tumor thickness is measured vertically from the top of the granular layer of the epidermis (or, if the surface overlying the entire dermal component is ulcerated, from the base of the ulcer) to the deepest invasive cell across the broad base of the tumor and should be recorded for all primary melanomas. It is discussed in greater detail under clinical staging. If there has been an incisional or partial biopsy of a melanoma (including shave or punch biopsy), only the findings of the initial biopsy are used for cT categorization purposes, whereas the maximum tumor thickness is used for pT categorization purposes.

Although not utilized for pT categorization purposes, under certain circumstances, the pathologist, after reviewing the initial partial biopsy and residual tumor in the reexcision specimen, may consider it valid and appropriate to estimate the true tumor thickness by adding the two thicknesses of tumor present in each of the biopsy specimens for clinical management purposes. For this strategy to be appropriate, the biopsy site reaction must be present above residual tumor in the reexcision specimen; the pathologist may estimate the true tumor thickness by adding the thickness of the tumor in the partial biopsy and that of the residual tumor (excluding overlying biopsy site reaction). In this instance, it should be clearly stated in the pathology report that the "estimated true tumor thickness" is based on review of both specimens and on adding the tumor thicknesses. Although this approach may more accurately reflect the true tumor thickness in certain instances and be useful for clinical purposes, it requires further validation before it is used for T pathological categorization and staging purposes.

Primary Tumor Ulceration

Ulceration represents full-thickness absence of an intact epidermis with associated host reaction above the primary

melanoma and is the second criterion for determining the T category. It is discussed in greater detail under clinical staging. If nontraumatic ulceration is present in either an initial partial biopsy or a reexcision specimen, then the tumor should be recorded as ulcerated for staging purposes.

Microsatellites

A microsatellite is a microscopic cutaneous and/or subcutaneous metastasis adjacent or deep to a primary melanoma on pathological examination of the primary tumor site (more commonly identified in wide excision specimens than punch or shave biopsy specimens). The metastatic tumor cells must be discontinuous from the primary tumor. If the tissue between the apparently separate nodule and the primary tumor is only fibrous scarring and/or inflammation, this does not indicate a microsatellite, because the aforementioned changes may represent regression of the intervening tumor. There is no minimal size threshold or distance from the primary tumor for defining microsatellites. However, because many primary melanomas do not have a circumscribed interface with adjacent tissues at their peripheral and/or deep margins, before diagnosing the presence of microsatellites, it is recommended that consideration be given to examining multiple sections cut from deeper levels within the tissue block to verify that the microsatellite is indeed discontinuous from the primary tumor. It is not uncommon for periadnexal extension of tumor that is contiguous with the main tumor to appear discontiguous on single sections. If this is not recognized, there is a risk of overdiagnosis of microsatellites. It is uncommon to identify microsatellites in partial biopsies, such as punch or shave biopsies or excision biopsies with narrow margins; they are identified more commonly in wide excision specimens.

The presence of microsatellites portends a relatively poor prognosis, comparable with that of patients with clinically detected satellite or in-transit metastases.[72] The presence of one or more microsatelites on the diagnostic biopsy in the absence of clinically involved lymph nodes or grossly visible satellite or in-transit metastases mandates a clinical Stage III designation.

Regional Lymph Node Metastasis

Clinically Occult versus Clinically Detected Regional Lymph Node Metastases

Patients without clinical or radiographic evidence of regional lymph node metastases but who have microscopically documented nodal metastases (usually detected by lymphatic mapping and SLN biopsy) are defined as having "clinically occult" (previously termed *microscopic* in the 7th Edition) disease, and represent the vast majority of patients who present with regional metastasis at diagnosis.[6,73] Patients with clinically occult metastases are designated as N1a, N2a, or N3a based on the number of tumor-involved nodes, unless microsatellites, satellites, or in-transit metastases are present. If they are, the patient is assigned N1c, N2c, or N3c according to the number of involved nodes. Patients who may undergo systemic treatment after needle biopsy of a clinically detected node or an SLN biopsy only are clinically staged as cN1 or greater. There is growing evidence that microscopic tumor burden in the sentinel node is prognostically significant.[74–86] Although this histopathologic characteristic is not proposed for the N category in the 8th Edition, documentation of sentinel node burden is an important factor that will be included in and likely guide future prognostic models and the development of clinical tools for patients with regional disease. Sentinel node tumor burden is discussed in detail in Additional Factors Recommended for Clinical Care.

In melanoma, there is no unequivocal evidence that there is a lower threshold of microscopically identifiable sentinel node tumor burden that should be used to define node-positive disease for staging purposes. A sentinel lymph node in which any metastatic tumor cells are identified, irrespective of how few the cells are or whether they are identified on hematoxylin and eosin (H&E) or immunostained sections, should be designated as a tumor-positive lymph node. This is unchanged from the 7th Edition. If melanoma cells are found within a lymphatic channel within or adjacent to a lymph node, that node is regarded as tumor-involved for staging purposes.

To determine the number of nodes involved for pathological staging, the number of tumor-positive sentinel nodes should be added to the number of tumor-positive nonsentinel nodes, if any, identified after completion lymph node dissection. Not all patients with a positive SLN biopsy undergo completion lymph node dissection. If a patient undergoes SLN biopsy that is positive for metastasis, the designation of pN1(sn) is appropriate and may be used. In the context of patients who undergo completion lymphadenectomy after SLN biopsy, the pN1 classification denotes that a completion lymph node dissection has been performed and the (sn) description is not used.

Patients who present with clinical evidence of regional disease are assigned as N1b, N2b, or N3b based on the number of nodes involved. If at least one node was clinically evident and there are additional involved nodes detected only on microscopic examination, the total number of involved nodes (e.g., both those clinically apparent and those detected only on microscopic examination of a complete lymphadenectomy specimen) should be recorded for N categorization. As noted for patients with clinically occult disease, those with clinically evident disease who also have microsatellites, satellites, or in-transit metastases at diagnosis are assigned as N1c, N2c, or N3c, based on the number of nodes involved by metastasis.

Patients with clinically occult regional disease have been shown to have better survival than patients with clinically

evident disease.[87–89] Overall, there is marked heterogeneity in prognosis among patients with Stage III regional node disease by N-category designation or by T category among patients with N+ disease. Although N category alone predicts outcome, more accurate prognostic estimation is obtained by also incorporating features of the primary tumor.

Melanoma metastases must be distinguished from benign nevus cells within lymph nodes (sometimes termed benign nevus rest cells), as the latter are not melanoma metastases and should not be assigned as such.

Extranodal Extension

The presence of extranodal extension (ENE) of tumor (also termed extranodal spread or extracapsular extension) is defined as the presence of a nodal metastasis extending through the lymph node capsule into adjacent tissues. It is characterized pathologically by the presence of tumor extending beyond the lymph node capsule, usually into peri-nodal adipose tissue. ENE usually occurs in association with large clinically detected nodal metastases that show gross effacement of normal nodal architecture, sometimes forming a mass of clinically matted nodes. Occasionally, ENE may be identified in association with smaller metastases, including those detected by SLN biopsy. ENE of tumor is an adverse prognostic parameter in melanoma patients. Matted nodes are two or more nodes that adhere to one another, identified at the time the specimen is examined macroscopically in the pathology laboratory, and their presence should be detailed in the macroscopic section of the pathology report.

Non-nodal Locoregional Metastases: Microsatellite, Satellite, and In-transit Metastases

The presence or absence of microsatellite, satellite, or in-transit metastases, regardless of the number of such lesions, represents an N-category criterion. They are thought to represent metastases that have occurred as a consequence of intra-lymphatic or possibly angiotropic tumor spread.[34,35] Satellite metastases are defined as grossly visible cutaneous and/or subcutaneous metastases occurring within 2 cm of the primary melanoma. Microsatellites are microscopic cutaneous and/or subcutaneous metastases found adjacent or deep to a primary melanoma on pathological examination (see detailed discussion in the pathological staging section). The metastatic tumor cells must be discontinuous from the primary tumor (but not separated only by fibrosis or inflammation, because this could signify regression of the intervening tumor). In-transit metastases are defined as clinically evident dermal and/or subcutaneous metastases identified at a distance >2 cm from the primary melanoma in the region between the primary and the first echelon of regional lymph nodes. The clinically or microscopically detected presence of satellite or in-transit metastases portends a relatively poor prognosis.[90–95] There was no substantial difference in survival outcome for these anatomically defined entities in the 8th Edition AJCC international melanoma database of contemporary patients, hence they are grouped together for staging purposes. Patients with microsatellite, satellite, and/or in-transit metastases are categorized as N1c, N2c, or N3c disease according to the number of positive regional lymph nodes (irrespective of whether they were clinically occult or clinically detected). N1c designates patients with microsatellite, satellite, and/or in-transit metastases but with no tumor-involved regional lymph nodes; N2c designates those with one involved node; and N3c designates those with two or more involved nodes.

Distant Metastatic Disease

In patients with distant metastases, the site(s) of metastases are used to delineate the M categories into four subcategories: M1a, M1b, M1c, and new to the 8th Edition, M1d.

Anatomic Site(s) of Distant Metastatic Disease

Patients with distant metastasis to the skin, subcutaneous tissue, muscle or distant lymph nodes are categorized as M1a; they have a relatively better prognosis compared with patients with distant metastases located in any other anatomic site.[86,96–100] Patients with metastasis to the lung (with or without concurrent metastasis in the skin, subcutaneous tissue, or distant lymph nodes) are categorized as M1b and have an "intermediate" prognosis when comparing survival. Patients with metastases to any other visceral sites (but without metastasis to the central nervous system) have a relatively worse prognosis and are designated as M1c. Patients with metastases to the CNS are designated as M1d (irrespective of the presence of metastases in other sites) and have the worst prognosis of any of the M categories.

In the 8th Edition of the AJCC melanoma staging system, serum level of LDH (discussed in detail in Additional Factors Recommended for Clinical Care) remains an important M-categorization factor. LDH level, however, no longer defines M1c disease. Instead, each of the M subcategories is modified based on whether LDH is elevated. Suffix descriptors have been added to all M1 subcategory designations that provide LDH values (designated as "0" for "not elevated" and "1" for "elevated" level) for all anatomic sites of distant disease. For example, skin/soft tissue/distant nodal metastasis with elevated LDH is now designated M1a(1), not M1c.

PROGNOSTIC FACTORS

Prognostic Factors Required for Stage Grouping

Beyond the factors used to assign T, N, or M categories, no additional prognostic factors are required for stage grouping.

47

Additional Factors Recommended for Clinical Care

Serum LDH

Although it generally is uncommon in staging classifications to include serum factors, an exception was made for elevated levels of serum LDH in the M category of the 7th Edition of the AJCC staging system for melanoma, as it was an independent predictor of survival outcome among patients presenting with or developing Stage IV disease.[6,27,101–104] Despite the introduction and widespread use of effective systemic drug therapies that prolong survival in advanced-stage melanoma, serum LDH remains one of the most influential factors associated with response, progression-free survival, and overall survival for patients receiving these drug therapies. In a pooled analysis of patients with BRAF-mutant advanced melanoma who received combination BRAF and MEK inhibition in randomized studies, baseline serum LDH was the most influential factor impacting progression-free survival and overall survival in hierarchical analysis; the 2-year overall survival for patients with a normal baseline LDH versus those with an elevated LDH was 67% versus 25%, respectively.[30,31] In clinical trials of immunotherapy, baseline serum LDH was independently associated with overall survival for ipilimumab[28] and anti–PD-1 therapy.[105] Similarly, patients with an elevated LDH were less likely to respond to anti–PD-1 therapy,[105] and no patients with an LDH greater than twice the upper limit of normal responded to ipilimumab.[29] AJCC Level of Evidence: I

Primary Tumor Mitotic Rate

Mitosis is a process in which a single cell divides into two daughter cells. During this process, the chromosomes in a cell nucleus are separated into two identical sets of chromosomes, and this separation can be recognized on pathological examination by light microscopy.

Mitotic rate is no longer used as a T-category criterion in the 8th Edition of the AJCC staging system, although it remains a major determinant of prognosis across its dynamic range in tumors of all thickness categories, and mitotic rate should be assessed and recorded in all primary invasive melanomas.[106] Mitotic rate was removed as a staging criterion for T1 tumors in the 8th Edition of the AJCC staging system because substratifying T1 tumors using a 0.8 mm cut point showed stronger associations with outcome than those obtained utilizing the presence or absence of mitoses (as in the 7th Edition). Mitotic rate likely will be an important parameter for the future development of prognostic models that will provide personalized prediction of prognosis for individual patients.

The recommended approach to enumerating mitoses is to first find the region in the dermis containing the most mitoses, the so-called hot spot or dermal hot spot. After counting the mitoses in the initial high-power field, the count is extended to immediately adjacent nonoverlapping fields until an area of tissue corresponding to 1 mm^2 is assessed. If no hot spot is found and mitoses are sparse and/or randomly scattered throughout the lesion, then a representative mitosis is chosen and, beginning with that field, the count is then extended to immediately adjacent nonoverlapping fields until an area corresponding to 1 mm^2 of tissue is assessed. The count then is expressed as the (whole) number of mitoses/mm^2. If the invasive component of the tumor involves an area <1 mm^2, the number of mitoses should be assessed and recorded as if they were found within a square millimeter. For example, if the entire dermal component of a tumor occupies 0.5 mm^2 and only one mitosis is identified, the mitotic rate should be recorded as 1/mm^2 (not 2/mm^2). The number of mitoses should be listed as a whole number per square millimeter. If no mitoses are identified, the mitotic rate may be recorded as "none identified" or "0/mm^2." This methodology for determining the mitotic rate of a melanoma has been shown to have excellent interobserver reproducibility, including among pathologists with widely differing experience in the assessment of melanocytic tumors.[107]

To obtain accurate measurement, calibration of individual microscopes is recommended by using a stage micrometer to determine the number of high-power fields that equates to a square millimeter.

The data that demonstrated the strong prognostic significance of mitotic rate were obtained from the melanoma pathology reports of routinely assessed H&E-stained sections.[106,108,109] It therefore is recommended that no additional sections be cut and examined in excess of those that would normally be used to report and diagnose the melanoma to determine the mitotic count (i.e., no additional sections should be cut and examined for the sole purpose of determining the mitotic rate, including in situations in which no mitoses are identified on the initial, routinely examined sections). Immunohistochemical stains for identifying mitoses are not used for determining mitotic rate for staging and/or reporting purposes. AJCC Level of Evidence: I

Level of Invasion

The level of invasion, as defined by Dr. Wallace Clark,[110] has been used for more than 40 years for various melanoma staging systems. Clark levels are defined as follows:

Level I: *Melanoma cells confined to the epidermis (melanoma in situ).*

Level II: *Melanoma cells invade into but do not fill or expand the papillary (superficial) dermis.*

Level III: *Melanoma cells fill and expand the papillary dermis with extension of tumor to the papillary–reticular dermal interface.* The boundary between the papillary and reticular dermis may be hard to identify, particularly if there is severe solar elastosis or in some sites such as the scalp, acral skin, and mucosal or anogenital regions. The papillary dermal collagen fibers are fine and oriented vertically, whereas the reticular dermal collagen bundles are coarse and have a more horizontal orientation; recognition of their

distinction may be enhanced by polarization microscopy, because dermal collagen is birefringent. Another useful landmark in separating the papillary and reticular dermis is the presence of a capillary plexus at the interface. Polypoid tumors that expand but do not fill the papillary dermis should be classified as level III.

Level IV: *Melanoma cells infiltrate into the reticular dermis.*

Level V: *Melanoma cells infiltrate into the subcutaneous fat.* Melanoma involving periadnexal adipose tissue (which represents extension of and is continuous with the subcutis) should not be interpreted as level V invasion.

Although Clark level of invasion has prognostic significance in univariate analysis, numerous publications have shown that the level of invasion is less reproducible among pathologists and does not reflect prognosis as accurately as tumor thickness.[37,39,88,90,111,112] Level of invasion was used in the AJCC Cancer Staging Manual, 6th Edition to define the specific subcategory of thin (T1) melanomas.[87,113–119] However, although level of invasion was an independent prognostic factor in more contemporary analyses, it had the lowest statistical correlation with survival compared with the other six independent prognostic variables in the 7th Edition melanoma staging database analysis.[6,27] Clark level is not used in the 8th Edition staging system but should be recorded as a primary tumor characteristic. AJCC Level of Evidence: I

Tumor-infiltrating Lymphocytes

Tumor-infiltrating lymphocytes (TILs) are defined as lymphocytes that infiltrate and disrupt tumor nests and/or directly oppose tumor cells. The degree of infiltration may be described by both the extent and the intensity of the TIL infiltrate.

The most commonly applied grading scheme for quantitating the presence of TILs is as follows:

1. Absent TIL infiltrate: no lymphocytes present or, if present, they do not interact with tumor cells. For example, a cuff of lymphocytes around the periphery of the tumor with no infiltration is considered absent. Furthermore, lymphocytes within the tumor nodule but in perivenular array or in fibrous bands in the tumor substance, without infiltration of the tumor itself, are considered absent.
2. Nonbrisk TIL infiltrate: focal areas of lymphocytic infiltration in the tumor. They may be isolated, multifocal, or segmental.
3. Brisk TIL infiltrate: TIL infiltration of the entire base of the tumor or diffuse permeation of the tumor.

Other systems for grading TIL infiltrates based on their density and distribution have been proposed, but these have not been independently validated.

Multiple studies demonstrated that TIL infiltration in primary cutaneous melanoma is a favorable prognostic factor.[120,121] Furthermore, TIL infiltrates in primary melanoma have been associated with less frequent SLN metastasis.[120,122–124] The presence and extent of TIL infiltrate using the grading scheme described should be recorded as a primary tumor characteristic. AJCC Level of Evidence: III

Lymphovascular Invasion

Lymphovascular invasion refers to the presence of melanoma cells within the lumina of blood vessels (termed vascular invasion) or lymphatics (termed lymphatic invasion), or both. Immunohistochemistry for vascular endothelial cell markers CD31 and CD34, nuclear transcription factor ERG, or the lymphatic marker D2-40 may assist in the identification of intravascular or intralymphatic tumor by highlighting vessel lumina. Lymphovascular invasion is recorded as present or absent. Lymphatic invasion and vascular invasion generally are regarded as adverse prognostic factors,[125–129] although this is not borne out in all studies. The identification of lymphatic invasion is enhanced in primary melanomas when double labeling of tumor cells and lymphatic endothelium is applied.[130] The presence or absence of lymphovascular invasion should be recorded as a primary tumor characteristic. AJCC Level of Evidence: III

Neurotropism

Neurotropism is defined as the presence of melanoma cells abutting nerve sheaths, usually circumferentially (perineural invasion) or within nerves (intraneural invasion). Occasionally, the tumor itself may form neuroid structures (termed neural transformation), and this also is regarded as neurotropism. Neurotropism is best identified at the periphery of the tumor; the presence of melanoma cells around nerves in the main tumor mass caused by entrapment of nerves in the expanding tumor does not represent neurotropism.

Neurotropism is recorded as present or absent.

Infiltration along nerve sheaths (or occasionally within the endoneurium) may be associated with an increased local recurrence rate (local persistence). Neurotropism is common in desmoplastic melanoma but may occur in other forms of melanoma. The presence of neurotropism has been associated with an increased risk of local recurrence and, in some cases, may be treated by wider excision margins and/or adjuvant radiotherapy. The presence or absence of neurotropism should be recorded as a primary tumor characteristic. AJCC Level of Evidence: III

Melanoma Tumor Burden and Location In Sentinel Nodes

Recent studies demonstrated that sentinel node tumor burden (defined by the extent and/or location of tumor deposits within sentinel nodes) correlates with the presence of positive nonsentinel nodes in completion lymphadenectomy specimens and predicts survival.[74–86] If there are only a few tumor cells in the subcapsular sinus of the sentinel node, the prognosis is very good and the chance of finding additional

metastatic disease in a completion lymphadenectomy specimen is extremely small. If, on the other hand, there are larger foci of tumor that involve the parenchyma of the sentinel node, the prognosis is much worse, and the chance of finding metastases in nonsentinel nodes in a completion lymphadenectomy specimen is increased.

Micromorphometric parameters of sentinel node metastases that have been assessed for quantitation of sentinel node tumor burden include the single largest maximum dimension of any discrete deposit, the maximum dimension of the largest metastasis, the maximum subcapsular depth of tumor extension (also termed tumor penetrative depth of the deposits and measured from the inner surface of the lymph node capsule to the deepest intranodal tumor cell), the microanatomic location of sentinel node tumor deposits (peripheral sinus vs. intraparenchymal location), the total percentage cross-sectional area of the sentinel node involved, the square area of the largest metastasis, and the presence of extranodal extension. However, assessing, classifying, and measuring these parameters may be difficult because the precise limits of tumor deposits for measurements may be difficult to discern and tumor deposits often are irregularly shaped. Furthermore, tumor size/burden depends to some degree on sectioning protocols, as more extensive sectioning may reveal additional tumor deposits or demonstrate a greater dimension of deposit(s) in the additional sections. Although methods used vary widely in reported studies (as does the likely level of precision of measurement), overall they demonstrate that increasing sentinel node tumor burden correlates with increased risk of nonsentinel node positivity and worse clinical outcome.[74–86] This suggests that sentinel node tumor burden is an important predictor of nonsentinel node positivity and patient outcome; the predictive value holds irrespective of whether it has been assessed with crude and imprecise methods or by using more sophisticated and precise means. The microanatomic location of tumor in tumor-involved sentinel nodes also is associated with nonsentinel node positivity and clinical outcome; metastases in a subcapsular location are less often associated with nonsentinel node involvement than those in nonsubcapsular locations. However, assessment of the precise localization of deposits may be difficult because the architecture of the sentinel node often is not clearly apparent in histopathologic sections.

In a multi-institution study evaluating interobserver reproducibility of the assessment of micromorphometric parameters of melanoma deposits in sentinel nodes, it was shown that assessment of quantifiable variables (maximum deposit size, maximum subcapsular depth and the percent of cross-sectional area of sentinel node involved by tumor) were highly reproducible.[131] However, assessment of microanatomic location and ENE was less reproducible.

Based on currently available evidence, the Melanoma Expert Panel recommends that as a minimum, the single largest maximum dimension (measured in millimeters to the nearest 0.1 mm using an ocular micrometer) of the largest discrete metastatic melanoma deposit in sentinel nodes be recorded in pathology reports. To be considered a discrete deposit, the tumor cells must be in direct continuity with adjacent tumor cells. In some instances, multiple small tumor aggregates may be dispersed within a lymph node and separated by lymphoid cells. In this circumstance, the size of the largest discrete single deposit (not the nodal area over which the multiple deposits are contained) should be recorded. The measurement may be made either on H&E-stained sections or on sections stained immunohistochemically for melanoma-specific markers. More detailed consideration of the topic of sentinel node tumor burden measurement will be forthcoming in a planned publication from the International Melanoma Pathology Study Group. Sentinel node tumor burden should be recorded in the pathology report. It will also will be incorporated into the development of future prognostic models and clinical tools. AJCC Level of Evidence: II

Extranodal Extension

The presence or absence of ENE of tumor should be documented. This parameter is discussed in detail in the pathological classification section. AJCC Level of Evidence: III

Number of Distant Metastases

The number of metastases at distant sites previously was documented as an important prognostic factor.[96,97,99,100] This also was confirmed by preliminary multivariate analyses using the 7th Edition AJCC Stage IV melanoma staging database. However, this feature was not incorporated into the staging system because of the significant variability in the deployment of diagnostic tests to comprehensively search for distant metastases. These may range from a chest X-ray in some centers to high-resolution double-contrast CT, PET/CT, and MR imaging in others. Until the indications and types of tests used are better standardized, the number of metastases cannot reliably or reproducibly be used for staging purposes. AJCC Level of Evidence: III

RISK ASSESSMENT MODELS

The AJCC recently established guidelines that will be used to evaluate published statistical prediction models for the purpose of granting endorsement for clinical use.[132] Although this is a monumental step toward the goal of precision medicine, this work was published only very recently. Four models[133–136] were evaluated for this cancer site by the Precision Medicine Core, but thus far, no existing melanoma model appears to have met the agreed-upon criteria.[137] A full list of the evaluated models and their adherence to the quality criteria is available on www.cancerstaging.org.

In the future, the statistical prediction models for this cancer site will be reevaluated, and those that meet all criteria will be endorsed.

DEFINITIONS OF TNM

Definition of Primary Tumor (T)

T Category	Thickness	Ulceration status
TX: primary tumor thickness cannot be assessed (e.g., diagnosis by curettage)	Not applicable	Not applicable
T0: no evidence of primary tumor (e.g., unknown primary or completely regressed melanoma)	Not applicable	Not applicable
Tis (melanoma *in situ*)	Not applicable	Not applicable
T1	≤1.0 mm	Unknown or unspecified
T1a	<0.8 mm	Without ulceration
T1b	<0.8 mm<br>0.8–1.0 mm	With ulceration<br>With or without ulceration
T2	>1.0–2.0 mm	Unknown or unspecified
T2a	>1.0–2.0 mm	Without ulceration
T2b	>1.0–2.0 mm	With ulceration
T3	>2.0-4.0 mm	Unknown or unspecified
T3a	>2.0–4.0 mm	Without ulceration
T3b	>2.0–4.0 mm	With ulceration
T4	>4.0 mm	Unknown or unspecified
T4a	>4.0 mm	Without ulceration
T4b	>4.0 mm	With ulceration

Definition of Regional Lymph Node (N)

N Category	Extent of regional lymph node and/or lymphatic metastasis: Number of tumor-involved regional lymph node	Presence of in-transit, satellite, and/or microsatellite metastases
NX	Regional nodes not assessed (e.g., SLN biopsy not performed, regional nodes previously removed for another reason)<br>**Exception**: pathological N category is not required for T1 melanomas, use cN.	No
N0	No regional metastases detected	No
N1	One tumor-involved node or in-transit, satellite, and/or microsatellite metastases with no tumor-involved nodes	
N1a	One clinically occult (i.e., detected by SLN biopsy)	No
N1b	One clinically detected	No
N1c	No regional lymph node disease	Yes
N2	Two or three tumor-involved nodes or in-transit, satellite, and/or microsatellite metastases with one tumor-involved node	
N2a	Two or three clinically occult (i.e., detected by SLN biopsy)	No
N2b	Two or three, at least one of which was clinically detected	No
N2c	One clinically occult or clinically detected	Yes
N3	Four or more tumor-involved nodes or in-transit, satellite, and/or microsatellite metastases with two or more tumor-involved nodes, or any number of matted nodes without or with in-transit, satellite, and/or microsatellite metastases	
N3a	Four or more clinically occult (i.e., detected by SLN biopsy)	No
N3b	Four or more, at least one of which was clinically detected, or presence of any number of matted nodes	No
N3c	Two or more clinically occult or clinically detected and/or presence of any number of matted nodes	Yes

Definition of Distant Metastasis (M)

M Category	M Criteria: Anatomic site	LDH level
M0	No evidence of distant metastasis	Not applicable
M1	Evidence of distant metastasis	See below
M1a	Distant metastasis to skin, soft tissue including muscle, and/or nonregional lymph node	Not recorded or unspecified
M1a(0)		Not elevated
M1a(1)		Elevated
M1b	Distant metastasis to lung with or without M1a sites of disease	Not recorded or unspecified
M1b(0)		Not elevated
M1b(1)		Elevated
M1c	Distant metastasis to non-CNS visceral sites with or without M1a or M1b sites of disease	Not recorded or unspecified
M1c(0)		Not elevated
M1c(1)		Elevated
M1d	Distant metastasis to CNS with or without M1a, M1b, or M1c sites of disease	Not recorded or unspecified
M1d(0)		Normal
M1d(1)		Elevated

Suffixes for M category: (0) LDH not elevated, (1) LDH elevated. No suffix is used if LDH is not recorded or is unspecified.

AJCC PROGNOSTIC STAGE GROUPS

Clinical (cTNM)

Clinical staging includes microstaging of the primary melanoma and clinical/radiologic/biopsy evaluation for metastases. By convention, clinical staging should be used after biopsy of the primary melanoma, with clinical assessment for regional and distant metastases. Note that pathological assessment of the primary melanoma is used for both clinical and pathological classification. Diagnostic biopsies to evaluate possible regional and/or distant metastasis also are included. Note there is only one stage group for clinical Stage III melanoma.

When T is...	And N is...	And M is...	Then the clinical stage group is...
Tis	N0	M0	0
T1a	N0	M0	IA
T1b	N0	M0	IB
T2a	N0	M0	IB
T2b	N0	M0	IIA
T3a	N0	M0	IIA
T3b	N0	M0	IIB
T4a	N0	M0	IIB
T4b	N0	M0	IIC
Any T, Tis	≥N1	M0	III
Any T	Any N	M1	IV

Pathological (pTNM)

Pathological staging includes microstaging of the primary melanoma, including any additional staging information from the wide-excision (surgical) specimen that constitutes primary tumor surgical treatment and pathological information about the regional lymph nodes after SLN biopsy or therapeutic lymph node dissection for clinically evident regional lymph node disease.

When T is...	And N is...	And M is...	Then the pathological stage group is...
Tis	N0	M0	0
T1a	N0	M0	IA
T1b	N0	M0	IA
T2a	N0	M0	IB
T2b	N0	M0	IIA
T3a	N0	M0	IIA
T3b	N0	M0	IIB
T4a	N0	M0	IIB
T4b	N0	M0	IIC
T0	N1b, N1c	M0	IIIB
T0	N2b, N2c, N3b or N3c	M0	IIIC
T1a/b–T2a	N1a or N2a	M0	IIIA
T1a/b–T2a	N1b/c or N2b	M0	IIIB
T2b/T3a	N1a–N2b	M0	IIIB
T1a–T3a	N2c or N3a/b/c	M0	IIIC
T3b/T4a	Any N ≥N1	M0	IIIC
T4b	N1a–N2c	M0	IIIC
T4b	N3a/b/c	M0	IIID
Any T, Tis	Any N	M1	IV

Pathological Stage 0 (melanoma *in situ*) and T1 do not require pathological evaluation of lymph nodes to complete pathological staging; use cN information to assign their pathological stage.

r/yc/yp Classification

For additional information on recurrent/retreatment (r) and/or posttherapy/post neoadjuvant therapy (yc/yp) staging, please refer to Chapter 1.

REGISTRY DATA COLLECTION VARIABLES

1. Breslow tumor thickness (xx.x mm)
2. Primary tumor ulceration (yes/no)
3. Mitotic rate (whole number per square millimeter [mm^2])
4. Microsatellites (pathologically detected satellites, not clinically apparent) (yes/no)
5. Tumor-infiltrating lymphocytes (absent, nonbrisk, or brisk)
6. Clark level of invasion (I–V)
7. Regression (yes/no)
8. Neurotropism (present or absent)
9. Lymphovascular invasion (present or absent)
10. In-transit and/or satellite metastasis (in-transit, satellite, both)
11. Regional lymph node clinically or radiologically detected (yes/no)
12. Microscopic confirmation of tumor metastasis in any regional lymph node that was clinically or radiologically detected (yes/no)
13. SLN biopsy performed (yes/no)
14. Number of nodes examined from sentinel node procedure (whole number)
15. Number of tumor-involved nodes from sentinel node procedure (whole number)
16. Sentinel node tumor burden (largest dimension of largest discrete deposit in xx.x mm)
17. ENE in any tumor-involved regional lymph node (sentinel or clinically detected) (present or absent)
18. Completion or therapeutic lymph node dissection performed (yes/no)

19. Number of lymph nodes examined from completion or therapeutic lymph node dissection (whole number)
20. Number of lymph nodes involved with tumor from completion or therapeutic lymph node dissection (whole number)
21. Matted nodes (yes/no)
22. Distant metastasis to skin, soft tissue, or distant nodes (yes/no)
23. Distant metastasis to lung (yes/no)
24. Distant metastasis to non-CNS viscera (yes/no)
25. Distant metastasis to CNS (yes/no)
26. Serum LDH level (xx,xxx U/L) and serum LDH level upper limit of normal from laboratory reference range (Note - serum LDH recorded for Stage IV only)

HISTOLOGIC GRADE (G)

Histologic grading is not used in the staging of melanoma.

HISTOPATHOLOGIC TYPE

The major melanoma subtypes defined by the World Health Organization (WHO)[138] are superficial spreading, nodular, lentigo maligna, acral lentiginous, and desmoplastic. This classification correlates with the epidemiologic characteristics of the patient populations and with the genomic status of the tumors. The data used to derive the TNM categories were based largely on melanomas of superficial spreading and nodular subtypes. There is evidence that melanomas of other subtypes, especially desmoplastic melanomas, but perhaps also lentigo maligna and acral lentiginous melanomas, have a different etiology and/or pathogenesis and natural history.[139–142] At present, the same staging criteria should be used for melanomas with any growth pattern.

Desmoplastic melanoma is a rare subtype of melanoma characterized by malignant spindle cells separated by prominent fibrocollagenous or fibromyxoid stroma. Primary melanomas may be entirely or almost entirely (>90% of dermal invasive tumor) desmoplastic ("pure" desmoplastic melanoma) or exhibit a desmoplastic component admixed with a nondesmoplastic component ("mixed" desmoplastic melanoma: 10–90% desmoplastic).[143] Improved disease-specific survival is observed in patients with pure desmoplastic melanoma, compared with patients with mixed desmoplastic melanoma and those with melanomas lacking a desmoplastic component.[144–146] Furthermore, regional nodal metastasis (including metastasis detected by SLN biopsy) is less common in patients presenting with clinically localized pure desmoplastic melanoma compared with those with mixed desmoplastic melanomas or conventional (nondesmoplastic) melanomas.[147–150] AJCC Level of Evidence: III

SURVIVAL DATA

Analysis of a completely new international melanoma database created at The University of Texas MD Anderson Cancer Center that builds upon myriad collaborative efforts of legacy AJCC Melanoma Staging Committees (renamed the Melanoma Expert Panel by the AJCC for the 8th Edition) and an expanding network of national and international academic melanoma clinician investigators representing institutions, cooperative groups, and tumor registries that was used to inform revisions to the AJCC melanoma staging system can be found in planned companion manuscripts.

ILLUSTRATIONS

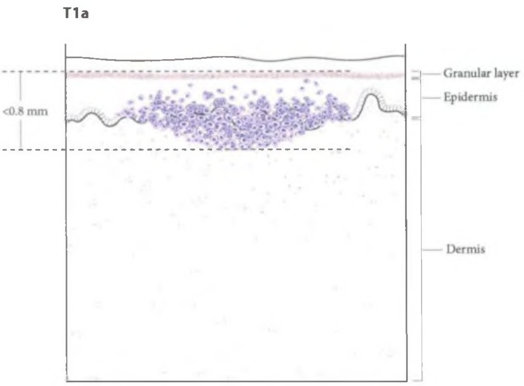

Fig. 47.1 T1a melanoma. T1a is defined as invasive melanoma <0.8 mm in thickness without ulceration. Tumor thickness is measured from the top of the granular layer of the epidermis to the deepest invasive cell across the broad base of the tumor

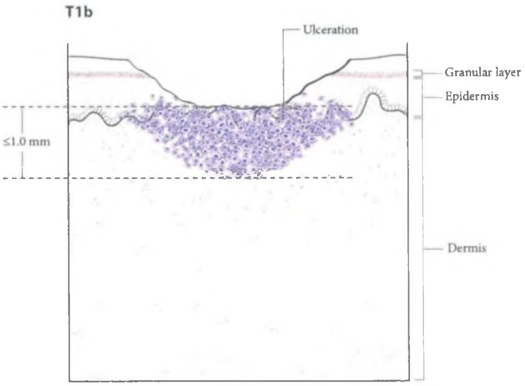

Fig. 47.2 T1b melanoma. T1b is defined as melanoma 0.8 to 1 mm in thickness regardless of ulceration status OR ulcerated melanoma <0.8 mm in thickness. Tumor thickness is measured from the top of the granular layer of the epidermis (or, if the surface overlying the entire dermal component is ulcerated, from the base of the ulcer) to the deepest invasive cell across the broad base of the tumor

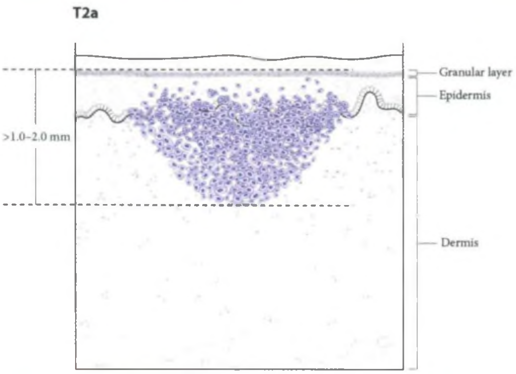

Fig. 47.3 T2a melanoma. T2a is defined as invasive melanoma >1.0 to 2.0 mm in thickness without ulceration. Tumor thickness is measured from the top of the granular layer of the epidermis to the deepest invasive cell across the broad base of the tumor

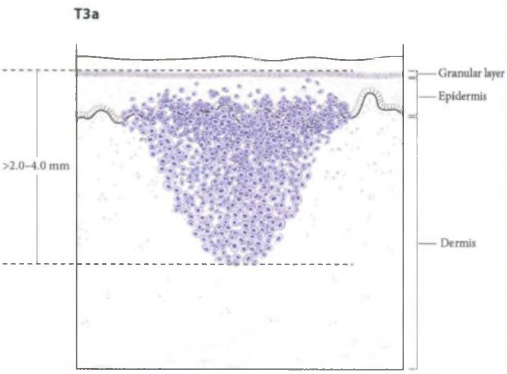

Fig. 47.5 T3a melanoma. T3a is defined as invasive melanoma >2.0 to 4.0 mm in thickness without ulceration. Tumor thickness is measured from the top of the granular layer of the epidermis to the deepest invasive cell across the broad base of the tumor

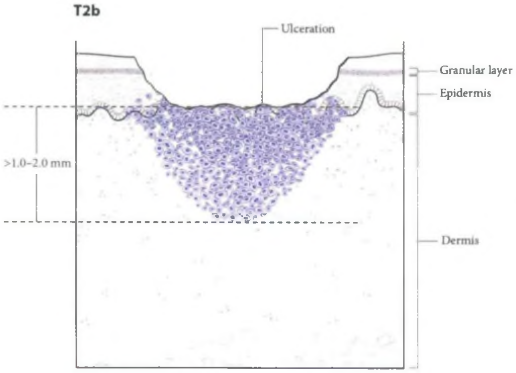

Fig. 47.4 T2b melanoma. T2b is defined as ulcerated melanoma >1.0 to 2.0 mm in thickness. Tumor thickness is measured from the base of the ulcer to the deepest invasive cell across the broad base of the tumor

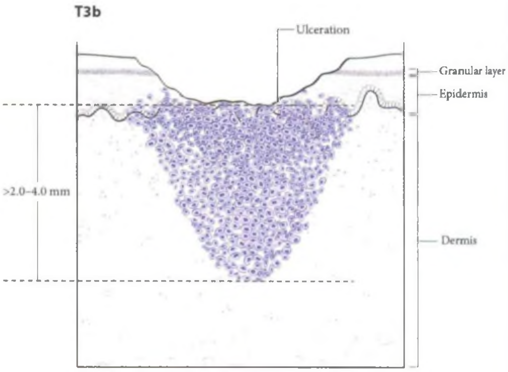

Fig. 47.6 T3b melanoma. T3b is defined as ulcerated melanoma >2.0 to 4.0 mm in thickness. Tumor thickness is measured from the base of the ulcer to the deepest invasive cell across the broad base of the tumor

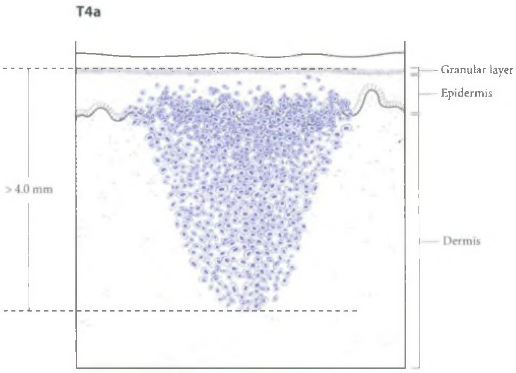

Fig. 47.7 T4a melanoma. T4a is defined as invasive melanoma >4.0 mm in thickness without ulceration. Tumor thickness is measured from the top of the granular layer of the epidermis to the deepest invasive cell across the broad base of the tumor

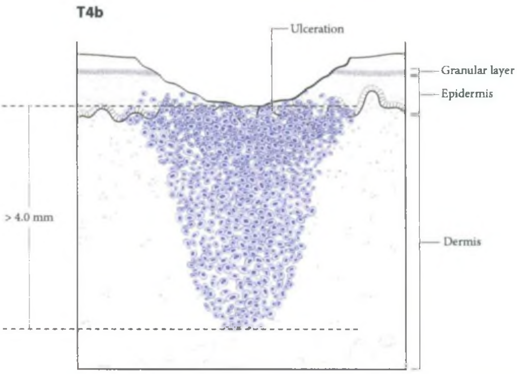

Fig. 47.8 T4b melanoma. T4b is defined as ulcerated melanoma >4.0 mm in thickness. Tumor thickness is measured from the base of the ulcer to the deepest invasive cell across the broad base of the tumor

ACKNOWLEDGMENTS

The AJCC 8th Edition Melanoma Expert Panel acknowledges the following institutions and associated individuals for their data contributions to the 8th Edition international melanoma database. Institutions are listed in alphabetical order as follows:

Fondazione IRCCS Istituto Nazionale dei Tumori, Milan, Italy (Mario Santinami MD and Andrea Maurichi MD)

Instituto Valenciano de Oncologia, Valencia, Spain (Eduardo Nagore MD)

John Wayne Cancer Institute, Santa Monica, CA (Mark Faries MD)

Melanoma Institute Australia, The University of Sydney, Sydney, Australia (John F. Thompson MD; Richard A. Scolyer MD; Serigne Lo PhD; Jonathan R. Stretch MBBS, DPhil(Oxon); Robyn R. P. Saw MB MS; and Andrew J. Spillane MD)

Melbourne Melanoma Project—Peter MacCallum Cancer Centre, Melbourne, Australia (Grant McArthur MBBS, PhD; David Gyorki MD; Michael Henderson MD; and Sonja Mailer BBc); Alfred Hospital Melbourne (John Kelly MBBS); and Austin Hospital Melbourne (Johnathon Cebon MBBS, PhD)

National and Kapodistrian University of Athens School of Medicine, Andreas Sygros Hospital, Athens, Greece (Alexander Stratigos MD)

National and Kapodistrian University of Athens School of Medicine, General Hospital of Athens, Laiko, Athens, Greece (Helen Gogas MD)

University of Texas MD Anderson Cancer Center, Houston, TX (Jeffrey E. Gershenwald MD; Lauren E. Haydu MIPH; and Julie M. Gardner MHA)

Veneto Institute of Oncology–IOV, Padova, Italy (Carlo Riccardo Rossi MD and Antonio Sommariva MD)

Winship Cancer Institute of Emory University, Atlanta, GA (Keith Delman MD)

The AJCC also acknowledges the Southwest Oncology Group (SWOG) for their contributions to the 8th Edition melanoma effort (Antoni Ribas MD, PhD; Lawrence Flaherty, MD; and James Moon, MS)

Bibliography

1. Siegel RL, Miller KD, Jemal A. Cancer statistics, 2016. *CA Cancer J Clin*. 2016;66:7–30.
2. Whiteman DC, Green AC, Olsen CM. The growing burden of invasive melanoma: projections of incidence rates and numbers of new cases in six susceptible populations through 2031. *J Invest Dermatol*. 2016;136:1161–1171.
3. Cancer Genome Atlas Network. Genomic classification of cutaneous melanoma. *Cell*. 2015;161:1681–1696.
4. Cust AE, Armstrong BK, Goumas C, et al. Sunbed use during adolescence and early adulthood is associated with increased risk of early-onset melanoma. *Int J Cancer*. 2011;128:2425–2435.
5. Ernst A, Grimm A, Lim HW. Tanning lamps: health effects and reclassification by the Food and Drug Administration. *JAMA Dermatol*. 2015;72:175–180.
6. Balch CM, Gershenwald JE, Soong SJ, et al. Final version of 2009 AJCC melanoma staging and classification. *J Clin Oncol*. 2009;27:6199–6206.
7. Gimotty PA, Elder DE, Fraker DL, et al. Identification of high-risk patients among those diagnosed with thin cutaneous melanomas. *J Clin Oncol*. 2007;25:1129–1134.
8. Gimotty PA, Guerry D, Ming ME, et al. Thin primary cutaneous malignant melanoma: a prognostic tree for 10-year metastasis is more accurate than American Joint Committee on Cancer staging. *J Clin Oncol*. 2004;22:3668–3676.

9. Green AC, Baade P, Coory M, Aitken JF, Smithers M. Population-based 20-year survival among people diagnosed with thin melanomas in Queensland, Australia. *J Clin Oncol.* 2012;30:1462–1467.
10. Hodi FS, O'Day SJ, McDermott DF, et al. Improved survival with ipilimumab in patients with metastatic melanoma. *N Engl J Med.* 2010;363:711–723.
11. Schadendorf D, Hodi FS, Robert C, et al. Pooled analysis of long-term survival data from phase II and phase III trials of ipilimumab in unresectable or metastatic melanoma. *J Clin Oncol.* 2015;33: 1889–1894.
12. Larkin J, Chiarion-Sileni V, Gonzalez R, et al. Combined nivolumab and ipilimumab or monotherapy in untreated melanoma. *N Engl J Med.* 2015;373:23–34.
13. Robert C, Long GV, Brady B, et al. Nivolumab in previously untreated melanoma without BRAF mutation. *N Engl J Med.* 2015; 372:320–330.
14. Robert C, Schachter J, Long GV, et al. Pembrolizumab versus ipilimumab in advanced melanoma. *N Engl J Med.* 2015;372: 2521–2532.
15. Chapman PB, Hauschild A, Robert C, et al. Improved survival with vemurafenib in melanoma with BRAF V600E mutation. *N Engl J Med.* 2011;364:2507–2516.
16. Flaherty KT, Puzanov I, Kim KB, et al. Inhibition of mutated, activated BRAF in metastatic melanoma. *N Engl J Med.* 2010; 363:809–819.
17. Falchook GS, Long GV, Kurzrock R, et al. Dabrafenib in patients with melanoma, untreated brain metastases, and other solid tumours: a phase 1 dose-escalation trial. *Lancet.* 2012;379(9829): 1893–1901.
18. Hauschild A, Grob JJ, Demidov LV, et al. Dabrafenib in BRAF-mutated metastatic melanoma: a multicentre, open-label, phase 3 randomised controlled trial. *Lancet.* 2012;380(9839):358–365.
19. Long GV, Trefzer U, Davies MA, et al. Dabrafenib in patients with Val600Glu or Val600Lys BRAF-mutant melanoma metastatic to the brain (BREAK-MB): a multicentre, open-label, phase 2 trial. *Lancet Oncol.* 2012;13:1087–1095.
20. Flaherty KT, Infante JR, Daud A, et al. Combined BRAF and MEK inhibition in melanoma with BRAF V600 mutations. *N Engl J Med.* 2012;367:1694–1703.
21. Larkin J, Ascierto PA, Dreno B, et al. Combined vemurafenib and cobimetinib in BRAF-mutated melanoma. *N Engl J Med.* 2014;371: 1867–1876.
22. Long GV, Stroyakovskiy D, Gogas H, et al. Combined BRAF and MEK inhibition versus BRAF inhibition alone in melanoma. *N Engl J Med.* 2014;371:1877–1888.
23. Robert C, Karaszewska B, Schachter J, et al. Improved overall survival in melanoma with combined dabrafenib and trametinib. *N Engl J Med.* 2015;372:30–39.
24. Long GV, Stroyakovskiy D, Gogas H, et al. Dabrafenib and trametinib versus dabrafenib and placebo for Val600 BRAF-mutant melanoma: a multicentre, double-blind, phase 3 randomised controlled trial. *Lancet.* 2015;386(9992):444–451.
25. Long GV, Menzies AM, Nagrial AM, et al. Prognostic and clinicopathologic associations of oncogenic BRAF in metastatic melanoma. *J Clin Oncol.* 2011;29:1239–1246.
26. Jakob JA, Bassett RL, Jr., Ng CS, et al. NRAS mutation status is an independent prognostic factor in metastatic melanoma. *Cancer.* 2012;118:4014–4023.
27. Edge SB, Byrd DR, Compton CC, Fritz AG, Greene FL, Trotti A III. AJCC Cancer Staging Manual. 7th ed. New York: Springer; 2010.
28. Kelderman S, Heemskerk B, van Tinteren H, et al. Lactate dehydrogenase as a selection criterion for ipilimumab treatment in metastatic melanoma. *Cancer Immunol Immunother.* 2014;63:449–458.
29. Larkin J, Chiarion Sileni V, Gonzalez R, et al. Efficacy and safety in key patient subgroups of nivolumab (NIVO) alone or combined with ipilimumab (IPI) versus IPI alone in treatment-naïve patients with advanced melanoma (MEL) (CheckMate 067). *Eur J Cancer.* 2015;51(Supp 3):S664-S665.
30. Long GV, Grob JJ, Davies MA, Lane S, Legenne P, Flaherty KT. Baseline and postbaseline characteristics associated with treatment benefit across dabrafenib and trametinib registration pooled data. *Pigment Cell Melanoma Res.* 2015;28:793.
31. Long GV, Weber JS, Infante JR, et al. Overall survival and durable responses in patients with BRAF V600-mutant metastatic melanoma receiving dabrafenib combined with trametinib. *J Clin Oncol.* 2016;34:871–878.
32. Menzies AM, Wilmott JS, Drummond M, et al. Clinicopathologic features associated with efficacy and long-term survival in metastatic melanoma patients treated with BRAF or combined BRAF and MEK inhibitors. *Cancer.* 2015;121:3826–3835.
33. El Ghissassi F, Baan R, Straif K, et al. A review of human carcinogens--part D: radiation. *Lancet Oncol.* 2009;10:751–752.
34. Van Es SL, Colman M, Thompson JF, McCarthy SW, Scolyer RA. Angiotropism is an independent predictor of local recurrence and in-transit metastasis in primary cutaneous melanoma. *Am J Surg Pathol.* 2008;32:1396–1403.
35. Wilmott J, Haydu L, Bagot M, et al. Angiotropism is an independent predictor of microscopic satellites in primary cutaneous melanoma. *Histopathology.* 2012;61:889–898.
36. Breslow A. Thickness, cross-sectional areas and depth of invasion in the prognosis of cutaneous melanoma. *Ann Surg.* 1970;172: 902–908.
37. Breslow A. Tumor thickness, level of invasion and node dissection in stage I cutaneous melanoma. *Ann Surg.* 1975;182:572–575.
38. Scolyer RA, Judge MJ, Evans A, et al. Data set for pathology reporting of cutaneous invasive melanoma: recommendations from the International Collaboration on Cancer Reporting (ICCR). *Am J Surg Pathol.* 2013;37:1797–1814.
39. Balch CM, Murad TM, Soong SJ, Ingalls AL, Halpern NB, Maddox WA. A multifactorial analysis of melanoma: prognostic histopathological features comparing Clark's and Breslow's staging methods. *Ann Surg.* 1978;188:732–742.
40. Balch CM, Soong SJ, Murad TM, Ingalls AL, Maddox WA. A multifactorial analysis of melanoma. II. Prognostic factors in patients with stage I (localized) melanoma. *Surgery.* 1979;86:343–351.
41. Balch CM, Wilkerson JA, Murad TM, Soong SJ, Ingalls AL, Maddox WA. The prognostic significance of ulceration of cutaneous melanoma. *Cancer.* 1980;45:3012–3017.
42. McGovern VJ, Shaw HM, Milton GW, McCarthy WH. Ulceration and prognosis in cutaneous malignant melanoma. *Histopathology.* 1982;6:399–407.
43. Spatz A, Cook MG, Elder DE, Piepkorn M, Ruiter DJ, Barnhill RL. Interobserver reproducibility of ulceration assessment in primary cutaneous melanomas. *Eur J Cancer.* 2003;39: 1861–1865.
44. Cormier JN, Xing Y, Feng L, et al. Metastatic melanoma to lymph nodes in patients with unknown primary sites. *Cancer.* 2006;106: 2012–2020.
45. Lee CC, Faries MB, Wanek LA, Morton DL. Improved survival after lymphadenectomy for nodal metastasis from an unknown primary melanoma. *J Clin Oncol.* 2008;26:535–541.
46. van der Ploeg AP, Haydu LE, Spillane AJ, et al. Melanoma patients with an unknown primary tumor site have a better outcome than those with a known primary following therapeutic lymph node dissection for macroscopic (clinically palpable) nodal disease. *Ann Surg Oncol.* 2014;21:3108–3116.
47. de Waal AC, Aben KK, van Rossum MM, Kiemeney LA. Melanoma of unknown primary origin: a population-based study in the Netherlands. *Eur J Cancer.* 2013;49:676–683.
48. Prens SP, van der Ploeg AP, van Akkooi AC, et al. Outcome after therapeutic lymph node dissection in patients with unknown primary melanoma site. *Ann Surg Oncol.* 2011;18:3586–3592.

49. Rutkowski P, Nowecki ZI, Dziewirski W, et al. Melanoma without a detectable primary site with metastases to lymph nodes. *Dermatol Surg.* 2010;36:868–876.
50. Weide B, Faller C, Elsasser M, et al. Melanoma patients with unknown primary site or nodal recurrence after initial diagnosis have a favourable survival compared to those with synchronous lymph node metastasis and primary tumour. *PLoS One.* 2013;8:e66953.
51. Kopf AW, Salopek TG, Slade J, Marghoob AA, Bart RS. Techniques of cutaneous examination for the detection of skin cancer. *Cancer.* 1995;75(2 Suppl):684–690.
52. Doepker MP, Thompson ZJ, Harb JN, et al. Dermal melanoma: a report on prognosis, outcomes, and the utility of sentinel lymph node biopsy. *J Surg Oncol.* 2016;113:98–102.
53. Swetter SM, Ecker PM, Johnson DL, Harvell JD. Primary dermal melanoma: a distinct subtype of melanoma. *Arch Dermatol.* 2004;140:99–103.
54. Teow J, Chin O, Hanikeri M, Wood BA. Primary dermal melanoma: a West Australian cohort. *ANZ J Surg.* 2015;85:664–667.
55. Davies MA, Liu P, McIntyre S, et al. Prognostic factors for survival in melanoma patients with brain metastases. *Cancer.* 2011;117:1687–1696.
56. Staudt M, Lasithiotakis K, Leiter U, et al. Determinants of survival in patients with brain metastases from cutaneous melanoma. *Br J Cancer.* 2010;102:1213–1218.
57. Margolin K, Ernstoff MS, Hamid O, et al. Ipilimumab in patients with melanoma and brain metastases: an open-label, phase 2 trial. *Lancet Oncol.* 2012;13:459–465.
58. Spagnolo F, Picasso V, Lambertini M, Ottaviano V, Dozin B, Queirolo P. Survival of patients with metastatic melanoma and brain metastases in the era of MAP-kinase inhibitors and immunologic checkpoint blockade antibodies: a systematic review. *Cancer Treat Rev.* 2016;45:38–45.
59. Sabel MS, Wong SL. Review of evidence-based support for pretreatment imaging in melanoma. *J Natl Compr Canc Netw.* 2009;7:281–289.
60. Chai CY, Zager JS, Szabunio MM, et al. Preoperative ultrasound is not useful for identifying nodal metastasis in melanoma patients undergoing sentinel node biopsy: preoperative ultrasound in clinically node-negative melanoma. *Ann Surg Oncol.* 2012;19:1100–1106.
61. Zager JS, Puleo CA, Sondak VK. What is the significance of the in transit or interval sentinel node in melanoma? *Ann Surg Oncol.* 2011;18:3232–3234. (Erratum to figure: Ann Surg Oncol. 2011:18(Suppl 3):317–318).
62. Chapman BC, Gleisner A, Kwak JJ, et al. SPECT/CT improves detection of metastatic sentinel lymph nodes in patients with head and neck melanoma. *Ann Surg Oncol.* 2016;23:2652–2657.
63. Brady MS, Akhurst T, Spanknebel K, et al. Utility of preoperative [(18)]F fluorodeoxyglucose-positron emission tomography scanning in high-risk melanoma patients. *Ann Surg Oncol.* 2006;13:525–532.
64. Reinhardt MJ, Joe AY, Jaeger U, et al. Diagnostic performance of whole body dual modality 18F-FDG PET/CT imaging for N- and M-staging of malignant melanoma: experience with 250 consecutive patients. *J Clin Oncol.* 2006;24:1178–1187.
65. Aloia TA, Gershenwald JE, Andtbacka RH, et al. Utility of computed tomography and magnetic resonance imaging staging before completion lymphadenectomy in patients with sentinel lymph node-positive melanoma. *J Clin Oncol.* 2006;24:2858–2865.
66. Rueth NM, Xing Y, Chiang YJ, et al. Is surveillance imaging effective for detecting surgically treatable recurrences in patients with melanoma? A comparative analysis of stage-specific surveillance strategies. *Ann Surg.* 2014;259:1215–1222.
67. Lewin JH, Sanelli A, Walpole I, et al. Surveillance imaging with FDG-PET in the follow-up of melanoma patients at high risk of relapse. *J Clin Oncol.* 2015;33:Suppl, Abstract #9003.
68. Xing Y, Bronstein Y, Ross MI, et al. Contemporary diagnostic imaging modalities for the staging and surveillance of melanoma patients: a meta-analysis. *J Natl Cancer Inst.* 2011;103:129–142.
69. Tyler DS, Onaitis M, Kherani A, et al. Positron emission tomography scanning in malignant melanoma. *Cancer.* 2000;89:1019–1025.
70. Damian DL, Fulham MJ, Thompson E, Thompson JF. Positron emission tomography in the detection and management of metastatic melanoma. *Melanoma Res.* 1996;6:325–329.
71. Pfannenberg C, Aschoff P, Schanz S, et al. Prospective comparison of 18F-fluorodeoxyglucose positron emission tomography/computed tomography and whole-body magnetic resonance imaging in staging of advanced malignant melanoma. *Eur J Cancer.* 2007;43:557–564.
72. Bartlett EK, Gupta M, Datta J, et al. Prognosis of patients with melanoma and microsatellitosis undergoing sentinel lymph node biopsy. *Ann Surg Oncol.* 2014;21:1016–1023.
73. Balch CM, Gershenwald JE, Soong SJ, et al. Multivariate analysis of prognostic factors among 2,313 patients with stage III melanoma: comparison of nodal micrometastases versus macrometastases. *J Clin Oncol.* 2010;28:2452–2459.
74. Cochran AJ, Wen DR, Huang RR, Wang HJ, Elashoff R, Morton DL. Prediction of metastatic melanoma in nonsentinel nodes and clinical outcome based on the primary melanoma and the sentinel node. *Mod Pathol.* 2004;17:747–755.
75. Dewar DJ, Newell B, Green MA, Topping AP, Powell BW, Cook MG. The microanatomic location of metastatic melanoma in sentinel lymph nodes predicts nonsentinel lymph node involvement. *J Clin Oncol.* 2004;22:3345–3349.
76. Egger ME, Bower MR, Czyszczon IA, et al. Comparison of sentinel lymph node micrometastatic tumor burden measurements in melanoma. *J Am Coll Surg.* 2014;218:519–528.
77. Fink AM, Weihsengruber F, Duschek N, et al. Value of micromorphometric criteria of sentinel lymph node metastases in predicting further nonsentinel lymph node metastases in patients with melanoma. *Melanoma Res.* 2011;21:139–143.
78. Francischetto T, Spector N, Neto Rezende JF, et al. Influence of sentinel lymph node tumor burden on survival in melanoma. *Ann Surg Oncol.* 2010;17:1152–1158.
79. Frankel TL, Griffith KA, Lowe L, et al. Do micromorphometric features of metastatic deposits within sentinel nodes predict nonsentinel lymph node involvement in melanoma? *Ann Surg Oncol.* 2008;15:2403–2411.
80. Gershenwald JE, Andtbacka RH, Prieto VG, et al. Microscopic tumor burden in sentinel lymph nodes predicts synchronous nonsentinel lymph node involvement in patients with melanoma. *J Clin Oncol.* 2008;26:4296–4303.
81. Ranieri JM, Wagner JD, Azuaje R, et al. Prognostic importance of lymph node tumor burden in melanoma patients staged by sentinel node biopsy. *Ann Surg Oncol.* 2002;9:975–981.
82. Scolyer RA, Li LX, McCarthy SW, et al. Micromorphometric features of positive sentinel lymph nodes predict involvement of nonsentinel nodes in patients with melanoma. *Am J Clin Pathol.* 2004;122:532–539.
83. Starz H, Balda BR, Kramer KU, Buchels H, Wang H. A micromorphometry-based concept for routine classification of sentinel lymph node metastases and its clinical relevance for patients with melanoma. *Cancer.* 2001;91:2110–2121.
84. van Akkooi AC, Nowecki ZI, Voit C, et al. Sentinel node tumor burden according to the Rotterdam criteria is the most important prognostic factor for survival in melanoma patients: a multicenter study in 388 patients with positive sentinel nodes. *Ann Surg.* 2008;248:949–955.

85. van der Ploeg AP, van Akkooi AC, Haydu LE, et al. The prognostic significance of sentinel node tumour burden in melanoma patients: an international, multicenter study of 1539 sentinel node-positive melanoma patients. *Eur J Cancer.* 2014;50:111–120.
86. van der Ploeg AP, van Akkooi AC, Rutkowski P, et al. Prognosis in patients with sentinel node-positive melanoma is accurately defined by the combined Rotterdam tumor load and Dewar topography criteria. *J Clin Oncol.* 2011;29:2206–2214.
87. Balch CM, Buzaid AC, Soong SJ, et al. Final version of the American Joint Committee on Cancer staging system for cutaneous melanoma. *J Clin Oncol.* 2001;19:3635–3648.
88. Balch CM, Soong S, Ross MI, et al. Long-term results of a multi-institutional randomized trial comparing prognostic factors and surgical results for intermediate thickness melanomas (1.0 to 4.0 mm). Intergroup Melanoma Surgical Trial. *Ann Surg Oncol.* 2000;7:87–97.
89. Cascinelli N, Belli F, Santinami M, et al. Sentinel lymph node biopsy in cutaneous melanoma: the WHO Melanoma Program experience. *Ann Surg Oncol.* 2000;7:469–474.
90. Buzaid AC, Ross MI, Balch CM, et al. Critical analysis of the current American Joint Committee on Cancer staging system for cutaneous melanoma and proposal of a new staging system. *J Clin Oncol.* 1997;15:1039–1051.
91. Cascinelli N, Bufalino R, Marolda R, et al. Regional non-nodal metastases of cutaneous melanoma. *Eur J Surg Oncol.* 1986;12:175–180.
92. Day CL, Jr., Harrist TJ, Gorstein F, et al. Malignant melanoma. Prognostic significance of "microscopic satellites" in the reticular dermis and subcutaneous fat. *Ann Surg.* 1981;194:108–112.
93. Harrist TJ, Rigel DS, Day CL, Jr, et al. "Microscopic satellites" are more highly associated with regional lymph node metastases than is primary melanoma thickness. *Cancer.* 1984;53:2183–2187.
94. Leon P, Daly JM, Synnestvedt M, Schultz DJ, Elder DE, Clark WH, Jr. The prognostic implications of microscopic satellites in patients with clinical stage I melanoma. *Arch Surg.* 1991;126:1461–1468.
95. Read RL, Haydu L, Saw RP, et al. In-transit melanoma metastases: incidence, prognosis, and the role of lymphadenectomy. *Ann Surg.* 2015;22:475–481.
96. Warso MA, Boddie AW. The natural history of melanoma, including the pattern of metastatic spread and the biological basis for metastases–staging of melanoma. *Cancer Treat Res.* 1993;65:141–160.
97. Barth A, Wanek LA, Morton DL. Prognostic factors in 1,521 melanoma patients with distant metastases. *J Am Coll Surg.* 1995;181:193–201.
98. Garrison M, Nathanson L. Prognosis and staging in melanoma. Semin Oncol. 1996;23:725–733.
99. Brand CU, Ellwanger U, Stroebel W, et al. Prolonged survival of 2 years or longer for patients with disseminated melanoma. An analysis of related prognostic factors. *Cancer.* 1997;79:2345–2353.
100. Cochran AJ, Bhuta S, Paul E, Ribas A. The shifting patterns of metastatic melanoma. *Clin Lab Med.* 2000;20:759–783.
101. Bedikian AY, Johnson MM, Warneke CL, et al. Prognostic factors that determine the long-term survival of patients with unresectable metastatic melanoma. *Cancer Invest.* 2008;26:624–633.
102. Keilholz U, Martus P, Punt CJ, et al. Prognostic factors for survival and factors associated with long-term remission in patients with advanced melanoma receiving cytokine-based treatments: second analysis of a randomised EORTC Melanoma Group trial comparing interferon-alpha2a (IFNalpha) and interleukin 2 (IL-2) with or without cisplatin. *Eur J Cancer.* 2002;38:1501–1511.
103. Manola J, Atkins M, Ibrahim J, Kirkwood J. Prognostic factors in metastatic melanoma: a pooled analysis of Eastern Cooperative Oncology Group trials. *J Clin Oncol.* 2000;18:3782–3793.
104. Sirott MN, Bajorin DF, Wong GY, et al. Prognostic factors in patients with metastatic malignant melanoma. A multivariate analysis. *Cancer.* 1993;72:3091–3098.
105. Weide B, Martens A, Hassel JC, et al. Baseline biomarkers for outcome of melanoma patients treated with pembrolizumab. *Clin Cancer Res.* 2016 May 16 [Epub ahead of print].
106. Thompson JF, Soong SJ, Balch CM, et al. Prognostic significance of mitotic rate in localized primary cutaneous melanoma: an analysis of patients in the multi-institutional American Joint Committee on Cancer melanoma staging database. *J Clin Oncol.* 2011;29:2199–2205.
107. Scolyer RA, Shaw HM, Thompson JF, et al. Interobserver reproducibility of histopathologic prognostic variables in primary cutaneous melanomas. *Am J Surg Pathol.* 2003;27:1571–1576.
108. Azzola MF, Shaw HM, Thompson JF, et al. Tumor mitotic rate is a more powerful prognostic indicator than ulceration in patients with primary cutaneous melanoma: an analysis of 3661 patients from a single center. *Cancer.* 2003;97:1488–1498.
109. Francken AB, Shaw HM, Thompson JF, et al. The prognostic importance of tumor mitotic rate confirmed in 1317 patients with primary cutaneous melanoma and long follow-up. *Ann Surg Oncol.* 2004;11:426–433.
110. Clark WH, Jr., From L, Bernardino EA, Mihm MC. The histogenesis and biologic behavior of primary human malignant melanomas of the skin. *Cancer Res.* 1969;29:705–727.
111. Breslow A. Problems in the measurement of tumor thickness and level of invasion in cutaneous melanoma. *Human Pathol.* 1977;8:1–2.
112. Prade M, Sancho-Garnier H, Cesarini JP, Cochran A. Difficulties encountered in the application of Clark classification and the Breslow thickness measurement in cutaneous malignant melanoma. *Int J Cancer.* 1980;26:159–163.
113. Buttner P, Garbe C, Bertz J, et al. Primary cutaneous melanoma. Optimized cutoff points of tumor thickness and importance of Clark's level for prognostic classification. *Cancer.* 1995;75:2499–2506.
114. Finley JW, Gibbs JF, Rodriguez LM, Letourneau R, Driscoll D, Kraybill W. Pathologic and clinical features influencing outcome of thin cutaneous melanoma: correlation with newly proposed staging system. *Am Surgeon.* 2000;66:527–531; discussion 531–522.
115. Mansson-Brahme E, Carstensen J, Erhardt K, Lagerlof B, Ringborg U, Rutqvist LE. Prognostic factors in thin cutaneous malignant melanoma. *Cancer.* 1994;73:2324–2332.
116. Marghoob AA, Koenig K, Bittencourt FV, Kopf AW, Bart RS. Breslow thickness and clark level in melanoma: support for including level in pathology reports and in American Joint Committee on Cancer Staging. *Cancer.* 2000;88:589–595.
117. Morton DL, Davtyan DG, Wanek LA, Foshag LJ, Cochran AJ. Multivariate analysis of the relationship between survival and the microstage of primary melanoma by Clark level and Breslow thickness. *Cancer.* 1993;71:3737–3743.
118. Salman SM, Rogers GS. Prognostic factors in thin cutaneous malignant melanoma. *J Dermatol Surg Oncol.* 1990;16:413–418.
119. Shaw HM, McCarthy WH, McCarthy SW, Milton GW. Thin malignant melanomas and recurrence potential. *Arch Surg.* 1987;122:1147–1150.
120. Azimi F, Scolyer RA, Rumcheva P, et al. Tumor-infiltrating lymphocyte grade is an independent predictor of sentinel lymph node status and survival in patients with cutaneous melanoma. *J Clin Oncol.* 2012;30:2678–2683.
121. Taylor RC, Patel A, Panageas KS, Busam KJ, Brady MS. Tumor-infiltrating lymphocytes predict sentinel lymph node positivity in patients with cutaneous melanoma. *J Clin Oncol.* 2007;25:869–875.
122. Burton AL, Roach BA, Mays MP, et al. Prognostic significance of tumor infiltrating lymphocytes in melanoma. *Am Surgeon.* 2011;77:188–192.
123. Schatton T, Scolyer RA, Thompson JF, Mihm MC, Jr. Tumor-infiltrating lymphocytes and their significance in melanoma prognosis. *Methods Mol Biol.* 2014;1102:287–324.

124. Thomas NE, Busam KJ, From L, et al. Tumor-infiltrating lymphocyte grade in primary melanomas is independently associated with melanoma-specific survival in the population-based genes, environment and melanoma study. *J Clin Oncol*. 2013;31:4252–4259.
125. Kashani-Sabet M, Sagebiel RW, Ferreira CM, Nosrati M, Miller JR 3rd. Vascular involvement in the prognosis of primary cutaneous melanoma. *Arch Dermatol*. 2001;137:1169–1173.
126. Nagore E, Oliver V, Botella-Estrada R, Moreno-Picot S, Insa A, Fortea JM. Prognostic factors in localized invasive cutaneous melanoma: high value of mitotic rate, vascular invasion and microscopic satellitosis. *Melanoma Res*. 2005;15:169–177.
127. Pasquali S, Montesco MC, Ginanneschi C, et al. Lymphatic and blood vasculature in primary cutaneous melanomas of the scalp and neck. *Head Neck*. 2015;37:1596–1602.
128. Storr SJ, Safuan S, Mitra A, et al. Objective assessment of blood and lymphatic vessel invasion and association with macrophage infiltration in cutaneous melanoma. *Modern Pathol*. 2012;25:493–504.
129. Straume O, Akslen LA. Independent prognostic importance of vascular invasion in nodular melanomas. *Cancer*. 1996;78:1211–1219.
130. Xu X, Chen L, Guerry D, et al. Lymphatic invasion is independently prognostic of metastasis in primary cutaneous melanoma. *Clin Cancer Res*. 2012;18:229–237.
131. Murali R, Cochran AJ, Cook MG, et al. Interobserver reproducibility of histologic parameters of melanoma deposits in sentinel lymph nodes: implications for management of patients with melanoma. *Cancer*. 2009;115:5026–5037.
132. Kattan MW, Hess KR, Amin MB, et al. American Joint Committee on Cancer acceptance criteria for inclusion of risk models for individualized prognosis in the practice of precision medicine. *CA Cancer J Clin*. Jan 19 2016 [Epub ahead of print].
133. Cadili A, Dabbs K, Scolyer RA, Brown PT, Thompson JF. Re-evaluation of a scoring system to predict nonsentinel-node metastasis and prognosis in melanoma patients. *J Am Coll Surg*. 2010;211:522–525.
134. Callender GG, Gershenwald JE, Egger ME, et al. A novel and accurate computer model of melanoma prognosis for patients staged by sentinel lymph node biopsy: comparison with the American Joint Committee on Cancer model. *J Am Coll Surg*. 2012;214:608–617; discussion 617–609.
135. Maurichi A, Miceli R, Camerini T, et al. Prediction of survival in patients with thin melanoma: results from a multi-institution study. *J Clin Oncol*. 2014;32:2479–2485.
136. Mitra A, Conway C, Walker C, et al. Melanoma sentinel node biopsy and prediction models for relapse and overall survival. *Br J Cancer*. 2010;103:1229–1236.
137. Mahar AL, Compton C, Halabi S, et al. Critical assessment of clinical prognostic tools in melanoma. *Ann Surg Oncol*. Apr 6 2016 [Epub ahead of print].
138. LeBoit PE, Burg G, Weedon D, Sarasin A, eds. WHO Classification of Tumours. Pathology and Genetics of Skin Tumours. Lyon: IARC Press; 2006.
139. Kuchelmeister C, Schaumburg-Lever G, Garbe C. Acral cutaneous melanoma in caucasians: clinical features, histopathology and prognosis in 112 patients. *Br J Dermatol*. 2000;143:275–280.
140. McGovern VJ, Shaw HM, Milton GW, Farago GA. Is malignant melanoma arising in a Hutchinson's melanotic freckle a separate disease entity? *Histopathology*. 1980;4:235–242.
141. Slingluff CL, Jr., Vollmer R, Seigler HF. Acral melanoma: a review of 185 patients with identification of prognostic variables. *J Surg Oncol*. 1990;45:91–98.
142. Urist MM, Balch CM, Soong SJ, et al. Head and neck melanoma in 534 clinical Stage I patients. A prognostic factors analysis and results of surgical treatment. *Ann Surg*. 1984;200:769–775.
143. Busam KJ, Mujumdar U, Hummer AJ, et al. Cutaneous desmoplastic melanoma: reappraisal of morphologic heterogeneity and prognostic factors. *Am J Surg Pathol*. 2004;28:1518–1525.
144. Han D, Han G, Zhao X, et al. Clinicopathologic predictors of survival in patients with desmoplastic melanoma. *PLoS One*. 2015;10:e0119716.
145. Hawkins WG, Busam KJ, Ben-Porat L, et al. Desmoplastic melanoma: a pathologically and clinically distinct form of cutaneous melanoma. *Ann Surg Oncol*. 2005;12:207–213.
146. Murali R, Shaw HM, Lai K, et al. Prognostic factors in cutaneous desmoplastic melanoma: a study of 252 patients. *Cancer*. 2010;116:4130–4138.
147. Pawlik TM, Ross MI, Prieto VG, et al. Assessment of the role of sentinel lymph node biopsy for primary cutaneous desmoplastic melanoma. *Cancer*. 2006;106:900–906.
148. Broer PN, Walker ME, Goldberg C, et al. Desmoplastic melanoma: a 12-year experience with sentinel lymph node biopsy. *Eur J Surg Oncol*. 2013;39:681–685.
149. Egger ME, Huber KM, Dunki-Jacobs EM, et al. Incidence of sentinel lymph node involvement in a modern, large series of desmoplastic melanoma. *J Am Coll Surg*. 2013;217:37–44; discussion 44–35.
150. Han D, Zager JS, Yu D, et al. Desmoplastic melanoma: is there a role for sentinel lymph node biopsy? *Ann Surg Oncol*. 2013;20:2345–2351.

Part XI

Breast

Members of the Breast Expert Panel
Sunil S. Badve, MD
Peter D. Beitsch, MD, FACS
Shikha Bose, MD
David R. Byrd, MD, FACS
Vivien W. Chen, PhD – Data Collection Core Representative
James L. Connolly, MD
Basak Dogan, MD
Carl J. D'Orsi, MD, FACR
Stephen B. Edge, MD, FACS – Editorial Board Liaison
Armando Giuliano, MD, FACS, FRCSEd – Co-Chair
Gabriel N. Hortobagyi, MD, FACP, FASCO – Co-Chair
Alyson L. Mahar, MSc – Precision Medicine Core Representatitive
Ingrid A. Mayer, MD, MSCI
Beryl McCormick, MD, FACR, FASTRO
Elizabeth A. Mittendorf, MD, PhD
Abram Recht, MD, FASTRO
Jorge S. Reis-Filho, MD, PhD, FRCPath
Hope S. Rugo, MD
Jean F. Simpson, MD – CAP Representative
Lawrence J. Solin, MD, FACR, FASTRO
W. Fraser Symmans, MD
Theresa M. Vallerand, BGS, CTR – Data Collection Core Representative
Liesbet J. Van Eycken, – UICC Representative
Donald L. Weaver, MD
David J. Winchester, MD, FACS

Breast

48

Gabriel N. Hortobagyi, James L. Connolly, Carl J. D'Orsi, Stephen B. Edge, Elizabeth A. Mittendorf, Hope S. Rugo, Lawrence J. Solin, Donald L. Weaver, David J. Winchester, and Armando Giuliano

CHAPTER SUMMARY

Cancers Staged Using This Staging System

Invasive (infiltrating) carcinoma of the breast, ductal carcinoma *in situ* of the breast

Cancers Not Staged Using This Staging System

These histopathologic types of cancer...	Are staged according to the classification for...	And can be found in chapter...
Breast sarcomas	Soft tissue sarcoma of the trunk and extremities	41
Phyllodes tumor	Soft tissue sarcoma – unusual histologies and sites	45
Breast lymphomas	Hematologic malignancies	79–81

Summary of Changes

Change	Details of Change	Level of Evidence
AJCC Anatomic and Prognostic Stage Groups	There are two stage group tables presented in this chapter:<br>1. Anatomic Stage Group table based solely on anatomic extent of cancer as defined by the T, N, and M categories.<br>2. Prognostic Stage Group table based on populations of persons with breast cancer that have been offered – and mostly treated with – appropriate endocrine and/or systemic chemotherapy, which includes anatomic T, N, and M plus tumor grade and the status of the biomarkers human epidermal growth factor receptor 2 (HER2), estrogen receptor (ER), and progesterone receptor (PR).	II
Selecting the Appropriate Stage Group Table	The Prognostic Stage Group table is preferred for patient care and is to be used for reporting of all cancer patients in the U.S.<br>The Anatomic Stage Group table is provided so that stage can be assigned in regions of the world where the biomarkers cannot be routinely obtained.	N/A
Definition of Primary Tumor (T)	Lobular carcinoma *in situ* (LCIS) is removed as a pTis category for T-categorization. Lobular carcinoma *in situ* is a benign entity and is removed from TNM staging.	I
Definition of Primary Tumor (T)	The general rules for rounding to the nearest millimeter do not apply for tumors between 1.0 and 1.5 mm, so as to not classify these cancers as microinvasive (T1mi) carcinomas (defined as invasive tumor foci 1.0 mm or smaller). Tumors >1 mm and <2 mm should be reported rounding to 2 mm.	II
Definition of Primary Tumor (T)	Confirmed that the maximum invasive tumor size (T) is a reasonable estimate of tumor volume. Small microscopic satellite foci of tumor around the primary tumor do not appreciably alter tumor volume and are not added to the maximum tumor size.	I

To access the AJCC cancer staging forms, please visit www.cancerstaging.org.

© American Joint Committee on Cancer 2017

M.B. Amin et al. (eds.), *AJCC Cancer Staging Manual, Eighth Edition*, DOI 10.1007/978-3-319-40618-3_48

Change	Details of Change	Level of Evidence
Definition of Primary Tumor (T)	Clarified the T categorization of multiple synchronous tumors. These are identified clinically and/or by macroscopic pathological examination and their presence documented using the (m) modifier for the T category. This new edition specifically continues using only the maximum dimension of the largest tumor for cT and pT; the size of multiple tumors is not added.	I
Definition of Primary Tumor (T)	Added a clear definition that satellite tumor nodules in the skin must be separate from the primary tumor and macroscopically identified to categorize as T4b. Skin and dermal tumor satellite nodules identified only on microscopic examination and in the absence of epidermal ulceration or skin edema (clinical peau d'orange) do not qualify as T4b. Such tumors should be categorized based on tumor size.	I
Definition of Regional Lymph Node (N)	The criteria for pathological measurement of lymph node metastases are clearly defined. The dimension of the area containing several or multiple tumor deposits is NOT used to determine pN category. The largest contiguous tumor deposit is used for pN; adjacent satellite tumor deposits are not added.	I
Definition of Regional Lymph Node (N)	The Expert Panel affirmed that cNX is not a valid category unless the node basin has been removed and cannot be examined by imaging or clinical examination; a cN0 category is to be assigned when any evaluation of the nodes is possible and the physical examination or imaging examination is negative.	I
Definition of Distant Metastasis (M)	The Expert Panel affirmed that pM0 is not a valid category. All cases should be categorized as either cM0 or cM1; however, if cM1 is subsequently microscopically confirmed, pM1 is used. See Chapter 1 as well.	I
Post Neoadjuvant Therapy Classification (ypTNM)	The Expert Panel clarified that the post-neoadjuvant therapy pathological T-category (ypT) is based on the largest focus of residual tumor, if present. Treatment-related fibrosis adjacent to residual invasive carcinoma is not included in the ypT maximum dimension. When multiple foci of residual tumor are present, the (m) modifier is included. The pathology report should include a description of the extent of residual tumor explaining the basis for the ypT categorization and, when possible, also should document the pretreatment cT category.	I
Post Neoadjuvant Therapy Classification (ypTNM)	The Expert Panel clarified that the largest focus of residual tumor in the lymph nodes, if present, is used for ypN categorization. Treatment-related fibrosis adjacent to residual nodal tumor deposits is not included in the ypN dimension and classification.	I
Complete Pathological Response	The Expert Panel affirmed that any residual invasive carcinoma detected by pathological examination in the breast or lymph nodes precludes posttreatment classification as a complete pathological response (pCR). If a cancer is categorized M1 (clinical or pathological) prior to therapy, the cancer is categorized as M1 following neoadjuvant therapy, regardless of the observed response to therapy.	I
Collection of Biomarkers (Hormone receptor assays and HER2 assay)	The Expert Panel determined that all invasive carcinomas should have estrogen receptor, progesterone receptor, and human epidermal growth factor receptor 2 (HER2) status determined by appropriate assays whenever possible.	I
Inclusion of Multigene Panels (when available) as Stage Modifiers – 21 Gene Recurrence Score (Oncotype Dx®)	For patients with hormone receptor-positive, HER2-negative, and lymph node-negative tumors, a 21-gene (Oncotype Dx®) recurrence score less than 11, regardless of T size, places the tumor into the same prognostic category as T1a–T1b N0 M0 and staged using the AJCC Prognostic Stage table as Stage I	I
Inclusion of Multigene Panels (when available) as Stage Modifiers – Mammaprint®	For patients with hormone receptor-positive, HER2-negative, and lymph node-negative tumors, a Mammaprint® low-risk score, regardless of T size, places the tumor into the same prognostic category as T1a–T1b N0 M0.	II
Inclusion of Multigene Panels (when available) as Stage Modifiers - EndoPredict®	For patients with hormone receptor-positive, HER2-negative, and lymph node-negative tumors, a 12-gene (EndoPredict) low-risk score, regardless of T size, places the tumor into the same prognostic category as T1a–T1b N0 M0.	II
Inclusion of Multigene Panels (when available) as Stage Modifiers – PAM 50® (Prosigna)	For patients with hormone receptor-positive, HER2-negative, and lymph node-negative tumors, a PAM50 risk of recurrence (ROR) score in the low range, regardless of T size, places the tumor into the same prognostic category as T1a–T1b N0 M0.	II
Inclusion of Multigene Panels (when available) as Stage Modifiers – Breast Cancer Index	For patients with hormone receptor-positive, HER2-negative, and lymph node-negative tumors, a Breast Cancer Index in the low-risk range, regardless of T size, places the tumor into the same prognostic category as T1a-T1b N0 M0.	II

ICD-O-3 Topography Codes

Code	Description
C50.0	Nipple
C50.1	Central portion of breast
C50.2	Upper-inner quadrant of breast
C50.3	Lower-inner quadrant of breast
C50.4	Upper-outer quadrant of breast
C50.5	Lower-outer quadrant of breast
C50.6	Axillary tail of breast
C50.8	Overlapping lesion of breast
C50.9	Breast, NOS

WHO Classification of Tumors

Code	Description
8500	Invasive carcinoma of no special type (NST)
8022	Pleomorphic carcinoma
8035	Carcinoma with osteoclast-like stromal giant cells
8520	Invasive lobular carcinoma
8211	Tubular carcinoma
8201	Cribriform carcinoma
8480	Mucinous carcinoma
8510	Medullary carcinoma
8513	Atypical medullary carcinoma
8500	Invasive carcinoma NST with medullary features
8507	Invasive micropapillary carcinoma
8575	Metaplastic carcinoma of no special type
8570	Low-grade adenosquamous carcinoma
8572	Fibromatosis-like metaplastic carcinoma
8070	Squamous cell carcinoma
8032	Spindle cell carcinoma
8571	Chondroid differentiation
8571	Osseous differentiation
8575	Other types of mesenchymal differentiation
8575	Mixed metaplastic carcinoma
8982	Myoepithelial carcinoma
8246	Neuroendocrine tumor, well-differentiated
8041	Neuroendocrine carcinoma, poorly differentiated (small cell carcinoma)
8574	Carcinoma with neuroendocrine differentiation
8502	Secretory carcinoma
8503	Invasive papillary carcinoma
8550	Acinic cell carcinoma
8430	Mucoepidermoid carcinoma
8525	Polymorphous carcinoma
8290	Oncocytic carcinoma
8314	Lipid-rich carcinoma
8315	Glycogen-rich clear cell carcinoma
8410	Sebaceous carcinoma
8983	Adenomyoepithelioma with carcinoma
8200	Adenoid cystic carcinoma
8500	Ductal carcinoma *in situ*
8503	Intraductal papilloma with ductal carcinoma *in situ*
8503	Intraductal papillary carcinoma
8504	Encapsulated papillary carcinoma
8504	Encapsulated papillary carcinoma with invasion
8509	Solid papillary carcinoma
8540	Paget disease of the nipple
8530	Inflammatory carcinoma

Lakhani SR, Ellis IO, Schnitt SJ, Hoon Tan P, van de Vijver MJ, eds. World Health Organization Classification of Tumours of the Breast. Lyon: IARC; 2012.

INTRODUCTION

This staging system for carcinoma of the breast applies to both invasive carcinoma (also designated infiltrating) and ductal carcinoma *in situ*, with or without microinvasion. Microscopic confirmation of the diagnosis is mandatory, and the histologic type and grade of carcinoma should be recorded. For all sites (T, N, M), clinical staging (c) is determined using information identified prior to surgery or neoadjuvant therapy. Pathological staging (p) includes information defined at surgery. Following neoadjuvant systemic therapy, posttherapy pathological staging is recorded using the "yp" designator. As discussed below, the benign entity termed "lobular carcinoma *in situ*" or "lobular neoplasia" is not included in this staging system.

Evolving knowledge of breast cancer biology and the increased validation of various biomarkers of prognosis and prediction of treatment benefit or resistance also suggest that several biomarkers should be documented at the time of initial diagnosis whenever this is possible. These biomarkers include histologic grade, hormone receptor status (estrogen receptor [ER] and progesterone receptor [PR]), human epidermal growth factor receptor-2 (HER2), a marker of proliferation (such as Ki-67 or a mitotic count), and for appropriate subgroups of tumors, a genomic prognostic panel (such as Oncotype Dx®, Mammaprint®, Endopredict, PAM50 (Prosigna), Breast Cancer Index, etc.), if available.

Codification of tumor staging into the TNM system by the American Joint Committee on Cancer (AJCC) started in 1959 (when the AJCC was operating as the American Joint Committee for Cancer Staging and End-Results Reporting). Since then, seven editions of the AJCC Cancer Staging Manual have been published, in which careful definitions of categories for the primary tumor (T), the status of the surrounding lymph nodes (N), and the presence of distant metastases have been refined to reflect updates in technology and clinical evidence.[1] During these five decades, changes to the TNM system in each revision were made cautiously, to reflect modern clinical approaches while maintaining connections with the past. As much as possible, changes were based on the highest level of evidence in the peer-reviewed literature.

Over the past decade, there have been fundamental changes in our understanding of the biology of breast cancer. We now think of breast cancer as a group of diseases with

different molecular characteristics (identified by gene expression profiling, immunohistochemistry, proteomics, next-generation sequencing, and other molecular techniques) that originate in breast epithelial tissue but have different prognoses, patterns of recurrence, and dissemination after primary multidisciplinary treatments and have different sensitivities to available therapies.[2] This enhanced knowledge has led to significant changes in diagnostic and therapeutic approaches, and such changes must be reflected in the current edition of the TNM classification, the AJCC Cancer Staging Manual, 8th Edition (8th Edition).

Rapid advances in both clinical and laboratory science and in translational research have raised questions about the ongoing relevance of TNM staging, especially in breast cancer. The TNM system was developed in 1959 in the absence of effective systemic therapy and based on limited understanding of the biology of breast cancer as well as the then-widely accepted paradigm of orderly progression for the tumor to regional nodes and thence to distant sites, which supported the use of the Halsted radical mastectomy introduced in the late 1800s. The TNM system was generated to reflect the risk of distant recurrence and death subsequent to local therapy, which at the time was almost universally aggressive surgery (radical mastectomy) and postoperative radiation to the chest wall. Therefore, the primary objective of TNM staging was to provide a standard nomenclature for prognosis of patients with newly diagnosed breast cancer, and its main clinical utility was to prevent apparently futile therapy in those patients who were destined to die rapidly in spite of aggressive local treatments.

Over the succeeding decades, remarkable progress challenged this Halstedian view of tumor progression with the understanding of the potential for distant systemic spread of all invasive cancers irrespective of node involvement and with demonstration of the value of adjuvant systemic therapy. This led to (1) more limited surgical management, with breast-conserving surgery being preferred for most patients with early-stage breast cancers and total mastectomy with axillary dissection for more advanced disease; (2) reduction in the extent of axillary staging, with sentinel lymph node biopsy becoming the leading approach for patients with clinically negative axillae; (3) dramatic improvements in the delivery and safety of radiation treatment; (4) the recognition that early (adjuvant) systemic therapy reduces the chance of recurrence and mortality; (5) the increasing implementation of preoperative (or neoadjuvant) systemic therapies for treatment of larger operable tumors and locally advanced breast cancer; and (6) a better understanding of biologic markers of prognosis and, perhaps more important, of prediction of response to selective categories of systemic therapy, such as those targeting cancer cells positive for ER and HER2 overexpression or amplification.[3] Heretofore, TNM staging based solely on the anatomic extent of disease has been used as a prognostic guide to select whether to apply systemic therapy. Based on such progress, biologic factors—such as grade, hormone receptor expression, HER2 overexpression/amplification, and genomic panels—have become as or more important than the anatomic extent of disease to define prognosis, select the optimal combination of systemic therapies,[3] and increasingly, influence the selection of locoregional treatments.[4]

Much of this biological information had started to appear at the time the 6th and 7th Editions were being developed, but published information with high enough level of evidence to incorporate biomarkers into the TNM classification was lacking or incomplete. As an example, it has been known for several decades that the expression of the ER in primary breast cancer conferred a more favorable prognosis than its absence to groups of patients in various clinical stages. However, precise analysis to demonstrate that within specific TNM stages, the presence of ER modified prognosis was not available. Similar statements can be made about grade, markers of proliferation, and HER2. Population-based registries have started to collect information about hormone receptors only within the past 10 to 15 years, and information about HER2 was not integrated into national databases (National Cancer Database [NCDB]; National Program of Cancer Registries [NPCR]; Surveillance, Epidemiology, and End Results [SEER]; and others) until 2010. In the meantime, clinical practice evolved rapidly, integrating modern biological knowledge into the selection of systemic treatments.[5] ER, PR, grade, and HER2 started to be collected by most clinical laboratories, and clinicians integrated these concepts into prognostication and selection of therapies. The widespread adoption of the concept of biologic intrinsic subtypes led to different treatment strategies for the three major biological subsets of breast cancer: (1) hormone receptor-positive (ER and/or PR positive), HER2-negative tumors (also referred to as luminal-type); (2) HER2-amplified or overexpressed breast cancers; and (3) breast cancers that do not express hormone receptors or HER2 (also known as triple-negative tumors).[3] More recently, it also was recognized that in the presence of HER2 overexpression/amplification, the presence or absence of hormone receptor expression was associated with different prognoses and responsiveness to anti-HER2 therapy. Based on that observation, the HER2-positive population is now approached differently based on the expression of hormone receptors. These advances raise two questions. (1) Is anatomic-based TNM staging still relevant for breast cancer? (2) What, exactly, is the objective of TNM staging for patients with this disease? The answer to the first question is twofold: The TNM staging classification based solely on anatomical/histological parameters is clearly relevant to that part of the world where that is the only information available to practitioners. It also remains useful as the foundational basis of staging classification for areas of the world where biological information is an integral part of the initial evaluation. However, in these regions, staging needs to expand to incorporate the prognostic and predictive value of biomarkers. The second question, on the objective of TNM staging, has three potential answers: (1) to provide continuity to breast cancer investigators, in regards to studying categories of patients that accurately reflect prior groupings over the last six decades, (2) to

permit current investigators in the field to communicate with one another using a standardized language that reflects disease burden and tumor biology, and (3) to improve individual patient care. The AJCC Breast Cancer Expert Panel has struggled with these questions for the past several editions and especially with the 8th Edition. The current Breast Cancer Expert Panel came to the conclusion that although the anatomy- and histology-based TNM staging system provides important insight into a patient's prognosis, the addition of various biomarkers refines the prognostic information and leads to better selection of systemic therapies and, therefore, better outcomes. For example, the ability to identify a group of patients with invasive breast cancer with prognosis that is so favorable that the patient might forego systemic therapy is an important feature of anatomical and biological staging. The ability to predict benefit from or resistance to specific treatments also is of critical importance.

Although anatomic T, N, and M still provide value in determining a patient's future outcome, the clinician today must take into account multiple factors that relate both to prognosis and prediction. For example, testing for ER and PR expression, as well as HER2 status, is now considered a prerequisite to treatment because it is factored into all prognostic and treatment decisions.[5] Although these factors have some limited intrinsic prognostic value in regards to the risk of subsequent recurrence for patients who do not receive systemic therapy, their main utility is prediction of benefit from therapy, guiding whether a patient should or should not receive adjuvant endocrine (anti-estrogen) or anti-HER2 (such as trastuzumab) therapy. The use of these factors as both prognostic and predictive markers is fundamentally important in evaluation and care of patients with newly diagnosed breast cancer, as well as for patients with metastatic breast cancer.

The situation has become even more complex with the availability of multigene expression assays.[6] One such assay, based on a 70-gene prognostic signature developed by investigators from Amsterdam,[7] has been cleared by the US Food and Drug Administration (FDA) for use in women who are younger than 61 years old and who have Stage I or II, node-negative breast cancer, explicitly to assess a patient's risk for distant metastases (http://www.fda.gov/ForConsumers/ConsumerUpdates/ucm048477.htm). The Tumor Marker Guidelines Committee of the American Society of Clinical Oncology (ASCO) has recommended that a second multigene assay, which is based on expression of 21 genes as determined by reverse transcriptase–polymerase chain reaction (RT-PCR) (designated the "21-gene recurrence score assay" or by its proprietary name, Oncotype Dx®) can be used to determine prognosis for patients with ER-positive breast cancer and uninvolved lymph nodes who will, at the least, receive adjuvant tamoxifen.[3] Similarly, the Breast Cancer Guideline Committee of the National Comprehensive Cancer Network (NCCN) states that, "The use of genomic/gene expression arrays which also incorporate additional prognostic/predictive biomarkers (e.g., Oncotype Dx® Recurrence Score) may provide additional prognostic and predictive information beyond anatomic staging and determination of ER, PR, and HER2 status."[5] Additional clinical validation of these assays continues to accumulate. As an example, the initial results of the ECOG-ACRIN Cancer Research Group-led *Trial Assigning Individualized Options for Treatment (Rx) (TAILORx)* trial were published in late 2015, documenting that the group of patients with hormone receptor-positive, HER2-negative, lymph node-negative breast cancer who had a Recurrence Score lower than 11 by the Oncotype Dx® assay had a 5-year distant recurrence-free survival of 99.3% with adjuvant endocrine therapy alone.[8] Two additional reports presented as abstracts at the 2015 European Society for Medical Oncology (ESMO) meeting confirmed the ability of the 21-gene genomic assay to identify the group of patients who can safely forgo chemotherapy and still have an excellent prognosis.[9,10]

The panel deliberated with the question of how these gene profile assays could be incorporated into the TNM staging system, because they portend future outcomes in several ways. Should they be used (1) as pure prognostic factors that serve as secondary modifiers of the basic TNM classification,[11,12] (2) as components of multifactorial prognostic models that calculate individual risk of recurrence and perhaps individual sensitivity to therapy,[13-17] or (3) as components of simple prognostic scoring systems that add to, but do not alter the basic structure of, the TNM staging classification?[18] Should the multiparameter prognostic assays (Oncotype Dx®, Mammaprint®, PAM50, EndoPredict, Breast Cancer Index, etc.) that appear to predict outcomes in newly diagnosed breast cancer patients be included in staging? Because their value may be as much a predictor of response to chemotherapy regardless of TNM stage as a prognostic factor, should an entirely new category related to prediction of benefit from systemic therapy be incorporated into the TNM staging system? Increasingly in the modern era, many treatment decisions for patients with newly diagnosed breast cancer are not based on anatomic TNM stage, and certainly not on stage alone. Large tumor size (T3 versus T1 or T2) and lymph node status (N1, N2, or N3 versus N0) influence decisions regarding whether radiation should be used after mastectomy or for directing the fields of radiation for women undergoing breast preservation and in recommendations for axillary dissection. However, in an era when many invasive cancers are detected at very small sizes due to breast screening, multicentricity and tumor margins appear to be as important as T or N in determining optimal local treatment approaches. In the past, recommendations for most systemic therapy, especially chemotherapy, have been based on nodal status, and in the absence of involved lymph nodes, tumor size.[19,20] Today, such decisions are largely reached based on the biologic characteristics of the primary tumor, rather than the extent of disease.

In 2013, 1.8 million women were diagnosed with breast cancer around the world and 471,000 died of this neoplasm.[21] Although the majority of breast cancers in the industrialized world are diagnosed in early stages and the great majority are cured, more than half of patients with breast cancer in the low- and middle-income countries (LMCs) are diagnosed in late

stages (III and IV), with the majority of them dying of metastatic breast cancer. It is projected that the annual global burden of new breast cancer cases will continue to increase and an ever-increasing fraction will be from LMCs.[22] Despite the common misconception that breast cancer is predominantly a problem of wealthy countries, the majority of breast cancer deaths each year in fact occur in LMCs.[21] LMCs simply may not be able to afford testing for individual molecular events or multiparameter profiles, nor will they be able to provide expensive therapies directed against ER, HER2, cdk 4/6, or other emerging targets. Tissue assays as basic as ER and PR may be unavailable in low-income settings, even when oral endocrine therapies can be provided. Thus, anatomic (TNM) staging remains a key aspect of cancer control in LMCs, because it directly reflects the degree to which early detection programs are working. In LMCs, anatomic staging will remain the cornerstone on which evaluation and treatment decisions of newly diagnosed breast cancer patients will be made.

Although the advances in molecular diagnosis have provided compelling new insights into cancer therapy, the Expert Panel understands that economic considerations limit the relevance of these observations to the societies in which resources permit widespread screening, molecular evaluation of tumor tissue, and application of cutting-edge biologically directed therapies. Nonetheless, as survival data continue to accumulate, these and other molecular assays must be incorporated into future updates of AJCC breast cancer staging.

After much deliberation, the Expert Panel determined that, in addition to modest adjustments to the T, N, and M categories for the 8th Edition, progress in biology, diagnostics, and therapeutics made incorporation of basic biomarkers into the TNM classification an absolute necessity. Therefore, the Breast Cancer Expert Panel made changes to the TNM staging system incorporating the basic biomarkers in widespread use today that have demonstrated clinical utility. These biomarkers are already being collected by the NCDB, NPCR, SEER, and other population-based registry databases in the United States. To preserve the relevance of the TNM staging classification for the entire world, the Expert Panel elected to integrate biomarkers as a second tier of prognostic modifiers, as have other tumor-specific Expert Panels within AJCC (e.g., esophagus and esophagogastric junction, prostate, gestational trophoblastic neoplasms, testis, and others).

At the same time, the Expert Panel agreed unanimously that the staging system must provide clinicians the ability to determine a purely anatomic-based stage. This allows usage around the world for patients who do not or cannot have the standard biomarker assays performed. It also provides for comparison of cases across time to continue to evaluate progress in breast cancer care on a population-wide basis.

The Expert Panel discussed at length the confounding effect of treatment on defining stage groupings. Data are not available on patients who do not receive treatment, a group historically considered the "gold standard" for a staging system. In reality, however, when the TNM staging classification was first developed, the great majority of breast cancers already were receiving treatment with definitive surgery and, if indicated, radiotherapy. Therefore, from its inception, the TNM classification reflected prognosis of patients who had received definitive locoregional therapy. Today, almost six decades later, few patients with breast cancer fail to receive therapy, except for those who refuse or those with comorbid conditions severe enough to preclude treatment. Therefore, TNM staging reflects the prognosis of patients treated with the current standard multimodality treatment. In that sense, the incorporation of biomarkers into the 8th Edition will refine the prognostic character of the classification and will guide the optimal selection of treatments for large subsets of patients with primary and/or metastatic breast cancer. It also will serve as the new platform to continue investigations that will lead to improvement of both prognostic and predictive ability in future editions. Updates for AJCC staging will depend upon the availability and validity of data of all predictors and prognosticators of breast cancer, both conventional TNM and molecular markers and genomic assays that influence survival.

More advanced AJCC staging models will undoubtedly reflect contemporary clinical and scientific data but will require adequate follow-up to accurately determine survival. As the complexity of survival predictions increase, stage groupings may evolve into calculation models to assign survival and stage. This progression of knowledge is likely to lead to more frequent modifications of survival-based stage assignments than has been experienced over the typical lifespan of the previous seven editions of the AJCC staging manuals. It is therefore anticipated that updates will be made on a more timely or "rolling" basis when relevant validated information is available, rather than the historical 6- to 8-year cycle of TNM revisions.

ANATOMY

Primary Site(s)

The mammary gland, situated on the anterior chest wall, is composed of glandular tissue with a dense fibrous stroma admixed with adipose tissue. The glandular tissue consists of lobules that group together into 8–15 lobes, occasionally more, arranged approximately in a spoke-like pattern. Multiple major and minor ducts connect the milk-secreting lobular units to the nipple. Small ducts course throughout the breast, converging into larger collecting ducts that open into the lactiferous sinuses at the base of the nipple. Each duct system has a unique anatomy: The smallest systems may comprise only a portion of a quadrant, whereas the largest systems may comprise more than a quadrant. The periphery of each system overlaps along their radial boundaries (Fig. 48.1). Most cancers form initially in the terminal duct lobular units of the breast. *In situ* carcinoma spreads along the duct system in the radial axis of the lobe; invasive carcinoma is more likely to spread in a centripetal orientation in

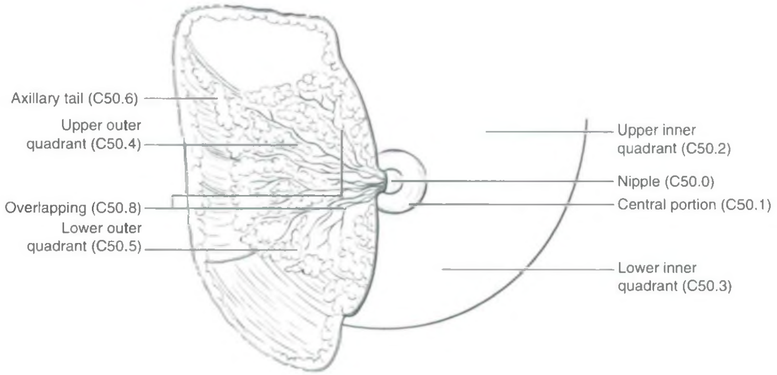

Fig. 48.1 Anatomic sites and subsites of the breast

the breast stroma from the initial locus of invasion, although opportunistic intraductal spread may be enhanced along the radial axes. Glandular tissue is more abundant in the upper outer portion of the breast; as a result, half of all breast cancers occur in this region.

Chest Wall

The chest wall includes ribs, intercostal muscles, and serratus anterior muscle, but not the pectoral muscles. Therefore, involvement of the pectoral muscle in the absence of invasion of these chest wall structures or skin does not constitute chest wall invasion, and such cancers are categorized on the basis of tumor size.

Regional Lymph Nodes

The breast lymphatics drain by way of three major routes: axillary, interpectoral, and internal mammary. Intramammary lymph nodes reside within breast tissue and are designated as axillary lymph nodes for staging purposes. Supraclavicular lymph nodes are categorized as regional lymph nodes for staging purposes. Metastases to any other lymph node, including cervical or contralateral internal mammary or contralateral axillary lymph nodes, are classified as distant metastases (M1) (Fig. 48.2).

The regional lymph nodes are as follows:

1. Axillary (ipsilateral): interpectoral (Rotter's) nodes and lymph nodes along the axillary vein and its tributaries that may be (but are not required to be) divided into the following levels:
   a. Level I (low-axilla): lymph nodes lateral to the lateral border of pectoralis minor muscle.
   b. Level II (mid-axilla): lymph nodes between the medial and lateral borders of the pectoralis minor muscle and the interpectoral (Rotter's) lymph nodes.
   c. Level III (apical axilla): lymph nodes medial to the medial margin of the pectoralis minor muscle and inferior to the clavicle. These are also known as apical or infraclavicular nodes. Metastases to these nodes portend a worse prognosis. Therefore, the infraclavicular designation will be used hereafter to differentiate these nodes from the remaining (Level I, II) axillary nodes. Level III infraclavicular nodes should be separately identified by the surgeon for microscopic evaluation.
2. Internal mammary (ipsilateral): lymph nodes in the intercostal spaces along the edge of the sternum in the endothoracic fascia.
3. Supraclavicular: lymph nodes in the supraclavicular fossa, a triangle defined by the omohyoid muscle and tendon (lateral and superior border), the internal jugular vein (medial border), and the clavicle and subclavian vein (lower border). Adjacent lymph nodes outside of this triangle are considered to be lower cervical nodes (M1).
4. Intramammary: lymph nodes within the breast; these are considered axillary lymph nodes for purposes of N categorization and staging.

Metastatic Sites

Tumor cells may be disseminated by either the lymphatic or the blood vascular system. The four most common sites of involvement are bone, lung, brain, and liver, but breast cancers also are capable of metastasizing to many other sites. Bone marrow micrometastases, circulating tumor cells (CTCs), and tumor deposits no larger than 0.2 mm detected inadvertently, such as in prophylactically removed ovarian tissue, are collectively known as microscopic disseminated tumor cells and clusters (DTCs). These deposits do not alone define or constitute metastatic disease, although data exist that demonstrate that, in early stage disease, DTCs correlate with recurrence and mortality risk, and in patients with established M1 disease, CTCs are prognostic for shorter survival.

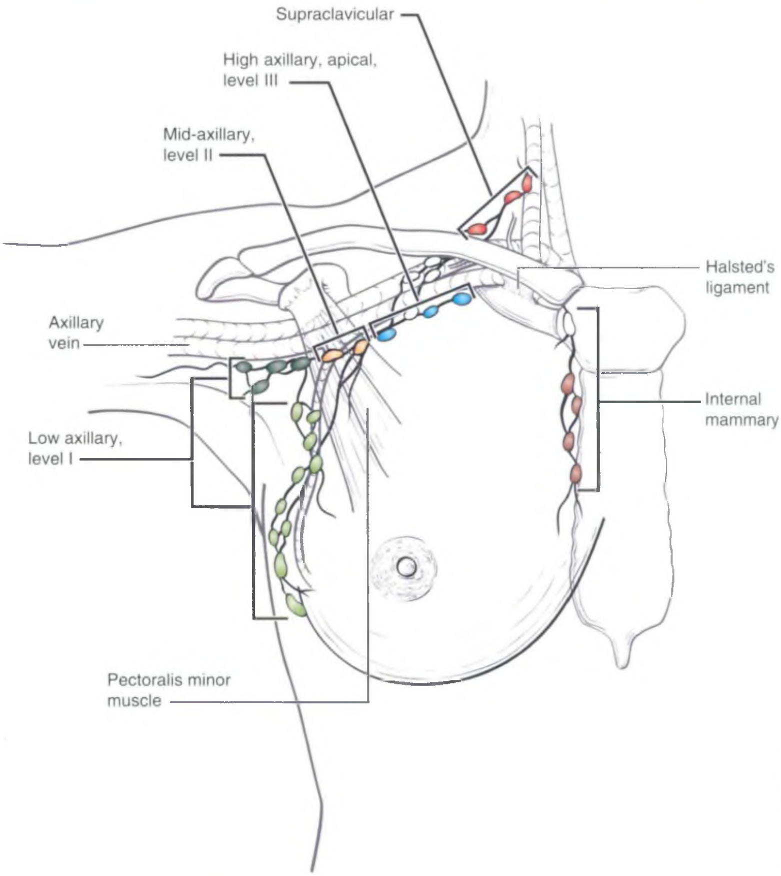

Fig. 48.2 Schematic diagram of the breast and regional lymph nodes

RULES FOR CLASSIFICATION

The anatomic TNM system is a method for coding extent of disease. This is done by assigning a category of extent of disease for the tumor (T), regional lymph nodes (N), and distant metastases (M). T, N, and M are assigned by clinical means and by adding surgical findings and pathological information to the clinical information (see Chapter 1). The documented prognostic impact of postneoadjuvant extent of disease and response to therapy warrant clear definitions of the use of the "yp" prefix and response to therapy. The use of neoadjuvant therapy does not change the clinical (pretreatment) stage. As per TNM rules, the clinical stage is identified with the prefix "c" (e.g., cT). In addition, clinical staging can include the use of fine needle aspiration (FNA) or core needle biopsy and sentinel lymph node biopsy before neoadjuvant therapy. These are denoted with the postscripts "f" and "sn," respectively. Nodal metastases confirmed by FNA or core needle biopsy are classified as macrometastases (cN1), regardless of the size of the tumor focus in the final pathological specimen. For example, if, prior to neoadjuvant systemic therapy, a patient with a 1 cm primary has no palpable nodes but has an ultrasound-guided FNA biopsy of an axillary lymph node that is positive, the patient will be categorized as cN1 (f) for clinical (pretreatment) staging and is assigned to Stage IIA. Likewise, if the patient has a positive axillary sentinel node identified prior to neoadjuvant systemic therapy, the tumor is categorized as cN1 (sn) (Stage IIA). As per TNM rules, in the absence of pathological T evaluation (removal of the primary tumor), which is identified with prefix "p" (e.g., pT), microscopic evaluation of nodes before neoadjuvant therapy, even by complete removal such as sentinel node biopsy, is still classified as clinical (cN).

Clinical Classification

Clinical categorization of a cancer is based on findings of history, physical examination, and any imaging studies that are done. Imaging studies are not required to assign clinical

categories or stage. Cases with a biopsy of lymph nodes or metastatic sites may be staged clinically, including the biopsy information.

Physical Examination

Physical examination includes careful inspection and palpation of the skin, mammary gland, and lymph nodes (axillary, supraclavicular, and cervical), imaging, and pathological examination of the breast or other tissues as appropriate to establish the diagnosis of breast carcinoma. The extent of tissue examined pathologically for clinical staging is not as great as that required for pathological staging (see "Pathological Classification" in this chapter).

Imaging

Imaging findings are considered elements of staging if they are collected within 4 months of diagnosis or through completion of surgery, whichever is longer in the absence of disease progression. Relevant imaging findings include the size of the primary invasive cancer and of chest wall invasion and the presence or absence of regional or distant metastases. Imaging and clinical findings obtained after a patient has been treated with neoadjuvant chemotherapy, hormonal therapy, immunotherapy, or radiation therapy are not considered elements of initial clinical staging. If recorded in the medical record, these should be denoted using the modifier prefix "yc."

Breast cancer clinical T, N, and M categorizations are based on a combination of clinical examination and imaging findings. Clinical findings are usually integrated with imaging to determine the size of primary tumor and the presence or absence of multiple synchronous lesions involving the same breast quadrant or different breast quadrants (i.e., multifocal or multicentric disease, respectively). The imaging modalities most commonly used to help determine T and N features are mammography and ultrasound. The routine use of breast magnetic resonance (MR) imaging in newly diagnosed cancer patients has not been shown to have significant benefit in obtaining clear surgical margins[23-26] and its effect on improving local recurrence and survival is under debate.[27,28] If MR imaging of the breast is performed, it should be done in consultation with the multidisciplinary treatment team, using a dedicated breast coil, and interpreted by a breast imaging team capable of performing MR imaging-guided biopsy. MR imaging is indicated in patients presenting with axillary breast cancer metastasis with no evident breast tumor on clinical, mammographic, and sonographic examination (occult breast primary) and may help facilitate breast-conserving therapy in this patient subgroup.

Primary Tumor (T) – Clinical and Pathological

The T category of the primary tumor is defined by the same criteria regardless of whether it is based on clinical or pathological criteria, or both. The T category is based primarily on the size of the invasive component of the cancer. See Figs. 48.3, 48.4 and 48.5 for illustrations of the T-categories.

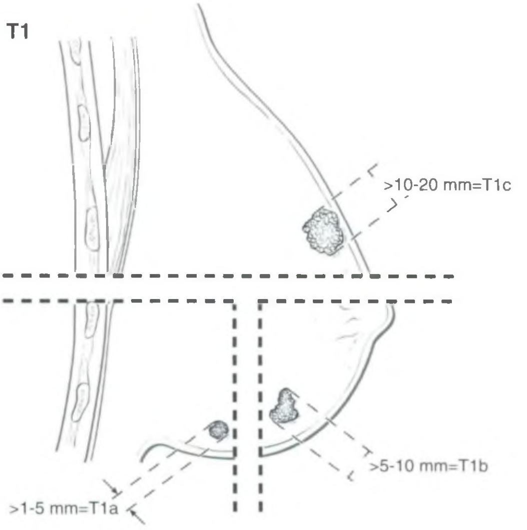

Fig. 48.3 T1 is defined as a tumor 20 mm or less in greatest dimension. T1mi is a tumor 1 mm or less in greatest diameter (not illustrated). T1a is defined as tumor more than 1 mm but not more than 5 mm in greatest dimension; T1b is defined as tumor more than 5 mm but not more than 10 mm in greatest dimension; T1c is defined as tumor more than 10 mm but not more than 20 mm in greatest dimension

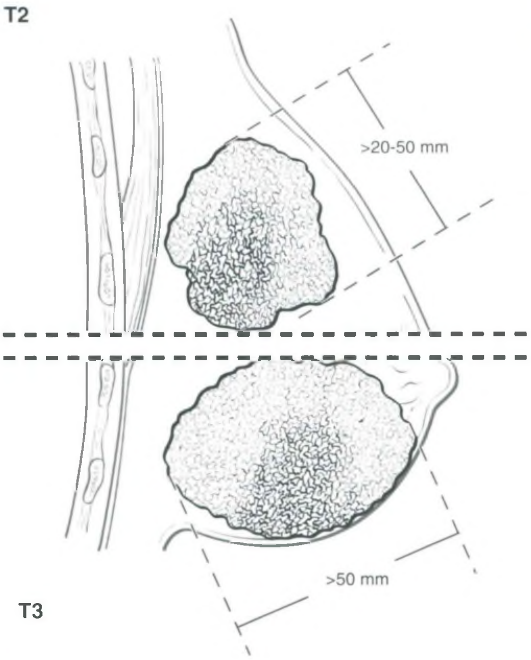

Fig. 48.4 T2 (above dotted line) is defined as tumor more than 20 mm but not more than 50 mm in greatest dimension, and T3 (below dotted line) is defined as tumor more than 50 mm in greatest dimension

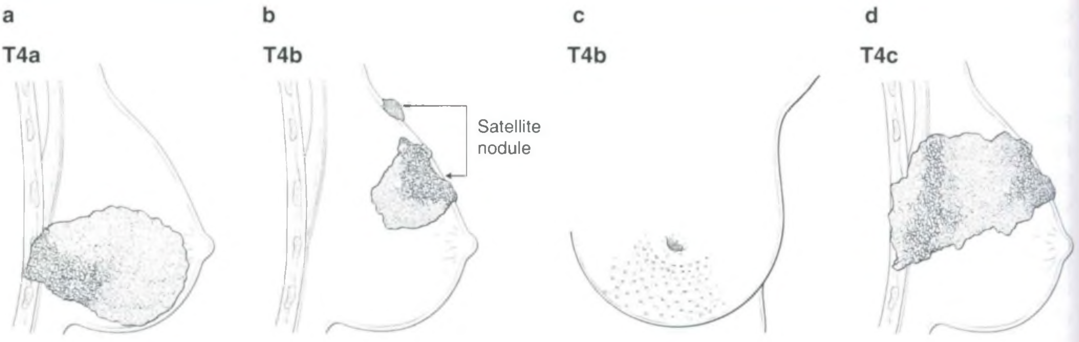

Fig. 48.5 T4 is defined as a tumor of any size with direct extension to chest wall and/or to the skin (ulceration or skin nodules). (**a**) T4a is extension to the chest wall. Adherence/invasion to the pectoralis muscle is NOT extension to the chest wall and is not categorized as T4. (**b**) T4b, illustrated here as satellite skin nodules, is defined as edema (including peau d'orange) of the skin, or ulceration of the skin of the breast, or satellite skin nodules confined to the same breast. These do not meet the criteria for inflammatory carcinoma. (**c**) T4b illustrated here as edema (including peau d'orange) of the skin. (**d**) T4c is defined as both T4a and T4b. T4d (not illustrated) is inflammatory cancer (see text for definition)

The maximum size of a tumor focus is used as an estimate of disease volume. The largest contiguous dimension of a tumor focus is used, and small satellite foci of noncontiguous tumor are not added to the size. The cellular fibrous reaction to invasive tumor cells is generally included in the measurement of a tumor prior to treatment; however, the dense fibrosis observed following neoadjuvant treatment is generally not included in the pathological measurement because its extent may overestimate the residual tumor volume.

Tumor Size

The clinical size of a primary tumor (T) can be measured based on clinical findings (physical examination and imaging modalities, such as mammography, ultrasound, and MR imaging) and pathological findings (gross and microscopic measurements). Clinical tumor size (cT) should be based on the clinical findings that are judged to be most accurate for a particular case, although it may still be somewhat inaccurate because the extent of some breast cancers is not always apparent with current imaging techniques and because tumors are composed of varying proportions of noninvasive and invasive disease, which these techniques are currently unable to distinguish.

Imaging Classification of Tumor (T)

The American College of Radiology (ACR) BI-RADS lexicon provides general guidelines for the reporting of mammography, breast ultrasound, and breast MR imaging studies.[29] All breast imaging reports should follow these guidelines. Information relevant to primary tumor size should be accurately measured in at least the longest diameter in the plane of measurement and should be included in the report body and the final impression sections. If the primary tumor also is associated with such features as calcifications or architectural distortion, this combined size should be provided in the report. If present, extension of the primary tumor to the ipsilateral nipple, overlying skin, or underlying chest wall should be clearly indicated. MR imaging is more accurate than ultrasound and mammography in confirming chest wall involvement by demonstrating abnormal enhancement within chest wall structures.[30] When more than one malignant lesion is identified on imaging, the size and description of their locations (i.e., quadrant and/or distance from the nipple and/or distance to the index tumor) should be defined in the imaging report. The same tumor may have different measurements using different modalities (e.g., mammography versus ultrasound versus MR imaging). If available, MR imaging measurements could be used based on prior studies demonstrating better correlation with overall tumor size. However, if index tumor size difference between different imaging modalities, including that of MR imaging, significantly affects T classification or overall clinical stage, imaging-guided biopsy could be considered to confirm disease extent. Imaging-guided tissue biopsy can similarly be considered for any additional lesions suspicious for multifocal or multicentric secondary lesions that affect clinical management.

Size should be measured to the nearest millimeter. If the tumor size is slightly less than or greater than a cutoff for a given T classification, the size should be rounded to the millimeter reading that is closest to the cutoff. For example, a reported size of 4.9 mm is reported as 5 mm, or a size of 2.04 cm is reported as 2.0 cm (20 mm). The exception to this rounding rule is for a breast tumor sized between 1.0 and 1.4 mm. These sizes are rounded up to 2 mm, because rounding down would result in the cancer's being categorized as microinvasive carcinoma (T1mi) defined as a size of 1.0 mm or less.

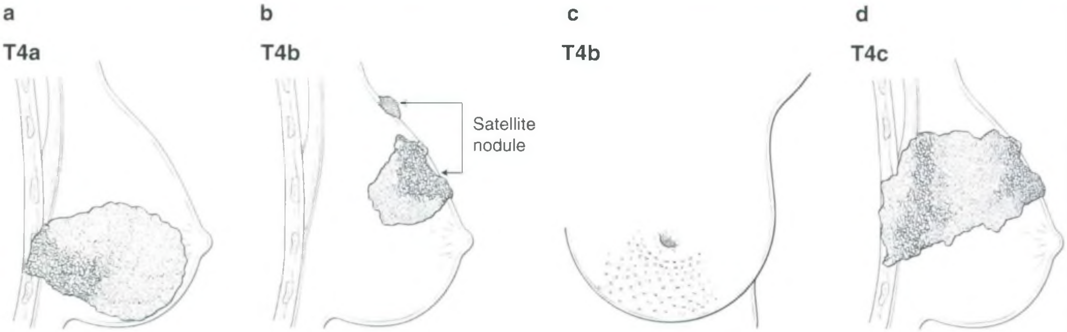

Fig. 48.5 T4 is defined as a tumor of any size with direct extension to chest wall and/or to the skin (ulceration or skin nodules). (**a**) T4a is extension to the chest wall. Adherence/invasion to the pectoralis muscle is NOT extension to the chest wall and is not categorized as T4. (**b**) T4b, illustrated here as satellite skin nodules, is defined as edema (including peau d'orange) of the skin, or ulceration of the skin of the breast, or satellite skin nodules confined to the same breast. These do not meet the criteria for inflammatory carcinoma. (**c**) T4b illustrated here as edema (including peau d'orange) of the skin. (**d**) T4c is defined as both T4a and T4b. T4d (not illustrated) is inflammatory cancer (see text for definition)

The maximum size of a tumor focus is used as an estimate of disease volume. The largest contiguous dimension of a tumor focus is used, and small satellite foci of noncontiguous tumor are not added to the size. The cellular fibrous reaction to invasive tumor cells is generally included in the measurement of a tumor prior to treatment; however, the dense fibrosis observed following neoadjuvant treatment is generally not included in the pathological measurement because its extent may overestimate the residual tumor volume.

Tumor Size

The clinical size of a primary tumor (T) can be measured based on clinical findings (physical examination and imaging modalities, such as mammography, ultrasound, and MR imaging) and pathological findings (gross and microscopic measurements). Clinical tumor size (cT) should be based on the clinical findings that are judged to be most accurate for a particular case, although it may still be somewhat inaccurate because the extent of some breast cancers is not always apparent with current imaging techniques and because tumors are composed of varying proportions of noninvasive and invasive disease, which these techniques are currently unable to distinguish.

Imaging Classification of Tumor (T)

The American College of Radiology (ACR) BI-RADS lexicon provides general guidelines for the reporting of mammography, breast ultrasound, and breast MR imaging studies.[29] All breast imaging reports should follow these guidelines. Information relevant to primary tumor size should be accurately measured in at least the longest diameter in the plane of measurement and should be included in the report body and the final impression sections. If the primary tumor also is associated with such features as calcifications or architectural distortion, this combined size should be provided in the report. If present, extension of the primary tumor to the ipsilateral nipple, overlying skin, or underlying chest wall should be clearly indicated. MR imaging is more accurate than ultrasound and mammography in confirming chest wall involvement by demonstrating abnormal enhancement within chest wall structures.[30] When more than one malignant lesion is identified on imaging, the size and description of their locations (i.e., quadrant and/or distance from the nipple and/or distance to the index tumor) should be defined in the imaging report. The same tumor may have different measurements using different modalities (e.g., mammography versus ultrasound versus MR imaging). If available, MR imaging measurements could be used based on prior studies demonstrating better correlation with overall tumor size. However, if index tumor size difference between different imaging modalities, including that of MR imaging, significantly affects T classification or overall clinical stage, imaging-guided biopsy could be considered to confirm disease extent. Imaging-guided tissue biopsy can similarly be considered for any additional lesions suspicious for multifocal or multicentric secondary lesions that affect clinical management.

Size should be measured to the nearest millimeter. If the tumor size is slightly less than or greater than a cutoff for a given T classification, the size should be rounded to the millimeter reading that is closest to the cutoff. For example, a reported size of 4.9 mm is reported as 5 mm, or a size of 2.04 cm is reported as 2.0 cm (20 mm). The exception to this rounding rule is for a breast tumor sized between 1.0 and 1.4 mm. These sizes are rounded up to 2 mm, because rounding down would result in the cancer's being categorized as microinvasive carcinoma (T1mi) defined as a size of 1.0 mm or less.

Inflammatory Carcinoma

Inflammatory carcinoma is a clinical-pathological entity characterized by diffuse erythema and edema (peau d'orange) involving approximately a third or more of the skin of the breast.[31] The tumor of inflammatory carcinoma is classified cT4d. It is important to remember that inflammatory carcinoma is primarily a clinical diagnosis. On imaging, there may be a detectable mass and characteristic thickening of the skin over the breast. An underlying mass may or may not be palpable. The skin changes may be due to lymphedema caused by tumor emboli within dermal lymphatics, which may or may not be obvious in a small skin biopsy. Therefore, the pathological finding of tumor in dermal lymphatics is not necessary to assign the diagnosis of inflammatory cancer. A tissue diagnosis is necessary to demonstrate an invasive carcinoma in the underlying breast parenchyma, or at least in the dermal lymphatics, and to determine biologic markers (ER, PR, HER2, and grade). Tumor emboli in dermal lymphatics without the clinical skin changes described above should be classified according to tumor size (T1, T2, or T3) and do not qualify as inflammatory carcinoma. Locally advanced breast cancers directly invading the dermis or ulcerating the skin without the clinical skin changes also do not qualify as inflammatory carcinoma. A characteristic of inflammatory carcinoma of the breast is its rapid evolution, from first symptom to diagnosis of less than 6 months.[31] Thus, the term *inflammatory carcinoma* should not be applied to a patient with neglected locally advanced cancer of the breast presenting late in the course of her disease.

Skin of Breast

Dimpling of the skin, nipple retraction, or any other skin change except those described under T4b and T4d may occur in T1, T2, or T3 tumors without changing the T classification.

The category should be made with the prefix "c" or "p" modifier to indicate whether the T category was determined by clinical information (physical examination with whatever breast imaging was done) or by clinical information supplemented by pathological measurements from surgical resection, respectively. In a few cases, such as for small tumors where the biopsy procedure may have removed a substantial portion of the tumor (e.g., vacuum-assisted core needle biopsy), such clinical information as imaging size and biopsy tumor dimension should be considered when assigning the final pathological size and category (pT).

Regional Lymph Nodes – Clinical (cN)

The definitions for clinical and pathological node categorization are different. See Fig. 48.6 for illustrations of the clinical categories for regional lymph nodes. Clinical categorization includes nodes detected by imaging studies (excluding lymphoscintigraphy) or by clinical examination and having characteristics highly suspicious for malignancy or a presumed histologic macrometastasis based on FNA biopsy, core needle biopsy, or sentinel node biopsy. Confirmation of clinically detected metastatic disease by fine needle aspiration or core needle biopsy is designated with an (f) suffix, for example, cN3a(f). Histologic confirmation in the absence of assignment of a pT (through surgical resection) is classified as cN, including excision of a node; for example, an axillary sentinel node biopsy with a macrometastasis is classified cN1a(sn) when primary tumor classification is clinical (cT). The method of confirmation of the nodal status should be designated as either clinical (cN), FNA/core biopsy (cN(f)), or sentinel node biopsy (cN(sn)).

Imaging studies are not necessary to categorize the regional nodes as negative. The designation cN0, not cNX, should be used for an axilla that is negative solely by physical examination. Even when regional lymph nodes have been previously removed, if no disease is identified in the nodal basin by imaging or clinical examination, it should be categorized as cN0.

For patients who are clinically node-positive, cN1 designates metastases to one or more movable ipsilateral Level I, II axillary lymph nodes. cN2a designates metastases to Level I, II axillary lymph nodes that are fixed to each other (matted) or to other structures, and cN3a indicates metastases to ipsilateral infraclavicular (Level III axillary) lymph nodes. Metastases to the ipsilateral internal mammary nodes detected by imaging studies (including computed tomography [CT] scan and ultrasound, but excluding lymphoscintigraphy) or by clinical examination are designated as cN2b when they do not occur in conjunction with metastases to the Level I, II axillary lymph nodes and cN3b when they occur in conjunction with axillary Level I, II metastases. Metastases to the ipsilateral supraclavicular lymph nodes are designated as cN3c regardless of the presence or absence of axillary or internal mammary nodal involvement. Because lymph nodes that are detected by clinical or imaging examination are frequently larger than 1.0 cm, the presence of tumor deposits should be confirmed by FNA or core needle biopsy, with cytologic/histologic examination if possible, but biopsy is not necessary to categorize as lymph node-positive. Lymph nodes classified as malignant by clinical or imaging characteristics alone, or only FNA cytology examination or core needle biopsy, and not by formal surgical dissection and pathological review, are presumed to contain macrometastases for purposes of clinical staging classification. When confirmed by FNA or core needle biopsy, the (f) modifier should be used to indicate cytologic/histologic confirmation, for example, cN2a(f). If a lymph node or nodes are removed by surgical excisional biopsy or sentinel lymph node biopsy and examined histopathologically, and the primary tumor has not been removed, the N category is recorded as clinical (cN).

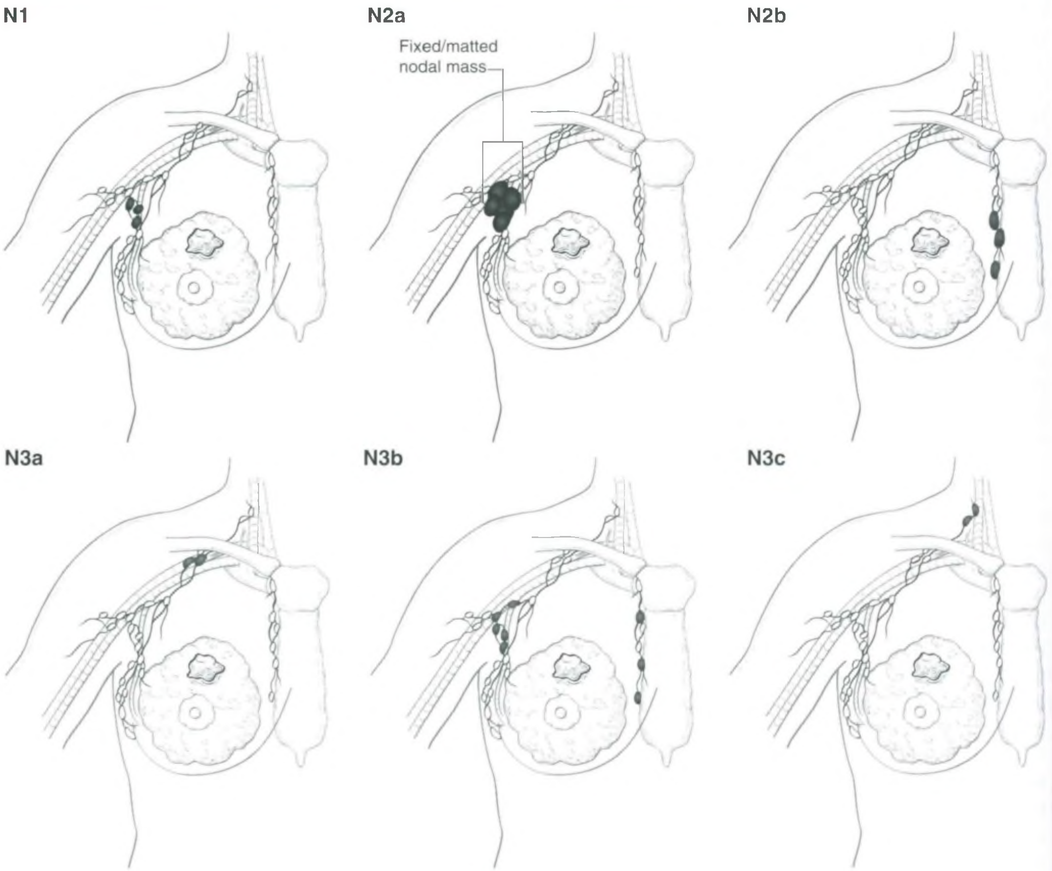

Fig. 48.6 Clinical Lymph Node Categories: cN1 is defined as metastasis in movable ipsilateral level I, II axillary lymph nodes. cN2a is defined as metastasis in ipsilateral level I, II axillary lymph nodes fixed to one another (matted). cN2b is defined as metastasis only in clinically detected ipsilateral internal mammary nodes and in the absence of clinically evident level I, II axillary lymph node metastasis. cN3a is defined as metastasis in ipsilateral infraclavicular (level III axillary) lymph node(s) with or without level I, II axillary lymph node involvement. cN3b is defined as metastasis in clinically detected ipsilateral internal mammary lymph node(s) and clinically evident axillary lymph node(s). cN3c is defined as metastasis in ipsilateral supraclavicular lymph node(s) with or without axillary or internal mammary lymph node involvement

Imaging Classification of Regional Lymph Nodes (N)

Imaging is not necessary to assign the clinical node category. Routine use of axillary ultrasound in breast cancer patients is controversial. Meta-analyses[32,33] suggest that among patients who prove to have positive nodes, clinically occult axillary nodal metastases can be detected in about half on preoperative ultrasound evaluation. In centers that routinely implement regional nodal ultrasound, imaging should include at least ipsilateral axillary levels I and II. Lymph node measurements are obtained by both long and short axis lengths on ultrasound. However, ultrasound measurements are operator- and technique-dependent. Ultrasound-guided needle biopsy of the index axillary node with clip placement should be considered in keeping with previously published guidelines.[19] Imaging or histopathological evidence of axillary Level I or II lymphadenopathy warrants an imaging investigation of Level III axillary, internal mammary chain, and supraclavicular lymph node involvement. Internal mammary nodes can be imaged using ultrasound.[34] Alternatively, they may be evident on breast MR imaging or chest CT if performed. Ultrasound, CT, or positron emission tomography (PET)-CT may be used to demonstrate any possible metastatic supraclavicular lymph nodes. Lymph node measurements are obtained by the length of their short axis on cross-sectional imaging.

Distant Metastasis (M)

Clinical assessment for distant metastases is by clinical history, physical examination, and imaging studies.

History and Physical Examination

Detection of metastatic disease by clinical exam should include a full history and physical examination, focusing on symptoms and radiographic findings. When appropriate, serial physical examinations based on evolving symptoms, physical findings, radiographic findings, and/or laboratory findings should be done on an iterative basis. Physical findings alone rarely will provide the basis for assigning cM1 category, and radiographic studies are almost always required. Whenever feasible, biopsy confirmation should be performed (pM1) and, if possible, tested for ER, PR, and HER2.

Imaging Classification of Metastases (M)

It is not necessary for the patient to have radiological evaluation of distant sites to be classified as clinically free of metastases (cM0). The indications for radiographic evaluation for the presence of metastases in the staging of breast cancer varies by T and N categorization. All guidelines stipulate that suspicious findings in the history or physical examination and/or elevated serologic tests for liver or bone function are indications to proceed with radiographic systemic imaging, such as bone or body scintigraphy or anatomic, cross-sectional imaging.[35] Most experts agree that systemic radiographic staging evaluation for metastases is not warranted in asymptomatic patients with normal blood tests who have T1–2, N0 breast cancer and, likewise, most experts agree that staging is appropriate for patients with Stage III disease (clinical or pathologic).[36] Recommendations are mixed for patients with T2N1. Whether radiographic staging should or should not be initiated might be influenced by the number of nodes, grade, and biomarker profile.

If imaging studies are indicated, these should focus on common sites of metastatic disease and/or sites indicated by symptoms or blood tests. Certain findings, such as multiple lesions with classical characteristics of metastases, and clear changes from earlier studies may provide a very high index of suspicion and result in M1 categorization. With radiographic screening or evaluation for another cause, false-positive staging studies in patients with newly diagnosed breast cancer are relatively common.

In patients with clinical Stage I and Stage II disease, routine use of imaging to detect occult distant metastasis is discouraged,[37] based on its previously demonstrated low yield and because of the risk of false-positive findings. For clinical Stages I–IIB, additional studies can be considered only if directed by the following signs or symptoms:

- Bone scan indicated if localized bone pain or elevated alkaline phosphatase
- Abdominal, with or without pelvic, diagnostic CT or MR imaging indicated if elevated alkaline phosphatase, abnormal liver function tests, abdominal symptoms, or abnormal physical examination of the abdomen or pelvis
- Chest diagnostic CT if pulmonary symptoms present[19]

For patients with clinical Stage IIIA and higher locoregional disease, the above diagnostic tests can be considered in the absence of clinical signs or symptoms of distant metastasis.[29] 18-Fluorodeoxyglucose-PET (18F-FDG-PET) can be used in the workup of patients with locally advanced breast cancer Stage IIIB and higher as a "screen" for distant disease. If one or more suspicious findings are detected, they can be further evaluated with CT and/or MR imaging depending on location. 18F-FDG-PET reports should include standardized uptake values (SUVs) of the identified lesions.

Cases in which no distant metastases are determined by clinical methods (history, physical examination, and imaging if indicated) are designated cM0, and cases in which one or more distant metastases are identified by clinical and/or radiographic methods are designated cM1. Positive supraclavicular lymph nodes are categorized as N3 (see previous discussion). A case is categorized as clinically free of metastases (cM0) unless evidence of metastases is documented by clinical means (cM1) or by biopsy of a metastatic site (pM1). M categorization of breast cancer refers to the classification of clinically significant distant metastases, which typically distinguishes whether or not there is a potential for long-term cure. The ascertainment of M categorization requires evaluations consisting of a review of systems and physical examination. It also may include radiographic imaging, blood tests, and tissue biopsy. The types of examinations needed in each case may vary and guidelines for these are available.[35] M categorization is based on best clinical and radiographic interpretation; pathological confirmation is recommended, although confirmation may not be possible for reasons of feasibility or safety. Whenever biopsy confirmation is possible and safe, repeat biomarker assessment (ER, PR, HER2) is recommended because differences in the biomarker profile of the metastases and the primary tumor affect treatment. Additionally, M category assessment may not yield a definitive answer on the initial set of evaluations, and follow-up studies may be needed, making the final determination a recursive and iterative process, assuming that the area of question was present at the time of diagnosis of the primary breast cancer. In these cases, the designated category should remain M0 unless a definitive designation is made that the patient truly had detectable metastases at the time of diagnosis, based on the guidelines that follow. Subsequent development of new metastases in areas not previously thought to be

suspicious does not change the patient's original classification and the patient would now be considered to have converted to recurrent Stage IV, which is considered recurrent disease without altering the original stage.

Pathological confirmation of suspected metastatic disease should be performed whenever feasible. The type of biopsy of a suspicious lesion should be guided by the location of the suspected metastases along with patient preference, safety, and the expertise and equipment available to the care team. FNA is adequate, especially for visceral lesions and with the availability of experienced cytopathologic interpretation. Negative FNA or cellular atypia might carry a significant risk of false-negative results, especially in bony or scirrhous lesions, so consideration of repeat FNA or other biopsy techniques, such as core needle or open surgical biopsy, may be warranted. Histopathologic examination should include standard hematoxylin and eosin (H&E) staining. In some cases, additional immunohistochemical staining or other specialized testing for confirmation of breast cancer or other cancer type is required. If adequate biomarker data (ER, PR, HER2) are not available from the primary tumor, these should be obtained on any other biopsy that shows cancer on H&E staining. Determination of biomarkers on the metastatic biopsy specimen is highly desirable, regardless of the availability of biomarker analysis on the primary tumor. Special caution should be taken with evaluation of tumor markers in tissue collected from bone biopsies. Decalcification procedures may create false-negative results for both immunohistochemistry (IHC) and fluorescent *in situ* hybridization (FISH). Incidentally detected cancer cells, clusters of cancer cells or foci ≤ 0.2 mm, or CTCs that are otherwise clinically and radiographically silent should not alone constitute M1 disease and are discussed in this chapter.

Laboratory Abnormalities

Patients with abnormal liver function tests should undergo liver imaging, whereas those with elevated alkaline phosphatase or calcium levels, or suggestive symptoms, should undergo bone imaging and/or scintigraphy. Unexplained anemia and other cytopenias require a full hematologic evaluation (e.g., examination of the peripheral smear, iron studies, B12/folate levels) and should be investigated with bone imaging and a bone marrow biopsy depending on the results of the evaluation. Other unexplained laboratory abnormalities, such as elevations in renal function, also should prompt appropriate imaging tests. Elevated tumor markers are known to be associated with variable degrees of false positivity and their use has not been shown to improve outcome. The routine ordering of these tests—such as cancer antigen (CA) 15-3, CA 27.29, carcinoembryonic antigen, and other protein-based markers—for staging is not indicated.[3]

Circulating Tumor Cells, Bone Marrow Micrometastases, and Disseminated Tumor Cells

The presence of CTCs in the blood or DTC clusters (≤ 0.2 mm) in the bone marrow or other nonregional nodal tissues does not constitute M1 in the absence of other apparent clinical and/or radiographic findings of metastases that correspond to pathological findings. However, an increasing number of studies are showing microscopic bone marrow and CTCs in M0 disease to be associated with adverse prognosis for recurrence or survival. Thus, denotation of histologically visible metastatic deposits ≤ 0.2 mm in bone marrow or other organs distant from the breast and regional lymph nodes should be denoted by the term cM0(i+). For breast cancer classified as cM1 (clinically detectable metastases), the enumeration of CTCs at the time of diagnosis of metastatic disease has been shown to strongly correlate with survival, but neither the presence nor the number of CTCs will change the overall classification.

When metastatic disease is confirmed by biopsy, the pM1 category may be used. When a biopsy fails to confirm M1 disease, the assignment of cM0 or cM1 is based on clinical and imaging data; pM0 is not a valid category for "M" (see Chapter 1).

Post Neoadjuvant Therapy Clinical Classification (yc)

Preoperative or "neoadjuvant systemic" therapy has been used for several decades for managing inflammatory and locally advanced breast cancer, and it is being used increasingly for managing earlier stages of the disease as well.[38]

Post Neoadjuvant Therapy ycT Classification

Clinical (pretreatment) T (cT) is defined by clinical and radiographic findings; clinical (posttreatment) T(ycT) is determined by the size and extent of disease on physical examination and imaging. The ycT is determined by measuring the largest single focus of residual tumor by examination or imaging.

If a cancer was classified as inflammatory (cT4d) before neoadjuvant chemotherapy, the cancer is classified as inflammatory breast cancer after therapy, even if complete resolution of the inflammatory findings is observed during treatment. The posttreatment clinical classification (ycT) should reflect the extent of identified residual disease on imaging. For example, a patient with several areas of residual disease measuring 2.0 mm to 9.0 mm in greatest dimension identified within a 2.2 cm^2 area of tumor bed previously involved is classified as ycT1b(m), and a patient with no residual disease identified is classified as ycT0.

Post Neoadjuvant Therapy ycN Classification

Clinical pretreatment and posttreatment node status (cN and ycN) is defined by clinical and radiographic findings with or without FNA, core needle biopsy, or sentinel node biopsy of a suspicious node or excision of a palpable node. If definitive resection of the primary tumor and/or nodes is performed, the pathological information for this category is ypN.

Post Neoadjuvant Therapy M Classification

The M category for patients treated with neoadjuvant therapy is the category assigned for pretreatment clinical stage, prior to initiation of neoadjuvant therapy. If a patient was designated as having detectable distant metastases (M1) before chemotherapy, the patient will be designated as M1 throughout. Identification of distant metastases after the start of therapy in cases where pretherapy evaluation showed no metastases is considered progression of disease.

Pathological Classification

Pathological staging includes all data used for clinical staging, plus data from surgical exploration and resection, as well as pathological examination (gross and microscopic) of the primary carcinoma, regional lymph nodes, and metastatic sites (if applicable); pathological examination must include excision of the primary carcinoma with no macroscopic tumor in any margin of resection. A cancer can be classified pT for pathological stage grouping if there is only microscopic, but not macroscopic, involvement at the margin. If macroscopic examination finds transected tumor in the margin of resection, the pathological size of the tumor may be estimated from available information, including imaging, but this is not necessarily the sum of the sizes of multiple resected pieces of tumor.

If the primary tumor is invasive, surgical evaluation of the axillary lymph nodes is usually performed. Exceptions may include microinvasive cancers, as well as some cases where the risk of axillary metastases is very low or where the presence of axillary metastases will not affect the use of systemic therapy (e.g., older women with small, hormone receptor-positive cancers). Evaluation of axillary nodes for pathological categorization requires surgical resection. Sentinel lymph node biopsy to remove one or more sentinel lymph nodes for pathological examination is commonly done for patients with clinically negative lymph nodes. The use of sentinel node biopsy is denoted by the "sn" modifier [e.g., pN(sn)]. Alternatively, dissection of the axillary lymph nodes may be performed. In women with clinically negative nodes, this entails resection of the nodal tissue located lateral to the lateral border of the pectoralis minor muscle (Level I) and beneath that muscle to its medial border (Level II).

When T data are otherwise sufficient for pathological staging, it is necessary to have microscopic analysis of at least one lymph node to classify the lymph node pathologically. This may be FNA, core needle biopsy, excisional node biopsy, or sentinel node biopsy. A case may be assigned a pathological N category if any lymph nodes are microscopically examined, irrespective of the number of nodes removed. However, the number of nodes removed should be reported. In most cases, lymph node dissection of Level I and Level II of the axilla includes 10 or more lymph nodes.

Certain histologic invasive cancer types [classic tubular carcinoma < 1 cm, classic mucinous carcinoma < 1 cm, and microinvasive carcinoma (pT1mi)] have a very low incidence of axillary lymph node metastases and may not require an axillary lymph node surgery, although sentinel lymph node biopsy may be considered. Invasive tumor nodules in the axillary fat adjacent to the breast, without histologic evidence of associated lymph node tissue, are classified as regional lymph node metastases (pN).

Pathological staging groups may be assigned if pathological information is available for T and N using the clinical category for M (pT pN cM0 or pT pN cM1), or the pathological category for M if metastases are biopsy proven (pT pN pM1). If surgery occurs after the patient has received neoadjuvant chemotherapy, hormonal therapy, immunotherapy, or radiation therapy, the prefix "yp" should be used with the TNM classification, for example, ypT ypN cM.

Pathological Characterization of the Primary Tumor (T)

Determining Tumor Size

Pathological tumor size (pT) based on gross measurement also may be somewhat inaccurate for the same reasons as discussed in the clinical classification. Microscopic assessment is preferred because it is able to distinguish fibrosis, noninvasive, and invasive carcinoma. Microscopically determined pT should be based on measuring only the invasive component. For small invasive tumors that can be submitted in one section or paraffin block, microscopic measurement is the most accurate way to determine pT. If an invasive tumor is too large to be submitted for microscopic evaluation in one tissue section or block, the gross measurement is the preferred method of determining pT. In some situations, systematic pathology evaluation allows microscopic reconstruction of the tumor; however, reconstruction measurements should be correlated with gross and imaging size before assigning pT. Whichever method is used, pT should be recorded to the nearest millimeter. The size of the primary tumor is measured for T categorization before any tissue is removed for special purposes, such as prognostic biomarkers or tumor banking. In patients who have undergone diagnostic core biopsies prior to surgical excision (particularly vacuum-assisted core needle biopsy

sampling), measuring only the residual tumor may result in underclassifying the T category and understaging the tumor, especially with smaller tumors. In such cases, the original invasive cancer size should be estimated and verified based on the best combination of imaging, gross, and microscopic histological findings. Adding the maximum invasive cancer dimension on the core needle biopsy to the residual invasive tumor in the excision is not recommended, because this method often overestimates maximum tumor dimension. In general, the maximum dimension in either the core needle biopsy or the excisional biopsy is used for T categorization unless imaging dimensions suggest a larger invasive cancer.

Posttreatment (ypT) size should be estimated based on the best combination of imaging, gross, and microscopic histological findings. The size of some invasive cancers, regardless of previous biopsy or chemotherapy, may not be apparent by any imaging modalities or gross pathological examination. In these cases, invasive cancer size can be estimated by carefully measuring and recording the relative positions of tissue samples submitted for microscopic evaluation and determining which contain invasive cancer (see "Post Neoadjuvant Therapy ypT Classification").

Tis Classification

Pure noninvasive carcinoma, or carcinoma *in situ*, is classified as Tis, with an additional parenthetical subclassification indicating the subtype. Two subtypes are currently recognized: ductal carcinoma *in situ* (DCIS) and Paget disease of the nipple with no underlying invasive cancer. These are categorized as Tis (DCIS) and Tis (Paget), respectively. "Intraductal carcinoma" is an outmoded term for DCIS that is still used occasionally, and tumors referred to in this manner (which is discouraged) should be categorized as Tis (DCIS). "Ductal intraepithelial neoplasia" (DIN) is a proposed, but uncommonly used, terminology encompassing both DCIS and atypical ductal hyperplasia (ADH), and only cases referred to as DIN containing DCIS (±ADH) should be classified as Tis (DCIS).[39,40] If both ductal and lobular *in situ* components (DCIS and LCIS) are present, the tumor currently is classified as Tis (DCIS). A recently published Cancer Protocol and Checklist from the College of American Pathology (CAP) provides much greater detail regarding definition and evaluation of *in situ* cancer of the breast (http://www.cap.org).[41]

Paget disease of the breast is characterized clinically by an exudate or crust of the nipple and areola caused by infiltration of the epidermis by noninvasive breast cancer epithelial cells. This condition usually occurs in one of the following three circumstances[42]:

1. Associated with an invasive carcinoma in the underlying breast parenchyma. The T classification should be based on the size of the invasive disease.
2. Associated with an underlying DCIS. T classification should be based on the underlying tumor as Tis (DCIS), accordingly. However, the presence of Paget disease associated with invasive or noninvasive carcinomas should still be recorded.
3. Paget disease without any associated identifiable underlying invasive or noninvasive disease is the only lesion classified as Tis (Paget). The very rare case of Paget Disease with LCIS in the breast parenchyma also is categorized as Tis (Paget).

The size of noninvasive (pTis) carcinomas does not change the T category. However, because tumor size may influence therapeutic decisions, an estimate of size should be provided based on the best combination of imaging, gross, and microscopic histological findings.[41] Recommendations for establishing and communicating the size of DCIS have been disseminated by CAP in its cancer protocols (www.cap.org).

LCIS, included in prior editions of the AJCC Cancer Staging Manual, is removed from the 8th Edition. LCIS is a benign condition and is not treated as a carcinoma. It is properly considered a proliferative disease with associated risk for developing a breast cancer in the future and, therefore, is no longer included in this cancer staging system.

One form of LCIS (often called "pleomorphic" LCIS or high-grade LCIS) has features overlapping DCIS, including high-grade nuclei and central necrosis, and some physicians believe it should be treated similarly to DCIS. Evidence is insufficient at present, primarily due to the low prevalence of this form of high-grade LCIS, to establish definitive recommendations for treatment. Thus, for the present, high-grade or pleomorphic LCIS also is not included in the pTis classification.

Microinvasive Carcinoma

Microinvasive carcinoma is defined as an invasive carcinoma with no focus measured larger than 1 mm. In cases with only one focus, its microscopic measurement should be provided. In cases with multiple foci, the pathologist should attempt to quantify the number of foci and the range of their sizes, including the largest. The sum of the sizes should not be reported or used for determining pT. If there are multiple foci, reporting of the number may be difficult. In these cases, it is recommended that an estimate of the number be provided or, alternatively, a note that the number of foci of microinvasion is too numerous to quantify, but that no identified focus is larger than 1.0 mm. Tumor foci larger than 1.0 mm should not be rounded down to 1.0 mm. If a registry system limits reporting to millimeter increments, those tumors that are larger than 1 mm but smaller than 2 mm should be reported as 2 mm. Microinvasive carcinoma is nearly always encountered in a setting of DCIS (or, infrequently, LCIS) where small foci of tumor cells have invaded through the basement membrane into the surrounding

stroma, although rare cases are encountered in the absence of noninvasive disease. The prognosis of microinvasive carcinoma is generally thought to be quite favorable, although the clinical impact of multifocal microinvasive disease is not well understood at this time.

Categories for pathological tumor (pT) are the same as for clinical (cT); see Definitions of AJCC TNM in this chapter.

Pathological Characterization of Regional Lymph Nodes (N)

Pathological classification (pN) is used only in conjunction with a pathological T assignment (surgical resection) (pT) and includes pathological evaluation of excised nodes from a sentinel lymph node biopsy and/or lymph node dissection. Classification based solely on sentinel lymph node biopsy with fewer than six nodes evaluated and without subsequent axillary lymph node dissection is designated (sn) for "sentinel node," for example, pN0(sn). Isolated tumor cell clusters (ITC) are defined as small clusters of cells not larger than 0.2 mm, or single tumor cells, or fewer than 200 cells in a single histologic cross-section. ITCs may be detected by routine histology or by IHC methods. Nodes containing only ITCs are excluded from the total positive node count for purposes of N categorization but should be included in the total number of nodes evaluated, and the number of nodes with only ITCs should be noted in the pathology report. When pT is assigned, the final pN classification may include clinical data; for example, when an ipsilateral internal mammary node is identified by imaging and meets criteria for cN3b and axillary or sentinel nodes have been removed for pathological evaluation, a pN3b classification may be assigned. See Figs. 48.10 and 48.11 for illustrations of the categories for pathological N (pN).

Macrometastases

Cases in which regional lymph nodes cannot be assessed (previously removed or not removed for pathological examination) are designated pNX. Cases in which no regional lymph node metastases are detected should be designated pN0.

The pN classification for breast carcinoma reflects the cumulative total regional lymph node burden of metastatic disease in the axillary, infraclavicular, supraclavicular, and ipsilateral internal mammary nodes. For patients who are pathologically node-positive with macrometastases, at least one node must contain a tumor deposit larger than 2 mm, and all remaining quantified nodes must contain tumor deposits larger than 0.2 mm (at least micrometastases); nodes containing only ITCs are excluded from the calculated positive node count for purposes of N categorization, but they should be recorded as additional ITC-involved nodes and should be included in the total nodes evaluated. Cases with one to three positive Level I/II axillary lymph nodes are classified pN1a; cases with four to nine positive axillary lymph nodes are classified pN2a; and cases with 10 or more positive axillary lymph nodes are classified pN3a.

Cases with histologically confirmed metastases to the internal mammary nodes, detected by sentinel lymph node dissection but not by clinical examination or imaging studies (excluding lymphoscintigraphy), are classified as pN1b if occurring in the *absence* of metastases to the axillary lymph nodes and as pN1c if occurring in the *presence* of metastases to one to three axillary lymph nodes. If four or more axillary lymph nodes are involved and internal mammary sentinel nodes are involved, the classification pN3b is used. Pathological classification is used when axillary nodes have been histologically examined and clinical involvement of the ipsilateral internal mammary nodes is detected by imaging studies (excluding lymphoscintigraphy); in the absence or presence of axillary nodal metastases, pN2b and pN3b classification is used, respectively. Histologic evidence of metastases in ipsilateral supraclavicular lymph node(s) is classified as pN3c. A classification of pN3, regardless of primary tumor size, is classified as Stage IIIC.

A case in which the categorization is based only on sentinel lymph node biopsy is given the additional designation (sn) for "sentinel node"—for example, pN1a(sn). For a case in which an initial categorization is based on a sentinel lymph node biopsy but a standard axillary lymph node dissection is subsequently performed, the categorization is based on the total results of both the axillary lymph node dissection and the sentinel node biopsy, and the (sn) modifier is removed. The (sn) modifier indicates that nodal categorization is based on less than an axillary dissection. When the combination of sentinel and nonsentinel nodes removed is less than a standard low axillary dissection (fewer than six nodes), the (sn) modifier is used. The number of quantified nodes for staging is generally the number of grossly identified, histologically confirmed lymph nodes. Care should be taken to avoid overcounting sectioned nodes or sectioned adipose tissue with no grossly apparent nodes.

The first priority in histologic evaluation of lymph nodes is to identify all macrometastases (metastases larger than 2.0 mm, see Fig. 48.7). The entire lymph node should be submitted for evaluation, and larger nodes should be bisected or thinly sliced no thicker than 2.0 mm. A single histologic section of each slice has a high probability of detecting all macrometastases present, although the largest dimension of the metastases may not be represented. More comprehensive evaluation of lymph node paraffin blocks is not required for categorization; however, such techniques as multilevel sectioning and IHC will identify additional tumor deposits, typically micrometastases and ITCs. It is recommended that nodal tissue that may contain a macrometastasis not be diverted for experimental or alternative testing, such as molecular analysis, if this diversion would potentially result in the pathologist's missing macrometastases detectable by routine microscopic examination.

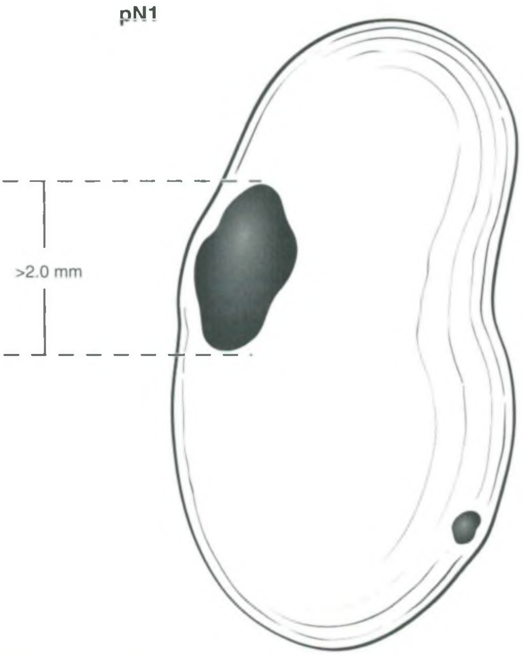

Fig. 48.7 Macrometastasis: pN1. At least one contiguous tumor deposit must be larger than 2.0 mm

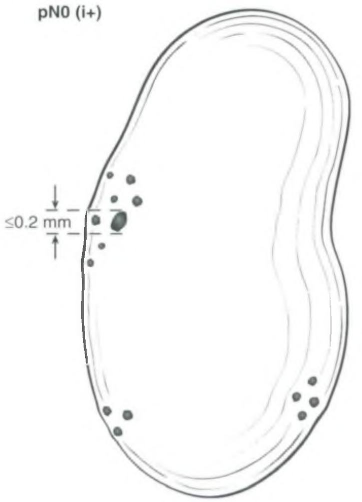

Fig. 48.8 Isolated tumor cell clusters (ITC); pN0(i+). The largest contiguous tumor deposit must be no larger than 0.2 mm. Multiple ITCs are often clustered and multiple foci are frequently present in a single node. The size of areas of noncontiguous adjacent ITCs are not added. When more than 200 single tumor cells are present in a single lymph node cross section, this signifies that the size of the deposit is likely greater than 0.2 mm and this should be classified as a micrometastasis

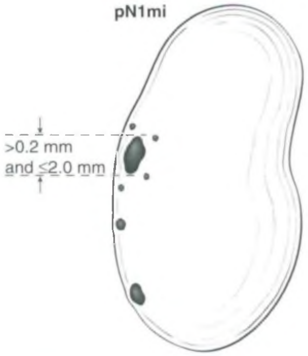

Fig. 48.9 Micrometastasis; pN1mi. At least one contiguous tumor deposit must be larger than 0.2 mm and the largest contiguous tumor deposit must be no larger than 2.0 mm. The sizes of noncontiguous adjacent tumor deposits are not added. Multiple micrometastases may be present in a single lymph node

Isolated Tumor Cell Clusters and Micrometastases

ITCs are defined as small clusters of cells not larger than 0.2 mm in largest dimension, or single cells, usually with little if any histologic stromal reaction. ITCs may be detected by routine histology or by IHC methods. When no single metastasis larger than 0.2 mm is identified, regardless of the number of nodes containing ITCs, the regional lymph nodes should be designated as pN0(i+) or pN0(i+)(sn), as appropriate, and the number of ITC-involved nodes should be noted. Multiple ITC clusters often are present, and only the size of the largest contiguous tumor cell cluster is used for pN category; neither the sum of the ITC cluster sizes nor the area in which the clusters are distributed is used for pN (Fig. 48.8).

A three-dimensional 0.2-mm cluster contains approximately 1,000 tumor cells. Thus, if more than 200 individual tumor cells are identified as single dispersed tumor cells or as a nearly confluent elliptical or spherical focus in a single histologic section of a lymph node, there is a high probability that more than 1,000 cells are present in the lymph node. In these situations, the node may be classified as containing micrometastasis (pN1mi). Cells in different lymph node cross- or longitudinal sections or levels of the block are not added together; the 200 cells must be in a single node profile even if the node has been thinly sectioned into multiple slices. It is recognized that there is substantial overlap between the upper limit of the ITC and the lower limit of the micrometastasis categories because of inherent limitations in pathological nodal evaluation and detection of minimal tumor burden in lymph nodes. Thus, the threshold of 200 cells in a single cross-section is a guideline to help pathologists distinguish between these two categories. The pathologist should use judgment regarding whether it is likely that the cluster of cells represents a true micrometastasis or is simply a group of isolated tumor cells.

Micrometastases are defined as tumor deposits larger than 0.2 mm but not larger than 2.0 mm in largest dimension (Fig. 48.9). Cases in which at least one micrometastasis

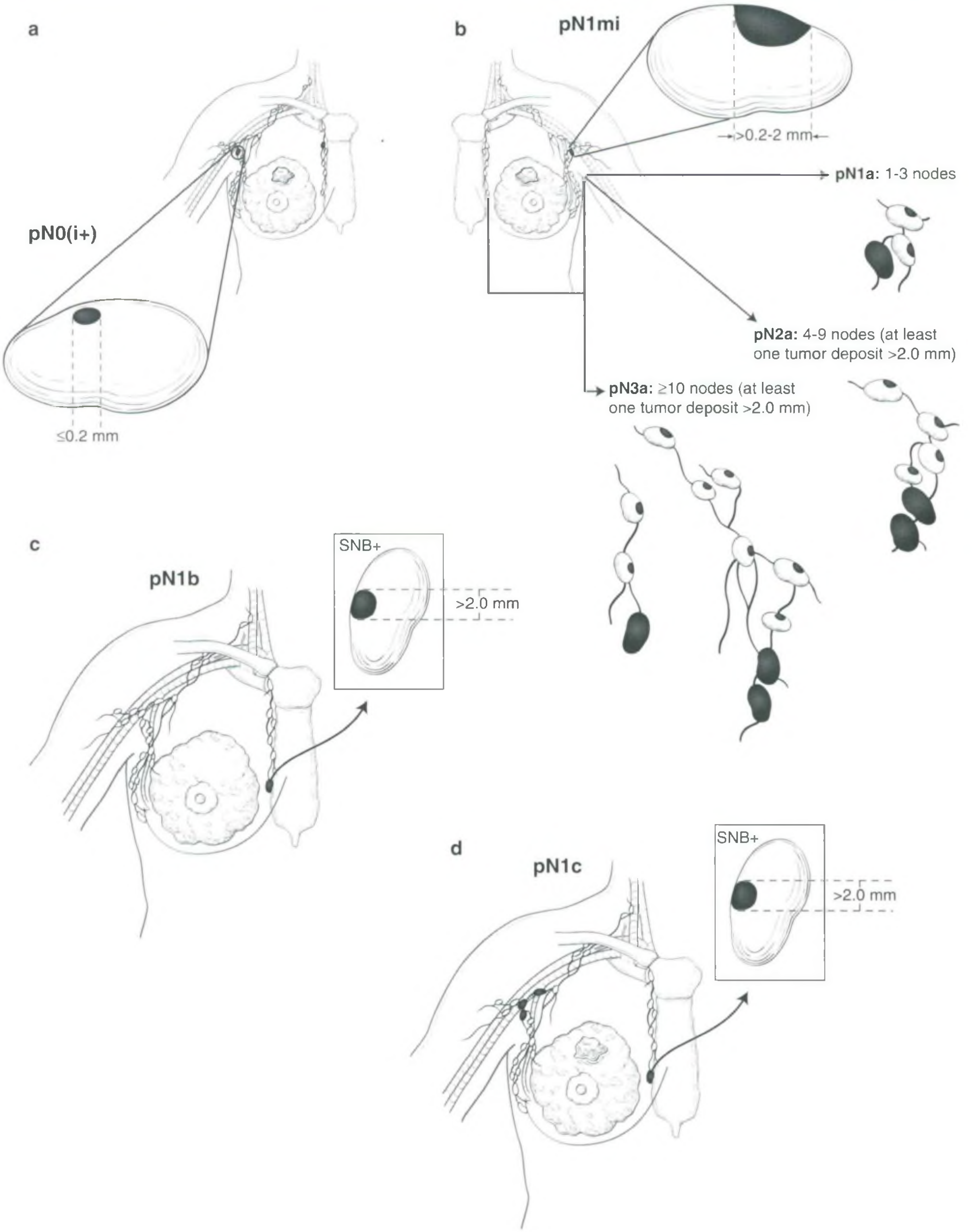

Fig. 48.10 Pathological nodal categories. (**a**) Isolated tumor cell clusters (ITC) are groups of tumor cells 0.2 mm or less and are categorized as pN0(i+). (**b**) The pN1 category includes pN1mi micrometases defined as node deposits of tumor cells 0.2 – 2mm; pN1a defined as 1 – 3 nodes with at least 1 node with a deposit greater than 2mm; pN2a is 4 – 9 positive nodes; pN3a is 10 or more positive nodes. (**c**) pN1b is assigned with a positive internal mammary sentinel node with a deposit greater than 0.2 mm in the absence of axillary node metastases. (**d**) pN1c is with combined pN1a and pN1b

48

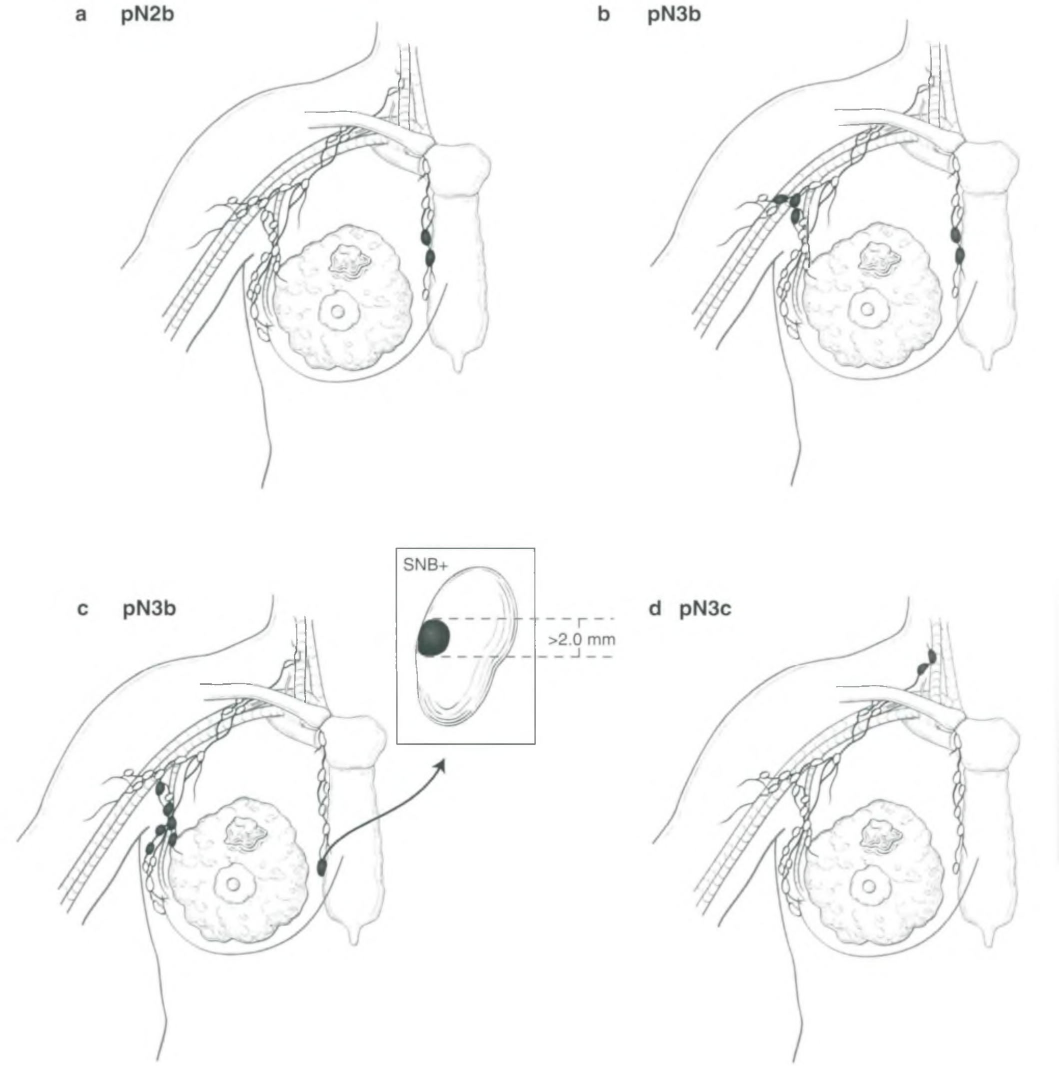

Fig. 48.11 Pathological Node Categories (continued). (**a**) pN2b is with clinically detected internal mammary nodes and negative axillary nodes; (**b** and **c**) pN3b is with pN1a or pN2a with clinically positive internal mammary nodes by imaging OR pN2a with pN1b; and (**d**) pN3c is metastases to ipsilateral supraclavicular lymph nodes with any other regional lymph node involvement

is detected but no metastases larger than 2 mm (macrometastases) are detected, regardless of the number of involved nodes, are classified pN1mi or pN1mi(sn), as appropriate, and the number of involved nodes should be noted.

The size of a tumor deposit is determined by measuring the largest dimension of any group of cells that are touching one another (confluent or contiguous tumor cells), regardless of whether the deposit is confined to the lymph node, extends outside the node (extranodal extension), is totally present outside the lymph node and invading adipose, or is present within a lymphatic channel adjacent to the node. When multiple tumor deposits are present in a lymph node, whether ITCs or micrometastases, the size of only the largest contiguous tumor deposit is used to classify the node, not the sum of all individual tumor deposits or the area in which the deposits are distributed. When a tumor deposit has induced a fibrous (desmoplastic) stromal reaction, the combined contiguous dimension of tumor cells and fibrosis determines the size of

the metastasis, except following neoadjuvant therapy. When a single case contains multiple positive lymph nodes and the largest tumor deposit in each node is categorically distinct, the number of nodes in each category (macrometastases, micrometastases, ITCs) should be recorded separately to facilitate N categorization as described previously.

If histologically negative lymph nodes are examined for evidence of unique tumor or epithelial cell markers using molecular methods (RT-PCR) and these markers are detected, the regional lymph nodes are classified as pN0(mol+) or pN0 (mol+)(sn), as appropriate. Sacrificing lymph node tissue for molecular analysis that would otherwise be available for histologic evaluation and staging is not recommended, particularly when the size of the sacrificed tissue is large enough to contain a macrometastasis. If data from molecular analyses are generated, they should be recorded by the registrar.

Pathological Characterization of Distant Metastases (M)

Categories for pathological (pM) are the same as for clinical (cM); see previous discussion of distant metastases characterization and Definitions of AJCC TNM in this chapter.

Post Neoadjuvant Therapy Pathological Classification (yp)

Post Neoadjuvant ypT Classification

Preoperative or neoadjuvant systemic therapy has been used for several decades for managing inflammatory and locally advanced breast cancer, and it is being used increasingly for managing earlier stages of the disease, as well.[38] Clinical (pretreatment) T (cT) is defined by clinical and radiographic findings; pathological (posttreatment) T (ypT) is determined by the pathological size and extent of disease. The ypT is determined by measuring the largest single focus of residual invasive tumor, with the modifier "m" indicating multiple foci of residual tumor. The measurement of the largest tumor focus should not include areas of fibrosis within the tumor bed. The inclusion of additional information in the pathology report—such as the distance over which tumor foci extend, the number of tumor foci present, or the number of slides/blocks in which tumor appears—may assist the clinician in estimating the extent of residual disease. A comparison of the tumor cellularity in the initial biopsy to that in the posttreatment specimen also may aid in the assessment of response. For example, residual tumor cellularity may be 30% compared to the pretreatment biopsy, or a 70% reduction in cellularity.

If a cancer was classified as inflammatory (cT4d) before neoadjuvant chemotherapy, the cancer is still classified as inflammatory breast cancer after therapy, even if complete resolution of the inflammatory findings is observed during treatment. The posttreatment pathological classification (ypT) should reflect the extent of identified residual disease, and the pathology report should note that the pretreatment classification was cT4d. For example, a patient with several foci of microscopically confirmed residual disease measuring 2–9 mm in greatest dimension identified within a 22 mm^2 area of tumor bed fibrosis is classified as ypT1b(m), and a patient with no residual disease identified is classified as ypT0.

Post Neoadjuvant Therapy ypN Classification

Clinical pretreatment node status (cN) is defined by clinical and radiographic findings with or without FNA, core needle biopsy, or sentinel node biopsy of a suspicious node or excision of a palpable node; pathological posttreatment N (ypN) is determined similar to pN. The "sn" modifier is used only if a sentinel node evaluation was performed after treatment and no axillary dissection has been performed. If no sentinel node or axillary dissection is performed, the (ypNX) classification is used.

The ypN categories are the same as those used for pN. Only the largest contiguous focus of residual tumor in the node evaluation is used for classification; any treatment-associated fibrosis is not included. Inclusion of additional information in the pathology report—such as the distance over which tumor foci extend and the number of tumor foci present—may assist the clinician in estimating the extent of residual disease.

Multiple prospective clinical trials determined the prognostic value of response to preoperative (neoadjuvant) therapy.[43,44] A pathological complete response (pCR) is associated with significantly improved disease-free and overall survival for individual patients. A recent meta-analysis confirmed the reproducible prognostic value of pCR, especially in patients with hormone receptor-negative breast cancer.[45]

Post Neoadjuvant Therapy M Classification

The M category for patients treated with neoadjuvant therapy is the category assigned for pretreatment clinical stage, prior to initiation of neoadjuvant therapy. If a patient was designated to have detectable distant metastases (M1) before chemotherapy, the patient will be designated as M1 throughout. Identification of distant metastases after the start of therapy in cases where pretherapy evaluation showed no metastases is considered progression of disease.

Other Rules for Classification – Functional Imaging, Multiple Primaries

Historically, TNM classification has been based on tumor morphology with size as the major indicator of prognosis and treatment efficacy. Although size is still the prime determinant in classification, the use of molecular breast

imaging, CT, PET and MR imaging with contrast enhancement brings up many more measurement possibilities other than anatomic size. This includes biologic functional imaging characteristics that may be more accurate than size alone to evaluate prognosis and treatment options. At the moment, validated data are insufficient to incorporate these findings into staging. When sufficient data are accumulated these factors may be introduced into the staging system.

For patients who receive neoadjuvant systemic or radiation therapy pretreatment, T is defined as clinical (cT). Pretreatment staging is clinical, and the clinical measurement defined from examination and imaging is recorded (cT).

Multiple Simultaneous Ipsilateral Primary Carcinomas

Multiple simultaneous ipsilateral primary carcinomas in the same breast, which are grossly or macroscopically distinct and measurable using available clinical and pathological techniques, are defined as invasive carcinomas. T category assignment in this setting should be based only on the largest tumor; the sum of the sizes should not be used. However, the presence and sizes of the smaller tumor(s) should be recorded and the "(m)" modifier, as defined by the staging rules in Chapter 1, should be appended to the T category.

Invasive cancers that are in close proximity, but are apparently separate grossly, may represent truly separate tumors, or one tumor with a complex shape, or intramammary spread of disease. Distinguishing these situations may require judgment and close correlation between pathological and clinical findings (especially imaging), and preference should be given to the modality thought to be the most accurate in a specific case. When macroscopically apparent distinct tumors are very close (e.g., less than 5 mm apart from each other), especially if they are similar histologically, they are most likely one tumor with a complex shape, and their T category—after consideration of imaging, macroscopic, and microscopic findings—may be based on the largest combined dimension. Careful and comprehensive microscopic evaluation often reveals subtle areas of continuity between tumor foci in this setting. However, contiguous uniform tumor density in the intervening tissue is needed to justify adding two grossly distinct masses. These criteria apply to multiple macroscopically identified and mea-

Table 48.1 Characterization of the Response to Neoadjuvant Therapy

Treatment Response Category	Description
Pathological Complete Response (pCR)<br>ypT0N0 or ypTisN0	Pathological complete response can only be determined by histopathologic evaluation and is defined by the absence of invasive carcinoma in the breast and lymph nodes.<br>The presence of *in situ* cancer after treatment in the absence of residual invasive disease, constitutes a pCR.<br>Patients with isolated tumor foci in lymph nodes are not classified as having a complete response. The presence of axillary nodal tumor deposits of any size, including cell clusters 0.2 mm or smaller, excludes a complete response. These patients will be categorized as ypN0(i+).
Clinical Partial Response (cPR)	A decrease in either or both the T or N category compared to the clinical (pretreatment) assignment, and with no increase in either T or N, represents a partial response. Assessing the degree of clinical partial response (cPR) is best defined by comparing pretreatment clinical categories for cT and cN with the clinical posttherapy categories (ycT and ycN). This comparison should be based on the clinical method that most clearly defined tumor dimensions before treatment. Defining the degree of pathological response that is less than a complete response is problematic, because in these cases there is no pretreatment pathological categorization for comparison.<br>Nodal involvement should be determined by physical examination or radiologic evaluation, if the nodes are palpable or visible before chemotherapy. If prechemotherapy microscopic lymph node involvement is demonstrated by FNA, core needle biopsy, or sentinel node biopsy, it should be recorded as such using cN. Nodal response will be evaluated by physical examination and imaging for ycN. Evaluation by microscopically examining resected nodes after chemotherapy will provide a pathological response (ypN).<br>Absence of posttreatment pathological nodal involvement should be used to document pathological complete response, and should be recorded, but does not necessarily represent a true "response" since one does not know whether lymph nodes removed surgically postchemotherapy were involved prior to chemotherapy.
No response (NR)	No apparent change in either the T or N categories compared to the clinical (pretreatment) assignment or an increase in the T or N category at the time of y pathological evaluation indicates no response to treatment. Clinical (pretreatment) T will be defined by clinical and radiographic findings. Posttreatment T will be determined by pathological size (ypT) in resectable tumors and by clinical exam and imaging in unresectable tumors (ycT).<br>For resectable tumors, the response category will be appended to the y stage description. For example: ypTisypN0cM0CR; ypT1ypN0cM0PR; ypT2ypN-1cM0NR.<br>Rarely the cancer grows or progresses during therapy. There is no specific category for this circumstance. In these situations, the code for "No Response" should be used for the registry.

surable tumors and do not apply to one macroscopic carcinoma associated with multiple separate microscopic (satellite) foci. Tumors along the same approximate radial axis frequently are related and have arisen in the same duct system.

Simultaneous Bilateral Primary Carcinomas

Each carcinoma is classified and staged as a separate primary carcinoma in a separate organ based on its own characteristics, including T category as specified in the staging rules (see Chapter 1). Each tumor should have a separate biomarker determination (ER, PR, HER2, and grade).

Biomarkers and Prognostic Factor-Based Breast Cancer Staging

From the start of the planning phase of the 8th Edition, the Breast Expert Panel discussed the importance of integrating biomarkers into TNM staging for this edition. In view of the challenges identified during the development of the 7th Edition, many of them persisting to date, a Methodology Task Force was created to advise the Breast Panel on how to accomplish the goal of integrating biomarkers into staging without compromising the ability of using the staging system if biomarker information was not available. The Methodology Task Force also reviewed appropriately validated multigene prognostic and predictive panels for consideration of integration into staging.

This issue was discussed in some detail in preparation of the 7th Edition. However it was determined that there were insufficient validated data to take that step. The discussion remains relevant for the current (8th) edition. If anything, the incorporation of biomarkers is a more pressing need now than at the time of the previous edition.

For the 8th Edition, a great deal of uncertainty remained about how to accurately integrate biomarkers and prognostic and predictive multigene panel results into the AJCC staging system. The large majority of the relevant data is retrospective in nature, with little prospective data available. Nonetheless, the clinical value of multigene panels for selecting treatment for certain subsets of patients has been demonstrated in a reproducible and convincing fashion. The value of multigene panels for managing patients has now progressed to the point where such panels are routinely incorporated into national guidelines and recommendations for treatment (e.g., NCCN and ASCO tumor marker guidelines).

Maintaining Anatomic Stage

The Expert Panel reached a strong consensus that each patient should be able to be assigned a purely anatomic stage even if prognostic staging is possible. It is recognized that prognostic staging is not appropriate for all subsets of patients and that in many situations biomarker determination and/or multigene panels are not routinely performed or available. This occurs most often in regions of the world with limited resources to pay for such testing. Furthermore, anatomic staging remains a valuable aspect of the staging process because it is a link to the past for comparison of studies and patient populations, as well as a common terminology for doctors regardless of country or available resources.

Breast Biomarkers

It is clear that in addition to the traditional tumor size, lymph node status, and presence of metastasis, tumor biology is vitally important in prognosis and response to therapy. The AJCC staging system has always applied to treated patients. Initially, the treatment was surgical, with or without radiation therapy. Over time the system has adapted from a classic tumor size, nodal status and the presence or absence of metastasis to include evaluation of sentinel lymph nodes only, post systemic therapy, and even findings at autopsy. There never was a group of "totally untreated" patients. To remain clinically relevant, it is critically important to gradually alter staging as new advances in the understanding and treatment of cancer develop.

A key proxy for the biologic character of a cancer is tumor differentiation. Tumor differentiation is reflected and assessed in many ways, including proliferative index, grade, hormone receptor status, expression of oncogenes, and gene expression profiles. The earliest attempts at evaluating tumor differentiation and prognosis were characterizing tumors by histologic or nuclear grade.[46-50] Different systems have been used, but the most reliable and widely used is the histologic grading system of Scarff, Bloom, and Richardson, as updated and standardized by the Nottingham group.[51-53] Tumors of high histologic grade or poorly differentiated tumors have a worse prognosis than low-grade or well-differentiated tumors without regard to hormonal or chemotherapy.

An analysis of data from the SEER Program of the National Cancer Institute has shown that histologic grade is an important prognostic factor, independent of the tumor size or number of positive lymph nodes.[54] Although the reproducibility of histologic grade among pathologists has been called into question,[55] the work of Elston and Ellis gives guidelines on how to reproducibly grade breast cancers.[52,53] They modified the Scarff–Bloom–Richardson (SBR) system with semiquantitative evaluations for tubules (glands), nuclear pleomorphism, and mitotic counts. Gland or tubule formation is judged over the entire tumor, as is nuclear pleomorphism. Mitotic counts are done in the most mitotic active area of carcinoma in 10 consecutive high-powered fields. The high-powered fields are standardized by measuring the diameter (and area) of the microscopic field and converting the mitotic counts in comparison to a standardized area.[52] This system has been endorsed by the Royal College of Pathologists' Working Group for the National Health Service Breast Screening Program, on Pathological Reporting. In

addition, it has been adopted by the Cancer Committee of CAP and is required by the Commission on Cancer (CoC) and the National Accreditation Program for Breast Centers (NAPBC). The guidelines for grading of breast cancers are available on the CAP website (www.cap.org).

High-grade and rapidly dividing tumor cells are more likely to respond to nontargeted chemotherapy. In the traditional histopathologic sense, the measure of dividing cells is the mitotic count. To attempt a more accurate picture of percent dividing cells, many pathologists use expression of Ki-67 measured by IHC.[56] Although there are no universally agreed-upon cut points for low, intermediate, or high Ki-67 values, and no standardized methodology is applied, it is clear that high Ki-67 levels reflect rapidly dividing tumor cells and predict response to anthracycline chemotherapy.[57]

It has been recognized since the late 1800s that hormonal manipulation can affect the growth of breast cancer.[58] More recently, ER assays have been standardized.[59] It has been shown that selective ER modulators, such as tamoxifen and other endocrine therapies, slow or stop progression of ER- and PR-positive tumors. The higher the level of expression of ER and PR, the greater the benefit.[60,61] The response rate is lower for tumors that are ER-positive and PR-negative, and lower still for ER-negative, PR-positive tumors. ER-negative, PR-negative tumors are very unlikely to respond to endocrine therapy.[60-62]

A number of oncogenes also have been linked to prognosis in breast cancer. The most studied is HER2.[63] The presence of HER2 positivity in untreated patients, either by gene amplification or protein overexpression, has been associated with a worse prognosis in both node-negative and node-positive patients.[64-66] HER2 positivity in breast cancers is associated with poor differentiation and, therefore, is very rarely seen with low-grade invasive ductal carcinomas or traditional invasive lobular carcinoma.[66] HER2 positivity, in addition to being associated with high-grade tumors, also is associated with high cell proliferation rates, DNA aneuploidy, and hormone receptor negativity.[67-69] ASCO and CAP have together issued guidelines for performing and evaluating HER2 testing.[70,71]

The development of HER2-targeting agents for the treatment of HER2-positive breast cancer has dramatically improved outcomes for patients with this disease. The monoclonal antibody trastuzumab and related agents, given in conjunction with various chemotherapeutic regimens, have been shown to be particularly effective in improving prognosis of HER2-positive patients.[72,73] There appear to be complex relationships between hormone receptor status and HER2 expression. It has been reported that patients with tumors that are HER2- and ER-positive are less responsive or resistant to single-agent tamoxifen.[74-76] Even in hormone receptor–positive tumors, the expression of HER2 appears to be inversely related to the expression of ER and PR.[77]

It is clear that breast cancer, like other cancers, is not a single disease; the cancers vary tremendously, not only in histologic appearance, grade, hormone receptor, and HER2 status, but also on a molecular/genetic basis. Genomic analysis of breast cancers identifies four groups,[78] similar to the intrinsic subtypes defined by gene expression profiling.[79-82] These subtypes—Luminal A, Luminal B, HER2 and Basal—have widely different gene expressions, natural histories, metastatic patterns, and sensitivity to existing therapies.[79,83,84]

Although gene expression profiling has become a more commonly used laboratory technique, and its cost has decreased significantly, it is still not broadly available as a validated diagnostic technique in most health care situations. Therefore, instead of gene expression—based molecular subtypes of breast cancer, clinically defined subtypes have been used to estimate prognosis and guide therapeutic decisions. These subtypes are based on the expression of ER, PR, and HER2, with the additional measurement of grade or a measure of proliferation, such as Ki-67 or mitotic count. The characteristics of each subtype are shown in Table 48.2.

Luminal A-type tumors are usually low-grade invasive ductal carcinomas (NOS type) or special types of carcinoma—such as tubular, cribriform, or mucinous—and have an excellent prognosis. These tumors generally have a poor response to traditional chemotherapy but have an excellent

Table 48.2 Clinically Defined Subtypes of Breast Cancer

**Clinically Defined – Treatment Oriented Subtypes of Breast Cancer**	
**LUMINAL LIKE**<br>Hormone receptor-positive and HER2-negative luminal disease as a spectrum:	**LUMINAL LIKE**<br>Hormone receptor-positive and HER2-negative luminal disease as a spectrum:
**(Luminal A-like)**<br>High receptor, low proliferation	Multiparameter molecular marker "favorable prognosis," if available; high ER/PR and clearly low proliferation rate (low Ki-67, low mitotic count); generally histological grade 1 or 2
**(Luminal B-like)**<br>Low receptor, high proliferation	Multiparameter molecular marker "unfavorable prognosis," if available; lower ER/PR with high proliferation rate (high Ki-67, high mitotic count); generally histological grade 3
**HER2 LIKE**<br>HER2-positive	HER2-positive and hormone receptor-negative *or* HER2-positive and hormone receptor-positive; generally histological grade 3
**BASAL LIKE**<br>Triple-negative	Negative ER, PR, and HER2; generally histological grade 3

(Modified with permission from Konecny et al. 2003[77] and Eiermann et al. 2013[83])

response to endocrine therapies. Luminal B tumors tend to be poorly differentiated, less likely to respond to endocrine therapy and more likely to respond to traditional chemotherapy. The HER2-like (or HER2-enriched) tumors, prior to the introduction of anti-HER2 therapy, were the most aggressive subtype and had the highest mortality rate and shortest survival. However, in current practice, when appropriately managed with anti-HER2 therapy, patients with these tumors have a much better prognosis. The basal-like tumors, which are thought to arise from myoepithelial cells, have the highest mortality and are most difficult to treat with adjuvant therapy.

Multigene Panels

Another consideration for adding biologic factors into breast cancer staging is to incorporate the findings from multigene panel testing. The multigene panels test for the levels of expression of a large number of genes in the breast cancer tissue, most often by some measure of the levels of message (RNA) present in the tumor. A number of such panels are in clinical use because of studies demonstrating their value in providing more specific prognostic information and in defining sensitivity to classes of systemic agents, especially chemotherapy.

One issue in assessing the use of multigene panels is that the panels currently in clinical use may simply represent a substitute for measuring proliferation. These panels often include significant numbers of proliferation genes and track closely with proliferation. The most widely used single marker of proliferation is Ki-67. As a single factor, Ki-67 was not considered a reliable factor for implementation in clinical practice, both because of the known lack of reproducibility (especially between different laboratories) as well as the lack of agreement on an optimal cut-point. Multigene panels have the advantage of being reproducible and reliable, but the disadvantage of substantial cost, at least at the present time.

As a consideration for integrating multigene marker panels into staging, the Expert Panel felt that a prerequisite to obtaining a multigene panel was to perform the required individual tumor markers, including at a minimum, ER, PR, and HER2. The strong recommendation was that prognostic and predictive models should not be part of the staging system without knowledge of ER, PR, and, in part because their use may be limited only to patients with specific breast cancer subtypes (e.g., hormone receptor–positive, HER2 negative). A second recommendation was that multigene panels should only be incorporated into the staging system for certain subsets of breast cancer. For example, multigene panels might be considered for hormone receptor–positive, HER2-negative, node-negative, Stage I/II subgroup of disease, but there was agreement that multigene panels would not be incorporated into staging for triple-negative tumors at this time because they have limited clinical value for these patients. Third, it was recognized that much of the literature on multigene panels was confounded by the impact of treatment with systemic therapy, and most reports do not include large, prospective cohorts of untreated patients.

A number of recent publications and abstracts have shown relevant data for integrating multigene panels into clinical staging. In the TAILORx study, patients were enrolled on a low-risk arm (Arm A; not randomized) based on the following criteria: hormone receptor–positive, HER2-negative, node-negative, invasive breast carcinoma, tumor size 1.1–5.0 cm (or 0.6 cm–1.0 cm with intermediate or high histologic or nuclear grade), and Oncotype Dx® Recurrence Score less than 11.[8] Systemic treatment was hormone therapy alone, without chemotherapy. At 5 years, the rate of invasive disease-free survival was 93.8%, the rate of freedom from recurrence of breast cancer at a distant site was 99.3%, the rate of freedom from recurrence was 98.7%, and the rate of overall survival was 98.0%.

Similar excellent results based on favorable Oncotype Dx® Recurrence Score results have been presented in two other studies published in abstract form only. First, a population-based study from Israel of 930 patients treated according to Recurrence Score has been reported as an abstract.[9] Of the 930 patients, 479 were classified as low risk based on the standard definition of Recurrence Score less than 18. Only 1% of this low-risk group received chemotherapy. At 5 years, the rate of breast cancer–specific survival was 99.8%, and the rate of distant recurrence was 0.5%. The analysis by Stemmer et al. was updated in abstract form with a larger cohort of patients at the 2015 San Antonio Breast Cancer Symposium.[85] This updated analysis was based on 1,594 patients with a 5.9-year median follow-up. The 5-year estimates for distant recurrence rate in patients with low and intermediate Recurrence Score results were 0.5% and 1.2%, respectively. Second, in a prospective German study of 3,198 patients, 348 were classified as low risk defined by the authors as a Recurrence Score less than 11 and were treated with endocrine therapy alone, without chemotherapy.[10] In this low-risk subgroup, the 3-year event-free survival was 98.3%. Real-life analysis evaluating 1,594 N0 or N1mi breast cancer patients for whom treatment decisions incorporated the 21-gene recurrence score result showed 5-year Kaplan-Meier estimates for breast cancer–specific survival with recurrence to be greater than 98% when score results were 30 or lower.

Investigators from Genomic Health, Inc., the company that developed the Oncotype Dx® assay, partnered with investigators at SEER and combined the data of patients who had the Oncotype Dx® recurrence score with clinical-pathological data available from the SEER database. An analysis based on 38,568 patients showed that 5-year breast cancer–specific survival for patients with a recurrence score less than 18 was 99.6%; for those with a recurrence score of 18–30, it was 98.6%.[86]

Drukker et al. reported results from 427 patients enrolled in the RASTER (microarRAy prognoSTics in breast cancER) from the Netherlands, which prospectively defined treatment based on the 70-gene signature (Mammaprint®), in addition to

clinical and pathological features. In the subset of 95 patients with low-risk clinical and molecular features (defined by Adjuvant! Online and the 70-gene signature, respectively), systemic therapy (chemotherapy and/or hormonal therapy) was given to less than 10% of these patients. At 5 years, the rate of distant disease-free survival was 94.3%, and the rate of distant recurrence-free survival was 95.3%.[87]

As a result of these recent publications and an exhaustive review of the literature, the ASCO Clinical Practice Guideline Committee updated its recent guideline regarding the use of biomarkers to guide decisions on adjuvant systemic therapy for patients with early-stage breast cancer.[3] This updated guideline was published online on February 8, 2016, and incorporates specific recommendations about the single biomarkers and multigene panels.[88]

In summary, comparison of the results from these studies demonstrates a consistently very low risk of recurrence of disease at 3–5 years in the low-risk subgroup of patients, as selected by low-risk molecular profiling in the context of clinically defined low-risk features. Caveats include that follow-up is short in these studies, with only 3- to 5-year results reported, differing clinical selection criteria, differing treatments used, differing molecular profiling tools used, and differing cut points used for selecting the low-risk subgroup of patients. Nonetheless, on balance, low-risk biology as identified by multigene molecular testing in reported studies to date is associated with a very favorable prognosis at 3–5 years.

Based on the best available evidence at this time, the Expert Panel determined that it was appropriate to incorporate multigene molecular profiling to incorporate the Oncotype Dx® score into staging for the subgroup of patients defined by Arm A of the TAILORx study (including Oncotype Dx® Recurrence Score less than or equal to 10). These patients should be staged according to the AJCC Prognostic Stage Groups. The findings for the Oncotype Dx® assay are supported by Level I Evidence (large-scale prospective clinical trial data). It is likely that other multigene panels provide the same information to allow assigning them to the Prognostic Stage Group I. However, the available data in 2016 is Level II evidence and therefore does not support assignment of this prognostic stage at this time.

For all patients, providers and registries should continue to collect and record ER, PR, HER2, and Ki-67 and should continue to collect and record multigene panel results in appropriate cases, if the markers and panels are performed.

Incorporating Biomarkers into TNM – Prognostic Stage Groups

Heretofore, large databases that have complete data on all biomarkers and sufficient follow-up have not been available, largely because HER2 was not routinely captured in population registries until 2010. However, with these biologic factors in mind, two members of the Breast Expert Panel for the 8th Edition analyzed large cohorts of patients to determine whether the incorporation of biologic markers would improve discrimination over the classic TNM system.

The first group conducting data analyses to demonstrate the value of biomarkers on prognosis and stage group assignment, led by Drs. Kelly K. Hunt and Elizabeth A. Mittendorf, used a large database from the University of Texas MD Anderson Cancer Center.[18] Invasive breast cancer patients treated at MD Anderson between January 1997 and December 2006 were included in the analysis if they had no known distant metastasis; had information about grade, ER, and PR status; had not received neoadjuvant chemotherapy; and had follow-up longer than 2 years: 3,728 patients fulfilled these criteria. Disease-specific survival (DSS) was calculated from the time of diagnosis to death due to breast cancer. Patients not experiencing this endpoint were censored at last follow-up. Pathological stage was then used to derive a prognostic model for DSS. Univariate and multivariate analyses were performed to identify factors associated with DSS. Factors evaluated included ER, PR, grade, and lymphovascular invasion. Independent predictors of DSS were assigned a prognostic score of 0 to 2, based on the hazard ratio (HR). For binary variables, the comparison group with a significant impact on DSS was assigned 1 point. For ordinal variables, comparison groups with a significant impact and an HR between 1.1 and 3 were assigned 1 point, and those between 3.1 and 6 were assigned 2 points. Six staging systems that included various combinations of biologic factors with pathological stage were evaluated, and the staging system that incorporated grade and ER status with pathological stage was determined to be the most precise, with a high C-index and low Akaike's information criterion (AIC). When compared to pathological stage alone, this novel staging system resulted in improved discrimination between stages with respect to DSS. These results were subsequently validated using the SEER data.

One limitation of this staging system is that its development predated the routine use of trastuzumab for patients with HER2-positive breast cancer. Recognizing this, the MD Anderson group updated the model using a cohort of 3,327 patients, including 306 patients with HER2-positive breast cancer, treated at their institution between January 2007 and December 2013. With this update, a multivariate analysis was again performed to identify factors associated with DSS. Factors evaluated included pathological stage, grade, ER status, PR status, and HER2 status. A score of 0 to 4 was assigned to each factor based on the HR. Factors with an HR of 1.1–3 were assigned 1 point, factors with a HR of 3.1–6 were assigned 2 points; those with an HR of 6.1–10 were assigned 3 points, and those with an HR greater than 10 were assigned 4 points (Table 48.3). An overall staging score was calculated by summing the

scores for the individual independent predictors of DSS. The staging system that included pathological stage, grade, ER, and HER2 had the highest C-index and lowest AIC. These results were validated using data from the California Cancer Registry.[89]

The analyses performed on these large databases from MD Anderson assumed proper multidisciplinary treatment with appropriate adjuvant chemotherapy and hormonal therapy. The data confirmed the prognostic significance of biologic factors to include grade, ER, and HER2 status and led to the development of a risk profile that can be used to further refine the prognostic information provided by the pathological stage. The risk profile is determined by assigning points as shown in Table 48.4.

The estimated 5-year DSS and overall survival for the MD Anderson cohort of patients treated from January 2007 to December 2013 (n = 3,327), based on the addition of the risk profile to the pathological stage, are shown in Table 48.5. The quantitative information about outcome will assist in the selection of postoperative adjuvant treatments.[89]

The other group, led by Dr. David J. Winchester and colleagues, studied the impact of prognostic factors on staging using the National Cancer Data Base (NCDB). The study used the conventional variables (TNM categories based upon 7th Edition stage groups), as well as tumor grade (Nottingham modification of the SBR system), ER status, PR status, and HER2 status. The analysis included 238,265 women diagnosed with invasive breast cancer in 2010-2011 with a median follow up of 37.6 months.[90] All patients had a complete set of variables. Survival calculations were performed for each prognostic subgroup based on 7th edition stage group, grade, HER2, ER and PR status combination. Patients with triple-negative tumors (all grades) and patients with grade 3 tumors that did not overexpress HER2 and did not express either ER or PR had decreased survival, comparable to patients at least one stage higher with 7th Edition criteria. Conversely, many subgroups with tumors expressing both ER and PR with or without HER2 overexpression had better survival than others with the same 7th Edition stage group. These findings were consistent with the point score developed in the MD Anderson model. Survival ranges of stage groups were defined using 7th Edition staging criteria to maintain consistency with previous stage survival expectations. Prognostic subgroups were assigned to a respective stage according to the calculated mean survival.

The NCDB analysis was used to establish Prognostic Stage Groups for the 8th Edition as included in this chapter. The inclusion of grade, HER2 and hormone receptor status

Table 48.3 Univariate and Multivariate Analyses of Prognostic Factors and Their Influence on Disease-Specific Survival (DSS). The last column shows the assignment of points based on the magnitude of the Hazard Ratios (HR). MD Anderson Analysis.

	5-year DSS	Univariate Analysis		Multivariate Analysis 2		Assigned Points
	(%)	HR	*p*	HR	*p*	
**Pathological stage**						
**I**	99.1	Referent		Referent		0
**IIA**	98.0	2.8	.002	2.3	.01	1
**IIB**	95.6	4.8	< .0001	4.0	< .0001	2
**IIIA**	95.4	6.8	< .0001	7.2	< .0001	3
**IIIC**	79.5	26.6	< .0001	19.9	< .0001	4
**Nuclear grade**						
**I**	99.8	Referent		Referent		0
**II**	98.9	5.0	.1	4.0	.2	0
**III**	95.3	25.0	.001	13.1	.01	1
**ER status**						
**Positive**	98.8	Referent		Referent		0
**Negative**	92.9	4.9	< .0001	2.5	.001	1
**PR status**						
**Positive**	98.8	Referent		Referent		
**Negative**	95.2	4.0	< .0001		NS	
**HER2 status**						
**Positive**	97.5	Referent		Referent		0
**Negative**	98.0	0.8	.5	2.2	.04	1

Table 48.4 Determination of the Risk Profile. MD Anderson Analysis.

Factor	0 points	1 point
**Grade**	Grade 1/2	Grade 3
**ER status**	ER positive	ER negative
**HER2 status**	HER2 positive	HER2 negative

Table 48.5 Overall Survival (OS) and Disease-Specific Survival, Determined by Adding the Risk Profile to the AJCC TNM Pathological Stage, MD Anderson Analysis.

Stage	Risk Profile	N	5-yr DSS	95% CI	5-yr OS	95% CI
I (IA and IB)	0	36	100%		97%	80.4%–99.6%
	1	1,173	99.4%	98.7%–99.7%	96.7%	95.4%–97.
	2	274	98.8%	96.4%–99.6%	94.6%	91.0%–96.8%
	3	119	96.6%	91.1%–98.7%	93.8%	87.5%–97.0%
IIA	0	31	100%		96.8%	79.2%–99.5%
	1	634	99.4%	97.5%–99.8%	97.1%	94.7%–98.4%
	2	236	97.5%	93.2%–99.1%	94.1%	88.7%–97.0%
	3	98	91.0%	81.8%–95.7%	88.2%	78.5%–93.8%
IIB	0	11	100%		100	
	1	309	96.9%	92.6%–98.8%	94.6%	89.6%–97.2%
	2	107	92.9%	83.6%–97.1%	89.3%	80.1%–94.4%
	3	40	91.5%	75.6%–97.2%	91.5%	75.6%–07.2%
IIIA	0	3	100%		100%	
	1	134	98.3%	88.2%–99.8%	91.5%	82.6–96.0%
	2	50	92.2%	77.2%–97.5%	90.3%	75.7%–96.3%
	3	7	68.6%	21.3%–91.2%	68.6%	21.3%–91.2%
IIIC	0	0				
	1	39	92.2%	72.1%–98.0%	84.4%	63.7%–93.9%
	2	16	80.8%	51.4%–93.4%	80.8%	51.4%–93.4%
	3	10	33.3%	6.3%–64.6%	33.3%	6.3%–64.6%

using this model resulted in stage reassignment for 41% of the patients to a stage group higher or lower than would otherwise be assigned using 7th Edition anatomic stage. It is important to note that in applying this table, survival and stage were derived from patients treated in approximately 1500 Commission on Cancer accredited hospitals, capturing over 70% of breast cancers diagnosed in the United States. The majority of the women in the NCDB were offered and treated with appropriate adjuvant endocrine and/or systemic chemotherapy. Stage and survival should be considered only in the consideration of appropriate therapy.

Multigene Panel - Specific Recommendation

Patients who meet the eligibility criteria for Arm A of the TAILORx study (ER positive, HER2 negative, lymph node negative, Oncotype Dx® Recurrence Score less than 11) with tumors 5 cm or smaller in size should be staged as Stage I, as found in AJCC Prognostic Stage Groups.

PROGNOSTIC FACTORS

Prognostic Factors Required for Stage Grouping

Estrogen Receptor Expression

ER expression is measured primarily by IHC. Any staining of 1% of cells or more is considered positive for both ER and PR.[61] AJCC Level of Evidence: I

Progesterone Receptor Expression

PR expression is measured primarily by IHC. Any staining of 1% of cells or more is considered positive for both ER and PR. AJCC Level of Evidence: I

Human Epidermal Growth Factor Receptor-2 (HER2)

The measurement of HER2 is primarily by either IHC to assess expression of the HER2 protein or by *in situ* hybridization (ISH) - most commonly by fluorescent labeled probes (FISH) or chromogenic labeled probes (CISH) to assess gene copy number. The 2013 American Society of Clinical Oncology/College of American Pathologists Guidelines provide standards for sequential performance of tests to accurately and efficiently determine HER2 status, most commonly starting with IHC and progressing to ISH testing if IHC is 2+ (equivocal). Below the standards are summarized. Users are referred to the full guideline for detailed information on HER2 testing and reporting.[70] AJCC Level of Evidence: I

IHC: Negative: 0 or 1+ staining
Equivocal: 2+ staining
Positive: 3+ staining

ISH: Possible negative results:

- HER2/CEP17 ratio < 2.0 **AND** HER2 copy number < 4

Possible equivocal results: (requires performing alternative ISH test to confirm equivocal)

- HER2/CEP17 ratio < 2.0 **AND** HER2 copy number ≥ 4 but < 6

Possible positive results:

- HER2/CEP17 ratio ≥ to 2.0 by ISH
- HER2 copy number ≥ to 6 regardless of ratio by ISH

The above summary is for dual probe ISH. Some laboratories may still use single probe. In that case, the cut points are:
Negative: < than 4 HER2 copies
Equivocal: ≥ to 4 HER2 copies but < 6 HER2 copies
Positive: 6 or more HER2 copies

Histological Grade (Scarff–Bloom–Richardson System – Nottingham Modification)

All invasive breast carcinomas should be assigned a histologic grade. The Nottingham combined histologic grade (Nottingham modification of the SBR grading system) is recommended.[48],[51],[52] The grade for a tumor is determined by assessing morphologic features (tubule formation, nuclear pleomorphism, and mitotic count), assigning a value from 1 (favorable) to 3 (unfavorable) for each feature, and totaling the scores for all three categories. A combined score of 3–5 points is designated as grade 1; a combined score of 6–7 points is grade 2; a combined score of 8–9 points is grade 3.

G	G Definition
GX	Grade cannot be assessed
G1	Low combined histologic grade (favorable), SBR score of 3–5 points
G2	Intermediate combined histologic grade (moderately favorable); SBR score of 6–7 points
G3	High combined histologic grade (unfavorable); BSR score of 8–9 points

Oncotype Dx®

Oncotype Dx® is a genomic test based on the assessment of 21 genes; the result is the outcome of a mathematical formula of the weighted expression of each gene combined into a single score. It is measured and reported by RT-PCR, with recurrence score of < 11 the most pertinent cutoff value.[14] Oncotype Dx® is required only for assigning prognostic stage group to patients with T1–2 N0 M0, ER-positive, HER2-negative cancers. AJCC Level of Evidence: I

Additional Factors Recommended for Clinical Care

Circulating Tumor Cells (CTC) and Method of Detection

(RT-PCR, immunomagnetic separation, other)

CTCs are cancer cells that detach from solid tumors and enter the blood stream. The presence of CTCs is an adverse prognostic factor for patients with primary and metastatic breast cancer. Multiple methods are available to identify and measure CTCs, but the only FDA-approved method is the CellSearch assay. A 7.5-mL sample of blood is centrifuged to separate solid blood components from plasma, then placed in the CELLTRACKS® AUTOPREP® system. Using ferrofluid nanoparticles with antibodies that target epithelial cell adhesion molecules, CTCs are magnetically separated from the bulk of other cells in the blood. CTCs are then stained with cytokeratin monoclonal antibodies, which are specific to epithelial cells. A monoclonal antibody stain is used to identify CD45, a marker specific to leukocytes, which identifies any leukocytes that may have contaminated the sample. A DNA stain called DAPI is added to highlight the nuclei of both CTCs and leukocytes. Cells are put in a magnet cartridge that applies a magnetic force that pulls the cells to a single focal depth. The cartridge containing stained CTCs is scanned by the CELLTRACKS ANALYZER II®, and the system displays tumor cell candidates that are positive for cytokeratin and DAPI. These candidate cells are presented to an operator for final review. For metastatic breast cancer, the cutoff for unfavorable prognosis is ≥ 5 cells/7.5 mL. For primary breast cancer, a cutoff of ≥ 1 cell/7.5 mL has been used. AJCC Level of Evidence: II

Disseminated tumor cells (DTC; bone marrow micrometastases) and Method of Detection

(RT-PCR, IHC, other)

DTCs in bone marrow (BM) might be used as a "liquid biopsy" to obtain information helpful to steer therapies in individual patients. There is an association between the presence of DTCs in BM at the time of initial tumor resection and postoperative metastatic relapse in patients with cancers of the breast. Cytokeratins are currently the standard markers for detecting epithelial tumor cells in mesenchymal organs, such as BM, blood, or lymph nodes. They are detected by IHC, and the pertinent cutoff value is ≥ 1 cell. AJCC Level of Evidence: I

Ki-67

Ki-67 is a nuclear protein associated with cellular proliferation.[55,91] The most prevalent analysis method of Ki-67 antigen is IHC; to date, however, no standard operating procedure or generally accepted cutoff definition for Ki-67 exists. AJCC Level of Evidence: III

Multigene Signature Scores

IHC4 combines the IHC assessment of ER, PR, HER2, and Ki-67.[82,92] The developers presented evidence to suggest that it has prognostic value similar to the Oncotype Dx® assay. The results are based on a multivariate model that uses semiquantitative information from IHC assessment of ER, PR, HER2, and Ki-67. IHC4 uses a mathematical formula that weighs the semiquantitative expression values and combines these into a single risk score. AJCC Level of Evidence: II

Table 48.6 Prognostic tools for breast cancer that met all AJCC quality criteria

Approved Prognostic Tool	Web Address	Factors Included in the Model
Adjuvant! Online	www.adjuvantonline.com/	Tumor size, number of positive lymph nodes, ER status, age, menopausal status, comorbidity, adjuvant therapy
PREDICT-Plus	www.predict.nhs.uk/predict.html	Age, number of positive lymph nodes, tumor size, tumor grade, mode of detection, chemotherapy, hormone therapy; separate models for ER-negative and ER-positive; HER2 added in PREDICT-Plus

Mammaprint®

Mammaprint® is a genomic test based on the level of expression of 70 genes associated with breast cancer recurrence.[6,7] It is measured and reported by gene expression profiling, with the pertinent cutoff value yielding binary results: low risk (< 10%) versus high risk of recurrence within 10 years. AJCC Level of Evidence: II

PAM50 (Prosigna)

PAM50 (Prosigna) is measured and reported in expression profiling as a single numerical score on a 0-to-100 scale that correlates with the probability of distant recurrence within 10 years.[80,82,91] AJCC Level of Evidence: II

Breast Cancer Index

Breast Cancer Index is measured and reported in gene expression profiling as a numerical result on a continuous curve (delineated by HIGH/LOW risk categories).[92] AJCC Level of Evidence: II

EndoPredict

EndoPredict is measured and reported in gene expression profiling as a numerical result on a continuous curve (from 0 to 15), with a score of 5 separating low risk from high risk.[93] AJCC Level of Evidence: II

RISK ASSESSMENT MODELS

Prognostic models will continue to play an important role in 21st century medicine for several reasons.[94] First, by identifying which factors predict outcomes, clinicians gain insight into the biology and natural history of the disease. Second, treatment strategies may be optimized based on the outcome risks of the individual patient. Third, because of the heterogeneity of disease in most cancers, prognostic models will play a critical role in the design, conduct, and analysis of clinical trials in oncology.[94] If developed and validated appropriately, these models will become part of routine patient care, decision-making trial design, and conduct.

The AJCC Precision Medicine Core (PMC) developed and published criteria for critical evaluation of prognostic tool quality,[95] which are presented and discussed in Chapter 4. Although developed independently by the PMC, the AJCC quality criteria correspond fully with the recently developed Cochrane CHARMS *CH*ecklist for critical *A*ppraisal and data extraction for systematic *R*eviews of prediction *M*odeling *S*tudies.[96]

Existing prognostic models for breast cancer meeting all of the AJCC inclusion/exclusion criteria and meriting AJCC endorsement are presented in this section. A full list of the evaluated models and their adherence to the quality criteria is available on www.cancerstaging.org.

The PMC performed a systematic search of literature for prognostic models/tools in breast cancer published from January 2011 to December 2015. The search strategy is provided in Chapter 4. The PMC defined "prognostic model" as a multivariable model where factors predict a clinical outcome that will occur in the future. Each tool identified was compared against the quality criteria developed by the PMC as guidelines for AJCC commendation for prognostication models (see Chapter 4).

Thirty prognostication tools for breast cancer were identified and reviewed against a checklist derived from the PMC guidelines. Only two tools, Adjuvant! Online[97,98] and PREDICT-Plus[99,100] were found to have met all predefined AJCC inclusion and none of the exclusion criteria. Table 48.6 presents information about these two models. One tool, CancerMath, looked promising, but not all the criteria could be evaluated with the available information in the scientific article and on the author's website.

Adjuvant! Online[97] is primarily a tool to assist in making decisions about adjuvant therapy for women with early-stage breast cancer. Outcome estimates are made from projections based on U.S. population-based SEER data, and adjuvant therapy efficacy estimates are from randomized trial overviews. These probability estimates are combined according to a proprietary system. Input data used to predict outcomes are periodically updated. PREDICT-Plus[100] was developed to predict outcome in women treated for early breast cancer in the United Kingdom. Estimates are based on a Cox proportional hazards regression model fit to data from a population-based registry. Both tools were externally validated with good calibration and acceptable levels of predictive accuracy.

DEFINITIONS OF AJCC TNM

Definition of Primary Tumor (T) – Clinical and Pathological

T Category	T Criteria
TX	Primary tumor cannot be assessed
T0	No evidence of primary tumor
Tis (DCIS)*	Ductal carcinoma *in situ*
Tis (Paget)	Paget disease of the nipple NOT associated with invasive carcinoma and/or carcinoma *in situ* (DCIS) in the underlying breast parenchyma. Carcinomas in the breast parenchyma associated with Paget disease are categorized based on the size and characteristics of the parenchymal disease, although the presence of Paget disease should still be noted.
T1	Tumor ≤ 20 mm in greatest dimension
T1mi	Tumor ≤ 1 mm in greatest dimension
T1a	Tumor > 1 mm but ≤ 5 mm in greatest dimension (round any measurement 1.0-1.9 mm to 2 mm).
T1b	Tumor > 5 mm but ≤ 10 mm in greatest dimension
T1c	Tumor > 10 mm but ≤ 20 mm in greatest dimension
T2	Tumor > 20 mm but ≤ 50 mm in greatest dimension
T3	Tumor > 50 mm in greatest dimension
T4	Tumor of any size with direct extension to the chest wall and/or to the skin (ulceration or macroscopic nodules); invasion of the dermis alone does not qualify as T4
T4a	Extension to the chest wall; invasion or adherence to pectoralis muscle in the absence of invasion of chest wall structures does not qualify as T4
T4b	Ulceration and/or ipsilateral macroscopic satellite nodules and/or edema (including peau d'orange) of the skin that does not meet the criteria for inflammatory carcinoma
T4c	Both T4a and T4b are present
T4d	Inflammatory carcinoma (see "Rules for Classification")

* Note: Lobular carcinoma *in situ* (LCIS) is a benign entity and is removed from TNM staging in the AJCC Cancer Staging Manual, 8th Edition.

Definition of Regional Lymph Nodes – Clinical (cN)

cN Category	cN Criteria
cNX*	Regional lymph nodes cannot be assessed (e.g., previously removed)
cN0	No regional lymph node metastases (by imaging or clinical examination)
cN1	Metastases to movable ipsilateral Level I, II axillary lymph node(s)
cN1mi**	Micrometastases (approximately 200 cells, larger than 0.2 mm, but none larger than 2.0 mm)
cN2	Metastases in ipsilateral Level I, II axillary lymph nodes that are clinically fixed or matted; *or* in ipsilateral internal mammary nodes in the absence of axillary lymph node metastases
cN2a	Metastases in ipsilateral Level I, II axillary lymph nodes fixed to one another (matted) or to other structures
cN2b	Metastases only in ipsilateral internal mammary nodes in the absence of axillary lymph node metastases
cN3	Metastases in ipsilateral infraclavicular (Level III axillary) lymph node(s) with or without Level I, II axillary lymph node involvement; *or* in ipsilateral internal mammary lymph node(s) with Level I, II axillary lymph node metastases; *or* metastases in ipsilateral supraclavicular lymph node(s) with or without axillary or internal mammary lymph node involvement
cN3a	Metastases in ipsilateral infraclavicular lymph node(s)
cN3b	Metastases in ipsilateral internal mammary lymph node(s) and axillary lymph node(s)
cN3c	Metastases in ipsilateral supraclavicular lymph node(s)

Note: (sn) and (f) suffixes should be added to the N category to denote confirmation of metastasis by sentinel node biopsy or fine needle aspiration/core needle biopsy respectively

*The cNX category is used sparingly in cases where regional lymph nodes have previously been surgically removed or where there is no documentation of physical examination of the axilla.

**cN1mi is rarely used but may be appropriate in cases where sentinel node biopsy is performed before tumor resection, most likely to occur in cases treated with neoadjuvant therapy.

Definition of Regional Lymph Nodes – Pathological (pN)

pN Category	pN Criteria
pNX	Regional lymph nodes cannot be assessed (e.g., not removed for pathological study or previously removed)
pN0	No regional lymph node metastasis identified or ITCs only
pN0(i+)	ITCs only (malignant cell clusters no larger than 0.2 mm) in regional lymph node(s)
pN0(mol+)	Positive molecular findings by reverse transcriptase polymerase chain reaction (RT-PCR); no ITCs detected
pN1	Micrometastases; or metastases in 1–3 axillary lymph nodes; and/or clinically negative internal mammary nodes with micrometastases or macrometastases by sentinel lymph node biopsy
pN1mi	Micrometastases (approximately 200 cells, larger than 0.2 mm, but none larger than 2.0 mm)
pN1a	Metastases in 1–3 axillary lymph nodes, at least one metastasis larger than 2.0 mm
pN1b	Metastases in ipsilateral internal mammary sentinel nodes, excluding ITCs
pN1c	pN1a and pN1b combined
pN2	Metastases in 4–9 axillary lymph nodes; or positive ipsilateral internal mammary lymph nodes by imaging in the absence of axillary lymph node metastases
pN2a	Metastases in 4–9 axillary lymph nodes (at least one tumor deposit larger than 2.0 mm)

48

pN2b	Metastases in clinically detected internal mammary lymph nodes with or without microscopic confirmation; with pathologically negative axillary nodes
pN3	Metastases in 10 or more axillary lymph nodes; *or* in infraclavicular (Level III axillary) lymph nodes; *or* positive ipsilateral internal mammary lymph nodes by imaging in the presence of one or more positive Level I, II axillary lymph nodes; *or* in more than three axillary lymph nodes and micrometastases or macrometastases by sentinel lymph node biopsy in clinically negative ipsilateral internal mammary lymph nodes; *or* in ipsilateral supraclavicular lymph nodes
pN3a	Metastases in 10 or more axillary lymph nodes (at least one tumor deposit larger than 2.0 mm); *or* metastases to the infraclavicular (Level III axillary lymph) nodes
pN3b	pN1a or pN2a in the presence of cN2b (positive internal mammary nodes by imaging); *or* pN2a in the presence of pN1b
pN3c	Metastases in ipsilateral supraclavicular lymph nodes

Note: (sn) and (f) suffixes should be added to the N category to denote confirmation of metastasis by sentinel node biopsy or FNA/core needle biopsy respectively, with NO further resection of nodes.

Definition of Distant Metastasis (M)

M Category	M Criteria
M0	No clinical or radiographic evidence of distant metastases*
cM0(i+)	No clinical or radiographic evidence of distant metastases in the presence of tumor cells or deposits no larger than 0.2 mm detected microscopically or by molecular techniques in circulating blood, bone marrow, or other nonregional nodal tissue in a patient without symptoms or signs of metastases
M1	Distant metastases detected by clinical and radiographic means (cM) and/or histologically proven metastases larger than 0.2 mm (pM)

* Note that imaging studies are not required to assign the cM0 category

AJCC ANATOMIC AND PROGNOSTIC STAGE GROUPS

There are two stage group tables: The Anatomic Stage Group table and the Prognostic Stage Group table. Cancer registries and clinicians in the United States must use the Prognostic Stage Group table for reporting. It is expected that grade, HER2, ER and PR are performed and reported on all cases of invasive cancer in the United States.

Cancer registries in the United States should use the Prognostic Stage Group table for case reporting. The Anatomic Stage Group table should only be used in regions of the world where tumor grading and/or biomarker testing for HER2, ER and PR are not routinely available. For worldwide comparison, the anatomic stage group can be back-calculated from U.S. registries from the recorded T, N, and M categories.

AJCC Anatomic Stage Groups

The Anatomic Stage Group table should only be used in global regions where biomarker tests are not routinely available.

Cancer registries in the U.S. must use the Prognostic Stage Group table for case reporting.

When T is...	And N is...	And M is...	Then the stage group is...
Tis	N0	M0	0
T1	N0	M0	IA
T0	N1mi	M0	IB
T1	N1mi	M0	IB
T0	N1	M0	IIA
T1	N1	M0	IIA
T2	N0	M0	IIA
T2	N1	M0	IIB
T3	N0	M0	IIB
T0	N2	M0	IIIA
T1	N2	M0	IIIA
T2	N2	M0	IIIA
T3	N1	M0	IIIA
T3	N2	M0	IIIA
T4	N0	M0	IIIB
T4	N1	M0	IIIB
T4	N2	M0	IIIB
Any T	N3	M0	IIIC
Any T	Any N	M1	IV

Notes for Anatomic Stage Grouping

- T1 includes T1mi.
- T0 and T1 tumors with nodal micrometastases only are excluded from Stage IIA and classified Stage IB.
- M0 includes M0(i+).
- The designation pM0 is not valid; any M0 is clinical.
- If a patient presents with M1 disease prior to neoadjuvant systemic therapy, the stage is Stage IV and remains Stage IV regardless of response to neoadjuvant therapy.
- Stage designation may be changed if postsurgical imaging studies reveal the presence of distant metasta-

ses, provided the studies are performed within 4 months of diagnosis in the absence of disease progression, and provided the patient has not received neoadjuvant therapy.

- Staging following neoadjuvant therapy is denoted with a "yc" or "yp" prefix to the T and N classification. No stage group is assigned if there is a complete pathological response (pCR) to neoadjuvant therapy, for example, ypT0ypN0cM0.

AJCC Prognostic Stage Groups

The Prognostic Stage Group table should be used in countries where these biomarker tests are routinely performed for patient care (U.S., Canada, etc.).

Cancer registries in the U.S. must use the Prognostic Stage Group table for case reporting. If biomarkers are not available, the cancer should be reported as unstaged.

When T is...	And N is...	And M is...	And G is...	And HER2 Status* is...	And ER Status is...	And PR Status is...	Then the Prognostic Stage Group is...
Tis	N0	M0	1-3	Any	Any	Any	0
T1	N0	M0	1	Positive	Any	Any	IA
T1	N0	M0	1-2	Negative	Positive	Positive	IA
T1	N0	M0	2	Positive	Positive	Positive	IA
T1	N0	M0	3	Positive	Positive	Any	IA
T0-1	N1mi	M0	1	Positive	Any	Any	IA
T0-1	N1mi	M0	1-2	Negative	Positive	Positive	IA
T0-1	N1mi	M0	2	Positive	Positive	Positive	IA
T0-1	N1mi	M0	3	Positive	Positive	Any	IA
MultiGene Panel** – Oncotype Dx® Recurrence Score Less Than 11							
T1-2	N0	M0	1-3	Negative	Positive	Any	IA
T1	N0	M0	1	Negative	Positive	Negative	IB
T1	N0	M0	1	Negative	Negative	Positive	IB
T1	N0	M0	2	Positive	Positive	Negative	IB
T1	N0	M0	2	Positive	Negative	Any	IB
T1	N0	M0	2	Negative	Negative	Positive	IB
T1	N0	M0	3	Positive	Negative	Any	IB
T1	N0	M0	3	Negative	Positive	Positive	IB
T0-1	N1mi	M0	1	Negative	Positive	Negative	IB
T0-1	N1mi	M0	1	Negative	Negative	Positive	IB
T0-1	N1mi	M0	2	Positive	Positive	Negative	IB
T0-1	N1mi	M0	2	Positive	Negative	Any	IB
T0-1	N1mi	M0	2	Negative	Negative	Positive	IB
T0-1	N1mi	M0	3	Positive	Negative	Any	IB
T0-1	N1mi	M0	3	Negative	Positive	Positive	IB
T2	N0	M0	1-3	Positive	Positive	Positive	IB
T2	N0	M0	1.2	Negative	Positive	Positive	IB
T1	N1	M0	1-3	Positive	Positive	Positive	IB
T1	N1	M0	1-2	Negative	Positive	Positive	IB
T2	N1	M0	1	Negative	Positive	Positive	IB***
T2	N1	M0	2	Positive	Positive	Positive	IB***
T0-2	N2	M0	1-2	Positive	Positive	Positive	IB***

When T is…	And N is…	And M is…	And G is…	And HER2 Status* is…	And ER Status is…	And PR Status is…	Then the Prognostic Stage Group is…
T3	N1-2	M0	1	Positive	Positive	Positive	IB***
T3	N1-2	M0	2	Positive	Positive	Positive	IB***
T1	N0	M0	1	Negative	Negative	Negative	IIA***
T1	N0	M0	2	Negative	Negative	Negative	IIA***
T1	N0	M0	3	Negative	Positive	Negative	IIA***
T1	N0	M0	3	Negative	Negative	Positive	IIA***
T1	N0	M0	3	Negative	Negative	Negative	IIA***
T0-1	N1mi	M0	1	Negative	Negative	Negative	IIA
T0-1	N1mi	M0	2	Negative	Negative	Negative	IIA
T0-1	N1mi	M0	3	Negative	Positive	Negative	IIA
T0-1	N1mi	M0	3	Negative	Negative	Positive	IIA
T0-1	N1mi	M0	3	Negative	Negative	Negative	IIA
T0-1	N1	M0	1	Positive	Positive	Negative	IIA
T0-1	N1	M0	1 - 2	Positive	Negative	Any	IIA
T0-1	N1	M0	1	Negative	Positive	Negative	IIA
T0-1	N1	M0	1	Negative	Negative	Positive	IIA
T0-1	N1	M0	3	Negative	Positive	Positive	IIA
T2	N0	M0	1	Positive	Positive	Negative	IIA
T2	N0	M0	1 - 2	Positive	Negative	Any	IIA
T2	N0	M0	1	Negative	Positive	Negative	IIA
T2	N0	M0	1	Negative	Negative	Positive	IIA
T2	N0	M0	3	Negative	Positive	Positive	IIA
T0-2	N2	M0	1	Negative	Positive	Positive	IIA***
T3	N1-2	M0	1	Negative	Positive	Positive	IIA
T0-1	N1	M0	1	Negative	Negative	Negative	IIB
T0-1	N1	M0	2	Positive	Positive	Negative	IIB
T0-1	N1	M0	2	Negative	Positive	Negative	IIB
T0-1	N1	M0	2	Negative	Negative	Positive	IIB
T0-1	N1	M0	3	Positive	Positive	Negative	IIB
T0-1	N1	M0	3	Positive	Negative	Any	IIB
T2	N0	M0	1	Negative	Negative	Negative	IIB
T2	N0	M0	2	Positive	Positive	Negative	IIB
T2	N0	M0	2	Negative	Positive	Negative	IIB
T2	N0	M0	2	Negative	Negative	Positive	IIB
T2	N0	M0	3	Positive	Positive	Negative	IIB
T2	N0	M0	3	Positive	Negative	Any	IIB
T2	N1	M0	1	Positive	Any	Any	IIB
T2	N1	M0	1	Negative	Negative	Positive	IIB
T0-2	N2	M0	2	Negative	Positive	Positive	IIB
T0-2	N2	M0	3	Positive	Positive	Positive	IIB
T3	N1-2	M0	2	Negative	Positive	Positive	IIB
T3	N1-2	M0	3	Positive	Positive	Positive	IIB
T0-1	N1	M0	2	Negative	Negative	Negative	IIIA***
T0-1	N1	M0	3	Negative	Positive	Negative	IIIA

When T is…	And N is…	And M is…	And G is…	And HER2 Status* is…	And ER Status is…	And PR Status is…	Then the Prognostic Stage Group is…
T0-1	N1	M0	3	Negative	Negative	Any	IIIA
T2	N0	M0	2	Negative	Negative	Negative	IIIA***
T2	N0	M0	3	Negative	Positive	Negative	IIIA***
T2	N0	M0	3	Negative	Negative	Any	IIIA***
T2	N1	M0	1	Negative	Positive	Negative	IIIA
T2	N1	M0	2	Positive	Negative	Negative	IIIA
T2	N1	M0	2	Negative	Positive	Negative	IIIA
T2	N1	M0	3	Positive	Positive	Negative	IIIA
T2	N1	M0	3	Positive	Negative	Negative	IIIA
T3	N0	M0	1	Negative	Positive	Negative	IIIA
T3	N0	M0	2	Positive	Negative	Negative	IIIA
T3	N0	M0	2	Negative	Positive	Negative	IIIA
T3	N0	M0	3	Positive	Positive	Negative	IIIA
T3	N0	M0	3	Positive	Negative	Negative	IIIA
T0-2	N2	M0	1	Positive	Positive	Negative	IIIA
T0-2	N2	M0	1	Positive	Negative	Any	IIIA
T0-2	N2	M0	1	Negative	Positive	Negative	IIIA
T0-2	N2	M0	1	Negative	Negative	Positive	IIIA
T0-2	N2	M0	2	Positive	Positive	Negative	IIIA
T0-2	N2	M0	2	Positive	Negative	Any	IIIA
T3	N1-2	M0	1	Positive	Positive	Negative	IIIA
T3	N1-2	M0	1	Positive	Negative	Any	IIIA
T3	N1-2	M0	1	Negative	Positive	Negative	IIIA
T3	N1-2	M0	1	Negative	Negative	Positive	IIIA
T3	N1-2	M0	2	Positive	Positive	Negative	IIIA
T3	N1-2	M0	2	Positive	Negative	Any	IIIA
T4	N0-2	M0	1	Negative	Positive	Positive	IIIA
Any	N3	M0	1	Negative	Positive	Positive	IIIA***
T2	N1	M0	1 - 2	Negative	Negative	Negative	IIIB***
T2	N1	M0	3	Negative	Positive	Negative	IIIB***
T3	N0	M0	1 - 2	Negative	Negative	Negative	IIIB
T3	N0	M0	3	Negative	Positive	Negative	IIIB
T0-2	N2	M0	2	Negative	Positive	Negative	IIIB
T0-2	N2	M0	2	Negative	Negative	Positive	IIIB
T0-2	N2	M0	3	Positive	Positive	Negative	IIIB
T0-2	N2	M0	3	Positive	Negative	Any	IIIB
T0-2	N2	M0	3	Negative	Positive	Positive	IIIB
T3	N1-2	M0	2	Negative	Positive	Negative	IIIB
T3	N1-2	M0	2	Negative	Negative	Positive	IIIB
T3	N1-2	M0	3	Positive	Positive	Negative	IIIB
T3	N1-2	M0	3	Positive	Negative	Any	IIIB
T3	N1-2	M0	3	Negative	Positive	Positive	IIIB
T4	N0-2	M0	1	Positive	Any	Any	IIIB
T4	N0-2	M0	2	Positive	Positive	Positive	IIIB

When T is...	And N is...	And M is...	And G is...	And HER2 Status* is...	And ER Status is...	And PR Status is...	Then the Prognostic Stage Group is...
T4	N0-2	M0	2	Negative	Positive	Positive	IIIB
T4	N0-2	M0	3	Positive	Positive	Positive	IIIB
Any	N3	M0	1	Positive	Any	Any	IIIB
Any	N3	M0	2	Positive	Positive	Positive	IIIB
Any	N3	M0	2	Negative	Positive	Positive	IIIB
Any	N3	M0	3	Positive	Positive	Positive	IIIB
T2	N1	M0	3	Negative	Negative	Any	IIIC***
T3	N0	M0	3	Negative	Negative	Any	IIIC
T0-2	N2	M0	2	Negative	Negative	Negative	IIIC***
T0-2	N2	M0	3	Negative	Positive	Negative	IIIC***
T0-2	N2	M0	3	Negative	Negative	Any	IIIC***
T3	N1-2	M0	2	Negative	Negative	Negative	IIIC***
T3	N1-2	M0	3	Negative	Positive	Negative	IIIC***
T3	N1-2	M0	3	Negative	Negative	Any	IIIC***
T4	N0-2	M0	1	Negative	Positive	Negative	IIIC
T4	N0-2	M0	1	Negative	Negative	Any	IIIC
T4	N0-2	M0	2	Positive	Positive	Negative	IIIC
T4	N0-2	M0	2	Positive	Negative	Any	IIIC
T4	N0-2	M0	2	Negative	Positive	Negative	IIIC
T4	N0-2	M0	2	Negative	Negative	Any	IIIC
T4	N0-2	M0	3	Positive	Positive	Negative	IIIC
T4	N0-2	M0	3	Positive	Negative	Any	IIIC
T4	N0-2	M0	3	Negative	Any	Any	IIIC
Any	N3	M0	1	Negative	Positive	Negative	IIIC
Any	N3	M0	1	Negative	Negative	Any	IIIC
Any	N3	M0	2	Positive	Positive	Negative	IIIC
Any	N3	M0	2	Positive	Negative	Any	IIIC
Any	N3	M0	2	Negative	Positive	Negative	IIIC
Any	N3	M0	2	Negative	Negative	Any	IIIC
Any	N3	M0	3	Positive	Positive	Negative	IIIC
Any	N3	M0	3	Positive	Negative	Any	IIIC
Any	N3	M0	3	Negative	Any	Any	IIIC
AnyT	AnyN	M1	1-3	Any	Any	Any	IV

* For cases where HER2 is determined to be "equivocal" by ISH (FISH or CISH) testing under the 2013 ASCO/CAP HER2 testing guidelines, HER2 "negative" category should be used for staging in the Prognostic Stage Group Table.[70,71]

**If OncotypeDx® is not performed, not available, or if the OncotypeDx® score is 11 or greater for patients with T1-2 N0 M0 HER2 negative ER positive cancer, then the Prognostic Stage Group is assigned based on the anatomic and biomarker categories shown above. OncotypeDx® is the only multigene panel included to classify Prognostic Stage because prospective Level I data supports this use for patients with a score <11. Future updates may include results from other multigene panels to assign cohorts of patients to prognostic stage groups when there are high level data to support these assignments.

***Denotes a Stage Group for which the use of grade and prognostic factors changed the group more than one stage group from the anatomic stage group (e.g. from Anatomic Stage Group IIB to Prognostic Stage Group IB).

Note: The prognostic value of these Prognostic Stage Groups is based on populations of persons with breast cancer that have been offered and mostly treated with appropriate endocrine and/or systemic chemotherapy.

The use of this prognostic table provides a marked improvement in grouping patients with similar prognosis. Compared to the anatomic stage groups, the application of the prognostic stage groups assigns 41% of cases to a different group with either a better or worse prognosis. The models used to generate these groups is based on analysis of survival of 238,265 patients using the National Cancer Data Base. Though the median survival is relatively short, the data are robust and clearly demonstrate findings consistent with the observations of clinicians in practice. While the application of this table in practice and cancer registries will be more complicated than the anatomic stage table, it is expected that electronic health record and cancer registry software systems will offer tools to generate the Prognostic Stage Group from the data entered for T, N, M, grade and prognostic factors. Regardless, the Expert Panel believes this is a necessary and positive step forward in breast cancer staging as it provides information more relevant to clinical practice that will better serve our patients.

It is recognized that in coming years, and potentially as soon as the next 1 – 3 years after publication of this Manual, additional data from the NCDB and other large populations of patients with full prognostic factor information and increasingly longer follow-up will become available. Based on analyses of these data, the Prognostic Stage Group Table may require revision. Further, it is likely that high level evidence related to multi-gene assays will also become available. The AJCC Breast Expert Panel will regularly review new data as they become available and make necessary revisions as needed in a more rapid fashion than the standard 5 – 7 year cycle for staging revision.

REGISTRY DATA COLLECTION VARIABLES

1. ER: positive versus negative; percent positive; Allred score, if available
2. PR: positive versus negative; percent positive; Allred score, if available
3. HER2—IHC: 0, 1+, 2+, 3+; or unknown or not performed
4. HER2—FISH: negative, positive; HER2:CEP17 ratio; and HER2 copy number, if available; or unknown or not performed
5. HER2: Overall result, negative, positive, unknown if done; not performed
6. Nottingham histologic grade: low (1), intermediate (2), high (3)
7. Ki-67, if available: percent positive
8. Oncotype Dx® recurrence score (numeric score preferred over risk level)
9. Oncotype Dx® DCIS recurrence score (numeric score preferred over risk level)
10. Mammaprint® (numeric score preferred over risk level)
11. PAM50 intrinsic subtypes and Risk of Recurrence score (ProSigna) (numeric score preferred over risk level)
12. Breast Cancer Index (numeric score preferred over risk level)
13. EndoPredict (numeric score preferred over risk level)
14. IHC4 (numeric score preferred over risk level)
15. Urokinase plasminogen activator (uPA) and plasminogen activator inhibitor type 1 (PAI-1)[101]
16. Response to treatment: CR, PR, NR

HISTOLOGIC GRADE (G)

All invasive breast carcinomas should be assigned a histologic grade. The Nottingham combined histologic grade (Nottingham modification of the SBR grading system) is recommended.[48,51,52] The grade for a tumor is determined by assessing morphologic features (tubule formation, nuclear pleomorphism, and mitotic count), assigning a value from 1 (favorable) to 3 (unfavorable) for each feature, and totaling the scores for all three categories. A combined score of 3–5 points is designated as grade 1; a combined score of 6–7 points is grade 2; a combined score of 8–9 points is grade 3.

48

G	G Definition
GX	Grade cannot be assessed
G1	Low combined histologic grade (favorable), SBR score of 3–5 points
G2	Intermediate combined histologic grade (moderately favorable); SBR score of 6–7 points
G3	High combined histologic grade (unfavorable); SBR score of 8–9 points

HISTOPATHOLOGIC TYPE

In situ Carcinomas

Ductal carcinoma *in situ*
Paget disease

Invasive Carcinomas

Not otherwise specified (NOS)
Ductal
Inflammatory
Medullary, NOS
Medullary with lymphoid stroma
Mucinous
Papillary (predominantly micropapillary pattern)

Tubular
Lobular
Paget disease and infiltrating
Undifferentiated
Squamous cell
Adenoid cystic
Secretory
Cribriform

Bibliography

1. Edge SB, Compton CC. *The AJCC Cancer Staging Manual*. 7th ed: Springer; 2009.
2. Prat A, Pineda E, Adamo B, et al. Clinical implications of the intrinsic molecular subtypes of breast cancer. *Breast*. Nov 2015;24 Suppl 2:S26–35.
3. Van Poznak C, Somerfield MR, Bast RC, et al. Use of biomarkers to guide decisions on systemic therapy for women with metastatic breast cancer: American Society of Clinical Oncology Clinical Practice Guideline. *Journal of Clinical Oncology*. 2015:JCO. 2015.2061. 1459.
4. Selz J, Stevens D, Jouanneau L, Labib A, Le Scodan R. Prognostic value of molecular subtypes, ki67 expression and impact of postmastectomy radiation therapy in breast cancer patients with negative lymph nodes after mastectomy. *International journal of radiation oncology, biology, physics*. Dec 1 2012;84(5):1123–1132.
5. Carlson RW, Allred DC, Anderson BO, et al. Breast cancer. Clinical practice guidelines in oncology. *Journal of the National Comprehensive Cancer Network : JNCCN*. Feb 2009;7(2):122–192.
6. van't Veer LJ, Paik S, Hayes DF. Gene expression profiling of breast cancer: a new tumor marker. *J Clin Oncol*. Mar 10 2005;23(8):1631–1635.
7. Buyse M, Loi S, van't Veer L, et al. Validation and clinical utility of a 70-gene prognostic signature for women with node-negative breast cancer. *Journal of the National Cancer Institute*. Sep 6 2006;98(17):1183–1192.
8. Sparano JA, Gray RJ, Makower DF, et al. Prospective Validation of a 21-Gene Expression Assay in Breast Cancer. *N Engl J Med*. Nov 19 2015;373(21):2005–2014.
9. Stemmer S, Steiner M, Rizel S, et al. 1963 First prospective outcome data in 930 patients with more than 5 year median follow up in whom treatment decisions in clinical practice have been made incorporating the 21-Gene Recurrence Score. *European journal of cancer*. 2015(51):S321.
10. Gluz O, Nitz U, Kreipe H, et al. 1937 Clinical impact of risk classification by central/local grade or luminal-like subtype vs. Oncotype DX®: First prospective survival results from the WSG phase III planB trial. *European journal of cancer*. 2015(51):S311.
11. Van De Vijver MJ, He YD, van't Veer LJ, et al. A gene-expression signature as a predictor of survival in breast cancer. *New England Journal of Medicine*. 2002;347(25):1999–2009.
12. Wang Y, Klijn JG, Zhang Y, et al. Gene-expression profiles to predict distant metastasis of lymph-node-negative primary breast cancer. *Lancet*. Feb 19-25 2005;365(9460):671–679.
13. Tang G, Cuzick J, Costantino JP, et al. Risk of recurrence and chemotherapy benefit for patients with node-negative, estrogen receptor-positive breast cancer: recurrence score alone and integrated with pathologic and clinical factors. *J Clin Oncol*. Nov 20 2011;29(33):4365–4372.
14. Paik S, Shak S, Tang G, et al. A multigene assay to predict recurrence of tamoxifen-treated, node-negative breast cancer. *N Engl J Med*. Dec 30 2004;351(27):2817–2826.
15. Dowsett M, Cuzick J, Wale C, et al. Prediction of risk of distant recurrence using the 21-gene recurrence score in node-negative and node-positive postmenopausal patients with breast cancer treated with anastrozole or tamoxifen: a TransATAC study. *Journal of Clinical Oncology*. 2010;28(11):1829–1834.
16. Paik S, Tang G, Shak S, et al. Gene expression and benefit of chemotherapy in women with node-negative, estrogen receptor-positive breast cancer. *J Clin Oncol*. Aug 10 2006;24(23):3726–3734.
17. Albain KS, Barlow WE, Shak S, et al. Prognostic and predictive value of the 21-gene recurrence score assay in postmenopausal women with node-positive, oestrogen-receptor-positive breast cancer on chemotherapy: a retrospective analysis of a randomised trial. *The lancet oncology*. Jan 2010;11(1):55–65.
18. Yi M, Mittendorf EA, Cormier JN, et al. Novel staging system for predicting disease-specific survival in patients with breast cancer treated with surgery as the first intervention: time to modify the current American Joint Committee on Cancer staging system. *Journal of Clinical Oncology*. 2011;29(35):4654–4661.
19. Gradishar WJ, Anderson BO, Balassanian R, et al. Breast Cancer Version 2.2015. *Journal of the National Comprehensive Cancer Network : JNCCN*. Apr 2015;13(4):448–475.
20. Goldhirsch A, Wood WC, Gelber RD, et al. Progress and promise: highlights of the international expert consensus on the primary therapy of early breast cancer 2007. *Ann Oncol*. Jul 2007;18(7):1133–1144.
21. Global Burden of Disease Cancer Collaboration, Fitzmaurice C, Dicker D, et al. The Global Burden of Cancer 2013. *JAMA oncology*. Jul 2015;1(4):505–527.
22. Anderson BO, Yip CH, Smith RA, et al. Guideline implementation for breast healthcare in low-income and middle-income countries: overview of the Breast Health Global Initiative Global Summit 2007. *Cancer*. Oct 15 2008;113(8 Suppl):2221–2243.
23. Turnbull L, Brown S, Harvey I, et al. Comparative effectiveness of MRI in breast cancer (COMICE) trial: a randomised controlled trial. *Lancet*. Feb 13 2010;375(9714):563–571.
24. Houssami N, Turner R, Morrow M. Preoperative magnetic resonance imaging in breast cancer: meta-analysis of surgical outcomes. *Annals of surgery*. Feb 2013;257(2):249–255.
25. Arnaout A, Catley C, Booth CM, et al. Use of Preoperative Magnetic Resonance Imaging for Breast Cancer: A Canadian Population-Based Study. *JAMA oncology*. 2015;1(9):1238–1250.
26. Vos EL, Voogd AC, Verhoef C, Siesling S, Obdeijn IM, Koppert LB. Benefits of preoperative MRI in breast cancer surgery studied in a large population-based cancer registry. *The British journal of surgery*. Dec 2015;102(13):1649–1657.
27. Houssami N, Turner R, Macaskill P, et al. An individual person data meta-analysis of preoperative magnetic resonance imaging and breast cancer recurrence. *J Clin Oncol*. Feb 10 2014;32(5):392–401.
28. Yi A, Cho N, Yang K-S, Han W, Noh D-Y, Moon WK. Breast cancer recurrence in patients with newly diagnosed breast cancer without and with preoperative MR Imaging: A matched cohort study. *Radiology*. 2015;276(3):695–705.
29. D'Orsi CJ, Radiology ACo, Committee B-R. *ACR BI-RADS Atlas: Breast Imaging Reporting and Data System*. 2013.
30. Morris EA, Schwartz LH, Drotman MB, et al. Evaluation of pectoralis major muscle in patients with posterior breast tumors on breast MR images: early experience. *Radiology*. Jan 2000;214(1):67–72.
31. Dawood S, Merajver SD, Viens P, et al. International expert panel on inflammatory breast cancer: consensus statement for standardized diagnosis and treatment. *Ann Oncol*. Mar 2011;22(3):515–523.
32. Houssami N, Diepstraten SC, Cody HS, 3rd, Turner RM, Sever AR. Clinical utility of ultrasound-needle biopsy for preoperative staging of the axilla in invasive breast cancer. *Anticancer research*. Mar 2014;34(3):1087–1097.

33. Diepstraten SC, Sever AR, Buckens CF, et al. Value of preoperative ultrasound-guided axillary lymph node biopsy for preventing completion axillary lymph node dissection in breast cancer: a systematic review and meta-analysis. *Annals of surgical oncology*. Jan 2014;21(1):51–59.
34. Dogan BE, Dryden MJ, Wei W, et al. Sonography and Sonographically Guided Needle Biopsy of Internal Mammary Nodes in Staging of Patients With Breast Cancer. *AJR. American journal of roentgenology*. Oct 2015;205(4):905–911.
35. Gradishar W, Anderson B, Blair S, Burstein H, Cyr A, Elias A. National comprehensive cancer network breast cancer panel. *Breast cancer version*.3:542–590.
36. Aebi S, Davidson T, Gruber G, Castiglione M, Group EGW. Primary breast cancer: ESMO Clinical Practice Guidelines for diagnosis, treatment and follow-up. *Ann Oncol*. May 2010;21 Suppl 5(suppl 5):v9–14.
37. Moy L, Newell MS, Mahoney MC, et al. ACR Appropriateness Criteria stage I breast cancer: initial workup and surveillance for local recurrence and distant metastases in asymptomatic women. *Journal of the American College of Radiology : JACR*. Dec 2014;11(12 Pt A):1160–1168.
38. Schwartz GF, Hortobagyi GN. Proceedings of the consensus conference on neoadjuvant chemotherapy in carcinoma of the breast, April 26-28, 2003, Philadelphia, Pennsylvania. *Cancer*. Jun 15 2004;100(12):2512–2532.
39. Tavassoli FA. Ductal carcinoma in situ: introduction of the concept of ductal intraepithelial neoplasia. *Modern pathology : an official journal of the United States and Canadian Academy of Pathology, Inc*. Feb 1998;11(2):140–154.
40. Tavassoli FA. Breast pathology: rationale for adopting the ductal intraepithelial neoplasia (DIN) classification. *Nat Clin Pract Oncol*. Mar 2005;2(3):116–117.
41. Lester SC, Bose S, Chen YY, et al. Protocol for the examination of specimens from patients with ductal carcinoma in situ of the breast. *Arch Pathol Lab Med*. Jan 2009;133(1):15–25.
42. Chen CY, Sun LM, Anderson BO. Paget disease of the breast: changing patterns of incidence, clinical presentation, and treatment in the US. *Cancer*. 2006;107(7):1448–1458.
43. Hortobagyi GN, Ames FC, Buzdar AU, et al. Management of stage III primary breast cancer with primary chemotherapy, surgery, and radiation therapy. *Cancer*. Dec 15 1988;62(12): 2507–2516.
44. Fisher B, Bryant J, Wolmark N, et al. Effect of preoperative chemotherapy on the outcome of women with operable breast cancer. *J Clin Oncol*. Aug 1998;16(8):2672–2685.
45. Cortazar P, Zhang L, Untch M, et al. Pathological complete response and long-term clinical benefit in breast cancer: the CTNeoBC pooled analysis. *Lancet*. Jul 12 2014;384(9938):164–172.
46. Greenough RB. Varying degrees of malignancy in cancer of the breast. *The Journal of Cancer Research*. 1925;9(4):453–463.
47. Patey D, Scarff R. The position of histology in the prognosis of carcinoma of the breast. *The Lancet*. 1928;211(5460):801–804.
48. Scarff R, Handley R. Prognosis in carcinoma of the breast. *The Lancet*. 1938;232(6001):582–583.
49. Bloom H, Richardson W. Histological grading and prognosis in breast cancer: a study of 1409 cases of which 359 have been followed for 15 years. *British journal of cancer*. 1957;11(3):359.
50. Scarff R, Torloni H. *Histological typing of breast tumors*. Geneva: WHO; 1968.
51. Black MM. Survival in breast cancer cases in relation to the structure of the primary tumor and regional lymphnodes. *Surg Gynecol Obstet*. 1955;100:543–551.
52. Elston CW, Ellis IO. Pathological prognostic factors in breast cancer. I. The value of histological grade in breast cancer: experience from a large study with long-term follow-up. *Histopathology*. Nov 1991;19(5):403–410.
53. Elston EW, Ellis IO. Method for grading breast cancer. *Journal of clinical pathology*. Feb 1993;46(2):189–190.
54. Schwartz AM, Henson DE, Chen D, Rajamarthandan S. Histologic grade remains a prognostic factor for breast cancer regardless of the number of positive lymph nodes and tumor size: a study of 161 708 cases of breast cancer from the SEER Program. *Arch Pathol Lab Med*. Aug 2014;138(8):1048–1052.
55. Gilchrist KW, Kalish L, Gould VE, et al. Interobserver reproducibility of histopathological features in stage II breast cancer. An ECOG study. *Breast cancer research and treatment*. 1985;5(1):3–10.
56. Gerdes J, Schwab U, Lemke H, Stein H. Production of a mouse monoclonal antibody reactive with a human nuclear antigen associated with cell proliferation. *Int J Cancer*. Jan 15 1983;31(1):13–20.
57. Coates AS, Winer EP, Goldhirsch A, et al. -Tailoring therapies-improving the management of early breast cancer: St Gallen International Expert Consensus on the Primary Therapy of Early Breast Cancer 2015. *Ann Oncol*. Aug 2015;26(8): 1533–1546.
58. Beatson G. ON THE TREATMENT OF INOPERABLE CASES OF CARCINOMA OF THE MAMMA: SUGGESTIONS FOR A NEW METHOD OF TREATMENT, WITH ILLUSTRATIVE CASES. 1. *The Lancet*. 1896;148(3802):104–107.
59. Hammond ME, Hayes DF, Dowsett M, et al. American Society of Clinical Oncology/College Of American Pathologists guideline recommendations for immunohistochemical testing of estrogen and progesterone receptors in breast cancer. *J Clin Oncol*. Jun 1 2010;28(16):2784–2795.
60. Group EBCTC. Relevance of breast cancer hormone receptors and other factors to the efficacy of adjuvant tamoxifen: patient-level meta-analysis of randomised trials. *The lancet*. 2011;378(9793):771–784.
61. Barnes DM, Harris WH, Smith P, Millis RR, Rubens RD. Immunohistochemical determination of oestrogen receptor: comparison of different methods of assessment of staining and correlation with clinical outcome of breast cancer patients. *Br J Cancer*. Nov 1996;74(9):1445–1451.
62. Hammond ME, Hayes DF, Dowsett M, et al. American Society of Clinical Oncology/College of American Pathologists guideline recommendations for immunohistochemical testing of estrogen and progesterone receptors in breast cancer (unabridged version). *Arch Pathol Lab Med*. Jul 2010;134(7):e48–72.
63. Schechter AL, Stern DF, Vaidyanathan L, et al. The neu oncogene: an erb-B-related gene encoding a 185,000-Mr tumour antigen. *Nature*. Dec 6-12 1984;312(5994):513–516.
64. Slamon DJ, Clark GM, Wong SG, Levin WJ, Ullrich A, McGuire WL. Human breast cancer: correlation of relapse and survival with amplification of the HER-2/neu oncogene. *Science*. Jan 9 1987;235(4785):177–182.
65. Ross JS, Slodkowska EA, Symmans WF, Pusztai L, Ravdin PM, Hortobagyi GN. The HER-2 receptor and breast cancer: ten years of targeted anti-HER-2 therapy and personalized medicine. *The oncologist*. Apr 2009;14(4):320–368.
66. Rosenthal SI, Depowski PL, Sheehan CE, Ross JS. Comparison of HER-2/neu oncogene amplification detected by fluorescence in situ hybridization in lobular and ductal breast cancer. *Appl Immunohistochem Mol Morphol*. Mar 2002;10(1):40–46.
67. Eccles SA. The role of c-erbB-2/HER2/neu in breast cancer progression and metastasis. *Journal of mammary gland biology and neoplasia*. Oct 2001;6(4):393–406.
68. Piccart M, Lohrisch C, Di Leo A, Larsimont D. The predictive value of HER2 in breast cancer. *Oncology*. 2001;61 Suppl 2(Suppl. 2):73–82.
69. Yarden Y. Biology of HER2 and its importance in breast cancer. *Oncology*. 2001;61 Suppl 2(Suppl. 2):1–13.

48

70. Wolff A, Hammond M, Hicks D, et al. Recommendations for human epidermal growth factor receptor 2 testing in breast cancer: American Society of Clinical Oncology/College of American Pathologists clinical practice guideline update. *Journal of clinical oncology: official journal of the American Society of Clinical Oncology.* 2013;31(31):3997–4013.
71. Wolff AC, Hammond MEH, Hicks DG, et al. Recommendations for human epidermal growth factor receptor 2 testing in breast cancer: American Society of Clinical Oncology/College of American Pathologists clinical practice guideline update. *Archives of Pathology and Laboratory Medicine.* 2013;138(2):241–256.
72. Slamon D, Eiermann W, Robert N, et al. Adjuvant trastuzumab in HER2-positive breast cancer. *N Engl J Med.* Oct 6 2011;365(14):1273–1283.
73. Moasser MM, Krop IE. The Evolving Landscape of HER2 Targeting in Breast Cancer. *JAMA oncology.* Nov 2015;1(8):1154–1161.
74. Dowsett M. Overexpression of HER-2 as a resistance mechanism to hormonal therapy for breast cancer. *Endocrine-related cancer.* 2001;8(3):191–195.
75. Muss HB. Role of adjuvant endocrine therapy in early-stage breast cancer. Paper presented at: Seminars in oncology2001.
76. Schmid P, Wischnewsky MB, Sezer O, Bohm R, Possinger K. Prediction of response to hormonal treatment in metastatic breast cancer. *Oncology.* 2002;63(4):309–316.
77. Konecny G, Pauletti G, Pegram M, et al. Quantitative association between HER-2/neu and steroid hormone receptors in hormone receptor-positive primary breast cancer. *Journal of the National Cancer Institute.* 2003;95(2):142–153.
78. Network CGA. Comprehensive molecular portraits of human breast tumours. *Nature.* 2012;490(7418):61–70.
79. Sorlie T, Perou CM, Tibshirani R, et al. Gene expression patterns of breast carcinomas distinguish tumor subclasses with clinical implications. *Proc Natl Acad Sci U S A.* Sep 11 2001;98(19):10869–10874.
80. Bastien RR, Rodriguez-Lescure A, Ebbert MT, et al. PAM50 breast cancer subtyping by RT-qPCR and concordance with standard clinical molecular markers. *BMC medical genomics.* 2012;5(1):44.
81. Bayraktar S, Royce M, Stork-Sloots L, de Snoo F, Glück S. Molecular subtyping predicts pathologic tumor response in early-stage breast cancer treated with neoadjuvant docetaxel plus capecitabine with or without trastuzumab chemotherapy. *Medical oncology.* 2014;31(10):1–7.
82. Dowsett M, Sestak I, Lopez-Knowles E, et al. Comparison of PAM50 risk of recurrence score with oncotype DX and IHC4 for predicting risk of distant recurrence after endocrine therapy. *J Clin Oncol.* Aug 1 2013;31(22):2783–2790.
83. Eiermann W, Rezai M, Kümmel S, et al. The 21-gene recurrence score assay impacts adjuvant therapy recommendations for ER-positive, node-negative and node-positive early breast cancer resulting in a risk-adapted change in chemotherapy use. *Annals of Oncology.* 2013;24(3):618–624.
84. Coates AS, Winer EP, Goldhirsch A, et al. Tailoring therapies-improving the management of early breast cancer: St Gallen International Expert Consensus on the Primary Therapy of Early Breast Cancer 2015. *Ann Oncol.* Aug 2015;26(8):1533–1546.
85. Stemmer S, Steiner M, Rizel S, et al. Real-life analysis evaluating 1594 N0/Nmic breast cancer patients for whom treatment decisions incorporated the 21-gene recurrence score result: 5-year KM estimate for breast cancer specific survival with recurrence score results≤ 30 is> 98%. *Cancer Research.* 2016;76(4 Supplement):P5-08-02-P05-08-02.
86. Shak S, Petkov V, Miller D, et al. Abstract P5-15-01: Breast cancer specific survival in 38,568 patients with node negative hormone receptor positive invasive breast cancer and oncotype DX recurrence score results in the SEER database. *Cancer Research.* 2016;76(4 Supplement):P5-15-01-P15-15-01.
87. Drukker CA, Bueno-de-Mesquita JM, Retel VP, et al. A prospective evaluation of a breast cancer prognosis signature in the observational RASTER study. *Int J Cancer.* Aug 15 2013;133(4):929-936.
88. Harris LN, Ismaila N, McShane LM, et al. Use of biomarkers to guide decisions on adjuvant systemic therapy for women with early-stage invasive breast cancer: American Society of Clinical Oncology Clinical Practice Guideline. *Journal of Clinical Oncology.* 2016:JCO652289.
89. Mittendorf EA. Personal Communication. In: Hortobagyi GN, ed2015.
90. Winchester DJ. Personal communication. In: Hortobagyi GN, ed2015.
91. Wallden B, Storhoff J, Nielsen T, et al. Development and verification of the PAM50-based Prosigna breast cancer gene signature assay. *BMC medical genomics.* 2015;8(1):54.
92. Sgroi DC, Sestak I, Cuzick J, et al. Prediction of late distant recurrence in patients with oestrogen-receptor-positive breast cancer: a prospective comparison of the breast-cancer index (BCI) assay, 21-gene recurrence score, and IHC4 in the TransATAC study population. *The lancet oncology.* Oct 2013;14(11):1067–1076.
93. Fitzal F, Filipits M, Rudas M, et al. The genomic expression test EndoPredict is a prognostic tool for identifying risk of local recurrence in postmenopausal endocrine receptor-positive, her-2neu-negative breast cancer patients randomised within the prospective ABCSG 8 trial. *British journal of cancer.* 2015;112(8):1405–1410.
94. Halabi S, Owzar K. The importance of identifying and validating prognostic factors in oncology. Paper presented at: Seminars in oncology2010.
95. Kattan MW, Hess KR, Amin MB, et al. American Joint Committee on Cancer acceptance criteria for inclusion of risk models for individualized prognosis in the practice of precision medicine. *CA: a cancer journal for clinicians.* Jan 19 2016.
96. Moons KG, de Groot JA, Bouwmeester W, et al. Critical appraisal and data extraction for systematic reviews of prediction modelling studies: the CHARMS checklist. *PLoS medicine.* Oct 2014;11(10):e1001744.
97. Ravdin PM, Siminoff LA, Davis GJ, et al. Computer program to assist in making decisions about adjuvant therapy for women with early breast cancer. *J Clin Oncol.* Feb 15 2001;19(4):980–991.
98. Olivotto IA, Bajdik CD, Ravdin PM, et al. Population-based validation of the prognostic model ADJUVANT! for early breast cancer. *J Clin Oncol.* Apr 20 2005;23(12):2716–2725.
99. Wishart GC, Azzato EM, Greenberg DC, et al. PREDICT: a new UK prognostic model that predicts survival following surgery for invasive breast cancer. *Breast cancer research : BCR.* 2010;12(1):R1.
100. Wishart GC, Bajdik CD, Dicks E, et al. PREDICT Plus: development and validation of a prognostic model for early breast cancer that includes HER2. *Br J Cancer.* Aug 21 2012;107(5):800–807.
101. Harbeck N, Schmitt M, Meisner C, et al. Ten-year analysis of the prospective multicentre Chemo-N0 trial validates American Society of Clinical Oncology (ASCO)-recommended biomarkers uPA and PAI-1 for therapy decision making in node-negative breast cancer patients. *European journal of cancer.* May 2013;49(8):1825–1835.

Part XII

Female Reproductive Organs

Members of the Female Reproductive Organs Expert Panel
Adriana Bermudez, MD, PhD – FIGO Representative
Priya R. Bhosale, MD
Robert K. Brookland, MD, FACR, FACRO – Editorial Board Liaison
Lee-may Chen, MD, FACS, FACOG
Larry J. Copeland, MD
Don S. Dizon, MD
Beth A. Erickson, MD
Randall K. Gibb, MD
Edward C. Grendys, MD, FACOG, FACS
Perry W. Grigsby, MD
Ian S. Hagemann, MD, PhD, FCAP
David G. Mutch, MD – Chair
Alexander B. Olawaiye, MD, FACS, MRCOG, FACOG – Vice Chair
Esther Oliva, MD
Christopher N. Otis, MD, FCAP – CAP Representative
Lorraine Portelance, MD
Matthew A. Powell, MD
Jaime Prat, MD, PhD, FRCPath – FIGO Representative
Aaron H. Wolfson, MD, FACR
Richard Zaino, MD

Introduction to Female Reproductive Organs

49

INTRODUCTION

The sites included in this section are cervix uteri, corpus uteri, ovary, vagina, vulva, fallopian tube, and gestational trophoblastic neoplasms. Cervix uteri and corpus uteri were among the first sites to be classified by the TNM system. The League of Nations stages for carcinoma of the cervix were first introduced more than 70 years ago, and since 1937, the Fédération Internationale de Gynécologie et d'Obstétrique (FIGO) has continued to modify these staging systems and to collect outcomes data from throughout the world. The TNM categories therefore have been defined to correspond to the FIGO stages. Some amendments have been made in collaboration with FIGO, and the classifications now published have the approval of FIGO, the American Joint Committee on Cancer (AJCC), and all other national TNM committees of the Union for International Cancer Control (UICC).

To access the AJCC cancer staging forms, please visit www.cancerstaging.org.

© American Joint Committee on Cancer 2017

M.B. Amin et al. (eds.), *AJCC Cancer Staging Manual, Eighth Edition*, DOI 10.1007/978-3-319-40618-3_49

Vulva

50

Randall K. Gibb, Alexander B. Olawaiye, Lee-may Chen,
Perry W. Grigsby, Ian S. Hagemann, Aaron H. Wolfson,
Richard Zaino, and David G. Mutch

CHAPTER SUMMARY

Cancers Staged Using This Staging System

All carcinomas of the vulva

Cancers Not Staged Using This Staging System

These histopathologic types of cancer	Are staged according to the classification for	And can be found in chapter
Melanoma of the vulva	Melanoma of the skin	47

Summary of Changes

Change	Details of Change	Level of Evidence
Histopathologic Type	Melanoma was removed from this chapter and is considered separately (Chapter 47)	I
Histopathologic Type	Classification of p16 status will be included if obtained but is not required	III

ICD-O-3 Topography Codes

Code	Description
C51.0	Labium majus
C51.1	Labium minus
C51.2	Clitoris
C51.8	Overlapping lesion of vulva
C51.9	Vulva, NOS

WHO Classification of Tumors

Code	Description
8070	Superficial invasive squamous cell carcinoma (SISCCA)
8013	Large cell neuroendocrine carcinoma
8041	Small cell carcinoma, NOS
8041	Small cell neuroendocrine carcinoma
8051	Squamous cell carcinoma, warty
8051	Squamous cell carcinoma, verrucous
8070	Squamous cell carcinoma
8071	Squamous cell carcinoma, keratinizing
8072	Squamous cell carcinoma, non-keratinizing
8083	Squamous cell carcinoma, basaloid
8090	Basal cell carcinoma
8120	Transitional cell carcinoma
8140	Adenocarcinoma
8140	Adenocarcinoma of Skene gland origin
8140	Adenocarcinoma of sweat gland type
8140	Adenocarcinoma of intestinal type
8200	Adenoid cystic carcinoma
8247	Merkel cell carcinoma
8500	Adenocarcinoma of mammary gland type
8542	Paget disease
8560	Adenosquamous carcinoma
9020	Phyllodes tumor, malignant
9071	Yolk sac tumor
9364	Ewing sarcoma

Kurman RJ, Carcangiu ML, Herrington CS, Young RH, eds. World Health Organization Classification of Tumours of the Female Reproductive System. Lyon: IARC; 2014.

To access the AJCC cancer staging forms, please visit www.cancerstaging.org.

© American Joint Committee on Cancer 2017
M.B. Amin et al. (eds.), *AJCC Cancer Staging Manual, Eighth Edition*, DOI 10.1007/978-3-319-40618-3_50

INTRODUCTION

The classification system for vulvar cancers has been modified for the AJCC Cancer Staging Manual, 8th Edition of TNM in accordance with changes adopted by the Fédération Internationale de Gynécologie et d'Obstétrique (FIGO) to remove melanoma as a histologic type for vulvar cancer and to classify it as though it were a cutaneous melanoma, and to further help delineate the appropriate anatomic classification of anterior perineal lesions.

ANATOMY

Primary Site(s)

The vulva is the anatomic area immediately external to the vagina. It includes the labia, clitoris, and perineum. The tumor may extend to involve the vagina, urethra, or anus (Fig. 50.1). It may be fixed to the pubic bone. Changes to the staging classification reflect a belief that tumor size independent of other factors (spread to adjacent structures, nodal metastases) is less important in predicting survival.

Regional Lymph Nodes

The femoral and inguinal nodes are the sites of regional spread (Fig. 50.2).

Metastatic Sites

The metastatic sites include any site beyond the area of the regional lymph nodes. Tumor involvement of pelvic lymph nodes, including internal iliac, external iliac, and common iliac lymph nodes, is considered distant metastasis.

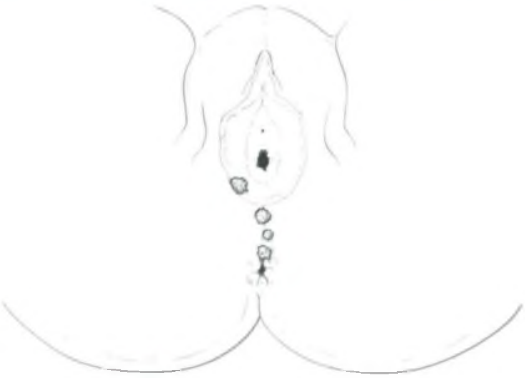

Fig. 50.1 Vulva and perineum lesions, from top to bottom: the lesion at the top is vulvar, the middle two lesions are perineal, and the lesion at the bottom is considered perianal

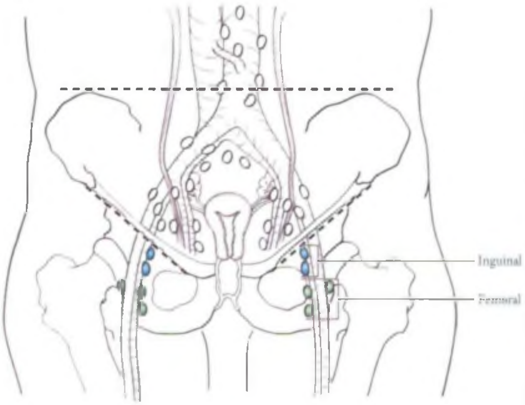

Fig. 50.2 Regional lymph nodes of the vulva

RULES FOR CLASSIFICATION

Clinical Classification

Cases should be classified as carcinoma of the vulva if the primary site of the growth is in the vulva. Tumors present on the vulva as secondary growths from either a genital or an extragenital site should be excluded. There should be histologic confirmation of the tumor.

Perineal lesions represent a challenging subset of cancers that may arise from either the vulva or the perianal mucosa. Anterior perineal lesions may be considered either vulvar or perianal, and the treatment plans may be quite dissimilar. For this reason, we recommend the following: Lesions that clearly arise from the vulva and extend onto the perineum and potentially involve the anus should be classified as vulvar. Similarly, lesions that clearly arise from the anus and extend onto the perineum should be classified as perianal. For lesions localized to the perineum that do not clearly arise from either the vulva or the anus, we recommend that the clinician denote their exact location as well as his or her favored impression regarding classification. We recommend the following terminology: "perineum favor vulva" and "perineum favor anus." We also recommend consultation with colleagues in gynecologic oncology and colorectal, general, or surgical oncology, as classification has a significant impact on treatment.

Rarely, lymph nodes are assessed by radiologic-guided fine-needle aspiration or by using imaging techniques such as computed tomography (CT), magnetic resonance (MR) imaging, or positron emission tomography (PET).

Single tumor cells or small clusters of cells not more than 0.2 mm in greatest diameter are classified as isolated tumor cells. These cells may be detected by routine histology or by immunohistochemical methods. They are designated N0(i+).

Imaging

MR imaging, CT, and PET/CT have been used in the staging of vulvar cancer. MR imaging, with its high soft tissue resolution, can stage the disease locally. CT and PET/CT may be used to assess for lymph node metastases and distant disease. Some reports suggest that PET/CT has greater sensitivity in assessing lymph node metastases in vulvar cancer.[1]

TNM Components of Tumor Staging

In stage T1 disease, multifocal lesions may be present. Currently, MR imaging is not used to assess the depth of invasion of the tumor into the dermal papilla. Assessing for depth of invasion of 1 mm is beyond the resolution of any currently available imaging modality. However, MR imaging may be used to measure a tumor's size, given its soft tissue resolution. If the tumor is 2 cm or smaller, it is category T1a. The disease may be visualized easier if vaginal gel is instilled and axial dynamic T1-weighted images are obtained. If the tumor is larger than 2 cm, it is category T1b. In category T2 disease, the tumor extends to the lower third of the vagina/urethra and the anus; it may be seen best on MR T2-weighted and dynamic sagittal T1-weighted sequences. In category T3 disease, the tumor may be any size and extends to the upper proximal two thirds of the urethra, the upper proximal two thirds of the vagina, the bladder mucosa, or the rectal mucosa, or it is fixed to pelvic bone. Skeletal involvement is seen best on MR non-fat-saturated T1-weighted sequences. However, involvement of other organs may be seen on MR sagittal T2-weighted and dynamic sagittal T1-weighted sequences.

Regional nodal metastases less than 5 mm (histologically) are considered N1 disease and cannot be assessed by cross-sectional imaging. Metastatic disease less than 5 mm is beyond the scope of any in vivo imaging modality. However, some N2 disease can be identified easily based on the number and size of nodes involved. The imaging criteria used to assess lymph node metastases is based on lymph node size, with abnormal being greater than 1 cm in the short axial dimension. However, because there may be false positive causes of enlarged nodes from benign disease, PET/CT is considered superior in assessing lymph node metastases. Metabolically active lymph nodes of any size on PET/CT are considered metastatic. Ulceration and immobility of the lymph nodes may be assessed better on physical examination.

The presence of pelvic lymph node metastases or lung, visceral, or bone metastases is considered M1 disease, and PET/CT is superior in assessing its extent. If PET/CT is not available, contrast-enhanced CT may be used.

Suggested Imaging Report Format

1. Primary tumor
   a. Size <2 cm or =2 cm
2. Local extent
   a. Involvement of the bladder and the rectum, vagina, and urethra
   b. Description of the site, number, and laterality of inguinal lymph nodes
3. Pelvic and distant lymph node involvement and extrapelvic disease

Pathological Classification

FIGO uses surgical/pathological staging for vulvar cancer. Stage should be assigned at the time of definitive surgical treatment before radiation or chemotherapy. If chemotherapy, radiation, or a combination of both treatment modalities is the initial mode of therapy, clinical staging should be used. The stage cannot be changed on the basis of disease progression or recurrence or on the basis of response to initial radiation or chemotherapy that precedes primary tumor resection.

For pN, histologic examination of regional lymphadenectomy specimens ordinarily includes six or more lymph nodes. For TNM staging, cases with fewer than six resected nodes should be classified using the TNM pathological classification based on the status of those nodes (e.g., pN0, pN1) according to the general rules of TNM. The number of resected and positive nodes should be recorded (note that FIGO classifies cases with less than six nodes resected as pNX). The concept of sentinel lymph node mapping, in which only one or two key nodes are removed, currently is being investigated. In most cases, regional lymph nodes are assessed surgically (via inguinal-femoral lymphadenectomy). Included in the 8th Edition is the opportunity to denote a micrometastatic lymph node using the N1mi or N2mi category. The current revisions to staging adopted reflect the recognition that the number and size of lymph node metastases more accurately reflect prognosis.

PROGNOSTIC FACTORS

Prognostic Factors Required for Stage Grouping

Beyond the factors used to assign T, N, or M categories, no additional prognostic factors are required for stage grouping.

Additional Factors Recommended for Clinical Care

Vulvar cancer is a surgically staged malignancy. Surgical/pathological staging provides specific information about primary tumor size and lymph node status, which are the most important prognostic factors in vulvar cancer. Other commonly evaluated items, such as histologic type, differentiation, DNA ploidy, and S-phase fraction analysis, as well as age, are not identified uniformly as important prognostic factors in vulvar cancer.

FIGO Stage
FIGO stage should be recorded. AJCC Level of Evidence: I

Femoral-Inguinal Nodal Status and Method of Assessment
Nodal spread identified on MR imaging, CT, or PET is prognostic and should be recorded and used in treatment planning. AJCC Level of Evidence: I

Pelvic Nodal Status and Method of Assessment
Nodal spread identified on MR imaging, CT, or PET is prognostic and should be recorded and used in treatment planning. AJCC Level of Evidence: I

p16
Essentially two pathways have been identified in the pathogenesis of invasive vulvar carcinoma. The first pathway is the classic progression of vulvar intraepithelial neoplasia (VIN), which is associated with high-grade human papillomavirus (HPV) infection (commonly HPV subtypes 16 and 18). The second pathway is referred to as differentiated VIN simplex, which is not associated with HPV infection but rather with vulvar dystrophy. In the classic presentation, the VIN tends to be multifocal and is more common in younger women, with a relatively low risk of progression into invasive squamous cell carcinoma of the vulva. It is diffusely positive for p16, a molecular marker that reflects the integration of the HPV genome into the cell. Some centers perform p16 staining on invasive vulvar carcinomas, and that information should be reported and collected for analysis to determine whether the presence of p16 might be used as a prognostic molecular marker in the future. AJCC Level of Evidence: III

RISK ASSESSMENT MODELS

The AJCC recently established guidelines that will be used to evaluate published statistical prediction models for the purpose of granting endorsement for clinical use.2 Although this is a monumental step toward the goal of precision medicine, this work was published only very recently. Therefore, the existing models that have been published or may be in clinical use have not yet been evaluated for this cancer site by the Precision Medicine Core of the AJCC. In the future, the statistical prediction models for this cancer site will be evaluated, and those that meet all AJCC criteria will be endorsed.

DEFINITIONS OF AJCC TNM

The definitions of the T categories correspond to the stages accepted by the Fédération Internationale de Gynécologie et d'Obstétrique (FIGO). Both systems are included for comparison.

Definition of Primary Tumor (T)

T Category	FIGO Stage	T Criteria
TX		Primary tumor cannot be assessed
T0		No evidence of primary tumor
T1	I	Tumor confined to the vulva and/or perineum<br>Multifocal lesions should be designated as such. The largest lesion or the lesion with the greatest depth of invasion will be the target lesion identified to address the highest pT stage. Depth of invasion is defined as the measurement of the tumor from the epithelial–stromal junction of the adjacent most superficial dermal papilla to the deepest point of invasion.
T1a	IA	Lesions 2 cm or less, confined to the vulva and/or perineum, and with stromal invasion of 1.0 mm or less
T1b	IB	Lesions more than 2 cm, or any size with stromal invasion of more than 1.0 mm, confined to the vulva and/or perineum
T2	II	Tumor of any size with extension to adjacent perineal structures (lower/distal third of the urethra, lower/distal third of the vagina, anal involvement)
T3	IVA	Tumor of any size with extension to any of the following—upper/proximal two thirds of the urethra, upper/proximal two thirds of the vagina, bladder mucosa, or rectal mucosa—or fixed to pelvic bone

Definition of Regional Lymph Node (N)

N category	FIGO Stage	N Criteria
NX		Regional lymph nodes cannot be assessed
N0		No regional lymph node metastasis
N0(i+)		Isolated tumor cells in regional lymph node(s) no greater than 0.2 mm
N1	III	Regional lymph node metastasis with one or two lymph node metastases each less than 5 mm, or one lymph node metastasis =5 mm
N1a*	IIIA	One or two lymph node metastases each less than 5 mm
N1b	IIIA	One lymph node metastasis =5 mm
N2		Regional lymph node metastasis with three or more lymph node metastases each less than 5 mm, or two or more lymph node metastases =5 mm, or lymph node(s) with extranodal extension
N2a*	IIIB	Three or more lymph node metastases each less than 5 mm
N2b	IIIB	Two or more lymph node metastases =5 mm
N2c	IIIC	Lymph node(s) with extranodal extension
N3	IVA	Fixed or ulcerated regional lymph node metastasis

*Includes micrometastasis, N1mi and N2mi.

Note: The site, size, and laterality of lymph node metastases should be recorded.

Definition of Distant Metastasis (M)

M Category	FIGO Stage	M Criteria
M0		No distant metastasis (no pathological M0; use clinical M to complete stage group)
M1	IVB	Distant metastasis (including pelvic lymph node metastasis)

AJCC PROGNOSTIC STAGE GROUPS

When T is...	And N is...	And M is...	Then the stage group is...
T1	N0	M0	I
T1a	N0	M0	IA
T1b	N0	M0	IB
T2	N0	M0	II
T1–T2	N1–N2c	M0	III
T1–T2	N1	M0	IIIA
T1–T2	N2a, N2b	M0	IIIB
T1–T2	N2c	M0	IIIC
T1–T3	N3	M0–M1	IV
T1–T2	N3	M0	IVA
T3	Any N	M0	IVA
Any T	Any N	M1	IVB

REGISTRY DATA COLLECTION VARIABLES

1. FIGO stage
2. Size of regional lymph node metastasis
3. Laterality of regional node metastasis
4. Femoral-inguinal nodal spread identified on imaging (yes or no)
5. Pelvic nodes identified on imaging (yes or no)
6. p16 (immunohistochemistry, yes/no; positive, yes/no)

HISTOLOGIC GRADE (G)

G	G Definition
GX	Grade cannot be assessed
G1	Well differentiated
G2	Moderately differentiated
G3	Poorly differentiated

HISTOPATHOLOGIC TYPE

Squamous cell carcinoma is the most frequent form of cancer of the vulva. This staging classification does not apply to melanoma.

The common histopathologic types are as follows:

Squamous cell carcinoma
Basal cell carcinoma
Invasive Paget disease/adenocarcinoma
Malignant Bartholin gland tumors
Adenocarcinoma of mammary gland type
Adenocarcinoma of Skene gland origin
Malignant sweat gland tumors
Adenocarcinomas of other types
Undifferentiated carcinoma

The presence or absence of lymphovascular space invasion should be noted in the pathology report.

ILLUSTRATIONS

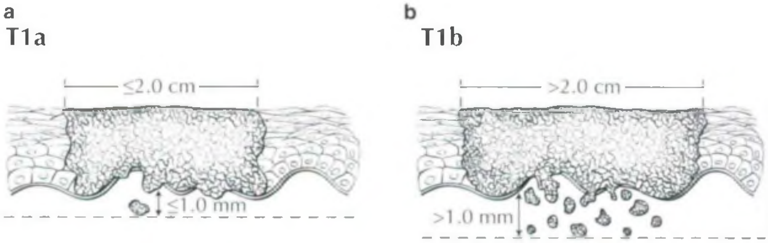

Fig. 50.3 (A) T1a is described as lesions 2 cm or less in size, confined to the vulva and/or perineum and with stromal invasion 1.0 mm or less. (B) T1b is described as lesions more than 2 cm in size or any size with stromal invasion more than 1.0 mm, confined to the vulva or perineum

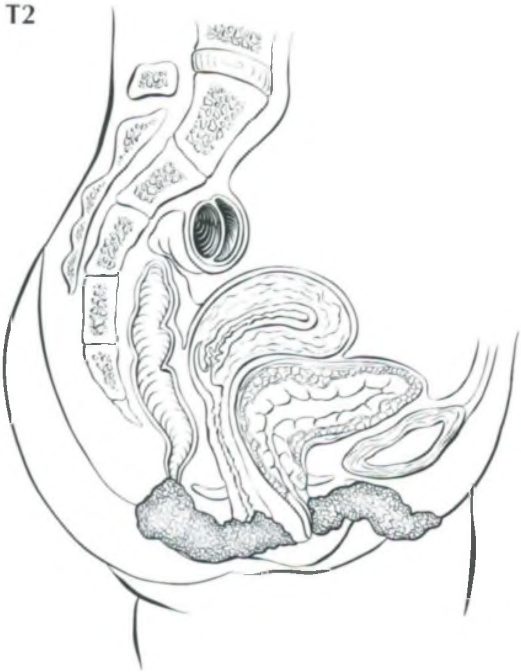

Fig. 50.4 Cross-sectional diagram showing spread of tumor into anus, lower vagina, and lower urethra. T2 is described as tumor of any size with extension to adjacent perineal structures (lower/distal third of urethra, lower/distal third of vagina, anal involvement)

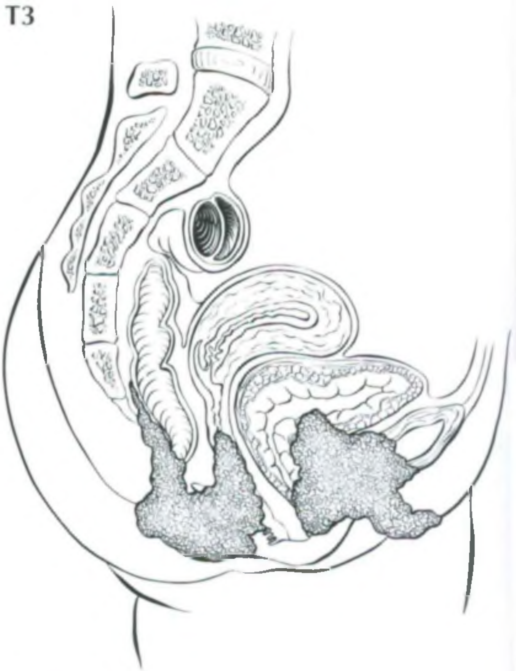

Fig. 50.5 T3 is described as tumor of any size with extension to any of the following: upper/promixal two-thirds of urethra, upper/proximal two-thirds of vagina, bladder mucosa, rectal mucosa, or fixed to pelvic bone

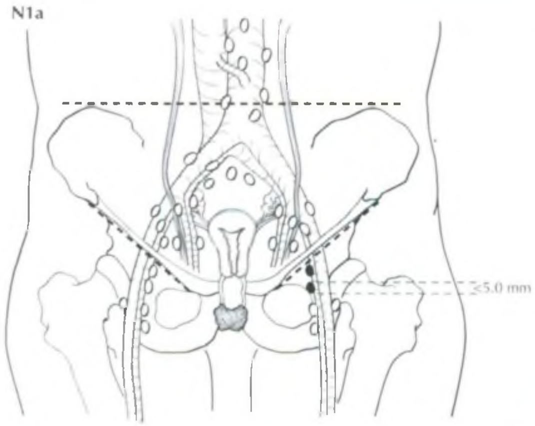

Fig. 50.6 N1a is described as one or two lymph nodes metastasis each less than 5 mm. Includes micrometastasis, N1mi and N2mi

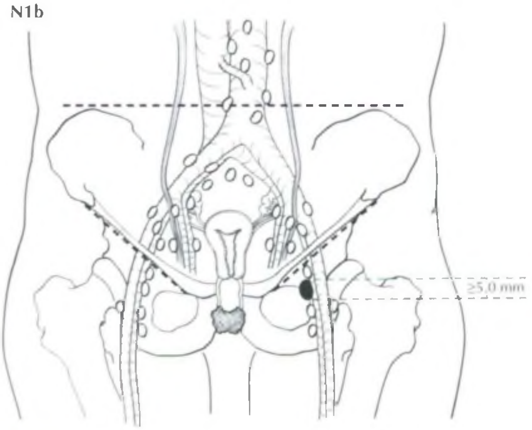

Fig. 50.7 N1b is described as one lymph node metastasis 5 mm or greater

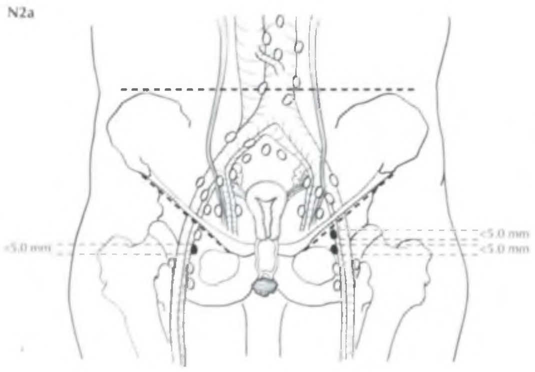

Fig. 50.8 N2a is described as three or more lymph node metastases each less than 5 mm. Includes micrometastasis, N1mi and N2mi

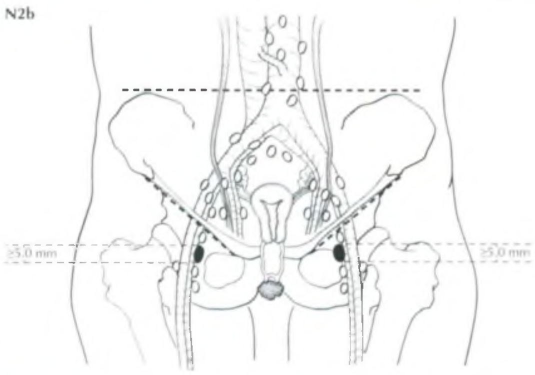

Fig. 50.9 N2b is described as two or more lymph node metastases 5 mm or greater

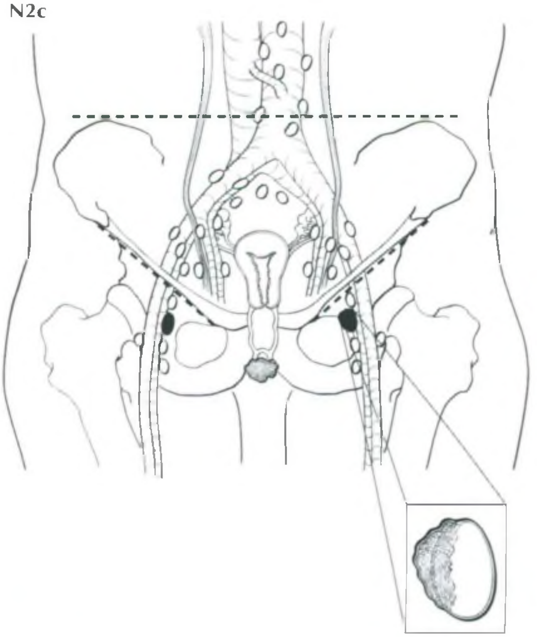

Fig. 50.10 N2c is described as lymph node(s) with extranodal extension

50

N3

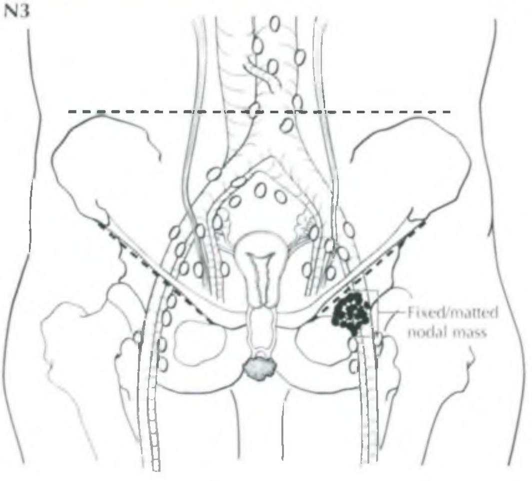

Fig. 50.11 N3 is described as fixed or ulcerated regional lymph node metastasis

M1

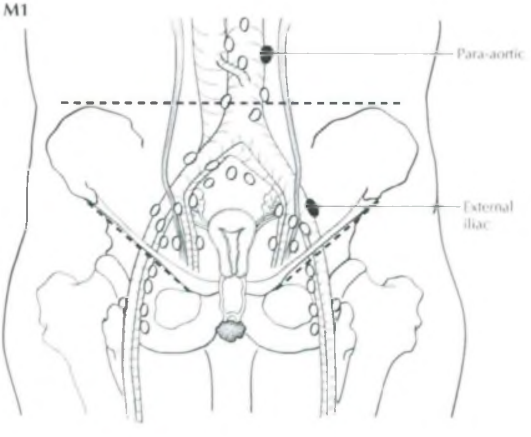

Fig. 50.12 These nodal metastases are considered M1

Bibliography

1. Expert Panel on Radiation O-G, Kidd E, Moore D, et al. ACR Appropriateness Criteria(R) management of locoregionally advanced squamous cell carcinoma of the vulva.*American journal of clinical oncology.*Aug 2013;36(4):415-422.
2. Kattan MW, Hess KR, Amin MB, et al. American Joint Committee on Cancer acceptance criteria for inclusion of risk models for individualized prognosis in the practice of precision medicine.*CA: a cancer journal for clinicians.*Jan 19 2016.
3. Beller U, Sideri M, Maisonneuve P, et al. Carcinoma of the vulva. *J Epidemiol Biostat.* 2001;6(1):155-173.
4. Chan JK, Sugiyama V, Pham H, et al. Margin distance and other clinico-pathologic prognostic factors in vulvar carcinoma: a multivariate analysis.*Gynecologic oncology.*Mar 2007;104(3):636-641.
5. Grendys EC, Jr., Fiorica JV. Innovations in the management of vulvar carcinoma.*Current opinion in obstetrics & gynecology.*Feb 2000;12(1):15-20.
6. Homesley HD, Bundy BN, Sedlis A, et al. Assessment of current International Federation of Gynecology and Obstetrics staging of vulvar carcinoma relative to prognostic factors for survival (a Gynecologic Oncology Group study).*Am J Obstet Gynecol.*Apr 1991;164(4):997-1003; discussion 1003-1004.
7. Magrina JF, Gonzalez-Bosquet J, Weaver AL, et al. Squamous cell carcinoma of the vulva stage IA: long-term results.*Gynecologic oncology.*Jan 2000;76(1):24-27.
8. McCluggage WG. Recent developments in vulvovaginal pathology.*Histopathology.*Jan 2009;54(2):156-173.
9. Moore DH, Thomas GM, Montana GS, Saxer A, Gallup DG, Olt G. Preoperative chemoradiation for advanced vulvar cancer: a phase II study of the Gynecologic Oncology Group.*International journal of radiation oncology, biology, physics.*Aug 1 1998;42(1):79-85.
10. Nash JD, Curry S. Vulvar cancer.*Surg Oncol Clin N Am.*Apr 1998;7(2):335-346.
11. Origoni M, Sideri M, Garsia S, Carinelli SG, Ferrari AG. Prognostic value of pathological patterns of lymph node positivity in squamous cell carcinoma of the vulva stage III and IVA FIGO.*Gynecologic oncology.*Jun 1992;45(3):313-316.
12. Paladini D, Cross P, Lopes A, Monaghan JM. Prognostic significance of lymph node variables in squamous cell carcinoma of the vulva.*Cancer.*Nov 1 1994;74(9):2491-2496.
13. van der Velden J, van Lindert AC, Lammes FB, et al. Extracapsular growth of lymph node metastases in squamous cell carcinoma of the vulva. The impact on recurrence and survival.*Cancer.*Jun 15 1995;75(12):2885-2890.
14. Moxley KM, Fader AN, Rose PG, et al. Malignant melanoma of the vulva: an extension of cutaneous melanoma?*Gynecologic oncology.*Sep 2011;122(3):612-617.

Vagina

51

Randall K. Gibb, Alexander B. Olawaiye, Lee-may Chen, Perry W. Grigsby, Ian S. Hagemann, Aaron H. Wolfson, Richard Zaino, and David G. Mutch

CHAPTER SUMMARY

Cancers Staged Using This Staging System

All carcinomas of the vagina

Cancers Not Staged Using This Staging System

These histopathologic types of cancer...	Are staged according to the classification for...	And can be found in chapter...
Mucosal melanoma of the vagina	No AJCC staging system	N/A

Summary of Changes

Change	Details of Change	Level of Evidence
Definition of Primary Tumor (T)	T1 and T2 subcategories were added to distinguish a tumor size cutoff of 2.0 cm for prospective data collection for studying prognostic significance.	III

ICD-O-3 Topography Codes

Code	Description
C52.9	Vagina, NOS

WHO Classification of Tumors

Code	Description
8070	Superficial invasive squamous cell carcinoma (SISCCA)
8013	Large cell neuroendocrine carcinoma
8020	Undifferentiated carcinoma
8041	Small cell neuroendocrine carcinoma
8051	Squamous cell carcinoma, warty
8051	Squamous cell carcinoma, verrucous
8052	Squamous cell carcinoma, papillary
8070	Squamous cell carcinoma
8071	Squamous cell carcinoma, keratinizing
8072	Squamous cell carcinoma, non-keratinizing
8083	Squamous cell carcinoma, basaloid
8098	Adenoid basal carcinoma
8310	Clear cell carcinoma
8380	Endometrioid carcinoma
8480	Mucinous carcinoma
8560	Adenosquamous carcinoma
8693	Paraganglioma
8933	Adenosarcoma
8980	Carcinosarcoma
9071	Yolk sac tumor
9110	Mesonephric carcinoma
9364	Ewing sarcoma

Kurman RJ, Carcangiu ML, Herrington CS, Young RH, eds. World Health Organization Classification of Tumours of the Female Reproductive System. Lyon: IARC; 2014.

To access the AJCC cancer staging forms, please visit www.cancerstaging.org.

© American Joint Committee on Cancer 2017

M.B. Amin et al. (eds.), *AJCC Cancer Staging Manual, Eighth Edition*, DOI 10.1007/978-3-319-40618-3_51

INTRODUCTION

Vaginal cancer is an uncommon gynecologic malignancy, with an overall incidence of 4,070 new cases, and accounted for about 910 deaths in 2015 in the United States.[1] The anatomic boundaries of the vagina include the vulva distally and the cervix proximally. Tumor involvement of the latter two sites must be excluded before a particular lesion may be considered a primary vaginal malignancy. The staging of this disease, as developed by the Fédération Internationale de Gynécologie et d'Obstétrique (FIGO) and the American Joint Committee on Cancer (AJCC), primarily is clinical. Squamous cell carcinoma is the most common histologic subtype of gynecologic cancers, including those of the cervix, vagina, and vulva. In fact, it comprises nearly 80% of all reported cases of primary vaginal cancer.[2]

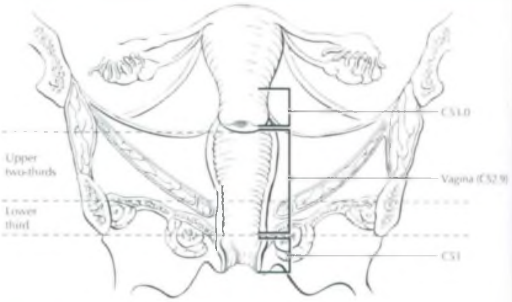

Fig. 51.1 Anatomic sites and subsites of the vagina

ANATOMY

Primary Site(s)

The vagina extends from the vulva upward to the uterine cervix. It is lined by squamous epithelium with only rare glandular structures (Fig. 51.1). The vagina is drained by lymphatics toward the pelvic nodes in its upper two thirds and toward the inguinal nodes in its lower third.

Regional Lymph Nodes

The upper two thirds of the vagina is drained by lymphatics to the pelvic nodes, including the following (Fig. 51.2):

- Parametrial
- Obturator
- Internal iliac (hypogastric)
- External iliac
- Sacral
- Presacral
- Common iliac
- Para-aortic
- Pelvic, NOS

The lower third of the vagina also drains to the groin nodes, including the following:

- Inguinal
- Femoral

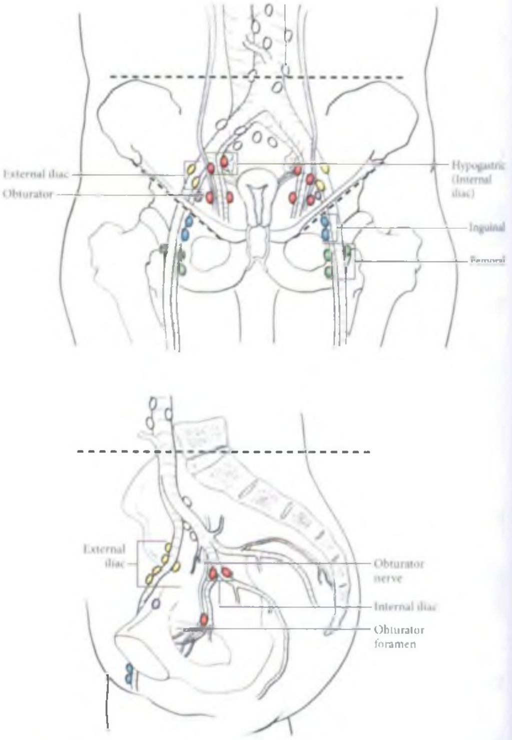

Fig. 51.2 Regional lymph nodes for the vagina

Metastatic Sites

The most common sites of distant spread include the aortic lymph nodes, lungs, and skeleton.

RULES FOR CLASSIFICATION

Clinical Classification

There should be histologic verification of the disease. The classification applies to primary carcinoma only. Cases should be classified as carcinoma of the vagina if the primary site of the growth is in the vagina. Tumors present in the vagina as secondary growths from either genital or extragenital sites should not be included. A growth involving the cervix, including the external os, should always be assigned to carcinoma of the cervix. A growth limited to the urethra should be classified as carcinoma of the urethra. Tumor involving the vulva and extending to the vagina should be classified as carcinoma of the vulva.

The Surveillance, Epidemiology, and End Results (SEER) database currently is being analyzed for clinical Stage I and II vaginal squamous cell carcinomas diagnosed between 2004 and 2012 with a minimum of 6 months of follow-up. The preliminary results suggest a prognostic impact of clinical tumor size on patient survival.3 This finding was the basis for the expert panel's recommendation to subdivide T1 and T2 into "a" and "b" categories to classify tumors ≤2 cm and those >2 cm, respectively.

FIGO uses clinical staging for cancer of the vagina. All data available before first definitive treatment should be used. The results of biopsy or fine-needle aspiration of inguinal/femoral or other nodes may be included in the clinical staging. The rules of staging are similar to those for carcinoma of the cervix.

Single tumor cells or small clusters of cells not more than 0.2 mm in greatest diameter are classified as isolated tumor cells. These may be detected by routine histology or by immunohistochemical methods. They are designated N0(i+).

Imaging

Magnetic resonance (MR) imaging currently is the preferred modality for local staging of vaginal cancer, given its superior soft tissue resolution. Computed tomography (CT) and positron emission tomography (PET)/CT may be used to assess for lymph node metastases and distant disease; however, PET/CT has greater sensitivity in assessing lymph node metastases. Information obtained from these higher-order imaging modalities are important for treatment planning but cannot be used to alter the assigned clinical stage, in accordance with FIGO's recommendations.

TNM Components of Tumor Staging

Stage 0 disease usually is not seen on imaging. In T1a disease, the tumor is visible on imaging, is confined to the vagina, and measures ≤2 cm, whereas in T1b disease, the tumor is >2 cm. Involvement of the paravaginal tissue of a tumor ≤2 cm constitutes stage T2a disease and can be well appreciated on MR imaging T2-weighted sequences; tumors >2 cm are considered T2b disease. T3 disease involves the pelvic sidewall and is best visualized on MR imaging T2-weighted and post-contrast T1-weighted sequences. T4a disease involves the bladder and rectum but does not extend beyond the pelvis, whereas T4b disease extends beyond the pelvis.

Cross-sectional imaging uses the size of >1 cm in the short axis to assess metastatic lymph nodes. CT and MR imaging perform equally well in evaluating lymph node size. Regional nodal metastasis is considered N1 disease. However, because there may be false positive causes of enlarged nodes from benign disease. PET/CT is considered superior in assessing lymph node metastases. Metabolically active lymph nodes of any size on PET/CT are considered metastatic.

The presence of lung, visceral, or bone metastases constitutes M1 disease, and PET/CT is superior in assessing the extent of disease in these cases. If PET/CT is not available, contrast-enhanced CT may be used.

Suggested Imaging Report Format

1. Primary tumor
   a. Size, <2 cm or >2 cm
2. Local extent
   a. Extravaginal involvement
   b. Parametrial extension
   c. Involvement of the bladder and rectum
3. Regional and distant lymph node involvement and extrapelvic disease

Pathological Classification

In addition to data used for clinical staging, information available from examination of the resected specimen, including pelvic and retroperitoneal lymph nodes, is to be used. The pT, pN, and c/pM categories correspond to the T, N, and M categories.

PROGNOSTIC FACTORS

Prognostic Factors Required for Stage Grouping

Beyond the factors used to assign T, N, or M categories, no additional prognostic factors are required for stage grouping., there are no additional prognostic factors required for staging.

Additional Factors Recommended for Clinical Care

The most significant prognostic factor is clinical anatomic staging, which reflects the extent of invasion into the surrounding tissue or the presence of metastatic spread.

FIGO Stage

FIGO stage should be documented. AJCC Level of Evidence: I

Pelvic Nodal Status and Method of Assessment

Nodal spread identified on MR imaging, CT, or PET does not change the stage but should be recorded and used in treatment planning. AJCC Level of Evidence: III

Superficial and Deep Inguinal Nodal Status and Method of Assessment

Nodal spread identified on MR imaging, CT, or PET does not change the stage but should be recorded and used in treatment planning. AJCC Level of Evidence: III

Para-aortic Nodal Status and Method of Assessment

Nodal spread identified on MR imaging, CT, or PET does not change the stage but should be recorded and used in treatment planning. AJCC Level of Evidence: III

Distant (Mediastinal, Scalene) Nodal Status and Method of Assessment

Nodal spread identified on MR imaging, CT, or PET does not change the stage but should be recorded and used in treatment planning. AJCC Level of Evidence: III

RISK ASSESSMENT MODELS

The AJCC recently established guidelines that will be used to evaluate published statistical prediction models for the purpose of granting endorsement for clinical use.[5] Although this is a monumental step toward the goal of precision medicine, this work was published only very recently. Therefore, the existing models that have been published or may be in clinical use have not yet been evaluated for this cancer site by the Precision Medicine Core of the AJCC. In the future, the statistical prediction models for this cancer site will be evaluated, and those that meet all AJCC criteria will be endorsed.

DEFINITIONS OF AJCC TNM

The definitions of the T categories correspond to the stages accepted by the Fédération Internationale de Gynécologie et d'Obstétrique (FIGO). Both systems are included for comparison.

Definition of Primary Tumor (T)

T Category	FIGO Stage	T Criteria
TX		Primary tumor cannot be assessed
T0		No evidence of primary tumor
T1	I	Tumor confined to the vagina
T1a	I	Tumor confined to the vagina, measuring ≤2.0 cm
T1b	I	Tumor confined to the vagina, measuring >2.0 cm
T2	II	Tumor invading paravaginal tissues but not to pelvic sidewall
T2a	II	Tumor invading paravaginal tissues but not to pelvic wall, measuring ≤2.0 cm
T2b	II	Tumor invading paravaginal tissues but not to pelvic wall, measuring >2.0 cm
T3	III	Tumor extending to the pelvic sidewall* and/or involving the lower third of the vagina and/or causing hydronephrosis or nonfunctioning kidney
T4	IVA	Tumor invading the mucosa of the bladder or rectum and/or extending beyond the true pelvis (bullous edema is not sufficient evidence to classify a tumor as T4)

**Pelvic sidewall* is defined as the muscle, fascia, neurovascular structures, or skeletal portions of the bony pelvis. On rectal examination, there is no cancer-free space between the tumor and pelvic sidewall.

Definition of Regional Lymph Node (N)

N Category	FIGO Stage	N Criteria
NX		Regional lymph nodes cannot be assessed
N0		No regional lymph node metastasis
N0(i+)		Isolated tumor cells in regional lymph node(s) no greater than 0.2 mm
N1	III	Pelvic or inguinal lymph node metastasis

Definition of Distant Metastasis (M)

M Category	FIGO Stage	M Criteria
M0		No distant metastasis
M1	IVB	Distant metastasis

AJCC PROGNOSTIC STAGE GROUPS

When T is...	And N is...	And M is...	Then the stage group is...
T1a	N0	M0	IA
T1b	N0	M0	IB
T2a	N0	M0	IIA
T2b	N0	M0	IIB
T1–T3	N1	M0	III
T3	N0	M0	III
T4	Any N	M0	IVA
Any T	Any N	M1	IVB

REGISTRY DATA COLLECTION VARIABLES

1. FIGO stage
2. Pelvic nodes identified on imaging (yes or no)
3. Para-aortic nodes identified on imaging (yes or no)
4. Distant (mediastinal, scalene) nodes identified on imaging (yes or no)

HISTOLOGIC GRADE (G)

G	G Definition
GX	Grade cannot be assessed
G1	Well differentiated
G2	Moderately differentiated
G3	Poorly differentiated

HISTOPATHOLOGIC TYPE

Squamous cell carcinoma is the most common type of cancer occurring in the vagina. Approximately 10% of vaginal cancers are adenocarcinoma; melanoma and sarcoma occur rarely.

SURVIVAL DATA

An ongoing study is analyzing the April 2015 release of SEER data regarding the diagnosis of Stages I and II vaginal squamous cell carcinomas from 2004 through 2012 based on AJCC Cancer Staging Manual, 6th Edition criteria. This analysis has yielded information on 529 patients with available tumor sizes; of these, 293 were classified as Stage I and 236 as Stage II. The median tumor size was 2.2 cm (range, 0.1–9.5 cm) in patients with Stage I and 4.0 cm (range, 0.4–8.2 cm) in those with Stage II disease. Statistical analyses including multivariate regression modeling demonstrated that tumor size—≤2.0 cm versus >2.0 cm—was most predictive of overall survival for both Stage I ($p = 0.011$) and II ($p = 0.033$) tumors. Kaplan–Meier curves by stage and tumor size for these patients are depicted in Figs. 51.3 and 51.4.

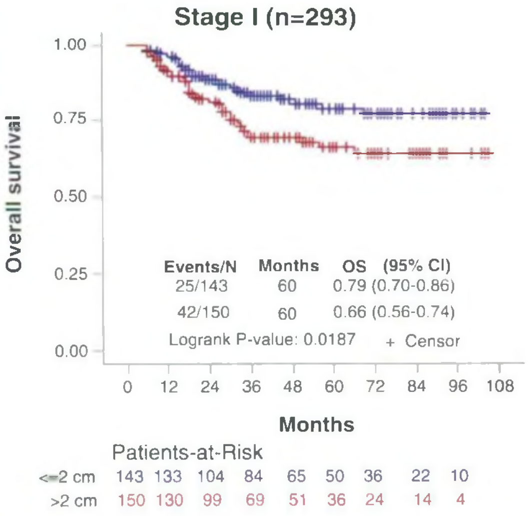

Fig. 51.3 Overall survival (OS) by tumor size among Stage I SEER study patients. CI, confidence interval

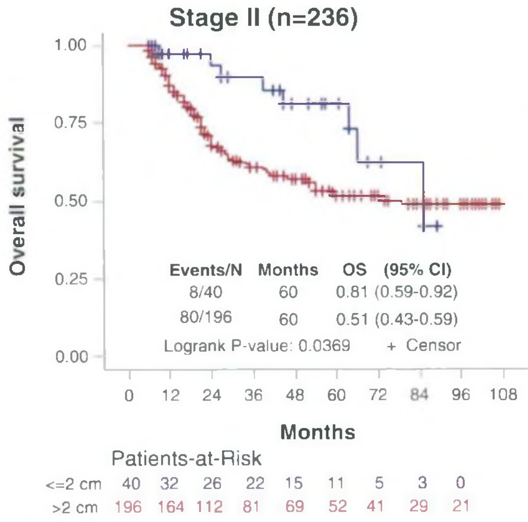

Fig. 51.4 Overall survival (OS) by tumor size among stage II SEER study patients. CI, confidence interval

51

ILLUSTRATIONS

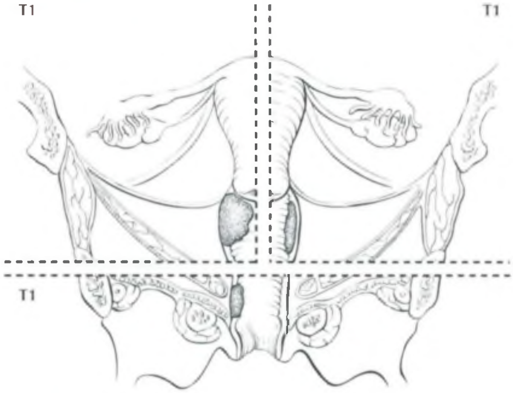

Fig. 51.5 T1 is tumor confined to vagina

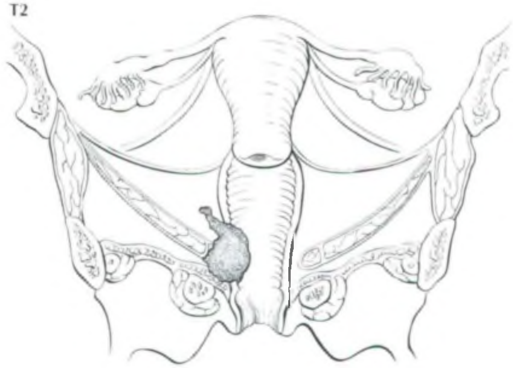

Fig. 51.6 T2 is tumor invading paravaginal tissues but not to pelvic wall

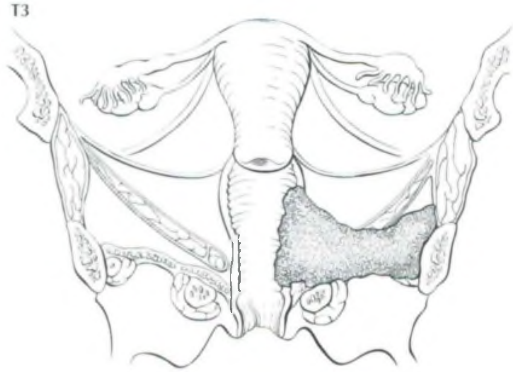

Fig. 51.7 T3 is tumor extending to the pelvic sidewall and/or involving the lower third of the vagina and/or causing hydronephrosis or nonfunctioning kidney. *Pelvic sidewall* is defined as the muscle, fascia, neurovascular structures, or skeletal portions of the bony pelvis

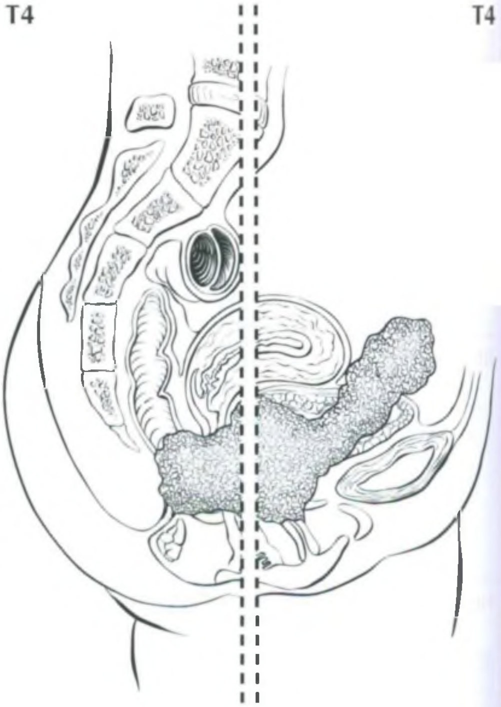

Fig. 51.8 T4 is tumor invading the mucosa of the bladder or rectum and/or extending beyond the true pelvis (bullous edema is not sufficient evidence to classify a tumor as T4)

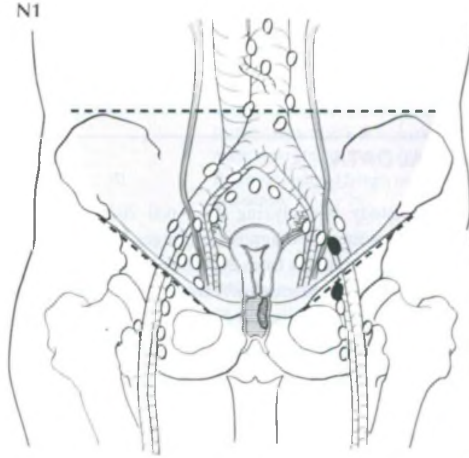

Fig. 51.9 N1 is pelvic or inguinal lymph node metastasis

Bibliography

1. Cancer Facts and Figures 2015. American Cancer Society http://www.cancer.org/cancer/vaginalcancer/detailedguide/vaginal-cancer-key-statistics. Accessed September 14, 2015http://www.cancer.org/cancer/vaginalcancer/detailedguide/vaginal-cancer-key-statistics
2. Creasman WT, Phillips JL, Menck HR. The National Cancer Data Base report on cancer of the vagina. Cancer. Sep 1 1998;83(5):1033–1040
3. Wolfson AH, Isildinha MR, Portelance L, Diaz DA, Zhao W, Gibb RK. Prognostic Impact of Clinical Tumor Size on Overall Survival for Subclassifying Stage I and II Vaginal Cancer: A SEER Analyses. 2016
4. Lee LJ, Jhingran A, Kidd E, et al. Acr appropriateness Criteria management of vaginal cancer. Oncology (Williston Park). Nov 2013;27(11):1166–1173
5. Kattan MW, Hess KR, Amin MB, et al. American Joint Committee on Cancer acceptance criteria for inclusion of risk models for individualized prognosis in the practice of precision medicine. CA: a cancer journal for clinicians. Jan 19 2016
6. Beller U, Sideri M, Maisonneuve P, et al. Carcinoma of the vagina. J Epidemiol Biostat. 2001;6(1):141–152
7. Foroudi F, Bull CA, Gebski V. Primary invasive cancer of the vagina: outcome and complications of therapy. Australasian radiology. Nov 1999;43(4):472–475
8. Goodman A. Primary vaginal cancer. Surg Oncol Clin N Am. Apr 1998;7(2):347–361
9. Pingley S, Shrivastava SK, Sarin R, et al. Primary carcinoma of the vagina: Tata Memorial Hospital experience. International journal of radiation oncology, biology, physics. Jan 1 2000;46(1):101–108
10. Stock RG, Chen AS, Seski J. A 30-year experience in the management of primary carcinoma of the vagina: analysis of prognostic factors and treatment modalities. Gynecologic oncology. Jan 1995;56(1):45–52
11. Sulak P, Barnhill D, Heller P, et al. Nonsquamous cancer of the vagina. Gynecologic oncology. Mar 1988;29(3):309–320

Cervix Uteri

52

Beth A. Erickson, Alexander B. Olawaiye,
Adriana Bermudez, Edward C. Grendys, Perry W. Grigsby,
Ian S. Hagemann, Esther Oliva, Lorraine Portelance,
Aaron H. Wolfson, and David G. Mutch

CHAPTER SUMMARY

Cancers Staged Using This Staging System

All malignancies arising primarily in the cervix

Summary of Changes

Change	Details of Change	Level of Evidence
FIGO Stage	N1 removed from FIGO Stage IIIB	N/A
Definition of Distant Metastasis (M)	Para-aortic nodes removed from M1 in AJCC stage	I

ICD-O-3 Topography Codes

Code	Description
C53.0	Endocervix
C53.1	Exocervix
C53.8	Overlapping lesion of cervix uteri
C53.9	Cervix uteri

WHO Classification of Tumors

Code	Description
8013	Large cell neuroendocrine carcinoma
8015	Glassy cell carcinoma
8020	Undifferentiated carcinoma
8041	Small cell neuroendocrine carcinoma
8051	Squamous cell carcinoma, warty
8051	Squamous cell carcinoma, verrucous
8052	Squamous cell carcinoma, papillary
8070	Squamous cell carcinoma, NOS
8071	Squamous cell carcinoma, keratinizing
8072	Squamous cell carcinoma, nonkeratinizing
8082	Squamous cell carcinoma, lymphoepithelioma-like
8083	Squamous cell carcinoma, basaloid
8098	Adenoid basal carcinoma
8120	Squamous cell carcinoma, squamotransitional
8140	Adenocarcinoma
8140	Endocervical adenocarcinoma, usual type
8144	Mucinous carcinoma, intestinal type
8200	Adenoid cystic carcinoma
8240	Carcinoid tumor
8249	Atypical carcinoid tumor
8263	Villoglandular carcinoma
8310	Clear cell carcinoma
8380	Endometrioid carcinoma
8441	Serous carcinoma
8480	Mucinous carcinoma, NOS
8482	Mucinous carcinoma, gastric type
8490	Mucinous carcinoma, signet-ring cell type
8560	Adenosquamous carcinoma
8574	Adenocarcinoma admixed with neuroendocrine carcinoma
8720	Malignant melanoma
8805	Undifferentiated endocervical sarcoma
8850	Liposarcoma
8890	Leiomyosarcoma

To access the AJCC cancer staging forms, please visit www.cancerstaging.org.

© American Joint Committee on Cancer 2017

M.B. Amin et al. (eds.), *AJCC Cancer Staging Manual, Eighth Edition*, DOI 10.1007/978-3-319-40618-3_52

Code	Description
8910	Rhabdomyosarcoma
8933	Adenosarcoma
8980	Carcinosarcoma
9110	Mesonephric carcinoma
9120	Angiosarcoma
9364	Ewing sarcoma
9540	Malignant peripheral nerve sheath tumor
9581	Alveolar soft part sarcoma

Kurman RJ, Carcangiu ML, Herrington CS, Young RH, eds. World Health Organization Classification of Tumours of the Female Reproductive System. Lyon: IARC; 2014.

INTRODUCTION

Cervical cancer is the third most common gynecologic cancer in the United States and the most common gynecologic cancer worldwide.[1]

The vast majority of women who develop cervical cancer are in developing countries, as it is predominantly a disease of those with poor access to surveillance and advanced imaging technology. As a result, the staging systems are based primarily on clinical examination and very basic imaging tests, such as chest radiography and intravenous pyelography, rather than more expensive and often unavailable imaging modalities, such as computed tomography (CT), magnetic resonance (MR) imaging, and positron emission tomography (PET). This spectrum of access to imaging, which now is highly developed and transformative where available, makes the staging system appear to lag behind the tools of technology in well-developed countries. However, there is no reason the information gained from these imaging studies, when available, should not be used to guide treatment management. Imaging findings, such as nodal and parametrial involvement, should be used to help determine whether additional treatment intervention, such as surgery, radiation, or chemotherapy, is needed. In addition, nodal involvement, along with tumor size and tumor volume, is prognostic and may affect treatment planning when this information is available.[2-7]

Response to treatment also may be assessed with additional imaging at key intervals, and salvage interventions, if needed, may be undertaken to improve outcome.[8]

ANATOMY

Primary Site(s)

The cervix is the lower third of the uterus. It is roughly cylindrical and projects into the upper vagina. The endocervical canal is lined by glandular or columnar epithelium and runs through the cervix; it is the passageway connecting the vagina with the uterine cavity. The vaginal portion of the cervix, known as the exocervix, is covered by squamous epithelium. The new squamocolumnar junction usually is located at the external cervical os, where the endocervical canal begins; the original squamocolumnar junction is located on the ectocervix, vaginal fornix, or upper vagina. The area between these two junctions is called the transformation zone. Cancer of the cervix may originate from the squamous epithelium of the exocervix or the glandular epithelium of the canal; however, the vast majority of these cancers arise in the transformation zone.

Regional Lymph Nodes

The cervix is drained by parametrial, cardinal, and uterosacral ligament routes into the following regional lymph nodes (Fig. 52.1):

- Parametrial
- Obturator
- Internal iliac (hypogastric)
- External iliac
- Sacral
- Presacral
- Common iliac
- Para-aortic

Metastatic Sites

The most common sites of distant spread include the mediastinal nodes, lungs, peritoneal cavity, and skeleton. Mediastinal or supraclavicular node involvement is considered distant metastasis and is assigned M1.

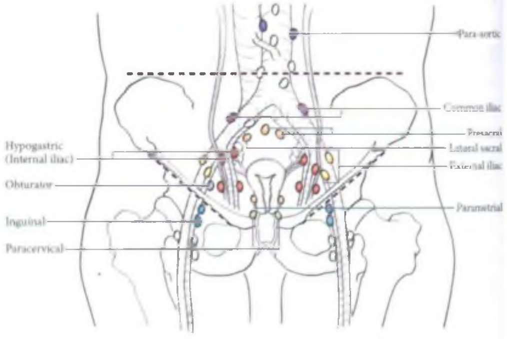

Fig. 52.1 Regional lymph nodes for the cervix uteri

RULES FOR CLASSIFICATION

Clinical Classification

The clinical stage should be determined before definitive therapy begins. The clinical stage must not be changed because of subsequent findings once treatment has started. If there is doubt regarding which stage a particular cancer should be allocated, the lesser stage should be selected. The classification applies only to carcinoma. There should be histologic confirmation of the disease.

Careful clinical examination should be performed in all cases, preferably by an experienced examiner and with the patient under anesthesia. A description of the cervical tumor size is important, especially for Stage I-II cancers, for which tumor size has shown prognostic utility. The 2009 Fédération Internationale de Gynécologie et d'Obstétrique (FIGO) staging classification has adopted T subclassifications based on tumor size (≤4 cm: T2a1; >4 cm: T2a2) for cervical carcinoma spreading beyond the cervix but not to the pelvic sidewall or lower one third of the vagina (T2 lesions).

The following examinations are recommended worldwide for staging purposes: palpation, inspection, colposcopy, endocervical curettage, hysteroscopy, cystoscopy, proctoscopy, intravenous urography, and X-ray examination of the lungs and skeleton. If available, CT, MR imaging, or PET may supplement or replace some of these more traditional tests. Suspected involvement of the bladder mucosa or rectal mucosa must be confirmed by biopsy and histology.

Lymph node status may be assessed by surgical means (radiologic-guided fine-needle aspiration, laparoscopic or extraperitoneal biopsy, or lymphadenectomy) or by imaging technologies (CT, MR imaging, or PET). The results of these additional examinations or procedures may not be used to determine clinical staging because these techniques are not universally available. However, they may be used to develop a treatment plan and may provide prognostic information. Single tumor cells or small clusters of cells smaller than 0.2 mm in greatest diameter are classified as isolated tumor cells (ITCs). These may be detected by routine histology or by immunohistochemical methods. They are designated as N0(i+).

If nodal metastases are identified, it is important to identify the extent of nodal involvement (pelvic lymph nodes and/or para-aortic lymph nodes) and the methodology by which the diagnosis was established (pathological or radiologic).

Nodal involvement found on imaging or at surgery will not change the clinical or pathological stage of the patient but should be used to affect treatment, as in the case of IB1 with positive pelvic lymph nodes. The location of the nodes, such as left internal iliac or right para-aortic nodes, also should be noted.

Imaging

MR imaging currently is the preferred modality for local assessment of cervical cancer.[9] Contrast-enhanced CT does not have the soft tissue resolution necessary to evaluate the local extent of the tumor and may not be helpful in assessing early disease, specifically in patients who want to undergo fertility-preserving trachelectomy. Determination of lymph node metastases on cross-sectional imaging is based on lymph node size, with abnormal being >1 cm in the short axial dimension. Hybrid PET/CT also may be used to determine lymph node status in patients who have locally advanced cancer. Metabolically active lymph nodes of any size on PET/CT are considered metastatic. PET/CT is considered superior to other modalities in evaluating for extrapelvic disease and bone metastases.

If available, the use of imaging to influence treatment is encouraged, although it is not officially incorporated in the FIGO or AJCC system. Modification of the TNM classification to account for imaging findings should be considered in the future—for example, upstaging IB patients to IIB if parametrial involvement is observed on MR imaging.[9]

TNM Components of Tumor Staging

Category T1a disease usually is not seen on imaging. In T1b disease, the tumor is visible on imaging and the T (i.e., size) component of the disease is assessed by measuring the greatest diameter of the tumor in any plane on the sequence in which it is most conspicuous. T2a disease involves the upper third of the vagina, which is visible on MR imaging. When cervical stroma is disrupted on the T2-weighted images, the T category remains unchanged, but the information should be used for treatment planning. When the tumor causes hydronephrosis or extends to the pelvic sidewall, it is considered to be clinical category T3b. Involvement of the adjacent organs is considered to be category T4a; however, the presence of bullous edema is not considered to be involvement of the bladder.

Regional nodal metastases are considered N1 disease and may be assessed easily on CT and MR imaging. This classification is based on the size criterion for abnormal being >1 cm in the short axial dimension. However, metabolically active lymph nodes of any size on fluorodeoxyglucose PET scans are considered metastatic.

The presence of nodes beyond the pelvis or paraaortic region, or bone metastases is considered M1 disease, and PET/CT is considered the best modality to assess its extent. If PET/CT is not available, contrast-enhanced CT may be used.

Suggested Imaging Report Format

a. Primary tumor
  - Size

b. Local extent
  - Involvement of the internal os
  - involvement of the vagina
  - Parametrial extension
  - Involvement of the bladder and rectum

c. Regional and distant lymph node involvement and extrapelvic disease

Pathological Classification

In cases treated surgically, the pathologist's findings in the removed tissues may be the basis for extremely accurate statements about the extent of disease. These findings should not be allowed to change the clinical staging, but they should be recorded in the manner described for the pathological staging of disease. The pTNM nomenclature is appropriate for this purpose and corresponds to the T, N, and M categories. Infrequently, hysterectomy is carried out in the presence of unsuspected invasive cervical carcinoma. Such cases cannot be clinically staged or included in therapeutic statistics; they should be reported separately.

For pN, histologic examination of regional lymphadenectomy specimens ordinarily includes six or more lymph nodes. For TNM staging, cases with fewer than six resected nodes should be classified using the TNM pathological classification based on the status of those nodes (e.g., pN0; pN1) according to the general rules of TNM. The number of resected and positive nodes should be recorded (note that FIGO classifies cases with less than six nodes resected as pNX).

PROGNOSTIC FACTORS

Prognostic Factors Required for Stage Grouping

Beyond the factors used to assign T, N, or M categories, no additional prognostic factors are required for stage grouping.

Additional Factors Recommended for Clinical Care

FIGO Stage

Tumor size is part of the current FIGO and AJCC systems for Stage I and II, with the designation of ≤4 cm or >4 cm. Additional data suggest that size may be even more important than this 4-cm cutoff reveals and is a prognostic variable not only clinically and on pathological examination, but also radiographically on MR imaging, CT, and PET.[10-12] Closely related to size is volume of disease, which incorporates all the dimensions of the tumor, not just the diameter.[13,14] In addition, closely tied to size and volume are the extensions of the tumor outside the cervix to the parametria, vagina, uterus, bladder, and rectum, which may be seen on MR imaging. There has been excellent correlation of MR findings with surgical specimen analysis, validating its use in assessing cervical cancers before treatment. Tumor volume at diagnosis and tumor regression throughout radiation therapy are powerful prognostic factors, even in the first weeks of treatment.[8] AJCC Level of Evidence: I

Pelvic Nodal Status and Method of Assessment

Pelvic node involvement is a potent predictor of outcome. Both the size and number of nodes are important. Involvement of the internal and external iliac lymph nodes puts the common iliac nodes at risk; likewise, the para-aortic nodes are at risk when the common iliac nodes are involved. Extranodal spread of disease also is prognostic.[5,6,15] AJCC Level of Evidence: I

Para-aortic Nodal Status and Method of Assessment

Increasingly, the use of CT and PET has revealed subclinical para-aortic nodal metastases that may be treated effectively with chemoradiation. The prognosis of patients with para-aortic nodal disease is better than that of patients with other distant sites of disease. The size and number of nodes have an impact on outcome.[16] AJCC Level of Evidence: I

Distant (Mediastinal, Scalene) Nodal Status and Method of Assessment

Patients with supraclavicular metastases sometimes do well with a combination of systemic chemotherapy and regional irradiation. Likewise, mediastinal disease may be approached with a combination of systemic and regional irradiation. AJCC Level of Evidence: I

Human Papillomavirus Status

Current data suggest that more than 90% of cervical cancers contain human papillomavirus (HPV) DNA, most frequently types 16 and 18. AJCC Level of Evidence: I

Histopathologic Type

In addition to extent or stage of disease, prognostic factors include histology and tumor differentiation. Small cell, neuroendocrine, and clear cell lesions have a worse

prognosis, as do poorly differentiated cancers. AJCC Level of Evidence: I

HIV Status

Women with cervical cancer who are infected with human immunodeficiency virus (HIV) have a very poor prognosis, often with rapidly progressive cancer. AJCC Level of Evidence: I

RISK ASSESSMENT MODELS

The AJCC recently established guidelines that will be used to evaluate published statistical prediction models for the purpose of granting endorsement for clinical use.[17] Although this is a monumental step toward the goal of precision medicine, this work was published only very recently. Therefore, the existing models that have been published or may be in clinical use have not yet been evaluated for this cancer site by the Precision Medicine Core of the AJCC. In the future, the statistical prediction models for this cancer site will be evaluated, and those that meet all AJCC criteria will be endorsed.

DEFINITIONS OF AJCC TNM

The definitions of the T categories correspond to the stages accepted by the Fédération Internationale de Gynécologie et d'Obstétrique (FIGO). Both systems are included for comparison.

Definition of Primary Tumor (T)

T Category	FIGO Stage	T Criteria
TX		Primary tumor cannot be assessed
T0		No evidence of primary tumor
T1	I	Cervical carcinoma confined to the uterus (extension to corpus should be disregarded)
T1a	IA	Invasive carcinoma diagnosed only by microscopy. Stromal invasion with a maximum depth of 5.0 mm measured from the base of the epithelium and a horizontal spread of 7.0 mm or less. Vascular space involvement, venous or lymphatic, does not affect classification.
T1a1	IA1	Measured stromal invasion of 3.0 mm or less in depth and 7.0 mm or less in horizontal spread
T1a2	IA2	Measured stromal invasion of more than 3.0 mm and not more than 5.0 mm, with a horizontal spread of 7.0 mm or less
T1b	IB	Clinically visible lesion confined to the cervix or microscopic lesion greater than T1a/IA2. Includes all macroscopically visible lesions, even those with superficial invasion.
T1b1	IB1	Clinically visible lesion 4.0 cm or less in greatest dimension
T1b2	IB2	Clinically visible lesion more than 4.0 cm in greatest dimension
T2	II	Cervical carcinoma invading beyond the uterus but not to the pelvic wall or to lower third of the vagina
T2a	IIA	Tumor without parametrial invasion
T2a1	IIA1	Clinically visible lesion 4.0 cm or less in greatest dimension
T2a2	IIA2	Clinically visible lesion more than 4.0 cm in greatest dimension
T2b	IIB	Tumor with parametrial invasion
T3	III	Tumor extending to the pelvic sidewall* and/or involving the lower third of the vagina and/or causing hydronephrosis or nonfunctioning kidney
T3a	IIIA	Tumor involving the lower third of the vagina but not extending to the pelvic wall
T3b	IIIB	Tumor extending to the pelvic wall and/or causing hydronephrosis or nonfunctioning kidney
T4	IVA	Tumor invading the mucosa of the bladder or rectum and/or extending beyond the true pelvis (bullous edema is not sufficient to classify a tumor as T4)

*The pelvic sidewall is defined as the muscle, fascia, neurovascular structures, and skeletal portions of the bony pelvis. On rectal examination, there is no cancer-free space between the tumor and pelvic sidewall.

Definition of Regional Lymph Node (N)

N Category	FIGO Stage	N Criteria
NX		Regional lymph nodes cannot be assessed
N0		No regional lymph node metastasis
N0(i+)		Isolated tumor cells in regional lymph node(s) no greater than 0.2 mm
N1		Regional lymph node metastasis

Definition of Distant Metastasis (M)

M Category	FIGO Stage	M Criteria
M0		No distant metastasis
M1	IVB	Distant metastasis (including peritoneal spread or involvement of the supraclavicular, mediastinal, or distant lymph nodes; lung; liver; or bone)

AJCC PROGNOSTIC STAGE GROUPS

When T is...	And N is...	And M is...	Then the stage group is...
T1	Any N	M0	I
T1a	Any N	M0	IA

(continued)

52

When T is...	And N is...	And M is...	Then the stage group is...
T1a1	Any N	M0	IA1
T1a2	Any N	M0	IA2
T1b	Any N	M0	IB
T1b1	Any N	M0	IB1
T1b2	Any N	M0	IB2
T2	Any N	M0	II
T2a	Any N	M0	IIA
T2a1	Any N	M0	IIA1
T2a2	Any N	M0	IIA2
T2b	Any N	M0	IIB
T3	Any N	M0	III
T3a	Any N	M0	IIIA
T3b	Any N	M0	IIIB
T4	Any N	M0	IVA
Any T	Any N	M1	IVB

REGISTRY DATA COLLECTION VARIABLES

1. FIGO stage
2. Pelvic nodal status and method of assessment (microscopic, CT, PET, MR imaging)
3. Para-aortic nodal status and method of assessment
4. Distant (mediastinal, scalene) nodal status and method of assessment
5. P16 status
6. HIV status

Histologic Grade (G)

G	G Definition
GX	Grade cannot be assessed
G1	Well differentiated
G2	Moderately differentiated
G3	Poorly differentiated

HISTOPATHOLOGIC TYPE

Cases should be classified as carcinoma of the cervix if the primary growth is in the cervix. All carcinomas should be included. Grading is encouraged but is not a basis for modifying the stage groupings. If surgery is the primary treatment, the histologic findings permit the case to have pathological staging, and the pTNM nomenclature is to be used. The histopathologic types are as follows:

- Squamous cell carcinoma
  - Invasive
  - Keratinizing
  - Nonkeratinizing
  - Verrucous
- Adenocarcinoma
- Endometrioid adenocarcinoma
- Clear cell adenocarcinoma
- Adenosquamous carcinoma
- Adenoid cystic carcinoma
- Adenoid basal cell carcinoma
- Small cell carcinoma
- Neuroendocrine
- Undifferentiated carcinoma

ILLUSTRATIONS

T1a1

Invasive carcinoma diagnosed only by microscopy

a

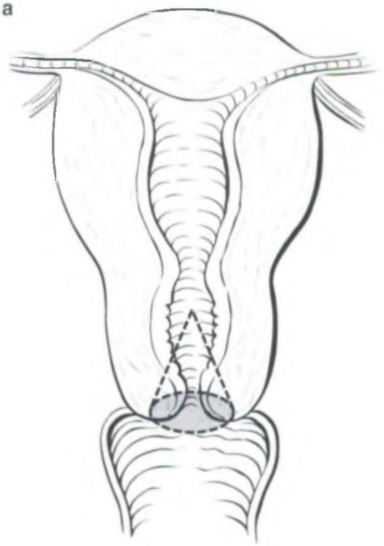

b

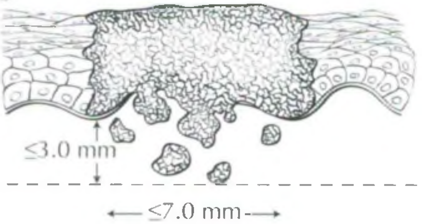

Fig. 52.2 T1a1 is measured stromal invasion 3.0 mm or less in depth and 7.0 mm or less in horizontal spread (B)

T1a2

Invasive carcinoma diagnosed only by microscopy

a

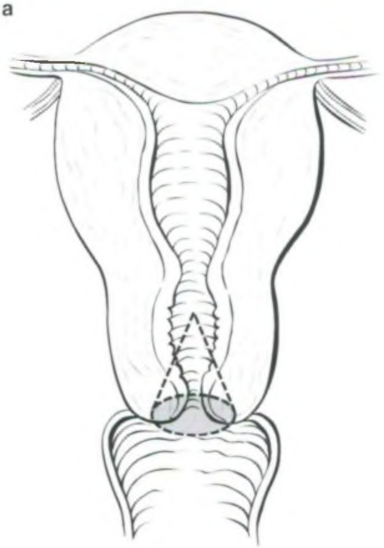

b

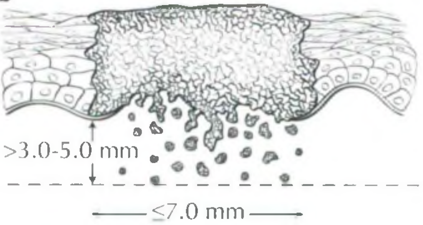

Fig. 52.3 T1a2 is measured stromal invasion more than 3.0 mm and not more than 5.0 mm with a horizontal spread 7.0 mm or less (B)

52

Fig. 52.4 T1b is clinically visible lesion confined to the cervix or microscopic lesion greater than T1a (B and C)

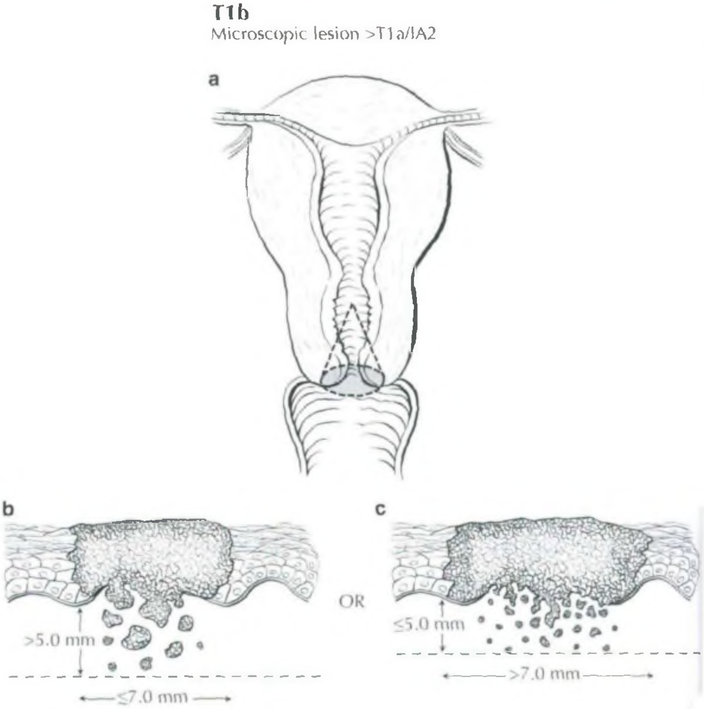

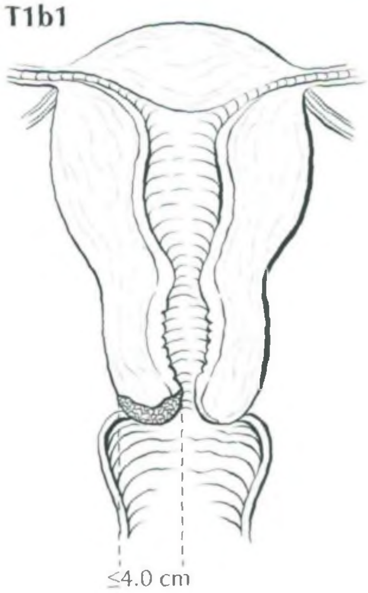

Fig. 52.5 T1b1 is clinically visible lesion 4.0 cm or less in greatest dimension

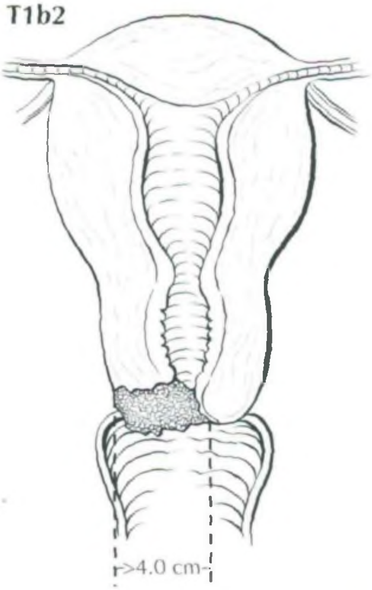

Fig. 52.6 T1b2 is clinically visible lesion more than 4.0 cm in greatest dimension

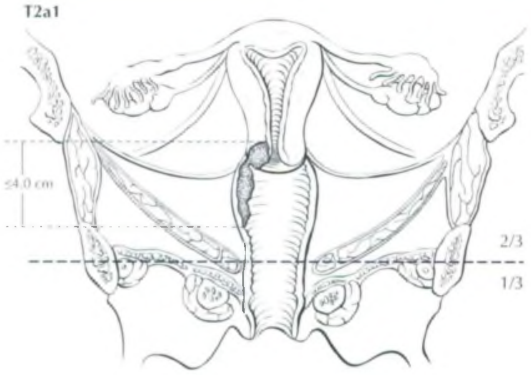

Fig. 52.7 T2a is tumor without parametrial invasion. T2a1 is clinically visible lesion 4.0 cm or less in greatest dimension

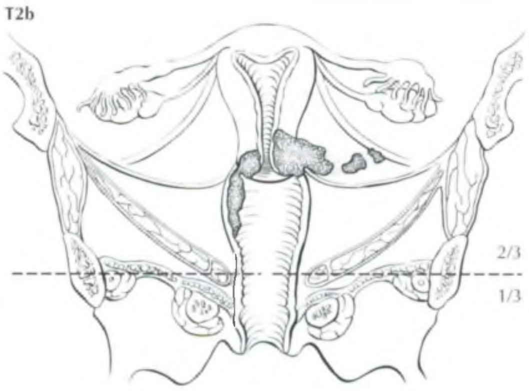

Fig. 52.9 T2b is tumor with parametrial invasion

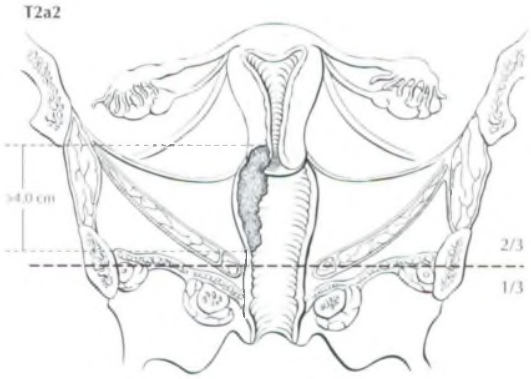

Fig. 52.8 T2a2 is clinically visible lesion more than 4.0 cm in greatest dimension

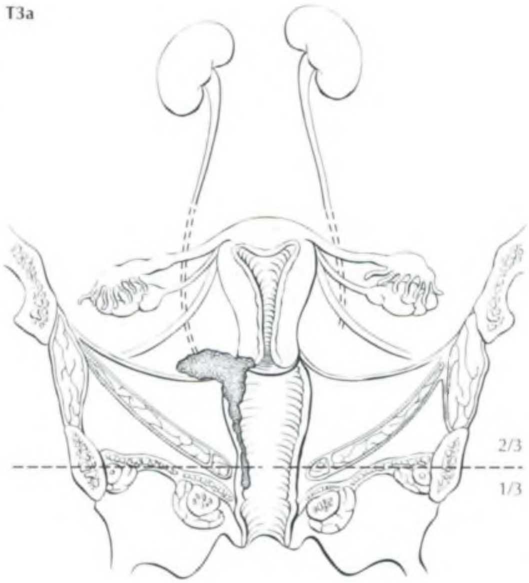

Fig. 52.10 T3a is tumor involves lower third of vagina but not extending to the pelvic wall

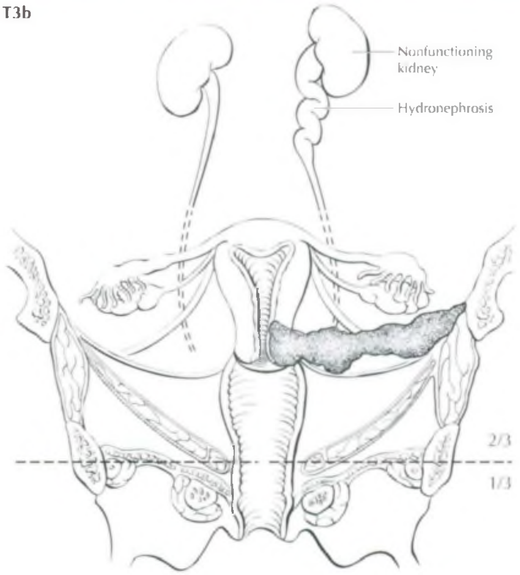

Fig. 52.11 T3b is tumor extends to pelvic wall and/or causes hydronephrosis or nonfunctioning kidney

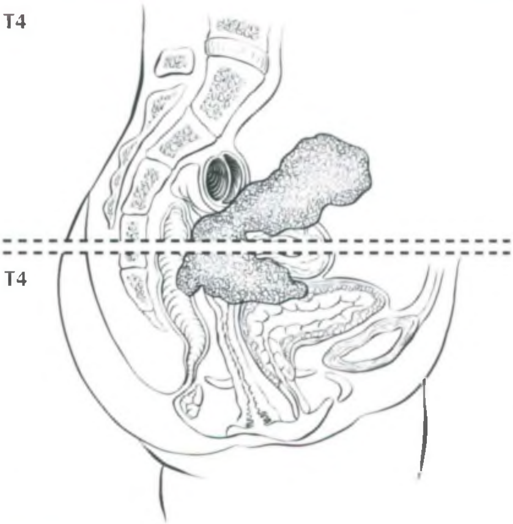

Fig. 52.12 T4 is tumor invading mucosa of bladder or rectum, and/or extends beyond true pelvis (bullous edema is not sufficient to classify a tumor as T4)

Bibliography

1. Ferlay J, Soerjomataram I, Dikshit R, et al. Cancer incidence and mortality worldwide: sources, methods and major patterns in GLOBOCAN 2012. *Int J Cancer*. Mar 1 2015;136(5):E359–386.
2. Cheng X, Cai S, Li Z, Tang M, Xue M, Zang R. The prognosis of women with stage IB1–IIB node-positive cervical carcinoma after radical surgery. *World journal of surgical oncology*. 2004;2:47.
3. Graflund M, Sorbe B, Karlsson M. Immunohistochemical expression of p53, bcl-2, and p21(WAF1/CIP1) in early cervical carcinoma: correlation with clinical outcome. *International journal of gynecological cancer : official journal of the International Gynecological Cancer Society*. May–Jun 2002;12(3):290–298.
4. Aoki Y, Sasaki M, Watanabe M, et al. High-risk group in node-positive patients with stage IB, IIA, and IIB cervical carcinoma after radical hysterectomy and postoperative pelvic irradiation. *Gynecologic oncology*. May 2000;77(2):305–309.
5. Sakuragi N, Satoh C, Takeda N, et al. Incidence and distribution pattern of pelvic and paraaortic lymph node metastasis in patients with Stages IB, IIA, and IIB cervical carcinoma treated with radical hysterectomy. *Cancer*. Apr 1 1999;85(7):1547–1554.
6. Benedetti-Panici P, Maneschi F, D'Andrea G, et al. Early cervical carcinoma: the natural history of lymph node involvement redefined on the basis of thorough parametrectomy and giant section study. *Cancer*. May 15 2000;88(10):2267–2274.
7. Hong JH, Tsai CS, Lai CH, et al. Risk stratification of patients with advanced squamous cell carcinoma of cervix treated by radiotherapy alone. *International journal of radiation oncology, biology, physics*. Oct 1 2005;63(2):492–499.
8. Mayr NA, Yuh WT, Jajoura D, et al. Ultra-early predictive assay for treatment failure using functional magnetic resonance imaging and clinical prognostic parameters in cervical cancer. *Cancer*. Feb 15 2010;116(4):903–912.
9. Bhosale P, Peungjesada S, Devine C, Balachandran A, Iyer R. Role of magnetic resonance imaging as an adjunct to clinical staging in cervical carcinoma. *Journal of computer assisted tomography*. Nov-Dec 2010;34(6):855–864.
10. Perez CA, Grigsby PW, Chao KS, Mutch DG, Lockett MA. Tumor size, irradiation dose, and long-term outcome of carcinoma of uterine cervix. *International journal of radiation oncology, biology, physics*. May 1 1998;41(2):307–317.
11. Perez CA, Grigsby PW, Nene SM, et al. Effect of tumor size on the prognosis of carcinoma of the uterine cervix treated with irradiation alone. *Cancer*. Jun 1 1992;69(11):2796–2806.
12. Eifel PJ, Morris M, Wharton JT, Oswald MJ. The influence of tumor size and morphology on the outcome of patients with FIGO stage IB squamous cell carcinoma of the uterine cervix. *International journal of radiation oncology, biology, physics*. Apr 30 1994;29(1):9–16.
13. Wagenaar HC, Trimbos JB, Postema S, et al. Tumor diameter and volume assessed by magnetic resonance imaging in the prediction of outcome for invasive cervical cancer. *Gynecologic oncology*. Sep 2001;82(3):474–482.
14. Mayr NA, Magnotta VA, Ehrhardt JC, et al. Usefulness of tumor volumetry by magnetic resonance imaging in assessing response to radiation therapy in carcinoma of the uterine cervix. *International journal of radiation oncology, biology, physics*. Jul 15 1996;35(5):915–924.
15. Horn LC, Hentschel B, Galle D, Bilek K. Extracapsular extension of pelvic lymph node metastases is of prognostic value in carcinoma of the cervix uteri. *Gynecologic oncology*. Jan 2008;108(1):63–67.
16. Petereit D, Hartenbach E, Thomas G. Para-aortic lymph node evaluation in cervical cancer: the impact of staging upon treatment

decisions and outcome. *International Journal of Gynecological Cancer*. 1998;8:353–364.
17. Kattan MW, Hess KR, Amin MB, et al. American Joint Committee on Cancer acceptance criteria for inclusion of risk models for individualized prognosis in the practice of precision medicine. *CA: a cancer journal for clinicians*. Jan 19 2016.
18. Benedet JL, Odicino F, Maisonneuve P, et al. Carcinoma of the cervix uteri. *J Epidemiol Biostat*. 2001;6(1):7–43.
19. Bodurka-Bevers D, Morris M, Eifel PJ, et al. Posttherapy surveillance of women with cervical cancer: an outcomes analysis. *Gynecologic oncology*. Aug 2000;78(2):187–193.
20. Coucke PA, Maingon P, Ciernik IF, Phuoc DOH. A survey on staging and treatment in uterine cervical carcinoma in the Radiotherapy Cooperative Group of the European Organization for Research and Treatment of Cancer. *Radiotherapy and oncology : journal of the European Society for Therapeutic Radiology and Oncology*. Mar 2000;54(3):221–228.
21. Koh W-J, Panwala K, Greer B. Adjuvant therapy for high-risk, early stage cervical cancer. Paper presented at: Seminars in radiation oncology 2000.
22. Pecorelli S. Revised FIGO staging for carcinoma of the vulva, cervix, and endometrium. *International journal of gynaecology and obstetrics: the official organ of the International Federation of Gynaecology and Obstetrics*. May 2009;105(2):103–104.
23. Siegel CL, Andreotti RF, Cardenes HR, et al. ACR Appropriateness Criteria® pretreatment planning of invasive cancer of the cervix. *Journal of the American College of Radiology*. 2012;9(6):395–402.
24. Zaino RJ. Glandular lesions of the uterine cervix. *Modern pathology : an official journal of the United States and Canadian Academy of Pathology, Inc*. Mar 2000;13(3):261–274.

Corpus Uteri – Carcinoma and Carcinosarcoma

53

Matthew A. Powell, Alexander B. Olawaiye, Adriana Bermudez, Perry W. Grigsby, Larry J. Copeland, Don S. Dizon, Beth A. Erickson, Ian S. Hagemann, Lorraine Portelance, Jaime Prat, and David G. Mutch

CHAPTER SUMMARY

Cancers Staged Using This Staging System

Uterine carcinomas and carcinosarcomas

Cancers Not Staged Using This Staging System

These histopathologic types of cancer…	Are staged according to the classification for…	And can be found in chapter…
Sarcomas: leiomyosarcomas, endometrial stromal sarcomas, adenosarcomas	Corpus uteri – sarcoma	54

Summary of Changes

Change	Details of Change	Level of Evidence
Histopathologic Type	Uterine sarcomas have been removed from this chapter and are considered separately (Chapter 54).	N/A
Definition of Primary Tumor (T)	Stage 0 and Tis (carcinoma *in situ*/pre-invasive carcinoma) have been removed.	N/A
Definition of Primary Tumor (T)	Endometrial intraepithelial carcinoma (EIC) should be considered a T1 cancer.	II
Histologic Grade (G)	Grade 4 has been eliminated and should be considered Grade 3.	I
Definition of Regional Lymph Node (N)	Lymph node micro-metastasis (< 2 mm in diameter) will be reported as N1mi and N2mi	I

ICD-O-3 Topography Codes

Code	Description
C54.0	Isthmus uteri
C54.1	Endometrium
C54.2	Myometrium
C54.3	Fundus uteri
C54.8	Overlapping lesion of corpus uteri
C54.9	Corpus uteri
C55.9	Uterus, NOS

WHO Classification of Tumors

Code	Description
8380	Endometrioid carcinoma
8570	Endometrioid carcinoma, squamous differentiation
8263	Endometrioid carcinoma, villoglandular
8382	Endometrioid carcinoma, secretory
8480	Mucinous carcinoma
8441	Serous carcinoma
8310	Clear cell carcinoma
8240	Carcinoid tumor
8041	Small cell neuroendocrine carcinoma
8013	Large cell neuroendocrine carcinoma
8323	Mixed cell adenocarcinoma
8020	Undifferentiated carcinoma
8980	Carcinosarcoma

Kurman RJ, Carcangiu ML, Herrington CS, Young RH, eds. World Health Organization Classification of Tumours of the Female Reproductive System. Lyon: IARC; 2014.

To access the AJCC cancer staging forms, please visit www.cancerstaging.org.

© American Joint Committee on Cancer 2017

M.B. Amin et al. (eds.), *AJCC Cancer Staging Manual, Eighth Edition*, DOI 10.1007/978-3-319-40618-3_53

INTRODUCTION

The classification for uterine cancers has been modified for the AJCC Cancer Staging Manual, 8th Edition TNM in accordance with changes adopted by the Fédération Internationale de Gynécologie et d'Obstétrique (FIGO) to have separate systems for endometrial adenocarcinomas and uterine sarcomas.

ANATOMY

Primary Site(s)

The upper two thirds of the uterus above the level of the internal cervical os is referred to as the uterine corpus. The oviducts (fallopian tubes) and the round ligaments enter the uterus at the upper and outer corners (cornu) of the pear-shaped organ. The portion of the uterus that is above a line connecting the tubo-uterine orifices is referred to as the uterine fundus. The lower third of the uterus is called the cervix and lower uterine segment (Fig. 53.1).

Regional Lymph Nodes

The regional lymph nodes are paired (right and left), and each of the paired sites may be evaluated to determine stage and prognosis and to help direct therapy (Fig. 53.2). The regional nodes are as follows:

- Parametrial
- Obturator
- Internal iliac (hypogastric)
- External iliac
- Sacral
- Presacral
- Common iliac
- Para-aortic

Metastatic Sites

The vagina and lung are the common metastatic sites. Intra-abdominal metastases to abdominal or pelvic peritoneal surfaces or the omentum are seen particularly with serous and clear cell tumors.

RULES FOR CLASSIFICATION

The significance of clinical compared with surgical/pathological staging is shown in Fig. 53.3. The prognosis for patients with clinical Stage I disease is similar to that for

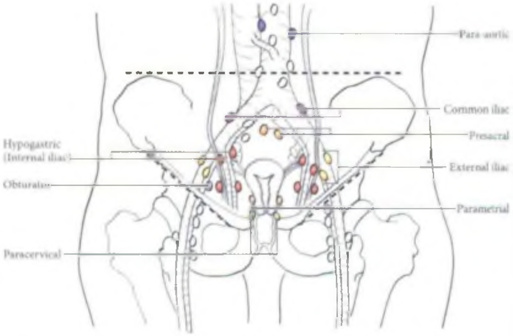

Fig. 53.2 Regional lymph nodes of the corpus uteri

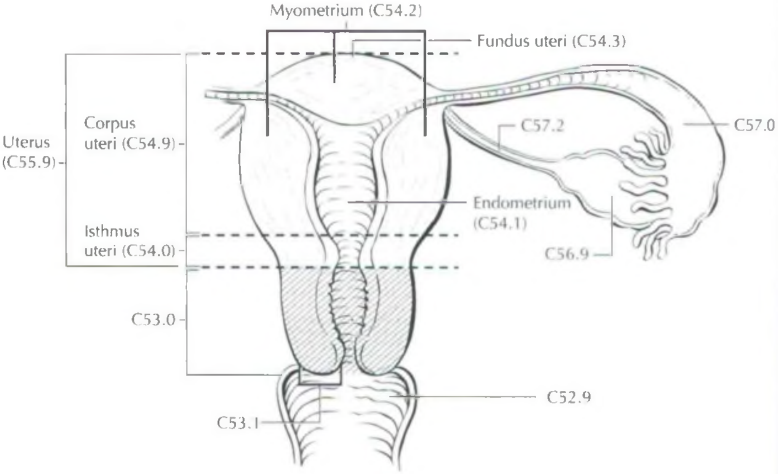

Fig. 53.1 Anatomic sites and subsites of the corpus uteri

women with surgical Stage III, and those with clinical Stage III cancers have the same prognosis as patients with surgical Stage IV lesions. These findings also emphasize the importance of clearly separating patients who are staged clinically from those who have the more accurate surgical/pathological staging recommended by AJCC and FIGO.

Clinical Classification

The classification applies only to carcinoma and carcinosarcoma (malignant mixed mesodermal tumors). There should be histologic verification and grading of the tumor.

Tumor involvement of the cervical stroma is prognostically important and affects staging (T2). The new staging system no longer recognizes endocervical mucosal/glandular involvement (formerly stage IIA), because this does not appear to effect prognosis. The location of the tumor must be evaluated carefully and recorded by the pathologist. The depth of tumor invasion into the myometrium also is of prognostic significance and should be included in the pathology report. Involvement of the ovaries by direct extension or metastases or penetration of tumor to the uterine serosa is important to identify, and the tumor should be classified as T3a.

Malignant cells in peritoneal cytology samples have been documented in approximately 10% of cases of presumed uterine-confined endometrial cancer cases. The prognostic importance of positive cytology has been debated. Depth of myometrial invasion, tumor grade, and presence of extrauterine disease are felt to be more prognostically significant; therefore the 2008 FIGO staging system stopped using peritoneal cytology for the purposes of staging (formerly T3a, FIGO stage IIIA). T3b lesions reflect regional extension of disease and include extension of the tumor through the myometrial wall of the uterus into the parametrium and/or extension/metastatic involvement of the vagina.

Distant metastasis (M1, FIGO IVB) includes metastases to inguinal lymph nodes, intraperitoneal disease, and metastases to the lung, liver, or bone. It excludes metastasis to the pelvic or para-aortic lymph nodes, vagina, uterine serosa, or adnexa.

Imaging

Magnetic resonance (MR) imaging currently is the preferred modality for local staging of endometrial cancer. Contrast-enhanced computed tomography (CT) scans do not have sufficient soft tissue resolution to identify the tumor in the uterine corpus or to assess the depth of myometrial invasion. Assessment of lymph node metastases on cross-sectional imaging is based on lymph node size, with nodes >1 cm in the short axial dimension considered abnormal. CT and MR imaging have been shown to perform equally well in assessing adenopathy. However, because there may be false positive causes of enlarged nodes from benign disease, positron emission tomography (PET)/CT is considered to be better in assessing lymph node metastases. Metabolically active lymph nodes of any size on PET/CT are considered metastatic. PET/CT is considered superior to other modalities in assessing for extrapelvic disease and bone metastases.

TNM Components of Tumor Staging

In category T1, the tumor is confined to the uterus. Category T1a involves <50% and category T1b involves >50% of the myometrium, which may be assessed on MR T1-weighted dynamic images. However, recent data suggest that the diffusion-weighted imaging (DWI) sequence also may be used to assess the depth of myometrial invasion. DWI also has shown promising results in assessing the depth of endometrial invasion. In category T2, the tumor invades the cervix and may be seen on contrast-enhanced T1-weighted sequences. In category T3, the tumor involves the serosa or adnexa, whereas in T3b there is vaginal involvement; this may be evaluated on contrast-enhanced T1- and T2-weighted MR imaging sequences.

Regional nodal metastases are considered to be category N1 and can be assessed easily on CT or MR imaging. This categorization is based on the size criterion for abnormality of >1 cm in the short axial dimension. Cases in which lymph node metastases are confined to the pelvis are considered IIIC1 disease; involvement above the inferior mesenteric artery (IMA) is considered IIIC2 disease.

Reporting Anatomic Staging of Tumor

Reports should describe the size and extent of the tumor and whether it involves the inner or outer half of the myometrium. Reporting cervical and vaginal involvement is crucial, as treatment differs in these cases. It is also important to report extension through the serosa of the uterus or involvement of the ovaries and adjacent organs.

Suggested Imaging Report Format

1. Primary tumor
   a. Size
2. Local extent
   a. Involvement of <50% or ≥50% of the myometrium
   b. Involvement of the vagina and cervix
   c. Extraserosal extension
   d. Involvement of the ovaries and adjacent organs
3. Regional and distant lymph node involvement and extrapelvic disease

53

Pathological Classification

FIGO uses surgical/pathological staging for corpus uteri cancer. Pathological stage is assigned at the time of definitive

surgical treatment, using histopathology and all clinical staging results. Clinical staging is assigned before radiation or chemotherapy if those are the initial modes of therapy. The stage should not be changed on the basis of disease progression or recurrence. If a patient receives radiation or chemotherapy before surgery, then a post-neoadjuvant therapy pathological (yp) stage is assigned (see Chapter 1). The depth of myometrial invasion (in millimeters) should be recorded, along with the thickness of the myometrium at that level (recorded as a percentage of myometrial invasion).

The presence of carcinoma in the regional lymph nodes is a clinically critical prognostic variable. Multiple studies confirmed the inaccuracy of clinical assessment of regional nodal metastasis in many anatomic sites. For this reason, surgical/pathological assessment of the regional lymph nodes is advocated for all patients with corpus uteri cancer; this is also the recommendation of FIGO. A therapeutic effect from nodal dissection was not found in two randomized controlled clinical trials[2] however, routine nodal dissection increased the frequency in which patients with node-involved disease were identified. Single-institution studies suggest that patients with small-volume, grade 1–2 endometrioid adenocarcinoma confined to the inner half of the myometrium have a negligible risk of nodal spread.[3] These uterine tumor features are being used increasingly in clinical practice to triage appropriate patients for lymphadenectomy versus no lymphadenectomy. Larger, prospective studies are still needed to rigorously assess this approach. If the surgeon feels that systematic regional lymph node sampling imposes an unfavorable risk-to-benefit ratio, clinical assessment of the pertinent node groups (obturator, para-aortic groups, internal iliac, common iliac, and external iliac) should be performed, specifically annotated in the operative report, and recorded as cN. Single tumor cells or small clusters of cells smaller than 0.2 mm in greatest diameter are classified as isolated tumor cells (ITCs). These may be detected by routine histology or by immunohistochemical methods and are designated as N0(i+).

For adequate evaluation of the regional lymph nodes, a representative evaluation of bilateral para-aortic and pelvic lymph nodes (including external iliac, internal iliac, and obturator nodes) should be documented in the operative and surgical pathology reports. Parametrial nodes are not detected commonly unless a radical hysterectomy is performed for cases with gross cervical stromal invasion.

For pN, histologic examination of regional lymphadenectomy specimens ordinarily includes six or more lymph nodes. For TNM staging, cases with fewer than six resected nodes should be classified using the TNM pathological classification based on the status of those nodes (e.g., pN0; pN1) according to the general rules of TNM. The number of resected and positive nodes should be recorded (note that FIGO classifies cases with fewer than six nodes resected and negative as pNX). Use of sentinel lymph node assessment for endometrial cancer has increased the identification of micrometastatic disease. N1mi and N2mi are new to the 8th Edition and represent nodal micro-metastasis to less than 2 mm of the pelvic and para-aortic lymph node regions, respectively.

Recently, sentinel nodes have been an important diagnostic factor. When a regional lymph node metastasis is identified by sentinel lymph node biopsy, and additional surgery in the form of a completion lymph node dissection is *not* performed, the N category is assigned with the addition of the (sn) suffix—for example, pN1a(sn). However, if the patient does undergo a completion lymph node dissection, the suffix is not used—for example, pN1a.

Fractional (endocervical) curettage is not adequate to establish cervical involvement or to distinguish between Stages I and II, given a high false-positive rate, and thus is not recommended for staging purposes. That distinction can be made best by histologic verification of clinically suspicious cervical involvement or histopathologic examination of the removed uterus.

The pT, pN, and c/pM categories correspond to the T, N, and M categories and are used to designate cases in which adequate pathological specimens are available for accurate stage groupings. If the surgical–pathological findings are insufficient, the clinical cT, cN, c/pM categories should be used on the basis of clinical evaluation.

PROGNOSTIC FACTORS

Prognostic Factors Required for Stage Grouping

Beyond the factors used to assign T, N, or M categories, no additional prognostic factors are required for stage grouping.

Additional Factors Recommended for Clinical Care

FIGO Stage

figo stage parallels AJCC stage, reflects prognosis, and is used worldwide for documenting patient status and outcome. AJCC Level of Evidence: I

Grade

The aggressiveness of the tumor appears to be related to the degree of differentiation of the glandular component. Clinicopathologic and immunohistochemical studies support classifying carcinosarcoma (malignant mixed mesodermal tumors) as high-grade (G3) malignancies of epithelial origin rather than as sarcomas with mixed epithelial and mesenchymal differentiation, as in earlier classification systems. AJCC Level of Evidence: I

Depth of Myometrial Invasion

Historically, the factors of tumor grade and depth of myometrial invasion have been recognized as important prognostic factors. In surgically staged patients, based on multivariate analysis, these factors are surrogates for the probability of nodal metastasis and treatment outcome. Preoperative endometrial sampling may not correlate accurately with final tumor grade and depth of myometrial invasion. Depth of myometrial invasion must be documented. AJCC Level of Evidence: I

Lymphovascular Space Invasion

The presence or absence of lymphovascular space involvement—also referred to as lymphovascular invasion (LVI)—of the myometrium is important in most, but not all, series. If present, LVI increases the probability of metastatic involvement of the regional lymph nodes. LVI should be recorded in the pathology report as present, not identified, or indeterminate. AJCC Level of Evidence: I

Peritoneal Cytology

The finding of tumor cells in peritoneal "washings" may have an adverse impact on prognosis, but this possibility remains controversial and requires further study. The newly adopted staging system[4] discontinued the use of positive cytology to alter stage, but AJCC still recommends the collection of this factor. AJCC Level of Evidence: I

Estrogen and Progesterone Receptor Status

Estrogen and progesterone receptor status should be recorded if clinically appropriate. AJCC Level of Evidence: II

Tumor Suppressor and Oncogene Expression

Molecular profiling is becoming increasingly available, but its exact clinical utility is not yet known. AJCC Level of Evidence: II

Pelvic Nodal Dissection with Number of Nodes Positive/Examined

The status of pelvic lymph nodes, if known, is a predictor of outcome. The number of nodes positive and examined should be recorded if known. AJCC Level of Evidence: I

Para-aortic Nodal Dissection with Number of Nodes Positive/Examined

The involvement of para-aortic nodes, if known, is a predictor of outcome. The number of para-aortic nodes positive and examined should be recorded if known. AJCC Level of Evidence: I

Percentage of Nonendometrioid Cell Type in Mixed-Histology Tumors

The significance of percentage of nonendometrioid cell type in mixed-histology tumors is unclear; however, collection of this information is important for future analysis of its impact on stage. AJCC Level of Evidence: II

Nodal Disease

The presence or absence of metastatic disease in the regional lymph nodes is the most important prognostic factor in carcinomas clinically confined to the uterus. The AJCC advocates the use of surgical/pathological assessment of nodal status whenever possible. Palpation of regional nodes is well recognized to be much less accurate than pathological evaluation of the nodes. AJCC Level of Evidence: I

Histopathologic Type

Serous and clear cell adenocarcinomas have a higher incidence of extrauterine disease at diagnosis than endometrioid adenocarcinomas. The risk of extrauterine disease does not correlate with the depth of myometrial invasion, because nodal or intraperitoneal metastases may be found even in the absence of myometrial invasion. For these reasons, they are classified as grade 3 tumors. Endometrial intraepithelial carcinoma (EIC) should be considered an invasive T1 cancer, as it has been associated with metastatic disease and is not a "precancerous" lesion. Up to two thirds of serous EICs may be associated with extrauterine disease. Level of Evidence: I

Omentectomy Performed

Omentectomy should be considered for higher-grade lesions. AJCC Level of Evidence: I

Morcellation

Intra-abdominal morcellation of a potentially cancerous organ should be avoided. However, with the increased use of minimally invasive techniques involving morcellation of the uterine specimen or subtotal removal, specimen integrity should be noted—for example, morcellated versus intact and total (including the cervix) versus subtotal (supracervical) hysterectomy specimen. AJCC Level of Evidence: I

RISK ASSESSMENT MODELS

The AJCC recently established guidelines that will be used to evaluate published statistical prediction models for the purpose of granting endorsement for clinical use.[5] Although this is a monumental step toward the goal of precision medicine, this work was published only very recently. Therefore, the existing models that have been published or may be in clinical use have not yet been evaluated for this cancer site by the Precision Medicine Core of the AJCC. In the future, the statistical prediction models for this cancer site will be evaluated, and those that meet all AJCC criteria will be endorsed.

DEFINITIONS OF AJCC TNM

The definitions of the T categories correspond to the stages accepted by the Fédération Internationale de Gynécologie et d'Obstétrique (FIGO). Both systems are included for comparison.

Definition of Primary Tumor (T)

T Category	FIGO Stage	T Criteria
TX		Primary tumor cannot be assessed
T0		No evidence of primary tumor
T1	I	Tumor confined to the corpus uteri, including endocervical glandular involvement
T1a	IA	Tumor limited to the endometrium or invading less than half the myometrium
T1b	IB	Tumor invading one half or more of the myometrium
T2	II	Tumor invading the stromal connective tissue of the cervix but not extending beyond the uterus. Does NOT include endocervical glandular involvement.
T3	III	Tumor involving serosa, adnexa, vagina, or parametrium
T3a	IIIA	Tumor involving the serosa and/or adnexa (direct extension or metastasis)
T3b	IIIB	Vaginal involvement (direct extension or metastasis) or parametrial involvement
T4	IVA	Tumor invading the bladder mucosa and/or bowel mucosa (bullous edema is not sufficient to classify a tumor as T4)

Definition of Regional Lymph Node (N)

N Category	FIGO Stage	N Criteria
NX		Regional lymph nodes cannot be assessed
N0		No regional lymph node metastasis
N0(i+)		Isolated tumor cells in regional lymph node(s) no greater than 0.2 mm
N1	IIIC1	Regional lymph node metastasis to pelvic lymph nodes
N1mi	IIIC1	Regional lymph node metastasis (greater than 0.2 mm but not greater than 2.0 mm in diameter) to pelvic lymph nodes
N1a	IIIC1	Regional lymph node metastasis (greater than 2.0 mm in diameter) to pelvic lymph nodes
N2	IIIC2	Regional lymph node metastasis to para-aortic lymph nodes, with or without positive pelvic lymph nodes
N2mi	IIIC2	Regional lymph node metastasis (greater than 0.2 mm but not greater than 2.0 mm in diameter) to para-aortic lymph nodes, with or without positive pelvic lymph nodes
N2a	IIIC2	Regional lymph node metastasis (greater than 2.0 mm in diameter) to para-aortic lymph nodes, with or without positive pelvic lymph nodes

Suffix (sn) is added to the N category when metastasis is identified only by sentinel lymph node biopsy.

Definition of Distant Metastasis (M)

M Category	FIGO Stage	M Criteria
M0		No distant metastasis
M1	IVB	Distant metastasis (includes metastasis to inguinal lymph nodes, intraperitoneal disease, lung, liver, or bone). (It excludes metastasis to pelvic or para-aortic lymph nodes, vagina, uterine serosa, or adnexa).

AJCC Prognostic Stage Groups

When T is...	And N is...	And M is...	Then the stage group is...
T1	N0	M0	I
T1a	N0	M0	IA
T1b	N0	M0	IB
T2	N0	M0	II
T3	N0	M0	III
T3a	N0	M0	IIIA
T3b	N0	M0	IIIB
T1-T3	N1/N1mi/N1a	M0	IIIC1
T1-T3	N2/N2mi/N2a	M0	IIIC2
T4	Any N	M0	IVA
Any T	Any N	M1	IVB

REGISTRY DATA COLLECTION VARIABLES

1. FIGO stage
2. Depth of myometrial invasion
3. Lymphovascular space invasion
4. Peritoneal cytology results (collected? [yes/no] positive or negative)
5. Estrogen and progesterone receptor status
6. Tumor suppressor and oncogene expression (yes/no)
7. Pelvic nodal dissection with number of nodes positive/examined
8. Para-aortic nodal dissection with number of nodes positive/examined
9. Percentage of nonendometrioid cell type in mixed-histology tumors
10. Omentectomy performed (yes/no)
11. Morcellation (yes/no)

HISTOLOGIC GRADE (G)

G	G Definition
GX	Grade cannot be assessed
G1	Well differentiated
G2	Moderately differentiated
G3	Poorly differentiated or undifferentiated

Histopathology: Degree of Differentiation

Cases of carcinoma of the corpus uteri should be grouped according to the degree of differentiation of the endometrioid adenocarcinoma:

G	G Definition
G1	5% or less of a nonsquamous or nonmorular solid growth pattern
G2	6-50% of a nonsquamous or nonmorular solid growth pattern
G3	More than 50% of a nonsquamous or nonmorular solid growth pattern.Papillary serous, clear cell, and carcinosarcoma are considered high grade.

Notes on Pathological Grading

1. Notable nuclear atypia exceeding that which is routinely expected for the architectural grade increases the tumor grade by 1 (i.e., 1 to 2 and 2 to 3).
2. Serous, clear cell, and mixed mesodermal tumors are *high risk* and considered grade 3.
3. Adenocarcinomas with benign squamous elements (squamous metaplasia) are graded according to the nuclear grade of the glandular component.

HISTOPATHOLOGIC TYPE

Endometrioid adenocarcinoma, not otherwise characterized
Endometrioid adenocarcinoma, variant (specify)
Mucinous adenocarcinoma
Serous adenocarcinoma
Clear cell adenocarcinoma
Mixed carcinoma (specify types and percentages)
Squamous cell carcinoma
Transitional cell carcinoma
Small cell carcinoma
Undifferentiated carcinoma
Carcinosarcoma

SURVIVAL DATA

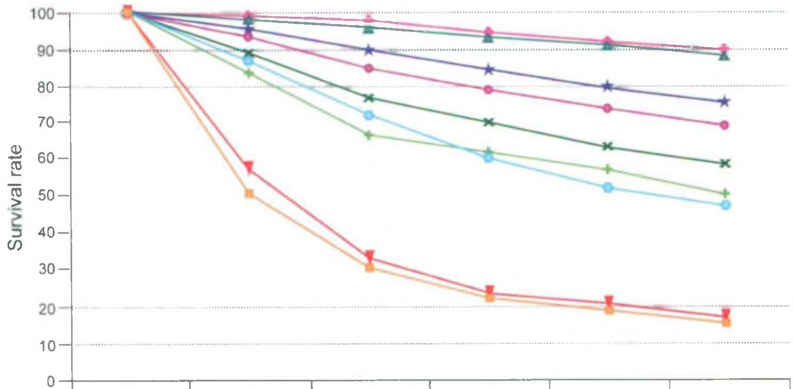

	0	1	2	3	4	5
0	100.0	98.8	97.7	94.6	92.3	90.1
IA	100.0	98.3	96.0	93.3	91.0	88.4
IB	100.0	95.6	89.8	84.4	79.4	75.1
II	100.0	93.3	85.0	78.4	73.4	68.9
IIIA	100.0	88.8	76.7	69.5	62.8	58.1
IIIB	100.0	83.0	66.5	61.6	56.4	49.9
IIIC	100.0	86.3	71.8	60.0	51.9	46.6
IVA	100.0	58.3	33.5	24.4	20.6	16.8
IVB	100.0	50.3	30.2	22.3	18.1	15.2

Years from diagnosis

Fig. 53.3 Observed survival rates for 21,904 cases with carcinoma of the corpus uterus. Data from the National Cancer Data Base (Commission on Cancer of the American College of Surgeons and the American Cancer Society), from diagnoses made from 2000 to 2002. Stage 0 includes 415 patients; Stage IA, 12,868; Stage IB, 2,559; Stage II, 2,098; Stage IIIA, 929; Stage IIIB, 91; Stage IIIC, 1,353; Stage IVA, 229; and Stage IVB, 1,362

ILLUSTRATIONS

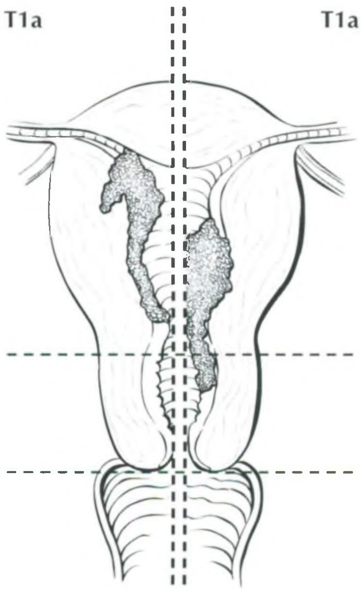

Fig. 53.4 For carcinomas T1a is tumor limited to the endometrium or invading less than half the myometrium

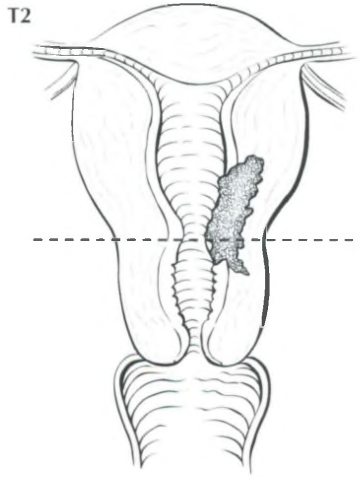

Fig. 53.6 For carcinomas T2 is tumor invading the stromal connective tissue of the cervix but not extending beyond the uterus. Does NOT include endocervical glandular involvement

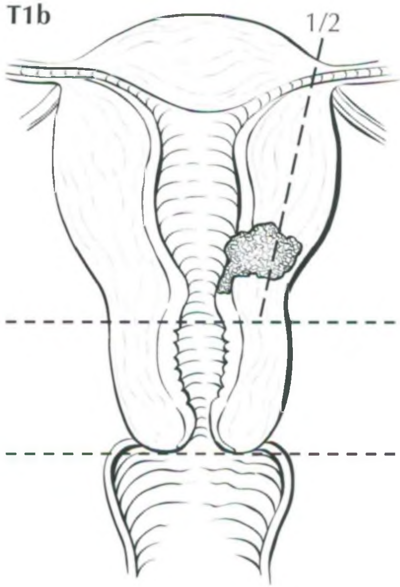

Fig. 53.5 For carcinomas T1b is tumor invading one half or more of the myometrium

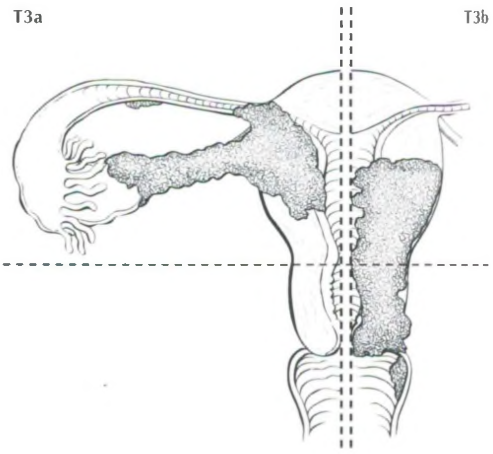

Fig. 53.7 For carcinomas T3a is tumor involving the serosa and/or adnexa (direct extension or metastasis). T3b is vaginal involvement (direct extension or metastasis) or parametrial involvement

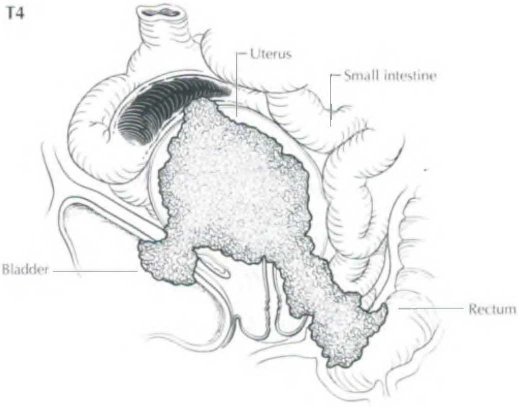

Fig. 53.8 For carcinomas T4 is tumor invading the bladder mucosa and/or bowel mucosa (bullous edema is not sufficient to classify a tumor as T4)

Bibliography

1. Lalwani N, Dubinsky T, Javitt MC, et al. ACR Appropriateness Criteria(R) pretreatment evaluation and follow-up of endometrial cancer. *Ultrasound Q*. Mar 2014;30(1):21–28.
2. Kitchener H, Swart AM, Qian Q, Amos C, Parmar MK. Efficacy of systematic pelvic lymphadenectomy in endometrial cancer (MRC ASTEC trial): a randomised study. *Lancet*. Jan 10 2009; 373(9658):125–136.
3. Mariani A, Webb MJ, Keeney GL, Aletti G, Podratz KC. Assessment of prognostic factors in stage IIIA endometrial cancer. *Gynecologic oncology*. Jul 2002;86(1):38–44.
4. Pecorelli S. Revised FIGO staging for carcinoma of the vulva, cervix, and endometrium. *International journal of gynaecology and obstetrics: the official organ of the International Federation of Gynaecology and Obstetrics*. May 2009;105(2):103–104.
5. Kattan MW, Hess KR, Amin MB, et al. American Joint Committee on Cancer acceptance criteria for inclusion of risk models for individualized prognosis in the practice of precision medicine. *CA: a cancer journal for clinicians*. Jan 19 2016.
6. Guidelines for referral to a gynecologic oncologist: rationale and benefits. The Society of Gynecologic Oncologists. *Gynecologic oncology*. Sep 2000;78(3 Pt 2):S1–13.
7. Cirisano FD, Robboy SJ, Dodge RK, et al. The outcome of stage I–II clinically and surgically staged papillary serous and clear cell endometrial cancers when compared with endometrioid carcinoma. *Gynecologic oncology*. 2000;77(1):55–65.
8. Colombi R. Sarcomatoid carcinomas of the female genital tract (malignant mixed mullerian tumors). Paper presented at: Seminars in diagnostic pathology1993.
9. Creasman WT, Odicino F, Maisonneuve P, et al. Carcinoma of the corpus uteri. *J Epidemiol Biostat*. 2001;6(1):47–86.
10. Creasman WT, Morrow CP, Bundy BN, Homesley HD, Graham JE, Heller PB. Surgical pathologic spread patterns of endometrial cancer. A Gynecologic Oncology Group Study. *Cancer*. Oct 15 1987;60(8 Suppl):2035–2041.
11. Creutzberg CL, van Putten WL, Koper PC, et al. Surgery and post-operative radiotherapy versus surgery alone for patients with stage-1 endometrial carcinoma: multicentre randomised trial. PORTEC Study Group. Post Operative Radiation Therapy in Endometrial Carcinoma. *Lancet*. Apr 22 2000;355(9213):1404–1411.
12. Kim CH, Khoury-Collado F, Barber EL, et al. Sentinel lymph node mapping with pathologic ultrastaging: a valuable tool for assessing nodal metastasis in low-grade endometrial cancer with superficial myoinvasion. *Gynecologic oncology*. Dec 2013;131(3):714–719.
13. Marth C, Windbichler G, Petru E, et al. Parity as an independent prognostic factor in malignant mixed mesodermal tumors of the endometrium. *Gynecologic oncology*. 1997;64(1):121–125.
14. Panici PB, Basile S, Maneschi F, et al. Systematic pelvic lymphadenectomy vs no lymphadenectomy in early-stage endometrial carcinoma: randomized clinical trial. *Journal of the National Cancer Institute*. 2008;100(23):1707–1716.
15. Prat J. FIGO staging for uterine sarcomas. *International journal of gynaecology and obstetrics: the official organ of the International Federation of Gynaecology and Obstetrics*. Mar 2009;104(3): 177–178.
16. Wheeler DT, Bell KA, Kurman RJ, Sherman ME. Minimal uterine serous carcinoma: diagnosis and clinicopathologic correlation. *The American journal of surgical pathology*. Jun 2000;24(6):797–806.
17. Zaino RJ, Kurman RJ, Diana KL, Paul Morrow C. The utility of the revised International Federation of Gynecology and Obstetrics histologic grading of endometrial adenocarcinoma using a defined nuclear grading system. A Gynecologic Oncology Group study. *Cancer*. 1995;75(1):81–86.
18. Zerbe MJ, Bristow R, Grumbine FC, Montz FJ. Inability of preoperative computed tomography scans to accurately predict the extent of myometrial invasion and extracorporal spread in endometrial cancer. *Gynecologic oncology*. Jul 2000;78(1):67–70.
19. Zheng W, Xiang L, Fadare O, Kong B. A proposed model for endometrial serous carcinogenesis. *The American journal of surgical pathology*. Jan 2011;35(1):e1–e14.
20. Corrigendum to "FIGO staging for uterine sarcomas". *International Journal of Gynecology & Obstetrics*. 2009;106:277.

53

Corpus Uteri - Sarcoma

54

Don S. Dizon, Alexander B. Olawaiye,
Robert K. Brookland, Beth A. Erickson, Ian S. Hagemann,
Matthew A. Powell, Jaime Prat, Aaron H. Wolfson,
and David G. Mutch

CHAPTER SUMMARY

Cancers Staged Using This Staging System

Sarcomas arising in the uterine corpus

Cancers Not Staged Using This Staging System

These histopathologic types of cancer…	Are staged according to the classification for…	And can be found in chapter…
Carcinosarcoma	Corpus uteri – carcinoma and carcinosarcoma	53

Summary of Changes

Change	Details of Change	Level of Evidence
New chapter	Uterine Sarcoma is a new chapter in AJCC Cancer Staging Manual, 8th Edition. The staging for leiomyosarcoma, endometrial sarcoma, and adenosarcoma was included in the corpus uteri chapter in previous editions.	N/A
Histologic Grade (G)	Grade is not a collected element in leiomyosarcoma, as all are high-grade tumors.	N/A

ICD-O-3 Topography Codes

Code	Description
C54.0	Isthmus uteri
C54.1	Endometrium
C54.2	Myometrium
C54.3	Fundus uteri
C54.8	Overlapping lesion of corpus uteri
C54.9	Corpus uteri
C55.9	Uterus, NOS

WHO Classification of Tumors

Code	Description
8714	Perivascular epithelioid cell tumor
8805	Undifferentiated uterine sarcoma
8890	Leiomyosarcoma
8891	Epithelioid leiomyosarcoma
8896	Myxoid leiomyosarcoma
8930	High-grade endometrial stromal sarcoma
8931	Low-grade endometrial stromal sarcoma
8933	Adenosarcoma

Kurman RJ, Carcangiu ML, Herrington CS, Young RH, eds. World Health Organization Classification of Tumours of the Female Reproductive System. Lyon: IARC; 2014.

To access the AJCC cancer staging forms, please visit www.cancerstaging.org.

© American Joint Committee on Cancer 2017

M.B. Amin et al. (eds.), *AJCC Cancer Staging Manual, Eighth Edition*, DOI 10.1007/978-3-319-40618-3_54

INTRODUCTION

Uterine sarcomas are rare tumors, comprising less than 10% of all malignancies of the uterus. They arise from the myometrium or connective tissue elements within the uterus. Primary uterine sarcomas include leiomyosarcoma (LMS), endometrial stromal sarcoma (ESS), and adenosarcoma. Much of what is known about the behavior of these tumors is based on what we know of LMS, which is the most common of these subtypes. The staging of uterine sarcomas mirrors that of the Féderation Internationale de Gynécologie et d'Obstétrique (FIGO), which uses one staging system for LMS and ESS and another for adenosarcomas, reflecting the presence of myometrial invasion, which is definitional in LMS and ESS but not typical of adenosarcoma.

The classification of uterine cancers has been subdivided for the 8th Edition TNM in accordance with changes adopted by FIGO.

Endometrial stromal tumors comprise 2% of all uterine tumors and fall into four categories: endometrial stromal nodule (ESN), low-grade endometrial stromal sarcoma (LGESS), high-grade endometrial stromal sarcoma (HGESS), and undifferentiated uterine sarcoma (UUS).[1] Adenosarcomas account for 6% of uterine sarcomas.[2]

By definition, LMS is a high-grade tumor characterized by brisk mitotitic activity (20 or more mitotic figures per 10 high-power fields [HPF]), cytologic atypia, and evidence of tumor cell necrosis. These tumors often are hormone receptor positive,[3] exhibit diffuse staining for p16,[4] and stain positive for fascin.[5] These tumors also stain for smooth muscle markers (e.g., h-caldesmon, smooth muscle actin, histone deacetylase 8), particularly if they are conventional rather than epithelioid or myxoid variants of LMS.[6]

ESSs are characterized by the presence of myometrial and/or vascular invasion. They frequently are hormone receptor positive and stain positively for CD10 but not for desmin or caldesmon.[7] Adenosarcomas are characterized by a stromal mitotic count of $\geq$2/10 HPF, marked stromal cellularity, and more than mild stromal nuclear atypia.[8] Although they may invade the myometrium, this is uncommon; however, myometrial invasion portends a worse prognosis.[9] Adenosarcomas commonly are hormone receptor positive and more than 70% stain for CD10 and Wilms tumor protein (WT1).[10]

One of the major breakthroughs in molecular characterization of uterine sarcomas has been the identification of chromosomal alterations for ESS. These include a translocation of the short arm of chromosome 7 and the long arm of chromosome 17 [t(7;17)], which results in the combination of two zinc finger genes (JAZF1/JJAZ1) and in the translation of a fusion protein.[11] This has been identified in up to 60% of ESSs, but it is not specific to ESS and may be demonstrated in the benign variant known as ESN.[12] Other rearrangements are a t(6;7), which results in the PHF1/JAZF1 fusion gene, t(6;10), which results in the EPC1/PHF1 fusion, and t(10;17), which recently was identified in a subset of ESS that appears to be of a higher grade and, clinically, more closely aligned with UUS.[13]

ANATOMY

Primary Site(s)

By definition, the upper two thirds of the uterus above the level of the internal cervical os is referred to as the uterine corpus. The oviducts (fallopian tubes) and the round ligaments enter the uterus at the upper and outer corners (cornu) of the pear-shaped organ. The portion of the uterus that is above a line connecting the tubo-uterine orifices is referred to as the uterine fundus. The lower third of the uterus is called the cervix and lower uterine segment (Fig. 54.1).

Regional Lymph Nodes

The regional lymph nodes are paired and include the following sites (Fig. 54.2):

Parametrial
Obturator
Internal iliac (hypogastric)
External iliac
Sacral
Presacral
Common iliac
Para-aortic

Metastatic Sites

The older literature on sarcoma does not provide enough information to understand the patterns of spread of uterine sarcoma, because these reports often included carcinosarcoma in their population under investigation.[14,15] Hence, much of what we know about the sites of spread for uterine sarcoma comes from a better understanding of the pattern of metastases from LMS. In one study in more than 100 patients, the lung, peritoneum, bone, and liver were the most common sites involved.[16] Even among patients with documented locoregional recurrence, the vast majority will develop concomitant distant disease.

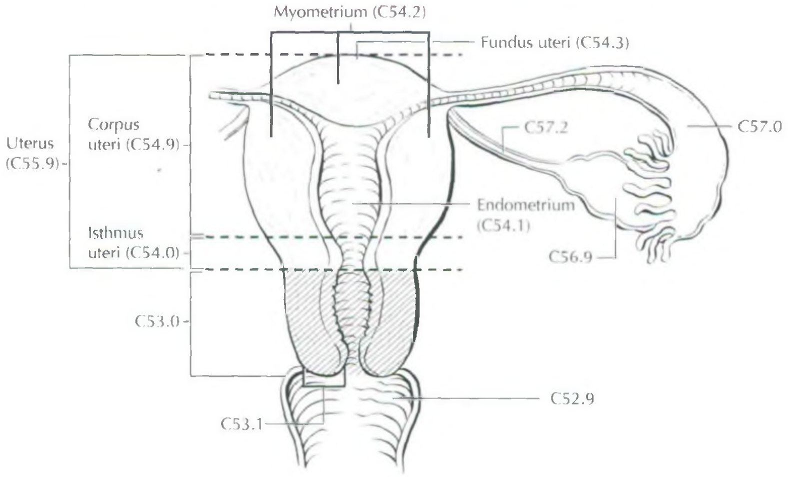

Fig. 54.1 Anatomic sites and subsites of the corpus uteri

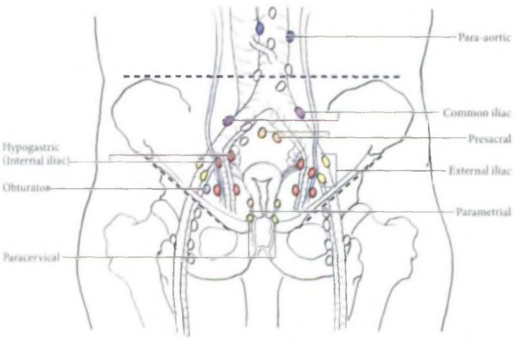

Fig. 54.2 Regional lymph nodes of the corpus uteri

RULES FOR CLASSIFICATION

Clinical Classification

The diagnosis of uterine sarcoma may be difficult to make based on clinical findings. There are no clinical examination findings or imaging characteristics that can readily distinguish these tumors from benign masses (e.g., leiomyoma). In general, the diagnosis often is made after myomectomy or hysterectomy. Rarely, it is made after endometrial sampling or diagnostic biopsy of a mass that protrudes through the cervix.

For patients with uterine LMS, tumor size is an important prognostic variable and is used in the AJCC staging system, using a cutoff of ≤5 cm to distinguish between T1a and T1b disease. Unlike endometrial carcinoma, myometrial invasion is definitional in LMS and ESS; however, myometrial invasion is not often present in adenosarcomas, although when it is, it is of prognostic significance. Tumor extension beyond the uterus is similar in all uterine sarcomas. Of note, peritoneal washings are not required in the staging of uterine sarcomas, but should be documented.

For patients with apparently uterine-confined disease and no palpable adenopathy at the time of surgery, the incidence of positively involved nodes may be as high as 10% in ESS,[17] although they rarely are encountered in patients with LMS.[18] Therefore, the histologic type of sarcoma (if known preoperatively) should be taken into account when decisions are made regarding the role of lymph node evaluation.

Clinical assessment of the pertinent node groups (obturator, para-aortic groups, internal iliac, common iliac, and external iliac) should be performed, specifically annotated in the operative report, and recorded as cN.

Single tumor cells or small clusters of cells not more than 0.2 mm in greatest diameter are classified as isolated tumor cells. These may be detected by routine histology or by immunohistochemical methods. They are designated N0(i+).

54

Imaging

Magnetic resonance (MR) imaging is thought to be useful in local staging of endometrial sarcoma, as it has better soft tissue resolution. The staging for adenosarcoma of the uterus is similar to that of endometrial cancer; staging for LMS and ESS is different.[19, 20]

TNM Components of Tumor Staging

In patients with LMS and ESS T1 disease, the tumor is confined to the uterus. MR imaging, with its superior soft tissue contrast resolution, allows assessment of tumor size on T2-weighted and contrast-enhanced T1-weighted images. Tumors ≤5 cm are categorized as T1a and those >5 cm as T1b disease. In category T2a, the tumor extends beyond the uterus; in T2b, it extends into the adnexa. In category T3 disease, the tumor infiltrates into the abdominal tissues. In category T4, the tumor involves the bladder or the rectum, which may be identified easily on T2-weighted and contrast-enhanced T1-weighted MR images.

The imaging criterion used to assess lymph node metastases is based on lymph node size, with abnormal being >1 cm in the short axial dimension on cross-sectional scans. Computed tomography (CT) and MR imaging have been shown to perform equally well in assessing adenopathy. However, because there may be false positive causes of enlarged nodes from benign disease, positron emission tomography (PET)/CT is considered to be better in assessing lymph node metastases. Metabolically active lymph nodes of any size on PET/CT are considered metastatic. PET/CT also is useful in assessing for extrapelvic metastatic disease.

Imaging Report

The size and extent of the tumor should be described, and whether it involves the inner or outer half of the myometrium should be documented for adenosarcomas. Extrauterine involvement, such as the adnexa, bladder, rectum, other pelvic tissues, and abdominal tissues should be reported. For ESS, the size of the tumor should be reported, as the cutoff point of >5 cm changes the stage from T1a to T1b.

Suggested Report Format

1. Primary tumor
   a. Size
2. Local extent
   a. Involvement of <50% or ≥50% of the myometrium (for adenosarcoma)
   b. Involvement of the vagina and cervix
   c. Extraserosal extension
   d. Involvement of the ovaries and adjacent organs
3. Regional and distant lymph node involvement and extrapelvic disease

Pathological Classification

As with endometrial carcinoma, FIGO uses surgical/pathological staging for uterine sarcoma. Stage should be assigned at the time of definitive surgical treatment. The stage should not be changed on the basis of disease progression or recurrence.

As in endometrial carcinoma, for TNM staging, cases with fewer than six resected nodes should be classified using the TNM pathological classification based on the status of those nodes (e.g., pN0; pN1) according to the general rules of TNM. The number of resected and positive nodes should be recorded (note that FIGO classifies cases with fewer than six nodes resected as pNX).

The pT, pN, and c/pM categories correspond to the T, N, and M categories and are used to designate cases in which adequate pathological specimens are available for accurate stage groupings. When there are insufficient surgical/pathological findings, the clinical cT, cN, and c/pM categories should be used on the basis of clinical evaluation.

PROGNOSTIC FACTORS

Prognostic Factors Required for Stage Grouping

Beyond the factors used to assign T, N, or M categories, no additional prognostic factors are required for stage grouping., there are no additional prognostic factors required for staging.

Additional Factors Recommended for Clinical Care

Sarcomatous Overgrowth (Adenosarcoma Only)

The presence of sarcomatous overgrowth in adenosarcoma portends a worse prognosis. In one single-institution experience with 100 patients, the presence of sarcomatous overgrowth was associated with significantly worse progression-free and overall survival, even among those with Stage I disease.[21] AJCC Level of Evidence: I

Lymphovascular Space Involvement

The presence or absence of lymphovascular space involvement of the myometrium is important in most, but not all, series. If present, lymphovascular space involvement increases the probability of metastatic involvement of the regional lymph nodes. The presence or absence of lymphovascular space involvement should be recorded in the pathology report. AJCC Level of Evidence: I

RISK ASSESSMENT MODELS

The AJCC recently established guidelines that will be used to evaluate published statistical prediction models for the purpose of granting endorsement for clinical use.[22] Although this is a monumental step toward the goal of precision medicine, this work was published only very recently. Therefore, the

existing models that have been published or may be in clinical use have not yet been evaluated for this cancer site by the Precision Medicine Core of the AJCC. In the future, the statistical prediction models for this cancer site will be evaluated, and those that meet all AJCC criteria will be endorsed.

DEFINITIONS OF AJCC TNM

Definition of Primary Tumor (T)

Leiomyosarcoma and Endometrial Stromal Sarcoma

T Category	FIGO Stage	T Criteria
TX		Primary tumor cannot be assessed
T0		No evidence of primary tumor
T1	I	Tumor limited to the uterus
T1a	IA	Tumor 5 cm or less in greatest dimension
T1b	IB	Tumor more than 5 cm
T2	II	Tumor extends beyond the uterus, within the pelvis
T2a	IIA	Tumor involves adnexa
T2b	IIB	Tumor involves other pelvic tissues
T3	III	Tumor infiltrates abdominal tissues
T3a	IIIA	One site
T3b	IIIB	More than one site
T4	IVA	Tumor invades bladder or rectum

Definition of Primary Tumor (T)

Adenosarcoma

T Category	FIGO Stage	T Criteria
TX		Primary tumor cannot be assessed
T0		No evidence of primary tumor
T1	I	Tumor limited to the uterus
T1a	IA	Tumor limited to the endometrium/endocervix
T1b	IB	Tumor invades to less than half of the myometrium
T1c	IC	Tumor invades more than half of the myometrium
T2	II	Tumor extends beyond the uterus, within the pelvis
T2a	IIA	Tumor involves adnexa
T2b	IIB	Tumor involves other pelvic tissues
T3	III	Tumor infiltrates abdominal tissues
T3a	IIIA	One site
T3b	IIIB	More than one site
T4	IVA	Tumor invades bladder or rectum

Definition of Regional Lymph Node (N)

All Uterine Sarcomas

N Category	FIGO Stage	N Criteria
NX		Regional lymph nodes cannot be assessed
N0		No regional lymph node metastasis
N0(i+)		Isolated tumor cells in regional lymph node(s) no greater than 0.2 mm
N1	IIIC	Regional lymph node metastasis

Definition of Distant Metastasis (M)

All Uterine Sarcomas

M Category	FIGO Stage	M Criteria
M0		No distant metastasis
M1	IVB	Distant metastasis (excluding adnexa, pelvic, and abdominal tissues)

AJCC PROGNOSTIC STAGE GROUPS

Leiomyosarcoma and Endometrial Stromal Sarcoma

When T is...	And N is...	And M is...	Then the stage group is...
T1	N0	M0	I
T1a	N0	M0	IA
T1b	N0	M0	IB
T1c	N0	M0	IC
T2	N0	M0	II
T3a	N0	M0	IIIA
T3b	N0	M0	IIIB
T1-3	N1	M0	IIIC
T4	Any N	M0	IVA
Any T	Any N	M1	IVB

Adenosarcoma

When T is...	And N is...	And M is...	Then the stage group is...
T1	N0	M0	I
T1a	N0	M0	IA
T1b	N0	M0	IB
T2	N0	M0	II
T3a	N0	M0	IIIA
T3b	N0	M0	IIIB
T1–3	N1	M0	IIIC
T4	Any N	M0	IVA
Any T	Any N	M1	IVB

REGISTRY DATA COLLECTION VARIABLES

1. Lymphovascular space involvement
2. Pelvic nodal dissection, with number of nodes positive/examined
3. Para-aortic nodal dissection, with number of nodes positive/examined
4. Omentectomy performed
5. Morcellation performed
6. Cytogenetic analysis (ESS only)
7. Presence of sarcomatous overgrowth (adenosarcoma only)
8. Peritoneal washings, if recorded

HISTOLOGIC GRADE (G)

G	G Definition
GX	Grade cannot be assessed
G1	Well differentiated
G2	Moderately differentiated
G3	Poorly differentiated or undifferentiated

HISTOPATHOLOGIC TYPE

Leiomyosarcoma
  Epithelioid leiomyosarcoma
  Myxoid leiomyosarcoma
Endometrial stromal and related tumors
  Low-grade endometrial stromal sarcoma
  Low-grade endometrial stromal sarcoma with:
    Smooth muscle differentiation
    Sex cord elements
    Glandular elements
  High-grade endometrial stromal sarcoma
  Undifferentiated uterine/endometrial sarcoma
Adenosarcoma
Adenosarcoma with:
  Rhabdomyoblastic differentiation
  Cartilagenous differentiation
  Osseous differentiation
  Other heterologous element (specify)
Adenosarcoma with sarcomatous overgrowth

SURVIVAL DATA

Observed survival difference for patients with stage II uterine leiomyosarcoma and endometrial stromal sarcoma after using the current (2008) FIGO staging system to retrospectively restage 83 patients who were originally staged with the 1988 FIGO staging system. Applying the 2008 system increased the number of patients with stage II disease from 0 to 12.5 % (Fig. 54.3).[23]

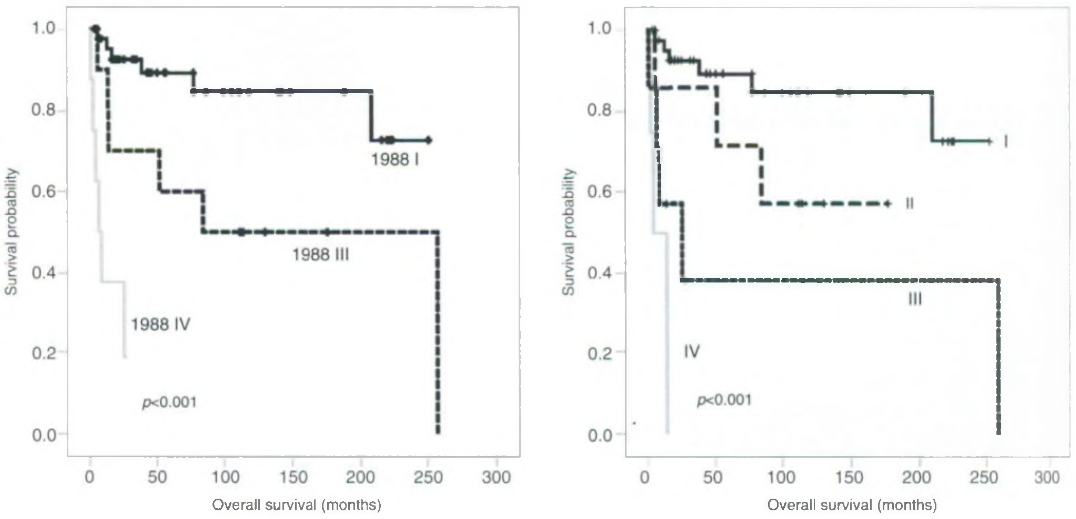

Fig. 54.3 Survival curves by stage (From Yim et al.,[23] with permission.)

ILLUSTRATIONS

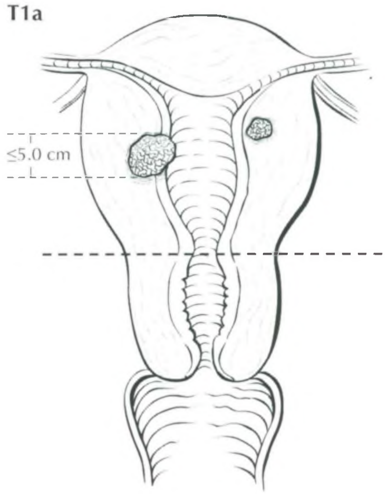

Fig. 54.4 T1 for leiomyosarcoma and endometrial stromal sarcoma is tumor limited to the uterus. T1a is tumor 5 cm or less in greatest dimension

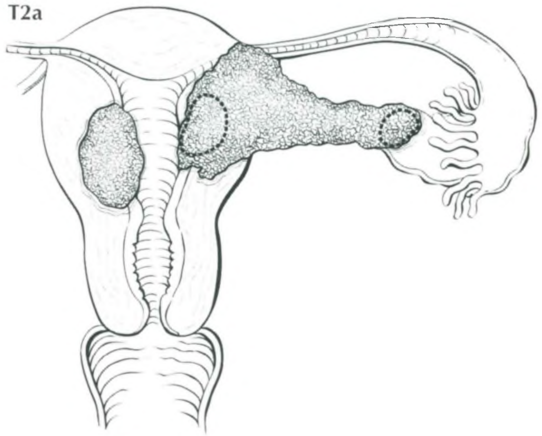

Fig. 54.6 T2 for leiomyosarcoma, endometrial stromal sarcoma, and adenosarcoma is tumor extending beyond the uterus, within the pelvis. T2a for sarcomas is tumor involving adnexa

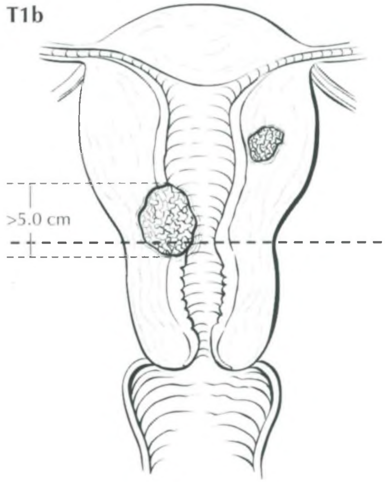

Fig. 54.5 T1 for leiomyosarcoma and endometrial stromal sarcoma is tumor limited to the uterus. T1b is tumor more than 5 cm

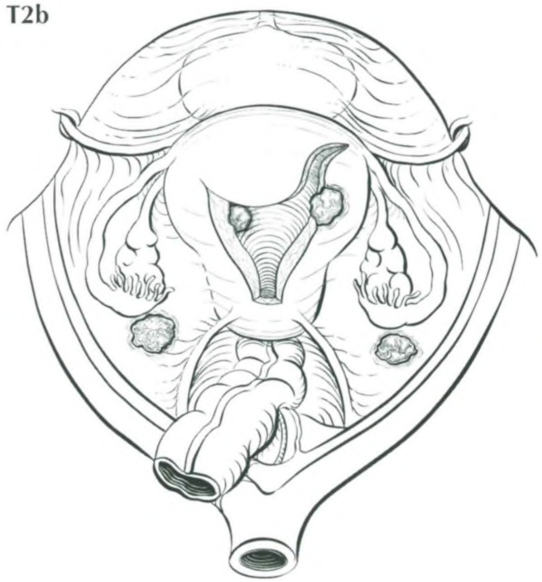

Fig. 54.7 T2b for leiomyosarcoma, endometrial stromal sarcoma, and adenosarcoma is tumor involving other pelvic tissues

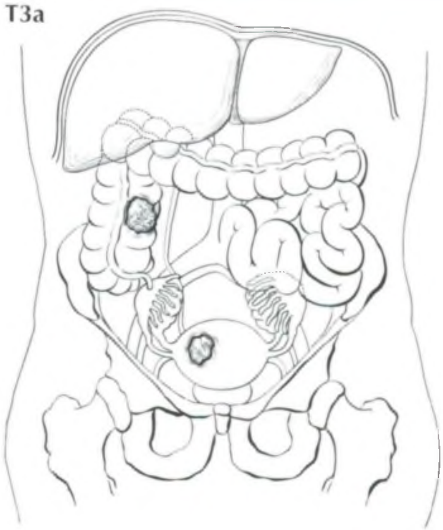

Fig. 54.8 T3 for leiomyosarcoma, endometrial stromal sarcoma, and adenosarcoma is tumor infiltrating abdominal tissues. T3a for sarcomas is one site

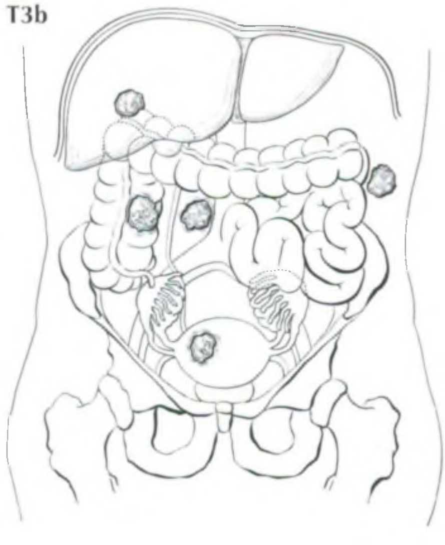

Fig. 54.9 T3 for leiomyosarcoma, endometrial stromal sarcoma, and adenosarcoma is tumor infiltrating abdominal tissues. T3b for sarcomas is more than one site

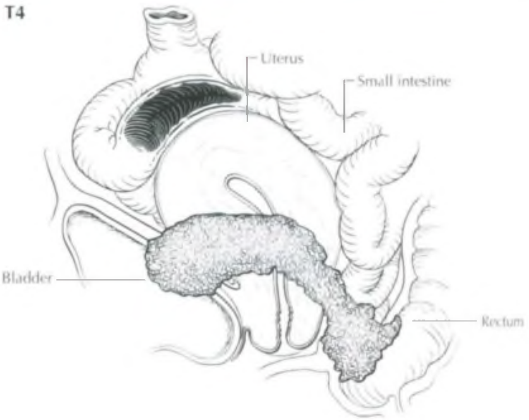

Fig. 54.10 T4 for leiomyosarcoma, endometrial stromal sarcoma, and adenosarcoma is tumor invades bladder or rectum

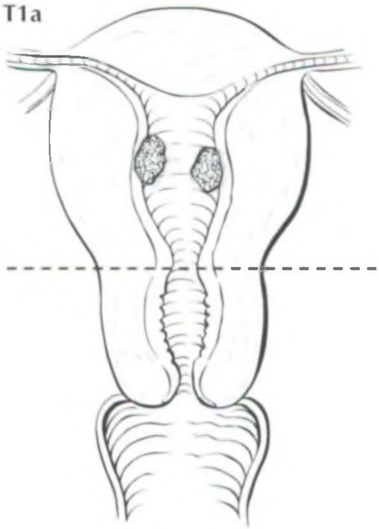

Fig. 54.11 T1 for adenosarcoma is tumor limited to the uterus. T1a is tumor limited to the endometrium/endocervix

18. Leitao MM, Sonoda Y, Brennan MF, Barakat RR, Chi DS. Incidence of lymph node and ovarian metastases in leiomyosarcoma of the uterus. *Gynecologic oncology*. Oct 2003;91(1):209–212.
19. Elshaikh MA, Yashar CM, Wolfson AH, et al. ACR Appropriateness Criteria® Advanced Stage Endometrial Cancer. *American journal of clinical oncology*. 2014;37(4):391–396.
20. Lalwani N, Dubinsky T, Javitt MC, et al. ACR Appropriateness Criteria® Pretreatment Evaluation and Follow-Up of Endometrial Cancer. *Ultrasound quarterly*. 2014;30(1):21–28.
21. Carroll A, Ramirez PT, Westin SN, et al. Uterine adenosarcoma: an analysis on management, outcomes, and risk factors for recurrence. *Gynecologic oncology*. Dec 2014;135(3):455–461.
22. Kattan MW, Hess KR, Amin MB, et al. American Joint Committee on Cancer acceptance criteria for inclusion of risk models for individualized prognosis in the practice of precision medicine. *CA: a cancer journal for clinicians*. Jan 19 2016.
23. Yim GW, Nam EJ, Kim SW, Kim YT. FIGO staging for uterine sarcomas: can the revised 2008 staging system predict survival outcome better? *Yonsei medical journal*. May 2014;55(3):563–569.
24. Corrigendum to "FIGO staging for uterine sarcomas". *International Journal of Gynecology & Obstetrics*. 2009;106:277.
25. Gallardo A, Prat J. Mullerian adenosarcoma: a clinicopathologic and immunohistochemical study of 55 cases challenging the existence of adenofibroma. *The American journal of surgical pathology*. Feb 2009;33(2):278–288.
26. D'Angelo E, Prat J. Uterine sarcomas: a review. *Gynecologic oncology*. Jan 2010;116(1):131–139.

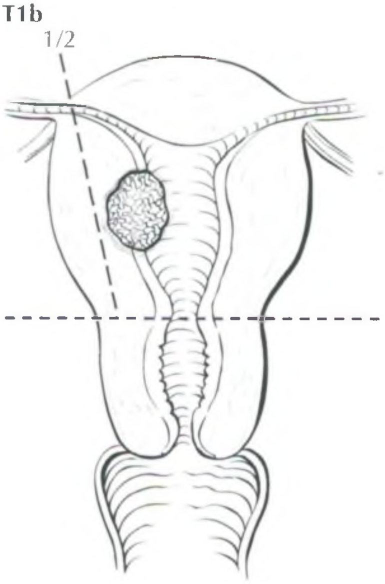

Fig. 54.12 T1 for adenosarcoma is tumor limited to the uterus. T1b is tumor invading less than half of the myometrium

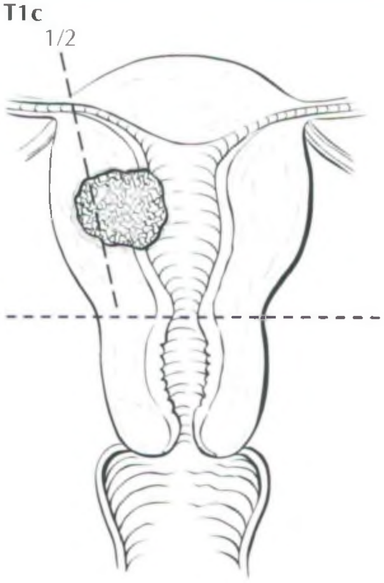

Fig. 54.13 T1 for adenosarcoma is tumor limited to the uterus. T1c is tumor invading more than half of the myometrium

Bibliography

1. Ali RH, Rouzbahman M. Endometrial stromal tumours revisited: an update based on the 2014 WHO classification. *Journal of clinical pathology*. May 2015;68(5):325–332.
2. Shi Y, Liu Z, Peng Z, Liu H, Yang K, Yao X. The diagnosis and treatment of Mullerian adenosarcoma of the uterus. *The Australian & New Zealand journal of obstetrics & gynaecology*. Dec 2008;48(6):596–600.
3. Bodner K, Laubichler P, Kimberger O, Czerwenka K, Zeillinger R, Bodner-Adler B. Oestrogen and progesterone receptor expression in patients with adenocarcinoma of the uterine cervix and correlation with various clinicopathological parameters. *Anticancer research*. 2010;30(4):1341–1345.
4. Lee CH, Turbin DA, Sung YC, et al. A panel of antibodies to determine site of origin and malignancy in smooth muscle tumors. *Modern pathology : an official journal of the United States and Canadian Academy of Pathology, Inc*. Dec 2009;22(12):1519–1531.
5. Kefeli M, Yildiz L, Kaya FC, Aydin O, Kandemir B. Fascin expression in uterine smooth muscle tumors. *Int J Gynecol Pathol*. Jul 2009;28(4):328–333.
6. Chiang S, Oliva E. Recent developments in uterine mesenchymal neoplasms. *Histopathology*. Jan 2013;62(1):124–137.
7. Rush DS, Tan J-y, Baergen RN, Soslow RA. h-Caldesmon, a novel smooth muscle-specific antibody, distinguishes between cellular leiomyoma and endometrial stromal sarcoma. *The American journal of surgical pathology*. 2001;25(2):253–258.
8. Manoharan M, Azmi MA, Soosay G, Mould T, Weekes AR. Mullerian adenosarcoma of uterine cervix: report of three cases and review of literature. *Gynecologic oncology*. Apr 2007;105(1):256–260.
9. Taçkın S, Bozacı EA, Sönmezer M, Ekinci C, Ortaç F. Late recurrence of uterine Mullerian adenosarcoma as heterologous sarcoma: Three recurrences in 8 months increasing in number and grade of sarcomatous components. *Gynecologic oncology*. 2006;101(1):179–182.
10. Soslow RA, Ali A, Oliva E. Mullerian adenosarcomas: an immunophenotypic analysis of 35 cases. *The American journal of surgical pathology*. Jul 2008;32(7):1013–1021.
11. Koontz JI, Soreng AL, Nucci M, et al. Frequent fusion of the JAZF1 and JJAZ1 genes in endometrial stromal tumors. *Proc Natl Acad Sci U S A*. May 22 2001;98(11):6348–6353.
12. Chiang S, Ali R, Melnyk N, et al. Frequency of known gene rearrangements in endometrial stromal tumors. *The American journal of surgical pathology*. Sep 2011;35(9):1364–1372.
13. Lee CH, Marino-Enriquez A, Ou W, et al. The clinicopathologic features of YWHAE-FAM22 endometrial stromal sarcomas: a histologically high-grade and clinically aggressive tumor. *The American journal of surgical pathology*. May 2012;36(5):641–653.
14. Rose PG, Piver MS, Tsukada Y, Lau T. Patterns of metastasis in uterine sarcoma. An autopsy study. *Cancer*. Mar 1 1989;63(5):935–938.
15. Goff BA, Rice LW, Fleischhacker D, et al. Uterine leiomyosarcoma and endometrial stromal sarcoma: lymph node metastases and sites of recurrence. *Gynecologic oncology*. Jul 1993;50(1):105–109.
16. Tirumani SH, Deaver P, Shinagare AB, et al. Metastatic pattern of uterine leiomyosarcoma: retrospective analysis of the predictors and outcome in 113 patients. *J Gynecol Oncol*. Oct 2014;25(4):306–312.
17. Dos Santos LA, Garg K, Diaz JP, et al. Incidence of lymph node and adnexal metastasis in endometrial stromal sarcoma. *Gynecologic oncology*. May 1 2011;121(2):319–322.

Ovary, Fallopian Tube, and Primary Peritoneal Carcinoma

55

Jaime Prat, Alexander B. Olawaiye, Adriana Bermudez, Lee-may Chen, Larry J. Copeland, Randall K. Gibb, Matthew A. Powell, and David G. Mutch

CHAPTER SUMMARY

Cancers Staged Using This Staging System

Malignant tumors arising in the ovary, fallopian tube, and primary peritoneum

Summary of Changes

Change	Details of Change	Level of Evidence
Cancers staged using this staging system	Fallopian tube carcinoma now shares the same staging system as ovary and primary peritoneal carcinoma.	N/A
AJCC Prognostic Stage Groups	Stage I: intraoperative rupture ("surgical spill"; Stage IC1) is separated from capsule ruptured before surgery (Stage IC2). Positive washings in the presence or absence of capsule rupture are considered indicative of Stage IC3.	II
AJCC Prognostic Stage Groups	Stage II: Tumors confined to the pelvis are substaged as Stage IIA (extension to and/or implants on the uterus and/or fallopian tubes and/or ovaries) or IIB (extension to other pelvic intraperitoneal tissues). Former substage IIC (i.e., IIA or IIB but with tumor on surface, capsule ruptured, or ascites or positive peritoneal washing) was thought to be redundant and therefore was eliminated.	III
AJCC Prognostic Stage Groups	Stage III: Spread to the retroperitoneal (pelvic and/or para-aortic) lymph nodes without extrapelvic peritoneal dissemination is Stage IIIA1, whereas microscopic extrapelvic (above the pelvic brim) peritoneal involvement with or without positive retroperitoneal lymph nodes is Stage IIIA2. In Stage IIIC, there is macroscopic peritoneal metastasis beyond the pelvis,>2 cm in size, with or without metastasis to the retroperitoneal lymph nodes. Tumor extension or metastasis to the liver and/or splenic capsule without parenchymal involvement is still Stage IIIC.	I
AJCC Prognostic Stage Groups	Stage III: IIIA1 is subdivided intoIIIA1(i)-metastasis up to 5 mm in greatest dimension-and IIIA1(ii)-metastasis more than 5 mm in greatest dimension	III
AJCC Prognostic Stage Groups	Stage IV: Parenchymal liver or splenic involvement by tumor extension or isolated metastasis is now Stage IVB and should be identified and distinguished from splenic or liver capsular involvement only. Status of splenic involvement as defined here is new with this edition. Transmural intestinal involvement is now Stage IVB.	I

ICD-O-3 Topography Codes

Code	Description
C56.9	Ovary
C57.0	Fallopian tube
C48.1	Specified parts of peritoneum (female only)
C48.2	Peritoneum (female only)
C48.8	Overlapping lesion of retroperitoneum and peritoneum (female only)

WHO Classification of Tumors

Code	Description
8020	Undifferentiated carcinoma
8041	Small cell carcinoma, pulmonary type
8044	Small cell carcinoma, hypercalcemic type
8070	Squamous cell carcinoma
8120	Transitional cell carcinoma
8140	Adenocarcinoma

To access the AJCC cancer staging forms, please visit www.cancerstaging.org.

© American Joint Committee on Cancer 2017
M.B. Amin et al. (eds.), *AJCC Cancer Staging Manual, Eighth Edition*, DOI 10.1007/978-3-319-40618-3_55

Code	Description
8240	Carcinoid
8243	Mucinous carcinoid
8260	Adult granulosa cell tumor
8310	Clear cell carcinoma
8313	Clear cell borderline tumor
8380	Endometrioid borderline tumor
8380	Endometrioid carcinoma
8410	Sebaceous carcinoma
8441	Serous tubal intraepithelial carcinoma
8442	Serous borderline tumor
8460	Low-grade serous carcinoma
8461	High-grade serous carcinoma
8472	Mucinous borderline tumor
8474	Seromucinous borderline tumor
8474	Seromucinous carcinoma
8480	Mucinous carcinoma
8542	Solid pseudopapillary neoplasm
8590	Sex cord-stromal tumor, NOS
8594	Mixed germ cell sex cord-stromal tumor, unclassified
8622	Juvenile granulosa cell tumor
8623	Sex cord tumor with annular tubules
8631	Sertoli-Leydig cell tumor, well differentiated
8631	Sertoli-Leydig cell tumor, moderately differentiated
8631	Sertoli-Leydig cell tumor, poorly differentiated
8633	Sertoli-Leydig cell tumor, retiform
8634	Sertoli-Leydig cell tumor, moderately differentiated with heterologous elements
8634	Sertoli-Leydig cell tumor, poorly differentiated with heterologous elements
8634	Sertoli-Leydig cell tumor, retiform with heterologous elements
8640	Sertoli cell tumor
8670	Steroid cell tumor, malignant
8806	Desmoplastic small round cell tumor
8810	Fibrosarcoma
8815	Solitary fibrous tumor
8822	Pelvic fibromatosis
8825	Inflammatory myofibroblastic tumor
8890	Leiomyomatosis peritonealis disseminata
8930	High-grade endometrioid stromal sarcoma
8931	Low-grade endometrioid stromal sarcoma
8933	Adenosarcoma
8960	Paraganglioma
8963	Extra-gastrointestinal stromal tumor
8980	Carcinosarcoma
9000	Borderline Brenner tumor
9000	Malignant Brenner tumor
9050	Mesothelioma
9052	Well-differentiated papillary mesothelioma
9060	Dysgerminoma
9070	Embryonal carcinoma
9071	Yolk sac tumor
9073	Gonadoblastoma, including gonadoblastoma with malignant germ cell tumor
9080	Immature teratoma
9085	Mixed germ cell tumor
9090	Struma ovarii, malignant
9091	Strumal carcinoid
9100	Nongestational choriocarcinoma
9110	Adenocarcinoma of rete ovarii
9110	Wolffian tumor

Kurman RJ, Carcangiu ML, Herrington CS, Young RH, eds. World Health Organization Classification of Tumours of the Female Reproductive System. Lyon: IARC; 2014.

INTRODUCTION

Ovarian cancer represents a heterogeneous group of distinct diseases. Approximately 90% of ovarian cancers are carcinomas (malignant epithelial tumors). Much less common are malignant germ cell tumors (3%) and potentially malignant sex cord-stromal tumors (1-2%). The most common type of epithelial ovarian cancer, high-grade serous carcinoma (HGSC), rarely may present as primary fallopian tube cancer or primary peritoneal cancer. Clinically, however, these three cancers are treated similarly, and a single staging system recently was proposed by the Fédération Internationale de Gynécologie et d'Obstétrique (FIGO). Whereas some HGSCs, mainly (breast cancer susceptibility gene-positive [BRCA+] cases, seem to originate in the fimbriated end of the fallopian tube, other cases most likely arise from embryonic progenitors in the peritoneum or the ovarian surface epithelium. High-grade serous tubal intraepithelial carcinoma (STIC) can metastasize and, therefore, cannot be considered carcinoma in situ. The staging of cancer of the ovary, fallopian tube, and peritoneum in the AJCC Cancer Staging Manual, 8th Edition mirrors that of FIGO: for Stage I tumors, intraoperative rupture ("surgical spill") is Stage IC1, capsule ruptured before surgery or tumor on ovarian or fallopian tube surface is Stage IC2, and positive peritoneal cytology with or without rupture is Stage IC3. The new staging preserves the separation between pelvic (Stage II) and extrapelvic (Stage III) spread but includes a revision of Stage III tumors; assignment to Stage IIIA1 is based on spread to the retroperitoneal (pelvic and/or para-aortic) lymph nodes without extrapelvic peritoneal dissemination (formerly classified as Stage IIIC). Extension and/or metastasis of

tumor to the liver or splenic parenchyma qualifies as Stage IVB and should be distinguished from splenic or liver capsular involvement only (Stage IIIC).

Ovarian cancer is the fifth most common cancer diagnosis among women in higher-resource regions.[1] Primary peritoneal cancer and primary fallopian tube cancer are rare malignancies, usually HGSCs—that is, identical to the most common histotype of ovarian cancer and the prototype tumor occurring in women with BRCA1 or BRCA2 germline mutations. Clinically, these three cancers are treated similarly,[2] and a single staging system has been adopted by FIGO.[3]

During the past 30 years, it has been recognized that ovarian cancer is not a homogeneous disease but rather a group of diseases—each with different morphology and biological behavior. Approximately 90% of ovarian cancers are carcinomas (malignant epithelial tumors), and based on histopathology, immunohistochemistry, and molecular genetic analysis, at least five main types are distinguished: HGSC (70%), endometrioid carcinoma (EC; 10%), clear cell carcinoma (CCC; 10%), mucinous carcinoma (MC; 3%), and low-grade serous carcinoma (LGSC; <5%). These tumor types, which account for 98% of ovarian carcinomas, can be reproducibly diagnosed by light microscopy and are inherently different diseases, as indicated by differences in epidemiologic and genetic risk factors, precursor lesions, patterns of spread, molecular genetic abnormalities, response to chemotherapy, and prognosis.[4-6] The vast majority of borderline tumors (formerly referred to as "tumors of low malignant potential") rarely recur or metastasize; however, up to 10% may recur. At the time of recurrence, some have the features of carcinoma, particularly of the serous and mucinous types. Much less common are malignant germ cell tumors (dysgerminomas, yolk sac tumors, and immature teratomas [3% of ovarian cancers] and potentially malignant sex cord–stromal tumors [1–2%, mainly granulosa cell tumors]). Ovarian cancers differ primarily based on histologic type.

Reproducible histopathologic diagnosis of tumor cell type is required for successful treatment. Different tumor histotypes respond differently to chemotherapy. Even if different patterns of dissemination would justify the use of separate staging systems for each type of ovarian carcinoma, such a complex classification would not be practical. For the sake of simplicity, a flexible staging system that takes into account the most relevant prognostic parameters shared by all tumor types should be used. Histologic type should be designated at staging (i.e., HGSC, EC, CCC, MC, LGSC, and borderline tumors; other or cannot be classified; and malignant germ cell tumors and potentially malignant sex cord–stromal tumors).

Patients with BRCA mutation (breast–ovarian cancer syndrome) undergoing risk-reducing salpingo-oophorectomy (RRSO) are found to have high-grade STIC, particularly in the fimbria.[7] Although STIC can metastasize and therefore cannot be considered carcinoma in situ, compelling evidence for a tubal origin of BRCA+ HGSC has accumulated during the past decade.[8,9] High-grade STIC also has been found in an undetermined number of advanced-stage sporadic HGSCs associated with ovarian tumor masses and in rare cases of primary tubal or peritoneal HGSCs without obvious ovarian involvement. The relative proportion of HGSCs of ovarian and tubal derivation is unknown, probably because in advanced-stage cancers, tumor growth conceals the primary site. Even in cases involving BRCA mutation, evidence of a tubal origin of HGSCs is incomplete and a multicentric origin of these tumors cannot be excluded.

Whereas asymptomatic BRCA+ women undergoing RRSO are found to have STIC in 6% of cases, symptomatic, rapidly progressive BRCA+ tumors discovered at advanced stage in younger patients are less likely to be associated with STIC. This paradox questions the effectiveness of salpingectomy alone in preventing HGSC in BRCA+ women.[10]

The aforementioned findings suggest that the fallopian tube is linked to only some HGSCs and that the remaining cases originate from the nearby peritoneum/ovarian surface epithelium. Recently, it was hypothesized that cytokeratin 7–positive embryonic/stem cells may be capable of Müllerian differentiation in cortical epithelial inclusion cysts resulting from ovarian surface epithelium (mesothelium) invaginations. Thus, embryonic progenitors may give rise to immunophenotypically distinct neoplastic progeny,[11] which would support the old concept of "Müllerian neometaplasia."

HGSCs and LGSCs are fundamentally different tumor types and, consequently, different diseases. HGSCs arethe most common ovarian carcinomas, and most patients (approximately 80%) present with advanced-stage disease; tumors confined to the ovary at diagnosis are distinctly uncommon (<10%). By contrast, LGSCs are much less common, usually contain a serous borderline component, and carry KRAS and BRAF mutations.[12,13] HGSCs are not associated with serous borderline tumors and typically exhibit TP53 mutations and BRCA abnormalities, resulting in chromosomal instability and widespread DNA copy number changes. This highly aberrant genome is the hallmark of HGSC and allows further evolution into different molecular subtypes associated with clinical outcome.

A putative tubal or peritoneal origin applies exclusively to HGSCs and not to the vast majority of ECs and CCCs, which

are thought to arise in the ovary from endometriosis. Although a significant number of HGSCs might not arise from the ovary, and the term ovarian cancer may not be pathogenically precise in every case, ovarian involvement is the rule in almost all cases. In view of the rarity of HGSCs associated with tubal tumor masses, it is unlikely that all HGSCs originate in the fallopian tube. The term HGSC of ovary should be kept until the different origins of ovarian tumors are better understood. Terms such as Müllerian or pelvic serous carcinoma are not recommended because they create confusion for patients, physicians, and medical investigators.[14]

ANATOMY

Primary Site(s)

The ovaries are a pair of solid, flattened ovoids 2 to 4 cm in diameter that are connected by a peritoneal fold to the broad ligament and by the infundibulopelvic ligament to the lateral wall of the pelvis. They are attachedmedially to the uterus by the utero-ovarian ligament (Fig. 55.1).

The fallopian tube extends from the posterior superior aspect of the uterine fundus laterally and anterior to the ovary. Its length is approximately 10 cm. The medial end arises in the cornual portion of the uterine cavity,and the lateral(fimbrial) end opens to the peritoneal cavity.

The peritoneum is the serous membrane of the abdominal cavity that lines the walls of the abdomen (parietal peritoneum) and covers the abdominal organs (visceral peritoneum). The pelvic peritoneum covers the fundus of the urinary bladder and the front of the rectum. In females, it lines the anterior and posterior surface of the uterus and the upper posterior vagina. There are two potential spaces posterior to the bladder (the uterovesical pouch) and posteriorto the uterus (the rectouterine pouch of Douglas).

On the anterior and posterior surfaces of the uterus, the peritoneum is reflected laterally to the pelvic sidewalls as the broad ligaments, containing the fallopian tubes.

Regional Lymph Nodes

The lymphatic drainage occurs by the infundibulopelvic and round ligament trunks and an external iliac accessory route into the following regional nodes (Fig. 55.2):

- External iliac
- Internal iliac (hypogastric)

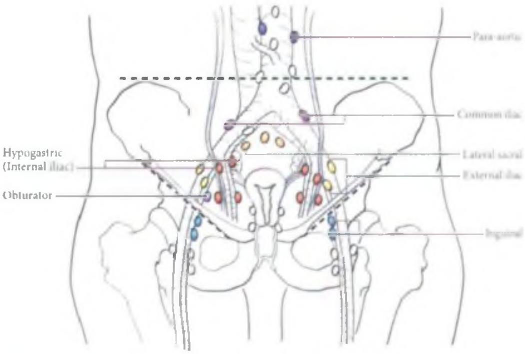

Fig. 55.2 Regional lymph nodes of ovary, fallopian tube and primary peritoneal carcinomas.

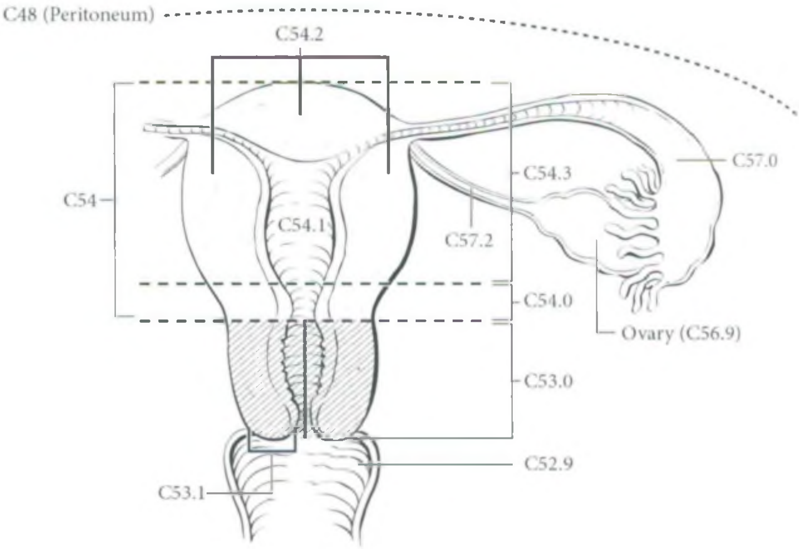

Fig. 55.1 Anatomic sites of the ovary (C56.9), fallopian tube (C57.0) and primary peritoneum (C48).

- Obturator
- Common iliac
- Para-aortic
- Pelvic, NOS
- Retroperitoneal, NOS

Regional Lymph Nodes

The peritoneum, including the omentum and the pelvic and abdominal visceral and parietal peritoneum, comprises common sites for seeding. Diaphragmatic and liver surface involvement also is common. However, to be consistent with FIGO staging, these implants within the abdominal cavity (T3) are not considered distant metastases. Extraperitoneal sites, including parenchymal liver, lung, spleen, and skeletal metastases, and inguinal, supraclavicular, and axillarynodes, are M1.

RULES FOR CLASSIFICATION

Clinical Classification

Ovarian, fallopian tube, and peritoneal cancer is surgically and pathologically staged. There should be histologic confirmation of the ovarian, fallopian tube, and peritoneal disease. Laparotomy or operative laparoscopy with resection of the ovarian mass, as well as hysterectomy, forms the basis for staging. Biopsies of all frequently involved sites, such as the omentum, mesentery, diaphragm, peritoneal surfaces, pelvic nodes, and para-aortic nodes, are required for ideal staging of early disease. For example, to stage a patient confidently as Stage IA (T1 N0 M0), negative biopsies of all of the aforementioned sites should be obtained to exclude microscopic metastases. Assignment to Stage IIIA1 is based on spread to the retroperitoneal lymph nodes without intraperitoneal dissemination, because an analysis of these patients indicates that their survival is significantly better than that of patients with intraperitoneal dissemination.[3,15-19] On the other hand, a single biopsy from an omental mass 2 cm or greater showing metastatic carcinoma is adequate to classify a patient as Stage IIIC, thus making other biopsies unnecessary from a staging standpoint. The final histologic and cytologic findings after surgery are to be considered in the staging. Operative findings before tumor debulking determine stage, which may be modified by histopathologic as well as clinical or radiologic evaluation (e.g., palpable supraclavicular node or pulmonary metastases on chest X-ray).

Although clinical assessments similar to those for other sites may be performed, surgical–pathological evaluation of the abdomen and pelvis is necessary to establish a definitive diagnosis of ovarian/fallopian tube/peritoneal cancer and to rule out other primary malignancies that may present with similar preoperative findings (e.g., bowel, uterine, and pancreatic cancers or, occasionally, lymphoma). Although laparotomy is the most widely accepted procedure for surgical–pathological staging, occasionally laparoscopy may be used. Occasionally, patients with advanced disease and/or women who are medically unsuitable candidates for surgery may be presumed to have ovarian cancer on the basis of cytology of ascites or pleural effusion showing typical carcinoma, combined with imaging studies demonstrating enlarged ovaries/fallopian tubes, and/or peritoneal involvement. Such patients usually are considered unstaged (TX), although positive cytology of a pleural effusion or supraclavicular lymph node occasionally allows designation of M1 or FIGO Stage IV disease. The presence of ascites does not affect staging unless malignant cells are present.

Imaging studies often are done in conjunction with definitive abdominal–pelvic surgery, and chest X-ray, bone scans, computed tomography (CT), or positron emission tomography (PET) may identify lung, bone, or brain metastases that should be considered in the final stage. Pleural effusions should be evaluated with cytology. In the future, pretreatment imaging will be more relevant to staging because of the increasing use of neoadjuvant chemotherapy in many women diagnosed with ovarian cancer.[20]

As with all gynecologic cancers, the final stage should be established at the time of initial treatment. It should not be modified or changed on the basis of subsequent findings.

Findings related to procedures such as laparoscopy or laparotomy after initial chemotherapy do not change the patient's original stage.

Imaging

CT currently is the preferred modality for the staging of ovarian cancer. Magnetic resonance (MR) imaging is excellent for characterizing adnexal masses. PET/CT is useful in evaluating distant disease but not for diagnosingprimary ovarian cancer.

TNM Components of Tumor Staging

In clinical category T1, disease is limited to the ovaries; either MR imaging or ultrasound may be helpful in diagnosing the malignant adnexal mass. Contrast-enhanced CT is useful in assessing peritoneal disease. If the disease is confined to the pelvis, it is clinical category T2. Category T3a/b includes retroperitoneal lymph node metastases. PET/CT has been advocated to assess for lymph node metastases, as it has better specificity than contrast-enhanced CT or MR imaging. Category T3c includes surface involvement of the liver and spleen without any parenchymal metastases, which can be assessed with contrast-enhanced CT. PET/CT, if available, may be used as a single modality to assess both peritoneal disease and lung parenchymal disease in patients with advanced cancer.

The imaging criteria used to assess lymph node metastases are based on node size, with abnormal being >1 cm in the short axial dimension on cross-sectional scans. CT and MR imaging are shown to perform equally well in assessing adenopathy. However, because there may be false positive causes of enlarged nodes from benign disease, PET/CT is considered superior for assessing lymph node metastases. Metabolically active lymph nodes of any size on PET/CT are considered metastatic.

Suggested Imaging Report Format

1. Primary tumor
   a. One or both ovaries
2. Local extent
   a. Ascites
   b. Indicate whether localized to the pelvis or whether extrapelvic disease is present
   c. Retroperitoneal adenopathy
   d. Liver or splenic surface disease or lung parenchymal disease
   e. Pleural effusion

Pathological Classification

Surgery and biopsy of all suspected sites of involvement provide the basis for staging. Histologic and cytologic data are required. This is the preferred method of staging for ovarian cancer. The operative notes and/or the pathology report should describe the location and size of metastatic lesions and the primary tumors for optimal staging. In addition, the size of the tumor outside the pelvis must be determined, and noted and documented in the operative report. Size is reported in centimeters and represents the largest implant, regardless of whether it was resected during surgical exploration.

Carcinoma of the fallopian tube almost always is HGSC, which may be accompanied by STIC. The tumor invades locally into the muscular wall of the tube and then into the peritubal soft tissue or adjacent organs, such as the uterus or ovary, or through the serosa of the tube into the peritoneal cavity. Metastatic tumor implants may be found throughout the peritoneal cavity. The tumor may obstruct the tubal lumen and present as a ruptured or unruptured hydrosalpinx or hematosalpinx. It has been suggested that carcinomas in the fimbriated end without invasion have a worse prognosisthan those invading the wall of the tube because of direct access to the peritoneal cavity.[15]

Examination of prophylactic salpingo-oophorectomy specimens from BRCA+ patients has shown thatmost early carcinomas detected in these samples occur in the tubal fimbria, and some of them are still confined to the mucosa in the form of STIC.[16,17] To detect these early carcinomas, serial longitudinal sections of the fallopian tube fimbria at 2- to 3-mm intervals should be obtained to examine most of the plicae surface.

Advanced invasive HGSC associated with STIC may be ovarian or tubal in origin without clinical relevance. For tumors limited to one ovary associated with STIC, there are three possibilities: a) STIC extending to one ovary, b) ovarian HGSC extending to the fallopian tube, and c) synchronous or metachronous tumor involving the ovary and fallopian tube. With regard to staging, these tumors are considered Stage IA ovarian carcinoma with STIC unless there is evidence of direct extension from STIC to the ovary, in which case they would be stage IIA fallopian tube carcinoma.

In some cases, an adenocarcinoma is primary in the peritoneum. The ovaries are not involved or are involved only with minimal surface implants. The clinical presentation, surgical therapy, chemotherapy, and prognosis of these peritoneal tumors mirror those of HGSC of the ovary. Patients who undergo prophylactic salpingo-oophorectomy for a familial history of ovarian cancer appear to retain a 1–2% chance of developing peritoneal adenocarcinoma,which is histopathologically and clinically similar to primary ovarian cancer. It is not possible to have Stage I peritoneal cancer.

Intranodal single tumor cells or small clusters of cells not more than 0.2 mm in greatest diameter are classified as isolated tumor cells. These may be detected by routine histology or by immunohistochemical methods. They are designated N0(i+).

For pN0, histologic examination should include both pelvic and para-aortic lymph nodes.

For patients receiving neoadjuvant therapy, it is important to record the clinical stage before treatment. Surgical staging after neoadjuvant therapy should be classified as "yp."

PROGNOSTIC FACTORS

Pathological Classification

Beyond the factors used to assign T, N, or M categories, no additional prognostic factors are required for stage grouping.

Additional Factors Recommended for Clinical Care

FIGO Stage

FIGO stage[3] is the strongest predictor of outcome in ovarian cancer. Whereas complete staging would be sufficient surgical treatment for tumors in Stages I and II, patients with advanced disease require cytoreductive surgery. Stage I ovarian cancer is confined to the ovaries, and fewer than 5% of HGSCs are Stage I tumors. Tumor rupture, ovarian surface involvement by tumor cells, or the presence of malignant cells in peritoneal washings or ascitic fluid warrants a Stage IC classification.

Fewer than 10% of HGSCs are found in Stage II, that is, extending or metastasizing to extraovarian/extratubal pelvic

organs or tissues. Stage II includes examples of direct extension to the tubes/ovaries and pelvic sidewall, as well as pelvic peritoneal metastases. Thus, it includes resectable and curable tumors that have extended to adjacent organs, as well as tumors that have seeded the pelvic peritoneum and are associated with a poor prognosis.

HGSC most commonly presents in Stage III, and the vast majority of these cases are Stage IIIC. These tumors typically spread along the abdominopelvic peritoneum, involving the omentum, serosa of the small and large bowel, mesentery, paracolic gutters, diaphragm, and peritoneal surfaces of the liver and spleen. Ascites is found in almost all cases, and positive lymph nodes are found in many patients who undergo node sampling or lymphadenectomy and in almost 80% of those with advanced-stage tumors.

The new FIGO staging system includes a revision of Stage III criteria. The designation of Stage IIIA1 is based on spread to the retroperitoneal lymph nodes without intraperitoneal dissemination, because an analysis of thesepatients indicates that their survival is significantly better than that of patients with intraperitoneal dissemination.[17-20,22] Nodal metastasis without peritoneal metastasis is relatively uncommon (about 9% of cases).[23] Most of these patients have positive para-aortic nodes. AJCC Level of Evidence: I

Histology and Grade

Histology and grade are important prognostic factors. Women with borderline tumors have an excellent prognosis, even when noninvasive extraovarian disease (noninvasive implants) is found. In patients with invasive ovarian cancer, low-grade tumors have a better prognosis than high-grade tumors, stage for stage. Histologic type, which includesthe histologic grade, also is extremely important. Some stromal tumors (granulosa and Sertoli–Leydig cell tumors) have an excellent prognosis, whereas malignant epithelial tumors in general have a less favorable outcome. For this reason, epithelial cell types generally are reported together and sex cord–stromal tumors and germ cell tumors are reported separately. Tumor cell type also helps guide the type of chemotherapy that is recommended. AJCC Level of Evidence: I

Residual Disease

In advanced disease, the most important prognostic factor is residual disease after initial surgical management. Even among patients with advanced-stage cancer, those with no gross residual disease after surgical debulking have a considerably better prognosis than those with minimal or extensive residual disease. Besides the size of the residual tumor, the number of sites of residual disease also appears to be important (tumor volume). AJCC Level of Evidence: I

Preoperative Cancer Antigen 125

The tumor marker cancer antigen 125 (CA-125) is useful for following the response to therapy in patients with epithelial ovarian cancer, who have elevated levels of this marker. The rate of regression during chemotherapy may have prognostic significance. Women with germ cell tumors also may have elevated serum tumor markers, namely α-fetoprotein or human chorionic gonadotropin. Other factors, such as growth factors and oncogene amplification, currently are under investigation. AJCC Level of Evidence: I

Gross Residual Tumor after Primary Cytoreductive Surgery

Gross residual tumor after primary cytoreductive surgery is a prognostic factor that has been demonstrated in several large studies. Whether patients undergo neoadjuvant chemotherapy or primary cytoreduction, the best prognostic category after surgery includes those who are left with no gross residual disease. Physicians should record the presence or absence of residual disease; if residual disease is observed, the size of the largest visible lesion should be documented. AJCC Level of Evidence: I

Residual Tumor Volume after Primary Cytoreductive Surgery

Although volume of residual disease is an important prognostic factor in most studies, it applies only to Stages IIIC and IV. The parameter that defines optimal cytoreduction is residual disease less than 1 cm. Physicians should record no gross tumor, tumor ≤1 cm, or tumor >1 cm. AJCC Level of Evidence: I

Residual Tumor Location following Primary Cytoreductive Surgery

Residual tumor location should be recorded in the operative notes. AJCC Level of Evidence:I

RISK ASSESSMENT MODELS

The AJCC recently established guidelines that will be used to evaluate published statistical predictionmodels for the purpose of granting endorsement for clinical use.[24] Although this is a monumental step toward the goal ofprecision medicine, this work was published only very recently. For this reason, the existing models that have been published or may be in clinical use have not yet been evaluated for this cancer site by the Precision Medicine Core of theAJCC. In the future, the statistical prediction models for this cancer site will be evaluated, and those that meet all AJCC criteria will be endorsed.

DEFINITIONS OF AJCC TNM

The definitions of the T categories correspond to the stages accepted by the FédérationInternationale de Gynécologie et d'Obstétrique (FIGO).[3] Both systems are included for comparison.

Definition of Primary Tumor (T)

T Category	FIGO Stage	T Criteria
TX		Primary tumor cannot be assessed
T0		No evidence of primary tumor
T1	I	Tumor limited to ovaries (one or both) or fallopian tube(s)
T1a	IA	Tumor limited to one ovary (capsule intact) or fallopian tube surface; no malignant cells in ascites or peritoneal washings
T1b	IB	Tumor limited to one or both ovaries (capsules intact) or fallopian tubes; no tumor on ovarian or fallopian tube surface; no malignant cells in ascites or peritoneal washings
T1c	IC	Tumor limited to one or both ovaries or fallopian tubes, with any of the following:
T1c1	IC1	Surgical spill
T1c2	IC2	Capsule ruptured before surgery or tumor on ovarian or fallopian tube surface
T1c3	IC3	Malignant cells in ascites or peritoneal washings
T2	II	Tumor involves one or both ovaries or fallopian tubes with pelvic extension below pelvic brim or primary peritoneal cancer
T2a	IIA	Extension and/or implants on the uterus and/or fallopian tube(s) and/or ovaries
T2b	IIB	Extension to and/or implants on other pelvic tissues
T3	III	Tumor involves one or both ovaries or fallopian tubes, or primary peritoneal cancer, with microscopically confirmed peritoneal metastasis outside the pelvis and/or metastasis to the retroperitoneal (pelvic and/ or para-aortic) lymph nodes
T3a	IIIA2	Microscopic extrapelvic (above the pelvic brim) peritonealinvolvement with or without positive retroperitoneal lymph nodes
T3b	IIIB	Macroscopic peritoneal metastasis beyond pelvis 2 cm or less in greatest dimension with or without metastasis to the retroperitoneallymph nodes
T3c	IIIC	Macroscopic peritoneal metastasis beyond the pelvis more than2 cm in greatest dimension with or without metastasis to the retroperitoneal lymph nodes (includes extension of tumor to capsule of liver and spleen without parenchymal involvement of either organ)

Definition of Regional Lymph Node (N)

N Category	FIGO Stage	N Criteria
NX		Regional lymph nodes cannot be assessed
N0		No regional lymph node metastasis
N0(i+)		Isolated tumor cells in regional lymph node(s) no greater than 0.2 mm
N1	IIIA1	Positive retroperitoneal lymph nodes only (histologically confirmed)
N1a	IIIA1i	Metastasis up to 10 mm in greatest dimension
N1b	IIIA1ii	Metastasis more than 10 mm in greatest dimension

Definition of Distant Metastasis (M)

M Category	FIGO Stage	M Criteria
M0		No distant metastasis
M1	IV	Distant metastasis, including pleural effusion with positive cytology; liver or splenic parenchymal metastasis; metastasis to extra-abdominal organs (including inguinal lymph nodes and lymph nodes outside the abdominal cavity); and transmural involvement of intestine
M1a	IVA	Pleural effusion with positive cytology
M1b	IVB	Liver or splenic parenchymal metastases; metastases to extra-abdominal organs (including inguinal lymph nodes and lymph nodes outside the abdominal cavity); transmural involvement of intestine

AJCC PROGNOSTIC STAGE GROUPS

When T is...	And N is...	And M is...	Then the stage group is...
T1	N0	M0	I
T1a	N0	M0	IA
T1b	N0	M0	IB
T1c	N0	M0	IC
T2	N0	M0	II
T2a	N0	M0	IIA
T2b	N0	M0	IIB
T1/T2	N1	M0	IIIA1
T3a	N0/N1	M0	IIIA2
T3b	N0/N1	M0	IIIB
T3c	N0/N1	M0	IIIC
Any T	Any N	M1	IV
Any T	Any N	M1a	IVA
Any T	Any N	M1b	IVB

REGISTRY DATA COLLECTION VARIABLES

1. FIGO stage
2. Preoperative CA-125 level
3. Gross residual tumor after primary cytoreductive surgery
4. Residual tumor volume after primary cytoreductive surgery
5. Residual tumor location following primary cytoreductive surgery

HISTOLOGIC GRADE (G)

G	G Definition
GX	Grade cannot be assessed
GB	Borderline tumor
G1	Well differentiated
G2	Moderately differentiated
G3	Poorly differentiated or undifferentiated

HISTOPATHOLOGIC TYPE

The AJCC endorses the histologic typing of malignant ovarian tumors as endorsed by the World Health Organization (WHO)[4] and recommends that all ovarian epithelial tumors be subdivided according to a simplified version of this classification. The three main histologic types, which include nearly all ovarian cancers, are malignant epithelial tumors, potentially malignant sex cord–stromal tumors, and primitive germ cell tumors. Nonepithelial primary ovarian cancers may be staged using this classification but should be reported separately.

1. Epithelial tumors
   a. Serous tumors
      i. Benign serous cystadenoma
      ii. Serous borderline tumor: serous cystadenoma with epithelial proliferation and nuclear atypia but with no destructive stromal invasion
      iii. Low-grade serous carcinoma
      iv. High-grade serous carcinoma
         1. Transitional cell variant
   b. Mucinous tumors
      i. Benign mucinous cystadenoma
      ii. Mucinous borderline tumor: mucinous cystadenoma with epithelial proliferation and nuclear atypia, but with no destructive stromal invasion
      iii. Mucinous carcinoma
   c. Endometrioid tumors
      i. Benign endometrioid cystadenoma
      ii. Endometrioid borderline tumor with epithelial proliferation and nuclear atypia but with no destructive stromal invasion
      iii. Endometrioid carcinoma
   d. Clear cell tumors
      i. Benign clear cell tumors
      ii. Borderline clear cell tumors with epithelial proliferation and nuclear atypia but with no destructive stromal invasion
      iii. Clear cell carcinoma
   e. Brenner tumors
      i. Borderline Brenner tumor
      ii. Malignant Brenner tumor
   f. Seromucinous tumors
      i. Borderline seromucinous tumor
      ii. Seromucinous carcinoma
   g. Undifferentiated carcinoma: a malignant tumor that is too poorly differentiated to be placed in any other group (most cases are thought to be HGSC)
   h. Mixed epithelial tumor: tumors composed (≥10%) of two or more of the five major cell types of common epithelial tumors (types should be specified)

Advanced invasive HGSC associated with STIC may be ovarian or tubal in origin without clinical relevance. For ovary-limited tumors associated with STIC, there are three possibilities: a) STIC extending to one ovary, b) ovarian HGSC extending to the fallopian tube, and c) synchronous or metachronous tumor involving the ovary and fallopian tube. With regard to staging, these tumors are considered Stage IA ovarian carcinoma with STIC unless there is evidence of direct extension from STIC to the ovary, in which case they would be stage IIA fallopian tube carcinoma.

Cases with intraperitoneal carcinoma in which the ovaries appear to be incidentally involved and not the primary origin should be labeled as extraovarian peritoneal carcinoma. They usually are staged based on the ovarian staging classification. Because the peritoneum essentially is always involved throughout the abdomen, peritoneal tumors usually fall within the Stage III (T3) or Stage IV (M1) categories.

ILLUSTRATIONS

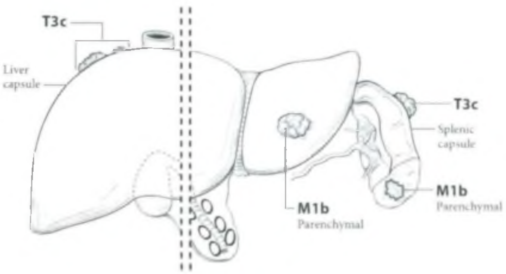

Fig. 55.3 T3c includes extension of tumor to capsule of liver and spleen without parenchymal involvement of either organ. M1b includes liver or splenic parenchymal metastases.

Bibliography

1. Ferlay J, Shin HR, Bray F, Forman D, Mathers C, Parkin DM. Estimates of worldwide burden of cancer in 2008: GLOBOCAN 2008.(*Int J Cancer.*)Dec 15 2010;127(12):2893-2917.
2. Ozols RF, Schwartz PE, Eifel PJ. Ovarian cancer, fallopian tube carcinoma, and peritoneal carcinoma.(*Cancer: Principles and Practice of Oncology, Sixth Edition. Philadelphia: Lippincott Williams & Wilkins.*)2001;2:1597-1632.
3. Prat J, FIGO Committee on Gynecologic Oncology. Staging classification for cancer of the ovary, fallopian tube, and peritoneum.(*International journal of gynaecology and obstetrics: the official organ of the International Federation of Gynaecology and Obstetrics.*)Jan 2014;124(1):1-5.
4. Kurman R, Carcangiu M, Herrington C, Young R.(*WHO classification of tumours of female reproductive organs.*) 4th ed. Lyon: International Agency for Research on Cancer; 2014.
5. Gilks CB, Prat J. Ovarian carcinoma pathology and genetics: recent advances.(*Human pathology.*)Sep 2009;40(9):1213-1223.
6. Prat J. Ovarian carcinomas: five distinct diseases with different origins, genetic alterations, and clinicopathological features.(*Virchows Arch.*)Mar 2012;460(3):237-249.
7. Piek JM, van Diest PJ, Zweemer RP, et al. Dysplastic changes in prophylactically removed Fallopian tubes of women predisposed to developing ovarian cancer.(*J Pathol.*)Nov 2001;195(4):451-456.
8. Callahan MJ, Crum CP, Medeiros F, et al. Primary fallopian tube malignancies in BRCA-positive women undergoing surgery for ovarian cancer risk reduction.(*J Clin Oncol.*)Sep 1 2007;25(25):3985-3990.
9. Kindelberger DW, Lee Y, Miron A, et al. Intraepithelial carcinoma of the fimbria and pelvic serous carcinoma: Evidence for a causal relationship.(*The American journal of surgical pathology.*)Feb 2007;31(2):161-169.
10. Howitt BE, Hanamornroongruang S, Lin DI, et al. Evidence for a dualistic model of high-grade serous carcinoma: BRCA mutation status, histology, and tubal intraepithelial carcinoma.(*The American journal of surgical pathology.*)Mar 2015;39(3):287-293.
11. Crum CP, Herfs M, Ning G, et al. Through the glass darkly: intraepithelial neoplasia, top?down differentiation, and the road to ovarian cancer.(*The Journal of pathology.*)2013;231(4):402-412.
12. Singer G, Oldt R, 3rd, Cohen Y, et al. Mutations in BRAF and KRAS characterize the development of low-grade ovarian serous carcinoma.(*Journal of the National Cancer Institute.*)Mar 19 2003;95(6):484-486.
13. Singer G, Stohr R, Cope L, et al. Patterns of p53 mutations separate ovarian serous borderline tumors and low- and high-grade carcinomas and provide support for a new model of ovarian carcinogenesis: a mutational analysis with immunohistochemical correlation.(*The American journal of surgical pathology.*)Feb 2005; 29(2):218-224.
14. Vaughan S, Coward JI, Bast RC, Jr., et al. Rethinking ovarian cancer: recommendations for improving outcomes.(*Nat Rev Cancer.*) Oct 2011;11(10):719-725.
15. Alvarado-Cabrero I, Young RH, Vamvakas EC, Scully RE. Carcinoma of the fallopian tube: a clinicopathological study of 105 cases with observations on staging and prognostic factors.(*Gynecologic oncology.*)Mar 1999;72(3):367-379.
16. Medeiros F, Muto MG, Lee Y, et al. The tubal fimbria is a preferred site for early adenocarcinoma in women with familial ovarian cancer syndrome.(*The American journal of surgical pathology.*)2006; 30(2):230-236.
17. Onda T, Yoshikawa H, Yasugi T, et al. Patients with ovarian carcinoma upstaged to stage III after systematic lymphadenctomy have similar survival to Stage I/II patients and superior survival to other Stage III patients.(*Cancer.*)1998;83(8):1555-1560.
18. Kanazawa K, Suzuki T, Tokashiki M. The validity and significance of substage IIIC by node involvement in epithelial ovarian cancer: impact of nodal metastasis on patient survival.(*Gynecologic oncology.*)May 1999;73(2):237-241.
19. Ferrandina G, Legge F, Petrillo M, Salutari V, Scambia G. Ovarian cancer patients with "node-positive-only" Stage IIIC disease have a more favorable outcome than Stage IIIA/B.(*Gynecologic oncology.*)2007; 107(1):154-156.
20. Baek S-J, Park J-Y, Kim D-Y, et al. Stage IIIC epithelial ovarian cancer classified solely by lymph node metastasis has a more favorable prognosis than other types of stage IIIC epithelial ovarian cancer.(*Journal of gynecologic oncology.*)2008;19(4):223-228.
21. Mitchell DG, Javitt MC, Glanc P, et al. ACR appropriateness criteria staging and follow-up of ovarian cancer.(*Journal of the American College of Radiology : JACR.*)Nov 2013;10(11):822-827.
22. Bakkar R, Gershenson D, Fox P, Vu K, Zenali M, Silva E. Stage IIIC ovarian/peritoneal serous carcinoma: a heterogeneous group of patients with different prognoses.(*Int J Gynecol Pathol.*)May 2014;33(3):302-308.
23. Cliby WA, Aletti GD, Wilson TO, Podratz KC. Is it justified to classify patients to Stage IIIC epithelial ovarian cancer based on nodal involvement only?(*Gynecologic oncology.*)2006;103(3): 797-801.
24. Kattan MW, Hess KR, Amin MB, et al. American Joint Committee on Cancer acceptance criteria for inclusion of risk models for individualized prognosis in the practice of precision medicine.(*CA: a cancer journal for clinicians.*)Jan 19 2016.

Gestational Trophoblastic Neoplasms

56

Lee-may Chen, Alexander B. Olawaiye, Priya R. Bhosale, Don S. Dizon, Beth A. Erickson, Perry W. Grigsby, Ian S. Hagemann, Lorraine Portelance, Christopher N. Otis, and David G. Mutch

CHAPTER SUMMARY

Cancers Staged Using This Staging System

Placental neoplasms

Summary of Changes

Change	Details of Change	Level of Evidence
WHO Classification of Tumors	Complete and partial hydatidiform tumors have been removed from the chapter. They are considered benign and are not tracked by tumor registries.	N/A

ICD-O-3 Topography Codes

Code	Description
C58.9	Placenta

WHO Classification of Tumors

Code	Description
9100	Invasive hydatidiform mole
9100	Choriocarcinoma
9104	Placental site trophoblastic tumor
9105	Epithelioid trophoblastic tumor

Kurman RJ, Carcangiu ML, Herrington CS, Young RH, eds. World Health Organization Classification of Tumours of the Female Reproductive System. Lyon: IARC; 2014.

INTRODUCTION

Gestational trophoblastic neoplasms (GTNs) are tumors arising from the placenta. The benign form of these tumors, referred to as gestational trophoblastic disease (GTD), usually arises from an accident in the developing egg in which the maternal chromosomes are lost, and either paternal chromosome duplication or dispermy result in a 46,XX or 46,XY tumor known as a complete hydatidiform mole. The tumor is composed of dilated, avascular, "grape-like" vesicles with no fetal parts. In some patients, the egg is fertilized by two sperm, resulting in a 69,XXX or 69,XXY complement. A trisomy fetus may develop, along with milder proliferation of trophoblastic tissue, resulting in a partial hydatidiform mole. Both complete mole and partial mole typically follow a benign course. Before ultrasound was available, molar pregnancies most frequently presented as vaginal bleeding in women carrying fetuses with a size greater than date, necessitating prompt uterine evacuation. Today, molar pregnancies most frequently are diagnosed through ultrasound, followed by suction and sharp dilatation and curettage, with pathological examination of the tissue. The incidence of molar pregnancy (GTD) in the United States is about 1 in 1,000 pregnancies. Approximately 20% of complete and 5% of partial moles persist locally, or metastasize, and thus are considered GTNs.

Much less frequently (about 1 in 20,000 pregnancies in the United States), a highly malignant, rapidly growing meta-

To access the AJCC cancer staging forms, please visit www.cancerstaging.org.

© American Joint Committee on Cancer 2017

M.B. Amin et al. (eds.), *AJCC Cancer Staging Manual, Eighth Edition*, DOI 10.1007/978-3-319-40618-3_56

static form of GTN called choriocarcinoma is encountered. This solid, anaplastic, vascular, and aggressively proliferative tumor is easily recognized microscopically and may present with symptoms of vaginal bleeding (as with a hydatidiform mole). However, metastatic lesions may be the first sign of this neoplasm, which may follow any pregnancy event, including an incomplete abortion or a full-term pregnancy.

The trophoblastic tissue that makes up these tumors usually produces a serum tumor marker, β-human chorionic gonadotropin (hCG), which is very helpful in the diagnosis and monitoring of therapy in these patients. GTNs are very responsive to chemotherapy, with cure rates approaching 100%.

Even less common than choriocarcinoma are the histologically distinctive placental site trophoblastic tumors (PSTTs) and epithelioid trophoblastic tumors. hCG is not a useful marker in these high-risk histologies.

ANATOMY

Primary Site(s)

By definition, GTNs arise from placental tissue in the uterus (Fig. 56.1). Although most of these tumors are noninvasive and are removed by uterine evacuation, local invasion of the myometrium may occur.

Regional Lymph Nodes

Nodal involvement in gestational trophoblastic neoplasia is uncommon (0.5%), but reportedly occurs in 6-16% of PSTTs.

Metastatic Sites

The common sites of distant metastasis of GTNs are the lung, liver, and brain. Other, less frequent metastatic sites include the kidney, gastrointestinal tract, and spleen.

RULES FOR CLASSIFICATION

Clinical Classification

Gestational trophoblastic neoplasia requires intervention, which may include chemotherapy or surgery for low-risk patients; high-risk patients may require more intensive chemotherapy to achieve remission. In 1991, the International Federation of Gynecology and Obstetrics (FIGO) added nonanatomic risk factors to the traditional staging system. Further modifications have been made in an attempt to merge several prognostic classification systems.

Unlike other gynecologic malignancies, GTNs are restaged by risk score if a change in chemotherapy is being considered. Depending on the extent of disease at restaging, recommendations are made for single-agent versus multiagent chemotherapy.

Women with complete and partial hydatidiform moles are at risk for gestational trophoblastic neoplasia. After uterine evacuation, hCG levels are measured weekly until they are normal. A plateau or rise in hCG confirms gestational trophoblatic neoplasia and prompts further clinical assessment to establish the extent of disease (staging) and the classification of low- versus high-risk disease. Initial imaging in patients with a rise or plateau in hCG includes chest X-ray

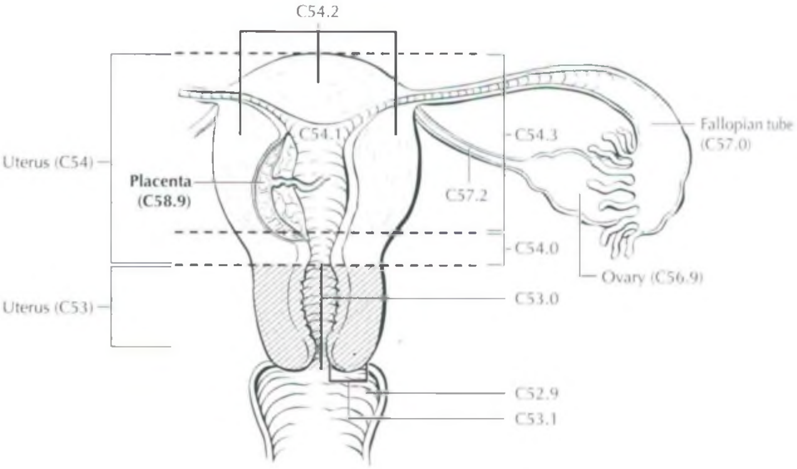

Fig. 56.1 Anatomic site of the placenta for gestational trophoblastic tumors.

and pelvic ultrasound. Additional imaging may include computed tomography (CT) scanning and/or magnetic resonance (MR) imaging.

Persistent gestational trophoblastic neoplasia after uterine evacuation is presumed to be the result of residual uterine disease, unless metastasis is identified through imaging. A hysterectomy specimen may confirm invasive hydatidiform mole, but this assessment is not performed commonly.

Indications for Treatment

The following criteria are suggested for the diagnosis of gestational trophoblastic neoplasia:

- Three or more values of hCG showing no significant change (a plateau) over 4 weeks, or
- A rise in hCG of 10% or greater for two values over 3 weeks or longer, or
- Persistence of elevated hCG 6 months after evacuation of molar pregnancy, or
- Histologic diagnosis of choriocarcinoma

GTNs confined to the uterus may be treated with hysterectomy, which also is the preferred management for PSTTs and epithelioid trophoblastic tumors. These histologies appear to be less chemoresponsive.

Diagnosis of Metastasis

For the diagnosis of lung metastasis, chest X-ray is appropriate and should be used to count metastases for risk scoring. Lung CT may be used but does not appear to alter outcome. AJCC Level of Evidence: I

For the diagnosis of intra-abdominal metastasis, CT scanning is preferred, although many institutions still use ultrasound to detect liver metastasis. MR imaging or positron emission tomography (PET)/CT also may be used.

For the diagnosis of brain metastasis, MR imaging is superior to CT.

The risk score may change based on prior treatment. An rTNM staging would indicate retreatment.

Imaging

Ultrasound is currently the modality used to diagnose and asses gestational trophoblastic tumors (GTD). MR imaging may be used to assess local extent of disease especially in patients with choriocarcinoma.

TNM Components of Tumor Staging

In patients with GTN T1 disease, the tumor is confined to the uterus and ultrasound currently is the modality of choice. MR imaging, with its superior soft tissue resolution, may be able to assess myometrial extension and extrauterine extension (T2 disease). Myometrial, ovarian, and vaginal involvement, as well as involvement of the broad ligament, may be evaluated on the sagittal and axial T2-weighted or contrast-enhanced sagittal T1-weighted MR imaging sequences.

Large lung metastases are visible on chest X-Ray (M1a). However, metastases 1 cm may not be seen on a plain chest radiograph (i.e., chest X-ray); therefore, chest CT may be the best modality to assess for pulmonary metastatic disease. Whether this improves clinical management, however, is unclear. For distant metastases, contrast-enhanced CT may be used. MR imaging is considered superior to CT for assessing brain metastases. The value of PET/CT has not been explored fully in this disease.

Imaging Report

The extent of tumor and whether it is confined to the uterus or extends beyond it should be described. Involvement of adjacent organs, such as the adnexa, bladder, rectum, or other pelvic and abdominal tissues, should be noted.

Suggested Imaging Report Format

1. Suggested Imaging Report Format
   a. Confined to the uterus
2. Local extent
   a. Involvement of the ovaries and the adjacent organs
3. Extrapelvic disease, lung metastases, and brain metastases

Pathological Classification

Unlike most gynecologic malignancies, GTNs are clinically staged. After an initial diagnosis by uterine evacuation, tumor markers (hCG level) and imaging establish disease burden and extent.

Any lymph node metastasis should be classified as metastatic (M1b) disease.

PSTTs and epithelioid trophoblastic tumors are managed surgically, with chemotherapy considered for advanced-stage disease.

PROGNOSTIC FACTORS

Prognostic Factors Required for Stage Grouping

Because of the responsiveness of GTNs to treatment and the accuracy of the serum tumor marker hCG in reflecting disease status, the traditional anatomic staging system used in most solid tumors has less prognostic significance than other well-defined prognostic factors. Beyond uterine evacuation (or hysterectomy), GTNs are clinically staged through the use of tumor markers and imaging, including ultrasound, X-ray, CT, and MR imaging. Pulmonary spread is quite common and does not count toward high-risk disease. AJCC Level of Evidence: I

Risk Factors

- Age
  - By year, at time of staging/restaging: cutoff point, age 40
- Antecedent pregnancy
  - Prior pregnancy categorized by molar pregnancy, abortion, or term pregnancy
- Interval in months from index pregnancy
  - By months, at the time of staging/restaging, from the time of initial uterine evacuation: cutoff points, < 4 months, 4–6 months, 7–12 months, > 12 months
- hCG level
  - By international units per milliliter, through laboratory values, at time of staging/restaging: cutoff points, <1,000 IU/mL, 1,000 to <10,000 IU/mL, 10,000 to 100,000 IU/mL, >100,000 IU/mL
- Tumor size
  - By centimeters, through imaging, at time of staging/restaging: cutoff points, <3 cm, 3–5 cm, >5 cm
- Site of metastases
  - By location, through imaging, at time of staging/restaging, categorized by lung, spleen/kidney, gastrointestinal tract, brain/liver
- Number of metastases
  - By number, through imaging, at time of staging/restaging: cutoff points, 1-4, 5-8, >8
- Previous failed chemotherapy

By number of drugs, categorized as one-prior, or two or more

Additional Factors Recommended for Clinical Care

FIGO Stage

FIGO staging depends on the anatomic location of the tumor for Stage I/II disease, the presence of lung metastases for Stage III disease, and the presence of distant metastases for Stage IV disease. Management most frequently is based on identification of high- versus low-risk disease. AJCC Level of Evidence: I

RISK ASSESSMENT MODELS

The AJCC recently established guidelines that will be used to evaluate published statistical prediction models for the purpose of granting endorsement for clinical use.[3] Although this is a monumental step toward the goal of precision medicine, this work was published only very recently. Therefore, the existing models that have been published or may be in clinical use have not yet been evaluated for this cancer site by the Precision Medicine Core of the AJCC. In the future, the statistical prediction models for this cancer site will be evaluated, and those that meet all AJCC criteria will be endorsed.

DEFINITIONS OF AJCC TNM

The definitions of the T categories correspond to the stages accepted by the Fédération Internationale de Gynécologie et d'Obstétrique (FIGO). Both systems are included for comparison.

Definition of Primary Tumor (T)

T Category	FIGO Stage	T Criteria
TX		Primary tumor cannot be assessed
T0		No evidence of primary tumor
T1	I	Tumor confined to uterus
T2	II	Tumor extends to other genital structures (ovary, tube, vagina, broad ligaments) by metastasis or direct extension

Definition of Distant Metastasis (M)

M Category	FIGO Stage	M Criteria
M0		No distant metastasis
M1		Distant metastasis
M1a	III	Lung metastasis
M1b	IV	All other distant metastases

Risk Score

The score on the FIGO-modified World Health Organization (WHO) Prognostic Scoring Index is used to stratify women with gestational trophoblastic neoplasia in addition to the stage group (Table 56.1). The risk score is appended to the anatomic stage.

Table 56.1 Prognostic scoring index for gestational trophoblastic tumors

Prognostic factor	Risk score			
	0	1	2	4
Age (years)	<40	≥40		
Antecedent pregnancy	Hydatidiform mole	Abortion	Term pregnancy	
Interval months from index pregnancy	<4	4-6	7-12	>12
Pretreatment hCG (IU/mL)	$<10^3$	10^3 to $<10^4$	10^4 to $<10^5$	$\geq 10^5$
Largest tumor size, including uterus (cm)	<3	3-5	>5	
Site of metastases	Lung	Spleen, kidney	Gastrointestinal tract	Brain, liver
Number of metastases identified		1-4	5-8	>8
Previous failed chemotherapy			Single drug	Two or more drugs
Total score				

AJCC PROGNOSTIC STAGE GROUPS

In 2000, FIGO combined its anatomic staging system with the modified WHO risk factor scoring system. In 2002, FIGO changed the WHO risk factor score cutoff for low-risk disease to <6, with high-risk disease >7, thus eliminating intermediate-risk disease. The current FIGO classification includes an anatomic stage designated by Roman numeral I, II, III, or IV, followed by the risk factor score expressed in Arabic numerals (e.g., Stage II: 4, Stage IV: 9).

When T is...	And M is...	Then stage is noted with risk score as follows...
T1	M0	I: (risk score)
T2	M0	II: (risk score)
Any T	M1a	III: (risk score)
Any T	M1b	IV: (risk score)

REGISTRY DATA COLLECTION VARIABLES

1. Risk score (see Table 56.1)
2. FIGO stage

HISTOLOGIC GRADE (G)

Histologic grade is not applicable to GTNs.

HISTOPATHOLOGIC TYPE

Invasive hydatidiform mole
Choriocarcinoma
PSTTs
Epithelioid trophoblastic tumors

ILLUSTRATIONS

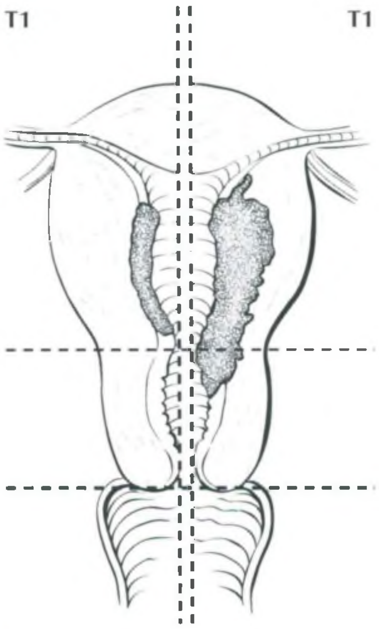

Fig. 56.2 T1 is tumor confined to uterus.

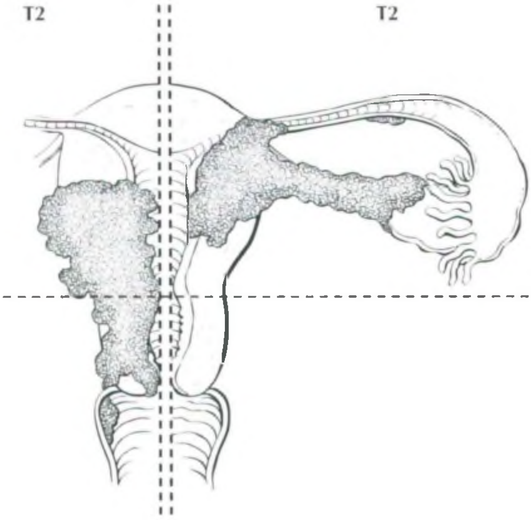

Fig. 56.3 T2 is tumor extends to other genital structures (ovary, tube, vagina, broad ligaments) by metastasis or direct extension.

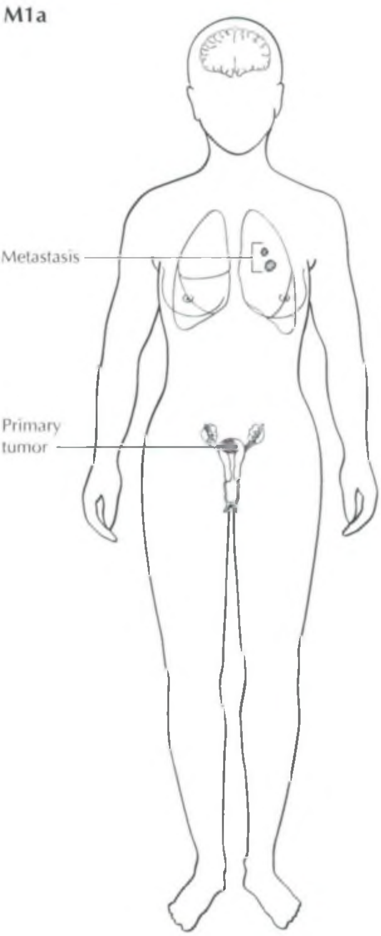

Fig. 56.4 M1a is lung metastasis.

M1b

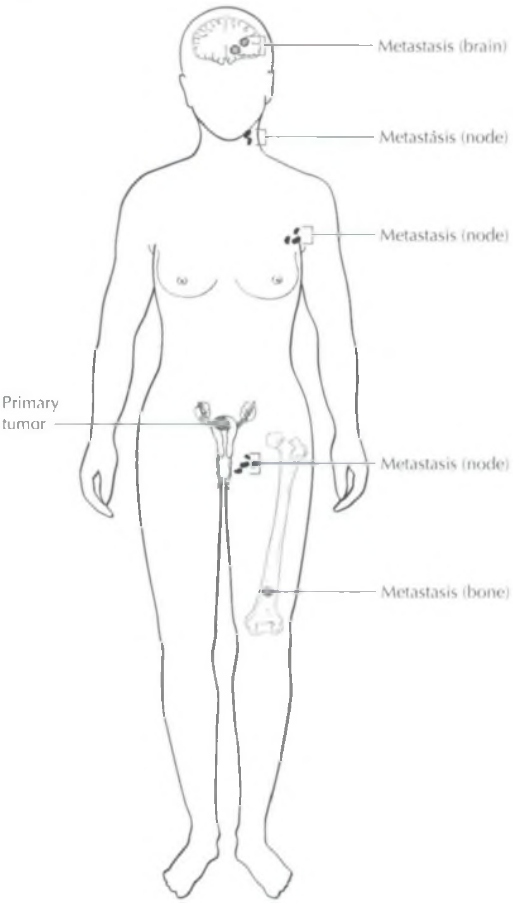

Fig. 56.5 M1b is all other distant metastasis.

Bibliography

1. Dhanda S, Ramani S, Thakur M. Gestational trophoblastic disease: a multimodality imaging approach with impact on diagnosis and management. *Radiology research and practice.* 2014;2014: 842751.
2. Mapelli P, Mangili G, Picchio M, et al. Role of 18F-FDG PET in the management of gestational trophoblastic neoplasia. *European journal of nuclear medicine and molecular imaging.* Apr 2013;40(4): 505-513.
3. Kattan MW, Hess KR, Amin MB, et al. American Joint Committee on Cancer acceptance criteria for inclusion of risk models for individualized prognosis in the practice of precision medicine. *CA: a cancer journal for clinicians.*
4. Darby S, Jolley I, Pennington S, Hancock BW. Does chest CT matter in the staging of GTN? *Gynecologic oncology.* 2009;112(1): 155-160.
5. Davis MR, Howitt BE, Quade BJ, et al. Epithelioid trophoblastic tumor: A single institution case series at the New England Trophoblastic Disease Center. *Gynecologic oncology.* Jun 2015; 137(3):456-461.
6. Lurain JR. Gestational trophoblastic disease I: epidemiology, pathology, clinical presentation and diagnosis of gestational trophoblastic disease, and management of hydatidiform mole. *Am J Obstet Gynecol.* Dec 2010;203(6):531-539.
7. Lurain JR. Gestational trophoblastic disease II: classification and management of gestational trophoblastic neoplasia. *Am J Obstet Gynecol.* Jan 2011;204(1):11-18.
8. Ngan HY, Odicino F, Maisonneuve P, et al. Gestational trophoblastic neoplasia. FIGO 26th Annual Report on the Results of Treatment in Gynecological Cancer. *International journal of gynaecology and obstetrics: the official organ of the International Federation of Gynaecology and Obstetrics.* Nov 2006;95 Suppl 1:S193-S203.

Part XIII

Male Genital Organs

Members of the Male Genital Organs Expert Panel
Mahul B. Amin, MD, FCAP – Editorial Board Liaison
Daniel A. Barocas, MD, MPH
Fadi Brimo, MD, FRCP(C)
Robert K. Brookland, MD, FACR, FACRO
Mark K. Buyyounouski, MD, MS
Peter L. Choyke, MD, FACR
Samson W. Fine, MD
Susan Halabi, PhD – Precision Medicine Core Representative
Daniel A. Hamstra, MD, PhD
Peter A. Humphrey, MD, PhD
Michael W. Kattan, PhD
Daniel W. Lin, MD – Chair
Malcolm D. Mason, MD – UICC Representative
Jesse K. McKenney, MD
William K. Oh, MD, FACP
Curtis A. Pettaway, MD
Charles J. Ryan, MD
Howard M. Sandler, MD, MS, FASTRO – Vice Chair
Oliver Sartor, MD
Maria J. Schymura, PhD – Data Collection Core Representative
John R. Srigley, MD, FRCPC, FCAP, FRCPath – CAP Representative
Karim A. Touijer, MD
Michael J. Zelefsky, MD

Penis

57

Curtis A. Pettaway, John R. Srigley, Robert K. Brookland, Peter L. Choyke, Charles J. Ryan, Peter A. Humphrey, Daniel A. Barocas, Mark K. Buyyounouski, Fadi Brimo, Antonio Cubilla, Samson W. Fine, Susan Halabi, Daniel A. Hamstra, Michael W. Kattan, Malcolm D. Mason, William K. Oh, Pheroze Tamboli, Karim A. Touijer, Michael J. Zelefsky, Howard M. Sandler, Daniel W. Lin, and Mahul B. Amin

CHAPTER SUMMARY

Cancers Staged Using This Staging System

Penile squamous carcinoma and associated histologic subtypes

Cancers Not Staged Using This System

These histopathologic types of cancer…	Are staged according to the classification for…	And can be found in chapter…
Urethral Carcinoma	Urethra	63
Sarcoma	Soft Tissue Sarcoma-Abdominal and Thoracic Visceral Organs	42
Melanoma	Melanoma of the Skin	47

Summary of Changes

Change	Details of Change	Level of Evidence
Histologic Grade (G)	The three-tiered World Health Organization (WHO)/ International Society of Urological Pathology (ISUP) grading system has been adopted. Any proportion of anaplastic cells is sufficient to categorize a tumor as grade 3.	III
Definition of Primary Tumor (T)	Ta definition is now broadened to include noninvasive localized squamous carcinoma.	II
Definition of Primary Tumor (T)	T1a and T1b have been separated by an additional prognostic indicator-the presence or absence or perineural invasion.	III
Definition of Primary Tumor (T)	T1a or T1b are described by the site where they occur on the penis and are designated glans, foreskin, or shaft. Anatomic layers invaded are described for the three locations.	I
Definition of Primary Tumor (T)	T2 definition includes corpus spongiosum invasion.	II
Definition of Primary Tumor (T)	T3 definition now involves corpora cavernosum invasion.	II
Definition of Regional Lymph Nodes (N)	pN1 is defined as ≤2 unilateral inguinal metastases, no extranodal extension.	II
Definition of Regional Lymph Nodes (N)	pN2 is defined as ≥3 unilateral inguinal metastases or bilateral metastases	II

To access the AJCC cancer staging forms, please visit www.cancerstaging.org.

© American Joint Committee on Cancer 2017

M.B. Amin et al. (eds.), *AJCC Cancer Staging Manual, Eighth Edition*, DOI 10.1007/978-3-319-40618-3_57

ICD-0-3 Topography Codse

Code	Description
C60.0	Prepuce
C60.1	Glans penis
C60.2	Body of penis
C60.8	Overlapping lesion of penis
C60.9	Penis, NOS

WHO Classification of Tumors

Code	Description
	**Non-HPV Related Squamous Cell Carcinomas**
8070	Squamous cell carcinoma
8052	Papillary, squamous cell carcinoma
8051	Verrucous carcinoma
8560	Adenosquamous carcinoma
8074	Sarcomatoid squamous cell carcinoma
8070	Mixed squamous cell carcinoma
8075	Pseudoglandular carcinoma
	**HPV-Related Squamous Cell Carcinomas**
8083	Basaloid squamous cell carcinoma
8054	Warty carcinoma
8084	Clear cell squamous cell carcinoma
8082	Lymphoepithelioma-like carcinoma

Moch H, Humphrey PA, Ulbright TM, Reuter VE, eds. World Health Organization Classification of Tumours of the Urinary System and Male Genital Organs. Lyon: IARC; 2016.

INTRODUCTION

Cancer of the penis is a disease seen mostly in men older than age 50.[1] The global incidence is variable, with fewer than 2,000 cases reported annually in the United States but reported more commonly in Africa, South America, and other parts of the developing world.[2,3] Exposure to the human papilloma virus (HPV) may increase the risk of acquiring this disease.[4] Risks also are increased in those with lichen sclerosus, patients with psoriasis exposed to ultraviolet therapy, and tobacco users.[4-7] Neonatal circumcision is virtually protective against invasive penile cancer.[8] It is hoped that vaccines that protect against infection with oncogenic HPV genotypes also may reduce the risk.[9]

The stage of penile cancer established at the time of diagnosis is a critical element in determining both optimum treatment and prognosis.[10] Accurate designation of the TNM stage categories requires integration of physical, imaging, and pathological findings.[11] This staging system applies to squamous cell carcinomas and associated subtypes. Although melanomas and sarcomas can arise in the penis and urethral cancer occurs in a penile structure, their staging is addressed in the chapters on urethral cancer, melanoma, and soft tissue sarcomas, respectively. There are certain limitations to staging, including the ability to clinically assess lymph node status even with the introduction of newer technologies, such as magnetic resonance (MR) imaging and positron emission tomography (PET) or computed tomography (CT) imaging. Despite these considerations, the American Joint Committee on Cancer (AJCC) staging classification remains a powerful tool for the evaluation of treatment results, clinical trials, and the exchange of information between researchers and clinicians.

ANATOMY

Primary Site(s)

The penis is composed of three cylindrical masses and is separated into three areas, termed the root (or penile base), the body (or shaft), and the glans penis (or head).[12,13]

The three cylinders are composed of two dorsal structures known as the corpora cavernosa, which are covered by a tough fibroelastic coat (termed the tunica albuginea) and internally composed of large central cavernous venous sinuses. The third cylinder lies ventrally and is termed the corpus spongiosum. (Fig. 57.1) It continues distally to form a bulbous expansion, termed the glans penis (also known as the head of the penis).[12,13] The corpus spongiosum is a smaller cavernous venous sinus structure that surrounds the urethra throughout its course from the genitourinary diaphragm proximally to the glans penis distally. The dorsally lying corpora cavernosa bodies separate at the base of the penis, and each is attached to the ipsilateral ischium of the pubic bone via dense fibrous attachments called the penile crura.[13] Distal to the point of separation, the corpora are attached to the front of the symphysis by the penile suspensory ligament.[13] The arterial supply of the penis emanates from branches of the internal pudendal artery, which include the corporal arteries, the bulbourethral artery, and the dorsal arteries of the penis that provide blood supply to all the penile corpora and the glans penis. The venous drainage of the penis is supplied via the cavernosal veins, as well as superficial and deep dorsal veins.[12] External to the corporal bodies, a layer of deep fascia (also known as the Buck's fascia) surrounds both the corpora cavernosa and spongiosum and binds them together as a unit. The deep dorsal vein, deep dorsal arteries, and nerves run within this layer.[12,13]

The skin of the penis shaft is quite elastic, without hair follicles or glands, except distally at the proximal rim of the glans penis, called the corona.[12] Here smegma-producing glands are located. Distally at the corona, the penile skin folds back upon itself to produce the foreskin (also known as the prepuce; Fig. 57.1). The ventral attachment of the foreskin below the urethral meatus is called the frenulum.[12,13]

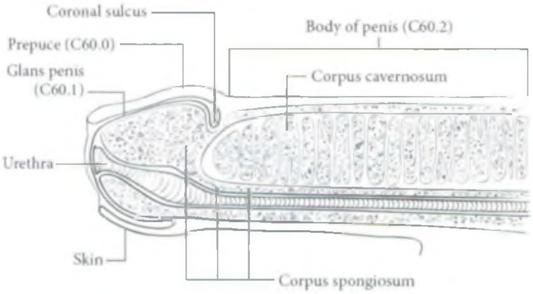

Fig. 57.1 Anatomy of the penis

The layers of tissue above the corpora are distinct in different regions of the penis.[12-14]

- At the level of the glans penis, the layers from outward to inward include the squamous epithelium, the lamina propria, and the underlying corpus spongiosum and corpora cavernosa.
- At the level of the foreskin covering the glans is an outer epidermis, a dermal layer, dartos fascia, lamina propria and an inner mucosal, nonkeratinized epithelial layer.
- At the level of the penile shaft proximal to the corona of the glans penis, the layers include epidermis, dermis, dartos fascia, Buck's fascia, and the underlying corpora.

The AJCC Cancer Staging Manual, 7th Edition (7th Edition) referred to tissue layers between the skin and corpora as "subepithelial connective tissue."[11] In this AJCC Cancer Staging Manual, 8th Edition (8th Edition), these areas are designated by their anatomical names to reflect the proper terminology and the levels of invasion prior to tumors' reaching corporal tissue.[12-14]

Primary Site Lymphatics

The lymphatics of the penile skin drain in an orderly fashion into the inguinal lymph nodes that are designated superficial and deep in relation to the fascia lata of the thigh.

The lymphatic vessels run along the dorsum of the penis toward the penile base. During transit, lymphatics from the right and left sides freely communicate. Cutaneous lymphatic vessels of the shaft skin from the right and left trunks drain into the superficial inguinal nodes, primarily of the superomedial inguinal group.[15,16]

Lymphatic drainage from the glans with tributaries from the urethra and cutaneous lymphatic vessels unite to form collecting trunks on the dorsal penile surface. These trunks run under the fascia in association with the deep dorsal vein. In the area of the penile suspensory ligament, the lymphatic trunks form a presymphyseal plexus, which then sends lymphatic trunks bilaterally to terminate in the superomedial inguinal nodes.[15,16]

The penile corporal lymphatic trunks also follow the deep dorsal vein to the symphysis pubis and either divide into discrete right or left trunks or anastomose with the presymphyseal lymphatic plexus, which then divides into lateral trunks.[15,16] The superomedial inguinal nodes also receive drainage from the corpora.

Regional Lymph Nodes

Regional lymph nodes are as follows:

- Superficial and deep inguinal lymph nodes
- Pelvic nodes (specified)
  - External iliac
  - Internal iliac (also called hypogastric)
  - Obturator
- Pelvic nodes (NOS)

The superficial inguinal lymph nodes are located above the fascia lata of the thigh and are separated into four quadrants and a central zone, divided by horizontal and vertical lines crossing at the saphenofemoral junction (Fig. 57.2).[17]

Lymph nodes primarily from the medial quadrants and the central zone receive penile afferent lymphatic vessels. Only occasionally do penile afferent lymphatic vessels drain into the superolateral quadrant and, rarely, into the inferolateral quadrant.[15,16]

The deep inguinal nodes lie beneath the fascia lata clustered about the femoral vessels within the femoral sheath. They also receive lymph drainage from the superficial inguinal nodes.[15,16]

Communication with the pelvic nodes occurs via the node of Cloquet, which lies within the femoral canal. The pelvic nodal field includes the lymph nodes associated with the iliac vessels from the aortic bifurcation to the inguinal ligament, including the hypogastric and obturator lymph node chains (Fig. 57.2).[15]

Metastatic Sites

Distant metastases are uncommon at initial presentation but are sometimes noted among patients with advanced regional metastases.[18] Sites include the following:

- Retroperitoneal lymph nodes (i.e., nodes outside the true pelvis)
- Lung
- Liver
- Cutaneous nodules distant from the primary site
- Bone

Fig. 57.2 Regional lymph nodes of the penis

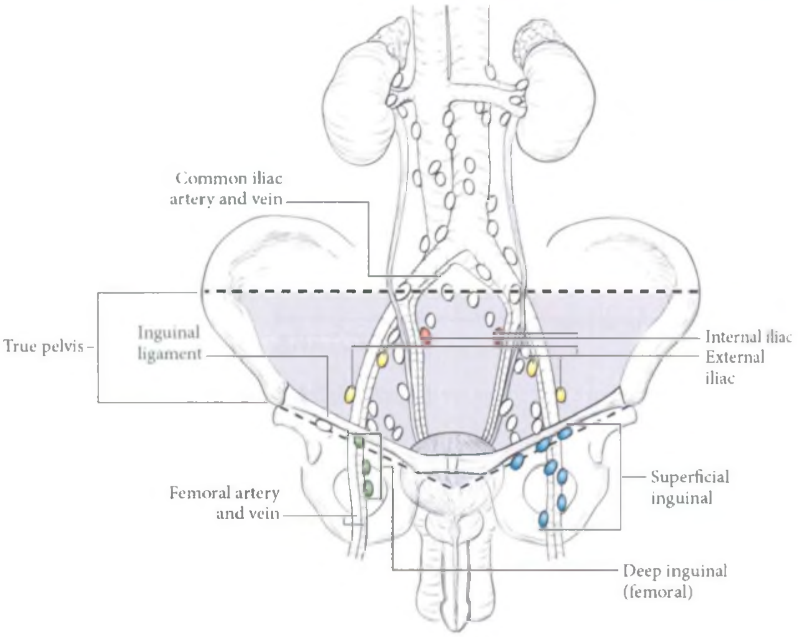

RULES FOR CLASSIFICATION

Clinical Classification

Clinical staging encompasses the diagnostic evaluation utilized to assign the extent of disease prior to definitive therapy. For penile cancer this includes the primary site, regional lymph nodes, and distant sites.

Primary tumor evaluation relies primarily on palpation to determine the anatomic structures involved, size, and mobility of the primary lesion (Figs. 57.3–57.9). Adequate incisional or excisional biopsy to sample the representative tumor depth is critical to evaluating the anatomic layers invaded, as well as determining the histologic type. An adequate biopsy also may reveal some of the prognostic factors related to tumor grade and the presence or absence of lymphovascular or perineural invasion. Given tumor heterogeneity, however, a biopsy may be inadequate to indicate all histologic parameters in the lesion.[19] Imaging can be helpful in certain scenarios as an adjunct to the physical examination in defining the extent of disease (see Imaging in this chapter).

Regional lymph node evaluation also requires a careful physical examination to assess the presence of unilateral or bilateral adenopathy, the number of palpable lymph nodes, or the presence of a fixed inguinal mass (Figs. 57.2, 57.10–57.14).[10] The presence of palpable adenopathy identifies a cohort of men at higher risk for adverse features when treated with surgery alone, including ≥3 positive nodes, extranodal extension (ENE) of cancer, and positive pelvic nodes.[20] The National Comprehensive Cancer Network (NCCN) guidelines identify patients with biopsy-proven metastases in multiple or bilateral nodes, a fixed mass, or pelvic adenopathy as candidates for neoadjuvant chemotherapy.[21]

Factors that can decrease the sensitivity of the inguinal examination include obesity and prior inguinal surgery. In such cases, cross-sectional imaging is useful in establishing the presence or absence of enlarged inguinal and pelvic lymph nodes.[10]

Distant metastases may be suspected by generalized malaise, weight loss, or bone pain. In addition, radiologic imaging studies also are indicated in the setting of obvious inguinal adenopathy to establish the extent of disease. These findings would be indicated as cM1. Biopsy of a metastatic site demonstrating tumor is included in clinical staging as pM1.

Imaging

Most superficial penile cancers (i.e., Tis-T1) do not require imaging. When the primary lesion is suspected of deeper invasion, penile MR imaging may be performed for local staging to differentiate invasion of the corpus spongiosum or corpora cavernosum (stage T2 versus T3).[22,23]

To determine local nodal status (N1-N3), palpation may be supplemented with MR imaging (obtained as part of a penile MR imaging) or CT of the pelvis, inclusive of the inguinal nodes, especially among patients who are obese or those in whom palpation may be unreliable. In patients with

no palpable inguinal nodes, sentinel node imaging (using a Tc99m-labeled nanocolloid injected around the primary tumor site) with excisional biopsy can be used as a minimally invasive tool to stage the inguinal nodal basin (also known as a dynamic sentinel lymph node biopsy [DSNB]).[24] Needle aspiration of enlarged or eccentric inguinal or pelvic nodes under ultrasound or CT guidance, respectively, can be useful in planning subsequent therapy. Conventional CT or MR imaging should be performed in all patients with palpable inguinal nodes to detect disease in pelvic nodes or distant nodal groups. Combined PET/CT may be helpful when CT or MR imaging are equivocal.[25,26]

A penile MR imaging report should distinguish T1 from T2 and T3 invasion (superficial versus invasion of corpora spongiosum versus invasion of corpora cavernosum). The pelvic MR imaging or CT should specify the number and laterality of enlarged nodes in the groin and note any enlarged nodes within the pelvis (N1, N2, N3).

Reports from radionuclide sentinel lymph node studies should describe the side, size, and number of visualized nodes.

Conventional staging CT or MR imaging should describe abnormalities suggestive of M1 disease (e.g., lung metastases, bone lesions). PET/CT reports should provide an overall assessment of the number of lesions and maximal standardized uptake value (SUV) of the fluorodeoxyglucose (FDG) activity.

A promising emerging technology is to perform sentinel lymph node imaging with a combination of an optical fluorophore with a radionuclide nanocolloid to provide intraoperative navigation for lymph node resection.[27]

Pathological Classification

Primary Tumor

Complete resection of the primary lesion with tumor-free margins provides the greatest certainty that all histologic parameters in terms of grade, anatomic structures involved, and the presence or absence of prognostic factors important in assigning AJCC TNM stage are characterized subsequent to microscopic evaluation.

The accurate description of histologic subtype and depth/structure invaded are important; for example, carcinoma in situ (penile intraepithelial neoplasia Tis) and pure verrucous carcinoma, although requiring control of the primary tumor site, do not metastasize.[28] Likewise, such tumors as basaloid and sarcomatoid squamous cell carcinoma are known to behave in an aggressive fashion. This pathological information is important for subsequent prognosis and management.

In the 7th Edition, category Ta referred to "noninvasive verrucous carcinoma." This term was misleading to some pathologists, who thought this would apply to all cases of verrucous carcinoma. The great majority of verrucous carcinomas are destructive, but the invading front is smooth and pushing, with the depth of invasion often difficult to assess. In the current classification, the Ta category is expanded and applies to both pure (well or completely sampled) verrucous carcinomas with no overt destructive invasion and noninvasive papillary, warty, basaloid, or mixed carcinomas (Fig. 57.3). These rare noninvasive surface-based tumors are somewhat analogous to noninvasive (pTa) papillary urothelial neoplasms.

Recent data have validated the AJCC TNM primary tumor pathological designations stage T1a and T1b as having different capacities for metastasis to inguinal nodes.[29] In a series reported by Sun et al., which included two cohorts, the incidence of inguinal metastases was 10.5-18.1% for pT1a tumors versus 33.3-50% for pT1b tumors. In addition to lymphovascular space involvement and high tumor grade, the presence of perineural invasion also has been shown to be significantly associated with inguinal lymph node metastasis and now is included as a criterion to define pT1b (Fig. 57.4).[30] According to both the European Association of Urology (EAU) and the NCCN guidelines, patients with proven noninvasive and superficially invasive lesions (i.e., Tis-T1) are candidates for organ-preserving surgical procedures, in addition to radiotherapy in select cases.[21,31]

In addition, recent studies provide a justification for the separation of tumors invading the corpus spongiosum from those invading the corpora cavernosa as pT2 and pT3 tumors respectively (Fig. 57.5–57.6). In two cohorts, invasion into the corpus spongiosum versus corpora cavernosa was associated with improved disease-specific survival ([77.7% versus 52.6%, respectively] and a lower incidence of inguinal lymph node metastasis [33-35% versus 48.6-52.5%, respectively]).[29,32]

Extensive tumors invading the scrotum and prostate (i.e., pT4; Figs. 57.7–57.9) are less common but may require either major amputative procedures, neoadjuvant chemotherapy prior to surgery, or palliative radiotherapy if unresectable.[31]

Regional Lymph Node

The presence and extent of lymph node metastasis dictate survival in penile cancer. Inguinal lymph node dissection provides the most accurate pathological assessment of the status of the inguinal nodes. To limit the morbidity associated with complete removal, DSNB has been refined and validated with sensitivities greater than 90% in some, but not all, centers to determine the presence of inguinal lymph node metastasis.[33,34]

Recent series have suggested refinements to the 7th Edition AJCC TNM lymph node definitions to better stratify the prognosis of patients with inguinal lymph node metastasis.[29,35] Data from Li, et al., revealed that the 3-year, disease-specific survival of patients with one or two unilateral inguinal metastases with no ENE was similar to patients with a single node of the same characteristics (89-90%, Fig. 57.15).[35]

Migrating patients with one or two nodes into the pN1 category places more patients into a low-risk category and avoids the need for adjuvant chemotherapy recommended for pN2 patients according to the EAU guidelines.[31]

Alternatively, patients with more than three or more positive unilateral inguinal nodes or bilateral metastasis (pN2, Figs. 57.16–57.17) have distinctly worse 3-year, disease-specific survival compared with those with one or two unilateral inguinal nodes (60% pN2 versus approximately 90% pN1).[35]

Based upon two series, the pN3 category (Figs. 57.18–57.20) remains defined as the presence of ENE or positive pelvic nodes. This group has an ominous 3–year cancer specific or relapse–free survival of at most 32-33%.[35,36] In this scenario, adjuvant chemotherapy is a strong consideration according to the EAU guidelines.[31]

PROGNOSTIC FACTORS

Prognostic Factors Required for Stage Grouping

Beyond the factors used to assign T, N, or M categories, no additional prognostic factors are required for stage grouping.

Additional Factors Recommended for Clinical Care

Tumor Grade

Tumor grade has traditionally been based on modifications of the Broder's grading system and consists of either a 3- or 4-grade system where grade 1 = well differentiated, 2 = moderately differentiated, and 3/4 = poorly differentiated/undifferentiated.[10,11,37,38] WHO has recently adopted the three-tiered WHO/ ISUP grading system.[38] Any proportion of anaplastic cells is sufficient to categorize a tumor as grade 3. Tumor grade has been a predictor of lymph node metastasis and outcome in many studies and has been shown to improve outcome prediction when added to tumor staging systems.[10,29,39] The grade 3 category, or presence of a sarcomatoid component, is important in separating stage T1b from T1a primary tumors, respectively. AJCC Level of Evidence: I

Lymphovascular Invasion (LVI)

LVI is a strong predictor of lymph node metastasis in several studies. It is characterized by the presence of tumor emboli seen within endothelium-lined spaces separated from the primary tumor at least one high power field.[10,40-42] The presence or absence of LVI is important in defining category T1b versus T1a primary tumors, respectively (see Fig. 57.4). AJCC Level of Evidence: I

Perineural Invasion (PNI)

PNI is characterized by tumor cells' infiltrating nerve branches separated from the main tumor mass. In one study, it was highly associated with lymph node metastasis.[30] The presence or absence of PNI is important in defining category T1b versus T1a primary tumors, respectively (see Fig. 57.4). AJCC Level of Evidence: III

Size of Largest Lymph Node Metastasis

The size of nodal metastasis will be reported as the maximum diameter (measured in millimeters) of the metastatic tumor focus in the node. A nodal mass should be counted as one node and the maximum diameter recorded. Inguinal lymph node metastasis size has been associated with pelvic lymph node metastasis.[43] AJCC Level of Evidence: III

Total Number of Lymph Nodes Removed

Total number of lymph nodes removed may serve as an index of the quality of surgical care for penile cancer and was shown by Johnson, et al., in a population-based study to be associated with enhanced 5 year survival when eight or more lymph nodes were removed, compared with having fewer than eight nodes removed.[44] In addition, this variable also is useful in calculating lymph node density (i.e., number of positive inguinal nodes/total nodes removed), which has been shown to be a strong prognostic factor for survival in several single-center series.[44,45] AJCC Level of Evidence: II

RISK ASSESSMENT MODELS

The AJCC recently established guidelines that will be used to evaluate published statistical prediction models for the purpose of granting endorsement for clinical use.[46] Although this is a monumental step toward the goal of precision medicine, this work was published only very recently. Therefore, the existing models that have been published or may be in clinical use have not yet been evaluated for this cancer site by the Precision Medicine Core of the AJCC. In the future, the statistical prediction models for this cancer site will be evaluated, and those that meet all AJCC criteria will be endorsed.

DEFINITIONS OF AJCC TNM

Definition of Primary Tumor (T)

T Category	T Criteria
TX	Primary tumor cannot be assessed
T0	No evidence of primary tumor
Tis	Carcinoma *in situ* (Penile intraepithelial neoplasia [PeIN])
Ta	Noninvasive localized squamous cell carcinoma

T Category	T Criteria
T1	Glans: Tumor invades lamina propria<br>Foreskin: Tumor invades dermis, lamina propria, or dartos fascia<br>Shaft: Tumor invades connective tissue between epidermis and corpora regardless of location<br>All sites with or without lymphovascular invasion or perineural invasion and is or is not high grade
T1a	Tumor is without lymphovascular invasion or perineural invasion and is not high grade (i.e., grade 3 or sarcomatoid)
T1b	Tumor exhibits lymphovascular invasion and/or perineural invasion or is high grade (i.e., grade 3 or sarcomatoid)
T2	Tumor invades into corpus spongiosum (either glans or ventral shaft) with or without urethral invasion
T3	Tumor invades into corpora cavernosum (including tunica albuginea) with or without urethral invasion
T4	Tumor invades into adjacent structures (i.e., scrotum, prostate, pubic bone)

Definition of Regional Lymph Node (N)

Clinical N (cN)

cN Category	cN Criteria
cNX	Regional lymph nodes cannot be assessed
cN0	No palpable or visibly enlarged inguinal lymph nodes
cN1	Palpable mobile unilateral inguinal lymph node
cN2	Palpable mobile ≥ 2 unilateral inguinal nodes or bilateral inguinal lymph nodes
cN3	Palpable fixed inguinal nodal mass or pelvic lymphadenopathy unilateral or bilateral

Pathological N (pN)

pN Category	pN Criteria
pNX	Lymph node metastasis cannot be established
pN0	No lymph node metastasis
pN1	≤2 unilateral inguinal metastases, no ENE
pN2	≥3 unilateral inguinal metastases or bilateral metastases
pN3	ENE of lymph node metastases or pelvic lymph node metastases

Definition of Distant Metastasis (M)

M Category	M Criteria
M0	No distant metastasis
M1	Distant metastasis present

AJCC PROGNOSTIC STAGE GROUPS

When T is...	And N is...	And M is...	Then the stage group is...
Tis	N0	M0	0is
Ta	N0	M0	0a
T1a	N0	M0	I
T1b	N0	M0	IIA
T2	N0	M0	IIA
T3	N0	M0	IIB
T1-3	N1	M0	IIIA
T1-3	N2	M0	IIIB
T4	Any N	M0	IV
Any T	N3	M0	IV
Any T	Any N	M1	IV

REGISTRY DATA COLLECTION VARIABLES

1. Histologic subtype
2. Size of largest nodal metastasis
3. Total number of lymph nodes removed
4. High-risk HPV expression
5. p16 immunohistochemical expression
6. Urethral mucosal invasion

HISTOLOGIC GRADE (G)

G	G Definition
GX	Grade cannot be assessed
G1	Well differentiated
G2	Moderately differentiated
G3	Poorly differentiated/high grade

HISTOPATHOLOGIC TYPE

Non-HPV Related Squamous Cell Carcinomas

Usual type
Pseudoglandular
Verrucous
Cuniculatum
Papillary, NOS
Pseudohyperplastic
Adenosquamous
Sarcomatoid
Mixed

HPV-Related Squamous Cell Carcinomas

Basaloid
Papillary-basaloid
Warty
Warty-basaloid
Clear cell

Rare Other Squamous Cell Carcinomas

ILLUSTRATIONS

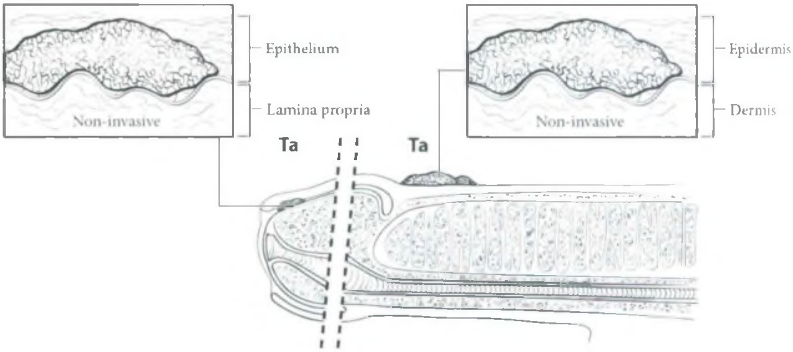

Fig. 57.3 Ta is defined as noninvasive localized squamous cell carcinoma

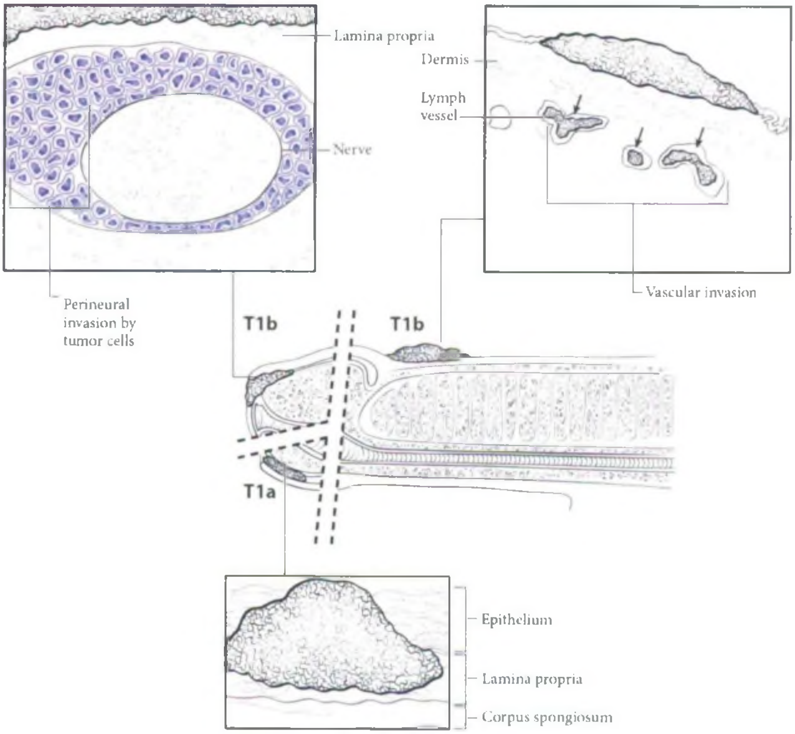

Fig. 57.4 T1a is defined as tumor without lymphovascular invasion or perineural invasion and is not high grade (i.e., grade 3 or sarcomatoid). T1b is defined as tumor that exhibits lymphovascular invasion and/or perineural invasion or is high grade (i.e., grade 3 or sarcomatoid)

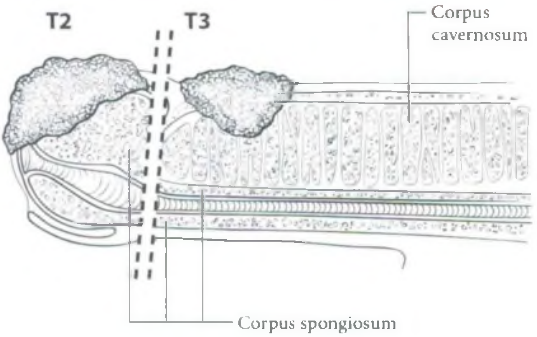

Fig. 57.5 T2 is defined as tumor that invades into corpus spongiosum (either glans or ventral shaft) with/without urethral invasion. T3 is defined as tumor that invades into corpora cavernosum (including tunica albuginea) with/without urethral invasion

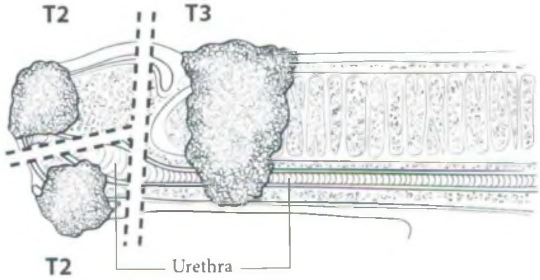

Fig. 57.6 T2 and T3 tumors with urethral invasion

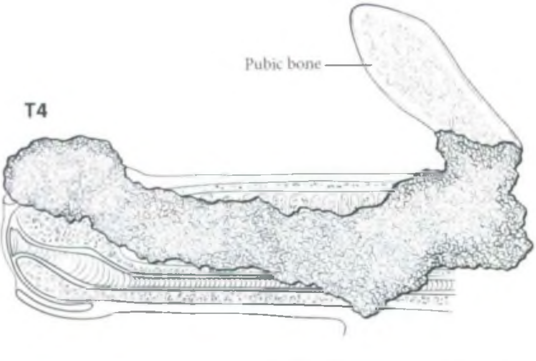

Fig. 57.7 T4 is defined as tumor that invades into adjacent structures such as scrotum, prostate, pubic bone (shown here)

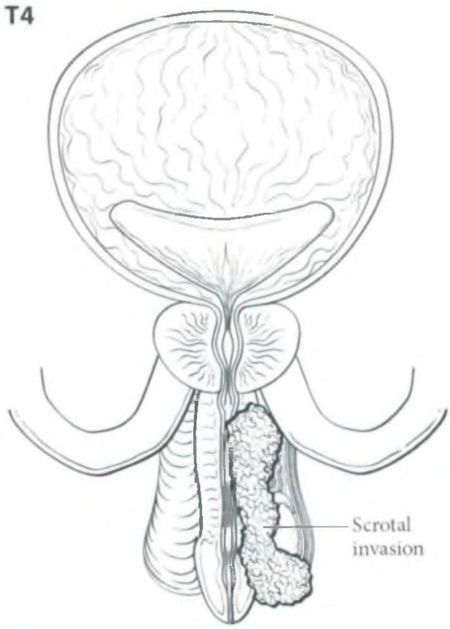

Fig. 57.8 T4 tumor with scrotal invasion

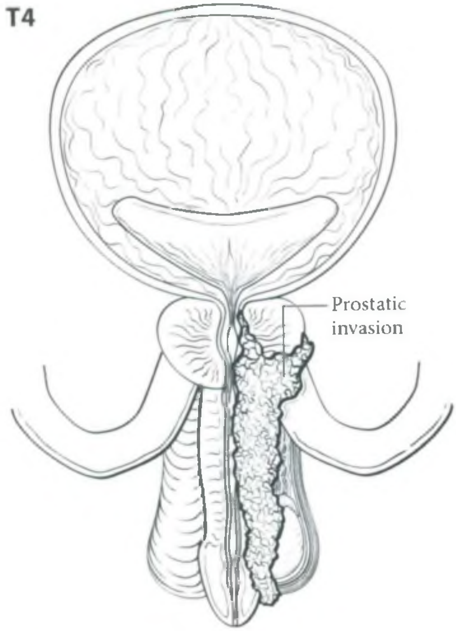

Fig. 57.9 T4 tumor with prostatic invasion

57

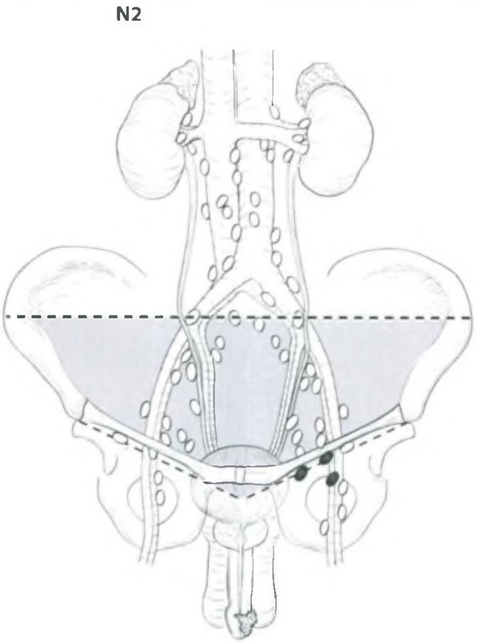

Fig. 57.10 Clinical N2 is defined as palpable mobile or bilateral lymph nodes

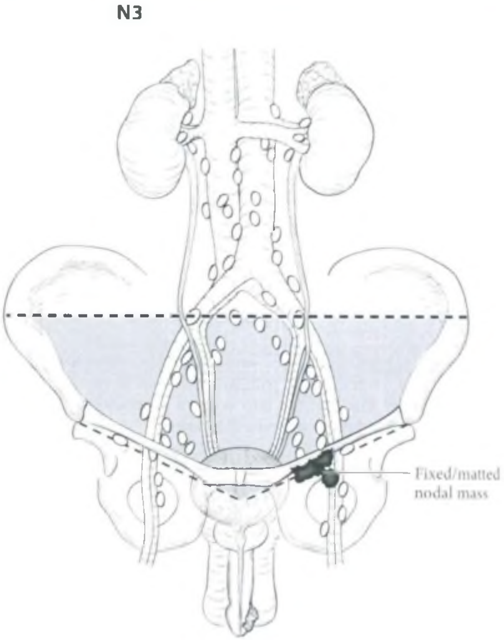

Fig. 57.12 Clinical N3 is defined as palpable fixed inguinal nodal mass or pelvic lymphadenopathy unilateral or bilateral

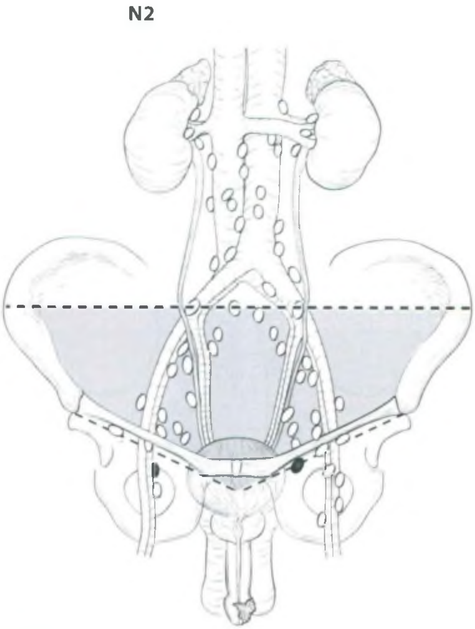

Fig. 57.11 Clinical N2 is defined as palpable mobile or bilateral lymph nodes

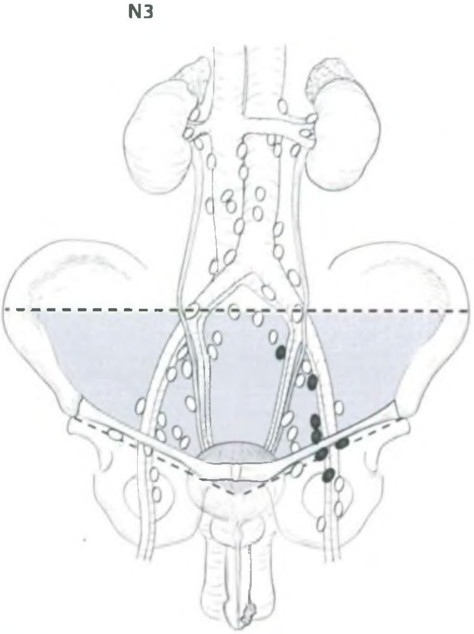

Fig. 57.13 Clinical N3 is defined as palpable fixed inguinal nodal mass or pelvic lymphadenopathy unilateral or bilateral

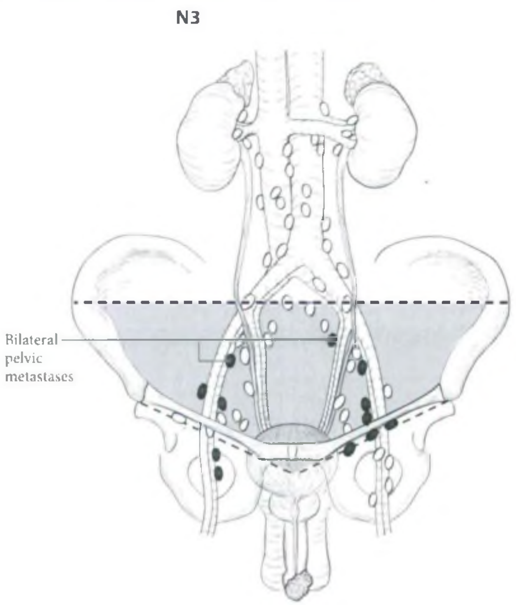

Fig. 57.14 Clinical N3 is defined as palpable fixed inguinal nodal mass or pelvic lymphadenopathy unilateral or bilateral

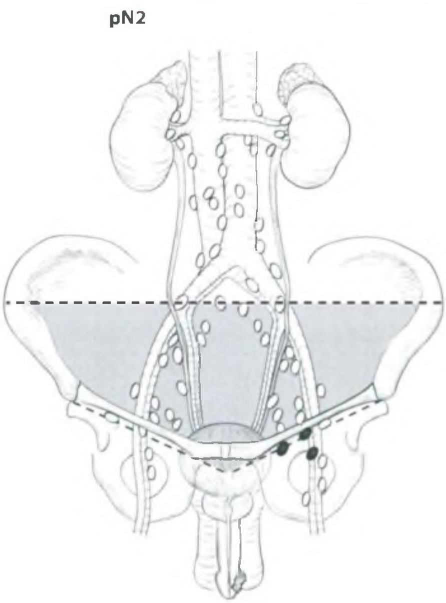

Fig. 57.16 pN2 is defined as three or more unilateral metastases or bilateral metastases

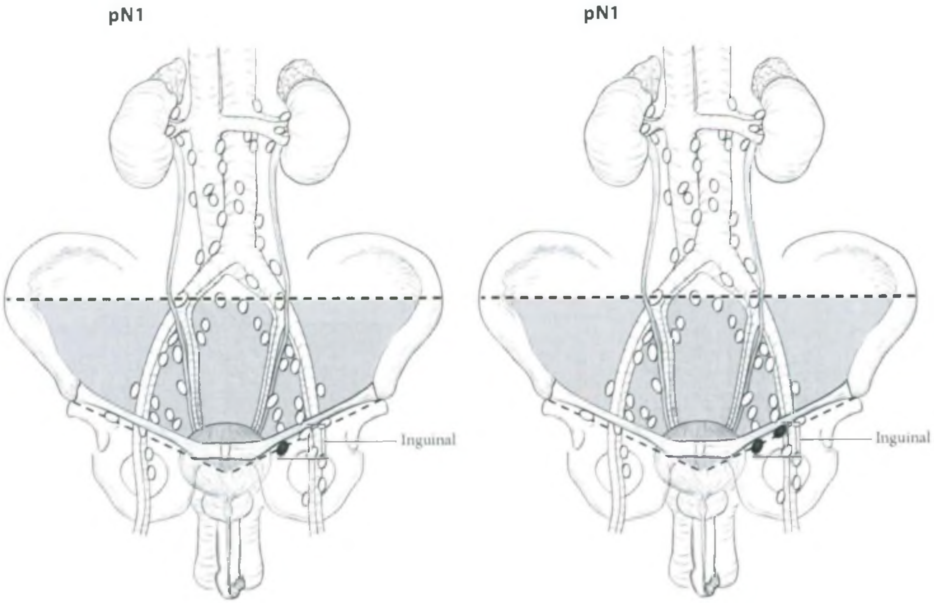

Fig. 57.15 pN1 is defined as one-two unilateral inguinal metastases, no extra nodal extension

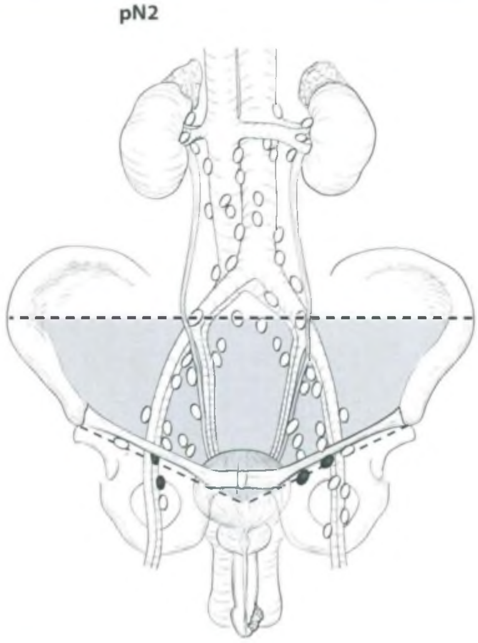

Fig. 57.17 pN2 is defined as three or more unilateral metastases or bilateral metastases

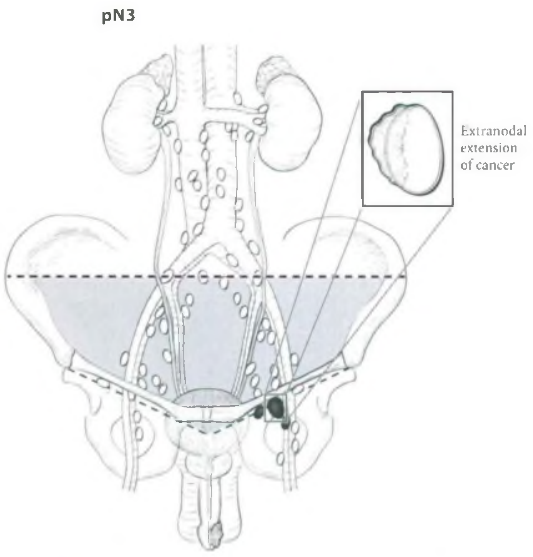

Fig. 57.18 pN3 is defined as extra nodal extension of lymph node metastases or pelvic lymph node metastases

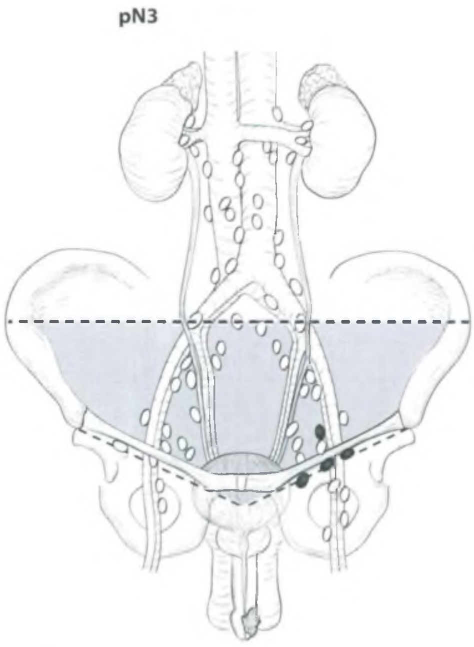

Fig. 57.19 pN3 is defined as extra nodal extension of lymph node metastases or pelvic lymph node metastases

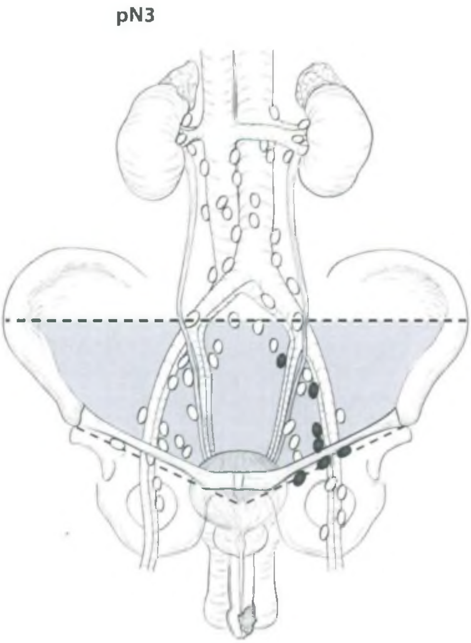

Fig. 57.20 pN3 is defined as extra nodal extension of lymph node metastases or pelvic lymph node metastases

Bibliography

1. Hernandez BY, Barnholtz-Sloan J, German RR, et al. Burden of invasive squamous cell carcinoma of the penis in the United States, 1998-2003. *Cancer.* Nov 15 2008;113(10 Suppl):2883–2891.
2. Siegel R, Ma J, Zou Z, Jemal A. Cancer statistics, 2014. *CA: a cancer journal for clinicians.* Jan-Feb 2014;64(1):9–29.
3. Derakhshani P, Neubauer S, Braun M, Bargmann H, Heidenreich A, Engelmann U. Results and 10-year follow-up in patients with squamous cell carcinoma of the penis. *Urol Int.* 1999;62(4):238–244.
4. Heidegger I, Borena W, Pichler R. The role of human papilloma virus in urological malignancies. *Anticancer research.* May 2015; 35(5):2513–2519.
5. Barbagli G, Palminteri E, Mirri F, Guazzoni G, Turini D, Lazzeri M. Penile carcinoma in patients with genital lichen sclerosus: a multicenter survey. *J Urol.* Apr 2006;175(4):1359–1363.
6. Archier E, Devaux S, Castela E, et al. Carcinogenic risks of psoralen UV-A therapy and narrowband UV-B therapy in chronic plaque psoriasis: a systematic literature review. *J Eur Acad Dermatol Venereol.* May 2012;26 Suppl 3:22–31.
7. Guimaraes GC, Rocha RM, Zequi SC, Cunha IW, Soares FA. Penile cancer: epidemiology and treatment. *Curr Oncol Rep.* Jun 2011; 13(3):231–239.
8. Schoen EJ, Oehrli M, Colby C, Machin G. The highly protective effect of newborn circumcision against invasive penile cancer. *Pediatrics.* Mar 2000;105(3):E36.
9. Giuliano AR, Palefsky JM, Goldstone S, et al. Efficacy of quadrivalent HPV vaccine against HPV Infection and disease in males. *N Engl J Med.* Feb 3 2011;364(5):401–411.
10. Pettaway C, Lance R, Davis J. Tumors of the Penis. In: Wein A, Kavoussi L, Novick A, Partin A, Peters C, eds. *Campbell-Walsh Urology.* 10th ed2012:901–933.
11. Edge S, Byrd D, Compton C. Penis. *AJCC Cancer Staging Manual.* 7th ed2010.
12. Chung B, Sommer G, Brooks J. Anatomy of the Lower Urinary Tract and Male Genitalia. In: Wein A, Kavoussi L, Novick A, Partin A, Peters C, eds. *Campbell-Walsh Urology 10th edition.* 10th ed: Elsevier-Saunders; 2012:64–66.
13. Clemente CD. *Anatomy, a regional atlas of the human body.* Urban & Schwarzenberg; 1987.
14. Fernández MJ, Sánchez DF, Cubilla AL. Pathology, Risk Factors, and HPV in Penile Squamous Cell Carcinoma. *Management of Penile Cancer:* Springer; 2014:21–46.
15. Corral DA PC. Atlas of Surgical Oncology. In: Contemporary Principles and Practice, Cancer of Penis and Urethra. 2001:791–812.
16. Rouviere H. Anatomy of the Human Lymphatic System: A Compendium Translated From the Original 'Anatomie des Lymphatiques de l'Homme'and Rearranged for the Use of Students and Practitioners by MJ Tobias. *Ann Arbor, Michigan, Edward Brothers.* 1938:218–226.
17. Daseler EH, Anson BJ, Reimann AF. Radical excision of the inguinal and iliac lymph glands; a study based upon 450 anatomical dissections and upon supportive clinical observations. *Surg Gynecol Obstet.* Dec 1948;87(6):679–694.
18. Pettaway CA, Pagliaro L, Theodore C, Haas G. Treatment of visceral, unresectable, or bulky/unresectable regional metastases of penile cancer. *Urology.* Aug 2010;76(2 Suppl 1):S58–65.
19. Velazquez EF, Barreto JE, Rodriguez I, Piris A, Cubilla AL. Limitations in the interpretation of biopsies in patients with penile squamous cell carcinoma. *Int J Surg Pathol.* Apr 2004;12(2): 139–146.
20. Graafland NM, van Boven HH, van Werkhoven E, Moonen LM, Horenblas S. Prognostic significance of extranodal extension in patients with pathological node positive penile carcinoma. *J Urol.* Oct 2010;184(4):1347–1353.
21. NCCN Clinical Practice Guidelines in Oncology: Penile Cancer V3.2015. 2015;http://www.nccn.org/professionals/physician_gls/pdf/penile.pdf.
22. Kirkham A. MRI of the penis. *Br J Radiol.* Nov 2012;85 Spec No 1:S86–93.
23. Suh CH, Baheti AD, Tirumani SH, et al. Multimodality imaging of penile cancer: what radiologists need to know. *Abdom Imaging.* Feb 2015;40(2):424–435.
24. Sadeghi R, Gholami H, Zakavi SR, Kakhki VR, Tabasi KT, Horenblas S. Accuracy of sentinel lymph node biopsy for inguinal lymph node staging of penile squamous cell carcinoma: systematic review and meta-analysis of the literature. *J Urol.* Jan 2012; 187(1):25–31.
25. Graafland NM, Teertstra HJ, Besnard AP, van Boven HH, Horenblas S. Identification of high risk pathological node positive penile carcinoma: value of preoperative computerized tomography imaging. *J Urol.* Mar 2011;185(3):881–887.
26. Graafland NM, Leijte JA, Valdes Olmos RA, Hoefnagel CA, Teertstra HJ, Horenblas S. Scanning with 18F-FDG-PET/CT for detection of pelvic nodal involvement in inguinal node-positive penile carcinoma. *Eur Urol.* Aug 2009;56(2):339–345.
27. Brouwer OR vdBN, Mathéron HM, van der Poel HG, van Rhijn BW, BexA, van Tinteren H, Valdés Olmos RA, van Leeuwen FW, Horenblas S. A hybrid radioactive and fluorescent tracer for sentinel node biopsy in penile carcinoma as a potential replacement for blue dye. *Eur Urol.* 2014;65(3):600–609.
28. Sanchez DF, Soares F, Alvarado-Cabrero I, et al. Pathological factors, behavior, and histological prognostic risk groups in subtypes of penile squamous cell carcinomas (SCC). *Seminars in diagnostic pathology.* May 2015;32(3):222–231.
29. Sun M, Djajadiningrat RS, Alnajjar HM, et al. Development and external validation of a prognostic tool for prediction of cancer-specific mortality after complete loco-regional pathological staging for squamous cell carcinoma of the penis. *BJU Int.* Nov 2015; 116(5):734–743.
30. Velazquez EF, Ayala G, Liu H, et al. Histologic grade and perineural invasion are more important than tumor thickness as predictor of nodal metastasis in penile squamous cell carcinoma invading 5 to 10 mm. *The American journal of surgical pathology.* Jul 2008;32(7):974–979.
31. Hakenberg OW, Comperat EM, Minhas S, et al. EAU guidelines on penile cancer: 2014 update. *Eur Urol.* Jan 2015;67(1):142–150.
32. Leijte JA, Gallee M, Antonini N, Horenblas S. Evaluation of current TNM classification of penile carcinoma. *J Urol.* Sep 2008; 180(3):933–938; discussion 938.
33. Kirrander P, Andrén O, Windahl T. Dynamic sentinel node biopsy in penile cancer: initial experiences at a Swedish referral centre. *BJU international.* 2013;111(3b):E48-E53.
34. Leijte JA, Hughes B, Graafland NM, et al. Two-center evaluation of dynamic sentinel node biopsy for squamous cell carcinoma of the penis. *J Clin Oncol.* Jul 10 2009;27(20):3325–3329.
35. Li ZS, Yao K, Chen P, et al. Modification of N staging systems for penile cancer: a more precise prediction of prognosis. *Br J Cancer.* May 26 2015;112(11):1766–1771.
36. Zhu Y, Ye DW, Yao XD, Zhang SL, Dai B, Zhang HL. New N staging system of penile cancer provides a better reflection of prognosis. *J Urol.* Aug 2011;186(2):518–523.
37. Broders A. Squamous cell epithelioma of the skin. *Annals of surgery.* 1921(73):141–143.
38. Cubilla AL AM, Ayala A, Ayala G, Chaux A, Corbishley C, Dillner J, Moch H, Sanchez DF, Soares FA, Tamboli P, Young RH, Moch H, Reuter V, Humphrey P, Ulbright T. Malignant epithelial tumours. In: WHO Classification of Tumours of the Urinary System and Male Genital Organs. *International Agency for Research on Cancer. Lyon, 2016* 2016:262–276.

39. Thuret R, Sun M, Abdollah F, et al. Tumor grade improves the prognostic ability of American Joint Committee on Cancer stage in patients with penile carcinoma. *J Urol*. Feb 2011;185(2):501–507.
40. Lopes A, Hidalgo GS, Kowalski LP, Torloni H, Rossi BM, Fonseca FP. Prognostic factors in carcinoma of the penis: multivariate analysis of 145 patients treated with amputation and lymphadenectomy. *J Urol*. Nov 1996;156(5):1637–1642.
41. Ornellas AA, Nobrega BL, Wei Kin Chin E, Wisnescky A, da Silva PC, de Santos Schwindt AB. Prognostic factors in invasive squamous cell carcinoma of the penis: analysis of 196 patients treated at the Brazilian National Cancer Institute. *J Urol*. Oct 2008;180(4): 1354–1359.
42. Slaton JW, Morgenstern N, Levy DA, et al. Tumor stage, vascular invasion and the percentage of poorly differentiated cancer: independent prognosticators for inguinal lymph node metastasis in penile squamous cancer. *J Urol*. Apr 2001;165(4):1138–1142.
43. Lughezzani G, Catanzaro M, Torelli T, et al. The relationship between characteristics of inguinal lymph nodes and pelvic lymph node involvement in penile squamous cell carcinoma: a single institution experience. *The Journal of urology*. 2014;191(4): 977–982.
44. Johnson TV, Hsiao W, Delman KA, Jani AB, Brawley OW, Master VA. Extensive inguinal lymphadenectomy improves overall 5-year survival in penile cancer patients. *Cancer*. 2010;116(12): 2960–2966.
45. Svatek RS, Munsell M, Kincaid JM, et al. Association between lymph node density and disease specific survival in patients with penile cancer. *The Journal of urology*. 2009;182(6):2721–2727.
46. Kattan MW, Hess KR, Amin MB, et al. American Joint Committee on Cancer acceptance criteria for inclusion of risk models for individualized prognosis in the practice of precision medicine. *CA: a cancer journal for clinicians*. Jan 19 2016.

Prostate

58

Mark K. Buyyounouski, Peter L. Choyke, Michael W. Kattan, Jesse K. McKenney, John R. Srigley, Daniel A. Barocas, Fadi Brimo, Robert K. Brookland, Jonathan I. Epstein, Samson W. Fine, Susan Halabi, Daniel A. Hamstra, Malcolm D. Mason, William K. Oh, Curtis A. Pettaway, Oliver Sartor, Maria J. Schymura, Karim A. Touijer, Michael J. Zelefsky, Howard M. Sandler, Mahul B. Amin, and Daniel W. Lin

CHAPTER SUMMARY

Cancers Staged Using This Staging System

This classification applies to adenocarcinomas and squamous carcinomas of the prostate gland.

Cancers Not Staged Using This Staging System

These histopathologic types of cancer…	Are staged according to the classification for…	And can be found in chapter…
Sarcomas	Soft Tissue Sarcoma of the Abdomen and Thoracic Visceral Organs	42
Urothelial cell carcinomas	Urethra (prostatic urethra)	63
Urothelial carcinoma of bladder involving prostate	Urinary Bladder	62

Summary of Changes

Change	Details of Change	Level of Evidence
Definition of Primary Tumor (T)	Pathologically organ-confined disease is considered pT2 and no longer subclassified by extent of involvement or laterality.	III[1]
Histologic Grade (G)	The Gleason score (2014 criteria) and the Grade Group should both be reported.	II[2]
AJCC Prognostic Stage Groups	Stage III includes select organ-confined disease tumors based on prostate-specific antigen (PSA) and Gleason/Grade Group status.	II

ICD-O-3 Topography Codes

Code	Description
C61.9	Prostate Gland

WHO Classification of Tumors

Code	Description
8140	Acinar adenocarcinoma
8480	Mucinous (colloid) acinar adenocarcinoma
8490	Signet ring-like cell acinar adenocarcinoma

To access the AJCC cancer staging forms, please visit www.cancerstaging.org.

© American Joint Committee on Cancer 2017
M.B. Amin et al. (eds.), *AJCC Cancer Staging Manual, Eighth Edition*, DOI 10.1007/978-3-319-40618-3_58

Code	Description
8572	Sarcomatoid acinar adenocarcinoma
8148	Prostatic intraepithelial neoplasia, high-grade
8500	Intraductal carcinoma
8500	Ductal adenocarcinoma
8201	Cribriform ductal adenocarcinoma
8260	Papillary ductal adenocarcinoma
8230	Solid ductal adenocarcinoma
8120	Urothelial carcinoma
8560	Adenosquamous carcinoma
8070	Squamous cell carcinoma
8147	Basal cell carcinoma
8574	Adenocarcinoma with neuroendocrine differentiation
8240	Well-differentiated neuroendocrine tumor
8041	Small cell neuroendocrine carcinoma
8013	Large cell neuroendocrine carcinoma

Moch H. Humphrey PA. Ulbright TM. Reuter VE, eds. World Health Organization Classification of Tumours of the Urinary System and Male Genital Organs. Lyon: IARC; 2016.

INTRODUCTION

Prostate cancer is the most common noncutaneous cancer in men. Earlier detection is possible through a screening blood test, prostate-specific antigen (PSA), and the diagnosis is generally made using transrectal ultrasound (TRUS) guided biopsy. Prostate cancer has a tendency to metastasize to bone.

The incidence of both clinical and latent carcinoma increases with age. However, this cancer is rarely diagnosed clinically in men under 40 years of age. There are substantial limitations in the ability of both digital rectal examination (DRE) and TRUS to precisely define the size or local extent of disease; DRE currently is the most common modality used to define the local stage. Heterogeneity within the T1c category resulting from inherent limitations of either DRE or imaging to quantify the cancer is balanced by the inclusion of other prognostic factors, such as histologic grade, PSA level, and extent of cancer on needle biopsies that contain cancer. Diagnosis of clinically suspicious areas of the prostate can be confirmed histologically by needle biopsy. Less commonly, prostate cancer may be diagnosed by histologic examination of the resected tissue from a transurethral resection of the prostate (TURP) for obstructive voiding symptoms.

TNM staging is regarded as the clinical "gold-standard" for prostate cancer and is used as the basis for guiding treatment decision-making.[3] For example, the classification of T3, non-organ confined disease, has implications for adjuvant radiotherapy after surgery or adjuvant hormone therapy with radiotherapy. Patients with N1 disease diagnosed either clinically or pathologically, are increasingly recognized as benefiting from radiotherapy.[4-6] Conversely, patients with M1 disease who are asymptomatic are at this time unproven to benefit from initial local therapy.

Stage grouping for prostate cancer was improved in the American Joint Committee on Cancer (AJCC) Cancer Staging Manual, 7th Edition, to include PSA and tumor grade (i.e., Gleason score) for the first time. This practice continues in the 8th Edition with revisions to be in greater alignment with clinical experience and practice guidelines.[3,7-9] With this goal in mind, together with the general nature of prostate cancer diagnoses in which approximately 95% of cancers are diagnosed while still clinically localized, the stage grouping approach is similar to that for liver, bone, and gastrointestinal stromal tumors (GIST) in that Group III may include organ-confined disease. The precedence in other solid tumor types that take into account the established impact of tumor grade (e.g., mitotic rate in GIST) and location/multifocality (e.g., multiple organ-confined tumors in hepatocellular carcinoma and discontinuous organ-confined tumors in bone site) are the primary reasons governing the Group III designation in prostate for Gleason 4+5, 5+4, or 5+5 (Gleason sum 9-10, Grade Group 5) and/or PSA > 20 ng/mL, despite an organ-confined primary tumor.

In this 8th Edition, some other major changes of AJCC staging for prostate cancer have been made. The subclassification of pT2 in the 7th Edition used a three-tier system based on the extent and laterality of disease (i.e., pT2a vs. pT2b vs. pT2c). However, several recent studies have failed to demonstrate prognostic value of either pathological tumor extent or laterality when disease is organ-confined.[1] Therefore, all pathological organ-confined disease now is classified as pT2. Clinical staging, however, retains the three-tier subclassification.

The 7th Edition described prognostic stage grouping categories based on TNM, pretreatment serum PSA, and histologic grade. In this 8th Edition, the categories have been revised to improve the prognostic value and to be more in harmony with treatment guidelines of the National Comprehensive Cancer Network (NCCN) and the American Urological Association (AUA), in addition to incorporation of Grade Groups recently endorsed by the International Society of Urologic Pathology (ISUP).

ANATOMY

Primary Site(s)

Adenocarcinoma of the prostate most commonly arises within the peripheral zone of the gland, where it may be amenable to detection by DRE (Fig. 58.1). A less common site of origin is the anteromedial prostate, the transition zone, which is remote from the rectal surface and is the site of origin of benign nodu-

lar hyperplasia. The central zone, which composes most of the base of the prostate, seldom is the source of cancer but often is invaded by the spread of larger cancers. Pathologically, cancers of the prostate often are multifocal; 80–85% arise from the peripheral zone, 10–15% from the transitional zone, and 5–10% from the central zone. Anterior cancers also occur and may be detected more easily with saturation biopsy or magnetic resonance (MR) imaging.

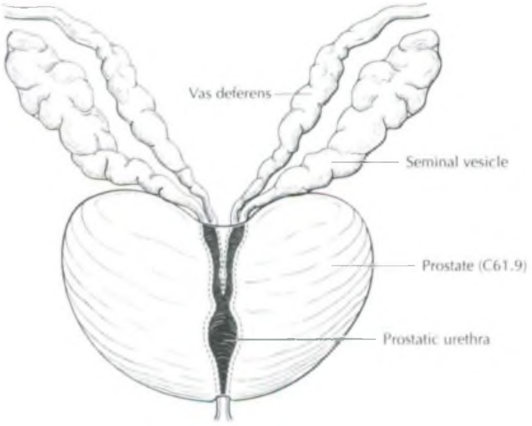

Fig. 58.1 Anatomy of the prostate

Regional Lymph Nodes

The regional lymph nodes are the nodes of the true pelvis, which essentially are the pelvic nodes below the bifurcation of the common iliac arteries (Fig. 58.2). They include the following groups:

- Pelvic, NOS
- Hypogastric
- Obturator
- Iliac (internal, external, or NOS)
- Sacral (lateral, presacral, promontory [Gerota's], or NOS)

Metastatic Sites

Distant lymph nodes lie outside the confines of the true pelvis. The distant lymph nodes include the following:

- Aortic (paraaortic lumbar)
- Common iliac
- Inguinal, deep
- Superficial inguinal (femoral)
- Supraclavicular
- Cervical
- Scalene
- Retroperitoneal, NOS

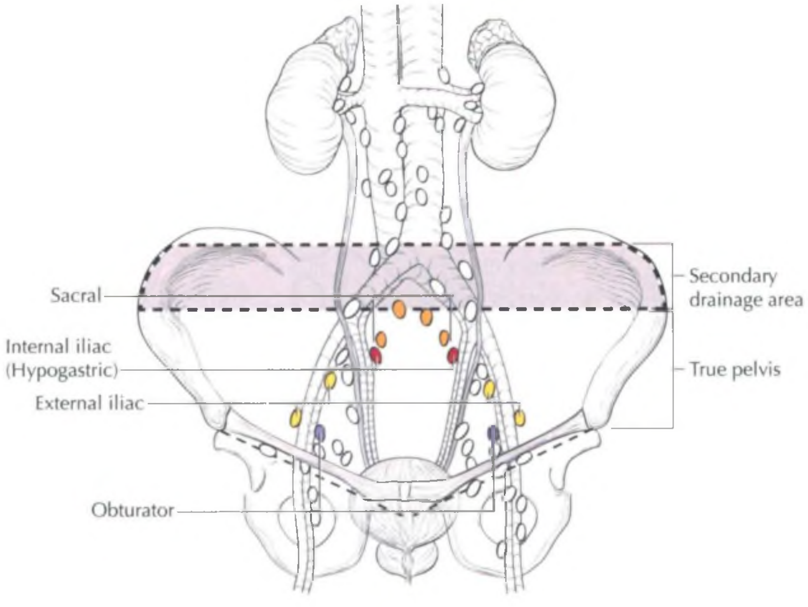

Fig. 58.2 Lymph nodes of the prostate. The shaded area represents distribution of regional lymph nodes. The non-shaded area indicates nodes outside of regional distribution

Osteoblastic metastases are the most common non-nodal osseous sites of prostate cancer metastasis. Lung and liver metastases usually are identified late in the course of the disease.

RULES FOR CLASSIFICATION

Clinical Classification

In healthy men suspected of having prostate cancer because of either an abnormal DRE or elevated PSA test, a transrectal needle biopsy of the prostate gland usually is performed, with ultrasound guidance, to obtain histologic or cytologic confirmation of prostate carcinoma. Alternatively, prostate cancer may be found in the tissue obtained during a TURP, where tissue is resected for relieving obstructive symptoms. In the situation where prostate cancer is incidentally found in less than 5% of the resected tissue, the T stage is designated as T1a; it also is accepted that T1a tumors would be Grade Group 1 (≤ 3+3) tumors. When cancer is found in more than 5% of the resected tissue and Grade Group 1 or any Grade Group 2-5 regardless of percentage tissue resected, then the T stage is designated as T1b (Fig. 58.3).

The primary clinical tumor assessment includes the information from the DRE of the prostate. Neither imaging information nor tumor laterality information from the prostate biopsy should be used for clinical staging. A tumor that is found in one or both sides by needle biopsy, but is not palpable or visible by imaging, is classified as T1c. Clinical T category should always reflect DRE findings only.

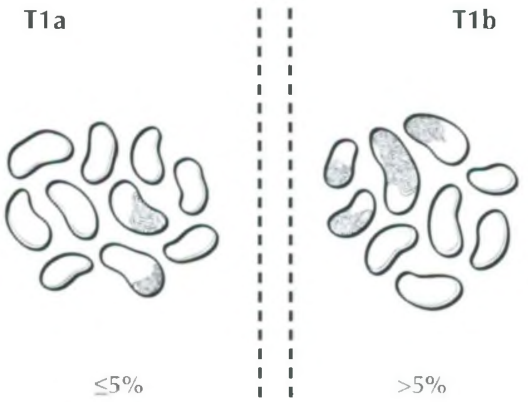

Fig. 58.3 Clinical T1a (left) is defined as a tumor with an incidental histologic finding in 5% or less of tissue resected. Clinical T1b (right) is defined as a tumor with an incidental histologic finding in more than 5% of tissue resected

Pathology reports of prostate biopsy also should include the number of positive biopsy regions, the biopsy core-length involvement, and both the Gleason score and Grade Group.

Lymph nodes can be imaged using ultrasound, computed tomography (CT), MR imaging, or lymphangiography. Although enlarged lymph nodes can occasionally be visualized on radiographic imaging, fewer patients are initially discovered with clinically evident metastatic disease. In lower risk patients, imaging tests have proven unhelpful. In lieu of imaging, risk tables often are used to determine individual patient risk of nodal involvement prior to therapy. Laterality of the regional nodes does not affect the N category. Involvement of distant lymph nodes is classified as M1a.

The histologic grade of the prostate cancer is important for prognosis. Based on published data from several thousand prostate cancers treated surgically and by radiation therapy, the World Health Organization (WHO) and ISUP formalized changes to Gleason scoring and the adoption of prognostically important Grade Groups for prostate cancer (ranging from 1-5) with Group 1 being ≤3+3 = 6 tumors; Group 2, 3+4 = 7; Group 3, 4+3 = 7; Group 4, Gleason sum 8; and Group 5, Gleason sum 9 and 10 tumors.[1,2,10] It is recommended that both the Gleason score and the Grade Group are reported together as both are used in the 8th Edition of the AJCC staging for prostate cancer.

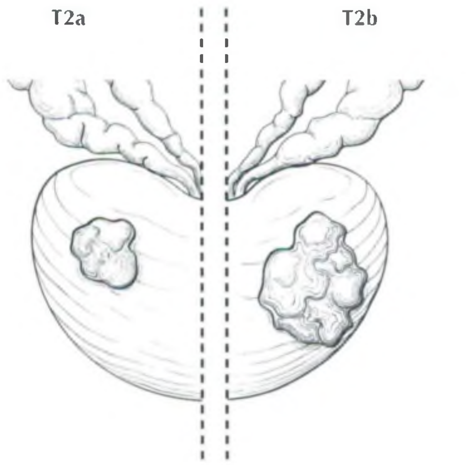

Fig. 58.4 Clinical T2 is defined as a tumor that is palpable and confined within the prostate. Clinical T2a (left) is defined as a tumor that involves one-half of one side or less, whereas clinical T2b (right) is defined as a tumor that involves more than one-half of one side but not both sides

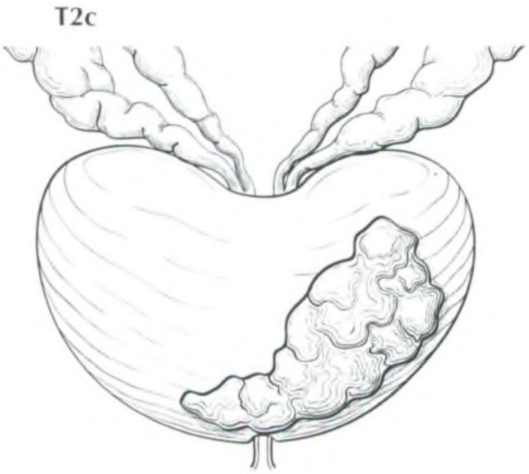

Fig. 58.5 Clinical T2c is defined as a tumor that involves both sides

Imaging

Although imaging could one day potentially improve clinical staging accuracy, interobserver reproducibility, issues with patient selection, and contradictory results have limited the utility of imaging in clinical staging, and imaging alone cannot replace the DRE as the clinical staging standard. Thus, for local T category assignment, no imaging test is explicitly required.

TRUS has not been proven to be satisfactory for predicting extracapsular/extraprostatic extension. Color Doppler and power Doppler identify increased vascularity, but have not yet been shown to improve staging accuracy. Similarly, contrast-enhanced and 3D ultrasound have not yet been adequately tested or shown to improve the delineation of the cancer and prostate capsule.

Three major MR imaging techniques that have been used to stage prostate cancer are T2-weighted MR imaging, MR spectroscopic imaging (MRSI), and dynamic contrast-enhanced MR imaging (DCE-MRI). None of these approaches has been proven to be consistently helpful in accurately staging the primary tumor.

MR imaging of the prostate has a relatively low predictive value for extraprostatic invasion, but MR imaging performs slightly better for seminal vesicle invasion. Although endorectal coil MR imaging provides high spatial resolution, there is controversy regarding the need for an endorectal coil, in particular depending on the strength of the imaging magnet; thus, the use of the endorectal coil is not routinely recommended. For N category, conventional MR imaging and CT are very insensitive for the detection of nodal abnormalities for most patients, as they rely on enlargement of the nodes, which carries both false negative and false positive errors. However, in high-risk patients, node enlargement can indicate the presence of nodal metastases. An enlarged node identified on MR imaging or CT can be targeted for biopsy or selective resection. For M category, Tc-99m bone scans are obtained to identify bone metastases in high-risk patients, typically defined by elevated PSA values (> 10 ng/mL).

Radiological reports of prostate MR imaging should localize the site of the primary cancer(s) and specify the presence of extraprostatic extension and/or seminal vesicle invasion. CT or MR imaging reports of the abdomen-pelvis should specify the number, size (bidimensional), and location of enlarged lymph nodes. Bone scans should report whether metastatic lesions are present or absent and if present, should specify the approximate number and location of the lesions. If correlative CT or MR imaging scans are available, the bone scan report also should include whether there is correspondence between the bone scan finding and the cross-sectional imaging.

Several emerging imaging methods have been suggested for prostate cancer staging. These include the sodium fluoride (NaF) positron emission tomography (PET)/CT scan that is more sensitive than a conventional bone scan and therefore, may be helpful in initial bone staging. A series of PET compounds targeting the prostate-specific membrane antigen (PSMA) appears to be highly sensitive for nodal and bony metastases and may emerge as a staging tool in the future. Other PET imaging agents such as C-11 choline, F-18 choline, and F-18 FACBC also have shown promise but cannot currently be recommended as a standard of care. Iron-oxide MR imaging also has shown promising results for nodal staging. None of these methods are widely available, and at this time, these methods are considered investigational.

Pathological Classification

Assigning the pathological T (pT) category is accomplished in radical prostatectomy specimens. In previous AJCC editions, organ-confined tumors (pT2) were subcategorized to maintain symmetry with clinical subcategories. However, given a lack of clinical relevance, the past subcategorization is not included in the 8th Edition. pT2 tumors are those confined to the prostate gland, while pT3 tumors are those with extension of tumor beyond the borders of the gland. The basic boundary of the prostate is a condensed fibromuscular layer of prostatic stroma, the "capsule", best recognized in posterior and posterolateral aspects of the gland, but not in the apex, anterior, or bladder neck regions. Even in regions with a well-defined capsule, tumor- or biopsy-related fibrous change may cause difficulty in the evaluation of extraprostatic extension.

Documenting and reporting pathological staging parameters in radical prostatectomy specimens is a key component in providing optimal management for patients. The College of American Pathologists provides guidelines on specimen handling.

In general, total prostatectomy, including regional lymph node dissection with full histologic evaluation, is required for complete pathological classification. Under certain circumstances, however, pathological T-classification can be determined with other means. For example, (1) positive biopsy of the rectum permits a pT4 classification without prostatectomy or a bladder transurethral resection demonstrating prostate cancer invasive into the bladder, and (2) a biopsy revealing carcinoma in extraprostatic soft tissue permits a pT3 classification, as does a biopsy revealing adenocarcinoma clearly infiltrating seminal vesicle smooth muscle tissue. There is no pT1 category.

Prostatectomy specimens should include Gleason score and Grade Group, and surgical margin status.

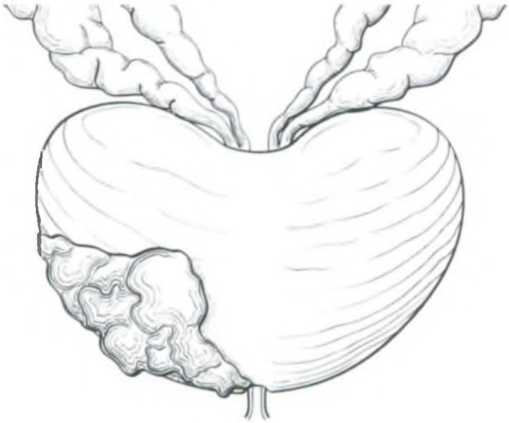

Fig. 58.6 Clinical and pathological T3a is defined as a tumor with unilateral extraprostatic extension

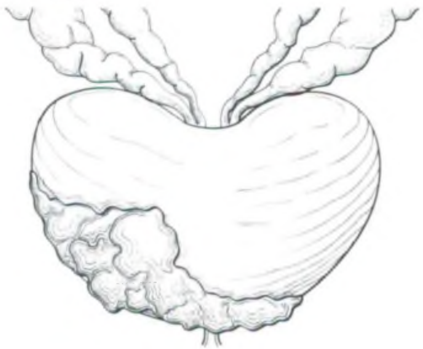

Fig. 58.7 Clinical and pathological T3a is defined as a tumor with bilateral extraprostatic extension

pT2

The 7th Edition of the AJCC TNM staging system subdivides pT2 disease into three categories: pT2a, pT2b, and pT2c, as determined by involvement of one-half of one side, more than one-half of one side, and involvement of both sides of the prostate gland. This system has been relied upon as a broad surrogate to describe cancer volume. Several retrospective outcome data analyses have challenged the utility of this subdivision. Sufficient evidence was found to justify collapsing pT2a, pT2b, and pT2c stages into a single category.[1] No data exist to allow correlation of pT2 stage subgroupings with survival in localized prostate cancer due to the indolent and prolonged clinical course of the disease.

pT3

The 8th Edition of the AJCC TNM staging system maintains the subdivision of pT3 disease into two categories: pT3a (Figs. 58.6 and 58.7) and pT3b (Fig. 58.8), as determined by the presence of extracapsular invasion in any location and the presence of seminal vesical invasion with or without extracapsular invasion, respectively.

The most easily recognizable sign of extraprostatic extension is tumor admixed with periprostatic fat. In the posterior and posterolateral prostate, a pT3a category also may be assigned to tumor identified within loose connective tissue and/or perineural spaces of the neurovascular bundles or to distinct tumor nodules within desmoplastic stroma that bulges beyond the prostatic contour. In the absence of clear histologic boundaries in the apex, anterior, and bladder neck regions, such evaluations are imprecise. Tumor detected in apex/distal margin sections is diagnosed as organ-confined (pT2). In the anterior prostate, the mingling of skeletal muscle, blood vessels, and medium-sized smooth muscle bundles among the anterior prostate and anterior extraprostatic space, make invasion into or at the level of adipose tissue the most reliable diagnostic feature of extraprostatic extension (pT3a). Some method of quantitation of extraprostatic extension is routinely reported, with the two most common approaches distinguishing "focal" (a few neoplastic glands just outside the prostate or extraprostatic tumor occupying less than 1 high-power field in no more than two sections) from "established" (more than focal). In the 7th Edition, microscopic bladder neck invasion (i.e., tumor detected in bladder neck/proximal margin sections) was reclassified as pT3a, rather than together with gross

invasion (pT4), a change that remains in the 8th Edition. Seminal vesicle invasion (pT3b) indicates tumor infiltration of the muscular wall of the seminal vesicle (Fig. 58.8). This finding should be distinguished from periseminal vesicle soft tissue invasion, which is staged as pT3a (extraprostatic extension). Pelvic lymph node dissection is the standard means for staging (pN) lymph node metastasis in prostate cancer. The number of lymph nodes identified and the number involved by tumor are routinely reported.

pT4

The 8th Edition of the AJCC TNM staging system also maintains the pT4 category to represent a tumor that is fixed or invades adjacent structures other than seminal vesicles such as external sphincter, rectum, bladder, levator muscles, and/or pelvic wall (Figs. 58.9 and 58.10).

Surgical Margin Status

Perhaps one of the more extensively debated aspects of pathological staging and risk stratification is one that technically is not an element of the current AJCC TNM staging system, namely the status of surgical resection margins in radical prostatectomy specimens. There is controversy regarding the "parameters or elements" to be reported in the case of identifying positive surgical margins in resected prostate glands. Although most agree that the pT category regardless of the margin status needs to be documented, there is no consensus on what aspects of surgical margin involvement are important to report.

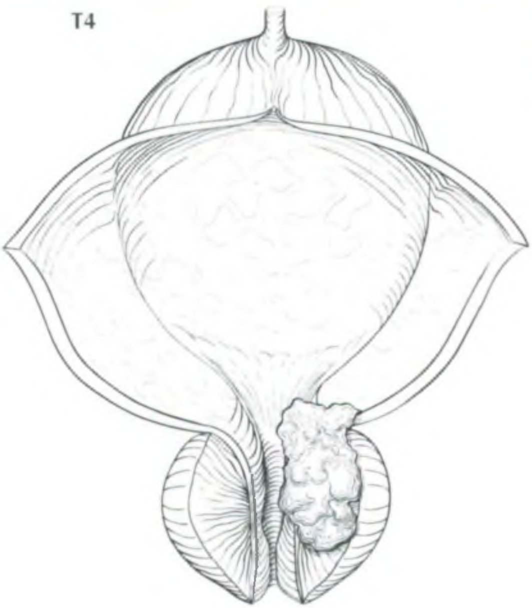

Fig. 58.9 Clinical and pathological T4 is defined as a tumor that is fixed or invades adjacent structures other than seminal vesicles such as bladder, as shown here, external sphincter, rectum, levator muscles, and/or pelvic wall

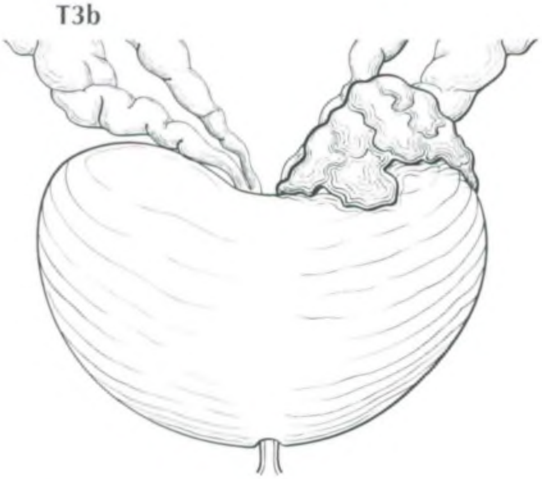

Fig. 58.8 Clinical and pathological T3b is defined as a tumor that invades seminal vesicle(s)

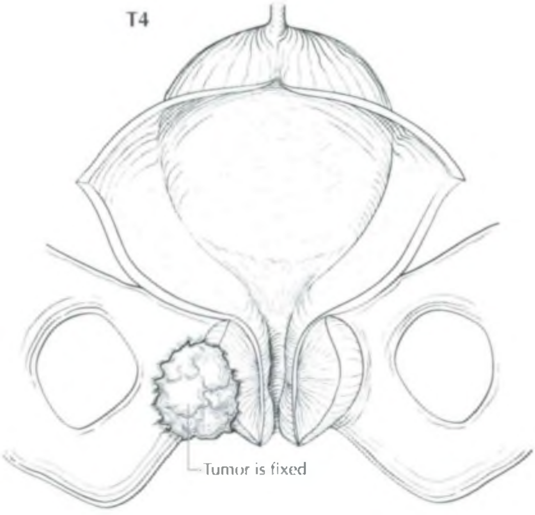

Fig. 58.10 Clinical and pathological T4 is defined as a tumor that is fixed to adjacent structures

PROGNOSTIC FACTORS

Prognostic Factors Required for Stage Grouping

Prostate-Specific Antigen (PSA)

PSA is a protein produced by cells of the prostate gland. The PSA level is measured PSA in blood. The results usually are reported as nanograms of PSA per milliliter (ng/mL) of blood. The higher a man's PSA level, the greater the risk of diagnosis of and mortality from prostate cancer.[11-15] In general, a PSA level less than 10 ng/mL is considered low, 10 to 20 ng/mL is intermediate, and greater than 20 ng/mL is high.[7] A PSA greater than 100 ng/mL without evidence of clinical metastasis is associated with much poorer survival.[16] AJCC Level of Evidence: I

Grade Group/Gleason Score

Grade Grouping is based on the histologic pattern of arrangement of carcinoma cells in hematoxylin and eosin-stained sections. Five basic grade patterns are used to generate a histologic Gleason score that ranges from 1 to 5. Grade Group is the stratification of histologic grade scores into prognostically relevant groups: Grade Group 1 (Gleason score ≤ 6), Grade Group 2 (Gleason score $3 + 4 = 7$), Grade Group 3 (Gleason score $4 + 3 = 7$), Grade Group 4 (Gleason score 8), and Grade Group 5 (Gleason score 9–10).[2,10,17-19] Grade Group is prognostic for PSA recurrence[2,17] as well as with prostate cancer mortality.[18] AJCC Level of Evidence: I

Additional Factors Recommended for Clinical Care

Surgical Margin Status

Although the status of surgical margins *per se* is not an element, it has potential impact for further postsurgical treatment and should be reported. In reporting pathological results of prostatectomy specimens, pT category should be reported along with margin status, and a positive surgical margin should be indicated by an R1 descriptor (residual microscopic disease). AJCC Level of Evidence: II

Histologic Type

The vast majority of prostate cancers are histologically described as acinar, microacinar, or conventional type. Histologic variants, including ductal carcinoma of prostate, signet ring-like cell adenocarcinoma of prostate, mucinous adenocarcinoma of prostate, adenosquamous carcinoma of prostate, small cell neuroendocrine carcinoma of prostate, and sarcomatoid carcinoma of prostate, are described and most of these histologic variants have an associated worse prognosis compared to the acinar or conventional adenocarcinoma; although due to their rarity of individual variants, stage matched studies to acinar adenocarcinoma are not available. AJCC Level of Evidence: III.

RISK ASSESSMENT MODELS

Prognostic models will continue to play an important role in 21st century medicine for several reasons.[20] First, by identifying which factors predict outcomes, clinicians gain insight into the biology and natural history of the disease. Second, treatment strategies may be optimized based on the outcome risks of the individual patient. Third, because of the heterogeneity of disease in most cancers, prognostic models will play a critical role in the design, conduct, and analysis of clinical trials in oncology.[20] If developed and validated appropriately, these models may become part of routine patient care and decision-making in trial design and conduct.

The AJCC Precision Medicine Core (PMC) developed and published criteria for critical evaluation of prognostic tool quality,[21] which are presented and discussed in Chapter 4. Although developed independently by the PMC, the AJCC quality criteria correspond fully with the recently developed Cochrane CHARMS tool for critical appraisal in systematic reviews of prediction modeling studies.[22]

Existing prognostic models for prostate cancer meeting all of the AJCC inclusion/exclusion criteria and meriting AJCC endorsement are presented in this section. A full list of the evaluated models and their adherence to the quality criteria is available on www.cancerstaging.org.

The PMC performed a systematic search of published literature for prognostic models/tools in prostate cancer from January 2011 to December 2015. The search strategy is provided in Chapter 4. The PMC defined "prognostic model" as a multivariable model where factors predict a clinical outcome that will occur in the future. Each tool identified was compared against the quality criteria developed by the PMC as guidelines for AJCC commendation for prognostication models (see Chapter 4).

Fifteen prognostication tools[23-37] for prostate cancer were identified: seven for patients with localized disease,[23-30] one for non-castrate patients with metastatic cancer,[31] and six for patients with metastatic castration-resistant prostate cancer.[32-37]

Of the 15 available models, 13 models were rejected based on the predefined criteria for exclusion.[23-31,33,34,36-38] For most of the models, the proportion of patients with missing data in the validation set was not stated.[23-29,31,34,38] Three of the models did not report on the follow-up status of the patients.[26,34,37]

Table 58.1 Prognostic tools for prostate cancer that met all AJCC quality criteria

Approved Prognostic Tool	Web Address	Factors Included in the Model
Metastatic castration-resistant prostate cancer[39]	https://www.cancer.duke.edu/Nomogram/firstlinechemotherapy.html	ECOG performance status, site of metastases, PSA, hemoglobin, albumin, alkaline phosphatase, LDH > 1 ULN, opioid analgesic use
Metastatic castration-resistant prostate cancer treated with second-line chemotherapy[35]	https://www.cancer.duke.edu/Nomogram/secondlinechemotherapy.html	ECOG performance status, visceral disease, progression on docetaxel, duration on hormone, measurable disease, pain, PSA, hemoglobin, alkaline phosphatase

One of the models for patients with localized disease met 11 out of the 14 criteria, although the endpoint was based on prostate cancer-specific survival rather than overall survival.[23] For this model, the equation was not readily available, and the number of events was unspecified.[23] Several models for patients with localized disease did not use overall survival as the outcome,[23-30] or were not validated (internally or externally), or did not provide the calibration plots. Hence, the PMC determined that these models were not readily available for use.[23-30] The model for non-castrate patients with metastatic disease lacked sufficient details on the number of events and on calibration, and was neither validated nor readily available for use.[31]

Among the six models for metastatic disease, two met all inclusion criteria[35,39] and are available online (Table 58.1). One model was for chemotherapy naive patients,[39] and the other model was for patients for whom first-line chemotherapy had failed.[35] Other models in the advanced metastatic setting met a subset of the inclusion criteria but did not include a calibration plot or were not readily available for use.[33,36-38] The sixth model was presented in a scientific meeting but has not been published at the time of this writing.[36] Nevertheless, a separate publication reported the results of the validation of this model.[34]

Fifteen prognostic models in prostate cancer were identified, but only two models for metastatic disease met all predefined AJCC inclusion and exclusion criteria and are, therefore, endorsed by the AJCC.[35,39] Both of the endorsed models were based on data from large phase III trials in metastatic patients and were externally validated.[35,39] In the models for patients with localized prostate cancer disease, an outcome other than overall survival was used.[23-30] Although another endpoint may be appropriate in this setting, the present AJCC guidelines[21] focus on the use of overall survival as the outcome of interest. It is expected that these guidelines will evolve over time and that other endpoints besides overall survival will be developed for patients with localized disease.

Recent guidelines on the reporting of prediction model development and validation have been published.[40,41] It is important to emphasize that validation will always be a fundamental step in prediction modeling.[42] Although external validation is considered ideal, model developers may not have access to external data.[20] Other validation approaches, such as bootstrapping, may be acceptable.[42,43] Two key missing criteria that were lacking in the majority of models identified in the AJCC review process were calibration plots and the tools to facilitate clinical utility. These should be, and are, easily addressable. In following these guidelines, authors will enhance the rigorous development, validation, and overall quality of future prognostic tools, resulting in a larger number of tools being endorsed by the AJCC.

DEFINITIONS OF AJCC TNM

Definition of Primary Tumor (T)

Clinical T (cT)

T Category	T Criteria
TX	Primary tumor cannot be assessed
T0	No evidence of primary tumor
T1	Clinically inapparent tumor that is not palpable
T1a	Tumor incidental histologic finding in 5% or less of tissue resected
T1b	Tumor incidental histologic finding in more than 5% of tissue resected
T1c	Tumor identified by needle biopsy found in one or both sides, but not palpable
T2	Tumor is palpable and confined within prostate
T2a	Tumor involves one-half of one side or less
T2b	Tumor involves more than one-half of one side but not both sides
T2c	Tumor involves both sides
T3	Extraprostatic tumor that is not fixed or does not invade adjacent structures
T3a	Extraprostatic extension (unilateral or bilateral)
T3b	Tumor invades seminal vesicle(s)
T4	Tumor is fixed or invades adjacent structures other than seminal vesicles such as external sphincter, rectum, bladder, levator muscles, and/or pelvic wall

Pathological T (pT)

T Category	T Criteria
T2	Organ confined
T3	Extraprostatic extension
T3a	Extraprostatic extension (unilateral or bilateral) or microscopic invasion of bladder neck
T3b	Tumor invades seminal vesicle(s)
T4	Tumor is fixed or invades adjacent structures other than seminal vesicles such as external sphincter, rectum, bladder, levator muscles, and/or pelvic wall

Note: There is no pathological T1 classification.
Note: Positive surgical margin should be indicated by an R1 descriptor, indicating residual microscopic disease.

Definition of Regional Lymph Node (N)

N Category	N Criteria
NX	Regional nodes were not assessed
N0	No positive regional nodes
N1	Metastases in regional node(s)

Definition of Distant Metastasis (M)

M Category	M Criteria
M0	No distant metastasis
M1	Distant metastasis
M1a	Nonregional lymph node(s)
M1b	Bone(s)
M1c	Other site(s) with or without bone disease

Note: When more than one site of metastasis is present, the most advanced category is used. M1c is most advanced.

Definition of Prostate-Specific Antigen (PSA)

PSA values are used to assign this category.

PSA values
<10
≥10 <20
<20
≥20
Any value

Definition of Histologic Grade Group (G)

Recently, the Gleason system has been compressed into so-called Grade Groups.[44]

Grade Group	Gleason Score	Gleason Pattern
1	≤6	≤3+3
2	7	3+4
3	7	4+3
4	8	4+4
5	9 or 10	4+5, 5+4, or 5+5

AJCC PROGNOSTIC STAGE GROUPS

When T is...	And N is...	And M is...	And PSA is...	And Grade Group is...	Then the stage group is...
cT1a-c, cT2a	N0	M0	< 10	1	I
pT2	N0	M0	< 10	1	I
cT1a-c, cT2a	N0	M0	≥ 10 < 20	1	IIA
cT2b-c	N0	M0	<20	1	IIA
T1-2	N0	M0	<20	2	IIB
T1-2	N0	M0	<20	3	IIC
T1-2	N0	M0	<20	4	IIC
T1-2	N0	M0	≥20	1–4	IIIA
T3-4	N0	M0	Any	1–4	IIIB
Any T	N0	M0	Any	5	IIIC
Any T	N1	M0	Any	Any	IVA
Any T	N0	M1	Any	Any	IVB

Note: When either PSA or Grade Group is not available, grouping should be determined by T category and/or either PSA or Grade Group as available.

REGISTRY DATA COLLECTION VARIABLES

1. Pretreatment serum PSA levels lab value (in tenths, highest value XXX.X, last prediagnosis value)
2. Grade Group for clinical stage
3. Gleason score for clinical stage
4. Gleason patterns for clinical stage
5. Grade Group for pathological stage
6. Gleason score for pathological stage
7. Gleason patterns for pathological stage
8. Tertiary Gleason pattern on prostatectomy
9. Number of cores examined
10. Number of cores positive
11. Needle core biopsies positive in one side, both sides, beyond prostate
12. Metastatic sites

HISTOPATHOLOGIC TYPE

This classification applies to adenocarcinomas and squamous carcinomas, but not to sarcoma or transitional cell (urothelial) carcinoma of the prostate. Adjectives used to describe histologic variants of adenocarcinomas of prostate include mucinous, signet ring cell, ductal, and neuroendocrine, including small cell carcinoma. There should be histologic confirmation of the disease.

58

LLUSTRATIONS

N1

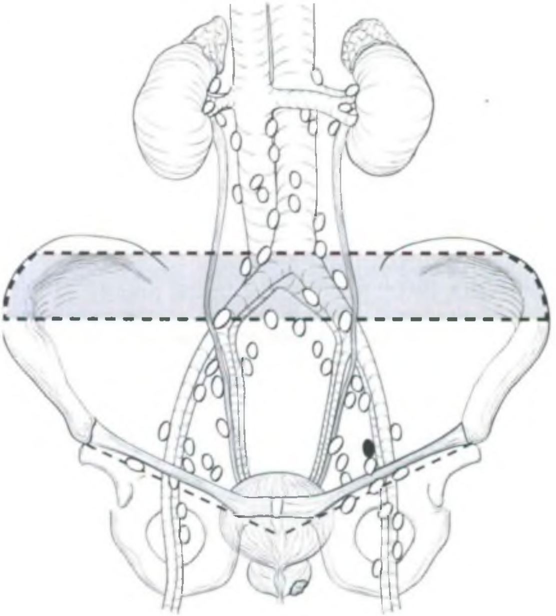

Fig. 58.11 N1 is metastasis in regional lymph nodes, shown here unilaterally

Bibliography

1. van der Kwast TH, Amin MB, Billis A, et al. International Society of Urological Pathology (ISUP) Consensus Conference on Handling and Staging of Radical Prostatectomy Specimens. Working group 2: T2 substaging and prostate cancer volume. Modern pathology : an official journal of the United States and Canadian Academy of Pathology, Inc. Jan 2011;24(1):16–25.
2. Epstein JI, Zelefsky MJ, Sjoberg DD, et al. A Contemporary Prostate Cancer Grading System: A Validated Alternative to the Gleason Score. Eur Urol. Mar 2016;69(3):428–435
3. Zaorsky NG, Li T, Devarajan K, Horwitz EM, Buyyounouski MK. Assessment of the American Joint Committee on Cancer staging (sixth and seventh editions) for clinically localized prostate cancer treated with external beam radiotherapy and comparison with the National Comprehensive Cancer Network risk-stratification method. Cancer. Nov 15 2012;118(22):5535–5543.
4. Abdollah F, Karnes RJ, Suardi N, et al. Impact of adjuvant radiotherapy on survival of patients with node-positive prostate cancer. J Clin Oncol. Dec 10 2014;32(35):3939–3947.
5. Briganti A, Karnes JR, Da Pozzo LF, et al. Two positive nodes represent a significant cut-off value for cancer specific survival in patients with node positive prostate cancer. A new proposal based on a two-institution experience on 703 consecutive N+ patients treated with radical prostatectomy, extended pelvic lymph node dissection and adjuvant therapy. Eur Urol. Feb 2009; 55(2):261–270.
6. Rusthoven CG, Carlson JA, Waxweiler TV, et al. The impact of definitive local therapy for lymph node-positive prostate cancer: a population-based study. International journal of radiation oncology, biology, physics. Apr 1 2014;88(5):1064–1073.
7. D'Amico AV, Whittington R, Malkowicz SB, et al. Biochemical outcome after radical prostatectomy, external beam radiation therapy, or interstitial radiation therapy for clinically localized prostate cancer. JAMA. Sep 16 1998;280(11):969–974.
8. Mohler JL, Kantoff PW, Armstrong AJ, et al. Prostate cancer, version 2.2014. Journal of the National Comprehensive Cancer Network : JNCCN. May 2014;12(5):686–718.
9. Partin AW, Kattan MW, Subong EN, et al. Combination of prostate-specific antigen, clinical stage, and Gleason score to predict pathological stage of localized prostate cancer. A multi-institutional update. JAMA. May 14 1997;277(18):1445–1451.
10. Moch H, Cubilla AL, Humphrey PA, Reuter VE, Ulbright TM. The 2016 WHO Classification of Tumours of the Urinary System and Male Genital Organs-Part A: Renal, Penile, and Testicular Tumours. Eur Urol. Feb 27 2016.
11. Roach M, 3rd, Weinberg V, McLaughlin PW, Grossfeld G, Sandler HM. Serum prostate-specific antigen and survival after external beam radiotherapy for carcinoma of the prostate. Urology. Apr 2003;61(4):730–735.
12. D'Amico AV, Chen MH, Roehl KA, Catalona WJ. Preoperative PSA velocity and the risk of death from prostate cancer after radical prostatectomy. N Engl J Med. Jul 8 2004;351(2):125–135.
13. D'Amico AV, Cote K, Loffredo M, Renshaw AA, Chen MH. Pretreatment predictors of time to cancer specific death after prostate specific antigen failure. J Urol. Apr 2003;169(4): 1320–1324.
14. Williams SG, Duchesne GM, Millar JL, Pratt GR. Both pretreatment prostate-specific antigen level and posttreatment biochemical failure are independent predictors of overall survival after radiotherapy for prostate cancer. International journal of radiation oncology, biology, physics. Nov 15 2004;60(4):1082–1087.
15. Kwan W, Pickles T, Duncan G, et al. PSA failure and the risk of death in prostate cancer patients treated with radiotherapy. International journal of radiation oncology, biology, physics. Nov 15 2004;60(4):1040–1046.
16. Ang M, Rajcic B, Foreman D, Moretti K, O'Callaghan ME. Men presenting with prostate-specific antigen (PSA) values of over 100 ng/mL. BJU Int. Feb 18 2016.
17. Berney D, Beltran L, Fisher G. Validation of contemporary prostate cancer grading system with long term outcome. British journal of cancer. (in press).
18. Berney D, Beltran L, Fisher G. Validation of contemporary prostate cancer grading system with long term outcome. British journal of cancer. (in press).
19. Kryvenko ON, Epstein JI. Changes in prostate cancer grading: Including a new patient-centric grading system. The Prostate. Apr 2016;76(5):427–433.
20. Halabi S, Owzar K. The importance of identifying and validating prognostic factors in oncology. Paper presented at: Seminars in oncology 2010.
21. Kattan MW, Hess KR, Amin MB, et al. American Joint Committee on Cancer acceptance criteria for inclusion of risk models for individualized prognosis in the practice of precision medicine. CA: a cancer journal for clinicians. Jan 19 2016.
22. Moons KG, de Groot JA, Bouwmeester W, et al. Critical appraisal and data extraction for systematic reviews of prediction modelling studies: the CHARMS checklist. PLoS medicine. Oct 2014;11(10): e1001744.
23. Eggener SE, Scardino PT, Walsh PC, et al. Predicting 15-year prostate cancer specific mortality after radical prostatectomy. J Urol. Mar 2011;185(3):869–875.
24. Brockman JA, Alanee S, Vickers AJ, et al. Nomogram Predicting Prostate Cancer-specific Mortality for Men with Biochemical Recurrence After Radical Prostatectomy. European urology. 2015;67(6):1160–1167.

25. Rajab R, Fisher G, Kattan MW, et al. An improved prognostic model for stage T1a and T1b prostate cancer by assessments of cancer extent. Modern pathology : an official journal of the United States and Canadian Academy of Pathology, Inc. Jan 2011;24(1):58–63.
26. Abdollah F, Boorjian S, Cozzarini C, et al. Survival following biochemical recurrence after radical prostatectomy and adjuvant radiotherapy in patients with prostate cancer: the impact of competing causes of mortality and patient stratification. European urology. 2013;64(4):557–564.
27. Giovacchini G, Incerti E, Mapelli P, et al. [11C] Choline PET/CT predicts survival in hormone-naive prostate cancer patients with biochemical failure after radical prostatectomy. European journal of nuclear medicine and molecular imaging. 2015;42(6):877–884.
28. Tollefson MK, Karnes RJ, Kwon ED, et al. Prostate cancer Ki-67 (MIB-1) expression, perineural invasion, and gleason score as biopsy-based predictors of prostate cancer mortality: the Mayo model. Paper presented at: Mayo Clinic Proceedings 2014.
29. Joniau S, Briganti A, Gontero P, et al. Stratification of high-risk prostate cancer into prognostic categories: a European multi-institutional study. Eur Urol. Jan 2015;67(1):157–164.
30. Abdollah F, Karnes RJ, Suardi N, et al. Predicting survival of patients with node-positive prostate cancer following multimodal treatment. European urology. 2014;65(3):554–562.
31. Gravis G, Boher JM, Fizazi K, et al. Prognostic Factors for Survival in Noncastrate Metastatic Prostate Cancer: Validation of the Glass Model and Development of a Novel Simplified Prognostic Model. Eur Urol. Sep 29 2014.
32. Halabi S, Lin C-Y, Kelly WK, et al. Updated prognostic model for predicting overall survival in first-line chemotherapy for patients with metastatic castration-resistant prostate cancer. Journal of Clinical Oncology. 2014;32(7):671–677.
33. Templeton AJ, Pezaro C, Omlin A, et al. Simple prognostic score for metastatic castration-resistant prostate cancer with incorporation of neutrophil-to-lymphocyte ratio. Cancer. Nov 1 2014;120(21):3346–3352.
34. Ravi P, Mateo J, Lorente D, et al. External validation of a prognostic model predicting overall survival in metastatic castrate-resistant prostate cancer patients treated with abiraterone. Eur Urol. Jul 2014;66(1):8–11.
35. Halabi S, Lin CY, Small EJ, et al. Prognostic model predicting metastatic castration-resistant prostate cancer survival in men treated with second-line chemotherapy. Journal of the National Cancer Institute. Nov 20 2013;105(22):1729–1737.
36. Chi KN, Kheoh TS, Ryan CJ, et al. A prognostic model for predicting overall survival (OS) in patients (pts) with metastatic castration-resistant prostate cancer (mCRPC) treated with abiraterone acetate (AA) after docetaxel. Paper presented at: ASCO Annual Meeting Proceedings 2013.
37. Fizazi K, Massard C, Smith M, et al. Bone-related parameters are the main prognostic factors for overall survival in men with bone metastases from castration-resistant prostate cancer. European urology. 2015;68(1):42–50.
38. Miyoshi Y, Noguchi K, Yanagisawa M, et al. Nomogram for overall survival of Japanese patients with bone-metastatic prostate cancer. BMC cancer. 2015;15(1):1.
39. Halabi S, Lin CY, Kelly WK, et al. Updated prognostic model for predicting overall survival in first-line chemotherapy for patients with metastatic castration-resistant prostate cancer. J Clin Oncol. Mar 1 2014;32(7):671–677.
40. Moons KG, Altman DG, Reitsma JB, et al. Transparent Reporting of a multivariable prediction model for Individual Prognosis or Diagnosis (TRIPOD): explanation and elaboration. Annals of internal medicine. Jan 6 2015;162(1):W1-73.
41. Collins GS, Reitsma JB, Altman DG, Moons KG. Transparent Reporting of a multivariable prediction model for Individual Prognosis or Diagnosis (TRIPOD): the TRIPOD statement. Annals of internal medicine. Jan 6 2015;162(1):55–63.
42. Harrell F. Regression modeling strategies: with applications to linear models, logistic and ordinal regression, and survival analysis. Springer; 2015.
43. Steyerberg EW, Moons KG, van der Windt DA, et al. Prognosis Research Strategy (PROGRESS) 3: prognostic model research. PLoS medicine. 2013;10(2):e1001381.
44. Epstein JI, Egevad L, Amin MB, et al. The 2014 International Society of Urological Pathology (ISUP) Consensus Conference on Gleason Grading of Prostatic Carcinoma: Definition of Grading Patterns and Proposal for a New Grading System. The American journal of surgical pathology. Feb 2016;40(2):244–252.

Testis

59

Fadi Brimo, John R. Srigley, Charles J. Ryan,
Peter L. Choyke, Peter A. Humphrey, Daniel A. Barocas,
Robert K. Brookland, Mark K. Buyyounouski,
Samson W. Fine, Susan Halabi, Daniel A. Hamstra,
Michael W. Kattan, Jesse K. McKenney,
Malcolm D. Mason, William K. Oh, Curtis A. Pettaway,
Karim A. Touijer, Michael J. Zelefsky, Howard M. Sandler,
Mahul B. Amin, and Daniel W. Lin

CHAPTER SUMMARY

Cancers Staged Using This Staging System

Postpubertal germ cell tumors of the testis and malignant sex cord-stromal tumors of the testis.

Cancers Not Staged Using This Staging System

These histopathologic types of cancer…	Are staged according to the classification for…	And can be found in chapter…
Spermatocytic tumor	No AJCC staging system	N/A
Nonmalignant sex cord-/gonadal-stromal tumors	No AJCC staging system	N/A
Prepubertal germ cell tumors	No AJCC staging system	N/A
Hematolymphoid tumors	Hematologic malignancies	79-82
Paratesticular neoplasms	No AJCC staging system	N/A

Summary of Changes

Change	Details of Change	Level of Evidence
Definition of Primary Tumor (T)	In pure seminoma, T1 is subclassified to T1a and T1b according to tumor's size using a 3 cm cutoff.	I
Definition of Primary Tumor (T)	Epididymal invasion is considered T2 rather than T1.	II
Definition of Primary Tumor (T)	Hilar soft tissue invasion is considered T2.	II
Definition of Distant Metastasis (M)	Discontinous involvement of the spermatic cord by vascular-lymphatic invasion represents M1 disease.	III

ICD-O-3 Topography Codes

Code	Description
C62.0	Undescended testis
C62.1	Descended testis
C62.9	Testis, NOS

WHO Classification of Tumors

Code	Description
9064	Germ cell neoplasia *in situ*
9061	Seminoma
9070	Embryonal carcinoma
9071	Yolk sac tumor, postpubertal type
9080	Teratoma, postpubertal type

To access the AJCC cancer staging forms, please visit www.cancerstaging.org.

© American Joint Committee on Cancer 2017

M.B. Amin et al. (eds.), *AJCC Cancer Staging Manual, Eighth Edition*, DOI 10.1007/978-3-319-40618-3_59

Code	Description
9084	Teratoma with somatic-type malignancies
9100	Choriocarcinoma
9104	Placental site trophoblastic tumor
9105	Epithelioid trophoblastic tumor
9085	Mixed germ cell tumor
8650	Malignant Leydig cell tumor
8640	Malignant Sertoli cell tumor
8591	Unclassified sex cord stromal tumor

Moch H, Humphrey PA, Ulbright TM, Reuter VE, eds. World Health Organization Classification of Tumours of the Urinary System and Male Genital Organs. Lyon: IARC; 2016.

INTRODUCTION

Cancer of the testis is nearly always of germ cell origin, usually affects young adults, and accounts for less than 1% of all malignancies in males. During the twentieth century, however, the incidence has more than doubled. Cryptorchidism is a predisposing condition, and the precursor lesion is germ cell neoplasia *in situ*. Postpubertal germ cell tumors of the testis are categorized into two main histologic types: seminoma and nonseminoma. The latter group is composed of either pure patterns or mixtures of histologic subtypes, including embryonal carcinoma, teratoma, choriocarcinoma, and yolk sac tumor. Seminoma components also may be found in a mixed tumor. The presence of elevation in serum tumor markers, including alpha-fetoprotein (AFP), human chorionic gonadotropin (hCG), and/or lactate dehydrogenase (LDH), is common in germ cell malignancies. Staging and prognostication are based on determination of the extent of disease and assessment of serum tumor markers. The TNM staging system for male germ cell tumors incorporates serum tumor marker elevation as a separate category (S) of staging information, which is unique to this organ site. Cancer of the testis is highly curable, even in cases with advanced metastatic disease. Therefore, clinical and pathological Stage IV are not included in this organ, and the highest stage is Stage IIIC.

In the American Joint Committee on Cancer (AJCC) Cancer Staging Manual, 8th Edition, we are addressing three aspects of clinical and/or pathological significance that were deficient or problematic in the 7th edition. First, the 7th edition did not take into account tumor size in the pT category, which now is assessed routinely by oncologists when considering adjuvant radiation therapy or carboplatin chemotherapy for seminoma.[1–8] In addition, the previous edition did not address the issue of hilar soft tissue invasion, a feature shown to be associated with higher stage disease in nonseminomatous germ cell tumors, and which is confused by pathologists with spermatic cord invasion resulting in variable assignments to pT1, pT2, or pT3 categories.[9, 10] Last, the pT1 and pT2 categories were not aligned from a histoanatomic standpoint with an understanding of the pathway of local tumor spread.

The 8th edition includes substantial changes and clarifications, particularly in the T category in which the recently reported and prognostically relevant pathological parameters are incorporated. It is important to note that large-scale studies of pathological factors in testicular germ cell tumors looking at tumor-specific survival are virtually impossible to conduct because of the excellent clinical outcomes, and therefore, studies using tumor recurrence have commonly been utilized. The pathological prognostic factors differ between seminoma and nonseminoma, and these differences are reflected here in the current staging system.

ANATOMY

Primary Site

The testes are composed of convoluted seminiferous tubules with a stroma containing functional endocrine interstitial cells. Both are encased in a dense capsule called the tunica albuginea, with fibrous septa extending into the testis and separating them into lobules. The tubules converge and exit at the hilum (mediastinum) of the testis into the rete testis and efferent ducts, which join a single duct. This duct, the epididymis, coils outside the upper and lower poles of the testicle and then joins the vas deferens, a muscular conduit that accompanies the vessels and lymphatic channels of the spermatic cord. The major route for tumor local extension is centrally through the hilum (mediastinum) of the testis, a region in direct continuity with the testicular parenchyma in which the tunica albuginea is absent. The hilar soft tissue is composed of fibroadipose tissue, lymphatic channels, and blood vessels of medium size. The tumor subsequently invades the epididymis and courses through the spermatic cord (Fig. 59.1). Extension of the tumor through the mesothelial covering of the tunica albuginea in the absence of hilar spread is very uncommon (< 5% of cases).

Regional Lymph Nodes

The following nodes are considered regional:

- Interaortocaval
- Paraaortic (periaortic)
- Paracaval
- Preaortic
- Precaval
- Retroaortic
- Retrocaval

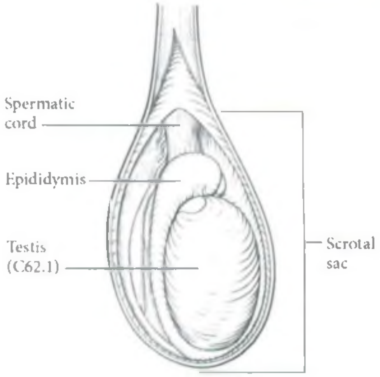

Fig. 59.1 Anatomy of the testis

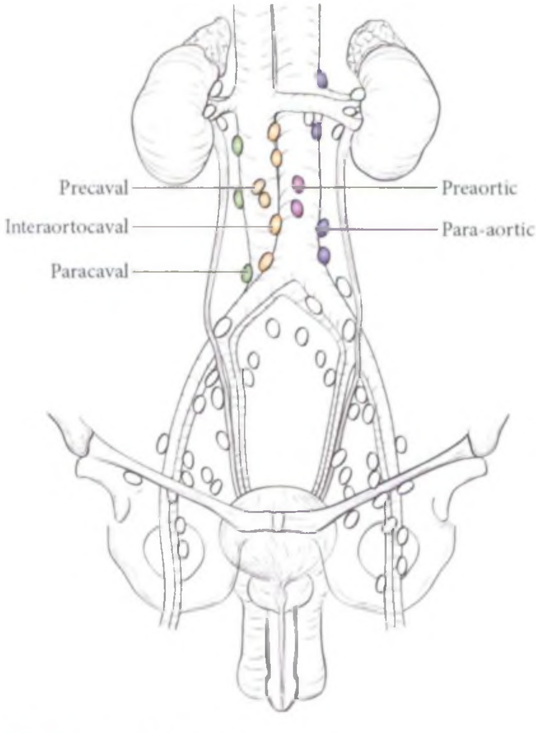

Fig. 59.2 Regional lymph nodes of the testis

The left and right testicles demonstrate predictable and different patterns of primary drainage that mirror the differences in venous drainage. The left testicle primarily drains to the paraaortic lymph nodes, and the right testicle primarily drains to the interaortocaval lymph nodes (Figs. 59.2 and 59.3). These areas usually are termed the "landing zones" of testicu-

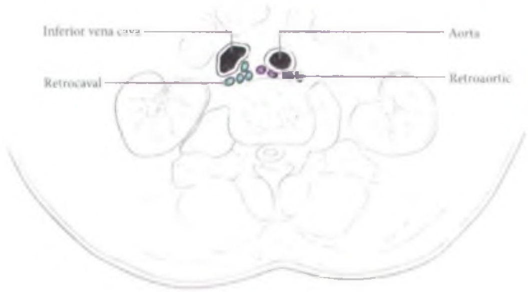

Fig. 59.3 Regional lymph nodes of the testis

lar tumors. The intrapelvic, external iliac, and inguinal nodes are considered regional only after scrotal or inguinal surgery prior to the presentation of the testis tumor. Nodes along the spermatic vein also are considered regional. All nodes outside the regional nodes are distant. Of note is that retroperitoneal nodes above the diaphragm are considered nonregional, and their involvement should be labeled as M1a.

Metastatic Sites

Distant spread of testicular tumors occurs most commonly to the lymph nodes, followed by metastases to the lung, liver, bone, and other visceral sites.

RULES FOR CLASSIFICATION

Clinical Classification

Staging of testis tumors includes determination of the T, N, M, and S categories. Clinical examination and histologic assessment are required for clinical staging. Radiographic assessment of the chest, abdomen, and pelvis is necessary to determine the N and M status of disease. Serum tumor markers—including AFP, hCG, and LDH—should be obtained prior to orchiectomy and are used to assign the clinical stage S category. These pretreatment markers also are needed for comparison when assigning the pathological stage S category, but levels after orchiectomy are used to complete the status of the serum tumor markers (S) for pathological staging.

Imaging

Although scrotal ultrasound is commonly used to diagnose testicular cancer, it is not used for local T-categorization. For nodal category assignment, computed tomography (CT) or magnetic resonance (MR) imaging of the abdomen is recom-

mended. For M category, a chest CT or chest radiograph also should be obtained.[11, 12]

Reports from CT or MR imaging studies performed for nodal assessment should specify the size (bi-dimensional) and location of abnormal nodes, which typically will be in the mid-retroperitoneum. Reports from studies performed for M assessment should specify the location of suspected metastases. Image-guided biopsy may be needed for confirmation of equivocal cross-sectional imaging findings.[11, 12]

Regional lymph nodes particularly relevant to testicular cancers are found near the termination of the testicular veins in the mid abdomen. The right testicular vein drains into the inferior vena cava, and the left testicular vein drains into the left renal vein.[11, 12] These areas should be evaluated carefully in imaging scans obtained for staging.

Pathological Classification

Histologic evaluation of the radical orchiectomy specimen must be used for the pT classification. The gross size of the tumor should be recorded. In the presence of multiple separated tumor nodules, the size of the largest tumor should be used for determining pT category. Careful gross examination should determine whether the tumor is intra- or extratesticular. It should be determined whether the tumor extends through the tunica albuginea and whether it invades the epididymis and/or hilar soft tissue and/or spermatic cord. Tissue sections should document these findings, and submitting routine sections from the hilar area is required. The tumor should be sampled extensively, including all grossly diverse areas (hemorrhagic, mucoid, solid, cystic, etc.). The junction of tumor and nonneoplastic testis and at least one block remote from the tumor should be obtained to determine whether germ cell neoplasia *in situ* (GCNIS) is present. These sections will allow assessment of either the presence or absence of vascular invasion. If possible, most tissue sections should include overlying tunica albuginea. Small tumors (2 cm or less) should be submitted *in toto*. For larger tumors, a sufficient amount of tissue should be sampled, generally one or two blocks per cm of maximum tumor diameter. If the sections show only a scar, possibly from regressed germ cell tumor, the entire lesion as well as multiple sections of the normal testis should be examined to rule out the presence of viable invasive tumor cells or GCNIS.

The clinical serum markers are needed for comparison when assigning the pathological stage S category, but levels after orchiectomy are used to complete the status of the serum tumor markers (S) for pathological staging. Serum tumor markers are measured immediately after orchiectomy and, if elevated, should be measured serially after orchiectomy to determine whether normal decay curves are followed and the absolute nadir levels of the tumor markers. The physiological half-life of AFP is 5–7 days, and the half-life of hCG is 24–48 hours. The presence of prolonged half-life times implies the presence of residual disease after orchiectomy. It should be noted that in some cases, tumor marker release may occur (e.g., in response to chemotherapy or handling of a primary tumor intraoperatively) and may cause transient and artefactual elevation of circulating tumor marker levels. The serum level of LDH has prognostic value in patients with metastatic disease and is included for staging, yet it is not unique to germ cell tumors. Stage grouping classification of Stage IS requires persistent elevation of serum tumor markers following orchiectomy after allowing adequate time for the markers to reach normal levels.

Subclassification of pT1

Several large studies of pure seminoma have identified that size is an independent predictor of disease recurrence and, therefore, tumor size is introduced as a defining parameter for substaging intratesticular pure seminomas, in congruence with the recommendations of the 2014 International Society of Urological Pathology consultation meeting.[1, 2, 5, 7] However, because of the variability in size cutoffs reported in the literature, it was agreed that a conservative approach is warranted and a relatively small size cutoff of 3 cm is used. It is important to note that the tumor size and substaging do not affect the overall stage grouping. Also, this subclassification only applies to pure seminomas, and nonseminomatous or mixed germ cell tumors are excluded. Furthermore, spermatocytic seminoma which is unrelated to the usual postpubertal germ cell tumors, recently has been renamed spermatocytic tumor because of its almost uniformly excellent prognosis, and it should be excluded from TNM staging.

Rete Testis Invasion

Although some studies suggest that involvement of the parenchyma of the rete testis (rete testis invasion and distinct from pagetoid involvement) is associated with a more advanced stage in pure seminoma, this observation was not confirmed in large contemporary cohorts and, therefore, it is not included as a pathological feature that necessitates change in staging.[2, 13] As such, the rete testis is considered part of the testis, and its involvement does not warrant a pT2 classification (Fig. 59.4).

Hilar Soft Tissue Versus Spermatic Cord Invasion

The hilar soft tissue invasion is categorized as pT2 (Fig. 59.5) and is defined as invasion of the adipose and loose fibrous connective tissue present in the hilar region beyond the boundaries of the rete testis and at the same plane of section as the testis parenchyma. The morphology of the spermatic cord may overlap with that of the hilar soft tissue, and

differentiating both is important in terms of tumor staging. Tumor grossly extending beyond the angle (notch) between the epididymis and spermatic cord proper is considered pT3 (Fig. 59.7). Microscopically, if the tumor surrounds or involves the vas deferens, then this is considered spermatic cord involvement (pT3). Correlation with the site from which sections were obtained also is important because the spermatic cord occasionally may be obliterated completely by tumor. Of note is that spermatic cord invasion refers to continuous involvement of the spermatic cord soft tissue by the primary tumor. Discontinuous involvement of the spermatic cord soft tissue via a vascular thrombus is better regarded as a metastatic deposit (pM1). Presence of only a intravascular tumor in the spermatic cord in the absence of parenchymal invasion is considered pT2.

Epididymal Invasion

Epididymal invasion is considered pT2 stage; it generally occurs secondary to invasion of the hilar soft tissue, but occasionally can be secondary to invasion through the tunica albuginea.

Lymphovascular Invasion

The presence of lymphovascular invasion (Fig. 59.6) changes the pathological T category of an intratesticular tumor. It is important to use strict criteria when assessing putative lymphovascular invasion because artifactual displacement of tumor cells (especially seminoma) and retraction artifacts are common and may lead to false positivity. Although lymphovascular invasion is better assessed in peritumoral locations, one can occasionally identify unequivocal intratumoral lymphovascular invasion.

Grading

Grading is not applicable in germ cell or sex cord-stromal tumors of the testis.

Lymph Nodes

The size cutoffs reported in the N category refer to the size of the involved lymph node on imaging or by histological examination and not the size of the metastatic deposit. The specimens from a defined node-bearing area (such as retroperitoneal lymph node dissection) must be used for the pN classification. All lymph nodes should be dissected, and the diameters of the largest nodes should be recorded, along with the number of lymph nodes involved by tumor. If present, extranodal soft tissue extension of disease should be noted. It is important to liberally sample and carefully examine the specimen, including cystic, fibrotic, hemorrhagic, necrotic, and solid areas. The aim is to identify viable germ cell tumor component or progression into non-germ cell somatic malignancy. Laterality does not affect the N category. In postchemotherapy specimens, it may be difficult to distinguish individual lymph nodes.

PROGNOSTIC FACTORS

Prognostic Factors Required for Stage Grouping

Serum Tumor Markers (S)

Testicular cancer is one of the few malignancies with associated serum tumor markers that guide both diagnosis and management of disease. These markers should be obtained at diagnosis, after orchiectomy, to monitor for response to treatment and for relapse in patients on surveillance. At the time of initial diagnosis, AFP levels are elevated in 50-70% of low stage (Stage Group I-IIB) and 60-80% of advanced (Stage Group IIB-IIIC) nonseminomatous germ cell tumor (NSGCT). It is important to note that patients with pure seminoma do not elaborate AFP; thus, patients who have pure seminoma in the orchiectomy specimen and have an elevated AFP are considered to have NSGCT. The half-life of AFP is 5–7 days. hCG levels are elevated in 20–40% of low stage NSGCT, 40–60% of advanced NSGCT, and 15% of pure seminomas. The half-life of hCG is 24–36 hours. LDH levels are elevated in approximately 20% of low stage and up to 50% of advanced stage germ cell tumors. The magnitude of LDH elevation directly correlates with tumor burden, but LDH is a nonspecific marker for testicular cancer. Patients suspected of having a GCT should have tumor marker levels obtained before orchiectomy, then serially afterwards to monitor for the expected decline in markers. Similarly, patients undergoing systemic therapy or radiation for advanced disease should have tumor markers obtained serially to monitor response to therapy. AJCC Level of Evidence: I

Additional Factors Recommended for Clinical Care

The International Germ Cell Consensus Classification Grouping (IGCCCG)

The IGCCCG system is widely used clinically to stratify patients into three risk categories based on the levels of serum markers and the presence of visceral metastasis or mediastinal location of the primary tumor.[14] This classification drives treatment decisions, in particular, the number of chemotherapy cycles. AJCC Level of Evidence: I

Lymphovascular Invasion

The presence of lymphovascular invasion changes the pathological T category of an intratesticular tumor. It is important to use strict criteria when assessing putative lymphovascular invasion because artifactual displacement of tumor cells (especially seminoma) and retraction artifacts are common and may lead to false positivity. Although lym-

Table 59.1 The International Germ Cell Consensus Classification for Advanced Disease. From Wilkinson et al., with permission.[14]

Prognosis (Risk Status)	Visceral Metastases or Mediastinal Primary	Serum Markers*			5-Year Progression-Free Survival	5-Year Overall Survival
		AFP	hCG	LDH		
Good	No	< 1,000	< 5,000	<1.5 × N**	89%	92%
Intermediate	No	1,000–10,000	5,000–50,000	1.5-10 × N**	75%	80%
Poor	Yes	> 10,000	> 50,000	>10 × N**	41%	48%

*Markers used for risk classification are post-orchiectomy.
**N indicates the upper limit of normal for the LDH assay.

phovascular invasion is better assessed in peritumoral locations, one can occasionally identify unequivocal intratumoral lymphovascular invasion. AJCC Level of Evidence: I

RISK ASSESSMENT MODELS

The AJCC recently established guidelines that will be used to evaluate published statistical prediction models for the purpose of granting endorsement for clinical use.[15] Although this is a monumental step toward the goal of precision medicine, this work was published only very recently. Therefore, the existing models that have been published or may be in clinical use have not yet been evaluated for this cancer site by the Precision Medicine Core of the AJCC. In the future, the statistical prediction models for this cancer site will be evaluated, and those that meet all AJCC criteria will be endorsed.

DEFINITIONS OF AJCC TNM

Definition of Primary Tumor (T)

Clinical T (cT)

cT Category	cT Criteria
cTX	Primary tumor cannot be assessed
cT0	No evidence of primary tumor
cTis	Germ cell neoplasia *in situ*
cT4	Tumor invades scrotum with or without vascular/lymphatic invasion

Note: Except for Tis confirmed by biopsy and T4, the extent of the primary tumor is classified by radical orchiectomy. TX may be used for other categories for clinical staging.

Pathological T (pT)

pT Category	pT Criteria
pTX	Primary tumor cannot be assessed
pT0	No evidence of primary tumor
pTis	Germ cell neoplasia *in situ*
pT1	Tumor limited to testis (including rete testis invasion) without lymphovascular invasion
pT1a*	Tumor smaller than 3 cm in size
pT1b*	Tumor 3 cm or larger in size
pT2	Tumor limited to testis (including rete testis invasion) with lymphovascular invasion<br>OR<br>Tumor invading hilar soft tissue or epididymis or penetrating visceral mesothelial layer covering the external surface of tunica albuginea with or without lymphovascular invasion
pT3	Tumor invades spermatic cord with or without lymphovascular invasion
pT4	Tumor invades scrotum with or without lymphovascular invasion

*Subclassification of pT1 applies only to pure seminoma.

Definition of Regional Lymph Node (N)

Clinical N (cN)

cN Category	cN Criteria
cNX	Regional lymph nodes cannot be assessed
cN0	No regional lymph node metastasis
cN1	Metastases with a lymph node mass 2 cm or smaller in greatest dimension<br>OR<br>Multiple lymph nodes, none larger than 2 cm in greatest dimension
cN2	Metastasis with a lymph node mass larger than 2 cm but not larger than 5 cm in greatest dimension<br>OR<br>Multiple lymph nodes, any one mass larger than 2 cm but not larger than 5 cm in greatest dimension
cN3	Metastasis with a lymph node mass larger than 5 cm in greatest dimension

Pathological N (pN)

pN Category	pN Criteria
pNX	Regional lymph nodes cannot be assessed
pN0	No regional lymph node metastasis
pN1	Metastasis with a lymph node mass 2 cm or smaller in greatest dimension and less than or equal to five nodes positive, none larger than 2 cm in greatest dimension

pN Category	pN Criteria
pN2	Metastasis with a lymph node mass larger than 2 cm but not larger than 5 cm in greatest dimension; or more than five nodes positive, none larger than 5 cm; or evidence of extranodal extension of tumor
pN3	Metastasis with a lymph node mass larger than 5 cm in greatest dimension

Definition of Distant Metastasis (M)

M Category	M Criteria
M0	No distant metastases
M1	Distant metastases
M1a	Non-retroperitoneal nodal or pulmonary metastases
M1b	Non-pulmonary visceral metastases

Definition of Serum Markers (S)

S Category	S Criteria
SX	Marker studies not available or not performed
S0	Marker study levels within normal limits
S1	LDH < 1.5 × N* *and* hCG (mIU/mL) < 5,000 *and* AFP (ng/mL) < 1,000
S2	LDH 1.5–10 × N* *or* hCG (mIU/mL) 5,000-50,000 *or* AFP (ng/mL) 1,000–10,000
S3	LDH > 10 × N* *or* hCG (mIU/mL) >50,000 *or* AFP (ng/mL) > 10,000

*N indicates the upper limit of normal for the LDH assay.

AJCC PROGNOSTIC STAGE GROUPS

When T is …	And N is …	And M is …	And S is …	Then the stage group is …
pTis	N0	M0	S0	0
pT1-T4	N0	M0	SX	I
pT1	N0	M0	S0	IA
pT2	N0	M0	S0	IB
pT3	N0	M0	S0	IB
pT4	N0	M0	S0	IB
Any pT/TX	N0	M0	S1–3	IS
Any pT/TX	N1–3	M0	SX	II
Any pT/TX	N1	M0	S0	IIA
Any pT/TX	N1	M0	S1	IIA
Any pT/TX	N2	M0	S0	IIB
Any pT/TX	N2	M0	S1	IIB
Any pT/TX	N3	M0	S0	IIC
Any pT/TX	N3	M0	S1	IIC
Any pT/TX	Any N	M1	SX	III
Any pT/TX	Any N	M1a	S0	IIIA
Any pT/TX	Any N	M1a	S1	IIIA
Any pT/TX	N1–3	M0	S2	IIIB
Any pT/TX	Any N	M1a	S2	IIIB
Any pT/TX	N1–3	M0	S3	IIIC
Any pT/TX	Any N	M1a	S3	IIIC
Any pT/TX	Any N	M1b	Any S	IIIC

59

REGISTRY DATA COLLECTION VARIABLES

1. Serum tumor markers (S) for both clinical and pathological stage grouping
2. Alpha fetoprotein (AFP) for both clinical and pathological stage grouping (xx,xxx ng/mL)
3. Human chorionic gonadotropin (hCG) for both clinical and pathological stage grouping (xx,xxx mIU/ml)
4. Lactate dehydrogenase (LDH) for both clinical and pathological stage grouping (xx,xxx U/L)

HISTOLOGIC GRADE (G)

Germ cell tumors are not graded.

HISTOPATHOLOGIC TYPE

Germ cell tumors may be either seminomatous or nonseminomatous. The tumor previously known as spermatocytic seminoma has been renamed spermatocytic tumor in the latest World Health Organization (WHO) Classification of Tumors. Anaplastic and classic seminoma are obsolete terms. Seminoma and seminoma with syncytiotrophoblast cells currently are the two recognized categories. Nonseminomatous germ cell tumors include embryonal carcinoma, yolk sac tumor, teratoma, and choriocarcinoma. Mixed germ cell tumors are tumors that contain more than one histological subtype, either nonseminomatous elements only or a mixture of seminomatous and nonseminomatous elements. Mixtures of different types (including seminoma) should be noted, starting with the most prevalent component and ending with the least represented. Similarly, gonadal stromal tumors should be classified according to the WHO classification, and the TNM staging should be applied only to malignant Leydig or Sertoli cell tumors and other malignant sex cord-stromal tumors. Tumors with benign or borderline malignant potential are not assigned a stage.

ILLUSTRATIONS

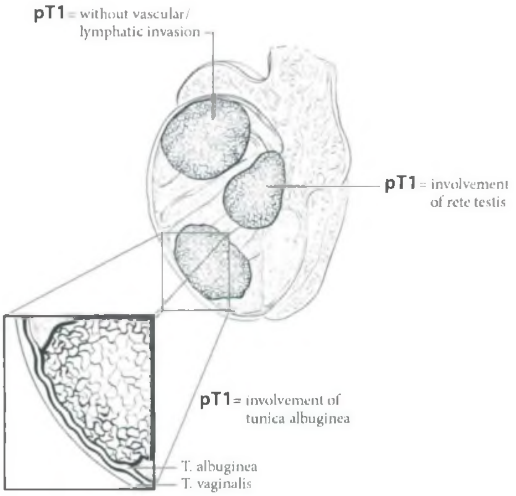

Fig. 59.4 pT1 is defined as tumor limited to testis (including rete testis invasion) without lymphovascular invasion. Tumor may invade into the tunica albuginea but not the tunica vaginalis

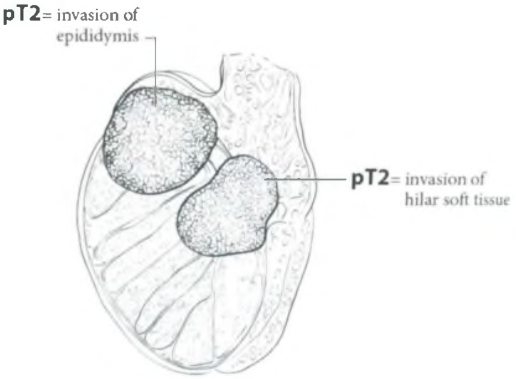

Fig. 59.5 pT2 is defined as tumor limited to testis (including rete testis invasion) with lymphovascular invasion or tumor invading hilar soft tissue (shown here), epididymis (shown here) or penetrating visceral mesothelial layer covering the external surface of tunica albuginea with or without lymphovascular invasion

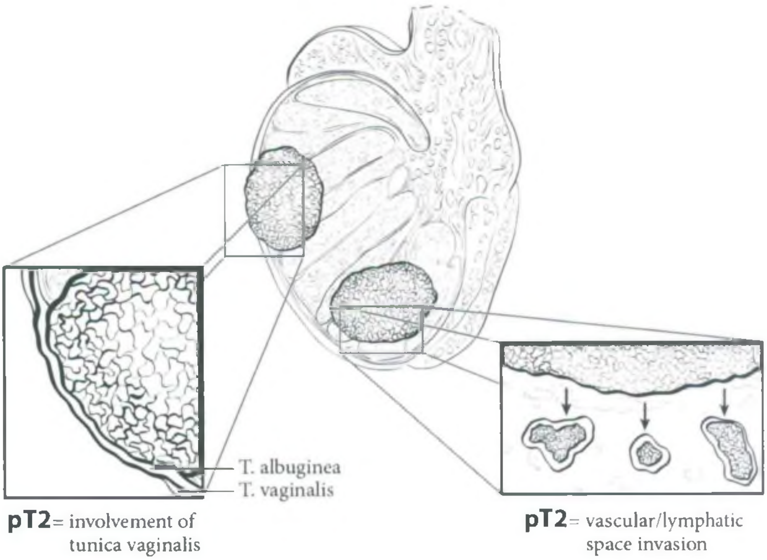

Fig. 59.6 pT2 is defined as tumor limited to testis (including rete testis invasion) with lymphovascular invasion (shown here) or tumor invading hilar soft tissue, epididymis or penetrating visceral mesothelial layer covering the external surface of tunica albuginea with or without lymphovascular invasion (shown here)

pT3

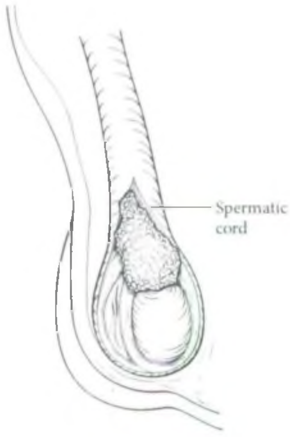

Fig. 59.7 A pT3 tumor invading the spermatic cord

pT4

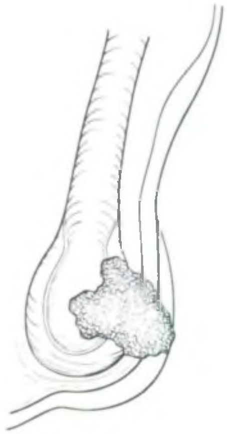

Fig. 59.8 A pT4 tumor invading the scrotum

Bibliography

1. Aparicio J. Maroto P. Garcia del Muro X. et al. Prognostic factors for relapse in stage I seminoma: a new nomogram derived from three consecutive, risk-adapted studies from the Spanish Germ Cell Cancer Group (SGCCG). *Ann Oncol.* Nov 2014;25(11):2173-2178.
2. Chung P. Daugaard G. Tyldesley S. et al. Evaluation of a prognostic model for risk of relapse in stage I seminoma surveillance. *Cancer medicine.* Jan 2015;4(1):155-160.
3. Cohn-Cedermark G. Stahl O. Tandstad T. Swenoteca. Surveillance vs. adjuvant therapy of clinical stage I testicular tumors - a review and the SWENOTECA experience. *Andrology.* Jan 2015;3(1):102-110.
4. Daugaard G. Gundgaard MG. Mortensen MS. et al. Surveillance for stage I nonseminoma testicular cancer: outcomes and long-term follow-up in a population-based cohort. *J Clin Oncol.* Dec 1 2014;32(34):3817-3823.
5. Kamba T. Kamoto T. Okubo K. et al. Outcome of different post-orchiectomy management for stage I seminoma: Japanese multi-institutional study including 425 patients. *International journal of urology : official journal of the Japanese Urological Association.* Dec 2010;17(12):980-987.
6. Krege S. Beyer J. Souchon R. et al. European consensus conference on diagnosis and treatment of germ cell cancer: a report of the second meeting of the European Germ Cell Cancer Consensus group (EGCCCG): part I. *Eur Urol.* Mar 2008;53(3):478-496.
7. Warde P. Specht L. Horwich A. et al. Prognostic factors for relapse in stage I seminoma managed by surveillance: a pooled analysis. *J Clin Oncol.* Nov 15 2002;20(22):4448-4452.
8. Zores T. Mouracade P. Duclos B. Saussine C. Lang H. Jacqmin D. Surveillance of stage I testicular seminoma: 20 years oncological results. *Prog Urol.* Apr 2015;25(5):282-287.
9. Berney DM. Algaba F. Amin M. et al. Handling and reporting of orchidectomy specimens with testicular cancer: areas of consensus and variation among 25 experts and 225 European pathologists. *Histopathology.* Sep 2015;67(3):313-324.
10. Yilmaz A. Cheng T. Zhang J. Trpkov K. Testicular hilum and vascular invasion predict advanced clinical stage in nonseminomatous germ cell tumors. *Modern pathology : an official journal of the United States and Canadian Academy of Pathology, Inc.* Apr 2013;26(4):579-586.
11. Coursey Moreno C. Small WC. Camacho JC. et al. Testicular Tumors: What Radiologists Need to Know-Differential Diagnosis, Staging, and Management. *Radiographics : a review publication of the Radiological Society of North America, Inc.* 2015;35(2):400-415.
12. Hedgire SS. Pargaonkar VK. Elmi A. Harisinghani AM. Harisinghani MG. Pelvic nodal imaging. *Radiol Clin North Am.* Nov 2012;50(6):1111-1125.
13. Vogt AP. Chen Z. Osunkoya AO. Rete testis invasion by malignant germ cell tumor and/or intratubular germ cell neoplasia: what is the significance of this finding? *Human pathology.* Sep 2010;41(9):1339-1344.
14. Wilkinson PM. Read G. International Germ Cell Consensus Classification: a prognostic factor-based staging system for metastatic germ cell cancers. International Germ Cell Cancer Collaborative Group. *Journal of Clinical Oncology.* 1997;15(2):594-603.
15. Kattan MW. Hess KR. Amin MB. et al. American Joint Committee on Cancer acceptance criteria for inclusion of risk models for individualized prognosis in the practice of precision medicine. *CA: a cancer journal for clinicians.* Jan 19 2016.

Part XIV

Urinary Tract

Members of the Urinary Tract Expert Panel
Mahul B. Amin, MD, FCAP – Editorial Board Liaison
Bernard H. Bochner, MD, FACS
Sam S. Chang, MD, FACS
Toni K. Choueiri, MD
Jason A. Efstathiou, MD, DPhil
Mary Gospodarowicz, MD, FRCPC, FRCR(Hon) – UICC Representative
Donna E. Hansel, MD, PhD
Patrick A. Kenney, MD
Badrinath R. Konety, MD
Jaime Landman, MD
Cheryl T. Lee, MD
Bradley C. Leibovich, MD, FACS
James M. McKiernan, MD – Vice Chair
Elizabeth R. Plimack, MD
Victor E. Reuter, MD
Brian I. Rini, MD, FACP
Srikala Sridhar, MD, MSc, FRCPC
Walter M. Stadler, MD, FACP – Chair
Satish K. Tickoo, MD
Raghunandan Vikram, MD
Ming Zhou, MD, PhD – CAP Representative

Kidney

60

Brian I. Rini, James M. McKiernan, Sam S. Chang, Toni K. Choueiri, Patrick A. Kenney, Jaime Landman, Bradley C. Leibovich, Satish K. Tickoo, Raghunandan Vikram, Ming Zhou, and Walter M. Stadler

CHAPTER SUMMARY

Cancers Staged Using This Staging System

Carcinomas arising in the kidney

Cancers Not Staged Using This Staging System

These histopathologic types of cancer…	Are staged according to the classification for…	And can be found in chapter…
Urothelial carcinoma	Renal pelvis and ureter	61
Lymphoma	Hodgkin and Non-Hodgkin lymphoma	79
Sarcoma	Soft tissue sarcoma of the abdomen and thoracic visceral organs	42
Wilms tumor	No AJCC staging system	N/A

Summary of Changes

Change	Details of Change	Level of Evidence
Definition of Primary Tumor (T)	For T3a disease: The word "grossly" was eliminated from the description of renal vein involvement, and "muscle containing" was changed to "segmental veins."	II
Definition of Primary Tumor (T)	For T3a disease: Invasion of the pelvicalyceal system was added.	II

ICD-O-3 Topography Codes

Code	Description
C64.9	Kidney, NOS

WHO Classification of Tumors

Code	Description
8310	Clear cell renal cell carcinoma
8316	Multilocular cystic renal neoplasm of low malignant potential
8260	Papillary renal cell carcinoma
8311	Hereditary leiomyomatosis renal cell carcinoma (HLRCC)-associated renal cell carcinoma
8323	Clear cell papillary renal cell carcinoma

To access the AJCC cancer staging forms, please visit www.cancerstaging.org.

© American Joint Committee on Cancer 2017

M.B. Amin et al. (eds.), *AJCC Cancer Staging Manual, Eighth Edition*, DOI 10.1007/978-3-319-40618-3_60

Code	Description
8317	Chromophobe renal cell carcinoma
8319	Collecting duct renal cell carcinoma
8510	Renal medullary carcinoma
8311	MiT family translocation renal cell carcinomas
8311	Succinate dehydrogenase (SDH) deficient renal cell carcinoma
8480	Mucinous tubular and spindle cell renal cell carcinoma
8316	Tubulocystic renal cell carcinoma
8316	Acquired cystic disease associated renal cell carcinoma
8312	Renal cell carcinoma, unclassified

Moch H. Humphrey PA, Ulbright TM, Reuter VE, eds. World Health Organization Classification of Tumours of the Urinary System and Male Genital Organs. Lyon: IARC; 2016.

INTRODUCTION

Cancers of the kidney account for 3% of all malignancies. Nearly all malignant tumors are carcinomas arising from the renal tubular epithelium. Tumors arising from the renal pelvis, sarcomas, lymphomas, and pediatric tumors, such as Wilms tumor, are covered in different chapters. Kidney cancers are more common in males by a 3:2 ratio. Most are sporadic, but 2–3% are hereditary. Pain and hematuria are potential presenting signs, and 3–5% of patients may present with evidence of vascular tumor thrombus. The majority of kidney tumors currently are detected incidentally in asymptomatic individuals. Staging depends on the size of the primary tumor, invasion of adjacent structures, and vascular extension, in addition to regional lymph node and distant spread.

Recent data also demonstrate that multiple adverse features act in a collaborative manner to further worsen the prognosis, and emerging algorithms are incorporating all of these parameters. These adverse features include perirenal fat invasion, tumor size as a continuous variable, size of the largest involved lymph node, and extranodal extension. It also is becoming clear that prognosis and outcome of different renal cancer histologic subtypes may differ. Finally, cancers of the kidney have a number of potential molecular prognostic factors, including genetic variables, proliferative markers, angiogenic parameters, growth factors and receptor, and adhesion molecules. Most of these factors have not been formally validated and are best still considered experimental. Ideally, future staging protocols would capture this information to facilitate individualized counseling and foster further progress in this field. Specific factors to be examined include degree of invasion, the presence/level of venous involvement, the presence and type of adrenal gland involvement, the type of grading system employed and grade determined, the presence/absence of sarcomatoid features, the presence/absence of lymphovascular invasion, the presence/absence of necrosis, and the molecular features of the primary tumor.

ANATOMY

Primary Site(s)

Encased by a fibrous capsule and surrounded by perirenal fat, the kidney consists of the cortex (glomeruli, convoluted tubules) and the medulla (Henle's loops, collecting ducts, and pyramids of converging tubules). Each papilla opens into the minor calyces; these, in turn, unite into the major calices and drain into the renal pelvis. At the hilum are the pelvis, ureter, and renal artery and vein. Gerota's fascia overlies the psoas and quadratus lumborum muscles. Renal cancer primary tumors can arise in any part of the renal unit. The anatomic sites and subsites of the kidney are illustrated in Fig. 60.1. One unique feature of renal cell carcinoma (RCC) is growth of the primary renal tumor into the draining renal vein, and sometimes into the inferior vena cava as high as the right atrium.

Regional Lymph Nodes

The regional lymph nodes, illustrated in Figs. 60.2 and 60.3, are as follows:

- Renal hilar
- Caval (precaval, interaortocaval, paracaval, and retrocaval)
- Aortic (preaortic, paraaortic, and retroaortic)

The primary landing zone for right-sided tumors is the interaortocaval zone and for left-sided tumors the aortic region. The more extended landing zones for RCC are analogous to those for right and left testicular tumors, respectively, although patterns of spread are somewhat more unpredictable.

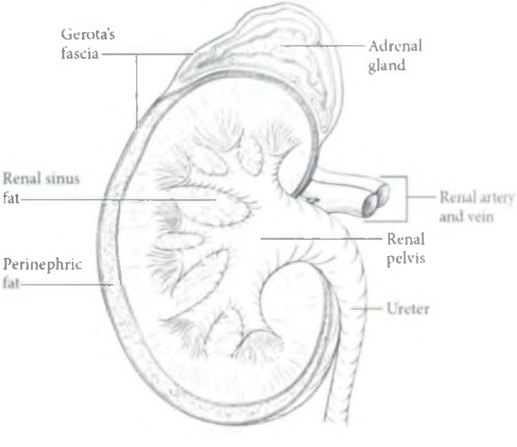

Fig. 60.1 Anatomic sites and subsites of the kidney.

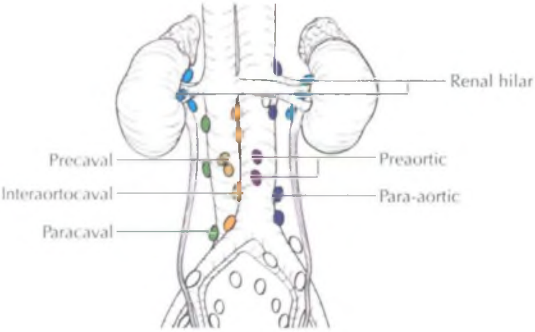

Fig. 60.2 Regional lymph nodes of the kidney.

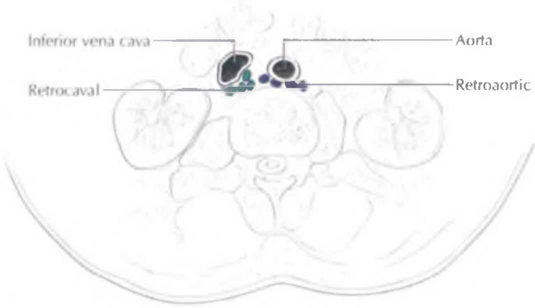

Fig. 60.3 Regional lymph nodes of the kidney.

Lymph nodes outside of these templates should be considered distal (metastatic), rather than regional.

Metastatic Sites

Common metastatic sites include the bone, liver, lung, brain, ipsilateral and contralateral adrenal glands, and distant lymph nodes. RCCs are known to metastasize to unusual sites (nasal sinuses, penis, skin, etc.) and for widespread dissemination.

RULES FOR CLASSIFICATION

Clinical Classification

Clinical examination, abdominal/pelvic computed tomography (CT) scanning, and other appropriate imaging techniques, such as magnetic resonance (MR) imaging of the primary tumor, are required for assessment of the tumor and its extensions, both local and distant (see below). Although percutaneous biopsy may not be necessary if surgical resection is planned, it is necessary if a non-renal cell cancer is suspected (e.g., lymphoma), or if an ablative rather than surgical extirpative procedure is planned, and can be performed safely. Extensive laboratory-based workup is generally not necessary, but should include a complete blood count, basic chemistries to assess renal and liver function, and calcium and lactate dehydrogenase (LDH) levels, which may be important for prognostication, at least in metastatic disease.

60

Imaging

Both CT and MR imaging are equally useful in local staging of renal cell carcinoma and can be considered as first line tests. However, CT has an advantage over MR imaging as it shows calcification and allows better visualization of other body parts, such as the chest, which may be used for staging. RCC is usually a solid, contrast-enhancing mass or cystic with solid components. Most contrast-enhancing renal masses tend to be malignant, and the odds ratio of malignancy increases with increasing size. Multiplanar imaging in MR or multiplanar reconstruction of CT images allows accurate measurement of renal tumors. However, small differences between imaging size measurements and measurements made on resected tumors postoperatively are common, but may not be clinically significant.[1,2] High-resolution CT using thin sections appears to improve detection of perinephric infiltration, although false positives are common.[3,4]

Presence of a pseudocapsule on MR imaging is useful in separating T1 or T2 tumors from T3a tumors in patients with RCC.[5] Involvement of renal sinus fat is more difficult to detect on preoperative imaging. Although CT and MR imaging have a high negative predictive value, detection of renal sinus fat invasion is often difficult with relatively low positive predictive value.[6]

Tumor thrombus in the renal vein or inferior vena cava (IVC) can be identified on the venous or delayed phase of contrast-enhanced CT or MR imaging. Signs suggestive of renal vein or caval thrombus include filling defects, enlargement of the vessel, and rim enhancement. Tumor thrombus in the segmental branches of the renal vein may be more difficult to detect than thrombus in the main renal vein and IVC.[7] Both CT and MR imaging have poor positive predictive value for distinguishing invasion from mere abutment with adjacent organs, such as adrenal glands, liver, diaphragm, psoas muscles, pancreas, and bowel. CT and MR imaging have a high sensitivity and nearly a 100% negative predictive value in detecting direct contiguous spread to the ipsilateral adrenal gland.[8,9]

Lymph nodes larger than 1 cm in short axis diameter or nodes that appear to have distorted architecture on imaging should raise suspicion for nodal metastasis. Although 10% of nodes that harbor metastases may be smaller than 1 cm, reactive hyperplasia is common and can be seen in up to 58% of enlarged nodes.[10]

The risk of metastasis depends on the size of renal tumors and other factors, such as subtype and sarcomatoid differentiation.[11-14] Distant metastases typically occur in lung, bone, liver, ipsilateral and contralateral adrenal glands, and brain.

Chest CT is the most sensitive test for detecting pulmonary metastasis, but a plain chest X-ray may be sufficient in low-risk patients.[12,15] Patients with advanced primary tumors with symptoms attributable to bone metastasis or patients with abnormal laboratory findings, such as elevated alkaline phosphatase, can be investigated with bone scan to detect bone metastasis.[16] Patients with localizing neurological signs should be investigated with MR imaging of the brain or a contrast-enhanced CT scan of the head to detect brain metastasis. Although no evidence justifies routine use of brain MR imaging, it can be used to detect asymptomatic occult brain metastasis in patients with advanced RCC.[17] Combined 18-F-fluorodeoxyglucose positron emission tomography/CT (18F-FDG PET-CT or PETCT) does not have an established role in the initial staging of renal cancer, in part because RCC lesions typically have low avidity for FDG relative to high background uptake and excretion in the kidneys.[18] Although low in sensitivity to detect distant metastasis, PET-CT has superior specificity and may have a complementary role as a problem-solving tool in cases that are equivocal by conventional imaging.[19,20]

Pathological Classification

Pathological staging requires surgical resection, which can be performed with either an open or minimally invasive approach. Resection of the primary tumor along with the overlying Gerota's fascia and perinephric fat is recommended. Partial nephrectomy is an acceptable treatment for localized tumors amenable to this approach and is the preferred form of management for clinical T1 tumors and when preservation of renal function is an issue.[12,21] Formal retroperitoneal lymph node dissection improves nodal staging accuracy; however, the impact of lymphadenectomy on oncologic outcome remains uncertain.[22-24] Adrenal gland involvement is categorized as M1 unless the mechanism is by direct extension from the renal tumor into the ipsilateral adrenal gland, which is category T4. En bloc resection of the ipsilateral adrenal gland is recommended if there is evidence of involvement by imaging or intraoperative findings, but is not necessary if the adrenal gland appears normal.[25-27]

For staging purposes, pathological tumor size is required. For large tumors, particularly those larger than 7 cm and those occurring in the region of the renal sinus, renal sinus invasion should be suspected and tumor sampling should be targeted to help make this determination. For tumor with extrarenal invasion, the greatest dimension of the tumor mass, including the extrarenal extension, should be measured. For tumor with intravascular extension, the tumor thrombus is excluded from the tumor size measurement. If a specimen contains multiple tumor nodules, a maximum of 5 nodules should be measured, provided all tumors have similar gross appearance and the largest is used to assign T-category with (m) used to indicate multiple tumors. Measurement should be taken for additional nodules if they have variable gross appearance. Tumors with differing histologies should have separate staging.

It is not uncommon that tumor involvement of the renal vein and, in particular, its branches is unrecognized at the time of gross examination of the specimen. This is even truer in partial nephrectomy specimens. Evaluation at microscopy not infrequently reveals such gross misses. Therefore, the word "grossly" has been excluded in the current pT3a staging. In addition, the diameter of a sinus vein or the quantity or the presence or absence of muscle in sinus veins is a poor indicator of the vein segment or its relationship to the main renal vein, and thus muscle does not need to be identified in a vein to classify it as a "renal vein segmental branch" and thus categorize the tumor as pT3a.[28] Note that circumscribed tumor nodules in the renal sinus fat likely represent vascular invasion.[28] Vascular invasion should be confirmed microscopically.

Perinephric/sinus fat invasion should be confirmed microscopically. Invasion into fat by tumor cells, with or without desmoplastic reaction, and vascular invasion in perinephric/sinus soft tissue are all evidence of perinephric/sinus invasion. It is reported as "present" or "absent."

Specimen Handling

The pathological specimen should be processed in a standardized fashion to allow for full pathological assessment. The International Society of Urological Pathology (ISUP) and College of American Pathologists (www.cap.org) have established practical guidelines for specimen processing.[29]

PROGNOSTIC FACTORS

Prognostic Factors Required for Stage Grouping

Beyond the factors used to assign T, N, or M categories, no additional prognostic factors are required for stage grouping.

Additional Factors Recommended for Clinical Care

Histologic Subtype

Histologic subtype is a strong prognostic factor and an increasingly important factor for treatment decisions, especially in the metastatic state. Histologic subtype should be categorized as discussed below. For example, type 1 papillary renal cancers tend to have a good prognosis, whereas

medullary and collecting duct carcinomas tend to have a poor prognosis.[30] AJCC Level of Evidence: I

World Health Organization (WHO)/ISUP nucleolar grade

Grade is a strong prognostic factor, especially for clear cell RCC and should be described as discussed in the section titled Histologic Grade (G). AJCC Level of Evidence: II

Sarcomatoid and rhabdoid features

Sarcomatoid differentiation in RCC consists of sheets and fascicles of malignant spindle cells, which can occur across all histologic subtypes. Occasionally, the sarcomatoid component resembles specific types of sarcoma, such as osteogenic sarcoma, chondrosarcoma, rhabdomyosarcoma, etc. Sarcomatoid differentiation can be seen in any of the RCC subtypes and is associated with a poor prognosis.

A minimum quantity of sarcomatoid component is not required to make the diagnosis; some experts require a low power field or a clear-cut area of sarcoma-like histology. The percentage of sarcomatoid component has been shown to correlate with cancer-specific mortality and an estimate of its quantity should be provided.[31] AJCC Level of Evidence: II

Rhabdoid differentiation is characterized by the presence of cells with abundant eosinophilic cytoplasm expanded by an eccentric granular eosinophilic inclusion and a large eccentrically placed nucleus and prominent eosinophilic nucleolus. Rhabdoid differentiation, like sarcomatoid differentiation, is a de-differentiation pathway common to all RCC subtypes. Both components may co-exist in the same tumor. The presence of rhabdoid differentiation in RCC is associated with a poor prognosis independent of histological subtypes, grade, and stage.[32] The presence of rhabdoid differentiation should be noted in the pathology report.[30] AJCC Level of Evidence: II

Histologic tumor necrosis

Coagulative necrosis is correlated with prognosis and, on microscopic examination, characterized by homogeneous clusters and sheets of degenerating and dead cells, or granular pink coagulum admixed with nuclear and cytoplasmic debris. Degenerative changes–such as hemorrhage, hyalinization and scar–and ischemic necrosis should not be mistaken for necrosis. Any amount of coagulative necrosis should be reported.[33] AJCC Level of Evidence: II

Microscopic angiolymphatic invasion

Microscopic lymphovascular invasion (LVI) is defined as the presence of tumor in the small vascular spaces within the host kidney. Its reported incidence is quite variable due to variable detection methods used (by immunohistochemical or standard hematoxylin and eosin stains). In the majority of the published studies, LVI has been shown to correlate with other prognostic parameters, including tumor size, grade, pT category, and the presence of lymph node and distant metastases. LVI also has been significantly associated with outcome determined as disease-free survival and cancer-specific survival. It is recommended to report LVI when identified on hematoxylin and eosin stains.[30] AJCC Level of Evidence: II

60

RISK ASSESSMENT MODELS

The American Joint Committee on Cancer (AJCC) recently has established guidelines that will be used to evaluate published statistical prediction models for the purpose of granting endorsement for clinical use.[34] Although this is a monumental step toward the goal of precision medicine, this work was published only very recently. For this reason, existing models that have been published or may be in clinical use have not yet been evaluated for this cancer site by the Precision Medicine core of the AJCC. In the future, the statistical prediction models for this cancer site will be evaluated, and those that meet all AJCC criteria will be endorsed.

DEFINITIONS OF AJCC TNM

Definition of Primary Tumor (T)

T Category	T Criteria
TX	Primary tumor cannot be assessed
T0	No evidence of primary tumor
T1	Tumor ≤ 7 cm in greatest dimension, limited to the kidney
T1a	Tumor ≤ 4 cm in greatest dimension, limited to the kidney
T1b	Tumor > 4 cm but ≤ 7 cm in greatest dimension limited to the kidney
T2	Tumor > 7 cm in greatest dimension, limited to the kidney
T2a	Tumor > 7 cm but ≤ 10 cm in greatest dimension, limited to the kidney
T2b	Tumor > 10 cm, limited to the kidney
T3	Tumor extends into major veins or perinephric tissues, but not into the ipsilateral adrenal gland and not beyond Gerota's fascia
T3a	Tumor extends into the renal vein or its segmental branches, or invades the pelvicalyceal system, or invades perirenal and/or renal sinus fat but not beyond Gerota's fascia
T3b	Tumor extends into the vena cava below the diaphragm
T3c	Tumor extends into the vena cava above the diaphragm or invades the wall of the vena cava
T4	Tumor invades beyond Gerota's fascia (including contiguous extension into the ipsilateral adrenal gland)

Definition of Regional Lymph Node (N)

N Category	N Criteria
NX	Regional lymph nodes cannot be assessed
N0	No regional lymph node metastasis
N1	Metastasis in regional lymph node(s)

Definition of Distant Metastasis (M)

M Category	M Criteria
M0	No distant metastasis
M1	Distant metastasis

AJCC PROGNOSTIC STAGE GROUPS

When T is...	And N is...	And M is...	Then the stage group is...
T1	N0	M0	I
T1	N1	M0	III
T2	N0	M0	II
T2	N1	M0	III
T3	N0	M0	III
T3	N1	M0	III
T4	Any N	M0	IV
Any T	Any N	M1	IV

REGISTRY DATA COLLECTION VARIABLES

1. Histologic subtype
2. WHO/ISUP grade
3. Tumor size
4. Invasion into perinephric fat or sinus tissues
5. Venous involvement with specific mention of intrarenal lymphovascular invasion, branches of renal vein in the renal sinus invasion, renal vein involvement, or IVC involvement
6. Lymphovascular invasion (LVI)
7. Adrenal gland involvement by direct extension (T4) or as a separate nodule (M1)
8. Sarcomatoid features; present or absent and percentage
9. Rhabdoid differentiation; present or absent
10. Histologic tumor necrosis

HISTOLOGIC GRADE (G)

The Fuhrman grading system, published in 1982, has been widely utilized. It is a four-tier system based on nuclear size, nuclear shape, and nucleolar prominence. Despite the widespread usage of Fuhrman grading, serious problems are associated with its implementation, reproducibility, and outcome prediction. As a result, a modified grading system has been proposed to be based on nucleolar prominence for the first three grading categories, while grade 4 is based on the presence of marked nuclear pleomorphism, which may include tumor giant cells or sarcomatoid and/or rhabdoid differentiation. Known as the WHO/ISUP grade, this grading system was validated for clear cell and papillary RCC, but was shown not to be useful for chromophobe RCC and has not been validated in other RCC histologic subtypes.[30]

G	G Definition
GX	Grade cannot be assessed
G1	Nucleoli absent or inconspicuous and basophilic at 400x magnification
G2	Nucleoli conspicuous and eosinophilic at 400x magnification, visible but not prominent at 100x magnification
G3	Nucleoli conspicuous and eosinophilic at 100x magnification
G4	Marked nuclear pleomorphism and/or multinucleate giant cells and/or rhabdoid and/or sarcomatoid differentiation

HISTOPATHOLOGIC TYPE

Clear cell renal cell carcinoma
Multilocular cystic renal neoplasm of low malignant potential
Papillary renal cell carcinoma
Hereditary leiomyomatosis renal cell carcinoma (HLRCC)-associated renal cell carcinoma
Clear cell papillary renal cell carcinoma
Chromophobe renal cell carcinoma
Collecting duct renal cell carcinoma
Renal medullary carcinoma
MiT Family translocation renal cell carcinomas
Succinate dehydrogenase (SDH) deficient renal cell carcinoma
Mucinous tubular and spindle cell renal cell carcinoma
Tubulocystic renal cell carcinoma
Acquired cystic disease associated renal cell carcinoma
Renal cell carcinoma, unclassified

ILLUSTRATIONS

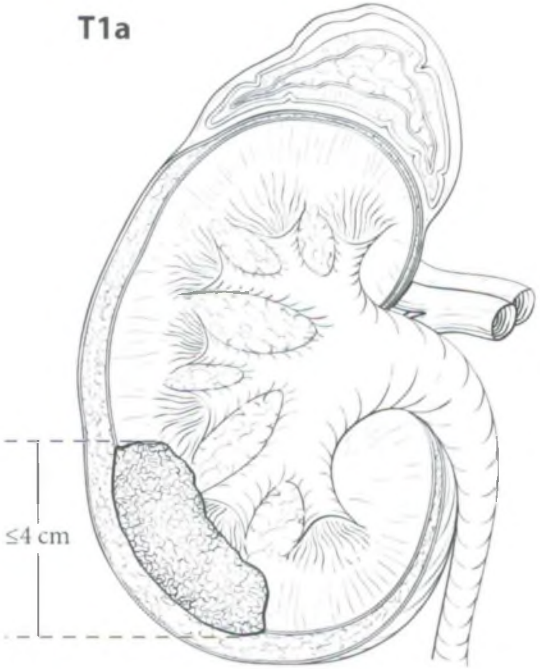

Fig. 60.4 T1a: Tumor 4 cm or smaller in greatest dimension, limited to the kidney.

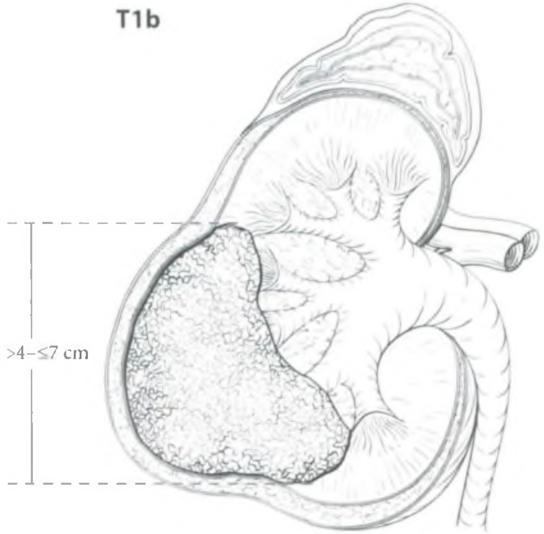

Fig. 60.5 T1b: Tumor larger than 4 cm but not larger than 7 cm in greatest dimension, limited to the kidney.

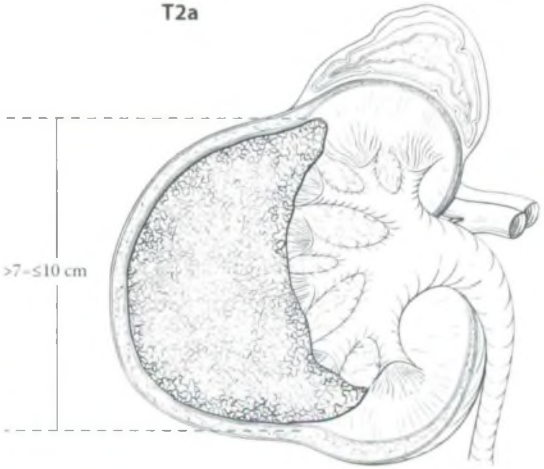

Fig. 60.6 T2a: Tumor larger than 7 cm in greatest dimension, but not larger than 10 cm, limited to the kidney.

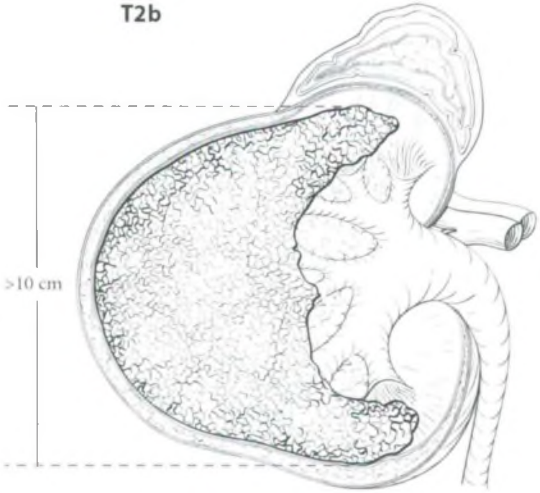

Fig. 60.7 T2b: Tumor larger than 10 cm in greatest dimension.

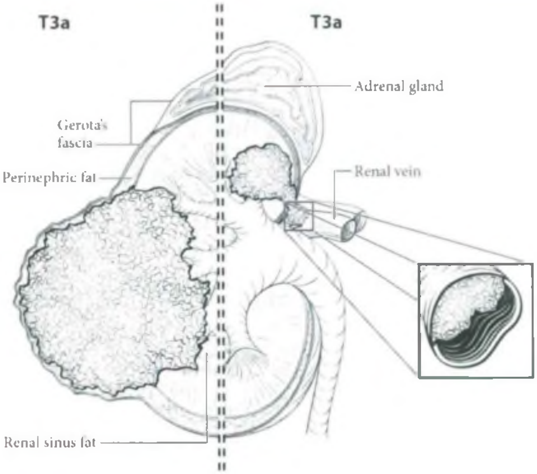

Fig. 60.8 (Left) T3a: Invasion into perirenal and/or renal sinus fat but not beyond Gerota's fascia. (Right) T3a: In addition to perirenal and/or renal sinus fat, tumor invades into the renal vein or segmental branches of renal vein in the renal sinus.

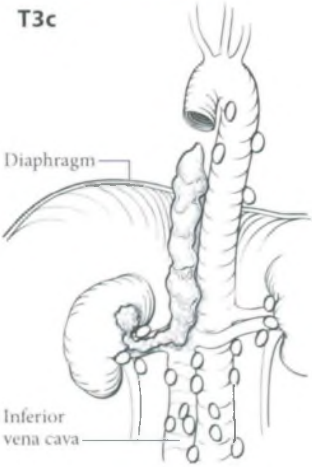

Fig. 60.10 T3c: Tumor extends into vena cava above diaphragm or invades the wall of the vena cava.

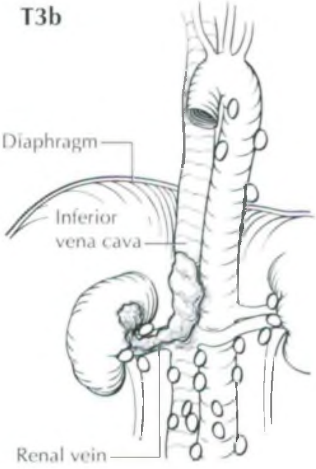

Fig. 60.9 T3b: Tumor extends into vena cava below the diaphragm.

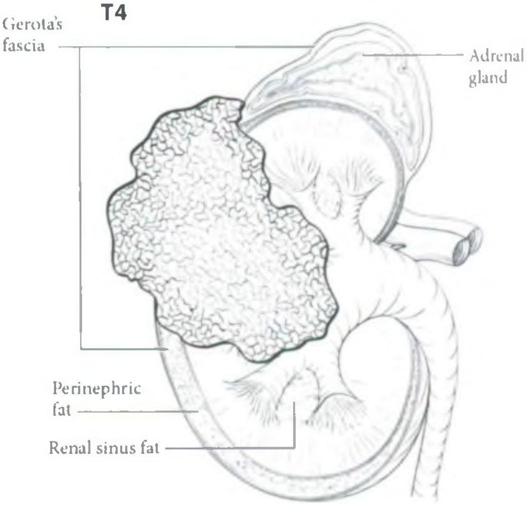

Fig. 60.11 T4: Invasion beyond Gerota's fascia.

11. Lee H, Lee JK, Kim K, et al. Risk of metastasis for T1a renal cell carcinoma. *World journal of urology.* Apr 2016;34(4):553–559.
12. Ljungberg B, Bensalah K, Canfield S, et al. EAU guidelines on renal cell carcinoma: 2014 update. *Eur Urol.* May 2015;67(5): 913–924.
13. Thompson RH, Hill JR, Babayev Y, et al. Metastatic renal cell carcinoma risk according to tumor size. *J Urol.* Jul 2009;182(1): 41–45.
14. Wunderlich H, Reichelt O, Schumann S, et al. Nephron sparing surgery for renal cell carcinoma 4 cm. or less in diameter: indicated or under treated? *J Urol.* May 1998;159(5):1465–1469.
15. Lim DJ, Carter MF. Computerized tomography in the preoperative staging for pulmonary metastases in patients with renal cell carcinoma. *J Urol.* Oct 1993;150(4):1112–1114.
16. Santini D, Procopio G, Porta C, et al. Natural history of malignant bone disease in renal cancer: final results of an Italian bone metastasis survey. *PloS one.* 2013;8(12):e83026.
17. Shuch B, La Rochelle JC, Klatte T, et al. Brain metastasis from renal cell carcinoma: presentation, recurrence, and survival. *Cancer.* Oct 1 2008;113(7):1641–1648.
18. Ozulker T, Ozulker F, Ozbek E, Ozpacaci T. A prospective diagnostic accuracy study of F-18 fluorodeoxyglucose-positron emission tomography/computed tomography in the evaluation of indeterminate renal masses. *Nuclear medicine communications.* Apr 2011;32(4):265–272.
19. Kang DE, White RL, Zuger JH, Sasser HC, Teigland CM. Clinical use of fluorodeoxyglucose F 18 positron emission tomography for detection of renal cell carcinoma. *The Journal of urology.* 2004;171(5):1806–1809.
20. Majhail NS, Urbain JL, Albani JM, et al. F-18 fluorodeoxyglucose positron emission tomography in the evaluation of distant metastases from renal cell carcinoma. *J Clin Oncol.* Nov 1 2003; 21(21):3995–4000.
21. Campbell SC, Novick AC, Belldegrun A, et al. Guideline for management of the clinical T1 renal mass. *J Urol.* Oct 2009;182(4): 1271–1279.
22. Blom JH, van Poppel H, Maréchal JM, et al. Radical nephrectomy with and without lymph-node dissection: final results of European Organization for Research and Treatment of Cancer (EORTC) randomized phase 3 trial 30881. *European urology.* 2009;55(1): 28–34.
23. Terrone C, Guercio S, De Luca S, et al. The number of lymph nodes examined and staging accuracy in renal cell carcinoma. *BJU Int.* Jan 2003;91(1):37–40.
24. Whitson JM, Harris CR, Reese AC, Meng MV. Lymphadenectomy improves survival of patients with renal cell carcinoma and nodal metastases. *J Urol.* May 2011;185(5):1615–1620.
25. Kutikov A, Piotrowski ZJ, Canter DJ, et al. Routine adrenalectomy is unnecessary during surgery for large and/or upper pole renal tumors when the adrenal gland is radiographically normal. *The Journal of urology.* 2011;185(4):1198–1203.
26. TSUI K-H, SHVARTS O, BARBARIC Z, FIGLIN R, de KERNION JB, BELLDEGRUN A. Is adrenalectomy a necessary component of radical nephrectomy? UCLA experience with 511 radical nephrectomies. *The Journal of urology.* 2000;163(2):437–441.
27. Weight CJ, Kim SP, Lohse CM, et al. Routine adrenalectomy in patients with locally advanced renal cell cancer does not offer oncologic benefit and places a significant portion of patients at risk for an asynchronous metastasis in a solitary adrenal gland. *European urology.* 2011;60(3):458–464.
28. Bonsib SM. Renal veins and venous extension in clear cell renal cell carcinoma. *Modern pathology : an official journal of the United States and Canadian Academy of Pathology, Inc.* Jan 2007;20(1): 44–53.
29. Trpkov K, Grignon DJ, Bonsib SM, et al. Handling and staging of renal cell carcinoma: the International Society of Urological Pathology Consensus (ISUP) conference recommendations. *The American journal of surgical pathology.* Oct 2013;37(10):1505–1517.
30. Delahunt B, Cheville JC, Martignoni G, et al. The International Society of Urological Pathology (ISUP) grading system for renal cell carcinoma and other prognostic parameters. *The American journal of surgical pathology.* Oct 2013;37(10):1490–1504.
31. Zhang BY, Thompson RH, Lohse CM, et al. A novel prognostic model for patients with sarcomatoid renal cell carcinoma. *BJU Int.* Mar 2015;115(3):405–411.
32. Przybycin CG, McKenney JK, Reynolds JP, et al. Rhabdoid differentiation is associated with aggressive behavior in renal cell carcinoma: a clinicopathologic analysis of 76 cases with clinical follow-up. *The American journal of surgical pathology.* Sep 2014;38(9):1260–1265.
33. Leibovich BC, Blute ML, Cheville JC, et al. Prediction of progression after radical nephrectomy for patients with clear cell renal cell carcinoma: a stratification tool for prospective clinical trials. *Cancer.* Apr 1 2003;97(7):1663–1671.
34. Kattan MW, Hess KR, Amin MB, et al. American Joint Committee on Cancer acceptance criteria for inclusion of risk models for individualized prognosis in the practice of precision medicine. *CA: a cancer journal for clinicians.* Jan 19 2016.

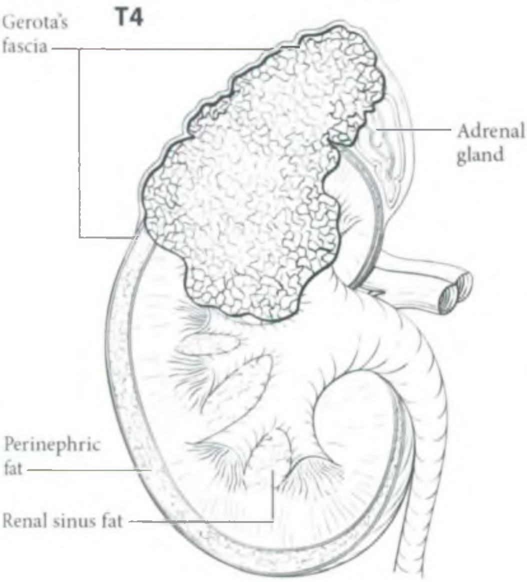

Fig. 60.12 T4: Invasion into ipsilateral adrenal gland.

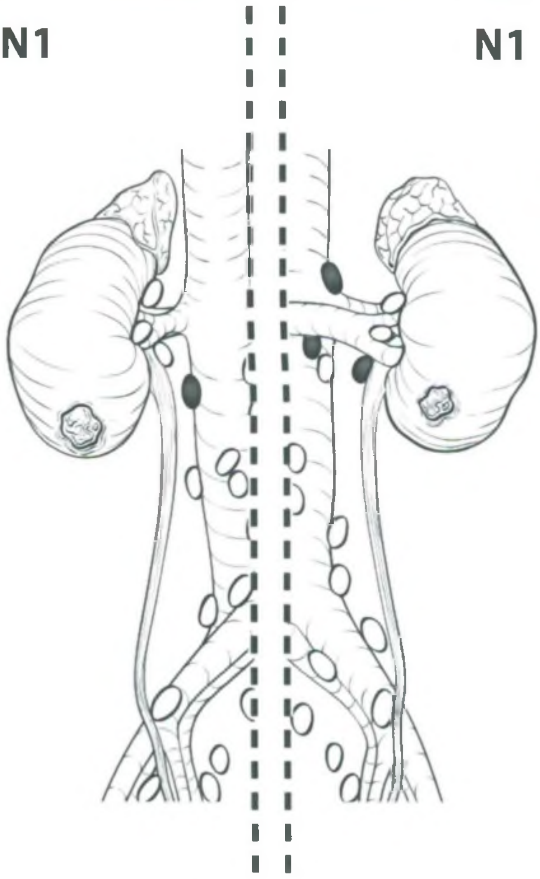

Fig. 60.13 N1 disease is defined as a single or multiple regional lymph node involvement.

Bibliography

1. Jeffery NN, Douek N, Guo DY, Patel MI. Discrepancy between radiological and pathological size of renal masses. *BMC urology.* 2011;11(1):2.
2. Roberts WW, Bhayani SB, Allaf ME, Chan TY, Kavoussi LR, Jarrett TW. Pathological stage does not alter the prognosis for renal lesions determined to be stage T1 by computerized tomography. *The Journal of urology.* 2005;173(3):713–715.
3. Catalano C, Fraioli F, Laghi A, et al. High-resolution multidetector CT in the preoperative evaluation of patients with renal cell carcinoma. *AJR. American journal of roentgenology.* May 2003;180(5):1271–1277.
4. Hallscheidt PJ, Bock M, Riedasch G, et al. Diagnostic accuracy of staging renal cell carcinomas using multidetector-row computed tomography and magnetic resonance imaging: a prospective study with histopathologic correlation. *Journal of computer assisted tomography.* May-Jun 2004;28(3):333–339.
5. Roy C, Sr., El Ghali S, Buy X, et al. Significance of the pseudocapsule on MRI of renal neoplasms and its potential application for local staging: a retrospective study. *AJR. American journal of roentgenology.* Jan 2005;184(1):113–120.
6. Kim C, Choi HJ, Cho KS. Diagnostic value of multidetector computed tomography for renal sinus fat invasion in renal cell carcinoma patients. *Eur J Radiol.* Jun 2014;83(6):914–918.
7. Hallscheidt P, Wagener N, Gholipour F, et al. Multislice computed tomography in planning nephron-sparing surgery in a prospective study with 76 patients: comparison of radiological and histopathological findings in the infiltration of renal structures. *Journal of computer assisted tomography.* Nov-Dec 2006;30(6):869–874.
8. Tsui KH, Shvarts O, Smith RB, Figlin RA, deKernion JB, Belldegrun A. Prognostic indicators for renal cell carcinoma: a multivariate analysis of 643 patients using the revised 1997 TNM staging criteria. *J Urol.* Apr 2000;163(4):1090-1095; quiz 1295.
9. Sawai Y, Kinouchi T, Mano M, et al. Ipsilateral adrenal involvement from renal cell carcinoma: retrospective study of the predictive value of computed tomography. *Urology.* Jan 2002;59(1):28–31.
10. Studer UE, Scherz S, Scheidegger J, et al. Enlargement of regional lymph nodes in renal cell carcinoma is often not due to metastases. *J Urol.* Aug 1990;144(2 Pt 1):243–245.

Renal Pelvis and Ureter

61

James M. McKiernan, Donna E. Hansel,
Bernard H. Bochner, Jason A. Efstathiou,
Badrinath R. Konety, Cheryl T. Lee, Elizabeth R. Plimack,
Victor E. Reuter, Srikala Sridhar, Raghunandan Vikram,
and Walter M. Stadler

CHAPTER SUMMARY

Cancers Staged Using This Staging System

Urothelial (transitional cell) carcinoma, including histologic variants micropapillary and nested subtypes

Cancers Not Staged Using This Staging System

These histopathologic types of cancer...	Are staged according to the classification for...	And can be found in chapter...
Renal cell carcinoma	Kidney	60
Renal medullary carcinoma	Kidney	60
Collecting duct carcinoma	Kidney	60
Lymphoma	Hodgkin and Non-Hodgkin Lymphoma	79
Mesenchymal tumors	Soft Tissue Sarcoma of the Abdomen and Thoracic Visceral Organs	42

Summary of Changes

Change	Details of Change	Level of Evidence
Definition of Regional Lymph Node (N)	The N3 category of a metastasis in a single lymph node larger than 5 cm in greatest dimension has been collapsed into the N2 category.	III

ICD-O-3 Topography Codes

Code	Description
C65.9	Renal pelvis
C65.9	Ureter

WHO Classification of Tumors

Code	Description
8120	Urothelial carcinoma
8131	Micropapillary urothelial carcinoma
8082	Lymphoepithelioma-like carcinoma
8122	Sarcomatoid urothelial carcinoma
8031	Giant cell urothelial carcinoma
8020	Poorly differentiated urothelial carcinoma (including those with osteoclast-like giant cells)
8130	Papillary urothelial carcinoma

To access the AJCC cancer staging forms, please visit www.cancerstaging.org.

© American Joint Committee on Cancer 2017

M.B. Amin et al. (eds.), *AJCC Cancer Staging Manual, Eighth Edition*, DOI 10.1007/978-3-319-40618-3_61

Code	Description
8140	Adenocarcinoma
8070	Squamous cell carcinoma
8041	Small cell neuroendocrine carcinoma

Moch H, Humphrey PA, Ulbright TM, Reuter VE, eds. World Health Organization Classification of Tumours of the Urinary System and Male Genital Organs. Lyon: IARC; 2016.

INTRODUCTION

This chapter describes the staging system for carcinoma of the renal pelvis and ureter. These cancers have a similar biology and histologic distribution as tumors of the urinary bladder and the reader is referred to the overview and details in the chapter for the urinary bladder.

Urothelial carcinoma may occur at any site within the upper urinary tract which spans the renal calyces at the proximal end to the ureterovesical junction at the distal end. Upper tract urothelial carcinoma occurs most commonly in adults but rarely in patients younger than 40 years of age. Incidence is two- to three-fold higher in men. Tumors may be unifocal or multifocal and are seen at a higher incidence in patients with a history of urothelial carcinoma of the bladder. In addition to cigarette smoking a number of analgesics (such as phenacetin) have been associated with this disease. Mutations in DNA mismatch repair genes are more common in upper tract urothelial tumors than in tumors arising in the urinary bladder. Local staging depends on the depth of invasion with unique landmarks present at the ureter and renal pelvis. A common staging system is used regardless of tumor location within the upper urinary collecting system except for category T3 which differs between the pelvis or calyceal system and the ureter.When present simultaneously ureteral and renal pelvic tumors should be staged separately; tumors of the renal pelvis and the major or minor calyceal system are considered to be tumors of the renal pelvis.

ANATOMY

Primary Site(s)

The renal pelvis and ureter form a single unit that is continuous with the collecting ducts of the renal pyramids and comprises the minor and major calyces which are continuous with the renal pelvis. The ureteropelvic junction is variable in position and location but serves as a "landmark" that separates the renal pelvis and the ureter continues caudad and traverses the wall of the urinary bladder as the intramural ureter opening in the trigone of the bladder at the ureteral orifice. The renal pelvis contains urothelial subepithelial connective tissue and muscularis layers that are surrounded by peripelvic fat and/or renal parenchyma depending upon the location. Whereas the intrarenal portion of the renal pelvis is surrounded by renal parenchyma the extrarenal pelvis is surrounded by perihilar fat where renal hilar lymph nodes are situated. Thickness of the muscularis and peripelvic fat may vary along the renal pelvis continuum. The histologic layers of the ureter include the epithelium (urothelium) subepithelial connective tissue muscularis and an outermost connective tissue adventitial layer. The ureter courses through the retroperitoneum adjacent to the parietal peritoneum and rests on the retroperitoneal musculature above the pelvic vessels. As it crosses the vessels and enters the deep pelvis the ureter is surrounded by pelvic fat until it traverses the bladder wall.

Regional Lymph Nodes

The absence of definitive mapping data challenges the definition of regional lymph nodes and makes it difficult to establish limitations or locations for the extent of surgical dissection. The regional lymph nodes for the renal pelvis are as follows (Fig. 61.1):

- Renal hilar
- Paracaval
- Aortic
- Retroperitoneal, NOS

The regional lymph nodes for the ureter are as follows (Fig. 61.2):

- Renal hilar
- Paracaval
- Iliac (common, internal [hypogastric], external)
- Periureteral
- Pelvic, NOS

Metastatic Sites

Distant spread is most commonly to lungs, lymph nodes, bone, or liver.

RULES FOR CLASSIFICATION

Clinical Classification

Primary tumor assessment includes radiographic imaging as described in the Imaging section. Ureteroscopic visualization of the tumor is desirable and tissue biopsy through the ureteroscope may be performed if feasible although the small tissue biopsies obtained may not always capture higher

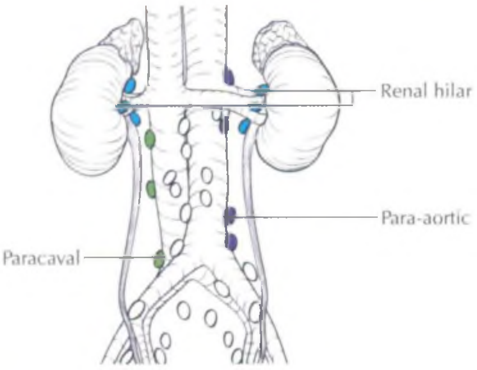

Fig. 61.1 The regional lymph nodes of the renal pelvis

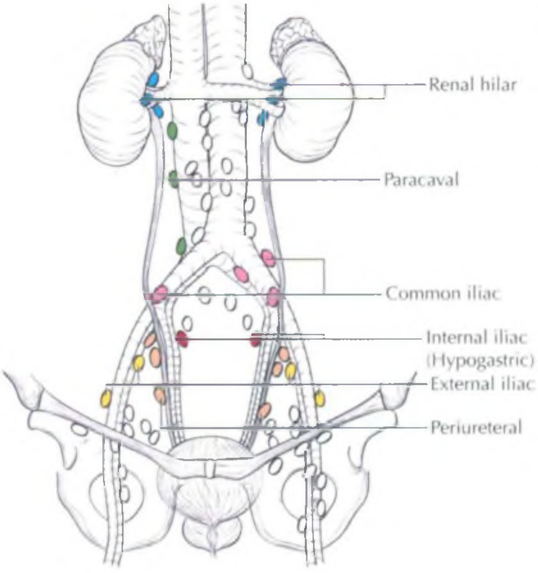

Fig. 61.2 The regional lymph nodes of the ureter

grade components or invasion present within larger tumors. Urine cytology may help determine tumor grade if tissue is not available. The relatively high incidence of concomitant bladder cancer in patients with renal pelvis and ureteral cancers necessitates evaluation and surveillance of these sites. Staging of tumors of the renal pelvis and ureter is not influenced by the presence of any concomitant bladder tumors; it may not be possible however to identify the true source of the primary tumor in the presence of metastases if both upper and lower tract tumors are present. In that situation the tumor ofhighest grade and/or stage is most likely to have contributed to the nodal or metastatic spread. Regional lymph node metastasis is a poor prognostic finding with an associated 33% 5-year cancer-free survival similar to that found in lymph node positive patients with urothelial cancer of the bladder. The therapeutic role of the lymphadenectomy in patients with upper tract urothelial carcinoma has yet to be fully defined.

Imaging

There is a paucity of information and minimal standard guidelines regarding the most appropriate imaging approach for clinical staging. The choice and extent of use of imaging for detecting nodal and distant disease should be based on tumor histology and underlying symptoms. CT scans magnetic resonance (MR) imaging and bone scintigraphy can be used to stage nodal disease and distant metastasis. A key feature to consider in primary tumor categorization of renal pelvic and ureter carcinomas is their multiplicity. Hence it is important to image the entire urinary tract. CT urogram (CTU) and MR urogram (MRU) have almost completely replaced the use of intravenous urography (IVU) as the imaging modality of choice for detecting lesions of the upper tracts. Both CTU and MRU are superior to IVU in detection of upper tract lesions and offer multiple advantages over conventional IVU in that the renal parenchyma and the remainder of the abdomen and pelvis also can be assessed to complete nodal staging and detect distant metastasis inthe abdomen.[1,2] CTU has a pooled sensitivity of 96% and specificity of 99% in detecting upper urinary tract urothelial carcinoma in comparison to the sensitivity for IVU of 50-75% for IVU.[1,3] MRU offers additional advantages over CTU which are valuable in patients who are unable to receive iodine-based contrast. With its superior tissue contrast resolution the standing column of urine in the upper tracts may be utilized to enhance visualization of the upper tract using heavily T2-weighted sequences in MRU. Addition of contrast improves the accuracy of MRU significantly.[4,5] Urothelial carcinomas are seen as small foci or mass lesions showing early enhancement on contrast-enhanced CT or MR imaging. In the excretory phase studies these are seen as filling defects in the upper tracts mass lesions or circumferential thickening of the pelvicalyceal system and ureter.[2,6,7] In MRU as in CTU excretory phase images are the key sequence used in detecting upper tract lesions and addition of a nephrographic phase improves the sensitivity for urothelial malignancy.[5]CT and MR imaging are of limited value in staging Ta-T2 tumors of the upper tract because they cannot accurately depict the depth of invasion.[2,8-11] However accuracy improves with advanced stage disease because of CTs and MR imaging's ability to demonstrate peripelvic and periureteric extension (Fig. 61.3). Gross extension of tumor into the periureteric fat renal sinus fat or abnormal enhancement of adjacent renal parenchyma is suggestive of T3

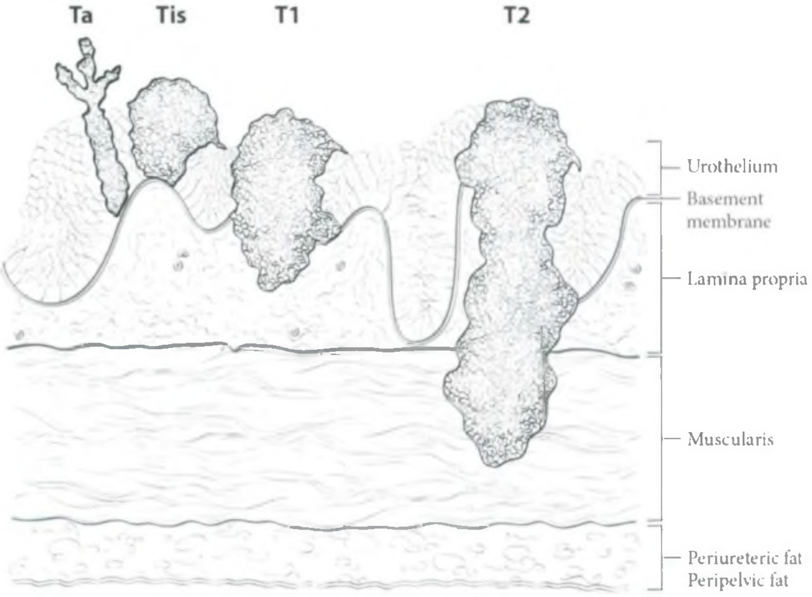

Fig. 61.3 Depth of invasion of Ta-T2 tumors

disease (Fig. 61.4). Presence of hydronephrosis in patients with ureteral tumors suggests advanced stage disease.[10] Periureteric edema superimposed infection and inflammatory changes can confound the findings and lead to erroneous staging.Both CT and MR imaging rely on size as the primary criterion for diagnosing metastatic lymph node involvement. However size can be an unreliable criterion because small lymph nodes can harbor metastasis.Metastatic disease is common in lungs liver and bones. Patients at risk of metastases can be investigated with the appropriate cross-sectional imaging of choice. Chest radiographs or chest CT may be considered to detect pulmonary metastasis depending on the degree of suspicion. CT is more sensitive than plain radiographs to detect pulmonary metastasis but with an added burden of costs and radiation exposure. In patients with symptoms suggestive of bone metastasis a radionuclide bone scan may be used. 18F-FDG positron emission tomography (PET) has been shown to be superior to conventional MR imaging and CT in detecting distant metastasis in transitional cell carcinoma.[12,13] Use of novel radiotracers including those targeted against specific cancer antigens is promising developments in the field of PET imaging which will likely improve staging in the near future.

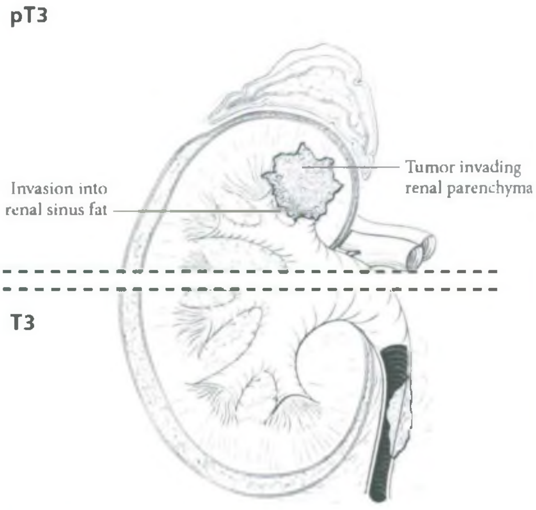

Fig. 61.4 T3 (for renal pelvis only, top of diagram): tumor invades beyond muscularis into peripelvic fat or the renal parenchyma. T3 (for ureter only, bottom of diagram): tumor invades beyond muscularis into periureteric fat

Pathological Classification

Pathological staging depends on histologic determination of either a noninvasive or *in situ* lesion or the extent of invasion by the primary tumor. Noninvasive papillary carcinomas and urothelial carcinoma can colonize underlying contiguous structures similar to that identified at other sites of the urinary tract. In the renal pelvis noninvasive lesions may spread into the collecting duct system leading to expansion

of renal tubules termed "intratubular spread" (involvement of renal collecting tubules without stromal invasion). In the absence of clear-cut interstitial invasion, this is considered to be noninvasive disease. The thickness of the subepithelial connective tissue and muscularis may vary in portions of the renal pelvis but this does not affect histologic criteria for staging. Differences in histology and tumor-related characteristics create several challenges to staging urothelial tumors of the renal pelvis depending on whether they are intrarenal or extrarenal. These have been discussed elsewhere.[14] Treatment frequently requires resection of the entire kidney, ureter, and a cuff of bladder surrounding the ureteral orifice. Surgical margins include the mucosal and soft tissue margins of the bladder cuff as well as resected soft tissue and vascular margins of the specimen. Appropriate regional nodes may be sampled. A more conservative surgical resection may be performed especially with distal ureteral tumors or in the presence of compromised renal function. Increasing evidence does suggest that a regional lymph node dissection not only improves staging but also may provide a benefit in cancer-specific outcome. No data exist to substantiate substratification of lymph node staging into three discrete categories. Therefore the N3 classification was collapsed into the N2 stage classification in an effort to remain consistent with the available evidence.

Endoscopic resection through a ureteroscope or a percutaneous approach may be used in some circumstances. Submitted tissue may be insufficient for accurate histologic examination and often will be insufficient for adequate pathological staging. Laser or electrocautery coagulation or vaporization of the tumor may be performed especially if the visible appearance is consistent with a low-grade and low-stage tumor. Under these circumstances there may be no material available for histologic review.

PROGNOSTIC FACTORS

Prognostic Factors Required for Stage Grouping

Beyond the factors used to assign T, N, or M categories no additional prognostic factors are required for stage grouping.

Additional Factors Recommended for Clinical Care

The prognostic factors that have routinely been associated with outcome in urothelial cancer of the bladder are important variables in outcome of urothelial cancers of the upper tract as well. These include concomitant carcinoma *in situ* in the setting of papillary disease, tumor size, variant histologic subtypes, surgical margin status, and tumor multifocality. In addition, ureteral location is associated with a worse outcome than renal pelvic location.[15] AJCC Level of Evidence: II

Pathologic Grade in pTa Disease

Noninvasive papillary carcinomas of the urinary tract are subdivided into low-grade and high-grade disease with the latter associated with an increased risk of invasive disease. Grading is based on microscopic assessment of cellular organization and nuclear features. High-grade tumors that appear noninvasive should be appropriately sampled to rule out invasion. Papilloma and papillary urothelial neoplasm of low malignant potential (PUNLMP) are excluded from this category. AJCC Level of Evidence: II

Lymphovascular Invasion

Lymphovascular invasion has been associated with diminished clinical outcomes in invasive tumors; although its overall value as an independent factor especially for T2 and T3 disease remains controversial. Assessment of lymphovascular invasion is performed using light microscopic analysis on invasive tumors of any stage. Immunohistochemistry to identify vascular or lymphatic spaces is currently not recommended. AJCC Level of Evidence: II

Concurrent Urothelial Carcinoma *In Situ*

The presence of urothelial carcinoma *in situ* has been associated with the presence of multifocal disease in the urinary tract and increased risk of invasive disease. Urothelial carcinoma *in situ* that occurs in association with high-grade papillary urothelial carcinoma should be reported and has been shown to be a negative prognostic indicator. In such cases care must be taken to distinguish the "shoulder" (lateral extension) of a high-grade papillary carcinoma from a separate focus of urothelial carcinoma *in situ*. AJCC Level of Evidence: II

RISK ASSESSMENT MODELS

The AJCC recently established guidelines that will be used to evaluate published statistical prediction models for the purpose of granting endorsement for clinical use.[16] Although this is a monumental step toward the goal of precision medicine this work was published only very recently. Therefore the existing models that have been published or may be in clinical use have not yet been evaluated for this cancer site by the Precision Medicine Core of the AJCC. In the future the statistical prediction models for this cancer site will be evaluated and those that meet all AJCC criteria will be endorsed.

DEFINITIONS OF AJCC TNM

Definition of Primary Tumor (T)

T Category	T Criteria
TX	Primary tumor cannot be assessed
T0	No evidence of primary tumor
Ta	Papillary noninvasive carcinoma
Tis	Carcinoma *in situ*
T1	Tumor invades subepithelial connective tissue
T2	Tumor invades the muscularis
T3	For renal pelvis only: Tumor invades beyond muscularis into peripelvic fat or into the renal parenchyma For ureter only:Tumor invades beyond muscularis into periureteric fat
T4	Tumor invades adjacent organs, or through the kidney into the perinephric fat

Definition of Regional Lymph Node (N)

N Category	N Criteria
NX	Regional lymph nodes cannot be assessed
N0	No regional lymph node metastasis
N1	Metastasis in a single lymph node, ≤2 cm in greatest dimension
N2	Metastasis in a single lymph node, >2 cm; or multiple lymph nodes

Definition of Distant Metastasis (M)

M Category	M Criteria
M0	No distant metastasis
M1	Distant metastasis

AJCC PROGNOSTIC STAGE GROUPS

When T is...	And N is...	And M is...	Then the stage group is...
Ta	N0	M0	0a
Tis	N0	M0	0is
T1	N0	M0	I
T2	N0	M0	II
T3	N0	M0	III
T4	N0	M0	IV
Any T	N1	M0	IV
Any T	N2	M0	IV
Any T	Any N	M1	IV

REGISTRY DATA COLLECTION VARIABLES

1. Presence or absence of extranodal extension
2. Size of the largest tumor deposit in the lymph nodes
3. Total number of lymph nodes dissected
4. Presence of urothelial carcinoma *in situ* (Tis) with other tumors
5. Presence of papillary noninvasive carcinoma (Ta) with other tumors
6. Lymphovascular invasion
7. World Health Organization/International Society of Urologic Pathology (WHO/ISUP) grade
8. Grade 1-3 for squamous and adenocarcinoma
9. Intratubular spread of Tis urothelial carcinoma (involvement of renal collecting tubules without stromal invasion)

HISTOLOGIC GRADE (G)

Urothelial Histologies

For urothelial histologies a low- and high-grade designation is used to match the current WHO/ISUP recommended grading system.

G	G Definition (Urothelial Carcinoma)
LG	Low grade
HG	High grade

Squamous Cell Carcinoma and Adenocarcinoma

For squamous cell carcinoma and adenocarcinoma, the following grading schema is recommended.

G	G Definition
GX	Grade cannot be assessed
G1	Well differentiated
G2	Moderately differentiated
G3	Poorly differentiated

HISTOPATHOLOGIC TYPE

Noninvasive carcinoma

- Low-grade papillary urothelial carcinoma
- High-grade papillary urothelial carcinoma
- Urothelial carcinoma *in situ*

Invasive carcinoma

- Conventional urothelial ("transitional cell") carcinoma
- Urothelial carcinoma variants
  - Urothelial carcinoma with divergent differentiation (squamous, glandular, and/or trophoblastic)
  - Nested urothelial carcinoma (including large nested carcinoma)
  - Microcystic urothelial carcinoma
  - Micropapillary urothelial carcinoma
  - Lymphoepithelioma-like urothelial carcinoma
  - Plasmacytoid urothelial carcinoma
  - Giant cell urothelial carcinoma
  - Lipid-rich urothelial carcinoma
  - Clear cell (glycogen-rich) urothelial carcinoma
  - Sarcomatoid urothelial carcinoma
  - Poorly differentiated urothelial carcinoma (including those with osteoclast-like giant cells)
- Squamous cell carcinoma
- Adenocarcinoma
- Small cell carcinoma

Bibliography

1. Jinzaki M, Matsumoto K, Kikuchi E, et al. Comparison of CT urography and excretory urography in the detection and localization of urothelial carcinoma of the upper urinary tract. *AJR. American journal of roentgenology*. May 2011;196(5):1102–1109
2. Vikram R, Sandler CM, Ng CS. Imaging and staging of transitional cell carcinoma: part 2, upper urinary tract. *AJR. American journal of roentgenology*. Jun 2009;192(6):1488–1493
3. Chlapoutakis K, Theocharopoulos N, Yarmenitis S, Damilakis J. Performance of computed tomographic urography in diagnosis of upper urinary tract urothelial carcinoma, in patients presenting with hematuria: Systematic review and meta-analysis. *Eur J Radiol*. Feb 2010;73(2):334–338
4. Takahashi N, Glockner JF, Hartman RP, et al. Gadolinium enhanced magnetic resonance urography for upper urinary tract malignancy. *J Urol*. Apr 2010;183(4):1330–1365
5. Takahashi N, Kawashima A, Glockner JF, et al. Small (<2-cm) upper-tract urothelial carcinoma: evaluation with gadolinium-enhanced three-dimensional spoiled gradient-recalled echo MR urography. *Radiology*. May 2008;247(2):451–457
6. Caoili EM, Cohan RH, Inampudi P, et al. MDCT urography of upper tract urothelial neoplasms. AJR. American journal of roentgenology. Jun 2005;184(6):1873–1881
7. Xu AD, Ng CS, Kamat A, Grossman HB, Dinney C, Sandler CM. Significance of upper urinary tract urothelial thickening and filling defect seen on MDCT urography in patients with a history of urothelial neoplasms. *AJR. American journal of roentgenology*. Oct 2010;195(4):959–965
8. Buckley JA, Urban BA, Soyer P, Scherrer A, Fishman EK. Transitional cell carcinoma of the renal pelvis: a retrospective look at CT staging with pathologic correlation. *Radiology*. Oct 1996;201(1):194–198
9. Hilton S, Jones LP. Recent advances in imaging cancer of the kidney and urinary tract. *Surg Oncol Clin N Am*. Oct 2014;23(4):863–910
10. Ng CK, Shariat SF, Lucas SM, et al. Does the presence of hydronephrosis on preoperative axial CT imaging predict worse outcomes for patients undergoing nephroureterectomy for upper-tract urothelial carcinoma? Paper presented at: Urologic Oncology: Seminars and Original Investigations 2011
11. Scolieri MJ, Paik ML, Brown SL, Resnick MI. Limitations of computed tomography in the preoperative staging of upper tract urothelial carcinoma. *Urology*. Dec 20 2000;56(6):930–934
12. Goodfellow H, Viney Z, Hughes P, et al. Role of fluorodeoxyglucose positron emission tomography (FDG PET)-computed tomography (CT) in the staging of bladder cancer. *BJU Int*. Sep 2014; 114(3):389–395
13. Kibel AS, Dehdashti F, Katz MD, et al. Prospective study of [18F] fluorodeoxyglucose positron emission tomography/computed tomography for staging of muscle-invasive bladder carcinoma. *J Clin Oncol*. Sep 10 2009;27(26):4314–4320
14. Gupta R, Paner GP, Amin MB. Neoplasms of the upper urinary tract: a review with focus on urothelial carcinoma of the pelvicalyceal system and aspects related to its diagnosis and reporting. *Adv Anat Pathol*. May 2008;15(3):127–139
15. Yafi FA, Novara G, Shariat SF, et al. Impact of tumour location versus multifocality in patients with upper tract urothelial carcinoma treated with nephroureterectomy and bladder cuff excision: a homogeneous series without perioperative chemotherapy. *BJU Int*. Jul 2012;110(2 Pt 2):E7–13
16. Kattan MW, Hess KR, Amin MB, et al. American Joint Committee on Cancer acceptance criteria for inclusion of risk models for individualized prognosis in the practice of precision medicine. *CA: a cancer journal for clinicians*. Jan 19 2016.

61

Urinary Bladder

62

Bernard H. Bochner, Donna E. Hansel, Jason A. Efstathiou, Badrinath Konety, Cheryl T. Lee, James M. McKiernan, Elizabeth R. Plimack, Victor E. Reuter, Srikala Sridhar, Raghunandan Vikram, and Walter M. Stadler

CHAPTER SUMMARY

Cancers Staged Using This Staging System

The typical epithelial cancer of the bladder is a urothelial carcinoma, previously termed transitional cell cancer. These cancers may include other histologic elements, including adenocarcinoma, squamous cell cancer, and small cell or neuroendocrine cancer, but should be classified as urothelial unless the cancer is composed entirely of the alternative histology.

Cancers Not Staged Using This Staging System

These histopathologic types of cancer…	Are staged according to the classification for…	And can be found in chapter…
Prostatic urothelial cancer	Urethra	63
Lymphoma	Hodgkin and Non-Hodgkin lymphoma	79
Sarcoma	Soft tissue sarcoma of the abdomen and thoracic visceral organs	42

Summary of Changes

Change	Details of Change	Level of Evidence
Definition of Regional Lymph Node (N)	Perivesical lymph node involvement is classified as N1.	II
Definition of Distant Metastasis (M)	M1 is subdivided into M1a and M1b.<br>M1a refers to a non-regional lymph node only.<br>M1b refers to non-lymph-node distant metastases.	II
AJCC Prognostic Stage Groups	Stage III is subdivided into IIIA and IIIB.<br>Stage IV is subdivided into IVA and IVB.	II

ICD-O-3 Topography Codes

Code	Description
C67.0	Trigone of bladder
C67.1	Dome of bladder
C67.2	Lateral wall of bladder
C67.3	Anterior wall of bladder
C67.4	Posterior wall of bladder
C67.5	Bladder neck
C67.6	Ureteric orifice
C67.7	Urachus
C67.8	Overlapping lesion of bladder
C67.9	Bladder, NOS

To access the AJCC cancer staging forms, please visit www.cancerstaging.org.

© American Joint Committee on Cancer 2017

M.B. Amin et al. (eds.), *AJCC Cancer Staging Manual, Eighth Edition*, DOI 10.1007/978-3-319-40618-3_62

WHO Classification of Tumors

Code	Description
8120	Infiltrating urothelial carcinoma
8131	Micropapillary urothelial carcinoma
8082	Lymphoepithelioma-like urothelial carcinoma
8031	Giant cell urothelial carcinoma
8020	Poorly differentiated urothelial carcinoma (including those with osteoclast-like giant cells)
8122	Sarcomatoid urothelial carcinoma
8130	Papillary urothelial carcinoma
8140	Adenocarcinoma
8070	Squamous cell carcinoma
8041	Small cell neuroendocrine carcinoma

Moch H, Humphrey PA, Ulbright TM, Reuter VE, eds. World Health Organization Classification of Tumours of the Urinary System and Male Genital Organs. Lyon: IARC; 2016.

INTRODUCTION

Urothelial cancer carcinogenesis is thought to be related to direct carcinogen exposure of the urothelium from substances present in the urine, especially smoking-related carcinogens. Thus, a field change is common based on the diffuse exposure and multiple tumors may develop in all locations of the urinary system, including the renal pelvis, ureters, bladder, and proximal urethra. Bladder cancer is 10 times more common than urothelial cancer of these other anatomical locations, perhaps due to more prolonged exposure of this site from carcinogens in the stored urine. Although urothelial cancer of these other anatomical locations is staged according to Chapters 61 and 63, overall staging rules and treatment approach are similar.

Urothelial carcinoma may present as a low- or high-grade papillary lesion, as urothelial carcinoma in situ involving the mucosal lining, or as an invasive cancer that can progress beyond the bladder wall and/or metastasize to regional lymph nodes. Non-invasive papillary lesions harbor a relatively low risk for progression to invasive disease; however, this risk is dependent on the grade of the lesion (i.e., high- versus low-grade). High-grade papillary carcinoma and urothelial carcinoma in situ may be associated with a progressive course, including invasion of the bladder wall and the subsequent development of regional and/or distant metastases. Distant metastases occur in the setting of invasive disease and are associated with increasing depth of invasion of the primary tumor into the bladder wall.

This chapter describes the staging system for tumors of the urinary bladder. All histologic cell types that are derived primarily from the urinary bladder epithelium should follow this staging system. In the male, direct involvement of the prostate or prostatic urethra is included as part of the primary stage of the bladder tumor. However, primary urothelial tumors of the prostate or prostatic urethra should be staged based on the established staging system for the male urethra as noted in Chapter 63.

Bladder cancers receive both clinical and pathological staging. Clinical staging of the primary tumor relies on physical examination, imaging, and pathologic information obtained from a biopsy, including evaluation of a transurethral biopsy. Pathological staging of the primary tumor relies on pathologic information obtained from a radical or partial cystectomy. Clinical staging of the regional lymph nodes and distant metastatic disease relies primarily on radiographic evaluation and may be confirmed by pathologic biopsy. Pathological staging of the regional lymph nodes relies on histologic review of the lymphadenectomy specimen.

ANATOMY

Primary Site(s)

The urinary bladder consists of three layers: the urothelium, the lamina propria (also referred to as subepithelial connective tissue), and the muscularis propria (detrusor muscle). The perivesical fat surrounding the bladder is lined by peritoneum along the superior surface and upper part of the bladder and is relevant for staging of extravesical bladder cancer. In the male, the bladder approximates the rectum and seminal vesicle posteriorly, the prostate inferiorly, and the pubis and peritoneum anteriorly. In the female, the vagina is located posteriorly to the bladder and the uterus superiorly. The bladder is located extraperitoneally. Cancers spread through direct extension through superficial to deep layers of the bladder wall, occasionally extending to adjacent organs in instances of advanced disease.

Regional Lymph Nodes

The regional lymph nodes draining the bladder include primary and secondary nodal drainage regions. Primary lymph nodes include the perivesical, internal and external iliac, and obturator basins. The sacral lymph nodes are classified as a primary drainage region; however, mapping studies have found this area to be a less frequent site of primary regional metastases. Primary nodal regions drain into the common iliac nodes, which constitute a secondary drainage region. It is thus of particular importance to distinguish common iliac lymph node involvement from internal and external iliac involvement.

Regional nodes include the following:

- Primary drainage
  - Perivesical pelvic, NOS
  - Iliac (internal, external)
  - Sacral (lateral, sacral promontory)
  - Obturator
- Secondary drainage
  - Common iliac

Metastatic Sites

Distant spread is most commonly to retroperitoneal lymph nodes, lung, bone, and liver. Lymph node involvement beyond the common iliac nodes (e.g., paracaval or intra-aortacaval) is considered metastatic (M1a).

RULES FOR CLASSIFICATION

Clinical Classification

Primary tumor assessment includes cystoscopic assessment, bimanual examination before and after endoscopic surgery (biopsy or transurethral resection), radiographic evaluation, and histologic verification of the presence or absence of tumor when indicated. All factors are important in determining a clinical stage of disease. Despite optimal evaluation, clinical understaging and overstaging remains a concern.

The definitions for Primary Tumor (T) are illustrated in (Figs. 62.1 and 62.2.) Cystoscopic evaluation should include the size, location, number, and growth characteristics of the tumor. The suffix "m" is added to denote multiple tumors. Cystoscopic biopsy and urinary cytology are key. Bimanual examination before and after endoscopic surgery is an indicator of clinical stage. The finding of bladder wall thickening, a mobile mass, or a fixed mass suggests the presence of T3 and/or T4 disease, respectively. After macroscopic complete endoscopic resection, the persistence of a palpable mobile mass suggests a cT3 tumor. The primary tumor may be noninvasive or invasive and can be partially or totally resected with sufficient tissue from the tumor base for evaluation of full depth of tumor invasion. It should be ensured that muscularis propria is adequately sampled, and if not, repeat sampling is required to confirm if muscularis propria is or is not involved. Repeat resection of early invasive tumors (T1) will provide optimal staging information, and multiple biopsies can be taken from other suspicious sites to rule out a field effect.

Regional lymph node staging is of significant prognostic importance given the negative impact on recurrence after treatment and long-term survival. Clinical staging of regional lymph node involvement by imaging often is inaccurate and does not necessarily provide information on the extent of disease within the nodes (see Imaging and Pathological Classification).

A number of studies have demonstrated that patients with metastatic disease limited to lymph nodes have a significantly better outcome than patients with visceral or bony metastases.[1-3] Although these patients generally are treated with systemic chemotherapy, approximately 10% experience a complete radiologic response, and an undefined fraction of these patients may be long-term survivors with or without additional primary tumor therapy. Patients with metastatic disease only in lymph nodes beyond the common iliacs thus should be classified as M1a, whereas all other metastatic disease should be classified as M1b. AJCC Level of Evidence: II[4,5]

Imaging

Imaging is recommended to stage and characterize most newly diagnosed bladder cancer. Published guidelines recommend pelvic and upper-tract evaluations for all patients with higher risk bladder tumors.[6,7] As most patients with bladder cancer present with hematuria, imaging evaluation of the upper urinary tract using Computed Tomography (CT) or magnetic resonance (MR) urography is recommended.[8] Intravenous pyelography (IVP), renal ultrasound, and retrograde pyelograms may be used if CT or MR imaging are contraindicated. Chest imaging with chest X-ray (CXR) or preferably CT may be indicated when muscle-invasive disease is present, and is indicated for clinical lymph node positive disease. Bone scintigraphy and brain MR imaging may be considered in individuals with relevant symptoms. 18F-fluorodeoxyglucose (18F-FDG) positron emission tomography (PET) has been shown to be superior to conventional MR imaging and CT in detecting distant metastasis.[9,10]

Imaging plays a complementary role to deep biopsy in local staging of bladder cancer. Although multidetector CT generally is used for detecting nodal and distant metastasis, it is inferior to MR imaging for local staging. High-resolution MR imaging can demonstrate the different layers of the bladder and hence can be exploited to stage bladder carcinomas. MR imaging consists of pre- and post-contrast T1-weighted images and multiplanar T2-weighted images. Diffusion-weighted images also may be obtained. It is important to image the urinary bladder before biopsy and intervention as post-treatment changes can cause inflammation and scarring, resulting in erroneous interpretation.

The detrusor muscle has low signal intensity on T2-weighted images and is interrupted in the case of muscle-invasive tumors. The overall staging accuracy of T2-weighted imaging is between 40% and 67%, with overstaging being the most common error.[11-14] Differential

62

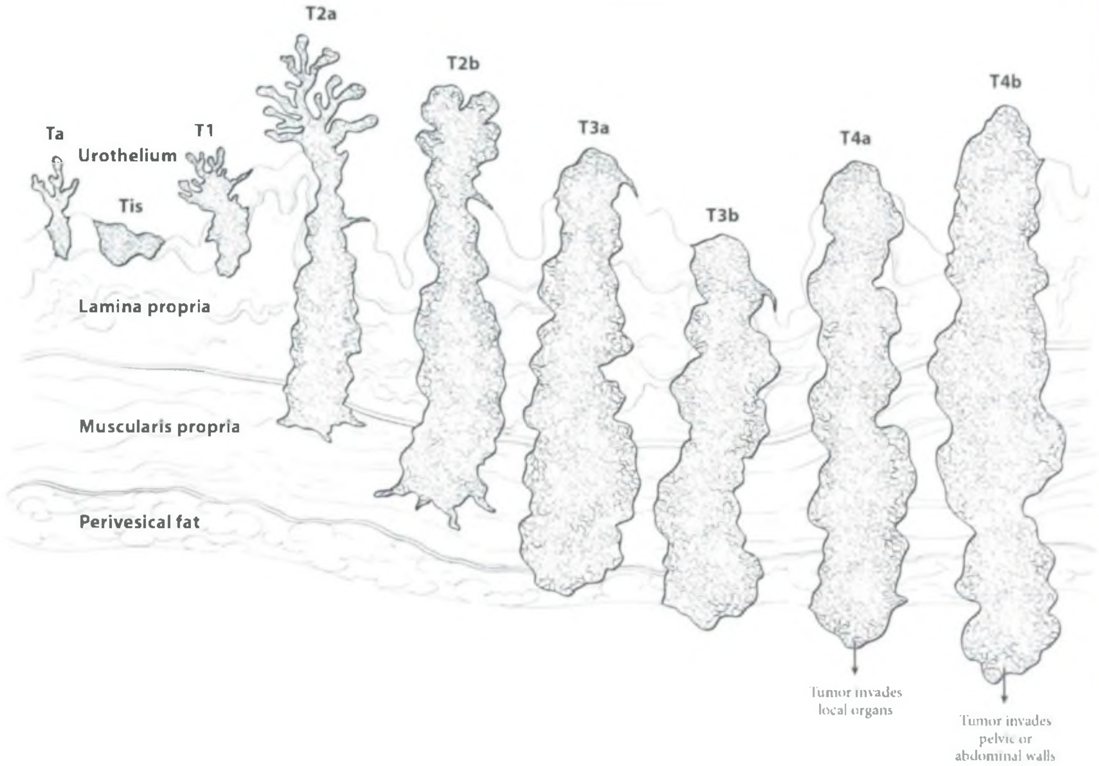

Fig. 62.1 Extent of primary bladder cancer

enhancement between the tumor and muscle layer in different phases of T1-weighted contrast studies sometimes can help identify muscle-invasive disease. Tumor enhances early, and muscle shows delayed enhancement and can be seen as an interrupted line in muscle-invasive disease. The overall staging accuracy of dynamic contrast-enhanced MR imaging is reported to be 52-85%.[11,15,16] Diffusion-weighted images may be useful in differentiating perivesical invasion versus inflammatory or reactive stranding.[14] Despite these purported advantages of MR imaging versus CT for T-staging, the relative clinical value remains to be determined.

MR imaging and CT are equally sensitive in detecting nodal metastasis when size criteria are used. However, these size criteria (>1 cm) frequently can lead to inaccurate conclusions, as metastatic nodes can be smaller than 1 cm. Use of ultrasmall superparamagnetic iron oxide particles in combination with T2 star imaging may improve nodal staging, but it is not always available and its clinical value is not yet clear.[17] Metastatic nodes show increased activity on 18F-FDG PET CT. However, the poor spatial resolution of PET can lead to false negative results. Moreover, nodes close to the urinary bladder may be obscured by activity of excreted radiotracer in the bladder.[18]

Pathological Classification

Pathological staging is performed on partial cystectomy and radical cystectomy specimens and is based on both gross and microscopic assessment. The College of American Pathologists (CAP) has generated a synoptic report for partial and radical cystectomy specimens that contains required and recommended elements to be included in all cancer-bearing cases (www.cap.org). Gross evaluation is critical in determining macroscopic extravesical extension of tumor and thus in influencing pT3 substaging, as well as ensuring that all areas of prior bladder resections have been sampled thoroughly. Visual inspection of any and all areas suspicious for extravesical extension should be examined microscopically for appropriate pathological stage evaluation. In cystoprostatectomy specimens, gross evaluation is required to determine whether a urinary

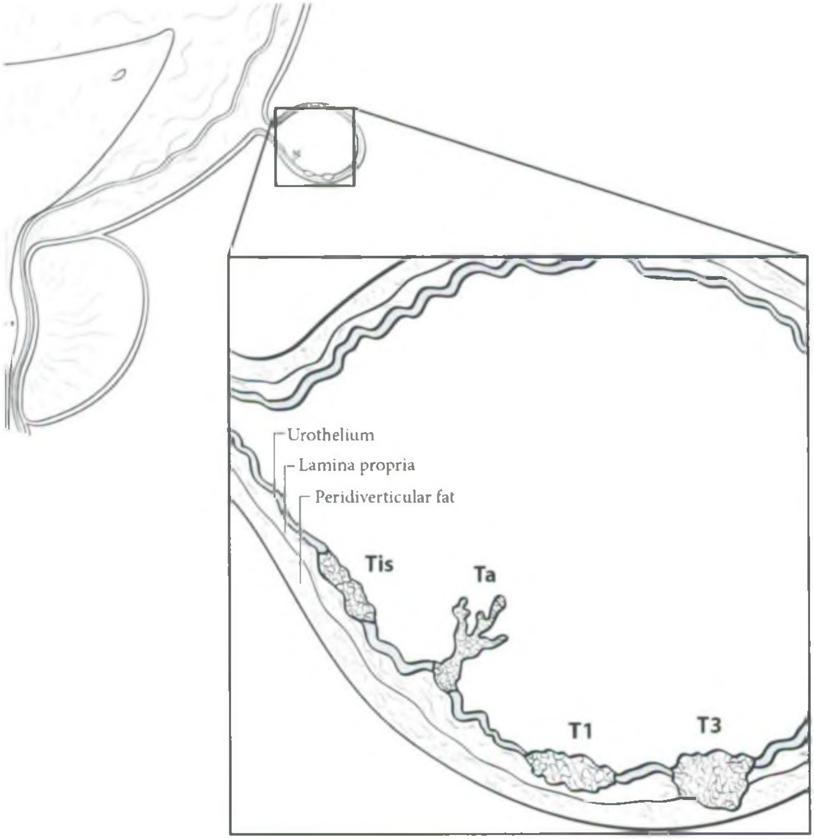

Fig. 62.2 Extent of Tis, Ta, T1, and T3

bladder carcinoma shows direct extravesical extension into the prostate and/or seminal vesicles. In addition, gross evaluation of the prostatic urethra is recommended to identify independent primary lesions involving the urethral lining.

Microscopic evaluation is required to confirm the presence or absence of non-invasive and invasive carcinoma and depth of invasive tumor cells for pathological staging, taking into account different staging paradigms for urinary bladder and urethral primary carcinomas. Microscopic assessment also is required to identify non-invasive or invasive disease at the surgical margins, including the urethral, ureteral, and soft tissue resection margins for radical cystectomy specimens and the mucosal margins for partial cystectomy specimens. Pathological staging should be specific to the cystectomy specimen at the time of surgery and should be assigned independently of previous clinical or biopsy information that is used for clinical stage assignment.

There is limited data on the best methodology to stage urothelial carcinoma that concurrently involves the urinary bladder and the prostatic urethra. Direct involvement of the prostate or prostatic urethra is included as part of the primary stage of the bladder tumor. Specifically, in patients in which a large urinary bladder carcinoma has invaded through the full thickness of the bladder wall and thereby secondarily involves the prostatic stroma, a pT4 category should be assigned per urinary bladder staging (Fig. 62.3). In other circumstances in which involvement by urothelial carcinoma is seen in both sites, separate urinary bladder and prostatic urethral staging should be assigned (see Chapter 63).

A pN status should be assessed regardless of the number of lymph nodes examined and irrespective of the laterality of the lymph nodes extracted. If no lymph nodes are evaluated, pNX status should be assigned. pN3 determination requires removal of the common iliac lymph nodes. Adequate nodal status (positive or negative) requires, at a minimum, removal of the primary lymph node regions that include the perivesical, left and right external iliac, internal iliac, and obturator nodes. Skip metastases to secondary drainage sites (common iliac nodes) may occur but are uncommon.[19] Based on contemporary mapping

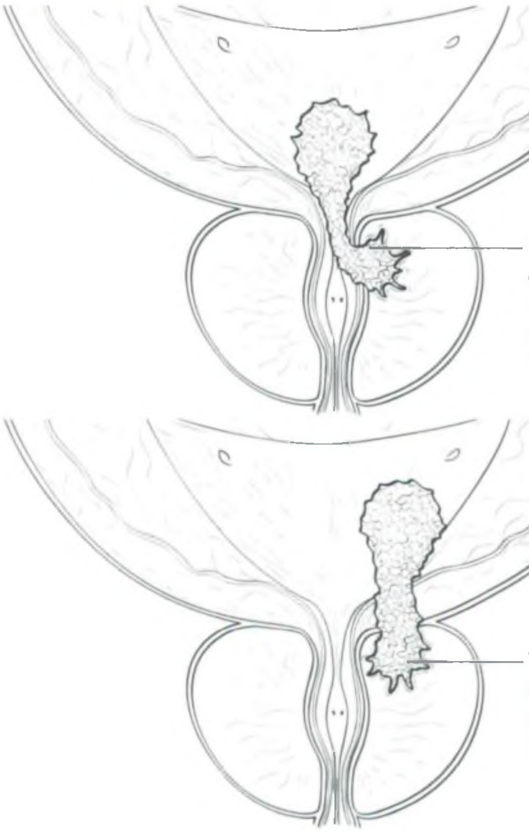

Fig. 62.3 Invasion of urethra and bladder tumors into prostratic stroma. Top: In prostatic urethra staging system (Chapters 63), T2 is defined as tumor invading the prostatic stroma surrounding ducts either by direct extension from the urothelial surface or by invasion from prostatic ducts. Bottom: In urinary bladder staging system, T4 is defined as extravesical tumor directly invading any of the following: prostatic stroma, seminal vesicles, uterus, vagina, pelvic wall, abdominal wall. Tumors that invade directly into prostatic stroma, as illustrated here, should be categorized as T4a

studies in which standard techniques were used to evaluate the pathologic specimen, excision of the primary nodal regions should result in an average of > 12 lymph nodes.[3,19,20] This should serve as a guide for the number of lymph nodes to be evaluated for optimized staging after radical cystectomy. However, the number of lymph nodes examined may be dependent on previous patient treatment and pathologic technique, with lymph node counts influenced by the presence of small lymph nodes, fatty lymph nodes, and matted lymph nodes. Improved lymph node counts have been shown to correlate with submission of individual lymph node packets for pathological assessment. Multiple series have demonstrated improved clinical outcomes with increasing number of lymph nodes removed. The optimal number of lymph nodes for diagnostic and therapeutic benefit has yet to be clearly defined. The number of lymph nodes examined from the operative specimen and the number of positive lymph nodes have been reported to be associated with survival; thus, these criteria should be recorded.

PROGNOSTIC FACTORS

Prognostic Factors Required for Stage Grouping

Beyond the factors used to assign T, N, or M categories, no additional prognostic factors are required for stage grouping.

Additional Factors Recommended for Clinical Care

Concurrent Urothelial Carcinoma in situ

The presence of urothelial carcinoma in situ has been associated with the presence of multifocal disease in the urinary tract and increased risk of invasive disease. Urothelial carcinoma *in situ* that occurs in association with high-grade papillary urothelial carcinoma should be reported and has been shown to be a negative prognostic indicator. In such cases, care must be taken to distinguish the "shoulder" (lateral extension) of a

high-grade papillary carcinoma from a separate focus of urothelial carcinoma *in situ*.[21–23] AJCC Level of Evidence: II

pT1 Categorization

Several experts have recommended substaging of pT1 disease, and numerous subcategories have been proposed. Although not formally endorsed in this staging system, pT1 categorization appears to have prognostic value, with early invasion ("microinvasive disease") into the lamina propria showing better outcomes than more advanced pT1 disease. The method of pT1 substaging has not been optimized, but microinvasive disease has been defined by different groups as invasive tumor of <1 high power field in content, greatest invasive tumor diameter of 1 mm, or invasive tumor above the muscularis mucosae extending to a depth of 2 mm or less. An attempt to categorize pT1 disease is strongly recommended using one of the above methods.[24–26] AJCC Level of Evidence: II

Extranodal ("Extracapsular") Extension

Extranodal extension is defined as invasion of tumor cells in a lymph node beyond the lymph node capsule and involving the perinodal fat. Deposits of tumor within fat without an associated lymph node should not be considered in this category; they should be reported separately. Whereas some studies have demonstrated an association between extranodal extension and reduced disease-specific survival and recurrence-free survival on multivariate analysis, other studies have failed to demonstrate prognostic significance.[27–29] AJCC Level of Evidence: III

Total Number of Lymph Nodes

The total number of lymph nodes resected has been associated with improved outcomes in patients undergoing radical cystectomy. In addition, the number of resected lymph nodes involved by metastatic disease has been associated with diminished outcomes, but neither factor has necessarily always been found to be an independent factor. Nevertheless, it is recommended that both total number of resected lymph nodes and number of lymph nodes involved by metastatic disease be reported.[19, 30] AJCC Level of Evidence: II

Lymphovascular Invasion

Lymphovascular invasion has been associated with diminished clinical outcomes in invasive tumors; however, its overall value as an independent factor remains controversial. Assessment of lymphovascular invasion is performed using light microscopic analysis on invasive tumors of any stage. Immunohistochemistry to identify vascular or lymphatic spaces currently is not recommended. AJCC Level of Evidence: III

Histopathologic Type

Histopathologic subtypes of bladder cancer (see Histopathologic Type) are reported as a diagnostic line and are included in the CAP synoptic protocol for bladder cancer. Although a number of subtypes are undergoing re-evaluation to determine association with prognosis, some subtypes, such as small cell carcinoma, plasmacytoid carcinoma, and sarcomatoid urothelial carcinoma, clearly have been recognized to be associated with reduced survival.[31] AJCC Level of Evidence: II

Margin Status

Tumor involvement of the resection margins, including the ureteral, urethral, soft tissue, and vaginal cuff margins, may indicate an increased likelihood of local cancer recurrence in the upper tract, penile urethra, or surrounding soft tissue. Additional margins should be adequately sampled and documented in anterior exenteration specimens, as appropriate.[32,33] AJCC Level of Evidence: II

62

RISK ASSESSMENT MODELS

The AJCC recently has established guidelines that will be used to evaluate published statistical prediction models for the purpose of granting endorsement for clinical use.[34] Although this is a monumental step toward the goal of precision medicine, this work was published only very recently. For this reason, the existing models that have been published or that may be in clinical use have not yet been evaluated for this cancer site by the Precision Medicine core of the AJCC. In the future, the statistical prediction models for this cancer site will be evaluated, and those that meet all AJCC criteria will be endorsed.

DEFINITIONS OF AJCC TNM

Definition of Primary Tumor (T)

T Category	T Criteria
TX	Primary tumor cannot be assessed
T0	No evidence of primary tumor
Ta	Non-invasive papillary carcinoma
Tis	Urothelial carcinoma *in situ*: "flat tumor"
T1	Tumor invades lamina propria (subepithelial connective tissue)
T2	Tumor invades muscularis propria
pT2a	Tumor invades superficial muscularis propria (inner half)
pT2b	Tumor invades deep muscularis propria (outer half)
T3	Tumor invades perivesical soft tissue
pT3a	Microscopically
pT3b	Macroscopically (extravesical mass)
T4	Extravesical tumor directly invades any of the following: prostatic stroma, seminal vesicles, uterus, vagina, pelvic wall, abdominal wall
T4a	Extravesical tumor invades directly into prostatic stroma, uterus, vagina
T4b	Extravesical tumor invades pelvic wall, abdominal wall

Definition of Regional Lymph Node (N)

N Category	N Criteria
NX	Lymph nodes cannot be assessed
N0	No lymph node metastasis
N1	Single regional lymph node metastasis in the true pelvis (perivesical, obturator, internal and external iliac, or sacral lymph node)
N2	Multiple regional lymph node metastasis in the true pelvis (perivesical, obturator, internal and external iliac, or sacral lymph node metastasis)
N3	Lymph node metastasis to the common iliac lymph nodes

Definition of Distant Metastasis (M)

M Category	M Criteria
M0	No distant metastasis
M1	Distant metastasis
M1a	Distant metastasis limited to lymph nodes beyond the common iliacs
M1b	Non-lymph-node distant metastases

AJCC PROGNOSTIC STAGE GROUPS

When T is...	And N is...	And M is...	Then the stage group is...
Ta	N0	M0	0a
Tis	N0	M0	0is
T1	N0	M0	I
T2a	N0	M0	II
T2b	N0	M0	II
T3a, T3b, T4a	N0	M0	IIIA
T1-T4a	N1	M0	IIIA
T1-T4a	N2, N3	M0	IIIB
T4b	N0	M0	IVA
Any T	Any N	M1a	IVA
Any T	Any N	M1b	IVB

REGISTRY DATA COLLECTION VARIABLES

1. Presence or absence of extranodal extension
2. Total number of lymph nodes examined pathologically and total number positive
3. Size of the largest tumor deposit in the lymph nodes
4. World Health Organization/International Society of Urologic Pathology (WHO/ISUP) grade
5. Presence of lymphovascular invasion
6. Concurrent/associated noninvasive papillary (Ta) with carcinoma *in situ* (Tis)
7. Concurrent/associated noninvasive papillary (Ta) and/or carcinoma *in situ* (Tis) with invasive cancers

HISTOLOGIC GRADE (G)

Urothelial Histologies

For urothelial histologies, a low- and high-grade designation is used to match the current WHO/ISUP recommended grading system.

G	G Definition
LG	Low-grade
HG	High-grade

Squamous Cell Carcinoma and Adenocarcinoma

For squamous cell carcinoma and adenocarcinoma, the following grading schema is recommended:

G	G Definition
GX	Grade cannot be assessed
G1	Well differentiated
G2	Moderately differentiated
G3	Poorly differentiated

Histopathologic Type

The predominant cancer is urothelial (transitional cell) carcinoma. The histologic subtypes are as follows:

Non-invasive carcinoma

- Low-grade papillary urothelial carcinoma
- High-grade papillary urothelial carcinoma
- Urothelial carcinoma *in situ*

Invasive carcinoma

- Conventional urothelial carcinoma
- Urothelial carcinoma variants
    - Urothelial carcinoma with divergent differentiation (squamous, glandular and/other)
    - Nested urothelial carcinoma (including large nested carcinoma)
    - Microcystic urothelial carcinoma
    - Micropapillary urothelial carcinoma
    - Lymphoepithelioma-like urothelial carcinoma
    - Plasmacytoid urothelial carcinoma
    - Giant cell urothelial carcinoma
    - Lipid-rich urothelial carcinoma
    - Clear cell (glycogen-rich) urothelial carcinoma
    - Sarcomatoid urothelial carcinoma
    - Poorly differentiated urothelial carcinoma (including those with osteoclast-like giant cells)

- Squamous cell carcinoma
- Adenocarcinoma
- Small cell neuroendocrine carcinoma

Bibliography

1. Ghoneim MA, Abdel-Latif M, el-Mekresh M., Radical cystectomy for carcinoma of the bladder: 2,720 consecutive cases 5 years later. J Urol. Jul 2008;180(1):121–127.
2. Stein JP, Lieskovsky G, Cote R., Radical cystectomy in the treatment of invasive bladder cancer: long-term results in 1,054 patients. J Clin Oncol. Feb 1 2001;19(3):666–675.
3. Tarin TV, Power NE, Ehdaie B., Lymph node-positive bladder cancer treated with radical cystectomy and lymphadenectomy: effect of the level of node positivity. Eur Urol. May 2012;61(5):1025–1030.
4. Bajorin DF, Dodd PM, Mazumdar M., Long-term survival in metastatic transitional-cell carcinoma and prognostic factors predicting outcome of therapy. J Clin Oncol. Oct 1999;17(10):3173–3181.
5. von der Maase H, Sengelov L, Roberts JT., Long-term survival results of a randomized trial comparing gemcitabine plus cisplatin, with methotrexate, vinblastine, doxorubicin, plus cisplatin in patients with bladder cancer. J Clin Oncol. Jul 20 2005;23(21):4602–4608.
6. Hall MC, Chang SS, Dalbagni G., Guideline for the management of nonmuscle invasive bladder cancer (stages Ta, T1, and Tis): 2007 update. J Urol. Dec 2007;178(6):2314–2330.
7. Babjuk M, Oosterlinck W, Sylvester R., EAU guidelines on non-muscle-invasive urothelial carcinoma of the bladder. Eur Urol. Aug 2008;54(2):303–314.
8. Davis R, Jones JS, Barocas DA., Diagnosis, evaluation and follow-up of asymptomatic microhematuria (AMH) in adults: AUA guideline. J Urol. Dec 2012;188(6 Suppl):2473–2481.
9. Goodfellow H, Viney Z, Hughes P., Role of fluorodeoxyglucose positron emission tomography (FDG PET)-computed tomography (CT) in the staging of bladder cancer. BJU Int. Sep 2014;114(3):389–395.
10. Kibel AS, Dehdashti F, Katz MD., Prospective study of [18F]fluorodeoxyglucose positron emission tomography/computed tomography for staging of muscle-invasive bladder carcinoma. J Clin Oncol. Sep 10 2009;27(26):4314–4320.
11. Tekes A, Kamel I, Imam K., Dynamic MRI of bladder cancer: evaluation of staging accuracy. AJR. American journal of roentgenology. Jan 2005;184(1):121–127.
12. El-Assmy A, Abou-El-Ghar ME, Mosbah A., Bladder tumour staging: comparison of diffusion- and T2-weighted MR imaging. European radiology. Jul 2009;19(7):1575–1581.
13. Saito W, Amanuma M, Tanaka J, Heshiki A. Histopathological analysis of a bladder cancer stalk observed on MRI. Magn Reson Imaging. May 2000;18(4):411–415.
14. Takeuchi M, Sasaki S, Naiki T., MR imaging of urinary bladder cancer for T-staging: a review and a pictorial essay of diffusion-weighted imaging. Journal of magnetic resonance imaging : JMRI. Dec 2013;38(6):1299–1309.
15. Takeuchi M, Sasaki S, Ito M., Urinary bladder cancer: diffusion-weighted MR imaging--accuracy for diagnosing T stage and estimating histologic grade. Radiology. Apr 2009;251(1):112–121.
16. Kim B, Semelka RC, Ascher SM, Chalpin DB, Carroll PR, Hricak H. Bladder tumor staging: comparison of contrast-enhanced CT, T1- and T2-weighted MR imaging, dynamic gadolinium-enhanced imaging, and late gadolinium-enhanced imaging. Radiology. Oct 1994;193(1):239–245.
17. Deserno WM, Harisinghani MG, Taupitz M., Urinary bladder cancer: preoperative nodal staging with ferumoxtran-10-enhanced MR imaging. Radiology. Nov 2004;233(2):449–456.
18. Jensen TK, Holt P, Gerke O., Preoperative lymph-node staging of invasive urothelial bladder cancer with 18F-fluorodeoxyglucose positron emission tomography/computed axial tomography and magnetic resonance imaging: correlation with histopathology. Scandinavian journal of urology and nephrology. Mar 2011;45(2):122–128.
19. Leissner J, Ghoneim MA, Abol-Enein H., Extended radical lymphadenectomy in patients with urothelial bladder cancer: results of a prospective multicenter study. J Urol. Jan 2004;171(1):139–144.
20. Vazina A, Dugi D, Shariat SF, Evans J, Link R, Lerner SP. Stage specific lymph node metastasis mapping in radical cystectomy specimens. J Urol. May 2004;171(5):1830–1834.
21. Gontero P, Sylvester R, Pisano F., Prognostic factors and risk groups in T1G3 non-muscle-invasive bladder cancer patients initially treated with Bacillus Calmette-Guerin: results of a retrospective multicenter study of 2451 patients. Eur Urol. Jan 2015;67(1):74–82.
22. Shariat SF, Palapattu GS, Karakiewicz PI., Concomitant carcinoma *in situ* is a feature of aggressive disease in patients with organ-confined TCC at radical cystectomy. Eur Urol. Jan 2007;51(1):152–160.
23. van Rhijn BW, Burger M, Lotan Y., Recurrence and progression of disease in non-muscle-invasive bladder cancer: from epidemiology to treatment strategy. Eur Urol. Sep 2009;56(3):430–442.
24. Brimo F, Wu C, Zeizafoun N., Prognostic factors in T1 bladder urothelial carcinoma: the value of recording millimetric depth of invasion, diameter of invasive carcinoma, and muscularis mucosa invasion. Human pathology. Jan 2013;44(1):95–102.
25. Hu Z, Mudaliar K, Quek ML, Paner GP, Barkan GA. Measuring the dimension of invasive component in pT1 urothelial carcinoma in transurethral resection specimens can predict time to recurrence. Annals of diagnostic pathology. Apr 2014;18(2):49–52.
26. van Rhijn BW, van der Kwast TH, Alkhateeb SS., A new and highly prognostic system to discern T1 bladder cancer substage. Eur Urol. Feb 2012;61(2):378–384.
27. Fleischmann A, Thalmann GN, Markwalder R, Studer UE. Extracapsular extension of pelvic lymph node metastases from urothelial carcinoma of the bladder is an independent prognostic factor. J Clin Oncol. Apr 1 2005;23(10):2358–2365.
28. Jensen JB, Ulhoi BP, Jensen KM. Evaluation of different lymph node (LN) variables as prognostic markers in patients undergoing radical cystectomy and extended LN dissection to the level of the inferior mesenteric artery. BJU Int. Feb 2012;109(3):388–393.
29. Stephenson AJ, Gong MC, Campbell SC, Fergany AF, Hansel DE. Aggregate lymph node metastasis diameter and survival after radical cystectomy for invasive bladder cancer. Urology. Feb 2010;75(2):382–386.
30. Bochner BH, Cho D, Herr HW, Donat M, Kattan MW, Dalbagni G. Prospectively packaged lymph node dissections with radical cystectomy: evaluation of node count variability and node mapping. J Urol. Oct 2004;172(4 Pt 1):1286–1290.
31. Amin MB, McKenney JK, Paner GP., ICUD-EAU International Consultation on Bladder Cancer 2012: Pathology. Eur Urol. Jan 2013;63(1):16–35.
32. Tollefson MK, Blute ML, Farmer SA, Frank I. Significance of distal ureteral margin at radical cystectomy for urothelial carcinoma. J Urol. Jan 2010;183(1):81–86.
33. Cho KS, Seo JW, Park SJ., The risk factor for urethral recurrence after radical cystectomy in patients with transitional cell carcinoma of the bladder. Urol Int. 2009;82(3):306–311.
34. Kattan MW, Hess KR, Amin MB., American Joint Committee on Cancer acceptance criteria for inclusion of risk models for individualized prognosis in the practice of precision medicine. CA: a cancer journal for clinicians. Jan 19 2016.

Urethra

63

Donna E. Hansel, Victor E. Reuter, Bernard H. Bochner, Jason A. Efstathiou, Badrinath R. Konety, Cheryl T. Lee, Elizabeth R. Plimack, Srikala Sridhar, Walter M. Stadler, Raghunandan Vikram, and James M. McKiernan

CHAPTER SUMMARY

Cancers Staged Using This Staging System

The classification applies to urothelial (transitional cell), squamous, and glandular carcinomas of the urethra and to urothelial (transitional cell) carcinomas of the prostate and prostatic urethra.

Cancers Not Staged Using This Staging System

These histopathologic types of cancer…	Are staged according to the classification for…	And can be found in chapter…
Squamous cell carcinoma of the penile foreskin	Penis	57
Primary urothelial carcinoma of the bladder with transmural involvement of the prostate	Urinary Bladder	62
Prostatic adenocarcinoma	Prostate	58
Lymphoma	Hodgkin and Non-Hodgkin Lymphoma	79
Mucosal melanoma of the urethra	No AJCC staging system	N/A
Sarcoma	Soft Tissue Sarcoma of the Abdomen and Thoracic Visceral Organs	42

Summary of Changes

Change	Details of Change	Level of Evidence
Definition of Primary Tumor (T)	In urothelial carcinoma of the prostate:<br>Carcinoma *in situ*, involvement of the prostatic urethra (previously designated as Tis pu) and Carcinoma *in situ*, involvement of the prostatic ducts and acini (previously designated as Tis pd) will be collapsed into a single stage designation:<br>Tis: carcinoma *in situ* involving the prostatic urethra, periurethral or prostatic ducts and acini without stromal invasion	III
Definition of Primary Tumor (T)	Clarification: For urothelial carcinoma of the prostate, T1 category is defined as tumors invading subepithelial connective tissue of prostatic urethra.	N/A
Definition of Primary Tumor (T)	Clarification: Extension of urethral cancer to the bladder wall proper will be classified as T4 disease to maintain consistency with bladder cancer staging in which direct extension to the prostate also is classified as T4. This applies to both urethral cancers in the female and urethral cancers arising in the male prostatic urethra or prostatic ducts.	N/A
Definition of Regional Lymph Node (N)	N1: Single regional lymph node metastasis in the inguinal region or true pelvis [perivesical, obturator, internal (hypogastric) and external iliac], or presacral lymph node	III
Definition of Regional Lymph Node (N)	N2: Multiple regional lymph node metastasis in the inguinal region or true pelvis [perivesical, obturator, internal (hypogastric) and external iliac], or presacral lymph node	III

To access the AJCC cancer staging forms, please visit www.cancerstaging.org.

© American Joint Committee on Cancer 2017

M.B. Amin et al. (eds.), *AJCC Cancer Staging Manual, Eighth Edition*, DOI 10.1007/978-3-319-40618-3_63

ICD-O-3 Topography Codes

Code	Description
C68.0	Urethra

WHO Classification of Tumors

Code	Description
8120	Infiltrating urothelial carcinoma
8131	Micropapillary urothelial carcinoma
8082	Lymphoepithelioma-like urothelial carcinoma
8031	Giant cell urothelial carcinoma
8020	Poorly differentiated urothelial carcinoma (including those with osteoclast-like giant cells)
8122	Sarcomatoid urothelial carcinoma
8130	Papillary urothelial carcinoma
8140	Adenocarcinoma
8070	Squamous cell carcinoma
8041	Small cell neuroendocrine carcinoma

Moch H, Humphrey PA, Ulbright TM, Reuter VE, eds. World Health Organization Classification of Tumours of the Urinary System and Male Genital Organs. Lyon: IARC; 2016.

INTRODUCTION

This chapter describes the staging system for carcinoma of the male and female urethra. Urothelial carcinoma of the urethra has a similar biology compared to tumors of the urinary bladder and the reader is referred to the overview and details in the chapter for the urinary bladder. Non-urothelial cancers have a unique biology. Cancers of the male prostatic, membranous, bulbar, and penile urethra are included in this chapter. Similarly, carcinoma of the distal and proximal female urethra are covered by the staging system described in this chapter.

Urethral carcinoma includes urothelium-derived carcinomas and those arising from periurethral glands and encompasses carcinomas of the prostatic urethra, the penile urethra, and the female urethra. Cancer of the urethra is a rare neoplasia that is found in both sexes but is more common in men when accounting for all three locations. The cancer may be associated in males with chronic stricture disease and in females with urethral diverticula. Distal urethral cancers of squamous origin, *in situ* or invasive, may occur synchronously or metachronously to squamous cell carcinoma of the penis. Squamous cell carcinoma of the penis, particularly involving the glans, may secondarily involve the distal urethra, and when tumors are large it is difficult to determine if they are penile or distal urethral cancers.

Carcinoma arising in the prostatic and penile urethra commonly consists of urothelial carcinoma or squamous cell carcinoma, respectively, although adenocarcinoma may be present in the penile urethra. Multiple studies have reported variance in the most common subtype of urethral carcinoma in women, where urothelial carcinoma, squamous cell carcinoma, and adenocarcinoma have all been reported. The prevalence of specific morphologies at each urethral site reflects the associated tissue elements and the unique biology of each specific location. In the setting of urothelial carcinoma, which often is multifocal, involvement of the male or female urethra may be the initial site of urothelial carcinogenesis or may occur following a prior diagnosis of urothelial carcinoma of the urinary bladder or upper urinary tract. Urothelial carcinoma is the most common carcinoma involving the prostatic urethra and may manifest with *in situ* or invasive disease; primary carcinoma involving the prostatic urethra is staged according to urethral guidelines and is distinct from transmural involvement of the prostate by a large bladder carcinoma (see Chapter 62 for urinary bladder staging guidelines). The penile urethra more commonly is involved by squamous cell carcinoma, although careful exclusion of secondary spread of a squamous cell carcinoma from the glans penis should be considered (see Chapter 57 for penile cancer staging guidelines). Finally, the periurethral glands will give rise to adenocarcinoma and thus represent a major diagnostic subtype of carcinoma in the penile and female urethra.

ANATOMY

Primary Site(s)

The male penile urethra contains an epithelial lining, subepithelial connective tissue, and surrounding corpus spongiosum. The meatal and parameatal urethra are lined with non-keratinizing squamous epithelium and the penile and bulbomembranous urethra with pseudostratified or stratified columnar epithelium. Scattered islands of stratified squamous epithelium and glands of Litté are liberally situated throughout the entire urethra distal to the membranous portion, which is associated with the presence of Cowper's glands. In contrast, the prostatic urethra is lined by urothelium.

The epithelium of the female urethra is lined by nonkeratinizing squamous epithelium in the distal two-thirds, whereas the proximal one-third is lined by urothelium. Subepithelial connective tissue underlies the epithelial lining and contains the periurethral glands of Skene, which are concentrated near the meatus but extend along the entire urethra. The periurethral glands are lined with pseudostratified and stratified columnar epithelium. The urethra is surrounded by a longitudinal layer of smooth muscle continuous with the bladder.

Regional Lymph Nodes

The regional lymph nodes are as follows (Fig. 63.1):

- Inguinal (superficial or deep)
- True pelvis (perivesical, obturator, or internal [hypogastric] and external iliac)
- Presacral
- Sacral, NOS
- Pelvic, NOS

Metastatic Sites

Distant spread is most commonly to retroperitoneal lymph nodes, lung, liver, or bone.

RULES FOR CLASSIFICATION

Clinical Classification

Radiographic imaging, cystourethroscopy, palpation, and biopsy or cytology of the tumor prior to definitive treatment are desirable. The site of origin should be confirmed to exclude metastatic disease or direct extension from an adjacent site.

Limited data are available on the best methodology to stage urothelial carcinoma that concurrently involves the urinary bladder and the prostatic urethra. In patients in which a large urinary bladder carcinoma has invaded through the full thickness of the bladder wall and thereby secondarily involves the prostatic stroma, a pT4 stage should be assigned per urinary bladder staging. In other circumstances in which involvement by urothelial carcinoma is seen in both sites, separate urinary bladder and prostatic urethral staging should be assigned.

The significance of regional lymph node metastasis in staging urethral cancer lies in the number and size, not in whether it is unilateral or bilateral. The distal urethra and meatus in the female and the membranous and pendulous urethra in the male drain primarily to the inguinal lymph nodes. The more proximal urethra in the female and the bulbar and prostatic urethra in the male drain predominantly to the obturator, iliac, and presacral lymph nodes.

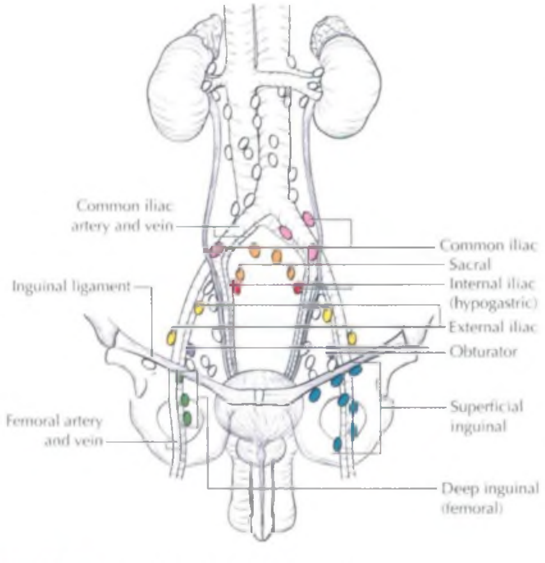

Fig. 63.1 Regional lymph nodes of the urethra

Imaging

Because of the rarity of urethral cancers, there is a paucity of information and minimal standard guidelines regarding the most appropriate imaging approach for clinical staging. The choice and extent of use of imaging for detecting nodal and distant disease should be based on tumor histology and underlying symptoms. Computed tomography (CT) scans, magnetic resonance (MR) imaging, and bone scintigraphy can be used to stage nodal disease and distant metastasis; in patients with urothelial carcinoma, they can be used for surveillance of the upper tracts.

For primary tumor categorization, high-resolution MR imaging has superior soft tissue contrast and is the imaging modality of choice to stage carcinoma of the urethra in both males and females.[1-3] Involvement of corpus spongiosum and cavernosum in males and anterior vaginal wall in females are well demonstrated on MR imaging. Invasion of other pelvic structures, such as rectum and prostate, also can be detected accurately using high-resolution MR imaging.[4,5] MR imaging protocol standardization is lacking and few large trials have studied its accuracy in staging. The typical sequence utilizes T1, T2, and post-gadolinium contrast in multiplanar orthogonal views utilizing a surface coil.[6] Tumors tend to show low signal intensity relative to that of the surrounding normal corporal tissue on T1- and T2-weighted images in males.[4] In females, the characteristic target appearance of the urethra is disrupted. Malignant lesions typically enhance with gadolinium.[3] Primary urethral carcinoma of the prostatic urethra is challenging to image because of the disruption of homogenous signal in the central gland surrounding the urethra caused by benign prostatic hyperplasia. Small studies have reported high accuracy of MR imaging in local staging of urethral tumors. Overestimation of tumor extent due to presence of associated inflammatory changes accounts for some of the inaccuracies of local staging.[5,7] MR imaging is an excellent modality for visualizing and evaluating urethral diverticulum,[8] within which carcinomas can

occur. The tumor within the diverticulum is seen as an enhancing soft tissue mass.

Inguinal and pelvic nodes are the common sites of regional nodal disease. Both CT and MR imaging rely on lymph node size as the primary criterion for suspecting metastatic involvement. Heterogeneous appearance and necrosis also are useful indicators of metastasis. The accuracy of CT and MR imaging in diagnosing lymph node metastasis in patients with urethral carcinoma has not been determined. Ultrasound with fine-needle aspiration cytology has a sensitivity and specificity of 93% and 100% in detecting inguinal nodal metastasis; however, its utility in the deeper pelvic nodes is limited.[6,9]

Distant metastases are found at the time of initial clinical presentation in up to 30% of patients with urethral cancer. Involvement of nonregional nodes, lung, liver, and bone is more common. Patients at risk of metastases can be investigated with the appropriate cross-sectional imaging of choice. Chest radiographs or chest CT may be considered to detect pulmonary metastasis depending on the degree of suspicion. CT is more sensitive than radiographs to detect pulmonary metastasis, but with an added burden of costs and radiation exposure. In patients with symptoms suggestive of bone metastasis, a radionuclide bone scan may be used. 18F-fluorodeoxyglucose (18F-FDG) positron emission tomography (PET) CT generally is not indicated for patients with primary urethral cancer, but it can be used as a problem-solving tool when metastasis is a concern.[6,10]

Pathological Classification

Urethral staging criteria are used to classify carcinomas arising from the urothelial, glandular, or squamous lining of the prostatic urethra, penile urethra, or female urethra. Stage is assigned on radical prostatectomy or cystoprostatectomy specimens (prostatic urethral carcinoma) and urethrectomy specimens (penile urethra or female urethra). A separate stage may be assigned to an independent primary prostatic urethral carcinoma identified on radical cystoprostatectomy for bladder cancer. The College of American Pathologists has generated a synoptic report for urethral cancer that contains required and recommended elements to be included in all cancer-bearing cases (http://www.cap.org).

The assignment of stage for urethral tumors is based on invasion into distinct regions, which is based on depth of invasion in the penile urethra and female urethra and into specified stromal elements in the prostatic urethra (Figs. 63.2 and 63.3). As in bladder cancer staging, extension to other organs, including extraprostatic extension of the bladder wall, should be categorized as T4 disease. Gross evaluation is critical to identify the macroscopic extent of tumor, especially when classifying large prostatic urethral tumors (to exclude direct extension from a bladder primary) and in penile urethra and female urethra specimens, where microscopic orientation of specimens may be challenging. Microscopic evaluation is important to establish histologic subtype of tumor and extent of invasion. Microscopic assessment also is required to assess *in situ* or invasive disease at the surgical margins, including the proximal and distal urethral margins, the periprostatic soft tissue resection margin, and the periurethral soft tissue resection margin. Pathological staging should be specific to the prostatectomy, cystoprostatectomy, or urethrectomy specimen at the time of surgery and should be assigned independently of previous clinical or biopsy information that is used for clinical stage assignment.

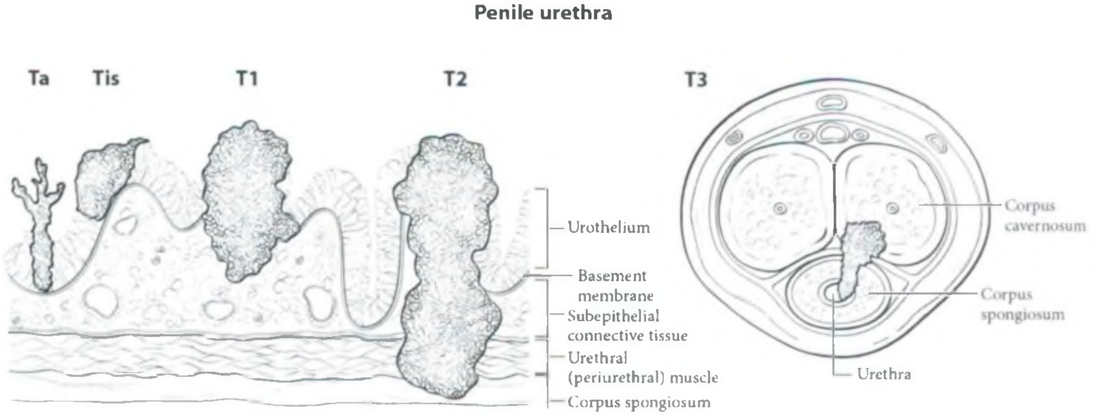

Fig. 63.2 Penile Urethra. Definition of primary tumor (T) for Ta, Tis, T1, T2, and T3 with depth of invasion ranging from the epithelium to the urogenital diaphragm

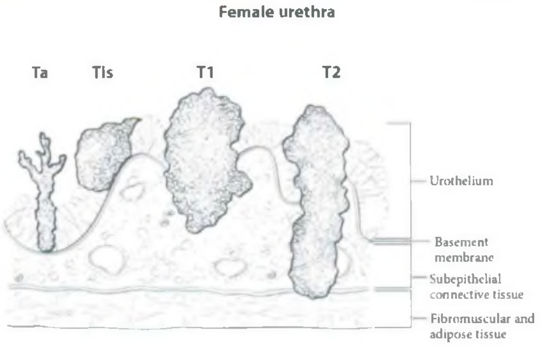

Fig. 63.3 Female Urethra. Definition of primary tumor (T) for Ta, Tis, T1, and T2 with depth of invasion ranging from the epithelium to the urogenital diaphragm

A pN status should be assessed regardless of the number of lymph nodes examined and irrespective of the laterality of the lymph nodes extracted. Location, rather than size, of lymph nodes has been recommended for pN categorization to maintain consistency with bladder cancer staging. If no lymph nodes are evaluated, pNX category should be assigned.

PROGNOSTIC FACTORS

Prognostic Factors Required for Stage Grouping

Beyond the factors used to assign T, N, or M categories, no additional prognostic factors are required for stage grouping.

Additional Factors Recommended for Clinical Care

See Chapter 62, Urinary Bladder. Many of the same factors are relevant for urothelial cancers of the urethra, but have not been independently studied or verified and may not be relevant for nonurothelial cancers. Prognostic factors that are specific to urethral cancers include histologic subtype, including presence of glandular features, clear cell histology, presence of a diverticulum, race, and female gender.[11]

World Health Organization/International Society of Urologic Pathology (WHO/ISUP) Pathological Grade in pTa Disease

Noninvasive papillary carcinomas of the urinary tract are subdivided into low-grade and high-grade disease, with the latter associated with an increased risk of invasive disease. Papilloma and papillary urothelial neoplasm of low malignant potential (PUNLMP) are excluded from this category. Grading is based on microscopic assessment of cellular organization and nuclear features. Level of evidence I.

RISK ASSESSMENT MODELS

The American Joint Committee on Cancer (AJCC) recently has established guidelines that will be used to evaluate published statistical prediction models for the purpose of granting endorsement for clinical use.[12] Although this is a monumental step toward the goal of precision medicine, this work was published only very recently. For this reason, existing models that have been published or may be in clinical use have not been evaluated yet for this cancer site by the Precision Medicine core of the AJCC. In the future, the statistical prediction models for this cancer site will be evaluated, and those that meet all AJCC criteria will be endorsed.

DEFINITIONS OF AJCC TNM

Definition of Primary Tumor (T)

Male Penile Urethra and Female Urethra

T Category	T Criteria
TX	Primary tumor cannot be assessed
T0	No evidence of primary tumor
Ta	Non-invasive papillary carcinoma
Tis	Carcinoma *in situ*
T1	Tumor invades subepithelial connective tissue
T2	Tumor invades any of the following: corpus spongiosum, periurethral muscle
T3	Tumor invades any of the following: corpus cavernosum, anterior vagina
T4	Tumor invades other adjacent organs (e.g., invasion of the bladder wall)

Prostatic Urethra

T Category	T Definition
Tis	Carcinoma *in situ* involving the prostatic urethra or periurethral or prostatic ducts without stromal invasion
T1	Tumor invades urethral subepithelial connective tissue immediately underlying the urothelium
T2	Tumor invades the prostatic stroma surrounding ducts either by direct extension from the urothelial surface or by invasion from prostatic ducts
T3	Tumor invades the periprostatic fat
T4	Tumor invades other adjacent organs (e.g., extraprostatic invasion of the bladder wall, rectal wall)

63

Definition of Regional Lymph Node (N)

N Category	N Criteria
NX	Regional lymph nodes cannot be assessed
N0	No regional lymph node metastasis
N1	Single regional lymph node metastasis in the inguinal region or true pelvis [perivesical, obturator, internal (hypogastric) and external iliac], or presacral lymph node
N2	Multiple regional lymph node metastasis in the inguinal region or true pelvis [perivesical, obturator, internal (hypogastric) and external iliac], or presacral lymph node

Definition of Distant Metastasis (M)

M Category	M Criteria
M0	No distant metastasis
M1	Distant metastasis

AJCC PROGNOSTIC STAGE GROUPS

When T is...	And N is...	And M is...	Then the stage group is...
Tis	N0	M0	0is
Ta	N0	M0	0a
T1	N0	M0	I
T1	N1	M0	III
T2	N0	M0	II
T2	N1	M0	III
T3	N0	M0	III
T3	N1	M0	III
T4	N0	M0	IV
T4	N1	M0	IV
Any T	N2	M0	IV
Any T	Any N	M1	IV

REGISTRY DATA COLLECTION VARIABLES

1. WHO/ISUP Grade
2. Grade 1–3 for squamous cell carcinoma and adenocarcinoma

HISTOLOGIC GRADE (G)

Urothelial Carcinoma

Grade is reported by the grade value. For urothelial histology, a low- and high-grade designation is used to match the current WHO/ISUP recommended grading system.

G	G Definition
LG	Low grade
HG	High grade

Squamous Cell Carcinoma and Adenocarcinoma

For squamous cell carcinoma and adenocarcinoma, the following grading schema is recommended.

G	G Definition
GX	Grade cannot be assessed
G1	Well differentiated
G2	Moderately differentiated
G3	Poorly differentiated

HISTOPATHOLOGIC TYPE

The classification applies to urothelial, squamous, and glandular carcinomas of the urethra and to urothelial carcinomas of the prostate and prostatic urethra. There should be histologic or cytologic confirmation of the disease.

Noninvasive carcinoma

- Low-grade papillary urothelial carcinoma
- High-grade papillary urothelial carcinoma
- Urothelial carcinoma *in situ*

Invasive carcinoma

- Conventional urothelial carcinoma
- Urothelial carcinoma variants
  - Urothelial carcinoma with divergent differentiation (squamous, glandular, and/or trophoblastic)
  - Nested urothelial carcinoma (including large nested carcinoma)
  - Microcystic urothelial carcinoma
  - Micropapillary urothelial carcinoma
  - Lymphoepithelioma-like urothelial carcinoma
  - Plasmacytoid urothelial carcinoma
  - Giant cell urothelial carcinoma
  - Lipid-rich urothelial carcinoma
  - Clear cell (glycogen-rich) urothelial carcinoma
  - Sarcomatoid urothelial carcinoma
  - Poorly differentiated urothelial carcinoma (including those with osteoclast-like giant cells)
- Squamous cell carcinoma
- Adenocarcinoma (including those arising from periurethral glands)
- Small cell carcinoma

SURVIVAL DATA

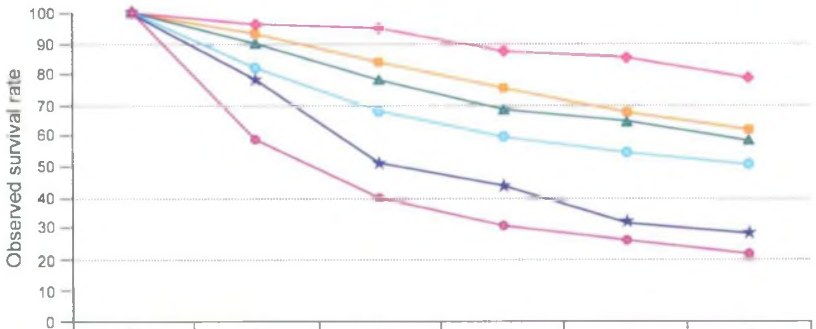

	0	1	2	3	4	5
I	100.0	96.8	95.1	87.9	86.0	79.3
II	100.0	93.2	84.0	75.6	67.2	61.6
III	100.0	90.2	78.6	68.8	64.8	59.0
IV	100.0	82.4	68.2	59.4	54.2	50.6
V	100.0	78.6	51.4	43.8	31.8	28.4
VI	100.0	58.9	40.0	31.0	25.9	21.8

Years from diagnosis

Fig. 63.4 Observed and overall survival rates for 1,278 patients with urethral cancer classified by the current AJCC staging classification. Data taken from the National Cancer Data Base (Commission on Cancer of the American College of Surgeons and the American Cancer Society) for the years 1998-2002. Stage 0a includes 129 patients; Stage 0is, 170; Stage I, 243; Stage II, 193; Stage III, 250; and Stage IV, 293

ILLUSTRATIONS

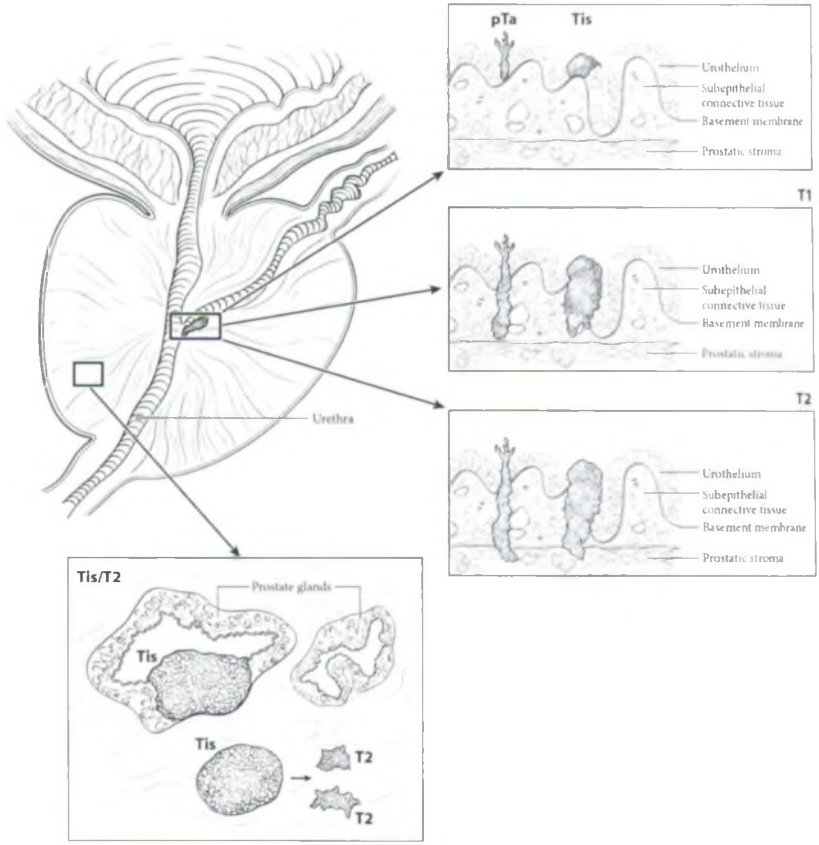

Fig. 63.5 Definition of primary tumor (T) for urothelial carcinoma of the prostate

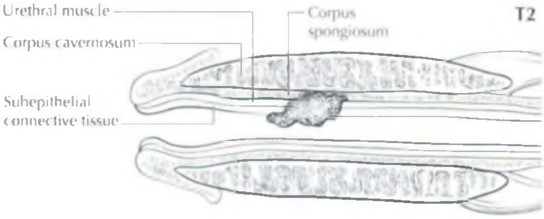

Fig. 63.6 In the male, T2 is defined as tumor with invasion of the corpus spongiosum

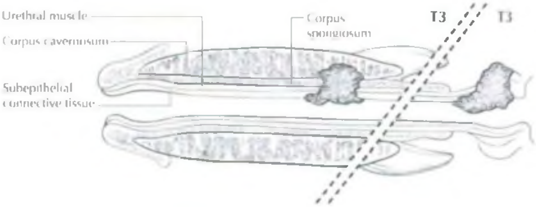

Fig. 63.7 In the male, T3 is defined as tumor with invasion of the corpus cavernosum (left) or the prostatic capsule (right)

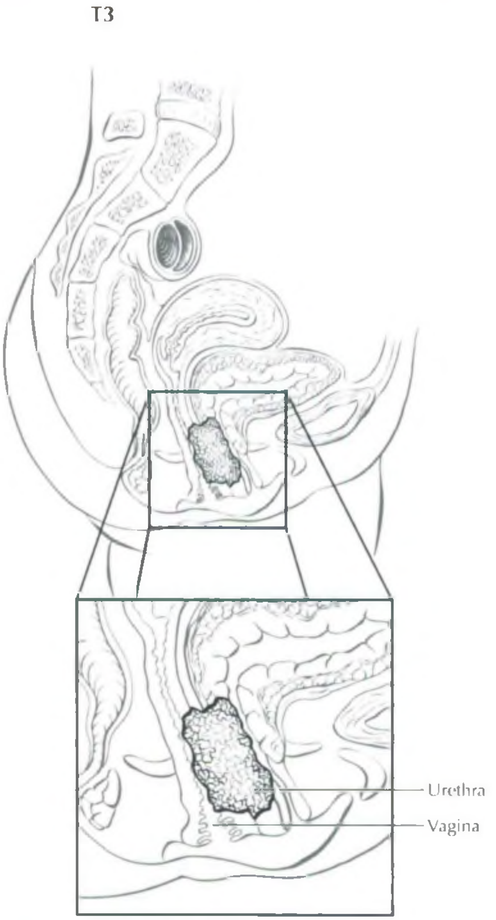

Fig. 63.8 In the female, T3 is defined as tumor that invades the anterior vagina

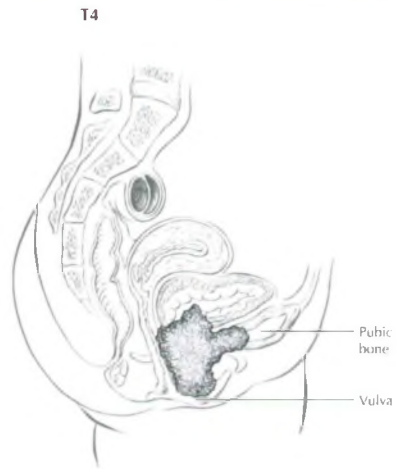

Fig. 63.9 In the female, T4 is defined as tumor that invades other adjacent organs (illustrated is invasion of the pubic bone and vulva)

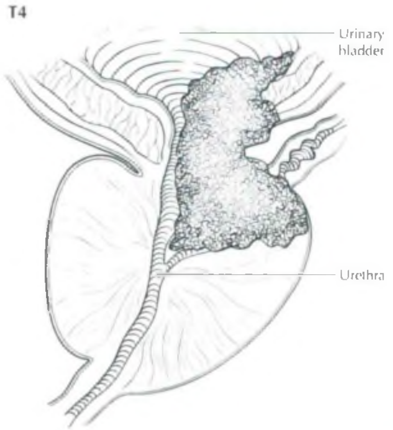

Fig. 63.10 T4 is defined as tumor that invades other adjacent organs (invasion of the bladder is illustrated)

Bibliography

1. Chaudhari VV, Patel MK, Douek M, Raman SS. MR imaging and US of female urethral and periurethral disease. *Radiographics : a review publication of the Radiological Society of North America, Inc*. Nov 2010;30(7):1857–1874.
2. Gakis G, Witjes JA, Comperat E, et al. EAU guidelines on primary urethral carcinoma. *Eur Urol*. Nov 2013;64(5):823–830.
3. Ryu J, Kim B. MR imaging of the male and female urethra. *Radiographics : a review publication of the Radiological Society of North America, Inc*. Sep-Oct 2001;21(5):1169–1185.
4. Kim B, Kawashima A, LeRoy AJ. Imaging of the male urethra. *Seminars in ultrasound, CT, and MR*. Aug 2007;28(4):258–273.
5. Gourtsoyianni S, Hudolin T, Sala E, Goldman D, Bochner B, Hricak H. MRI at the completion of chemoradiotherapy can accurately evaluate the extent of disease in women with advanced urethral carcinoma undergoing anterior pelvic exenteration. *Clinical radiology*. 2011;66(11):1072–1078.
6. Stewart SB, Leder RA, Inman BA. Imaging tumors of the penis and urethra. *Urol Clin North Am*. Aug 2010;37(3):353–367.
7. Hricak H, Secaf E, Buckley DW, Brown JJ, Tanagho EA, McAninch JW. Female urethra: MR imaging. *Radiology*. Feb 1991;178(2):527–535.
8. Dwarkasing RS, Dinkelaar W, Hop WC, Steensma AB, Dohle GR, Krestin GP. MRI evaluation of urethral diverticula and differential diagnosis in symptomatic women. *AJR. American journal of roentgenology*. Sep 2011;197(3):676–682.
9. Hall TB, Barton DP, Trott PA, et al. The role of ultrasound-guided cytology of groin lymph nodes in the management of squamous cell carcinoma of the vulva: 5-year experience in 44 patients. *Clin Radiol*. May 2003;58(5):367–371.

63

10. Leijte JA, Graafland NM, Valdes Olmos RA, van Boven HH, Hoefnagel CA, Horenblas S. Prospective evaluation of hybrid 18F-fluorodeoxyglucose positron emission tomography/computed tomography in staging clinically node-negative patients with penile carcinoma. *BJU Int.* Sep 2009;104(5):640–644.
11. Champ CE, Hegarty SE, Shen X, et al. Prognostic factors and outcomes after definitive treatment of female urethral cancer: a population-based analysis. *Urology.* Aug 2012;80(2):374–381.
12. Kattan MW, Hess KR, Amin MB, et al. American Joint Committee on Cancer acceptance criteria for inclusion of risk models for individualized prognosis in the practice of precision medicine. *CA: a cancer journal for clinicians.* Jan 19 2016.

Part XV

Ophthalmic Sites

Members of the Ophthalmic Sites Expert Panel

Daniel M. Albert, MD, MS
Anush G. Amiryan, MD
Claudia Auw-Hädrich, MD
Diane Baker, CTR – Data Collection Core Representative
Raymond Barnhill, MD, MSc
José M. Caminal, MD, PhD
William L. Carroll, MD
Nathalie Cassoux, MD, PhD
Jaume Catala-Mora, MD
Guillermo Chantada, MD
Patricia Chévez-Barrios, MD
R. Max Conway, MD, PhD, FRANZCO
Sarah E. Coupland, MBBS, PhD, FRCPath – Vice Chair
Bertil E. Damato, MD, PhD
Hakan Demirci, MD
Laurence G. Desjardins, MD
François Doz, MD, MSc
Jonathan J. Dutton, MD, PhD, FACS
Bita Esmaeli, MD, FACS
Paul T. Finger, MD, FACS – Chair
Brenda L. Gallie, MD, FACSC
Gerardo F. Graue, MD
Hans E. Grossniklaus, MD – CAP Representative
Steffen Heegaard, MD
Leonard M. Holbach, MD
Santosh G. Honavar, MD, FACS
Martine J. Jager, MD, PhD
Tero Kivelä, MD, FEBO
Emma Kujala, MD
Livia Lumbroso-Le Rouic, MD
Ashwin C. Mallipatna, MBBS, MS, DNB
Giulio M. Modorati, MD
Francis L. Munier, MD
Timothy G. Murray, MD, MBA, FACS
Anna C. Pavlick, MS, DO
Jacob Pe'er, MD
David E. Pelayes, MD, PhD
Gaelle Pierron
Victor G. Prieto, MD, PhD
Manuel Jorge Rodrigues, MD
Svetlana Saakyan, MD
Wolfgang A.G. Sauerwein, MD, PhD
Ekaterina Semenova, MD
Stefan Seregard, MD
Carol Shields, MD
E. Rand Simpson, MD, FRCS(C)
Arun D. Singh, MD
Shigenobu Suzuki, MD, PhD
Mary Kay Washington, MD, PhD – Editorial Board Liaison
Valerie A. White, MD, MHSc, FRCPC
Michelle Williams, MD
Matthew W. Wilson, MD, FACS
Christian W. Wittekind, MD – UICC Representative
Vivian Yin, MD, MPH, FRCSC

Eyelid Carcinoma

64

Bita Esmaeli, Jonathan J. Dutton, Gerardo F. Graue,
Leonard M. Holbach, Valerie A. White, Sarah E. Coupland,
and Paul T. Finger

CHAPTER SUMMARY

Cancers Staged Using This Staging System

All primary carcinomas of the eyelid, including basal cell carcinoma (BCC), squamous cell carcinoma (SCC), sebaceous carcinoma, and other rare carcinomas, such as all varieties of sweat gland carcinoma (e.g., eccrine carcinoma), are classified using this staging system.

Cancers Not Staged Using This Staging System

Carcinomas of the head and neck region from another anatomic site (other than the eyelids and orbital area) but that secondarily extend to the eyelids should be classified under head and neck skin cancer. Metastases to the eyelid are not covered by this staging system.

Eyelid melanomas, which may arise from lesions within the conjunctiva (posterior eyelid lamella) or from lesions within the eyelid skin (anterior eyelid lamella) are not included in this chapter. Please refer to the chapters on conjunctival melanoma or skin melanoma for melanomas affecting the ocular adnexa.

Eyelid Merkel cell carcinoma is not staged using this system but instead is staged using the Merkel cell carcinoma staging system (Chapter 46). We encourage those who care for these patients to work with their registries to collect the ophthalmic items listed under Registry Data Collection Variables in the Merkel cell carcinoma chapter to help inform local control and treatment-related side effects, as well as potential biomarkers for metastatic disease going forward.

These histopathologic types of cancer…	Are staged according to the classification for…	And can be found in chapter…
Carcinomas of the head and neck with direct extension to eyelid	Cutaneous squamous cell carcinoma of the head and neck	15
Merkel cell carcinoma of the eyelid	Merkel cell carcinoma	46
Melanoma of the eyelid	Melanoma of the skin	47

Summary of Changes

Change	Details of Change	Level of Evidence
Definition of Primary Tumor (T)	T1, T2, T3, and T4 category definitions have been revised based on anatomic extent of primary tumor rather than subjective descriptions, such as "surgical resectability" or "need to do enucleation."	III
Definition of Regional Lymph Node (N)	N1 category has been divided into N1 and N2 based on size and location of positive lymph node(s).	III

To access the AJCC cancer staging forms, please visit www.cancerstaging.org.

© American Joint Committee on Cancer 2017

M.B. Amin et al. (eds.), *AJCC Cancer Staging Manual, Eighth Edition*, DOI 10.1007/978-3-319-40618-3_64

Change	Details of Change	Level of Evidence
AJCC Prognostic Stage Groups	Stage groups have been modified to incorporate new T and N categories; Stage III is defined as node-positive disease and Stage IV as distant metastases.	III

ICD-O-3 Topography Codes

Code	Description
C44.1	Eyelid

WHO Classification of Tumors

Code	Description
8090	Basal cell carcinoma
8070	Squamous cell carcinoma
8390	Skin appendage carcinoma
8480	Mucinous adenocarcinoma
8940	Pleomorphic adenoma
8940	Mixed tumor, NOS
8940	Mixed tumor, salivary gland type, NOS
8940	Chondroid syringoma
8940	Mixed tumor, malignant, NOS
8940	Mixed tumor, salivary gland type, malignant
8940	Malignant chondroid syringoma
8940	Carcinoma in pleomorphic adenoma
8980	Carcinosarcoma (NOS)
8410	Sebaceous carcinoma
8413	Eccrine adenocarcinoma
8401	Apocrine adenocarcinoma
8200	Adenoid cystic carcinoma

International Agency for Research on Cancer, World Health Organization. International Classification of Diseases for Oncology. ICD-O-3-Online. http://codes.iarc.fr/home. Accessed May 15, 2016.

INTRODUCTION

Eyelid carcinomas are the most common malignancies of the eyelid region. A wide variety of carcinomas may involve either the skin or the tarsal and conjunctival layer of the eyelid. Critical size criteria defining the T category for eyelid carcinomas are inherently different from those of other anatomic sites because of the unique anatomic considerations in the eyelid region. In this edition, we further refine the definitions of T and N categories for eyelid carcinoma. Management of eyelid carcinomas consists of surgery in most instances, as well as the judicious use of adjuvant radiation therapy and topical chemotherapy in select patients. In addition, new targeted, systemic treatments aim to preserve the eye and its function in patients with locally advanced disease that previously was treated by orbital exenteration. The functional and anatomic considerations in the eyelid and periocular region make carcinomas of the eyelid unique in terms of AJCC prognostic criteria, outcome reporting, patient selection for clinical trials, and the choice of appropriate treatments.

The TNM classification of eyelid carcinomas reflects both morbidity and mortality risks in order to provide useful guidelines for patient management.[1–5] These relate to survival but also to local control as outcome measures. The tumor biology of primary eyelid carcinoma encompasses a broad spectrum of behaviors, from indolent low-grade tumors, such as nodular BCCs, to highly aggressive sebaceous and Merkel cell carcinomas.

Primary eyelid carcinomas mainly include BCCs (90%) which rarely are metastatic but also include other carcinomas with a more pronounced metastatic potential, such as SCC, sebaceous carcinoma, Merkel cell carcinoma, and less common histopathologic types.[6–16]

Melanomas involving the eyelid are addressed in other chapters (see chapters Conjunctival Melanoma and Melanoma of the Skin).

ANATOMY

Primary Site(s)

The eyelid is composed of anterior and posterior lamellae, which divide along the mucocutaneous lid margin. From anterior to posterior, the eyelid is composed of skin, orbicularis muscle, tarsus, and conjunctiva. The levator aponeurosis and Müller's muscle are attached at the superior aspect of the tarsus, with analogous retractors in the lower eyelid. There is a rich supply of sebaceous, eccrine, and apocrine sweat glands; accessory lacrimal and neuroendocrine glandular elements are diffused within the eyelid, caruncle, and periorbital tissues. Sebaceous glands are concentrated in the tarsus, in the eyelash margin, and within smaller pilosebaceous units that cover the eyelid and caruncle. Accessory lacrimal glands are located at the upper edge of the tarsus (accessory lacrimal glands of Wolfring) and in the fornix (accessory lacrimal glands of Krause). Glandular elements and skin are the precursor cell types for carcinoma of the eyelid.

Regional Lymph Nodes

The eyelids are supplied with lymphatics that drain into the preauricular, intraparotid, submandibular, and other cervical

lymph node basins (Fig. 64.1). Recent investigations have shown that all areas of the eyelids may drain into the parotid nodes, in addition to the medial lids draining into the submandibular nodes.[12]

Metastatic Sites

Metastatic potential is highly dependent on histopathologic tumor type and grade. Eyelid carcinomas metastasize via lymphatic channels and, less frequently, by hematogenous spread. Distant metastatic sites may include lung, liver, other viscera, and brain.

RULES FOR CLASSIFICATION

Clinical Classification

Staging of eyelid carcinoma begins with a comprehensive ophthalmic, orbital, and periorbital clinical examination. This approach includes a slit lamp or equivalent biomicroscopic evaluation, neuro-ophthalmic examination for evidence of perineural invasion, and regional assessment of the head and neck to include lymphatic drainage basins. Preoperative photography of the extent of disease is recommended.

Carcinoma of the eyelid may extend directly into adjacent structures through mechanisms of direct contiguous invasion, perineural or intravascular spread, and mucosal invasion. Sites of local invasion include the lacrimal drainage system, orbital soft tissue and bone, globe, face, nasal cavity and paranasal sinuses, orbital apex, base of the skull, and central nervous system.

With the exception of BCC, which only rarely metastasizes to the regional lymph nodes, all eyelid carcinomas have the propensity for lymph node metastasis. Historically, it is known that for head and neck squamous carcinomas, lymph node metastasis is a vital independent prognostic factor.[13,17]

A clinically positive lymph node should be biopsied, and if positive, additional surgery and/or radiation therapy should be offered to achieve regional control.[13]

The decision to do careful and frequent lymph node evaluation and/or possible biopsy of the draining lymph nodes for eyelid carcinomas should be based on tumor size, histopathologic type, and tumor grade. Analogous to the experience with ocular adnexal melanomas, in the past 15 years, new insights have been gained regarding the value of sentinel lymph node (SLN) biopsy as a staging method for eyelid carcinomas, specifically eyelid Merkel cell and sebaceous carcinomas.[18-24]

It has been shown that standard clinical and imaging assessment of the regional lymph nodes fails to detect lymph node metastasis in up to 25% of cases of head and neck squamous carcinomas and 32% of head and neck Merkel cell carcinomas. Studies suggest that lymph node dissection in all patients is not justified because of significant morbidity, surgical risk, and low yield for positive nodes. However, SLN biopsy may prove useful for nodal staging, particularly when sampling first-order lymph nodes.[22] When positive, SLN biopsy provides critical staging information leading to the selection of patients who may benefit from additional treatments.[18,21-23]

Technetium-99m lymphoscintigraphy followed by SLN biopsy requires modest adaptation for use in patients with eyelid carcinoma.[25,26] The volume of radioactive isotope is reduced to match the reduced thickness and size of the eyelid tissues. Step serial sectioning and immunohistochemical staining improve the sensitivity of this sampling technique.[26,27] Since the AJCC Cancer Staging Manual, 7th Edition, several manuscripts have attempted to assess the risk of nodal metastasis as a function of AJCC T category for various histologic subtypes of eyelid carcinoma. Recent research suggests that the 7th Edition clinical stage T2b or greater for eyelid sebaceous carcinomas, squamous carcinomas, and Merkel cell carcinomas may be associated with increased risk of nodal metastasis.[6,8,14,16,27]

Distant metastasis associated with eyelid carcinomas is most likely to occur in very large tumors (T3 or greater) and in the more aggressive histologies, such as Merkel cell carcinoma, sebaceous carcinoma, and microcystic adnexal carcinoma.[14,28,29]

Imaging

The requirement for imaging modalities, including computed tomography (CT), magnetic resonance (MR) imaging, and ultrasonography, is highly dependent on the histopathologic type of eyelid carcinoma and clinical extent of the primary eyelid tumor.

For locally advanced eyelid carcinomas, an orbital CT scan with axial and coronal views with contrast, with both tissue and bone window settings, should be obtained initially to evaluate the extent of tumor into the orbital soft tissue or periorbital structures, such as the paranasal sinuses or nasal cavity, or posteriorly or superiorly into the skull base.

For the more aggressive tumor types with the potential for lymph node metastasis, such as sebaceous carcinomas and squamous carcinomas of the eyelid that are ≥T2b, a baseline imaging study of the head and neck should be obtained. This study might be a CT scan with axial and coronal cuts to include the regional lymph nodes. Ultrasonography of the regional lymph nodes also is quite good at detecting abnormal lymph nodes that may then be biopsied with fine-needle aspiration. SLN biopsy should be considered only in patients who have had negative find-

ings on clinical examination and on imaging studies such as CT and ultrasound.[25,26,29]

MR imaging with gadolinium enhancement is the imaging study of choice if overt clinically symptomatic invasion of large nerves is suspected in association with a previously treated or recurrent locally advanced eyelid carcinoma. If metastatic spread is suspected, as in the case of a large eyelid Merkel cell carcinoma, a positron emission tomography (PET)/CT scan may be helpful in evaluating for widespread metastatic disease and for response to treatment.

Pathological Classification

Precise macroscopic description is essential: the surgical nature of the excisional surgical specimen (i.e., excisional biopsy, wide local excision, or radical excision including exenteration), as well as its size, should be noted. Incisional biopsies performed to establish the pathological diagnosis are considered part of the clinical classification. The specimen should be oriented carefully and inked for margin evaluation. Pathological classification is based on the specific tumor type, its differentiation (grade), and the completeness of tumor removal. Greatest tumor dimension and evaluation of the surgical specimen margins are mandatory.

PROGNOSTIC FACTORS

Prognostic Factors Required for Stage Grouping

Beyond the factors used to assign T, N, or M categories, no additional prognostic factors are required for stage grouping.

Additional Factors Recommended for Clinical Care

Muir–Torre Syndrome

The diagnosis of sebaceous carcinoma should prompt consideration of a workup to exclude Muir–Torre syndrome, a phenotypic variant of hereditary nonpolyposis colon cancer, or Lynch syndrome. Sebaceous neoplasia (including adenoma, epithelioma, and carcinoma) and keratoacanthomas are cutaneous manifestations of Muir–Torre syndrome, together with visceral malignancies, especially colorectal and endometrial carcinomas. The disorder has been linked to mutations in *MLH1* and *MSH2*.[30–33] AJCC Level of Evidence: III

Mismatch Repair Testing: Microsatelllite Instability or Immunohistochemistry

Defective mismatch repair is important for detection of Lynch syndrome and, accordingly, is a feature of tumors arising in Muir–Torre syndrome.[30–32] Tumor DNA may be tested for microsatellite instability by polymerase chain reaction technology using a panel of at least five microsatellite markers assessing for changes in the length of the microsatellite loci. In addition, immunohistochemistry may be performed for parallel assessment for retained expression of mismatch repair proteins (MLH1, MSH2, MSH6, PMS2) in formalin-fixed, paraffin-embedded tissue. Intact expression of all four proteins indicates that mismatch repair enzymes are intact but does not entirely exclude Lynch syndrome, as missense mutations may lead to a nonfunctional protein with retained immunogenicity. AJCC Level of Evidence: III

RISK ASSESSMENT MODELS

The AJCC recently established guidelines that will be used to evaluate published statistical prediction models for the purpose of granting endorsement for clinical use.[34] Although this is a monumental step toward the goal of precision medicine, this work was published only very recently. Therefore, the existing models that have been published or may be in clinical use have not yet been evaluated for this cancer site by the Precision Medicine Core of the AJCC. In the future, the statistical prediction models for this cancer site will be evaluated, and those that meet all AJCC criteria will be endorsed.

DEFINITIONS OF AJCC TNM

Definition of Primary Tumor (T)

T Category	T Criteria
TX	Primary tumor cannot be assessed
T0	No evidence of primary tumor
Tis	Carcinoma *in situ*
T1	Tumor ≤10 mm in greatest dimension
T1a	Tumor does not invade the tarsal plate or eyelid margin
T1b	Tumor invades the tarsal plate or eyelid margin
T1c	Tumor involves full thickness of the eyelid
T2	Tumor >10 mm but ≤20 mm in greatest dimension
T2a	Tumor does not invade the tarsal plate or eyelid margin
T2b	Tumor invades the tarsal plate or eyelid margin

(continued)

T Category	T Criteria
T2c	Tumor involves full thickness of the eyelid
T3	Tumor >20 mm but ≤30 mm in greatest dimension
T3a	Tumor does not invade the tarsal plate or eyelid margin
T3b	Tumor invades the tarsal plate or eyelid margin
T3c	Tumor involves full thickness of the eyelid
T4	Any eyelid tumor that invades adjacent ocular, orbital, or facial structures
T4a	Tumor invades ocular or intraorbital structures
T4b	Tumor invades (or erodes through) the bony walls of the orbit or extends to the paranasal sinuses or invades the lacrimal sac/nasolacrimal duct or brain

Definition of Regional Lymph Node (N)

N Category	N Criteria
NX	Regional lymph nodes cannot be assessed
N0	No evidence of lymph node involvement
N1	Metastasis in a single ipsilateral regional lymph node, ≤3 cm in greatest dimension
N1a	Metastasis in a single ipsilateral lymph node based on clinical evaluation or imaging findings
N1b	Metastasis in a single ipsilateral lymph node based on lymph node biopsy
N2	Metastasis in a single ipsilateral lymph node, >3 cm in greatest dimension, or in bilateral or contralateral lymph nodes
N2a	Metastasis documented based on clinical evaluation or imaging findings
N2b	Metastasis documented based on microscopic findings on lymph node biopsy

Definition of Distant Metastasis (M)

M Category	M Criteria
M0	No distant metastasis
M1	Distant metastasis

AJCC PROGNOSTIC STAGE GROUPS

When T is...	And N is...	And M is...	Then the stage group is...
Tis	N0	M0	0
T1	N0	M0	IA
T2a	N0	M0	IB
T2b–d, T3	N0	M0	IIA
T4	N0	M0	IIB
Any T	N1	M0	IIIA
Any T	N2	M0	IIIB
Any T	Any N	M1	IV

REGISTRY DATA COLLECTION VARIABLES

1. Tumor size (greatest dimension in millimeters)
2. Specific anatomic location (e.g., upper eyelid, lower eyelid, both eyelids, medial canthus, lateral canthus)
3. Tumor thickness (depth of invasion)
4. Presence/absence of perineural invasion
5. Presence/absence of lymphovascular invasion
6. Mitotic figures per square millimeter
7. Microsatellite instability markers for sebaceous carcinoma
8. Sentinel node biopsy status and number of sentinel nodes (if applicable)
9. History of HIV infection
10. History of solid organ transplant
11. History of Muir–Torre syndrome
12. History of xeroderma pigmentosum

64

HISTOLOGIC GRADE (G)

A histologic grading system is used predominantly for SCCs and sebaceous carcinomas. It is not used for Merkel cell carcinoma or BCC.

G	G Definition
GX	Grade cannot be assessed
G1	Well differentiated
G2	Moderately differentiated
G3	Poorly differentiated
G4	Undifferentiated

HISTOPATHOLOGIC TYPE

Squamous cell carcinoma
Basal cell carcinoma
Sebaceous carcinoma
Mucoepidermoid cancer
Primary eccrine adenocarcinoma
Microcystic adnexal carcinoma
Primary apocrine adenocarcinoma
Adenoid cystic carcinoma

ILLUSTRATIONS

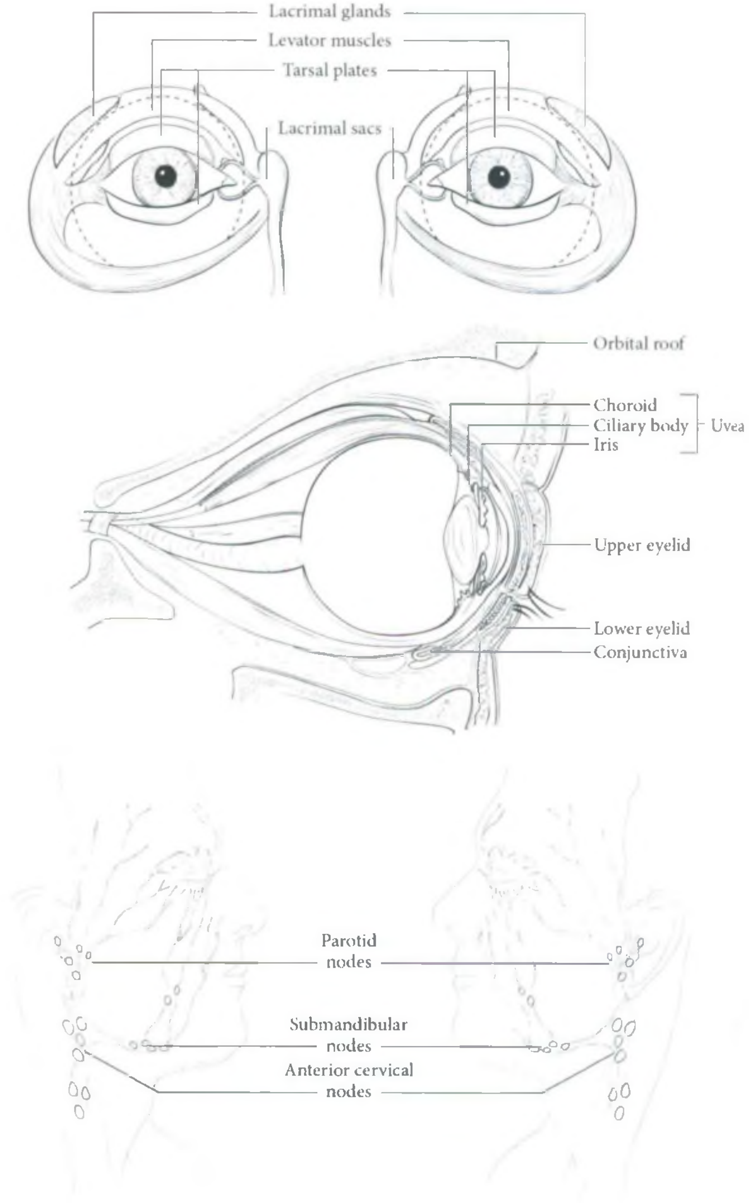

Fig. 64.1 Anatomic sites and regional lymph nodes for ophthalmic sites

Bibliography

1. Ainbinder DJ, Esmaeli B, Groo SC, Finger PT, Brooks JP. Introduction of the 7th edition eyelid carcinoma classification system from the American Joint Committee on Cancer-International Union Against Cancer staging manual. *Arch Pathol Lab Med*. Aug 2009;133(8):1256–1261.
2. Breuninger H. Seventh edition American Joint Committee on Cancer staging of cutaneous non-melanoma skin cancer. *American journal of clinical dermatology*. Jun 1 2011;12(3):155.
3. Crawford C, Fernelius C, Young P, Groo S, Ainbinder D. Application of the AJCC 7th edition carcinoma of the eyelid staging system: a medical center pathology based, 15-year review. *Clinical ophthalmology*. 2011;5:1645–1648.
4. Droll L, Seigler D, Esmaeli B. Prospective collection of data using the 7th edition of the AJCC Cancer Staging Manual for cancers of the eyelid, orbit, and conjunctiva. *Ophthalmic plastic and reconstructive surgery*. Mar-Apr 2011;27(2):142.
5. Shinder R, Ivan D, Seigler D, Dogan S, Esmaeli B. Feasibility of using American Joint Committee on Cancer Classification criteria for staging eyelid carcinomas. *Orbit*. Oct 2011;30(5):202–207.
6. Choi YJ, Jin HC, Lee MJ, Kim N, Choung HK, Khwarg SI. Prognostic value of clinical and pathologic T stages defined by the American Joint Committee on Cancer for eyelid sebaceous carcinoma in Korea. *Japanese journal of ophthalmology*. Jul 2014; 58(4):327–333.
7. Conway RM, Themel S, Holbach LM. Surgery for primary basal cell carcinoma including the eyelid margins with intraoperative frozen section control: comparative interventional study with a minimum clinical follow up of 5 years. *The British journal of ophthalmology*. Feb 2004;88(2):236–238.
8. Esmaeli B, Nasser QJ, Cruz H, Fellman M, Warneke CL, Ivan D. American Joint Committee on Cancer T category for eyelid sebaceous carcinoma correlates with nodal metastasis and survival. *Ophthalmology*. May 2012;119(5):1078–1082.
9. Faustina M, Diba R, Ahmadi MA, Esmaeli B. Patterns of regional and distant metastasis in patients with eyelid and periocular squamous cell carcinoma. *Ophthalmology*. Oct 2004;111(10):1930–1932.
10. Herbert HM, Sun MT, Selva D, et al. Merkel cell carcinoma of the eyelid: management and prognosis. *JAMA ophthalmology*. Feb 2014;132(2):197–204.
11. Nasser QJ, Roth KG, Warneke CL, Yin VT, El Sawy T, Esmaeli B. Impact of AJCC 'T' designation on risk of regional lymph node metastasis in patients with squamous carcinoma of the eyelid. *The British journal of ophthalmology*. Apr 2014;98(4):498–501.
12. Nijhawan N, Marriott C, Harvey JT. Lymphatic drainage patterns of the human eyelid: assessed by lymphoscintigraphy. *Ophthalmic plastic and reconstructive surgery*. Jul-Aug 2010;26(4):281–285.
13. Ross GL, Shoaib T, Soutar DS, et al. The First International Conference on Sentinel Node Biopsy in Mucosal Head and Neck Cancer and adoption of a multicenter trial protocol. *Annals of surgical oncology*. May 2002;9(4):406–410.
14. Sniegowski MC, Warneke CL, Morrison WH, et al. Correlation of American Joint Committee on Cancer T category for eyelid carcinoma with outcomes in patients with periocular Merkel cell carcinoma. *Ophthalmic plastic and reconstructive surgery*. Nov-Dec 2014;30(6):480–485.
15. Sun MT, Andrew NH, O'Donnell B, McNab A, Huilgol SC, Selva D. Periocular Squamous Cell Carcinoma: TNM Staging and Recurrence. *Ophthalmology*. Jul 2015;122(7):1512–1516.
16. Watanabe A, Sun MT, Pirbhai A, Ueda K, Katori N, Selva D. Sebaceous carcinoma in Japanese patients: clinical presentation, staging and outcomes. *The British journal of ophthalmology*. Nov 2013;97(11):1459–1463.
17. LeBlanc KG, Jr., Monheit GD. Understanding and use of the American Joint Committee on Cancer seventh edition guidelines for cutaneous squamous cell carcinoma: a survey of dermatologic surgeons. *Dermatologic surgery : official publication for American Society for Dermatologic Surgery [et al.]*. May 2014;40(5):505–510.
18. Esmaeli B, Naderi A, Hidaji L, Blumenschein G, Prieto VG. Merkel cell carcinoma of the eyelid with a positive sentinel node. *Archives of ophthalmology*. May 2002;120(5):646–648.
19. Ho VH, Ross MI, Prieto VG, Khaleeq A, Kim S, Esmaeli B. Sentinel lymph node biopsy for sebaceous cell carcinoma and melanoma of the ocular adnexa. *Archives of otolaryngology--head & neck surgery*. Aug 2007;133(8):820–826.
20. Maalouf TJ, George J-L. Reply re:"Sentinel Lymph Node Biopsy in Patients With Conjunctival and Eyelid Cancers: Experience in 17 Patients". *Ophthalmic Plastic & Reconstructive Surgery*. 2012; 28(6):471–472.
21. Nijhawan N, Ross MI, Diba R, Gutstein BF, Ahmadi MA, Esmaeli B. Experience with sentinel lymph node biopsy for eyelid and conjunctival malignancies at a cancer center. *Ophthalmic Plastic & Reconstructive Surgery*. 2004;20(4):291–295.
22. Pfeiffer ML, Savar A, Esmaeli B. Sentinel lymph node biopsy for eyelid and conjunctival tumors: what have we learned in the past decade? *Ophthalmic Plastic & Reconstructive Surgery*. 2013; 29(1):57–62.
23. Savar A, Oellers P, Myers J, et al. Positive sentinel node in sebaceous carcinoma of the eyelid. *Ophthalmic plastic and reconstructive surgery*. Jan-Feb 2011;27(1):e4–6.
24. Schwartz JL, Griffith KA, Lowe L, et al. Features predicting sentinel lymph node positivity in Merkel cell carcinoma. *Journal of Clinical Oncology*. Mar 10 2011;29(8):1036–1041.
25. Amato M, Esmaeli B, Ahmadi MA, et al. Feasibility of preoperative lymphoscintigraphy for identification of sentinel lymph nodes in patients with conjunctival and periocular skin malignancies. *Ophthalmic plastic and reconstructive surgery*. Mar 2003;19(2):102–106.
26. Esmaeli B. Sentinel node biopsy as a tool for accurate staging of eyelid and conjunctival malignancies. *Current opinion in ophthalmology*. 2002;13(5):317–323.
27. Allen PJ, Busam K, Hill AD, Stojadinovic A, Coit DG. Immunohistochemical analysis of sentinel lymph nodes from patients with Merkel cell carcinoma. *Cancer*. Sep 15 2001;92(6): 1650–1655.
28. Gupta SG, Wang LC, Penas PF, Gellenthin M, Lee SJ, Nghiem P. Sentinel lymph node biopsy for evaluation and treatment of patients with Merkel cell carcinoma: The Dana-Farber experience and meta-analysis of the literature. *Archives of Dermatology*. Jun 2006; 142(6):685–690.
29. Warner RE, Quinn MJ, Hruby G, Scolyer RA, Uren RF, Thompson JF. Management of merkel cell carcinoma: the roles of lymphoscintigraphy, sentinel lymph node biopsy and adjuvant radiotherapy. *Annals of surgical oncology*. Sep 2008;15(9):2509–2518.
30. Gaskin BJ, Fernando BS, Sullivan CA, Whitehead K, Sullivan TJ. The significance of DNA mismatch repair genes in the diagnosis and management of periocular sebaceous cell carcinoma and Muir-Torre syndrome. *The British journal of ophthalmology*. Dec 2011;95(12):1686–1690.
31. Goldberg M, Rummelt C, Foja S, Holbach LM, Ballhausen WG. Different genetic pathways in the development of periocular sebaceous gland carcinomas in presumptive Muir-Torre syndrome patients. *Hum Mutat*. Feb 2006;27(2):155–162.
32. Holbach LM, von Moller A, Decker C, Junemann AG, Rummelt-Hofmann C, Ballhausen WG. Loss of fragile histidine triad (FHIT) expression and microsatellite instability in periocular sebaceous gland carcinoma in patients with Muir-Torre syndrome. *American journal of ophthalmology*. Jul 2002;134(1):147–148.
33. John AM, Schwartz RA. Muir-Torre syndrome (MTS): An update and approach to diagnosis and management. *Journal of the American Academy of Dermatology*. Mar 2016;74(3):558–566.
34. Kattan MW, Hess KR, Amin MB, et al. American Joint Committee on Cancer acceptance criteria for inclusion of risk models for individualized prognosis in the practice of precision medicine. *CA: a cancer journal for clinicians*. Jan 19 2016.

64

Conjunctival Carcinoma

65

R. Max Conway, Gerardo F. Graue, David Pelayes,
Jacob Pe'er, Matthew W. Wilson, Christian W. Wittekind,
Sarah E. Coupland, and Paul T. Finger

CHAPTER SUMMARY

Cancers Staged Using This Staging System

The classification applies only to carcinoma of the conjunctiva. Other tumors of the conjunctiva, including secondary conjunctival tumors (e.g., intraocular tumors extending through the conjunctiva, such as uveal melanoma or uveal non-Hodgkin lymphoma, and orbital tumors extending into the conjunctiva, such as rhabdomyosarcoma), are not classified using this schema.

Cancers Not Staged Using This Staging System

These histopathologic types of cancer ...	Are staged according to the classification for ...	And can be found in chapter ...
Conjunctival lymphoma	Ocular adnexal lymphoma	71
Conjunctival melanoma	Conjunctival melanoma	66

Summary of Changes

Change	Details of Change	Level of Evidence
Definition of Primary Tumor (T)	T1 and T2 definitions have been revised to include invasion of the conjunctival basement membrane.	II
Prognostic Factors	Expanded Emerging Prognostic Factors list	II and III

ICD-O-3 Topography Codes

Code	Description
C69.0	Conjunctiva

WHO Classification of Tumors

Code	Description
8010	Carcinoma *in situ*, NOS, intraepithelial carcinoma
8010	Carcinoma, NOS, epithelial tumor, malignant
8070	Squamous carcinoma *in situ*, NOS
8070	Squamous cell carcinoma, NOS
8072	Squamous cell carcinoma, large cell, nonkeratinizing
8073	Squamous cell carcinoma, small cell, nonkeratinizing
8074	Squamous cell carcinoma, spindle cell
8075	Squamous cell carcinoma, adenoid

To access the AJCC cancer staging forms, please visit www.cancerstaging.org.

© American Joint Committee on Cancer 2017

M.B. Amin et al. (eds.), *AJCC Cancer Staging Manual, Eighth Edition*, DOI 10.1007/978-3-319-40618-3_65

Code	Description
8076	Squamous cell carcinoma *in situ* with questionable stromal invasion
8076	Squamous cell carcinoma, microinvasion
8090	Basal cell carcinoma, NOS
8410	Sebaceous adenocarcinoma
8430	Mucoepidermoid carcinoma
8560	Adenosquamous carcinoma

International Agency for Research on Cancer, World Health Organization. International Classification of Diseases for Oncology. ICD-O-3-Online. http://codes.iarc.fr/home. Accessed May 15, 2016.

INTRODUCTION

The AJCC staging system for conjunctival carcinoma remains largely unchanged from the AJCC Cancer Staging Manual, 7th Edition, apart from a more precisely defined disease extent for early invasive lesions (T1 and T2). This change has implications for management, as emerging studies support adjuvant treatment in some of these patients.

It is acknowledged that the TNM staging system for these tumors would benefit greatly from the establishment of multicenter registries, prospective randomized trials, and prospective data mining to consolidate and expand the definitions of prognostic significance and to incorporate biomarkers.

Similarly, it is noted that data from a large registry would help provide significance to the currently applied subdivisions of intraepithelial disease (i.e., conjunctival squamous intraepithelial neoplasia, I–III). However, these data currently do not exist.

AJCC staging for conjunctival carcinoma is becoming more important, because the incidence of conjunctival squamous cell carcinoma (SCC) associated with HIV is increasing, especially in younger individuals in developing countries with high incidences of HIV. Such tumors behave more aggressively.

This chapter clearly defines the clinical term *ocular squamous surface neoplasia* and explains why a histopathologic tissue diagnosis is needed for TNM staging.

This staging system applies to conjunctival carcinoma comprising predominantly SCC and corneal squamous intraepithelial neoplasia, along with other histologic subtypes (see ICD-O-3 Histology Codes). Nonepithelial tumors of the conjunctiva are not staged using these criteria. Biopsy is required for tumor staging.

Risk factors for the disease are exposure to sun and ultraviolet B light, as well as light-colored skin. Other risk factors include radiation exposure, smoking, human papillomavirus (HPV) infection, chemical exposures, immunosuppression, and particular syndromes (e.g., xeroderma pigmentosum). In developed countries, this condition is more common in men, with a peak incidence in the seventh decade of life. At diagnosis, it is typically localized to the corneal limbus. Conjunctival carcinoma also is associated with HIV infection, and this association is particularly prevalent in developing countries, where it may be considered an AIDS-defining illness, especially in younger patients.[1–10]

Ocular surface squamous neoplasia is a clinical term encompassing the continuum of disease from mild epithelial dysplasia to SCC. Because this term includes overlapping histopathologic grades and entities, it is imprecise and should be avoided in histopathology reports. The precise morphologic changes should be documented using terminology applied in pathology.[11,12]

After clinical and pathological diagnosis, excisional treatment typically is supplemented by adjunctive double freeze–thaw cryotherapy to the conjunctival margins and sclera base at the time of primary resection.[13] Additional adjuvant and/or alternative treatments include topical chemotherapy (mitomycin C, 5-fluorouracil, or interferon alfa-2b). Radiation therapy (teletherapy or brachytherapy) may be used when complete resection is not possible or as salvage treatment to avoid orbital exenteration.[14–20]

ANATOMY

Primary Site(s)

Anatomically, the conjunctiva consists of stratified epithelium that contains mucus-secreting goblet cells. These cells are most numerous in the fornices. Palpebral conjunctiva lines the eyelid; bulbar conjunctiva covers the eyeball. Conjunctival epithelium merges with that of the cornea at the limbus.[2] Conjunctival squamous carcinomas are most likely to arise from the exposed bulbar limbal location.

Conjunctival squamous intraepithelial neoplasia embraces all forms of intraepithelial dysplasia, including SCC *in situ*. Spread occurs initially by direct local extension radially into the adjacent conjunctiva and cornea, and ultimately vertically into the conjunctival stroma, Tenon's capsule, and the sclera. Perineural invasion may occur at an earlier stage, typically in more aggressive histologic subtypes (e.g., mucoepidermoid carcinoma), leading to orbital extension. Larger lesions may develop the capacity for metastatic spread to regional lymph nodes following invasion into lymphatic vessels in the conjunctiva. Intraocular spread may occur through the sclera, particularly if weakened by prior surgery.[2,4,5,21–28]

Advanced tumors may directly invade the eyelid, eye, nasolacrimal system, orbit, adjacent paranasal sinus structures, or brain.[26,28,29]

Regional Lymph Nodes

The regional lymph nodes are preauricular (parotid), submandibular, and cervical nodes (Fig. 65.1).

Metastatic Sites

In addition to spreading via regional lymphatics, tumors of the conjunctiva also may metastasize hematogenously, although this is rare. Sites of metastasis include the parotid and submandibular gland, lungs, and bone.[29]

RULES FOR CLASSIFICATION

Clinical Classification

Initial and subsequent clinical assessments of conjunctival carcinoma are based on inspection, slit lamp examination, and palpation of the regional lymph nodes. All conjunctival surfaces should be inspected, measured, documented, and photographed (including eversion of the upper eyelid) (Fig. 65.2). Tumor photography should pay particular attention to the lesion margins, evidence of pagetoid spread, corneal epithelial extension, and involvement of the punctum. Gonioscopy should be performed with photography (for limbal disease), particularly when intraocular extension is suspected. Examination of the ipsilateral sinuses is indicated (particularly if punctal involvement has been noted).

The diagnosis of conjunctival squamous carcinoma typically requires excisional biopsy because histopathologic examination allows for the assessment of vertical tumor extent, which is needed to determine the levels required for TNM staging. In contrast, cytologic techniques have been found to be useful in determining cytologic atypia. Cytology has been particularly helpful as an adjunct to clinical diagnosis and in evaluation for recurrent disease.[11]

Imaging

Anterior segment ultrasound imaging and optical coherence tomography are useful for measuring tumor thickness and evaluating invasion of adjacent structures (e.g., the sclera, uvea, and anterior orbit). Ultrasound biomicroscopy (UBM) findings suspicious for intraocular invasion include angle blunting and uveal thickening.[30] Low-frequency posterior segment ultrasonography also may be used to evaluate for choroidal and orbital invasion.[31]

Radiologic evaluation may be necessary to stage locally invasive and nodal disease and may include computed tomography (CT), magnetic resonance imaging, and positron emission tomography/CT. Metastatic survey typically includes a physical examination as well as hematologic screening and radiologic evaluations of the head, chest, and abdomen.

Pathological Classification

For pathological staging, complete resection of the primary site is indicated, if possible.

To obtain the best histopathologic information, it is important to send the conjunctival specimen to the laboratory, spread evenly on a piece of filter paper with orientation marks on the paper. These measures prevent the specimen from curling and enable orthogonal sections to be taken from the tissue, allowing assessment of the depth of tumor penetration. Histopathologic evaluation for negative peripheral or deep margins should be performed. If mapping biopsy samples are sent to the laboratory, they should be placed in separate containers labeled with the appropriate anatomic location. Sentinel lymph node biopsy is investigational.[32]

As stated earlier, the clinical term *ocular squamous surface neoplasia* is imprecise and may even include benign growths; thus, it should be avoided in surgical pathology reports.[12]

For pN, histologic examination of regional lymphadenectomy specimens, if performed, will include one or more regional lymph nodes.[28,32]

PROGNOSTIC FACTORS

Prognostic Factors Required for Stage Grouping

Beyond the factors used to assign T, N, or M categories, no additional prognostic factors are required for stage grouping.

Additional Factors Recommended for Clinical Care

- Presence or absence of subepithelial invasion, as determined by histopathologic examination (AJCC Level of Evidence: II)
- Tumor size as determined by clinical measurement, clock hour evaluation, and UBM (AJCC Level of Evidence: II)
- Local invasion as assessed by gonioscopy, ultrasound, and radiologic testing (AJCC Level of Evidence: II)

65

RISK ASSESSMENT MODELS

The AJCC recently established guidelines that will be used to evaluate published statistical prediction models for the purpose of granting endorsement for clinical use.[33] Although this is a monumental step toward the goal of precision medicine, this work was published only very recently. Therefore, the existing models that have been published or may be in clinical use have not yet been evaluated for this cancer site by the Precision Medicine Core of the AJCC. In the future, the statistical prediction models for this cancer site will be evaluated, and those that meet all AJCC criteria will be endorsed.

DEFINITIONS OF AJCC TNM

Adjacent structures include the cornea (3, 6, 9, or 12 clock hours), intraocular compartments, forniceal conjunctiva (lower and/or upper), palpebral conjunctiva (lower and/or upper), tarsal conjunctiva (lower and/or upper), lacrimal punctum and canaliculi (lower and/or upper), plica, caruncle, posterior eyelid lamella, anterior eyelid lamella, and/or eyelid margin (lower and/or upper); see Fig. 65.2.

Definition of Primary Tumor (T)

T Category	T Criteria
TX	Primary tumor cannot be assessed
T0	No evidence of primary tumor
Tis	Carcinoma *in situ*
T1	Tumor (≤5 mm in greatest dimension) invades through the conjunctival basement membrane without invasion of adjacent structures
T2	Tumor (>5 mm in greatest dimension) invades through the conjunctival basement membrane without invasion of adjacent structures
T3	Tumor invades adjacent structures (excluding the orbit)
T4	Tumor invades the orbit with or without further extension
T4a	Tumor invades orbital soft tissues without bone invasion
T4b	Tumor invades bone
T4c	Tumor invades adjacent paranasal sinuses
T4d	Tumor invades brain

Definition of Regional Lymph Node (N)

N Category	N Criteria
NX	Regional lymph nodes cannot be assessed
N0	No regional lymph node metastasis
N1	Regional lymph node metastasis

Definition of Distant Metastasis (M)

M Category	M Criteria
M0	No distant metastasis
M1	Distant metastasis

AJCC PROGNOSTIC STAGE GROUPS

There is no proposal for anatomic stage and prognostic groups for conjunctival carcinoma.

REGISTRY DATA COLLECTION VARIABLES

1. Ki-67 growth fraction, reported as percentage of positive tumor cells by immunohistochemistry

HISTOLOGIC GRADE (G)

G	G Definition
GX	Grade cannot be assessed
G1	Well differentiated
G2	Moderately differentiated
G3	Poorly differentiated
G4	Undifferentiated

HISTOPATHOLOGIC TYPE

Conjunctival intraepithelial neoplasia, including SCC *in situ*
Squamous cell carcinoma
Mucoepidermoid carcinoma
Spindle cell carcinoma
Sebaceous gland carcinoma including pagetoid (conjunctival) spread
Basal cell carcinoma

ILLUSTRATIONS

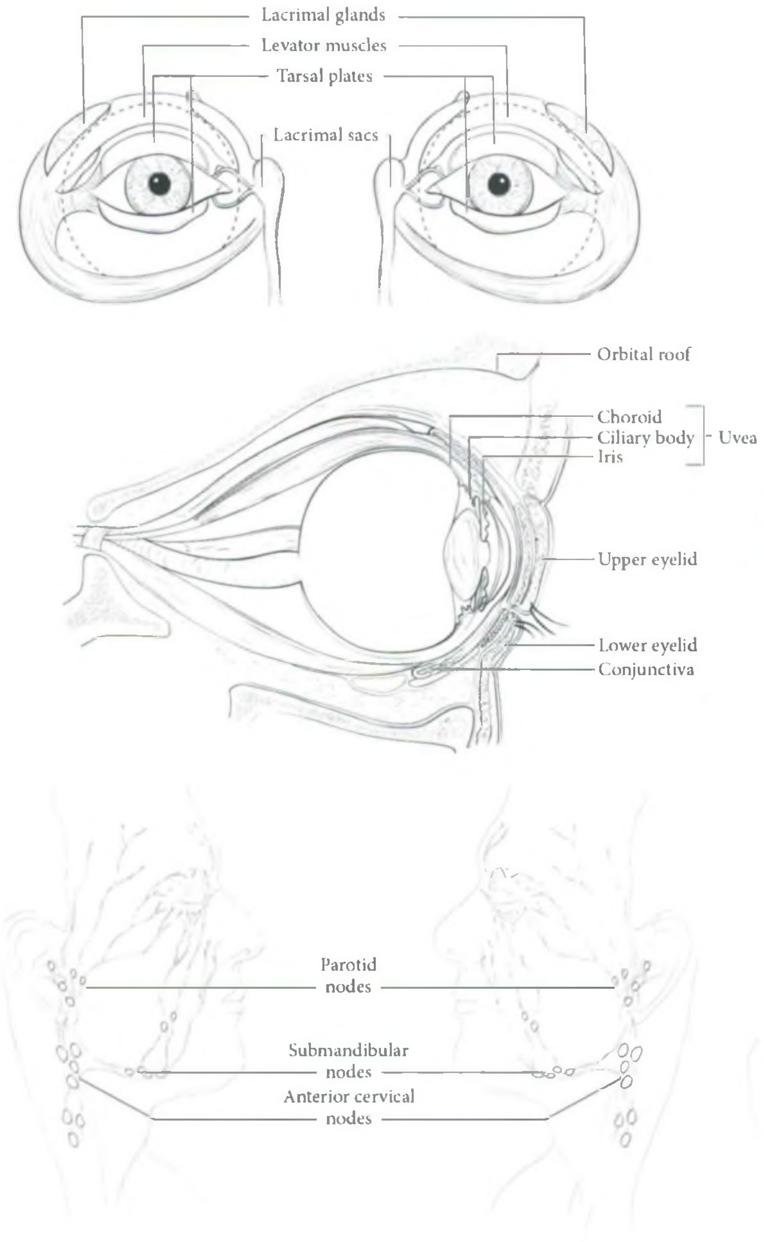

Fig. 65.1 Anatomic sites and regional lymph nodes for ophthalmic sites

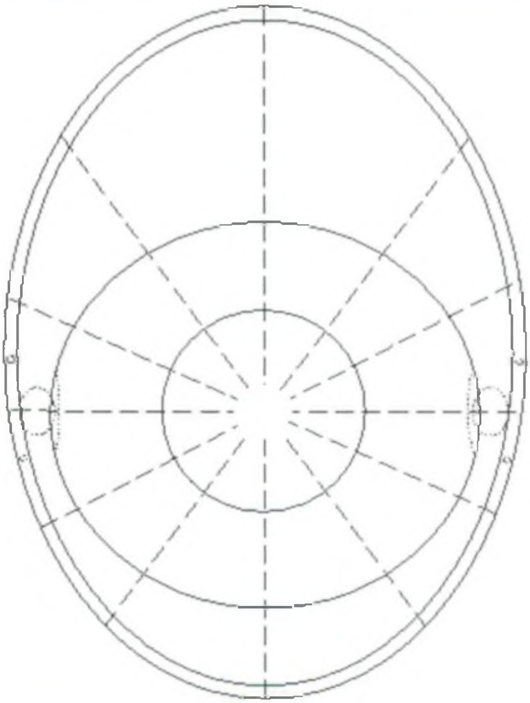

Fig. 65.2 Clinical mapping system for conjunctival carcinoma. The map displays the entire conjunctiva as a flat surface, with the central point located at the center of the cornea and concentric regions, such as the limbus, bulbar conjunctiva, fornix, palpebral conjunctiva, and eyelid, considered progressively more peripheral. Radial lines represent clock hours (Modified from Damato and Coupland[14])

Bibliography

1. Mehta M, Fay A. Squamous cell carcinoma of the eyelid and conjunctiva. *International ophthalmology clinics*. 2009;49(1):111–121.
2. Eagle RC. *Eye pathology: an atlas and text*. Lippincott Williams & Wilkins; 2012.
3. Ramberg I, Heegaard S, Prause JU, Sjo NC, Toft PB. Squamous cell dysplasia and carcinoma of the conjunctiva. A nationwide, retrospective, epidemiological study of Danish patients. *Acta ophthalmologica*. Nov 2015;93(7):663–666.
4. McKelvie PA, Daniell M, McNab A, Loughnan M, Santamaria JD. Squamous cell carcinoma of the conjunctiva: a series of 26 cases. *The British journal of ophthalmology*. Feb 2002;86(2):168–173.
5. Seitz B, Fischer M, Holbach LM, Naumann GO. [Differential diagnosis and prognosis of 112 excised epibulbar epithelial tumors]. *Klinische Monatsblätter für Augenheilkunde*. Oct 1995;207(4):239–246.
6. Lee GA, Hirst LW. Ocular surface squamous neoplasia. *Survey of ophthalmology*. May-Jun 1995;39(6):429–450.
7. Kenawy N, Garrick A, Heimann H, Coupland SE, Damato BE. Conjunctival squamous cell neoplasia: the Liverpool Ocular Oncology Centre experience. *Graefe's archive for clinical and experimental ophthalmology = Albrecht von Graefes Archiv fur klinische und experimentelle Ophthalmologie*. Jan 2015;253(1):143–150.
8. Kamal S, Kaliki S, Mishra DK, Batra J, Naik MN. Ocular Surface Squamous Neoplasia in 200 Patients: A Case-Control Study of Immunosuppression Resulting from Human Immunodeficiency Virus versus Immunocompetency. *Ophthalmology*. Aug 2015;122(8):1688–1694.
9. Rogena EA, Simbiri KO, De Falco G, Leoncini L, Ayers L, Nyagol J. A review of the pattern of AIDS defining, HIV associated neoplasms and premalignant lesions diagnosed from 2000–2011 at Kenyatta National Hospital, Kenya. *Infectious agents and cancer*. 2015;10(1):1–7.
10. Yin VT, Merritt HA, Sniegowski M, Esmaeli B. Eyelid and ocular surface carcinoma: diagnosis and management. *Clin Dermatol*. Mar-Apr 2015;33(2):159–169.
11. Semenova EA, Milman T, Finger PT, et al. The diagnostic value of exfoliative cytology vs histopathology for ocular surface squamous neoplasia. *American journal of ophthalmology*. Nov 2009;148(5):772–778 e771.
12. Margo C, White A. Ocular surface squamous neoplasia: terminology that is conceptually friendly but clinically perilous. *Eye*. 2014;28(5):507.
13. Finger PT. "Finger-tip" cryotherapy probes: treatment of squamous and melanocytic conjunctival neoplasia. *The British journal of ophthalmology*. Aug 2005;89(8):942–945.
14. Buuns DR, David TT, Folberg R. Microscopically controlled excision of conjunctival squamous cell carcinoma. *American journal of ophthalmology*. 1994;117(1):97–102.
15. Li AS, Shih CY, Rosen L, Steiner A, Milman T, Udell IJ. Recurrence of Ocular Surface Squamous Neoplasia Treated With Excisional Biopsy and Cryotherapy. *American journal of ophthalmology*. Aug 2015;160(2):213–219 e211.
16. Wilson MW, Czechonska G, Finger PT, Rausen A, Hooper ME, Haik BG. Chemotherapy for eye cancer. *Survey of ophthalmology*. Mar-Apr 2001;45(5):416–444.
17. Arepalli S, Kaliki S, Shields CL, Emrich J, Komarnicky L, Shields JA. Plaque radiotherapy in the management of scleral-invasive conjunctival squamous cell carcinoma: an analysis of 15 eyes. *JAMA ophthalmology*. Jun 2014;132(6):691–696.
18. Graue GF, Tena LB, Finger PT. Electron beam radiation for conjunctival squamous carcinoma. *Ophthalmic plastic and reconstructive surgery*. Jul-Aug 2011;27(4):277–281.
19. Pe'er J. Ocular surface squamous neoplasia: evidence for topical chemotherapy. *International ophthalmology clinics*. Winter 2015;55(1):9–21.
20. Walsh-Conway N, Conway RM. Plaque brachytherapy for the management of ocular surface malignancies with corneoscleral invasion. *Clinical & experimental ophthalmology*. Aug 2009;37(6):577–583.
21. Brownstein S. Mucoepidermoid carcinoma of the conjunctiva with intraocular invasion. *Ophthalmology*. Dec 1981;88(12):1226–1230.
22. Cohen BH, Green WR, Iliff NT, Taxy JB, Schwab LT, de la Cruz Z. Spindle cell carcinoma of the conjunctiva. *Archives of ophthalmology*. Oct 1980;98(10):1809–1813.
23. Grossniklaus HE, Green WR, Luckenbach M, Chan CC. Conjunctival lesions in adults. A clinical and histopathologic review. *Cornea*. 1987;6(2):78–116.
24. Grossniklaus HE, Martin DF, Solomon AR. Invasive conjunctival tumor with keratoacanthoma features. *American journal of ophthalmology*. Jun 15 1990;109(6):736–738.
25. Husain SE, Patrinely JR, Zimmerman LE, Font RL. Primary basal cell carcinoma of the limbal conjunctiva. *Ophthalmology*. Nov 1993;100(11):1720–1722.
26. Rao NA, Font RL. Mucoepidermoid carcinoma of the conjunctiva: a clinicopathologic study of five cases. *Cancer*. Oct 1976;38(4):1699–1709.

27. Shields JA, Demirci H, Marr BP, Eagle RC, Jr., Stefanyszyn M, Shields CL. Conjunctival epithelial involvement by eyelid sebaceous carcinoma. The 2003 J. Howard Stokes lecture. *Ophthalmic plastic and reconstructive surgery*. Mar 2005;21(2): 92–96.
28. Johnson TE, Tabbara KF, Weatherhead RG, Kersten RC, Rice C, Nasr AM. Secondary squamous cell carcinoma of the orbit. *Archives of ophthalmology*. Jan 1997;115(1):75–78.
29. Tabbara KF, Kersten R, Daouk N, Blodi FC. Metastatic squamous cell carcinoma of the conjunctiva. *Ophthalmology*. Mar 1988;95(3):318–321.
30. Garcia JP, Jr., Spielberg L, Finger PT. High-frequency ultrasound measurements of the normal ciliary body and iris. *Ophthalmic Surg Lasers Imaging*. 2011;42(4):321–327.
31. Conway RM, Chew T, Golchet P, Desai K, Lin S, O'Brien J. Ultrasound biomicroscopy: role in diagnosis and management in 130 consecutive patients evaluated for anterior segment tumours. *The British journal of ophthalmology*. Aug 2005;89(8):950–955.
32. Mendoza PR, Grossniklaus HE. Sentinel Lymph Node Biopsy for Eyelid and Conjunctival Tumors: What is the Evidence? *International ophthalmology clinics*. 2015;55(1):123–136.
33. Kattan MW, Hess KR, Amin MB, et al. American Joint Committee on Cancer acceptance criteria for inclusion of risk models for individualized prognosis in the practice of precision medicine. *CA: a cancer journal for clinicians*. Jan 19 2016.
34. Damato B, Coupland SE. Clinical mapping of conjunctival melanomas. *The British journal of ophthalmology*. Nov 2008; 92(11):1545–1549.

Conjunctival Melanoma

66

Sarah E. Coupland, Raymond Barnhill, R. Max Conway, Bertil E. Damato, Bita Esmaeli, Daniel M. Albert, Claudia Auw-Hädrich, Patricia Chévez-Barrios, Hans E. Grossniklaus, Steffen Heegaard, Leonard M. Holbach, Tero Kivelä, Anna C. Pavlick, Jacob Pe'er, Carol Shields, Arun D. Singh, Christian W. Wittekind, Michelle D. Williams, Victor G. Prieto, and Paul T. Finger

CHAPTER SUMMARY

Cancers Staged Using This Staging System

Melanomas arising from the bulbar and palpebral conjunctiva and from the caruncle

Cancers Not Staged Using This Staging System

These histopathologic types of cancer...	Are staged according to the classification for...	And can be found in chapter...
Primary eyelid melanomas	Melanoma of the skin	47
Secondary involvement of the conjunctiva by extraocular uveal melanoma	Uveal melanoma	67

Summary of Changes

Change	Details of Change	Level of Evidence
Definition of Primary Tumor (T)	Criteria of T categories have changed to describe circumferential extent.	III
Definition of Regional Lymph Node (N)	Criteria of N category have changed to describe whether a biopsy was performed.	III
Histologic Grade (G)	Histologic grade has been removed.	II

ICD-O-3 Topography Codes

Code	Description
C69.0	Conjunctiva

WHO Classification of Tumors

Code	Description
8720	Melanoma *in situ*
8721	Melanoma, NOS
8723	Melanoma, regressing
8730	Amelanotic melanoma
8740	Melanoma arising from a nevus: specify cell type
8741	Melanoma arising in atypical melanocytosis; specify type
8743	Melanoma with adjacent intraepithelial component
8745	Desmoplastic melanoma
8770	Invasive melanoma, cellular type: specify

International Agency for Research on Cancer, World Health Organization. International Classification of Diseases for Oncology. ICD-O-3-Online. http://codes.iarc.fr/home. Accessed May 15, 2016.

To access the AJCC cancer staging forms, please visit www.cancerstaging.org.

© American Joint Committee on Cancer 2017
M.B. Amin et al. (eds.), *AJCC Cancer Staging Manual, Eighth Edition*, DOI 10.1007/978-3-319-40618-3_66

INTRODUCTION

This chapter addresses invasive conjunctival melanoma and its precursor lesions. Conjunctival melanoma, which constitutes about 2% of all primary ocular malignancies and 5% of all ocular melanomas,[1–4] is the second most frequent primary malignant neoplasm of the conjunctiva after squamous cell carcinoma (see Conjunctival Carcinoma, Chapter 65), with an incidence of 0.2 to 0.8 per million individuals per year in Caucasians; only rare cases are reported in non-Caucasian races.[5] Presentation is rare before the age of 40 years and peaks around 60 years. Although some studies described equal incidence in men and women, others have reported a higher frequency in males.[6,7]

Conjunctival melanoma arises either de novo, from benign nevi, or from conjunctival melanoma *in situ*. The percentages vary in the literature, but it is suggested that 5% of conjunctival melanoma cases arise de novo, 25% from nevi, and the remainder from melanoma *in situ*.[8–11] It is uncertain whether the origin of the malignant tumor influences the prognosis.

In contrast to uveal melanomas, approximately 40% of conjunctival melanomas have *BRAF* mutations.[12–15] Thus, patients with metastatic *BRAF*-mutated conjunctival melanoma have been treated with BRAF inhibitors, with limited response.[16] Other mutations described in conjunctival melanomas are similar to those described in skin melanomas and include *NRAS*, *c-Kit*, and *TERT* promoter mutations.[14,17,18] The presence of *TERT* promoter mutations with ultraviolet (UV) light signatures in conjunctival melanomas supports a UV-induced pathogenesis.[3,19]

The diagnosis of conjunctival melanoma—and any associated atypical melanocytic intraepithelial neoplasia—is based on both clinical and histomorphologic features. Pathological analysis may be obtained by excisional biopsy of the main tumor (if possible) and/or mapping biopsies of the peritumoral tissue. Primary excision without adjuvant therapy is associated with a high risk of recurrence.[9,20–23] Therefore, it typically is supplemented with adjunctive therapy in the form of cryotherapy, plaque brachytherapy, or proton beam radiotherapy, and/or topical chemotherapy (e.g., mitomycin C or interferon).[9,20–23]

Metastatic disease develops in 20–30% of conjunctival melanoma patients, and carries a poor prognosis.[4,22,24–27]

Conjunctival melanoma is a rare, poorly understood tumor that is treated with a variety of methods. TNM staging is important for conjunctival melanoma because of the need for standardized reporting, improved understanding of histopathologic risk factors, and evaluations of treatment. Because of a paucity of medical evidence, the AJCC recognizes the need to collect statistically significant outcome data of this rare tumor that may be used to create evidence-based stage groups.

ANATOMY

Primary Site(s)

The conjunctiva is a thin and transparent mucous membrane that secretes mucous material from goblet cells, which contributes to the tear film. Beyond that, it serves to protect the eye by forming an incomplete sac that provides an anatomic and cellular barrier to pathogens.

The conjunctiva may be subdivided into three distinct zones. The palpebral, or tarsal, conjunctiva starts at the inner border of the eyelid and is firmly attached to the posterior tarsal plates. The forniceal conjunctiva comprises the loose and redundant folds between the palpebral conjunctive and bulbar conjunctiva, the latter of which covers the anterior sclera and transists into the corneal epithelium at the limbus.

Although the conjunctiva is a continuous membrane, its histologic features differ among the three different zones. The conjunctival stroma (substantia propria) contains small blood vessels and lymphatics and consists of loose connective tissue. The accessory lacrimal glands of Krause and Wolfring are located within the stroma and typically drain into the fornix. A modified nonkeratinized, stratified squamous epithelium covers the lid margin and the bulbar conjunctiva, whereas the tarsal and forniceal conjunctivas are covered by a thinner stratified cuboidal epithelium. Goblet cells are located within the epithelium and have varying densities among the conjunctival zones. They are scarce near the lid margin and at the limbus.

Melanocytes typically are present only in the basal layer of normal conjunctival epithelium. These melanocytes are typically found singly and widely spaced along the basement membrane, and their dendrites pass between the conjunctival keratinocytes and goblet cells. Like other cells, they may undergo reactive or degenerative change, as well as benign and/or malignant neoplastic transformation.

A spectrum of conditions arise from the conjunctival melanocytes, ranging from benign conjunctival nevi, benign "acquired melanosis," atypical intraepithelial melanocytic hyperplasia/proliferation/neoplasia, and conjunctival melanoma *in situ* to frank invasive conjunctival melanoma.

The terminology of the conjunctival intraepithelial melanocytic lesions has been (and remains) controversial.[28–31] The most common clinical term used for acquired melanosis of the conjunctiva is *primary acquired melanosis* (PAM). Histologically, PAM has been divided into two subgroups—PAM with and without atypia; PAM with atypia is subdivided further into grades of mild, moderate, and severe.

On histomorphologic examination, PAM without atypia is characterized by (1) excessive melanin production from

normal melanocytes with accumulation of granules within the cytoplasm, and/or (2) normal or increased numbers of typical conjunctival melanocytes (i.e., a true cellular proliferation) in the basal epithelial layer without any evidence of cellular atypia. In contrast, PAM with atypia demonstrates a proliferation of melanocytes that no longer are limited to the basal conjunctival (basilar) epithelial layer, but instead involve the suprabasal epithelium, and demonstrate increasing degrees of cytologic atypia. Pagetoid spread and epithelioid cell cytomorphology of the atypical melanocytes also may be seen.[8,30–32] The distinction between PAM with severe atypia and conjunctival melanoma *in situ* is ill-defined. The concept of a conjunctival melanoma *in situ* (pTis) stage was introduced for the first time in the AJCC Cancer Staging Manual, 7th Edition TNM staging system in an attempt to address this. According to the 7th Edition TNM staging system, PAM with atypia or melanoma confined to the epithelium (mild, moderate, or severe) is termed *conjunctival melanoma in situ*, or pTis. This chapter recommends that the term *primary acquired melanosis* be used *only* as a clinical description and that the histomorphologic examiner should precisely report the underlying pathological process(es)—that is, melanin overproduction and melanocytic proliferation (i.e., melanosis and/or melanocytosis, respectively) or both—as well as the extent of these changes.

Invasive conjunctival melanomas arise most often in the lateral bulbar conjunctiva, with a significant minority involving the palpebral conjunctiva. Clinically, they are distinguished further by color (pigmented, amelanotic); shape (nodular, diffuse, mixed); location (bulbar, palpebral, plica, caruncular); size (largest basal diameter and thickness); and focality (unifocal, multifocal).

Conjunctival melanomas tend to be hypervascular, may have recruited posterior feeder vessels, and may be affixed to the sclera and extend onto the cornea. For tumors affixed to the eye wall, high-frequency ultrasound examination and gonioscopy may be used to look for scleral and intraocular invasion. Conjunctival melanoma may invade the globe, eyelids, nasolacrimal system, orbit, maxillofacial sinuses, and, rarely, the central nervous system.[33]

If possible, an excisional biopsy of conjunctival melanomas should be performed, preferably in a center with experience in diagnosing and treating these rare tumors. Invasive conjunctival melanoma is histologically characterized by a destruction of the conjunctival epithelial basement membrane and infiltration of the lamina propria by atypical melanocytes. These usually occur in tumor nests and often are composed of epithelioid cells with scattered mitoses.

Reported morphologic features of invasive conjunctival melanoma associated with a poor prognosis include the following: tumor thickness >2 mm; ulceration; a mitotic figure count $>1/mm^2$; epithelioid cell morphology; and the presence of extravascular matrix patterns, lymphatic invasion, high microvascular density, microscopic satellites, and lymphocytic or macrophage infiltration.[24,34–37] Conjunctival melanoma *in situ* adjacent to or distant from the main tumor is associated with high rates of tumor recurrence following therapy and a poorer prognosis.[24,25,36–38]

Regional Lymph Nodes

Lymphatic metastases occur in the regional lymph nodes—that is, the preauricular (parotid), postauricular, submandibular, and cervical lymph nodes (Fig. 66.1).

The parotid nodes consist of three groups: superficial, intraparotid, and deep nodes. Lymphatic drainage from the conjunctiva/eyelid drains primarily into the superficial group but also may involve the intraparotid nodes.[39–44]

Metastatic Sites

Metastatic spread of conjunctival melanoma may occur via both the lymphatic and vascular systems (Fig. 66.1). Almost any site may be affected by metastases, but the primary targets include the lungs, liver, brain, and bones.[39,45,46]

RULES FOR CLASSIFICATION

Clinical Classification

Clinical staging typically is performed after slit-lamp examination of the eyelids, puncta, and all conjunctival surfaces (Fig. 66.2). Palpation of regional lymph nodes and histologic assessment are described separately.

Nodular or well-defined conjunctival melanomas are categorized according to the regional location of their posterior and anterior margins (i.e., cornea, limbus, bulbar conjunctiva, fornix, palpebral conjunctiva, plica, caruncle, or eyelid skin) and by their circumferential extent, in clock minutes, in each of these regions. A "quadrant" comprises 15 clock minutes regardless of the meridian locations of the lateral tumor margins (Fig. 66.2).

The entire bulbar and palpebral conjunctiva, including the fornices, should be inspected and photographed. In defining the palpebral conjunctiva, tarsal conjunctiva should be distinguished from nontarsal conjunctiva, near the fornix, as the prognostic implications may be different for these two locations. Supplemental drawings should be used to depict the entire tumor surface, with documenta-

tion of the radial and circumferential location as well as the extent of any nodular tumors and melanotic areas. Invasion of the corneal epithelium and recruited vascularity should be noted. Photographs also should capture the whole tumor, demonstrating its margins as well as any apparent intraepithelial disease and punctal, plical, or caruncular involvement.

The tumor should be assessed for attachment to the sclera and for intraocular invasion. Palpation through the eyelid or with a cotton-tipped applicator may demonstrate tumor adherence. Anterior segment infiltration may be visualized by gonioscopy (with or without photography). In these cases, anterior segment ultrasound imaging may demonstrate scleral infiltration, angle blunting, and uveal thickening. Deeper invasion (e.g., of the globe and/or orbit and/or sinuses) should prompt posterior segment ultrasound and/or radiographic imaging. Computed tomography (CT) or magnetic resonance (MR) imaging may detect extension into the orbits and sinuses. Evaluations for metastatic disease typically include radiographic imaging of the head, chest, abdomen, and/or bones.

Treatment approaches vary and are heavily influenced by the customs and practice at each eye cancer center. Treatments are selected according to the size and local distribution of the conjunctival melanoma, as well as any associated intraepithelial disease.[15,20–22,24,36] Nodular tumors typically are excised. Centers have suggested using the so-called "no-touch" technique to prevent surgical instrument–generated tumor dissemination.[11,22,30] Incisional or needle biopsy of conjunctival melanomas has been avoided, as this procedure may be associated with seeding and tumor recurrence. Impression cytology also is not recommended for either conjunctival melanocytic intraepithelial lesions or invasive melanoma.

However, for diffuse intraepithelial disease, mapping biopsies may be required to determine the extent of conjunctival involvement, particularly for amelanotic lesions. Examination of the nasal passages and lacrimal sac should be considered to exclude secondary spread of medial conjunctival melanomas located near the nasolacrimal duct. Topical chemotherapy (e.g., mitomycin and interferon alfa), cryotherapy, plaque brachytherapy, or proton beam radiotherapy may be used as adjunctive therapy.

Each excisional or mapping specimen should be placed on a separate paper card to avoid scrolling and sent in buffered formalin for histopathologic examination, with one specimen per container.[47] The use of a cassette further prevents scrolling of the specimen. Larger specimens should be oriented on the card (i.e., nasal, temporal, superior, inferior). Care should be taken to avoid crush artifact.

Imaging

Conjunctival imaging techniques include color photography, anterior segment ultrasound imaging, and optical coherence tomography, whereas deep invasion may be assessed with posterior segment ultrasonography, MR imaging, and/or PET/CT.[48–51]

Pathological Classification

Four types of specimen are likely to be received from patients suspected of having conjunctival melanocytic lesions: excisional biopsy, incisional biopsy, and multiple incisional mapping biopsy samples and, for advanced conjunctival melanoma, orbital exenterations. The latter may be complete or limited, with complete orbital exenteration comprising the eyelids, globe, optic nerve, extraocular muscles, orbital fat, and periosteum.[52]

Pathological examination should provide a precise histologic diagnosis of the pathological process(es) (i.e., "melanosis"/hypermelanosis, conjunctival melanocytic intraepithelial proliferation/neoplasia, melanoma in situ, and/or invasive melanoma), describing the relationship to anatomic structures and surgical margins.

With respect to *invasive conjunctival melanoma*, the following histomorphologic features should be detailed in the report:

- Tumor thickness: infiltration depth (measured in millimeters) into the substantia propria from the surface of the conjunctival epithelium
- Cytomorphology: presence/absence of epithelioid cells
- Mitotic count: number of mitoses per square millimeter
- Presence/absence of surface ulceration
- Presence/absence of growth regression
- Presence/absence of vessel invasion: blood or lymphatic invasion
- Presence/absence of perineural invasion
- Status of all surgical margins (i.e., whether tumor extends to the lateral and deep margins)
- Presence/absence of adjacent conjunctival melanoma *in situ*, including status within surgical margins
- Presence/absence of coexisting nevus
- Presence/absence of microsatellites: Microscopic satellites are discrete micronodules of melanoma near the primary melanoma (within 1 to 2 mm), at least 0.05 mm in diameter, and separated from the primary melanoma by at least 0.3 mm of uninvolved connective tissue (absence of scar, reparative changes, or significant inflammation), usually in the substantia propria.

The role of sentinel lymph node (SLN) biopsy with morphologic and immunohistochemical examination has been investi-

gated during the past several years.[39–44,53] Several prospective and retrospective reports have demonstrated the safety and efficacy of SLN biopsy in identifying microscopically positive SLNs in patients with invasive conjunctival melanoma that are not found with conventional imaging techniques, such as MR imaging, CT, or ultrasound, or on physical examination. Although there is no consensus requirement for SLN biopsy at this time, it might be considered for conjunctival melanomas that are thicker than 2 mm or show evidence of ulceration.

PROGNOSTIC FACTORS

Prognostic Factors Required for Stage Grouping

Beyond the factors used to assign T, N, or M categories, no additional prognostic factors are required for stage grouping.

Additional Factors Recommended for Clinical Care

BRAF

BRAF mutational status of tumor cells should be considered with the view that the patient may develop metastases and be eligible for treatment with BRAF inhibitors. BRAF mutational status may be determined by using either molecular or immunohistologic techniques.[16] AJCC Level of Evidence: III

RISK ASSESSMENT MODELS

The AJCC recently established guidelines that will be used to evaluate published statistical prediction models for the purpose of granting endorsement for clinical use.[54] Although this is a monumental step toward the goal of precision medicine, this work was published only very recently. Therefore, the existing models that have been published or may be in clinical use have not yet been evaluated for this cancer site by the Precision Medicine Core of the AJCC. In the future, the statistical prediction models for this cancer site will be evaluated, and those that meet all AJCC criteria will be endorsed.

DEFINITIONS OF AJCC TNM

Definition of Primary Tumor (T)

Clinical Tumor (cT)

cT Category	cT Criteria
TX	Primary tumor cannot be assessed
T0	No evidence of primary tumor
T1	Tumor of the bulbar conjunctiva
T1a	<1 quadrant
T1b	≥1 to <2 quadrants
T1c	≥2 to <3 quadrants
T1d	≥3 quadrants
T2	Tumor of the nonbulbar (forniceal, palpebral, tarsal) conjunctiva, and tumor involving the caruncle
T2a	Noncaruncular, and ≤1 quadrant of the nonbulbar conjunctiva involved
T2b	Noncaruncular, and >1 quadrant of the nonbulbar conjunctiva involved
T2c	Caruncular, and ≤1 quadrant of the nonbulbar conjunctiva involved
T2d	Caruncular, and >1 quadrant of the nonbulbar conjunctiva involved
T3	Tumor of any size with local invasion
T3a	Globe
T3b	Eyelid
T3c	Orbit
T3d	Nasolacrimal duct and/or lacrimal sac and/or paranasal sinuses
T4	Tumor of any size with invasion of the central nervous system

Pathological Tumor (pT)

pT Category	pT Criteria
TX	Primary tumor cannot be assessed
T0	No evidence of primary tumor
Tis	Melanoma confined to the conjunctival epithelium
T1	Tumor of the bulbar conjunctiva
T1a	Tumor of the bulbar conjunctiva with invasion of the substantia propria, not more than 2.0 mm in thickness
T1b	Tumor of the bulbar conjunctiva with invasion of the substantia propria, more than 2.0 mm in thickness
T2	Tumor of the nonbulbar (forniceal, palpebral, tarsal) conjunctiva, and tumor involving the caruncle
T2a	Tumor of the nonbulbar conjunctiva with invasion of the substantia propria, not more than 2.0 mm in thickness
T2b	Tumor of the nonbulbar conjunctiva with invasion of the substantia propria, more than 2.0 mm in thickness
T3	Tumor of any size with local invasion
T3a	Globe
T3b	Eyelid
T3c	Orbit
T3d	Nasolacrimal duct and/or lacrimal sac and/or paranasal sinuses
T4	Tumor of any size with invasion of the paranasal sinuses and/or central nervous system

Definition of Regional Lymph Node (N)

N Category	N Criteria
NX	Regional lymph nodes cannot be assessed
N0	No regional lymph node metastasis
N1	Regional lymph node metastasis

Definition of Distant Metastasis (M)

M Category	M Criteria
M0	No distant metastasis
M1	Distant metastasis

AJCC PROGNOSTIC STAGE GROUPS

There is no proposal for anatomic stage and prognostic groups for conjunctival melanoma.

REGISTRY DATA COLLECTION VARIABLES

Invasive Conjunctival Melanoma

1. Tumor thickness: infiltration depth (measured in millimeters) into the substantia propria from the surface of the conjunctival epithelium
2. Cytomorphology: presence/absence of epithelioid cells
3. Mitotic count: number of mitosis per square millimiter
4. Presence/absence of surface ulceration
5. Presence/absence of growth regression
6. Presence/absence of vessel invasion: blood or lymphatic invasion
7. Presence/absence of perineural invasion
8. Status of all surgical margins (i.e., whether tumor extends to the lateral and deep margins)
9. Presence/absence of adjacent conjunctival melanoma *in situ*, including status within surgical margins
10. Presence/absence of coexisting nevus
11. Presence/absence of microsatellites

Lymph Node Metastases

The presence or absence of microscopic satellites/satellite in-transit metastases, which may be considered for future pathologic staging of pN level, as in the case of cutaneous melanoma.*

*Satellite in-transit metastasis: discrete micronodule/nodule of melanoma <1 mm to several millimeters in diameter in subepithelial tissue close to but clearly separated from the primary melanoma by at least 1 to 2 mm or more of uninvolved connective tissue.[55,56] Both these types of metastasis usually are angiotropic and may be solitary or often multiple.[57]

HISTOLOGIC GRADE (G)

In accordance with melanomas at other anatomic sites, grading is not performed for conjunctival melanoma.

HISTOPATHOLOGIC TYPE

This categorization applies only to melanoma of the conjunctiva.

Spindle cell melanoma
Epithelioid melanoma
Anaplastic melanoma

ILLUSTRATIONS

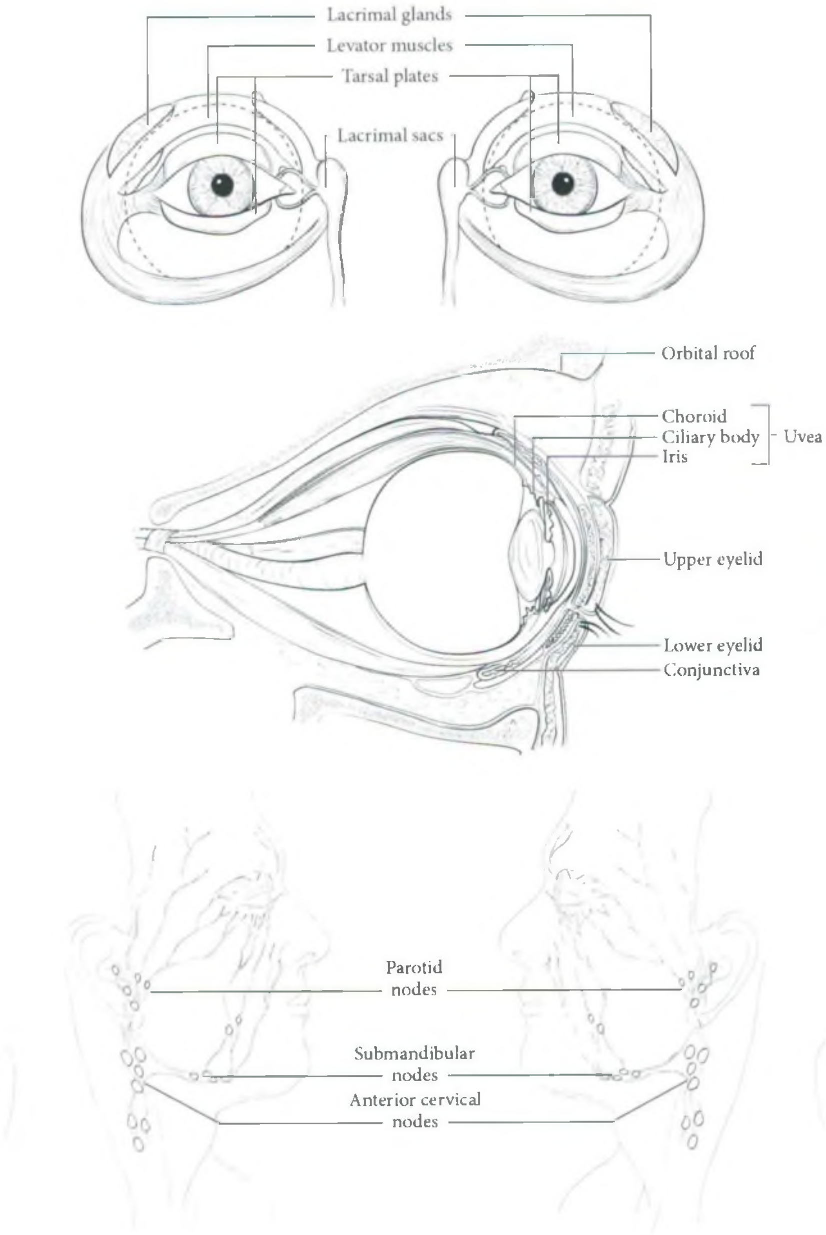

Fig. 66.1 Anatomic sites and regional lymph nodes for ophthalmic sites

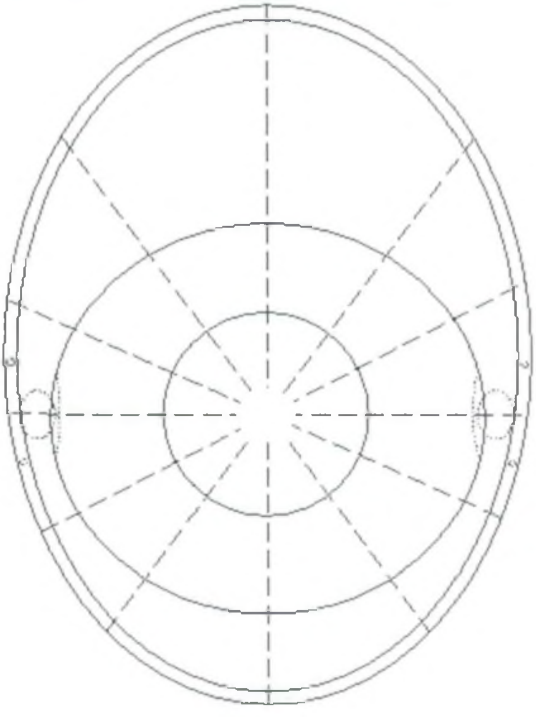

Fig. 66.2 Clinical mapping system for conjunctival melanoma. The map displays the entire conjunctiva as a flat surface, with the central point located at the center of the cornea and concentric regions such as the limbus, bulbar conjunctiva, fornix, palpebral conjunctiva, and eyelid considered progressively more peripheral. Radial lines represent clock hours (Modified from Damato and Coupland).[47]

Bibliography

1. Seregard S. Conjunctival melanoma. *Survey of ophthalmology*. Jan-Feb 1998;42(4):321–350.
2. Brownstein S. Malignant melanoma of the conjunctiva. *Cancer control : journal of the Moffitt Cancer Center*. Sep-Oct 2004;11(5):310–316.
3. Jovanovic P, Mihajlovic M, Djordjevic-Jocic J, Vlajkovic S, Cekic S, Stefanovic V. Ocular melanoma: an overview of the current status. *Int J Clin Exp Pathol*. 2013;6(7):1230–1244.
4. Missotten GS, Keijser S, De Keizer RJ, De Wolff-Rouendaal D. Conjunctival melanoma in the Netherlands: a nationwide study. *Investigative Ophthalmology and Visual Science* Jan 2005;46(1): 75–82.
5. Grossniklaus HE, Green WR, Luckenbach M, Chan CC. Conjunctival lesions in adults. A clinical and histopathologic review. *Cornea*. 1987;6(2):78–116.
6. McLaughlin CC, Wu XC, Jemal A, Martin HJ, Roche LM, Chen VW. Incidence of noncutaneous melanomas in the US. *Cancer*. 2005;103(5):1000–1007.
7. Yu G-P, Hu D-N, McCormick S, Finger PT. Conjunctival melanoma: is it increasing in the United States? *American journal of ophthalmology*. 2003;135(6):800–806.
8. Folberg R, McLean IW, Zimmerman LE. Conjunctival melanosis and melanoma. *Ophthalmology*. Jun 1984;91(6):673–678.
9. Shildkrot Y, Wilson MW. Conjunctival melanoma: pitfalls and dilemmas in management. *Current Opinion in Ophthalmology* Sep 2010;21(5):380–386.
10. Shields CL, Markowitz JS, Belinsky I, et al. Conjunctival melanoma: outcomes based on tumor origin in 382 consecutive cases. *Ophthalmology*. Feb 2011;118(2):389–395 e381–382.
11. Shields CL, Kaliki S, Al-Dahmash SA, Lally SE, Shields JA. American Joint Committee on Cancer (AJCC) clinical classification predicts conjunctival melanoma outcomes. *Ophthalmic plastic and reconstructive surgery*. Sep-Oct 2012;28(5):313–323.
12. Gear H, Williams H, Kemp EG, Roberts F. BRAF mutations in conjunctival melanoma. *Investigative Ophthalmology and Visual Science* Aug 2004;45(8):2484–2488.
13. Lake SL, Jmor F, Dopierala J, Taktak AF, Coupland SE, Damato BE. Multiplex ligation-dependent probe amplification of conjunctival melanoma reveals common BRAF V600E gene mutation and gene copy number changes. *Investigative Ophthalmology and Visual Science* Jul 2011;52(8):5598–5604.
14. Griewank KG, Westekemper H, Murali R, et al. Conjunctival melanomas harbor BRAF and NRAS mutations and copy number changes similar to cutaneous and mucosal melanomas. *Clin Cancer Res*. Jun 15 2013;19(12):3143–3152.
15. Larsen AC, Dahmcke CM, Dahl C, et al. A Retrospective Review of Conjunctival Melanoma Presentation, Treatment, and Outcome and an Investigation of Features Associated With BRAF Mutations. *JAMA ophthalmology*. Nov 2015;133(11):1295–1303.
16. Chapman PB, Hauschild A, Robert C, et al. Improved survival with vemurafenib in melanoma with BRAF V600E mutation. *New England Journal of Medicine* Jun 30 2011;364(26):2507–2516.
17. El-Shabrawi Y, Radner H, Muellner K, Langmann G, Hoefler G. The role of UV-radiation in the development of conjunctival malignant melanoma. *Acta Ophthalmol Scand*. Feb 1999;77(1): 31–32.
18. Beadling C, Jacobson-Dunlop E, Hodi FS, et al. KIT gene mutations and copy number in melanoma subtypes. *Clin Cancer Res*. Nov 1 2008;14(21):6821–6828.
19. Griewank K, Murali R, Schilling B, et al. TERT promoter mutations in ocular melanoma distinguish between conjunctival and uveal tumours. *British journal of cancer*. 2013;109(2):497–501.
20. Finger PT. "Finger-tip" cryotherapy probes: treatment of squamous and melanocytic conjunctival neoplasia. *The British journal of ophthalmology*. Aug 2005;89(8):942–945.
21. Finger PT. Topical mitomycin chemotherapy for malignant conjunctival and corneal neoplasia. *The British journal of ophthalmology*. Jul 2006;90(7):807–809.
22. Damato B, Coupland SE. An audit of conjunctival melanoma treatment in Liverpool. *Eye*. Apr 2009;23(4):801–809.
23. Wong JR, Nanji AA, Galor A, Karp CL. Management of conjunctival malignant melanoma: a review and update. *Expert Review in Ophthalmology* Jun 2014;9(3):185–204.
24. Damato B, Coupland SE. Management of conjunctival melanoma. *Expert review of anticancer therapy*. Sep 2009;9(9):1227–1239.
25. Anastassiou G, Heiligenhaus A, Bechrakis N, Bader E, Bornfeld N, Steuhl KP. Prognostic value of clinical and histopathological parameters in conjunctival melanomas: a retrospective study. *The British journal of ophthalmology*. Feb 2002;86(2):163–167.
26. Tuomaala S, Eskelin S, Tarkkanen A, Kivela T. Population-based assessment of clinical characteristics predicting outcome of conjunctival melanoma in whites. *Investigative Ophthalmology and Visual Science* Nov 2002;43(11):3399–3408.
27. Shields CL, Shields JA, Gündüz K, et al. Conjunctival melanoma: risk factors for recurrence, exenteration, metastasis, and death in

150 consecutive patients. *Archives of ophthalmology.* 2000;118(11): 1497–1507.

28. Ackerman A, Sood R, Koenig M. Primary acquired melanosis of the conjunctiva is melanoma in situ. *Modern pathology: an official journal of the United States and Canadian Academy of Pathology, Inc.* 1991;4(2):253–263.
29. Folberg R, Jakobiec F, McLean I, Zimmerman L. Is primary acquired melanosis of the conjunctiva equivalent to melanoma in situ? *Modern pathology: an official journal of the United States and Canadian Academy of Pathology, Inc.* 1992;5(1):2.
30. Damato B, Coupland SE. Conjunctival melanoma and melanosis: a reappraisal of terminology, classification and staging. *Clinical & experimental ophthalmology.* Nov 2008;36(8):786–795.
31. Chowers I, Livni N, Solomon A, et al. MIB-1 and PC-10 immunostaining for the assessment of proliferative activity in primary acquired melanosis without and with atypia. *British journal of ophthalmology.* 1998;82(11):1316–1319.
32. Shields JA, Shields CL, De Potter P. Surgical management of conjunctival tumors. The 1994 Lynn B. McMahan Lecture. *Archives of ophthalmology.* Jun 1997;115(6):808–815.
33. Shields JA, Shields CL, Gunduz K, Cater J. Clinical features predictive of orbital exenteration for conjunctival melanoma. *Ophthalmic plastic and reconstructive surgery.* May 2000;16(3):173–178.
34. Werschnik C, Lommatzsch PK. Long-term follow-up of patients with conjunctival melanoma. *American journal of clinical oncology.* Jun 2002;25(3):248–255.
35. Heindl LM, Hofmann-Rummelt C, Adler W, et al. Prognostic significance of tumor-associated lymphangiogenesis in malignant melanomas of the conjunctiva. *Ophthalmology.* Dec 2011;118(12): 2351–2360.
36. Lim LA, Madigan MC, Conway RM. Conjunctival melanoma: a review of conceptual and treatment advances. *Clinical ophthalmology.* 2013;6:521–531.
37. Sheng X, Li S, Chi Z, et al. Prognostic factors for conjunctival melanoma: a study in ethnic Chinese patients. *The British journal of ophthalmology.* Jul 2015;99(7):990–996.
38. Yousef YA, Finger PT. Predictive value of the seventh edition American Joint Committee on Cancer staging system for conjunctival melanoma. *Archives of ophthalmology.* May 2012;130(5):599–606.
39. Tuomaala S, Kivela T. Metastatic pattern and survival in disseminated conjunctival melanoma: implications for sentinel lymph node biopsy. *Ophthalmology.* Apr 2004;111(4):816–821.
40. Tuomaala S, Kivela T. Sentinel lymph node biopsy guidelines for conjunctival melanoma. *Melanoma research.* Jun 2008;18(3):235.
41. Nijhawan N, Marriott C, Harvey JT. Lymphatic drainage patterns of the human eyelid: assessed by lymphoscintigraphy. *Ophthalmic plastic and reconstructive surgery.* Jul-Aug 2010;26(4):281–285.
42. Cohen VM, Tsimpida M, Hungerford JL, Jan H, Cerio R, Moir G. Prospective study of sentinel lymph node biopsy for conjunctival melanoma. *The British journal of ophthalmology.* Dec 2013;97(12): 1525–1529.
43. Pfeiffer ML, Savar A, Esmaeli B. Sentinel lymph node biopsy for eyelid and conjunctival tumors: what have we learned in the past decade? *Ophthalmic Plastic & Reconstructive Surgery.* 2013;29(1):57–62.
44. Mendoza PR, Grossniklaus HE. Sentinel Lymph Node Biopsy for Eyelid and Conjunctival Tumors: What is the Evidence? *International ophthalmology clinics.* 2015;55(1):123–136.
45. Esmaeli B, Wang X, Youssef A, Gershenwald JE. Patterns of regional and distant metastasis in patients with conjunctival melanoma: experience at a cancer center over four decades. *Ophthalmology.* 2001;108(11):2101–2105.
46. Esmaeli B, Roberts D, Ross M, et al. Histologic features of conjunctival melanoma predictive of metastasis and death (an American Ophthalmological thesis). *Transactions of the American Ophthalmological Society* Dec 2012;110:64–73.
47. Damato B, Coupland SE. Clinical mapping of conjunctival melanomas. *The British journal of ophthalmology.* Nov 2008;92(11): 1545–1549.
48. Finger PT, Tran HV, Turbin RE, et al. High-frequency ultrasonographic evaluation of conjunctival intraepithelial neoplasia and squamous cell carcinoma. *Archives of ophthalmology.* Feb 2003; 121(2):168–172.
49. Ho VH, Prager TC, Diwan H, Prieto V, Esmaeli B. Ultrasound biomicroscopy for estimation of tumor thickness for conjunctival melanoma. *Journal of clinical ultrasound : JCU.* Nov-Dec 2007;35(9): 533–537.
50. Kurli M, Chin K, Finger PT. Whole-body 18 FDG PET/CT imaging for lymph node and metastatic staging of conjunctival melanoma. *The British journal of ophthalmology.* Apr 2008;92(4):479–482.
51. Nanji AA, Sayyad FE, Galor A, Dubovy S, Karp CL. High-Resolution Optical Coherence Tomography as an Adjunctive Tool in the Diagnosis of Corneal and Conjunctival Pathology. *The Ocular Surface.* 2015.
52. Paridaens AD, McCartney AC, Minassian DC, Hungerford JL. Orbital exenteration in 95 cases of primary conjunctival malignant melanoma. *The British journal of ophthalmology.* Jul 1994;78(7): 520–528.
53. Savar A, Esmaeli B, Ho H, Liu S, Prieto VG. Conjunctival melanoma: local-regional control rates, and impact of high-risk histopathologic features. *J Cutan Pathol.* Jan 2011;38(1):18–24.
54. Kattan MW, Hess KR, Amin MB, et al. American Joint Committee on Cancer acceptance criteria for inclusion of risk models for individualized prognosis in the practice of precision medicine. *CA: a cancer journal for clinicians.* Jan 19 2016.
55. Zbytek B, Carlson JA, Granese J, Ross J, Mihm MC, Jr., Slominski A. Current concepts of metastasis in melanoma. *Expert review of dermatology.* Oct 2008;3(5):569–585.
56. Barnhill RL LS, Lévy-Gabriel C, Rodrigues M, Desjardins L, Dendale R, Vincent-Salomon A, Roman-Roman S, Lugassy C, Cassoux N. . Satellite in Transit Metastases in Rapidly Fatal Conjunctival Melanoma: Implications for Angiotropism and Extravascular Migratory Metastasis (Description of a Murine Model for Conjunctival Melanoma). *Pathology Case Reviews.* 2015.
57. Lugassy C, Zadran S, Bentolila LA, et al. Angiotropism, pericytic mimicry and extravascular migratory metastasis in melanoma: an alternative to intravascular cancer dissemination. *Cancer Microenviron.* Dec 2014;7(3):139–152.

Uveal Melanoma

67

Tero Kivelä, E. Rand Simpson, Hans E. Grossniklaus, Martine J. Jager, Arun D. Singh, José M. Caminal, Anna C. Pavlick, Emma Kujala, Sarah E. Coupland, and Paul T. Finger

CHAPTER SUMMARY

Cancers Staged Using This Staging System

Malignant melanoma of the iris, ciliary body, and choroid

Cancers Not Staged Using This Staging System

These histopathologic types of cancer…	Are staged according to the classification for…	And can be found in chapter…
Cutaneous melanoma metastatic to the iris, ciliary body, and choroid	Melanoma of the skin	47
Secondary intraocular extension of conjunctival melanoma	Conjunctival melanoma	66

Summary of Changes

Change	Details of Change	Level of Evidence
Definition of Primary Tumor (T)	T2a–b: Iris melanomas without secondary glaucoma are assigned to subcategories T2a and T2b so that they may be distinguished from those with glaucoma.	III
Definition of Primary Tumor (T)	New category T2a for iris melanoma: tumor confluent with or extending into the ciliary body but not into the choroid, without secondary glaucoma	III
Definition of Primary Tumor (T)	New category T2b for iris melanoma: tumor confluent with or extending into the ciliary body and choroid, without secondary glaucoma	III
Definition of Primary Tumor (T)	Changed T2c category for iris melanoma: tumor confluent with or extending into the ciliary body, choroid, or both, with secondary glaucoma	III
Definition of Primary Tumor (T)	Deleted T3a for iris melanoma: Subcategory T3a (with secondary glaucoma) was removed because of the very small number of T3 iris melanomas.	III
Definition of Regional Lymph Node (N)	A new N1b subcategory was added for choroidal and ciliary body melanoma.<br>No regional lymph nodes are involved, but tumor deposits exist in the orbit that are not contiguous to the eye with the primary tumor.<br>The AJCC Cancer Staging Manual, 7th Edition did not differentiate between patients with an extrascleral extension, typically adherent to the eye, and those in whom the tumor has spread regionally to orbital areas not contiguous with the eye with the primary tumor	IV

To access the AJCC cancer staging forms, please visit www.cancerstaging.org.

© American Joint Committee on Cancer 2017
M.B. Amin et al. (eds.), *AJCC Cancer Staging Manual, Eighth Edition*, DOI 10.1007/978-3-319-40618-3_67

ICD-O-3 Topography Codes

Code	Description
C69.3	Choroid
C69.4	Ciliary body and iris

WHO Classification of Tumors

Code	Description
8720	Melanoma
8770	Mixed epithelioid and spindle cell melanoma
8771	Epithelioid cell melanoma
8772	Spindle cell melanoma
8773	Spindle cell melanoma, type A
8774	Spindle cell melanoma, type B

International Agency for Research on Cancer, World Health Organization. International Classification of Diseases for Oncology. ICD-O-3-Online. http://codes.iarc.fr/home. Accessed May 15, 2016.

INTRODUCTION

This chapter addresses malignant melanoma of the uvea, the most common intraocular cancer in adults. It predominantly affects white Caucasians and Hispanic people, as compared with Asian and African populations. It is a cancer that shows a high propensity to metastasize hematogenously to the liver. Staging of uveal melanoma is divided in two systems, one for anteriorly located iris melanomas and the other for posteriorly located ciliary body and choroidal melanomas, because these two types differ not only in anatomic location but also in prognosis. Both systems are based on assessment of the anatomic extent of the tumor. The system for ciliary body and choroidal melanoma was revised extensively for the AJCC Cancer Staging Manual, 7th Edition; it is evidence-based and has since been externally validated. Only minor adjustments are introduced in the AJCC Cancer Staging Manual, 8th Edition. Most uveal melanomas are managed conservatively, mainly with radiotherapy, although select large tumors continue to be treated with enucleation.[1] Prognostic biopsies of conservatively treated uveal melanomas that allow analysis of their cytogenetic, gene expression, and molecular genetic features are increasingly common. However, evidence for a long-term association between these characteristics and survival according to the anatomic extent of the tumor is still incomplete.

Uveal melanoma may occur in the iris, ciliary body, or choroid. Of these sites, choroidal melanoma is the most common, with an estimated 8,000 new cases per year. The original AJCC Cancer Staging Manual, 2nd to 5th Edition staging system for uveal melanoma was based on definitions from one epidemiological study.[2] In 2003, in an effort to be relevant to the size categories widely in use at that time, the AJCC Cancer Staging Manual, 6th Edition staging was reconciled with that of the Collaborative Ocular Melanoma Study (COMS).[2] For these editions, there was no foundation of clinical evidence available from which to create an accurate system.

In contrast, the 7th Edition AJCC staging system for uveal melanoma was evidence based; it was empirically derived from a collaborative database of 7,369 patients.[3] The previous T categories, based on cut points of tumor thickness and largest tumor basal diameter, were replaced by redefined T categories based on blocks representing 3×3-mm size fractions (Fig. 67.1). In addition, this edition took into account involvement of the ciliary body and extrascleral tissues, the other predominant and independent clinical predictors of survival in uveal melanoma. For the first time, staging was defined so that categories of anatomic extent with mutually similar survival were assigned to the same stage. Empirical data were insufficient to propose a major revision of the AJCC staging system for iris melanomas.

During the past 5 years, the 7th Edition staging system for choroidal and ciliary body melanomas has been independently validated. Ten-year survival rates for the seven stages—I, IIA–B, IIIA–C, and IV—in the 5,403 patients in the original European dataset used to formulate the stages,[3] and in a later international validation study of 3,217 patients,[4] are clearly distinct between stages and consistent in these two large studies, especially with regard to 5-year survival (Table 67.1). Follow-up was shorter in the validation study, which likely explains the moderate differences in 10-year survival rates, especially in higher-stage categories, which contained fewer patients; however, the confidence intervals overlap. Several single-center studies and series of specific groups of patients, such as children and young adults, also have supported the validity of staging on the basis of anatomic extent.[5–7]

The past decade has seen a surge in cytogenetic and molecular genetic data on uveal melanoma. It is widely agreed that uveal melanomas that show a combination of monosomy 3 and gains in chromosome 8q, which often occur together,[8,9] are associated with the highest risk for systemic metastasis.[10,11] Tumors with either monosomy 3 or gains in chromo-

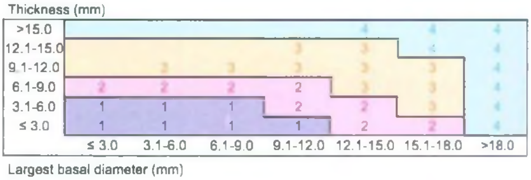

Fig. 67.1 Classification of ciliary body and choroid uveal melanoma based on thickness and diameter

Table 67.1 Stage-specific 5- and 10-year survival rates in the original and validation studies of staging for nonmetastatic primary choroidal and ciliary body melanomas[3,4]

	5-Year survival rate		10-Year survival rate	
Stage	Original study (%)	Validation study (%)	Original study (%)	Validation study (%)
I	96 (94–97)	97 (95–98)	88 (84–91)	94 (91–96)
IIA	89 (87–91)	89 (86–91)	80 (76–83)	84 (80–88)
IIB	81 (78–84)	79 (75–83)	67 (62–71)	70 (62–76)
IIIA	66 (62–70)	67 (59–73)	45 (39–51)	60 (51–68)
IIIB	45 (39–52)	50 (33–65)	27 (19–36)	50 (33–65)
IIIC	26 (13–40)	25 (4–53)	N/A	N/A

N/A, not available because of a very small number of patients surviving; numbers in parentheses are 95% confidence intervals.

some 8q carry intermediate risk, whereas those with disomy 3 carry the lowest risk for metastasis.[10] Other recurrent chromosomal aberrations also have been found in uveal melanomas, and their type and number may modify prognosis.[12] The risk for metastasis from uveal melanoma also may be determined by using gene expression profiling (GEP), which differentiates tumors—for example, into low-risk class 1A, intermediate-risk class 1B, and high-risk class 2.[13,14] More recently, mutations in the *BAP1* gene on chromosome 3 were shown to be associated with a high risk for metastasis. Conversely, a mutation in either *EIF1AX* or *SF3B1* predicts a low risk, and the wild type of these two genes predicts an intermediate risk for metastasis.[15–18] The latter two mutations generally are mutually exclusive with those in *BAP1*.[15–17]

Although it would be ideal to implement cytogenetic, GEP, and molecular genetic prognosticators in the AJCC staging system for uveal melanoma, the aforementioned observations are too recent to allow that with confidence. Specifically, the average follow-up in patients with identified changes is less than 5 years, and the number of patients in many AJCC T size categories is still very small. Preliminary observations suggest that most patients with disomy 3 and probably those with the most favorable GEP class—1A—have a very small risk of metastasis, one that approaches the risk in Stage I or less.[6] Conversely, patients in AJCC Stages IIA to IIIC have increasing proportions of uveal melanomas that show monosomy 3 and chromosome 8 changes, or represent GEP class 2. These patients likely are responsible for most of the variation seen in survival, by anatomic extent, in AJCC Stages IIA to IIIC.[6] The question of how patients with a genetic signature of intermediate risk fit into this scheme is still open. A genetic amendment to the AJCC staging system eventually will reassign patients to stages according to a combination of genetic data and anatomic extent. As in the 7th and 8th Editions, such changes must be evidence based. Time will allow for the emergence of the most reliable and cost-effective genetic biomarkers to be included in the AJCC staging system for uveal melanoma. Lastly, it is important to note that AJCC anatomic staging continues to be invaluable when cytogenetic prognostication is unavailable or not offered to the patient.

ANATOMY

Primary Site(s)

The uveal tract is the middle layer of the eye, situated between the sclera externally and the retina and analogous neuroepithelial tissues internally. The uveal tract is divided into three regions: the iris, ciliary body, and choroid. Uveal melanomas arise most commonly in the choroid, less frequently in the ciliary body, and least often in the iris.[4]

The uveal tract is a highly vascular structure that comprises blood vessels and intervening stroma. The stroma contains variable numbers of melanocytes of neural crest origin, from which uveal melanomas arise. The uvea has no basal membrane comparable with that of the skin to be breached; therefore, uveal melanoma is immediately in contact with blood vessels.[19] Moreover, because there is no traditional lymphatic drainage within the eye and orbit, uveal melanomas metastasize almost exclusively hematogenously. Most uveal melanomas are slow-growing tumors,[20,21] so clinical metastases also may grow slowly, only to appear decades after successful treatment of the primary tumor.[22,23]

Choroidal melanomas extend commonly into the subretinal space through Bruch's membrane, which separates the choroid from the retina. More rarely, they further extend through the retina to reach the vitreous cavity. Choroidal melanomas also may extend through the sclera into the orbit, either by growing along intrascleral blood vessels and nerves or by directly invading the sclera, whereas iris and ciliary body melanomas may extend to the conjunctiva, either along aqueous outflow channels or by direct invasion.[24] Choroidal melanomas occasionally invade the optic nerve by direct extension,[25] and rare retinoinvasive melanomas[26] may invade it by disseminating along the retina or through the vitreous.

Regional Lymph Nodes

In the rare event that uveal melanoma metastasizes to the regional lymph nodes, it occurs after extraocular spread

and invasion of conjunctival or adnexal lymphatics.[27] This category consequently applies only to uveal melanomas with anterior extrascleral extension.

The relevant regional lymph nodes include the following:

- Preauricular (parotid)
- Submandibular
- Cervical

Metastatic Sites

Uveal melanomas metastasize hematogenously to various viscera in about half of all patients, and such dissemination may be delayed for up to three decades.[22] However, the risk of metastasis for an individual patient varies widely based on the clinical, histomorphologic, and genetic features of his or her uveal melanoma. The liver is by far the most common initial site of metastasis in over 90% of patients.[28–30] It often is the only site of clinically detectable metastasis, even at the time of death. Less common, and typically late secondary sites of metastasis include the lung, subcutaneous tissues, bone, and brain.[31–34]

RULES FOR CLASSIFICATION

Clinical Classification

Iris Melanomas

Iris melanomas typically are visible through the cornea. They originate from, and are predominantly located in, this region of the uvea. If less than half the tumor volume is located within the iris and it involves the chamber angle, the tumor likely originated in the ciliary body and should be classified accordingly.

Ciliary body involvement may be evaluated and measured by slit-lamp examination, ophthalmoscopy, gonioscopy, transillumination, and anterior segment optical coherence tomography. However, calipers and anterior segment ultrasound imaging are used for more accurate assessment. Extension through the sclera is evaluated visually before and during surgery and preoperatively with ultrasonography, computed tomography (CT), or magnetic resonance (MR) imaging.

Pigmented iris tumors that demonstrate intrinsic vascularity; measure greater than 3 clock hours, are greater than 1 mm in thickness, and are associated with sentinel vessels, sector cataract, dispersion of melanocytic tumor cells, secondary glaucoma, and extrascleral extension are more likely iris melanomas than benign melanocytic proliferations.[35]

Of iris melanomas, 56% are classified as T1, 34% as T2, 2% as T3, and 1% as T4. Kaplan–Meier estimates of survival at 5 years for categories T1, T2, and higher were about 100%, 80%, and 50%, respectively.[35]

Choroidal and Ciliary Body Melanomas

Choroidal and ciliary body melanomas commonly are diagnosed, measured, and assessed based on clinical examination, including slit-lamp examination, direct and indirect ophthalmoscopy, and ultrasonography. Additional methods, such as anterior segment ultrasound imaging, optical coherence tomography, fundus autofluorescence, standard and wide-field fundus photography, fluorescein and indocyanine green angiography, CT, positron emission tomography (PET)/CT, and MR imaging may enhance the accuracy of appraisal, especially in atypical cases.

The large prospective randomized COMS demonstrated 99.6% accuracy in the clinical diagnosis of medium-sized and large choroidal melanomas. However, smaller melanomas typically exhibit fewer diagnostic characteristics, making them more difficult to diagnose.[36–38] A multitude of clinical findings have been used to predict the risk of growth of small choroidal melanocytic lesions. Choroidal melanocytic tumors with orange pigment, subretinal fluid, and thickness greater than 2 mm should always be referred for evaluation. A low internal reflectivity on ultrasound examination also suggests the diagnosis of uveal melanoma. Currently, small uveal melanocytic lesions often are either observed for growth or biopsied before being defined and treated as uveal melanomas. When a needle aspiration or vitreous cutter biopsy is pursued, a nonmalignant result will not exclude the possibility of uveal melanoma because of potential sampling error, technical error, or tumor heterogeneity.[39,40]

The T categories describe the anatomic extent of choroidal and ciliary body melanomas, expressed as tumor size and involvement of the ciliary body and extrascleral tissues.[3] The category thresholds are defined in a nonrectangular, tabular format to achieve homogeneous survival within each category (Fig. 67.1). Category T4 also includes larger extrascleral extensions. Survival worsens not only with increasing T category,[7] but also with the subcategories within each T category.[3] Staging takes this variation into account and should always be used for reporting survival. Stages I to IIIC are confined to uveal melanoma patients who have no evidence of metastases, either at regional or distant sites, based on clinical, radiologic, and laboratory evaluation. Stage IV indicates metastatic disease or noncontiguous intraorbital invasion. Staging is not implemented for iris melanomas.

Of iris, ciliary body, and choroidal melanomas, 21–32% are classified as Stage I, 32–34% as Stage IIA, 22–23% as

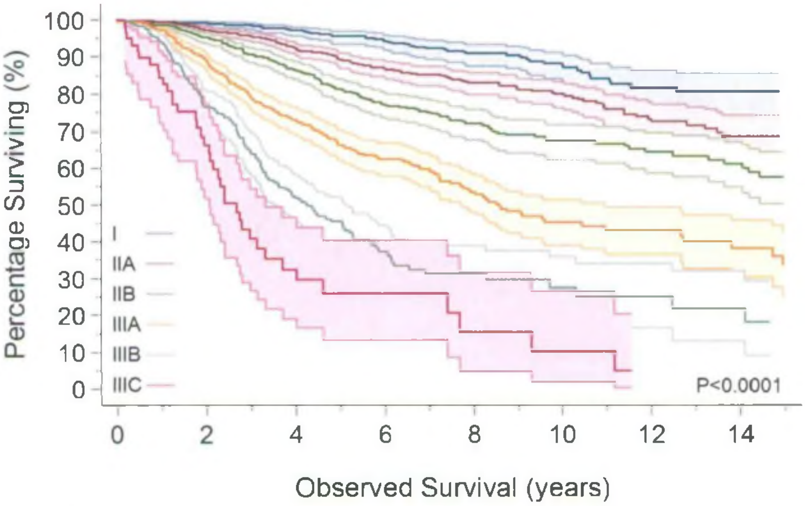

Number at risk

I	1119	721	500	333	222	145	82	47
IIA	1705	1167	720	435	281	175	105	64
IIB	1217	814	488	274	171	123	75	54
IIIA	913	571	303	176	100	53	31	20
IIIB	383	197	93	41	23	12	8	6
IIIC	66	30	11	6	3	2	0	0

Fig. 67.2 Observed melanoma-related overall Kaplan–Meier survival rates for nonmetastatic primary choroidal and ciliary body melanomas[3]

Stage IIB, 9–16% as Stage IIIA, 3–7% as Stage IIIB, 1% as Stage IIIC, and 2–3% as Stage IV.[3,4] Kaplan–Meier stage-specific estimates of survival at 5 and 10 years are shown in Table 67.1, and survival is plotted for 15 years in Fig. 67.2.

Metastases

At the time of diagnosis of the intraocular tumor, lymph node metastases are found in fewer than 1% of patients and systemic metastases in only 2–3% of patients.[41] Liver imaging and chest radiography are recommended to exclude both hepatic metastasis and a nonocular primary tumor metastatic to the uvea.[31,41] Local tumor recurrence is associated with a higher risk of metastasis.[42] Sentinel lymph node biopsy is not practiced.

No international consensus has been reached regarding posttreatment review guidelines and surveillance techniques for early detection of metastatic uveal melanoma. Although national guidelines exist,[43] there is no existing objective evidence that any surveillance improves prognosis.[44] Available evidence includes the COMS finding that physical examination and liver function tests are inadequate,[45] although the latter are useful for excluding diffuse hepatic metastasis. Largely dependent on customs and practice, as well as knowledge that metastases typically present in the liver, it is common to examine patients one to two times per year with abdominal imaging (e.g., ultrasound, MR imaging, and CT).[46] Some eye cancer centers use PET/CT for initial staging and subsequent surveillance of patients at high risk for metastasis, in part because whole-body scanning is more likely to detect nonhepatic metastases (e.g., to skin and bone) as well as second, nonocular primary cancers.[41]

M1 was divided into three subcategories in the previous edition based on the largest diameter of the largest metastasis. This measure has been shown to correlate strongly with survival after diagnosis of metastases. The divisions were based on a European collaborative dataset of 249 patients with metastatic uveal melanoma. Median survival times for subcategories M1a to M1c were 17.5 months, 9.6 months, and 5.0 months, respectively (Fig. 67.3), and they included 47%, 45%, and 8% of patients, respectively. These subcategories are retained in the 8th Edition.

Patients with Stage IV uveal melanoma have clinical or radiologic evidence of regional (N1a–b) or systemic (M1a–c) metastases, or positive diagnostic biopsies of these regional nodes or distant metastases. Suspected orbital invasion, regional lymph node involvement, and systemic metastasis are confirmed by needle biopsy or resection.

Because staging of metastatic uveal melanoma is evolving and depends on several factors in addition to diameter of the largest metastasis, especially liver enzyme levels and performance status,[28,47] no substaging currently is proposed.

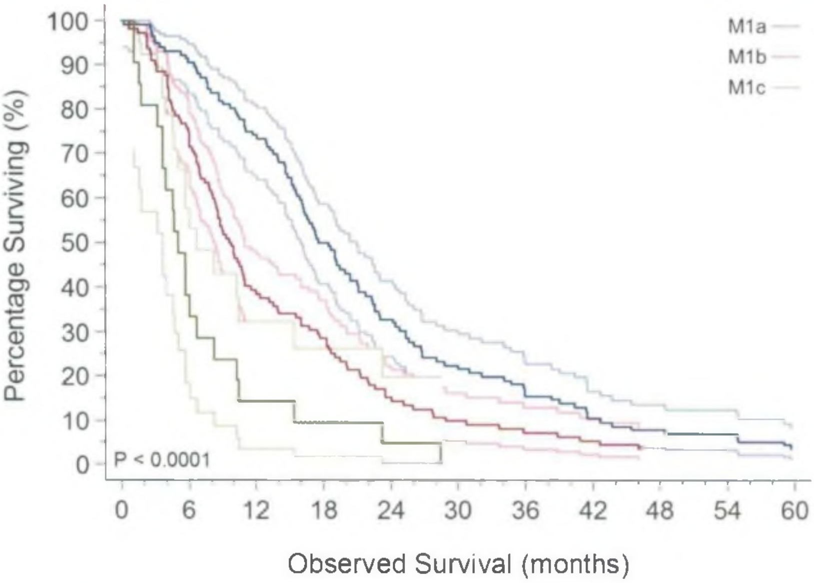

Number at risk

M1a	116	105	86	58	38	26	18	12	9	8	4
M1b	112	84	43	32	17	11	8	6	4	4	4
M1c	21	8	3	2	1	0	0	0	0	0	0

Fig. 67.3 Observed melanoma-related overall Kaplan–Meier survival rates for metastatic primary choroidal and ciliary body melanomas

Pathological Classification

Resection of the primary tumor by iridectomy, iridocyclectomy, local resection, or enucleation is required for complete pathological staging; then, the extent of the tumor measured in clock hours of involvement, basal dimensions, tumor thickness, and margins of resection may be assessed. It is uncommon in current practice to resect small choroidal melanomas because these tumors may be controlled effectively with nonsurgical treatment, such as plaque brachytherapy or proton beam therapy.

The T categories of anatomic extent are the same for clinical and pathological classification. When histopathologic measurements are recorded after fixation, however, tumor diameter and thickness may be underestimated because of tissue shrinkage. On the other hand, assessment of ciliary body involvement and extrascleral extension often is more accurate if a resection specimen is available for histopathologic evaluation.

Suspected orbital invasion, regional lymph node involvement, and systemic metastasis are confirmed by needle biopsy or resection.

Uveal melanomas exhibit marked variation in cytologic composition. They display a spectrum of cell types ranging from slender spindle cells through plump spindle cells to epithelioid cells. Many tumors contain some admixture of these different types.

Today, DNA- or RNA-based genetic profiling and GEP, if available and combined with data on tumor size and histopathology, may enhance accuracy in providing a prognosis for an individual patient as compared with clinical and histopathologic data alone. Consideration should be given to harvesting tumor tissue for this purpose during the histopathologic examination.

Regional lymphadenectomy ordinarily includes six or more regional lymph nodes. Because of the rarity of regional lymph node metastasis, sentinel lymph node biopsy is not practiced.

PROGNOSTIC FACTORS

Prognostic Factors Required for Stage Grouping

Beyond the factors used to assign T, N, or M categories, no additional prognostic factors are required for stage grouping.

Additional Factors Recommended for Clinical Care

Size

The size of uveal melanoma is strongly associated with risk for metastasis (AJCC Level of Evidence: I).[3,4,48,49] Although it generally is accepted that largest basal tumor diameter is the predominant clinical predictor of prognosis, tumor thickness is an independent prognostic indicator, even when ciliary

body involvement and extrascleral extension are taken into account.[3] The largest tumor basal diameter may be estimated in optic disk diameters (DD; average: 1 DD = 1.5 mm), and tumor thickness may be estimated in diopters (average: 2.5 diopters = 1 mm). Evaluation and comparison of fundus photography and ultrasonography provide more accurate measurements.

Location

Intraocular location of a uveal melanoma relative to the ciliary body is independently associated with metastatic risk (AJCC Level of Evidence: I).[3,50] Ciliary body involvement is evaluated by slit-lamp examination, ophthalmoscopy, gonioscopy, transillumination, and anterior segment optical coherence tomography. Anterior segment ultrasound imaging provides a more accurate assessment. Tumors confined to the iris carry the most favorable prognosis, followed by those confined in the choroid; ciliary body involvement carries the least favorable prognosis.

Extraocular Extension

The presence of extraocular extension is independently associated with higher metastatic risk (AJCC Level of Evidence: II).[3,51] Anterior extension through the sclera is evaluated visually, measured, and photographed. Posterior extension may be measured with ultrasonography, CT, or MR imaging.

Cell Type

Cell type is independently associated with metastatic risk (AJCC Level of Evidence: I).[49,52] Spindle cells have ovoid nuclei and tend to grow in a compact cohesive fashion. Epithelioid cells are larger, more irregularly contoured, and pleomorphic, and contain abundant, typically eosinophilic cytoplasm. Their nuclei and nucleoli are larger, and they grow less cohesively than spindle cells. No consensus has been reached regarding what proportions of spindle and epithelioid cells qualify a uveal melanoma as being of mixed and epithelioid type. Some ophthalmic pathologists primarily record the presence or absence of epithelioid cells and do not try to subclassify uveal melanomas with epithelioid cells into mixed or epithelioid categories. Spindle cell melanomas predict the longest and epithelioid cell melanomas the shortest survival times.

Chromosomal Analysis

Monosomy 3, especially if combined with a frequently coexisting gain in chromosome 8q, is independently associated with metastatic risk (AJCC Level of Evidence: II).[10,11,18,51,53–56] The clinical prognostic significance of other recurring chromosomal changes is not as firmly established. Chromosome 3 and 8 status may be determined with karyotyping or fluorescent *in situ* hybridization, but these studies do not identify isodisomy 3. Array comparative genomic hybridization, DNA polymorphism analysis (e.g., single-nucleotide polymorphism and microsatellite), and multiplex ligation-dependent probe amplification provide more reliable results. Droplet polymerase chain reaction allows copy number assessment of small tissue samples. Disomy 3 predicts the longest (partial) survival, monosomy 3 or chromosome 8q gain alone predicts an intermediate survival, and the combination of monosomy 3 and chromosome 8q gain predicts the shortest survival time. However, determination of chromosome status may be affected by prior irradiation.[57]

Gene Expression Profiling

GEP class 2 (high grade) or equivalent is independently associated with higher metastatic risk (AJCC Level of Evidence: II).[13,14] The profile is determined by using RNA extracted from the tumor and a gene chip. Class 1A predicts the longest survival, class 1B an intermediate survival, and class 2 the shortest survival time.

Mitotic Count

Mitotic count is independently associated with metastatic risk (AJCC Level of Evidence: II).[55] The number of mitotic figures is counted under light microscopy per 40 high-power fields (HPF; 40×), which typically have a field area of 0.15–0.19 mm^2. The College of American Pathologists Protocol for the Examination of Specimens from Patients with Uveal Melanoma (www.cap.org) recommends using a field area of 0.152 mm^2 for standardization of reporting. Higher counts are associated with shorter survival. Mitotic cells also may be identified by immunohistochemical methods.[58]

Extravascular Matrix Loops and Networks

The presence of certain types of extravascular matrix patterns is independently associated with risk of metastasis (AJCC Level of Evidence: II).[52,59,60] This is documented conclusively for individual loops and for loops forming networks consisting of at least three back-to-back loops. The patterns are assessed with light microscopy under a dark green filter after staining with periodic acid–Schiff stain without counterstain. Absence of both loops and networks is associated with the longest survival and presence of loops forming networks is associated with the shortest survival time.

Microvascular Density

Microvascular density is independently associated with metastatic risk (AJCC Level of Evidence: II).[61] The number of immunopositive elements is labeled with a marker for vascular endothelial cells (e.g., CD34 epitope, CD31 epitope, factor VIII–related antigen) and counted from areas of densest vascularization (typical field area, 0.31 mm^2). Higher counts are associated with shorter survival.

Tumor-infiltrating Macrophages

The number of tumor-infiltrating macrophages is independently associated with metastatic risk (AJCC Level of Evidence: II).[54,62,63] The number of immunopositive elements labeled with a marker for macrophages (e.g., CD68 epitope, CD163 epitope) is semiquantitatively graded as few, moderate, or many, for example, against published standard photographs.[62] Higher numbers are associated with shorter survival.

RISK ASSESSMENT MODELS

The AJCC recently established guidelines that will be used to evaluate published statistical prediction models for the purpose of granting endorsement for clinical use.[64] Although this is a monumental step toward the goal of precision medicine, this work was published only very recently. Therefore, the existing models that have been published or may be in clinical use have not yet been evaluated for this cancer site by the Precision Medicine Core of the AJCC. In the future, the statistical prediction models for this cancer site will be evaluated, and those that meet all AJCC criteria will be endorsed.

DEFINITIONS OF AJCC TNM

Definition of Primary Tumor (T)

Iris Melanomas

T Category	T Criteria
T1	Tumor limited to the iris
T1a	Tumor limited to the iris, not more than 3 clock hours in size
T1b	Tumor limited to the iris, more than 3 clock hours in size
T1c	Tumor limited to the iris with secondary glaucoma
T2	Tumor confluent with or extending into the ciliary body, choroid, or both
T2a	Tumor confluent with or extending into the ciliary body, without secondary glaucoma
T2b	Tumor confluent with or extending into the ciliary body and choroid, without secondary glaucoma
T2c	Tumor confluent with or extending into the ciliary body, choroid, or both, with secondary glaucoma
T3	Tumor confluent with or extending into the ciliary body, choroid, or both, with scleral extension
T4	Tumor with extrascleral extension
T4a	Tumor with extrascleral extension ≤5 mm in largest diameter
T4b	Tumor with extrascleral extension >5 mm in largest diameter

Note: Iris melanomas originate from, and are predominantly located in, this region of the uvea. If less than half the tumor volume is located within the iris, the tumor may have originated in the ciliary body, and consideration should be given to classifying it accordingly.

Choroidal and Ciliary Body Melanomas

T Category	T Criteria
T1	Tumor size category 1
T1a	Tumor size category 1 without ciliary body involvement and extraocular extension
T1b	Tumor size category 1 with ciliary body involvement
T1c	Tumor size category 1 without ciliary body involvement but with extraocular extension ≤5 mm in largest diameter
T1d	Tumor size category 1 with ciliary body involvement and extraocular extension ≤5 mm in largest diameter
T2	Tumor size category 2
T2a	Tumor size category 2 without ciliary body involvement and extraocular extension
T2b	Tumor size category 2 with ciliary body involvement
T2c	Tumor size category 2 without ciliary body involvement but with extraocular extension ≤5 mm in largest diameter
T2d	Tumor size category 2 with ciliary body involvement and extraocular extension ≤5 mm in largest diameter
T3	Tumor size category 3
T3a	Tumor size category 3 without ciliary body involvement and extraocular extension
T3b	Tumor size category 3 with ciliary body involvement
T3c	Tumor size category 3 without ciliary body involvement but with extraocular extension ≤5 mm in largest diameter
T3d	Tumor size category 3 with ciliary body involvement and extraocular extension ≤5 mm in largest diameter
T4	Tumor size category 4
T4a	Tumor size category 4 without ciliary body involvement and extraocular extension
T4b	Tumor size category 4 with ciliary body involvement
T4c	Tumor size category 4 without ciliary body involvement but with extraocular extension ≤5 mm in largest diameter
T4d	Tumor size category 4 with ciliary body involvement and extraocular extension ≤5 mm in largest diameter
T4e	Any tumor size category with extraocular extension >5 mm in largest diameter

Notes:
1. Primary ciliary body and choroidal melanomas are classified according to the four tumor size categories defined in Fig. 67.1
2. In clinical practice, the largest tumor basal diameter may be estimated in optic disc diameters (DD; average: 1 DD = 1.5 mm), and tumor thickness may be estimated in diopters (average: 2.5 diopters = 1 mm). Ultrasonography and fundus photography are used to provide more accurate measurements.
3. When histopathologic measurements are recorded after fixation, tumor diameter and thickness may be underestimated because of tissue shrinkage.

Definition of Regional Lymph Node (N)

N Category	N Criteria
N1	Regional lymph node metastases or discrete tumor deposits in the orbit
N1a	Metastasis in one or more regional lymph node(s)

(continued)

N Category	N Criteria
N1b	No regional lymph nodes are positive, but there are discrete tumor deposits in the orbit that are not contiguous to the eye.

Definition of Distant Metastasis (M)

M Category	M Criteria
M0	No distant metastasis by clinical classification
M1	Distant metastasis
M1a	Largest diameter of the largest metastasis ≤3.0 cm
M1b	Largest diameter of the largest metastasis 3.1–8.0 cm
M1c	Largest diameter of the largest metastasis ≥8.1 cm

AJCC PROGNOSTIC STAGE GROUPS

Choroidal and Ciliary Body Melanomas

When T is…	And N is…	And M is…	Then the stage group is…
T1a	N0	M0	I
T1b–d	N0	M0	IIA
T2a	N0	M0	IIA
T2b	N0	M0	IIB
T3a	N0	M0	IIB
T2c–d	N0	M0	IIIA
T3b–c	N0	M0	IIIA
T4a	N0	M0	IIIA
T3d	N0	M0	IIIB
T4b–c	N0	M0	IIIB
T4d–e	N0	M0	IIIC
Any T	N1	M0	IV
Any T	Any N	M1a–c	IV

REGISTRY DATA COLLECTION VARIABLES

1. Tumor site: ciliary body or iris (ICD code lacks specificity)
2. Largest basal diameter and thickness of tumor
3. Ciliary body involvement
4. Extraocular extension
5. Histologic type
6. Chromosome 3 and 8 loss or gain
7. Gene expression profile
8. Mitotic count (number of mitoses per 40 HPF, determined by using a 40× objective with a field area of 0.152 mm^2)
9. Extravascular matrix patterns (extracellular closed loops and networks, defined as at least three back-to-back closed loops, is associated with death from metastatic disease)
10. Microvascular density

HISTOLOGIC GRADE (G)

G	G Definition
GX	Grade cannot be assessed
G1	Spindle cell melanoma (>90% spindle cells)
G2	Mixed cell melanoma (>10% epithelioid cells and <90% spindle cells)
G3	Epithelioid cell melanoma (>90% epithelioid cells)

Note: Because of the lack of universal agreement regarding which proportion of epithelioid cells classifies a tumor as mixed or epithelioid, some ophthalmic pathologists currently combine grades 2 and 3 (non-spindle, i.e. epithelioid cells detected) and contrast them with grade 1 (spindle, i.e. no epithelioid cells detected).

HISTOPATHOLOGIC TYPE

Mixed epithelioid and spindle cell melanoma
Epithelioid cell melanoma
Spindle cell melanoma
Spindle cell melanoma, type A
Spindle cell melanoma, type B

67

ILLUSTRATIONS

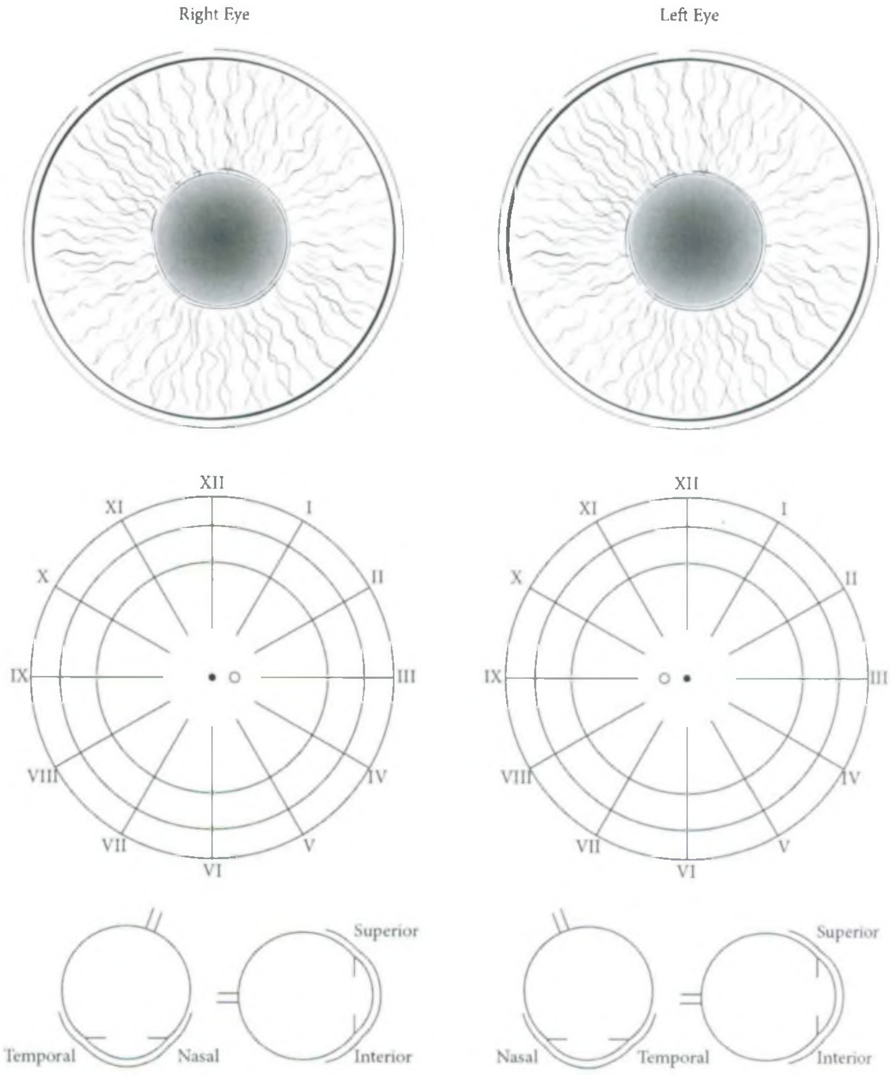

Fig. 67.4 Uveal melanoma staging diagram

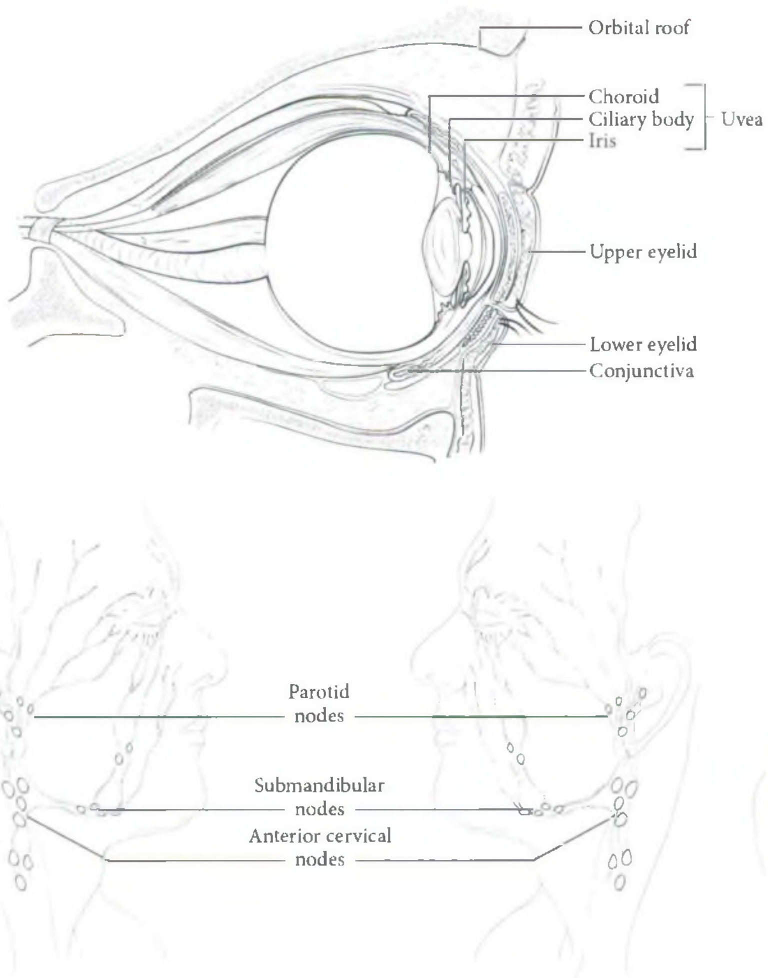

Fig. 67.5 Anatomic sites and regional lymph nodes for ophthalmic sites

67

Bibliography

1. American Brachytherapy Society – Ophthalmic Oncology Task Force. The American Brachytherapy Society consensus guidelines for plaque brachytherapy of uveal melanoma and retinoblastoma. *Brachytherapy.* Jan-Feb 2014;13(1):1–14.
2. Kujala E, Kivelä T. Tumor, node, metastasis classification of malignant ciliary body and choroidal melanoma evaluation of the 6th edition and future directions. *Ophthalmology.* Jun 2005;112(6):1135–1144.
3. Kujala E, Damato B, Coupland SE, et al. Staging of ciliary body and choroidal melanomas based on anatomic extent. *J Clin Oncol.* Aug 1 2013;31(22):2825–2831.
4. AJCC Ophthalmic Oncology Task Force. International validation of the American Joint Committee on Cancer's 7th Edition classification of uveal melanoma. *JAMA Ophthalmology.* Apr 2015;133(4):376–383.
5. Al-Jamal RT, Cassoux N, Desjardins L, et al. The Pediatric Choroidal and Ciliary Body Melanoma Study: A survey by the European Ophthalmic Oncology Group. *Ophthalmology.* Apr 2016;123(4):898–907.
6. Bagger M, Andersen MT, Andersen KK, Heegaard S, Andersen MK, Kiilgaard JF. The prognostic effect of American Joint Committee on Cancer staging and genetic status in patients with choroidal and ciliary body melanoma. *Invest Ophthalmol Vis Sci.* Jan 2015;56(1):438–444.
7. Shields CL, Kaliki S, Furuta M, Fulco E, Alarcon C, Shields JA. American Joint Committee on Cancer classification of uveal melanoma (anatomic stage) predicts prognosis in 7,731 patients: The 2013 Zimmerman Lecture. *Ophthalmology.* Jun 2015;122(6):1180–1186.
8. Hammond DW, Al-Shammari NS, Danson S, Jacques R, Rennie IG, Sisley K. High-resolution array CGH analysis identifies regional deletions and amplifications of chromosome 8 in uveal melanoma. *Invest Ophthalmol Vis Sci.* Jun 2015;56(6):3460–3466.
9. Sisley K, Rennie IG, Parsons MA, et al. Abnormalities of chromosomes 3 and 8 in posterior uveal melanoma correlate with prognosis. *Genes Chromosomes Cancer.* May 1997;19(1):22–28.
10. Caines R, Eleuteri A, Kalirai H, et al. Cluster analysis of multiplex ligation-dependent probe amplification data in choroidal melanoma. *Mol Vis.* 2015;21:1–11.
11. Cassoux N, Rodrigues MJ, Plancher C, et al. Genome-wide profiling is a clinically relevant and affordable prognostic test in

posterior uveal melanoma. *Br J Ophthalmol.* Jun 2014;98(6): 769–774.

12. van Engen-van Grunsven AC, Baar MP, Pfundt R, et al. Whole-genome copy-number analysis identifies new leads for chromosomal aberrations involved in the oncogenesis and metastastic behavior of uveal melanomas. *Melanoma Res.* Jun 2015;25(3):200–209.
13. Onken MD, Worley LA, Char DH, et al. Collaborative Ocular Oncology Group report number 1: prospective validation of a multi-gene prognostic assay in uveal melanoma. *Ophthalmology.* Aug 2012;119(8):1596–1603.
14. Onken MD, Worley LA, Ehlers JP, Harbour JW. Gene expression profiling in uveal melanoma reveals two molecular classes and predicts metastatic death. *Cancer Res.* Oct 2004;64(20):7205–7209.
15. Harbour JW, Onken MD, Roberson ED, et al. Frequent mutation of BAP1 in metastasizing uveal melanomas. *Science.* Dec 2010;330(6009):1410–1413.
16. Martin M, Masshofer L, Temming P, et al. Exome sequencing identifies recurrent somatic mutations in EIF1AX and SF3B1 in uveal melanoma with disomy 3. *Nat Genet.* Aug 2013;45(8):933–936.
17. Harbour JW, Roberson ED, Anbunathan H, Onken MD, Worley LA, Bowcock AM. Recurrent mutations at codon 625 of the splicing factor SF3B1 in uveal melanoma. *Nat Genet.* Feb 2013;45(2):133–135.
18. Ewens KG, Kanetsky PA, Richards-Yutz J, et al. Chromosome 3 status combined with BAP1 and EIF1AX mutation profiles are associated with metastasis in uveal melanoma. *Invest Ophthalmol Vis Sci.* Aug 2014;55(8):5160–5167.
19. Folberg R. Tumor progression in ocular melanomas. *J Invest Dermatol.* Mar 1993;100(3):326S–331S.
20. Char DH, Kroll S, Phillips TL. Uveal melanoma. Growth rate and prognosis. *Arch Ophthalmol.* Aug 1997;115(8):1014–1018.
21. Augsburger JJ, Gonder JR, Amsel J, Shields JA, Donoso LA. Growth rates and doubling times of posterior uveal melanomas. *Ophthalmology.* Dec 1984;91(12):1709–1715.
22. Kujala E, Mäkitie T, Kivelä T. Very long-term prognosis of patients with malignant uveal melanoma. *Invest Ophthalmol Vis Sci.* Nov 2003;44(11):4651–4659.
23. Eskelin S, Pyrhönen S, Summanen P, Hahka-Kemppinen M, Kivelä T. Tumor doubling times in metastatic malignant melanoma of the uvea: tumor progression before and after treatment. *Ophthalmology.* Aug 2000;107(8):1443–1449.
24. Coupland SE, Campbell I, Damato B. Routes of extraocular extension of uveal melanoma: risk factors and influence on survival probability. *Ophthalmology.* Oct 2008;115(10):1778–1785.
25. Lindegaard J, Isager P, Prause JU, Heegaard S. Optic nerve invasion of uveal melanoma. *APMIS.* Jan 2007;115(1):1–16.
26. Kivelä T, Summanen P. Retinoinvasive malignant melanoma of the uvea. *Br J Ophthalmol.* Aug 1997;81(8):691–697.
27. Tojo D, Wenig BL, Resnick KI. Incidence of cervical metastasis from uveal melanoma: implications for treatment. *Head Neck.* Mar-Apr 1995;17(2):137–139.
28. Eskelin S, Pyrhönen S, Hahka-Kemppinen M, Tuomaala S, Kivelä T. A prognostic model and staging for metastatic uveal melanoma. *Cancer.* Jan 2003;97(2):465–475.
29. Collaborative Ocular Melanoma Study Group. Assessment of metastatic disease status at death in 435 patients with large choroidal melanoma in the Collaborative Ocular Melanoma Study (COMS): COMS report no. 15. *Archives of ophthalmology.* May 2001;119(5):670–676.
30. Rietschel P, Panageas KS, Hanlon C, Patel A, Abramson DH, Chapman PB. Variates of survival in metastatic uveal melanoma. *J Clin Oncol.* Nov 2005;23(31):8076–8080.
31. Eskelin S, Summanen P, Pyrhönen S, Tarkkanen A, Kivelä T. Screening for metastatic uveal melanoma revisited. *Cancer.* Mar 1999;85:1151–1159.
32. Diener-West M, Reynolds SM, Agugliaro DJ, et al. Development of metastatic disease after enrollment in the COMS trials for treatment of choroidal melanoma: Collaborative Ocular Melanoma Study Group Report No. 26. *Arch Ophthalmol.* Dec 2005;123(12):1639–1643.
33. Lorigan JG, Wallace S, Mavligit GM. The prevalence and location of metastases from ocular melanoma: imaging study in 110 patients. *AJR Am J Roentgenol.* Dec 1991;157(6):1279–1281.
34. Cerbone L, van Ginderdeuren R, van den Oord J, et al. Clinical presentation, pathological features and natural course of metastatic uveal melanoma, an orphan and commonly fatal disease. *Oncology.* 2014;86(3):185–189.
35. Khan S, Finger PT, Yu GP, et al. Clinical and pathologic characteristics of biopsy-proven iris melanoma: a multicenter international study. *Arch Ophthalmol.* Jan 2012;130(1):57–64.
36. Finger PT. Ocular Malignancies: Choroidal Melanoma, Retinoblastoma, Ocular Adnexal Lymphoma and Eyelid Cancer In: O'Sullivan B, Brierley J, D'Cruz AK, et al., eds. *UICC Manual of Clinical Oncology* 9th ed. Oxford, United Kingdom: Wiley Blackwell; 2015:726–744.
37. Finger PT. Intraocular Melanoma. In: DeVita JVT, Lawrence TS, Rosenberg SA, eds. *Cancer: Principles and Practice of Oncology.* 10th ed. Philadelphia, PA: Wolters Kluwer, Lippincott, Williams and Wilkins; 2014:1770–1779.
38. Kivelä T. Diagnosis of uveal melanoma. *Developments in ophthalmology.* 2012;49:1–15.
39. Shields JA, Shields CL, Ehya H, Eagle RC, Jr., De Potter P. Fine-needle aspiration biopsy of suspected intraocular tumors. The 1992 Urwick Lecture. *Ophthalmology.* Nov 1993;100(11):1677–1684.
40. Bagger M, Andersen MT, Heegaard S, Andersen MK, Kiilgaard JF. Transvitreal retinochoroidal biopsy provides a representative sample from choroidal melanoma for detection of chromosome 3 aberrations. *Invest Ophthalmol Vis Sci.* Sep 2015;56(10): 5917–5924.
41. Freton A, Chin KJ, Raut R, Tena LB, Kivelä T, Finger PT. Initial PET/CT staging for choroidal melanoma: AJCC correlation and second nonocular primaries in 333 patients. *European journal of ophthalmology.* Mar-Apr 2012;22(2):236–243.
42. The Ophthalmic Oncology Task Force. Local recurrence significantly increases the risk of metastatic uveal melanoma. *Ophthalmology.* Jan 2016;123(1):86–91.
43. Nathan P, Cohen V, Coupland S, et al. Uveal Melanoma UK National Guidelines. *Eur J Cancer.* Nov 2015;51(16):2404–2412.
44. Augsburger JJ, Correa ZM, Trichopoulos N. Surveillance testing for metastasis from primary uveal melanoma and effect on patient survival. *Am J Ophthalmol.* Jul 2011; 152(1):5–9 e1.
45. Diener-West M, Reynolds SM, Agugliaro DJ, et al. Screening for metastasis from choroidal melanoma: the Collaborative Ocular Melanoma Study Group Report 23. *J Clin Oncol.* Jun 2004;22(12):2438–2444.
46. Gomez D, Wetherill C, Cheong J, et al. The Liverpool uveal melanoma liver metastases pathway: outcome following liver resection. *J Surg Oncol.* May 2014;109(6):542–547.
47. Valpione S, Moser JC, Parrozzani R, et al. Development and external validation of a prognostic nomogram for metastatic uveal melanoma. *PloS one.* 2015;10(3):e0120181.
48. Shields CL, Furuta M, Thangappan A, et al. Metastasis of uveal melanoma millimeter-by-millimeter in 8033 consecutive eyes. *Arch Ophthalmol.* Aug 2009;127(8):989–998.
49. Damato B, Coupland SE. A reappraisal of the significance of largest basal diameter of posterior uveal melanoma. *Eye.* Dec 2009;23(12):2152–2160.
50. Li W, Gragoudas ES, Egan KM. Metastatic melanoma death rates by anatomic site after proton beam irradiation for uveal melanoma. *Arch Ophthalmol.* Aug 2000;118(8):1066–1070.

51. van Beek JG, Koopmans AE, Vaarwater J, et al. The prognostic value of extraocular extension in relation to monosomy 3 and gain of chromosome 8q in uveal melanoma. *Invest Ophthalmol Vis Sci.* Mar 2014;55(3):1284–1291.
52. Mäkitie T, Summanen P, Tarkkanen A, Kivelä T. Microvascular loops and networks as prognostic indicators in choroidal and ciliary body melanomas. *J Natl Cancer Inst.* Feb 1999;91(4):359–367.
53. Shields CL, Ganguly A, Bianciotto CG, Turaka K, Tavallali A, Shields JA. Prognosis of uveal melanoma in 500 cases using genetic testing of fine-needle aspiration biopsy specimens. *Ophthalmology.* Feb 2011;118(2):396–401.
54. Maat W, Ly LV, Jordanova ES, de Wolff-Rouendaal D, Schalij-Delfos NE, Jager MJ. Monosomy of chromosome 3 and an inflammatory phenotype occur together in uveal melanoma. *Invest Ophthalmol Vis Sci.* Feb 2008;49(2):505–510.
55. Damato B, Dopierala JA, Coupland SE. Genotypic profiling of 452 choroidal melanomas with multiplex ligation-dependent probe amplification. *Clin Cancer Res.* Dec 2010;16(24):6083–6092.
56. Versluis M, de Lange MJ, van Pelt SI, et al. Digital PCR validates 8q dosage as prognostic tool in uveal melanoma. *PloS one.* 2015;10(3):e0116371.
57. Dogrusoz M, Kroes WG, van Duinen SG, et al. Radiation treatment affects chromosome testing in uveal melanoma. *Invest Ophthalmol Vis Sci.* Sep 2015;56(10):5956–5964.
58. Angi M, Damato B, Kalirai H, Dodson A, Taktak A, Coupland SE. Immunohistochemical assessment of mitotic count in uveal melanoma. *Acta Ophthalmol.* Mar 2011;89(2):e155–160.
59. Mäkitie T, Summanen P, Tarkkanen A, Kivelä T. Microvascular density in predicting survival of patients with choroidal and ciliary body melanoma. *Invest Ophthalmol Vis Sci.* Oct 1999;40(11):2471–2480.
60. Foss AJ, Alexander RA, Jefferies LW, Hungerford JL, Harris AL, Lightman S. Microvessel count predicts survival in uveal melanoma. *Cancer Res.* Jul 1996;56(13):2900–2903.
61. Chen X, Maniotis AJ, Majumdar D, Pe'er J, Folberg R. Uveal melanoma cell staining for CD34 and assessment of tumor vascularity. *Invest Ophthalmol Vis Sci.* Aug 2002;43(8):2533–2539.
62. Mäkitie T, Summanen P, Tarkkanen A, Kivelä T. Tumor-infiltrating macrophages (CD68(+) cells) and prognosis in malignant uveal melanoma. *Invest Ophthalmol Vis Sci.* Jun 2001;42(7):1414–1421.
63. Bronkhorst IH, Ly LV, Jordanova ES, et al. Detection of M2-macrophages in uveal melanoma and relation with survival. *Invest Ophthalmol Vis Sci.* Feb 2011;52(2):643–650.
64. Kattan MW, Hess KR, Amin MB, et al. American Joint Committee on Cancer acceptance criteria for inclusion of risk models for individualized prognosis in the practice of precision medicine. *CA Cancer J Clin.* Jan 19 2016.

Retinoblastoma

68

Ashwin C. Mallipatna, Brenda L. Gallie, Patricia Chévez-Barrios, Livia Lumbroso-Le Rouic, Guillermo L. Chantada, François Doz, Hervé J. Brisse, Francis L. Munier, Daniel M. Albert, Jaume Català-Mora, Laurence Desjardins, Shigenobu Suzuki, William L. Carroll, Sarah E. Coupland, and Paul T. Finger

CHAPTER SUMMARY

Cancers Staged Using This Staging System

Retinoblastoma

Cancers Not Staged Using This Staging System

These histopathologic types of cancer …	Are staged according to the classification for …	And can be found in chapter …
Central nervous system component of "trilateral retinoblastoma"	Brain and spinal cord	72
Retinoma (or retinocytoma)	No AJCC staging system	N/A
Medulloepithelioma	No AJCC staging system	N/A

Summary of Changes

Change	Details of Change	Level of Evidence
Definition of Primary Tumor (T)	Changes to the definitions of cT1 to cT4	II
Definition of Primary Tumor (T)	Changes to the definitions of pT1 to pT4	I
Definition of Regional Lymph Node (N)	Changes to the definition of N1	II
Definition of Distant Metastasis (M)	Changes to the definition of M1	II
Definition of Heritable Trait (H)	Introduction of the definition of H1 to indicate a heritable trait	I
AJCC Prognostic Stage Groups	Introduction of stage groups for clinical and pathological staging based on cT1–cT4 and pT1–pT4	II

ICD-O-3 Topography Codes

Code	Description
C69.2	Retina

WHO Classification of Tumors

Code	Description
9510	Retinoblastoma, NOS
9511	Retinoblastoma, differentiated

To access the AJCC cancer staging forms, please visit www.cancerstaging.org.

© American Joint Committee on Cancer 2017

M.B. Amin et al. (eds.), *AJCC Cancer Staging Manual, Eighth Edition*, DOI 10.1007/978-3-319-40618-3_68

Code	Description
9512	Retinoblastoma, undifferentiated
9513	Retinoblastoma, diffuse

International Agency for Research on Cancer, World Health Organization. International Classification of Diseases for Oncology. ICD-O-3-Online. http://codes.iarc.fr/home. Accessed May 15, 2016.

INTRODUCTION

Retinoblastoma is a childhood cancer that arises in the developing retina when there is a defect in both alleles of the *RB1* gene. Retinoblastoma classifications changed as the primary treatment evolved from radiation to combinations of chemotherapy and focal therapy. Further, management protocols are changing rapidly to include more localized chemotherapy and new protocols for treating extraocular disease. Most treatment protocols are awaiting multicenter clinical trials.

A universal classification system is needed to standardize accrual and facilitate outcome analysis for multicenter trials. Currently used classifications are based on the authors' experience or small group consensus, and they have not been validated or universally accepted.

The retinoblastoma chapter of the AJCC Cancer Staging Manual, 8th Edition is based on outcome evidence from previous retinoblastoma classification systems, current literature, and deliberations and consensus of the AJCC Retinoblastoma Committee. The Ophthalmic Oncology Task Force (OOTF) International Survey of 1,728 eyes with retinoblastoma that were diagnosed up to the end of 2011 (Fig. 68.1), provides additional evidence for retinoblastoma staging for the 8th Edition.

The evidence and international consensus behind the 8th Edition AJCC staging system leads to optimism that it will be the universally accepted standard to define the extent of disease at diagnosis and to predict eye survival, metastatic risk, and patient survival. This staging system will provide a foundation to empower retinoblastoma research and prospective multicenter studies.

Retinoblastoma is a rare and unique cancer arising in the immature retina. It is the most common childhood intraocular cancer, with India and China having the most new cases per year because of their population growth rate.[1] Retinoblastoma tumors are caused by the inactivation of both alleles of the *RB1* gene, a tumor suppressor located at chromosome 13q14.2.[2] Both copies of the *RB1* gene are impaired in the tumor, whereas one copy of the gene is mutant in blood of patients with a germline predisposition to the tumor.

This is a cancer for which survival of all affected children is within reach. The earlier the children are diagnosed and treated, the better the outcomes. Initially, tumors tend to remain intraocular, and the most common presenting sign is a white glow from the tumor through the pupil of the eye, termed *leukocoria*. Various social and cultural factors, however, may lead to a delay in diagnosis and affect children's survival. These factors are complicated to address in clinical staging systems.[3] If diagnosis is timely (i.e., if the general public and health workers are aware) and treatment is prompt (with access to expert care), eye and life salvage are achievable.[1] Therefore, cancer staging for retinoblastoma must include the features that define the extent of intraocular tumor that may be used to predict ocular salvage and the risk of extraocular extension—T category—for each eye. Retinoblastoma staging also requires the standard node (N) and metastasis (M) categories. Each eye may have a different extent of intraocular tumor, scored separately, but for overall staging, the T category of the worst eye is counted in the overall TNM stage.

Research and clinical care in retinoblastoma have lagged behind those in other pediatric cancers, in part because of the multiple intraocular staging systems used worldwide. For example, the first staging system successfully predicted eye salvage after external beam radiation therapy.[4] However, external beam radiotherapy no longer is used for primary retinoblastoma. In 2005, the International Intraocular Retinoblastoma Classification (IIRC)[5] was introduced to predict eye salvage and high-risk disease (see a summary of the survey results of the international retinoblastoma staging working group later in this chapter), with a modification (the International Classification of Retinoblastoma; ICRB) published in 2006.[6] Significant differences in these systems altered the ability to predict risk.[1] An extraocular retinoblastoma staging system was proposed in 2006,[7] and later in the AJCC Cancer Staging Manual, 7th Edition staging for retinoblastoma.[8]

Evidence-based data derived from published literature and from the retrospective survey conducted by the OOTF were used to support the changes in this edition. Wide adoption of the classification is an opportunity to replace the present confusion in reporting the outcome of retinoblastoma with a complete intraocular and extraocular staging system that may be used to achieve high-quality multicenter studies.

This chapter also includes a new category, heritable trait, denoted by the capital letter *H*. Knudson's two-hit hypothesis[9] proposed that children with bilateral disease were genetically predisposed to developing the tumors in both eyes. This hypothesis led to the discovery of the *RB1* gene,[2] the first tumor suppressor gene. Having a germline *RB1* mutation predisposes strongly to retinoblastoma (with more than 95% penetrance) and other cancers affecting patient survival, and has an impact on the health of the whole family. The germline predisposition significantly affects patient outcomes, including cancer-related morbidity and mortality. A similar constitutional predisposition for a specific cancer is a feature of other tumor suppressor genes, such as *BRCA1* and *BRCA2* in breast and ovarian cancer, *TP53* in Li-Fraumeni syndrome, *CDH1* in gastric cancer, *APC* in colon cancers,

and *MSH2* in pancreatic cancer. Other tumor suppressor genes are being added rapidly to this growing list. Classifying other cancers for a predisposing constitutional germline mutation (i.e., heritable trait) is anticipated.

ANATOMY

Primary Site(s)

The retina is composed of neurons and glial cells. The precursors of the neuronal elements give rise to retinoblastoma, whereas the glial cells give rise to astrocytoma. The internal limiting membrane separates the retina from the vitreous cavity internally. Externally, the retina is separated from the choroid by the retinal pigment epithelium and Bruch's membrane, the latter of which act as a natural barrier to extension of retinal tumors into the choroid. The continuation of the retina with the optic nerve allows direct extension of retinoblastoma tumor cells into the optic nerve and then to the subarachnoid space. Because the retina has no lymphatics, spread of retinal tumors is either by direct extension into adjacent structures (vitreous uvea, sclera, optic nerve, anterior chamber, orbit, and brain) or by distant metastasis through hematogenous routes. Local extension may result in a mass pushing the eye out (proptosis) to protrude between the lids, and/or soft tissue involvement of the face. Posterior extension results in retinoblastoma extending into the orbit and/or through the optic nerve into the brain.

Regional Lymph Nodes

There are no known intraocular lymphatics; this category of staging applies only to extraocular extension involving ocular adnexal tissues with lymphatic supply (e.g., conjunctiva). The regional lymph nodes that might be involved with extraocular retinoblastoma are the preauricular (parotid), submandibular, and cervical nodes (Fig. 68.3).

Metastatic Sites

Any retinoblastoma outside the eyeball predicts poor survival. Retinoblastomas metastasize through the hematogenous route to various sites, most commonly to the bone marrow and bone. The tumor might spread from within the optic nerve or subarachnoid space into the intracranial space (Fig. 68.3). The prognosis for central nervous system (CNS) involvement of retinoblastoma currently is worse than for spread to bone marrow, because the blood–brain barrier may inhibit systemic chemotherapy from effectively reaching intracranial tumor cells.

RULES FOR CLASSIFICATION

Clinical Classification

The most common signs of retinoblastoma at presentation are a white pupil (leukocoria) and strabismus.[10] Small tumors appear as creamy white lesions within the retina. In the data analyzed by the International Retinoblastoma Staging Working Group, tumors larger than 3 mm (approximately measuring two disc diameters) and those closer than 1.5 mm (approximately measuring one optic disc diameter) from the center of the fovea or margin of the optic disc reflected worse ocular survival. Some very small tumors that are not visible on clinical examination or photography may be detected on retinal imaging with optical coherence tomography (OCT), usually in children predicted to be predisposed to retinoblastoma because of a family history of retinoblastoma.[16] Calcification is pathognomonic of retinoblastoma, and contributes to the diagnosis if detected by ultrasonography or radiologic imaging. Tumors may be associated with an exudative retinal detachment.

Retinoblastoma tumors may progress to "seeds" that disseminate below the retina in an exudative retinal detachment (called "subretinal seeds") or may breach the inner limiting membrane of the retina to access the vitreous (called "vitreous seeds"). The morphology of seeds in the vitreous has been described as dust, clouds, or pearl-like spheres.[11,12] The morphologic type of vitreous seeding may predict the duration of treatment of vitreous disease. Extensive tumor necrosis concomitant with intraocular tissue necrosis may cause ocular inflammation, aseptic orbital cellulitis, or shrinkage of the whole eye (phthisis bulbi).[13] The signs of dangerously advanced disease include orbital cellulitis, glaucoma, enlargement of the eye and proptosis, intraocular hemorrhage, and anterior chamber involvement. Orbital and CNS involvement may be suspected or unsuspected in clinical examination, so pretreatment brain and orbit imaging is especially important in all children with cT3 disease.

Unique to the 8th Edition TNM for retinoblastoma is the inclusion of germline cancer predisposition, which incurs a high risk for new postdiagnosis retinoblastoma tumors and second primary tumors (such as osteosarcoma and cutaneous melanoma), thus affecting overall patient survival.[14] The impact of germline predisposition supports avoidance of unnecessary exposure to diagnostic or therapeutic radiation (including X-rays or computed tomography [CT] scans). With this chapter, we introduce the stage category H to indicate the germline status of the *RB1* gene (H1), inferred clinically by bilateral (synchronous or metachronous) retinoblastoma, with or without intracranial CNS midline embryonic tumor (commonly a pinealoblastoma, i.e., trilateral retinoblastoma), a family history of retinoblastoma, or high-quality molecular evidence of a constitutional *RB1*

mutation. In a unilaterally affected eye, multiple tumors are not sufficient evidence of a germline *RB1* mutation, because they cannot be clinically distinguished reliably from tumor seeded in the subretinal space or vitreous from the primary tumor.[15] Clear OCT evidence of multiple small tumors that are intraretinal, involve the inner nuclear layer of the retina, and are not preretinal or subretinal seeding may categorize the child as H1.[16]

If clinical evidence of heritable trait is not present (or available), molecular evidence is required before designating a child in category H1. Clear demonstration of the sensitivity and quality of the laboratory *RB1* tests is important. For true positive (H1) there must be solid evidence supporting the mutation, and variants of unknown significance should be categorized as HX. If residual (false negative) risk for a mutation is less than 1% or at population risk (0.007%) in a laboratory with demonstrated sensitivity greater than 97%, H0 may be assigned. Some unilateral tumors occurring in children at age 4 to 5 months were found to have no *RB1* mutation, but significant amplification of the *MYCN* oncogene.[17] These unilateral "$RB1^{+/+}$MYCN^{A}" retinoblastomas were associated with unique histologic features. This distinct tumor type is not known to be hereditary and therefore may be assigned an H0 status if both normal *RB1* gene and high-level *MYCN* amplification in the tumor has been documented.

To define the full clinical extent of disease, all children suspected of having retinoblastoma require a complete clinical examination under anesthesia. After the intraocular pressure in each eye is ascertained and the anterior segment visualized (with a handheld slit lamp or operating microscope if available), a detailed fundus examination is performed with an indirect ophthalmoscope using scleral indentation to visualize the retina completely. The pupil must be pharmacologically well-dilated for a complete examination. The fundus findings are documented with detailed fundus drawings and wide-field fundus photography. Fundus fluorescein angiography is useful to detect subtle iris neovascularization.[18] OCT detects small tumors in the inner nuclear layer of the retina before they are visible on ophthalmoscopy or photography. This is particularly relevant to children predisposed to retinoblastoma by an *RB1* germline mutation, because treatment of these "invisible" tumors may be initiated.[16] OCT also is useful in evaluating tumors abutting the optic nerve margin, differentiating tumors that invade the optic nerve.[19] The authors acknowledge that although wide-field fundus photography, fluorescein angiography, ultrasound biomicroscopy, and OCT may add value to our clinical assessment and potentially improve clinical decisions and patient survival, they are not essential to tumor staging.

Imaging

The diagnosis of retinoblastoma relies on a thorough clinical examination. In differentiating retinoblastoma from other diagnoses (such as Coats' disease), ultrasound may demonstrate the near-pathognomonic calcification of retinoblastoma. Imaging tools are especially useful in diagnosing retinoblastoma with atypical presentations, such as in children presenting with buphthalmos, hyphema, or orbital cellulitis. These investigations also are required to determine the full extent of retinoblastoma, including optic nerve, orbital, and intracranial involvement. They also aid in the early detection of intracranial CNS midline embryonic tumors, which often arise in the pineal gland (pinealoblastoma) or the parasellar region.[20] CT scanning no longer is recommended as the first choice for supporting the diagnosis or staging disease with a clinical diagnosis of retinoblastoma.[14,20]

Ocular Ultrasonography

A heterogeneous hyperechoic intraocular mass arising from the retina, with highly reflective shadowing suggesting calcification, is pathognomonic of retinoblastoma.[21] Ultrasound can determine the extent and size of larger tumors, and their relationship to the optic nerve head. Imaging the retrobulbar optic nerve sometimes is possible with appropriate equipment and a skilled examiner, but most often the resolution is better with magnetic resonance (MR) imaging. High-frequency anterior segment ultrasonography (such as with the ultrasound biomicroscope [UBM]) can ascertain the extent of the tumor in relation to the ciliary body, anterior hyaloid face, and posterior surface of the iris.[22,23] Although not universally available, UBM examination can determine whether the uveal stroma is involved. Tumor invasion (i.e., intraciliary tumor involvement) of the pars plana and ciliary body would be classified as cT3b, but tumor or seeds at the hyaloid face overlying the pars plana (i.e., epiciliary involvement) would not be considered cT3b. UBM also is useful in determining the mechanism of glaucoma, differentiating angle-closure glaucoma (which responds to chemotherapy for a large tumor) from neovascular glaucoma (which is an ominous sign for safely salvaging an eye).[24]

MR Imaging and CT

Radiologic imaging determines the extent of the tumor into the optic nerve, orbit, and CNS. MR imaging using high-resolution protocols currently is considered the most accurate and valuable tool in pretreatment staging of retinoblastoma.[25-29] High-resolution images (defined as a pixel resolution $\leq 0.5 \times 0.5$ mm^2 and a slice thickness ≤ 2 mm) may be obtained by using surface coils with the 1.5 Tesla machines or a multichannel head coil with the 3 Tesla machines. The diagnostic value of CT scans in detecting metastatic risk factors is limited.[26,30] A more major concern is radiation exposure, which might increase the risk of second primary tumors developing in children with germline disease.[14] We still suggest, however, that contrast-enhanced CT is performed when MR imaging is not available. CT scans may help diagnose (preclinical) optic nerve thickening as well as scleral and extrascleral tumor invasion.[26]

mutation. In a unilaterally affected eye, multiple tumors are not sufficient evidence of a germline *RB1* mutation, because they cannot be clinically distinguished reliably from tumor seeded in the subretinal space or vitreous from the primary tumor.[15] Clear OCT evidence of multiple small tumors that are intraretinal, involve the inner nuclear layer of the retina, and are not preretinal or subretinal seeding may categorize the child as H1.[16]

If clinical evidence of heritable trait is not present (or available), molecular evidence is required before designating a child in category H1. Clear demonstration of the sensitivity and quality of the laboratory *RB1* tests is important. For true positive (H1) there must be solid evidence supporting the mutation, and variants of unknown significance should be categorized as HX. If residual (false negative) risk for a mutation is less than 1% or at population risk (0.007%) in a laboratory with demonstrated sensitivity greater than 97%, H0 may be assigned. Some unilateral tumors occurring in children at age 4 to 5 months were found to have no *RB1* mutation, but significant amplification of the *MYCN* oncogene.[17] These unilateral "$RB1^{+/+}$MYCN^{A}" retinoblastomas were associated with unique histologic features. This distinct tumor type is not known to be hereditary and therefore may be assigned an H0 status if both normal *RB1* gene and high-level *MYCN* amplification in the tumor has been documented.

To define the full clinical extent of disease, all children suspected of having retinoblastoma require a complete clinical examination under anesthesia. After the intraocular pressure in each eye is ascertained and the anterior segment visualized (with a handheld slit lamp or operating microscope if available), a detailed fundus examination is performed with an indirect ophthalmoscope using scleral indentation to visualize the retina completely. The pupil must be pharmacologically well-dilated for a complete examination. The fundus findings are documented with detailed fundus drawings and wide-field fundus photography. Fundus fluorescein angiography is useful to detect subtle iris neovascularization.[18] OCT detects small tumors in the inner nuclear layer of the retina before they are visible on ophthalmoscopy or photography. This is particularly relevant to children predisposed to retinoblastoma by an *RB1* germline mutation, because treatment of these "invisible" tumors may be initiated.[16] OCT also is useful in evaluating tumors abutting the optic nerve margin, differentiating tumors that invade the optic nerve.[19] The authors acknowledge that although wide-field fundus photography, fluorescein angiography, ultrasound biomicroscopy, and OCT may add value to our clinical assessment and potentially improve clinical decisions and patient survival, they are not essential to tumor staging.

Imaging

The diagnosis of retinoblastoma relies on a thorough clinical examination. In differentiating retinoblastoma from other diagnoses (such as Coats' disease), ultrasound may demonstrate the near-pathognomonic calcification of retinoblastoma. Imaging tools are especially useful in diagnosing retinoblastoma with atypical presentations, such as in children presenting with buphthalmos, hyphema, or orbital cellulitis. These investigations also are required to determine the full extent of retinoblastoma, including optic nerve, orbital, and intracranial involvement. They also aid in the early detection of intracranial CNS midline embryonic tumors, which often arise in the pineal gland (pinealoblastoma) or the parasellar region.[20] CT scanning no longer is recommended as the first choice for supporting the diagnosis or staging disease with a clinical diagnosis of retinoblastoma.[14,20]

Ocular Ultrasonography

A heterogeneous hyperechoic intraocular mass arising from the retina, with highly reflective shadowing suggesting calcification, is pathognomonic of retinoblastoma.[21] Ultrasound can determine the extent and size of larger tumors, and their relationship to the optic nerve head. Imaging the retrobulbar optic nerve sometimes is possible with appropriate equipment and a skilled examiner, but most often the resolution is better with magnetic resonance (MR) imaging. High-frequency anterior segment ultrasonography (such as with the ultrasound biomicroscope [UBM]) can ascertain the extent of the tumor in relation to the ciliary body, anterior hyaloid face, and posterior surface of the iris.[22,23] Although not universally available, UBM examination can determine whether the uveal stroma is involved. Tumor invasion (i.e., intraciliary tumor involvement) of the pars plana and ciliary body would be classified as cT3b, but tumor or seeds at the hyaloid face overlying the pars plana (i.e., epiciliary involvement) would not be considered cT3b. UBM also is useful in determining the mechanism of glaucoma, differentiating angle-closure glaucoma (which responds to chemotherapy for a large tumor) from neovascular glaucoma (which is an ominous sign for safely salvaging an eye).[24]

MR Imaging and CT

Radiologic imaging determines the extent of the tumor into the optic nerve, orbit, and CNS. MR imaging using high-resolution protocols currently is considered the most accurate and valuable tool in pretreatment staging of retinoblastoma.[25–29] High-resolution images (defined as a pixel resolution $\leq 0.5 \times 0.5$ mm^2 and a slice thickness ≤ 2 mm) may be obtained by using surface coils with the 1.5 Tesla machines or a multichannel head coil with the 3 Tesla machines. The diagnostic value of CT scans in detecting metastatic risk factors is limited.[26,30] A more major concern is radiation exposure, which might increase the risk of second primary tumors developing in children with germline disease.[14] We still suggest, however, that contrast-enhanced CT is performed when MR imaging is not available. CT scans may help diagnose (preclinical) optic nerve thickening as well as scleral and extrascleral tumor invasion.[26]

Retinoblastoma appears as an intraocular hyperintense mass on T1-weighted MR images and a hypointense mass on T2-weighted images (compared with vitreous) and shows contrast enhancement. Calcifications also may be demonstrated in the tumor.[31,32] Contrast enhancement of the optic nerve in continuity with the intraocular mass[28,33,34] and thickening of the retrobulbar optic nerve[26,34] suggest tumor invasion of the retrobulbar optic nerve. Tumor invasion of the orbit and orbital inflammation are important to differentiate, but could be challenging. Inflammatory changes appear as ill-defined areas with high signal intensity in soft tissues (orbital fat, muscles, eyelids) on T2-weighted images, with diffuse enhancement after contrast injection on T1-weighted images, and without a delineated "mass." Tumor invasion is either limited to the optic nerve or involves the orbital fat, as a delineated mass crossing the sclera and having the same signal intensity as the primary tumor (i.e., low signal intensity compared with fat on T2). Interruption of the linear enhancement pattern of the choroid or focal thickening raises suspicion of choroidal invasion. A recent study showed that tumor size might predict retrolaminar optic nerve invasion and (to a lesser extent) massive choroidal invasion.[35] Concomitant retrolaminar optic nerve invasion and massive choroidal invasion were seen only in tumors larger than 1.4 cm^3 in volume or longer than 16.5 mm in the largest diameter of axial MR images. However, we emphasize that despite advanced MR imaging technologies available, predicting optic nerve and choroidal invasion in pathology through radiology is not fail-proof, especially in the early stages.[25,33]

If palpable preauricular lymph nodes are present, we suggest imaging the regions of the submandibular and cervical lymph nodes in an attempt to detect the full extent of lymph node involvement. Baseline brain (preferably MR) imaging should always be performed to rule out a concurrent intracranial CNS midline embryonic tumor (e.g., a tumor involving the pineal gland or parasellar/suprasellar regions).[20]

Pathological Classification

Enucleation of the eye is required for pathological staging. The entire eye should be submitted for histopathologic examination. Guidelines for gross examination and sampling are available from the College of American Pathologists (www.cap.org)[46] and the Royal College of Pathologists (www.rcpath.org). Fresh tumor material may be collected from the enucleated eye for clinical genetic testing (and research) before formalin fixation. Identification of *RB1* mutations and other genetic studies in tumor tissue are difficult with formalin-fixed tissue.

Histopathologic features of retinoblastoma include small round cells staining blue on hematoxylin and eosin. Flexner–Wintersteiner rosettes (typical for retinoblastoma) and Homer Wright rosettes (characteristic of neuroectodermal tumors) both occur. Tumors with many Flexner–Wintersteiner rosettes are graded as moderately differentiated. Some tumors show photoreceptor-like differentiation (fleurettes) or neuronal differentiation without mitoses or apoptosis, which is evidence of an underlying premalignant lesion: retinoma.[36,37] Tumors that show no fleurettes or rosettes are graded as poorly differentiated. The nuclei of poorly differentiated tumors may show anaplasia.[38] Rarely, unilateral retinoblastoma tumors show a loose cellular pattern with round nuclei and prominent multiple nucleoli indicative of *MYCN* amplification and normal *RB1* alleles.[39] Retinoblastoma undergoes pathognomonic dystrophic calcification. Small tumors initially are limited by the retinal boundaries (Bruch's membrane and the inner limiting membrane). As the tumor grows, it spreads into the adjacent vitreous, subretinal space, underlying choroid, optic nerve, or anterior segment (iris, trabecular meshwork, or Schlemm's canal).

Invasive tumors that breach Bruch's membrane spread into the vascular choroid, facilitating hematogenous metastasis.[40,41] Tumors that extend outside the eye (episclera, cut end of optic nerve, or subconjunctival tissues) frequently become metastatic and have the worst prognosis.[22] These features are found most commonly in patients with a delayed diagnosis. Some studies, including a prospective trial from the Children's Oncology Group (ARET0332), demonstrated that the amount of tumor in the choroid is important. Retinoblastomas with focal invasion of the choroid (<3 mm) that does not reach the sclera have a 0–1% risk of metastatic relapse after enucleation alone, even in the presence of concomitant prelaminar optic nerve invasion.[41,42] A maximum diameter of tumor in choroid of greater than 3 mm (or multiple foci of tumor in the choroid measuring a total of >3 mm) is associated with a 3% risk of metastatic relapse if no concomitant postlaminar optic nerve invasion is present. This risk may increase 5–8% if the tumor reaches the sclera.[41–43]

Alternatively, the tumor may spread through the lamina cribrosa into the optic nerve or subarachnoid space, accessing cerebrospinal fluid and the CNS. Invasion into the sclera may follow massive choroidal invasion directly or through emissary channels.[44] Tumor spreading through sclera may invade into the orbit and result in systemic and preauricular lymph node metastasis.

Although pathology provides histologic verification of retinoblastoma, a clinical diagnosis alone is considered adequate to initiate treatment. Clinicians and pathologists should be warned that pre-enucleation chemotherapy alters the histopathologic findings and may significantly downstage disease severity.[45] To make that distinction, the prefix *y* indicates neoadjuvant therapy.

Palpable lymph nodes require fine-needle aspiration biopsy or open biopsy to confirm the presence of malignant cells. To pathologically stage for distant metastasis, cerebrospinal fluid and bone marrow analysis are required.

68

Histopathologic screening for metastasis should be considered if high-risk features (pT3, pT4) on histopathologic examination of the enucleated eye. If eye preservation will be attempted, cerebrospinal fluid and bone marrow analysis is advised before starting chemotherapy, as chemotherapy may downstage the disease.[45] Lumbar puncture for cytologic analysis of the cerebrospinal fluid after cytocentrifugation is advised if there are any signs of optic nerve involvement or if the optic disc cannot be visualized on clinical examination. Bone marrow biopsy from bilateral sites is advisable in all children presenting with advanced intraocular disease (cT3) or extraocular disease (cT4).

PROGNOSTIC FACTORS

Prognostic Factors Required for Stage Grouping

RB1 Gene Germline Mutation Status

This chapter is unique in its attempt to recognize the importance of a heritable cancer trait in predicting the overall patient survival for this cancer, suggested by the new H category. This feature has level I evidence to support it. Clinical features that support the presence of a heritable trait are bilateral disease, a family history that suggests a hereditary trait for retinoblastoma, and the presence of a concomitant CNS midline embryonic tumor (commonly in the pineal region: i.e.; pinealoblastoma). Children with any of those features will be assigned the H1 status, even without molecular testing. When discrete clinical evidence of heritable trait is not present, high-quality molecular evidence is mandatory before designating a child as H1. A clear demonstration of the sensitivity and quality of the laboratory *RB1* tests is important. Variants of unknown significance should be categorized as HX. If residual (false negative) risk for a mutation is less than 1% or at population risk (0.007%) in a laboratory with demonstrated sensitivity greater than 97%, H0 may be assigned.

Additional Factors Recommended for Clinical Care

- Age at diagnosis and at treatment initiation
- Vision at diagnosis and visual potential

RISK ASSESSMENT MODELS

The AJCC recently established guidelines that will be used to evaluate published statistical prediction models for the purpose of granting endorsement for clinical use.[47] Although this is a monumental step toward the goal of precision medicine, this work was published only very recently. Therefore, the existing models that have been published or may be in clinical use have not yet been evaluated for this cancer site by the Precision Medicine Core of the AJCC. In the future, the statistical prediction models for this cancer site will be evaluated, and those that meet all AJCC criteria will be endorsed.

DEFINITIONS OF AJCC TNM

Clinical Classification (cTNM)

Definition of Primary Tumor (cT)

Each eye is scored for cT criteria, and the cT category for the patient is based on the most advanced eye.

cT Category	cT Criteria
cTX	Unknown evidence of intraocular tumor
cT0	No evidence of intraocular tumor
cT1	Intraretinal tumor(s) with subretinal fluid ≤5 mm from the base of any tumor
cT1a	Tumors ≤3 mm and further than 1.5 mm from disc and fovea
cT1b	Tumors >3 mm or closer than 1.5 mm from disc or fovea
cT2	Intraocular tumor(s) with retinal detachment, vitreous seeding, or subretinal seeding
cT2a	Subretinal fluid >5 mm from the base of any tumor
cT2b	Vitreous seeding and/or subretinal seeding
cT3	Advanced intraocular tumor(s)
cT3a	Phthisis or pre-phthisis bulbi
cT3b	Tumor invasion of choroid, pars plana, ciliary body, lens, zonules, iris, or anterior chamber
cT3c	Raised intraocular pressure with neovascularization and/or buphthalmos
cT3d	Hyphema and/or massive vitreous hemorrhage
cT3e	Aseptic orbital cellulitis
cT4	Extraocular tumor(s) involving orbit, including optic nerve
cT4a	Radiologic evidence of retrobulbar optic nerve involvement or thickening of optic nerve or involvement of orbital tissues
cT4b	Extraocular tumor clinically evident with proptosis and/or an orbital mass

Definition of Regional Lymph Node (cN)

cN Category	cN Criteria
cNX	Regional lymph nodes cannot be assessed
cN0	No regional lymph node involvement
cN1	Evidence of preauricular, submandibular, and cervical lymph node involvement

Definition of Distant Metastasis (M)

cM Category	cM Criteria
cM0	No signs or symptoms of intracranial or distant metastasis
cM1	Distant metastasis without microscopic confirmation
cM1a	Tumor(s) involving any distant site (e.g., bone marrow, liver) on clinical or radiologic tests
cM1b	Tumor involving the CNS on radiologic imaging (not including trilateral retinoblastoma)
pM1	Distant metastasis with microscopic confirmation
pM1a	Pathological evidence of tumor at any distant site (e.g., bone marrow, liver, or other)
pM1b	Pathological evidence of tumor in the cerebrospinal fluid or CNS parenchyma

Definition of Heritable Trait (H)

H Category	H Criteria
HX	Unknown or insufficient evidence of a constitutional *RB1* gene mutation.
H0	Normal *RB1* alleles in blood tested with demonstrated high-sensitivity assays
H1	Bilateral retinoblastoma, retinoblastoma with an intracranial primitive neuroectodermal tumor (i.e., trilateral retinoblastoma), patient with family history of retinoblastoma, **or** molecular definition of a constitutional *RB1* gene mutation

Pathological Classification (pTNM)

Definition of Primary Tumor (pT)

pT Category	pT Criteria
pTX	Unknown evidence of intraocular tumor
pT0	No evidence of intraocular tumor
pT1	Intraocular tumor(s) without any local invasion, focal choroidal invasion, or pre- or intralaminar involvement of the optic nerve head
pT2	Intraocular tumor(s) with local invasion
pT2a	Concomitant focal choroidal invasion and pre- or intralaminar involvement of the optic nerve head
pT2b	Tumor invasion of stroma of iris and/or trabecular meshwork and/or Schlemm's canal
pT3	Intraocular tumor(s) with significant local invasion
pT3a	Massive choroidal invasion (>3 mm in largest diameter, or multiple foci of focal choroidal involvement totalling >3 mm, or any full-thickness choroidal involvement)
pT3b	Retrolaminar invasion of the optic nerve head, not involving the transected end of the optic nerve
pT3c	Any partial-thickness involvement of the sclera within the inner two thirds
pT3d	Full-thickness invasion into the outer third of the sclera and/or invasion into or around emissary channels
pT4	Evidence of extraocular tumor: tumor at the transected end of the optic nerve, tumor in the meningeal spaces around the optic nerve, full-thickness invasion of the sclera with invasion of the episclera, adjacent adipose tissue, extraocular muscle, bone, conjunctiva, or eyelids

Histopathologic screening for metastasis should be considered if high-risk features (pT3, pT4) on histopathologic examination of the enucleated eye. If eye preservation will be attempted, cerebrospinal fluid and bone marrow analysis is advised before starting chemotherapy, as chemotherapy may downstage the disease.[45] Lumbar puncture for cytologic analysis of the cerebrospinal fluid after cytocentrifugation is advised if there are any signs of optic nerve involvement or if the optic disc cannot be visualized on clinical examination. Bone marrow biopsy from bilateral sites is advisable in all children presenting with advanced intraocular disease (cT3) or extraocular disease (cT4).

PROGNOSTIC FACTORS

Prognostic Factors Required for Stage Grouping

RB1 Gene Germline Mutation Status

This chapter is unique in its attempt to recognize the importance of a heritable cancer trait in predicting the overall patient survival for this cancer, suggested by the new H category. This feature has level I evidence to support it. Clinical features that support the presence of a heritable trait are bilateral disease, a family history that suggests a hereditary trait for retinoblastoma, and the presence of a concomitant CNS midline embryonic tumor (commonly in the pineal region: i.e.: pinealoblastoma). Children with any of those features will be assigned the H1 status, even without molecular testing. When discrete clinical evidence of heritable trait is not present, high-quality molecular evidence is mandatory before designating a child as H1. A clear demonstration of the sensitivity and quality of the laboratory *RB1* tests is important. Variants of unknown significance should be categorized as HX. If residual (false negative) risk for a mutation is less than 1% or at population risk (0.007%) in a laboratory with demonstrated sensitivity greater than 97%, H0 may be assigned.

Additional Factors Recommended for Clinical Care

- Age at diagnosis and at treatment initiation
- Vision at diagnosis and visual potential

RISK ASSESSMENT MODELS

The AJCC recently established guidelines that will be used to evaluate published statistical prediction models for the purpose of granting endorsement for clinical use.[47] Although this is a monumental step toward the goal of precision medicine, this work was published only very recently. Therefore, the existing models that have been published or may be in clinical use have not yet been evaluated for this cancer site by the Precision Medicine Core of the AJCC. In the future, the statistical prediction models for this cancer site will be evaluated, and those that meet all AJCC criteria will be endorsed.

DEFINITIONS OF AJCC TNM

Clinical Classification (cTNM)

Definition of Primary Tumor (cT)

Each eye is scored for cT criteria, and the cT category for the patient is based on the most advanced eye.

cT Category	cT Criteria
cTX	Unknown evidence of intraocular tumor
cT0	No evidence of intraocular tumor
cT1	Intraretinal tumor(s) with subretinal fluid ≤5 mm from the base of any tumor
cT1a	Tumors ≤3 mm and further than 1.5 mm from disc and fovea
cT1b	Tumors >3 mm or closer than 1.5 mm from disc or fovea
cT2	Intraocular tumor(s) with retinal detachment, vitreous seeding, or subretinal seeding
cT2a	Subretinal fluid >5 mm from the base of any tumor
cT2b	Vitreous seeding and/or subretinal seeding
cT3	Advanced intraocular tumor(s)
cT3a	Phthisis or pre-phthisis bulbi
cT3b	Tumor invasion of choroid, pars plana, ciliary body, lens, zonules, iris, or anterior chamber
cT3c	Raised intraocular pressure with neovascularization and/or buphthalmos
cT3d	Hyphema and/or massive vitreous hemorrhage
cT3e	Aseptic orbital cellulitis
cT4	Extraocular tumor(s) involving orbit, including optic nerve
cT4a	Radiologic evidence of retrobulbar optic nerve involvement or thickening of optic nerve or involvement of orbital tissues
cT4b	Extraocular tumor clinically evident with proptosis and/or an orbital mass

Definition of Regional Lymph Node (cN)

cN Category	cN Criteria
cNX	Regional lymph nodes cannot be assessed
cN0	No regional lymph node involvement
cN1	Evidence of preauricular, submandibular, and cervical lymph node involvement

Definition of Distant Metastasis (M)

cM Category	cM Criteria
cM0	No signs or symptoms of intracranial or distant metastasis
cM1	Distant metastasis without microscopic confirmation
cM1a	Tumor(s) involving any distant site (e.g., bone marrow, liver) on clinical or radiologic tests
cM1b	Tumor involving the CNS on radiologic imaging (not including trilateral retinoblastoma)
pM1	Distant metastasis with microscopic confirmation
pM1a	Pathological evidence of tumor at any distant site (e.g., bone marrow, liver, or other)
pM1b	Pathological evidence of tumor in the cerebrospinal fluid or CNS parenchyma

Definition of Heritable Trait (H)

H Category	H Criteria
HX	Unknown or insufficient evidence of a constitutional *RB1* gene mutation.
H0	Normal *RB1* alleles in blood tested with demonstrated high-sensitivity assays
H1	Bilateral retinoblastoma, retinoblastoma with an intracranial primitive neuroectodermal tumor (i.e., trilateral retinoblastoma), patient with family history of retinoblastoma, **or** molecular definition of a constitutional *RB1* gene mutation

Pathological Classification (pTNM)

Definition of Primary Tumor (pT)

pT Category	pT Criteria
pTX	Unknown evidence of intraocular tumor
pT0	No evidence of intraocular tumor
pT1	Intraocular tumor(s) without any local invasion, focal choroidal invasion, or pre- or intralaminar involvement of the optic nerve head
pT2	Intraocular tumor(s) with local invasion
pT2a	Concomitant focal choroidal invasion and pre- or intralaminar involvement of the optic nerve head
pT2b	Tumor invasion of stroma of iris and/or trabecular meshwork and/or Schlemm's canal
pT3	Intraocular tumor(s) with significant local invasion
pT3a	Massive choroidal invasion (>3 mm in largest diameter, or multiple foci of focal choroidal involvement totalling >3 mm, or any full-thickness choroidal involvement)
pT3b	Retrolaminar invasion of the optic nerve head, not involving the transected end of the optic nerve
pT3c	Any partial-thickness involvement of the sclera within the inner two thirds
pT3d	Full-thickness invasion into the outer third of the sclera and/or invasion into or around emissary channels
pT4	Evidence of extraocular tumor: tumor at the transected end of the optic nerve, tumor in the meningeal spaces around the optic nerve, full-thickness invasion of the sclera with invasion of the episclera, adjacent adipose tissue, extraocular muscle, bone, conjunctiva, or eyelids

Definition of Regional Lymph Node (pN)

pN Category	pN Criteria
pNX	Regional lymph node involvement cannot be assessed
pN0	No lymph node involvement
pN1	Regional lymph node involvement

Definition of Distant Metastasis (M)

M Category	M Criteria
cM0	No signs or symptoms of intracranial or distant metastasis
cM1	Distant metastasis without microscopic confirmation
cM1a	Tumor(s) involving any distant site (e.g., bone marrow, liver) on clinical or radiologic tests
cM1b	Tumor involving the CNS on radiologic imaging (not including trilateral retinoblastoma)
pM1	Distant metastasis with histopathologic confirmation
pM1a	Histopathologic confirmation of tumor at any distant site (e.g., bone marrow, liver, or other)
pM1b	Histopathologic confirmation of tumor in the cerebrospinal fluid or CNS parenchyma

AJCC PROGNOSTIC STAGE GROUPS

Clinical Stage (cTNM)

When cT is..	And N is ...	And M is ...	And H is ...	Then the clinical stage group is ...
cT1, cT2, cT3	cN0	cM0	Any	I
cT4a	cN0	cM0	Any	II
cT4b	cN0	cM0	Any	III
Any	cN1	cM0	Any	III
Any	Any	cM1 or pM1	Any	IV

Pathological Stage (pTNM)

When pT is..	And N is ...	And M is ...	And H is ...	Then the pathological stage group is ...
pT1, pT2, pT3	pN0	cM0	Any	I
pT4	pN0	cM0	Any	II
Any	pN1	cM0	Any	III
Any	Any	cM1 or pM1	Any	IV

REGISTRY DATA COLLECTION VARIABLES

The authors have not noted any additional factors for registry data collection.

HISTOLOGIC GRADE (G)

G	G Definition
GX	Grade cannot be assessed
G1	Tumor with areas of retinoma (fleurettes or neuronal differentiation)
G2	Tumor with many rosettes (Flexner–Wintersteiner or Homer Wright)
G3	Tumor with occasional rosettes (Flexner–Wintersteiner or Homer Wright)
G4	Tumor with poorly differentiated cells without rosettes and/or with extensive areas (more than half of tumor) of anaplasia

HISTOPATHOLOGIC TYPE

This classification applies only to retinoblastoma. The central nervous system component of "trilateral retinoblastoma" would be required in addition to this staging, using the section pertinent to brain and spinal cord tumors (Chap. 72).

SURVIVAL DATA

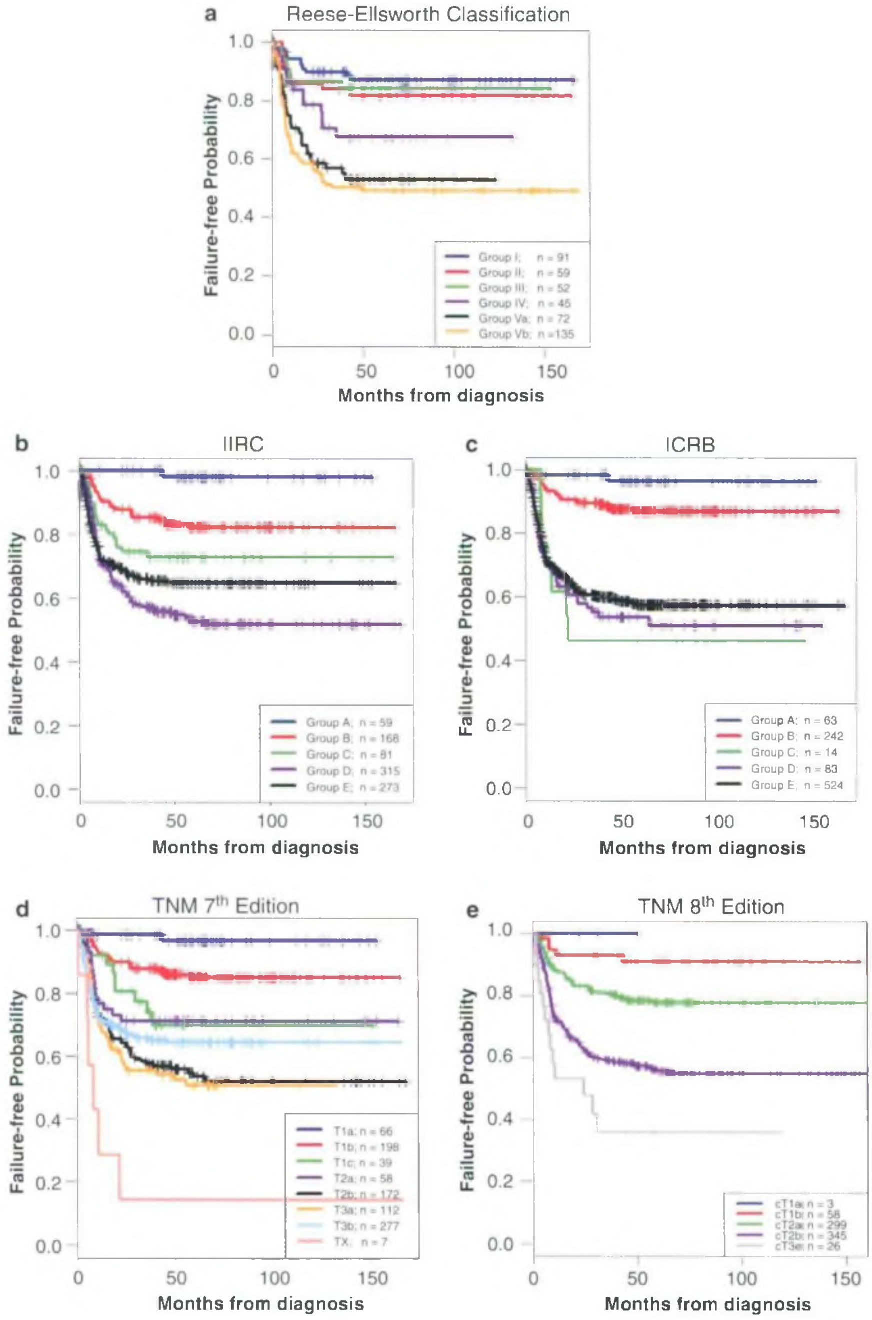

Fig. 68.1 The Ophthalmic Oncology Task Force (OOTF) Multicenter International Survey collected data required to stage eye(s) with retinoblastoma by five different schemes. With research ethics board approvals by the University Health Network (lead center for the survey) and each of the participating sites, clinical features of 1,728 eyes with retinoblastoma diagnosed between 2001 and 2011 were collected. Kaplan–Meier analyses of the proportion of eyes salvaged without external beam irradiation is shown for six different ocular staging systems: (**a**) Reese–Ellsworth,[4] (**b**) IIRC,[5] (**c**) ICRB,[6] (**d**) 7th Edition of TNM,[8] and (**e**) 8th Edition TNM (2016). The features for 2016 cT3a–d did not match the survey data so cannot be included

68

ILLUSTRATIONS

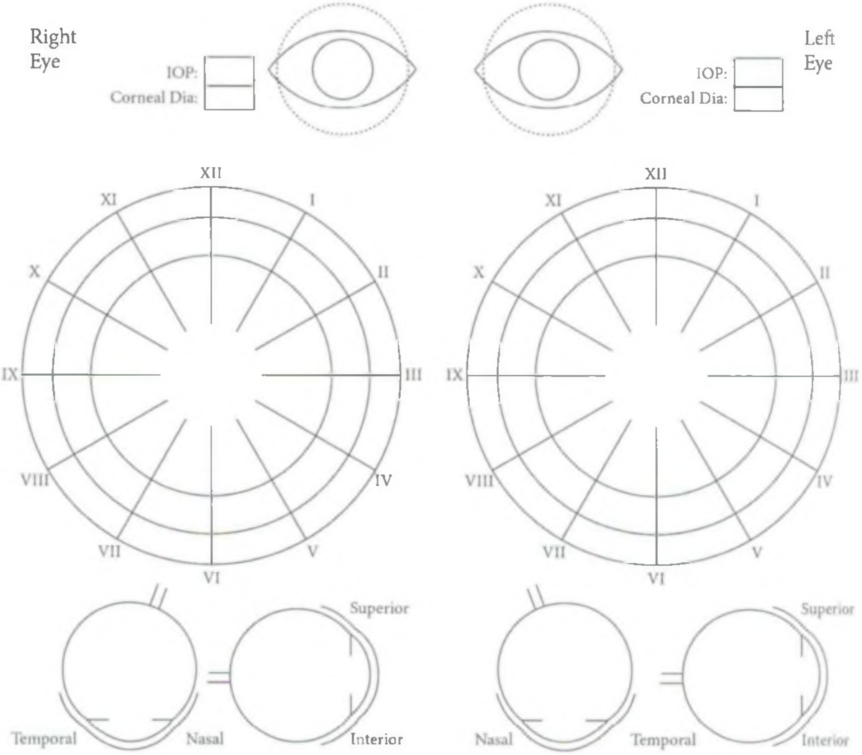

Fig. 68.2 Retinoblastoma staging diagram for clinical use

Clinical Extent:

	cT0	cT1a	cT1b	cT2a	cT2b	cT3a	cT3b	cT3c	cT3d	cT3e	cT4a	cT4b
Right Eye												
Left Eye												

Imaging Extent:

	No Tumor	Intraocular Tumor	Orbital Optic Nerve Involved	Pre-Chiasmatic Optic Nerve	Chiasmatic Involvement	Leptomengial Disease
Right Eye						
Left Eye						

Hereditary Trait:

Pinealoblastoma: N | Y

Family History: N | Y

Constitutional RB1 Mutation: N | Y

Metastasis:

Systemic:

pM0	cM0	pM1a	cM1a	pM1b	cM1b

Lymph Node:

N0	cN1	pN1

Pathology:

	pTX	pT0	pT1	pT2a	pT2b	pT3a	pT3b	pT3c	pT3d	pT4
Right Eye										
Left Eye										

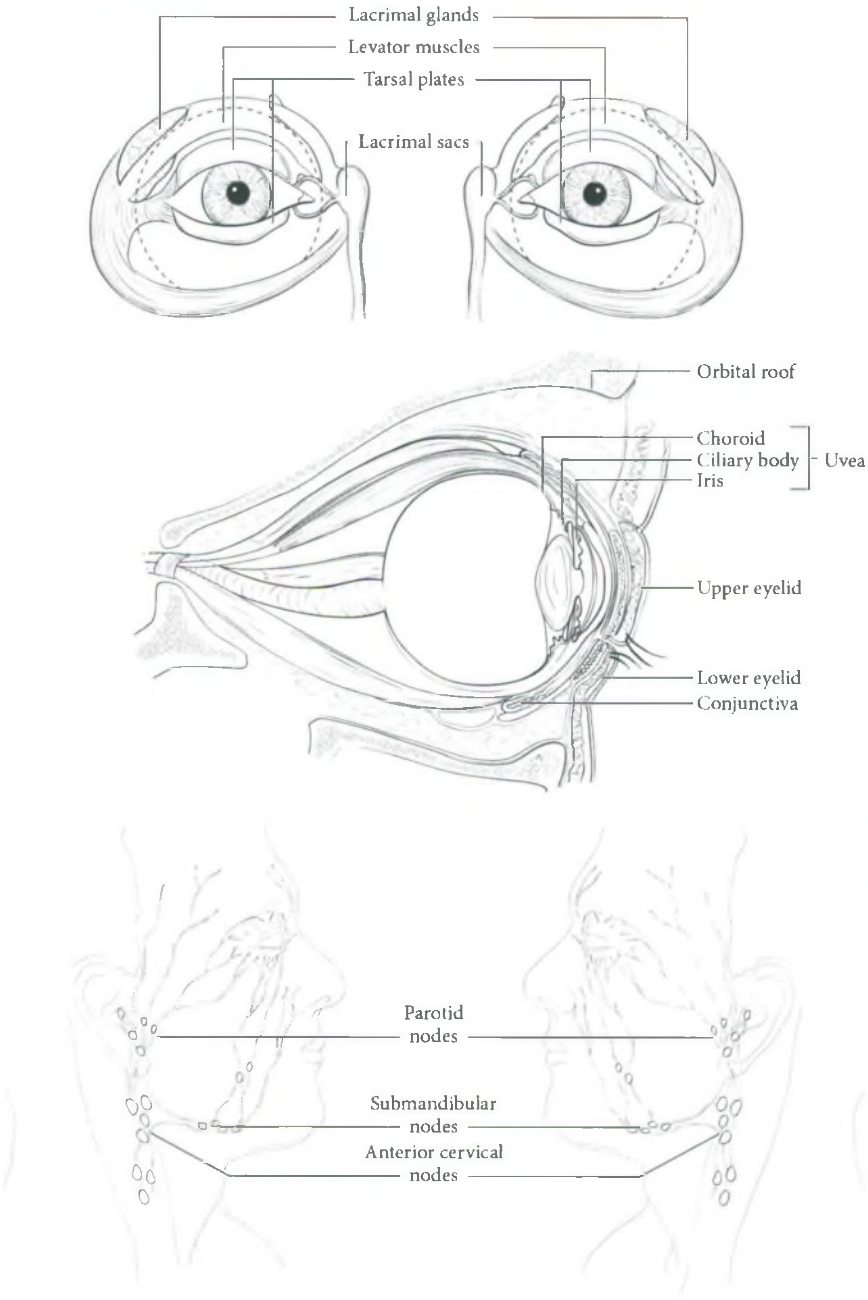

Fig. 68.3 Anatomic sites and regional lymph nodes for ophthalmic sites

Bibliography

1. Dimaras H. Retinoblastoma genetics in India: From research to implementation. *Indian J Ophthalmol*. Mar 2015;63(3): 219–226.
2. Friend SH, Bernards R, Rogelj S, et al. A human DNA segment with properties of the gene that predisposes to retinoblastoma and osteosarcoma. *Nature*. 1986;323(6089):643–646.
3. Finger PT, Harbour JW, Karcioglu ZA. Risk factors for metastasis in retinoblastoma. *Survey of ophthalmology*. Jan-Feb 2002;47(1):1–16.
4. Reese AB, Ellsworth RM. The evaluation and current concept of retinoblastoma therapy. *Transactions – American Academy of Ophthalmology and Otolaryngology. American Academy of Ophthalmology and Otolaryngology*. Mar-Apr 1963;67:164–172.
5. Murphree A. Intraocular retinoblastoma: the case for a new group classification. *Ophthalmology clinics of North America*. 2005;18(1): 41–53.

6. Shields CL, Shields JA. Basic understanding of current classification and management of retinoblastoma. *Curr Opin Ophthalmol.* Jun 2006;17(3):228–234.
7. Chantada G, Doz F, Antoneli CB, et al. A proposal for an international retinoblastoma staging system. *Pediatric blood & cancer.* Nov 2006;47(6):801–805.
8. The AJCC Ophthalmic Oncology Task Force. Retinoblastoma. In: Edge S, Byrd D, Compton C, eds. *AJCC Cancer Staging Manual.* 7th ed. New York, NY: Springer-Verlag; 2010:561–568.
9. Knudson AG, Jr. Mutation and cancer: statistical study of retinoblastoma. *Proc Natl Acad Sci U S A.* Apr 1971;68(4):820–823.
10. Zhao J, Li S, Shi J, Wang N. Clinical presentation and group classification of newly diagnosed intraocular retinoblastoma in China. *Br J Ophthalmol.* 2011;95(10):1372–1375.
11. Francis JH, Abramson DH, Gaillard MC, Marr BP, Beck-Popovic M, Munier FL. The classification of vitreous seeds in retinoblastoma and response to intravitreal melphalan. *Ophthalmology.* Jun 2015;122(6):1173–1179.
12. Munier FL. Classification and Management of Seeds in RetinoblastomaEllsworth Lecture Ghent August 24th 2013. *Ophthalmic genetics.* 2014;35(4):193–207.
13. Chong E-M, Coffee RE, Chintagumpala M, Hurwitz RL. Extensively necrotic retinoblastoma is associated with high-risk prognostic factors. *Archives of pathology & laboratory medicine.* 2006;130(11):1669.
14. MacCarthy A, Bayne AM, Brownbill PA, et al. Second and subsequent tumours among 1927 retinoblastoma patients diagnosed in Britain 1951–2004. *Br J Cancer.* Jun 25 2013;108(12):2455–2463.
15. Lohmann D, Gallie BL. Retinoblastoma. In: Pagon RA, Adam MP, Ardinger HH, Wallace SE, Amemiya A, Bean LGH, Bird TD, Fong C-T, Mefford HC, Smith RJH, and Stephens K., editor. GeneReviews™ [Internet] Seattle (WA): University of Washington, Seattle; 1993-2015 Available at http://wwwncbinlmnihgov/books/NBK1452/2015. Accessed Jul 31, 2016.
16. Rootman DB, Gonzalez E, Mallipatna A, et al. Hand-held high-resolution spectral domain optical coherence tomography in retinoblastoma: clinical and morphologic considerations. *The British journal of ophthalmology.* Jan 2013;97(1):59–65.
17. Rushlow DE, Mol BM, Kennett JY, et al. Characterisation of retinoblastomas without RB1 mutations: genomic, gene expression, and clinical studies. *The lancet oncology.* Apr 2013;14(4):327–334.
18. Kim JW, Ngai LK, Sadda S, Murakami Y, Lee DK, Murphree AL. Retcam fluorescein angiography findings in eyes with advanced retinoblastoma. *The British journal of ophthalmology.* Dec 2014;98(12):1666–1671.
19. Mallipatna A, Vinekar A, Jayadev C, et al. The use of handheld spectral domain optical coherence tomography in pediatric ophthalmology practice: Our experience of 975 infants and children. *Indian journal of ophthalmology.* 2015;63(7):586.
20. de Jong MC, Kors WA, de Graaf P, Castelijns JA, Kivela T, Moll AC. Trilateral retinoblastoma: a systematic review and meta-analysis. *The lancet oncology.* Sep 2014;15(10):1157–1167.
21. Marr BP, Singh AD. Retinoblastoma: Evaluation and Diagnosis. *Clinical Ophthalmic Oncology*: Springer; 2015:1–11.
22. Finger PT, Meskin SW, Wisnicki HJ, Albekioni Z, Schneider S. High-frequency ultrasound of anterior segment retinoblastoma. *American journal of ophthalmology.* May 2004;137(5):944–946.
23. Moulin AP, Gaillard MC, Balmer A, Munier FL. Ultrasound biomicroscopy evaluation of anterior extension in retinoblastoma: a clinicopathological study. *The British journal of ophthalmology.* Mar 2012;96(3):337–340.
24. Vasquez LM, Giuliari GP, Halliday W, Pavlin CJ, Gallie BL, Heon E. Ultrasound biomicroscopy in the management of retinoblastoma. *Eye.* Feb 2011;25(2):141–147.
25. de Jong MC, de Graaf P, Brisse HJ, et al. The potential of 3T high-resolution magnetic resonance imaging for diagnosis, staging, and follow-up of retinoblastoma. *Survey of ophthalmology.* Jul-Aug 2015;60(4):346–355.
26. de Jong MC, de Graaf P, Noij DP, et al. Diagnostic performance of magnetic resonance imaging and computed tomography for advanced retinoblastoma: a systematic review and meta-analysis. *Ophthalmology.* May 2014;121(5):1109–1118.
27. Brisse HJ, de Graaf P, Galluzzi P, et al. Assessment of early-stage optic nerve invasion in retinoblastoma using high-resolution 1.5 Tesla MRI with surface coils: a multicentre, prospective accuracy study with histopathological correlation. *European radiology.* 2014;25(5):1443–1452.
28. Sirin S, Schlamann M, Metz KA, et al. High-resolution MRI using orbit surface coils for the evaluation of metastatic risk factors in 143 children with retinoblastoma: Part 1: MRI vs. histopathology. *Neuroradiology.* Aug 2015;57(8):805–814.
29. Sirin S, Schlamann M, Metz KA, et al. High-resolution MRI using orbit surface coils for the evaluation of metastatic risk factors in 143 children with retinoblastoma: Part 2: new vs. old imaging concept. *Neuroradiology.* Aug 2015;57(8):815–824.
30. Brisse HJ, Guesmi M, Aerts I, et al. Relevance of CT and MRI in retinoblastoma for the diagnosis of postlaminar invasion with normal-size optic nerve: a retrospective study of 150 patients with histological comparison. *Pediatric radiology.* Jul 2007;37(7):649–656.
31. Galluzzi P, Hadjistilianou T, Cerase A, De Francesco S, Toti P, Venturi C. Is CT still useful in the study protocol of retinoblastoma? *American Journal of Neuroradiology.* 2009;30(9):1760–1765.
32. Rodjan F, de Graaf P, van der Valk P, et al. Detection of calcifications in retinoblastoma using gradient-echo MR imaging sequences: comparative study between in vivo MR imaging and ex vivo high-resolution CT. *American Journal of Neuroradiology.* 2015;36(2):355–360.
33. Brisse HJ, de Graaf P, Galluzzi P, et al. Assessment of early-stage optic nerve invasion in retinoblastoma using high-resolution 1.5 Tesla MRI with surface coils: a multicentre, prospective accuracy study with histopathological correlation. *European radiology.* May 2015;25(5):1443–1452.
34. de Graaf P, Barkhof F, Moll AC, et al. Retinoblastoma: MR imaging parameters in detection of tumor extent. *Radiology.* Apr 2005;235(1):197–207.
35. de Jong M, al. E. The diagnostic accuracy of intraocular tumor size measured by magnetic resonance imaging to predict postlaminar optic nerve invasion and massive choroidal invasion of retinoblastoma. *Radiology.* 2015.
36. Gallie BL, Ellsworth RM, Abramson DH, Phillips RA. Retinoma: spontaneous regression of retinoblastoma or benign manifestation of the mutation? *Br J Cancer.* Apr 1982;45(4):513–521.
37. Dimaras H, Khetan V, Halliday W, et al. Loss of RB1 induces non-proliferative retinoma: increasing genomic instability correlates with progression to retinoblastoma. *Hum Mol Genet.* May 15 2008;17(10):1363–1372.
38. Mendoza PR, Specht CS, Hubbard GB, et al. Histopathologic grading of anaplasia in retinoblastoma. *American journal of ophthalmology.* Apr 2015;159(4):764–776.
39. Rushlow DE, Mol BM, Kennett JY, et al. Characterisation of retinoblastomas without RB1 mutations: genomic, gene expression, and clinical studies. *The lancet oncology.* 2013;14(4):327–334.
40. Chantada GL, Dunkel IJ, de Davila MT, Abramson DH. Retinoblastoma patients with high risk ocular pathological features: who needs adjuvant therapy? *The British journal of ophthalmology.* Aug 2004;88(8):1069–1073.
41. Aerts I, Sastre-Garau X, Savignoni A, et al. Results of a multicenter prospective study on the postoperative treatment of unilateral retinoblastoma after primary enucleation. *J Clin Oncol.* Apr 10 2013;31(11):1458–1463.
42. Bosaleh A, Sampor C, Solernou V, et al. Outcome of children with retinoblastoma and isolated choroidal invasion. *Archives of ophthalmology.* Jun 2012;130(6):724–729.

43. Sastre X, Chantada GL, Doz F, et al. Proceedings of the consensus meetings from the International Retinoblastoma Staging Working Group on the pathology guidelines for the examination of enucleated eyes and evaluation of prognostic risk factors in retinoblastoma. *Archives of Pathology and Laboratory Medicine* Aug 2009;133(8):1199–1202.
44. Chantada G, Luna-Fineman S, Sitorus RS, et al. SIOP-PODC recommendations for graduated-intensity treatment of retinoblastoma in developing countries. *Pediatric blood & cancer.* May 2013; 60(5):719–727.
45. Zhao J, Dimaras H, Massey C, et al. Pre-enucleation chemotherapy for eyes severely affected by retinoblastoma masks risk of tumor extension and increases death from metastasis. *J Clin Oncol.* Mar 1 2011;29(7):845–851.
46. Grossniklaus HE, Finger PT, Harbour JW, Kivela T. Protocol for the examination of specimens from patients with retinoblastoma. *CAP Cancer Protocol Templates* 2016: http://www.cap.org/ShowProperty?nodePath=/UCMCon/Contribution%20Folders/WebContent/pdf/cp-retinoblast-16protocol-3200.pdf. Accessed March 14, 2016, 2016.
47. Kattan MW, Hess KR, Amin MB, et al. American Joint Committee on Cancer acceptance criteria for inclusion of risk models for individualized prognosis in the practice of precision medicine. *CA: a cancer journal for clinicians.* Jan 19 2016.

68

Lacrimal Gland Carcinoma

69

Valerie A. White, Bita Esmaeli, Jonathan J. Dutton, Steffen Heegaard, Vivian Yin, Wolfgang A.G. Sauerwein, Sarah E. Coupland, and Paul T. Finger

CHAPTER SUMMARY

Cancers Staged Using This Staging System

Carcinomas of the lacrimal gland

Cancers Not Staged Using This Staging System

These histopathologic types of cancer...	Are staged according to the classification for...	And can be found in chapter...
Carcinomas of nasolacrimal sac	No AJCC staging system	N/A
Lymphoma	Ocular adnexal lymphomas	71

Summary of Changes

Change	Details of Change	Level of Evidence
Definition of Primary Tumor (T)	T1–T3 category definitions now are based on size, with three modifiers for each category:<br>a: no periosteal or bone involvement<br>b: periosteal involvement only<br>c: periosteal and bone involvement	III
Definition of Primary Tumor (T)	T4 tumors (those extending outside the orbit) are now subdivided based on size:<br>a: ≤2 cm<br>b: >2cm and ≤4 cm<br>c: >4 cm	III

ICD-O-3 Topography Codes

Code	Description
C69.5	Lacrimal gland (excluding lacrimal sac)

WHO Classification of Tumors

Code	Description
8941	Carcinoma ex pleomorphic adenoma
8525	Polymorphous low-grade carcinoma
8430	Mucoepidermoid carcinoma
8562	Epithelial–myoepithelial carcinoma
8440	Cystadenocarcinoma and papillary cystadenocarcinoma
8550	Acinic cell carcinoma
8147	Basal cell adenocarcinoma
8480	Mucinous adenocarcinoma
8200	Adenoid cystic carcinoma
8140	Adenocarcinoma, NOS
8500	Ductal adenocarcinoma
8070	Squamous cell carcinoma
8410	Sebaceous adenocarcinoma

To access the AJCC cancer staging forms, please visit www.cancerstaging.org.

© American Joint Committee on Cancer 2017

M.B. Amin et al. (eds.), *AJCC Cancer Staging Manual, Eighth Edition*, DOI 10.1007/978-3-319-40618-3_69

Code	Description
8982	Myoepithelial carcinoma
8082	Lymphoepithelial carcinoma
8980	Carcinosarcoma

International Agency for Research on Cancer, World Health Organization. International Classification of Diseases for Oncology. ICD-O-3-Online. http://codes.iarc.fr/home. Accessed May 15, 2016.

INTRODUCTION

This chapter describes an updated TNM classification for primary carcinomas of the lacrimal gland, which is significantly changed from the AJCC Cancer Staging Manual, 7th Edition (7th Edition) staging system. The AJCC Cancer Staging Manual, 8th Edition lacrimal gland carcinoma staging system is designed to determine which factor—tumor size or periosteal or bone invasion—is the most important factor in determining outcome for these malignancies. This chapter does not apply to mesenchymal, hematologic, or melanocytic tumors of the lacrimal gland, nor does it apply to carcinomas of the lacrimal sac or nasolacrimal duct system.

The retrospective study of 265 epithelial tumors of the lacrimal gland conducted by the Armed Forces Institute of Pathology (AFIP) improved our understanding of the histologic classification and clinical behavior of epithelial tumors of the lacrimal gland.[1] The historic works of Forrest (1954)[2] and Zimmerman (1962)[3] alleviated confusion by applying to epithelial tumors of the lacrimal gland the histopathologic classification of salivary gland tumors. The histologic classification used herein is a modification of the World Health Organization (WHO) classification of salivary gland tumors[4] and is similar to that used in the most recent AFIP fascicle on tumors of the eye and ocular adnexa (2006).[5] This classification was confirmed in recent large series of epithelial lacrimal gland tumors.[6,7]

The use of the TNM classification for lacrimal gland neoplasms is in its infancy, with only two major studies.[8,9] A change introduced in the 7th Edition to combine periosteal and bone involvement resulted in the upstaging of many tumors.[10,11] This was not found to correlate with survival, however, so they were separated again in the 8th Edition. While preparing this edition, we found that only about 50% of lacrimal tumors in the National Cancer Data Base had been staged and could be used for assessment of outcome. This severely limits the usefulness of the database for this ophthalmic site and indicates that greater diligence is necessary in recording these data in patient charts for abstraction by registrars.

Recent molecular investigations of lacrimal gland adenoid cystic carcinomas revealed that they share similar genetic abnormalities with more widely studied tumors from the salivary glands. These abnormalities include the presence of the *MYB–NFIB* fusion gene transcript, increased expression of the MYB protein and MYB target proteins, and rearrangements of the *MYB* gene. Although these anomalies do not correlate with outcome, they provide potential targets for future therapeutic interventions.[12]

The treatment strategies for lacrimal gland malignancies are varied, are not universally accepted, and do not necessarily prevent future recurrence of distant metastases.[13–15] The most widely practiced approach for managing lacrimal gland malignancies is radical, multidisciplinary surgical treatment, including orbital exenteration. Historically, orbital exenteration has been the most commonly performed surgical procedure for lacrimal gland carcinoma. A major reason for this historical trend is the difficulty of achieving adequately wide excision margins without removing or significantly damaging the eye or vital orbital contents, such as extraocular muscles and nerves. Tse et al. in 2006 reported encouraging preliminary results of intra-arterial cytoreductive chemotherapy followed by orbital exenteration followed by adjuvant radiation therapy and several cycles of adjuvant intravenous chemotherapy in patients with lacrimal gland adenoid cystic carcinoma.[16] In 2013, the same authors reported long-term results of this treatment approach in 19 patients and found a potential benefit in local disease control and in increasing disease-free survival in about half the patients.[17] However, this approach remains controversial, and more conservative, eye-sparing techniques have been introduced. Others have proposed orbital-sparing surgery followed by adjuvant radiotherapy, thus avoiding exenteration-related loss of vision and the eye.[13,18] About 50% of adenoid cystic carcinomas have oncogenic mutations, and gene-targeted therapies might be available in the future.[19]

ANATOMY

Primary Site(s)

The lacrimal gland is situated in the anterior superotemporal orbit under the orbital rim. It is not clinically palpable unless enlarged or prolapsed into the upper eyelid. The gland is anatomically divided into two lobes, separated by the lateral horn of the levator aponeurosis. The orbital lobe lies behind the orbital septum, in the extraconal orbital space within the bony lacrimal gland fossa; it is about 20 mm long, 12 mm wide, and 5 mm thick. The smaller palpebral lobe is situated more superficially and inferiorly, behind the levator aponeurosis, where it projects into the lateral portion of the upper eyelid behind the palpebral conjunctiva. The gland lacks a true capsule, but portions of it are covered by a connective tissue layer

continuous with the periorbita. This layer is surgically distinct and important in the management of lacrimal gland tumors. Arterial supply is from the lacrimal branch of the ophthalmic artery, and venous drainage is via the lacrimal vein into the superior ophthalmic vein into the cavernous sinus.

The tumors initially enlarge locally and may grow posteriorly into the orbit, where they may not be evident clinically until quite enlarged. They generally cause proptosis and may compress the globe. Tumors may invade the periosteum and, subsequently, the overlying bone. Many lacrimal neoplasms tend to spread locally via perineural invasion, leading to indistinct margins and poor local control.

Regional Lymph Nodes

Recent investigations identified abundant lymphatic channels in the lacrimal gland. These drain forward into the eyelid lymphatic drainage basin to regional lymph nodes. These nodes include:

Preauricular (parotid)
Superficial
Intraparotid
Submandibular
Anterior cervical

Metastatic Sites

The lung is the most common metastatic site of lacrimal gland carcinomas, followed by bone and liver.[20]

RULES FOR CLASSIFICATION

Clinical Classification

Clinical staging includes a complete history (with emphasis on duration of symptoms, pain, or dysesthesia) and physical examination (including globe displacement or distortion, palpation, and sensory and motor examination). Imaging of the orbit (measuring the largest dimension of the tumor) should be performed as described under the Imaging section to provide critical diagnostic and staging data.

Orbital imaging should evaluate size, shape, extent, and invasion of adjacent structures, including the periosteum, bone, skull base, and periorbital areas. The lateral orbital wall and roof often are involved with adenoid cystic carcinoma of the lacrimal gland; thus, en bloc excision of these orbital walls may be indicated when the bony walls appear either radiographically or clinically (intraoperatively) involved.[14,15] Evaluation of the cervical lymph nodes, lungs, and bone should be included to stage disease (Fig. 69.1).

Fine-needle or incisional biopsy usually is performed to determine the histopathologic tumor type and to guide treatment planning.

Imaging

At a minimum, fine-cut orbital computed tomography (CT) scans (including the skull base) with axial and coronal views, with both tissue and bone window settings, should be obtained initially to evaluate the tumor characteristics necessary for initial staging. Magnetic resonance (MR) imaging with gadolinium enhancement may be used to further define tissue characteristics as indicated.[21,22] If metastatic spread is suspected, a positron emission tomography/CT scan may be helpful at baseline and to follow the treatment progress.[23,24]

TNM information extracted from imaging studies include maximum tumor dimensions, location within the orbit, involvement of orbital walls, and extension into adjacent periorbital sites, including the cranial cavity, sinuses, and infratemporal and temporal fossae.

Emerging imaging modalities may provide additional helpful information.[25] In a preliminary study, echo-planar diffusion-weighted MR imaging could distinguish malignant from benign orbital tumors, with an accuracy of 93%, and revealed a significant difference between well- and poorly differentiated malignancies.[26]

69

Pathological Classification

Complete resection of lacrimal gland carcinomas is indicated. The resection specimen should be sampled thoroughly for evaluation of histologic type and grade of tumor, size, possible presence of a preexistent pleomorphic adenoma, and surgical margins (including the periosteum). For carcinomas arising in pleomorphic adenoma, the extent of invasion beyond the capsule of the pleomorphic adenoma should be described. Approximate percentage of the basaloid pattern present on pathological examination should be reported for adenoid cystic carcinoma. In addition, perineural spread, most characteristic of adenoid cystic carcinoma, may result in a clinical underestimation of the true anatomic extent of disease. All bone removed during surgical treatment should be fully examined pathologically for evidence of involvement by carcinoma.

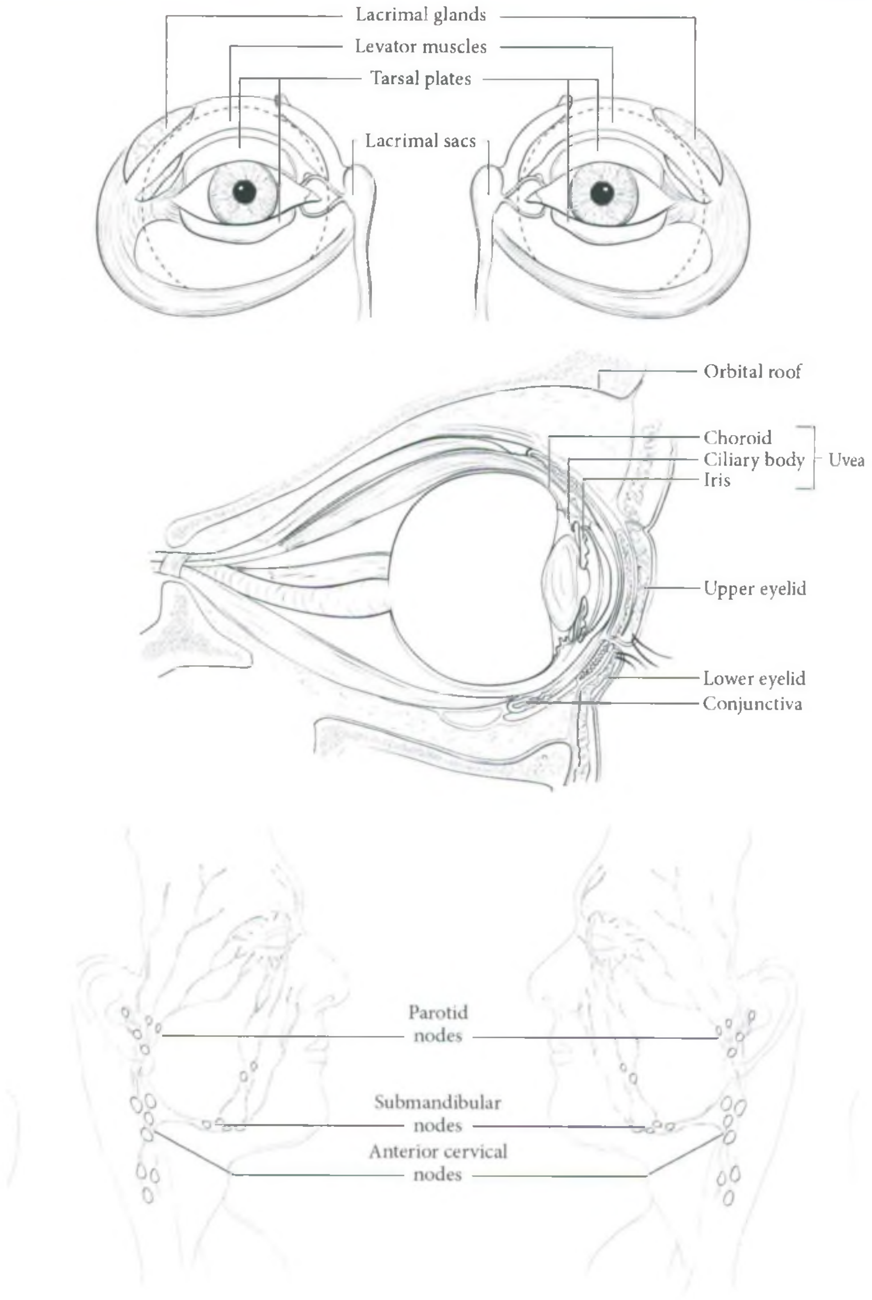

Fig. 69.1 Anatomic sites and regional lymph nodes for ophthalmic sites.

PROGNOSTIC FACTORS

Prognostic Factors Required for Stage Grouping

Beyond the factors used to assign T, N, or M categories, no additional prognostic factors are required for stage grouping.

Additional Factors Recommended for Clinical Care

The authors have not noted any additional factors for clinical care.

RISK ASSESSMENT MODELS

The AJCC recently established guidelines that will be used to evaluate published statistical prediction models for the purpose of granting endorsement for clinical use.[27] Although this is a monumental step toward the goal of precision medicine, this work was published only very recently. For this reason, the existing models that have been published or may be in clinical use have not yet been evaluated for this cancer site by the Precision Medicine Core of the AJCC. In the future, the statistical prediction models for this cancer site will be evaluated, and those that meet all AJCC criteria will be endorsed.

DEFINITIONS OF AJCC TNM

Definition of Primary Tumor (T)

T Category	T Criteria
TX	Primary tumor cannot be assessed
T0	No evidence of primary tumor
T1	Tumor ≤2 cm in greatest dimension with or without extraglandular extension into the orbital soft tissue
T1a	No periosteal or bone involvement
T1b	Periosteal involvement only
T1c	Periosteal and bone involvement
T2	Tumor >2 cm and ≤4 cm in greatest dimension
T2a	No periosteal or bone involvement
T2b	Periosteal involvement only
T2c	Periosteal and bone involvement
T3	Tumor >4 cm in greatest dimension
T3a	No periosteal or bone involvement
T3b	Periosteal involvement only
T3c	Periosteal and bone involvement
T4	Involvement of adjacent structures, including sinuses, temporal fossa, pterygoid fossa, superior orbital fissure, cavernous sinus, or brain
T4a	Tumor ≤2 cm in greatest dimension
T4b	Tumor >2 cm and ≤4 cm in greatest dimension
T4c	Tumor >4 cm in greatest dimension

Definition of Regional Lymph Node (N)

N Category	N Criteria
NX	Regional lymph nodes cannot be assessed
N0	No regional lymph node metastasis
N1	Regional lymph node metastasis

Definition of Distant Metastasis (M)

M Category	M Criteria
M0	No distant metastasis
M1	Distant metastasis

AJCC PROGNOSTIC STAGE GROUPS

No stage groupings are currently recommended for lacrimal gland carcinomas.

REGISTRY DATA COLLECTION VARIABLES

Pathology Related

1. Tumor location (lacrimal gland or lacrimal sac—ICD code lacks specificity)
2. Greatest diameter of the tumor
3. Histopathologic type
4. Perineural invasion present on pathological examination
5. Ki-67 growth fraction (percentage of tumor cells positive for Ki-67 on immunohistochemistry)
6. For carcinoma ex pleomorphic adenoma, extent of invasion beyond capsule of pleomorphic adenoma
7. For adenoid cystic carcinoma, approximate percentage of basaloid pattern present on pathological examination
8. Tumor grade
9. Presence of high-grade transformation in any tumor type
10. Regional lymph node involvement present on any evaluation modality
11. Presence of distant metastases
12. Involvement of periosteum only or periosteum and bone

Treatment Related

1. Globe-sparing surgery performed
2. Exenteration performed
3. Orbital bone removed
4. Postoperative radiotherapy
5. Preoperative chemotherapy (intra-arterial vs. systemic)
6. Postoperative chemotherapy
7. Concurrent chemoradiation

HISTOLOGIC GRADE (G)

Grade is defined largely by the histopathologic type.

G	G Definition
GX	Grade cannot be assessed
G1	Well differentiated
G2	Moderately differentiated: includes adenoid cystic carcinoma without basaloid (solid) pattern
G3	Poorly differentiated: includes adenoid cystic carcinoma with basaloid (solid) pattern
G4	Undifferentiated

HISTOPATHOLOGIC TYPE

Low Grade

Carcinoma ex pleomorphic adenoma (in which the carcinoma is noninvasive or minimally invasive, as defined by the WHO classification [extension ≤1.5 mm beyond the capsule, into surrounding tissue])
Polymorphous low-grade carcinoma
Mucoepidermoid carcinoma, grades 1 and 2
Epithelial–myoepithelial carcinoma
Cystadenocarcinoma and papillary cystadenocarcinoma
Acinic cell carcinoma
Basal cell adenocarcinoma
Mucinous adenocarcinoma

High Grade

Carcinoma ex pleomorphic adenoma (malignant mixed tumor) that includes adenocarcinoma and adenoid cystic carcinoma arising in a pleomorphic adenoma (in which the carcinoma is invasive, as defined by the WHO classification [extension >1.5 mm beyond the capsule, into surrounding tissue])
Adenoid cystic carcinoma, NOS
Adenocarcinoma, NOS
Mucoepidermoid carcinoma, grade 3
Ductal adenocarcinoma
Squamous cell carcinoma
Sebaceous adenocarcinoma
Myoepithelial carcinoma
Lymphoepithelial carcinoma
Carcinosarcoma
Other rare and unclassifiable carcinomas

Bibliography

1. Font R, Gamel J. Epithelial tumors of the lacrimal gland: an analysis of 265 cases. *Ocular and adnexal tumors*: Aesculapius Birmingham; 1978:787–805.
2. Forrest A. Epithelial lacrimal gland tumors: pathology as a guide to prognosis. *Transactions-American Academy of Ophthalmology and Otolaryngology. American Academy of Ophthalmology and Otolaryngology*. 1953;58(6):848–866.
3. Zimmerman LE, Sanders TE, Ackerman LV. Epithelial tumors of the lacrimal gland: prognostic and therapeutic significance of histologic types. *International ophthalmology clinics*. 1962;2(2):337–367.
4. Barnes L, Eveson JW, Reichart P, Sidransky D, eds. *World Health Organization Classification of Tumours Pathology and Genetics of Head and Neck Tumours*. 3rd ed. Lyon: IARC Press; 2005.
5. Schoenfield L. AFIP Atlas of Tumor Pathology: Tumors of the Eye and Ocular Adnexa. LWW; 2008.
6. von Holstein SL, Coupland SE, Briscoe D, Le Tourneau C, Heegaard S. Epithelial tumours of the lacrimal gland: a clinical, histopathological, surgical and oncological survey. *Acta ophthalmologica*. May 2013;91(3):195–206.
7. Weis E, Rootman J, Joly TJ, et al. Epithelial lacrimal gland tumors: pathologic classification and current understanding. *Archives of ophthalmology*. Aug 2009;127(8):1016–1028.
8. Ahmad SM, Esmaeli B, Williams M, et al. American Joint Committee on Cancer classification predicts outcome of patients with lacrimal gland adenoid cystic carcinoma. *Ophthalmology*. Jun 2009;116(6):1210–1215.
9. Skinner HD, Garden AS, Rosenthal DI, et al. Outcomes of malignant tumors of the lacrimal apparatus: the University of Texas MD Anderson Cancer Center experience. *Cancer*. Jun 15 2011;117(12):2801–2810.
10. El-Sawy T, Savar A, Williams MD, De Monte F, Esmaeli B. Prognostic accuracy of the seventh edition vs sixth edition of the American Joint Committee on Cancer tumor classification for adenoid cystic carcinoma of the lacrimal gland. *Archives of ophthalmology*. May 2012;130(5):664–666.
11. Rootman J, White VA. Changes in the 7th edition of the AJCC TNM classification and recommendations for pathologic analysis of lacrimal gland tumors. *Arch Pathol Lab Med*. Aug 2009;133(8):1268–1271.
12. von Holstein SL, Fehr A, Persson M, et al. Adenoid cystic carcinoma of the lacrimal gland: MYB gene activation, genomic imbalances, and clinical characteristics. *Ophthalmology*. Oct 2013;120(10):2130–2138.
13. Finger PT. Radiation therapy for orbital tumors: concepts, current use, and ophthalmic radiation side effects. *Survey of ophthalmology*. 2009;54(5):545–568.
14. Schwarcz RM, Coupland SE, Finger PT. Cancer of the orbit and adnexa. *American journal of clinical oncology*. Apr 2013;36(2): 197–205.
15. Woo KI, Yeom A, Esmaeli B. Management of Lacrimal Gland Carcinoma: Lessons From the Literature in the Past 40 Years. *Ophthalmic plastic and reconstructive surgery*. Jan-Feb 2016; 32(1):1–10.
16. Tse DT, Benedetto P, Dubovy S, Schiffman JC, Feuer WJ. Clinical analysis of the effect of intraarterial cytoreductive chemotherapy in the treatment of lacrimal gland adenoid cystic carcinoma. *American journal of ophthalmology*. 2006;141(1):44–53, e41.
17. Tse DT, Kossler AL, Feuer WJ, Benedetto PW. Long-term outcomes of neoadjuvant intra-arterial cytoreductive chemotherapy for lacrimal gland adenoid cystic carcinoma. *Ophthalmology*. 2013;120(7):1313–1323.
18. Holliday EB, Esmaeli B, Pinckard J, et al. A Multidisciplinary Orbit-Sparing Treatment Approach That Includes Proton Therapy for Epithelial Tumors of the Orbit and Ocular Adnexa. *International journal of radiation oncology, biology, physics*. 2016;95(1): 344–352.
19. Bell D, Sniegowski MC, Wani K, Prieto V, Esmaeli B. Mutational landscape of lacrimal gland carcinomas and implications for treatment. *Head & neck*. 2016;38(Suppl 1):E724–729.
20. Esmaeli B, Ahmadi MA, Youssef A, et al. Outcomes in patients with adenoid cystic carcinoma of the lacrimal gland. *Ophthalmic plastic and reconstructive surgery*. Jan 2004;20(1):22–26.
21. Heran F, Berges O, Blustajn J, et al. Tumor pathology of the orbit. *Diagn Interv Imaging*. Oct 2014;95(10):933–944.

22. Tailor TD, Gupta D, Dalley RW, Keene CD, Anzai Y. Orbital neoplasms in adults: clinical, radiologic, and pathologic review. *Radiographics : a review publication of the Radiological Society of North America, Inc.* Oct 2013;33(6):1739–1758.
23. Bhagat N, Zuckier LS, Hameed M, Cathcart C, Baredes S, Ghesani NV. Detection of recurrent adenoid cystic carcinoma with PET-CT. *Clinical nuclear medicine.* Jul 2007;32(7):574–577.
24. Choi M, Koo JS, Yoon JS. Recurred Adenoid Cystic Carcinoma of Lacrimal Gland with Aggressive Local Invasion to the Maxillary Bone Marrow without Increased Uptake in PET-CT. *Korean Journal of Ophthalmology.* 2015;29(1):68–70.
25. Hricak H. Oncologic imaging: a guiding hand of personalized cancer care. *Radiology.* Jun 2011;259(3):633–640.
26. Razek AAKA, Elkhamary S, Mousa A. Differentiation between benign and malignant orbital tumors at 3-T diffusion MR-imaging. *Neuroradiology.* 2011;53(7):517–522.
27. Kattan MW, Hess KR, Amin MB, et al. American Joint Committee on Cancer acceptance criteria for inclusion of risk models for individualized prognosis in the practice of precision medicine. *CA Cancer J Clin.* 2016 Jan 19. doi: 10.3322/caac.21339 [Epub ahead of print].

69

Orbital Sarcoma

70

Jonathan J. Dutton, Bita Esmaeli, Valerie A. White, Christian W. Wittekind, Wolfgang A.G. Sauerwein, Hakan Demirci, Sarah E. Coupland, and Paul T. Finger

CHAPTER SUMMARY

Cancers Staged Using This Staging System

Sarcomas

Cancers Not Staged Using This Staging System

These histopathologic types of cancer...	Are staged according to the classification for...	And can be found in chapter...
Osseous and cartilaginous tumors arising in bone	Bone	38
Secondary tumors that arise in adjacent periorbital sites with orbital invasion	Primary site of tumor	N/A
Lacrimal gland carcinoma	Lacrimal gland carcinoma	69
Lacrimal gland sarcomas	Soft tissue sarcoma of the head and neck	40

Summary of Changes

Change	Details of Change	Level of Evidence
T1 and T2 categories	Cutoff between T1 and T2 changed from 15 mm to 20 mm	IV

ICD-O-3 Topography Codes

Code	Description
C69.0	Conjunctiva
C69.1	Cornea, NOS
C69.2	Retina
C69.3	Choroid
C69.4	Ciliary body
C69.5	Lacrimal gland
C69.6	Orbit, NOS
C69.8	Overlapping lesion of eye and adnexa
C69.9	Eye, NOS
C72.3	Optic nerve

WHO Classification of Tumors

Code	Description
8804	Epithelioid sarcoma
8806	Desmoplastic small round cell tumor
8810	Fibrosarcoma
8811	Myxoinflammatory fibroblastic sarcoma/atypical myxoinflammatory fibroblastic tumor
8811	Myxofibrosarcoma
8814	Infantile fibrosarcoma
8815	Solitary fibrous tumor, malignant
8821	Ectomesenchymoma
8825	Inflammatory myofibroblastic tumor

To access the AJCC cancer staging forms, please visit www.cancerstaging.org.

© American Joint Committee on Cancer 2017
M.B. Amin et al. (eds.), *AJCC Cancer Staging Manual, Eighth Edition*, DOI 10.1007/978-3-319-40618-3_70

Code	Description
8825	Low-grade myofibroblastic sarcoma
8832	Dermatofibrosarcoma protuberans
8840	Low-grade fibromyxoid sarcoma
8840	Sclerosing epithelioid fibrosarcoma
8850	Liposarcoma, NOS
8852	Myxoid liposarcoma
8854	Pleomorphic liposarcoma
8858	Dedifferentiated liposarcoma
8890	Leiomyosarcoma
8901	Pleomorphic rhabdomyosarcoma
8902	Alveolar rhabdomyosarcoma
8910	Embryonal rhabdomyosarcoma
8912	Spindle cell/sclerosing rhabdomyosarcoma
8963	Extrarenal rhabdoid tumor
9040	Synovial sarcoma
9120	Angiosarcoma
9133	Epithelioid hemangioendothelioma
9240	Extraskeletal mesenchymal chondrosarcoma
9264	Extraskeletal Ewing sarcoma
9540	Malignant peripheral nerve sheath tumor
9580	Malignant granular cell tumor
9581	Alveolar soft part sarcoma

Fletcher CDM, Bridge JA, Hogendoorn P, Mertens F, eds. World Health Organization Classification of Tumours of Soft Tissue and Bone. Fourth Edition. Lyon: IARC; 2013.

INTRODUCTION

This chapter covers primary soft tissue sarcomas of the orbit. Such tumors arise from a variety of orbital mesenchymal or precursor tissues, including skeletal and smooth muscle, adipose, fibroblastic, vascular, and nerve sheath tissues. Some tumors of uncertain differentiation, such as synovial sarcoma and alveolar soft part sarcoma also are included in this chapter.

Sarcomas are extremely rare tumors, representing about 1% of all solid malignancies in adults, only 0.4% of which occur in the orbit. They include a wide variety of neoplasms that show differentiation toward components of the connective (soft) tissue. Except for rhabdomyosarcoma, most are lesions with fewer than 40 to 50 cases described in the literature; therefore, evidence-based data frequently are inadequate or absent. Because of this, recommendations regarding treatment for this diverse group of malignancies are based largely on our experience with sarcomas elsewhere in the body. Response to chemotherapy and radiotherapy varies according to histologic type.[1–3] Given the small size of the orbit and the desire to preserve visual function, ocular motility, and cosmetic appearance if possible, surgery with clear margins, combined with appropriate adjuvant and neoadjuvant therapy, is recommended in most cases.[4]

The classification in this chapter applies to more than 30 different sarcomas[5,6] but does not include cartilaginous/osseous neoplasms, such as osteogenic sarcoma and chrondrosarcoma, which are covered in other chapters. In addition, it does not apply to sarcomas that secondarily involve the orbit, including those arising from the globe, conjunctiva, nasal and paranasal sinus mucosa, dura, and brain. Metastatic tumors are discussed in chapters dealing with the primary lesions. However, because extension of primary orbital tumors into adjacent sites does influence outcome, staging should specify secondary involvement of periorbital regions.

As with systemic sarcomas, histologic type and grade are important parameters for adequate staging. Histologic grade is added here to include features such as subtype, degree of differentiation, necrosis, and mitotic activity.

ANATOMY

Primary Site(s)

The orbit is a cone-shaped cavity surrounded by seven bones, with a volume of approximately 30 cc (Fig. 70.1). The globe constitutes about 7 cc of this total and is positioned centrally and anteriorly within the orbital cavity. Numerous anatomic systems that support the globe and periorbital tissues are crowded within the orbit or traverse the orbit around the globe.[7] These structures include the optic nerve and its meninges, lacrimal gland, extraocular muscles, fascial connective tissue, orbital fat, cranial and autonomic nerves, and blood vessels. Any of these tissues may be the site of origin for a wide variety of primary sarcomas. Secondary tumors from adjacent structures, such as the paranasal sinuses, conjunctiva, and globe, as well as metastatic tumors from distant locations, are encountered in the orbit. In addition, because of their immediate proximity, primary orbital tumors may invade into the central nervous system, nasal cavity, and paranasal sinuses.

The orbit is said to have two unique histopathologic features that may have some influence on tumor dissemination to and from this location: the absence of lymphatics and the absence of venous valves. Although the human orbit was long considered to be devoid of lymphatics, they have been identified behind the orbital septum; sparsely in the lacrimal gland, optic nerve dura, and extraocular muscles; and in the orbital apex.[8] Similarly, orbital veins have been considered to be valveless, but a recent study demonstrated valves in the superior ophthalmic vein and its major facial tributaries, with valves oriented for blood flow back toward the cavernous sinus.[9]

Primary orbital soft tissue sarcomas may show differentiation toward fat (liposarcoma), striated muscle (rhabdomyosarcoma), smooth muscle (leiomyosarcoma), fibroconnective tissue (fibrosarcoma), vascular tissues (angiosarcoma, hemangiopericytoma), and peripheral nerve (peripheral nerve sheath tumor), as well as being of uncertain derivation.

Regional Lymph Nodes

Although there are very limited diffuse and poorly organized lymphatics behind the orbital septum, their role in orbital tumor dissemination remains unknown (Fig. 70.1). For orbital tumors involving the lacrimal gland or extending forward to the eyelids and conjunctiva, lymphatic drainage is into the parotid (preauricular), submandibular, and cervical lymph nodes. Recent investigations have shown that all areas of the eyelids may drain into the parotid nodes, in addition to the medial lids draining into the submandibular nodes.[10] The parotid nodes consist of three groups: superficial, intraparotid, and deep nodes. Lymphatic drainage from the eyelids flows primarily into the superficial group but also may involve the intraparotid nodes (Fig. 70.1). A recent study showed that intraglandular nodes may be present in 30% of deep parotid gland lobes.[11] There also is considerable variation in eyelid lymphatic drainage patterns, and some channels may bypass expected nodes to reach nodes in the lower neck. For orbital tumors extending to the lacrimal gland, conjunctiva, or eyelids, dissemination is possible via direct access to the facial lymphatic drainage system. The regional lymph nodes include the following:

- Preauricular (parotid)
  - Superficial
  - Intraparotid
- Submandibular
- Cervical

Metastatic Sites

Metastatic spread from the orbit occurs primarily by hematogenous dissemination, but spread via local periobital lymphatics may occur if the eyelids, conjunctiva, and lacrimal gland are involved. Almost any site may be affected with metastases, but the lungs are a primary target.

RULES FOR CLASSIFICATION

Clinical Classification

Clinical classification should be based on the medical history, physical examination, diagnostic incisional biopsies, and imaging studies. Symptoms and signs related to loss of vision and visual field deficits, degree of global displacement, loss or limitation of extraocular motility, a palpable mass, degree of compressive optic neuropathy, eyelid edema, or ptosis should be recorded along with the regional node evaluation. The direction of globe displacement should be noted, as it is a clue to the anatomic location of the tumor within the orbit. Results of surgical exploration or biopsy may be included. Diagnostic tests should include perimetry, optic nerve function, ocular motility, routine clinical imaging, and specific studies, such as angiography, if indicated. The histopathology results of biopsy, fine-needle aspiration, or complete excision should be noted.

Evidence of tumor size, as well as involvement of orbital structures such as the extraocular muscles, lacrimal gland, globe, and orbital bones, must be assessed to assign clinical staging and TNM category. Primary orbital malignancies may extend directly into adjacent periorbital structures (T4), including the conjunctiva, eyelids, paranasal sinuses, infratemporal and temporal fossae, and intracranial cavity. These also should be recorded.

Imaging

At a minimum, an orbital computed tomography (CT) scan with axial and coronal views, with both tissue and bone window settings, should be obtained initially to evaluate the tumor characteristics necessary for initial staging. Magnetic resonance (MR) imaging with gadolinium enhancement may be performed to further define tissue characteristics as indicated.[12,13] For anterior orbital lesions, B-scan echography sometimes is useful in evaluating size, consistency, and vascularity. A-scan ultrasonography may be useful for characterizing gross histologic structure. If metastatic spread is suspected, a positron emission tomography/CT scan may be helpful at baseline and in following the progress of treatment.[14]

TNM information extracted from imaging studies would include maximum tumor dimensions, location within the orbit, involvement of orbital walls, and extension into adjacent periorbital sites, including the cranial cavity, sinuses, and eyelids.

Emerging modalities may provide some additional helpful information.[15] In a preliminary study, echo-planar diffusion-weighted MR imaging could distinguish malignant from benign orbital tumors with an accuracy of 93% and showed a significant difference between well- and poorly differentiated malignancies.[16]

Pathological Classification

Pathological classification is based on the specific histopathologic type or subtype of the tumor, its differentiation (grade), and the extent of removal (evaluation of its excisional margins). In total excision specimens, evaluation of the surgical margins is mandatory. Tumor size should be recorded in at least two dimensions based on surgical or pathological measurements. In circumstances in which direct tumor measurement is not possible, radiologic assessment should be performed. A pathologist should assign the histologic grade.

For pathological evaluation of node involvement (pN), the examination of a biopsy or regional lymphadenectomy specimen ordinarily would include one or more lymph node(s).

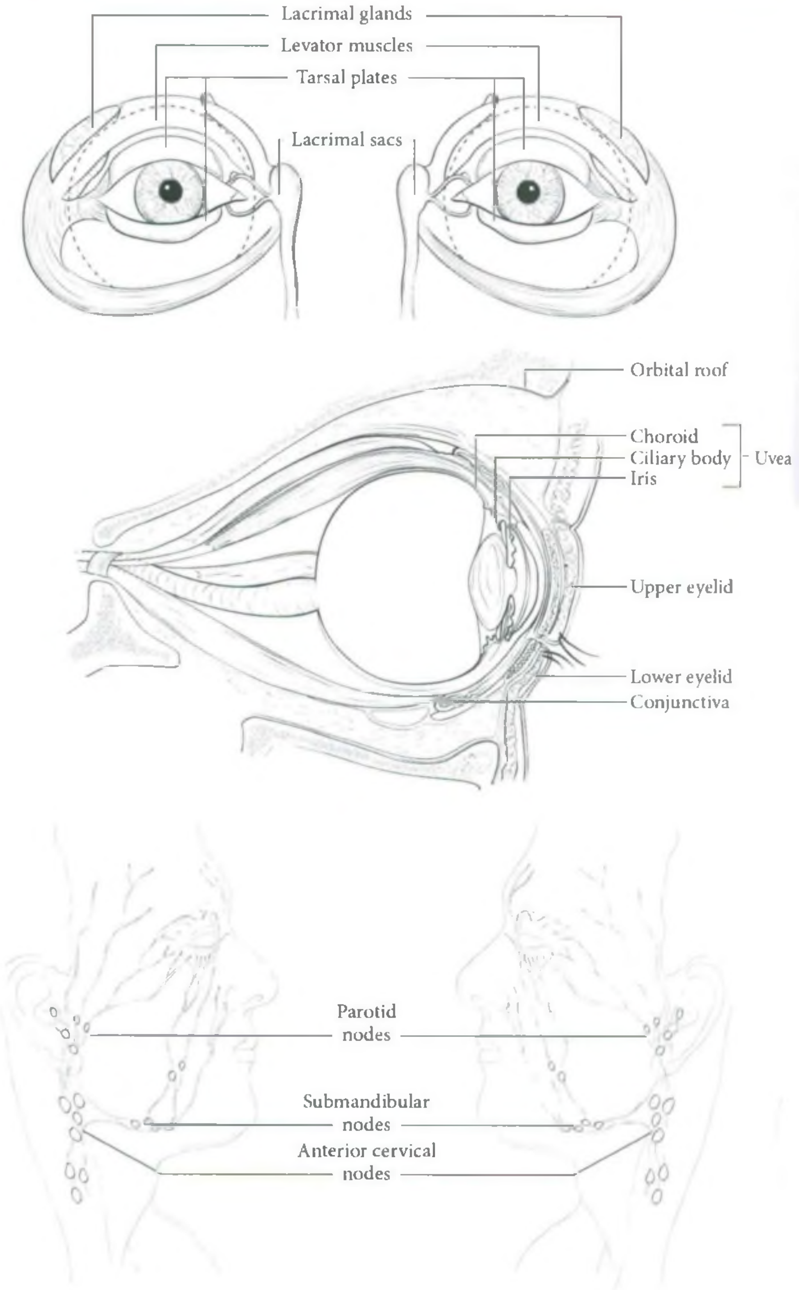

Fig. 70.1 Anatomic sites and regional lymph nodes for ophthalmic sites

PROGNOSTIC FACTORS

Prognostic Factors Required for Stage Grouping

Beyond the factors used to assign T, N, or M categories, no additional prognostic factors are required for stage grouping.

Additional Factors Recommended for Clinical Care

- Tumor histologic type
- Tumor grade
- Tumor maximum dimension
- Invasion of orbital structures such as the globe, lacrimal gland, extraocular muscles, and bony walls
- Invasion of adjacent periorbital structures such as the eyelid, conjunctiva, sinuses, and brain
- Regional lymph node metastasis
- Distant metastasis
- Tumor recurrence
- Mitotic count, assessed as 10 successive high-power fields (HPF; at 400× magnification = 0.1734 mm^2) using a 40× objective

RISK ASSESSMENT MODELS

The AJCC recently established guidelines that will be used to evaluate published statistical prediction models for the purpose of granting endorsement for clinical use.[17] Although this is a monumental step toward the goal of precision medicine, this work was published only very recently. Therefore, the existing models that have been published or may be in clinical use have not yet been evaluated for this cancer site by the Precision Medicine Core of the AJCC. In the future, the statistical prediction models for this cancer site will be evaluated, and those that meet all AJCC criteria will be endorsed.

DEFINITIONS OF AJCC TNM

Definition of Primary Tumor (T)

T Category	T Criteria
TX	Primary tumor cannot be assessed
T0	No evidence of primary tumor
T1	Tumor ≤2 cm in greatest dimension
T2	Tumor >2 cm in greatest diameter without invasion of bony walls or globe
T3	Tumor of any size with invasion of bony walls
T4	Tumor of any size with invasion of globe or periorbital structures, including eyelid, conjunctiva, temporal fossa, nasal cavity, paranasal sinuses, and/or central nervous system

Definition of Regional Lymph Node (N)

N Category	N Criteria
NX	Regional lymph nodes cannot be assessed
N0	No regional lymph node metastasis
N1	Regional lymph node metastasis

Definition of Distant Metastasis (M)

M Category	M Criteria
M0	No distant metastasis
M1	Distant metastasis

AJCC PROGNOSTIC STAGE GROUPS

There is no proposal for anatomic stage and prognostic groups at this time.

REGISTRY DATA COLLECTION VARIABLES

None

HISTOLOGIC GRADE (G)

Currently, the preferred system for grading of sarcomas is the one proposed by the French Federation of Cancer Centers Sarcoma Group (FNCLCC), otherwise known as the French grading system.[18] It uses three independent prognostic factors to determine the grade: mitotic activity, necrosis, and degree of differentiation of the primary tumor. Each feature is scored separately, and the three scores are added to obtain the grade. Grade 1 is defined as a total score of 2 or 3, grade 2 as a total score of 4 or 5, and grade 3 as a total score of 6 to 8. To enhance the reproducibility of the system, the parameters are defined as precisely as possible. The main value of the grading is to determine risk of distant metastases and overall survival, rather than local recurrence, which depends more on adequate surgical margins.

Mitotic Count

In the most mitotically active area of the sarcoma, 10 successive high-power fields (HPF; one HPF at 400× magnification = 0.1734 mm^2) are assessed using a 40× objective.

Mitotic Count Score	Definition
1	0–9 mitoses per 10 HPF
2	10–19 mitoses per 10 HPF
3	≥20 mitoses per 10 HPF

Tumor Necrosis

Tumor necrosis is evaluated on gross examination and validated with histologic sections. Necrosis related to previous surgery or to ulceration is not be taken into account, nor is hemorrhage or hyalinization.

Necrosis Score	Definition
0	No necrosis
1	<50% tumor necrosis
2	≥50% tumor necrosis

Tumor Differentiation

Tumor differentiation is histology specific and is a mixture of histologic type and subtype and/or true differentiation.

Differentiation Score	Definition
1	Sarcomas closely resembling normal adult mesenchymal tissue (e.g., low-grade leiomyosarcoma)
2	Sarcomas for which histologic typing is certain (e.g., myxoid/round cell liposarcoma)
3	Embryonal and undifferentiated sarcomas, sarcomas of doubtful type, synovial sarcomas, soft tissue osteosarcoma, Ewing sarcoma /primitive neuroectodermal tumor (PNET) of soft tissue

FNCLCC Histologic Grade

G	G Definition
GX	Grade cannot be assessed
G1	Total differentiation, mitotic count and necrosis score 2 or 3
G2	Total differentiation, mitotic count and necrosis score 4 or 5
G3	Total differentiation, mitotic count and necrosis score 6, 7, or 8

HISTOPATHOLOGIC TYPE

Malignancies of the orbit primarily include a broad spectrum of malignant soft tissue tumors[19]:

- Skeletal muscle tumors
  - Rhabdomyosarcoma[20, 21]
    - Embryonal
    - Alveolar
    - Spindle cell/sclerosing
    - Pleomorphic
- Adipocytic
  - Myxoid liposarcoma[22]
  - Pleomorphic liposarcoma[23]
  - Dedifferentiated liposarcoma[24]
  - Liposarcoma, NOS[25]
- Fibroblastic
  - Malignant
    - Adult fibrosarcoma[26]
    - Myxofibrosarcoma[27]
    - Low-grade fibromyxoid sarcoma[28]
    - Sclerosing epithelioid fibrosarcoma[29]
  - Intermediate (rarely metastasizing)
    - Dermatofibrosarcoma protuberans[30]
    - Solitary fibrous tumor, malignant/hemangiopericytoma[31]
    - Inflammatory myofibroblastic tumor[32]
    - Low-grade myofibroblastic sarcoma[33]
    - Myxoinflammatory fibroblastic sarcoma/atypical myxoinflammatory fibroblastic tumor[34]
    - Infantile fibrosarcoma[35]
- Vascular
  - Epithelioid hemangioendothelioma[36]
  - Angiosarcoma of soft tissue[37]
- Nerve sheath tumors
  - Malignant peripheral nerve sheath tumor[38]
  - Ectomesenchymoma[39]
  - Malignant granular cell tumor[40]
- Smooth muscle tumors
  - Leiomyosarcoma[41]
- Extraskeletal mesenchymal chondrosarcoma[42]
- Tumors of uncertain differentiation
  - Synovial sarcoma[43]
  - Epithelioid sarcoma[44]
  - Alveolar soft part sarcoma[45]
  - Extraskeletal Ewing sarcoma[46]
  - Extrarenal rhabdoid tumor[47]
  - Desmoplastic small round cell tumor[48]
- Undifferentiated/unclassified sarcomas

Bibliography

1. Finger PT. Radiation therapy for orbital tumors: concepts, current use, and ophthalmic radiation side effects. *Survey of ophthalmology*. 2009;54(5):545–568.
2. Schoffski P, Cornillie J, Wozniak A, Li H, Hompes D. Soft tissue sarcoma: an update on systemic treatment options for patients with advanced disease. *Oncol Res Treat*. 2014;37(6):355–362.
3. Potter BO, Sturgis EM. Sarcomas of the head and neck. *Surg Oncol Clin N Am*. Apr 2003;12(2):379–417.
4. Savar A, Trent J, Al-Zubidi N, et al. Efficacy of adjuvant and neoadjuvant therapies for adult orbital sarcomas. *Ophthalmic plastic and reconstructive surgery*. May-Jun 2010;26(3):185–189.
5. Doyle LA. Sarcoma classification: an update based on the 2013 World Health Organization Classification of Tumors of Soft Tissue and Bone. *Cancer*. Jun 15 2014;120(12):1763–1774.
6. Fletcher CD. The evolving classification of soft tissue tumours–an update based on the new 2013 WHO classification. *Histopathology*. 2014;64(1):2–11.
7. Dutton JJ. Atlas of Clinical and Surgical Orbital Anatomy, 2nd edition. 2011.
8. Dickinson A, Gausas R. Orbital lymphatics: do they exist? *Eye (London, England)*. 2006;20(10):1145–1148.
9. Zhang J, Stringer MD. Ophthalmic and facial veins are not valveless. *Clinical & experimental ophthalmology*. 2010;38(5):502–510.
10. Nijhawan N, Marriott C, Harvey JT. Lymphatic drainage patterns of the human eyelid: assessed by lymphoscintigraphy. *Ophthalmic plastic and reconstructive surgery*. Jul-Aug 2010;26(4):281–285.
11. Ergün SS, Gayretli Ö, Büyükpınarbaşılı N, et al. Determining the number of intraparotid lymph nodes: Postmortem examination. *Journal of Cranio-Maxillofacial Surgery*. 2014;42(5):657–660.
12. Heran F, Berges O, Blustajn J, et al. Tumor pathology of the orbit. *Diagn Interv Imaging*. Oct 2014;95(10):933–944.
13. Tailor TD, Gupta D, Dalley RW, Keene CD, Anzai Y. Orbital neoplasms in adults: clinical, radiologic, and pathologic review. *Radiographics : a review publication of the Radiological Society of North America, Inc*. Oct 2013;33(6):1739–1758.
14. Hui KH, Pfeiffer ML, Esmaeli B. Value of positron emission tomography/computed tomography in diagnosis and staging of primary ocular and orbital tumors. *Saudi journal of ophthalmology : official journal of the Saudi Ophthalmological Society*. Oct 2012;26(4):365–371.
15. Hricak H. Oncologic imaging: a guiding hand of personalized cancer care. *Radiology*. Jun 2011;259(3):633–640.
16. Razek AAKA, Elkhamary S, Mousa A. Differentiation between benign and malignant orbital tumors at 3-T diffusion MR-imaging. *Neuroradiology*. 2011;53(7):517–522.
17. Kattan MW, Hess KR, Amin MB, et al. American Joint Committee on Cancer acceptance criteria for inclusion of risk models for individualized prognosis in the practice of precision medicine. *CA: a cancer journal for clinicians*. Jan 19 2016.
18. Neuville A, Chibon F, Coindre JM. Grading of soft tissue sarcomas: from histological to molecular assessment. *Pathology*. Feb 2014;46(2):113–120.
19. Fletcher CDM, Bridge JA, Hogendoorn P, Mertens F, eds. *World Health Organization Classification of Tumours of Soft Tissue and Bone*. 4th ed. Lyon: IARC; 2013.
20. Karcioglu ZA, Hadjistilianou D, Rozans M, DeFrancesco S. Orbital rhabdomyosarcoma. *Cancer control : journal of the Moffitt Cancer Center*. Sep-Oct 2004;11(5):328–333.
21. Shields JA, Shields CL. Rhabdomyosarcoma: Review for the Ophthalmologist. *Survey of ophthalmology*. 2003;48(1):39–57.
22. Gire J, Weinbreck N, Labrousse F, Denis D, Adenis JP, Robert PY. Myxofibrosarcoma of the orbit: case report and review of literature. *Ophthalmic plastic and reconstructive surgery*. Jan-Feb 2012;28(1):e9–e11.
23. Wang L, Ren W, Zhou X, Sheng W, Wang J. Pleomorphic liposarcoma: a clinicopathological, immunohistochemical and molecular cytogenetic study of 32 additional cases. *Pathol Int*. Nov 2013;63(11):523–531.
24. Saeed MU, Chang BY, Atherley C, Khandwala M, Merchant DW, Liddington M. A rare diagnosis of dedifferentiated liposarcoma of the orbit. *Orbit*. Mar 2007;26(1):43–45.
25. Madge SN, Tumuluri K, Strianese D, et al. Primary orbital liposarcoma. *Ophthalmology*. Mar 2010;117(3):606–614.
26. Scruggs BA, Ho ST, Valenzuela AA. Diagnostic challenges in primary orbital fibrosarcoma: a case report. *Clinical ophthalmology*. 2014;8:2319–2323.
27. Wang M, Khurana RN, Parikh JG, Hidayat AA, Rao NA. Myxofibrosarcoma of the orbit: an underrecognized entity? Case report and review of the literature. *Ophthalmology*. Jul 2008;115(7):1237–1240 e1232.
28. Papadimitriou JC, Ord RA, Drachenberg CB. Head and neck fibromyxoid sarcoma: clinicopathological correlation with emphasis on peculiar ultrastructural features related to collagen processing. *Ultrastruct Pathol*. Jan-Feb 1997;21(1):81–87.
29. Hasan Z, Clark JR, Fowler A. A facial dismasking approach for resection of an infratemporal fossa sclerosing epithelioid fibrosarcoma. *ANZ journal of surgery*. Dec 2011;81(12):947–948.
30. Rahman T, Bhattacharjee K, Sarma JD, Dey D, Kuri G. Primary dermatofibrosarcoma protuberans of orbit--a rare entity. *Orbit*. Apr 2013;32(2):127–129.
31. Tenekeci G, Sari A, Vayisoglu Y, Serin O. Giant Solitary Fibrous Tumor of Orbit. *J Craniofac Surg*. Jul 2015;26(5):e390–392.
32. Cramer SK, Skalet A, Mansoor A, Wilson DJ, Ng JD. Inflammatory myofibroblastic tumor of the orbit: a case report. *Ophthalmic plastic and reconstructive surgery*. Jan-Feb 2015; 31(1):e22–23.
33. Takahama A, Jr., Nascimento AG, Brum MC, Vargas PA, Lopes MA. Low-grade myofibroblastic sarcoma of the parapharyngeal space. *International journal of oral and maxillofacial surgery*. Oct 2006;35(10):965–968.
34. Kato M, Tanaka T, Ohno T. Myxoinflammatory Fibroblastic Sarcoma: A Radiographical, Pathological, and Immunohistochemical Report of Rare Malignancy. *Case Rep Orthop*. 2015;2015:620923.
35. Weiner JM, Hidayat AA. Juvenile fibrosarcoma of the orbit and eyelid. A study of five cases. *Archives of ophthalmology*. Feb 1983;101(2):253–259.
36. Kiratli H, Tarlan B, Ruacan S. Epitheloid hemangioendothelioma of the palpebral lobe of the lacrimal gland. *Orbit*. Apr 2013;32(2):120–123.
37. Siddens JD, Fishman JR, Jackson IT, Nesi FA, Tsao K. Primary orbital angiosarcoma: a case report. *Ophthalmic plastic and reconstructive surgery*. Nov 1999;15(6):454–459.
38. Miller NR. Primary tumours of the optic nerve and its sheath. *Eye*. Nov 2004;18(11):1026–1037.
39. Paikos P, Papathanassiou M, Stefanaki K, Fotopoulou M, Grigorios S, Tzortzatou F. Malignant ectomesenchymoma of the orbit in a child: Case report and review of the literature. *Survey of ophthalmology*. Jul-Aug 2002;47(4):368–374.
40. Morgenstern C, Lipman H, Gruntzig J. [Granular cell tumor of the orbits. Diagnosis and therapy]. *Laryngol Rhinol Otol (Stuttg)*. Dec 1986;65(12):691–692.
41. Meekins BB, Dutton JJ, Proia AD. Primary orbital leiomyosarcoma. A case report and review of the literature. *Archives of ophthalmology*. Jan 1988;106(1):82–86.
42. Kaur A, Kishore P, Agrawal A, Gupta A. Mesenchymal chondrosarcoma of the orbit: a report of two cases and review of the literature. *Orbit*. 2008;27(1):63–67.

43. Liu K, Duan X, Yang L, Yu Y, Liu B. Primary synovial sarcoma in the orbit. *J AAPOS*. Dec 2012;16(6):582–584.
44. Jurdy LL, Blank LE, Bras J, Saeed P. Orbital Epithelioid Sarcoma: A Case Report. *Ophthalmic plastic and reconstructive surgery*. Mar-Apr 2016;32(2):e47–48.
45. Kim HJ, Wojno T, Grossniklaus HE, Shehata BM. Alveolar soft-part sarcoma of the orbit: report of 2 cases with review of the literature. *Ophthalmic plastic and reconstructive surgery*. Nov-Dec 2013;29(6):e138–142.
46. Pang NK, Bartley GB, Giannini C. Primary Ewing sarcoma of the orbit in an adult. *Ophthalmic plastic and reconstructive surgery*. Mar-Apr 2007;23(2):153–154.
47. Mulay K, Honavar SG. Primary, orbital, malignant extra-renal, non-cerebral rhabdoid tumour. *Orbit*. Aug 2014;33(4):292–294.
48. Yoon M, Desai K, Fulton R, et al. Desmoplastic small round cell tumor: a potentially lethal neoplasm manifesting in the orbit with associated visual symptoms. *Archives of ophthalmology*. Apr 2005;123(4):565–567.

Ocular Adnexal Lymphoma

71

Steffen Heegaard, Patricia Chevez-Barrios,
Valerie A. White, Sarah E. Coupland, and Paul T. Finger

CHAPTER SUMMARY

Cancers Staged Using This Staging System

The lymphomas staged according to this system are primary lymphomas arising in the ocular adnexa—namely, the conjunctiva, eyelids, lacrimal gland, lacrimal drainage apparatus, and other orbital tissues surrounding the eye.

Cancers Not Staged Using This Staging System

These histopathologic types of cancer…	Are staged according to the classification for…	And can be found in chapter…
Secondary ocular adnexal lymphomas	Hodgkin and non-Hodgkin lymphoma	79
Intraocular lymphomas	Hodgkin and non-Hodgkin lymphoma	79

Summary of Changes

Change	Details of Change	Level of Evidence
Definition of Primary Tumor (T)	T categories have been revised and are now based on anatomic extent of disease instead of tumor size. Subcategories for T1, T2, T3, and T4 have been removed.	II
Definition of Regional Lymph Node (N)	N categories have been revised to reflect site of nodal involvement.	III

ICD-O-3 Topography Codes

Code	Description
C44.1	Eyelid
C69.0	Conjunctiva
C69.5	Lacrimal gland
C69.6	Orbit, NOS

WHO Classification of Tumors

Code	Description
9590	Malignant lymphoma, NOS
9590	Lymphoma, NOS
9591	Malignant lymphoma, diffuse, NOS
9591	Malignant lymphoma, non–cleaved cell, NOS
9591	B-cell lymphoma, NOS
9591	Malignant lymphoma, non-Hodgkin, NOS
9591	Non-Hodgkin lymphoma, NOS
9591	Malignant lymphoma, small cell, noncleaved, diffuse
9591	Malignant lymphoma, undifferentiated cell type, NOS
9591	Malignant lymphoma, undifferentiated cell, non-Burkitt
9591	Malignant lymphoma, lymphocytic, intermediate differentiation, nodular
9591	Malignant lymphoma, small cleaved cell, diffuse
9591	Malignant lymphoma, lymphocytic, poorly differentiated, diffuse

To access the AJCC cancer staging forms, please visit www.cancerstaging.org.

© American Joint Committee on Cancer 2017
M.B. Amin et al. (eds.), *AJCC Cancer Staging Manual, Eighth Edition*, DOI 10.1007/978-3-319-40618-3_71

Code	Description
9591	Malignant lymphoma, small cleaved cell, NOS
9591	Malignant lymphoma, cleaved cell, NOS
9591	Hairy cell leukemia variant
9596	Composite Hodgkin and non-Hodgkin lymphoma
9596	B-cell lymphoma, unclassifiable, with features intermediate between diffuse large B-cell lymphoma and classical Hodgkin lymphoma
9597	Primary cutaneous follicle center lymphoma
9702	Mature T-cell lymphoma, NOS
9702	Peripheral T-cell lymphoma, NOS
9702	T-cell lymphoma, NOS
9702	Peripheral T-cell lymphoma, pleomorphic small cell
9702	Peripheral T-cell lymphoma, pleomorphic medium and large cell
9702	Peripheral T-cell lymphoma, large cell
9702	T-zone lymphoma
9702	Lymphoepithelioid lymphoma
9702	Lennert lymphoma
9702	Anaplastic large cell lymphoma, ALK (anaplastic lymphoma kinase)
9705	Angioimmunoblastic T-cell lymphoma
9705	Peripheral T-cell lymphoma, AILD (angioimmunoblastic lymphadenopathy with dysproteinemia)
9705	Angioimmunoblastic lymphoma

Swerdlow SH, Campo E, Harris NL, Jaffe ES, Pileri SA, Stein H, Thiele J, Vardiman J, eds. World Health Organization Classification of Tumours of Haematopoietic and Lymphoid Tissues. Lyon: IARC; 2008.

INTRODUCTION

Ocular adnexal lymphomas (OALs) originate in the conjunctiva, eyelids, lacrimal gland, lacrimal drainage apparatus, and other orbital tissues surrounding the eye. The anatomic site plays an important role, along with the histologic lymphoma subtype. The Ann Arbor system is widely used for clinical staging of nodal lymphomas but is not ideally suited to extranodal diseases such as OAL. The results of larger collaborative, international studies on OAL are now being published, and the participation of more than 1,000 patients has made it possible to form evidence-based conclusions regarding OAL and this AJCC Cancer Staging Manual, 8th Edition revision.[1,2] The following revised TNM staging system for OAL addresses these limitations.

Almost all OALs are extranodal non-Hodgkin lymphomas, most commonly extranodal marginal zone B-cell lymphomas (EMZLs) of the mucosa-associated lymphoid tissue (MALT) type as well as other small B-cell lymphomas, according to the World Health Organization (WHO) Classification of Tumors of the Hematopoietic and Lymphoid Tissues.[3–7] Although rare, T/NK-cell lymphomas also arise in the ocular adnexa.[8,9]

Although anatomic site plays an important role, equally significant is the histologic lymphoma subtype according to the WHO lymphoma classification.[10] The Ann Arbor system is widely used for clinical staging of nodal lymphomas but is not ideally suited to extranodal disease.[11,12] For example, among OALs, eyelid lymphomas have a worse prognosis than conjunctival lymphomas, but both are categorized as Stage I.[7,13,14] The following TNM staging system for OALs addresses these limitations. It must be emphasized that this system should not be used for secondary lymphomatous involvement of the ocular adnexa or for the intraocular lymphomas.[15]

ANATOMY

Primary Site(s)

Eyelid

The eyelids consist of eight layers: skin, subcutaneous connective tissue, orbicularis oculi muscle, orbital septum, levator muscle, tarsal plate, Müller's muscle, and conjunctiva (Fig. 71.1). Accessory eyelid structures include the plica semilunaris and the caruncle. OAL is defined as involving the eyelid if it infiltrates preseptal tissues, such as the dermis or orbicularis muscle of the anterior eyelid skin.[16]

Conjunctiva

The conjunctiva lines the posterior eyelid surface and the anterior surface of the eye, with these two areas meeting at the fornix. It is a mucous membrane overlying the substantia propria, which contains a sparse population of lymphoid cells.

Orbit

The orbit is a bony cavity containing the eye, lacrimal gland, lacrimal sac, nasolacrimal duct, extraocular muscles, fat, arteries, veins, and nerves, with no lymphatics. The orbit is adjacent to the ethmoid sinuses medially, the frontal sinus and cranial cavity superiorly and posteriorly, the maxillary sinus inferiorly, and the temporalis fossa laterally.

Lacrimal Gland

The lacrimal gland is situated immediately posterior to the superotemporal orbital rim. It is an exocrine gland secreting tears containing IgA and other protective agents. Several tiny accessory glands of Krause and Wolfring are located in the region of the fornices. The lacrimal drainage system comprises the upper and lower canaliculi, the lacrimal sac, and the nasolacrimal duct.

Branches of the internal and external carotid arteries provide the arterial blood supply. Venous drainage from pretarsal tissues is via the angular vein medially and the superficial temporal vein laterally. Post–arsal tissue drainage is into the orbital veins and the deeper branches of the anterior facial vein and pterygoid plexus. Lymphatic drainage from the medial conjunctiva and medial eyelids is to the submandibular nodes, with lateral areas of these tissues draining to preauricular lymph nodes and then into the deeper cervical nodes.

Code	Description
9591	Malignant lymphoma, small cleaved cell, NOS
9591	Malignant lymphoma, cleaved cell, NOS
9591	Hairy cell leukemia variant
9596	Composite Hodgkin and non-Hodgkin lymphoma
9596	B-cell lymphoma, unclassifiable, with features intermediate between diffuse large B-cell lymphoma and classical Hodgkin lymphoma
9597	Primary cutaneous follicle center lymphoma
9702	Mature T-cell lymphoma, NOS
9702	Peripheral T-cell lymphoma, NOS
9702	T-cell lymphoma, NOS
9702	Peripheral T-cell lymphoma, pleomorphic small cell
9702	Peripheral T-cell lymphoma, pleomorphic medium and large cell
9702	Peripheral T-cell lymphoma, large cell
9702	T-zone lymphoma
9702	Lymphoepithelioid lymphoma
9702	Lennert lymphoma
9702	Anaplastic large cell lymphoma, ALK (anaplastic lymphoma kinase)
9705	Angioimmunoblastic T-cell lymphoma
9705	Peripheral T-cell lymphoma, AILD (angioimmunoblastic lymphadenopathy with dysproteinemia)
9705	Angioimmunoblastic lymphoma

Swerdlow SH, Campo E, Harris NL, Jaffe ES, Pileri SA, Stein H, Thiele J, Vardiman J, eds. World Health Organization Classification of Tumours of Haematopoietic and Lymphoid Tissues. Lyon: IARC; 2008.

INTRODUCTION

Ocular adnexal lymphomas (OALs) originate in the conjunctiva, eyelids, lacrimal gland, lacrimal drainage apparatus, and other orbital tissues surrounding the eye. The anatomic site plays an important role, along with the histologic lymphoma subtype. The Ann Arbor system is widely used for clinical staging of nodal lymphomas but is not ideally suited to extranodal diseases such as OAL. The results of larger collaborative, international studies on OAL are now being published, and the participation of more than 1,000 patients has made it possible to form evidence-based conclusions regarding OAL and this AJCC Cancer Staging Manual, 8th Edition revision.[1,2] The following revised TNM staging system for OAL addresses these limitations.

Almost all OALs are extranodal non-Hodgkin lymphomas, most commonly extranodal marginal zone B-cell lymphomas (EMZLs) of the mucosa-associated lymphoid tissue (MALT) type as well as other small B-cell lymphomas, according to the World Health Organization (WHO) Classification of Tumors of the Hematopoietic and Lymphoid Tissues.[3–7] Although rare, T/NK-cell lymphomas also arise in the ocular adnexa.[8,9]

Although anatomic site plays an important role, equally significant is the histologic lymphoma subtype according to the WHO lymphoma classification.[10] The Ann Arbor system is widely used for clinical staging of nodal lymphomas but is not ideally suited to extranodal disease.[11,12] For example, among OALs, eyelid lymphomas have a worse prognosis than conjunctival lymphomas, but both are categorized as Stage I.[7,13,14] The following TNM staging system for OALs addresses these limitations. It must be emphasized that this system should not be used for secondary lymphomatous involvement of the ocular adnexa or for the intraocular lymphomas.[15]

ANATOMY

Primary Site(s)

Eyelid

The eyelids consist of eight layers: skin, subcutaneous connective tissue, orbicularis oculi muscle, orbital septum, levator muscle, tarsal plate, Müller's muscle, and conjunctiva (Fig. 71.1). Accessory eyelid structures include the plica semilunaris and the caruncle. OAL is defined as involving the eyelid if it infiltrates preseptal tissues, such as the dermis or orbicularis muscle of the anterior eyelid skin.[16]

Conjunctiva

The conjunctiva lines the posterior eyelid surface and the anterior surface of the eye, with these two areas meeting at the fornix. It is a mucous membrane overlying the substantia propria, which contains a sparse population of lymphoid cells.

Orbit

The orbit is a bony cavity containing the eye, lacrimal gland, lacrimal sac, nasolacrimal duct, extraocular muscles, fat, arteries, veins, and nerves, with no lymphatics. The orbit is adjacent to the ethmoid sinuses medially, the frontal sinus and cranial cavity superiorly and posteriorly, the maxillary sinus inferiorly, and the temporalis fossa laterally.

Lacrimal Gland

The lacrimal gland is situated immediately posterior to the superotemporal orbital rim. It is an exocrine gland secreting tears containing IgA and other protective agents. Several tiny accessory glands of Krause and Wolfring are located in the region of the fornices. The lacrimal drainage system comprises the upper and lower canaliculi, the lacrimal sac, and the nasolacrimal duct.

Branches of the internal and external carotid arteries provide the arterial blood supply. Venous drainage from pretarsal tissues is via the angular vein medially and the superficial temporal vein laterally. Post–arsal tissue drainage is into the orbital veins and the deeper branches of the anterior facial vein and pterygoid plexus. Lymphatic drainage from the medial conjunctiva and medial eyelids is to the submandibular nodes, with lateral areas of these tissues draining to preauricular lymph nodes and then into the deeper cervical nodes.

Regional Lymph Nodes

The regional lymph nodes of the ocular adnexa include the submandibular, preauricular, and cervical lymph nodes (Fig. 71.1). Distant nodes include "central" nodes, located in the trunk (e.g., mediastinal and para-aortic nodes), and "peripheral" nodes at other distant sites not draining the ocular adnexa.

Metastatic Sites

The most common metastatic sites of OAL are other extranodal tissues that are noncontiguous with the ocular adnexa, including organs such as the salivary glands, gastrointestinal tract, lung, and liver. Bone marrow infiltration may be micronodular, paratrabecular, or diffuse interstitial.

RULES FOR CLASSIFICATION

Clinical Classification

Clinical classification includes a complete history and ophthalmic examination including but not limited to exophthalmometry, color vision testing, inspection and palpation of the eyelids and orbit, evaluation of ocular motility, and examination of the entire conjunctiva (with eversion of the upper eyelids). Intraocular pressure measurements and findings on dilated ophthalmoscopy may indicate compressive ocular disease.

Imaging

Ultrasonography may be used in the clinical setting to evaluate the orbit. Systemic physical examination should be performed, along with radiographic imaging of the orbits and sinuses, chest, abdomen, and pelvis. These examinations may be performed using computer tomography (CT) and/or magnetic resonance imaging. CT scans are preferable when it is necessary to visualize bony structures. OAL has a homogeneous signal distribution and is hyperdense compared with orbital fat. The borders of the lesion may be sharply delineated but normally are feathery. Some centers now use whole-body positron emission tomography/CT to stage patients with OAL.[17,18]

Pathological Classification

As defined by the AJCC and UICC,[19,20] the suffix descriptor *m* and prefix descriptor *r* or *a* may be used. These descriptors indicate, respectively, multiple tumors in one ocular adnexal structure, recurrent disease, and autopsy classification for a tumor identified only at autopsy and not suspected. For example, T1a(m) indicates multiple bulbar conjunctival (extralimbal) tumors in one eye.

PROGNOSTIC FACTORS

Prognostic Factors Required for Stage Grouping

Beyond the factors used to assign T, N, or M categories, no additional prognostic factors are required for stage grouping.

Additional Factors Recommended for Clinical Care

Prognostic Indices

The proposed TNM classification of OAL defines the anatomic extent of disease in greater detail, which has been considered of prognostic value in the literature.[21–24] As with nodal lymphomas, the International Prognostic Index (IPI)[25] should be applied to subdivide patients with primary diffuse large B-cell lymphomas (DLBCLs) of the ocular adnexa according to prognosis, thereby enhancing individual patient care. Similarly, the Follicular Lymphoma International Prognostic Index (FLIPI),[26] which includes age, Ann Arbor stage, number of nodal sites, serum lactate dehydrogenase (LDH) level, and hemoglobin level to build a three-category index, should be applied in patients with primary ocular adnexal follicular lymphomas (see Chapter 79, Hodgkin and non-Hodgkin Lymphomas). The Mantle Cell International Prognostic Index (MIPI) should be applied for mantle cell lymphoma.[27] AJCC Level of Evidence: II

Tumor Cell Growth Fraction (Ki-67, MIB-1)

Tumor cell growth fraction should be assessed by counting the number of tumor cells with clear nuclear positivity for Ki-67 per 5 × 100 tumor cells using the 40× objective. A percentage value therefore is obtained: for example, a Ki-67 tumor cell growth fraction of 15%. Reactive cells should not be included if possible. For example, the germinal center in MALT lymphomas should not be included in the assessment. AJCC Level of Evidence: III

Serum LDH

The serum LDH value should be assessed at the time of diagnosis. AJCC Level of Evidence: III

RISK ASSESSMENT MODELS

The AJCC recently established guidelines that will be used to evaluate published statistical prediction models for the purpose of granting endorsement for clinical use.[28] Although this is a monumental step toward the goal of precision medicine, this work was published only very recently. Therefore, the

existing models that have been published or may be in clinical use have not yet been evaluated for this cancer site by the Precision Medicine Core of the AJCC. In the future, the statistical prediction models for this cancer site will be evaluated, and those that meet all AJCC criteria will be endorsed.

DEFINITIONS OF AJCC TNM

Definition of Primary Tumor (T)

T Category	T Criteria
TX	Lymphoma extent not specified
T0	No evidence of lymphoma
T1	Lymphoma involving the conjunctiva alone without eyelid or orbital involvement
T2	Lymphoma with orbital involvement with or without conjunctival involvement
T3	Lymphoma with preseptal eyelid involvement with or without orbital involvement and with or without conjunctival involvement
T4	Orbital adnexal lymphoma and extraorbital lymphoma extending beyond the orbit to adjacent structures, such as bone, maxillofacial sinuses, and brain.

Definition of Regional Lymph Node (N)

N Category	N Criteria
NX	Involvement of lymph nodes not assessed
N0	No evidence of lymph node involvement
N1	Involvement of lymph node region or regions draining the ocular adnexal structures and superior to the mediastinum (preauricular, parotid, submandibular, and cervical nodes)
N1a	Involvement of a single lymph node region superior to the mediastinum
N1b	Involvement of two or more lymph node regions, superior to the mediastinum
N2	Involvement of lymph node regions of the mediastinum
N3	Diffuse or disseminated involvement of peripheral and central lymph node regions

Definition of Distant Metastasis (M)

M Category	M Criteria
M0	No evidence of involvement of other extranodal sites
M1a	Noncontiguous involvement of tissues or organs external to the ocular adnexa (e.g., parotid glands, submandibular gland, lung, liver, spleen, kidney, breast)
M1b	Lymphomatous involvement of the bone marrow
M1c	Both M1a and M1b involvement

AJCC PROGNOSTIC STAGE GROUPS

There is no prognostic stage grouping for OAL.

REGISTRY DATA COLLECTION VARIABLES

1. History of rheumatoid arthritis
2. History of Sjögren's syndrome
3. History of connective tissue disease
4. History of recurrent dry eye syndrome (sicca syndrome)
5. History of IgG4 ocular adnexal disease
6. Any evidence of previous or current infection with hepatitis B, hepatitis C, or HIV
7. Any evidence of *Helicobacter pylori* infection
8. Any evidence of an infection caused by *Chlamydia psittaci*
9. Presence or absence of an *A20* deletion
10. IGH-locus translocation or somatic mutation pattern (EMZL)
11. Concordant /discordant bone marrow involvement (DLBCL)
12. Centroblastic/immunoblastic (DLBCL)

HISTOLOGIC GRADE (G)

Grade is assigned only to follicular lymphomas, as described by the 2008 WHO classification[10,18] for malignant lymphomas as follows. For data collection purposes, WHO grade 3a is collected as G3 and WHO grade 3b as G4.

G	G Definition
GX	Grade cannot be assessed
G1	1–5 centroblasts per 10 high-power fields (HPF)
G2	Between 5 and 15 centroblasts per 10 HPF
G3	More than 15 centroblasts per 10 HPF but with admixed centrocytes
G4	More than 15 centroblasts per 10 HPF but without centrocytes

HISTOPATHOLOGIC TYPE

The main primary OAL subtypes include the following:

Extranodal marginal zone B-cell lymphoma (MALT lymphoma)
Diffuse large B-cell lymphoma
Follicular lymphoma

ILLUSTRATIONS

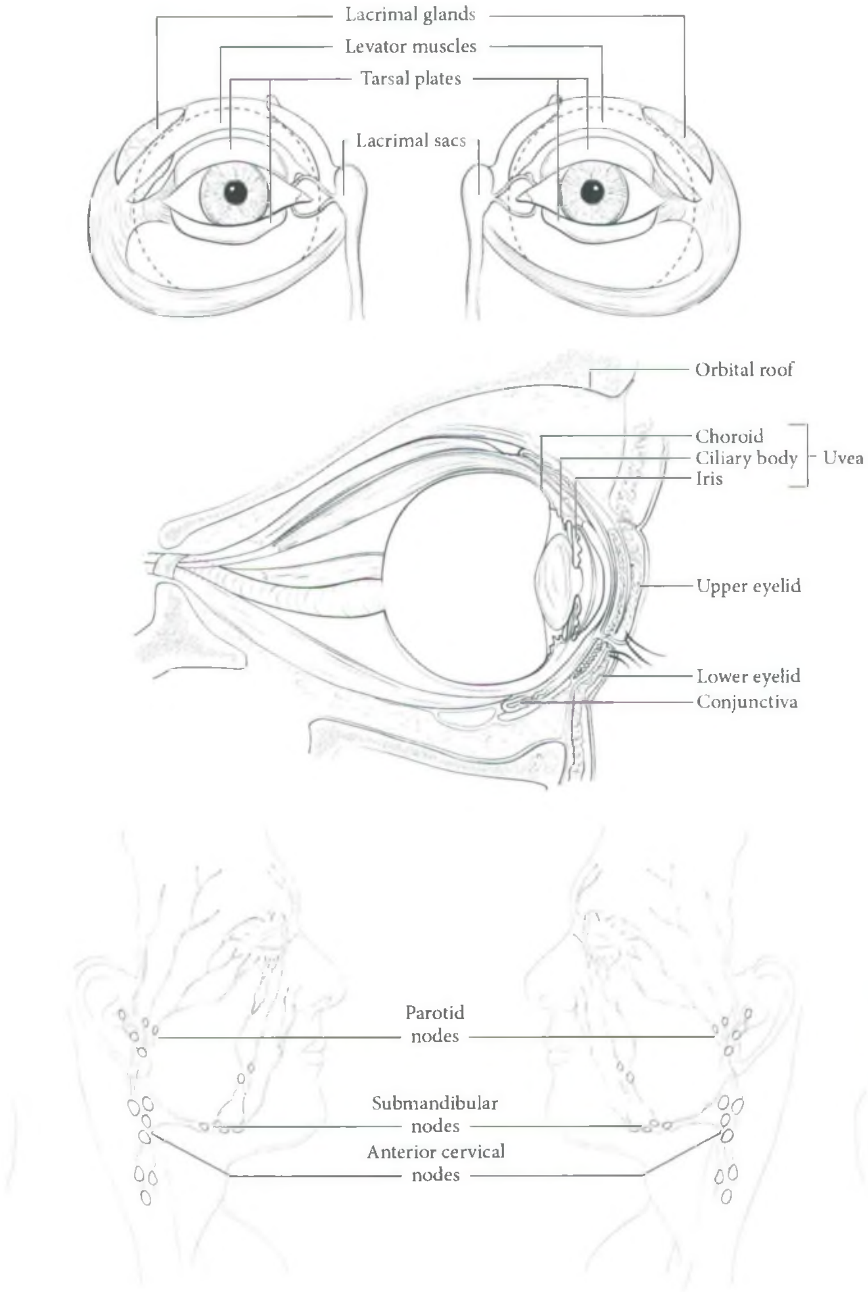

Fig. 71.1 Anatomic sites and regional lymph nodes for ophthalmic sites

Bibliography

1. Munch-Petersen HD, Rasmussen PK, Coupland SE, et al. Ocular adnexal diffuse large B-cell lymphoma: a multicenter international study. *JAMA ophthalmology*. Feb 2015;133(2):165–173.
2. Rasmussen PK, Coupland SE, Finger PT, et al. Ocular adnexal follicular lymphoma: a multicenter international study. *JAMA ophthalmology*. Jul 2014;132(7):851–858.
3. Coupland SE, Krause L, Delecluse HJ, et al. Lymphoproliferative lesions of the ocular adnexa. Analysis of 112 cases. *Ophthalmology*. Aug 1998;105(8):1430–1441.
4. Jenkins C, Rose GE, Bunce C, et al. Histological features of ocular adnexal lymphoma (REAL classification) and their association with patient morbidity and survival. *British journal of ophthalmology*. 2000;84(8):907–913.
5. Decaudin D, de Cremoux P, Vincent-Salomon A, Dendale R, Rouic LL. Ocular adnexal lymphoma: a review of clinicopathologic features and treatment options. *Blood*. Sep 1 2006;108(5):1451–1460.
6. Ferry JA, Fung CY, Zukerberg L, et al. Lymphoma of the ocular adnexa: A study of 353 cases. *The American journal of surgical pathology*. Feb 2007;31(2):170–184.
7. Jakobiec FA. Ocular adnexal lymphoid tumors: progress in need of clarification. *American journal of ophthalmology*. Jun 2008;145(6):941–950.
8. Coupland SE, Foss HD, Assaf C, et al. T-cell and T/natural killer-cell lymphomas involving ocular and ocular adnexal tissues: a clinicopathologic, immunohistochemical, and molecular study of seven cases. *Ophthalmology*. Nov 1999;106(11):2109–2120.
9. Woog JJ, Kim YD, Yeatts RP, et al. Natural killer/T-cell lymphoma with ocular and adnexal involvement. *Ophthalmology*. Jan 2006;113(1):140–147.
10. Vardiman J, Bennett J, Bain B, Baumann I, Thiele J, Orazi A. WHO classification of tumours of haematopoietic and lymphoid tissues. *International Angency for Research on Cancer (IARC), Lyon*. 2008:32–37.
11. Ruskone-Fourmestraux A, Dragosics B, Morgner A, Wotherspoon A, De Jong D. Paris staging system for primary gastrointestinal lymphomas. *Gut*. Jun 2003;52(6):912–913.
12. Kim YH, Willemze R, Pimpinelli N, et al. TNM classification system for primary cutaneous lymphomas other than mycosis fungoides and Sezary syndrome: a proposal of the International Society for Cutaneous Lymphomas (ISCL) and the Cutaneous Lymphoma Task Force of the European Organization of Research and Treatment of Cancer (EORTC). *Blood*. 2007;110(2):479–484.
13. Auw-Haedrich C, Coupland SE, Kapp A, Schmitt-Graff A, Buchen R, Witschel H. Long term outcome of ocular adnexal lymphoma subtyped according to the REAL classification. Revised European and American Lymphoma. *The British journal of ophthalmology*. Jan 2001;85(1):63–69.
14. Johnson TE, Tse DT, Byrne GE, Jr., et al. Ocular-adnexal lymphoid tumors: a clinicopathologic and molecular genetic study of 77 patients. *Ophthalmic plastic and reconstructive surgery*. May 1999;15(3):171–179.
15. Coupland SE, Damato B. Understanding intraocular lymphomas. *Clinical & experimental ophthalmology*. Aug 2008;36(6):564–578.
16. Knowles DM. *Neoplastic hematopathology*. Lippincott Williams & Wilkins; 2001.
17. Rasmussen PK, Ralfkiaer E, Prause JU, et al. Diffuse large B-cell lymphoma of the ocular adnexal region: a nation-based study. *Acta ophthalmologica*. Mar 2013;91(2):163–169.
18. Rasmussen PK, Ralfkiaer E, Prause JU, et al. Follicular lymphoma of the ocular adnexal region: a nation-based study. *Acta ophthalmologica*. Mar 2015;93(2):184–191.
19. Finger PT. Eye: choroidal melanoma, retinoblastoma, ocular adnexal lymphoma and eyelid cancers. *UICC Manual of Clinical Oncology, 9*. 2015:726–744.
20. Greene FL. *AJCC cancer staging manual*. Vol 1: Springer Science & Business Media; 2002.
21. Sniegowski MC, Roberts D, Bakhoum M, et al. Ocular adnexal lymphoma: validation of American Joint Committee on Cancer seventh edition staging guidelines. *The British journal of ophthalmology*. Sep 2014;98(9):1255–1260.
22. Rath S, Connors JM, Dolman PJ, Rootman J, Rootman DB, White VA. Comparison of American Joint Committee on Cancer TNM-based staging system (7th edition) and Ann Arbor classification for predicting outcome in ocular adnexal lymphoma. *Orbit*. Feb 2014;33(1):23–28.
23. Graue GF, Finger PT, Maher E, et al. Ocular adnexal lymphoma staging and treatment: American Joint Committee on Cancer versus Ann Arbor. *European journal of ophthalmology*. 2012;23(3):344–355.
24. Coupland SE, White VA, Rootman J, Damato B, Finger PT. A TNM-based clinical staging system of ocular adnexal lymphomas. *Arch Pathol Lab Med*. Aug 2009;133(8):1262–1267.
25. Shipp M. A predictive model for aggressive non-Hodgkin's lymphoma. *N Engl J Med*. 1993;329:987–994.
26. Solal-Celigny P, Roy P, Colombat P, et al. Follicular lymphoma international prognostic index. *Blood*. Sep 1 2004;104(5):1258–1265.
27. Hoster E, Dreyling M, Klapper W, et al. A new prognostic index (MIPI) for patients with advanced-stage mantle cell lymphoma. *Blood*. Jan 15 2008;111(2):558–565.
28. Kattan MW, Hess KR, Amin MB, et al. American Joint Committee on Cancer acceptance criteria for inclusion of risk models for individualized prognosis in the practice of precision medicine. *CA: a cancer journal for clinicians*. Jan 19 2016.

Part XVI

Central Nervous System

Members of the Central Nervous System Expert Panel
Melissa L. Bondy, PhD
Daniel J. Brat, MD, PhD – CAP Representative
Henry Brem, MD
Susan M. Chang, MD
Rivka R. Colen, MD
Walter J. Curran Jr., MD – Vice Chair
Laurie E. Gaspar, MD, FASTRO, FACR, MBA – Editorial Board Liaison
Edward R. Laws Jr., MD, FACS – Chair
M. Beatriz Lopes, MD, PhD
David N. Louis, MD
Malcolm D. Mason, MD – UICC Representative
Michael D. Prados, MD
David Schiff, MD
Theresa M. Vallerand, BGS, CTR – Data Collection Core Representative
Patrick Y. Wen, MD
Maria Werner-Wasik, MD

Brain and Spinal Cord

72

Edward R. Laws Jr., Walter J. Curran Jr., Melissa L. Bondy,
Daniel J. Brat, Henry Brem, Susan M. Chang,
Rivka R. Colen, M. Beatriz Lopes, David N. Louis,
Michael D. Prados, David Schiff, Theresa M. Vallerand,
Patrick Y. Wen, and Maria Werner-Wasik

CHAPTER SUMMARY

Cancers staged using this staging system

All tumors of the central nervous system

Summary of Changes

Change	Details of Change	Level of Evidence
WHO Classification of Tumors	Completely revised by the World Health Organization (2016)	I
Prognostic Factors	"Integrated diagnosis" incorporating histologic and molecular advances was added.	II
Biomarkers	New genetic and molecular markers under investigation	II
Recommendations for Clinical Trial Stratification	New parameters and candidates: radiotherapy/radiosurgery, chemotherapy, immunotherapy, antiangiogenesis, biologic therapies	II

ICD-O-3 Topography Codes

Code	Description
C70.0	Cerebral meninges
C70.1	Spinal meninges
C70.9	Meninges, NOS
C71.0	Cerebrum
C71.1	Frontal lobe
C71.2	Temporal lobe
C71.3	Parietal lobe
C71.4	Occipital lobe
C71.5	Ventricle, NOS
C71.6	Cerebellum, NOS
C71.7	Brainstem
C71.8	Overlapping lesion of brain
C71.9	Brain, NOS
C72.0	Spinal cord
C72.1	Cauda equina
C72.2	Olfactory nerve
C72.3	Optic nerve
C72.4	Acoustic nerve
C72.5	Cranial nerve, NOS
C72.8	Overlapping lesion of brain and central nervous system
C72.9	Nervous system, NOS
C75.1	Pituitary gland
C75.2	Craniopharyngeal duct
C75.3	Pineal gland

WHO Classification of Tumors

Code	Description
	Diffuse astrocytic and oligodendroglial tumors
9400	Diffuse astrocytoma, IDH mutant
9411	Gemistocytic astrocytoma, IDH mutant
9400	Diffuse astrocytoma, IDH wild type
9400	Diffuse astrocytoma, NOS
9401	Anaplastic astrocytoma, IDH mutant

To access the AJCC cancer staging forms, please visit www.cancerstaging.org.

© American Joint Committee on Cancer 2017
M.B. Amin et al. (eds.), *AJCC Cancer Staging Manual, Eighth Edition*, DOI 10.1007/978-3-319-40618-3_72

INTRODUCTION

Attempts to develop a TNM-based classification and staging system for central nervous system (CNS) tumors have been neither practical nor pertinent. Early editions of this manual proposed a system that was used with poor compliance and was not useful as a predictor of outcome, neither in practice nor in clinical trials for patients with primary CNS tumors. The reasons for this are several.

1. Tumor size is significantly less relevant than tumor histology and the location of the tumor; the T category is less important than the inherent biologic nature of the tumor.
2. Because the brain and spinal cord have no lymphatic system, the N category does not apply, as there are no lymph nodes to identify or count.
3. An M category is not pertinent to most CNS neoplasms because of the inherent biology that favors local recurrence and regional spread.

The CNS expert panel continues to recommend that a formal TNM-based classification not be attempted. We continue to incorporate the World Health Organization (WHO) CNS tumor nomenclature and classification, which were revised recently (2016), and the ICD topography system for location of the lesions.

This chapter attempts to highlight what is known and recommended with regard to prognostic factors and biomarkers in CNS tumors. We also review the current and proposed strategies used for the diagnosis and management of primary tumors of the CNS, particularly the malignant gliomas.[1]

DESCRIPTIVE EPIDEMIOLOGY

Summary: Incidence of CNS Tumors (Fig. 72.1)

- The overall average annual age-adjusted incidence rate for 2008 to 2012 for all primary brain and CNS tumors was 21.97 per 100,000 population. The overall incidence rate was 5.57 per 100,000 population for children and adolescents aged 0 to 19 years, 5.37 per 100,000 population for children aged 0 to 14 years, and 28.57 per 100,000 population for adults aged ≥20 years.[2]
- Brain and CNS tumors are the most common cancer among those aged 0 to 19 years, with an average annual age-adjusted incidence rate of 5.57 per 100,000 population.[3]
- Overall, 42.1% of all tumors diagnosed between 2008 and 2012 occurred in males (150,271 tumors) and 57.9% in females (206,565 tumors); 55.0% of malignant tumors occurred in males (65,056 tumors) and 45% in females (51,967 tumors). Approximately 36.0% of the nonmalignant tumors occurred in males (85,616 tumors) and 64% in females (154,219 tumors).
- The broad category of glioma represents approximately 27% of all tumors and 80% of malignant tumors. The most frequently reported histology of all brain and CNS tumors is meningioma (36.4%), followed by tumors of the pituitary (15.5%) and glioblastoma (GBM; 15.1%).

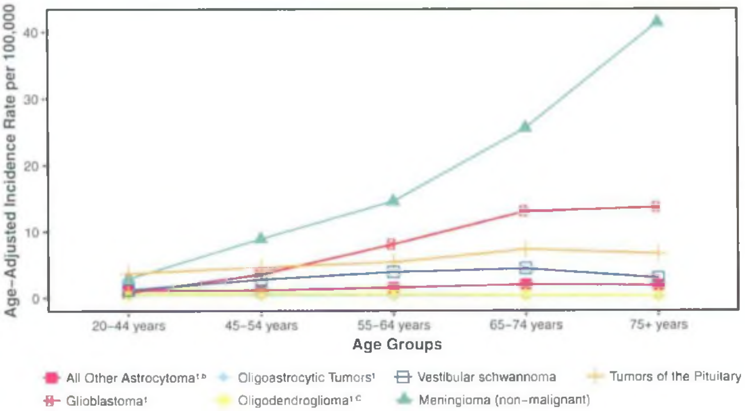

Fig. 72.1 Relative Age Adjusted Incidence of Brain Tumors (from Ostrom et al.[2] with permission)

† All or some of this histology are included in the CBTRUS definition of gliomas, including ICD-O-3 histology codes 9380-9384, 9391-9460 (Table 2a). a. Rates per 100,000 and age-adjusted to the 2000 United States standard population. b. ICD-O-3 Histology Codes: 9381, 9384, 9424, 9400, 9401, 9410, 9411, 9420. c. ICD-O-3 Histology Codes: 945, 9451, 9460. d. ICD-O-3 Code: 9560. e. ICD-O-3 Histology Codes: 9530/0,9530/1, 9531/0, 9532/0, 9533/0, 9534/0, 9537/0, 9538/1, 9539/1.

72

Code	Description
9401	Anaplastic astrocytoma, IDH wild type
9401	Anaplastic astrocytoma, NOS
9440	Glioblastoma, IDH wild type
9441	Giant cell glioblastoma
9442	Gliosarcoma
9440	Epithelioid glioblastoma
9445	Glioblastoma, IDH mutant
9440	Glioblastoma, NOS
9385	Diffuse midline glioma, H3 K27M mutant
9450	Oligodendroglioma, IDH mutant and 1p/19q codeleted
9450	Oligodendroglioma, NOS
9451	Anaplastic oligodendroglioma, IDH mutant and 1p/19q codeleted
9451	Anaplastic oligodendroglioma, NOS
9382	Oligoastrocytoma, NOS
9382	Anaplastic oligoastrocytoma, NOS
	Other astrocytic tumors
9425	Pilomyxoid astrocytoma
9424	Pleomorphic xanthoastrocytoma
9424	Anaplastic pleomorphic xanthoastrocytoma
	Ependymal tumors
9391	Ependymoma
9393	Papillary ependymoma
9391	Clear cell ependymoma
9391	Tanycytic ependymoma
9396	Ependymoma, RELA fusion positive
9392	Anaplastic ependymoma
	Other gliomas
9430	Astroblastoma
	Choroid plexus tumors
9390	Choroid plexus carcinoma
	Neuronal and mixed neuronal–glial tumors
9505	Anaplastic ganglioglioma
	Tumors of the pineal region
9362	Pineal parenchymal tumor of intermediate differentiation
9362	Pineoblastoma
9395	Papillary tumor of the pineal region
	Embryonal tumors
9475	Medulloblastoma, WNT activated
9476	Medulloblastoma, SHH activated and TP53 mutant
9471	Medulloblastoma, SHH activated and TP53 wild type
9477	Medulloblastoma, non-WNT/non-SHH
9470	Medulloblastoma, classic
9471	Medulloblastoma, desmoplastic/nodular
9471	Medulloblastoma with extensive nodularity
9474	Medulloblastoma, large cell/anaplastic
9470	Medulloblastoma, NOS
9478	Embryonal tumor with multilayered rosettes, C19MC altered
9478	Embryonal tumor with multilayered rosettes, NOS
9501	Medulloepithelioma
9500	CNS neuroblastoma
9490	CNS ganglioneuroblastoma
9473	CNS embryonal tumor, NOS
9508	Atypical teratoid/rhabdoid tumor
9508	CNS embryonal tumor with rhabdoid features
	Tumors of the cranial and paraspinal nerves
9540	Malignant peripheral nerve sheath tumor (MPNST)
9540	Epithelioid MPNST
9540	MPNST with perineural differentiation
	Meningiomas
9538	Papillary meningioma
9538	Rhabdoid meningioma
9530	Anaplastic (malignant) meningioma
	Mesenchymal, nonmeningothelial tumors
8815	Solitary fibrous tumor/hemangiopericytoma, grade 3
9133	Epithelioid hemangioendothelioma
9120	Angiosarcoma
9140	Kaposi sarcoma
9360	Ewing sarcoma/peripheral primitive neuroectodermal tumor
8850	Liposarcoma
8810	Fibrosarcoma
8802	Undifferentiated pleomorphic sarcoma (UPS)/ malignant fibrous histiocytoma (MFH)
8890	Leiomyosarcoma
8900	Rhabdomyosarcoma
9220	Chondrosarcoma
9180	Osteosarcoma
	Melanocytic lesions
8720	Meningeal melanoma
8728	Meningeal melanomatosis
	Lymphomas
9680	Diffuse large B-cell lymphoma (DLBCL) of the CNS
9714	Anaplastic large cell lymphoma, ALK positive
9702	Anaplastic large cell lymphoma, ALK negative
9712	Intravascular large B-cell lymphoma
9699	MALT lymphoma of the dura
	Histiocytic tumors
9571	Langerhans cell histiocytosis
9750	Erdheim–Chester disease
9755	Histiocytic sarcoma
	Germ cell tumors
9064	Germinoma
9070	Embryonal carcinoma
9071	Yolk sac tumor
9100	Choriocarcinoma
9080	Immature teratoma
9084	Teratoma with malignant transformation
9085	Mixed germ cell tumor

Louis DN, Ohgaki H, Wiestler OD, Cavenee WK, Ellison DW, Figarella-Branger D, Perry A, Reifenberger G, von Deimling A. World Health Organization Classification of Tumours of the Central Nervous System, Revised 4th Edition. Lyon: IARC; 2016.

CNS TUMOR RISK FACTORS

Currently, the only established environmental risk factors for brain tumors are ionizing radiation exposure and family history. Some studies investigated the role of chemical exposures (pesticides, heavy metals, and nitroso compounds), physical factors (electromagnetic fields, including mobile phones, and head trauma), infectious agents (viruses), and immunologic conditions (allergies, asthma, eczema, autoimmune diseases, and diabetes), but none of these factors has been definitively established to be involved in glioma risk. Most significantly, there has been a consistent decrease across studies regarding risk by history of allergies or atopic disease(s). Direct evidence for inherited predisposition to gliomas is provided by several rare, inherited cancer syndromes, such as Turcot and Li–Fraumeni syndromes, and neurofibromatosis. However, even collectively, these diseases account for little of the twofold increased risk of glioma seen in first-degree relatives of glioma patients. Support for polygenic susceptibility to glioma has come from genome-wide association studies (GWASs) that have identified single-nucleotide polymorphisms (SNPs) at eight loci influencing glioma risk—3q26.2 (near *TERC*), 5p15.33 (near *TERT*), 7p11.2 (near *EGFR*), 8q24.21 (near *CCDC26*), 9p21.3 (near *CDKN2A/CDKN2B*), 11q23.3 (near *PHLDB1*), 17p13.1 (*TP53*), and 20q13.33 (near *RTEL1*). Perhaps not surprisingly, there is variability in genetic effects on glioma by histology, with subtype-specific associations at 5p15.33, 20q13.33, and 7p11.2 for GBM and at 11q23.3 and 8q24 for non-GBM glioma. Recently, five new risk loci for GBM at 12q23.33 and non-GBM at 10q25.2, 11q23.2, 12q21.2 and 15q24.[4] As more research is published we will gain a better understanding of the role of genes and other glioma risk factors.

MENINGIOMA RISK FACTORS

Although most meningiomas are encapsulated and benign tumors with limited numbers of genetic aberrations, their intracranial location often leads to serious and potentially lethal consequences. At present, the primary environmental risk factor identified for meningioma is exposure to ionizing radiation, with risks from 6- to 10-fold reported. Exposure to some dental X-rays performed in the past, when radiation exposure was greater than in the current era, appears to be associated with an increased risk of intracranial meningioma. At high dose levels, data exist for atomic bomb survivors showing a greatly increased risk for meningioma. Inherited susceptibility to meningioma is suggested both by family history and candidate gene studies in DNA repair genes. People with certain mutations in the neurofibromatosis gene (*NF2*) have an increased risk for meningioma. Because women are twice as likely as men to develop a meningioma, and these tumors harbor hormone receptors, an etiologic role for hormones (both endogenous and exogenous) has been hypothesized. In a comprehensive meta-analysis of 12 studies, Niedermaier et al[5] found a moderate increase in the risk of meningioma in overweight individuals and a substantial increase in meningioma risk in obese individuals as compared with their normal-weight peers. In addition, we observed a moderate decrease in the risk of meningioma with high versus low physical activity levels. Many of these modifiable risk factors might be a benefit to patients.

IMAGING

The mainstay of imaging for CNS tumors involving the head, the spine, and the peripheral nerves has been magnetic resonance (MR) imaging. This modality, with magnet strengths from 1.5 to 3.0 T, has provided excellent anatomic detail along with the imaging aspects of physiologic changes associated with these tumors. The former primary imaging modality, namely computed tomography (CT), is occasionally useful for tumors containing significant degrees of calcification, such as oligodendrogliomas, craniopharyngiomas, and some meningiomas. Because CT shows such precise bony detail, it is often useful for tumors that involve the skull or the spinal segments, and for some types of computerized image guidance, it may be superior to MR imaging. Angiography is often an important part of the evaluation of tumors of the CNS, and the development of strategies for their surgical management. In addition to traditional angiography, the new methods of MR and CT angiography have become quite helpful in this regard.

The administration of contrast agents has provided important information both for MR imaging and for CT. In general, contrast agents are most commonly used to delineate areas of relative breakdown of the blood–brain barrier (BBB), usually a feature of the more malignant tumors, such as GBMs, that affect the CNS. In addition to the features elicited by contrast, new and effective MR imaging sequences continue to develop. Comparisons between T1 and T2 imaging can define the presence of edema, the location and extent of cystic changes, and areas of hemorrhage within the CNS and within its tumors. Sequences such as T2 fluid attenuated inversion recovery (FLAIR) can demonstrate secondary changes produced by tumors in exquisite detail, and may even be used to track infiltrating tumor within the substance of the brain. Other sequences may be used to analyze vascular perfusion of the brain and brain tumors. Anatomic features of the brain and the distortions produced by tumors may be elicited by MR tractography, and diffusion-weighted or diffusion tensor imaging and FIESTA (Fast Imaging Employing Steady-state Acquisition) sequences can differentiate various secondary changes in the brain affected by

CNS tumors. Functional MR (fMR) imaging can locate the so-called eloquent areas of brain involved with language, motor movements, and sensation. These functional analyses of CNS localization are extremely useful in preserving normal and important brain and spinal cord function.

Many variations of intraoperative MR (iMR) imaging units have developed, and the current standard for iMR imaging is a 3.0-T magnet that can image the head during an operation, provide information on the extent of resection, and allow immediate reoperation if indicated. Postoperative contrast-enhanced MR imaging done within the first 24 hours has become a standard measure of extent of resection for GBMs and other primary malignant tumors of the brain.

MR imaging can determine whether tumors of the brain or spine are multifocal. MR imaging with contrast can help guide stereotactic biopsy to the most important areas of the brain most likely to be diagnostic. A new form of imaging called MR elastography (MRe) is being evaluated as a method of determining the consistency of a tumor before surgery.[6] MR imaging in the postoperative patient provides information essential for determining adjunctive therapy, as well as for measuring its effect. Important in this postoperative analysis is the differentiation of residual tumor or recurrent tumor from necrotic changes subsequent to radiation or chemotherapy. Later, it also becomes important to differentiate these changes from progression of tumor, as opposed to "pseudoprogression" related to treatment-induced changes, a critical factor in assessing the results of clinical trials. These changes have been codified as the Revised Assessment in Neuro-Oncology (RANO) criteria.[7]

Metabolic "nuclear" imaging also plays a role in the evaluation of CNS tumors. Positron emission tomography (PET) scans using 2-deoxyglucose can detect tumors and areas of tumor that have a high metabolic rate. This can effectively guide stereotactic biopsy and improve the accuracy of pathological and molecular diagnosis and classification of tumors. PET scans using other isotopes can help distinguish necrosis from active tumor, bacterial infections from neoplasms, and the degree of protein synthesis and turnover present in tumor cells.

These modalities of CNS tumor imaging are constantly improving and are becoming more precise and ever more informative. Clearly, they are instrumental in improving outcomes for our patients, as well as in assisting with the safety, effectiveness, and accuracy of our surgical and adjunctive therapies.

PRINCIPLES OF NEURO-ONCOLOGY

Surgery

Surgery for brain tumors in general, and for primary malignant gliomas (GBMs) in particular, is usually first-line therapy. Surgical management offers relief from increased intracranial pressure and progressive neurologic deficits. It provides histologic confirmation, as well as tissue for classification and investigation of the biological markers pertinent to the tumor. This includes genetic features, tumor markers, and other molecular studies that might clarify the pathogenesis of the tumor and the postsurgical management of the patient. Surgery provides the basic goal of cytoreduction, decreasing the tumor burden, and selectively sparing eloquent areas of the brain. For patients in whom seizures are part of the presenting symptoms, surgery may offer seizure control in some cases. Resection of the lesion may provide increased tumor-free survival and improved quality of life.

For many decades, there has been a continuing controversy as to the actual benefit of aggressive surgery for maximal tumor resection. Although the preponderance of studies show a survival benefit that correlates with increasing extent of resection, the confounding issues of selection bias and imperfections in tumor classification and staging have been of concern. During the past 5 to 10 years, numerous studies, benefiting from technological advances in surgery and in neuroimaging, have convincingly demonstrated that extent of resection is a major prognostic factor for both high-grade and low-grade gliomas.[1] This, along with new molecular and genetic data, has resulted in major efforts to provide safe, extensive resections of these primary brain tumors.

More precise and informative diagnostic imaging has been of primary importance. Computer-assisted image guidance is routine for determining the location and boundaries of these tumors. Many of the operations for gliomas are now done with the patient under awake sedation so that he or she can be tested repeatedly during surgery. This has allowed relatively secure methods of preserving speech and language, higher cortical function, vision, motor function, and sensation. Preoperative analysis with fMR imaging and the intraoperative use of electrophysiology are integral parts of this effort to obtain maximal resection without damaging important functions of the brain.[8] Newer techniques, such as fluorescent tumor detection and MR spectroscopy, offer further potential for detection and eradication of these lesions. Extent of resection has become an important part of postoperative prognostic staging.

Radiation Oncology

Radiation therapy (RT) plays a prominent role in the management of most CNS neoplasms, significantly prolonging survival in patients with high-grade primary CNS tumors.[9]

General principles of RT for gliomas and ependymomas include partial brain treatment, limited to the gross tumor (or resection cavity) as seen on the MR imaging scan of the brain obtained no later than 2 to 3 days after biopsy or resection, with a 1- to 2.5-cm expansion margin to account for

microscopic tumor spread.[10] External beam photon (X-ray) irradiation with energies of 6 to 10 MV is standard, and particle (proton) irradiation is being investigated. Modern RT techniques use either three-dimensional (3D) conformal RT or intensity-modulated RT (IMRT). Multiple radiation beams in 3D RT allow for the concentration of the high-dose RT region to the tumor itself, with a relative sparing of normal brain tissue, which receives low-dose RT only. IMRT, through modulation of the beam fluence (or intensity), allows for a higher level of sparing of critical organs such as the optic chiasm, optic nerves, eyes, and brainstem.

A common approach in RT for high-grade gliomas is to use a "shrinking field" technique, whereby the nonenhancing T2-signal region receives a dose of 44 to 45 Gy, followed by a boost to the enhancing tumor/resection cavity to 59.4 to 60 Gy, delivered with concurrent temozolomide chemotherapy in GBM.[11] In poorly performing or elderly patients, a shortened, accelerated RT course (2–4 weeks) may be equivalent to the standard 6-week RT course.

In general, low-grade gliomas receive total doses of 45 to 54 Gy.[12, 13] Similar doses are used for pituitary adenomas and spinal cord tumors.

Common benign CNS tumors, such as grade 1 meningiomas, may be treated with RT if a complete surgical resection is not possible or desirable, or upon postsurgical tumor recurrence. Higher-grade meningiomas (WHO grade 3) have a high propensity for local recurrence, requiring postoperative RT immediately after surgery.[14, 15] RT is considered for resected or incompletely resected WHO grade 2 or incompletely resected grade 1 tumors. Meningiomas diagnosed by radiographic criteria may receive either single-fraction radiosurgery or fractionated RT, depending on location and size.[16]

RT dose to the whole brain in primary CNS lymphoma is evolving, driven by a concern regarding cognitive decline with the prior standard dose of 45 Gy in 1.8-Gy fractions given after methotrexate-based systemic chemotherapy. The current recommendation is either to administer a dose of 36 Gy in 1.2-Gy twice-daily fractions for all patients or to use 23.4 Gy in those with a complete response to chemotherapy, with a boost to 45 Gy for those with a partial response.[17, 18]

For tumors at high risk for CNS dissemination, such as medulloblastomas and primitive neuroectodermal tumors (PNETs), initial craniospinal irradiation (CSI) is performed, followed by a boost to the primary tumor site, most often with systemic chemotherapy, either concurrent and/or sequential.[19]

Chemotherapy and Other Forms of Systemic Therapy

The treatment of brain tumors with chemotherapy faces several challenges, some of which are unique to the CNS. First is the presence of the BBB; brain capillary endothelial cells have tight intercellular junctions, no fenestrations, and few pinocytic vesicles. Thus, diffusion represents the principal mechanism for drugs to cross the BBB; moreover, coverage of the endothelial basement membranes by astrocytic foot processes further decreases surface area. Only low molecular weight lipophilic drugs freely cross the BBB. Some small hydrophilic compounds gain access through carrier-mediated and receptor-mediated transport. Furthermore, the endothelial cells of many brain tumors (and in some cases, the tumor cells themselves) express membrane transporter proteins, such as P-glycoprotein and breast cancer resistance protein (BCRP), that further restrict access of chemotherapy to tumor cells. High intratumoral interstitial pressure contributes to limited drug delivery. Intrinsic tumor cell resistance to chemotherapy via the presence of DNA damage repair proteins is another problem, as epitomized in the resistance to the lipid-soluble alkylating agent temozolomide in

Table 72.1 RT doses in primary brain tumors

Tumor type	Grade	RT dose, Gy	Daily fraction size, Gy	Tumor type	Grade / response to chemotherapy	RT dose, Gy	Fraction size, Gy
Glioma	2	45–54	1.8–2	Meningioma	1	54	1.8–2
Glioma	3	59.4	1.8	Meningioma	2	54–60	1.8–2
Glioma	4 (GB)	60	2	Meningioma	3	59.4–60	1.8–2
Ependymoma (localized)	2	54–59.4	2	Primary CNS lymphoma	Complete response	23.4	1.8
Pituitary adenoma		46–50.4	1.8–2	Primary CNS lymphoma	Partial response	36<br>45 (boost)	1.2 BID<br>1.8
Pituitary adenoma				Medulloblastoma / PNET	All patients	CSI 30.6–36, boost 55.8	1.8

Abbreviation: BID, twice daily.

GBMs expressing alkyl-guanine alkyltransferase (the product of the gene *MGMT*).[20] Acquired drug resistance is also a factor, as is seen in the "hypermutator phenotype" of GBMs and other gliomas treated with temozolomide.

Despite these challenges, cytotoxic chemotherapy is of dramatic benefit in some CNS malignancies and of modest benefit in others. For example, primary CNS lymphoma is highly sensitive to methotrexate-based chemotherapy, although the methotrexate must be given in high doses to achieve tumoricidal levels. The addition of chemotherapy to craniospinal RT in medulloblastoma has increased the cure rate; similarly, CNS germinoma usually is exquisitely sensitive to platinum-based chemotherapy. These examples emphasize that the BBB in CNS tumors is often incomplete, as reflected in the tendency of many tumors to "enhance" on MR imaging or CT with the administration of intravenous iodinated or gadolinium contrast agents. Chemotherapy is of more modest benefit in gliomas; the most active cytotoxic agents are lipid-soluble alkylating drugs such as temozolomide, carmustine, and lomustine.

Targeting angiogenesis, a cardinal feature of GBM, gained traction following the anecdotal observation of dramatic improvement in contrast enhancement and vasogenic edema following administration of the anti–vascular epithelial growth factor (VEGF) monoclonal antibody bevacizumab in recurrent tumors. Bevacizumab received approval by the US Food and Drug Adminstration (FDA) in 2009 for use in recurrent GBM; however, subsequent studies adding bevacizumab to standard RT and temozolomide in newly diagnosed patients failed to show prolongation of overall survival. Other attempts to target VEGF and its receptor are ongoing but have not yielded success to date.

Numerous other strategies are under study to try to improve the outcome for malignant brain tumors, most notably in GBM, the most common and malignant primary CNS tumor; a necessarily incomplete listing follows. Small molecule inhibitors of critical targets such as the epidermal growth factor receptor (EGFR), the PI3K/Akt/mTOR pathway, and the MAPK kinase pathway identified through The Cancer Genome Atlas (TCGA) analysis of GBMs have been tried singly and in combination. Vaccine approaches include the use of a fragment of the EGFRvIII mutation found in 30% of GBMs and other attempts with common GBM-associated antigens with or without dendritic cells. Other immunologic approaches include the use of immune checkpoint inhibitors, which recently entered GBM clinical trials. Viral strategies have used viruses to deliver therapeutic transgenes that locally convert prodrugs to chemotherapeutics, and replication-competent tumor-specific viruses have been developed to cause oncolysis and/or trigger an antitumor immune response. Another novel approach has been the use of very low-intensity electrical fields beamed at the tumor through battery-attached scalp electrodes (the Optune device; Novocure, Portsmouth, NH); Optune has garnered FDA approval for recurrent GBM and is under study for newly diagnosed tumors.[1]

PROGNOSTIC FACTORS

Prognostic Factors Required for Stage Grouping

CNS tumors are not staged; therefore, there are no prognostic factors required for stage grouping.

Additional Factors Recommended for Clinical Care

Histopathology

1. Pathological WHO grade (required) and accuracy of diagnosis
2. Presence and extent of mitoses, pleomorphism, necrosis, endothelial proliferation, oligodendroglial component, gemistocytes
3. Proliferative fraction (Ki-67, MIB1; required)

Age of the Patient

Young favorable, >65 years unfavorable

Location of Tumor

1. Unifocal or multifocal
2. Eloquent or noneloquent brain area

Functional Neurologic Status

Karnofsky score, quality of life

Symptoms at Presentation and Duration before Diagnosis

Seizures and long duration of symptoms are favorable.

Tumor

Primary or recurrent

Extent of Resection

Biopsy, subtotal, radical, complete (gross)

Molecular Aspects

1. *IDH* mutation status required for gliomas for clinical care: level I
2. 1p, 19q deletions required for gliomas for clinical care: level I
3. MGMT methylation status required for gliomas for clinical care: level I

CNS tumor classification and grading follows the WHO system. The 2016 WHO Classification of Tumours of the Central Nervous System (2016 CNS WHO) provides two related approaches to prognosis: classification and grading, with grades more closely linked to classification than in most tumors of other organ systems.

Tumor Classification and "Integrated" Diagnoses

One of the most clinically meaningful prognostic factors for brain and spinal cord tumors is the primary classification as determined by histology, immunophenotype, and molecular profile. The 2016 CNS WHO breaks with the tradition of prior CNS WHO classifications in that it uses pure histologic diagnoses as well as "integrated" diagnoses, that is, those that incorporate both histologic and molecular parameters in assigning a diagnosis.[21] Examples of such integrated diagnoses are *Glioblastoma, IDH mutant* and *Glioblastoma, IDH wild type*—tumors with different biological and clinical features despite similar histologic findings. Notably, many of the genetic parameters may be assessed by using widely available techniques, such as immunohistochemistry or fluorescence *in situ* hybridization. Some centers may not have the ability to carry out molecular analyses, and some molecular results may not be conclusive. As a result, a diagnostic designation of "not otherwise specified" (NOS) has been added to the 2016 classification in certain places to indicate that there is insufficient information to assign a more specific diagnosis. In this context, NOS includes tumors that have not been tested for the genetic parameter(s) as well as those that have been tested but do not show the diagnostic genetic alterations.

Grading

CNS WHO classifications use a grading scheme that is a "malignancy scale" ranging across a wide variety of neoplasms rather than a strict histologic grading system that can be applied equally to all tumor types. The grades are I through IV, given in Roman numerals. Grade I lesions generally are circumscribed, with low proliferative potential and the possibility of cure after surgical resection. Grade II tumors typically are infiltrative in nature and have a relatively high likelihood of recurrence, with some grade II lesions also having a propensity for malignant progression. Grade III tumors demonstrate histologic evidence of malignancy and often follow a malignant course. Grade IV lesions are histologically malignant and follow an aggressive clinical course, including a propensity for spread within the brain and craniospinal spaces in some instances. In this system, grade is based on studies of natural history rather than the expected clinical course following therapy. Thus, a grade IV assigned to a medulloblastoma reflects the expectedly aggressive course in the absence of therapy, rather than the potentially favorable course expected for some medulloblastomas in the setting of current therapies.

In nearly all instances within the CNS WHO classification, each entity is assigned to a specific WHO grade. For example, without exception, pilocytic astrocytoma corresponds to a grade I, and GBM to a grade IV. This approach reduces grading flexibility in the CNS WHO classifications as compared with non–nervous system tumors. It also is important to note that the natural histories of entities recently defined by combined histologic–molecular approaches may not be well defined. For example, IDH-mutant diffuse astrocytic tumors have better prognoses than histologically defined diffuse astrocytic tumors of the past, yet the understanding of the natural history of these entities and their optimal risk stratification is incomplete. (Given that the presence of particular genetic alterations may shift prognostic estimates markedly, sometimes overriding the prognostic strength of histologic grade itself, the basic principle of grade as a reflection of natural history likely will require revision in the future.)

Prognostic Factors for Gliomas

MGMT

MGMT (O^6-methylguanine-DNA methyltransferase) is a prognostic and predictive marker in high-grade gliomas. The current standard therapy for GBM includes radiation and chemotherapy with temozolomide, which acts as a DNA crosslinker by alkylating multiple sites, including the O^6 position of guanine.[11] Because DNA crosslinking at the O^6 position of guanine is reversed by MGMT, low levels of *MGMT* expression are associated with an enhanced response to alkylating agents. MGMT status typically is evaluated by the level of methylation involving the gene's promoter. "Epigenetic silencing" caused by *MGMT* promoter methylation occurs in 40–50% of GBMs and may be determined by methylation-specific polymerase chain reaction–based tests of genomic DNA. Most investigations have shown that epigenetic gene silencing of *MGMT* is a strong predictor of prolonged survival, independent of other clinical factors or treatment.[22] It also has been demonstrated that *MGMT* promoter methylation is associated with prolonged progression-free and overall survival in patients with GBM treated with chemotherapy and RT.[20]

Isocitrate Dehydrogenase 1 and 2 (IDH1 and 2)

Isocitrate dehydrogenase (IDH) is a diagnostic and prognostic marker among gliomas. Mutations in *IDH1* are frequent (70–80%) in WHO grade II and III astrocytomas and a small subset (5–10%) of GBMs (WHO grade IV).[23] Mutations in *IDH2* are much less frequent. Oligodendrogliomas are *IDH*-mutant gliomas that also show codeletion of chromosomes 1p and 19q. IDH normally catalyzes the reaction of isocitrate to α-ketoglutarate, whereas the mutant forms of IDH1 and IDH2 lead to production of the oncometabolite 2-hydroxyglutarate, which inhibits the function of numerous α-ketoglutarate–dependent enzymes and leads to high levels of DNA methylation, which has been referred to as the CpG

island methylator phenotype (G-CIMP).[24, 25] *IDH* mutations identify a subset of gliomas with a slower rate of growth and a substantially improved prognosis, grade for grade, compared with gliomas that are *IDH* wild type. More than 90% of *IDH1* mutations in gliomas occur at a specific site and are characterized by a base exchange of guanine to adenine within codon 132, resulting in an amino acid change from arginine to histidine (R132H). A monoclonal antibody to the mutant protein has been developed, allowing its use in paraffin-embedded specimens (mIDH1R132H).[26] The ability of the antibody to detect a small number of cells as mutant may make this method more sensitive than sequencing in identifying *R132H*-mutant gliomas. However, mutations in *IDH2* and other mutations in *IDH1* will not be detected using immunohistochemistry with this antibody.

1p/19q Codeletion

Codeletion of chromosomes 1p and 19q, a diagnostic, prognostic, and predictive marker among the gliomas, is best known for its strong association with the oligodendroglioma phenotype.[27] The combination of *IDH* mutation and 1p/19q loss is considered the molecular signature of oligodendroglioma.[28, 29] The finding of 1p/19q codeletion is associated with enhanced response to radiochemotherapy and prolonged survival as compared with gliomas without this finding. Codeletion of 1p/19q occurs by an unbalanced translocation in which only one copy of the short arm of chromosome 1 and one copy of the long arm of chromosome 19 remain and der(1;19)(q10;p10) is produced. Solitary or focal losses of 1p or 19q also are noted occasionally within infiltrating gliomas, but are not as strongly linked to the oligodendroglioma histology, and are not predictive of enhanced response to therapy nor of prolonged survival. Oligodendrogliomas of grades II and III that are *IDH* mutant and 1p/19q codeleted also have a high frequency of *TERT* promoter mutations, *FUBP1* mutations on the remaining chromosome 1p allele, and *CIC* mutation on the remaining 19q allele.[30]

Ki-67

Ki-67, a prognostic marker among grade II and III diffuse gliomas, also is widely used as a marker of biological potential in other CNS neoplasms. Ki-67 is a nuclear antigen expressed in cells actively engaged in the cell cycle but not expressed in the resting phase, G0.[31] Results are expressed as a percentage of positive-staining tumor cell nuclei (Ki-67 labeling index). Numerous investigations have demonstrated a positive correlation between Ki-67 indices and histologic grade for the diffuse gliomas.[32] Among grade II and III diffuse gliomas, the Ki-67 index provides prognostic value, as there is a strong inverse relationship to survival on multivariate analysis. In contrast, investigations of Ki-67 proliferation and patient outcome in GBM, WHO grade IV, have consistently concluded that it does not provide prognostic value.[33] One potential shortcoming of Ki-67 as a marker is the high degree of variability in tissue processing, immunohistochemical staining, and quantization techniques among laboratories, making it difficult to standardize proliferation indices.[34] Large variations in proliferation rates within a single tumor also may be noted. Nonetheless, if interpreted uniformly within a given laboratory, the Ki-67 proliferation index provides prognostic value and may be helpful in histologically borderline cases, such as those on the border between grades II–III and grades III–IV. A high labeling index in this setting may indicate a more aggressive neoplasm.

Other Diagnostic Markers for Gliomas

TP53

TP53 mutation is a diagnostic marker among the gliomas. Nearly all *IDH*-mutant gliomas that do not harbor 1/19q codeletions will demonstrate mutations in *TP53*. In this regard, *TP53* mutation is a marker of astrocytoma lineage in the setting of *IDH* mutation and occurs in infiltrative astrocytomas, grade II; anaplastic astrocytomas, grade III; and GBM, WHO grade IV.[23] *TP53* mutations are extremely rare in oligodendrogliomas with *IDH* mutation and 1p/19q codeletion. Nuclear immunohistochemical reactivity for the p53 protein is often used as a marker for astrocytic differentiation in gliomas, because the mutant protein is degraded more slowly and accumulates in the nucleus of tumor cells with *TP53* mutations. However, this immunostain reacts with both the normal and mutant forms of p53, and therefore is not entirely specific for *TP53* mutations. Tumors with *IDH* mutations and ATRX loss by immunohistochemistry typically show strong nuclear p53 immunoreactivity in >10% of tumor cells.

ATRX

ATRX (alpha thalassemia/mental retardation syndrome X-linked) is a diagnostic marker among the gliomas. Inactivating alterations in *ATRX*, a gene that encodes a protein involved in chromatin remodeling, are strongly associated with *IDH1* mutation and *TP53* mutation in infiltrating gliomas.[35] As such, in the setting of *IDH*-mutant gliomas, *ATRX* mutation or deletion is a marker of astrocytic lineage and is mutually exclusive with 1p/19q codeletion. Nearly all gliomas with *IDH* and *ATRX* mutations also harbor *TP53* mutation, and are associated with the alternative lengthening of telomeres (ALT) phenotype.[36] Immunohistochemistry for ATRX demonstrates a loss of protein expression in neoplastic cells that harbor inactivating mutations, whereas expression is retained in nonneoplastic cells within the sample (e.g., endothelial cells).

H3 K27M

H3 K27M is a diagnostic marker among the gliomas. *H3F3A* encodes for H3.3, a histone variant that is normally recruited to DNA via the ATRX–DAXX heterodimer. Mutations are

most frequent in pediatric high-grade gliomas, yet occasionally may be identified in adults.[37–39] Approximately 40% of pediatric GBMs harbor *H3F3A* mutations, and the vast majority of these also harbor *ATRX* mutations. Mutations lead to decreased methylation of H3 histones and usually involve amino acid substitutions at K27 and G34. The site of *H3F3A* mutations in high-grade gliomas is associated with patient age and tumor location. Those with mutations at K27 tend to occur in young children and in midline locations, predominantly the pons and thalamus, but also involving other brainstem locations, the hypothalamus, and the spinal cord. Those with mutations at G34 occur in teenagers and young adults, and arise most frequently within the cerebral hemispheres. An antibody to the mutant form of the H3.3 K27M protein can identify tumors with this mutation, classified by the 2016 CNS WHO as diffuse midline glioma, H3 K27M mutant. Because of their location in deep midline structures, these gliomas often are biopsied or incompletely resected, which may result in a histologic grade that does not reflect their aggressive biologic potential. The finding of a mutant H3 K27M protein by immunohistochemistry identifies the tumor as a biologically aggressive form of glioma.

Diagnostic and Prognostic Embryonal Tumor Markers

Medulloblastomas are primitive embryonal neoplasms of the cerebellum, generally arising in childhood, whose molecular genetic alterations are now well defined. In addition to the histologically defined variants (see Table 72.1), four robust, clinically relevant transcriptional subgroups have been established: WNT, sonic hedgehog (SHH), "group 3," and "group 4."[40] The 2016 CNS WHO recognizes WNT, SHH, and non-WNT/non-SHH (groups 3 and 4) medulloblastoma in its classification. Biomarkers and their prognostic relevance are provided as follows:

WNT medulloblastomas display monosomy 6, and most also show nuclear accumulation of the WNT pathway protein β-catenin, the latter serving as a useful immunohistochemical screen for this group. Medulloblastomas with >50% nuclear staining for β-catenin have been shown to have WNT pathway activation, *CTNNB1* mutations, and monosomy 6, whereas those with only focal nuclear staining do not. The overall survival rates for WNT pathway medulloblastomas are dramatically longer than those of the other subtypes, and clinical practices are changing in light of this.

SHH medulloblastomas often show a nodular/desmoplastic histology and are associated with a better prognosis in younger children and infants. 9q deletion is characteristic of the SHH group. GAB1 is expressed in the cytoplasm of nearly all SHH medulloblastomas, but not in other groups, and may be detected immunohistochemically, making it a valuable SHH-group marker. SHH medulloblastomas that harbor *TP53* mutations have a worse prognosis than those that do not; therefore, WHO recognizes as distinct entities *medulloblastoma, SHH activated and TP53 mutant* and *medulloblastoma, SHH activated and TP53 wild type*. MYCN amplification, although uncommon in this subset, is associated with a poor prognosis. Targeted therapies have been developed for SHH medulloblastoma.[41] Non-WNT/non-SHH medulloblastoma consists of transcriptional group 3, which has the worst overall prognosis, and group 4, which also has a poor prognosis but is more variable. Clinically, these tumors are defined by the absence of WNT and SHH markers. Group 3 contains the vast majority of *MYC*-amplified tumors, whereas group 4 tumors (along with a small subset of SHH tumors) contain *MYCN* amplifications. *MYC* and *MYCN* amplifications are strong prognostic factors associated with aggressive clinical behavior, although they occur in only a small percentage of cases.

RISK ASSESSMENT MODELS

The AJCC recently established guidelines that will be used to evaluate published statistical prediction models for the purpose of granting endorsement for clinical use.[42] Although this is a monumental step toward the goal of precision medicine, this work was published only very recently. Therefore, the existing models that have been published or may be in clinical use have not yet been evaluated for this cancer site by the Precision Medicine Core of the AJCC. In the future, the statistical prediction models for this cancer site will be evaluated, and those that meet all AJCC criteria will be endorsed.

REGISTRY DATA COLLECTION VARIABLES

Gliomas

1. IDH mutation
2. WHO grade classification
3. Ki-67/MIB1 labeling index (LI): brain
4. Functional neurologic status—e.g., Karnofsky performance scale (KPS)
5. Methylation of MGMT
6. Chromosome 1p: loss of heterozygosity (LOH)
7. Chromosome 19q: LOH
8. Extent of surgical resection
9. Unifocal versus multifocal tumor

HISTOLOGIC GRADE (G)

CNS WHO tumor grades are used in histologic grading. This provides uniformity of classification and categorization of CNS tumors (Table 72.2).

G	G Definition
I	Circumscribed tumors of low proliferative potential associated with the possibility of cure following resection
II	Infiltrative tumors with low proliferative potential with increased risk of recurrence
III	Tumors with histologic evidence of malignancy, including nuclear atypia and mitotic activity, associated with an aggressive clinical course
IV	Tumors that are cytologically malignant, mitotically active, and associated with rapid clinical progression and potential for dissemination

Table 72.2 WHO grading system for selected tumors of the CNS[43]

Tumor Group	Tumor Type	Grade I	Grade II	Grade III	Grade IV
Astrocytic tumors	Diffuse astrocytoma		X		
	Anaplastic astrocytoma			X	
	Glioblastoma				X
	Pilocytic astrocytoma	X			
	Pilomyxoid astrocytoma		X		
	Subependymal giant cell astrocytoma	X			
	Pleomorphic xanthoastrocytoma		X		
	Anaplastic pleomorphic xanthoastrocytoma			X	
Oligodendrogliomas	Oligodendroglioma		X		
	Anaplastic oligodendroglioma			X	
Ependymal tumors	Ependymoma		X		
	Anaplastic ependymoma			X	
	Subependymoma	X			
	Myxopapillary ependymoma	X			
Choroid plexus tumors	Choroid plexus papilloma	X			
	Atypical choroid plexus papilloma		X		
	Choroid plexus carcinoma			X	
Other gliomas	Angiocentric glioma	X			
	Chordoid glioma of the third ventricle		X		
Neuronal–glial tumors	Gangliocytoma	X			
	Desmoplastic infantile ganglioglioma/ astrocytoma (DIG/DIA)	X			
	Dysembryoplastic neuroepithelial tumor (DNET)	X			
	Ganglioglioma	X			
	Anaplastic ganglioglioma			X	
	Central neurocytoma		X		
	Extraventricular neurocytoma		X		
	Cerebellar liponeurocytoma		X		
	Papillary glioneuronal tumor (PGNT)	X			
	Rosette-forming glioneuronal tumor of the fourth ventricle (RGNT)	X			
	Paraganglioma	X			
Pineal parenchymal tumors	Pineocytoma	X			
	Pineal parenchymal tumor of intermediate differentiation		X	X	
	Pineoblastoma				X
	Papillary tumor of the pineal region		X	X	
Embryonal tumors	Medulloblastoma				X
	Embryonal tumor with multilayered rosettes				X
	Medulloepithelioma				X
	CNS neuroblastoma				X
	CNS ganglioneuroblastoma				X
	CNS embryonal tumor				X
	Atypical teratoid/rhabdoid tumor				X

Table 72.2 (continued)

Tumor Group	Tumor Type	Grade I	Grade II	Grade III	Grade IV
Cranial and peripheral nerve tumors	Schwannoma	X			
	Neurofibroma	X			
	Perineurioma	X	X	X	
	Malignant peripheral nerve sheath tumor (MPNST)		X	X	X
Meningeal tumors	Meningioma	X			
	Atypical meningioma		X		
	Clear cell meningioma		X		
	Chordoid meningioma		X		
	Anaplastic meningioma			X	
	Papillary meningioma			X	
	Rhabdoid meningioma			X	
Mesenchymal tumors	*(Named as soft tissue counterpart)*	X	X	X	X
	Solitary fibrous tumor/hemangiopericytoma	X	X	X	
	Hemangioblastoma	X			
Tumors of the sellar region	Craniopharyngioma	X			
	Pituicytoma	X			
	Granular cell tumor	X			
	Spindle cell oncocytoma	X			
	Pituitary adenoma	X			

Bibliography

1. Weller M, Wick W, Brada M, et al. Glioma. *Nature Reviews Disease Primers*. 2015;1:1–18.
2. Ostrom QT, Gittleman H, Fulop J, et al. CBTRUS Statistical Report: Primary Brain and Central Nervous System Tumors Diagnosed in the United States in 2008-2012. *Neuro-oncology*. Oct 2015;17 Suppl 4(suppl 4):iv1–iv62.
3. Bondy ML, Scheurer ME, Malmer B, et al. Brain tumor epidemiology: consensus from the Brain Tumor Epidemiology Consortium. *Cancer*. Oct 1 2008;113(7 Suppl):1953–1968.
4. Kinnersley B, Labussiere M, Holroyd A, et al. Genome-wide association study identifies multiple susceptibility loci for glioma. *Nat Commun*. 2015;6:8559.
5. Niedermaier T, Behrens G, Schmid D, Schlecht I, Fischer B, Leitzmann MF. Body mass index, physical activity, and risk of adult meningioma and glioma: A meta-analysis. *Neurology*. Oct 13 2015;85(15):1342–1350.
6. Murphy MC, Huston J, 3rd, Jack CR, Jr., et al. Measuring the characteristic topography of brain stiffness with magnetic resonance elastography. *PloS one*. 2013;8(12):e81668.
7. Huang RY, Rahman R, Ballman KV, et al. The Impact of T2/FLAIR Evaluation per RANO Criteria on Response Assessment of Recurrent Glioblastoma Patients Treated with Bevacizumab. *Clin Cancer Res*. Feb 1 2016;22(3):575–581.
8. Hervey-Jumper SL, Li J, Lau D, et al. Awake craniotomy to maximize glioma resection: methods and technical nuances over a 27-year period. *J Neurosurg*. Aug 2015;123(2):325–339.
9. Walker MD, Green SB, Byar DP, et al. Randomized comparisons of radiotherapy and nitrosoureas for the treatment of malignant glioma after surgery. *N Engl J Med*. Dec 4 1980;303(23):1323–1329.
10. Kelly PJ, Daumas-Duport C, Kispert DB, Kall BA, Scheithauer BW, Illig JJ. Imaging-based stereotaxic serial biopsies in untreated intracranial glial neoplasms. *J Neurosurg*. Jun 1987;66(6):865–874.
11. Stupp R, Mason WP, van den Bent MJ, et al. Radiotherapy plus concomitant and adjuvant temozolomide for glioblastoma. *N Engl J Med*. Mar 10 2005;352(10):987–996.
12. Karim AB, Maat B, Hatlevoll R, et al. A randomized trial on dose-response in radiation therapy of low-grade cerebral glioma: European Organization for Research and Treatment of Cancer (EORTC) Study 22844. *International journal of radiation oncology, biology, physics*. Oct 1 1996;36(3):549–556.
13. Shaw E, Arusell R, Scheithauer B, et al. Prospective randomized trial of low- versus high-dose radiation therapy in adults with supratentorial low-grade glioma: initial report of a North Central Cancer Treatment Group/Radiation Therapy Oncology Group/Eastern Cooperative Oncology Group study. *J Clin Oncol*. May 1 2002;20(9):2267–2276.
14. Aghi MK, Carter BS, Cosgrove GR, et al. Long?Term Recurrence Rates of Atypical Meningiomas After Gross Total Resection With or Without Postoperative Adjuvant Radiation. *Neurosurgery*. 2009;64(1):56–60.
15. Hug EB, Devries A, Thornton AF, et al. Management of atypical and malignant meningiomas: role of high-dose, 3D-conformal radiation therapy. *Journal of neuro-oncology*. Jun 2000;48(2):151–160.
16. Kondziolka D, Mathieu D, Lunsford LD, et al. Radiosurgery as definitive management of intracranial meningiomas. *Neurosurgery*. 2008;62(1):53–60.
17. DeAngelis LM, Seiferheld W, Schold SC, Fisher B, Schultz CJ, Radiation Therapy Oncology Group S. Combination chemotherapy and radiotherapy for primary central nervous system lymphoma: Radiation Therapy Oncology Group Study 93-10. *J Clin Oncol*. Dec 15 2002;20(24):4643–4648.
18. Shah GD, Yahalom J, Correa DD, et al. Combined immunochemotherapy with reduced whole-brain radiotherapy for newly diagnosed primary CNS lymphoma. *J Clin Oncol*. Oct 20 2007;25(30):4730–4735.
19. Padovani L, Sunyach MP, Perol D, et al. Common strategy for adult and pediatric medulloblastoma: a multicenter series of 253 adults. *International journal of radiation oncology, biology, physics*. Jun 1 2007;68(2):433–440.
20. Rivera AL, Pelloski CE, Gilbert MR, et al. MGMT promoter methylation is predictive of response to radiotherapy and prognostic in the absence of adjuvant alkylating chemotherapy for glioblastoma. *Neuro-oncology*. 2010;12(2):116–121.

21. Louis DN, Perry A, Burger P, et al. International Society of Neuropathology-Haarlem consensus guidelines for nervous system tumor classification and grading. *Brain pathology*. 2014;24(5):429–435.
22. Hegi ME, Diserens AC, Gorlia T, et al. MGMT gene silencing and benefit from temozolomide in glioblastoma. *N Engl J Med*. Mar 10 2005;352(10):997–1003.
23. Yan H, Parsons DW, Jin G, et al. IDH1 and IDH2 mutations in gliomas. *N Engl J Med*. Feb 19 2009;360(8):765–773.
24. Noushmehr H, Weisenberger DJ, Diefes K, et al. Identification of a CpG island methylator phenotype that defines a distinct subgroup of glioma. *Cancer cell*. 2010;17(5):510–522.
25. Turcan S, Rohle D, Goenka A, et al. IDH1 mutation is sufficient to establish the glioma hypermethylator phenotype. *Nature*. 2012;483(7390):479–483.
26. Capper D, Weissert S, Balss J, et al. Characterization of R132H mutation-specific IDH1 antibody binding in brain tumors. *Brain Pathol*. Jan 2010;20(1):245–254.
27. Cairncross JG, Ueki K, Zlatescu MC, et al. Specific genetic predictors of chemotherapeutic response and survival in patients with anaplastic oligodendrogliomas. *Journal of the National Cancer Institute*. Oct 7 1998;90(19):1473–1479.
28. Cancer Genome Atlas Research Network, Brat DJ, Verhaak RG, et al. Comprehensive, Integrative Genomic Analysis of Diffuse Lower-Grade Gliomas. *N Engl J Med*. Jun 25 2015; 372(26):2481–2498.
29. Eckel-Passow JE, Lachance DH, Molinaro AM, et al. Glioma Groups Based on 1p/19q, IDH, and TERT Promoter Mutations in Tumors. *N Engl J Med*. Jun 25 2015;372(26):2499–2508.
30. Killela PJ, Reitman ZJ, Jiao Y, et al. TERT promoter mutations occur frequently in gliomas and a subset of tumors derived from cells with low rates of self-renewal. *Proc Natl Acad Sci U S A*. Apr 9 2013;110(15):6021–6026.
31. Brat DJ, Prayson RA, Ryken TC, Olson JJ. Diagnosis of malignant glioma: role of neuropathology. *Journal of neuro-oncology*. Sep 2008;89(3):287–311.
32. Giannini C, Scheithauer BW, Burger PC, et al. Cellular proliferation in pilocytic and diffuse astrocytomas. *Journal of neuropathology and experimental neurology*. Jan 1999;58(1):46–53.
33. Moskowitz SI, Jin T, Prayson RA. Role of MIB1 in predicting survival in patients with glioblastomas. *Journal of neuro-oncology*. Jan 2006;76(2):193–200.
34. Grzybicki DM, Liu Y, Moore SA, et al. Interobserver variability associated with the MIB-1 labeling index: high levels suggest limited prognostic usefulness for patients with primary brain tumors. *Cancer*. Nov 15 2001;92(10):2720–2726.
35. Wiestler B, Capper D, Holland-Letz T, et al. ATRX loss refines the classification of anaplastic gliomas and identifies a subgroup of IDH mutant astrocytic tumors with better prognosis. *Acta Neuropathol*. Sep 2013;126(3):443–451.
36. Nguyen DN, Heaphy CM, de Wilde RF, et al. Molecular and morphologic correlates of the alternative lengthening of telomeres phenotype in high-grade astrocytomas. *Brain Pathol*. May 2013;23(3):237–243.
37. Gajjar A, Pfister SM, Taylor MD, Gilbertson RJ. Molecular insights into pediatric brain tumors have the potential to transform therapy. *Clinical Cancer Research*. 2014;20(22):5630–5640.
38. Reuss DE, Sahm F, Schrimpf D, et al. ATRX and IDH1-R132H immunohistochemistry with subsequent copy number analysis and IDH sequencing as a basis for an "integrated" diagnostic approach for adult astrocytoma, oligodendroglioma and glioblastoma. *Acta neuropathologica*. 2015;129(1):133–146.
39. Sturm D, Bender S, Jones DT, et al. Paediatric and adult glioblastoma: multiform (epi)genomic culprits emerge. *Nat Rev Cancer*. Feb 2014;14(2):92–107.
40. MacDonald TJ, Aguilera D, Castellino RC. The rationale for targeted therapies in medulloblastoma. *Neuro-oncology*. Jan 2014;16(1):9–20.
41. Rudin CM, Hann CL, Laterra J, et al. Treatment of medulloblastoma with hedgehog pathway inhibitor GDC-0449. *N Engl J Med*. Sep 17 2009;361(12):1173–1178.
42. Kattan MW, Hess KR, Amin MB, et al. American Joint Committee on Cancer acceptance criteria for inclusion of risk models for individualized prognosis in the practice of precision medicine. *CA: a cancer journal for clinicians*. Jan 19 2016.
43. Louis DN, Ohgaki H, Wiestler OD, et al. World Health Organization Classification of Tumours of the Central Nervous System. Revised 4th Edition. Lyon: IARC; 2016.

Part XVII

Endocrine System

Members of the Endocrine System Expert Panel

Elliot A. Asare, MD
James D. Brierley, BSc, MB, FRCP, FRCR, FRCP(C) – UICC Representative
David R. Byrd, MD, FACS
Herbert Chen, MD, FACS – Vice Chair
Kimberly DeWolfe, MS, CTR – Data Collection Core Representative
Frederick L. Greene, MD, FACS – Editorial Board Liaison
Raymon H. Grogan, MD
Robert Haddad, MD
Bryan R. Haugen, MD
Jennifer L. Hunt, MD, MEd
Camilo Jimenez, MD
Christine S. Landry, MD
Steven K. Libutti, MD, FACS
Ricardo V. Lloyd, MD, PhD
Rana R. McKay, MD
Lilah F. Morris, MD
Nancy D. Perrier, MD, FACS – Chair
Alexandria T. Phan, MD, FACP
John A. Ridge, MD, PhD, FACS
Eric Rohren, MD, PhD
Jennifer E. Rosen, MD, FACS
Raja R. Seethala, MD – CAP Representative
Jatin P. Shah, MD, PhD(Hon), FACS, FRCS(Hon)
Julie A. Sosa, MD
Rathan M. Subramaniam, MD, PhD, MPH
R. Michael Tuttle, MD
Tracy S. Wang, MD, MPH, FACS
Lori J. Wirth, MD

Thyroid – Differentiated and Anaplastic Carcinoma

73

R. Michael Tuttle, Lilah F. Morris, Bryan R. Haugen,
Jatin P. Shah, Julie A. Sosa, Eric Rohren,
Rathan M. Subramaniam, Jennifer L. Hunt,
and Nancy D. Perrier

CHAPTER SUMMARY

Cancers Staged Using This Staging System

Papillary thyroid carcinoma, follicular thyroid carcinoma, Hurthle cell thyroid carcinoma, poorly differentiated thyroid carcinoma, anaplastic (undifferentiated) carcinomas

Cancers Not Staged Using This Staging System

These histopathologic types of cancer…	Are staged according to the classification for…	And can be found in chapter…
Medullary thyroid cancer	Thyroid—medullary	74
Thyroid lymphoma	Hodgkin and Non-Hodgkin Lymphoma	79–80
Thyroid cancer arising from thyroglossal duct cyst	No AJCC staging system	N/A
Thyroid cancer in malignant struma ovarii	No AJCC staging system	N/A

Summary of Changes

Change	Details of Change	Level of Evidence
Prognostic Factors Required for Stage Grouping	The age at diagnosis cutoff used for staging was increased from 45 years to 55 years.	I
Definition of Primary Tumor (T)	Minor extrathyroidal extension was removed from the definition of T3 disease. As a result, minor extrathyroidal extension does not affect either T category or overall stage.	I
Definition of Primary Tumor (T)	T3a is a new category and refers to a tumor >4 cm in greatest dimension limited to the thyroid gland.	I
Definition of Primary Tumor (T)	T3b is a new category and is defined as a tumor of any size with gross extrathyroidal extension invading only strap muscles (sternohyoid, sternothyroid, thyrohyoid, or omohyoid muscles).	I
Definition of Regional Lymph Node (N)	The definition of central neck (N1a) was expanded to include both level VI and level VII (upper mediastinal) lymph node compartments. Previously, level VII lymph nodes were classified as lateral neck lymph nodes (N1b).	II
Definition of Regional Lymph Node (N)	The pN0 designation is clarified as one or more cytologically or histologically confirmed benign lymph node(s).	II

To access the AJCC cancer staging forms, please visit www.cancerstaging.org.

© American Joint Committee on Cancer 2017
M.B. Amin et al. (eds.), *AJCC Cancer Staging Manual, Eighth Edition*, DOI 10.1007/978-3-319-40618-3_73

Change	Details of Changes	Level of Evidence
AJCC Prognostic Stage Groups	The definition of Stages I, II, III, IV was changed for patients older than 55 years at diagnosis.	I
AJCC Prognostic Stage Groups	Stage I now includes T1 and T2 tumors if N0/NX and M0 in patients older than 55 years at diagnosis.	I
AJCC Prognostic Stage Groups	Stage II now includes T1 and T2 tumors if N1 and T3a/T3b tumors with any N if M0 in patients older than 55 years at diagnosis.	I
AJCC Prognostic Stage Groups	Stage III now includes only T4a with any N, if M0 in patients older than 55 years at diagnosis.	I
AJCC Prognostic Stage Groups	Stage IV now includes T4b with any N, any M and M1 with any T or N in patients older than 55 years at diagnosis.	I
Definition of Primary Tumor (T)	Unlike previous editions where all anaplastic tumors were classified as having T4 disease, the T category for anaplastic thyroid cancers will now use the same definitions used for differentiated thyroid cancers.	II
AJCC Prognostic Stage Groups	With anaplastic carcinoma, intrathyroidal disease is stage IVA, gross extrathyroidal extension or cervical node metastases is stage IVB, and distant metastases is stage IVC	II
Histologic Grade (G)	GX–G4 grading system was removed.	II

ICD-O-3 Topography Codes

Code	Description
C73.9	Thyroid gland

WHO Classification of Tumors

Code	Description
8050	Papillary carcinoma
8341	Papillary microcarcinoma
8340	Follicular variant
8230	Solid variant
8290	Hurthle cell variant
8330	Follicular carcinoma
8331	Encapsulated noninvasive
8335	Minimally invasive
8350	Widely invasive
8290	Hurthle cell carcinoma
8337	Poorly differentiated carcinoma (used for insular carcinoma as a subtype of poorly differentiated)
8021	Anaplastic carcinoma

DeLellis RA, Lloyd RV, Heitz PU, Eng C, eds. World Health Organization Classification of Tumours Pathology and Genetics of Tumours of Endocrine Organs. Lyon: IARC; 2004.

INTRODUCTION

This chapter provides prognostic information and recommendations with regard to staging for thyroid cancers arising from thyroid follicular cells. Staging recommendations are provided for papillary thyroid cancer (PTC), follicular thyroid cancer (FTC), anaplastic thyroid cancer, poorly differentiated thyroid cancers, and their various subtypes. In addition, prognostic information without specific staging recommendations is provided for thyroid cancers arising from thyroglossal duct remnants and from struma ovarii. Information regarding staging and prognosis in medullary thyroid cancer and thyroid lymphoma is provided in Chapters 74 and 79–80, respectively.

The term *thyroid cancer* encompasses several distinct histologies that arise from thyroid follicular or parafollicular C cells. Papillary thyroid cancers and FTCs (and their respective variants) are classified as differentiated thyroid cancers that arise from thyroid follicular cells and generally have an excellent prognosis, with 10-year survival rates that exceed 90–95%. Papillary thyroid cancer is the most common thyroid cancer, accounting for more than 90% of all thyroid cancers.

Poorly differentiated thyroid cancers probably arise from either PTCs or FTCs and have a poorer prognosis, with 10-year survival rates approximating 50%. Conversely, anaplastic thyroid carcinoma is an aggressive undifferentiated tumor of thyroid follicular cell origin and, in most series, is associated with 5-year survival rates of less than 10%.

The past 20 years have seen a dramatic increase in the incidence of thyroid cancer, now one of the most rapidly increasing cancer diagnoses in the United States.[1] The increased incidence is predominantly the result of an increase in the diagnosis of relatively small (<2-cm) PTCs, with a much smaller increase in larger tumors.[2]

A variety of staging systems have been used to predict disease-specific mortality in differentiated thyroid cancers.[3] Each of these staging systems relies on a relatively small set of clinicopathologic variables available at the time of initial therapy, including age at diagnosis, histology, tumor size, the presence/absence of gross extrathyroidal extension, and distant metastases. Regional lymph node metastases are considered prognostically significant in some, but not all, of the staging systems.[3] Although staging

for cancers in other head and neck sites is based entirely on the anatomic extent of disease, it is not possible to follow this pattern for the unique group of malignant tumors that arise in the thyroid gland. Both the histologic diagnosis and the age of the patient are of such importance in the behavior and prognosis of thyroid cancer that these factors are included in this staging system.

Although none of the staging systems has been proven to be clearly superior to the others, the AJCC TNM system demonstrates one of the highest proportions of variance explained (a statistical measure of how well a staging system predicts the outcome of interest) and is the staging system recommended by the American Thyroid Association (ATA) and National Comprehensive Cancer Network (NCCN) guidelines.[3–5] In addition to initial staging with the AJCC TNM system, the ATA also recommends (1) the use of additional staging systems designed to predict clinical outcomes other than disease-specific mortality (e.g., risk of recurrence, risk of persistent disease) and (2) a method to modify risk estimates over time as a function of response to therapy and the biological behavior of the cancer.[3,4]

Although risk stratification traditionally has been considered a static estimate obtained at the time of initial risk stratification, the current management approach emphasizes the use of data obtained after initial therapy to individualize and modify initial risk estimates.[4] Factors such as the serum thyroglobulin value obtained 4 to 6 weeks after initial surgery, calculations of the thyroglobulin doubling time, radioactive iodine (RAI) and fluorodeoxyglucose (FDG) avidity of metastatic lesions, and identification of new or progressive structural disease during follow-up may have important prognostic significance.[3,4,6]

Differentiated Thyroid Cancers Arising outside the Thyroid Gland

This section is included for informational purposes. These cancers are not staged using this staging system.

Thyroid Cancer Arising from Thyroglossal Duct Cyst

Thyroglossal duct cysts, present in up to 7% of the adult population, often contain an epithelial lining of stratified squamous, pseudostratified, ciliated columnar epipthelial cells and ectopic thyroid cells.[7–10] More than 90–95% of PTCs arising in thyroglossal duct cysts are confined to the cyst, without evidence of local invasion or metastatic spread, and are usually diagnosed after surgical removal of what was presumed to be a benign thyroglossal duct cyst.[11,12]

Following complete surgical resection (usually a Sistrunk procedure with or without thyroidectomy), prognosis in PTCs arising in these remnants is excellent, with very low recurrence rates and 10-year survival rates exceeding 95%.[12] Squamous cell carcinomas appear to have a significantly worse prognosis.[11] Because fewer than 300 cases of thyroglossal duct remnant carcinomas have been reported in the literature, it is difficult to confidently identify specific prognostic features. However, Plaza et al.[13] proposed classifying tumors confined to the remnant as low-risk tumors that may be managed with a Sistrunk procedure alone. Although the prognosis in thyroglossal duct remnant PTC appears to be very similar to that of PTC arising in the thyroid primary, the pattern of lymphatic drainage may differ, as level I lymph nodes may be involved more commonly than would be expected in thyroid cancers arising within the thyroid gland proper.[14] Although not proven conclusively, it seems likely that a poorer prognosis would be expected in the few patients who demonstrate gross extension of the tumor outside the cyst, regional or distant metastases, or a more aggressive histology (e.g., squamous cell carcinoma).

Thyroid Cancer Arising in Malignant Struma Ovarii

Struma ovarii is a monodermal type of ovarian mature teratoma containing thyroid tissue either predominantly or exclusively.[15] Thyroid cancers arising in struma ovarii may be difficult to diagnosis, as they tend to be very well-differentiated PTCs or FTCs.[16,17] Fewer than 200 cases have been reported in the medical literature.[18–20] Although the primary tumor size may range from 1 to 200 mm, 80% of tumors appear to be localized to the ovary at diagnosis.[21] Metastases are uncommon but may develop in intra-abdominal locations, lungs, or bones.[16]

Overall survival rates after initial therapy range from 89–94% at 10 years and 84–85% at 20 years,[21,22] with only one disease-specific death over a median of 8 years of follow-up reported in an analysis of 68 patients from the Surveillance, Epidemiology, and End Results (SEER) database.[21] Although no staging system for malignant struma ovarii has been widely accepted, Yassa et al.[23] suggested that patients with thyroid carcinomas confined to the ovary without worrisome histologic features and less than 2 cm may be considered low risk, whereas patients with larger tumors, extension of disease outside the ovary, metastatic spread, or more aggressive histologies may be considered high risk.

ANATOMY

Primary Site(s)

The thyroid gland ordinarily is composed of a right and a left lobe lying adjacent and lateral to the upper trachea and esophagus (Fig. 73.1). An isthmus connects the two lobes, and in some cases, a pyramidal lobe is present, extending cephalad anterior to the thyroid cartilage.

Rarely, thyroid cancer may arise from thyroid follicular cells located outside the thyroid gland in locations such as

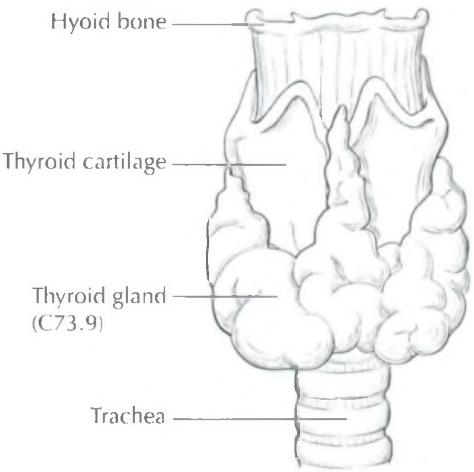

Fig. 73.1 Anatomy of the thyroid gland

the thyroglossal duct remnants, thyroid rests in the neck/upper mediastinum (thyrothymic tract), and ovaries (malignant struma ovarii).

Regional Lymph Nodes

A seven-compartment nomenclature is commonly used to define anatomic lymph node compartment boundaries (Fig. 73.2).[24,25] The term *central neck* usually refers to levels VI and VII, whereas the *lateral neck* includes levels I, II, III, IV, and V. The first echelon of nodal metastasis most commonly includes the paralaryngeal, paratracheal, and prelaryngeal (Delphian) nodes adjacent to the thyroid gland.

Metastases also may involve the high (level IIA), mid- (level III), and lower jugular (level IV), and the supraclavicular (level V) and (less commonly) the upper deep jugular and spinal accessory lymph nodes (level IIB). Lymph node metastasis to submandibular and submental lymph nodes (level I) is rare. Upper mediastinal (level VII) nodal spread occurs frequently, both anteriorly and posteriorly. Retropharyngeal nodal metastasis may be seen, usually in the presence of extensive lateral cervical metastases. Bilateral nodal spread is common.

Metastatic Sites

Distant metastases are seen at diagnosis in 2–5% of patients presenting with differentiated thyroid carcinoma.[26,27] Lung parenchyma is the most common site of distant metastases (80–85%), followed less commonly by bone (5–10%) and brain (1%). Metastases may uncommonly be identified in the liver, kidney, adrenal gland, pituitary gland, or skin.

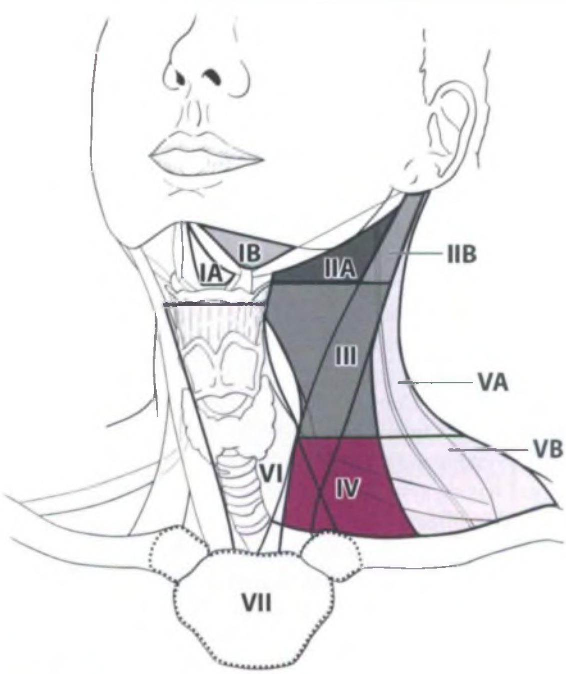

Fig. 73.2 Location of the lymph node levels in the neck

RULES FOR CLASSIFICATION

Clinical Classification

Most thyroid cancer patients present with asymptomatic nodules in the thyroid in the setting of normal thyroid function tests. Symptoms such as changes in the voice, dysphagia, or upper airway problems suggest more aggressive local disease. Distant metastases usually are identified as asymptomatic pulmonary nodules in high-risk patients but also may present as painful bone metastases or as masses causing local neurologic or vascular compromise.

Detailed guidance in preoperative assessments and management is provided in guidelines from the NCCN and the ATA.[4,5] Preoperative staging for thyroid cancer typically includes neck ultrasound to evaluate the thyroid gland and central and lateral neck lymph node compartments. Fine-needle aspiration of suspicious thyroid nodules and/or abnormal-appearing lymph nodes should be undertaken preoperatively to obtain a definitive diagnosis and allow for appropriate surgical planning. FDG positron emission tomography (PET) scanning or neck computed tomography (CT)/magnetic resonance (MR) imaging is not recommended, except in patients in whom there is clinical suspicion of gross extrathyroidal extension or extensive, clinically apparent, cervical/mediastinal lymphadenopathy.

For staging purposes, the treatment date for most patients with differentiated thyroid cancer should be the date of thyroidectomy, because thyroid surgery is almost always the first step in treatment. Rarely, patients may receive external beam irradiation, chemotherapy, metastasectomy, or other neoadjuvant therapy as their initial treatment (before thyroid surgery or in patients who never undergo thyroid surgery). In these cases, the treatment date would correspond to the initiation of these other therapies, provided they begin before thyroid surgery.

The date of diagnosis should correspond to the first date of cytologic or histologic confirmation of thyroid cancer.

With regard to sizing of the primary tumor (T), this is almost always done based on the size of the largest differentiated thyroid cancer nodule within the thyroid gland, determined when the thyroid surgical specimen is processed for histologic examination. In situations in which the thyroid cancer is not surgically removed, the size of the primary lesion may be obtained by correlating cross-sectional imaging studies with biopsy results (by cytology or histology). These situations may become more common, as the 2015 ATA guidelines allow for an active surveillance management approach (observation instead of immediate surgery) in subcentimeter thyroid nodules that are either cytologically confirmed PTC or presumed to be thyroid cancer based on highly suspicious ultrasound characteristics.[4] Although a highly suspicious ultrasound pattern carries a >70–90% likelihood that thyroid cancer is present,[28–30] cytologic or histologic proof of disease is required before staging.

Extrathyroidal extension refers to the involvement of perithyroidal soft tissues by direct extension from the thyroid primary. Invasion outside the thyroid that can be identified by imaging or intraoperative findings ranges from T3b disease (gross extrathyroidal extension involving only strap muscles) to T4a disease (demonstrating gross extrathyroidal extension invading the subcutaneous soft tissues, larynx, trachea, esophagus, muscle, or recurrent laryngeal nerve) to T4b disease (demonstrating gross extrathyroidal extension invading the prevertebral fascia or encasing the carotid artery or mediastinal vessels). The four infrahyoid muscles that either originate from or insert on the hyoid are often referred to as the strap muscles (including the sternohyoid, sternothyroid, thyrohyoid, and omohyoid muscles). Lesser degrees of extrathyroidal extension (minor) that are not clinically appreciated can be identified by microscopy as the tumor involves perithyroidal adipose tissue, strap muscles, nerves, or small vascular structures. Because the fibrous capsule of the thyroid is often incomplete, it is often difficult to determine whether the the boundary between thyroid cancer and fibroadipose tissue reflects an invasive process or simply the absence of a well-defined thyroid capsule in that area. For these reasons, and based on the lack of prognostic significance, the presence of minor extrathyroidal extension involving perithyroidal adipose tissue, strap muscles, nerves, or small vascular structures detected only by microscopy (not grossly evident) does not constitute T3b disease.

Clinical N1 disease (cN1) includes clinically apparent lymph node metastases (palpable or seen on imaging) that are either cytologically confirmed or highly suspicious for metastatic disease. Likewise, M1 status can be confirmed by cytologic/histologic assessment, documentation of RAI avidity of the metastatic lesion, or other imaging findings highly suspicious for distant metastasis in the proper clinical setting (e.g., inappropriately elevated serum thyroglobulin, ATA high-risk patient).

Imaging

Pretherapy Imaging

Preoperative neck ultrasonography to evaluate the thyroid gland as well as central and lateral neck lymph node chains is usually recommended as the initial staging procedure. Additional cross-sectional imaging with CT or MR imaging of the neck or distant sites is usually reserved for patients demonstrating clinical features of advanced disease, such as locally invasive primary tumor, clinically apparent multiple or bulky lymph node metastases, symptoms of distant metastases, or anaplastic thyroid cancers. Preoperative FDG-PET scanning is not routinely recommended but may be considered as part of initial staging in cases in which there is a reasonable likelihood of distant metastatic disease, such as in cases of poorly differentiated thyroid cancers, Hurthle cell cancers, and anaplastic thyroid cancers.[4,5]

Because of the high likelihood of both regional and distant metastases in anaplastic thyroid cancer, initial staging usually includes neck ultrasound; cross-sectional imaging of the head, neck, chest, abdomen, and pelvis with either CT or MR imaging; and/or FDG-PET scanning. At sites where PET scanning is performed using optimized PET/CT, the CT portion of the scan may supplant the need for additional anatomic imaging.[4,5]

These preoperative imaging examinations form the primary basis for preoperative clinical staging. Clinical T stage is based on the size of the primary tumor and an assessement of whether imaging demonstrates invasion of the tumor into the strap muscles, subcutaneous soft tissues, larynx, trachea, esophagus, recurrent laryngeal nerve, or prevertebral fascia or whether the tumor encases either the carotid artery or mediastinal vessels. The location of metastatic lymph nodes is used to define the clinical N stage (central neck vs. lateral neck disease). Most patients will be clinical MX, as routine use of cross-sectional or functional (RAI) imaging beyond the neck is not routinely performed, except in patients with locally advanced or anaplastic thyroid cancers.

One of the challenges with clinical staging is that nonspecific cervical lymphadenopathy is commonly found on routine ultrasonographic imaging and cannot be confidently classified as cN0 or cN1. In clinical practice,

ultrasound-guided fine-needle aspiration of sonographically suspicious lymph nodes ≥8 mm in smallest dimension is often performed if the results of the biopsy would alter initial management.[4] Likewise, nonspecific pulmonary nodules also are quite common in the general population and usually cannot be confidently classified as benign or malignant findings before thyroid surgery.

Posttherapy Imaging

Many patients undergo RAI scanning several weeks after thyroid surgery and at various time points during follow-up. These scans take advantage of the unique ability of most thyroid cells (both thyroid cancer and normal thyroid cells) to concentrate iodine. Although a focus of RAI uptake on the scan outside the thyroid bed usually indicates the presence of persistent or recurrent thyroid cancer, false positives do occur, which means that the RAI scans must be interpreted within the context of serum thyroglobulin and other patient risk factors for recurrence.

In most patients, neck ultrasonography is the primary imaging modality, with the testing interval based on initial risk stratification and the patient's response to therapy. Patients at high risk of regional or distant metastases also may be evaluated with cross-sectional imaging or FDG-PET scanning, depending on the serum thyroglobulin level and response to therapy classification.[4]

Because of the very high risk of recurrence and distant metastases, patients with anaplastic thyroid cancer require more frequent and extensive imaging. Cross-sectional imaging of the brain, neck, chest, abdomen, and pelvis is often performed at 1- to 3-month intervals for the first year of follow-up and then at 4- to 6-month intervals for an additional year. In addition, FDG-PET scanning is also considered at 3 to 6 months after initial therapy to identify persistent or recurrent disease.[31]

Radioiodine imaging is typically performed with either iodine-123 or iodine-131. Both are imaged with a conventional gamma camera, typically 24 to 48 hours after administration of the RAI. There is increasing interest in radioiodine imaging with an isotope of iodine that emits positrons, allowing the use of PET scanning for imaging. Iodine-124 has a half-life of 4.18 days, allowing for delayed imaging. PET scanning has a high sensitivity for detecting small-volume disease, and coacquisition with CT provides anatomic localization for therapy planning purposes. The quantitiative nature of PET scanning also allows for dosimetric therapy planning for radioiodine treatment using iodine-131, maximizing dose delivery to the tumor while limiting toxicity to bone marrow and other organs. This imaging technique has not yet achieved widespread acceptance, but it is being actively investigated at several sites.[32]

Pathological Classification

Pathological staging requires the use of all information obtained during clinical staging, as well as histologic study of the surgically resected specimen. The surgeon's description of gross extrathyroidal extension must also be included.

In this edition, the presence of minor extrathyroidal extension identified only on histologic examination and not apparent clinically is not used as a risk factor for staging. No distinction is made between tumors with and those without minor extrathyroidal extension. However, gross extrathyroidal extension that can be identified clearly by imaging or intraoperative findings is classified as T3b disease (gross extrathyroidal extension involving only strap muscles), T4a disease (gross extrathyroidal extension invading the subcutaneous soft tissues, larynx, trachea, esophagus, muscle, or recurrent laryngeal nerve), or T4b disease (gross extrathyroidal extension invading the prevertebral fascia or encasing the carotid artery or mediastinal vessels). Furthermore, because of the poorer survival outcomes associated with gross extrathyroidal extension, patients older than 55 years at diagnosis with T3b disease are classified as Stage II, those with T4a disease are classified as Stage III, and those with T4b disease are classified as Stage IV.

For staging purposes, "any N" includes pN0, pN1, pNX, cN0, or cN1 disease. Pathological confirmation of lymph node status is not required for staging purposes. Rather, patients with pNX disease who are cN0 are classified as "cN0/pNX" in the staging tables. As detailed in the section on the impact of regional lymph mode metastasis on prognosis in differentiated thyroid cancer, subclinical (cN0) small-volume pN1 disease has little prognostic significance and is associated with outcomes that are very similar to those of pN0 disease. Because there is no requirement for a minimum number of lymph nodes to be sampled, pathological confirmation of one or more benign lymph nodes mandates a pN0 designation.

Complete assessment of N/M status may not be possible until after the RAI scans are complete, which often happens 1 to 3 months after initial surgery. Therefore, identification of metastatic disease (by any modality) within the first 4 months of thyroid surgery should be used to refine the N and M status.

Consistent with AJCC staging rules, the formal stage established during the first 4 months of follow-up does not change over time, even if the cancer progresses or recurs. However, the cancer may be "restaged" as new data become available during follow-up using the same approach and definitions applied during the initial staging. The lower case *r* is used to designate the restaging. In differentiated thyroid cancer, clinicians recognize both structural disease recurrence/progression (structural or functional evidence of disease) and biochemical disease recurrence/progression (abnormal

thyroglobulin without structural or functional evidence of disease). Consistent with the approach to initial staging, restaging should be based only on the identification of structurally or functionally identifiable disease and not on the basis of abnormal biomarkers of disease (serum thyroglobulin or thyroglobulin antibodies).

PROGNOSTIC FACTORS

Prognostic Factors Required for Stage Grouping

Age at Diagnosis

Unlike with most malignancies, age at diagnosis of thyroid cancer is almost always identified as an independent predictor of disease-specific survival (DSS) in the published staging systems. Poor outcomes in differentiated thyroid cancer were reported as early as 1979 in patients older than 45 years at diagnosis.[33] The AJCC TNM staging system has incorporated a 45-year age cutoff as a major determinant of DSS since the AJCC Cancer Staging Manual, 2nd Edition was published in 1983. Most of the other clinicopathologic staging systems use an age cut point of between 40 and 50 years in their models.[3] The MACIS system, designed as a postoperative risk stratification system, uses age as a continuous variable in patients more than 40 years old at diagnosis.[34]

Multiple studies confirmed that mortality from PTC increases progressively with advancing age, beginning at about age 35.[35–43] Unfortunately, there is no single age cutoff that discretely allocates patients into separate risk categories. Many authors have recommended using nomograms,[37,44] mathematical models,[34,45] or multiple age categories[37,46] to better reflect the continuous nature of the relationship between age at diagnosis and disease-specific mortality. Other authors have endorsed using an age cutoff of 55 years as the optimal single time point for prognostic models.[47–51]

A recent international multicenter retrospective study demonstrated that by moving the age cut point from 45 to 55 years, 17% of the patient population was downstaged to a lower risk category.[37,52] Overall, 10% of patients who would have been classified as having advanced-stage disease based on the 45-year-old cut point (Stage III/IV) were downstaged to Stage I/II when a 55-year-old cut point was used, without affecting the survival curves in the lower risk categories. Furthermore, an age cutoff of 55 years produced a wider distribution in survival among the risk groups, ranging from 99.6% in Stage I to 70% in Stage IV, compared with the corresponding values of 99.6% and 79% when 45 years was used as the age cut point. Likewise, Ito et al.[49] demonstrated effective risk stratification when comparing the iStage modifications with the Union for International Cancer Control (UICC) TNM system. Therefore, although it seems unlikely that raising the age will have a significant impact on the performance of the staging system, it does have the significant clinical benefit of preventing upstaging based only on age of diagnosis between 45 and 55 years in patients who otherwise would be considered low risk (Stage I or II).

Histologies

The specific histologies are described in the pathology report. There are no pertinent cutoff values. AJCC Level of Evidence: I

Additional Factors Recommended for Clinical Care

Extrathyroidal Extension

Extrathyroidal extension may range from gross extrathyroidal extension involving major structures (T3b, T4a, T4b) to minor extension through the thyroid capsule identified only on histologic examination.

Gross extrathyroidal extension is documented in the operative report, whereas minor extrathyroidal extension is found in the pathology report, described as extension of the primary tumor through the thyroid capsule and into surrounding structures.

Gross extrathyroidal extension (see definitions for T3b, T4a, and T4b disease) identified either preoperatively or intraoperatively is an important factor for staging, whereas minor extension through the thyroid capsule seen only on histologic examination is not used for staging. AJCC Level of Evidence: I

Gross extrathyroidal extension in differentiated thyroid cancer increases disease persistence/recurrence and decreases survival.[53–55] Most differentiated thyroid cancer staging systems incorporate gross extrathyroidal extension as a predictor of recurrence and/or death (AMES, MACIS, AJCC, UICC).[3]

The AJCC Cancer Staging Manual, 6th Edition first distinguished between minimal and gross extrathyroidal extension. Authors downstaged to T3 "any tumor with minimal extrathyroidal extention (e.g., extention to sternothyroid muscle or perithyroidal soft tissue)." Since this delineation in 2002, pathological and clinical thyroid cancer studies have attempted to define its relevance.

Pathologically, the thyroid has an incomplete capsule. The thyroid gland may contain adipose tissue and skeletal muscle under normal circumstances. According to the College of American Pathologists, "defining (minimal) extrathyroidal extension may be problematic and subjective." Ghossein and colleagues[57,58] warned that the presence of adipose tissue, and muscle in some circumstances, in association with thyroid carcinoma should not be mistaken for extrathyroidal extension.[56,57]

During the past decade of clinical studies, nuances have been identified in the spectrum between minimal and gross extrathyroidal extension. Several recent studies demonstrated that microscopic extrathyroidal extension is not an independent prognostic factor for persistent/recurrent disease; disease-free survival is equivalent in patients with microscopic extrathyroidal extension and those with completely intrathyroidal tumors.[58–62] A study of T1/T2 well-differentiated thyroid carcinoma showed no difference in 10-year DSS or recurrence-free survival in those with microscopic extrathyroidal extension (who would have been upstaged on the basis of extrathyroidal extension alone).[63] There appears to be agreement that minimal/microscopic extrathyroidal extension in small differentiated thyroid cancer portends an outcome equivalent to that seen with completely intrathyroidal tumors.

However, several retrospective studies suggest an association between minimal extrathyroidal extension and the presence of lymph node metastases/extranodal extension,[59,61,64,65] concluding that minimal extrathyroidal extension is an indicator of disease biology in PTC. None demonstrated minimal extrathyroidal extension as an independent predictor of persistence/recurrence or survival. A recent large clinicohistopathologic analysis demonstrated that the presence of extrathyroidal extension in PTC is not associated with extranodal extension, whereas the number of positive lymph nodes is.[66]

Margin positivity in differentiated thyroid cancer may be considered similarly to extrathyroidal extension. There appears to be no difference in outcomes in patients with an R0 resection (microscopically negative margin) compared with those with an R1 resection (microscopically positive margin).[60,67] However, patients with a grossly positive margin (R2, or incomplete, resection) have a significantly higher risk of recurrence and disease-specific death.

Presence/Absence of Lymph Node Metastases

Clinical staging information is found in preoperative imaging and clinical examination reports, whereas pathological staging information is found in the pathology report. There are no pertinent cutoff values. AJCC Level of Evidence: I

The combination of high-resolution imaging, extensive neck dissection, and meticulous histological examination results in the identification of regional lymph node metastases in up to 80% of patients with PTC.[68] In many cases, the lymph node metastases are quite small (<1 cm), but up to 35% of patients may present with larger lymph node metastases.[68,69] Regional lymph node metastases are also common in medullary and anaplastic thyroid cancers but are seen less often in FTCs and Hurthle cell thyroid cancers.

Most studies,[59,70–77] but not all,[34,78,79] have suggested that regional lymph node metastases have prognostic significance in differentiated thyroid cancer. The impact of lymph node metastasis on survival is most evident in older patients.[59,72–74,76,77] Based on these data, previous AJCC staging systems used the presence of N1 disease to influence stage in patients older than 45 years at diagnosis.

The impact of lymph node metastases in younger patients has remained more controversial. However, recent studies based on the SEER and National Cancer Data Base (NCDB) datasets provided strong evidence that lymph node metastasis in patients younger than 45 years at diagnosis has a statistically significant impact on overall survival.[70,76] However, this statistically significant difference translates to 20-year adjusted survival rates of 97% without lymph node metastasis and 96% with lymph node metastasis in patients less than 45 years of age at diagnosis.[70]

Unlike the data available with regard to the risk of structural disease recurrence, data correlating lymph node characteristics with survival are less well developed. Several studies demonstrated that lateral neck lymph node metastases are associated with compromised survival,[74–76,80] which forms the basis for differentiating risk based on location of regional metastases (central vs. lateral neck compartments). However, because prophylactic neck dissections seldom are performed in the lateral neck, these observations are confounded by the fact that pathologically involved lateral neck lymph nodes are usually much larger than metastatic lymph nodes identified in the central neck (often incidentally removed or identified only by prophylactic neck dissection). Therefore, it is very difficult to differentiate the effect of location (central vs. lateral neck) from the effect of size and number of lymph node metastases in these retrospective datasets. Some[70] but not all[71,75] have suggested that the number of involved lymph nodes may be related to survival. Using both the SEER and NCDB databases, controlling for important confounders, and restricting the analysis to patients younger than 45 years at diagnosis, Adam et al.[70] demonstrated a statistically significant association between the number of involved lymph nodes and survival. In their analysis, mortality increased incrementally up to six involved lymph nodes, after which no further increase in mortality was seen with additional metastatic lymph nodes.

Multiple studies confirmed the association between extranodal extension and persistent/recurrent disease[68,80–82] and demonstrated that extranodal extension is not infrequently seen in subcentimeter lymph node metastases.[83] Some studies,[64] but not all,[66] demonstated a correlation between extranodal extension and extrathyroidal extension. Although several publications demonstrated statistically significant associations between extranodal extension and DSS,[48,80,82,84,85] each of these studies was single center, rather small, and lacked long-term follow-up. Furthermore, the impact of extranodal extension on survival appears to be dependent on the clinical context, with more significant impacts seen in association with *BRAF*-mutant tumors[82] or in the setting of lateral neck disease.[48] Therefore, although the data strongly suggest that extranodal extension may have a strong association with DSS in differentiated thyroid cancer, currently available evidence does not rise to a level that justifies its inclusion as an independent survival variable.

Other investigators examined the impact of metastatic lymph node ratio (number of metastatic nodes/total number of nodes harvested) on prognosis. Metastatic lymph node ratio (>0.42) was associated with compromised DSS, but this significance was lost when lateral neck lymph node metastases were excluded.[75] Similarly, metastatic lymph node ratio was not a significant predictor of survival in either young patients or older patients with metastatic lymph node involvement, even when the analysis was restricted to patients with six or more lymph nodes examined.[71]

No studies have adequately evaluated the impact of metastatic lymph node size on survival. Further complicating this analysis is the observation that some pathologists report the overall size of the lymph node, whereas others report the size of the metastatic foci within the lymph node. However, several clinical observations suggest that small-volume regional metastases likely have very little impact on overall survival. Extensive lymph node dissections can identify regional lymph node metastases in up to 80% of patients.[68] Despite having a DSS rate of >99%, prophylactic neck dissections in cN0 papillary microcarcinoma patients may identify central neck regional metastases in 40–50% and lateral neck regional metastases disease in 45%.[86–89] Lymph node metastasis identified in prophylactic neck dissections usually represents small-volume disease (95% <1 cm, a mean of two to three involved nodes).[87,88] These data demonstrate that many patients classified as having N0 disease would prove to have small-volume pN1 disease if extensive neck dissections had been performed since staging is commonly based on histologic examination of three or fewer cervical lymph nodes[71,75] or without histologic examination of any lymph nodes.[70,77] Hence, very similar recurrence risks and mortality outcomes are seen in patients classified as having cN0, pN0, or small volume pN1. When these observations are combined with the data showing low recurrence rates with small-volume lymph node metastases (five or fewer subcentimeter metastatic lymph nodes),[68] it is clear that small-volume lymph node metastases have little prognostic impact on survival in differentiated thyroid cancer.

In summary, regional lymph node metastases appear to have strong prognostic significance in most differentiated thyroid cancer. Although statistically significant in all adult age groups, the clinical impact of N1 disease on survival is most apparent in older patients, but the magnitude of effect on overall survival appears much smaller if the N1 disease is not associated with T4a, T4b, or M1 disease. With regard to lymph node characteristics, lateral neck regional node disease carries a worse prognosis than central neck regional node disease, although it is not clear whether the poorer outcomes are related to location or to the size/number of metastatic lymph nodes. It seems likely that the number of involved lymph nodes also may correlate with survival, but additional studies are needed, particularly in the older population. Small-volume lymph node metastases have little impact on the risk of structural disease recurrence and DSS.

Using Age at Diagnosis, Extrathyroidal Extension, and Regional Lymph Node Status to Define Prognostic Stage Groups

Previous editions of the AJCC thyroid cancer staging systems provided suboptimal separation with regard to the risk for disease-specific mortality between Stage I and Stage II disease. Furthermore, less than 20% of patients classified as having Stage III or IV disease will actually die of thyroid cancer. In most series, 5- to 10-year survival (variously reported as overall survival or relative survival) approximates 97–100% in Stage I and II disease and 88–95% in Stage III disease. Stage IV disease consistently predicts 10-year survival rates in the 50–75% range.[52,90–92] However, within the AJCC Cancer Staging Manual, 7th Edition Stage IV group, significantly worse outcomes are seen with Stage IVc (M1 disease, any N, any T) compared with Stage IVa/b (T4a or T1–2, N1b, M0), with 10-year survival rates of approximately 50% and 70%, respectively.[76,92]

In the 7th Edition, identification of central neck cervical lymph node metastases upstaged patients to Stage III if the primary tumor was T1–T3, whereas the presence of lateral neck disease (N1b) upstaged patients to Stage IVa if the primary tumor was T1–T4a. This stratification was based on previous studies demonstrating poorer survival in older patients with N1 disease and on data suggesting that N1b disease has a worse outcome than N1a disease.[59,70–77,79] In most of these series, 10-year overall survival for all N0 patients was approximately 80–85%, whereas for N1 patients, it was 75–80%.[72,77] The difference in overall survival between N0 and N1 patients is much smaller in young patients (<1–2%) than in older patients (5–10%).[52,70,77,90–93]

Furthermore, much of the mortality risk associated with N1 disease in older patients may be attributed to the common co-ocurrence of M1 and/or T4a/b disease in the setting of clinically significant N1b disease. For example, in older patients, 99% DSS is seen in N0 disease (only 1.5% have concurrent M1 disease), whereas a DSS of 92% is observed in patients with N1 disease in levels II to VA (5% M1) and 85% DSS is seen in patients with Vb/VII nodes involved (10% M1).[93] Studies analyzing the impact of lymph node metastases in patients without M1 or T4b disease demonstrated that the mortality risk associated with T1–3N1bM0 disease or T4aN1M0 disease is minimal.[39,52,74,94–97] For example, N1bM0 was associated with 85% 15-year relative survival.[92] Additionally 96–97% DSS was reported in patients with T1-3N1M0, whereas much poorer outcomes were seen in T4a, any N, M0 (82% DSS) and T4aN1b disease (70% DSS).[52,74,89,94]

Although the preponderance of the data demonstrates that lymph node metastases may convey a statistically measurable increase in the risk of disease-specific mortality in the elderly, and probably to a lesser extent in younger adult patients, the actual magnitude of this risk is much smaller than the risk conveyed by either T4a/b or M1 disease. Therefore, in the AJCC Cancer Staging Manual, 8th Edition staging system, N1 disease is classified as Stage I in patients less than 55 years old at

diagnosis and reclassified as Stage II disease in older patients. Because the survival of older patients with N1 disease in the absence of T4a/M1 disease should approximate 85–95%, these changes should define a cohort of patients who have outcomes worse than those of Stage I patients.

With regard to the 7th Edition Stage IV grouping, it is clear that two separate subgroups can be defined: patients with distant metastasis (IVC patients) and those with gross extrathyroidal extension (T4a/T4b patients) regardless of the lymph node status. Because all N1 disease is now classified as Stage II in older patients (but still as Stage I in younger patients), the previous Stage III would include only T3N0 disease, which would not be expected to have significant mortality. Therefore, the 8th Edition Stage III includes only the older T4a, any N, M0 patients and is expected to have a survival curve very similar to the 7th Edition Stage IVA curve. Finally, older patients with T4b, any N, M0, and all M1 disease (any T, any M) will define the 8th Edition Stage IV disease.

To validate the 8th Edition staging system, the proposed changes were analyzed using the previously published dataset from Memorial Sloan Kettering Cancer Center (re-analysis by Ian Ganly and Jatin Shah, Head and Neck Surgery, MSKCC).[52]

Location of Involved Lymph Nodes (N1a vs. N1b)

The location of metastatic lymph nodes, referring to either central (N1a) or lateral (N1b) cervical lymph node chains. The location of metastatic lymph nodes may be found in the operative notes and the pathology report. There are no pertinent cutoff values. AJCC Level of Evidence: II

Number of Involved Lymph Nodes

The number of lymph nodes histologically confirmed to have a metastatic foci of thyroid cancer. The number of involved lymph nodes is described in the pathology report. There are no pertinent cutoff values. AJCC Level of Evidence: II

Number of Lymph Nodes Sampled

The number of lymph nodes histologically examined (including both metastatic and benign lymph nodes). The number of involved lymph nodes is described in the pathology report. There are no pertinent cutoff values. AJCC Level of Evidence: II

Size of the Largest Involved Lymph Node

The maximum diameter of the largest metastatic lymph node in millimeters. This will be described in the pathology report. There are no pertinent cutoff values. AJCC Level of Evidence: II

Size of Metastatic Foci within Involved Lymph Nodes

The maximum diameter of the metastatic tumor foci within a lymph node in millimeters. This will be described in the pathology report. There are no pertinent cutoff values. AJCC Level of Evidence: II

Extranodal Extension

Microscopic or gross extension of the metastatic lymph node tumor foci outside the lymph node capsule. This will be described in the pathology report. There are no pertinent cutoff values. AJCC Level of Evidence: II

Vascular Invasion

Vascular invasion is defined as invasion of the thyroid cancer into vascular structures. This will be described in the pathology report. There are no pertinent cutoff values. AJCC Level of Evidence: II

Postoperative Serum Thyroglobulin

A serum tumor marker specific for thyroid tissue (benign or malignant). It is measured by a clinical laboratory using a variety of methods and is reported in the clinical record in nanograms per milliliter. There are no pertinent cutoff values. AJCC Level of Evidence: II

Completeness of Resection

A description of whether the surgeon provided a complete resection of all grossly visible disease. This is reported in the operative note and staged using residual tumor (R) classification. There are no pertinent cutoff values. AJCC Level of Evidence: II

Histologic Features

In addition to the specific histologic subtypes defined by the WHO Classification of Tumors table, several other histologic features may have prognostic importance, including perineural invasion, multifocality, and high mitotic index.[4] These features are documented on the pathology report. There are no pertinent cutoff values. AJCC Level of Evidence: II

Follicular cell–derived thyroid cancers display a spectrum of biological behavior ranging from clinically insignificant (papillary microcarcinomas in the elderly) to highly lethal (anaplastic thyroid cancer). When matched for stage, the prognosis of PTCs and FTCs are usually very similar. Poorer prognosis may be seen in some of the more aggressive variants, such as the poorly differentiated thyroid cancers, tall cell variants, hobnail variants, and columnar cell variants. Conversely, better prognosis may be seen in noninvasive encapsulated FTC and noninvasive encapsulated follicular variants of PTC.[98,99] Hurthle cell carcinomas are classified as a variant of follicular carcinoma by the World Health Organization (WHO), but newer data suggesting differences in biological behavior and unique genetic alterations indicate that Hurthle cell cancers may be best classified as a distinct histologic tumor (rather than a subtype of FTC).[100] Although anaplastic thyroid cancer is associated with 1-year survival rates of less than 10–20%, small, incidentally discovered anaplastic thyroid cancers that are completely resected may display a much better prognosis.[31,101]

RISK ASSESSMENT MODELS

The AJCC recently established guidelines that will be used to evaluate published statistical prediction models for the purpose of granting endorsement for clinical use.[102] Although this is a monumental step toward the goal of precision medicine, this work was published only very recently. Therefore, the existing models that have been published or may be in clinical use have not yet been evaluated for this cancer site by the Precision Medicine Core of the AJCC. In the future, the statistical prediction models for this cancer site will be evaluated, and those that meet all AJCC criteria will be endorsed.

DEFINITIONS OF AJCC TNM

Definition of Primary Tumor (T)

Papillary, Follicular, Poorly Differentiated, Hurthle Cell and Anaplastic Thyroid Carcinoma

T Category	T Criteria
TX	Primary tumor cannot be assessed
T0	No evidence of primary tumor
T1	Tumor ≤2 cm in greatest dimension limited to the thyroid
T1a	Tumor ≤1 cm in greatest dimension limited to the thyroid
T1b	Tumor >1 cm but ≤2 cm in greatest dimension limited to the thyroid
T2	Tumor >2 cm but ≤4 cm in greatest dimension limited to the thyroid
T3	Tumor >4 cm limited to the thyroid, or gross extrathyroidal extension invading only strap muscles
T3a	Tumor >4 cm limited to the thyroid
T3b	Gross extrathyroidal extension invading only strap muscles (sternohyoid, sternothyroid, thyrohyoid, or omohyoid muscles) from a tumor of any size
T4	Includes gross extrathyroidal extension
T4a	Gross extrathyroidal extension invading subcutaneous soft tissues, larynx, trachea, esophagus, or recurrent laryngeal nerve from a tumor of any size
T4b	Gross extrathyroidal extension invading prevertebral fascia or encasing the carotid artery or mediastinal vessels from a tumor of any size

Note: All categories may be subdivided: (s) solitary tumor and (m) multifocal tumor (the largest tumor determines the classification).

Definition of Regional Lymph Node (N)

N Category	N Criteria
NX	Regional lymph nodes cannot be assessed
N0	No evidence of locoregional lymph node metastasis
N0a	One or more cytologically or histologically confirmed benign lymph nodes
N0b	No radiologic or clinical evidence of locoregional lymph node metastasis
N1	Metastasis to regional nodes
N1a	Metastasis to level VI or VII (pretracheal, paratracheal, or prelaryngeal/Delphian, or upper mediastinal) lymph nodes. This can be unilateral or bilateral disease.
N1b	Metastasis to unilateral, bilateral, or contralateral lateral neck lymph nodes (levels I, II, III, IV, or V) or retropharyngeal lymph nodes

Definition of Distant Metastasis (M)

M Category	M Criteria
M0	No distant metastasis
M1	Distant metastasis

AJCC PROGNOSTIC STAGE GROUPS

Differentiated

When age at diagnosis is...	And T is...	And N is...	And M is...	Then the stage group is...
<55 years	Any T	Any N	M0	I
<55 years	Any T	Any N	M1	II
≥55 years	T1	N0/NX	M0	I
≥55 years	T1	N1	M0	II
≥55 years	T2	N0/NX	M0	I
≥55 years	T2	N1	M0	II
≥55 years	T3a/T3b	Any N	M0	II
≥55 years	T4a	Any N	M0	III
≥55 years	T4b	Any N	M0	IVA
≥55 years	Any T	Any N	M1	IVB

Anaplastic

When T is…	And N is…	And M is…	Then the stage group is…
T1–T3a	N0/NX	M0	IVA
T1–T3a	N1	M0	IVB
T3b	Any N	M0	IVB
T4	Any N	M0	IVB
Any T	Any N	M1	IVC

REGISTRY DATA COLLECTION VARIABLES

1. Histology
2. Age at diagnosis
3. Number of involved lymph nodes
4. Maximum diameter of involved lymph nodes
5. Size of largest metastatic foci within an involved lymph node

HISTOLOGIC GRADE (G)

There is no formal grading system for thyroid cancers.

HISTOPATHOLOGIC TYPE

Papillary carcinoma
Papillary microcarcinoma
Follicular variant
Solid variant
Hurthle cell variant
Follicular carcinoma
Encapsulated noninvasive
Minimally invasive
Widely Invasive
Hurthle cell carcinoma
Poorly differentiated carcinoma (used for insular carcinoma as a subtype of poorly differentiated)
Anaplastic carcinoma

SURVIVAL DATA

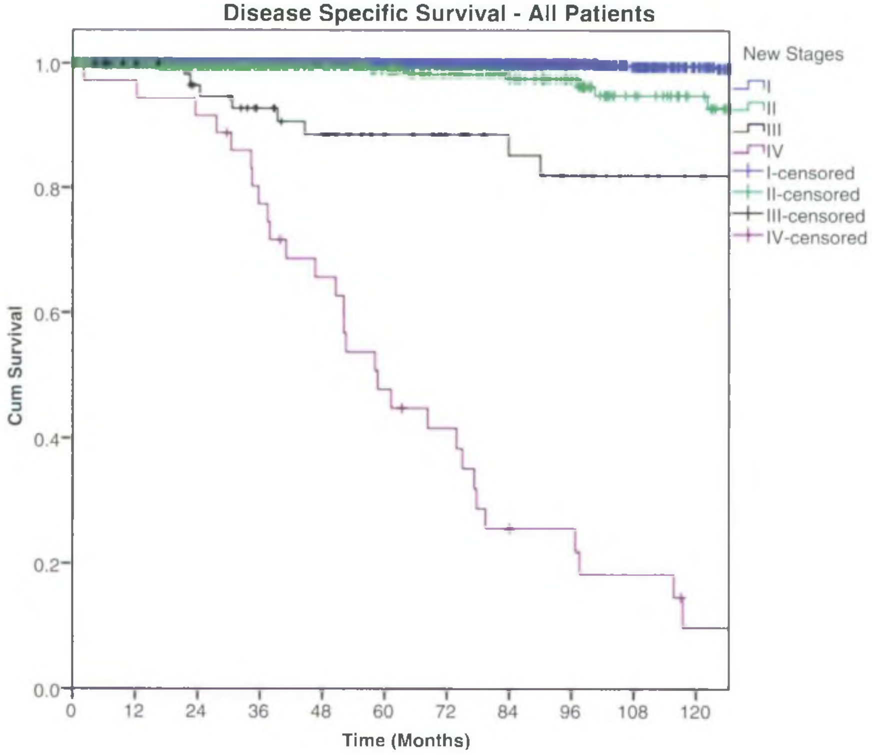

Fig. 73.3 Disease-specific survival: all patients, MSKCC data, 8th Edition

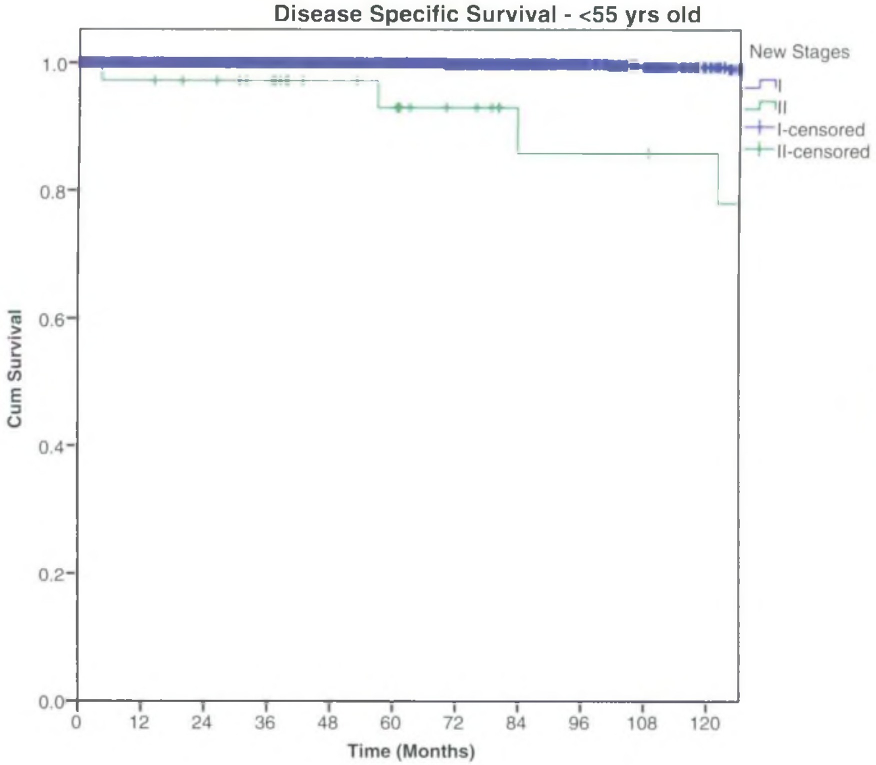

Fig. 73.4 Disease-specific survival: patients <55 years old, MSKCC data, 8th Edition

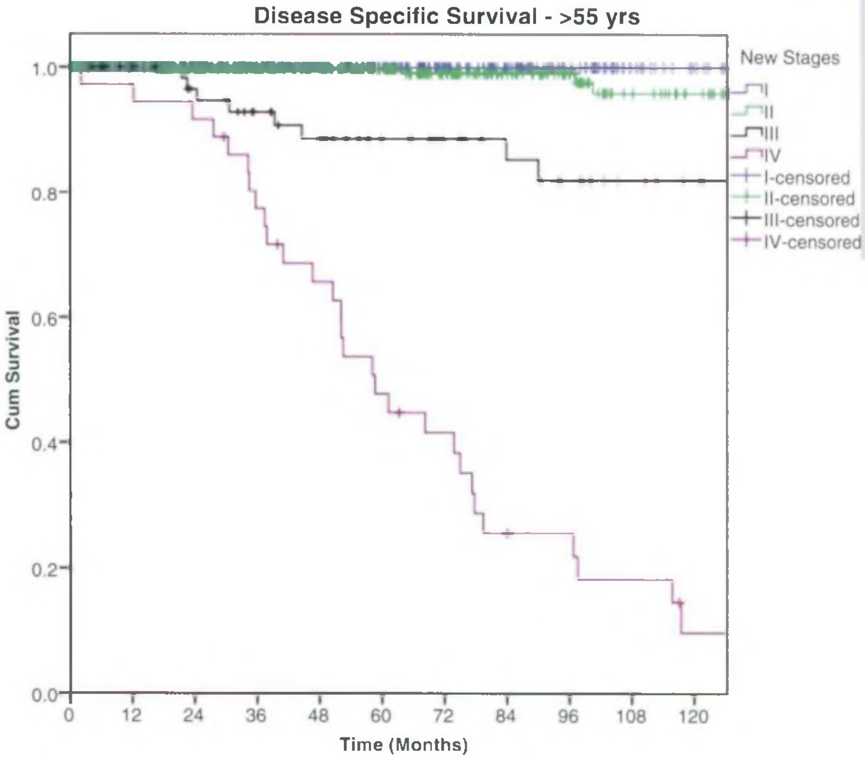

Fig. 73.5 Disease-specific survival: all patients >55 years old. MSKCC data. 8th Edition

ILLUSTRATIONS

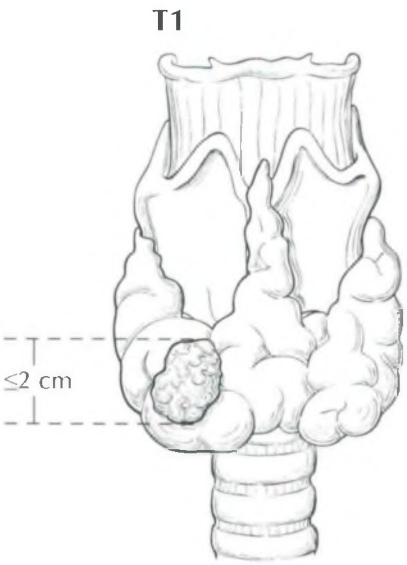

Fig. 73.6 T1 is defined as tumor 2 cm or less in greatest dimension limited to the thyroid. T1a, tumor 1cm or less, limited to the thyroid. T1b, tumor more than 1 cm but not more than 2 cm in greatest dimension, limited to the thyroid

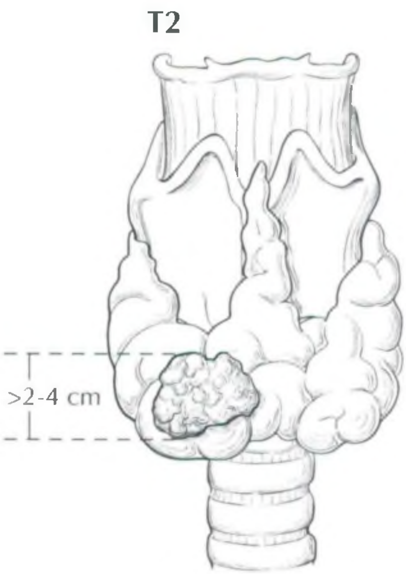

Fig. 73.7 T2 is defined as tumor more than 2 cm but not more than 4 cm in greatest dimension limited to the thyroid

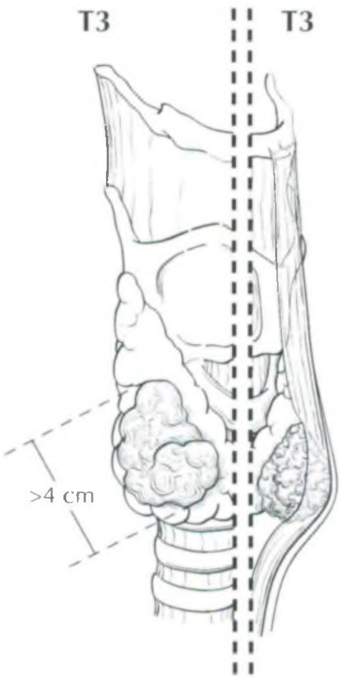

Fig. 73.8 Two views of T3: on the left, tumor more than 4 cm in greatest dimension limited to the thyroid (categorized as T3a); on the right, a tumor of any size with gross extrathyroidal extension invading only strap muscles (sternohyoid, sternothyroid, thyrohyoid or omohyoid muscles) (categorized as T3b)

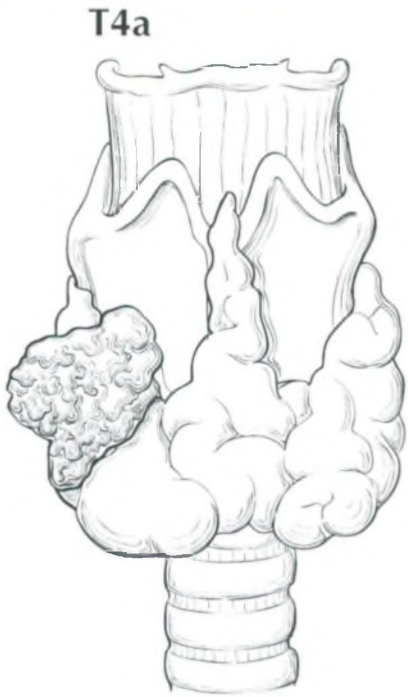

Fig. 73.9 T4a is defined as gross extrathyroidal extension invading subcutaneous soft tissues, larynx, trachea, esophagus, or recurrent laryngeal nerve from a tumor of any size

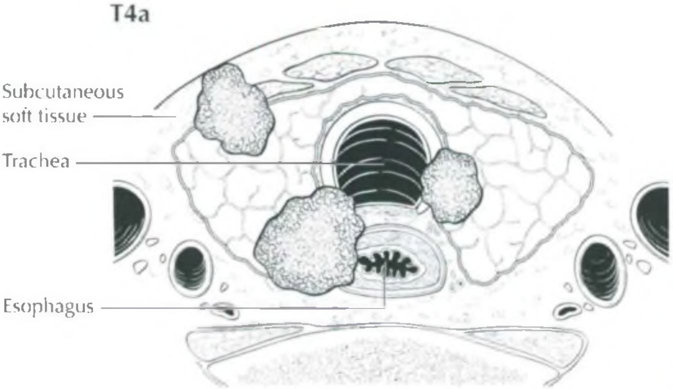

Fig. 73.10 Cross-sectional diagram of three different parameters of T4a: tumor invading subcutaneous soft tissues; tumor invading trachea; tumor invading esophagus

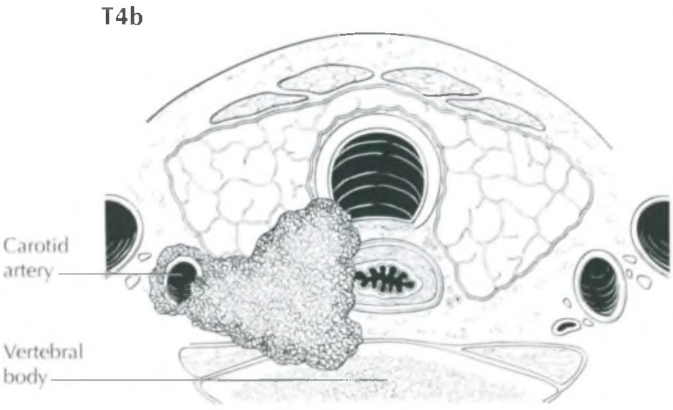

Fig. 73.11 T4b is defined as gross extrathyroidal extension invading prevertebral fascia or encasing the carotid artery or mediastinal vessels from a tumor of any size. Cross-sectional diagram of two different parameters of T4b: tumor encases carotid artery; tumor invades vertebral body

Bibliography

1. Davies L, Welch HG. Current thyroid cancer trends in the United States. *JAMA otolaryngology--head & neck surgery*. Apr 2014;140(4):317–322.
2. Davies L, Welch HG. Increasing incidence of thyroid cancer in the United States, 1973–2002. *JAMA*. May 10 2006;295(18):2164–2167.
3. Momesso DP, Tuttle RM. Update on differentiated thyroid cancer staging. *Endocrinology and metabolism clinics of North America*. Jun 2014;43(2):401–421.
4. Haugen BR, Alexander EK, Bible KC, et al. 2015 American Thyroid Association Management Guidelines for Adult Patients with Thyroid Nodules and Differentiated Thyroid Cancer: The American Thyroid Association Guidelines Task Force on Thyroid Nodules and Differentiated Thyroid Cancer. *Thyroid : official journal of the American Thyroid Association*. Jan 2016;26(1): 1–133.
5. Tuttle R, Ball D, Byrd D, Dickson P, Duh Q, Farrar W. NCCN Clinical Practice Guidelines in Oncology: Thyroid Carcinoma. Version 1.2015. *NCCN Guidelines*. 2015.
6. Miyauchi A, Kudo T, Miya A, et al. Prognostic impact of serum thyroglobulin doubling-time under thyrotropin suppression in patients with papillary thyroid carcinoma who underwent total thyroidectomy. *Thyroid : official journal of the American Thyroid Association*. Jul 2011;21(7):707–716.
7. Choi YM, Kim TY, Song DE, et al. Papillary thyroid carcinoma arising from a thyroglossal duct cyst: a single institution experience. *Endocrine journal*. 2013;60(5):665–670.
8. Wei S, LiVolsi VA, Baloch ZW. Pathology of thyroglossal duct: an institutional experience. *Endocrine pathology*. Mar 2015;26(1):75–79.
9. Gordini L, Podda F, Medas F, et al. Tall cell carcinoma arising in a thyroglossal duct cyst: A case report. *Annals of medicine and surgery*. Jun 2015;4(2):129–132.
10. Warner E, Ofo E, Connor S, Odell E, Jeannon JP. Mucoepidermoid carcinoma in a thyroglossal duct remnant. *Int J Surg Case Rep*. 2015;13:43–47.
11. Carter Y, Yeutter N, Mazeh H. Thyroglossal duct remnant carcinoma: beyond the Sistrunk procedure. *Surgical oncology*. Sep 2014;23(3):161–166.
12. Patel SG, Escrig M, Shaha AR, Singh B, Shah JP. Management of well-differentiated thyroid carcinoma presenting within a thyroglossal duct cyst. *Journal of surgical oncology*. Mar 2002;79(3):134–139; discussion 140–131.
13. Plaza CP, Lopez ME, Carrasco CE, Meseguer LM, Perucho Ade L. Management of well-differentiated thyroglossal remnant thyroid carcinoma: time to close the debate? Report of five new cases and proposal of a definitive algorithm for treatment. *Annals of surgical oncology*. May 2006;13(5):745–752.
14. Dzodic R, Markovic I, Stanojevic B, et al. Surgical management of primary thyroid carcinoma arising in thyroglossal duct cyst: an experience of a single institution in Serbia. *Endocrine journal*. 2012;59(6):517–522.
15. Kurman R, Carcangiu M, Herrington C, Young R. *WHO classification of tumours of female reproductive organs*. 4th ed. Lyon: International Agency for Research on Cancer; 2014.
16. Garg K, Soslow RA, Rivera M, Tuttle MR, Ghossein RA. Histologically bland "extremely well differentiated" thyroid carcinomas arising in struma ovarii can recur and metastasize. *International Journal of Gynecologic Pathology*. 2009;28(3):222–230.
17. Park MJ, Kim MA, Shin MK, Min HS. Follicular proliferative lesion arising in struma ovarii. *Journal of pathology and translational medicine*. May 2015;49(3):262–266.
18. Leite I, Cunha TM, Figueiredo JP, Felix A. Papillary carcinoma arising in struma ovarii versus ovarian metastasis from primary thyroid carcinoma: a case report and review of the literature. *J Radiol Case Rep*. Oct 2013;7(10):24–33.
19. Marcy PY, Thariat J, Benisvy D, Azuar P. Lethal, malignant, metastatic struma ovarii. *Thyroid : official journal of the American Thyroid Association*. Sep 2010;20(9):1037–1040.
20. Salman WD, Singh M, Twaij Z. A case of papillary thyroid carcinoma in struma ovarii and review of the literature. *Patholog Res Int*. 2010;2010:352476.
21. Goffredo P, Sawka AM, Pura J, Adam MA, Roman SA, Sosa JA. Malignant struma ovarii: a population-level analysis of a large series of 68 patients. *Thyroid : official journal of the American Thyroid Association*. Feb 2015;25(2):211–215.
22. Robboy SJ, Shaco-Levy R, Peng RY, et al. Malignant struma ovarii: an analysis of 88 cases, including 27 with extraovarian spread. *Int J Gynecol Pathol*. Sep 2009;28(5):405–422.
23. Yassa L, Sadow P, Marqusee E. Malignant struma ovarii. *Nature clinical practice. Endocrinology & metabolism*. Aug 2008;4(8):469–472.
24. American Thyroid Association Surgery Working G, American Association of Endocrine S, American Academy of O-H, et al. Consensus statement on the terminology and classification of central neck dissection for thyroid cancer. *Thyroid : official journal of the American Thyroid Association*. Nov 2009;19(11):1153–1158.
25. Stack BC, Jr., Ferris RL, Goldenberg D, et al. American Thyroid Association consensus review and statement regarding the anatomy, terminology, and rationale for lateral neck dissection in differentiated thyroid cancer. *Thyroid : official journal of the American Thyroid Association*. May 2012;22(5):501–508.
26. Hay ID. Papillary thyroid carcinoma. *Endocrinology and metabolism clinics of North America*. Sep 1990;19(3):545–576.
27. Mazzaferri EL, Kloos RT. Clinical review 128: Current approaches to primary therapy for papillary and follicular thyroid cancer. *The Journal of clinical endocrinology and metabolism*. Apr 2001;86(4):1447–1463.
28. Horvath E, Majlis S, Rossi R, et al. An ultrasonogram reporting system for thyroid nodules stratifying cancer risk for clinical management. *The Journal of clinical endocrinology and metabolism*. May 2009;94(5):1748–1751.
29. Ito Y, Amino N, Yokozawa T, et al. Ultrasonographic evaluation of thyroid nodules in 900 patients: comparison among ultrasonographic, cytological, and histological findings. *Thyroid : official journal of the American Thyroid Association*. Dec 2007;17(12):1269–1276.
30. Tae HJ, Lim DJ, Baek KH, et al. Diagnostic value of ultrasonography to distinguish between benign and malignant lesions in the management of thyroid nodules. *Thyroid : official journal of the American Thyroid Association*. May 2007;17(5):461–466.
31. Smallridge RC, Ain KB, Asa SL, et al. American Thyroid Association guidelines for management of patients with anaplastic thyroid cancer. *Thyroid : official journal of the American Thyroid Association*. Nov 2012;22(11):1104–1139.
32. Grewal RK, Ho A, Schoder H. Novel Approaches to Thyroid Cancer Treatment and Response Assessment. *Semin Nucl Med*. Mar 2016;46(2):109–118.
33. Byar DP, Green SB, Dor P, et al. A prognostic index for thyroid carcinoma. A study of the E.O.R.T.C. Thyroid Cancer Cooperative Group. *European journal of cancer*. Aug 1979;15(8):1033–1041.
34. Hay ID, Bergstralh EJ, Goellner JR, Ebersold JR, Grant CS. Predicting outcome in papillary thyroid carcinoma: development of a reliable prognostic scoring system in a cohort of 1779 patients surgically treated at one institution during 1940 through 1989. *Surgery*. Dec 1993;114(6):1050–1057; discussion 1057–1058.
35. Banerjee M, Muenz DG, Chang JT, Papaleontiou M, Haymart MR. Tree-based model for thyroid cancer prognostication. *The Journal of clinical endocrinology and metabolism*. Oct 2014;99(10):3737–3745.
36. Bischoff L, Curry J, Ahmed I, Pribitkin E, Miller J. Is above age 45 appropriate for upstaging well-differentiated papillary thyroid cancer? *Endocrine Practice*. 2013;19(6):995–997.

37. Ganly I, Nixon IJ, Wang LY, et al. Survival from Differentiated Thyroid Cancer: What Has Age Got to Do with It? *Thyroid : official journal of the American Thyroid Association*. Oct 2015;25(10):1106–1114.
38. Krook KA, Fedewa SA, Chen AY. Prognostic indicators in well-differentiated thyroid carcinoma when controlling for stage and treatment. *The Laryngoscope*. Apr 2015;125(4):1021–1027.
39. Lang BH, Chow SM, Lo CY, Law SC, Lam KY. Staging systems for papillary thyroid carcinoma: a study of 2 tertiary referral centers. *Annals of surgery*. Jul 2007;246(1):114–121.
40. Orosco RK, Hussain T, Brumund KT, Oh DK, Chang DC, Bouvet M. Analysis of age and disease status as predictors of thyroid cancer-specific mortality using the surveillance, epidemiology, and end results database. *Thyroid : official journal of the American Thyroid Association*. 2015;25(1):125–132.
41. Oyer SL, Fritsch VA, Lentsch EJ. Comparison of survival rates between papillary and follicular thyroid carcinomas among 36,725 patients. *Annals of Otology, Rhinology & Laryngology*. 2014;123(2):94–100.
42. Oyer SL, Smith VA, Lentsch EJ. Reevaluating the prognostic significance of age in differentiated thyroid cancer. *Otolaryngology--head and neck surgery : official journal of American Academy of Otolaryngology-Head and Neck Surgery*. Aug 2012;147(2):221–226.
43. Yang L, Shen W, Sakamoto N. Population-based study evaluating and predicting the probability of death resulting from thyroid cancer and other causes among patients with thyroid cancer. *J Clin Oncol*. Feb 1 2013;31(4):468–474.
44. Pathak KA, Mazurat A, Lambert P, Klonisch T, Nason RW. Prognostic nomograms to predict oncological outcome of thyroid cancers. *The Journal of clinical endocrinology and metabolism*. Dec 2013;98(12):4768–4775.
45. Gimm O, Ukkat J, Dralle H. Determinative factors of biochemical cure after primary and reoperative surgery for sporadic medullary thyroid carcinoma. *World journal of surgery*. Jun 1998;22(6):562–567; discussion 567–568.
46. Jonklaas J, Nogueras-Gonzalez G, Munsell M, et al. The impact of age and gender on papillary thyroid cancer survival. *The Journal of clinical endocrinology and metabolism*. Jun 2012;97(6):E878–887.
47. Hendrickson-Rebizant J, Sigvaldason H, Nason R, Pathak K. Identifying the most appropriate age threshold for TNM stage grouping of well-differentiated thyroid cancer. *European Journal of Surgical Oncology (EJSO)*. 2015.
48. Ito Y, Fukushima M, Tomoda C, et al. Prognosis of patients with papillary thyroid carcinoma having clinically apparent metastasis to the lateral compartment. *Endocrine journal*. 2009;56(6):759–766.
49. Ito Y, Ichihara K, Masuoka H, et al. Establishment of an intraoperative staging system (iStage) by improving UICC TNM classification system for papillary thyroid carcinoma. *World journal of surgery*. Nov 2010;34(11):2570–2580.
50. Kim SJ, Myong JP, Suh H, Lee KE, Youn YK. Optimal Cutoff Age for Predicting Mortality Associated with Differentiated Thyroid Cancer. *PloS one*. 2015;10(6):e0130848.
51. Mazurat A, Torroni A, Hendrickson-Rebizant J, Benning H, Nason RW, Pathak KA. The age factor in survival of a population cohort of well-differentiated thyroid cancer. *Endocrine connections*. 2013;2(3):154–160.
52. Nixon IJ, Kuk D, Wreesmann V, et al. Defining a Valid Age Cutoff in Staging of Well-Differentiated Thyroid Cancer. *Annals of surgical oncology*. Feb 2016;23(2):410–415.
53. Andersen PE, Kinsella J, Loree TR, Shaha AR, Shah JP. Differentiated carcinoma of the thyroid with extrathyroidal extension. *American journal of surgery*. Nov 1995;170(5):467–470.
54. Bellantone R, Lombardi CP, Boscherini M, et al. Prognostic factors in differentiated thyroid carcinoma: a multivariate analysis of 234 consecutive patients. *Journal of surgical oncology*. Aug 1998;68(4):237–241.
55. Ito Y, Tomoda C, Uruno T, et al. Prognostic significance of extrathyroid extension of papillary thyroid carcinoma: massive but not minimal extension affects the relapse-free survival. *World journal of surgery*. 2006;30(5):780–786.
56. Ghossein R. Problems and controversies in the histopathology of thyroid carcinomas of follicular cell origin. *Arch Pathol Lab Med*. May 2009;133(5):683–691.
57. Mete O, Asa SL. Pathological definition and clinical significance of vascular invasion in thyroid carcinomas of follicular epithelial derivation. *Modern pathology : an official journal of the United States and Canadian Academy of Pathology, Inc*. Dec 2011;24(12):1545–1552.
58. Arora N, Turbendian HK, Scognamiglio T, et al. Extrathyroidal extension is not all equal: implications of macroscopic versus microscopic extent in papillary thyroid carcinoma. *Surgery*. 2008;144(6):942–948.
59. Leboulleux S, Rubino C, Baudin E, et al. Prognostic factors for persistent or recurrent disease of papillary thyroid carcinoma with neck lymph node metastases and/or tumor extension beyond the thyroid capsule at initial diagnosis. *The Journal of clinical endocrinology and metabolism*. Oct 2005;90(10):5723–5729.
60. Radowsky JS, Howard RS, Burch HB, Stojadinovic A. Impact of degree of extrathyroidal extension of disease on papillary thyroid cancer outcome. *Thyroid : official journal of the American Thyroid Association*. Feb 2014;24(2):241–244.
61. Shin JH, Ha TK, Park HK, et al. Implication of minimal extrathyroidal extension as a prognostic factor in papillary thyroid carcinoma. *International journal of surgery*. 2013;11(9):944–947.
62. Woo CG, Sung CO, Choi YM, et al. Clinicopathological Significance of Minimal Extrathyroid Extension in Solitary Papillary Thyroid Carcinomas. *Annals of surgical oncology*. Dec 2015;22 Suppl 3:728–733.
63. Nixon IJ, Ganly I, Patel S, et al. The impact of microscopic extrathyroid extension on outcome in patients with clinical T1 and T2 well-differentiated thyroid cancer. *Surgery*. Dec 2011;150(6):1242–1249.
64. Clain JB, Scherl S, Dos Reis L, et al. Extrathyroidal extension predicts extranodal extension in patients with positive lymph nodes: an important association that may affect clinical management. *Thyroid : official journal of the American Thyroid Association*. Jun 2014;24(6):951–957.
65. Moon HJ, Kim EK, Chung WY, Yoon JH, Kwak JY. Minimal extrathyroidal extension in patients with papillary thyroid microcarcinoma: is it a real prognostic factor? *Annals of surgical oncology*. Jul 2011;18(7):1916–1923.
66. Machens A, Dralle H. Breach of the thyroid capsule and lymph node capsule in node-positive papillary and medullary thyroid cancer: Different biology. *European journal of surgical oncology : the journal of the European Society of Surgical Oncology and the British Association of Surgical Oncology*. Jun 2015;41(6):766–772.
67. Wang LY, Ghossein R, Palmer FL, et al. Microscopic Positive Margins in Differentiated Thyroid Cancer Is Not an Independent Predictor of Local Failure. *Thyroid : official journal of the American Thyroid Association*. Sep 2015;25(9):993–998.
68. Randolph GW, Duh QY, Heller KS, et al. The prognostic significance of nodal metastases from papillary thyroid carcinoma can be stratified based on the size and number of metastatic lymph nodes, as well as the presence of extranodal extension. *Thyroid : official journal of the American Thyroid Association*. Nov 2012;22(11):1144–1152.
69. Tufano RP, Clayman G, Heller KS, et al. Management of recurrent/persistent nodal disease in patients with differentiated thyroid cancer: a critical review of the risks and benefits of surgical intervention versus active surveillance. *Thyroid : official journal of the American Thyroid Association*. Jan 2015;25(1):15–27.
70. Adam MA, Pura J, Goffredo P, et al. Presence and Number of Lymph Node Metastases Are Associated With Compromised

Survival for Patients Younger Than Age 45 Years With Papillary Thyroid Cancer. *J Clin Oncol*. Jul 20 2015;33(21):2370–2375.

71. Beal SH, Chen SL, Schneider PD, Martinez SR. An evaluation of lymph node yield and lymph node ratio in well-differentiated thyroid carcinoma. *The American surgeon*. Jan 2010;76(1):28–32.
72. Hughes CJ, Shaha AR, Shah JP, Loree TR. Impact of lymph node metastasis in differentiated carcinoma of the thyroid: a matched-pair analysis. *Head & neck*. Mar-Apr 1996;18(2):127–132.
73. McHenry CR, Rosen IB, Walfish PG. Prospective management of nodal metastases in differentiated thyroid cancer. *American journal of surgery*. Oct 1991;162(4):353–356.
74. Nixon IJ, Wang LY, Palmer FL, et al. The impact of nodal status on outcome in older patients with papillary thyroid cancer. *Surgery*. Jul 2014;156(1):137–146.
75. Schneider DF, Chen H, Sippel RS. Impact of lymph node ratio on survival in papillary thyroid cancer. *Annals of surgical oncology*. Jun 2013;20(6):1906–1911.
76. Tran Cao HS, Johnston LE, Chang DC, Bouvet M. A critical analysis of the American Joint Committee on Cancer (AJCC) staging system for differentiated thyroid carcinoma in young patients on the basis of the Surveillance, Epidemiology, and End Results (SEER) registry. *Surgery*. Aug 2012;152(2):145–151.
77. Zaydfudim V, Feurer ID, Griffin MR, Phay JE. The impact of lymph node involvement on survival in patients with papillary and follicular thyroid carcinoma. *Surgery*. Dec 2008;144(6):1070–1077; discussion 1077–1078.
78. Bhattacharyya N. A population-based analysis of survival factors in differentiated and medullary thyroid carcinoma. *Otolaryngology--head and neck surgery : official journal of American Academy of Otolaryngology-Head and Neck Surgery*. Jan 2003;128(1):115–123.
79. Podnos YD, Smith D, Wagman LD, Ellenhorn JD. The implication of lymph node metastasis on survival in patients with well-differentiated thyroid cancer. *The American surgeon*. Sep 2005;71(9):731–734.
80. Wu MH, Shen WT, Gosnell J, Duh QY. Prognostic significance of extranodal extension of regional lymph node metastasis in papillary thyroid cancer. *Head & neck*. Sep 2015;37(9):1336–1343.
81. Lango M, Flieder D, Arrangoiz R, et al. Extranodal extension of metastatic papillary thyroid carcinoma: correlation with biochemical endpoints, nodal persistence, and systemic disease progression. *Thyroid : official journal of the American Thyroid Association*. Sep 2013;23(9):1099–1105.
82. Ricarte-Filho J, Ganly I, Rivera M, et al. Papillary thyroid carcinomas with cervical lymph node metastases can be stratified into clinically relevant prognostic categories using oncogenic BRAF, the number of nodal metastases, and extra-nodal extension. *Thyroid : official journal of the American Thyroid Association*. Jun 2012;22(6):575–584.
83. Alpert EH, Wenig BM, Dewey EH, Su HK, Dos Reis L, Urken ML. Size distribution of metastatic lymph nodes with extranodal extension in patients with papillary thyroid cancer: a pilot study. *Thyroid : official journal of the American Thyroid Association*. Feb 2015;25(2):238–241.
84. Yamashita H, Noguchi S, Murakami N, Kawamoto H, Watanabe S. Extracapsular invasion of lymph node metastasis is an indicator of distant metastasis and poor prognosis in patients with thyroid papillary carcinoma. *Cancer*. Dec 15 1997;80(12):2268–2272.
85. Yamashita H, Noguchi S, Murakami N, et al. Extracapsular invasion of lymph node metastasis. A good indicator of disease recurrence and poor prognosis in patients with thyroid microcarcinoma. *Cancer*. Sep 1 1999;86(5):842–849.
86. Ito Y, Tomoda C, Uruno T, et al. Ultrasonographically and anatomopathologically detectable node metastases in the lateral compartment as indicators of worse relapse-free survival in patients with papillary thyroid carcinoma. *World journal of surgery*. Jul 2005;29(7):917–920.
87. Roh JL, Kim JM, Park CI. Central cervical nodal metastasis from papillary thyroid microcarcinoma: pattern and factors predictive of nodal metastasis. *Annals of surgical oncology*. Sep 2008;15(9):2482–2486.
88. Vergez S, Sarini J, Percodani J, Serrano E, Caron P. Lymph node management in clinically node-negative patients with papillary thyroid carcinoma. *European journal of surgical oncology : the journal of the European Society of Surgical Oncology and the British Association of Surgical Oncology*. Aug 2010;36(8):777–782.
89. Wada N, Duh QY, Sugino K, et al. Lymph node metastasis from 259 papillary thyroid microcarcinomas: frequency, pattern of occurrence and recurrence, and optimal strategy for neck dissection. *Annals of surgery*. Mar 2003;237(3):399–407.
90. Hundahl SA, Fleming ID, Fremgen AM, Menck HR. A National Cancer Data Base report on 53,856 cases of thyroid carcinoma treated in the U.S., 1985–1995 [see commetns]. *Cancer*. Dec 15 1998;83(12):2638–2648.
91. Mankarios D, Baade P, Youl P, et al. Validation of the QTNM staging system for cancer-specific survival in patients with differentiated thyroid cancer. *Endocrine*. Jun 2014;46(2):300–308.
92. Verburg FA, Mader U, Tanase K, et al. Life expectancy is reduced in differentiated thyroid cancer patients >/= 45 years old with extensive local tumor invasion, lateral lymph node, or distant metastases at diagnosis and normal in all other DTC patients. *The Journal of clinical endocrinology and metabolism*. Jan 2013;98(1):172–180.
93. Smith VA, Sessions RB, Lentsch EJ. Cervical lymph node metastasis and papillary thyroid carcinoma: does the compartment involved affect survival? Experience from the SEER database. *Journal of surgical oncology*. Sep 15 2012;106(4):357–362.
94. Wada N, Masudo K, Nakayama H, et al. Clinical outcomes in older or younger patients with papillary thyroid carcinoma: impact of lymphadenopathy and patient age. *European journal of surgical oncology : the journal of the European Society of Surgical Oncology and the British Association of Surgical Oncology*. Feb 2008;34(2):202–207.
95. Fukushima M, Ito Y, Hirokawa M, Miya A, Shimizu K, Miyauchi A. Prognostic impact of extrathyroid extension and clinical lymph node metastasis in papillary thyroid carcinoma depend on carcinoma size. *World journal of surgery*. Dec 2010;34(12):3007–3014.
96. Ito Y, Higashiyama T, Takamura Y, et al. Risk factors for recurrence to the lymph node in papillary thyroid carcinoma patients without preoperatively detectable lateral node metastasis: validity of prophylactic modified radical neck dissection. *World journal of surgery*. Nov 2007;31(11):2085–2091.
97. Wada N, Nakayama H, Suganuma N, et al. Prognostic value of the sixth edition AJCC/UICC TNM classification for differentiated thyroid carcinoma with extrathyroid extension. *The Journal of clinical endocrinology and metabolism*. Jan 2007;92(1):215–218.
98. Ghossein R. Encapsulated malignant follicular cell-derived thyroid tumors. *Endocrine pathology*. Dec 2010;21(4):212–218.
99. Xu B, Ghossein R. Encapsulated Thyroid Carcinoma of Follicular Cell Origin. *Endocrine pathology*. Sep 2015;26(3):191–199.
100. Ganly I, Ricarte Filho J, Eng S, et al. Genomic dissection of Hurthle cell carcinoma reveals a unique class of thyroid malignancy. *The Journal of clinical endocrinology and metabolism*. May 2013;98(5):E962–972.
101. Kebebew E, Greenspan FS, Clark OH, Woeber KA, McMillan A. Anaplastic thyroid carcinoma. Treatment outcome and prognostic factors. *Cancer*. Apr 1 2005;103(7):1330–1335.
102. Kattan MW, Hess KR, Amin MB, et al. American Joint Committee on Cancer acceptance criteria for inclusion of risk models for individualized prognosis in the practice of precision medicine. *CA: a cancer journal for clinicians*. Jan 19 2016.

Thyroid – Medullary

74

Jennifer E. Rosen, Ricardo V. Lloyd, James D. Brierley, Raymon H. Grogan, Robert Haddad, Jennifer L. Hunt, John A. Ridge, Raja R. Seethala, Julia A. Sosa, Rathan M. Subramaniam, Tracy S. Wang, Lori J. Wirth, and Nancy D. Perrier

CHAPTER SUMMARY

Cancers Staged Using This Staging System

Medullary thyroid cancer

Cancers Not Staged Using This Staging System

These histopathologic types of cancer…	Are staged according to the classification for…	And can be found in chapter…
Differentiated and anaplastic thyroid	Thyroid—Differentiated and Anaplastic Carcinoma	73

Summary of Changes

Change	Details of Change	Level of Evidence
New chapter	Medullary thyroid cancer is now a stand-alone chapter.	N/A
Prognostic Factors	Tumor mutation was added as a new category.	I
Prognostic Factors	Calcitonin and carcinoembryonic antigen levels were added.	II
Definition of Regional Lymph Node (N)	N1a category now includes level VII (upper mediastinal) nodes.	II

ICD-O-3 Topography Codes

Code	Description
C73.9	Thyroid gland

WHO Classification of Tumors

Code	Description
8345	Medullary thyroid carcinoma

DeLellis RA, Lloyd RV, Heitz PU, Eng C, eds. World Health Organization Classification of Tumours Pathology and Genetics of Tumours of Endocrine Organs. Lyon: IARC; 2004.

INTRODUCTION

This chapter provides prognostic information and recommendations with regard to staging for thyroid cancers arising from thyroid parafollicular cells. Staging recommendations are provided for medullary thyroid cancer. In addition, prognostic information, without specific staging recommendations, is provided.

Medullary thyroid cancer (MTC) accounts for 1–2% of thyroid cancers in the United States.[1] In contrast to other types of thyroid cancer, MTC originates from the neural crest–derived parafollicular C cells of the thyroid gland.[2–4] The past 10 years have seen a decline in the relative incidence of MTC.

To access the AJCC cancer staging forms, please visit www.cancerstaging.org.

© American Joint Committee on Cancer 2017

M.B. Amin et al. (eds.), *AJCC Cancer Staging Manual, Eighth Edition*, DOI 10.1007/978-3-319-40618-3_74

most likely because of the marked increase in the incidence of papillary thyroid cancer. MTC may occur sporadically or in a hereditary form. A patient may prove to be the proband for the familial form, or be identified during screening of related family members. The most common hereditary cancers are components of the multiple endocrine neoplasia (MEN) type 2 syndromes, including MEN2A and MEN2B, as well as familial MTC (FMTC). The *RET* protooncogene, located on chromosome 10q11, is found to contain a germline mutation in virtually all patients with MEN2A, MEN2B, and FMTC and a somatic mutation in approximately 50% of patients with sporadic MTC.[5–9] Multiple RET mutations may be seen in several different exons. A variety of staging systems have been used to predict disease-specific mortality in MTC. Most of these rely on a common set of clinicopathologic variables, including tumor size, the presence/absence of gross extrathyroidal extension, locoregional lymph node metastases, and distant metastases. Prognosis in MTC may be related to the genetic mutation, if present. In addition, secretory products of the C cells, including calcitonin and carcinoembryonic antigen (CEA), have been recognized as potentially valuable tumor markers.[10,11] There is no current consensus surrounding the proper timing and collection of tumor markers and their application to staging.[12–17] Few studies have been published on the integration of genetic mutations or circulating secretory products into the current AJCC TNM staging guidelines and how they affect prognosis. Currently, including genetic mutations and measurements of circulating tumor markers in the staging of MCT would be premature. These features should be recorded systematically in the cancer registry database to permit their future incorporation.

ANATOMY

Primary Site(s)

The thyroid gland is ordinarily composed of a right and left lobe connected by an isthmus, which lies anterior to the upper trachea and esophagus (Fig. 74.1). In some cases, a pyramidal lobe is present and extends cephalad anterior to the thyroid cartilage.

Regional Lymph Nodes

The cervical lymph node stations most commonly are divided into seven compartments (Fig. 74.2). The "central neck" usually refers to levels VI and VII, whereas the "lateral neck" includes levels II, III, IV, and V.

Regional lymph nodes are the central compartment, lateral cervical, and upper mediastinal lymph nodes (levels I, II, III, IV, V, VI, and VII).

Lymph node metastases most commonly spread to central nodes (level VI) followed by mid- (level III) and lower (level IV) jugular nodes, and less commonly to high (level IIA) jugular and supraclavicular (level V) nodes. The

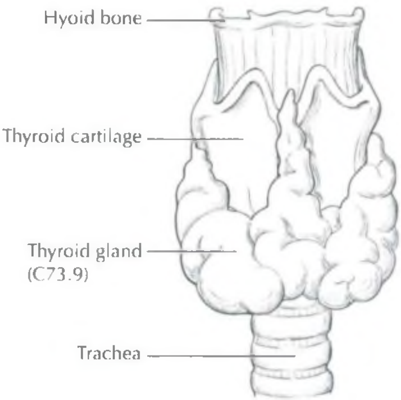

Fig. 74.1 Anatomy of the thyroid gland

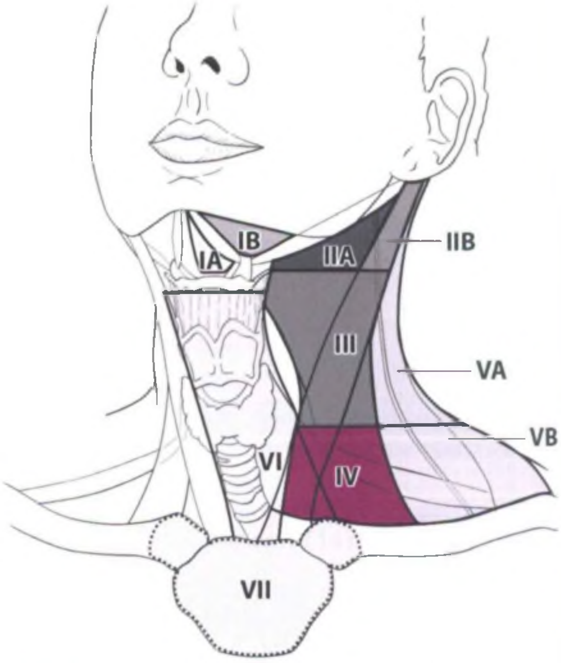

Fig. 74.2 Location of the lymph node levels in the neck

upper deep jugular and spinal accessory lymph nodes (level IIB) are affected less commonly, and lymph node metastasis to submandibular and submental lymph nodes (level I) and the retropharyngeal region are rare. Upper mediastinal (level VII) nodal spread may occur but is unusual without level VI lymph node spread.

Metastatic Sites

Clinically apparent metastases are present at initial diagnosis in 1–5% of patients with MTC.[18] Common locations include the liver[19] and lung/mediastinum.[20] Less common sites for MTC metastases include bone,[21–25] brain,[26–28] and skin.[29]

RULES FOR CLASSIFICATION

Clinical Classification

Patients may present with a thyroid nodule or palpable nodal metastases, have an elevated calcitonin or CEA level detected on screening, or have disease detected incidentally on chest or neck imaging or as a result of screening in a family carrying a germline mutation. Change in voice, dysphagia or odynophagia, and upper airway obstructive symptoms suggest advanced local disease. Initial distant metastases are usually identified in the liver or lung.[19,20] Patients with elevated calcitonin levels may present with diarrhea. Those whose tumors produce adrenocorticotropic hormone (ACTH) may present with signs and symptoms of Cushing's syndrome, including diarrhea.[30]

The diagnosis of MTC should be excluded for any patient who develops a thyroid nodule and/or elevated calcitonin and/or CEA levels in the setting of FMTC or MEN disease.

Detailed guidance as to the appropriate preoperative assessment and management may be found in guidelines from the National Comprehensive Cancer Network (NCCN)[31] and the American Thyroid Association (ATA).[32] Preoperative assessment of suspicious thyroid nodules typically includes ultrasound-guided fine-needle aspiration of the nodule with cytologic examination. Assessment of calcitonin, chromogranin, and CEA can verify the diagnosis of MTC (as well as the relative lack of thyroglobulin staining). In patients with a preoperative diagnosis of MTC, staging should include bilateral lateral neck ultrasound or neck computed tomography (CT) to evaluate the central and lateral neck lymph node compartments, as well as measurement of serum CEA and calcitonin. Cytologic confirmation of suspicious lateral lymph nodes often is performed to help decide whether more extensive lymph node dissection is needed in surgery.

For the purpose of staging, the date of diagnosis should be the date of histologic proof; this should be the date of the cytologic or histologic confirmation of MTC. Most patients undergo thyroidectomy as the first step in treatment. Occasionally, patients undergo neoadjuvant therapy including external beam irradiation, chemotherapy, or other treatments before or in place of surgery. The treatment date should be the date of thyroidectomy or the initiation of induction therapy in rare patients.

Categorization of the primary tumor should be based on the dimensions of the largest malignant nodule within the thyroid gland, measured by histologic examination. Cross-sectional imaging may be used in patients whose thyroid cancer is not surgically removed.

Extrathyroidal extension or *gross advanced disease* refers to the involvement by direct extension from the thyroid primary into the perithyroidal soft tissue. Invasion sometimes can be identified either by preoperative imaging or by intraoperative findings.

Clinical N1 disease should include patients with detectable lymph node metastases that are highly suspicious for metastatic disease based on physical examination and/or radiologic imaging. Cytologic confirmation may confirm clinical N1 status.

Lymph node metastases most commonly spread to central (level VI) nodes, followed by mid- (level III) and lower (level IV) jugular nodes, and less commonly to high (level IIA) jugular and supraclavicular (level V) nodes. The upper deep jugular and spinal accessory lymph nodes (level IIB) are affected less commonly, and lymph node metastasis to submandibular and submental lymph nodes (level I) is rare. Upper mediastinal (level VII) nodal spread may occur but is unusual without level VI lymph node spread.

Patients who present with a newly detected thyroid nodule and histologic confirmation of MTC should have a physical examination and measurement of their basal serum calcitonin and CEA levels. MTC readily metastasizes to lymph nodes within the neck.[33,34] The cervical lymph node stations most commonly are divided into seven compartments. The "central neck" usually refers to levels VI and VII, whereas the "lateral neck" includes levels II, III, IV, and V. Ultrasound evaluation of the neck should be performed to assess the central and lateral compartment lymph nodes. Patients with a palpable thyroid nodule have a high rate of lymph node metastases that may not be detected either by preoperative imaging evaluation or by intraoperative inspection and palpation. The overwhelming majority of MTC patients have nodal metastases.[33–35] Most patients with lateral

neck disease have involved central compartment nodes as well. Patients with extensive central compartment lymph node involvement also are likely to have lateral lymph node involvement.[36,37]

Distant metastases can be confirmed either by cytologic/histologic assessment or by documentation of imaging findings highly suspicious for distant metastasis in patients who also have elevated tumor markers, including calcitonin and CEA.

Imaging

Pretherapy Imaging

Primary Tumor

If MTC is clinically suspected, ultrasound is the imaging modality of choice for characterizing a primary thyroid nodule or lesion. MTC primary lesions usually are hypoechoic. Microcalcifications, increased vascularity, and ill-defined margins are ultrasonic features for malignancy. However, these features do not distinguish MTC from differentiated thyroid cancers.

Cross-sectional imaging with CT or magnetic resonance (MR) imaging is indicated only if there is clinical evidence of local invasion.

Neck Nodal Metastases

Imaging is very useful to identify neck nodal metastases, especially if they are clinically suspected or if the calcitonin level is elevated. Ultrasound is useful to identify metastases in the central or lateral neck compartments. An abnormally round shape, loss of fatty hilum, blurred capsule, microcalcification, and increased vascularity are features of metastatic lymph nodes. Limitations of ultrasound include operator dependency and limited evaluation of upper mediastinal nodes or retropharyngeal nodes.

Cross-sectional imaging with CT or MR is useful to identify upper mediastinal and retropharyngeal nodes. In addition, CT or MR imaging may be performed if there is clinical suspicion of lateral neck compartment nodal metastases, to map out the surgical planning of lateral neck dissection.

Distant Metastases

Additional imaging with CT of the chest and abdomen (for liver metastases), radionuclide bone scanning (for skeletal metastases), MR imaging of the brain (for brain metastses), or fluorodeoxyglucose (FDG) positron emission tomography (PET)/CT (for whole-body imaging) may be useful if distant metastases are suspected or the calcitonin level is >150 pg/mL. The sensitivity of the FDG PET/CT examination for systemic staging is >70% if the calcitonin level is >1,000 pg/mL.[38]

Posttherapy Imaging

Posttherapy imaging is indicated only if there is clinical suspicion of metastases or recurrence, or the calcitonin or CEA level is persistently elevated, especially if >150 pg/mL.[32,39] The imaging modality of choice depends on the suspected site of recurrence: ultrasound for neck nodes, chest CT for lung metastases, radionuclide bone scan for skeletal metastases, and abdominal CT or liver MR imaging for liver metastases. FDG PET/CT is useful for systemic evaluation, but its sensitivity is low, especially for calcitonin levels <500 pg/mL.

Postoperative assessment of MTC also may be done using indium-111 diethylenetriaminepentaacetic acid (^{111}In-DTPA) octreotide, technetium-99m dimercaptosuccinic acid (^{99m}Tc-DMSA), or iodine-131 metaiodobenzylguanidine (^{131}I-MIBG) scintigraphy. MIBG also offers the prospect of targeted therapy in tumor-positive cases.[40]

Pathological Classification

Pathological staging should include use the information obtained in the clinical staging as well as the reported histologic study of any surgically resected specimens. The surgeon's description of intraoperative findings and any worrisome lymph nodes identified during the procedure (extrathyroidal extension or overall completeness of resection) should be included.

MTC usually arises in the lateral upper two thirds of the thyroid gland, which is where the highest concentrations of C cells are located. The appearance of MTC on aspiration cytology may vary. The cells may be dyshesive or weakly cohesive, spindle-shaped, plasmacytoid, or epithelioid. One may see a dispersed cell pattern of triangular or polygonal cells, cytoplasmic granules that are azurophilic, and nuclei placed eccentrically with coarse granular chromatin and amyloid.[41] The tumors typically are composed of epithelioid and spindle cells.[42] The tumor stroma usually contains endocrine amyloid (procalcitonin), but about 25% of MTCs, especially very small tumors, do not contain amyloid. MTC may appear either as a solitary unilateral tumor or multicentric and bilateral. Sporadic MTC tends to be unifocal, and inherited MTC more often tends to be multifocal. There are several histologic variants of MTC, including a small cell variant, a giant cell variant, a papillary variant, and an oncocytic, squamoid, and clear cell variant; therefore, immunohistochemical (IHC) staining with calcitonin and general neuroendocrine markers, such as chromogranin A and synaptophysin, are needed to establish the diagnosis. Other biomarkers that may be present include CEA, vasoactive intestinal peptide, neural cell adhesion molecule, and somatostatin. Up to about 40% of MTCs contain mucin, most of which is extracellular, but intracellular mucin may be seen in a small percentage of cases.

The risk of lymph node metastases likely increases with the preoperative basal calcitonin level. The C cells of the thyroid gland secrete several hormones, including ACTH, calcitonin, CEA, chromogranin, histaminase, neurotensin, somatostatin, and B-melanocyte–stimulating hormone.[43–48] Calcitonin and CEA are the most relevant tumor markers in patients with MTC, because their serum concentrations are known to be directly related to the C-cell mass. In a study of 300 patients with MTC treated by total thyroidectomy and compartment-oriented lymph node dissections, when the calcitonin level was less than 20 pg/mL, there was virtually no risk of lymph node metastasis.[49] No distant metastases were detected if the baseline serum calcitonin level was less than 500 pg/mL. This finding for the predictive value of calcitonin is not entirely consistent and likely is related to additional patient factors.[49,50] Patients whose basal serum calcitonin level is normal (<10 pg/mL) have a 98% survival at 10 years following an attempt at complete lymph node dissection; only 3% will have a biochemical recurrence within 7.5 years. Preoperative CEA elevation also is associated with the presence of cervical node metastases but is less useful because of variable baseline values.[51]

Lymph node metastases most commonly spread to central (level VI) nodes, followed by mid- (level III) and lower (level IV) jugular nodes, and less commonly to high (level IIA) jugular and supraclavicular (level V) nodes. The upper deep jugular and spinal accessory lymph nodes (level IIB) are affected less commonly, and lymph node metastasis to submandibular, retropharyngeal, and submental lymph nodes (level I) is rare. Upper mediastinal (level VII) nodal spread may occur but is unusual without level VI lymph node spread.

Histologic confirmation by nodal dissection defines the pathological N1 category. In patients who undergo resection, this may be identified either preoperatively or intraoperatively, but histologic confirmation is required for these patients to confer pathological N1 category.

The definition of nodal metastasis includes both micro- and macrometastatic nodal involvement. There is no specific number of lymph nodes to be sampled to define nodal metastatic disease, although details regarding the number of lymph nodes sampled, the number of lymph nodes involved, extranodal involvement and size of the lymph nodes containing metastatic disease, and the size of the metastatic focus of the disease are suggested for recording as prognostic factors to be classified.

Postoperative serum calcitonin levels >150 pg/mL are associated with identifiable metastatic sites.[52] Patients with a preoperative calcitonin level >500 pg/mL warrant imaging and evaluation for distant disease before undergoing resection.[49]

IHC staining should include cytokeratins, thyroid transcription factor 1, and chromogranin A, as well as calcitonin and CEA.[53] In patients with hereditary MTC, C-cell hyperplasia may be identified with abnormal or atypical C-cell hyperplasia with a desmoplastic stromal response.

Complete assessment of N/M status may not be possible until after surgical resection. Therefore, identification of metastatic disease in the 12 to 16 weeks after thyroid surgery should be used to refine the N and M status. Consistent with the AJCC staging rules, the formal stage established during the first 4 months of follow-up does not change over time (even if the cancer progresses or recurs). However, the cancer may be "restaged" as new data become available during follow-up by using the same approach and definitions used for the initial staging. The lower case *r* is used to designate the restaging. In MTC, clinicians recognize both structural disease recurrence/progression (structural or functional evidence of disease) and biochemical disease recurrence/progression (abnormal CEA or calcitonin without structural or functional evidence of disease). Consistent with the approach to initial staging, restaging should be based only on the identification of structurally or functionally identifiable disease and not on the basis of abnormal biomarkers of disease.

The most important pathological prognostic factors include tumor pattern, amyloid content, necrosis, and mitotic activity.[54] Encapsulated tumors, tumors with abundant amyloid, and tumors with uniform cytology usually have a better prognosis. However, in multivariate analysis, the prognostic significance of any one or more of these variables was uncertain; therefore, we have not incorporated them into our primary staging scheme.[55]

PROGNOSTIC FACTORS

Prognostic Factors Required for Stage Grouping

Beyond the factors used to assign T, N, or M categories, no additional prognostic factors are required for stage grouping.

Additional Factors Recommended for Clinical Care

Number of Involved Lymph Nodes, Size of Involved Lymph Nodes, and Measurement of Metastatic Focus

Regional lymph node metastases are common in patients with MTC. Many studies are emerging that suggest that regional lymph node metastases have prognostic significance. Some studies have suggested that there is a significant association between the number of involved lymph nodes and survival, as well as the extent of lymph node metastasis within the examined node. Lymph nodes should be measured in three dimensions—length, width, and thickness—and the presence and extent of the metastatic focus, whether it encompasses part or all of the gland, and whether any

extranodal extension is present should be noted. There are no pertinent cutoff values. AJCC Level of Evidence: II

Completeness of Resection

In the operative report, the surgeon notes whether he or she was able to provide a complete resection of all grossly visible disease. Occasionally, this information also is found within the pathology report if the surgeon performed a biopsy for assessment of the surgical margin and includes a note regarding whether it was positive or negative. There are no pertinent cutoff values. AJCC Level of Evidence: II

Biochemical Parameters

Preoperative and postoperative measurement of secretory products of MTC, including calcitonin, measured in picograms per milliliter (normal is 0.0–5.0 pg/mL); CEA, measured in nanograms per milliliter (normal, 0.0–3.0 ng/mL); and ACTH, measured in picograms per milliliter (normal, <46 pg/mL).

The C cells of the thyroid gland secrete several hormones or biogenic amines. Calcitonin and CEA, in particular, are tumor markers in patients with MTC and may be measured both pre- and postoperatively to guide surgical management and postoperative care. In particular, calcitonin levels may be increased in patients with other medical diseases and cause false positive elevations. There also is variability in the assay and reference range by gender, as well as variation among different assays, in younger versus older age, in the use of provocative testing, and in the time it takes for normalization of serum calcitonin levels.[56–58] CEA, in comparison, is not a specific biomarker and may sometimes be useful for evaluating disease progression and for postoperative monitoring. Postoperative measurements of calcitonin and CEA may aid in determining whether surgery was curative.[59] MTC growth rate over multiple time points may be performed by measuring serum levels of calcitonin or CEA to determine doubling time for values.[18,60,61] AJCC Level of Evidence: II

Genetic Mutation Analysis

The Ret protooncogene is expressed in cells derived from the neural crest, the branchial arches, and the urogenital system.[62,63] It is located on chromosome 10q11 and encodes a transmembrane receptor of the tyrosine kinase family. Nearly all patients with MEN2A, MEN2B, or FMTC have *RET* germline mutations; approximately half of patients with sporadic MTCs have somatic *RET* mutations.[6–9,64,65] An additional portion of patients with sporadic MTCs may have somatic mutations of *HRAS*, *KRAS*, or, rarely, *NRAS*.[66–68] In particular, the somatic *RET* codon mutation M918T[69,70] is associated with an aggressive clinical course and confers a poor prognosis. In one study of 160 patients with sporadic MTC, the prevalence varied with tumor size, ranging from 11.3% in 53 patients with tumors <1 cm and 58.8% in 17 patients with tumors >3 cm.[71] The North American Neuroendocrine Tumor Society, the NCCN, and the ATA have all published guidelines on the management of patients with MTC, including recommendations based on the specific *RET* mutations and for screening of family members when a familial proband is first identified.[72–75] The highest-risk category included patients with MEN2B and the *RET* codon M918T mutation, the high-risk category included patients with *RET* codon C634 mutations and the *RET* codon A883F, and the moderate-risk category included patients with mutations other than those listed here. There are no pertinent cutoff values. AJCC Level of Evidence: II

Sporadic or Medullary MTC

The prognosis and clinical behavior of patients with sporadic MTC are less predictable than in patients with hereditary MTC. Molecular genetic testing should be performed in patients with familial MEN2A, and mutations that are identified in the *RET* codon M918T or A883F or from entire *RET* coding sequencing should be included in the data collection. Patients with sporadic MTC may have hereditary disease up to 25% of the time and therefore usually are advised to undergo genetic counseling and direct DNA analysis. There are no pertinent cutoff values. AJCC Level of Evidence: II

RISK ASSESSMENT MODELS

The AJCC recently established guidelines that will be used to evaluate published statistical prediction models for the purpose of granting endorsement for clinical use.[76] Although this is a monumental step toward the goal of precision medicine, this work was published only very recently. Therefore, the existing models that have been published or may be in clinical use have not yet been evaluated for this cancer site by the Precision Medicine Core of the AJCC. In the future, the statistical prediction models for this cancer site will be evaluated, and those that meet all AJCC criteria will be endorsed.

DEFINITIONS OF AJCC TNM

Definition of Primary Tumor (T)

T Category	T Criteria
TX	Primary tumor cannot be assessed
T0	No evidence of primary tumor
T1	Tumor ≤2 cm in greatest dimension limited to the thyroid
T1a	Tumor ≤1 cm in greatest dimension limited to the thyroid
T1b	Tumor >1 cm but ≤2 cm in greatest dimension limited to the thyroid
T2	Tumor >2 cm but <4 cm in greatest dimension limited to the thyroid
T3	Tumor ≥4 cm or with extrathyroidal extension
T3a	Tumor ≥4 cm in greatest dimension limited to the thyroid
T3b	Tumor of any size with gross extrathyroidal extension invading only strap muscles (sternohyoid, sternothyroid, thyrohyoid or omohyoid muscles)

(continued)

T Category	T Criteria
T4	Advanced disease
T4a	Moderately advanced disease; tumor of any size with gross extrathyroidal extension into the nearby tissues of the neck, including subcutaneous soft tissue, larynx, trachea, esophagus, or recurrent laryngeal nerve
T4b	Very advanced disease; tumor of any size with extension toward the spine or into nearby large blood vessels, invading the prevertebral fascia, or encasing the carotid artery or mediastinal vessels

Definition of Regional Lymph Node (N)

N Category	N Criteria
NX	Regional lymph nodes cannot be assessed
N0	No evidence of locoregional lymph node metastasis
N0a	One or more cytologically or histologically confirmed benign lymph nodes
N0b	No radiologic or clinical evidence of locoregional lymph node metastasis
N1	Metastasis to regional nodes
N1a	Metastasis to level VI or VII (pretracheal, paratracheal, or prelaryngeal/Delphian, or upper mediastinal) lymph nodes. This can be unilateral or bilateral disease.
N1b	Metastasis to unilateral, bilateral, or contralateral lateral neck lymph nodes levels I, II, III, IV, or V) or retropharyngeal lymph nodes

Definition of Distant Metastasis (M)

M Category	M Criteria
M0	No distant metastasis
M1	Distant metastasis

AJCC PROGNOSTIC STAGE GROUPS

When T is...	And N is...	And M is...	Then the stage group is...
T1	N0	M0	I
T2	N0	M0	II
T3	N0	M0	II
T1–3	N1a	M0	III
T4a	Any N	M0	IVA
T1–3	N1b	M0	IVA
T4b	Any N	M0	IVB
Any T	Any N	M1	IVC

REGISTRY DATA COLLECTION VARIABLES

1. Age at diagnosis
2. Gender
3. Race
4. Histology
5. Size of primary tumor
6. Number of involved lymph nodes
7. Presence of extranodal extension
8. Size of the involved lymph nodes
9. Size of the metastatic focus in the involved lymph nodes
10. Completeness of resection
11. Preoperative calcitonin
12. Preoperative CEA
13. Genetic mutations, including specific codon information for mutations in the *RET* protooncogene, including the method of measurement, if available. Other mutations to be documented are in the *RAS* (*HRAS*, *KRAS*, or *NRAS*) group.
14. Whether the patient has MTC that is sporadic or hereditary, if known

HISTOLOGIC GRADE (G)

Grade is not used in the staging for MTC.

HISTOPATHOLOGIC TYPE

Medullary thyroid carcinoma

ILLUSTRATIONS

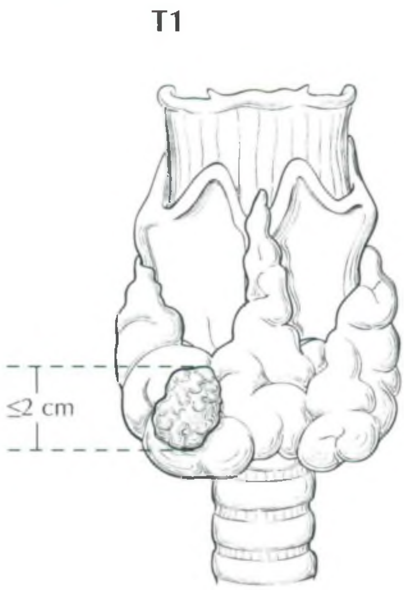

Fig. 74.3 T1 is defined as tumor 2 cm or less in greatest dimension limited to the thyroid. T1a, tumor 1cm or less, limited to the thyroid. T1b, tumor more than 1 cm but not more than 2 cm in greatest dimension, limited to the thyroid

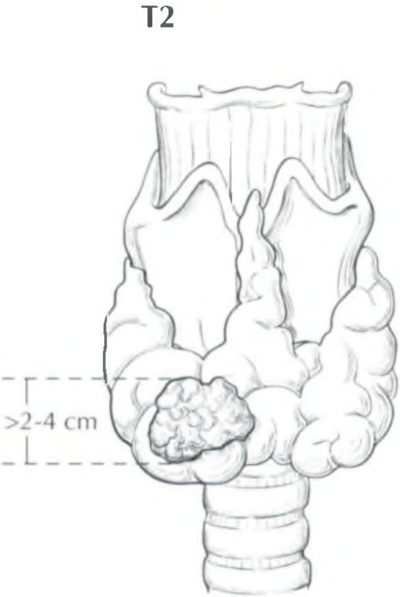

Fig. 74.4 T2 is defined as tumor more than 2 cm but not more than 4 cm in greatest dimension limited to the thyroid

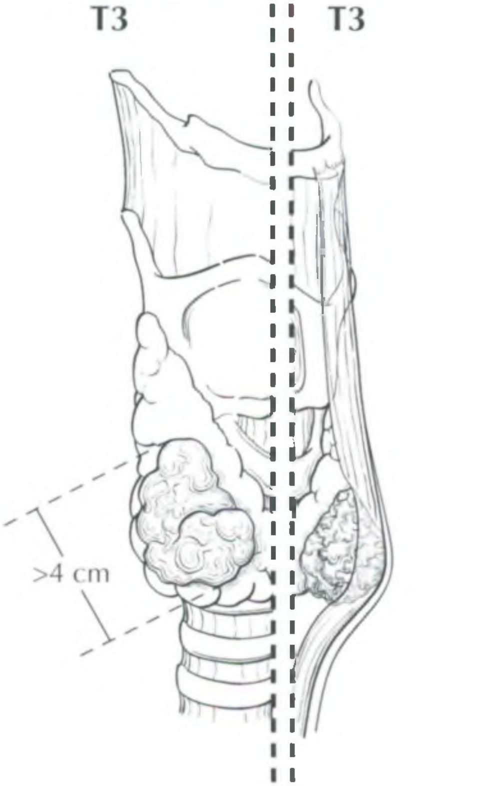

Fig. 74.5. Two views of T3: on the left, tumor more than 4 cm in greatest dimension limited to the thyroid (categorized as T3a); on the right, a tumor of any size with gross extrathyroidal extension invading only strap muscles (sternohyoid, sternothyroid, thyrohyoid or omohyoid muscles) (categorized as T3b)

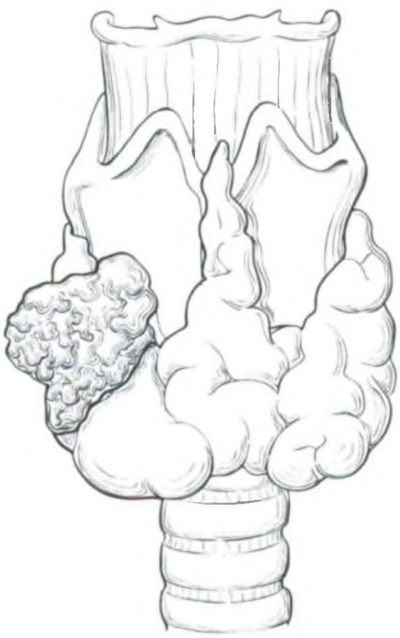

Fig. 74.6 T4a is defined as moderately advanced disease; tumor of any size with gross extrathyroidal extension into the nearby tissues of the neck, including subcutaneous soft tissue, larynx, trachea, esophagus, or recurrent laryngeal nerve

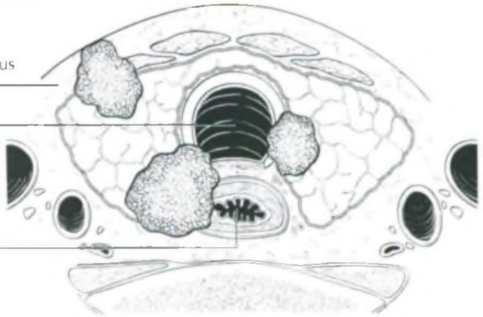

Fig. 74.7 Cross-sectional diagram of three different parameters of T4a: tumor invading subcutaneous soft tissues; tumor invading trachea; tumor invading esophagus

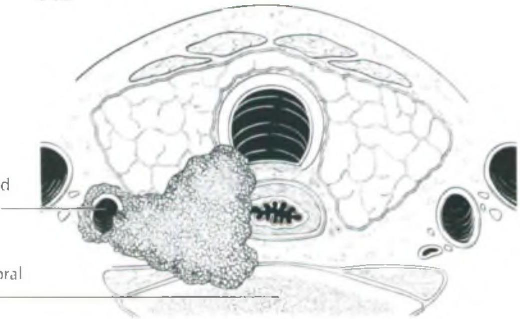

Fig. 74.8 T4b is defined as very advanced disease: tumor of any size with extension toward the spine or into nearby large blood vessels, invading the prevertebral fascia, or encasing the carotid artery or mediastinal vessels. Cross-sectional diagram of two different parameters of T4b: tumor encases carotid artery; tumor invades vertebral body

Bibliography

1. Davies L, Morris LG, Haymart M, et al. American Association of Clinical Endocrinologists and American College of Endocrinology Disease State Clinical Review: The Increasing Incidence of Thyroid Cancer. *Endocr Pract.* Jun 2015;21(6):686–696.
2. J J. Virchows Arch Pathol Anat Physiol. 1906.
3. Hazard JB, Hawk WA, Crile G, Jr. Medullary (solid) carcinoma of the thyroid; a clinicopathologic entity. *The Journal of clinical endocrinology and metabolism.* Jan 1959;19(1):152–161.
4. Williams ED. Histogenesis of medullary carcinoma of the thyroid. *Journal of clinical pathology.* Mar 1966;19(2):114–118.
5. Takahashi M, Ritz J, Cooper GM. Activation of a novel human transforming gene, ret, by DNA rearrangement. *Cell.* Sep 1985; 42(2):581–588.
6. Donis-Keller H, Dou S, Chi D, et al. Mutations in the RET proto-oncogene are associated with MEN 2A and FMTC. *Hum Mol Genet.* Jul 1993;2(7):851–856.
7. Mulligan LM, Kwok JB, Healey CS, et al. Germ-line mutations of the RET proto-oncogene in multiple endocrine neoplasia type 2A. *Nature.* Jun 3 1993;363(6428):458–460.
8. Carlson KM, Dou S, Chi D, et al. Single missense mutation in the tyrosine kinase catalytic domain of the RET protooncogene is associated with multiple endocrine neoplasia type 2B. *Proc Natl Acad Sci U S A.* Feb 15 1994;91(4):1579–1583.
9. Hofstra RM, Landsvater RM, Ceccherini I, et al. A mutation in the RET proto-oncogene associated with multiple endocrine neoplasia type 2B and sporadic medullary thyroid carcinoma. *Nature.* Jan 27 1994;367(6461):375–376.
10. Tashjian AH, Jr., Melvin EW. Medullary carcinoma of the thyroid gland. Studies of thyrocalcitonin in plasma and tumor extracts. *N Engl J Med.* Aug 8 1968;279(6):279–283.

11. Wells SA, Jr., Baylin SB, Linehan WM, Farrell RE, Cox EB, Cooper CW. Provocative agents and the diagnosis of medullary carcinoma of the thyroid gland. *Annals of surgery.* Aug 1978;188(2):139–141.
12. Preissner CM, Dodge LA, O'Kane DJ, Singh RJ, Grebe SK. Prevalence of heterophilic antibody interference in eight automated tumor marker immunoassays. *Clin Chem.* Jan 2005;51(1):208–210.
13. Toledo SP, Lourenco DM, Jr., Santos MA, Tavares MR, Toledo RA, Correia-Deur JE. Hypercalcitoninemia is not pathognomonic of medullary thyroid carcinoma. *Clinics (Sao Paulo).* 2009;64(7):699–706.
14. Leboeuf R, Langlois MF, Martin M, Ahnadi CE, Fink GD. "Hook effect" in calcitonin immunoradiometric assay in patients with metastatic medullary thyroid carcinoma: case report and review of the literature. *The Journal of clinical endocrinology and metabolism.* Feb 2006;91(2):361–364.
15. Basuyau JP, Mallet E, Leroy M, Brunelle P. Reference intervals for serum calcitonin in men, women, and children. *Clin Chem.* Oct 2004;50(10):1828–1830.
16. Wells SA, Jr., Haagensen DE, Jr., Linehan WM, Farrell RE, Dilley WG. The detection of elevated plasma levels of carcinoembryonic antigen in patients with suspected or established medullary thyroid carcinoma. *Cancer.* Sep 1978;42(3 Suppl):1498–1503.
17. Frank-Raue K, Machens A, Leidig-Bruckner G, et al. Prevalence and clinical spectrum of nonsecretory medullary thyroid carcinoma in a series of 839 patients with sporadic medullary thyroid carcinoma. *Thyroid : official journal of the American Thyroid Association.* Mar 2013;23(3):294–300.
18. Laure Giraudet A, Al Ghulzan A, Auperin A, et al. Progression of medullary thyroid carcinoma: assessment with calcitonin and carcinoembryonic antigen doubling times. *European journal of endocrinology / European Federation of Endocrine Societies.* Feb 2008;158(2):239–246.
19. Tung WS, Vesely TM, Moley JF. Laparoscopic detection of hepatic metastases in patients with residual or recurrent medullary thyroid cancer. *Surgery.* Dec 1995;118(6):1024–1029; discussion 1029–1030.
20. Tsutsui H, Kubota M, Yamada M, et al. Airway stenting for the treatment of laryngotracheal stenosis secondary to thyroid cancer. *Respirology.* 2008;13(5):632–638.
21. Wexler JA. Approach to the thyroid cancer patient with bone metastases. *The Journal of clinical endocrinology and metabolism.* Aug 2011;96(8):2296–2307.
22. Quan GM, Pointillart V, Palussiere J, Bonichon F. Multidisciplinary treatment and survival of patients with vertebral metastases from thyroid carcinoma. *Thyroid : official journal of the American Thyroid Association.* Feb 2012;22(2):125–130.
23. Frassica DA. General principles of external beam radiation therapy for skeletal metastases. *Clinical orthopaedics and related research.* Oct 2003(415 Suppl):S158–164.
24. Vitale G, Fonderico F, Martignetti A, et al. Pamidronate improves the quality of life and induces clinical remission of bone metastases in patients with thyroid cancer. *Br J Cancer.* Jun 15 2001;84(12):1586–1590.
25. Abrahamsen B, Eiken P, Eastell R. Subtrochanteric and diaphyseal femur fractures in patients treated with alendronate: a register-based national cohort study. *J Bone Miner Res.* Jun 2009;24(6):1095–1102.
26. Borcek P, Asa SL, Gentili F, Ezzat S, Kiehl TR. Brain metastasis from medullary thyroid carcinoma. *BMJ Case Rep.* 2010;2010.
27. Kim IY, Kondziolka D, Niranjan A, Flickinger JC, Lunsford LD. Gamma knife radiosurgery for metastatic brain tumors from thyroid cancer. *Journal of neuro-oncology.* Jul 2009;93(3):355–359.
28. McWilliams RR, Giannini C, Hay ID, Atkinson JL, Stafford SL, Buckner JC. Management of brain metastases from thyroid carcinoma: a study of 16 pathologically confirmed cases over 25 years. *Cancer.* Jul 15 2003;98(2):356–362.
29. Santarpia L, El-Naggar AK, Sherman SI, et al. Four patients with cutaneous metastases from medullary thyroid cancer. *Thyroid : official journal of the American Thyroid Association.* Aug 2008;18(8):901–905.
30. Bhansali A, Walia R, Rana SS, et al. Ectopic Cushing's syndrome: experience from a tertiary care centre. *The Indian journal of medical research.* Jan 2009;129(1):33–41.
31. Haddad RI. New developments in thyroid cancer. *Journal of the National Comprehensive Cancer Network : JNCCN.* May 2013;11(5 Suppl):705–707.
32. Wells SA, Jr., Asa SL, Dralle H, et al. Revised American Thyroid Association guidelines for the management of medullary thyroid carcinoma. *Thyroid : official journal of the American Thyroid Association.* Jun 2015;25(6):567–610.
33. Moley JF, DeBenedetti MK. Patterns of nodal metastases in palpable medullary thyroid carcinoma: recommendations for extent of node dissection. *Annals of surgery.* Jun 1999;229(6):880–887; discussion 887–888.
34. Weber T, Schilling T, Frank-Raue K, et al. Impact of modified radical neck dissection on biochemical cure in medullary thyroid carcinomas. *Surgery.* Dec 2001;130(6):1044–1049.
35. Kazaure HS, Roman SA, Sosa JA. Medullary thyroid microcarcinoma: a population-level analysis of 310 patients. *Cancer.* Feb 1 2012;118(3):620–627.
36. Machens A, Holzhausen HJ, Dralle H. Contralateral cervical and mediastinal lymph node metastasis in medullary thyroid cancer: systemic disease? *Surgery.* Jan 2006;139(1):28–32.
37. Machens A, Hauptmann S, Dralle H. Prediction of lateral lymph node metastases in medullary thyroid cancer. *The British journal of surgery.* May 2008;95(5):586–591.
38. Ong SC, Schoder H, Patel SG, et al. Diagnostic accuracy of 18F-FDG PET in restaging patients with medullary thyroid carcinoma and elevated calcitonin levels. *Journal of nuclear medicine : official publication, Society of Nuclear Medicine.* Apr 2007;48(4):501–507.
39. Giraudet AL, Vanel D, Leboulleux S, et al. Imaging medullary thyroid carcinoma with persistent elevated calcitonin levels. *The Journal of clinical endocrinology and metabolism.* Nov 2007;92(11):4185–4190.
40. Gao Z, Biersack HJ, Ezziddin S, Logvinski T, An R. The role of combined imaging in metastatic medullary thyroid carcinoma: 111In-DTPA-octreotide and 131I/123I-MIBG as predictors for radionuclide therapy. *Journal of cancer research and clinical oncology.* 2004;130(11):649–656.
41. Papaparaskeva K, Nagel H, Droese M. Cytologic diagnosis of medullary carcinoma of the thyroid gland. *Diagn Cytopathol.* Jun 2000;22(6):351–358.
42. DeLellis RA. *Pathology and genetics of tumours of endocrine organs.* Vol 8: IARC; 2004.
43. Abe K, Adachi I, Miyakawa S, et al. Production of calcitonin, adrenocorticotropic hormone, and beta-melanocyte-stimulating hormone in tumors derived from amine precursor uptake and decarboxylation cells. *Cancer Res.* Nov 1977;37(11):4190–4194.
44. Baylin SB, Beaven MA, Engelman K, Sjoerdsma A. Elevated histaminase activity in medullary carcinoma of the thyroid gland. *N Engl J Med.* Dec 3 1970;283(23):1239–1244.
45. Ishikawa N, Hamada S. Association of medullary carcinoma of the thyroid with carcinoembryonic antigen. *Br J Cancer.* Aug 1976;34(2):111–115.
46. Zeytinoglu FN, Gagel RF, Tashjian AH, Jr., Hammer RA, Leeman SE. Characterization of neurotensin production by a line of rat medullary thyroid carcinoma cells. *Proc Natl Acad Sci U S A.* Jun 1980;77(6):3741–3745.
47. Mato E, Matias-Guiu X, Chico A, et al. Somatostatin and somatostatin receptor subtype gene expression in medullary thyroid carcinoma. *The Journal of clinical endocrinology and metabolism.* Jul 1998;83(7):2417–2420.

48. Costante G, Durante C, Francis Z, Schlumberger M, Filetti S. Determination of calcitonin levels in C-cell disease: clinical interest and potential pitfalls. *Nature clinical practice. Endocrinology & metabolism.* Jan 2009;5(1):35–44.
49. Machens A, Dralle H. Biomarker-based risk stratification for previously untreated medullary thyroid cancer. *The Journal of clinical endocrinology and metabolism.* Jun 2010;95(6):2655–2663.
50. Dralle H, Machens A. Surgical management of the lateral neck compartment for metastatic thyroid cancer. *Current opinion in oncology.* Jan 2013;25(1):20–26.
51. Machens A, Ukkat J, Hauptmann S, Dralle H. Abnormal carcinoembryonic antigen levels and medullary thyroid cancer progression: a multivariate analysis. *Archives of surgery.* Mar 2007;142(3):289–293; discussion 294.
52. Brierley JD, Tsang RW. External beam radiation therapy for thyroid cancer. *Endocrinology and metabolism clinics of North America.* Jun 2008;37(2):497–509, xi.
53. Trimboli P, Cremonini N, Ceriani L, et al. Calcitonin measurement in aspiration needle washout fluids has higher sensitivity than cytology in detecting medullary thyroid cancer: a retrospective multicentre study. *Clin Endocrinol (Oxf).* Jan 2014;80(1):135–140.
54. Schroder S, Bocker W, Baisch H, et al. Prognostic factors in medullary thyroid carcinomas. Survival in relation to age, sex, stage, histology, immunocytochemistry, and DNA content. *Cancer.* Feb 15 1988;61(4):806–816.
55. Dottorini ME, Assi A, Sironi M, Sangalli G, Spreafico G, Colombo L. Multivariate analysis of patients with medullary thyroid carcinoma. Prognostic significance and impact on treatment of clinical and pathologic variables. *Cancer.* Apr 15 1996;77(8):1556–1565.
56. Franc S, Niccoli-Sire P, Cohen R, et al. Complete surgical lymph node resection does not prevent authentic recurrences of medullary thyroid carcinoma. *Clin Endocrinol (Oxf).* Sep 2001;55(3):403–409.
57. Ismailov SI, Piulatova NR. Postoperative calcitonin study in medullary thyroid carcinoma. *Endocrine-related cancer.* Jun 2004;11(2):357–363.
58. Elisei R, Pinchera A. Advances in the follow-up of differentiated or medullary thyroid cancer. *Nature reviews. Endocrinology.* Aug 2012;8(8):466–475.
59. Engelbach M, Gorges R, Forst T, et al. Improved diagnostic methods in the follow-up of medullary thyroid carcinoma by highly specific calcitonin measurements. *The Journal of clinical endocrinology and metabolism.* May 2000;85(5):1890–1894.
60. Barbet J, Campion L, Kraeber-Bodere F, Chatal JF, Group GTES. Prognostic impact of serum calcitonin and carcinoembryonic antigen doubling-times in patients with medullary thyroid carcinoma. *The Journal of clinical endocrinology and metabolism.* Nov 2005;90(11):6077–6084.
61. Miyauchi A, Onishi T, Morimoto S, et al. Relation of doubling time of plasma calcitonin levels to prognosis and recurrence of medullary thyroid carcinoma. *Annals of surgery.* Apr 1984;199(4):461–466.
62. Pachnis V, Mankoo B, Costantini F. Expression of the c-ret proto-oncogene during mouse embryogenesis. *Development.* Dec 1993;119(4):1005–1017.
63. Zordan P, Tavella S, Brizzolara A, et al. The immediate upstream sequence of the mouse Ret gene controls tissue-specific expression in transgenic mice. *Int J Mol Med.* Oct 2006;18(4):601–608.
64. Eng C, Smith DP, Mulligan LM, et al. Point mutation within the tyrosine kinase domain of the RET proto-oncogene in multiple endocrine neoplasia type 2B and related sporadic tumours. *Hum Mol Genet.* Feb 1994;3(2):237–241.
65. Marsh DJ, Learoyd DL, Andrew SD, et al. Somatic mutations in the RET proto-oncogene in sporadic medullary thyroid carcinoma. *Clin Endocrinol (Oxf).* Mar 1996;44(3):249–257.
66. Moura MM, Cavaco BM, Pinto AE, Leite V. High prevalence of RAS mutations in RET-negative sporadic medullary thyroid carcinomas. *The Journal of clinical endocrinology and metabolism.* May 2011;96(5):E863–868.
67. Boichard A, Croux L, Al Ghuzlan A, et al. Somatic RAS mutations occur in a large proportion of sporadic RET-negative medullary thyroid carcinomas and extend to a previously unidentified exon. *The Journal of clinical endocrinology and metabolism.* Oct 2012;97(10):E2031–2035.
68. Ciampi R, Mian C, Fugazzola L, et al. Evidence of a low prevalence of RAS mutations in a large medullary thyroid cancer series. *Thyroid : official journal of the American Thyroid Association.* Jan 2013;23(1):50–57.
69. Schilling T, Burck J, Sinn HP, et al. Prognostic value of codon 918 (ATG→ACG) RET proto-oncogene mutations in sporadic medullary thyroid carcinoma. *Int J Cancer.* Jan 20 2001;95(1):62–66.
70. Elisei R, Cosci B, Romei C, et al. Prognostic significance of somatic RET oncogene mutations in sporadic medullary thyroid cancer: a 10-year follow-up study. *The Journal of clinical endocrinology and metabolism.* Mar 2008;93(3):682–687.
71. Raue F, Frank-Raue K. Genotype-phenotype correlation in multiple endocrine neoplasia type 2. *Clinics (Sao Paulo).* 2012;67 Suppl 1:69–75.
72. Brandi ML, Gagel RF, Angeli A, et al. Guidelines for diagnosis and therapy of MEN type 1 and type 2. *The Journal of clinical endocrinology and metabolism.* Dec 2001;86(12):5658–5671.
73. Tuttle RM, Ball DW, Byrd D, et al. Medullary carcinoma. *Journal of the National Comprehensive Cancer Network : JNCCN.* May 2010;8(5):512–530.
74. Chen H, Sippel RS, O'Dorisio MS, et al. The North American Neuroendocrine Tumor Society consensus guideline for the diagnosis and management of neuroendocrine tumors: pheochromocytoma, paraganglioma, and medullary thyroid cancer. *Pancreas.* Aug 2010;39(6):775–783.
75. American Thyroid Association Guidelines Task F, Kloos RT, Eng C, et al. Medullary thyroid cancer: management guidelines of the American Thyroid Association. *Thyroid : official journal of the American Thyroid Association.* Jun 2009;19(6):565–612.
76. Kattan MW, Hess KR, Amin MB, et al. American Joint Committee on Cancer acceptance criteria for inclusion of risk models for individualized prognosis in the practice of precision medicine. *CA: a cancer journal for clinicians.* Jan 19 2016.

Parathyroid

75

Christine S. Landry, Tracy S. Wang, Elliot A. Asare, Raymon H. Grogan, Jennifer L. Hunt, John A. Ridge, Eric Rohren, Jatin P. Shah, Rathan M. Subramaniam, James D. Brierley, Raja R. Seethala, and Nancy D. Perrier

CHAPTER SUMMARY

Cancers Staged Using This Staging System

Parathyroid carcinoma

Summary of Changes

This is a new chapter for the AJCC Cancer Staging Manual.

ICD-O-3 Topography Codes

Code	Description
C75.0	Parathyroid gland

WHO Classification of Tumors

Code	Description
8000	Neoplasm, malignant
8001	Tumor cells, malignant
8005	Malignant tumor, clear cell type
8010	Carcinoma, NOS
8140	Adenocarcinoma, NOS
8290	Oxyphilic adenocarcinoma
8310	Clear cell adenocarcinoma, NOS
8322	Water-clear cell adenocarcinoma

DeLellis RA, Lloyd RV, Heitz PU, Eng C, eds. World Health Organization Classification of Tumours Pathology and Genetics of Tumours of Endocrine Organs. Lyon: IARC; 2004.

INTRODUCTION

Parathyroid carcinoma accounts for fewer than 1% of cases of primary hyperparathyroidism, and data are limited. Few studies have been published on parathyroid carcinoma, and most are retrospective reviews from individual institutions or large databases such as the Surveillance, Epidemiology, and End Results (SEER) database and the National Cancer Data Base (NCDB). There is no generally accepted staging system, and identification of significant prognostic factors has been challenging because of the wide variation among existing studies. As a result, the panel feels that proposing a staging system at this time would be premature. Instead, this chapter includes recommendations for recording specific variables in the cancer registry to be used to develop a formal staging system in future AJCC manuals.

To access the AJCC cancer staging forms, please visit www.cancerstaging.org.

© American Joint Committee on Cancer 2017

M.B. Amin et al. (eds.), *AJCC Cancer Staging Manual, Eighth Edition*, DOI 10.1007/978-3-319-40618-3_75

ANATOMY

Primary Site(s)

The parathyroid glands are composed of chief cells, oxyphil cells, and clear cells, and are usually located in the neck adjacent to the thyroid gland. Because the parathyroid glands descend during embryologic development, the location of each gland varies. The superior parathyroid glands, derived from the fourth branchial pouch, descend with the thyroid gland, and are commonly located posterior to the upper third of the thyroid lobe.[1] The inferior parathyroid glands descend with the thymus from the third branchial pouch, and may be located anywhere from the superior thyroidal poles to the anterior mediastinum.[1,2] Parathyroid glands also may be located within the thyroid gland.[3] Although the exact number of parathyroid glands varies among individuals, most people have two superior and two inferior parathyroid glands (Fig. 75.1).

The average weight of an individual gland is 30 to 50 mg.[1,2] These glands are usually contained within a thin connective tissue capsule.[1] If the capsule is absent, there may be nests of ectopic epithelial parathyroid cells mixed with fatty tissue near the gland.[1]

Parathyroid carcinoma develops within the parathyroid gland itself, and the location of the carcinoma is dependent on the embryologic descent and location of the affected gland.

Regional Lymph Nodes

Parathyroid carcinoma has been shown to metastasize to locoregional lymph nodes. Occasionally, parathyroid cancer metastasizes to lymph nodes in the central compartment of the neck (level VI or VII) near the primary tumor.[4] Rarely, parathyroid carcinoma metastasizes to lymph nodes in the lateral neck (levels II, III, IV, and V) (Fig. 75.2).

Metastatic Sites

Evidence of disease beyond the neck should be considered distant metastasis. Parathyroid carcinoma metastasizes to the lung in most cases.[5–7] Among patients who develop metastatic disease, as many as 15% have bony metastases.[7–9] Other described sites of metastases include the brain, skin, liver, mediastinum, adrenal glands, and pancreas.[2,5,7]

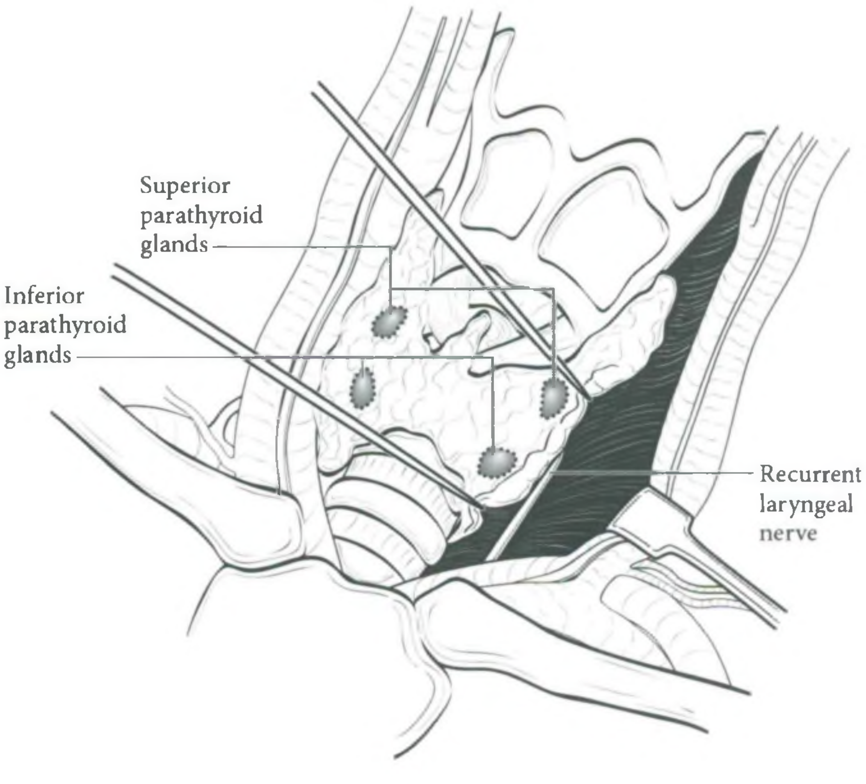

Fig. 75.1 Anatomy of the parathyroid gland

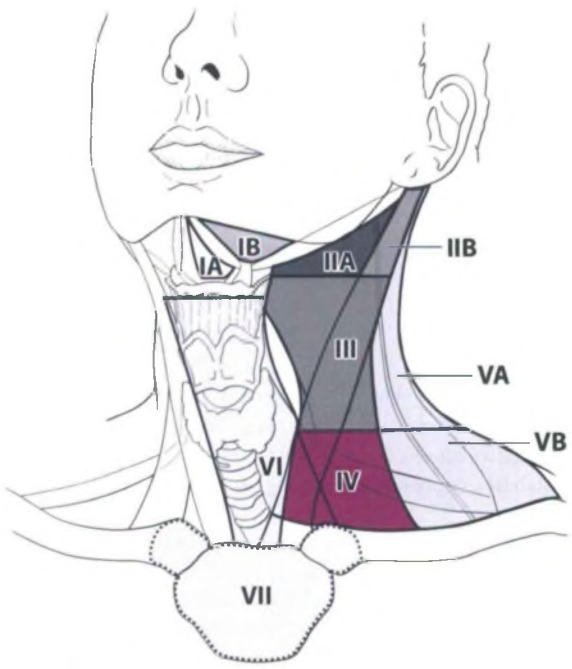

Fig. 75.2 Lymph node levels in the neck

RULES FOR CLASSIFICATION

Clinical Classification

Patients who present with a palpable neck mass, a serum calcium level greater than 14 mg/dL, and significantly elevated parathyroid hormone (PTH) levels should be suspected of harboring parathyroid carcinoma. Symptoms are similar to those in patients with severe primary hyperparathyroidism, including fatigue, cognitive deficits (difficulty with sleep, concentration, memory, multitasking, depression), bone/joint pain, fragility fractures, osteoporosis, pancreatitis, and kidney stones.[5,6,8,10] Some patients with parathyroid carcinoma may develop weight loss and thromboembolic disease.[2] Rarely, patients may present with neck pain.[5] Some parathyroid carcinomas do not overproduce PTH, and these patients may be asymptomatic, with normal calcium levels.[11]

The diagnosis of parathyroid carcinoma should be considered in any patient with significantly elevated calcium and PTH levels. Preoperative biopsy of patients with suspected parathyroid carcinoma is not recommended because of the risk of seeding the needle track with tumor. Because patients with parathyroid carcinoma often present with the same symptoms as patients with benign, sporadic primary hyperparathyroidism, the diagnosis is often assigned intraoperatively.[10] Unlike a parathyroid adenoma, a parathyroid carcinoma appears as a firm, white-gray mass often adhering to surrounding tissues.

Consideration should be given to *en bloc* resection of the parathyroid with the ipsilateral thyroid lobe and/or locoregional lymph nodes if necessary for complete tumor resection.

Often, parathyroid carcinoma is not recognized at the first operation, and scar tissue at the second operation may increase the difficulty of recognizing parathyroid cancer. In these cases, persistent disease (defined as biochemical evidence of inappropriately elevated calcium 6 months after the first operation) and time to recurrence should be considered.

Microscopic and macroscopic classifications of the primary tumor have not been standardized. Whether size or extent of invasion affects overall survival remains controversial. Until more is known about prognostic features associated with the primary tumor in parathyroid carcinoma, the expert panel recommends classifying the primary tumor according to both size and extent of invasion.

The prognostic significance of regional lymph node involvement is unclear, and studies are conflicting.[7,8,11–15] Until more is known about the prognostic significance of regional lymph node metastasis, the panel recommends collecting the type of lymph node dissection, the number of lymph nodes removed, and the number of lymph nodes with metastatic disease.

Parathyroid carcinoma is usually sporadic and may be associated with primary, secondary, or tertiary hyperparathyroidism. Patients with previous neck irradiation or certain hereditary syndromes may be at increased risk for developing parathyroid carcinoma. For instance, hyperparathyroidism–jaw tumor syndrome (HPT-JT) is a rare autosomal dominant disease caused by a mutation in the *CDC73* (*HRPT2*) gene located on chromosome 1q25-32.[16] Patients with this syndrome have ossifying fibromas of the jaw and various tumors/cysts of the kidneys, as well as Mullerian tract tumors in females. While 90% of patients develop primary hyperparathyroidism during their lifetime, as many as 15% develop parathyroid carcinoma.[2]

Familial isolated primary hyperparathyroidism is a rare autosomal dominant disease thought to be a variant of multiple endocrine neoplasia type I. This disease is also associated with an increased risk of developing parathyroid carcinoma.[17]

Imaging

Patients with parathyroid carcinoma often have imaging characteristics similar to those of patients with parathyroid adenomas. However, patients who are found to have a large mass, complex cystic lesions with internal septations, central necrosis, and/or compression of adjacent tissues should raise the preoperative suspicion of parathyroid carcinoma.[10]

Preoperative imaging studies may be performed in patients with a biochemical diagnosis of primary hyperparathyroidism to facilitate performance of a minimally invasive parathyroidectomy. This approach involves a focused resection of an abnormal parathyroid gland in conjunction with intraoperative PTH monitoring.[18,19] If there is clinical suspicion for parathyroid carcinoma at operation, then en bloc resection of adjacent tissue (i.e. thyroid) and/or adjacent lymph nodes should be performed. Preoperative imaging is also essential in patients undergoing reoperative parathyroidectomy.[20–24]

The most commonly used imaging modalities include ultrasonography, which allows concurrent examination of the thyroid; technetium-99 sestamibi scanning (in conjunction with single-photon emission computed tomography [CT] and/or X-ray based CT); and four-dimensional CT, which uses thin-section dynamic scanning techniques with multiplanar reconstruction capabilities.[25–29] In addition, fludeoxyglucose positron emission tomography may be helpful for the detection of parathyroid carcinoma, particularly for recurrence of distant metastatic disease; however, current data on this imaging modality are limited. Several preoperative imaging algorithms have been proposed; the optimal algorithm is informed by institutional capabilities and knowledge of one's institutional resources, capabilities, reported sensitivities, and operative outcomes.[30]

Pathological Classification

The diagnosis of parathyroid carcinoma is based on a combination of clinical and histologic findings on the resected parathyroid gland. The most reliable criteria for the diagnosis of parathyroid carcinoma are the presence of vascular or perineural invasion, invasion into adjacent soft tissues, and/or regional and distant metastasis.[1,2,31] Supportive findings include the presence of broad fibrous bands, necrosis, mitotic figures, and trabecular growth; although without the criteria noted earlier, these features are insufficient for a diagnosis of carcinoma.[1,2] Similarly, although the Ki-67 proliferation index (PI) is often elevated (>5%) in parathyroid carcinoma, this finding is not always conclusive because it may be elevated in benign disease.[1]

PROGNOSTIC FACTORS

Prognostic Factors Required for Stage Grouping

Beyond the factors used to assign T, N, or M categories, no additional prognostic factors are required for stage grouping.

Additional Factors Recommended for Clinical Care

Age

Defined as age at initial diagnosis. Among nine retrospective studies, three identified older age as a risk factor for decreased overall survival.[5–8,11–14,32] Most of the studies showing that age is not predictive of survival were limited in statistical value because of small sample sizes. AJCC Level of Evidence: III

Gender

Defined as male or female. Most of the studies evaluating gender as a prognostic factor showed that it was not predictive of survival.[6,8,11,12,14] However, most these studies were limited because of small sample sizes. Studies from large databases or meta-analyses suggest that men have a worse prognosis.[7,13,32] AJCC Level of Evidence: III

Size of Primary Tumor

Defined as measurement of the longest axis of the primary tumor in millimeters. Studies evaluating size as a prognostic factor are conflicting.[5,7,8,12–14,32] Likewise, no analysis has been performed to determine whether there is a significant cutoff value in terms of prognosis. AJCC Level of Evidence: III

Extent of Invasion of Primary Tumor

Few studies have been published regarding whether the extent of invasion of the primary tumor affects overall prognosis, and the results are conflicting.[7,8] Comparison among studies is difficult, because classification according to extent of the primary tumor was not standardized before publication of this chapter. AJCC Level of Evidence: III

Location of Primary Tumor

Defined as left or right and superior (upper) or inferior (lower)

Lymph Node Metastasis

Several analyses showed lymph node metastases to be predictive of outcome, but some of the large database studies did not identify lymph node metastasis as a prognostic factor.[7,8,11–14,32] This discrepancy may be related to how the survival analysis was performed (e.g., disease-specific vs. overall survival, exclusion of patients with more than one primary tumor). AJCC Level of Evidence: III

Distant Metastasis

Distant metastasis is defined as evidence of disease beyond the central and lateral neck. Several published studies demonstrated that the presence of distant metastasis portends a worse survival.[8,11–13] In fact, the presence of distant metastasis

is the only consistent factor across the literature that is predictive of overall survival. AJCC Level of Evidence: I

Preoperative Calcium at Diagnosis

Defined as highest preoperative serum calcium level at diagnosis before the first operation. Few studies evaluated preoperative calcium as a predictive factor, and the results are conflicting.[5,7,8,11] Likewise, these analyses were limited because of small sample sizes. AJCC Level of Evidence: III

Preoperative PTH Level

Defined as highest PTH level at diagnosis before the first operation. Two studies concluded that preoperative PTH is not predictive of survival, but both were limited because of small sample sizes.[7,8] Recorded PTH level should include the assay's limits of normal. AJCC Level of Evidence: III

Lymphovascular Invasion

Defined as tumor in a lymphatic or vascular space. Few studies evaluated this factor as a prognostic variable, and the results are conflicting.[7,8] AJCC Level of Evidence: III

Mitotic Rate

Defined as the number of mitoses per high-power field.2 Few institutions have evaluated the number of mitotic figures as a prognostic factor, and the results are limited because of small sample sizes and a lack of uniform methodology for assessment.[7,8,33] AJCC Level of Evidence: III

Weight of Primary Tumor

Defined as the weight of the primary tumor in milligrams. AJCC Level of Evidence: IV

Cytonuclear Grade

Defined as low grade or high grade. Only a few studies attempted to define grade in parathyroid carcinomas.[33,34] Few data exist regarding grade as a prognostic factor for parathyroid cancer.[14] AJCC Level of Evidence: III

Time to Recurrence

Defined as time after first operation to time of first recurrence in months. There is not sufficient evidence that time to recurrence is predictive of outcome.[11] AJCC Level of Evidence: III

RISK ASSESSMENT MODELS

The AJCC recently established guidelines that will be used to evaluate published statistical prediction models for the purpose of granting endorsement for clinical use.[35] Although this is a monumental step toward the goal of precision medicine, this work was published only very recently. Therefore, the existing models that have been published or may be in clinical use have not yet been evaluated for this cancer site by the Precision Medicine Core of the AJCC. In the future, the statistical prediction models for this cancer site will be evaluated, and those that meet all AJCC criteria will be endorsed.

DEFINITIONS OF AJCC TNM

Definition of Primary Tumor (T)

T Category	T Criteria
TX	Primary tumor cannot be assessed
T0	No evidence of primary tumor
Tis	Atypical parathyroid neoplasm (neoplasm of uncertain malignant potential)*
T1	Localized to the parathyroid gland with extension limited to soft tissue
T2	Direct invasion into the thyroid gland
T3	Direct invasion into recurrent laryngeal nerve, esophagus, trachea, skeletal muscle, adjacent lymph nodes, or thymus
T4	Direct invasion into major blood vessel or spine

*Defined as tumors that are histologically or clinically worrisome but do not fulfill the more robust criteria (i.e., invasion, metastasis) for carcinoma. They generally include tumors that have two or more concerning features, such as fibrous bands, mitotic figures, necrosis, trabecular growth, or adherence to surrounding tissues intraoperatively.[31,36] Atypical parathyroid neoplasms usually have a smaller dimension, weight, and volume than carcinomas and are less likely to have coagulative tumor necrosis.[10]

Definition of Regional Lymph Node (N)

N Category	N Criteria
NX	Regional nodes cannot be assessed
N0	No regional lymph node metastasis
N1	Regional lymph node metastasis
N1a	Metastasis to level VI (pretracheal, paratracheal, and prelaryngeal/Delphian lymph nodes) or superior mediastinal lymph nodes (level VII)
N1b	Metastasis to unilateral, bilateral, or contralateral cervical (level I, II, III, IV, or V) or retropharyngeal nodes

Definition of Distant Metastasis (M)

M Category	M Criteria
M0	No distant metastasis
M1	Distant metastasis

AJCC PROGNOSTIC STAGE GROUPS

There are not enough data to propose anatomic stage and prognostic groups for parathyroid carcinoma.

REGISTRY DATA COLLECTION VARIABLES

1. Age at diagnosis
2. Gender
3. Race
4. Size of primary tumor in millimeters
5. Location of primary tumor: left or right and superior (upper) or inferior (lower)
6. Invasion into surrounding tissue (present or absent)
7. Distant metastasis
8. Number of lymph nodes removed (by level)
9. Number of lymph nodes positive (by level)
10. Highest preoperative calcium (number in tenths in milligrams per deciliter [e.g., 11.5 mg/dL])
11. Highest preoperative PTH (whole number in picograms per milliliter [e.g., 350 pg/mL])
12. Lymphovascular invasion (present or absent)
13. Grade (LG or HG)
14. Weight of primary tumor (in milligrams)
15. Mitotic rate
16. Time to recurrence (months)

HISTOLOGIC GRADE (G)

Cytonuclear grade is defined as low grade or high grade. Low-grade tumors consist of round monomorphic nuclei with only mild to moderate nuclear size variation, indistinct nucleoli, and chromatin characteristics resembling those of normal parathyroid or of adenoma. High-grade tumors have more pleomorphism, with a nuclear size variation greater than 4:1; prominent nuclear membrane irregularities; chromatin alterations, including hyperchromasia or margination of chromatin; and prominent nucleoli. Unlike the random "endocrine atypia" seen even in normal parathyroid glands, high-grade tumors show several discrete confluent areas with nuclear changes.

G	G Definition
LG	Low grade: round monomorphic nuclei with only mild to moderate nuclear size variation, indistinct nucleoli, and chromatin characteristics resembling those of normal parathyroid or of adenoma.
HG	High grade: more pleomorphism, with a nuclear size variation greater than 4:1; prominent nuclear membrane irregularities; chromatin alterations, including hyperchromasia or margination of chromatin; and prominent nucleoli. High-grade tumors show several discrete confluent areas with nuclear changes.

HISTOPATHOLOGIC TYPE

Tumor cells may be arranged in trabecular, sheet-like, or rosette-like patterns. Occasionally the nodular structures have central calcifications with necrosis. Nuclear morphology varies between pleomorphism with clumped chromatin and enlarged nucleoli. Proof of vascular invasion or invasion into adjacent organs is a defining hallmark. Terms that may be used include invasive parathyroid neoplasm, neoplasm of undetermined malignant significance, parathyroid neoplasm with locally invasive or atypical features, and parathyroid neoplasm with invasion into connective tissue.

SURVIVAL DATA

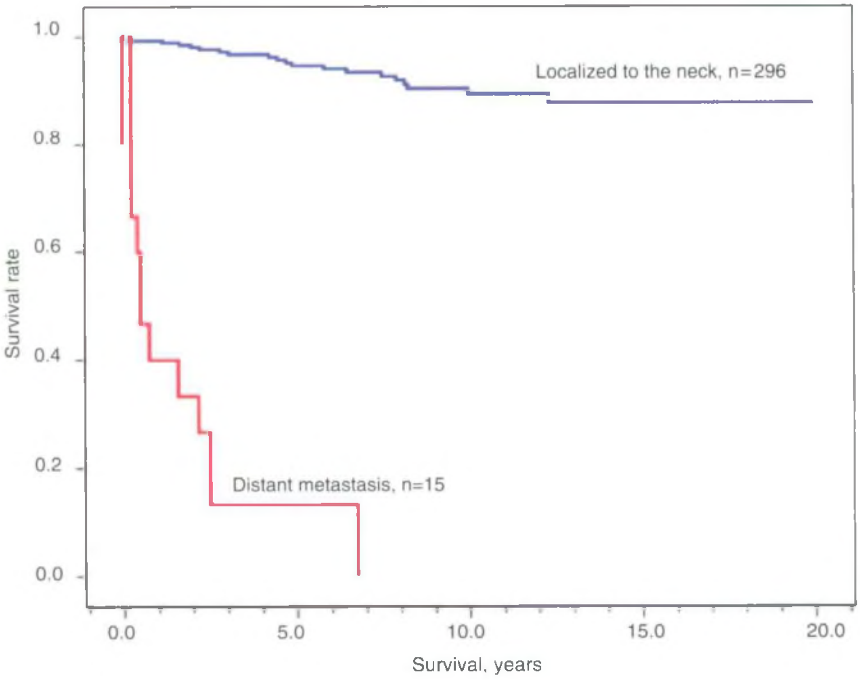

Fig. 75.3 Cancer-specific survival comparing patients with disease localized to the neck versus patients with distant metastasis. Data was obtained from the SEER program for patients diagnosed from 1973 to 2012 (patients with multiple primary tumors excluded)

Bibliography

1. Lloyd RV. *Endocrine Pathology:: Differential Diagnosis and Molecular Advances*. Springer Science & Business Media; 2010.
2. V-SR HM. Atlas of Endocrine Neoplasia. Houston: The University of Texas M.D. Anderson Cancer Center. 2006.
3. Mazeh H. Kouniavsky G. Schneider DF. et al. Intrathyroidal parathyroid glands: small, but mighty (a Napoleon phenomenon). *Surgery*. Dec 2012;152(6):1193–1200.
4. Schulte KM. Talat N. Miell J. Moniz C. Sinha P. Diaz-Cano S. Lymph node involvement and surgical approach in parathyroid cancer. *World journal of surgery*. Nov 2010;34(11):2611–2620.
5. Busaidy NL. Jimenez C. Habra MA. et al. Parathyroid carcinoma: a 22-year experience. *Head & neck*. Aug 2004;26(8):716–726.
6. Iihara M. Okamoto T. Suzuki R. et al. Functional parathyroid carcinoma: Long-term treatment outcome and risk factor analysis. *Surgery*. Dec 2007;142(6):936–943; discussion 943 e931.
7. Talat N. Schulte KM. Clinical presentation, staging and long-term evolution of parathyroid cancer. *Annals of surgical oncology*. Aug 2010;17(8):2156–2174.
8. Villar-del-Moral J. Jimenez-Garcia A. Salvador-Egea P. et al. Prognostic factors and staging systems in parathyroid cancer: a multicenter cohort study. *Surgery*. Nov 2014;156(5):1132–1144.
9. Li M. Lu H. Gao Y. FDG-anorectic parathyroid carcinoma with FDG-avid bone metastasis on PET/CT images. *Clinical nuclear medicine*. Nov 2013;38(11):916–918.
10. Quinn CE. Healy J. Lebastchi AH. et al. Modern experience with aggressive parathyroid tumors in a high-volume New England referral center. *Journal of the American College of Surgeons*. Jun 2015;220(6):1054–1062.
11. Harari A. Waring A. Fernandez-Ranvier G. et al. Parathyroid carcinoma: a 43-year outcome and survival analysis. *The Journal of clinical endocrinology and metabolism*. Dec 2011;96(12):3679–3686.
12. Hsu K-T. Sippel RS. Chen H. Schneider DF. Is central lymph node dissection necessary for parathyroid carcinoma? *Surgery*. 2014;156(6):1336–1341.
13. Lee PK. Jarosek SL. Virnig BA. Evasovich M. Tuttle TM. Trends in the incidence and treatment of parathyroid cancer in the United States. *Cancer*. May 1 2007;109(9):1736–1741.
14. Sadler C. Gow KW. Beierle EA. et al. Parathyroid carcinoma in more than 1,000 patients: A population-level analysis. *Surgery*. Dec 2014;156(6):1622–1629; discussion 1629–1630.
15. Hundahl SA. Fleming ID. Fremgen AM. Menck HR. Two hundred eighty-six cases of parathyroid carcinoma treated in the U.S. between 1985-1995: a National Cancer Data Base Report. The American College of Surgeons Commission on Cancer and the American Cancer Society. *Cancer*. Aug 1 1999;86(3):538–544.
16. Carpten JD. Robbins CM. Villablanca A. et al. HRPT2, encoding parafibromin, is mutated in hyperparathyroidism-jaw tumor syndrome. *Nature genetics*. Dec 2002;32(4):676–680.
17. Udelsman R. Akerstrom G. Biagini C. et al. The surgical management of asymptomatic primary hyperparathyroidism: proceedings of the Fourth International Workshop. *The Journal of clinical endocrinology and metabolism*. Oct 2014;99(10):3595–3606.
18. Siperstein A. Berber E. Barbosa GF. et al. Predicting the success of limited exploration for primary hyperparathyroidism using ultrasound, sestamibi, and intraoperative parathyroid hormone: analysis of 1158 cases. *Annals of surgery*. Sep 2008;248(3):420–428.
19. Udelsman R. Lin Z. Donovan P. The superiority of minimally invasive parathyroidectomy based on 1650 consecutive patients

75

with primary hyperparathyroidism. *Annals of surgery.* Mar 2011;253(3):585–591.

20. Chen H, Wang TS, Yen TW, et al. Operative failures after parathyroidectomy for hyperparathyroidism: the influence of surgical volume. *Annals of surgery.* Oct 2010;252(4):691–695.
21. Hessman O, Stalberg P, Sundin A, et al. High success rate of parathyroid reoperation may be achieved with improved localization diagnosis. *World journal of surgery.* May 2008;32(5):774–781; discussion 782–773.
22. Mortenson MM, Evans DB, Lee JE, et al. Parathyroid exploration in the reoperative neck: improved preoperative localization with 4D-computed tomography. *Journal of the American College of Surgeons.* May 2008;206(5):888–895; discussion 895–886.
23. Udelsman R, Donovan PI. Remedial parathyroid surgery: changing trends in 130 consecutive cases. *Annals of surgery.* Sep 2006;244(3): 471–479.
24. Yen TW, Wang TS, Doffek KM, Krzywda EA, Wilson SD. Reoperative parathyroidectomy: an algorithm for imaging and monitoring of intraoperative parathyroid hormone levels that results in a successful focused approach. *Surgery.* Oct 2008;144(4): 611–619; discussion 619–621.
25. Harari A, Allendorf J, Shifrin A, DiGorgi M, Inabnet WB. Negative preoperative localization leads to greater resource use in the era of minimally invasive parathyroidectomy. *American journal of surgery.* Jun 2009;197(6):769–773.
26. Lubitz CC, Hunter GJ, Hamberg LM, et al. Accuracy of 4-dimensional computed tomography in poorly localized patients with primary hyperparathyroidism. *Surgery.* Dec 2010;148(6): 1129–1137; discussion 1137–1128.
27. Rodgers SE, Hunter GJ, Hamberg LM, et al. Improved preoperative planning for directed parathyroidectomy with 4-dimensional computed tomography. *Surgery.* Dec 2006;140(6):932–940; discussion 940–931.
28. Solorzano CC, Carneiro-Pla DM, Irvin GL, 3rd. Surgeon-performed ultrasonography as the initial and only localizing study in sporadic primary hyperparathyroidism. *Journal of the American College of Surgeons.* Jan 2006;202(1):18–24.
29. Starker LF, Mahajan A, Björklund P, Sze G, Udelsman R, Carling T. 4D parathyroid CT as the initial localization study for patients with de novo primary hyperparathyroidism. *Annals of surgical oncology.* 2011;18(6):1723–1728.
30. Wang TS, Cheung K, Farrokhyar F, Roman SA, Sosa JA. Would scan, but which scan? A cost-utility analysis to optimize preoperative imaging for primary hyperparathyroidism. *Surgery.* Dec 2011;150(6):1286–1294.
31. Seethala RR OJ, Virji M. Pathology of the Parathyroid Glands. In: Barnes EL, ed. Surgical Pathology of the Head and Neck. New York. *Informa healthcare.* 2008:1429–1473.
32. Asare EA, Sturgeon C, Winchester DJ, et al. Parathyroid Carcinoma: An Update on Treatment Outcomes and Prognostic Factors from the National Cancer Data Base (NCDB). *Annals of surgical oncology.* Nov 2015;22(12):3990–3995.
33. Bondeson L, Sandelin K, Grimelius L. Histopathological variables and DNA cytometry in parathyroid carcinoma. *The American journal of surgical pathology.* Aug 1993;17(8):820–829.
34. Yip L, Seethala RR, Nikiforova MN, et al. Loss of heterozygosity of selected tumor suppressor genes in parathyroid carcinoma. *Surgery.* Dec 2008;144(6):949–955; discussion 954–945.
35. Kattan MW, Hess KR, Amin MB, et al. American Joint Committee on Cancer acceptance criteria for inclusion of risk models for individualized prognosis in the practice of precision medicine. *CA: a cancer journal for clinicians.* Jan 19 2016.
36. McCoy KL, Seethala RR, Armstrong MJ, et al. The clinical importance of parathyroid atypia: is long-term surveillance necessary? *Surgery.* Oct 2015;158(4):929–935; discussion 935–926.

Adrenal Cortical Carcinoma

76

Alexandria T. Phan, Raymon H. Grogan, Eric Rohren, and Nancy D. Perrier

CHAPTER SUMMARY

Cancers Staged Using This Staging System

Adrenal cortical carcinoma

Cancers Not Staged Using This Staging System

These histopathologic types of cancer…	Are staged according to the classification for…	And can be found in chapter…
Adrenal medullary compartment, such as pheochromocytoma	Adrenal neuroendocrine	77
Neuroblastic tumors of the adrenal gland	No AJCC staging system	N/A

Summary of Changes

Change	Details of Change	Level of Evidence
Definition of Primary Tumor (T)	To be congruent with the European Network for the Study of Adrenal Tumors (ENSAT), T4 is now defined as tumor of any size that invades surrounding organs or large vessels (renal vein or vena cava).	I
AJCC Prognostic Stage Groups	To be congruent with ENSAT, Stage III now includes T3N0–1M0 and T4N0–1M0 as well as T1–2N0–1M0. T3–4 lesions, regardless of node status, are part of the Stage III grouping.	I
AJCC Prognostic Stage Groups	To be congruent with ENSAT, the Stage IV grouping is restricted to distant metastatic disease.	I

ICD-O-3 Topography Codes

Code	Description
C74.0	Cortex of the adrenal gland

WHO Classification of Tumors

Code	Description
8370	Adrenal cortical adenocarcinoma
8370	Adrenal cortical carcinoma
8370	Adrenal cortical tumor, malignant
8290	Carcinoma, oncocytic

DeLellis RA, Lloyd RV, Heitz PU, Eng C, eds. World Health Organization Classification of Tumours Pathology and Genetics of Tumours of Endocrine Organs. Lyon: IARC; 2004.

To access the AJCC cancer staging forms, please visit www.cancerstaging.org.

© American Joint Committee on Cancer 2017

M.B. Amin et al. (eds.), *AJCC Cancer Staging Manual, Eighth Edition*, DOI 10.1007/978-3-319-40618-3_76

INTRODUCTION

The International Union Against Cancer (UICC) TNM staging system for adrenal cortical carcinoma (ACC) was first proposed in 2004. It has not changed significantly since that time, and it was adopted by the AJCC for the first time as the definition of TNM Stage Grouping for ACC in the AJCC Cancer Staging Manual, 7th Edition (7th Edition). Since the release of the 7th Edition, there were at least two large "validation" studies of the AJCC/UICC staging system. The results of these studies support the conclusion that the AJCC staging system did not adequately distinguish between Stage II and Stage III tumors. Specifically, the studies found that tumor invasion into large vessels was not accounted for in the 7th Edition, and Stage IV disease was not reserved strictly for distant metastatic disease. To address these deficiencies, changes are made in this edition. T4 is now defined as tumor of any size that invades surrounding organs or large vessels (renal vein or vena cava). Stage IV grouping is restricted to distant metastatic disease; therefore, T3 and T4 lesions, regardless of node status, are part of the Stage III grouping.

The adrenal gland may be thought of as two distinct organs embryologically and functionally: the adrenal cortex, which produces the steroid hormones aldosterone, cortisol, and testosterone, and the adrenal medulla, which produces catecholamines. Tumors of the adrenal gland remain relatively uncommon. The advancement of therapy for ACC has been limited by many barriers related to its being a rare malignancy. One of the most significant barriers to meaningful clinical research or the development of widely accepted treatment guidelines for ACC has been the lack of a common language for prognosticating the disease. A staging system for adrenal cortical cancers was first introduced in the 7th Edition. This staging system was limited to the adrenal cortex and addressed only ACC. The AJCC Cancer Staging Manual, 8th Edition staging system also includes, separately, tumors of the adrenal medullary compartment, such as pheochromocytoma. More unusual tumors, such neuroblastic tumors of the adrenal gland, which primarily are tumors of the pediatric population, are not included. The cortical staging system is based on information and data primarily from adult populations.

The previously proposed staging system used known anatomic prognostic features, such as size of the primary tumor, local invasion, and the presence or absence of invasion into adjacent organs. The AJCC Cancer Staging Manual, 8th Edition (8th Edition) now uses the presence or absence of large vessel (renal vein or vena cava) vascular invasion to further distinguish between Stage II and Stage III and restricts Stage IV to distant metastatic disease. These changes/updates are based on two large "validation" studies of the AJCC/UICC staging system performed after the release of the 7th Edition. The initial validation study was carried out in a cohort of 492 patients from the German ACC Registry in 2009, which came from the European Network for the Study of Adrenal Tumors (ENSAT) proposing the first changes. A second follow-up study to confirm the ENSAT findings was performed in 2010 by a group at the University of Montreal using a cohort of 573 cases from the Surveillance, Epidemiology, and End Results (SEER) database. Applying the ENSAT changes to the SEER cohort allowed the ability to distinguish between Stages II and III. Both these large studies had small populations of Stage I patients; therefore, there were insufficient data for further delineation of Stages I and II in the 8th Edition. This prompted a study of the National Cancer Data Base (NCDB) data to address this limitation by adding an age cutoff to Stage I and II disease. Age above and below 55 years was used as the cutoff, because this was the median age of the cohort being studied from the NCDB. The data show that using this age cutoff in Stage I and Stage II separates the two stages better. These findings have not been validated in a second large cohort. Age has long been known to be a factor in outcomes with ACC, but at this time, it remains unclear how to best use age in the staging systems. This will require future validation.

Tumor grade has been a significant part of ACC diagnosis and prognosis since the Weiss criteria were first described in the 1980s. However, although useful for diagnosis, the Weiss score is not a reliable tool for prognosis. Of the Weiss variables, mitotic count seems to have the most prognostic validity. A modification of the current TNM staging system to incorporate mitotic count was proposed, but thus far no large studies have been reported to validate mitotic count in staging for ACC. Studies were performed to evaluate the role of other characteristics, such as disease functionality, size, and Ki-67, as prognostic factors. However, similar to age, these other factors do not have a role yet in the current TNM staging system.

Overall, the changes in the 8th Edition are now consistent with the 2008 ENSAT staging system, allowing wider acceptance of the AJCC staging system for ACC. The TNM staging system remains vitally important for the current management of ACC. Because of the rarity of this malignancy, efforts on a global scale are necessary to advance knowledge, to develop relevant clinical research, and to increase meaningful therapeutic options. As a result, having a widely accepted TNM staging system is a necessary foundation of global collaboration. With an improved basic science understanding of adrenal cortical cancer, and results

of ongoing large validating studies, future versions of TNM staging will have more robust, validated prognostic factors, from both a clinical and molecular perspective. Additionally, with more advanced imaging techniques, adrenal cortical neoplasms are being discovered at much smaller limits, and often are incidentally discovered. As more information becomes available on these incidentally detected tumors, the staging system may need to be modified. Validation and publication of additional results from multi-institutional collaborative efforts and population registries are encouraged.

ANATOMY

Primary Site(s)

The adrenal glands sit in a suprarenal location (retroperitoneal) surrounded by connective tissue and a layer of adipose tissue (Fig. 76.1). They are intimately associated with the kidneys and are enclosed within the renal (Gerota's) fascia. Each gland has an outer cortex, which is lipid rich and on gross examination appears bright yellow surrounding an inner "gray-white" medullary compartment composed of chromaffin cells. There is a rich vascular supply derived from the aorta, inferior phrenic arteries, and renal arteries. Veins emerge from the hilum of the glands. The shorter right central vein drains into the inferior vena cava (IVC), and the left central vein opens into the renal vein.

Regional Lymph Nodes

Adrenal cortical tumors regionally involve the aortic (para-aortic, periaortic) and retroperitoneal basins. Nodal spread above the diaphragm is not commonly seen, but is considered distant disease extension.

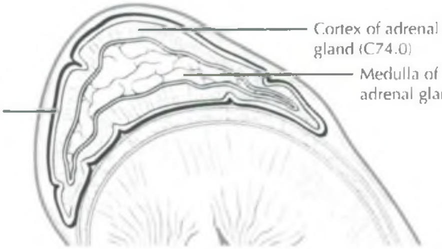

Fig. 76.1 Anatomy of the adrenal gland

Metastatic Sites

Common distant metastatic sites include the liver, lung, bone, and peritoneum.[1] Metastases to bone, brain, and skin are uncommon. Brain metastases are observed more commonly in children with ACC; they are reported very infrequently in the literature among adults.[2,3]

RULES FOR CLASSIFICATION

Clinical Classification

The classification applies only to ACC. Adenoma is excluded, as are pheochromocytoma and neuroblastic tumors. Like its previous verison, the currently proposed staging system is based on information from studies of adult ACC. ACC in the pediatric population appears to have a better prognosis overall than pathologically identical tumors in the adult population. The staging system for pediatric ACC used by most pediatric oncology groups, however, is based on the same data, and the stage of disease appears to be the most relevant prognostic factor in this group of patients.

Clinical examination and radiographic imaging are required to assess the size of the primary tumor and the extent of disease, both local and distant. Biochemical studies should be performed to evaluate the functional status of the tumor. Although functional status of ACC is important in clinical staging, affecting the management of the disease and its symptoms, at present there is not enough supporting evidence to incorporate functional status of disease into TNM stage grouping.

Regional disease involvement of adjacent organs or vasculature depends on the tumor size. Invasion of adjacent contiguous organs, such as the kidney and liver, may be seen with larger tumors and is considered T4. Typical vascular invasion or extension to the renal veins and vena cava is now considered T4, previously considered M1 in the AJCC 7th Edition.

Imaging

There are several imaging features that should increase the suspicion of ACC if an adrenal mass is encountered on imaging[1,4–6]: tumor size >4 cm, irregular tumor margins, central intratumoral necrosis or hemorrhage, heterogeneous enhancement, invasion into adjacent structures, venous extension (renal vein or IVC), and calcification.

On computed tomography (CT), ACC typically is a large heterogenous but well-defined suprarenal mass that displaces adjacent structures as it grows.[7] Adrenal lesions

76

with an attenuation value >10 Hounsfield units (HU) on unenhanced CT or an enhancement washout of <40% (by relative percentage washout at 15-minute delayed enhanced CT) and a delayed attenuation of more than 35 HU (on 10- to 15-minute delayed enhanced CT) are suspicious for malignancy, both primary and secondary.[8] However, because ACCs arise from the cortex like adenomas, they sometimes are reported to contain foci of intracellular lipid, which although more often in a patchy heterogenous distribution, might lead to their being mistaken for an adenoma on noncontrast imaging. Some metastatic lesions, including adrenal metastases from clear cell cancer and hepatocellular cancer, should be kept in mind because they also may contain intracellular lipid, mimicking adenomas. CT is of value in demonstrating the local and distant spread of ACC.[9] Preservation of fat planes around the tumor indicates that there is no local invasion. Metastases often are found at presentation; common sites of metastasis are the regional and para-aortic lymph nodes, lungs, liver, and bone.[4,10,11] Hepatic metastases typically are hypervascular and best identified on arterial phase imaging following intravenous contrast administration.

ACCs typically appear heterogeneous on both T1- and T2-weighted images because of the presence of internal hemorrhage and necrosis.[12] Enhancement following gadolinium is distinct, and washout is usually slow. Hemorrhagic byproducts, predominantly methemoglobin, may result in areas of high signal intensity within the lesion on T1-weighted images; areas of necrosis have high signal intensity on T2-weighted images.[13] Again, ACC may contain foci of intracytoplasmic lipid, which results in a loss of signal intensity on out-of-phase images, mimicking an adenoma.[12,14] Large adrenal carcinomas may invade the adrenal vein and IVC. Magnetic resonance (MR) imaging has been demonstrated to be superior to CT in delineating the presence and extent of IVC invasion.[15,16]

2-Deoxy-2-[fluorine-18]fluoro-D-glucose (^{18}F-FDG) positron emission tomography (PET)/CT is of some use in the evaluation of ACC. One large study[17] was aimed at differentiating benign from malignant adrenal tumors in 81 cancer patients by using the adrenal tumor-to-liver ratio. Using this ratio proved problematic, however; some benign adrenal lesions also demonstrate a moderate to high ^{18}F-FDG uptake, including up to 5% of adenomas as well as benign and malignant pheochromocytomas, which generally have a moderate to high ^{18}F-FDG uptake. PET is most useful in its ability to identify distant metastases, which is of particular relevance as up to one third of patients with ACC have metastatic disease at presentation.[18–20]

Chest CT, as well as CT or MR imaging of the abdomen (if not already done), usually is performed preoperatively to exclude metastases.

Standard TNM staging for ACC classifies the tumor based on tumor size (greater or less than 5 cm) as well as local invasion and/or invasion of adjacent organs. The tumors are classified further on the basis of whether there is regional or metastatic nodal involvement. The presence or absence of metastasis aids in further classification.

CT and MR imaging are useful in assessing for local extent of the tumor, as well as for nodal involvement and distant spread. As previously discussed, MR imaging is superior to CT in determining whether there is IVC invasion/involvement. ^{18}F-FDG PET is useful in assessing for nodal involvement and distant spread. The Radiology Society of North America (RSNA) advocates the use of structured reporting templates for describing adrenal masses. In terms of the primary tumor, the RSNA recommends reporting the site and size of the mass, the unenhanced appearance, the unenhanced attenuation, the parenchymal phase attenuation, and the delayed phase attenuation. Absolute washout also should be documented.

Early results from MR spectroscopy are promising. Faria et al.[21] identified 60 patients with adrenal masses. Using spectral analysis, they classified adrenal mass lesions as adenoma, pheochromocytoma, ACC, or metastasis. Further research into this area is necessary.

Another emerging modality for adrenal imaging is carbon-11 metomidate PET. Metomidate binds to adrenal 11β-hydroxylase and therefore is an excellent tool to distinguish lesions of adrenocortical origin from other lesions.[22–27] It is of particular use in characterizing potential metastatic disease in ACC; however, it currently is not widely available in clinical practice.

Pathological Classification

Pathological staging requires the use of all information obtained in the clinical staging, as well as histologic study of the surgically resected specimen. The surgeon's description of gross tumor extension also must be included. ACCs usually are large and generally weigh more than 750 g.[1] However, some carcinomas with proven metastases may weigh less than 50 g. Tumor size covers a wide range, from 3 to 40 cm.[1,5,6] The gross appearance of the tumor shows varying degrees of nodularity, and the color may vary from yellow-tan to pink depending on the lipid content. Foci of necrosis, hemorrhage, and calcification may be seen grossly depending on the tumor size. Invasion of adjacent contiguous organs, such as the kidney and liver, may be seen with larger tumors and are important features for staging. Specifically, a tumor of any size that invades adjacent organs (kidney, diaphragm, pancreas, spleen, or liver) or large blood vessels (renal vein or vena cava) now is defined as T4.

The microscopic patterns of the carcinoma may vary from solid to alveolar and trabecular, and more commonly a mixture of these patterns is present in large tumors. Areas of necrosis are common in large tumors. Pseudoglandular spindle cell and pseudopapillary patterns of growth may be present. Some tumors may be associated with myxoid features, and recent studies suggest that myxoid adrenocortical tumors are usually malignant.[7] Mitotic activity usually is prominent and is a useful feature for grading these carcinomas.[8] Oncocytic ACCs are uncommon, and the criteria for malignancy of these lesions are somewhat different from those for conventional ACCs.[9] The criteria for diagnosis of ACCs in pediatric patients is somehat different from those for tumors in adults.[11,12] Some authors propose using designations of low risk, intermediate risk, and high risk for pediatric adrenal cortical tumors, because histologic features of malignancy and clinical behavior are more difficult to correlate than with adrenal cortical tumors in adults.[12]

PROGNOSTIC FACTORS

Prognostic Factors Required for Stage Grouping

Beyond the factors used to assign T, N, or M categories, no additional prognostic factors are required for stage grouping.

Additional Factors Recommended for Clinical Care

In ACC, disease-free and overall survival rates appear to correlate strongly with stage grouping based on TNM classification. Evidence supporting stage as prognostic comes mainly from two large validation studies: the German ACC Registry of 492 patients and the Montreal SEER analysis of 573 patients.

Many physicians also consider other factors to be important modifiers of stage and use them in medical decision making; however, these factors are not part of TNM because of inconclusive data. These factors include:

- Age at diagnosis (>50 or <50) in adults with Stage I or Stage II disease; AJCC Level of Evidence II
- Tumor grade (based on mitotic counts per 50 high-power fields [HPF]) in adults with Stage I or Stage II disease; AJCC Level of Evidence II
- Functional status; AJCC Level of Evidence II
- Size of primary tumor (measured in millimeters); AJCC Level of Evidence II

Additional factors have not yet become standard of practice for medical decision making but have sufficient evidence to support their consideration as physicians make plans for treatment.

RISK ASSESSMENT MODELS

The AJCC recently established guidelines that will be used to evaluate published statistical prediction models for the purpose of granting endorsement for clinical use. Although this is a monumental step toward the goal of precision medicine, this work was published only very recently. For this reason, the existing models that have been published or may be in clinical use have not yet been evaluated for this cancer site by the Precision Medicine Core of the AJCC. In the future, the statistical prediction models for this cancer site will be evaluated, and those that meet all AJCC criteria will be endorsed.

DEFINITIONS OF AJCC TNM

Definition of Primary Tumor (T)

T Category	T Criteria
TX	Primary tumor cannot be assessed
T0	No evidence of primary tumor
T1	Tumor ≤5 cm in greatest dimension, no extra-adrenal invasion
T2	Tumor >5 cm, no extra-adrenal invasion
T3	Tumor of any size with local invasion but not invading adjacent organs
T4	Tumor of any size that invades adjacent organs (kidney, diaphragm, pancreas, spleen, or liver) or large blood vessels (renal vein or vena cava)

Definition of Regional Lymph Node (N)

N Category	N Criteria
NX	Regional lymph nodes cannot be assessed
N0	No regional lymph node metastasis
N1	Metastasis in regional lymph node(s)

Definition of Distant Metastasis (M)

M Category	M Criteria
M0	No distant metastasis
M1	Distant metastasis

AJCC PROGNOSTIC STAGE GROUPS

When T is…	And N is…	And M is…	Then the stage group is…
T1	N0	M0	I
T2	N0	M0	II
T1	N1	M0	III
T2	N1	M0	III
T3	Any N	M0	III
T4	Any N	M0	III
Any T	Any N	M1	IV

REGISTRY DATA COLLECTION VARIABLES

1. Tumor weight in grams
2. Vascular invasion
3. Mitotic count
4. Ki-67 proliferative indext
5. Weiss score

HISTOLOGIC GRADE (G)

G	G Definition
LG	Low grade (≤20 mitoses per 50 HPF)
HG	High grade (>20 mitosis per 50 HPF); *TP53* or *CTNNB* mutation

HISTOPATHOLOGIC TYPE

The most common histopathologic variant of ACC is called the oncocytic variant, because the predominant cell type in this variant is an oncocyte, which is defined as a cell with abundant granular cytoplasm related to accumulation of mitochondria and endoplasmic reticulum. The other significant ACC variant is called the myxoid variety because of the production of abundant extracellular myxoid substances. Finally, sarcomotoid ACCs (carcinosarcomas) also have been described as they have for most other carcinoma types. The development of a sarcomatoid histology, although rare, generally portends aggressive tumor behavior. Mixed histology is always more aggressive.

ILLUSTRATIONS

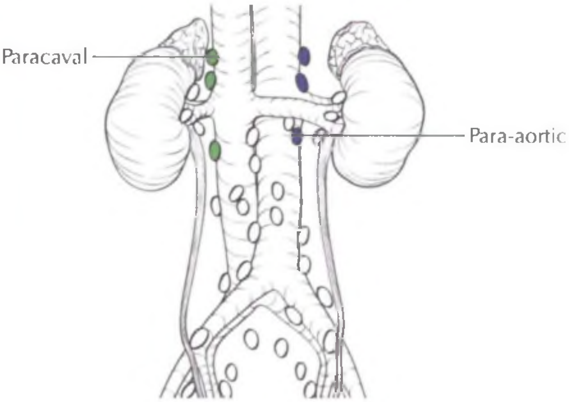

Fig. 76.2 Regional lymph nodes of the adrenal gland

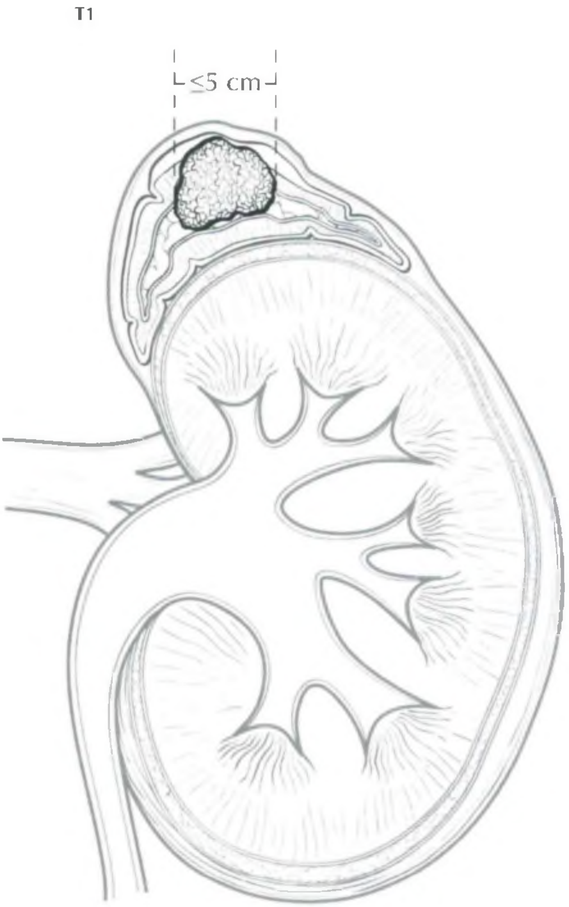

Fig. 76.3 T1 is defined as tumor ≤5 cm in greatest dimension, no extra-adrenal invasion

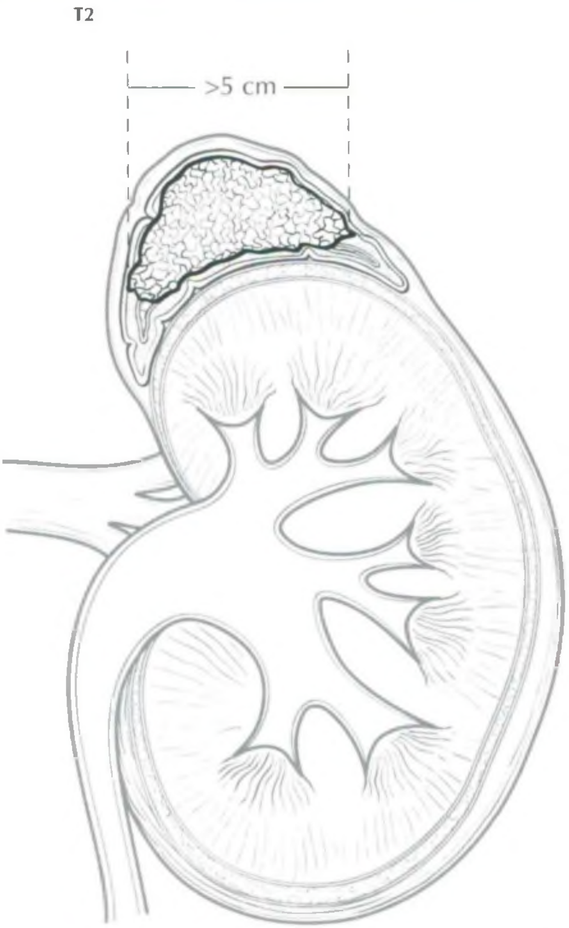

Fig. 76.4 T2 is defined as tumor >5 cm, no extra-adrenal invasion

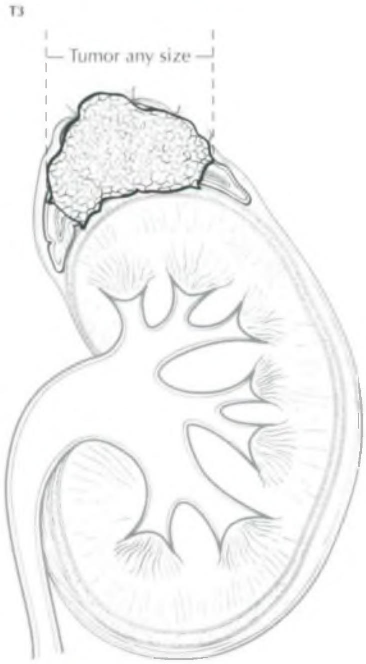

Fig. 76.5 T3 is defined as tumor of any size with local invasion but not invading adjacent organs

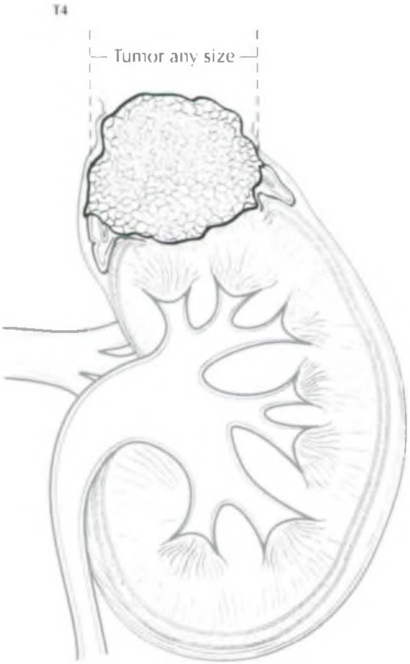

Fig. 76.6 T4 is defined as tumor of any size that invades adjacent organs (kidney, diaphragm, pancreas, spleen, or liver) or large blood vessels (renal vein or vena cava)

N1

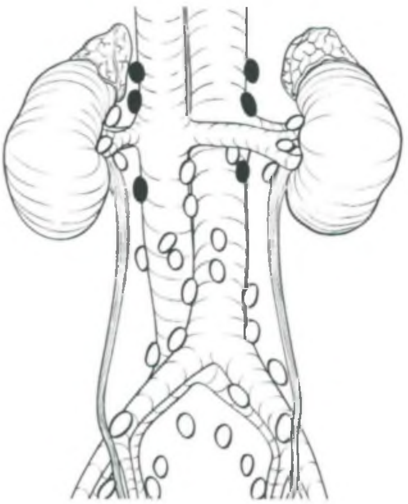

Fig. 76.7 N1 is defined as involvement of regional lymph nodes

Bibliography

1. Hussain S, Belldegrun A, Seltzer SE, Richie JP, Gittes RF, Abrams HL. Differentiation of malignant from benign adrenal masses: predictive indices on computed tomography. *AJR. American journal of roentgenology.* Jan 1985;144(1):61–65.
2. Gulack BC, Rialon KL, Englum BR, et al. Factors associated with survival in pediatric adrenocortical carcinoma: An analysis of the National Cancer Data Base (NCDB). *Journal of pediatric surgery.* Jan 2016;51(1):172–177.
3. McAteer JP, Huaco JA, Gow KW. Predictors of survival in pediatric adrenocortical carcinoma: a Surveillance, Epidemiology, and End Results (SEER) program study. *Journal of pediatric surgery.* May 2013;48(5):1025–1031.
4. Reznek RH, Narayanan P. Primary adrenal malignancy. *Husband & Reznek's Imaging in Oncology.* 3 ed. London, UK: Informa Healthcare; 2010:280–298.
5. Ilias I, Sahdev A, Reznek RH, Grossman AB, Pacak K. The optimal imaging of adrenal tumours: a comparison of different methods. *Endocrine-related cancer.* Sep 2007;14(3):587–599.
6. Rockall AG, Babar SA, Sohaib SA, et al. CT and MR imaging of the adrenal glands in ACTH-independent cushing syndrome. *Radiographics : a review publication of the Radiological Society of North America, Inc.* Mar-Apr 2004;24(2):435–452.
7. Fishman EK, Deutch BM, Hartman DS, Goldman SM, Zerhouni EA, Siegelman SS. Primary adrenocortical carcinoma: CT evaluation with clinical correlation. *AJR. American journal of roentgenology.* Mar 1987;148(3):531–535.
8. Lee MJ, Hahn PF, Papanicolaou N, et al. Benign and malignant adrenal masses: CT distinction with attenuation coefficients, size, and observer analysis. *Radiology.* May 1991;179(2):415–418.
9. Bharwani N, Rockall AG, Sahdev A, et al. Adrenocortical carcinoma: the range of appearances on CT and MRI. *AJR. American journal of roentgenology.* Jun 2011;196(6):W706–714.
10. Nagase LL, Semelka RC, Armao D. Adrenal glands. In: Semelka RC, ed. *Abdominal-pelvic MRI.* New York, NY: Wiley-Liss; 2002: 695–740.
11. Ng L, Libertino JM. Adrenocortical carcinoma: diagnosis, evaluation and treatment. *J Urol.* Jan 2003;169(1):5–11.
12. Schlund JF, Kenney PJ, Brown ED, Ascher SM, Brown JJ, Semelka RC. Adrenocortical carcinoma: MR imaging appearance with current techniques. *Journal of magnetic resonance imaging : JMRI.* Mar-Apr 1995;5(2):171–174.
13. Elsayes KM, Mukundan G, Narra VR, et al. Adrenal masses: mr imaging features with pathologic correlation. *Radiographics : a review publication of the Radiological Society of North America, Inc.* Oct 2004;24 Suppl 1:S73–86.
14. Mackay B, el-Naggar A, Ordonez NG. Ultrastructure of adrenal cortical carcinoma. *Ultrastruct Pathol.* Jan-Apr 1994;18(1–2): 181–190.
15. Hricak H, Amparo E, Fisher MR, Crooks L, Higgins CB. Abdominal venous system: assessment using MR. *Radiology.* Aug 1985; 156(2):415–422.
16. Soler R, Rodriguez E, Lopez MF, Marini M. MR imaging in inferior vena cava thrombosis. *Eur J Radiol.* Jan 1995;19(2):101–107.
17. Caoili EM, Korobkin M, Brown RK, Mackie G, Shulkin BL. Differentiating adrenal adenomas from nonadenomas using (18)F-FDG PET/CT: quantitative and qualitative evaluation. *Academic radiology.* Apr 2007;14(4):468–475.
18. Nader S, Hickey RC, Sellin RV, Samaan NA. Adrenal cortical carcinoma. A study of 77 cases. *Cancer.* Aug 15 1983;52(4):707–711.
19. Luton JP, Cerdas S, Billaud L, et al. Clinical features of adrenocortical carcinoma, prognostic factors, and the effect of mitotane therapy. *N Engl J Med.* Apr 26 1990;322(17):1195–1201.
20. Fassnacht M, Kenn W, Allolio B. Adrenal tumors: how to establish malignancy? *Journal of endocrinological investigation.* 2004;27(4): 387–399.
21. Faria JF, Goldman SM, Szejnfeld J, et al. Adrenal masses: characterization with in vivo proton MR spectroscopy—initial experience. *Radiology.* Dec 2007;245(3):788–797.
22. Allolio B, Fassnacht M. Clinical review: Adrenocortical carcinoma: clinical update. *The Journal of clinical endocrinology and metabolism.* Jun 2006;91(6):2027–2037.
23. Bergstrom M, Juhlin C, Bonasera TA, et al. PET imaging of adrenal cortical tumors with the 11beta-hydroxylase tracer 11C-metomidate. *Journal of nuclear medicine:official publication, Society of Nuclear Medicine.* Feb 2000;41(2):275–282.
24. Eriksson B, Bergstrom M, Sundin A, et al. The role of PET in localization of neuroendocrine and adrenocortical tumors. *Ann N Y Acad Sci.* Sep 2002;970:159–169.
25. Khan TS, Sundin A, Juhlin C, Langstrom B, Bergstrom M, Eriksson B. 11C-metomidate PET imaging of adrenocortical cancer. *European journal of nuclear medicine and molecular imaging.* Mar 2003;30(3):403–410.
26. Minn H, Salonen A, Friberg J, et al. Imaging of adrenal incidentalomas with PET using (11)C-metomidate and (18)F-FDG. *Journal of nuclear medicine:official publication, Society of Nuclear Medicine.* Jun 2004;45(6):972–979.
27. Zettinig G, Mitterhauser M, Wadsak W, et al. Positron emission tomography imaging of adrenal masses: (18)F-fluorodeoxyglucose and the 11beta-hydroxylase tracer (11)C-metomidate. *European journal of nuclear medicine and molecular imaging.* Sep 2004; 31(9):1224–1230.

Adrenal – Neuroendocrine Tumors

77

Camilo Jimenez, Steven K. Libutti, Christine S. Landry, Ricardo V. Lloyd, Rana R. McKay, Eric Rohren, Raja R. Seethala, Tracy S. Wang, Herbert Chen, and Nancy D. Perrier

CHAPTER SUMMARY

Cancers Staged Using This Staging System

Pheochromocytoma and paraganglioma

Cancers Not Staged Using This Staging System

These histopathologic types of cancer…	Are staged according to the classification for…	And can be found in chapter…
Neuroendocrine tumor of the pancreas	Neuroendocrine tumors of the pancreas	34
Carotid body tumors	Not staged	N/A

Summary of Changes

This is a new chapter for the AJCC Cancer Staging Manual.

ICD-O-3 Topography Codes

Code	Description
C74.1	Adrenal medulla
C75.5	Aortic body and other paraganglioma

WHO Classification of Tumors

Code	Description
8680	Paraganglioma, malignant
8693	Extra-adrenal paraganglioma, malignant
8693	Nonchromaffin paraganglioma, malignant
8700	Pheochromocytoma, malignant
8700	Adrenal medullary paraganglioma, malignant

DeLellis RA, Lloyd RV, Heitz PU, Eng C, eds. World Health Organization Classification of Tumours Pathology and Genetics of Tumours of Endocrine Organs. Lyon: IARC; 2004.

INTRODUCTION

Pheochromocytomas (PHs) and paragangliomas (PGs) are rare neuroendocrine tumors originating in the paraganglia. The paraganglia is a group of neuroendocrine cells that during embryonic life migrate to give origin to the different components of the autonomous nervous system. PHs originate in the adrenal medulla and are sympathetic tumors. PGs may originate either in parasympathetic or sympathetic autonomous nervous system ganglia.

Sympathetic PGs (SPGs) and PHs frequently secrete catecholamines, such as noradrenaline and/or adrenaline, predisposing to cardiovascular disease, gastrointestinal complications, and other endocrine problems. Unlike SPGs, many PHs secrete adrenaline. Approximately 30% of PH/PGs have a hereditary predisposition. At the end of the 20th century, several hereditary

To access the AJCC cancer staging forms, please visit www.cancerstaging.org.

© American Joint Committee on Cancer 2017

M.B. Amin et al. (eds.), *AJCC Cancer Staging Manual, Eighth Edition*, DOI 10.1007/978-3-319-40618-3_77

disorders with a relatively obvious phenotype were identified as predisposing diseases for PH/PGs, including von Hippel–Lindau (VHL) disease, multiple endocrine neoplasia type 2 syndrome (MEN2A and B), and neurofibromatosis type 1 (NF1).[1] At the beginning of the 21st century, new germline mutations were identified in the genes that code for the different subunits of the mitochondrial enzymatic complex 2 or succinate dehydrogenase enzyme. These mutations predispose to PH/PGs, kidney cancer, and gastrointestinal stromal tumors. These syndromes are called paraganglioma syndromes 1 through 4. Most recently, mutations in the fumarase, malate dehydrogenase, *MAX*, and *TMEM127* genes also were associated with the development of rare hereditary PH/PGs.[2–4]

Malignant PH/PGs account for 14–17% of PH/PGs.[5] The incidence of malignant PH/PGs is less than 1 per million people per year, and malignant PH/PGs account for less than 1% of all endocrine tumors. Unlike most other tumors, no molecular or histologic markers exist for determining whether a PH/PG is malignant. Vascular invasion, mitotic activity, cellular atypia, and even local recurrence without soft tissue or lymph node tumor involvement cannot be used to definitively identify and differentiate tumors with metastatic potential.[6]

The current TNM means of staging patients with PH/PG is challenging because currently it is impossible to differentiate benign from malignant tumors from a histologic perspective, and because there are no molecular, biochemical, or genetic markers that can absolutely predict risk of distant spread. Nevertheless, TNM staging may help in the follow-up and treatment of patients with PH/PG; therefore, this staging system should be based on the recognition of clinical predictors of metastases and survival in the context of metastatic disease.

ANATOMY

Primary Site(s)

The adrenal glands are in a retroperitoneal position above the kidneys and are surrounded by connective and adipose tissues. These glands are enclosed within the renal (Gerota's) fascia. Each gland has an outer cortex that is lipid rich and an inner component, known as the adrenal medulla, composed of chromaffin cells. These glands are very vascular organs; their vascular supply is derived from the aorta and the renal and inferior phrenic arteries. Veins emerge from the hilus of the glands. The shorter right central vein drains into the inferior vena cava, and the left central vein drains into the renal vein.

The name *pheochromocytoma* is given to tumors arising from the adrenal medulla. *Paragangliomas* are tumors arising from the autonomic nervous system ganglia (paraganglia outside the adrenal medulla). PGs may occur in the head, neck, chest, abdomen, or pelvis.

PGs of the head and neck areas are classified as parasympathetic PGs (PPGs); PGs located in the thorax, abdomen, and pelvis usually are SPGs. PPGs almost never secrete catecholamines. These tumors may be locally invasive; however, they rarely develop metastases. SPGs frequently secrete catecholamines, such as dopamine and/or noradrenaline, leading to hormonal syndromes characterized by cardiovascular, gastrointestinal, and constitutional symptoms. They do not secrete adrenaline. Occasionally, these tumors do not secrete catecholamines, and their clinical manifestations are derived from location and tumor burden. Currently, malignancy is defined only by the presence of metastasis.

Regional Lymph Nodes

PG/PHs can metastasize to locoregional lymph nodes. For abdominal and pelvic PGs, the regional lymph nodes are aortic (para-aortic, periaortic) and retroperitoneal. For thoracic PGs, regional lymph nodes are usually located in the posterior mediastinum.

Metastatic Sites

The most common locations of metastases are the lymph nodes (80%), skeleton (72%), liver (50%), and lungs (50%).[7] The liver, pancreas, and kidneys may be infiltrated because of adjacent vicinity.[8] Patients occasionally have presented with metastases in the skin or breasts. Malignant potential appears to be associated with the tumor genotype. Malignant tumors are rare in patients with MEN2 and PGL3 and have not yet been described in patients with *TMEM127* mutations and PGL2.[9–11] Approximately 5% of patients with PH/PG associated with VHL or NF1 present with metastases.[1] Metastatic PH/PG may occur in 3% of patients with PGL1.[12] Conversely, up to 50% of PH/PG patients with PGL4 may have metastatic disease.[13]

RULES FOR CLASSIFICATION

Clinical Classification

Patients who present with a PH larger than 5 cm, a SPG, and mutations in the succinate dehydrogenase subunit B gene (*SDHB*) should be suspected of harboring a malignant PH/PG.[5] From a clinical perspective, the hormonal manifestations are similar to those observed in patients with benign tumors (e.g., hypertension, throbbing headaches, palpitations, diaphoresis). Constipation has been observed more

often in patients with malignant disease.[14] Some patients with malignant PH/PGs associated with an excessive secretion of catecholamines have minimal or no symptoms related to the excessive hormonal secretion.

Preoperative biopsy of patients with suspected PH/PGs is not recommended because of the increased risk of a catecholamine crisis, tumor rupture, and seeding.[15]

Evaluation of biochemical function is critical if there is clinical suspicion of the disease. In fact, the presence of plasma or urinary fractionated metanephrine concentrations higher than three times the upper limit of normal suggests the diagnosis of these tumors before they are removed.[16] The final diagnosis usually is assigned once the primary tumor has been removed.

The prognostic significance of regional lymph node involvement is unclear. There are no current studies addressing this issue. Until more is known about the prognostic significance of regional lymph node metastases, data pertaining to the extent of lymph node dissection, number of lymph nodes removed, and number of lymph nodes with metastatic disease should be recorded. Malignant tumors may not be associated with locoregional lymph node metastases.[7]

Imaging

Both computed tomography (CT) and magnetic resonance (MR) imaging provide useful information for assessing PH and PG, and either may be an appropriate first imaging test. MR provides T2-weighted imaging information on adrenal and extra-adrenal masses and does not involve the use of iodinated contrast media. Abdominal CT scanning has an accuracy of 85–95% for detecting adrenal masses with a spatial resolution of 1 cm or greater but is less accurate for lesions smaller than 1 cm. Differentiating an adenoma from a PH is difficult using CT scanning. Although most PHs have CT attenuation of greater than 10 Hounsfield units (HU), they very rarely contain sufficient intracellular fat to have an attenuation less than 10 HU.[17] Although it has been thought that the use of intravenous contrast poses a risk of inducing hypertensive crisis in patients with PH, a controlled prospective study in patients receiving low-osmolar CT scan contrast[18] and a retrospective review in patients who received nonionic contrast[19] concluded that the use of intravenous contrast is safe, even in patients not receiving α- or β-blockers. When contrast is given, PHs usually are more hypervascular than adenomas on early postcontrast imaging. Approximately one third show washout-like adenomas on delayed-washout analysis.

MR imaging is preferred for detection of PH in children and in pregnant or lactating women. MR imaging has a reported sensitivity of up to 100% in detecting adrenal PHs, does not require contrast, and does not expose the patient to ionizing radiation. MR imaging also is superior to CT scanning for detecting PGs. In approximately 70% of cases, PHs appear hyperintense on T2-weighted images because of their high water content.[20] Similar to what is observed with CT, the tumor is hypervascular post contrast, unless it has undergone mass degeneration. Even in such cases, the more peripheral nondegenerated regions of the mass usually continue to enhance.

Imaging with iodine-123 (^{123}I)–labeled metaiodobenzylguanidine (MIBG) is reserved for cases in which a PH is confirmed biochemically but CT scanning or MR imaging does not demonstrate a tumor. MIBG is a substrate for the norepinephrine transporter and concentrates within adrenal or extra-adrenal PHs. MIBG scanning frequently is used in cases of familial PH syndromes, recurrent PH, or malignant PH. Estimates of the sensitivity and specificity of ^{123}I-MIBG vary widely; the reported sensitivity ranges from 53–94% and the specificity from 82–92%.[21–23]

Positron emission tomography (PET) scanning with 18-fluoro-2-deoxyglucose (^{18}F-FDG) has been demonstrated to detect occult PHs. ^{18}F-FDG is selectively concentrated as part of the abnormal metabolism of many neoplasms. PHs usually show increased uptake on FDG-PET scanning.[24]

Usually, CT or MR is the initial imaging modality of choice. MIBG imaging is considered if there is concern regarding metastases/multifocality or if the diagnosis of PH/PG is unclear. MR angiography/MR venography is performed if there is concern regarding vascular invasion.

The Radiology Society of North America (RSNA) advocates the use of structured reporting templates for describing adrenal masses (http://www.radreport.org). In terms of the primary tumor, the recommendation includes reporting the site and size of the mass, the unenhanced appearance, the unenhanced attenuation, the parenchymal phase attenuation, and the delayed phase attenuation. Absolute washout also should be documented.

CT and MR are useful in assessing for local extent of the tumor, as well as nodal involvement and distant spread. As previously mentioned, MR imaging is superior to CT in assessing for extra-adrenal PH. Dedicated adrenal CT is excellent for adrenal medulla tumors; MIBG imaging may also be of benefit for this indication. ^{18}F-FDG PET is useful in assessing for nodal involvement and distant spread.

In terms of emerging imaging modalities, there are several promising options. Regarding scintigraphy, impressive results to date have been observed with 6-[^{18}F]-fluorodopamine (FDOPA) PET scanning.[22] Studies suggest that scans performed with this radioisotope are extremely useful in the detection and localization of PHs. Further results from studies with this agent are eagerly awaited.

Recent studies demonstrated high diagnostic accuracy with gallium-68 DOTA,1-Nal(3)-octreotide (^{68}Ga-DOTANOC) PET/CT in patients in whom PH is suspected.[26,27] A study of 62 patients found that ^{68}Ga-DOTANOC PET/CT was superior to ^{131}I-MIBG imaging for this purpose. The best results of

^{68}Ga-DOTANOC PET/CT were seen in patients with MEN2-associated and malignant PH.[26]

Initial studies suggest that MR spectroscopy may be used to distinguish PHs from other adrenal masses.[28,29] Specifically, a resonance signature of 6.8 ppm appears to be unique to PHs; the signature apparently is attributable to the catecholamines and catecholamine metabolites present in PHs.[29]

Pathological Classification

Pathological classification of PH and extra-adrenal PG requires total tumor removal. If adjacent tissue is involved, extirpation should be complete. Tumors may vary in color from gray-white to pink-tan, with foci of congestion.[30] Larger tumors may contain areas of fibrosis or may show focal cystic degeneration. Familial PHs are usually bilateral and multicentric, and the adjacent medulla may appear hyperplastic. Tumors are composed of medium-sized to large polygonal cells, which may be arranged in various trabecular or solid patterns. The nuclei are usually round to ovoid. Large tumors may show hemorrhage and necrosis, and the stroma may show myxoid change. In some cases, amyloid may be present in the stroma. Foci of capsular and vascular invasion may be observed, but these features do not correlate with malignant behavior.

Immunohistochemical stains are usually positive for chromogranin A and synaptophysin. S-100 protein usually stains the sustentacular cells, although these cells may be decreased or lost in malignant tumors. The diagnosis of malignant PH or PG is confirmed in the presence of metastatic disease. Recent studies used morphologic and biochemical features to assist in the diagnosis.[31,32] These systems include the Pheochromocytoma of the Adrenal Gland Scaled Score (PASS)[31] and the Grading System for Adrenal Pheochromocytomas and Paragangliomas (GAPP system) from Japan,[32] which incorporates Ki-67 labeling index and epinephrine and norepinephrine secretion into the factors needed to make a diagnosis. However, these systems are difficult to reproduce by different pathologists who study the same tumor sample. Therefore, they cannot be used to confirm the diagnosis of malignancy.

PROGNOSTIC FACTORS

Prognostic Factors Required for Stage Grouping

Beyond the factors used to assign T, N, or M categories, no additional prognostic factors are required for stage grouping.

Additional Factors Recommended for Clinical Care

Clinical Predictors of Malignancy and Overall Survival

Many patients with malignant PH/SPGs present with apparently benign tumors (no evidence of metastasis at the time of diagnosis); however, some of these patients will later develop metastatic disease (metachronous metastasis).[5] Historically speaking, most of these patients have not had adequate follow-up; consequently, by the time malignancy is recognized most have extensive, unresectable disease. It is then important to recognize clinical predictors of metastasis to determine which patients need long-term follow-up so that metastatic recurrence could be identified early and treated, preventing further spread and/or complications. Age, gender, and histologic or biochemical characteristics do not predict the risk of metastases in patients with PH/PGS.[5] Currently, there are only three well-recognized clinical predictors of metastasis: 1) primary tumor size, 2) primary tumor location (adrenal vs. extra-adrenal), and 3) germline mutations of *SDHB*.[5,13]

Primary Tumor Size

The size of the primary tumor is defined as the measurement of the longest axis of the primary tumor in millimeters. Retrospective studies evaluated size as a prognostic factor. A PH larger than 5 cm is associated with an increased risk of metastasis and shorter overall survival (OS). Metastatic disease in PHs smaller than 5 cm, although possible, is uncommon (<5% of cases).[5,33] AJCC Level of Evidence: II

Primary Tumor Location

A PG located in the abdominal (e.g., Zuckerkandl organ, para-aortic, and perirenal), pelvic (bladder), and thoracic cavities are frequently malignant, and metastatic disease may be observed in 40–70% of cases. Although in most metastatic SPGs, the primary tumor is larger than 5 cm, in up to 20% of cases, the primary tumor is smaller than 5 cm, and distant metastases have been described in patients with tumors as small as 1 cm. An extra-adrenal location is associated with twice the risk of death from disease compared with a primary tumor larger than 5 cm, making an extra-adrenal location a stronger predictor of aggressiveness, metastasis, and decreased survival than primary tumor size. Nevertheless, metastatic PHs and SPGs have similar OS, suggesting that their oncopathogenesis overlaps.[5] (Fig. 77.1A and B). AJCC Level of Evidence: II

SDHB Mutations

Metastatic disease and decreased OS are also observed in approximately 50% of patients with PH/SPG associated with *SDHB* mutations.[13] Although most *SDHB* tumors are SPGs,

several metastatic SPGs are not associated with *SDHB* mutations. Therefore, the higher prevalence of malignancy in SPG cannot be explained in every case by an association between the genetic background and tumor site alone.[8] AJCC Level of Evidence: III

A retrospective study with a limited population of patients compared the OS of patients with metastatic *SDHB* PH/PGs with that of patients with sporadic metastic tumors. The study suggested that *SDHB* carriers may have a lower OS[34] (Fig. 77.2). AJCC Level of Evidence: III

Prognostic Factors at the Time of Diagnosis of Metastases

The natural history of metastatic PH/SPG has not been described yet. As for most well-differentiated neuroendocrine tumors, the prognosis of metastatic tumors is characterized by their heterogeneity. The consequences of excess catecholamine on blood pressure increase, cardiovascular function, and other organ physiology (e.g., gastrointestinal tract) as well as the tumor extent should be actively characterized. Indeed, reported causes of death in these patients include hypertensive crisis, cardiac dysfunction, colonic obstruction, and/or tumor burden and progression. Only four studies looked for prognostic parameters in patients with metastatic disease.

Tumor Burden versus Catecholamine Secretion

Timmers et al.[35] evaluated the impact of excessive catecholamine secretion and the tumor extent on clinical outcome in patients with PH/SPG. Although the excessive secretion of catecholamines was associated with substantial morbidity, the tumor burden was the main determinant of survival. This study, however, did not clearly define tumor burden. AJCC Level of Evidence: III

Extent of Invasion of Primary Tumor

No studies have addressed whether the extent of invasion of the primary tumor affects overall prognosis. AJCC Level of Evidence: IV

Lymph Node Metastasis

No studies have evaluated whether the presence of lymph node metastases might predict outcome. AJCC Level of Evidence: IV

Distant Metastases

Distant metastasis is defined as evidence of disease in organs in which chromaffin cells are not supposed to exist, including the liver, lungs, and bones. Several studies demonstrated that the presence of distant metastases portends worse survival. In fact, only 60% of patients with distant metastases are alive 5 years after initial diagnosis.[8] AJCC Level of Evidence: I

Timing of Metastases: Synchronous versus Metachronous

Ayala-Ramirez et al.[5] found that approximately 50% of patients with malignant PH/SPG present with metastasis ≥6 months after the initial time of diagnosis and/or resection of the primary tumor (metachronous metastasis). As expected, timing of metastasis is an important determinant of prognosis, as patients with metachronous metastasis exhibit better OS than those with synchronous metastasis (diagnosis of metastases at the time of or <6 months after diagnosis of the primary tumor). AJCC Level of Evidence: I

Location of Distant Metastases

In another study by Ayala-Ramirez et al.,[7] patients who manifested malignancy exclusively with skeletal metastasis (20%) exhibited a significantly longer OS that those with or without skeletal metastasis but with metastasis to other organs, such as the liver and lungs (12 years vs. 5 years vs. 7.5 years, respectively; log-rank test p value = 0.005; Fig. 77.3). AJCC Level of Evidence: II

RISK ASSESSMENT MODELS

The AJCC recently established guidelines that will be used to evaluate published statistical prediction models for the purpose of granting endorsement for clinical use.[36] Although this is a monumental step toward the goal of precision medicine, this work was published only very recently. Therefore, the existing models that have been published or may be in clinical use have not yet been evaluated for this cancer site by the Precision Medicine Core of the AJCC. In the future, the statistical prediction models for this cancer site will be evaluated, and those that meet all AJCC criteria will be endorsed.

DEFINITIONS OF AJCC TNM

Definition of Primary Tumor (T)

T Category	T Criteria
TX	Primary tumor cannot be assessed
T1	PH <5 cm in greatest dimension, no extra-adrenal invasion
T2	PH ≥ 5 cm or PG-sympathetic of any size, no extra-adrenal invasion
T3	Tumor of any size with invasion into surrounding tissues (e.g., liver, pancreas, spleen, kidneys)

PH: within adrenal gland
PG Sympathetic: functional
PG Parasympathetic: nonfunctional, usually in the head and neck region
Note: Parasympathetic Paragaglioma are not staged because they are largely benign.

Definition of Regional Lymph Node (N)

N Category	N Criteria
NX	Regional lymph nodes cannot be assessed
N0	No lymph node metastasis
N1	Regional lymph node metastasis

Definition of Distant Metastasis (M)

M Category	M Criteria
M0	No distant metastasis
M1	Distant metastasis
M1a	Distant metastasis to only bone
M1b	Distant metastasis to only distant lymph nodes/liver or lung
M1c	Distant metastasis to bone plus multiple other sites

AJCC PROGNOSTIC STAGE GROUPS

Pheochromocytoma/Sympathetic Paraganglioma

When T is...	And N is...	And M is...	Then the stage group is...
T1	N0	M0	I
T2	N0	M0	II
T1	N1	M0	III
T2	N1	M0	III
T3	Any N	M0	III
Any T	Any N	M1	IV

REGISTRY DATA COLLECTION VARIABLES

1. Primary tumor size (measured in centimeters)
2. Primary tumor location: PH, PG (specific location: e.g., aortic bifurcation, mediastinum)
3. Regional lymph node metastases
4. Location of distant metastases
5. Hormonal function: 24-hour urinary fractionated metanephrines/plasma metanephrines
6. Chromogranin A
7. Mitotic count
8. Germline mutation status
9. Plasma methoxytyramine

HISTOLOGIC GRADE (G)

There is no recommended histologic grading system at this time.

Definition of Regional Lymph Node (N)

N Category	N Criteria
NX	Regional lymph nodes cannot be assessed
N0	No lymph node metastasis
N1	Regional lymph node metastasis

Definition of Distant Metastasis (M)

M Category	M Criteria
M0	No distant metastasis
M1	Distant metastasis
M1a	Distant metastasis to only bone
M1b	Distant metastasis to only distant lymph nodes/liver or lung
M1c	Distant metastasis to bone plus multiple other sites

AJCC PROGNOSTIC STAGE GROUPS

Pheochromocytoma/Sympathetic Paraganglioma

When T is...	And N is...	And M is...	Then the stage group is...
T1	N0	M0	I
T2	N0	M0	II
T1	N1	M0	III
T2	N1	M0	III
T3	Any N	M0	III
Any T	Any N	M1	IV

REGISTRY DATA COLLECTION VARIABLES

1. Primary tumor size (measured in centimeters)
2. Primary tumor location: PH, PG (specific location: e.g., aortic bifurcation, mediastinum)
3. Regional lymph node metastases
4. Location of distant metastases
5. Hormonal function: 24-hour urinary fractionated metanephrines/plasma metanephrines
6. Chromogranin A
7. Mitotic count
8. Germline mutation status
9. Plasma methoxytyramine

HISTOLOGIC GRADE (G)

There is no recommended histologic grading system at this time.

SURVIVAL DATA

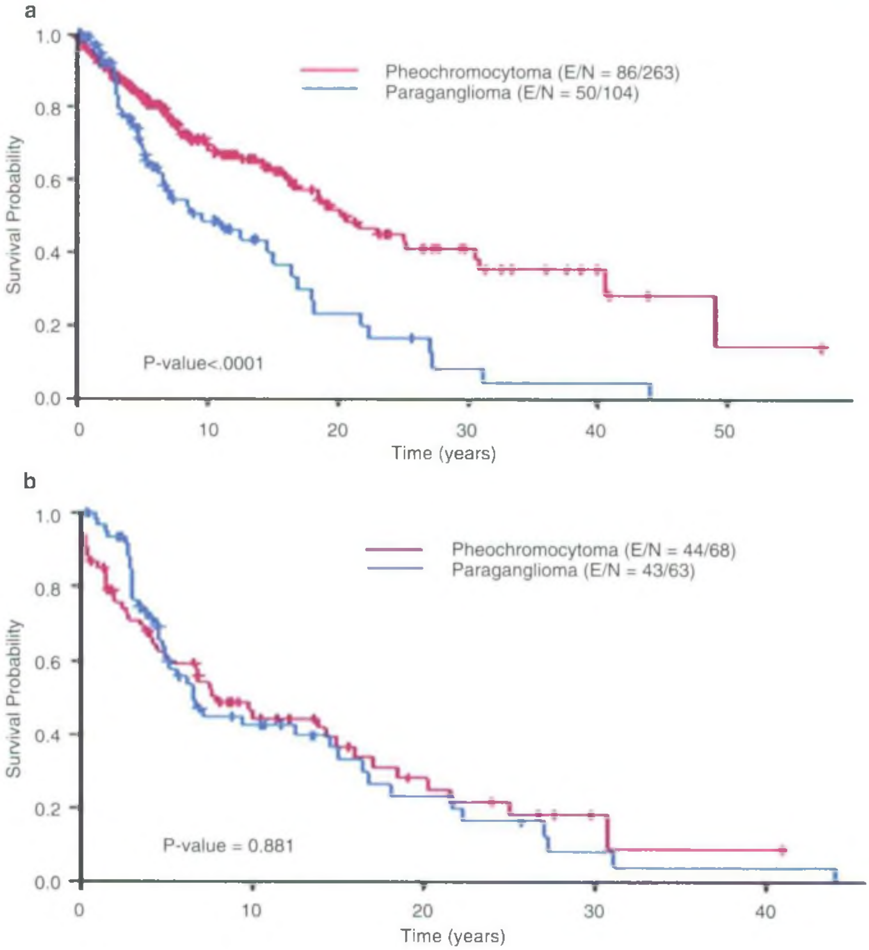

Fig. 77.1 A. Overall survival in patients with PHs and SPGs. B. Overall survival in patients with metastatic PHs and SPGs. E/N. number of events/total number of patients. From Ayala-Ramirez et. al. 2011 with permission

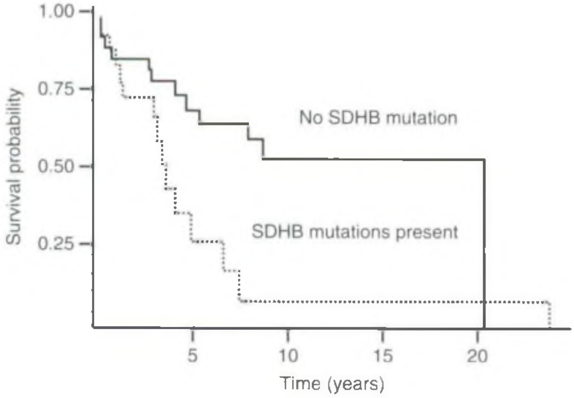

Fig. 77.2 Probability of survival according to mutation status. From Amar et. al. 2007 with permission

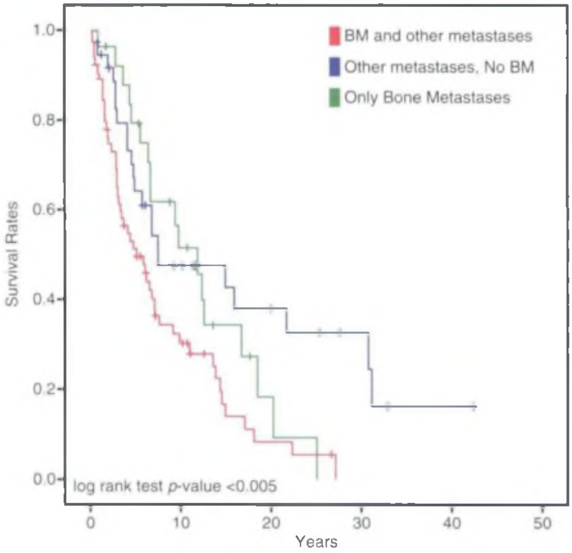

Fig. 77.3 Median OS in patients with bone metastases (BM) and other metastases, those without BM, and those with only BM. From Ayala-Ramirez et. al. 2013 with permission

Bibliography

1. Jiménez C, Cote G, Arnold A, Gagel RF. Should patients with apparently sporadic pheochromocytomas or paragangliomas be screened for hereditary syndromes? *The Journal of Clinical Endocrinology & Metabolism*. 2006;91(8):2851–2858.
2. Dahia PL. Pheochromocytoma and paraganglioma pathogenesis: learning from genetic heterogeneity. *Nat Rev Cancer*. Feb 2014;14(2):108–119.
3. Cascón A, Comino-Méndez I, Currás-Freixes M, et al. Whole-Exome Sequencing Identifies MDH2 as a New Familial Paraganglioma Gene. *Journal of the National Cancer Institute*. 2015;107(5):djv053.
4. Favier J, Amar L, Gimenez-Roqueplo AP. Paraganglioma and phaeochromocytoma: from genetics to personalized medicine. *Nature reviews. Endocrinology*. Feb 2015;11(2):101–111.
5. Ayala-Ramirez M, Feng L, Johnson MM, et al. Clinical risk factors for malignancy and overall survival in patients with pheochromocytomas and sympathetic paragangliomas: primary tumor size and primary tumor location as prognostic indicators. *The Journal of clinical endocrinology and metabolism*. Mar 2011;96(3):717–725.
6. Wu D, Tischler AS, Lloyd RV, et al. Observer variation in the application of the Pheochromocytoma of the Adrenal Gland Scaled Score. *The American journal of surgical pathology*. Apr 2009;33(4):599–608.
7. Ayala-Ramirez M, Palmer JL, Hofmann MC, et al. Bone metastases and skeletal-related events in patients with malignant pheochromocytoma and sympathetic paraganglioma. *The Journal of clinical endocrinology and metabolism*. Apr 2013;98(4):1492–1497.
8. Jimenez C, Rohren E, Habra MA, et al. Current and future treatments for malignant pheochromocytoma and sympathetic paraganglioma. *Curr Oncol Rep*. Aug 2013;15(4):356–371.
9. Thosani S, Ayala-Ramirez M, Palmer L, et al. The characterization of pheochromocytoma and its impact on overall survival in multiple endocrine neoplasia type 2. *The Journal of Clinical Endocrinology & Metabolism*. 2013;98(11):E1813–E1819.
10. Rich T, Jackson M, Roman-Gonzalez A, Shah K, Cote GJ, Jimenez C. Metastatic sympathetic paraganglioma in a patient with loss of the SDHC gene. *Fam Cancer*. Dec 2015;14(4):615–619.
11. Toledo SP, Lourenco DM, Jr., Sekiya T, et al. Penetrance and clinical features of pheochromocytoma in a six-generation family carrying a germline TMEM127 mutation. *The Journal of clinical endocrinology and metabolism*. Feb 2015;100(2):E308–318.
12. Timmers HJ, Pacak K, Bertherat J, et al. Mutations associated with succinate dehydrogenase D-related malignant paragangliomas. *Clin Endocrinol (Oxf)*. Apr 2008;68(4):561–566.
13. Amar L, Bertherat J, Baudin E, et al. Genetic testing in pheochromocytoma or functional paraganglioma. *J Clin Oncol*. Dec 1 2005;23(34):8812–8818.
14. Thosani S, Ayala-Ramirez M, Roman-Gonzalez A, et al. Constipation: an overlooked, unmanaged symptom of patients with pheochromocytoma and sympathetic paraganglioma. *European journal of endocrinology / European Federation of Endocrine Societies*. Sep 2015;173(3):377–387.
15. Rafat C, Zinzindohoue F, Hernigou A, et al. Peritoneal implantation of pheochromocytoma following tumor capsule rupture during surgery. *The Journal of clinical endocrinology and metabolism*. Dec 2014;99(12):E2681–2685.
16. Lenders JW, Eisenhofer G, Mannelli M, Pacak K. Phaeochromocytoma. *Lancet*. Aug 20-26 2005;366(9486):665–675.
17. Szolar DH, Korobkin M, Reittner P, et al. Adrenocortical carcinomas and adrenal pheochromocytomas: mass and enhancement loss evaluation at delayed contrast-enhanced CT. *Radiology*. Feb 2005;234(2):479–485.
18. Baid SK, Lai EW, Wesley RA, et al. Brief communication: radiographic contrast infusion and catecholamine release in patients with pheochromocytoma. *Annals of internal medicine*. Jan 6 2009;150(1):27–32.
19. Bessell-Browne R, O'Malley ME. CT of pheochromocytoma and paraganglioma: risk of adverse events with i.v. administration of nonionic contrast material. *AJR. American journal of roentgenology*. Apr 2007;188(4):970–974.

20. Blake MA, Kalra MK, Maher MM, et al. Pheochromocytoma: an imaging chameleon. *Radiographics:a review publication of the Radiological Society of North America, Inc*. Oct 2004;24 Suppl 1:S87–99.
21. Wiseman GA, Pacak K, O'Dorisio MS, et al. Usefulness of 123I-MIBG scintigraphy in the evaluation of patients with known or suspected primary or metastatic pheochromocytoma or paraganglioma: results from a prospective multicenter trial. *Journal of nuclear medicine:official publication, Society of Nuclear Medicine*. Sep 2009;50(9):1448–1454.
22. Fottner C, Helisch A, Anlauf M, et al. 6-18F-fluoro-L-dihydroxyphenylalanine positron emission tomography is superior to 123I-metaiodobenzyl-guanidine scintigraphy in the detection of extraadrenal and hereditary pheochromocytomas and paragangliomas: correlation with vesicular monoamine transporter expression. *The Journal of clinical endocrinology and metabolism*. Jun 2010; 95(6):2800–2810.
23. Jacobson AF, Deng H, Lombard J, Lessig HJ, Black RR. 123I-meta-iodobenzylguanidine scintigraphy for the detection of neuroblastoma and pheochromocytoma: results of a meta-analysis. *The Journal of clinical endocrinology and metabolism*. Jun 2010;95(6):2596–2606.
24. Taieb D, Sebag F, Barlier A, et al. 18F-FDG avidity of pheochromocytomas and paragangliomas: a new molecular imaging signature? *Journal of nuclear medicine : official publication, Society of Nuclear Medicine*. May 2009;50(5):711–717.
25. Yamamoto S, Hellman P, Wassberg C, Sundin A. 11C-hydroxyephedrine positron emission tomography imaging of pheochromocytoma: a single center experience over 11 years. *The Journal of clinical endocrinology and metabolism*. Jul 2012; 97(7):2423–2432.
26. Sharma P, Dhull VS, Arora S, et al. Diagnostic accuracy of (68) Ga-DOTANOC PET/CT imaging in pheochromocytoma. *European journal of nuclear medicine and molecular imaging*. Mar 2014; 41(3):494–504.
27. Naswa N, Sharma P, Nazar AH, et al. Prospective evaluation of (6) (8)Ga-DOTA-NOC PET-CT in phaeochromocytoma and paraganglioma: preliminary results from a single centre study. *European radiology*. Mar 2012;22(3):710–719.
28. Faria JF, Goldman SM, Szejnfeld J, et al. Adrenal masses: characterization with in vivo proton MR spectroscopy--initial experience. *Radiology*. Dec 2007;245(3):788–797.
29. Kim S, Salibi N, Hardie AD, et al. Characterization of adrenal pheochromocytoma using respiratory-triggered proton MR spectroscopy: initial experience. *AJR. American journal of roentgenology*. Feb 2009;192(2):450–454.
30. DeLellis RA. *Pathology and genetics of tumours of endocrine organs*. Vol 8: IARC; 2004.
31. Thompson LD. Pheochromocytoma of the Adrenal gland Scaled Score (PASS) to separate benign from malignant neoplasms: a clinicopathologic and immunophenotypic study of 100 cases. *The American journal of surgical pathology*. May 2002;26(5): 551–566.
32. Kimura N, Takayanagi R, Takizawa N, et al. Pathological grading for predicting metastasis in phaeochromocytoma and paraganglioma. *Endocrine-related cancer*. Jun 2014;21(3):405–414.
33. Plouin PF, Fitzgerald P, Rich T, et al. Metastatic pheochromocytoma and paraganglioma: focus on therapeutics. *Hormone and metabolic research = Hormon- und Stoffwechselforschung = Hormones et metabolisme*. May 2012;44(5):390–399.
34. Amar L, Baudin E, Burnichon N, et al. Succinate dehydrogenase B gene mutations predict survival in patients with malignant pheochromocytomas or paragangliomas. *The Journal of clinical endocrinology and metabolism*. Oct 2007;92(10):3822–3828.
35. Timmers H, Brouwers F, Hermus A, et al. Metastases but not cardiovascular mortality reduces life expectancy following surgical resection of apparently benign pheochromocytoma. *Endocrine-related cancer*. 2008;15(4):1127–1133.
36. Kattan MW, Hess KR, Amin MB, et al. American Joint Committee on Cancer acceptance criteria for inclusion of risk models for individualized prognosis in the practice of precision medicine. *CA: a cancer journal for clinicians*. Jan 19 2016.

Part XVIII

Hematologic Malignancies

Members of the Hematologic Malignancies Expert Panel

Ranjana H. Advani, MD
Serhan Alkan, MD
Daniel A. Arber, MD
P. Leif Bergsagel, MD
William L. Carroll, MD
Elihu H. Estey, MD
Mary Gospodarowicz, MD, FRCPC, FRCR(Hon) – UICC Representative
Michael M. Graham, MD, PhD
Nathan C. Hall, MD, PhD
Nancy Lee Harris, MD
Richard T. Hoppe, MD
Elaine S. Jaffe, MD – Vice Chair
Joseph Khoury, MD – CAP Representative
Ola Landgren, MD, PhD
John P. Leonard, MD – Chair
Michael P. Link, MD
Soheil Meshinchi, MD
Attilio Orazi, MD
Pat Pekatos – Data Collection Core Representative
LoAnn C. Peterson, MD
Jerald P. Radich, MD
Vincent Rajkumar, MD
Steven T. Rosen, MD
Richard L. Schilsky, MD, FACP, FASCO – Editorial Board Liaison
Andrew D. Zelenetz, MD, PhD

Introduction to Hematologic Malignancies

78

INTRODUCTION

Hematologic malignancies are a diverse group of disorders. These malignancies share derivation from B cells, T cells, and NK cells, but they vary widely in presentation, clinical course, and response to therapy. The incidence of hematologic malignancies is significant and increasing. Non-Hodgkin lymphomas occur in more than 71,000 new individuals each year and have been increasing in incidence over the past several decades. Hodgkin lymphoma occurs in approximately 9,000 new individuals each year in the United States, and its incidence generally is stable. Approximately 27,000 new cases of multiple myeloma and more than 20,000 new cases of lymphoid leukemia occur annually in the United States.[1]

PATHOLOGY

Hematologic malignancies include Hodgkin lymphoma (Hodgkin disease), non-Hodgkin lymphoma, plasma cell myeloma (formerly referred to as multiple myeloma), and leukemia. An arbitrary distinction between lymphoma and leukemia often is artificial, as many lymphoid neoplasms may have circulating neoplastic cells. However, significant differences exist between lymphoid neoplasms derived from mature lymphoid cells, most often considered lymphoma, and neoplasms derived from precursor lymphoid cells, which frequently are leukemic and considered with the acute leukemias. Plasma cell neoplasms, including plasma cell myeloma and plasmacytoma, are derived from terminally differentiated B cells and therefore are included in the classification of lymphoid neoplasms.

To access the AJCC cancer staging forms, please visit www.cancerstaging.org.

© American Joint Committee on Cancer 2017

M.B. Amin et al. (eds.), *AJCC Cancer Staging Manual, Eighth Edition*, DOI 10.1007/978-3-319-40618-3_78

Table 78.1 Updated World Health Organization classification of tumors of the hematopoietic and lymphoid tissues (2016)[2,3]

**Myeloproliferative neoplasms**
Chronic myeloid leukemia, *BCR-ABL1* positive
Chronic neutrophilic leukemia
Polycythemia vera
Primary myelofibrosis
Essential thrombocythemia
Chronic eosinophilic leukemia, not otherwise specified (NOS)
Myeloproliferative neoplasm, unclassifiable
**Mastocytosis**
Cutaneous mastocytosis
Systemic mastocytosis
Mast cell sarcoma
**Myeloid/lymphoid neoplasms with eosinophilia and rearrangements of *PDGFRA*, *PDGFRB*, or *FGFR1*, or with *PCM1-JAK2***
Myeloid/lymphoid neoplasms with *PDGFRA* rearrangement
Myeloid/lymphoid neoplasms with *PDGFRB* rearrangement
Myeloid/lymphoid neoplasms with *FGFR1* rearrangement
Myeloid/lymphoid neoplasms with PCM1-JAK2
**Myelodysplastic/myeloproliferative neoplasms**
Chronic myelomonocytic leukemia
Chronic myelomonocytic leukemia 0
Chronic myelomonocytic leukemia 1
Chronic myelomonocytic leukemia 2
Atypical chronic myeloid leukemia, *BCR-ABL1* negative
Juvenile myelomonocytic leukemia
Myelodysplastic/myeloproliferative neoplasm with ring sideroblasts and thrombocytosis
Myelodysplastic/myeloproliferative neoplasm, unclassifiable
**Myelodysplastic syndromes/neoplasms**
Myelodysplastic syndrome with single-lineage dysplasia
Myelodysplastic syndrome with ring sideroblasts
Myelodysplastic syndrome with ring sideroblasts and single-lineage dysplasia
Myelodysplastic syndrome with ring sideroblasts and multilineage dysplasia
Myelodysplastic syndrome with multilineage dysplasia
Myelodysplastic syndrome with excess blasts
Myelodysplastic syndrome with excess blasts 1
Myelodysplastic syndrome with excess blasts 2
Myelodysplastic syndrome with isolated del(5q)
Myelodysplastic syndrome, unclassifiable
Childhood myelodysplastic syndrome
Refractory cytopenia of childhood

Table 78.1 (continued)

**Familial myelodysplastic syndrome/acute myeloid leukemia and related predisposition syndromes**
**Acute myeloid leukemia and related precursor neoplasms**
Acute myeloid leukemia with recurrent genetic abnormalities
Acute myeloid leukemia with t(8;21)(q22;q22.1); *RUNX1-RUNX1T1*
Acute myeloid leukemia with inv(16)(p13.1q22) or t(16;16)(p13.1;q22); *CBFB-MYH11*
Acute promyelocytic leukemia with *PML-RARA*
Acute myeloid leukemia with t(9;11)(p21.3;q23.3); *KMT2A/MLL-MLLT3*
Acute myeloid leukemia with t(6;9)(p23;q34.1); *DEK-NUP214*
Acute myeloid leukemia with inv(3)(q21.3q26.2) or (3;3)(q21.3;q26.2); *GATA2, MECOM (EVI1)*
Acute myeloid leukemia (megakaryoblastic) with t(1;22)(p13.3;q13.1); *RBM15-MKL1*
Acute myeloid leukemia with BCR-ABL1
Acute myeloid leukemia with gene mutations
Acute myeloid leukemia with mutated *NPM1*
Acute myeloid leukemia with biallelic mutations of *CEBPA*
Acute myeloid leukemia with mutated RUNX1
Acute myeloid leukemia with myelodysplasia-related changes
Therapy-related myeloid neoplasms
Acute myeloid leukemia, NOS
Acute myeloid leukemia with minimal differentiation
Acute myeloid leukemia without maturation
Acute myeloid leukemia with maturation
Acute myelomonocytic leukemia
Acute monoblastic and monocytic leukemia
Acute erythroid leukemia
Acute megakaryoblastic leukemia
Acute basophilic leukemia
Acute panmyelosis with myelofibrosis
Myeloid sarcoma
Myeloid proliferations related to Down syndrome
Transient abnormal myelopoiesis associated with Down syndrome
Myeloid leukemia associated with Down syndrome
**Blastic plasmacytoid dendritic cell neoplasm**
**Acute leukemias of ambiguous lineage**
Acute undifferentiated leukemia
Mixed phenotype acute leukemia with t(9;22)(q34.1;q11.2); *BCR-ABL1*
Mixed phenotype acute leukemia with t(v;11q23.3); *KMT2A* rearranged
Mixed phenotype acute leukemia, B/myeloid, NOS
Mixed phenotype acute leukemia, T/myeloid, NOS
Mixed phenotype acute leukemia, NOS: rare types
Acute leukemias of ambiguous lineage, NOS

(continued)

Table 78.1 (continued)

**Precursor lymphoid neoplasms**
B-lymphoblastic leukemia/lymphoma, NOS
B-lymphoblastic leukemia/lymphoma with recurrent genetic abnormalities
B-lymphoblastic leukemia/lymphoma with t(9;22)(q34.1;q11.2); *BCR-ABL1*
B-lymphoblastic leukemia/lymphoma with t(v;11q23.3); *KMT2A* rearranged
B-lymphoblastic leukemia/lymphoma with t(12;21)(p13.2;q22.1); *ETV6-RUNX*
B-lymphoblastic leukemia/lymphoma with hyperdiploidy
B-lymphoblastic leukemia/lymphoma with hypodiploidy (hypodiploid ALL)
B-lymphoblastic leukemia/lymphoma with t(5;14)(q31.1;q32.1); *IGH/IL3*
B-lymphoblastic leukemia/lymphoma with t(1;19)(q23;p13.3); *TCF3-PBX*
B-lymphoblastic leukemia/lymphoma, BCR-ABL1-like
B-lymphoblastic leukemia/lymphoma with iAMP21
T-lymphoblastic leukemia/lymphoma
Early T-cell precursor acute lymphoblastic leukemia
Natural killer (NK) cell lymphoblastic leukemia/lymphoma
**Mature B-cell neoplasms**
Chronic lymphocytic leukemia/small lymphocytic lymphoma
Monoclonal B-cell lymphocytosis
B-cell prolymphocytic leukemia
Splenic marginal zone lymphoma
Hairy cell leukemia
Splenic B-cell lymphoma/leukemia, unclassifiable
Splenic diffuse red pulp small B-cell lymphoma
Hairy cell leukemia–variant
Lymphoplasmacytic lymphoma
Waldenström macroglobulinemia
Monoclonal gammopathy of undetermined significance (MGUS), IgM
Mu heavy chain disease
Gamma heavy chain disease
Alpha heavy chain disease
MGUS, IgG/A
Plasma cell myeloma
Solitary plasmacytoma of bone
Extraosseous plasmacytoma
Monoclonal immunoglobulin deposition diseases
Extranodal marginal zone lymphoma of mucosa-associated lymphoid tissue (MALT lymphoma)
Nodal marginal zone lymphoma
Pediatric nodal marginal zone lymphoma
Follicular lymphoma
in situ follicular neoplasia
Duodenal-type follicular lymphoma
Pediatric-type follicular lymphoma
Large B-cell lymphoma with IRF4 rearrangement
Primary cutaneous follicle center lymphoma
Mantle cell lymphoma
in situ mantle cell neoplasia
Diffuse large B-cell lymphoma (DLBCL), NOS
Germinal center B-cell type
Activated B-cell type
T-cell/histiocyte-rich large B-cell lymphoma
Primary DLBCL of the central nervous system
Primary cutaneous DLBCL, leg type
Epstein–Barr virus (EBV)-positive DLBCL, NOS
EBV-positive mucocutaneous ulcer
DLBCL associated with chronic inflammation
Lymphomatoid granulomatosis
Primary mediastinal (thymic) large B-cell lymphoma
Intravascular large B-cell lymphoma
Anaplastic lymphoma kinase (ALK)-positive large B-cell lymphoma
Plasmablastic lymphoma
Primary effusion lymphoma
HHV8-positive DLBCL, NOS
Burkitt lymphoma
Burkitt-like lymphoma with 11q aberration
High-grade B-cell lymphoma, with *MYC* and *BCL2* and/or *BCL6* rearrangements
High-grade B-cell lymphoma, NOS
B-cell lymphoma, unclassifiable, with features intermediate between DLBCL and classical Hodgkin lymphoma

Table 78.1 (continued)

**Mature T and NK neoplasms**
T-cell prolymphocytic leukemia
T-cell large granular lymphocytic leukemia
Chronic lymphoproliferative disorder of NK cells
Aggressive NK-cell leukemia
Systemic EBV-positive T-cell lymphoma of childhood
Hydroa vacciniforme-like lymphoproliferative disorder
Adult T-cell leukemia/lymphoma
Extranodal NK/T-cell lymphoma, nasal type
Enteropathy-associated T-cell lymphoma
Monomorphic epitheliotropic intestinal T-cell lymphoma
Indolent T-cell lymphoproliferative disorder of the gastrointestinal tract
Hepatosplenic T-cell lymphoma
Subcutaneous panniculitis-like T-cell lymphoma
Mycosis fungoides
Sézary syndrome
Primary cutaneous CD30-positive T-cell lymphoproliferative disorders
Lymphomatoid papulosis
Primary cutaneous anaplastic large cell lymphoma
Primary cutaneous gamma-delta T-cell lymphoma
Primary cutaneous CD8-positive aggressive epidermotropic cytotoxic T-cell lymphoma
Primary cutaneous acral CD8-positive T-cell lymphoma
Primary cutaneous CD4-positive small/medium T-cell lymphoproliferative disorder
Peripheral T-cell lymphoma, NOS
Angioimmunoblastic T-cell lymphoma
Follicular T-cell lymphoma
Nodal peripheral T-cell lymphoma with T follicular helper (TFH) phenotype
Anaplastic large cell lymphoma, ALK positive
Anaplastic large cell lymphoma, ALK negative
Breast implant–associated anaplastic large cell lymphoma
**Hodgkin lymphoma**
Nodular lymphocyte-predominant Hodgkin lymphoma
Classical Hodgkin lymphoma
Nodular sclerosis classical Hodgkin lymphoma
Lymphocyte-rich classical Hodgkin lymphoma
Mixed-cellularity classical Hodgkin lymphoma
Lymphocyte-depleted classical Hodgkin lymphoma
**Posttransplant lymphoproliferative disorders (PTLDs)**
Plasmacytic hyperplasia PTLD
Infectious mononucleosis PTLD
Florid follicular hyperplasia PTLD
Polymorphic PTLD
Monomorphic PTLD (B- and T/NK-cell types)
Classical Hodgkin lymphoma PTLD
**Histiocytic and dendritic cell neoplasms**
Histiocytic sarcoma
Langerhans cell histiocytosis
Langerhans cell sarcoma
Indeterminate dendritic cell tumor
Interdigitating dendritic cell sarcoma
Follicular dendritic cell sarcoma
Fibroblastic reticular cell tumor
Disseminated juvenile xanthogranuloma
Erdheim–Chester disease

Note: Provisional entities are listed in italics.

Bibliography

1. Siegel RL, Miller KD, Jemal A. Cancer statistics, 2015. *CA: a cancer journal for clinicians*. 2015;65(1):5–29.
2. Swerdlow SH, Campo E, Pileri SA, et al. The 2016 revision of the World Health Organization (WHO) classification of lymphoid neoplasms. *Blood*. Mar 15 2016.
3. Arber DA, Orazi A, Hasserjian RP. The 2016 revision to the World Health Organization (WHO) classification of myeloid neoplasms and acute leukemia. *Blood*. 2016.

Hodgkin and Non-Hodgkin Lymphomas

79

Andrew D. Zelenetz, Elaine S. Jaffe, Ranjana H. Advani, Nancy Lee Harris, Richard T. Hoppe, Michael P. Link, Steven T. Rosen, and John P. Leonard

CHAPTER SUMMARY

Cancers Staged Using This Staging System

Adult Hodgkin and non-Hodgkin lymphomas

Cancers Not Staged Using This Staging System

These histopathologic types of cancer…	Are staged according to the classification for…	And can be found in chapter…
Ocular adnexal lymphoma	Ocular adnexal lymphoma	71
Pediatric lymphoma	Pediatric lymphoma	80
Primary cutaneous Lymphoma	Primary cutaneous lymphoma	81
Multiple myeloma	Plasma cell myeloma	82

Summary of Changes

Change	Details of Change	Level of Evidence
Ann Arbor staging	The Cotswold modification[1] of the Ann Arbor staging system[2,3] has been updated to the Lugano classification[4]	I
A and B Classification (Symptoms)	B symptoms were eliminated for non-Hodgkin lymphoma (retained for Hodgkin lymphoma).	I
X subscript	X subscript for bulk was eliminated. The diameter of the largest mass must be recorded.	I
Stage III	The extension of disease into extralymphatic sites (E lesions) was eliminated from Stage III; any extralymphatic involvement with nodal disease above and below the diaphragm is Stage IV.	I
Stage IIIS	Involvement of the spleen no longer part of stage grouping.	I
Stage II	Although four staging categories are retained, the concept of the Lugano classification is to divide patients into limited and advanced stages. Stage II bulky is variably categorized as limited- or advanced-stage based on the histology and prognostic factors.	I
Imaging	Posteroanterior chest X-ray is no longer required for the determination of bulk in Hodgkin or non-Hodgkin lymphoma.	I

To access the AJCC cancer staging forms, please visit www.cancerstaging.org.

© American Joint Committee on Cancer 2017
M.B. Amin et al. (eds.), *AJCC Cancer Staging Manual, Eighth Edition*, DOI 10.1007/978-3-319-40618-3_79

ICD-O-3 Topography Codes

Code	Description
C00–C14	Lip, oral cavity, and pharynx
C15–C26	Digestive organs
C30-C39	Respiratory system and intrathoracic organs
C40–C41	Bones, joints, and articular cartilage
C42.0	Blood
C42.1	Bone marrow
C42.2	Spleen
C42.3	Reticuloendothelial system, NOS
C42.4	Hematopoietic system, NOS
C44	Skin, excluding C44.1 eyelid
C47	Peripheral nerves and autonomic nervous system
C48	Retroperitoneum and peritoneum
C49	Connective, subcutaneous, and other soft tissues
C50	Breast
C51–C58	Female genital organs
C60–C63	Male genital organs
C64–C68	Urinary tract
C69.1	Cornea, NOS
C69.2	Retina
C69.3	Choroid
C69.4	Ciliary body
C73–C74	Thyroid and adrenal glands
C76	Other and ill-defined sites
C77.0	Lymph nodes of head, face, and neck
C77.1	Intrathoracic lymph nodes
C77.2	Intra-abdominal lymph nodes
C77.3	Lymph nodes of axilla or arm
C77.4	Lymph nodes of inguinal region or leg
C77.5	Pelvic lymph nodes
C77.8	Lymph nodes of multiple regions
C77.9	Lymph node, NOS
C80.9	Unknown primary site

WHO Classification of Tumors

Code	Description
9590	Malignant lymphoma, NOS
9591	Malignant lymphoma, non-Hodgkin, NOS
9596	Composite Hodgkin and non-Hodgkin lymphoma
9597	Primary cutaneous follicle center lymphoma
9650	Hodgkin lymphoma, NOS
9651	Hodgkin lymphoma, lymphocyte-rich
9652	Hodgkin lymphoma, mixed cellularity, NOS
9653	Hodgkin lymphoma, lymphocyte depletion, NOS
9654	Hodgkin lymphoma, lymphocyte depletion, diffuse fibrosis
9655	Hodgkin lymphoma, lymphocyte depletion, reticular
9659	Hodgkin lymphoma, nodular lymphocyte predominance
9661	Hodgkin granuloma [obs]
9662	Hodgkin sarcoma [obs]
9663	Hodgkin lymphoma, nodular sclerosis, NOS
9664	Hodgkin lymphoma, nodular sclerosis, cellular phase
9665	Hodgkin lymphoma, nodular sclerosis, grade 1
9667	Hodgkin lymphoma, nodular sclerosis, grade 2
9670	Malignant lymphoma, small B lymphocytic, NOS (see also M-9823)
9671	Malignant lymphoma, lymphoplasmacytic (see also M-9761)
9673	Mantle cell lymphoma (Includes all variants: blastic, pleomorphic, small cell)
9675	Malignant lymphoma, mixed small and large cell, diffuse (see also M-9690) [obs]
9678	Primary effusion lymphoma
9679	Mediastinal (thymic) large B-cell lymphoma
9680	Malignant lymphoma, large B-cell, diffuse, NOS
9684	Malignant lymphoma, large B-cell, diffuse, immunoblastic, NOS
9687	Burkitt lymphoma, NOS (Includes all variants, see also M-9826)
9688	T-cell–rich large B-cell lymphoma
9689	Splenic marginal zone B-cell lymphoma
9690	Follicular lymphoma, NOS (see also M-9675)
9691	Follicular lymphoma, grade 2
9695	Follicular lymphoma, grade 1
9698	Follicular lymphoma, grade 3
9699	Marginal zone B-cell lymphoma, NOS
9702	Mature T-cell lymphoma, NOS
9705	Angioimmunoblastic T-cell lymphoma
9708	Subcutaneous panniculitis-like T-cell lymphoma
9709	Cutaneous T-cell lymphoma, NOS
9712	Intravascular large B-cell lymphoma
9714	Anaplastic large cell lymphoma, T-cell and Null-cell type
9716	Hepatosplenic T-cell lymphoma
9717	Intestinal T-cell lymphoma
9718	Primary cutaneous CD30+ T-cell lymphoproliferative disorder
9719	NK/T-cell lymphoma, nasal and nasal type
9720	Precursor cell lymphoblastic lymphoma
9724	Systemic Epstein–Barr virus (EBV)-positive T-cell lymphoproliferative disease of childhood
9725	Hydroa vacciniforme–like lymphoma
9726	Primary cutaneous gamma–delta T-cell lymphoma
9727	Precursor cell lymphoblastic lymphoma, NOS (see also M-9835)
9728	Precursor B-cell lymphoblastic lymphoma (see also M-9836)
9729	Precursor T-cell lymphoblastic lymphoma (see also M-9837/3)
9735	Plasmablastic lymphoma
9737	ALK-positive large B-cell lymphoma
9738	Large B-cell lymphoma arising in human herpesvirus 8 (HHV8)-associated multicentric Castleman disease
9761	Waldenström macroglobulinemia (C42.0) (see also M-9671)
9823	B-cell lymphocytic leukemia/small lymphocytic lymphoma (see also M-9670)

Code	Description
9826	Burkitt cell leukemia (see also M-9687)
9827	Adult T-cell leukemia/lymphoma (human T-cell lymphotropic virus 1 [HTLV1]-positive) (includes all variants)
9835	Precursor cell lymphoblastic leukemia, NOS (see also M-9727)
9836	Precursor B-cell lymphoblastic leukemia (see also M-9728)
9837	Precursor T-cell lymphoblastic leukemia (see also M-9729/3)

Swerdlow SH, Campo E, Harris NL, Jaffe ES, Pileri SA, Stein H, Thiele J, Vardiman J, eds. World Health Organization Classification of Tumours of Haematopoietic and Lymphoid Tissues. Lyon: IARC; 2008.

INTRODUCTION

All newly diagnosed patients with malignant lymphomas should have formal documentation of the anatomic disease extent before the initial therapeutic intervention; that is, clinical stage must be assigned and recorded. Although patients with recurrent disease generally do not have a new clinical stage assigned at the time of relapse, some prognostic models include stage at the time of second-line therapy, particularly in Hodgkin lymphoma (HL) and diffuse large B-cell lymphoma (DLBCL), with intent to proceed with high-dose therapy and autologous stem cell rescue.[5–8] In all cases, recording of the anatomic disease extent at the time of relapse is recommended.

Lugano Classification Modification of the Ann Arbor Staging System

Anatomic staging of lymphomas traditionally has been based on the Ann Arbor classification system, which was originally developed more than 30 years ago for HL. It was based on the relatively predictable pattern of spread of HL and improved the ability to determine which patients might be suitable candidates for radiation therapy.[2] It was updated as the "Cotswold system" to address some of the issues present in the original staging system and to accommodate newer diagnostic techniques, including computed tomography (CT) scan.[1] It subsequently was applied to non-Hodgkin lymphoma (NHL) as well, despite the fact that the pattern of spread is less predictable than that of HL. The Ann Arbor classification has been accepted as the best means of describing the anatomic disease extent and has been useful as a universal system for a variety of lymphomas; therefore, it was adopted by the AJCC and the Union for International Cancer Control (UICC) as the official system for classifying the anatomic extent of disease in HL and NHL, with the exception of cutaneous lymphomas (e.g., mycosis fungoides), which are discussed later in this chapter. However, advances in diagnostics and therapy provided the impetus to review and modernize the evaluation and staging of lymphoma. Workshops were held at the 11th and 12th International Conference on Malignant Lymphoma to study areas in need of clarification or updating and then to review the proposed changes. The Lugano classification was published and forms the basis for revised recommendations regarding anatomic staging and evaluation of disease before and after therapy.[4] This staging system is adopted by the AJCC.

For the purposes of coding and staging, lymph nodes, Waldeyer's ring, thymus, and spleen are considered *nodal* or *lymphatic* sites. *Extranodal* or *extralymphatic* sites include the adrenal glands, blood, bone, bone marrow, central nervous system (CNS; leptomeningeal and parenchymal brain disease), gastrointestinal (GI) tract, gonads, kidneys, liver, lungs, skin, ocular adnexae (conjunctiva, lacrimal glands, and orbital soft tissue), skin, uterus, and others. HL rarely presents in an extranodal site alone, but about 25% of NHLs are extranodal at presentation. The frequency of extranodal presentation varies dramatically among different lymphomas, however, with some (e.g., mycosis fungoides and mucosa-associated lymphoid tissue [MALT] lymphomas) being virtually always extranodal, except in advanced stages of the diseases, and some (e.g., follicular lymphoma) seldom being extranodal, except for bone marrow involvement.

The Lugano classification includes an E suffix for lymphomas with either localized extralymphatic presentation (Stage IE) or by contiguous spread from nodal disease (Stage IIE). For example, lymphoma presenting in the thyroid gland with cervical lymph node involvement should be staged as IIE. However, in a change from the Cotswold modification of the Ann Arbor staging system, E lesions do not apply to patients with Stage III nodal disease; any patient with nodal disease above and below the diaphragm with concurrent contiguous extralymphatic involvement is Stage IV (previously Stage IIIE). Frequently, extensive lymph node involvement is associated with extranodal extension of disease that also may directly invade other organs. Such extension should be described with the E suffix if the nodal disease is on one side of the diaphragm. For example, mediastinal lymph nodes with adjacent lung extension should be classified as Stage IIE disease. Other examples of Stage IIE diseases include extension into the anterior chest wall and into the pericardium from a large mediastinal mass (two areas of extralymphatic involvement) and no nodal involvement below the diaphragm; involvement of the iliac bone in the presence of adjacent iliac lymph node involvement and no nodal involvement above the diaphragm; involvement of a lumbar vertebral body in conjunction with para-aortic lymph node involvement and no nodal involvement above the diaphragm; and involvement of the pleura or chest wall as an extension from adjacent internal mammary nodes. A pleural or pericar-

79

dial effusion with negative (or unknown) cytology is not an E lesion. Liver involvement is an exception; any liver involvement by contiguous or noncontiguous spread should be recorded as Stage IV.

The definition of disease bulk varies according to lymphoma histology. In HL, the extent of mediastinal disease is defined as the ratio between the maximum diameter of the mediastinal mass and maximal intrathoracic diameter based on CT imaging in the Lugano classification. In HL, bulk at other sites is defined as a mass >10 cm. A recent analysis has suggested that in early stage disease, masses > 7 cm (at any site) may dictate the inclusion of radiation to provide optimal outcomes. For NHL, the recommended definitions of bulk vary by lymphoma subtype. In follicular lymphoma, 6 cm has been suggested based on the Follicular Lymphoma International Prognostic Index, version 2 (FLIPI-2) and its validation.[9,10] In DLBCL, cutoffs ranging from 5 to 10 cm have been used, although 10 cm is recommended.[11]

Lymph Node Regions

The staging classification for lymphoma uses the term *lymph node region*. The lymph node regions were defined at the Rye Symposium in 1965 and have been used in the Ann Arbor classification; this is unchanged in the Lugano classification (Fig. 79.1). They are not based on any physiologic principles but rather have been agreed upon by convention. The currently accepted classification of core nodal regions is as follows:

- Right cervical nodes (including cervical, supraclavicular, occipital, and preauricular lymph nodes)
- Left cervical nodes
- Right axillary nodes
- Left axillary nodes
- Right infraclavicular nodes
- Left infraclavicular lymph nodes
- Mediastinal lymph nodes
- Right hilar lymph nodes
- Left hilar lymph nodes
- Para-aortic lymph nodes
- Mesenteric lymph nodes
- Right pelvic lymph nodes
- Left pelvic lymph nodes
- Right inguinofemoral lymph nodes
- Left inguinofemoral lymph nodes

In addition to these core regions, HL and NHLs may involve epitrochlear lymph nodes, popliteal lymph nodes, internal mammary lymph nodes (considered mediastinal by convention), occipital lymph nodes, submental lymph nodes, preauricular lymph nodes (all considered cervical, Fig. 79.1), and many other small nodal areas. Clinical prognostic models may include specific definitions of nodal regions. For exam-

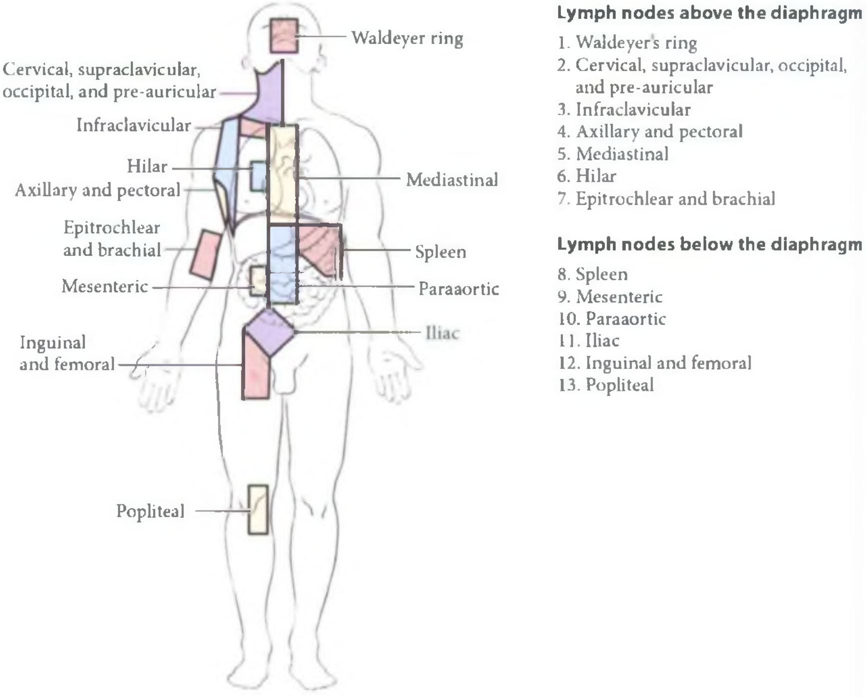

Fig. 79.1 Lymph nodes above and below the diaphragm (Ann Arbor/Lugano classification)

ple, in follicular lymphoma, the FLIPI-2 uses a different definition of nodal regions (see prognostic factors for follicular lymphoma). This is also the case in determination of favorable versus unfavorable early-stage HL as proposed by the German Hodgkin Study Group (GHSG) and the European Organisation for Research and Treatment of Cancer (EORTC; see prognostic factors for HL).

A and B Classification (Symptoms)

For HL, each stage should be classified as either A or B according to the absence or presence of defined constitutional symptoms. The designation A or B is not included in the revised staging of NHL,[4] although clinicians are encouraged to record the presence of these symptoms in the medical record. The symptoms are as follows:

1. *Fevers.* Unexplained fever with temperature above 38°C
2. *Night sweats.* Drenching sweats (e.g., those that require change of bedclothes)
3. *Weight loss.* Unexplained weight loss of more than 10% of the usual body weight in the 6 months prior to diagnosis

Other symptoms, such as chills, pruritus, alcohol-induced pain, and fatigue, are not included in the A or B designation but are recorded in the medical record, as the reappearance of these symptoms may be a harbinger of recurrence.

Criteria for Organ Involvement

Lymph Node Involvement

Lymph node involvement is demonstrated by enlargement of a node detected clinically or by imaging when alternative pathology may reasonably be ruled out. Imaging criteria include demonstration of fludeoxyglucose (FDG) avidity on FDG positron emission tomography (FDG-PET) or unexplained node enlargement on CT. Suspicious nodes should always be biopsied if treatment decisions are based on their involvement, preferably with an excisional biopsy; fine-needle aspirations are strongly discouraged because of the potential for false negatives or misdiagnosis because of loss of lymph node architecture. Core needle biopsy may be able to provide adequate material for diagnosis, particularly of a secondary site.

Spleen Involvement

Spleen involvement is suggested by unequivocal palpable splenomegaly and demonstrated by radiologic confirmation (FDG-PET or CT). Positive findings on FDG-PET include diffuse uptake, a solitary mass, miliary lesions, or nodules and those on CT include enlargement of >13 cm in cranial–caudal dimension, a mass, or nodules that are neither cystic nor vascular.

Liver Involvement

Liver involvement is demonstrated on FDG-PET by diffuse uptake or mass lesions and on CT by nodules that are neither cystic nor vascular. Clinical enlargement alone, with or without abnormalities of liver function tests, is not adequate. Liver biopsy may be used to confirm the presence of liver involvement in a patient with abnormal liver function tests or when imaging assessment is equivocal, if treatment will be altered on the basis of those results.

Lung Involvement

Lung involvement is demonstrated by FDG-avid pulmonary nodules on FDG-PET and evidence of parenchymal involvement on CT in the absence of other likely causes, especially infection. Lung biopsy may be required to clarify equivocal cases.

Bone Involvement

Bone involvement is demonstrated in FDG-avid lymphoma by avid lesions on FDG-PET. It is quite common for FDG-PET to demonstrate more sites of bone involvement than CT imaging. A bone biopsy from an involved area of bone may be necessary for a precise diagnosis, if treatment decisions depend on the findings.

CNS Involvement

CNS involvement often is heralded by symptoms and is demonstrated by (a) a spinal intradural deposit or spinal cord or meningeal involvement, which may be diagnosed on the basis of the clinical history and findings supported by plain radiology, cerebrospinal fluid (CSF) examination with flow cytometry, CT, and/or magnetic resonance (MR) imaging (spinal extradural deposits should be carefully assessed because they may be the result of soft tissue disease that represents extension from bone metastasis or disseminated disease) and (b) parenchymal brain disease demonstrated on CT and/or MR imaging, which may be confirmed by biopsy.

Bone Marrow Involvement

Bone marrow involvement is assessed by an aspiration and bone marrow biopsy. In HL, it is rare to have bone marrow involvement in the absence FDG-avid bone site. Therefore, if FDG-PET/CT is performed as part of the staging evaluation, routine bone marrow aspiration and biopsy is not required for staging of HL. In DLBCL, the presence of FDG-avid skeletal lesions precludes the need for a bone marrow aspiration and biopsy. However, the procedure generally should be done in the absence of FDG-avid bone disease because of the risk of identifying discordant bone marrow involvement by a small cell lymphoma. For the indolent B-cell lymphoma, bone marrow aspiration and

biopsy remains the standard for evaluation; however, it may be deferred in patients who are candidates for initial observation. Immunohistochemistry (IHC) and/or flow cytometry may be useful adjuncts to histologic interpretation to determine whether a lymphocytic infiltrate is malignant.

RULES FOR CLASSIFICATION

Clinical Classification

Clinical staging includes careful recording of medical history and physical examination; imaging of chest, abdomen, and pelvis; blood chemistry determination; complete blood count; erythrocyte sedimentation rate (ESR; in HL); and bone marrow biopsy (if indicated) (Table 79.1).

The basic staging investigation in HL and NHLs includes physical examination; complete blood count; lactate dehydrogenase (LDH) measurement; liver function tests; FDG-PET (for FDG-avid lymphomas); contrast-enhanced CT scan of the neck, chest, abdomen, and pelvis; and bone marrow aspiration and biopsy in selected cases. In the Lugano classification, both FDG-PET and diagnostic contrast-enhanced CT scanning are recommended. However, in clinical practice, FDG-PET (for FDG-avid lymphomas) may suffice for diagnosis and response evaluation; however, at the end of treatment, contrast-enhanced CT may be useful if imaging is planned during follow up. In patients presenting with extranodal lymphoma, imaging of the presenting area with either CT or MR is required to define local disease extent. In patients at high risk for occult CNS involvement (see Table 79.1), CSF cytology is performed, preferably with flow cytometry. Biopsies of any suspicious lesions also may be conducted as part of the initial clinical staging, especially if this would alter stage assignment. Bone marrow aspiration and biopsy is recommended in indolent lymphoma and aggressive NHL if there is no FDG-avid skeletal disease. It is not routinely necessary for staging of HL. Liver biopsy is not required as part of clinical staging, unless abnormal liver function occurs in the presence of otherwise limited-stage disease. Clinical staging is repeated at the end of therapy and forms the basis for defining response.

Table 79.1 Recommendations for the diagnostic evaluation of patients with lymphoma

A. Mandatory procedures
   1. Biopsy (preferably excisional), with interpretation by a qualified pathologist. Diagnosis from a core biopsy should be based on multiple core biopsies. Appropriate IHC and ancillary studies should be performed to establish a diagnosis.[12]
   2. History, with special attention to the presence and duration of fever, night sweats, and unexplained loss of 10% or more of body weight in the previous 6 months (B symptoms should be recorded in the medical record; in HL, they are part of staging)
   3. Physical examination
   4. Laboratory evaluation
      a. Complete blood cell count and platelet count with differential and slide review
      b. ESR (HL patients)
      c. Comprehensive metabolic panel (electrolytes, blood urea nitrogen [BUN], creatinine, calcium, aspartate transaminase [AST; serum glutamic oxaloacetic transaminase (SGOT)], alanine transaminase [ALT; serum glutamic-pyruvic transaminase (SGPT)], bilirubin, total protein, albumin, alkaline phosphatase
      d. LDH, phosphorus, uric acid
      e. HIV testing
      f. Hepatitis B core antibody and hepatitis B surface antigen, especially in patients being considered for anti-CD20 therapy
   5. Radiographic examination
      a. CT of neck, chest, abdomen, and pelvis with intravenous (IV) contrast (if safe)
      b. Functional (metabolic) imaging with FDG-PET
   6. Bone marrow aspiration and biopsy in selected cases
B. Examples of ancillary procedures
   1. Plain bone radiographs and/or MR imaging in patients with bone disease on functional imaging to evaluate for fracture
   2. Esophagogastroduodenoscopy (EGD), colonoscopy, and/or GI series in patients with GI presentations
   3. MR imaging of the spine in patients with suspected leptomeningeal disease
   4. MR imaging of the brain in patients with cranial nerve palsy or suspicion of leptomeningeal or parenchymal brain disease
   5. CSF cytology with flow cytometry in patients with
      a. DLBCL
         i. CNS risk score*[13] 4–6
         ii. CNS risk score 2–3 and double expressor (BCL2 and MYC by IHC)[13]
         iii. HIV infection
         iv. Testicular involvement[14]
         v. Breast involvement[15]
         vi. Children
      b. Burkitt lymphoma

*CNS risk score: 1 point for each of the following risk factors: age >60 years; performance status (PS) ≤2; LDH greater than upper limit of normal; two or more extralymphatic sites; Stage III/IV; adrenal or renal involvement

Additionally, baseline evaluation of HIV status should be done in all cases, as this may have an impact on treatment. Evaluation of hepatitis B serology is essential if anti-CD20 therapy is contemplated and is recommended in all cases because of the risk of reactivation of occult hepatitis B with chemotherapy alone. In patients receiving anti-CD20 therapy and for patients with small lymphocytic lymphoma (SLL)/chronic lymphocytic leukemia (CLL), baseline quantitative immunoglobulins are helpful to evaluate for the presence of hypogammaglobulinemia.

Pathological Classification

The use of the term pathological staging is reserved for patients who undergo staging laparotomy with an explicit intent to assess the presence of abdominal disease or to define histologic microscopic disease extent in the abdomen. As a result of improved diagnostic imaging, staging laparotomy and pathological staging generally are no longer performed.

PROGNOSTIC FACTORS

Stage is only one component in clinical prognostication. Clinical prognostic models have become the cornerstone for categorization of patients into various risk groups and in some cases, to guide therapy. However, the clinical prognostic models vary by lymphoma histology. In addition, important insights have been gained into the biology of the lymphoid neoplasms that also have had a profound impact on outcome and an emerging impact on therapy. These important factors for determination of clinical risk are discussed by disease entity.

Diffuse Large B Cell Lymphoma

Prognostic Factors Required for Stage Grouping

The Lugano classification includes no prognostic variables required for determination of stage.

Additional Factors Recommended for Clinical Care

Several factors have emerged that have a reproducible impact on the outcome of patients with DLBCL (see Table 79.2).

Table 79.2 DLBCL additional factors recommended for clinical care

Factor	Definition	Clinical significance	Level of evidence
International Prognostic Index (IPI)/age-adjusted IPI (aaIPI)[16]	Five clinical factors predict outcome (0–1, low risk; 2, low-intermediate risk; 3, intermediate-high risk; 4–5, high risk): age (>60 years), PS (≤2), LDH (>ULN), extranodal sites (≥2), and CS (III/IV) are adverse. aaIPI does not include age; risk groups are 0–1, 2, 3, and 4 risk factors (RFs)	Robust predictor of outcome, even in the rituximab era	II
Revised IPI (R-IPI)[17]	IPI RFs grouped into three risk groups: good, 0 RF; intermediate, 1–2 RFs; poor, 3–5 RFs	Better performance than IPI in rituximab era (although IPI continues to work)	III
NCCN-IPI[18]	IPI RFs restratified: age (41–60, 1 point; 61–75, 2 points; >75, 3 points); stage (III/IV, 1 point); PS (2–4, 1 point); extranodal sites (bone marrow, CNS, liver, lung, GI–yes, 1 point); LDH (>1–3 × ULN, 1 point; >3 × ULN, 2 points). Total 8 points: low risk, 0–1; low-intermediate risk, 2–3; intermediate-high risk, 4–5; high risk, ≥6	Better discrimination of low- and high-risk patients than IPI in rituximab era	I
Stage-modified IPI[36]	Stage-modified IPI RFs: age >60; LDH > ; PS (2–4); Stage II. Adverse risk: ≥1 RF	Discriminates RFs for patients with early-stage DLBCL (Stage I–II)	II
Cell of origin[22–25]	Determine the COO of DLBCL by best available means* (minimally, IHC for CD10, BCL6, IRF4/MUM1)	In retrospective studies, differential outcome with standard therapy and targeted therapy (e.g., ibrutinib and lenalidomide).[37–41]	II
Double-hit(MYC/BCL2 or MYC/BCL6)/triple-hit (MYC/BCL2/BCL6) lymphoma[4,27–31]	Restricted to GC-DLBCL phenotype: FISH for translocations of MYC with BCL2 and/or BCL6 translocation	Double-/triple-hit lymphoma has a very poor outcome with conventional therapy.	I
Double-expressor (MYC/BCL2) lymphoma[32–35]	Concurrent expression of BCL2 and MYC by IHC	Tumors expressing MYC and BCL2 have an inferior prognosis; identified additional tumors that are not double hit	II

*Determination of COO is evolving. There are several emerging platforms that are superior to IHC, including the Lymph2CX (NanoString Technologies, Seattle, WA)[25] and Fluidigm (South San Francisco, CA) assays[26]

International Prognostic Index (IPI) and Related Indices. The International Non-Hodgkin Lymphoma Prognostic Factors Project used pretreatment prognostic factors in a sample of several thousand patients with aggressive lymphomas treated with doxorubicin-based combination chemotherapy to develop a predictive model of outcome for aggressive NHL.[16] A multivariate analysis identified five pretreatment characteristics as independent, statistically significant prognostic factors: age in years (≤60 vs. >60); Stage I or II (localized) versus III or IV (advanced); number of extranodal sites of involvement (0–1 vs. ≥2); patient's performance status (Eastern Cooperative Oncology Group [ECOG] 0 or 1 vs. ≥2); and serum LDH level (normal vs. abnormal). With the use of these five pretreatment risk factors, patients could be assigned to one of the four risk groups on the basis of the number of presenting risk factors: low (0 or 1), low intermediate (2), high intermediate (3), and high (4 or 5). When patients were analyzed by risk factors, they were found to have very different outcomes with regard to complete response (CR), relapse-free survival (RFS), and overall survival (OS). The outcomes indicated that the low-risk patients had an 87% CR rate and an OS rate of 73% at 5 years, in contrast to a 44% CR rate and 26% 5-year survival in patients in the high-risk group. A similar pattern of decreasing survival with several adverse factors was observed when only younger patients were considered (age-adjusted IPI), as well as when only limited stage (I–II) patients were considered (stage-modified IPI).

The IPI has remained reliable in the rituximab era; however, the delineation among risk groups is not as robust. This has led to suggestions to modify the grouping in the IPI. The revised IPI (R-IPI) restratified the same five clinical factors[17] into three categories: good risk, zero factors; intermediate risk, one to two factors; and poor risk (three to five factors). This risk stratification performed better in patients treated in the rituximab era but has not been universally reproduced or adopted.

The National Comprehensive Cancer Network IPI (NCCN-IPI)[18] uses the same five clinical factors; however, the key difference is the recognition that age and LDH are continuous variables for which simple dichotomization is inappropriate. Age is divided into three groups: 41–60 years, 1 point; 61–75 years, 2 points, and >75 years, 3 points. LDH is divided into three groups: within normal limits, 0 points; greater than one to three times the upper limit of normal (ULN), 1 point; and greater than three times the ULN, 2 points. A point is given for any extralymphatic involvement of bone marrow, CNS, liver, lung, and/or GI tract. The NCCN-IPI has a total of 8 points, and the groups are stratified as follows: low risk, 0–1 points; low-intermediate risk, 2–3 points; intermediate-high risk, 4–5 points; and high risk, ≥6 points. Compared with the IPI, the NCCN-IPI had better discrimination between the low- and high-risk groups with respect to 5-year OS: NCCN-IPI low 96% versus high 33%; IPI low 90% versus high 54%. This index has been validated independently, albeit with the suggestion that addition of albumin and β_2-microglobulin may further improve prognostication.[19]

Cell of Origin (COO). The heterogeneity of DLBCL has been appreciated on morphologic grounds since the introduction of the REAL and subsequent World Health Organization (WHO) classifications.[20,21] Gene expression profiling confirmed this heterogeneity and found that DLBCL, not otherwise specified, in large part could be divided into tumors derived from germinal center B cells (GC-DLBCL) or activated B cells (ABC-DLBCL).[22] COO is a strong correlate of outcome in retrospective analyses in the rituximab era, with ABC-DLBCL having an inferior outcome compared with GC-DLBCL.[23] Furthermore, several novel therapies, including ibrutinib and lenalidomide, have differential activity based on COO; both these agents are more active in ABC-DLBCL.

Distinction of DLBCL based on COO was incorporated into the 4th edition of the WHO classification, but its use in clinical practice was optional.[12] In the upcoming revision, the recommendation is that COO be determined by the best available means. Although the Hans algorithm[24] using IHC for CD10, BCL6, and MUM1/IRF4 has been the most commonly used method for determining COO, its limitations have been widely reported. The gold standard, gene expression profiling of tumor RNA on high-density gene arrays, was still a discovery tool unsuitable for clinical application. Recently, several assays were developed that use RNA extracted from formalin-fixed paraffin-embedded tissue and provide reproducible information about the COO that correlates with the gold standard and robustly predicts clinical outcome; however, these test have not yet been approved by the Clinical Laboratory Improvement Amendments (CLIA).[25,26] As of this writing, IHC is still the most commonly available tool for determining COO, and this needs to be recorded by the registrar as reflected in the pathology report.

Double-hit and Double-expressor DLBCL. The double/triple-hit lymphomas (simultaneous translocation of BCL2 or BCL6 and MYC or translocation of BCL2/BCL6 and MYC) have been reported to have very poor outcomes. Biologically these tumors are GC-DLBCL but have outcomes inferior to those that do not have the dual translocations.[4,27–31] It may be impractical to perform fluorescence *in situ* hybridization (FISH) on all cases of GC-DLBCL. IHC for MYC expression may serve as a screening tool to identify cases in which FISH should be performed. IHC for BCL2 or BCL6 is not useful in predicting translocations in these genes, however.

All cases of DLBCL should be evaluated by IHC for expression of BCL2 (≥70% of neoplastic cells is positive), BCL6 (≥50% is positive), and MYC (≥40% is positive) because patients with double expression (BCL2/MYC or

BCL6/MYC) or triple expression (BCL2/BCL6/MYC) have an inferior outcome.[32–35] Compared with patients with double or triple translocations, expression (without translocation) carries an intermediate prognosis. Unlike the double/triple-hit lymphomas, double expression is not restricted to GC-DLBCL, and double expression of BCL2 and MYC in the absence of translocation is more often seen in ABC-DLBCL.[34]

Special Clinical Considerations Affecting Therapy. Several relatively common clinical scenarios have an impact on therapeutic decisions, namely primary mediastinal large B cell lymphoma (PMBL); testicular lymphoma; and DLBCL associated with HIV. PMBL is a clinicopathologic diagnosis facilitated by communication of the clinical picture by the clinician to the pathologist. This diagnosis may have an impact on treatment choice. In the case of testicular lymphoma, there is a risk of disease in the contralateral testis, necessitating inclusion of scrotal radiation into the treatment plan. Furthermore, there is a risk of late parenchymal brain involvement, which may be affected by prophylactic CNS-directed therapy during initial treatment. Association of DLBCL with HIV infection also affects the choice of therapy and the decision to include antiviral therapy.

Mantle Cell Lymphoma

Prognostic Factors Required for Stage Grouping

The Lugano classification includes no prognostic factors necessary for determining stage.

Additional Factors Recommended for Clinical Care

It is increasingly recognized that the clinical course of mantle cell lymphoma (MCL) may be highly variable. Some patients are candidates for observation,[42] whereas others have a very aggressive course. Several prognostic factors have emerged that help categorize clinical behavior (Table 79.3).

Proliferation Index. Gene expression profiling of MCL identified a gene signature associated with cellular proliferation that was strongly correlated with clinical outcome.[43] However, determination of the proliferation signature is not a clinically validated test. Several studies demonstrated that the proliferation index (% positive/total tumor cells) estimated by the percentage of tumor cell expressing MKI67, as detected by either Ki-67 or MIB1, is an excellent surrogate for the gene expression proliferation signature and predicts clinical outcomes.[44–47] Although several cutoffs have been suggested, a proliferation index greater than 30% is most commonly used to identify patients with a more aggressive clinical course. In some studies, a proliferation index less than 10% was associated with a very indolent course. The proliferation index (% of positivity with either the Ki-67 or MIB1 monoclonal antibody) should be recorded.

MCL International Prognostic Index (MIPI). The IPI can predict the outcome of patients with MCL; however, the risk groups are not well distributed, limiting its utility for clinical prognostication and use in clinical trials. Therefore, the German Low Grade Lymphoma Study Group (GLSG) developed a prognostic model based on analysis of pooled data from several randomized trials conducted by GLSG and the European MCL Network.[48] The resulting prognostic model was termed the MIPI and identified several clinical factors associated with outcome: age, ECOG performance status, LDH, and white blood cell (WBC) count. A second model included the proliferative index when available ($MIPI_b$). The resulting model is weighted and is calculated by the following formula:

[0.03535 × age (years)] + 0.6978 (if ECOG > 1) + [1.367 × $\log_{10}$(LDH/ULN)] + [0.9393 × $\log_{10}$(white cells/μL blood)].

The formula for the $MIPI_b$ includes an additional term: + [0.02142 × Ki-67 (%)]. Given the complexity of the calculation, online calculators are available (see URLs in Table 79.3). The prognostic cutoffs for the MIPI are as follows: low risk, <5.7 (5-year OS, 60%); intermediate risk, 5.7 to <6.2 (median OS, 51 months); and high risk, ≥6.2 (median OS, 21 months).

Table 79.3 MCL additional factors recommended clinical care

Factor	Definition	Clinical significance	Level of evidence
Proliferation index (PI)[44–47]	Percent (in deciles) of tumor cell expressing MIB1/Ki-67 by IHC	PI is one of the key factors dictating disease prognosis.	I
MCL International Prognostic Index (MIPI)[48]	Clinical RFs (age, ECOG status, LDH, WBC count) calculated by [0.03535 × age (years)] + 0.6978 (if ECOG status >1) + [1.367 × $\log_{10}$(LDH/ULN)] + [0.9393 × logEmphasis$_{10}$(white cells/μL blood)] (URL for calculator: http://bloodref.com/lymphoid/lymphoma/mipi). Low risk, <5.7; intermediate risk, 5.7 to <6.2; high risk, ≥6.2.	Predictor of outcome; has not been universally useful	II
$MIPI_b$[48]	If PI is available: clinical RFs (age, ECOG status, LDH, WBC count, PI) calculated by [0.03535 × age (years)] + 0.6978 (if ECOG >1) + [1.367 × $\log_{10}$(LDH/ULN)] + [0.9393 × $\log_{10}$(WBC count)] + [0.02142 × Ki-67 (%)] (URL for calculator: http://bloodref.com/lymphoid/lymphoma/mipib). Low risk, <5.7; intermediate risk, 5.7 to <6.5; high risk, ≥ 6.5.	Predictor of outcome; has not been universally useful	II

The prognostic cutoffs for the MIPI$_b$ are as follows: low risk, <5.7 (5-year OS, 72%); intermediate risk, 5.7 to <6.5 (median OS, 50 months); and high risk, ≥6.5 (median OS, 36 months). The MIPI has been evaluated in several settings: it predicted neither the outcome of patients treated with R-HyperCVAD/R-MA at MD Anderson Cancer Center nor that of patients treated with high-dose therapy/stem cell rescue at Memorial Sloan Kettering Cancer Center.[47,49] However, it was validated in an analysis of a population-based cohort in the Netherlands and in a retrospective analysis of immunochemotherapy and high-dose therapy/stem cell rescue trials of the European Mantle Cell Network.[50,51] The variability in validation of the MIPI has limited its adoption in the United States as a basis for treatment selection.

Follicular Lymphoma

Prognostic Factors Required for Stage Grouping

The Lugano classification includes no prognostic factors necessary for determining stage.

Additional Factors Recommended for Clinical Care

The extent of disease and tumor-related symptoms as assessed by the Groupe d'Etude des Lymphomes Folliculaires (GELF) criteria and the follicular lymphoma prognostic index are clinical prognostic factors that may influence treatment and outcomes (Table 79.4).

GELF Criteria. The GELF cooperative group defined patients with high tumor burden as having at least one of the following: disease-related symptoms, cytopenia, leukemic-phase disease, disease-related symptoms, organ compression by adenopathy, a single mass >7 cm, or three or more nodal masses >3 cm.[52] The absence of these factors defines a group of patients with an indolent natural history, in whom initial therapy may be deferred. The presence of one or more of these factors has been used in many trials to indicate a need for therapy. Tumor disease burden (high [one or more factors] vs. low [0 factors]) based on the presence or absence of GELF criteria should be recorded.

Follicular Lymphoma Prognostic Index (FLIPI). The IPI was less useful in follicular lymphomas; therefore, the FLIPI was proposed. Factors included in the index are the number of nodal sites (four or fewer vs. more than four), serum LDH (normal vs. elevated), age (with 60 years as the cutoff), stage (I–II vs. III–IV), and serum hemoglobin (Hgb) concentration (≥12 vs. <12 g/dL). The three risk groups identified were zero or one adverse factor, two factors, and three or more factors. Ten-year survival was 71% in patients with low-risk disease, 51% in those with intermediate-risk disease, and only 36% in those with high-risk disease. A prospectively determined model, the FLIPI-2, also was proposed, but in general the original FLIPI (now referred to as FLIPI-1) is more widely adopted and recommended for clinical use. The registrar should record the FLIPI (as FLIPI-1 or FLIPI-2) if it is available in the medical record.

Marginal Zone Lymphoma

Prognostic Factors Required for Stage Grouping

The Lugano classification includes no prognostic factors necessary for determining stage.

Additional Factors Recommended for Clinical Care

Marginal zone lymphoma includes three distinct entities: extranodal marginal zone lymphoma (ENMZL), splenic marginal zone lymphoma (SMZL), and nodal marginal zone lymphoma (Table 79.5). ENMZL may involve anatomic sites, although the most common site of involvement is the stomach. Gastric ENMZL associated with *Helicobacter pylori* infection often is responsive to antibiotics. However, the presence of the t(11;18)(q21;q21) translocation juxtaposing the *MALT1* and *API2* genes predicts resistance to antibiotic therapy.[57–60] This translocation may be detected by FISH or reverse transcription polymerase chain reaction (RT-PCR); it should be evaluated in cases of *H. pylori*–associated gastric ENMZL.

SMZL may be associated with hepatitis C virus (HCV) infection; in these cases, it is common for patients to

Table 79.4 Follicular lymphoma additional factors recommended for clinical care

Factor	Definition	Clinical significance	Level of evidence
GELF criteria[52]	Criteria include three or more nodal masses >3 cm, a single mass >7 cm, splenomegaly, organ compression, ascites or pleural effusion, disease-related symptoms, cytopenia, and leukemic-phase disease	Patients with low tumor burden and no symptoms may defer treatment without adverse impact on survival[52-55]	I
FLIPI-1 and FLIPI-2[10,56]	Clinical factors in FLIPI-1: number of nodal groups (≥3), LDH (>ULN), age (>60), Stage III/VI, Hgb <12 g/dL; in FLIPI-2: longest diameter of single site (>6 cm), 03B2$_2$-macroglobulin >ULN, bone marrow involvement (yes), age >60, Hgb <12 g/dL	Clinical course, including response to therapy, is related to the FLIPI; patients presenting with high-risk FLIPI may have a high risk of transformation to an aggressive DLBCL. Although FLIPI-2 was developed prospectively, FLIPI-1 is used more widely.	I

Table 79.5 Marginal zone lymphoma additional factors recommended for clinical care

Factor	Definition	Clinical significance	Level of evidence
t(11;18) (q21;q21)[57–60]	Chromosomal translocation of *MALT1* and *API2* detected by FISH or RT-PCR	In gastric ENMZL, translocation is associated with resistance to antibiotic treatment in *H. pylori*–positive cases.	I
HCV[61]	HCV viral load	SMZL associated with HCV may be responsive to HCV-directed therapy.	II
H. pylori in gastric ENMZL	Evaluation for *H. pylori* should be performed in all cases of gastric ENMZL by best available means.	This has a direct bearing on the choice of initial therapy.	I

have detectable cryoglobulins. Several small case series demonstrated regression of HCV-associated SMZL with interferon therapy,[61] and although the data are limited, the association is widely accepted. All patients with SMZL should be evaluated for HCV.

Chronic Lymphocytic Leukemia / Small Lymphocytic Lymphoma

Clinical Classification

CLL and SLL are a clinical continuum of a single entity characterized by clonal expansion of small B cells involving the bone marrow, the peripheral blood, and often the lymph nodes. The neoplastic cells express CD5, CD19, CD20, and CD23 on their surface and have low levels of surface immunoglobulin. The diagnosis of CLL requires demonstration of more than 5,000 clonal B lymphocytes per microliter of peripheral blood, with or without demonstrated adenopathy. If there is adenopathy with fewer than 5,000 clonal B lymphocytes per microliter of peripheral blood, the diagnosis is SLL. Fewer than 5,000 clonal CLL-phenotype cells in the peripheral blood in the absence of adenopathy is monoclonal B cell lymphocytosis (MBL). CLL/SLL has a variable clinical course, ranging from an asymptomatic disease requiring no treatment for many years to a rapidly progressive disease necessitating prompt and repeated treatment.

CLL most often is diagnosed in asymptomatic individuals, often when a complete blood count (CBC) is obtained as part of an annual physical examination. Modest lymphocytosis may be the only presenting abnormality and often is overlooked unless it is very high. An initial evaluation should include a clinical history and physical examination, including careful assessment for adenopathy or organomegaly. The diagnosis of CLL/SLL is established by flow cytometry analysis of a peripheral blood sample and/or results of a lymph node biopsy. A bone marrow examination is not required to establish the diagnosis.

Monoclonal B lymphocytosis (MBL)

MBL is defined as the presence of monoclonal B-cell populations in the peripheral blood of up to 5×10^9/L, usually with the phenotype of CLL, in the absence of lymphomatous features diagnostic of SLL. Found in up to 12% of healthy individuals, in some it may be an extremely small population, but in others it may be associated with clinically evident lymphocytosis. MBL precedes virtually all cases of CLL/SLL. Current practice distinguishes between "low count" MBL, defined as a peripheral blood CLL count of $<0.5 \times 10^9$/L, from "high count" MBL. Low-count MBL has an extremely small chance of progression and does not require active clinical monitoring. High-count MBL requires routine/yearly follow-up and has phenotypic and genetic/molecular features very similar to those of Rai stage 0 CLL, although *IGHV*-mutated cases are more frequent in MBL. Recently, a nodal equivalent of MBL was described with a variable rate of progression to CLL/SLL, correlating with nodal size and presence of growth centers. Non–CLL-type MBLs, at least some of which may be closely related to SMZL, are less common, although they recently were recognized.

Prognostic Factors Required for Stage Grouping

Although SLL and CLL represent different clinical presentations of the same disease, traditionally they have been staged by using distinct staging systems (Table 79.6). SLL generally was staged with Ann Arbor staging and now is staged with the Lugano classification. For CLL, two staging systems are commonly used: the Rai (Table 79.7) and Binet (Table 79.8) staging systems. Both are based on physical examination and CBC measurement. CT scans are *not* required for application of these staging systems. However, in clinical practice, CT scans commonly are performed. In most cases, this information is not used to determine CLL stage but may indicate more extensive disease than appreciated by physical examination and may influence treatment decisions. CT may be used to evaluate the spleen and peripheral nodes in an obese patient if the physical examination is limited. A finding of retroperitoneal or mesenteric adenopathy on CT does *not* alter stage. The modified Rai system is predominant in North America, whereas the Binet system is in wide use outside the United States. These staging systems provide prognostic information and also guide decisions regarding the start of therapy.

Table 79.6 SLL prognostic factors required for staging

Factor	Definition	Clinical significance	Level of evidence
Absolute lymphocyte count (ALC)	ALC >5,000 cells/μL	SLL with ALC >5,000 cells/μL is CLL and should be staged as such (see Tables 79.10 and 79.11)	I
Adenopathy	Presence of lymph nodes >1.5 cm on physical examination (PE)	Defines stage	I
Organomegaly	Enlarged liver and/or spleen on PE	Defines stage	I
Anemia	Hgb <11.0 g/dL	Defines stage	I
Thrombocytopenia	Platelets (Plt) <100,000/μL	Defines stage	I

Table 79.7 Modified Rai staging system

Stage	Risk	Findings	Survival (mo)
0	Low	Lymphocytosis only	>120
I	+ Intermediate	+ Adenopathy	95
II	Intermediate	+ Enlarged spleen and/or liver	72
III	High	Lymphocytosis + Hgb <11 g/dL	30
IV	High	Lymphocytosis + Plt <100,000/μL	30

Table 79.8 Binet staging system

Stage	Findings	Survival (mo)
A	Lymphocytosis only	>120
B	+ Adenopathy	95
C	+ Enlarged spleen and/or liver	72

Additional Factors Recommended for Clinical Care

The modern era of prognostic markers in SLL/CLL is based on two key findings: recurrent cytogenetic abnormalities detected by FISH and the correlation of outcome with *IGHV* mutation status. Karyotypic analysis of SLL/CLL classically has been associated with limited results, because metaphases have been obtained in only 40–50% of cases secondary to the low mitotic rate. A landmark study evaluating a panel of FISH probes identified four recurrent abnormalities in SLL/CLL: del(11q), del(13q), trisomy 12, and del(17p).[62] The del(13q) was the most common abnormality (occurring in 18–54% of cases) and is associated with a favorable OS (median, 11–15 years). Patients with trisomy 12 (occurring in 14–19%) had a median OS similar to that of patients with a normal FISH study (median, 10 years). Patients with del(11q) (11–20%) had a median OS of 6 to 9 years. Those with del(17p) (6–16% of patients) had the worst outcome, with a median OS of 2 to 4 years. In addition to del(17p), *TP53* mutation was shown to be prognostic in CLL, as was mutation of *ATM*, which is almost invariably lost in del(11q). Neither trisomy 12 nor del(13q) as sole abnormalities drive treatment decisions and therefore are not included in Table 79.9.

IGHV Mutation Status. The role of *IGHV* mutation status is well established in SLL/CLL.[63,64] This reflects the fact that some CLLs develop directly from nave B cells and express a germline *IGHV* whereas others are derived from B cells that have transited through the germinal center (*IGHV* mutated). Evaluation of ZAP70 expression by flow cytometry has been suggested as a surrogate for *IGHV* testing. However, the results of ZAP70 flow cytometry may vary from laboratory to laboratory, and *IHGV* mutation testing is now readily available commercially. The cutoff for mutation is 2%, which reliably identifies a favorable *IGHV*-mutant group with a superior OS (median, >20 years) compared with the *IGHV* germline patients (median, 7–10 years). *IGHV* is invariant, thus needs to be tested only once during the course of the disease. It is an important predictor of time for first therapy.[65] Notably, CLL with a rearranged VH3-21 has an aggressive course if the gene is germline or mutated.[66]

del(17p)/ TP53 Mutation. As noted earlier, the finding of del(17p) by FISH is associated with a poor OS. Furthermore, this finding has been associated with a short time to initial therapy, short progression-free survival (PFS) and chemotherapy resistance.[67–69] The incidence of del(17p) and *TP53* mutation increases in relapsed/refractory disease, and reassessment of the tumor is appropriate if therapy is being considered. *TP53* mutations also independently predict adverse outcome in CLL, including short time to initial therapy, PFS, OS, and chemotherapy resistance.[70–73] New therapies approved for treatment of SLL/CLL, namely idelalisib and ibrutinib, both B-cell pathway inhibitors, have demonstrated activity in the treatment of cases with del(17p) and *TP53* mutation.[74,75] Additional agents (including venetoclax) have been identified that are active in patients with del(17p) and/or *TP53* mutations. Therefore, obtaining this information is essential for treatment selection.

del(11q)/ ATM Mutation. Cases of SLL/CLL with del(11q) have a poor prognosis and are associated with extensive peripheral, abdominal, and mediastinal adenopathy.[76] Cases with del(11q) are more likely to be associated

Table 79.9 SLL additional factors recommended for clinical care

Factor	Definition	Clinical significance	Level of evidence
IGHV mutation status[63,64]	Determination by DNA sequencing whether the *IGHV* is germline (unfavorable) or mutated (favorable)	Tumors with mutated *IGHV* are post germinal center and have a favorable outcome.	I
del(17p)[62,71,72,76]	Identification of del(17p) by FISH	Long-term outcome is generally worse in patients with del(17p); however, it is not adequate as a sole basis for treatment. Associated with resistance to genotoxic therapy.	I
TP53 mutation[71–73]	Identification of *TP53* mutation by DNA sequencing	Long-term outcome is generally worse in patients with *TP53* mutation; however, it is not adequate as a sole basis for treatment.	I
del(11q)[71,76]	Identification of del(11q) by FISH (*ATM* deletion)	Associated with inferior outcome; outcome improved by addition of an alkylator to therapy	I
CIRS score	Fit (≤6) or unfit (>6)	Contributes to choice of therapy	I

with germline *IGHV*. The del(11q) almost invariably involves deletion of the *ATM* gene, and mutation of *ATM* also has been associated with poor outcome.[77] Cases with del(11q) are relatively resistant to treatment with fludarabine.[68] In the LRF CLL4 trial, the addition of cyclophosphamide to fludarabine significantly increased OS compared with fludarabine alone (47% vs. 18.5% at 2 years).[78] Furthermore, the addition of rituximab to fludarabine and cyclophosphamide significantly enhanced overall response rate, PFS, and OS in del(11q) patients in the CLL8 trial.[71] As with del(17p), the incidence of del(11q) increases in patients with relapsed/refractory disease and must be evaluated before planning a course of therapy.

Cumulative Illness Rating Scale (CIRS). Clinical outcome in CLL/SLL is influenced by patient fitness. A measure of fitness that has been adopted in the management of patients with CLL/SLL is the CIRS score.[79] Several studies used a CIRS score cutoff of >6 to indicate lack of fitness for aggressive chemoimmunotherapy. This scale is used for the selection of therapy.

Peripheral T-Cell Lymphoma

Prognostic Factors Required for Stage Grouping

The Lugano classification includes no prognostic factors necessary for determining stage.

Additional Factors Recommended for Clinical Care

Mature T- and NK-cell lymphomas, including peripheral T-cell lymphoma (PTCL), comprise a heterogeneous group of neoplasms characterized by diverse clinical presentations, an aggressive clinical course, and poor response to conventional chemotherapy.[80]

Identification of clinical prognostic factors in PTCL is an area of ongoing investigation (Table 79.10). Unlike aggressive B-cell NHL, for which the IPI and its variants provide a robust clinical prognostic model, its utility in PTCL is limited because of the clinical and biological heterogeneity of the various subtypes. Therefore, adjusted prognostic models with better predictive accuracy than the IPI have been proposed for subtypes of T-cell lymphomas, including peripheral T-cell NHL (PTLC) NOS (Prognostic Index for PTLC NOS [PIT] model), angioimmunoblastic T-cell lymphoma (AITL), nasal-type natural killer(NK)/T-cell lymphoma, and enteropathy-associated T-cell lymphoma (EATL).[81–84] A retrospective study of PTCL identified 8,802 patients between 2000 and 2010.[85] Multivariate analysis demonstrated that PTCL subtypes of hepatosplenic, enteropathy-associated, and extranodal NK/T-cell histologies; age ≥55 years; black race; and advanced stage were significant ($p < 0.0001$ each). Based on these factors, a prognostic model was constructed and validated in an independent cohort, with an OS ranging from 9 months (highest-risk group) to 120 months (lowest-risk group).

Clinical Risk Assessment. In PTCL, the main entity for which IPI factors into treatment recommendations is ALK-positive anaplastic T-cell lymphoma (ALCL). The FFS for patients with zero/one, two, three, and four/five IPI risk factors is 80%, 60%, 40%, and 25%, respectively, suggesting that standard anthracycline-based therapy alone may not be adequate for patients with higher-risk disease.[86] Additionally, age >40 years has been determined to be one of the strongest prognostic factors in ALCL.[87] Patients with ALK-negative ALCL who were <40 years old had outcomes similarly favorable to those of the ALK-positive patients in this age group. Thus, patients younger than 40 years or with advanced IPI ALK-positive ALCL may warrant more aggressive therapy, that is, consideration of autologous stem cell transplant consolidation.[88]

PTCL NOS. Currently 50% of PTCL cases are not further classifiable and thus are termed PTCL NOS. It is a clinically and morphologically heterogeneous entity; by definition PTCL NOS comprises entities that do not fulfill the diagnostic criteria of other well-defined subtypes, and generally they are associated with poor survival.[89]

To better define the clinical outcome of T-cell lymphomas grouped within the broad category of PTCL NOS, the

Table 79.10 T-cell lymphoma additional factors recommended for clinical care

Factor	Definition	Clinical significance	Level of evidence
ALK in ALCL	Positive test on IHC	ALK+ associated with better outcomes; may help direct therapy with ALK inhibitors	I
EBV viral load (extranodal NK/T cells)	Detection in plasma	Level correlates with outcome	I
EBV viral load during treatment, at end of treatment, and during surveillance	Detection in plasma	Levels associated with outcomes	I
CD30 expression	Positive test on IHC	May help direct choice of targeted therapy; however, cutoffs are very controversial	II

Intergruppo Italiano Linfomi (IIL) proposed a new prognostic model for PTCL NOS called PIT.[81] Four factors (age >60, ECOG PS ≥2, LDH >ULN, and bone marrow involvement) independently predicted survival. Four risk groups were identified that predicted 5-year and 10-year OS: group 1, no adverse factors (62.3% and 54.9%, respectively); group 2, one adverse factor (52.9% and 38.8%, respectively); group 3, two adverse factors, (32.9% and 18.0%, respectively), and group four, three to four adverse factors, (18.3 and 12.6%, respectively). This model meets level III evidence.

AITL. AITL is the second most common PTCL subtype worldwide. It has a characteristic clinical presentation, often manifesting features of immune dysregulation.[80] This PTCL subtype is one of the more common ones to exhibit increased numbers of latent Epstein–Barr virus (EBV)–infected B cells (>80% of cases), likely because of T-cell dysfunction, which may give rise to clonal B-cell proliferations and overt B-cell lymphomas. It is characterized by an aggressive course and dismal outcome with current therapies. As an alternative, the Prognostic Index for AITL (PIAI), has been proposed; it includes age >60 years, ECOG PS ≥2, more than one extranodal site, B symptoms, and platelet count <150 × 10^9/L. The simplified PIAI has a low-risk group with zero to one adverse factors that has a 5-year survival of 44% and a high-risk group with two to five adverse factors having a 5-year survival of 24% ($p = .0065$).[90]

EATL. EATL is a rare intestinal NHL with a poor, although variable outcome. The new EATL prognostic index (EPI) consists of three risk groups: a high-risk group characterized by the presence of B symptoms (median OS of 2 months]; an intermediate-risk group comprising patients without B symptoms and with an IPI score ≥2 (median OS of 7 months), and a low-risk group representing patients without B symptoms and with an IPI score of 0 to 1 (median OS, 34 months). Internal validation showed stability of statistical significance and prognostic discrimination. In contrast with the IPI and PIT, the EPI better classified patients in risk groups according to their clinical outcome.[84] This index meets level III evidence.

Extranodal NK-cell Lymphoma (ENKL): ENKL is a predominantly extranodal lymphoma, mainly occurring in the nasal/paranasal area, skin/soft tissue, or GI tract. The clinical course is aggressive, and the prognosis is poor. Ki-67 expression has been reported to be prognostic in patients with Stage I/II ENKL, nasal type. High Ki-67 expression (≥65%) was associated with a shorter OS and disease-free survival (DFS).[91] In multivariate analysis, Ki-67 expression and primary site of involvement were found to be independent prognostic factors for both OS and DFS.

A Korean group developed a prognostic model specifically for extranodal NK/T-cell lymphoma (Korean Prognostic Index [KPI]) based on the presence of B symptoms, Stage III/IV, LDH >ULN, and regional lymph nodes.[82] Four different risk groups were identified based on the number of adverse risk factors: group 1, no risk factors; group 2, on risk factor; group 3, two risk factors; and group 4, three or four risk factors. The KPI had a balanced distribution of patients: group 1, 27% of patients, 5-year OS 81%; group 2, 31% of patients, 5-year OS 64%; group 3, 20% of patients, 5-year OS 34%; group 4, 22% of patients, 5-year OS 7%. The KPI showed better prognostic discrimination than the IPI. Expression of CD30 by ENKL further divides patients with KPI scores of 0 to 1 into two subgroups, with significant differences in OS and PFS.[92] The 5-year PFS and OS rates in the CD30-positive group were both significantly lower than those in the CD30-negative group (34.1% vs. 64.4%, $p = 0.002$, for 5-year OS; 26.0% vs. 66.7%, $p < 0.001$, for 5 year-PFS). A Chinese study investigated the prognostic role of pretreatment Hgb level in patients with Stage I/II ENKTL. Patients with an Hgb level <120 g/L had significantly inferior PFS and OS than those with an Hgb level ≥120 g/L. In a multivariate Cox regression model, the IPI risk factors and a pretreatment Hgb level <120 g/L were all independent prognostic factors for PFS and OS ($p < 0.05$). Using these five parameters, a modified international prognostic index (mIPI) model was constructed that categorized three groups (no, one to two, and three or more adverse factors) with significantly different PFS and OS (both $p < 0.0001$) in both the training set and the validation set.[93] Recently, a prognostic nomogram for extranodal NK/T-cell lymphoma, nasal type (ENKTCL) has been proposed based on stage, age, PS, LDH, and primary tumor invasion.[94] The C index of the nomogram for OS prediction was 0.72 and was superior to the predictive power (range, 0.56–0.64) of stage, IPI, and KPI in the primary and validation cohorts. This nomogram meets level I evidence.

EBV infection, a hallmark of ENKL, can be detected by means of *in situ* hybridization (ISH) or Southern blotting. Because EBV has a transforming activity on lymphocytes, it is believed to play an important role in lymphomagenesis. EBV is present in lymphoma cells of almost all patients, accounting for the pathogenesis of ENKL. A negative EBER-ISH result should prompt hematopathologic review for an alternative diagnosis. Fragmented EBV DNA is released from tumor cells and may be detected in the peripheral blood of patients, and it serves as a surrogate biomarker of lymphoma load.[95] EBV DNA copy numbers are associated with tumor burden and predict the prognosis of ENKL at diagnosis, during therapy, and for disease monitoring.[96] Because this lymphoma occurs in regions with the highest seroprevalence rate of EBV infection, long-lived circulating memory B cells contain EBV, introducing unpredictable errors if whole blood is used for EBV DNA quantification. These limitations may be overcome by quantification of cell-free plasma EBV DNA.[95] Detection of circulating EBV DNA is prognostic across multiple studies, including those that incorporate L-asparaginase–based therapy.[94,97]

ALCL. ALCL, ALK-positive (ALK+) remains the only PTCL to date defined by recurrent chromosomal rearrangements. These involve the *ALK* gene located on chromosome 2p23. The nucleophosmin gene, *NPM*, on 5q35 is the most common translocation partner, resulting in t(2;5)(p23;q35) in 55–85% of cases, whereas variant translocations involving *ALK* and other partner genes are detected in the remainder. The translocation t(2;5)(p23;q35) results in the fusion protein NPM–ALK, leading to constitutive activation of the ALK tyrosine kinase and alterations in signaling, metabolic, and prosurvival pathways. Determination of ALK expression by IHC is essential in the selection of care. Patients with ALK expression have good outcomes with conventional chemotherapy, whereas those lacking ALK expression may require additional high-dose therapy and stem cell rescue (HDT/ASCR).

79

Hodgkin Lymphoma

Prognostic Factors Required for Stage Grouping

The Lugano classification includes B symptoms as a prognostic variable for determining stage only in HL. The presence (B) or absence (A) of symptoms must be recorded to determine stage (Table 79.11).

Additional Factors Recommended for Clinical Care

The current therapeutic approach to HL is based on the concept of early and advanced disease. Early disease is divided further into early favorable and early unfavorable. Although the concept is now universally recognized, the definitions for these different prognostic groups vary in different study groups and guidelines (Table 79.13). The clinical prognostic factors (Table 79.12) are discussed as they apply to determination of early- (favorable vs. unfavorable) and advanced-stage disease.

Early-stage Disease. Several risk factors are of prognostic importance in early-stage HL, namely B symptoms, tumor

Table 79.11 HL prognostic factors required for staging

Factor	Definition	Clinical significance	Level of evidence
B symptoms[2]	Presence of fever (>38°C, frequently in Pel–Ebstein [98] pattern), drenching night sweats, or weight loss (>10% of baseline within 6 months)	Important for determining stage (presence [B] or absence [A] of symptoms)	I

Table 79.12 HL additional factors recommended for clinical care

Factor	Definition	Clinical significance	Level of evidence
Tumor bulk	Mediastinal, measured on a PA CXR (>.33 of maximal thoracic diameter; >10 on CT	Factor for determining early-stage prognosis	I
ESR	Measure in serum. Adverse: ≥50 mm/h (A symptoms), ≥30 mm/h (B symptoms)	Factor for determining early-stage prognosis	I
Number of nodal sites	Distinct nodal regions involved (differ based on risk model—GSHG, EORTC, or NCCN—see Table 79.13)	Factor for determining early-stage prognosis	I
IPS[106]	Prognostic score based on 7 factors: stage (IV); age (>45); sex (male); WBC (>15); albumin (<4); lymphopenia (ALC <0.4)	Predicts outcome with chemotherapy, increased risk of treatment failure with each additional RF	I
PET-2[100–103]	Evaluation of PET activity after 2 cycles of chemotherapy, interpreted according to the 5-point scale[104,105]	Residual FDG avidity (>2 or >3 on 5-point scale, depending on the treatment) is associated with a poor prognosis, which may be improved by change in therapy*	I

*The interim PET-2 is not a staging study but has a major influence on therapy. If possible, the result of the interim PET-2 should be recorded

Table 79.13 Risk factors in early-stage HL (unfavorable early stage if one or more risk factors are present

	**Study group**		
**Risk Factor**	**GHSG**	**EORTC**	**NCCN**
Bulk Mediastinal mass on CT (mass/thoracic diameter ratio)	≥1/3	≥0.35	≥1/3
Largest mass on CT			≥10 cm
ESR	≥50	≥50	≥50
A	≥30	≥30	≥50
B			
Nodal areas	≥3 (of 11 GHSG areas)	≥4 (5 supradiaphragm EOTC areas)	≥4 (of 17 AA regions)
Other	≥1 extranodal lesion	Age >50	B symptoms

bulk, ESR, the number of involved nodal regions, the presence of extralymphatic disease, and age. There are three commonly used risk models for distinguishing early favorable from unfavorable: those of the GHSG, EORTC, and NCCN (Table 79.13). In each of these models, the presence of a single risk factor is sufficient to define the patient as having early unfavorable disease. Patients with favorable disease have none of these additional risk factors. Although very similar, the risk models differ in some details regarding definition of bulk, cutoffs for ESR, and number and definition of nodal areas of involvement. In interpreting and applying clinical trial data, it is very important to know which risk model was used to generate the results. Thus, application of the results of GHSG trials in early stage HL should be based on GHSG risk models.

Tumor Bulk. In the Lugano classification, tumor bulk is now measured by CT rather than posteroanterior (PA) chest X-ray (CXR). The definitions given in Table 79.12 are based on CXR; the close concordance between the determination of bulk on CXR and CT supports using the same definition.

Nodal Regions. Favorable versus unfavorable early-stage disease also is dictated by the number of nodal regions involved on imaging. The three prognostic models use a different number of sites to define poor risk, as well as distinct regional nodal maps. In the GHSG model, involvement of three or more nodal regions is an unfavorable risk factor. The GHSG map of nodal regions is shown in Fig. 79.2. The EORTC risk model defines four or more nodal sites as unfavorable, with the nodal grouping shown in Fig. 79.3. Note that the EORTC model is applicable only to supradiaphragmatic disease. The NCCN risk model also considers four or more nodal areas as adverse, although this is based on Ann Arbor regions (Fig. 79.1).

Other Factors. Each of the models includes a unique factor. In the GHSG model, involvement of an extralymphatic site is unfavorable. In the EORTC model, age over 50 is unfavorable. Finally, the presence of B symptoms is an unfavorable risk factor in the NCCN model.

Advanced Stage. Advanced-stage HL includes Stage III(A/B) and Stage IV(A/B) disease. However, for GHSG studies, advanced stage includes Stage IIB with bulk or extralymphatic disease (Table 79.14). In other studies, any patient with Stage IIB disease is considered advanced stage. Again, when interpreting and applying clinical trial data to

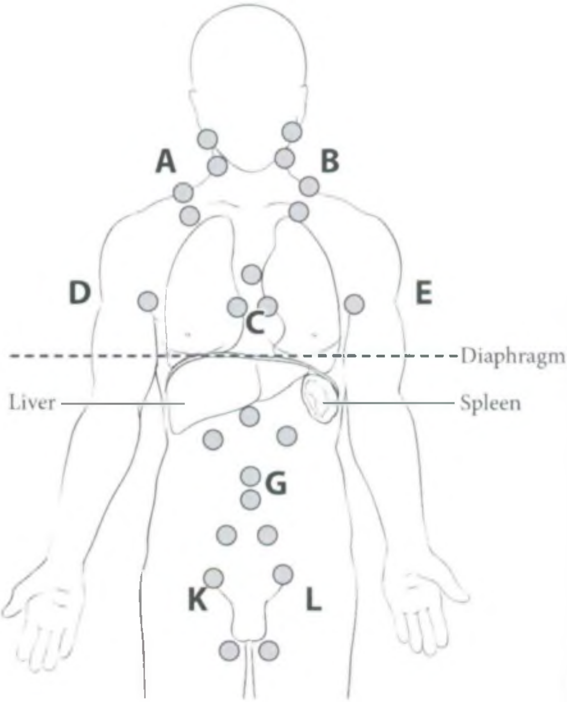

Fig. 79.2 Alternative nodal site map used by the GHSG. The GHSG map defines 11 nodal regions designated A through L

practice, it is important to be aware of the definition of *advanced stage disease* used in a particular study.

International Prognostic Score (IPS). The IPS was developed by using pooled clinical data from more than 5,000 patients treated with combination chemotherapy for advanced-stage HL (using the GHSG definition of advanced stage). The model includes seven factors that predict outcome based on the following adverse factors: serum albumin <4 g/dL, Hgb concentration <10.5 g/dL, male sex, age ≥45 years, Stage IV disease, WBC count ≥15,000/mm3, and lymphocytopenia <600/mm3 or <8%. The rate of freedom from progression by risk category was as follows: no factors, 84%; one factor, 77%; two factors, 67%; three factors, 60%; four factors, 51%; five or more factors, 42%. Other factors of note in HL have included the number of disease sites and the ESR. Several trials selected

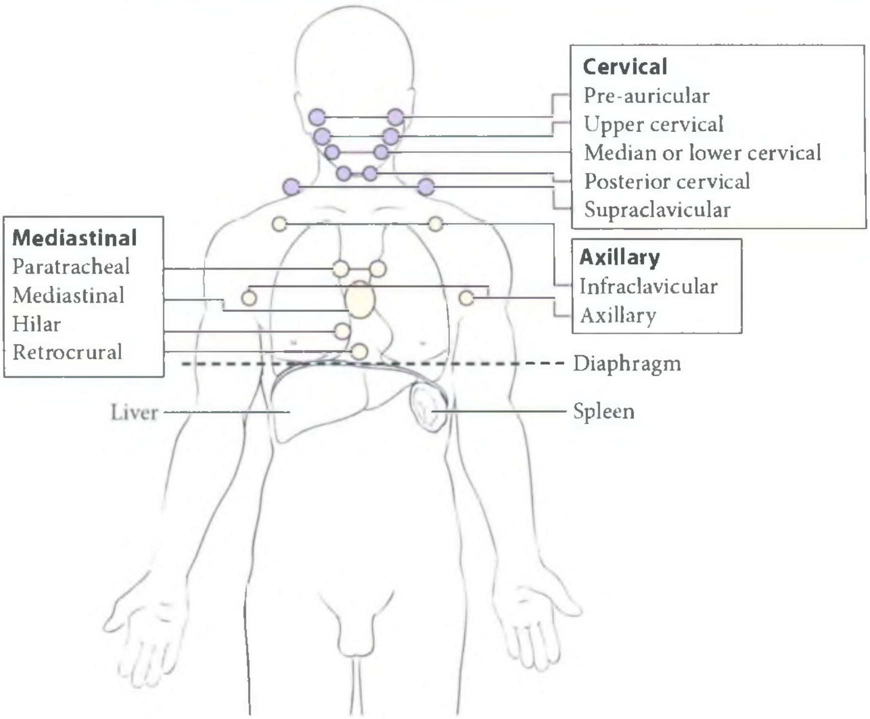

Fig. 79.3 Alternative nodal site map used the EORTC. The EORTC map defines five supradiaphragmatic nodal regions. The cervical includes two regions (right and left) as does the axillary (right and left)

Table 79.14 Classification of risk based on stage

Risk Factor	IA, IB, IIA	IIB	IIIA, IIIB	IVA, IVB
None	Early Favorable			
>3 lymph node areas (GHSG)				
ESR A ≥50 B ≥30		?	Advanced stage	
Mediastinal mass >1/3 of thoracic diameter	Early unfavorable			
Extralymphatic disease				

Note: The areas shaded in orange represent early unfavorable disease according to the GHSG. However, in some studies, any patient with CS IIB disease is categorized as having advanced-stage disease; the area in question is indicated by the "?".

patients for more intensive therapy based on an unfavorable IPS. In a 2012 reevaluation of the IPS by the Vancouver group, 19% of the patients had four or more risk factors.[99] There was a significantly inferior FFTF and OS for patients with four or more risk factors compared with others treated with ABVD-based therapy. Thus, the IPS has retained its prognostic value. Risk stratification of initial therapy based on the IPI is suggested as an option in the NCCN guidelines for HL; however, the evidence for this is less well established.

Interim PET scan. Interim PET scan after two cycles of combination chemotherapy (PET-2) has been shown to be prognostic in both early- and advanced-stage HL.[100–103] The

original report demonstrated very dramatic differences in 2-year failure-free survival: 96% for PET-2 negative and 6% for PET-2 positive. Although the direction of benefit was confirmed in many studies, the magnitude of the difference is much less in subsequent reports. In a follow-up prospective study, 3-year PFS for patients with a PET-2–negative scan was 95%, compared with 28% for a positive scan. An important lesson from the literature is that scans must be interpreted in a standardized fashion.[104] Applying various standards for interpretation, the frequency of positive scans may vary from 29% using International Harmonization Criteria (IHP) to 12% using the Deauville 5-point scale criteria; correspondingly, a positive scan may have no relation to PFS (IHP) or a major correlation with PFS (5-point scale), depending on criteria. The Lugano classification recommends the use of the 5-point scan as the standard for interpreting interim PET scans.[105] One additional subtlety of the 5-point scale is that the cutoff for a positive result may be variable. In general, when treatment is being limited, values of 3 to 5 are considered positive. In studies of full-course therapy, values of 4 to 5 are considered positive. In early-stage disease the PET positivity rate may range from 25% (3–5 as positive) to 9% (4–5 as positive). In most studies of advanced-stage disease, approximately 15–20% of patients are PET-2 positive based on a definition of 4 to 5 as positive. Recent studies evaluated PET-2 as a means for risk-adapting treatment. Two studies that were presented but not yet published suggest that patients with PET-2 positivity may have improved outcomes if their treatment is changed from ABVD to escalated BEACOPP (international RATHL study and SWOG intergroup presented at the 12th International Conference of Malignant Lymphoma). Interim PET is not part of the staging but is valuable for treatment; therefore, the PET-2 results should be recorded.

RISK ASSESSMENT MODELS

Risk assessment models and prognostic tools play an important role in cancer medicine because they provide a mechanism to integrate disparate data elements into a process that leads to decreased prognostic heterogeneity. Such processes are useful for (1) identifying and characterizing important prognostic factors, (2) improving prognostic predictions for individual patients, and (3) designing, conducting, and analyzing clinical trials.[107] The most common type of prognostic tool is a prognostic calculator that provides time-specific outcome (e.g., 5-year OS) probability predictions for individual patients based on their demographic, clinical, and tumor characteristics. The prognostic nomogram developed by Yang et al[94] is an example of a risk calculator. Another type of prognostic tool is a prognostic classifier that places patients into ordered prognostic risk classes (either directly or based on cutoffs for individual probability estimates). The remaining tools referenced in this chapter (e.g., IPI, MIPI, FLIPI, and CLL-IPI) are prognostic classifiers. The AJCC Precision Medicine Core (PMC) developed and published criteria for critical evaluation of prognostic calculators,[108] which are presented and discussed in Chapter 4. The prognostic nomogram developed by Yang et al[94] meets all but one of the AJCC PMC criteria because it lacks discussion of how missing data were treated.

AJCC PROGNOSTIC STAGE GROUPS

Lugano Classification for Hodgkin and Non-Hodgkin Lymphoma[4]

Stage	Stage description
Limited stage	
I	Involvement of a single lymphatic site (i.e., nodal region, Waldeyer's ring, thymus, or spleen)
IE	Single extralymphatic site in the absence of nodal involvement (rare in Hodgkin lymphoma)
II	Involvement of two or more lymph node regions on the same side of the diaphragm
IIE	Contiguous extralymphatic extension from a nodal site with or without involvement of other lymph node regions on the same side of the diaphragm
*II bulky**	*Stage II with disease bulk***
Advanced stage	
III	Involvement of lymph node regions on both sides of the diaphragm; nodes above the diaphragm with spleen involvement
IV	Diffuse or disseminated involvement of one or more extralymphatic organs, with or without associated lymph node involvement;<br>or *noncontiguous* extralymphatic organ involvement in conjunction with nodal Stage II disease<br>or *any* extralymphatic organ involvement in nodal Stage III disease<br>Stage IV includes *any* involvement of the CSF, bone marrow, liver, or lungs (other than by direct extension in Stage IIE disease)

* Stage II bulky may be considered either early or advanced stage based on lymphoma histology and prognostic factors (see discussion of Hodgkin lymphoma prognostic factors).

* * The definition of disease bulk varies according to lymphoma histology. In the Lugano classification,[4] bulk in Hodgkin lymphoma is defined as a mass greater than one third of the thoracic diameter on CT of the chest or a mass >10 cm. For NHL, the recommended definitions of bulk vary by lymphoma histology. In follicular lymphoma, 6 cm has been suggested based on the Follicular Lymphoma International Prognostic Index-2 (FLIPI-2) and its validation.[9, 10] In DLBCL, cutoffs ranging from 5 to 10 cm have been used, although 10 cm is recommended.[11]

Note: Hodgkin lymphoma uses A or B designation with stage group. A/B is no longer used in NHL.

Chronic Lymphocytic Leukemia/ Small Lymphocytic Lymphoma

Modified Rai staging system (mainly used in North America)			
**Stage**	**Risk**	**Findings**	**Survival (mo)**
0	Low	Lymphocytosis only	>120
I	Intermediate	+ Adenopathy	95
II	Intermediate	+ Enlarged spleen and/or liver	72
III	High	Lymphocytosis + Hgb <11 g/dL	30
IV	High	Lymphocytosis + Plt <100,000/μL	30

Binet staging system		
**Stage**	**Findings**	**Survival (mo)**
A	Lymphocytosis only	>120
B	+ Adenopathy	95
C	+ Enlarged spleen and/or liver	72

REGISTRY DATA COLLECTION VARIABLES

1. For all lymphomas: size of the largest mass in millimeters for all stages; essential for Stages I and II
2. DLCBL:
   a. NCCN IPI points (08)
   b. IHC-determined COO
3. Mantle cell lymphoma: proliferation index (% of positivity with either the Ki-67 or MIB1 monoclonal antibodies
4. Follicular lymphoma:
   a. Tumor disease burden (high [one or more factors] vs. low [0 factors]) based on the presence or absence of GELF criteria
   b. FLIPI (as FLIPI-1 or FLIPI-2)
5. CLL/SLL: (should always be abstracted as lymphoma per expert panel)
   a. ALC >5,000 cells/μL
   b. Adenopathy: presence of lymph nodes >1.5 cm on PE
   c. Organomegaly: enlarged liver and/or spleen on PE
   d. Anemia: Hgb <11.0 g/dL
   e. Thrombocytopenia: Plt <100,000/μL
6. HL:
   a. A or B designation for symptoms must be part of the stage
   b. IPS

Bibliography

1. Lister TA, Crowther D, Sutcliffe SB, et al. Report of a committee convened to discuss the evaluation and staging of patients with Hodgkin's disease: Cotswolds meeting. *J Clin Oncol*. Nov 1989; 7(11):1630–1636.
2. Carbone PP, Kaplan HS, Musshoff K, Smithers DW, Tubiana M. Report of the Committee on Hodgkin's Disease Staging Classification. *Cancer Res*. Nov 1971;31(11):1860–1861.
3. Rosenberg SA. Validity of the Ann Arbor staging classification for the non-Hodgkin's lymphomas. *Cancer Treat Rep*. Sep 1977;61(6): 1023–1027.
4. Cheson BD, Fisher RI, Barrington SF, et al. Recommendations for initial evaluation, staging, and response assessment of Hodgkin and non-Hodgkin lymphoma: the Lugano classification. *J Clin Oncol*. Sep 20 2014;32(27):3059–3068.
5. Hamlin PA, Zelenetz AD, Kewalramani T, et al. Age-adjusted International Prognostic Index predicts autologous stem cell transplantation outcome for patients with relapsed or primary refractory diffuse large B-cell lymphoma. *Blood*. Sep 15 2003;102(6): 1989–1996.
6. Moskowitz CH, Kewalramani T, Nimer SD, Gonzalez M, Zelenetz AD, Yahalom J. Effectiveness of high dose chemoradiotherapy and autologous stem cell transplantation for patients with biopsy-proven primary refractory Hodgkin's disease. *British journal of haematology*. Mar 2004;124(5):645–652.
7. Moskowitz CH, Matasar MJ, Zelenetz AD, et al. Normalization of pre-ASCT, FDG-PET imaging with second-line, non-cross-resistant, chemotherapy programs improves event-free survival in patients with Hodgkin lymphoma. *Blood*. Feb 16 2012;119(7): 1665–1670.
8. Perales MA, Jenq R, Goldberg JD, et al. Second-line age-adjusted International Prognostic Index in patients with advanced non-Hodgkin lymphoma after T-cell depleted allogeneic hematopoietic SCT. *Bone Marrow Transplant*. Sep 2010;45(9):1408–1416.
9. Arcaini L, Rattotti S, Gotti M, Luminari S. Prognostic assessment in patients with indolent B-cell lymphomas. *ScientificWorldJournal*. 2012;2012:107892.
10. Federico M, Bellei M, Marcheselli L, et al. Follicular lymphoma international prognostic index 2: a new prognostic index for follicular lymphoma developed by the international follicular lymphoma prognostic factor project. *J Clin Oncol*. Sep 20 2009;27(27):4555–4562.
11. Pfreundschuh M, Ho AD, Cavallin-Stahl E, et al. Prognostic significance of maximum tumour (bulk) diameter in young patients with good-prognosis diffuse large-B-cell lymphoma treated with CHOP-like chemotherapy with or without rituximab: an exploratory analysis of the MabThera International Trial Group (MInT) study. *The lancet oncology*. May 2008;9(5):435–444.
12. Swerdlow SH, International Agency for Research on Cancer., World Health Organization. *WHO classification of tumours of haematopoietic and lymphoid tissues*. Lyon, France: International Agency for Research on Cancer; 2008.
13. Savage KJ, Zeynalova S, Kansara RR, et al. Validation of a Prognostic Model to Assess the Risk of CNS Disease in Patients with Aggressive B-Cell Lymphoma. *Blood*. 2014;124(21):Abract 394.
14. Vitolo U, Chiappella A, Ferreri AJ, et al. First-line treatment for primary testicular diffuse large B-cell lymphoma with rituximab-CHOP, CNS prophylaxis, and contralateral testis irradiation: final results of an international phase II trial. *J Clin Oncol*. Jul 10 2011;29(20):2766–2772.
15. Hosein PJ, Maragulia JC, Salzberg MP, et al. A multicentre study of primary breast diffuse large B-cell lymphoma in the rituximab era. *British journal of haematology*. May 2014;165(3):358–363.
16. Shipp M, Harrington D, Anderson J, et al. A predictive model for aggressive non-Hodgkin's lymphoma. *New England Journal of Medicine*. 1993;329(14):987–994.
17. Sehn LH, Berry B, Chhanabhai M, et al. The revised International Prognostic Index (R-IPI) is a better predictor of outcome than the standard IPI for patients with diffuse large B-cell lymphoma treated with R-CHOP. *Blood*. Mar 1 2007;109(5):1857–1861.
18. Zhou Z, Sehn LH, Rademaker AW, et al. An enhanced International Prognostic Index (NCCN-IPI) for patients with diffuse large

79

B-cell lymphoma treated in the rituximab era. *Blood*. Feb 6 2014;123(6):837–842.

19. Melchardt T, Troppan K, Weiss L, et al. A modified scoring of the NCCN-IPI is more accurate in the elderly and is improved by albumin and beta2 -microglobulin. *British journal of haematology*. Jan 2015;168(2):239–245.
20. Harris NL, Jaffe ES, Stein H, et al. A revised European-American classification of lymphoid neoplasms: a proposal from the International Lymphoma Study Group. *Blood*. Sep 1 1994;84(5): 1361–1392.
21. Jaffe ES, World Health Organization. *Pathology and genetics of tumours of haematopoietic and lymphoid tissues*. Lyon Oxford: IARC Press : Oxford University Press (distributor); 2001.
22. Alizadeh AA, Eisen MB, Davis RE, et al. Distinct types of diffuse large B-cell lymphoma identified by gene expression profiling. *Nature*. Feb 3 2000;403(6769):503–511.
23. Lenz G, Wright GW, Emre NC, et al. Molecular subtypes of diffuse large B-cell lymphoma arise by distinct genetic pathways. *Proc Natl Acad Sci U S A*. Sep 9 2008;105(36):13520–13525.
24. Hans CP, Weisenburger DD, Greiner TC, et al. Confirmation of the molecular classification of diffuse large B-cell lymphoma by immunohistochemistry using a tissue microarray. *Blood*. Jan 1 2004;103(1):275–282.
25. Scott DW, Mottok A, Ennishi D, et al. Prognostic Significance of Diffuse Large B-Cell Lymphoma Cell of Origin Determined by Digital Gene Expression in Formalin-Fixed Paraffin-Embedded Tissue Biopsies. *J Clin Oncol*. Sep 10 2015;33(26):2848–2856.
26. Xue X, Zeng N, Gao Z, Du MQ. Diffuse large B-cell lymphoma: sub-classification by massive parallel quantitative RT-PCR. *Lab Invest*. Jan 2015;95(1):113–120.
27. Kanungo A, Medeiros LJ, Abruzzo LV, Lin P. Lymphoid neoplasms associated with concurrent t(14;18) and 8q24/c-MYC translocation generally have a poor prognosis. *Modern pathology : an official journal of the United States and Canadian Academy of Pathology, Inc*. Jan 2006;19(1):25–33.
28. Kramer MH, Hermans J, Wijburg E, et al. Clinical relevance of BCL2, BCL6, and MYC rearrangements in diffuse large B-cell lymphoma. *Blood*. Nov 1 1998;92(9):3152–3162.
29. Le Gouill S, Talmant P, Touzeau C, et al. The clinical presentation and prognosis of diffuse large B-cell lymphoma with t(14;18) and 8q24/c-MYC rearrangement. *Haematologica*. Oct 2007;92(10): 1335–1342.
30. Cheah CY, Oki Y, Westin JR, Turturro F. A clinician's guide to double hit lymphomas. *British journal of haematology*. Mar 2015; 168(6):784–795.
31. Petrich AM, Gandhi M, Jovanovic B, et al. Impact of induction regimen and stem cell transplantation on outcomes in double-hit lymphoma: a multicenter retrospective analysis. *Blood*. Oct 9 2014;124(15):2354–2361.
32. Green TM, Young KH, Visco C, et al. Immunohistochemical double-hit score is a strong predictor of outcome in patients with diffuse large B-cell lymphoma treated with rituximab plus cyclophosphamide, doxorubicin, vincristine, and prednisone. *J Clin Oncol*. Oct 1 2012;30(28):3460–3467.
33. Horn H, Ziepert M, Becher C, et al. MYC status in concert with BCL2 and BCL6 expression predicts outcome in diffuse large B-cell lymphoma. *Blood*. Mar 21 2013;121(12):2253–2263.
34. Hu S, Xu-Monette ZY, Tzankov A, et al. MYC/BCL2 protein coexpression contributes to the inferior survival of activated B-cell subtype of diffuse large B-cell lymphoma and demonstrates high-risk gene expression signatures: a report from The International DLBCL Rituximab-CHOP Consortium Program. *Blood*. May 16 2013;121(20):4021–4031; quiz 4250.
35. Johnson NA, Slack GW, Savage KJ, et al. Concurrent expression of MYC and BCL2 in diffuse large B-cell lymphoma treated with rituximab plus cyclophosphamide, doxorubicin, vincristine, and prednisone. *J Clin Oncol*. Oct 1 2012;30(28):3452–3459.
36. Miller TP. The limits of limited stage lymphoma. *J Clin Oncol*. Aug 1 2004;22(15):2982–2984.
37. Hernandez-Ilizaliturri FJ, Deeb G, Zinzani PL, et al. Higher response to lenalidomide in relapsed/refractory diffuse large B-cell lymphoma in nongerminal center B-cell-like than in germinal center B-cell-like phenotype. *Cancer*. Nov 15 2011;117(22): 5058–5066.
38. Nowakowski GS, LaPlant B, Macon WR, et al. Lenalidomide combined with R-CHOP overcomes negative prognostic impact of non-germinal center B-cell phenotype in newly diagnosed diffuse large B-Cell lymphoma: a phase II study. *J Clin Oncol*. Jan 20 2015;33(3):251–257.
39. Vitolo U, Chiappella A, Franceschetti S, et al. Lenalidomide plus R-CHOP21 in elderly patients with untreated diffuse large B-cell lymphoma: results of the REAL07 open-label, multicentre, phase 2 trial. *The lancet oncology*. Jun 2014;15(7):730–737.
40. Wilson WH, Young RM, Schmitz R, et al. Targeting B cell receptor signaling with ibrutinib in diffuse large B cell lymphoma. *Nature medicine*. Aug 2015;21(8):922–926.
41. Younes A, Thieblemont C, Morschhauser F, et al. Combination of ibrutinib with rituximab, cyclophosphamide, doxorubicin, vincristine, and prednisone (R-CHOP) for treatment-naive patients with CD20-positive B-cell non-Hodgkin lymphoma: a non-randomised, phase 1b study. *The lancet oncology*. Aug 2014;15(9):1019–1026.
42. Martin P, Chadburn A, Christos P, et al. Outcome of deferred initial therapy in mantle-cell lymphoma. *J Clin Oncol*. Mar 10 2009;27(8):1209–1213.
43. Rosenwald A, Wright G, Wiestner A, et al. The proliferation gene expression signature is a quantitative integrator of oncogenic events that predicts survival in mantle cell lymphoma. *Cancer Cell*. Feb 2003;3(2):185–197.
44. Determann O, Hoster E, Ott G, et al. Ki-67 predicts outcome in advanced-stage mantle cell lymphoma patients treated with anti-CD20 immunochemotherapy: results from randomized trials of the European MCL Network and the German Low Grade Lymphoma Study Group. *Blood*. Feb 15 2008;111(4):2385–2387.
45. Geisler CH, Kolstad A, Laurell A, et al. Long-term progression-free survival of mantle cell lymphoma after intensive front-line immunochemotherapy with in vivo-purged stem cell rescue: a non-randomized phase 2 multicenter study by the Nordic Lymphoma Group. *Blood*. Oct 1 2008;112(7):2687–2693.
46. Klapper W, Hoster E, Determann O, et al. Ki-67 as a prognostic marker in mantle cell lymphoma-consensus guidelines of the pathology panel of the European MCL Network. *J Hematop*. Jul 2009;2(2):103–111.
47. Schaffel R, Hedvat CV, Teruya-Feldstein J, et al. Prognostic impact of proliferative index determined by quantitative image analysis and the International Prognostic Index in patients with mantle cell lymphoma. *Ann Oncol*. Jan 2010;21(1):133–139.
48. Hoster E, Dreyling M, Klapper W, et al. A new prognostic index (MIPI) for patients with advanced-stage mantle cell lymphoma. *Blood*. Jan 15 2008;111(2):558–565.
49. Shah JJ, Fayad L, Romaguera J. Mantle Cell International Prognostic Index (MIPI) not prognostic after R-hyper-CVAD. *Blood*. Sep 15 2008;112(6):2583; author reply 2583–2584.
50. van de Schans SA, Janssen-Heijnen ML, Nijziel MR, Steyerberg EW, van Spronsen DJ. Validation, revision and extension of the Mantle Cell Lymphoma International Prognostic Index in a population-based setting. *Haematologica*. Sep 2010;95(9):1503–1509.
51. Hoster E, Klapper W, Hermine O, et al. Confirmation of the mantle-cell lymphoma International Prognostic Index in randomized trials of the European Mantle-Cell Lymphoma Network. *J Clin Oncol*. May 1 2014;32(13):1338–1346.
52. Brice P, Bastion Y, Lepage E, et al. Comparison in low-tumor-burden follicular lymphomas between an initial no-treatment policy, prednimustine, or interferon alfa: a randomized study from the Groupe d'Etude des Lymphomes Folliculaires. Groupe d'Etude

des Lymphomes de l'Adulte. *J Clin Oncol*. Mar 1997;15(3): 1110–1117.
53. Ardeshna KM, Qian W, Smith P, et al. Rituximab versus a watch-and-wait approach in patients with advanced-stage, asymptomatic, non-bulky follicular lymphoma: an open-label randomised phase 3 trial. *The lancet oncology*. Apr 2014;15(4):424–435.
54. Ardeshna KM, Smith P, Norton A, et al. Long-term effect of a watch and wait policy versus immediate systemic treatment for asymptomatic advanced-stage non-Hodgkin lymphoma: a randomised controlled trial. *Lancet*. Aug 16 2003;362(9383): 516–522.
55. Young RC, Longo DL, Glatstein E, Ihde DC, Jaffe ES, DeVita VT, Jr. The treatment of indolent lymphomas: watchful waiting v aggressive combined modality treatment. *Seminars in hematology*. Apr 1988;25(2 Suppl 2):11–16.
56. Solal-Celigny P, Roy P, Colombat P, et al. Follicular lymphoma international prognostic index. *Blood*. Sep 1 2004;104(5): 1258–1265.
57. Auer IA, Gascoyne RD, Connors JM, et al. t(11;18)(q21;q21) is the most common translocation in MALT lymphomas. *Ann Oncol*. Oct 1997;8(10):979–985.
58. Leroux D, Seite P, Hillion J, et al. t(11;18)(q21;q21) may delineate a spectrum of diffuse small B-cell lymphoma with extranodal involvement. *Genes Chromosomes Cancer*. May 1993;7(1):54–56.
59. Liu H, Ruskon-Fourmestraux A, Lavergne-Slove A, et al. Resistance of t(11;18) positive gastric mucosa-associated lymphoid tissue lymphoma to Helicobacter pylori eradication therapy. *Lancet*. Jan 6 2001;357(9249):39–40.
60. Zhang W, Garces J, Dong HY. Detection of the t(11;18) API2/MALT1 translocation associated with gastric MALT lymphoma in routine formalin-fixed, paraffin-embedded small endoscopic biopsy specimens by robust real-time RT-PCR. *Am J Clin Pathol*. Dec 2006;126(6):931–940.
61. Hermine O, Lefrere F, Bronowicki JP, et al. Regression of splenic lymphoma with villous lymphocytes after treatment of hepatitis C virus infection. *N Engl J Med*. Jul 11 2002;347(2):89–94.
62. Dohner H, Stilgenbauer S, Benner A, et al. Genomic aberrations and survival in chronic lymphocytic leukemia. *N Engl J Med*. Dec 28 2000;343(26):1910–1916.
63. Damle RN, Wasil T, Fais F, et al. Ig V gene mutation status and CD38 expression as novel prognostic indicators in chronic lymphocytic leukemia. *Blood*. Sep 15 1999;94(6):1840–1847.
64. Hamblin TJ, Davis Z, Gardiner A, Oscier DG, Stevenson FK. Unmutated Ig V(H) genes are associated with a more aggressive form of chronic lymphocytic leukemia. *Blood*. Sep 15 1999; 94(6):1848–1854.
65. Wierda WG, O'Brien S, Wang X, et al. Multivariable model for time to first treatment in patients with chronic lymphocytic leukemia. *J Clin Oncol*. Nov 1 2011;29(31):4088–4095.
66. Thorselius M, Krober A, Murray F, et al. Strikingly homologous immunoglobulin gene rearrangements and poor outcome in VH3–21-using chronic lymphocytic leukemia patients independent of geographic origin and mutational status. *Blood*. Apr 1 2006;107(7):2889–2894.
67. Dohner H, Fischer K, Bentz M, et al. p53 gene deletion predicts for poor survival and non-response to therapy with purine analogs in chronic B-cell leukemias. *Blood*. Mar 15 1995;85(6):1580–1589.
68. Byrd JC, Gribben JG, Peterson BL, et al. Select high-risk genetic features predict earlier progression following chemoimmunotherapy with fludarabine and rituximab in chronic lymphocytic leukemia: justification for risk-adapted therapy. *J Clin Oncol*. Jan 20 2006;24(3):437–443.
69. Wattel E, Preudhomme C, Hecquet B, et al. p53 mutations are associated with resistance to chemotherapy and short survival in hematologic malignancies. *Blood*. Nov 1 1994;84(9):3148–3157.
70. Dicker F, Herholz H, Schnittger S, et al. The detection of TP53 mutations in chronic lymphocytic leukemia independently predicts rapid disease progression and is highly correlated with a complex aberrant karyotype. *Leukemia*. Jan 2009;23(1):117–124.
71. Hallek M, Fischer K, Fingerle-Rowson G, et al. Addition of rituximab to fludarabine and cyclophosphamide in patients with chronic lymphocytic leukaemia: a randomised, open-label, phase 3 trial. *Lancet*. Oct 2 2010;376(9747):1164–1174.
72. Seiffert M, Dietrich S, Jethwa A, Glimm H, Lichter P, Zenz T. Exploiting biological diversity and genomic aberrations in chronic lymphocytic leukemia. *Leuk Lymphoma*. Jun 2012: 53(6):1023–1031.
73. Zenz T, Vollmer D, Trbusek M, et al. TP53 mutation profile in chronic lymphocytic leukemia: evidence for a disease specific profile from a comprehensive analysis of 268 mutations. *Leukemia*. Dec 2010;24(12):2072–2079.
74. Furman RR, Sharman JP, Coutre SE, et al. Idelalisib and rituximab in relapsed chronic lymphocytic leukemia. *N Engl J Med*. Mar 13 2014;370(11):997–1007.
75. O'Brien S, Jones JA, Coutre S, et al. Efficacy and safety of ibrutinib in patients with relapsed or refractory chronic lymphocytic leukemia or small lymphocytic leukemia with 17p deletion: Results from the phase II RESONATE™-17 trial. *Blood*. 2014;124(21):327–327.
76. Dohner H, Stilgenbauer S, James MR, et al. 11q deletions identify a new subset of B-cell chronic lymphocytic leukemia characterized by extensive nodal involvement and inferior prognosis. *Blood*. Apr 1 1997;89(7):2516–2522.
77. Rossi D, Gaidano G. ATM and chronic lymphocytic leukemia: mutations, and not only deletions, matter. *Haematologica*. Jan 2012; 97(1):5–8.
78. Oscier D, Wade R, Davis Z, et al. Prognostic factors identified three risk groups in the LRF CLL4 trial, independent of treatment allocation. *Haematologica*. Oct 2010;95(10):1705–1712.
79. Linn BS, Linn MW, Gurel L. Cumulative illness rating scale. *Journal of the American Geriatrics Society*. May 1968;16(5):622–626.
80. Vose J, Armitage J, Weisenburger D, International TCLP. International peripheral T-cell and natural killer/T-cell lymphoma study: pathology findings and clinical outcomes. *J Clin Oncol*. Sep 1 2008;26(25):4124–4130.
81. Gallamini A, Stelitano C, Calvi R, et al. Peripheral T-cell lymphoma unspecified (PTCL-U): a new prognostic model from a retrospective multicentric clinical study. *Blood*. Apr 1 2004;103(7): 2474–2479.
82. Lee J, Suh C, Park YH, et al. Extranodal natural killer T-cell lymphoma, nasal-type: a prognostic model from a retrospective multicenter study. *J Clin Oncol*. Feb 1 2006;24(4):612–618.
83. Tokunaga T, Shimada K, Yamamoto K, et al. Retrospective analysis of prognostic factors for angioimmunoblastic T-cell lymphoma: a multicenter cooperative study in Japan. *Blood*. Mar 22 2012;119(12):2837–2843.
84. de Baaij LR, Berkhof J, van de Water JM, et al. A New and Validated Clinical Prognostic Model (EPI) for Enteropathy-Associated T-cell Lymphoma. *Clin Cancer Res*. Jul 1 2015;21(13):3013–3019.
85. Petrich AM, Helenowski IB, Bryan LJ, Rozell SA, Galamaga R, Nabhan C. Factors predicting survival in peripheral T-cell lymphoma in the USA: a population-based analysis of 8802 patients in the modern era. *British journal of haematology*. Mar 2015;168(5):708–718.
86. Savage KJ, Harris NL, Vose JM, et al. ALK- anaplastic large-cell lymphoma is clinically and immunophenotypically different from both ALK+ ALCL and peripheral T-cell lymphoma, not otherwise specified: report from the International Peripheral T-Cell Lymphoma Project. *Blood*. Jun 15 2008;111(12):5496–5504.
87. Sibon D, Fournier M, Briere J, et al. Long-term outcome of adults with systemic anaplastic large-cell lymphoma treated within the Groupe d'Etude des Lymphomes de l'Adulte trials. *J Clin Oncol*. Nov 10 2012;30(32):3939–3946.
88. Moskowitz AJ, Lunning MA, Horwitz SM. How I treat the peripheral T-cell lymphomas. *Blood*. Apr 24 2014;123(17):2636–2644.

79

89. Weisenburger DD, Savage KJ, Harris NL, et al. Peripheral T-cell lymphoma, not otherwise specified: a report of 340 cases from the International Peripheral T-cell Lymphoma Project. *Blood*. Mar 24 2011;117(12):3402–3408.
90. Federico M, Rudiger T, Bellei M, et al. Clinicopathologic characteristics of angioimmunoblastic T-cell lymphoma: analysis of the international peripheral T-cell lymphoma project. *J Clin Oncol*. Jan 10 2013;31(2):240–246.
91. Kim SJ, Kim BS, Choi CW, et al. Ki-67 expression is predictive of prognosis in patients with stage I/II extranodal NK/T-cell lymphoma, nasal type. *Ann Oncol*. Aug 2007;18(8):1382–1387.
92. Li P, Jiang L, Zhang X, Liu J, Wang H. CD30 expression is a novel prognostic indicator in extranodal natural killer/T-cell lymphoma, nasal type. *BMC cancer*. 2014;14:890.
93. Wang L, Xia ZJ, Lu Y, et al. A modified international prognostic index including pretreatment hemoglobin level for early stage extranodal natural killer/T cell lymphoma. *Leuk Lymphoma*. Nov 2015;56(11):3038–3044.
94. Yang Y, Zhang YJ, Zhu Y, et al. Prognostic nomogram for overall survival in previously untreated patients with extranodal NK/T-- cell lymphoma, nasal-type: a multicenter study. *Leukemia*. Jul 2015;29(7):1571–1577.
95. Tse E, Kwong YL. Management of advanced NK/T-cell lymphoma. *Current hematologic malignancy reports*. Sep 2014;9(3): 233–242.
96. Kwong YL, Anderson BO, Advani R, et al. Management of T-cell and natural-killer-cell neoplasms in Asia: consensus statement from the Asian Oncology Summit 2009. *The lancet oncology*. Nov 2009;10(11):1093–1101.
97. Kwong YL, Pang AW, Leung AY, Chim CS, Tse E. Quantification of circulating Epstein-Barr virus DNA in NK/T-cell lymphoma treated with the SMILE protocol: diagnostic and prognostic significance. *Leukemia*. Apr 2014;28(4):865–870.
98. Reimann HA. Periodic (Pel-Ebstein) fever of lymphomas. *Annals of clinical and laboratory science*. Jan-Feb 1977;7(1):1–5.
99. Moccia AA, Donaldson J, Chhanabhai M, et al. International Prognostic Score in advanced-stage Hodgkin's lymphoma: altered utility in the modern era. *J Clin Oncol*. Sep 20 2012;30(27): 3383–3388.
100. Gallamini A, Barrington SF, Biggi A, et al. The predictive role of interim positron emission tomography for Hodgkin lymphoma treatment outcome is confirmed using the interpretation criteria of the Deauville five-point scale. *Haematologica*. Jun 2014;99(6): 1107–1113.
101. Gallamini A, Rigacci L, Merli F, et al. The predictive value of positron emission tomography scanning performed after two courses of standard therapy on treatment outcome in advanced stage Hodgkin's disease. *Haematologica*. Apr 2006;91(4): 475–481.
102. Hutchings M, Loft A, Hansen M, et al. FDG-PET after two cycles of chemotherapy predicts treatment failure and progression-free survival in Hodgkin lymphoma. *Blood*. Jan 1 2006;107(1):52–59.
103. Zinzani PL, Tani M, Fanti S, et al. Early positron emission tomography (PET) restaging: a predictive final response in Hodgkin's disease patients. *Ann Oncol*. Aug 2006;17(8):1296–1300.
104. Le Roux PY, Gastinne T, Le Gouill S, et al. Prognostic value of interim FDG PET/CT in Hodgkin's lymphoma patients treated with interim response-adapted strategy: comparison of International Harmonization Project (IHP), Gallamini and London criteria. *European journal of nuclear medicine and molecular imaging*. Jun 2011;38(6):1064–1071.
105. Meignan M, Gallamini A, Meignan M, Gallamini A, Haioun C. Report on the First International Workshop on Interim-PET-Scan in Lymphoma. *Leuk Lymphoma*. Aug 2009;50(8): 1257–1260.
106. Hasenclever D, Diehl V. A prognostic score for advanced Hodgkin's disease. International Prognostic Factors Project on Advanced Hodgkin's Disease. *N Engl J Med*. Nov 19 1998;339(21): 1506–1514.
107. Halabi S, Owzar K. The importance of identifying and validating prognostic factors in oncology. Paper presented at: Seminars in oncology2010.
108. Kattan MW, Hess KR, Amin MB, et al. American Joint Committee on Cancer acceptance criteria for inclusion of risk models for individualized prognosis in the practice of precision medicine. *CA: a cancer journal for clinicians*. Jan 19 2016.
109. Kumar A, Burger IA, Zhang Z, et al. Definition of bulky disease in early stage Hodgkin lymphoma in computed tomography era: prognostic significance of measurements in the coronal and transverse planes. *Haematologica*. 2016.

Pediatric Hodgkin and Non-Hodgkin Lymphomas

80

Michael P. Link, Elaine S. Jaffe, and John P. Leonard

CHAPTER SUMMARY

Cancers Staged Using This Staging System

Hodgkin lymphoma and non-Hodgkin lymphomas of childhood. It is noteworthy that different systems are used for staging Hodgkin lymphoma and non-Hodgkin lymphoma in children. These are described separately in this chapter.

Cancers Not Staged Using This Staging System

These histopathologic types of cancer…	Are staged according to the classification for…	And can be found in chapter…
Pediatric lymphoid leukemias, including acute lymphoblastic leukemia	Leukemia	83

Summary of Changes

Change	Details of Change	Level of Evidence
AJCC Prognostic Stage Groups	The Ann Arbor staging system has been found to be inappropriate for staging of the non-Hodgkin lymphomas (NHLs) of childhood. The St. Jude staging system[1] has been widely accepted for more than three decades and remains the recommended staging system for NHLs.<br>Staging of childhood Hodgkin lymphoma is the same as for the adult counterpart. See Chapters 79 for a summary of modifications to the Ann Arbor staging system as adopted in the Lugano classification.[2-5]	I
Emerging Factors for Clinical Care	Recently, a multidisciplinary expert panel proposed a revised International Pediatric Non-Hodgkin Lymphoma Staging System (INHLSS) to address some of the shortcomings of the St. Jude staging classification.[6] As this system gains more acceptance, AJCC will consider using this revised staging classification in the future for uniformity in management of children with NHL and will facilitate comparisons of study results.	II

To access the AJCC cancer staging forms, please visit www.cancerstaging.org.

© American Joint Committee on Cancer 2017

M.B. Amin et al. (eds.), *AJCC Cancer Staging Manual, Eighth Edition*, DOI 10.1007/978-3-319-40618-3_80

ICD-O-3 Topography Codes

Code	Description
C00–C14	Lip, oral cavity, and pharynx
C15–C26	Digestive organs
C30–C39	Respiratory system and intrathoracic organs
C40–C41	Bones, joints, and articular cartilage
C42.0	Blood
C42.1	Bone marrow
C42.2	Spleen
C42.3	Reticuloendothelial system, NOS
C42.4	Hematopoietic system, NOS
C44	Skin, excluding C44.1, eyelid
C47	Peripheral nerves and autonomic nervous system
C48	Retroperitoneum and peritoneum
C49	Connective, subcutaneous, and other soft tissues
C50	Breast
C51–C58	Female genital organs
C60–C63	Male genital organs
C64–C68	Urinary tract
C69.1	Cornea, NOS
C69.2	Retina
C69.3	Choroid
C69.4	Ciliary body
C73–C74	Thyroid and adrenal gland
C76	Other and ill-defined sites
C77.0	Lymph nodes of head, face, and neck
C77.1	Intrathoracic lymph nodes
C77.2	Intra-abdominal lymph nodes
C77.3	Lymph nodes of axilla or arm
C77.4	Lymph nodes of inguinal region or leg
C77.5	Pelvic lymph nodes
C77.8	Lymph nodes of multiple regions
C77.9	Lymph node, NOS
C80.9	Unknown primary site

WHO Classification of Tumors

Code	Description
9679	Mediastinal (thymic) large B-cell lymphoma
9680	Malignant lymphoma, large B-cell, diffuse, NOS
9687	Burkitt lymphoma, NOS
9714	Anaplastic large cell lymphoma, T-cell and Null-cell type
9728	Precursor B-cell lymphoblastic lymphoma
9729	Precursor T-cell lymphoblastic lymphoma
9650	Hodgkin lymphoma, NOS
9651	Hodgkin lymphoma, lymphocyte-rich
9652	Hodgkin lymphoma, mixed cellularity, NOS
9653	Hodgkin lymphoma, lymphocyte depletion, NOS
9654	Hodgkin lymphoma, lymphocyte depletion, diffuse fibrosis
9655	Hodgkin lymphoma, lymphocyte depletion, reticular
9659	Hodgkin lymphoma, nodular lymphocyte predominance

Swerdlow SH, Campo E, Harris NL, Jaffe ES, Pileri SA, Stein H, Thiele J, Vardiman J, eds. World Health Organization Classification of Tumours of Haematopoietic and Lymphoid Tissues. Lyon: IARC; 2008.

INTRODUCTION

The lymphoid neoplasms of childhood together comprise between one third and one half of all the cancers encountered among children and adolescents, respectively. Of these, acute lymphoblastic leukemias (ALLs) account for approximately 30% of childhood cancers, whereas the malignant lymphomas (Hodgkin lymphoma and non-Hodgkin lymphoma [NHL]) account for 10% of all cancers diagnosed in children younger than 15 years and 15% of all malignancies among children and adolescents younger than 20 years.[7] Progress in the management of affected children is one of the success stories of modern medicine. Ninety percent of children with ALL and more than 80% of children with Hodgkin lymphoma and with the commonly seen subtypes of NHL are cured with modern treatment regimens. As a result, late complications of therapy are a key concern, because children cured of cancer have a lifetime ahead of them to manifest the long-term toxicities of therapy. Thus, although many of the lymphoid malignancies that occur in children also are observed in adults, the focus of modern therapies for affected children may differ from the approach appropriate for adult patients, particularly if the effects of therapies on growing and developing individuals are substantial. Preserving the excellent cure rates while minimizing late effects of therapy, which may have long latency, often drives the therapeutic strategies adopted for children. Staging systems appropriate for adult lymphomas may be suitable for children as well, although therapeutic options chosen for children may differ from those recommended for adults with the same stage of disease[8-10] (e.g., minimizing radiation dose and volume in children with Hodgkin lymphoma, and minimizing exposure to alkylating agents and anthracyclines in all lymphoma subtypes).

Hodgkin Lymphoma

There is little to suggest that the clinical behavior of Hodgkin lymphoma in children differs markedly from that seen in adults. Nodular lymphocyte-predominant Hodgkin lymphoma is more common in young children, who usually present with early-stage disease.[11] The mixed-Cellularity variant (often with evidence of Epstein-Barr virus) is commonly observed in young children (<10 years old), with a marked male preponderance.[11] Otherwise, the disease in children is felt to follow the same course as in adults, and staging studies recommended are the same (see Chapter 79). As in adults, determination of disease extent and assignment of clinical stage are critical for selecting the appropriate treatment. In general, staging studies divide patients into those with early-stage, favorable presentations, for whom cure is likely and the focus of treatment is to minimize acute and late complications of therapy, and those with more

advanced-stage, less favorable presentations, for whom more intensive therapies may be required for cure. The utility of the Ann Arbor staging classification and subsequent modifications for risk stratification and choice of therapy for children with Hodgkin lymphoma are the same as for adults (Table 80.3). The application of the International Prognostic Index (IPI)[12] to children is problematic in view of the importance of age in that system; however, the other elements of the IPI generally are considered in risk stratification of children. Other prognostic scoring systems have been proposed but have not been widely accepted.[9] Efforts are under way to harmonize the various risk stratification schemes adopted by pediatric oncologists to facilitate comparisons among studies of Hodgkin lymphoma in children and adolescents. In general, the excellent outcome of children with Hodgkin lymphoma has made accurate outcome prediction from initial staging and other studies less useful; rapidity of response to therapy (assessed early in therapy by resolution of hypermetabolic sites on positron emission tomography [PET] scan with or without measure of anatomic response by computed tomography [CT] or magnetic resonance [MR] imaging) has emerged as the most reliable predictor of outcome, and has been used to introduce treatment modifications (intensification of therapy and/or addition or omission of radiotherapy depending on early response) tailored to the individual patient.[8]

A and B Classification (Symptoms)

For Hodgkin lymphoma, each stage should be classified as either A or B according to the absence or presence of defined constitutional symptoms. The designation A or B is not included in the revised staging of NHL,[5] although clinicians are encouraged to record the presence of these symptoms in the medical record. They are as follows:

1. *Fevers.* Unexplained fever with temperature above 38°C
2. *Night sweats.* Drenching sweats (e.g., those that require change of bedclothes)
3. *Weight loss.* Unexplained weight loss of more than 10% of the usual body weight in the 6 months prior to diagnosis

Other symptoms, such as chills, pruritus, alcohol-induced pain, and fatigue, are not included in the A or B designation but are recorded in the medical record, as the reappearance of these symptoms may be a harbinger of recurrence.

Non-Hodgkin Lymphoma

Most of the histologic subtypes of NHL that occur in adults rarely are observed in children. When encountering children with such unusual lymphoma subtypes, pediatric oncologists depend on guidelines for staging and treatment developed for adult patients with these lymphomas. Follicular lymphomas occur rarely in children, often present with localized disease, most often do not express bcl2, and follow an indolent course.[13,14] Follicular lymphomas in adolescents commonly resemble the adult counterpart.

Forty percent to 50% of pediatric NHLs derive from B cells and morphologically are Burkitt lymphomas or diffuse large B-cell lymphomas (the latter primarily of germinal center cell subtype).[15] Fewer than 5% of mature B-cell lymphomas of childhood are cases of primary mediastinal (thymic) large B-cell lymphomas, which are associated with an adverse outcome when treated with regimens that have proven successful for the other common mature B-cell lymphomas of childhood.[16] Ten percent to 15% of childhood NHLs are systemic anaplastic large cell lymphomas (ALCLs) characterized by expression of CD30 as well as ALK. The remaining pediatric NHLs are lymphoblastic lymphomas, most of which derive from immature T cells expressing immunophenotypes of immature thymocytes and appear to be closely related to T-Cell ALLs. A few of these lymphoblastic lymphomas derive from B-cell progenitors and are closely related to the B-progenitor ALLs but, by definition, present with involvement of peripheral sites rather than with primary bone marrow involvement.[17]

Pediatric NHLs present with rapidly evolving clinical symptoms and associated masses that may produce life-threatening complications. Thus, staging studies designed to determine the extent of disease, as well as other laboratory and clinical evaluations that may have prognostic value, should be initiated as soon as the diagnosis of NHL has been confirmed. However, occult disseminated disease is presumed to exist in all patients, and meticulous staging studies are neither indicated nor wise. Studies that delay the institution of specific therapy should be avoided

Recommended staging studies (Table 80.1) include careful physical examination with attention to all sites of palpable disease and complete blood count with platelet count and differential. Level of serum lactate dehydrogenase (LDH) has been found to correlate strongly with tumor burden and with prognosis in children with some subtypes of NHL and is an important factor (along with stage) in assessing relapse risk and thus treatment assignment.[18–21] Assessment of renal function and levels of electrolytes, calcium, phosphorus, and uric acid is essential, because children with bulky tumors may present with biochemical derangements that may be exacerbated by the institution of therapy (tumor lysis syndrome), leading to life-threatening complications in patients with curable disease. PET/CT and/or PET/MR imaging have replaced other radiologic technologies for assessing extent of disease in most patients, although radionuclide bone scan may be useful to detect unsuspected bone lesions. Involvement of the bone marrow is an important criterion for staging and defines patients with more advanced disease.

Table 80.1 Recommendation for the diagnostic evaluation of children with NHL

A. Mandatory procedures
  1. Biopsy (preferably excisional), with interpretation by a qualified pathologist. Diagnosis from a core biopsy should be based on multiple core biopsies. Appropriate IHC and ancillary studies to establish a diagnosis.[17]
  2. Physical examination
  3. Laboratory evaluation
     a. Complete blood cell count and platelet count with differential and slide review
     b. Comprehensive metabolic panel (electrolytes, blood urea nitrogen, creatinine, calcium, phosphorus, uric acid, aspartate transaminase (AST; serum glutamic oxaloacetic transaminase [SGOT]), alanine transaminase (ALT; serum glutamic-pyruvic transaminase [SGPT]), bilirubin, total protein, albumin, alkaline phosphatase
     c. LDH
     d. HIV testing
     e. Hepatitis B core antibody and hepatitis B surface antigen in patients being considered for anti-CD20 therapy
  4. Radiographic examination
     a. CT of neck, chest, abdomen, and pelvis with intravenous contrast (if safe)
     b. Functional (metabolic) imaging with fludeoxyglucose (FDG)-PET
     c. FDG-PET in conjunction with CT or MR imaging is preferred
     d. Head CT or MR imaging, or spine MR imaging if symptoms suggest CNS involvement
  5. Bilateral bone marrow aspirate and biopsy
  6. CSF evaluation

Because extent of marrow involvement is based on enumeration of lymphoma cells in the marrow, morphologic assessment of marrow aspirates is required. Current staging classifications acknowledge that the pediatric ALLs and NHLs are at the ends of the same disease spectrum. For many patients, marrow involvement may simply represent further clinical evolution of lymphoma; the distinction between ALL and NHL with marrow involvement is somewhat arbitrary and based on the percentage of blast cells in the marrow. Patients with bone marrow aspirates containing 5–25% malignant blast cells by standard morphologic assessment are considered to have NHL with marrow involvement (Stage IV), whereas those with >25% blasts in the marrow are considered to have ALL. Treatment strategies for children with NHL have evolved to acknowledge the close relationship between NHL and ALL, and that the distinction between NHL and ALL is at best ill-defined. There is little evidence that within a single histologic or immunologic subgroup, patients with leukemia and those with NHL require different therapies. Current trends in clinical practice reflect this. For most entities, the treatments of NHL and ALL of similar morphology and immunophenotype (e.g., T-Cell lymphoblastic lymphoma and T-Cell ALL) are comparable. Involvement of the central nervous system (CNS) is common in childhood NHL at presentation, and the CNS is a frequent site of disease recurrence; CNS involvement at diagnosis has important prognostic significance. Cranial nerve palsy and mass lesions are frequent manifestations of CNS involvement. Lumbar puncture with microscopic examination of a cytocentrifuge preparation of cerebrospinal fluid (CSF) is important for proper staging.

The application of sophisticated technology (flow cytometry and molecular techniques) has vastly increased the sensitivity and specificity for detecting malignant cells in the marrow (and peripheral blood), at levels in the range of 0.01-0.001% in morphologically normal-appearing bone marrow specimens from children with NHL (so-called minimal detectable disease [MDD]).[22–27] Patients with all the common histologies of NHL in childhood with detectable MDD in the marrow at diagnosis have a significantly worse outcome than those without MDD. Similarly, application of molecular or immunologic techniques to CSF assessment has proven more sensitive for detection of occult CSF involvement.

The goal of staging studies is to assess the extent of disease to determine prognosis, assess the risk of treatment failure, and assign appropriate therapy. The Ann Arbor staging system with subsequent refinements, which has been widely accepted for its utility in the management of Hodgkin lymphoma, is not suitable for children with NHL.[1] The progression of disease in children with NHL does not follow an orderly and predictable pattern. For example, children with T-cell lymphoblastic lymphoma apparently localized to the mediastinum rapidly develop dissemination to the bone marrow and meninges and, without intensive therapy, have an unfavorable prognosis despite the limited initial sites of involvement. The frequency of extranodal involvement in childhood NHL likewise makes strict application of the Ann Arbor system awkward.

The clinical staging system proposed at St. Jude Children's Research Hospital more than 35 years ago (Table 80.4)[1] has proven to be more suitable for childhood NHL and has been widely accepted. Primary site and disease extent are considered in assigning clinical stage. Patients with a mediastinal tumor (regardless of other involved sites), as well as those with a massive unresectable abdominal tumor, are considered to have advanced-stage disease (Stage III), reflecting the adverse prognosis associated with these presentations, whereas patients with more limited disease (including those with a completely resectable gastrointestinal primary tumor) fall into a more favorable prognostic group. Stage IV is reserved for children who present with involvement of the bone marrow (as defined morphologically) or CNS.

Recently, a multidisciplinary expert panel proposed a revised International Pediatric Non-Hodgkin Lymphoma Staging System (INHLSS) to address some of the shortcomings of the St. Jude staging classification.[6] Notably, the St.

Jude staging system was designed primarily for children with Burkitt or lymphoblastic lymphoma; newer subtypes of childhood NHL have different patterns of organ involvement not well accounted for by the St. Jude system. Moreover, newer methods to detect involvement of the bone marrow and CNS have emerged in the three decades since the St. Jude system was proposed. The INHLSS specifies primary site of involvement (nodal, extranodal skin, extranodal bone) and a change from the St. Jude system by classification of multiple organ involvement as Stage III, regardless of whether there is involvement on both sides of the diaphragm. In addition, explicit designation of organ involvement is included. Whether Stage IV results from marrow or CNS involvement (or both) also is specified, particularly because CNS involvement carries a much more negative prognostic implication than does marrow involvement. By what means bone marrow or CNS involvement is determined also is part of the INHLSS. Key features of the St. Jude staging system are retained; in particular, definition of Stage IV continues to be based on standard morphologic criteria, regardless of whether minimal marrow or CNS involvement can be ascertained by newer, more sensitive techniques. Broad acceptance of the INHLSS would facilitate comparisons among the various treatment regimens now in use for children with NHL.

Other Prognostic Factors (Table 80.2)

As noted above, serum LDH has been found to correlate with tumor burden, but also has prognostic value. It now is used for risk stratification and treatment selection in children with B-cell NHLs. The detection of MDD in otherwise normal bone marrow and peripheral blood has been found to predict inferior outcome in all the common histologies of childhood NHL.[23,26,28] By analogy to childhood ALL, flow cytometric methods using combinations of blast-specific markers or molecular methods using polymerase chain reaction (PCR) to amplify specific genetic abnormalities or clonal T-Cell receptor or immunoglobulin gene rearrangements may be used to detect MDD in lymphoblastic lymphomas. Analogous molecular techniques may be used to detect MDD in children with Burkitt lymphoma; detection of MDD in the marrow at diagnosis is associated with an inferior outcome. Finally, in ALCL, MDD can be detected by molecular methods focused on the *NPM-ALK* rearrangement found in most cases or by detection of ALK protein overexpression with immunohistochemistry (IHC).

Cytogenetic and molecular studies of malignant blast cells have revealed the heterogeneity of ALL, resulting in the division of the disease into multiple subsets with differing genetic mutations driving the leukemia, each with a different prognosis and response to therapy.[29,30] Modern therapies stratify patients with ALL based on risk of treatment failure, defined by cytogenetic and molecular characterization of malignant cells. The close relationship between lymphoblastic lymphomas and lymphoblastic leukemias suggests that

Table 80.2 Pediatric Non-Hodgkin Lymphoma: additional factors recommended for clinical care

Factor	Definition	Clinical significance	Level of evidence
Serum LDH	Compared with laboratory normal	Elevation >2× normal limit is highly prognostic in B-cell lymphomas. Necessary for proper treatment selection in B-cell lymphomas.	I
Minimal Detectable Disease (MDD) in marrow and/or peripheral blood	Use of molecular and/or immunophenotypic strategies to detect low levels of involvement not detectable by standard morphologic analysis	Detection of MDD in morphologically normal bone marrow and peripheral blood has been found to predict inferior outcome in all of the common histologies of childhood NHL.	I

the molecular characteristics of malignant cells in lymphomas should likewise provide important prognostic information. Preliminary studies suggest that this is the case. Certain cytogenetic rearrangements already were shown to adversely affect outcome in children with Burkitt lymphoma.[31,32] Children with the early T-Cell precursor subtype of ALL fare poorly on regimens successful in most other children with T-ALL.[33] It is likely that children with T-Cell lymphoblastic lymphoma with blast cells expressing the early T-Cell precursor subtype might also be expected to fare less well. The small volumes of tissue often obtained in biopsies from children with NHL limit the studies that can be performed to fully characterize the malignant cells. However, information from detailed studies of malignant blast cells in lymphomas likely will contribute substantially to risk stratification and therapy selection and therefore should be encouraged.

PROGNOSTIC FACTORS

Prognostic factors for Hodgkin lymphoma may be found in Chapter 79.

Prognostic Factors Required for Stage Grouping

There are no additional prognostic factors required for stage grouping.

Additional Factors Recommended for Clinical Care

AJCC PROGNOSTIC STAGE GROUPS

Pediatric Hodgkin Lymphomas

Table 80.3 Lugano classification applicable for pediatric Hodgkin lymphomas

Stage	Stage description
Limited stage	
I	Involvement of a single lymphatic site (i.e., nodal region, Waldeyer's ring, thymus, or spleen)
IE	Single extralymphatic site in the absence of nodal involvement (rare in Hodgkin lymphoma)
II	Involvement of two or more lymph node regions on the same side of the diaphragm
IIE	Contiguous extralymphatic extension from a nodal site with or without involvement of other lymph node regions on the same side of the diaphragm
*II bulky**	*Stage II with disease bulk* **
Advanced stage	
III	Involvement of lymph node regions on both sides of the diaphragm; nodes above the diaphragm with spleen involvement
IV	Diffuse or disseminated involvement of one or more extralymphatic organs, with or without associated lymph node involvement;<br>or *noncontiguous* extralymphatic organ involvement in conjunction with nodal Stage II disease<br>or *any* extralymphatic organ involvement in nodal Stage III disease<br>Stage IV includes *any* involvement of the bone marrow, liver, or lungs (other than by direct extension in Stage IIE disease)

* Stage II bulky may be considered either early or advanced stage based on lymphoma histology and prognostic factors (see discussion of Hodgkin lymphoma prognostic factors)

* * The definition of disease bulk varies according to lymphoma histology. In the Lugano classification,[5] bulk in Hodgkin lymphoma is defined as a mass greater than one third of the thoracic diameter on CT of the chest or a mass >10 cm

Note: Hodgkin lymphoma uses A or B designation with stage group

Pediatric Non-Hodgkin Lymphomas

Table 80.4 St. Jude Children's Research Hospital staging system for non-hodgkin lymphoma

Stage	Stage description
I	A single tumor (extranodal) or single anatomic area (nodal), with the exclusion of the mediastinum or abdomen
II	A single tumor (extranodal) with regional node involvement<br>Two or more nodal areas on the same side of the diaphragm<br>Two or more nodal areas on the same side of the diaphragm<br>Two single (extranodal) tumors with or without regional node involvement on the same side of the diaphragm<br>A primary gastrointestinal tract tumor, usually in the ileocecal area, with or without involvement of associated mesenteric nodes only*
III	Two single tumors (extranodal) on opposite sides of the diaphragm<br>Two or more nodal areas above and below the diaphragm<br>All the primary intrathoracic tumors (mediastinal, pleural, and thymic)<br>All extensive primary intra-abdominal disease*<br>All paraspinal or epidural tumors, regardless of other tumor site(s)
IV	Any of the above with initial CNS and/or bone marrow involvement* *

* A distinction is made between apparently localized gastrointestinal tract lymphoma versus more extensive intra-abdominal disease because of their quite different patterns of survival after appropriate therapy. Stage II disease typically is limited to a segment of the gut plus or minus the associated mesenteric nodes only, and the primary tumor can be completely removed grossly by segmental excision Stage III disease typically exhibits spread to para-aortic and retroperitoneal areas by implants and plaques in mesentery or peritoneum, or by direct infiltration of structures adjacent to the primary tumor. Ascites may be present, and complete resection of all gross tumor is not possible

* * If marrow involvement is present initially, the number of abnormal cells must be ≤25% in an otherwise normal marrow aspirate with a normal peripheral blood picture.

Modified from Murphy SB[1]

Bibliography

1. Murphy SB. Classification, staging and end results of treatment of childhood non-Hodgkin's lymphomas: dissimilarities from lymphomas in adults. *Semin Oncol.* Sep 1980;7(3):332–339.
2. Carbone PP, Kaplan HS, Musshoff K, Smithers DW, Tubiana M. Report of the Committee on Hodgkin's Disease Staging Classification. *Cancer Res.* Nov 1971;31(11):1860–1861.
3. Rosenberg SA. Validity of the Ann Arbor staging classification for the non-Hodgkin's lymphomas. *Cancer Treat Rep.* Sep 1977;61(6):1023–1027.
4. Lister TA, Crowther D, Sutcliffe SB, et al. Report of a committee convened to discuss the evaluation and staging of patients with Hodgkin's disease: Cotswolds meeting. *J Clin Oncol.* Nov 1989;7(11):1630–1636.
5. Cheson BD, Fisher RI, Barrington SF, et al. Recommendations for initial evaluation, staging, and response assessment of Hodgkin and non-Hodgkin lymphoma: the Lugano classification. *J Clin Oncol.* Sep 20 2014;32(27):3059–3068.
6. Rosolen A, Perkins SL, Pinkerton CR, et al. Revised International Pediatric Non-Hodgkin Lymphoma Staging System. *J Clin Oncol.* Jun 20 2015;33(18):2112–2118.
7. Percy CL, Smith MA, Linet M, Gloeckler Ries LA, Friedman DL. Lymphomas and Reticuloendothelial Neoplasms. In: Ries LAG, Smith MA, Gurney JG, et al., eds. *Cancer Incidence and Survival among Children and Adolescents: United States SEER Program 1975–1995.* Bethesda, MD: National Cancer Institute, SEER Program. NIH Pub. No. 99-4649; 1999:35–49.
8. Kelly KM. Hodgkin lymphoma in children and adolescents: improving the therapeutic index. *Blood.* Nov 26 2015;126(22):2452–2458.
9. Mauz-Korholz C, Metzger ML, Kelly KM, et al. Pediatric Hodgkin Lymphoma. *J Clin Oncol.* Sep 20 2015;33(27):2975-2985.
10. Weinstein HJ, Hudson MM, Link MP. *Pediatric lymphomas.* Berlin; New York: Springer; 2007.

11. Cleary SF, Link MP, Donaldson SS. Hodgkin's disease in the very young. *International journal of radiation oncology, biology, physics.* Jan 1 1994;28(1):77–83.
12. Shipp M, Harrington D, Anderson J, et al. A predictive model for aggressive non-Hodgkin's lymphoma. *New England Journal of Medicine.* 1993;329(14):987–994.
13. Lorsbach RB, Shay-Seymore D, Moore J, et al. Clinicopathologic analysis of follicular lymphoma occurring in children. *Blood.* Mar 15 2002;99(6):1959–1964.
14. Louissaint A, Jr., Ackerman AM, Dias-Santagata D, et al. Pediatric-type nodal follicular lymphoma: an indolent clonal proliferation in children and adults with high proliferation index and no BCL2 rearrangement. *Blood.* Sep 20 2012;120(12):2395–2404.
15. Deffenbacher KE, Iqbal J, Sanger W, et al. Molecular distinctions between pediatric and adult mature B-cell non-Hodgkin lymphomas identified through genomic profiling. *Blood.* Apr 19 2012; 119(16):3757–3766.
16. Gerrard M, Waxman IM, Sposto R, et al. Outcome and pathologic classification of children and adolescents with mediastinal large B-cell lymphoma treated with FAB/LMB96 mature B-NHL therapy. *Blood.* Jan 10 2013;121(2):278–285.
17. Swerdlow SH. International Agency for Research on Cancer., World Health Organization. *WHO classification of tumours of haematopoietic and lymphoid tissues.* Lyon, France: International Agency for Research on Cancer; 2008.
18. Cairo MS, Sposto R, Gerrard M, et al. Advanced stage, increased lactate dehydrogenase, and primary site, but not adolescent age (>/= 15 years), are associated with an increased risk of treatment failure in children and adolescents with mature B-cell non-Hodgkin's lymphoma: results of the FAB LMB 96 study. *J Clin Oncol.* Feb 1 2012;30(4):387–393.
19. Murphy SB, Fairclough DL, Hutchison RE, Berard CW. Non-Hodgkin's lymphomas of childhood: an analysis of the histology, staging, and response to treatment of 338 cases at a single institution. *J Clin Oncol.* Feb 1989;7(2):186–193.
20. Patte C, Auperin A, Michon J, et al. The Societe Francaise d'Oncologie Pediatrique LMB89 protocol: highly effective multiagent chemotherapy tailored to the tumor burden and initial response in 561 unselected children with B-cell lymphomas and L3 leukemia. *Blood.* Jun 1 2001;97(11):3370–3379.
21. Reiter A, Schrappe M, Tiemann M, et al. Improved treatment results in childhood B-cell neoplasms with tailored intensification of therapy: A report of the Berlin-Frankfurt-Munster Group Trial NHL-BFM 90. *Blood.* Nov 15 1999;94(10):3294–3306.
22. Coustan-Smith E, Sandlund JT, Perkins SL, et al. Minimal disseminated disease in childhood T-Cell lymphoblastic lymphoma: a report from the children's oncology group. *J Clin Oncol.* Jul 20 2009;27(21):3533–3539.
23. Damm-Welk C, Busch K, Burkhardt B, et al. Prognostic significance of circulating tumor cells in bone marrow or peripheral blood as detected by qualitative and quantitative PCR in pediatric NPM-ALK-positive anaplastic large-cell lymphoma. *Blood.* Jul 15 2007; 110(2):670–677.
24. Damm-Welk C, Schieferstein J, Schwalm S, Reiter A, Woessmann W. Flow cytometric detection of circulating tumour cells in nucleophosmin/anaplastic lymphoma kinase-positive anaplastic large cell lymphoma: comparison with quantitative polymerase chain reaction. *British journal of haematology.* Aug 2007;138(4):459–466.
25. Mussolin L, Pillon M, Conter V, et al. Prognostic role of minimal residual disease in mature B-cell acute lymphoblastic leukemia of childhood. *J Clin Oncol.* Nov 20 2007;25(33):5254–5261.
26. Mussolin L, Pillon M, d'Amore ES, et al. Prevalence and clinical implications of bone marrow involvement in pediatric anaplastic large cell lymphoma. *Leukemia.* Sep 2005;19(9):1643–1647.
27. Shiramizu B, Goldman S, Kusao I, et al. Minimal disease assessment in the treatment of children and adolescents with intermediate-risk (Stage III/IV) B-cell non-Hodgkin lymphoma: a children's oncology group report. *British journal of haematology.* Jun 2011; 153(6):758–763.
28. Mussolin L, Pillon M, d'Amore ES, et al. Minimal disseminated disease in high-risk Burkitt's lymphoma identifies patients with different prognosis. *J Clin Oncol.* May 1 2011;29(13):1779–1784.
29. Pui CH, Relling MV, Downing JR. Acute lymphoblastic leukemia. *N Engl J Med.* Apr 8 2004;350(15):1535–1548.
30. Hunger SP, Mullighan CG. Acute Lymphoblastic Leukemia in Children. *N Engl J Med.* Oct 15 2015;373(16):1541–1552.
31. Nelson M, Perkins SL, Dave BJ, et al. An increased frequency of 13q deletions detected by fluorescence in situ hybridization and its impact on survival in children and adolescents with Burkitt lymphoma: results from the Children's Oncology Group study CCG-5961. *British journal of haematology.* Feb 2010;148(4):600–610.
32. Poirel HA, Cairo MS, Heerema NA, et al. Specific cytogenetic abnormalities are associated with a significantly inferior outcome in children and adolescents with mature B-cell non-Hodgkin's lymphoma: results of the FAB/LMB 96 international study. *Leukemia.* Feb 2009;23(2):323–331.
33. Coustan-Smith E, Mullighan CG, Onciu M, et al. Early T-Cell precursor leukaemia: a subtype of very high-risk acute lymphoblastic leukaemia. *The lancet oncology.* Feb 2009;10(2):147–156.
34. Arcaini L, Rattotti S, Gotti M, Luminari S. Prognostic assessment in patients with indolent B-cell lymphomas. *ScientificWorldJournal.* 2012;2012:107892.
35. Federico M, Bellei M, Marcheselli L, et al. Follicular lymphoma international prognostic index 2: a new prognostic index for follicular lymphoma developed by the international follicular lymphoma prognostic factor project. *J Clin Oncol.* Sep 20 2009;27(27):4555–4562.

Primary Cutaneous Lymphomas

81

Steven T. Rosen, Andrew D. Zelenetz, Elaine S. Jaffe, and John P. Leonard

CHAPTER SUMMARY

Cancers Staged Using This Staging System

Cutaneous lymphoma, mycosis fungoides, Sézary syndrome

Cancers Not Staged Using This Staging System

These histopathologic types of cancer…	Are staged according to the classification for…	And can be found in chapter…
Eyelid skin	Ocular adnexal lymphoma	71

Summary of Changes

Change	Details of Change	Level of Evidence
Prognostic Factors	Addition of prognostic tables	I
AJCC Prognostic Stage Groups	TNM staging for non-mycosis fungoides/Sézary syndrome	II

ICD-O-3 Topography Codes

Code	Description
C44.0	Skin of lip, NOS
C44.2	External ear
C44.3	Skin of other and unspecified parts of face
C44.4	Skin of scalp and neck
C44.5	Skin of trunk
C44.6	Skin of upper limb and shoulder
C44.7	Skin of lower limb and hip
C44.8	Overlapping lesion of skin
C44.9	Skin, NOS
C51.0	Labium majus
C51.1	Labium minus
C51.2	Clitoris
C51.8	Overlapping lesion of vulva
C51.9	Vulva, NOS
C60.0	Prepuce
C60.1	Glans penis
C60.2	Body of penis
C60.8	Overlapping lesion of penis
C60.9	Penis, NOS
C63.2	Scrotum, NOS

WHO Classification of Tumors

Code	Description
9597	Primary cutaneous follicle center lymphoma
9680	Primary cutaneous diffuse large B-cell lymphoma, leg type
9700	Mycosis fungoides
9701	Sézary syndrome
9708	Subcutaneous panniculitis-like T-cell lymphoma
9709	Cutaneous lymphoma, NOS
9709	Primary cutaneous CD8+ aggressive epidermotropic cytotoxic T-cell lymphoma
9709	Primary cutaneous CD4+ small/medium T-cell lymphoma
9712	Intravascular large B-cell lymphoma
9718	Primary cutaneous CD30+ T-cell lymphoproliferative disorder (C44._)
9718	Lymphomatoid papulosis (C44._)
9718	Primary cutaneous anaplastic large cell lymphoma (C44._)
9718	Primary cutaneous CD30+ large T-cell lymphoma (C44._)
9719	Extranodal NK/T-cell lymphoma, nasal type
9726	Primary cutaneous gamma-delta T-cell lymphoma

To access the AJCC cancer staging forms, please visit www.cancerstaging.org.

© American Joint Committee on Cancer 2017

M.B. Amin et al. (eds.), *AJCC Cancer Staging Manual, Eighth Edition*, DOI 10.1007/978-3-319-40618-3_81

Code	Description
9727	Precursor hematologic neoplasm: CD4+/CD56+ hematodermic neoplasm, blastic NK-cell lymphoma

Swerdlow SH, Campo E, Harris NL, Jaffe ES, Pileri SA, Stein H, Thiele J, Vardiman J, eds. World Health Organization Classification of Tumours of Haematopoietic and Lymphoid Tissues. Lyon: IARC; 2008.

INTRODUCTION

MF and its variants represent the most common form of cutaneous T-cell lymphoma (CTCL). The malignant cell is derived from a post-thymic T cell that typically bears a CD4+ helper/memory antigen profile. The disease is characterized by erythematous patches (usually in sun-protected areas) that progress to plaques or tumors. Initial evaluation should include delineation of skin involvement with photographs, skin biopsy (histopathology, immunophenotyping, and T-cell receptor [TCR] gene analysis); complete blood count with differential, Sézary cell count (peripheral blood); chemistry panel with lactate dehydrogenase and, in select instances, peripheral blood flow cytometric analysis of T-cell subsets (CD4/CD8 ratio); TCR gene analysis on peripheral blood; lymph node biopsy and bone marrow biopsies (histopathology, immunophenotyping, and TCR gene analysis); computed tomography/positron emission tomography scans; and serologic tests (HTLV1 and HIV). Skin-directed and systemic therapies are determined by the patient's stage and symptoms. Prognosis is stage dependent.

Sézary Syndrome

SS, the aggressive leukemic and erythrodermic form of CTCL, is characterized by circulating atypical, malignant T lymphocytes with cerebriform nuclei (Sézary cells) and lymphadenopathy. The Sézary cells also have a mature memory T-cell phenotype (CD3+, CD4+) with loss of CD7 and CD26.

Primary Cutaneous CD30+ Lymphoproliferative Disorders

Primary cutaneous CD30+ lymphoproliferative disorders are the second most common group of CTCLs. This spectrum of diseases includes lymphomatoid papulosis, anaplastic large cell lymphoma, and borderline cases. The distinction among these entities may be challenging and requires correlation with clinical behavior. Lymphomatoid papulosis represents a benign, chronic, recurrent, self-healing, papulonodular, and papulonecrotic CD4+, CD30+ skin eruption. Primary cutaneous anaplastic large cell lymphoma typically presents with solitary or localized nodules. It is ALK negative.

Primary Cutaneous T-Cell Lymphoma (Miscellaneous Non-MF/SS)

A variety of other primary cutaneous T-cell lymphomas have been described, including NK/T-cell lymphoma, panniculitic T-cell lymphoma, T-cell lymphoma NOS, and gamma-delta T-cell lymphoma. Their presentations and histologic appearances vary.

Follicle Center Cell Lymphoma

Follicle center cell lymphoma is the most common cutaneous B-cell lymphoma (CBCL). Erythematous nodules or plaques are composed of a proliferation of centrocytes (small to large cleaved cells) and centroblasts (large round cells with prominent nuclei). The clinical course usually is indolent, even if the infiltrate is composed predominantly of large cells.

Marginal Zone Lymphoma

Marginal zone lymphoma is an indolent CBCL. It has the histologic appearance of a mucosa-associated lymphoid tissue (MALT) lymphoma and shows a nodular or diffuse dermal infiltrate with a heterogeneous cellular infiltrate of small lymphocytes, lymphoplasmacytoid cells, plasma cells, intranuclear inclusions (Dutcher bodies), and reactive germinal centers that may be infiltrated by neoplastic cells. It often is localized and usually follows an indolent course.

Large B-Cell Lymphoma of the Leg

Large B-cell lymphoma of the leg is an aggressive lymphoma most commonly seen in older patients, with a female predominance. Patients present with tumors that may ulcerate. The histologic evaluation shows a diffuse dermal infiltrate composed predominantly of centroblasts, often with multilobulated

nuclei. The standard of care is treatment with chemoimmunotherapy with or without involved-site or region radiation.

PROGNOSTIC FACTORS

Mycosis Fungoides and Sézary Syndrome

Prognostic Factors Required for Stage Grouping

Factor	Definition	Clinical significance	Level of evidence
Blood	Extent involvement<br>B1: >5%<br>B2: >1,000/μL<br>(flow cytometry)	Overall survival	I

Additional Factors Recommended for Clinical Care

Factor	Definition	Clinical significance	Level of evidence
Skin	Patch, plaque, tumor, erythroderma, extent involvement (i.e., <10%)	Therapy consideration, overall survival	I
Nodes	Extent involvement (pathology)	Overall survival	I
Visceral	Location (pathology)	Overall survival	I
Folliculotropic MF	Pathology	Therapy consideration, overall survival	I
Large cell transformation	Pathology	Overall survival, treatment selection (a subset expresses CD30 and is responsive to brentuximab vedotin)	I

Primary Cutaneous B-Cell/T-Cell Lymphoma (Non-MF/SS) Lymphoma

Prognostic Factors Required for Stage Grouping

Beyond the factors used to assign T, N, or M categories, no additional prognostic factors are required for stage grouping.[2]

Additional Factors Recommended for Clinical Care

The Lugano classification includes no prognostic variable for determination of stage. A separate TNM staging system is used for non-MF/SS lymphomas.

Factor	Definition	Clinical significance	Level of evidence
Skin	Extent involvement; solitary, regional, generalized (clinical)	Therapy consideration, overall survival	I
Nodes	Involvement of draining lymph node, nondraining or central (pathology)	Overall survival	I
Metastases	Visceral organ (pathology)	Overall survival	I
Pathology	Histologic type, immunophenotype	Affects choice of therapy and overall survival	I

DEFINITIONS OF AJCC TNM

Mycosis Fungoides and Sézary Syndrome

Definition of Primary Tumor (T)

ISCL/EORTC revision to the classification of mycosis fungoides and Sézary Syndrome

Skin

T Category	T Criteria
T1	Limited patches,* papules, and/or plaques** covering < 10% of the skin surface
T1a	T1a (patch only)
T1b	T1b (plaque ± patch)
T2	Patches, papules, or plaques covering ≥10% of the skin surface
T2a	T2a (patch only)
T2b	T2b (plaque ± patch)
T3	One or more tumors*** (≥ cm in diameter)
T4	Confluence of erythema covering ≥80% of body surface area

*For skin, *patch* indicates any size skin lesion without significant elevation or induration. Presence/absence of hypo- or hyperpigmentation, scale, crusting, and/or poikiloderma should be noted.

**For skin, *plaque* indicates any size skin lesion that is elevated or indurated. Presence/absence of scale, crusting, and/or poikiloderma should be noted. Histologic features such as folliculotropism, large cell transformation (>25% large cells), and CD30 positivity or negativity, as well as clinical features such as ulceration, are important to document.

***For skin, *tumor* indicates at least one 1-cm diameter solid or nodular lesion with evidence of depth and/or vertical growth. Note the total number of lesions, total volume of lesions, largest size lesion, and region of body involved. Also note whether there is histologic evidence of large cell transformation. Phenotyping for CD30 is encouraged.

Fig. 81.1 Regional percent of body surface area in the adult (From Olsen et al.,[1] with permission)

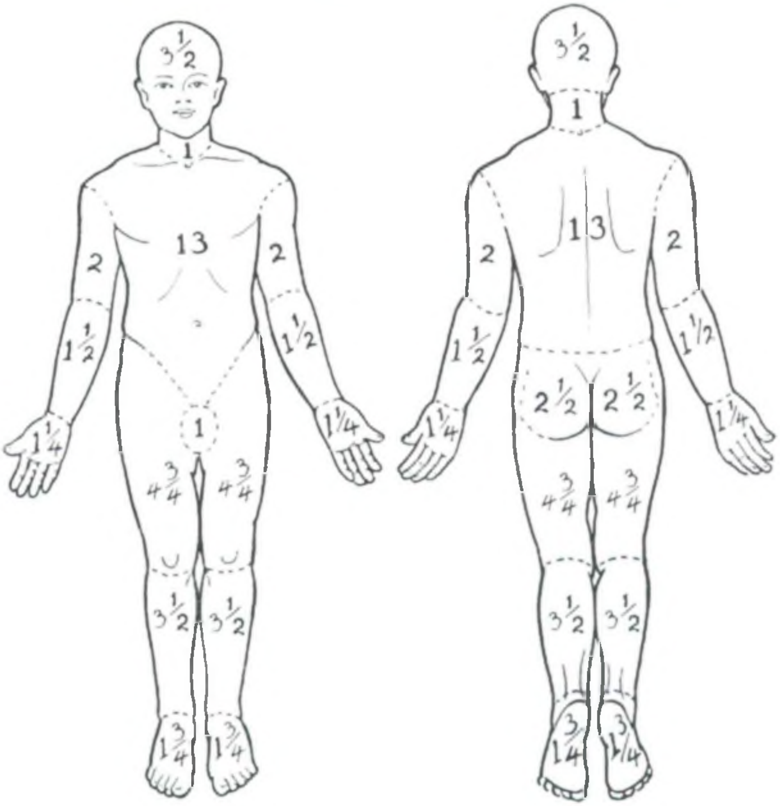

Definition of Regional Lymph Node (N)

Node

N Category	N Criteria
NX	Clinically abnormal peripheral lymph nodes; no histologic confirmation
N0	No clinically abnormal peripheral lymph nodes*; biopsy not required
N1	Clinically abnormal peripheral lymph nodes; histopathology Dutch grade 1 or National Cancer Institute (NCI) LN0-2
N1a	Clone negative**
N1b	Clone positive**
N2	Clinically abnormal peripheral lymph nodes; histopathology Dutch grade 2 or NCI LN3
N2a	Clone negative**
N2b	Clone positive**
N3	Clinically abnormal peripheral lymph nodes; Histopathology Dutch grades 3-4 or NCI LN4; clone positive or negative

*For node, *abnormal peripheral lymph node(s)* indicates any palpable peripheral node that on physical examination is firm, irregular, clustered, fixed or ≥1.5 cm in diameter. Node groups examined on physical examination include cervical, supraclavicular, epitrochlear, axillary, and inguinal. Central nodes, which generally are not amenable to pathological assessment, currently are not considered in the nodal classification unless used to establish N3 histopathologically.

**A T-cell clone is defined by polymerase chain reaction (PCR) or Southern blot analysis of the TCR gene.

Definition of Distant Metastasis (M)

Visceral

M Category	M Criteria
M0	No visceral organ involvement
M1	Visceral involvement (must have pathology confirmation,* and organ involved should be specified)

*For viscera, spleen and liver may be diagnosed by imaging criteria.

Peripheral Blood Involvement (B)

B Category	B Criteria
B0	Absence of significant blood involvement: ≥5% of peripheral blood lymphocytes are atypical (Sézary) cells*
B0a	Clone negative**
B0b	Clone positive**
B1	Low blood tumor burden: >5% of peripheral blood lymphocytes are atypical (Sézary) cells, but does not meet the criteria of B2

(continued)

B Category	B Criteria
B1a	Clone negative**
B1b	Clone positive**
B2	High blood tumor burden: ≥1,000/μL Sézary cells* with positive clone**

*For blood, Sézary cells are defined as lymphocytes with hyperconvoluted cerebriform nuclei. If Sézary cells cannot be used to determine tumor burden for B2, then one of the following modified ISCL criteria, along with a positive clonal rearrangement of the TCR, may be used instead: (1) expanded CD4+ or CD3+ cells with a CD4/CD8 ratio of ≥10, or (2) expanded CD4+ cells with abnormal immunophenotype, including loss of CD7 or CD26.
**A T-cell clone is defined by PCR or Southern blot analysis of the TCR gene.
From Olsen et al., with permission from the American Society of Hematology.[1]

Table 81.1 Histopathologic staging of lymph nodes in *mycosis fungoides* and Sézary syndrome

EORTC classification	Dutch system	NCI-VA classification
N1	Grade 1: dermatopathic lymphadenopathy (DL)	LN0: no atypical lymphocytes<br>LN1: occasional and isolated atypical lymphocytes (not arranged in clusters)<br>LN2: many atypical lymphocytes or lymphocytes in 3-6-cell clusters
N2	Grade 2: DL; early involvement by MF (presence of cerebriform nuclei <7.5 μm)	LN3: aggregates of atypical lymphocytes; nodal architecture preserved
N3	Grade 3: partial effacement of lymph node architecture; many atypical cerebriform mononuclear cells<br>Grade 4: complete effacement	LN4: partial/complete effacement of nodal architecture byatypical lymphocytes or franklyneoplastic cells

From Olsen et al.,[1] with permission from the American Society of Hematology.

AJCC PROGNOSTIC STAGE GROUPS

Mycosis Fungoides and Sézary Syndrome

ISCL/EORTC revision to the staging of mycosis fungoides and Sézary syndrome

When T is...	And N is...	And M is...	And peripheral blood involvement (B) is...	Then the stage group is...
T1	N0	M0	B0,1	IA
T2	N0	M0	B0,1	IB
T1,2	N1,2	M0	B0,1	IIA
T3	N0-2	M0	B0,1	IIB
T4	N0-2	M0	B0,1	III
T4	N0-2	M0	B0	IIIA
T4	N0-2	M0	B1	IIIB
T1-4	N0-2	M0	B2	IVA1
T1-4	N3	M0	B0-2	IVA2
T1-4	N0-3	M1	B0-2	IVB

(continued)

From Olsen et al.,[1] with permission from the American Society of Hematology.

REGISTRY DATA COLLECTION VARIABLES

Mycosis Fungoides and Sézary Syndrome

1. Peripheral blood involvement

DEFINITIONS OF AJCC TNM

Primary Cutaneous B-Cell/T-Cell Lymphoma (Non-MF/SS) Lymphoma

Definition of Primary Tumor (T)

T Category	T Criteria
T1	Solitary skin involvement
T1a	Solitary lesion <5 cm
T1b	Solitary lesion ≥5 cm
T2	Regional skin involvement: multiple lesions limited to one body region or two contiguous body regions
T2a	All disease encompassing in a <15-cm circular area
T2b	All disease encompassing in a ≥15-cm and <30-cm circular area
T2c	All disease encompassing in a ≥30-cm circular area
T3	Generalized skin involvement

Definition of Regional Lymph Node (N)

N Category	N Criteria
NX	Regional lymph nodes cannot be assessed
N0	No clinical or pathological lymph node involvement
N1	Involvement of one peripheral node region that drains an area of current or prior skin involvement
N2	Involvement of two or more peripheral node regions or involvement of any lymph node region that does not drain an area of current or prior skin involvement
N3	Involvement of cenrowal nodes

81

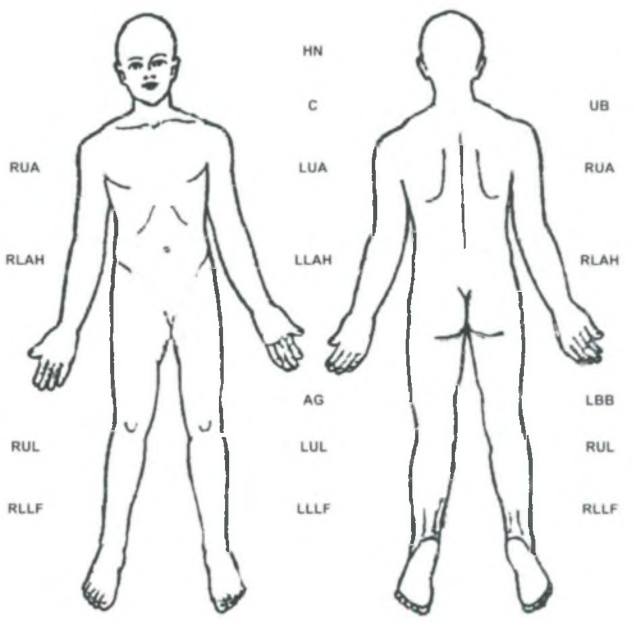

HN	Head & Neck
C	Chest
LUA	Left Upper Arm
LLAH	Left Lower Arm & Hand
AG	Abdominal & Genital
LUL	Left Upper Leg
LLLF	Left Lower Leg & Feet
RUA	Right Upper Arm
RLAH	Right Lower Arm & Hand
RUL	Right Upper Leg
RLLF	Right Lower Leg & Feet
UB	Upper Back
LBB	Lower Back & Buttock

Fig. 81.2 Body regions as defined in the proposed TNM system for designating T (skin involvement) category. Left and right extremities are assessed as separate body regions. The designation of these body regions are based on regional lymph node drainage patterns (From Kim et al.,[2] with permission)

Definition of Distant Metastasis (M)

M Category	M Criteria
M0	No evidence of extracutaneous non-lymph node disease
M1	Extracutaneous non-lymph node disease present

AJCC PROGNOSTIC STAGE GROUPS

Primary Cutaneous B-Cell/T-Cell Lymphoma (Non-MF/SS) Lymphoma

There is no stage group for other primary cutaneous lymphomas – including cutaneous T-cell, B-cell, NK cell and non-MF/SS lymphoma – at this time.

Bibliography

1. Olsen E, Vonderheid E, Pimpinelli N, et al. Revisions to the staging and classification of mycosis fungoides and Sezary syndrome: a proposal of the International Society for Cutaneous Lymphomas (ISCL) and the cutaneous lymphoma task force of the European Organization of Research and Treatment of Cancer (EORTC). *Blood*. Sep 15 2007;110(6):1713–1722.
2. Kim YH, Willemze R, Pimpinelli N, et al. TNM classification system for primary cutaneous lymphomas other than mycosis fungoides and Sezary syndrome: a proposal of the International Society for Cutaneous Lymphomas (ISCL) and the Cutaneous Lymphoma Task Force of the European Organization of Research and Treatment of Cancer (EORTC). *Blood*. Jul 15 2007;110(2):479–484.

Plasma Cell Myeloma and Plasma Cell Disorders

82

P. Leif Bergsagel, Elaine S. Jaffe, and John P. Leonard

CHAPTER SUMMARY

Cancers Staged Using This Staging System

Plasma cell myeloma

Cancers Not Staged Using This Staging System

These histopathologic types of cancer…	Are staged according to the classification for…	And can be found in chapter…
Smoldering multiple myeloma	No AJCC staging system	N/A
Monoclonal gammopathy of undetermined significance	No AJCC staging system	N/A
Waldenström's macroglobulinemia	No AJCC staging system	N/A

Summary of Changes

Details of Change	Rationale	Level of Evidence
New classification of plasma cell myeloma, also known as multiple myeloma (MM): ultra-high-risk smoldering MM (SMM) is now classified as MM	Adds as myeloma-defining events three biomarkers associated with an ~80% risk of end-organ damage within 2 years	I
Updated definition of SMM	Automatic, given the change in MM definition	I
Updated classification of monoclonal gammopathy of undetermined significance	Classification is important because the progression events are different	I
Updated definition of solitary plasmacytoma	Divides this heterogeneous entity into two clinically distinct groups with markedly different risks of progression	I
Updated staging system for MM	Incorporates markers of aggressive disease biology	I

ICD-O-3 Topography Codes

Code	Description
C42.1	Bone marrow
C31	Accessory sinuses: maxillary, ethmoid, frontal, sphenoid, accessory sinus NOS
C40-C41	Bones, joints, and articular cartilage
C44	Skin

WHO Classification of Tumors

Code	Description
9732	Plasma cell myeloma
9731	Solitary plasmacytoma of bone
9734	Extraosseous plasmacytoma

Swerdlow SH, Campo E, Harris NL, Jaffe ES, Pileri SA, Stein H, Thiele J, Vardiman J, eds. World Health Organization Classification of Tumours of Haematopoietic and Lymphoid Tissues. Lyon: IARC; 2008.

To access the AJCC cancer staging forms, please visit www.cancerstaging.org.

© American Joint Committee on Cancer 2017

M.B. Amin et al. (eds.), *AJCC Cancer Staging Manual, Eighth Edition*, DOI 10.1007/978-3-319-40618-3_82

INTRODUCTION

Plasma cell myeloma, also known as multiple myeloma (MM), is a neoplastic disorder characterized by the proliferation of a single clone of plasma cells derived from B cells. This clone of plasma cells grows in the bone marrow and frequently invades the adjacent bone, producing skeletal destruction that results in bone pain and fractures. Other common clinical findings include anemia, hypercalcemia, and renal insufficiency. Recurrent bacterial infections and bleeding may occur, but the hyperviscosity syndrome is rare. The clone of plasma cells produces monoclonal (M) protein of IgG or IgA (rarely IgD, IgE, or IgM) or free monoclonal light chains (kappa or lambda; Bence Jones protein).

RULES FOR CLASSIFICATION

Clinical Classification

The criteria for the diagnosis of plasma cell myeloma and related plasma cell proliferative disorders are provided in Table 82.1. The diagnosis requires ≥10% clonal plasma cells in the bone marrow or presence of a biopsy-proven plasmacytoma *plus* any one or more myeloma-defining events (MDEs). Metastatic carcinoma, lymphoma, leukemia, and connective tissue disorders must be excluded. In addition, monoclonal gammopathy of undetermined significance (MGUS), smoldering multiple myeloma (SMM), and solitary plasmacytoma must be excluded.

Table 82.1 Diagnostic criteria for plasma cell myeloma and related plasma cell disorders

Disorder	Disease definition
Plasma cell myeloma	Both of the following criteria must be met:<br>• Clonal bone marrow plasma cells ≥10% or biopsy-proven bony or extramedullary plasmacytoma<br>• One or more of the following myeloma-defining events:<br>○ Evidence of end-organ damage that may be attributed to the underlying plasma cell proliferative disorder, specifically:<br>▪ Hypercalcemia: serum calcium >0.25 mmol/L (>1 mg/dL) higher than the upper limit of normal or >2.75 mmol/L (>11 mg/dL)<br>▪ Renal insufficiency: creatinine clearance < 40 mL/min or serum creatinine >177 μmol/L (>2 mg/dL)<br>▪ Anemia: hemoglobin value of >2 g/dL below the lower limit of normal, or a hemoglobin value <a10 g/dL<br>▪ Bone lesions: one or more osteolytic lesions on computed tomography (CT) at least 5 mm in size<br>○ Clonal bone marrow plasma cell percentage ≥60%<br>○ Involved: uninvolved serum free light chain (FLC) ratio ≥100 (involved FLC level must be ≥100 mg/L)<br>○ Two or more focal lesions on magnetic resonance (MR) imaging or positron emission tomography (PET)/CT studies (at least 5 mm in size)
Non-IgM MGUS	All three of the following criteria must be met:<br>• Serum monoclonal protein (non-IgM type) <3 g/dL<br>• Clonal bone marrow plasma cells <10%*<br>• Absence of end-organ damage, such as hypercalcemia, renal insufficiency, anemia, and bone lesions (CRAB), that can be attributed to the plasma cell proliferative disorder, and absence of myeloma-defining events
IgM MGUS	All three of the following criteria must be met:<br>• Serum IgM monoclonal protein <3 g/dL<br>• Bone marrow lymphoplasmacytic infiltration <10%<br>• No evidence of anemia, constitutional symptoms, hyperviscosity, lymphadenopathy, or hepatosplenomegaly that can be attributed to the underlying lymphoproliferative disorder, and absence of myeloma-defining events
Light chain MGUS	All of the following criteria must be met:<br>• Abnormal FLC ratio (<0.26 or >1.65)<br>• Increased level of the appropriate involved light chain (increased kappa FLC in patients with ratio >1.65 and increased lambda FLC in patients with ratio <0.26)<br>• No immunoglobulin heavy chain expression on immunofixation<br>• Absence of end-organ damage that can be attributed to the plasma cell proliferative disorder, and absence of myeloma-defining events<br>• Clonal bone marrow plasma cells <10%<br>• Urinary monoclonal protein <500 mg/24 h
SMM	Both of the following criteria must be met:<br>• Serum monoclonal protein (IgG or IgA) ≥3 g/dL, or urinary monoclonal protein ≥500 mg/24 h and/or clonal bone marrow plasma cells 10–60%<br>• Absence of myeloma-defining events or amyloidosis

Table 82.1 (continued)

Disorder	Disease definition
Solitary plasmacytoma	All four of the following criteria must be met:<br>• Biopsy-proven solitary lesion of bone or soft tissue with evidence of clonal plasma cells<br>• Normal bone marrow with no evidence of clonal plasma cells<br>• Normal MR imaging (or CT) of spine and pelvis or whole-body PET/CT (except for the primary solitary lesion)<br>• Absence of end-organ damage, such as CRAB, that can be attributed to a lymphoplasma cell proliferative disorder, and absence of myeloma-defining events
Solitary plasmacytoma with minimal marrow involvement**	All four of the following criteria must be met:<br>• Biopsy-proven solitary lesion of bone or soft tissue with evidence of clonal plasma cells<br>• Clonal bone marrow plasma cells <10%<br>• Normal MR imaging (or CT) of spine and pelvis or whole-body PET/CT (except for the primary solitary lesion)<br>• Absence of end-organ damage, such as CRAB, that can be attributed to a lymphoplasma cell proliferative disorder, and absence of myeloma-defining events

Reproduced from Rajkumar et al.[1]

*A bone marrow biopsy may be deferred in patients with low-risk MGUS (IgG type, M protein <15 g/L, normal FLC ratio) in whom there are no clinical features concerning for myeloma.

**Solitary plasmacytoma with ≥10% clonal plasma cells is considered plasma cell myeloma.

Monoclonal Gammopathy of Undetermined Significance

The prevalence of monoclonal gammopathy of undetermined significance (MGUS), including light chain MGUS, is 4% in persons 50 years or older, 9% in those over 80 years of age, and is higher in men than women; African-Americans have a 2-fold higher prevalence of MGUS than whites.[2] The rate of progression is approximately 0.5% to 1% per year. [The level of monoclonal protein and the subtype (i.e., IgA and IgM are at greater risk) along with the serum free light chain (FLC) level are important prognostic features.] Patients must continue to be observed throughout their life because the risk of progression persists.[3,4]

SMM

SMM is characterized by the presence of M protein ≥3 g/dL and/or 10–60% clonal plasma cells in the bone marrow but no myeloma-defining events. Based on retrospective registry data, the risk of progression to plasma cell myeloma or AL amyloidosis is 10% per year for the first 5 years, approximately 3% per year for the next 5 years, and then 1% per year for the following 10 years.

Waldenström's Macroglobulinemia

Waldenström's macroglobulinemia (WM) is a clonal lymphoplasmacytic neoplasm associated with the secretion of an IgM monoclonal protein. The diagnostic criteria include IgM protein spike (of any size), ≥10% bone marrow lymphoplasmacytic infiltration (usually intertrabecular) by small lymphocytes that exhibit plasmacytoid or plasma cell differentiation, and a typical immunophenotype (e.g., surface IgM+, CD5+/–, CD10–, CD19+, CD20+, CD23–) that satisfactorily excludes other lymphoproliferative disorders, including chronic lymphocytic leukemia and mantle cell lymphoma. Patients who meet criteria for WM who do not have anemia, constitutional symptoms, hyperviscosity, lymphadenopathy, or hepatosplenomegaly that can be attributed to the underlying lymphoproliferative disorder are considered to have smoldering WM. In contrast to plasma cell myeloma, no immunoglobulin heavy chain translocations are found. Most patients have a recurrent somatic mutation, L265P, involving the *MYD88* gene. Median survival is approximately 6 years. (Gender; age; hemoglobin, neutrophil, and platelet levels; serum albumin; and β_2-microglobulin are all prognostic features.)

Solitary Plasmacytoma (Solitary Myeloma) of Bone

The diagnosis of solitary plasmacytoma of bone depends on histologic proof of a solitary plasma cell tumor (Table 82.1). Solitary bone plasmacytoma (normal bone marrow) has a risk of progression of approximately 10% within 3 years. In contrast, solitary bone plasmacytoma with minimal marrow involvement (clonal bone marrow cells present but <10%) is associated with a risk of progression of approximately 60% within 3 years. The persistence of a serum monoclonal protein ≥0.5 g/dL 1 to 2 years after diagnosis and an abnormal FLC ratio at the time of diagnosis indicate a high risk for disease progression.

Solitary Extramedullary Plasmacytoma

Solitary extramedullary plasmacytoma is a plasma cell tumor that arises outside the bone marrow. The upper respiratory tract is involved in approximately 80% of cases. As with solitary bone plasmacytoma, the risk of progression varies depending on whether the bone marrow is involved. Solitary extramedullary

this work was published only very recently. Therefore, the existing models that have been published or may be in clinical use have not yet been evaluated for this cancer site by the Precision Medicine Core of the AJCC. In the future, the statistical prediction models for this cancer site will be evaluated, and those that meet all AJCC criteria will be endorsed.

AJCC PROGNOSTIC STAGE GROUPS

RISS stage group	Factors
Stage I	Serum β_2-microglobulin <3.5 mg/L<br>*and*<br>serum albumin ≥3.5 g/dL<br>*and*<br>no high-risk cytogenetics*<br>*and*<br>Normal LDH
Stage II	Not stage I or III
Stage III	Serum β_2-microglobulin ≥5.5 mg/L<br>*and*<br>high-risk cytogenetics*<br>*and/or*<br>high LDH

*High-risk cytogenetics consist of one or more of the following: del17p, t(4;14), or t(14;16).

Note: The following variables must be collected at the time of diagnosis for staging of plasma cell myeloma according to the RISS: serum β2-microglobulin, serum albumin, serum LDH, and FISH results from the bone marrow specimen for t(4;14), t(14;16), and del17p.

Adapted from Palumbo et al.[6]

REGISTRY DATA COLLECTION VARIABLES

1. RISS stage group (Table 83.3)
2. ISS stage group (Table 83.2)
3. Imaging elements: bone disease demonstrated on imaging, plain film (skeletal survey), CT, MR imaging, PET/CT
4. Number of bone lesions identified on imaging: none, one, or more than one
5. Hemoglobin: all measurements are pretreatment
6. Serum β_2-microglobulin in milligrams per liter, xx.x; all measurements are pretreatment
7. Serum albumin in grams per deciliter, x.x; all measurements are pretreatment
8. Serum calcium in milligrams per deciliter, xx.x; all measurements are pretreatment
9. Serum creatinine in milligrams per deciliter, x.x; all measurements are pretreatment
10. LDH, normal or above normal, xx,xxx units per liter; all measurements are pretreatment
11. IgG in milligrams per deciliter, xx,xxx; all measurements are pretreatment
12. IgA in milligrams per deciliter, xx,xxx; all measurements are pretreatment
13. IgM in milligrams per deciliter, xx,xxx; all measurements are pretreatment
14. Monoclonal protein levels in serum and urine (M spike): grams per deciliter for serum, xx.x; grams for 24-hour urine, xx.x; all measurements are pretreatment
15. Serum free kappa light chain levels in grams per liter, xx,xxx (milligrams per deciliter × 10 to convert to grams per liter); all measurements are pretreatment
16. Serum free lambda light chain levels in grams per liter, xx,xxx (milligrams per deciliter × 10 to convert to grams per liter); all measurements are pretreatment
17. Cytogenetics: t(4;14), t(14;16), t(14;20), t(11;14), t(6;14), add1q, del1p, del17p, trisomy 3, trisomy 5, trisomy 7, trisomy 9, trisomy 11, trisomy 15, trisomy 19, trisomy 21

Bibliography

1. Rajkumar SV, Dimopoulos MA, Palumbo A, et al. International Myeloma Working Group updated criteria for the diagnosis of multiple myeloma. *The lancet oncology.* 2014;15(12):e538–548.
2. Landgren O, Graubard BI, Katzmann JA, et al. Racial disparities in the prevalence of monoclonal gammopathies: a population-based study of 12,482 persons from the National Health and Nutritional Examination Survey. *Leukemia.* 2014;28(7):1537–1542.
3. Sigurdardottir EE, Turesson I, Lund SH, et al. The Role of Diagnosis and Clinical Follow-up of Monoclonal Gammopathy of Undetermined Significance on Survival in Multiple Myeloma. *JAMA oncology.* 2015;1(2):168–174.
4. Turesson I, Kovalchik SA, Pfeiffer RM, et al. Monoclonal gammopathy of undetermined significance and risk of lymphoid and myeloid malignancies: 728 cases followed up to 30 years in Sweden. *Blood.* 2014;123(3):338–345.
5. Mihailovic J, Goldsmith SJ. Multiple myeloma: 18F-FDG-PET/CT and diagnostic imaging. *Semin Nucl Med.* 2015;45(1):16–31.
6. Palumbo A, Avet-Loiseau H, Oliva S, et al. Revised International Staging System for Multiple Myeloma: A Report From International Myeloma Working Group. *J Clin Oncol.* 2015;33(26):2863–2869.
7. Kattan MW, Hess KR, Amin MB, et al. American Joint Committee on Cancer acceptance criteria for inclusion of risk models for individualized prognosis in the practice of precision medicine. CA: *a cancer journal for clinicians.* 2016.

82

plasmacytoma (normal bone marrow) has a risk of progression of approximately 10% within 3 years. In contrast, solitary extramedullary plasmacytoma with minimal marrow involvement (clonal bone marrow cells present but <10%) is associated with a risk of progression of approximately 20% within 3 years.

Imaging

The imaging test relevant for diagnosis and assessment of treatment response in plasma cell myeloma is either MR imaging or low-dose whole-body CT or PET/CT, with the choice depending on availability and local expertise. In the past, planar X-rays were used for a skeletal survey to look for lytic lesions. Currently, this is regarded as an outdated approach and should be used only if the newer imaging modalities are not available.

MR imaging is most useful for evaluating marrow lesions.[5] CT imaging usually is done without iodinated intravenous contrast. The imaging findings sought with CT are lytic bone lesions. CT is more sensitive than planar X-rays but less sensitive than MR imaging. Note that if a PET/CT scan is performed, there is no need to order a separate CT scan.

MR and CT imaging should include the skull, spine, sternum, clavicles, shoulders, proximal humeri, pelvis, and proximal femora. PET/CT imaging is done with fluorine-18 fluorodeoxyglucose (FDG) from the top of the head to mid-thigh. The PET/CT findings that indicate active plasma cell myeloma are focal areas of increased FDG uptake (hot spots) in marrow or in soft tissue masses. The hot spots may be difficult to detect in the presence of diffuse marrow involvement. PET/CT assessment of marrow involvement is less accurate than MR imaging, because normal marrow uptake may be heterogeneous and may be markedly elevated in patients treated with granulocyte colony-stimulating factor.[5]

PROGNOSTIC FACTORS

The median survival of patients with plasma cell myeloma varies considerably and is affected by host factors such as age, comorbidities, performance status, and renal function. It also is influenced by tumor burden and disease biology. Tumor burden historically has been assessed by either the Durie–Salmon staging system (DSS) or the International Staging System (ISS) (Table 82.2). Disease biology is best assessed by cytogenetic abnormalities and by factors such as lactate dehydrogenase (LDH) level and circulating plasma cells. Recently, the Revised International Staging System (RISS) was adopted by the International Myeloma Working Group; it combines elements of tumor burden and disease biology into one powerful prognostic tool (Table 82.3). The presence of t(4;14), t(14;16), or del 17p by fluorescence *in situ* hybridization (FISH) is a predictor of poor outcome. Elevated LDH and the presence of high numbers of circulating plasma cells in the peripheral blood also are associated with more aggressive disease. The proteasome inhibitor bortezomib has shown promise in partially overcoming these adverse prognostic factors for conventional and high-dose therapies.

Prognostic Factors Required for Stage Grouping

Table 82.2 International Staging System (ISS)

Factor	Stage group	Patients, %	5-Year overall survival, %	Level of evidence
Serum β_2-microglobulin <3.5 mg/L and serum albumin ≥3.5 g/dL	ISS 1	38	77	II
Not ISS 1 or ISS 3	ISS 2	38	62	II
Serum β_2-microglobulin ≥5.5 mg/L	ISS 3	23	47	II

Adapted from Palumbo et al.[6]

Table 82.3 Revised International Staging System (RISS)

Factor	Stage group	Patients, %	5-Year overall survival, %	Level of evidence
ISS 1 *and* no high-risk cytogenetics,* *and* Normal LDH	I	28	82	III
Not Stage I or III	II	62	62	III
ISS 3 *and* high-risk cytogenetics* *or* high LDH	III	10	40	III

*High-risk cytogenetics consist of one or more of the following: del17p, t(4;14), or t(14;16).

Note: The following variables must be collected at the time of diagnosis for staging of plasma cell myeloma according to the RISS: serum β_2-microglobulin, serum albumin, serum LDH, and FISH results from the bone marrow specimen for t(4;14), t(14;16), and del17p.

Adapted from Palumbo et al.[6]

Additional Factors Recommended for Clinical Care

Factor	Definition	Clinical significance	Level of evidence
Serum calcium	Elevation above normal	Adverse prognostic factor	I
Serum creatinine	Elevation above normal	Adverse prognostic factor	I

RISK ASSESSMENT MODELS

The AJCC recently established guidelines that will be used to evaluate published statistical prediction models for the purpose of granting endorsement for clinical use.[7] Although this is a monumental step toward the goal of precision medicine,

Leukemia

83

Jerald P. Radich, Soheil Meshinchi, Elihu H. Estey,
William L. Carroll, Ryan Cassaday, Steven Coutre,
Elaine S. Jaffe, and John P. Leonard

CHAPTER SUMMARY

Cancers Staged Using This Staging System

Myeloid and lymphoid leukemia

Cancers Not Staged Using This Staging System

These histopathologic types of cancer…	Are staged according to the classification for…	And can be found in chapter…
Multiple myeloma	Multiple myeloma	82
Myelodysplastic syndrome	No AJCC staging system	N/A
Myeloproliferative neoplasm	No AJCC staging system	N/A

Summary of Changes

Leukemia is a new malignancy for the AJCC cancer staging system.

ICD-O-3 Topography Codes

Code	Description
C42.1	Bone marrow

WHO Classification of Tumors

Code	Description
9875	Chronic myelogenous leukemia, BCR-ABL1 positive
9945	Chronic myelomonocytic leukemia
9876	Atypical chronic myeloid leukemia, BCR-ABL1 negative
9896	Acute myeloid leukemia (AML) with t(8;21)(q22;q22); RUNX1-RUNX1T1
9871	AML with inv(16) (p13.1q22) or t(16;16)(p13.1;q22); CBFB-MYH11
9866	Acute promyelocytic leukemia with t(15;17)(q22;q12); PML-RARA
9897	AML with t(9;11)(p22;q23); MLLT3-MLL
9865	AML with t(6;9)(p23;q34); DEK-NUP214
9869	AML with inv(3)(q21q26.2) or t(3;3)(q21;q26.2); RPN1-EVI1
9911	AML (megakaryoblastic) with t(1;22)(p13;q13); RBM15-MKL1
9861	AML with mutated NPM1
9861	AML with mutated CEBPA
9895	AML with myelodysplasia-related changes
9920	Therapy-related myeloid neoplasm
9861	AML, NOS
9872	AML with minimal differentiation
9873	AML without maturation
9874	AML with maturation
9867	Acute myelomonocytic leukemia
9891	Acute monoblastic and monocytic leukemia
9840	Acute erythroid leukemia
9910	Acute megakaryoblastic leukemia
9870	Acute basophilic leukemia
9931	Acute panmyelosis with myelofibrosis
9930	Myeloid sarcoma
9898	Myeloid leukemia associated with Down syndrome
9727	Blastic plasmacytoid dendritic cell neoplasm
	ACUTE LEUKEMIAS OF AMBIGUOUS LINEAGE

To access the AJCC cancer staging forms, please visit www.cancerstaging.org.

© American Joint Committee on Cancer 2017

M.B. Amin et al. (eds.), *AJCC Cancer Staging Manual, Eighth Edition*, DOI 10.1007/978-3-319-40618-3_83

Code	Description
9801	Acute undifferentiated leukemia
9806	Mixed phenotype acute leukemia with t(9;22)(q34;q11.2); BCR-ABL1
9807	Mixed phenotype acute leukemia with t(v;11q23); mixed lineage leukemia (MLL) rearranged
9808	Mixed phenotype acute leukemia, B/myeloid, NOS
9809	Mixed phenotype acute leukemia, T/myeloid, NOS
	**PRECURSOR LYMPHOID NEOPLASMS**
9811	B-lymphoblastic leukemia/lymphoma, NOS
9812	B-lymphoblastic leukemia/lymphoma with t(9;22)(q34;q11.2); BCR-ABL1
9813	B-lymphoblastic leukemia/lymphoma with t(v;11q23); MLL rearranged
9814	B-lymphoblastic leukemia/lymphoma with t(12;21)(p13;q22); TEL-AML1 (ETV6-RUNX1)
9815	B-lymphoblastic leukemia/lymphoma with hyperdiploidy
9816	B-lymphoblastic leukemia/lymphoma with hypodiploidy (hypodiploid acute lymphoblastic leukemia [ALL])
9817	B-lymphoblastic leukemia/lymphoma with t(5;14)(q31;q32); IL3-IGH
9818	B-lymphoblastic leukemia/lymphoma with t(1;19)(q23;p13.3); E2A-PBX1 (TCF3-PBX1)
9837	T-lymphoblastic leukemia/lymphoma
	**MATURE B-CELL NEOPLASMS**
9823	Chronic lymphocytic leukemia/small lymphocytic lymphoma
9833	B-cell prolymphocytic leukemia
9940	Hairy cell leukemia
9591	Splenic B-cell lymphoma/leukemia, unclassifiable
9591	Hairy cell leukemia variant
	**MATURE T-CELL AND NK-CELL NEOPLASMS**
9834	T-cell type prolymphocytic leukemia
9831	T-cell large granular lymphocytic leukemia
9831	Chronic lymphoproliferative disorder of NK cells
9948	Aggressive NK-cell leukemia
9724	Systemic Epstein–Barr virus–positive T-cell lymphoproliferative disease of childhood
9827	Adult T-cell lymphoma/leukemia

Swerdlow SH, Campo E, Harris NL, Jaffe ES, Pileri SA, Stein H, Thiele J, Vardiman J, eds. World Health Organization Classification of Tumours of Haematopoietic and Lymphoid Tissues. Lyon: IARC; 2008.

INTRODUCTION

Leukemia is a cancer of the hematopoietic system. The disease is subdivided by the tempo of disease (acute vs. chronic) as well as the primary lineage involved (myeloid vs. lymphoid). In general, acute leukemia is characterized by a block in cell differentiation, resulting in a proliferation of immature cells (blasts). In chronic leukemia, the cells at diagnosis appear more normally differentiated but generally are very high in number in the bone marrow and peripheral blood. When leukemia is fatal, it generally is because normal hematopoietic function has been usurped by the leukemia, resulting in thrombocytopenia and neutropenia, leading to death from bleeding or infections.

Further subclassification of each leukemia may depend on specific genetic abnormalities [e.g., the t(9;22) "Philadelphia (Ph) chromosome" in acute lymphoblastic leukemia (ALL)] or cell phenotype type (e.g., B- vs. T-cell ALL). In addition, because the acute leukemias more often involve a larger age spectrum than the chronic leukemias (which are rare in younger people), attempts have been made to subclassify these leukemias into pediatric, adult, and elderly. These age boundaries have changed over the years, and there are no established cutoffs.

Because of the nature of the disease, leukemia does not easily fit into the TNM staging system. Rather, clinically meaningful staging is defined differently for each type of leukemia.

ACUTE MYELOID LEUKEMIA

Rules for Classification

Clinical Classification

Acute myeloid leukemia (AML) is a hematopoietic malignancy that represents the culmination of genetic and epigenetic alterations in hematopoietic stem/progenitor cells (HSPCs), leading to dysregulation of critical signal transduction pathways and resulting in the expansion of undifferentiated myeloid cells.[1] AML may be divided broadly into two general categories, de novo AML and secondary AML. De novo AML develops without obvious antecedent. *Secondary AML* refers to the evolution of AML after exposure to cytotoxic therapy (t-AML) or to an antecedent hematologic disorder (AHD; e.g., myelodysplastic syndrome [MDS]) associated with distinct karyotypic and molecular alterations, including *MLL* translocations after exposure to topoisomerase inhibitors or complex karyotypes after treatment with alkylating agents.[2] However, it now is apparent that some patients with seemingly de novo AML have genomic alterations reminiscent of those seen in secondary AML. In these patients, the clinical picture typically is similar to that of secondary AML.[3]

Many somatic karyotypic and molecular alterations have been identified in AML. However, despite the association of many of these alterations with distinct clinical phenotypes, most have no prognostic value, nor do they identify a specific target or a distinct pathway that can be readily exploited for therapeutic intervention.[1] The paucity of targets is more notable in childhood AML, particularly because the observed age-associated evolution of molecular alterations reveals a distinct profile for younger children with AML compared with older children and adolescents with AML. Furthermore, the landscape of genetic alterations differs markedly from AML in adults.[4,5]

AML is diagnosed in very young children and comprises nearly 25% of pediatric leukemias; however, it is far more prevalent in adults and generally is considered a disease of older adults, whose median age at diagnosis is nearly 70 years. The incidence of AML is better appreciated by close evaluation of the most recent Surveillance, Epidemiology, and End Results (SEER) data (http://seer.cancer.gov/statfacts/html/amyl.html). These data demonstrate a low incidence of AML in children and young adults, with a significant increase in older adults.[6] However, closer examination demonstrates that the highest incidence of AML in younger patients (<40 years) occurs in infancy, with an incidence of 1.6 cases per 100,000, similar to that in the fourth decade of life. After infancy, there is a declining incidence of approximately 0.12 cases per 100,000 per year in the first decade of life to an incidence of 0.4 per 100,000 by age 10. In the following three decades, there is a steady increase in AML incidence of approximately 0.02 cases per 100,000 per year to a rate of 1.3 cases per 100,000 per year by age 45, nearly equivalent to that seen in infants. A substantial rise in the incidence of AML occurs in the fifth decade, reaching nearly 10 times the observed rate in the previous three decades to an incidence of 6.2 per 100,000 by age 65. After age 65, AML diagnosis again increases substantially, more than 30-fold higher than that seen in younger patients (age 10–40). Seemingly spontaneously arising AML in patients over age 60 to 65 often is characterized by complex cytogenetic changes that are very similar to those seen in patients with MDS or in AML arising after receipt of chemotherapy for other conditions.[7] This of course suggests that these "spontaneous" AMLs reflect evolution from an underlying MDS and/or the cumulative effect of lifetime toxin exposure. It has been demonstrated that the small minority (5–10%) of patients who receive chemotherapy for other diseases and subsequently develop AML have specific polymorphisms for genes involved in detoxifying carcinogens.[8–10]

It is becoming increasingly apparent that aging is accompanied by development of aberrations in genes that are also abnormal in AML, such as *DNMT3a* and *ASXL*.[11] This observation led to the suggestion that acquisition of AML, like other cancers, is a multistep process that may take years to complete. Indeed, the genetic landscape of AML is complex. An average of 13 coding mutations have been reported in de novo AML, with, on average, five of these occurring in genes that are recurrently mutated, consistent with a pathogenic role in AML.[1] Most mutations appear to be stochastically acquired events in normal HSPCs, and these are retained after the HSPC acquires a relatively small number of "driver" mutation(s). However, this small number ignores observations that during AML evolution, multiple new critical abnormalities may be acquired that will then act as active drivers in disease progression.[12] These additional mutations may be downstream of the initial (founding) mutation(s), or they may bypass these founding mutations by using a parallel cellular pathway. These processes may be hastened by AML chemotherapy. In this fashion, the founding clone frequently gives rise to a variety of subclones potentially resistant to therapy. These clones may predominate at relapse, making AML, molecularly at least, a "progressive" disease.

Laboratory Features

Unlike cancers of solid organs, AML is widely disseminated at diagnosis; hence, a TNM-type staging system is of little practical value. For many years, classification of AML was almost exclusively morphologic and was dominated by the French–American–British (FAB) system.[13] AML was diagnosed only if the marrow or blood contained >30% myeloid blasts, identified primarily by histochemical stains specific to, for example, the granulocytic (peroxidase) or monocytic lineages (esterase). Various FAB subtypes, M0 through M7, were recognized. A problem with the FAB system was that its clinical relevance was limited, with the very important exception of subtype M3, acute promyelocytic leukemia (APL), which unlike other types of AML, is routinely curable with all-trans retinoic acid and arsenic trioxide with or without an anthracycline such as idarubicin. Furthermore, as more was learned about the prognostic significance of cytogenetics and various molecular abnormalities, it became obvious that any classification system needed to incorporate this information. This recognition led to development of the World Health Organization (WHO) system, currently the most widely accepted means of classifying AML.[14]

Unlike the FAB system, the WHO criterion for a diagnosis of AML is <20% blasts, with the exceptions noted here. This change from <30% reflected observations that after accounting for various prognostic factors, such as cytogenetics or whether AML was de novo or secondary (unfavorable), patients with 21–30% blasts fared identically to those with <30% blasts when treated identically.[15] Some experts have contended, however, that any minimum blast criterion (many cooperative groups use 10%) is too simplistic and ignores the complex medical decision making inherent in AML treatment. Rather than relying on histochemical stains to identify blasts of myeloid lineage, cell surface antigens now serve this purpose (Table 83.1).

The great majority of patients with AML fall into one of the first four WHO categories: AML with recurrent genetic changes, AML with myelodysplasia-related changes, therapy-related AML, or AML not otherwise specified (NOS) (Table 83.2).

AML with Recurrent Genetic Changes

Patients with the inv(16), t(16;16), or t(8;21) cytogenetic abnormality are lumped together into a "core-binding factor" (CBF) group. *CBF* refers to regulators of transcription

Table 83.1 Expression of cell-surface and cytoplasmic markers for the diagnosis of AML and mixed phenotype acute leukemia (MPAL)

**Diagnosis of AML***	
Precursor stage	CD34, CD38, CD117, CD133, HLA-DR
Granulocytic markers	CD13, CD15, CD16, CD33, CD65, cytoplasmic myeloperoxidase
Monocytic markers	Nonspecific esterase (NSE), CD11c, CD14, CD64, lysozyme, CD4, CD11b, CD36, NG2 homologue**
Megakaryocytic markers	CD41 (glycoprotein IIb/IIIa), CD61 (glycoprotein IIIa), CD42 (glycoprotein Ib)
Erythroid marker	CD235a (glycophorin A)
**Diagnosis of MPAL*****	
Myeloid lineage	MPO or evidence of monocytic differentiation (at least two of the following: NSE, CD11c, CD14, CD64, lysozyme)
B lineage	CD19 (strong) with at least one of the following: CD79a, cCD22, CD10
	Or CD19 (weak) with at least two of the following: CD79a, cCD22, CD10
T lineage	cCD3 or surface CD3

*For the diagnosis of AML, the table provides a list of selected markers rather than a mandatory marker panel.
**Most cases with 11q23 abnormalities express the NG2 homologue (encoded by *CSPG4*) reacting with the monoclonal antibody 7.1.
***Requirements for assigning more than one lineage to a single blast population are adopted from the WHO classification.30 Note that the requirement for assigning myeloid lineage in MPAL is more stringent than for establishing a diagnosis of AML. Note also that MPAL can be diagnosed if there are separate populations of lymphoid and myeloid blasts.

that have a β subunit and three α subunits. RUNX1 and RUNX1T1 t(8;21) affects one of the α subunits, whereas CBFB-MYH11 affects the β subunit. Depending on the age of the population in question, CBF AML comprises 5–15% of cases and is relatively sensitive to cytosine arabinoside (ara-C), thus benefiting from "high" doses of this drug and having a relatively good prognosis.[16] Despite being considered a single group, there are important clinical differences between t(8;21) and inv(16). For example, the latter is much more likely to have multiple remissions and thus longer survival.[17]

The t(15;17) abnormality is characteristic of FAB subtype M3, as discussed earlier. About 5% of cases of APL have normal cytogenetics, thus it is critical to assess for the PML-RARα aberration associated with the t(15;17).[18] The abnormal juxtaposition of the PML and RARα genes results in the impaired promyelocytic differentiation that is the hallmark of APL. The abnormal positioning of PML leads to a characteristic pattern of nuclear immunofluorescence, demonstration of which is the quickest way to diagnose APL.[19]

Abnormalities in the mixed lineage leukemia (MLL) gene are associated with deletions or translocations in the long arm of chromosome 11 (11q). The clinical patterns associated with different MLL partners vary considerably. For example, pairing of MLL with MLLT3, resulting in t(9;11), leads to a prognosis better than that seen when MLL is paired with other partners.[20] Thus, as with CBF, classifying an AML as "MLL AML" is itself a gross oversimplification. MLL abnormalities are particularly important to recognize because a class of drugs (DOT1L inhibitors) appear to target MLL and have produced (mainly minor) responses in clinical trials.[21]

The t(6;9) (which transposes the *DEK* and *NUP214* genes) often is associated with basophilia but is perhaps mainly of interest because 80% of cases have an internal tandem duplication (ITD) of the *FLT3* gene; t (6;9) is the AML subtype most frequently associated with *FLT3* ITD.[22] The 2008 version of WHO system does not recognize *FLT3* ITD as falling in the category of recurrent genetic abnormalities (Table 83.2). This is because in perhaps 30% of cases, patients who present with an *FLT3* ITD are either not *FLT3* ITD positive at relapse or vice versa.[23] Hence, it would appear that *FLT3* ITDs are not foundational abnormalities. In contrast, a change in *NPM1* or *CEBPA* mutation status between diagnosis and relapse is believed to be less common, leading to inclusion of these in the WHO system.[24] Nonetheless, *FLT3* ITD, *NPM1* mutations, and *CEBPA* double mutations all have major prognostic importance.[25] *NPM1* mutations and double (biallelic) *CEBPA* mutations convey a relatively favorable prognosis, whereas the opposite is true of *FLT3* ITDs; mutations in the tyrosine kinase domain (TKD) of *FLT3* are of less prognostic relevance. Of course, the significance of these aberrations depends on the context in which they occur. For example, patients in whom <50% of alleles have an *FLT3* ITD have a poorer prognosis than patients with lower allelic burdens, whereas patients with *NPM1* mutations and *FLT3* ITDs do worse than those with only *NPM1* mutations.[26]

It is also worth noting that patients with RUNX1-RUNX1T1 [i.e., t(8;21)] or CBFB-MYH11 [i.e., inv16 or t(16;16)] are considered by WHO to have AML regardless of blast count.[14] This can readily be justified on clinical grounds, given that these patients have responses to AML therapy similar to those of patients with <20% blasts.[15] It is likely that blast count eventually will be considered immaterial to a

Table 83.2 AML and related precursor neoplasms, and acute leukemias of ambiguous lineage[3,30]

**Acute myeloid leukemia with recurrent genetic abnormalities**
AML with t(8;21)(q22;q22.1); RUNX1-RUNX1T1
AML with inv(16)(p13.1q22) or t(16;16)(p13.1;q22); CBFB-MYH11
**APL with PML-RARA***
AML with t(9;11)(p22.3;q23.3); MLLT3-KMT2A**
AML with t(6;9)(p23;q34.1); DEK-NUP214
AML with inv(3)(q21.3q26.2) or t(3;3)(q21.3;q26.2)
AML (megakaryoblastic) with t(1;22)(p13.3;q13.1); RBM15-MKL1
AML with mutated NPM1
AML with biallelic mutations of CEBPA
Provisional entity: AML with BCR-ABL1
Provisional entity: AML with mutated RUNX1
**Acute myeloid leukemia with myelodysplasia-related changes*****
**Therapy-related myeloid neoplasms********
**Acute myeloid leukemia, not otherwise specified (NOS)**
Acute myeloid leukemia with minimal differentiation
AML without maturation
AML with maturation
Acute myelomonocytic leukemia
Acute monoblastic/monocytic leukemia
Pure erythroid leukemia
Acute megakaryoblastic leukemia
Acute basophilic leukemia
Acute panmyelosis with myelofibrosis (syn.: acute myelofibrosis; acute myelosclerosis)
**Myeloid sarcoma (syn.: extramedullary myeloid tumor; granulocytic sarcoma; chloroma)**
**Myeloid proliferations related to Down syndrome**
Transient abnormal myelopoiesis (syn.: transient myeloproliferative disorder)
Myeloid leukemia associated with Down syndrome
**Blastic plasmacytoid dendritic cell neoplasm**
Acute leukemias of ambiguous lineage
**Acute undifferentiated leukemia**
Mixed phenotype acute leukemia with t(9;22)(q34;q11.2); BCR-ABL1*****
Mixed phenotype acute leukemia with t(v;11q23); *KMT2A* rearranged
Mixed phenotype acute leukemia, B/myeloid, NOS
Mixed phenotype acute leukemia, T/myeloid, NOS
Provisional entity: Natural killer (NK) cell lymphoblastic leukemia/lymphoma

Adapted from Lindsley et al.[3] For a diagnosis of AML, a marrow blast count of ≥20% is required, except for AML with the recurrent genetic abnormality t(15;17), t(8;21), inv(16), or t(16;16) and some cases of erythroleukemia.

*Other recurring translocations involving *RARA* should be reported accordingly: e.g., AML with t(11;17)(q23;q12)/ZBTB16-RARA; AML with t(11;17)(q13;q12); NUMA1-RARA; AML with t(5;17)(q35;q12); NPM1-RARA; or AML with STAT5B-RARA (the latter having a normal chromosome 17 on conventional cytogenetic analysis).

**Other translocations involving *KMT2A* (MLL) should be reported accordingly: e.g., AML with t(6;11)(q27;q23); MLLT4-MLL; AML with t(11;19)(q23;p13.3); MLL-MLLT1; AML with t(11;19)(q23;p13.1); MLL-ELL; AML with t(10;11)(p12;q23); MLLT10-MLL.

***More than 20% blood or marrow blasts AND any of the following: previous history of MDS or myelodysplastic/myeloproliferative neoplasm (MDS/MPN), myelodysplasia-related cytogenetic abnormality (see following list); multilineage dysplasia in ≥50% of cells in at least two cell lines; AND absence of both prior cytotoxic therapy for unrelated disease and aforementioned recurring genetic abnormalities. Cytogenetic abnormalities sufficient to diagnose AML with myelodysplasia-related changes are:

- complex karyotype (defined as three or more chromosomal abnormalities)
- unbalanced changes: -7 or del(7q);del(5q) or unbalanced t(5q); i(17q) or t(17p); -13 or del(13q); del(11q); del(12p) or t(12p); idic(X)(q13)
- balanced changes: t(11;16)(q23.3;p13.3); t(3;21)(q26.2;q22.1); t(1;3)(p36.3;q21.2); t(2;11)(p21;q23.3); t(5;12)(q32;p13.2); t(5;7)(q32;q11.2); t(5;17)(q32;p13.2); t(5;10)(q32;q21); t(3;5)(q25.3;q35.1)

****Cytotoxic agents implicated in therapy-related hematologic neoplasms: alkylating agents, ionizing radiation therapy, topoisomerase II inhibitors, and others.

*****BCR-ABL1–positive leukemia may present as MPAL but should be treated as BCR-ABL1–positive ALL.

diagnosis of AML in patients with *NPM1* mutations and/or *CEBPA* double mutations.

AML with Myelodysplasia-related Changes

Patients fall into this category if they have ≥20% blasts and either morphologic features of myelodysplasia (MDS), a history of MDS, or a myeloproliferative neoplasm (MPN), or MDS-related cytogenetic abnormalities without the specific genetic abnormalities characteristic of AML, as noted in Table 83.2. MDS-related cytogenetic abnormalities are complex karyotypes (at least three distinct clonal abnormalities), monosomy of or deletion of the long arm of chromosomes 5 and/or 7 (-5,del 5q, -7, del 7q), t(3;5)(q25;q34) and changes in chromosome 11q not involving MLL.

This formulation has several problems. For example, concordance is far from perfect among pathologists in describing *dysplasia*. The prognostic relevance of dysplasia is in doubt.[27] Finally, as noted earlier, the distinction between de novo and secondary AML (e.g., arising on a background of MDS or MPN) is probably better made based on genomics than clinical history.[3]

Therapy-related AML (t-AML)

t-AMLs generally are divided into those associated with use of alkylating agents (e.g., cyclophosphamide or melphalan) and those associated with use of topoisomerase II–reactive drugs (e.g., anthracyclines or etoposide).[2] The former are characterized by a 5- to 10-year latency period and complex cytogenetics. The latter more often present with abnormalities of chromosome 11q or balanced translocations, such as t(15;17) or t(8;21). Such cases generally are regarded as "AML with recurrent genetic abnormalities."[14] This is reasonable from a prognostic standpoint, as cases of t-AML with t(15;17), t(8;21), or inv(16) have prognoses similar to those of cases in which these abnormalities arise seemingly spontaneously.[28]

Assignment of a case to the t-AML category is not always straightforward, such as when the latency period is <10 years after administration of alkylating agents. Here again, defining secondary AML based on genomic patterns likely is better than doing so based on clinical history.

AML NOS

These cases fall outside the other types of AML specified in Table 83.2. They comprise about 25% of all cases. Essentially, they are classified using FAB nomenclature. There likely will be fewer cases as more genetic subtypes of AML are recognized by the WHO.

Using data from 5,848 patients with AML NOS treated in studies by the Southwest Oncology Group (SWOG), MD Anderson Cancer Center, U.K. Medical Research Council/National Cancer Research Institute (MRC/NCRI), and Dutch–Belgian Cooperative Trial Group for Hematology/Oncology and the Swiss Group for Clinical Cancer Research (HOVON/SAKK). Walter et al.[29] studied the prognostic effect of different AML NOS subtypes, such as AML with minimal differentiation (M0), AML without maturation (M1), and AML with maturation (M2). After multivariate adjustment, FAB M0 was independently associated with a significantly lower likelihood of achieving complete remission and inferior relapse-free and overall survival compared with M1, M2, M4, M5, and M6, with inconclusive data regarding M7. However, when attention was restricted to known *NPM1*-negative patients, FAB M0 was no longer associated with worse outcomes; restricting attention to patients known to be *NPM1* negative/*CEPBA* negative (i.e., excluding the provisional entities of "AML with mutated *NPM1*" and "AML with mutated *CEBPA*"; Table 83.2) did not affect this result. Hence, FAB subclassification of AML NOS does not provide prognostic information if data on *NPM1* and *CEBPA* mutations are available.

Prognostic Factors

Prognostic Factors Required for Clinical Care

The prognostic factors in this section are required for diagnosis of acute myeloid leukemia.

Factor	Definition	Clinical Significance	Level of Evidence
Age	chronologic	Older age independently associated with more treatment related mortality(TRM) and resistance to therapy	I
Zubrod performance status(PS)	0 or 1 = minimal symptoms; 4 = bed ridden, 3 =in bed 50–100% of time, 2= between 1 and 3	PS 3-4 independently associated with TRM;	I
Hematopoietic cell transplantation comorbidity index (HCT-CI)	See Hunger et al.[31]	Determinant of risk of TRM after hematopoietic cell transplant (HCT)	I
Cytogenetics (20 metaphase)	Categorized as "favorable", "intermediate" or "adverse" as per Dohner et al.[25]	Remains most important predictor of resistance to therapy; adverse and many intermediate patients are candidates for HCT and/or trials of new therapies	I

Factor	Definition	Clinical Significance	Level of Evidence
Status of NPM,FLT3 and CEBPA genes	a) NPM1 mutation in absence FLT3 internal tandem duplication<br>b) bi allelic CEBPA mutation<br>c) FLT3 internal tandem duplication	a) Associated with sufficiently low risk of relapse to obviate need for HCT<br>b) as for a)<br>c) Associated with sufficiently high risk of relapse as to justify HCT; patients are candidates for combinations of chemotherapy and "FLT3" inhibitors, particularly multitargeted ones such as midostaurin	I

Additional Factors Recommended for Clinical Care

Factor	Definition	Clinical Significance	Level of Evidence
Measurable residual disease (MRD) after completion of induction or post remission chemotherapy	Assessed by multiparameter flow cytometry and/or, molecular testing for selected mutations such as NPM1, CBFB-MYH11, RUNX1-RUNX1T1, PML-RARA	Presence of MRD associated with increased probability of relapse and probably more important in ths regard than staging factors noted above	I
ASXL, TET2, and TP53 genes	Assessed for mutations	Associated with sufficiently high risk of relapse to justify HCT;	I[26]

ACUTE LYMPHOBLASTIC LEUKEMIA IN CHILDREN

Introduction

Acute lymphoblastic leukemia (ALL) is the most common malignancy of children, representing close to 30% of all malignancies. Fortunately, the cure rate has risen dramatically during the past four to five decades, with cure rates of 15–20% in the 1960s to 80–90% today.[31] These remarkable improvements have been achieved by the application of combination chemotherapy with progressive advancements gained through highly disciplined cooperative group clinical trials, the use of presymptomatic or prophylactic central nervous system (CNS) therapy, intensification of commonly used agents, and the introduction of risk-stratified treatment based on prognostically important clinical and laboratory variables. The goal of therapy is to maximize cure rates while minimizing short- and long-term side effects in the growing child.

The clinical and biological features of leukemia in children differ from those noted in adults, although several studies show a better outcome for adolescents and young adults when treated on pediatric-inspired protocols.

Rules for Classification

Clinical Classification

Common symptoms of ALL include fatigue, fever, bruising/petechiae, and bone/joint pain. An unexplained limp should alert clinicians to a possible diagnosis of leukemia. About one third to one half of children have lymphadenopathy and/or hepatosplenomegaly, which are observed more commonly in T-ALL versus B-ALL. Often there is associated anemia (50% with hemoglobin <8 g/dL), neutropenia, and/or thrombocytopenia (75–80% with platelet count $\leq 100 \times 10^9/L$), but peripheral blood counts may be normal in the setting of complete replacement of the bone marrow by blasts.[32] Similarly, blasts may be detected on a peripheral blood smear, but a definitive diagnosis based on morphology alone may be ambiguous. Bone marrow aspiration and biopsy are definitive for the diagnosis in most cases. The distinction between ALL and lymphoblastic lymphoma (LLy; either B or T) is somewhat arbitrary, and treatment is similar, although LLy therapy often is of shorter duration. The definition usually is based on the Murphy staging system in children who have Stage IV LLy with <25% marrow blasts and those who have ALL with ≥25% marrow blasts.[33]

About 5% of children present with overt CNS leukemia, and these patients may have cranial nerve involvement, with or without blasts detected in the spinal fluid.[32] CNS3 disease (see Prognostic Factors for a cytologic definition) is associated with an adverse outcome.[34] These patients require additional doses of intrathecal therapy and cranial irradiation. Likewise, a very small number (<1%) of males present with testicular involvement, and this does not affect prognosis.[35] Nonetheless, such patients may require additional therapy, especially if their clinical response to induction therapy is poor.

Although several clinical variables have been used to risk stratify ALL, age appears to be the most important, and initial risk-stratified therapy is based on age and white blood cell (WBC) count. These are continuous variables, but international consensus has led to the definition of standard risk (SR: age 1 year to <10 years, initial WBC count <50,000 μL, 5-year

83

Table 83.3 Recurrent cytogenetic abnormalities in childhood ALL and their prognostic significance

Cytogenetic abnormality	Estimated incidence (%)	Prognostic significance
**B-ALL**		
High hyperdiploidy	30	Favorable
t(12;21) *EVT6/RUNX1*	25	Favorable
t(1;19) TCF3(*E2A*)/*PBX1*	6	Neutral
Hypodiploidy (<44)	1	Poor
t(9;22)(q34;q11) *BCR/ABL1*	2	Poor*
MLL (11q23) rearrangements	6	Poor
iAMP21	1	Poor
**T-ALL**		
TAL1 rearrangements t(1;14)(p32;q11) and del1(p32) interstitial *SCL-TAL1*	3–10	Uncertain
TLX1(*HOX11*) fusions t(7;10)(q34;q24); t(10;14)(q24;q11)	7	Uncertain
t(5;14)(q35;q32) *TLX3*(*HOX11L2*) fusion	20	Equivocal

*Pre-imatinib era.

survival <90%) and high risk (HR: age ≥10 years and/or WBC count ≥50,000 μL, 5-year survival <80%).[36] Infants form a distinct biological subgroup because most of these cases display rearrangements of the *MLL* gene, usually t(4;11)(q21;q23) *MLL-AF4*.[37] Patients with CNS3 disease also fall into a very high-risk group. Gender is prognostic, with girls doing better than boys, and although this does not affect risk stratification, in some protocols therapy duration is shorter for girls.

Laboratory Features

Several laboratory features have an impact on prognosis, most notably WBC count. By definition, the bone marrow must contain ≥25% WBCs to establish the diagnosis of ALL. A uniform morphologic classification scheme was developed by the French–American–British (FAB) Working Group.[13] L1 is the most common subtype, accounting for 85–90% of cases (uniform cells with scant cytoplasm and indistinct nucleoli). L2 accounts for 10–15% of cases, and these cells vary in size, with more abundant cytoplasm and more prominent nucleoli. They may be confused with myeloblasts based on morphology alone. The L3 variant (1% of ALL cases) is characterized by large cells with a basophilic cytoplasm and vacuoles. This subtype almost always carries a *MYC* translocation and is identical to Burkitt lymphoma cells. There is no prognostic significance associated with the L1 versus L2 subtypes. *MYC*-rearranged L3 variants are treated on protocols specific for Burkitt lymphoma.

Among lymphoblastic leukemia cases, about 15% may be classified as T-ALL (cytoplasmic [c]CD3, TdT, and two or more of the following: CD5, CD7, CD8, CD4, CD2, CD1a) and 85% as B-ALL (CD19, cCD22, CCD79a, CD10, and TdT). T-ALL is associated with higher-risk features, such as older age and higher WBC counts. (Note: two thirds of B-ALL cases are classified as National Cancer Institute (NCI) SR based on age and WBCs, but only one third of T-ALL cases are classified as NCI SR). T-cell immunophenotype also carries independent prognostic significance, and in general these patients are treated on more augmented protocols distinct from B-ALL. The outcome for T-ALL has improved considerably.[38] Early T-cell precursor (ETP) ALL is a recently described subset characterized by distinct immunophenotypic features (CD1a–, CD8–, CD5^{dim}, and one or more stem cell or myeloid antigens [CD13, CD33, CD34, CD117, or HLA-DR]).[38,39] Although historically ETP ALL is associated with a very poor outcome, recent evidence indicates that these patients do as well as non-ETP ALL patients when treated according to risk-stratified therapy incorporating minimal residual disease (MRD) testing.[40] Rare cases of mixed phenotype acute leukemia (MPAL) may be seen, and the diagnosis rests on the classification scheme noted earlier for adult ALL. In general, these cases have a better outcome if treated on ALL as opposed to AML regimens.

In addition to immunophenotype, pathological classification includes the genetic subgroup. Although treatment usually starts based on immunophenotype (T vs. B) and NCI risk status (SR vs. HR), cytogenetic subgroup assignment plays a major role in refining risk-adapted therapy. The spectrum of cytogenetic subgroups in pediatric ALL is distinctly different from that seen in adults, and this accounts in part for the substantially better outcome seen in children. The favorable cytogenetic subgroups of hyperdiploidy and t(21;21)(p13;q22) *EVT6/RUNX1* account for over 50% of B-ALLs, compared with less than 10% in adults, and the t(9;22)(q34;q11.2) *BCR-ABL1* accounts for only 2% of childhood ALLs, compared with ≥20% in adults.[41,42]

Prognostic Factors

The integration of prognostic factors into treatment decisions has been the hallmark of modern ALL therapy in childhood. Typically, age and WBC count at diagnosis (NCI risk group), CNS status, cytogenetics and molecular genetics of the blast,

and early response to therapy as assessed by MRD at defined time points form the basis for stratification into low-, standard-, high-, and very high-risk groups, with approximate projected event-free survivals of >95%, 90–95%, 85–90%, and <80%, respectively, depending on the specifics of the approach used.[43]

Initial therapy usually is based on treatment assignment based on age and WBC count. A consensus conference led to the definition of SR ALL (age 1 year to <10 years and WBCs <50,000/μL) and HR ALL (≥10 years or WBCs ≥50,000 μL).[36] Depending on the induction protocol used, high-risk patients may have more augmented therapy. In general, patients with T-ALL have a poorer prognosis than those with B-ALL, although outcomes with intensified therapy have improved considerably.[31]

The presence of extramedullary disease also may have an impact on outcome. CNS status is defined by the presence of blasts in the spinal fluid at the onset of therapy: CNS3 (>5 WBCs/μL or cranial nerve findings), CNS2 (<5 WBCs/μL but blasts on Cytospin centrifugation [Thermo Scientific, Waltham, MA]), and CNS1 (<5 WBCs/μL and no blasts). Formulas exist to determine true CNS involvement from traumatic lumbar puncture, although some studies indicate that this procedure also is associated with an inferior relapse-free survival. CNS3 status is strongly associated with an inferior outcome, and many patients will require cranial irradiation, although very recent protocols indicate that intensifying systemic therapy may allow complete abandonment of cranial irradiation with erosion of event-free survival (EFS).[34,44,45] Most studies also show a lower EFS associated with CNS2 status, and many protocols now mandate additional intrathecal therapy for such patients. Testicular disease does not appear to affect the outcome of treatment based on contemporary protocols, but patients may require additional therapy, such as irradiation, if clinical findings do not resolve with induction therapy.[35,46]

Blast cytogenetics play a profound role in risk assignment in children, and they remain prognostic in multivariate analysis. Since the 1980s, ALL cases involving children with high hyperdiploidy, characterized by >50 to 67 chromosomes, have been noted to have an unusually good prognosis.[47] There is ongoing debate as to whether it is the modal chromosome number or the gain of certain trisomies that influences survival. For example, analysis of legacy Pediatric Oncology Group and Children's Cancer Group protocols demonstrated a powerful impact of trisomies 4, 10, and 17, whereas Medical Research Council trials showed that trisomy 18 was the most important predictor of outcome.[48,49] Other studies show that the modal chromosome number was most critical.[50] Likewise, numerous studies have shown that the t(12;21) *EVT6/RUNX1* (formally *TEL/AML1*) fusion is associated with an excellent prognosis.[51,52] This is usually the result of a cryptic translocation determined by fluorescence *in situ* hybridization (FISH) or reverse transcription polymerase chain reaction (RT-PCR).

In contrast, three cytogenetic subgroups are associated with a poor prognosis and are routinely included in risk classification. Hypodiploidy (<44 chromosomes) is associated with a very poor prognosis. Patients with low hypodiploid karyotypes (30–39 chromosomes) and near haploid (25–29 chromosomes) fare especially poorly and may carry germline p53 mutations. MLL gene (11q23) rearrangements occur in 2–5% of childhood ALLs and are common in infants younger than 1 year, in whome they portend an inferior outcome.[37] There are a variety of fusion partners, but the most common translocations are t(4;11)(q21;q23) (*MLL-AFF1(AF9)*), t(9;11)(p22;q23) (*MLL-MLLT3(AF9)*), and t(11;19)(q23;p13.3) (*MLL-MLLT1*).[53] The prognostic impact of *MLL* rearrangements in children older than 1 year is less certain; some cooperative groups stratify all such patients into intensified protocols, others stratify only those with certain MLL partners [e.g. t(4;11)] into intensified protocols, and still others do not factor this variable into clinical decision making.[48,54] Note that 11q23 deletions usually do not affect the *MLL* gene and therefore should not be used to stratify therapy, and *MLL*-rearranged T-ALL [t(11;19)] patients do well. iAMP21 refers to three extra copies of the *RUNX1* gene and is usually discovered by FISH performed to determine the *EVT6/RUNX1* status. Historically, such patients have had a very poor outcome, but they fare better on more intensified treatment regimens.[55,56]

The *BCR-ABL1* fusion (Ph+), a product of the t(9;22)(q34;q11) translocation, is observed in 3–5% of childhood ALLs, in contrast to 25% of adult ALLs. Ph+ ALL was associated with a marked inferior outcome of 31% EFS at 7 years.[57] Although high-dose chemotherapy with hematopoietic stem cell transplantation (HSCT) appeared to benefit these patients, most of them had a relapse. However, the integration of tyrosine kinase inhibitors (TKIs) has had a profound impact on outcome for Ph+ ALL, with a 7-year EFS of 71% for chemotherapy plus imatinib without HSCT.[58,59]

In contrast to B-ALL, cytogenetic abnormalities usually do not factor into risk-based classification for T-ALL, because most studies do not show an independent prognostic impact once early response is considered. Most T-ALLs have constitutive activation of NOTCH signaling with mutation of *NOTCH* itself (50%) or of *FBXW7* (20%), which encodes a protein that degrades NOTCH.[60] Rarely, a t(7;9)(q34;q34.3) activates NOTCH. Reports vary regarding the prognostic importance of NOTCH activation, but it is not being used routinely for risk-based classification.[61]

More recently, a "Ph-like" subset of patients has been described that displays a gene expression signature like Ph+ ALL but lacks the BCR-ABL fusion.[62,63] This subset may comprise 15% of adolescent and 25% of young adult ALLs. Most, but not all, studies demonstrate a worse outcome for these patients, and Ph-like ALL remains an independent prognostic factor, even when accounting for early response.

This subgroup is defined by translocation and genomic rearrangements that lead to fusion genes such as BCR/ABL1 (e.g., fusions involving *ABL1* [non-*BCR*], *ABL2*, *CSF1R*, and *PDGFRB*), which may be targeted by US Food and Drug Administration–approved TKIs, or mutations/rearrangements that activate the Janus kinase (JAK) signaling pathway, which can be targeted by JAK inhibitors such as ruxolitinib.[64] Clinical trials are underway to assess the effectiveness of targeted therapy when given on a chemotherapy backbone.

Deregulated expression of the cytokine receptor *CRLF2* was associated with a poor outcome in many studies.[65,66] *CRLF2* overexpression has been associated with translocations involving *CRLF2* located in the pseudoautosomal region (PAR1) of the X or Y chromosome and the IgH locus or through an interstitial deletion in PAR1 that brings the *P2RY8* promoter in juxtaposition to *CRLF2* (seen frequently in patients with Down syndrome). However, a substantial number of *CRLF2*-overexpressing cases do not have such genomic lesions.[67] As mentioned, controversy exists regarding the independent prognostic significance of *CRLF2* overexpression, because it is associated with many other genomic alterations, such as activation of the JAK pathway via mutations or arrangements (as seen in Ph-like ALL) and *IKZF1* deletions.[67] It is not used routinely to stratify patients but might be important in determining subgroups that are candidates for targeted therapy with JAK inhibitors.

Many recurring submicroscopic amplifications and deletions characterize ALL.[68] Deletions (and mutations) of the B-cell development gene *IKZF1* is observed in 15% of ALLs and have been associated with an increased risk of relapse.[62] However, in other studies they were not shown to be an independent prognostic factor, possibly because of the cooperative effect of other genomic alterations.[67]

The most important factor determining outcome, and therefore of great use for risk stratification, is response to therapy. Early studies showed that morphologic response (e.g., regression of blasts) in peripheral blood during the first week of induction and/or in bone marrow on days 7 to 14 was highly prognostic.[69,70] More-sensitive methods for detecting MRD have been developed for disease assessment at many times during therapy. Although 98% of patients with ALL enter remission based on standard morphologic assessment, about 30% have disease in the bone marrow detected by using more sensitive techniques. Two basic methods are used to detect MRD: PCR amplification of clonal antigen receptor rearrangements or fusion transcripts and flow cytometric determination of aberrant leukemia-associated immunophenotype. PCR can detect MRD to 10^{-5}, whereas flow cytometry routinely detects MRD to 10^{-4}. Thus, PCR is more sensitive but is technically more complex and has a longer turnaround time. Flow cytometry–based methods can be completed in 24 hours, allowing for rapid clinical decision making.

End-induction MRD was shown to be the most important prognostic factor in multivariate analysis for childhood ALL. Based on flow-based MRD, patients who have <0.01% MRD at end induction have a 5-year EFS of 88%, compared with 59%, 49%, and 30% for MRDs <0.01 to ≤0.1%, <0.1% to ≤1.0%, and >1.0%, respectively.[71] The use of a later time point (TP) may refine the prognosis of end-induction MRD-positive cases. Patients who are both end-induction and end-consolidation positive have a 5-year EFS of 25–56%, depending on other risk factors. Additional studies using PCR-based methods showed similar trends. B-ALL patients who are MRD negative at both end induction and end consolidation have a 5-year EFS of 92%, compared with 77.6% for those positive at end induction and negative at end consolidation (<0.01%). Patients positive at end induction and end consolidation have an EFS of 50.1%.[72]

End-consolidation MRD status is especially important for T-ALL, because most T-ALL patients are MRD positive at end induction. In the largest study to date, MRD was determined by PCR at TP1 (day 33, negative $<10^{-4}$) and TP2 (day 78, negative $<10^{-3}$). The outcome for the three groups (negative at both TPs, positive/negative, and positive at both TPs) was 91.1%, 80.6%, and 49.8%.[73]

Next-generation sequencing of antigen receptor rearrangements is now available and has been shown to be quite reliable and sensitive (10^{-5} to 10^{-6}).[74] It also has the advantage of identifying clonal evolution missed by PCR and flow-based methods. Whether these advantages can outweigh potential differences in cost remains to be determined.

Prognostic Factors Required for Clinical Care

The prognostic factors in this section are required for diagnosis of acute lymphoblastic leukemia in children.

Factor	Definition	Clinical significance	Level of evidence
Age	1 to <10 years; >10 years	Infants with an inferior outcome based on unique biology (e.g., MLL rearrangements); older patients with an inferior outcome	I
WBC count at diagnosis	<50,000 to ≥50,000 µL	Lower WBCs with better outcome	I
T immunophenotype	CD5, CD7, CD8, CD4, CD2, or CD1a	Worse outcome	I
CNS involvement	Blasts on Cytospin	Worse outcome	I
Hyperdiploidy	>50–67 chromosomes or specific trisomies (e.g., 4 and 10)	Better outcome	I

Factor	Definition	Clinical significance	Level of evidence
t(12;21) (p13:q22) *EVT6/RUNX1*	Cryptic translocation detected by FISH, RT-PCR	Better outcome	I
Hypodiploidy	<44 chromosomes by karyotype	Worse outcome	I
MLL rearrangements	Karyotype or FISH (>100 fusion partners defined)	Worse outcome	I (infants) II (age >1 year)
iAMP21	Three or more extra copies of *RUNX1* on an abnormal chromosome 21	Worse outcome	I
t(9;22)(q24:q11) Ph+	FISH or karyotype	Targeted therapy with TKI	I
MRD	Flow cytometry or antigen receptor/fusion gene PCR	Detectable levels at end induction and end consolidation associated with worse outcome	I

Additional Factors Recommended for Clinical Care

Factor	Definition	Clinical significance	Level of evidence
Early T precursor (ETP)	CD1a–, CD8–, CD5^{dim} with myeloid/stem cell antigens	Worse outcome	III
Testicular involvement	Testicular mass or blasts on biopsy	May require additional therapy without resolution	II
Ph-like ALL	Gene expression	Worse outcome; candidate for targeted therapy	II
CRLF2 expression	Overexpression by RT-PCR	Worse outcome	II
IKZF1 deletion/ mutation	Single nucleotide polymorphism arrays, comparative genomic hybridization, multiplex ligation-dependent probe amplification, sequencing	Worse outcome	II

Risk Assessment Models

Many classification models are in use for risk stratification of childhood ALL. Although the exact details may vary somewhat among the major cooperative groups worldwide, nearly all use age, WBCs, immunophenotype, and CNS status at diagnosis, as well as blast cytogenetics and molecular genetics and early response to therapy, to define risk groups with a very favorable prognosis, for whom future clinical trials may focus on lowering the burden of therapy, as well as very high-risk groups, for whom HCST and/or novel therapy are indicated to improve survival. As overall outcome improves, it is anticipated that some prognostic factors may lose significance while targeted therapy for biological subgroups such as Ph-like ALL is evolving.

ACUTE LYMPHOCYTIC LEUKEMIA IN ADULTS

Rules for Classification

Clinical Classification

Acute lymphocytic leukemia is also known as acute lymphoid leukemia and acute lymphoblastic leukemia, but all are commonly abbreviated ALL. ALL is a high-grade malignancy of lymphoid precursors. According to registry data, ALL is diagnosed in about 6,000 people per year in the United States, making it about one third as common as AML.[75] It is substantially more common in children, with a median age at diagnosis of 14 years. However, ALL may affect individuals throughout their lifespan, with one to two cases per 100,000 people diagnosed consistently in those between the ages of 20 and 80 years. Age at diagnosis often is used to subcategorize adults with ALL; patients younger than 40 years are considered young adults, whereas those older than 40 are categorized as older adults. Further, patients older than 60 years often are labeled "elderly." Although these distinctions are somewhat arbitrary, they often are used for stratification in clinical trials, which leads to differences in treatment paradigms.

Bone marrow and peripheral blood involvement is the norm in ALL, although features more typical of mature lymphoid neoplasms (e.g., lymphadenopathy, hepatosplenomegaly) are not uncommon. There is a particular tropism for ALL to the CNS. Testicular involvement in men also must be considered. Both the CNS and the testes represent potential "sanctuary sites," because both are protected by a blood–brain/testes barrier. In reality, virtually every tissue and organ system in the body may be affected. The diagnosis rests on the identification of abnormal clonal lymphoblasts involving one or more of these sites.

ALL generally is considered to be disseminated at presentation in virtually all cases. Therefore, an anatomically based staging system is not applicable to ALL. However, some patients may have more focal or isolated involvement of nodal or extramedullary sites. In these circumstances, the diagnosis of lymphoblastic lymphoma (LBL) is applied. Here, the Ann Arbor staging system for lymphoma may be utilized.[76] Alternatively, the Murphy staging system is used primarily in children and relies not only on the distribution of affected sites but also on the specific anatomic sites primarily

83

Table 83.4 WHO classification of precursor lymphoid neoplasms[14,30]

**B-lymphoblastic leukemia/lymphoma**
B-lymphoblastic leukemia/lymphoma, NOS
B-lymphoblastic leukemia/lymphoma with recurrent genetic abnormalities
B-lymphoblastic leukemia/lymphoma with t(9;22)(q34;q11.2);*BCR-ABL1*
B-lymphoblastic leukemia/lymphoma with t(v;11q23);*KMT2A* (*MLL*) rearranged
B-lymphoblastic leukemia/lymphoma with t(12;21)(p13;q22) *TEL-AML1* (*ETV6-RUNX1*)
B-lymphoblastic leukemia/lymphoma with hyperdiploidy
B-lymphoblastic leukemia/lymphoma with hypodiploidy
B-lymphoblastic leukemia/lymphoma with t(5;14)(q31;q32) *IL3-IGH*
B-lymphoblastic leukemia/lymphoma with t(1;19)(q23;p13.3);*TCF3-PBX1*
Provisional entity: B-lymphoblastic leukemia/lymphoma with translocations involving receptor tyrosine kinases or cytokine receptors ("BCR-ABL1-like ALL")
Provisional Entity: B-lymphoblastic leukemia/lymphoma with intrachromosomal amplification of chromosome 21 (iAMP21)
**T-Lymphoblastic Leukemia/Lymphoma**

involved (e.g., mediastinum).[33] However, it is unclear whether treatment can be altered or risk-stratified using these systems in adults, thus limiting their utility in making management decisions. Patients with CNS involvement, either in isolation or concurrent with systemic disease, often are considered a distinct entity, as treatment often must be modified to some degree to ensure that adequate therapy is delivered across the blood–brain barrier. Similarly, disease in the testes may not respond as readily to systemic therapy, in which case local therapy, such as radiation, should be considered to ensure its eradication. However, the presence of CNS or testicular disease at diagnosis does not have a consistent prognostic impact.

Laboratory Features

Historically, the distinction between ALL and LBL has hinged on the degree of bone marrow involvement, with ≥25% blasts in the marrow classified as ALL.[14] However, such definitions are somewhat arbitrary. In fact, the most recent iterations of the WHO classification of hematopoietic disorders defines this entity as lymphoblastic leukemia/lymphoma,[77] hinting at the substantial overlap between these two disorders (Table 83.4). Genomic studies suggest some distinct features between ALL and LBL,[78] although for practical purposes in the current era, these two diseases generally are managed similarly in adults.

Considering ALL and LBL as one entity, the most common pathological subclassification used is based on the lineage (i.e., T cell or B cell), which is determined primarily by the immunophenotype of the abnormal blasts (Table 83.4). B lineage typically is defined by strong expression of CD19 along with CD10, CD79a, and cytoplasmic CD22; T lineage is assigned in the presence of CD3. ALL is more commonly of B lineage, whereas LBL is far more often of T lineage. Rarely, blasts may harbor features of both lymphoid and myeloid lineages, or even both B- and T-cell lineages. In these circumstances, a diagnosis of mixed phenotype acute leukemia (MPAL) may be appropriate. Little is understood about how best to classify and manage these uncommon entities.

The other major pathological classification system used in ALL is based on cytogenetic abnormalities. These may be identified by metaphase preparations or *in situ* FISH. There are several recurrent cytogenetic abnormalities in ALL, some of which are more applicable to adults (Tables 83.4 and 83.5). By far the single most important cytogenetic abnormality in adult ALL is the Philadelphia (Ph) chromosome, which (as in chronic myeloid leukemia) is a reciprocal translocation involving the *BCR* gene at 22q11 and the *ABL1* gene at 9q34. It is increasingly common with older age, present in less than 5% of children but representing approximately 20% or more of adult cases, with a greater proportion in older adults.[79,80]

Historically, the morphologic appearance of blasts was used in the French–American–British (FAB) classification of ALL. However, as in AML, these distinctions are no longer routinely used.

Prognostic Factors

Identification of prognostic factors in adult ALL is an area of intense investigation. Unlike in pediatric ALL, it is not yet possible to incorporate this information into the initial chemotherapy approach. Instead, this information typically is used to guide decisions regarding the role of hematopoietic cell transplantation (HCT), an intervention often considered for adults with ALL in first complete remission but perceived to be at high risk of relapse.

Historically, risk stratification in adults with ALL has relied primarily on three factors determined at the time of diagnosis: age, WBC count, and cytogenetics. Generally, the presence of any one of these factors is felt to confer unfavorable risk. Regarding age, higher-risk patients are those older than 35 years. WBC count has different thresholds depending on the cell lineage: >30,000/μL is an adverse feature in

B-cell ALL, whereas >100,000/µL is the level used for T-cell ALL. Cytogenetic risk categories are desribed in Table 83.5. The presence of the Ph+ chromosome is associated with a poor prognosis, but it also predicts response to ABL kinase inhibitors, which have had a substantial effect on the expected outcomes for this subtype of ALL. Although it is not fully known what impact these agents have had in terms of altering the risk profile of Ph+ ALL, it is fair to say that outcomes for these patients are not nearly as dismal as they once were. Nevertheless, most experts still feel that Ph+ remains an unfavorable risk factor. In pediatric ALL, intrachromosomal amplification of chromosome 21 (iAMP21) has been identified as a high-risk cytogenetic abnormality.[81] Present in approximately 2% of cases, it is defined as three or more extra copies of the *RUNX1* gene (i.e., a total of five or more *RUNX1* signals per cell by FISH). However, it is unclear what significance this finding has in adult ALL.

There have been several recent advances in defining risk groups based on molecular or genomic profiling. In B-cell ALL, alterations in the *IKZF1* gene on chromosome 7p12.2 (which encodes the lymphoid transcription factor IKAROS) were first identified as a powerful prognostic feature in pediatric ALL,[62] but subsequent work has shown its importance in adults as well.[86] Biologically, loss of function of the *IKZF1* gene is what leads to the unfavorable risk profile, which may be mediated by focal monoallelic deletions that produce a dominant-negative gene product or broader genomic instability that downregulates both alleles. Loss of *IKZF1* function also appears to be associated with another evolving poor-risk subgroup for B-cell ALL: so-called Ph-like or *BCR-ABL1*–like ALL. This interesting subgroup harbors gene expression profiles that are highly analogous to those observed from cases harboring the classic *BCR-ABL1* translocation. This similar biology is thought to be mediated by alternate gene rearrangements involving *ABL1*, *JAK2*, *PDGFRB*, and several other oncogenes.[64] This signature may be identified in about 25% of young adults with B-cell ALL, and it confers a poor prognosis if present. However, it may be less common in older adults, and its prognostic significance in this age group has yet to be defined.[87] Regardless of age, it remains to be seen how this information can be utilized in routine clinical practice, although studies are under way to address this question.

In T-cell ALL, genetic lesions with prognostic significance also have been described. Investigators from the French Group for Research in Adult Acute Lymphoblastic Leukemia (GRAALL) identified a genomic risk classification system for T-ALL.[88] To summarize, about 50% of their study population harbored mutations in either *NOTCH* or *FBXW7* without mutations with *KRAS*, *NRAS*, or *PTEN*, and these individuals had the most favorable outcome, even after adjustment for other established risk factors, such as age and WBC count at diagnosis in multivariable models. Assays to establish these mutations are not widely available clinically, so it is practically challenging in the current era to apply this knowledge broadly in routine practice.

Also significant for T-cell ALL is the recognition of the early T-cell precursor (ETP) immunophenotype. Initially described in pediatric cases, it is defined by the lack of expression (<5% positive lymphoblasts) of CD1a and CD8, with weak expression (<75% positive lymphoblasts) of CD5, in the presence (>25% positive lymphoblasts) of one or more stem cell or myeloid antigens (e.g., CD34, CD117).[39] About 13% of children with T-cell ALL treated in two large prospective studies were retrospectively identified as having ETP-ALL, and their outcomes were generally poor. Smaller studies also suggested a similarly poor prognosis in adults with T-cell ALL.[89] Because this subgroup can be identified by using routine multiparameter flow cytometry (MFC), it should be recognizable in the course of routine clinical evaluation. How this immunophenotypic signature relates to any of the aforementioned high-risk cytogenetic or genomic factors has yet to be elucidated.

Although these pretreatment factors may provide important information for risk stratification, response to treatment may supersede many of them. Approximately 90% of adults with ALL can achieve morphologic complete remission (CR; i.e., <5% blasts on a manual differential of a bone marrow specimen) with standard induction therapy, but the relatively small remainder with primary refractory disease historically have done very poorly.[90] For the overwhelming majority who do achieve a morphologic CR, posttreatment risk stratification is based primarily on the measurement of minimal residual disease (MRD). Several studies in adults with ALL have demonstrated the prognostic and predictive value of MRD in the context of both initial chemotherapy and HCT.[91] Consensus guidelines have been developed that define, among other things, three categories of MRD status: complete MRD response, MRD persistence, and MRD reappearance or relapse.[92] There also is a time-dependent element to MRD assessments, with achievement of complete MRD response earlier in treatment predicting a more favorable outcome with certain chemotherapy regimens.[93,94]

MRD can be assessed using two major platforms. The most widely used is polymerase chain reaction (PCR), which can detect and quantify levels of leukemia-specific fusion transcripts (e.g., *BCR-ABL1*) or clone-specific immunoglobulin (Ig) or T-cell receptor (TCR) genes. The other approach uses MFC. In general, PCR is more sensitive than MFC and is more readily standardized. However, a baseline sample is required to confirm detection of the amplification product, and (in the case of Ig- and TCR-based PCR methods) false negatives may occur in the event of clonal evolution. On the other hand, MFC typically provides results more rapidly, although discriminating leukemic blasts from normal lymphoid progenitors (i.e., hematogones) may pose a diagnostic challenge. A third and very exciting methodology for MRD detection described recently uses next-generation sequencing (NGS) techniques to detect clone-specific Ig and TCR

Table 83.5 Recurrent cytogenetic abnormalities in adult ALL and their prognostic significance[79,80,82–85]

Cytogenetic abnormality	Estimated incidence (%)	Prognostic significance
t(9;22)	15–28	Poor
KMT2A (MLL)/11q23 rearrangement	4–9	Poor
+8	3–8	Poor
Complex*	5–7	Poor
–7	2–6	Poor
Low hypodiploid/near triploid**	3–4	Poor
Del 9p	1–12	Equivocal
t(1;19)	1–3	Equivocal
t(10;14)	2	Equivocal
t(5;14)	<2	Equivocal
High hyperdiploid***	7–10	Favorable
t(12;21)	<2	Favorable

**Complex karyotype* is defined as five or more chromosomal abnormalities in the absence of an established cytogenetic subgroup.
***Low hypodiploidy* is defined as 30 to 39 chromosomes, whereas *near-triploidy* is defined as 60 to 78 chromosomes. These two categories are felt to represent manifestations of the same biology, with near-triploidy deriving from duplication of low hypodiploidy.
****High hyperdiploidy* is defined as 51 to 65 chromosomes.

sequences. Early studies based on retrospective or post hoc comparisons suggest that this approach may be better than standard PCR or MFC at discriminating among risk groups.[95–98] However, prospective comparisons of these techniques have not yet been reported, making it difficult to determine how best to incorporate NGS-based assays into routine clinical practice.

Prognostic Factors Required for Clinical Care

The prognostic factors in this section are required for diagnosis of acute lymphocytic leukemia in adults.

Factor	Definition	Clinical significance	Level of evidence
CNS involvement	Presence of blasts in cerebrospinal fluid	Additional CNS-directed therapy required; no clear impact on prognosis	I
Testicular involvement	Testicular mass or swelling; presence of blasts on biopsy	Inadequate response to systemic therapy requires additional local therapy (e.g., radiation); no clear impact on prognosis	II

Additional Factors Recommended for Clinical Care

Factor	Definition	Clinical significance	Level of evidence
Age at diagnosis	>35 years	Worse outcome	I
WBC count at diagnosis	>30,000/μL (B-cell); >100,000/μL (T-cell)	Worse outcome	I
Cytogenetics	See Table 83.2	See Table 83.2	I
Complete MRD response (by MFC or PCR)	MRD negativity on ≥2 independent samples	Better outcome	I
MRD persistence (by MFC or PCR)	Quantifiable MRD positivity for ≥2 time points	Worse outcome	I
MRD reappearance/ relapse (by MFC or PCR)	Conversion to MRD; positivity after MRD negativity	Worse outcome	I
IKZF1 deletions	Focal monoallelic deletions; biallelic gene disruption	Worse outcome	II
Ph-like/*BCR-ABL1*–like signature	Gene expression profiling in specialized laboratories	Worse outcome	II
ETP immunophenotype	Lack of CD1a and CD8, weak CD5, with stem cell or myeloid antigen(s)	Worse outcome	II

Risk Assessment Models

Although the anatomic distribution of disease at diagnosis does not have clear prognostic significance, baseline characteristics such as age, WBC count, cytogenetics, and other molecular/genetic factors allow pretreatment risk stratification. After treatment, the depth of response (as determined by assessment of MRD) provides additional and powerful prognostic and predictive information. Presently, the information gained from the presence or absence of these factors is used

primarily to identify the patients most likely to relapse following initial chemotherapy and thus most likely to benefit from HCT in first remission. However, methods to integrate these multiple pre- and posttreatment factors are needed.

CHRONIC MYELOID LEUKEMIA

Rules for Classification

Clinical Classification

Chronic myeloid leukemia (CML) is a malignant disorder of hematopoiesis resulting from the clonal expansion of a primitive hematopoietic cell. At the outset of disease, this malignant stem cell retains the capacity to differentiate, leading to marked marrow hyperplasia and increased numbers of myeloid cells and platelets in the peripheral blood. The natural history of untreated CML is a relatively benign chronic phase (CP) lasting, on average, approximately 3 years, followed by an accelerated phase (AP) lasting several months and eventually terminating in a rapidly fatal blast crisis (BC). CML was the first malignant disease found to be consistently associated with a specific cytogenetic abnormality, in this case the Philadelphia (Ph) chromosome, and was one of the first diseases clearly curable by transplantation. The advent of the tyrosine kinase inhibitors (imatinib, dasatinib, nilotinib, bosutinib, and ponatinib) has changed the landscape of CML therapy and has moved transplantation from the upfront therapy of choice to an option for salvage therapy.

The diagnosis of CML often is made during a routine physical, with an elevated white blood cell count. At diagnosis, splenomegaly often is present. With progression to AP and BC, increasing splenomegaly often is accompanied by fever. However, disease staging, which is important for prognosis and for choosing treatment approaches, is based on bone marrow morphology and cytogenetic changes.

Initial evaluation of patients with CML should include a history and physical examination, including palpation of spleen, complete blood count (CBC) with differential, chemistry profile, bone marrow aspiration, and biopsy. Conventional bone marrow cytogenetics should be performed to confirm the diagnosis of Ph-positiv CML at initial workup. If collection of bone marrow is not feasible, fluorescence *in situ* hybridization (FISH) on a peripheral blood specimen with dual probes for *BCR* and *ABL1* genes is an acceptable method for confirming the diagnosis of CML.

Several prognostic scoring systems are available for risk stratification in CML; the most commonly used are those of Sokal[99] and Hasford.[100] Both these scoring systems stratify patients into three risk groups (low, intermediate, and high) and have been used for risk stratification in clinical trials evaluating TKIs. The Sokal score is based on the patient's age, spleen size, platelet count, and percentage of blasts in the peripheral blood.[99] The Hasford model includes eosinophils and basophils in the peripheral blood in addition to the same clinical variables used in the Sokal model.[100]

Laboratory Features

CML is characterized by the presence of the Ph chromosome, resulting from a reciprocal translocation between chromosomes 9 and 22 [t(9;22)]. Translocation t(9;22) results in the head-to-tail fusion of the breakpoint cluster region (*BCR*) gene on chromosome 22 at band q11 and the Abelson murine leukemia (*ABL1*) gene located on chromosome 9 at band q34.[101] The product of the *BCR-ABL* fusion gene (p210), a fusion protein with deregulated tyrosine kinase activity, plays a central role in the pathogenesis of CML. Another fusion protein, p190, also is produced, usually in the setting of Ph-positiv acute lymphoblastic leukemia (ALL). p190 is detected in only 1% of patients with CML.[102]

Various definitions have been used for AP and blast crisis BC CML.[77,103–107] The clinical trials of TKIs largely have used the modified MD Anderson Cancer Center AP criteria of 15–29% peripheral blood or bone marrow blasts, ≥30% peripheral blood blasts and promyelocytes, ≥20% peripheral blood or bone marrow basophils, a platelet count ≤100 × 10^9/L unrelated to therapy, and clonal evolution (the presence of Ph and additional cytogenetic abnormalities).[106] These criteria are in contrast to the WHO criteria, which define AP as the presence of any of the following features: 10–19% of blasts in the peripheral blood or bone marrow, ≥20% of basophils in the peripheral blood, persistent thrombocytopenia (>100 × 10^9/L) unrelated to therapy or persistent thrombocytosis (>1,000 × 10^9/L) unresponsive to therapy, increasing spleen size, and increasing WBC count unresponsive to therapy.[77]

Approximately 50% of all blast phase cases are of the myeloid subtype, 25% are of the lymphoid subtype, and the rest are undifferentiated. According to the International Bone Marrow Transplant Registry (IBMTR), BC is defined as ≥30% blasts in the blood, bone marrow, or both, or as the presence of extramedullary disease.[108] In the WHO criteria, BC is defined as ≥20% blast cells in the peripheral blood or bone marrow, the presence of extramedullary blast proliferation, and large foci or clusters of blasts in the bone marrow biopsy sample.[77]

Prognostic Factors

Prognostic Factors Required for Clinical Care

Factor	Definition	Clinical significance	Level of evidence
Bone marrow	Blast count	Defines stage	I
Cytogenetics	Ph chromosome	Defines disease	I
Cytogenetics	Additional clonal changes	Defines stage of disease (AP)	I

Additional Prognostic Factors Recommended for Clinical Care

Factor	Definition	Clinical significance	Level of evidence
FISH (PB or BM)	BCR-ABL	Defines disease if bone marrow cytogenetics unavailable	I
BCR-ABL RT-PCR (PB)	BCR-ABL transcript level	Allows baseline for montoring during therapy	I
ABL mutation	Point mutations in the ABL binding domain	In AP and BC, defines potential resistance mutations and thus helps pick the correct TKI	II

Risk Assessment Models

All therapies (TKI, transplantation) are more effective in CP than advanced-phase (AP, BC) CML. Thus, the staging of CML phase by blast count and morphology is the primary risk stratification measure. Other clinical systems (Sokal, Hasford, Euro) are helpful in accessing risk and thus in choosing a TKI (e.g., a CP patient with a high risk score might be better served by a more potent second generation TKI).

CHRONIC LYMPHOCYTIC LEUKEMIA

The staging systems for CLL are discussed in the chapter on Hodgkin and non-Hodgkin lymphoma (Chapter 79).

Bibliography

1. Cancer Genome Atlas Research Network. Genomic and epigenomic landscapes of adult de novo acute myeloid leukemia. *N Engl J Med.* May 30 2013;368(22):2059–2074.
2. Churpek JE, Larson RA. The evolving challenge of therapy-related myeloid neoplasms. *Best practice & research. Clinical haematology.* Dec 2013;26(4):309–317.
3. Lindsley RC, Mar BG, Mazzola E, et al. Acute myeloid leukemia ontogeny is defined by distinct somatic mutations. *Blood.* Feb 26 2015;125(9):1367–1376.
4. Kolb EA, Meshinchi S. Acute myeloid leukemia in children and adolescents: identification of new molecular targets brings promise of new therapies. *Education Program of the American Society of Hematology. American Society of Hematology. Education Program.* Dec 5 2015;2015(1):507–513.
5. Tarlock K, Meshinchi S. Pediatric acute myeloid leukemia: biology and therapeutic implications of genomic variants. *Pediatr Clin North Am.* Feb 2015;62(1):75–93.
6. Altekruse S, Kosary C, Krapcho M, et al. SEER Cancer Statistics Review, 1975–2007, National Cancer Institute. Bethesda, MD. *From* http://seer.cancer.gov/csr/1975_2007/, *based on November 2009 SEER data submission, posted to the SEER website, 2010.* 2010.
7. Kayser S, Dohner K, Krauter J, et al. The impact of therapy-related acute myeloid leukemia (AML) on outcome in 2853 adult patients with newly diagnosed AML. *Blood.* Feb 17 2011;117(7):2137–2145.
8. Churpek JE, Marquez R, Neistadt B, et al. Inherited mutations in cancer susceptibility genes are common among survivors of breast cancer who develop therapy-related leukemia. *Cancer.* Jan 15 2016;122(2):304–311.
9. Knight JA, Skol AD, Shinde A, et al. Genome-wide association study to identify novel loci associated with therapy-related myeloid leukemia susceptibility. *Blood.* May 28 2009;113(22):5575–5582.
10. Larson RA, Wang Y, Banerjee M, et al. Prevalence of the inactivating 609C→T polymorphism in the NAD(P)H:quinone oxidoreductase (NQO1) gene in patients with primary and therapy-related myeloid leukemia. *Blood.* Jul 15 1999;94(2):803–807.
11. Abkowitz JL. Clone wars–the emergence of neoplastic blood-cell clones with aging. *N Engl J Med.* Dec 25 2014;371(26):2523–2525.
12. Welch JS, Ley TJ, Link DC, et al. The origin and evolution of mutations in acute myeloid leukemia. *Cell.* Jul 20 2012;150(2):264–278.
13. Bennett JM, Catovsky D, Daniel MT, et al. Proposals for the classification of the acute leukaemias. French-American-British (FAB) co-operative group. *British journal of haematology.* Aug 1976;33(4):451–458.
14. Vardiman JW, Thiele J, Arber DA, et al. The 2008 revision of the World Health Organization (WHO) classification of myeloid neoplasms and acute leukemia: rationale and important changes. *Blood.* Jul 30 2009;114(5):937–951.
15. Estey E, Thall P, Beran M, Kantarjian H, Pierce S, Keating M. Effect of diagnosis (refractory anemia with excess blasts, refractory anemia with excess blasts in transformation, or acute myeloid leukemia [AML]) on outcome of AML-type chemotherapy. *Blood.* Oct 15 1997;90(8):2969–2977.
16. Sinha C, Cunningham LC, Liu PP. Core Binding Factor Acute Myeloid Leukemia: New Prognostic Categories and Therapeutic Opportunities. *Seminars in hematology.* Jul 2015;52(3):215–222.
17. Marcucci G, Mrozek K, Ruppert AS, et al. Prognostic factors and outcome of core binding factor acute myeloid leukemia patients with t(8;21) differ from those of patients with inv(16): a Cancer and Leukemia Group B study. *J Clin Oncol.* Aug 20 2005;23(24):5705–5717.
18. Sanz MA, Grimwade D, Tallman MS, et al. Management of acute promyelocytic leukemia: recommendations from an expert panel on behalf of the European LeukemiaNet. *Blood.* Feb 26 2009;113(9):1875–1891.
19. Dyck JA, Warrell RP, Jr., Evans RM, Miller WH, Jr. Rapid diagnosis of acute promyelocytic leukemia by immunohistochemical localization of PML/RAR-alpha protein. *Blood.* Aug 1 1995;86(3):862–867.
20. Bernt KM, Armstrong SA. Targeting epigenetic programs in MLL-rearranged leukemias. *Hematology / the Education Program of the American Society of Hematology. American Society of Hematology. Education Program.* 2011;2011:354–360.
21. Chen CW, Armstrong SA. Targeting DOT1L and HOX gene expression in MLL-rearranged leukemia and beyond. *Exp Hematol.* Aug 2015;43(8):673–684.
22. Tarlock K, Alonzo TA, Moraleda PP, et al. Acute myeloid leukaemia (AML) with t(6;9)(p23;q34) is associated with poor outcome in childhood AML regardless of FLT3-ITD status: a report from the Children's Oncology Group. *British journal of haematology.* Jul 2014;166(2):254–259.
23. Ottone T, Zaza S, Divona M, et al. Identification of emerging FLT3 ITD-positive clones during clinical remission and kinetics of disease relapse in acute myeloid leukaemia with mutated nucleophosmin. *British journal of haematology.* May 2013;161(4):533–540.

24. Palmisano M, Grafone T, Ottaviani E, Testoni N, Baccarani M, Martinelli G. NPM1 mutations are more stable than FLT3 mutations during the course of disease in patients with acute myeloid leukemia. *Haematologica*. Sep 2007;92(9):1268–1269.
25. Dohner H, Estey EH, Amadori S, et al. Diagnosis and management of acute myeloid leukemia in adults: recommendations from an international expert panel, on behalf of the European LeukemiaNet. *Blood*. Jan 21 2010;115(3):453–474.
26. Patel JP, Gonen M, Figueroa ME, et al. Prognostic relevance of integrated genetic profiling in acute myeloid leukemia. *N Engl J Med*. Mar 22 2012;366(12):1079–1089.
27. Miesner M, Haferlach C, Bacher U, et al. Multilineage dysplasia (MLD) in acute myeloid leukemia (AML) correlates with MDS-related cytogenetic abnormalities and a prior history of MDS or MDS/MPN but has no independent prognostic relevance: a comparison of 408 cases classified as "AML not otherwise specified" (AML-NOS) or "AML with myelodysplasia-related changes" (AML-MRC). *Blood*. Oct 14 2010;116(15):2742–2751.
28. Quesnel B, Kantarjian H, Bjergaard JP, et al. Therapy-related acute myeloid leukemia with t(8;21), inv(16), and t(8;16): a report on 25 cases and review of the literature. *J Clin Oncol*. Dec 1993; 11(12):2370–2379.
29. Walter RB, Othus M, Burnett AK, et al. Significance of FAB subclassification of "acute myeloid leukemia, NOS" in the 2008 WHO classification: analysis of 5848 newly diagnosed patients. *Blood*. Mar 28 2013;121(13):2424–2431.
30. Arber DA, Orazi A, Hasserjian RP. The 2016 revision to the World Health Organization (WHO) classification of myeloid neoplasms and acute leukemia. *Blood*. 2016.
31. Hunger SP, Lu X, Devidas M, et al. Improved survival for children and adolescents with acute lymphoblastic leukemia between 1990 and 2005: a report from the children's oncology group. *J Clin Oncol*. May 10 2012;30(14):1663–1669.
32. Moricke A, Zimmermann M, Reiter A, et al. Prognostic impact of age in children and adolescents with acute lymphoblastic leukemia: data from the trials ALL-BFM 86, 90, and 95. *Klin Padiatr*. Nov-Dec 2005;217(6):310–320.
33. Murphy SB. Classification, staging and end results of treatment of childhood non-Hodgkin's lymphomas: dissimilarities from lymphomas in adults. *Semin Oncol*. Sep 1980;7(3):332–339.
34. Pui CH, Howard SC. Current management and challenges of malignant disease in the CNS in paediatric leukaemia. *The lancet oncology*. Mar 2008;9(3):257–268.
35. Hijiya N, Liu W, Sandlund JT, et al. Overt testicular disease at diagnosis of childhood acute lymphoblastic leukemia: lack of therapeutic role of local irradiation. *Leukemia*. Aug 2005;19(8): 1399–1403.
36. Smith M, Arthur D, Camitta B, et al. Uniform approach to risk classification and treatment assignment for children with acute lymphoblastic leukemia. *J Clin Oncol*. Jan 1996;14(1):18–24.
37. Pieters R. Infant acute lymphoblastic leukemia: Lessons learned and future directions. *Current hematologic malignancy reports*. Jul 2009;4(3):167–174.
38. Wood B, Winter SS, Dunsmore KP, et al. T-lymphoblastic leukemia (T-ALL) shows excellent outcome, lack of significance of early thymic precursor (ETP) immunophenotype, and validation of end-induction minimal residual disease (MRD) in Children's Oncology Group (COG) study AALL0434. *Blood*. Dec. 2014; 124(21):1.
39. Coustan-Smith E, Mullighan CG, Onciu M, et al. Early T-cell precursor leukaemia: a subtype of very high-risk acute lymphoblastic leukaemia. *The lancet oncology*. Feb 2009;10(2): 147–156.
40. Patrick P, Wade R, Goulden N, et al. Characteristics and Outcome of children and Young Adults with Early T-Precursor (ETP) ALL Treated On UKALL 2003. *Blood*. Nov 2013;122(21):58.
41. Pui CH, Carroll WL, Meshinchi S, Arceci RJ. Biology, risk stratification, and therapy of pediatric acute leukemias: an update. *J Clin Oncol*. Feb 10 2011;29(5):551–565.
42. Graux C, Cools J, Michaux L, Vandenberghe P, Hagemeijer A. Cytogenetics and molecular genetics of T-cell acute lymphoblastic leukemia: from thymocyte to lymphoblast. *Leukemia*. Sep 2006;20(9):1496–1510.
43. Teachey DT, Hunger SP. Predicting relapse risk in childhood acute lymphoblastic leukaemia. *British journal of haematology*. Sep 2013;162(5):606–620.
44. Pui CH, Campana D, Pei D, et al. Treating childhood acute lymphoblastic leukemia without cranial irradiation. *N Engl J Med*. Jun 25 2009;360(26):2730–2741.
45. Burger B, Zimmermann M, Mann G, et al. Diagnostic cerebrospinal fluid examination in children with acute lymphoblastic leukemia: significance of low leukocyte counts with blasts or traumatic lumbar puncture. *J Clin Oncol*. Jan 15 2003;21(2): 184–188.
46. Sirvent N, Suciu S, Bertrand Y, Uyttebroeck A, Lescoeur B, Otten J. Overt testicular disease (OTD) at diagnosis is not associated with a poor prognosis in childhood acute lymphoblastic leukemia: results of the EORTC CLG Study 58881. *Pediatric blood & cancer*. Sep 2007;49(3):344–348.
47. Paulsson K, Johansson B. High hyperdiploid childhood acute lymphoblastic leukemia. *Genes Chromosomes Cancer*. Aug 2009;48(8):637–660.
48. Moorman AV, Ensor HM, Richards SM, et al. Prognostic effect of chromosomal abnormalities in childhood B-cell precursor acute lymphoblastic leukaemia: results from the UK Medical Research Council ALL97/99 randomised trial. *The lancet oncology*. May 2010;11(5):429–438.
49. Sutcliffe MJ, Shuster JJ, Sather HN, et al. High concordance from independent studies by the Children's Cancer Group (CCG) and Pediatric Oncology Group (POG) associating favorable prognosis with combined trisomies 4, 10, and 17 in children with NCI Standard-Risk B-precursor Acute Lymphoblastic Leukemia: a Children's Oncology Group (COG) initiative. *Leukemia*. May 2005;19(5):734–740.
50. Dastugue N, Suciu S, Plat G, et al. Hyperdiploidy with 58-66 chromosomes in childhood B-acute lymphoblastic leukemia is highly curable: 58951 CLG-EORTC results. *Blood*. Mar 28 2013;121(13):2415–2423.
51. Schultz KR, Pullen DJ, Sather HN, et al. Risk- and response-based classification of childhood B-precursor acute lymphoblastic leukemia: a combined analysis of prognostic markers from the Pediatric Oncology Group (POG) and Children's Cancer Group (CCG). *Blood*. Feb 1 2007;109(3):926–935.
52. Harrison CJ, Haas O, Harbott J, et al. Detection of prognostically relevant genetic abnormalities in childhood B-cell precursor acute lymphoblastic leukaemia: recommendations from the Biology and Diagnosis Committee of the International Berlin-Frankfurt-Munster study group. *British journal of haematology*. Oct 2010; 151(2):132–142.
53. Krivtsov AV, Armstrong SA. MLL translocations, histone modifications and leukaemia stem-cell development. *Nat Rev Cancer*. Nov 2007;7(11):823–833.
54. Moorman AV. The clinical relevance of chromosomal and genomic abnormalities in B-cell precursor acute lymphoblastic leukaemia. *Blood Rev*. May 2012;26(3):123–135.
55. Harrison CJ, Moorman AV, Schwab C, et al. An international study of intrachromosomal amplification of chromosome 21 (iAMP21): cytogenetic characterization and outcome. *Leukemia*. May 2014;28(5):1015–1021.
56. Heerema NA, Carroll AJ, Devidas M, et al. Intrachromosomal amplification of chromosome 21 is associated with inferior outcomes in children with acute lymphoblastic leukemia treated in

contemporary standard-risk children's oncology group studies: a report from the children's oncology group. *J Clin Oncol*. Sep 20 2013;31(27):3397–3402.

57. Arico M, Schrappe M, Hunger SP, et al. Clinical outcome of children with newly diagnosed Philadelphia chromosome-positive acute lymphoblastic leukemia treated between 1995 and 2005. *J Clin Oncol*. Nov 1 2010;28(31):4755–4761.
58. Schultz KR, Bowman WP, Aledo A, et al. Improved early event-free survival with imatinib in Philadelphia chromosome-positive acute lymphoblastic leukemia: a children's oncology group study. *J Clin Oncol*. Nov 1 2009;27(31):5175–5181.
59. Schultz KR, Carroll A, Heerema NA, et al. Long-term follow-up of imatinib in pediatric Philadelphia chromosome-positive acute lymphoblastic leukemia: Children's Oncology Group study AALL0031. *Leukemia*. Jul 2014;28(7):1467–1471.
60. Aifantis I, Raetz E, Buonamici S. Molecular pathogenesis of T-cell leukaemia and lymphoma. *Nature reviews. Immunology*. May 2008;8(5):380–390.
61. Zuurbier L, Homminga I, Calvert V, et al. NOTCH1 and/or FBXW7 mutations predict for initial good prednisone response but not for improved outcome in pediatric T-cell acute lymphoblastic leukemia patients treated on DCOG or COALL protocols. *Leukemia*. Dec 2010;24(12):2014–2022.
62. Mullighan CG, Su X, Zhang J, et al. Deletion of IKZF1 and prognosis in acute lymphoblastic leukemia. *N Engl J Med*. Jan 29 2009;360(5):470–480.
63. Den Boer ML, van Slegtenhorst M, De Menezes RX, et al. A subtype of childhood acute lymphoblastic leukaemia with poor treatment outcome: a genome-wide classification study. *The lancet oncology*. Feb 2009;10(2):125–134.
64. Roberts KG, Li Y, Payne-Turner D, et al. Targetable kinase-activating lesions in Ph-like acute lymphoblastic leukemia. *N Engl J Med*. Sep 11 2014;371(11):1005–1015.
65. Russell LJ, Capasso M, Vater I, et al. Deregulated expression of cytokine receptor gene, CRLF2, is involved in lymphoid transformation in B-cell precursor acute lymphoblastic leukemia. *Blood*. Sep 24 2009;114(13):2688–2698.
66. Harvey RC, Mullighan CG, Chen IM, et al. Rearrangement of CRLF2 is associated with mutation of JAK kinases, alteration of IKZF1, Hispanic/Latino ethnicity, and a poor outcome in pediatric B-progenitor acute lymphoblastic leukemia. *Blood*. Jul 1 2010; 115(26):5312–5321.
67. Chen IM, Harvey RC, Mullighan CG, et al. Outcome modeling with CRLF2, IKZF1, JAK, and minimal residual disease in pediatric acute lymphoblastic leukemia: a Children's Oncology Group study. *Blood*. Apr 12 2012;119(15):3512–3522.
68. Mullighan CG, Goorha S, Radtke I, et al. Genome-wide analysis of genetic alterations in acute lymphoblastic leukaemia. *Nature*. Apr 12 2007;446(7137):758–764.
69. Schrappe M, Reiter A, Zimmermann M, et al. Long-term results of four consecutive trials in childhood ALL performed by the ALL-BFM study group from 1981 to 1995. Berlin-Frankfurt-Munster. *Leukemia*. Dec 2000;14(12):2205–2222.
70. Nachman JB, Sather HN, Sensel MG, et al. Augmented post-induction therapy for children with high-risk acute lymphoblastic leukemia and a slow response to initial therapy. *N Engl J Med*. Jun 4 1998;338(23):1663–1671.
71. Borowitz MJ, Devidas M, Hunger SP, et al. Clinical significance of minimal residual disease in childhood acute lymphoblastic leukemia and its relationship to other prognostic factors: a Children's Oncology Group study. *Blood*. Jun 15 2008;111(12):5477–5485.
72. Conter V, Bartram CR, Valsecchi MG, et al. Molecular response to treatment redefines all prognostic factors in children and adolescents with B-cell precursor acute lymphoblastic leukemia: results in 3184 patients of the AIEOP-BFM ALL 2000 study. *Blood*. Apr 22 2010;115(16):3206–3214.
73. Schrappe M, Valsecchi MG, Bartram CR, et al. Late MRD response determines relapse risk overall and in subsets of childhood T-cell ALL: results of the AIEOP-BFM-ALL 2000 study. *Blood*. Aug 25 2011;118(8):2077–2084.
74. Faham M, Zheng J, Moorhead M, et al. Deep-sequencing approach for minimal residual disease detection in acute lymphoblastic leukemia. *Blood*. Dec 20 2012;120(26):5173–5180.
75. Howlader N, Noone AM, Krapcho M, et al. SEER Cancer Statistics Review, 1975-2012 National Cancer Institute. Bethesda, MD. http://seer.cancer.gov/csr/1975_2012/, based on November 2014 SEER data submission, posted to the SEER web site, April 2015. Accessed 2/19/16.
76. Lister TA, Crowther D, Sutcliffe SB, et al. Report of a committee convened to discuss the evaluation and staging of patients with Hodgkin's disease: Cotswolds meeting. *J Clin Oncol*. Nov 1989;7(11):1630–1636.
77. Swerdlow SH, Campo E, Harris NL, et al. *WHO classification of tumours of haematopoietic and lymphoid tissues*. 4 ed. Lyon, France: IARC; 2008.
78. Raetz EA, Perkins SL, Bhojwani D, et al. Gene expression profiling reveals intrinsic differences between T-cell acute lymphoblastic leukemia and T-cell lymphoblastic lymphoma. *Pediatric blood & cancer*. Aug 2006;47(2):130–140.
79. Moorman AV, Harrison CJ, Buck GA, et al. Karyotype is an independent prognostic factor in adult acute lymphoblastic leukemia (ALL): analysis of cytogenetic data from patients treated on the Medical Research Council (MRC) UKALLXII/Eastern Cooperative Oncology Group (ECOG) 2993 trial. *Blood*. Apr 15 2007;109(8):3189–3197.
80. Wetzler M, Dodge RK, Mrozek K, et al. Prospective karyotype analysis in adult acute lymphoblastic leukemia: the cancer and leukemia Group B experience. *Blood*. Jun 1 1999;93(11):3983–3993.
81. Moorman AV, Robinson H, Schwab C, et al. Risk-directed treatment intensification significantly reduces the risk of relapse among children and adolescents with acute lymphoblastic leukemia and intrachromosomal amplification of chromosome 21: a comparison of the MRC ALL97/99 and UKALL2003 trials. *J Clin Oncol*. Sep 20 2013;31(27):3389–3396.
82. Charrin C, Thomas X, Ffrench M, et al. A report from the LALA-94 and LALA-SA groups on hypodiploidy with 30 to 39 chromosomes and near-triploidy: 2 possible expressions of a sole entity conferring poor prognosis in adult acute lymphoblastic leukemia (ALL). *Blood*. Oct 15 2004;104(8):2444–2451.
83. Mancini M, Scappaticci D, Cimino G, et al. A comprehensive genetic classification of adult acute lymphoblastic leukemia (ALL): analysis of the GIMEMA 0496 protocol. *Blood*. May 1 2005;105(9):3434–3441.
84. Moorman AV, Richards SM, Martineau M, et al. Outcome heterogeneity in childhood high-hyperdiploid acute lymphoblastic leukemia. *Blood*. Oct 15 2003;102(8):2756–2762.
85. Moorman AV, Chilton L, Wilkinson J, Ensor HM, Bown N, Proctor SJ. A population-based cytogenetic study of adults with acute lymphoblastic leukemia. *Blood*. Jan 14 2010;115(2):206–214.
86. Beldjord K, Chevret S, Asnafi V, et al. Oncogenetics and minimal residual disease are independent outcome predictors in adult patients with acute lymphoblastic leukemia. *Blood*. Jun 12 2014;123(24):3739–3749.
87. Herold T, Baldus CD, Gokbuget N. Ph-like acute lymphoblastic leukemia in older adults. *N Engl J Med*. Dec 4 2014;371(23):2235.
88. Trinquand A, Tanguy-Schmidt A, Ben Abdelali R, et al. Toward a NOTCH1/FBXW7/RAS/PTEN-based oncogenetic risk classification of adult T-cell acute lymphoblastic leukemia: a Group for Research in Adult Acute Lymphoblastic Leukemia study. *J Clin Oncol*. Dec 1 2013;31(34):4333–4342.
89. Jain P, Kantarjian H, Ravandi F, et al. The combination of hyper-CVAD plus nelarabine as frontline therapy in adult T-cell acute lym-

phoblastic leukemia and T-lymphoblastic lymphoma: MD Anderson Cancer Center experience. *Leukemia.* Apr 2014;28(4):973–975.
90. Rowe JM, Buck G, Burnett AK, et al. Induction therapy for adults with acute lymphoblastic leukemia: results of more than 1500 patients from the international ALL trial: MRC UKALL XII/ECOG E2993. *Blood.* Dec 1 2005;106(12):3760–3767.
91. Bruggemann M, Raff T, Kneba M. Has MRD monitoring superseded other prognostic factors in adult ALL? *Blood.* Nov 29 2012;120(23):4470–4481.
92. Bruggemann M, Schrauder A, Raff T, et al. Standardized MRD quantification in European ALL trials: proceedings of the Second International Symposium on MRD assessment in Kiel, Germany, 18–20 September 2008. *Leukemia.* Mar 2010;24(3):521–535.
93. Bassan R, Spinelli O, Oldani E, et al. Improved risk classification for risk-specific therapy based on the molecular study of minimal residual disease (MRD) in adult acute lymphoblastic leukemia (ALL). *Blood.* Apr 30 2009;113(18):4153–4162.
94. Bruggemann M, Raff T, Flohr T, et al. Clinical significance of minimal residual disease quantification in adult patients with standard-risk acute lymphoblastic leukemia. *Blood.* Feb 1 2006;107(3):1116–1123.
95. Logan AC, Vashi N, Faham M, et al. Immunoglobulin and T cell receptor gene high-throughput sequencing quantifies minimal residual disease in acute lymphoblastic leukemia and predicts post-transplantation relapse and survival. *Biology of blood and marrow transplantation : journal of the American Society for Blood and Marrow Transplantation.* Sep 2014;20(9):1307–1313.
96. Sala Torra O, Othus M, Williamson DW, et al. Minimal Residual Disease Detection By Next Generation Sequencing in Adult B-Cell Acute Lymphoblastic Leukemia (ALL) Patients Treated on SWOG Trial S0333. Paper presented at: Blood2014.
97. Wu D, Emerson RO, Sherwood A, et al. Detection of minimal residual disease in B lymphoblastic leukemia by high-throughput sequencing of IGH. *Clin Cancer Res.* Sep 1 2014;20(17):4540–4548.
98. Wu D, Sherwood A, Fromm JR, et al. High-throughput sequencing detects minimal residual disease in acute T lymphoblastic leukemia. *Sci Transl Med.* May 16 2012;4(134):134ra163.
99. Sokal JE, Cox EB, Baccarani M, et al. Prognostic discrimination in "good-risk" chronic granulocytic leukemia. *Blood.* Apr 1984;63(4):789–799.
100. Hasford J, Pfirrmann M, Hehlmann R, et al. A new prognostic score for survival of patients with chronic myeloid leukemia treated with interferon alfa. Writing Committee for the Collaborative CML Prognostic Factors Project Group. *Journal of the National Cancer Institute.* Jun 3 1998;90(11):850–858.
101. Faderl S, Talpaz M, Estrov Z, O'Brien S, Kurzrock R, Kantarjian HM. The biology of chronic myeloid leukemia. *N Engl J Med.* Jul 15 1999;341(3):164–172.
102. Verma D, Kantarjian HM, Jones D, et al. Chronic myeloid leukemia (CML) with P190 BCR-ABL: analysis of characteristics, outcomes, and prognostic significance. *Blood.* Sep 10 2009;114(11):2232–2235.
103. Kantarjian HM, Deisseroth A, Kurzrock R, Estrov Z, Talpaz M. Chronic myelogenous leukemia: a concise update. *Blood.* Aug 1 1993;82(3):691–703.
104. Savage DG, Szydlo RM, Chase A, Apperley JF, Goldman JM. Bone marrow transplantation for chronic myeloid leukaemia: the effects of differing criteria for defining chronic phase on probabilities of survival and relapse. *British journal of haematology.* Oct 1997;99(1):30–35.
105. Sokal JE, Baccarani M, Russo D, Tura S. Staging and prognosis in chronic myelogenous leukemia. *Seminars in hematology.* Jan 1988;25(1):49–61.
106. Talpaz M, Silver RT, Druker BJ, et al. Imatinib induces durable hematologic and cytogenetic responses in patients with accelerated phase chronic myeloid leukemia: results of a phase 2 study. *Blood.* Mar 15 2002;99(6):1928–1937.
107. Cortes JE, Talpaz M, O'Brien S, et al. Staging of chronic myeloid leukemia in the imatinib era: an evaluation of the World Health Organization proposal. *Cancer.* Mar 15 2006;106(6):1306–1315.
108. Druker BJ. Chronic Myelogenous Leukemia In: DeVita VT, Lawrence TS, Rosenburg SA, eds. *DeVita, Hellman, and Rosenberg's Cancer: Principles & Practice of Oncology.* Vol 2. 8 ed: Lippincott, Williams and Wilkins; 2007:2267–2304.

Additional Contributors

Content Harmonization Core
Elliot A. Asare MD
James D. Brierley BSc, MB, FRCP, FRCR, FRCP(C)
David R. Byrd MD, FACS
Carolyn C. Compton MD, PhD, FCAP
Stephen B. Edge MD, FACS
Jeffrey E. Gershenwald MD, FACS - Chair
Frederick L. Greene MD, FACS
Donna M. Gress RHIT, CTR – Vice Chair
Kenneth R. Hess PhD
J. Milburn Jessup MD
Martin Madera
Laura R. Meyer, CAPM
Mary Kay Washington MD, PhD

Evidence Based Medicine and Statistics Core
William E. Barlow PhD
Carolyn C. Compton MD, PhD, FCAP
Jeffrey E. Gershenwald MD, FACS
Phyllis A. Gimotty PhD
Lauren E. Haydu
Kenneth R. Hess PhD - Chair
Hemant Ishwaran PhD
Michael W. Kattan PhD
Michael LeBlanc PhD
Serban Negoita MD, DrPH
Andrew J. Vickers

Precision Medicine Core
Ulysses G.J. Balis MD, FCAP, FASCP
Donald A. Berry PhD
Carolyn C. Compton MD, PhD, FCAP - Chair
Jeffrey E. Gershenwald MD, FACS
Phyllis A. Gimotty PhD
Justin Guinney PhD
Susan Halabi PhD
Kenneth R. Hess PhD
J. Milburn Jessup MD
Michael W. Kattan PhD – Vice Chair
George A. Komatsoulis PhD
Alexander J. Lazar MD, PhD, FACP
Michael LeBlanc PhD
Ying Lu PhD, FASA
Alyson L. Mahar MSc
Angela Mariotto PhD
Karel GM Moons Prof., PhD, MSc
Snehal G. Patel MD
Daniel J. Sargent PhD
Martin R. Weiser MD

Imaging Core
Priya R. Bhosale MD
Peter L. Choyke MD, FACR
Rivka R. Colen MD
Basak Dogan MD
Carl J. D'Orsi MD, FACR
Jeremy J. Erasmus MD
Jean-Francois H. Geschwind MD, FSIR
Ritu R. Gill MD, MPH
Christine M. Glastonbury MBBS
Michael M. Graham MD, PhD
John E. Madewell MD
Suresh K. Mukherji MD, FACR
Mark D. Murphey MD, FACR
David M. Panicek MD, FACR
Eric Rohren MD, PhD
Daniel I. Rosenthal MD, FACR
Lawrence H. Schwartz MD
Rathan M. Subramaniam MD, PhD, MPH
Daniel C. Sullivan MD - Chair
Bachir Taouli MD
Raghunandan Vikram MD
Richard L. Wahl MD
Chadwick L. Wright MD, PhD

© American Joint Committee on Cancer 2017

M.B. Amin et al. (eds.), *AJCC Cancer Staging Manual, Eighth Edition*, DOI 10.1007/978-3-319-40618-3

Data Collection Core
Diane Baker CTR
Vivien W. Chen PhD
Carolyn C. Compton MD, PhD, FCAP
Kathleen A. Cronin PhD
Kimberly DeWolfe MS, CTR
Stephen B. Edge MD, FACS
Jeffrey E. Gershenwald MD, FACS
Frederick L. Greene MD, FACS
Donna M. Gress RHIT, CTR – Vice Chair
Kenneth R. Hess PhD
J. Milburn Jessup MD - Chair
Shirley Jordan Seay PhD, OCN, CTR
Martin Madera
Laura R. Meyer, CAPM
Sarah J. Manson RHIT, CTR
Pat Pekatos
Cathy Rimmer BA, MDIV, CTR
Frances E. Ross CTR
Maria J. Schymura PhD
Paige S. Tedder RHIT, CTR
Kathleen Thoburn BA, CTR
Theresa M. Vallerand BGS, CTR

National Cancer Data Base (NCDB) Staff
Ashley Loomis, MPH
Katherine Mallin PhD
Bryan E. Palis
Greer Gay, PhD
Ryan M. McCabe, PhD

Illustrator
Alice Chen

Copyeditors
Georgette Forgione
The Scientific Consulting Group, Inc. – Alicia Rosov, Beverly Campbell, Margaret Christoph, Joanna Mandecki, Maria Osvald

Acknowledgement and Sponsorship
American Cancer Society
American College of Surgeons

The AJCC wishes to acknowledge the Centers for Disease Control and Prevention for its collaboration for improving and promoting standardized cancer staging under cooperative agreement DP13-1310-4 awarded to the American Joint Committee on Cancer. The findings and conclusions in this report are those of the author(s) and do not necessarily represent the official position of the Centers for Disease Control and Prevention.

The AJCC wishes to acknowledge the generosity of Dr. Carolyn Compton, professor with the Arizona State University School of Life Sciences, in sponsoring the foundational meeting of the Precision Medicine Core committee in January 2015, Phoenix, AZ. This meeting was supported by professional funds provided to Dr. Compton by the Office of the President at ASU.

Index

A

Abdominal and thoracic visceral organs
  AJCC prognostic stage groups, 520
  clinical classification, 519
  distant metastasis (M), 520
  histologic grade (G), 521
  histopathologic type, 521
  ICD-O-3 topography codes, 518
  imaging, 519–520
  metastatic sites, 519
  pathological classification, 520
  primary site(s), 519
  primary tumor (T), 517, 520
  prognostic factors, 520
  regional lymph node (N), 519, 520
  registry data collection variables, 520
  risk assessment models, 520
  staging system, 517
  WHO tumors classification
    adipocytic tumors, 518
    chondro-osseous tumors, 518
    fibroblastic/myofibroblastic tumors, 518
    fibrohistiocytic tumors, 518
    nerve sheath tumors, 519
    pericytic (perivascular) tumors, 518
    skeletal muscle tumors, 518
    smooth muscle tumors, 518
    vascular tumors, soft tissue, 518
ACC. *See* Adrenal cortical carcinoma (ACC)
ACTH. *See* Adrenocorticotropic hormone (ACTH)
Acute lymphoblastic leukemia (ALL)
  in adults
    clinical classification, 989–990
    laboratory features, 990
    prognostic factors, 990–992
    risk assessment models, 992–993
  in children
    clinical and biological features, 985
    clinical classification, 985–986
    laboratory features, 986
    prognostic factors, 986–989
    risk assessment models, 989
Acute myeloid leukemia (AML), 933
  AML NOS, 984
  clinical classification, 980–981
  laboratory features, 981
  myelodysplasia-related changes, 983, 984
  prognostic factors, 984–985
  recurrent genetic changes, 981–984
  t-AML, 984
Adenocarcinoma
  esophagus, 185, 197–199
  appendix, 238
  colon and rectum (*see* Colon and rectal carcinoma)
  of mammary gland type, 638
  of Skene gland origin, 638
  of small intestine (*see* Small intestinal cancer)
  vulva, 637
Adenocarcinoma *in situ* (AIS), 444
Adenosquamous carcinoma, 196
Adipocytic tumors, 532, 846
Adrenal cortical carcinoma (ACC)
  AJCC prognostic stage groups, 916
  clinical classification, 913
  CT and MR imaging, 914
  distant metastasis (M), 915
  ^{18}F-FDG PET/CT, 914
  histologic grade (G), 916
  histopathologic type, 916
  metastatic sites, 913
  N1, regional lymph nodes involvement, 918
  pathological classification, 914–915
  primary site(s), 913
  primary tumor (T), 915
  prognostic factors, stage grouping, 915
  recommended factors, clinical care, 915
  regional lymph node (N), 913, 915, 916
  registry data collection variables, 916
  risk assessment models, 915
  T1, T2, T3 and T4, 916–917
Adrenal-neuroendocrine tumors
  clinical classification, 920–921
  CT imaging, 921
  histologic grade (G), 924
  metastatic sites, 920
  MR imaging, 921
  MR spectroscopy, 922
  pathological classification, 922
  PET, 921
  primary site(s), 920
  prognostic factors
    distant metastasis, 923
    lymph node metastasis, 923
    malignancy and overall survival, 922

© American Joint Committee on Cancer 2017

M.B. Amin et al. (eds.), *AJCC Cancer Staging Manual, Eighth Edition*, DOI 10.1007/978-3-319-40618-3

Adrenal-neuroendocrine tumors (*cont.*)
metastases, 923
primary tumor location, 922
primary tumor size, 922
SDHB mutations, 922–923
stage grouping, 922
synchronous *vs.* metachronous, 923
tumor burden *vs.* catecholamine secretion, 923
regional lymph nodes, 920
registry data collection variables, 924
risk assessment models, 923
survival data, 925–926
Adrenocorticotropic hormone (ACTH), 408
AJCC. *See* American Joint Committee on Cancer (AJCC)
AJCC Breast Cancer Expert Panel, 593
anatomic stage grouping, 620–621
prognostic stage groups, 621–625
AJCC Cancer Staging Manual, 6, 312, 313
publication year for, 3, 4
8th Edition, 34
cancer staging data form, 37
chapter outline for, 32, 33
clinical trial stratification, 37
imaging, 35–36
levels of evidence, 32–33, 35
prognostic factors, 36
project plan, development of, 31–32
risk assessment models, 36
vision for, 31
TNM staging system (*see* Tumor node metastasis (TNM) system)
AJCC Soft Tissue Sarcoma Expert Panel, 508
ALL. *See* Acute lymphoblastic leukemia (ALL)
Alpha-fetoprotein (AFP), 291, 728, 733
Alpha thalassemia/mental retardation syndrome X-linked (ATRX), 865
American Cancer Society, 338
American College of Chest Physicians (ACCP), 442
American College of Radiology (ACR), 5
American Joint Committee on Cancer (AJCC), 369, 376
American Society of Clinical Oncology (ASCO) Guidelines, 5
American Thyroid Association (ATA), 875
AML. *See* Acute myeloid leukemia (AML)
Ampulla of Vater
AJCC prognostic stage groups, 344
clinical classification, 339–340
distant metastasis (M), 344
and duodenum, NETs (*see* Duodenum and ampulla of Vater)
histologic grade (G), 344–345
histologic type, 345
ICD-O-3 topography codes, 338
imaging, 340
metastatic sites, 338–339
pathological classification, 341–343
primary site(s), 338
primary tumor (T), 344
prognostic factors, 343–344
regional lymph node (N), 338, 344
registry data collection variables, 344
risk assessment models, 344
WHO tumors classification, 338
Anal cancer
adenocarcinoma, 277
BCCs, 277
clinical classification, 279
distant metastasis (M), 282
histologic grade (G), 282
histopathologic type, 283
ICD-O-3 topography codes, 276
imaging, 279–280
metastatic sites, 279
pathological classification, 280
perianalvulvar, 276
primary site(s), 277–278
primary tumor (T), 282
prognostic factors
gender, 281
grade, 282
HIV status, 281
HPV infection, 281
HPV status and p16/p18 expression, 282
NCDB, 280
overall and disease-free survival, 281
stage grouping, 281
tumor location, 281
regional lymph node (N), 279, 282
registry data collection variables, 282
risk assessment models, 282
SCCs, 276–277
verrucous carcinoma, 277
WHO tumors classification, 276
Anaplastic large cell lymphomas (ALCLs), 949, 951, 961
Anaplastic melanoma, 800
Anaplastic thyroid cancer, 874
ANCHOR trial, 276
Angioimmunoblastic T-cell lymphoma (AITL), 950
Ann Arbor system, 850
Aortic lymph nodes
kidney, 740
prostate, 718
renel pelvis, 750
Appendiceal carcinomas. *See* Carcinomas
Appendiceal neuroendocrine tumors
AJCC prognostic stage groups, 389, 392
clinical classification, 391
distant metastasis (M), 392
histologic grade (G), 393
histopathologic type, 393
ICD-O-3 topography codes, 389
imaging, 391
metastatic sites, 391
pathological classification, 391
primary site(s), 390
primary tumor (T), 392
prognostic factors
Ki-67 proliferative index, 391
mitotic count, 391
plasma CgA level, 392
plasma serotonin level, 391
plasma/urinary 5-HIAA level, 392
regional lymph node (N), 390, 392
registry data collection variables, 393
restaging, 391
risk assessment models, 392
survival data, 393
WHO tumors classification, 389
Appendicular skeleton
chondrosarcoma, 477
Ewing sarcoma/PNET, 478
osteosarcoma, 479
trunk, skull and facial bones, 483
Application Programming Interface (API), 32
Axillary ipsilateral lymph nodes, 595

B
Barcelona Clinic Liver Cancer (BCLC), 290
Basal cell carcinomas (BCCs), 172, 175, 276, 277, 781
Batts–Ludwig system, 290
B-cell lymphomas
  AJCC prognostic stage groups, 971
  distant metastasis (M), 972
  primary tumor (T), 971
  prognostic factors, 969
  regional lymph node (N), 972
Bilateral primary carcinomas, 611
Binet staging system, 948, 955
Bismuth–Corlette type IV, 313
Bone cancer
  AJCC prognostic stage groups, 471, 504
  anatomic subsites, 483
  biopsy, 474–475
  chondrosarcoma, 472
  clinical classification, 473
  distant metastasis (M), 477
  Ewing sarcoma, 472
  histologic grade (G), 477
  histopathologic type, 477–478
  ICD-O-3 topography codes, 471–472
  imaging, 473–474
  metastatic sites, 473
  osteosarcoma, 472
  pathological classification, 475
  primary site(s), 472
  primary tumor (T), 476–477
  prognostic factors, 475–476
  regional lymph node (N), 472, 477
  registry data collection variables, 477
  risk assessment models, 476
  staging system, 471
  survival data, 478
  WHO tumors classification, 472
BRAF, 164, 252, 266–267, 683, 796, 799
BRAF V600E mutation, 266, 267
Brain and spinal cord
  computed tomography (CT), 860
  descriptive epidemiology, 859
  diffusion tensor imaging, 860
  diffusion-weighted imaging, 860
  FIESTA, 860
  FLAIR, 860
  fMR imaging, 861
  histologic grading (G), 866–868
  iMR imaging, 861
  magnetic resonance (MR) imaging, 861
  meningioma risk factors, 860
  metabolic "nuclear" imaging, 861
  MR elastography (MRe), 861
  MR tractography, 860
  neuro-oncology
    chemotherapy, 862–863
    radiation therapy, 861–862
    surgery, 861
  positron emission tomography (PET), 861
  prognostic factors, staging grouping, 863
  recommended factors
    functional neurologic status, 863
    grading, 864
    histopathology, 863
    molecular aspects, 863–864
    patient age, 863
    primary/recurrent tumor, 863
    resection, 863
    seizures and long duration of symptoms, 863
    tumor classification and "integrated" diagnoses, 864
    tumor location, 863
  registry data collection variables, 866
  risk assessment models, 866
  risk factors, 860
  WHO grading system, 857–858
Breast
  aggressive local treatments, 592
  AJCC breast cancer staging, 594
  anatomic stage/prognostic groups, 611
  biologic intrinsic subtypes, 592
  biomarkers, 611–613
  bone marrow micrometastases, 602
  BRCA1 and *BRCA2*, 820
  chest wall, 595
  clinical categorization, 596–597
  clinically defined subtypes, 612
  clinical stage I and stage II disease, 601
  CTCs, 602
  disseminated tumor cells, 602
  distant metastases (M), 601–602
  histologic grade (G), 625
  histopathologic type, 625–626
  history and physical examination, 601
  imaging classification, 597–598
  imaging findings, 597
  inflammatory carcinoma, 598
  laboratory abnormalities, 602
  locoregional treatments, 592
  macrometastases, 596
  metastatic sites, 595
  molecular characteristics, 592
  multigene marker panels, 613–614
  nodal metastases, 596, 605
  pathological classification
    axillary lymph nodes, 603
    distant metastases (M), 609
    histologic invasive cancer types, 603
    primary carcinoma, 603
    primary tumor (T), 603–605
    regional lymph nodes (N), 605–609
    staging groups, 603
  physical examination, 597
  posttreatment M classification, 603, 609
  posttreatment ycN classification, 603
  posttreatment ycT classification, 602
  posttreatment ypN classification, 609
  posttreatment ypt classification, 609
  primary site, 594, 595
  primary tumor (T), 417, 423–426, 597
  prognostic factors, 611, 616–618
  prognostic tools, 618
  regional lymph nodes (N), 416, 424, 595, 598–600
  registry data collection variables, 625
  risk assessment models, 618
  risk profile determination, 615
  SEER Program, 40–42
  skin, 598
  TNM prognostic stage groups, 614–616
  TNM staging classification, 592, 594
  tumor size, 597
  univariate and multivariate analyses of prognostic factors, 615

Breast Cancer Index, 618
Breast cancer resistance protein (BCRP), 862
Breast–ovarian cancer syndrome, 683
Brenner tumors, 689

C
Cancer antigen 125 (CA-125), 687
Cancer of the Liver Italian Program (CLIP), 290
Cancer Research and Biostatistics (CRAB), 424, 458
Cancer staging data form, 37
Cancer survival analysis
  actuarial method, 40
  cause-adjusted survival, 42
  competing risks, 42
  confidence interval, 43
  cumulative incidence method, 42
  Kaplan–Meier method, 39, 41
  life table method, 39–41
  log-rank test, 43
  observed survival, 42
  patient, disease, and treatment specific survival, 41–42
  regression model, 43
  relative survival, 42
  SEER Program
    breast cancer patients, 40–42
    lung cancer patients, 40–41
  standard error, 43
  starting time, 44
  survival probability and curve, 39
  time interval, 44
  uncensored and censored cases, 39
  vital status, 44
CAP Cancer Protocols, 5
Carcinoembryonic antigen (CEA), 210, 227, 263
Carcinoid syndrome, 413
Carcinomas
  appendix
    AJCC prognostic stage groups, 238, 228
    clinical classification, 239–240
    distant metastasis, 237, 243
    histologic grade, 241
    histopathologic type, 242
    ICD-O-3 topography codes, 238
    metastatic sites, 239
    pathological classification, 238, 240–241
    primary sites, 239
    primary tumor, 237, 243, 245–247
    prognostic factor, 241–242
    regional lymph nodes, 239, 243, 248, 249
    registry data collection variables, 243
    residual tumor (R), 244
    risk assessment models, 242–243
    survival data, 244–245
    WHO classification, 238
  breast, 591
  colon and rectum (*see* Colon and rectal carcinoma)
  fallopian tube, 682
Carney–Stratakis syndrome, 525
Caval lymph nodes, 729
CEA. *See* Carcinoembryonic antigen (CEA)
Cell of origin (COO), 944
Central nervous system (CNS) tumors
  incidence, 859
  risk factors, 860
Cervical esophagus, 187–188
Cervical lymph node metastasis
  AJCC prognostic stage groups, 75
  clinical classification, 70
    imaging, 72–73
    occult primary tumor, 70, 72
  HPV-negative cancers, ENE in, 67–68
  ICD-O-3 topography codes, 68–69
  overall survival data, 75–77
  pathological classification, 69, 73–74
  regional lymph nodes, 67, 70–72, 74–75
  registry data collection variables, 75
  WHO classification, 69
Cervical lymph nodes, 717
Cervix uteri
  clinical stage, 651–652
  distant (mediastinal, scalene) nodal status, 652
  FIGO stage, 652
  gynecologic cancer, 650
  histopathologic type, 652–654
  HIV status, 653
  human papillomavirus status, 652
  lymph node status, 651
  metastatic sites, 650
  MR imaging, 651
  nodal metastases, 651
  para-aortic nodal status, 652
  pathological classification, 652
  pelvic node involvement, 652
  primary site(s), 650
  primary tumor, 655–658
  prognostic factors, 652–653
  pTNM nomenclature, 652
  regional lymph nodes, 650
  risk assessment models, 653
  staging systems, 650
  tumor staging, 651
Child–Pugh scoring system, 289
Chinese University Prognostic Index (CUPI) scoring system, 290
Cholelithiasis, 304
Chondrosarcoma
  appendicular skeleton, 478
  pelvis, 479
  spine, 480
Choriocarcinoma, 692
  GTNs, 691
  histologic diagnosis, 693
  histopathologic type, 695
Choroidal melanoma
  AJCC prognostic stage groups, 813
  clinical classification, 806, 808–809
  Kaplan–Meier stage-specific estimates, 808, 809
  metastatic, 809
  nonmetastatic, 807
  primary tumor (T), 812
Chromogranin A (CgA), 354, 378, 379, 392, 398–399
Chronic lymphocytic leukemia (CLL), 994
  Binet staging system, 948, 955
  CIRS, 949
  clinical classification, 947–949
  del(11q)/ ATM mutation, 949–950
  IGHV mutation status, 949
  MBL, 947
  Rai staging system, 948, 955
Chronic myeloid leukemia (CML)
  clinical classification, 993
  laboratory features, 993
  prognostic factors, 993–994
  risk assessment models, 994

Ciliary body melanoma
AJCC prognostic stage groups, 813
clinical classification, 806, 808–809
Kaplan–Meier stage-specific estimates, 808, 809
primary tumor (T), 812
Circulating tumor cells (CTCs), 602, 617
Circumferential resection margin (CRM), 263–265, 269
Clear cell tumors, 662, 689
Clinical Laboratory Improvement Amendments (CLIA), 354, 368, 379, 391, 399
CLL. *See* Chronic lymphocytic leukemia (CLL)
CML. *See* Chronic myeloid leukemia (CML)
Codification of tumor staging into the TNM system, 591
Collaborative Ocular Melanoma Study (COMS), 806
College of American Pathology (CAP), 4, 354, 379, 399
Colon and rectal carcinoma, 36, 226, 821
AJCC prognostic stage groups, 269
anastomotic recurrence, 262
BRAF mutation, 262, 263, 266–267
circumferential resection margin, 263–265
clinical classification, 255–256
distant metastasis, 251, 268–269
histologic grade, 269
histopathologic type, 269
ICD-O-3 topography codes, 252
isolated tumor cells, 261
KRAS and NRAS mutation, 262, 263, 266
lymphovascular invasion, 263, 265
metastasis, 262
micrometastases, 261
microsatellite instability, 263, 265–266
NETs
AJCC prognostic stage groups, 392
clinical classification, 391
distant metastasis (M), 392, 402–404
histologic grade (G), 399–400
histopathologic type, 400
ICD-O-3 topography codes, 395
imaging, 398
metastatic sites, 397
pathological classification, 398
primary site(s), 396
primary tumor (T), 392, 402–404
prognostic factors, 398–399
regional lymph node (N), 390, 392, 402–404
registry data collection variables, 355
restaging, 398
risk assessment models, 399
staging system, 395
survival data, 400–401
WHO tumors classification, 395
perineural invasion, 241, 265
primary sites, 252–254
primary tumor, 256–260, 268
regional lymph node, 251, 254–255, 260, 261, 268–271
registry data collection variables, 269
risk assessment models, 267–268
serum CEA levels, 263
survival data, 270
total mesorectal excision, 260
tumor deposits, 261–263
tumor regression, 260, 263–264
WHO classification, 252
Common iliac lymph nodes, 642, 662, 672
cervix uteri, 650
fallopian tube, 684
ovary, 684
prostate, 718
urethra, 768
urinary bladder, 759
Complete hydatidiform mole, 691
Complexity Index in Sarcoma (CINSARC), 496
Component Content Management System (CCMS), 32
Conjunctival carcinoma
AJCC prognostic stage groups, 790
AJCC TNM staging system, 788, 790
clinical classification, 789
clinical mapping system, 792
distant metastasis (M), 790
histologic grade (G), 790
histopathologic type, 790
imaging, 789
metastatic sites, 789
ocular surface squamous neoplasia, 788
ophthalmic sites, 791
pathological classification, 789
primary site(s), 788–789
primary tumor (T), 790
prognostic factors, 789
regional lymph node (N), 789, 790
registry data collection variables, 790
risk assessment models, 790
Conjunctival melanoma
AJCC prognostic stage groups, 800
clinical classification, 797–798, 802
conjunctival imaging techniques, 798
diagnosis of, 796
distant metastasis (M), 800
histologic grade (G), 800
histopathologic type, 800
invasive conjunctival melanoma, 800
lymph node metastases, 800
metastatic sites, 797
pathological analysis, 796
pathological classification, 798–799
primary site(s), 796–797
primary tumor (T), 799
prognostic factors, stage grouping, 799
regional lymph node (N), 797, 800, 801
risk assessment models, 799
Content Harmonization Core (CHC), 4
Core-binding factor (CBF), 981
Corpus uteri
carcinoma and carcinosarcoma
AJCC Prognostic Stage Groups, 666
anatomic sites, 662
anatomic staging, tumor, 663
clinical classification, 663
distant metastasis (M), 666
FIGO stage, 664
grade, 664
histologic grade (G), 666
histopathologic type, 665, 666
histopathology, 667
magnetic resonance (MR) imaging, 663
metastatic sites, 662
myometrial invasion, 665
pathological grading, 667
peritoneal cytology, 663
primary site(s), 662
primary tumor (T), 666
prognostic factors, 664–665
regional lymphadenectomy specimens, 664
regional lymph nodes (N), 662, 666

Corpus uteri (*cont.*)
regional nodal metastases, 663
risk assessment models, 665
sentinel lymph node assessment, 664
surgical/pathological staging, 663–664
tumor staging, 663
sarcoma
clinical classification, 673–674
distant metastasis (M), 675
histopathologic type, 676
magnetic resonance (MR) imaging, 673
metastatic sites, 672
pathological classification, 674
primary sites, 672
primary tumor (T), 675, 677–679
prognostic factors, 674
regional lymph nodes (N), 672, 675
risk assessment models, 674–675
tumor staging, 674
Couinaud's segmental anatomy, liver, 288
Cox proportional hazards regression model, 43
CRAB. *See* Cancer Research And Biostatistics (CRAB)
CRM. *See* Circumferential resection margin (CRM)
Crohn's disease, 227
CSCC. *See* Cutaneous squamous cell carcinoma (CSCC)
Cumulative Illness Rating Scale (CIRS), 949
Cumulative incidence method, 42
Cushing's syndrome, 408, 413
Cutaneous squamous cell carcinoma (CSCC)
AJCC prognostic stage groups, 179
clinical classification, 173–174
histologic grade, 179
histopathologic type, 179
ICD-O-3 topography codes, 171
incidence of, 172
metastatic sites, 173
pathological classification, 174
primary sites, 173
primary tumor, 178
prognostic factor
anatomic site, 176
bony structures, 176
comorbidity, 177
desmoplasia, 176
extranodal extension, 175
histopathologic grade/differentiation, 176
immunosuppression and advanced disease, 177
lifestyle factors, 177
nodal disease, 176–177
overall health, 177
perineural invasion, 176
tobacco use, 177
tumor depth, 175
tumor diameter, 175
regional lymph nodes, 173, 178
registry data collection variables, 179
risk assessment models, 177–178
WHO/IARC classification, 171–172
Zubrod/ECOG performance scale, 177

D

Deep lymph nodes
prostate, 718
urethra, 769
Depth of invasion (DOI), 80, 85
Desmoplasia, 176
Desmoplastic melanoma, 579
Diffuse large B-cell lymphoma, 852
COO, 944
DLBCL additional factors, 943
double-hit and double-expressor DLBCL, 945
IPI and related indices, 944
prognostic factors, stage grouping, 945
Disseminated peritoneal adenomucinosis (DPAM), 238, 242
Disseminated tumor cells (DTCs), 602, 617
Distal bile duct
adjacent organs, invasion of, 322
AJCC prognostic stage groups, 333
clinical classification, 319
distant metastasis (M), 333
histologic grade (G), 334
histopathologic type, 334
ICD-O-3 topography codes, 318
imaging, 320–321
metastatic sites, 318
pathological classification, 321–322
primary site(s), 318
primary tumor (T), 333
prognostic factors
adjacent organs, invasion of, 322
histologic parameters, 322
resection, extent of, 322
stage grouping, 322
tumor markers CEA and CA 19-9, 322
regional lymph node (N), 318, 333
registry data collection items, 334
risk assessment models, 333
staging system, 317
survival data, 324
TNM classification, 318
WHO tumors classification, 317, 318
Distant metastasis (M), 744
abdominal and thoracic visceral organs, 520
ACC, 915
adenocarcinoma, 197
adrenal-neuroendocrine tumors, 923
appendix, carcinomas, 237, 243
breast, 609, 620
cervix uteri, 654
colon and rectal carcinoma, 252, 269
conjunctival carcinoma, 790
conjunctival melanoma, 797
corpus uteri-carcinoma and carcinosarcoma, 667
corpus uteri-sarcoma, 675
GIST, 527
GTNs, 692, 695
head and neck, 504
HPV-OPSCC, 114
jejunum and ileum, 380
lacrimal gland carcinomas, 837
larynx, 152, 157
lip and oral cavity cancers, 91
location, 922
malignant pleural mesothelioma, 466
MF, 970
MTC, 897
nasal cavity and paranasal sinuses, 143
nasopharyngeal carcinoma, 106, 107
ocular adnexal lymphomas, 849, 850
orbital sarcoma, 841
ovary and primary peritoneal carcinoma, 681
pancreatic neuroendocrine tumors, 416
parathyroid, 907, 908

penis, 704, 707
primary cutaneous B-cell, 972
prostate, 723–724
retinoblastoma, 825
retroperitoneal soft tissue sarcoma, 536
skin melanoma, 573
small intestinal cancer, 227
squamous cell carcinoma, 198
testis, 733
thymic tumor, 428
thyroid-differentiated and anaplastic carcinoma, 883
TNM system, 10
trunk and extremities, STS, 511
uveal melanoma, 813
vagina, 644
vulva, 637
Duodenum and ampulla of Vater
AJCC prognostic stage groups, 361, 369
anatomic sites, 364
clinical classification, 366
distant metastasis (M), 369
gangliocytic paraganglioma, 363
gastrinomas, 366
histologic grade (G), 370
histopathologic type, 370
ICD-O-3 topography codes, 362
imaging, 366–367
metastatic sites, 366
neurofibromatosis type 1, 363
pancreaticoduodenectomy, 368
pathological classification, 367
primary site(s), 364–365
primary tumor (T), 369
prognostic factors
associated genetic syndrome, 368
CgA, 368
Ki-67 labeling index, 368
location, 368
mitotic rate, 367–368
stage grouping, 367
regional lymph node (N), 365, 367, 369
registry data collection variables, 369–370
risk assessment models, 368–369
somatostatinoma, 366
staging system, 361
survival data, 370–371
Von Hippel–Lindau syndrome, 363
WHO tumors classification, 362
ZES, 362
Durie–Salmo staging system (DSS), 976

E
EDC. *See* Electronic data capture (EDC)
Electronic data capture (EDC), 437, 458
Endometrial stromal sarcoma, 675, 695
Endometrioid adenocarcinoma, 667
Endometrioid tumors, 689
EndoPredict, 618
Endoscopic mucosal resection (EMR), 362
Endoscopic ultrasound-guided fine-needle aspiration (EUS-FNA), 525
ENE. *See* Extranodal extension (ENE)
ENETS. *See* European Neuroendocrine Tumor Society (ENETS)
Enterochromaffin-like (ECL), 352
Enteropathy-associated T-cell lymphoma (EATL), 951
Epithelioid melanoma, 797
Epithelioid trophoblastic tumors, 692
Esophageal wall, 187, 189
Esophagus and esophagogastric junction, 105
adenocarcinoma (*see* Adenocarcinoma)
adjacent structures, 187
celiac lymph node, 191
cervical esophagus, 187
clinical classification, 191–192
esophageal wall, 187, 189
extranodal extension, 196–197
HER2, adenocarcinoma, 197
histoviability, 196
ICD-O-3 topography codes, 186
lower thoracic, 188–189
lymphovascular invasion, 196
metastatic sites, 190
middle thoracic, 188
pathological classifications, 194
regional lymph nodes, 189–190
risk assessment models, 197
squamous cell carcinoma (*see* Squamous cell carcinomas (SCCs))
surgical margin (R category), 196
tumor length, 196
upper thoracic, 187–188
WHO classification, 186
Ethmoid sinus
anatomical subsites, 138
T4a tumors, 142, 145
T4b tumors, 142, 147
T1 tumors, 142, 145
T2 tumors, 142, 146
T3 tumors, 142, 146
European Network for the Study of Adrenal Tumors (ENSAT), 911, 912
European Neuroendocrine Tumor Society (ENETS), 363, 376, 396, 408
European Organisation for Research and Treatment of Cancer (EORTC), 281, 532
European Society of Thoracic Surgeons (ESTS), 442
Ewing sarcoma/PNET
appendicular skeleton, 483
pelvis, 482
spine, 482
Exocrine pancreas
AJCC prognostic stage groups, 344
clinical classification, 339
distant metastasis (M), 343
histologic grade (G), 344–345
histologic type, 345
ICD-O-3 topography codes, 338
imaging
CT, 339
endoscopic ultrasound, 340
MR imaging, 339
radiology report format, 341
TNM categories of staging, 340–341
metastatic sites, 338–339
pathological classification, 341–343
primary site(s), 338
primary tumor (T), 344
prognostic factors
completeness of resection, 343
histologic features, 344
metastatic disease, regional lymph nodes, 343
preoperative CA 19-9 levels, 343
stage grouping, 343
tumor regression, neoadjuvant therapy, 343–344
tumor size, 342
visceral arteries, 343

- Exocrine pancreas (*cont.*)
  - regional lymph node (N), 344
  - regional lymph nodes, 338
  - registry data collection variables, 344
  - risk assessment models, 344
  - staging system, 337
  - survival data, 345
  - WHO tumors classification, 338
- External iliac lymph nodes, 642, 662, 672
  - cervix uteri, 650
  - fallopian tube, 685
  - ovary, 685
  - penis, 706
  - urethra, 768
- Extranodal extension (ENE), 81, 573, 882
  - cervical lymph node, 67–68, 71, 75, 77
  - esophagus and esophagogastric junction, 196
  - head and neck cancers, 57
  - larynx, 150, 153
  - lip and oral cavity cancers
    - clinical/radiographic ENE, 83–84
    - histopathologic designations, 85
    - metastatic carcinoma, 85
    - pathological assessment of, 85
  - major salivary glands, 96, 97, 99
  - nasal cavity and paranasal sinuses, 141
  - p16-oropharyngeal cancers, 123, 124
- Extranodal marginal zone B-cell lymphomas (EMZLs), 850, 852, 946
- Extraskeletal mesenchymal chondrosarcoma, 846
- Extrathyroidal extension, 877, 879–882, 893
- Eyelid carcinoma
  - anatomic sites and regional lymph nodes, 784
  - clinical and imaging assessment, 781
  - clinical classification, 781
  - distant metastasis (M), 781, 783
  - histologic grading system, 783
  - histopathologic type, 783
  - imaging, 781–782
  - melanomas, 781
  - metastatic potential, 781
  - microsatelllite instability/immunohistochemistry, 782
  - mismatch repair testing, 782
  - Muir–Torre syndrome, 782
  - pathological classification, 782
  - primary eyelid carcinoma, 780
  - primary site(s), 780
  - primary tumor (T), 782
  - prognostic factors, stage grouping, 782
  - regional lymph nodes (N), 781, 783
  - registry data collection variables, 783
  - risk assessment models, 782
  - technetium-99m lymphoscintigraphy, 781
  - TNM classification, 780

F

- Fallopian tube
  - anatomic sites, 684
  - clinical classification, 685–686
  - histologic typing, 689
  - histology and grade, 687, 689
  - imaging, 685, 686
  - pathological classification, 686
  - primary site(s), 684
  - primary tumors (T), 690
  - prognostic factors, 686–687
  - regional lymph nodes, 684–685
  - risk assessment models, 687
  - surgical management, 687
- Familial myelodysplastic syndrome, 933
- Fast Imaging Employing Steady-state Acquisition (FIESTA), 860
- Fédération Internationale de Gynécologie et d'Obstétrique (FIGO), 631, 686–687
- Flexner–Wintersteiner rosettes, 823
- Fluid attenuated inversion recovery (FLAIR), 860
- Fluorescence in situ hybridization (FISH), 313
- Fluorodeoxyglucose positron emission tomography (^{18}F-FDG PET) scans, 541, 601, 742, 760, 914
- FNCLCC. *See* French Federation of Cancer Centers Sarcoma Group (FNCLCC)
- Follicular lymphoma, 852
  - FLIPI, 946
  - GELF criteria, 946
  - prognostic factors, stage grouping, 946
- Follicular Lymphoma International Prognostic Index (FLIPI), 851, 946
- Follicular thyroid cancer (FTC), 874
- French-American-British (FAB) system., 981
- French Federation of Cancer Centers Sarcoma Group (FNCLCC), 520, 533, 542
- Fuhrman grading system, 744

G

- Gallbladder
  - anatomic stage and prognostic groups, 308
  - clinical classification, 304–306
  - distant metastasis (M), 308
  - histologic grade (G), 308
  - histologic type, 308
  - ICD-O-3 topography codes, 303
  - imaging, 305
  - metastatic sites, 304
  - pathological classification, 306–307
  - primary site(s), 304, 305
  - primary tumor (T), 307
  - prognostic factors
    - histologic grade, 307
    - histologic subtype, 307
    - lymphovascular invasion, 307
    - resection margin and extent, 307
    - stage grouping, 307
  - regional lymph node (N), 304, 308
  - registry data collection variables, 308
  - risk assessment models, 307
  - staging system, 303
  - WHO tumors classification, 303–304
- Gastric cancer, 205, 208, 210, 213–216, 820
- "Gastrinoma triangle," 362
- Gastroenteropancreatic NETs (GEP-NETs), 376
- Gastroesophageal reflux disease (GERD), 362
- Gastrointestinal stromal tumors (GISTs), 228, 541
  - AJCC prognostic stage groups, 527–529
  - Carney triad/dyad, 525
  - clinical classification, 525–526
  - distant metastasis (M), 527
  - histologic grade (G), 528
  - histopathologic type, 528
  - ICD-O-3 topography codes, 523–524
  - imaging, 526
  - KIT/PDGFRA, 524
  - metastatic sites, 525

mitotic rate, 527
pathological classification
distant metastasis, 527
mitotic rate, 526–527
tumor size, 526
primary site(s), 525
primary tumor (T), 527
prognostic factors, 527
regional lymph node (N), 525, 527
registry data collection variables, 528
risk assessment models, 527
staging system, 523
survival data, 529
tyrosine kinase inhibitors, 524
WHO tumors classification, 524
Gastroscopy, 353
Gene expression profiling (GEP), 807
Genetic mutation analysis, 896
Genome-wide association studies (GWASs), 860
German Low Grade Lymphoma Study Group (GLSG), 945
Germ cell tumors, 683, 687, 689
Gerota's fascia, 746
Gestational trophoblastic disease (GTD). *See* Gestational trophoblastic neoplasms (GTNs)
Gestational trophoblastic neoplasms (GTNs)
AJCC prognostic stage groups, 695
clinical classification, 692–693
distant metastasis (M), 692, 694
FIGO staging, 694
histologic grade (G), 695
histopathologic type, 695
imaging, 693
lung metastasis, diagnosis, 693
nodal involvement, 692
pathological classification, 693
primary site(s), 692
primary tumor (T), 694, 696–697
prognostic factors, 693
prognostic scoring index, 694
regional lymph nodes (N), 692
risk assessment models, 694
risk factors
age, 694
antecedent pregnancy, 694
hCG level, 694
site of metastases, 694
tumor size, 694
risk score, 694
treatment indications, 693
trophoblastic tissue, 692
tumor staging, 693
GISTs. *See* Gastrointestinal stromal tumors (GISTs)
Gleason score, 716, 724
Gliomas
prognostic factors
ATRX, 865
diagnostic and prognostic embryonal tumor markers, 866
H3 K27M, 865–866
IDH1 and 2, 864–865
Ki-67, 865
MGMT, 864
1p/19q codeletion, 865
TP53, 865
registry data collection variables, 866
Glottis, 149, 155, 159–160
Goblet cell carcinoids, 238

H
Head and neck cancers
CSCC (*see* Cutaneous squamous cell carcinoma (CSCC))
HPV-OPSCC (*see* Human papillomavirus (HPV)-related squamous cell carcinoma of the oropharynx (HPV-OPSCC))
larynx (*see* Larynx)
lip and oral cavity (*see* Lip and oral cavity cancers)
major salivary glands (*see* Major salivary gland tumors)
mucosal melanoma (*see* Mucosal melanoma (MM))
nasal cavity and paranasal sinuses (*see* Nasal cavity and paranasal sinuses)
nasopharynx (*see* Nasopharyngeal carcinoma (NPC))
staging
clinical classification, 56–58
distant metastases (M), 61
pathological classification, 56, 58
primary tumor (T), 56–57
prognosis factors, 58
regional lymph node (N), 57–61
STS
adipocytic tumors, 500
AJCC prognostic stage groups, 504
chondro-osseous tumors, 501
clinical classification, 502–503
distant metastasis (M), 504
fibroblastic/myofibroblastic tumors, 501
fibrohistiocytic tumors, 501
FNCLCC histologic grade, 504
histologic grade (G), 499, 503, 504
histopathologic type, 505
ICD-O-3 topography codes, 500
imaging, 503
metastatic sites, 502
nerve sheath tumors, 501
new classification, 499
pathological classification, 503
pericytic (perivascular) tumors, 501
primary site(s), 502
primary tumor (T), 499, 503
prognostic factors, 503
regional lymph node (N), 499, 502, 504
registry data collection variables, 504
risk assessment models, 503
skeletal muscle tumors, 501
smooth muscle tumors, 501
staging system, 499
tumor staging, TNM categories of, 502
vascular tumors, soft tissue, 501
survival data, 61–64
Helicobacter pylori, 366, 413
Hematologic malignancies
acute leukemias of ambiguous lineage, 933
acute myeloid leukemia, 933
blastic plasmacytoid dendritic cell neoplasm, 933
familial myelodysplastic syndrome, 933
histiocytic and dendritic cell neoplasms, 935
Hodgkin lymphoma, 935
mastocytosis, 932
mature B-cell neoplasms, 934
mature T and NK neoplasms, 935
myelodysplastic syndromes/neoplasms, 932
myeloid/lymphoid neoplasms, 932
myeloproliferative neoplasms, 932
pathology, 931
precursor lymphoid neoplasms, 934
predisposition syndromes, 933
PTLDs, 935

Hepatocellular carcinoma (HCC), 288
High-grade appendiceal mucinous neoplasm (HAMN), 240
High-grade dysplasia, 240, 256
High-grade serous carcinoma (HGSC), 682
High-grade squamous intraepithelial lesion (HSIL), 276
High-power fields (HPF), 354, 378, 527
High-risk HPV (HR-HPV)-associated cancers, 56, 114
Hilar soft tissue *versus* spermatic cord invasion, 730–731
Histiocytic and dendritic cell neoplasms, 935
Histologic grade (G)
  abdominal and thoracic visceral organs, 520–521
  adenocarcinoma, 195-196, 198
  ampulla of Vater, 334
  appendiceal neuroendocrine tumors, 393
  bone cancer, 477
  carcinomas, appendix, 243
  cervix uteri, 654
  colon and rectal carcinoma, 269, 399–400
  CSCC, 179
  distal bile duct, 323
  duodenum and ampulla of Vater, 370
  exocrine pancreas, 345
  gallbladder, 307
  GIST, 528
  head and neck, 499, 504
  HPV-OPSCC, 120
  intrahepatic bile ducts, 299
  jejunum and ileum, NETs, 380
  larynx, 156
  lip and oral cavity cancers, 92
  liver, 291
  lung, 451
  major salivary gland tumors, 97
  MCC, 556
  MPM, 466
  mucosal melanoma, 168
  nasal cavity and paranasal sinuses, 143
  oropharynx, 130
  pancreatic neuroendocrine tumors, 416–417
  perihilar bile ducts, 315
  renel pelvis, 754–755
  retroperitoneal soft tissue sarcoma, 536
  small intestinal cancer, 236
  squamous cell carcinoma, 195, 198
  stomach, 212, 355–356
  STS,496
  thymic tumor, 428
  trunk and extremities, 511–512
  urethra, 772
  urinary bladder, 764–765
Hodgkin lymphoma (HL), 935
  advanced-stage HL, 368, 953
  Ann Arbor classification, 939
  A and B classification (symptoms), 941
  clinical classification, 942–943
  Cotswold system, 939
  early-stage HL, 951, 952
  extranodal/extralymphatic sites, 940
  factors, 952
  interim PET scan, 953–954
  IPS, 953
  Lugano classification, 940, 954
  lymph node region, 940–941
  nodal/lymphatic sites, 940
  nodal regions, 952
  organ involvement, 941–942
  pathological classification, 943
  pediatric
    AJCC prognostic stage groups, 964
    A and B classification (symptoms), 961
    prognostic factors, 964
  prognostic factors, stage grouping, 952
  registry data collection variables, 955
  risk assessment models, 954
  tumor bulk, 952
Homer Wright rosettes, 823
HSIL. *See* High-grade squamous intraepithelial lesion (HSIL)
Human chorionic gonadotropin (hCG), 687
Human epidermal growth factor receptor 2 (HER2), 616–617
Human papillomavirus (HPV), 34, 276, 653
Human papillomavirus (HPV)-related squamous cell carcinoma of the oropharynx (HPV-OPSCC), 56
  AJCC prognostic stage groups, 119–120
  clinical classification, 115–116
  distant metastasis (M), 120
  histologic grade (G), 120
  histopathologic type, 120
  HR-HPV, 113, 114
  ICD-O-3 topography codes, 113
  metastatic sites, 115
  pathological classification, 117–119
  primary sites, 115
  primary tumor (T), 120
  prognostic factors, 119
  regional lymph nodes (N), 115, 120
  registry data collection variables, 120
  risk assessment models, 119
  synonyms for, 114
  WHO classification, 119
  Zubrod/ECOG performance scale, 119
Hydrosalpinx, 686
Hyperparathyroidism–jaw tumor syndrome (HPT-JT), 905
Hypogastric lymph nodes, 642, 662, 672
  cervix uteri, 651
  penis, 707
  prostate, 717
Hypopharynx. *See also* Oropharynx
anatomical sites and subsites, 105
  clinical classification, 126
  distant metastases, 130
  primary sites, 125
  regional lymph nodes, 126, 127, 129
  T4a tumor, 129, 134
  T4b tumor, 129, 135
  T1 tumor, 129, 132, 133
  T2 tumor, 129, 133, 134
  T3 tumor, 129, 134

I

IASLC. *See* International Association for the Study of Lung Cancer (IASLC)
Iliac lymph nodes
  ureter, 758
  urethra, 769
  urinary bladder, 760
Inflammatory carcinoma, 598
Inguinal lymph nodes, 642
  prostate, 717
  urethra, 769

Interaortocaval lymph nodes
kidney, 740
testis, 729
Intermediate tumors, 846
Internal iliac lymph nodes, 642, 662, 672
cervix uteri, 650
fallopian tube, 684
ovary, 684
penis, 703
urethra, 761
Internal tandem duplication (ITD), 982
International Association for the Study of Lung Cancer (IASLC), 431, 462
International Classification of Diseases for Oncology (ICD-O) 3rd edition, 5
International Collaboration for Oropharyngeal Cancer Network for Staging (ICON-S), 115
International Germ Cell Consensus Classification Grouping (IGCCCG), 731
International Intraocular Retinoblastoma Classification (IIRC), 820
International Melanoma Pathology Study Group, 576
International Mesothelioma Interest Group (IMIG), 458
International Non-Hodgkin Lymphoma Prognostic Factors Project, 944
International Prognostic Index (IPI), 851, 944
International Prognostic Score (IPS), 952–953
International Staging System (ISS), 976
International Thymic Malignancies Interest Group (ITMIG), 427
International Union Against Cancer (UICC), 631
Intrahepatic bile ducts
AJCC prognostic stage groups, 300
cancer staging system, 295
clinical classification, 297, 298
distant metastasis (M), 300
extrahepatic adenocarcinomas, 296
histologic grade (G), 300
histopathologic type, 301
ICD-O-3 topography codes, 295
imaging, 297, 299
intrahepatic cholangiocarcinoma, 296
metastatic sites, 297
pathological classification, 299
pN classification, 299
primary hepatobiliary malignancy, 296
primary site(s), 296
primary tumor (T), 300
prognostic factors
clinical care, 299
nontumoral hepatic parenchymal fibrosis/cirrhosis, 299
primary sclerosing cholangitis, 300
risk assessment models, 300
serum carbohydrate antigen 19-9 level, 300
stage grouping, 299
pT classification, 299
radiologic imaging, 299
regional lymph node (N), 296, 297, 300
registry data collection variables, 300
WHO tumor classification, 295–296
Intramucosal adenocarcinoma (IMC), 240
Intramucosal carcinoma, 256
Ipsilateral lymph nodes, 595
Iris melanomas
clinical classification, 808
primary tumor (T), 812
Ishak scoring system, 296
Isocitrate dehydrogenase (IDH), 864–865

J

Japan Integrated Staging (JIS) system, 290
Jejunum and ileum
AJCC prognostic stage groups, 375, 380
carcinoid syndrome, 376
classification rules
CgA, 379
clinical classification, 377–378
echocardiogram, 379
imaging, 378
pathological classification, 378
plasma pancreastatin level, 379
plasma serotonin level, 379
plasma/urinary 5-HIAA level, 379
restaging, 378
distant metastasis (M), 380
histologic grade (G), 380
histopathologic type, 380
ICD-O-3 topography codes, 376
metastatic sites, 377
primary site(s), 228, 375, 376
primary tumor (T), 379
prognostic factors, 375
Ki-67 proliferative index, 378
mitotic count, 378–379
regional lymph node (N), 228, 375, 376, 380
registry data collection variables, 380
risk assessment models, 379
small intestine, 377
staging system, 375
survival data, 380–386
WHO tumors classification, 375

K

Kaplan–Meier method, 39, 41
Kidney
angiolymphatic invasion, 743
clinical classification, 741
coagulative necrosis, 743
CT and MR imaging, 741–742
Gerota's fascia, 746
histologic subtype, 742–743
histologic tumor necrosis, 744
histopathologic type, 744
integrated algorithms, 740
ipsilateral adrenal gland, 747
ISUP nucleolar grade, 743
LVI, 743
metastatic sites, 741
N1 disease, 747
pain and hematuria, 740
pathological specimen handling, 742
pathological staging, 742
perirenal/renal sinus fat, 746
primary site(s), 740
primary tumor (T), 743, 747
prognostic factors, stage grouping, 742
regional lymph nodes, 740–741
rhabdoid differentiation, 743
risk assessment models, 743
sarcomatoid differentiation, 743
tumor size, 745, 746
WHO, 743

L

Lacrimal gland carcinoma, 841
- AJCC prognostic stage groups, 837
- anatomic sites and regional lymph nodes, 836
- clinical classification, 835
- computed tomography (CT), 835
- distant metastasis (M), 837
- echo-planar diffusion-weighted MR imaging, 835
- histologic grade (g), 838
- histopathologic type, 838
- magnetic resonance (MR) imaging, 835
- metastatic site, 835
- pathological classification, 835
- primary site(s), 834–835
- primary tumor (T), 837
- prognostic factors, stage grouping, 837
- recommended factors, clinical care, 837
- regional lymph node (N), 835, 837
- registry data collection variables, 837
- risk assessment models, 837
- treatment strategies, 834

Larynx
- AJCC prognostic stage groups, 156
- clinical classification, 152
- distant metastasis, 152, 156
- glottis, 151, 155, 159–160
- histologic grade, 156
- histopathologic type, 156
- ICD-O-3 topography codes, 150
- pathological classification, 153
- primary sites, 155, 150–151
- prognostic factor, 153
- regional lymph nodes, 151–152, 155
- registry data collection variables, 156
- risk assessment models, 154
- subglottis, 151, 155, 160–161
- supraglottis, 151, 155, 157–159
- WHO classification, 150
- Zubrod/ECOG performance scale, 154

Leiomyosarcoma, 675–676, 695

Leukemia
- ALL (*See* Acute lymphoblastic leukemia (ALL))
- AML
  - AML NOS, 984
  - clinical classification, 980–981
  - laboratory features, 981
  - myelodysplasia-related changes, 984
  - prognostic factors, 984–985
  - recurrent genetic changes, 981–982, 984
  - t-AML, 984
- CLL, 994
- CML
  - clinical classification, 993
  - laboratory features, 993
  - prognostic factors, 993–994
  - risk assessment models, 994

Life table method, 39–41

Li-Fraumeni syndrome, 860

Lip and oral cavity cancers
- AJCC prognostic stage groups, 91
- biopsy, 82
- clinical/radiographic ENE, 83–84
- distant metastasis, 91
- exophytic, ulcerated and endophytic, 92
- histologic grade, 91
- histopathologic type, 91–92
- history and physical examination, 82
- ICD-O-3 topography codes, 80
- imaging, 84
- metastatic sites, 82
- pathological classification, 84–85
- primary sites, 79, 81
- primary tumor, 79, 90, 92–93
- prognostic factors
  - comorbidity, 88
  - depth of invasion, 85, 87
  - extranodal extension, 85, 86
  - lymphovascular invasion, 88
  - overall health, 88
  - perineural invasion, 88, 89
  - resection margin, 85
  - tobacco and alcohol abuse, 90
  - worst pattern of invasion, 85, 88
  - Zubrod/ECOG performance scale, 90
- regional lymph nodes, 79–80, 82, 90
- registry data collection variables, 91
- risk assessment models, 90
- survival data, 62
- WHO classification, 82

Liver cancer
- AJCC prognostic stage groups, 291
- cancer staging system, 287
- cirrhosis, 289
- clinical classification, 289
- distant metastasis (M), 291
- HCC, 288
- histologic grade (G), 291
- histopathologic type, 291–292
- ICD-O-3 topography codes, 287
- imaging, 289–290
- metastatic sites, 288–289
- pathological classification, 290
- primary site(s), 288
- primary tumor (T), 291
- prognostic factors
  - cirrhosis, 290
  - α-Fetoprotein (AFP), 291
  - fibrosis score, 290
  - MELD, 291
  - stage grouping, 290
- regional lymph node (N), 288, 291
- registry data collection variables, 291
- risk assessment models, 291
- survival data, 292
- TNM staging, 288
- WHO tumors classification, 287

Lobar atrophy, 314

Log-rank test, 43

Low-grade appendiceal mucinous neoplasm (LAMN), 239, 240

Luminal A-type tumors, 612–613

Lung cancer
- AJCC prognostic stage groups, 432, 448
- clinical classification
  - diffuse pneumonic-type adenocarcinoma, 439
  - extrathoracic metastatic disease, 437
  - IASLC database, 436
  - intrapulmonary metastases, 438, 445
  - intrathoracic metastases, 437
  - lymph node stations, 438
  - multifocal lung adenocarcinomas, 438–439
  - second primary tumors, 438, 445
- distant metastasis (M), 448

histologic grade (G), 450
histopathologic type, 450
ICD-O-3 topography codes, 432
imaging
chest X-rays, 440
complex investigations, 440
CT, 441
EBUS-FNA, 442
invasive procedures, 440
invasive staging, 442
medical history and physical examination, 440
MR imaging, 441
PET, 441, 442
pleural fluid, 442
posteroanterior and lateral chest radiography, 440
sputum cytology, 441–442
SUV_{max}, 441
metastatic sites, 433
pathological classification
AIS, 444
ground-glass/lepidic lung adenocarcinoma, 445
MIA, 444
pleural lavage cytology, 444
pneumonic-type adenocarcinoma, 445
primary tumor, 443
systematic nodal dissection, 443
UICC, 443
visceral pleural invasion, 443
primary site(s), 433
T1 tumor, 450
T2 tumor, 451
T3 tumor, 451, 453
T4 tumor, 452, 453
prognostic factors, 446–447
regional lymph node (N), 433, 448
registry data collection variables, 450
risk assessment models, 447
SEER Program, 40–41
staging system, 431
survival data, 449
TNM classification, 434
WHO tumors classification, 432
Lymphoid neoplasms, 931, 932, 934, 943, 960
Lymphovascular invasion (LVI), 29, 196, 227, 740, 743
colon and rectal carcinoma, 263, 265
lip and oral cavity, 88
penis, 706
vulva, 637
Lynch syndrome, 227, 782

M

MACIS system, 879
Magnetic resonance cholangiopancreatography (MRCP), 297, 313, 319, 329
Major salivary gland tumors
AJCC prognostic stage groups, 100
clinical classification, 97
distant metastases, 97, 100
histologic grade, 101
histopathologic type, 101
parotid, submandibular, and sublingual glands, 97, 107
pathological classification, 98
primary tumor, 96, 99
prognostic factors, 99, 98–99
regional lymph nodes, 95–97, 99
registry data collection variables, 100–101
risk assessment models, 99
WHO classification, 96
Zubrod/ECOG performance scale, 99
Malignant Bartholin gland tumors, vulva, 638
Malignant fibrous histiocytoma, 541
Malignant peripheral nerve sheath tumor (MPNST), 503, 533, 858, 868
Malignant pleural mesothelioma (MPM)
AJCC prognostic stage groups, 466
AJCC TNM definitions
distant metastasis (M), 466
primary tumor (T), 457, 465
regional lymph node (N), 457, 465
anatomy
metastatic sites, 463
primary site(s), 463
regional lymph nodes, 463
classification rules
clinical classification, 464
imaging, 464
histologic grade (G), 466
histopathologic type, 466
ICD-O-3 topography codes, 458
M categories, 461
N-category analyses, 460
pathological classification, 464–465
prognostic factors, 465
registry data collection variables, 466
risk assessment models, 465
second IASLC MPM database, 458–459
stage groupings, 461–462
staging system, 457
statistical methods, 459
strengths and limitations, 462
WHO tumors classification, 458
Malignant soft tissue tumors
adipocytic, 846
anatomic stage and prognostic groups, 845
extraskeletal mesenchymal chondrosarcoma, 846
fibroblastic, 846
intermediate, 846
nerve sheath tumors, 846
skeletal muscle tumors, 846
smooth muscle tumors, 846
tumors differentiation, 846
undifferentiated/unclassified sarcomas, 846
vascular, 846
Malignant sweat gland tumors, vulva, 638
Mammaprint®, 618
Mantle Cell International Prognostic Index (MIPI), 851, 945, 954
Mantle cell lymphoma (MCL), 945
Marginal zone lymphoma
ENMZL, 946–947
indolent CBCL, 968
nodal marginal zone lymphoma, 946
prognostic factors, stage grouping, 946
SMZL, 946–947
Mastocytosis, 932
Mature B-cell neoplasms, 934
Maxillary sinus
anatomical subsites, 138
T4a tumors, 142, 145
T4b tumors, 142, 145
T1 tumors, 142, 144
T2 tumors, 142, 144
T3 tumors, 142, 144

Medullary thyroid cancer (MTC)
anatomic stage and prognostic groups, 897
clinical classification, 893–894
histologic grade (G), 897
histopathologic type, 897
metastatic sites, 893
pathological classification, 894–895
posttherapy imaging, 894
pretherapy imaging, 894
primary site(s), 892
prognostic factors
biochemical parameters, 896
completeness of resection, 896
genetic mutation analysis, 896
lymph node number, 895
lymph node size, 895
metastatic focus, 895–896
sporadic/medullary MTC, 896
stage grouping, 895
regional lymph nodes, 892–893
registry data collection variables, 897
risk assessment models, 896
T4a and T4b tumors, 898
T1, T2 and T3 tumors, 897–898
Merkel cell carcinoma (MCC)
AJCC prognostic stage groups, 549, 556
AJCC TNM definitions, 556
anatomy
metastatic sites, 552
primary site(s), 551, 559–561
regional lymph nodes, 551
classification rules
clinical classification, 552
imaging, 552
pathological classification, 553
histologic grade (G), 556
histopathologic type, 556
ICD-O-3 topography codes, 550
immunohistochemistry, 550
prognostic factors
immunosuppression, 555
NCDB data, 554
primary tumor size, 554
regional nodal tumor burden, 554
stage grouping, 553
tumor thickness, 554
unknown primary tumor, 554
registry data collection variables, 556
risk assessment models, 555
staging system, 549
survival data, 557–559
WHO tumors classification, 550
Merkel cell polyomavirus (MCPyV), 50
Metachronous primary cancers, 28
Metastatic castration-resistant prostate cancer, 722–723
METAVIR score, 290
Microsatellite instability (MSI), 211
colon and rectal carcinoma, 263, 266
HNPCC, 266
prognostic and predictive factor, 251
Microscopic lymphovascular invasion (LVI), 29, 743
Minimally invasive adenocarcinoma (MIA), 440, 444, 445
Mismatch repair (MMR), 227, 255, 750, 782
Mixed lineage leukemia (MLL), 980, 982
Monoclonal B lymphocytosis (MBL), 947
Monoclonal gammopathy of undetermined significance (MGUS), 974, 975
MPM. *See* Malignant pleural mesothelioma (MPM)
MR elastography (MRe), 861
MTC. *See* Medullary thyroid cancer (MTC)
Mucinous adenocarcinoma, 238, 239, 242, 667
Mucosal melanoma (MM)
clinical classification, 165
histologic grade, 167
histopathologic type, 167
ICD-O-3 topography codes, 163–164
metastatic sites, 165
pathological classification, 165
primary sites, 164
primary tumor, 166, 168–169
prognostic factors, 165–166
regional lymph nodes, 164, 167
registry data collection variables, 167
risk assessment models, 166
survival data, 167
WHO classification, 165
Zubrod/ECOG performance scale, 166
Muir-Torre syndrome, 782, 783
Multiple endocrine neoplasia (MEN), 352, 892
Multiple endocrine neoplasia type 1 (MEN1), 352, 363, 365, 366, 368, 370, 409, 410, 415
Multiple myeloma
AJCC prognostic stage groups, 977
clinical classification
MGUS, 975
SMM, 975
solitary extramedullary plasmacytoma, 975–976
solitary plasmacytoma of bone, 975
WM, 975
CT imaging, 976
ISS, 976
MR imaging, 976
PET/CT imaging, 976
recommended factors, 976
registry data collection variables, 976, 977
risk assessment models, 977
RISS, 976
Multiple simultaneous ipsilateral primary carcinomas, 610–611
Mycosis fungoides (MF), 968
AJCC prognostic stage groups, 972
AJCC TNM, 969–971
histopathologic staging, 971
prognostic factors, 969
registry data collection variables, 971
Myelodysplastic syndromes (MDS), 933, 980, 984
Myeloproliferative neoplasms (MPN), 932, 984

N

Nasal cavity and paranasal sinuses
AJCC prognostic stage groups, 143
clinical classification, 139–140
distant metastasis, 156
ethmoid sinus, 138, 145–147
histologic grade, 143
histopathologic type, 144
ICD-O-3 topography codes, 138
maxillary sinus, 138, 144–145
metastatic sites, 139
pathological classification, 140

primary sites, 137, 138
primary tumor, 142
prognostic factor, 138, 141–142
regional lymph nodes, 137, 139, 142–143
registry data collection variables, 143
risk assessment models, 142
WHO classification, 138
Zubrod/ECOG performance scale, 141
Nasopharyngeal carcinoma (NPC)
AJCC prognostic stage groups, 103, 108
clinical classification, 106
distant metastasis, 106, 107
epithelial tumors, 103
ICD-O-3 topography codes, 104
infratemporal fossa, 104
management of, 104
pathological classification, 106
primary sites, 105
primary tumor, 103, 107
prognostic factors, 106–107
regional lymph nodes, 103, 105–106
risk assessment models, 107
survival data, 109–110
WHO classification, 104, 108, 109
Zubrod/ECOG performance scale, 107
National Cancer Database (NCDB), 62, 63, 76, 204, 213, 216, 222, 226, 241, 242, 244, 245, 280, 298, 462, 478–482, 550–554, 557–559, 592, 594, 615, , 880, 903, 912
National Comprehensive Cancer Network (NCCN), 5, 23, 164, 256, 266, 339, 367, 368, 376, 379, 392, 396, 398, 415, 593, 611, 704, 705, 716, 875, 876, 893, 896, 952, 953
Neuroendocrine tumors (NETs)
appendiceal NETs (*see* Appendiceal neuroendocrine tumors)
colon and rectum (*see* Colon and rectal carcinoma)
duodenum and ampulla of Vater (*see* Duodenum and ampulla of Vater)
gastric NETs, 352
jejunum and ileum (*see* Jejunum and ileum)
pancreas (*see* Pancreatic neuroendocrine tumors)
stomach (*see* Stomach)
Neurokinin A (NKA), 378, 397
Neuro-oncology
chemotherapy, 862–863
radiation therapy, 861–862
Non Hodgkin lymphoma (NHL)
Ann Arbor classification, 939
A and B classification (symptoms), 941
Cotswold system, 939
extranodal or extralymphatic sites, 939
Lugano classification, 940, 954
lymph node region, 940–941
nodal or lymphatic sites, 939
organ involvement, 941–942
pediatric
AJCC prognostic stage groups, 964
ALCLs, 961
Ann Arbor staging system, 962
clinical staging system, 962
follicular lymphomas, 961
INHLSS, 962
minimal detectable disease, 962
PET/CT and PET/MR imaging, 961
prognostic factors, 963–964
recommended staging studies, 962
serum lactate dehydrogenase, 961
sophisticated technology, 962
Nonmelanoma skin carcinoma (NMSC), 172, 173, 175, 176
North American Neuroendocrine Tumor Society (NANETS), 376, 396
NPC. *See* Nasopharyngeal carcinoma (NPC)

O

OALs. *See* Ocular adnexal lymphomas (OALs)
Obturator lymph nodes, 642, 672
cervix uteri, 650
Corpus Uteri, 672
fallopian tube, 685
ovary and primary peritoneal carcinoma, 684
penis, 703
prostate, 717
urinary bladder, 759
Octreoscan™ imaging, 353, 354, 378, 391, 398, 413
Ocular adnexal lymphomas (OALs)
anatomic sites and regional lymph nodes, 853
anatomic stage and prognostic groups, 852
Ann Arbor system, 850
clinical classification, 851
computed tomography (CT), 851
distant metastasis (M), 852
histologic grade (G), 852
histopathologic type, 852
metastatic site, 851
pathological classification, 851
positron emission tomography, 851
primary site(s), 850
primary tumor (T), 852
prognostic factors, stage grouping, 851
recommended factors, clinical care, 851
regional lymph nodes, 851, 852
registry data collection variables, 852
risk assessment models, 851–852
ultrasonography, 851
Ocular surface squamous neoplasia, 788, 789
Okuda staging system, 290
Omentectomy, 665, 666, 676
Oncotype Dx®, 590, 614, 617, 625
Ophthalmic Oncology Task Force (OOTF) International Survey, 820, 827
Oral cavity cancer. *See* Lip and oral cavity cancers
Orbital sarcoma
anatomic sites and regional lymph nodes, 844
A-scan ultrasonography, 843
B-scan echography, 843
clinical classification, 843
computed tomography (CT), 843
distant metastasis (M), 845
echo-planar diffusion-weighted MR imaging, 843
histologic grade (G)
FNCLCC histologic grade, 846
mitotic count, 846
tumor differentiation, 846
tumor necrosis, 846
histopathologic type, 846
magnetic resonance (MR) imaging, 843
metastatic site, 843
pathological classification, 843
primary site(s), 842
primary tumor (T), 845
prognostic factors, stage grouping, 845
recommended factors, clinical care, 845
regional lymph node (N), 843, 845
registry data collection variables, 845
risk assessment models, 845

Oropharynx
- anatomical sites and subsites, 83
- HPV-mediated carcinogenesis (*see* Human papillomavirus (HPV)-related squamous cell carcinoma of the oropharynx (HPV-OPSCC))
- p16-negative squamous carcinomas
  - AJCC prognostic stage groups, 130
  - clinical classification, 126
  - distant metastases, 126, 130
  - ENE, 127
  - histologic grade, 130
  - histopathologic type, 130
  - ICD-O-3 topography codes, 124
  - pathological classification, 127
  - primary sites, 123, 125
  - prognostic factor, 127–128
  - regional lymph nodes, 123–126, 128–129
  - registry data collection variables, 130
  - risk assessment models, 128
  - T4a tumor, 129, 131
  - T4b tumor, 129, 131
  - T1 tumors, 128, 130
  - T2 tumors, 128, 131
  - T3 tumors, 128, 131
  - WHO classification, 124
  - Zubrod/ECOG performance scale, 128

Osteosarcoma
- appendicular skeleton, 478
- pelvis, 479
- spine, 479

Ovary and primary peritoneal carcinoma
- AJCC prognostic stage groups, 688
- anatomic sites, 684
- BRCA mutation, 683
- clinical classification, 685
- distant metastasis (M), 688
- epithelial tumors, 689
- HGSCs, 683
- histologic grade (G), 689
- histologic typing, 689
- imaging, 685
- Müllerian differentiation, 683
- nonepithelial tumors, 689
- pathological classification, 686
- primary site(s), 684
- primary tumors (T), 688
- prognostic factors, 686–687
- putative tubal/peritoneal origin, 683–684
- regional lymph nodes, 684–685, 688
- reproducible histopathologic diagnosis, 683
- risk assessment models, 687
- surgical management, 687
- tumor staging, 685–686

P

Paget disease, 277, 604, 619

Pancreatic neuroendocrine tumors
- AJCC prognostic stage groups, 407, 416
- AJCC TNM definitions
  - distant metastasis (M), 416
  - primary tumor (T), 407, 415
  - regional lymph node (N), 416
- anatomy
  - metastatic sites, 412
  - primary site(s), 411–412
  - regional lymph nodes, 412
- classification rules
  - clinical classification, 412–413
  - imaging, 413
  - pathological classification, 413–414
  - restaging, 414
- clinical features of, 409
- ENETS staging system, 410
- histologic grade (G), 416–417
- histopathologic type, 417
- ICD-O-3 topography codes, 408
- MEN1, 409
- multifocal disease, 409–410
- prognostic factors
  - associated genetic syndrome, 415
  - CgA, 415
  - functionality, 415
  - Ki-67 labeling index, 414
  - mitotic count, 414
- registry data collection variables, 416
- risk assessment models, 415
- staging system, 407
- survival data, 417
- WHO tumors classification, 408

Papillary thyroid cancer (PTC), 874, 875, 882

Para-aortic lymph nodes, 642, 662, 672
- cervix uteri, 650
- fallopian tube, 685
- kidney, 740
- ovary and primary peritoneal carcinoma, 684

Paracaval lymph nodes
- kidney, 740
- renel pelvis, 750
- testis, 728
- ureter, 750

Paragangliomas (PGs). *See* Adrenal-neuroendocrine tumors

Parametrial lymph nodes, 642, 672
- cervix uteri, 650
- corpus uteri, 662

Paranasal sinuses and nasal cavity. *See* Nasal cavity and paranasal sinuses

Parasympathetic paragangliomas (PPGs), 920

Parathyroid carcinoma
- anatomic stage and prognostic groups, 908
- clinical classification, 905–906
- distant metastasis (M), 907
- histologic grade (G), 908
- histopathologic type, 908
- metastatic sites, 904
- pathological classification, 906
- preoperative imaging studies, 906
- primary site(s), 904
- primary tumor (T), 907
- prognostic factors, 906–907
- regional lymph node (N), 904, 907
- registry data collection variables, 908
- risk assessment models, 907
- survival data, 909

Parathyroid hormone (PTH), 410, 413, 905, 907

Pelvic lymph nodes, 642, 665
- fallopian tube, 685
- ovary and primary peritoneal carcinoma, 685
- prostate, 717
- ureter, 750
- urethra, 769

Penis
- AJCC prognostic stage groups, 707
- AJCC TNM, 706–707
- clinical staging, 704–705

distant metastases, 703
histologic grade, 707
histopathologic type, 707
HPV, 702
imaging, 704–705
pathological classification, 705–706
primary site(s), 702–703
primary tumors, 705, 708–709
prognostic factors, 706
regional lymph nodes, 703
risk assessment models, 706
Peptic ulcer disease (PUD), 362, 366
Perihilar bile ducts
AJCC prognostic stage groups, 311, 316
AJCC TNM definitions, 312, 315
anatomy
metastatic sites, 312
primary site(s), 312
regional lymph nodes, 312
classification rules
clinical classification, 313–314
imaging, 313–314
pathological classification, 314
histologic grade (G), 316
histopathologic type, 316
ICD-O-3 topography codes, 312
papillary histology, 316
primary sclerosing cholangitis, 316
registry data collection variables, 316
risk assessment models, 315
stage grouping, 314
staging system, 311
tumor location and extent, 316
WHO tumors classification, 312
Perineural invasion (), 176
colon and rectal carcinoma, 263, 265
lip and oral cavity, 88, 89
penis, 706
Peripheral T-cell lymphoma
AITL, 950
ALCL, 951
clinical risk assessment, 949
EATL, 950–951
factors, 949
prognostic factors, stage grouping, 949
PTCL NOS, 949–950
Peutz–Jeghers syndrome, 227
Pheochromocytomas (PHs). *See* Adrenal-neuroendocrine tumors
"Philadelphia (Ph) chromosome," 980, 990, 993
Plasma cell disorders
AJCC prognostic stage groups, 977
clinical classification
MGUS, 975
SMM, 975
solitary extramedullary plasmacytoma, 975–976
solitary plasmacytoma of bone, 975
WM, 975
CT imaging, 976
ISS, 976
MR imaging, 976
PET/CT imaging, 976
recommended factors, 976
registry data collection variables, 976, 977
risk assessment models, 976–977
RISS, 976
Posttransplant lymphoproliferative disorders (PTLDs), 935
PPI. *See* Proton-pump inhibitor (PPI)
Primary acquired melanosis (PAM), 796
Primary cutaneous lymphomas
B-cell lymphomas
AJCC prognostic stage groups, 972
AJCC TNM, 971–972
prognostic factors, 969
CD30+ lymphoproliferative disorders, 968
follicle center cell lymphoma, 968
large B-cell lymphoma, leg, 968–969
marginal zone lymphoma, 968
mycosis fungoides (MF), 968
AJCC prognostic stage groups, 971
AJCC TNM, 969–971
histopathologic staging, 971
prognostic factors, 969
registry data collection variables, 971
Sézary syndrome (SS), 968
AJCC prognostic stage groups, 971
AJCC TNM, 969–971
histopathologic staging, 971
prognostic factors, 969
registry data collection variables, 971
T-cell lymphomas, 969
AJCC TNM, 971–972
prognostic factors, 969
Primary tumor (T), 9
abdominal and thoracic visceral organs, 517, 520
ACC, 915
adenocarcinoma, 197
adrenal-neuroendocrine tumors, 923
ampulla of Vater, 327, 333
appendiceal adenocarcinomas, 237, 243, 245–247
appendiceal neuroendocrine tumors, 392
bone cancer, 471, 476–477
breast
categorisation, 619
clinical and pathological, 619
microinvasive carcinoma, 604–605
pathological tumor size (pT), 603–604
posttreatment (ypT) size, 604
Tis classification, 604
cervix uteri, 653
choroidal and ciliary body melanomas, 812
clinical T (cT), 723
colon and rectal carcinoma, 256–260, 268
colonic and rectal neuroendocrine tumors, 399, 402–404
conjunctival carcinoma, 790
conjunctival melanoma, 799
CSCC, 178
distal bile duct, 317, 323
duodenum and ampulla of Vater, 361, 369
exocrine pancreas, 337, 344
extent of invasion of, 923
eyelid carcinoma, 782–783
fallopian tube, 689
gallbladder, 303, 307
GIST, 527
GTNs, 694, 696–697
head and neck, 56–57, 503, 511
HPV-OPSCC, 116–117, 120
hypopharynx, 126–127, 132–135
intrahepatic bile ducts, 295, 300
iris melanomas, 812
jejunum and ileum, NETs, 379
kidney, 743, 747
lacrimal gland carcinomas, 837
larynx, 155

Primary tumor (T) (*cont.*)
- lip and oral cavity, 79, 90, 92–93
- liver, 287, 291
- location, 922
- lung, 431–432, 447
- major salivary gland, 96, 99
- MCC, 555–556
- MF, 969
- MPM, 457, 465–467
- MTC, 896–897
- mucosal melanoma, 166, 168–169
- nasal cavity and paranasal sinuses, 142
- nasopharyngeal carcinoma, 103, 105, 107
- ocular adnexal lymphomas, 852
- orbital sarcoma, 845
- oropharynx, 126–129, 131, 132
- ovary and primary peritoneal carcinoma, 688
- pancreatic neuroendocrine tumors, 407, 415
- parathyroid, 907
- pathological classification, breast, 603–605
- penis, 706–707
- perihilar bile ducts, 311, 315
- primary cutaneous B-Cell, 971
- prostate, 723–724
- renel pelvis and ureter, 752
- retinoblastoma, 825
- retroperitoneal soft tissue sarcoma, 531, 535–536
- size, 922
- small intestinal cancer, 221, 227–229
- squamous cell carcinoma, 197
- stomach, 208, 209, 211, 217–219
- stomach, NETs, 355–358
- STS, 490
- testis, 732, 734, 735
- thymic tumor, 428
- thyroid-differentiated and anaplastic carcinoma, 883
- trunk and extremities, STS, 507
- urethra, 774, 775
- urinary bladder, 761
- vagina, 644
- vulva, 636, 638–640

Prognostication models
- AJCC PMC
  - checklist, 51
  - evaluation process, 48
  - exclusion criteria, 51
  - inclusion criteria, 49–51
  - prediction model, 48–49
  - risk calculator/model, 48, 49
  - TRIPOD, 51
- multivariable statistical regression models, 48

Prognostication tools
- breast cancer, 47, 618
- colorectal cancer, 47, 267, 268
- lung cancer, 47
- melanoma, 47
- prostate cancer, 47, 722
- search strategy for_, 48

Prognostic scoring index, 695

Prostate cancer
- AJCC prognostic stage groups, 724
- AJCC quality criteria, 723
- anatomic stage/prognostic groups, 716–718
- clinical classification, 718–719
- distant lymph nodes, 718
- distant metastasis (M), 724
- histologic grade, 718
- histologic type, 722
- histopathologic type, 725
- imaging, 719
- metastatic sites, 717
- pathological T (pT) category, 720
- primary site, 716–717
- primary tumor (T), 723–724
- prognostic factors, 722
- pT2 disease, 720
- pT3 disease, 720–721
- pT4 disease, 721
- regional lymph nodes, 717
- risk assessment models, 722–723
- surgical margin status, 721

Prostate-specific antigen (PSA), 716, 722, 724

Proton-pump inhibitor (PPI), 352, 366, 378, 397, 413

Pseudomyxoma peritonei, 237, 239

R

Radiation Therapy Oncology Group (RTOG), 277, 280

Radiology Society of North America (RSNA), 6, 36, 914, 921

Rai staging system, 948, 955

Rectal carcinoma. *See* Colon and rectal carcinoma

Regional lymph node (N), 9, 744
- abdominal and thoracic visceral organs, 520
- ACC, 915, 913, 914
- adenocarcinoma, 203
- adrenal-neuroendocrine tumors, 924
- ampulla of Vater, 329
- anal cancer, 279
- anterior cervical, 835
- appendiceal adenocarcinomas, 239, 243, 248, 249
- appendiceal neuroendocrine tumors, 390
- bone cancer, 472
- breast
  - clinical (cN), 619
  - isolated tumor cell clusters, 606
  - macrometastases, 605–606
  - micrometastases, 606
  - pathological (pN), 619–620
  - pathological classification, breast, 606–609
  - pathological nodal categories, 607
- cervical lymph node, 67, 71–72, 76, 808, 843
- cervix Uteri, 654
- clinical N (cN), 707
- colon and rectal carcinoma, 252, 255, 259, 261, 268–270
- colonic and rectal neuroendocrine tumors,
- conjunctival carcinoma, 789
- conjunctival melanoma, 800
- CSCC, 173, 179
- definition, 845
- distal bile duct, 319
- duodenum and ampulla of Vater, 365
- exocrine pancreas, 338
- eyelid carcinoma, 781, 784
- fallopian tube, 684
- gallbladder, 305
- GIST, 525
- GTNs, 692
- head and neck, 57–61, 499, 504
- HPV-OPSCC, 115, 119
- hypopharynx, 126, 129–130
- intrahepatic bile ducts, 298
- intraparotid, 835, , 843
- jejunum and ileum, NETs, 375, 380
- kidney, 740–741

lacrimal gland carcinomas, 837
larynx, 150–152, 155–156
lip and oral cavity, 79–82, 92
liver, 288
lung, 434
major salivary glands, 95, 96, 100
MCC, 551–552
MF, 971
MPM, 459, 465
MTC, 897
mucosal melanoma, 164, 167
nasal cavity and paranasal sinuses, 137, 139–140, 142–143
nasopharyngeal carcinoma, 103, 105
in neck, 904–905
ocular adnexal lymphomas, 852, 853
oropharynx, 123–125, 129–130
ovary and primary peritoneal carcinoma, 684–685, 689
pancreatic neuroendocrine tumors, 416
parathyroid, 908
pathological N (pN), 707
penis, 703–706
perihilar bile ducts, 313
preauricular (parotid), 808, 835, 843
primary cutaneous B-cell, 995
prognostic significance, 905, 921
prostate, 717–718, 724
renel pelvis, 750
renel pelvis and ureter, 750–751
retinoblastoma, 825
retroperitoneal soft tissue sarcoma, 536
small intestinal cancer, 222–224, 227
squamous cell carcinoma, 197
stomach, 203, 206–210, 352, 353
STS, 490
submandibular, 808, 835, 843
superficial, 835, 843
testis, 729, 733
thymic tumor, 428
thyroid-differentiated and anaplastic carcinoma, 883
trunk and extremities, STS, 512
urethra, 769
urinary bladder, 759
uveal melanoma, 813
vagina, 645
vulva, 634, 637, 639, 640

Renel pelvis and ureter
AJCC prognostic stage groups, 754
AJCC TNM, 754
clinical classification, 750–751
CT and MR imaging, 752
histologic distribution, 750
histologic grade (G), 754
histopathologic type, 754–755
imaging, 751–753
lymphovascular invasion, 753
metastatic disease, 752
metastatic sites, 751
pathological staging, 753
primary site(s), 750
primary tumor, 751, 752
prognostic factors, stage grouping, 753
regional lymph nodes, 750
registry data collection variables, 754
risk assessment models, 754
urothelial carcinoma, 750

Retinoblastoma
anatomic sites and regional lymph nodes, 829
anatomic stage and prognostic groups, 826
clinical classification
fundus fluorescein angiography, 822
MR imaging and CT, 822–823
ocular ultrasonography, 822
subretinal seeds, 821
ultrasound, 822
vitreous seeds, 821
clinical classification (cTNM)
distant metastasis (M), 825
heritable trait (H), 825
primary tumor (cT), 825
regional lymph node (cN), 825
enucleated retinoblastoma eye, 824
histologic grade (G), 826
histopathologic type, 826
Knudson's two-hit hypothesis, 820–821
metastatic sites, 821
pathological classification, 823–824
distant metastasis (M), 825
primary tumor (pT), 825
regional lymph node (pN), 826
primary site(s), 821
prognostic factors, stage grouping, 824
regional lymph nodes, 821
registry data collection variables, 826
risk assessment models, 824
staging diagram, 828
survival data, 827

Retroperitoneal soft tissue sarcoma
AJCC prognostic stage groups, 536
AJCC TNM definitions
distant metastasis (M), 536
histologic grade (G), 536
primary tumor (T), 531, 535
regional lymph node (N), 539
classification rules
clinical classification, 533–534
imaging, 534
pathological classification, 534
histologic grade (G), 536
histopathologic type, 536
ICD-O-3 topography codes, 532
metastatic sites, 533
primary site(s), 533
prognostic factors, 534–535
regional lymph nodes, 533
registry data collection variables, 536
risk assessment models, 531, 535
staging system, 531
TNM staging, 532
WHO tumors classification, 532

Reverse transcriptase-polymerase chain reaction (RT-PCR), 593, 947, 987
Revised International Staging System (RISS), 976, 977
Rotter's nodes, 595

S

Salivary gland cancers. *See* Major salivary gland tumors
Scarff–Bloom–Richardson (SBR) system, 611, 617
SCCs. *See* Squamous cell carcinomas (SCCs)
Scrotal ultrasound, 729–730
SEER. *See* Surveillance Epidemiology and End Results (SEER)

Sentinel lymph node biopsy (SLNB), 551–555
Serum tumor markers, 729–731, 733
Sex–cord stromal tumors, 689
Sézary syndrome (SS), 968
  AJCC prognostic stage groups, 971
  AJCC TNM, 969–971
  histopathologic staging, 971
  prognostic factors, 969
  registry data collection variables, 971
Skeletal muscle tumors, 846
Skin melanoma
  AJCC prognostic stage groups, 564, 578
  anatomy
    metastatic sites, 564, 567
    primary site(s), 564, 566
    regional lymph nodes, 564, 566–567
  anti–CTLA-4 and anti–PD-1, 566
  armamentarium, 565
  BRAF and BRAF/MEK, 566
  Breslow thickness, 567–568, 571
  clinical classification, 567
  distant metastasis, 569–570, 573
  epidermal components, 569
  histologic type, 579
  ICD-O-3 topography codes, 565
  imaging, 570–571
  immunotherapy, 565
  indeterminate melanomas, 568
  LDH level, 566
  metastatic melanoma, 569
  microsatellites, 569, 572
  molecularly targeted therapy, 565
  multiple primary melanomas, 568
  nonnodal locoregional metastases, 573
  pathological classification, 571
  primary tumor ulceration, 568–569, 571–572
  prognostic factors
    Clark's level of invasion, 574–575
    distant metastases, number of, 576
    extranodal extension, 576
    lymphovascular invasion, 575
    neurotropism, 575
    primary tumor mitotic rate, 574
    sentinel nodes, 575–576
    serum LDH, 574
    stage grouping, 573
    TILs, 575
  regional lymph node metastasis
    clinically occult *vs.* clinically detected, 572–573
    ENE, 573
  registry data collection variables, 578–579
  risk assessment models, 576
  SLN biopsy, 565
  staging system, 563
  survival data, 579
  TNM definitions, 577
  WHO tumors classification, 565
SLL. *See* Small lymphocytic leukemia (SLL)
Small intestinal cancer
  AJCC prognostic stage groups, 221,
  clinical classification, 224–226
  distant metastasis, 228
  histologic grade, 228
  histopathologic type, 228
  ICD-O-3 topography codes, 222
  incidence of, 222
  metastatic sites, 223
  pathological classification, 226
  primary sites, 222–223
  primary tumor, 221, 227–228
  prognostic factor, 226–227
  regional lymph nodes, 221, 223, 224, 228
  registry data collection variables, 228
  risk assessment models, 227
  small bowel adenocarcinoma, 22
  survival data, 230–232
  WHO classification, 22
Small lymphocytic leukemia (SLL)
  Binet staging system, 947, 948
  CIRS, 949
  clinical classification, 947
  del(11q)/ ATM mutation, 948–949
  IGHV mutation status, 948
  MBL, 947
  prognostic factors, 948
  Rai staging system, 947, 948
  recommended factors, 948
Smoldering multiple myeloma (SMM), 974
Soft tissue sarcoma (STS)
  AJCC TNM definitions
    primary tumor (T), 490
    regional lymph node (N), 490
  anatomy
    metastatic sites, 493, 541–542
    primary site(s), 492, 541
    regional lymph nodes, 492, 541
  aneuploid sarcomas, 541
  areas, 490, 540
  classification rules
    clinical classification, 493, 542–544
    imaging, 495, 542
    pathological classification, 495–496, 542
  depth, 493
  FNCLCC histologic grade, 494–495
  GIST, 492, 541
  grade, 493–494
  head and neck sarcoma staging system, 490
  histologic grade (G), 493, 544–545
  histopathologic type, 493, 545
  ICD-O-3 topography codes, 490, 540
  mitotic count, 494
  nodal disease, 493
  prognostic factors, 496, 542
  proposed risk categories, 495
  registry data collection variables, 496, 542
  retroperitoneal sarcoma staging system, 490
  risk assessment models, 544
  site groups, 492
  staging system, 489, 539
  tumor
    definition of, 493
    differentiation, 494
    staging, TNM categories of, 493
  tumor assessment, genetic techniques, 541
  tumor necrosis, 494
  visceral sarcoma staging system, 490
  WHO tumors classification, 540
    adipocytic tumors, 491
    chondro-osseous tumors, 491
    fibroblastic/myofibroblastic tumors, 491

fibrohistiocytic tumors, 491
gastrointestinal stromal tumors, 491
nerve sheath tumors, 491
pericytic (perivascular) tumors, 491
skeletal muscle tumors, 491
smooth muscle tumors, 491
unncertain differentiation, tumors of, 491
unndifferentiated/unclassified sarcomas, 491
vascular tumors, soft tissue, 491
Spindle cell melanoma, 800
Splenic marginal zone lymphoma (SMZL), 946–947
Squamous cell carcinoma *in situ* (SCIS), 444
Squamous cell carcinomas (SCCs)
AJCC prognostic stage groups, 185, 197
anal cancer, 276–277
distant metastasis, 197
histologic grade, 195, 197
histopathologic cell type, 195
location, 195, 197
primary sites, 185
primary tumor, 197
regional lymph nodes, 197
registry data collection variables, 199
vulva, 637
Staging forms
abdominal and thoracic visceral organs, 520
adenocarcinoma, 185, 198
adrenal cortical carcinoma, 911
adrenal–neuroendocrine tumors, 924
ampulla of Vater, 333
appendiceal neuroendocrine tumors, 389, 392
bone cancer, 471, 504
breast, 611
carcinomas, 238, 243
cervical lymph node, 75
colon and rectal carcinoma, 269
colonic and rectal neuroendocrine tumors, 399
conjunctival carcinoma, 790
conjunctival melanoma, 800
CSCC, 179
distal bile duct, 323
duodenum and ampulla of Vater, 361, 369
exocrine pancreas, 344
eyelid carcinoma, 782
GIST, 527–528
head and neck, STS, 504
Hodgkin and non-Hodgkin lymphomas, 954, 964
HPV-OPSCC, 120
intrahepatic bile ducts, 300
jejunum and ileum, NETs, 375, 380
kidney, 740
lacrimal gland carcinoma, 837
larynx, 156
lip and oral cavity, 91
liver cancer, 291
major salivary glands, 100
MCC, 549, 556
MPM, 466
multiple myeloma and plasma cell disorders, 977
nasal cavity and paranasal sinuses, 143
nasopharyngeal carcinoma, 103, 108
NETs, 351, 355
ocular adnexal lymphoma, 852
orbital sarcoma, 845
oropharynx, 130
ovary and primary peritoneal carcinoma, 685
parathyroid, 908
perihilar bile ducts, 311, 316
primary cutaneous lymphomas, 972
prostate, 724
renel pelvis and ureter, 751, 752
retinoblastoma, 826
retroperitoneal soft tissue sarcoma, 536
skin melanoma, 564, 578
small intestinal cancer, 221, 228
squamous cell carcinoma, 185, 197
stomach, 203, 211
thymic tumor, 428
thyroid–differentiated and anaplastic carcinoma, 883
thyroid-medullary, 897
TNM system, 7, 10, 25–26
trunk and extremities, STS, 507, 511
urethra, 769, 770
uveal melanoma, 813
vagina, 643–644
Stomach
AJCC prognostic stage groups, 203, 211–212
AJCC TNM definition, 211
clinical classification, 206–209
histologic grade, 212
histopathologic type, 212
ICD-O-3 topography codes, 204
metastatic sites, 206
NETs, 352
AJCC prognostic stage groups, 351, 355
AJCC TNM definitions, 354–358
clinical classification, 352–353
histologic grade (G), 355–356
histologic type, 356
ICD-O-3 topography codes, 351
imaging, 353–354
metastatic sites, 352
pathological classification, 354
primary site(s), 352
prognostic factors, 354
regional lymph nodes, 352, 353
registry data collection variables, 355
restaging, 354
risk assessment models, 354–355
staging system, 351
survival data, 356
WHO tumors classification, 351
pathological classification, 209–210
post-neoadjuvant therapy classification, 210
primary sites, 203, 205, 206
prognostic factors, 210
regional lymph nodes, 203, 205–206
registry data collection variables, 212
risk assessment models, 211
survival data, 213–216
T1a tumor, 217
T1b tumor, 217
T2 tumor, 217
T3 tumor, 217, 218
T4a tumor, 218, 219
T4b tumor, 219
WHO classification, 204
Struma ovarii, 875
STS. *See* Soft tissue sarcoma (STS)
Subglottis, 155, 159–161

Succinate dehydrogenase (SDH), 524, 525, 744
Supraglottis, 155, 158–159
Surveillance Epidemiology and End Results (SEER), 277, 338, 352, 364, 376, 396, 408, 643
  breast cancer patients, 40–42
  lung cancer patients, 40
  small intestinal cancer, 227
Sympathetic PGs (SPGs), 919
Synchronous primary cancers, 27–28

T

T-cell (non-MF/SS) lymphoma
  AJCC TNM, 971–972
  prognostic factors, 969
Technetium scintigraphy, 474
Testis
  AJCC prognostic stage groups, 733
  clinical classification, 729
  clinical serum markers, 730
  epididymal invasion, 731
  grading, 731
  hilar soft tissue *versus* spermatic cord invasion, 731
  histopathologic type, 733
  imaging, 729–730
  lymphovascular invasion, 731
  metastatic sites, 729
  pathological classification, 730
  primary site, 728
  prognostic factors, 731–732
  regional lymph nodes, 729, 732, 733
  rete testis invasion, 731
  risk assessment models, 732
  scrotal ultrasound, 729
  subclassification of pT1, 730
The Cancer Genome Atlas (TCGA), 31, 863
Therapy-related AML (t-AML), 981
Thymic tumor
  AJCC prognostic stage groups, 428
  AJCC TNM definitions
    distant metastasis (M), 428
    primary tumor (T), 428
    regional lymph node (N), 428
  anatomy
    metastatic sites, 425
    primary site(s), 424
    regional lymph nodes, 424–425
  classification rules
    clinical classification, 425–426
    imaging, 426
    pathological classification, 426–427
  histologic grade (G), 428
  histopathologic type, 428
  ICD-O-3 topography codes, 423
  prognostic factors, 427
  registry data collection variables, 428
  risk assessment models, 428
  staging system, 423
  survival data, 429
  WHO tumors classification, 423
Thyroglossal duct cysts, 875
Thyroid-differentiated and anaplastic carcinoma
  anatomic stage and prognostic groups, 883
  clinical classification, 877
  distant metastasis (M), 883
  histologic grade (G), 884
  histolopathologic type, 884
  metastatic sites, 876
  pathological classification, 878–879
  posttherapy imaging, 878
  pretherapy imaging, 877–878
  primary site(s), 876
  primary tumor (T), 883
  prognostic factors
    age at diagnosis, 879, 881–882
    completeness of resection, 882
    extranodal extension, 882
    extrathyroidal extension, 879–882
    histologic features, 882
    histologies, 879
    lymph node, 880–882
    postoperative serum thyroglobulin, 883
    vascular invasion, 882
  regional lymph node (N), 883, 876
  registry data collection variables, 884
  risk assessment models, 883
  struma ovarii, 875
  survival data, 885–886
  T4a and T4b, 887
  thyroglossal duct cysts, 875
  T1, T2 and T3, 886–887
TILs. *See* Tumor-infiltrating lymphocytes (TILs)
TNM staging system. *See* Tumor node metastasis (TNM) system
Total mesorectal excision (TME), 260, 261
Transparent reporting of a multivariable prediction model for individual prognosis or diagnosis (TRIPOD), 51
Transrectal ultrasound (TRUS) guided biopsy, 716
Transurethral resection of the prostate (TURP), 716
Trial Assigning Individualized Options for Treatment (Rx) (TAILORx) trial, 593
Trunk and extremities, STS
  AJCC prognostic stage groups, 507, 512
  AJCC TNM definitions
    distant metastasis (M), 511
    grade (G), 511
    primary tumor (T), 507
    regional lymph node (N), 511
  anatomy
    metastatic sites, 509
    primary site(s), 509
    regional lymph nodes, 508
  classification rules
    clinical classification, 509
    grade, 509–510
    imaging, 510
    nodal disease, 509
    tumor staging, TNM categories of, 509
  histologic grade (G), 512
  histopathologic type, 512
  ICD-O-3 topography codes, 507
  prognostic factors
    FNCLCC grade, 511
    neurovascular and bone invasion, 511
    validation, 511
  registry data collection variables, 512
  risk assessment models, 511
  staging system, 507
  survival data, 512–514
  WHO tumors classification
    adipocytic tumors, 508
    chondro-osseous tumors, 508
    fibroblastic/myofibroblastic tumors, 508
    fibrohistiocytic tumors, 508

nerve sheath tumors, 508
pericytic (perivascular) tumors, 508
skeletal muscle tumors, 508
smooth muscle tumors, 508
vascular tumors, soft tissue, 508
Tumor-infiltrating lymphocytes (TILs), 575
Tumor node metastasis (TNM) system
AJCC prognostic stage groups, 7, 10, 25–26
anatomic staging and nonanatomic factors, 5
autopsy classification, 7, 13, 24
clinical classification, 6, 12–17
distant metastasis, 9
general rules, 6, 10–12
histopathologic grade, 28–29
histopathologic type, 28
lymphovascular invasion, 29
managing physician, role of, 4
multiple primary tumors, 27–28
N suffixes, 26, 27
pathologic classification, 6, 12, 18–22
philosophy of, 3–4
posttherapy/post-neoadjuvant therapy, 6, 13, 23–24
primary tumor, 9
prognostic factor categories, 7–9
prognostic stage group tables, 8
recurrence/retreatment classification, 6, 13, 24
regional lymph nodes, 9
related publications, 4–5
residual tumor and surgical margins, 29–30
response to neoadjuvant therapy, 30
stage groupings, 10
stage recording, 7–8
staging nomenclature, comprehensive analysis of, 4
staging windows, 6
T, N and M, definition, 7
unknown primary tumor, 28
unknown X designation, 9–10
Tumor-specific mesorectal excision (TSME), 260
Turcot syndrome, 860

U
Ultrasound biomicroscopy (UBM), 789
Ultraviolet (UV) radiation, 171, 550, 565, 796
Union for International Cancer Control (UICC), 3, 4, 10, 32, 55, 104, 124, 164, 410, 443, 458, 879
Upper thoracic esophageal cancers, 187–189
Urethral carcinoma
clinical care, 771
clinical classification, 769–770
corpus spongiosum, 774
distant metastasis (M), 772
female, 770, 771
histologic grade (G), 772
histopathologic type, 772–773
male and female urethra, 768
male penile and female, 771
metastatic sites, 769
pathological classification, 770–771
penile, 770
primary site(s), 768–769
primary tumor (T), 774
prognostic factors, stage grouping, 771
prognostic stage groups, 772
prostatic, 771
pTa disease, 771
regional lymph nodes, 769, 772
registry data collection variables, 772
risk assessment models, 771
WHO/ISUP, 771
Urinary bladder
AJCC criteria, 763
anatomic stage/prognostic group, 758
clinical and pathological staging, 758
clinical classification, 759
extranodal extension, 763
histologic cell types, 758
histologic grade (G), 764–765
histopathologic subtypes, 763
lymphovascular invasion, 763
margin status, 763
metastatic sites, 759
microscopic evaluation, 761
MR imaging and CT, 759–760
non-invasive papillary lesions, 758
pathological staging, 760–762
primary site(s), 758–759
primary tumor assessment, 759
prognostic factors, stage grouping, 762
pT1 categorization, 763
regional lymph nodes, 758–759
registry data collection variables, 764
risk assessment models, 763
total number of lymph nodes, 763
tumor involvement, 763
urothelial cancer carcinogenesis, 758
urothelial carcinoma *in situ*, 762–763
Uveal melanoma
anatomic sites and regional lymph nodes, 815
anatomic stage and prognostic groups, 813
BAP1 gene, 807
classification, 806
distant metastasis (M), 813
histologic grade (G), 813
histopathologic type, 813
iris melanomas, 808
metastases, 809–810
metastatic sites, 808
occurence, 806
pathological classification, 810
primary site(s), 807
primary tumor (T), 812
prognostic biopsies, 806
prognostic factors
cell type, 811
chromosomal analysis, 811
extraocular extension, 811
extravascular matrix loops and networks, 811
GEP, 811
location, 811
microvascular density, 811
mitotic count, 811
size, 810–811
stage grouping, 810
tumor-infiltrating macrophages, 812
regional lymph node (N), 807–808, 812–813
registry data collection variables, 813
risk assessment models, 812
staging diagram, 814
survival rates, 807

V

Vaginal cancer
- AJCC TNM, 644
- anatomic sites, 642
- clinical classification, 643
- cross-sectional imaging, 643
- histologic grade (G), 645
- histopathologic type, 645
- magnetic resonance (MR) imaging, 643
- metastatic sites, 642
- pathological classification, 643
- primary site(s), 642
- primary tumor, 646
- prognostic factors, 643–644
- regional lymph nodes, 642
- risk assessment models, 644
- squamous cell carcinoma, 645
- tumor staging, 643

Von Hippel–Lindau syndrome, 364, 409
von Recklinghausen's disease, 409
Vulvar cancer
- classification system, 634
- clinical classification, 634–635
- histologic grade (G), 637
- histopathologic type, 637–638
- imaging, 635
- metastatic sites, 634
- nodal metastases, 640
- and perineum lesions, 634
- primary site(s), 634
- primary tumors, 638–640
- prognostic factors, 636
- regional lymph nodes, 634
- risk assessment models, 636
- surgical/pathological staging, 635
- TNM components of tumor staging, 635

W

Waldenström's macroglobulinemia (WM), 975
Whipple procedure, 410
World Health Organization (WHO), 5, 281, 354, 363, 379, 391, 398, 408, 432, 579, 981
- abdominal and thoracic visceral organs, 518–519
- ampulla of Vater, 328
- anal cancer, 276
- appendiceal adenocarcinomas, 238
- appendiceal neuroendocrine tumors, 390
- bone cancer, 472
- cervical lymph node metastasis, 69
- colon and rectal carcinoma, 252
- colonic and rectal neuroendocrine tumors, 395
- CSCC, 171–172
- distal bile duct, 317, 318
- duodenum and ampulla of Vater, 362
- esophagus and esophagogastric junction, 186
- exocrine pancreas, 338
- gallbladder, 304
- GIST, 524
- head and neck, STS, 500–501
- HPV-OPSCC, 114
- intrahepatic bile ducts, 298
- jejunum and ileum, NETs, 376
- larynx, 150
- liver, 287
- lung, 432
- major salivary gland tumors, 96
- MCC, 550
- MPM, 458
- mucosal melanoma, 164
- nasal cavity and paranasal sinuses, 14
- nasopharyngeal carcinoma, 104, 108, 109
- oropharynx, 124
- pancreatic neuroendocrine tumors, 408
- perihilar bile ducts, 312
- retroperitoneal soft tissue sarcoma, 532
- skin melanoma, 565
- small intestinal cancer, 222
- stomach, 204
- stomach, NETs, 351
- STS, 491
- thymic tumor, 423
- trunk and extremities, STS, 508

Worst pattern of invasion (WPOI), 85, 88, 89

Z

Zollinger-Ellison syndrome (ZES), 352, 362, 410, 413
Zubrod/ECOG performance scale
- CSCC, 177
- HPV-OPSCC, 119
- larynx, 154
- lip and oral cavity, 90
- major salivary glands, 99
- mucosal melanoma, 166
- nasal cavity and paranasal sinuses, 141
- nasopharyngeal carcinoma, 107
- oropharynx, 128
